# Supplementary material for: Unravelling the Complex Duplication History of Deuterostome Glycerol Transporters
Source: Cells. 2020 Jul 10;9(7):1663. doi: 10.3390/cells9071663 (PMC7408487; doi:10.3390/cells9071663)
Supplement: Supplementary file 1 [file cells-09-01663-s001.pdf]

Fig. S1

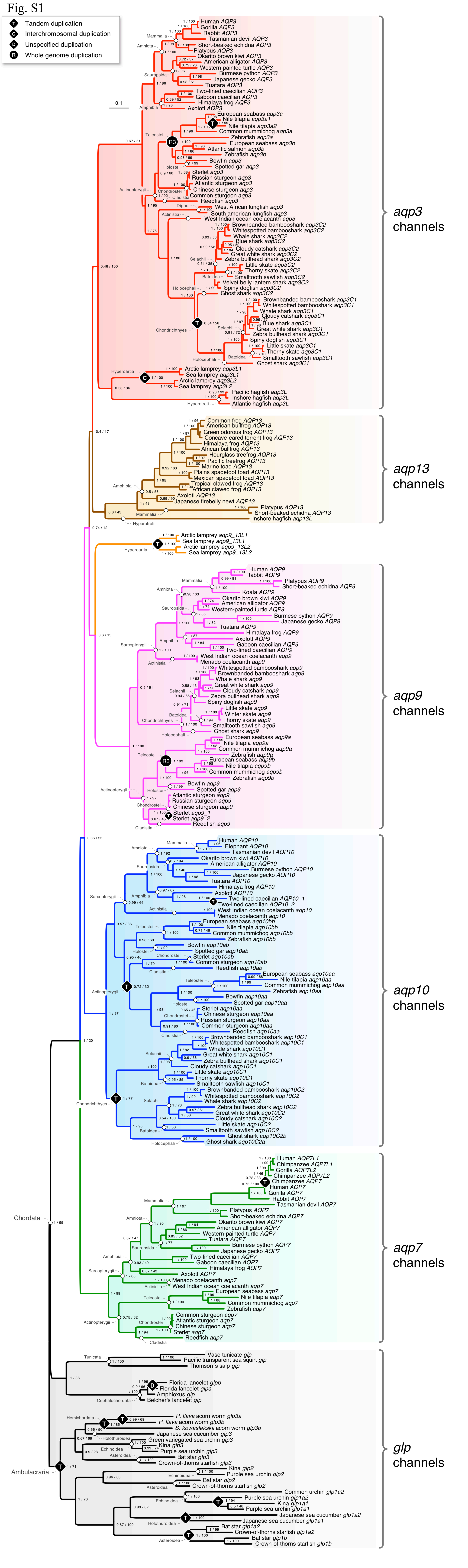

Tandem duplication
Interchromosomal duplication
Unspecified duplication
Whole genome duplication
0.1
aqp3 channels
aqp13 channels
aqp9 channels
aqp10 channels
aqp7 channels
glp channels
Chordata
Ambulacraria

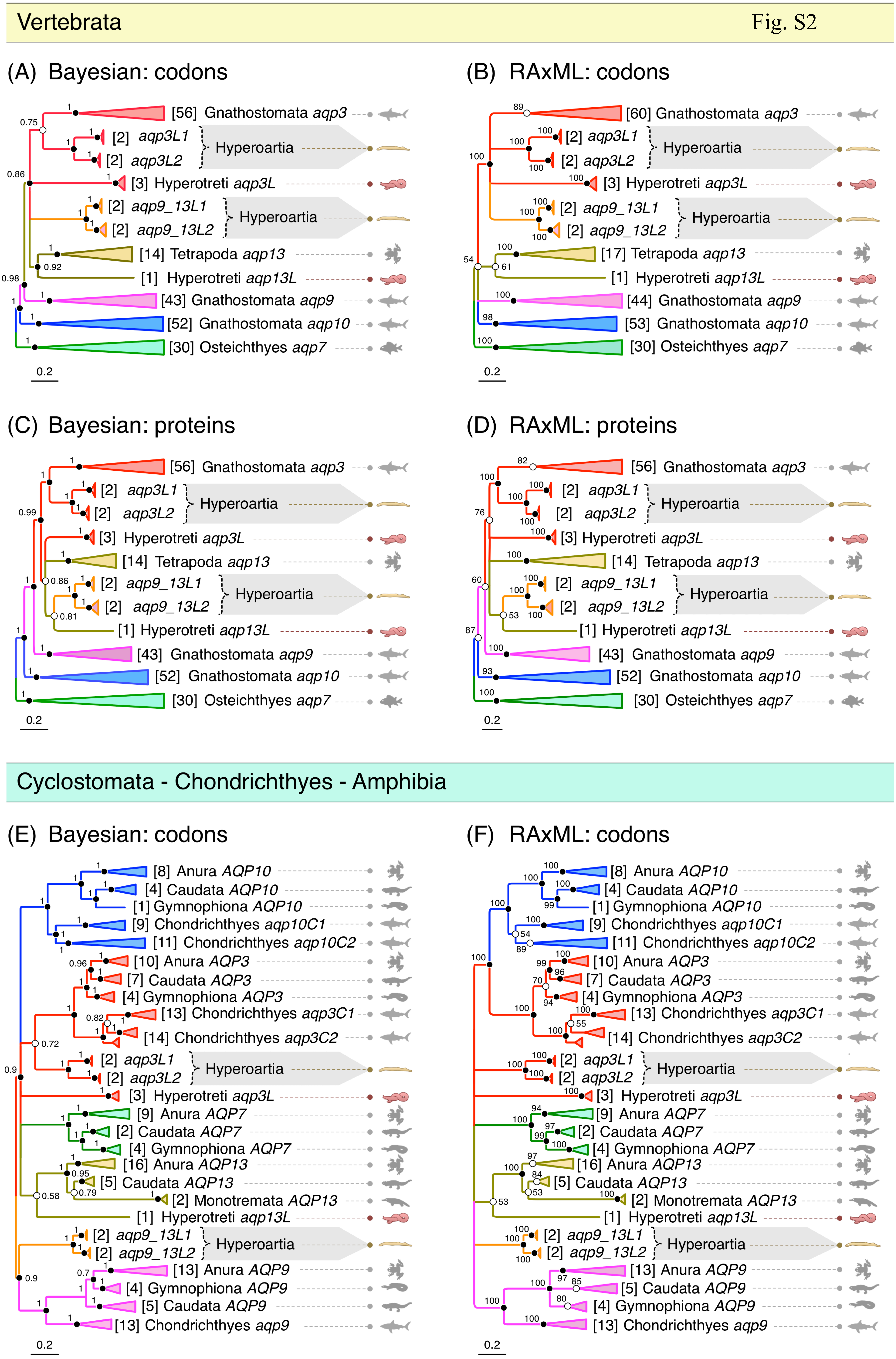
Vertebrata
Fig. S2
(A) Bayesian: codons
[56] Gnathostomata aqp3
[2] aqp3L1
[2] aqp3L2
Hyperoartia
[3] Hyperotreti aqp3L
[2] aqp9_13L1
[2] aqp9_13L2
Hyperoartia
[14] Tetrapoda aqp13
[1] Hyperotreti aqp13L
[43] Gnathostomata aqp9
[52] Gnathostomata aqp10
[30] Osteichthyes aqp7
0.2
(B) RAxML: codons
[60] Gnathostomata aqp3
[2] aqp3L1
[2] aqp3L2
Hyperoartia
[3] Hyperotreti aqp3L
[2] aqp9_13L1
[2] aqp9_13L2
Hyperoartia
[17] Tetrapoda aqp13
[1] Hyperotreti aqp13L
[44] Gnathostomata aqp9
[53] Gnathostomata aqp10
[30] Osteichthyes aqp7
0.2
(C) Bayesian: proteins
[56] Gnathostomata aqp3
[2] aqp3L1
[2] aqp3L2
Hyperoartia
[3] Hyperotreti aqp3L
[14] Tetrapoda aqp13
[2] aqp9_13L1
[2] aqp9_13L2
Hyperoartia
[1] Hyperotreti aqp13L
[43] Gnathostomata aqp9
[52] Gnathostomata aqp10
[30] Osteichthyes aqp7
0.2
(D) RAxML: proteins
[56] Gnathostomata aqp3
[2] aqp3L1
[2] aqp3L2
Hyperoartia
[3] Hyperotreti aqp3L
[14] Tetrapoda aqp13
[2] aqp9_13L1
[2] aqp9_13L2
Hyperoartia
[1] Hyperotreti aqp13L
[43] Gnathostomata aqp9
[52] Gnathostomata aqp10
[30] Osteichthyes aqp7
0.2
Cyclostomata - Chondrichthyes - Amphibia
(E) Bayesian: codons
[8] Anura AQP10
[4] Caudata AQP10
[1] Gymnophiona AQP10
[9] Chondrichthyes aqp10C1
[11] Chondrichthyes aqp10C2
[10] Anura AQP3
[7] Caudata AQP3
[4] Gymnophiona AQP3
[13] Chondrichthyes aqp3C1
[14] Chondrichthyes aqp3C2
[2] aqp3L1
[2] aqp3L2
Hyperoartia
[3] Hyperotreti aqp3L
[9] Anura AQP7
[2] Caudata AQP7
[4] Gymnophiona AQP7
[16] Anura AQP13
[5] Caudata AQP13
[2] Monotremata AQP13
[1] Hyperotreti aqp13L
[2] aqp9_13L1
[2] aqp9_13L2
Hyperoartia
[13] Anura AQP9
[4] Gymnophiona AQP9
[5] Caudata AQP9
[13] Chondrichthyes aqp9
0.2
(F) RAxML: codons
[8] Anura AQP10
[4] Caudata AQP10
[1] Gymnophiona AQP10
[9] Chondrichthyes aqp10C1
[11] Chondrichthyes aqp10C2
[10] Anura AQP3
[7] Caudata AQP3
[4] Gymnophiona AQP3
[13] Chondrichthyes aqp3C1
[14] Chondrichthyes aqp3C2
[2] aqp3L1
[2] aqp3L2
Hyperoartia
[3] Hyperotreti aqp3L
[9] Anura AQP7
[2] Caudata AQP7
[4] Gymnophiona AQP7
[16] Anura AQP13
[5] Caudata AQP13
[2] Monotremata AQP13
[1] Hyperotreti aqp13L
[2] aqp9_13L1
[2] aqp9_13L2
Hyperoartia
[13] Anura AQP9
[5] Caudata AQP9
[4] Gymnophiona AQP9
[13] Chondrichthyes aqp9
0.2

Fig. S3

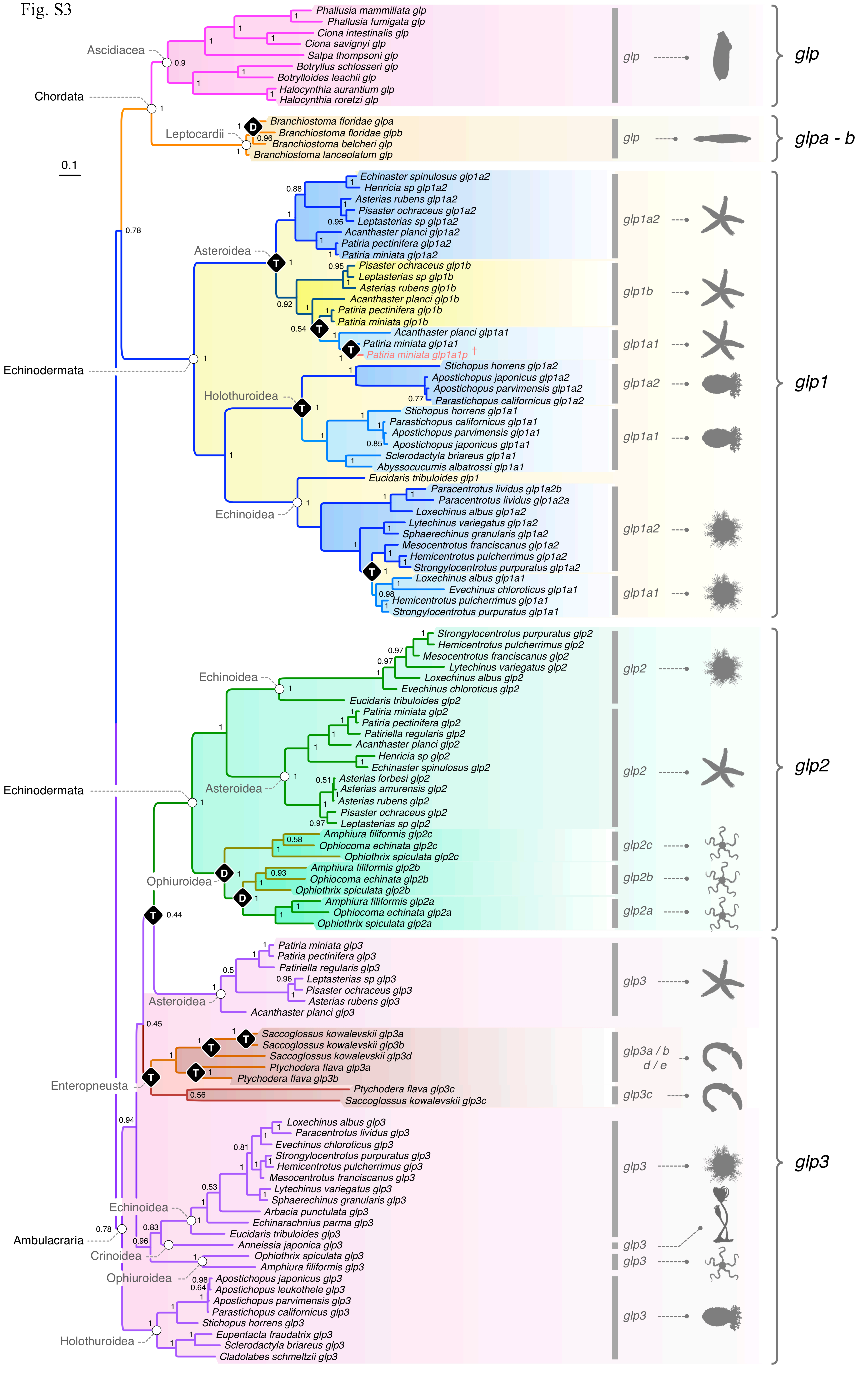

Phallusia mammillata glp
Phallusia fumigata glp
Ciona intestinalis glp
Ciona savignyi glp
Salpa thompsoni glp
Botryllus schlosseri glp
Botrylloides leachii glp
Halocynthia aurantium glp
Halocynthia roretzi glp
Ascidiacea
Chordata
Branchiostoma floridae glpa
Branchiostoma floridae glpb
Branchiostoma belcheri glp
Branchiostoma lanceolatum glp
Leptocardii
0.1
glp
glp
glpa - b
Echinaster spinulosus glp1a2
Henricia sp glp1a2
Asterias rubens glp1a2
Pisaster ochraceus glp1a2
Leptasterias sp glp1a2
Acanthaster planci glp1a2
Patiria pectinifera glp1a2
Patiria miniata glp1a2
Asteroidea
Pisaster ochraceus glp1b
Leptasterias sp glp1b
Asterias rubens glp1b
Acanthaster planci glp1b
Patiria pectinifera glp1b
Patiria miniata glp1b
Acanthaster planci glp1a1
Patiria miniata glp1a1
Patiria miniata glp1a1p †
Echinodermata
Stichopus horrens glp1a2
Apostichopus japonicus glp1a2
Apostichopus parvimensis glp1a2
Parastichopus californicus glp1a2
Holothuroidea
Stichopus horrens glp1a1
Parastichopus californicus glp1a1
Apostichopus parvimensis glp1a1
Apostichopus japonicus glp1a1
Sclerodactyla briareus glp1a1
Abyssocucumis albatrossi glp1a1
Eucidaris tribuloides glp1
Paracentrotus lividus glp1a2b
Paracentrotus lividus glp1a2a
Loxechinus albus glp1a2
Echinoidea
Lytechinus variegatus glp1a2
Sphaerechinus granularis glp1a2
Mesocentrotus franciscanus glp1a2
Hemicentrotus pulcherrimus glp1a2
Strongylocentrotus purpuratus glp1a2
Loxechinus albus glp1a1
Evechinus chloroticus glp1a1
Hemicentrotus pulcherrimus glp1a1
Strongylocentrotus purpuratus glp1a1
glp1a2
glp1b
glp1a1
glp1a2
glp1a1
glp1a2
glp1a1
glp1
Strongylocentrotus purpuratus glp2
Hemicentrotus pulcherrimus glp2
Mesocentrotus franciscanus glp2
Lytechinus variegatus glp2
Loxechinus albus glp2
Evechinus chloroticus glp2
Echinoidea
Eucidaris tribuloides glp2
Patiria miniata glp2
Patiria pectinifera glp2
Patiriella regularis glp2
Acanthaster planci glp2
Henricia sp glp2
Echinaster spinulosus glp2
Asterias forbesi glp2
Asterias amurensis glp2
Asterias rubens glp2
Pisaster ochraceus glp2
Leptasterias sp glp2
Asteroidea
Echinodermata
Amphiura filiformis glp2c
Ophiocoma echinata glp2c
Ophiothrix spiculata glp2c
Amphiura filiformis glp2b
Ophiocoma echinata glp2b
Ophiothrix spiculata glp2b
Ophiuroidea
Amphiura filiformis glp2a
Ophiocoma echinata glp2a
Ophiothrix spiculata glp2a
glp2
glp2
glp2c
glp2b
glp2a
glp2
Patiria miniata glp3
Patiria pectinifera glp3
Patiriella regularis glp3
Leptasterias sp glp3
Pisaster ochraceus glp3
Asterias rubens glp3
Asteroidea
Acanthaster planci glp3
Saccoglossus kowalevskii glp3a
Saccoglossus kowalevskii glp3b
Saccoglossus kowalevskii glp3d
Ptychodera flava glp3a
Ptychodera flava glp3b
Enteropneusta
Ptychodera flava glp3c
Saccoglossus kowalevskii glp3c
Loxechinus albus glp3
Paracentrotus lividus glp3
Evechinus chloroticus glp3
Strongylocentrotus purpuratus glp3
Hemicentrotus pulcherrimus glp3
Mesocentrotus franciscanus glp3
Lytechinus variegatus glp3
Sphaerechinus granularis glp3
Arbacia punctulata glp3
Echinoidea
Echinarachnius parma glp3
Eucidaris tribuloides glp3
Ambulacraria
Anneissia japonica glp3
Crinoidea
Ophiothrix spiculata glp3
Amphiura filiformis glp3
Ophiuroidea
Apostichopus japonicus glp3
Apostichopus leukothele glp3
Apostichopus parvimensis glp3
Parastichopus californicus glp3
Stichopus horrens glp3
Holothuroidea
Eupentacta fraudatrix glp3
Sclerodactyla briareus glp3
Cladolabes schmeltzii glp3
glp3
glp3a / b
d / e
glp3c
glp3
glp3
glp3
glp3
glp3

Fig. S4

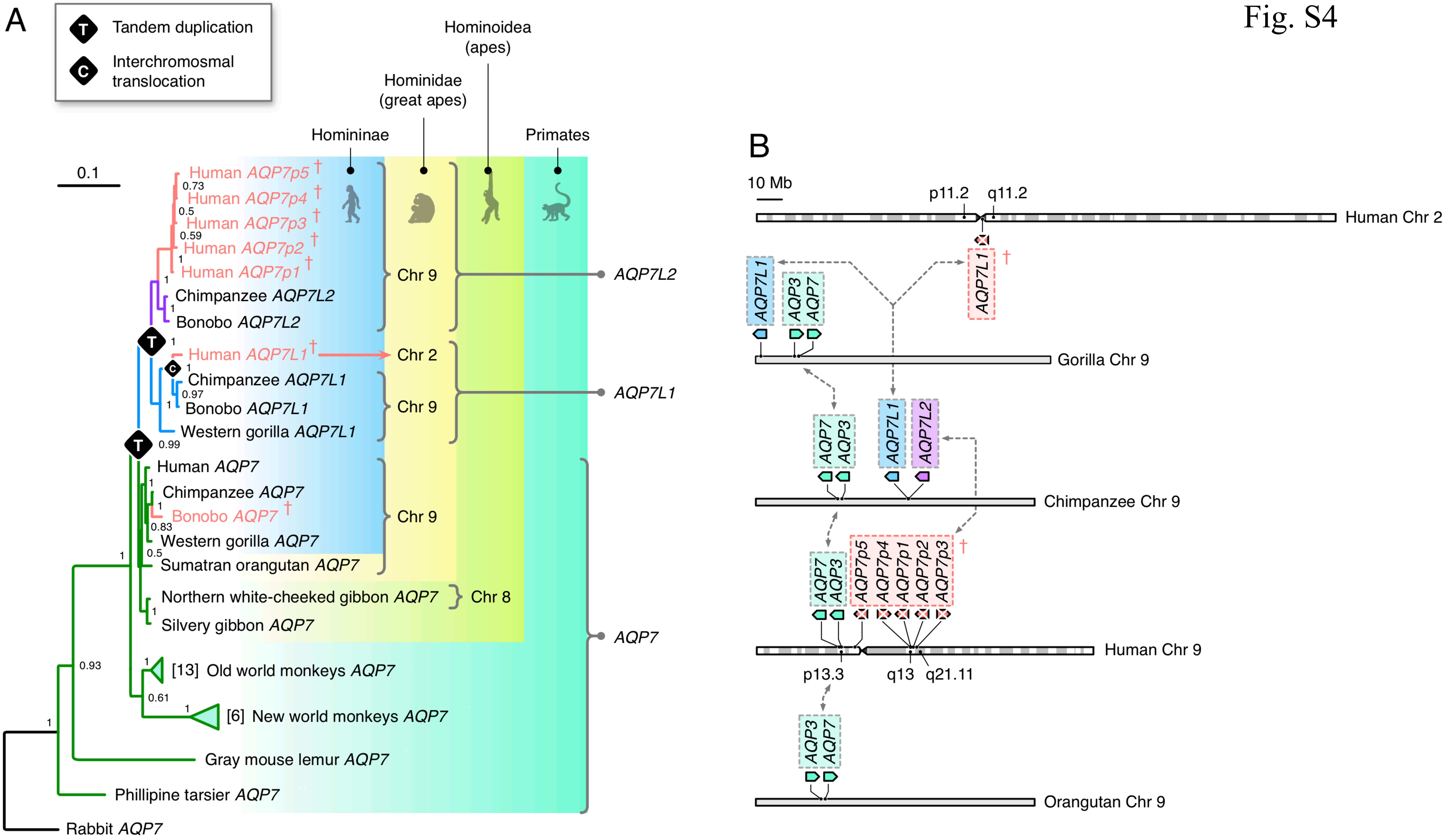
A
Tandem duplication
Interchromosmal translocation
Hominoidea (apes)
Hominidae (great apes)
Homininae
Primates
0.1
Human AQP7p5
Human AQP7p4
Human AQP7p3
Human AQP7p2
Human AQP7p1
Chimpanzee AQP7L2
Bonobo AQP7L2
Human AQP7L1
Chimpanzee AQP7L1
Bonobo AQP7L1
Western gorilla AQP7L1
Human AQP7
Chimpanzee AQP7
Bonobo AQP7
Western gorilla AQP7
Sumatran orangutan AQP7
Northern white-cheeked gibbon AQP7
Silvery gibbon AQP7
[13] Old world monkeys AQP7
[6] New world monkeys AQP7
Gray mouse lemur AQP7
Phillipine tarsier AQP7
Rabbit AQP7
Chr 9
Chr 2
Chr 8
AQP7L2
AQP7L1
AQP7
B
10 Mb
p11.2
q11.2
Human Chr 2
Gorilla Chr 9
Chimpanzee Chr 9
Human Chr 9
Orangutan Chr 9
p13.3
q13
q21.11
AQP7L1
AQP3
AQP7
AQP7L2
AQP7p5
AQP7p4
AQP7p1
AQP7p2
AQP7p3

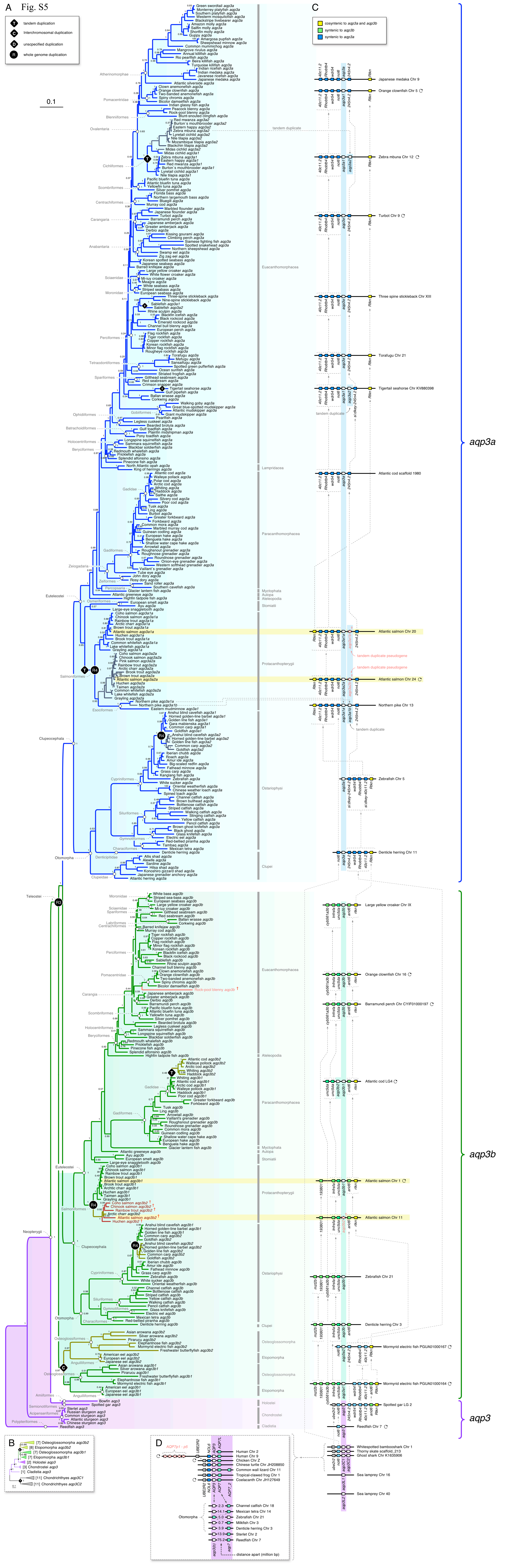
A
Fig. S5
tandem duplication
Interchromosomal duplication
unspecified duplication
whole genome duplication
0.1
C
cosyntenic to aqp3a and aqp3b
syntenic to aqp3b
syntenic to aqp3a
aqp3a
aqp3b
aqp3
B
D
Japanese medaka Chr 9
Orange clownfish Chr 5
Zebra mbuna Chr 12
Turbot Chr 9
Three spine stickleback Chr XIII
Torafugu Chr 21
Tigertail seahorse Chr KV880398
Atlantic cod scaffold 1980
Atlantic salmon Chr 20
Atlantic salmon Chr 24
Northern pike Chr 13
Zebrafish Chr 5
Denticle herring Chr 11
Large yellow croaker Chr IX
Orange clownfish Chr 16
Atlantic cod LG4
Atlantic salmon Chr 1
Atlantic salmon Chr 11
Zebrafish Chr 21
Denticle herring Chr 3
Spotted gar LG 2
Reedfish Chr 7
Sea lamprey Chr 16
Sea lamprey Chr 40
Human Chr 2
Human Chr 9
Chicken Chr Z
distance apart (million bp)

A Fig. S6

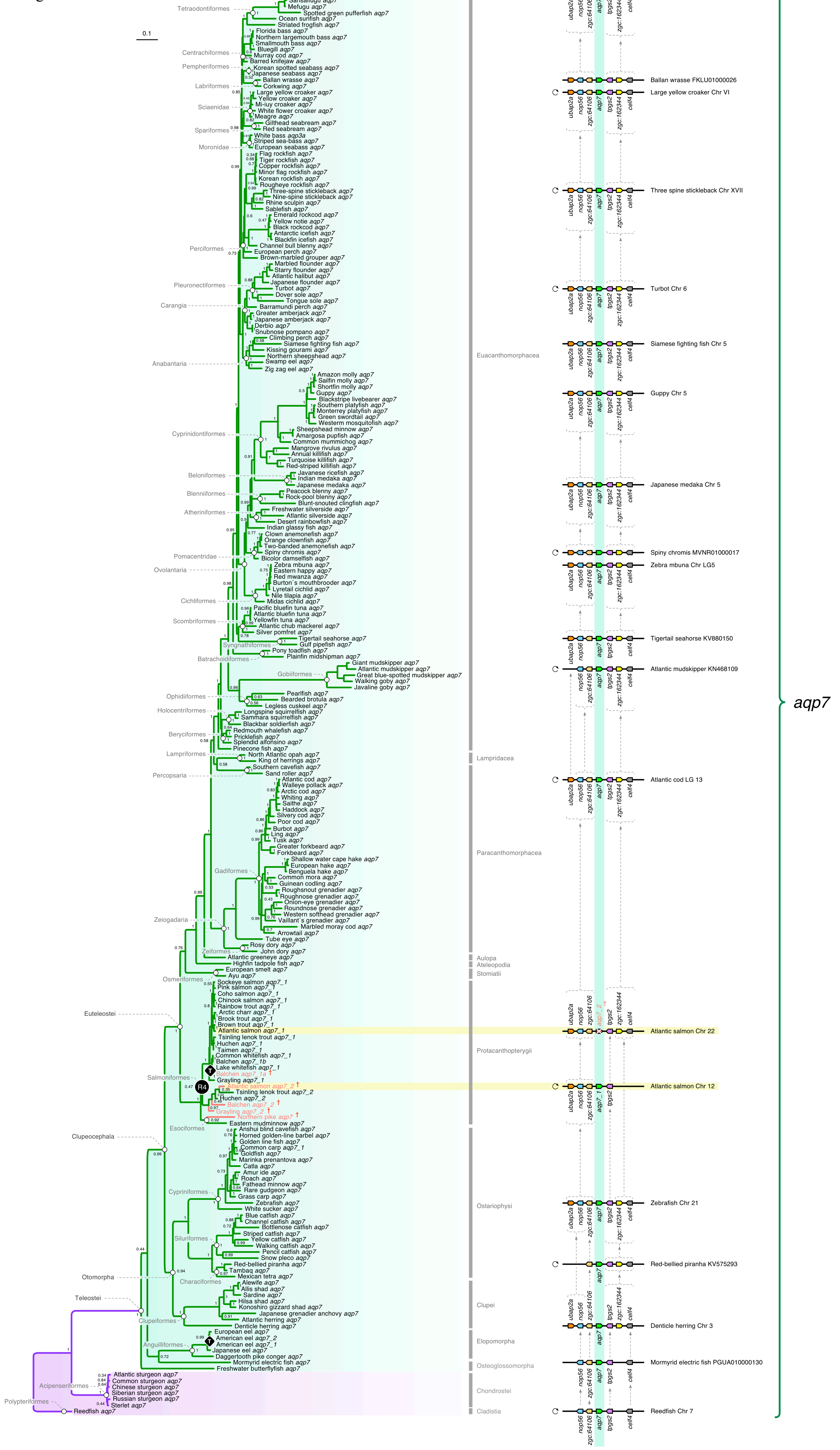

B
0.1
Tetraodontiformes
Centrachiformes
Pempheriformes
Labriformes
Sciaenidae
Spariformes
Moronidae
Perciformes
Pleuronectiformes
Carangia
Anabantaria
Cyprinidontiformes
Beloniformes
Blenniiformes
Atheriniformes
Pomacentridae
Ovolantaria
Cichliformes
Scombriformes
Syngnathiformes
Batrachoidiformes
Gobiiformes
Ophidiiformes
Holocentriformes
Beryciformes
Lampriformes
Percopsaria
Gadiformes
Zeiogadaria
Zeiformes
Osmeriformes
Euteleostei
Salmoniformes
Esociformes
Clupeocephala
Cypriniformes
Siluriformes
Characiformes
Otomorpha
Teleostei
Clupeiformes
Anguilliformes
Acipenseriformes
Polypteriformes
Euacanthomorphacea
Lampridacea
Paracanthomorphacea
Aulopa
Ateleopodia
Stomiatii
Protacanthopterygii
Ostariophysi
Clupei
Elopomorpha
Osteoglossomorpha
Chondrostei
Cladistia
R4
T
ubap2a
nop56
zgc:64106
aqp7
tpgs2
zgc:162344
cell4
Torafugu Chr 19
Ballan wrasse FKLU01000026
Large yellow croaker Chr VI
Three spine stickleback Chr XVII
Turbot Chr 6
Siamese fighting fish Chr 5
Guppy Chr 5
Japanese medaka Chr 5
Spiny chromis MVNR01000017
Zebra mbuna Chr LG5
Tigertail seahorse KV880150
Atlantic mudskipper KN468109
Atlantic cod LG 13
Atlantic salmon Chr 22
Atlantic salmon Chr 12
Zebrafish Chr 21
Red-bellied piranha KV575293
Denticle herring Chr 3
Mormyrid electric fish PGUA010000130
Reedfish Chr 7
aqp7

Fig. S7

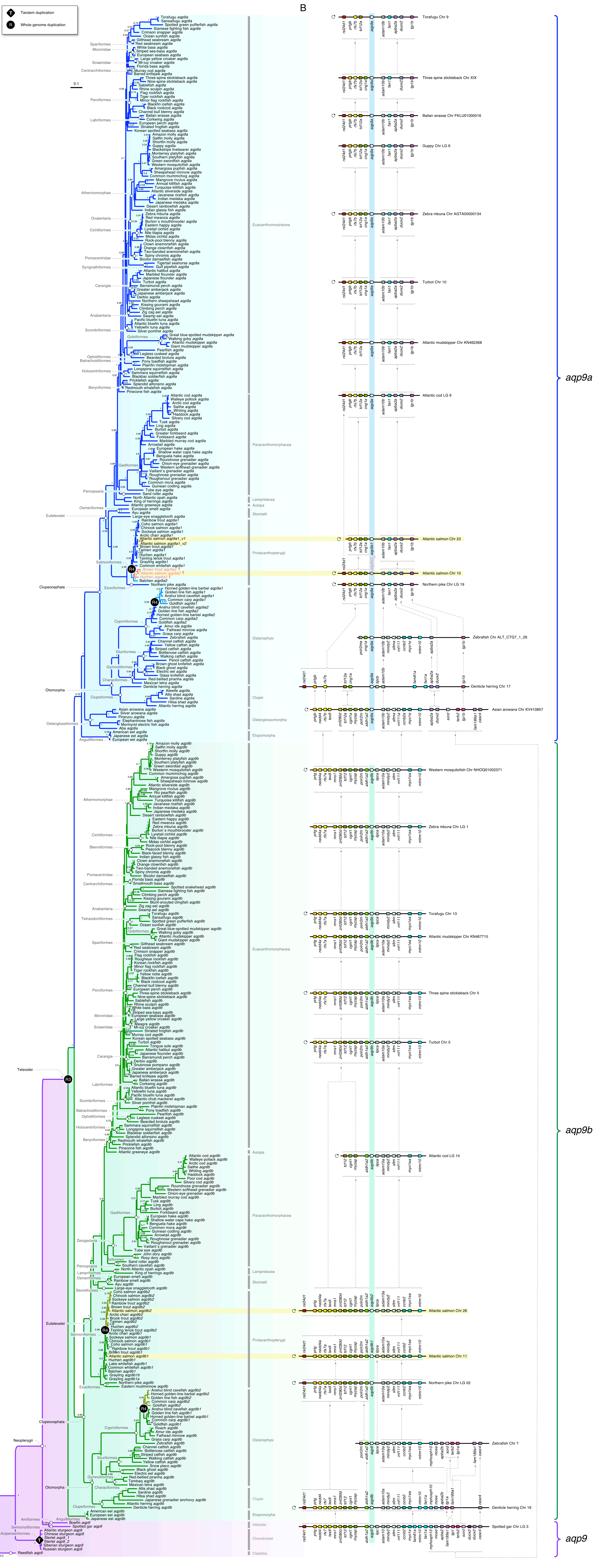
A
B
Tandem duplication
Whole genome duplication
aqp9a
aqp9b
aqp9

A Fig. S8

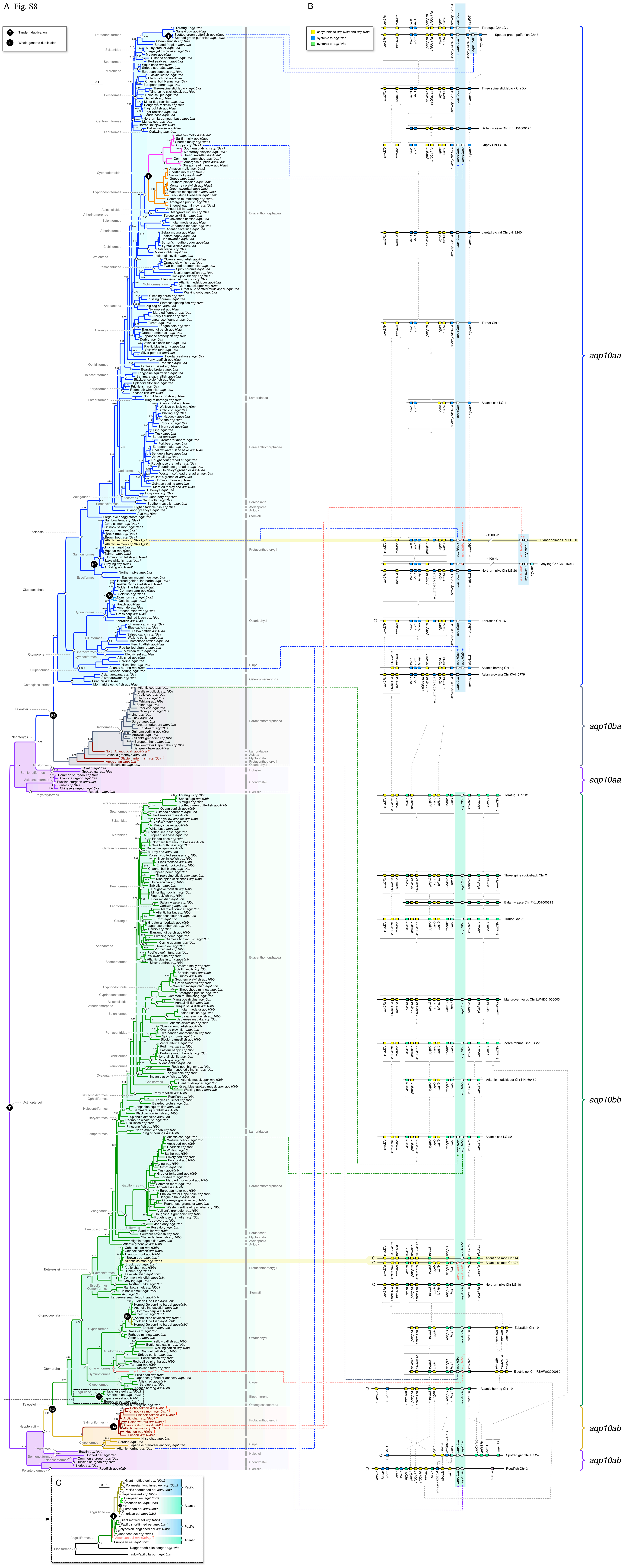
B
C
Tandem duplication
Whole genome duplication
cosyntenic to aqp10aa and aqp10bb
syntenic to aqp10aa
syntenic to aqp10bb
aqp10aa
aqp10ba
aqp10aa
aqp10bb
aqp10ab
aqp10ab
Euacanthomorphacea
Paracanthomorphacea
Protacanthopterygii
Ostariophysi
Clupei
Osteoglossomorpha
Lampridacea
Stomiati
Holostei
Chondrostei
Cladistia
Elopomorpha
Actinopterygii
Neopterygii
Teleostei
Atlantic salmon Chr LG 20
Atlantic salmon Chr 14
Atlantic salmon Chr 27
Pacific
Atlantic

Fig. S9

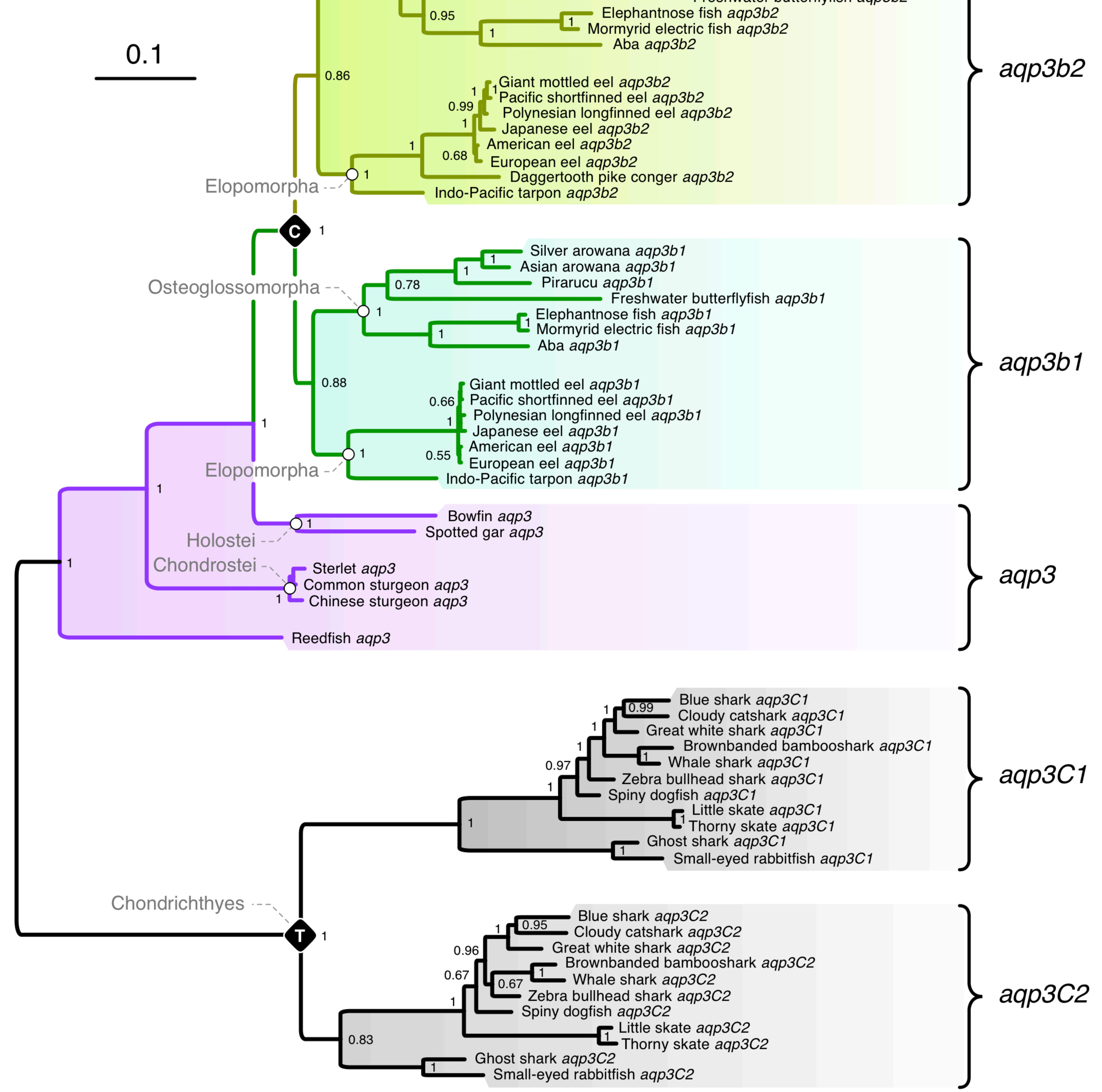
0.1
Osteoglossomorpha
Elopomorpha
Holostei
Chondrostei
Chondrichthyes
C
T
Silver arowana aqp3b2
Asian arowana aqp3b2
Pirarucu aqp3b2
Freshwater butterflyfish aqp3b2
Elephantnose fish aqp3b2
Mormyrid electric fish aqp3b2
Aba aqp3b2
Giant mottled eel aqp3b2
Pacific shortfinned eel aqp3b2
Polynesian longfinned eel aqp3b2
Japanese eel aqp3b2
American eel aqp3b2
European eel aqp3b2
Daggertooth pike conger aqp3b2
Indo-Pacific tarpon aqp3b2
Silver arowana aqp3b1
Asian arowana aqp3b1
Pirarucu aqp3b1
Freshwater butterflyfish aqp3b1
Elephantnose fish aqp3b1
Mormyrid electric fish aqp3b1
Aba aqp3b1
Giant mottled eel aqp3b1
Pacific shortfinned eel aqp3b1
Polynesian longfinned eel aqp3b1
Japanese eel aqp3b1
American eel aqp3b1
European eel aqp3b1
Indo-Pacific tarpon aqp3b1
Bowfin aqp3
Spotted gar aqp3
Sterlet aqp3
Common sturgeon aqp3
Chinese sturgeon aqp3
Reedfish aqp3
Blue shark aqp3C1
Cloudy catshark aqp3C1
Great white shark aqp3C1
Brownbanded bambooshark aqp3C1
Whale shark aqp3C1
Zebra bullhead shark aqp3C1
Spiny dogfish aqp3C1
Little skate aqp3C1
Thorny skate aqp3C1
Ghost shark aqp3C1
Small-eyed rabbitfish aqp3C1
Blue shark aqp3C2
Cloudy catshark aqp3C2
Great white shark aqp3C2
Brownbanded bambooshark aqp3C2
Whale shark aqp3C2
Zebra bullhead shark aqp3C2
Spiny dogfish aqp3C2
Little skate aqp3C2
Thorny skate aqp3C2
Ghost shark aqp3C2
Small-eyed rabbitfish aqp3C2
aqp3b2
aqp3b1
aqp3
aqp3C1
aqp3C2

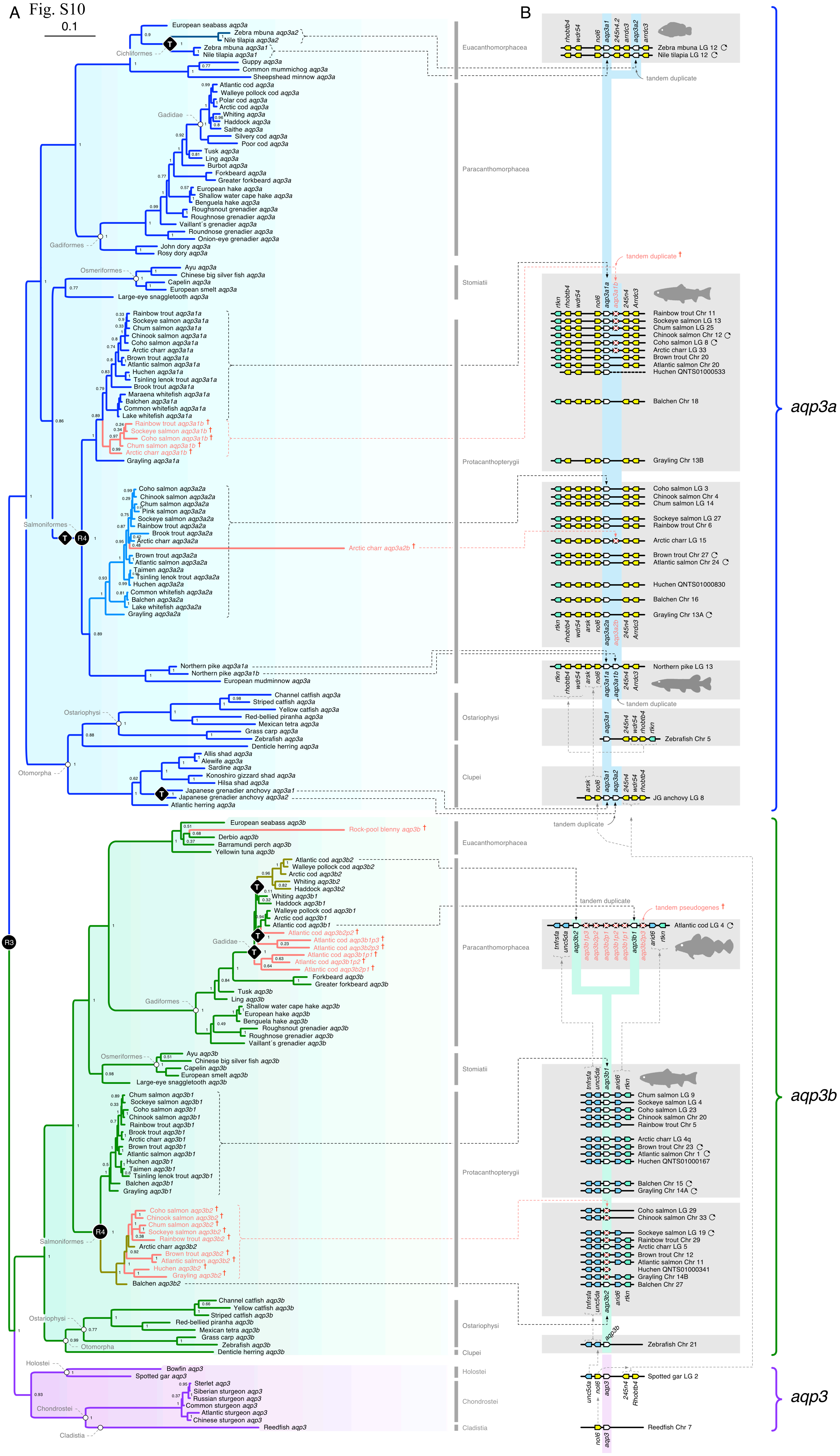
A
0.1
B
European seabass aqp3a
Zebra mbuna aqp3a2
Nile tilapia aqp3a2
Zebra mbuna aqp3a1
Nile tilapia aqp3a1
Cichliformes
Guppy aqp3a
Common mummichog aqp3a
Sheepshead minnow aqp3a
Atlantic cod aqp3a
Walleye pollock cod aqp3a
Polar cod aqp3a
Arctic cod aqp3a
Whiting aqp3a
Haddock aqp3a
Saithe aqp3a
Gadidae
Silvery cod aqp3a
Poor cod aqp3a
Tusk aqp3a
Ling aqp3a
Burbot aqp3a
Forkbeard aqp3a
Greater forkbeard aqp3a
European hake aqp3a
Shallow water cape hake aqp3a
Benguela hake aqp3a
Roughsnout grenadier aqp3a
Roughnose grenadier aqp3a
Vaillant´s grenadier aqp3a
Roundnose grenadier aqp3a
Onion-eye grenadier aqp3a
Gadiformes
John dory aqp3a
Rosy dory aqp3a
Osmeriformes
Ayu aqp3a
Chinese big silver fish aqp3a
Capelin aqp3a
European smelt aqp3a
Large-eye snaggletooth aqp3a
Rainbow trout aqp3a1a
Sockeye salmon aqp3a1a
Chum salmon aqp3a1a
Chinook salmon aqp3a1a
Coho salmon aqp3a1a
Arctic charr aqp3a1a
Brown trout aqp3a1a
Atlantic salmon aqp3a1a
Huchen aqp3a1a
Tsinling lenok trout aqp3a1a
Brook trout aqp3a1a
Maraena whitefish aqp3a1a
Balchen aqp3a1a
Common whitefish aqp3a1a
Lake whitefish aqp3a1a
Rainbow trout aqp3a1b †
Sockeye salmon aqp3a1b †
Coho salmon aqp3a1b †
Chum salmon aqp3a1b †
Arctic charr aqp3a1b †
Grayling aqp3a1a
Coho salmon aqp3a2a
Chinook salmon aqp3a2a
Chum salmon aqp3a2a
Pink salmon aqp3a2a
Sockeye salmon aqp3a2a
Rainbow trout aqp3a2a
Brook trout aqp3a2a
Arctic charr aqp3a2a
Arctic charr aqp3a2b †
Brown trout aqp3a2a
Atlantic salmon aqp3a2a
Taimen aqp3a2a
Tsinling lenok trout aqp3a2a
Huchen aqp3a2a
Common whitefish aqp3a2a
Balchen aqp3a2a
Lake whitefish aqp3a2a
Grayling aqp3a2a
Salmoniformes
T
R4
Northern pike aqp3a1a
Northern pike aqp3a1b
European mudminnow aqp3a
Channel catfish aqp3a
Striped catfish aqp3a
Yellow catfish aqp3a
Red-bellied piranha aqp3a
Mexican tetra aqp3a
Grass carp aqp3a
Zebrafish aqp3a
Denticle herring aqp3a
Ostariophysi
Allis shad aqp3a
Alewife aqp3a
Sardine aqp3a
Konoshiro gizzard shad aqp3a
Hilsa shad aqp3a
Japanese grenadier anchovy aqp3a1
Japanese grenadier anchovy aqp3a2
Atlantic herring aqp3a
Otomorpha
R3
European seabass aqp3b
Rock-pool blenny aqp3b †
Derbio aqp3b
Barramundi perch aqp3b
Yellowtin tuna aqp3b
Atlantic cod aqp3b2
Walleye pollock cod aqp3b2
Arctic cod aqp3b2
Whiting aqp3b2
Haddock aqp3b2
Whiting aqp3b1
Haddock aqp3b1
Walleye pollock cod aqp3b1
Arctic cod aqp3b1
Atlantic cod aqp3b1
Atlantic cod aqp3b2p2 †
Atlantic cod aqp3b1p3 †
Atlantic cod aqp3b2p3 †
Atlantic cod aqp3b1p1 †
Atlantic cod aqp3b1p2 †
Atlantic cod aqp3b2p1 †
Gadidae
Forkbeard aqp3b
Greater forkbeard aqp3b
Tusk aqp3b
Ling aqp3b
Shallow water cape hake aqp3b
European hake aqp3b
Benguela hake aqp3b
Roughsnout grenadier aqp3b
Roughnose grenadier aqp3b
Vaillant´s grenadier aqp3b
Gadiformes
Osmeriformes
Ayu aqp3b
Chinese big silver fish aqp3b
Capelin aqp3b
European smelt aqp3b
Large-eye snaggletooth aqp3b
Chum salmon aqp3b1
Sockeye salmon aqp3b1
Coho salmon aqp3b1
Chinook salmon aqp3b1
Rainbow trout aqp3b1
Brook trout aqp3b1
Arctic charr aqp3b1
Brown trout aqp3b1
Atlantic salmon aqp3b1
Huchen aqp3b1
Taimen aqp3b1
Tsinling lenok trout aqp3b1
Balchen aqp3b1
Grayling aqp3b1
Coho salmon aqp3b2 †
Chinook salmon aqp3b2 †
Chum salmon aqp3b2 †
Sockeye salmon aqp3b2 †
Rainbow trout aqp3b2 †
Arctic charr aqp3b2
Brown trout aqp3b2 †
Atlantic salmon aqp3b2 †
Huchen aqp3b2 †
Grayling aqp3b2 †
Balchen aqp3b2
Salmoniformes
Channel catfish aqp3b
Yellow catfish aqp3b
Striped catfish aqp3b
Red-bellied piranha aqp3b
Mexican tetra aqp3b
Grass carp aqp3b
Zebrafish aqp3b
Denticle herring aqp3b
Ostariophysi
Otomorpha
Holostei
Bowfin aqp3
Spotted gar aqp3
Sterlet aqp3
Siberian sturgeon aqp3
Russian sturgeon aqp3
Common sturgeon aqp3
Atlantic sturgeon aqp3
Chinese sturgeon aqp3
Chondrostei
Reedfish aqp3
Cladistia
Euacanthomorphacea
Paracanthomorphacea
Stomiatii
Protacanthopterygii
Ostariophysi
Clupei
Euacanthomorphacea
Paracanthomorphacea
Stomiatii
Protacanthopterygii
Ostariophysi
Clupei
Holostei
Chondrostei
Cladistia
rhobtb4 wdr54 nol6 aqp3a1 245n4.2 arrdc3 aqp3a2 arrdc3
Zebra mbuna LG 12
Nile tilapia LG 12
tandem duplicate
tandem duplicate †
rtkn rhobtb4 wdr54 nol6 aqp3a1a aqp3a1b 245n4 Arrdc3
Rainbow trout Chr 11
Sockeye salmon LG 13
Chum salmon LG 25
Chinook salmon Chr 12
Coho salmon LG 8
Arctic charr LG 33
Brown trout Chr 20
Atlantic salmon Chr 20
Huchen QNTS01000533
Balchen Chr 18
Grayling Chr 13B
Coho salmon LG 3
Chinook salmon Chr 4
Chum salmon LG 14
Sockeye salmon LG 27
Rainbow trout Chr 6
Arctic charr LG 15
Brown trout Chr 27
Atlantic salmon Chr 24
Huchen QNTS01000830
Balchen Chr 16
Grayling Chr 13A
rtkn rhobtb4 wdr54 arsk nol6 aqp3a2a aqp3a2b 245n4 Arrdc3
Northern pike LG 13
rtkn rhobtb4 wdr54 arsk nol6 aqp3a1a aqp3a1b 245n4 Arrdc3
tandem duplicate
aqp3a1 245n4 wdr54 rhobtb4 rtkn
Zebrafish Chr 5
arsk nol6 aqp3a1 aqp3a2 245n4 wdr54 rhobtb4
JG anchovy LG 8
tandem duplicate
tandem duplicate
tandem pseudogenes †
Atlantic cod LG 4
tnfrsfa unc5da aqp3b2 aqp3b1p3 aqp3b2p2 aqp3b2p1 aqp3b1p2 aqp3b1p1 aqp3b1 aqp3b2p3 arid6 rtkn
tnfrsfa unc5da aqp3b1 arid6 rtkn
Chum salmon LG 9
Sockeye salmon LG 4
Coho salmon LG 23
Chinook salmon Chr 20
Rainbow trout Chr 5
Arctic charr LG 4q
Brown trout Chr 23
Atlantic salmon Chr 1
Huchen QNTS01000167
Balchen Chr 15
Grayling Chr 14A
Coho salmon LG 29
Chinook salmon Chr 33
Sockeye salmon LG 19
Rainbow trout Chr 29
Arctic charr LG 5
Brown trout Chr 12
Atlantic salmon Chr 11
Huchen QNTS01000341
Grayling Chr 14B
Balchen Chr 27
tnfrsfa unc5da aqp3b2 arid6 rtkn
aqp3b
Zebrafish Chr 21
Spotted gar LG 2
unc5da nol6 aqp3 245n4 Rhobtb4
Reedfish Chr 7
nol6 aqp3
aqp3a
aqp3b
aqp3

Fig. S10

Fig. S11

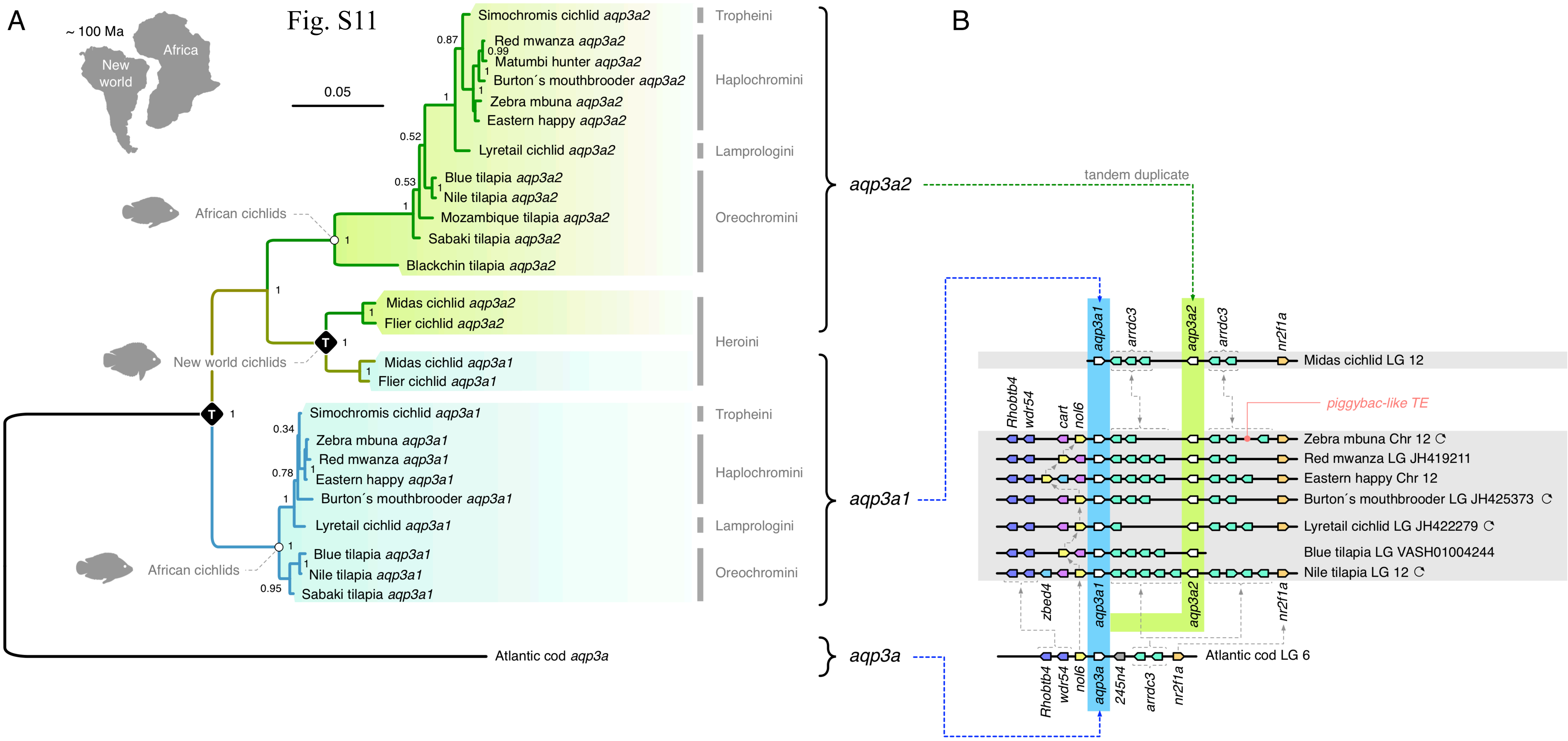

A
~ 100 Ma
Africa
New world
0.05
Simochromis cichlid aqp3a2
Red mwanza aqp3a2
Matumbi hunter aqp3a2
Burton´s mouthbrooder aqp3a2
Zebra mbuna aqp3a2
Eastern happy aqp3a2
Lyretail cichlid aqp3a2
Blue tilapia aqp3a2
Nile tilapia aqp3a2
Mozambique tilapia aqp3a2
Sabaki tilapia aqp3a2
Blackchin tilapia aqp3a2
Midas cichlid aqp3a2
Flier cichlid aqp3a2
Midas cichlid aqp3a1
Flier cichlid aqp3a1
Simochromis cichlid aqp3a1
Zebra mbuna aqp3a1
Red mwanza aqp3a1
Eastern happy aqp3a1
Burton´s mouthbrooder aqp3a1
Lyretail cichlid aqp3a1
Blue tilapia aqp3a1
Nile tilapia aqp3a1
Sabaki tilapia aqp3a1
Atlantic cod aqp3a
African cichlids
New world cichlids
Tropheini
Haplochromini
Lamprologini
Oreochromini
Heroini
aqp3a2
aqp3a1
aqp3a
B
tandem duplicate
piggybac-like TE
Midas cichlid LG 12
Zebra mbuna Chr 12
Red mwanza LG JH419211
Eastern happy Chr 12
Burton´s mouthbrooder LG JH425373
Lyretail cichlid LG JH422279
Blue tilapia LG VASH01004244
Nile tilapia LG 12
Atlantic cod LG 6
Rhobtb4
wdr54
cart
nol6
zbed4
arrdc3
nr2f1a
245n4

A Fig. S12

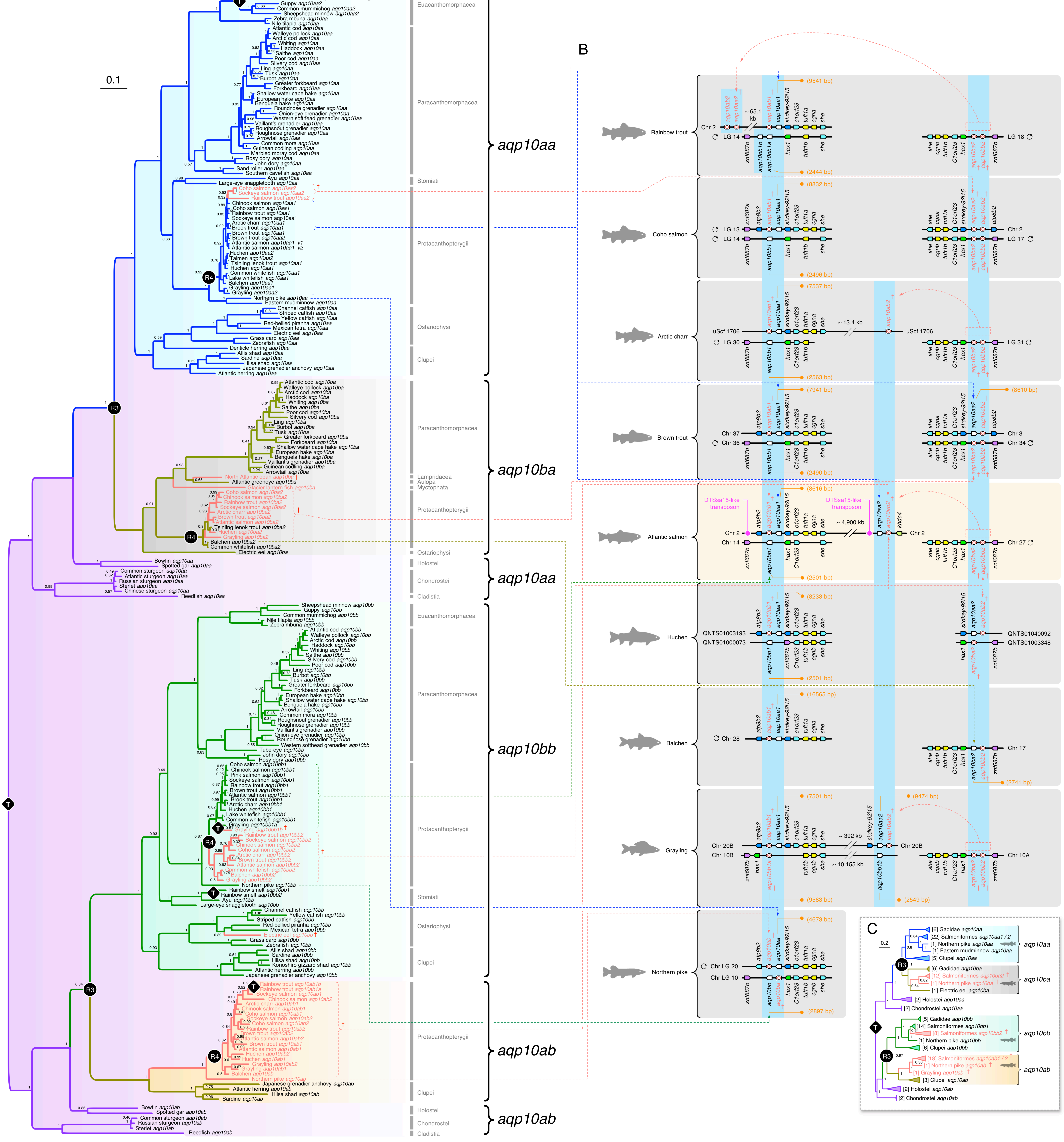

0.1
aqp10aa
aqp10ba
aqp10aa
aqp10bb
aqp10ab
aqp10ab
Euacanthomorphacea
Paracanthomorphacea
Stomiatii
Protacanthopterygii
Ostariophysi
Clupei
Lampridacea
Aulopa
Myctophata
Holostei
Chondrostei
Cladistia
B
Rainbow trout
Coho salmon
Arctic charr
Brown trout
Atlantic salmon
Huchen
Balchen
Grayling
Northern pike
DTSsa15-like transposon
C
aqp10aa
aqp10ba
aqp10bb
aqp10ab

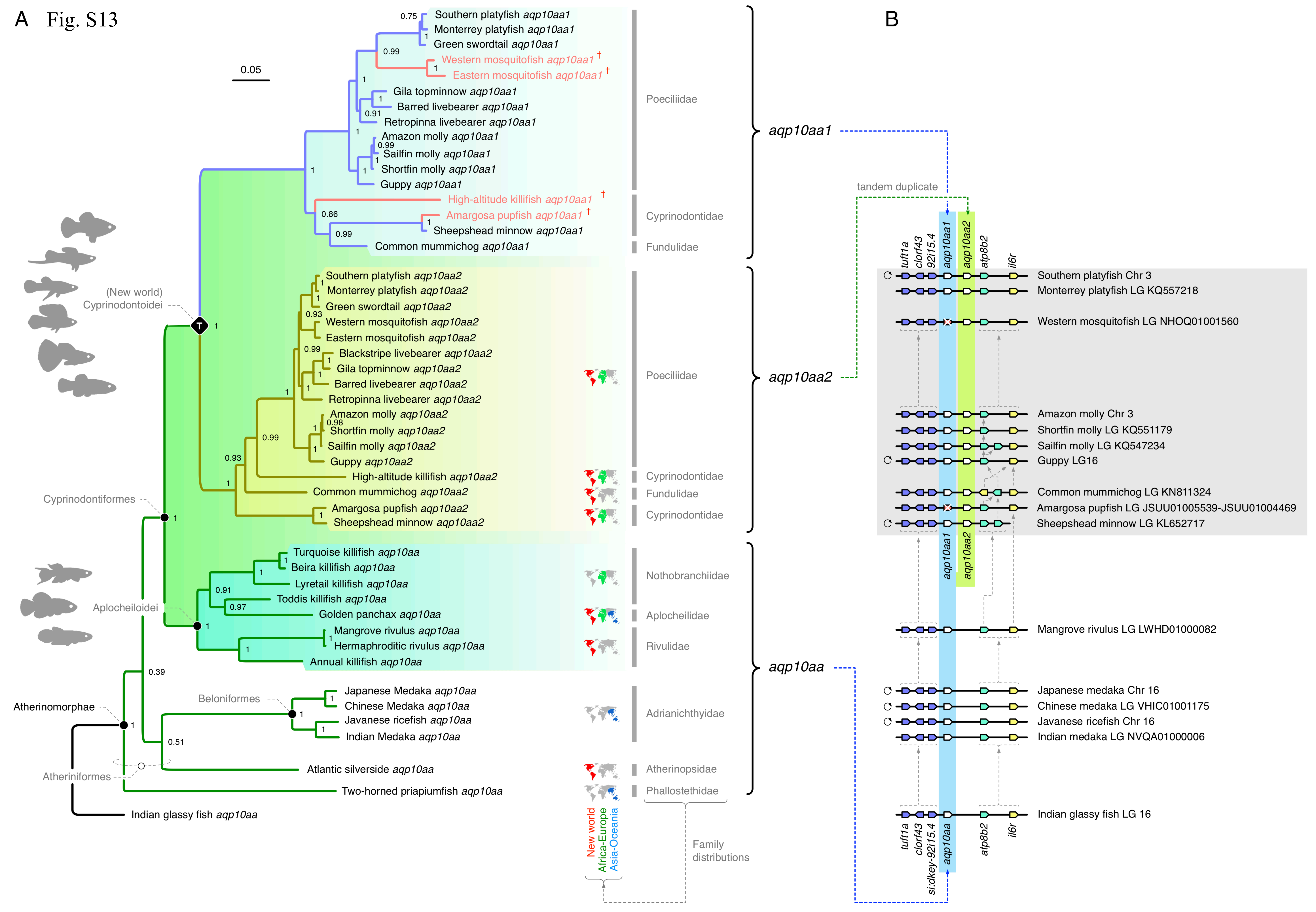

A
Fig. S13
B
0.05
Southern platyfish aqp10aa1
Monterrey platyfish aqp10aa1
Green swordtail aqp10aa1
Western mosquitofish aqp10aa1 †
Eastern mosquitofish aqp10aa1 †
Gila topminnow aqp10aa1
Barred livebearer aqp10aa1
Retropinna livebearer aqp10aa1
Amazon molly aqp10aa1
Sailfin molly aqp10aa1
Shortfin molly aqp10aa1
Guppy aqp10aa1
High-altitude killifish aqp10aa1 †
Amargosa pupfish aqp10aa1 †
Sheepshead minnow aqp10aa1
Common mummichog aqp10aa1
Southern platyfish aqp10aa2
Monterrey platyfish aqp10aa2
Green swordtail aqp10aa2
Western mosquitofish aqp10aa2
Eastern mosquitofish aqp10aa2
Blackstripe livebearer aqp10aa2
Gila topminnow aqp10aa2
Barred livebearer aqp10aa2
Retropinna livebearer aqp10aa2
Amazon molly aqp10aa2
Shortfin molly aqp10aa2
Sailfin molly aqp10aa2
Guppy aqp10aa2
High-altitude killifish aqp10aa2
Common mummichog aqp10aa2
Amargosa pupfish aqp10aa2
Sheepshead minnow aqp10aa2
Turquoise killifish aqp10aa
Beira killifish aqp10aa
Lyretail killifish aqp10aa
Toddis killifish aqp10aa
Golden panchax aqp10aa
Mangrove rivulus aqp10aa
Hermaphroditic rivulus aqp10aa
Annual killifish aqp10aa
Japanese Medaka aqp10aa
Chinese Medaka aqp10aa
Javanese ricefish aqp10aa
Indian Medaka aqp10aa
Atlantic silverside aqp10aa
Two-horned priapiumfish aqp10aa
Indian glassy fish aqp10aa
(New world) Cyprinodontoidei
Cyprinodontiformes
Aplocheiloidei
Beloniformes
Atherinomorphae
Atheriniformes
Poeciliidae
Cyprinodontidae
Fundulidae
Poeciliidae
Cyprinodontidae
Fundulidae
Cyprinodontidae
Nothobranchiidae
Aplocheilidae
Rivulidae
Adrianichthyidae
Atherinopsidae
Phallostethidae
New world
Africa-Europe
Asia-Oceania
Family distributions
aqp10aa1
aqp10aa2
aqp10aa
tandem duplicate
tuft1a
clorf43
92i15.4
aqp10aa1
aqp10aa2
atp8b2
il6r
Southern platyfish Chr 3
Monterrey platyfish LG KQ557218
Western mosquitofish LG NHOQ01001560
Amazon molly Chr 3
Shortfin molly LG KQ551179
Sailfin molly LG KQ547234
Guppy LG16
Common mummichog LG KN811324
Amargosa pupfish LG JSUU01005539-JSUU01004469
Sheepshead minnow LG KL652717
Mangrove rivulus LG LWHD01000082
Japanese medaka Chr 16
Chinese medaka LG VHIC01001175
Javanese ricefish Chr 16
Indian medaka LG NVQA01000006
Indian glassy fish LG 16
tuft1a
clorf43
si:dkey-92i15.4
aqp10aa
atp8b2
il6r

Fig. S14

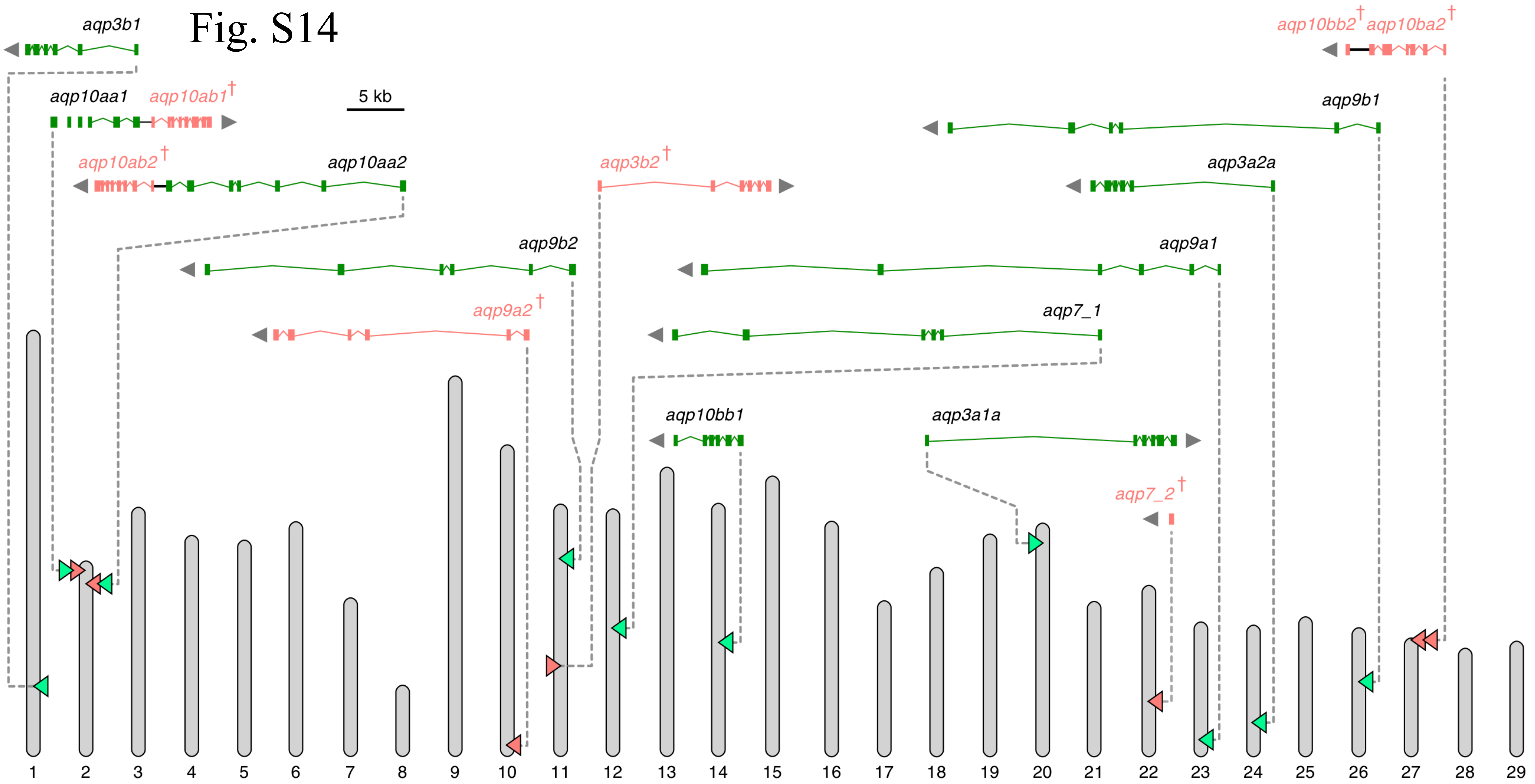

aqp3b1
aqp10bb2† aqp10ba2†
5 kb
aqp10aa1 aqp10ab1†
aqp9b1
aqp10ab2†
aqp10aa2
aqp3b2†
aqp3a2a
aqp9b2
aqp9a1
aqp9a2†
aqp7_1
aqp10bb1
aqp3a1a
aqp7_2†
1 2 3 4 5 6 7 8 9 10 11 12 13 14 15 16 17 18 19 20 21 22 23 24 25 26 27 28 29

Supplementary Table S1

List of primers used in this study

Primer name	Primer Sequence	GC %	Tm °C	bp
aqp3a1a_Fw	gatatcACCTACCTACCTGCTGTCAC	50	62.2	1040
*aqp3a1a*_Rv	actagtTCAAGGAGACCATACTGTACCC	46.6	63.3	
*aqp3a2a*_Fw	gatatcCCCTACCTACCTACTGTCACC	51.9	62.9	1048
*aqp3a2a*_Rv	actagtAGTGAAGGCTGCTGGAGAGT	50	64.6	
*aqp3b1*_Fw	gatatcGCATCATGGGAAAACAGAAGGC	46.4	64.5	908
*aqp3b1*_Rv	actagtTCATGCACTGTCCTTGGTAGTT	42.9	64	
*aqp7_1*_Fw	gatatcAGGACTGTAAACTTGAGTGTGACT	40	62.6	1062
*aqp7_1*_Rv	actagtCCAGTTACTTTGGGTGCAGC	50	64.8	
*aqp9a1_v1*_Fw	gatatcAGCCCATGTGAACCCTGC	61.1	60	101
*aqp9a1_v1*_Rv	actagtAAGCACCAAGGAACTGAGCG	55	60.9	
*aqp9a1_v2*_Fw	gatatcGTGTCAGATGCGTTCATGGACT	50	60.9	87
*aqp9a1_v2*_Rv	actagtAGGGTAAGAGGCAAAGATGTGAC	47.8	60.3	
*aqp9b1*_Fw	gatatcCCATGCTCACACTCACACTC	50	63.1	985
*aqp9b1*_Rv	actagtGCCAAGCCTCAGTACCATTG	50	64.6	
*aqp9b2*_Fw	gatatcACTCTTCAACTGTCTGTCCTGA	42.9	62.1	961
*aqp9b2*_Rv	actagtTGCCAAGCCTCAGTACCAT	48	63.8	
*aqp10aa1*_v1_Fw	gatatcCAGAGGTCAGAAAGATGGGG	50	62.2	1073
*aqp10aa1*_v1_Rv	actagtGGGTCACATCTAGTAGGAGTCT	46.4	63.5	
*aqp10bb1*_Fw	gatatcGAGAGTTCACTGTCCAAGTTACG	44.8	63.2	1046
*aqp10bb1*_Rv	actagtATAGCCCTTTAGGATATAACCGTT	36.7	61.1	
*aqp10aa1*_v2-Fw	gatatcGGTTTAGGCAGATGCGTATAGAT	41.4	62.6	1107
*aqp10aa1*_v2-Rv	actagtTCTCACTTCAACAGTCGGC	56	63.9	

Supplementary Table S2

Calibration times

		Hard minimum age (Ma)	95% Soft maximum age (Ma)
Ostariophysi	--> Salmoniformes	220	235
Salmoniformes	--> Stomiatii	185	192
Stomiatii	--> Zeogadaria	178	196
Zeogadaria	--> Lampriformes	145	150
Lampriformes	--> Carangaria	142	145
Percopsaria	--> Zeogadaria	125	140
Scombriformes	--> Carangaria	105	108
Carangaria	--> Cichliformes	100	105
Zeogadaria	--> Gadiformes	85	90
Merlucida	--> Gadidae	60	65

File S1: Alignment for Fig. 1
>Human_AQP3
---ATGGGTCGA
CAGAAG---GAGCTGGTGTCCCGCTGCGGGGAGATGCTC------CACATCCGCTACCGG
CTGCTCCGACAGGCGCTGGCCGAGTGCCTGGGGACCCTCATCCTGGTGATGTTTGGCTGT
GGCTCCGTGGCCCAGGTTGTGCTCAGCCGG------GGCACCCACGGTGGTTTCCTCACC
ATCAACCTGGCCTTTGGCTTTGCTGTCACTCTGGGCATCCTCATCGCTGGCCAGGTCTCT
GGGGCCCACCTGAACCCTGCCGTGACCTTTGCCATGTGCTTCCTGGCTCGTGAGCCCTGG
ATCAAGCTGCCCATCTACACCCTGGCACAGACGCTGGGAGCCTTCTTGGGTGCTGGAATA
GTTTTT---GGGCTGTATTATGATGCAATCTGGCACTTCGCCGAC---AACCAGCTTTTT
GTTTCGGGCCCCAAT---GGCACAGCCGGCATCTTTGCTACCTACCCCTCTGGACACTTG
GATATGATCAATGGCTTCTTTGACCAGTTCATAGGCACAGCCTCCCTTATCGTGTGTGTG
CTGGCCATTGTTGACCCCTACAACAACCCCGTCCCCCGAGGCCTGGAGGCCTTCACCGTG
GGCCTGGTGGTCCTGGTCATTGGCACCTCCATGGGCTTCAACTCCGGCTATGCCGTCAAC
CCTGCCCGGGACTTT---GGCCCCCGCCTTTTTACAGCCCTTGCGGGCTGGGGCTCTGCA
GTCTTC---ACG------ACCGGCCAGCATTGGTGGTGGGTGCCCATCGTGTCCCCACTC
CTGGGCTCCATTGCGGGTGTCTTCGTGTACCAGCTGATGATCGGCTGCCACCTGGAGCAG
---------------CCCCCACCCTCCAAC------------------------------
------------------------GAGGAA------------------------------
------------------------GAGAAT------------GTGAAGCTGGCCCATGTG
AAGCACAAGGAGCAGATC--

>Gorilla_AQP3
---ATGGGTCGA
CAGAAG---GAGCTGGTGTCCCGCTGCGGGGAGATGCTC------CACATCCGCTACCGG
CTGCTCCGACAGGCGCTGGCCGAATGCCTGGGGACCCTCATCCTCGTGATGTTTGGCTGT
GGCTCCGTGGCCCAGGTTGTGCTCAGCCGG------GGCACCCACGGTGGTTTCCTCACC
ATCAACCTGGCCTTTGGCTTTGCTGTCACTCTGGGCATCCTCATCGCTGGCCAGGTCTCT
GGGGCCCACCTGAACCCTGCCGTGACCTTTGCCATGTGCTTCCTGGCTCGTGAGCCCTGG
ATCAAGCTGCCCATCTACACCCTGGCACAGACACTGGGAGCCTTCTTGGGTGCTGGAATA
GTTTTT---GGGCTGTATTATGATGCAATCTGGCGCTTTGCCGAC---AACCAGCTTTTT
GTTTCGGGCCCCAAT---GGCACAGCTGGCATCTTTGCTACCTACCCCTCTGGACACTTG
GATATGATCAATGGCTTCTTTGACCAGTTCATAGGCACAGCCTCCCTTATCGTGTGTGTG
CTGGCCATTGTTGACCCCTACAACAACCCCGTCCCCCGAGGCCTGGAGGCCTTCACCGTG
GGCCTGGTGGTCCTGGTCATTGGCACCTCCATGGGCTTCAACTCCGGCTATGCCGTCAAC
CCTGCCCGGGACTTT---GGCCCCCGCCTGTTTACAGCCCTTGCGGGCTGGGGCTCTGCA
GTCTTC---ACG------ACCGGCCAGCATTGGTGGTGGGTGCCCATCGTGTCCCCACTC
CTGGGCTCCATTGCGGGTGTCTTCGTGTACCAGCTGATGATCGGCTGCCACCTGGAGCAG
---------------CCCCCACCCTCCACC------------------------------
------------------------GAGGAA------------------------------
------------------------GAGAAT------------GTGAAGCTGGCCCATGTG
AAGCACAAGGAGCAGATC--

>Rabbit_AQP3
---ATGGGTCGA
CAGAAG---GAGCTGGTGTCCCGCTGCGGGGAGATGCTC------CACATCCGCTACCGG
CTGCTACGCCAGGCGCTGGCCGAGTGCCTGGGGACCCTCATCCTCGTGATGTTCGGCTGT
GGCTCCGTCGCCCAGGTCGTGCTCAGCCGG------GGCACCCACGGAGGTTTCCTCACC
ATCAACCTGGCCTTTGGCTTTGCAGTCACCCTGGGCATCCTGGTCGCTGGCCAGGTCTCT
GGGGCCCACCTGAACCCTGCCGTGACCTTTGCCATGTGCTTCCTGGCACGTGAGCCCTGG
ATCAAGCTGCCCATCTACACCTTGGCACAGACGCTCGGAGCCTTCTTGGGTGCTGGGATC
GTTTTC---GGGCTATATTATGATGCAATCTTGAGCTTTGCCAAC---AATGAGTTTATA
GTCTCGGGCCCCAAT---GGCACAGCAGGCATCTTTGCCACCTACCCCTCTGGACACCTG
GACATGGTCAATGGCTTCTTCGACCAGTTCATAGGCACAGCTTCCCTCATCGTGTGCGTG
TTGGCCATTGTGGACCCTTACAACAACCCTGTCCCCCGCGGCCTGGAGGCCTTCACCGTG
GGCCTGGTGGTCCTGGTCATTGGCACCTCTATGGGCTTCAACTCTGGCTACGCTGTCAAC
CCTGCCCGGGATTTC---GGCCCCCGTCTTTTCACTGCCATCGCGGGCTGGGGCTCTGAG

GTCTTC---ACG------ACTGGCCGGCACTGGTGGTGGGTGCCCATCGTCTCTCCCCTG
CTGGGCTCCATTGCGGGCGTCTTCGTGTACCAGCTCATGATCGGCTGCCACCTGGAGCAG
---------------CCTCCTCCCTCCACC------------------------------
------------------------GAGTCG------------------------------
------------------------GAGAAC------------GTGAAGCTGGCCCACGTG
AAGCACAAGGAGCAGATC--

>Tasmanian_devil_AQP3
---ATGGGCCGT
CAGAAG---GAGCTAGTGTCGCGCTGCGGGGACTTGCTG------CGGGTCCGGTACCGG
CTGCTGCGCCAGGCTCTCGCTGAATGCCTCGGGACGCTCATCCTGGTGATGTTTGGCTGT
GGCTCTGTGGCCCAGGTTGTGTTGAGCAGA------GGAACCCATGGTGGCTTTCTTACC
ATCAACCTGGCTTTTGGCTTTGCAGTCACGCTTGGTATCCTTATTGCTGGCCAAGTGTCT
GGGGCCCATCTAAATCCAGCTGTGACCTTTGCTCTGTGCTTCCTGGCAAGGGAGCCCTGG
ATCAAGCTGCCCATCTACACATTGGCCCAGACTGTGGGAGCCTTCCTAGGAGCTGGAATT
GTCTTT---GGGCTGTACTATGATGCAATTTGGTCCTTTGCTGGT---AATGAACTTTTT
GTCTCTGGCCCCAAT---GGTACTGCCGGCATCTTTGCTACCTATCCCTCTGGCCACTTG
GACATGGTCAATGGCTTCTTTGACCAGTTCATTGGTACAGCATCCCTCATCGTCTGTGTG
TTGGCTATTGTGGATCCTCACAACAACCCCGTTCCCCGAGGTCTGGAAGCCTTCACTGTT
GGCCTTGTGGTTCTGGTCATTGGTACATCCATGGGCTTCAACTCTGGATATGCTGTTAAC
CCAGCTCGAGATTTT---GGTCCCCGCCTCTTCACTGCCATTGCTGGCTGGGGTTCTGAG
GTCTTC---ACA------ACGGGCCGGCACTGGTGGTGGGTGCCCATCGTTTCACCACTT
CTTGGTTCCATTTGCGGAGTTTGTGTCTATCAGCTCATGATTGGATTTCACCTGGAGCCT
---------------CCACCTCCTTCCACA------------------------------
------------------------GAGCAG------------------------------
------------------------GAGAAT------------GTCAAGCTGGCTCACGTG
AAACACAAAGAACAGATC--

>Platypus_AQP3
---ATGGGCCGG
CAGAAG---GAGCTGGTGTCGCGCTGCGGGGAGATGCTG------AGGATCCGCTACAAG
CTGTTGCGGCAGGCCCTGGCCGAGTGTCTGGGTACCCTCATCCTCGTGATGTTCGGCTGC
GGCTCCGTGGCCCAGGTGATACTTAGTCGT------GGCACCCACGGCGACTTCCTCACC
ATCAACCTGGCCTTTGGCTTTGCTGTCACCCTGGGAATCCTCATTGCCGGTCAGGTCTCA
GGTGCCCACTTGAACCCGGCCGTGACATTTGCACTGTGTTTTCTGGCCCGGGAACCCTGG
ATCAAGCTGCCCATCTATGCCTTGGCACAGACCCTAGGTGCGTTCCTGGGTGCTGGCATC
ATCTTC---GGCCTCTACTATGATGCCATCTGGGCCTTTGCGGAC---AACCAGCTGATT
GTGTCCGGCCCCAAC---GGCACTGCTGGGATCTTTGCAACCTACCCGTCCGGTCACTTG
GACACCCTCAACGGCTTCTTTGACCAGTTCATCGGCACGGCCTCCCTCATCGTCTGTGTC
CTGGCCATCGTGGATCCTTACAACAACCCCGTCCCCAGGGGCCTGGAGGCCTTCACCGTT
GGTTTTGTCGTCTTGGTCATCGGCACCTCCATGGGCTTTAACTCTGGCTATGCCGTCAAT
CCCGCCCGAGACTTC---GGCCCCCGCCTCTTCACCGCCATCGCTGGATGGGGCTCTGAG
GTCTTC---ACG------ACGGGCAAGCACTGGTGGTGGGTGCCCATCGTCTCACCCCTT
CTGGGCTCCATCGCCGGAGTCTTCGTCTACCAGCTGATGATTGGCTGCCACATGGAGGCC
---------------CCGCCCCCATCCACC------------------------------
------------------------GAGCAG------------------------------
------------------------GAGAAC------------GTCAAGTTGGCCAACGTC
AAGCACAAGGAGCGGATC--

>Echidna_AQP3
---ATGGGGCGG
CAGAAG---GAGCTGGTTTCGCGCTGCGGGGAGATGCTG------AGGATCCGCTACAAG
CTGCTGCGCCAGGCCCTGGCAGAGTGTCTGGGCACCCTCATCCTCGTGATGTTCGGTTGT
GGTTCTGTGGCCCAGGTCATACTTAGTCGT------GGTACCCACGGCGGCTTCCTCACC
ATCAACCTGGCCTTTGGCTTTGCTGTCATGATGGGAATCCTCATTGCCGGTCAGGTCTCA

```
GGCGCCCACTTGAACCCGGCTGTGACATTTGCCCTGTGTTTCCTGGCCCGGGAACCCTGG
ATCAAGCTGCCCATCTATACCCTGGCACAGACCATAGGTGCGTTCCTGGGTGCTGCCATC
ATCTTC---GGCCTCTACTATGATGCCATCTTGGCCTTTGCGGAC---AATCAGCTGATT
GTGTCTGGACCCAAC---GGCACGGCTGGGATCTTTGCAACTTACCCATCCGGTCACTTG
GACACCGTCAACGGCTTCTTTGACCAGTTCATCGGCACGGCCTCCCTCATCGTCTGTGTC
CTGGCCATCGTGGATCCCTACAACAACCCCGTCCCCAGGGGCCTGGAGGCCTTCACCGTC
GGTTTTGTCGTCTTGGTCATCGGCACCTCCATGGGCTTTAACTCCGGCTATGCCGTCAAT
CCCGCCCGGGACTTC---GGCCCCCGCCTCTTCACCGCCATCGCCGGCTGGGGCTCTGAG
GTCTTC---ACG------ACGGGCAGGCACTGGTGGTGGGTGCCCATCGTCTCACCCTTC
CTGGGTGCCATCGCCGGAGTGTTGGTCTATCAGCTGATGATTGGCTGCCACATGGAGGCC
---------------CCGCCCCCGTCCACT------------------------------
------------------------GAGCAG------------------------------
------------------------GAGAAT------------GTCAAGTTGGCCAACGTC
AAGCACAAGGAGCGGATC------------------------------------------
------------------------------------------------------------
------------------------------------------------------------
---------------------
>Kiwi_AQP3
---------------------------------------------------ATGGGGAGG
CAAAAG---GACATCCTCGCTACTATTGGGGAGCACCTG------AGAATCAGAAACAAA
CTAGTGCGGCAAGCGCTGGCCGAGTGCCTGGGGACGCTGATTCTGGTGCTGTTTGGCTGC
GGCTCTGTTGCACAGACTGTGCTCAGCAGA------GGGTCTCATGGAGTCTTCCTGACT
GTTAACTTGGCCTTCGGCTTCGCGGTGACACTCGGCATTTTGATCTCAGGACAGGTGTCA
GGTGGACACTTGAACCCAGCTGTCACTTTTGCCATGTGCTTCTTGGCCCGAGAGCCCTGG
ATCAAGCTACCAATTTATGCCCTGGCCCAGACACTGGGGGCTTTCCTAGGAGCTGGCATT
GTCTTT---GGGCTGTACTATGATGCTATCTGGGCTTTTGGCAAA---AACCAGCTGTAT
GTAACAGGACAGAAT---GGCACTGCTGGTATCTTTGCCACCTACCCGTCTGAGCATTTG
AACGTTGTGAATGGATTCTTTGACCAGTTCATTGGCACTGCCTCCCTGATTGTTTGTGTC
TTGGCTATTGTGGATCCCTACAACAATCCCGTTCCCATAGGGCTGGAAGCTTTCACAGTT
GGTTTTGTTGTCCTTGTTATTGGAACCTCCATGGGCTTCAACTCTGGCTATGCTGTCAAC
CCTGCCAGGGACTTT---GGGCCTCGTCTCTTCACAGCCATCGCTGGATGGGGCACTGAA
GTGTTC---TGG------ACTGGTAGGCAGTGGTGGTGGGTCCCAATTGTTGCTCCTTTC
CTTGGCGCCATTGCAGGAGTGGTGGTCTATCAACTGATGATTGGATGTCATGATGAACCT
---------------TCTCCACCTGCCTCT------------------------------
------------------------GAGCAG------------------------------
------------------------GAAACA------------GTCAAGCTGGCTAATGTG
AAGCACAAGGAAAGGGTC------------------------------------------
------------------------------------------------------------
------------------------------------------------------------
---------------------
>Alligator_AQP3
---------------------------------------------------ATGGGCAGG
CAAAAG---GAGCTTCTTTCTAGGATCGACGGGCTGCTG------AGGATCCGGAGCAAG
CTACTGAGGCAAGCGCTGGCCGAATGCCTGGGGACTCTGGTCCTAGTGATGTTTGGCTGC
GGCTCCGTCGCTCAGATCGTGCTCAGCAAA------GGGACCCACGGAGGCTTCCTGACG
GTTAACCTGGCCTTCGGCTTTGCCGTGACTCTTGGCATCCTCATCGCGGGACAGGTGTCA
GGCGGCCACTTGAACCCAGCGGTAACTTTTGCCATGTGCTTCTTGGCTCGTGAGCCCTGG
ATTAAGCTGCCGGTTTATGCGCTTGCACAAACCCTGGGGTCTTTCCTAGGAGCAGGCATT
GTCTTC---GGTCTGTACTATGATGCCATCTGGGCTTTTGGCCAA---AACAAGCTGATC
GTATCAGGAGAGAAT---GCCACCGCCGGCATTTTTGCCACGTATCCCTCCGAGCATCTG
GATGTAGTGAACGGATTTTTTGACCAGTTCATTGGCACTGCAGCCCTGATAGTTTGTGTC
TTGGCTATCGTGGATCCTTACAACAACCCTGTCCCACGTGGCCTGGAGGCTTTCACCGTC
GGCTTTGTGGTCCTCGTTATTGGGACCTCCATGGGCTTCAACTCCGGCTATGCCGTCAAC
CCAGCTAGGGACTTT---GGGCCTCGCCTGTTCACTTCCATTGCTGGCTGGGGCTCCGAA
GTATTC---AGT------ACCGGGAAGCATTGGTGGTGGGTGCCAATTATCGCCCCTTTC
CTTGGGGCCGTCGGAGGAGTGATGGTTTACCAGCTGATGATCGGGTGCCACGACGAGTGT
---------------CCTCCACCAGCCACG------------------------------
------------------------GAGCAG------------------------------
------------------------GAAACC------------GTCAAACTGTCCAATGTG
AAACACAAGGAAAGGGTC------------------------------------------
------------------------------------------------------------
```


>Turtle_AQP3
--ATGGGGAGG
CAAAAG---GAAGTTCTCGCTAGAATTGGAGAGATGCTG------AGGATCAGAAACAAA
CTAGTGAGGCAAGCACTGGCCGAGTGCCTGGGGACCCTGATTCTGGTGTTGTTTGGCTGT
GGCTCGGTCGCTCAGATCGTGCTCAGTAGA------GGAACCCACGGAGGCTTCTTGACT
GTGAACCTGGCCTTCGGCTTTGCTGTTACACTTGGAATTCTGATAGCAGGGCAAGTGTCA
GGTGGCCACTTGAATCCTGCTGTAACTTTTGCTCTGTGCCTCATGGCCCGGGAGCCGTGG
ATTAAGCTGCCTATTTACATCCTGGCACAAACCATCGGTGCTTTCCTAGGAGCGGGCATC
GTCTTT---GGCTTGTACTTTGATGCCATATGGGCTTTTGGCAAC---AACCAGTTGTTT
GTATTGGGACCGAAT---GGCACCGCTGGAATTTTTGCCACATACCCGTCTGATCATCTG
AATGTATTGAATGGATTCTTTGACCAGTTCATTGGCACGGCATCCTTGATTGTTTGCGTC
TTGGCTATTGTGGATCCCTACAACAACCCTATCCCCCGGGGCCTAGAGGCGTTCACTGTT
GGCTTTGTGGTCCTGGTTATCGGGACGTCCATGGGCTTCAACTCCGGATACGCCGTCAAC
CCAGCCAGGGACTTC---GGGCCTCGTCTTTTCACCTCCATTGCTGGCTGGGGCACAGAA
GTATTT---TCG------ACTGGCATGCAGTGGTGGTGGGTCCCGATCGTGGCCCCTTTC
CTCGGGGCAGTTGCTGGAGTGCTGGTTTACCAGCTGATGATTGGATGCCATGACGAGCCG
---------------TCTCCACCATCCACT------------------------------
------------------------GAGCAG------------------------------
------------------------GAAAAT------------GTCAGGTTGGCCAACGTG
AAGCATAAGGAAAGGGTC--

>Tuatara_AQP3
--ATGGGGCGG
CAAAAG---GAGTTGGGTGCTAGAATTGGGGGCATGCTG------AGGATCAGAAATAAG
CTTCTACGGCAGGCTTTGGCTGAGTGCTTGGGGACCCTGATCTTGGTGCTCTTTGGCTGT
GGCTCAGTTGCACAGGTCATCCTCAGCAGA------GGGACCCATGGAGGCTTCTTGACC
ATTAACCTGGCTTTCGGCTTCGCTGTGACGCTGGGAATTCTGATAGCGGGGCAGGTGTCA
GGTGCCCACCTGAACCCAGCTGTGACGTTTGCCATGTGCCTGTTGGCTCGGGAACCCTGG
ATCAAATTTCCGGTATATACGCTGGCACAAACCCTCGGGGCCTTCCTAGGAGCGGGTATT
GTCTAC---GGCTTGTACTTTGATGCCATCTGGGCTTTTGGCGAT---AACCAACTCTTG
GTAGCGGGACCGAAC---GGCACTGCTGGTATCTTTGCCACGTTTCCCTCTGGCCATCTG
AACTCTCTCAATGGATTCTTTGACCAGTTCATTGGAACCGCGTCATTGATCGTTTGCATC
CTCGCTATCGTGGATCCCTATAACAACCCCGTCCCCACAGGCCTGGAAGCTTTCACCGTT
GGCTTTGTGGTCTTGGTCATTGGAACATCCATGGGATTCAATTCTGGCTACGCCGTCAAC
CCAGCCAGGGACTTT---GGGCCACGCCTCTTCACGGCCATCGCCGGTTGGGGCACTGAA
GTTTTC---ACA------ACTGGGAGGCAGTGGTGGTGGATCCCGATAATTGCTCCCTTG
CTTGGGGCTGTGGCTGGCATCCTGGTTTACCAGCTGATGATTGGATTCCACGATGAGGCT
---------------CCTCCCCCAGCCAAC------------------------------
------------------------GAGCAG------------------------------
------------------------GAAATG------------GTGAAGCTGTCCAACGTG
AAGCAGAGAGAGAGAGTT--

>Python_AQP3
--ATGGGGAGG
CAGAGA---GAAGTGCTGACGGTCCTTGGACAGAGGCTG------AGGATCAGGAATAAG
CTCCTCCGGCAGGCTCTGGCTGAATGCCTGGGCACGCTGATCTTGGTGATGTTCGGTTGT
GGGTCCGTGGCCCAGATCGTGCTCAGCAGA------GGGAGCCATGGACGTTTCCTGACG
GTCAACCTGGCGTTTGGTTTTGCCGTGACGCTTGGAATTCTGATTGCTGGACAAATCTCA
GGTGGTCATTTGAACCCTGCCGTCACCTTTGCTATGTGCTTTATGGCCCGGGAACCCTGG
ATCAAGCTTCCGATTTATGCCCTAGCCCAGACCCTTGGGGCTTTTCTAGGGGCAGGCATC
GTGTAC---GGCTTA---TACGATGCCATCTGGGCTTTTGGTGAC---AACCAACTCTTC
GTATTGGGCCCCAAT---GGGACTGCTGGCATCTTTGCTACCTACCCATCTGAGCATCTG
AATTCTGTGAATGGATTTTTGACCAGTTCATTGGCACTGCAGCATTGCTGGTTTGTGTT
CTGGCCATTGTGGATCCTTACAACAACCCTGTCCCAAGAGGGTTGGAAGCATTTACTGTT
GGCTTCGTCATCTTGGTTATTGGAACATCCATGGGGTTCAATTCTGGTTACGCTGTCAAC

```
CCAGCCCGAGACTTA---GGGCCCCGTCTCTTCACAGCCATTGCTGGCTGGGGTACCGAA
GTTTTC------------ACTGGTAGGCACTGGTGGTGGATTCCCATCGTTGCTCCTTTC
CTTGGTGCCATCGCAGGTGTTATGGTCTACCAACTGATGATAGGGTGCCATGATGAACCA
---------------CCTCCCGAGACCAGT------------------------------
------------------------GACGAG------------------------------
------------------------GAAAAC------------GTCAGGCTGTCCAAGGTG
AAGCAGAGGGACAATGTC------------------------------------------
------------------------------------------------------------
------------------------------------------------------------
---------------------
>Gecko_AQP3
---------------------------------------------------ATGGGCAGG
CAAAAG---GAGGTGCTGTCAGTCATGGGACAGAGGCTG------AGGATCAGGAATAAA
CTCATCCGGCAAGCTCTGGCCGAATGCCTGGGGACCTTGGTGCTGGTGTTGTTCGGCTGT
GGTTCAGTGGCTCAAATCGTCCTCAGCAGA------GGAAGCCACGGAGCTTTCCTGACA
GTCAATCTGGCGTTCGGTTTTGCGGTGACGCTGGGAATTCTCATCGCTGGACAGATCTCA
GGTGGCCACTTGAATCCAGCCGTCACCTTCGCCATGTGCTTTATGGCTCGGGAACCTTGG
ATAAAGTTGCCTGTATATGCTTTAGCACAGACCCTCGGCGCGTTCTTGGGAGCTGGCATC
GTCTTC---GGCTTGTACTATGATGCCATTTGGGCCTTCGGTGGG---AACCAGCTGTTT
GTCGTCGGCGAGAAT---GCCACGGCCGGGATCTTCGCCACGTACCCCTCAGAGCACTTG
AACTCCGTGAATGGATTTTTGACCAGTTTATTGGCACAGCAGCCCTGCTTGTCTGCGTC
TTGGCGATCGTCGACCCCCACAACAACCCGGTCCCCCGGGGTCTGGAGGCCTTCACCGTC
GGCTTTGTCATCTTGGTGATTGGAACGTCCATGGGCTTCAACTCCGGCTACGCCGTCAAC
CCGGCCAGAGATTTC---GGGCCCCGCCTCTTCACGGCCATCGCTGGGTGGGGCTCGGAA
GTTTTC---TCG------ACGGGCCGGCAGTGGTGGTGGGTCCCAGTCATTGCCCCGTTC
CTCGGGGCCATTGCCGGCGTGGGGGTCTACCAGCTGATGGTCGGCTGCCACGACGAGCCG
---------------CCCCCCGAGGCGAAT------------------------------
------------------------GATGAG------------------------------
------------------------GAGACC------------GTCAAGCTGTCCAATGTG
AAGCAGAGGGACAACGTT------------------------------------------
------------------------------------------------------------
------------------------------------------------------------
---------------------
>Himalaya_frog_AQP3
---------------------------------------------------ATGGGCCGA
CAGAAG---GAATTTATCAACCGCATCAACGCCGCGCTG------AGGATCCGCAACAAG
CTGCTGAGGCAAGCGCTCTCCGAGTGCCTGGGGACCCTCATTCTGGTGATGTTCGGCTGT
GGCTCTGTGGCTCAGGTGGTCCTTAGTAAA------GGATCCCATGGTCAGTTCTTGACT
GTCAATCTGGCTTTCGGTTTTGCTTGCATGTTGGGTATTCTCATATCCGGACAAGTTTCA
GGTGGACATCTCAACCCCGCTGTGACGTTTGCACTATGTATCATAGCACGAGAACCATGG
ATCAAGTTTCCTGTATACACCTTGGCACAGACCCTGGGTGCCTTCCTCGGAGCGGGCATC
GTCTAT---GGCTTGTATTATGATGCCATCTGGTTTTTCGCCAAC---GACCAGCTCTAC
GTAATGGGCCCCAAC---GGCACTGCTGGGATTTTCGCCACATTCCCAACAGATCATCTG
ACCCTCATGAATGGTTTTTTGACCAGTTTATTGGCACAGCTGCTTTGGTGGTCTGTGTC
TTGGCCATCGTGGACCCTTACAATAACCCCATCCCCCGTGGTTTGGAGGCCTTCACTGTT
GGCTTCGTCGTCCTTGTCATTGGATTGTCCATGGGCTTCAACTCCGGATATGCCGTCAAC
CCCGCCAGGGACTTC---GGACCACGTCTGTTCACAGCTCTGGCTGGATGGGGTTCAGAA
GTTTTC---TGG------GCTGGAGGTCAGTGGTGGTGGGTCCCCATCGTGTCGCCTTTA
CTGGGTGCCTTTGCCGGAGTCCTGGTCTACCAGCTGATGCTTGGCTGCCACCTCCAACCT
---------------CCACCTGAATCCACC------------------------------
------------------------GAACAA------------------------------
------------------------GAAAAT------------GTCAAGCTGGCCAACGTC
AAACAAAAAGAGAGAATC------------------------------------------
------------------------------------------------------------
------------------------------------------------------------
---------------------
>Axolotl_AQP3
---------------------------------------------------ATGGGTCGG
CAGAAG---GAACTGGTCAACAAAATCGGGGACATGTTG------AGGATCCAGAACAAG
CTGCTGAGGCAGGCGCTGGCCGAGTGCCTGGGGACCCTCATCCTCGTGATGTTTGGCTGC
GGGTCGGTGGCCCAGGTCGTTCTCAGCAAG------GGCTCCCATGGACAGTTCTTGACA
```

GTGAACATGGCCTTTGGTTTCGCTGTGATGCTTGGCATCATTATCTGCGGGCAGGTGTCC
GGCGGCCACCTGAATCCGGCAGTCACGTTTGCTTTATGCTTCCTGGCCCGGGAGCCTTGG
ATCAAGTTCCCCATTTACACATTGGCACAGACACTGGGAGCGTTCCTGGGCGCTGGCATT
ATCTAC---GGATTGTACTATGATGCTATTTGGGCTTTTGCTCAG---GATCAACTCTAC
GTCATGGGGCCCAAT---GGGACAGCCGGGATTTTTGCAACCTACCCGACCGAGCATCTG
ACGCTTATGAATGGATTCTTCGACCAGTTCATTGGCACCGCAGCCTTGGTGGTCTGTGTC
CTCGCCATTGTGGACCCAAACAACAACCCAATCCCCAAGGGCCTTGAGGCCTTCACTGTT
GGATTTGTGGTTCTCGTCATTGGACTTTCTATGGGTTTCAACTCTGGATATGCCGTCAAC
CCGGCCAGGGACTTT---GGACCACGTCTCTTCACGGCAATTGCTGGCTGGGGCTCTGAA
GTCTTC---TCG------GCTGGAGAACAGTGGTGGTGGGTGCCAATCGTTTCCCCCTTT
CTTGGATCTGTCGGCGGAGTTTTAGTCTACCAGCTGATGATCGGGTTCCATATCTTGCCT
---------------ATCCCAGAGTCCACG------------------------------
------------------------GAGCAG------------------------------
------------------------GAGAAC------------GTCAAGTTGGCCAATGTC
AAGCACAAGGAGCGGATC--

>2Lcaecilian_AQP3
---ATGGGTCGG
CAGAAG---GAGTTGGTTAACCGGTGCGGGGAGATGCTG------CGAATCCACAACAAG
CTGATGAGACAGGCTCTGGCGGAGTGCCTGGGGACCTTGATCCTGGTGATGTTTGGCTGT
GGTTCTGTGGCTCAGGTGGTTCTGAGCAGG------GGATCGCATGGACAGTTCTTGACT
GTCAACCTGGCCTTTGGTTTTGCCGTGATGCTCGGTATCATAATATCGGGCCAGATATCA
GGTGGCCACCTCAATCCTGCTGTGACCTTTGCAACGTGCTTGTTGGCAAGAGAGCCGTGG
ATTAAATTCCCGATTTATACCTTGGCACAGACCCTCGGGGCTTTTCTTGGAGCAGGCATT
GTCTAT---GGCTTGTACTATGATGCCATATGGATTTTCGGTGGT---GATCAGCTGTAC
GTAACGGGAGAAAAT---GCCACAGCAGGGATCTTCGCAACCTTCCCGACTGACCATTTG
ACCCTAATGAATGGATTCTTTGATCAGGTAATTGGTACGGCGGCACTGGTGGTCTGTGTC
CTTGCTATTATAGATCCATACAATAATCCAATTCCCCGGGGCCTCGAGGCATTCACCGTT
GGATTTGTGGTCCTGGTCATTGGACTTTCCATGGGCTTCAACTCCGGATATGCTGTTAAC
CCTGCTAGGGATTTT---GGACCTCGTCTCTTCACGTCTATTGCTGGTTGGGGCACTGAG
GTTTTC---AGC------GCTGGAGGCCAGTGGTGGTGGGTGCCGATAGTTTCCCCGTTG
CTCGGGGCGGTGGGCGGCATCTTGGTTTACCAGCTCATGATTGGCTTTCACATCGAGCCC
---------------CCGCCGCAGTCCACC------------------------------
------------------------GAGCAA------------------------------
------------------------GAAAAC------------GTCAAGCTAGCCAACGTG
AAGCAGAAAGAGAGGATC--

>Gaboon_caecilian_AQP3
---ATGGGTCGT
CAAAAG---GAATTAGTGAACCGGTGTGGGGAGATGCTC------CGAATCCACAACAAA
CTGATAAGACAATTCCTCTCGGAGTGCCTCGGGACGCTGATCCTGGTGATGTTTGGCTGC
GGATCTGTGGCTCAGGTGGTTCTCAGCAAG------GGATCCCATGGACAGTTCTTGACT
GTCAACCTGGCCTTTGGTTTTGCCGTGATGCTTGGTATTCTGATATCAGGGCAGGTGTCA
GGTGGTCACCTCAATCCTGCTGTCACTTTTGCATTTTGCTTGTTGGCAAAGGAACCATGG
ATTAAATTCCCCATTTATACCTTGGCACAAACCCTTGGGGCTTTTCTTGGAGCTGGCATC
GTCTAC---GGCTTGTACTACGATGCCATATGGTATTTTGCGAAT---GATCAACTGTAT
GTGATGGGACCCAAC---GGCACAGCAGGGATCTTTGCGACTTACCCAACTGAACATTTG
ACATTAATGAATGGATTCTTTGATCAGTTCATTGGTACAGCAGCCCTAGTGGTCTGTGTC
CTGGCTATTGTGGATCCGTACAATAATCCAGTTCCTAGGGGCCTCGAGGCTTTCACCGTT
GGGTTTGTTGTCTTGGTCATTGGACTCTCCATGGGCTTCAACTCCGGCTATGCTGTCAAC
CCTGCCAGGGACTTT---GGACCTCGCCTTTTCACATCCATTGCTGGTTGGGGCACGGAG
GTTTTC---AGT------GCTGGAGACCAATGGTGGTGGGTGCCGATCGTTTCACCATTA
CTCGGCGCAGTGGCTGGCGTTTTGGTGTACGAGTTAATGATTGGGTTTCACGTTGAGCCT
---------------CCACCACCGTCCACC------------------------------
------------------------GAGCAA------------------------------
------------------------GAAAAC------------GTCAAGCTGTCAAACGTG
AAGCACAAAGACAGGATC--

>European_seabass_Aqp3a
---ATGGGCAGA
CATAAG---GTGTATCTGGACAAACTGTCCCGGTTCTTC------CAGATCCGTCACCTG
CTGCTTCGTCAGGCCCTGGCAGAGTGTCTTGGCACCCTCATCCTTGTGATGTTTGGCTGC
GGTGCTGTGGCCCAGCTAGTGTTGAGCGGT------GGTTCCCATGGCATGTTCCTAACG
GTCAACTTTGCCTTCGGCTTCGCTGCCACCTTAGGCATCCTGGTCTGTGGCCAGGTATCA
GGTGGCCATCTGAACCCTGCAGTGACCTTTGCACTGTGTCTGCTTGGAAGAGAGCGCTGG
AGAAAGTTCCCCATGTACTTCCTCTTTCAGACAATCGGTGCTTTTTTTGGTGCTGCAATC
ATTTTT---GGCATGTACTACGATGCCCTGTGGGACCACCCTGGA------TGTTTCAAT
GTGACTGGACCTAAT---GCCACAGCTGGCATCTTTGCTACCTACCCTGGAAAACATCTC
ACCCTTGTCAATGGCTTCTTTGATCAGATAATTGGCACAGCAGCACTGATAGTTTGTATT
CTGGCTATTGTGGATCCATACAACAACCCCATCCCCCAAGGGCTGGAGGCCTTCACTGTG
GGCTTTGTGGTTCTGGTGATTGGATTGTCTATGGGCTTTAACTCTGGCTATGCTGTGAAT
CCTGCCAGAGACCTT---GGACCACGTATTTTCACAGCTTTGGCTGGGTGGGGCAGCGAT
GTTTTC---ACG------GTTAGAAATGGCTGGTTCCTGGTGCCAGTTTGTGCCCCATTC
CTTGGCACCATCATCGGTGTGATGATCTACCAGTTAATGGTTGGTTTCCATGTGGAGGGA
---------------GAAGTACGTGACCAA---------------------AAG------
------------AGCAGA------GACCAG------------------------------
------------------------GAGAAT------------GTCCGACTCACTAATGTC
TCCAGCAACGACAACTCCAAAGAAGAT---------------------------------
---------ACCAAAGAAATGCAC------------------------------------

>Nile_tilapia_Aqp3a1
---ATGGGCTGG
CAAAAG---CATTATCTGGATAAACTGTCCCGCTTCTTC------CAGATCCGCAACCTG
CTGCTTCGTCAAGCCCTGGCAGAGTGTCTTGGCACTCTCATCCTTGTGATGTTTGGCTGT
GGTTCTGTGGCCCAGTTGGTGTTGAGCGGT------GGTTCTCATGGTATGTTCCTTACT
GTCAACTTTGCCTTCGGCTTTGCTGCTACGTTAGGCATCTTGGTCTGTGGCCAAATATCA
GGCGGCCATCTCAACCCAGCAGTAACTTTTGCCTTGTGTCTGCTTGGAAGAGAGCGCTGG
AGAAAGTTTCCCATGTACTTCCTCTTTCAGACAATTGGTGCTTTTTTTGGTGCTGCTATT
ATTTTT---GGCATGTACTATGATGCCCTGTGGGACCATCCTGGA------AGTTTTAAT
GTGACTGGACCTGAC---GCCACAGCTGGCATCTTTGCTACTTATCCTGGAACCCATCTC
ACCCTTGTCAATGGTTTCTTTGATCAGATTATTGGCACAGCAGCACTGATAGTTTGCATT
CTGGCCATTGTGGATCCTTACAACAACCCCATCCCTCAAGGGCTGGAGGCCTTCACTGTG
GGATTTGTGGTTCTGGTCATTGGATTGTCTATGGGCTTTAATTCTGGCTATGCAGTCAAT
CCTGCCAGAGACCTC---GGACCACGTCTTTTCACCGCTATAGCTGGTTGGGGGAGTGAG
GTTTTC---ACG------GCTAGCCCAGGCTGGTTCCTGGTCCCTGTTTTTGCTCCATTC
CTTGGTACCCTCATTGGTGTAATGATCTACCAGCTGATGGTTGGTTTCCACATGGAAGGG
---------------GAAGTACGTGACAGA---------------------AAA------
------------GAGAGC---ACAGAGCAG------------------------------
------------------------GAGACT------------GTCCGACTCACCAATGTG
ACTTCCAAAGACAACTCCAGAGAGGCT---------------------------------
---------GTGAAAGAAAGAAATGAGTGT------------------------------

>Nile_tilapia_Aqp3a2
---ATGGGCAGA
CAAAAG---GAGTATCTGGATAAACTGTCCCGCTTCTTC------CAGATCCGCAACCTG
CTGCTTCGTCAAGCCTTAGCAGAGTGTCTTGGCACGCTCATCCTTGTAATGTTTGGCTGT
GGCGCTGTGGCCCAGCGGGTGTTGAGCGGT------GGTTCCCATGGCTTGTTCCTTACT
GTCAACTTTGCCTTTGGCTTTGCTGCCATGTTAGGCATCTTGGTCTGTGGCCAAGTGTCA
GGCGGCCATCTCAACCCAGCAGTAACTTTTGCCTTGTGTCTGCTTGGAAGAGAGCGCTGG
AGAAAGTTTCCCATGTACTTCCTCTTTCAGACAATTGGTGCTTTTTTTGGTTCTGCTATC
ATTTTT---GGCATGTACTATGATGCTTTGTTGCTCCGTCCTGGA------AGTTTTAAT
TTGACTAGCACTAAC---AACACGGCTGGCATATTTGCAACTTACCCTGCGAGGCATCTC
ACACTTGTCAATGGTTTCTTTGATCAGATTATTGGCACAACAGCACTCATAGTTTGTGTT
CTGGCCATTGTGGATCCTTACAACAACCCCATCCCCCAAGGGCTGGAGGCCTTCACTGTG

```
GGATTTGTGGTTCTGGTCATTGGATTGTCTATGGGTTTTAATTCTGGCTATGCAGTCAAT
CCTGCCAGAGACTTT---GGACCACGTCTTTTCACCTCTATGTCTGGGTGGGGGGGTGCT
GTTTTC---ACG------GCTAGAGACTGCTGGTTCCTGGTCCCTATTTTTGCCCCATTC
CTTGGCGCCATTCTTGGTGTGCTGATTTACCAGTTGATGGTTGGTTTCCACACAGAGGGA
---------------GAAGCACGTGACAAG---------------------AAG------
------------CAGGGG---ACAGTCCAG------------------------------
------------------------GAGAAC------------CTCCAACTCGCCAATGTT
GCTTCCTCAAACAACTCTAAAGAGGCT---------------------------------
---------ACCAAAGAAATTTAC------------------------------------
------------------------------------------------------------
---------------------
>Mummichog_Aqp3a
---------------------------------------------------ATGGGTCGA
CAGAAG---ATTTATTTGGACAAACTGGCCCGCTTCTTT------CAGATCCGTCACCTG
TTGCTCCGCCAGGCCCTGGCAGAGTGCCTTGGCACTCTCATCCTTGTGATGTTTGGCTGT
GGCGCTGTGGCCCAAAGCGTGCTGAGTCGT------GGTTCCCATGGCAGGTTCCTCACT
ATCAACTTTGCCTTTGGCTTTGGCGCCACCTTAGGGATCCTGGTCTGTGGCCAAGTATCA
GGTGGCCATCTGAACCCTGCAGTGACCTTTTCACTTTGCCTGCTTGGAAGAGAGCGCTGG
AGAAAGTTCCCCATGTACTTCCTCTTTCAGACAATCGGAGCATTTTTCGGCGCTGCTATT
ATCTTT---GGCATGTATTATGATGCCTTTTGGGACTTTCCCGGG------TGCTTTAAT
GTTACTGGGGATTCT---TCCACTGCTGGCGTCTTTGCCACCTACCCCGGAAAGCATCTT
ACTCTTGTGAATGGATTCTTTGATCAGATTATTGGCACGGCAGCCCTCATAGTGTGCATT
CTGGCAATTGTGGACCCATACAACAACCCCATTCCCCAAGGGTTGGAGGCCTTCACTGTG
GGATTTGTGGTTTTGGTCATTGGATTGTCCATGGGCTTCAATTCCGGTTATGCTGTTAAC
CCTGCCAGAGACATC---GGACCACGCCTTTTCACTGCTATAGCTGGCTGGGGAACTGAT
GTTTTT---ACG------GCTAGAAATGGCTGGTTCTTGGTGCCACTCTGCGTCCCATTC
CTTGGTGCCCTTATTGGCACGATAATTTATCAGCTGATGGTTGGTTTCCATGTGGAAGGA
---------------GAAGCACGTGATCAG---------------------AAA------
------------AAGAAC---ACG---GAA------------------------------
------------------------GAGAGT------------CTACAACTTACCAACCCC
GACTCAAAATCTAACGCCAACGGC------------------------------------
------------AAAGAGGCTAAC---TGC------------------------------
------------------------------------------------------------
---------------------
>Zebrafish_Aqp3a
---------------------------------------------------ATGGGTTGG
CAGAAA---AGCGTTCTGGATAAGCTTGCGCAGACTTTC------CAAATCCGCAACAAG
TTACTGCGCCAGGGATTGGCTGAATGCTTAGGAACTCTCATCCTGGTGATGTTTGGCTGT
GGTTCATTGGCCCAGTTGAAACTAAGCGAA------GGTTCTCATGGTCTCTTTCTCACT
GCAAACCTTGCTTTTGGGTTTGGTGCTACTCTTGGAATCTTGGTTTGCGGCCAGGTGTCA
GGCGGACATTTAAATCCTGCTGTTACATTTGCTCTCTGCCTCTTGGGAAGAGAAAAATGG
AGAAAGTTTCCTGTGTACTTTCTGTTCCAAACACTCGGATCCTTCTTGGGTGCTGCTATT
ATCTTT---GCCGAATACCATGACGCAATTTATGATTATGCTGGAGAAAATGAGTTGCTT
GTACTGGGTGAAAAA---GAAACAGCTGGGATTTTTGCTACATACCCAAGCAAATATCTC
ACCCCCCTAAATGGATTTTTTGACCAGGTGATAGGCACAGCATCCCTGATTGTGTGCATC
CTGGCCATTGTGGACCCCTACAACAACCCGATCCCTCAAGGTCTTGAGGCCTTCACAGTG
GGATTCAGCGTCCTTATCATTGGTCTCTCCATGGGCTTCAATTCTGGCTATGCAGTAAAC
CCAGCTCGAGATTTT---GGACCTCGTCTTTTTACTGCCATGGCTGGTTGGGGTAGTGAA
GTCTTC---ACA------GCCAGGGATTATTGGTTTTTGGTGCCCATCTTTGCTCCGTTC
ATTGGAGCCGTTATTGGTGTGATTGTGTACCAGCTGATGGTGGGATGGCATGTGGAAGGA
---------------GAGGCACGAGATAAG---------------------AAA------
------------GCTAAA---GCTAGAGAG------------------------------
------------------------GAGGTG------------ATGAACCTCAATGACGTC
GCCAGCAAGGAA------------------------------------------------
------------------------------------------------------------
------------------------------------------------------------
---------------------
>European_seabass_Aqp3b
---------------------------------------------------ATGGGAAGA
CAAAAC---AACATTTTGGAGAAACTGGCAGAGACCTTT------CAGATCCGTCATGTG
CTCCTCCGCCAGGCCCTGGCAGAGTGCCTGGGAACCCTGATTCTGGTGATGTTTGGTTGT
```

GGTGCAGTAGCACAGATGGTCCTAAGCGGG------GGCACCCATGGTACGTTCCTCACC
GTCAACTTTGCTTTTGGTTTTGCCGCAACTTTGGGAATCCTTGTGAGTGGGCAGATCTCA
GGTGGTCATCTGAACCCAGCGGTGACATTCACACTGTGTTTACTTGGAAGAGAGCCTTGG
AGGAAGTTCCCGTTGTTCTTTTTGTTCCAGACTCTTGGAGCCTTTCTGGGTGCAGCGATT
GTATTT---GCCATGTATTTTGATGCATTATCGGACTTCAGCCAG---GGGGAGCTGATG
GTGGTGGGGAAGAAT---GCCACAGCTGGGATTTTTGCCACATATCCTTCTAAACATCTC
ACAATGATTAATGGATTCTTTGATCAGATAATTGGAACAGCAGCACTGATAGTGTGCATC
CTGGCCATAGTGGATCCACACAACAATCCAATCCCTAGAGGTCTGGAGCCCTTCACTGTT
GGTTTTGTGGTGCTGGTCATCGGGCTGTCGATGGGCTTCAATTCTGGCTATGCTGTCAAC
CCAGCCAGGGACCTG---GGACCGCGCATCTTCACGGCTCTTGCTGGTTGGGGTGGAGAG
GTTTTC---ACG------GCACACACCTACTGGTTCTTTGTGCCCATCTGCGCCCCCTTC
CTGGGAGCAGTGGTTGGTGTGCTGATGTACCAGCTAATGATCGGATATCATTTGGAAAGA
---------------GAAGTGCAAGAGAAG---------------------CAG------
------------AAGAAG---GAGGAGGAG------------------------------
------------------------GACAGA------------TTTGAACTTTCCAATATT
ACAACCAATGAAGATGCA--

>Atlantic_salmon_Aqp3b1
---ATGGGAAAA
CAGAAG---GCGCTTATGGACAAGCTCGCAAGGAACTTT------CAGATCCGTCACATG
CTGCTACGCCAGGCTCTGGCAGAGTGCCTGGGAACCCTGATACTGGTGATGTTTGGCTGT
GGTGCGGTGGCCCAGTTGGTGCTAAGTGGT------GGCTCCCATGGAATGTTCCTCACC
GTCAACTTTGCCTTTGGCTTCGCTGCTACACTGGGCATCCTGGTCAGCGGACAGATCTCA
GGAGGCCACCTGAACCCAACGGTGACCTTCGCCCTCTGTCTATTGGGGAGAGAACCTTGG
AGGAAATTCCCTGTGTTTTTCTTGTTCCAGACTATAGGGGCCTTCCTGGGAGCTGGGATC
ATATTT---GGCATGTATTTTGATGCGTTGTGGGACTACGGCCAA---GGAACACTGATC
GTTGTGGGGGAGAAT---GCCACTGCTGGGATCTTTGCCACATATCCCTCCAAACATCTC
ACTCTGGTTAATGGATTCTTTGACCAGATTATAGGCACAGCAGCTCTGATCGTGTGTATC
CTGGCCATCGTGGACCCCTACAACAACCCCATCCCCCGGGGCCTGGAGGCCTTCACGGTG
GGGTTCGTGGTGCTGGTCATCGGCCTGTCAATGGGCTTCAACTCGGGCTATGCTGTCAAC
CCTGCCAGGGACCTG---GGCCCACGCATCTTTACTGCCCTGGCTGGCTGGGGTGGTGAG
GTCTTC---ACG------GTTAATGCCTACTGGTTCTTTGTGCCAATCTTTGCCCCCTTC
ATTGGTGCGGTGTTGGGGGTGATGGTGTACCAGCTGATGGTTGGGTACCATGTGGAGGGA
---------------GAAGCACGGGAGAAG---------------------AAG------
GAAGAGGAGGAGAAGGAG---GAGCAAGAT------------------------------
------------------------GAAAGG------------CTCAAACTGTCCAGCCTA
ACTACCAAGGACAGTGCA--

>Zebrafish_Aqp3b
---ATGGGAAGA
CAGAAG---GTAATCCTGGAAAAGATGGCTCGGATCTTT------CAGATTCGGAACATG
CTGATGAGACAAGCACTGGCAGAATGCCTGGGCACCCTCATTCTAGTGATGTTTGGTTGT
GGTGCTCTTGCCCAGCATATTTTAAGCGGA------GGCTCTCATGGAATGTTTCTGACA
GTGAATTTTGCATTTGGATTCGCTGCTACATTGGGAATCCTGGTTTGTGGGCAAGTCTCA
GGAGGTCACATAAACCCTACTGTGACCTTTTCTCTCTGTTTGTTGGGGAGGGAGCCCTGG
AGGAAATTTCCCGTTTACTTTCTGGCCCAGACTGTGGGGGCTTTTCTTGGAGCTGGAATA
ATATTT---GGCATGTATTTTGATGCAATTTGGAAATTTGGACAA---GGTTCTCTTGAT
GTTGATGGGGTAAAT---GCAACTGCTGGAATCTTTGCTACGTACCCTTCTAAACACCTT
ACTTTGCTAAATGGATTCTTTGATCAGATGATCGGCACGGCAGCTCTGATCGTGTGTATC
CTTGCCATTGTTGACCCTTACAATAACCCCATCCCGCAAGGACTGGAGGCCTTCACTGTG
GGCTTTGTGGTTCTGGTGATTGGTCTGTCTATGGGATTTAACTCAGGCTATGCTGTAAAC
CCAGCCAGAGACTTG---GGACCACGGATCTTCACTGCAATTGCTGGATGGGGCTCAAAA
GTGTTC---TCA------GCGGAGTCTTACTGGTCCTTTGTGCCAGTCTTTGCCCCATTC
ATTGGTGCTGTGTTCGGTGTGATGGTGTATCAGTTGATGGTGGGATGCCATGTGAAAGGA
---------------GAAGAAAGAGATAAA---------------------AGA------
GAAGCGGTG---GAAAGA---GAAGAGAAG------------------------------
------------------------GAAAGA------------CTCAAACTATCTGCCGTT

TCTGATAAAGATGCAGCA--

>Bowfin_Aqp3
---ATGGGCAGA
CAGAAG---TTTTACCTGGACAAGCTGGCTCGCACCTTC------CAGGTCAGGAACATG
CTCATACGCCAGTTTCTGGCTGAGTGTCTGGGGACCCTCATTCTTGTGATGTTCGGCTGT
GGTGGCGTTGCCCAGGTGATCCTCAGTGGT------GGCACACACGGCAGGTTCTTGACT
GTCAACTTTGCTTTCGGCTTTGGAGCCACCCTAGGCATCCTCATCTGCGGACAAGTTTCA
GGAGGGCACCTGAACCCCGCTGTGACCTTTGCCCAGTGCCTGCTAGGGAGAGACCCCTGG
AAGAAGTTCCCAGTTTACTTTGCAGCTCAGACCCTGGGTTCCTTTCTGGGCGCTGGTATC
ATCTTT---GGCCTGTACTTTGATGCCATTTGGGGCTTTGGCCAA---GGACAGCTGTTT
GTGGTTGGCGAAAAC---GCCACTGCAGGCATTTTTGCCACCTACCCATCTGACCATCTG
ACCCTCCTCAATGGCTTGTTTGACCAGCTGATCGGTACTGCCGCCCTGGTGGTATGTATC
CTGGCTATCGTTGATCCGTACAACAACCCCATCCCACGTGGGTTGGAGGCCTTCACAGTG
GGCTTCGTGGTGCTGGTCATTGGCCTGTCCATGGGCTTCAACTCTGGCTACGCTGTCAAC
CCTGCCAGGGACCTG---GGGCCCCGTCTCTTCACTGCCATAGCTGGCTGGGGTGGTGAG
GTGTTT---ACT------GCCAAAAACTACTGGTTCTTTGTGCCAATCTTCGCCCCCTTC
CTGGGTTCTTTTGTGGGCGTATTGGTATACCAGCTAATGGTGGGATACCATGTGGAGGGG
---------------GAGGCCCGGGAGAAG---------------------GCA------
------------AAGCAG---GATGGAGAG------------------------------
------------------------GATAGG------------ATCAAACTGTCCAACACT
AACAAGGAGCTCCTG---

>Gar_Aqp3
---ATGGGCAGG
CAGAAG---TACTACATTGACAAGCTGGCGCGCGCCTTC------CAGATCAGGAACCTG
CTGCTGCGCCAGGCCCTGGCAGAGTGCCTGGGGACCCTCATTCTCGTGATGTTCGGCTGT
GGTGCAGTGGCTCAGCTGGTGCTTAGCGGT------GGCTCTCATGGTATGTTCATGACC
GTGAACTTTGCTTTCGGCTTTGCGGCTACCCTAGGAATCCTGATCTGCGGACAGGTGTCG
GGAGGACATCTTAACCCGGCGGTGACTTTTGCCCTGTGCCTGCTTGGGAGGGATCCCTGG
AGGAAACTCCCTGCTTACTTTCTGGCCCAGACACTGGGAGCCTTCCTGGGCTCTGGGATC
ATCTTC---GGCCTGTACTTTGATGCCCTGTGGGATTTCGCTGGT---GGGGATCTGCTG
GTGACCGGAACAAAT---GCGACTGCTGGCATCTTCGCCACCTACCCCTCCAACCACCTC
ACCCTGATCAACGGCTTATTTGACCAGCTGATTGGTACTGCCGCCCTGGTGGTGTGTATC
CTGGCTATCGTGGACCCCTACAACAACCCCATCCCACGCGGGCTGGAAGCCTTCACGGTG
GGCTTCGTGGTGCTGGTTATCGGCCTGTCCATGGGCTTCAACTCCGGCTATGCAGTCAAC
CCTGCCAGAGACCTC---GGCCCGCGGCTCTTCACCGCCATAGCTGGCTGGGGCAGCGAG
GTCTTC---ACG------GCTAATGACTACTGGTTCTTTGTGCCCATCTTCGCCCCCTTC
ATTGGCTCGTTTGTGGGTGTGGTGGTGTACCAGCTGATGGTTGGCTACCACGTGGAGGGA
---------------GAGGTTCGGGACAGA---------------------GAG------
------------GAGAGA---GGCGGGGAG------------------------------
------------------------GAGGGA------------GCCAAGCTGTCCAACTTC
AACCCCAAGGAGACGGCG--

>Chinese_sturgeon_Aqp3
---ATGGGCAGA
CAGAAG---CAGGCTCTGACCAGGATCTCTGAGCTGTTT------TATATCCAGAACTTG
ATGCTGAGGCAAGCTCTGGCAGAATGTCTGGGGACCCTCGTTCTAGTGATGTTCGGCTGT
GGCTCTGTCGCTCAGCTAGTTCTCAGTGGT------GGATCTCATGGACAGTTCCTGACT
GTGAACTTCGCTTTCGGCTTTGCTGCGACCCTGGGTGTCCTGGTGTCTGGGCAGGTGTCA
GGTGGCCATCTGAACCCGGCTCTGACCTTTGCCCTCTGTCTGCTGGGGCGGGAGCCGTGG
CTCAAGATGCCGGTGTACTTCTTGGCGCAGACGGTGGGTGCCTTCCTGGGGGCTGGGGTC
ATCTTC---GGAATGTACTTCGATGCCTTGTGGATGTTTGGGAAT---AACGATCTGTTA
GTGTCAGGAGCAAAC---AGCACCGCCGGGATCTTCGCTACCTATCCATCTCAGCACTTG
ACTCTGCTCAATGGCTTCTTTGACCAGATGATTGGTACTGCTGCCCTGATCGTGTGCCTC

CTGGCCATCGTGGACCCCTACAACAACCCCATCCCCAGGGGTCTGGAGGCCTTTACTGTG
GGCTTCGTCGTCTTGGTCATCGGTCTGTCCATGGGCTTCAACTCTGGCTACGCCGTCAAC
CCTGCCAGAGACTTT---GGTCCTCGCCTCTTCACCGCCATAGCCGGCTGGGGCACAGAG
GTCTTC---ACC------ACTGGATCTCACTGGTGGTTGGTGCCCATCTTTGCCCCCTTC
CTGGGTGCCTTCGTCGGGGTTCTGGTGTACCAGTTCATGGTGGGCTTCCACACGGAGGGC
---------------GAGGCCAAAGACAAA---------------------GCA------
------------AGGGAG---GACAATGAG------------------------------
------------------------GAGAAC------------GTCAAGCTGTCCAACATC
CACTCTAAACAGATCGCT--

>Atlantic_sturgeon_Aqp3
---ATGGGCAGA
CAGAAG---CAGGCTCTGACCAGGATCTCTGAGATGTTT------TACATCCAGAACTTG
CTGATTAGGCAAGCTCTGGCAGAATGTCTGGGGACCCTCGTTCTAGTGATGTTTGGCTGT
GGCTCTGTCGCTCAGCTAGTTCTCAGTGGT------GGATCTCATGGACAGTTCCTGACT
GTGAACTTCGCTTTCGGCTTTGCTGCGACCCTGGGTGTCCTGGTGTCTGGGCAGGTGTCA
GGTGGCCATCTGAACCCGGCTCTGACCTTTGCCCTCTGTCTGCTGGGGCGGGAGCGGTGG
CTCAAGCTGCCGGTGTACTTCTTGGCGCAGACGGTGGGTGCCTTCCTGGGGGCTGGGGTC
ATCTTC---GGAATGTACTTCGATGCCTTGTGGATGTTTGCGAAT---AACGATCTGTTA
GTGACAGGAAAAAAC---AGTACCGCTGGGATCTTCGCTACCTATCCATCTCAGCACTTG
ACCCTGCTCAATGGCTTCTTTGACCAGATGATTGGTACTGCTGCCCTGATCGTGTGCCTC
CTGGCCATCGTGGACCCCTTCAACAACCCCATCCCCAGGGGTCTGGAGGCCTTTACTGTG
GGCTTCGCCGTCTTGGTCATCGGTCTGTCCATGGGCTTCAACTCTGGCTATGCCGTCAAC
CCTGCCAGAGACTTT---GGTCCTCGCCTCTTCACCGCCTTAGCCGGCTGGGGCACAGAG
GTCTTC---ACC------ACTGGATCTCACTGGTGGTTGGTGCCCATCTTTGCCCCCTTC
CTGGGTGCCTTCATCGGGGTTCTGGTGTACCAGTTCATGGTGGGCTTCCACACGGAGGGC
---------------GAGGCCAAAGACAGC---------------------GCA------
------------AGGGAG---GACAATGAG------------------------------
------------------------GAGAAC------------GTCAAGCTGTCCAACATC
CACTCTAAACAGATCGCT--

>Russian_sturgeon_Aqp3
---ATGGGCAGA
CAGAAG---CAGGCTCTGGCCAGGATCTCTGAGCTGTTT------TATATCCAGAACTTG
CTGCTGAGGCAGGCTCTGGCAGAATGTCTGGGGACCCTCGTTCTAGTGATGTTCGGCTGT
GGCTCTGTCGCTCAGCTAGTTCTCAGTGGT------GGATCTCATGGACAGTTCCTGACT
GTGAACTTCGCTTTCGGCTTTGCTGCGACCCTGGGTGTCCTGGTGTCTGGGCAGGTGTCA
GGTGGCCATCTGAACCCGGCTCTGACCTTTGCCCTCTGTCTGCTGGGGCGGGAGCCGTGG
CGCAAGATGCCGGTGTACTTCTTGGCGCAGACGGTGGGTGCCTTCCTGGGGGCTGGGGTC
ATCTTT---GGAATGTACTTCGATGCCTTGTGGTTCTTTGGGAAT---GACAATCTGTTA
GTG------TCAAAC---GGTACCGCCGGGATCTTTGCTACCTATCCATCTCAGCACTTG
ACCCTGCTCAATGGCTTCTTTGACCAGATGATTGGTACTGCTGCCCTGATCGTGTGCCTC
CTGGCCATCGTGGACCCCTACAACAACCCCATCCCCAGGGGTCTGGAGGCCTTTACTGTG
GGCTTTGTCGTCTTGGTCATCGGTCTGTCCATGGGCTTCAACTCTGGCTACGCCGTCAAC
CCTGCCAGAGACTTT---GGTCCTCGCCTCTTCACCGCCATAGCCGGCTGGGGCACAGAG
GTCTTC---ACC------ACTGGACCTCACTGGTGGTTGGTGCCCATCTTTGCCCCCTTC
CTGGGTGCCTTCATCGGGGTTCTGGTGTACCAGTTCATGGTGGGCTTCCACACTGAGGGC
---------------GAGGCCAAAGACCGC---------------------GCA------
------------AGGGAG---GACAATGAG------------------------------
------------------------GAGAAC------------GTCAAGCTGTCCAACATT
CACTCCAAACAGACAGCT--

>Sterlet_Aqp3
---ATGGGCAGA
CAGAAG---CAGGCTCTGACCAGGATCTCTGAGCTGTTT------TATATCCAGAACTTG

CTGCTGAGGCAAGCTCTGGCAGAATGTCTGGGGACCCTCGTTCTAGTGATGTTCGGCTGT
GGCTCTGTCGCTCAGCTAGTTCTCAGTGGT------GGATCTCATGGACAGTTCCTGACT
GTGAACTTCGCTTTCGGCTTTGCTGCGACCCTGGGTGTCCTGGTGTCTGGGCAGGTGTCA
GGTGGCCATCTGAACCCGGCTCTGACCTTTGCCCTCTGTCTGCTGGGGCGGGAGCCGTGG
CGCAAGATGCCGGTGTACTTCTTGGCGCAGACGGTGGGTGCCTTCCTGGGGGCTGGGGTC
ATCTTT---GGAATGTACTTCGATGCCTTGTGGTTCTTTGGGAAT---GACAATCTGTTA
GTG------TCAAAC---GGTACCGCCGGGATCTTTGCTACCTATCCATCTCAGCACTTG
ACCCTGCTCAATGGCTTCTTTGACCAGATGATTGGTACTGCTGCCCTGATCGTGTGCCTC
CTGGCCATCGTGGACCCCTACAACAACCCCATCCCCAGGGGTCTGGAGGCCTTTACTGTG
GGCTTTGTCGTCTTGGTCATCGGTCTGTCCATGGGCTTCAACTCTGGCTACGCCGTCAAC
CCTGCCAGAGACTTT---GGTCCTCGCCTCTTCACCGCCATAGCCGGCTGGGGCACAGAG
GTCTTC---ACG------ACTGGACCTCACTGGTGGTTGGTGCCCATCTTTGCCCCCTTC
CTGGGTGCCGTCATCGGGGTTCTGGTGTACCAGTTCATGGTGGGCTTCCACACTGAGGGC
---------------GAGGCCAAAGACCGC---------------------GCA------
------------AGGGAG---GACAATGAG------------------------------
------------------------GAGAAC------------GTCAAGCTGTCCAACATT
CACTCCAAACAGACAGCT--

>Common_sturgeon_Aqp3
---ATGGGCAGA
CAGAAG---CAGGCTCTGACCAGGATCTCTGAGCTGTTT------TATATCCAGAACTTG
CTGCTGAGGCAAGCTCTGGCAGAATGTCTGGGGACCCTCGTTCTAGTGATGTTCGGCTGT
GGCTCTGTCGCTCAGCTAGTTCTCAGTGGT------GGATCTCATGGACAGTTCCTGACT
GTGAACTTCGCTTTCGGCTTTGCTGCGACCCTGGGTGTCCTGGTGTCTGGGCAGGTGTCA
GGTGGCCATCTGAACCCGGCTCTGACCTTTGCCCTCTGTCTGCTGGGGCGGGAGCCGTGG
CGCAAGATGCCGGTGTACTTCTTGGCGCAGACGGTGGGTGCCTTCCTGGGGGCTGGGGTC
ATCTTT---GGAATGTACTTCGATGCCTTGTGGTTCTTTGGGAAT---GACAATCTGTTA
GTG------TCAAAC---GGTACCGCCGGGATCTTTGCTACCTATCCATCTCAGCACTTG
ACCCTGCTCAATGGCTTCTTTGACCAGATGATTGGTACTGCTGCCCTGATCGTGTGCCTC
CTGGCCATCGTGGACCCCTACAACAACCCCATCCCCAGGGGTCTGGAGGCCTTTACTGTG
GGCTTTGTCGTCTTGGTCATCGGTCTGTCCATGGGCTTCAACTCTGGCTACGCCGTCAAC
CCTGCCAGAGACTTT---GGTCCTCGCCTCTTCACCGCCATAGCCGGCTGGGGCACAGAG
GTCTTC---ACC------ACTGGATCTCACTGGTGGTGGGTGCCCATCTTTGCCCCCTTC
CTGGGTGCCTTCATCGGGGTTCTGGTGTACCAGTTCATGGTGGGCTTCCACACGGAGGGT
---------------GAGGCCAAAGACAAA---------------------GCA------
------------AGGGAG---GACAATGAG------------------------------
------------------------GAGAAC------------GTCAAGCTGTCCAACATC
CACTCTAAACAGATCGCT--

>Reedfish_Aqp3
---ATGGAAAGA
CAAAAG---CGTTTCCTGGCCTGGATGGGACAGATGTTC------TACATTAAGAATAAA
CTTTTAAGGCAATCACTAGCTGAGTGTTTGGGCACCCTAATCTTGGTGATGTTTGGCTGT
GGCTCTGTGGCTCAGCTGGTCCTTAGTGGT------GGCACCCATGGCCAATTTCTTACA
GTCAACTTTGCATTTGGTTTTGCAGTCACACTCGGCATACTTATTTCTGGACAGGTGTCA
GGTGGACATCTCAACCCTGCTGTGACCTTTTCTCTTTGCCTTCTGGGACGTGAGCCTTGG
ATCAAATTCCCAGTGTACTTTCTGGCTCAGACCCTAGGATCCTTCCTTGGGGCTGGAATA
ATCTTT---GGACTGTACTTTGATGCCCTTTGGGCCTATGGAGGT---AATCATCTCCTT
GTGACTGGGTCAAAC---GCCACTGCAGGAATCTTTGCTACCTTCCCCTCAGAGCATCTT
ACTCTTGTAAATGGTTTTTTTGACCAGGTAATTGGCACTGCTGCTCTCATTGTGTGTATC
TTGGCAATTGTTGACCCATACAACAACCCCATTCCACAAGGCCTGGAGGCCTTCACTGTG
GGTTTTGTAGTTCTTGTTATTGGACTTTCAATGGGCTTCAACTCTGGATATGCAGTTAAC
CCAGCCAGGGACTTT---GGGCCAAGACTGTTCACTTCAATTGCAGGCTGGGGTTCCGAG
GTCTTC---ACG------TCTGGAAATAATTGGTGGTGGGTACCAATTCTTGCCCCCTTC
TTCGGAGCCTTCGCTGGAGTAATTGTGTATCAGTTCATGGTTGGCTTTCATGTGGAAGGG
---------------ATGTCAAAGCAAAAG---------------------AAA------
----------------------CAAAGTGAA------------------------------

------------------------GAAAAC------------ATCAAATTGTCCAATACA
AGCTCTAAAGATATGGCA--

>WA_lungfish_Aqp3
---ATGGGAATG
CAAAAG---GAATGTCTCTCAAAAGTTGGCAATTTCTTA------AGAATCAAAAATAAA
CTGTTAAGGCAAGCTTTGGCAGAATGTTTGGGGACGTTAATACTTGTGATGTTTGGCTGT
GGATCAGTAGCACAAGTTGTCCTTAGTGAG------GGTCAAAAGGGACGTTTCCTAACA
GTCAACTTAGCCTTTGGATTTGCTGTGACCCTTGGGATCCTTGTATGTGGACAAGTATCT
GGTGGTCATCTAAATCCAGCTGTGACCTTTGCTTTATGCCTACTTGGTCGTGACAGGTGG
CGCAAATTCCCAATATATTTCTTTGCTCAGACAGTAGGCTCATTTATTGCCGCTGCTATC
ATCTAT---GGACTGTACTTTGATGCTCTGATGAACTATGGAGGT---GGTCAACTGAGA
GTAACAGGAGCTAAT---TCAACAGCTGGAATCTTTGCAACCTATCCAGGAGAACATCTC
ACAACGCTAAATGGATTCTTTGATCAGCTCATTGGCACAGCAGCTCTGATTGTCTGTATC
CTTGCGATTGTCGATCCTTACAACAACCCTATTCCCAGACATCTAGAGGCATTCACTGTT
GGATTTGTAGTCCTGGTCATCGGTCTTTCAATGGGACTGAATTCTGGATATGCTGTTAAT
CCTGCTAGGGACTTT---GGACCCCGACTTTTCACAGCAATGGCTGGATGGGGCTCAGAG
GTTTTC---ACT------GCTGGATCACACTGGTGGTGGGTACCTATAATTTCCCCATTT
ATTGGAACAGTTATTGGAACAGCAGTATACCAGATAATGATTGGTATCCATGTTGAACTC
---------------GATGTTCCTGCAAGC---------------------CCT------
---------------AGC---ACCCCAGAA------------------------------
------------------------GAAGCT------------GTCAAGCTGGCAAAAATA
AAGGCAAATGAAACTGCA--

>SA_lungfish_Aqp3
---ATGGGAGTG
CAAAAG---GACTGTCTTACAAAAATTGGCAATTTCCTC------AGAATCAAAAATAAA
TTGTTAAGGCAGGCTCTGGCAGAATGTTTGGGGACTTTAATTCTTGTGATGTTTGGCTGT
GGATCAGTAGCACAGGTTATCCTTAGTGAA------GGAGCGAAAGGACGTTTCCTAACT
GTTAACTTAGCCTTTGGATTTGCTGTTACCCTTGGGATCCTTGTATGTGGACAAGTATCT
GGTGGTCACCTAAATCCAGCTGTAACCTTTGCTTTGTGCCTACTTGGTCGGGAGAGGTGG
CGTAAATTCCCAGTGTATTTCTTTGCTCAGACATTAGGTGCATTTGTTGCATCTGCCATC
ATCTAT---GGGCTGTACTATGATGCTTTGATGCATTATGGAGGT---GGCCAGCTGTCA
GTACTTGGAAACAAT---GCAACAGCTGGAATCTTTGCAACCTATCCAGGAGAACATCTC
ACAATGCTAAATGGATTCTTTGATCAGATCATAGGCACTGCAGCATTGATTGTCTGTATC
CTTGCGATTGTGGACCCTTACAACAACCCCATTCCCAGACATCTAGAGGCATTCACTGTT
GGATTTGTAGTCCTGGTCATTGGTCTCTCAATGGGACTGAATTCTGGATATGCTGTTAAC
CCTGCTAGGGACTTC---GGACCTCGACTTTTCACAGCAATAGCTGGATGGGGCACAGAA
GTTTTC---ACT------GCTGGATCTCATTGGTGGTGGGTACCTATAACTGCTCCCTTC
ATTGGCACAGTTTGCGGAACTTTAGTATACCAGATAATGATCGGCATCCATGTCGAACTT
---------------GATGTCCCTCAAAGC---------------------CCT------
---------------AGC---TCTCCAGAA------------------------------
------------------------GAAAAT------------GTCAAGCTGGCAAATATA
AAGAAAAATGAGACTGCA--

>Coelacanth_Aqp3
---ATGGGAAAA
CAAAAA---GAAATGCTCACTAAAATGTTCCGACTGTTT------AAAATTAGAAACAAG
CTGCTGAGAGAAGCTTTGGCAGAATGTCTGGGCACTTTAATTTTGGTGTTGTTCGGCTGC
GGTGCTTTAGCCCAGGTGGTTCTCAGCAAT------GGCTCCCACGGACAGTTCTTGACT
GTCAATCTTGCCTTTGGTTTTGCGGCAACTCTCGGGGTTCTGATTGCAGGGCAGATCTCA
GGTGCCCATCTTAATCCTGCTGTCACCTTTGCAGTGTGCCTTCTTGCCCGCGAGCCATGG
ATTAAATTCCCCGTTTACTCCTTAGCTCAAATCCTGGGTGGATTTCTGGCATCGGGCATC
ATCTTT---GCCATGTACTTTGATGCTATTTGGCTCTATGGCAAC---AACCAGCTGATA
GTAATGGGGCCCAAA---GGCACTGCGGGAATCTTTGCCACTTACCCTTCTGAACACCTG

ACACTCCTCAACGGATTCTTTGATCAGTTAATCGGCACTGCTGCCCTGGTTCTCTGCATC
CTTGCCATTGTGGACCCTCATAACAATCCCATCCCCAGGGGCCTGGAGGCCTTCACCATT
GGCTTTGTGGTCCTAAACATCGGGCTCGCCATGGGCTTCAATTCTGGCTACGCCGTCAAT
CCCGCCCGAGACTTT---GGGCCCCGTCTGTTCACAGCCGTTGCTGGCTGGGGCACAGAG
GTTTTC---AGT------GCTGGTGGACATTGGTGGTGGGTGCCGATAGTCGCGCCACTC
CTAGGAGCAGTACTGGGAGTTCTGGTGTACCAGCTGATGATTGGAGTCCACTTTGAACCC
---------------GAGACCCCATCCACC------------------------------
------------------------GCACCA------------------------------
------------------------GAAAAT------------GTCAATCTAACTAAAGTA
GACACCATAGAGAAACCT--

>B_bambooshark_Aqp3C2
---ATGGGAAAA
CAAAAG---GCAATAATCAGAAAAATTGAAGACTCATTC------AGAATAAGAAATCTA
TTGCTCAGACAATGTCTTGCTGAATGTTTAGGAACATTAATTCTTGTGTTGTTTGGGTGT
GGAGCACTAGCACAAGTTACCCTCAGCAGG------GGTACACATGGACGGTTTTTGACT
GTCAATTTTGCTTTTGGATTTGCAGTAATGCTCGGTGTACTAATAGCTGGCCAAGTGTCA
GGTGCTCACCTGAACCCTGCTGTGACCTTTGCTATGTGCTTACTTGCTCGCGAACCTTGG
ATAAAATTTCCCCTTTACTCTCTGGCACAAATATTAGGTGGCTTCATTGGATCTGGTATC
ATTTTT---GGTTTGTATTTTGATGCCATGTGGGACTTCAGTGGTCAAAATAAACTGTTG
GTATATGGCCCCAAT---GCAACTGCTGGCATATTTGCTACTTACCCATCTGCACACTTA
ACTCCACTCAATGGCTTTTTTGATCAGCTGATTGGAACGAGTGCTCTGATAGTTTGCATC
CTTGCTATTGTGGATAAGTACAATAACCCAGTGCCAAAGGGTGTGGAGGCATTTACTGTT
GGCTTTACCGTACTGGTTATTGGCTTGTCAATGGGTTTCAACTCTGGGTATGCTGTGAAC
CCTGCCAGAGACTTT---GGACCTCGTTTGTTTACGGCATTGGCTGGCTGGGGAGCTGAA
GTTTTC---AGT------GCCGGACACTACTGGTTTTGGATCCCAATTTTTGCCCCCCTC
CTTGGTTCTTTACTGGGCATTCTGATATATCAACTAATGATTGGGATACATCTGGAGCCT
---------------GTAAACCACAATTCA---------------------TCC------
---------------------CCTAGAGAA------------------------------
------------------------GAAAAT------------GTAAAACTGGCTAATGTG
AACTTAAGAGAAAGTTCC--

>W_bambooshark_Aqp3C2
---ATGGGAAAA
CAAAAG---GCAATAATCAGAAAAATTGAAGACTCTTTC------AGAATAAGAAATCTA
TTGCTCAGACAATGTCTTGCTGAATGTTTAGGAACATTAATTCTTGTGTTGTTTGGGTGT
GGAGCACTGGCACAAATTACCCTCAGCAGG------GGTACACATGGACGGTTTTTGACT
GTCAATTTTGCTTTTGGATTTGCAGTAATGCTTGGTGTACTAATAGCTGGCCAAGTGTCA
GGTGCTCACCTGAACCCTGCTGTGACCTTTGCTATGTGCTTACTTGCTCGTGAACCTTGG
ATAAAATTTCCCCTTTACTCTCTGGCACAAATACTAGGTGGCTTCATTGGATCTGGTATC
ATTTTT---GGTTTGTATTTTGATGCCATGTGGGACTTCAGTGGTCAAAATAAACTGTTG
GTATATGGCCCCAAT---GCAACTGCTGGCATATTTGCTACTTACCCATCTGCACACTTA
ACTCCACTCAATGGCTTTTTTGATCAGCTGATTGGAACGAGTGCTCTGATAGTTTGCATC
CTTGCTATTGTGGATAAGTACAATAACCCAGTGCCAAAGGGTGTGGAGGCATTTACCGTT
GGCTTTACCGTACTGGTTATTGGCTTGTCAATGGGTTTCAACTCTGGGTATGCTGTGAAC
CCTGCCAGAGACTTT---GGACCTCGTTTGTTTACGGCATTGGCTGGCTGGGGAGCTGAA
GTTTTC---ACG------GCTGGACACTACTGGTTTTGGATCCCAATTTTTGCCCCCCTC
CTTGGTTCTTTACTGGGCATTCTGATATATCAACTAATGATTGGGATACATCTGGAGCCT
---------------GTAAACCACAATTCA---------------------TCC------
---------------------ACTAGAGAA------------------------------
------------------------GAAAAT------------GTAAAACTGGCTAATGTG
AACTTAAGAGAAAGTTCC--

>Whale_shark_Aqp3C2
---ATGGGAAAA

```
CAAAAG---GCAATAATCAGAAAAATTGAAGATTCATTC------AGAGTAAGAAATCTA
TTGCTAAGGCAATGTCTTGCTGAATGTTTAGGAACATTGATTCTTGTGTTGTTTGGGTGT
GGAGCGCTGGCACAAGTTACCCTCAGCAGG------GGCACACATGGAAGGTTTTTGACT
GTCAATTTTGCTTTTGGATTTGCAGCAATGCTTGGTGTACTATTAGCTGGCCAAGTGTCA
GGTGCTCACCTGAACCCTGCTGTGACATTTGCTATGTGCTTACTTGCTCGCGAACCCTGG
ATAAAATTTCCCCTTTACTCTTTGGCACAAATACTAGGCGGCTTCCTTGGATCTGGTATC
ATTTTT---GGTTTGTATTTTGATGCCATGTGGGACTTCAGTGGTCAAAATAAACTGTTG
GTATATGGCCCCAAT---GCAACTGCTGGTATATTTGCTACTTACCCATCTGCACATCTA
ACTCCACTCAATGGCTTTTTGATCAGCTGATTGGAACTAGCGCTCTGATAGTTTGCATC
CTTGCTATTGTGGATAAATTCAATAACCCAGTGCCAAAGGGTGTGGAGGCATTTACAGTT
GGCTTTACTGTACTGGTCATTGGCTTGTCAATGGGCTTCAACTCTGGATATGCTGTGAAC
CCTGCCAGAGATTTT---GGACCTCGTTTGTTTACAGCATTGGCCGGCTGGGGAGCTGAA
GTTTTC---AGT------GCTGGACACTACTGGTTTTGGATTCCAATTTTTGCCCCCCTC
CTTGGTTCTTTACTTGGCACTCTAGTATATCAACTAATGATTGGGATACATCTCGAGCCT
---------------GTAAACCATAATTTA---------------------CCC------
---------------------ACTAGAGAA------------------------------
------------------------GAAAAT------------GTAAAACTGGCTCATGTA
AATTTAAAAGAAAGCTCC------------------------------------------
------------------------------------------------------------
------------------------------------------------------------
---------------------
>Zebra_bullheadshark_Aqp3C2
---------------------------------------------------ATGGGAAAG
CAAAAG---GCAATTATCAGAAAAATTGAAGATTCATTC------AGAATAAGAAATCTA
TTGCTAAGACAATGTCTTGCTGAATGTTTAGGAACATTGATTCTTGTGTTGTTTGGCTGT
GGAGCATTGGCACAAGTTACCCTCAGCAGG------GGTACACATGGACTGTTTTTGACT
GTTAATTTTGCCTTCGGATTTGCAGTGATGCTCGGTGTACTAGTAGCTGGCCAAGTGTCA
GGAGCTCACCTGAATCCTGCTGTGACCTTTGCTATGTGCTTACTTGCTCGCGAACCCTGG
ATAAAATTTCCCCTTTACTCTTTGGCACAAATACTAGGTGGCTTCCTTGGATCTGGTATC
ATTTTC---GGTTTGTATTTTGATGCCATGTGGGACTTCAGTGGACAAAACCAACTGTTA
GTACATGGGCCCAAT---GCCACTGCTGGTATATTCGCCACATACCCATCTGTGCACTTA
ACTCCACTCAATGGCTTTTTGATCAGCTGATTGGAACTACTGCTCTCATAGTTTGCATC
CTTGCCATTGTGGATAAATTCAATAACCCAGTGCCAAAGGGTCTGGAGGCATTTACTGTT
GGCTTTACCGTCCTGGTAATCGGCTTGTCAATGGGTTTCAACTCCGGGTATGCTGTAAAC
CCTGCCAGAGACTTT---GGACCTCGTTTGTTTACAGCATTGGCTGGCTGGGGAGCTGAA
GTTTTC---ATT------GCTGGAAACTACTGGTTTTGGATCCCTATTTTTGCCCCACTC
CTTGGTTCTGTGCTTGGTGTTCTGGTTTATCAACTAATGATTGGAATACATATTGAGCCC
---------------ATAAACCATAGTTCA---------------------CCT------
---------------------ATTGAAGAA------------------------------
------------------------GAAAAT------------GTAAAACTGGCTAATGTA
AAATTAAGAGAAAGTTCA------------------------------------------
------------------------------------------------------------
------------------------------------------------------------
---------------------
>Cloudy_catshark_Aqp3C2
---------------------------------------------------ATGGGAAAA
CAAAAG---GCAATAATCAGGAAAATCGAAGATTCGTTC------AGAATAAGAAATTTA
TTGGTCAGACAATGTCTTGCTGAATGTTTAGGAACATTGGTTCTTGTGTTATTTGGCTGT
GGAGCAGTGGCACAAGTTACCCTCAGCAGA------GGAACCCATGGACTGTTTTTGACT
GTTAATTTTGCTTTTGGATTTGCAGTGATGCTGGGTGCACTAATAGCTGGCCAAGTGTCA
GGAGCTCACCTGAACCCTGCTGTGACCTTTGCTATGTGTATACTTGCTCGCGAGCCCTGG
ATAAAATTTCCCCTCTACTCTTTCGCACAAATATTAGGCGGTTTCCTTGGGTCTGGTGTC
ATTTTT---GGTTTGTACTTTGATGCCATGTGGGGCTTCAGTGGCCAAAACAAACTCTTA
GTACATGGCCCCAAT---GCGACTGCTGGGATATTTGCTACATACCCAGCTCTGCACTTA
ACTCCAGTAAATGGCTTTTTGATCAGCTGATTGGGACTGCAGCTCTCATAGTTTGCATC
CTAGCCATTGTGGATAAATTCAACAACCCAGTGCCAAAGGGACTGGAGGCATTTAGTGTT
GGCTTTGCCGTGCTGGTAATTGGCATGTCAATGGGTTTCAATTCTGGGTACGCTATAAAC
CCTGCCAGAGACTTT---GGACCTCGTCTGTTTACAGCACTGGCCGGCTGGGGAGCTGAG
GTTTTC---AGT------GCTGGAAACTACTGGTTTTGGATTCCCATCGTTGCCCCACTC
CTTGGTTCTGTATTTGGTATTTTGGTATATCAACTAATGGTTGGAATACACCTTGAGCCC
---------------ATAAACCACAGTTCA---------------------ACC------
```

---------------------ACGGAAGAG------------------------------
------------------------GAAAAT------------GTAAAACTTGCGAATGTT
AAATTAAGAGAAAGTTCC--

>Blue_shark_Aqp3C2
---ATGGGAAAA
CAAAAG---GCAATGATTAGAAAAATCGAAGATTCGTTC------AGAATCAGAAACCTG
TTGTTAAGACAATGTCTTGCTGAATGTTTAGGTACATTGATACTTGTGTTGTTTGGCTGT
GGAGCAGTGGCACAAGTTACCCTCAGCAGG------GGTACCCATGGACAGTTTTTGACT
GTTAATTTTGCTTTTGGGTTTGCAGTGATGCTTGGTGTCCTGATAGCTGGCCAAGTGTCA
GGTGCTCACCTGAACCCTGCTGTGACCTTTGCTATGTGTTTGCTTGCTCGTGAGCCCTGG
ATAAAATTTCCTCTCTACTCTTTGGCCCAAATATTTGGTGGCTTCCTTGGATCTGGTGTC
ATTTTT---GGCTTGTACTTTGATGCCATTTGGGGCTTCAGTGGCCAAAACAAACTCTTA
GTACATGGTCCCAAT---GCGACTGCTGGTATATTTGCTACATACCCATCTGCGCACTTA
ACTCCAGTAAATGGCTTTTTTGATCAGCTGATTGGAACTACTGCTCTCATAGTTTGCATC
CTTGCCATTGTGGATAAGTTCAACAACCCAGTGCCAAAGGGACTGGAGGCATTTACTGTT
GGTTTTGTCATATTGGTAATTGGCCTGTCAATGGGTTTCAACTCTGGGTATGCTGTGAAC
CCTGCCAGAGACTTT---GGACCTCGCTTGTTTACAGCACTGGCTGGTTGGGGAGCTGAA
GTTTTC---AGT------GCCGGAAGCTACTGGTTTTGGGTTCCCATTTTTGCCCCACTC
CTTGGTTCTGTACTTGGTGTTCTGATATACCAGCTAATGATTGGAATTCACCTTGAGCCC
---------------ATAAAGCCCAGTTCA---------------------CCC------
---------------------GCTGGAGAA------------------------------
------------------------GATAAT------------GTAAAACTGGCTAATGTA
AAATCAAGAGAAAGTTGC--

>Great_white_shark_Aqp3C2
---ATGGGAAAA
CAAAAA---GCACTGATCAGAAAAATTGAACATTCATTC------AGAATAAGAAATCTG
TTGGTAAGAGAATGTCTTGCTGAATGTTTAGGAACATTAATTCTTGTGTTGTTTGGCTGT
GGAGCATTGGCACAAGTTACTCTCAGCAGA------GGTACACATGGAATGTTTTTGACT
GTTAATTTTGCTTTTGGATTTGCAGTGATGCTCGGTGTACTAATAGCTGGCCAAGTGTCA
GGAGCTCACCTGAATCCTGCCGTGACATTTGCCATGTGTTTACTTGCTCGTGAGCCCTGG
ATAAAATTTCCCCTCTACTCTTTGGCACAAATATTAGGTGGTTTCCTTGGATCTGGTGTC
ATTTTT---GGTTTGTATTTTGATGCCATGTGGGGTTTTAGTGGCCAAAACAAACTCTTA
GTACATGGCCCCAAT---GCGACTGCTGGTATATTTGCTACATACCCATCTGTGCACTTA
ACACCAGTAAATGGCTTTTTTGATCAGCTGATTGGGACGACCGCTCTCATAATTTGCATC
CTTGCGATTGTGGATAAACTCAATAACCCAGTGCCAAAGGGACTGGAGGCATTTACCATT
GGCTTTGCCGTGCTGGTAATTGGCCTGTCAATGGGTTTCAACTCTGGGTACGCTGTGAAC
CCTGCCAGAGACTTT---GGACCTCGCTTGTTTACAGCATTGGCTGGCTGGGGAGCTGAG
GTTTTC---AGT------GCTGGAAACTACTGGTTTTGGATTCCCATTTTTGCCCCACTC
CTTGGTTCTGTATTTGGTATTCTGGTATATCAGCTAATGATTGGAATACACCTCGATCCC
---------------ATAAACTGCAGTTCA---------------------CCC------
---------------------TCTGGAGAA------------------------------
------------------------GAAAAT------------GTAAAACTGGCTAATGTA
AAATTAAGAGAAAGTTCC--

>VB_lantern_shark_Aqp3C2
---ATGGGAAAA
CAAAAA---GCAATAATCAGAAAAATTGAAGATTCATTC------CGAATAAGAAATCTG
TTGGTAAGACAATGTCTTGCTGAATGTTTAGGAACGTTGATTCTTGTGTTGTTTGGCTGT
GGAGCATTGGCACAAATTACCCTCAGCAGG------GGTACACATGGACAGTTTTTGACT
GTTAATTTTGCCTTCGGATTTGCAGTGATGCTCGGTGTACTCCTGGCTGGCCAAGTCTCA
GGAGCGCACTTGAATCCTGCCGTGACCTTTGCTATGTGCTTACTTGCTCGCGAACCCTGG
TTAAAATTTCCCCTTTACTCTTTGGCACAAATACTAGGCGGCTTCCTTGGATCTGGTATC
ATTTTC---GGTTTGTATTTTGATGCCATGTGGGACTTTAGTGGTCAAAACCAACTGTTA

GTACATGGCCCCAAT---GCTACTGCTGGTATATTCGCTACCTACCCATCTGTGCACTTA
ACTCCA------------TTTGATCAGCTGATTGGAACTGCCGCTCTCATAGTTTGCATC
CTTGGCATTGTGGATAAATTCAATAACCCGGTGCCAAAAGGACTGGAGGCATTTACTGTT
GGCTTTACTGTCCTGGTAATTGGCTTATCAATGGGTTTCAACTCTGGGTATGCCGTGAAC
CCTGCCAGAGACTTT---GGACCTCGTTTGTTTACAGCATTGGCTGGCTGGGGAGCTGAG
GTTTTC---GTT------GCTGGAAACTACTGGTTTTGGATCCCTATTTTTGCCCCACTC
CTTGGTTCTGTGCTTGGTATTCTGGTATATCAACTAATGATTGGAATACACCTCGAGCCC
---------------GAAAACCACAATTCA---------------------CCC------
---------------------ATTGGAGAA------------------------------
------------------------GAGAAT------------GTAAAACTGGCTGATGTA
AAATTAAGAGAAAGTTCC--

>Spiny_dogfish_Aqp3C2

---AGAATAAGAAATCTG
TTGGTAAGACAATGTCTTGCTGAATGTTTAGGAACGTTGATTCTTGTGTTGTTTGGCTGT
GGAGCATTGGCACAAATGACCCTCAGTAGG------GGTACACACGGACAGTTTTTGACT
GTTAATTTTGCCTTCGGATTTGCAGTGATGCTCGGTGTACTCCTGGCTGGCCAAGTCTCA
GGAGCGCACTTGAATCCTGCCGTGACCTTTGCTATGTGCTTACTTGCTCGCGAACCCTGG
TTAAAATTTCCACTTTACTCTTTGGCACAAATACTAGGCGGCTTCCTTGGCTCTGGTATC
ATTTTC---GGTTTGTATTTTGATGCCATGTGGGACTTTAGTGGTCAAAACAAACTGTTA
ATATATGGCCCCAAT---GCCACTGCTGGTATATTCGCTACATACCCATCTGTGCACTTA
ACTCCACTCAATGGCTTTTTTGATCAGCTGATTGGAACTGCCGCTCTCATAGTTTGCATC
CTTAGCATTGTGGATAAATTCAATAACCCGGTGCCAAAGGGACTGGAGGCATTTACTGTT
GGCTTTACCGTCCTGGTAATTGGCTTGTCTATGGGTTTCAACTCTGGGTATGCCGTGAAC
CCTGCCAGAGACTTT---GGACCTCGTTTGTTTACATCATTGGCTGGCTGGGGAGCTGAG
GTTTTC---ATT------GCTGGAAACTACTGGTTTTGGATCCCTATTTTTGCCCCACTC
CTTGGTTCTGTACTTGGTATTCTGGTATATCAGCTTATGATTGGAATACACCTCGAGCCC
---------------GAAAACCACAATTCA---------------------CCC------
---------------------ATTGGAGAA------------------------------
------------------------GAAAAT------------GTAAAACTGGCTAATGTA
AAATTAAGAGAAAGTTCC--

>Little_skate_Aqp3C2
---ATGGGAAAA
CAGAAA---GCAATGATCAAAAAAATTGAAAATCATTGC------CGAATAGAAAATCTG
TTGGGAAGACAGTGTCTTGCTGAATGTCTAGGAACATTGATTCTTGTGTTATTTGGTTGT
GGAGCAGTGGCACAAGTTACCCTCAGCAGG------GGTACACATGGACAATTTTTGACA

GGAGCTCATCTGAACCCCGCCGTGACCTTTGCCATGTGCTTGCTTGCCCGTGAGCCCTGG
ATAAAGTTTCCCCTTTACTCTTTGGCGCAAACACTTGGTGCCTTCCTAGCATCGTGTGTT
ATTTTT---GGTTTGTATTATGATGCGTTTTGGGACTTCAGTGGTCAAAACCAACTACTA
GTTTACGGTCCTAAT---GCCACGGCTGGAATTTTTGCAACGTATCCATCTGCACATTTA
GGTTCATCCAATGGTTTCTTTGATCAGGTGATTGGGACAGCTGCTCTCATAGTGTGCATC
CTGGCCATTGTGGACAAATGGAATACCCCAGTGCCTAACGGACTAGAGGCATTTACTGTT
GGCTTTACCGTGCTTGTAATCGGTTTGTCAATGGGTTTCAACTCTGGGTATGCGGTGAAC
CCAGCCAGAGATCTT---GGACCCCGTTTGTTTACATCATTAGCTGGCTGGGGAACTGAG
GTTTTC---ACG------GCTGGAAACTACTGGTTTTGGATCCCCATTTTTGCCCCACTT
CTTGGTTCTATACTTGGTATTCTGGTATATCAATTAATGATCGGCATACACTTTGAGCCT
---------------AAAGAGCAGAGTTCA---------------------CCC------
---------------------GCTGGTGAA------------------------------
------------------------GAAAAT------------GTAAAACTCGCAAATGTG
AAATTAAGAGATGGTTGC--

>Thorny_skate_Aqp3C2

```
---------------------------------------------------------ATGGGAAAA
CAGAAA---GCAATGATCAAAAAAATGGAAAGTTATTGC------CGAATACAAAATCTG
TTGGGAAGACAGTGTCTTGCTGAATGTCTAGGAACATTGATTCTTGTGTTATTTGGTTGT
GGAGCAGTGGCACAAGTTACCCTCAGCAGG------GGTTCACATGGACAATTTTTGACA
GTTAATTTTGCATTCGGATTTTCAGTGATGCTTGGTGTGCTCATTGCTGGCAAAGTATCA
GGAGCTCATCTGAACCCCGCCGTGACCTTTGCCATGTGCTTACTTGCCCGCGAGCCCTGG
ATAAAGTTTCCCCTTTACTCTTTGGCGCAAACACTTGGCGCCTTCCTAGCATCGTGTATT
ATTTTT---GGTTTGTATTATGATGCATTTTGGGACTTCAGTGGTCAACACCAACTATTA
GTTTACGGTCCTAAT---GCCACGGCTGGTATTTTCGCAACGTATCCATCTGCACATTTA
GGTTCATCCAATGGTTTCTTTGATCAGGTGATTGGGACAGCTGCTCTCATAGTGTGCATC
TTGGCTATTGTGGACAAATGGAATACCCCAGTGCCTAACGGACTAGAGGCATTCACAGTT
GGCTTTACCGTGCTTGTAATCGGCTTGTCAATGGGTTTCAACTCTGGGTATGCGGTGAAC
CCAGCCAGAGATCTT---GGACCGCGTTTGTTTACATCATTAGCTGGCTGGGGAACTGAG
GTTTTC---ACG------GCTGGAAACTACTGGTTTTGGATCCCCATTTTTGCCCCACTT
CTTGGTTCTATACTTGGTATTCTGGTATATCAATTAATGATCGGCATACACCTTGAGCCT
---------------AAAGAGCAGAGTTCA---------------------CCC------
---------------------GTTGGGGAA------------------------------
------------------------GAAAAT------------CTAAAACTCGCAAATGTG
AAATTAAGAGAAGGTTGC------------------------------------------
------------------------------------------------------------
------------------------------------------------------------
---------------------
>Smalltooth_sawfish_Aqp3C2
---------------------------------------------------------ATGGGAAAA
CAGAAG---GCAATGATCAAAAAACTTGAAAATTCATTC------AGAATACAAAATATG
TTGGGAAGACAATGTCTTGCTGAATGTTTAGGGACATTGATTCTTGTGTTATTTGGTTGT
GGAGCACTGGCCCAAGTTACCCTCAGCAGG------GGTACACATGGACGTTTTTTGACT
GTTAATTTTGCCTTCGGATTTTCAGTGATGCTCGGTGTGCTCATAGCTGGCCAAGTGTCA
GGAGCACACCTGAATCCTGCCGTGACCTTTGCCATGTGCTTACTTGCCCGTGAACCCTGG
ATAAAGTTTCCCCTTTACTCTTTGTCACAAATACTAGGTGCCTTCCTAGGTTCGGGCGTT
ATCTTT---GGTTTGTATTTTGATGCTATGTGGGACTTCAGTGGTCAAAATCAACTATTA
GTTTATGGTCCTAAT---GCCACAGCTGGTATTTTTGCTACATATCCATCTCCACACTTA
AGTTCATCCAATGGCTTTTTTGATCAGATGATTGGGACGGCTGCTCTCATAGTGTGCATC
CTTACTATTGTGGACAAATGGAACAACCCAGTGCCAAAGGGACTGGAGGCATTTACTATT
GGATTTACGGTGCTGGTAATTGGCTTGTCGATGGGTTTCAACTCTGGGTATGCTGTGAAC
CCTGCAAGAGACCTT---GGACCTCGTTTGTTTACATCAGTAGCTGGCTGGGGATCTGAG
GTTTTC---ACG------GCTGGAAACTACTGGTTTTGGATCCCTATTTTTGCCCCACTC
CTTGGTTCTGTTCTTGGTATTCTGGTATATCAACTAATGATTGGCATACACCTTGAGCCT
---------------GAAGACCAGAGTTCA---------------------CCC------
---------------------ATTGGAGAA------------------------------
------------------------GAAAAT------------GTAAAACTGGCAAATGTA
AAATTAAGAGAAAGTTGC------------------------------------------
------------------------------------------------------------
------------------------------------------------------------
---------------------
>Ghost_shark_Aqp3C2
---------------------------------------------------------ATGGGGAAA
CAGAAA---GAAACCATCAGGAAAATTCAAGACTTATTC------CGTATAAGAAACATG
CTGGTAAAACAATGCCTTGCTGAATGTCTGGGGACTTTAATTCTCGTGCTGTTTGGCTGC
GGGGCACTGGCACAGATCACTCTCAGTCGG------GGAACGCACGGAGCCTTTCTAACA
GTCAACTTAGCCTTCGGATTTGCGGTGACTCTTGGTGTGCTTGTGGCCGGTCAAGTGTCG
GGAGCTCACCTGAATCCTGCTGTGACCTTTGCTTTGTGTCTACTTGCTCGTGAGCCCTGG
ATCAAATTTCCCCTTTTCTCTTTGGCACAAATATTGGGTGGATTTCTTGGAGCAGGTATC
ATCTTT---GGATTATATTTTGATGCAATTTGGCTCCATGGTAAT---AACCATCTGATA
GTAATGGGACCTAAT---GCTACTGCTGGGATATTTGCGACTTACCCGTCTGAACACTTG
ACTTTAATCAATGGTTTCTTTGATCAGCTGATTGGCACAGCAGCTCTCATCGTCTGTATC
CTTGCCATTGTGGATCCATTCAACAACCCGGTGCCAAAGGGAGTGGAGGCCTTCACTATT
GGCTTTGTGGTTCTGGTAATCGGCTTGGCAATGGGCTTTAACTGTGGCTACGCTGTGAAC
CCAGCCAGAGATTTT---GGACCTCGCTTGTTTACCTCACTGGCTGGCTGGGGCACCAAA
GTCTTC---AGT------GCTGGAAACTACTGGTTTTGGGTGCCCATTCTAGCCCCGCTC
CTTGGTGCAGTGCTTGGTATTCTGGTTTACCAGCTGATGGTTGGCATACACACCGAACCC
```

----------------GAAGAAAACCATTCA-----------------------TCC------
----------------------AGTGCAGAA-------------------------------
-------------------------GAAAGA-------------ATAAAACTGGCCAACATG
AAGCCGAAAGAAAACTGC--

>B_bambooshark_Aqp3C1
---ATGGAAAGA
CAAAAA---GAAATCATCAGAAATATGACAACTATCCTG------AAAGTCCGAAGTCTT
CTGTTGAAACAATGCCTTGCTGAATGTTTAGGAACTTTGATTCACACGATGCTTAGCTGT
GGAGGAGTAGCACAATTTACTCTCAGTTAT------GGCACACACAAAGAATTCTTGACT
GTTACAATTGCTGGTGGATTTGCAGTAGCTCTGGGTATATTGGTAACTAGTAAAGTCTCA
GGAGCTCACCTGAATCCTGCAGTGACCTTTGCTTTGTGCTTGCTTGCTTGTGAACCTTGG
TTAAAATTTCCTTTATTCTTTTTGGCACAAACGTTTGGTGCCTTTCTTGGATCAGGAATA
ATGTTT---GGTTTGTACTATGATACATTGTGGCATTATGGCAAT---AAACAGCTAACA
GTAATTGGAGCAAAT---TCTACTGCTGGAATATTTACAACTTATCCACATGAACATTTG
AGCGCAGTCAATGGCATTTTTAATCAGGCAATTGGGACTGCAGCACTTATACTTTGTATC
CTTATCATTGTGGATCCATTGAACGATGCTATACCAACAGGACTAGAAGCTTTTACAATT
GGCTTTGTGGTTTTGCTAATTGGCTGGTCAATGGGTTCCAATTCTCAGTACTCATTAAAT
CCTGCCAGAGATATT---GGACCTCGTCTGTTTACTGCAATTGCTGGTTGGGGAACTGAA
GCTTTC---ACG------GCTGGCAATTATTGGTTTTGGATCCCAGTTGCCAGTCCAATC
ATTGGTGCCATATTTGGTGTTCTCCTGTACAAGTTCATTGTTGGATTGCGTGCTGAAGCA
----------------AGAACCAGCTGCCCA-----------------------TCC------
----------------------AGTGCAGAA-------------------------------
-------------------------CAAAAT-------------ATAAAGTTAATGAACCAA
AAACCAAAGAGGAGGCTC--

>W_bambooshark_Aqp3C1
---ATGGAAAGA
CAAAAA---GAAATCATCAGAAATATGACAACTATCCTG------AAAGTCCAAAGTCTT
CTGTTGAAACAATGCCTTGCTGAATGTTTAGGAACTTTGATTCACACGATGCTTAGCTGT
GGAGGAGTTGCACAATTTACTCTCAGTTAT------GGCACACACAAAGAATTCTTGACT
GTTACAATTGCTGGTGGATTTGCAGTAGCTCTGGGTATATTGGTAACTAGTAAAGTCTCA
GGAGCTCATCTGAATCCTGCAGTGACCTTTGCTTTGTGCTTGCTTGCTTGTGAACCTTGG
TTAAAATTTCCTTTATTCTTTTTGGCACAAACGTTAGGTGCCTTTCTTGGATCAGGAATA
ATGTTT---GGTTTGTACTATGATACATTGTGGCATTATGGCAAT---AAACAGCTAACA
GTAATTGGAGCAAAT---TCTACTGCTGGAATATTTACAACTTATCCACATGAACATTTG
AGCGCAGTCAATGGCATTTTTAATCAGGCAATTGGGACTGCAGCACTTATACTTTGTATC
CTTATCATTGTGGATCCATTGAATGATGCTATTCCAACAGGACTAGAAGCTTTTACAATT
GGCTTTGTGGTTTTGCTAATTGGCTGGTCAATGGGTTCCAATTCTCAGTACTCATTAAAT
CCTGCCAGAGATATT---GGACCTCGCCTGTTTACTGCAATTGCTGGTTGGGGAACTGAA
GCTTTC---ACG------GCTGGCAATTATTGGTTTTGGATCCCAGTTGCCAGTCCAATC
ATTGGTGCTATATTTGGTGTTCTCCTGTACAAGTTCATTGTTGGATTGCGTGCTGAAGCA
----------------AGAACCAGCTGCCCA-----------------------TCC------
----------------------AATGCAGAA-------------------------------
-------------------------CAAAAT-------------ATAAAGTTAATGAACCAA
AAACCAAAGAGGAGGCTC--

>Whale_shark_Aqp3C1
---ATGGAAAAA
CAAAAA---GAAATCATCAGAAAAATGACAACTGTCCTG------AAAGTCCGAAGTCTT
CTGATGAAACAATGCCTTGCTGAATGTTTAGGAACTTTGATTCACACAATGCTTAGCTGT
GGAGCAGTAGCACAATTTACTCTCAGTTAT------GGCACACACAAAGAATTCTTGACT
GTTACAATTGCTGGTGGATTTGCAGTAGCTCTGGGTATATTGGTAACTAGTAAAGTCTCA
GGAGCTCACCTGAATCCTGCAGTGACCTTTGCTTTGTGCTTGCTTGCTAGTGAACCTTGG
TTAAAATTTCCATTATTCTTTTTGGCACAAACATTAGGTGCCTTTCTTGGATCAGGAATA

ATGTTT---GGTTTGTATTATGATGCATTGTGGCATTATGGTAAT---AAACAGCTAACA
GTAATTGGAGCAAAT---TCTACTGCTGGAATATTTGCTACTTATCCACATGAACATTTG
AGTGCAGTCAATGGCATTTTTGATCAGGCAATTGGGACTGCAGCACTTATACTTTGTATC
CTTATCATTGTGGATCCATTAAACAACTCAGTACGGACAGGACTAGAAGCTTTTACAATT
GGCTTTGTGGTTTTGCTAATTGGCTGGTCAATGGGTTCAAATTCCCAATACTCATTAAAT
CCTGCCAGAGATATT---GGACCTCGCCTGTTTACTGCAATTGCTGGTTGGGGAACTGAA
ACTTTC---ACG------GCTGGTAATTATTGGTTTTGGATTCCAATTGCCAGTCCAATC
ATTGGTGCCATATTTGGTGTCCTACTGTATCAGTTCATTGTTGGATTGCGTGCTGAAGCA
----------------AGAACCAGCCGCCCA----------------------ACT------
----------------------AATGCAGAA-------------------------------
-------------------------GAAAAT------------GTAAAGTTAATGAGTCAG
AAACCAAAGGGGAGGCTCCGA---------------------------------------

>Cloudy_catshark_Aqp3C1
--ATGGAAAAA
CAAAAA---GAAATCCTGCGAAAAATGACTACAATGCTA------AAGGTCCGAAGCCTT
CTGATGAAACAATGCCTTGCTGAATGTTTAGGGACTTTAATTCATACAATGCTGAGCTGT
GGAGCGACAGCGCAGTTTGTTCTGAGTTGT------GGTGCACACAAAGACTTTTTGACT
GTTACTTTTGCTGGTGGATTTGCAGTGGCTCTGGGTATATTGGTAACTAGTAAAGTCTCA
GGAGCTCACCTGAATCCCGCAGTGACCTTTGCTTTGTGCTTACTCGCCTGTGAACCCTGG
TTAAAATTTCCTTTCTTCTTTTTGGCACAAACATTAGGTGCCTTCCTTGGATCAGGAATA
ATGTTT---GGTTTGTATTACGATAAATTGTGGCATTATGGTAAT---AAACAGCTGACA
GTATTTGGAGCAAAC---TCTACTGCTGGAATATTTGCTACTTATCCACATGAACATTTG
AGTGCAGTAAATGGCATTTTTGAACAGGCAATTGGGACTGCGGCACTTATACTTTGCATC
CTCATCATTGTGGACCCATTGAACAACTTAGTGCCAACAGGACTGGAAGCTTTTACCATT
GGCTGTGTGGTTCTGATGATTGGCTGGTCAATGGGTTTAAATTCCCAGTACTCATTAAAT
CCCGCCAGAGATATT---GGACCTCGCTTGTTTACTGCAATTGCTGGTTGGGGATCTGAA
GTTTTC---AGT------GCTGGCAACTATTGGTTTTGGATCCCAGTTGCCAGCCCAATC
ATTGGTGCCATACTTGGTGTTCTGATCTATCGTTTCATTGTTGGATTGCGAGCTGAAGCA
----------------AAAACCAGTTGCTCA----------------------CCC------
----------------------AATGCAGAA-------------------------------
-------------------------CAAAAT------------GTAAAGTTATTGAGCCAG
AAACCAAAGGGGAGGTGC--

>Blue_shark_Aqp3C1
--ATGGAGAAA
CAAAAA---GGAATCCGCAGAAAAATGACGATTATGCTG------AAGGTTCGAAGTCTT
CTAATGAAGCAATGCCTTGCTGAATGTTTAGGAACTTTAATTCATACAATGCTTAGCTGT
GGAGCAGGAGCACAATATGTTCTCAGTTAT------GGGACACACAAAGACTTTTTGACT
GTTACTTTTGCCAGTGGATTTGCAGTGGCTCTGGGTATATTGGTAACTAATAAAGTCTCA
GGAGCCCACCTGAATCCTGCAGTGACCTTTGCTTTGTGCTTACTCGCTTGTGAACCTTGG
TTAAAATTTCCTTTCTTCTTTTTGGCACAAACATTAGGTGCCTTTCTTGGATCAGGAATA
ATGTTT---GGTTTGTATTATGATAAACTGTGGCATTATGGTAAT---AAACAGCTAACA
GTAATTGGAGCAAAC---GCTACTGCTGGAATATTTACTACTTACCCACATGAACATTTG
AGTGCAGTAAATGGCATTTTTGATCAGGCAATTGGGACGGCAGCACTTATACTTTGCATC
CTTATCATTGTGGACCCATTGAACAATTCAGTGCCAACAGGGCTGGAAGCTTTTACCATT
GGCTGTGTAGTTCTGATGATTGGCTGGTCAATGGGTTCAAACTCCCAGTACTCATTAAAT
CCTGCCAGAGACATT---GGACCCCGCTTGTTCACTGCAATTGCTGGTTGGGGATCTGAA
GTTTTC---ACT------GCTGGCAATTATTGGTTTTGGATCCCAGTTGCCAGCCCAATC
ATTGGTGCCACATTTGGTGTTCTGGTCTACAAGTTCATTGTTGGACTGCGAGATGAAGCA
----------------AGAACCAGCTGCCCA----------------------TCC------
----------------------AATGGAGAA-------------------------------
-------------------------CGAAAT------------GAAAAGTTAATGAGCCAA
AAACCAAAGGGAAGGCGC--


```
>Great_white_shark_Aqp3C1
---------------------------------------------------ATGGAAAAA
---AAA---GAAATCCTCAGAAAAATGACGACTATACTG------AAGGTCCAAAGTCTT
CTGATGAAACAATGCCTTGCTGAATGTTTAGGAACTTTAATTCATACCATGCTTAGCTGT
GGAGTGATAGCACAATTTGTTCTCAGTTAT------GGTACGCACAAAGAGTTTTTGACT
GTTACTTTTGCCAGTGGATTTGCAGTAGCTCTGGGTATATTGGTAACGAGTAAAGTCTCA
GGAGCTCACCTGAATCCTGCAGTGACCTTTGCTTTCTGCTTACTTGCTTGTGAACCCTGG
TTAAAATTTCCCTTCTTCTTTTTGGCACAAACATTAGGTGCCTTTCTTGGATCAGGAATA
ATGTTT---GGTTTGTATTATGATAAATTGTGGCATTATGGTAAT---AAACAGCTAACA
GTAATTGGAGCAAAC---TCTACTGCTGGAATATTTACTACTTATCCACATGAACATTTG
AATGTAGTAAATGGCATTTTTGATCAGGCAATTGGGACTGCAGCGCTTATACTTTGTATC
CTTATCATTGTGGACCCATTGAACAACTCAGTGCCAACAGGACTGGAAGCTTTTACCATT
GGCTTTGTGGTTCTGATAATTGGCTGGTCAATGGGTTCAAATTCCCAGTACTCATTAAAT
CCTGCCAGAGATATT---GGACCTCGCTTGTTTACTGCAATTGCTGGTTGGGGATCTGAA
GTTTTC---AGT------GCTGGCAGCTATTGGTTTTGGATCCCCGTTGCCAGCCCAATC
ATTGGTGCCATATTTGGTGTCCTGATGTATCAGTTCATTGTTGGATTGCGAACTGAGGCA
---------------AGAACCAACTGCTCA---------------------CCC------
---------------------AATGCAGAA------------------------------
------------------------GAAAAT------------GTAAAGTTAATGAGCCAG
AAACCAAAGGGGAGGTGC------------------------------------------
------------------------------------------------------------
------------------------------------------------------------
---------------------
>Zebra_bullheadshark_Aqp3C1
---------------------------------------------------ATGGAAAAA
CAAAAA---GAAATGCTCAGAAAAATGACAACTATACTG------AAAGTCCGAAGCCTT
CTGCTGAAACAATGTCTTGCTGAATGTTTAGGAACTTTAATTCATACAATGCTTAGCTGT
GGAGCAGTGGCACAATTTACTCTCAGTTAT------GGTACACAGAAAGAATTCTTGACT
GTTACTTTTGCCATTGGATTTGCAGTAGCTCTGGGTATATTGGTAACTAGTAAAGTGTCA
GGAGCTCACCTGAATCCTGCAGTGACCTTTGCTTTGTGCTTGCTTGCTTGTGAGCCTTGG
TTAAAATTCCCCTTCTTCTTTGTGGCACAAACATTCGGTGCCTTTCTTGGATCAGGAATA
ATGTTT---GGTTTGTGTTACGATAATTTGTGGCATTATGGTAAT---AAACAGCTAACA
GTAATTGGACCAAAC---TCTACTGCTGGAGTATTTACTACTTATCCACATGAACATTTG
AGTGCAGTAAATGGCATTTTTGATCAGGCAATTGGGACTGCAGCTCTTATACTTTGTATC
CTTATCATTGTGGATCCATTGAACAACTCAGTGCCAACAGGACTGGAAGCCTTTACCATT
GGCTTTGTGGTTCTGATAATTGGTTGGTCAATGGGTTCAAATTCCCAGTACTCATTAAAT
CCTGCCAGAGATATT---GGACCTCGCTTGTTTACTGCAATTGCTGGTTGGGGATCTAAG
GTTTTC---ACT------GCTGGCAACTATTGGTTTTGGATCCCAATTGTCAGCCCAATC
ATTGGTGCCATTTTTGGTGTTCTGATGTATCAGTTCATTATTGGATTGCGTATTGAAGCA
---------------AGGCCCAGCTGCTCA---------------------CCC------
---------------------ATTGCAGAA------------------------------
---------------------------AAT------------GTAATGTTAATGAGCCAG
AAACCAAAGGGGAGACGCCGA---------------------------------------
------------------------------------------------------------
------------------------------------------------------------
---------------------
>Spiny_dogfish_Aqp3C1
---------------------------------------------------ATGGGAAAA
CAAAAA---GAGATCCTCAGAAAAATGACAACGACACTG------AAAGTCCGAAGTATT
CTGGTGAAACAATGTCTTGCTGAATGTTTAGGAACTCTAATTCATACAATGCTTAGCTGT
GGAGCAATAGCACAATTTACTCTCGGTTAT------GGTACACACAAAGAATTTTTGACG
GTTACTTTTGCCATCGGATTTGCAGTAGCTCTGGGTATAATGGTAACTAGTAAAGTGTCA
GGAGCTCACCTGAATCCTGCAGTGACCTTTGCTTTGTGCTTGCTTGCTTGTGAGCCTTGG
TTAAAATTCCCCTTCTTCTTTTTGGCACAAACATTCGGTGCCTTTCTTGGATCAGGAATA
ATGTTT---GGTTTGTATTACGATAAATTGTGGCATTATGGTAAT---AAACAGCTAACA
GTAATGGGACCAAAC---TCTACTGCTGGAATATTTACTACTTATCCGCCAGAACATTTG
AGTGCAGTCAGTGGCATTTTTGATCAGGCAATTGGGACGGCAGCTCTGATACTTTGTATC
CTTATCATTGTGGATCCAATGAACAAGCCAGTGCCAACAGGACTGGAAGCCTTTACCATT
GGCTTTGTGGTTCTGATAATTGGCTGGTCAATGGATTCAAATTCCCAGTACTCATTAAAT
CCTGCCAGAGATATT---GGACCTCGCTTGTTTACTGCAATTGCTGGTTGGGGATCTGAA
GTTTTC---ACT------GCTGGAAACTATTGGTTTTGGATCCCACTTGTCAGCCCAATC
```

ATTGGTGCCATTTTTGGTGTTCTGATGTATCTATTCATTGTTGGATTGCGTGTTGAAGCA
---------------AGAAGCGGCTGCTCA---------------------CCC------
---------------------AATGCAGAA------------------------------
------------------------CAAAAT------------GTAAAGTTAATGAGCCAG
AAACCAAAGGGGAGGCGC--

>Little_skate_Aqp3C1
---ATGGGGAAA
CAAGAG---GAGATCCTCAGAAAATTGGCAATTCTGTTG------AGAATTCGAAGTATT
CTAGTAAAGCAATGCCTGGCCGAATGCTTGGGAACATTAATTCATACCATGCTTAGCTGT
GGAGCAATAGCACAGTTCACACTTAACTGT------GGCACACACAGTCAATTTCTGTCT
GTCACCTTTGCCATAGGATTTGCAGTAGCTCTGGGCATATTGGTAACCAGCAAAGTGTCA
GGAGCTCACCTGAATCCAGCAGTGACCTTTGCTTTGTGCTTACTTGCTTGTGAGCCTTGG
TTAAAATTCCCTTTCTTCTTTTTGGCACAAACAGTGGGTGCCTTTCTTGGATCAGGAATA
ATGTTT---GGTTTGTATTATGATAAATTGTGGCTTCATGGAAAT---AAACAGCTAACG
GTAATTGGACCAAAC---TCAACTGCTGGGATATTTACAAGTTTTCCACTTGAACATGTG
AGTGCACTCAATGGCATTTTTGATCAGGTGATTGGGACTGCAGCTCTCATATTGTGTATC
CTTATCATTGTGGATCCTTTACACACCCCAGTGCAGACGGGACTGGAAGCTTTCACCATC
GGTGTAGTGGTTCTTATTATTGGCTGGTCAATGGGATCAAATTCCCAGTACTCATTAAAT
CCTGCCAGGGATATT---GGACCTCGCTTGTTTACGGCTATTGCTGGTTGGGGATTTGAA
GTTTTC---ACG------GCTGGAAGCTATTGGTTTTGGATTCCACTTGTCAGCCCAATC
ATTGGTGCTATTTTGGGTGTTCTGATGTACCAATTCACTGTGGGATTACGTGTTGATACA
---------------AGAGATGACTCCTCA---------------------TCC------
---------------------AATGCAGAG------------------------------
------------------------CAAAAT------------GTAAAATTAATGGGCCAG
GAAACAAAGGGAAGGTGC--

>Thorny_skate_Aqp3C1
---ATGGGGAAA
CAAGAG---GAGATCCTCAGAAAAATGGCAATTCTGTTG------AGAATTCGAAGTATT
CTAGTAAAGCAATGCCTGGCCGAATGCTTGGGAACATTAATTCATACCATGCTTAGCTGT
GGAGCAATAGCACAGTTCACACTTAACTGT------GGCACACACAGTCAATTTCTGTCT
GTCACCTTTGCCATAGGATTTGCAGTAGCTCTGGGCATATTGGTAACCAGCAAAGTGTCA
GGAGCTCACCTGAATCCAGCAGTGACCTTTGCTTTGTGCTTACTTGCTTGTGAGCCTTGG
TTAAAATTCCCTTTCTTCTTTTTGGCACAAACAGTGGGTGCCTTTCTTGGATCAGGGATA
ATGTTT---GGTTTGTATTACGATAAATTGTGGCTTCATGGAAAT---AAACAACTAACG
GTAATTGGACCAAAC---TCAACTGCTGGGATATTTACAAGTTTTCCACTTGAACATGTG
AGTGCACTCAATGGCATTTTTGATCAGGCAATTGGGACTGCAGCTCTCATATTGTGTATC
CTTATCATTGTGGATCCTTTACACACCCCAGTGCAGACGGGGCTGGAAGCTTTCACCATC
GGTGGTGTGGTTCTTATTATTGGCTGGTCAATGGGATCAAATTCCCAGTACTCATTAAAT
CCTGCCAGGGATATT---GGACCTCGCTTGTTTACGGCTATTGCTGGTTGGGGATCTGAA
GTTTTC---ACG------GCTGGAAGCTATTGGTTTTGGATTCCACTTGTCAGCCCAATC
ATTGGTGCTATTTTGGGCGTTCTGATGTACCAATTCACTGTGGGATTACGTGTTGATACA
---------------AGAAATGACTCCTCA---------------------TCC------
---------------------AATGTAGAG------------------------------
------------------------CAAAAT------------GTAAAATTAATGGGCCAG
GAAACAAAGGGAAGGTGC--

>Smalltooth_sawfish_Aqp3C1
---ATGGGGAAA
CAAGAG---AAGATCCTCAGGAAAATGACGATTGTGCTG------AGAATTCGAAGTATT
CTGATAAAGCAATGCCTGGCTGAATGTTTGGGAACTCTAATTCATACAATGCTTAGCTGT
GGAGCAATAGCACAATTTACACTCAACTGT------GGCACACACAAAGAATTTCAGTCC
GTAACTTTTGTAATTGGATTTGCTGTAGCACTGGGCATATTGGTAACCAGTAAAGTGTCA
GGAGCTCACCTGAATCCAGCAGTGACCTTTGCTTTGTGCTTGCTTGCTTGTGAGCCTTGG

```
TTAAAATTCCCCTTCTTCTTTATGGCCCAAACAATAGGTGCCTTTCTTGGATCAGGAATA
ATGTTT---GGATTGTTATTAGATAAATTGTGGCTTCATGATAAT---AAACAGCTAACA
GTTATTGGACCAAAC---TCGACTGCTGGGATATTTACAACTTTTCCACTTGAACATGTG
AGTGCACTCAATGGCATTTTTGATCAGGCAACTGGAACTGCAGCTCTCATATTTTGTATC
CTTATCATTGTGGATCCTTTGCACACCCCAGTGCGAACAGGGCTGGAAGCCTTTACCATT
GGCTTAGTAGTTCTGATTATTGGCTGGTCAATGGGTTCAAACTCCCAGTACTCACTAAAT
CCTGCCAGGGATATT---GGACCTCGCTTGTTTACAGCAATTGCTGGTTGGGGATCTGAA
GTTTTC---ACG------GCTGGAAGCCATTGGTTTTGGATCCCGCTTGTCAGCCCAATC
ATTGGTGCCATTTTGGGTGTTTTGATGTACCAGTTCATTGTTGGATTACGTGTTGATGCA
---------------AGAAATGGCTGCTCA---------------------TCC------
---------------------AATGCAGAA------------------------------
------------------------CAAAAT------------GAAAAATCAGTGAGCCAG
AAAACGAAGGGAAGGTGC------------------------------------------
------------------------------------------------------------
------------------------------------------------------------
---------------------
>Ghost_shark_Aqp3C1
----------------------------------------------------ATGGGATA
CAAAGA---GAAACCCTGAGAAGGATGGCAACGACATTC------AGAGTGCGGAGCGTG
CTGGCAAAGCAATGCCTGGCGGAATGTTTGGGAACGTTGATCCACACGATGCTCAGCTGT
GGAGCAATGGCACAATACACCCTCAGTCGC------GGTGAGCAGAGAGAGTTTTTGACT
CTTACTTTCACCCTTGGCTTTGCGGTGGCTCTTGGTATACTTGTAACCGGTAAAGTGTCA
GGAGCTCACTTGAATCCTGCTGTGACCTTTGCTTTGTGCTTACTTGCTTGTGAACCCTGG
ATAAAATTCCCCTTCTTCTTTGTGGCACAAACATTGGGAGCATTTTTTGGATCTGGGATA
ATGTTT---GGCTTGTATCACGATGCAATAATGCACTATGGTAAC---AATCAACTCACA
ATCATTGGACCCAAT---TCCACTGCTGCTATATTTACCACATACCCATCCGAAGATTTG
ACTGTGGTGAATGGGTTTCTTGACCAGGTCATCGGCACGGCCGCTCTCATATTCTGTATC
CTGACCATCGCGGATCCACTGAAAAGACCGGTGGCCACAGGGCTGGAGGCTTTCACCATA
GGCTTTGTGGTTCTAATCGTTGGCTTGTCCATGGGCTTCAACTCCAAGTATTGCTTAAAT
CCTGCCAGAGACATT---GGACCTCGTTTGTTCAGCGCCATGGCTGGCTGGGGATATGAC
GTTTTC---AGC------ACAGGGAACTATTGGTTTTGGGTGCCAGTTCTCAGCCCAATC
GTCGGGGCCATGTTTGGCGTTCTGGTCTATCAGTTCATGGTGGGTCTGCAGGTTGAGACA
---------------AAAAGTAACTGCACG---------------------TCC------
---------------------CATTCAGAG------------------------------
------------------------GAAACC------------GTAAAGTTAACCAGCGTG
AAAGCGAAGGGCAGGCGT------------------------------------------
------------------------------------------------------------
------------------------------------------------------------
---------------------
>Sea_lamprey_Aqp3L1
---------------------------------------------------ATGCCGGTG
GTAGAG---GGGCGAATTAAGAGGCTGCAGCGGAGGCTG------TTCATCCGAAATACG
CTTGTCTGCGAGGCGATGGCAGAGTTTGTGGGGACGTACATTCTGGTTTTGTTTGGGTGT
GGCTCGGTGGCGCAGGTTGAATTGAGTGGA------GGACTCAAGGGGCAGTTCCTCACG
ATCAACCTCGCCTTTGGGTTTGCGGTCACCATGGGACTGCACGCCGCTGCAGGCGTTTCA
GGTGGACACCTGAACCCAGCCGTTACATTTGCGTTTGCCGTTCTGGGTCGCTTCGAATGG
CACAAGTTACCTCTCTACATGCTGGCTCAGCTCCTCGGAGCTTTCATGGGCGCAGGCACC
GTGTTC---GGACTGTATTATGATGCCTTCCTGTTCGCCGCCAAT---GGGAACTACACA
ATT---------------CAGCTGGCTGGAGTTTTTGCCACTTACCCGTCAGAGCACCTG
TCTCTGGGAAATGGATTTGTGGATCAGCTGATTGGCACTGCAGCCCTGCTGGTGTGCATC
ATGGCGGTGATAGACAAGCGTAACAACCCGGCTCCAAAGGGCATGCAGCCCTACATAATA
GGCCTCGTCGTCGTCCTCATTGGCCTCTCCATGGGCTTCAACGCGGGGTACGCCGTGAAC
CCGGCCCGTGACCTC---GGCCCGCGTCTCTTCACGTCCATTGCTGGATGGGGTTGGGCA
GTCTTT---TCG------TCCGGTAACTACTGGAGCTGGGTGCCTGTGGTGGCACCCATG
ATCGGTGGGGTGCTCGGTGCGTTTTTTTACGAGCTGTTCGTTGGATTTCATCTTATGGAG
---------------GCGTCACTCACGCCA---------------------GCG------
------------GTGGAG---CCCCCACCG------------------------TCT---
---------CCACGA---------GAAAAA------------AAGAGCCTCCTCAAGCTC
CAACCGGAAAGCATGGCTGATGTCATGTGC------------------------------
------------------------------------------------------------
------------------------------------------------------------
```

```
---------------------
>Arctic_lamprey_Aqp3L1
---------------------------------------------------ATGCCGGTG
CAAGAG---GGGCGAATGAAGAGGCTGCAGCGGAGGCTG------TTCATCCGAAATAGG
CTGGTCTGCGAGGCGATGGCAGAGTTTGTGGGGACGTACATTCTGGTTTTGTTTGGGTGT
GGCTCGGTGGCGCAGGTTGAATTGAGTGGA------GGACTCAAGGGGCAGTTCCTCACG
ATCAACCTCGCCTTTGGGTTTGCGGTCACCATGGGACTCCACGCCGCTGCAGGCGTTTCA
GGTGGACACCTGAACCCAGCCGTTACATTTGCGTTTGCCGTTCTGGGTCGCTTCGAATGG
CACAAGTTACCTCTCTACATGCTGGCTCAGCTCCTCGGAGCTTTCATGGGCGCAGGCACC
GTGTTC---GGACTGTATTATGATGCCTTCCTGTACGCCGGCAAT---GGGAACTACACA
ATT---------------CAGCTGGCTGGAGTTTTTGCCACTTACCCCTCAGAGCACCTG
TCTCTGGGAAATGGATTTGTGGATCAGCTGATTGGCACTGCAGCCCTGCTGGTGTGCATC
ATGGCGGTGATAGACAAGCGAAACAACCCAGCTCCAAAGGGCATGCAGCCCTACATAATA
GGCCTCGTCGTCGTGCTCATCGGGCTCTCCATGGGCTTCAACGCGGGGTACGCCGTGAAC
CCGGCCCGTGACCTC---GGCCCGCGTCTCTTCACGTCCATTGCTGGATGGGGTTGGAAA
GTATTT---TCG------TCCGGTGGCTACTGGAGCTGGGTGCCTGTGGTGGCACCCATG
ATCGGTGGGGTGCTCGGTGCGTTTGTTTACGAGCTGTTCGTTGGATTTCATCTTGTGGAG
---------------GCGTCACTCCCGCCG---------------------GCG------
------------GTGGAG---CCCCCACCG-----------------------TCT---
---------CCACGA---------GAAAAA------------AAGGGGCTCCTCAAGCTC
CAGCCAGAAAGCATGGCTGACGTCATGTGC------------------------------
------------------------------------------------------------
------------------------------------------------------------
---------------------
>Sea_lamprey_Aqp3L2
---------------------------------------------------ATGCCGTCG
CACGAT---GGTCGCTTCAAGCAGCTGGAGCGCAAGCTG------CACGTGAAGAACGTG
ATGATCCGTGAGGCCTTGGCCGAGTTCATGGGAACCTTCCTTCTCGTCCTGTTTGGCTGC
GGATCGGTGGCGCAGGTGGAGCTGAGCGAC------GGCACCAAGGGGCGGTTCCTCACC
ATCAACCTCGCCTTCGGCTTCGCCGTCACCATGGGCGCCTACTGTGCGGCCGGCGTCTCC
GGCGCGCACTTGAACCCGGCGGTGTCGATGGCGCTGGCGGTGCTCGGCCGCTTCTCTTGG
AGCAAGTTCCCCTTGTACGTCACGGCTCAGCTGCTCGGGGCCTTCATGGGCGCCGGCACC
GTCTTC---GGCCTCTACTACGACGCCTTCATGTACGTCTCCAAA---GGGAACCTGACG
CTG---------------CAGCTCGCAGGGGTCTTCGCCACCTTCCCCTCGCCGCATCTC
TCCATCGGAAACGGATTTGTGGATCAGCTGATCGGCACGGCGGCGCTGCTCGTGTGCATC
CTCGCCGTGATCGACAAGCGCAACAACCCGGCGCCGCGCGGCATGCAGCCCTTCCTCATC
GGCCTCGTCGTCGTGCTCATCGGCCTTTCCATGGGCTTCAACGCCGGCTACGCCGTGAAC
CCCGCGCGCGACCTC---GGCCCCCGCATCTTCACCGCCCTCGCCGGCTGGGGCTGGCAG
GTCTTC---TCG------GCGGGCAACTACTGGAGCTGGGTGCCCGTGGTGGCCCCCATG
CTGGGCGGCGTGCTGGGGGCCTTCATCTACGAGATCTTCATTGGCCTGCACCTCCCCGAA
---------------GAGCCGGCCTGCTCC---------------------GGA------
------------CCCGAG---CCCGGGACC------------------------------
---------CCGCGG---------TCGCAC------------CAGGAGCTCGCCAAGCTG
CAGTCC---AGCATGGCAGACGCTATGTGC------------------------------
------------------------------------------------------------
------------------------------------------------------------
---------------------
>Arctic_lamprey_Aqp3L2
---------------------------------------------------ATGCCGTCG
CACGAT---GGTCGCTTCAAGCAGCTGGAGCGCAAGCTG------CACGTGAAGAACGTG
ATGATCCGTGAGGCCTTGGCGGAGTTCATGGGGACCTTCCTTCTCGTCCTGTTTGGCTGC
GGCTCAGTGGCGCAGGTGGAGCTGAGCGAC------GGCACCAAGGGACGGTTCCTCACC
ATCAACCTCGCCTTCGGCTTCGCCGTCACCATGGGCGCCTACTGTGCCGCCGGCGTCTCC
GGCGCGCACTTGAACCCGGCGGTGTCGATGGCGCTGGCGGTGCTCGGCCGCTTCTCTTGG
AGCAAGTTCCCCTTGTACGTCACCGCTCAGCTGCTCGGGGCCTTCATGGGCGCCGGCACC
GTCTTC---GGCCTCTACTACGACGCCTTCATGTACGTCTCCAAA---GGGAACCTGACG
CTG---------------CAGCTCGCAGGAGTCTTCGCCACCTTCCCGTCACCGCATCTC
TCCATCGGAAACGGATTTGTTGATCAGGTGATCGGAACTGCGGCGCTGCTCGTGTGTATC
CTCGCCGTGATCGACAAGCGCAACAACCCGGCGCCCCGCGGCATGCAGCCCTTCCTCATC
GGCCTCATCGTCGTGCTCATCGGCCTCTCCATGGGCTTCAACGCCGGCTACGCAGTGAAC
CCCGCACGCGACCTC---GGCCCCCGCATCTTCACCGCCCTCGCCGGCTGGGGCTGGCAG
```

GTCTTC---TCG------GCGGGCAACTACTGGAGCTGGGTGCCTGTGGTGGCGCCCATG
CTGGGCGGCGTGCTGGGCGCCTTCATCTACGAGATCTTCATCGGCCTGCACCTCCCCGAA
---------------GAGCCGGCCAACTCC---------------------GGG------
------------CCCGAG---CCCGGGACC------------------------------
---------CCGCGG---------TCGCAC------------CAGGAGCTCGCCAAGCTG
CAGGCC---AACATGGCAGACGCCATGTGC------------------------------

>Inshore_hagfish_Aqp3L
---ATGTCGACA
GGAGGG---AGCAAGCTGCGGGTCCTCGCACAGCGAATG------CGCGTCACGAACCCC
CTAATCCGAGAAGCGTTCGCCGAAGGTTTGGGAACATTTGTTTTGGTGCTATTTGGTTGC
GGATCCGTAGCACAGATGGTCTTT---CAT------GGCCCGGGTGTTCCTTTCCTGTCT
GTCAATCTGGCTTTTGGTTTGGCAGTCACGATGGGATGCTACATCGCTGGTGGAGTGTCT
GGAGCTCATCTGAACCCGGCGGTTTCTCTGGCGATGGTTGTGCTGGGCCGTCTGACTTTG
CTCAAGATGTTGGTCTACTGGGTTGCTCAGCTGCTTGGAGCCTTCATCGGAGCCGCCATG
GTCTTT---CTCATTTATCTGGACGCTGAGAAGAAACATTCGAGA---CCC---TGGAGC
ATG---------------GAGACTGCGGGAATATATGCCACTTATCCGAACACCCACTTG
TCCACCGGAGGTGGTTTCTTTGATCAGGTTATGGGAACTGCTGCACTACTTCTGTGCATA
CTTGCCTTGCTCGACAAGAAAAACACAGCACCACCTGATGGGGTGACACCTGTAATTATT
GGGCTGGTTGTTGCCGTGATTGGAATGGCTATGGGGCACAACTGCGGCTACGCCATCAAT
CCTGCACGCGACCTC---GGCCCTCGACTGTTCACCCTTATTGCGGGTTGGGGACGGGCT
GTCTTT---ACG------CACGGTAACTACTGGTTTTGGGTTCCAATCATCGCACCACTG
CTCGGCGGAGTGCTCGGCGCACTCACTTACATCTTGTTTGTGGAGTTGCATCATCCCTCT
---------------TCGTCTGACACCGGC---------------------ACC------
------------AATACC---AAAGAGGAC------------------------GCA---
---GAG------------------AAGCAT------------TATGACCTGCGAAAATTT
GATTCT---CACGCTGCCGAAACGGTGGCC------------------------------

>Pacific_hagfish_Aqp3L
---ATGTCAACA
GGAGGG---AGCAAGCTGCGGGTCCTCGCACAGCGAATG------CGCGTCACGAATCCC
CTTATCCGAGAAGCGTTCGCCGAAGGTTTGGGAACATTTGTTTTGGTGCTATTTGGTTGT
GGATCTGTAGCACAGATGGTCTTT---CAA------GGCCCGGGTGTTCCTTTCCTGTCT
GTCAATCTGGCTTTTGGTTTGGCAGTCACGATGGGATGCTACATCGCTGGTGGAGTGTCT
GGAGCTCATCTGAACCCGGCGGTTTCTCTGGCGATGGTTGTGCTGGGCCGTCTGACTTTG
CTCAAGATGTTGGTCTACTGGGTTGCTCAGCTGCTTGGAGCCTTCATCGGAGCCGCCATG
GTCTTT---CTCATTTATCTGGACGCAGAGAAGCAACATTCGAGA---CCC---TGGAGC
ATG---------------GAGACCGCGGGAATATATGCCACTTATCCGAACGCCCACTTG
TCCACCGGAGGTGGTTTCTTTGATCAGGTTATGGGCACCGCTGCACTACTTCTGTGCATA
CTTGCCTTGCTCGACAAGAAAAACACAGCACCACCTGACGGGGTGACACCTGTAATTGTT
GGGCTGGTTGTTGCCGTGATTGGAATGGCTATGGGGCACAACTGCGGCTACGCCATCAAT
CCTGCACGCGACCTC---GGCCCTCGACTGTTCACCCTTATTGCGGGTTGGGGACGGGCT
GTCTTT---ACG------CACGGTAACTACTGGTTTTGGGTTCCAATCATCGCACCACTG
CTCGGCGGAGTGCTCGGCGCACTCACTTACGTCTTGTTTGTGGAGTTGCATCATCCCTCT
---------------TCGTCTGACACCGGC---------------------ACC------
------------AATAAC---AAAGAGGAC------------------------GCA---
---GAA------------------AAGCAT------------TATGACCTGCGAAAATTT
GATTCT---CACGCCGCCGAAACGGTGGCC------------------------------

>Atlantic_hagfish_Aqp3L
---ATGTCGACC
GGAGGG---AGCAAGTTACGGGTCCTCGCGCAGCGTCTA------CGCGTCACGAGACCC
CTCATTCGTGAAGCGTTCGCCGAAGGTTTGGGAACTTTCGTGCTGGTGCTATTTGGTTGT
GGATCTGTAGCACAGATGGTCTTT---CGT------GGCCCAGGTGTTCCTTTCCTGTCT
GTCAACCTGGCTTTTGGTTTGGCAGTCACCATGGGATGCTACATCGCTGGTGGAGTCTCT

GGAGCTCATCTGAATCCGGCGGTTTCTCTGGCTATGGTTGTCCTGGGCCGGTTGACTTTG
CTCAAGATGTTGGTCTACTGCGTTGCTCAGCTGGTTGGAGCCTTCCTTGGAGCCGCCATG
GTCTTT---CTCATTTATCTGGATGCTGAGAAGAAACACTCTAAA---CCC---TGGAGC
ATG---------------GAGACTGCTGGAATATATGCAACTTACCCTGGCCCTCATTTG
TCCACCGGAGGTGGTTTCTTTGATCAGGTTATGGGCACCGCCGCATTACTTCTGTGCATA
CTTGCCTTGCTCGACAAGAAAAACTCAGCACCACCTGACGGGGTGACGCCTCTGATTGTC
GGGCTGGTGGTCACCGTGATTGGAATGGCCATGGGGCACAACTGCGGCTATGCCATCAAC
CCCGCACGCGACCTC---GGCCCTCGGCTGTTCACCCTTATTGCGGGTTGGGGGCGGCCT
GTCTTT---ACG------CACAGGAACTACTGGTTTTGGGTTCCGATCACCGCGCCATTT
CTCGGTGGAGTGCTCGGCGCACTCACTTACGTCTTGTTTGTGGAGTTGCATCATCCCTCC
---------------TCATCCGACTCCGGC---------------------ACC------

>cetFrog_AQP13
---ATGGACCTG
CATACT---CTGATCCTAACAACATTGAAAATGAAGTTG------CGAACGAATAATCAG
TACGTGCGCTGCGGCCTGGCTGAGTTACTGGGGACTCTCATATTGATTCTCTTTGGCTGT
GGTGCGGTGGCTCAGATGGAGTTGAGCGGT------CTTGCGAAGGGGCAGTTCCTGAGT
GTCAACATGGCATTTGGGTTTGCAGTGACTGCTGGGGCCTACATCTGTGCTGGAGTGTCA
GGTGCCCACCTCAACCCAGCCGTGTCCCTTTCCATGTACCTCCTCCAGAGGATGACTTGT
AGGATGATGCTGGTCTACTTCTTGGCCCAGTTCATTGGATGTTTCATTGGGGCTGCTCTG
GTCTTC---GCTCTTTATTTTGATGCTCTTCATGTGTACAGTGGA---GGTAACTGGACG
GTTTATGGACCCCAA---GCAACAGCAGGGATATTTGCATCGTACCCATCGGAACACCTG
AGTGTTTTTAATGGCCTTTCAGATCAGGTGATTGCCACTGCTGCTTTGATGGTCAGTATC
CTGGCAGTAATAGATGAAGCCAACAATGCTGCTCCTCGAGGTCTCCAGCCTTTTGTGATT
GGTCTTGTGGTTCTTCTGGTTGGTCTATCAATGGGCTTCAACTGTGGGTACCCAATAAAT
CCTGCCAGAGACTTG---GCACCTCGTATCTTCACTGCAATGGCTGGCTGGGGCCTGGAA
GTCTTC---AGG------GCTGGTGGGCACTGGTGGTGGGTGCCAGTGCTGGGACCAATG
GTAGGGGCAGTGGTGGGGACGCTGACTTATGAACTGCTGGTTGGAATTCACCACCCTGTG
---------------CTCCACGAGAAGGAT---------------------GAA------
------------CCTGAA---GAGGATGGT------------------------GAG---
---GAGAACCATGCC---------ACACAG------------TATGAGTTGGTTCAGTCT
CATGCT--

>goFrog_AQP13
---ATGGACCTG
CATACT---CTGATCCTAAAAACATTGAAAATGAAGTTG------CGAACGAATAATCAG
TATGTGCGCTGCGGGCTGGCTGAGTTCCTGGGGACTCTCATATTGATTCTCTTTGGCTGC
GGTGCTGTGGCTCAGATGGAGTTGAGCGGT------CTTGCGAAGGGGCAGTTCCTGAGC
GTAAACATGGCGTTTGGGTTTGCAGTGACTGCTGGGGCCTACATCTGTGCTGGAGTGTCA
GGTGCCCACCTCAACCCAGCCGTGTCCCTTTCCATGTACCTCCTCCAGAGGATGACTTGT
AGGATGATGCTGGTCTACTTCTTGGCCCAGTTCATTGGATGTTTCATTGGGGCTGCTCTG
GTCTTC---GCTCTTTATTTTGATGCTCTTCATGTGTACGGTGGA---GGTAACTGGACG
GTGTCTGGACCCCAA---GCAACAGCAGGGATATTTGCATCGTACCCATCGGAACACCTG
AGTGTTTTTAATGGCCTTTCAGATCAGGTGATTGCCACTGCTGCCTTGATG---------

---GGCCTGGAA
GTCTTC---AGG------GCTGGTGGGCACTGGTGGTGGGTGCCAGTGCTGGGACCAATG
GTAGGGGCAGTGGTGGGGACGCTGACTTATGAACTGCTGGTTGGAATTCACCACCCTGTG
---------------CTCCACGAGAAGGAT---------------------GAA------
------------CCTGAA---GAGGATGGT------------------------GAG---
---GAGAACCATGCC---------CCACAG------------TATGAGTTGGTTCAGTCT
CATGCT--

>Common_frog_AQP13
---ATG
TATACT---CTCTTCCTAAAAACAATGAGAATGAAGTTG------CGAACGAATAATCAG
TACGTGCGCTGCGGGCTGGCCGAATTGCTGGGGACTCTCATATTGATTCTCTTTGGCTGT
GGCGCTGTAGCTCAGATGGAGTTGAGCGGT------CTTGCTAAGGGGCAGTTCCTGAGT
GTCAACATGGCGTTTGGGTTTGCAGTGACTGCTGGGGCCTACATCTGTGCTGGAGTGTCA
GGTGCCCACCTCAACCCAGCCGTGTCTCTATCCATGTACCTCCTCCAGAGGATGACTTGT
AGGATGATGCTGGTCTACTTCTTGGCCCAGTTCATTGGATGTTTCATTGGGGCTGCTCTG
GTCTTT---GCTCTTTATTTTGATGCTCTTCATGTGTACAGTGGA---GGTAACTGGACG
GTGTCAGGAACCCAA---GCAACAGCAGGGATATTTGCATCCTACCCATCGGAACACCTG
AGCGTTTTAAATGGACTTTCAGATCAGGTGATTGCCACGGCTGCCTTGATGGTCTGTATC
CTGGCAGTAGTAGATGAAGCCAACAATGCTGCTCCTCGAGGACTCCAGCCTTTTGTGATT
GGCCTTGTGGTTCTTCTGGTTGGTCTATCAATGGGCTTCAACTGTGGGTACCCAATAAAT
CCTGCCAGAGACTTG---GCACCTCGTATCTTCACTGCAATGGCTGGCTGGGGCCTGGAA
GTCTTC---AGG------GCGGGTGGGCACTGGTGGTGGGTGCCAGTGCTGGGACCAATG
CTAGGGGCAGTGTTGGGGACGTTTACTTATGAACTGTTGGTTGGAATTCACCACCCTATG
---------------CTTCACGAGAAGGAT---------------------GAA------
------------CCTGAA---GAGGATGCT------------------------GGG---
---GAGAACCACACC---------CCACAA------------TATGAATTGGTTCAGTCT
CATGCT--

>American_bullfrog_AQP13
---ATG
TATGCT---CTCTTCCTAAAAACACTGAAAATGAAGTTG------CGAACGAATAATCAG
TATGTGCGCTGCGGGCTGGCCGAGTTCCTGGGGACTCTCATATTGATTCTATTTGGCTGT
GGTGCTGTAGCTCAGATGGAGTTGAGCGGT------CTTGCTAAGGGGCAGTTCCTGAGT
GTCAACATGGCGTTTGGGTTTGCAGTGACTGCTGGAGCCTACATCTGTGCTGGGGTGTCA
GGTGCCCACCTCAACCCAGCCGTGTCTCTATCCATGTACCTCCTCCAGAGGATGAATTGT
AGGATGATGCTGGTCTACTTCTTGGCCCAGTTCATTGGATGCTTCATTGGGGCTGCTCTG
GTCTTT---GCTCTTTATTTTGATGCCCTTCATGTGTACAGCGGA---GGTAACTGGACA
GTGTCTGGAACCCAA---GCAACAGCAGGGATATTTGCATCATACCCATCAGAACACCTG
AGTGTTTTTAATGGACTTTCAGATCAGGTGATTGCCACTGCTGCCTTGATGATCTGTATC
CTGGCAGTAGTAGATGAAGCCAACAATGCTGCTCCTCGAGGACTCCAGCCTTTTGTGATT
GGTCTTGTGGTTCTTCTGGTTGGTCTATCGATGGGCTTCAACTGTGGGTACCCAATAAAT
CCTGCCAGAGACTTG---GCACCTCGTATCTTCACTGCAATGGCTGGCTGGGGCCTGGAA
GTCTTC---AGG--

>Himalaya_frog_AQP13
---ATGGACCTG
TATACT---CTCATCCTAAAAAAGCTGAAAATGAAGCTG------CGGACGGATAATCAA
TATGTGCGCTGCGGGCTGGCTGAATTCCTGGGGACCCTCATATTGATTCTGTTTGGTTGT
GGCTCTGTGGCTCAGATGGAACTGAGTGGC------TATGCTAAGGCGCAATTTTTGAGT
GTCAACATGGCGTTTGGGTTTGCTGTGACCGCTGGGGCCTACGTCTGTGCTGGAGTGTCA
GGTGCCCACCTCAACCCGGCTGTGTCTCTTTCAATGTACCTCCTCCAGAGGATGACCTGT
AGAATGATGTTGGTCTACTTCTTGGCTCAGTTCCTTGGAGCTTTTATTGGGGCTGCTCTG
GTCTTT---GCTCTGTATATTGATGCTCTTCATGCGTACAGTGGA---GGTAACTGGACG
GTGTCTAGCACCCAG---GCAACAGCGGGGATATTTGCTTCGTACCCATCCGAACACTTG
ACCATTCTTAATGGACTCACAGATCAGGTAATTGCCACTGCTGCCTTAATGATCTGTATA
CTAGCAGTAATAGATGAATCCAACAATGCTGCTCCTCGAGGCCTTCAGCCATTTGTGATT
GGTCTTGTGGTTCTTCTGGTTGGTCTATCAATGGGCTTCAACTGTGGGTACCCAATAAAT

CCTGCCAGAGACTTG---GCACCTCGTATCTTCACTGCAATGGCTGGCTGGGGCCTAGAA
GTCTTC---AGG------GCTGGTAATCACTGGTGGTGGGTGCCAGTGCTGGGACCAATG
ATAGGAGGAGTGATAGGGACCCTGATTTATGAACTGCTGGTTGGAATTCACAGCCGTACT
---------------CTCCATGAGGAGGAT---------------------GAA------
------------CCTGAA---GAGGACGTT------------------------GAG---
---GAGAACCATCCC---------CGACAG------------TATGAGTTAGTTCAGTCT
AATGCT--

>African_bullfrog_AQP13
ATG------------------------------------ATGAAAGTTCACATGAACATG
TTTGCA---TTGCTCTTACAGAAACTCAGCATGAAGTTA------CAGACAAAAAATCTG
TATGTGCGCTGCGGGCTGGCTGAGTTCCTGGGGACCCTCATATTGATTCTGTTTGGCTGT
GGCTCTGTAGCTCAGATGGAATTGAGTGGT------TTTGCAAAAGCGCAGTTCCTGAGT
GTCAACATGGCGTTTGGATTTGCAGTGACGGCTGGGGCCTACGTCTGCGCTGGAGTGTCA
GGCGCCCATCTCAACCCAGCGGTGTCGCTTTCCATGTATCTCCTCCAGAAGATGACTTGT
AGGATGATGCTGGTATACTTCTTGGCTCAGTTCCTTGGTTCCTTCATTGGGGCAGGTCTG
GTCTTT---GCTCTGTATTTTGATGCTCTGCATGCATACAGTGGC---GGTAACTGGACA
GTGTCTGGGACCCAA---GCAACAGCGGGGATATTTGCTTCGTACCCCTCAGAACATCTA
AGTGTTCTTAATGGATTCACAGATCAGGTAATTGCCACCGCTGCCTTGATGGTCTGTATA
CTTGCCATAATAGATGAATCAAACAATGCTGCTCCTCGAGGTCTTCAACCATTTGTGATT
GGGTTGGTGGTTCTTCTGGTTGGTCTATCAATGGGTTTCAACTGTGGGTACCCAATCAAT
CCTGCCAGAGACCTG---GCACCTCGTATCTTCACTGCAATGGCTGGCTGGGGCCTGGAA
GTCTTT---AGG------GCTGGTGGATGCTGGTGGTGGGTGCCAGTGCTGGGACCAATG
GTAGGAGGAGTGGTGGGTACCCTGATTTATGAACTGCTCATTGGAATTCATCACCCTGCT
---------------CAACACCAGACAGAA---------------------GAA------
------------CCAGTA---GAGGATGGT------------------------------
---GGGAACCACCAC---------AGACAC------------TATGAACTGGTTCAGTCT
CAAGCT--

>msToad_AQP13
---ATGGAGGCT
CGTGTC---ATGCTGCTGAAAAAGCTGAAGCTTAGGTTG------CGGACGGAAAACCTT
TATGTACGATGCGGGCTGGCCGAGTTCCTGGGGACGCTTATTTTAATTCTGTTCGGCTGT
GGGTCTGTGGCTCAGATGGAGTTGAGTGGT------TTCGCTAAAGCCCAGTTCCTGAGT
GTGAATATGGCGTTCGGCTTCGCTGTCACTGCTGGAGCCTATGTGTGTGCCGGAGTATCG
GGTGCCCATCTGAACCCTGCTGTGTCTCTCTCCATGTACTGTCTGAGGAAGCTGAGTGGG
AGACTTATGTTGGTCTACTGGTTGGCTCAGTTCCTAGGAGCATTCTTTGGAGCCGCATTG
GTCTTT---GCCTTGTATTACGATGCCCTGTGTGTGTACAGCGGT---GGGAATTTCACA
GTGACTGGATCTCAG---GCCACAGCCGGCATATTTGCTTCATACCCTTCGGAGCATCTG
AGTGCGATCAATGGATTTACAGACCAGGTGATTGCTACAGCGGCCTTGATGATCGCTATT
CTGGCCATACTGGATGAAGAAAACAACGCGCCTCCTCGTAGTATCCAGCCCTTCATAATT
GGAATATCTGTGTTACTGGTCGGTCTGTCAATGGGCTTCAACTGTGGTTACCCCATAAAT
CCTGCCCGGGACCTA---TCTCCTCGCATCTTCACAGCTATGGCTGGCTGGGGTCTTGAT
GTGTTC---AGG------GCTGGAGATAACTGGTGGTGGGTGCCGGTGATTGGACCTCTA
GTAGGAGCACTGGTTGGCGTGCTGATCTATGAACTTTTTATTGAAATCCATCACCCGGCG
---------------AGCAAACAGAAAGAT---------------------GAC------
------------CAAGAG---GCGGAAGAA------------------------GAG---
---CAGACCCATCCA---------CCTCAG------------TATGAGCTGGTTCAATCG
ACCGCA--

>psToad_AQP13
---ATGGAGGCT
CGTGTC---ATGCTGCTGAAAAAGTTGAAGCTTAAGTTG------CGGACTGAAAACCTT
TATGTGCGATGCGGGCTGGCTGAGTTCCTGGGAACGCTTATTTTAATTCTGTTTGGCTGT
GGGTCTGTGGCTCAGATGGAGTTGAGTGGT------TTCGCTAAAGCCCAGTTCCTGAGT

GTGAACATGGCGTTCGGCTTCGCTGTCACTGCTGGAGCCTATGTGTGTGCCGGAGTATCG
GGTGCCCATCTGAACCCTGCTGTGTCTCTCTCAATGTACTGTCTGAGGAAGCTGAGTGGG
AGACTTATGTTGGTCTACTGGTTGGCTCAGTTCCTAGGAGCATTCTTTGGAGCCGCATTG
GTCTTT---GCCTTGTACTACGATGCCCTGTGTGTGTACAGCGGT---GGGAATTTCACA
GTGACTGGATCTCAG---GCCACAGCTGGCATATTTGCTTCATACCCTTCAGAACATCTG
AGTGCGATCAATGGATTTACAGACCAGGTGATTGCTACAGCGGCCCTGATGATCGCTATT
CTGGCCATACTGGATGAAGAAAACAATGCGCCTCCTCGTAGTATTCAGCCCTTCATAATT
GGAATATCTGTGTTACTGGTCGGCTTGTCCATGGGCTTCAACTGTGGTTACCCCATAAAT
CCTGCCCGGGACCTA---TCTCCTCGCATCTTCACAGCTATGGCTGGCTGGGGTCTTGAT
GTGTTC---AGG------GCTGGAGATAACTGGTGGTGGGTGCCGGTGATCGGACCTCTA
GTAGGAGCACTGGTTGGTGTCCTGATCTATGAACTTTTTATTGAAATCCATCACCCGGTG
---------------AGCAAACAGAAAGAT---------------------GCC------
------------CAAGAG---GAGGAAGAG------------------------------
---CAGACCCATCCA---------CCTCAG------------TATGAGCTGGTTCAATCA
ACCGCG--

>P_treefrog_Aqp13
---ATGGACGCC
TATTCT---CTGCTGATCAAAAAGTGGAAGTTAAAGTTC------AGGACGAGCAATCGC
TATGTGCGCTGTGGGCTGGCGGAGTTCTTGGGGACGCTCATATTAATCCTATTTGGCTGT
GGGTCTGTGGCACAGATGGAATTAAGCGGT------TTTGCCAAAGCCCAGTTTCTAAGC
GTTAATATGGCATTCGGCTTTGCGGTCACTGCTGGAGCCTATGTCTGTGCCGGAGTCTCA
GGCGCCCACCTCAACCCGGCTGTGTCTCTCTCCATGTTCCTCCTCCAGAAGATGACGGGG
AGGCTAATGCTWGTSTACTGTGTGGCACAATTCCTCGGCTCTTTTGTTGGCGCGGCACTG
GTATTT---GCCCTGTATTTCGACTCCCTTCATGTGTACAGTGGA---GGCAACTGGACA
GTATACGGACCC--
---------------------------------GCCACCGCGGCCCTGATGATCTGCATC
CTGGCCATCATCGATGAAGACAATAACGCAGCCCCCCGGGGTCTTCAGCCATTTATCATT
GGACTGATAGTTCTTCTAGTTGGTCTTTCTATGGGATTCAACTGTGGATACCCCATTAAT
CCAGCCCGGGACCTG---GCACCTCGAATCTTCACATCATTAGCCGGCTGGGGCCTAGAA
GTCTTC---AGG------GCCGGTGACAACTGGTGGTGGGTGCCAGTCCTGGGCCCCATG
ATTGGAGCGGTTGTCGGGACAATCCTTTATGAGCTGCTTATTGGGATCCATCACACGTCT
---------------GCCCAGCAGAAGGAA---------------------GAA------
---------------------CATGATGGA------------------------GAG---
---GAGCGTCACCCC---------CCTGAG------------TATGAGTTGGTTCACTCA
ACAGCA--

>Marine_toad_AQP13
---ATGGATGCT
TATGCT---CTGCTCGTGAAAAAGCTCAAGTTAAGGTTT------AGGACGAGCAATCGC
TATGTGCGCTGTGGGCTGGCTGAGTTTTTGGGAACTGTCATATTGATTTTGTTTGGTTGC
GGTTCTGTGGCTCAGATGGAATTAAGTGGT------TTTGCCAAAGCTCAGTTTCTGAGT
GTCAATATGGCGTTTGGTTTTGCAGTCACTGCTGGAGCTTATGTATGCGCTGGAGTCTCA
GGTGCCCACCTCAATCCAGCCGTCTCGCTCTCCATGTTCCTCCTCCAGAAGATGACCGGG
AGGCTGATGCTAGTCTACTGCTTGGCACAATTCCTCGGCTGTTTTGTTGGCGCCAGCCTG
GTATTT---GCTCTGTATTTTGATGCCCTTCATGTGTACGGTGGT---GGCAACTGGACA
GTGTATGGACCACAG---GCGACTGCTGGGATATTCGCTTCCTATCCATCAGAACATCTG
AGTGTGATCAATGGACTCTCAGATCAGGTAATTGCCACCGCTGCCTTGATGATCTGCATT
TTGGCCATCATTGATGAAGACAATAATGCAGCCCCCCGAGGACTTCAGCCATTTGTCATT
GGTCTTATAGTTCTCCTAGTTGGTCTCTCTATGGGCTTCAACTGTGGCTACCCCATTAAT
CCAGCCCGCGACCTG---GCACCTCGAATTTTCACAGGAATTGCTGGCTGGGGTCTAGAA
GTCTTC---AGG------GCTGGTGACAACTGGTGGTGGGTTCCAGTATTGGGCCCCATG
ATTGGTGGGGTCATTGGAACATTTATTTATGAAATGCTCGTTGGAATTCATCACTCAGCA
---------------GAACAGAAGAAAGAG---------------------GAA------
------------GATGAT---GAAGATAGC------------------------GAG---
---GAGCATCACCCC---------CCGGAG------------TACGAGTTGGTTCAGTCA
ACGGCA--

```
------------------------------------------------------------
------------------------------------------------------------
---------------------
>HG_treefrog_AQP13
---------------------------------------------------ATGGAGGCT
TATTCT---CAATTGCTCAAAAAGTCCAAGCTAAAGTGT------AGGACAAGCAATCGC
TATGTGCGCTGCGGGATGGCGGAGTTTTTGGGGACTCTCATATTAATTTTATTTGGCTGT
GGGTCTGTAGCTCAGATGGAATTAAGTGGT------TTTGCCAAAGCCCAGTTCCTAAGC
GTTAATATGGCGTTTGGCTTTGCGGTCACTGCTGGGGCCTATGTGTGCGCTGGGGTCTCA
GGTGCCCACCTCAATCCGGCTGTGTCTCTCTCCATGTTCCTCCTCCAGAAGATGAATGGG
AGGCTTTTGCTAGTCTACTGTTTGGCACAATTCCTTGGCTCCTTTATCGGTGCTTGCCTG
GTGTTT---GCCCTGTATTTTGACGCCCTTCATGTATACAGTGGA---GGCAACTGGACA
GTATATGGACCACAG---GCGACTGCTGGTATATTTGCCTCCTATCCGTCAGAACATCTG
AGTGCAATTAATGGATTCACAGATCAGGTGATCGCCACTGCTGCCTTGATGATCTGCATT
CTGGCCATTATCGATGAAGACAATAACGCAGCCCCCCGAGGTCTTCAGCCGTTTGTTATT
GGACTTGTAGTTTTATTAGTTGGTCTTTCTATGGGATTCAACTGCGGATACCCCATTAAT
CCAGCACGGGACCTG---GCGCCTCGACTGTTCACTGCCTTAGCTGGCTGGGGCCTAGAA
GTCTTC---AGG------GCTGGTGGAAACTGGTGGTGGGTGCCAGTGCTGGGCCCCATG
ATTGGAGGGGTTATTGGGACAACTATTTATGAGGTATTCATTGGGATCCATCATTCACCT
---------------AGCCAGAAGGAGGAA---------------------CAA------
------------GATGAA------------------------------------GAG---
---GAGCATTACCCC---------CCTGAA------------TACGAGTTGGTTCACTCC
ACAGCA------------------------------------------------------
------------------------------------------------------------
------------------------------------------------------------
---------------------
>WC_frog_AQP13
---------------------------------------------------ATGGTCAGC
CATTTA---GCTTTCCTGAAAAACATGAAGATTAAGCTG------AGGACCCAGAACCAG
TATGTGCGCTGCGGGCTGGCAGAGTTCCTGGGCACGCTCATTCTAATCCTGTTTGGCTGT
GGTTCTGTGGCGCAGATGGAGCTGAGTGGT------TTTGCCAAAGCCCAGTTTCTGAGC
GTTAATATGGCGTTTGGCTTCGCAGTCACTGCTGGAGCTTACGTATGCGCCGGTGTGTCG
GGTGCCCATCTGAATCCTGCCGTGTCCCTCGCCATGTTCATACTGAAGAAGCTGAGCTGG
AGGCTGCTCCTTACCTACTGCCTGGCACAGTTCCTCGGGGCTTTCATTGGAGCCGCTCTG
GTCTTT---TCTCTTTATTATGATGCCTTGCACGTGTACAGCAGT---GGGAACTGGACA
GTCTATGGCCCCCAG---GCTACAGCAGGGATATTTGCTTCCTACCCCTCCGAGCACCTC
AGTGTCATAAATGGCTTTACAGACCAGGTGATTGCCACAGCGGCTCTGCTGATCTGTATC
CTGGCTATACTGGATGAAGCCAACAACGCAGCACCCAGGGGCCTACAGCCTTTTCTAATT
GGCATTGTGGTGCTGCTGGTTGGGCTAGCAATGGGGTTTAACTGCGGGTACCCCATAAAT
CCGGCTCGAGACCTC---GCACCGCGCTTCTTCACGGCTATTGCAGGCTGGGGCTCCGAG
GTCTTC---AGC------GCCGGAGGCCACTGGTGGTGGGTGCCGGTTATAGGACCTTTG
GTAGGTGGGGTGCTCGGCGTGGTAATCTATGAAGTATTCATCGAATTCCACCATCCTCCT
---------------GCCAATCAAAAGCAA---------------------GAA------
------------TCCGAG---GAACCCACT------------------------GAG---
---GGAACCCATCGT---------CCTCAC------------TATGAGCTTGTTCAGTCC
TCTGCT------------------------------------------------------
------------------------------------------------------------
------------------------------------------------------------
---------------------
>AC_frog_Aqp13
---------------------------------------------------ATGGTCAGT
CATTTA---GCTTTCCTGAAAAACATGAAGCTTAAGCTG------AGGACTCAGAACTTG
TATGTGCGCTGTGGGCTGGCTGAGTTCCTGGGCACCCTCATTTTAATTCTGTTTGGCTGC
GGTTCTGTGGCCCAGATGGAGCTGAGTGGT------TTTGCCAAGGCTCAGTTTCTGAGT
GTGAATATGGCATTTGGCTTTGCAGTCACCGCTGGAGCTTATGTATGTGCTGGAGTCTCA
GGTGCTCATCTGAATCCTGCTGTGTCCCTCGCCATGTTCATACTGAAGAAGCTGAGCTGG
AAGCTGTTCCTGATCTACTGTTTGGCACAGTTCCTCGGGGCTTTTATTGGAGCCGCTCTG
GTCTTT---TCTCTGTATTATGATGCCTTGCACGTGTACAGCAAT---GGCAACTGGACT
GTCTATGGCCCCCAG---TCGACAGCAGGGATATTTGCTTCCTACCCTTCAGAGCACCTC
AGTGCCATAAACGGCTTTACAGACCAGGTGATTGCCACGGCAGCTCTGCTGATCTGTATA
CTGGCCATACTGGATGAAGCCAACAACGCAGCACCCAGGGGCCTGCAGCCTTTTCTCATT
```

GGCATTATGGTGCTGCTGGTTGGGCTAGCAATGGGGTTCAACTGCGGGTACCCCATCAAT
CCAGCTCGTGACCTT---GCACCCCGCTTCTTCACTGCTATTGCAGGCTGGGGGTCAGAA
GTCTTC---AGT------GCTGGAGGCCACTGGTGGTGGGTGCCAGTTCTAGGACCTTTG
GTAGGTGGGGTGGTTGGTGCGGTCATCTATGAAGTCTTCATCGAATTCCACCATCCTTCT
---------------CCCAATCAAAAGCAA---------------------GAA------
------------TCTGAG---GAACCCACT-----------------------GAA---
---GGAATCAATCGT---------CCTCAC------------TATGAGCTGGTTCAGTCC
TCTGCT--

>JF_newt_Aqp13

---AGT
GTCAACATGGCGTTTGGATATGCAGTCACTGCGGGAGCCTACGTATGTGCT---GTATCG
GGCGCT---CTG---CCGGCTGTATCCCTCGCCATGTTCCTCTTGAGAAGGATGACTTTT
AAGCTGATGCTGGTCTATTGCCTTGCCCAGTTCCTGGGGGCCTTTTTTGGAGCAGCCATT
GTTTTT---GCGCTTTACTTTGATGCTCTGCATGTGTACAGTGGC---GGCAACTGGACC
GTGGTGGGACCTCAA---GCTACAGCTGGGATCTTTGCATCCTACCCTTCGGAACACCTC
AGTGTTATCAATGGATTTACAGACCAGGTGATTGCTACTGCTGCTCTGCTGATCTGCATT
CTGGCTGTTGTGGATGAGCGGAACAATGCTGCTCCACCGGGACTGCAGCCATTTGTGATT
GGTCTAATGGTGTTGCTGGTTGGCCTTGCTATGGGATTCAACTGTGGCTATCCCATCAAC
CCAGCCAGAGACCTG---GGGCCACGCCTCTTCACTGCCATAGCTGGATGGGGCCTTGAA
GTGTTT---AGG------GCTGGGAACCACTGGTGGTGGGTACCAGTTTTGGGGCCCCTT
GTGGGTGGCCTGATTGGCGCCATTATCTATGAGCTGTTCGTGGAAATCCATCACCCGCTG
---------------GATCAGGTAAAAGAG---------------------GTC------
------------CTAGAT---GCCAACTGT------------------------------
---CCTGATCACCCAACTCGT---CCCCAA------------TATGAACTTGTTCAGAGC
ACAGCA--

>Axolotl_AQP13
---ATGGACGGC
CACGCC---GCCCTGCTGAAGTACTTGAAGCTCAAGTTC------AGAACTCGAAGTCTC
CACGTGCGCTGTGGGCTGGCAGAGTTTTTGGGAACGTTCATTTTAATTCTGTTTGGCTGT
GGTTCCGTGGCCCAGATGGAGCTGAGTGGC------TTCGCAAAAGCACAGTTCCTTAGT
GTGAACATGGCCTTTGGATTTGCCGTCACCGCCGGTGCCTATGTGTGTGCCGGAGTATCA
GGTGCTCACTTGAACCCGGCTGTGTCGCTCGCAATGTTCCTCTTGAGACGGATGTCATGG
AAGCTGCTTCTTGTCTACTGCCTTTCCCAGTTCTTGGGAGCGTTCTTTGGTGCTGCCATT
GTTTTT---TCACTTTACTTTGATGCTTTGCATGCGTTTAGTGGT---GGAAACTGGACT
GTGGCCGGCCCTCAA---GCGACTGCTGGGATCTTCGCCTCCTACCCTTCAGAACACCTC
AGTGTTATCAATGGATTTACAGACCAGGTGATTGCCACAGCTGCTCTGTTGATCTGCATC
CTGGCCATTGTGGATGAGAAAAATAACGCTGCCCCGGCTGGATTGCAGCCATTTGTGATT
GGTCTAATGGTGCTGCTGGTAGGCCTTGGCATGGGATTCAACTGTGGCTATCCCATCAAC
CCAGCCAGGGACCTA---GGGCCACGCCTCTTCACTGCCATAGCTGGATGGGGCCTTGAA
GTATTT---ACC------GCTGGAAACCACTGGTGGTGGGTCCCAGTGTTGGGGCCCCTC
GTGGGAGGCTTAATCGGCGCCATAATCTACGAGCTGTTTGTGGAATTCCATCATCCGCTG
---------------GATTCTGAGAAAGAG---------------------AAC------
------------ACCGAA---AACGACAGC------------------------------
---GTTGATCATCCAAATCGTTGTCCCCAT------------TACAAACTAGTTCAGACG
ACCGCAGCC---

>Platypus_AQP13
---ATGGCCGGA
CTCAAC---CCCTGGCCGGAAGGGCCGGTCTGGGGTGCA------------CAGAGTGCC
CACCTGCGCTGTGGCCTGGCTGAGTTCTGGGGGACTTTTGTGCTGATTGTACTCGGCTGC

GGCTGGGTGGCCCAGGCTGAGCTGCGAGGC------CTGGCCCACGTGAGACTTCTCGGC
GCGGACAAGCCCCTCCGGTTCATGGTCTCTGCGGGAGCCTACATAGGCTCCGGAGCCTCA
GGCACTCATCTGAACCCCGCCGTGTCCCTGGCCATGTGTTTCCTGCGGCGGCTGGACTGG
AACCTCCTGCCCACTTTGTGCTTAGCCCAGCTGGCCGGGGCCTTCTGCGGCGCAGCCACC
GTCTTC---GTCTGGCACTACGACGGTCTCCAGGCCCTGAGCGCT---GGTGCCTGGACA
GTGGCGGGACCCAAT---GCCACAGCTGGGATCTTTGCTTCCTACCCCTCCGGGCAGCAG
AGTTCACTCAGCTGCTTTGCTGACCAGGTGATGGCGTCGGCAGCTTTTCTCACCTGTGTC
CTGGCCGTGCTGGGCGAGGGAGTCCCAGCCCCCCGCCACAAGCTGCAGCCCCCCGCCCAG
CGGCTGGCCCTGTTCCTAGTAGGCTCGGCCTTGGGCTCGAGCTGTGGGTGCCCCATCAAC
CCCGCCGAGGACCTG---GGCCCGCGGGCGTTCGCGGCAGTGGCCGGCTGGGGGCTGGAA
GTCTTC---AGG------GTTGGAAACCACTGGTGGTGGATCCCCGTCCTGGGGCCCCTG
GTGGGCGCCCTGCTCGGGGCCTCCGTGTACCACCATGCCCTGGCCCTACCTGTTCTGCTC
---------------TCGGGGAGAAAGAAA---------------------ACT------
---------------------GCGGCC---------------------------------

>Echidna_AQP13
--ATGGCCGGA
CTTAGC---CCCTGGCCAGAAGGGCCGGTCTGGGGC---------AAA---CAAAGTGTC
CACCTGCGCTGTGGCCTGGCTGAGTTCTGGGGGACTTTTGTGCTGATTCTGCTCGGCTGT
GGCTGGGTGGCCCAGGCTGAGCTGCGAGGC------CTGGCCCACATGCGACTTCTCGGC
ATGGACAAGCCCCTCCGGTTCGTGGTCTCCGCAGGAGCCTACATAGGCTCCGGAGCCTCA
GGTACTCACCTGAACCCCGCGGTGTCCCTGGCCATGTGCTTCCTGCGGCGACTGGACTGG
AACCTCCTGCCCACATTGTGCCTAGCCCAACTGGCCGGGGCCTTCTGCGGCGCGGCCACC
GTCTTC---GCCTGGCACTATGACGCTCTCCAAGCCTTCAGCGCT---GGTGCCTGGGCA
GTGGCCGGACCCAAT---GCCACAGCTGGGATCTTTGCTTCCTACCCCTCCGGGCAGCAG
AGTTCACTCAGCAGCTTTGCTGACCAGGTGATGGCGTCGGCAGCGTTCCTCACCTGCGTC
CTGGCCGTGCTGGGAGAGGGAGCCCCAGCCCCCCGCCACAAGCTGCAGCCCCCCGCCCGG
CGGCTGGCCCTGTTCCTCATTGGCTCTGCCTTGGGCTCGAGCTGTGGGTGCCCCATCAAC
CCCGCCGAGGACCTG---GGCCCAAGGGTGTTCGCGGCAGTGGCTGGCTGGGGGCTGGAA
GTCTTC---AGG------GTTGGAAACCACTGGTGGTGGATCCCAGTCCTGGGGCCCCTG
GTGGGTGCCCTGCTCGGGGCCTCCGTGTACCACCATGCCCTGGCCCTGCCTGCTCTGCTC
---------------TCGGGAAGAAAGAAA---------------------GCT------
---------------------GCGGCC---------------------------------

>Inshore_hagfish_Aqp13
ATG------GAGTCATGC----------------------------AGCAAGTTGGCTGAC
CTCCTG---AGAGATGCCGCAAAGATGCTCAACAATCTG------AGGACCAGCAATGAG
TCCATACGTTTGTTTATGGCGGAGCTGCTTGCGACAGCAATGTTGGTGCTCATTGGTTGT
GCAGCTGTTGCTCAGGGGGTGTTGTCAGGT------GGTGGTGTTGCTGCCACTCTGAGC
ATTAATTTGGCCTTTGGATTGGCTGTGATGATGTCTGCGCACATCGCAATGGGAGTTTCA
GGTGCTCACATGAATCCTGCGGTTACCCTGGCGATGTGCATCTTGGACCGATTTCCATGG
AAACGGCTTCCTCTCTATTGGATCTCACAGTTCTTGGGTGCATTCATTGGTGCTCTAGGG
GTCTAT---GCCCTGTATCATGAGGGGATCCAAGCATACGAAGGT---GGGGAGCTGACT
GTTGTCGGGCCTCAT---GCCACGGCCGGAATATTTGCCACCTACCCCAACTCTCACCTC
TCCACTGGAGGAGGAGTTATTGATCAGGTGATTGGAACCGCTATTTTGCTTTGCCTCATT
CTTGCACTTACGGACGAATCGAACTGTGCAATGCCACGCTATCTGCAACCCTTGGGTTTT
GGTTTAGTGGTGGTTGCTATCGGGCAAGCTACGAACTTCAACTGTGGTTATGCCATTAAT
CCAGCCCGGGATTTG---GGGCCCAGGATTTTCAGCGCGCTGGTTGGATATGGATCTGAG
GTGTTT---TGG------GCGAAGGACTGTTGGTGGTGGGTCCCTGTGCTCGCTCCTCTC
ATTGGATCTGTTCTCGGATCTTTGTGTTACCAACTCCTGATCGCACTCCATTACCCGAGG
---------------AGCACTGACAGGGAA---------------------AAT------
------------GAGAAA---CCACCTTCGCTTCTGCAATGCCATGATAACCTCGATGGT
TCACAGGTGGACAAGATGTCT---GAAGTT------------CACGAGATTTCTGAGGAA

TTG---

>Human_AQP9
ATG------CAGCCT------------------------------------GAGGGA---
GCAGAA---AAG---GGAAAAAGCTTCAAGCAGAGACTG------GTCTTGAAGAGCAGC
TTAGCGAAAGAAACCCTCTCTGAGTTCTTGGGCACGTTCATCTTGATTGTCCTTGGATGT
GGCTGTGTTGCCCAAGCTATTCTCAGTCGA------GGACGTTTTGGAGGGGTCATCACT
ATCAATGTTGGATTTTCAATGGCAGTTGCAATGGCCATTTATGTGGCTGGCGGTGTCTCT
GGTGGTCACATCAACCCAGCTGTGTCTTTAGCAATGTGTCTCTTTGGACGGATGAAATGG
TTCAAATTGCCATTTTATGTGGGAGCCCAGTTCTTGGGAGCCTTTGTGGGGGCTGCAACC
GTCTTT---GGCATTTACTATGATGGACTTATGTCCTTTGCTGGT---GGAAAACTGCTG
ATCGTGGGAGAAAAT---GCAACAGCACACATTTTTGCAACATACCCAGCTCCGTATCTA
TCTCTGGCGAACGCATTTGCAGATCAAGTGGTGGCCACCATGATACTCCTCATAATCGTC
TTTGCCATCTTTGACTCCAGAAACTTGGGAGCCCCCAGAGGCCTAGAGCCCATTGCCATC
GGCCTCCTGATTATTGTCATTGCTTCCTCCCTGGGACTGAACAGTGGCTGTGCCATGAAC
CCAGCTCGAGACCTG---AGTCCCAGACTTTTCACTGCCTTGGCAGGCTGGGGGTTTGAA
GTCTTC---AGA------GCTGGAAACAACTTCTGGTGGATTCCTGTAGTGGGCCCTTTG
GTTGGTGCTGTCATTGGAGGCCTCATCTATGTTCTTGTCATTGAAATCCACCATCCAGAG
---------------CCTGACTCAGTCTTT---------------------AAG------
------------ACAGAA---CAATCTGAG------------------------GAC---
---------AAACCA---------GAGAAA------------TATGAACTCAGTGTCATC
ATG---

>Rabbit_AQP9
ATG------CGGTCC------------------------------------GAGAAC---
GGACAG---AAA---ACGAAGAGCTTCAAACAGAGACTG------GTCTTGAAGAACAGC
TTGGCTAAAGAAGCGCTCTCCGAGTTCTTGGGCACATTTATAATGATCGTTCTTGGATGT
GGCTCTGTTGCCCAGGCTGTCCTGAGTCGA------GGACATTTTGGAGGAATTGTCACT
CTCAATGTTGGATTTGCTATGTCAGTTGCAATGGCCATTTATGTGACTGGAGGTGTCTCT
GGTGGTCACATCAACCCAGCTGTGTCTTTCGCAATGTGTCTCTTTGGACGAATGAAATGG
TTCAAATTTCCATTTTATGTGGGAGCCCAGTTCTTAGGAGCTTTTGTAGGGGCTGCAACC
CTCTTT---GGCATTTACTATGATGGATTCATGTCTTTTGCTGGT---GGAAAATTGCTC
ATCGTGGGAGAAAAT---GCAACAGCACATATTTTTGCAACATACCCGGCTCCATATCTG
TCTCTGACGAATGCATTTGCAGACCAAGTAATGTCCACCATGTTTCTCCTCATACTTGTG
TTTGCCATTTTTGACTCCAAAAATTTGGGGGTGCCCAGAGGCCTAGAGCCTATTGTCATT
GGCCTCTTGATTATTGTCCTCTCTGGCTCTTTGGGGCTGAACAGTGGCTGTGCCATGAAC
CCAGCTCGAGACCTG---AGTCCCAGACTTTTCACAGCATTGGCAGGATGGGGATTTGAA
GTCTTC---ACA------GTCGGAAATAATTTCTGGTGGATTCCTGTTGTGGGCCCTTTG
GTTGGTGCTGTCATTGGAGGCCTCGTCTATGTTCTTCTCATTGAAATTCACCACCCAAAC
---------------CCTAACCCAGACTTC---------------------GAG------
------------GTAGAA---CAGTCTGAG------------------------GAC---
---------AAGCCA---------GAGAAA------------TATGAATTTAGTGTTATA
ATG---

>Koala_AQP9
ATGATT---CAGGCT------------------------------------ATGGAC---
AGAAAG---ACC---GAAAAAACCTTCAAAGAGAAAATC------ATCCTGAAGAACTCA
TTGGCCAAAGAAGTGATTGGAGAATTCCTGGGGACATTCACATTGGTTGTCCTTGGATGT
GGCTCTGTAGCCCAAGCCGTCCTTAGCCGA------GGGGCTTCAGGTGGAATTGTGACC
ATAAATATTGGCTTTTCTGCGGCTGTTGCAATGGCTGTTTATGTGACAGGAGGCATCTCT
GGTGGTCACATTAACCCAGCAGTGTCCTTTGCTATGTGTCTCTATGGACGGATGAAATGG
TTCAAATTCCCTTTCTATGCGGGGGCCCAGTTCCTGGGAGCTTTTCTAGGGGCTGCAGCC
CTCTTT---GGAGTTTACCATGACGCACTTAGGTCCTTTGCTGAG---GGAAAACTGATC
GTCACAGGAGAAAAT---GCAACAGCCCATATTTTTGCAACTTATCCAATTAAATACCTG
ACTGTAACAAATGAAATTGCAGACCAAGTGATGTCTTCCGCATTCCTTCTCCTGGTAGTA

TTTGCCGTTTTTGATGAGAAAAACTGGAGGGTCCCCAAGGGCCTAGAACCCATTGTCCTT
GGCCTACTGATCCTTGTCNTTTCTTCCTCATTGGGAATGAACAGTGGTTGTGCCATGAAT
CCAGCTAGAGACCTG---GGTCCCAGAGTATTCACAGCTCTGGCAGGGTGGGGGCTTGAA
GTCTTC---ACA------GCTGGAAGTTACTTCTGGTGGATCCCAGTCGTGGGGCCTTTC
CTTGGTGGCGCCTTAGGAGGCTTCATCTATATTTACGGCATTGAAATCCACCATACAGAC
---------------CCAGACTCAGATGAA---------------------AAG------
------------AGGAAA---CAAGTGGAG------------------------------
------------------------GAGAAA------------CATGAACTTAGTGTGATA
ATG---

>Platypus_AQP9
ATG------AAGGAC------------------------------------TCGGAG---
GGGAAG---AGC---GAAAGGACCTTCAAGGAGAGGCTC------GTCCTCAAGAGCCGC
CTGGCGAAGGAGAGTCTCTCCGAGTTCCTGGGGACCCTCATCATGATCGTGCTCGGGTGT
GGGTCCGTGGCCCAAGCCGTCCTCAGCCGG------GGGCAGCTGGGCAACATAGTGACC
ATCAATGTGGGTTTTGCCATGGCCGTGGTCATGGCCATTTATGTGACCGGGGGCATATCC
GGCGGCCACATCAACCCGGCCGTGTCCTTCGCCATGTGCCTCTCTGGCCGGATGTCTTGG
CTCAAGCTGCCATTCTACGTGGCGGCCCAGTTCCTCGGGGCTTTCACCGGGGCCGCCTTG
CTCTTC---GGCATCTACTACGATGCCCTCATGGCCTTCGCCGAA---GGAGAGCTACTC
ATCACCGGAGAAAAT---GCCACGGCCTTCATCTTTGCCACATACCCGGCCGGGTACCTG
TCCCCGGCAAACGCGTTCGTGGACCAAGTGGCGTCCACCACTTTCCTGATCCTGGTGATC
TTCGCCGTCTTCGACACCAGGAACCTGGGGGTGCCCAGGGGCCTGGAGCCGATCGTCATC
GGACTGCTCATCGTTGTTCTTGCGTCCTCTCTGGGGCTGAACAGCGGCTGTGCCATGAAC
CCGGCCCGGGACCTG---GGCCCCAGGGTCTTCACCGCGGTGGCCGGGTGGGGGCTGGAG
GTCTTC---ACG------GCTGGAAACCACTTCTGGTGGATCCCGGTGGTGGGGCCGATG
GTGGGAGCAGTCTTGGGCGTGCTGGTCTACCAGCTGTGCATCGCCATCCAGCTCCCCCAG
---------------CCGGAGGCGGAGCAG---------------------GAG------
------------GGACAG---GTCATGACC------------------------------
---------AAGATG---------GAGAAA------------TACGAACTCAGCGTCATG
ATG---

>Echidna_653412_AQP9
ATG------CAGGAC------------------------------------TCGGAG---
GGGAAG---AGC---AAGAGGACTTTCAAGGAGAAGCTC------GTCCTCAAGAGCCGC
CTGGCGAAGGAGAGTCTCTCTGAGTTCCTGGGGACCCTCATCCTCATCGTGCTCGGATGT
GGGTCCGTGGCCCAAGCCGTCCTCAGCCGG------GGGCAGCTGGGCAGCATAGTGACT
ATCAATGTTGGCTTTGCCATGGCCGTGGTCATGGCCATTTATGTGGCCGGGGGCATGTCC
GGTGGCCACATCAACCCTGCCGTGTCCTTCGCCATGTGCCTCTCCGGCCGGATGACGTGG
CTCAAGCTGCCGTTCTACATGGTGGCCCAGTTTCTCGGGGCTTTCACCGGGGCCGCCCTG
CTCTTT---GGCATCTACTATGATGCCCTCATGGCCTTTGCCGGG---GGAGAGCTACTC
ATCACAGGAGAAAAT---GCCACGGCCTTCATCTTTGCCACCTATCCAGCCGGGTATCTG
TCCTTGGCAAACGCATTTGTGGACCAAGTGGCGTCCACCACGTTCCTGATCCTGGTAATC
TTAGCCATCTTCGACACCAGGAACCTGGGGGTGCCCAGGGGCCTGGAGCCGGCGGTCATC
GGGCTGCTCATCGTTGCCCTCGCGTGCTCCCTGGGGCTGAACAGCGGCTGCGCCATGAAC
CCGGCCCGAGACCTG---GGCCCCAGGGTCTTCACCGCTGTGGCCGGGTGGGGGTTGGAG
GTCTTC---ACG------GCCGGAAATCACTTCTGGTGGATCCCAGTGGTGGGGCCGATG
GTGGGAGCCGTCTTGGGTGTGCTGGTCTACCAGCTGTGCATCTCGATCCAGCTCCCCAGG
---------------CCGGAGGCGGAGCAG---------------------GAG------
------------GGACAG---GTCACGACC------------------------------
---------AAGGTG---------GAGAAA------------TACGAACTCAGCGTCATG
ATG---

>Kiwi_AQP9
---ATGAGC---
CGGAAA---AAC---AAAAGGAGTTTAAAGGAGAAATTT------GCCTTGAAGAACAGC

TTGGTTAAAGAAGCCCTCTCAGAATTCCTGGGAACTTTTATATTGATAGCACTCGGATGT
GGGTGTGTGGGGCAGGCTGTCCTTAGCCGA------GGAGCCCATGGTGGGCCCATGACA
GTATCTGTTGGATTTGCAGTGGCAGTCACCATGGCAGTGTACGTGTCTGGGGGGGTTTCT
GGTGGTCACATAAACCCAGCAGTTTCATTAGCCATGTGCGTGACTGGAAGATTAAAATGG
ACCAAATTACCAATTTACATATTAGCACAATTCCTGGGAGCATTTGTTGGAGCAGCGGCT
GTCTTT---GGGATTTATTATGATGCCTTTATGGATTATAGCAAT---GGAACACTTGAA
GTCACAGGACCTAAT---GCAACGGCACATATCTTTGCAACATACCCAGCTCCGTATCTG
TCCCTCATAAATGGATTTGCAGATCAGGTGATGTCAACAGCTGTTCTTCTTCTGGCTATA
TTTGCTATTTTTGACTCAAAAAATAACGGAGTACCAAAGGGCCTGGAGCCAATTGCAGTA
GGACTCCTTATAATTGTACTTACCTGCTCCTTGGGAATGAACAGCGGCTGTGCCATGAAC
CCAGCCAGGGACCTA---GGCCCAAGGCTCTTCACAGCCATTGCAGGATGGGGAATGGAA
GTATTC---ACG------GCTGGAAATAATTGGTGGTGGGTCCCTATAGTTGCACCTATG
CTGGGGGGAGTACTTGGAGCTGTCACCTATATAGTTTTTATTGAAATTCATCATCCAGAT
---------------ACTCAGTCAGGA---------------------------------
---------------------GAAGAAAAT------------------------GAC---
---------ATACAT---------GACAAA------------TATGAACTTACTAATATG
GAG---

>Alligator_AQP9
--ATGAGC---
AAAAAA---GGC---AAAAGAAGTTTCAAGGAAAAATTT------GCCTTGAAGAACAGC
CTGATTAAAGAAGCCCTCTCAGAGTTCCTGGGAACTTTTGTATTGATAGCGCTCGGATGT
GGGTCTGTTGCCCAAACTGTTCTTAGCCAT------GGGGCCATGGGTGAGTCTATAACA
ATATACGTCGGGTTTGCAGTGGCAGTCACCATAGCAATCTATGTATCCGGAGGCGTGTCT
GGCGGTCACATTAACCCAGCAGTTTCATTAGCCATGTGTGTGACTGGAAGGTTGAAATGG
ATCAAATTACCGGTCTACATATTAGCCCAACACCTGGGGGCATTTATTGGAGCAGTAGCT
GTCTTT---AGTGTTAATTATGATGCGCTTATGCAGTACAGTGAT---GGAGAACTTACA
GTCACAGGACCCAAT---GCAACTGCACATATTTTTGCAACATATCCAGCTCCACATCTG
TCACTGATAAATGGATTTGTTGAGCAGGTGATGTCAACAGCTTTTCTTCTTTTGGGTGTA
TTTGCCATTTTTGACACCAAAAACATAGGAGTACCCAAGGGCTTGGAACCAATTGCAGTT
GGACTTCTTATCTTGGCACTTACTTGCTCCTTGGCAATGAACAGTGGCTGTGCCATGAAC
CCAGCCAGGGACCTA---GGCCCAAGGCTCTTCACATTAATAGCAGGATGGGGAACAGAA
GTATTT---ACG------GCTGGAAATAACTGGTGGTGGGTTCCTATAGTTGCACCTATG
ATGGGAGGAGTAATTGGAGCCCTTATTTATGTTCTTTTTATTGAAATCCACCATTCAGAT
---------------ACTCAGCCAGAA---------------------------------
---------------------CAAGACACT------------------------GAC---
---------ATGTAT---------ACCAAA------------TATGAACTTACCAACGTG
GGG---

>Turtle_AQP9
--ATGAAC---
GGGCAA---AGC---AAAAGAACATTCAAGGAGAAATTT------GCCTTGAAGAACAGC
TTTATAAAAGAAGCCCTGTCCGAATTCCTGGGAACATTTGTAATGATAGTACTCGGATGT
GGGTCTGTTGCCCAAGCCGTCCTTAGCCGA------GGGGCCATGGGTGGAACCGTAACA
ATATCTGTTGGATTTGCAATGGCAGTCACTATGGCAGTGTATGTCTCTGGGGGCGTGTCT
GGTGGTCACATAAACCCAGCCGTTTCATTAGCCATGTGTGCAACTGGAAGGTTGAAATGG
ACCAAATTACCTTTTTATATATTAGCACAATTCCTGGGAGCATTCGTTGGAGCAGCAGCT
GTCTTT---GGGATTTATTATGATGCACTCATGAACTACAGTGGT---GGAGAGCTTATA
GTTACAGGATCTAAT---GCAACCGCACATATTTTTGCAACTTATCCAGCTCCACATCTG
TCCCTGATAAGTGGATTTGCAGATCAAGTAATGTCAACAGCTCTTCTTGTTCTGGTTGTC
TTTGCTATTTTTGACACCAAAAATATGGGTGTACCAAAGGGATTGGAACCAATTGCAGTT
GGACTTCTTATCCTTCTGCTCACTTGTTCCTTGGGAATGAACAGTGGCTGTGCCATGAAC
CCAGCCAGGGACCTA---AGCCCAAGGATTTTCACAGCTATAGCCGGATGGGGTTTCGAA
GTATTC---ACG------GCTGGAAATAACTGGTGGTGGGTTCCTACAGTTGCACCTATG
ATGGGAGGCGTAATTGGAGCCATTACCTATATTATTTTTATTGAAATTCATCATTCAGAT
---------------ACACAGCTGGAA---------------------------------
---------------------GAAGACACT------------------------GAT---

---------ATGTAT---------GAAAAA------------TATGAACTTACCAATATG

>Tuatara_AQP9
--ATGAAC---
AAGAAA---AGC---GAAAGAAGCTTGAAGGAGAAATTT------GCCCTGAAGAACAGC
CTGATTAAAGAAGCCCTGGCAGAATTCTTGGGAACATTTATATTGATAGTACTTGGATGC
GGGTGTGTGGCCCAAGCTGTCCTTAGCAGA------GGGGAGCTGGGCGGAGTGATTACC
ATATCCGTTGGTTTCGCAATGGCCGTCACTATAGCAATATATGTAGCCGGGGGCGTGTCT
GGTGGTCACATAAACCCAGCTGTTTCACTTGCCATGTGTGTCACTGGAAAGATGAAATGG
GCCAAATTACCTGTTTACATATTAGCACAATTCCTGGGATCCTTTGTTGGAGCAGCAGCC
CTCTTT---GGGATCAATTATGATGCATTTATGGACTACACTGGG---GGAGAGTTGAAT
GTCACAGGACCAAGG---AAGACTGCACATATTTTTGCAACTTATCCAGCTCCATATTTG
TCCTTGGCAAATGGATTTGCAGATCAAGTGATGTCAACAGCTATTCTTGTCCTGGCTGCC
TTTGCTATTTTTGACACAAAAAATCTATGGGTACCAAAGGGATTGGAGCCCATTGCAGTT
GGACTTCTTATCATATTACTCACT---TGTATGGGATTGAACAGTGGCTGTGCCATGAAC
CCAGCCAGAGACTTA---GCCCCAAGGATCTTCACAGCCATAGCAGGATGGGGGATGGAA
GTGTTC---ACG------GCTGGAAACCACTGGTGGTGGATCCCCACAGTTGCACCTATG
CTGGGCGGTGTTCTCGGAGCTGTCACCTATATGCTTTTTATTGAAATTCACCATTTGGAT
---------------GTGCAGGTGGAA---------------------------------
---------------------CAAGACATG------------------------GAC---
---------TCATCT---------GGCAAA------------TATGAACTCACCAATATG

>Python_AQP9
--ATGACA---
AAGAAA---GCA---GGAAAGAGCCTGAGAGAAAAGTTT------GCCCTGAAGAACAAT
TTGCTGAAGGAAGGGCTGGCAGAACTCCTGGGAACCTTCATCTTGATCGTACTGGGCTGT
GGGAGCGTGGCGCAAACAGTCCTGAGCAGA------GGAGCTCTGGGAGGAGCACTGATG
ATCTCTGTTGGCTTCGCCATGTCCGTCACCCTCGCCGTCTACGTTGCGGGAGGCGTTTCT
GGGGGTCACATCAATCCAGCCGTTTCTTTTGCCATGTGCCTGACTGGGAAGATGAAATGG
GTCAAATTTCCCGTTTATGTCTTTGCGCAATGTTTTGGTGCATTTCTTGGAGCGGCCATG
GTGTTC---GGGATCAACTACGATGCGCTTATGTTCCACACTCAT---GGAGTCTTCACT
GTCACGGGGCCCAAC---GCCACAGCACATATTTTTGCAACATATCCCCAGGAATATTTG
TCCCTGACCACTGGCTTTGTAGACCAGGTCGTATCCACGGCCTTCCTTATCCTGGGTATC
TTCGCCATCTTCGACACGGACAATTTGGGTGTCCCAAAGGGCCTGGAGCCCATCGCCATC
GGCCTCCTCATCATCCTGCTGACCTCTTCTATGGCCCTGAACAGTGGCTGCGCCATGAAC
CCTGCCCGAGATCTG---GCCCCGCGTGTCTTCACATACCTGGCGGGATGGGGGGCCGAA
GTCTTT---ACG------GCTGGCAGTCACTGGTGGTGGGTCCCCGTTGTCGGCCCGATG
GTTGGTGCGGCTGTCGGAACTGCCACCTACATGCTCTTCATTGAACTTCACCACGCTCCC
---------------GTGCCCCTCTGCCAG---------------------AAG------
---------------------AATGCCCCT------------------------GAC---
---------ACCCAC---------CACGAG------------TATGAGCTTACCCACCTG
GAAGAAAGGAAA--

>Gecko_AQP9
--ATGGCC---
AAGAGG---AAC---GGGAGGAACTGGAAGGAGAGCTTT------GCCCTGAAGAACAGC
CTGGTGAAGGAAGCCCTCTCCGAGTTCCTGGGGACCTTCGTGATGGTAACCCTGGGATGC
GGGTGCGTGGCCCAGTCGGTGCTGAGCGGG------GAAGCGCTGGGAGGAGCGCAGATG
GTCTCCGTGGGCTTCGCCATGGCCGTCACCATCGCCATCTACGTCGCAGGAGGCGTCTCC
GGGGGCCACATCAACCCGGCCGTTTCGTTTGCCATGTGCATCACCGGCAAGATGACGTGG
GCCAAGTTCCCCGCCTACGTCCTCACGCAGTTTCTCGGAGCATTCGTGGGAGCAGCCGCC
GTCTAC---GGGGTCAACTACGATGCACTGATGTCCTACACCGGG---GGGAACTTCACC
GTCACAGGTGACATC---CGCACCGCCCATATCTTCGCCACGTACCCATCAGAATACCTC

```
TCTGTGACCAACGGATTTGCCGACCAGGTGATCTCAACGGCCTTCCTCCTCGTGGGCGTC
TTTGCCATCTTCGACCAGGGGAACCTGGGGGTCCCGAAGGGGCTGGAGCCCATTGGGGTG
GGTCTTCTGATCATCTTGCTGACCTCCTCCCTGTCCATGAACAGCGGCTGCGCCATGAAC
CCCGCTCGGGACCTC---AGCCCACGCCTCTTCACGCTCCTGGCCGGGTGGGGTCCAGAG
GTCTTC---ACG------GCTGGCAACCACTGGTGGTGGGTGCCGATCGTTGGTCCCATG
GTTGGGGCCGGTTTTGGGGCCGCTATCTACATGCTGTTTATCGAACTGCACCATGTGCAG
---------------CCGCCGCCGCCA---------------------------------
---------------------CAGAGCAAC------------------------GCC---
---------GCTCAC---------GACAAA------------TACGAACTCACCAGCATG
------------------------------------------------------------
------------------------------------------------------------
------------------------------------------------------------
---------------------
>Himalaya_frog_AQP9
------------------------------------------------------------
ATGCAA---AAG---GACAGAAGCTGTTTAGGAAAACTG------AAACTAAGGAACAGC
CTGGCGAAGGAAACCCTGTCTGAGTTTTTCGGCACATGTTTGTTGATTGTATTAGGCTGT
TCGTGTGTAGCATCTTCTGTTCTCAGTGGG------GGAAAGGCTGGAGGTCACTTAACT
AATAATCTTGGCTTTGCAATGGCAGTCGCTATGGCAGTCTACGCTACTGGAGGAGTGTCT
GGGGGCCACATTAATCCTGCAGTTTCATTTGCCATGTGTTTAACTGGAAGATTAGAATGG
GTCAAGCTCCCCTTCTATGTGAGTGCACAGTTTGTGGGTGCCATTACTGGGTCTGCTTTA
GTTTTT---GGTGTTTATTATGAAGCAATAATCAAATATTCTGGT---GGAGTTCTTACA
GTAGATGGTCCAAAT---GCCACAGCTCATATTTTTGCAACCTATCCTGCTCCATATCTG
TCAACAATAAATGGACTTGGAGATCAGATACTGTCTACTGCACTTCTGCTCATTTTGGTA
TTTGCCATATTTGACAAGAAAAACATTGCAGCCCCTAAGGGATTGGAGCCGATTGCAATT
GGCCTTCTGATTATGCTTCTAGGATTAGCTCTGGGAATGAATTGTGGCGGTGCTATGAAC
CCAGCTAGAGACTTA---GGTCCACGAATCTTCACAGCAGTGGCTGGATGGGGTTATGAT
GTTTTC---ACG------GCTGGCAATAACTTTTGGTGGATTCCTGTAGTTGGCCCAATG
GCTGGTGGAGCACTTGGAGCTTACATATATATTCTCTGCATAGAAGCTCATCATCACAAT
---------------GGACAAGATAGCATG---------------------CAT------
------------CCTGTT---GATACTGAT------------------------------
---------CAGTAT---------GAAAAG------------CATGAACTTACCAATATG
GCA---------------------------------------------------------
------------------------------------------------------------
------------------------------------------------------------
---------------------
>Axolotl_AQP9
---------------------------------------------------ATGACC---
CAAACA---ACC---CATAAGAGCTTGCGAGACAGGCTG------GCACTGAAGAACAGC
CTGTCAAAGGAGATCGTCGCTGAGTTCCTGGCCACATTTGTGTTAATTGTACTTGGATGT
GGAAGTGTTGCCCAAGCTGTCCTGAGCAGA------GGAGTTGCTGGCAATGCTGTAACC
ATAAATGCTGGGTTTGCGATGGCAGTCACTATGGCCGTCTACGTCGCAGGTGGGGTTTCT
GGGGGCCATGTAAATCCTGCCATTTCTTTGGCCATGTGTGCAACTGGAAAGATGAGGTGG
TGTAAATTCCCTTTTTATGTCTGTGCACAGATGCTGGGAGCCATGGCTGGGGCAGCAGCC
ATTTTT---GGAATATATTATGATGCATTCATGTCCTTTGCTGAG---GGGACGCTTGCA
GTCTCAGGTCCAAAT---GCAACAGCACATATATTTGCCACATACCCAGCATCGTTTCTT
TCCACCACAAATGGATTTGCAGAGCAAGTGGCATCGACTGCTCTCTTGGTTATTTCTGTA
TTTGCCATTTTCGACAACAAAAACATAGGAGTGCCTGCAGGGCTGGAACCGGTGGTAGTT
GGCCTGCTCATTTTTCTTTTAAGTTGTTCTTTGGGGATGAACTCTGGCTGCGCTATGAAT
CCAGCAAGGGACCTA---GGTCCAAGGATGTTCACCGCTGTGGCAGGATGGGGTCTTGAA
GTCTTC---ACT------GCTGGTAATAATTGGTGGTGGGTTCCCGTAGTGGCCCCGATG
ATTGGAGGACTTATTGGTGCTTATGTCTACATCCTGGCCATCGAATTGCATCATCAGAAA
---------------CCGCTAGAAGACACA---------------------AAG------
------------TCAGAA---CAAGAGGCC------------------------GAAGAT
---GGATCACTATAT---------GGGAAA------------TATGAACTTACGAATAGT
GTA---------------------------------------------------------
------------------------------------------------------------
------------------------------------------------------------
---------------------
>2Lcaecilian_AQP9
---------------------------------------------------ATGAAT---
```

```
GCAAAG---AGC---AGAAGAAGCTTGAAAGATAAGATT------TCACTTAAGAACAGC
CTGGTAAAAGAAACCCTGTCCGAGTTCTTTGCAACTTCTTTACTGATTGTACTTGGGTGC
GGCTGTATTGCTCAGTCTGTCCTGAGTCAA------GGGGCTGCTGGGAATATTATCACG
ATAAGTGCTGGGTTTGCAATGGCAGTGACTATGGCAGTGTATGTAGCAGGAGGTGTATCT
GGTGGTCACATAAACCCAGCGGTTTCATTTGCTATGTGCTTAACTGGAAAGTTAAAATGG
AGAAAATTTCCTTTTTATGTATTGGCGCAAATGTTGGGTGCCATGGCTGGAGCAGCAGCA
GTTTTC---GGGGTCTATTATGATGCATTGATGCATTACACAGGT---GGAAATCTTACC
GTCAGTGGTCCAAAT---GCAACAGCCCAGATTTTTGCAACATACCCTGCTCCGTTCCTG
TCCACCATGAATGGATTTGTAGACCAAGTGATGGCCACGGCTTTGTTAGTAATTGCTATT
TTTGCTATCTTTGATAGCAAAAATATAGGTGTACCCAAGGGGTTGGAACCAGTTGCAGTG
GGTTTACTCATTATGCTTCTATGTTTCTCCCTGGGGCTTAACTCAGGCTGTGCCATAAAC
CCAGCTAGAGATCTA---GGGCCAAGGATCTTCACCGCAGCAGCAGGATGGGGACTTGAT
GTATTC---AGG------GCTGGAAATAGTTGGTGGTGGATTCCTGTTGTTGGACCTATG
ATTGGAGCTGCTATTGGAGCATTTGCCTATATGATGTGCATTGAAATTCACCATAGTAAA
---------------CAATCTGAAGATGTA---------------------AAG------
------------TCAGAA---CAAAATGAT------------------------GGA---
---------ATGTAT---------GAAAAA------------TCTGAACTGACCTCTATG
------------------------------------------------------------
------------------------------------------------------------
------------------------------------------------------------
---------------------
>Gaboon_caecilian_AQP9
---------------------------------------------------ATGACC---
ACAGCA---AGC---AGACGAAGCTTGAGAGACAGAATT------GCGCTGAAGAACAGC
CTTACAAAAGAAGTCTTGGCTGAGTTCTTTGCGACGTCGTTACTGATTGTTTTTGGATGT
GGTTGCGTTGCTCAGGCTGTTCTGAGTCGA------GGAGCTGCTGGGAATATTGTATCA
ATCAGTGCTGGGTTTGCAATGGCAGTAACTATGGCAGTGTATGTAGCAGGCGGCGTATCT
GGTGGTCACGTAAACCCAGCTGTATCATTTGCTATGTGCTTAATGGGAAAGCTTCCATGG
AGAAAACTTCCTTTCTACATACTGGCACAGTTGCTGGGTGCCATAGCTGGATCAGCAGTG
GTCTTT---GGTGTCTATCATGATGCACTGATGGAATACACGGGT---GGAGAACTTACT
ACCAGCGGTCCGAAT---GCAACAGCTCATATCTTTGCCACATATCCATCTCAATTCCTC
TCTACTATGAATGGATTTGCAGACCAAGTAATAGGAACGGCTCTGTTGGTGATTGCTATT
TTTGCTATTTTTGATAGCAAAAATATAGGCGCACCCCGAGGGTTGGAACCAATTGCAGTC
GGTCTGCTCGTTATGCTTCTAAGCTTCTCGATGGGGCTAAACTCAGGCTGTGCCGTAAAT
CCAGCCAGAGATCTA---GGGCCAAGGCTCTTCACAGCAGCAGCAGGATGGGGACTTGAT
GTATTC---AGA------GCTGGAAATAGCTGGTGGTGGATTCCTGTTGTGGCACCTATG
GTTGGAGGAGCCGTTGGCGCTTTCGTCTACATGATATGCATCGAAGTCCACCACAGTAAA
---------------CAATCAGACAATAAG---------------------AAG------
------------TCAGAA---CACAATTCT------------------------GAC---
---------ATGTAT---------GAAAAA------------TGTGAACTGACCAATATG
------------------------------------------------------------
------------------------------------------------------------
------------------------------------------------------------
---------------------
>Coelacanth_Aqp9
---------------------------------------------------ATGGAT---
GCAAGG---GAC---AAGAAGAGCTTCAAAGACAAGTTC------GTCTTAAGGAATAGA
CTGGTTAAGGAATCATTATCAGAGTTTCTAGGGACATGTCTATTAATAGTTTTTGGATGT
GGTTCAATTGCTCAGTCTGTACTCAGTCAT------GAGAGTATGGGTGGGTTTCTAACA
ATAAACATTGCTTTTCCAATGGCAGTGACTATGGCTATTTATGTAGGAGGGGGAGTATCA
GGTGCGCACATAAACCCAGCTGTTTCGTTTGCTATGTGTATTGTGGGGAGACTTAACTGG
TTGAAGCTGCCATTCTATATTGTAGCTCAGATGCTGGGTGCATTTGTTGGCGCAGGTGCA
GTTTTC---GGAATTTATTACGAAGCTTTAATGACCTACACAGGG---GGAGTGCTTACA
GTCACTGGTCCCAAT---GCAACAGCACAGATATTTGCCACTTATCCTTCTCCGTTCTTG
TCTATTGCAAGTGGGCTTATAGACCAAATTATAGGAACATCTTTACTGTTAGTTTGCATC
TTTGGTATTTTTGACAATAAAAATAATGGTGTGCCAAAGGGATTGGAACCAATAGCAGTT
GGCCTCTGTATTATGGTGCTTGGGTTGTCCATGACATTAAACTGTGGCTGTGCCATCAAC
CCAGCACGGGATCTA---GGTCCAAGAATGTTTACAGCAGTGGCAGGATGGGGTACTGAA
GTTTTC---AGT------GCTGGAAATAACTGGTGGTGGGTTCCTGTTGTCGGCCCTATG
ATTGGAGCAGCTGTTGGATGTTTCATTTACGTTATGTTTATTGAACTTCACCACCTAGAC
---------------CCGCTTGATGAATCA---------------------ACA------
```

```
---------------------GGCGACAAT------------------------GAC---
---------TCAAAT---------GAGAAA------------TGTGAAATAATTGCAATG
AAG---------------------------------------------------------
------------------------------------------------------------
------------------------------------------------------------
---------------------
>mCoelacanth_Aqp9
---------------------------------------------------ATGGAT---
GCAAGG---GAC---AAGAAGAGCTTCAAAGACAAGTTC------GTCTTAAGGAATAGA
CTGGTTAAGGAATCATTATCAGAGTTTCTAGGGACATGTCTATTAATAGTTTTTGGATGT
GGTTCAATTGCTCAGTCTGTACTCAGTCAT------GAGAGTATGGGTGGGTTTCTAACA
ATAAACATTGCTTTTCCAATGGCAGTGACTATGGCTATTTATGTAGGAGGGGGAGTATCA
GGTGCGCACATAAACCCAGCTGTTTCGTTTGCTATGTGTATTGTGGGAGACTTAACTGG
TTGAAGCTGCCATTCTATATTGTAGCTCAGATGCTGGGTGCATTTGTTGGCGCAGGTGCA
GTTTTC---GGAATTTATTACGAAGCTTTAATGACCTACACAGGG---GGAGTGCTTACA
GTCACTGGTCCCAAT---GCAACAGCACAGATATTTGCCACTTATCCTTCTCCGTTCTTG
TCTATTGCAAATGGGCTTATAGACCAAATTATAGGAACATCTTTACTGTTAGTTTGCATC
TTTGGTATTTTTGACAATAAAAATAATGGTGTGCCAAAGGGATTGGAACCAATAGCAGTT
GGCCTCTGTATTATGGTGCTTGGGTTGTCCATGACATTAAACTGTGGCTGTGCCATCAAC
CCAGCACGGGATCTA---GGTCCAAGAATCTTTACAGCAGTGGCAGGATGGGGTACTGAA
GTTTTC------------------------------------------------------
------------------------------------------------------------
------------------------------------------------------------
------------------------------------------------------------
------------------------------------------------------------
------------------------------------------------------------
------------------------------------------------------------
------------------------------------------------------------
---------------------
>European_seabass_Aqp9a
------------------------------------------------------GAA---
ACACAA---CGC---AGGAGAAGCATGAGGCAACACTGT------GCTCTCAAACATGGA
ATATTTAAGGAATTCCTGGCAGAATTCCTAGGAACATTTGTCTTGGTTCTGTTTGGCTGC
GGCTCAGTCGCTCAGACCGTCCTCAGTCGA------AACAGCCTGGGTGAACCTCTCACC
GTCCACATCGGCTTCTCTGTGGGACTGATGATGGCAGCGTATGTGGCTGGTGGAGTGTCA
GGGGGCCATGTGAACCCTGCTGTGTCTCTGGCCATGGTGATTCTGGGAAAACTGAAGATC
TGGAAGTTTCCCTTCTATGTCATCGCTCAGTTTCTTGGTGCTTTTGTTGGCGCTGCTGCA
GTCTTT---GGGTTGTACTATGATGCTTTCATGGACTTCACCAGT---GGGATTCTGTCA
GTGACGGGAATCAAT---GCAACAGGTCACATTTTTGCTTCCTACCCTGCGAGACACCTG
TCAGTCATCGGCGGCTTCATCGATCAGGTGATAGGGACGGGTATGCTGGTCTTGTGTATA
CTGGCTATCATTGATGGTGGAAATATCGGAGCTCCCAAAGGCGTGGAACCGCTTGCCATT
GGTCTGATCATCATGGCCATCGGTGTGTCCATGGGGCTGAACTGTGGCTACCCCCTGAAC
CCTGCCAGAGACCTG---GGACCCCGACTGTTCACAGCTTTAGCAGGATGGGGAATGGAG
GTCTTC---AGG------ACTGCAGACTACTGGTGGTGGATCCCTGTGGCGGGGCCCATG
GTGGGGGGCGTGGTCGCAGCCATCATCTACTACCTGTTTATTGAGCTGCACCACCACCGT
GATGAGCCCGAGAACCCCCACGAGGAGGAG---------------------GAAGAAGAG
GAGGAAGATGAAGAGGAC---GAGGACAGC------------------------AGT---
---------CTGAAG---------GACAAA------------TATGAGATGATCACCATG
AGC---------------------------------------------------------
------------------------------------------------------------
------------------------------------------------------------
---------------------
>Nile_tilapia_Aqp9a
---------------------------------------------------ATGGAA---
GTACAA---AAC---AAGAGAAGCATGAGGCAACACTGT------GCTCTAAAACATGGA
ATATTCAAGGAATTCCTGGCAGAATTCCTGGGAACCTTTGTCTTGGTTCTGTTTGGCTGT
GGCTCCGTTGCTCAGACCGTCCTCAGTCGA------AACACCCTGGGCGAACCCCTCACC
GTCCACATCGGCTTCTCTGTAGGCCTGATGATGGCAGCGTATGTGGCCGGTGGAGTTTCA
GGGGGCCATGTGAACCCCGCTGTGTCTTTGGCCATGGTAATTTTGGGCAAACTGAAGATC
TGGAAGTTTCCGTTCTACATCATCGCTCAGTTTCTTGGTGCTTTTGCAGGAGCTGCTGCA
GTCTTT---GGATTATACTATGATGCCTTCATGGACTTCACCAGT---GGGATTCTGTCA
```

```
GTGACGGGAATCAAT---GCAACGGGCCACATTTTTGCCTCATACCCCGCCAGACACCTG
TCAGTCCTCGGCGGCTTCATTGATCAGGTGGTGGGAACAGGTATGCTGGTTTTGTGCATT
CTTGCTATCACTGACAGCGGAAACATCGGAGCTCCTAAAGGCATCGAGCCGCTGGCCATC
GGCCTGATCATCATGGCCATCGGCGTGTCCATGGGGCTGAACTGTGGCTACCCCCTAAAC
CCAGCCAGAGACCTG---GGACCCAGACTGTTCACAGCTGTGGCAGGATGGGGGATGGAG
GTCTTT---AGG------ACCGCAGACAACTGGTGGTGGATCCCAGTGGCGGGACCGATG
GTGGGGGGAGTGGTCGCGGCCGTCATCTACTACCTGTTCATCGAGCTGCACCACCCCCAC
GCCGAGCACGAGAAGCCCCAGGAGGAAGAG---------------------GAGGAGGAC
GAGGAAGACGAAGAGGAT---GATGACAGC------------------------AGT---
---------CTGAAG---------GACAAA------------TATGAGATGATCACGATG
AGT---------------------------------------------------------
------------------------------------------------------------
------------------------------------------------------------
---------------------
>Mummichog_Aqp9a
------------------------------------------------------------
------------------AGAAACATGAGGCAGCACTGC------GCGCTCAAACATGGA
ATATTCAAGGAATTCCTGGCTGAGTTTCTCGGGACTTTTGTCTTGGTATTGTTTGGCTGT
GGCTCAGTTGCTCAGACCGTCCTGAGTCGA------AACACGCTGGGTGAGCCGCTCACC
ATCCACATCGGCTTCTCTGTGGGCCTCATGATGGCAGTGTACGTGTCTGGTGGGGTGTCA
GGAGGCCATGTGAACCCCGCTGTGTCTCTGGCCATGGTGATTCTGGGCAAACTGAAAATC
TGGAAGTTTCCCTTCTACGTCATCGCTCAGTTTCTTGGTGCTTTTGCAGGAGCTGCGGCG
GTCTTC---GGATTATATTACGATGCTTTCATGGACTTCACAAGT---GGTATTTTGTCA
GTGACAGGAATCAAT---GCGACAGGTCACATTTTTGCCTCTTACCCCGCCAGACACCTG
TCAGTCCTCGGAGGCTTCATTGATCAGGTGGTGGGACGGGCATGCTGGTTCTCTGTATC
CTGGCCATCATAGACGGCGGCAACATCGGCGCTCCTAAAGGCGTGGAGCCGCTGGCCATC
GGCCTGATCATCATGGCCATCGGCGTGTCCATGGGACTCAACTGCGGCTACCCCCTGAAC
CCCGCCAGAGACTTC---GGACCCCGACTGTTCACCGCAGTGGCAGGATGGGGGATGGAG
GTCTTC---AGG------ACTGGAGGCTACTGGTGGTGGATCCCGGTGGCGGGGCCCATG
GTGGGAGGCGTGGTGGCCGCCGTCCTCTACTTCCTGCTCATCGAGCTTCACCACCCCCAC
GACGAGGATGAGAAGGCCCACGAGGTGGAG---------------------GAAGAGGAA
GAAGAGGAGGAAGACGAC---GAGGACAGC------------------------AGT---
---------CTTAAG---------GACAAA------------TACGAGATGATCGCCATG
AGT---------------------------------------------------------
------------------------------------------------------------
------------------------------------------------------------
---------------------
>Zebrafish_Aqp9a
------------------------------------------------------------
------------------------ATGAAGCAGCACTGC------GCGCTCAAACAGCGG
CTCTTCAAGGAGTTTCTGGCCGAGTTTTTGGGGACCTTCGTGCTGGTGCTGTTCGGCTGT
GGGTCGGTGGCTCAGACAGTGTTGAGCAGA------AACACACTCGGTGAACCGCTGACC
ATCCACATCGGCTTCAGCACCGGCCTCATGATGGGCGTCTATGTGTCCGGCGGCGTCTCA
GGCGGGCACCTGAACCCGGCCGTGTCTCTGGCGATGGTGATTCTGGGGAAGCTGAAGATC
TGGAAGTTCCCGGTGTACGTGATCGCGCAGATGCTCGGAGCGTTTGCAGGAGCGGCCGCT
GTGTTC---GGCCTGTACTACGACGCCTTCATGGAGTTCACCAGC---GGGATCCTGTCA
GTGACGGGCATTAAC---GCCACAGGACACATCTTCTCCTCGTACCCGGGCAGACACCTG
ACGGTCCTGGGCGGGTTCGTGGATCAGGTGGTGGGCACAGGGATGCTGGTGCTCTGTATT
CTCGCTATAGTGGACGGCAGGAACATCGGCGCCCCCAGAGGTGTGGAGCCGCTGGCTGTG
GGTGTGGTGCTGCTGGGCATCAGCGTCTCCATGGGCCTGAACTGCGGATACCCCCTGAAC
CCGGCCCGAGACCTG---GGGCCCAGACTCTTCACCGCACTCGCCGGATGGGGGATGGAG
GTGTTC---AGC------ACTGCAGATTACTGGTGGTGGATCCCAGTTGCCGGGCCGCTG
GTGGGGGGTGTCGTCGGAGCGGTGATCTACTTCCTGTTAATCGAGCTTCATCACTCCAAT
---CACAACGACACACCACAGGAGGAGCCT---------------------GAGGAGGAG
GAGGATGAGGATGAAGAA---GAGGACAGC------------------------AGC---
---------CTGAAG---------GACAAA------------TACGAGATGATCAACATG
AGC---------------------------------------------------------
------------------------------------------------------------
------------------------------------------------------------
---------------------
>European_seabass_Aqp9b
```

```
---------------------------------------------------ATGGAA---
AATGAG---AGC---AAGAGGAAGATGAAGGAGAGATTT------GGCCTGAGGCGGGAC
ATTTTTAAGGAGTTCCTGGCGGAATTTCTGGGGATATTTGTCCTGATACTCTTTGGATGT
GGTTCAGTGGCCCAGACAGTGCTGAGTAAA------GGGGCTCTAGGGGAGCCCTTGACC
ATCCACGTTGGTTTCACTCTGGGAGTCATGATGGCTGTCTACATGGCAGGGGGAGTGTCA
GGAGCCCACGTTAACCCTGCAGTCTCTCTGGCCATGGTGATCCTGGGGAAGCTTCCTCTG
AAGAAGTTCCCTGTGTATGTGATGGCACAGTTCCTGGGGGCTTTTGCTGGATCCTGTGCT
GTCTAT---GGGTTGTATTACGATGCTTTGATGGAATACACTGAT---GGAGAATTTGTT
GTTACTGGTGCAAAT---GCTACAGCCAACATATTTGCATCTTATCCTGCAAAACATCTC
TCAGTCCTTAATGGGTTTGTTGATCAGGTAATTGCAACTGGTGCGCTGATCCTGTGCATC
CTGGCCATCACTGACAGGAAGAATATCGGTGCTCCAAAAGGTATGGAGCCTCTGTGCATC
GGCCTGATCATTATGGCCATTGGAGTGTCCATGGGTCTTAACTGCGGCTATCCAATCAAC
CCAGCGCGAGACCTC---GGCCCACGGTTCTTCACGGCTGTGGCTGGGTGGGGCATGGAC
GTATTC---AGG------GCTGGAGGTTGCTGGTGGTGGATCCCTGTGGCAGGACCCATG
GTGGGCGGAGCAGTTGGAGCAGGCGTTTACTTCCTCTTCATTGAGTTGCACCACCCTGAG
---------------CCTGAAAAACAG---------------------------------
---------------------GAGGAGAAC-----------------------AAT---
---------GTTCAG---------GACAAA------------TATGAAATGATAACTATG
AGT---------------------------------------------------------
------------------------------------------------------------
------------------------------------------------------------
---------------------
>Nile_tilapia_Aqp9b
---------------------------------------------------ATGGAA---
AACGAA---TAC---AGGAGGAAGATGAAAGAGAAACTT------GGCCTGAGGCGAGAC
ATCTTTAAGGAGTTCCTGGCGGAGTTTCTGGGGATATTTTTCCTGATACTCTTTGGATGC
GGTTCGGTGGCCCAGATGGTCCTGAGCAGA------GCAGGTCCTGGTGACATACTGAGC
GTCCACATTGGTTTCACTCTTGGAGTCATGATGGCCGTCTATATTGCAGGGGGAGTGTCA
GGAGCCCACGTGAACCCGGCAGTCTCTCTGGCCATGCTGATCCTGGGGAAGCTGCCTCTA
AAGAAGTTCCCCATATACGTGGCAGCACAGTTCCTGGGGGCTTTTGCTGGATCATGTGCC
GTCTAT---GGATTGTATTATGATGCTTTGATGGACTTTACCAAA---GGAGAATTTATT
GTTACAGGTGAAAAT---GCCACAGCCACCATATTTGCAAGTTATCCTGCAAAACATCTG
TCTGTCCTAAACGGGCTTGGTGATCAGGTAATTGCAACCGCTGCACTGGTCATATGCATC
CTGGCTATCACTGACAGGAAGAATATTGGCGCTCCAAAGGGCATGGAGCCTCTGTGCATT
GGCCTGATCATCGCGGCCATTGGAGTGTCCATGAATCTGAACTGTGGCTATCCAATCAAC
CCGGCTCGAGACCTC---GGCCCCCGTTTCTTCACGGCTTTGGCCGGATGGGGCATGGAA
GTTTTC---AGG------GCTGGAGGCTGCTGGTGGTGGATTCCTGTGGTAGGACCCATG
GTGGGTGGCGCAGTTGGAGCAGGTGTTTACCTTATCTTCATTGAGCTGCACCACCCTGAA
---------------CCTGAAAAACAA---------------------------------
---------------------GAGGAGAAC-----------------------AAT---
---------GTCCAG---------GACAAA------------TATGAGATTGTAACTATG
ACT---------------------------------------------------------
------------------------------------------------------------
------------------------------------------------------------
---------------------
>Mummichog_Aqp9b
---------------------------------------------------ATGGAA---
GATGAG---AGG---AGGAGGAAGATGAAGGAGAAATTT------GGCCTGAGGCGGGAC
GTCCTGAAGGAGTTCCTGGCGGAGTTTCTGGGGATCTTTGTCCTGATACTCTTTGGATGC
GGTTCAGTGGCCCAGACGGTGCTGAGCAAA------GGGGCTCTGGGAGAGCCGCTCACC
ATCCACATCGGTTTCACCCTGGGAGTCATGATGGCCGTCTACATGGCGGGGGGAGTGTCA
GGAGCCCATGTGAACCCGGCCGTCTCTTTGGCCATGGTGATTCTGGGGAAGCTACCTGTG
AAGAAGTTCCCGGTGTATGTGGTGGCACAGTTCCTCGGAGCATTTGCTGGCTCCTGTGCT
GTCTTT---GGGTTGTATTACGATGCTTTGATGGAGTACACCGGT---GGGGTGCTTGCA
GTTACTGGTGAAAAT---GCAACGGCCAACATATTTGCCTCTTATCCTGCAAAACATCTC
TCGGTGCTAAATGGATTTGTTGACCAGGTCATTGCAACCGGCGCTCTGATTCTGTGCATC
CTGGCCATCACTGACAGGAAGAACATCGGCGCCCCAAAAGGCATGGAGCCGCTGTGCATC
GGCTTGATCATCATGGCCATCGGCGTCTCCATGGGTCTGAACTGCGGCTATCCCATCAAC
CCGGCGCGAGACCTC---GGCCCACGGTTCTTCACTGCCGTGGCCGGGTGGGGCATGGAG
GTGTTC---AGG------GCCGGAGACTGCTGGTGGTGGATCCCTGTGGCGGGACCGATG
GTGGGTGGAGCGGTTGGAGCTGGGATTTACTTTCTCTTCATTGACTTGCACCAACCTGAG
```

```
---------------CCTCAGAAACAC---------------------------------
---------------------GAGGACAAC------------------------AAC---
---------GTTCAG---------GACAAA------------TATGACATCATTACTATG
ACT---------------------------------------------------------
------------------------------------------------------------
------------------------------------------------------------
---------------------
>Zebrafish_Aqp9b
---------------------------------------------------ATGGAGTAT
CTCGAG---AAC---ATCCGAAATCTGAGGGGGAGATGC------GTCCTGAGGCGCGAC
ATCATCCGAGAGTTTCTGGCAGAATTACTCGGGACATTCGTGTTAATACTTTTCGGTTGC
GGTTCAGTGGCCCAGACTGTCCTCAGCAGA------GAAGCAAAAGGACAGCTTCTCACC
ATCCATTTTGGCTTTACTCTAGGGGTGATGCTGGCCGTCTACATGGCAGGAGGCGTGTCA
GGAGGACATGTGAACCCTGCTGTTTCTTTGGCTATGGTTGTCCTGAGGAAACTCCCACTA
AAGAAGTTCCCTGTGTATGTGTTGGCCCAATTTCTAGGTGCCTTTTTTGGGTCTTGTGCC
GTCTAC---TGTCTTTACTATGATGCCTTTACAGAATTTGCTAAT---GGAGAGCTAGCT
GTAACTGGCCCAAAT---GTCACAGCAGGTATCTTTGCATCATATCCACGTGAAGGACTC
TCATTGTTAAATGGATTCATTGATCAGGTGATTGGTGCAGGTGCCCTGGTCCTCTGTATT
TTAGCTGTTGTAGATAAGAAGAACATTGGAGCACCTAAAGGAATGGAGCCTCTGCTTGTC
GGTCTGAGCATCCTGGCTATTGGAGTGTCAATGGCACTAAACTGTGGATATCCTATAAAC
CCTGCCAGAGACTTG---GGACCTCGGCTGTTCACTGCCATTGCAGGATGGGGATTAACG
GTGTTC---AGT------GCTGGCAATGGCTGGTGGTGGGTTCCAGTGGTGGGGCCAATG
GTGGGCGGAGTGGTTGGTGCTGCTATCTACTTCCTGATGATCGAGATGCATCACCCTGAG
---------------AACGACAAGAACCTG---------------------GAA------
---------------------GACGACAAC------------------------AGC---
---------CTTAAA---------GACAAA------------TATGAGCTGAACACCGTC
AAC---------------------------------------------------------
------------------------------------------------------------
------------------------------------------------------------
---------------------
>Bowfin_Aqp9
---------------------------------------------------ATGGAG---
AGGAAA---AAT---AAGAGGAACCTGAAGGAGCGATTT------GCCATGAGAAACAGC
ATCGTCAAGGAGGCTTTGGCAGAGTTTCTGGGGACATTCGTGCTCATTCTCTTTGGCTGC
GGGTCGGTGGCCCAGACGGTTCTGAGCCGG------GGTGCCGTGGGAGAGGTGCTGACG
ATCCACATTGGCTTCACTCTGGGAGTGACAATGGCGGTGTATGTGGCTGGTGGAGTGTCA
GGGGCTCATGTTAACCCAGCAGTCTCCCTGGCCATGCTGGTCCTGGGCAAGCTGAAGCTG
GTGAAGTTCCCAGTGTACGTAGTGGCCCAGTTTCTTGGGGCGTTTGCTGGAGCGGCCGCC
GTGTAC---GGGCTCTACTATGATGCCTTTATGGATTATACCAAC---GGCATTCTGACT
GTTACTGGCCCCAAT---GCTACTGCACAGATTTTTGCTTCGTACCCAGGAAGACATCTG
TCAATCCTGAATGGCTTCATCGATCAGGTCATAGGTACTGGTGCCTTGGTTCTCTGTATT
CTGGCCATCTTGGACGGAAAGAACATTGGGGCCCCCAAAGGCATGGAGCCCCTGGTGATT
GGCCTGATCATCATGGCTATCGGCGTCTCCATGAACCTGAACTGCGGATACCCCATCAAC
CCCGCCCGTGACCTG---GGGCCGCGGCTTTTCACCGCAGTGGCAGGGTGGGGCACCGCT
GTCTTC---AGT------GCTGGCAATAACTGGTGGTGGATTCCCGTGGCCGGGCCCTTG
GTGGGGGGGATCGTGGGAGCTGTGCTGTACCTGCTCTTCATCGAGCTACACCACACTGAG
---------------CCGCAGAAACACTTG---------------------GAG------
---------------------GAGGAGAAC------------------------AAC---
---------GTCAAG---------GACAAG------------TACGAAATGATCACCATG
AGT---------------------------------------------------------
------------------------------------------------------------
------------------------------------------------------------
---------------------
>Gar_Aqp9
---------------------------------------------------ATGGAG---
AGGAAA---ACT---AGGAGGAACCTGCGGGAGCGCTTC------GCCCTGAAGAACAGC
ATTGTCAAGGAAGCCTTGGCAGAATTCCTCGGGACATTCGTACTCATCCTTTTCGGCTGT
GGGTCAGTTGCACAGACCGTTTTGAGTCGC------GGTGCCGTGGGAGAGATGCTGACC
ATCCACATCGGATTCACTCTGGGTGTGACCATGGCGGTGTACGTGGCCGGAGGAGTGTCA
GGGGCACACGTGAACCCCGCGGTGTCCCTCGCCATGCTGGTGCTGGGCAAGTTGAAGCTG
GTGAAGTTCCCGGTGTACGTGCTCGCACAGTTCCTCGGCGCCTTTGCCGGGGCTGCAGCC
```

```
GTGTAC---GGGCTGTATTACGATGCCTTTATGGATTACACCAAT---GGAATACTAATT
GTTACTGGTCCCAAC---GCTACAGCACATATCTTTGCATCATATCCGGGAAGACACTTG
TCAATCCTAAATGGATTCATAGATCAGGTCATAGGGACGGGCGCCCTGGTGCTTTGTATT
CTGGCCATTGTGGATGGGAAGAACAATGGGGCTCCCAAAGGAATGGAGCCTTTAGTGATT
GGGCTGATCATCATGGCTATTGGGGTCTCCATGGGACTGAACTGTGGGTACCCTATAAAT
CCTGCCCGGGACCTC---GGCCCACGCCTGTTCACTGCAGTGGCAGGATGGGGAAGAGAT
GTCTTC---AGC------GCTGGGAGGAACTGGTGGTGGATTCCAGTGTCTGGCCCGTTG
GTGGGCGGGGTTGTAGGAGCTGTGTTGTACCTGTTGTTCATTGAGCTGCACCACGCCGAG
---------------CCGCAGAAGCACCTG---------------------GAA------
---------------------GAAGAGAAC------------------------AGC---
---------GTCAAG---------GAAAAG------------TATGAAATGATA---ATG
AGC---------------------------------------------------------
------------------------------------------------------------
------------------------------------------------------------
---------------------
>Russian_sturgeon_Aqp9
---------------------------------------------------ATGGAA---
AATGAA---AAC---AAGAAGAGCATTAAAGAGAGGTTT------GCTCTGAGGAACAGC
CTGGTGAAGGAAGCATTAGCAGAATTCCTTGGGACATTTCTGCTCATTCTTTTCGGCGTC
GGGTCAGTTGCCCAAACTGTCCTGAGCAGA------GGTTCCATGGGAGATCCACTCACC
ATACACATTGGCTTTACACTCGCAGTCACCATGGCTGTGTATGTGGCGGGAGGAGTTTCA
GGCGCTCACATAAACCCTGCTGTCTCTCTGGCGATGTGTGTGCTGGGGAGGCTGAGCCTC
CGCAAATTCCCAGTCTACGCCGTGTCTCAGTTCCTTGGTGCTTTTATTGGCGCCGCAGCA
GTCTAT---GGACTTTATTATGATGCCTTCCTGGATTATACAGGT---GGAGTGCTGACA
GTTACTGGCCCCAAT---GCAACAGCACACATTTTTCATCCTACCCTGGTAAACATCTT
TCAATCTTAAATGGATTTATAGATCAGGTTATAGGAACTGGTGCATTGCTCCTCTGCATC
CTTGCCATCTTGGACAACAAGAACAAGGGCGCCCCGAAAGGAATGGAGCCCTTGATGATT
GGTCTCATCATCATGGTTATCGGGGTCTCCATGGGCCTGAACTGCGGCTATCCAATCAAC
CCAGCGCGTGACCTC---GGCCCCAGGCTCTTCACTGCCATAGCAGGGTGGGGCACTGAA
GTTTTC---AGC------GCTGGAAGCCACTGGTGGTGGATTCCTGTGGCTGGGCCGCTG
GTTGGAGGGATGGTCGGTGCTGTGATCTATGTGCTCTTCATCGAGCTTCACCACGCGGAC
---------------CCTCAGAAGACTCCA---------------------GAA------
---------------------GAAGAAAAC------------------------AGC---
---------ATGAAG---------GATAAA------------TACGAAATGATTGCAATG
AGC---------------------------------------------------------
------------------------------------------------------------
------------------------------------------------------------
---------------------
>Atlantic_sturgeon_Aqp9
---------------------------------------------------ATGGAA---
AATGAA---AAC---AAGAAGAGCATTAAAGAGAGGTTT------GCTCTGAGGAACAGC
CTGGTGAAGGAAGCCTTAGCAGAATTCCTTGGGACATTTCTGCTCATTCTTTTCGGCGTC
GGGTCAGTTGCCCAGACTGTCCTGAGCAGA------GGTTCCATGGGAGATCCACTCACC
ATACACATTGGCTTTACACTCGCAGTCACCATGGCTGTGTATGTGGCGGGAGGAGTTTCA
GGCGCTCACATAAACCCTGCTGTCTCTCTGGCGATGTGTGTGCTGGGGAGGCTGAGCCTC
TGCAAATTCCCAGTCTACGCCGTGTCTCAGTTCCTTGGTGCTTTTATTGGCGCCGCAGCA
GTCTAT---GGACTTTATTATGATGCCTTCCTGGATTTTACAGGT---GGAGTGTTCACA
GTTACTGGCCCCAAT---GCAACAGCACATATTTTTCATCCTACCCTGGTAAACATCTT
TCAATCTTAAATGGATTTATAGATCAGGTTATAGGAACTGGTGCATTGCTCCTCTGCATC
CTTGCCATCTTGGACAACAAGAACAAGGGCACCCCGAAAGGAATGGAGCCCTTGATGATT
GGTCTCATCATCATGGTTATCGGGGTCTCCATGGGCCTGAACTGCGGCTACCCAATCAAC
CCAGCGCGTGATCTC---GGCCCCAGGCTCTTCACTGCCATAGCAGGGTGGGGCACTGAA
GTTTTC---AGC------GCTGGAAGTCACTGGTGGTGGATTCCTGTGGCTGGGCCGCTG
GTTGGAGGGATGGCCGGAGCTGTGATCTATGTGCTCTTCATCGAGCTTCACCACGCGGAC
---------------CCTCAGAAGACTCCA---------------------GAA------
---------------------GAAGAAAAC------------------------AGC---
---------ATGAAG---------GATAAA------------TACGAGATGATTGCAATG
AGC---------------------------------------------------------
------------------------------------------------------------
------------------------------------------------------------
---------------------
```

```
>Chinese_sturgeon_Aqp9
-------------------------------------------------------ATGGAA---
AATGAA---AAC---AACAAGAGAATTAAAGAGAGGTTT------GCTCTGAGGAACAGC
CTGGTGAAGGAAGCCTTAGCAGAATTCCTTGGGACATTTCTGCTCATTCTTTTCGGCGTC
GGGTCAGTTGCCCAGACTGTCCTGAGCAGA------GGTTCCATGGGAGATCCACTCACC
ATACACATTGGCTTTACCCTCGCAGTCACCATGGCTGTGTATGTGGCGGGAGGAGTTTCA
GGCGCTCACATAAACCCTGCTGTCTCTCTGGCGATGTGTGTGCTGGGGAGGCTGAGCCTC
TGTAAATTCCCAGTCTACGCTGTGTCTCAGTTCCTTGGTGCTTTTATTGGCGCCGCAGCA
GTCTAT---GGACTTTATTATGATGCCTTCCTGGATTATACAGGT---GGAGTGCTGACA
GTTACTGGCCCCAAT---GCAACAGCACACATTTTTTCATCCTACCCTGGTAAACATCTT
TCAATCTTAAATGGATTTATAGACCAGGTTATAGGAACTGGTGCATTGCTCCTCTGCATC
CTTGCCATCTTGGACAACAAGAACAAGGGCGCCCCGAAAGGAATGGAGCCCTTGATGATT
GGTCTCATCATCATGGTTATCGGGGTCTCCATGGGCCTGAACTGCGGCTACCCAATTAAC
CCAGCACGTGATCTC---GGCCCCAGGCTCTTCACTGCCATAGCAGGGTGGGGCACTGAA
GTTTTC---AGC------GCTGGAAGCCACTGGTGGTGGATTCCTGTGGCTGGGCCGCTG
GTTGGAGGGATGGTCGGAGCTGTGATCTATGTGCTCTTCATCGAGCTTCACCACGCGGAC
----------------CCTCAGAAGACTCCA----------------------GAA------
----------------------GAAGAAAAC-------------------------AGC---
---------ATGAAG---------GATAAA------------TATGAAATTATTGCAATG
AGC---------------------------------------------------------
------------------------------------------------------------
------------------------------------------------------------
---------------------
>Sterlet_Aqp9_2
-------------------------------------------------------ATGGAA---
AATGAA---AAC---AAGAAGAGCATTAAAGAGAGGTTT------GCTCTGAGGAACAGC
CTGGTGAAGGAAGCATTAGCAGAATTCCTTGGAACATTTCTGCTCATTCTTTTCGGCGTC
GGGTCAGTTGCCCAAACTGTCCTGAGCAGA------GGTTCCATGGGAGATCCACTCACT
ATACACATTGGCTTTACACTCGCAGTCACCATGGCTGTGTATGTGGCGGGAGGAGTTTCA
GGCGCTCACATAAACCCTGCTGTCTCTCTGGCGATGTGTGTGCTGGGGAGGCTGAGCCTC
TGTAAATTCCCAGTCTACGTCGTGTCTCAGTTCCTTGGTGCTTTTATTGGTGCCGCAGCA
GTCTAT---GGACTTTATTATGATGCCTTCCTGGATTATACAGGT---GGAGTGCTGACA
GTTACTGGCCCCAAT---GCAACAGCACACATTTTTTCATCCTACCCTGGTAAACATCTT
TCAATCTTAAATGGATTTATAGATCAGGTTATAGGAACTGGTGCATTGCTCCTCTGCATC
CTTGCCATCTTGGACAACAAGAACAAGGGCGCCCCGAAAGGAATGGAGCCCTTGATGATT
GGTCTCATCATCATGGTTATCGGGGTCTCCATGGGCCTGAACTGCGGCTACCCAGTCAAC
CCAGCGCGTGATCTC---GGCCCCAGGCTCTTCACTGCCATAGCAGGGTGGGGCACTGAA
GTTTTC---AGG------GCTGGAAGCCACTGGTGGTGGATTCCTGTGGCTGGGCCGCTG
GTTGGAGGGATGGTCGGAGCTGTGATCTATGTGCTCTTCATCGAGCTTCACCACGCGGAC
----------------CCTCAGAAGACTCCA----------------------GAA------
----------------------GCAGAAAAC-------------------------AGC---
---------ATGAAG---------GATAAA------------TATGAAATGATTGCAATG
AGC---------------------------------------------------------
------------------------------------------------------------
------------------------------------------------------------
---------------------
>Sterlet_Aqp9_1
-------------------------------------------------------ATGGAA---
AATGAA---AAC---AAGAAGAGCATTAAAGAGAGGGTT------GCTCTGAGGAACAGC
CTGGTGAAGGAAGCCTTAGCAGAATTCCTTGGGACATTTCTGCTCATTCTTTTCGGCGTC
GGGTCAGTTGCCCAGACTGTCCTGAGCAGA------GGTTCCATGGGAGATCCACTCACT
ATACACATTGGCTTTACACTCGCAGTCACCATGGCTGTGTATGTGGCGGGAGGAGTTTCA
GGCGCTCACATAAACCCTGCTGTCTCTCTGGCGATGTGTGTGCTGGGGAGGCTGAGCCTC
TGCAAATTCCCAGTCTACGCCGTGTGTCAGTTCCTTGGTGCTTTCATTGGTGCCGCAGCA
GTCTAT---GGACTTTATTATGATGCCTTCCTGGATTATACAGGT---GGAGTGCTGACA
GTTACTGGCCCCAAT---GCAACAGCACACATTTTTTCATCCTACCCTGGTAAACATCTT
TCAATCTTAAATGGATTTATAGATCAGGTTATAGGAACTGGTGCATTGCTCCTCTGCATC
CTTGCCATCTTGGACAACAAGAACAAGGGCGCCCCGAAAGGAATAGAGCCCTTGATGATT
GGTCTCATCATCATGGTTATCGGGGTCTCCATGGGCCTGAACTGCGGCTACCCAGTCAAC
CCAGCGCGTGATCTC---GGCCCCAGGCTCTTCACTGCCATAGCAGGGTGGGGCACTGAA
GTTTTC---AGG------GCTGGAAGCCACTGGTGGTGGATTCCTGTGGCTGGGCCGCTA
```

GTTGGAGGGATGGTCGGAGCTGTGATCTATGTGCTTTTCATCGAGCTTCACCACGTGGAC
---------------CCTCAGAAGACTCCA---------------------GAA------
---------------------GAAGAAAAC------------------------AGC---
---------ATGAAG---------GATAAA------------TACGAAATGATTGCAATG
AGC---

>Reedfish_Aqp9
---ATGGAA---
---GCG---GAG---AATAAAAGTTTTAAGAAGAGAATT------GCTGTGAGAAGCAAA
ATCCTAAGGGAAGCGCTCGCAGAATTCCTTGGAACATTTATACTAATTATTTTGGCTGT
GGCTCAGTCGCCCAGACCGTCCTCAGCAGC------GGGGCTTTTGGTGAGATGCTGACT
ATTCACATTGGCTTCACTATTGGTGTCATAATGGCAGTATACGTGGCAGGGGGGATTTCA
GGTGCACATGTGAATCCTGCAGTTTCTCTTGCCATGTGTGTTACGGGCAGGTTGAGCTTC
TTGAAGTTCCCAGTCTATGTCCTGTCTCAGTTCCTTGGGGCTTTCACTGGTGCAGCAGCA
GTGTAT---GGACTCTACTATGACGCATTTCTGAACTACACAGGA---GGCGTGCTCACT
GTCACCGGCCCTAAT---GCAAGTGCACAGATATTTGCAACTTACCCTGCAACATATCTT
TCAATATTTAATGGCTTCATGGATCAGGTGGTAAGTACCGGAGCTTTGGTGCTCTGCATC
TTTGCCATCACTGACAAGAAGAACAACGGTGCCCCAAAAGGAATGGAGCCACTTCTCATT
GGTTTAATTATCATGGTAATTGGGGTCTCTATGGGCCTGAACTGTGGCTACCCCATCAAT
CCAGCTCGAGATCTT---GGCCCTCGCCTTTTTACCTTCATTGCAGGGTGGGGTGTTGAA
GTTTTC---AGG------GCTGGGAATAACTGGTGGTGGGTCCCTGTGGCAGGCCCAATG
GTTGGTGGGATTGTTGGTGCCTTGATCTACGTTGCATTTATTGAGCTGCACCATCACGAC
---------------CCACAGAAGCAATCT---------------------GAA------
---------------------GAAGATCAC------------------------AAT---
---------GTCCAA---------GATAAA------------TATGAGATGATTACAATG
AAC---

>B_bambooshark_Aqp9
---ATGGAGACA
CAGAGC---CAG---AAGCACAGCCTGATAGACAAATGC------AAACTAAAGAATAGA
TGGATTAAAGAAGGGCTCGCCGAATTTTTGGGAACATTTATACTGATTCTGTTTGGTTGT
GGTTCACTTGCCCAATCAATACTGAGCAGA------GGAGTATATGGCAACATGCTGACA
GTTGCCATTGGCTTTCCATTTGGAATCACCATAGCTGTTTATGCTACCATAGGAGTATCA
GGAGCACATCTGAATCCTGCTATTTCCCTTTCTATGTGTGTTCTTGGACGGCTTCAGTGG
TTGAAACTGCCGGTCTACTGTTTTTCTCAGCTTGTCGGGGCCTTTGTTGGTTCAGCAGCT
GTTTTT---GGACTTTACTATGATGCGTTCATGGCTTTTGATGAT---GGGAACTTCGCA
ATAACTGGTCAAAAT---GCAACAGCACAAATCTTCTCCTCTTATCCAGCCTCACATTTG
TCGTTTGCAAATGGCTTCGCAGACCAGGTAGTGGGTACTGCTGCCCTTCTCTTCTCCATT
CTGGCCATTTTAGACTCTAAGAATAATTGCGTCCCCAAAGGTTTGGAGCCAGTGGTGATT
GGCCTCATCATCATGGTCATTGGTCTTTCGATGGGGTACAACTGTGGTGGTCCCATCAAC
CCAGCTCGTGATCTT---GGACCACGACTCTTCACAGCCGTGGCTGGATGGGGACTGGAG
GTTTTC---ACG------GCTGGTAATGGATGGTGGTGGGTTCCTGTCATCGCACCACTG
ATTGGAGGAGTTCTCGGGACTGCCATTTATGTACTGATAATTGAGCTGCACCACATGGAC
---------------ACAACCCTGAAGGAG---------------------AAC------
---CACTGTACCTCAGAA---CAACAGGCG------------------------GAA---
---------GGGAAG---------GCAAAA------------TATGAAATGATTACAATA
CAGTGCAACAAA--

>W_bambooshark_Aqp9
---ATGGAGACA
CAGAGC---CAG---AAGCACAGCCTGATAGACAAATGC------AAACTAAAGAATAGA
TGGATTAAAGAAGGGCTCGCCGAATTTTTGGGAACATTTATACTGATTCTGTTTGGTTGT
GGTTCACTTGCCCAATCAATACTGAGCAGA------GGAGTATATGGCAACATGCTGACA
GTTGCCATTGGCTTTCCATTTGGAATCACCATAGCTGTTTATGCTACCATAGGAGTATCA
GGAGCACATCTGAATCCTGCTATTTCCCTTTCTATGTGTGTTCTTGGACGGCTTCAGTGG

TTGAAACTGCCGGTCTACTGTTTTTCTCAGCTTGTCGGGGCCTTTGTTGGTTCAGCAGCT
GTGTTT---GGACTTTACTATGATGCGTTCATGGCTTTTGATGAT---GGGAACTTCGCA
ATAACTGGTCAAAAT---GCAACAGCACAAATCTTCTCCTCTTATCCAGCCTCACATTTG
TCGTTTGCAAATGGCTTCGCAGACCAGATGATGGGTACTGCTGCCCTTCTCTTCTCCATT
CTGGCCATTTTAGACTCTAAGAATAATTGCGTCCCCAAAGGTTTGGAGCCAGTGGTGATT
GGCCTCATCATCATGGTCATTGGTCTTTCGATGGGGTACAACTGTGGTGGTCCCATCAAC
CCAGCTCGTGATCTT---GGACCACGACTCTTCACAGCCGTGGCTGGATGGGGACTGGAG
GTTTTT---ACG------GCTGGTAATGGATGGTGGTGGGTTCCTGTCATCGCACCACTG
ATTGGAGGAGTTCTCGGGACTGCCATTTATGTTCTGATAATTGAGCTGCACCACATGGAC
---------------ACAACCCTGAAGGAG---------------------AAT------
---CACTGTACCTCAGAA---CAACAGGCG------------------------GAA---
---------GGGAAG---------GCAAAA------------TATGAAATGATTACAATA
CAGTGCAACAAA--

>Whale_shark_Aqp9
---ATGGAGACA
CAGGCT---CAG---AAGCACAGCCTGATAGACAAATGC------AAATTAAAGAATAGA
TGGATCAAAGAAGGGCTCGCCGAATTTCTGGGAACATTTATACTGATTCTATTTGGTTGT
GGTTCACTTGCCCAATCAATACTGAGCAGA------GGAGTAAATGGCAACATGCTGACA
GTTGCCATTGGCTTTCCATTTGGAATCACCATAGCTGTTTATGCTACCATAGGAGTATCA
GGAGCACATCTGAATCCTGCTATTTCCCTTTCTATGTGTGTTCTTGGACGGCTTCAGTGG
TTGAAACTGCCAGTCTACTGCTTTTCTCAGCTTGTCGGGGCTTTTATTGGCTCAGCAGCC
GTTTTT---GGACTTTACTATGATGCGTTCATGGCTTTTGATGGT---GGGAACTTGGCA
ATAACTGGTCAAAAT---GCAACAGCACAAATTTTCTCCTCTTATCCAGCTCCGCATTTG
TCTTTTGCAAATGGCTTTGTAGACCAGGTAGTGGGTACTGCTGCTCTACTCTTCTCCATT
CTGGCTATTTTGGACTCTAAGAATGATTGCGTCCCCAAAGGTTTGGAGCCAGTGGTGATT
GGCCTCATCATCATGGTCATTGGTCTTTCGATGGGGTTCAACTGTGGTGGCCCCATCAAT
CCAGCGCGTGATCTT---GGACCACGACTCTTCACAGCTGTGGCTGGATGGGGATTTGAG
GTTTTC---ACG------GCTGGTGATGGATGGTGGTGGGTCCCTATCATCGCACCACTG
ATTGGAGGAGTTCTCGGGACTGCCATTTATGTTCTGATAATTGAGCTTCACCACAAGGAG
---------------ACAGCCCCAAAGGAG---------------------AAC------
---CACTGTACCTCAGAA---CAACAGATG------------------------GAG---
---------GGGAAG---------GCAAAA------------TATGAAATGACTTCAATA
CAGTGCAACAAA--

>Zebra_bullhead_shark_Aqp9
---ATGGAAACA
CAGAAG---CAG---AAACAGAGTCTGGTCGACAAATGC------AAATTAAAGAATAGC
TGGATTAAAGAAGGACTCGCCGAATTTCTGGGAACATTTATACTGATTCTGTTTGGTTGT
GGTTCACTTGCCCAATCAATACTGAGTAGA------GGAGTAAGTGGCAACATGCTGACT
GTATCCATTGGCTTTTCATTTGGAATCACCATAGCTGTTTATGCTACCATAGGAGTATCA
GGGGCACATCTGAATCCTGCTATTTCCCTCTCTATGTGTGTGCTGGGTCGGCTTCAATGG
TTGAAACTTCCCATCTACTGTGTTGCTCAGTTTGTCGGGGCCTTTGTTGGTTCAGCAGCA
GTTTTT---GGGCTTTATTATGATGCATTCATGGCTTTTGATGGT---GGGAACTTGGCA
ATAACTGGTCAAAAT---GCAACCGCGCAGATCTTCTCCTCTTATCCAGCTCCACATTTG
TCTTTTGCGAATGGCTTTGCAGACCAGATTGTGGGTGCTGCTGCACTGCTCTTCTCTATC
CTGGCCATTCTGGACTCGAAGAATGATTGTGTACCCAAAGGTTTGGAGCCAGTGGTGATT
GGCCTGATCCTCATGGTTATCAGTCTTTCAATGGGCTTCAACTGCGGTGGTCCTATAAAC
CCAGCTCGTGATCTC---GGACCCAGGCTCTTTACAGCTGTGGCTGGATGGGGATTTGAG
GTTTTC---AGG------GCTGGTGATGGATGGTGGTGGGTTCCTGTCATCGCACCAATG
ATTGGCGGAGTTCTCGGGACGGCCATTTACGTTCTGATTATTGAGCTGCACCATAAAGAC
---------------ATAACGCCAAAGGAG---------------------ATC------
---CGCTGTACTTCAGAACCCCCGCAAAGT------------------------GAA---
---------GGGAAG---------GCAAAA------------TATGAAATGATTTCAGTA
CAATGTAACAAA--


```
---------------------
>Cloudy_catshark_Aqp9
---------------------------------------------------ATGGAAACA
GAACCC---CAG---AAACACAGCCTGACAGACAAATAC------AAGTTAAAGAACAGC
TGGATAAAACAAGGGCTCGCCGAGTTCGTGGGGACCTTTATGTTGATTCTCTTTGGTTGC
GGTTCACTTGCCCAATCAGTACTGAGCAGA------GGCGTTAGTGGCAACATGCTGACT
GGTTCCATTGGCTTCTCATTTGGACTCACCATAGCTGTTTATGCTACCATAGGAGTGTCA
GGGGCACATCTGAATCCTGCTATTTCCCTTTCCATGTGTGTGTTGGGAAGGCTCCAATGG
TTGAAACTGCCTGTCTACTGCATTGCTCAGTTTATTGGAGCCTTTGTCGGTTCAGCGACA
GTCTTT---GGGCTTTATTACGATGCATTCATGGCTTTTGATGGC---GGAAACTTGGCA
ATAACGGGCCAAAAT---GCAACAGCGCAGATTTTCTCCTCTTATCCAGCTCCACATTTA
TCCTTTGCGAATGGCTTTGCAGACCAGATCATAGGTACGGCAGCTCTGCTCTTCTCCATT
CTGGCCATCTTGGACTCGAAGAATGACGGTGTACCCAAGGGTTTGGAGCCGGTGGTCATT
GGCCTGATCATCATGGTCATCGGTTTTTCAATGAGCTTCAACTGTGGTGGTCCTATCAAC
CCAGCTCGTGACCTT---GCGCCGCGGCTCTTCACAGCCATGGCGGGGTGGGGATTTGGG
GTATTC---AGG------GCCGGCGACGGGTGGTGGTGGGTTCCTGTCATCGCACCAATG
ATCGGCGGAGTTGTTGGGACTCTTGTTTATGTCCTGATCATCGAGCTGCACCACAACGAG
---------------ACAGTCCCAAAGGAG---------------------AGG------
---TCCTGTACCTCAGAA---CAGCAACTG------------------------GAG---
---------GCGAAG---------GCAAAA------------TACGAAATGATTTCTGTG
CAGTGTAACAAA------------------------------------------------
------------------------------------------------------------
------------------------------------------------------------
---------------------
>Great_white_shark_Aqp9
---------------------------------------------------ATGGAAACG
CAGATT---CAG---AAGCACAGCCTGATAGACAAGTGC------AAATTAAAGAACAGC
TGGATAAAACAAGGGCTCGCCGAATTTCTGGGGACATTTGCATTGATTCTGTTTGGTTGT
GGTTCAGTTGCCCAAGCAGTATTGAGCAGA------GGAGTAAGTGGCAACCTGCTGACT
GTTTCCATTGGCTTTCCATTTGGAATCACCATAGCTGCTTATGCTACCATAGGAGTATCA
GGGGCACATCTGAATCCTGCTATTTCCTTTTCTATGTGTGTGCTGGGACGGCTTCAATGG
TTGAAACTGCCTGTCTACTGCCTTGCTCAGCTTGTAGGAGCCTTTGCTGGTTCAGCAGCA
GTTTTT---GGGCTTTATTATGATGCATTCATGGCTTTTGATGGT---GGGAACTTGGCA
ATAACTGGTGAAAAT---GCAACAGCGCAGATTTTCTCCTCTTATCCAGCTCCACATTTG
TCTCTTGCAAATGGCTTTGCAGACCAGATCGTGGGCACTGCTGCTCTGCTCTTCGCTATC
CTTGCCATTTTGGACTCCAAGAATAATGCAGTGCCCAAAGGTTTGGAGCCAGTGGTGATT
GGCCTGATCATCATGGTTATCAGTCTTTCAATGGGCTTCAACTGTGGCTGTCCTATCAAC
CCAGCTCGTGACCTT---GGGCCGCGGCTCTTCACAGCTGTGGCTGGATGGGGATTTGAG
GTTTTC---AGG------GTCAGCGATGGGTGGTGGTGGGTTCCTGTCATCGCACCAATG
ATCGGTGGAGTCCTTGGGACCGCCATTTATGTTCTGACAATCGAGCTGCACCATGAAGAG
---------------ACAACTCCAAAGGAG---------------------AGC------
---CACACTACCTCAGGA---CAGCAAGTG------------------------GAA---
---------GGGAAG---------GCAAAA------------TATGAAATGATTTCAGTG
CAGAGTAACAAA------------------------------------------------
------------------------------------------------------------
------------------------------------------------------------
---------------------
>Spiny_dogfish_Aqp9
---------------------------------------------------ATGGAGCCG
CAGATC---CAG---AAGAAGAGTCTGATAGACAGATGC------AAATTAAAGAACCGC
TTCCTAAAAGAAGGGCTGGCCGAATTTTTGGGAACATTCATATTGATTCTGTTTGGTTGT
GGTTCACTCGCCCAATCAATACTGAGCAGA------GGAGTAAGTGGTAATATACTGACC
TCTTCCATTGGCTTTCCATTTGGAATTACCATAGCTGTTTATGCTACAATGGGAGTTTCA
GGGGGACACCTGAATCCTGCGATTTCCCTTTCTATGTGTGTGCTGGGACGGCTTCAATGG
TTGAAACTTCCTGTCTACTGTGTTGCTCAGCTTTTCGGAGCCTTTATCGGTTCAGCGGCA
GTTTTT---GGGCTTTATTACGATGCCTTCATGGCTTTCGATGAC---GGGAACTTGACA
ATAACCGGTCAAAAT---GCAACAGCGCAGATTTTCTCCTCTTATCCAAGTCCACATTTA
TCATTTGCGAATGGCTTTGCAGATCAGATTGTGGGCGCTGCTGCACTGCTCTTCTCTATT
CTGGCCATTTTGGACTCTAAGAATGACGGTGTACCCAAAGGTTTGGAGCCAGTGGTGATT
GGCCTGATCGTCATGGTTATCGGTGTTTCAATGGGCTACAACTGTGGTTGTCCCATAAAC
CCAGCTCGTGACCTT---GGACCCCGGCTCTTTACAGCTGTGGCCGGATGGGGATTTGAG
```

GTTTTC---AGT------GCGGGCGATGGCTGGTGGTGGGTTCCTGTCATCGCACCAATG
ATTGGAGGAATGCTCGGGACTTCCATTTATGTTCTGATAATTGAGCTGCACCACAAAGAG
---------------TCGATCCCAGAGGAG---------------------AGC------
---CGCTGTATTCCAGAA---CCGCAAATC------------------------GAA---
---------GGGAAG---------GCAAAA------------TATGAAATGATTTCAATA
CAGTGTAACAAA--

>Little_skate_Aqp9
---ATGGAAGCT
CTGGTC---CAG---AAGAAGTCTTTGGTGGATAAATGC------AAGCTGAAGAACAGC
TGGATCAAAGAAGGGCTCGCCGAATTTCTCGGGACATTTATTCTAATTTTGTTTGGTTGC
GGTTCTATAGCTCAGTCAGTGCTGAGCAGA------GGAGCGAGTGGCAATATGCTGACC
TCTTCTATTGGTTTTCCATTTGGTGTTACGATCGGTGTTTATGCAACAATAGGGGTTTCA
GGGGCGCACCTGAATCCTGCAATTTCGCTGTCAATGTGTGTACTGGGCCGTCTTCAGTGG
TTGAAACTTCCCATCTACTGCACTGCTCAGCTCATAGGAGCTTTTATTGGTGCCGCAGCA
GTATTT---GGTCTTTATTATGATGCCTTCATGAGTTATGATGGC---GGAAACTTGACA
ATCACCGGTCAAAAT---GCAACTGCACACATCTTCTCCTCTTATCCTGCTCCACATCTA
TCCTTTGCAAATGGATTT------CAGATTGTGGGCACGGCCGCGCTACTTTTCTCCATT
TTGGCCATTTTGGACTCCAAGAACGATCGTGTGCCCAAGGGTCTGGAGCCGGTGGTGATC
GGTCTGATCCTCATGGTGATCGGACTATCGATGGGATACAACTGCGGTGGTCCCATCAAC
CCAGCGCGTGACCTG---GGTCCTCGGCTCTTCACTGCTGTGGCCGGATGGGGAATTGAG
GTTTTC---AGG------GCTGGTAATGGCTGGTGGTGGGTCCCTGTCATTGCGCCTTTC
ATCGGGGGAGTCCTGGGCACTGCCATTTACGTTCTGTTCATTGAACTACATCACAAAGAG
---------------ACGGTCTTGGAGGAG---------------------GCC------
---CGGTGCGTTAAAGAA---CAGCAGGCG------------------------GAG---
---------GGGAAA---------ACAAAA------------TACGAAATGATTGATATA
CAGATAAACAAA--

>Winter_skate_Aqp9
---ATGGAAGCT
CTGGTC---CAG---AAGAAGTCTTTGGTGGATAAATGC------AAACTGAAGAACAGC
TGGATCAAAGAAGGGCTCGCCGAATTTCTCGGGACATTTATTCTAATTTTGTTTGGTTGC
GGTTCTATAGCTCAGTCAGTGCTGAGCAGA------GGAGCGAGTGGCAATATGCTGACC
TCTTCTATTGGTTTTCCATTTGGTGTTACCATCGGTGTTTATGCAACAATAGGGGTGTCA
GGGGCGCACCTGAATCCTGCAATTTCGCTGTCAATGTGTGTACTGGGCCGGCTTCAGTGG
TTGAAACTTCCCATCTACTGCACTGCTCAGCTCATAGGAGCTTTTATTGGTTCTGCAGCA
GTATTT---GGCCTTTATTATGATGCCTTCATGAGTTATGATGGC---GGAAACTTGACA
ATCACCGGCCAAAAT---GCAACTGCACACATCTTCTCCTCTTATCCTGCTCCACATCTA
TCCTTTGCAAATGGATTTGCCGACCAGATTGTGGGCACGGCCGCGCTACTTTTCTCCATT
TTGGCCATTTTGGACTCCAAGAACGATCGTGTGCCCAAGGGTCTGGAGCCGGTGGTGATC
GGTCTGATCCTCATGGTGATCGGACTATCGATGGGCTACAACTGCGGTGGTCCCATCAAC
CCAGCGCGTGACCTG---GGTCCTCGGCTCTTCACTGCTGTGGCCGGATGGGGAATTGAG
GTTTTC---AGA------GCTGGTAATGGCTGGTGGTGGGTTCCTGTCATTGCGCCTTTC
ATCGGGGGAGTCCTGGGCACTGCCATTTACGTTCTGTTCATTGAACTACATCACAAAGAG
---------------ACGGTCTTGGAGGAG---------------------GCC------
---CGGTGCGTTAAAGAA---CAGCAGGTG------------------------GAG---
---------GGGAAA---------ACAAAA------------TATGAAATGATTGATATA
CAGATAAACAAA--

>Thorny_skate_Aqp9
---ATGGAAGCT
CAGATC---CAG---AGGAAGACTTTGGTGGATAAATGC------AAACTGAAGAACAGC
TGGATCAAAGAAGGGCTCGCCGAATTTCTGGGGACATTTATTCTAATCTTGTTTGGTTGC
GGATCTATAGCTCAGTCAGTGCTGAGCAGA------GGAACGAGTGGCAATATGTTGACC
TCTTCTATTGGTTTTCCATTTGGAGTTACCATCGGTGCTTATGCGACAATAGGGGTGTCA

GGGGCGCACCTGAATCCTGCAATTTCGCTGTCAATGTGTGTACTGGGCCGGCTTCAGTGG
TTGAAACTTCCCATCTACTGCACTGCTCAGCTCATAGGAGCTTTTATTGGTTCCGCAGCA
GTATTT---GGCCTTTATTATGATGCCTTCATGAGTTATGATGGC---GGAAACTTGACA
ATCACCGGTCAAAAT---GCAACTGCACACATCTTCTCCTCTTATCCTGCTCCACATCTA
TCCTTTGCAAATGGATTTGCGGACCAGATTGTGGGCACGGCCGCTCTACTTTTCTCCATT
TTGGCCATTTTGGACTCCAAGAACGATCGTGTGCCCAAGGGTCTGGAGCCGGTGGTGATC
GGTCTGATCCTCATGGTGATCGGACTATCGATGGGCTACAACTGCGGTGGTCCCATCAAC
CCAGCACGTGACCTG---GGTCCTCGGCTCTTCACTGCTGTGGCCGGATGGGGATTTGAG
GTTTTC---AGG------GCTGGTAATGGCTGGTGGTGGGTCCCTGTCATTGCGCCTTTC
ATCGGGGGAGTCCTGGGCACTGCCATTTACGTTCTGTTCATTGAACTACATCACAAAGAG
---------------ACGGTCTTGGAGGAG---------------------GCC------
---CGGTGCGTTAAAGAA---CAGCAGGCG------------------------GAG---
---------GGGAAA---------ACAAAA------------TATGAAATGATTGATATA
CAGTGTAACAAA--

>Smalltooth_sawfish_Aqp9
---ATGCAATCA
CAGATC---CAG---AAGAAGAGTTTGGTAGATACATGC------AAACTCAAGAACAGC
TGGATAAAAGAAGGGCTCGCCGAATTTTTGGGAACATTTATTCTGATTCTGTTTGGTTGT
GGGTCTATCGCCCAGTCAGTGCTGAGCAGA------GGAGTAAGTGGCAACATGTTGACT
TCTTCTATTGGCTTTCCATTTGGCGTTACCATTGCTGTTTATGCTACAATAGGAGTGTCA
GGGGGACACCTGAACCCTGCTATTTCACTGTCAATGTGTGTGCTGGGGCGGCTTCAGTGG
CTGAAACTTCCTGTCTACTGCTTCGCTCAGTTCGTCGGAGCTTTTATTGGTTCAGCGGCA
GTTTTT---GGTCTTTATTATGATGCCTTCATGACTTTTGATGGT---GGGAACTTGACA
ATAACTGGTCAAAAT---GCAACAGCGCAGATTTTCTCCTCTTATCCAGCTCCACATCTG
TCCTTTGTAAATGGATTTGCAGACCAGATTGTGGGCACAGCCGCACTGCTTTTCTCCATT
TTGGCCATCTTGGACTCCAAGAACAATCGTGTTCCCAAAGGTCTCGAGCCAGTAGTGATT
GGTCTGATCATTATGGTTATCAGTCTTTCAATGGGCTTCAACTGCGGTGGTCCCATAAAT
CCAGCTCGTGACCTG---GGACCACGTCTCTTCACTGCTGTAGCCGGATGGGGAATTGAG
GTTTTC---AGG------GCTGGTGACGGCTGGTGGTGGGTTCCCGTCATTGCACCCTTG
ATCGGGGGCATCCTGGGCGCTTCCATATATATTCTGTTCATTGAACTTCACCACAAAGAA
---------------AAGGTCCTAGAGGAG---------------------GTC------
---CGCTGTACTAAAGAG---CAGCAAGCC------------------------GAG---
---------GGAAAG---------TCAAAA------------TATGAAATGATTTCTGTA
CACTGTAACAAA--

>Ghost_shark_Aqp9
---ATG------
GAGAAA---CAA---AAGACAAGTTGGAAAGAGAAATGT------AAATTAAAGAACGGT
TTGTTAAAAGAAGGGCTTGCAGAGTGCTTCGGGACATTCATACTAATTCTCTTTGGTTGT
AGCTCAAATGCCCAAACAGTGCTGAGCAGA------GGAGTGAATGGTAACATAATTACC
TCTTCCGTTGGCTTTTCATTTGGAATAACCATTGCTGCTTATGCAACAATAGGAGTATCA
GGGGCACACCTGAATCCTGCTATTTCTCTTTCTATGTGTGTGCTGGGTCAGCTTCAATGG
TTGAAGTTTCCAATTTACTGCATTGCTCAGTTTGTCGGAGCCTTTGTGGGTTCAGCAGCT
GTCTTT---GGGCTCTATTATGACGCTTTCATGGCATTCGATGGT---GGAAACCTGACA
ATTACCGGTGAAAAT---GCGACAGCACAAATCTTCTCTTCTTACCCGTCTCCGCATTTA
TCCTTCGTGAATGGCTTTGCAGACCAGATAGTTGGCACAGCTGCACTGCTCTTCTCCGTG
CTAGCCATTCTGGACTCCAAAAACGACAGGGTGCCGAAAGGCTTGGAGCCGGTGGTGATT
GGAATCATCATTTTGGTCCTCGGCTGTTCAATGAGCTTCAACTGCGGTGGCTCCATCAAT
CCAGCTCGCGACCTT---GGGCCACGCCTCTTCACGGCAGTGGCAGGATGGGGGCTGGAG
GTGTTC---AGG------------GATGGCTGGTGGTGGGTTCCTGTCTTGGCACCGATG
ATTGGTGGAGTTGTCGGCACTTCATTTTACCTTCTCATCATTGAACTCCACCACAAGGAA
---------------TCAGTCACTGAGCAG------------------------------
------TGTGCTATAGAA---CCACAACAA------------------------TCC---
---------CCGAAG---------AAAAAC------------TACGAACTGGTTGCCATA
AAGGGCAACATT--

>Arctic_lamprey_Aqp9_13L1
ATG------TGC--TCCCCCGG
CAGCTC---CACGCTCCGACGCGCCTGGCGGCCCGGATC------AAGGCATCGAACCCG
ATCGTGAGGGAGGCTCTGGCTGAGTTCCTCGGCACGTTCGTGCTCATCGTGTTCGGATGT
GGTTCCGTGGCTCAGGTAGAACTGAGCCAC------CACTCCGCCGGAGAGACTCTCACC
ATCAACCTCGCCTTCGCATTTGGGGTGGTCATGGGCGCTTACCTGTCCTGGGGCATCTCA
GGTGCACACCTGAACCCCGCCGTCTCGTTCTCCATGTCGCTGATCGGACGCTTCCACTGG
TGGAAGCTGCCCATTTTCTGCCTCGCGCAGTTCCTGGGCGCGTTCACAGCGGCCGCCACC
GTCTAC---GGCCTCTACCACGAGGCCCTCATGGCGTTCAACGGT---GGGAACCTGACT
GTGACTGGCCCCGGT---GCCACGGCCGCGATATTCGCCACCTATCCCTCGGAACACCTG
TCCATCGCCGGTGGCTTCCTTGACCAGGTGCTGGGCACGGCGCTCCTCCTGCTGTGCGTG
ATGGCGCTGCTGGACCCCAAGAACAACGCGGTGCCGCGCGGCCTCGAGCCACTGCTCGTG
GGGCTCGTGGTGCTCGTCATCGGCCTCTCCATGGGCTTCAACGCCGGCTATGCCATCAAC
CCCGCGCGCGACCTC---GGCCCCCGCGTCTTCACCGCCATGGTCGGATACGGCAGCGAA
GTGTTC---ACG------ACGGGCCCCCACTGGTGGTGGGTTCCCGTGGTGGCCCCCCTC
GTGGGGGGCCCGCTGGGCACAATGTGCTACATGTTCTTCGTGGACCTGCACCACCCCGCC
---------------CCGCCGGAGCTGTCCACCGGCAAAGCCAAGCCGCTGGTG------
------------GACAGC---GACAGCGAC------------------------CAC---
---------CCCAAG---------GCCAAC------------TACGCCGTGGCGGTGAAG
ATGGAGCAGCAGGCA---

>Sea_lamprey_Aqp9_13L1
ATG------TGC--TCCCCCGG
CACCTC---CACGCTCCGCCGCGCCTGGCGGCCCGGATC------AAGGCGTCAAACCCG
ATCGTGAGGGAGGCTCTGGCTGAGTTCCTCGGCACGTTCGTGCTCATCGTGTTCGGATGT
GGTTCCGTGGCTCAGGTGGAGCTGAGCCAC------CACTCCGCCGGAGAGACTCTCACC
ATCAACCTCGCCTTCGCATTTGGGGTGGTCATGGGCGCTTACCTATCCTGGGGCATCTCA
GGTGCACACCTGAACCCCGCCGTCTCCTTCTCCATGTCGCTCATCGGACGCTTTCACTGG
TGGAAGCTGCCCATTTTCTGCCTCGCTCAGTTCCTGGGCGCGTTCACAGCAGCCGCCACC
GTCTAT---GGCCTCTACCACGAGGCCCTCATGGCGTTCAACGGT---GGGAACCTGACG
GTGACCGGCCCCGGC---GCCACGGCCGCGATCTTCGCCACCTATCCCTCGGAACACCTG
TCCATCGCCGGTGGCTTCCTTGACCAGGTGCTGGGTACGGCGCTCCTCCTGCTGTGCGTG
ATGGCGCTGCTGGACCCCAAGAACAACGCGGTGCCGCGCGGCCTCGAGCCACTGCTCGTG
GGGCTCGTGGTGCTCGTCATCGGCCTCTCCATGGGCTTCAACGCCGGCTATGCCATCAAC
CCCGCGCGCGACCTC---GGCCCCCGCGTCTTCACCGCCATGGTCGGATACGGCAGCGAA
GTGTTC---ACG------ATGGGGCCCCACTGGTGGTGGGTCCCCGTAGTGGCCCCCCTC
GTGGGGGGCCCGCTGGGCACAATGTGCTACATATTCTTCGTGGACCTGCACCACCCCGCC
---------------CCGCCGGAGCTGTCCACCGGCAAAGCCAAGTCGCTGGTG------
------------ACCAGC---GACAGCGAG------------------------CAC---
---------GCCAAG---------GCCAAC------------TATGCCGTGGTGGTGAAG
ATGGAGCAGCAGGCA---

>Arctic_lamprey_Aqp9_13L2
--ATGGGCTCC
CCAGGG---CCG---CTCTACTCGCGCCTGTCGCGGGTC------AAAGTGTCCAATCCC
GTGCTGCGAGAAGCCCTCGCGGAGTTCATCGGCGTCTTTATTCTCATCCTGTTCGGCTGC
GGCTCAGTGGCCCAGGTGCATCTGAGCCGC------CACTCCGCCGGGCAGACTCTCGGC
ATCAACTTCGCCTTCGCCTTCGGGGTGGTCATGGGGGCCCACCTGGCCTGGGGAGTCTCG
GGTGCGCACCTGAACCCCGCCGTCTCGCTCTCCATGTCGCTGCTCGGCCGCTTACCCTGG
AGGAAGCTCCCCGTGTTCTGCCTCGCGCAGTTCCTGGGCTCGTTCGTGGCGGCTGCCACC
GTCTAC---GGCCTCTACTACGATGCCCTCATGGCGTTCAACGGC---GGGAACCTGACG
GTGACCGGCCCCGGT---GCCACGGCCGCGATCTTCGCCACTTATCCCTCGGAACACCTG
TCCATCGCCGGCGGCTTCTTTGACCAGGTGCTGGGCACGGCGGCGCTGCTGCTGTGCATC
ATGGCACTCCTCGATTCCAAGAACAACGCCGTGCCGCGCGGCCTGGAGCCGCTGCTCATC
GGCCTGGCGGTGTTCGGCATCAGCCTCTCCATGGCCTTCAACTCGGGGTGCGCCATCAAC

CCCGCGCGAGACTTC---GGCCCCCGCCTCTTCACCGCCATGGCGGGATACGGCAGCGAG
GTGTTC---ACG------GTGGGCCCCCATTGGTGGTGGGTCCCCATCATCGCCCCCCTC
GTGGGGGGCCCGCTGGGTGCACTGTGCTACATTTTGTTCGTGGACCTGCATCACATCGCC
----------------CCGCCGGAGCTGCCCCCAATGGCGCTGGAGCCGCTGGTG------
------------TCCGAC---GAGAACCAC------------------------CAC---
---------AGCAAG---------GGCAGC------------TACGTCGCCGTG---AAG
ACGGGGCAGCAGCCA---

>Sea_lamprey_Aqp9_13L2
---ATGGGCTAC
CCAGGG---CCG---CTCTACTCCCGCCTGTCGCGGGTC------AAAAGTGTCAATCCG
TGTTGCGAGAAGCTCTCGCGCGAGTTCATCGGCGTCTTTATTCTAATCCTGTTCGGCTGC
GGTTCAGTGGCCCAGGTGCATCTGAGCCGC------CACTCCCCGCGGCAGACTCTTGGC
ATCAACTTCGCCTTCGCCTTCGGGGTGGTCATGGGGGCATACCTCGCCTGGGGG------
GGTGCGCACCTGAACCCCGCCGTTTCGCTCTCCATGTCGCTGCTCGGCCGCTTACCCTGG
AGGAAGCTCCCCGCGTTCTGCCTCGCTCAGTTCCTCGGCTCGTTTTGGCGGCTGCCACC
GTCTAC---GGCCTCTACTACGAGGCCCTCATGGCGTTCAACGGT---GGGAACCTGACG
GTGACCGGCCCCGGC---GCCACGGCCGCGATCTTCGCCACCTACCCCTCTGAACACCTG
TCCATCGCCGGTGGCTTCTTTGACCAGGTGCTGGGCACGGCGGCGCTGCTGCTGTGCATC
ATGGCGCTCCTCGACTCCAAGAACAACGCCGTGCCGCGCGGCCTGGAGCCGCTACTCATC
GGCCTGGCGGTGTTCGGCATCAGCCTCTCCATGGCCTTCAACTCCGGGTGTGCCATCAAC
CCCGCGCGAGACTTC---GGCCCCCGCCTCTTCACTGCCATGGCGGGATACGGCAGAGAA
GTGTTC---ACG------GTGGGCCCCCACTGGTGGTGGGTCCCCATCATTGCCCCCCTC
ATGGGGGGCCCGCTGGGTGCCCTGTGCTACATATTATTCGTGGACCTGCATCACCTCGCC
----------------CCGCCGGAGCTGCCCGCAATAGTGCTGGAGCCGCTGGTG------
------------TCCGAC---GAGAACAAC------------------------CAC---
---------AGCAAG---------GGCAGC------------TACGTCACAGTG---AAG
ACGGAG--

>Human_AQP10
---ATGGTCTTC
ACTCAG---GCC---CCGGCTGAAATCATGGGCCACCTC------CGGATACGCAGCCTC
CTGGCCCGGCAGTGCCTGGCAGAGTTTCTGGGTGTGTTTGTACTCATGCTCCTCACCCAA
GGAGCTGTGGCCCAGGCTGTCACCAGTGGA------GAAACCAAAGGCAACTTCTTCACC
ATGTTTCTGGCTGGCTCTCTGGCCGTTACGATAGCCATCTACGTGGGTGGTAACGTCTCA
GGGGCCCACCTGAATCCAGCCTTCTCCCTGGCCATGTGCATCGTTGGACGCCTCCCCTGG
GTCAAGCTCCCCATTTACATCTTGGTGCAGTTGCTGTCTGCTTTCTGTGCTTCGGGAGCC
ACCTAT---GTTCTCTACCATGATGCCCTACAGAACTATACAGGT---GGGAACCTGACA
GTGACTGGCCCCAAG---GAGACAGCCTCCATTTTTGCCACCTATCCTGCCCCCTATCTG
TCCCTGAACAATGGCTTCCTGGATCAGGTTCTGGGCACTGGGATGCTGATTGTGGGGCTC
TTGGCCATCCTGGACAGACGGAACAAGGGAGTCCCTGCGGGTCTGGAGCCTGTGGTGGTG
GGGATGCTGATCCTGGCCCTCGGGTTATCCATGGGTGCCAACTGCGGGATTCCACTCAAC
CCTGCCCGGGACCTG---GGCCCACGTCTCTTCACCTACGTGGCTGGCTGGGGTCCTGAA
GTCTTC---AGT------GCTGGTAATGGCTGGTGGTGGGTGCCTGTGGTGGCCCCTCTG
GTGGGGGCCACCGTTGGCACAGCCACTTACCAGCTGTTGGTGGCTCTGCACCACCCTGAG
----------------GGCCCAGAGCCAGCT---------------------CAG------
------------GATCTG---GTGTCTGCT------------------------CAA---
------------CAC---------AAAGCC------------TCAGAGTTGGAAACTCCT
GCCTCAGCTCAGATGCTGGAGTGTAAGCTA------------------------------

>Elephant_AQP10
---ATGACCTAC
ACTCAG---CTC---CTGGCCAGATTTGGGGGTCGGTTC------CAAATTCGTAGTGTC
CTGGCCCGACAGTGCCTAGCAGAGTTTCTCGGTGTGTTTGTGCTCTTGCTCCTCACTCAA
GGGGCTGTAGCCCGGGCTGTCACCAGTGGA------GAAACCAAAGGCAACCTCTTCACC

```
ATGTTTCTGGCTGGCTCTTTGGCTGTTACCATAGCCATCTACGTGGGTGGCAATGTCTCA
GGGGCCCACCTGAATCCAGCCTTCTCTCTGGCCATGTGCCTCCTGGGACGCTTCCCCTGG
GCCAAGCTCCCCATTTACTCCTTGGTGCAGCTGCTGGCTGCTTTCTGTGCTTCAGGAGCC
ACCTAT---GTCCTCTACTATGATGCCTTACAGAATTATACAGGT---GGAAACCTGACA
GTGACCGGCCCCAAG---GAGACAGCCTCCATCTTTGCGACCTACCCTGCGCCCTATCTG
TCCCTGAACAATGGCTTCCTGGATCAGGTTATGGGCACTGGAATGCTGATTGTGGGGCTC
TTGGCCATCTTGGACACACGGAACAAAGGAGTGCCTGCAGGTCTGGAGCCTGTGGCAGTG
GGGCTGCTGATCCTCACCCTTGCGCTATCCATGGGTGTTAACTGTGGGTTGCCACTCAAT
CCTGCTCGGGACTTG---GGCCCACGACTTTTCACCTATGTGGCTGGCTGGGGCCCTGAA
GTCTTC---AGT------ACTGGTAATGGCTGGTGGTGGGTGCCTGTGGTGGCCCCCCTG
GTGGGGGCCACGCTTGGCACAGCCACATACCAGCTGCTGATAGCTCTGCACCACCCTGAG
---------------GAGTCAGAGCCAGCT---------------------CAG------
------------AATCTG---GAGTTAGCC------------------------CAA---
------------CAC---------AAAGCC------------TCAGACTTGGAAACTCCT
GCCTCAGCTCAGATGCCGGTGTGTAAGCTA------------------------------
------------------------------------------------------------
------------------------------------------------------------
---------------------
>Wallaby_AQP10
---------------------------------------------------ATGGTTTGT
---------------ATTCCTGGCCCAGGTCAGAGCCTG------CGAATCCGCAACCTC
CTAGCCCGACAGTGCTTGGCTGAATTTTTGAGTGTCTTCGTGTTTATGCTCATCATACAG
GGAGCAGGGGCTCAGGCTGTTACCAGTGAA------GAAACCAAAGGCAACTTCTTCACA
ATGTTCCTGGCAAGTGCCCTGGCTGTGACAGTGGCCATCTACGTGGGAGGCAATGTCTCA
GGGGCGCACCTGAATCCTGCCTTCTCCTTGTCCATGTGTCTCGTGGGTCGCCTACCTTGG
GCCAAACTTCCCATCTATATACTGGTACAGCTGCTTTCTGCCTTCTCTGCTTCAGGAGCA
ACCTAT---GTCCTCTATTATGATGCTCTGCAGAATTATACTGGT---GGAAACCTGACA
GTGACTGGCCCCAAG---GAGACAGCTTCTATCTTTGCCACCTACCCAGCCCCTTATCTG
TCCCTGGAAAATGGCTTCCTGGACCAGGTTCTAGGCACTGCTGTGATGATTGTGGGCATC
TTTGCCATCACAGACACCAAGAATAAGGGGGTGCCTGCAGGATTGGAGCCAGTGGCAATT
GGGCTGATGATTCTGGCCATTAGCTTCTCCATGGGTGCCAACTGTGGTTACCCACTCAAC
CCTGCTCGAGACTTG---GGACCTCGGCTCTTCACCTACGTGGCCGGCTGGGGATCTGAG
GTCTTC---AGT------GCTGGCAACGGTTGGTGGTGGGTGCCTGTGGTGGCTCCCATG
GTGGGTGCTGTGTTGGGGACTGCCACGTACCAGCTGCTGGTGGGTCTGCATCATCCTGAT
---------------GATCTGGAGCCTGCT---------------------CCA------
------------GAGAAA---GCCTCTGAA------------------------------
------------------------AAGGTC---------------------------CCC
ATCTTACCTCAAAACCTTGAGTGCAAACTG------------------------------
------------------------------------------------------------
------------------------------------------------------------
---------------------
>Kiwi_AQP10
---------------------------------------------------ATGGTCTCC
GCTCCC---TTC---TTGAAGAGGGCCCGGGCCCTGCTC------CGCATCCGCAACCAG
CTGGCGCGGGAATGCCTGGCCGAGCTGCTGGCCGTCTTCGTGCTCATCCTCATCACCCTG
GGCGGGTCGGCGCAGACGGTCACCAGCTCG------GGCACCAAGGGCGACCTCCTCACC
TCCTACCTGGCCGGGGCCCTGGCCGTTATGGTGGCGATTTACATGGCCGGCGGCGTCTCA
GGGGCCCACCTGAACCCGGCTTTTTCCCTCGCCCTGTGCACGCTGGCGCAGTTCCCCTGG
TGGAAATTTCCMATCTTCCTCACTGTGCAGACCCTGGGAGCTTTCCTCTCCGCGGGCGCC
GTCTAC---GCCCTCTACTATGACGCCATCCAGCACTACAGCAAC---GGCACCCTGGCC
GTCTCCGGCCCCAGG---GAAACCGCCTCCATCTTCGCCACCTACCCGGCCGACTACCTC
TCGCTCCGCAACGGCTTCCTCGACCAGGTGATGGGCACCGGGCTGCTGGTGGCCGGCGTC
CTGGCCATCGGGGACCCCCGCAACAACGGCATCCCCAGGGGCCTGGAGCCGGTGGCCRTG
GCCCTGCTGGTCTTCTCCATCGAGCTCTCCATGGGCTCCAACTGCGGCTGCCCCATCAAC
CCGGCGCGGGATTTC---GGGCCCCGGCTCTTCACCTACGTGGCGGGCTGGGGCTCCGAG
GTGTTC---AGC------AGGGGCAACGGCTGGTGGTGGGTGCCCGTGGTGGCCCCGCTG
CTGGGCGCCGTGGCCGGCTCGGCCCTTTACCAGCTCTTCGTGGCCTTTCACCACCCGGCG
---------------GAGGAGAGCGTGGCC---------------------CCG------
------------GCCTGC---GACGGCCCC------------------GGCGTCGAG---
------------------------------------------------------------
------------------------------------------------------------
```

```
------------AAGAACATCATGATCCCCATCGCCACCAGGACGCCC------------
------------------------------------------------------------
---------------------
>Alligator_Aqp10
---------------------------------------------------ATGGTCTCT
GCACGC---TTC---CTGGCAAAGGCTGGGACTCTGCTC------CATGTCCGCAGCCGC
TTGGTCCGGGAGTGCCTGGCTGAGTCGCTTGCTGTCTTTGTGCAGATGCTGATAACCCTG
GGCGCCGCGGCACAGACCATCACCAGCCAG------GGCGCGAAGGGTGGGTATGTCTCC
TCCTGCCTGGCCGGGGCCGTGGCCGTCATGGTGGCCATCTACATTGCTGGCGGAGTCTCA
GGTGCCCACCTGAACCCTGCCTTCTCATTGGCCATGTGTGTGCTGGCCCGGTGCCCCTGG
TGGAAGCTGCCCATCTTCACTGTCGTGCAGACCCTAGCAGCCTTCCTGGGTGCCGGTGCT
GTCTAT---GCCCTCTATTATGATGCCATCCAACACTACAGTAAT---GGGACCCTTGCA
GTCACGGGTCCCTGG---GAAACTGCCTCCATCTTTGCCACCTACCCCGCACCCTACCTG
TCCCTCCAGAGTGGCTTCCTGGACCAGGTGATGGGCACAGCACTGCTGCTGGTGGGCATC
CTGGCCATCGTGGACACCCAGAACAAGGGCATCCCAAAGGGGCTGGAGCCGGTGGCCGTG
GCCATGCTGGTGCTGGCCCTCAGTCTCTCTGTAGGCTCCAACTGCAGCTGCGCTATCAAC
CCTGCCCGTGACTTT---GGGCCACGGCTTTTCACCTATGTGGCAGGCTGGGGCCCCCAG
GTCTTC---AGG------GCTGGCCATGGCTGGTGGTGGGTGCCGGTAGTGGCTCCCCTG
GTAGGGGCCGTGGTGGGCTCCATGCTCTACCAGGTGCTGGTGGCATTTCACCACTCGCCT
---------------GCCCCAGGAAGCGCC---------------------CAT------
------------GAACTG---GAGGTGGAG------------------------------
------------------------------------------------------------
------------------------------------------------------------
------------AAGCAGAAGGAGATCCCCATCTTCATCATCAGCCCTCAGGCTGAAGAG
CAGCTCAAGAAGGCCACAGTCCTGGAGGAACCCGGGCCCAGTACAGACAGAGAGGGGTCC
---TCTCCCCGCCACGCTATG
>Tuatara_AQP10
---------------------------------------------------ATGGTCTCC
GCGCGC---TTC---CTGGCCCGGGCCAGCTCTTGGCTC------CGTATCCGCAGCCTC
CTGGTGCGGGAGTGCCTGGCGGAGCTCCTGGCTGTTTTCGTGCTGATTTTGATCACGCTG
AGCTCGGCGGCCCAGACGGTCACTAGTGAG------GGTGCGAAGGGCGGGTACCTCAGT
TCCTGCCTGGCTGGTGGCATCGCCGTCATGGTGGCGATTTACGTTTCAGGCGGTGTCTCA
GGAGGGCACCTCAACCCTGCCTTCTCGCTGGCTATGTGCTTGCTGGGGCGCTGCCCGTGG
TGGAAGCTGCCTCTCTTTGCCGCTGTGCAGACCGTGGCTGCCTTCGTGGGGGCCGGCACC
GTCTAC---GCCTTGTATTACGACGCCATCCATCATTACAGCAAC---GGGAGCCTGGAT
GTCACTGGCCCCCAA---GAAACGGCCTCCATCTTCGCCACCTACCCAGCCGCCTACCTG
TCCCTGCGGAATGGCTTCCTTGACCAGGTGCTGGGTACGGCTATGCTCCTGGTGGGCGTC
CTGGCCATCATTGACACCCGCAACAAGGGCATCCCCAAGGGCCTGGAGCCGGTGGCTGTG
GGGCTGCTGGTGCTCTCCCTTACCCTGGCCATGGGCGCCAACTGCAGCTGTGCCATCAAC
CCTGCCCGCGACCTG---GGCCCCCGGCTCTTCACCTACGTGGCCGGCTGGGGCCCCGAG
GTGTTC---AGC------GCCGGGAACAGCTGGTGGTGGGTGCCCGTCCTGGCCCCGATG
GTGGGGGCATCGACTGGCACGGCCTTGTATCAGCTGTTTGTGGGGCTTCACCATCCGGCT
---------------GCCGAAGAGCAGGGC---------------------CCC------
------------TCTGGA---GCCGCCGCC---GCCGCGCTCCAGCCGGTCCCAGAG---
------------------------------------------------------------
------------------------------------------------------------
------------AAGGACGTGGAGATCCCGATCTTTGCCATGAACCCGTACGCGGAAAGC
TGGGCAGAGCCAGGGCGGGGAGGCTGCCTCAGC---------------------------
---------------------
>Python_AQP10
---------------------------------------------------ATGACTTAT
TCTCGC---TTA---TTGGCCAGGGTGCAAGCCAGCTGT------CAGCTGCGCAGTGAC
CTCCTACGGCAATGCCTCGCGGAGTTCCTGGGCGTCTTTATTTTAATTGTGATCACCCTC
AGCGCGGTTGCGCAAGTGGTCACCAGCGAA------GGCATCAAGGGTAACTATCTTAGC
TCCTCCTTGGCCAGCGGGATCGCCGTGATGGTAGCCGTCCACGTCTCAGGCGGCGTCTCA
GGAGGACACCTGAACCCAGCCTTCTCGCTGTCCATGTGCCTGCTGGCTCAGCTCCCCTGG
TGGAAACTCCCCATTTACCTGGCCATCCAAATCCTGGGGGCCTTTATGGGAGCTGGCACC
GTCTAC---ACCCTTTATTACGATGCCATCTACAGCTACAGCAAC---GGGACACTCAGG
GTTACGGGGCCCCGT---GAAACTGCCTCCATCTTTGCCACCTACCCAGCCCCTTACCTG
TCACTACGCAATGGGTTTTTTGACCAGGTACTGGGAACGGGGGTGCTGGTGCTGGGCATC
CTCGCCCTCCTTGATGCCCGGAACAAGCGCATCCCGCAGGGCTTAGAACCCTTCGGTGTC
```

GGCCTTCTGGTGTCCACCCTCAGCTTAGCTATGGGAGCCAACAGCGGCTGTGCCATCAAC
CCGGCCCGGGACCTG---GGGCCCCGGCTGTTCACCTACGTAGCTGGGTGGGGTCCCCAG
GTGTTC---AGG------GCTGGAGAAAACTGGTGGTGGATACCCGTTGTGGCCCCAATG
TTGGGGGCCACCATCGGCACGGTGGTGTATCAGCTCGGAGTGGAGTTCCATCACCCGGCA
---------------GATCAGAAGGAGCAG---------------------CAG------
------------CGCTCG---GTTCCCGAA---AAGCATTGC---CCGGGACACGAG---

------------AAAGACATGGACCTTCCTGTCTACGCTGTGAACGTGTATGCAGAGTCG
TGGATAGGTGCCAGTCCGGATGCCCGC---TCT---------------------------
---GCCGGCGAACACCCAAAA
>Gecko_AQP10
---ATGATTTAC
GCCCGC---TTC---TTCGCCAAGGCACGGGGCTGCCTC------CGTCTCCGCAACCTC
CTCCTTCGCCAGTGCCTAGCGGAACTCCTGGGGGTCTTTGTGCTGATCGTGATAACGGTC
AGCGCTTCGGCCCAGTCTGTCACCAGCGAG------GGGACAAAGGGTGGCTATATGAGC
TCGGCCCTGGCCAGCGGCATCAGCGTGATGGTAGCTATTCACGTTTCAGGCGGCATCTCA
GGAGGGCACCTGAACCCGGCCTTCTCCCTAGCCATGTGCCTCCTGAGCCAGTGTCCGTGG
TGGAAGCTGCCCATTTTCTCCACTTTTCAAATCCTGGGGGCTTTCCTGGGCTCGGCCACC
GTCTAC---GCCCTGTACTACGATGCCATCCAACACTATACCAAT---GGGACCTTGACA
GTCACCGGCCCTCGG---GAAACGGCGTCCATCTTTGCCACCTATCCAGCCCCCTACCTG
TCCCTGCACAATGGCTTCTTGGACCAGGTGTTGGGCACCGGGATGCTGCTGTTGGGCATT
CTAGCGTTCATCGATTCCCGAAACAAGAACATCCCGAAAGGTTTGGAGCCTCTGGCCGTG
GGTCTGCTGGTCCTCTCCCTCAGCCTGGCCATGGGGGCCAACTGCAGCTGCGCCATCAAC
CCCGCCAGGGACTTG---GGCCCCCGGCTCTTCACCTACGTGGCCGGATGGGGCCCTCAG
GTTTTC---AGT------GCTGGAAACTACTGGTGGTGGGTTCCGATCGTGGCACCGATG
GTGGGCTCGGCCACCGGCACTGCCGTCTACCAGCTCTGCGTGGAGTTCCACCACCCTCCA
---------------GAGGAGGAGGAGGAG---------------------GCA------
------------TTCACC---ATGCCTGCCAATGAGAAGGTCCACCCAGGGACTGAA---

------------AAAGAGGTGGACATTCCGATTTATGTCGTCAACAAGTATGTGGACACG
TGGCTGGACGGGAACTCCGTGGGCAAC---CCG---------------------------
---GCCACAGAGCATGGCAAA
>Himalaya_frog_AQP10
---ATGGCACGC
TGGCCT---GCC---CGGGAGAGGTTGCAGAACAGGCTG------AGACTCAGGAACACT
CTAGCCCGGGAATGCCTCGCCGAATTTCTTGGAGTTTTCGTCCTGCTTTTGATCACGGTT
GCGGCCACTGCGCAGGGGGTCACAAGCCAA------AACACCAAGGGCAACTTCTTTTGC
ATGTATCTGGGCGGAGCCATTGGTGTCACTATGGCGATTTATGTGTCAGGGGGTGTGTCA
GGTGGGCATCTAAATCCAGCATACTCCCTCAGCCTGTGTGTCCTGGGCCGATTCCCATGG
TGGAAATTGCCCATCTATGTCATCATCCAAATGGTGGGGTCTTTTGCTGGAGCAGCAGCT
GTGTTC---GCGCTGTACTACGATGCCATCCAGAATTACACCGGC---GGCAACCTGACT
GTGCATGGACCACGA---GAGACGGCCTCCATATTTTCTTCGTACCCCGCCCCCTACCTG
AGCATTGCAAACGGATTTCTGGATCAGGTGATGGGCACAGCAATGTTGATGATTGGCATC
TTGGCCATTATTGATTCCAGCAACAAGCCAGTCCCAAAGGGTCTTGAGCCAATCGCGGTG
GGGATGCTGGTCTTCTCCATCGGCCTTTCCATGGGGGCAAACTGCGGCTATCCGATCAAC
CCAAGTCGAGACCTG---GGGCCACGGCTCTTCACCTATGTGGCGGGATGGGGCTCAGAC
GTCTTC---AGG------GCCGGGAACAACTGGTGGTGGGTCCCCATCGTAGCACCATGC
TTTGGGGGCGTTTTTGGCACGCTGCTGTACCAGATCTTGGTGGCGATCCACCACCCAGCA
---------------CAGGAAACCAGAGCC---------------------GAG------
------------GAGGCC---ACCAATGTC-------------------------GAG---
------------------------ACCCCA------------------GCGGAG------

------------AAAATGAAGGAGATTCCGGTCTTCACAATA------------------
---CATTTGGACAACTCT
TTGTCTCATCGGCTT------
>Axolotl_AQP10
---ATGGCCCGC
---------GCC---ATCCACCTTGCCAGGGCCCTGCTG------CGCATAGAGCACCCC
CTGGCCCGCGAGTGCATGGCAGAGTTCCTGGGTGTCTTTGTGCTGATTCTGATAACCAAT

GGCGCGACGGCGCAGGCTGTGACCAGCTTA------CAAACAAAGGGCGGCTATTTCACT
ATGTACCTGGGAGGTGCCCTGGCGGTTACCGTAGCAATCTACGTCTCGGGTGGCGTCTCA
GGAGGGCACCTGAACCCGGCCTACACGCTCAGCATGTGCCTTCTTGGC---TTCCAGTGG
AGGAAGATGCCGCTATTCGTCTTCATCCAGATCCTCGCCTCCTTTGCAGCTGCCGGAACT
GCATAC---GCTCTTTATTACGATGCAATCCAGCAGTATTGCAAT---GGAAGCTTGACT
GTAACCGGCCCTAGG---GAGACTGCATCAATCTTCGCCACATACCCTGCCGACTACCTC
ACCTTATGGAACGGCTTCCTGGACCAGGTGATTGGGACGGCCATGCTGCTTGTTGGCATC
TTGGCAATCGTGGACTCTAAGAACAAACCAGTGCCAAAAGGCCTGGAGCCGCTCCTGGTG
GGCTTACTCGTTCTCTCCATCGGCCTTTCTATGGGCTCCAACTGTGGCTATCCCATCAAC
CCTGCTCGGGATCTT---GGCCCGCGTCTATTTACGTTTGTGGCA---------------
---TTC---AGT------GCAGGGAACAATTGGTGGTGGATACCGATCGTAGCACCGCTA
CTTGGCGCAGTCATCGGATCAGTCGTATATGAGGTCTTTGTAGAGTTCCATCATCCTGAT
---------------AACCCCTCTGAACCT---------------------TTG------
------------GCTTCG---TCAACCAGC------------------------AAA---
------------------------GACCAC------------TTGTACGTGGAGAAGTGG
ACACCGGAAACTAAGAAG--
---------AAGAAACTAGAGATGGAGCCGGTCTTTACTGTGGACACATACATGGAGGGA
TGCACAGAGAGATCCAGG---------------------------GAGACAGAGGGCTAC
TTGAGCCACAGGTTG------
>2Lcaecilian_AQP10_2
---ATGGCCAAG
GTTCCC---ACA---CTAGACCAAGCCAGGCCCCTGCTG------CGAATCGAAAATGCC
ACTGTTCGGCAGTGCCTGGCTGAGTTCCTGGGGGTGTTTCTGCTGATCCTAATCACCATT
GGTGCAACGGCTCAAAGCGTGACCAGCTTT------GACAGGAAGGGTGGCTACTTCCCC
ATGTGCCTGGCAGGAGCCCTTGCTGTTACCATGGCAATTTATGTCTCAGGAGGAGTCTCA
GGGGGCCACCTGAACCCTGCCTACTCTCTGAGCCTGTGCCTGCTGGGGCGCTTCCAGTGG
ACAAAGCTACCCTTGTTCTTTCTTGTCCAGACTTCGGCAGCCTTTCTCGCTGCAGCGGGG
GCCTAC---GCCCTGTATTACGATGCGATCCACAACTATTGCAGT---GGGAATCTGACT
GTCACTGGTCCCCGG---GAAACTGCCTCCATTTTTGCCACCTACCCTGCCAGTTACCTC
TCAGCTTGGAATGGATTCCTGGATCAGGTGATTGGAACCGCCACCCTCCTGCTCAGCATC
CTGAGTTTGGTGGACTCCAAGAACAAGCCTGTCCCAAAAGGCCTGGAGCCCGTGGTTGTG
GGCATGGTGGTTCTCTCCATTGGCCTTTCGATGGGCTCCAACTGTGGTTACCCCATCAAC
CCAGCCCGGGACCTG---GGGCCCAGACTTTTCACGTGGCTGGCTGGCTGGGGCCCGGAG
GTTTTC---AGG------GCTGGCAATAACTGGTGGTGGATACCTATAGTGGCACCGTTA
GTAGGCGCTGTTATTGGGTCAACTTTGTATGAGCTGATGATCGAGTTCCACCATCCGGAA
---------------ACCCAGAGTGAGCTG---------------------ACG------
------------GACGCC---GGCAAGGAG------------------------AGC---
------------------------CACTGC------------TTGGAGGCAGAGAAGGGA
CGGCCTCCTGTGGAGGCC--
---------ATAAAAGCTGGAGAAGTGCCAGTTTTCACCATTGACACATACATGGAGGGC
TTCATGCAAAGGGAGGGA---------------------------CACAGGAAGAAAGTT
ATAAGTCACAGGCTG------
>2Lcaecian_AQP10_1
---ATGGCCAAG
GTTCCC---ACA---CTAGACCAAGCCAGGCCCCTGCTG------CGAATCGAAAACGTC
ACTGTTCGGCAGTGCCTGGCTGAGTTCCTGGGGGTGTTTCTGCTGATCCTAATCACCATT
GGTGCAACGGCTCAAAGCGTGACCAGCTTT------GACAGGAAGGGTGGCTACTTCCCC
ATGTGCCTGGCAGGAGCCCTTGCTGTTACCATGGCAATTTATGTCTCAGGAGGAGTCTCA
GGGGGCCACCTGAACCCTGCCTACTCTCTGAGCCTGTGCCTGCTGGGGCGCTTCCAGTGG
ACAAAGCTACCCTTGTTCTTTCTTGTCCAGACTTCGGCAGCCTTTCTCGCTGCAGCGGGG
GCCTAC---GCCCTGTATTACGATGCGATCCACAACTATTGCAGT---GGGAATCTGACT
GTCACTGGTCCCCGG---GAAACTGCCTCCATTTTTGCCACCTACCCTGCCAGTTACCTC
TCAGCTTGGAATGGATTCCTGGATCAGGTG------------------CTGCTCAGCATC
CTGAGTTTGGTGGACTCCAAGAACAAGCCTGTCCCAAAGGCCTGGAGCCCGTGGTTGTGG
GCATGGTGGTTCTCTCCATTGGCCTTTCGATGGGCTCCAACTGTGGTTACCCCCATCAAC
CCAGCCCGGGACCTG---GGGCCCAGACTTTTCACGTGGCTGGCTGGCTGGGGCCCGGAG
GTTTTC---AGG------GCTGGCAATAACTGGTGGTGGATACCTATAGTGGCACCGTTA
GTAGGCGCTGTTATTGGGTCAACTTTGTATGAGCTGATGATCGAGTTACACCATCCGGAA
---------------AACCAAGAG------------------------------------

>Coelacanth_Aqp10
---ATGGGAAAG
GATCTT---GTG---ACAGAGAGAATTAGAGCCTTATTT------CGAACAAGAAGCTCA
TTGATACGAGAATGCCTAGCTGAACTTTTTGGTACCTATGTGATGGTTGTGTTTTGCTTG
GGTACGACAGCTCAGATGATAATCAGCAGC------ACTGAAAAGGGAGACTTCACGGCC
ACTAATGTTGCCTGTGGTTTGGGGGTTGCCTTTGGAATTTACATTGGCAGTGGCGTTTCA
GGGGGTCACCTGAGCCCAGCGGTGTCCCTAGCTATGTACCTGACAGGGAAGCTTTGCTGG
TGGAAATATCCCTTCTATGCTGTGTTCCAGTTTTTCGGGGCCTTTATGTCATCGGTGACT
GTGTAT---ATTCTTTACTATGATGCCATCCATTCTTATAGTGGA---GGGAATCTGACA
GTCACAGGACCATCA---GAAACTGCTTCTATCTTTGCAACTTACCCAGCTCCCTACCTC
ACGATTAGGAATGGCTTTATTGACCAGGTCATAGGAACTGCAATCCTGATCATCTGTATA
TTGACTATTGTAGACTCCCAGAATAGCCAGGTGTTAAAGAGTCTGGAACCACTGTTGGTG
GGACTGGTAGTGATGACCATCGGAATGTCCATGGGTGTCAACAGCGGGTATGCCATGAAT
CCGGCCAGGGACCTA---GCACCACGCCTCTTTACTTTTTTTGCAGGCTGGGGACCAGAA
GTCTTC---GTG------GCTGGCAATAGTTGGTGGTGGGTTCCTGTTGTTGCCCCTCTG
TTTGGATCTGGCATTGGGACCTTCTTGTACCTGGTTTTTGTGCAGTTCCATCACCCAGAC

>mCoelacanth_Aqp10
---ATGGGAAAG
GATCTT---GTG---ACAGAGAGAATTAGAGCCTTATTT------CGAACAAGGAGCTCA
TTGATACGAGAATGCCTAGCTGAACTTTTTGGTACCTATGTGATGGTTGTGTTTTGCTTG
GGTACGACAGCTCAGATGATAATCAGCAGC------ACTGAAAAGGGAGACTTCACGGCC
ACTAATGTTGCCTGTGGTTTGGGGGTTGCCTTTGGAATTTACATTGGCAGTGGCGTTTCA
GGGGGTCACCTGAGCCCAGCGGTGTCCCTAGCTATGTACCTGACAGGGAAGCTTTGCTGG
TGGAAATATCCCTTCTATGCTGTGTTCCAGTTTTTCGGGGCCTTTATGTCATCGGTGACT
GTGTAT---ATTCTTTACTATGATGCCATCCATTCTTATAGTGGA---GGGAATCTGACA
GTCACAGGACCATCA---GAAACTGCTTCTATCTTTGCAACTTACCCAGCTCCCTACCTC
ACGATTAGGAATGGCTTTATTGACCAGGTCATAGGAACTGCAATCCTGATCATCTGTATA
TTGACTATTGTAGACTCCCAGAATAGCCAGGTGTTAAAGAGTCTGGAACCACTGTTGGTG
GGACTGGTAGTGATGACCATCGGAATGTCCATGGGTGTCAACAGCGGGTATGCCATGAAT
CCGGCCAGGGACCTA---GCACCACGCCTCTTTACTTTTTTTGCAGGCTGGGGACCAGAA
GTCTTC---GTG------GCTGGCAATAGTTGGTGGTGGGTTCCTGTTGTTGCCCCTCTG
TTTGGATCTGGCATTGGGACCTTCTTGTACCTGGTTTTTGTGCAGTTCCATCACCCAGAC

>European_seabass_Aqp10bb

---------------ATGGAGAGGCTGCTGAGGAAATGT------CAGATCAGAAACCAA
CTGGTCAGGGAGTGCTTGGCTGAATGCCTTGGAGTCTACGTTCTGATTCTGTTTGGATGC
GGCTCCGTTGCCCAAGTGACTACAACTCAA------GATAAGAAGGGGCAGTACCTGTCA
ATCAATCTGGGTTTTGCTCTGGGAGTAACATTTGGGGTGTTTGTGTCTCGTGGAGTCTCA
GGTGCCCATCTGAACCCAGCTGTATCTCTGAGCTTGTGCGTTTTGGGCAGACATCCCTGG
ATAAAGCTACCTTTCTATATCTTCTTCCAAGTGTTTGGGGCCTTTCTGGCTGCAGCCACC
GTTGGC---CTGCAGTACTATGATGCCATTCAGGCGTACAGCGGA---GGTGAGCTGACG
GTGACGGGCCCCACT---GCCACAGCAGGCATATTCTCCACCTACCCAGCTGACTACCTC
AGCGTGTGGGGAGGTGTCGTGGACCAGGTAATAGGCACAGCTGCACTGCTGCTGTGTGTC

CTGGCTCTTGGGGACCAGAGGAACAGCTCCCTCCCTGATGGTCTTCAGCCTGTACTGGTG
GGAGCAGCTGTGCTTGTTATTGGCATCTCGATGGGCGCCAACAGCGGCTACGCACTTAAC
CCGGCCAGGGATTTA---GGACCTCGGTTGTTCACGTACATTGGCGGCTGGGGAGTCGAT
GTTTTC---AAG------GCTGGAGGTGGTTGGTGGTGGGTGCCCATTGTGGCTCCCTGT
GTCGGGGCGCTGTTGGGAACACTGATTTACGAGCTGATGATTGAAGTCCACCATCCTGCC
---------------AGTCCGCCGGAGCTC---------------------CAG------
------------ACCTCATGTCAGGAGGCC---------------------ACTGAG---
---------GGCAAG---------ACGGGA------------CTGGAGCTGGAGGGGGTG
GAGCAAGACTGTGAAAAACCCAGTAGTAAAATG---------------------------

>Nile_tilapia_Aqp10bb

---------------ATGGAGAGGCTGCTGAAGAAATGT------CAGATCAGAAACCAG
CTGATCAGGGAGTGCATGGCTGAATGTCTCGGCGTCTACGTCCTGATTCTGTTTGGATGT
GGCTCTGTTGCCCAAGTGGTCACAACTGAA------GACAAAAAGGGCCAGTACCTCTCA
ATTAATCTGGGTTTTGCTCTGGGAGTAACATTTGGGGTCTTTGTGTCTCGTGGAGTATCG
GGTGCTCATTTAAACCCGGCTGTGTCTCTGAGCTTGTGTGTTTTGGGCCGCCACTCATGG
ATGAAACTGCCTTTCTACATCTTCTTCCAAGTGTTGGGAGCCTTTCTGGCTGCGGCTACA
GTTGCT---CTGCAGTACTATGACGCCATCCAGGCGTACAGCGGA---GGCGATCTGACA
GTGACGGGTCCCAAA---GCCACAGCAGGCATATTCTGCACTTACCCCGCTGACTACCTG
AGTGTGTGGGGAGGTATCGTGGACCAGGTGATTGGCACGGCCGCTCTGCTGCTGTGCGTC
CTGGCTCTCGGAGACCAGAGGAACAGCTCCATCCCTCATTATCTTCAGCCTGTCTTAGTG
GGAGCAGTAGTGCTGGTCATTGGCATCTCAATGGGCTCAAACAGCGGATATGCACTCAAC
CCGGCAAGAGATTTT---GGACCTCGGTTGTTCACCTACATTGCCGGTTGGGGGGCGGAT
GTTTTC---AAG------GCCGGAAGTGGCTGGTGGTGGGTGCCCATAGTGGCTCCTTGT
GTTGGAGCGCTTCTGGGAACACTGATCTACGAGCTGATGGTTGAAGTCCACCATCCTTCA
---------------GAGCAGTCCGAATCC---------------------CAG------
------------GCCTCGTGTCCGGAGAAT---------------------ACAAAT---
---------GACAAG---------ATGGGG------------GTTGAGCTGGAGGGGGTG
GAAGCAGACCGTGAAAAGCCAACT------------------------------------

>Mummichog_Aqp10bb

---------------ATGGAGAAGCTGCTGAGGAGGTGC------CGGGTCAGAAGCCTC
CTGGTGCGGGAGTGCCTGGCTGAATGTCTGGGAGTCTACGTCCTGATTTTGTTCGGATGT
GGCTCTGTTGCACAGGTGACCACGTCTGAG------GAAAAAAACGGCCAGTATCTGTCC
ATCAACCTTGGCTTTGCTTTGGGGGTAACATTTGGGGTCTTTGTGTCCCGCGGCGTCTCA
GGTGCTCACCTGAACCCCGCAGTTACTCTGAGCTTGTGTGTTTTGGGCAGACATCCATGG
GTGAAACTTCCCTTATACGCCTTCTTCCAGGTGCTCGGAGCCTTCCTGGCTGCAGCCACG
GTTGGT---CTGCAGTACTACGACGCCATCCAGAAGTACAGCGGC---GGTCAGCTGACG
GTGACGGGTCCGACA---GCCACAGCAGGAATATTCTGCACTTATCCGGCTGAATACCTC
AGTTTGTGGGGCGGCATCGTGGACCAGGTGATCGGCACCGCCGCCTTGCTGCTGTGTATC
CTGGCCATAGGGGACCGGAGGAACACCTCCATACCAGACTATCTCCAGCCACTCCTGGCT
GGAGCGTCGGTGCTGGTCATCGGCATCTCCATGGGCTCAAACAGCGGATACGCCCTGAAC
CCGGCGAGGGATTTG---GGACCCCGGTTCTTCACTTACATCGCTGGCTGGGGAGACGAA
GTTTTC---AAG------GCTGGAGGCGGGTGGTGGTGGGTCCCGTTGGTGGCTCCCTGC
ATCGGCGCGCTGGTAGGGACTCTGATCTACCTACTGATGGTTGAAGTCCACCATCCGCCC
---------------CTCTCTCCGGAGATC---------------------CAG------
------------CCGTCGTGTCAGGAAGCC---------------------ACGGAG---
---------GGCAAG---------ACT---------------CTGGAGCTGGAGGGAGTG
GAACCAGACGGTGGAAAACCTGCC------------------------------------

>Zebrafish_Aqp10bb

---------------ATGGACCGTCTGCTGAGGAGATGC------CGAATCAAGAGTCGT

CTGCCCAGAGAATGTCTGGCGGAGTTTTTCGGAGTCTATGTTTTAATACTGTTCGGGTGT
GGATCAGTGGCTCAGGTCACCACCTCTCAG------AATACCAAGGGAGAGTACCTGTCA
ATCAACCTGGGCTTCGCACTGGGAACCACATTTGGCATCTACATTGCAAAAGGAGTGTCA
GGAGCTCACCTGAATCCAGCGGTTTCCCTCAGTTTGTGTGTTTTGGGCAGGTTTTCGTGG
ACTCGTCTTCCTTTCTACGTGTGTTCGCAGCTCTTCGGTGCATTTCTGGCTGCAGCAACG
GTTGCC---CTGCAGTATTATGACGCCATAATGGATTTCACTGGA---GGGCATCTGACA
GTCAGTGGTGCCACG---GCTACAGCGGGCATCTTCTCAACTTATCCAGCAGATTATCTG
AGTCTGTGGGGAGGAGTGGTAGACCAGATCATTGGCACGGCTGCTCTGCTGGTGTGTGTT
CTTGCATTGGGAGATGCTCATAACACACCTGCACCTGCAGGTCTGGAGCCTGTACTTGTC
GGAGCCGCCGTGCTGGTGATCGGGATCTCCATGGGATCTAACAGCGGATATGCCATCAAT
CCAGCCAGAGACTTC---GGCCCAAGACTCTTCTCCTACATCGCAGGCTGGGGAGACGAG
GTGTTC---AGG------GCTGGACATGGATGGTGGTGGGTGCCTATAATCGTAACGTGT
GTCGGGGCTCTTCTGGGATCATTACTGTACGAGCTGCTGATTGGAGTTCATCATCCTGAC
---------------TCAGAGGCAGTGGAT---------------------CAT------
------------GAAGAC---CCGACAGCG------------------------GCG---
---------CTCCAG---------CAAACT------------GTCGAGATGGAGGGTGCG
CAGAGTTTTGACACCATTAAAGAAAAC---------------------------------
---------AAAAAGAGC--
---GGGATCTTTTCT
ATAACCTCAGCAGACGTAGGG
>Bowfin_Aqp10ab

---------------ATGGACAGAGCACGCCGGCTGTGC------CGAGTCAGGAGCCAG
CTGCTCCGGGAGTGTCTGGCCGAGTCTCTGGGCACCTACATAATGATTCTGTTCGGCTGC
GGTGGGGTTGCCCAGGTGACAACGAGCAGC------GAGACAAAGGGGCAGTACCTGTCG
ATCAACCTGGCCTTCGCCCTGGGGGTCACATTCGCCGTGTATGCCTCCCGCGGTGTCTCA
GGGGCCCACCTGAACCCTGCTGTCTCTCTCAGCCTGTGTGTTCTGGGCAAGCACCAGTGG
AAACGCCTGCCGTTCTATGTCTTCTTCCAACTGCTGGGGTCCTTCCTTGCCGCGGCAACA
GTATAT---GCTTTGTACTATGAGGCGATAATGGAATACAGTCAG---GGCAATCTGACA
GTGTCTGGCCCCAGA---GCCACGGCTGGGATCTTCGCCACCTACCCCTCTGACTACCTC
AGCATCTGGAGTGGTTTCCTGGATCAGGTGGTGGGCACTGCGGCACTGTTGCTGTGTGTG
CTGGCAGTAGGGGATGAGCGGAACAGCGCCCCCCCCGCAGGGCTGGAGCCGGTGCTGGTG
GGCCTGGCAGTGCTGGGCATTGGCATCTCCATGGGGGGCAACTGTGGGTACCCCCTGAAC
CCCGCCCGTGACCTG---GGGCCCCGCCTTTTCACGTTCATCGCAGGCTGGGGGGGACAG
GTCTTC---ACG------GCGGGTGGGGGCTGGTGGTGGGTGCCTCTGGTGGCCCCCCTG
CTTGGCGCTCTGACGGGCACAGCACTGTACCAGCTGCTGGTGGAGTTCCATCACCCCCCC
---------------CATGAACTCGACCCC---------------------CCT------
------------GGCACA---CAGGCCAAT------------------------GAA---
---------GTGGGC---------CTAGCG------------CTGGAGGTGGAAAGGGAG
GCCGGGGAGGAGAAGGGGGTCAGAAAT---------------------------------
---------GGGGGGGAGGTG---------------------------------------
---GGCGACGAGCGC
ATAATCAACCGCCTC------
>Gar_Aqp10ab

---------------ATGGAGAAGGTACAGAGACTGTTC------CAGGTACAGAACCAG
CTGCTCCGGCAGTGCCTGGCCGAGCTGCTGGGGACCTACGTCATGATGGTGTTTGGCTGC
GGTGCAGTCGCCCAGGTGACGACGAGTGGT------GAGAAAAAGGGTGATTTCCTGTCC
ATCAACCTGGCTTTTGCCCTCGGTGTCACCTTTGCTGTACACGTCTCTGGAGGAGTGTCA
GGAGCCCATCTGAATCCTGCGGTGTCCCTGAGTCTTTGTGTTCTGGGCAGGCACCAGTGG
AGACGCCTTCCACTGTACATCCTCTCCCAGGTGACGGGGGCCTTCCTTGCCGCGGCAACC
ATCTAT---GTGGTCTACTACGAGGCCATCATGGAGTACTGCGGA---GGGAATCTCACA
GCGACTGGCCCAAAC---GCCACTGCTGGCATTTTCGCCACCTACCCCTCGTCCTACCTC
ACCCTCTGGAACGGGTTTGTGGACCAGGTAATAGGCACTGGGATGCTGTTGCTGTGTGTT
CTGGCTCTGGGTGATGTCAATAACAGCCCAGCCCCCCCGGGTCTGGAGCCGGTGTTGGTG
GGATTGGTAGTTCTGGCGATCGGCATCTCCTTTGGGAGTAATTGTGGGTACCCCCTGAAC
CCCGCCCGTGACCTG---GGGCCCCGACTTTTCACCTACATTGCTGGCTGGGGGAGCGAA
GTCTTC---AGG------GCTGGGGGAGGCTGGTGGTGGGTGCCTCTGCTGGGCCCCCTG
GTGGGCGCGCTGACAGGCACGGCACTGTACCAGCTGCTCATCGAGCTCCACCACCCCGGG
---------------CCGGGGCTCGGCTCG---------------------GGC------
------------GACACC---GCCAGCGGG------------------------CAG---

---------GCCCCT---------CCCCCG------------GAGGAGGCAGAGCTGGGG
CTGCCGGGCGAGAAGGGGGACGAGAGG---------------------------------
---------CAGGACGAG--
---GGCGAGGCCTGC
GTCACCCACCGCCTC------
>Sterlet_Aqp10ab

---------------ATGGACAAAGTGAAGGAGCTGCTG------AGAGTGAGGAACGCT
CTGGTGAGGGAATGCTTCGCAGAATTCCTGGGCATTTACATCCTGATCATTTTCGGGTGC
GGCGGCGTGGCGCAGGTCACGACGAGCAGT------CTCACCAGAGGAGAGTATCTCTCC
ATCAACCTGGCTTTTGCATTCGGGGTGACGTTTGGCGTGCACGTCTCCCGCGGCGTTTCA
GGAGCCCACCTCAACCCCGCGGTTTCCCTGGCCATGTGCTTGCTGGGCCGCCTGCCCTGG
CGGAAGCTGCCGGTCTACGCGCGCTTTCAGCTGCTCGGGGCGTTCGTTGCTGCCGCCACG
GTGTAC---GCGCAGTACCGCGACGCCATCATGCAGTACAGCGGG---GGCAACCTCACA
GTCACGGGGCCCTTA---GAGACGGCCTCTATCTTCGCTACCTACCCCGCACCCCATCTG
TCCATTTCCAATGGATTTGTGGATCAGGTGATTGGCACAGCCGCTCTGCTTGTGTGTGTT
CTGGCGCTGGGTGACTCTCAGAACAGTCCCGCTCCGAAGGGGCTGGAGCCGGTTCTGGTG
GGGATGTCGGTGCTGGTGATCGGACTGGCCATGGGCTCCAACTGCGGGTACGCCATTAAC
CCCGCCCGCGACCTG---GGACCCCGACTCTTCACCTGCGTGGGGGGCTGGGGTGTGGAG
GTGTTT---AGG------GCTGGGAGACACTGGTGGTGGGTTCCCTTGCTCGGTCCCATG
GTGGGCTCTTTGGTCGGGACCGCCGTGTATTGGCTTCTTATCGAACTGCACCACCCAGCC
---------------CAGGAGGGGGCGCTA---------------------GAG------
------------GACTGC---CAGACCGTG------------------------AAA---
---------AACAAA---------TACGCC------------ATCGAACTGGAGGAGGGG
GCTTCTAAACCCGGAGGGGGGGGGGCA---------------------------------
---------AGGATGGAGAGCACGAGGGAG---------------------AGGGAAGGG
--GAGGGGGAGGGGGAGGAGTGC
ACCGGCAAGCGGCTG------
>Common_sturgeon_Aqp10ab

---------------ATGGACAAAGTGAAGGAGCTGTTG------AGAGTGAGGAACGCT
GTGGTGCGGGAATGCTTCGCAGAATTCCTGGGTATTTACATCCTGATCATTTTCGGGTGC
GGCGGCGTGGCGCAGGTCACGACGAGCAGT------CTCACCAGAGGAGAGTATCTCTCC
ATCAACCTGGCTTTTGCATTCGGGGTGACGTTTGGCGTGCACGTCTCCCGCGGCGTTTCA
GGAGCTCACCTCAACCCCGCGGTTTCCCTGGCCATGTGCTTGCGGGGCCGCCTGCCCTGG
TGGAAGCTGCCGGTCTACGCGCTCTTTCAGCTGCTCGGGGCGTTCGTTGCCGCCGCCACG
GTGTAC---GCGCAGTACCGGGACGCCATCATGCAGTACAGCGGG---GGCAACCTCACA
GTGACTGGGCCCTTC---GAGACGGCCTCTATCTTCGCTACCTACCCCGCACCCCATCTG
TCCATTTTAAATGGATTTGTGGATCAGGTGATTGGCACAGCCGCTCTGCTTGTGTGTGTT
CTGGCGCTGGGTGACTCTCAGAACAGTCCCGCTCCGAAGGGGCTGGAGCCGGTTCTGGTG
GGGATGTCGGTGCTGGTGATCGGACTGGCCATGGGCTCCAACTGCGGGTACGCCATTAAC
CCCGCCCGCGACCTG---GGACCCCGACTCTTCACCTGCGTGGGGGGCTGGGGTGTGGAG
GTGTTT---AGT------GCTGGGAGACACTGGTGGTGGGTTCCCTTGCTCGGTCCCATG
GTGGGCTCTCTGGTCGGGACCGCCGTGTATTGGCTTCTTATCGAACTGCACCACCCAGCC
---------------CAGGAGGGGGCGCTA---------------------GAG------
------------GACTGC---CAGACCGTG------------------------AAA---
---------AACAAA---------TCCGCC------------ATCGAACTGGAGGAGGGG
GCTTCCAAACCCGGAGGGGGGGGGCCA---------------------------------
---------AGGACGGAGAGCACAGGG---------------------------------

>Reedfish_Aqp10ab

---------------ATGGAGAAAGTGTTCCAAGTCCTC------AGGGTGAAGAACCAG
CTTTTTAGGGAATGTCTTGCTGAACTTTTGGGTGATTACGTGCTGATAATTTTTGGCTGT
GGAGCAGTGGCTCAGGTCACCACAAGCTAT------GATACAAAGGGGCAGTTTCTTTCC
ATCAACCTGGCATTTGCTATTGGCATCACTTTTGGTGTTCACATTGCTCATGGAATTTCA
GGTGCCCATTTGAACCCTGCAGTCTCTCTTGCTTTTTGTTTAATTGGTCGCTTCCAATGG
AGCAAGCTTCCCTTCTACGTGTTATCTCAGATTATTGGTGCATACCTGGGTGCTGCCACT
GTGTTT---GCACAGTATTATGATGCTATCATGAGTTATGGTGGT---GGCAATCTGACT
GTCACCGGCCCTACA---GCAACAGCCTATATCTTTGCAACATACCCTGCAGCATACCTG

ACAACAGCAAATGGATTTGTAGACCAGGTGGTTGGCACTGGCGCTCTGCTTCTCTGTCTT
ATGGCTCTTGTCGACACTCGTAATTCACCTGCTCCAAAGGGACTGGAACCTGCATTAGTG
GGAACAATTGTTCTTGTAATTGGAATTGCAATGGGTTCCAATTGCGGTTATGCTATCAAT
CCAGCCCGTGACCTG---GGACCACGGCTCTTCACATATACAGCTGGCTGGGGTTTGGAT
GTTTTC---ACG------GCAGGAAATTACTGGTGTTGGGTGCCTCTGGTTGCTCCTTTG
GTTGGTGCAATTTTGGGATGTATCATTTATCTGCTCTTCATTGAAATTCACCATTCAAGA
---------------AAAGAGGATGAAAAA---------------------CAG------
------------ACAAAT---GAGAGAGGC------------------------AAT---
---------ACTGAA---------TATGAC------------ATTGATCTGGATGAATGG
AAGCCCAACAAAAGGAATCTAACAACA---------------------------------
---------CTTACAGCATCTGACAAAGTACACATCATCAACAACCCCTTTCAGGAAGGA
CAGTCAGAGAACTCTGATTGGAAGAAG------------TCATCGTGTAATGAAGAATGT
ATTGTCAATAGATTT------
>European_seabass_Aqp10aa

---------------ATGTTAAAGCTA---CGTGCTCTG------AGAGTGAGAAATGCT
CTGGTGCGAGAGTGCATGGCTGAGTTCTTGGGAACCTTTGTCTTGTTGCTCTTTGGCTGC
TCTGCCGCAGCGCAGGTAAAAACAAGCAGG------GAGACTAAAGGCCAGTTCCTGTCG
GTTAACATGGCCTTCTCTGTGGGCGTGATGTCAGCTATGTACCTCACCAAGGGCATCACA
GGTGCTCATCTGAACCCAGCGGTGACTCTGAGTTTCTGTGTGTTGGGCCAGGTGCCCTGG
GGACGGCTGGTTCCCTACTGCCTCTCCCAGCTGCTGGGGGCTTACATGGCATCAGCGCTT
GTCTAC---CTGGTGTACTATGATGCTATAATGGACTTTAGTGGA---GGAGTATTGACT
GTATATGGCCCAAAT---GAGACAGCATCTATATTTGCCACATATCCCTCAGAGTACATA
ACTTTGGGCAGAAGTTTCCTCGACCAGGTCGTGGGCACTGGCATGCTGATGTTGTGCATC
CTGTGTTTGGGGGAAAAGAGGAATACCCCAGCTCCCTCAGAGCTGATTCCTCCTATAGTG
GCAGTGATCGTCCTGGGGATCTCCATGTCAATGTCAGGTAACTGTGGTGCTGCAATAAAT
CCTGCTCGGGACCTG---GGGCCACGCCTCTTTACGCTGACGGCAGGCTGGGGCACAGAG
GTCTTC---ACG------TGTTACAACTACTGGTTCTGGGTACCCCTGGTAGCCCCACCT
ATCGGAGGTGTCATAGGTACTTTCATGTATTTGATCTTCATCGACTGGCACCTGCCTGAC
---------------CCGGACCAGCCTGAA---------------------AGC------
------------CTCTCC---ATTCTATCC------------------------ACC---
---------ATCAGT---------GACAAA------------ATCCAGCAGCCCGGCACA
ACGTGGGACAAAGGAACAGGGTTAAAG---------------------------------

---ACTGCACATTTC------
>Nile_tilapia_Aqp10aa

---------------ATGTTGAAGCTA---CGTGGAATG------ACGGTGAGGTGTCCC
CTCGTGAGAGAGTGCATGGCCGAATTCTTGGGAACTTTCGTCTTAATGCTCTTTGGCTGC
GCTGCTGCTGCACAAGTAAAGACCAGCAAT------GAGACTAAAGGCCAGTTTCTGTCT
GGCAACATGGCCTTCTCTGTGGGTGTTATGTCAGCCATGTATCTCACCAAGGGCATCTCA
GGTGCCCATCTGAACCCAGCGGTGACCCTGAGTTTCTGTGTTTTGGGGAAGGTGTCCTGG
TCGAGGCTCGTGCCCTATTCCCTCTCCCAGGTGGTGGGGGCTTTCGTGGCGTCGGGGGTT
GTCTAT---CTGGTCTACTACGATGCTATAATGACTTTTAGTGGA---GGAGTGTTGACT
GTATACGGCAGAAAT---GAGACTGCCTCGATATTTGCCACATACCCCTCTGACTACCTT
TCTGTGGGCAGAAGTTTCTTTGACCAAGTAGTGGGCACCAGCATGTTGCTGTTGTGTATC
CTGGGTTTGGAGGAAAAGAGGAACACCCCCGCTCCCTCTGAGCTGGTTCCTGTTGTAGTG
GCAGGTGTCGTTCTGGGGATCTCCATTTCAATGTCGGCTAACTGTGGTGCTGCCATAAAT
CCTGCTCGGGACCTG---GGGCCTCGCCTCTTTACCCTAGCTGCAGGCTGGGGGACTGAG
GTCTTC---ACG------TGTTACAACTACTGGTTCTGGGTACCAGTGGTGGCTCCGCTT
GTTGGAGGCGTTTTCGGTAGTTTGATGTATCTCATCTTTATCGACTGGCAGCTGCCTGAC
---------------CCAGACCAACTTGAG---------------------AGG------
------------ATTTCC---ACCATCAGT------------------------GAA---
------------AAA---------CTTGCA------------AAAAAGCAGCCGGTCACC
ACATTGGAAAAAGGAGATGAGTTAAAG---------------------------------

---GCTATGCATTTT------
>Mummuchog_Aqp10aa

---------------ATGTTGAAGCTG---CGTACGCTC------AGGCTGAGGGCTGCC
CTGGTGCGAGAATGTATGGCTGAGTTCCTGGGAACATTTGTCTTGATGCTGTTCGGTTGC
TCTGCAGCAGCCCAGGTAAAAACCAGTCAA------GACACCAAAGGCCAGTATCTGTCG
GTCAACATGTCCTTCTCCGTAGGGGTGATGTCAGCCATGTACCTGACCAAGGGGATCTCA
GGTGCCCACCTGAACCCTGCAGTGACTCTAAGTTTATGCGTTTTGAGGAAAGTGAGGTGG
AGAAGTCTGCTACCCTTCTGCCTCTCCCAGGTGCTCGGCGCATATGTGGCATCGGCAGTG
GTCTAT---ATGGTCTACTATGATGCTATAATGGATTTTAGTGGA---GGCGTGTTGACC
GTTTATGGCCCAAAT---GAAACAGCATCTATATTTGCCACGTATCCATCTGAGTACCTC
ACGTTAGGCAGGAGTTTCCTCGATCAAGTGGTGGGGACTGGCACATTGATGCTGTGCATC
CTAAGTTTGGATGAAAAGAGGAACACGCCTGCTCCCTCTGGTCTGATACCTCCCATAGTT
GCCACGATTGTCCTGGGGATCTCCATGTCCATGTCAGCTAACTGTGGTGCTGCAATAAAT
CCTGCTCGCGACCTT---GGACCACGCCTCTTCACCCTGACTGCAGGCTGGGGGACTGAA
GTCTTT---ACG------TGTAAAAATTACTGGTTCTGGGTCCCACTCGTGGCGCCCCTA
ATTGGAGGCCCTTTAGGCTCTTTCCTTTATGTGGTCTTCATCGAATGGCAGCTGCCTGAT
---------------CCAGAACCCCCACAG---------------------AAC------
------------CTCTCA---ACAGTTTCC------------------------ACC---
---------AACAGC---------GACAGC------------ATCAAAAAGCCTGCCACC
AGGTGGGGGACAGGAGGCGATTTAAAG---------------------------------

---TCCACATATTTT------
>Zebrafish_Aqp10a
---ATGAGTCAG
---------------ATGAAGAAGATCATGAAGAGGATG------AAGGTGAAAAATGAA
CTGGCACGACAGATTATGGGAGAGATCTTGGGCACTTTTGTTCTTCTGTTGTTTGGTTGT
GCTGCAGCGGCTCAGGTGAAAACCAGCAGA------GAAACAAAGGGGCAGTTTCTGTCT
GGTAACATCGCCTTCTCTGTAGGTGTCATGTCTGCCATGTACCTCTGCAGGGCAGTATCA
GGAGCTCATCTAAACCCAGCTGTGTCTCTGAGTTTCTGTGTATTGGGAGACCTGGCCTGG
ATAAAGCTGCTACCATATTCTCTCGCTCAAATTTTAGGGGCTTACCTTGCTTCAGGGCTT
GTCTAT---CTCATCTACCATGATGCCATCATGGAGTTCAGTGGT---GGAGTTCTGACC
GTATTTGGCCCTAAT---GAAACAGCCAGTATCTTCGCCACTTACCCAACCGATGTAGTA
TCAGTGCAGACCAATTTCCTGGATCAGGTGGTTGGCACAGCCATGTTGATGCTGTGCATT
TTGCCTCTGAATGATAAGAGAAACGCCCCAGCTCCTGAAGCGCTGCTCCCACCCATTGTA
GCCACTGTTGTTCTAGGGATTTCCATCTCAATGTCTGCTAATTGTGGAGCAGCCATAAAT
CCAGCACGTGATCTT---GGTCCACGACTCTTTACCTTTACAGCAGGCTGGGGCACTGAA
GTCTTT---ACG------TGCTATGACTACTTCTTTTGGATCCCATTGGTGGCTCCTATG
GTAGGGGGTGTCCTGGGCTCCATCATTTATTTGGTTTTCATCCAGTGGCATCTGCCTGAG
---------------CTTGAAGATGAATCT---------------------GAA------
------------TCTGAG---GAGATGAAT------------------------GAT---
---------CAAACA---------AAAGTC------------ATGGAG------------
CACAACAACAAAAAAGATGAGATATAC---------------------------------

---CTT
AAAATGTCTTCAATT------
>Bowfin_Aqp10aa
---ATGTTG---
---------------ATGGAGCGAGCCAGGCGGGGGCTG------AGGCTGAGCAGCCCG
CTGGCAAGAGAGTGCCTTAGTGAGTTTATGGGCACGTTTGTCCTGCTGCTGTTTGGCTGT
GCAGCCTCTGCGCAGGTGAGGACGACGGGG------GGTGAGAAGGGACAGTACCTGTCC
ATCAACCTGGCATTCTCTGTAGGCGTGATGTCGGCCATGTACCTGTGCATGGGGGTCTCA
GGTGCTCACCTGAACCCTGCAGTGTCCCTGAGTTTCTGTGTGCTGGGGCGGCTGCAGTGG
CCCCGGCTGCCCTGCTATGCTGTGGTCCAGGTGCTGGGCGCCTTCACAGCCTCGGCCGTG
GTCTTC---CTGCAGTACTACGACGCGATCATGGATTTCTCGGGG---GGCAACTTGACA
GTGACTGGCCCCACA---GAGACGGCGTCCATCTTCGCCACCTACCCCTCCCCCCACCTC
AGCCTGTCTAGCAGCTTCATCGACCAGGTGATCGGCACGGCTACGCTGTTGCTCTGCATC
CTGCCCCTTGATGACAAGCTGAACAGCCCTGCCCCCCCCGGCCTGGTGCCCCCACTGGCT
GCCGTGGTGGTGCTGGGCATCTCCATGTCGATGAGTGCCAACTGCGGGGCCGCCATTAAC
CCGGCGCGTGACCTG---GGGCCCCGGCTGTTCACCCTGACCGCAGGCTGGGGGCCTGAG
GTTTTC---ACG------GCATACTCTCACTGGTGGTGGGTACCATTAGTCGCCCCAATG
CTTGGTGGAGTTCTGGGCTCACTGCTTTATCTCGTGTTCATCCAGTGGCACCTCCTTGAC
---------------CCGGACAGTCACCCT---------------------GGC------

------------ACAGAA---CCAGAACCC------------------GGGTCTGAA---
---CCCATGGCCAAAGGAGTG---GGGAAG------------ATGGAGAGAGAG------

---AGTATAAAGCTG------
>Gar_Aqp10aa

---------------------------GTGGTTGGCACGGCGACGCTGTTGCTGTGCATC
CTGCCCTTGGACGACAGGCTCAACAGCCCCGCCCCCAAGGGCCTGGTGCCGCCATTGGTT
GGCCTGGTCGTCCTGGGGATCTCCATGGCGATGAGCTCCAACTGCGGGGCCGCCATCAAC
CCGGCCCGCGACCTC---GGGCCGCGGCTGTTCACGCTGACCGCGGGCTGGGGGACCGAG
GTGTTC---ACG------GCGTACCGACACTGGTGGTGGGTACCTGTGGTCGCCCCGATG
CTGGGCTCGGTCCTGGGCAGCTGCCTGTACCTCACCTTCATCCAGTGGCACCTTCCCGAC
---------------CCGCAGGGGGGCCCG---------------------AGC------
------------CCAGAC---GCCGCCTCC------------------------AGA---
---------ACCAAG--------------------------ATCCAGGCCCCCTCAGCC
CCAGCCCCGCACAGCGACTCCGAGACCATCGCC---------------------------

--ATGCCCAGCTGG
GACCCC---------------
>Russian_sturgeon_Aqp10aa

--TTGTTTGGTTGC
GCGGCAGCAGCGCAGGTCAAGACCACCAAC------GAGGTGAAGGGTCAGTACCTCTCC
GTCAACCTGGCGTTCGCTGTGGGCGTCGTCTCCGCCGTCTACATCTCCCGAGGAGTCTCC
GGAGCGCACCTGAACCCCGCCGTCTCTCTCAGCTTCTGTGTGCTGGGGAGGTTCCAGTGG
AAACGGTTGCCTTTTTACACCCTCTTCCAAATCATGGGGGCCTTCGCGGCGTCCGGAACG
GTCTTC---ATTCAATACTACGACGCCATCATGCAGTACAGCGGG---GGTAACCTCACA
GTCACCGGGCCCTTA---GAGACGGCCTCTATCTTCGCTACCTACCCCAGTGAATATCTC
TCGTTTTCAAATGCGTTCGTGGACCAGGTGATCGGCACAGCCGCCCTGCTCCTGTGCATC
CTCCCCCTGGATGATTCCCGGAACAGCCCGGCTCCCGAGGGTCTGCAGCCCCTGCTGGTT
GGCTTCGTGGTCCTGGGGATTGGCATGGCCATGAGCTCCAACTGCGGGGGGGCCATCAAC
CCCGCCAGGGACCTG---GGGCCCCGCCTGTTCACCCTGGCAGCGGGCTGGGGCCCCGAA
GTCTTC---ACG------GCGTTTGAGAACTGGTGGTGGATTCCCGTTGTGGCCCCGCTG
GTCGGTGGAGTGACGGGGGCTTCACTGTATCAGCTTTTCATTGAGTTTCATCACAAGGAC
---------------CCGGAGACAGACCAG---------------------CCC------
------------GAGGCC---AAGACGCGA------------------------GAG---
---------AACGGG---------GCGTCCCAA---------ACCAATCTGGGG------

---GACTCGGGTTCATAC
CTGACCAGCAAGTTT------
>Sterlet_Aqp10aa
---ATGGTCCCT
---------------ATTATGAAACTCAAAGACAAACTC------AAACTGAAAAATAGG
CTTCTGAGAGAATGCTTAGCGGAGTTTTTGGGAACATTTGTGCTCATGTTGTTTGGTTGC
GCGGCAGCAGCGCAGGTCAAGACCACCAAC------GAGGTGAAGGGTCAGTACCTCTCC
GTCAACCTGGCGTTCGCTGTGGGCGTCGTCTCCGCCGTCTACATCTCCCGAGGAGTCTCC
GGAGCGCACCTGAACCCCGCCGTCTCTCTCAGCTTCTGTGTGCTGGGGAGGTTCCAGTGG
AAACGGTTGCCTTTTTACACCCTCTTCCAAATCATGGGGGCCTTCGCGGCGTCCGGAACG
GTCTTC---ATTCAATACTACGACGCCATCATGCAGTACAGCGGG---GGCAACCTCACA

```
GTCACTGGGCCCTTA---GAGACGGCCTCTATCTTCGCTACCTACCCCAGTGAATATCTC
TCGTTTTCAAATGCGTTCGTGGACCAGGTGATCGGCACAGCCGCCCTGCTCCTGTGCATC
CTCCCCCTGGATGATTCCCGGAACAGCCCGGCTCCCGAGGGTCTGCAGCCCCTGCTGGTT
GGCTTCGTGGTCCTGGGGATTGGCATGGCCATGAGCTCCAACTGCGGGGGGGCCATCAAC
CCCGCCAGGGACCTG---GGGCCCCGCCTGTTCACCCTGGCAGCGGGCTGGGGCCCCGAA
GTCTTC---ACG------GCGTTTGAGAACTGGTGGTGGATTCCCGTTGTGGCCCCTCTG
ATCGGTGGAGTGACGGGGGCGTCACTGTATCAGCTTTTCATTGAGTTTCATCACGAGGAC
---------------CCGGAGACAGACCAG---------------------CCC------
------------GAGGCG---AAGACGCGA------------------------GAG---
---------AACGGG---------ACGTCCCAA---------ACCAGC------------
------------------------------------------------------------
------------------------------------------------------------
------------------------------------------------------------
---------AAGTTT------
>Common_sturgeon_Aqp10aa
---------------------------------------------------ATGTTCCCT
---------------ATTATGAAACTCAAAGACAAACTC------AAACTGAAAAACAGG
CTCCTGAGGGAATGCTTAGCGGAGTTTTTGGGAACATTTGTGCTCATGTTGTTTGGTTGC
GCGGCAGCAGCGCAGGTCAAGACCACCAGC------GAGGTGAAGGGTCAGTACCTCTCC
GTCAACCTGGCGTTCGCTGTGGGCGTCGTCTCCGCCGTCTACATCTCCCGAGGAGTCTCC
GGAGCGCACCTGAACCCCGCCGTCTCTCTCAGCTTCTGTGTGCTGGGGAGGTTCCAGTGG
AAACGGTTGCCTTTTTACATCCTCTTCCAAATCATGGGGGCCTTCGCGGCGTCCGGAACG
GTCTTC---ATTCAATACTACGACGCCATCATGCAGTACAGCGGG---GGCAACCTCACA
GTCACTGGGCCCTTA---GAGACGGCCTCTATCTTCGCTACCTACCCCAGTGAATATCTC
TCGTTTTCAAATGCGTTCGTGGACCAGGTGATCGGCACAGCCACCCTGCTCCTGTGCATC
CTCCCCCTGGATGATTCCCTGAACAGCCCGGCTCCCGAGGGTCTGCAGCCCCTGCTGGTC
GGCTTCGTGGTCCTGGGGATTGGCATGGCCATGAGCTCCAACTGCGGGGGGGCCATCAAC
CCCGCCAGGGACCTG---GGGCCCCGCCTGTTCACCCTGGCAGCGGGCTGGGGCCCCGAA
GTCTTC---ACG------GCGTTTGAGAGCTGGTGGTGGATTCCCGTTGTGGCCCCGCTG
GTCGGTGGAGTGACGGGGGCTTCACTGTATCAGCTTTTCATTGAGTTTCATCACGAGGAC
---------------CCGGAGACAGACCAG---------------------CCC------
------------GAGGCC---AAGACGCGA------------------------GAG---
---------AACGGG---------GCGTCCCAA---------ACCAATCTGGGG------
------------------------------------------------------------
------------------------------------------------------------
----------------------------------------------GACTCGGGTTCATAC
CTGACCAGCAAGTTT------
>Chinese_sturgeon_Aqp10aa
------------------------------------------------------------
------------------------------------------------------------
------------------------------------------------TTGTTTGGTTGC
GCGGCAGCAGCGCAGGTCAAGACCACCAAC------GAGGTGAAGGGTCAGTACCTCTCC
GTCAACCTGGCGTTCGCTGTGGGAGTCGTCTCCGCCGTCTACATCTCCCGAGGAGTCTCC
GGTGCGCACCTGAACCCCGCCGTCTCTCTCAGCTTCTGTGTGCTGGGGAGGCTCCAGTGG
AAACGGTTGCCTTTTTACGCCCTCTTCCAAATCATGGGGGCCTTCGCGGCGTCTGGAACG
GTCTTC---ATTCAATACTACGACGCCATCATGCAGTACAGCGGG---GGCAACCTCACA
GTGACTGGGCCCTTA---GAGACGGCCTCTATCTTCGCTACCTACCCCAGTGAATATCTC
TCGTTTTCAAATGCGTTCGTGGACCAGGTGATCGGCACAGCCGCCCTGCTCCTGTGCATC
CTCCCCCTGGATGATTCCCGGAACAGCCCGGCTCCCGAGGGTCTGCAGCCCCTGCTGGTC
GGCCTCGTGGTCCTGGGGATCGGCATGGCCATGAGCTCCAACTGCGGGGGGGCAATCAAC
CCCGCCAGGGACCTG---GGGCCCCGCCTGTTCACCCTGGCAGCGGGCTGGGGCCCCGAA
GTCTTC---ACG------GCGTTTGGGAACTGGTGGTGGATTCCTGTTGTAGCTCCGCTG
ATCGGAGGAGTGACGGGGGCTTCACTGTATCAGCTTTTCATTGAGTTTCATCACGAGGAC
---------------ACGGAGACAGACCAG---------------------CCC------
------------GAGGCG---AAGACGCGA------------------------GAG---
---------GACGGG---------GCGTCCCAA---------AACAATCTGGGG------
------------------------------------------------------------
------------------------------------------------------------
----------------------------------------------GACTCGGGTTCACAC
CTGACCAGCAAGTTT------
>Reedfish_Aqp10aa
```

---------------ATGCAGAAACTAAGACATCTTCTC------AGACTGAAGAACAAA
CTTCTGAAGGAATGCTTAGCAGAGTTTTCTGGGAACATTTGTTCTGTTCTGTTTGGATGT
TCTGCTGCTGCTCAAGTGAAAACAAGTCAT------GAGACTAAAGGCCAGTATTTATCT
ATCAATATGGCCTTTGCTATCGGAGTCATTTTTGCTATGTACATCTCACGAGGAGTTTCA
GGAGCACACCTAAACCCAGCAGTTTCCCTCAGTTTTTGCATGATTGGCCGATTTCCTTGG
ACCAAAGTACCTATTTATATATTTTTTCAGATATTTGGTGCATATATGGCTTCTGCTACT
GTATTT---GCCCTTTATTATGATGCTATAATGAATTACAGTGGT---GGTAATCTGACA
GTTACTGGATCCCAA---GAAACTGCCTCCATCTTTGCTACCTATCCATCTGACTACCTG
TCTCAAGCAAACGCATTTTTTGATCAGGTAGTAGGAACAGCTGCTTTGCTGCTCTGTATT
CTGCCAATTGAAGATGCCCACAATAGCCCTGCTCCGAAAGGCTTTGAACCAGTACTTGTT
GGATTTCTGGTACTAGGAATAAGTATGTCGATGAGTTCAAACTGTGGTGCTGCCATCAAT
CCAGCACGTGACCTG---GGCCCAAGACTCTTCACATGGTCTGCAGGCTGGGGGACAGCG
GTGTTC---ACG------GCCTTTGATAACTGGTGGTGGATCCCTATTGTTGCTCCTATG
TTAGGAGGGGTGATTGGTGCTTATATTTATCTGCTTTTCATTGAATTTCATCATGAAGAT
---------------TCTGAC---------------------------------------

--TGGGGCTATCAC
ATCAGTAGCCGGCTT------
>B_bambooshark_Aqp10C1
---ATGGGTAGA
GCAGCC---ACAATCCTGGTCAAAGTCCACGATGCGTTT------CGATTGAAGAACAAA
CTCTTCAGAGAGTGTCTGGCAGAGTTCCTGGGGGTCTGCATGTTGATTCTGTTTGGCTGT
GGAGCTGTAGCGCAGATGGTAGTCAGTAAC------ACGACACGTGGTGAATTCCTGTCG
GTCAATCTCGGCTTTGGACTCGGGGCAACGTTTGGAATCTACATCTCCGGAGGGATCTCA
GGT---

------------------------CAGTTGATTGGCACCGGCACCCTCCTCCTGTGCATC
TTCGCGGTGGTGGACTCCCAGAACTACGGCGCTCCCAAGATCCTTCAGCCCATCTTCATC
GGCCTTTCGGTGGTGGGCATCGGGATGTCCATGGGCTCCAATTCTGGTTACGCCATCAAT
CCCGCCCGGGACTTC---GGGCCACGTCTGCTCACCCTGGCCGCGGGATGGGGCACCGAG
GTCTTC---ACG------GCCGGGAACGGTTGGTGGTGGATCCCCATTGTGGCGCCCATG
GTGGGCGCGGTGCTGGGGGCCCTGGTCTACGAGTTGCTGGTGGAGCTGCACCATCTGGAG
---------------GCCAAGTCAGGTCCC---------------------ACC------
------------AACTAC---GCCGATGAG------------------------GAG---
---------ACG------------CTGAAG---------------------GGGACGGAT
GCCAGGGGCAAGGACGGCCCGGCCATA---------------------------------
---------CGGAGGGACAAGGCCGGAGCA------------------------------
--GATGACCAGTTC
GTCATGGCGATG---------
>W_bambooshark_Aqp10C1
---ATGGGGAGA
GCAGCC---ACAATCCTGGTCAAAGTCCACGATGCATTT------CGATTGAAGAACAAA
CTCTTCAGAGAGTGTCTGGCAGAGTTCCTGGGGGTCTGCATGTTGATTCTGTTTGGATGT
GGAGCTGTCGCGCAGATGGTCGTCAGTAAC------ACGACACGTGGTGAATTCCTGTCG
GTCAATCTCGGCTTCGGACTCGGGGCAACGTTCGGAATTTACATCTCCGGAGGGATCTCA
GGGGGCCATCTGAATCCGGCCGTGTCCTTCAGTCTGTGCCTGCTTGGCCGGTTCCAATGG
AAGAAGTTGCCCTTTTACATGTTCTTCCAGACCCTGGGGGGGTTTGTCGGAGCGGCTGTA
GTCTAT---GGGGTGCATCACGATGGGATCCATGCTGTGAACAAC---GGGACTTTGTCT
GTCACCGGGCCACGT---GCCACCGCCTTCATTTTTGGCACCTACCCTGCACCGTTCCTC
ACCCTTCCCAACGGCTTTATAGACCAGTTGATTGGCACCGGCACCCTCCTCCTGTGCATC
TTCGCGGTGGTGGACTCCCAGAACTACGGCGCTCCCAAGATCCTTCAGCCCATCTTCATC
GGCCTGTCGGTGGTGGGCATCGGGATGTCCATGGGCTCCAATTCCGGTTACGCCATCAAT
CCCGCCCGGGACTTC---GGGCCGCGTCTGCTCACCCTGGCCGCGGGATGGGGCACCGAG
GTCTTC---ACG------GCCGGGAACGGTTGGTGGTGGATCCCCATTGTGGCGCCCATG
GTGGGCGCGGTGCTGGGGGCCCTGGTCTACGAGTTGCTGGTGGAGCTGCACCACCTGGAG

---------------GCCAAGTCGGGTGCC---------------------GCC------
------------AACTAC---GCCGACGAG-----------------------GAG---
---------ACG------------CTGAAG---------------------GGGACGGGT
GCCGGGGGCAAGGACGGCCCGGCCGTG---------------------------------
---------CGGAGGGACAAGGCCGGAGCA------------------------------
--GATGACCAGTTC
GTCATGGCGATG---------
>Whale_shark_Aqp10C1
---ATGGGCCAA
GCAGCC---ACAATCCAAGAGAAGGTCCTTGATGCGTTT------CGACTGAAGAATCAA
CTGTTCAGAGAATGTTTGGCTGAGTTTCTGGGAGTCTGCATGTTGATCCTGTTTGGGTGT
GGGGCAGTGGCACAGATGGTGGTCAGTAAC------ACAACACGTGGTGAATTTCTGTCG
GTCAATCTCGGCTTCGGGCTTGGTGCCACGTTTGGGATCTACATCTCCGGCGGGATCTCA
GGGGGCCATCTGAACCCGGCTGTGTCCTTCAGTCTGTGTTTACTCGGGAGGTTCCAGTGG
AAGAAATTGCCCTTTTACATGTTCTTCCAGACCCTGGGAGGGTTTGTCGGAGCTGCTGTA
GTCTAT---GGGGTACATCAC---------------------------------------

------------------GCCGGGAACAGTTGGTGGTGGGTCCCCATCGTGGCTCCCATG
GTGGGCGCGGTGCTGGGGGCATTGGTGTACGAGCTCCTGGTGGAACTACACCATCTGCAG
---------------GCCAAGGCAGTGCCC---------------------ACA------
------------GACGAC---GACATGATG-----------------------GAG---
---------CAGAAG---------CTGAAG------------GAGGTCGGGGGCCGGGGC
ACAGCGGGCAAGGATGGCCCGTCGGTG---------------------------------
---------CGGAACGACAAGGCCAGAGCG------------------------------
--GACGACGAATTT
GTCATGGCAATG---------
>Great_white_shark_Aqp10C1
---ATGGGGACA
ACCGAG---ATGGTGTTACAAAAGGTGCATGGTGCACTG------CGATTGAAGAATCAC
CTCGTGAGGGAGTGCTTCGCCGAGTTTCTGGGAGTCTGCATGCTTATCCTCTTCGGATGT
GGTGCCGTGGCACAAATGGTGGTGAGTAAG------ACAACCCGCGGTGAATTCCTGTCC
GTCAACCTGGGCTTCGGACTCGGAGCCACGTTTGGCATCTACATTTCGGGCGGCATCTCT
GGTGGCCATCTGAACCCAGCTGTATCCTTCAGCTTGTGTTTACTCGGACGGTTCCCATGG
AAGAAGCTCCCATTTTACATGTTCTTCCAAACTCTGGGAGGATTTGTTGGTGCTGCCATC
GTCTAC---GGAGTTCATCACGATGGGATACACTCTGTTGACAAT---GGGACCTTATCT
GTCACCGGACCACGA---GCCACTGCATTTATCTTTGGCACCTACCCCGCACCGTTCCTC
AGCCTTTCGAACGGCTTCATTGACCAGGTAATTGGCACTGGCACCTTGCTCCTGTGCATC
TTCGCCGTGGTGGACACCCAGAACTATGGGGCCCCCAAGATCTTGCAGCCCATCTTCATC
GGCCTGTCGATCGTGGGCATCGGGATGTCGATGGGATCCAACTCCGGCTATGCCATCAAC
CCCGCCCGGGATTTC---GGGCCCCGCCTGTTGACCCTCGCTGCCGGCTGGGGGACCGAG
GTTTTC---ACG--

>Zebra_bullhead_shark_Aqp10C1
---ATGGGGAGA
GTAGAG---ACGTTTTTCAGAGCACTACATGATGGGTTG------CAGTTGAAGAATCGC
CTCTTCAGGGAATGCTTAGCGGAGTTTCTAGGAGTTTGCATGCTGATTTTGTTTGGCTGT
GGTGCCGTGGCACAGATGGTGACGAGCCAC------ACAACCCGCGGAGAATTCCTGTCT
GTCAACCTAGGATTCGGCCTTGGCGCAACCTTTGGCATCTACATCTCAGGTGGCATCTCG
GGTGGCCATCTGAACCCGGCCGTGTCCTTCAGTTTGTGTTTGCTGGGAAGGTTCCAGTGG
AAGAAGCTGCCATTCTACATGTTCTTCCAGACACTTGGGGGTTTTGTTGGTGCTGCCATA

GTCTTT---GGGGTCCACCATGATGGTATCTACGCTGTTGATAAC---GGGACCTTGTCC
GTCACCGGACCGCGG---GCCACTGCATTTATTTTGGCACGTATCCCGCAGCATACCTC
ACCCTTCCGAATGGCTTCATTGACCAGCTGATTGGCACCGGCACTCTGCTCCTCTGTATT
TTTGCCGTGGTGGATGCCCGGAACTACGGAGCCCCGAAGATCTTACAGCCCATCTTCATC
GGTCTGTCGGTGGTTGGCATCGGGATGTCCATGGGCTCCAACTCTGGCTATGCCATCAAC
CCCGCCCGTGATTTT---GGGCCCCGCCTGTTAACCCTGGCCGCCGGCTGGGGCACCGAA
GTCTTT---ACG------GCTGGAGGCGGGTGGTGGTGGATCCCCATCGTGGCGCCGATG
GTTGGCGCGGTCCTTGGCGCCCTGGCCTACGAGCTGCTGATCGAGCTTCACCACCTGCAG
---------------GCCAGGGACGGGCCC---------------------AGC------
------------GAGAGG---GACGTGACG------------------------GAG---
---------CAGAAG---------CTGAAG------------GAGATGGGCAATCCGGCC
GCCGGCTGCAAGGAGGGCCCGGCCTGG---------------------------------
---------CGGAACGAGGCGGCGGGCGCG------------------------------
--GGTGACCAGTTC
GCCGTGGCGATG---------
>Cloudy_catshark_Aqp10C1
---ATGGGGAGA
GCTGCA---GCAATAGCACAGACGGTGCACAATGCTCTG------CAGCTGAGGAACCGT
CTCTTTCGCGAATGCTTGGCTGAGTTTCTGGGGGTGTGCATGCTGATCCTGTTCGGGTGC
GGGGCCGTGGCACAGATGGTGGTCAGCAAC------ACTACCCGTGGGGAATTCCTCTCC
GTCAACCTGGGCTTTGGACTGGGCGCCACGTTCGGCATCTACATCGCTGGTGGGATCTCA
GGT---

---------------------GATGGGATTCACGCTGTCAATAAC---GGGACCTTATCT
GTCACTGGGCCACGA---GCAACTGCCTTCATCTTTGGCACCTATCCAGCACCATTCCTC
ACCCTTTCGAATGGCTTCATTGACCAGCTAATCGGCACCGCCACTCTACTGCTTTGCATC
TTCGCTGTCCTGGACGGCAAGAACAACGGCGCCCCCAAAGTCCTGCAGCCTATCTTCATT
GGCCTGTCGGTTGTCGGCATCGGAATGTCCATGGGATCCAACTCCGGCTACGCCATCAAC
CCCGCCCGAGATTTC---GGACCCCGCCTGCTGACCCTCGCCGCGGGCTGGGGGACTGAG
GTCTTC---ACG------GCTGGCAACTCCTGGTGGTGGATACCCATCGTGGCGCCCATG
GTGGGCGCCGTCCTGGGCGCCCTGGCGTACGAGCTGCTGGTTGAACTGCACCACCTGCAG
---------------GCCCAGGACGAGTGC---------------------GAG------
------------GGGAGG---GAGCAGACG------------------------GAC---
---------CAGAAG---------CTGAAG------------GAGGTGGGC---------
---GGCTGCAAGGAGGGCCCCGCCTGC---------------------------------
---------CGGAGAGACCCGGACAGCTCG------------------------------
--GGGGACCAGTTC
GTCATGGCGATG---------
>Little_skate_Aqp10C1
---ATGGAGCAG
GTCCGG---GCCATCTGCAGCTCGGTTCGCGAATCACTA------AAGCTGAAGAACCGC
CTGTTCCGTGAGTGTTTGGCTGAATTTCTGGGAGTTTGCGTTTTGATT------------

GGTGCACATCTGAATCCAGCCGTGTCCTTCAGTCTGTGTCTGCTGGGAAGATTCCAATGG
AAGAAACTACCGTTTTACATGTTCTTCCAGACACTCGGAGGGTTTGTTGGAGCTGCCATA
GTCTAC---GGAGTCCACCAC---------------------------------------

-------------------------CAGCTGATCGGCACGGGAACGCTGCTGCTCTGTATC
CTGGCTGTGGTGGACAGCAAGAACAATGGGGCTCCGAAGGCCCTTCAGCCCATCTTCATA
GGACTGTCCGTGCTGGCCATCGGGATGTCCATGGGATCCAACTCGGGATACGCCATCAAT
CCGGCCCGAGACTTC---GGACCACGGCTGCTCACACTCGCTGCTGGGTGGGGAACTGAG
GTCTTC---ACG--


```
>Thorny_skate_Aqp10C1
---------------------------------------------------ATGGAGCGG
GTCCGG---GCCATCTTCAGCCCGGTTCGCGAATCACTA------AAGCTGAAGAACCGC
CTGTTCCGTGAGTGTTTGGCTGAATTTCTGGGAGTTTGCATTTTGATTTTATTTGGCTGT
GGGGCAGTGGCCCAGATGGTCACCAGCCAC------ACCACCCGCGGGGAATTCCTCTCC
GTCAACCTCGGCTTCGGGATCGGAGCCACATTCGGAGTTTGTATCGCGGGAGAAATCTCC
GGAGCGCATCTGAACCCAGCCGTGTCCTTCAGCCTGTGTCTGCTCGGCCGGTTCCAATGG
AAGAAACTACCTTTCTACATGTTCTTCCAGACGCTCGGAGGGTTTGTTGGAGCTGCCATA
GTCTAC---GGAGTCCACCACGATGGGATACATGCCGTCAACAAC---GGAACTCTCTCT
GTCACTGGCCCCAAT---GCCACTGCT---------------------------------
------------------------------------------------------------
------------------------------------------------------------
------------------------------------------------------------
------------------------------------------------------------
------------------------------------------------------------
------------------------------------------------------------
------------------------------------------------------------
------------------------------------------------------------
------------------------------------------------------------
------------------------------------------------------------
------------------------------------------------------------
------------------------------------------------------------
---------------------
>Smalltooth_sawfish_Aqp10C1
---------------------------------------------------ATGGAGAGG
GTCGGG---GCACTGGGGAGAAGTTTGCACCGAGCTCTG------CAGCTCAAGAGTCGC
CTCTTCAGGGAATGTTTGGCCGAGTTTCTTGGAGTCTGCATTCTGGTTCTGTTCGGCTGC
GGGGCCGTCGCGCAGATGGTCACCAGTCAC------ACCAACGCCGGGAAGTTCCTGTCC
GTCAACCTGGGATTCGGGCTGGGAGCCACCTTCGGGGTCTACATCGCCGGGGGAATCTCC
GGAGCCCATCTGAATCCAGCCGTGTCCTTCAGCATGTGTTTGCTGGGAAGGTTCCAGTGG
AGGAAGCTGCCGTTTTATATGTTCTTCCAGACCCTCGGGGGATTCGTTGGCGCTGCGGTC
GTGTAC---GGTGTGCATCACGATGGCATCTACGCGGTGAACAAT---GGCACCCTCTCT
GTCACCGGCCCAAGA---GCCACCGCCTTCATTTTCGGCACCTACCCTGCGCCCTTCCTG
ACCCTCCTGAATGGCTTCATTGACCAGCTGATCGGGACGGGGACGCTTCTGCTCTGTATC
CTGGCCGTGGGGGACACCCAGAACAACGGAGCTCCCAAGGTGCTTCAGCCCATCTTCATC
GGCCTATCCATCGTGGCCATCGGAATGTCCATGGGCTCCAACTCAGGATACGCCATCAAC
CCCGCCCGAGACCTT---GGACCTCGCCTCCTGACCCTGGTGGCGGGTTGGGGGACAGAA
GTCTTC---ACG------GCCGGGGGAGGCTGGTGGTGGGTGCCGATCGTGGCGCCGATG
GTGGGGGCCATCCTGGGCTCCGCGGTCTACGAGCTGCTGATCGAGTTCCACCACTTGGAG
---------------GACCAGAGGAGGGCC---------------------GAG------
------------GAGCTG---GGTCTGAAG------------------------GAG---
---------GCACGG---------AGGCAG------------AGGTCGGAGGGCGAGGAG
GCGAGGCAGCCGCAGACGGTCGTCTAC---------------------------------
---------AAGAAGGAGGAGGAGGAGGGG------------------------------
------------------------------------------------GGGCACTACATG
---------------------
>B_bambooshark_Aqp10C2
------------------------------------------------------------
---------------ATGAGGAGCCTCCGTCAGAAGCTG------CAGATCAGGAACAGG
CTGGCCCGGGAATGCCTGGCCGAATTCTTCGGGGAATACATGCTCATTCTCATGGGCACA
GCGGCAGTGGCTCAAGTGGTGACAAACTTC------GATCAGAAAGGGACCTATTTATCG
ATTAACATTGGCTATGCTGCTGGAGTTCTGTTTGGGATCTACGCCTCAGTTGGAGTCTCA
GGGGCTCACCTGAACCCGGCGGTGACCTTCAGCCTGTGCGTCCTGGGACGGTTTCCCTGG
AAAAAGCTTCCGTTCTACACCATCTCCGAGTGCCTGGGGTCATTCGTCGCCTCGGCAACC
ACGTTC---ACCCTCTACTACGACGCCATCCAAGAGTTCTCTGGT---GGCAATCTGACC
GTGCGTGGGCCGAGG---GGAACAGCTGGCATCTTTGCGACGTATCCCGTGGAGTATCTG
TCTGTCCGCAACGGCTTCATAACTGAGGTAATTGGCACCGCTGTTTTGTTGATTTGCGTC
TTGAGTGTTGGCGACGCCAAAAATGCTGGTGCCCCGCCTTTCATCCAGCCGTTGCTGATC
TCGGTCTCGGTACTCGTCATCGGTGCTGCTATGGGTGCCAACACCGGCTACGCCATCAAC
CCAGCGAGAGACCTC---GGACCCAGACTCTTCACCTTCGTGGCTGGCTGGGGGACTGAA
GTTTTC---AAG------GCCGGGAATGGCTGGTGGTGGATCCCTATCGTCGCACCCCTG
```

ATTGGCGGTGTACTAGGCAGCCTGGCCTACACGCTCCTCATCGACCTGCACCACGCGGAG
---------------CCCGTCTCGGCCAAA---------------------GAG------
------------GAGGTG---AAGGACGTC------------------------AAG---
---------GCTGAA---------CCTCAA------------GCTGAGTCAGAGGAGGCC
GCGGAGGAGCCTGTT---

>W_bambooshark_Aqp10C2

---------------ATGAGGAGTCTCCGTCAGAAGCTG------CAGATCAGGAACAGG
CTGGCCCGGGAATGCCTGGCCGAATTCCTCGGGGAATACATGCTCATTCTCATGGGCACA
GCGGCAGTGGCTCAGGTGGTGACAAACTTC------GATCAGAAAGGGACCTATTTATCG
ATTAACATTGGCTATGCTGCTGGAGTTCTGTTTGGGATCTACGCCTCAGTTGGAGTCTCA
GGGGCTCACCTGAACCCGGCGGTGACCTTCAGCCTGTGCGTCCTGGGCCGGTTTCCCTGG
AGAAAGCTTCCGTTCTACACCATCTCCGAGTGCCTGGGGTCATTCGTCGCCTCGGCAACC
ACGTTC---ACCCTCTACTACGACGCCATCCACGAGTTCTCTGGT---GGCAATCTGACC
GTGCGTGGGCCGAGG---GGAACAGCTGGCATCTTTGCGACGTATCCCGTCGAGTACCTG
TCTGTCCGCAACGGCTTCATAACTGAGGTAATTGGCACCGCTGTTTTGTTGATTTGCGTC
TTGAGTGTCGGCGACGCCAAAAATGCTGGTGCCCCGGCTTTCCTCCAGCCGTTGCTGATC
TCGGTCTCGGTACTCGTCATCGGTGCTGCTATGGGTGCCAACACCGGCTATGCCATCAAC
CCAGCGAGAGACCTT---GGACCCAGACTCTTCACATTCGTGGCTGGCTGGGGGACTGAA
GTTTTC---AAG------GCCGGGAACGGCTGGTGGTGGATCCCTATCGTCGCGCCCCTG
ATTGGCGGCGTAATAGGCAGCCTGGCCTACACGCTCCTCATCGAACTGCACCACGCGGAA
---------------CCCGTGTCGGCCAAA---------------------GAG------
------------GAGGTG---AAGGACGTC------------------------AAG---
---------GCCGAA---------CCTCAA------------GCTGAAACAGAGGAGGCC
GGGGAGGAGCCTGTT---

>Whale_shark_Aqp10C2

---------------ATGAAGTCCCTCCGTCGGAAGCTG------CAGATCCGGAACCAG
CTCGCCCGGGAATGCCTCGCCGAATTCCTCGGGGAATACATGCTGATT------------

---------------------GATGCCATCCAGGAATTTTCCGGG---GGCAATCTGACC
GTGCGTGGACCAAGG---GGAACGGCCGGAATCTTCGCAACGTATCCTGTTGAGTACCTG
TCCATCCGGAATGGGTTCCTG---CAGTTGATTGCCACCGCTGTTTTGTTGATCTGCATC
ATGTGTGTCGGCGACGCCAAAAACGCCGGTGCTCCGGCTTTCCTGCACCCCCTGCTGGTC
TCGGTCGCCGTGCTCGTCATCGGTGCCACCATGGGTGCTAACACCGGCTATGCCATCAAC
CCCGCACGGGACCTT---GGACCCAGGCTCTTCACCTTCATAGCTGGATGGGGCACTGAG
GTGTTC---AGG--

>Zebra_bullhead_shark_Aqp10C2

---TCA
GGTGCTCACTTGAACCCTGCCGTCAGTCTATGCCTCTGCGTGCTCGGCCGCTTCCCGTGG

```
AAGAAGCTGCCGTTTTACACCTTGGCCGAGTGTCTGGGGTCCTTCACCGCCGCGGCCACC
ACCTTC---TGCCTCTATTACGATGCAATACAGGAATTTTCTCAA---GGGAATTTAACT
GTTCGTGGCCCAAGA---GGCACAGCAGGTTTATTCGCCACCTACCCTGTTGAAAACCTC
AGCGTTCGTAATGGATTCATCACGGAGGTGATAGGCACCGCTGTTTTGCTAATCTGTATT
CTGGCTATCGGTGATGCCAAAAACGCGGGCGCTCCCGCTTTCCTGCAGCCCCCCCTGGTC
GCCATTTCGGTTTTCGCCATTGGCGTTGGGTTGGGTGCCAACACTGGCTATGCCATCAAC
CCAGCCAGAGACATA---GGA---------------------------------------
------------------------------------------------------------
------------------------------------------------------------
------------------------------------------------------------
------------------------------------------------------------
------------------------------------------------------------
------------------------------------------------------------
------------------------------------------------------------
------------------------------------------------------------
---------------------
>Cloudy_catshark_Aqp10C2
------------------------------------------------------------
---------------ATGAACAGTTTGAAGAAAAAACTC------CAGATTAAGAACCTG
CTCGTACGACAGTGTCTGGCTGAGTTTCTGGGTGTTTACTTGCTTATGTTGATGGGGACA
GCATCTGTGGCTCAGGTTGTAACAAACTTT------GACCAGAAGGGAACATTCTTATCT
ATTAACTTTGGCTATGCTGCCGGAGTCCTGTTTGGCGTCTACGCATCAGTCGGTGTCTCA
GGTGCCCATTTGAACCCCGCCGTCTCGTTGAGCCTGTGCATAATTGGCCGGTTCCCGTGG
AAGAAGCTGCCTTTCTACATCATCGCCGAGTGCCTGGGGGCCTTTATTGCCGCGGCTACC
ACCTTC---TGCCTTTACTACGATGCGATACACGAATTCTCTCGA---GGGAATTTCACG
GTCCGAGGTCCGCGG---GGCACAGCCGGGATCTTCGCCACCTACCCCGTTGAATACCTC
TCGACCAGCAATGGCTTCATCACCGAGGTGATTGCCACCGCCGTTTTGCTAATCTGCATC
CTGTGCGTTTTGGACAGCAAAAACGCGGCTGTGCCGCCATTTTTACAGCCGCCCATGATC
GCGGTGTCAGTGCTGGTCATTGGACTTTCCATGGGCGCCAACACTGGCTACGCCATCAAT
CCAGCCAGAGATTTC---GGCCCGAGGATGTTCACCTTTGTGGCTGGGTGGGGCTCCGAG
GTTTTC---ACG------GCTGGTCACGGATGGTGGTGGGTTCCGATCGTCGCCCCCATG
ATCGGAGGTGTCGCCGGTACCCTAGTCTACATGCTCCTCATCGATCTGCACCACGAGGAT
---------------ACCAAGGAGCTTGAA---------------------TCA------
------------GAAGCC---AAATTGATC------------------------AAG---
---------CAGGAC---------ACGGAA------------GATGAGCCCGAACCGATC
AGCGAGGGGCCTGTG---------------------------------------------
------------------------------------------------------------
------------------------------------------------------------
---------------------
>Great_white_shark_Aqp10C2
------------------------------------------------------------
---------------ATGAAGAGACTTCGGAAGAAACTC------CGGATTAAGAACAAG
CTCATTCGGGAATGCCTGGCAGAATTCTTCGGCGTTTACCTGCTGATTTTGATGGGCTCG
GCGTCAGTGGCACAGGTTGTATTGTTTTTC------GACCGGAAGGGCGAGTACCTGTCA
ATTGCCTTTGGATACGCTTGCGGGGTCCTGTTCGGCATCTATGCATCAAGAGGAATCTCA
GGTGCTCATTTGAACCCCGCCGTCACCTTCAGTCTGTGCCTGCTGGGCCGGTGCCCATGG
AAGAAGCTGCCTTTTTACACCATCGCTGAGTGCCTGGGCTCCTTCACTGCCGCAGCGACC
ACCTTC---TGCCTTTATTAC---------------------------------------
------------------------------------------------------------
------------------------CAGATCATTGGTACCGCTGTTTTGTTAATCTGCATC
CTGTGCGTTGGGGATGCCAAAAATGCTGGGGCTCCGGCATTCTTACAACCCCCTCTTGTC
GCCACCTCAGTGTTTATCATTGGCATTTGCATGGGTGCCAACACTGGTTACGCCATCAAC
CCAGCAAGGGACTTC---GGACCTCGCTTGTTTACCTATGTAGCTGGGTGGGGCACTGAG
GTGTTC---ACG------GCCGGCAACAATTGGTGGTGGATCCCGATCGTCGCCCCGGTC
CTGGGGGGAGCGCTGGGCAGCCTGGCCTACGTTCTCCTCATCGAGATGCACCACGAGGAC
---------------CCCAAGCCACTCAAA---------------------GAT------
---------------GTC---AAGGACGAG------------------------GCA---
---------GTCGAT---------------------------GACGCG------------
------------------------------------------------------------
------------------------------------------------------------
------------------------------------------------------------
```

```
---------------------
>Little_skate_Aqp10C2
---------------------------------------------------ATGAACCCG
CTGAGC---GGGGATGGGTCGCGGTGAGGAGGAAACTA------CGCGTGAAGAACAGG
CTCCTGCGCGAATGTATGGCCGAGTTCATCGGAGAATACATGCTGATCTTGTTCGGGTCA
GCGGCTGTTGCCCAGGTGGTCACCAACTAC------GACAGGAAGGGGATGTATCTCTCC
ATCAACCTGGGATACGCCATTGGCGTGCTGTTTGGCATTTACGTCTCAGCCGGTGTGTCA
GGGGGTCATTTGAACCCAGCCGTGACGTTCTGCTTGTGTGTTCTGGGCCGCTGCCCGTGG
TATAAGCTGCCCCTCTACACGTTGTCCGAGTGCCTGGGGTCTTTCATGGCAGCGGCTACC
ACCTTC---GCCGTGTACTAC----------------------------------------
------------------------------------------------------------
------------------------------------------------------------
------------------------------------------------------------
------------------------------------------------------------
------------------------------------------------------------
------------------GCTGGTAATGGCTGGTGGTGGGTCCCTGTCATTGCGCCTTTC
ATCGGGGGAGTCCTGGGCACTGCCATTTACGTTCTGTTCATTGAACTACATCACAAAGAG
---------------ACGGTC---TTG---------------------------------
------------------------------------------------------------
------------------------------------------------------------
------------------------------------------------------------
------------------------------------------------------------
------------------------------------------------------------
---------------------
>Smalltooth_sawfish_Aqp10C2
ATG------GAGGATCGA---------GCTTCGACCCCAGTCCAACGGGCGCTAGACAAG
GCGGCG---ACGGGGGTGAAGGCGTTCCGGAGGCACCTA------CGGGTGAAGAACGAG
CTACTGCGACAGTGCCTGGCCGAATTCTTGGGGGAGTACATACTGATCTTAATGGGATCG
GCGACTGTGGCACAGGTCATCACCAACTAT------GACCGGAAAGGGACCTACCTGTCG
ATCAACATGGGCTATGCCATCGGAGTCCTGTTCGGAATCTACATGTCAAGTGGAGTCTCA
GGTGCACACCTGAATCCGGCGGTCACCTTGAGCCTGTGCGCCCTGGGTCGATTCCCCTGG
CAGAAGATGCCCTTCTACACGCTGGCGGAATGCCTGGGCTCCTTCGTGGCAGCGGCCACC
ACCTAC---GCTCTTTACTACGATTCTATCCACGAGTTTTCCAAC---GGGAGTCTGACT
GTGCTGGGACCCAGG---GGGACGGCCGGAATCTTCGCCACCTACCCCGCGGAACACATC
ACCACCCGCAACGGCTTCATCACCGAGGTGATAGCCACCGGGGTGTTGTTGATCTGCATC
CTGGCGATCGGCGATTCCCGGAACGCCATGTTGCCCGACTTCCTCAGGCCGCTGGTGACC
TCCATCTGCGTGCTCACCATCGGGATGGGCATGGGAGCCAACACTGGCTACGCCATTAAC
CCCGCGAGGGACATC---GGGCCACGCATGTTCACCTTCGTCGCTGGCTGGGGCTCCGAA
GTATTC---ACG------GCTGGGAACAGCTGGTGGTGGATCCCACTGGTCGCCCCGATG
TTGGGGGGTCTCCTGGGGACGCTGATCTACGAGCTGCTCATTGAGTTCCACCACGAGGAC
---------------GTGCCGCACCCCGGA---------------------GAG------
------------GCCAGG---GCGGCCGAG------------------------GAG---
------------------------------------------GAGGCGCGGCTG---GCA
CAGGAAGATCCCGTG---------------------------------------------
------------------------------------------------------------
------------------------------------------------------------
---------------------
>Ghost_shark_Aqp10C2b
ATG------AACTCCAAT---------------------------CCGACCCTGAGGAGC
CTCCAG---GCTTTGCCTGTCCAGATGCAGAGGAAACTC------CACCTGAAGAACAAG
CTCCTGCGGGAATGTTTGGCCGAATTTCTGGGTGTCTTCCTGTTGATTTTTATCGGAGGT
GCGGCTGTAGCTCAGGTCCAGACCACA------------GGAAAAGGTTCGTATCTGTCC
ATCAACATCGGTTACGGTATCGGTGTTATGTTCGCCATTTACGCAGCCGGAGGCGTCTCA
GGAGCTCACCTGAACCCGGCCGTTTCCATCTCTTTCTGTGTTTTGGGGAAACTGATTTGG
TGGAAAGTCCCATTTTATATCTTCTCCCAAACGTTCGGAGCCTTCACCGCGGCGGCCGTC
ATCTTC---ACCATGTACTACGATTCCATCATGCACTTCACGGGG---GGGCAGCTGATC
GCCGATGGGGGAAACCTGGCAACGGGGGGAATATTCGCCACATATCCGGCCTCGTTCCTC
ACCACACGGAACGGCTTCATTGACCAGATCGTGGCGACGGGGATCTTGCTCCTGGTTATC
CTGAGCCTGAACGACTCGCGGAACAATGAGCCCCCGGACTTCCTGAAGCCCCTCCTGGTG
GGGGCTCTGGTGCTGGTGATCGGAGTGGCCATGGGGTCCAATTGCGGTTACCCCATCAAC
CCGGCCCGCGACATC---GGCCCCCGACTCTTCTCCTACCTGGCGGGGTACGGACCCCAA
```

GTCTTC---ACG------GCTGGTGATCACTGGTGGTGGGTCCCCATTGTAGCCCCAGTT
ATTGGGGGACTCGTTGGTTGTTTCTTCTACAAGATCCTGATCGAGATCCACCACGACGAC
----------------TTCGAGGTGGAGGAG----------------------ATA------
-------------GAACTA---GAGAAAGAC-------------------------------
---CCCGAGAGACCCCGAGCC
GACAATTCATCTGAAGAGAAA---------------------------------------

>Ghost_shark_Aqp10C2a
---ATGGCCAGT
GTGTCT---GCCATCCTGAAACAAGTGAAGAGGAGGCTC------CATTTGAAGAACTGT
CTCGTGCGGGAATGCTTGGCGGAGTTCCTGGGAACTTTCCTGGTTATTTTTATAGGAGGA
GCAGCGGTTGCTCAGGTACAGACCACG------------AGCAAAGGTGCATACCTGTCC
ATTAACATTGGCTATGGTGTGGGCGTCATGTTTGGCATTTATGCAGCTGGAGGCGTCTCA
GGTGCTCACTTGAACCCAGCCATCTCCATCTGCTTCTGTGTGTTGGGGAAGCTGCCGTGG
TGGAAGCTGCCATTTTACATCCTGTCTCAGACCTTTGGAGCTTTCGTAGGTGCAGCTGTG
ATCTTT---ACCATGTATTACGATTCCATCATGCACTTCACTGGG---GGCGAGCTGATA
GCTGACGGTGCAACGCTGGCTACTGGGGGGATATTTGCCACCTACCCTGCCGTGTACCTC
ACCACACGTAATGGCTTCATTGACCAGGTTGTGGCCACTGCAGTTCTGCTGCTTGTCATC
CTGAGTTTAAACGACTCCCGGAATAATGAGCCCCCGGCGTTCCTCAAGCCCATCCTGATT
GGGACGCTGGTACTTGTGATAGGTGTGGCCATGGGCTCCAACTGTGGCTATGCCATCAAT
CCCGCCCCGAGACTTC---GGCCCACGCCTCTTCTCCTATCTTGTTGGTTATGGCAGTCAG
GTATTC---ACG------GCTGGGGACAGCTGGTGGTGGGTACCTATCGTGGCCCCGGTG
TTGGGAGGGCTGCTTGGCTGTTTCTTGTACAAGATCCTAATCGAGATCCACCACGATGAC
----------------CCTGAGGAGCATAGC----------------------CAG------
-------------CAGCCA---GCCGCAGAC-------------------------------
---CTCGAACTGCCAACCATA
GACAGTTCATCCAGAGAGCAG---------------------------------------

>Human_AQP7
ATG------GGCTCGGGC---CACTGTCTTAGGTCCACCCGTGGCTCCAAAATGGTCTCC
TGGTCC---GTG---ATAGCAAAGATCCAGGAAATACTG------------CAGAGGAAG
ATGGTGCGAGAGTTCCTGGCCGAGTTCATGAGCACATATGTCATGATGGTATTCGGCCTT
GGTTCCGTGGCCCATATGGTTCTAAAT---------AAAAAATATGGGAGCTACCTTGGT
GTCAACTTGGGTTTTGGCTTCGGAGTCACCATGGGAGTGCACGTGGCAGGCCGCATCTCT
GGAGCCCACATGAACGCAGCTGTGACCTTTGCTAACTGTGCGCTGGGCCGCGTGCCCTGG
AGGAAGTTTCCGGTCTATGTGCTGGGGCAGTTCCTGGGCTCCTTCCTGGCGGCTGCCACC
ATCTAC---AGTCTCTTCTACACGGCCATTCTCCACTTTTCGGGT---GGACAGCTGATG
GTGACCGGTCCCGTC---GCTACAGCTGGCATTTTTGCCACCTACCTTCCTGATCACATG
ACATTGTGGCGGGGCTTCCTGAATGAGGCGTGGCTGACCGGGATGCTCCAGCTGTGTCTC
TTCGCCATCACGGACCAGGAGAACAACCCAGCACTGCCAGGAACAGAGGCGCTGGTGATA
GGCATCCTCGTGGTCATCATCGGGGTGTCCCTTGGCATGAACACAGGATATGCCATCAAC
CCGTCCCGGGACCTG---CCCCCCCGCATCTTCACCTTCATTGCTGGTTGGGGCAAACAG
GTCTTC---AGC------AATGGGGAGAACTGGTGGTGGGTGCCAGTGGTGGCACCACTT
CTGGGTGCCTATCTAGGTGGCATCATCTACCTGGTCTTCATTGGCTCCACCATCCCACGG
----------------GAGCCCCTGAAATTG----------------------GAG------
-------------GATTCT---GTGGCGTAT-------------------------GAA---
-------------------------GACCACGGG------ATAACCGTATTGCCCAAGATG
GGATCTCATGAACCCACGATCTCTCCCCTCACCCCC----------------GTCTCTGTG
AGCCCTGCCAACAGATCTTCAGTCCACCCTGCCCCACCC---------------------
--TTACATGAATCCATG
GCCCTAGAGCACTTC------
>Chimpanzee_AQP7
ATG------GGCTCGGGC---CACTGTCTCAGGTCCACCCGTGGCTCCAAAATGGTCTCC
TGGTCC---GTG---ATAGCAAAGATCCAGGAAATACTG------------CAGAGGAAG
ATGGTGCGAGAGTTCCTGGCCGAGTTCATGAGCACATATGTCATGATGGTATTCGGCCTT
GGTTCCGTGGCCCATATGGTTCTAAAT---------AAAACATATGGGAGCTACCTTGGT
GTCAACTTGGGTTTTGGCTTCGGAGTCACCATGGGAGTGCACGTGGCAGGCCGCATCTCT

GGAGCCCACATGAATGCAGCTGTGACCTTTGCTAACTGTGCGCTGGGCCGCGTGCCCTGG
AGGAAGTTTCCGGTCTATGTGCTGGGGCAGTTCCTGGGCTCCTTCCTGGCGGCTGCCACC
ATCTAC---AGTCTCTTCTACACGGCCATTCTCCACTTTTCGGGT---GGAGAGCTGATG
GTGACCGGTCCCGTC---GCTACAGCTGGCATTTTTGCCACCTACCTTCCTGATCACATG
ACATTGTGGCGGGGCTTCCTGAATGAGGTGTGGCTGACCGGGATGCTCCAGCTGTGTCTC
TTCGCCATCACGGACCAGGAGAACAACCCAGCACTGCCAGGAACAGAGGCGCTGGTGATA
GGCATCCTCGTGGTCATCATCGGGGTGTCCCTTGGCATGAACACAGGATATGCCATCAAC
CCATCCCGGGACCTG---CCCCCCCGCATCTTCACCTTCGTTGCTGGTTGGGGCAAACAG
GTCTTC---AGC------AATGGGGAGAACTGGTGGTGGGTGCCAGTGGTGGCACCACTT
CTGGGTGCCTATCTAGGTGGCATCATCTACCTGGTCTTCATTGGCTCCACCATCCCACGG
---------------GAGCCCCTGAAATTG---------------------GAG------
------------GACTCT---GTGGCATAT------------------------GAA---
------------------------GACCACGGG------ATAACCGTATTGCCCAAGATG
GGATCTCATGAACCCACGATCTCTCCCCTCACCCCC---------------GTCTCCGTG
AGCCCTGCCAACAGATCTTCAGTCCACCCTGCCCCACCC---------------------
---TTACATGAATCCATG
GCCCTAGAGCACTTC------
>Gorilla_AQP7
---------------------CACTGTCTCAGGTCCACCCGTGGCTCCAAAATGGTCTCC
TGGTCC---GTG---ATAGCAAAGATCCAGGAAATACTG------------CAGAGGAAG
ATGGTGCGAGAGTTCCTGGCTGAGTTCATGAGCACATATGTCATGATGGTATTCGGCCTT
GGTTCCGTGGCCCATATGGTTCTAAAT---------AAAAAATATGGGAGCTACCTTGGT
GTCAACTTGGGTTTTGGCTTCGGAGTCACCATGGGAGTGCACGTGGCAGGCCGCATCTCT
GGAGCCCACATGAATGCAGCTGTGACCTTTGCTAACTGTGCGCTGGGCCGCGTGCCCTGG
AGGAAGTTTCCGGTCTATGTGCTGGGGCAGTTCCTGGGCTCCTTCCTGGCGGCTGCCACC
ATCTAC---AGTCTCTTCTACAGTGCCATTCTCCACTTTTCGGGT---GGACAGCTGACG
GTGACCGGTCCCGTC---GCTACAGCTGGCATTTTTGCCACCTACCTTCCTGATCACATG
ACATTGTGGCGGGGCTTCCTGAATGAGGTCTGGCTGACCGGGATGCTCCAGCTGTGTCTC
TTCGCCATCACGGACCAGGAGAACAACCCAGCACTGCCAGGAACAGAGGCGCTGGTGATA
GGCATCCTCGTGGTCATCATCGGGGTGTCCCTTGGCATGAACACAGGATATGCCATCAAC
CCGTCCCGGGACCTG---CCCCCTCGCATCTTCACCTTCATTGCTGGTTGGGGCAAACAG
GTCTTC---AGC------AATGGGGAGAACTGGTGGTGGGTGCCAGTGGTGGCACCACTT
CTGGGTGCCTATCTAGGTGGCATCATCTACCTGGTCTTCATTGGCTCCACCATCCCACGG
---------------GAGCCCCTGAAATTG---------------------GAG------
------------GACTCT---GTGGCGTAT------------------------GAA---
------------------------GACCACGGG------ATAACCGTATTGCCCAAGATG
GGATCTCATGAACCCACGATCTCTCCCCTCACCCCC---------------GTCTCTGTG
AGCCCTGCCAACAGATCTTCAGTCCACCCTGCCCCACCC---------------------
---TTACATGAATCCATG
GCCCTAGAGCACTTC------
>Human_AQP7L1
ATGGTTCAAGCATCTGGG---CAC---AGGCGGTCCACCCGTGGCTCCAAAATGGTCTCC
TGGTCC---GTG---ATAGCAAAGATCCAGGAAATATGGTGCGAGGAAGATGAGAGGAAG
ATGGTGCGAGAGTTCTTGGCCGAGTTCATGAGCACATATGTCATGATGGTATTCGGCCTT
GGTTCTGTGGCCCATATGGTTCTAAAT---------AAAACATATGGGAGCTACCTTGGT
GTCAACTTGGGTTTTGGCTTCGGGGTCACCATGGGAGTCCACGTGGCAGGCCGCATCTCT
GGAGCCCACATGAATGCAGCTGTGACCTTCACTAACTGTGCGCTGGGCCGCGTGCCCTGG
AGGAAGTTTCCAGTCCATGTGCTGGGGCAGTTCCTGGGCTCCTTCCTGGCAGCTGCCACC
ATCTACATCTGTGTCTCCGCAGCGGCCATTCTCCACTTTTCGGGT---GGAGAGCTGATG
GTGACCGGTCCCTTT---GCTACAGCTGGCATTTTTGCCACCTACCTTCCTGATCACATG
ACATTGTGGCGGGGCTTCCTGAATGAGGAGTGGCTGACCAGGATGCTCCAGCTGTGTCTC
TTCACCATCACGGACCAGGAGAACAACCCAGCACTGCCAGGAACACACGCGCTGGTGATA
AGCATCCTCGTGGTCATCATCAGGGTGTCCCATGGCATAAACACAGGATATGCCATCAAT
CCATCCCGGGACCCG---CCCCCCAGCATCTTCACCTTCATTGCTGGCTGGGGCAAACAG
GTCTTC---AGC------GATGGGGAGAACTGGTGGTGGGTGCCAGTGGTGGCACCACTT
CTGGGTGCCTCTCTAGGTGGCATCATCTACCTGGTCTTCATTGGCTCCACCATCCCACGG
---------------GAGCCCCTGAAATTG---------------------GAG------
------------GACTCT---GTGGCGTAT------------------------GAA---
------------------------GACCACGGG------ATAACCGTATTGCCCAAGATG
GGATCTCATGAACCCATGATCTCTCCCCTCACCCTC---------------ATCTCCGTG
AGCCTTGCCAACAGATCTTCAGTCCACTCTGCCCCACCC---------------------

```
---------------------------------------------TTACATGAATCCATG
GCCCTAGAGCACTTC------
>Chimpanzee_AQP7L1
ATGGTTCAAGCATCTGGG---CAC---AGGCAGTCCACCCGTGGCTCCAAAATGGTCTCC
TCATCC---GTG---ATAGCAAAGATCCAGGAAATATGGTGCGAGGAAGATGAGAGGAAG
ATGGTGCGAGAGTTCCTGGCCGAGTTCATGAGCACATATGTCATGATGATATTCGGCCTT
GGTTCTGTGGCCCATATGGTTCTAAAT---------AAAACATATGGGAGCTACCTTGGT
GTCAACTTGGGTTTTGGCTTCGGAGTCACCATGGGAGTCCACGTGGCAGGCCGCATCTCT
GGAGCCCACATGAATGCAGCTGTGACCTTCACTAACTGTGCACTGGGCCGCGTGCCC---
AGGAAGTTTCCAGTCTATGTGCTGGGGCAGTTCCTGGGCTCCTTCCTGGTGGCTGCCACC
ATCTACATCTGTGTCTCCGCAGCGGCCATTCTCCACTTTTTGGGT---GGAGAGCTGATG
GTGACCGGTCCCTTT---GCTACAGCTGGCATTTTTGCCACCTACCTTCCTGATCACATG
ACATTGTGGCGGGGCTTCCTGAATGAGGAGTGGCTGACCAGGGTGCTCCAGCTGTGTCTC
TTCACCATCACGGACCAGGAGAACAACCCAGCACTGCCAGGAACACACGCGCTGGTGATA
AGCATCCTCGTGGTCATCATCGGGGTGTCCCATGGCATAAACACAGGATATGCCATCAAT
CCATCCCGGGACCCA---CCCCCCAGCATCTTCACCTTCATTGCTGGTTGGGGCAAACAG
GTCTTC---AGC------GATGGGGAGAACTGGTGGTGGGTGCCAGTGGTGGCACCACTT
CTGGGTGCCTCTCTAGGTGGCATCATCTACCTGGTCTTCATTGGCTCCACCATCCCACGG
---------------GAGCCCCTGAAATTG---------------------GAG------
------------GACTCT---GTGGTGTAT------------------------GAA---
------------------------GACCAGGGG------TTAACCGTATTGCCCAAGATG
GGATCTCATGAACCCATGATCTCTCCCCTCACCCTC---------------ATCTCCGTG
AGCCTTGCCAACAGATCTTCAGTCCACTCTGCCCCACCC---------------------
---------------------------------------------TTACATGAATCCATG
GCCCTAGAGCACTTC------
>Gorilla_AQP7L1
ATG------GGCTCGGGC---CACTGTATCTGGTCCACCCGTGGCTCCAAAATGGTCTCC
TGGTCC---GTG---ATAGCAAAGATCCAGGAAATATGGTGCGAGGAAGATGAGAGGAAG
ATGGTGCGAGAGTTCCTGGCTGAGTTCATGAGCACATATGTCATGATGGTATTCGGCCTT
GGTTCCGTGGCCCATATGGTTCTAAAT---------AAAAAATATGGGAGCTACCTTGGT
GTCAACTTGGGTTTTGGCTTTGGAGTCACCATGGGATTGCACATGGCAGGCCGCATCTCT
GGAGCCCACATGAACACAGCTGTGAGCTTCGCTAACTGTGCACTGGGCCACGTGCCCTGG
AGGAAGTTTCCAGTCTATGTGCTGGGGCAGTTCCTGGGCTCCTTCCTGGCGGCTGCCACC
ATC------TGTGTCTCCACAGAGGCCATTCTCCACTTTTCGGGT---GGAGAGCTGATG
GTGACCGGTCCCGTT---GCTACAGCTGGCATTTTCGCCACCTACCTTCCTGATCACATG
ACATTGTGGCGGGGCTTCCTGAATGAGGAGTGGCTGACCGGGATGCTCCAGCTGTGTCTC
TTCGCCATCACGGACCAGGAGAACAATGCAGCACTGCCAGGAACACAAGCACTGGTGATA
GGCATCCTCGTGGTCATCATCGGGGTGTCCCATGGCATAAACACAGGATATGCCATCAAT
CCGTCCTGGGACCTG---CCCCCCAGCACCTTCACCTTCATTGCTGGTTGGGGCAAACAG
GTCTTC---AGC------GATGCGGAGAACTGGTGGTGGGTGCCAGTGGTGGCACCACTT
CTGGGTGCCTCTCTAGGTGGCATCATCTACCTGGTCTTCATTGGCTCCACCATCCCACGG
---------------GAGCCCCTGAAATTG---------------------GAG------
------------GACTCT---GTGGTGGAA------------------------------
------------------------GACCACGGG------ATAACCGTATTGCCCAAGATG
GGATCTCATGAACCCATGATCTCTCCCCTCACCCTC---------------ATCTCCGTG
AGCCTTGCCAACAGATCTTCAGTCCACTCTGCCCCACCC---------------------
---------------------------------------------TTACATGAATCCATG
GCCCTAGAGCACTTC------
>Chimpanzee_AQP7L2
ATG------GGCTCGGGC---CACTGTATCAGGTCCACCCGTGGCTCCAAAATGGTCTCC
TGGTCC---ATG---ATAGCAAAGATCCAGGAAATATGG------------------AAG
ATAGCGCGAGAGTTCCTGGCCGAGTTCATGAGCACATATGTCATGATGGTATTCGGCCTT
GGTTCCGTGGCCCATATGCTTCTAAAT---------AAAACATTTGGGAGCTACCTTGGT
GTCAACTTGGGTTTCGGCTTCGGAGTCACCATGGGAGTGCACGTGGCAGGCTGCATCTCT
GGAGCCCACATGAACGCAGCTGTGAGCTTCACTAACTGTGCACTGGGCCGTGTGCCCTGG
AGGAAGTTTCCAGTCTATGTGCTGGGGCAGTTCCTGGGCTCCTTCCTGGCGGCTGCCACC
ATCTAC---AGTCTCTTCTACACGGCCATTCTCCACTTTTCGGGT---GGAGAGCTGATG
GTGACCGGTCCCATT---GCTACAGCTGGCATTTTTGCCACCTACCTTCCTGATCACATG
ACATTGTGGCGGGGCTTCCTGAATGAGGAGTGGCTGACCGGGATGCTCCAGCTGTGTCTC
TTTGCCATCACGGACCAGGAGAACAACCCAGCACTGCCAGGAACACACACGCTGGTGATA
GGCATCCTCGTGGTCATTATCAGGGTGTCCCATGGCATGAACACAGGATATGCCATCAAT
```

CCGTCCTGGGACCTGCCCCCCCCCCGCATCTTCACCTTCATTGCTGGTTGGGGCAAACAG
GTCTTC---AGC------GATGGGGAGAACTTGTGGTGGGTGCCAGTGGTGGCACCACTT
CTGGGTGCCTCTCTAGGTGGCATCATCTACCTGGTCTTCATTGGCTCCACCATCCCACGG
---------------GAGCCCCTGAAATTG---------------------GAG------
------------GACTCT---GTGGCATAT------------------------GAA---
------------------------GACCACGGG------ATAACCGTATTGCCCAAGATG
GGATCTCATGAACCCATGATCTCTCCCCTTACCCTC---------------ATCTCCGTG
AGCCCTGCCAACGGATCTTCAGTCCACCCTGCCCCACCC---------------------
---TTACATGAATCCATG
GCCCTAGAGCACTTC------
>Rabbit_AQP7
ATG------GGCTCGGGC---TGCTGCTTCAGGTCCCCCTGCAGCTCCCAGATGGTCTCC
TGGCCT---GTG---CTAGTGCGGATCCACGCAGTCCTG------------CAGAAGGAG
ACGGTGCGAGAGTTCCTGGCAGAGTTCCTGAGCACGTACGTCATGATGGTGTTTGGTCTT
GGTTCTGTGGCCCATATGGTTCTAGGAGGC------CAAAAATTCGGGAGCTTCCTCGCT
GTCAACTTGGGTTTTGGCTTTGGAGTCACCATGGGCGTGCACGTGGCAGGCAACATCTCT
GGGGCCCACATGAACGCTGCCGTGAGCTTCACCGCCTGCGCGCTCGGCCGCATGTCCTGG
AAGAAGTTTCCCGTGTACGTGTTGGGTCAGTTCCTGGGCTCCTTCACCGCGGCTGCCACC
ATCTAC---GGCCTCTTCTACACGGCCATTCTGCACTTCTCCGGC---GGACATCTGGCA
GTGACCGGGCCCACA---GCCACTGCGAACATTTTTGCCACCTATCTTCCTGACCACATG
ACGCTGTGGTGGGGCTTCCTCAATGAGGTGGTACTGACGGGGATGCTCCAGCTGTGTCTC
TTAGCCATCACCGATAAGGAGAATAACCCAGCGCTGCCGGGGACACAGGCCCTGGTCACC
GGCATCCTTGTTGTCATCATTGGGGCATCCCTGGGCATGAACACAGGATATGCTATCAAC
CCATCTCGGGACCTG---CCTCCCCGCTTCTTCACCTTCATTGCCGGCTGGGGCAAACAG
GTGTTC---AGG------GCTGGGGAGAACTGGTGGTGGGTGCCAGTGGTGGCACCAATT
GTAGGTGCCTACCTGGGAGGAATCGTCTATCTGGTCTTCATTGGCTCCGGCATCCCACGG
---------------GAGCCCCAGGAATCA---------------------GAG------
------------GACTCG---GCCATGAAT------------------------GAA---
------------------------GAACACAGG------ATAACCACATTGTCCAAGACA
AGTGCTCACCTATCCGTGACCTCTCACCTCGACCCT---------------GTCTCCAGA
GTCCCTGCCAACACATCTTCAGTCCAGCCTGTCCCACCT---------------------
---GTGAATGGCTCTATA
CTCTTAGAGAGCTTC------
>Tasmanian_devil_AQP7

---------------ATGCAGCGCATGCAAGATGCTTTG------------CGGGGAGAC
ATGATGCGAGAATTTTTGGCTGAATTCATAAGTACATATGTCATGATGGTGTTTGGTTTG
GGATCTGTGGCCCAACAAGTTCTTGGGGAG------AATAATTATGGGACATATCTCAGC
ATCAACTTGGGATTTGGCTTTGGTGTTGTAATGGGGGTCCATGTGGCTGGGGGAATCTCT
GGTGCCCATATGAACAGTGCGCTTACCTTCACAAGCTGTGTCCTGGGTCAGATGCCCTGG
AAGAAGTTTCCTGTCTACACAGTGGCACAGTGCTTTGGATCCTTTTTAGCAGCAGCTACC
ATATAT---GGTCTTTTCTATCAGGCACTCCTACGGTACACAGAT---GGGAATCTGACA
GTAACCGGTCCCCGG---GCAACTGCAGGCATCTTTGCTACTTACCCTGCCTCCTATATG
ACTCTGTGGAGGGGATTCGTAGATGAGATATTCTTAACTGGGATACTCCAGGTATGCCTG
TTGGCCATCAATGACAAGAAGAACTGCCCGGCTCTGCAAGGGACCCATCCTTTGGTCATA
GGTGTCCTTGTGATTACAATAGGATTGTCACTGGGCATGAACACTGGTTATGCCATCAAT
CCATCCAGAGACCTG---CCACCAAGGATCTTTACAAGCATAGCAGGCTGGGGAAATGAA
GTCTTT---ACA------GCAGCAGAGAGCTGGTGGTGGGTCCCTGTGGTTGCCCCGCCA
TTAGGCTCCCTCATGGGAGCTATTGTCTACCTACTCCTTATTGGATCCAGTCATCGACAT
---------------CAGAACGACCGAAAT---------------------GCA------
------------GAGGTT---GCAACGACA------------------------CCT---
------------------------AAGCCT------------CCTCGAGTCCTGCAGTCA
CTGCAGCCCCAGCAGTCCCTGAAGCAGAAG---------------------GTGATTGTG
CCCTCGTCTCATCATTCATCTATGTTTCCCCTGTCTTCT---------------------
--CAC---TCA---

>Platypus_AQP7
ATG---
---------GCC---GTCCAGAAGTTCTTGCTATCGTTC------CTGACGGAGAACAAG
CTGACCAGAGAGTTCCTGGCAGAAATGCTGAGCACATTTGTCCTCATGGTGTTCGGGCTG
GGGTCTGTGGCGCAGGTGGTGCTGGGAAAG------CGGGAGTTCGGGGATTTTCTGAGC

```
ATTAACTTGGGCTTTGGCTTCGGCGTCACCATGGGCATACATGTGGCTGGAGGCATCTCG
GGTGCTCATATGAATGCTGCCATCACCTTTGCCTCCTGTGTCCTGGGCCAGCTACCATGG
AAGAAGTTACCCGTCTATGTGCTGGGCCAGTTTCTGGGTTCTTTCTTGGCTGCTGGCACC
GTCTAC---TTGCTCTACAAAGATGCTCTCTATTCCTTCTCGGGA---GGAAACTTGACG
GTGACGGGGCCTAAT---TCAACAGCGGGGATCTTCTCAACTTACCCTGCTCCTTACATG
GACCTGCTTGGGGGATTTGTGAATGAGCTCATCGCCACGGCAATGCTCCAGCTCTGCATT
CTCGCCATTACGGACAAGAAGAACAGTGCTGCGCTGGATGGCACGCAGGCCCTCATCATC
GGCCTCCTGGTGGCCGTCATTGGCATGTCCCTGGGCATGAACACGGGTTACGCCATCAAT
CCTTCCCGGGACTTG---CCACCCCGCATCTTCACCGCCATTGCGGGCTGGGGCATGGAT
GTCTTT---CGG------GCCGGCAATAGCTGGTGGTGGGTGCCCTTGATTGCCCCGACG
ATTGGCAGCGTCCTTGGAGCTCTGATTTACAAGATCCTCATCGACCATCACAACCGCCCG
---------------GCCCCACAGCCGGAG---------------------TCC------
------------TGCATG---ACCTCGCCG------------------------GCA---
------------------------GAGCCG------------GAGCCGGAGGCCACCTGC
TTAGGCATGGAGATGAAGGCG---------------------------------------
------------------------------------------------------------
------------------------------------------------------------
---------------------
>Echidna_AQP7
ATG---------------------------------------------------------
---------GCC---GCCCAGAAGTTCTTGCTGTCGGTC------CTGACAGAGAACAAG
CTGACCAGAGAGTTCCTGGCAGAGCTGCTGAGCACATTTGTCCTCATGATGTTTGGACTC
GGTTCTGTGGCCCAGGTGGTGCTGGGAAAG------CAGGACTTTGGGGATTTTCTGAGC
ATTAACTTGGGCTTCGGCTTCGGCGTCACCATGGGCATTCATGTGGCTGGAGGCATCTCG
GGTGCTCATATGAATGCCGCCATCACCTTCACCTCCTGTGTCCTGGGCCAGCTACCATGG
AAGAAGTTACCCGTCTATGTGCTGGGCCAGTTTCTGGGTTCTTTCCTGGCCTCTGGCACC
GTCTTC---TTGCTCTACAAAGATGCTCTCTATTCCTTCTCGGGA---GGAAACCTGACC
GTGACGGGGCCTACT---TCAACAGCTGGGATCTTCTCAACCTACCCTGCTCCTTACATG
GACCTGCTTGGGGGATTTGTGAATGAGCTCATCGCCACGGGAATGCTCCAGCTCTGCATT
CTCGCCATTACGGACAAGAAGAACAGTGCCGCGCTGGATGGCACGCAGGCCCTTGTCATC
GGCCTCCTGGTAGCCATCATTGGCATGTCCATGGGCATGAACACAGGCTATGCCATCAAT
CCTTCCCGGGACTTG---CCACCCCGCATCTTCACGGCCATCGCGGGCTGGGGCATGGAT
GTCTTC---CGG------GCTAGCAATAGCTGGTGGTGGGTGCCTTTGATCGCCCCGACA
ATTGGCAGCGTCATTGGCGCTCTGATTTACAAGATCCTCATCGACCACCACAACCGCTCG
---------------GCCCCGCAGCCGGAG---------------------TCC------
------------CATCTG---ACCTTGCCG------------------------TCA---
------------------------GAGCCA------------GAGCCCGAGGCCACCTGC
ATAGGCTTGGAGATGAAGGCA---------------------------------------
------------------------------------------------------------
------------------------------------------------------------
---------------------
>Kiwi_AQP7
------------------------------------------------------------
---------ATG---CTGGAGAAAATTCGGAAAGCACTC------ACGGTTCGTAACAGG
ACCATCAGGGAGCTGCTGGCAGAAGCACTGGGGATGTTCATCCTGATGCTCATTGGCTTG
TCTTCTGCTGCTCAAGTGACTTTAGGACGA------GGAGAATTTGGGCAATATCTGAGC
ATCAATTTGGGATTTGGCATTGGTGTCACCTTGGGAATCCATGCAGCTGGAGGAATCTCT
GGAGCTCATCTGAATGCCTCCATTACCCTCACACACTGCCTTTTAGGGAACCTTCCCTGG
AAAAAGCTCCCAGTTTATCTGCTTGGCCAGTTCCTGGGCTCCTTTCTGGCAGCAGCTACT
GTTTTT---GGCATCTACTATGATGCACTGTACGACTATACCAAG---GGGAACTTTACA
GTGACGGGTCCAACT---GCCACGGCGGGGATCTTCTCCACTTACCCTGCTCCCTATATG
TCCTTGATAGGGGGCTTCTTCACAGAGTTTATAGCAACGATGATGCTGTTCTTGGGCATT
CTAGTCATCCATGATGAGAAAAATAATGGCGCCCTGAAGGGCACACAGGCCCTGCTCACG
GGTATCCTGGTCCTAGGCATTGGCCTGGCGATGGGGATGAACACAGGCTATTCCATGAAC
CCCTCCCGGGACCTG---CCCCCAAGGGTCTTCACTGCCATTGCCGGCTGGGGAGTGGAC
GTCTTC---ACG------GCTGGACATGGCTGGTGGTGGATCCCACTCGTAGCTCCAACA
CTGGGAAGTCTTTTTGGTGTTTTAATCCACAAACTCTTTATTGATTTTCACAATCAGCCT
---------------GTCCTGGAAAGTGGA---------------------AAT------
------------GAGAAA---AGACAGCCA------------------------GAC---
------------------------GTGGAG------------ATTTCTAGGATG------
------------------------------------------------------------
```

```
------------------------------------------------------------
------------------------------------------------------------
---------------------
>Alligator_AQP7
------------------------------------------------------------
---------ATG---CTGGAGAAAATGCAGAAGATCCTG------ACGGTCCGCAACGAG
CTCGTCCGGGAGGCACTGGCCGAGGCGCTGGGCACCTTTCTGCTGATGGTACTTGGCTTA
GGGTCCGTTGCCCAGGTGAATCTAGGAGGA------GGAAAGTTTGGGGAATACCTGAGC
ATCAACCTGTCATTCGGACTGGGCGTTGCTTTGGGCATCCATGCAGCTGGCGGGATCTCA
GGAGCTCACATGAATGCCTCCATTACCTTCACCTACTGCCTCCTGGGGAAGCTGCCCTGG
AAGAAGCTCCCGGCCTACGTGCTGGGCCAGTTCCTGGGGTCCTTCGTGGCAGCAGCCACG
ATCTTT---GGCTTGTATTACGATGCACTTTACCTCTACACTGGA---GGGAACTTCACC
GTGACGGGACCCAAT---GCCACGGCCGGGTTCTTCGCCACCTACCCAGCCCCTTATATG
TCCCTGACAGGGGCATTTTTCAACGAGTTTACTGGCACGGCAGTGCTGCTTTTGGGCATA
CTGATCATCCACGACGAGAAGAATAATCCAGCCTTAAAGGGAACCCAGGCAGTGGTCACG
GGGCTCCTGGTCTTTGTGATCGGCATGTCAATGGGAATCAACACAGGATATGCCATCAAC
CCCTCGAGAGACCTG---CCTCCCCGGATCTTCACTGCCATAGCCGGCTGGGGACTGGAG
GTCTTC---AGG------GCTGGAAATAACTGGTGGTGGGTCCCTATCGTAGCTCCGACG
TTGGGAAGTCTGTTTGGTGTTTTAGTCCACAAACTCTTCATTGACCTCCATAATCAGACG
---------------GCTACAGACAGTGGA---------------------AAT------
------------GAGAAA---GAGAAGTAC------------------------ACT---
------------------------TTGGAG------------AGTGCTCGGATG------
------------------------------------------------------------
------------------------------------------------------------
------------------------------------------------------------
---------------------
>Turtle_AQP7
------------------------------------------------------------
---------ATG---CTGGAGAAAATTAAGAAAGCGCTC------GCGATCAAAAACAGA
ACCGTCCGGGAGGCGCTGGCTGAGGCCTTGGGGACGTTCCTCCTGATGTTCTTTGGTATA
GGTGGTGTTGCTCAGGTGATATTAGGAAAA------GGAGAATTTGGGAAGTATCTGAGC
ATCAACTTGGCATTTGGAATTGGCGTGACTATGGGGATTCATGCAGCCGGCGGCATCTCT
GGAGCTCATATGAATGCCTCAATAAGCGTCACACACTGCGTCTTAGGAAATCTTCCCTGG
AGAAAGCTGCCAGCTTACATAATCGGCCAGTTCCTGGGCTCCTTCTTGGCAGCATCCCTA
GTGTTC---TGCATGTACTACGATGCGCTGTGTGAATACTCGGAC---GGACACTTCATT
GTGACGGGACCTAAT---GGCACAGCTGGGATCTTCGCCACCTACCCTGCTCCGTATATG
ACTCTGCTGGGAGGGTTTGTAAATGAGTTCCTGGCCACAGCGGTGCTCATGTTGTGCATT
CTTGCCATCTATGACAAGAAGAATAATGGAGCCCTAGAGGGCACTCAGCCTGTGATCACT
GGGCTCCTGGTGTTAGTGATCGGCATGACAATGGGAATAAACACTGGATATGCAATAAAC
CCCTCCAGGGACCTG---CCTCCAAGGATCTTTACTGCAATAGCAGGGTGGGGAATTGAA
GTCTTT---AGG------GCTGGAGATTATTGGTGGTGGGTCCCACTTGTAGCGCCAACC
CTGGGAAGTCTTGCTGGTGCCTTAGCCTACAAACTCCTAATTGACTTTCACAATCAGACT
---------------GCACTGGAAGGCGGA---------------------GAT------
------------GAGAAA---GGAAAGGAC------------------------GAT---
------------------------TTGCAG------------ACTAACAGTGTG------
------------------------------------------------------------
------------------------------------------------------------
------------------------------------------------------------
---------------------
>Python_AQP7
------------------------------------------------------------
---------ATG---TTGGAAAAAATTAATCAACTCGGTG------ACGGTCCGGAATGAA
ACTGTCCGCCAGACTCTGGCAGAAGCCCTGGCGACCTTCCTCTTGATGGTTTTTGGCCTC
GGCTCCGTTGCGCAAGTCGTGTTGGGAAGG------AAAAACTTTGGAGAGTATTTGAGC
ATCAATCTAGGATTTGGGTTTGGTGTCATGTTGGGGATCCATGCTGCTGGTGGAATCTCA
GGTGCTCACATGAACGCTTCCATAACTTTCGCCAACTGTGTGGTAGGAAAACTTCTCTGG
CGGAAACTTCCGGCGTATGTGATTGGTCAGTTCGTGGGATCGTTTGCAGCGTCGGCTGTG
ATTTTT---CTTTTGTATTATGAAGCACTACAGAATTACACGGGA---GGAAACCTGACT
GTGACTGGGCCCACA---GCCACTGCGGGGATATTTGCCACCTACCCTGCCCCTTACATG
TCTCTGTGGAGTGGATTTCTACAGGAGTTTATTGCAACCAGCTTGCTTATGATCGGTGTC
CTTGCCATCAGTGACATGAAGAATGCAGGTGCCCTGCCGGGCACCAACGCCTTCATCACT
```

GGGCTGTTGGTTGTGGTAATTGGCATGTCTATGGGGATGAACACCGGCTACGCCATCAAC
CCTTCCAGAGATCTG---CCCCCCAGGATTTTCACTGCTCTTGCTGGATGGGGGTTAGAG
GTGTTC---CGG------GCAGCCAACTGTTGGTGGTGGGTCCCAGTGGTGGCCCCAACG
CTGGGGAGCCTTCTTGGGATCTTCATCTACAACATCCTGATCGACTTCCACAATCGCCCC
---------------CCACCAGATCCAGAA---------------------TCT------
------------TGGAAG---CAAGACCCA------------------------GAG---
------------------------GCCGAG------------ATCACGTCTCCCATG---

>Gecko_AQP7

---------ATG---CTGGAGAAATTCACCAGCCGACTG------GCGATCAAGAATGCA
ACTGTCCGAGAGGCCCTGGCCGAGGCCCTGGCGACATTCCTCTTAATGCTCTTTGGCACG
GGCTCCGTTGCCCAGGTGGTGCTGGGAAAG------CAAATGTATGGAACGTTCTTGAGC
ATCAACCTGGGGTATGGATTCGGGGTCATGATGGGCATCCATGCGGCTGGTGGCATCTCA
GGTGCTCACATGAACGCAGCGGTAACCTTTGCAAACTGTGCGATTGGAAACCTCCCCTGG
TGCAAGCTTCCAGCATACGTAATCGGCCAGTTCCTGGGATCGTTCATTGCATCTGCTAGT
GTTTTC---TTAATGTACTATGACGCTCTGCAGGATTACACCGGA---GGAAACCTGACT
GTTATAGGGCCGACT---GCTACAGCTGGGATCTTCGCCACATACCCGGCTCCATACATG
TCTGTGTGGAGAGGGTTCATCCAAGAGTTCATTGCCACCGCTGTGCTGGTCATTGGCATC
CTGGCCATCAATGACAAGAAGAATGCAGCGGCCCTTCCGGGCACCAACGCCTTCATCATT
GGACTTCTGGTTACTGCGATCGGCATATCACTGGGCATGAACACCGGCTATGCCATCAAC
CCTTCCCGGGATCTG---CCACCGAGGATCTTCACGGCTATAGCTGGCTGGGGCCTGGAA
GTCTTC---AGG--
---------------GGAATCTTCGTGTACAACATCTTGATCGACTTCCACAATCGCCCT
---------------TCGCTGGAACCCGGC---------------------AGC------
------------AGCGAC---GAGAGCGCG------------------------AAG---
------------------------AAAAGC------------TTTGAGACGGTGGTGGCC
ACTGAACTCCAAAATAGTGTG---------------------------------------

>Tuatara_AQP7

---------ATGCGGGTGGAT---GTCAGAAAATGGCTT------ACAATCCATAACAGC
ATCGTCCGGGAGTCGTTGGCTGAAGCTCTGGGCACGTTTGTCATGATGCTTCTTGGGTTG
GGTTCCTGTGCCCAGGTGGTTTTGGGACGA------GGAGAATTCGGGATGTACCTGAGC
ATCAATCTGTCGTTTGGACTGGGCGTCACGATGGGGATTCACACGGCCGGCGGAATCTCC
GGTGCTCACATGAATGCTGCCATCTCCTTCATAAGCTGTGTCTTGGGACGGCTCCCCTGG
AAGAAGCTGCCAGCCTACATCGCCGGCCAGTTCTTGGGCTCCTTCCTAGCTGCAGCCACC
GTGTTC---TGCATTTACTACGATGCTCTGCACAGCTACACCGCG---GGGAACCTCACC
GTTTCCGGGCCGACT---GGCACCGCCGGGATCTTTGCCACGTACCCGGCTCCGTATATG
AGCTTGCTGGGCGGATTTATCAATGAGCTTATCTGCACGGCAATGCTGGTGCTGTGCGTC
CTCGCCATCTACGACAAGAACAACAACGCGGCCCTCGAGGGCACCCAGGCTCTTCTCACG
GGGCTGCTGGTTGTGGTGCTCGGCATGACCATGGGCATGAACACTGGCTACGCCATCAAT
CCATCCAGGGACCTC---CCGCCAAGGCTCTTCACTGCCATCGCCGGCTGGGGGCTGGAG
GTCTTC---AGG------GCGGGAAATTACTGGTGGTGGGTCCCCATCGTAGCTCCAACA
CTGGGCAGTCTTCTTGGGGCCTCGGTCTACAAGATCTGTGTCGACTTCCACAATCCGCCA
---------------CGTCCAGAAAGAAAA---------------------GCC------
------------AGCGAG---GATAAAGAA------------------------ATG---
------------------------ACCGAG------------TCGCGGACGGCGATATGC
AACATCTCCCAGGAGAGACTGCGAAAG---------------------------------
------------GAAGACCAGATGGATCCCGTGCCTCCA---------------------
---GCTAGGCGC------
CTGTCTCGGCAGCTG------
>Himalaya_frog_AQP7
---ATGAAGATG
GGGACA---TCCTTCATCCAATCGATCCACGCCGCCATC------TCCATCCGCAACCAA
TGGGCTCGGGAGGCCATGGCCGAGATGCTGTCCACCTTCATTATGATGCTGTTTGGTTTG

GGCTCAGTGGCGCAGGTGGTTCTGGGTAAG------AAGCACTATGGACAATACTTCAGC
ATTAACCTTTCCTTTGGAATCGGTGTCACGATGGGGATCCATGTGGCCGGGGGGGTATCC
GGAGCTCACATGAACTGGGCCGTGTCCTTAACGAATTGCGTCTTGGGGAATCTTCCATGG
AAGAAACTTCCCCTTTATATGGTATCACAGGTCGCCGGATCCTTCCTGGCCGCTGCGGTG
GTGTTC---TTCGTCTACTATGAGGCCCTGGACAACTACTGCGGG---GGGAATTTCACT
GTAACTGGAGATTTG---GAAACCGCCAGTATATTTGCCACCTACCCACAGCCGTACCTG
TCTATAGGGGGCGGATTCCTGGACCAGGTGGTGGGAACGGCGGCACTTATGTTATGTATT
TTGGCCATCGGTGATAAGAAGAACAGCCCAGCCCTGGATGGCATGCAGGCCGTGGTAGTC
GGGCTCTTGGTAACCGTCATTGGAATGTCTATGGGCATGAATTCCGGATACGCCATCAAC
CCTGCCAGGGACCTG---GGACCCCGAATCTTCACTGCCGCTGCAGGGTGGGGGCTGGAA
GTGTTC---AGG------GCTGGGAACTACTGGTTCTGGATACCTATTGTGGCCCCTCTA
GTGGGCGCCCTGACCGGGGCTTTCCTGTACAAGCTGCTGGTGGGACTTCACCATCAGAAA
---------------GACCAGGGGGAGAAA---------------------AAG------
------------TTACAG---GAGGTGGAG------------------------AAG---
------------------------ACGAAC------------ACAGAGATGGTGAACTGT
GAACACATG---

>Axolotl_AQP7
ATG------ACG---------------------------------------ACAGCCGGC
GAGAAG---ATGCGC---GCCAAAATCAGGACTCGCCTG------AGGATCGGCAACCAG
GCCGCGCGGGAAGCCGCCGCCGAGCTGCTCTCCACCTTCGTCATGATGACTTTTGGATTA
GGTTCGGTTGCTCAAGCGGTTCTGGGCAGA------AAAGAATATGGGCAGAATTTAAGC
ATCAATCTGTCTTTCGGATTTGGCGTGACAATGGGGATCCATGTAGCTGGAGGGGTTTCA
GGAGCGCATATGAATACTTCTGTATCCTTAACAAACTGCATCCTGGGAAACCTTCCATGG
AGAAAGCTGCCATTGTATGCACTGGCACAGTGCCTGGGTTCGTTCCTCGCAGCTGTTGTG
GTATAC---TGTCTCTATCATGAAGCATTGTTGGACTACTGTGGG---GGGAACCTCACA
GTAACAGGCCCTTGC---GCTACAGCCGGCATATTTGCAACATACCCAGCACCGTATTTG
TCTATCGGAGGCGGGTTTGTAGACCAGGTGATCGGCACAGCAGCACTCTTGCTCTGCATT
CTTGCGATCAATGACAAGAGGAACAGCCCGGCCTTGAACGGGACTCAGGGCCTTCTGGTT
GGGATCCTAGTGGTGCTAATTGGTATGTCTATGGGTATGAATTCTGGATATGCCATTAAC
CCAGCCAGAGATCTA---CCTCCAAGGATATTCACCGCCATGGCAGGCTGGGGACTGGAA
GTATTT---TGG------GCTGGTAATAACTGGTGGTGGGTTCCAGTGGTTGCTCCTTTT
GTTGGGAGTGTCATAGGAGCGTTCATCTACAAACTCCTCATCGAATTACACCATCCACCC
---------------GAGCCAGAAGAAACA---------------------AAG------
------------TGTCAA---CTATCCAAG------------------------GAC---
------------------------ATTCAA------------GACAATGACGTTATGTGT
CAACATATG---

>2Lcaecilian_AQP7
---ATGGCA---
AAAAAG---ATACGGCTCATGAAAATAAAACAAAAATGC------ATAATAAGAAACGGA
ACTGTCAGGGAGACGCTGGCTGAGGCTCTGGCGACCTTTGTCATGATGAGTTTTGGTTTA
GGTTCTGTTGCCCAGGTCGTCCTAGGAAAA------AAGGAATACGGGGAGTATTTGAGC
ATCAATCTCGCTTTTGGATTTGGTGTTACCATGGGCATTCACGTGGCTGGAAACATTTCC
GGAGCCCACATGAATACTTCTGTTTCGCTTACAAACTGCATCTTGGGTTACCTGCCTTGG
AGAAAACTGCCATGTTACGCCTTGGGACAGTTCATTGGCTCCTTTCTCGCAGCTGCGTTG
GTATTC---TGTTTATATTATGAGGCGTTGTACGACTATTGTGGC---GGGAACCTGACT
GTCACAGGGCCTTAT---GCTACAGCAGGGATATTTTCCACCTATCCTGCTCCTCATATG
TCTGCAGGAGGAGGATTTCTAGCTGAGGTTGTTGCCACGGGAATGCTTCTGCTGTGCATC
CTTGCCATTACCGACAAGAAGAACAACGCAGCCTTGGACGGGACGCAGGCTCTTCTGGTT
GGAATCCTTGTTATTGTGATTGGTTTGGCGATGGGAATGAACACCGGCTATGCCATAAAC
CCAGCCAGGGACCTT---CCTCCCAGGATCTTCACTGCAATCGCAGGATGGGGCCTGGAG
GTCTTC---AGG------GCTGGGAATTACTGGTGGTGGATCCCAGTAGTAGCGCCATTC
ATAGGAAGTATCTGCGGTGCCTTAATCTATAAACTTTTAATAGCCTTGCACAATAAGATC
---------------GAGCCAGAAGATGTG---------------------ATC------
------------GGCGAA---GACGTGAAA------------------------GGA---
------------------------AATCAA------------GAAAAAGATCAAGAC---

CAATATATG---

>Gaboon_caecilian_AQP7
---ATGAAGACA
GAAAGG---AAGCAGTTTATGAAAATCAAACAAAAATGC------ATCCTCAAAAATGGA
ACTGCCAGGGAGATGCTGGCGGAGGCTCTGGGGACCTTTGTAATGATGAGTTTTGGTTTA
GGTTCTGTTGCACAGGTTGTCCTAGGAAAA------AAGGAGTACGGACAGTATTTGAGT
ATTAATCTCTCATTTGGATTTGGTGTCACCATGGGAATTCATGTAGCCGGAGGAATCTCT
GGAGCCCACATGAACACTTCTGTATCACTGACAAACTGCATTTTGGGACACCTGCCCTGG
AGAAAATTACCATTTTACGCCTTAGGACAATTCACTGGCTCCTTCCTCGCAGCCGCGTTG
GTTTAC---TGTGTGTATTATGATGCATTGTATGATTACTGTGGC---GGTAACCTGACT
GTAACAGGGCCTTAT---GCAACAGCGGGGATATTTTCCACCTATCCTGCGGCCTATATG
ACTCCGGGAGGAGGATTTCTAACTCAGTTTGTTGCCACTGGAATGCTTTTGCTTTGTATC
CTTGCCATCAACGACAAGAAGAACTTTGCCGCCCTGGATGGAACTCAAGCTCTTCTGGTT
GGAATCCTTGTTATAGTAATTGGTATGGCGATGGGAATGAATACCGGATATGCCATAAAT
CCAGCCAGGGATCTT---CCTCCCAGAATCTTTACTGCAATAGCAGGTTGGGGAGTTGAG
GTCTTC---AGG------GCTGGAAATTATTGGTGCTGGATTCCAGTAGTGGCTCCATTG
GCAGGAAGTATTACTGGTGCCTTTATCTATAAACTCCTAATAGCACTGCACAATCAGGCC
---------------GTGCCAGATGATGTG---------------------AAA------
------------TGTGAA---GATACGAAA------------------------GAA---
------------------------AATCCA------------GATATTGTCATGACTGGT
CAGTATATG---

>Coelacanth_Aqp7
---ATGAAGGGG
AGGAAA---GTA---GTAGCAAAAGTCAAAACGTTTCTA------AGGATAAAAAATAAA
ACCTTGCGAGAAGCATTAGCAGAGGTTCTGGGGACCTTCATAATGATGCTGTTTGGTTTA
GGTTCAGTGGCCCAAGTGATTATCGGAGGT------GGAAAAAATGGCGAGTACCTGAGC
ATAAACTTGTCCTTTGCCATGGGTGTCACCATGGGGATCCACATGGCTGGTGGCGTATCT
GGAGCCCATTTGAATACCGCTGTTTCTTTCACAATGTGTATACTGGGAAAGCTTTGCTGG
AGGAAACTGCCTATTTACACCCTGGCACAGTTCTTCGGTTCCTTCCTGGCAGCTGGCCTG
GTATAT---TTCCTTTACTATGATGCTTTGCACGAATACTGCGGC---GGAAACCTCACA
GTGACTGGACCCAAG---GCTACAGCAGAGATATTCTCTACCTACCCTGCTCCTTACCTC
ACTCTCAGCAATGGATTTTTAGACCAGGTGGTTGGCACTGCAGTCCTCTTGATAGGCATT
CTTGCCATCAATGACCAGAAGAATAACCCAGCACTCAATGGCACTCAGGGGCTCAGTGTT
GGTCTCCTGGTGTTAGTTATTGGAATGTCAATGGGGATGAACTGTGGATATGCCATAAAC
CCAGCTAGAGATCTA---CCTCCCAGGATCTTCACGGCTATAGCTGGCTGGGGACTAGAA
GTATTC---AGA------GCAGGAAACAACTGGTGGTGGGTCCCTATTGTAGCTCCCTTG
GTAGGAAGCGTCATTGGTGCCTGCATCTATCAGATCTTCATTGAAGGCCATCACAAGCCT
---------------GAACCGGAAGGCAAT---------------------TCC------
------------TCCATC---GAATTCGAA------------------------TCC---
------------------------GGAAAA------------ATGGAAAACACAATTAAT
ATT---

>mCoelacanth_Aqp7
---ATGAAGGGG
AGGAAA---GTA---GTAGCAAAAGTCAAAACGTTTCTA------AGGATAAAAAATAAA
ACCTTGCGAGAAGCATTAGCAGAGGTTCTGGGGACCTTCATAATGATGCTGTTTGGTTTA
GGTTCAGTGGCCCAAGTGATTATCGGAGGT------GGAAAAAATGGCGAGTACCTGAGC
ATAAACTTGTCCTTTGCCATGGGTGTCACCATGGGGATCCACATGGCTGGTGGCGTATCT
GGAGCCCATTTGAATACCGCTGTTTCTTTCACAATGTGTATACTGGGAAAGCTTTGCTGG
AGGAAACTGCCTATTTACACCCTGGCACAGTTCTTCGGTTCCTTCCTGGCAGCTGGCCTG
GTATAT---TTCCTTTACTATGATGCTTTGCACGAATACTGCGGC---GGAAACCTCACA
GTGACTGGACCCAAG---GCTACAGCAGAGATATTCTCTACCTACCCTGCTCCTTACCTC
ACTCTCAGCAATGGATTTTTAGACCAGGTGGTTGGCACTGCAGTCCTCTTGATAGGCATT

CTTGCCATCAATGACCAGAAGAATAACCCAGCCCTCAATGGCACTCAGGGGCTCAGCGTT
GGTCTCCTGGTGTTAGTTATTGGAATGTCAATGGGGATGAACTGTGGATATGCCATAAAC
CCAGCTAGAGATCTA---CCTCCCAGGATCTTCACGGCTATAGCTGGCTGGGGACTAGAA
GTATTC---AGA------GCAGGAAACAACTGGTGGTGGGTCCCTATTGTAGCTCCCTTG
GTAGGAAGCGTCATTGGTGCCTGCATCTATCAGATCTTCATTGAAGGCCATCACAAGCCT
---------------GAACCGGAAGGCAAT---------------------TCC------
------------TCCATC---GAATTAGAA------------------------TCC---
------------------------GGAAAA------------ATGGAAAACACAATTAAT
ATT---

>European_seabass_Aqp7
ATG------AAGGAC------------TTAATGCAGTCAGTAGAACTAGGAGTCTCTCAG
CGGAAA---GGA---GTTAACGTAACTGGACCAAAAGTT------TGGCTAAAGAATGAA
GTGGTTCGTGTGGGACTTGCTGAATCCCTTTGCACATATGTCATGATGGTGTTTGGCCTG
GGGTCTGTGGCCCAGGTAGTGACAGGACAG------GGAGCGTTCGGAGAGTACCTCAGC
ATCAACCTGGGTTTTGGACTGGGTGTTGCAATGGGGGTTCATGTTGGAGGGAAGGTCTCA
GGGGCTCATATGAATGCGGCAGTGTCTCTCACAATGTGCACATTTGGCCGCCTTGCATGG
AAGATGCTGCCTGTGTATGTTTTTGCACAGCTATTGGGGTCATTTCTGGCAGCGGGGACA
ATTTAT---GCTGTCTATTATGAAGCCATTTATGACTATTGTGGA---GGAAACCTGACT
GTGACTGGTGTAAAG---GCCACAGCTGGTATCTTTGCCACCTATCCTGCACCGTACCTC
TCCTTGCTGGCTGGATTCATTGACCAGGTGTTTGGCACAGCTATGCTGCTGCTGTGCCTG
ATGGCTCTGTCCGACCAGAAGAACAAACCGGCCGCAGCAGGCAGTGAGCCTGTTGCAGTG
GGTCTCCTAGTGCTACTCATCGGCATTTCCCTAGGCAGCAACAGTGGCTATGCCATCAAC
CCCACCAGAGACATC---GCACCGAGGGTCTTCACTGCCATAGCAGGCTGGGGGTCCGAT
GTGTTC---AGG------GCTGGAAATGGGTGGTGGTGGGTGCCTCTGGCTGCACCTCCC
ATTGGTGGAGTATTGGGTGCGGGGCTCTACAAGGCCTTGGTGGAAATGCACCACCCACCC
---------------CTCTCTGAACAGGGT---------------------GAG------
------------GGGCTG---CTCAAGGAG------------------------------
------------------------GAGACT------------GCCCCTTTGAGGAAACAA
GAAAACATCTGTGCTAATGTATGTGTT---------------------------------

>Nile_tilapia_Aqp7
ATG------AAGGAC------------TTGGCGCAGTCAGTAGAAGTTGGGGTGTTTCAG
CAGAGG---GGA---GGTAAAGTAACTCGACCAAAAGTT------TGGCTAAAGAATGAA
CTCATTCGTGTGGGACTTGCTGAATCACTTAGCACATATGTCATGATGTCATTGGGCTTG
GGGTCTGTGGCCCAGGTAGTGACCGGTCAG------GGAGCTTTTGGACAGTACCTCAGC
ATCAACCTGGGTTTTGGACTGGCTGTTGCCATGGGGTCTCATGTTGGAGGGAAGATCTCG
GGGGCTCATATGAACGGAGCCGTATCATTCACAATGTGTGTGTTTCGCCGCCTCCCGTGG
AAGATGCTACCTCTTTATATTTCGGCACAGCTATTGGGGTCATTTCTGGCAGCAGGGACA
ATTTAT---GCTGTCTACTATGAAGCCATTCATGACTACTGTGGA---GGGAACCTGACT
GTGACTGGTGAGAAG---GCCACAGCTGGTATCTTTGCCACCTATCCTGCTCCATACCTC
TCTCTGATAGCTGGATTTTTTGACCAGGTATTTGGCACAGCGATGCTACTACTGTGCCTT
ATGGCTCTATCCGACCAGAAGAACAAACCAGCGCCAGCAGGAAGTGAGCCTGCATTTGTG
GGTTTCCTGGTGCTTCTCATTGGCATTTCTTTGGGTAGCAACAGTGGCTATGCCATCAAC
CCCACCAGAGACATC---GCACCCAGGGTTTTCACTGCCATGGCAGGCTGGGGAACTGAT
GTGTTC---AGG------GTTGGAAATGGATGGTGGTGGGTGCCTCTAGTTGCAACCCCT
ATTGGAGGAGTCCTTGGAGCAGGGCTATACAAGGCTGTTGTGGAACTGCAACACCCACAC
---------------CTCTCTGAAGCAGGT---------------------GGA------
------------GAGATG---GTTGAAGAA------------------------GCT---
--GTCCCTCTGGATAAAGAG
ATAAATACCAGTGAAAATGTGTGTGTG---------------------------------

>Mummichog_Aqp7
ATG------AAGGAC------------TTTGTAGAGTCAGTGGAACTGGGGGTCTCCCAG
CGGAGC---GCA---GTGGAGCTAAAACGACCCAAAGTT------TGGCTGAGGAGTGAA

CTCTCACGAGTGGGACTCGCTGAATTCCTTTCAACATATGTGATGATGGCATTAGGTCTT
GGTTCTGTGGCCCAGGTCGTGACCGGTCAA------GGAGTATTCGGACAGTACCTTAGC
ATCAACCTTGGCTTTGGGCTGGCTGTCGCAATGGGGGTTCATGTTGGAGGAAATGTCTCT
GGAGCTCATATGAACGGAGCAGTCTCCTTCACTATGTGTGTTTTTGGCCGCCTACAGTGG
AAGAGGCTGCCCTTGTATATTTTCGTACAGCTTTGCGGTTCGTTTCTTGCAGCAGCAACA
ATTTAT---GCTATTTACTACGAGGCCATTTATGACTATTGTGGG---GGAAACCTGACT
GTAAGTGGTGAAAGG---GCCACAGCTGGGATCTTTGCCACATATCCGGCACCATACCTG
TCCTTGATTGGTGGCTTTGTTGACCAGGTGTTTGGCACAGCCATGCTGCTGCTGTGCCTG
ATGGCTCTGTCTGACCAAAAGAACAAACCAGCAGCCAGGGGGGCTGAACCCATTGCTGTG
GGTCTCCTGGTGGTTCTCATTGGCCTTTCTTTGGGCAGCAACAGTGGTTATGCTATCAAC
CCCACCCGAGACATC---GCACCAAGGGTCTTCACTGCTATTGCAGGCTGGGGATGGGAA
GTGTTC---AGG------TCTGGACATGGGTGGTGGTGGGTCCCTCTAGTAGCTACTCCC
ATTGGAGGAGTGCTGGGAGCAGGGTTGTACAAGGCATTTGTAGAAATGCACCACCCCCTC
---------------TTCGCTGGACGGGAT---------------------AGG------
------------GAGCCC---AGTGAGGAG------------------------------
------------------------GAGTCT------------CTCCCGCTGAAGAAAGAG
CGAAACATCTGCTCTGATGTATGTGTGAAACCTGACACAAATGGAAACAAC---------

>Zebrafish_Aqp7
---------------------------------------ATGGAAGATGGCAGCATTCAA
GGCCGC---ATG---GCTCCAAATGTTGGATCCATGTTG------AAGATCAAGAATGAA
TACATTCGAGTGGCTTTGGCAGAAAGCCTCTGCACATTCATCATGATGGTGTTTGGCCTT
GGCACTGTTGCACAAGTGGTTACAGGAGAA------GGTTATTTTGGTGAATATCTCAGC
ATTAATATAGGCTTTGGGCTGGCAGTGGCTATGGGTGTGCATGTTGGTGGAAAAGTGTCA
GGAGCTCATATGAACGCAGCTGTTTCATTCACAATGTGCGTGTTTGGCCGATTGCGCTGG
AAGATGCTGCCGCTGTATGCTTTCGCTCAGTTTCTGGGTTCATTCCTTGCCGCCGGGACC
ATTTTT---TCACTTTATTATGATGCCATAAATCATTTCTGCGGG---GGTAATTTGACT
GTGTCCGGACCCAAA---GCAACAGCTGGGATCTTCGCCACATATCCAGCACCCTATATC
TCAGTCTACACTGGATTCTTTGATCAGGTTGCTGGCACGGGCCTGCTGTTGTTGTGTCTG
ATGGCTCTGTCAGACCAAAGGAACCAGCCGCTGGTGTCTGGAGGTGAAGCCGTCGGTGTG
GGGCTTCTAGTGATGCTCATCGGCATCTCTATGGGGAGCAACAGCGGTTACGCCATCAAT
CCCACACGGGACCTG---GGGCCACGGCTCTTCACACTCATAGCAGGATGGGGCACAGAG
GTTTTT---AGG------GCAGGCAATTGCTGGTGGTGGGTACCCTTGGTGGCTCCTTTT
ATTGGAGGAGTTTTAGGGGCTTTAATCTACAAAGCACTTGTAGAACTACACCACCCTGAT
---------------CTTAAAAACACTACA---------------------ACA------
------------CGGCCA---GCAGTAGAT------------------------CCT---
------------------------GAATGC------------ATTCCTCTGGACAAGTGC
AAGAACGGCAGAATAGAGATACCTGTG---------------------------------

>Atlantic_sturgeon_Aqp7
---ATGAAGCTG
GACAAG---CAGTTTGTATCCAATGTAAAGAAATCCATT------CGAATCCGAAACGAA
TACCTGCGAGAGGCTCTGGCTGAGATACTCAGCACCTTTGTCATGATGGTTTTCGGTCTG
GGCTCGGTAGCCCAGGTGGTCATGGGAGGT------GGTTCCTATGGTGACTACTTCAGT
ATAAATTTGGGCTTCGGCCTTGGGGTCACCATGGGCATTCACATCGCTGGGGGCGTCTCA
GGAGCTCATATGAACACTGCAGTCACCTTCTCCATGTGTGTGCTGGGGAGCCTGAGCTGG
AGGAAGCTGCCGGTCTACGCTGCTGCCCAGTTCCTCGGCTCCTTTATGGCTGCGGTCACC
GTCTTC---TGGGTCTACTATGATGCTCTGTTTGAGTTCTGCAGA---GGGAACTTCACA
GTGACGGGCCCAAGA---GCAACTGCTGGGATCTTCGCTACCTACCCGGCGCCATACCTG
TCAGTGGGAGGAGGCTTTCTGGACCAGGTGCTGGGCACTGCCATGCTGCTGCTGTGTATC
CTGGCGCTGAATGACCACAGGAACAGCCCGGCGCTCAGAGGCACGCAGCCTCTGCTCATC
GGCCTCCTGGTGGTGGTGATTGGCATCTCTCTGGGCAGCAACAGCGGGTACGCCATCAAC
CCCGCCCGCGACCTG---CCCCCACGCTTCTTCACCTCCATGGCAGGCTGGGGTCCTGAC
GTCTTC---AGT------GCTGGTAATGGCTGGTGGTGGATCCCTGTGGTGGCGCCCATG
GTTGGCAGTGTGACTGGCTCTCTGCTCTACAAACTCTTCATCGAGTATCACCATCTGGCT
---------------GAGGAGCATCTAGAG---------------------GAG------
------------GGGCTG---------GGG------------------------GGA---

```
-------------------------GTCTGC------------TCTGATCAAAAACACTGC
TTCCGCTTT---------------------------------------------------
------------------------------------------------------------
------------------------------------------------------------
---------------------
>Common_sturgeon_Aqp7
------------------------------------------------------------
------------------------------------------------------------
------------------------------------------------------------
------------------------------------------------------------
------------------------------------------------------------
------------------------------------TGTGTGCTGGGGAGCCTGAGCTGG
AGGAAGCTGCCGGTCTACGCTGCTGCCCAGTTCCTCGGCTCCTTTATGGCTGCGGTCACT
GTCTTC---TGGGTCTACTATGATGCTCTGTTTGAGTTCTGCGGA---GGGAACTTCACA
GTGACGGGCCCGAGA---GCAACTGCTGGGATCTTCGCTACCTACCCGGCACCATACCTG
TCAGTGGGAGGAGGCTTTCTGGACCAGGTGCTGGGTACTGCCATGCTGCTGCTGTGTATC
CTGGCGCTGAATGACCACAGGAACAGCCCGGCGCTCAGCGGCACGCAGCCTCTGCTCATC
GGCCTCCTGGTGGTGGTGATTGGCATCTCTCTGGGCAGCAACAGCGGGTACGCCATCAAC
CCCGCCCGCGACCTG---CCCCCGCGCTTCTTCACCTCCATGGCAGGCTGGGGTCCTGGC
GTCTTC---AGT------GCTGGTAATGGCTGGTGGTGGATCCCTGTGGTGGCGCCCATG
GTTGGCAGTGTGACTGGCTCTCTGCTCTACAAACTCTTCATCGAGTATCACCATCCGGCT
---------------GAGGAGCATCTAGAG---------------------GAG------
------------GGGCTG---------GGG-------------------------GGA---
-------------------------GTCTGC------------TCTGATCAAAAACACTGC
TTCCGCTTT---------------------------------------------------
------------------------------------------------------------
------------------------------------------------------------
---------------------
>Chinese_sturgeon_Aqp7
---------------------------------------------------ATGAAGCTG
GACAAG---CAGTTTGTATCCAACGTAAAGAAAGCCATT------CGAATCCGAAACGAA
TACCTGCGAGAGGCTCTGGCTGAGATACTCAGCACCTTTGTCATGATGGTTTTCGGTCTG
GGCTCGGTAGCCCAGGTGGTCATGGGAGGT------GGTTCCTATGGTGACTACTTCAGT
ATAAATTTGGGCTTCGGCCTTGGGGTCACCATGGGTATTCACATCGCTGGGGGCGTCTCA
GGAGCTCATATGAACACTGCAGTCACCTTCTCCATGTGTGTGCTGGGGATCCTGAGCTGG
AGGAAGCTGCCGGTCTACGCTGCTGCCCAGTTCCTCGGCTCCTTTATGGCTGCGGTCACC
GTCTTC---TGGGTCTACTATGATGCTCTGTTTGAGTTCTGCGGA---GGGAACTTCACA
GTGACGGGCCCGAGA---GCAACTGCTGGGATCTTCGCTACCTACCCGGCACCATACCTG
TCAGTGGGAGGAGGCTTTCTGGACCAGGTGCTGGGCACTGCCATGCTGCTGCTGTGTATC
CTGGCGCTGAATGACCACAGGAACAGCCCGGCGCTCAGCGGCACGCAGCCTCTGCTCATC
GGCCTCCTGGTGGTGGTGATTGGCATCTCTCTGGGCAGCAACAGCGGGTACGCCATCAAC
CCCGCCCGCGACCTG---CCCCCGCGCTTCTTCACCTCCGTGGCAGGCTGGGGTCCTGAC
GTCTTC---AGT------GCTGGTAATGGCTGGTGGTGGATCCCTGTGGTGGCGCCCATG
GTTGGCAGTGTGACTGGCTCTCTGCTCTACAAACTCTTCATTGAGTATCACCATCCGGCT
---------------GAGGAGCATCTAGAG---------------------GAG------
------------GGGCTG---------GGG-------------------------GGA---
-------------------------GTCTGC------------TCTGATCAAAAACACTGC
TTCCGCTTT---------------------------------------------------
------------------------------------------------------------
------------------------------------------------------------
---------------------
>Sterlet_Aqp7
---------------------------------------------------ATGAAGCTG
GACAAG---CAGTTTGTATCCAATGTAAAGAAAGCCATT------CGAATCCGA------
------------------------------------------------GTTTTCGGTCTG
GGCTCGGTAGCCCAGGTGGTCATGGGAGGT------GGTTCCTATGGTGACTACTTCAGT
ATAAATTTGGGCTTCGGCCTTGGGGTCACCATGGGTATTCACATCGCTGGGGGCGTCTCA
GGAGCTCATATGAACACTGCAGTCACCTTCTCCATGTGTGTTCTGGGGAGCCTGAGCTGG
AGGAAGCTGCCGGTCTACGCTGCTGCCCAGTTCCTCGGCTCCTTTATGGCTGCGGTCACC
GTCTTC---TGGGTCTACTATGATGCTCTGTTTGAGTTCTGCGGA---GGGAACTTCACA
GTGACTGGCCCGAGA---GCAACTGCTGGGATCTTCGCTACCTACCCGGCACCATACCTG
```

TCAGTGGGAGGAGGCTTTCTGGACCAGGTGCTGGGCACTGCCATGCTGCTGCTGTGTATC
CTGGCGCTGAATGACCACAGGAACAGCCCGGCGCTCAGCGGCACGCAGCCTCTGCTCATC
GGCCTCCTGGTGGTGGTGATTGGCATCTCTCTGGGCAGCAACAGCGGGTACGCCATCAAC
CCCGCCCGTGACCTG---CCCCCGCGCTTCTTCACCTCCATGGCAGGCTGGGGTCCTGAC
GTCTTC---AGG------GCTGGTAATGGCTGGTGGTGGATCCCTGTGGTGGCACCCATG
GTTGGCAGTGTGACTGGCTCTCTGCTCTACAAACTCTTCATCGAGTATCACCATCCATCT
---------------GAGGAGCATCTAGAG---------------------GAG------
------------GGGCTG---------GGG------------------------GGA---
------------------------GTCTGC------------TCTGATCAAAAACACTGC
TTCCGCTTT---

>Reedfish_Aqp7

-------------------------------------ATG------AAGATCACAAATCAA
TATATCCGAGAGTTTCTTTCAGAATTCTTAAGCACTTATATCATGATGCTCTTTGGCTTA
GGCTCAGTTGCTCAGGTGGTCATGGGAGGT------GATTCCTATGGTAACTACTTAAGT
ATAAACCTGGGCTTTGGTCTTGGAGTCACCATGGGCATTCACATTGGTGGTGGGGTCTCA
GGAGCTCACATGAACACTTCAGTGACATTTGCAATGTGCCTAATAGGAAATCTGTTTTGG
AAGAAGCTGCCAGTATATGCACTGGGACAATTACTGGGCTCCTTTTTGGCTGCAGTCACC
ATCTTC---TGGCTTTATTATGATGCCTTACAAGACTACTGTGGT---GGAAACTTCACG
GTGACTGGACCCAAA---GCCACAGCGGGGATCTTTGCTACATATCCAGCTCCTTACTTG
TCTGTAGCAGGAGGTTTTATAGACCAGGTGGTAGGAACAGCGGTACTGCTGCTATGTATT
CAAGCTATCAATGATCAGAAGAACTGCTCTGCTCTCAGTGGGACATCTCCATTGGTGACT
GGCCTGTTGGTGGCACTTATTGGTATCTCCTTGGGTAGTAACAGTGGCTATCCTATCAAT
CCAGCCAGAGATCTT---CCTCCCAGGATCTTCACTGCTATGGCAGGTTGGGGGACTACT
GTTTTC---AGT------GCAGGAAATAACTGGTGGTGGATCCCTGTTGTTGCACCAATG
TTTGGCAGTGTGACTGGAGTCCTAATCTACAAAGTGTTCATTGAAATGCATCATCCTTCA
---------------GTTGAGCAGCAGAAG---------------------AAA------
------------CAACTT---CAAGAAAAA------------------------AGC---
------------------------CCAAGT------------ACAGATCTTTCTCTTTGC
TTT---

>vTunicate_Glp
---ATGGAAAAA
TCTTCGACGTCTTGGAGAAAAAAAGTCTCATCTTTAGTT------CGAATTGAAAACGTC
CTTTTGAGGGAAATGTTGGCGGAGTTTCTGGGGACGTTCATACTGCTTGTTTTTGGCAAT
GGAGCGGTCGCACAAAAAGTATTAAGCAGA------GACACGTTGGGCACCACACTTTCA
ATAAACTGGGCGTATGGTTTTGGTGTCACTATGGCAGTATATGTTACTGGAAAAGTTTCA
GGTGCACATATAAACCCAGCTGTATCGGTAGCACAATGTGCATTTGGGAACTTACCATTA
TACAAGCTGCCTTGTTACATTTTCTCACAAGTCTTTGGTGGTTTTGTTTCCGGGGCGGCT
GTTTAT---TCAATCTATTATGAGGCACTTAACGCTTTTGACGGT---GGACAACGTTCT
GTTTTGGGTCCAAAT---GGAACCGGGGGTATCTTCGCAACATACCCGCAAGATTACCTT
TCCATTAACAACGGTCTTTGGGACCAGGTCTTCGGCACTGCACTACTAGTTGGTATTATA
TTTGCTGTTACTGACAACAAAAACAATACAATTGCTGATGGTCTTACTCCTATAATAATC
GGCCTGCTTGTCTTTATACTAGGTACATCGTTCGGACTCAACTGTGGCTATGCTATCAAC
CCGGCTCGCGATTTT---GGACCGCGCTTATTTACTTTTGCCGCTGGCTGGGGCCCGGGA
GTCTTT---ACAGAGCCAAATGGCATGTCGTGGTGGTGGGTGCCTATCGTGGGCCCTATT
ATAGGCGGGTTGACTGGAGCAATATTGTATAAGTTAATGGTTGGAACTCATTTACCATCG
---------------GGAAGTCAAAGTGTT---------------------ATA------
------------ACCGAC---GAGGAAGAC------------------------GAC---
--TTCGAGATTTGTTCACAC
ACTTCGGACAACAGAGAAAAATTGTGTAAAGGA---------------------------

---TTGACCAAGCTT------
>Csavignyi_Glp

--ATCAAAAACGTC
TTGATTAGAGAAATGTTGGCGGAATTTTTCGGCACATTTATACTACTGGTTTTCGGAAAT
GGGGCTGTTGCCCAAAAAGTGCTAAGCAGA------GAAACTTTAGGCACTACACTGTCG
ATAAACTGGGCATACGGTTTGGGCGTTACAATGGCAATTTATGTAACTGGCAAAGTATCA
GGAGCGCACATTAATCCTGCTGTCTCCGTCGCTTTATGTGCATTTGGAAAACTTCCTCTG
TACAAACTTCCTTGCTATGTAATTTCCCAGGTCTTTGGTGGTTTCGTATCGGGTGCCGCT
GTTTAT---TCGGTTTATTACGATGCAATAAACGAATTCGACGGT---GGTAAACGTTAT
GTTCTGGGACCAAAC---GGCACTGGCGGGATATTTGCTACATACCCACAAGATTACCTT
TCACTTGGAAGCGGTTTGTGGGATCAGGTGTTCGGAACAGCTCTACTTGTTGGAATTATC
TTCGCTGTAACGGATGACAAAAATAATACAATTGCTGATGGTTTAACTCCCATTATTATA
GGACTACTGGTATTTATTTTAGGAACTTCATTTGGACTGAACTGCGGATATGCTATCAAT
CCTGCGCGAGATTTA---GGGCCGAGGTTGTTTACTTTTGCCGCTGGGTGGGGCACAGAA
GTTTTT---ACGGAGCCAAATGGAATGTCATGGTGGTGGGTTCCAATTGTTGGACCTATT
CTCGGGGGGCTAACAGGCGCAGTCATATACAAGTTATTTGTGGGCACTCATTTGCCATCA
---------------GAT--

>SalThom_Glp1
---ATGACTTAC
GAGAAATTTTCTTTCATGGAACGAACGCTGGATCGACTG------AAAGTAGAAAATTCC
CTAATCCGAGAAATGTTTGCCGAATTTCTCGGTGTCCTCATCTTAGTGACGTTTGGAAAT
GGAGCAGTTGCACAAAGTGTGTTGAGTAAG------AAGTCGCAAGGTGAAACTATTTCC
ATCAATTGGGCTTACGGAATGGGAGTTACAATGGCAATCTATGTGGCAGGAAACGTATCA
GGTGCACACTTGAATCCTGCCATTTCAATCCTCTTCTCAGTCATGGGTCGTCTCTCGTGG
CTAAAACTCCCTTTTTATATTTTGGCCCAGATGTTGGGCGGATTTGTCTCCGGTGCGGTT
GTGTAC---TCCACTTACTACGATGCATTGAACAGTTTTGACGGA---GGGGTACGGAGT
GTATACGGAGTAAAC---GCCACAGCTGGAATATTCGCCACATATCCACAAGATTATCTC
TCCATAACAAACGGATTGTGGGATCAGGTGGTCGGTACAGCTTTACTGGCCAGCATGCTC
CTAGCCATCACGTCGGAT------AACAAGATAGTCCATGGATTTGTGCCCTTATTAGTT
GGACTGTTGGTGTTTTCGATTGGCTTGTCATATGGGTTCAATTGTGGATATGCGATTAAT
CCCGCACGTGACATG---GGTCCTCGACTCTTCACGTTTGCTGTTGGATACGGAAGTGGT
GTTTTC---ACGGAGCCGAACGGTTTGTGCTGGTGCTGGGTGCCTGTCGTCGGGCCAATA
GTGGGCGCTCTTGTGGGTGGACTCATCTACAAGATGTTGGTTGGTCATCATCTTCCCGAA
---------------GAGGAGGAAGTCTGC---------------------GAG------
------------GATCTT---TCAAACAAA------------------------ACA---
------------------------ACAAAA------------CTT---------------

>Amx_Glp1
---ATGGGTAAA
CCTTCG------TGGACGGAGCGAGTTGTGAGCGCTCTT------CGGGTGAGGAGATTT
GTAATCCGGGCGACGTTGGCAGAGCTACTCGGCACATTTCTCCTGGTGACGTTTGGAAAC
GGATCGGTGGCGCAGGTGGTGCTGAGTCGT------GAGGACAAGGGAACGTTCTTATCC
ATCAACTGGGCGTACGGCATCGGGGTGGTCATCGGCGTGTACGCCTCCTGGGGTGTCTCA
GGAGCCCATCTGAACCCTGCGGTATCCCTGACCATGGCAGTCCTGGGGAAGTTACGCTGG
GTGTACCTGCCGTGTTACGTCATGGCTCAGATGATAGGCGCCTTCTTGTCAGCAGTTTGT
GTGTAC---ATTGTGTACTATGATGCATTGGTGAACTTTGACGGC---GGGACCCGGGCA
GTGTTGGGTGTGAAC---GGGACCGGAGGAATCTTCTGTACGTACCCGCAAGACTACCTG
TCTATCGGCAGTGGAGTCCTTGACCAGGTAGTGGGCACCGCTCTGTTACTCTGCGGTGTC
CTGGCTCTGACTGACGCTAAGAACAACAAGGTAACGGCGGGGATGGAGCCGCTGCTGGTC
GGCCTGCTGGTGTTCGCCATCGGGACGTCCTTCGGGTTCAACTGCGGATACGCCATCAAC
CCTGCCCGAGACCTG---GGCCCGAGGATCTTCACGGCGATGGCAGGCTGGGGCCTGGAG
GTCTTC---AGG------GCGGGAAACCATTGGTGGTGGGTGCCGATAGTCGGACCGTTG
ATCGGCGGGCTGGTCGGGGGTTTGGTCTACACGCTGATGGTGGCGCTGCATCATCCAGAG
---------------GAAGAAGATAACGAG---------------------TCA------

------------GTACAC---GGGTCCCCG------------------------GAGCTG
------------------------CAGGAC------------GTAAAGGTTCCAGAAGGT
GGCGTTGCTAACGCTGCCATGGAG------------------------------------
---------------------GACTTACATAGCAATACTATAGTG---------------

>Bfloridae_Glp1
---ATGGGTAAA
CCATCG------TGGACGGAGCGAGTTGTAGGCGCTCTT------CGGGTGAGGAGGTTC
GTATTCCGGGCGGCGCTGGCTGAACTACTCGGCACATTTCTGCTGGTGACGATGGGAAAC
GGATCCGTGGCGCAGGTCGTGCTGAGTCGG------GAGGACAAAGGAACGTTTTTATCC
ATTAACTGGGGGTACGGCATCGGGGTAGTCATCGGAGTCTACGCCTCCTGGGGTGTCTCA
GGAGCCCATCTAAACCCTGCGGTATCCTTAACCATGGCAGTCCTGGGGAAGCTGCGCTGG
GTCTATCTGCCTTGTTACGTCTTGGCGCAGATGTTAGGGGCCTTCTTGTCAGCAGTTTGT
GTGTAC---ATAGTGTACTATGATGCATTAGCAAACTTTGACGGC---GGGACCCGGGCA
GTTTTAGGTGTGAAC---GGGACCGGAGGAATCTTCAGTACGTACCCGCAAGACTACCTG
TCTATTGGCAGTGGAGTCCTGGACCAGGTGGTGGGCACTGGCCTGTTACTCTGCGGTGTT
CTGGCCCTGACTGACTCCAGGAACAACAAGGTAACGGCGGGGATGGAGCCGCTGCTTGTC
GGCCTGCTGGTGCTCGCTATCGGCACGTCCTTCGGGTTTAACTGCGGCTACGCCATCAAC
CCAGCCCGAGACCTG---GGCCCGAGAATCTTCACGGCAATGGCAGGTTGGGGCATAGAA
GTCTTC---AGG------GCGGGAAACCATTGGTGGTGGGTGCCGATAGTCGGACCGTTG
ATCGGCGGGCTGGTTGGAGGTCTGGTCTACACGCTTATGGTGGCGCTGCATCATCCGGAG
---------------GAAGAGGATGACGAG---------------------CCA------
------------GTACAC---GGATCACCG------------------------GAGCTG
------------------------CAGGAC------------GTAAAGGTTCCAGAAGGT
GGCGTTGCTAACGCTGCTATGGAA------------------------------------
---------------------GACTTACCAAGCAACACTATCGTG---------------

>Bbelcheri_Glp1
---ATGGGTAAG
CCGACC------TGGACCGAGCGAGTTGTGAGCGCTCTT------CGGGTGAGGAGGTTC
TTGGTTCGGGCGGCATTGGCTGAACTTCTCGGCACATTTCTCCTGGTGACGATCGGAAAC
GGCTCCGTGGCGCAGGTCGTGTTGAGTCGG------GAGGCTAAGGGGACGTTCTTGTCC
ATCAACTGGGCGTACGGCATCGGGGTAGTCATCGGCGTGTACGCCTCCTGGGGGGTCTCA
GGAGCCCATCTGAACCCTGCGGTCTCCCTGACCATGGCAGTCCTGGGTAAACTACGCTGG
GTGTACCTGCCGTGTTACGTCATGGCTCAGATGATAGGGGCCTTCTTGTCAGCGGTTTGT
GTGTAC---ATTGTGTACTATGATGCATTGGGTAACTTTGACGGT---GGTACCCGAGCA
GTTGTAGGTGTGAAC---GGGACGGGAGGCATCTTCAGTACCTACCCGCAAGACTATCTG
TCTATCGGCAGTGGAGTGCTGGACCAGGTGGTGGGCACTGCCCTGTTGCTCTGCGGTGTT
CTGGCTCTGACGGACTCCAAGAACAACAAGGTGGCGGCCGGGATGGAGCCTCTCCTGGTC
GGCCTGCTGGTGTTCGCCATCGGCACGTCCTTCGGGTTCAACTGCGGGTACGCCATCAAC
CCTGCCCGAGACCTG---GGCCCGAGACTCTTCACGGCGATGGCAGGCTGGGGCCTGGAA
GTCTTC---AGG------GCGGGAAACCACTGGTGGTGGGTGCCGATAGTCGGCCCGTTA
ATCGGCGGGCTGGTCGGAGGGTTGGTCTACACGCTGATGGTGGCGCTGCATCACCCGGAG
---------------GATGAAGACGACAAG---------------------CCA------
------------GTACAC---GAGTCACCG------------------------GAGCTG
------------------------CAGGAC------------GTCAAGGTTCCAGGTGGT
GGCGTTGCTAACGCTGCCATGGAG------------------------------------
---------------------GACTTGCCCAGTAACACTGCGGTG---------------

>Bfloridae_Glp2
---ATGGGTACA
CCATCG------TGGACAGAACGAGTTGTGGGCACTCTT------CGGGTGAAGAGGTTC
ATAGTCCGGGCAGCTATGGCAGAAATGCTCGGCACATTCCTCCTGATGACGATAGGAAAC
GGTTCCGTGGCACAGGTCGTGCTGAGTCGG------GAGGACAAGGGAACGTTCTTTACA
ATCAACTGGGGGTACGGTATCGGGGTGGTCATCGGAGCATACGCCTCCTGGGGTGTCTCA
GGAGCCCATCTAAACCCTGCCGTATCCCTGACCATGGCAGTCCTGGGGAAGCTACGCTGG
GTCTACCTGCCGTGTTACGTCATGGCACAGATGATAGGGGCCTTCCTGTCAGCAGCCTGT
GTGTAT---GCTGTGTACTATGATGCGTTAGCAAACTTTGACGGT---GGGACCCGGGCA

GTTTTAGGTGTTAAC---GGGACCGGAGGCATCTTCAGTACGTACCCCCAAGACTACCTG
TCTATTGGCAGTGGAGTGCTGGACCAGGTGGTGGGCACTGGCCTGTTACTCTGCGGTGTT
CTGGCCCTGACTGACTCAAGGAACAACAAGGTAACAGCAGGGATGGAGCCGCTGCTTGTC
GGCCTACTGGTGTTCGCTATTGGCACGTCCTTCGGGCTCAACTGTGGGTACCCTATCAAC
CCAGCCCGGGACCTG---GGGCCGAGAATATTCACGGCAATGGCAGGCTGGGGCCTGGAT
GTCTTC---AGG------GCTGGAAACCATTGGTGGTGGGTCCCTATAGTCGGACCATTG
ATTGGTGGGCCGGTCGGAGGTTTGGTATACACCCTGATGGTGGGGCTGCACCATCCAGAG
---------------GAAGATGATGACAAG---------------------CCA------
------------ATATAC---GGATCAACG-----------------------GAGCTG
------------------------CAGGAC------------CCAAAGGTTCCAGAAGGT
GGCGTTGCTAATGCTGCCATGGAGCTT---------------------------------
------------------GATGACATTCCAAACAATACTATTGTACACAGGAAC------
---------------------------------ATGCAAACCTTGCCCAAGGCTTGGGAT
GTTGAGGAT------------
>PtyFlava_Glp3a
--ATGGGAGCC
TACGCC---AACTTTACCAACAAGATTATCAAAGCCATC------AGAATCCGTAATCAG
CTGGTCAGGGAGTCGCTGGCAGAGTTCATCGGGACATTCATACTCATCGTTTTGGGGAT
GGTTCTGTGGCTCAAAGTGTCCTCAGCGAC------AAAGCATATGGTGAATTTTTGTCA
ATCAACTGGGCCTGGGGTGTTGGCGTCACCATGGCCGTTTACTTTGCAAACGGGGTCTCA
GGTGCACATATAAACCCAGCAGTGACCTTGGCCATGGCGATAGTCGGACGATTTCCCTGG
TACAAAGTAATCTTTTACTGGTTGATGCAGTTTCTTGGCGCCTTTGCTGCCGCCGCCTGT
GTCTTT---GGAGTTTATTACGATGCTATCAATGACTTCGATGGA---GGTCAAAGACAG
GTCACTGGACCGAAT---GCGACGGCGGGCATTTTTGCGACGTACCCGGCTGATTTTTG
AGAATTGAAAGTGGACTCGGAGATCAGATATTCGCCACCATGTTATTGGTGGCTTGCATC
ATGGCAATCACCGATAAAAGGAACACCAAACCACCGGCGGGAATGGAGCCTTTCCTCATA
GGTTTGGTGGTGTTCGTCATCGGCTTGGCCTTCGGTTTCAACTGCGGCTACGCCATCAAC
CCGGCCCGAGACTTC---TCACCAAGGCTCTTCACGGCCATTGCAGGCTGGGGCTCTGAA
GTCTGG---ACT---CCGAATGGTATGAGCTGGTGGTGGGTTCCTATAGCGGGACCTTTC
ATCGGGGGAATATGTGGAGCGTTTGTCTACATCGTGTTCATCGAGGCTCACCACCCACCA
---------------GCACAGGAGTATGAT---------------------CTT------
------------GAACGA---CCTCCAGAC------------------------------
------------------------TCGGAA------------TACACTATGAAACCGGTC
GGGGACGAGGTTGTGGACAAGAATGGT---------------------------------
ACAGCTGGCGAACAGAACAAAAACTTCGAAGGCGAT------------------------

>PtyFlava_Glp3b
--ATGGGCGCA
TATACG---AAATTCATCAACAAAATCACCAATGCTATC------GCGATCAAGAATCGT
CTGGTTCGGGAGTCCTTAGCGGAATTCATCGGAACTTTCATACTTATGATTTTTGGAAAT
GGATCTGAAGCCCAAAGCATTCTTAGCCGT------GGAGCGTACGGCGAATATCTTTCC
GTCAACTGGGCCTGGGGTATCGCCGTTACCATGGGCATTCACTTTGCGTCGAGTGTCTCA
GGTGCTCACATTAATCCGGCGGTGACGTTAGCTAAAGCAGTCGTTGGTAGATTCCCATGG
TACAAAGTTATCCCCTACTGGATTATGCAGTGCTTGGGGGCGTTCGCTGCCTCGGCATGT
GTCTAC---GGAGTCTATTATGACGCCATCAATGCCTTCGACGAC---GGTATAAGACAA
GTCACCGGTCCAAAC---GCAACGGCCGCTATCTTTGCGACATATCCGGGCCAGTATCTG
AGTATAGCGAGCGGACTTGGAGATCAGATTGTGGGCACGATGCTATTGCTGTCTTGCATA
TTTGCTATCATCGATGAAAGAAACGCCAAGCCACCGACTGGAATGGAACCATTCCTGATT
GGCTTAGCGGTGTTTGTGATCGGTTTGTCGTTCGGCGCAAACTGTGGCTACCCGTTGAAT
CCGGCCCGTGATTTC---CCGCCGAGACTGTTCTCGTACATGGTCGGCTACCCTGACGAA
GTCTGG---ACT---CCCAATGGTGTACACTGGTGGTGGGTTCCCATAGTTGGACCTTTC
ATCGGTGGGATTTGTGGAGCTGTAGTTTACATCATTCTCATTGAAGCTCATCATCCCAAA
---------------GTTAATGAACTTGGT---------------------GGG------
------------ACTGTG---ACAACCAAT-----------------------GAT---
------------------------GAAGAT------------GTGGAGATGAAGCGTGTC
GGGAATACGGACCGAGACCTGGTAGAGAGCGATCAAGGC---------------------
ACTTCCGGCACGGAAAACAAGTCTTTCAATGCTGAG------------------------

>SK_Acon_worm_Glp3b

```
-------------------------------------------------------ATGGGGGCG
TACAGT---AACTTTGTGAACAAG---ATTAAAGCTATT------GGTATTCGGAATCAG
GTTGTCCGTGAATCGTTGGCTGAGTTCATTGGCACGTTTATTCTTATTACATTCGGAGAT
GGTTCGGTGGCACAGTCGGTTCTCAGTCGA------GGTGAAAAAGGCGAATATCTTTCC
ATCAACTGGGGATGGGGTATAGCTGTTGTTATGGGAGTACATTTCGCCTCTGGCGTATCA
GGAGCTCACATCAATCCAGCTGTGACTTTAGCTATGGCAACTATCGGAAGATTTGATTGG
GTAAAAGTTCCCCTTTATTGGTTGATGCAATTTCTCGGTTCATTTGCAGCGTGTGGATGT
TTATAC---GGAGTATATTATGATGCAATAGAGGATTTTGATGGC---GGCGAACGACAA
GTATATGGTCCTAAC---GCCACTGCCGCTATATGGGCAACGTATCCACAAGATTATGTT
ACCATAGAAACAGGTTTAGGCGATCAGATTTTTGGAACCATGTTGCTGCTGGGTTGTATA
ATGGCAATAGTGGACACGAGAAACAACAAGCCGCCACATGGTATGGAGCCTCTGCTGATT
GGGTTGGCAGTCTTTGTTATAGGATTGGCTTTTGGTCATAACTGTGACTATGCTATCAAC
CCAGCAAGGGATTTG---GCACCACGTACTTTCTCATACTGGGTCGGATATGGGGCAGAG
GTTTGG---ACG---CCTAATGGTATAAACTGGTGGTGGGTGCCAGTTGTAGGTCCACTA
ATAGGAGGTGTATGTGGGGCGCTTATTTATATTGTATTCGTAGAAGCCCATCATCCTGAT
---------------GACAAGGATTCGCAT---------------------AGT------
------------CTGGAT---CAGAACGAG------------------------TTT---
------------------------ACCCTA------------AAAGATTTAACACAGGAA
GAAGGTACAGATCAAGCAACATGTACTGTGAGCAAATCC---------------------
AATGCCGGATTTGAAGAAGAA---------------------------------------
------------------------------------------------------------
---------------------
>jsCucumber_Glp3
-------------------------------------------------------ATG---GCG
GAGAGT---AAGAAGATATCGAATTTTATAGACAAGTTC------AGAATCAGGAATCAT
CTCGCTAGGGAAATCTTGGCAGAATTTATTGGAACATGGATTCTTCTGGTATTTGGAGAC
GGTGTAGTCGCTCAAACAGTACTCAGTCGT------GGGGCTAATGGGTCTGCTCTCTCC
ATTAATTGGGCGTGGGGCATGGCAGTGGTGATGGGTATCTATTTCGCTGGTGGTATATCA
GGAGCACATATAAATCCCGCAGTGACGGTAACCATGGCAACGATTGGACGGTTTCCCTGG
AAGAAAGTTCCTTTCTATATTATAGCACAATTTCTAGGCGCATTCATGGCAGCCGCGTGT
GTATTT---GGAGTTTATCATGATGCAATCCAGAACTATGACGGA---GGCGAAAGACAA
GTATATGGACCCAAC---GCTACTGCTGGAATATTTGCCACTTATCCACAAGACTTTCTT
TCAGTTGGAAGTGGCTTTGCTGATCAGGTATTTGGTACTGCTCTTCTTCTGGCGTGTATT
CTAGCCATCTCCGATTCGCGGAATGGTCCTCCACCACCAGGCATGGGTGCGCTGATGGTC
GGTCTCGTGGTATTCGTCATTGGAATGACATTTGGCTTCAACTGCGGCTACGCTATCAAT
CCTGCCAGGGACTTT---GGTCCTCGAGTCTTTACAGCTATGGCTGGATATGGGCAGGAA
GTTTGGTTCACG---CGCGATGGCAAGCATTGGTGGTGGGTGCCTATATTAGGCCCCATC
GTAGGGGGTATCTGTGGTGCTCTCATGTACATTGTCTTCGTGGAAATGCACCATGAACCA
---------------GAGAAGGTAGAGAAT---------------------GGC------
------------AAAAGA---AAACGACTT------------------------GAA---
------------------------AACAAC------------CATGAGGAAACACCCATG
AATGATAACAAGGTT---------------------------------------------
------------------------------------------------------------
------------------------------------------------------------
---------------------
>Kina_Glp3
-------------------------------------------------------ATGACC---
---------AACGTTGTGGAGAGGGTGTTGAGCCCGTTG------CAGATTCGCAACAAG
CTAGCGAAGGAGATCTTAGCAGAAGCAATCGGGACCTACATTCTGATCGTGTTTGGCGAT
GCCTCGGTAGCACAGTCGGTGCTGAGTAAA------GGAGAGAATGGCGGGTTTCTCTCT
ATCAACTGGGGCTGGGGTGTCGGTGTTACACTTGCTGTTTACTTCGCATCTGGTGTATCA
GGAGCTCACATTAACCCTGCAGTAACACTAGCCTTCGCATGCCTTGGACGGTTTCCATGG
AAGAAGGTCCCCTTTTACATGTTGGCCCAGATGGTAGGAGCTTTCGTTGCGGCAGCCTGT
GTCTTT---GGGGTGTATTCAGATGCCATCAATGACTTTGACGGT---GGAACTCGAGCC
GTCCTTGGGGAGAAT---GGGACGGCTGGCATTTTTGCGACCTATCCGAAGGACTTCCTA
TCAATTTGGAGTGGATTTGGTGATCAGATATTAGGTACTGCTCTTTTGATGTCCTGTATA
TTGGCTATCACCGACAAGAGAAACAACTCGCCCCCGAATGGTATGGAACCCCTTCTAATC
GGCTTCATCGTATTCAACATCGGTATTTGCTTCGGCTACAATTGCGGCTACGCTATCAAC
CCTGCCAGAGATCTG---GGGCCGAGGTTATTCACCGCATGTGCGGGTTACGGCCAAGAT
GTGTGG---ACT---CCAAATGGTATGCATTGGTGGTGGGTGCCCATAGTAGGACCTTGT
CTTGGCGCCATCTTGGGAGGCTATCTCTACGTGCTCGCCATCGAACTCCACCACGACACC
```

---------------GAAACGGCATCGGCA---------------------AGA------
------------GATGAA---GACGGCGGA------------------------------
------------------------GGAGACTAC---AGGATGGCTGATCTCTCAGGTAAA
GGGGAAGACAACCAGGCAGTTCAGAACCATGGAGATGTG---------------------

>gvUrchin_Glp3

------------------------------------CTTTCACTCCAGGTATTTGGTGAC
GCATCGGTAGCACAGTCTGTTCTGAGTAGC------GGGGCTAATGGCGGCTTTCTCTCA
ATCAATTGGGGATGGGGAATCGCCGTCACACTCGCCGTTTATTTCGCATCTGGTGTGTCA
GGTGCCCACATAAACCCAGCAGTGACCTTGGCCTTTGCCTGCTTGGGACGGTTTCCTTGG
AAGAAGGTCCCGTTTTACATCTTGGCTCAGATGGTCGGTGCCTTTGTCGCAGCTGCCTGT
GTGTTC---GGAGTGTATTCAGATGCTATCAATGCCTTTGACGGT---GGAACTCGAGCA
GTACTCGGCGAAAAC---GGGACAGCTGGTATTTTTGCAACCTATCCGAAGGACTTCCTT
TCTATCTGGAGTGGACTCGGCGATCAGATCCTAGGCACTGCTCTTTTGATGTCCTGCATA
TTGGCTATCACGGACAAGAGAAACAATTCGCCTCCTAACGGGATGGAACCCCTTCTCATT
GGTCTCGTTGTGTTCAATATTGGTATTTGCTTCGGTTTCAACTGCGGCTACGCCATTAAC
CCAGCCAGGGACCTG---GGCCCTAGGTTATTCACCGCCTGCGCAGGTTATGGTCAAGAT
GTATGG---ACGAGTCCCAATGGTATGCATTGGTGGTGGGTGCCCATAGTAGGACCCTGT
GTGGGTGCCATCTTGGGTGGATACCTCTACGTGTTCACTGTCGAGCTTCATCACGACACA
---------------GAAACAGCATCGGCG---------------------AGA------
------------GACGAA---GATGGAGGA------------------------------
------------------------GGAGACTAC------ATGGCTGATTTAACGGGAAAA
GGCGAAGACAACCCAGCTATTCAGAACCACGGCGAAGCA---------------------

>pUrchin_Glp3
---ATGCCG---
---------ACCGCTTTGGACAGGCTGCTGAGCCCGCTG------CAAATTCGCAACAAG
CTTGCGAAGGAGATCTTAGCCGAGGCAATTGCGACCTTCATTCTCATTGTGTTTGGCGAT
GCCTCGGTAGCACAGTCGGTGCTAAGCAAT------GGAGCTAATGGTGGTTTTCTCTCC
ATCAACTGGGGATGGGGTGTCGGTGTTATACTTGGTGTTTACTTCGCAGCAGGTGTATCA
GGAGCCCACCTCAACCCTGCGGTGACCTTGGCCTTCGCTTGCTTAGGACGGTTCCCGTGG
AAGAAGGTCCCCTTCTACATCTTAGCCCAGATGGTCGGTGCCTTCATAGCTGCAGCCTGT
GTATTT---GGGGTGTATTCGGATGCTATTAATGATTTTGACGGT---GGAGTTCGAGCT
GTACTCGGGGAGAAT---GGGACAGCCGGCATCTTTGCTACCTATCCGAAGGACTTTCTC
TCAATCTGGAGTGGACTTGGAGATCAGATATTAGGCACTGCTCTGTTGATGTCCTGTATA
TTGGCCATCACTGACAAGAGAAACAACTCTCCTCCTAACGGCATGGAACCCCTTCTTATC
GGTCTCGTTGTATTCAACATCGGTATTTGCTTCGGATACAACTGCGGCTATGCCATCAAC
CCTGCCAGGGATCTG---GGGCCGAGGTTGTTCACCGCCTGCGCGGGTTACGGCCAAGAT
GTTTGG---ACT---CCCAATGGTATGCATTGGTGGTGGGTGCCTATAGTGGGACCGATA
TTGGGCGCCATCTTGGGCGGATATCTTTACTTGTTCGCTATCGAACTCCACCATGACACG
---------------GACACGGCATCGGCG---------------------AGA------
------------GACGAA---GACGGAGGA------------------------------
------------------------GGAGATTAC---ATGATGGCTGAATTGTCAGGAAAA
GGTGAAGATAACCAAGCGGTTCAGAATCATGGACAGGCA---------------------

>cotStar_Glp3
---ATGGTG---
------------------------TGGACGCGGCATCTC------AGGATAAGGAACCAG
TGGGTGCGGGAATTCTTGGCAGAATTTCTGGGGACGTTTATTTTGATGGTTTTTGGAGAT
GGCAACGTAGCGCAGAGCGTCCTCAGCAGC------AGTGCTGCCGGTGAATTCCTCTCC
ATCAACGTTGGCTGGTGGGTTGCAGTTACCATGGGCGTGTATGCGTCGGCCGGAGTCTCT
GGTGGCCACATCAATCCGGCCGTGTCTTTAGCCCTGGCTGTGGTGGGCAAGTTCAGTTGG
ATCAAGCTTCCCATGTACGTGTTGGCCCAGTTCCTCGGCTCTTTTGCCGCATCAGCTTGT

```
CTCTAC---GGAGTCTACCTCGACGCACTGAATGCTTATGATGGA---GGCAATCGCACG
GTAATAGGTCCTAAA---GGCACTGCCGGCATCTGGGCCACTTATCCCCAAGATTTCCTT
TCACTGCAGGGCGGCCTAGGAGACCAGATTTTTGCCACGGGGCTGCTTCTGGTCTGTGTG
CTGGCCATCACGGACAAGAGGAACGACGGGGCACCCAGCGGTATGGCAGCCATCATGGTT
GGTCTGAGCGTGCTTGGAATCGGTGTGAGCTTCGGTTTCAACTGCGGCTACGCCATCAAT
CCAGCGCGAGACTTC---CCGCCGAGACTCTTCACCTACTGCGCTGGTTGGGGCGTTGAT
GTTTGG---GTC---CCCAACGGAATGCATTGGTGGTGGGTGCCGATCGTTGGGCCCTGC
ATTGGAGCGGTACTGGGCGCCCTGTTGTACATTCTCTGTGTGGAGGCCCATCATACCCCG
---------------GAAGACGAATTGCCA---------------------ATC------
------------ACCGAA---GGGGATGCA---------------------TATGCTGTT
------------------TCT---GAAGAT------------GTTGTGATGAAACGCCGA
GAGGAAGACGACTCTCTC------------------------------------------
------------------------------------------------------------
------------------------------------------------------------
---------------------
>bStar_Glp3
---------------------------------------------------ATGGCG---
------------------------TGGACGCGGCATCTG------AGGATCAAGAACCAG
TGGGTCAAGGAATTCTTGGCGGAGTTTCTAGGGACTTTTGTTTTGATGTTATTTGGAGAT
GGCAACGTAGCACAAAGCGTCCTGAGCAGA------GAGGCATACGGGGACTTTCTCTCT
ATCAACCTGGGATGGTGGGTGGCCGTCACCATGGGAATCTACGCGTCGGCTGGGATATCT
GGGGGTCACATCAATCCAGCCGTCTCTCTGGCCCTGGCCGTGGTGGGCAAGTTTAGCTGG
AAGAAACTGCCCGTGTATGTGCTGGCCCAGTTCATTGGCGCGTTTGCTGCCTCGGCCTGT
CTTTAC---GGAGTCTACCTTGATGCACTGAACGCTTTTGACGGT---GGTAATCGGATG
GTGTTAGGCGTTAAT---GCCTCCGCTATGATTTGGGCAACATACCCCCAGGAATATCTC
ACCCTACAGGGCGGCTTGGGAGACCAGATTCTTGGTACGGCGCTACTAATGCTTAGCGTG
TTGGCCATCACCGATAAGCGGAACAATGGTGCACCAGCAGGGATGGTGGCTATCATGGTG
GGGTTGAGCGTGCTTGGTATCGGCTTGAGCTTCGGGTCCAATTGCGGCTACGCTATCAAT
CCGGCACGTGACTTC---CCTCCCAGGCTCTTTACCTACTGCGCCGGTTGGGGTACTGAA
GTTTGG---ACC---CCCCGCGGTATGCACTGGTGGTTTGTGCCGATCGTTGGGCCGTGC
ATCGGAGCGATAGGCGGCGCCCTGTTGTACATCCTATGCGTGGAGGCCCATCACACACCG
---------------GAACATGAAATGCCG---------------------ATC------
------------ACTGAC---CAGGAGGAA------------------------AATATT
------------------TCT---GATGAC------------ATTATTATGAAGCGTCGA
GATGAAGACAGCAACTCCCTC---------------------------------------
------------------------------------------------------------
------------------------------------------------------------
---------------------
>Kina_Glp2
------------------------------------------------------------
------------------------------------CTT------CAAATCAACAATTGG
TGGGTCAAAGTGTTTCTAGCTGAGCTCATTGGAACTTTCACTTTAGTGTTTGTAACAGAT
GGCGCTATAGCAAGAACGGTTCTTAGCGAT------GGGGCAGCGGGGGACGCTCTATCC
GCCAACATTGGTGCCGCCTTTGCTGTCACTGTTGGAATCTACATAAGCGGGGGAGTTTCA
GGTGGTCATATAAATCCGGCCGTGACTCTATCGATGTGTATTATGGGTCGGCTGAGATGG
CTAGCTCTACCCGTCTATTGGGTTGCTCAGTTTATTGGTGCTTTCCTTGGGGCTACTGTA
GTGTAT---GGTATTTACCTGGATGGTATAAACAGCGTTGAAGGC---GGGCCTTCTAAC
CGAACATTA---------GCCACAGCAGGAATTTTCGCAACATACCCCGCACCTTTTGTA
ACTATACCGGGAGAAGTCATGGACCAGCTTGTAGGTACAGCGCTCCTGGTTGCCGGTATT
TTCGCCATCTTTGATAAGCACAACTTCAAACCACCAACGGGTCTAGAACCAATATCAGTT
GGTCTACTTCTTCTTGTCGTCAATATATCATATGGATACAATGCTGGCGCAGCAGTCAAC
CCTGCTAGAGATTTT---AGTCCTAGATTATTCACCGCTCTGGTAGGCTACGGGGAGGAC
ATTTGGATCACC---CCATCTGGTGACCATTTCTGGTGGATACCACTGCTTGTCCCTTTT
ATCGGTGGCCCTATAGGTGCCTGGCTATACTACCTTACTATAGAAGTTCACCATGGTTAT
---------------ACTGGCAGAGCAGAG------------------------------
------------------------------------------------------------
------------------------GAATCA------------TTACCGCTTGTGCAAAGC
ATGTCAAAGGATGATGAT------------------------------------------
------------------------------------------------------------
------------------------------------------------------------
---------------------
```

```
>pUrchin_Glp2
---------------------------------------------------ATGGCGAGG
---------------GACACACCTTTCAAAGAAAGGCTT------CACATCGGTAACTGG
TGGATGAGGGTATTTCTATCCGAACTTTTTGGAACTTTCATGTTGGTGTTTATTACAGAT
GGCGCTGTAGCAAGAACCGTTCTGAGCCGT------GGAGCAGCGGGTGGTGCTCTGTCA
CTTAACATTGGTGCCTCTCTTGCTGTCACCGTGTGTATTTACATGACCGGGGGAGTGTCA
GGTGGTCACATAAATCCGGCCGTAACTCTCTCGATGTGTAGCCTTGGCCGTCTGAGATGG
TTAGCTCTGCCTGTCTACTGGTTTGCTCAGTTTATCGGTGCTTTCCTTGGTGCTGCTGTA
GTCTAT---GGTATCTACCTAGATGGTATCAACAGTTTTGAAGGA---GGGCCTTCTAAT
CGATCTCTA---------GCCACCGCAGTAATCTTTGCCACATACCCCAATGGATACCTA
AGTGTACCAGGAGGAGTCATGGACCAGCTTGTGGGTTCGGCCCTTCTCGTTGGCGGTATT
TTCGCCATCTTTGATAAACACAACATCAAACCACCTGCGGGGCTTGAACCAATAGCAGTA
GGTCTACTTCTTCTCGTCGTTAATATAGCATACGGCTACAACGCGGGTGCAGCGGTCAAC
CCTGCAAGGGATTTC---AGCCCGAGACTGTTCACCGCTTGTGCTGGATACGGAAAGGAC
ATCTGGGTGACT---CCATCTGGTGACCATTTTTGGTGGATACCGCTGTTTGTACCCTTG
GTCGGGGGCCCGATAGGAGGATGGGTCTACTATCTCACTATAGAAGTCCATCATCCACAT
---------------AAAATTAGTAGCAGA---------------------AAA------
------------GCTAAA---GGGGAGTCT------------------------------
------------------------------------------TTGCATCTTATACGAGAC
GCCCCAGCGGTCGAGATA------------------------------------------
------------------------------------------------------------
------------------------------------------------------------
---------------------
>bStar_Glp2
---------------------------------------------------ATGTCGGAG
GGTTGGTTCATGCACGTGGACAAGTTCCTGGACAACCTC------CGTCTCAGGAACGAG
CTTATCCGTCAATTTTGCGCCGAGCTCCTGGGGACGTTCATGCTAGTGTTGATTGGTGAT
GGAGCCATTGCGCAGATGAAGTTCTTCGGTGGGACAGGCACTGGGGCAGGTTTTCTGAAC
GTCAACATCGGTTATGCCTTCGGCTTGATGCTGGGGGTGTATTTCACAGCATCAGTGTCA
GGTGGTCATTTGAATCCGGCGGTGTCACTTGCCTTCTGCACCTTGGGCAAGCTGAAGTGG
CTTGCCTTCCCGGTCTACATGTTGGCTCAGTTCATCGGTGCCTTCCTGGCCGCGGCCATG
GTCTTC---GCCGTCTATTACGATTCCATTGACAAAGAAGACCAC------AATAGGACG
GTGGTGTTACCGACC---AGTACGGCGGGCATTTTTGCTACTTACCCCACACCGGGGCTG
TCCTGGGGTGTCGGATTCGTCGACCAGCTCTTGGGCACAGCCCTCCTGATGGGTGGCATC
ATGGCCGTGACAGACAGCAAGAACTCCAAGCCACCCAGCGGTCTGGAACCCATCTTCGTT
GCCTTGACTTTCTTCGCTGTGGGCATCAGCTTCGGCTACAACTTTGGCTATGGCATCAAC
CCTGCCAGGGACTTC---GGACCGCGTGTCTTCACCGCCCTTGCCGGCTATGGGTCCGAT
GTCTGG---ACA---CAAGCTGGGGTTCACTGGTGGATCATCCCGACCTTTGTGCCCTTC
CTCGGGGCACCGATAGGAGCCTGGGTGTACTACCTCGGTATCGGCATGCACTGCGAGTCG
---------------GCCTACAAACGACAC---------------------GAG------
------------GACAAC---GCGCAGCTA------------------------------
------------------------------------------CTGGAGTCTCAACCACCG
ATGACGGAAGACAAATCCATG---------------------------------------
------------------------------------------------------------
------------------------------------------------------------
---------------------
>cotStar_Glp2
---------------------------------------------------ATGTCGGAG
GGTTGGTTCACGCACGTAGACAAGTTCCTGGACAACCTC------CGTCTCAGAAATGAA
CTCGTGCGCCAGTTCTTTGCCGAGCTCCTGGGAACTTTTATGCTGGTGTTGATCGGTGAT
GGAGCTATTGCGCAGATGCAGTTCTTTGGCGGAACAGCATTAGGGGCTGGCTTTCTGAAT
GTGAACATCGGGTATGCCTTCGGGTTGATGCTTGGGGTATATTTTACAGCTTCAGTATCA
GGTGGTCACTTGAATCCGGCCGTTTCACTGGCCTTCTGTACCTTGGGGAAGTTGAGATGG
ATCGCCTTCCCTGTCTACATGCTTGCCCAGTTTATCGGAGCCTTCCTGGCGGCTGTCATG
GTGTTT---GCTGTCTATTATGATGCCATCGACAAGCATGACCAC------AACAGGACG
GTGGTAGGA---------GGTACGGCGGGCATATTCGCCACCTACCCCAAAGCAGAGCTG
TCCTGGGGAGTTGGATTCGTGGACCAGCTCTTGGGCACGGCCCTCTTGATGGCGGGCATC
ATGGCGGTGACGGACAACAAGAACTCCAAGCCCCCCAACGGTCTGGAGCCCATCTTCGTG
GCCCTGACGTTCTTCGCCGTGGGCATCAGCTTCGGCTACAACTTCGGATACGGCATCAAC
CCAGCGAGAGACTTC---GGCCCTCGGGTCTTTACTGCTATAGCTGGCTACGGATCTGAC
GTTTGG---ACG---CTGAACGGGATTCAGTGGTGGATCATCCCCACATTCGTGCCTTTC
```

```
CTGGGGGCACCGGTTGGCGCCTGGGTCTACTACCTGGCGATCGGCATGCACTGCGAGTCG
---------------CCCCACCGAGGTCAG---------------------GGG------
------------GATCAC---GCGGAGCTG------------------------------
------------------------------------------CTGGAAGCGCAGGCGCCG
GACGCCGCTGAGAAATCCATG---------------------------------------
------------------------------------------------------------
------------------------------------------------------------
---------------------
>jsCucumber_Glp1a1
---------------------------------------------------ATGCCTACT
GAA------CAATTAGTGAAGAAATTGAAGTGTAAGCTC------CGAGTGAAGAGCGAT
TTAATACGATGTCTCATGGCCGAATTCATCGGGACTTTTGTGTTGGTTGCTCTTGTAGAT
GGCGGCGGGGCAGGGGTGATAACTAGTAGATTAAGTGGTTTG---GAGAACTACCTTTGG
TTGTCTTTCGGATCTGGATTTGGCGTTGGCTTTGGCATCTGGACTGCGTATGGTATTTCA
GGTGGTCACGTGAATCCAGCGGTAACTCTTGGTTTGACAATGACAGGAAAGTTTCCATGG
CGACGAGTACCATTTTACTTCCTGGCTCAACTATGTGGCGCTTTCTGCGCATGTGTAGTT
TGTTTC---CTTGTCTACTTTGATGGTATTAACAACATTGACGGA---GGTAGGATAGCG
GTCGACCATCCCAAC---GCGACTTGTGGTATCTTTACCACGTTCCCTGCTGCATACGTA
TCTCTCAGTACTGGATTCTTTGAACAGATCGTGAATACTGGTATTATGCTCGGTCTTATC
CACGTGATCGGTGACATGCGCAATGCAGGCCCTCCATTCAACCTTGCCCCTCTCTTCTAC
GGGCTGATTGTCTTCTCCGTTATCCTGTCATATGGTACGAATGCGGGCGCTCCTCTAAAC
CCCGCCCGCGACTTC---GCCGGTAGACTGATGTGCGCTATCGCTGGATATGGACCAAAA
GTTTGG---GCA---CCGTACGGTGTCCAGTGGTGGTGGATACCAACCTTTGGTCCCCTG
GTAGGGGCAGCTGTCGGTTCATTGGTCTATCTGTTCCTCATTGAAATTCACCATCCACCG
---------------GAAGAGAAACCAAAG---------------------GAT------
------------GACTTT---GGAGAGCTC---------------------GTCGAG---
---GATATCACAAAT---------AAAACACAAAACGGAGGAGCTTCTGCTGAGGAAGTT
AAAGTT------------------------------------------------------
------------------------------------------------------------
------------------------------------------------------------
---------------------
>jsCucumber_Glp1a2
---------------------------------------------------ATGTCTATC
GAA------AACTTTGTCGAGAATTTGAAGGGCAAGTTG------GCGATACAAAATGAC
CTATTCAGATGTCTATTGTCAGAATTTCTGGGGACATTTATCTTAGTGGTACTTGCAGAT
GGCGCTCTTGCAAGTCTTGCCATTAGTGGA------------TCCGATAATCATCTGTGG
GCGGCTTTTGGAGTTGGGTTTGCGGTGGCGTTTGGTATTTGGACTGCTTTAGGAGTGTCA
GGTGGACACGTAAACCCAGCCGTAAGTCTAGGCCTGGCTGTTACTGGTAAATTCCCCATG
AAGCGATTTTTACCTTATTGTTTAGCCCAATGCTCAGGTGCGTTCGTAGCCAGCGTCGTG
TGTTAC---TCTGTATACTATGATGGAGTAAAAAACGCT------------CCATTAGAC
GATGTTAACCATACA---GCAGCATGTGGCATCTTCACCACATTCCCTGGAGAGAGTGTA
GGGTATACAACTGCCTTTTTTGATCAGATAATTACCACTGGGCTGTTGATTGGACTTCTT
CTCGCATTGTTAGACAAACGGAATATGGCACCTGACCTCACCCTCGTACCCTTGTCTGCT
GGCATCCTTGTTTCTAGTATCATTCTGTCCTATGGTATGAACACTAGTGCACCTCTTAAT
CCAGCACGTGATTTC---GCAGGACGAATCATGTGCACACTGGCTAACTACGGACCGGAC
GTATGG---CTG---GATGACGGAAAACATTGGTGGTTTATTCCTACATTTGGTCCATTG
GTTGGTTCTGTAGTCGGTGCCTGGACATATATGCTCTGTATTGAGATCCACCATCCTAAC
---------------GAAGAAACAGAGGAG---------------------GCT------
------------AGCCGA---AAAGAAATT---------------------GGTGAA---
---GATGACAAAAAT---------GAAAATTTCGGCTTCAACGTTGATGTCGAAGAACCA
AAAACGGAACAGAGCGGTGTT---------------------------------------
------------------------------------------------------------
------------------------------------------------------------
---------------------
>Kina_Glp1a1
------------------------------------------------------------
---------------------------------ATGATA------ACAGTTCGAAATGAG
CTTGCACGACACCTCATCTGCGAATTCTATGCGACTTGCGTTTTCGCGCTATTCGTACGT
GGAGCCATCGCTCAGGATGTCACATCGGGG------------ACGGGATCAACGTTCAAC
ATCGCTATCGCCGTCGGTATGGCGGCCTGTTTTGGCATCTATCAAGGCTTTGGGGTCTCA
GGTGGTCACGTCAACCTGTCTATTACGATAGGCCTAGCTGCAATAGGGTTATTTCCCTGG
```

AAAAGAGTACCTATCTATTTCGTGATTCAAGTAGCTGCTAGCTTCGTCGCCACTTTGTTC
GTCTAT---CTCGTTTACGAAGACGCTTTCGATTATATTGATGGT---GGAACACGAGTG
GCGTTTGGTGAGAAC---GGAACGGCGGGAATCTTCGCTTCCTTTCCGCAACCATACTTG
TCTGTTACGACCGGCTTTTTCGAGCAGGTATTGAACACTGGTCTGCTCTTAGCATGTGTA
GGGGCGATGTTGGATAGTCGTAACAACCCACCCCCACTCAGCCTAGCACCTTTCTTCTTC
GGTTTGATTATCTTCACAATCATACTGACCTTTGGTCATAACGCTGGAGCACCTCTGAAT
CCTTCTCTAGACCTA---GCTGGTCGTCTTCTGATGTCTGCAGTCGGGTATGACAAGGAG
GTCTGG---GTA---CCCGAAGGTATTCATTGGTGGTGGATACCTATCGTAGGACCTTCT
GTTGGGAGTGCTCTAGGATCGTGGGCCTATTACCTGTCAGTCACCTTACACCATCCTCCT
---------------CTAGAAGGCAAGGGA---------------------GAT------
------------ACAACC---GACAACGGG------------------------------
--TCAGAGTTTGGGGAGAAA
GATGGAATGACCAACAATATCTTGATG---------------------------------
---GATTCGATGGACACG
AGAGGACGAACATCATCTGTTGAAGCACTCCACCGTAAATCATTAGAAAACGAGTCGACT
TCTGATATACATTTG------
>pUrchin_Glp1a1
ATG------TGTTTTACGGGAGAGGTCCTATTTGGAATACAGGATAAAAACATGGAACG
GGTAAGATATCTTGGAAGAAGAAGATACTAAATTCGTTA------ACAATTCGGAATGAG
CTTGGACGATATCTCATCTGCGAGTTCTACTCGACGTTCATTGTATCGGCCTTCGTACAT
GCGGCTGTCGCTCAGGATGTGGTATCTAGG------------ACAGGATCGGGATTCAAC
GTCGCCTTGGCCGCCGGTATGGGGGTCATGTTCGGCATCTACTCCGGGTTCGGTGTCTCA
GGGGGCCACGTCAACCTGGCTCTATCGATAGGAATAGCTGCTATAGGAATATTCCCTTGG
AGAAGGATTCCTCTCTATTTCTTAGCCGAACTAGCGGGCGGGTTCGCCGGAGCCTGGGTC
GTCTAT---CGAGTTTACGAAGACGCCTTTGATCATTTTGACGGT---GGAATGCGTCAA
GCGTTTGGCGAGAAC---GGCACGGCGGGGATCTTTGCCACGTTCCCACAACCTTACCTG
TCTGTTACGACCGGGTTCCTCGAACAGGTATTGAACACGGCGCTATTATTAGCCGTTATA
GGTGCGATGATGGACCGACGAAACAACCCTCCTCCTCTCAGCTTCGCACCTTTCTTCTTT
GGTTTGATTGTATTCACCATCCTCATGAGCTATGGATATAATGCAGGAGCACCCCTGAAT
CCTTCGATAGACCTA---TCAGGACGTCTTCTTCTGGCTGCAATGGGCTACGGCAAGGAG
GTCTGG---ACA---CCCGGTGGTGTTCATTGGTGGTGGATACCCATCTTAGGACCCGCC
ATCGGGAGTGCTCTCGGATCCTGGGCCTACTACCTCGCTATCTCCATACACCATCCTCCT
---------------CTTGATGTGAAAGAG---------------------AAT------
------------GCAACA---GACTACAAC------------------------------
------------------------GAAAAC------------ATAGAGCTTGGCAATAAA
GATGGAATGACTAACAACCACACTGCGGAGCAAAATGGTTTAACGAACTCGACGACGATC
ACATTGAGAAGAAGGTCATCGGACGTCGACCAGGAG------AATGATATCATGGATGAC
ATGTCGAGACCGCTATCTGTTGACGCCCTCTGCCATAATTCATTACAAAACGATGCCACG
AATGATATATATTTG------
>pUrchin_Glp1a2
---ATGGGGATGGATAAA
ATGAAA---ACTTGGATGAAGAAAATACTGAGTGTGATA------ACAATTCGGAGTGAC
CTCGGACGATGTCTCATGGCCGAGTTCTACTCGACGTTCATGCTATCGGTCTTCTTGCGT
GCGGCCATCGCTCAGGATCTCACTTCAGGA------------ACGGGATCAGTATTCAAC
ATCGCTTTCACCGCCGGTATGGGGGTCGCTTTCGCCATCTACTCCAGTTTCGGCGTCTCA
GGGGGTCACATCAACCTCGCCGTATCGATAGGAGTAGCTGTTTTAGGAAAATTACCATGG
AAAAGGGTTCCTGTCTATTACTTAGCCCAACTAGCGGGCGGGTTTGTCGGAGCTGTGCTT
GTCTAT---GCTGTTTACCAAGATGCCTTCGATGATTTCGACGGT---GGAGAGCGTCAG
GCGTTTGGCCCGAAT---GGGACGGCAGCGATATTTGCTACATTCCCCCAACTGTATCTG
TCTGTGACGACCGGGTTTTTCGAACAGGTATTGAGCACAGCGCTATTTTTAGCCATCATA
GGCGCAGTGTTGGACCATCGCAACGTCCCGCCCCCTCTCAACTGGGCCCCTTTCTTCTTT
GGTTTGATTATTTTAGCCCTCGTCATGACCTTTGGTCATAATGCAGGGGCACCCCTGAAT
CCTTCGATAGACCTT---TCGGGACGTCTTTGTCTGGGTGTCATGGGGTACGGCGCGGAG
GTCTGG---GTGCTACACGATGGTGTTCATTGGTGGTTGATACCCATCTTTGGACCCGCC
ATTGGGGGCGCTGTGGGATCCTGGGCCTACTACCTCGCAATCGAACTCCACCATCCTCCT
---------------GTTGATGAGAAAGAA---------------------GAT------
------------GCAACA---GACTACAAA------------------------------
------------------------GAAAAG------------ATAGAGCTTGAAAAGAAA
GATGGAATGCCCAAAACCCACGCTGCAGAGATTCAATATCCCCTGTTACCG---------

---GAGAGAGACTCC---

---CAGACATCCGTG------
>cUrchin_Glp1a2
ATG------GAGAATACA----------------------------------TGTGCTGAG
AAACCA---TCATGGAAATCAAAAATGGTGGACAAAATG------AGAATAAGAAATGAT
CTGGTCAGATGTTGTGTTGCTGAATGTTATGGAACATTTCTCATGGCGTCTTTCACACGT
GGAGCTCTCGCCCAGGATACCGTATCTCGA------------ACAGGATCAACGTTTAAC
ATCTCAATCACAATTGGTTTAGGATTAATGTTTTCAATCTATTCAGCATTCGGAGTATCG
GGGGGTCATGTAAACCCGTCTATCTCAATCGGCTTAGCAGCGGGAGGATTGTTTCCATGG
AAACGAGTACCAATATACATCATCTCTCAAGTCACTGGAGGATTTTTAGGAGCTGCCTGT
GTGTAT---GGAATTTATCAAGAAACTTTTGATGAGTTTGATGGA---GGTACGAGACAG
GCGTTCGGTGAGAAT---GGAACGGCGGGAATTTTTGCTACATTCCCTCAACCATATATC
TCATTCACTACAGGCTTCCTTGAACAGATTTTCAACACAGGTCTCATGCTCGCCCTCACC
CTAGCTGTATCAGATCAGAGGAACAGTGCCCCACCTCCATACGTTCAACCAGTCCTATTT
GGTATCAGTGTTTTCATCATCTTAATGAGTTATTCATTCAATGCCGGATCTCCTCTTAAC
CCATCTCTTGATCTT---TCTGGAAGAACACTCTTAGCTATTGCTGGATATGGAAAAGAG
GCTTGG---AAA---CCTGGTGATCGTCACTGGTGGTGGATACCTCACTTAGGACCAGCC
ATAGGGAGCGCACTAGGGTCTTGGTCTTACGTACTCGCTATAAGACTACATCATGTTCCC
---------------GATCATCATCATCCT---------------------GCC------
------------TCCACA---GAGACAAGA------------------------CAA---
--AGTGGTATAGACAACCCA
TCAACAATTGATCCATCATTGGAAGGTCCTGTGCCC------------------------

---------------------------------------ACAGTG---TGTGAT------
---GATACAAGCTTA------
>cotStar_Glp1a2
---ATGGGT---
TTGCCT---CTGGTTCCGAAGAGGGCGCGGAACTGCATC------ACGGTTAAGAATGAG
ATCGTACGGGGAATGCTTGCAGAAATGGCTGGGATATTCGTACTCGTACTCTTTGTTGAC
GGGGGTCTCGCCGCGGGTATCGTTAGTGGA------TGGATTTCAGGGACACCGGTGTCT
ACTGCCGTCACGTCCGGAATGGGCGTTGCATTCGGGATATACACCGGCGTGGCTGTATCA
GGTGGTCACGTGAATCCCTCGATTACAGTCGGCTTAGCGTCCGTCGGCAAGTTTCCATGG
CGACGGGTACCACTCTGGATCCTTGCTCAACTCGTTGGCGCCTTGTGCGGTGCTTCGGTT
GTCTTT---GCAATATACTATCAGGGCATCAATGCGCTGGATGGG---GGTAACAGGACT
GTCTTCGGTGACACG---GGCACCGGTGGAATCTTCTGTACTTACCCGCAGGAGTACTTC
GGGATGGGTCCTGGATTTGTGGAGCAGATCATTAACACGGGCCTCCTCCTGCACTGCACC
TTAATGTTTTTCGACGAGAAGAACGGCAAACCGACCAAAGGGATGGAGCCGTTCTTCGTG
GGTCTGTCAGTGGCTATGATTATCCTAGCGTGGGGCCACAACGCAGGCTCACCCATGAAC
CCAGCCAGGGACTTC---GCTGGCAGGTTTCTTACATGGATAGCTGGCTACGGATCTGAA
GTCTGG---GTA---CCTCGGGGAGTCCACTGGTGGTGGATTCCCACGTTCGTCCCGCCC
CTTGGGGGAATCGTCGGGGCCAATTTCTACTACCTGTTCGTCGAGCTGCACCACCCGAGC
---------------GATAGCGAGGTGGTA---------------------GCT------
------------GATCAC---GGGAACTCG------------------------GAGCTC
AGACAGATCCAGAAT---------GAGGAC------------GTGAAGCTGGTCGGTAAG
TCATCATATATCAAAGTTGCGACTGAACCCATTTCTGAG---------------------

>bStar_Glp1a2
---ATGGGT---
TTGCCA---CTGGTGCCGAAGAAGGTGCGCAGCTGCATC------ACGGTGAAGCATGAG
ATAGCCAGGGGAATGCTCGCCGAAATGGCTGGAATATTTGTCCTCGTACTCTTCGTGGAT
GGCGGTCTTGCCGGGGGTATCGTCAGCGGA------TGGATCTCGGGCACTCCAGTCTCG
ACCGCCTTCTCCTCCGGAATGGGGGTGGCATTCGGCATATACACCGGTCTTGCAGTGTCG
GGTGGTCACGTGAACCCGGCGATTACCGTGGGCTTAGCGTCCGTTGGTAAGTTTCCATGG
CGACGGGTGCCACTCTGGATCCTGGCTCAACTTATCGGAGCCTTCTGCGGCGCTTCGGTC
GTCTTC---GGTATTTACTACCAGAGCATCGATGCGCTCGATGGA---GGTAACCGAACG
GTCTTTGGACCCACA---GGGACCGGCGGGATCTTCTGTACCTACCCACAGGAGTATTTC
GGCATGGGTCCTGGATTTGTGGAACAGATCATAAACACAGGACTACTCTTGCACTGCACC
CTGATGTTTTTCGACGAGAGAAACGGCAAACCGACCAAAGGGATGGAGCCGTTCTTTGTG
GGTCTGTCTGTGGCCGTGATCATCTTGGCCTGGGGACACAACGCCGGG------------

>cotStar_Glp1b
--ATG------
---------CCAGTCTGCGATTCGATACGTAGGCATTTA------GCCATTAAAAGTAAC
TTGGCACGTGGAATTCTCGCTGAGATGGCCGGAACCTTCATACTTGTTTTATTCGTGGAT
GGCGGATTAGCCCAAGCCGCAGTGAGCGGC------TACACATCCGGCACACGTGTCTCC
ACCGCGCTGACCTCAGGCATGGGCGTGGCATTTGGGATCTACACTGGTGTAGGGGTGTCA
GGAGGCCACGTGAATCCATCAATTACTGTGGGCCTGGCATCGGTTGGCAAGTTTGCGTGG
CGACGGGCCCCACTCTGGATAGCAGCTCAATTCATCGGCGCATTCGTCGCAGCATATGTG
GTGTAT---GGGATATACTACCCTGGGCTGGATCATTTTGACAAC---GGGAACCGGACA
GTTTTTGGACCTACT---GGGACAGGAGGGATCTTCTGCACCTATCCGCAGCCATACTTT
GGGCTCTGGGTAGGTCTCGCTGAACAGATTATTAACACCGGTCTCTTGCTGCACTGCACC
CTGATGTTTTTCGACGAGCGAAATGGTAAACCGACTAAAGGGATGGAGCCGTTCTTTGTG
GGTCTGTCCGTGGCAGTTATCATTCTGGCATGGGGACACAACGCTGGAGCGCCCATGAAT
CCAGCCAGGGACTTG---GCCGGTCGGTTGCTGTCCTGGCAGGTCGGATACGGAGCTGAT
GTGTGG---CAG---CCTCATGGAATGCACTGGTGGTGGGTGCCAACCTTTGTACCGCTA
CTAGGGGGGCTGTGCGGGGCCAACTTTTACTTCTTATTCGTGGAAATGCACCACCCAAAG
---------------AATGTCGGCGAAAGT------------------------------
------------TCT------GCCAACGAG------------------------GGG---
--ACACCTTTAGAAGAGACA
GGTGCT--

>bStar_Glp1b
--ATG------
---------CCGGTCTGCACCTCGATACGCCGGCATCTG------GCCGTCAGGAGCGAC
ATAGCACGAGGCATGCTGGCTGAAATGGCAGGAACCTTCATCCTGGTTTTATTCGTGGAT
GGCGGACTAGCCCAAGGTATCGTCAGTGGC------TGGACATCCGGGACACCCGTTTCC
ACCGCACTGACCTCAGGCATGGGCGTGGCCTTCGGTATCTACACAGGTGTAGGGGTATCA
GGAGGGCACGTGAATCCATCCATTACTCTGGGCTTGGCTTCTGTCGGCAAGTTTGCCTGG
CGAAGGGCTCCGCTGTGGATACTCGCCCAGTTTCTGGGCGCATTTGCTGCAGCGTCTGTT
GTATTT---GGAATATACTACCCTGGGTTGGATAATTTCGACGGA---GGCAACCGGACG
GTTTTCGGGCCGACC---GGGACGGGAGGGATTTTTTGCACTTATCCGCAGCCGTACTTT
GGGCTCTGGGCCGGCCTGGCTGAACAGATACTCAACACGGGTCTCCTGTTGCACTGCACG
ATGATGTTTTTCGACGAGAGAAACGGCAAACCGACCAAAGGGATGGAGCCGTTCTTTGTG
GGTCTGTCTGTGGCCGTGATCATCCTGGCCTGGGGACACAACGCCGGGGCGCCCATGAAC
CCAGCCAGGGACTTG---GCTGGTAGGTTCCTGTCATGGCTGGTCGGCTACGGAAGCGAA
GTGTGG---ATG---CCTTACAAAGCGCACTGGTGGTTGGTTTCTACCTTCGTGCCGCTA
GCCGGCGGAATCTGCGGTGCCTACTTTTACGTTTTGTTCGTTAAGAGGCATCACCCGGGC
---------------AATCTCGCCCAGGAC---------------------TAC------
------------CAGAAA---GTCCAACGT------------------------AGC---
--CCACCTGCTGACGAGCTC
GGAACGTCACACACAGTTTGTCAAACGGAGCTGGATGATATATTG---------------

File S2: Alignment for Fig. S2A-D
>Human_AQP3
ATG-------------------------------GGTCGACAG------------------
----------------------AAGGAGCTGGTGTCCCGCTGCGGGGAGATGCTC------
CACATCCGCTACCGGCTGCTCCGACAGGCGCTGGCCGAGTGCCTGGGGACCCTCATCCTG

GTGATGTTTGGCTGTGGCTCCGTGGCCCAGGTTGTGCTCAGCCGGGGCACCCACGGTGGT
TTCCTCACCATCAACCTGGCCTTTGGCTTTGCTGTCACTCTGGGCATCCTCATCGCTGGC
CAGGTCTCTGGGGCCCACCTGAACCCTGCCGTGACCTTTGCCATGTGCTTCCTGGCTCGT
GAGCCCTGGATCAAGCTGCCCATCTACACCCTGGCACAGACGCTGGGAGCCTTCTTGGGT
GCTGGAATAGTTTTT---GGGCTGTATTATGATGCAATCTGGCACTTCGCC---GACAAC
CAGCTTTTTGTTTCGGGCCCCAAT---GGCACAGCCGGCATCTTTGCTACCTACCCCTCT
GGACACTTGGATATGATCAATGGCTTCTTTGACCAGTTCATAGGCACAGCCTCCCTTATC
GTGTGTGTGCTGGCCATTGTTGACCCCTACAACAACCCCGTCCCCCGAGGCCTGGAGGCC
TTCACCGTGGGCCTGGTGGTCCTGGTCATTGGCACCTCCATGGGCTTCAACTCCGGCTAT
GCCGTCAACCCTGCCCGGGACTTTGGCCCCCGCCTTTTTACAGCCCTTGCGGGCTGGGGC
TCTGCAGTCTTCACGACCGGCCAGCATTGGTGGTGGGTGCCCATCGTGTCCCCACTCCTG
GGCTCCATTGCGGGTGTCTTCGTGTACCAGCTGATGATCGGCTGCCACCTGGAGCAGCCC
CCACCCTCCAAC--
------------GAGGAA---------GAGAATGTGAAGCTGGCC---CATGTGAAGCAC
AAGGAGCAGATC--

>Gorilla_AQP3
ATG-------------------------------GGTCGACAG-----------------
----------------------AAGGAGCTGGTGTCCCGCTGCGGGGAGATGCTC------
CACATCCGCTACCGGCTGCTCCGACAGGCGCTGGCCGAATGCCTGGGGACCCTCATCCTC
GTGATGTTTGGCTGTGGCTCCGTGGCCCAGGTTGTGCTCAGCCGGGGCACCCACGGTGGT
TTCCTCACCATCAACCTGGCCTTTGGCTTTGCTGTCACTCTGGGCATCCTCATCGCTGGC
CAGGTCTCTGGGGCCCACCTGAACCCTGCCGTGACCTTTGCCATGTGCTTCCTGGCTCGT
GAGCCCTGGATCAAGCTGCCCATCTACACCCTGGCACAGACACTGGGAGCCTTCTTGGGT
GCTGGAATAGTTTTT---GGGCTGTATTATGATGCAATCTGGCGCTTTGCC---GACAAC
CAGCTTTTTGTTTCGGGCCCCAAT---GGCACAGCTGGCATCTTTGCTACCTACCCCTCT
GGACACTTGGATATGATCAATGGCTTCTTTGACCAGTTCATAGGCACAGCCTCCCTTATC
GTGTGTGTGCTGGCCATTGTTGACCCCTACAACAACCCCGTCCCCCGAGGCCTGGAGGCC
TTCACCGTGGGCCTGGTGGTCCTGGTCATTGGCACCTCCATGGGCTTCAACTCCGGCTAT
GCCGTCAACCCTGCCCGGGACTTTGGCCCCCGCCTGTTTACAGCCCTTGCGGGCTGGGGC
TCTGCAGTCTTCACGACCGGCCAGCATTGGTGGTGGGTGCCCATCGTGTCCCCACTCCTG
GGCTCCATTGCGGGTGTCTTCGTGTACCAGCTGATGATCGGCTGCCACCTGGAGCAGCCC
CCACCCTCCACC--
------------GAGGAA---------GAGAATGTGAAGCTGGCC---CATGTGAAGCAC
AAGGAGCAGATC--

>Rabbit_AQP3
ATG-------------------------------GGTCGACAG-----------------
----------------------AAGGAGCTGGTGTCCCGCTGCGGGGAGATGCTC------
CACATCCGCTACCGGCTGCTACGCCAGGCGCTGGCCGAGTGCCTGGGGACCCTCATCCTC
GTGATGTTCGGCTGTGGCTCCGTCGCCCAGGTCGTGCTCAGCCGGGGCACCCACGGAGGT
TTCCTCACCATCAACCTGGCCTTTGGCTTTGCAGTCACCCTGGGCATCCTGGTCGCTGGC
CAGGTCTCTGGGGCCCACCTGAACCCTGCCGTGACCTTTGCCATGTGCTTCCTGGCACGT
GAGCCCTGGATCAAGCTGCCCATCTACACCTTGGCACAGACGCTCGGAGCCTTCTTGGGT
GCTGGGATCGTTTTC---GGGCTATATTATGATGCAATCTTGAGCTTTGCC---AACAAT
GAGTTTATAGTCTCGGGCCCCAAT---GGCACAGCAGGCATCTTTGCCACCTACCCCTCT
GGACACCTGGACATGGTCAATGGCTTCTTCGACCAGTTCATAGGCACAGCTTCCCTCATC
GTGTGCGTGTTGGCCATTGTGGACCCTTACAACAACCCTGTCCCCCGCGGCCTGGAGGCC
TTCACCGTGGGCCTGGTGGTCCTGGTCATTGGCACCTCTATGGGCTTCAACTCTGGCTAC
GCTGTCAACCCTGCCCGGGATTTCGGCCCCCGTCTTTTCACTGCCATCGCGGGCTGGGGC
TCTGAGGTCTTCACGACTGGCCGGCACTGGTGGTGGGTGCCCATCGTCTCTCCCCTGCTG
GGCTCCATTGCGGGCGTCTTCGTGTACCAGCTCATGATCGGCTGCCACCTGGAGCAGCCT
CCTCCCTCCACC--
------------GAGTCG---------GAGAACGTGAAGCTGGCC---CACGTGAAGCAC
AAGGAGCAGATC--

>Tasmanian_devil_AQP3
ATG-------------------------------GGCCGTCAG-----------------

---------------------AAGGAGCTAGTGTCGCGCTGCGGGGACTTGCTG------
CGGGTCCGGTACCGGCTGCTGCGCCAGGCTCTCGCTGAATGCCTCGGGACGCTCATCCTG
GTGATGTTTGGCTGTGGCTCTGTGGCCCAGGTTGTGTTGAGCAGAGGAACCCATGGTGGC
TTTCTTACCATCAACCTGGCTTTTGGCTTTGCAGTCACGCTTGGTATCCTTATTGCTGGC
CAAGTGTCTGGGGCCCATCTAAATCCAGCTGTGACCTTTGCTCTGTGCTTCCTGGCAAGG
GAGCCCTGGATCAAGCTGCCCATCTACACATTGGCCCAGACTGTGGGAGCCTTCCTAGGA
GCTGGAATTGTCTTT---GGGCTGTACTATGATGCAATTTGGTCCTTTGCT---GGTAAT
GAACTTTTTGTCTCTGGCCCCAAT---GGTACTGCCGGCATCTTTGCTACCTATCCCTCT
GGCCACTTGGACATGGTCAATGGCTTCTTTGACCAGTTCATTGGTACAGCATCCCTCATC
GTCTGTGTGTTGGCTATTGTGGATCCTCACAACAACCCCGTTCCCCGAGGTCTGGAAGCC
TTCACTGTTGGCCTTGTGGTTCTGGTCATTGGTACATCCATGGGCTTCAACTCTGGATAT
GCTGTTAACCCAGCTCGAGATTTTGGTCCCCGCCTCTTCACTGCCATTGCTGGCTGGGGT
TCTGAGGTCTTCACAACGGGCCGGCACTGGTGGTGGGTGCCCATCGTTTCACCACTTCTT
GGTTCCATTTGCGGAGTTTGTGTCTATCAGCTCATGATTGGATTTCACCTGGAGCCTCCA
CCTCCTTCCACA--
------------GAGCAG---------GAGAATGTCAAGCTGGCT---CACGTGAAACAC
AAAGAACAGATC--

>Platypus_AQP3
ATG--------------------------------GGCCGGCAG------------------
---------------------AAGGAGCTGGTGTCGCGCTGCGGGGAGATGCTG------
AGGATCCGCTACAAGCTGTTGCGGCAGGCCCTGGCCGAGTGTCTGGGTACCCTCATCCTC
GTGATGTTCGGCTGCGGCTCCGTGGCCCAGGTGATACTTAGTCGTGGCACCCACGGCGAC
TTCCTCACCATCAACCTGGCCTTTGGCTTTGCTGTCACCCTGGGAATCCTCATTGCCGGT
CAGGTCTCAGGTGCCCACTTGAACCCGGCCGTGACATTTGCACTGTGTTTTCTGGCCCGG
GAACCCTGGATCAAGCTGCCCATCTATGCCTTGGCACAGACCCTAGGTGCGTTCCTGGGT
GCTGGCATCATCTTC---GGCCTCTACTATGATGCCATCTGGGCCTTTGCG---GACAAC
CAGCTGATTGTGTCCGGCCCCAAC---GGCACTGCTGGGATCTTTGCAACCTACCCGTCC
GGTCACTTGGACACCCTCAACGGCTTCTTTGACCAGTTCATCGGCACGGCCTCCCTCATC
GTCTGTGTCCTGGCCATCGTGGATCCTTACAACAACCCCGTCCCCAGGGGCCTGGAGGCC
TTCACCGTTGGTTTTGTCGTCTTGGTCATCGGCACCTCCATGGGCTTTAACTCTGGCTAT
GCCGTCAATCCCGCCCGAGACTTCGGCCCCCGCCTCTTCACCGCCATCGCTGGATGGGGC
TCTGAGGTCTTCACGACGGGCAAGCACTGGTGGTGGGTGCCCATCGTCTCACCCCTTCTG
GGCTCCATCGCCGGAGTCTTCGTCTACCAGCTGATGATTGGCTGCCACATGGAGGCCCCG
CCCCCATCCACC--
------------GAGCAG---------GAGAACGTCAAGTTGGCC---AACGTCAAGCAC
AAGGAGCGGATC--

>Zebra_finch_Aqp3
ATG--------------------------------GGGCGGCAA------------------
---------------------AAGGATGTTCTTGCTACCATTGAGGAACACCTG------
AGGATCAGAAACAAATTAGTGCGGCAAGCACTGGCTGAGTGCTTGGGAACACTGATCCTG
GTGCTCTTTGGCTGTGGCTCTGTTGCCCAGATCATTCTCAGCAGAGGGACTCATGGGGGT
TTCCTGACTGTCAACCTGGCCTTCGGCTTCGCTGTAACGCTCGGCATTTTGATCGCAGGA
CAAGTATCAGGTGGACATCTGAACCCAGCTGTCACTTTTGCCATGTGCTTATTGGCCCGG
GAGCCCTGGATCAAGCTACCAATTTATGCACTTGCACAAACCCTGGGGGCTTTCCTTGGA
GCTGGTATAGTCTTT---GGGCTGTACTATGATGCTATATGGGCTTTTGAC---AATAAC
CAGCTCACTGTTATAGGAAAGAAT---GCCACTGCTGGTATTTTTGCCACCTACCCATCT
GAGCATCTGAATGTTGTGAATGGCTTCTTTGACCAGTTCATTGGCACTGCCTCCCTGATT
GTTTGTGTCTTGGCTATTGTTGATCCCTACAACAACCCCGTCCCCACTGGGCTGGAGGCT
TTCACAGTTGGTTTTGTAGTCCTCGTTATTGGAACCTCCATGGGCTTCAACTCTGGCTAT
GCTGTCAACCCTGCCAGGGACTTTGGGCCTCGTCTCTTCACAGCCATTGCTGGCTGGGGC
ATGGAAGTGTTCCGGGTTGGTAAGCACTGGTGGTGGGTTCCAGTTGTCGCTCCTTTCCTT
GGGGCAGTGGCGGGAGTGATTGTCTATCAGCTGACGATTGGATGTCATGATGAGCCTTCT
CCGCCTGCCTCT--
------------GAGCAG---------GAAACAGTCAAGCTGGCT---AATGTGAAGCAC
AAGGAGAGGGTC--


```
>Alligator_AQP3
ATG-------------------------------GGCAGGCAA------------------
----------------------AAGGAGCTTCTTTCTAGGATCGACGGGCTGCTG------
AGGATCCGGAGCAAGCTACTGAGGCAAGCGCTGGCCGAATGCCTGGGGACTCTGGTCCTA
GTGATGTTTGGCTGCGGCTCCGTCGCTCAGATCGTGCTCAGCAAAGGGACCCACGGAGGC
TTCCTGACGGTTAACCTGGCCTTCGGCTTTGCCGTGACTCTTGGCATCCTCATCGCGGGA
CAGGTGTCAGGCGGCCACTTGAACCCAGCGGTAACTTTTGCCATGTGCTTCTTGGCTCGT
GAGCCCTGGATTAAGCTGCCGGTTTATGCGCTTGCACAAACCCTGGGGTCTTTCCTAGGA
GCAGGCATTGTCTTC---GGTCTGTACTATGATGCCATCTGGGCTTTTGGC---CAAAAC
AAGCTGATCGTATCAGGAGAGAAT---GCCACCGCCGGCATTTTTGCCACGTATCCCTCC
GAGCATCTGGATGTAGTGAACGGATTTTTTGACCAGTTCATTGGCACTGCAGCCCTGATA
GTTTGTGTCTTGGCTATCGTGGATCCTTACAACAACCCTGTCCCACGTGGCCTGGAGGCT
TTCACCGTCGGCTTTGTGGTCCTCGTTATTGGGACCTCCATGGGCTTCAACTCCGGCTAT
GCCGTCAACCCAGCTAGGGACTTTGGGCCTCGCCTGTTCACTTCCATTGCTGGCTGGGGC
TCCGAAGTATTCAGTACCGGGAAGCATTGGTGGTGGGTGCCAATTATCGCCCCTTTCCTT
GGGGCCGTCGGAGGAGTGATGGTTTACCAGCTGATGATCGGGTGCCACGACGAGTGTCCT
CCACCAGCCACG------------------------------------------------
------------GAGCAG---------GAAACCGTCAAACTGTCC---AATGTGAAACAC
AAGGAAAGGGTC------------------------------------------------
------------------------------------------------------------
------------------------------------------------------------
>Turtle_AQP3
ATG-------------------------------GGGAGGCAA------------------
----------------------AAGGAAGTTCTCGCTAGAATTGGAGAGATGCTG------
AGGATCAGAAACAAACTAGTGAGGCAAGCACTGGCCGAGTGCCTGGGGACCCTGATTCTG
GTGTTGTTTGGCTGTGGCTCGGTCGCTCAGATCGTGCTCAGTAGAGGAACCCACGGAGGC
TTCTTGACTGTGAACCTGGCCTTCGGCTTTGCTGTTACACTTGGAATTCTGATAGCAGGG
CAAGTGTCAGGTGGCCACTTGAATCCTGCTGTAACTTTTGCTCTGTGCCTCATGGCCCGG
GAGCCGTGGATTAAGCTGCCTATTTACATCCTGGCACAAACCATCGGTGCTTTCCTAGGA
GCGGGCATCGTCTTT---GGCTTGTACTTTGATGCCATATGGGCTTTTGGC---AACAAC
CAGTTGTTTGTATTGGGACCGAAT---GGCACCGCTGGAATTTTTGCCACATACCCGTCT
GATCATCTGAATGTATTGAATGGATTCTTTGACCAGTTCATTGGCACGGCATCCTTGATT
GTTTGCGTCTTGGCTATTGTGGATCCCTACAACAACCCTATCCCCCGGGGCCTAGAGGCG
TTCACTGTTGGCTTTGTGGTCCTGGTTATCGGGACGTCCATGGGCTTCAACTCCGGATAC
GCCGTCAACCCAGCCAGGGACTTCGGGCCTCGTCTTTTCACCTCCATTGCTGGCTGGGGC
ACAGAAGTATTTTCGACTGGCATGCAGTGGTGGTGGGTCCCGATCGTGGCCCCTTTCCTC
GGGGCAGTTGCTGGAGTGCTGGTTTACCAGCTGATGATTGGATGCCATGACGAGCCGTCT
CCACCATCCACT------------------------------------------------
------------GAGCAG---------GAAAATGTCAGGTTGGCC---AACGTGAAGCAT
AAGGAAAGGGTC------------------------------------------------
------------------------------------------------------------
------------------------------------------------------------
>Python_AQP3
ATG-------------------------------GGGAGGCAG------------------
----------------------AGAGAAGTGCTGACGGTCCTTGGACAGAGGCTG------
AGGATCAGGAATAAGCTCCTCCGGCAGGCTCTGGCTGAATGCCTGGGCACGCTGATCTTG
GTGATGTTCGGTTGTGGGTCCGTGGCCCAGATCGTGCTCAGCAGAGGGAGCCATGGACGT
TTCCTGACGGTCAACCTGGCGTTTGGTTTTGCCGTGACGCTTGGAATTCTGATTGCTGGA
CAAATCTCAGGTGGTCATTTGAACCCTGCCGTCACCTTTGCTATGTGCTTTATGGCCCGG
GAACCCTGGATCAAGCTTCCGATTTATGCCCTAGCCCAGACCCTTGGGGCTTTTCTAGGG
GCAGGCATCGTGTAC---GGCTTA---TACGATGCCATCTGGGCTTTTGGT---GACAAC
CAACTCTTCGTATTGGGCCCCAAT---GGGACTGCTGGCATCTTTGCTACCTACCCATCT
GAGCATCTGAATTCTGTGAATGGATTTTTTGACCAGTTCATTGGCACTGCAGCATTGCTG
GTTTGTGTTCTGGCCATTGTGGATCCTTACAACAACCCTGTCCCAAGAGGGTTGGAAGCA
TTTACTGTTGGCTTCGTCATCTTGGTTATTGGAACATCCATGGGGTTCAATTCTGGTTAC
GCTGTCAACCCAGCCCGAGACTTAGGGCCCCGTCTCTTCACAGCCATTGCTGGCTGGGGT
ACCGAAGTTTTC---ACTGGTAGGCACTGGTGGTGGATTCCCATCGTTGCTCCTTTCCTT
GGTGCCATCGCAGGTGTTATGGTCTACCAACTGATGATAGGGTGCCATGATGAACCACCT
CCCGAGACCAGT------------------------------------------------
------------GACGAG---------GAAAACGTCAGGCTGTCC---AAGGTGAAGCAG
AGGGACAATGTC------------------------------------------------
```


>Gecko_AQP3
ATG------------------------------GGCAGGCAA------------------
---------------------AAGGAGGTGCTGTCAGTCATGGGACAGAGGCTG------
AGGATCAGGAATAAACTCATCCGGCAAGCTCTGGCCGAATGCCTGGGGACCTTGGTGCTG
GTGTTGTTCGGCTGTGGTTCAGTGGCTCAAATCGTCCTCAGCAGAGGAAGCCACGGAGCT
TTCCTGACAGTCAATCTGGCGTTCGGTTTTGCGGTGACGCTGGGAATTCTCATCGCTGGA
CAGATCTCAGGTGGCCACTTGAATCCAGCCGTCACCTTCGCCATGTGCTTTATGGCTCGG
GAACCTTGGATAAAGTTGCCTGTATATGCTTTAGCACAGACCCTCGGCGCGTTCTTGGGA
GCTGGCATCGTCTTC---GGCTTGTACTATGATGCCATTTGGGCCTTCGGT---GGGAAC
CAGCTGTTTGTCGTCGGCGAGAAT---GCCACGGCCGGGATCTTCGCCACGTACCCCTCA
GAGCACTTGAACTCCGTGAATGGATTTTTTGACCAGTTTATTGGCACAGCAGCCCTGCTT
GTCTGCGTCTTGGCGATCGTCGACCCCCACAACAACCCGGTCCCCCGGGGTCTGGAGGCC
TTCACCGTCGGCTTTGTCATCTTGGTGATTGGAACGTCCATGGGCTTCAACTCCGGCTAC
GCCGTCAACCCGGCCAGAGATTTCGGGCCCCGCCTCTTCACGGCCATCGCTGGGTGGGGC
TCGGAAGTTTTCTCGACGGGCCGGCAGTGGTGGTGGGTCCCAGTCATTGCCCCGTTCCTC
GGGGCCATTGCCGGCGTGGGGGTCTACCAGCTGATGGTCGGCTGCCACGACGAGCCGCCC
CCCGAGGCGAAT--
------------GATGAG---------GAGACCGTCAAGCTGTCC---AATGTGAAGCAG
AGGGACAACGTT--

>Himalaya_frog_AQP3
ATG------------------------------GGCCGACAG------------------
---------------------AAGGAATTTATCAACCGCATCAACGCCGCGCTG------
AGGATCCGCAACAAGCTGCTGAGGCAAGCGCTCTCCGAGTGCCTGGGGACCCTCATTCTG
GTGATGTTCGGCTGTGGCTCTGTGGCTCAGGTGGTCCTTAGTAAAGGATCCCATGGTCAG
TTCTTGACTGTCAATCTGGCTTTCGGTTTTGCTTGCATGTTGGGTATTCTCATATCCGGA
CAAGTTTCAGGTGGACATCTCAACCCCGCTGTGACGTTTGCACTATGTATCATAGCACGA
GAACCATGGATCAAGTTTCCTGTATACACCTTGGCACAGACCCTGGGTGCCTTCCTCGGA
GCGGGCATCGTCTAT---GGCTTGTATTATGATGCCATCTGGTTTTTCGCC---AACGAC
CAGCTCTACGTAATGGGCCCCAAC---GGCACTGCTGGGATTTTCGCCACATTCCCAACA
GATCATCTGACCCTCATGAATGGTTTTTTTGACCAGTTTATTGGCACAGCTGCTTTGGTG
GTCTGTGTCTTGGCCATCGTGGACCCTTACAATAACCCCATCCCCCGTGGTTTGGAGGCC
TTCACTGTTGGCTTCGTCGTCCTTGTCATTGGATTGTCCATGGGCTTCAACTCCGGATAT
GCCGTCAACCCCGCCAGGGACTTCGGACCACGTCTGTTCACAGCTCTGGCTGGATGGGGT
TCAGAAGTTTTCTGGGCTGGAGGTCAGTGGTGGTGGGTCCCCATCGTGTCGCCTTTACTG
GGTGCCTTTGCCGGAGTCCTGGTCTACCAGCTGATGCTTGGCTGCCACCTCCAACCTCCA
CCTGAATCCACC--
------------GAACAA---------GAAAATGTCAAGCTGGCC---AACGTCAAACAA
AAAGAGAGAATC--

>Axolotl_AQP3
ATG------------------------------GGTCGGCAG------------------
---------------------AAGGAACTGGTCAACAAAATCGGGGACATGTTG------
AGGATCCAGAACAAGCTGCTGAGGCAGGCGCTGGCCGAGTGCCTGGGGACCCTCATCCTC
GTGATGTTTGGCTGCGGGTCGGTGGCCCAGGTCGTTCTCAGCAAGGGCTCCCATGGACAG
TTCTTGACAGTGAACATGGCCTTTGGTTTCGCTGTGATGCTTGGCATCATTATCTGCGGG
CAGGTGTCCGGCGGCCACCTGAATCCGGCAGTCACGTTTGCTTTATGCTTCCTGGCCCGG
GAGCCTTGGATCAAGTTCCCCATTTACACATTGGCACAGACACTGGGAGCGTTCCTGGGC
GCTGGCATTATCTAC---GGATTGTACTATGATGCTATTTGGGCTTTTGCT---CAGGAT
CAACTCTACGTCATGGGGCCCAAT---GGGACAGCCGGGATTTTTGCAACCTACCCGACC
GAGCATCTGACGCTTATGAATGGATTCTTCGACCAGTTCATTGGCACCGCAGCCTTGGTG
GTCTGTGTCCTCGCCATTGTGGACCCAAACAACAACCCAATCCCCAAGGGCCTTGAGGCC
TTCACTGTTGGATTTGTGGTTCTCGTCATTGGACTTTCTATGGGTTTCAACTCTGGATAT
GCCGTCAACCCGGCCAGGGACTTTGGACCACGTCTCTTCACGGCAATTGCTGGCTGGGGC
TCTGAAGTCTTCTCGGCTGGAGAACAGTGGTGGTGGGTGCCAATCGTTTCCCCCTTTCTT
GGATCTGTCGGCGGAGTTTTAGTCTACCAGCTGATGATCGGGTTCCATATCTTGCCTATC
CCAGAGTCCACG--

------------GAGCAG---------GAGAACGTCAAGTTGGCC---AATGTCAAGCAC
AAGGAGCGGATC--

>2Lcaecilian_Aqp3
ATG-------------------------------GGTCGGCAG-----------------
---------------------AAGGAGTTGGTTAACCGGTGCGGGGAGATGCTG------
CGAATCCACAACAAGCTGATGAGACAGGCTCTGGCGGAGTGCCTGGGGACCTTGATCCTG
GTGATGTTTGGCTGTGGTTCTGTGGCTCAGGTGGTTCTGAGCAGGGGATCGCATGGACAG
TTCTTGACTGTCAACCTGGCCTTTGGTTTTGCCGTGATGCTCGGTATCATAATATCGGGC
CAGATATCAGGTGGCCACCTCAATCCTGCTGTGACCTTTGCAACGTGCTTGTTGGCAAGA
GAGCCGTGGATTAAATTCCCGATTTATACCTTGGCACAGACCCTCGGGGCTTTTCTTGGA
GCAGGCATTGTCTAT---GGCTTGTACTATGATGCCATATGGATTTTCGGT---GGTGAT
CAGCTGTACGTAACGGGAGAAAAT---GCCACAGCAGGGATCTTCGCAACCTTCCCGACT
GACCATTTGACCCTAATGAATGGATTCTTTGATCAGGTAATTGGTACGGCGGCACTGGTG
GTCTGTGTCCTTGCTATTATAGATCCATACAATAATCCAATTCCCCGGGGCCTCGAGGCA
TTCACCGTTGGATTTGTGGTCCTGGTCATTGGACTTTCCATGGGCTTCAACTCCGGATAT
GCTGTTAACCCTGCTAGGGATTTTGGACCTCGTCTCTTCACGTCTATTGCTGGTTGGGGC
ACTGAGGTTTTCAGCGCTGGAGGCCAGTGGTGGTGGGTGCCGATAGTTTCCCCGTTGCTC
GGGGCGGTGGGCGGCATCTTGGTTTACCAGCTCATGATTGGCTTTCACATCGAGCCCCCG
CCGCAGTCCACC--
------------GAGCAA---------GAAAACGTCAAGCTAGCC---AACGTGAAGCAG
AAAGAGAGGATC--

>Gaboon_Caecian_AQP3
ATG-------------------------------GGTCGTCAA-----------------
---------------------AAGGAATTAGTGAACCGGTGTGGGGAGATGCTC------
CGAATCCACAACAAACTGATAAGACAATTCCTCTCGGAGTGCCTCGGGACGCTGATCCTG
GTGATGTTTGGCTGCGGATCTGTGGCTCAGGTGGTTCTCAGCAAGGGATCCCATGGACAG
TTCTTGACTGTCAACCTGGCCTTTGGTTTTGCCGTGATGCTTGGTATTCTGATATCAGGG
CAGGTGTCAGGTGGTCACCTCAATCCTGCTGTCACTTTTGCATTTTGCTTGTTGGCAAAG
GAACCATGGATTAAATTCCCCATTTATACCTTGGCACAAACCCTTGGGGCTTTTCTTGGA
GCTGGCATCGTCTAC---GGCTTGTACTACGATGCCATATGGTATTTTGCG---AATGAT
CAACTGTATGTGATGGGACCCAAC---GGCACAGCAGGGATCTTTGCGACTTACCCAACT
GAACATTTGACATTAATGAATGGATTCTTTGATCAGTTCATTGGTACAGCAGCCCTAGTG
GTCTGTGTCCTGGCTATTGTGGATCCGTACAATAATCCAGTTCCTAGGGGCCTCGAGGCT
TTCACCGTTGGGTTTGTTGTCTTGGTCATTGGACTCTCCATGGGCTTCAACTCCGGCTAT
GCTGTCAACCCTGCCAGGGACTTTGGACCTCGCCTTTTCACATCCATTGCTGGTTGGGGC
ACGGAGGTTTTCAGTGCTGGAGACCAATGGTGGTGGGTGCCGATCGTTTCACCATTACTC
GGCGCAGTGGCTGGCGTTTTGGTGTACGAGTTAATGATTGGGTTTCACGTTGAGCCTCCA
CCACCGTCCACC--
------------GAGCAA---------GAAAACGTCAAGCTGTCA---AACGTGAAGCAC
AAAGACAGGATC--

>European_seabass_Aqp3a
ATG-------------------------------GGCAGACAT-----------------
---------------------AAGGTGTATCTGGACAAACTGTCCCGGTTCTTC------
CAGATCCGTCACCTGCTGCTTCGTCAGGCCCTGGCAGAGTGTCTTGGCACCCTCATCCTT
GTGATGTTTGGCTGCGGTGCTGTGGCCCAGCTAGTGTTGAGCGGTGGTTCCCATGGCATG
TTCCTAACGGTCAACTTTGCCTTCGGCTTCGCTGCCACCTTAGGCATCCTGGTCTGTGGC
CAGGTATCAGGTGGCCATCTGAACCCTGCAGTGACCTTTGCACTGTGTCTGCTTGGAAGA
GAGCGCTGGAGAAAGTTCCCCATGTACTTCCTCTTTCAGACAATCGGTGCTTTTTTTGGT
GCTGCAATCATTTTT---GGCATGTACTACGATGCCCTGTGGGACCACCCT------GGA
TGTTTCAATGTGACTGGACCTAAT---GCCACAGCTGGCATCTTTGCTACCTACCCTGGA
AAACATCTCACCCTTGTCAATGGCTTCTTTGATCAGATAATTGGCACAGCAGCACTGATA
GTTTGTATTCTGGCTATTGTGGATCCATACAACAACCCCATCCCCCAAGGGCTGGAGGCC
TTCACTGTGGGCTTTGTGGTTCTGGTGATTGGATTGTCTATGGGCTTTAACTCTGGCTAT
GCTGTGAATCCTGCCAGAGACCTTGGACCACGTATTTTCACAGCTTTGGCTGGGTGGGGC
AGCGATGTTTTCACGGTTAGAAATGGCTGGTTCCTGGTGCCAGTTTGTGCCCCATTCCTT

GGCACCATCATCGGTGTGATGATCTACCAGTTAATGGTTGGTTTCCATGTGGAGGGAGAA
GTACGTGACCAAAAG---
------AGCAGAGACCAG---------GAGAATGTCCGACTCACT---AATGTCTCCAGC
AACGACAACTCCAAAGAAGATACCAAAGAAATGCAC------------------------

>Nile_tilapia_Aqp3a1
ATG-------------------------------GGCTGGCAA------------------
---------------------AAGCATTATCTGGATAAACTGTCCCGCTTCTTC------
CAGATCCGCAACCTGCTGCTTCGTCAAGCCCTGGCAGAGTGTCTTGGCACTCTCATCCTT
GTGATGTTTGGCTGTGGTTCTGTGGCCCAGTTGGTGTTGAGCGGTGGTTCTCATGGTATG
TTCCTTACTGTCAACTTTGCCTTCGGCTTTGCTGCTACGTTAGGCATCTTGGTCTGTGGC
CAAATATCAGGCGGCCATCTCAACCCAGCAGTAACTTTTGCCTTGTGTCTGCTTGGAAGA
GAGCGCTGGAGAAAGTTTCCCATGTACTTCCTCTTTCAGACAATTGGTGCTTTTTTTGGT
GCTGCTATTATTTTT---GGCATGTACTATGATGCCCTGTGGGACCATCCT------GGA
AGTTTTAATGTGACTGGACCTGAC---GCCACAGCTGGCATCTTTGCTACTTATCCTGGA
ACCCATCTCACCCTTGTCAATGGTTTCTTTGATCAGATTATTGGCACAGCAGCACTGATA
GTTTGCATTCTGGCCATTGTGGATCCTTACAACAACCCCATCCCTCAAGGGCTGGAGGCC
TTCACTGTGGGATTTGTGGTTCTGGTCATTGGATTGTCTATGGGCTTTAATTCTGGCTAT
GCAGTCAATCCTGCCAGAGACCTCGGACCACGTCTTTTCACCGCTATAGCTGGTTGGGGG
AGTGAGGTTTTCACGGCTAGCCCAGGCTGGTTCCTGGTCCCTGTTTTTGCTCCATTCCTT
GGTACCCTCATTGGTGTAATGATCTACCAGCTGATGGTTGGTTTCCACATGGAAGGGGAA
GTACGTGACAGAAAA---
---GAGAGCACAGAGCAG---------GAGACTGTCCGACTCACC---AATGTGACTTCC
AAAGACAACTCCAGAGAGGCTGTGAAAGAAAGAAATGAGTGT------------------

>Nie_tilapia_Aqp3a2
ATG-------------------------------GGCAGACAA------------------
---------------------AAGGAGTATCTGGATAAACTGTCCCGCTTCTTC------
CAGATCCGCAACCTGCTGCTTCGTCAAGCCTTAGCAGAGTGTCTTGGCACGCTCATCCTT
GTAATGTTTGGCTGTGGCGCTGTGGCCCAGCGGGTGTTGAGCGGTGGTTCCCATGGCTTG
TTCCTTACTGTCAACTTTGCCTTTGGCTTTGCTGCCATGTTAGGCATCTTGGTCTGTGGC
CAAGTGTCAGGCGGCCATCTCAACCCAGCAGTAACTTTTGCCTTGTGTCTGCTTGGAAGA
GAGCGCTGGAGAAAGTTTCCCATGTACTTCCTCTTTCAGACAATTGGTGCTTTTTTTGGT
TCTGCTATCATTTTT---GGCATGTACTATGATGCTTTGTTGCTCCGTCCT------GGA
AGTTTTAATTTGACTAGCACTAAC---AACACGGCTGGCATATTTGCAACTTACCCTGCG
AGGCATCTCACACTTGTCAATGGTTTCTTTGATCAGATTATTGGCACAACAGCACTCATA
GTTTGTGTTCTGGCCATTGTGGATCCTTACAACAACCCCATCCCCCAAGGGCTGGAGGCC
TTCACTGTGGGATTTGTGGTTCTGGTCATTGGATTGTCTATGGGTTTTAATTCTGGCTAT
GCAGTCAATCCTGCCAGAGACTTTGGACCACGTCTTTTCACCTCTATGTCTGGGTGGGGG
GGTGCTGTTTTCACGGCTAGAGACTGCTGGTTCCTGGTCCCTATTTTTGCCCCATTCCTT
GGCGCCATTCTTGGTGTGCTGATTTACCAGTTGATGGTTGGTTTCCACACAGAGGGAGAA
GCACGTGACAAGAAG---
---CAGGGGACAGTCCAG---------GAGAACCTCCAACTCGCC---AATGTTGCTTCC
TCAAACAACTCTAAAGAGGCTACCAAAGAAATTTAC------------------------

>Mummichog_Aqp3a
ATG-------------------------------GGTCGACAG------------------
---------------------AAGATTTATTTGGACAAACTGGCCCGCTTCTTT------
CAGATCCGTCACCTGTTGCTCCGCCAGGCCCTGGCAGAGTGCCTTGGCACTCTCATCCTT
GTGATGTTTGGCTGTGGCGCTGTGGCCCAAAGCGTGCTGAGTCGTGGTTCCCATGGCAGG
TTCCTCACTATCAACTTTGCCTTTGGCTTTGGCGCCACCTTAGGGATCCTGGTCTGTGGC
CAAGTATCAGGTGGCCATCTGAACCCTGCAGTGACCTTTTCACTTTGCCTGCTTGGAAGA
GAGCGCTGGAGAAAGTTCCCCATGTACTTCCTCTTTCAGACAATCGGAGCATTTTTCGGC
GCTGCTATTATCTTT---GGCATGTATTATGATGCCTTTTGGGACTTTCCC------GGG
TGCTTTAATGTTACTGGGGATTCT---TCCACTGCTGGCGTCTTTGCCACCTACCCCGGA
AAGCATCTTACTCTTGTGAATGGATTCTTTGATCAGATTATTGGCACGGCAGCCCTCATA
GTGTGCATTCTGGCAATTGTGGACCCATACAACAACCCCATTCCCCAAGGGTTGGAGGCC
TTCACTGTGGGATTTGTGGTTTTGGTCATTGGATTGTCCATGGGCTTCAATTCCGGTTAT

GCTGTTAACCCTGCCAGAGACATCGGACCACGCCTTTTCACTGCTATAGCTGGCTGGGGA
ACTGATGTTTTTACGGCTAGAAATGGCTGGTTCTTGGTGCCACTCTGCGTCCCATTCCTT
GGTGCCCTTATTGGCACGATAATTTATCAGCTGATGGTTGGTTTCCATGTGGAAGGAGAA
GCACGTGATCAGAAA---
------AAGAACACGGAA---------GAGAGTCTACAACTTACCATCAACCCCGACTCA
AAATCTAACGCCAACGGC------AAAGAGGCTAAC---TGC------------------

>Zebrafish_Aqp3a
ATG-------------------------------GGTTGGCAG-----------------
---------------------AAAAGCGTTCTGGATAAGCTTGCGCAGACTTTC------
CAAATCCGCAACAAGTTACTGCGCCAGGGATTGGCTGAATGCTTAGGAACTCTCATCCTG
GTGATGTTTGGCTGTGGTTCATTGGCCCAGTTGAAACTAAGCGAAGGTTCTCATGGTCTC
TTTCTCACTGCAAACCTTGCTTTTGGGTTTGGTGCTACTCTTGGAATCTTGGTTTGCGGC
CAGGTGTCAGGCGGACATTTAAATCCTGCTGTTACATTTGCTCTCTGCCTCTTGGGAAGA
GAAAAATGGAGAAAGTTTCCTGTGTACTTTCTGTTCCAAACACTCGGATCCTTCTTGGGT
GCTGCTATTATCTTT---GCCGAATACCATGACGCAATTTATGATTATGCTGGATCAAAT
GAGTTGCTTGTACTGGGTGAAAAA---GAAACAGCTGGGATTTTTGCTACATACCCAAGC
AAATATCTCACCCCCCTAAATGGATTTTTTGACCAGGTGATAGGCACAGCATCCCTGATT
GTGTGCATCCTGGCCATTGTGGACCCCTACAACAACCCGATCCCTCAAGGTCTTGAGGCC
TTCACAGTGGGATTCAGCGTCCTTATCATTGGTCTCTCCATGGGCTTCAATTCTGGCTAT
GCAGTAAACCCAGCTCGAGATTTTGGACCTCGTCTTTTTACTGCCATGGCTGGTTGGGGT
AGTGAAGTCTTCACAGCCAGGGATTATTGGTTTTTGGTGCCCATCTTTGCTCCGTTCATT
GGAGCCGTTATTGGTGTGATTGTGTACCAGCTGATGGTGGGATGGCATGTGGAAGGAGAG
GCACGAGATAAGAAA---
---GCTAAAGCTAGAGAG---------GAGGTGATGAACCTCAAT---GACGTCGCCAGC
AAGGAA--

>European_seabass_Aqp3b
ATG-------------------------------GGAAGACAA-----------------
---------------------AACAACATTTTGGAGAAACTGGCAGAGACCTTT------
CAGATCCGTCATGTGCTCCTCCGCCAGGCCCTGGCAGAGTGCCTGGGAACCCTGATTCTG
GTGATGTTTGGTTGTGGTGCAGTAGCACAGATGGTCCTAAGCGGGGGCACCCATGGTACG
TTCCTCACCGTCAACTTTGCTTTTGGTTTTGCCGCAACTTTGGGAATCCTTGTGAGTGGG
CAGATCTCAGGTGGTCATCTGAACCCAGCGGTGACATTCACACTGTGTTTACTTGGAAGA
GAGCCTTGGAGGAAGTTCCCGTTGTTCTTTTTGTTCCAGACTCTTGGAGCCTTTCTGGGT
GCAGCGATTGTATTT---GCCATGTATTTTGATGCATTATCGGACTTCAGC---CAGGGG
GAGCTGATGGTGGTGGGGAAGAAT---GCCACAGCTGGGATTTTTGCCACATATCCTTCT
AAACATCTCACAATGATTAATGGATTCTTTGATCAGATAATTGGAACAGCAGCACTGATA
GTGTGCATCCTGGCCATAGTGGATCCACACAACAATCCAATCCCTAGAGGTCTGGAGCCC
TTCACTGTTGGTTTTGTGGTGCTGGTCATCGGGCTGTCGATGGGCTTCAATTCTGGCTAT
GCTGTCAACCCAGCCAGGGACCTGGGACCGCGCATCTTCACGGCTCTTGCTGGTTGGGGT
GGAGAGGTTTTCACGGCACACACCTACTGGTTCTTTGTGCCCATCTGCGCCCCCTTCCTG
GGAGCAGTGGTTGGTGTGCTGATGTACCAGCTAATGATCGGATATCATTTGGAAAGAGAA
GTGCAAGAGAAGCAG---
---AAGAAGGAGGAGGAG---------GACAGATTTGAACTTTCC---AATATTACAACC
AATGAAGATGCA--

>Atlantic_salmon_Aqp3b1
ATG-------------------------------GGAAAACAG-----------------
---------------------AAGGCGCTTATGGACAAGCTCGCAAGGAACTTT------
CAGATCCGTCACATGCTGCTACGCCAGGCTCTGGCAGAGTGCCTGGGAACCCTGATACTG
GTGATGTTTGGCTGTGGTGCGGTGGCCCAGTTGGTGCTAAGTGGTGGCTCCCATGGAATG
TTCCTCACCGTCAACTTTGCCTTTGGCTTCGCTGCTACACTGGGCATCCTGGTCAGCGGA
CAGATCTCAGGAGGCCACCTGAACCCAACGGTGACCTTCGCCCTCTGTCTATTGGGGAGA
GAACCTTGGAGGAAATTCCCTGTGTTTTTCTTGTTCCAGACTATAGGGGCCTTCCTGGGA
GCTGGGATCATATTT---GGCATGTATTTTGATGCGTTGTGGGACTACGGC---CAAGGA
ACACTGATCGTTGTGGGGGAGAAT---GCCACTGCTGGGATCTTTGCCACATATCCCTCC
AAACATCTCACTCTGGTTAATGGATTCTTTGACCAGATTATAGGCACAGCAGCTCTGATC

GTGTGTATCCTGGCCATCGTGGACCCCTACAACAACCCCATCCCCCGGGGCCTGGAGGCC
TTCACGGTGGGGTTCGTGGTGCTGGTCATCGGCCTGTCAATGGGCTTCAACTCGGGCTAT
GCTGTCAACCCTGCCAGGGACCTGGGCCCACGCATCTTTACTGCCCTGGCTGGCTGGGGT
GGTGAGGTCTTCACGGTTAATGCCTACTGGTTCTTTGTGCCAATCTTTGCCCCCTTCATT
GGTGCGGTGTTGGGGGTGATGGTGTACCAGCTGATGGTTGGGTACCATGTGGAGGGAGAA
GCACGGGAGAAGAAG----------------------------------GAAGAG------
GAGGAGAAGGAGGAGCAAGAT------GAAAGGCTCAAACTGTCC---AGCCTAACTACC
AAGGACAGTGCA--

>Zebrafish_Aqp3b
ATG------------------------------GGAAGACAG------------------
---------------------AAGGTAATCCTGGAAAAGATGGCTCGGATCTTT------
CAGATTCGGAACATGCTGATGAGACAAGCACTGGCAGAATGCCTGGGCACCCTCATTCTA
GTGATGTTTGGTTGTGGTGCTCTTGCCCAGCATATTTTAAGCGGAGGCTCTCATGGAATG
TTTCTGACAGTGAATTTTGCATTTGGATTCGCTGCTACATTGGGAATCCTGGTTTGTGGG
CAAGTCTCAGGAGGTCACATAAACCCTACTGTGACCTTTTCTCTCTGTTTGTTGGGGAGG
GAGCCCTGGAGGAAATTTCCCGTTTACTTTCTGGCCCAGACTGTGGGGGCTTTTCTTGGA
GCTGGAATAATATTT---GGCATGTATTTTGATGCAATTTGGAAATTTGGA---CAAGGT
TCTCTTGATGTTGATGGGGTAAAT---GCAACTGCTGGAATCTTTGCTACGTACCCTTCT
AAACACCTTACTTTGCTAAATGGATTCTTTGATCAGATGATCGGCACGGCAGCTCTGATC
GTGTGTATCCTTGCCATTGTTGACCCTTACAATAACCCCATCCCGCAAGGACTGGAGGCC
TTCACTGTGGGCTTTGTGGTTCTGGTGATTGGTCTGTCTATGGGATTTAACTCAGGCTAT
GCTGTAAACCCAGCCAGAGACTTGGGACCACGGATCTTCACTGCAATTGCTGGATGGGGC
TCAAAAGTGTTCTCAGCGGAGTCTTACTGGTCCTTTGTGCCAGTCTTTGCCCCATTCATT
GGTGCTGTGTTCGGTGTGATGGTGTATCAGTTGATGGTGGGATGCCATGTGAAAGGAGAA
GAAAGAGATAAAAGA----------------------------------GAAGCG------
GTGGAAAGAGAAGAGAAG---------GAAAGACTCAAACTATCT---GCCGTTTCTGAT
AAAGATGCAGCA--

>Bowfin_Aqp3
ATG------------------------------GGCAGACAG------------------
---------------------AAGTTTTACCTGGACAAGCTGGCTCGCACCTTC------
CAGGTCAGGAACATGCTCATACGCCAGTTTCTGGCTGAGTGTCTGGGGACCCTCATTCTT
GTGATGTTCGGCTGTGGTGGCGTTGCCCAGGTGATCCTCAGTGGTGGCACACACGGCAGG
TTCTTGACTGTCAACTTTGCTTTCGGCTTTGGAGCCACCCTAGGCATCCTCATCTGCGGA
CAAGTTTCAGGAGGGCACCTGAACCCCGCTGTGACCTTTGCCCAGTGCCTGCTAGGGAGA
GACCCCTGGAAGAAGTTCCCAGTTTACTTTGCAGCTCAGACCCTGGGTTCCTTTCTGGGC
GCTGGTATCATCTTT---GGCCTGTACTTTGATGCCATTTGGGGCTTTGGC---CAAGGA
CAGCTGTTTGTGGTTGGCGAAAAC---GCCACTGCAGGCATTTTTGCCACCTACCCATCT
GACCATCTGACCCTCCTCAATGGCTTGTTTGACCAGCTGATCGGTACTGCCGCCCTGGTG
GTATGTATCCTGGCTATCGTTGATCCGTACAACAACCCCATCCCACGTGGGTTGGAGGCC
TTCACAGTGGGCTTCGTGGTGCTGGTCATTGGCCTGTCCATGGGCTTCAACTCTGGCTAC
GCTGTCAACCCTGCCAGGGACCTGGGGCCCCGTCTCTTCACTGCCATAGCTGGCTGGGGT
GGTGAGGTGTTTACTGCCAAAAACTACTGGTTCTTTGTGCCAATCTTCGCCCCCTTCCTG
GGTTCTTTTGTGGGCGTATTGGTATACCAGCTAATGGTGGGATACCATGTGGAGGGGGAG
GCCCGGGAGAAGGCA---
---AAGCAGGATGGAGAG---------GATAGGATCAAACTGTCC---AACACTAAC---
AAGGAGCTCCTG--

>Gar_Aqp3
ATG------------------------------GGCAGGCAG------------------
---------------------AAGTACTACATTGACAAGCTGGCGCGCGCCTTC------
CAGATCAGGAACCTGCTGCTGCGCCAGGCCCTGGCAGAGTGCCTGGGGACCCTCATTCTC
GTGATGTTCGGCTGTGGTGCAGTGGCTCAGCTGGTGCTTAGCGGTGGCTCTCATGGTATG
TTCATGACCGTGAACTTTGCTTTCGGCTTTGCGGCTACCCTAGGAATCCTGATCTGCGGA
CAGGTGTCGGGAGGACATCTTAACCCGGCGGTGACTTTTGCCCTGTGCCTGCTTGGGAGG
GATCCCTGGAGGAAACTCCCTGCTTACTTTCTGGCCCAGACACTGGGAGCCTTCCTGGGC
TCTGGGATCATCTTC---GGCCTGTACTTTGATGCCCTGTGGGATTTCGCT---GGTGGG

GATCTGCTGGTGACCGGAACAAAT---GCGACTGCTGGCATCTTCGCCACCTACCCCTCC
AACCACCTCACCCTGATCAACGGCTTATTTGACCAGCTGATTGGTACTGCCGCCCTGGTG
GTGTGTATCCTGGCTATCGTGGACCCCTACAACAACCCCATCCCACGCGGGCTGGAAGCC
TTCACGGTGGGCTTCGTGGTGCTGGTTATCGGCCTGTCCATGGGCTTCAACTCCGGCTAT
GCAGTCAACCCTGCCAGAGACCTCGGCCCGCGGCTCTTCACCGCCATAGCTGGCTGGGGC
AGCGAGGTCTTCACGGCTAATGACTACTGGTTCTTTGTGCCCATCTTCGCCCCCTTCATT
GGCTCGTTTGTGGGTGTGGTGGTGTACCAGCTGATGGTTGGCTACCACGTGGAGGGAGAG
GTTCGGGACAGAGAG---
---GAGAGAGGCGGGGAG---------GAGGGAGCCAAGCTGTCC---AACTTCAACCCC
AAGGAGACGGCG--

>Chinese_sturgeon_Aqp3
ATG------------------------------GGCAGACAG------------------
---------------------AAGCAGGCTCTGACCAGGATCTCTGAGCTGTTT------
TATATCCAGAACTTGATGCTGAGGCAAGCTCTGGCAGAATGTCTGGGGACCCTCGTTCTA
GTGATGTTCGGCTGTGGCTCTGTCGCTCAGCTAGTTCTCAGTGGTGGATCTCATGGACAG
TTCCTGACTGTGAACTTCGCTTTCGGCTTTGCTGCGACCCTGGGTGTCCTGGTGTCTGGG
CAGGTGTCAGGTGGCCATCTGAACCCGGCTCTGACCTTTGCCCTCTGTCTGCTGGGGCGG
GAGCCGTGGCTCAAGATGCCGGTGTACTTCTTGGCGCAGACGGTGGGTGCCTTCCTGGGG
GCTGGGGTCATCTTC---GGAATGTACTTCGATGCCTTGTGGATGTTTGGG---AATAAC
GATCTGTTAGTGTCAGGAGCAAAC---AGCACCGCCGGGATCTTCGCTACCTATCCATCT
CAGCACTTGACTCTGCTCAATGGCTTCTTTGACCAGATGATTGGTACTGCTGCCCTGATC
GTGTGCCTCCTGGCCATCGTGGACCCCTACAACAACCCCATCCCCAGGGGTCTGGAGGCC
TTTACTGTGGGCTTCGTCGTCTTGGTCATCGGTCTGTCCATGGGCTTCAACTCTGGCTAC
GCCGTCAACCCTGCCAGAGACTTTGGTCCTCGCCTCTTCACCGCCATAGCCGGCTGGGGC
ACAGAGGTCTTCACCACTGGATCTCACTGGTGGTTGGTGCCCATCTTTGCCCCCTTCCTG
GGTGCCTTCGTCGGGGTTCTGGTGTACCAGTTCATGGTGGGCTTCCACACGGAGGGCGAG
GCCAAAGACAAAGCA---
---AGGGAGGACAATGAG---------GAGAACGTCAAGCTGTCC---AACATCCACTCT
AAACAGATCGCT--

>Atlantic_sturgeon_Aqp3
ATG------------------------------GGCAGACAG------------------
---------------------AAGCAGGCTCTGACCAGGATCTCTGAGATGTTT------
TACATCCAGAACTTGCTGATTAGGCAAGCTCTGGCAGAATGTCTGGGGACCCTCGTTCTA
GTGATGTTTGGCTGTGGCTCTGTCGCTCAGCTAGTTCTCAGTGGTGGATCTCATGGACAG
TTCCTGACTGTGAACTTCGCTTTCGGCTTTGCTGCGACCCTGGGTGTCCTGGTGTCTGGG
CAGGTGTCAGGTGGCCATCTGAACCCGGCTCTGACCTTTGCCCTCTGTCTGCTGGGGCGG
GAGCGGTGGCTCAAGCTGCCGGTGTACTTCTTGGCGCAGACGGTGGGTGCCTTCCTGGGG
GCTGGGGTCATCTTC---GGAATGTACTTCGATGCCTTGTGGATGTTTGCG---AATAAC
GATCTGTTAGTGACAGGAAAAAAC---AGTACCGCTGGGATCTTCGCTACCTATCCATCT
CAGCACTTGACCCTGCTCAATGGCTTCTTTGACCAGATGATTGGTACTGCTGCCCTGATC
GTGTGCCTCCTGGCCATCGTGGACCCCTTCAACAACCCCATCCCCAGGGGTCTGGAGGCC
TTTACTGTGGGCTTCGCCGTCTTGGTCATCGGTCTGTCCATGGGCTTCAACTCTGGCTAT
GCCGTCAACCCTGCCAGAGACTTTGGTCCTCGCCTCTTCACCGCCTTAGCCGGCTGGGGC
ACAGAGGTCTTCACCACTGGATCTCACTGGTGGTTGGTGCCCATCTTTGCCCCCTTCCTG
GGTGCCTTCATCGGGGTTCTGGTGTACCAGTTCATGGTGGGCTTCCACACGGAGGGCGAG
GCCAAAGACAGCGCA---
---AGGGAGGACAATGAG---------GAGAACGTCAAGCTGTCC---AACATCCACTCT
AAACAGATCGCT--

>Russian_Sturgeon_Aqp3
ATG------------------------------GGCAGACAG------------------
---------------------AAGCAGGCTCTGGCCAGGATCTCTGAGCTGTTT------
TATATCCAGAACTTGCTGCTGAGGCAGGCTCTGGCAGAATGTCTGGGGACCCTCGTTCTA
GTGATGTTCGGCTGTGGCTCTGTCGCTCAGCTAGTTCTCAGTGGTGGATCTCATGGACAG
TTCCTGACTGTGAACTTCGCTTTCGGCTTTGCTGCGACCCTGGGTGTCCTGGTGTCTGGG
CAGGTGTCAGGTGGCCATCTGAACCCGGCTCTGACCTTTGCCCTCTGTCTGCTGGGGCGG

GAGCCGTGGCGCAAGATGCCGGTGTACTTCTTGGCGCAGACGGTGGGTGCCTTCCTGGGG
GCTGGGGTCATCTTT---GGAATGTACTTCGATGCCTTGTGGTTCTTTGGG---AATGAC
AATCTGTTAGTGTCA------AAC---GGTACCGCCGGGATCTTTGCTACCTATCCATCT
CAGCACTTGACCCTGCTCAATGGCTTCTTTGACCAGATGATTGGTACTGCTGCCCTGATC
GTGTGCCTCCTGGCCATCGTGGACCCCTACAACAACCCCATCCCCAGGGGTCTGGAGGCC
TTTACTGTGGGCTTTGTCGTCTTGGTCATCGGTCTGTCCATGGGCTTCAACTCTGGCTAC
GCCGTCAACCCTGCCAGAGACTTTGGTCCTCGCCTCTTCACCGCCATAGCCGGCTGGGGC
ACAGAGGTCTTCACCACTGGACCTCACTGGTGGTTGGTGCCCATCTTTGCCCCCTTCCTG
GGTGCCTTCATCGGGGTTCTGGTGTACCAGTTCATGGTGGGCTTCCACACTGAGGGCGAG
GCCAAAGACCGCGCA---
---AGGGAGGACAATGAG---------GAGAACGTCAAGCTGTCC---AACATTCACTCC
AAACAGACAGCT--

>Sterlet_Aqp3
ATG-------------------------------GGCAGACAG-----------------
---------------------AAGCAGGCTCTGACCAGGATCTCTGAGCTGTTT------
TATATCCAGAACTTGCTGCTGAGGCAAGCTCTGGCAGAATGTCTGGGGACCCTCGTTCTA
GTGATGTTCGGCTGTGGCTCTGTCGCTCAGCTAGTTCTCAGTGGTGGATCTCATGGACAG
TTCCTGACTGTGAACTTCGCTTTCGGCTTTGCTGCGACCCTGGGTGTCCTGGTGTCTGGG
CAGGTGTCAGGTGGCCATCTGAACCCGGCTCTGACCTTTGCCCTCTGTCTGCTGGGGCGG
GAGCCGTGGCGCAAGATGCCGGTGTACTTCTTGGCGCAGACGGTGGGTGCCTTCCTGGGG
GCTGGGGTCATCTTT---GGAATGTACTTCGATGCCTTGTGGTTCTTTGGG---AATGAC
AATCTGTTAGTGTCA------AAC---GGTACCGCCGGGATCTTTGCTACCTATCCATCT
CAGCACTTGACCCTGCTCAATGGCTTCTTTGACCAGATGATTGGTACTGCTGCCCTGATC
GTGTGCCTCCTGGCCATCGTGGACCCCTACAACAACCCCATCCCCAGGGGTCTGGAGGCC
TTTACTGTGGGCTTTGTCGTCTTGGTCATCGGTCTGTCCATGGGCTTCAACTCTGGCTAC
GCCGTCAACCCTGCCAGAGACTTTGGTCCTCGCCTCTTCACCGCCATAGCCGGCTGGGGC
ACAGAGGTCTTCACGACTGGACCTCACTGGTGGTTGGTGCCCATCTTTGCCCCCTTCCTG
GGTGCCGTCATCGGGGTTCTGGTGTACCAGTTCATGGTGGGCTTCCACACTGAGGGCGAG
GCCAAAGACCGCGCA---
---AGGGAGGACAATGAG---------GAGAACGTCAAGCTGTCC---AACATTCACTCC
AAACAGACAGCT--

>Common_sturgeon_Aqp3
ATG-------------------------------GGCAGACAG-----------------
---------------------AAGCAGGCTCTGACCAGGATCTCTGAGCTGTTT------
TATATCCAGAACTTGCTGCTGAGGCAAGCTCTGGCAGAATGTCTGGGGACCCTCGTTCTA
GTGATGTTCGGCTGTGGCTCTGTCGCTCAGCTAGTTCTCAGTGGTGGATCTCATGGACAG
TTCCTGACTGTGAACTTCGCTTTCGGCTTTGCTGCGACCCTGGGTGTCCTGGTGTCTGGG
CAGGTGTCAGGTGGCCATCTGAACCCGGCTCTGACCTTTGCCCTCTGTCTGCTGGGGCGG
GAGCCGTGGCGCAAGATGCCGGTGTACTTCTTGGCGCAGACGGTGGGTGCCTTCCTGGGG
GCTGGGGTCATCTTT---GGAATGTACTTCGATGCCTTGTGGTTCTTTGGG---AATGAC
AATCTGTTAGTGTCA------AAC---GGTACCGCCGGGATCTTTGCTACCTATCCATCT
CAGCACTTGACCCTGCTCAATGGCTTCTTTGACCAGATGATTGGTACTGCTGCCCTGATC
GTGTGCCTCCTGGCCATCGTGGACCCCTACAACAACCCCATCCCCAGGGGTCTGGAGGCC
TTTACTGTGGGCTTTGTCGTCTTGGTCATCGGTCTGTCCATGGGCTTCAACTCTGGCTAC
GCCGTCAACCCTGCCAGAGACTTTGGTCCTCGCCTCTTCACCGCCATAGCCGGCTGGGGC
ACAGAGGTCTTCACCACTGGATCTCACTGGTGGTGGGTGCCCATCTTTGCCCCCTTCCTG
GGTGCCTTCATCGGGGTTCTGGTGTACCAGTTCATGGTGGGCTTCCACACGGAGGGTGAG
GCCAAAGACAAAGCA---
---AGGGAGGACAATGAG---------GAGAACGTCAAGCTGTCC---AACATCCACTCT
AAACAGATCGCT--

>Reedfish_Aqp3
ATG-------------------------------GAAAGACAA-----------------
---------------------AAGCGTTTCCTGGCCTGGATGGGACAGATGTTC------
TACATTAAGAATAAACTTTTAAGGCAATCACTAGCTGAGTGTTTGGGCACCCTAATCTTG
GTGATGTTTGGCTGTGGCTCTGTGGCTCAGCTGGTCCTTAGTGGTGGCACCCATGGCCAA

```
TTTCTTACAGTCAACTTTGCATTTGGTTTTGCAGTCACACTCGGCATACTTATTTCTGGA
CAGGTGTCAGGTGGACATCTCAACCCTGCTGTGACCTTTTCTCTTTGCCTTCTGGGACGT
GAGCCTTGGATCAAATTCCCAGTGTACTTTCTGGCTCAGACCCTAGGATCCTTCCTTGGG
GCTGGAATAATCTTT---GGACTGTACTTTGATGCCCTTTGGCCTATGGA---GGTAAT
CATCTCCTTGTGACTGGGTCAAAC---GCCACTGCAGGAATCTTTGCTACCTTCCCCTCA
GAGCATCTTACTCTTGTAAATGGTTTTTTGACCAGGTAATTGGCACTGCTGCTCTCATT
GTGTGTATCTTGGCAATTGTTGACCCATACAACAACCCCATTCCACAAGGCCTGGAGGCC
TTCACTGTGGGTTTTGTAGTTCTTGTTATTGGACTTTCAATGGGCTTCAACTCTGGATAT
GCAGTTAACCCAGCCAGGGACTTTGGGCCAAGACTGTTCACTTCAATTGCAGGCTGGGGT
TCCGAGGTCTTCACGTCTGGAAATAATTGGTGGTGGGTACCAATTCTTGCCCCCTTCTTC
GGAGCCTTCGCTGGAGTAATTGTGTATCAGTTCATGGTTGGCTTTCATGTGGAAGGGATG
TCAAAGCAAAAGAAA---------------------------------------------
---------CAAAGTGAA---------GAAAACATCAAATTGTCC---AATACAAGCTCT
AAAGATATGGCA------------------------------------------------
------------------------------------------------------------
------------------------------------------------------------
>WA_lungfish_Aqp3
ATG------------------------------GGAATGCAA------------------
---------------------AAGGAATGTCTCTCAAAAGTTGGCAATTTCTTA------
AGAATCAAAAATAAACTGTTAAGGCAAGCTTTGGCAGAATGTTTGGGGACGTTAATACTT
GTGATGTTTGGCTGTGGATCAGTAGCACAAGTTGTCCTTAGTGAGGGTCAAAAGGGACGT
TTCCTAACAGTCAACTTAGCCTTTGGATTTGCTGTGACCCTTGGGATCCTTGTATGTGGA
CAAGTATCTGGTGGTCATCTAAATCCAGCTGTGACCTTTGCTTTATGCCTACTTGGTCGT
GACAGGTGGCGCAAATTCCCAATATATTTCTTTGCTCAGACAGTAGGCTCATTTATTGCC
GCTGCTATCATCTAT---GGACTGTACTTTGATGCTCTGATGAACTATGGA---GGTGGT
CAACTGAGAGTAACAGGAGCTAAT---TCAACAGCTGGAATCTTTGCAACCTATCCAGGA
GAACATCTCACAACGCTAAATGGATTCTTTGATCAGCTCATTGGCACAGCAGCTCTGATT
GTCTGTATCCTTGCGATTGTCGATCCTTACAACAACCCTATTCCCAGACATCTAGAGGCA
TTCACTGTTGGATTTGTAGTCCTGGTCATCGGTCTTTCAATGGGACTGAATTCTGGATAT
GCTGTTAATCCTGCTAGGGACTTTGGACCCCGACTTTTCACAGCAATGGCTGGATGGGGC
TCAGAGGTTTTCACTGCTGGATCACACTGGTGGTGGGTACCTATAATTTCCCCATTTATT
GGAACAGTTATTGGAACAGCAGTATACCAGATAATGATTGGTATCCATGTTGAACTCGAT
GTTCCTGCAAGCCCT---------------------------------------------
------AGCACCCCAGAA---------GAAGCTGTCAAGCTGGCA---AAAATAAAGGCA
AATGAAACTGCA------------------------------------------------
------------------------------------------------------------
------------------------------------------------------------
>SA_lungfish_Aqp3
ATG------------------------------GGAGTGCAA------------------
---------------------AAGGACTGTCTTACAAAAATTGGCAATTTCCTC------
AGAATCAAAAATAAATTGTTAAGGCAGGCTCTGGCAGAATGTTTGGGGACTTTAATTCTT
GTGATGTTTGGCTGTGGATCAGTAGCACAGGTTATCCTTAGTGAAGGAGCGAAAGGACGT
TTCCTAACTGTTAACTTAGCCTTTGGATTTGCTGTTACCCTTGGGATCCTTGTATGTGGA
CAAGTATCTGGTGGTCACCTAAATCCAGCTGTAACCTTTGCTTTGTGCCTACTTGGTCGG
GAGAGGTGGCGTAAATTCCCAGTGTATTTCTTTGCTCAGACATTAGGTGCATTTGTTGCA
TCTGCCATCATCTAT---GGGCTGTACTATGATGCTTTGATGCATTATGGA---GGTGGC
CAGCTGTCAGTACTTGGAAACAAT---GCAACAGCTGGAATCTTTGCAACCTATCCAGGA
GAACATCTCACAATGCTAAATGGATTCTTTGATCAGATCATAGGCACTGCAGCATTGATT
GTCTGTATCCTTGCGATTGTGGACCCTTACAACAACCCCATTCCCAGACATCTAGAGGCA
TTCACTGTTGGATTTGTAGTCCTGGTCATTGGTCTCTCAATGGGACTGAATTCTGGATAT
GCTGTTAACCCTGCTAGGGACTTCGGACCTCGACTTTTCACAGCAATAGCTGGATGGGGC
ACAGAAGTTTTCACTGCTGGATCTCATTGGTGGTGGGTACCTATAACTGCTCCCTTCATT
GGCACAGTTTGCGGAACTTTAGTATACCAGATAATGATCGGCATCCATGTCGAACTTGAT
GTCCCTCAAAGCCCT---------------------------------------------
------AGCTCTCCAGAA---------GAAAATGTCAAGCTGGCA---AATATAAAGAAA
AATGAGACTGCA------------------------------------------------
------------------------------------------------------------
------------------------------------------------------------
>Coelacanth_Aqp3
ATG------------------------------GGAAAACAA------------------
---------------------AAAGAAATGCTCACTAAAATGTTCCGACTGTTT------
```

AAAATTAGAAACAAGCTGCTGAGAGAAGCTTTGGCAGAATGTCTGGGCACTTTAATTTTG
GTGTTGTTCGGCTGCGGTGCTTTAGCCCAGGTGGTTCTCAGCAATGGCTCCCACGGACAG
TTCTTGACTGTCAATCTTGCCTTTGGTTTTGCGGCAACTCTCGGGGTTCTGATTGCAGGG
CAGATCTCAGGTGCCCATCTTAATCCTGCTGTCACCTTTGCAGTGTGCCTTCTTGCCCGC
GAGCCATGGATTAAATTCCCCGTTTACTCCTTAGCTCAAATCCTGGGTGGATTTCTGGCA
TCGGGCATCATCTTT---GCCATGTACTTTGATGCTATTTGGCTCTATGGC---AACAAC
CAGCTGATAGTAATGGGGCCCAAA---GGCACTGCGGGAATCTTTGCCACTTACCCTTCT
GAACACCTGACACTCCTCAACGGATTCTTTGATCAGTTAATCGGCACTGCTGCCCTGGTT
CTCTGCATCCTTGCCATTGTGGACCCTCATAACAATCCCATCCCCAGGGGCCTGGAGGCC
TTCACCATTGGCTTTGTGGTCCTAAACATCGGGCTCGCCATGGGCTTCAATTCTGGCTAC
GCCGTCAATCCCGCCCGAGACTTTGGGCCCCGTCTGTTCACAGCCGTTGCTGGCTGGGGC
ACAGAGGTTTTCAGTGCTGGTGGACATTGGTGGTGGGTGCCGATAGTCGCGCCACTCCTA
GGAGCAGTACTGGGAGTTCTGGTGTACCAGCTGATGATTGGAGTCCACTTTGAACCCGAG
ACCCCATCCACC--
------------GCACCA---------GAAAATGTCAATCTAACT---AAAGTAGACACC
ATAGAGAAACCT--

>B_bambooshark_Aqp3C2
ATG--------------------------------GGAAAACAA------------------
----------------------AAGGCAATAATCAGAAAAATTGAAGACTCATTC------
AGAATAAGAAATCTATTGCTCAGACAATGTCTTGCTGAATGTTTAGGAACATTAATTCTT
GTGTTGTTTGGGTGTGGAGCACTAGCACAAGTTACCCTCAGCAGGGGTACACATGGACGG
TTTTTGACTGTCAATTTTGCTTTTGGATTTGCAGTAATGCTCGGTGTACTAATAGCTGGC
CAAGTGTCAGGTGCTCACCTGAACCCTGCTGTGACCTTTGCTATGTGCTTACTTGCTCGC
GAACCTTGGATAAAATTTCCCCTTTACTCTCTGGCACAAATATTAGGTGGCTTCATTGGA
TCTGGTATCATTTTT---GGTTTGTATTTTGATGCCATGTGGGACTTCAGTGGTCAAAAT
AAACTGTTGGTATATGGCCCCAAT---GCAACTGCTGGCATATTTGCTACTTACCCATCT
GCACACTTAACTCCACTCAATGGCTTTTTTGATCAGCTGATTGGAACGAGTGCTCTGATA
GTTTGCATCCTTGCTATTGTGGATAAGTACAATAACCCAGTGCCAAAGGGTGTGGAGGCA
TTTACTGTTGGCTTTACCGTACTGGTTATTGGCTTGTCAATGGGTTTCAACTCTGGGTAT
GCTGTGAACCCTGCCAGAGACTTTGGACCTCGTTTGTTTACGGCATTGGCTGGCTGGGGA
GCTGAAGTTTTCAGTGCCGGACACTACTGGTTTTGGATCCCAATTTTTGCCCCCCTCCTT
GGTTCTTTACTGGGCATTCTGATATATCAACTAATGATTGGGATACATCTGGAGCCTGTA
AACCACAATTCATCC---
---------CCTAGAGAA---------GAAAATGTAAAACTGGCT---AATGTGAACTTA
AGAGAAAGTTCC--

>w_bambooshark_Aqp3C2
ATG--------------------------------GGAAAACAA------------------
----------------------AAGGCAATAATCAGAAAAATTGAAGACTCTTTC------
AGAATAAGAAATCTATTGCTCAGACAATGTCTTGCTGAATGTTTAGGAACATTAATTCTT
GTGTTGTTTGGGTGTGGAGCACTGGCACAAATTACCCTCAGCAGGGGTACACATGGACGG
TTTTTGACTGTCAATTTTGCTTTTGGATTTGCAGTAATGCTTGGTGTACTAATAGCTGGC
CAAGTGTCAGGTGCTCACCTGAACCCTGCTGTGACCTTTGCTATGTGCTTACTTGCTCGT
GAACCTTGGATAAAATTTCCCCTTTACTCTCTGGCACAAATACTAGGTGGCTTCATTGGA
TCTGGTATCATTTTT---GGTTTGTATTTTGATGCCATGTGGGACTTCAGTGGTCAAAAT
AAACTGTTGGTATATGGCCCCAAT---GCAACTGCTGGCATATTTGCTACTTACCCATCT
GCACACTTAACTCCACTCAATGGCTTTTTTGATCAGCTGATTGGAACGAGTGCTCTGATA
GTTTGCATCCTTGCTATTGTGGATAAGTACAATAACCCAGTGCCAAAGGGTGTGGAGGCA
TTTACCGTTGGCTTTACCGTACTGGTTATTGGCTTGTCAATGGGTTTCAACTCTGGGTAT
GCTGTGAACCCTGCCAGAGACTTTGGACCTCGTTTGTTTACGGCATTGGCTGGCTGGGGA
GCTGAAGTTTTCACGGCTGGACACTACTGGTTTTGGATCCCAATTTTTGCCCCCCTCCTT
GGTTCTTTACTGGGCATTCTGATATATCAACTAATGATTGGGATACATCTGGAGCCTGTA
AACCACAATTCATCC---
---------ACTAGAGAA---------GAAAATGTAAAACTGGCT---AATGTGAACTTA
AGAGAAAGTTCC--

>Whale_shark_Aqp3C2

ATG-------------------------------GGAAAACAA------------------
----------------------AAGGCAATAATCAGAAAAATTGAAGATTCATTC------
AGAGTAAGAAATCTATTGCTAAGGCAATGTCTTGCTGAATGTTTAGGAACATTGATTCTT
GTGTTGTTTGGGTGTGGAGCGCTGGCACAAGTTACCCTCAGCAGGGGCACACATGGAAGG
TTTTTGACTGTCAATTTTGCTTTTGGATTTGCAGCAATGCTTGGTGTACTATTAGCTGGC
CAAGTGTCAGGTGCTCACCTGAACCCTGCTGTGACATTTGCTATGTGCTTACTTGCTCGC
GAACCCTGGATAAAATTTCCCCTTTACTCTTTGGCACAAATACTAGGCGGCTTCCTTGGA
TCTGGTATCATTTTT---GGTTTGTATTTTGATGCCATGTGGGACTTCAGTGGTCAAAAT
AAACTGTTGGTATATGGCCCCAAT---GCAACTGCTGGTATATTTGCTACTTACCCATCT
GCACATCTAACTCCACTCAATGGCTTTTTTGATCAGCTGATTGGAACTAGCGCTCTGATA
GTTTGCATCCTTGCTATTGTGGATAAATTCAATAACCCAGTGCCAAAGGGTGTGGAGGCA
TTTACAGTTGGCTTTACTGTACTGGTCATTGGCTTGTCAATGGGCTTCAACTCTGGATAT
GCTGTGAACCCTGCCAGAGATTTTGGACCTCGTTTGTTTACAGCATTGGCCGGCTGGGGA
GCTGAAGTTTTCAGTGCTGGACACTACTGGTTTTGGATTCCAATTTTTGCCCCCCTCCTT
GGTTCTTTACTTGGCACTCTAGTATATCAACTAATGATTGGGATACATCTCGAGCCTGTA
AACCATAATTTACCC---
---------ACTAGAGAA---------GAAAATGTAAAACTGGCT---CATGTAAATTTA
AAAGAAAGCTCC--

>Zebra_bullhead_shark_Aqp3C2
ATG-------------------------------GGAAAGCAA------------------
----------------------AAGGCAATTATCAGAAAAATTGAAGATTCATTC------
AGAATAAGAAATCTATTGCTAAGACAATGTCTTGCTGAATGTTTAGGAACATTGATTCTT
GTGTTGTTTGGCTGTGGAGCATTGGCACAAGTTACCCTCAGCAGGGGTACACATGGACTG
TTTTTGACTGTTAATTTTGCCTTCGGATTTGCAGTGATGCTCGGTGTACTAGTAGCTGGC
CAAGTGTCAGGAGCTCACCTGAATCCTGCTGTGACCTTTGCTATGTGCTTACTTGCTCGC
GAACCCTGGATAAAATTTCCCCTTTACTCTTTGGCACAAATACTAGGTGGCTTCCTTGGA
TCTGGTATCATTTTC---GGTTTGTATTTTGATGCCATGTGGGACTTCAGTGGACAAAAC
CAACTGTTAGTACATGGGCCCAAT---GCCACTGCTGGTATATTCGCCACATACCCATCT
GTGCACTTAACTCCACTCAATGGCTTTTTTGATCAGCTGATTGGAACTACTGCTCTCATA
GTTTGCATCCTTGCCATTGTGGATAAATTCAATAACCCAGTGCCAAAGGGTCTGGAGGCA
TTTACTGTTGGCTTTACCGTCCTGGTAATCGGCTTGTCAATGGGTTTCAACTCCGGGTAT
GCTGTAAACCCTGCCAGAGACTTTGGACCTCGTTTGTTTACAGCATTGGCTGGCTGGGGA
GCTGAAGTTTTCATTGCTGGAAACTACTGGTTTTGGATCCCTATTTTTGCCCCACTCCTT
GGTTCTGTGCTTGGTGTTCTGGTTTATCAACTAATGATTGGAATACATATTGAGCCCATA
AACCATAGTTCACCT---
---------ATTGAAGAA---------GAAAATGTAAAACTGGCT---AATGTAAAATTA
AGAGAAAGTTCA--

>Cloudy_catshark_Aqp3C2
ATG-------------------------------GGAAAACAA------------------
----------------------AAGGCAATAATCAGGAAAATCGAAGATTCGTTC------
AGAATAAGAAATTTATTGGTCAGACAATGTCTTGCTGAATGTTTAGGAACATTGGTTCTT
GTGTTATTTGGCTGTGGAGCAGTGGCACAAGTTACCCTCAGCAGAGGAACCCATGGACTG
TTTTTGACTGTTAATTTTGCTTTTGGATTTGCAGTGATGCTGGGTGCACTAATAGCTGGC
CAAGTGTCAGGAGCTCACCTGAACCCTGCTGTGACCTTTGCTATGTGTATACTTGCTCGC
GAGCCCTGGATAAAATTTCCCCTCTACTCTTTCGCACAAATATTAGGCGGTTTCCTTGGG
TCTGGTGTCATTTTT---GGTTTGTACTTTGATGCCATGTGGGGCTTCAGTGGCCAAAAC
AAACTCTTAGTACATGGCCCCAAT---GCGACTGCTGGGATATTTGCTACATACCCAGCT
CTGCACTTAACTCCAGTAAATGGCTTTTTTGATCAGCTGATTGGGACTGCAGCTCTCATA
GTTTGCATCCTAGCCATTGTGGATAAATTCAACAACCCAGTGCCAAAGGGACTGGAGGCA
TTTAGTGTTGGCTTTGCCGTGCTGGTAATTGGCATGTCAATGGGTTTCAATTCTGGGTAC
GCTATAAACCCTGCCAGAGACTTTGGACCTCGTCTGTTTACAGCACTGGCCGGCTGGGGA
GCTGAGGTTTTCAGTGCTGGAAACTACTGGTTTTGGATTCCCATCGTTGCCCCACTCCTT
GGTTCTGTATTTGGTATTTTGGTATATCAACTAATGGTTGGAATACACCTTGAGCCCATA
AACCACAGTTCAACC---
---------ACGGAAGAG---------GAAAATGTAAAACTTGCG---AATGTTAAATTA
AGAGAAAGTTCC--

>Blue_shark_Aqp3C2
ATG------------------------------GGAAAACAA------------------
---------------------AAGGCAATGATTAGAAAAATCGAAGATTCGTTC------
AGAATCAGAAACCTGTTGTTAAGACAATGTCTTGCTGAATGTTTAGGTACATTGATACTT
GTGTTGTTTGGCTGTGGAGCAGTGGCACAAGTTACCCTCAGCAGGGGTACCCATGGACAG
TTTTTGACTGTTAATTTTGCTTTTGGGTTTGCAGTGATGCTTGGTGTCCTGATAGCTGGC
CAAGTGTCAGGTGCTCACCTGAACCCTGCTGTGACCTTTGCTATGTGTTTGCTTGCTCGT
GAGCCCTGGATAAAATTTCCTCTCTACTCTTTGGCCCAAATATTTGGTGGCTTCCTTGGA
TCTGGTGTCATTTTT---GGCTTGTACTTTGATGCCATTTGGGGCTTCAGTGGCCAAAAC
AAACTCTTAGTACATGGTCCCAAT---GCGACTGCTGGTATATTTGCTACATACCCATCT
GCGCACTTAACTCCAGTAAATGGCTTTTTTGATCAGCTGATTGGAACTACTGCTCTCATA
GTTTGCATCCTTGCCATTGTGGATAAGTTCAACAACCCAGTGCCAAAGGGACTGGAGGCA
TTTACTGTTGGTTTTGTCATATTGGTAATTGGCCTGTCAATGGGTTTCAACTCTGGGTAT
GCTGTGAACCCTGCCAGAGACTTTGGACCTCGCTTGTTTACAGCACTGGCTGGTTGGGGA
GCTGAAGTTTTCAGTGCCGGAAGCTACTGGTTTTGGGTTCCCATTTTTGCCCCACTCCTT
GGTTCTGTACTTGGTGTTCTGATATACCAGCTAATGATTGGAATTCACCTTGAGCCCATA
AAGCCCAGTTCACCC---
---------GCTGGAGAA---------GATAATGTAAAACTGGCT---AATGTAAAATCA
AGAGAAAGTTGC--

>Great_white_shark_Aqp3C2
ATG------------------------------GGAAAACAA------------------
---------------------AAAGCACTGATCAGAAAAATTGAACATTCATTC------
AGAATAAGAAATCTGTTGGTAAGAGAATGTCTTGCTGAATGTTTAGGAACATTAATTCTT
GTGTTGTTTGGCTGTGGAGCATTGGCACAAGTTACTCTCAGCAGAGGTACACATGGAATG
TTTTTGACTGTTAATTTTGCTTTTGGATTTGCAGTGATGCTCGGTGTACTAATAGCTGGC
CAAGTGTCAGGAGCTCACCTGAATCCTGCCGTGACATTTGCCATGTGTTTACTTGCTCGT
GAGCCCTGGATAAAATTTCCCCTCTACTCTTTGGCACAAATATTAGGTGGTTTCCTTGGA
TCTGGTGTCATTTTT---GGTTTGTATTTTGATGCCATGTGGGGTTTTAGTGGCCAAAAC
AAACTCTTAGTACATGGCCCCAAT---GCGACTGCTGGTATATTTGCTACATACCCATCT
GTGCACTTAACACCAGTAAATGGCTTTTTTGATCAGCTGATTGGGACGACCGCTCTCATA
ATTTGCATCCTTGCGATTGTGGATAAACTCAATAACCCAGTGCCAAAGGGACTGGAGGCA
TTTACCATTGGCTTTGCCGTGCTGGTAATTGGCCTGTCAATGGGTTTCAACTCTGGGTAC
GCTGTGAACCCTGCCAGAGACTTTGGACCTCGCTTGTTTACAGCATTGGCTGGCTGGGGA
GCTGAGGTTTTCAGTGCTGGAAACTACTGGTTTTGGATTCCCATTTTTGCCCCACTCCTT
GGTTCTGTATTTGGTATTCTGGTATATCAGCTAATGATTGGAATACACCTCGATCCCATA
AACTGCAGTTCACCC---
---------TCTGGAGAA---------GAAAATGTAAAACTGGCT---AATGTAAAATTA
AGAGAAAGTTCC--

>vblShark_Aqp3C2
ATG------------------------------GGAAAACAA------------------
---------------------AAAGCAATAATCAGAAAAATTGAAGATTCATTC------
CGAATAAGAAATCTGTTGGTAAGACAATGTCTTGCTGAATGTTTAGGAACGTTGATTCTT
GTGTTGTTTGGCTGTGGAGCATTGGCACAAATTACCCTCAGCAGGGGTACACATGGACAG
TTTTTGACTGTTAATTTTGCCTTCGGATTTGCAGTGATGCTCGGTGTACTCCTGGCTGGC
CAAGTCTCAGGAGCGCACTTGAATCCTGCCGTGACCTTTGCTATGTGCTTACTTGCTCGC
GAACCCTGGTTAAAATTTCCCCTTTACTCTTTGGCACAAATACTAGGCGGCTTCCTTGGA
TCTGGTATCATTTTC---GGTTTGTATTTTGATGCCATGTGGGACTTTAGTGGTCAAAAC
CAACTGTTAGTACATGGCCCCAAT---GCTACTGCTGGTATATTCGCTACCTACCCATCT
GTGCACTTAACTCCA------------TTTGATCAGCTGATTGGAACTGCCGCTCTCATA
GTTTGCATCCTTGGCATTGTGGATAAATTCAATAACCCGGTGCCAAAAGGACTGGAGGCA
TTTACTGTTGGCTTTACTGTCCTGGTAATTGGCTTATCAATGGGTTTCAACTCTGGGTAT
GCCGTGAACCCTGCCAGAGACTTTGGACCTCGTTTGTTTACAGCATTGGCTGGCTGGGGA
GCTGAGGTTTTCGTTGCTGGAAACTACTGGTTTTGGATCCCTATTTTTGCCCCACTCCTT
GGTTCTGTGCTTGGTATTCTGGTATATCAACTAATGATTGGAATACACCTCGAGCCCGAA
AACCACAATTCACCC---
---------ATTGGAGAA---------GAGAATGTAAAACTGGCT---GATGTAAAATTA

```
AGAGAAAGTTCC------------------------------------------------
------------------------------------------------------------
------------------------------------------------------------
>Spiny_dogfish_Aqp3C2
------------------------------------------------------------
------------------------------------------------------------
AGAATAAGAAATCTGTTGGTAAGACAATGTCTTGCTGAATGTTTAGGAACGTTGATTCTT
GTGTTGTTTGGCTGTGGAGCATTGGCACAAATGACCCTCAGTAGGGGTACACACGGACAG
TTTTTGACTGTTAATTTTGCCTTCGGATTTGCAGTGATGCTCGGTGTACTCCTGGCTGGC
CAAGTCTCAGGAGCGCACTTGAATCCTGCCGTGACCTTTGCTATGTGCTTACTTGCTCGC
GAACCCTGGTTAAAATTTCCACTTTACTCTTTGGCACAAATACTAGGCGGCTTCCTTGGC
TCTGGTATCATTTTC---GGTTTGTATTTTGATGCCATGTGGGACTTTAGTGGTCAAAAC
AAACTGTTAATATATGGCCCCAAT---GCCACTGCTGGTATATTCGCTACATACCCATCT
GTGCACTTAACTCCACTCAATGGCTTTTTTGATCAGCTGATTGGAACTGCCGCTCTCATA
GTTTGCATCCTTAGCATTGTGGATAAATTCAATAACCCGGTGCCAAAGGGACTGGAGGCA
TTTACTGTTGGCTTTACCGTCCTGGTAATTGGCTTGTCTATGGGTTTCAACTCTGGGTAT
GCCGTGAACCCTGCCAGAGACTTTGGACCTCGTTTGTTTACATCATTGGCTGGCTGGGGA
GCTGAGGTTTTCATTGCTGGAAACTACTGGTTTTGGATCCCTATTTTTGCCCCACTCCTT
GGTTCTGTACTTGGTATTCTGGTATATCAGCTTATGATTGGAATACACCTCGAGCCCGAA
AACCACAATTCACCC---------------------------------------------
---------ATTGGAGAA---------GAAAATGTAAAACTGGCT---AATGTAAAATTA
AGAGAAAGTTCC------------------------------------------------
------------------------------------------------------------
------------------------------------------------------------
>Little_skate_Aqp3C2
ATG-------------------------------GGAAAACAG------------------
----------------------AAAGCAATGATCAAAAAAATTGAAAATCATTGC------
CGAATAGAAAATCTGTTGGGAAGACAGTGTCTTGCTGAATGTCTAGGAACATTGATTCTT
GTGTTATTTGGTTGTGGAGCAGTGGCACAAGTTACCCTCAGCAGGGGTACACATGGACAA
TTTTTGACA---------------------------------------------------
---------GGAGCTCATCTGAACCCCGCCGTGACCTTTGCCATGTGCTTGCTTGCCCGT
GAGCCCTGGATAAAGTTTCCCCTTTACTCTTTGGCGCAAACACTTGGTGCCTTCCTAGCA
TCGTGTGTTATTTTT---GGTTTGTATTATGATGCGTTTTGGGACTTCAGTGGTCAAAAC
CAACTACTAGTTTACGGTCCTAAT---GCCACGGCTGGAATTTTTGCAACGTATCCATCT
GCACATTTAGGTTCATCCAATGGTTTCTTTGATCAGGTGATTGGGACAGCTGCTCTCATA
GTGTGCATCCTGGCCATTGTGGACAAATGGAATACCCCAGTGCCTAACGGACTAGAGGCA
TTTACTGTTGGCTTTACCGTGCTTGTAATCGGTTTGTCAATGGGTTTCAACTCTGGGTAT
GCGGTGAACCCAGCCAGAGATCTTGGACCCCGTTTGTTTACATCATTAGCTGGCTGGGGA
ACTGAGGTTTTCACGGCTGGAAACTACTGGTTTTGGATCCCCATTTTTGCCCCACTTCTT
GGTTCTATACTTGGTATTCTGGTATATCAATTAATGATCGGCATACACTTTGAGCCTAAA
GAGCAGAGTTCACCC---------------------------------------------
---------GCTGGTGAA---------GAAAATGTAAAACTCGCA---AATGTGAAATTA
AGAGATGGTTGC------------------------------------------------
------------------------------------------------------------
------------------------------------------------------------
>Thorny_skate_Aqp3C2
ATG-------------------------------GGAAAACAG------------------
----------------------AAAGCAATGATCAAAAAAATGGAAAGTTATTGC------
CGAATACAAAATCTGTTGGGAAGACAGTGTCTTGCTGAATGTCTAGGAACATTGATTCTT
GTGTTATTTGGTTGTGGAGCAGTGGCACAAGTTACCCTCAGCAGGGGTTCACATGGACAA
TTTTTGACAGTTAATTTTGCATTCGGATTTTCAGTGATGCTTGGTGTGCTCATTGCTGGC
AAAGTATCAGGAGCTCATCTGAACCCCGCCGTGACCTTTGCCATGTGCTTACTTGCCCGC
GAGCCCTGGATAAAGTTTCCCCTTTACTCTTTGGCGCAAACACTTGGCGCCTTCCTAGCA
TCGTGTATTATTTTT---GGTTTGTATTATGATGCATTTTGGGACTTCAGTGGTCAACAC
CAACTATTAGTTTACGGTCCTAAT---GCCACGGCTGGTATTTTCGCAACGTATCCATCT
GCACATTTAGGTTCATCCAATGGTTTCTTTGATCAGGTGATTGGGACAGCTGCTCTCATA
GTGTGCATCTTGGCTATTGTGGACAAATGGAATACCCCAGTGCCTAACGGACTAGAGGCA
TTCACAGTTGGCTTTACCGTGCTTGTAATCGGCTTGTCAATGGGTTTCAACTCTGGGTAT
GCGGTGAACCCAGCCAGAGATCTTGGACCGCGTTTGTTTACATCATTAGCTGGCTGGGGA
ACTGAGGTTTTCACGGCTGGAAACTACTGGTTTTGGATCCCCATTTTTGCCCCACTTCTT
GGTTCTATACTTGGTATTCTGGTATATCAATTAATGATCGGCATACACCTTGAGCCTAAA
```

GAGCAGAGTTCACCC--
---------GTTGGGGAA---------GAAAATCTAAAACTCGCA---AATGTGAAATTA
AGAGAAGGTTGC---

>Smalltooth_sawfish_Aqp3C2
ATG------------------------------GGAAAACAG------------------
---------------------AAGGCAATGATCAAAAAACTTGAAAATTCATTC------
AGAATACAAAATATGTTGGGAAGACAATGTCTTGCTGAATGTTTAGGGACATTGATTCTT
GTGTTATTTGGTTGTGGAGCACTGGCCCAAGTTACCCTCAGCAGGGGTACACATGGACGT
TTTTTGACTGTTAATTTTGCCTTCGGATTTTCAGTGATGCTCGGTGTGCTCATAGCTGGC
CAAGTGTCAGGAGCACACCTGAATCCTGCCGTGACCTTTGCCATGTGCTTACTTGCCCGT
GAACCCTGGATAAAGTTTCCCCTTTACTCTTTGTCACAAATACTAGGTGCCTTCCTAGGT
TCGGGCGTTATCTTT---GGTTTGTATTTTGATGCTATGTGGGACTTCAGTGGTCAAAAT
CAACTATTAGTTTATGGTCCTAAT---GCCACAGCTGGTATTTTTGCTACATATCCATCT
CCACACTTAAGTTCATCCAATGGCTTTTTTGATCAGATGATTGGGACGGCTGCTCTCATA
GTGTGCATCCTTACTATTGTGGACAAATGGAACAACCCAGTGCCAAAGGGACTGGAGGCA
TTTACTATTGGATTTACGGTGCTGGTAATTGGCTTGTCGATGGGTTTCAACTCTGGGTAT
GCTGTGAACCCTGCAAGAGACCTTGGACCTCGTTTGTTTACATCAGTAGCTGGCTGGGGA
TCTGAGGTTTTCACGGCTGGAAACTACTGGTTTTGGATCCCTATTTTTGCCCCACTCCTT
GGTTCTGTTCTTGGTATTCTGGTATATCAACTAATGATTGGCATACACCTTGAGCCTGAA
GACCAGAGTTCACCC--
---------ATTGGAGAA---------GAAAATGTAAAACTGGCA---AATGTAAAATTA
AGAGAAAGTTGC---

>Ghost_shark_Aqp3C2
ATG------------------------------GGGAAACAG------------------
---------------------AAAGAAACCATCAGGAAAATTCAAGACTTATTC------
CGTATAAGAAACATGCTGGTAAAACAATGCCTTGCTGAATGTCTGGGGACTTTAATTCTC
GTGCTGTTTGGCTGCGGGGCACTGGCACAGATCACTCTCAGTCGGGGAACGCACGGAGCC
TTTCTAACAGTCAACTTAGCCTTCGGATTTGCGGTGACTCTTGGTGTGCTTGTGGCCGGT
CAAGTGTCGGGAGCTCACCTGAATCCTGCTGTGACCTTTGCTTTGTGTCTACTTGCTCGT
GAGCCCTGGATCAAATTTCCCCTTTTCTCTTTGGCACAAATATTGGGTGGATTTCTTGGA
GCAGGTATCATCTTT---GGATTATATTTTGATGCAATTTGGCTCCATGGT---AATAAC
CATCTGATAGTAATGGGACCTAAT---GCTACTGCTGGGATATTTGCGACTTACCCGTCT
GAACACTTGACTTTAATCAATGGTTTCTTTGATCAGCTGATTGGCACAGCAGCTCTCATC
GTCTGTATCCTTGCCATTGTGGATCCATTCAACAACCCGGTGCCAAAGGGAGTGGAGGCC
TTCACTATTGGCTTTGTGGTTCTGGTAATCGGCTTGGCAATGGGCTTTAACTGTGGCTAC
GCTGTGAACCCAGCCAGAGATTTTGGACCTCGCTTGTTTACCTCACTGGCTGGCTGGGGC
ACCAAAGTCTTCAGTGCTGGAAACTACTGGTTTTGGGTGCCCATTCTAGCCCCGCTCCTT
GGTGCAGTGCTTGGTATTCTGGTTTACCAGCTGATGGTTGGCATACACACCGAACCCGAA
GAAAACCATTCATCC--
---------AGTGCAGAA---------GAAAGAATAAAACTGGCC---AACATGAAGCCG
AAAGAAAACTGC---

>B_bambooshark_Aqp3C1
ATG------------------------------GAAAGACAA------------------
---------------------AAAGAAATCATCAGAAATATGACAACTATCCTG------
AAAGTCCGAAGTCTTCTGTTGAAACAATGCCTTGCTGAATGTTTAGGAACTTTGATTCAC
ACGATGCTTAGCTGTGGAGGAGTAGCACAATTTACTCTCAGTTATGGCACACACAAAGAA
TTCTTGACTGTTACAATTGCTGGTGGATTTGCAGTAGCTCTGGGTATATTGGTAACTAGT
AAAGTCTCAGGAGCTCACCTGAATCCTGCAGTGACCTTTGCTTTGTGCTTGCTTGCTTGT
GAACCTTGGTTAAAATTTCCTTTATTCTTTTTGGCACAAACGTTTGGTGCCTTTCTTGGA
TCAGGAATAATGTTT---GGTTTGTACTATGATACATTGTGGCATTATGGC---AATAAA
CAGCTAACAGTAATTGGAGCAAAT---TCTACTGCTGGAATATTTACAACTTATCCACAT
GAACATTTGAGCGCAGTCAATGGCATTTTTAATCAGGCAATTGGGACTGCAGCACTTATA
CTTTGTATCCTTATCATTGTGGATCCATTGAACGATGCTATACCAACAGGACTAGAAGCT
TTTACAATTGGCTTTGTGGTTTTGCTAATTGGCTGGTCAATGGGTTCCAATTCTCAGTAC
TCATTAAATCCTGCCAGAGATATTGGACCTCGTCTGTTTACTGCAATTGCTGGTTGGGGA

ACTGAAGCTTTCACGGCTGGCAATTATTGGTTTTGGATCCCAGTTGCCAGTCCAATCATT
GGTGCCATATTTGGTGTTCTCCTGTACAAGTTCATTGTTGGATTGCGTGCTGAAGCAAGA
ACCAGCTGCCCATCC---
---------AGTGCAGAA---------CAAAATATAAAGTTAATG---AACCAAAAACCA
AAGAGGAGGCTC--

>W_bambooshark_Aqp3C1
ATG-------------------------------GAAAGACAA-----------------
---------------------AAAGAAATCATCAGAAATATGACAACTATCCTG------
AAAGTCCAAAGTCTTCTGTTGAAACAATGCCTTGCTGAATGTTTAGGAACTTTGATTCAC
ACGATGCTTAGCTGTGGAGGAGTTGCACAATTTACTCTCAGTTATGGCACACACAAAGAA
TTCTTGACTGTTACAATTGCTGGTGGATTTGCAGTAGCTCTGGGTATATTGGTAACTAGT
AAAGTCTCAGGAGCTCATCTGAATCCTGCAGTGACCTTTGCTTTGTGCTTGCTTGCTTGT
GAACCTTGGTTAAAATTTCCTTTATTCTTTTTGGCACAAACGTTAGGTGCCTTTCTTGGA
TCAGGAATAATGTTT---GGTTTGTACTATGATACATTGTGGCATTATGGC---AATAAA
CAGCTAACAGTAATTGGAGCAAAT---TCTACTGCTGGAATATTTACAACTTATCCACAT
GAACATTTGAGCGCAGTCAATGGCATTTTTAATCAGGCAATTGGGACTGCAGCACTTATA
CTTTGTATCCTTATCATTGTGGATCCATTGAATGATGCTATTCCAACAGGACTAGAAGCT
TTTACAATTGGCTTTGTGGTTTTGCTAATTGGCTGGTCAATGGGTTCCAATTCTCAGTAC
TCATTAAATCCTGCCAGAGATATTGGACCTCGCCTGTTTACTGCAATTGCTGGTTGGGGA
ACTGAAGCTTTCACGGCTGGCAATTATTGGTTTTGGATCCCAGTTGCCAGTCCAATCATT
GGTGCTATATTTGGTGTTCTCCTGTACAAGTTCATTGTTGGATTGCGTGCTGAAGCAAGA
ACCAGCTGCCCATCC---
---------AATGCAGAA---------CAAAATATAAAGTTAATG---AACCAAAAACCA
AAGAGGAGGCTC--

>Whale_shark_Aqp3C1
ATG-------------------------------GAAAAACAA-----------------
---------------------AAAGAAATCATCAGAAAAATGACAACTGTCCTG------
AAAGTCCGAAGTCTTCTGATGAAACAATGCCTTGCTGAATGTTTAGGAACTTTGATTCAC
ACAATGCTTAGCTGTGGAGCAGTAGCACAATTTACTCTCAGTTATGGCACACACAAAGAA
TTCTTGACTGTTACAATTGCTGGTGGATTTGCAGTAGCTCTGGGTATATTGGTAACTAGT
AAAGTCTCAGGAGCTCACCTGAATCCTGCAGTGACCTTTGCTTTGTGCTTGCTTGCTAGT
GAACCTTGGTTAAAATTTCCATTATTCTTTTTGGCACAAACATTAGGTGCCTTTCTTGGA
TCAGGAATAATGTTT---GGTTTGTATTATGATGCATTGTGGCATTATGGT---AATAAA
CAGCTAACAGTAATTGGAGCAAAT---TCTACTGCTGGAATATTTGCTACTTATCCACAT
GAACATTTGAGTGCAGTCAATGGCATTTTTGATCAGGCAATTGGGACTGCAGCACTTATA
CTTTGTATCCTTATCATTGTGGATCCATTAAACAACTCAGTACGGACAGGACTAGAAGCT
TTTACAATTGGCTTTGTGGTTTTGCTAATTGGCTGGTCAATGGGTTCAAATTCCCAATAC
TCATTAAATCCTGCCAGAGATATTGGACCTCGCCTGTTTACTGCAATTGCTGGTTGGGGA
ACTGAAACTTTCACGGCTGGTAATTATTGGTTTTGGATTCCAATTGCCAGTCCAATCATT
GGTGCCATATTTGGTGTCCTACTGTATCAGTTCATTGTTGGATTGCGTGCTGAAGCAAGA
ACCAGCCGCCCAACT---
---------AATGCAGAA---------GAAAATGTAAAGTTAATG---AGTCAGAAACCA
AAGGGGAGGCTCCGA---

>Cloudy_catshark_Aqp3C1
ATG-------------------------------GAAAAACAA-----------------
---------------------AAAGAAATCCTGCGAAAAATGACTACAATGCTA------
AAGGTCCGAAGCCTTCTGATGAAACAATGCCTTGCTGAATGTTTAGGGACTTTAATTCAT
ACAATGCTGAGCTGTGGAGCGACAGCGCAGTTTGTTCTGAGTTGTGGTGCACACAAAGAC
TTTTTGACTGTTACTTTTGCTGGTGGATTTGCAGTGGCTCTGGGTATATTGGTAACTAGT
AAAGTCTCAGGAGCTCACCTGAATCCCGCAGTGACCTTTGCTTTGTGCTTACTCGCCTGT
GAACCCTGGTTAAAATTTCCTTTCTTCTTTTTGGCACAAACATTAGGTGCCTTCCTTGGA
TCAGGAATAATGTTT---GGTTTGTATTACGATAAATTGTGGCATTATGGT---AATAAA
CAGCTGACAGTATTTGGAGCAAAC---TCTACTGCTGGAATATTTGCTACTTATCCACAT
GAACATTTGAGTGCAGTAAATGGCATTTTTGAACAGGCAATTGGGACTGCGGCACTTATA
CTTTGCATCCTCATCATTGTGGACCCATTGAACAACTTAGTGCCAACAGGACTGGAAGCT

TTTACCATTGGCTGTGTGGTTCTGATGATTGGCTGGTCAATGGGTTTAAATTCCCAGTAC
TCATTAAATCCCGCCAGAGATATTGGACCTCGCTTGTTTACTGCAATTGCTGGTTGGGGA
TCTGAAGTTTTCAGTGCTGGCAACTATTGGTTTGGATCCCAGTTGCCAGCCCAATCATT
GGTGCCATACTTGGTGTTCTGATCTATCGTTTCATTGTTGGATTGCGAGCTGAAGCAAAA
ACCAGTTGCTCACCC---
---------AATGCAGAA---------CAAAATGTAAAGTTATTG---AGCCAGAAACCA
AAGGGGAGGTGC--

>Blue_shark_Aqp3C1
ATG------------------------------GAGAAACAA------------------
---------------------AAAGGAATCCGCAGAAAAATGACGATTATGCTG------
AAGGTTCGAAGTCTTCTAATGAAGCAATGCCTTGCTGAATGTTTAGGAACTTTAATTCAT
ACAATGCTTAGCTGTGGAGCAGGAGCACAATATGTTCTCAGTTATGGGACACACAAAGAC
TTTTTGACTGTTACTTTTGCCAGTGGATTTGCAGTGGCTCTGGGTATATTGGTAACTAAT
AAAGTCTCAGGAGCCCACCTGAATCCTGCAGTGACCTTTGCTTTGTGCTTACTCGCTTGT
GAACCTTGGTTAAAATTTCCTTTCTTCTTTTTGGCACAAACATTAGGTGCCTTTCTTGGA
TCAGGAATAATGTTT---GGTTTGTATTATGATAAACTGTGGCATTATGGT---AATAAA
CAGCTAACAGTAATTGGAGCAAAC---GCTACTGCTGGAATATTTACTACTTACCCACAT
GAACATTTGAGTGCAGTAAATGGCATTTTTGATCAGGCAATTGGGACGGCAGCACTTATA
CTTTGCATCCTTATCATTGTGGACCCATTGAACAATTCAGTGCCAACAGGGCTGGAAGCT
TTTACCATTGGCTGTGTAGTTCTGATGATTGGCTGGTCAATGGGTTCAAACTCCCAGTAC
TCATTAAATCCTGCCAGAGACATTGGACCCCGCTTGTTCACTGCAATTGCTGGTTGGGGA
TCTGAAGTTTTCACTGCTGGCAATTATTGGTTTGGATCCCAGTTGCCAGCCCAATCATT
GGTGCCACATTTGGTGTTCTGGTCTACAAGTTCATTGTTGGACTGCGAGATGAAGCAAGA
ACCAGCTGCCCATCC---
---------AATGGAGAA---------CGAAATGAAAAGTTAATG---AGCCAAAAACCA
AAGGGAAGGCGC--

>Great_white_shark_Aqp3C1
ATG------------------------------GAAAAA---------------------
---------------------AAAGAAATCCTCAGAAAAATGACGACTATACTG------
AAGGTCCAAAGTCTTCTGATGAAACAATGCCTTGCTGAATGTTTAGGAACTTTAATTCAT
ACCATGCTTAGCTGTGGAGTGATAGCACAATTTGTTCTCAGTTATGGTACGCACAAAGAG
TTTTTGACTGTTACTTTTGCCAGTGGATTTGCAGTAGCTCTGGGTATATTGGTAACGAGT
AAAGTCTCAGGAGCTCACCTGAATCCTGCAGTGACCTTTGCTTTCTGCTTACTTGCTTGT
GAACCCTGGTTAAAATTTCCCTTCTTCTTTTTGGCACAAACATTAGGTGCCTTTCTTGGA
TCAGGAATAATGTTT---GGTTTGTATTATGATAAATTGTGGCATTATGGT---AATAAA
CAGCTAACAGTAATTGGAGCAAAC---TCTACTGCTGGAATATTTACTACTTATCCACAT
GAACATTTGAATGTAGTAAATGGCATTTTTGATCAGGCAATTGGGACTGCAGCGCTTATA
CTTTGTATCCTTATCATTGTGGACCCATTGAACAACTCAGTGCCAACAGGACTGGAAGCT
TTTACCATTGGCTTTGTGGTTCTGATAATTGGCTGGTCAATGGGTTCAAATTCCCAGTAC
TCATTAAATCCTGCCAGAGATATTGGACCTCGCTTGTTTACTGCAATTGCTGGTTGGGGA
TCTGAAGTTTTCAGTGCTGGCAGCTATTGGTTTGGATCCCCGTTGCCAGCCCAATCATT
GGTGCCATATTTGGTGTCCTGATGTATCAGTTCATTGTTGGATTGCGAACTGAGGCAAGA
ACCAACTGCTCACCC---
---------AATGCAGAA---------GAAAATGTAAAGTTAATG---AGCCAGAAACCA
AAGGGGAGGTGC--

>Zebra_bullhead_shark_Aqp3C1
ATG------------------------------GAAAAACAA------------------
---------------------AAAGAAATGCTCAGAAAAATGACAACTATACTG------
AAAGTCCGAAGCCTTCTGCTGAAACAATGTCTTGCTGAATGTTTAGGAACTTTAATTCAT
ACAATGCTTAGCTGTGGAGCAGTGGCACAATTTACTCTCAGTTATGGTACACAGAAAGAA
TTCTTGACTGTTACTTTTGCCATTGGATTTGCAGTAGCTCTGGGTATATTGGTAACTAGT
AAAGTGTCAGGAGCTCACCTGAATCCTGCAGTGACCTTTGCTTTGTGCTTGCTTGCTTGT
GAGCCTTGGTTAAAATTCCCCTTCTTCTTTGTGGCACAAACATTCGGTGCCTTTCTTGGA
TCAGGAATAATGTTT---GGTTTGTGTTACGATAATTTGTGGCATTATGGT---AATAAA
CAGCTAACAGTAATTGGACCAAAC---TCTACTGCTGGAGTATTTACTACTTATCCACAT

GAACATTTGAGTGCAGTAAATGGCATTTTTGATCAGGCAATTGGGACTGCAGCTCTTATA
CTTTGTATCCTTATCATTGTGGATCCATTGAACAACTCAGTGCCAACAGGACTGGAAGCC
TTTACCATTGGCTTTGTGGTTCTGATAATTGGTTGGTCAATGGGTTCAAATTCCCAGTAC
TCATTAAATCCTGCCAGAGATATTGGACCTCGCTTGTTTACTGCAATTGCTGGTTGGGGA
TCTAAGGTTTTCACTGCTGGCAACTATTGGTTTTGGATCCCAATTGTCAGCCCAATCATT
GGTGCCATTTTTGGTGTTCTGATGTATCAGTTCATTATTGGATTGCGTATTGAAGCAAGG
CCCAGCTGCTCACCC---
---------ATTGCAGAA------------AATGTAATGTTAATG---AGCCAGAAACCA
AAGGGGAGACGCCGA---

>Spiny_dogfish_Aqp3C1
ATG------------------------------GGAAAACAA------------------
----------------------AAAGAGATCCTCAGAAAAATGACAACGACACTG------
AAAGTCCGAAGTATTCTGGTGAAACAATGTCTTGCTGAATGTTTAGGAACTCTAATTCAT
ACAATGCTTAGCTGTGGAGCAATAGCACAATTTACTCTCGGTTATGGTACACACAAAGAA
TTTTTGACGGTTACTTTTGCCATCGGATTTGCAGTAGCTCTGGGTATAATGGTAACTAGT
AAAGTGTCAGGAGCTCACCTGAATCCTGCAGTGACCTTTGCTTTGTGCTTGCTTGCTTGT
GAGCCTTGGTTAAAATTCCCCTTCTTCTTTTTGGCACAAACATTCGGTGCCTTTCTTGGA
TCAGGAATAATGTTT---GGTTTGTATTACGATAAATTGTGGCATTATGGT---AATAAA
CAGCTAACAGTAATGGGACCAAAC---TCTACTGCTGGAATATTTACTACTTATCCGCCA
GAACATTTGAGTGCAGTCAGTGGCATTTTTGATCAGGCAATTGGGACGGCAGCTCTGATA
CTTTGTATCCTTATCATTGTGGATCCAATGAACAAGCCAGTGCCAACAGGACTGGAAGCC
TTTACCATTGGCTTTGTGGTTCTGATAATTGGCTGGTCAATGGATTCAAATTCCCAGTAC
TCATTAAATCCTGCCAGAGATATTGGACCTCGCTTGTTTACTGCAATTGCTGGTTGGGGA
TCTGAAGTTTTCACTGCTGGAAACTATTGGTTTTGGATCCCACTTGTCAGCCCAATCATT
GGTGCCATTTTTGGTGTTCTGATGTATCTATTCATTGTTGGATTGCGTGTTGAAGCAAGA
AGCGGCTGCTCACCC---
---------AATGCAGAA---------CAAAATGTAAAGTTAATG---AGCCAGAAACCA
AAGGGGAGGCGC--

>Little_skate_Aqp3C1
ATG------------------------------GGGAAACAA------------------
----------------------GAGGAGATCCTCAGAAAATTGGCAATTCTGTTG------
AGAATTCGAAGTATTCTAGTAAAGCAATGCCTGGCCGAATGCTTGGGAACATTAATTCAT
ACCATGCTTAGCTGTGGAGCAATAGCACAGTTCACACTTAACTGTGGCACACACAGTCAA
TTTCTGTCTGTCACCTTTGCCATAGGATTTGCAGTAGCTCTGGGCATATTGGTAACCAGC
AAAGTGTCAGGAGCTCACCTGAATCCAGCAGTGACCTTTGCTTTGTGCTTACTTGCTTGT
GAGCCTTGGTTAAAATTCCCTTTCTTCTTTTTGGCACAAACAGTGGGTGCCTTTCTTGGA
TCAGGAATAATGTTT---GGTTTGTATTATGATAAATTGTGGCTTCATGGA---AATAAA
CAGCTAACGGTAATTGGACCAAAC---TCAACTGCTGGGATATTTACAAGTTTTCCACTT
GAACATGTGAGTGCACTCAATGGCATTTTTGATCAGGTGATTGGGACTGCAGCTCTCATA
TTGTGTATCCTTATCATTGTGGATCCTTTACACACCCCAGTGCAGACGGGACTGGAAGCT
TTCACCATCGGTGTAGTGGTTCTTATTATTGGCTGGTCAATGGGATCAAATTCCCAGTAC
TCATTAAATCCTGCCAGGGATATTGGACCTCGCTTGTTTACGGCTATTGCTGGTTGGGGA
TTTGAAGTTTTCACGGCTGGAAGCTATTGGTTTTGGATTCCACTTGTCAGCCCAATCATT
GGTGCTATTTTGGGTGTTCTGATGTACCAATTCACTGTGGGATTACGTGTTGATACAAGA
GATGACTCCTCATCC---
---------AATGCAGAG---------CAAAATGTAAAATTAATG---GGCCAGGAAACA
AAGGGAAGGTGC--

>Thorny_skate_Aqp3C1
ATG------------------------------GGGAAACAA------------------
----------------------GAGGAGATCCTCAGAAAAATGGCAATTCTGTTG------
AGAATTCGAAGTATTCTAGTAAAGCAATGCCTGGCCGAATGCTTGGGAACATTAATTCAT
ACCATGCTTAGCTGTGGAGCAATAGCACAGTTCACACTTAACTGTGGCACACACAGTCAA
TTTCTGTCTGTCACCTTTGCCATAGGATTTGCAGTAGCTCTGGGCATATTGGTAACCAGC
AAAGTGTCAGGAGCTCACCTGAATCCAGCAGTGACCTTTGCTTTGTGCTTACTTGCTTGT
GAGCCTTGGTTAAAATTCCCTTTCTTCTTTTTGGCACAAACAGTGGGTGCCTTTCTTGGA

TCAGGGATAATGTTT---GGTTTGTATTACGATAAATTGTGGCTTCATGGA---AATAAA
CAACTAACGGTAATTGGACCAAAC---TCAACTGCTGGGATATTTACAAGTTTTCCACTT
GAACATGTGAGTGCACTCAATGGCATTTTTGATCAGGCAATTGGGACTGCAGCTCTCATA
TTGTGTATCCTTATCATTGTGGATCCTTTACACACCCCAGTGCAGACGGGGCTGGAAGCT
TTCACCATCGGTGGTGTGGTTCTTATTATTGGCTGGTCAATGGGATCAAATTCCCAGTAC
TCATTAAATCCTGCCAGGGATATTGGACCTCGCTTGTTTACGGCTATTGCTGGTTGGGGA
TCTGAAGTTTTCACGGCTGGAAGCTATTGGTTTTGGATTCCACTTGTCAGCCCAATCATT
GGTGCTATTTTGGGCGTTCTGATGTACCAATTCACTGTGGGATTACGTGTTGATACAAGA
AATGACTCCTCATCC---
---------AATGTAGAG---------CAAAATGTAAAATTAATG---GGCCAGGAAACA
AAGGGAAGGTGC--

>Smalltooth_sawfish_Aqp3C1
ATG-------------------------------GGGAAACAA------------------
---------------------GAGAAGATCCTCAGGAAAATGACGATTGTGCTG------
AGAATTCGAAGTATTCTGATAAAGCAATGCCTGGCTGAATGTTTGGGAACTCTAATTCAT
ACAATGCTTAGCTGTGGAGCAATAGCACAATTTACACTCAACTGTGGCACACACAAAGAA
TTTCAGTCCGTAACTTTTGTAATTGGATTTGCTGTAGCACTGGGCATATTGGTAACCAGT
AAAGTGTCAGGAGCTCACCTGAATCCAGCAGTGACCTTTGCTTTGTGCTTGCTTGCTTGT
GAGCCTTGGTTAAAATTCCCCTTCTTCTTTATGGCCCAAACAATAGGTGCCTTTCTTGGA
TCAGGAATAATGTTT---GGATTGTTATTAGATAAATTGTGGCTTCATGAT---AATAAA
CAGCTAACAGTTATTGGACCAAAC---TCGACTGCTGGGATATTTACAACTTTTCCACTT
GAACATGTGAGTGCACTCAATGGCATTTTTGATCAGGCAACTGGAACTGCAGCTCTCATA
TTTTGTATCCTTATCATTGTGGATCCTTTGCACACCCCAGTGCGAACAGGGCTGGAAGCC
TTTACCATTGGCTTAGTAGTTCTGATTATTGGCTGGTCAATGGGTTCAAACTCCCAGTAC
TCACTAAATCCTGCCAGGGATATTGGACCTCGCTTGTTTACAGCAATTGCTGGTTGGGGA
TCTGAAGTTTTCACGGCTGGAAGCCATTGGTTTTGGATCCCGCTTGTCAGCCCAATCATT
GGTGCCATTTTGGGTGTTTTGATGTACCAGTTCATTGTTGGATTACGTGTTGATGCAAGA
AATGGCTGCTCATCC---
---------AATGCAGAA---------CAAAATGAAAAATCAGTG---AGCCAGAAAACG
AAGGGAAGGTGC--

>Ghost_shark_Aqp3C1
ATG-------------------------------GGGATACAA------------------
---------------------AGAGAAACCCTGAGAAGGATGGCAACGACATTC------
AGAGTGCGGAGCGTGCTGGCAAAGCAATGCCTGGCGGAATGTTTGGGAACGTTGATCCAC
ACGATGCTCAGCTGTGGAGCAATGGCACAATACACCCTCAGTCGCGGTGAGCAGAGAGAG
TTTTTGACTCTTACTTTCACCCTTGGCTTTGCGGTGGCTCTTGGTATACTTGTAACCGGT
AAAGTGTCAGGAGCTCACTTGAATCCTGCTGTGACCTTTGCTTTGTGCTTACTTGCTTGT
GAACCCTGGATAAAATTCCCCTTCTTCTTTGTGGCACAAACATTGGGAGCATTTTTTGGA
TCTGGGATAATGTTT---GGCTTGTATCACGATGCAATAATGCACTATGGT---AACAAT
CAACTCACAATCATTGGACCCAAT---TCCACTGCTGCTATATTTACCACATACCCATCC
GAAGATTTGACTGTGGTGAATGGGTTTCTTGACCAGGTCATCGGCACGGCCGCTCTCATA
TTCTGTATCCTGACCATCGCGGATCCACTGAAAAGACCGGTGGCCACAGGGCTGGAGGCT
TTCACCATAGGCTTTGTGGTTCTAATCGTTGGCTTGTCCATGGGCTTCAACTCCAAGTAT
TGCTTAAATCCTGCCAGAGACATTGGACCTCGTTTGTTCAGCGCCATGGCTGGCTGGGGA
TATGACGTTTTCAGCACAGGGAACTATTGGTTTTGGGTGCCAGTTCTCAGCCCAATCGTC
GGGGCCATGTTTGGCGTTCTGGTCTATCAGTTCATGGTGGGTCTGCAGGTTGAGACAAAA
AGTAACTGCACGTCC---
---------CATTCAGAG---------GAAACCGTAAAGTTAACC---AGCGTGAAAGCG
AAGGGCAGGCGT--

>Sea_lamprey_Aqp3L1
ATG-------------------------------CCGGTGGTA------------------
---------------------GAGGGGCGAATTAAGAGGCTGCAGCGGAGGCTG------
TTCATCCGAAATACGCTTGTCTGCGAGGCGATGGCAGAGTTTGTGGGGACGTACATTCTG
GTTTTGTTTGGGTGTGGCTCGGTGGCGCAGGTTGAATTGAGTGGAGGACTCAAGGGGCAG
TTCCTCACGATCAACCTCGCCTTTGGGTTTGCGGTCACCATGGGACTGCACGCCGCTGCA

GGCGTTTCAGGTGGACACCTGAACCCAGCCGTTACATTTGCGTTTGCCGTTCTGGGTCGC
TTCGAATGGCACAAGTTACCTCTCTACATGCTGGCTCAGCTCCTCGGAGCTTTCATGGGC
GCAGGCACCGTGTTC---GGACTGTATTATGATGCCTTCCTGTTCGCCGCC---AATGGG
AACTACACAATT---------------CAGCTGGCTGGAGTTTTTGCCACTTACCCGTCA
GAGCACCTGTCTCTGGGAAATGGATTTGTGGATCAGCTGATTGGCACTGCAGCCCTGCTG
GTGTGCATCATGGCGGTGATAGACAAGCGTAACAACCCGGCTCCAAAGGGCATGCAGCCC
TACATAATAGGCCTCGTCGTCGTCCTCATTGGCCTCTCCATGGGCTTCAACGCGGGGTAC
GCCGTGAACCCGGCCCGTGACCTCGGCCCGCGTCTCTTCACGTCCATTGCTGGATGGGGT
TGGGCAGTCTTTTCGTCCGGTAACTACTGGAGCTGGGTGCCTGTGGTGGCACCCATGATC
GGTGGGGTGCTCGGTGCGTTTTTTTACGAGCTGTTCGTTGGATTTCATCTTATGGAGGCG
TCACTCACGCCAGCG---------------------------------GTGGAG------
CCCCCACCGTCTCCACGA---------GAAAAAAAGAGCCTCCTC---AAGCTCCAACCG
GAAAGCATGGCTGATGTCATGTGC------------------------------------

>Arctic_lamprey_Aqp3L1
ATG------------------------------CCGGTGCAA------------------
---------------------GAGGGGCGAATGAAGAGGCTGCAGCGGAGGCTG------
TTCATCCGAAATAGGCTGGTCTGCGAGGCGATGGCAGAGTTTGTGGGGACGTACATTCTG
GTTTTGTTTGGGTGTGGCTCGGTGGCGCAGGTTGAATTGAGTGGAGGACTCAAGGGGCAG
TTCCTCACGATCAACCTCGCCTTTGGGTTTGCGGTCACCATGGGACTCCACGCCGCTGCA
GGCGTTTCAGGTGGACACCTGAACCCAGCCGTTACATTTGCGTTTGCCGTTCTGGGTCGC
TTCGAATGGCACAAGTTACCTCTCTACATGCTGGCTCAGCTCCTCGGAGCTTTCATGGGC
GCAGGCACCGTGTTC---GGACTGTATTATGATGCCTTCCTGTACGCCGGC---AATGGG
AACTACACAATT---------------CAGCTGGCTGGAGTTTTTGCCACTTACCCCTCA
GAGCACCTGTCTCTGGGAAATGGATTTGTGGATCAGCTGATTGGCACTGCAGCCCTGCTG
GTGTGCATCATGGCGGTGATAGACAAGCGAAACAACCCAGCTCCAAAGGGCATGCAGCCC
TACATAATAGGCCTCGTCGTCGTGCTCATCGGGCTCTCCATGGGCTTCAACGCGGGGTAC
GCCGTGAACCCGGCCCGTGACCTCGGCCCGCGTCTCTTCACGTCCATTGCTGGATGGGGT
TGGAAAGTATTTTCGTCCGGTGGCTACTGGAGCTGGGTGCCTGTGGTGGCACCCATGATC
GGTGGGGTGCTCGGTGCGTTTGTTTACGAGCTGTTCGTTGGATTTCATCTTGTGGAGGCG
TCACTCCCGCCGGCG---------------------------------GTGGAG------
CCCCCACCGTCTCCACGA---------GAAAAAAAGGGGCTCCTC---AAGCTCCAGCCA
GAAAGCATGGCTGACGTCATGTGC------------------------------------

>Sea_lamprey_Aqp3L2
ATG------------------------------CCGTCGCAC------------------
---------------------GATGGTCGCTTCAAGCAGCTGGAGCGCAAGCTG------
CACGTGAAGAACGTGATGATCCGTGAGGCCTTGGCCGAGTTCATGGGAACCTTCCTTCTC
GTCCTGTTTGGCTGCGGATCGGTGGCGCAGGTGGAGCTGAGCGACGGCACCAAGGGGCGG
TTCCTCACCATCAACCTCGCCTTCGGCTTCGCCGTCACCATGGGCGCCTACTGTGCGGCC
GGCGTCTCCGGCGCGCACTTGAACCCGGCGGTGTCGATGGCGCTGGCGGTGCTCGGCCGC
TTCTCTTGGAGCAAGTTCCCCTTGTACGTCACGGCTCAGCTGCTCGGGGCCTTCATGGGC
GCCGGCACCGTCTTC---GGCCTCTACTACGACGCCTTCATGTACGTCTCC---AAAGGG
AACCTGACGCTG---------------CAGCTCGCAGGGGTCTTCGCCACCTTCCCCTCG
CCGCATCTCTCCATCGGAAACGGATTTGTGGATCAGCTGATCGGCACGGCGGCGCTGCTC
GTGTGCATCCTCGCCGTGATCGACAAGCGCAACAACCCGGCGCCGCGCGGCATGCAGCCC
TTCCTCATCGGCCTCGTCGTCGTGCTCATCGGCCTTTCCATGGGCTTCAACGCCGGCTAC
GCCGTGAACCCCGCGCGCGACCTCGGCCCCCGCATCTTCACCGCCCTCGCCGGCTGGGGC
TGGCAGGTCTTCTCGGCGGGCAACTACTGGAGCTGGGTGCCCGTGGTGGCCCCCATGCTG
GGCGGCGTGCTGGGGGCCTTCATCTACGAGATCTTCATTGGCCTGCACCTCCCCGAAGAG
CCGGCCTGCTCCGGA---------------------------------CCCGAG------
---CCCGGGACCCCGCGG---------TCGCACCAGGAGCTCGCC---AAGCTGCAGTCC
---AGCATGGCAGACGCTATGTGC------------------------------------

>Arctic_lamprey_Aqp3L2
ATG------------------------------CCGTCGCAC------------------
---------------------GATGGTCGCTTCAAGCAGCTGGAGCGCAAGCTG------
CACGTGAAGAACGTGATGATCCGTGAGGCCTTGGCGGAGTTCATGGGGACCTTCCTTCTC

GTCCTGTTTGGCTGCGGCTCAGTGGCGCAGGTGGAGCTGAGCGACGGCACCAAGGGACGG
TTCCTCACCATCAACCTCGCCTTCGGCTTCGCCGTCACCATGGGCGCCTACTGTGCCGCC
GGCGTCTCCGGCGCGCACTTGAACCCGGCGGTGTCGATGGCGCTGGCGGTGCTCGGCCGC
TTCTCTTGGAGCAAGTTCCCCTTGTACGTCACCGCTCAGCTGCTCGGGGCCTTCATGGGC
GCCGGCACCGTCTTC---GGCCTCTACTACGACGCCTTCATGTACGTCTCC---AAAGGG
AACCTGACGCTG---------------CAGCTCGCAGGAGTCTTCGCCACCTTCCCGTCA
CCGCATCTCTCCATCGGAAACGGATTTGTTGATCAGGTGATCGGAACTGCGGCGCTGCTC
GTGTGTATCCTCGCCGTGATCGACAAGCGCAACAACCCGGCGCCCCGCGGCATGCAGCCC
TTCCTCATCGGCCTCATCGTCGTGCTCATCGGCCTCTCCATGGGCTTCAACGCCGGCTAC
GCAGTGAACCCCGCACGCGACCTCGGCCCCCGCATCTTCACCGCCCTCGCCGGCTGGGGC
TGGCAGGTCTTCTCGGCGGGCAACTACTGGAGCTGGGTGCCTGTGGTGGCGCCCATGCTG
GGCGGCGTGCTGGGCGCCTTCATCTACGAGATCTTCATCGGCCTGCACCTCCCCGAAGAG
CCGGCCAACTCCGGG---------------------------------CCCGAG------
---CCCGGGACCCCGCGG---------TCGCACCAGGAGCTCGCC---AAGCTGCAGGCC
---AACATGGCAGACGCCATGTGC------------------------------------

>Inshore_hagfish_Aqp3
ATG------------------------------TCGACAGGA------------------
---------------------GGGAGCAAGCTGCGGGTCCTCGCACAGCGAATG------
CGCGTCACGAACCCCCTAATCCGAGAAGCGTTCGCCGAAGGTTTGGGAACATTTGTTTTG
GTGCTATTTGGTTGCGGATCCGTAGCACAGATGGTCTTT---CATGGCCCGGGTGTTCCT
TTCCTGTCTGTCAATCTGGCTTTTGGTTTGGCAGTCACGATGGGATGCTACATCGCTGGT
GGAGTGTCTGGAGCTCATCTGAACCCGGCGGTTTCTCTGGCGATGGTTGTGCTGGGCCGT
CTGACTTTGCTCAAGATGTTGGTCTACTGGGTTGCTCAGCTGCTTGGAGCCTTCATCGGA
GCCGCCATGGTCTTT---CTCATTTATCTGGACGCTGAGAAGAAACATTCG---AGACCC
---TGGAGCATG---------------GAGACTGCGGGAATATATGCCACTTATCCGAAC
ACCCACTTGTCCACCGGAGGTGGTTTCTTTGATCAGGTTATGGGAACTGCTGCACTACTT
CTGTGCATACTTGCCTTGCTCGACAAGAAAAACACAGCACCACCTGATGGGGTGACACCT
GTAATTATTGGGCTGGTTGTTGCCGTGATTGGAATGGCTATGGGGCACAACTGCGGCTAC
GCCATCAATCCTGCACGCGACCTCGGCCCTCGACTGTTCACCCTTATTGCGGGTTGGGGA
CGGGCTGTCTTTACGCACGGTAACTACTGGTTTTGGGTTCCAATCATCGCACCACTGCTC
GGCGGAGTGCTCGGCGCACTCACTTACATCTTGTTTGTGGAGTTGCATCATCCCTCTTCG
TCTGACACCGGCACC---------------------------------AATACC------
AAAGAGGACGCAGAGAAG------------CATTATGACCTGCGA---AAATTTGATTCT
---CACGCTGCCGAAACGGTGGCC------------------------------------

>Pacific_hagfish_Aqp3
ATG------------------------------TCAACAGGA------------------
---------------------GGGAGCAAGCTGCGGGTCCTCGCACAGCGAATG------
CGCGTCACGAATCCCCTTATCCGAGAAGCGTTCGCCGAAGGTTTGGGAACATTTGTTTTG
GTGCTATTTGGTTGTGGATCTGTAGCACAGATGGTCTTT---CAAGGCCCGGGTGTTCCT
TTCCTGTCTGTCAATCTGGCTTTTGGTTTGGCAGTCACGATGGGATGCTACATCGCTGGT
GGAGTGTCTGGAGCTCATCTGAACCCGGCGGTTTCTCTGGCGATGGTTGTGCTGGGCCGT
CTGACTTTGCTCAAGATGTTGGTCTACTGGGTTGCTCAGCTGCTTGGAGCCTTCATCGGA
GCCGCCATGGTCTTT---CTCATTTATCTGGACGCAGAGAAGCAACATTCG---AGACCC
---TGGAGCATG---------------GAGACCGCGGGAATATATGCCACTTATCCGAAC
GCCCACTTGTCCACCGGAGGTGGTTTCTTTGATCAGGTTATGGGCACCGCTGCACTACTT
CTGTGCATACTTGCCTTGCTCGACAAGAAAAACACAGCACCACCTGACGGGGTGACACCT
GTAATTGTTGGGCTGGTTGTTGCCGTGATTGGAATGGCTATGGGGCACAACTGCGGCTAC
GCCATCAATCCTGCACGCGACCTCGGCCCTCGACTGTTCACCCTTATTGCGGGTTGGGGA
CGGGCTGTCTTTACGCACGGTAACTACTGGTTTTGGGTTCCAATCATCGCACCACTGCTC
GGCGGAGTGCTCGGCGCACTCACTTACGTCTTGTTTGTGGAGTTGCATCATCCCTCTTCG
TCTGACACCGGCACC---------------------------------AATAAC------
AAAGAGGACGCAGAAAAG------------CATTATGACCTGCGA---AAATTTGATTCT
---CACGCCGCCGAAACGGTGGCC------------------------------------

>Atlantic_hagfish_Aqp3
ATG------------------------------TCGACCGGA------------------

----------------------GGGAGCAAGTTACGGGTCCTCGCGCAGCGTCTA------
CGCGTCACGAGACCCCTCATTCGTGAAGCGTTCGCCGAAGGTTTGGGAACTTTCGTGCTG
GTGCTATTTGGTTGTGGATCTGTAGCACAGATGGTCTTT---CGTGGCCCAGGTGTTCCT
TTCCTGTCTGTCAACCTGGCTTTTGGTTTGGCAGTCACCATGGGATGCTACATCGCTGGT
GGAGTCTCTGGAGCTCATCTGAATCCGGCGGTTTCTCTGGCTATGGTTGTCCTGGGCCGG
TTGACTTTGCTCAAGATGTTGGTCTACTGCGTTGCTCAGCTGGTTGGAGCCTTCCTTGGA
GCCGCCATGGTCTTT---CTCATTTATCTGGATGCTGAGAAGAAACACTCT---AAACCC
---TGGAGCATG---------------GAGACTGCTGGAATATATGCAACTTACCCTGGC
CCTCATTTGTCCACCGGAGGTGGTTTCTTTGATCAGGTTATGGGCACCGCCGCATTACTT
CTGTGCATACTTGCCTTGCTCGACAAGAAAAACTCAGCACCACCTGACGGGGTGACGCCT
CTGATTGTCGGGCTGGTGGTCACCGTGATTGGAATGGCCATGGGGCACAACTGCGGCTAT
GCCATCAACCCCGCACGCGACCTCGGCCCTCGGCTGTTCACCCTTATTGCGGGTTGGGGG
CGGCCTGTCTTTACGCACAGGAACTACTGGTTTTGGGTTCCGATCACCGCGCCATTTCTC
GGTGGAGTGCTCGGCGCACTCACTTACGTCTTGTTTGTGGAGTTGCATCATCCCTCCTCA
TCCGACTCCGGCACC---

>cetFrog_AQP13
ATG--------------------------------GACCTGCAT------------------
----------------------ACTCTGATCCTAACAACATTGAAAATGAAGTTG------
CGAACGAATAATCAGTACGTGCGCTGCGGCCTGGCTGAGTTACTGGGGACTCTCATATTG
ATTCTCTTTGGCTGTGGTGCGGTGGCTCAGATGGAGTTGAGCGGTCTTGCGAAGGGGCAG
TTCCTGAGTGTCAACATGGCATTTGGGTTTGCAGTGACTGCTGGGGCCTACATCTGTGCT
GGAGTGTCAGGTGCCCACCTCAACCCAGCCGTGTCCCTTTCCATGTACCTCCTCCAGAGG
ATGACTTGTAGGATGATGCTGGTCTACTTCTTGGCCCAGTTCATTGGATGTTTCATTGGG
GCTGCTCTGGTCTTC---GCTCTTTATTTTGATGCTCTTCATGTGTACAGT---GGAGGT
AACTGGACGGTTTATGGACCCCAA---GCAACAGCAGGGATATTTGCATCGTACCCATCG
GAACACCTGAGTGTTTTTAATGGCCTTTCAGATCAGGTGATTGCCACTGCTGCTTTGATG
GTCAGTATCCTGGCAGTAATAGATGAAGCCAACAATGCTGCTCCTCGAGGTCTCCAGCCT
TTTGTGATTGGTCTTGTGGTTCTTCTGGTTGGTCTATCAATGGGCTTCAACTGTGGGTAC
CCAATAAATCCTGCCAGAGACTTGGCACCTCGTATCTTCACTGCAATGGCTGGCTGGGGC
CTGGAAGTCTTCAGGGCTGGTGGGCACTGGTGGTGGGTGCCAGTGCTGGGACCAATGGTA
GGGGCAGTGGTGGGGACGCTGACTTATGAACTGCTGGTTGGAATTCACCACCCTGTGCTC
CACGAGAAGGATGAA----------------------------------CCTGAA------
GAGGATGGTGAGGAGAACCATGCC---ACACAGTATGAGTTGGTT---CAGTCTCATGCT

>goFrog_AQP13
ATG--------------------------------GACCTGCAT------------------
----------------------ACTCTGATCCTAAAAACATTGAAAATGAAGTTG------
CGAACGAATAATCAGTATGTGCGCTGCGGGCTGGCTGAGTTCCTGGGGACTCTCATATTG
ATTCTCTTTGGCTGCGGTGCTGTGGCTCAGATGGAGTTGAGCGGTCTTGCGAAGGGGCAG
TTCCTGAGCGTAAACATGGCGTTTGGGTTTGCAGTGACTGCTGGGGCCTACATCTGTGCT
GGAGTGTCAGGTGCCCACCTCAACCCAGCCGTGTCCCTTTCCATGTACCTCCTCCAGAGG
ATGACTTGTAGGATGATGCTGGTCTACTTCTTGGCCCAGTTCATTGGATGTTTCATTGGG
GCTGCTCTGGTCTTC---GCTCTTTATTTTGATGCTCTTCATGTGTACGGT---GGAGGT
AACTGGACGGTGTCTGGACCCCAA---GCAACAGCAGGGATATTTGCATCGTACCCATCG
GAACACCTGAGTGTTTTTAATGGCCTTTCAGATCAGGTGATTGCCACTGCTGCCTTGATG

---GGC
CTGGAAGTCTTCAGGGCTGGTGGGCACTGGTGGTGGGTGCCAGTGCTGGGACCAATGGTA
GGGGCAGTGGTGGGGACGCTGACTTATGAACTGCTGGTTGGAATTCACCACCCTGTGCTC
CACGAGAAGGATGAA----------------------------------CCTGAA------
GAGGATGGTGAGGAGAACCATGCC---CCACAGTATGAGTTGGTT---CAGTCTCATGCT


```
>Common_frog_AQP13
------------------------------------ATGTAT------------------
---------------------ACTCTCTTCCTAAAAACAATGAGAATGAAGTTG------
CGAACGAATAATCAGTACGTGCGCTGCGGGCTGGCCGAATTGCTGGGGACTCTCATATTG
ATTCTCTTTGGCTGTGGCGCTGTAGCTCAGATGGAGTTGAGCGGTCTTGCTAAGGGGCAG
TTCCTGAGTGTCAACATGGCGTTTGGGTTTGCAGTGACTGCTGGGGCCTACATCTGTGCT
GGAGTGTCAGGTGCCCACCTCAACCCAGCCGTGTCTCTATCCATGTACCTCCTCCAGAGG
ATGACTTGTAGGATGATGCTGGTCTACTTCTTGGCCCAGTTCATTGGATGTTTCATTGGG
GCTGCTCTGGTCTTT---GCTCTTTATTTTGATGCTCTTCATGTGTACAGT---GGAGGT
AACTGGACGGTGTCAGGAACCCAA---GCAACAGCAGGGATATTTGCATCCTACCCATCG
GAACACCTGAGCGTTTTAAATGGACTTTCAGATCAGGTGATTGCCACGGCTGCCTTGATG
GTCTGTATCCTGGCAGTAGTAGATGAAGCCAACAATGCTGCTCCTCGAGGACTCCAGCCT
TTTGTGATTGGCCTTGTGGTTCTTCTGGTTGGTCTATCAATGGGCTTCAACTGTGGGTAC
CCAATAAATCCTGCCAGAGACTTGGCACCTCGTATCTTCACTGCAATGGCTGGCTGGGGC
CTGGAAGTCTTCAGGGCGGGTGGGCACTGGTGGTGGGTGCCAGTGCTGGGACCAATGCTA
GGGGCAGTGTTGGGGACGTTTACTTATGAACTGTTGGTTGGAATTCACCACCCTATGCTT
CACGAGAAGGATGAA---------------------------------CCTGAA------
GAGGATGCTGGGGAGAACCACACC---CCACAATATGAATTGGTT---CAGTCTCATGCT
------------------------------------------------------------
------------------------------------------------------------
------------------------------------------------------------
>American_bullfrog_AQP13
------------------------------------ATGTAT------------------
---------------------GCTCTCTTCCTAAAAACACTGAAAATGAAGTTG------
CGAACGAATAATCAGTATGTGCGCTGCGGGCTGGCCGAGTTCCTGGGGACTCTCATATTG
ATTCTATTTGGCTGTGGTGCTGTAGCTCAGATGGAGTTGAGCGGTCTTGCTAAGGGGCAG
TTCCTGAGTGTCAACATGGCGTTTGGGTTTGCAGTGACTGCTGGAGCCTACATCTGTGCT
GGGGTGTCAGGTGCCCACCTCAACCCAGCCGTGTCTCTATCCATGTACCTCCTCCAGAGG
ATGAATTGTAGGATGATGCTGGTCTACTTCTTGGCCCAGTTCATTGGATGCTTCATTGGG
GCTGCTCTGGTCTTT---GCTCTTTATTTTGATGCCCTTCATGTGTACAGC---GGAGGT
AACTGGACAGTGTCTGGAACCCAA---GCAACAGCAGGGATATTTGCATCATACCCATCA
GAACACCTGAGTGTTTTTAATGGACTTTCAGATCAGGTGATTGCCACTGCTGCCTTGATG
ATCTGTATCCTGGCAGTAGTAGATGAAGCCAACAATGCTGCTCCTCGAGGACTCCAGCCT
TTTGTGATTGGTCTTGTGGTTCTTCTGGTTGGTCTATCGATGGGCTTCAACTGTGGGTAC
CCAATAAATCCTGCCAGAGACTTGGCACCTCGTATCTTCACTGCAATGGCTGGCTGGGGC
CTGGAAGTCTTCAGG---------------------------------------------
------------------------------------------------------------
------------------------------------------------------------
------------------------------------------------------------
------------------------------------------------------------
------------------------------------------------------------
------------------------------------------------------------
>Himalaya_frog_AQP13
ATG------------------------------GACCTGTAT------------------
---------------------ACTCTCATCCTAAAAAAGCTGAAAATGAAGCTG------
CGGACGGATAATCAATATGTGCGCTGCGGGCTGGCTGAATTCCTGGGGACCCTCATATTG
ATTCTGTTTGGTTGTGGCTCTGTGGCTCAGATGGAACTGAGTGGCTATGCTAAGGCGCAA
TTTTTGAGTGTCAACATGGCGTTTGGGTTTGCTGTGACCGCTGGGGCCTACGTCTGTGCT
GGAGTGTCAGGTGCCCACCTCAACCCGGCTGTGTCTCTTTCAATGTACCTCCTCCAGAGG
ATGACCTGTAGAATGATGTTGGTCTACTTCTTGGCTCAGTTCCTTGGAGCTTTTATTGGG
GCTGCTCTGGTCTTT---GCTCTGTATATTGATGCTCTTCATGCGTACAGT---GGAGGT
AACTGGACGGTGTCTAGCACCCAG---GCAACAGCGGGGATATTTGCTTCGTACCCATCC
GAACACTTGACCATTCTTAATGGACTCACAGATCAGGTAATTGCCACTGCTGCCTTAATG
ATCTGTATACTAGCAGTAATAGATGAATCCAACAATGCTGCTCCTCGAGGCCTTCAGCCA
TTTGTGATTGGTCTTGTGGTTCTTCTGGTTGGTCTATCAATGGGCTTCAACTGTGGGTAC
CCAATAAATCCTGCCAGAGACTTGGCACCTCGTATCTTCACTGCAATGGCTGGCTGGGGC
CTAGAAGTCTTCAGGGCTGGTAATCACTGGTGGTGGGTGCCAGTGCTGGGACCAATGATA
GGAGGAGTGATAGGGACCCTGATTTATGAACTGCTGGTTGGAATTCACAGCCGTACTCTC
CATGAGGAGGATGAA---------------------------------CCTGAA------
GAGGACGTTGAGGAGAACCATCCC---CGACAGTATGAGTTAGTT---CAGTCTAATGCT
------------------------------------------------------------
```

```
------------------------------------------------------------
------------------------------------------------------------
>African_bullfrog_AQP13
ATG------------------------------AACATGTTT------------------
---------------------GCATTGCTCTTACAGAAACTCAGCATGAAGTTA------
CAGACAAAAAATCTGTATGTGCGCTGCGGGCTGGCTGAGTTCCTGGGGACCCTCATATTG
ATTCTGTTTGGCTGTGGCTCTGTAGCTCAGATGGAATTGAGTGGTTTTGCAAAAGCGCAG
TTCCTGAGTGTCAACATGGCGTTTGGATTTGCAGTGACGGCTGGGGCCTACGTCTGCGCT
GGAGTGTCAGGCGCCCATCTCAACCCAGCGGTGTCGCTTTCCATGTATCTCCTCCAGAAG
ATGACTTGTAGGATGATGCTGGTATACTTCTTGGCTCAGTTCCTTGGTTCCTTCATTGGG
GCAGGTCTGGTCTTT---GCTCTGTATTTTGATGCTCTGCATGCATACAGT---GGCGGT
AACTGGACAGTGTCTGGGACCCAA---GCAACAGCGGGGATATTTGCTTCGTACCCCTCA
GAACATCTAAGTGTTCTTAATGGATTCACAGATCAGGTAATTGCCACCGCTGCCTTGATG
GTCTGTATACTTGCCATAATAGATGAATCAAACAATGCTGCTCCTCGAGGTCTTCAACCA
TTTGTGATTGGGTTGGTGGTTCTTCTGGTTGGTCTATCAATGGGTTTCAACTGTGGGTAC
CCAATCAATCCTGCCAGAGACCTGGCACCTCGTATCTTCACTGCAATGGCTGGCTGGGGC
CTGGAAGTCTTTAGGGCTGGTGGATGCTGGTGGTGGGTGCCAGTGCTGGGACCAATGGTA
GGAGGAGTGGTGGGTACCCTGATTTATGAACTGCTCATTGGAATTCATCACCCTGCTCAA
CACCAGACAGAAGAA---------------------------------CCAGTA------
GAGGATGGTGGGAACCACCAC------AGACACTATGAACTGGTT---CAGTCTCAAGCT
------------------------------------------------------------
------------------------------------------------------------
------------------------------------------------------------
>Pacific_treefrog_AQP13
ATG------------------------------GACGCCTAT------------------
---------------------TCTCTGCTGATCAAAAAGTGGAAGTTAAAGTTC------
AGGACGAGCAATCGCTATGTGCGCTGTGGGCTGGCGGAGTTCTTGGGGACGCTCATATTA
ATCCTATTTGGCTGTGGGTCTGTGGCACAGATGGAATTAAGCGGTTTTGCCAAAGCCCAG
TTTCTAAGCGTTAATATGGCATTCGGCTTTGCGGTCACTGCTGGAGCCTATGTCTGTGCC
GGAGTCTCAGGCGCCCACCTCAACCCGGCTGTGTCTCTCTCCATGTTCCTCCTCCAGAAG
ATGACGGGGAGGCTAATGCTWGTSTACTGTGTGGCACAATTCCTCGGCTCTTTTGTTGGC
GCGGCACTGGTATTT---GCCCTGTATTTCGACTCCCTTCATGTGTACAGT---GGAGGC
AACTGGACAGTATACGGACCC---------------------------------------
------------------------------------------GCCACCGCGGCCCTGATG
ATCTGCATCCTGGCCATCATCGATGAAGACAATAACGCAGCCCCCCGGGGTCTTCAGCCA
TTTATCATTGGACTGATAGTTCTTCTAGTTGGTCTTTCTATGGGATTCAACTGTGGATAC
CCCATTAATCCAGCCCGGGACCTGGCACCTCGAATCTTCACATCATTAGCCGGCTGGGGC
CTAGAAGTCTTCAGGGCCGGTGACAACTGGTGGTGGGTGCCAGTCCTGGGCCCCATGATT
GGAGCGGTTGTCGGGACAATCCTTTATGAGCTGCTTATTGGGATCCATCACACGTCTGCC
CAGCAGAAGGAAGAA---------------------------------------------
CATGATGGAGAGGAGCGTCACCCC---CCTGAGTATGAGTTGGTT---CACTCAACAGCA
------------------------------------------------------------
------------------------------------------------------------
------------------------------------------------------------
>Marine_toad_AQP13
ATG------------------------------GATGCTTAT------------------
---------------------GCTCTGCTCGTGAAAAAGCTCAAGTTAAGGTTT------
AGGACGAGCAATCGCTATGTGCGCTGTGGGCTGGCTGAGTTTTTGGGAACTGTCATATTG
ATTTTGTTTGGTTGCGGTTCTGTGGCTCAGATGGAATTAAGTGGTTTTGCCAAAGCTCAG
TTTCTGAGTGTCAATATGGCGTTTGGTTTTGCAGTCACTGCTGGAGCTTATGTATGCGCT
GGAGTCTCAGGTGCCCACCTCAATCCAGCCGTCTCGCTCTCCATGTTCCTCCTCCAGAAG
ATGACCGGGAGGCTGATGCTAGTCTACTGCTTGGCACAATTCCTCGGCTGTTTTGTTGGC
GCCAGCCTGGTATTT---GCTCTGTATTTTGATGCCCTTCATGTGTACGGT---GGTGGC
AACTGGACAGTGTATGGACCACAG---GCGACTGCTGGGATATTCGCTTCCTATCCATCA
GAACATCTGAGTGTGATCAATGGACTCTCAGATCAGGTAATTGCCACCGCTGCCTTGATG
ATCTGCATTTTGGCCATCATTGATGAAGACAATAATGCAGCCCCCCGAGGACTTCAGCCA
TTTGTCATTGGTCTTATAGTTCTCCTAGTTGGTCTCTCTATGGGCTTCAACTGTGGCTAC
CCCATTAATCCAGCCCGCGACCTGGCACCTCGAATTTTCACAGGAATTGCTGGCTGGGGT
CTAGAAGTCTTCAGGGCTGGTGACAACTGGTGGTGGGTTCCAGTATTGGGCCCCATGATT
GGTGGGGTCATTGGAACATTTATTTATGAAATGCTCGTTGGAATTCATCACTCAGCAGAA
CAGAAGAAAGAGGAA---------------------------------GATGAT------
```

GAAGATAGCGAGGAGCATCACCCC---CCGGAGTACGAGTTGGTT---CAGTCAACGGCA

>hgtFrog_AQP13
ATG-------------------------------GAGGCTTAT------------------
----------------------TCTCAATTGCTCAAAAAGTCCAAGCTAAAGTGT------
AGGACAAGCAATCGCTATGTGCGCTGCGGGATGGCGGAGTTTTTGGGGACTCTCATATTA
ATTTTATTTGGCTGTGGGTCTGTAGCTCAGATGGAATTAAGTGGTTTTGCCAAAGCCCAG
TTCCTAAGCGTTAATATGGCGTTTGGCTTTGCGGTCACTGCTGGGGCCTATGTGTGCGCT
GGGGTCTCAGGTGCCCACCTCAATCCGGCTGTGTCTCTCTCCATGTTCCTCCTCCAGAAG
ATGAATGGGAGGCTTTTGCTAGTCTACTGTTTGGCACAATTCCTTGGCTCCTTTATCGGT
GCTTGCCTGGTGTTT---GCCCTGTATTTTGACGCCCTTCATGTATACAGT---GGAGGC
AACTGGACAGTATATGGACCACAG---GCGACTGCTGGTATATTTGCCTCCTATCCGTCA
GAACATCTGAGTGCAATTAATGGATTCACAGATCAGGTGATCGCCACTGCTGCCTTGATG
ATCTGCATTCTGGCCATTATCGATGAAGACAATAACGCAGCCCCCCGAGGTCTTCAGCCG
TTTGTTATTGGACTTGTAGTTTTATTAGTTGGTCTTTCTATGGGATTCAACTGCGGATAC
CCCATTAATCCAGCACGGGACCTGGCGCCTCGACTGTTCACTGCCTTAGCTGGCTGGGGC
CTAGAAGTCTTCAGGGCTGGTGGAAACTGGTGGTGGGTGCCAGTGCTGGGCCCCATGATT
GGAGGGGTTATTGGGACAACTATTTATGAGGTATTCATTGGGATCCATCATTCACCTAGC
CAGAAGGAGGAACAA----------------------------------GATGAA------
---------GAGGAGCATTACCCC---CCTGAATACGAGTTGGTT---CACTCCACAGCA

>WC_frog_AQP113
ATG-------------------------------GTCAGCCAT------------------
----------------------TTAGCTTTCCTGAAAAACATGAAGATTAAGCTG------
AGGACCCAGAACCAGTATGTGCGCTGCGGGCTGGCAGAGTTCCTGGGCACGCTCATTCTA
ATCCTGTTTGGCTGTGGTTCTGTGGCGCAGATGGAGCTGAGTGGTTTTGCCAAAGCCCAG
TTTCTGAGCGTTAATATGGCGTTTGGCTTCGCAGTCACTGCTGGAGCTTACGTATGCGCC
GGTGTGTCGGGTGCCCATCTGAATCCTGCCGTGTCCCTCGCCATGTTCATACTGAAGAAG
CTGAGCTGGAGGCTGCTCCTTACCTACTGCCTGGCACAGTTCCTCGGGGCTTTCATTGGA
GCCGCTCTGGTCTTT---TCTCTTTATTATGATGCCTTGCACGTGTACAGC---AGTGGG
AACTGGACAGTCTATGGCCCCCAG---GCTACAGCAGGGATATTTGCTTCCTACCCCTCC
GAGCACCTCAGTGTCATAAATGGCTTTACAGACCAGGTGATTGCCACAGCGGCTCTGCTG
ATCTGTATCCTGGCTATACTGGATGAAGCCAACAACGCAGCACCCAGGGGCCTACAGCCT
TTTCTAATTGGCATTGTGGTGCTGCTGGTTGGGCTAGCAATGGGGTTTAACTGCGGGTAC
CCCATAAATCCGGCTCGAGACCTCGCACCGCGCTTCTTCACGGCTATTGCAGGCTGGGGC
TCCGAGGTCTTCAGCGCCGGAGGCCACTGGTGGTGGGTGCCGGTTATAGGACCTTTGGTA
GGTGGGGTGCTCGGCGTGGTAATCTATGAAGTATTCATCGAATTCCACCATCCTCCTGCC
AATCAAAAGCAAGAA----------------------------------TCCGAG------
GAACCCACTGAGGGAACCCATCGT---CCTCACTATGAGCTTGTT---CAGTCCTCTGCT

>AC_frog_AQP13
ATG-------------------------------GTCAGTCAT------------------
----------------------TTAGCTTTCCTGAAAAACATGAAGCTTAAGCTG------
AGGACTCAGAACTTGTATGTGCGCTGTGGGCTGGCTGAGTTCCTGGGCACCCTCATTTTA
ATTCTGTTTGGCTGCGGTTCTGTGGCCCAGATGGAGCTGAGTGGTTTTGCCAAGGCTCAG
TTTCTGAGTGTGAATATGGCATTTGGCTTTGCAGTCACCGCTGGAGCTTATGTATGTGCT
GGAGTCTCAGGTGCTCATCTGAATCCTGCTGTGTCCCTCGCCATGTTCATACTGAAGAAG
CTGAGCTGGAAGCTGTTCCTGATCTACTGTTTGGCACAGTTCCTCGGGGCTTTTATTGGA
GCCGCTCTGGTCTTT---TCTCTGTATTATGATGCCTTGCACGTGTACAGC---AATGGC
AACTGGACTGTCTATGGCCCCCAG---TCGACAGCAGGGATATTTGCTTCCTACCCTTCA
GAGCACCTCAGTGCCATAAACGGCTTTACAGACCAGGTGATTGCCACGGCAGCTCTGCTG
ATCTGTATACTGGCCATACTGGATGAAGCCAACAACGCAGCACCCAGGGGCCTGCAGCCT
TTTCTCATTGGCATTATGGTGCTGCTGGTTGGGCTAGCAATGGGGTTCAACTGCGGGTAC
CCCATCAATCCAGCTCGTGACCTTGCACCCCGCTTCTTCACTGCTATTGCAGGCTGGGGG
TCAGAAGTCTTCAGTGCTGGAGGCCACTGGTGGTGGGTGCCAGTTCTAGGACCTTTGGTA

GGTGGGGTGGTTGGTGCGGTCATCTATGAAGTCTTCATCGAATTCCACCATCCTTCTCCC
AATCAAAAGCAAGAA---------------------------------TCTGAG------
GAACCCACTGAAGGAATCAATCGT---CCTCACTATGAGCTGGTT---CAGTCCTCTGCT

>JF_newt_AQP13

------AGTGTCAACATGGCGTTTGGATATGCAGTCACTGCGGGAGCCTACGTATGTGCT
---GTATCGGGCGCTCTG------CCGGCTGTATCCCTCGCCATGTTCCTCTTGAGAAGG
ATGACTTTTAAGCTGATGCTGGTCTATTGCCTTGCCCAGTTCCTGGGGGCCTTTTTTGGA
GCAGCCATTGTTTTT---GCGCTTTACTTTGATGCTCTGCATGTGTACAGT---GGCGGC
AACTGGACCGTGGTGGGACCTCAA---GCTACAGCTGGGATCTTTGCATCCTACCCTTCG
GAACACCTCAGTGTTATCAATGGATTTACAGACCAGGTGATTGCTACTGCTGCTCTGCTG
ATCTGCATTCTGGCTGTTGTGGATGAGCGGAACAATGCTGCTCCACCGGGACTGCAGCCA
TTTGTGATTGGTCTAATGGTGTTGCTGGTTGGCCTTGCTATGGGATTCAACTGTGGCTAT
CCCATCAACCCAGCCAGAGACCTGGGGCCACGCCTCTTCACTGCCATAGCTGGATGGGGC
CTTGAAGTGTTTAGGGCTGGGAACCACTGGTGGTGGGTACCAGTTTTGGGGCCCCTTGTG
GGTGGCCTGATTGGCGCCATTATCTATGAGCTGTTCGTGGAAATCCATCACCCGCTGGAT
CAGGTAAAAGAGGTC---------------------------------CTAGAT------
GCCAACTGTCCTGATCACCCAACTCGTCCCCAATATGAACTTGTT---CAGAGCACAGCA

>Axolotl_AQP13
ATG-----------------------------GACGGCCAC------------------
---------------------GCCGCCCTGCTGAAGTACTTGAAGCTCAAGTTC------
AGAACTCGAAGTCTCCACGTGCGCTGTGGGCTGGCAGAGTTTTTGGGAACGTTCATTTTA
ATTCTGTTTGGCTGTGGTTCCGTGGCCCAGATGGAGCTGAGTGGCTTCGCAAAAGCACAG
TTCCTTAGTGTGAACATGGCCTTTGGATTTGCCGTCACCGCCGGTGCCTATGTGTGTGCC
GGAGTATCAGGTGCTCACTTGAACCCGGCTGTGTCGCTCGCAATGTTCCTCTTGAGACGG
ATGTCATGGAAGCTGCTTCTTGTCTACTGCCTTTCCCAGTTCTTGGGAGCGTTCTTTGGT
GCTGCCATTGTTTTT---TCACTTTACTTTGATGCTTTGCATGCGTTTAGT---GGTGGA
AACTGGACTGTGGCCGGCCCTCAA---GCGACTGCTGGGATCTTCGCCTCCTACCCTTCA
GAACACCTCAGTGTTATCAATGGATTTACAGACCAGGTGATTGCCACAGCTGCTCTGTTG
ATCTGCATCCTGGCCATTGTGGATGAGAAAAATAACGCTGCCCCGGCTGGATTGCAGCCA
TTTGTGATTGGTCTAATGGTGCTGCTGGTAGGCCTTGGCATGGGATTCAACTGTGGCTAT
CCCATCAACCCAGCCAGGGACCTAGGGCCACGCCTCTTCACTGCCATAGCTGGATGGGGC
CTTGAAGTATTTACCGCTGGAAACCACTGGTGGTGGGTCCCAGTGTTGGGGCCCCTCGTG
GGAGGCTTAATCGGCGCCATAATCTACGAGCTGTTTGTGGAATTCCATCATCCGCTGGAT
TCTGAGAAAGAGAAC---------------------------------ACCGAA------
AACGACAGCGTTGATCATCCAAATCGTCCCCATTACAAACTAGTT---CAGACGACCGCA
GCC---

>Platypus_AQP13

------CAGAGTGCCCACCTGCGCTGTGGCCTGGCTGAGTTCTGGGGGACTTTTGTGCTG
ATTGTACTCGGCTGCGGCTGGGTGGCCCAGGCTGAGCTGCGAGGC---------------
------------------------TGGTTCATGGTCTCTGCGGGAGCCTACATAGGCTCC
GGAGCCTCAGGCACTCATCTGAACCCCGCCGTGTCCCTGGCCATGTGTTTCCTGCGGCGG
CTGGACTGGAACCTCCTGCCCACTTTGTGCTTAGCCCAGCTGGCCGGGGCCTTCTGCGGC
GCAGCCACCGTCTTC---GTCTGGCACTACGACGGTCTCCAGGCCCTGAGC---GCTGGT
GCCTGGACAGTGGCGGGACCCAAT---GCCACAGCTGGGATCTTTGCTTCCTACCCCTCC
GGGCAGCAGAGTTCACTCAGCTGCTTTGCTGACCAGGTGATGGCGTCGGCAGCTTTTCTC
ACCTGTGTCCTGGCCGTGCTGGGCGAGGGAGTCCCAGCCCCCCGCCACAAGCTGCAGCCC
CCCGCCCAGCGGCTGGCCCTGTTCCTAGTAGGCTCGGCCTTGGGCTCGAGCTGTGGGTGC

CCCATCAACCCCGCCGAGGACCTGGGCCCGCGGGCGTTCGCGGCAGTGGCCGGCTGGGGG
CTGGAAGTCTTCAGGGTTGGAAACCACTGGTGGTGGATCCCCGTCCTGGGGCCCCTGGTG
GGCGCCCTGCTCGGGGCCTCCGTGTAC---------------------------------

>Arctic_lamprey_Aqp9_13L1
ATG------------------------------TGCTCCCCCCGG---------------
------------------CAGCTCCACGCTCCGACGCGCCTGGCGGCCCGGATC------
AAGGCATCGAACCCGATCGTGAGGGAGGCTCTGGCTGAGTTCCTCGGCACGTTCGTGCTC
ATCGTGTTCGGATGTGGTTCCGTGGCTCAGGTAGAACTGAGCCACCACTCCGCCGGAGAG
ACTCTCACCATCAACCTCGCCTTCGCATTTGGGGTGGTCATGGGCGCTTACCTGTCCTGG
GGCATCTCAGGTGCACACCTGAACCCCGCCGTCTCGTTCTCCATGTCGCTGATCGGACGC
TTCCACTGGTGGAAGCTGCCCATTTTCTGCCTCGCGCAGTTCCTGGGCGCGTTCACAGCG
GCCGCCACCGTCTAC---GGCCTCTACCACGAGGCCCTCATGGCGTTCAAC---GGTGGG
AACCTGACTGTGACTGGCCCCGGT---GCCACGGCCGCGATATTCGCCACCTATCCCTCG
GAACACCTGTCCATCGCCGGTGGCTTCCTTGACCAGGTGCTGGGCACGGCGCTCCTCCTG
CTGTGCGTGATGGCGCTGCTGGACCCCAAGAACAACGCGGTGCCGCGCGGCCTCGAGCCA
CTGCTCGTGGGGCTCGTGGTGCTCGTCATCGGCCTCTCCATGGGCTTCAACGCCGGCTAT
GCCATCAACCCCGCGCGCGACCTCGGCCCCCGCGTCTTCACCGCCATGGTCGGATACGGC
AGCGAAGTGTTCACGACGGGCCCCCACTGGTGGTGGGTTCCCGTGGTGGCCCCCCTCGTG
GGGGGCCCGCTGGGCACAATGTGCTACATGTTCTTCGTGGACCTGCACCACCCCGCCCCG
CCGGAGCTGTCCACCGGCAAAGCCAAGCCG---------------CTGGTGGAC------
AGCGACAGC---GACCACCCCAAG---GCCAACTACGCCGTGGCGGTGAAGATGGAGCAG
CAGGCA--

>Sea_lamprey_Aqp9_13L1
ATG------------------------------TGCTCCCCCCGG---------------
------------------CACCTCCACGCTCCGCCGCGCCTGGCGGCCCGGATC------
AAGGCGTCAAACCCGATCGTGAGGGAGGCTCTGGCTGAGTTCCTCGGCACGTTCGTGCTC
ATCGTGTTCGGATGTGGTTCCGTGGCTCAGGTGGAGCTGAGCCACCACTCCGCCGGAGAG
ACTCTCACCATCAACCTCGCCTTCGCATTTGGGGTGGTCATGGGCGCTTACCTATCCTGG
GGCATCTCAGGTGCACACCTGAACCCCGCCGTCTCCTTCTCCATGTCGCTCATCGGACGC
TTTCACTGGTGGAAGCTGCCCATTTTCTGCCTCGCTCAGTTCCTGGGCGCGTTCACAGCA
GCCGCCACCGTCTAT---GGCCTCTACCACGAGGCCCTCATGGCGTTCAAC---GGTGGG
AACCTGACGGTGACCGGCCCCGGC---GCCACGGCCGCGATCTTCGCCACCTATCCCTCG
GAACACCTGTCCATCGCCGGTGGCTTCCTTGACCAGGTGCTGGGTACGGCGCTCCTCCTG
CTGTGCGTGATGGCGCTGCTGGACCCCAAGAACAACGCGGTGCCGCGCGGCCTCGAGCCA
CTGCTCGTGGGGCTCGTGGTGCTCGTCATCGGCCTCTCCATGGGCTTCAACGCCGGCTAT
GCCATCAACCCCGCGCGCGACCTCGGCCCCCGCGTCTTCACCGCCATGGTCGGATACGGC
AGCGAAGTGTTCACGATGGGGCCCCACTGGTGGTGGGTCCCCGTAGTGGCCCCCCTCGTG
GGGGGCCCGCTGGGCACAATGTGCTACATATTCTTCGTGGACCTGCACCACCCCGCCCCG
CCGGAGCTGTCCACCGGCAAAGCCAAGTCG---------------CTGGTGACC------
AGCGACAGC---GAGCACGCCAAG---GCCAACTATGCCGTGGTGGTGAAGATGGAGCAG
CAGGCA--

>Arctic_lamprey_Aqp9_13L2
ATG------------------------------GGCTCCCCA------------------
---------------------GGGCCGCTCTACTCGCGCCTG---TCGCGGGTC------
AAAGTGTCCAATCCCGTGCTGCGAGAAGCCCTCGCGGAGTTCATCGGCGTCTTTATTCTC
ATCCTGTTCGGCTGCGGCTCAGTGGCCCAGGTGCATCTGAGCCGCCACTCCGCCGGGCAG
ACTCTCGGCATCAACTTCGCCTTCGCCTTCGGGGTGGTCATGGGGGCCCACCTGGCCTGG
GGAGTCTCGGGTGCGCACCTGAACCCCGCCGTCTCGCTCTCCATGTCGCTGCTCGGCCGC
TTACCCTGGAGGAAGCTCCCCGTGTTCTGCCTCGCGCAGTTCCTGGGCTCGTTCGTGGCG
GCTGCCACCGTCTAC---GGCCTCTACTACGATGCCCTCATGGCGTTCAAC---GGCGGG
AACCTGACGGTGACCGGCCCCGGT---GCCACGGCCGCGATCTTCGCCACTTATCCCTCG
GAACACCTGTCCATCGCCGGCGGCTTCTTTGACCAGGTGCTGGGCACGGCGGCGCTGCTG

CTGTGCATCATGGCACTCCTCGATTCCAAGAACAACGCCGTGCCGCGCGGCCTGGAGCCG
CTGCTCATCGGCCTGGCGGTGTTCGGCATCAGCCTCTCCATGGCCTTCAACTCGGGGTGC
GCCATCAACCCCGCGCGAGACTTCGGCCCCCGCCTCTTCACCGCCATGGCGGGATACGGC
AGCGAGGTGTTCACGGTGGGCCCCCATTGGTGGTGGGTCCCCATCATCGCCCCCCTCGTG
GGGGGCCCGCTGGGTGCACTGTGCTACATTTTGTTCGTGGACCTGCATCACATCGCCCCG
CCGGAGCTGCCCCCAATGGCGCTGGAGCCG---------------CTGGTGTCC------
GACGAGAAC---CACCACAGCAAG---GGCAGCTACGTCGCCGTG---AAGACGGGGCAG
CAGCCA--

>Sea_lamprey_Aqp9_13L2
ATG------------------------------GGCTACCCA------------------
---------------------GGGCCGCTCTACTCCCGCCTG---TCGCGGGTC------
AAAAGTGTCAATCCGTGTTGCGAGAAGCTCTCGCGCGAGTTCATCGGCGTCTTTATTCTA
ATCCTGTTCGGCTGCGGTTCAGTGGCCCAGGTGCATCTGAGCCGCCACTCCCCGCGGCAG
ACTCTTGGCATCAACTTCGCCTTCGCCTTCGGGGTGGTCATGGGGGCATACCTCGCCTGG
GGG------GGTGCGCACCTGAACCCCGCCGTTTCGCTCTCCATGTCGCTGCTCGGCCGC
TTACCCTGGAGGAAGCTCCCCGCGTTCTGCCTCGCTCAGTTCCTCGGCTCGTTTTTGGCG
GCTGCCACCGTCTAC---GGCCTCTACTACGAGGCCCTCATGGCGTTCAAC---GGTGGG
AACCTGACGGTGACCGGCCCCGGC---GCCACGGCCGCGATCTTCGCCACCTACCCCTCT
GAACACCTGTCCATCGCCGGTGGCTTCTTTGACCAGGTGCTGGGCACGGCGGCGCTGCTG
CTGTGCATCATGGCGCTCCTCGACTCCAAGAACAACGCCGTGCCGCGCGGCCTGGAGCCG
CTACTCATCGGCCTGGCGGTGTTCGGCATCAGCCTCTCCATGGCCTTCAACTCCGGGTGT
GCCATCAACCCCGCGCGAGACTTCGGCCCCCGCCTCTTCACTGCCATGGCGGGATACGGC
AGAGAAGTGTTCACGGTGGGCCCCCACTGGTGGTGGGTCCCCATCATTGCCCCCCTCATG
GGGGGCCCGCTGGGTGCCCTGTGCTACATATTATTCGTGGACCTGCATCACCTCGCCCCG
CCGGAGCTGCCCGCAATAGTGCTGGAGCCG---------------CTGGTGTCC------
GACGAGAAC---AACCACAGCAAG---GGCAGCTACGTCACAGTG---AAGACGGAG---

>Inshore_hagfish_Aqp13
TTG------------------------------GCTGACCTC------------------
------------------CTGAGAGATGCCGCAAAGATGCTC---AACAATCTG------
AGGACCAGCAATGAGTCCATACGTTTGTTTATGGCGGAGCTGCTTGCGACAGCAATGTTG
GTGCTCATTGGTTGTGCAGCTGTTGCTCAGGGGGTGTTGTCAGGTGGTGGTGTTGCTGCC
ACTCTGAGCATTAATTTGGCCTTTGGATTGGCTGTGATGATGTCTGCGCACATCGCAATG
GGAGTTTCAGGTGCTCACATGAATCCTGCGGTTACCCTGGCGATGTGCATCTTGGACCGA
TTTCCATGGAAACGGCTTCCTCTCTATTGGATCTCACAGTTCTTGGGTGCATTCATTGGT
GCTCTAGGGGTCTAT---GCCCTGTATCATGAGGGGATCCAAGCATACGAA---GGTGGG
GAGCTGACTGTTGTCGGGCCTCAT---GCCACGGCCGGAATATTTGCCACCTACCCCAAC
TCTCACCTCTCCACTGGAGGAGGAGTTATTGATCAGGTGATTGGAACCGCTATTTTGCTT
TGCCTCATTCTTGCACTTACGGACGAATCGAACTGTGCAATGCCACGCTATCTGCAACCC
TTGGGTTTTGGTTTAGTGGTGGTTGCTATCGGGCAAGCTACGAACTTCAACTGTGGTTAT
GCCATTAATCCAGCCCGGGATTTGGGGCCCAGGATTTTCAGCGCGCTGGTTGGATATGGA
TCTGAGGTGTTTTGGGCGAAGGACTGTTGGTGGTGGGTCCCTGTGCTCGCTCCTCTCATT
GGATCTGTTCTCGGATCTTTGTGTTACCAACTCCTGATCGCACTCCATTACCCGAGGAGC
ACTGACAGGGAAAATGAGAAACCACCTTCG---------------CTTCTGCAA---TGC
AATGACCATGATAACCTCGAT------GGTTCACAGGTGGACAAG---ATGTCTGAAGTT
CACGAGATTTCTGAGGAATTG---------------------------------------

>Human_AQP9
ATG------------------------------CAGCCTGAG------------------
------------------GGAGCAGAAAAGGGAAAAAGCTTCAAGCAGAGACTG------
GTCTTGAAGAGCAGCTTAGCGAAAGAAACCCTCTCTGAGTTCTTGGGCACGTTCATCTTG
ATTGTCCTTGGATGTGGCTGTGTTGCCCAAGCTATTCTCAGTCGAGGACGTTTTGGAGGG
GTCATCACTATCAATGTTGGATTTTCAATGGCAGTTGCAATGGCCATTTATGTGGCTGGC
GGTGTCTCTGGTGGTCACATCAACCCAGCTGTGTCTTTAGCAATGTGTCTCTTTGGACGG
ATGAAATGGTTCAAATTGCCATTTTATGTGGGAGCCCAGTTCTTGGGAGCCTTTGTGGGG
GCTGCAACCGTCTTT---GGCATTTACTATGATGGACTTATGTCCTTTGCT---GGTGGA

AAACTGCTGATCGTGGGAGAAAAT---GCAACAGCACACATTTTTGCAACATACCCAGCT
CCGTATCTATCTCTGGCGAACGCATTTGCAGATCAAGTGGTGGCCACCATGATACTCCTC
ATAATCGTCTTTGCCATCTTTGACTCCAGAAACTTGGGAGCCCCCAGAGGCCTAGAGCCC
ATTGCCATCGGCCTCCTGATTATTGTCATTGCTTCCTCCCTGGGACTGAACAGTGGCTGT
GCCATGAACCCAGCTCGAGACCTGAGTCCCAGACTTTTCACTGCCTTGGCAGGCTGGGGG
TTTGAAGTCTTCAGAGCTGGAAACAACTTCTGGTGGATTCCTGTAGTGGGCCCTTTGGTT
GGTGCTGTCATTGGAGGCCTCATCTATGTTCTTGTCATTGAAATCCACCATCCAGAGCCT
GACTCAGTCTTTAAG---------------------------------ACAGAA------
CAATCTGAGGACAAACCA---------GAGAAATATGAACTCAGT---GTCATCATG---

>Rabbit_AQP9
ATG-----------------------------CGGTCCGAG------------------
------------------AACGGACAGAAAACGAAGAGCTTCAAACAGAGACTG------
GTCTTGAAGAACAGCTTGGCTAAAGAAGCGCTCTCCGAGTTCTTGGGCACATTTATAATG
ATCGTTCTTGGATGTGGCTCTGTTGCCCAGGCTGTCCTGAGTCGAGGACATTTTGGAGGA
ATTGTCACTCTCAATGTTGGATTTGCTATGTCAGTTGCAATGGCCATTTATGTGACTGGA
GGTGTCTCTGGTGGTCACATCAACCCAGCTGTGTCTTTCGCAATGTGTCTCTTTGGACGA
ATGAAATGGTTCAAATTTCCATTTTATGTGGGAGCCCAGTTCTTAGGAGCTTTTGTAGGG
GCTGCAACCCTCTTT---GGCATTTACTATGATGGATTCATGTCTTTTGCT---GGTGGA
AAATTGCTCATCGTGGGAGAAAAT---GCAACAGCACATATTTTTGCAACATACCCGGCT
CCATATCTGTCTCTGACGAATGCATTTGCAGACCAAGTAATGTCCACCATGTTTCTCCTC
ATACTTGTGTTTGCCATTTTTGACTCCAAAAATTTGGGGGTGCCCAGAGGCCTAGAGCCT
ATTGTCATTGGCCTCTTGATTATTGTCCTCTCTGGCTCTTTGGGGCTGAACAGTGGCTGT
GCCATGAACCCAGCTCGAGACCTGAGTCCCAGACTTTTCACAGCATTGGCAGGATGGGGA
TTTGAAGTCTTCACAGTCGGAAATAATTTCTGGTGGATTCCTGTTGTGGGCCCTTTGGTT
GGTGCTGTCATTGGAGGCCTCGTCTATGTTCTTCTCATTGAAATTCACCACCCAAACCCT
AACCCAGACTTCGAG---------------------------------GTAGAA------
CAGTCTGAGGACAAGCCA---------GAGAAATATGAATTTAGT---GTTATAATG---

>Koala_AQP9
ATG-----------------------------GAC------------------------
---------------------AGAAAGACCGAAAAAACCTTCAAAGAGAAAATC------
ATCCTGAAGAACTCATTGGCCAAAGAAGTGATTGGAGAATTCCTGGGGACATTCACATTG
GTTGTCCTTGGATGTGGCTCTGTAGCCCAAGCCGTCCTTAGCCGAGGGGCTTCAGGTGGA
ATTGTGACCATAAATATTGGCTTTTCTGCGGCTGTTGCAATGGCTGTTTATGTGACAGGA
GGCATCTCTGGTGGTCACATTAACCCAGCAGTGTCCTTTGCTATGTGTCTCTATGGACGG
ATGAAATGGTTCAAATTCCCTTTCTATGCGGGGGCCCAGTTCCTGGGAGCTTTTCTAGGG
GCTGCAGCCCTCTTT---GGAGTTTACCATGACGCACTTAGGTCCTTTGCT---GAGGGA
AAACTGATCGTCACAGGAGAAAAT---GCAACAGCCCATATTTTTGCAACTTATCCAATT
AAATACCTGACTGTAACAAATGAAATTGCAGACCAAGTGATGTCTTCCGCATTCCTTCTC
CTGGTAGTATTTGCCGTTTTTGATGAGAAAAACTGGAGGGTCCCCAAGGGCCTAGAACCC
ATTGTCCTTGGCCTACTGATCCTTGTCNTTTCTTCCTCATTGGGAATGAACAGTGGTTGT
GCCATGAATCCAGCTAGAGACCTGGGTCCCAGAGTATTCACAGCTCTGGCAGGGTGGGGG
CTTGAAGTCTTCACAGCTGGAAGTTACTTCTGGTGGATCCCAGTCGTGGGGCCTTTCCTT
GGTGGCGCCTTAGGAGGCTTCATCTATATTTACGGCATTGAAATCCACCATACAGACCCA
GACTCAGATGAAAAGAGGAAA---------------------------------------
CAAGTGGAG------------------GAGAAACATGAACTTAGT---GTGATAATG---

>Platypus_AQP9
ATG---------------------------AAGGACTCGGAG------------------
---------------------GGGAAGAGCGAAAGGACCTTCAAGGAGAGGCTC------
GTCCTCAAGAGCCGCCTGGCGAAGGAGAGTCTCTCCGAGTTCCTGGGGACCCTCATCATG
ATCGTGCTCGGGTGTGGGTCCGTGGCCCAAGCCGTCCTCAGCCGGGGGCAGCTGGGCAAC
ATAGTGACCATCAATGTGGGTTTTGCCATGGCCGTGGTCATGGCCATTTATGTGACCGGG
GGCATATCCGGCGGCCACATCAACCCGGCCGTGTCCTTCGCCATGTGCCTCTCTGGCCGG

ATGTCTTGGCTCAAGCTGCCATTCTACGTGGCGGCCCAGTTCCTCGGGGCTTTCACCGGG
GCCGCCTTGCTCTTC---GGCATCTACTACGATGCCCTCATGGCCTTCGCC---GAAGGA
GAGCTACTCATCACCGGAGAAAAT---GCCACGGCCTTCATCTTTGCCACATACCCGGCC
GGGTACCTGTCCCCGGCAAACGCGTTCGTGGACCAAGTGGCGTCCACCACTTTCCTGATC
CTGGTGATCTTCGCCGTCTTCGACACCAGGAACCTGGGGGTGCCCAGGGGCCTGGAGCCG
ATCGTCATCGGACTGCTCATCGTTGTTCTTGCGTCCTCTCTGGGGCTGAACAGCGGCTGT
GCCATGAACCCGGCCCGGGACCTGGGCCCCAGGGTCTTCACCGCGGTGGCCGGGTGGGGG
CTGGAGGTCTTCACGGCTGGAAACCACTTCTGGTGGATCCCGGTGGTGGGGCCGATGGTG
GGAGCAGTCTTGGGCGTGCTGGTCTACCAGCTGTGCATCGCCATCCAGCTCCCCCAGCCG
GAGGCGGAGCAGGAGGGA--
CAGGTCATGACCAAGATG---------GAGAAATACGAACTCAGC---GTCATGATG---

>Zebra_finch_AQP9
ATG------------------------------AGC------------------------
---------------------CGGAAAAACAAAAGGAGTTTAAAGGAGAAATTT------
TCCTTGAAGAACAGCCTGGTTAAAGAAGCCCTCTCAGAGTTCCTGGGAACTTTTATACTG
ATAGCTCTTGGCTGTGGCTGTGTGGGGCAGGCCGTGCTCAGCCCGCGGAGCGCATGGAGGG
CCCATGACGGTGTCGGTGGGCTTTGCCATGGCAGTCACCATAGCAGTGTACGTGTCTGGG
GGCGTTTCTGGTGGTCACATAAACCCAGCTGTTTCATTAGCCATGTGCGTGACTGGAAGA
TTAAAATGGACCAAATTACCAATTTACATATTAGCACAATTCCTGGGAGCATTCATTGGA
GCAGCGGCTGTCTTT---GGGATTTATTACAATGCCTTTATGGAGTATAGT---GATGGA
AAACTTGAAGTCACAGGACCAAAT---GCAACAGCACAAATCTTTGCAACATATCCAGCT
CCTTATTTGTCCCTTGTAAATGGATTTGCAGATCAGGTGATGTCAACAGCTGTTCTTCTT
CTGGCTATATTTGCTATTTTTGACACCAAAAATAACAGCGTACCCAAGGGCCTGGAGCCA
ATTGCAGTAGGACTTCTTATAATTGTTCTTACTTGCTCTTTGGGAATGAACAGTGGCTGT
GCTATGAACCCAGCCAGGGATCTTGGCCCAAGGCTCTTCACGGCTGTTGCAGGATGGGGG
ATGGAAGTATTCACGGCTGGTAATAATTGGTGGTGGGTTCCTATAGTTGCACCTCTGCTG
GGAGGTGTACTTGGAGCAATGACCTATGTAATTTTTATTGAAATTCATCACTCAGATCCT
CAGTCAGGA---
GAAGACAAAGATGTGCAT---------ACTAAATATGAATTGACC---AATATGGCA---

>Alligator_AQP9
ATG------------------------------AGC------------------------
---------------------AAAAAAGGCAAAAGAAGTTTCAAGGAAAAAATTT------
GCCTTGAAGAACAGCCTGATTAAAGAAGCCCTCTCAGAGTTCCTGGGAACTTTTGTATTG
ATAGCGCTCGGATGTGGGTCTGTTGCCCAAACTGTTCTTAGCCATGGGGCCATGGGTGAG
TCTATAACAATATACGTCGGGTTTGCAGTGGCAGTCACCATAGCAATCTATGTATCCGGA
GGCGTGTCTGGCGGTCACATTAACCCAGCAGTTTCATTAGCCATGTGTGTGACTGGAAGG
TTGAAATGGATCAAATTACCGGTCTACATATTAGCCCAACACCTGGGGGCATTTATTGGA
GCAGTAGCTGTCTTT---AGTGTTAATTATGATGCGCTTATGCAGTACAGT---GATGGA
GAACTTACAGTCACAGGACCCAAT---GCAACTGCACATATTTTTGCAACATATCCAGCT
CCACATCTGTCACTGATAAATGGATTTGTTGAGCAGGTGATGTCAACAGCTTTTCTTCTT
TTGGGTGTATTTGCCATTTTTGACACCAAAAACATAGGAGTACCCAAGGGCTTGGAACCA
ATTGCAGTTGGACTTCTTATCTTGGCACTTACTTGCTCCTTGGCAATGAACAGTGGCTGT
GCCATGAACCCAGCCAGGGACCTAGGCCCAAGGCTCTTCACATTAATAGCAGGATGGGGA
ACAGAAGTATTTACGGCTGGAAATAACTGGTGGTGGGTTCCTATAGTTGCACCTATGATG
GGAGGAGTAATTGGAGCCCTTATTTATGTTCTTTTTATTGAAATCCACCATTCAGATACT
CAGCCAGAA---
CAAGACACTGACATGTAT---------ACCAAATATGAACTTACC---AACGTGGGG---

>Turtle_AQP9
ATG------------------------------AAC------------------------
---------------------GGGCAAAGCAAAAGAACATTCAAGGAGAAATTT------
GCCTTGAAGAACAGCTTTATAAAAGAAGCCCTGTCCGAATTCCTGGGAACATTTGTAATG
ATAGTACTCGGATGTGGGTCTGTTGCCCAAGCCGTCCTTAGCCGAGGGGCCATGGGTGGA

ACCGTAACAATATCTGTTGGATTTGCAATGGCAGTCACTATGGCAGTGTATGTCTCTGGG
GGCGTGTCTGGTGGTCACATAAACCCAGCCGTTTCATTAGCCATGTGTGCAACTGGAAGG
TTGAAATGGACCAAATTACCTTTTTATATATTAGCACAATTCCTGGGAGCATTCGTTGGA
GCAGCAGCTGTCTTT---GGGATTTATTATGATGCACTCATGAACTACAGT---GGTGGA
GAGCTTATAGTTACAGGATCTAAT---GCAACCGCACATATTTTTGCAACTTATCCAGCT
CCACATCTGTCCCTGATAAGTGGATTTGCAGATCAAGTAATGTCAACAGCTCTTCTTGTT
CTGGTTGTCTTTGCTATTTTTGACACCAAAAATATGGGTGTACCAAAGGGATTGGAACCA
ATTGCAGTTGGACTTCTTATCCTTCTGCTCACTTGTTCCTTGGGAATGAACAGTGGCTGT
GCCATGAACCCAGCCAGGGACCTAAGCCCAAGGATTTTCACAGCTATAGCCGGATGGGGT
TTCGAAGTATTCACGGCTGGAAATAACTGGTGGTGGGTTCCTACAGTTGCACCTATGATG
GGAGGCGTAATTGGAGCCATTACCTATATTATTTTTATTGAAATTCATCATTCAGATACA
CAGCTGGAA---
GAAGACACTGATATGTAT---------GAAAAATATGAACTTACC---AATATG------

>Python_AQP9
ATG------------------------------ACA------------------------
---------------------AAGAAAGCAGGAAAGAGCCTGAGAGAAAAGTTT------
GCCCTGAAGAACAATTTGCTGAAGGAAGGGCTGGCAGAACTCCTGGGAACCTTCATCTTG
ATCGTACTGGGCTGTGGGAGCGTGGCGCAAACAGTCCTGAGCAGAGGAGCTCTGGGAGGA
GCACTGATGATCTCTGTTGGCTTCGCCATGTCCGTCACCCTCGCCGTCTACGTTGCGGGA
GGCGTTTCTGGGGGTCACATCAATCCAGCCGTTTCTTTTGCCATGTGCCTGACTGGGAAG
ATGAAATGGGTCAAATTTCCCGTTTATGTCTTTGCGCAATGTTTTGGTGCATTTCTTGGA
GCGGCCATGGTGTTC---GGGATCAACTACGATGCGCTTATGTTCCACACT---CATGGA
GTCTTCACTGTCACGGGGCCCAAC---GCCACAGCACATATTTTTGCAACATATCCCCAG
GAATATTTGTCCCTGACCACTGGCTTTGTAGACCAGGTCGTATCCACGGCCTTCCTTATC
CTGGGTATCTTCGCCATCTTCGACACGGACAATTTGGGTGTCCCAAAGGGCCTGGAGCCC
ATCGCCATCGGCCTCCTCATCATCCTGCTGACCTCTTCTATGGCCCTGAACAGTGGCTGC
GCCATGAACCCTGCCCGAGATCTGGCCCCGCGTGTCTTCACATACCTGGCGGGATGGGGG
GCCGAAGTCTTTACGGCTGGCAGTCACTGGTGGTGGGTCCCCGTTGTCGGCCCGATGGTT
GGTGCGGCTGTCGGAACTGCCACCTACATGCTCTTCATTGAACTTCACCACGCTCCCGTG
CCCCTCTGCCAGAAG---
AATGCCCCTGACACCCAC---------CACGAGTATGAGCTTACC---CACCTGGAAGAA
AGGAAA--

>Gecko_AQP9
ATG------------------------------GCC------------------------
---------------------AAGAGGAACGGGAGGAACTGGAAGGAGAGCTTT------
GCCCTGAAGAACAGCCTGGTGAAGGAAGCCCTCTCCGAGTTCCTGGGGACCTTCGTGATG
GTAACCCTGGGATGCGGGTGCGTGGCCCAGTCGGTGCTGAGCGGGGAAGCGCTGGGAGGA
GCGCAGATGGTCTCCGTGGGCTTCGCCATGGCCGTCACCATCGCCATCTACGTCGCAGGA
GGCGTCTCCGGGGGCCACATCAACCCGGCCGTTTCGTTTGCCATGTGCATCACCGGCAAG
ATGACGTGGGCCAAGTTCCCCGCCTACGTCCTCACGCAGTTTCTCGGAGCATTCGTGGGA
GCAGCCGCCGTCTAC---GGGGTCAACTACGATGCACTGATGTCCTACACC---GGGGGG
AACTTCACCGTCACAGGTGACATC---CGCACCGCCCATATCTTCGCCACGTACCCATCA
GAATACCTCTCTGTGACCAACGGATTTGCCGACCAGGTGATCTCAACGGCCTTCCTCCTC
GTGGGCGTCTTTGCCATCTTCGACCAGGGGAACCTGGGGGTCCCGAAGGGGCTGGAGCCC
ATTGGGGTGGGTCTTCTGATCATCTTGCTGACCTCCTCCCTGTCCATGAACAGCGGCTGC
GCCATGAACCCCGCTCGGGACCTCAGCCCACGCCTCTTCACGCTCCTGGCCGGGTGGGGT
CCAGAGGTCTTCACGGCTGGCAACCACTGGTGGTGGGTGCCGATCGTTGGTCCCATGGTT
GGGGCCGGTTTTGGGGCCGCTATCTACATGCTGTTTATCGAACTGCACCATGTGCAGCCG
CCGCCGCCA---
CAGAGCAACGCCGCTCAC---------GACAAATACGAACTCACC---AGCATG------

>Himalaya_frog_AQP9
ATG---
---------------------CAAAAG---GACAGAAGCTGTTTAGGAAAACTG------

AAACTAAGGAACAGCCTGGCGAAGGAAACCCTGTCTGAGTTTTTCGGCACATGTTTGTTG
ATTGTATTAGGCTGTTCGTGTGTAGCATCTTCTGTTCTCAGTGGGGAAAGGCTGGAGGT
CACTTAACTAATAATCTTGGCTTTGCAATGGCAGTCGCTATGGCAGTCTACGCTACTGGA
GGAGTGTCTGGGGGCCACATTAATCCTGCAGTTTCATTTGCCATGTGTTTAACTGGAAGA
TTAGAATGGGTCAAGCTCCCCTTCTATGTGAGTGCACAGTTTGTGGGTGCCATTACTGGG
TCTGCTTTAGTTTTT---GGTGTTTATTATGAAGCAATAATCAAATATTCT---GGTGGA
GTTCTTACAGTAGATGGTCCAAAT---GCCACAGCTCATATTTTTGCAACCTATCCTGCT
CCATATCTGTCAACAATAAATGGACTTGGAGATCAGATACTGTCTACTGCACTTCTGCTC
ATTTGGTATTTGCCATATTTGACAAGAAAAACATTGCAGCCCCTAAGGGATTGGAGCCG
ATTGCAATTGGCCTTCTGATTATGCTTCTAGGATTAGCTCTGGGAATGAATTGTGGCGGT
GCTATGAACCCAGCTAGAGACTTAGGTCCACGAATCTTCACAGCAGTGGCTGGATGGGGT
TATGATGTTTTCACGGCTGGCAATAACTTTTGGTGGATTCCTGTAGTTGGCCCAATGGCT
GGTGGAGCACTTGGAGCTTACATATATATTCTCTGCATAGAAGCTCATCATCACAATGGA
CAAGATAGCATGCAT---CCT
GTTGATACTGATCAGTAT---------GAAAAGCATGAACTTACC---AATATGGCA---

>Axolotl_AQP9
ATG------------------------------ACC------------------------
----------------------CAAACAACCCATAAGAGCTTGCGAGACAGGCTG------
GCACTGAAGAACAGCCTGTCAAAGGAGATCGTCGCTGAGTTCCTGGCCACATTTGTGTTA
ATTGTACTTGGATGTGGAAGTGTTGCCCAAGCTGTCCTGAGCAGAGGAGTTGCTGGCAAT
GCTGTAACCATAAATGCTGGGTTTGCGATGGCAGTCACTATGGCCGTCTACGTCGCAGGT
GGGGTTTCTGGGGGCCATGTAAATCCTGCCATTTCTTTGGCCATGTGTGCAACTGGAAAG
ATGAGGTGGTGTAAATTCCCTTTTTATGTCTGTGCACAGATGCTGGGAGCCATGGCTGGG
GCAGCAGCCATTTTT---GGAATATATTATGATGCATTCATGTCCTTTGCT---GAGGGG
ACGCTTGCAGTCTCAGGTCCAAAT---GCAACAGCACATATATTTGCCACATACCCAGCA
TCGTTTCTTTCCACCACAAATGGATTTGCAGAGCAAGTGGCATCGACTGCTCTCTTGGTT
ATTTCTGTATTTGCCATTTTCGACAACAAAAACATAGGAGTGCCTGCAGGGCTGGAACCG
GTGGTAGTTGGCCTGCTCATTTTTCTTTTAAGTTGTTCTTTGGGGATGAACTCTGGCTGC
GCTATGAATCCAGCAAGGGACCTAGGTCCAAGGATGTTCACCGCTGTGGCAGGATGGGGT
CTTGAAGTCTTCACTGCTGGTAATAATTGGTGGTGGGTTCCCGTAGTGGCCCCGATGATT
GGAGGACTTATTGGTGCTTATGTCTACATCCTGGCCATCGAATTGCATCATCAGAAACCG
CTAGAAGACACAAAG---------------------------------TCAGAACAAGCC
GAAGATGGATCACTATAT---------GGGAAATATGAACTTACG---AATAGTGTA---

>2Lcaecilian_AQP9
ATG------------------------------AAT------------------------
----------------------GCAAAGAGCAGAAGAAGCTTGAAAGATAAGATT------
TCACTTAAGAACAGCCTGGTAAAAGAAACCCTGTCCGAGTTCTTTGCAACTTCTTTACTG
ATTGTACTTGGGTGCGGCTGTATTGCTCAGTCTGTCCTGAGTCAAGGGGCTGCTGGGAAT
ATTATCACGATAAGTGCTGGGTTTGCAATGGCAGTGACTATGGCAGTGTATGTAGCAGGA
GGTGTATCTGGTGGTCACATAAACCCAGCGGTTTCATTTGCTATGTGCTTAACTGGAAAG
TTAAAATGGAGAAAATTTCCTTTTTATGTATTGGCGCAAATGTTGGGTGCCATGGCTGGA
GCAGCAGCAGTTTTC---GGGGTCTATTATGATGCATTGATGCATTACACA---GGTGGA
AATCTTACCGTCAGTGGTCCAAAT---GCAACAGCCCAGATTTTTGCAACATACCCTGCT
CCGTTCCTGTCCACCATGAATGGATTTGTAGACCAAGTGATGGCCACGGCTTTGTTAGTA
ATTGCTATTTTTGCTATCTTTGATAGCAAAAATATAGGTGTACCCAAGGGGTTGGAACCA
GTTGCAGTGGGTTTACTCATTATGCTTCTATGTTTCTCCCTGGGGCTTAACTCAGGCTGT
GCCATAAACCCAGCTAGAGATCTAGGGCCAAGGATCTTCACCGCAGCAGCAGGATGGGGA
CTTGATGTATTCAGGGCTGGAAATAGTTGGTGGTGGATTCCTGTTGTTGGACCTATGATT
GGAGCTGCTATTGGAGCATTTGCCTATATGATGTGCATTGAAATTCACCATAGTAAACAA
TCTGAAGATGTAAAG---------------------------------TCAGAA------
CAAAATGATGGAATGTAT---------GAAAAATCTGAACTGACC---TCTATG------

>Gaboon_caecilian_AQP9

ATG------------------------------ACC------------------------
---------------------ACAGCAAGCAGACGAAGCTTGAGAGACAGAATT------
GCGCTGAAGAACAGCCTTACAAAAGAAGTCTTGGCTGAGTTCTTTGCGACGTCGTTACTG
ATTGTTTTTGGATGTGGTTGCGTTGCTCAGGCTGTTCTGAGTCGAGGAGCTGCTGGGAAT
ATTGTATCAATCAGTGCTGGGTTTGCAATGGCAGTAACTATGGCAGTGTATGTAGCAGGC
GGCGTATCTGGTGGTCACGTAAACCCAGCTGTATCATTTGCTATGTGCTTAATGGGAAAG
CTTCCATGGAGAAAACTTCCTTTCTACATACTGGCACAGTTGCTGGGTGCCATAGCTGGA
TCAGCAGTGGTCTTT---GGTGTCTATCATGATGCACTGATGGAATACACG---GGTGGA
GAACTTACTACCAGCGGTCCGAAT---GCAACAGCTCATATCTTTGCCACATATCCATCT
CAATTCCTCTCTACTATGAATGGATTTGCAGACCAAGTAATAGGAACGGCTCTGTTGGTG
ATTGCTATTTTTGCTATTTTTGATAGCAAAAATATAGGCGCACCCCGAGGGTTGGAACCA
ATTGCAGTCGGTCTGCTCGTTATGCTTCTAAGCTTCTCGATGGGGCTAAACTCAGGCTGT
GCCGTAAATCCAGCCAGAGATCTAGGGCCAAGGCTCTTCACAGCAGCAGCAGGATGGGGA
CTTGATGTATTCAGAGCTGGAAATAGCTGGTGGTGGATTCCTGTTGTGGCACCTATGGTT
GGAGGAGCCGTTGGCGCTTTCGTCTACATGATATGCATCGAAGTCCACCACAGTAAACAA
TCAGACAATAAGAAG---------------------------------TCAGAA------
CACAATTCTGACATGTAT---------GAAAAATGTGAACTGACC---AATATG------

>Coelacanth_Aqp9
ATG------------------------------GAT------------------------
---------------------GCAAGGGACAAGAAGAGCTTCAAAGACAAGTTC------
GTCTTAAGGAATAGACTGGTTAAGGAATCATTATCAGAGTTTCTAGGGACATGTCTATTA
ATAGTTTTTGGATGTGGTTCAATTGCTCAGTCTGTACTCAGTCATGAGAGTATGGGTGGG
TTTCTAACAATAAACATTGCTTTTCCAATGGCAGTGACTATGGCTATTTATGTAGGAGGG
GGAGTATCAGGTGCGCACATAAACCCAGCTGTTTCGTTTGCTATGTGTATTGTGGGGAGA
CTTAACTGGTTGAAGCTGCCATTCTATATTGTAGCTCAGATGCTGGGTGCATTTGTTGGC
GCAGGTGCAGTTTTC---GGAATTTATTACGAAGCTTTAATGACCTACACA---GGGGGA
GTGCTTACAGTCACTGGTCCCAAT---GCAACAGCACAGATATTTGCCACTTATCCTTCT
CCGTTCTTGTCTATTGCAAGTGGGCTTATAGACCAAATTATAGGAACATCTTTACTGTTA
GTTTGCATCTTTGGTATTTTTGACAATAAAAATAATGGTGTGCCAAAGGGATTGGAACCA
ATAGCAGTTGGCCTCTGTATTATGGTGCTTGGGTTGTCCATGACATTAAACTGTGGCTGT
GCCATCAACCCAGCACGGGATCTAGGTCCAAGAATGTTTACAGCAGTGGCAGGATGGGGT
ACTGAAGTTTTCAGTGCTGGAAATAACTGGTGGTGGGTTCCTGTTGTCGGCCCTATGATT
GGAGCAGCTGTTGGATGTTTCATTTACGTTATGTTTATTGAACTTCACCACCTAGACCCG
CTTGATGAATCAACA---
GGCGACAATGACTCAAAT---------GAGAAATGTGAAATAATT---GCAATGAAG---

>mCoelacanth_Aqp9
ATG------------------------------GAT------------------------
---------------------GCAAGGGACAAGAAGAGCTTCAAAGACAAGTTC------
GTCTTAAGGAATAGACTGGTTAAGGAATCATTATCAGAGTTTCTAGGGACATGTCTATTA
ATAGTTTTTGGATGTGGTTCAATTGCTCAGTCTGTACTCAGTCATGAGAGTATGGGTGGG
TTTCTAACAATAAACATTGCTTTTCCAATGGCAGTGACTATGGCTATTTATGTAGGAGGG
GGAGTATCAGGTGCGCACATAAACCCAGCTGTTTCGTTTGCTATGTGTATTGTGGGGAGA
CTTAACTGGTTGAAGCTGCCATTCTATATTGTAGCTCAGATGCTGGGTGCATTTGTTGGC
GCAGGTGCAGTTTTC---GGAATTTATTACGAAGCTTTAATGACCTACACA---GGGGGA
GTGCTTACAGTCACTGGTCCCAAT---GCAACAGCACAGATATTTGCCACTTATCCTTCT
CCGTTCTTGTCTATTGCAAATGGGCTTATAGACCAAATTATAGGAACATCTTTACTGTTA
GTTTGCATCTTTGGTATTTTTGACAATAAAAATAATGGTGTGCCAAAGGGATTGGAACCA
ATAGCAGTTGGCCTCTGTATTATGGTGCTTGGGTTGTCCATGACATTAAACTGTGGCTGT
GCCATCAACCCAGCACGGGATCTAGGTCCAAGAATCTTTACAGCAGTGGCAGGATGGGGT
ACTGAAGTTTTC--


```
------------------------------------------------------------
>European_seabass_Aqp9a
---------------------------------GAA------------------------
---------------------ACACAACGCAGGAGAAGCATGAGGCAACACTGT------
GCTCTCAAACATGGAATATTTAAGGAATTCCTGGCAGAATTCCTAGGAACATTTGTCTTG
GTTCTGTTTGGCTGCGGCTCAGTCGCTCAGACCGTCCTCAGTCGAAACAGCCTGGGTGAA
CCTCTCACCGTCCACATCGGCTTCTCTGTGGGACTGATGATGGCAGCGTATGTGGCTGGT
GGAGTGTCAGGGGGCCATGTGAACCCTGCTGTGTCTCTGGCCATGGTGATTCTGGGAAAA
CTGAAGATCTGGAAGTTTCCCTTCTATGTCATCGCTCAGTTTCTTGGTGCTTTTGTTGGC
GCTGCTGCAGTCTTT---GGGTTGTACTATGATGCTTTCATGGACTTCACC---AGTGGG
ATTCTGTCAGTGACGGGAATCAAT---GCAACAGGTCACATTTTTGCTTCCTACCCTGCG
AGACACCTGTCAGTCATCGGCGGCTTCATCGATCAGGTGATAGGGACGGGTATGCTGGTC
TTGTGTATACTGGCTATCATTGATGGTGGAAATATCGGAGCTCCCAAAGGCGTGGAACCG
CTTGCCATTGGTCTGATCATCATGGCCATCGGTGTGTCCATGGGGCTGAACTGTGGCTAC
CCCCTGAACCCTGCCAGAGACCTGGGACCCCGACTGTTCACAGCTTTAGCAGGATGGGGA
ATGGAGGTCTTCAGGACTGCAGACTACTGGTGGTGGATCCCTGTGGCGGGGCCCATGGTG
GGGGGCGTGGTCGCAGCCATCATCTACTACCTGTTTATTGAGCTGCACCACCACCGTGAT
GAGCCCGAGAACCCCCACGAGGAGGAGGAAGAAGAGGAGGAAGATGAAGAGGAC------
GAGGACAGCAGTCTGAAG---------GACAAATATGAGATGATC---ACCATGAGC---
------------------------------------------------------------
------------------------------------------------------------
------------------------------------------------------------
>Nile_tilapia_Aqp9a
ATG------------------------------GAA------------------------
---------------------GTACAAAACAAGAGAAGCATGAGGCAACACTGT------
GCTCTAAAACATGGAATATTCAAGGAATTCCTGGCAGAATTCCTGGGAACCTTTGTCTTG
GTTCTGTTTGGCTGTGGCTCCGTTGCTCAGACCGTCCTCAGTCGAAACACCCTGGGCGAA
CCCCTCACCGTCCACATCGGCTTCTCTGTAGGCCTGATGATGGCAGCGTATGTGGCCGGT
GGAGTTTCAGGGGGCCATGTGAACCCCGCTGTGTCTTTGGCCATGGTAATTTTGGGCAAA
CTGAAGATCTGGAAGTTTCCGTTCTACATCATCGCTCAGTTTCTTGGTGCTTTTGCAGGA
GCTGCTGCAGTCTTT---GGATTATACTATGATGCCTTCATGGACTTCACC---AGTGGG
ATTCTGTCAGTGACGGGAATCAAT---GCAACGGGCCACATTTTTGCCTCATACCCCGCC
AGACACCTGTCAGTCCTCGGCGGCTTCATTGATCAGGTGGTGGGAACAGGTATGCTGGTT
TTGTGCATTCTTGCTATCACTGACAGCGGAAACATCGGAGCTCCTAAAGGCATCGAGCCG
CTGGCCATCGGCCTGATCATCATGGCCATCGGCGTGTCCATGGGGCTGAACTGTGGCTAC
CCCCTAAACCCAGCCAGAGACCTGGGACCCAGACTGTTCACAGCTGTGGCAGGATGGGGG
ATGGAGGTCTTTAGGACCGCAGACAACTGGTGGTGGATCCCAGTGGCGGGACCGATGGTG
GGGGGAGTGGTCGCGGCCGTCATCTACTACCTGTTCATCGAGCTGCACCACCCCCACGCC
GAGCACGAGAAGCCCCAGGAGGAAGAGGAGGAGGACGAGGAAGACGAAGAGGAT------
GATGACAGCAGTCTGAAG---------GACAAATATGAGATGATC---ACGATGAGT---
------------------------------------------------------------
------------------------------------------------------------
------------------------------------------------------------
>Mummichog_Aqp9a
------------------------------------------------------------
---------------------------------AGAAACATGAGGCAGCACTGC------
GCGCTCAAACATGGAATATTCAAGGAATTCCTGGCTGAGTTTCTCGGGACTTTTGTCTTG
GTATTGTTTGGCTGTGGCTCAGTTGCTCAGACCGTCCTGAGTCGAAACACGCTGGGTGAG
CCGCTCACCATCCACATCGGCTTCTCTGTGGGCCTCATGATGGCAGTGTACGTGTCTGGT
GGGGTGTCAGGAGGCCATGTGAACCCCGCTGTGTCTCTGGCCATGGTGATTCTGGGCAAA
CTGAAAATCTGGAAGTTTCCCTTCTACGTCATCGCTCAGTTTCTTGGTGCTTTTGCAGGA
GCTGCGGCGGTCTTC---GGATTATATTACGATGCTTTCATGGACTTCACA---AGTGGT
ATTTTGTCAGTGACAGGAATCAAT---GCGACAGGTCACATTTTTGCCTCTTACCCCGCC
AGACACCTGTCAGTCCTCGGAGGCTTCATTGATCAGGTGGTGGGGACGGGCATGCTGGTT
CTCTGTATCCTGGCCATCATAGACGGCGGCAACATCGGCGCTCCTAAAGGCGTGGAGCCG
CTGGCCATCGGCCTGATCATCATGGCCATCGGCGTGTCCATGGGACTCAACTGCGGCTAC
CCCCTGAACCCCGCCAGAGACTTCGGACCCCGACTGTTCACCGCAGTGGCAGGATGGGGG
ATGGAGGTCTTCAGGACTGGAGGCTACTGGTGGTGGATCCCGGTGGCGGGGCCCATGGTG
GGAGGCGTGGTGGCCGCCGTCCTCTACTTCCTGCTCATCGAGCTTCACCACCCCCACGAC
GAGGATGAGAAGGCCCACGAGGTGGAGGAAGAGGAAGAAGAGGAGGAAGACGAC------
GAGGACAGCAGTCTTAAG---------GACAAATACGAGATGATC---GCCATGAGT---
```


>Zebrafish_Aqp9a

---------------------------------------ATGAAGCAGCACTGC------
GCGCTCAAACAGCGGCTCTTCAAGGAGTTTCTGGCCGAGTTTTTGGGGACCTTCGTGCTG
GTGCTGTTCGGCTGTGGGTCGGTGGCTCAGACAGTGTTGAGCAGAAACACACTCGGTGAA
CCGCTGACCATCCACATCGGCTTCAGCACCGGCCTCATGATGGGCGTCTATGTGTCCGGC
GGCGTCTCAGGCGGGCACCTGAACCCGGCCGTGTCTCTGGCGATGGTGATTCTGGGGAAG
CTGAAGATCTGGAAGTTCCCGGTGTACGTGATCGCGCAGATGCTCGGAGCGTTTGCAGGA
GCGGCCGCTGTGTTC---GGCCTGTACTACGACGCCTTCATGGAGTTCACC---AGCGGG
ATCCTGTCAGTGACGGGCATTAAC---GCCACAGGACACATCTTCTCCTCGTACCCGGGC
AGACACCTGACGGTCCTGGGCGGGTTCGTGGATCAGGTGGTGGGCACAGGGATGCTGGTG
CTCTGTATTCTCGCTATAGTGGACGGCAGGAACATCGGCGCCCCCAGAGGTGTGGAGCCG
CTGGCTGTGGGTGTGGTGCTGCTGGGCATCAGCGTCTCCATGGGCCTGAACTGCGGATAC
CCCCTGAACCCGGCCCGAGACCTGGGGCCCAGACTCTTCACCGCACTCGCCGGATGGGGG
ATGGAGGTGTTCAGCACTGCAGATTACTGGTGGTGGATCCCAGTTGCCGGGCCGCTGGTG
GGGGGTGTCGTCGGAGCGGTGATCTACTTCCTGTTAATCGAGCTTCATCACTCCAATCAC
AACGACACA---CCACAGGAGGAGCCTGAGGAGGAGGAGGATGAGGATGAAGAA------
GAGGACAGCAGCCTGAAG---------GACAAATACGAGATGATC---AACATGAGC---

>European_seabass_Aqp9b
ATG------------------------------GAA------------------------
---------------------AATGAGAGCAAGAGGAAGATGAAGGAGAGATTT------
GGCCTGAGGCGGGACATTTTTAAGGAGTTCCTGGCGGAATTTCTGGGGATATTTGTCCTG
ATACTCTTTGGATGTGGTTCAGTGGCCCAGACAGTGCTGAGTAAAGGGGCTCTAGGGGAG
CCCTTGACCATCCACGTTGGTTTCACTCTGGGAGTCATGATGGCTGTCTACATGGCAGGG
GGAGTGTCAGGAGCCCACGTTAACCCTGCAGTCTCTCTGGCCATGGTGATCCTGGGGAAG
CTTCCTCTGAAGAAGTTCCCTGTGTATGTGATGGCACAGTTCCTGGGGGCTTTTGCTGGA
TCCTGTGCTGTCTAT---GGGTTGTATTACGATGCTTTGATGGAATACACT---GATGGA
GAATTTGTTGTTACTGGTGCAAAT---GCTACAGCCAACATATTTGCATCTTATCCTGCA
AAACATCTCTCAGTCCTTAATGGGTTTGTTGATCAGGTAATTGCAACTGGTGCGCTGATC
CTGTGCATCCTGGCCATCACTGACAGGAAGAATATCGGTGCTCCAAAAGGTATGGAGCCT
CTGTGCATCGGCCTGATCATTATGGCCATTGGAGTGTCCATGGGTCTTAACTGCGGCTAT
CCAATCAACCCAGCGCGAGACCTCGGCCCACGGTTCTTCACGGCTGTGGCTGGGTGGGGC
ATGGACGTATTCAGGGCTGGAGGTTGCTGGTGGTGGATCCCTGTGGCAGGACCCATGGTG
GGCGGAGCAGTTGGAGCAGGCGTTTACTTCCTCTTCATTGAGTTGCACCACCCTGAGCCT
GAAAAACAG---
GAGGAGAACAATGTTCAG---------GACAAATATGAAATGATA---ACTATGAGT---

>Nile_tilapia_Aqp9b
ATG------------------------------GAA------------------------
---------------------AACGAATACAGGAGGAAGATGAAAGAGAAACTT------
GGCCTGAGGCGAGACATCTTTAAGGAGTTCCTGGCGGAGTTTCTGGGGATATTTTTCCTG
ATACTCTTTGGATGCGGTTCGGTGGCCCAGATGGTCCTGAGCAGAGCAGGTCCTGGTGAC
ATACTGAGCGTCCACATTGGTTTCACTCTTGGAGTCATGATGGCCGTCTATATTGCAGGG
GGAGTGTCAGGAGCCCACGTGAACCCGGCAGTCTCTCTGGCCATGCTGATCCTGGGGAAG
CTGCCTCTAAAGAAGTTCCCCATATACGTGGCAGCACAGTTCCTGGGGGCTTTTGCTGGA
TCATGTGCCGTCTAT---GGATTGTATTATGATGCTTTGATGGACTTTACC---AAAGGA
GAATTTATTGTTACAGGTGAAAAT---GCCACAGCCACCATATTTGCAAGTTATCCTGCA
AAACATCTGTCTGTCCTAAACGGGCTTGGTGATCAGGTAATTGCAACCGCTGCACTGGTC
ATATGCATCCTGGCTATCACTGACAGGAAGAATATTGGCGCTCCAAAGGGCATGGAGCCT
CTGTGCATTGGCCTGATCATCGCGGCCATTGGAGTGTCCATGAATCTGAACTGTGGCTAT
CCAATCAACCCGGCTCGAGACCTCGGCCCCCGTTTCTTCACGGCTTTGGCCGGATGGGGC
ATGGAAGTTTTCAGGGCTGGAGGCTGCTGGTGGTGGATTCCTGTGGTAGGACCCATGGTG
GGTGGCGCAGTTGGAGCAGGTGTTTACCTTATCTTCATTGAGCTGCACCACCCTGAACCT

GAAAAACAA---
GAGGAGAACAATGTCCAG---------GACAAATATGAGATTGTA---ACTATGACT---

>Mummichog_Aqp9b
ATG--------------------------------GAA------------------------
---------------------GATGAGAGGAGGAGGAAGATGAAGGAGAAATTT------
GGCCTGAGGCGGGACGTCCTGAAGGAGTTCCTGGCGGAGTTTCTGGGGATCTTTGTCCTG
ATACTCTTTGGATGCGGTTCAGTGGCCCAGACGGTGCTGAGCAAAGGGGCTCTGGGAGAG
CCGCTCACCATCCACATCGGTTTCACCCTGGGAGTCATGATGGCCGTCTACATGGCGGGG
GGAGTGTCAGGAGCCCATGTGAACCCGGCCGTCTCTTTGGCCATGGTGATTCTGGGGAAG
CTACCTGTGAAGAAGTTCCCGGTGTATGTGGTGGCACAGTTCCTCGGAGCATTTGCTGGC
TCCTGTGCTGTCTTT---GGGTTGTATTACGATGCTTTGATGGAGTACACC---GGTGGG
GTGCTTGCAGTTACTGGTGAAAAT---GCAACGGCCAACATATTTGCCTCTTATCCTGCA
AAACATCTCTCGGTGCTAAATGGATTTGTTGACCAGGTCATTGCAACCGGCGCTCTGATT
CTGTGCATCCTGGCCATCACTGACAGGAAGAACATCGGCGCCCCAAAAGGCATGGAGCCG
CTGTGCATCGGCTTGATCATCATGGCCATCGGCGTCTCCATGGGTCTGAACTGCGGCTAT
CCCATCAACCCGGCGCGAGACCTCGGCCCACGGTTCTTCACTGCCGTGGCCGGGTGGGGC
ATGGAGGTGTTCAGGGCCGGAGACTGCTGGTGGTGGATCCCTGTGGCGGGACCGATGGTG
GGTGGAGCGGTTGGAGCTGGGATTTACTTTCTCTTCATTGACTTGCACCAACCTGAGCCT
CAGAAACAC---
GAGGACAACAACGTTCAG---------GACAAATATGACATCATT---ACTATGACT---

>Zebrafish_Aqp9b
ATG--------------------------------GAGTAT---------------------
---------------------CTCGAGAACATCCGAAATCTGAGGGGGAGATGC------
GTCCTGAGGCGCGACATCATCCGAGAGTTTCTGGCAGAATTACTCGGGACATTCGTGTTA
ATACTTTTCGGTTGCGGTTCAGTGGCCCAGACTGTCCTCAGCAGAGAAGCAAAAGGACAG
CTTCTCACCATCCATTTTGGCTTTACTCTAGGGGTGATGCTGGCCGTCTACATGGCAGGA
GGCGTGTCAGGAGGACATGTGAACCCTGCTGTTTCTTTGGCTATGGTTGTCCTGAGGAAA
CTCCCACTAAAGAAGTTCCCTGTGTATGTGTTGGCCCAATTTCTAGGTGCCTTTTTTGGG
TCTTGTGCCGTCTAC---TGTCTTTACTATGATGCCTTTACAGAATTTGCT---AATGGA
GAGCTAGCTGTAACTGGCCCAAAT---GTCACAGCAGGTATCTTTGCATCATATCCACGT
GAAGGACTCTCATTGTTAAATGGATTCATTGATCAGGTGATTGGTGCAGGTGCCCTGGTC
CTCTGTATTTTAGCTGTTGTAGATAAGAAGAACATTGGAGCACCTAAAGGAATGGAGCCT
CTGCTTGTCGGTCTGAGCATCCTGGCTATTGGAGTGTCAATGGCACTAAACTGTGGATAT
CCTATAAACCCTGCCAGAGACTTGGGACCTCGGCTGTTCACTGCCATTGCAGGATGGGGA
TTAACGGTGTTCAGTGCTGGCAATGGCTGGTGGTGGGTTCCAGTGGTGGGGCCAATGGTG
GGCGGAGTGGTTGGTGCTGCTATCTACTTCCTGATGATCGAGATGCATCACCCTGAGAAC
GACAAGAACCTG--------------------------------------GAA------
GACGACAACAGCCTTAAA---------GACAAATATGAGCTGAAC---ACCGTCAAC---

>Bowfin_Aqp9
ATG--------------------------------GAG------------------------
---------------------AGGAAAAATAAGAGGAACCTGAAGGAGCGATTT------
GCCATGAGAAACAGCATCGTCAAGGAGGCTTTGGCAGAGTTTCTGGGGACATTCGTGCTC
ATTCTCTTTGGCTGCGGGTCGGTGGCCCAGACGGTTCTGAGCCGGGGTGCCGTGGGAGAG
GTGCTGACGATCCACATTGGCTTCACTCTGGGAGTGACAATGGCGGTGTATGTGGCTGGT
GGAGTGTCAGGGGCTCATGTTAACCCAGCAGTCTCCCTGGCCATGCTGGTCCTGGGCAAG
CTGAAGCTGGTGAAGTTCCCAGTGTACGTAGTGGCCCAGTTTCTTGGGGCGTTTGCTGGA
GCGGCCGCCGTGTAC---GGGCTCTACTATGATGCCTTTATGGATTATACC---AACGGC
ATTCTGACTGTTACTGGCCCCAAT---GCTACTGCACAGATTTTTGCTTCGTACCCAGGA
AGACATCTGTCAATCCTGAATGGCTTCATCGATCAGGTCATAGGTACTGGTGCCTTGGTT
CTCTGTATTCTGGCCATCTTGGACGGAAAGAACATTGGGGCCCCCAAAGGCATGGAGCCC
CTGGTGATTGGCCTGATCATCATGGCTATCGGCGTCTCCATGAACCTGAACTGCGGATAC
CCCATCAACCCCGCCCGTGACCTGGGGCCGCGGCTTTTCACCGCAGTGGCAGGGTGGGGC

ACCGCTGTCTTCAGTGCTGGCAATAACTGGTGGTGGATTCCCGTGGCCGGGCCCTTGGTG
GGGGGGATCGTGGGAGCTGTGCTGTACCTGCTCTTCATCGAGCTACACCACACTGAGCCG
CAGAAACACTTG---------------------------------------GAG------
GAGGAGAACAACGTCAAG---------GACAAGTACGAAATGATC---ACCATGAGT---

>Gar_Aqp9
ATG------------------------------GAG------------------------
---------------------AGGAAAACTAGGAGGAACCTGCGGGAGCGCTTC------
GCCCTGAAGAACAGCATTGTCAAGGAAGCCTTGGCAGAATTCCTCGGGACATTCGTACTC
ATCCTTTTCGGCTGTGGGTCAGTTGCACAGACCGTTTTGAGTCGCGGTGCCGTGGGAGAG
ATGCTGACCATCCACATCGGATTCACTCTGGGTGTGACCATGGCGGTGTACGTGGCCGGA
GGAGTGTCAGGGGCACACGTGAACCCCGCGGTGTCCCTCGCCATGCTGGTGCTGGGCAAG
TTGAAGCTGGTGAAGTTCCCGGTGTACGTGCTCGCACAGTTCCTCGGCGCCTTTGCCGGG
GCTGCAGCCGTGTAC---GGGCTGTATTACGATGCCTTTATGGATTACACC---AATGGA
ATACTAATTGTTACTGGTCCCAAC---GCTACAGCACATATCTTTGCATCATATCCGGGA
AGACACTTGTCAATCCTAAATGGATTCATAGATCAGGTCATAGGGACGGGCGCCCTGGTG
CTTTGTATTCTGGCCATTGTGGATGGGAAGAACAATGGGGCTCCCAAAGGAATGGAGCCT
TTAGTGATTGGGCTGATCATCATGGCTATTGGGGTCTCCATGGGACTGAACTGTGGGTAC
CCTATAAATCCTGCCCGGGACCTCGGCCCACGCCTGTTCACTGCAGTGGCAGGATGGGGA
AGAGATGTCTTCAGCGCTGGGAGGAACTGGTGGTGGATTCCAGTGTCTGGCCCGTTGGTG
GGCGGGGTTGTAGGAGCTGTGTTGTACCTGTTGTTCATTGAGCTGCACCACGCCGAGCCG
CAGAAGCACCTG---------------------------------------GAA------
GAAGAGAACAGCGTCAAG---------GAAAAGTATGAAATGATA------ATGAGC---

>Russian_sturgeon_Aqp9
ATG------------------------------GAA------------------------
---------------------AATGAAAACAAGAAGAGCATTAAAGAGAGGTTT------
GCTCTGAGGAACAGCCTGGTGAAGGAAGCATTAGCAGAATTCCTTGGGACATTTCTGCTC
ATTCTTTTCGGCGTCGGGTCAGTTGCCCAAACTGTCCTGAGCAGAGGTTCCATGGGAGAT
CCACTCACCATACACATTGGCTTTACACTCGCAGTCACCATGGCTGTGTATGTGGCGGGA
GGAGTTTCAGGCGCTCACATAAACCCTGCTGTCTCTCTGGCGATGTGTGTGCTGGGGAGG
CTGAGCCTCCGCAAATTCCCAGTCTACGCCGTGTCTCAGTTCCTTGGTGCTTTTATTGGC
GCCGCAGCAGTCTAT---GGACTTTATTATGATGCCTTCCTGGATTATACA---GGTGGA
GTGCTGACAGTTACTGGCCCCAAT---GCAACAGCACACATTTTTTCATCCTACCCTGGT
AAACATCTTTCAATCTTAAATGGATTTATAGATCAGGTTATAGGAACTGGTGCATTGCTC
CTCTGCATCCTTGCCATCTTGGACAACAAGAACAAGGGCGCCCCGAAAGGAATGGAGCCC
TTGATGATTGGTCTCATCATCATGGTTATCGGGGTCTCCATGGGCCTGAACTGCGGCTAT
CCAATCAACCCAGCGCGTGACCTCGGCCCCAGGCTCTTCACTGCCATAGCAGGGTGGGGC
ACTGAAGTTTTCAGCGCTGGAAGCCACTGGTGGTGGATTCCTGTGGCTGGGCCGCTGGTT
GGAGGGATGGTCGGTGCTGTGATCTATGTGCTCTTCATCGAGCTTCACCACGCGGACCCT
CAGAAGACTCCA---------------------------------------GAA------
GAAGAAAACAGCATGAAG---------GATAAATACGAAATGATT---GCAATGAGC---

>Atlantic_sturgeon_Aqp9
ATG------------------------------GAA------------------------
---------------------AATGAAAACAAGAAGAGCATTAAAGAGAGGTTT------
GCTCTGAGGAACAGCCTGGTGAAGGAAGCCTTAGCAGAATTCCTTGGGACATTTCTGCTC
ATTCTTTTCGGCGTCGGGTCAGTTGCCCAGACTGTCCTGAGCAGAGGTTCCATGGGAGAT
CCACTCACCATACACATTGGCTTTACACTCGCAGTCACCATGGCTGTGTATGTGGCGGGA
GGAGTTTCAGGCGCTCACATAAACCCTGCTGTCTCTCTGGCGATGTGTGTGCTGGGGAGG
CTGAGCCTCTGCAAATTCCCAGTCTACGCCGTGTCTCAGTTCCTTGGTGCTTTTATTGGC
GCCGCAGCAGTCTAT---GGACTTTATTATGATGCCTTCCTGGATTTTACA---GGTGGA
GTGTTCACAGTTACTGGCCCCAAT---GCAACAGCACATATTTTTTCATCCTACCCTGGT
AAACATCTTTCAATCTTAAATGGATTTATAGATCAGGTTATAGGAACTGGTGCATTGCTC
CTCTGCATCCTTGCCATCTTGGACAACAAGAACAAGGGCACCCCGAAAGGAATGGAGCCC

TTGATGATTGGTCTCATCATCATGGTTATCGGGGTCTCCATGGGCCTGAACTGCGGCTAC
CCAATCAACCCAGCGCGTGATCTCGGCCCCAGGCTCTTCACTGCCATAGCAGGGTGGGGC
ACTGAAGTTTTCAGCGCTGGAAGTCACTGGTGGTGGATTCCTGTGGCTGGGCCGCTGGTT
GGAGGGATGGCCGGAGCTGTGATCTATGTGCTCTTCATCGAGCTTCACCACGCGGACCCT
CAGAAGACTCCA---------------------------------------GAA------
GAAGAAAACAGCATGAAG---------GATAAATACGAGATGATT---GCAATGAGC---

>Chinese_sturgeon_Aqp9
ATG------------------------------GAA------------------------
---------------------AATGAAAACAACAAGAGAATTAAAGAGAGGTTT------
GCTCTGAGGAACAGCCTGGTGAAGGAAGCCTTAGCAGAATTCCTTGGGACATTTCTGCTC
ATTCTTTTCGGCGTCGGGTCAGTTGCCCAGACTGTCCTGAGCAGAGGTTCCATGGGAGAT
CCACTCACCATACACATTGGCTTTACCCTCGCAGTCACCATGGCTGTGTATGTGGCGGGA
GGAGTTTCAGGCGCTCACATAAACCCTGCTGTCTCTCTGGCGATGTGTGTGCTGGGGAGG
CTGAGCCTCTGTAAATTCCCAGTCTACGCTGTGTCTCAGTTCCTTGGTGCTTTTATTGGC
GCCGCAGCAGTCTAT---GGACTTTATTATGATGCCTTCCTGGATTATACA---GGTGGA
GTGCTGACAGTTACTGGCCCCAAT---GCAACAGCACACATTTTTTCATCCTACCCTGGT
AAACATCTTTCAATCTTAAATGGATTTATAGACCAGGTTATAGGAACTGGTGCATTGCTC
CTCTGCATCCTTGCCATCTTGGACAACAAGAACAAGGGCGCCCCGAAAGGAATGGAGCCC
TTGATGATTGGTCTCATCATCATGGTTATCGGGGTCTCCATGGGCCTGAACTGCGGCTAC
CCAATTAACCCAGCACGTGATCTCGGCCCCAGGCTCTTCACTGCCATAGCAGGGTGGGGC
ACTGAAGTTTTCAGCGCTGGAAGCCACTGGTGGTGGATTCCTGTGGCTGGGCCGCTGGTT
GGAGGGATGGTCGGAGCTGTGATCTATGTGCTCTTCATCGAGCTTCACCACGCGGACCCT
CAGAAGACTCCA---------------------------------------GAA------
GAAGAAAACAGCATGAAG---------GATAAATATGAAATTATT---GCAATGAGC---

>Sterlet_Aqp9_2
ATG------------------------------GAA------------------------
---------------------AATGAAAACAAGAAGAGCATTAAAGAGAGGTTT------
GCTCTGAGGAACAGCCTGGTGAAGGAAGCATTAGCAGAATTCCTTGGAACATTTCTGCTC
ATTCTTTTCGGCGTCGGGTCAGTTGCCCAAACTGTCCTGAGCAGAGGTTCCATGGGAGAT
CCACTCACTATACACATTGGCTTTACACTCGCAGTCACCATGGCTGTGTATGTGGCGGGA
GGAGTTTCAGGCGCTCACATAAACCCTGCTGTCTCTCTGGCGATGTGTGTGCTGGGGAGG
CTGAGCCTCTGTAAATTCCCAGTCTACGTCGTGTCTCAGTTCCTTGGTGCTTTTATTGGT
GCCGCAGCAGTCTAT---GGACTTTATTATGATGCCTTCCTGGATTATACA---GGTGGA
GTGCTGACAGTTACTGGCCCCAAT---GCAACAGCACACATTTTTTCATCCTACCCTGGT
AAACATCTTTCAATCTTAAATGGATTTATAGATCAGGTTATAGGAACTGGTGCATTGCTC
CTCTGCATCCTTGCCATCTTGGACAACAAGAACAAGGGCGCCCCGAAAGGAATGGAGCCC
TTGATGATTGGTCTCATCATCATGGTTATCGGGGTCTCCATGGGCCTGAACTGCGGCTAC
CCAGTCAACCCAGCGCGTGATCTCGGCCCCAGGCTCTTCACTGCCATAGCAGGGTGGGGC
ACTGAAGTTTTCAGGGCTGGAAGCCACTGGTGGTGGATTCCTGTGGCTGGGCCGCTGGTT
GGAGGGATGGTCGGAGCTGTGATCTATGTGCTCTTCATCGAGCTTCACCACGCGGACCCT
CAGAAGACTCCA---------------------------------------GAA------
GCAGAAAACAGCATGAAG---------GATAAATATGAAATGATT---GCAATGAGC---

>Sterlet_Aqp9_1
ATG------------------------------GAA------------------------
---------------------AATGAAAACAAGAAGAGCATTAAAGAGAGGGTT------
GCTCTGAGGAACAGCCTGGTGAAGGAAGCCTTAGCAGAATTCCTTGGGACATTTCTGCTC
ATTCTTTTCGGCGTCGGGTCAGTTGCCCAGACTGTCCTGAGCAGAGGTTCCATGGGAGAT
CCACTCACTATACACATTGGCTTTACACTCGCAGTCACCATGGCTGTGTATGTGGCGGGA
GGAGTTTCAGGCGCTCACATAAACCCTGCTGTCTCTCTGGCGATGTGTGTGCTGGGGAGG
CTGAGCCTCTGCAAATTCCCAGTCTACGCCGTGTGTCAGTTCCTTGGTGCTTTCATTGGT
GCCGCAGCAGTCTAT---GGACTTTATTATGATGCCTTCCTGGATTATACA---GGTGGA
GTGCTGACAGTTACTGGCCCCAAT---GCAACAGCACACATTTTTTCATCCTACCCTGGT

AAACATCTTTCAATCTTAAATGGATTTATAGATCAGGTTATAGGAACTGGTGCATTGCTC
CTCTGCATCCTTGCCATCTTGGACAACAAGAACAAGGGCGCCCCGAAAGGAATAGAGCCC
TTGATGATTGGTCTCATCATCATGGTTATCGGGGTCTCCATGGCCTGAACTGCGGCTAC
CCAGTCAACCCAGCGCGTGATCTCGGCCCCAGGCTCTTCACTGCCATAGCAGGGTGGGGC
ACTGAAGTTTTCAGGGCTGGAAGCCACTGGTGGTGGATTCCTGTGGCTGGGCCGCTAGTT
GGAGGGATGGTCGGAGCTGTGATCTATGTGCTTTTCATCGAGCTTCACCACGTGGACCCT
CAGAAGACTCCA---------------------------------------GAA------
GAAGAAAACAGCATGAAG---------GATAAATACGAAATGATT---GCAATGAGC---

>Reedfish_Aqp9
ATG------------------------------GAA------------------------
---------------------GCGGAGAAT---AAAAGTTTTAAGAAGAGAATT------
GCTGTGAGAAGCAAAATCCTAAGGGAAGCGCTCGCAGAATTCCTTGGAACATTTATACTA
ATTATTTTTGGCTGTGGCTCAGTCGCCCAGACCGTCCTCAGCAGCGGGGCTTTTGGTGAG
ATGCTGACTATTCACATTGGCTTCACTATTGGTGTCATAATGGCAGTATACGTGGCAGGG
GGGATTTCAGGTGCACATGTGAATCCTGCAGTTTCTCTTGCCATGTGTGTTACGGGCAGG
TTGAGCTTCTTGAAGTTCCCAGTCTATGTCCTGTCTCAGTTCCTTGGGGCTTTCACTGGT
GCAGCAGCAGTGTAT---GGACTCTACTATGACGCATTTCTGAACTACACA---GGAGGC
GTGCTCACTGTCACCGGCCCTAAT---GCAAGTGCACAGATATTTGCAACTTACCCTGCA
ACATATCTTTCAATATTTAATGGCTTCATGGATCAGGTGGTAAGTACCGGAGCTTTGGTG
CTCTGCATCTTTGCCATCACTGACAAGAAGAACAACGGTGCCCCAAAAGGAATGGAGCCA
CTTCTCATTGGTTTAATTATCATGGTAATTGGGGTCTCTATGGGCCTGAACTGTGGCTAC
CCCATCAATCCAGCTCGAGATCTTGGCCCTCGCCTTTTTACCTTCATTGCAGGGTGGGGT
GTTGAAGTTTTCAGGGCTGGGAATAACTGGTGGTGGGTCCCTGTGGCAGGCCCAATGGTT
GGTGGGATTGTTGGTGCCTTGATCTACGTTGCATTTATTGAGCTGCACCATCACGACCCA
CAGAAGCAATCT---------------------------------------GAA------
GAAGATCACAATGTCCAA---------GATAAATATGAGATGATT---ACAATGAAC---

>B_bambooshark_Aqp9
ATG------------------------------GAGACA---------------------
---------------------CAGAGCCAGAAGCACAGCCTGATAGACAAATGC------
AAACTAAAGAATAGATGGATTAAAGAAGGGCTCGCCGAATTTTTGGGAACATTTATACTG
ATTCTGTTTGGTTGTGGTTCACTTGCCCAATCAATACTGAGCAGAGGAGTATATGGCAAC
ATGCTGACAGTTGCCATTGGCTTTCCATTTGGAATCACCATAGCTGTTTATGCTACCATA
GGAGTATCAGGAGCACATCTGAATCCTGCTATTTCCCTTTCTATGTGTGTTCTTGGACGG
CTTCAGTGGTTGAAACTGCCGGTCTACTGTTTTTCTCAGCTTGTCGGGGCCTTTGTTGGT
TCAGCAGCTGTTTTT---GGACTTTACTATGATGCGTTCATGGCTTTTGAT---GATGGG
AACTTCGCAATAACTGGTCAAAAT---GCAACAGCACAAATCTTCTCCTCTTATCCAGCC
TCACATTTGTCGTTTGCAAATGGCTTCGCAGACCAGGTAGTGGGTACTGCTGCCCTTCTC
TTCTCCATTCTGGCCATTTTAGACTCTAAGAATAATTGCGTCCCCAAAGGTTTGGAGCCA
GTGGTGATTGGCCTCATCATCATGGTCATTGGTCTTTCGATGGGGTACAACTGTGGTGGT
CCCATCAACCCAGCTCGTGATCTTGGACCACGACTCTTCACAGCCGTGGCTGGATGGGGA
CTGGAGGTTTTCACGGCTGGTAATGGATGGTGGTGGGTTCCTGTCATCGCACCACTGATT
GGAGGAGTTCTCGGGACTGCCATTTATGTACTGATAATTGAGCTGCACCACATGGACACA
ACCCTGAAGGAGAACCAC------------------------TGTACCTCAGAA------
CAACAGGCGGAAGGGAAG---------GCAAAATATGAAATGATT---ACAATACAGTGC
AACAAA--

>W_bambooshark_Aqp9
ATG------------------------------GAGACA---------------------
---------------------CAGAGCCAGAAGCACAGCCTGATAGACAAATGC------
AAACTAAAGAATAGATGGATTAAAGAAGGGCTCGCCGAATTTTTGGGAACATTTATACTG
ATTCTGTTTGGTTGTGGTTCACTTGCCCAATCAATACTGAGCAGAGGAGTATATGGCAAC
ATGCTGACAGTTGCCATTGGCTTTCCATTTGGAATCACCATAGCTGTTTATGCTACCATA
GGAGTATCAGGAGCACATCTGAATCCTGCTATTTCCCTTTCTATGTGTGTTCTTGGACGG
CTTCAGTGGTTGAAACTGCCGGTCTACTGTTTTTCTCAGCTTGTCGGGGCCTTTGTTGGT

TCAGCAGCTGTGTTT---GGACTTTACTATGATGCGTTCATGGCTTTTGAT---GATGGG
AACTTCGCAATAACTGGTCAAAAT---GCAACAGCACAAATCTTCTCCTCTTATCCAGCC
TCACATTTGTCGTTTGCAAATGGCTTCGCAGACCAGATGATGGGTACTGCTGCCCTTCTC
TTCTCCATTCTGGCCATTTTAGACTCTAAGAATAATTGCGTCCCCAAAGGTTTGGAGCCA
GTGGTGATTGGCCTCATCATCATGGTCATTGGTCTTTCGATGGGGTACAACTGTGGTGGT
CCCATCAACCCAGCTCGTGATCTTGGACCACGACTCTTCACAGCCGTGGCTGGATGGGGA
CTGGAGGTTTTTACGGCTGGTAATGGATGGTGGTGGGTTCCTGTCATCGCACCACTGATT
GGAGGAGTTCTCGGGACTGCCATTTATGTTCTGATAATTGAGCTGCACCACATGGACACA
ACCCTGAAGGAGAATCAC------------------------TGTACCTCAGAA------
CAACAGGCGGAAGGGAAG---------GCAAAATATGAAATGATT---ACAATACAGTGC
AACAAA--

>Whale_shark_Aqp9
ATG--------------------------------GAGACA---------------------
----------------------CAGGCTCAGAAGCACAGCCTGATAGACAAATGC------
AAATTAAAGAATAGATGGATCAAAGAAGGGCTCGCCGAATTTCTGGGAACATTTATACTG
ATTCTATTTGGTTGTGGTTCACTTGCCCAATCAATACTGAGCAGAGGAGTAAATGGCAAC
ATGCTGACAGTTGCCATTGGCTTTCCATTTGGAATCACCATAGCTGTTTATGCTACCATA
GGAGTATCAGGAGCACATCTGAATCCTGCTATTTCCCTTTCTATGTGTGTTCTTGGACGG
CTTCAGTGGTTGAAACTGCCAGTCTACTGCTTTTCTCAGCTTGTCGGGGCTTTTATTGGC
TCAGCAGCCGTTTTT---GGACTTTACTATGATGCGTTCATGGCTTTTGAT---GGTGGG
AACTTGGCAATAACTGGTCAAAAT---GCAACAGCACAAATTTTCTCCTCTTATCCAGCT
CCGCATTTGTCTTTTGCAAATGGCTTTGTAGACCAGGTAGTGGGTACTGCTGCTCTACTC
TTCTCCATTCTGGCTATTTTGGACTCTAAGAATGATTGCGTCCCCAAAGGTTTGGAGCCA
GTGGTGATTGGCCTCATCATCATGGTCATTGGTCTTTCGATGGGGTTCAACTGTGGTGGC
CCCATCAATCCAGCGCGTGATCTTGGACCACGACTCTTCACAGCTGTGGCTGGATGGGGA
TTTGAGGTTTTCACGGCTGGTGATGGATGGTGGTGGGTCCCTATCATCGCACCACTGATT
GGAGGAGTTCTCGGGACTGCCATTTATGTTCTGATAATTGAGCTTCACCACAAGGAGACA
GCCCCAAAGGAGAACCAC------------------------TGTACCTCAGAA------
CAACAGATGGAGGGGAAG---------GCAAAATATGAAATGACT---TCAATACAGTGC
AACAAA--

>Zebra_bullhead_shark_Aqp9
ATG--------------------------------GAAACA---------------------
----------------------CAGAAGCAGAAACAGAGTCTGGTCGACAAATGC------
AAATTAAAGAATAGCTGGATTAAAGAAGGACTCGCCGAATTTCTGGGAACATTTATACTG
ATTCTGTTTGGTTGTGGTTCACTTGCCCAATCAATACTGAGTAGAGGAGTAAGTGGCAAC
ATGCTGACTGTATCCATTGGCTTTTCATTTGGAATCACCATAGCTGTTTATGCTACCATA
GGAGTATCAGGGGCACATCTGAATCCTGCTATTTCCCTCTCTATGTGTGTGCTGGGTCGG
CTTCAATGGTTGAAACTTCCCATCTACTGTGTTGCTCAGTTTGTCGGGGCCTTTGTTGGT
TCAGCAGCAGTTTTT---GGGCTTTATTATGATGCATTCATGGCTTTTGAT---GGTGGG
AACTTGGCAATAACTGGTCAAAAT---GCAACCGCGCAGATCTTCTCCTCTTATCCAGCT
CCACATTTGTCTTTTGCGAATGGCTTTGCAGACCAGATTGTGGGTGCTGCTGCACTGCTC
TTCTCTATCCTGGCCATTCTGGACTCGAAGAATGATTGTGTACCCAAAGGTTTGGAGCCA
GTGGTGATTGGCCTGATCCTCATGGTTATCAGTCTTTCAATGGGCTTCAACTGCGGTGGT
CCTATAAACCCAGCTCGTGATCTCGGACCCAGGCTCTTTACAGCTGTGGCTGGATGGGGA
TTTGAGGTTTTCAGGGCTGGTGATGGATGGTGGTGGGTTCCTGTCATCGCACCAATGATT
GGCGGAGTTCTCGGGACGGCCATTTACGTTCTGATTATTGAGCTGCACCATAAAGACATA
ACGCCAAAGGAGATCCGC------------------------TGTACTTCAGAA---CCC
CCGCAAAGTGAAGGGAAG---------GCAAAATATGAAATGATT---TCAGTACAATGT
AACAAA--

>Cloudy_catshark_Aqp9
ATG--------------------------------GAAACA---------------------
----------------------GAACCCCAGAAACACAGCCTGACAGACAAATAC------
AAGTTAAAGAACAGCTGGATAAAACAAGGGCTCGCCGAGTTCGTGGGGACCTTTATGTTG
ATTCTCTTTGGTTGCGGTTCACTTGCCCAATCAGTACTGAGCAGAGGCGTTAGTGGCAAC
ATGCTGACTGGTTCCATTGGCTTCTCATTTGGACTCACCATAGCTGTTTATGCTACCATA

GGAGTGTCAGGGGCACATCTGAATCCTGCTATTTCCCTTTCCATGTGTGTGTTGGGAAGG
CTCCAATGGTTGAAACTGCCTGTCTACTGCATTGCTCAGTTTATTGGAGCCTTTGTCGGT
TCAGCGACAGTCTTT---GGGCTTTATTACGATGCATTCATGGCTTTTGAT---GGCGGA
AACTTGGCAATAACGGGCCAAAAT---GCAACAGCGCAGATTTTCTCCTCTTATCCAGCT
CCACATTTATCCTTTGCGAATGGCTTTGCAGACCAGATCATAGGTACGGCAGCTCTGCTC
TTCTCCATTCTGGCCATCTTGGACTCGAAGAATGACGGTGTACCCAAGGGTTTGGAGCCG
GTGGTCATTGGCCTGATCATCATGGTCATCGGTTTTTCAATGAGCTTCAACTGTGGTGGT
CCTATCAACCCAGCTCGTGACCTTGCGCCGCGGCTCTTCACAGCCATGGCGGGGTGGGGA
TTTGGGGTATTCAGGGCCGGCGACGGGTGGTGGTGGGTTCCTGTCATCGCACCAATGATC
GGCGGAGTTGTTGGGACTCTTGTTTATGTCCTGATCATCGAGCTGCACCACAACGAGACA
GTCCCAAAGGAGAGGTCC------------------------TGTACCTCAGAA------
CAGCAACTGGAGGCGAAG---------GCAAAATACGAAATGATT---TCTGTGCAGTGT
AACAAA--

>Great_white_shark_Aqp9
ATG------------------------------GAAACG---------------------
---------------------CAGATTCAGAAGCACAGCCTGATAGACAAGTGC------
AAATTAAAGAACAGCTGGATAAAACAAGGGCTCGCCGAATTTCTGGGGACATTTGCATTG
ATTCTGTTTGGTTGTGGTTCAGTTGCCCAAGCAGTATTGAGCAGAGGAGTAAGTGGCAAC
CTGCTGACTGTTTCCATTGGCTTTCCATTTGGAATCACCATAGCTGCTTATGCTACCATA
GGAGTATCAGGGGCACATCTGAATCCTGCTATTTCCTTTTCTATGTGTGTGCTGGGACGG
CTTCAATGGTTGAAACTGCCTGTCTACTGCCTTGCTCAGCTTGTAGGAGCCTTTGCTGGT
TCAGCAGCAGTTTTT---GGGCTTTATTATGATGCATTCATGGCTTTTGAT---GGTGGG
AACTTGGCAATAACTGGTGAAAAT---GCAACAGCGCAGATTTTCTCCTCTTATCCAGCT
CCACATTTGTCTCTTGCAAATGGCTTTGCAGACCAGATCGTGGGCACTGCTGCTCTGCTC
TTCGCTATCCTTGCCATTTTGGACTCCAAGAATAATGCAGTGCCCAAAGGTTTGGAGCCA
GTGGTGATTGGCCTGATCATCATGGTTATCAGTCTTTCAATGGGCTTCAACTGTGGCTGT
CCTATCAACCCAGCTCGTGACCTTGGGCCGCGGCTCTTCACAGCTGTGGCTGGATGGGGA
TTTGAGGTTTTCAGGGTCAGCGATGGGTGGTGGTGGGTTCCTGTCATCGCACCAATGATC
GGTGGAGTCCTTGGGACCGCCATTTATGTTCTGACAATCGAGCTGCACCATGAAGAGACA
ACTCCAAAGGAGAGCCAC------------------------ACTACCTCAGGA------
CAGCAAGTGGAAGGGAAG---------GCAAAATATGAAATGATT---TCAGTGCAGAGT
AACAAA--

>Spiny_dogfish_Aqp9
ATG------------------------------GAGCCG---------------------
---------------------CAGATCCAGAAGAAGAGTCTGATAGACAGATGC------
AAATTAAAGAACCGCTTCCTAAAAGAAGGGCTGGCCGAATTTTTGGGAACATTCATATTG
ATTCTGTTTGGTTGTGGTTCACTCGCCCAATCAATACTGAGCAGAGGAGTAAGTGGTAAT
ATACTGACCTCTTCCATTGGCTTTCCATTTGGAATTACCATAGCTGTTTATGCTACAATG
GGAGTTTCAGGGGGACACCTGAATCCTGCGATTTCCCTTTCTATGTGTGTGCTGGGACGG
CTTCAATGGTTGAAACTTCCTGTCTACTGTGTTGCTCAGCTTTTCGGAGCCTTTATCGGT
TCAGCGGCAGTTTTT---GGGCTTTATTACGATGCCTTCATGGCTTTCGAT---GACGGG
AACTTGACAATAACCGGTCAAAAT---GCAACAGCGCAGATTTTCTCCTCTTATCCAAGT
CCACATTTATCATTTGCGAATGGCTTTGCAGATCAGATTGTGGGCGCTGCTGCACTGCTC
TTCTCTATTCTGGCCATTTTGGACTCTAAGAATGACGGTGTACCCAAAGGTTTGGAGCCA
GTGGTGATTGGCCTGATCGTCATGGTTATCGGTGTTTCAATGGGCTACAACTGTGGTTGT
CCCATAAACCCAGCTCGTGACCTTGGACCCCGGCTCTTTACAGCTGTGGCCGGATGGGGA
TTTGAGGTTTTCAGTGCGGGCGATGGCTGGTGGTGGGTTCCTGTCATCGCACCAATGATT
GGAGGAATGCTCGGGACTTCCATTTATGTTCTGATAATTGAGCTGCACCACAAAGAGTCG
ATCCCAGAGGAGAGCCGC------------------------TGTATTCCAGAA------
CCGCAAATCGAAGGGAAG---------GCAAAATATGAAATGATT---TCAATACAGTGT
AACAAA--

>Little_skate_Aqp9
ATG------------------------------GAAGCT---------------------
---------------------CTGGTCCAGAAGAAGTCTTTGGTGGATAAATGC------
AAGCTGAAGAACAGCTGGATCAAAGAAGGGCTCGCCGAATTTCTCGGGACATTTATTCTA

ATTTTGTTTGGTTGCGGTTCTATAGCTCAGTCAGTGCTGAGCAGAGGAGCGAGTGGCAAT
ATGCTGACCTCTTCTATTGGTTTTCCATTTGGTGTTACGATCGGTGTTTATGCAACAATA
GGGGTTTCAGGGGCGCACCTGAATCCTGCAATTTCGCTGTCAATGTGTGTACTGGGCCGT
CTTCAGTGGTTGAAACTTCCCATCTACTGCACTGCTCAGCTCATAGGAGCTTTTATTGGT
GCCGCAGCAGTATTT---GGTCTTTATTATGATGCCTTCATGAGTTATGAT---GGCGGA
AACTTGACAATCACCGGTCAAAAT---GCAACTGCACACATCTTCTCCTCTTATCCTGCT
CCACATCTATCCTTTGCAAATGGATTT------CAGATTGTGGGCACGGCCGCGCTACTT
TTCTCCATTTTGGCCATTTTGGACTCCAAGAACGATCGTGTGCCCAAGGGTCTGGAGCCG
GTGGTGATCGGTCTGATCCTCATGGTGATCGGACTATCGATGGGATACAACTGCGGTGGT
CCCATCAACCCAGCGCGTGACCTGGGTCCTCGGCTCTTCACTGCTGTGGCCGGATGGGGA
ATTGAGGTTTTCAGGGCTGGTAATGGCTGGTGGTGGGTCCCTGTCATTGCGCCTTTCATC
GGGGGAGTCCTGGGCACTGCCATTTACGTTCTGTTCATTGAACTACATCACAAAGAGACG
GTCTTGGAGGAGGCCCGG------------------------TGCGTTAAAGAA------
CAGCAGGCGGAGGGGAAA---------ACAAAATACGAAATGATT---GATATACAGATA
AACAAA--

>Winter_skate_Aqp9
ATG------------------------------GAAGCT---------------------
---------------------CTGGTCCAGAAGAAGTCTTTGGTGGATAAATGC------
AAACTGAAGAACAGCTGGATCAAAGAAGGGCTCGCCGAATTTCTCGGGACATTTATTCTA
ATTTTGTTTGGTTGCGGTTCTATAGCTCAGTCAGTGCTGAGCAGAGGAGCGAGTGGCAAT
ATGCTGACCTCTTCTATTGGTTTTCCATTTGGTGTTACCATCGGTGTTTATGCAACAATA
GGGGTGTCAGGGGCGCACCTGAATCCTGCAATTTCGCTGTCAATGTGTGTACTGGGCCGG
CTTCAGTGGTTGAAACTTCCCATCTACTGCACTGCTCAGCTCATAGGAGCTTTTATTGGT
TCTGCAGCAGTATTT---GGCCTTTATTATGATGCCTTCATGAGTTATGAT---GGCGGA
AACTTGACAATCACCGGCCAAAAT---GCAACTGCACACATCTTCTCCTCTTATCCTGCT
CCACATCTATCCTTTGCAAATGGATTTGCCGACCAGATTGTGGGCACGGCCGCGCTACTT
TTCTCCATTTTGGCCATTTTGGACTCCAAGAACGATCGTGTGCCCAAGGGTCTGGAGCCG
GTGGTGATCGGTCTGATCCTCATGGTGATCGGACTATCGATGGGCTACAACTGCGGTGGT
CCCATCAACCCAGCGCGTGACCTGGGTCCTCGGCTCTTCACTGCTGTGGCCGGATGGGGA
ATTGAGGTTTTCAGAGCTGGTAATGGCTGGTGGTGGGTTCCTGTCATTGCGCCTTTCATC
GGGGGAGTCCTGGGCACTGCCATTTACGTTCTGTTCATTGAACTACATCACAAAGAGACG
GTCTTGGAGGAGGCCCGG------------------------TGCGTTAAAGAA------
CAGCAGGTGGAGGGGAAA---------ACAAAATATGAAATGATT---GATATACAGATA
AACAAA--

>Thorny_skate_Aqp9
ATG------------------------------GAAGCT---------------------
---------------------CAGATCCAGAGGAAGACTTTGGTGGATAAATGC------
AAACTGAAGAACAGCTGGATCAAAGAAGGGCTCGCCGAATTTCTGGGGACATTTATTCTA
ATCTTGTTTGGTTGCGGATCTATAGCTCAGTCAGTGCTGAGCAGAGGAACGAGTGGCAAT
ATGTTGACCTCTTCTATTGGTTTTCCATTTGGAGTTACCATCGGTGCTTATGCGACAATA
GGGGTGTCAGGGGCGCACCTGAATCCTGCAATTTCGCTGTCAATGTGTGTACTGGGCCGG
CTTCAGTGGTTGAAACTTCCCATCTACTGCACTGCTCAGCTCATAGGAGCTTTTATTGGT
TCCGCAGCAGTATTT---GGCCTTTATTATGATGCCTTCATGAGTTATGAT---GGCGGA
AACTTGACAATCACCGGTCAAAAT---GCAACTGCACACATCTTCTCCTCTTATCCTGCT
CCACATCTATCCTTTGCAAATGGATTTGCGGACCAGATTGTGGGCACGGCCGCTCTACTT
TTCTCCATTTTGGCCATTTTGGACTCCAAGAACGATCGTGTGCCCAAGGGTCTGGAGCCG
GTGGTGATCGGTCTGATCCTCATGGTGATCGGACTATCGATGGGCTACAACTGCGGTGGT
CCCATCAACCCAGCACGTGACCTGGGTCCTCGGCTCTTCACTGCTGTGGCCGGATGGGGA
TTTGAGGTTTTCAGGGCTGGTAATGGCTGGTGGTGGGTCCCTGTCATTGCGCCTTTCATC
GGGGGAGTCCTGGGCACTGCCATTTACGTTCTGTTCATTGAACTACATCACAAAGAGACG
GTCTTGGAGGAGGCCCGG------------------------TGCGTTAAAGAA------
CAGCAGGCGGAGGGGAAA---------ACAAAATATGAAATGATT---GATATACAGTGT
AACAAA--

>Smalltooth_sawfish_Aqp9
ATG------------------------------CAATCA---------------------

---------------------CAGATCCAGAAGAAGAGTTTGGTAGATACATGC------
AAACTCAAGAACAGCTGGATAAAAGAAGGGCTCGCCGAATTTTTGGGAACATTTATTCTG
ATTCTGTTTGGTTGTGGGTCTATCGCCCAGTCAGTGCTGAGCAGAGGAGTAAGTGGCAAC
ATGTTGACTTCTTCTATTGGCTTTCCATTTGGCGTTACCATTGCTGTTTATGCTACAATA
GGAGTGTCAGGGGGACACCTGAACCCTGCTATTTCACTGTCAATGTGTGTGCTGGGGCGG
CTTCAGTGGCTGAAACTTCCTGTCTACTGCTTCGCTCAGTTCGTCGGAGCTTTTATTGGT
TCAGCGGCAGTTTTT---GGTCTTTATTATGATGCCTTCATGACTTTTGAT---GGTGGG
AACTTGACAATAACTGGTCAAAAT---GCAACAGCGCAGATTTTCTCCTCTTATCCAGCT
CCACATCTGTCCTTTGTAAATGGATTTGCAGACCAGATTGTGGGCACAGCCGCACTGCTT
TTCTCCATTTTGGCCATCTTGGACTCCAAGAACAATCGTGTTCCCAAAGGTCTCGAGCCA
GTAGTGATTGGTCTGATCATTATGGTTATCAGTCTTTCAATGGGCTTCAACTGCGGTGGT
CCCATAAATCCAGCTCGTGACCTGGGACCACGTCTCTTCACTGCTGTAGCCGGATGGGGA
ATTGAGGTTTTCAGGGCTGGTGACGGCTGGTGGTGGGTTCCCGTCATTGCACCCTTGATC
GGGGGCATCCTGGGCGCTTCCATATATATTCTGTTCATTGAACTTCACCACAAAGAAAAG
GTCCTAGAGGAGGTCCGC----------------------TGTACTAAAGAG------
CAGCAAGCCGAGGGAAAG---------TCAAAATATGAAATGATT---TCTGTACACTGT
AACAAA--

>Ghost_shark_Aqp9
ATG---
---------------------GAGAAACAAAAGACAAGTTGGAAAGAGAAATGT------
AAATTAAAGAACGGTTTGTTAAAAGAAGGGCTTGCAGAGTGCTTCGGGACATTCATACTA
ATTCTCTTTGGTTGTAGCTCAAATGCCCAAACAGTGCTGAGCAGAGGAGTGAATGGTAAC
ATAATTACCTCTTCCGTTGGCTTTTCATTTGGAATAACCATTGCTGCTTATGCAACAATA
GGAGTATCAGGGGCACACCTGAATCCTGCTATTTCTCTTTCTATGTGTGTGCTGGGTCAG
CTTCAATGGTTGAAGTTTCCAATTTACTGCATTGCTCAGTTTGTCGGAGCCTTTGTGGGT
TCAGCAGCTGTCTTT---GGGCTCTATTATGACGCTTTCATGGCATTCGAT---GGTGGA
AACCTGACAATTACCGGTGAAAAT---GCGACAGCACAAATCTTCTCTTCTTACCCGTCT
CCGCATTTATCCTTCGTGAATGGCTTTGCAGACCAGATAGTTGGCACAGCTGCACTGCTC
TTCTCCGTGCTAGCCATTCTGGACTCCAAAAACGACAGGGTGCCGAAAGGCTTGGAGCCG
GTGGTGATTGGAATCATCATTTTGGTCCTCGGCTGTTCAATGAGCTTCAACTGCGGTGGC
TCCATCAATCCAGCTCGCGACCTTGGGCCACGCCTCTTCACGGCAGTGGCAGGATGGGGG
CTGGAGGTGTTCAGG------GATGGCTGGTGGTGGGTTCCTGTCTTGGCACCGATGATT
GGTGGAGTTGTCGGCACTTCATTTTACCTTCTCATCATTGAACTCCACCACAAGGAATCA
GTCACTGAGCAG------------------------------TGTGCTATAGAA------
CCACAACAATCCCCGAAG---------AAAAACTACGAACTGGTT---GCCATAAAGGGC
AACATT--

>Human_AQP10
ATG------------------------------GTCTTC---------------------
---------------------ACTCAGGCCCCGGCTGAAATCATGGGCCACCTC------
CGGATACGCAGCCTCCTGGCCCGGCAGTGCCTGGCAGAGTTTCTGGGTGTGTTTGTACTC
ATGCTCCTCACCCAAGGAGCTGTGGCCCAGGCTGTCACCAGTGGAGAAACCAAAGGCAAC
TTCTTCACCATGTTTCTGGCTGGCTCTCTGGCCGTTACGATAGCCATCTACGTGGGTGGT
AACGTCTCAGGGGCCCACCTGAATCCAGCCTTCTCCCTGGCCATGTGCATCGTTGGACGC
CTCCCCTGGGTCAAGCTCCCCATTTACATCTTGGTGCAGTTGCTGTCTGCTTTCTGTGCT
TCGGGAGCCACCTAT---GTTCTCTACCATGATGCCCTACAGAACTATACA---GGTGGG
AACCTGACAGTGACTGGCCCCAAG---GAGACAGCCTCCATTTTTGCCACCTATCCTGCC
CCCTATCTGTCCCTGAACAATGGCTTCCTGGATCAGGTTCTGGGCACTGGGATGCTGATT
GTGGGGCTCTTGGCCATCCTGGACAGACGGAACAAGGGAGTCCCTGCGGGTCTGGAGCCT
GTGGTGGTGGGGATGCTGATCCTGGCCCTCGGGTTATCCATGGGTGCCAACTGCGGGATT
CCACTCAACCCTGCCCGGGACCTGGGCCCACGTCTCTTCACCTACGTGGCTGGCTGGGGT
CCTGAAGTCTTCAGTGCTGGTAATGGCTGGTGGTGGGTGCCTGTGGTGGCCCCTCTGGTG
GGGGCCACCGTTGGCACAGCCACTTACCAGCTGTTGGTGGCTCTGCACCACCCTGAGGGC
CCAGAGCCAGCTCAG----------------------------------GATCTG------
GTGTCTGCTCAACAC------------AAAGCCTCAGAGTTGGAA---ACTCCTGCCTCA
GCTCAGATGCTGGAGTGTAAGCTA------------------------------------


```
>Elephant_AQP10
ATG------------------------------ACCTAC---------------------
---------------------ACTCAGCTCCTGGCCAGATTTGGGGGTCGGTTC------
CAAATTCGTAGTGTCCTGGCCCGACAGTGCCTAGCAGAGTTTCTCGGTGTGTTTGTGCTC
TTGCTCCTCACTCAAGGGGCTGTAGCCCGGGCTGTCACCAGTGGAGAAACCAAAGGCAAC
CTCTTCACCATGTTTCTGGCTGGCTCTTTGGCTGTTACCATAGCCATCTACGTGGGTGGC
AATGTCTCAGGGGCCCACCTGAATCCAGCCTTCTCTCTGGCCATGTGCCTCCTGGGACGC
TTCCCCTGGGCCAAGCTCCCCATTTACTCCTTGGTGCAGCTGCTGGCTGCTTTCTGTGCT
TCAGGAGCCACCTAT---GTCCTCTACTATGATGCCTTACAGAATTATACA---GGTGGA
AACCTGACAGTGACCGGCCCCAAG---GAGACAGCCTCCATCTTTGCGACCTACCCTGCG
CCCTATCTGTCCCTGAACAATGGCTTCCTGGATCAGGTTATGGGCACTGGAATGCTGATT
GTGGGGCTCTTGGCCATCTTGGACACACGGAACAAAGGAGTGCCTGCAGGTCTGGAGCCT
GTGGCAGTGGGGCTGCTGATCCTCACCCTTGCGCTATCCATGGGTGTTAACTGTGGGTTG
CCACTCAATCCTGCTCGGGACTTGGGCCCACGACTTTTCACCTATGTGGCTGGCTGGGGC
CCTGAAGTCTTCAGTACTGGTAATGGCTGGTGGTGGGTGCCTGTGGTGGCCCCCCTGGTG
GGGGCCACGCTTGGCACAGCCACATACCAGCTGCTGATAGCTCTGCACCACCCTGAGGAG
TCAGAGCCAGCTCAG---------------------------------AATCTG------
GAGTTAGCCCAACAC------------AAAGCCTCAGACTTGGAA---ACTCCTGCCTCA
GCTCAGATGCCGGTGTGTAAGCTA------------------------------------
------------------------------------------------------------
------------------------------------------------------------
>Wallaby_AQP10
ATG------------------------------GTTTGT---------------------
---------------------ATTCCTGGCCCA---------GGTCAGAGCCTG------
CGAATCCGCAACCTCCTAGCCCGACAGTGCTTGGCTGAATTTTTGAGTGTCTTCGTGTTT
ATGCTCATCATACAGGGAGCAGGGGCTCAGGCTGTTACCAGTGAAGAAACCAAAGGCAAC
TTCTTCACAATGTTCCTGGCAAGTGCCCTGGCTGTGACAGTGGCCATCTACGTGGGAGGC
AATGTCTCAGGGGCGCACCTGAATCCTGCCTTCTCCTTGTCCATGTGTCTCGTGGGTCGC
CTACCTTGGGCCAAACTTCCCATCTATATACTGGTACAGCTGCTTTCTGCCTTCTCTGCT
TCAGGAGCAACCTAT---GTCCTCTATTATGATGCTCTGCAGAATTATACT---GGTGGA
AACCTGACAGTGACTGGCCCCAAG---GAGACAGCTTCTATCTTTGCCACCTACCCAGCC
CCTTATCTGTCCCTGGAAAATGGCTTCCTGGACCAGGTTCTAGGCACTGCTGTGATGATT
GTGGGCATCTTTGCCATCACAGACACCAAGAATAAGGGGGTGCCTGCAGGATTGGAGCCA
GTGGCAATTGGGCTGATGATTCTGGCCATTAGCTTCTCCATGGGTGCCAACTGTGGTTAC
CCACTCAACCCTGCTCGAGACTTGGGACCTCGGCTCTTCACCTACGTGGCCGGCTGGGGA
TCTGAGGTCTTCAGTGCTGGCAACGGTTGGTGGTGGGTGCCTGTGGTGGCTCCCATGGTG
GGTGCTGTGTTGGGGACTGCCACGTACCAGCTGCTGGTGGGTCTGCATCATCCTGATGAT
CTGGAGCCTGCTCCA------------------------------------GAG------
---------------------------AAAGCCTCTGAA---AAG---GTCCCCATCTTA
CCTCAAAACCTTGAGTGCAAACTG------------------------------------
------------------------------------------------------------
------------------------------------------------------------
>Zebra_finch_AQP10
ATG------------------------------GGCACC---------------------
---------------------ACTTCTTTCCTGACGAGGGCCCAGAGTCTGCTC------
CGCGTCCGGAACCAGCTGGTGCGGGAATGCCTGGGCGAGGTGCTGTCCACCTTTGTGATG
ATGACAATCACCCTGAGCAGTGCTGCACAGAAGATTGCCTTCTTTGAGACGAAGGGGAAC
CTCATCACCAGCTACCTGGGAGGCGCCCTGGGTGTCATGGCAGGCATCTACACGGCAGGG
GGAATCTCTGGGGCCCACATGAACCCGGCGTTCTCCTTAGCCATGTGCCTGACAGAGCAG
TTTCCCTGGTGGAAATTTCCCATCTTTGTGCTTGTGCAGACCTTGGGATCTTTCATATCT
GCTGGAGCTGTTTAC---ATCCTCTACTATGATGCCATCTGGCACCATAGC---AATGGG
ACCCTTACTGTCACTGGCCCCCAA---GAAACTGCCTCCATCTTCGCCACTTACCCAGCT
GACTTTGTGTCCATTGCCAATGGCTTCTTGGACCAGGTGATCGGCACAGGGGTGCTGATA
ATTGCTGTCATGGGCATCATGGACGCCCGCAACAAGCCTGTCCCCAAGGGCCTGGAACCA
GTGGTTGTGGCTCTCTTAGTGCTCTCCATTGAGTGCTCCATGGGGGCCAACTGTGGCTGC
CCCCTGAACCCTGCCCGTGACATCGGGCCCCGGCTCTTCACCTACCTGGCAGGTTGGGGC
CCAGAGGTCTTCAGCAGGGGCAATGGGTGGTGGTGGGTGCCACTGGTAGCACCGCTGCTG
GGGGCCGCCGTGGGCACGTACCTGTACCAACTCTTCGTGGCTTTCCACTACCCGAATGAG
GACAGCGAATACGTG---------------------------------GCAGAG------
CACGGCTCCATCGTTCTA---------GTCAACACCGCCATCGAC---ACAGACATTGGG
ATGTCACCCAAGGAAAAGGACACTGGGGAGACAGTGCCTGCCGGGCACCCCCCAACACCA
```

CACACC---------------------------ATCAGCACAGTCTCTCCCACATTCACT
GACACACCATTAAAAGAGTCC---------------------------------------
>Alligator_AQP10
ATG------------------------------GTCTCT---------------------
---------------------GCACGCTTCCTGGCAAAGGCTGGGACTCTGCTC------
CATGTCCGCAGCCGCTTGGTCCGGGAGTGCCTGGCTGAGTCGCTTGCTGTCTTTGTGCAG
ATGCTGATAACCCTGGGCGCCGCGGCACAGACCATCACCAGCCAGGGCGCGAAGGGTGGG
TATGTCTCCTCCTGCCTGGCCGGGGCCGTGGCCGTCATGGTGGCCATCTACATTGCTGGC
GGAGTCTCAGGTGCCCACCTGAACCCTGCCTTCTCATTGGCCATGTGTGTGCTGGCCCGG
TGCCCCTGGTGGAAGCTGCCCATCTTCACTGTCGTGCAGACCCTAGCAGCCTTCCTGGGT
GCCGGTGCTGTCTAT---GCCCTCTATTATGATGCCATCCAACACTACAGT---AATGGG
ACCCTTGCAGTCACGGGTCCCTGG---GAAACTGCCTCCATCTTTGCCACCTACCCCGCA
CCCTACCTGTCCCTCCAGAGTGGCTTCCTGGACCAGGTGATGGGCACAGCACTGCTGCTG
GTGGGCATCCTGGCCATCGTGGACACCCAGAACAAGGGCATCCCAAAGGGGCTGGAGCCG
GTGGCCGTGGCCATGCTGGTGCTGGCCCTCAGTCTCTCTGTAGGCTCCAACTGCAGCTGC
GCTATCAACCCTGCCCGTGACTTTGGGCCACGGCTTTTCACCTATGTGGCAGGCTGGGGC
CCCCAGGTCTTCAGGGCTGGCCATGGCTGGTGGTGGGTGCCGGTAGTGGCTCCCCTGGTA
GGGGCCGTGGTGGGCTCCATGCTCTACCAGGTGCTGGTGGCATTTCACCACTCGCCTGCC
CCAGGAAGCGCCCAT---------------------------------GAACTG------
-------------------------------------GAGGTGGAG---------------
------------------------AAGCAGAAGGAGATCCCCATCTTCATCATCAGCCCT
CAGGCTGAAGAGCAGCTCAAGAAGGCCACAGTCGTGACTTCTCTGCCTGGGCCCAGTACA
GACAGAGAGGGGTCCTCTCCCCGCCACGCTATG---------------------------
>Python_AQP10
ATG------------------------------ACTTAT---------------------
---------------------TCTCGCTTATTGGCCAGGGTGCAAGCCAGCTGT------
CAGCTGCGCAGTGACCTCCTACGGCAATGCCTCGCGGAGTTCCTGGGCGTCTTTATTTTA
ATTGTGATCACCCTCAGCGCGGTTGCGCAAGTGGTCACCAGCGAAGGCATCAAGGGTAAC
TATCTTAGCTCCTCCTTGGCCAGCGGGATCGCCGTGATGGTAGCCGTCCACGTCTCAGGC
GGCGTCTCAGGAGGACACCTGAACCCAGCCTTCTCGCTGTCCATGTGCCTGCTGGCTCAG
CTCCCCTGGTGGAAACTCCCCATTTACCTGGCCATCCAAATCCTGGGGGCCTTTATGGGA
GCTGGCACCGTCTAC---ACCCTTTATTACGATGCCATCTACAGCTACAGC---AACGGG
ACACTCAGGGTTACGGGGCCCCGT---GAAACTGCCTCCATCTTTGCCACCTACCCAGCC
CCTTACCTGTCACTACGCAATGGGTTTTTTGACCAGGTACTGGGAACGGGGGTGCTGGTG
CTGGGCATCCTCGCCCTCCTTGATGCCCGGAACAAGCGCATCCCGCAGGGCTTAGAACCC
TTCGGTGTCGGCCTTCTGGTGTCCACCCTCAGCTTAGCTATGGGAGCCAACAGCGGCTGT
GCCATCAACCCGGCCCGGGACCTGGGGCCCCGGCTGTTCACCTACGTAGCTGGGTGGGGT
CCCCAGGTGTTCAGGGCTGGAGAAAACTGGTGGTGGATACCCGTTGTGGCCCCAATGTTG
GGGGCCACCATCGGCACGGTGGTGTATCAGCTCGGAGTGGAGTTCCATCACCCGGCAGAT
CAGAAGGAGCAGCAG---------------------------------CGCTCG------
GTTCCCGAAAAGCATTGC---------CCGGGACACGAG---------------------
------------------------AAAGACATGGACCTTCCTGTCTACGCTGTGAACGTG
TATGCAGAGTCGTGGATAGGTGCCAGTCCGGAT------------------------GCC
CGCTCTGCCGGCGAACACCCAAAA------------------------------------
>Gecko_AQP10
ATG------------------------------ATTTAC---------------------
---------------------GCCCGCTTCTTCGCCAAGGCACGGGGCTGCCTC------
CGTCTCCGCAACCTCCTCCTTCGCCAGTGCCTAGCGGAACTCCTGGGGGTCTTTGTGCTG
ATCGTGATAACGGTCAGCGCTTCGGCCCAGTCTGTCACCAGCGAGGGGACAAAGGGTGGC
TATATGAGCTCGGCCCTGGCCAGCGGCATCAGCGTGATGGTAGCTATTCACGTTTCAGGC
GGCATCTCAGGAGGGCACCTGAACCCGGCCTTCTCCCTAGCCATGTGCCTCCTGAGCCAG
TGTCCGTGGTGGAAGCTGCCCATTTTCTCCACTTTTCAAATCCTGGGGGCTTTCCTGGGC
TCGGCCACCGTCTAC---GCCCTGTACTACGATGCCATCCAACACTATACC---AATGGG
ACCTTGACAGTCACCGGCCCTCGG---GAAACGGCGTCCATCTTTGCCACCTATCCAGCC
CCCTACCTGTCCCTGCACAATGGCTTCTTGGACCAGGTGTTGGGCACCGGGATGCTGCTG
TTGGGCATTCTAGCGTTCATCGATTCCCGAAACAAGAACATCCCGAAAGGTTTGGAGCCT
CTGGCCGTGGGTCTGCTGGTCCTCTCCCTCAGCCTGGCCATGGGGGCCAACTGCAGCTGC
GCCATCAACCCCGCCAGGGACTTGGGCCCCCGGCTCTTCACCTACGTGGCCGGATGGGGC
CCTCAGGTTTTCAGTGCTGGAAACTACTGGTGGTGGGTTCCGATCGTGGCACCGATGGTG
GGCTCGGCCACCGGCACTGCCGTCTACCAGCTCTGCGTGGAGTTCCACCACCCTCCAGAG
GAGGAGGAGGAGGCA---------------------------------TTCACC------

ATGCCTGCCAATGAGAAGGTCCAC---CCAGGGACTGAA---------------------
-------------------------AAAGAGGTGGACATTCCGATTTATGTCGTCAACAAG
TATGTGGACACGTGGCTGGACGGGAACTCCGTG------------------------GGC
AACCCGGCCACAGAGCATGGCAAAAGGGAAACGGCTCCTTCACCC---------------
>Himalaya_frog_AQP10
ATG------------------------------GCACGC---------------------
----------------------TGGCCTGCCCGGGAGAGGTTGCAGAACAGGCTG------
AGACTCAGGAACACTCTAGCCCGGGAATGCCTCGCCGAATTTCTTGGAGTTTTCGTCCTG
CTTTTGATCACGGTTGCGGCCACTGCGCAGGGGGTCACAAGCCAAAACACCAAGGGCAAC
TTCTTTTGCATGTATCTGGGCGGAGCCATTGGTGTCACTATGGCGATTTATGTGTCAGGG
GGTGTGTCAGGTGGGCATCTAAATCCAGCATACTCCCTCAGCCTGTGTGTCCTGGGCCGA
TTCCCATGGTGGAAATTGCCCATCTATGTCATCATCCAAATGGTGGGGTCTTTTGCTGGA
GCAGCAGCTGTGTTC---GCGCTGTACTACGATGCCATCCAGAATTACACC---GGCGGC
AACCTGACTGTGCATGGACCACGA---GAGACGGCCTCCATATTTTCTTCGTACCCCGCC
CCCTACCTGAGCATTGCAAACGGATTTCTGGATCAGGTGATGGGCACAGCAATGTTGATG
ATTGGCATCTTGGCCATTATTGATTCCAGCAACAAGCCAGTCCCAAAGGGTCTTGAGCCA
ATCGCGGTGGGGATGCTGGTCTTCTCCATCGGCCTTTCCATGGGGGCAAACTGCGGCTAT
CCGATCAACCCAAGTCGAGACCTGGGGCCACGGCTCTTCACCTATGTGGCGGGATGGGGC
TCAGACGTCTTCAGGGCCGGGAACAACTGGTGGTGGGTCCCCATCGTAGCACCATGCTTT
GGGGGCGTTTTTGGCACGCTGCTGTACCAGATCTTGGTGGCGATCCACCACCCAGCACAG
GAAACCAGAGCCGAG-----------------------------------GAGGCC------
---ACCAATGTCGAG------------ACCCCAGCGGAG---------------------
-------------------------AAAATGAAGGAGATTCCGGTCTTCACAATACAT---
---TTG
GACAACTCTTTGTCTCATCGGCTT------------------------------------
>Axolotl_AQP10
ATG---
----------------------GCCCGCGCCATCCACCTTGCCAGGGCCCTGCTG------
CGCATAGAGCACCCCCTGGCCCGCGAGTGCATGGCAGAGTTCCTGGGTGTCTTTGTGCTG
ATTCTGATAACCAATGGCGCGACGGCGCAGGCTGTGACCAGCTTACAAACAAAGGGCGGC
TATTTCACTATGTACCTGGGAGGTGCCCTGGCGGTTACCGTAGCAATCTACGTCTCGGGT
GGCGTCTCAGGAGGGCACCTGAACCCGGCCTACACGCTCAGCATGTGCCTTCTTGGC---
TTCCAGTGGAGGAAGATGCCGCTATTCGTCTTCATCCAGATCCTCGCCTCCTTTGCAGCT
GCCGGAACTGCATAC---GCTCTTTATTACGATGCAATCCAGCAGTATTGC---AATGGA
AGCTTGACTGTAACCGGCCCTAGG---GAGACTGCATCAATCTTCGCCACATACCCTGCC
GACTACCTCACCTTATGGAACGGCTTCCTGGACCAGGTGATTGGGACGGCCATGCTGCTT
GTTGGCATCTTGGCAATCGTGGACTCTAAGAACAAACCAGTGCCAAAAGGCCTGGAGCCG
CTCCTGGTGGGCTTACTCGTTCTCTCCATCGGCCTTTCTATGGGCTCCAACTGTGGCTAT
CCCATCAACCCTGCTCGGGATCTTGGCCCGCGTCTATTTACGTTTGTGGCA---------
---------TTCAGTGCAGGGAACAATTGGTGGTGGATACCGATCGTAGCACCGCTACTT
GGCGCAGTCATCGGATCAGTCGTATATGAGGTCTTTGTAGAGTTCCATCATCCTGATAAC
CCCTCTGAACCTTTG-----------------------------------GCTTCG------
TCAACCAGCAAAGACCAC---------TTGTACGTGGAGAAGTGG---ACACCGGAAACT
AAGAAG----------------AAGAAACTAGAGATGGAGCCGGTCTTTACTGTGGACACA
TACATGGAGGGATGCACAGAGAGAGATCCAGGGAG------------------------ACA
GAGGGCTACTTGAGCCACAGGTTG------------------------------------
>2Lcaecilian_Aqp10_2
ATG------------------------------GCCAAG---------------------
----------------------GTTCCCACACTAGACCAAGCCAGGCCCCTGCTG------
CGAATCGAAAATGCCACTGTTCGGCAGTGCCTGGCTGAGTTCCTGGGGGTGTTTCTGCTG
ATCCTAATCACCATTGGTGCAACGGCTCAAAGCGTGACCAGCTTTGACAGGAAGGGTGGC
TACTTCCCCATGTGCCTGGCAGGAGCCCTTGCTGTTACCATGGCAATTTATGTCTCAGGA
GGAGTCTCAGGGGGCCACCTGAACCCTGCCTACTCTCTGAGCCTGTGCCTGCTGGGGCGC
TTCCAGTGGACAAAGCTACCCTTGTTCTTTCTTGTCCAGACTTCGGCAGCCTTTCTCGCT
GCAGCGGGGGCCTAC---GCCCTGTATTACGATGCGATCCACAACTATTGC---AGTGGG
AATCTGACTGTCACTGGTCCCCGG---GAAACTGCCTCCATTTTTGCCACCTACCCTGCC
AGTTACCTCTCAGCTTGGAATGGATTCCTGGATCAGGTGATTGGAACCGCCACCCTCCTG
CTCAGCATCCTGAGTTTGGTGGACTCCAAGAACAAGCCTGTCCCAAAAGGCCTGGAGCCC
GTGGTTGTGGGCATGGTGGTTCTCTCCATTGGCCTTTCGATGGGCTCCAACTGTGGTTAC
CCCATCAACCCAGCCCGGGACCTGGGGCCCAGACTTTTCACGTGGCTGGCTGGCTGGGGC
CCGGAGGTTTTCAGGGCTGGCAATAACTGGTGGTGGATACCTATAGTGGCACCGTTAGTA

GGCGCTGTTATTGGGTCAACTTTGTATGAGCTGATGATCGAGTTCCACCATCCGGAAACC
CAGAGTGAGCTGACG---------------------------------GACGCC------
GGCAAGGAGAGCCACTGC---------TTGGAGGCAGAGAAGGGA---CGGCCTCCTGTG
GAGGCC----------------ATAAAAGCTGGAGAAGTGCCAGTTTTCACCATTGACACA
TACATGGAGGGCTTCATGCAAAGGGAGGGACAC------------------------AGG
AAGAAAGTTATAAGTCACAGGCTG------------------------------------
>2Lcaecilian_Aqp10_1
ATG-------------------------------GCCAAG---------------------
---------------------GTTCCCACACTAGACCAAGCCAGGCCCCTGCTG------
CGAATCGAAAACGTCACTGTTCGGCAGTGCCTGGCTGAGTTCCTGGGGGTGTTTCTGCTG
ATCCTAATCACCATTGGTGCAACGGCTCAAAGCGTGACCAGCTTTGACAGGAAGGGTGGC
TACTTCCCCATGTGCCTGGCAGGAGCCCTTGCTGTTACCATGGCAATTTATGTCTCAGGA
GGAGTCTCAGGGGGCCACCTGAACCCTGCCTACTCTCTGAGCCTGTGCCTGCTGGGGCGC
TTCCAGTGGACAAAGCTACCCTTGTTCTTTCTTGTCCAGACTTCGGCAGCCTTTCTCGCT
GCAGCGGGGGCCTAC---GCCCTGTATTACGATGCGATCCACAACTATTGC---AGTGGG
AATCTGACTGTCACTGGTCCCCGG---GAAACTGCCTCCATTTTTGCCACCTACCCTGCC
AGTTACCTCTCAGCTTGGAATGGATTCCTGGATCAGGTG------------------CTG
CTCAGCATCCTGAGTTTGGTGGACTCCAAGAACAAGCCTGTCCCAAAGGCCTGGAGCCCG
TGGTTGTGGGCATGGTGGTTCTCTCCATTGGCCTTTCGATGGGCTCCAACTGTGGTTACC
CCCATCAACCCAGCCCGGGACCTGGGGCCCAGACTTTTCACGTGGCTGGCTGGCTGGGGC
CCGGAGGTTTTCAGGGCTGGCAATAACTGGTGGTGGATACCTATAGTGGCACCGTTAGTA
GGCGCTGTTATTGGGTCAACTTTGTATGAGCTGATGATCGAGTTACACCATCCGGAAAAC
CAA---GAG---

>Coelacanth_Aqp10
ATG-------------------------------GGAAAG---------------------
---------------------GATCTTGTGACAGAGAGAATTAGAGCCTTATTT------
CGAACAAGAAGCTCATTGATACGAGAATGCCTAGCTGAACTTTTTGGTACCTATGTGATG
GTTGTGTTTTGCTTGGGTACGACAGCTCAGATGATAATCAGCAGCACTGAAAAGGGAGAC
TTCACGGCCACTAATGTTGCCTGTGGTTTGGGGGTTGCCTTTGGAATTTACATTGGCAGT
GGCGTTTCAGGGGGTCACCTGAGCCCAGCGGTGTCCCTAGCTATGTACCTGACAGGGAAG
CTTTGCTGGTGGAAATATCCCTTCTATGCTGTGTTCCAGTTTTTCGGGGCCTTTATGTCA
TCGGTGACTGTGTAT---ATTCTTTACTATGATGCCATCCATTCTTATAGT---GGAGGG
AATCTGACAGTCACAGGACCATCA---GAAACTGCTTCTATCTTTGCAACTTACCCAGCT
CCCTACCTCACGATTAGGAATGGCTTTATTGACCAGGTCATAGGAACTGCAATCCTGATC
ATCTGTATATTGACTATTGTAGACTCCCAGAATAGCCAGGTGTTAAAGAGTCTGGAACCA
CTGTTGGTGGGACTGGTAGTGATGACCATCGGAATGTCCATGGGTGTCAACAGCGGGTAT
GCCATGAATCCGGCCAGGGACCTAGCACCACGCCTCTTTACTTTTTTTGCAGGCTGGGGA
CCAGAAGTCTTCGTGGCTGGCAATAGTTGGTGGTGGGTTCCTGTTGTTGCCCCTCTGTTT
GGATCTGGCATTGGGACCTTCTTGTACCTGGTTTTTGTGCAGTTCCATCACCCAGAC---

>mCoelacanth_Aqp10
ATG-------------------------------GGAAAG---------------------
---------------------GATCTTGTGACAGAGAGAATTAGAGCCTTATTT------
CGAACAAGGAGCTCATTGATACGAGAATGCCTAGCTGAACTTTTTGGTACCTATGTGATG
GTTGTGTTTTGCTTGGGTACGACAGCTCAGATGATAATCAGCAGCACTGAAAAGGGAGAC
TTCACGGCCACTAATGTTGCCTGTGGTTTGGGGGTTGCCTTTGGAATTTACATTGGCAGT
GGCGTTTCAGGGGGTCACCTGAGCCCAGCGGTGTCCCTAGCTATGTACCTGACAGGGAAG
CTTTGCTGGTGGAAATATCCCTTCTATGCTGTGTTCCAGTTTTTCGGGGCCTTTATGTCA
TCGGTGACTGTGTAT---ATTCTTTACTATGATGCCATCCATTCTTATAGT---GGAGGG
AATCTGACAGTCACAGGACCATCA---GAAACTGCTTCTATCTTTGCAACTTACCCAGCT
CCCTACCTCACGATTAGGAATGGCTTTATTGACCAGGTCATAGGAACTGCAATCCTGATC
ATCTGTATATTGACTATTGTAGACTCCCAGAATAGCCAGGTGTTAAAGAGTCTGGAACCA
CTGTTGGTGGGACTGGTAGTGATGACCATCGGAATGTCCATGGGTGTCAACAGCGGGTAT

GCCATGAATCCGGCCAGGGACCTAGCACCACGCCTCTTTACTTTTTTGCAGGCTGGGGA
CCAGAAGTCTTCGTGGCTGGCAATAGTTGGTGGTGGGTTCCTGTTGTTGCCCCTCTGTTT
GGATCTGGCATTGGGACCTTCTTGTACCTGGTTTTTGTGCAGTTCCATCACCCAGAC---

>European_seabass_Aqp10bb

------------------------------ATGGAGAGGCTGCTGAGGAAATGT------
CAGATCAGAAACCAACTGGTCAGGGAGTGCTTGGCTGAATGCCTTGGAGTCTACGTTCTG
ATTCTGTTTGGATGCGGCTCCGTTGCCCAAGTGACTACAACTCAAGATAAGAAGGGGCAG
TACCTGTCAATCAATCTGGGTTTTGCTCTGGGAGTAACATTTGGGGTGTTTGTGTCTCGT
GGAGTCTCAGGTGCCCATCTGAACCCAGCTGTATCTCTGAGCTTGTGCGTTTTGGGCAGA
CATCCCTGGATAAAGCTACCTTTCTATATCTTCTTCCAAGTGTTTGGGGCCTTTCTGGCT
GCAGCCACCGTTGGC---CTGCAGTACTATGATGCCATTCAGGCGTACAGC---GGAGGT
GAGCTGACGGTGACGGGCCCCACT---GCCACAGCAGGCATATTCTCCACCTACCCAGCT
GACTACCTCAGCGTGTGGGGAGGTGTCGTGGACCAGGTAATAGGCACAGCTGCACTGCTG
CTGTGTGTCCTGGCTCTTGGGGACCAGAGGAACAGCTCCCTCCCTGATGGTCTTCAGCCT
GTACTGGTGGGAGCAGCTGTGCTTGTTATTGGCATCTCGATGGGCGCCAACAGCGGCTAC
GCACTTAACCCGGCCAGGGATTTAGGACCTCGGTTGTTCACGTACATTGGCGGCTGGGGA
GTCGATGTTTTCAAGGCTGGAGGTGGTTGGTGGTGGGTGCCCATTGTGGCTCCCTGTGTC
GGGGCGCTGTTGGGAACACTGATTTACGAGCTGATGATTGAAGTCCACCATCCTGCCAGT
CCGCCGGAGCTCCAG--------------------------------ACCTCATGTCAG
GAGGCCACTGAGGGCAAG---------ACGGGACTGGAGCTGGAG---GGGGTGGAGCAA
GACTGTGAAAAACCCAGTAGT---AAAATG------------------------------

>Nile_tilapia_Aqp10bb

------------------------------ATGGAGAGGCTGCTGAAGAAATGT------
CAGATCAGAAACCAGCTGATCAGGGAGTGCATGGCTGAATGTCTCGGCGTCTACGTCCTG
ATTCTGTTTGGATGTGGCTCTGTTGCCCAAGTGGTCACAACTGAAGACAAAAAGGGCCAG
TACCTCTCAATTAATCTGGGTTTTGCTCTGGGAGTAACATTTGGGGTCTTTGTGTCTCGT
GGAGTATCGGGTGCTCATTTAAACCCGGCTGTGTCTCTGAGCTTGTGTGTTTTGGGCCGC
CACTCATGGATGAAACTGCCTTTCTACATCTTCTTCCAAGTGTTGGGAGCCTTTCTGGCT
GCGGCTACAGTTGCT---CTGCAGTACTATGACGCCATCCAGGCGTACAGC---GGAGGC
GATCTGACAGTGACGGGTCCCAAA---GCCACAGCAGGCATATTCTGCACTTACCCCGCT
GACTACCTGAGTGTGTGGGGAGGTATCGTGGACCAGGTGATTGGCACGGCCGCTCTGCTG
CTGTGCGTCCTGGCTCTCGGAGACCAGAGGAACAGCTCCATCCCTCATTATCTTCAGCCT
GTCTTAGTGGGAGCAGTAGTGCTGGTCATTGGCATCTCAATGGGCTCAAACAGCGGATAT
GCACTCAACCCGGCAAGAGATTTTGGACCTCGGTTGTTCACCTACATTGCCGGTTGGGGG
GCGGATGTTTTCAAGGCCGGAAGTGGCTGGTGGTGGGTGCCCATAGTGGCTCCTTGTGTT
GGAGCGCTTCTGGGAACACTGATCTACGAGCTGATGGTTGAAGTCCACCATCCTTCAGAG
CAGTCCGAATCCCAG--------------------------------GCCTCGTGTCCG
GAGAATACAAATGACAAG---------ATGGGGGTTGAGCTGGAG---GGGGTGGAAGCA
GACCGTGAAAAGCCAACT--

>Mummichog_Aqp10bb

------------------------------ATGGAGAAGCTGCTGAGGAGGTGC------
CGGGTCAGAAGCCTCCTGGTGCGGGAGTGCCTGGCTGAATGTCTGGGAGTCTACGTCCTG
ATTTTGTTCGGATGTGGCTCTGTTGCACAGGTGACCACGTCTGAGGAAAAAAACGGCCAG
TATCTGTCCATCAACCTTGGCTTTGCTTTGGGGGTAACATTTGGGGTCTTTGTGTCCCGC
GGCGTCTCAGGTGCTCACCTGAACCCCGCAGTTACTCTGAGCTTGTGTGTTTTGGGCAGA
CATCCATGGGTGAAACTTCCCTTATACGCCTTCTTCCAGGTGCTCGGAGCCTTCCTGGCT
GCAGCCACGGTTGGT---CTGCAGTACTACGACGCCATCCAGAAGTACAGC---GGCGGT
CAGCTGACGGTGACGGGTCCGACA---GCCACAGCAGGAATATTCTGCACTTATCCGGCT
GAATACCTCAGTTTGTGGGGCGGCATCGTGGACCAGGTGATCGGCACCGCCGCCTTGCTG

CTGTGTATCCTGGCCATAGGGGACCGGAGGAACACCTCCATACCAGACTATCTCCAGCCA
CTCCTGGCTGGAGCGTCGGTGCTGGTCATCGGCATCTCCATGGGCTCAAACAGCGGATAC
GCCCTGAACCCGGCGAGGGATTTGGGACCCCGGTTCTTCACTTACATCGCTGGCTGGGGA
GACGAAGTTTTCAAGGCTGGAGGCGGGTGGTGGTGGGTCCCGTTGGTGGCTCCCTGCATC
GGCGCGCTGGTAGGGACTCTGATCTACCTACTGATGGTTGAAGTCCACCATCCGCCCCTC
TCTCCGGAGATCCAG---------------------------------CCGTCGTGTCAG
GAAGCCACGGAGGGCAAG---------ACT---CTGGAGCTGGAG---GGAGTGGAACCA
GACGGTGGAAAACCTGCC--

>Zebrafish_Aqp10bb

------------------------------ATGGACCGTCTGCTGAGGAGATGC------
CGAATCAAGAGTCGTCTGCCCAGAGAATGTCTGGCGGAGTTTTTCGGAGTCTATGTTTTA
ATACTGTTCGGGTGTGGATCAGTGGCTCAGGTCACCACCTCTCAGAATACCAAGGGAGAG
TACCTGTCAATCAACCTGGGCTTCGCACTGGGAACCACATTTGGCATCTACATTGCAAAA
GGAGTGTCAGGAGCTCACCTGAATCCAGCGGTTTCCCTCAGTTTGTGTGTTTTGGGCAGG
TTTTCGTGGACTCGTCTTCCTTTCTACGTGTGTTCGCAGCTCTTCGGTGCATTTCTGGCT
GCAGCAACGGTTGCC---CTGCAGTATTATGACGCCATAATGGATTTCACT---GGAGGG
CATCTGACAGTCAGTGGTGCCACG---GCTACAGCGGGCATCTTCTCAACTTATCCAGCA
GATTATCTGAGTCTGTGGGGAGGAGTGGTAGACCAGATCATTGGCACGGCTGCTCTGCTG
GTGTGTGTTCTTGCATTGGGAGATGCTCATAACACACCTGCACCTGCAGGTCTGGAGCCT
GTACTTGTCGGAGCCGCCGTGCTGGTGATCGGGATCTCCATGGGATCTAACAGCGGATAT
GCCATCAATCCAGCCAGAGACTTCGGCCCAAGACTCTTCTCCTACATCGCAGGCTGGGGA
GACGAGGTGTTCAGGGCTGGACATGGATGGTGGTGGGTGCCTATAATCGTAACGTGTGTC
GGGGCTCTTCTGGGATCATTACTGTACGAGCTGCTGATTGGAGTTCATCATCCTGACTCA
GAGGCAGTGGATCAT------------------------------------GAAGAC------
CCGACAGCGGCGCTCCAG---------CAAACTGTCGAGATGGAG---GGTGCGCAGAGT
TTTGACACCATTAAAGAAAACAAAAAGAGC------------------------------
---GGG
ATCTTTTCTATAACCTCAGCAGACGTAGGG------------------------------
>Bowfin_Aqp10ab

------------------------------ATGGACAGAGCACGCCGGCTGTGC------
CGAGTCAGGAGCCAGCTGCTCCGGGAGTGTCTGGCCGAGTCTCTGGGCACCTACATAATG
ATTCTGTTCGGCTGCGGTGGGGTTGCCCAGGTGACAACGAGCAGCGAGACAAAGGGGCAG
TACCTGTCGATCAACCTGGCCTTCGCCCTGGGGGTCACATTCGCCGTGTATGCCTCCCGC
GGTGTCTCAGGGGCCCACCTGAACCCTGCTGTCTCTCTCAGCCTGTGTGTTCTGGGCAAG
CACCAGTGGAAACGCCTGCCGTTCTATGTCTTCTTCCAACTGCTGGGGTCCTTCCTTGCC
GCGGCAACAGTATAT---GCTTTGTACTATGAGGCGATAATGGAATACAGT---CAGGGC
AATCTGACAGTGTCTGGCCCCAGA---GCCACGGCTGGGATCTTCGCCACCTACCCCTCT
GACTACCTCAGCATCTGGAGTGGTTTCCTGGATCAGGTGGTGGGCACTGCGGCACTGTTG
CTGTGTGTGCTGGCAGTAGGGGATGAGCGGAACAGCGCCCCCCCCGCAGGGCTGGAGCCG
GTGCTGGTGGGCCTGGCAGTGCTGGGCATTGGCATCTCCATGGGGGGCAACTGTGGGTAC
CCCCTGAACCCCGCCCGTGACCTGGGGCCCCGCCTTTTCACGTTCATCGCAGGCTGGGGG
GGACAGGTCTTCACGGCGGGTGGGGGCTGGTGGTGGGTGCCTCTGGTGGCCCCCCTGCTT
GGCGCTCTGACGGGCACAGCACTGTACCAGCTGCTGGTGGAGTTCCATCACCCCCCCCAT
GAACTCGACCCCCCT---------------------------------GGCACA------
CAGGCCAATGAAGTGGGC---------CTAGCGCTGGAGGTGGAA---AGGGAGGCCGGG
GAGGAGAAGGGGGTCAGAAATGGGGGGGAGGTG---------------------------
---GGC
GACGAGCGCATAATCAACCGCCTC------------------------------------
>Gar_Aqp10ab

------------------------------ATGGAGAAGGTACAGAGACTGTTC------
CAGGTACAGAACCAGCTGCTCCGGCAGTGCCTGGCCGAGCTGCTGGGGACCTACGTCATG
ATGGTGTTTGGCTGCGGTGCAGTCGCCCAGGTGACGACGAGTGGTGAGAAAAAGGGTGAT
TTCCTGTCCATCAACCTGGCTTTTGCCCTCGGTGTCACCTTTGCTGTACACGTCTCTGGA
GGAGTGTCAGGAGCCCATCTGAATCCTGCGGTGTCCCTGAGTCTTTGTGTTCTGGGCAGG
CACCAGTGGAGACGCCTTCCACTGTACATCCTCTCCCAGGTGACGGGGGCCTTCCTTGCC
GCGGCAACCATCTAT---GTGGTCTACTACGAGGCCATCATGGAGTACTGC---GGAGGG

AATCTCACAGCGACTGGCCCAAAC---GCCACTGCTGGCATTTTCGCCACCTACCCCTCG
TCCTACCTCACCCTCTGGAACGGGTTTGTGGACCAGGTAATAGGCACTGGGATGCTGTTG
CTGTGTGTTCTGGCTCTGGGTGATGTCAATAACAGCCCAGCCCCCCCGGGTCTGGAGCCG
GTGTTGGTGGGATTGGTAGTTCTGGCGATCGGCATCTCCTTTGGGAGTAATTGTGGGTAC
CCCCTGAACCCCGCCCGTGACCTGGGGCCCCGACTTTTCACCTACATTGCTGGCTGGGGG
AGCGAAGTCTTCAGGGCTGGGGGAGGCTGGTGGTGGGTGCCTCTGCTGGGCCCCCTGGTG
GGCGCGCTGACAGGCACGGCACTGTACCAGCTGCTCATCGAGCTCCACCACCCCGGGCCG
GGGCTCGGCTCGGGC---------------------------------GACACC------
GCCAGCGGGCAGGCCCCT---------CCCCCGGAGGAGGCAGAG---CTGGGGCTGCCG
GGCGAGAAGGGGGACGAGAGGCAGGACGAG------------------------------
---GGC
GAGGCCTGCGTCACCCACCGCCTC------------------------------------
>Sterlet_Aqp10ab

------------------------------ATGGACAAAGTGAAGGAGCTGCTG------
AGAGTGAGGAACGCTCTGGTGAGGGAATGCTTCGCAGAATTCCTGGGCATTTACATCCTG
ATCATTTTCGGGTGCGGCGGCGTGGCGCAGGTCACGACGAGCAGTCTCACCAGAGGAGAG
TATCTCTCCATCAACCTGGCTTTTGCATTCGGGGTGACGTTTGGCGTGCACGTCTCCCGC
GGCGTTTCAGGAGCCCACCTCAACCCCGCGGTTTCCCTGGCCATGTGCTTGCTGGGCCGC
CTGCCCTGGCGGAAGCTGCCGGTCTACGCGCGCTTTCAGCTGCTCGGGGCGTTCGTTGCT
GCCGCCACGGTGTAC---GCGCAGTACCGCGACGCCATCATGCAGTACAGC---GGGGGC
AACCTCACAGTCACGGGGCCCTTA---GAGACGGCCTCTATCTTCGCTACCTACCCCGCA
CCCCATCTGTCCATTTCCAATGGATTTGTGGATCAGGTGATTGGCACAGCCGCTCTGCTT
GTGTGTGTTCTGGCGCTGGGTGACTCTCAGAACAGTCCCGCTCCGAAGGGGCTGGAGCCG
GTTCTGGTGGGGATGTCGGTGCTGGTGATCGGACTGGCCATGGGCTCCAACTGCGGGTAC
GCCATTAACCCCGCCCGCGACCTGGGACCCCGACTCTTCACCTGCGTGGGGGGCTGGGGT
GTGGAGGTGTTTAGGGCTGGGAGACACTGGTGGTGGGTTCCCTTGCTCGGTCCCATGGTG
GGCTCTTTGGTCGGGACCGCCGTGTATTGGCTTCTTATCGAACTGCACCACCCAGCCCAG
GAGGGGGCGCTAGAG---------------------------------GACTGC------
CAGACCGTGAAAAACAAA---------TACGCCATCGAACTGGAG---GAGGGGGCTTCT
AAACCCGGAGGGGGGGGGGCAAGGATGGAGAGCACGAGGGAG------------------
---AGGGAAGGGGAGGGGGAG------------------------------------GGG
GAGGAGTGCACCGGCAAGCGGCTG------------------------------------
>Common_sturgeon_Aqp10ab

------------------------------ATGGACAAAGTGAAGGAGCTGTTG------
AGAGTGAGGAACGCTGTGGTGCGGGAATGCTTCGCAGAATTCCTGGGTATTTACATCCTG
ATCATTTTCGGGTGCGGCGGCGTGGCGCAGGTCACGACGAGCAGTCTCACCAGAGGAGAG
TATCTCTCCATCAACCTGGCTTTTGCATTCGGGGTGACGTTTGGCGTGCACGTCTCCCGC
GGCGTTTCAGGAGCTCACCTCAACCCCGCGGTTTCCCTGGCCATGTGCTTGCGGGGCCGC
CTGCCCTGGTGGAAGCTGCCGGTCTACGCGCTCTTTCAGCTGCTCGGGGCGTTCGTTGCC
GCCGCCACGGTGTAC---GCGCAGTACCGGGACGCCATCATGCAGTACAGC---GGGGGC
AACCTCACAGTGACTGGGCCCTTC---GAGACGGCCTCTATCTTCGCTACCTACCCCGCA
CCCCATCTGTCCATTTTAAATGGATTTGTGGATCAGGTGATTGGCACAGCCGCTCTGCTT
GTGTGTGTTCTGGCGCTGGGTGACTCTCAGAACAGTCCCGCTCCGAAGGGGCTGGAGCCG
GTTCTGGTGGGGATGTCGGTGCTGGTGATCGGACTGGCCATGGGCTCCAACTGCGGGTAC
GCCATTAACCCCGCCCGCGACCTGGGACCCCGACTCTTCACCTGCGTGGGGGGCTGGGGT
GTGGAGGTGTTTAGTGCTGGGAGACACTGGTGGTGGGTTCCCTTGCTCGGTCCCATGGTG
GGCTCTCTGGTCGGGACCGCCGTGTATTGGCTTCTTATCGAACTGCACCACCCAGCCCAG
GAGGGGGCGCTAGAG---------------------------------GACTGC------
CAGACCGTGAAAAACAAA---------TCCGCCATCGAACTGGAG---GAGGGGGCTTCC
AAACCCGGAGGGGGGGGGCCAAGGACGGAGAGCACAGGG---------------------

>Reedfish_Aqp10ab

------------------------------ATGGAGAAAGTGTTCCAAGTCCTC------
AGGGTGAAGAACCAGCTTTTTAGGGAATGTCTTGCTGAACTTTTGGGTGATTACGTGCTG
ATAATTTTTGGCTGTGGAGCAGTGGCTCAGGTCACCACAAGCTATGATACAAAGGGGCAG
TTTCTTTCCATCAACCTGGCATTTGCTATTGGCATCACTTTTGGTGTTCACATTGCTCAT
GGAATTTCAGGTGCCCATTTGAACCCTGCAGTCTCTCTTGCTTTTTGTTTAATTGGTCGC

TTCCAATGGAGCAAGCTTCCCTTCTACGTGTTATCTCAGATTATTGGTGCATACCTGGGT
GCTGCCACTGTGTTT---GCACAGTATTATGATGCTATCATGAGTTATGGT---GGTGGC
AATCTGACTGTCACCGGCCCTACA---GCAACAGCCTATATCTTTGCAACATACCCTGCA
GCATACCTGACAACAGCAAATGGATTTGTAGACCAGGTGGTTGGCACTGGCGCTCTGCTT
CTCTGTCTTATGGCTCTTGTCGACACTCGTAATTCACCTGCTCCAAAGGGACTGGAACCT
GCATTAGTGGGAACAATTGTTCTTGTAATTGGAATTGCAATGGGTTCCAATTGCGGTTAT
GCTATCAATCCAGCCCGTGACCTGGGACCACGGCTCTTCACATATACAGCTGGCTGGGGT
TTGGATGTTTTCACGGCAGGAAATTACTGGTGTTGGGTGCCTCTGGTTGCTCCTTTGGTT
GGTGCAATTTTGGGATGTATCATTTATCTGCTCTTCATTGAAATTCACCATTCAAGAAAA
GAGGATGAAAAACAG----------------------------------ACAAAT------
GAGAGAGGCAATACTGAA---------TATGACATTGATCTGGAT---GAATGGAAGCCC
AACAAAAGGAATCTAACAACACTTACAGCATCTGACAAAGTACACATCATCAACAACCCC
TTTCAGGAAGGACAGTCAGAGAACTCTGAT---AAGTCATCGTGT------------AAT
GAAGAATGTATTGTCAATAGATTT------------------------------------
>European_seabass_Aqp10aa

------------------------------ATGTTAAAG---CTACGTGCTCTG------
AGAGTGAGAAATGCTCTGGTGCGAGAGTGCATGGCTGAGTTCTTGGGAACCTTTGTCTTG
TTGCTCTTTGGCTGCTCTGCCGCAGCGCAGGTAAAAACAAGCAGGGAGACTAAAGGCCAG
TTCCTGTCGGTTAACATGGCCTTCTCTGTGGGCGTGATGTCAGCTATGTACCTCACCAAG
GGCATCACAGGTGCTCATCTGAACCCAGCGGTGACTCTGAGTTTCTGTGTGTTGGGCCAG
GTGCCCTGGGGACGGCTGGTTCCCTACTGCCTCTCCCAGCTGCTGGGGGCTTACATGGCA
TCAGCGCTTGTCTAC---CTGGTGTACTATGATGCTATAATGGACTTTAGT---GGAGGA
GTATTGACTGTATATGGCCCAAAT---GAGACAGCATCTATATTTGCCACATATCCCTCA
GAGTACATAACTTTGGGCAGAAGTTTCCTCGACCAGGTCGTGGGCACTGGCATGCTGATG
TTGTGCATCCTGTGTTTGGGGGAAAAGAGGAATACCCCAGCTCCCTCAGAGCTGATTCCT
CCTATAGTGGCAGTGATCGTCCTGGGGATCTCCATGTCAATGTCAGGTAACTGTGGTGCT
GCAATAAATCCTGCTCGGGACCTGGGGCCACGCCTCTTTACGCTGACGGCAGGCTGGGGC
ACAGAGGTCTTCACGTGTTACAACTACTGGTTCTGGGTACCCCTGGTAGCCCCACCTATC
GGAGGTGTCATAGGTACTTTCATGTATTTGATCTTCATCGACTGGCACCTGCCTGACCCG
GACCAGCCTGAAAGC----------------------------------CTCTCC------
ATTCTATCCACCATCAGT---------GACAAAATCCAGCAGCCC---GGCACAACGTGG
GACAAAGGAACAGGG------TTAAAGACTGCACATTTC---------------------

>Nile_tilapia_Aqp10aa

------------------------------ATGTTGAAG---CTACGTGGAATG------
ACGGTGAGGTGTCCCCTCGTGAGAGAGTGCATGGCCGAATTCTTGGGAACTTTCGTCTTA
ATGCTCTTTGGCTGCGCTGCTGCTGCACAAGTAAAGACCAGCAATGAGACTAAAGGCCAG
TTTCTGTCTGGCAACATGGCCTTCTCTGTGGGTGTTATGTCAGCCATGTATCTCACCAAG
GGCATCTCAGGTGCCCATCTGAACCCAGCGGTGACCCTGAGTTTCTGTGTTTTGGGGAAG
GTGTCCTGGTCGAGGCTCGTGCCCTATTCCCTCTCCCAGGTGGTGGGGGCTTTCGTGGCG
TCGGGGGTTGTCTAT---CTGGTCTACTACGATGCTATAATGACTTTTAGT---GGAGGA
GTGTTGACTGTATACGGCAGAAAT---GAGACTGCCTCGATATTTGCCACATACCCCTCT
GACTACCTTTCTGTGGGCAGAAGTTTCTTTGACCAAGTAGTGGGCACCAGCATGTTGCTG
TTGTGTATCCTGGGTTTGGAGGAAAAGAGGAACACCCCCGCTCCCTCTGAGCTGGTTCCT
GTTGTAGTGGCAGGTGTCGTTCTGGGGATCTCCATTTCAATGTCGGCTAACTGTGGTGCT
GCCATAAATCCTGCTCGGGACCTGGGGCCTCGCCTCTTTACCCTAGCTGCAGGCTGGGGG
ACTGAGGTCTTCACGTGTTACAACTACTGGTTCTGGGTACCAGTGGTGGCTCCGCTTGTT
GGAGGCGTTTTCGGTAGTTTGATGTATCTCATCTTTATCGACTGGCAGCTGCCTGACCCA
GACCAACTTGAGAGG----------------------------------ATTTCC------
ACCATCAGTGAAAAACTT---------GCAAAA---AAGCAGCCG---GTCACCACATTG
GAAAAAGGAGATGAG------TTAAAGGCTATGCATTTT---------------------

>Mummichog_Aqp10aa2

------------------------------ATGTTGAAG---CTGCGTACGCTC------
AGGCTGAGGGCTGCCCTGGTGCGAGAATGTATGGCTGAGTTCCTGGGAACATTTGTCTTG
ATGCTGTTCGGTTGCTCTGCAGCAGCCCAGGTAAAAACCAGTCAAGACACCAAAGGCCAG

TATCTGTCGGTCAACATGTCCTTCTCCGTAGGGGTGATGTCAGCCATGTACCTGACCAAG
GGGATCTCAGGTGCCCACCTGAACCCTGCAGTGACTCTAAGTTTATGCGTTTTGAGGAAA
GTGAGGTGGAGAAGTCTGCTACCCTTCTGCCTCTCCCAGGTGCTCGGCGCATATGTGGCA
TCGGCAGTGGTCTAT---ATGGTCTACTATGATGCTATAATGGATTTTAGT---GGAGGC
GTGTTGACCGTTTATGGCCCAAAT---GAAACAGCATCTATATTTGCCACGTATCCATCT
GAGTACCTCACGTTAGGCAGGAGTTTCCTCGATCAAGTGGTGGGGACTGGCACATTGATG
CTGTGCATCCTAAGTTTGGATGAAAAGAGGAACACGCCTGCTCCCTCTGGTCTGATACCT
CCCATAGTTGCCACGATTGTCCTGGGGATCTCCATGTCCATGTCAGCTAACTGTGGTGCT
GCAATAAATCCTGCTCGCGACCTTGGACCACGCCTCTTCACCCTGACTGCAGGCTGGGGG
ACTGAAGTCTTTACGTGTAAAAATTACTGGTTCTGGGTCCCACTCGTGGCGCCCCTAATT
GGAGGCCCTTTAGGCTCTTTCCTTTATGTGGTCTTCATCGAATGGCAGCTGCCTGATCCA
GAACCCCCACAGAAC---------------------------------CTCTCA------
ACAGTTTCCACCAACAGC---------GACAGCATCAAAAAGCCT---GCCACCAGGTGG
GGGACAGGAGGCGAT------TTAAAGTCCACATATTTT---------------------

>Zebrafish_Aqp10aa

---------------------ATGAGTCAGATGAAGAAGATCATGAAGAGGATG------
AAGGTGAAAAATGAACTGGCACGACAGATTATGGGAGAGATCTTGGGCACTTTTGTTCTT
CTGTTGTTTGGTTGTGCTGCAGCGGCTCAGGTGAAAACCAGCAGAGAAACAAAGGGGCAG
TTTCTGTCTGGTAACATCGCCTTCTCTGTAGGTGTCATGTCTGCCATGTACCTCTGCAGG
GCAGTATCAGGAGCTCATCTAAACCCAGCTGTGTCTCTGAGTTTCTGTGTATTGGGAGAC
CTGGCCTGGATAAAGCTGCTACCATATTCTCTCGCTCAAATTTTAGGGGCTTACCTTGCT
TCAGGGCTTGTCTAT---CTCATCTACCATGATGCCATCATGGAGTTCAGT---GGTGGA
GTTCTGACCGTATTTGGCCCTAAT---GAAACAGCCAGTATCTTCGCCACTTACCCAACC
GATGTAGTATCAGTGCAGACCAATTTCCTGGATCAGGTGGTTGGCACAGCCATGTTGATG
CTGTGCATTTTGCCTCTGAATGATAAGAGAAACGCCCCAGCTCCTGAAGCGCTGCTCCCA
CCCATTGTAGCCACTGTTGTTCTAGGGATTTCCATCTCAATGTCTGCTAATTGTGGAGCA
GCCATAAATCCAGCACGTGATCTTGGTCCACGACTCTTTACCTTTACAGCAGGCTGGGGC
ACTGAAGTCTTTACGTGCTATGACTACTTCTTTTGGATCCCATTGGTGGCTCCTATGGTA
GGGGGTGTCCTGGGCTCCATCATTTATTTGGTTTTCATCCAGTGGCATCTGCCTGAGCTT
GAAGATGAATCTGAA---------------------------------TCTGAG------
GAGATGAATGATCAAACA---------AAAGTCATGGAGCACAAC---------------
AACAAAAAAGATGAGATATACCTTAAAATGTCTTCAATT---------------------

>Bowfin_Aqp10aa

---------------------ATG---TTGATGGAGCGAGCCAGGCGGGGGCTG------
AGGCTGAGCAGCCCGCTGGCAAGAGAGTGCCTTAGTGAGTTTATGGGCACGTTTGTCCTG
CTGCTGTTTGGCTGTGCAGCCTCTGCGCAGGTGAGGACGACGGGGGGTGAGAAGGGACAG
TACCTGTCCATCAACCTGGCATTCTCTGTAGGCGTGATGTCGGCCATGTACCTGTGCATG
GGGGTCTCAGGTGCTCACCTGAACCCTGCAGTGTCCCTGAGTTTCTGTGTGCTGGGGCGG
CTGCAGTGGCCCCGGCTGCCCTGCTATGCTGTGGTCCAGGTGCTGGGCGCCTTCACAGCC
TCGGCCGTGGTCTTC---CTGCAGTACTACGACGCGATCATGGATTTCTCG---GGGGGC
AACTTGACAGTGACTGGCCCCACA---GAGACGGCGTCCATCTTCGCCACCTACCCCTCC
CCCCACCTCAGCCTGTCTAGCAGCTTCATCGACCAGGTGATCGGCACGGCTACGCTGTTG
CTCTGCATCCTGCCCCTTGATGACAAGCTGAACAGCCCTGCCCCCCCCGGCCTGGTGCCC
CCACTGGCTGCCGTGGTGGTGCTGGGCATCTCCATGTCGATGAGTGCCAACTGCGGGGCC
GCCATTAACCCGGCGCGTGACCTGGGGCCCCGGCTGTTCACCCTGACCGCAGGCTGGGGG
CCTGAGGTTTTCACGGCATACTCTCACTGGTGGTGGGTACCATTAGTCGCCCCAATGCTT
GGTGGAGTTCTGGGCTCACTGCTTTATCTCGTGTTCATCCAGTGGCACCTCCTTGACCCG
GACAGTCACCCTGGC---------------------------------ACAGAA------
CCAGAACCCGGGTCTGAACCCATGGCCGGGAAGATGGAGAGAGAG---AGTATAAAGCTG

>Gar_Aqp10aa

------------------------------------GTGGTTGGCACGGCGACGCTGTTG
CTGTGCATCCTGCCCTTGGACGACAGGCTCAACAGCCCCGCCCCCAAGGGCCTGGTGCCG
CCATTGGTTGGCCTGGTCGTCCTGGGGATCTCCATGGCGATGAGCTCCAACTGCGGGGCC
GCCATCAACCCGGCCCGCGACCTCGGGCCGCGGCTGTTCACGCTGACCGCGGGCTGGGGG
ACCGAGGTGTTCACGGCGTACCGACACTGGTGGTGGGTACCTGTGGTCGCCCCGATGCTG
GGCTCGGTCCTGGGCAGCTGCCTGTACCTCACCTTCATCCAGTGGCACCTTCCCGACCCG
CAGGGGGGCCCGAGC---------------------------------CCAGAC------
GCCGCCTCCAGAACC----------------AAGATCCAGGCCCCC---TCAGCCCCAGCC
CCGCACAGCGACTCCGAGACCATCGCCATGCCCAGCTGGGACCCC---------------

>Russian_sturgeon_Aqp10aa

---TTGTTTGGTTGCGCGGCAGCAGCGCAGGTCAAGACCACCAACGAGGTGAAGGGTCAG
TACCTCTCCGTCAACCTGGCGTTCGCTGTGGGCGTCGTCTCCGCCGTCTACATCTCCCGA
GGAGTCTCCGGAGCGCACCTGAACCCCGCCGTCTCTCTCAGCTTCTGTGTGCTGGGGAGG
TTCCAGTGGAAACGGTTGCCTTTTTACACCCTCTTCCAAATCATGGGGGCCTTCGCGGCG
TCCGGAACGGTCTTC---ATTCAATACTACGACGCCATCATGCAGTACAGC---GGGGGT
AACCTCACAGTCACCGGGCCCTTA---GAGACGGCCTCTATCTTCGCTACCTACCCCAGT
GAATATCTCTCGTTTTCAAATGCGTTCGTGGACCAGGTGATCGGCACAGCCGCCCTGCTC
CTGTGCATCCTCCCCCTGGATGATTCCCGGAACAGCCCGGCTCCCGAGGGTCTGCAGCCC
CTGCTGGTTGGCTTCGTGGTCCTGGGGATTGGCATGGCCATGAGCTCCAACTGCGGGGGG
GCCATCAACCCCGCCAGGGACCTGGGGCCCCGCCTGTTCACCCTGGCAGCGGGCTGGGGC
CCCGAAGTCTTCACGGCGTTTGAGAACTGGTGGTGGATTCCCGTTGTGGCCCCGCTGGTC
GGTGGAGTGACGGGGGCTTCACTGTATCAGCTTTTCATTGAGTTTCATCACAAGGACCCG
GAGACAGACCAGCCC---------------------------------GAGGCC------
AAGACGCGAGAGAACGGG---------GCGTCCCAAACCAATCTG---GGGGACTCGGGT
TCA---

------TACCTGACCAGCAAGTTT------------------------------------
>Sterlet_Aqp10aa

---------------------ATGGTCCCTATTATGAAACTCAAAGACAAACTC------
AAACTGAAAAATAGGCTTCTGAGAGAATGCTTAGCGGAGTTTTTGGGAACATTTGTGCTC
ATGTTGTTTGGTTGCGCGGCAGCAGCGCAGGTCAAGACCACCAACGAGGTGAAGGGTCAG
TACCTCTCCGTCAACCTGGCGTTCGCTGTGGGCGTCGTCTCCGCCGTCTACATCTCCCGA
GGAGTCTCCGGAGCGCACCTGAACCCCGCCGTCTCTCTCAGCTTCTGTGTGCTGGGGAGG
TTCCAGTGGAAACGGTTGCCTTTTTACACCCTCTTCCAAATCATGGGGGCCTTCGCGGCG
TCCGGAACGGTCTTC---ATTCAATACTACGACGCCATCATGCAGTACAGC---GGGGGC
AACCTCACAGTCACTGGGCCCTTA---GAGACGGCCTCTATCTTCGCTACCTACCCCAGT
GAATATCTCTCGTTTTCAAATGCGTTCGTGGACCAGGTGATCGGCACAGCCGCCCTGCTC
CTGTGCATCCTCCCCCTGGATGATTCCCGGAACAGCCCGGCTCCCGAGGGTCTGCAGCCC
CTGCTGGTTGGCTTCGTGGTCCTGGGGATTGGCATGGCCATGAGCTCCAACTGCGGGGGG
GCCATCAACCCCGCCAGGGACCTGGGGCCCCGCCTGTTCACCCTGGCAGCGGGCTGGGGC
CCCGAAGTCTTCACGGCGTTTGAGAACTGGTGGTGGATTCCCGTTGTGGCCCCTCTGATC
GGTGGAGTGACGGGGGCGTCACTGTATCAGCTTTTCATTGAGTTTCATCACGAGGACCCG
GAGACAGACCAGCCC---------------------------------GAGGCG------
AAGACGCGAGAGAACGGG---------ACGTCCCAAACCAGC------------------

------------------AAGTTT------------------------------------
>Common_sturgeon_Aqp10aa

---------------------ATGTTCCCTATTATGAAACTCAAAGACAAACTC------
AAACTGAAAAACAGGCTCCTGAGGGAATGCTTAGCGGAGTTTTTGGGAACATTTGTGCTC
ATGTTGTTTGGTTGCGCGGCAGCAGCGCAGGTCAAGACCACCAGCGAGGTGAAGGGTCAG
TACCTCTCCGTCAACCTGGCGTTCGCTGTGGGCGTCGTCTCCGCCGTCTACATCTCCCGA
GGAGTCTCCGGAGCGCACCTGAACCCCGCCGTCTCTCTCAGCTTCTGTGTGCTGGGGAGG
TTCCAGTGGAAACGGTTGCCTTTTTACATCCTCTTCCAAATCATGGGGGCCTTCGCGGCG
TCCGGAACGGTCTTC---ATTCAATACTACGACGCCATCATGCAGTACAGC---GGGGGC
AACCTCACAGTCACTGGGCCCTTA---GAGACGGCCTCTATCTTCGCTACCTACCCCAGT
GAATATCTCTCGTTTTCAAATGCGTTCGTGGACCAGGTGATCGGCACAGCCACCCTGCTC
CTGTGCATCCTCCCCCTGGATGATTCCCTGAACAGCCCGGCTCCCGAGGGTCTGCAGCCC
CTGCTGGTCGGCTTCGTGGTCCTGGGGATTGGCATGGCCATGAGCTCCAACTGCGGGGGG
GCCATCAACCCCGCCAGGGACCTGGGGCCCCGCCTGTTCACCCTGGCAGCGGGCTGGGGC
CCCGAAGTCTTCACGGCGTTTGAGAGCTGGTGGTGGATTCCCGTTGTGGCCCCGCTGGTC
GGTGGAGTGACGGGGGCTTCACTGTATCAGCTTTTCATTGAGTTTCATCACGAGGACCCG
GAGACAGACCAGCCC--------------------------------GAGGCC------
AAGACGCGAGAGAACGGG---------GCGTCCCAAACCAATCTG---GGGGACTCGGGT
TCA---

------TACCTGACCAGCAAGTTT------------------------------------
>Chinese_sturgeon_Aqp10aa

---TTGTTTGGTTGCGCGGCAGCAGCGCAGGTCAAGACCACCAACGAGGTGAAGGGTCAG
TACCTCTCCGTCAACCTGGCGTTCGCTGTGGGAGTCGTCTCCGCCGTCTACATCTCCCGA
GGAGTCTCCGGTGCGCACCTGAACCCCGCCGTCTCTCTCAGCTTCTGTGTGCTGGGGAGG
CTCCAGTGGAAACGGTTGCCTTTTTACGCCCTCTTCCAAATCATGGGGGCCTTCGCGGCG
TCTGGAACGGTCTTC---ATTCAATACTACGACGCCATCATGCAGTACAGC---GGGGGC
AACCTCACAGTGACTGGGCCCTTA---GAGACGGCCTCTATCTTCGCTACCTACCCCAGT
GAATATCTCTCGTTTTCAAATGCGTTCGTGGACCAGGTGATCGGCACAGCCGCCCTGCTC
CTGTGCATCCTCCCCCTGGATGATTCCCGGAACAGCCCGGCTCCCGAGGGTCTGCAGCCC
CTGCTGGTCGGCCTCGTGGTCCTGGGGATCGGCATGGCCATGAGCTCCAACTGCGGGGGG
GCAATCAACCCCGCCAGGGACCTGGGGCCCCGCCTGTTCACCCTGGCAGCGGGCTGGGGC
CCCGAAGTCTTCACGGCGTTTGGGAACTGGTGGTGGATTCCTGTTGTAGCTCCGCTGATC
GGAGGAGTGACGGGGGCTTCACTGTATCAGCTTTTCATTGAGTTTCATCACGAGGACACG
GAGACAGACCAGCCC--------------------------------GAGGCG------
AAGACGCGAGAGGACGGG---------GCGTCCCAAAACAATCTG---GGGGACTCGGGT
TCA---

------CACCTGACCAGCAAGTTT------------------------------------
>Reedfish_Aqp10aa

------------------------------ATGCAGAAACTAAGACATCTTCTC------
AGACTGAAGAACAAACTTCTGAAGGAATGCTTAGCAGAGTTTTCTGGGAACATTTGTTCT
GTTCTGTTTGGATGTTCTGCTGCTGCTCAAGTGAAAACAAGTCATGAGACTAAAGGCCAG
TATTTATCTATCAATATGGCCTTTGCTATCGGAGTCATTTTTGCTATGTACATCTCACGA
GGAGTTTCAGGAGCACACCTAAACCCAGCAGTTTCCCTCAGTTTTTGCATGATTGGCCGA
TTTCCTTGGACCAAAGTACCTATTTATATATTTTTTCAGATATTTGGTGCATATATGGCT
TCTGCTACTGTATTT---GCCCTTTATTATGATGCTATAATGAATTACAGT---GGTGGT
AATCTGACAGTTACTGGATCCCAA---GAAACTGCCTCCATCTTTGCTACCTATCCATCT
GACTACCTGTCTCAAGCAAACGCATTTTTTGATCAGGTAGTAGGAACAGCTGCTTTGCTG
CTCTGTATTCTGCCAATTGAAGATGCCCACAATAGCCCTGCTCCGAAAGGCTTTGAACCA
GTACTTGTTGGATTTCTGGTACTAGGAATAAGTATGTCGATGAGTTCAAACTGTGGTGCT
GCCATCAATCCAGCACGTGACCTGGGCCCAAGACTCTTCACATGGTCTGCAGGCTGGGGG
ACAGCGGTGTTCACGGCCTTTGATAACTGGTGGTGGATCCCTATTGTTGCTCCTATGTTA
GGAGGGGTGATTGGTGCTTATATTTATCTGCTTTTCATTGAATTTCATCATGAAGATTCT
GACTGGGGCTATCAC--------------------------------ATCAGT------
---AGCCGGCTT--

>B_bambooshark_Aqp10C1
ATG---------------------------------GGTAGAGCA------------------
----------------------GCCACAATCCTGGTCAAAGTCCACGATGCGTTT------
CGATTGAAGAACAAACTCTTCAGAGAGTGTCTGGCAGAGTTCCTGGGGGTCTGCATGTTG
ATTCTGTTTGGCTGTGGAGCTGTAGCGCAGATGGTAGTCAGTAACACGACACGTGGTGAA
TTCCTGTCGGTCAATCTCGGCTTTGGACTCGGGGCAACGTTTGGAATCTACATCTCCGGA
GGGATCTCAGGT--

---------------------------------CAGTTGATTGGCACCGGCACCCTCCTC
CTGTGCATCTTCGCGGTGGTGGACTCCCAGAACTACGGCGCTCCCAAGATCCTTCAGCCC
ATCTTCATCGGCCTTTCGGTGGTGGGCATCGGGATGTCCATGGGCTCCAATTCTGGTTAC
GCCATCAATCCCGCCCGGGACTTCGGGCCACGTCTGCTCACCCTGGCCGCGGGATGGGGC
ACCGAGGTCTTCACGGCCGGGAACGGTTGGTGGTGGATCCCCATTGTGGCGCCCATGGTG
GGCGCGGTGCTGGGGGCCCTGGTCTACGAGTTGCTGGTGGAGCTGCACCATCTGGAGGCC
AAGTCAGGTCCCACC----------------------------------AACTAC------
GCCGATGAGGAGACG------------CTGAAGGGGACGGATGCC---AGG---------
GGCAAGGACGGCCCGGCCATACGGAGGGACAAGGCCGGA---------------------
---GCA
GATGACCAGTTCGTCATGGCGATG------------------------------------
>W_bambooshark_Aqp10C1
ATG---------------------------------GGGAGAGCA------------------
----------------------GCCACAATCCTGGTCAAAGTCCACGATGCATTT------
CGATTGAAGAACAAACTCTTCAGAGAGTGTCTGGCAGAGTTCCTGGGGGTCTGCATGTTG
ATTCTGTTTGGATGTGGAGCTGTCGCGCAGATGGTCGTCAGTAACACGACACGTGGTGAA
TTCCTGTCGGTCAATCTCGGCTTCGGACTCGGGGCAACGTTCGGAATTTACATCTCCGGA
GGGATCTCAGGGGGCCATCTGAATCCGGCCGTGTCCTTCAGTCTGTGCCTGCTTGGCCGG
TTCCAATGGAAGAAGTTGCCCTTTTACATGTTCTTCCAGACCCTGGGGGGGTTTGTCGGA
GCGGCTGTAGTCTAT---GGGGTGCATCACGATGGGATCCATGCTGTGAAC---AACGGG
ACTTTGTCTGTCACCGGGCCACGT---GCCACCGCCTTCATTTTTGGCACCTACCCTGCA
CCGTTCCTCACCCTTCCCAACGGCTTTATAGACCAGTTGATTGGCACCGGCACCCTCCTC
CTGTGCATCTTCGCGGTGGTGGACTCCCAGAACTACGGCGCTCCCAAGATCCTTCAGCCC
ATCTTCATCGGCCTGTCGGTGGTGGGCATCGGGATGTCCATGGGCTCCAATTCCGGTTAC
GCCATCAATCCCGCCCGGGACTTCGGGCCGCGTCTGCTCACCCTGGCCGCGGGATGGGGC
ACCGAGGTCTTCACGGCCGGGAACGGTTGGTGGTGGATCCCCATTGTGGCGCCCATGGTG
GGCGCGGTGCTGGGGGCCCTGGTCTACGAGTTGCTGGTGGAGCTGCACCACCTGGAGGCC
AAGTCGGGTGCCGCC----------------------------------AACTAC------
GCCGACGAGGAGACG------------CTGAAGGGGACGGGTGCC---GGG---------
GGCAAGGACGGCCCGGCCGTGCGGAGGGACAAGGCCGGA---------------------
---GCA
GATGACCAGTTCGTCATGGCGATG------------------------------------
>Whale_shark_Aqp10C1
ATG---------------------------------GGCCAAGCA------------------
----------------------GCCACAATCCAAGAGAAGGTCCTTGATGCGTTT------
CGACTGAAGAATCAACTGTTCAGAGAATGTTTGGCTGAGTTTCTGGGAGTCTGCATGTTG
ATCCTGTTTGGGTGTGGGGCAGTGGCACAGATGGTGGTCAGTAACACAACACGTGGTGAA
TTTCTGTCGGTCAATCTCGGCTTCGGGCTTGGTGCCACGTTTGGGATCTACATCTCCGGC
GGGATCTCAGGGGGCCATCTGAACCCGGCTGTGTCCTTCAGTCTGTGTTTACTCGGGAGG
TTCCAGTGGAAGAAATTGCCCTTTTACATGTTCTTCCAGACCCTGGGAGGGTTTGTCGGA
GCTGCTGTAGTCTAT---GGGGTACATCAC------------------------------

---------------GCCGGGAACAGTTGGTGGTGGGTCCCCATCGTGGCTCCCATGGTG
GGCGCGGTGCTGGGGGCATTGGTGTACGAGCTCCTGGTGGAACTACACCATCTGCAGGCC
AAGGCAGTGCCCACA----------------------------------GACGAC------
GACATGATGGAGCAGAAG---------CTGAAGGAGGTCGGGGGC---CGGGGCACAGCG

GGCAAGGATGGCCCGTCGGTGCGGAACGACAAGGCCAGA--------------------
---GCG
GACGACGAATTTGTCATGGCAATG------------------------------------
>Great_white_shark_Aqp10C1
ATG------------------------------GGGACAACC------------------
---------------------GAGATGGTGTTACAAAAGGTGCATGGTGCACTG------
CGATTGAAGAATCACCTCGTGAGGGAGTGCTTCGCCGAGTTTCTGGGAGTCTGCATGCTT
ATCCTCTTCGGATGTGGTGCCGTGGCACAAATGGTGGTGAGTAAGACAACCCGCGGTGAA
TTCCTGTCCGTCAACCTGGGCTTCGGACTCGGAGCCACGTTTGGCATCTACATTTCGGGC
GGCATCTCTGGTGGCCATCTGAACCCAGCTGTATCCTTCAGCTTGTGTTTACTCGGACGG
TTCCCATGGAAGAAGCTCCCATTTTACATGTTCTTCCAAACTCTGGGAGGATTTGTTGGT
GCTGCCATCGTCTAC---GGAGTTCATCACGATGGGATACACTCTGTTGAC---AATGGG
ACCTTATCTGTCACCGGACCACGA---GCCACTGCATTTATCTTTGGCACCTACCCCGCA
CCGTTCCTCAGCCTTTCGAACGGCTTCATTGACCAGGTAATTGGCACTGGCACCTTGCTC
CTGTGCATCTTCGCCGTGGTGGACACCCAGAACTATGGGGCCCCCAAGATCTTGCAGCCC
ATCTTCATCGGCCTGTCGATCGTGGGCATCGGGATGTCGATGGGATCCAACTCCGGCTAT
GCCATCAACCCCGCCCGGGATTTCGGCCCCGCCTGTTGACCCTCGCTGCCGGCTGGGGG
ACCGAGGTTTTCACG---

>Zebra_bullhead_shark_Aqp10C1
ATG------------------------------GGGAGAGTA------------------
---------------------GAGACGTTTTTCAGAGCACTACATGATGGGTTG------
CAGTTGAAGAATCGCCTCTTCAGGGAATGCTTAGCGGAGTTTCTAGGAGTTTGCATGCTG
ATTTTGTTTGGCTGTGGTGCCGTGGCACAGATGGTGACGAGCCACACAACCCGCGGAGAA
TTCCTGTCTGTCAACCTAGGATTCGGCCTTGGCGCAACCTTTGGCATCTACATCTCAGGT
GGCATCTCGGGTGGCCATCTGAACCCGGCCGTGTCCTTCAGTTTGTGTTTGCTGGGAAGG
TTCCAGTGGAAGAAGCTGCCATTCTACATGTTCTTCCAGACACTTGGGGGTTTTGTTGGT
GCTGCCATAGTCTTT---GGGGTCCACCATGATGGTATCTACGCTGTTGAT---AACGGG
ACCTTGTCCGTCACCGGACCGCGG---GCCACTGCATTTATTTTTGGCACGTATCCCGCA
GCATACCTCACCCTTCCGAATGGCTTCATTGACCAGCTGATTGGCACCGGCACTCTGCTC
CTCTGTATTTTTGCCGTGGTGGATGCCCGGAACTACGGAGCCCCGAAGATCTTACAGCCC
ATCTTCATCGGTCTGTCGGTGGTTGGCATCGGGATGTCCATGGGCTCCAACTCTGGCTAT
GCCATCAACCCCGCCCGTGATTTGGGCCCCGCCTGTTAACCCTGGCCGCCGGCTGGGGC
ACCGAAGTCTTTACGGCTGGAGGCGGGTGGTGGTGGATCCCCATCGTGGCGCCGATGGTT
GGCGCGGTCCTTGGCGCCCTGGCCTACGAGCTGCTGATCGAGCTTCACCACCTGCAGGCC
AGGGACGGGCCCAGC---------------------------------GAGAGG------
GACGTGACGGAGCAGAAG---------CTGAAGGAGATGGGCAAT---CCGGCCGCCGGC
TGCAAGGAGGGCCCGGCCTGGCGGAACGAGGCGGCGGGC---------------------
---GCG
GGTGACCAGTTCGCCGTGGCGATG------------------------------------
>Cloudy_catshark_Aqp10C1
ATG------------------------------GGGAGAGCT------------------
---------------------GCAGCAATAGCACAGACGGTGCACAATGCTCTG------
CAGCTGAGGAACCGTCTCTTTCGCGAATGCTTGGCTGAGTTTCTGGGGGTGTGCATGCTG
ATCCTGTTCGGGTGCGGGGCCGTGGCACAGATGGTGGTCAGCAACACTACCCGTGGGGAA
TTCCTCTCCGTCAACCTGGGCTTTGGACTGGGCGCCACGTTCGGCATCTACATCGCTGGT
GGGATCTCAGGT--

------------------------------GATGGGATTCACGCTGTCAAT---AACGGG
ACCTTATCTGTCACTGGGCCACGA---GCAACTGCCTTCATCTTTGGCACCTATCCAGCA
CCATTCCTCACCCTTTCGAATGGCTTCATTGACCAGCTAATCGGCACCGCCACTCTACTG
CTTTGCATCTTCGCTGTCCTGGACGGCAAGAACAACGGCGCCCCCAAAGTCCTGCAGCCT
ATCTTCATTGGCCTGTCGGTTGTCGGCATCGGAATGTCCATGGGATCCAACTCCGGCTAC
GCCATCAACCCCGCCCGAGATTTCGGACCCCGCCTGCTGACCCTCGCCGCGGGCTGGGGG
ACTGAGGTCTTCACGGCTGGCAACTCCTGGTGGTGGATACCCATCGTGGCGCCCATGGTG
GGCGCCGTCCTGGGCGCCCTGGCGTACGAGCTGCTGGTTGAACTGCACCACCTGCAGGCC

CAGGACGAGTGCGAG---------------------------------GGGAGG------
GAGCAGACGGACCAGAAG---------CTGAAGGAGGTGGGCGGC---------------
TGCAAGGAGGGCCCCGCCTGCCGGAGAGACCCGGACAGC---------------------
---TCG
GGGGACCAGTTCGTCATGGCGATG------------------------------------
>Little_skate_Aqp10C1
ATG------------------------------GAGCAGGTC------------------
---------------------CGGGCCATCTGCAGCTCGGTTCGCGAATCACTA------
AAGCTGAAGAACCGCCTGTTCCGTGAGTGTTTGGCTGAATTTCTGGGAGTTTGCGTTTTG
ATT---

---------GGTGCACATCTGAATCCAGCCGTGTCCTTCAGTCTGTGTCTGCTGGGAAGA
TTCCAATGGAAGAAACTACCGTTTTACATGTTCTTCCAGACACTCGGAGGGTTTGTTGGA
GCTGCCATAGTCTAC---GGAGTCCACCAC------------------------------

---------------------------------CAGCTGATCGGCACGGGAACGCTGCTG
CTCTGTATCCTGGCTGTGGTGGACAGCAAGAACAATGGGGCTCCGAAGGCCCTTCAGCCC
ATCTTCATAGGACTGTCCGTGCTGGCCATCGGGATGTCCATGGGATCCAACTCGGGATAC
GCCATCAATCCGGCCCGAGACTTCGGACCACGGCTGCTCACACTCGCTGCTGGGTGGGGA
ACTGAGGTCTTCACG---

>Thorny_skate_Aqp10C1
ATG------------------------------GAGCGGGTC------------------
---------------------CGGGCCATCTTCAGCCCGGTTCGCGAATCACTA------
AAGCTGAAGAACCGCCTGTTCCGTGAGTGTTTGGCTGAATTTCTGGGAGTTTGCATTTTG
ATTTTATTTGGCTGTGGGGCAGTGGCCCAGATGGTCACCAGCCACACCACCCGCGGGGAA
TTCCTCTCCGTCAACCTCGGCTTCGGGATCGGAGCCACATTCGGAGTTTGTATCGCGGGA
GAAATCTCCGGAGCGCATCTGAACCCAGCCGTGTCCTTCAGCCTGTGTCTGCTCGGCCGG
TTCCAATGGAAGAAACTACCTTTCTACATGTTCTTCCAGACGCTCGGAGGGTTTGTTGGA
GCTGCCATAGTCTAC---GGAGTCCACCACGATGGGATACATGCCGTCAAC---AACGGA
ACTCTCTCTGTCACTGGCCCCAAT---GCCACTGCT------------------------

>Smalltooth_sawfish_Aqp10C1
ATG------------------------------GAGAGGGTC------------------
---------------------GGGGCACTGGGGAGAAGTTTGCACCGAGCTCTG------
CAGCTCAAGAGTCGCCTCTTCAGGGAATGTTTGGCCGAGTTTCTTGGAGTCTGCATTCTG
GTTCTGTTCGGCTGCGGGGCCGTCGCGCAGATGGTCACCAGTCACACCAACGCCGGGAAG
TTCCTGTCCGTCAACCTGGGATTCGGGCTGGGAGCCACCTTCGGGGTCTACATCGCCGGG
GGAATCTCCGGAGCCCATCTGAATCCAGCCGTGTCCTTCAGCATGTGTTTGCTGGGAAGG
TTCCAGTGGAGGAAGCTGCCGTTTTATATGTTCTTCCAGACCCTCGGGGGATTCGTTGGC
GCTGCGGTCGTGTAC---GGTGTGCATCACGATGGCATCTACGCGGTGAAC---AATGGC
ACCCTCTCTGTCACCGGCCCAAGA---GCCACCGCCTTCATTTTCGGCACCTACCCTGCG
CCCTTCCTGACCCTCCTGAATGGCTTCATTGACCAGCTGATCGGGACGGGGACGCTTCTG
CTCTGTATCCTGGCCGTGGGGGACACCCAGAACAACGGAGCTCCCAAGGTGCTTCAGCCC
ATCTTCATCGGCCTATCCATCGTGGCCATCGGAATGTCCATGGGCTCCAACTCAGGATAC
GCCATCAACCCCGCCCGAGACCTTGGACCTCGCCTCCTGACCCTGGTGGCGGGTTGGGGG

ACAGAAGTCTTCACGGCCGGGGGAGGCTGGTGGTGGGTGCCGATCGTGGCGCCGATGGTG
GGGGCCATCCTGGGCTCCGCGGTCTACGAGCTGCTGATCGAGTTCCACCACTTGGAGGAC
CAGAGGAGGGCCGAG----------------------------------GAGCTG------
GGTCTGAAGGAGGCACGG---------AGGCAGAGGTCGGAGGGC---GAGGAGGCGAGG
CAGCCGCAGACGGTCGTCTACAAGAAGGAGGAGGAGGAG---------------------

GGGGGGCACTAC---ATG--
>B_bambooshark_Aqp10C2

------------------------------ATGAGGAGCCTCCGTCAGAAGCTG------
CAGATCAGGAACAGGCTGGCCCGGGAATGCCTGGCCGAATTCTTCGGGGAATACATGCTC
ATTCTCATGGGCACAGCGGCAGTGGCTCAAGTGGTGACAAACTTCGATCAGAAAGGGACC
TATTTATCGATTAACATTGGCTATGCTGCTGGAGTTCTGTTTGGGATCTACGCCTCAGTT
GGAGTCTCAGGGGCTCACCTGAACCCGGCGGTGACCTTCAGCCTGTGCGTCCTGGGACGG
TTTCCCTGGAAAAAGCTTCCGTTCTACACCATCTCCGAGTGCCTGGGGTCATTCGTCGCC
TCGGCAACCACGTTC---ACCCTCTACTACGACGCCATCCAAGAGTTCTCT---GGTGGC
AATCTGACCGTGCGTGGGCCGAGG---GGAACAGCTGGCATCTTTGCGACGTATCCCGTG
GAGTATCTGTCTGTCCGCAACGGCTTCATAACTGAGGTAATTGGCACCGCTGTTTTGTTG
ATTTGCGTCTTGAGTGTTGGCGACGCCAAAAATGCTGGTGCCCCGCCTTTCATCCAGCCG
TTGCTGATCTCGGTCTCGGTACTCGTCATCGGTGCTGCTATGGGTGCCAACACCGGCTAC
GCCATCAACCCAGCGAGAGACCTCGGACCCAGACTCTTCACCTTCGTGGCTGGCTGGGGG
ACTGAAGTTTTCAAGGCCGGGAATGGCTGGTGGTGGATCCCTATCGTCGCACCCCTGATT
GGCGGTGTACTAGGCAGCCTGGCCTACACGCTCCTCATCGACCTGCACCACGCGGAGCCC
GTCTCGGCCAAAGAG----------------------------------GAGGTG------
AAGGACGTCAAGGCTGAA---------CCTCAAGCTGAGTCAGAG---GAGGCCGCGGAG
GAGCCTGTT---

>W_bambooshark_Aqp10C2

------------------------------ATGAGGAGTCTCCGTCAGAAGCTG------
CAGATCAGGAACAGGCTGGCCCGGGAATGCCTGGCCGAATTCCTCGGGGAATACATGCTC
ATTCTCATGGGCACAGCGGCAGTGGCTCAGGTGGTGACAAACTTCGATCAGAAAGGGACC
TATTTATCGATTAACATTGGCTATGCTGCTGGAGTTCTGTTTGGGATCTACGCCTCAGTT
GGAGTCTCAGGGGCTCACCTGAACCCGGCGGTGACCTTCAGCCTGTGCGTCCTGGGCCGG
TTTCCCTGGAGAAAGCTTCCGTTCTACACCATCTCCGAGTGCCTGGGGTCATTCGTCGCC
TCGGCAACCACGTTC---ACCCTCTACTACGACGCCATCCACGAGTTCTCT---GGTGGC
AATCTGACCGTGCGTGGGCCGAGG---GGAACAGCTGGCATCTTTGCGACGTATCCCGTC
GAGTACCTGTCTGTCCGCAACGGCTTCATAACTGAGGTAATTGGCACCGCTGTTTTGTTG
ATTTGCGTCTTGAGTGTCGGCGACGCCAAAAATGCTGGTGCCCCGGCTTTCCTCCAGCCG
TTGCTGATCTCGGTCTCGGTACTCGTCATCGGTGCTGCTATGGGTGCCAACACCGGCTAT
GCCATCAACCCAGCGAGAGACCTTGGACCCAGACTCTTCACATTCGTGGCTGGCTGGGGG
ACTGAAGTTTTCAAGGCCGGGAACGGCTGGTGGTGGATCCCTATCGTCGCGCCCCTGATT
GGCGGCGTAATAGGCAGCCTGGCCTACACGCTCCTCATCGAACTGCACCACGCGGAACCC
GTGTCGGCCAAAGAG----------------------------------GAGGTG------
AAGGACGTCAAGGCCGAA---------CCTCAAGCTGAAACAGAG---GAGGCCGGGGAG
GAGCCTGTT---

>Whale_shark_Aqp10C2

------------------------------ATGAAGTCCCTCCGTCGGAAGCTG------
CAGATCCGGAACCAGCTCGCCCGGGAATGCCTCGCCGAATTCCTCGGGGAATACATGCTG
ATT---

------------------------------GATGCCATCCAGGAATTTTCC---GGGGGC
AATCTGACCGTGCGTGGACCAAGG---GGAACGGCCGGAATCTTCGCAACGTATCCTGTT
GAGTACCTGTCCATCCGGAATGGGTTCCTG---CAGTTGATTGCCACCGCTGTTTTGTTG
ATCTGCATCATGTGTGTCGGCGACGCCAAAAACGCCGGTGCTCCGGCTTTCCTGCACCCC

CTGCTGGTCTCGGTCGCCGTGCTCGTCATCGGTGCCACCATGGGTGCTAACACCGGCTAT
GCCATCAACCCCGCACGGGACCTTGGACCCAGGCTCTTCACCTTCATAGCTGGATGGGGC
ACTGAGGTGTTCAGG---

>Zebra_bullhead_shark_Aqp10C2

------TCAGGTGCTCACTTGAACCCTGCCGTCAGTCTATGCCTCTGCGTGCTCGGCCGC
TTCCCGTGGAAGAAGCTGCCGTTTTACACCTTGGCCGAGTGTCTGGGGTCCTTCACCGCC
GCGGCCACCACCTTC---TGCCTCTATTACGATGCAATACAGGAATTTTCT---CAAGGG
AATTTAACTGTTCGTGGCCCAAGA---GGCACAGCAGGTTTATTCGCCACCTACCCTGTT
GAAAACCTCAGCGTTCGTAATGGATTCATCACGGAGGTGATAGGCACCGCTGTTTTGCTA
ATCTGTATTCTGGCTATCGGTGATGCCAAAAACGCGGGCGCTCCCGCTTTCCTGCAGCCC
CCCCTGGTCGCCATTTCGGTTTTCGCCATTGGCGTTGGGTTGGGTGCCAACACTGGCTAT
GCCATCAACCCAGCCAGAGACATAGGA---------------------------------

>Cloudy_catshark_Aqp10C2

------------------------------ATGAACAGTTTGAAGAAAAAACTC------
CAGATTAAGAACCTGCTCGTACGACAGTGTCTGGCTGAGTTTCTGGGTGTTTACTTGCTT
ATGTTGATGGGGACAGCATCTGTGGCTCAGGTTGTAACAAACTTTGACCAGAAGGGAACA
TTCTTATCTATTAACTTTGGCTATGCTGCCGGAGTCCTGTTTGGCGTCTACGCATCAGTC
GGTGTCTCAGGTGCCCATTTGAACCCCGCCGTCTCGTTGAGCCTGTGCATAATTGGCCGG
TTCCCGTGGAAGAAGCTGCCTTTCTACATCATCGCCGAGTGCCTGGGGGCCTTTATTGCC
GCGGCTACCACCTTC---TGCCTTTACTACGATGCGATACACGAATTCTCT---CGAGGG
AATTTCACGGTCCGAGGTCCGCGG---GGCACAGCCGGGATCTTCGCCACCTACCCCGTT
GAATACCTCTCGACCAGCAATGGCTTCATCACCGAGGTGATTGCCACCGCCGTTTTGCTA
ATCTGCATCCTGTGCGTTTTGGACAGCAAAAACGCGGCTGTGCCGCCATTTTTACAGCCG
CCCATGATCGCGGTGTCAGTGCTGGTCATTGGACTTTCCATGGGCGCCAACACTGGCTAC
GCCATCAATCCAGCCAGAGATTTCGGCCCGAGGATGTTCACCTTTGTGGCTGGGTGGGGC
TCCGAGGTTTTCACGGCTGGTCACGGATGGTGGTGGGTTCCGATCGTCGCCCCCATGATC
GGAGGTGTCGCCGGTACCCTAGTCTACATGCTCCTCATCGATCTGCACCACGAGGATACC
AAGGAGCTTGAATCA---------------------------------GAAGCC------
AAATTGATCAAGCAGGAC---------ACGGAAGATGAGCCCGAA---CCGATCAGCGAG
GGGCCTGTG---

>Great_white_shark_Aqp10C2

------------------------------ATGAAGAGACTTCGGAAGAAACTC------
CGGATTAAGAACAAGCTCATTCGGGAATGCCTGGCAGAATTCTTCGGCGTTTACCTGCTG
ATTTTGATGGGCTCGGCGTCAGTGGCACAGGTTGTATTGTTTTTCGACCGGAAGGGCGAG
TACCTGTCAATTGCCTTTGGATACGCTTGCGGGGTCCTGTTCGGCATCTATGCATCAAGA
GGAATCTCAGGTGCTCATTTGAACCCCGCCGTCACCTTCAGTCTGTGCCTGCTGGGCCGG
TGCCCATGGAAGAAGCTGCCTTTTTACACCATCGCTGAGTGCCTGGGCTCCTTCACTGCC
GCAGCGACCACCTTC---TGCCTTTATTAC------------------------------

---------------------------------CAGATCATTGGTACCGCTGTTTTGTTA
ATCTGCATCCTGTGCGTTGGGGATGCCAAAAATGCTGGGGCTCCGGCATTCTTACAACCC
CCTCTTGTCGCCACCTCAGTGTTTATCATTGGCATTTGCATGGGTGCCAACACTGGTTAC
GCCATCAACCCAGCAAGGGACTTCGGACCTCGCTTGTTTACCTATGTAGCTGGGTGGGGC
ACTGAGGTGTTCACGGCCGGCAACAATTGGTGGTGGATCCCGATCGTCGCCCCGGTCCTG
GGGGGAGCGCTGGGCAGCCTGGCCTACGTTCTCCTCATCGAGATGCACCACGAGGACCCC
AAGCCACTCAAA------------------------------------GATGTC------
AAGGACGAGGCAGTCGAT------------GACGCG------------------------

>Little_skate_Aqp10C2
ATG------------------------------AACCCGCTG------------------
---------------------AGCGGGGGATGGGTCGCGGTGAGGAGGAAACTA------
CGCGTGAAGAACAGGCTCCTGCGCGAATGTATGGCCGAGTTCATCGGAGAATACATGCTG
ATCTTGTTCGGGTCAGCGGCTGTTGCCCAGGTGGTCACCAACTACGACAGGAAGGGGATG
TATCTCTCCATCAACCTGGGATACGCCATTGGCGTGCTGTTTGGCATTTACGTCTCAGCC
GGTGTGTCAGGGGGTCATTTGAACCCAGCCGTGACGTTCTGCTTGTGTGTTCTGGCCGC
TGCCCGTGGTATAAGCTGCCCCTCTACACGTTGTCCGAGTGCCTGGGGTCTTTCATGGCA
GCGGCTACCACCTTC---GCCGTGTACTAC------------------------------

---------------GCTGGTAATGGCTGGTGGTGGGTCCCTGTCATTGCGCCTTTCATC
GGGGGAGTCCTGGGCACTGCCATTTACGTTCTGTTCATTGAACTACATCACAAAGAGACG
GTC---TTG---

>Smalltooth_sawfish_Aqp10C2
ATGGAGGATTCGACCCCAGTCCAACGGGCGCTAGACAAGGCG------------------
---------------------GCGACGGGGGTGAAGGCGTTCCGGAGGCACCTA------
CGGGTGAAGAACGAGCTACTGCGACAGTGCCTGGCCGAATTCTTGGGGGAGTACATACTG
ATCTTAATGGGATCGGCGACTGTGGCACAGGTCATCACCAACTATGACCGGAAAGGGACC
TACCTGTCGATCAACATGGGCTATGCCATCGGAGTCCTGTTCGGAATCTACATGTCAAGT
GGAGTCTCAGGTGCACACCTGAATCCGGCGGTCACCTTGAGCCTGTGCGCCCTGGGTCGA
TTCCCCTGGCAGAAGATGCCCTTCTACACGCTGGCGGAATGCCTGGGCTCCTTCGTGGCA
GCGGCCACCACCTAC---GCTCTTTACTACGATTCTATCCACGAGTTTTCC---AACGGG
AGTCTGACTGTGCTGGGACCCAGG---GGGACGGCCGGAATCTTCGCCACCTACCCCGCG
GAACACATCACCACCCGCAACGGCTTCATCACCGAGGTGATAGCCACCGGGGTGTTGTTG
ATCTGCATCCTGGCGATCGGCGATTCCCGGAACGCCATGTTGCCCGACTTCCTCAGGCCG
CTGGTGACCTCCATCTGCGTGCTCACCATCGGGATGGGCATGGGAGCCAACACTGGCTAC
GCCATTAACCCCGCGAGGGACATCGGGCCACGCATGTTCACCTTCGTCGCTGGCTGGGGC
TCCGAAGTATTCACGGCTGGGAACAGCTGGTGGTGGATCCCACTGGTCGCCCCGATGTTG
GGGGGTCTCCTGGGGACGCTGATCTACGAGCTGCTCATTGAGTTCCACCACGAGGACGTG
CCGCACCCCGGA------------------------------------GAGGCC------
AGG------GCGGCCGAG---------GAGGAGGCGCG----------CTGGCACAGGAA
GATCCCGTG---

>Ghost_shark_Aqp10C2b
ATGAAC---TCCAATCCG---------ACCCTGAGGAGCCTC------------------
---------------------CAGGCTTTGCCTGTCCAGATGCAGAGGAAACTC------
CACCTGAAGAACAAGCTCCTGCGGGAATGTTTGGCCGAATTTCTGGGTGTCTTCCTGTTG
ATTTTTATCGGAGGTGCGGCTGTAGCTCAGGTCCAGACCACA------GGAAAAGGTTCG
TATCTGTCCATCAACATCGGTTACGGTATCGGTGTTATGTTCGCCATTTACGCAGCCGGA
GGCGTCTCAGGAGCTCACCTGAACCCGGCCGTTTCCATCTCTTTCTGTGTTTTGGGGAAA
CTGATTTGGTGGAAAGTCCCATTTTATATCTTCTCCCAAACGTTCGGAGCCTTCACCGCG

GCGGCCGTCATCTTC---ACCATGTACTACGATTCCATCATGCACTTCACG---GGGGGG
CAGCTGATCGCCGATGGGGGAAACCTGGCAACGGGGGGAATATTCGCCACATATCCGGCC
TCGTTCCTCACCACACGGAACGGCTTCATTGACCAGATCGTGGCGACGGGGATCTTGCTC
CTGGTTATCCTGAGCCTGAACGACTCGCGGAACAATGAGCCCCCGGACTTCCTGAAGCCC
CTCCTGGTGGGGGCTCTGGTGCTGGTGATCGGAGTGGCCATGGGGTCCAATTGCGGTTAC
CCCATCAACCCGGCCCGCGACATCGGCCCCCGACTCTTCTCCTACCTGGCGGGGTACGGA
CCCCAAGTCTTCACGGCTGGTGATCACTGGTGGTGGGTCCCCATTGTAGCCCCAGTTATT
GGGGGACTCGTTGGTTGTTTCTTCTACAAGATCCTGATCGAGATCCACCACGACGACTTC
GAGGTGGAG---GAGATA------
GAACTAGAGAAAGACCCC---------GAGAGACCCCGAGCCGAC---AATTCATCTGAA
GAGAAA--

>Ghost_shark_Aqp10C2a
ATG------------------------------GCCAGTGTG------------------
---------------------TCTGCCATCCTGAAACAAGTGAAGAGGAGGCTC------
CATTTGAAGAACTGTCTCGTGCGGGAATGCTTGGCGGAGTTCCTGGGAACTTTCCTGGTT
ATTTTTATAGGAGGAGCAGCGGTTGCTCAGGTACAGACCACG------AGCAAAGGTGCA
TACCTGTCCATTAACATTGGCTATGGTGTGGGCGTCATGTTTGGCATTTATGCAGCTGGA
GGCGTCTCAGGTGCTCACTTGAACCCAGCCATCTCCATCTGCTTCTGTGTGTTGGGGAAG
CTGCCGTGGTGGAAGCTGCCATTTTACATCCTGTCTCAGACCTTTGGAGCTTTCGTAGGT
GCAGCTGTGATCTTT---ACCATGTATTACGATTCCATCATGCACTTCACT---GGGGGC
GAGCTGATAGCTGACGGTGCAACGCTGGCTACTGGGGGGATATTTGCCACCTACCCTGCC
GTGTACCTCACCACACGTAATGGCTTCATTGACCAGGTTGTGGCCACTGCAGTTCTGCTG
CTTGTCATCCTGAGTTTAAACGACTCCCGGAATAATGAGCCCCCGGCGTTCCTCAAGCCC
ATCCTGATTGGGACGCTGGTACTTGTGATAGGTGTGGCCATGGGCTCCAACTGTGGCTAT
GCCATCAATCCCGCCCGAGACTTCGGCCCACGCCTCTTCTCCTATCTTGTTGGTTATGGC
AGTCAGGTATTCACGGCTGGGGACAGCTGGTGGTGGGTACCTATCGTGGCCCCGGTGTTG
GGAGGGCTGCTTGGCTGTTTCTTGTACAAGATCCTAATCGAGATCCACCACGATGACCCT
GAGGAGCATAGCCAG---
CAGCCAGCCGCAGACCTC---------GAACTGCCAACCATAGAC---AGTTCATCCAGA
GAGCAG--

>Human_AQP7
ATG------------------------------GGCTCGGGCCACTGTCTTAGGTCCACC
CGTGGCTCCAAAATGGTCTCCTGGTCCGTGATAGCAAAGATCCAGGAAATACTG------
------CAGAGGAAGATGGTGCGAGAGTTCCTGGCCGAGTTCATGAGCACATATGTCATG
ATGGTATTCGGCCTTGGTTCCGTGGCCCATATGGTTCTAAATAAA---AAATATGGGAGC
TACCTTGGTGTCAACTTGGGTTTTGGCTTCGGAGTCACCATGGGAGTGCACGTGGCAGGC
CGCATCTCTGGAGCCCACATGAACGCAGCTGTGACCTTTGCTAACTGTGCGCTGGGCCGC
GTGCCCTGGAGGAAGTTTCCGGTCTATGTGCTGGGGCAGTTCCTGGGCTCCTTCCTGGCG
GCTGCCACCATCTAC---AGTCTCTTCTACACGGCCATTCTCCACTTTTCG---GGTGGA
CAGCTGATGGTGACCGGTCCCGTC---GCTACAGCTGGCATTTTTGCCACCTACCTTCCT
GATCACATGACATTGTGGCGGGGCTTCCTGAATGAGGCGTGGCTGACCGGGATGCTCCAG
CTGTGTCTCTTCGCCATCACGGACCAGGAGAACAACCCAGCACTGCCAGGAACAGAGGCG
CTGGTGATAGGCATCCTCGTGGTCATCATCGGGGTGTCCCTTGGCATGAACACAGGATAT
GCCATCAACCCGTCCCGGGACCTGCCCCCCCGCATCTTCACCTTCATTGCTGGTTGGGGC
AAACAGGTCTTCAGCAATGGGGAGAACTGGTGGTGGGTGCCAGTGGTGGCACCACTTCTG
GGTGCCTATCTAGGTGGCATCATCTACCTGGTCTTCATTGGCTCCACCATCCCACGGGAG
CCCCTGAAATTGGAG---------------------------------GATTCT------
GTGGCGTATGAAGACCAC---------GGGATAACCGTATTGCCC---AAGATGGGATCT
CATGAACCCACGATCTCTCCC---------------------------------------
---------------------------------CTCACCCCCGTCTCTGTGAGCCCTGCC
AACAGATCTTCAGTCCACCCTGCCCCACCCTTACATGAATCCATGGCCCTAGAGCACTTC
>Chimpanzee_AQP7
ATG------------------------------GGCTCGGGCCACTGTCTCAGGTCCACC
CGTGGCTCCAAAATGGTCTCCTGGTCCGTGATAGCAAAGATCCAGGAAATACTG------
------CAGAGGAAGATGGTGCGAGAGTTCCTGGCCGAGTTCATGAGCACATATGTCATG
ATGGTATTCGGCCTTGGTTCCGTGGCCCATATGGTTCTAAATAAA---ACATATGGGAGC
TACCTTGGTGTCAACTTGGGTTTTGGCTTCGGAGTCACCATGGGAGTGCACGTGGCAGGC

CGCATCTCTGGAGCCCACATGAATGCAGCTGTGACCTTTGCTAACTGTGCGCTGGGCCGC
GTGCCCTGGAGGAAGTTTCCGGTCTATGTGCTGGGGCAGTTCCTGGGCTCCTTCCTGGCG
GCTGCCACCATCTAC---AGTCTCTTCTACACGGCCATTCTCCACTTTTCG---GGTGGA
GAGCTGATGGTGACCGGTCCCGTC---GCTACAGCTGGCATTTTTGCCACCTACCTTCCT
GATCACATGACATTGTGGCGGGGCTTCCTGAATGAGGTGTGGCTGACCGGGATGCTCCAG
CTGTGTCTCTTCGCCATCACGGACCAGGAGAACAACCCAGCACTGCCAGGAACAGAGGCG
CTGGTGATAGGCATCCTCGTGGTCATCATCGGGGTGTCCCTTGGCATGAACACAGGATAT
GCCATCAACCCATCCCGGGACCTGCCCCCCCGCATCTTCACCTTCGTTGCTGGTTGGGGC
AAACAGGTCTTCAGCAATGGGGAGAACTGGTGGTGGGTGCCAGTGGTGGCACCACTTCTG
GGTGCCTATCTAGGTGGCATCATCTACCTGGTCTTCATTGGCTCCACCATCCCACGGGAG
CCCCTGAAATTGGAG---------------------------------GACTCT------
GTGGCATATGAAGACCAC---------GGGATAACCGTATTGCCC---AAGATGGGATCT
CATGAACCCACGATCTCTCCC---------------------------------------
---------------------------------CTCACCCCCGTCTCCGTGAGCCCTGCC
AACAGATCTTCAGTCCACCCTGCCCCACCCTTACATGAATCCATGGCCCTAGAGCACTTC
>Gorilla_AQP7
--CACTGTCTCAGGTCCACC
CGTGGCTCCAAAATGGTCTCCTGGTCCGTGATAGCAAAGATCCAGGAAATACTG------
------CAGAGGAAGATGGTGCGAGAGTTCCTGGCTGAGTTCATGAGCACATATGTCATG
ATGGTATTCGGCCTTGGTTCCGTGGCCCATATGGTTCTAAATAAA---AAATATGGGAGC
TACCTTGGTGTCAACTTGGGTTTTGGCTTCGGAGTCACCATGGGAGTGCACGTGGCAGGC
CGCATCTCTGGAGCCCACATGAATGCAGCTGTGACCTTTGCTAACTGTGCGCTGGGCCGC
GTGCCCTGGAGGAAGTTTCCGGTCTATGTGCTGGGGCAGTTCCTGGGCTCCTTCCTGGCG
GCTGCCACCATCTAC---AGTCTCTTCTACAGTGCCATTCTCCACTTTTCG---GGTGGA
CAGCTGACGGTGACCGGTCCCGTC---GCTACAGCTGGCATTTTTGCCACCTACCTTCCT
GATCACATGACATTGTGGCGGGGCTTCCTGAATGAGGTCTGGCTGACCGGGATGCTCCAG
CTGTGTCTCTTCGCCATCACGGACCAGGAGAACAACCCAGCACTGCCAGGAACAGAGGCG
CTGGTGATAGGCATCCTCGTGGTCATCATCGGGGTGTCCCTTGGCATGAACACAGGATAT
GCCATCAACCCGTCCCGGGACCTGCCCCCTCGCATCTTCACCTTCATTGCTGGTTGGGGC
AAACAGGTCTTCAGCAATGGGGAGAACTGGTGGTGGGTGCCAGTGGTGGCACCACTTCTG
GGTGCCTATCTAGGTGGCATCATCTACCTGGTCTTCATTGGCTCCACCATCCCACGGGAG
CCCCTGAAATTGGAG---------------------------------GACTCT------
GTGGCGTATGAAGACCAC---------GGGATAACCGTATTGCCC---AAGATGGGATCT
CATGAACCCACGATCTCTCCC---------------------------------------
---------------------------------CTCACCCCCGTCTCTGTGAGCCCTGCC
AACAGATCTTCAGTCCACCCTGCCCCACCCTTACATGAATCCATGGCCCTAGAGCACTTC
>Human_AQP7L1
ATGGTT------------------------CAAGCATCTGGGCAC---AGGCGGTCCACC
CGTGGCTCCAAAATGGTCTCCTGGTCCGTGATAGCAAAGATCCAGGAAATATGGTGCGAG
GAAGATGAGAGGAAGATGGTGCGAGAGTTCTTGGCCGAGTTCATGAGCACATATGTCATG
ATGGTATTCGGCCTTGGTTCTGTGGCCCATATGGTTCTAAATAAA---ACATATGGGAGC
TACCTTGGTGTCAACTTGGGTTTTGGCTTCGGGGTCACCATGGGAGTCCACGTGGCAGGC
CGCATCTCTGGAGCCCACATGAATGCAGCTGTGACCTTCACTAACTGTGCGCTGGGCCGC
GTGCCCTGGAGGAAGTTTCCAGTCCATGTGCTGGGGCAGTTCCTGGGCTCCTTCCTGGCA
GCTGCCACCATCTACATCTGTGTCTCCGCAGCGGCCATTCTCCACTTTTCG---GGTGGA
GAGCTGATGGTGACCGGTCCCTTT---GCTACAGCTGGCATTTTTGCCACCTACCTTCCT
GATCACATGACATTGTGGCGGGGCTTCCTGAATGAGGAGTGGCTGACCAGGATGCTCCAG
CTGTGTCTCTTCACCATCACGGACCAGGAGAACAACCCAGCACTGCCAGGAACACACGCG
CTGGTGATAAGCATCCTCGTGGTCATCATCAGGGTGTCCCATGGCATAAACACAGGATAT
GCCATCAATCCATCCCGGGACCCGCCCCCCAGCATCTTCACCTTCATTGCTGGCTGGGGC
AAACAGGTCTTCAGCGATGGGGAGAACTGGTGGTGGGTGCCAGTGGTGGCACCACTTCTG
GGTGCCTCTCTAGGTGGCATCATCTACCTGGTCTTCATTGGCTCCACCATCCCACGGGAG
CCCCTGAAATTGGAG---------------------------------GACTCT------
GTGGCGTATGAAGACCAC---------GGGATAACCGTATTGCCC---AAGATGGGATCT
CATGAACCCATGATCTCTCCC---------------------------------------
---------------------------------CTCACCCTCATCTCCGTGAGCCTTGCC
AACAGATCTTCAGTCCACTCTGCCCCACCCTTACATGAATCCATGGCCCTAGAGCACTTC
>Chimpanzee_AQP7L1
ATGGTT------------------------CAAGCATCTGGGCAC---AGGCAGTCCACC
CGTGGCTCCAAAATGGTCTCCTCATCCGTGATAGCAAAGATCCAGGAAATATGGTGCGAG
GAAGATGAGAGGAAGATGGTGCGAGAGTTCCTGGCCGAGTTCATGAGCACATATGTCATG

ATGATATTCGGCCTTGGTTCTGTGGCCCATATGGTTCTAAATAAA---ACATATGGGAGC
TACCTTGGTGTCAACTTGGGTTTTGGCTTCGGAGTCACCATGGGAGTCCACGTGGCAGGC
CGCATCTCTGGAGCCCACATGAATGCAGCTGTGACCTTCACTAACTGTGCACTGGGCCGC
GTGCCC---AGGAAGTTTCCAGTCTATGTGCTGGGGCAGTTCCTGGGCTCCTTCCTGGTG
GCTGCCACCATCTACATCTGTGTCTCCGCAGCGGCCATTCTCCACTTTTTG---GGTGGA
GAGCTGATGGTGACCGGTCCCTTT---GCTACAGCTGGCATTTTTGCCACCTACCTTCCT
GATCACATGACATTGTGGCGGGGCTTCCTGAATGAGGAGTGGCTGACCAGGGTGCTCCAG
CTGTGTCTCTTCACCATCACGGACCAGGAGAACAACCCAGCACTGCCAGGAACACACGCG
CTGGTGATAAGCATCCTCGTGGTCATCATCGGGGTGTCCCATGGCATAAACACAGGATAT
GCCATCAATCCATCCCGGGACCCACCCCCCAGCATCTTCACCTTCATTGCTGGTTGGGGC
AAACAGGTCTTCAGCGATGGGGAGAACTGGTGGTGGGTGCCAGTGGTGGCACCACTTCTG
GGTGCCTCTCTAGGTGGCATCATCTACCTGGTCTTCATTGGCTCCACCATCCCACGGGAG
CCCCTGAAATTGGAG---------------------------------GACTCT------
GTGGTGTATGAAGACCAG---------GGGTTAACCGTATTGCCC---AAGATGGGATCT
CATGAACCCATGATCTCTCCC---------------------------------------
---------------------------------CTCACCCTCATCTCCGTGAGCCTTGCC
AACAGATCTTCAGTCCACTCTGCCCCACCCTTACATGAATCCATGGCCCTAGAGCACTTC
>Gorilla_AQP7L1
ATG------------------------------GGCTCGGGCCACTGTATCTGGTCCACC
CGTGGCTCCAAAATGGTCTCCTGGTCCGTGATAGCAAAGATCCAGGAAATATGGTGCGAG
GAAGATGAGAGGAAGATGGTGCGAGAGTTCCTGGCTGAGTTCATGAGCACATATGTCATG
ATGGTATTCGGCCTTGGTTCCGTGGCCCATATGGTTCTAAATAAA---AAATATGGGAGC
TACCTTGGTGTCAACTTGGGTTTTGGCTTTGGAGTCACCATGGGATTGCACATGGCAGGC
CGCATCTCTGGAGCCCACATGAACACAGCTGTGAGCTTCGCTAACTGTGCACTGGGCCAC
GTGCCCTGGAGGAAGTTTCCAGTCTATGTGCTGGGGCAGTTCCTGGGCTCCTTCCTGGCG
GCTGCCACCATC------TGTGTCTCCACAGAGGCCATTCTCCACTTTTCG---GGTGGA
GAGCTGATGGTGACCGGTCCCGTT---GCTACAGCTGGCATTTTCGCCACCTACCTTCCT
GATCACATGACATTGTGGCGGGGCTTCCTGAATGAGGAGTGGCTGACCGGGATGCTCCAG
CTGTGTCTCTTCGCCATCACGGACCAGGAGAACAATGCAGCACTGCCAGGAACACAAGCA
CTGGTGATAGGCATCCTCGTGGTCATCATCGGGGTGTCCCATGGCATAAACACAGGATAT
GCCATCAATCCGTCCTGGGACCTGCCCCCCAGCACCTTCACCTTCATTGCTGGTTGGGGC
AAACAGGTCTTCAGCGATGCGGAGAACTGGTGGTGGGTGCCAGTGGTGGCACCACTTCTG
GGTGCCTCTCTAGGTGGCATCATCTACCTGGTCTTCATTGGCTCCACCATCCCACGGGAG
CCCCTGAAATTGGAG---------------------------------GACTCT------
GTGGTG---GAAGACCAC---------GGGATAACCGTATTGCCC---AAGATGGGATCT
CATGAACCCATGATCTCTCCC---------------------------------------
---------------------------------CTCACCCTCATCTCCGTGAGCCTTGCC
AACAGATCTTCAGTCCACTCTGCCCCACCCTTACATGAATCCATGGCCCTAGAGCACTTC
>Chimpanzee_AQP7L2
ATG------------------------------GGCTCGGGCCACTGTATCAGGTCCACC
CGTGGCTCCAAAATGGTCTCCTGGTCCATGATAGCAAAGATCCAGGAAATATGG------
------------AAGATAGCGCGAGAGTTCCTGGCCGAGTTCATGAGCACATATGTCATG
ATGGTATTCGGCCTTGGTTCCGTGGCCCATATGCTTCTAAATAAA---ACATTTGGGAGC
TACCTTGGTGTCAACTTGGGTTTCGGCTTCGGAGTCACCATGGGAGTGCACGTGGCAGGC
TGCATCTCTGGAGCCCACATGAACGCAGCTGTGAGCTTCACTAACTGTGCACTGGGCCGT
GTGCCCTGGAGGAAGTTTCCAGTCTATGTGCTGGGGCAGTTCCTGGGCTCCTTCCTGGCG
GCTGCCACCATCTAC---AGTCTCTTCTACACGGCCATTCTCCACTTTTCG---GGTGGA
GAGCTGATGGTGACCGGTCCCATT---GCTACAGCTGGCATTTTTGCCACCTACCTTCCT
GATCACATGACATTGTGGCGGGGCTTCCTGAATGAGGAGTGGCTGACCGGGATGCTCCAG
CTGTGTCTCTTTGCCATCACGGACCAGGAGAACAACCCAGCACTGCCAGGAACACACACG
CTGGTGATAGGCATCCTCGTGGTCATTATCAGGGTGTCCCATGGCATGAACACAGGATAT
GCCATCAATCCGTCCTGGGACCTGCCCCCCCGCATCTTCACCTTCATTGCTGGTTGGGGC
AAACAGGTCTTCAGCGATGGGGAGAACTTGTGGTGGGTGCCAGTGGTGGCACCACTTCTG
GGTGCCTCTCTAGGTGGCATCATCTACCTGGTCTTCATTGGCTCCACCATCCCACGGGAG
CCCCTGAAATTGGAG---------------------------------GACTCT------
GTGGCATATGAAGACCAC---------GGGATAACCGTATTGCCC---AAGATGGGATCT
CATGAACCCATGATCTCTCCC---------------------------------------
---------------------------------CTTACCCTCATCTCCGTGAGCCCTGCC
AACGGATCTTCAGTCCACCCTGCCCCACCCTTACATGAATCCATGGCCCTAGAGCACTTC
>Rabbit_AQP7
ATG------------------------------GGCTCGGGCTGCTGCTTCAGGTCCCCC

```
TGCAGCTCCCAGATGGTCTCCTGGCCTGTGCTAGTGCGGATCCACGCA------------
GTCCTGCAGAAGGAGACGGTGCGAGAGTTCCTGGCAGAGTTCCTGAGCACGTACGTCATG
ATGGTGTTTGGTCTTGGTTCTGTGGCCCATATGGTTCTAGGAGGCCAAAAATTCGGGAGC
TTCCTCGCTGTCAACTTGGGTTTTGGCTTTGGAGTCACCATGGGCGTGCACGTGGCAGGC
AACATCTCTGGGGCCCACATGAACGCTGCCGTGAGCTTCACCGCCTGCGCGCTCGGCCGC
ATGTCCTGGAAGAAGTTTCCCGTGTACGTGTTGGGTCAGTTCCTGGGCTCCTTCACCGCG
GCTGCCACCATCTAC---GGCCTCTTCTACACGGCCATTCTGCACTTCTCC---GGCGGA
CATCTGGCAGTGACCGGGCCCACA---GCCACTGCGAACATTTTTGCCACCTATCTTCCT
GACCACATGACGCTGTGGTGGGGCTTCCTCAATGAGGTGGTACTGACGGGGATGCTCCAG
CTGTGTCTCTTAGCCATCACCGATAAGGAGAATAACCCAGCGCTGCCGGGGACACAGGCC
CTGGTCACCGGCATCCTTGTTGTCATCATTGGGGCATCCCTGGGCATGAACACAGGATAT
GCTATCAACCCATCTCGGGACCTGCCTCCCCGCTTCTTCACCTTCATTGCCGGCTGGGGC
AAACAGGTGTTCAGGGCTGGGGAGAACTGGTGGTGGGTGCCAGTGGTGGCACCAATTGTA
GGTGCCTACCTGGGAGGAATCGTCTATCTGGTCTTCATTGGCTCCGGCATCCCACGGGAG
CCCCAGGAATCAGAG---------------------------------GACTCG------
GCCATGAATGAAGAACAC---------AGGATAACCACATTGTCC---AAGACAAGTGCT
CACCTATCCGTGACCTCTCAC---------------------------------------
---------------------------------CTCGACCCTGTCTCCAGAGTCCCTGCC
AACACATCTTCAGTCCAGCCTGTCCCACCTGTGAATGGCTCTATACTCTTAGAGAGCTTC
>Tasmanian_devil_AQP7
------------------------------------------------------------
------------------------------ATGCAGCGCATGCAAGAT------------
GCTTTGCGGGGAGACATGATGCGAGAATTTTTGGCTGAATTCATAAGTACATATGTCATG
ATGGTGTTTGGTTTGGGATCTGTGGCCCAACAAGTTCTTGGGGAGAATAATTATGGACA
TATCTCAGCATCAACTTGGGATTTGGCTTTGGTGTTGTAATGGGGGTCCATGTGGCTGGG
GGAATCTCTGGTGCCCATATGAACAGTGCGCTTACCTTCACAAGCTGTGTCCTGGGTCAG
ATGCCCTGGAAGAAGTTTCCTGTCTACACAGTGGCACAGTGCTTTGGATCCTTTTTAGCA
GCAGCTACCATATAT---GGTCTTTTCTATCAGGCACTCCTACGGTACACA---GATGGG
AATCTGACAGTAACCGGTCCCCGG---GCAACTGCAGGCATCTTTGCTACTTACCCTGCC
TCCTATATGACTCTGTGGAGGGGATTCGTAGATGAGATATTCTTAACTGGGATACTCCAG
GTATGCCTGTTGGCCATCAATGACAAGAAGAACTGCCCGGCTCTGCAAGGGACCCATCCT
TTGGTCATAGGTGTCCTTGTGATTACAATAGGATTGTCACTGGGCATGAACACTGGTTAT
GCCATCAATCCATCCAGAGACCTGCCACCAAGGATCTTTACAAGCATAGCAGGCTGGGGA
AATGAAGTCTTTACAGCAGCAGAGAGCTGGTGGTGGGTCCCTGTGGTTGCCCCGCCATTA
GGCTCCCTCATGGGAGCTATTGTCTACCTACTCCTTATTGGATCCAGTCATCGACATCAG
AACGACCGAAATGCA---------------------------------GAGGTT------
GCAACGACACCTAAGCCT------------CCTCGAGTCCTG------CAGTCACTGCAG
CCCCAGCAGTCCCTGAAGCAG---------------------------------------
-----------------------------------------AAGGTGATTGTGCCC---
---TCGTCTCATCATTCATCTATGTTTCCCCTGTCT---TCTCACTCA------------
>Platypus_AQP7
ATG---------------------------------------------------------
---------------------------GCCGTCCAGAAGTTCTTGCTATCGTTC------
CTGACGGAGAACAAGCTGACCAGAGAGTTCCTGGCAGAAATGCTGAGCACATTTGTCCTC
ATGGTGTTCGGGCTGGGGTCTGTGGCGCAGGTGGTGCTGGGAAAGCGGGAGTTCGGGGAT
TTTCTGAGCATTAACTTGGGCTTTGGCTTCGGCGTCACCATGGGCATACATGTGGCTGGA
GGCATCTCGGGTGCTCATATGAATGCTGCCATCACCTTTGCCTCCTGTGTCCTGGGCCAG
CTACCATGGAAGAAGTTACCCGTCTATGTGCTGGGCCAGTTTCTGGGTTCTTTCTTGGCT
GCTGGCACCGTCTAC---TTGCTCTACAAAGATGCTCTCTATTCCTTCTCG---GGAGGA
AACTTGACGGTGACGGGGCCTAAT---TCAACAGCGGGGATCTTCTCAACTTACCCTGCT
CCTTACATGGACCTGCTTGGGGGATTTGTGAATGAGCTCATCGCCACGGCAATGCTCCAG
CTCTGCATTCTCGCCATTACGGACAAGAAGAACAGTGCTGCGCTGGATGGCACGCAGGCC
CTCATCATCGGCCTCCTGGTGGCCGTCATTGGCATGTCCCTGGGCATGAACACGGGTTAC
GCCATCAATCCTTCCCGGGACTTGCCACCCCGCATCTTCACCGCCATTGCGGGCTGGGGC
ATGGATGTCTTTCGGGCCGGCAATAGCTGGTGGTGGGTGCCCTTGATTGCCCCGACGATT
GGCAGCGTCCTTGGAGCTCTGATTTACAAGATCCTCATCGACCATCACAACCGCCCGGCC
CCACAGCCGGAGTCC---------------------------------TGCATG------
ACCTCGCCGGCAGAGCCG---------GAGCCGGAGGCCACCTGCTTAGGCATGGAGATG
AAGGCG------------------------------------------------------
------------------------------------------------------------
------------------------------------------------------------
```

```
>Zebra_finch_AQP7
------------------------------------------------------------
---------------------------ATGTTGGAGAAGACTAAGAAGGTGTTC------
AGGATTCGTAACACTGCTATAAGGCAGATGCTGGCAGAAGCACTGGGAACATTCATCGTC
ATGGTTTTTGGCTTGTCCTCTGTGGCACAAGTGGTATTGGGAAAAGGAAAAAATGGCCAA
TATCTGAGCATCAATATCGCATTTGGCATTGGCGTTACCTTGGGCATATATGCTGCTGGA
GGAATATCTGGAGCTCATCTGAATGCTGCCATCACCATTACACAATGTGTTTTAGGAAAC
ATCTCCTGGACAACGGCTATAGCTTATATAATCGGCCAGTTCCTGGGCTCCTTTTTGGCA
GCAGCCACTGTCTTT---GCTCTCTACTATGATGCTATCTATGCCTACAGC---AATGGG
AACCTTACAGTGTCAGGACCAAAT---GCCACAGCAATGATCTTCTCTACGTATCCTGCT
CCCAATGTGTCCTTGCAGGGAGCCTTCTTTACAGAGTTTACAGCAACAGTTATGCTGATC
CTGGGTATTCTAGTCATCCATGATGAGAAAAACAATGCAGCAATCAAAGGCGCTCAGCCT
GTGCTCACGGGTTTGCTGGTCCTGGGCATTGGTCTGGGAATGAGGCTAAACACAGGCTAT
GCCATAAACCCCTCCAGGGACCTGCCCCCCCGGATCTTCATGGCACTTGCTGGCTGGGGA
ATGGCTGTCTTCACGGGTGACCATTCCTGGTGGTGGGTCCCAGTCACAGCTCCAGTTCTG
GGAAGTCTTTTTGGTGTTTTCCTCTACAAACTCTGCATTGACTTTCACAACCAACCTAGC
CATGAAACTGAACAT---------------------------------------------
------------GAGAAA---------GAACAGGCAGGCATGGAG---ACTTCC------
------------------------------------------------------------
------------------------------------------------------------
------------------------------------------------------------
>Alligator_AQP7
------------------------------------------------------------
---------------------------ATGCTGGAGAAAATGCAGAAGATCCTG------
ACGGTCCGCAACGAGCTCGTCCGGGAGGCACTGGCCGAGGCGCTGGGCACCTTTCTGCTG
ATGGTACTTGGCTTAGGGTCCGTTGCCCAGGTGAATCTAGGAGGAGGAAAGTTTGGGGAA
TACCTGAGCATCAACCTGTCATTCGGACTGGGCGTTGCTTTGGGCATCCATGCAGCTGGC
GGGATCTCAGGAGCTCACATGAATGCCTCCATTACCTTCACCTACTGCCTCCTGGGGAAG
CTGCCCTGGAAGAAGCTCCCGGCCTACGTGCTGGGCCAGTTCCTGGGGTCCTTCGTGGCA
GCAGCCACGATCTTT---GGCTTGTATTACGATGCACTTTACCTCTACACT---GGAGGG
AACTTCACCGTGACGGGACCCAAT---GCCACGGCCGGGTTCTTCGCCACCTACCCAGCC
CCTTATATGTCCCTGACAGGGGCATTTTTCAACGAGTTTACTGGCACGGCAGTGCTGCTT
TTGGGCATACTGATCATCCACGACGAGAAGAATAATCCAGCCTTAAAGGGAACCCAGGCA
GTGGTCACGGGGCTCCTGGTCTTTGTGATCGGCATGTCAATGGGAATCAACACAGGATAT
GCCATCAACCCCTCGAGAGACCTGCCTCCCCGGATCTTCACTGCCATAGCCGGCTGGGGA
CTGGAGGTCTTCAGGGCTGGAAATAACTGGTGGTGGGTCCCTATCGTAGCTCCGACGTTG
GGAAGTCTGTTTGGTGTTTTAGTCCACAAACTCTTCATTGACCTCCATAATCAGACGGCT
ACAGACAGTGGAAAT---------------------------------------------
------------GAGAAA---------GAGAAGTACACTTTGGAG---AGTGCTCGGATG
------------------------------------------------------------
------------------------------------------------------------
------------------------------------------------------------
>Turtle_AQP7
------------------------------------------------------------
---------------------------ATGCTGGAGAAAATTAAGAAAGCGCTC------
GCGATCAAAAACAGAACCGTCCGGGAGGCGCTGGCTGAGGCCTTGGGGACGTTCCTCCTG
ATGTTCTTTGGTATAGGTGGTGTTGCTCAGGTGATATTAGGAAAAGGAGAATTTGGGAAG
TATCTGAGCATCAACTTGGCATTTGGAATTGGCGTGACTATGGGGATTCATGCAGCCGGC
GGCATCTCTGGAGCTCATATGAATGCCTCAATAAGCGTCACACACTGCGTCTTAGGAAAT
CTTCCCTGGAGAAAGCTGCCAGCTTACATAATCGGCCAGTTCCTGGGCTCCTTCTTGGCA
GCATCCCTAGTGTTC---TGCATGTACTACGATGCGCTGTGTGAATACTCG---GACGGA
CACTTCATTGTGACGGGACCTAAT---GGCACAGCTGGGATCTTCGCCACCTACCCTGCT
CCGTATATGACTCTGCTGGGAGGGTTTGTAAATGAGTTCCTGGCCACAGCGGTGCTCATG
TTGTGCATTCTTGCCATCTATGACAAGAAGAATAATGGAGCCCTAGAGGGCACTCAGCCT
GTGATCACTGGGCTCCTGGTGTTAGTGATCGGCATGACAATGGGAATAAACACTGGATAT
GCAATAAACCCCTCCAGGGACCTGCCTCCAAGGATCTTTACTGCAATAGCAGGGTGGGGA
ATTGAAGTCTTTAGGGCTGGAGATTATTGGTGGTGGGTCCCACTTGTAGCGCCAACCCTG
GGAAGTCTTGCTGGTGCCTTAGCCTACAAACTCCTAATTGACTTTCACAATCAGACTGCA
CTGGAAGGCGGAGAT---------------------------------------------
------------GAGAAA---------GGAAAGGACGATTTGCAG---ACTAACAGTGTG
------------------------------------------------------------
```

```
------------------------------------------------------------
------------------------------------------------------------
>Python_AQP7
------------------------------------------------------------
---------------------------ATGTTGGAAAAATTAATCAACTCGGTG------
ACGGTCCGGAATGAAACTGTCCGCCAGACTCTGGCAGAAGCCCTGGCGACCTTCCTCTTG
ATGGTTTTTGGCCTCGGCTCCGTTGCGCAAGTCGTGTTGGGAAGGAAAAACTTTGGAGAG
TATTTGAGCATCAATCTAGGATTTGGGTTTGGTGTCATGTTGGGGATCCATGCTGCTGGT
GGAATCTCAGGTGCTCACATGAACGCTTCCATAACTTTCGCCAACTGTGTGGTAGGAAAA
CTTCTCTGGCGGAAACTTCCGGCGTATGTGATTGGTCAGTTCGTGGGATCGTTTGCAGCG
TCGGCTGTGATTTTT---CTTTTGTATTATGAAGCACTACAGAATTACACG---GGAGGA
AACCTGACTGTGACTGGGCCCACA---GCCACTGCGGGGATATTTGCCACCTACCCTGCC
CCTTACATGTCTCTGTGGAGTGGATTTCTACAGGAGTTTATTGCAACCAGCTTGCTTATG
ATCGGTGTCCTTGCCATCAGTGACATGAAGAATGCAGGTGCCCTGCCGGGCACCAACGCC
TTCATCACTGGGCTGTTGGTTGTGGTAATTGGCATGTCTATGGGGATGAACACCGGCTAC
GCCATCAACCCTTCCAGAGATCTGCCCCCCAGGATTTTCACTGCTCTTGCTGGATGGGGG
TTAGAGGTGTTCCGGGCAGCCAACTGTTGGTGGTGGGTCCCAGTGGTGGCCCCAACGCTG
GGGAGCCTTCTTGGGATCTTCATCTACAACATCCTGATCGACTTCCACAATCGCCCCCCA
CCAGATCCAGAATCT---------------------------------------------
------------TGGAAG---------CAAGACCCAGAGGCCGAGATCACGTCTCCCATG
------------------------------------------------------------
------------------------------------------------------------
------------------------------------------------------------
>Gecko_AQP7
------------------------------------------------------------
---------------------------ATGCTGGAGAAATTCACCAGCCGACTG------
GCGATCAAGAATGCAACTGTCCGAGAGGCCCTGGCCGAGGCCCTGGCGACATTCCTCTTA
ATGCTCTTTGGCACGGGCTCCGTTGCCCAGGTGGTGCTGGGAAAGCAAATGTATGGAACG
TTCTTGAGCATCAACCTGGGGTATGGATTCGGGGTCATGATGGGCATCCATGCGGCTGGT
GGCATCTCAGGTGCTCACATGAACGCAGCGGTAACCTTTGCAAACTGTGCGATTGGAAAC
CTCCCCTGGTGCAAGCTTCCAGCATACGTAATCGGCCAGTTCCTGGGATCGTTCATTGCA
TCTGCTAGTGTTTTC---TTAATGTACTATGACGCTCTGCAGGATTACACC---GGAGGA
AACCTGACTGTTATAGGGCCGACT---GCTACAGCTGGGATCTTCGCCACATACCCGGCT
CCATACATGTCTGTGTGGAGAGGGTTCATCCAAGAGTTCATTGCCACCGCTGTGCTGGTC
ATTGGCATCCTGGCCATCAATGACAAGAAGAATGCAGCGGCCCTTCCGGGCACCAACGCC
TTCATCATTGGACTTCTGGTTACTGCGATCGGCATATCACTGGGCATGAACACCGGCTAT
GCCATCAACCCTTCCCGGGATCTGCCACCGAGGATCTTCACGGCTATAGCTGGCTGGGGC
CTGGAAGTCTTCAGG---------------------------------------------
------------GGAATCTTCGTGTACAACATCTTGATCGACTTCCACAATCGCCCTTCG
CTGGAACCCGGCAGC---------------------------AGCGACGAGAGC------
GCGAAGAAAAGCTTTGAG---------ACGGTGGTGGCCACTGAACTCCAAAATAGTGTG
------------------------------------------------------------
------------------------------------------------------------
------------------------------------------------------------
>Himalaya_frog_AQP7
ATGAAG------------------------------ATGGGG------------------
---------------------ACATCCTTCATCCAATCGATCCACGCCGCCATC------
TCCATCCGCAACCAATGGGCTCGGGAGGCCATGGCCGAGATGCTGTCCACCTTCATTATG
ATGCTGTTTGGTTTGGGCTCAGTGGCGCAGGTGGTTCTGGGTAAGAAGCACTATGGACAA
TACTTCAGCATTAACCTTTCCTTTGGAATCGGTGTCACGATGGGGATCCATGTGGCCGGG
GGGGTATCCGGAGCTCACATGAACTGGGCCGTGTCCTTAACGAATTGCGTCTTGGGGAAT
CTTCCATGGAAGAAACTTCCCCTTTATATGGTATCACAGGTCGCCGGATCCTTCCTGGCC
GCTGCGGTGGTGTTC---TTCGTCTACTATGAGGCCCTGGACAACTACTGC---GGGGGG
AATTTCACTGTAACTGGAGATTTG---GAAACCGCCAGTATATTTGCCACCTACCCACAG
CCGTACCTGTCTATAGGGGGCGGATTCCTGGACCAGGTGGTGGGAACGGCGGCACTTATG
TTATGTATTTTGGCCATCGGTGATAAGAAGAACAGCCCAGCCCTGGATGGCATGCAGGCC
GTGGTAGTCGGGCTCTTGGTAACCGTCATTGGAATGTCTATGGGCATGAATTCCGGATAC
GCCATCAACCCTGCCAGGGACCTGGGACCCCGAATCTTCACTGCCGCTGCAGGGTGGGGG
CTGGAAGTGTTCAGGGCTGGGAACTACTGGTTCTGGATACCTATTGTGGCCCCTCTAGTG
GGCGCCCTGACCGGGGCTTTCCTGTACAAGCTGCTGGTGGGACTTCACCATCAGAAAGAC
CAGGGGGAGAAAAAG--------------------------------TTACAG------
```

GAGGTGGAGAAGACGAAC---------ACAGAGATGGTGAACTGT---GAA---CACATG

>Axolotl_AQP7
ATGACG---------------------------ACAGCCGGC------------------
---------------------GAGAAGATGCGCGCCAAAATCAGGACTCGCCTG------
AGGATCGGCAACCAGGCCGCGCGGGAAGCCGCCGCCGAGCTGCTCTCCACCTTCGTCATG
ATGACTTTTGGATTAGGTTCGGTTGCTCAAGCGGTTCTGGGCAGAAAAGAATATGGGCAG
AATTTAAGCATCAATCTGTCTTTCGGATTTGGCGTGACAATGGGGATCCATGTAGCTGGA
GGGGTTTCAGGAGCGCATATGAATACTTCTGTATCCTTAACAAACTGCATCCTGGGAAAC
CTTCCATGGAGAAAGCTGCCATTGTATGCACTGGCACAGTGCCTGGGTTCGTTCCTCGCA
GCTGTTGTGGTATAC---TGTCTCTATCATGAAGCATTGTTGGACTACTGT---GGGGGG
AACCTCACAGTAACAGGCCCTTGC---GCTACAGCCGGCATATTTGCAACATACCCAGCA
CCGTATTTGTCTATCGGAGGCGGGTTTGTAGACCAGGTGATCGGCACAGCAGCACTCTTG
CTCTGCATTCTTGCGATCAATGACAAGAGGAACAGCCCGGCCTTGAACGGGACTCAGGGC
CTTCTGGTTGGGATCCTAGTGGTGCTAATTGGTATGTCTATGGGTATGAATTCTGGATAT
GCCATTAACCCAGCCAGAGATCTACCTCCAAGGATATTCACCGCCATGGCAGGCTGGGGA
CTGGAAGTATTTTGGGCTGGTAATAACTGGTGGTGGGTTCCAGTGGTTGCTCCTTTTGTT
GGGAGTGTCATAGGAGCGTTCATCTACAAACTCCTCATCGAATTACACCATCCACCCGAG
CCAGAAGAAACAAAG---------------------------------TGTCAA------
CTATCCAAGGACATTCAA---------GACAATGACGTTATGTGT---CAA---CATATG

>2Lcaecilian_AQP7
ATGGCA---------------------------------AAA------------------
---------------------AAGATACGGCTCATGAAAATAAAACAAAAATGC------
ATAATAAGAAACGGAACTGTCAGGGAGACGCTGGCTGAGGCTCTGGCGACCTTTGTCATG
ATGAGTTTTGGTTTAGGTTCTGTTGCCCAGGTCGTCCTAGGAAAAAAGGAATACGGGGAG
TATTTGAGCATCAATCTCGCTTTTGGATTTGGTGTTACCATGGGCATTCACGTGGCTGGA
AACATTTCCGGAGCCCACATGAATACTTCTGTTTCGCTTACAAACTGCATCTTGGGTTAC
CTGCCTTGGAGAAAACTGCCATGTTACGCCTTGGGACAGTTCATTGGCTCCTTTCTCGCA
GCTGCGTTGGTATTC---TGTTTATATTATGAGGCGTTGTACGACTATTGT---GGCGGG
AACCTGACTGTCACAGGGCCTTAT---GCTACAGCAGGGATATTTTCCACCTATCCTGCT
CCTCATATGTCTGCAGGAGGAGGATTTCTAGCTGAGGTTGTTGCCACGGGAATGCTTCTG
CTGTGCATCCTTGCCATTACCGACAAGAAGAACAACGCAGCCTTGGACGGGACGCAGGCT
CTTCTGGTTGGAATCCTTGTTATTGTGATTGGTTTGGCGATGGGAATGAACACCGGCTAT
GCCATAAACCCAGCCAGGGACCTTCCTCCCAGGATCTTCACTGCAATCGCAGGATGGGGC
CTGGAGGTCTTCAGGGCTGGGAATTACTGGTGGTGGATCCCAGTAGTAGCGCCATTCATA
GGAAGTATCTGCGGTGCCTTAATCTATAAACTTTTAATAGCCTTGCACAATAAGATCGAG
CCAGAAGATGTGATC---------------------------------GGCGAA------
GACGTGAAAGGAAATCAA---------GAAAAAGATCAAGAC------CAA---TATATG

>Gaboon_caecilian_AQP7
ATGAAG---------------------------ACA---GAA------------------
---------------------AGGAAGCAGTTTATGAAAATCAAACAAAAATGC------
ATCCTCAAAAATGGAACTGCCAGGGAGATGCTGGCGGAGGCTCTGGGGACCTTTGTAATG
ATGAGTTTTGGTTTAGGTTCTGTTGCACAGGTTGTCCTAGGAAAAAAGGAGTACGGACAG
TATTTGAGTATTAATCTCTCATTTGGATTTGGTGTCACCATGGGAATTCATGTAGCCGGA
GGAATCTCTGGAGCCCACATGAACACTTCTGTATCACTGACAAACTGCATTTTGGGACAC
CTGCCCTGGAGAAAATTACCATTTTACGCCTTAGGACAATTCACTGGCTCCTTCCTCGCA
GCCGCGTTGGTTTAC---TGTGTGTATTATGATGCATTGTATGATTACTGT---GGCGGT
AACCTGACTGTAACAGGGCCTTAT---GCAACAGCGGGGATATTTTCCACCTATCCTGCG
GCCTATATGACTCCGGGAGGAGGATTTCTAACTCAGTTTGTTGCCACTGGAATGCTTTTG
CTTTGTATCCTTGCCATCAACGACAAGAAGAACTTTGCCGCCCTGGATGGAACTCAAGCT
CTTCTGGTTGGAATCCTTGTTATAGTAATTGGTATGGCGATGGGAATGAATACCGGATAT
GCCATAAATCCAGCCAGGGATCTTCCTCCCAGAATCTTTACTGCAATAGCAGGTTGGGGA
GTTGAGGTCTTCAGGGCTGGAAATTATTGGTGCTGGATTCCAGTAGTGGCTCCATTGGCA

GGAAGTATTACTGGTGCCTTTATCTATAAACTCCTAATAGCACTGCACAATCAGGCCGTG
CCAGATGATGTGAAA---------------------------------TGTGAA------
GATACGAAAGAAAATCCA---------GATATTGTCATGACTGGT---CAG---TATATG

>Coelacanth_Aqp7
ATGAAG---------------------------------GGG------------------
---------------------AGGAAAGTAGTAGCAAAAGTCAAAACGTTTCTA------
AGGATAAAAAATAAAACCTTGCGAGAAGCATTAGCAGAGGTTCTGGGGACCTTCATAATG
ATGCTGTTTGGTTTAGGTTCAGTGGCCCAAGTGATTATCGGAGGTGGAAAAAATGGCGAG
TACCTGAGCATAAACTTGTCCTTTGCCATGGGTGTCACCATGGGGATCCACATGGCTGGT
GGCGTATCTGGAGCCCATTTGAATACCGCTGTTTCTTTCACAATGTGTATACTGGGAAAG
CTTTGCTGGAGGAAACTGCCTATTTACACCCTGGCACAGTTCTTCGGTTCCTTCCTGGCA
GCTGGCCTGGTATAT---TTCCTTTACTATGATGCTTTGCACGAATACTGC---GGCGGA
AACCTCACAGTGACTGGACCCAAG---GCTACAGCAGAGATATTCTCTACCTACCCTGCT
CCTTACCTCACTCTCAGCAATGGATTTTTAGACCAGGTGGTTGGCACTGCAGTCCTCTTG
ATAGGCATTCTTGCCATCAATGACCAGAAGAATAACCCAGCACTCAATGGCACTCAGGGG
CTCAGTGTTGGTCTCCTGGTGTTAGTTATTGGAATGTCAATGGGGATGAACTGTGGATAT
GCCATAAACCCAGCTAGAGATCTACCTCCCAGGATCTTCACGGCTATAGCTGGCTGGGGA
CTAGAAGTATTCAGAGCAGGAAACAACTGGTGGTGGGTCCCTATTGTAGCTCCCTTGGTA
GGAAGCGTCATTGGTGCCTGCATCTATCAGATCTTCATTGAAGGCCATCACAAGCCTGAA
CCGGAAGGCAATTCC---------------------------------TCCATC------
GAATTCGAATCCGGAAAA---------ATGGAAAACACAATTAAT---ATT---------

>mCoelacanth_Aqp7
ATGAAG---------------------------------GGG------------------
---------------------AGGAAAGTAGTAGCAAAAGTCAAAACGTTTCTA------
AGGATAAAAAATAAAACCTTGCGAGAAGCATTAGCAGAGGTTCTGGGGACCTTCATAATG
ATGCTGTTTGGTTTAGGTTCAGTGGCCCAAGTGATTATCGGAGGTGGAAAAAATGGCGAG
TACCTGAGCATAAACTTGTCCTTTGCCATGGGTGTCACCATGGGGATCCACATGGCTGGT
GGCGTATCTGGAGCCCATTTGAATACCGCTGTTTCTTTCACAATGTGTATACTGGGAAAG
CTTTGCTGGAGGAAACTGCCTATTTACACCCTGGCACAGTTCTTCGGTTCCTTCCTGGCA
GCTGGCCTGGTATAT---TTCCTTTACTATGATGCTTTGCACGAATACTGC---GGCGGA
AACCTCACAGTGACTGGACCCAAG---GCTACAGCAGAGATATTCTCTACCTACCCTGCT
CCTTACCTCACTCTCAGCAATGGATTTTTAGACCAGGTGGTTGGCACTGCAGTCCTCTTG
ATAGGCATTCTTGCCATCAATGACCAGAAGAATAACCCAGCCCTCAATGGCACTCAGGGG
CTCAGCGTTGGTCTCCTGGTGTTAGTTATTGGAATGTCAATGGGGATGAACTGTGGATAT
GCCATAAACCCAGCTAGAGATCTACCTCCCAGGATCTTCACGGCTATAGCTGGCTGGGGA
CTAGAAGTATTCAGAGCAGGAAACAACTGGTGGTGGGTCCCTATTGTAGCTCCCTTGGTA
GGAAGCGTCATTGGTGCCTGCATCTATCAGATCTTCATTGAAGGCCATCACAAGCCTGAA
CCGGAAGGCAATTCC---------------------------------TCCATC------
GAATTAGAATCCGGAAAA---------ATGGAAAACACAATTAAT---ATT---------

>European_seabass_Aqp7
ATGAAGGAC---------TTAATGCAGTCAGTAGAACTAGGAGTC---------------
---------------TCTCAGCGGAAAGGAGTTAACGTAACTGGACCAAAAGTT------
TGGCTAAAGAATGAAGTGGTTCGTGTGGGACTTGCTGAATCCCTTTGCACATATGTCATG
ATGGTGTTTGGCCTGGGGTCTGTGGCCCAGGTAGTGACAGGACAGGGAGCGTTCGGAGAG
TACCTCAGCATCAACCTGGGTTTTGGACTGGGTGTTGCAATGGGGGTTCATGTTGGAGGG
AAGGTCTCAGGGGCTCATATGAATGCGGCAGTGTCTCTCACAATGTGCACATTTGGCCGC
CTTGCATGGAAGATGCTGCCTGTGTATGTTTTTGCACAGCTATTGGGGTCATTTCTGGCA
GCGGGGACAATTTAT---GCTGTCTATTATGAAGCCATTTATGACTATTGT---GGAGGA
AACCTGACTGTGACTGGTGTAAAG---GCCACAGCTGGTATCTTTGCCACCTATCCTGCA
CCGTACCTCTCCTTGCTGGCTGGATTCATTGACCAGGTGTTTGGCACAGCTATGCTGCTG
CTGTGCCTGATGGCTCTGTCCGACCAGAAGAACAAACCGGCCGCAGCAGGCAGTGAGCCT
GTTGCAGTGGGTCTCCTAGTGCTACTCATCGGCATTTCCCTAGGCAGCAACAGTGGCTAT

GCCATCAACCCCACCAGAGACATCGCACCGAGGGTCTTCACTGCCATAGCAGGCTGGGGG
TCCGATGTGTTCAGGGCTGGAAATGGGTGGTGGTGGGTGCCTCTGGCTGCACCTCCCATT
GGTGGAGTATTGGGTGCGGGGCTCTACAAGGCCTTGGTGGAAATGCACCACCCACCCCTC
TCTGAACAGGGTGAG---------------------------------GGGCTG------
CTCAAGGAGGAGACTGCCCCTTTGAGGAAACAAGAAAACATCTGT---GCTAATGTATGT
GTT---

>Nile_tilapia_Aqp7
ATGAAGGAC---------TTGGCGCAGTCAGTAGAAGTTGGGGTG---------------
---------------TTTCAGCAGAGGGGAGGTAAAGTAACTCGACCAAAAGTT------
TGGCTAAAGAATGAACTCATTCGTGTGGGACTTGCTGAATCACTTAGCACATATGTCATG
ATGTCATTGGGCTTGGGGTCTGTGGCCCAGGTAGTGACCGGTCAGGGAGCTTTTGGACAG
TACCTCAGCATCAACCTGGGTTTTGGACTGGCTGTTGCCATGGGGTCTCATGTTGGAGGG
AAGATCTCGGGGGCTCATATGAACGGAGCCGTATCATTCACAATGTGTGTGTTTCGCCGC
CTCCCGTGGAAGATGCTACCTCTTTATATTTCGGCACAGCTATTGGGGTCATTTCTGGCA
GCAGGGACAATTTAT---GCTGTCTACTATGAAGCCATTCATGACTACTGT---GGAGGG
AACCTGACTGTGACTGGTGAGAAG---GCCACAGCTGGTATCTTTGCCACCTATCCTGCT
CCATACCTCTCTCTGATAGCTGGATTTTTTGACCAGGTATTTGGCACAGCGATGCTACTA
CTGTGCCTTATGGCTCTATCCGACCAGAAGAACAAACCAGCGCCAGCAGGAAGTGAGCCT
GCATTTGTGGGTTTCCTGGTGCTTCTCATTGGCATTTCTTTGGGTAGCAACAGTGGCTAT
GCCATCAACCCCACCAGAGACATCGCACCCAGGGTTTTCACTGCCATGGCAGGCTGGGGA
ACTGATGTGTTCAGGGTTGGAAATGGATGGTGGTGGGTGCCTCTAGTTGCAACCCCTATT
GGAGGAGTCCTTGGAGCAGGGCTATACAAGGCTGTTGTGGAACTGCAACACCCACACCTC
TCTGAAGCAGGTGGA---------------------------------GAGATG------
---GTTGAAGAAGCTGTCCCTCTGGATAAAGAGATAAATACCAGT---GAAAATGTGTGT
GTG---

>Mummichog_Aqp7
ATGAAGGAC---------TTTGTAGAGTCAGTGGAACTGGGGGTC---------------
---------------TCCCAGCGGAGCGCAGTGGAGCTAAAACGACCCAAAGTT------
TGGCTGAGGAGTGAACTCTCACGAGTGGGACTCGCTGAATTCCTTTCAACATATGTGATG
ATGGCATTAGGTCTTGGTTCTGTGGCCCAGGTCGTGACCGGTCAAGGAGTATTCGGACAG
TACCTTAGCATCAACCTTGGCTTTGGGCTGGCTGTCGCAATGGGGGTTCATGTTGGAGGA
AATGTCTCTGGAGCTCATATGAACGGAGCAGTCTCCTTCACTATGTGTGTTTTTGGCCGC
CTACAGTGGAAGAGGCTGCCCTTGTATATTTTCGTACAGCTTTGCGGTTCGTTTCTTGCA
GCAGCAACAATTTAT---GCTATTTACTACGAGGCCATTTATGACTATTGT---GGGGGA
AACCTGACTGTAAGTGGTGAAAGG---GCCACAGCTGGGATCTTTGCCACATATCCGGCA
CCATACCTGTCCTTGATTGGTGGCTTTGTTGACCAGGTGTTTGGCACAGCCATGCTGCTG
CTGTGCCTGATGGCTCTGTCTGACCAAAAGAACAAACCAGCAGCCAGGGGGGCTGAACCC
ATTGCTGTGGGTCTCCTGGTGGTTCTCATTGGCCTTTCTTTGGGCAGCAACAGTGGTTAT
GCTATCAACCCCACCCGAGACATCGCACCAAGGGTCTTCACTGCTATTGCAGGCTGGGGA
TGGGAAGTGTTCAGGTCTGGACATGGGTGGTGGTGGGTCCCTCTAGTAGCTACTCCCATT
GGAGGAGTGCTGGGAGCAGGGTTGTACAAGGCATTTGTAGAAATGCACCACCCCCTCTTC
GCTGGACGGGATAGG---------------------------------GAGCCC------
AGTGAGGAGGAGTCTCTCCCGCTGAAGAAAGAGCGAAACATCTGC---TCTGATGTATGT
GTGAAACCTGACACAAATGGAAACAAC---------------------------------

>Zebrafish_Aqp7
ATGGAA------------------------------GATGGCAGC---------------
---------------ATTCAAGGCCGCATGGCTCCAAATGTTGGATCCATGTTG------
AAGATCAAGAATGAATACATTCGAGTGGCTTTGGCAGAAAGCCTCTGCACATTCATCATG
ATGGTGTTTGGCCTTGGCACTGTTGCACAAGTGGTTACAGGAGAAGGTTATTTTGGTGAA
TATCTCAGCATTAATATAGGCTTTGGGCTGGCAGTGGCTATGGGTGTGCATGTTGGTGGA
AAAGTGTCAGGAGCTCATATGAACGCAGCTGTTTCATTCACAATGTGCGTGTTTGGCCGA
TTGCGCTGGAAGATGCTGCCGCTGTATGCTTTCGCTCAGTTTCTGGGTTCATTCCTTGCC
GCCGGGACCATTTTT---TCACTTTATTATGATGCCATAAATCATTTCTGC---GGGGGT
AATTTGACTGTGTCCGGACCCAAA---GCAACAGCTGGGATCTTCGCCACATATCCAGCA
CCCTATATCTCAGTCTACACTGGATTCTTTGATCAGGTTGCTGGCACGGGCCTGCTGTTG

TTGTGTCTGATGGCTCTGTCAGACCAAAGGAACCAGCCGCTGGTGTCTGGAGGTGAAGCC
GTCGGTGTGGGGCTTCTAGTGATGCTCATCGGCATCTCTATGGGGAGCAACAGCGGTTAC
GCCATCAATCCCACACGGGACCTGGGGCCACGGCTCTTCACACTCATAGCAGGATGGGGC
ACAGAGGTTTTTAGGGCAGGCAATTGCTGGTGGTGGGTACCCTTGGTGGCTCCTTTTATT
GGAGGAGTTTTAGGGGCTTTAATCTACAAAGCACTTGTAGAACTACACCACCCTGATCTT
AAAAACACTACAACA--------------------------------CGGCCA------
GCAGTAGATCCTGAATGC---------ATTCCTCTGGACAAGTGC---AAGAACGGCAGA
ATAGAGATACCTGTG---

>Atalntic_sturgeon_Aqp7
ATGAAG------------------------------CTGGAC------------------
---------------------AAGCAGTTTGTATCCAATGTAAAGAAATCCATT------
CGAATCCGAAACGAATACCTGCGAGAGGCTCTGGCTGAGATACTCAGCACCTTTGTCATG
ATGGTTTTCGGTCTGGGCTCGGTAGCCCAGGTGGTCATGGGAGGTGGTTCCTATGGTGAC
TACTTCAGTATAAATTTGGGCTTCGGCCTTGGGGTCACCATGGGCATTCACATCGCTGGG
GGCGTCTCAGGAGCTCATATGAACACTGCAGTCACCTTCTCCATGTGTGTGCTGGGGAGC
CTGAGCTGGAGGAAGCTGCCGGTCTACGCTGCTGCCCAGTTCCTCGGCTCCTTTATGGCT
GCGGTCACCGTCTTC---TGGGTCTACTATGATGCTCTGTTTGAGTTCTGC---AGAGGG
AACTTCACAGTGACGGGCCCAAGA---GCAACTGCTGGGATCTTCGCTACCTACCCGGCG
CCATACCTGTCAGTGGGAGGAGGCTTTCTGGACCAGGTGCTGGGCACTGCCATGCTGCTG
CTGTGTATCCTGGCGCTGAATGACCACAGGAACAGCCCGGCGCTCAGAGGCACGCAGCCT
CTGCTCATCGGCCTCCTGGTGGTGGTGATTGGCATCTCTCTGGGCAGCAACAGCGGGTAC
GCCATCAACCCCGCCCGCGACCTGCCCCCACGCTTCTTCACCTCCATGGCAGGCTGGGGT
CCTGACGTCTTCAGTGCTGGTAATGGCTGGTGGTGGATCCCTGTGGTGGCGCCCATGGTT
GGCAGTGTGACTGGCTCTCTGCTCTACAAACTCTTCATCGAGTATCACCATCTGGCTGAG
GAGCATCTAGAGGAG---------------------------------GGGCTG------
------GGGGGAGTCTGC---------TCTGATCAAAAACACTGC---TTCCGCTTT---

>Common_sturgeon_Aqp7

---TGTGTGCTGGGGAGC
CTGAGCTGGAGGAAGCTGCCGGTCTACGCTGCTGCCCAGTTCCTCGGCTCCTTTATGGCT
GCGGTCACTGTCTTC---TGGGTCTACTATGATGCTCTGTTTGAGTTCTGC---GGAGGG
AACTTCACAGTGACGGGCCCGAGA---GCAACTGCTGGGATCTTCGCTACCTACCCGGCA
CCATACCTGTCAGTGGGAGGAGGCTTTCTGGACCAGGTGCTGGGTACTGCCATGCTGCTG
CTGTGTATCCTGGCGCTGAATGACCACAGGAACAGCCCGGCGCTCAGCGGCACGCAGCCT
CTGCTCATCGGCCTCCTGGTGGTGGTGATTGGCATCTCTCTGGGCAGCAACAGCGGGTAC
GCCATCAACCCCGCCCGCGACCTGCCCCCGCGCTTCTTCACCTCCATGGCAGGCTGGGGT
CCTGGCGTCTTCAGTGCTGGTAATGGCTGGTGGTGGATCCCTGTGGTGGCGCCCATGGTT
GGCAGTGTGACTGGCTCTCTGCTCTACAAACTCTTCATCGAGTATCACCATCCGGCTGAG
GAGCATCTAGAGGAG---------------------------------GGGCTG------
------GGGGGAGTCTGC---------TCTGATCAAAAACACTGC---TTCCGCTTT---

>Chinese_sturgeon_Aqp7
ATGAAG------------------------------CTGGAC------------------
---------------------AAGCAGTTTGTATCCAACGTAAAGAAAGCCATT------
CGAATCCGAAACGAATACCTGCGAGAGGCTCTGGCTGAGATACTCAGCACCTTTGTCATG
ATGGTTTTCGGTCTGGGCTCGGTAGCCCAGGTGGTCATGGGAGGTGGTTCCTATGGTGAC
TACTTCAGTATAAATTTGGGCTTCGGCCTTGGGGTCACCATGGGTATTCACATCGCTGGG
GGCGTCTCAGGAGCTCATATGAACACTGCAGTCACCTTCTCCATGTGTGTGCTGGGGATC
CTGAGCTGGAGGAAGCTGCCGGTCTACGCTGCTGCCCAGTTCCTCGGCTCCTTTATGGCT
GCGGTCACCGTCTTC---TGGGTCTACTATGATGCTCTGTTTGAGTTCTGC---GGAGGG

AACTTCACAGTGACGGGCCCGAGA---GCAACTGCTGGGATCTTCGCTACCTACCCGGCA
CCATACCTGTCAGTGGGAGGAGGCTTTCTGGACCAGGTGCTGGGCACTGCCATGCTGCTG
CTGTGTATCCTGGCGCTGAATGACCACAGGAACAGCCCGGCGCTCAGCGGCACGCAGCCT
CTGCTCATCGGCCTCCTGGTGGTGGTGATTGGCATCTCTCTGGGCAGCAACAGCGGGTAC
GCCATCAACCCCGCCCGCGACCTGCCCCCGCGCTTCTTCACCTCCGTGGCAGGCTGGGGT
CCTGACGTCTTCAGTGCTGGTAATGGCTGGTGGTGGATCCCTGTGGTGGCGCCCATGGTT
GGCAGTGTGACTGGCTCTCTGCTCTACAAACTCTTCATTGAGTATCACCATCCGGCTGAG
GAGCATCTAGAGGAG----------------------------------GGGCTG------
------GGGGGAGTCTGC---------TCTGATCAAAAACACTGC---TTCCGCTTT---

>Sterlet_Aqp7
ATGAAG------------------------------CTGGAC------------------
----------------------AAGCAGTTTGTATCCAATGTAAAGAAAGCCATT------
CGAATCCGA---
---GTTTTCGGTCTGGGCTCGGTAGCCCAGGTGGTCATGGGAGGTGGTTCCTATGGTGAC
TACTTCAGTATAAATTTGGGCTTCGGCCTTGGGGTCACCATGGGTATTCACATCGCTGGG
GGCGTCTCAGGAGCTCATATGAACACTGCAGTCACCTTCTCCATGTGTGTTCTGGGGAGC
CTGAGCTGGAGGAAGCTGCCGGTCTACGCTGCTGCCCAGTTCCTCGGCTCCTTTATGGCT
GCGGTCACCGTCTTC---TGGGTCTACTATGATGCTCTGTTTGAGTTCTGC---GGAGGG
AACTTCACAGTGACTGGCCCGAGA---GCAACTGCTGGGATCTTCGCTACCTACCCGGCA
CCATACCTGTCAGTGGGAGGAGGCTTTCTGGACCAGGTGCTGGGCACTGCCATGCTGCTG
CTGTGTATCCTGGCGCTGAATGACCACAGGAACAGCCCGGCGCTCAGCGGCACGCAGCCT
CTGCTCATCGGCCTCCTGGTGGTGGTGATTGGCATCTCTCTGGGCAGCAACAGCGGGTAC
GCCATCAACCCCGCCCGTGACCTGCCCCCGCGCTTCTTCACCTCCATGGCAGGCTGGGGT
CCTGACGTCTTCAGGGCTGGTAATGGCTGGTGGTGGATCCCTGTGGTGGCACCCATGGTT
GGCAGTGTGACTGGCTCTCTGCTCTACAAACTCTTCATCGAGTATCACCATCCATCTGAG
GAGCATCTAGAGGAG----------------------------------GGGCTG------
------GGGGGAGTCTGC---------TCTGATCAAAAACACTGC---TTCCGCTTT---

>Reedfish_Aqp7

---ATG------
AAGATCACAAATCAATATATCCGAGAGTTTCTTTCAGAATTCTTAAGCACTTATATCATG
ATGCTCTTTGGCTTAGGCTCAGTTGCTCAGGTGGTCATGGGAGGTGATTCCTATGGTAAC
TACTTAAGTATAAACCTGGGCTTTGGTCTTGGAGTCACCATGGGCATTCACATTGGTGGT
GGGGTCTCAGGAGCTCACATGAACACTTCAGTGACATTTGCAATGTGCCTAATAGGAAAT
CTGTTTTGGAAGAAGCTGCCAGTATATGCACTGGGACAATTACTGGGCTCCTTTTTGGCT
GCAGTCACCATCTTC---TGGCTTTATTATGATGCCTTACAAGACTACTGT---GGTGGA
AACTTCACGGTGACTGGACCCAAA---GCCACAGCGGGGATCTTTGCTACATATCCAGCT
CCTTACTTGTCTGTAGCAGGAGGTTTTATAGACCAGGTGGTAGGAACAGCGGTACTGCTG
CTATGTATTCAAGCTATCAATGATCAGAAGAACTGCTCTGCTCTCAGTGGGACATCTCCA
TTGGTGACTGGCCTGTTGGTGGCACTTATTGGTATCTCCTTGGGTAGTAACAGTGGCTAT
CCTATCAATCCAGCCAGAGATCTTCCTCCCAGGATCTTCACTGCTATGGCAGGTTGGGGG
ACTACTGTTTTCAGTGCAGGAAATAACTGGTGGTGGATCCCTGTTGTTGCACCAATGTTT
GGCAGTGTGACTGGAGTCCTAATCTACAAAGTGTTCATTGAAATGCATCATCCTTCAGTT
GAGCAGCAGAAGAAA----------------------------------CAACTT------
CAAGAAAAAAGCCCAAGT---------ACAGATCTTTCTCTTTGC---TTT---------

File S3: Alignment for Fig. S2E-F
>Japanese_treefrog_AQP3
ATGGGGCGCCAGAAGGAGGTTCTCAACAGCATCAGCGGCATGCTGCGCATCCGCAACAAG
CTCATCCGCCAGGCGCTGGCCGAATGTCTGGGCACTCTCATACTCGTTATGTTTGGCTGT
GGTTCTGTAGCTCAGGTGGTCCTCAGTAAAGGTTCCCATGGTCTCTTCTTGACTGTCAAT
CTGGCTTTTGGATTTGCCGTCATGCTGGGTATCCTCATCGCCGGACAGGTTTCAGGCGGC

CACCTGAATCCAGCTGTAACTTTTGCCTTGTGTATCATGGCAAGAGAACCTTGGATCAAG
TTCCCTGTGTACACCCTGGCACAGACCCTAGGAGCGTTCCTTGGAGCGGGCATTGTTTAT
GGCTTGTACTATGACGCCATCTGGTATTTTGCAAAC---GACCAGCTCTATGTGATGGGT
CCCAAT---GGCACAGCCGGAATCTTCGCCACTTACCCTACAGAGCATCTCACCCTAATG
AATGGCTTCTTTGACCAGTTTATTGGTACTGCAGCTCTAGTCGTGTGTGTCTTGGCCATT
GTTGACCCCTACAACAACCCCATTCCCCGTGGTCTTGAGGCTTTCACTGTTGGGTTTGTC
GTCCTCGTCATTGGATTGTCCATGGGCTTTAACTCTGGCTACGCTGTCAACCCCGCTAGG
GACTTTGGTCCACGACTCTTCACCGCCTTGGCTGGCTGGGGCACAGAAGTCTTTAGTGCT
GGCGGGCAGTGGTGGTGGGTCCCCATCGTCTCACCTTTGCTGGGAGCTTTTGCCGGAGTT
CTGGTCTACCAACTAATGATCGGTTGCCACATTGAGCCTGCACCAGAGTCGACCGAACAA

------------------GAGAATGTCAAGTTG---TCTAATGTCAAACACAAAGAGAGG
ATC---

>Southern_gray_treefrog_AQP3
ATGGGGCGCCAGAAGGAGGTTCTCAACAGCATGAGCGGCATGCTGCGCATCCGCAACAAG
CTCATCCGCCAGGCGCTGGCCGAATGTCCGGGCACCCTCATACTCGTTATGTTTGGCTGT
GGTTCTGTGGCTCAGGTGGTCCTCAGTAAAGGCTCCCATGGTCTCTTCTTGACTGTCAAT
CTGGCTTTTGGATTTGCCGTCATGCTGGGAATCCTCATCGCCGGACAGGTGTCAGGCGGC
CATCTGAATCCGGCTGTAACTTTTGCCTTGTGTATCATGGCAAGAGAACCTTGGATCAAG
TTCCCTGTGTACACCCTGGCACAGACCCTAGGAGCGTTCCTTGGAGCGGGCATTGTTTAT
GGCTTGTACTATGACGCCATCTGGTATTTTGCAAAC---GACCAGCTCTATGTGATGGGT
CCCAAT---GGCACAGCCGGAATCTTCGCCACTTACCCTACAGAGCATCTCACCCTGATG
AATGGCTTCTTTGACCAGTTTATTGGTACTGCAGCTCTAGTCGTGTGTGTCTTGGCCATT
GTTGACCCCTACAACAACCCCATTCCCCGTGGTCTTGAGGCTTTCACTGTTGGGTTTGTC
GTCCTCGTCATTGGATTGTCCATGGGCTTTAACTCTGGCTACGCTGTCAACCCCGCTAGG
GACTTTGGTCCACGACTCTTCACCGCCTTGGCTGGCTGGGGCACAGAAGTCTTTAGTGCT
GGCGGGCAGTGGTGGTGGGTCCCCATCGTCTCACCTTTGCTTGGAGCTTTTGCCGGAGTT
CTGGTCTACCAACTAATGATCGGCTGCCACATTGAGCCTGCACCAGAGTCGACCGAACAA

------------------GAGAATGTCAAGTTG---TCTAATGTCAAACACAAAGAGAGG
ATC---

>Hourglass_treefrog_AQP3
ATGGGGCGCCAGAAGGAGATCCTCAACAGCATGTCTGGCATGCTGCGCATCCGCAACAAG
CTGATCCGCCAGGCGCTCGCCGAATGTCTGGGGACTCTCATACTTGTCATGTTCGGCTGC
GGCTCGGTGGCTCAGGTGGTCCTCAGTAAAGGCTCCCATGGTCTCTTCCTGACTGTCAAT
CTGGCTTTCGGATTCGCCTGCATGCTGGGAATTCTCATTGCCGGACAGGTTTCAGGCGGC
CATCTGAATCCAGCTGTAACTTTTGCCTTGTGTATCATGGCAAGAGAACCTTGGATCAAG
TTCCCAGTGTATACCCTGGCACAGACCCTAGGAGCGTTCCTCGGAGCTGGCATCGTCTAT
GGCTTGTACTATGACGCCATCTGGTATTTCGCAAAT---GACCAGCTGTATGTGACCGGA
GCGAAT---GGCACAGCCGGTATCTTCGCTACTTACCCTACGGAACATCTCACTCTCATG
AACGGCTTCTTTGACCAGTTTATCGGTACTGCCGCTCTGATCGTGTGTGTCTTGGCCATC
GTTGACCCCTACAATAACCCCATTCCTCGTGGGCTCGAGGCTTTCACTGTTGGGTTTGTC
GTCCTTGTCATTGGATTGTCCATGGGCTTCAACTCTGGCTATGCCGTCAACCCTGCTAGG
GACTTTGGGCCGCGACTTTTCACCGCCTTGGCCGGCTGGGGCACAGAGGTCTTCAGGGCT
GGCGGGCAGTGGTGGTGGGTCCCCATCGTCTCACCCTTGCTGGGAGCCTTTGCCGGAGTA
CTGGTCTACCAACTGATGATCGGCTGCCATATTGAGAATAAACCAGAGTCTACCGAACAA

------------------GAGAACGTCAAGTTG---TCCAACGTCAAACACAAAGAGAGG
ATC---

>Himalaya_frog_AQP3
ATGGGCCGACAGAAGGAATTTATCAACCGCATCAACGCCGCGCTGAGGATCCGCAACAAG
CTGCTGAGGCAAGCGCTCTCCGAGTGCCTGGGGACCCTCATTCTGGTGATGTTCGGCTGT
GGCTCTGTGGCTCAGGTGGTCCTTAGTAAAGGATCCCATGGTCAGTTCTTGACTGTCAAT
CTGGCTTTCGGTTTTGCTTGCATGTTGGGTATTCTCATATCCGGACAAGTTTCAGGTGGA
CATCTCAACCCCGCTGTGACGTTTGCACTATGTATCATAGCACGAGAACCATGGATCAAG
TTTCCTGTATACACCTTGGCACAGACCCTGGGTGCCTTCCTCGGAGCGGGCATCGTCTAT
GGCTTGTATTATGATGCCATCTGGTTTTTCGCCAAC---GACCAGCTCTACGTAATGGGC
CCCAAC---GGCACTGCTGGGATTTTCGCCACATTCCCAACAGATCATCTGACCCTCATG

AATGGTTTTTTTGACCAGTTTATTGGCACAGCTGCTTTGGTGGTCTGTGTCTTGGCCATC
GTGGACCCTTACAATAACCCCATCCCCCGTGGTTTGGAGGCCTTCACTGTTGGCTTCGTC
GTCCTTGTCATTGGATTGTCCATGGGCTTCAACTCCGGATATGCCGTCAACCCCGCCAGG
GACTTCGGACCACGTCTGTTCACAGCTCTGGCTGGATGGGGTTCAGAAGTTTTCTGGGCT
GGAGGTCAGTGGTGGTGGGTCCCCATCGTGTCGCCTTTACTGGGTGCCTTTGCCGGAGTC
CTGGTCTACCAGCTGATGCTTGGCTGCCACCTCCAACCTCCACCTGAATCCACCGAACAA

------------------GAAAATGTCAAGCTG---GCCAACGTCAAACAAAAAGAGAGA
ATC---

>American_bullfrog_AQP3
ATGGGACGACAGAAGGATTTTATCAACAAAGTCAATTCTATGCTAAGGATCAGGAGCAAA
CTGCTGAGGCAAGCCCTCGCCGAGTGCCTAGGGACACTCATTCTGGTGATGTTTGGCTGT
GGCTCTGTGGCTCAGGTGGTCCTTAGTAAAGGCTCTCATGGTCAGTTCTTGACTGTTAAT
CTGGCTTTTGGTTTCGCTGTCATGCTGGGGATTCTGATTAGTGGACAAGTTTCAGGTGGA
CATTTGAACCCAGCTGTGACCTTTGCCCTGTGTATCATGGCACGGGAACCATGGATCAAG
TTTCCTGTATACACCTTGGCACAAACCCTAGGTGCCTTCCTTGGAGCAGGCATCGTTTAT
GGCTTGTATTATGATGCCATCTGGTTTTTCGCCAAT---GACCAACTCTACGTAATGGGA
CCTAAC---GGCACTGCTGGAATTTTCGCCACCTTCCCAACAGAGCATCTGACCCTCATG
AATGGTTTCTTTGACCAGTTCATTGGTACAGCTGCTTTGGTGGTCTGTGTCTTGGCCATC
GTTGACCCTTACAATAACCCCATCCCCCGTGGTTTGGAGGCCTTCACTGTTGGCTTTGTT
GTCCTCGTCATTGGATTGTCCATGGGCTTCAACTCTGGATACGCTGTCAACCCTGCCAGG
GACTTCGGACCGCGTCTGTTTACATCTATAGCTGGATGGGGTTCAGAAGTCTTCTGGGCC
GGCGATCAGTGGTGGTGGGTCCCCATCGTGTCACCTTTACTGGGAGCTTTCGCCGGAGTC
TTGGTCTATCAGCTGATGATTGGCTGTCACATCCAACCCCCACCCGAGTCCTCCGAACAA

------------------GAAAACGTCAAGCTG---GCCAATGTTAAACAGAAAGAGAGA
ATC---

>African_bullfrog_AQP3
ATGGGTCGACAGAAGGATTTTATCAGCAGCGTCAATTCCATGCTGAGGATCCGGAACAAG
CTGCTGAGACAAGCGCTCTCCGAATGCCTGGGGACACTCATCCTGGTGATGTTCGGCTGT
GGTTCCGTGGCTCAGGTGGTCCTTAGTAAAGGCTCCCATGGTCTGTTCTTGACTGTTAAT
CTGGCTTTCGGTTTTGCTTGCATGCTGGGCATTCTCATTGCAGGACAAATTTCAGGAGGA
CATTTGAACCCAGCTGTGACTTTTGCTTTGTGTATTTTGGCACGAGAACCATGGATCAAA
TTTCCTATTTATACTTTGGCACAGACCGTGGGTGCCTTCCTTGGAGCGGGCATCGTTTAT
GGCTTGTATTATGATGCCATCTGGTTTTTTGCCAAC---GATCAGCTCTACGTAACGGGC
CCCAAT---GGCACTGCTGGGATATTCGCCACTTACCCCACAGAACATCTGACCCTTATG
AATGGTTTCTTTGATCAGTTCATTGGCACTGCAGCTTTGGTGGTCTGTGTGTTGGCCATC
GTTGACCCTTACAATAACCCCATCCCTCGTGGTCTGGAGGCTTTCACCGTTGGCTTCGTC
GTCCTCGTCATTGGGTTGTCCATGGGCTTCAACTCCGGATATGCTGTCAACCCTGCCAGG
GACTTTGGACCACGTCTGTTCACGGCTTTGGCTGGATGGGGTTCAGAAGTCTTCTGGGCC
TATGGCCAATGGTGGTGGGTCCCCATTGTATCACCCTTACTGGGTGCCTTCGCTGGAGTC
TTGGTCTACCAGCTGATGATTGGTTGCCACATCGAACCCGCACCCGAGTCCACCGAACAA

------------------GAAAACGTCAAGCTG---GCCAACGTCAAACACAAAGAAAGA
ATC---

>Eastern_banjo_frog_AQP3
ATGGGGAGACAGAAGGAGCTTCTCAACAGCCTGTCAGGGATGCTGCGTATCCGAAACATG
CTGCTGAGGCAGGCGCTATCCGAATGCCTGGGGACACTCATTCTTGTGATGTTCGGCTGT
GGCTCCGTGGCTCAAGTGGTTCTCAGTAAAGGGTCACACGGTCAGTTCTTGACTATTAAC
TTGGCTTTCGGATTTGCTGTCACGCTTGGCATTCTAATCGCAGGACAGGTTTCAGGTGGT
CATCTGAATCCAGCCGTAACATTCGCCTTGTGCATTATGGCAAGGGAACCTTGGATTAAG
TTTCCTATATACACCCTGGCACAGACTGTGGGCGCCTTCCTTGGAGCGGGAATCGTTTAT
GGCTTATATTATGACGCCAACTGGTTTTTCGCAAAT---GACAGTCTCTACGTGACAGGA
CCTAAC---GGCACCGCAGGGATTTTTGCCACATTTCCCACTGAACATCTGACTCTCATG
AATGGATTTTTTGACCAGTTTATAGGCACAGCTGCTTTGATTGTCTGTGTCTTGGCCATT
GTTGACCCTAACAACAACCCCATTCCCCGTGGGTTGGAAGCCTTCACTGTTGGGTTTGTG
GTCTTAGTCATTGGACTGTCCATGGGTTTCAACTCTGGTTATGCCGTGAACCCTGCAAGG
GACTTTGGACCCCGTTTGTTTACCTCACTGGCTGGCTGGGGCACAGAAGTCTTTTGGGCT

GGTGATCAGTGGTGGTGGGTCCCAATCGTCTCACCCTTACTAGGGGCATTTGCTGGAGTC
TTAGTCTATCAGTTGATGATCGGATGTCACATTGAACCTGCACCTCAATCCACCGAACAA

------------------GAGAACGTCAAGTTG---GCCAACGTCAAACACAAAGAGAGA
ATC---

>Argentine_toad_AQP3
ATGGGGCGCCAGAAGGAGCTTATCAACAGCATCAACTCCATGCTGCGCATCAGGAGCAAG
CTGCTCAGGCAGGCGCTGGCCGAATGTCTGGGCACCCTCATACTTGTCATGTTCGGCTGT
GGCTCCGTGGCTCAGGTCGTGCTCAGTAAAGGCTCCCATGGTCAGTTCTTGACCGTTAAT
CTGGCTTTTGGATTTGCCGTCATGCTGGGTATCCTTATCGCCGGACAGGTTTCAGGTGGA
CATCTGAATCCAGCTGTAACTTTTGCCTTGTGTCTTATGGCAAGAGAACCATGGATCAAG
TTACCAGTGTACACCATTGCACAGACTCTAGGAGCGTTCCTTGGAGCCGGCATCGTTTAT
GGCCTGTACTTTGACGCCATCTGGTATTTTGCAAAT---GACAATCTCTATGTAACCGGT
CCAAAC---GGCACAGCCGGTATTTTTGCCACTTACCCAACCGAGCATCTCACCCTGATG
AATGGCTTCTTTGACCAGTTTATTGGCACTGCAGCCCTGATCGTCTGTGTCCTGGCCATT
GTTGACCCCTACAACAACCCCATCCCTCGTGGACTTGAAGCCTTCACTGTTGGGTTTGTT
GTCCTTGTCATTGGACTGTCAATGGGTTTCAACTCTGGTTATGCCGTCAACCCCGCCAGG
GACTTTGGACCACGTCTCTTCACTGCTTTGGCTGGCTGGGGCACAGGAGTTTTCACTTCT
GGAGGTCACTGGTGGTGGGTCCCCATTGTCTCACCGTTACTGGGAGCTGTTGCTGGAGTT
ATGGTCTACCAACTGATGATCGGATGCCATGTTGAACCTAGGCCAGAGTCTACTGAACAA

------------------GAAAACGTCAAGTTG---GCCAATGTCAAACACAAAGAAAGG
ATC---

>LaPaz_rubber_frog_AQP3
ATGGGGCGCCAGAAGGAGGTTCTCAGCCGCATGAGTGCCATGCTGCGCATCAGGAACAAG
CTGCTCAGGCAGGCGCTGTCCGAATGTCTGGGCACCCTCATACTTGTCATGTTCGGCTGT
GGCTCGGTGGCTCAGGTGGTCCTCAGTAAAGGCTCCCATGGTCTCTTCTTGACTGTTAAT
CTGGCTTTTGGATTTGCCGTCATGCTGGGTATCCTCATTGCCGGACAGGTGTCAGGTGGC
CATTTGAATCCAGCTGTAACATTTGCCTTGTGTATTATGGCACGAGAACCTTGGATCAAG
TTCCCGGTGTACACCCTGGCACAGACTCTCGGAGCGTTCCTTGGTGCTGGCATCGTTTAT
GGCTTGTATTATGATGCTATCTGGTTCTTTGCAAAC---GACAGTCTTTATGTAACCGGT
CCGAAC---GGCACAGCCGGCATTTTCGCCACTTACCCTACGGACCATCTCACGCTGATG
AATGGCTTCTTTGACCAGTTCATTGGCACCGCAGCTCTGATCGTCTGCGTCTTGGCCATT
GTTGACCCTTACAACAACCCCATTCCTCGGGGACTTGAGGCCTTCACTGTTGGTTTTGTC
GTCCTTGTCATTGGATTGTCTATGGGCTTCAACTCTGGTTATGCCGTCAACCCCGCTAGG
GACTTTGGACCACGTCTCTTTACTGCCTTGGCCGGCTGGGGAACAGAAGTTTTCACTGCT
GGTGGGCAGTGGTGGTGGGTCCCCATCGTCTCGCCTTTTCTGGGAGCCTTTGCCGGAGTT
CTGGTCTACCAACTATTTATCGGCTGCCACATCGAGCCTGCACCAGAGTCTACTGAACAA

------------------GAGAATGTCAAGCTA---TCCAACGTCAAACACAAAGAGAGG
ATC---

>Ornate_chorus_frog_AQP3
ATGGGCCGCCAGAAGGATTGCCTCAACAGCGTCTACTCCATGCTGAGGATCCGCAACAAG
CTCATGAGGCAGGCGCTGGCCGAGTGTCTGGGCACCCTCATCCTCGTGATGTTCGGCTGC
GGCTCGGTGGCTCAGGTGGTCCTCAGTAAAGGCTCCCACGGTCAGTTCTTGACTGTCAAC
CTGGCTTTTGGCTTTGCCGTCATGCTGGGCATTCTCATAGCCGGACAAATCTCAGGCGGA
CATTTGAACCCAGCAGTGACCTTCGCCTTGTGTATCATGGCACGGGAACCTTGGATCAAG
CTTCCGGTATACACGCTGGCACAGACACTGGGCGCCTTCCTTGGAGCCGGTATTGTTTAC
GGCTTGTATTATGATGCCATCTGGGCATTCGCCAGT---GACAGCCTTTACGTCACAGGG
CCCAAC---GGCACCGCCGGCATCTTTGCCACCTTCCCAACTGAGCATCTAACCCTCATG
AATGGATTCTTTGACCAGTTTATCGGGACAGCCGCTTTGATCGTCTGCGTCTTGGCCATT
GTCGACCCCTACAATAACCCCATTCCCCGAGGTCTGGAAGCCTTCACCGTTGGCTTTGTC
GTCCTGGTCATTGGAACATCCATGGGCTTCAACTCCGGATACGCCGTCAACCCAGCTAGG
GACTTTGGACCTCGTCTGTTCACCTCTCTGGCTGGATGGGGAAGTGAGGTCTTCTCAGCC
GGTGACCAGTGGTGGTGGGTCCCCATCGTGTCTCCCTTACTGGGCTCCTTCGCCGGAGTC
CTTGTCTACCAGTTTATGATCGGCTGCCATATTGAACCCCCACCCCAATCCACCGAACAG

------------------GAAAACATCAAGCTG---TCCAACGTCAAACACAAAGATATG

AACCTA--

>Chinese_salamander_AQP3
ATGGGGAGGCAAAAGGAGATGGTGACCCGAATTGAGGGGATGCTGAGAATCCGGAACATG
CTGCTGAGGCAGGCTCTGGCCGAGTGCCTGGGGACTCTCATTCTGGTGATGTTTGGCTGT
GGGTCGGTTGCGCAGGTGGTTCTCAGCAAAGGATCCCATGGACAATTCTTCTCCGTCAAC
ATGGCCTTTGGTTTTGCTGTGATGCTGGGCATTCTGATCTGTGGCCAGGTGTCAGGTGGC
CACCTGAATCCCGCAGTAACGTTTGCTTTATGTCTAATGGCACGGGAACCATGGATCAAG
TTCCCTGTTTACACGCTGGCACAAACCATTGGCGCTTTCCTTGGCGCAGGAATTATCTAC
GGCTTGTATTTCGATGCCATCTGGGCTTTTGCTGAA---GACCAACTCATTGTCATGGGG
CCCAAC---GGCACGGCTGGCATCTTTGCTACCTATCCGACCGAACATCTGACGCTTATC
AATGGATTCTTTGACCAGTTCATTGGCACGGCAGCCTTGATCGTGTGCGTCCTCGCCATT
GTGGATCCGAACAACAATCCCATCCCCAGAGGCCTCGAGGCTTTCACTGTTGGGTTTGTG
GTTCTTTGCATTGGACTGTCCATGGGCTTCAACTCCGGATATGCCGTCAACCCTGCCAGG
GACTTCGGACCACGCCTTTTCACGGCTATTGCTGGTTGGGGTTCTGAAGTTTTCTCGGCC
GGAGGACAGTGGTGGTGGGTGCCAATCGTTTCCCCCCTGCTTGGGTCCGTTGCAGGCATC
CTGGTCTATCAACTGATGATCGGAACCCACATTGTGCCTGCTCCACAGTCCACCCCGCAG

------------------GAGAATGTCAAGTTG---GCCAACGTCAAGCACAAGGAGATG
ATC---

>Hokkaido_salamander_AQP3
ATGGGGCGGCAAAAGGAGATGGTGACCCGAATTGAGGGGATGCTGAGAATCCGTAACATG
CTCCTGAGGCAGGCTCTGGCCGAGTGCCTGGGGACTCTCATTCTGGTGATGTTTGGCTGT
GGGTCGGTGGCGCAGGTGGTTCTCAGCAAAGGATCCCATGGACAATTCTTCTCCGTCAAC
ATGGCCTTTGGTTTTGCTGTGATGCTGGGCATTCTGATCTGTGGCCAGGTGTCAGGCGGC
CATCTGAATCCCGCAGTAACGTTTGCTTTATGTCTAATGGCACGGGAACCATGGATCAAG
TTCCCTGTTTACACGCTGGCCCAAACCATTGGCGCTTTCCTTGGCGCAGGAATTATCTAC
GGCTTGTATTTCGATGCCATCTGGGCTTTTGCTGAA---GACCAACTCATTGTCATGGGG
CCCAAC---GGCACGGCTGGCATCTTTGCTACCTATCCGACCGAACATCTGACGCTCGTC
AATGGATTCTTTGACCAGTTCATTGGCACGGCAGCCTTGATCGTGTGCGTCCTTGCCATT
GTGGATCCGAACAACAATCCCATCCCCAGAGGCCTGGAGGCTTTCACTGTTGGGTTTGTG
GTTCTTGTCATTGGACTGTCCATGGGCTTCAACTCCGGATATGCCGTCAACCCTGCCAGG
GACTTCGGACCACGCCTTTTCACAGCTATTGCTGGTTGGGGCTCTGAAGTTTTCTCGGCC
GGAGGACAGTGGTGGTGGGTGCCAATCGTTTCCCCCCTGCTTGGGTCCGTCGCAGGCATC
CTGGTCTACCAACTGATGATCGGAACCCACATTGTGCCTGCTCCACAGTCCACCCCGCAG

------------------GAGAATGTCAAGTTG---GCCAACGTCAAGCACAAGGAGATG
ATC---

>Fire_salamander_AQP3
ATGGGGCGACAGAAGGACGTGGTGAACAAGATCGGGGACATGCTGAGACTGAGGAGCAAG
CTGATGAGGCAGGCCCTGGCCGAGTGCCTGGGGACCCTCATCCTGGTGATGTTTGGCTGC
GGGTCGGTGGCGCAGGTCGTCCTCAGCAAGGGGTCCCACGGACAGTTCTTAACTGTGAAC
ATGGCCTTCGGTTTTGCTGTCATGCTTGGTATTATTGTCGCTGGGCAGGTGTCAGGTGGC
CATCTGAATCCGGCAGTAACGTTTGCTTTATGCTTGCTGGCACGAGAACCGTGGATCAAG
TTCCCCATTTACACGTTGGCACAAACTCTTGGCGCTTTCCTTGGCGCTGGCATTGTCTAC
GGCTTGTACTATGATGCTATTTGGGCTTTTGCTTCT---GAACAACTCTACGTCATGGGG
CCCAAT---GGTACGGCTGGAATATTTGCAACCTACCCGACGGACCATCTTACACTCATC
AACGGGTTCTTTGACCAGTTCATTGGCACCGCAGCCCTCATCGTCTGTGTCCTCGCCATC
GTGGACCCGAACAATAACCCAATCCCCCGGGGTCTGGAGGCATTCACCGTTGGGTTTGTG
GTGCTCGTCATTGGACTGTCCATGGGCTTCAACTCTGGATATGCTGTCAACCCGGCCAGG
GACTTTGGCCCACGCCTGTTCACCGCCATTGCTGGTTGGGGCACCGAAGTTTTCTCGGCC
GGCGGACAGTGGTGGTGGGTGCCAATTGTTTCCCCCTTGCTTGGATCAGTCGCCGGCATT
TTGGTCTACCAGTTGATGATTGGGTTCCACATTGAGCCTGTGCCACAGTCGACCGAGCAG

------------------GAAAATGTCAAGTTG---GCCAACGTCAAGCACAAGGAGAGG
ATC---

>Rough_skinned_newt_AQP3
ATGGGGCGCCAAAAGGAACTGGTGAACAAAATTGGGGACATGCTGAGGCTCCGGAGCAAG

TTGATGAGACAGGCTCTGGCCGAGTGCCTGGGGACCCTCATACTGGTGATGTTCGGCTGC
GGGTCGGTGGCGCAGGTGGTCCTCAGCAGGGGCTCCCACGGGCAGTTCTTGACTGTAAAC
ATGGCCTTCGGCTTCGCTGTCATGCTTGGGATTCTTATCTCAGGGCAGGTGTCAGGTGGC
CATCTGAATCCGGCAGTAACATTTGCTTTATGTTTGCTGGCACGAGAACCGTGGATCAAG
TTCCCCGTTTACACGTTGGCACAAACTCTCGGCGCTTTTCTTGGCGCTGGCATAGTCTAT
GGCTTGTATTATGATGCTATTTGGGCCTTTGCTGGT---GAACAGCTCGTCGTCATGGGG
CCCAAT---GATACAGCTGGAATATTTGCGACCTTCCCGACGGACCACCTGACGCTCCTC
AACGGGTTCTTTGACCAGTTCATTGGCACCGCAGCCCTGGTCGTCTGTGTCCTCGCCATC
GTGGACCCGAACAATAACCCAATCCCCGGGGCCTGGAGGCCTTCACAGTTGGGTTTGTG
GTGCTTGTCATTGGACTGTCCATGGGATTCAACTCTGGATACGCTGTCAACCCGGCCAGG
GACTTTGGGCCGCGCCTGTTCACGGCCATCGCTGGTTGGGGCACCGAAGTTTTTCGGCC
GGCGGACAGTGGTGGTGGGTGCCAATTGTGTCCCCCTTACTTGGATCAATCGCCGGCATT
TTGGTCTACCAGCTAATGATCGGGTTCCACATTGAGCCTCTGCCTCAGTCCACTGAGCAG

------------------GAAAACGTCAAGTTG---GCCAATGTCAAGCACAAGGAGAGG
ATC---

>Wenxian_knobby_newt_AQP3
ATGGGGCGTCAGAAGGATCTGGTGAACAAGATCGGGGACATGCTGAGGATCCGGAGCAAA
CTGATGAGGCAGGCTCTGGCCGAGTGCCTGGGGACCCTCATCCTGGTGATGTTCGGCTGC
GGGTCGGTGGCGCAGGTCGTCCTCAGCAAGGGCTCCCACGGACAGTTCATGACTGTAAAC
ATGGCCTTCGGCTTCGCCGTCATGCTTGGGATTCTTATCGCAGGGCAGGTGTCAGGTGGC
CATCTGAATCCGGCAGTGACATTTGCTTTATGCCTCCTGGCGCGAGAACCGTGGATCAAA
TTCCCCATTTACACGTTGGCGCAAACTCTTGGTGCTTTTCTTGGCGCTGGCGTTGTCTAC
GGCTTGTACTATGATGCTATTTGGGCCTTTGCTTCA---GAACAGCTCTTCGTCATGGGG
CCCAAT---GGTACGGCTGGAATATTTGCGACCTACCCGACGGAACATCTGACACTCCTC
AACGGGTTCTTTGACCAGTTCATCGGCACCGCAGCCCTGATCGTCTGTGTCCTCGCCATC
GTGGACCCGAACAACAACCCAATCCCTCGGGGTCTGGAGGCATTCACCGTTGGGTTTGTG
GTGCTTGTCATTGGACTGTCCATGGGCTTCAACTCTGGATACGCTGTCAACCCGGCCAGG
GACTTTGGGCCACGCCTGTTTACGGCTATCGCGGGTTGGGGCACCGAAGTTTCTCGGCC
GGTGGACAGTGGTGGTGGGTGCCAATTGTGTCCCCCTTACTTGGATCAGTCGCCGGCATT
TTGGTCTACCAGCTAATGATCGGGTTCCATATTGTGCCTGTGCCTCAGTCCACCGAGCAG

------------------GAAAACGTCAAGTTG---GCCAACGTCAAGCACAAGGAGAGG
ATC---

>ymSalamander_AQP3
ATGGGCCGCCAAAAGGAGCTGGTGAACAAGATCGGCCGAGCCCTGCAGATCCATAGCTTG
CTGCTGCGCCAGGCGCTGGCCGAGTGCCTGGGCACGCTCATCCTCGTGATGTTCGGCTGC
GGCTCGGTGGCGCAGGTGGTGCTCAGCAGAGGCTCCCACGGACAGTTCTTGACGGTGAAC
ATGGCCTTCGGCTTCGCTGTCATGCTTGGGATCATTATCGCCGGACAGGTGTCAGGCGGG
CACCTGAATCCGGCAGTGACGTTTGCGCTTTGCTTCCTGGCCCGCGAGCCATGGATCAAG
TTCCCGGTGTACACGATAGCGCAGACTCTCGGCGCCTTCCTTGGTGCCGGAATTGTTTAC
GGCTTGTACTTTGATGCAATATGGGCCTTTGCCGGA---GAGCAGCTCTTTGTCATGGGG
CCTAAC---GGAACGGCCGGAATATTTGCAACTTACCCAACAGATCATCTGACCCTCGTC
AACGGATTCTTTGACCAGTTCATCGGCACCGCAGCCCTGGTGGTGTGTGTTCTCGCCATT
GTTGACCCCAATAACAATCCCATTCCCAGGGGTCTTGAGGCATTCACCGTTGGATTCGTG
GTTCTTGTCATTGGACTGTCCATGGGTTTCAACTCAGGATACGCTGTTAACCCGGCCAGG
GACTTCGGACCACGTCTCTTCACGGCCATAGCTGGTTGGGGGTCTGAGGTCTTCTCGGCC
GGAGGACAGTGGTGGTGGGTGCCCATCGTTTCCCCATTGCTCGGGTCAGTCGCCGGCATC
TTGGTCTACCAGCTGTTGATTGGATTCCACATTGAGGGTCCTCCACCGTCTACAGAACAG

------------------GAGAACGTCAAGCTG---GCTAACGTCAAGCACAAGGAGAGG
GTC---

>Axolotl_AQP3
ATGGGTCGGCAGAAGGAACTGGTCAACAAAATCGGGGACATGTTGAGGATCCAGAACAAG
CTGCTGAGGCAGGCGCTGGCCGAGTGCCTGGGGACCCTCATCCTCGTGATGTTTGGCTGC
GGGTCGGTGGCCCAGGTCGTTCTCAGCAAGGGCTCCCATGGACAGTTCTTGACAGTGAAC
ATGGCCTTTGGTTTCGCTGTGATGCTTGGCATCATTATCTGCGGGCAGGTGTCCGGCGGC
CACCTGAATCCGGCAGTCACGTTTGCTTTATGCTTCCTGGCCCGGGAGCCTTGGATCAAG

TTCCCCATTTACACATTGGCACAGACACTGGGAGCGTTCCTGGGCGCTGGCATTATCTAC
GGATTGTACTATGATGCTATTTGGGCTTTTGCTCAG---GATCAACTCTACGTCATGGGG
CCCAAT---GGGACAGCCGGGATTTTTGCAACCTACCCGACCGAGCATCTGACGCTTATG
AATGGATTCTTCGACCAGTTCATTGGCACCGCAGCCTTGGTGGTCTGTGTCCTCGCCATT
GTGGACCCAAACAACAACCCAATCCCCAAGGGCCTTGAGGCCTTCACTGTTGGATTTGTG
GTTCTCGTCATTGGACTTTCTATGGGTTTCAACTCTGGATATGCCGTCAACCCGGCCAGG
GACTTTGGACCACGTCTCTTCACGGCAATTGCTGGCTGGGGCTCTGAAGTCTTCTCGGCT
GGAGAACAGTGGTGGTGGGTGCCAATCGTTTCCCCCTTTCTTGGATCTGTCGGCGGAGTT
TTAGTCTACCAGCTGATGATCGGGTTCCATATCTTGCCTATCCCAGAGTCCACGGAGCAG

------------------GAGAACGTCAAGTTG---GCCAATGTCAAGCACAAGGAGCGG
ATC---

>2Lcaecilian_Aqp3
ATGGGTCGGCAGAAGGAGTTGGTTAACCGGTGCGGGGAGATGCTGCGAATCCACAACAAG
CTGATGAGACAGGCTCTGGCGGAGTGCCTGGGGACCTTGATCCTGGTGATGTTTGGCTGT
GGTTCTGTGGCTCAGGTGGTTCTGAGCAGGGGATCGCATGGACAGTTCTTGACTGTCAAC
CTGGCCTTTGGTTTTGCCGTGATGCTCGGTATCATAATATCGGGCCAGATATCAGGTGGC
CACCTCAATCCTGCTGTGACCTTTGCAACGTGCTTGTTGGCAAGAGAGCCGTGGATTAAA
TTCCCGATTTATACCTTGGCACAGACCCTCGGGGCTTTTCTTGGAGCAGGCATTGTCTAT
GGCTTGTACTATGATGCCATATGGATTTTCGGTGGT---GATCAGCTGTACGTAACGGGA
GAAAAT---GCCACAGCAGGGATCTTCGCAACCTTCCCGACTGACCATTTGACCCTAATG
AATGGATTCTTTGATCAGGTAATTGGTACGGCGGCACTGGTGGTCTGTGTCCTTGCTATT
ATAGATCCATACAATAATCCAATTCCCCGGGGCCTCGAGGCATTCACCGTTGGATTTGTG
GTCCTGGTCATTGGACTTTCCATGGGCTTCAACTCCGGATATGCTGTTAACCCTGCTAGG
GATTTTGGACCTCGTCTCTTCACGTCTATTGCTGGTTGGGGCACTGAGGTTTTCAGCGCT
GGAGGCCAGTGGTGGTGGGTGCCGATAGTTTCCCCGTTGCTCGGGGCGGTGGGCGGCATC
TTGGTTTACCAGCTCATGATTGGCTTTCACATCGAGCCCCCGCCGCAGTCCACCGAGCAA

------------------GAAAACGTCAAGCTA---GCCAACGTGAAGCAGAAAGAGAGG
ATC---

>Gaboon_caecilian_AQP3
ATGGGTCGTCAAAAGGAATTAGTGAACCGGTGTGGGGAGATGCTCCGAATCCACAACAAA
CTGATAAGACAATTCCTCTCGGAGTGCCTCGGGACGCTGATCCTGGTGATGTTTGGCTGC
GGATCTGTGGCTCAGGTGGTTCTCAGCAAGGGATCCCATGGACAGTTCTTGACTGTCAAC
CTGGCCTTTGGTTTTGCCGTGATGCTTGGTATTCTGATATCAGGGCAGGTGTCAGGTGGT
CACCTCAATCCTGCTGTCACTTTTGCATTTTGCTTGTTGGCAAAGGAACCATGGATTAAA
TTCCCCATTTATACCTTGGCACAAACCCTTGGGGCTTTTCTTGGAGCTGGCATCGTCTAC
GGCTTGTACTACGATGCCATATGGTATTTTGCGAAT---GATCAACTGTATGTGATGGGA
CCCAAC---GGCACAGCAGGGATCTTTGCGACTTACCCAACTGAACATTTGACATTAATG
AATGGATTCTTTGATCAGTTCATTGGTACAGCAGCCCTAGTGGTCTGTGTCCTGGCTATT
GTGGATCCGTACAATAATCCAGTTCCTAGGGGCCTCGAGGCTTTCACCGTTGGGTTTGTT
GTCTTGGTCATTGGACTCTCCATGGGCTTCAACTCCGGCTATGCTGTCAACCCTGCCAGG
GACTTTGGACCTCGCCTTTTCACATCCATTGCTGGTTGGGGCACGGAGGTTTTCAGTGCT
GGAGACCAATGGTGGTGGGTGCCGATCGTTTCACCATTACTCGGCGCAGTGGCTGGCGTT
TTGGTGTACGAGTTAATGATTGGGTTTCACGTTGAGCCTCCACCACCGTCCACCGAGCAA

------------------GAAAACGTCAAGCTG---TCAAACGTGAAGCACAAAGACAGG
ATC---

>tcCaecian_AQP3
ATGGGTCGCCAGAAGGAATTGGTTAACCGGTGTGGGGAGATGCTACGGATTCAGAACAAA
CTGCTGAGACAGGCTCTAGCAGAGTGCCTGGGGACGCTAATCCTAGTGATGTTTGGCTGT
GGTTCTGTGGCTCAGGTGGTTCTCAGCAAGGGATCCCATGGACAGTTCTTGACTGTTAAC
CTAGCTTTTGGTTTCGCTGTGACGCTTGGCATTCTGATAGCAGGGCAGGTGTCAGGTGGC
CATCTCAATCCTGCAGTCACTTTTGCATTGTGCTTGTTGGCAAAGGAGCCGTGGGTTAAA
TTCCCCATCTATTGCTTGGCACAAACCCTCGGGGCTTTTCTCGGAGCTGGCATAGTCTAC
GGCTTGTATTATGATGCCATATGGATGTTTGCGAAT---GATCGACTGTATGTATTGGGA
GAAAAC---GGCACGGCAGGGATCTTTGCAACTTACCCAACTGACCATTTGACGTTAATG
AATGGATTCTTTGATCAGTTCATTGGTACGGCGGCCCTGGTGGTCTGTGTCCTGGCTATC

ATAGATCCATACAATAATCCCGTCCCAAGGGGCCTTGAGGCTTTCACCGTTGGGTTTGTC
GTCTTGGTCATCGGACTCTCCATGGGCTTCAACTCCGGCTATGCTGTTAACCCTGCGAGG
GACTTCGGACCTCGTCTTTTCACAGCCATTGCTGGTTGGGGCACTGAGGTTTTCAGCGCT
GGAGACCAGTGGTGGTGGGTGCCAATCGTTTCACCATTACTTGGTGCAGTGGCCGGCATT
TTGGTTTACCAGCTCATGGTTGGGTTTCACTTTGAACCTCCACCACAGTCAACCGAGCAA

------------------GAAAACGTCAAGTTA---TCAAATGTGAAGCACAAAGAGAGG
ATC---

>bCaecilian_AQP3
ATGGGTCGCCAAAAGGAGTTCATGAACCGCATTGGGGACATGCTACGAATCCACAACAAA
CTCATGAGACAGTTTCTAGCGGAGTGCCTCGGGACTCTCATCCTAGTGATGTTCGGCTGT
GGTTCTGTGGCGCAGGTGGTTCTCAGCAAGGGATCCCATGGACAGTTCTTGACTGTTAAC
CTGGCCTTTGGTTTTGCGGTGACGCTTGGTATTCTGATAGCAGGGCAGGTGTCAGGTGGT
CACCTCAATCCTGCTGTCACATTTGCATTGTGCTTGCTGTCCTGGGAACCATGGATTAAA
CTCCCCATTTACATCTTGGCACAAACCCTTGGGGCTTTTCTGGGAGCTGGCATCGTCTAT
GGCTTGTACTTCGATGCCATATGGTTTTTCGCAAAT---GATCAGCTATATGTAATGGGA
CCCAAC---GACACGGCAGGGATATTTGCAACCTACCCGACTGAACATTTGACACTAATG
AATGGATTCTTTGATCAGTTCATTGGCACGGCGGCCCTCGTGGTCTGTGTCCTGGCTATC
GTAGATCCGAACAATAATCCAGTCCCTAGAGGCCTCGAGGCTTTCACCGTGGGGTTTGTC
GTGCTGGTCATTGGACTGTCCATGGGCTTCAACTCTGGCTATGCCGTGAACCCTGCCAGG
GACTTTGGACCTCGGCTTTTCACATCCATCGCTGGTTGGGGCACTGAGGTTTTCAGTGCT
GGAGGCCAGTGGTGGTGGGTGCCGATCGTTTCACCATTACTCGGCGCAGTGGCCGGGGTC
TTGGTTTACCAGCTAATGATTGGAGTTCACATTGAACACCCACCACCATCCACCGAGCAA

------------------GAAAATGTCAAGCTC---TCAAATATGAAGCACAAAGAGAGG
ATT---

>B_bambooshark_Aqp3C2
ATGGGAAAACAAAAGGCAATAATCAGAAAAATTGAAGACTCATTCAGAATAAGAAATCTA
TTGCTCAGACAATGTCTTGCTGAATGTTTAGGAACATTAATTCTTGTGTTGTTTGGGTGT
GGAGCACTAGCACAAGTTACCCTCAGCAGGGGTACACATGGACGGTTTTTGACTGTCAAT
TTTGCTTTTGGATTTGCAGTAATGCTCGGTGTACTAATAGCTGGCCAAGTGTCAGGTGCT
CACCTGAACCCTGCTGTGACCTTTGCTATGTGCTTACTTGCTCGCGAACCTTGGATAAAA
TTTCCCCTTTACTCTCTGGCACAAATATTAGGTGGCTTCATTGGATCTGGTATCATTTTT
GGTTTGTATTTTGATGCCATGTGGGACTTCAGTGGTCAAAATAAACTGTTGGTATATGGC
CCCAAT---GCAACTGCTGGCATATTTGCTACTTACCCATCTGCACACTTAACTCCACTC
AATGGCTTTTTTGATCAGCTGATTGGAACGAGTGCTCTGATAGTTTGCATCCTTGCTATT
GTGGATAAGTACAATAACCCAGTGCCAAAGGGTGTGGAGGCATTTACTGTTGGCTTTACC
GTACTGGTTATTGGCTTGTCAATGGGTTTCAACTCTGGGTATGCTGTGAACCCTGCCAGA
GACTTTGGACCTCGTTTGTTTACGGCATTGGCTGGCTGGGGAGCTGAAGTTTTCAGTGCC
GGACACTACTGGTTTTGGATCCCAATTTTTGCCCCCCTCCTTGGTTCTTTACTGGGCATT
CTGATATATCAACTAATGATTGGGATACATCTGGAGCCTGTAAACCACAATTCATCCCCT
AGAGAA--
------------------GAAAATGTAAAACTG---GCTAATGTGAACTTAAGAGAAAGT
TCC---

>W_bambooshark_Aqp3C2
ATGGGAAAACAAAAGGCAATAATCAGAAAAATTGAAGACTCTTTCAGAATAAGAAATCTA
TTGCTCAGACAATGTCTTGCTGAATGTTTAGGAACATTAATTCTTGTGTTGTTTGGGTGT
GGAGCACTGGCACAAATTACCCTCAGCAGGGGTACACATGGACGGTTTTTGACTGTCAAT
TTTGCTTTTGGATTTGCAGTAATGCTTGGTGTACTAATAGCTGGCCAAGTGTCAGGTGCT
CACCTGAACCCTGCTGTGACCTTTGCTATGTGCTTACTTGCTCGTGAACCTTGGATAAAA
TTTCCCCTTTACTCTCTGGCACAAATACTAGGTGGCTTCATTGGATCTGGTATCATTTTT
GGTTTGTATTTTGATGCCATGTGGGACTTCAGTGGTCAAAATAAACTGTTGGTATATGGC
CCCAAT---GCAACTGCTGGCATATTTGCTACTTACCCATCTGCACACTTAACTCCACTC
AATGGCTTTTTTGATCAGCTGATTGGAACGAGTGCTCTGATAGTTTGCATCCTTGCTATT
GTGGATAAGTACAATAACCCAGTGCCAAAGGGTGTGGAGGCATTTACCGTTGGCTTTACC
GTACTGGTTATTGGCTTGTCAATGGGTTTCAACTCTGGGTATGCTGTGAACCCTGCCAGA
GACTTTGGACCTCGTTTGTTTACGGCATTGGCTGGCTGGGGAGCTGAAGTTTTCACGGCT
GGACACTACTGGTTTTGGATCCCAATTTTTGCCCCCCTCCTTGGTTCTTTACTGGGCATT

```
CTGATATATCAACTAATGATTGGGATACATCTGGAGCCTGTAAACCACAATTCATCCACT
AGAGAA------------------------------------------------------
------------------GAAAATGTAAAACTG---GCTAATGTGAACTTAAGAGAAAGT
TCC---------------------------------------------------------
---------------
>Whale_shark_Aqp3C2
ATGGGAAAACAAAAGGCAATAATCAGAAAAATTGAAGATTCATTCAGAGTAAGAAATCTA
TTGCTAAGGCAATGTCTTGCTGAATGTTTAGGAACATTGATTCTTGTGTTGTTTGGGTGT
GGAGCGCTGGCACAAGTTACCCTCAGCAGGGGCACACATGGAAGGTTTTTGACTGTCAAT
TTTGCTTTTGGATTTGCAGCAATGCTTGGTGTACTATTAGCTGGCCAAGTGTCAGGTGCT
CACCTGAACCCTGCTGTGACATTTGCTATGTGCTTACTTGCTCGCGAACCCTGGATAAAA
TTTCCCCTTTACTCTTTGGCACAAATACTAGGCGGCTTCCTTGGATCTGGTATCATTTTT
GGTTTGTATTTTGATGCCATGTGGGACTTCAGTGGTCAAAATAAACTGTTGGTATATGGC
CCCAAT---GCAACTGCTGGTATATTTGCTACTTACCCATCTGCACATCTAACTCCACTC
AATGGCTTTTTTGATCAGCTGATTGGAACTAGCGCTCTGATAGTTTGCATCCTTGCTATT
GTGGATAAATTCAATAACCCAGTGCCAAAGGGTGTGGAGGCATTTACAGTTGGCTTTACT
GTACTGGTCATTGGCTTGTCAATGGGCTTCAACTCTGGATATGCTGTGAACCCTGCCAGA
GATTTTGGACCTCGTTTGTTTACAGCATTGGCCGGCTGGGGAGCTGAAGTTTTCAGTGCT
GGACACTACTGGTTTTGGATTCCAATTTTTGCCCCCCTCCTTGGTTCTTTACTTGGCACT
CTAGTATATCAACTAATGATTGGGATACATCTCGAGCCTGTAAACCATAATTTACCCACT
AGAGAA------------------------------------------------------
------------------GAAAATGTAAAACTG---GCTCATGTAAATTTAAAAGAAAGC
TCC---------------------------------------------------------
---------------
>Zebra_bullhead_Aqp3C2
ATGGGAAAGCAAAAGGCAATTATCAGAAAAATTGAAGATTCATTCAGAATAAGAAATCTA
TTGCTAAGACAATGTCTTGCTGAATGTTTAGGAACATTGATTCTTGTGTTGTTTGGCTGT
GGAGCATTGGCACAAGTTACCCTCAGCAGGGGTACACATGGACTGTTTTTGACTGTTAAT
TTTGCCTTCGGATTTGCAGTGATGCTCGGTGTACTAGTAGCTGGCCAAGTGTCAGGAGCT
CACCTGAATCCTGCTGTGACCTTTGCTATGTGCTTACTTGCTCGCGAACCCTGGATAAAA
TTTCCCCTTTACTCTTTGGCACAAATACTAGGTGGCTTCCTTGGATCTGGTATCATTTTC
GGTTTGTATTTTGATGCCATGTGGGACTTCAGTGGACAAAACCAACTGTTAGTACATGGG
CCCAAT---GCCACTGCTGGTATATTCGCCACATACCCATCTGTGCACTTAACTCCACTC
AATGGCTTTTTTGATCAGCTGATTGGAACTACTGCTCTCATAGTTTGCATCCTTGCCATT
GTGGATAAATTCAATAACCCAGTGCCAAAGGGTCTGGAGGCATTTACTGTTGGCTTTACC
GTCCTGGTAATCGGCTTGTCAATGGGTTTCAACTCCGGGTATGCTGTAAACCCTGCCAGA
GACTTTGGACCTCGTTTGTTTACAGCATTGGCTGGCTGGGGAGCTGAAGTTTTCATTGCT
GGAAACTACTGGTTTTGGATCCCTATTTTTGCCCCACTCCTTGGTTCTGTGCTTGGTGTT
CTGGTTTATCAACTAATGATTGGAATACATATTGAGCCCATAAACCATAGTTCACCTATT
GAAGAA------------------------------------------------------
------------------GAAAATGTAAAACTG---GCTAATGTAAAATTAAGAGAAAGT
TCA---------------------------------------------------------
---------------
>Cloudy_catshark_Aqp3C2
ATGGGAAAACAAAAGGCAATAATCAGGAAAATCGAAGATTCGTTCAGAATAAGAAATTTA
TTGGTCAGACAATGTCTTGCTGAATGTTTAGGAACATTGGTTCTTGTGTTATTTGGCTGT
GGAGCAGTGGCACAAGTTACCCTCAGCAGAGGAACCCATGGACTGTTTTTGACTGTTAAT
TTTGCTTTTGGATTTGCAGTGATGCTGGGTGCACTAATAGCTGGCCAAGTGTCAGGAGCT
CACCTGAACCCTGCTGTGACCTTTGCTATGTGTATACTTGCTCGCGAGCCCTGGATAAAA
TTTCCCCTCTACTCTTTCGCACAAATATTAGGCGGTTTCCTTGGGTCTGGTGTCATTTTT
GGTTTGTACTTTGATGCCATGTGGGGCTTCAGTGGCCAAAACAAACTCTTAGTACATGGC
CCCAAT---GCGACTGCTGGGATATTTGCTACATACCCAGCTCTGCACTTAACTCCAGTA
AATGGCTTTTTTGATCAGCTGATTGGGACTGCAGCTCTCATAGTTTGCATCCTAGCCATT
GTGGATAAATTCAACAACCCAGTGCCAAAGGGACTGGAGGCATTTAGTGTTGGCTTTGCC
GTGCTGGTAATTGGCATGTCAATGGGTTTCAATTCTGGGTACGCTATAAACCCTGCCAGA
GACTTTGGACCTCGTCTGTTTACAGCACTGGCCGGCTGGGGAGCTGAGGTTTTCAGTGCT
GGAAACTACTGGTTTTGGATTCCCATCGTTGCCCCACTCCTTGGTTCTGTATTTGGTATT
TTGGTATATCAACTAATGGTTGGAATACACCTTGAGCCCATAAACCACAGTTCAACCACG
GAAGAG------------------------------------------------------
------------------GAAAATGTAAAACTT---GCGAATGTTAAATTAAGAGAAAGT
TCC---------------------------------------------------------
```

```
---------------
>Blue_shark_Aqp3C2
ATGGGAAAACAAAAGGCAATGATTAGAAAAATCGAAGATTCGTTCAGAATCAGAAACCTG
TTGTTAAGACAATGTCTTGCTGAATGTTTAGGTACATTGATACTTGTGTTGTTTGGCTGT
GGAGCAGTGGCACAAGTTACCCTCAGCAGGGGTACCCATGGACAGTTTTTGACTGTTAAT
TTTGCTTTTGGGTTTGCAGTGATGCTTGGTGTCCTGATAGCTGGCCAAGTGTCAGGTGCT
CACCTGAACCCTGCTGTGACCTTTGCTATGTGTTTGCTTGCTCGTGAGCCCTGGATAAAA
TTTCCTCTCTACTCTTTGGCCCAAATATTTGGTGGCTTCCTTGGATCTGGTGTCATTTTT
GGCTTGTACTTTGATGCCATTTGGGGCTTCAGTGGCCAAAACAAACTCTTAGTACATGGT
CCCAAT---GCGACTGCTGGTATATTTGCTACATACCCATCTGCGCACTTAACTCCAGTA
AATGGCTTTTTTGATCAGCTGATTGGAACTACTGCTCTCATAGTTTGCATCCTTGCCATT
GTGGATAAGTTCAACAACCCAGTGCCAAAGGGACTGGAGGCATTTACTGTTGGTTTTGTC
ATATTGGTAATTGGCCTGTCAATGGGTTTCAACTCTGGGTATGCTGTGAACCCTGCCAGA
GACTTTGGACCTCGCTTGTTTACAGCACTGGCTGGTTGGGGAGCTGAAGTTTTCAGTGCC
GGAAGCTACTGGTTTTGGGTTCCCATTTTTGCCCCACTCCTTGGTTCTGTACTTGGTGTT
CTGATATACCAGCTAATGATTGGAATTCACCTTGAGCCCATAAAGCCCAGTTCACCCGCT
GGAGAA------------------------------------------------------
------------------GATAATGTAAAACTG---GCTAATGTAAAATCAAGAGAAAGT
TGC---------------------------------------------------------
---------------
>Great_white_shark_Aqp3C2
ATGGGAAAACAAAAAGCACTGATCAGAAAAATTGAACATTCATTCAGAATAAGAAATCTG
TTGGTAAGAGAATGTCTTGCTGAATGTTTAGGAACATTAATTCTTGTGTTGTTTGGCTGT
GGAGCATTGGCACAAGTTACTCTCAGCAGAGGTACACATGGAATGTTTTTGACTGTTAAT
TTTGCTTTTGGATTTGCAGTGATGCTCGGTGTACTAATAGCTGGCCAAGTGTCAGGAGCT
CACCTGAATCCTGCCGTGACATTTGCCATGTGTTTACTTGCTCGTGAGCCCTGGATAAAA
TTTCCCCTCTACTCTTTGGCACAAATATTAGGTGGTTTCCTTGGATCTGGTGTCATTTTT
GGTTTGTATTTTGATGCCATGTGGGGTTTTAGTGGCCAAAACAAACTCTTAGTACATGGC
CCCAAT---GCGACTGCTGGTATATTTGCTACATACCCATCTGTGCACTTAACACCAGTA
AATGGCTTTTTTGATCAGCTGATTGGGACGACCGCTCTCATAATTTGCATCCTTGCGATT
GTGGATAAACTCAATAACCCAGTGCCAAAGGGACTGGAGGCATTTACCATTGGCTTTGCC
GTGCTGGTAATTGGCCTGTCAATGGGTTTCAACTCTGGGTACGCTGTGAACCCTGCCAGA
GACTTTGGACCTCGCTTGTTTACAGCATTGGCTGGCTGGGGAGCTGAGGTTTTCAGTGCT
GGAAACTACTGGTTTTGGATTCCCATTTTTGCCCCACTCCTTGGTTCTGTATTTGGTATT
CTGGTATATCAGCTAATGATTGGAATACACCTCGATCCCATAAACTGCAGTTCACCCTCT
GGAGAA------------------------------------------------------
------------------GAAAATGTAAAACTG---GCTAATGTAAAATTAAGAGAAAGT
TCC---------------------------------------------------------
---------------
>vblShark_Aqp3C2
ATGGGAAAACAAAAAGCAATAATCAGAAAAATTGAAGATTCATTCCGAATAAGAAATCTG
TTGGTAAGACAATGTCTTGCTGAATGTTTAGGAACGTTGATTCTTGTGTTGTTTGGCTGT
GGAGCATTGGCACAAATTACCCTCAGCAGGGGTACACATGGACAGTTTTTGACTGTTAAT
TTTGCCTTCGGATTTGCAGTGATGCTCGGTGTACTCCTGGCTGGCCAAGTCTCAGGAGCG
CACTTGAATCCTGCCGTGACCTTTGCTATGTGCTTACTTGCTCGCGAACCCTGGTTAAAA
TTTCCCCTTTACTCTTTGGCACAAATACTAGGCGGCTTCCTTGGATCTGGTATCATTTTC
GGTTTGTATTTTGATGCCATGTGGGACTTTAGTGGTCAAAACCAACTGTTAGTACATGGC
CCCAAT---GCTACTGCTGGTATATTCGCTACCTACCCATCTGTGCACTTAACTCCA---
---------TTTGATCAGCTGATTGGAACTGCCGCTCTCATAGTTTGCATCCTTGGCATT
GTGGATAAATTCAATAACCCGGTGCCAAAAGGACTGGAGGCATTTACTGTTGGCTTTACT
GTCCTGGTAATTGGCTTATCAATGGGTTTCAACTCTGGGTATGCCGTGAACCCTGCCAGA
GACTTTGGACCTCGTTTGTTTACAGCATTGGCTGGCTGGGGAGCTGAGGTTTTCGTTGCT
GGAAACTACTGGTTTTGGATCCCTATTTTTGCCCCACTCCTTGGTTCTGTGCTTGGTATT
CTGGTATATCAACTAATGATTGGAATACACCTCGAGCCCGAAAACCACAATTCACCCATT
GGAGAA------------------------------------------------------
------------------GAGAATGTAAAACTG---GCTGATGTAAAATTAAGAGAAAGT
TCC---------------------------------------------------------
---------------
>Spiny_dogfish_Aqp3C2
---------------------------------------------AGAATAAGAAATCTG
TTGGTAAGACAATGTCTTGCTGAATGTTTAGGAACGTTGATTCTTGTGTTGTTTGGCTGT
```

```
GGAGCATTGGCACAAATGACCCTCAGTAGGGGTACACACGGACAGTTTTTGACTGTTAAT
TTTGCCTTCGGATTTGCAGTGATGCTCGGTGTACTCCTGGCTGGCCAAGTCTCAGGAGCG
CACTTGAATCCTGCCGTGACCTTTGCTATGTGCTTACTTGCTCGCGAACCCTGGTTAAAA
TTTCCACTTTACTCTTTGGCACAAATACTAGGCGGCTTCCTTGGCTCTGGTATCATTTTC
GGTTTGTATTTTGATGCCATGTGGGACTTTAGTGGTCAAAACAAACTGTTAATATATGGC
CCCAAT---GCCACTGCTGGTATATTCGCTACATACCCATCTGTGCACTTAACTCCACTC
AATGGCTTTTTGATCAGCTGATTGGAACTGCCGCTCTCATAGTTTGCATCCTTAGCATT
GTGGATAAATTCAATAACCCGGTGCCAAAGGGACTGGAGGCATTTACTGTTGGCTTTACC
GTCCTGGTAATTGGCTTGTCTATGGGTTTCAACTCTGGGTATGCCGTGAACCCTGCCAGA
GACTTTGGACCTCGTTTGTTTACATCATTGGCTGGCTGGGGAGCTGAGGTTTTCATTGCT
GGAAACTACTGGTTTTGGATCCCTATTTTTGCCCCACTCCTTGGTTCTGTACTTGGTATT
CTGGTATATCAGCTTATGATTGGAATACACCTCGAGCCCGAAAACCACAATTCACCCATT
GGAGAA------------------------------------------------------
------------------GAAAATGTAAAACTG---GCTAATGTAAAATTAAGAGAAAGT
TCC---------------------------------------------------------
---------------
>Little_skate_Aqp3C2
ATGGGAAAACAGAAAGCAATGATCAAAAAAATTGAAAATCATTGCCGAATAGAAAATCTG
TTGGGAAGACAGTGTCTTGCTGAATGTCTAGGAACATTGATTCTTGTGTTATTTGGTTGT
GGAGCAGTGGCACAAGTTACCCTCAGCAGGGGTACACATGGACAATTTTTGACA------
------------------------------------------------------GGAGCT
CATCTGAACCCCGCCGTGACCTTTGCCATGTGCTTGCTTGCCCGTGAGCCCTGGATAAAG
TTTCCCCTTTACTCTTTGGCGCAAACACTTGGTGCCTTCCTAGCATCGTGTGTTATTTTT
GGTTTGTATTATGATGCGTTTTGGGACTTCAGTGGTCAAAACCAACTACTAGTTTACGGT
CCTAAT---GCCACGGCTGGAATTTTTGCAACGTATCCATCTGCACATTTAGGTTCATCC
AATGGTTTCTTTGATCAGGTGATTGGGACAGCTGCTCTCATAGTGTGCATCCTGGCCATT
GTGGACAAATGGAATACCCCAGTGCCTAACGGACTAGAGGCATTTACTGTTGGCTTTACC
GTGCTTGTAATCGGTTTGTCAATGGGTTTCAACTCTGGGTATGCGGTGAACCCAGCCAGA
GATCTTGGACCCCGTTTGTTTACATCATTAGCTGGCTGGGGAACTGAGGTTTTCACGGCT
GGAAACTACTGGTTTTGGATCCCCATTTTTGCCCCACTTCTTGGTTCTATACTTGGTATT
CTGGTATATCAATTAATGATCGGCATACACTTTGAGCCTAAAGAGCAGAGTTCACCCGCT
GGTGAA------------------------------------------------------
------------------GAAAATGTAAAACTC---GCAAATGTGAAATTAAGAGATGGT
TGC---------------------------------------------------------
---------------
>Thorny_skate_Aqp3C2
ATGGGAAAACAGAAAGCAATGATCAAAAAAATGGAAAGTTATTGCCGAATACAAAATCTG
TTGGGAAGACAGTGTCTTGCTGAATGTCTAGGAACATTGATTCTTGTGTTATTTGGTTGT
GGAGCAGTGGCACAAGTTACCCTCAGCAGGGGTTCACATGGACAATTTTTGACAGTTAAT
TTTGCATTCGGATTTTCAGTGATGCTTGGTGTGCTCATTGCTGGCAAAGTATCAGGAGCT
CATCTGAACCCCGCCGTGACCTTTGCCATGTGCTTACTTGCCCGCGAGCCCTGGATAAAG
TTTCCCCTTTACTCTTTGGCGCAAACACTTGGCGCCTTCCTAGCATCGTGTATTATTTTT
GGTTTGTATTATGATGCATTTTGGGACTTCAGTGGTCAACACCAACTATTAGTTTACGGT
CCTAAT---GCCACGGCTGGTATTTTCGCAACGTATCCATCTGCACATTTAGGTTCATCC
AATGGTTTCTTTGATCAGGTGATTGGGACAGCTGCTCTCATAGTGTGCATCTTGGCTATT
GTGGACAAATGGAATACCCCAGTGCCTAACGGACTAGAGGCATTCACAGTTGGCTTTACC
GTGCTTGTAATCGGCTTGTCAATGGGTTTCAACTCTGGGTATGCGGTGAACCCAGCCAGA
GATCTTGGACCGCGTTTGTTTACATCATTAGCTGGCTGGGGAACTGAGGTTTTCACGGCT
GGAAACTACTGGTTTTGGATCCCCATTTTTGCCCCACTTCTTGGTTCTATACTTGGTATT
CTGGTATATCAATTAATGATCGGCATACACCTTGAGCCTAAAGAGCAGAGTTCACCCGTT
GGGGAA------------------------------------------------------
------------------GAAAATCTAAAACTC---GCAAATGTGAAATTAAGAGAAGGT
TGC---------------------------------------------------------
---------------
>Smalltooth_sawfish_Aqp3C2
ATGGGAAAACAGAAGGCAATGATCAAAAAACTTGAAAATTCATTCAGAATACAAAATATG
TTGGGAAGACAATGTCTTGCTGAATGTTTAGGGACATTGATTCTTGTGTTATTTGGTTGT
GGAGCACTGGCCCAAGTTACCCTCAGCAGGGGTACACATGGACGTTTTTTGACTGTTAAT
TTTGCCTTCGGATTTTCAGTGATGCTCGGTGTGCTCATAGCTGGCCAAGTGTCAGGAGCA
CACCTGAATCCTGCCGTGACCTTTGCCATGTGCTTACTTGCCCGTGAACCCTGGATAAAG
TTTCCCCTTTACTCTTTGTCACAAATACTAGGTGCCTTCCTAGGTTCGGGCGTTATCTTT
```

GGTTTGTATTTTGATGCTATGTGGGACTTCAGTGGTCAAAATCAACTATTAGTTTATGGT
CCTAAT---GCCACAGCTGGTATTTTTGCTACATATCCATCTCCACACTTAAGTTCATCC
AATGGCTTTTTTGATCAGATGATTGGGACGGCTGCTCTCATAGTGTGCATCCTTACTATT
GTGGACAAATGGAACAACCCAGTGCCAAAGGGACTGGAGGCATTTACTATTGGATTTACG
GTGCTGGTAATTGGCTTGTCGATGGGTTTCAACTCTGGGTATGCTGTGAACCCTGCAAGA
GACCTTGGACCTCGTTTGTTTACATCAGTAGCTGGCTGGGGATCTGAGGTTTTCACGGCT
GGAAACTACTGGTTTTGGATCCCTATTTTTGCCCCACTCCTTGGTTCTGTTCTTGGTATT
CTGGTATATCAACTAATGATTGGCATACACCTTGAGCCTGAAGACCAGAGTTCACCCATT
GGAGAA--
------------------GAAAATGTAAAACTG---GCAAATGTAAAATTAAGAGAAAGT
TGC---

>Ghost_shark_Aqp3C2
ATGGGGAAACAGAAAGAAACCATCAGGAAAATTCAAGACTTATTCCGTATAAGAAACATG
CTGGTAAAACAATGCCTTGCTGAATGTCTGGGGACTTTAATTCTCGTGCTGTTTGGCTGC
GGGGCACTGGCACAGATCACTCTCAGTCGGGGAACGCACGGAGCCTTTCTAACAGTCAAC
TTAGCCTTCGGATTTGCGGTGACTCTTGGTGTGCTTGTGGCCGGTCAAGTGTCGGGAGCT
CACCTGAATCCTGCTGTGACCTTTGCTTTGTGTCTACTTGCTCGTGAGCCCTGGATCAAA
TTTCCCCTTTTCTCTTTGGCACAAATATTGGGTGGATTTCTTGGAGCAGGTATCATCTTT
GGATTATATTTTGATGCAATTTGGCTCCATGGTAAT---AACCATCTGATAGTAATGGGA
CCTAAT---GCTACTGCTGGGATATTTGCGACTTACCCGTCTGAACACTTGACTTTAATC
AATGGTTTCTTTGATCAGCTGATTGGCACAGCAGCTCTCATCGTCTGTATCCTTGCCATT
GTGGATCCATTCAACAACCCGGTGCCAAAGGGAGTGGAGGCCTTCACTATTGGCTTTGTG
GTTCTGGTAATCGGCTTGGCAATGGGCTTTAACTGTGGCTACGCTGTGAACCCAGCCAGA
GATTTTGGACCTCGCTTGTTTACCTCACTGGCTGGCTGGGGCACCAAAGTCTTCAGTGCT
GGAAACTACTGGTTTTGGGTGCCCATTCTAGCCCCGCTCCTTGGTGCAGTGCTTGGTATT
CTGGTTTACCAGCTGATGGTTGGCATACACACCGAACCCGAAGAAAACCATTCATCCAGT
GCAGAA--
------------------GAAAGAATAAAACTG---GCCAACATGAAGCCGAAAGAAAAC
TGC---

>Smalleyed_rabbitfish_Aqp3C2
ATGGGGAAACAGAAAGAAACCATCAGGAAAATTCAAGAGTTATTCCGTATTCGAAACGAG
TTGGTAAAACAGTGCCTTGCTGAATGCCTAGGGACGTTAATTCTCGTGCTGTTTGGCTGT
GGGGCAGTGGCCCAGGTTACACTCAGTCGGGGAACACATGGAGTCTTTCTAACAGTCAAT
TTAGCCTTCGGATTTGCAGTGACTCTTGGCGTGCTGATGGCTGGTCAGGTGTCAGGAGCT
CACCTGAATCCTGCTGTGACCTTTGCTATGTGCCTACTTGCTCGCGAGCCCTGGATCAAA
TTTCCCCTTTACTCTTTGGCACAAATATTGGGTGGTTTTCTCGGAGCTGGTATCATCTTT
GGATTGTATTTTGATGCAATTTGGCTCCATGCCAAT---AACCATCTGGTAGTAATGGGA
CCTAAC---GCTACTGCTGGGATATTTGCCACCTACCCATCTGAACACTTGACTTTAGTC
AATGGTTTCTTTGATCAGCTGATTGGCACGGCGGCCCTCATCGTGTGTATCCTCGCCATT
GTGGATCCGTACAACAACCCGGTGCCAAAGGGAGTGGAGGCCTTCACTATTGGCTTTGTG
GTGCTGGTAATTGGTTTGTCAATGGGCTTCAACTGTGGCTACGCTGTGAACCCAGCCAGA
GATTTTGGACCTCGGCTGTTTACGTCTATGGCTGGTTGGGGGTCCAGTGTCTTTAGGGCT
GGAAACTACTGGTTTTGGGTCCCAATCCTAGCTCCCCTCCTGGGTGCAGTGCTTGGTATT
CTGGTTTACCAGCTGATGATTGGCATACACACTGAGCCCGAAGACCACCATTCATCCAGC
ACAGAA--
------------------GAAAGAATAAAACTG---TCCAACGTGAAGCCAAAAGAAAAC
TGC---

>B_bambooshark_Aqp3C1
ATGGAAAGACAAAAAGAAATCATCAGAAATATGACAACTATCCTGAAAGTCCGAAGTCTT
CTGTTGAAACAATGCCTTGCTGAATGTTTAGGAACTTTGATTCACACGATGCTTAGCTGT
GGAGGAGTAGCACAATTTACTCTCAGTTATGGCACACACAAAGAATTCTTGACTGTTACA
ATTGCTGGTGGATTTGCAGTAGCTCTGGGTATATTGGTAACTAGTAAAGTCTCAGGAGCT
CACCTGAATCCTGCAGTGACCTTTGCTTTGTGCTTGCTTGCTTGTGAACCTTGGTTAAAA
TTTCCTTTATTCTTTTTGGCACAAACGTTTGGTGCCTTTCTTGGATCAGGAATAATGTTT
GGTTTGTACTATGATACATTGTGGCATTATGGCAAT---AAACAGCTAACAGTAATTGGA
GCAAAT---TCTACTGCTGGAATATTTACAACTTATCCACATGAACATTTGAGCGCAGTC
AATGGCATTTTTAATCAGGCAATTGGGACTGCAGCACTTATACTTTGTATCCTTATCATT
GTGGATCCATTGAACGATGCTATACCAACAGGACTAGAAGCTTTTACAATTGGCTTTGTG

GTTTTGCTAATTGGCTGGTCAATGGGTTCCAATTCTCAGTACTCATTAAATCCTGCCAGA
GATATTGGACCTCGTCTGTTTACTGCAATTGCTGGTTGGGGAACTGAAGCTTTCACGGCT
GGCAATTATTGGTTTTGGATCCCAGTTGCCAGTCCAATCATTGGTGCCATATTTGGTGTT
CTCCTGTACAAGTTCATTGTTGGATTGCGTGCTGAAGCAAGAACCAGCTGCCCATCCAGT
GCAGAA--
------------------CAAAATATAAAGTTA---ATGAACCAAAAACCAAAGAGGAGG
CTC---

>W_bambooshark_Aqp3C1
ATGGAAAGACAAAAAGAAATCATCAGAAATATGACAACTATCCTGAAAGTCCAAAGTCTT
CTGTTGAAACAATGCCTTGCTGAATGTTTAGGAACTTTGATTCACACGATGCTTAGCTGT
GGAGGAGTTGCACAATTTACTCTCAGTTATGGCACACACAAAGAATTCTTGACTGTTACA
ATTGCTGGTGGATTTGCAGTAGCTCTGGGTATATTGGTAACTAGTAAAGTCTCAGGAGCT
CATCTGAATCCTGCAGTGACCTTTGCTTTGTGCTTGCTTGCTTGTGAACCTTGGTTAAAA
TTTCCTTTATTCTTTTTGGCACAAACGTTAGGTGCCTTTCTTGGATCAGGAATAATGTTT
GGTTTGTACTATGATACATTGTGGCATTATGGCAAT---AAACAGCTAACAGTAATTGGA
GCAAAT---TCTACTGCTGGAATATTTACAACTTATCCACATGAACATTTGAGCGCAGTC
AATGGCATTTTTAATCAGGCAATTGGGACTGCAGCACTTATACTTTGTATCCTTATCATT
GTGGATCCATTGAATGATGCTATTCCAACAGGACTAGAAGCTTTTACAATTGGCTTTGTG
GTTTTGCTAATTGGCTGGTCAATGGGTTCCAATTCTCAGTACTCATTAAATCCTGCCAGA
GATATTGGACCTCGCCTGTTTACTGCAATTGCTGGTTGGGGAACTGAAGCTTTCACGGCT
GGCAATTATTGGTTTTGGATCCCAGTTGCCAGTCCAATCATTGGTGCTATATTTGGTGTT
CTCCTGTACAAGTTCATTGTTGGATTGCGTGCTGAAGCAAGAACCAGCTGCCCATCCAAT
GCAGAA--
------------------CAAAATATAAAGTTA---ATGAACCAAAAACCAAAGAGGAGG
CTC---

>Whale_shark_Aqp3C1
ATGGAAAAACAAAAAGAAATCATCAGAAAAATGACAACTGTCCTGAAAGTCCGAAGTCTT
CTGATGAAACAATGCCTTGCTGAATGTTTAGGAACTTTGATTCACACAATGCTTAGCTGT
GGAGCAGTAGCACAATTTACTCTCAGTTATGGCACACACAAAGAATTCTTGACTGTTACA
ATTGCTGGTGGATTTGCAGTAGCTCTGGGTATATTGGTAACTAGTAAAGTCTCAGGAGCT
CACCTGAATCCTGCAGTGACCTTTGCTTTGTGCTTGCTTGCTAGTGAACCTTGGTTAAAA
TTTCCATTATTCTTTTTGGCACAAACATTAGGTGCCTTTCTTGGATCAGGAATAATGTTT
GGTTTGTATTATGATGCATTGTGGCATTATGGTAAT---AAACAGCTAACAGTAATTGGA
GCAAAT---TCTACTGCTGGAATATTTGCTACTTATCCACATGAACATTTGAGTGCAGTC
AATGGCATTTTTGATCAGGCAATTGGGACTGCAGCACTTATACTTTGTATCCTTATCATT
GTGGATCCATTAAACAACTCAGTACGGACAGGACTAGAAGCTTTTACAATTGGCTTTGTG
GTTTTGCTAATTGGCTGGTCAATGGGTTCAAATTCCCAATACTCATTAAATCCTGCCAGA
GATATTGGACCTCGCCTGTTTACTGCAATTGCTGGTTGGGGAACTGAAACTTTCACGGCT
GGTAATTATTGGTTTTGGATTCCAATTGCCAGTCCAATCATTGGTGCCATATTTGGTGTC
CTACTGTATCAGTTCATTGTTGGATTGCGTGCTGAAGCAAGAACCAGCCGCCCAACTAAT
GCAGAA--
------------------GAAAATGTAAAGTTA---ATGAGTCAGAAACCAAAGGGGAGG
CTCCGA--

>Cloudy_catshark_Aqp3C1
ATGGAAAAACAAAAAGAAATCCTGCGAAAAATGACTACAATGCTAAAGGTCCGAAGCCTT
CTGATGAAACAATGCCTTGCTGAATGTTTAGGGACTTTAATTCATACAATGCTGAGCTGT
GGAGCGACAGCGCAGTTTGTTCTGAGTTGTGGTGCACACAAAGACTTTTTGACTGTTACT
TTTGCTGGTGGATTTGCAGTGGCTCTGGGTATATTGGTAACTAGTAAAGTCTCAGGAGCT
CACCTGAATCCCGCAGTGACCTTTGCTTTGTGCTTACTCGCCTGTGAACCCTGGTTAAAA
TTTCCTTTCTTCTTTTTGGCACAAACATTAGGTGCCTTCCTTGGATCAGGAATAATGTTT
GGTTTGTATTACGATAAATTGTGGCATTATGGTAAT---AAACAGCTGACAGTATTTGGA
GCAAAC---TCTACTGCTGGAATATTTGCTACTTATCCACATGAACATTTGAGTGCAGTA
AATGGCATTTTTGAACAGGCAATTGGGACTGCGGCACTTATACTTTGCATCCTCATCATT
GTGGACCCATTGAACAACTTAGTGCCAACAGGACTGGAAGCTTTTACCATTGGCTGTGTG
GTTCTGATGATTGGCTGGTCAATGGGTTTAAATTCCCAGTACTCATTAAATCCCGCCAGA
GATATTGGACCTCGCTTGTTTACTGCAATTGCTGGTTGGGGATCTGAAGTTTTCAGTGCT
GGCAACTATTGGTTTTGGATCCCAGTTGCCAGCCCAATCATTGGTGCCATACTTGGTGTT
CTGATCTATCGTTTCATTGTTGGATTGCGAGCTGAAGCAAAAACCAGTTGCTCACCCAAT

GCAGAA--
------------------CAAAATGTAAAGTTA---TTGAGCCAGAAACCAAAGGGGAGG
TGC---

>Blue_shark_Aqp3C1
ATGGAGAAACAAAAAGGAATCCGCAGAAAAATGACGATTATGCTGAAGGTTCGAAGTCTT
CTAATGAAGCAATGCCTTGCTGAATGTTTAGGAACTTTAATTCATACAATGCTTAGCTGT
GGAGCAGGAGCACAATATGTTCTCAGTTATGGGACACACAAAGACTTTTTGACTGTTACT
TTTGCCAGTGGATTTGCAGTGGCTCTGGGTATATTGGTAACTAATAAAGTCTCAGGAGCC
CACCTGAATCCTGCAGTGACCTTTGCTTTGTGCTTACTCGCTTGTGAACCTTGGTTAAAA
TTTCCTTTCTTCTTTTTGGCACAAACATTAGGTGCCTTTCTTGGATCAGGAATAATGTTT
GGTTTGTATTATGATAAACTGTGGCATTATGGTAAT---AAACAGCTAACAGTAATTGGA
GCAAAC---GCTACTGCTGGAATATTTACTACTTACCCACATGAACATTTGAGTGCAGTA
AATGGCATTTTTGATCAGGCAATTGGGACGGCAGCACTTATACTTTGCATCCTTATCATT
GTGGACCCATTGAACAATTCAGTGCCAACAGGGCTGGAAGCTTTTACCATTGGCTGTGTA
GTTCTGATGATTGGCTGGTCAATGGGTTCAAACTCCCAGTACTCATTAAATCCTGCCAGA
GACATTGGACCCCGCTTGTTCACTGCAATTGCTGGTTGGGGATCTGAAGTTTTCACTGCT
GGCAATTATTGGTTTTGGATCCCAGTTGCCAGCCCAATCATTGGTGCCACATTTGGTGTT
CTGGTCTACAAGTTCATTGTTGGACTGCGAGATGAAGCAAGAACCAGCTGCCCATCCAAT
GGAGAA--
------------------CGAAATGAAAAGTTA---ATGAGCCAAAAACCAAAGGGAAGG
CGC---

>Great_white_shark_Aqp3C1
ATGGAAAAA---AAAGAAATCCTCAGAAAAATGACGACTATACTGAAGGTCCAAAGTCTT
CTGATGAAACAATGCCTTGCTGAATGTTTAGGAACTTTAATTCATACCATGCTTAGCTGT
GGAGTGATAGCACAATTTGTTCTCAGTTATGGTACGCACAAAGAGTTTTTGACTGTTACT
TTTGCCAGTGGATTTGCAGTAGCTCTGGGTATATTGGTAACGAGTAAAGTCTCAGGAGCT
CACCTGAATCCTGCAGTGACCTTTGCTTTCTGCTTACTTGCTTGTGAACCCTGGTTAAAA
TTTCCCTTCTTCTTTTTGGCACAAACATTAGGTGCCTTTCTTGGATCAGGAATAATGTTT
GGTTTGTATTATGATAAATTGTGGCATTATGGTAAT---AAACAGCTAACAGTAATTGGA
GCAAAC---TCTACTGCTGGAATATTTACTACTTATCCACATGAACATTTGAATGTAGTA
AATGGCATTTTTGATCAGGCAATTGGGACTGCAGCGCTTATACTTTGTATCCTTATCATT
GTGGACCCATTGAACAACTCAGTGCCAACAGGACTGGAAGCTTTTACCATTGGCTTTGTG
GTTCTGATAATTGGCTGGTCAATGGGTTCAAATTCCCAGTACTCATTAAATCCTGCCAGA
GATATTGGACCTCGCTTGTTTACTGCAATTGCTGGTTGGGGATCTGAAGTTTTCAGTGCT
GGCAGCTATTGGTTTTGGATCCCCGTTGCCAGCCCAATCATTGGTGCCATATTTGGTGTC
CTGATGTATCAGTTCATTGTTGGATTGCGAACTGAGGCAAGAACCAACTGCTCACCCAAT
GCAGAA--
------------------GAAAATGTAAAGTTA---ATGAGCCAGAAACCAAAGGGGAGG
TGC---

>Zebra_bullhead_shark_Aqp3C1
ATGGAAAAACAAAAAGAAATGCTCAGAAAAATGACAACTATACTGAAAGTCCGAAGCCTT
CTGCTGAAACAATGTCTTGCTGAATGTTTAGGAACTTTAATTCATACAATGCTTAGCTGT
GGAGCAGTGGCACAATTTACTCTCAGTTATGGTACACAGAAAGAATTCTTGACTGTTACT
TTTGCCATTGGATTTGCAGTAGCTCTGGGTATATTGGTAACTAGTAAAGTGTCAGGAGCT
CACCTGAATCCTGCAGTGACCTTTGCTTTGTGCTTGCTTGCTTGTGAGCCTTGGTTAAAA
TTCCCCTTCTTCTTTGTGGCACAAACATTCGGTGCCTTTCTTGGATCAGGAATAATGTTT
GGTTTGTGTTACGATAATTTGTGGCATTATGGTAAT---AAACAGCTAACAGTAATTGGA
CCAAAC---TCTACTGCTGGAGTATTTACTACTTATCCACATGAACATTTGAGTGCAGTA
AATGGCATTTTTGATCAGGCAATTGGGACTGCAGCTCTTATACTTTGTATCCTTATCATT
GTGGATCCATTGAACAACTCAGTGCCAACAGGACTGGAAGCCTTTACCATTGGCTTTGTG
GTTCTGATAATTGGTTGGTCAATGGGTTCAAATTCCCAGTACTCATTAAATCCTGCCAGA
GATATTGGACCTCGCTTGTTTACTGCAATTGCTGGTTGGGGATCTAAGGTTTTCACTGCT
GGCAACTATTGGTTTTGGATCCCAATTGTCAGCCCAATCATTGGTGCCATTTTTGGTGTT
CTGATGTATCAGTTCATTATTGGATTGCGTATTGAAGCAAGGCCCAGCTGCTCACCCATT
GCAGAA--
---------------------AATGTAATGTTA---ATGAGCCAGAAACCAAAGGGGAGA
CGCCGA--

>Spiny_dogfish_Aqp3C1
ATGGGAAAACAAAAAGAGATCCTCAGAAAAATGACAACGACACTGAAAGTCCGAAGTATT
CTGGTGAAACAATGTCTTGCTGAATGTTTAGGAACTCTAATTCATACAATGCTTAGCTGT
GGAGCAATAGCACAATTTACTCTCGGTTATGGTACACACAAAGAATTTTTGACGGTTACT
TTTGCCATCGGATTTGCAGTAGCTCTGGGTATAATGGTAACTAGTAAAGTGTCAGGAGCT
CACCTGAATCCTGCAGTGACCTTTGCTTTGTGCTTGCTTGCTTGTGAGCCTTGGTTAAAA
TTCCCCTTCTTCTTTTTGGCACAAACATTCGGTGCCTTTCTTGGATCAGGAATAATGTTT
GGTTTGTATTACGATAAATTGTGGCATTATGGTAAT---AAACAGCTAACAGTAATGGGA
CCAAAC---TCTACTGCTGGAATATTTACTACTTATCCGCCAGAACATTTGAGTGCAGTC
AGTGGCATTTTTGATCAGGCAATTGGGACGGCAGCTCTGATACTTTGTATCCTTATCATT
GTGGATCCAATGAACAAGCCAGTGCCAACAGGACTGGAAGCCTTTACCATTGGCTTTGTG
GTTCTGATAATTGGCTGGTCAATGGATTCAAATTCCCAGTACTCATTAAATCCTGCCAGA
GATATTGGACCTCGCTTGTTTACTGCAATTGCTGGTTGGGGATCTGAAGTTTTCACTGCT
GGAAACTATTGGTTTTGGATCCCACTTGTCAGCCCAATCATTGGTGCCATTTTTGGTGTT
CTGATGTATCTATTCATTGTTGGATTGCGTGTTGAAGCAAGAAGCGGCTGCTCACCCAAT
GCAGAA--
------------------CAAAATGTAAAGTTA---ATGAGCCAGAAACCAAAGGGGAGG
CGC---

>Little_skate_Aqp3C1
ATGGGGAAACAAGAGGAGATCCTCAGAAAATTGGCAATTCTGTTGAGAATTCGAAGTATT
CTAGTAAAGCAATGCCTGGCCGAATGCTTGGGAACATTAATTCATACCATGCTTAGCTGT
GGAGCAATAGCACAGTTCACACTTAACTGTGGCACACACAGTCAATTTCTGTCTGTCACC
TTTGCCATAGGATTTGCAGTAGCTCTGGGCATATTGGTAACCAGCAAAGTGTCAGGAGCT
CACCTGAATCCAGCAGTGACCTTTGCTTTGTGCTTACTTGCTTGTGAGCCTTGGTTAAAA
TTCCCTTTCTTCTTTTTGGCACAAACAGTGGGTGCCTTTCTTGGATCAGGAATAATGTTT
GGTTTGTATTATGATAAATTGTGGCTTCATGGAAAT---AAACAGCTAACGGTAATTGGA
CCAAAC---TCAACTGCTGGGATATTTACAAGTTTTCCACTTGAACATGTGAGTGCACTC
AATGGCATTTTTGATCAGGTGATTGGGACTGCAGCTCTCATATTGTGTATCCTTATCATT
GTGGATCCTTTACACACCCCAGTGCAGACGGGACTGGAAGCTTTCACCATCGGTGTAGTG
GTTCTTATTATTGGCTGGTCAATGGGATCAAATTCCCAGTACTCATTAAATCCTGCCAGG
GATATTGGACCTCGCTTGTTTACGGCTATTGCTGGTTGGGGATTTGAAGTTTTCACGGCT
GGAAGCTATTGGTTTTGGATTCCACTTGTCAGCCCAATCATTGGTGCTATTTTGGGTGTT
CTGATGTACCAATTCACTGTGGGATTACGTGTTGATACAAGAGATGACTCCTCATCCAAT
GCAGAG--
------------------CAAAATGTAAAATTA---ATGGGCCAGGAAACAAAGGGAAGG
TGC---

>Thorny_skate_Aqp3C1
ATGGGGAAACAAGAGGAGATCCTCAGAAAAATGGCAATTCTGTTGAGAATTCGAAGTATT
CTAGTAAAGCAATGCCTGGCCGAATGCTTGGGAACATTAATTCATACCATGCTTAGCTGT
GGAGCAATAGCACAGTTCACACTTAACTGTGGCACACACAGTCAATTTCTGTCTGTCACC
TTTGCCATAGGATTTGCAGTAGCTCTGGGCATATTGGTAACCAGCAAAGTGTCAGGAGCT
CACCTGAATCCAGCAGTGACCTTTGCTTTGTGCTTACTTGCTTGTGAGCCTTGGTTAAAA
TTCCCTTTCTTCTTTTTGGCACAAACAGTGGGTGCCTTTCTTGGATCAGGGATAATGTTT
GGTTTGTATTACGATAAATTGTGGCTTCATGGAAAT---AAACAACTAACGGTAATTGGA
CCAAAC---TCAACTGCTGGGATATTTACAAGTTTTCCACTTGAACATGTGAGTGCACTC
AATGGCATTTTTGATCAGGCAATTGGGACTGCAGCTCTCATATTGTGTATCCTTATCATT
GTGGATCCTTTACACACCCCAGTGCAGACGGGGCTGGAAGCTTTCACCATCGGTGGTGTG
GTTCTTATTATTGGCTGGTCAATGGGATCAAATTCCCAGTACTCATTAAATCCTGCCAGG
GATATTGGACCTCGCTTGTTTACGGCTATTGCTGGTTGGGGATCTGAAGTTTTCACGGCT
GGAAGCTATTGGTTTTGGATTCCACTTGTCAGCCCAATCATTGGTGCTATTTTGGGCGTT
CTGATGTACCAATTCACTGTGGGATTACGTGTTGATACAAGAAATGACTCCTCATCCAAT
GTAGAG--
------------------CAAAATGTAAAATTA---ATGGGCCAGGAAACAAAGGGAAGG
TGC---

>Smalltooth_sawfish_Aqp3C1
ATGGGGAAACAAGAGAAGATCCTCAGGAAAATGACGATTGTGCTGAGAATTCGAAGTATT
CTGATAAAGCAATGCCTGGCTGAATGTTTGGGAACTCTAATTCATACAATGCTTAGCTGT
GGAGCAATAGCACAATTTACACTCAACTGTGGCACACACAAAGAATTTCAGTCCGTAACT

TTTGTAATTGGATTTGCTGTAGCACTGGGCATATTGGTAACCAGTAAAGTGTCAGGAGCT
CACCTGAATCCAGCAGTGACCTTTGCTTTGTGCTTGCTTGCTTGTGAGCCTTGGTTAAAA
TTCCCCTTCTTCTTTATGGCCCAAACAATAGGTGCCTTTCTTGGATCAGGAATAATGTTT
GGATTGTTATTAGATAAATTGTGGCTTCATGATAAT---AAACAGCTAACAGTTATTGGA
CCAAAC---TCGACTGCTGGGATATTTACAACTTTTCCACTTGAACATGTGAGTGCACTC
AATGGCATTTTTGATCAGGCAACTGGAACTGCAGCTCTCATATTTTGTATCCTTATCATT
GTGGATCCTTTGCACACCCCAGTGCGAACAGGGCTGGAAGCCTTTACCATTGGCTTAGTA
GTTCTGATTATTGGCTGGTCAATGGGTTCAAACTCCCAGTACTCACTAAATCCTGCCAGG
GATATTGGACCTCGCTTGTTTACAGCAATTGCTGGTTGGGGATCTGAAGTTTTCACGGCT
GGAAGCCATTGGTTTTGGATCCCGCTTGTCAGCCCAATCATTGGTGCCATTTTGGGTGTT
TTGATGTACCAGTTCATTGTTGGATTACGTGTTGATGCAAGAAATGGCTGCTCATCCAAT
GCAGAA--
-------------------CAAAATGAAAAATCA---GTGAGCCAGAAAACGAAGGGAAGG
TGC---

>Ghost_shark_Aqp3C1
ATGGGGATACAAAGAGAAACCCTGAGAAGGATGGCAACGACATTCAGAGTGCGGAGCGTG
CTGGCAAAGCAATGCCTGGCGGAATGTTTGGGAACGTTGATCCACACGATGCTCAGCTGT
GGAGCAATGGCACAATACACCCTCAGTCGCGGTGAGCAGAGAGAGTTTTTGACTCTTACT
TTCACCCTTGGCTTTGCGGTGGCTCTTGGTATACTTGTAACCGGTAAAGTGTCAGGAGCT
CACTTGAATCCTGCTGTGACCTTTGCTTTGTGCTTACTTGCTTGTGAACCCTGGATAAAA
TTCCCCTTCTTCTTTGTGGCACAAACATTGGGAGCATTTTTTGGATCTGGGATAATGTTT
GGCTTGTATCACGATGCAATAATGCACTATGGTAAC---AATCAACTCACAATCATTGGA
CCCAAT---TCCACTGCTGCTATATTTACCACATACCCATCCGAAGATTTGACTGTGGTG
AATGGGTTTCTTGACCAGGTCATCGGCACGGCCGCTCTCATATTCTGTATCCTGACCATC
GCGGATCCACTGAAAAGACCGGTGGCCACAGGGCTGGAGGCTTTCACCATAGGCTTTGTG
GTTCTAATCGTTGGCTTGTCCATGGGCTTCAACTCCAAGTATTGCTTAAATCCTGCCAGA
GACATTGGACCTCGTTTGTTCAGCGCCATGGCTGGCTGGGGATATGACGTTTTCAGCACA
GGGAACTATTGGTTTTGGGTGCCAGTTCTCAGCCCAATCGTCGGGGCCATGTTTGGCGTT
CTGGTCTATCAGTTCATGGTGGGTCTGCAGGTTGAGACAAAAAGTAACTGCACGTCCCAT
TCAGAG--
-------------------GAAACCGTAAAGTTA---ACCAGCGTGAAAGCGAAGGGCAGG
CGT---

>Smalleyed_rabbitfish_Aqp3C1
ATGGGGATACAAAGAGAAACCCTGAGAAGGATGGCAACCACGTTCAAAGTGCGGAGCGTG
CTGGCAAAGCAATGCCTGGCAGAATGTTTGGGAACCTTGATACACACGATGCTGAGCTGT
GGAGCAGTGGCACAATATACCCTCAGCCGTGGCGAGCGGAGAGAGTTTTTGACTCTTACT
TTCACCCTTGGCTTTGCGGTGGCTCTCGGTATAATTGTAACTGGTAAAGTATCAGGAGCT
CACTTGAATCCTGCGGTGACCTTTGCTTTGTGCTTGCTTGCTTGTGAACCCTGGATAAAA
TTCCCCTTCTTCTTTGTGGCACAAACAGTGGGAGCCTTTTTTGGATCTGGGATAATGTTT
GGTTTGTATCACGAAGCAATAATGGACTATGGCAAT---AATCAACTCACAGTCATTGGA
CCGAAT---TCCACCGCCGGTATATTCACCACATACCCATCCGAAGATGTGACTGTGGTG
AATGGATTTCTTGACCAGGTCATTGGCACGGCCGCTCTCATATTCTGCATCCTGACCATC
GTGGATCCACTGAACAGACCCGTGGCCACAGGGCTGGAGGCTTTCACCATTGGCTTCATG
GTTCTAATCGTTGGCCTATCCATGGGCTTCAACTCCAAGTATTGCTTAAACCCTGCCAGA
GACATAGGACCACGCTTGTTCAGCGCCATGGCTGGCTGGGGATATGATGTTTTCAGCACA
GGGAACTATTGGTTTTGGGTGCCAGTGCTTAGCCCAATCGTCGGAGCCATGTTTGGTGTG
CTGGTCTATCAATTCATGGTGGGTCTGCAGGTTGAGACAAAAAATAACTGCGCGTCCCAT
TCAGAG--
-------------------GAAACCGTGAAGTCA---ACCAGCCTGAAAGCAAAAGGGAGG
CAT---

>Sea_lamprey_Aqp3L1
ATGCCGGTGGTAGAGGGGCGAATTAAGAGGCTGCAGCGGAGGCTGTTCATCCGAAATACG
CTTGTCTGCGAGGCGATGGCAGAGTTCGTGGGGACGTACATTCTGGTTTTGTTTGGGTGT
GGCTCGGTGGCGCAGGTTGAATTGAGTGGAGGACTCAAGGGGCAGTTCCTCACGATCAAC
CTCGCCTTTGGGTTTGCGGTCACCATGGGACTGCACGCCGCTGCAGGCGTTTCAGGTGGA
CACCTGAACCCAGCCGTTACATTTGCGTTTGCCGTTCTGGGTCGCTTCGAATGGCACAAG
TTACCTCTCTACATGCTGGCTCAGCTCCTCGGAGCTTTCATGGGCGCAGGCACCGTGTTC
GGACTGTATTATGATGCCTTCCTGTTCGCCGCCAAT---GGGAACTACACAATT------

---------CAGCTGGCTGGAGTTTTTGCCACTTACCCGTCAGAGCACCTGTCTCTGGGA
AATGGATTTGTGGATCAGCTGATTGGCACTGCAGCCCTGCTGGTGTGCATCATGGCGGTG
ATAGACAAGCGTAACAACCCGGCTCCAAAGGGCATGCAGCCCTACATAATAGGCCTCGTC
GTCGTCCTCATTGGCCTCTCCATGGGCTTCAACGCGGGGTACGCCGTGAACCCGGCCCGT
GACCTCGGCCCGCGTCTCTTCACGTCCATTGCTGGATGGGGTTGGGCAGTCTTTTCGTCC
GGTAACTACTGGAGCTGGGTGCCTGTGGTGGCACCCATGATCGGTGGGGTGCTCGGTGCG
TTTTTTTACGAGCTGTTCGTTGGATTTCATCTTATGGAGGCGTCACTCACGCCAGCGGCG
GAGCCCCCACCGTCTCCACGA---------------------------------------
------------------GAAAAAAAGAGCCTC---CTCAAGCTCCAGCCGGAAAGTATG
GCTGATGTCATGTGC---

>Arctic_lamprey_Aqp3L1
ATGCCGGTGCAAGAGGGGCGAATGAAGAGGCTGCAGCGGAGGCTGTTCATCCGAAATAGG
CTGGTCTGCGAGGCGATGGCAGAGTTTGTGGGGACGTACATTCTGGTTTTGTTTGGGTGT
GGCTCGGTGGCGCAGGTTGAATTGAGTGGAGGACTCAAGGGGCAGTTCCTCACGATCAAC
CTCGCCTTTGGGTTTGCGGTCACCATGGGACTCCACGCCGCTGCAGGCGTTTCAGGTGGA
CACCTGAACCCAGCCGTTACATTTGCGTTTGCCGTTCTGGGTCGCTTCGAATGGCACAAG
TTACCTCTCTACATGCTGGCTCAGCTCCTCGGAGCTTTCATGGGCGCAGGCACCGTGTTC
GGACTGTATTATGATGCCTTCCTGTACGCCGGCAAT---GGGAACTACACAATT------
---------CAGCTGGCTGGAGTTTTTGCCACTTACCCCTCAGAGCACCTGTCTCTGGGA
AATGGATTTGTGGATCAGCTGATTGGCACTGCAGCCCTGCTGGTGTGCATCATGGCGGTG
ATAGACAAGCGAAACAACCCAGCTCCAAAGGGCATGCAGCCCTACATAATAGGCCTCGTC
GTCGTGCTCATCGGGCTCTCCATGGGCTTCAACGCGGGGTACGCCGTGAACCCGGCCCGT
GACCTCGGCCCGCGTCTCTTCACGTCCATTGCTGGATGGGGTTGGAAAGTATTTTCGTCC
GGTGGCTACTGGAGCTGGGTGCCTGTGGTGGCACCCATGATCGGTGGGGTGCTCGGTGCG
TTTGTTTACGAGCTGTTCGTTGGATTTCATCTTGTGGAGGCGTCACTCCCGCCGGCGGTG
GAGCCCCCACCGTCTCCACGA---------------------------------------
------------------GAAAAAAAGGGGCTC---CTCAAGCTCCAGCCAGAAAGCATG
GCTGACGTCATGTGC---

>Sea_lamprey_Aqp3L2
ATGCCGTCGCACGATGGTCGCTTCAAGCAGCTGGAGCGCAAGCTGCACGTGAAGAACGTG
ATGATCCGTGAGGCCTTGGCCGAGTTCATGGGAACCTTCCTTCTCGTCCTGTTTGGCTGC
GGATCGGTGGCGCAGGTGGAGCTGAGCGACGGCACCAAGGGGCGGTTCCTCACCATCAAC
CTCGCCTTCGGCTTCGCCGTCACCATGGGCGCCTACTGTGCAGCCGGCGTCTCCGGCGCG
CACTTGAACCCGGCGGTGTCGATGGCGCTGGCGGTGCTCGGCCGCTTCTCTTGGAGCAAG
TTCCCCTTGTACGTCACGGCTCAGCTGCTCGGGGCCTTCATGGGCGCCGGCACCGTCTTC
GGCCTCTACTACGACGCCTTCATGTACGTCTCCAAA---GGGAACCTGACGCTG------
---------CAGCTCGCAGGGGTCTTCGCCACCTTCCCCTCACCGCATCTCTCCATCGGA
AACGGATTTGTGGATCAGCTGATCGGCACGGCAGCGCTGCTCGTGTGCATCCTCGCCGTG
ATCGACAAGCGCAACAACCCGGCGCCGCGCGGCATGCAGCCCTTCCTCATCGGCCTCGTC
GTCGTGCTCATCGGCCTTTCCATGGGCTTCAACGCCGGCTACGCCGTGAACCCCGCGCGC
GACCTCGGCCCCCGCATCTTCACCGCCCTCGCCGGCTGGGGCTGGCAGGTCTTCTCGGCG
GGCAACTACTGGAGCTGGGTGCCCGTGGTGGCCCCCATGCTGGGCGGCGTGCTGGGGGCC
TTCATCTACGAGATCTTCATTGGCCTGCACCTCCCCGAAGAGCCGGCCTGCTCCGGACCC
GAGCCC---GGGACCCCGCGG---------------------------------------
------------------TCGCACCAGGAGCTC---GCCAAGCTGCAGTCCAGC---ATG
GCAGACGCTATGTGC---

>Arctic_lamprey_Aqp3L2
ATGCCGTCGCACGATGGTCGCTTCAAGCAGCTGGAGCGCAAGCTGCACGTGAAGAACGTG
ATGATCCGTGAGGCCTTGGCGGAGTTCATGGGGACCTTCCTTCTCGTCCTGTTTGGCTGC
GGCTCAGTGGCGCAGGTGGAGCTGAGCGACGGCACCAAGGGACGGTTCCTCACCATCAAC
CTCGCCTTCGGCTTCGCCGTCACCATGGGCGCCTACTGTGCCGCCGGCGTCTCCGGCGCG
CACTTGAACCCGGCGGTGTCGATGGCGCTGGCGGTGCTCGGCCGCTTCTCTTGGAGCAAG
TTCCCCTTGTACGTCACCGCTCAGCTGCTCGGGGCCTTCATGGGCGCCGGCACCGTCTTC
GGCCTCTACTACGACGCCTTCATGTACGTCTCCAAA---GGGAACCTGACGCTG------
---------CAGCTCGCAGGAGTCTTCGCCACCTTCCCGTCACCGCATCTCTCCATCGGA
AACGGATTTGTTGATCAGGTGATCGGAACTGCGGCGCTGCTCGTGTGTATCCTCGCCGTG
ATCGACAAGCGCAACAACCCGGCGCCCCGCGGCATGCAGCCCTTCCTCATCGGCCTCATC
GTCGTGCTCATCGGCCTCTCCATGGGCTTCAACGCCGGCTACGCAGTGAACCCCGCACGC

GACCTCGGCCCCCGCATCTTCACCGCCCTCGCCGGCTGGGGCTGGCAGGTCTTCTCGGCG
GGCAACTACTGGAGCTGGGTGCCTGTGGTGGCGCCCATGCTGGGCGGCGTGCTGGGCGCC
TTCATCTACGAGATCTTCATCGGCCTGCACCTCCCCGAAGAGCCGGCCAACTCCGGGCCC
GAGCCC---GGGACCCCGCGG---------------------------------------
------------------TCGCACCAGGAGCTC---GCCAAGCTGCAGGCCAAC---ATG
GCAGACGCCATGTGC---

>Inshore_hagfish_Aqp3L
ATGTCGACAGGAGGGAGCAAGCTGCGGGTCCTCGCACAGCGAATGCGCGTCACGAACCCC
CTAATCCGAGAAGCGTTCGCCGAAGGTTTGGGAACATTTGTTTTGGTGCTATTTGGTTGC
GGATCCGTAGCACAGATGGTCTTT---CATGGCCCGGGTGTTCCTTTCCTGTCTGTCAAT
CTGGCTTTTGGTTTGGCAGTCACGATGGGATGCTACATCGCTGGTGGAGTGTCTGGAGCT
CATCTGAACCCGGCGGTTTCTCTGGCGATGGTTGTGCTGGGCCGTCTGACTTTGCTCAAG
ATGTTGGTCTACTGGGTTGCTCAGCTGCTTGGAGCCTTCATCGGAGCCGCCATGGTCTTT
CTCATTTATCTGGACGCTGAGAAGAAACATTCGAGA---CCC---TGGAGCATG------
---------GAGACTGCGGGAATATATGCCACTTATCCGAACACCCACTTGTCCACCGGA
GGTGGTTTCTTTGATCAGGTTATGGGAACTGCTGCACTACTTCTGTGCATACTTGCCTTG
CTCGACAAGAAAAACACAGCACCACCTGATGGGGTGACACCTGTAATTATTGGGCTGGTT
GTTGCCGTGATTGGAATGGCTATGGGGCACAACTGCGGCTACGCCATCAATCCTGCACGC
GACCTCGGCCCTCGACTGTTCACCCTTATTGCGGGTTGGGGACGGGCTGTCTTTACGCAC
GGTAACTACTGGTTTTGGGTTCCAATCATCGCACCACTGCTCGGCGGAGTGCTCGGCGCA
CTCACTTACATCTTGTTTGTGGAGTTGCATCATCCCTCTTCGTCTGACACCGGCACCAAT
ACCAAA---------------GAGGACGCAGAG---------------------------
------------------AAGCATTATGACCTG---CGAAAATTTGATTCTCACGCTGCC
GAAACGGTGGCC--

>Pacific_hagfish_Aqp3L
ATGTCAACAGGAGGGAGCAAGCTGCGGGTCCTCGCACAGCGAATGCGCGTCACGAATCCC
CTTATCCGAGAAGCGTTCGCCGAAGGTTTGGGAACATTTGTTTTGGTGCTATTTGGTTGT
GGATCTGTAGCACAGATGGTCTTT---CAAGGCCCGGGTGTTCCTTTCCTGTCTGTCAAT
CTGGCTTTTGGTTTGGCAGTCACGATGGGATGCTACATCGCTGGTGGAGTGTCTGGAGCT
CATCTGAACCCGGCGGTTTCTCTGGCGATGGTTGTGCTGGGCCGTCTGACTTTGCTCAAG
ATGTTGGTCTACTGGGTTGCTCAGCTGCTTGGAGCCTTCATCGGAGCCGCCATGGTCTTT
CTCATTTATCTGGACGCAGAGAAGCAACATTCGAGA---CCC---TGGAGCATG------
---------GAGACCGCGGGAATATATGCCACTTATCCGAACGCCCACTTGTCCACCGGA
GGTGGTTTCTTTGATCAGGTTATGGGCACCGCTGCACTACTTCTGTGCATACTTGCCTTG
CTCGACAAGAAAAACACAGCACCACCTGACGGGGTGACACCTGTAATTGTTGGGCTGGTT
GTTGCCGTGATTGGAATGGCTATGGGGCACAACTGCGGCTACGCCATCAATCCTGCACGC
GACCTCGGCCCTCGACTGTTCACCCTTATTGCGGGTTGGGGACGGGCTGTCTTTACGCAC
GGTAACTACTGGTTTTGGGTTCCAATCATCGCACCACTGCTCGGCGGAGTGCTCGGCGCA
CTCACTTACGTCTTGTTTGTGGAGTTGCATCATCCCTCTTCGTCTGACACCGGCACCAAT
AACAAA---------------GAGGACGCAGAA---------------------------
------------------AAGCATTATGACCTG---CGAAAATTTGATTCTCACGCCGCC
GAAACGGTGGCC--

>Atlantic_hagfish_Aqp3L
ATGTCGACCGGAGGGAGCAAGTTACGGGTCCTCGCGCAGCGTCTACGCGTCACGAGACCC
CTCATTCGTGAAGCGTTCGCCGAAGGTTTGGGAACTTTCGTGCTGGTGCTATTTGGTTGT
GGATCTGTAGCACAGATGGTCTTT---CGTGGCCCAGGTGTTCCTTTCCTGTCTGTCAAC
CTGGCTTTTGGTTTGGCAGTCACCATGGGATGCTACATCGCTGGTGGAGTCTCTGGAGCT
CATCTGAATCCGGCGGTTTCTCTGGCTATGGTTGTCCTGGGCCGGTTGACTTTGCTCAAG
ATGTTGGTCTACTGCGTTGCTCAGCTGGTTGGAGCCTTCCTTGGAGCCGCCATGGTCTTT
CTCATTTATCTGGATGCTGAGAAGAAACACTCTAAA---CCC---TGGAGCATG------
---------GAGACTGCTGGAATATATGCAACTTACCCTGGCCCTCATTTGTCCACCGGA
GGTGGTTTCTTTGATCAGGTTATGGGCACCGCCGCATTACTTCTGTGCATACTTGCCTTG
CTCGACAAGAAAAACTCAGCACCACCTGACGGGGTGACGCCTCTGATTGTCGGGCTGGTG
GTCACCGTGATTGGAATGGCCATGGGGCACAACTGCGGCTATGCCATCAACCCCGCACGC
GACCTCGGCCCTCGGCTGTTCACCCTTATTGCGGGTTGGGGGCGGCCTGTCTTTACGCAC
AGGAACTACTGGTTTTGGGTTCCGATCACCGCGCCATTTCTCGGTGGAGTGCTCGGCGCA
CTCACTTACGTCTTGTTTGTGGAGTTGCATCATCCCTCCTCATCCGACTCCGGCACC---

>cetFrog_AQP13
ATGGACCTGCATACTCTGATCCTAACAACATTGAAAATGAAGTTGCGAACGAATAATCAG
TACGTGCGCTGCGGCCTGGCTGAGTTACTGGGGACTCTCATATTGATTCTCTTTGGCTGT
GGTGCGGTGGCTCAGATGGAGTTGAGCGGTCTTGCGAAGGGGCAGTTCCTGAGTGTCAAC
ATGGCATTTGGGTTTGCAGTGACTGCTGGGGCCTACATCTGTGCTGGAGTGTCAGGTGCC
CACCTCAACCCAGCCGTGTCCCTTTCCATGTACCTCCTCCAGAGGATGACTTGTAGGATG
ATGCTGGTCTACTTCTTGGCCCAGTTCATTGGATGTTTCATTGGGGCTGCTCTGGTCTTC
GCTCTTTATTTTGATGCTCTTCATGTGTACAGTGGA---GGTAACTGGACGGTTTATGGA
CCCCAA---GCAACAGCAGGGATATTTGCATCGTACCCATCGGAACACCTGAGTGTTTTT
AATGGCCTTTCAGATCAGGTGATTGCCACTGCTGCTTTGATGGTCAGTATCCTGGCAGTA
ATAGATGAAGCCAACAATGCTGCTCCTCGAGGTCTCCAGCCTTTTGTGATTGGTCTTGTG
GTTCTTCTGGTTGGTCTATCAATGGGCTTCAACTGTGGGTACCCAATAAATCCTGCCAGA
GACTTGGCACCTCGTATCTTCACTGCAATGGCTGGCTGGGGCCTGGAAGTCTTCAGGGCT
GGTGGGCACTGGTGGTGGGTGCCAGTGCTGGGACCAATGGTAGGGGCAGTGGTGGGGACG
CTGACTTATGAACTGCTGGTTGGAATTCACCACCCTGTGCTCCACGAGAAGGATGAACCT
GAAGAG------------GATGGTGAGGAGAACCATGCC---------------------
------------------ACACAGTATGAGTTG---GTTCAGTCTCATGCT---------

>goFrog_AQP13
ATGGACCTGCATACTCTGATCCTAAAAACATTGAAAATGAAGTTGCGAACGAATAATCAG
TATGTGCGCTGCGGGCTGGCTGAGTTCCTGGGGACTCTCATATTGATTCTCTTTGGCTGC
GGTGCTGTGGCTCAGATGGAGTTGAGCGGTCTTGCGAAGGGGCAGTTCCTGAGCGTAAAC
ATGGCGTTTGGGTTTGCAGTGACTGCTGGGGCCTACATCTGTGCTGGAGTGTCAGGTGCC
CACCTCAACCCAGCCGTGTCCCTTTCCATGTACCTCCTCCAGAGGATGACTTGTAGGATG
ATGCTGGTCTACTTCTTGGCCCAGTTCATTGGATGTTTCATTGGGGCTGCTCTGGTCTTC
GCTCTTTATTTTGATGCTCTTCATGTGTACGGTGGA---GGTAACTGGACGGTGTCTGGA
CCCCAA---GCAACAGCAGGGATATTTGCATCGTACCCATCGGAACACCTGAGTGTTTTT
AATGGCCTTTCAGATCAGGTGATTGCCACTGCTGCCTTGATG------------------

---------------------------------------GGCCTGGAAGTCTTCAGGGCT
GGTGGGCACTGGTGGTGGGTGCCAGTGCTGGGACCAATGGTAGGGGCAGTGGTGGGGACG
CTGACTTATGAACTGCTGGTTGGAATTCACCACCCTGTGCTCCACGAGAAGGATGAACCT
GAAGAG------------GATGGTGAGGAGAACCATGCC---------------------
------------------CCACAGTATGAGTTG---GTTCAGTCTCATGCT---------

>Common_frog_AQP13
------ATGTATACTCTCTTCCTAAAAACAATGAGAATGAAGTTGCGAACGAATAATCAG
TACGTGCGCTGCGGGCTGGCCGAATTGCTGGGGACTCTCATATTGATTCTCTTTGGCTGT
GGCGCTGTAGCTCAGATGGAGTTGAGCGGTCTTGCTAAGGGGCAGTTCCTGAGTGTCAAC
ATGGCGTTTGGGTTTGCAGTGACTGCTGGGGCCTACATCTGTGCTGGAGTGTCAGGTGCC
CACCTCAACCCAGCCGTGTCTCTATCCATGTACCTCCTCCAGAGGATGACTTGTAGGATG
ATGCTGGTCTACTTCTTGGCCCAGTTCATTGGATGTTTCATTGGGGCTGCTCTGGTCTTT
GCTCTTTATTTTGATGCTCTTCATGTGTACAGTGGA---GGTAACTGGACGGTGTCAGGA
ACCCAA---GCAACAGCAGGGATATTTGCATCCTACCCATCGGAACACCTGAGCGTTTTA
AATGGACTTTCAGATCAGGTGATTGCCACGGCTGCCTTGATGGTCTGTATCCTGGCAGTA
GTAGATGAAGCCAACAATGCTGCTCCTCGAGGACTCCAGCCTTTTGTGATTGGCCTTGTG
GTTCTTCTGGTTGGTCTATCAATGGGCTTCAACTGTGGGTACCCAATAAATCCTGCCAGA
GACTTGGCACCTCGTATCTTCACTGCAATGGCTGGCTGGGGCCTGGAAGTCTTCAGGGCG
GGTGGGCACTGGTGGTGGGTGCCAGTGCTGGGACCAATGCTAGGGGCAGTGTTGGGGACG
TTTACTTATGAACTGTTGGTTGGAATTCACCACCCTATGCTTCACGAGAAGGATGAACCT
GAAGAG------------GATGCTGGGGAGAACCACACC---------------------
------------------CCACAATATGAATTG---GTTCAGTCTCATGCT---------

>American_bullfrog_AQP13

------ATGTATGCTCTCTTCCTAAAAACACTGAAAATGAAGTTGCGAACGAATAATCAG
TATGTGCGCTGCGGGCTGGCCGAGTTCCTGGGGACTCTCATATTGATTCTATTTGGCTGT
GGTGCTGTAGCTCAGATGGAGTTGAGCGGTCTTGCTAAGGGGCAGTTCCTGAGTGTCAAC
ATGGCGTTTGGGTTTGCAGTGACTGCTGGAGCCTACATCTGTGCTGGGGTGTCAGGTGCC
CACCTCAACCCAGCCGTGTCTCTATCCATGTACCTCCTCCAGAGGATGAATTGTAGGATG
ATGCTGGTCTACTTCTTGGCCCAGTTCATTGGATGCTTCATTGGGGCTGCTCTGGTCTTT
GCTCTTTATTTTGATGCCCTTCATGTGTACAGCGGA---GGTAACTGGACAGTGTCTGGA
ACCCAA---GCAACAGCAGGGATATTTGCATCATACCCATCAGAACACCTGAGTGTTTTT
AATGGACTTTCAGATCAGGTGATTGCCACTGCTGCCTTGATGATCTGTATCCTGGCAGTA
GTAGATGAAGCCAACAATGCTGCTCCTCGAGGACTCCAGCCTTTTGTGATTGGTCTTGTG
GTTCTTCTGGTTGGTCTATCGATGGGCTTCAACTGTGGGTACCCAATAAATCCTGCCAGA
GACTTGGCACCTCGTATCTTCACTGCAATGGCTGGCTGGGGCCTGGAAGTCTTCAGG---

>Himalaya_frog_AQP13
ATGGACCTGTATACTCTCATCCTAAAAAAGCTGAAAATGAAGCTGCGGACGGATAATCAA
TATGTGCGCTGCGGGCTGGCTGAATTCCTGGGGACCCTCATATTGATTCTGTTTGGTTGT
GGCTCTGTGGCTCAGATGGAACTGAGTGGCTATGCTAAGGCGCAATTTTTGAGTGTCAAC
ATGGCGTTTGGGTTTGCTGTGACCGCTGGGGCCTACGTCTGTGCTGGAGTGTCAGGTGCC
CACCTCAACCCGGCTGTGTCTCTTTCAATGTACCTCCTCCAGAGGATGACCTGTAGAATG
ATGTTGGTCTACTTCTTGGCTCAGTTCCTTGGAGCTTTTATTGGGGCTGCTCTGGTCTTT
GCTCTGTATATTGATGCTCTTCATGCGTACAGTGGA---GGTAACTGGACGGTGTCTAGC
ACCCAG---GCAACAGCGGGGATATTTGCTTCGTACCCATCCGAACACTTGACCATTCTT
AATGGACTCACAGATCAGGTAATTGCCACTGCTGCCTTAATGATCTGTATACTAGCAGTA
ATAGATGAATCCAACAATGCTGCTCCTCGAGGCCTTCAGCCATTTGTGATTGGTCTTGTG
GTTCTTCTGGTTGGTCTATCAATGGGCTTCAACTGTGGGTACCCAATAAATCCTGCCAGA
GACTTGGCACCTCGTATCTTCACTGCAATGGCTGGCTGGGGCCTAGAAGTCTTCAGGGCT
GGTAATCACTGGTGGTGGGTGCCAGTGCTGGGACCAATGATAGGAGGAGTGATAGGGACC
CTGATTTATGAACTGCTGGTTGGAATTCACAGCCGTACTCTCCATGAGGAGGATGAACCT
GAAGAG------------GACGTTGAGGAGAACCATCCC---------------------
------------------CGACAGTATGAGTTA---GTTCAGTCTAATGCT---------

>African_bullfrog_AQP13m
ATGAACATGTTTGCATTGCTCTTACAGAAACTCAGCATGAAGTTACAGACAAAAAATCTG
TATGTGCGCTGCGGGCTGGCTGAGTTCCTGGGGACCCTCATATTGATTCTGTTTGGCTGT
GGCTCTGTAGCTCAGATGGAATTGAGTGGTTTTGCAAAAGCGCAGTTCCTGAGTGTCAAC
ATGGCGTTTGGATTTGCAGTGACGGCTGGGGCCTACGTCTGCGCTGGAGTGTCAGGCGCC
CATCTCAACCCAGCGGTGTCGCTTTCCATGTATCTCCTCCAGAAGATGACTTGTAGGATG
ATGCTGGTATACTTCTTGGCTCAGTTCCTTGGTTCCTTCATTGGGGCAGGTCTGGTCTTT
GCTCTGTATTTTGATGCTCTGCATGCATACAGTGGC---GGTAACTGGACAGTGTCTGGG
ACCCAA---GCAACAGCGGGGATATTTGCTTCGTACCCCTCAGAACATCTAAGTGTTCTT
AATGGATTCACAGATCAGGTAATTGCCACCGCTGCCTTGATGGTCTGTATACTTGCCATA
ATAGATGAATCAAACAATGCTGCTCCTCGAGGTCTTCAACCATTTGTGATTGGGTTGGTG
GTTCTTCTGGTTGGTCTATCAATGGGTTTCAACTGTGGGTACCCAATCAATCCTGCCAGA
GACCTGGCACCTCGTATCTTCACTGCAATGGCTGGCTGGGGCCTGGAAGTCTTTAGGGCT
GGTGGATGCTGGTGGTGGGTGCCAGTGCTGGGACCAATGGTAGGAGGAGTGGTGGGTACC
CTGATTTATGAACTGCTCATTGGAATTCATCACCCTGCTCAACACCAGACAGAAGAACCA
GTAGAG------------GATGGTGGG---AACCACCAC---------------------
------------------AGACACTATGAACTG---GTTCAGTCTCAAGCT---------

>Eastern_spadefoot_toad_AQP13
ATGGAGGCCTATGCCGTGCTACTGAAAAAGCTGAGGCTTAAACTGAGGACCGGGAACATT
TATGTGCGATGCGGGCTGGCCGAGTTCCTGGGTACACTCATCCTTATTCTGTTTGGCTGT
GGGTCTGTGGCTCAGATGGAGTTGAGTGGTTTTGCTAAAGCCCAGTTCCTGAGTGTGAAT
ATGGCATTCGGCTTTGCGGTTACTGCTGGAGCCTATGTGTGTGCAGGAGTATCGGGTGCC

CATCTGAACCCTGCTGTGTCTCTCTCCATGTACTGTCTGAGGAAGCTTAGTGGGAGACTT
ATGTTG--
------------GATGCCCTGTATTGCTATAGCGGT---GGGAACTTCACGGTGTCTGGA
CCACAG---GCTACGGCCGGGATATTTGCCTCCTACCCTTCCGAACATCTGAGTGTGATC
AATGGATTCACAGACCAGGTAATCGCTACATCGGCCTTGATGATCACTATTCTGGCCATA
CTGGATGAAGAAAACAATGCGCCTCCTCGTGGTCTTCAACCCTTTCTAATTGGAATATCT
GTGCTACTAGTCGGTCTGTCAATGGGCTTCAACTGCGGCTACCCCATAAATCCTGCCCGG
GACCTGTCACCTCGGATCTTCACGGCTATGGCTGGCTGGGGTATTGAGGTGTTCAGGGCT
GGAAACAACTGGTGGTGGGTGCCAGTGACTGGACCCCTAGTAGGTGCGTTGGTTGGAGTG
CTGATCTATGAACTGTTTATTGCAATCCATCACCCACTGAGTGAGCAGAAAGGCGACCAA
GAGGAA------------GATGAAGAGGAGACGCATCCA---------------------
------------------CCTCATTATGAGTTG---GTTCAATCTACAGCA---------

>Couch_spadefoot_toad_AQP13

--CTATTTGGCTGC
GGGTCTGTGGCTCAGATGGAGTTGAGTGGTTTTGCTAAAGCCCAGTTCCTGAGTGTGAAT
ATGGCATTCGGCTTCGCGGTTACTGCTGGGGCCTACGTGTGTGCAGGAGTATCGGGTGCC
CATCTGAACCCGGCTGTGTCTCTCTCCATGTACTGTCTGAGGAAGCTAAGTGGGAGACTT
ATGTTGGTCTACTGGTTGGCTCAGTTCCTGGGGGCC------------------------

---GCTATTCTGGCCATA
CTGGATGAGGAAAACAATGCACCTCCTCGTGGTCTTCAACCCTTTATAATTGGAATATCT
GTGCTACTAGTCGGCCTGTCAATGGGCTTCAACTGTGGCTACCCCATAAATCCTGCCCGG
GACCTGTCTCCTCGCATCTTCACGGCTATGGCTGGCTGGGGTATTGAGGTGTTCAGGGCT
GGAAACAACTGGTGGTGGGTGCCAGTGACTGGACCCCTAGTAGGTGCGTTGGTTGGGGTG
CTGATCTATGAACTATTTATTGCAATCCATCACCCACTGATTGAGCAGAAAGGCGACCAA
GAAGATGGTGGA------GATGAAGAGGAGACGCATCCA---------------------
------------------CCACAGTATGAGTTG---GTTCAATCTACCGCC---------

>Mexican_spadefoot_toad_AQP13
ATGGAGGCTCGTGTCATGCTGCTGAAAAAGCTGAAGCTTAGGTTGCGGACGGAAAACCTT
TATGTACGATGCGGGCTGGCCGAGTTCCTGGGGACGCTTATTTTAATTCTGTTCGGCTGT
GGGTCTGTGGCTCAGATGGAGTTGAGTGGTTTCGCTAAAGCCCAGTTCCTGAGTGTGAAT
ATGGCGTTCGGCTTCGCTGTCACTGCTGGAGCCTATGTGTGTGCCGGAGTATCGGGTGCC
CATCTGAACCCTGCTGTGTCTCTCTCCATGTACTGTCTGAGGAAGCTGAGTGGGAGACTT
ATGTTGGTCTACTGGTTGGCTCAGTTCCTAGGAGCATTCTTTGGAGCCGCATTGGTCTTT
GCCTTGTATTACGATGCCCTGTGTGTGTACAGCGGT---GGGAATTTCACAGTGACTGGA
TCTCAG---GCCACAGCCGGCATATTTGCTTCATACCCTTCGGAGCATCTGAGTGCGATC
AATGGATTTACAGACCAGGTGATTGCTACAGCGGCCTTGATGATCGCTATTCTGGCCATA
CTGGATGAAGAAAACAACGCGCCTCCTCGTAGTATCCAGCCCTTCATAATTGGAATATCT
GTGTTACTGGTCGGTCTGTCAATGGGCTTCAACTGTGGTTACCCCATAAATCCTGCCCGG
GACCTATCTCCTCGCATCTTCACAGCTATGGCTGGCTGGGGTCTTGATGTGTTCAGGGCT
GGAGATAACTGGTGGTGGGTGCCGGTGATTGGACCTCTAGTAGGAGCACTGGTTGGCGTG
CTGATCTATGAACTTTTTATTGAAATCCATCACCCGGCGAGCAAACAGAAAGATGACCAA
GAGGCG------------GAAGAAGAGCAGACCCATCCA---------------------
------------------CCTCAGTATGAGCTG---GTTCAATCGACCGCA---------

>Plains_spadefoot_toad_AQP13
ATGGAGGCTCGTGTCATGCTGCTGAAAAAGTTGAAGCTTAAGTTGCGGACTGAAAACCTT
TATGTGCGATGCGGGCTGGCTGAGTTCCTGGGAACGCTTATTTTAATTCTGTTTGGCTGT
GGGTCTGTGGCTCAGATGGAGTTGAGTGGTTTCGCTAAAGCCCAGTTCCTGAGTGTGAAC
ATGGCGTTCGGCTTCGCTGTCACTGCTGGAGCCTATGTGTGTGCCGGAGTATCGGGTGCC
CATCTGAACCCTGCTGTGTCTCTCTCAATGTACTGTCTGAGGAAGCTGAGTGGGAGACTT
ATGTTGGTCTACTGGTTGGCTCAGTTCCTAGGAGCATTCTTTGGAGCCGCATTGGTCTTT
GCCTTGTACTACGATGCCCTGTGTGTGTACAGCGGT---GGGAATTTCACAGTGACTGGA
TCTCAG---GCCACAGCTGGCATATTTGCTTCATACCCTTCAGAACATCTGAGTGCGATC

AATGGATTTACAGACCAGGTGATTGCTACAGCGGCCCTGATGATCGCTATTCTGGCCATA
CTGGATGAAGAAAACAATGCGCCTCCTCGTAGTATTCAGCCCTTCATAATTGGAATATCT
GTGTTACTGGTCGGCTTGTCCATGGGCTTCAACTGTGGTTACCCCATAAATCCTGCCCGG
GACCTATCTCCTCGCATCTTCACAGCTATGGCTGGCTGGGGTCTTGATGTGTTCAGGGCT
GGAGATAACTGGTGGTGGGTGCCGGTGATCGGACCTCTAGTAGGAGCACTGGTTGGTGTC
CTGATCTATGAACTTTTTATTGAAATCCATCACCCGGTGAGCAAACAGAAAGATGCCCAA
GAGGAG---------------GAAGAGCAGACCCATCCA---------------------
------------------CCTCAGTATGAGCTG---GTTCAATCAACCGCG---------

>Leishan_spiny_toad_AQP13
ATGTCAGGCTACGGCGTGCTGCTGGAGAAGCTGAGGTTCGCGCTTCGGACTGAAAACCAG
TATGTGCGCTGCGGGCTGGCGGAGTTTTTGGGGACCCTCATATTAATCCTCTTCGGCTGC
GGATCGGTGGCTCAGATGGAGTTGAGTGGTTTTGCTAAAGCGCAATTCCTGAGCGTTAAC
ATGGCGTTTGGATTTGCAGTGACTACCGGGGCTTACACATGCGCAGGAGTGTCCGGAGCC
CACCTGAACCCTGCGGTGTCTCTATCGATGTGCGTTCTGAACAGGCTCAGCAAGAGGATG
ATGCTGGTTTACTGGTTGGCACAATTCTTGGGTTCTTTCGTTGGTGCCTCCTTGGTCTTC
TCATTGTACTATGATGCCATTTATGTGTATAGTGGT---GGGAACTTCACGGTGACTGGA
CCACAG---GCTACGGCAGGGATATTCGCTTCTTACCCTTCTGAACACCTGAGCGCCATC
AATGGCTTTACAGACCAGGTGATCGCCACGGCTGCCCTGATGATCTCTATCCTGGCCATA
GTGGACGAAGCCAACAACGCACCTCCTCGTGGCATGCAGCCATTTATGATTGGCATTGTG
GTCTTGCTTGTTGGGTTGTCAATGGGCTTTAATTGCGGCTACCCCATCAATCCTGCACGG
GACCTGGCTCCCCGGATCTTCACTGCAATGGCTGGTTGGGGCCTTGAGGTTTTCAGGGCT
GGAGGAAACTGGTGGTGGGTGCCTGTAATTGGACCAATGGTCGGGGCACTAGTTGGGGTG
TTCATCTACATGGTATTGATTGAAATCCATCACCCGATGGTCAGGCATAAAGATGATCAT
GAGGAG------------GAAGCCAATGAGGGCAGAAAC---------------------
------------------CCTCATTATGAACTG---GTCTCGACTACAGCG---------

>Pacific_treefrog_Aqp13
ATGGACGCCTATTCTCTGCTGATCAAAAAGTGGAAGTTAAAGTTCAGGACGAGCAATCGC
TATGTGCGCTGTGGGCTGGCGGAGTTCTTGGGGACGCTCATATTAATCCTATTTGGCTGT
GGGTCTGTGGCACAGATGGAATTAAGCGGTTTTGCCAAAGCCCAGTTTCTAAGCGTTAAT
ATGGCATTCGGCTTTGCGGTCACTGCTGGAGCCTATGTCTGTGCCGGAGTCTCAGGCGCC
CACCTCAACCCGGCTGTGTCTCTCTCCATGTTCCTCCTCCAGAAGATGACGGGGAGGCTA
ATGCTWGTSTACTGTGTGGCACAATTCCTCGGCTCTTTTGTTGGCGCGGCACTGGTATTT
GCCCTGTATTTCGACTCCCTTCATGTGTACAGTGGA---GGCAACTGGACAGTATACGGA
CCC---
------------------------GCCACCGCGGCCCTGATGATCTGCATCCTGGCCATC
ATCGATGAAGACAATAACGCAGCCCCCCGGGGTCTTCAGCCATTTATCATTGGACTGATA
GTTCTTCTAGTTGGTCTTTCTATGGGATTCAACTGTGGATACCCCATTAATCCAGCCCGG
GACCTGGCACCTCGAATCTTCACATCATTAGCCGGCTGGGGCCTAGAAGTCTTCAGGGCC
GGTGACAACTGGTGGTGGGTGCCAGTCCTGGGCCCCATGATTGGAGCGGTTGTCGGGACA
ATCCTTTATGAGCTGCTTATTGGGATCCATCACACGTCTGCCCAGCAGAAGGAAGAACAT
------------------GATGGAGAGGAGCGTCACCCC---------------------
------------------CCTGAGTATGAGTTG---GTTCACTCAACAGCA---------

>Marine_toad_AQP13
ATGGATGCTTATGCTCTGCTCGTGAAAAAGCTCAAGTTAAGGTTTAGGACGAGCAATCGC
TATGTGCGCTGTGGGCTGGCTGAGTTTTTGGGAACTGTCATATTGATTTTGTTTGGTTGC
GGTTCTGTGGCTCAGATGGAATTAAGTGGTTTTGCCAAAGCTCAGTTTCTGAGTGTCAAT
ATGGCGTTTGGTTTTGCAGTCACTGCTGGAGCTTATGTATGCGCTGGAGTCTCAGGTGCC
CACCTCAATCCAGCCGTCTCGCTCTCCATGTTCCTCCTCCAGAAGATGACCGGGAGGCTG
ATGCTAGTCTACTGCTTGGCACAATTCCTCGGCTGTTTTGTTGGCGCCAGCCTGGTATTT
GCTCTGTATTTTGATGCCCTTCATGTGTACGGTGGT---GGCAACTGGACAGTGTATGGA
CCACAG---GCGACTGCTGGGATATTCGCTTCCTATCCATCAGAACATCTGAGTGTGATC
AATGGACTCTCAGATCAGGTAATTGCCACCGCTGCCTTGATGATCTGCATTTTGGCCATC
ATTGATGAAGACAATAATGCAGCCCCCCGAGGACTTCAGCCATTTGTCATTGGTCTTATA
GTTCTCCTAGTTGGTCTCTCTATGGGCTTCAACTGTGGCTACCCCATTAATCCAGCCCGC
GACCTGGCACCTCGAATTTTCACAGGAATTGCTGGCTGGGGTCTAGAAGTCTTCAGGGCT

GGTGACAACTGGTGGTGGGTTCCAGTATTGGGCCCCATGATTGGTGGGGTCATTGGAACA
TTTATTTATGAAATGCTCGTTGGAATTCATCACTCAGCAGAACAGAAGAAAGAGGAAGAT
GATGAA------------GATAGCGAGGAGCATCACCCC---------------------
------------------CCGGAGTACGAGTTG---GTTCAGTCAACGGCA---------

>Hourglass_treefrog_AQP13
ATGGAGGCTTATTCTCAATTGCTCAAAAAGTCCAAGCTAAAGTGTAGGACAAGCAATCGC
TATGTGCGCTGCGGGATGGCGGAGTTTTTGGGGACTCTCATATTAATTTTATTTGGCTGT
GGGTCTGTAGCTCAGATGGAATTAAGTGGTTTTGCCAAAGCCCAGTTCCTAAGCGTTAAT
ATGGCGTTTGGCTTTGCGGTCACTGCTGGGGCCTATGTGTGCGCTGGGGTCTCAGGTGCC
CACCTCAATCCGGCTGTGTCTCTCTCCATGTTCCTCCTCCAGAAGATGAATGGGAGGCTT
TTGCTAGTCTACTGTTTGGCACAATTCCTTGGCTCCTTTATCGGTGCTTGCCTGGTGTTT
GCCCTGTATTTTGACGCCCTTCATGTATACAGTGGA---GGCAACTGGACAGTATATGGA
CCACAG---GCGACTGCTGGTATATTTGCCTCCTATCCGTCAGAACATCTGAGTGCAATT
AATGGATTCACAGATCAGGTGATCGCCACTGCTGCCTTGATGATCTGCATTCTGGCCATT
ATCGATGAAGACAATAACGCAGCCCCCCGAGGTCTTCAGCCGTTTGTTATTGGACTTGTA
GTTTTATTAGTTGGTCTTTCTATGGGATTCAACTGCGGATACCCCATTAATCCAGCACGG
GACCTGGCGCCTCGACTGTTCACTGCCTTAGCTGGCTGGGGCCTAGAAGTCTTCAGGGCT
GGTGGAAACTGGTGGTGGGTGCCAGTGCTGGGCCCCATGATTGGAGGGGTTATTGGGACA
ACTATTTATGAGGTATTCATTGGGATCCATCATTCACCTAGCCAGAAGGAGGAACAAGAT
GAAGAG---------------------GAGCATTACCCC---------------------
------------------CCTGAATACGAGTTG---GTTCACTCCACAGCA---------

>WC_frog_AQP13
ATGGTCAGCCATTTAGCTTTCCTGAAAAACATGAAGATTAAGCTGAGGACCCAGAACCAG
TATGTGCGCTGCGGGCTGGCAGAGTTCCTGGGCACGCTCATTCTAATCCTGTTTGGCTGT
GGTTCTGTGGCGCAGATGGAGCTGAGTGGTTTTGCCAAAGCCCAGTTTCTGAGCGTTAAT
ATGGCGTTTGGCTTCGCAGTCACTGCTGGAGCTTACGTATGCGCCGGTGTGTCGGGTGCC
CATCTGAATCCTGCCGTGTCCCTCGCCATGTTCATACTGAAGAAGCTGAGCTGGAGGCTG
CTCCTTACCTACTGCCTGGCACAGTTCCTCGGGGCTTTCATTGGAGCCGCTCTGGTCTTT
TCTCTTTATTATGATGCCTTGCACGTGTACAGCAGT---GGGAACTGGACAGTCTATGGC
CCCCAG---GCTACAGCAGGGATATTTGCTTCCTACCCCTCCGAGCACCTCAGTGTCATA
AATGGCTTTACAGACCAGGTGATTGCCACAGCGGCTCTGCTGATCTGTATCCTGGCTATA
CTGGATGAAGCCAACAACGCAGCACCCAGGGGCCTACAGCCTTTTCTAATTGGCATTGTG
GTGCTGCTGGTTGGGCTAGCAATGGGGTTTAACTGCGGGTACCCCATAAATCCGGCTCGA
GACCTCGCACCGCGCTTCTTCACGGCTATTGCAGGCTGGGGCTCCGAGGTCTTCAGCGCC
GGAGGCCACTGGTGGTGGGTGCCGGTTATAGGACCTTTGGTAGGTGGGGTGCTCGGCGTG
GTAATCTATGAAGTATTCATCGAATTCCACCATCCTCCTGCCAATCAAAAGCAAGAATCC
GAGGAA------------CCCACTGAGGGAACCCATCGT---------------------
------------------CCTCACTATGAGCTT---GTTCAGTCCTCTGCT---------

>AC_frog_AQP13
ATGGTCAGTCATTTAGCTTTCCTGAAAAACATGAAGCTTAAGCTGAGGACTCAGAACTTG
TATGTGCGCTGTGGGCTGGCTGAGTTCCTGGGCACCCTCATTTTAATTCTGTTTGGCTGC
GGTTCTGTGGCCCAGATGGAGCTGAGTGGTTTTGCCAAGGCTCAGTTTCTGAGTGTGAAT
ATGGCATTTGGCTTTGCAGTCACCGCTGGAGCTTATGTATGTGCTGGAGTCTCAGGTGCT
CATCTGAATCCTGCTGTGTCCCTCGCCATGTTCATACTGAAGAAGCTGAGCTGGAAGCTG
TTCCTGATCTACTGTTTGGCACAGTTCCTCGGGGCTTTTATTGGAGCCGCTCTGGTCTTT
TCTCTGTATTATGATGCCTTGCACGTGTACAGCAAT---GGCAACTGGACTGTCTATGGC
CCCCAG---TCGACAGCAGGGATATTTGCTTCCTACCCTTCAGAGCACCTCAGTGCCATA
AACGGCTTTACAGACCAGGTGATTGCCACGGCAGCTCTGCTGATCTGTATACTGGCCATA
CTGGATGAAGCCAACAACGCAGCACCCAGGGGCCTGCAGCCTTTTCTCATTGGCATTATG
GTGCTGCTGGTTGGGCTAGCAATGGGGTTCAACTGCGGGTACCCCATCAATCCAGCTCGT
GACCTTGCACCCCGCTTCTTCACTGCTATTGCAGGCTGGGGGTCAGAAGTCTTCAGTGCT
GGAGGCCACTGGTGGTGGGTGCCAGTTCTAGGACCTTTGGTAGGTGGGGTGGTTGGTGCG
GTCATCTATGAAGTCTTCATCGAATTCCACCATCCTTCTCCCAATCAAAAGCAAGAATCT
GAGGAA------------CCCACTGAAGGAATCAATCGT---------------------
------------------CCTCACTATGAGCTG---GTTCAGTCCTCTGCT---------

>JF_newt_Aqp13

---AGTGTCAAC
ATGGCGTTTGGATATGCAGTCACTGCGGGAGCCTACGTATGTGCT---GTATCGGGCGCT
CTG------CCGGCTGTATCCCTCGCCATGTTCCTCTTGAGAAGGATGACTTTTAAGCTG
ATGCTGGTCTATTGCCTTGCCCAGTTCCTGGGGGCCTTTTTGGAGCAGCCATTGTTTTT
GCGCTTTACTTTGATGCTCTGCATGTGTACAGTGGC---GGCAACTGGACCGTGGTGGGA
CCTCAA---GCTACAGCTGGGATCTTTGCATCCTACCCTTCGGAACACCTCAGTGTTATC
AATGGATTTACAGACCAGGTGATTGCTACTGCTGCTCTGCTGATCTGCATTCTGGCTGTT
GTGGATGAGCGGAACAATGCTGCTCCACCGGGACTGCAGCCATTTGTGATTGGTCTAATG
GTGTTGCTGGTTGGCCTTGCTATGGGATTCAACTGTGGCTATCCCATCAACCCAGCCAGA
GACCTGGGGCCACGCCTCTTCACTGCCATAGCTGGATGGGGCCTTGAAGTGTTTAGGGCT
GGGAACCACTGGTGGTGGGTACCAGTTTTGGGGCCCCTTGTGGGTGGCCTGATTGGCGCC
ATTATCTATGAGCTGTTCGTGGAAATCCATCACCCGCTGGATCAGGTAAAAGAGGTCCTA
GATGCC---------------AACTGTCCTGATCACCCAACTCGT---------------
------------------CCCCAATATGAACTT---GTTCAGAGCACAGCA---------

>Axolotl_AQP13
ATGGACGGCCACGCCGCCCTGCTGAAGTACTTGAAGCTCAAGTTCAGAACTCGAAGTCTC
CACGTGCGCTGTGGGCTGGCAGAGTTTTTGGGAACGTTCATTTTAATTCTGTTTGGCTGT
GGTTCCGTGGCCCAGATGGAGCTGAGTGGCTTCGCAAAAGCACAGTTCCTTAGTGTGAAC
ATGGCCTTTGGATTTGCCGTCACCGCCGGTGCCTATGTGTGTGCCGGAGTATCAGGTGCT
CACTTGAACCCGGCTGTGTCGCTCGCAATGTTCCTCTTGAGACGGATGTCATGGAAGCTG
CTTCTTGTCTACTGCCTTTCCCAGTTCTTGGGAGCGTTCTTTGGTGCTGCCATTGTTTTT
TCACTTTACTTTGATGCTTTGCATGCGTTTAGTGGT---GGAAACTGGACTGTGGCCGGC
CCTCAA---GCGACTGCTGGGATCTTCGCCTCCTACCCTTCAGAACACCTCAGTGTTATC
AATGGATTTACAGACCAGGTGATTGCCACAGCTGCTCTGTTGATCTGCATCCTGGCCATT
GTGGATGAGAAAAATAACGCTGCCCCGGCTGGATTGCAGCCATTTGTGATTGGTCTAATG
GTGCTGCTGGTAGGCCTTGGCATGGGATTCAACTGTGGCTATCCCATCAACCCAGCCAGG
GACCTAGGGCCACGCCTCTTCACTGCCATAGCTGGATGGGGCCTTGAAGTATTTACCGCT
GGAAACCACTGGTGGTGGGTCCCAGTGTTGGGGCCCCTCGTGGGAGGCTTAATCGGCGCC
ATAATCTACGAGCTGTTTGTGGAATTCCATCATCCGCTGGATTCTGAGAAAGAGAACACC
GAAAAC---------------GACAGCGTTGATCATCCAAATCGT---------------
---------------TGTCCCCATTACAAACTA---GTTCAGACGACCGCAGCC------

>Platypus_AQP13
ATGGCCGGACTCAAC---------------------CCGGTCTGGGGTGCACAGAGTGCC
CACCTGCGCTGTGGCCTGGCTGAGTTCTGGGGGACTTTTGTGCTGATTGTACTCGGCTGC
GGCTGGGTGGCCCAGGCTGAGCTGCGAGGCCTGGCCCACGTGAGACTTCTCGGCGCGGAC
AAGCCCCTCCGGTTCATGGTCTCTGCGGGAGCCTACATAGGCTCCGGAGCCTCAGGCACT
CATCTGAACCCCGCCGTGTCCCTGGCCATGTGTTTCCTGCGGCGGCTGGACTGGAACCTC
CTGCCCACTTTGTGCTTAGCCCAGCTGGCCGGGGCCTTCTGCGGCGCAGCCACCGTCTTC
GTCTGGCACTACGACGGTCTCCAGGCCCTGAGCGCT---GGTGCCTGGACAGTGGCGGGA
CCCAAT---GCCACAGCTGGGATCTTTGCTTCCTACCCCTCCGGGCAGCAGAGTTCACTC
AGCTGCTTTGCTGACCAGGTGATGGCGTCGGCAGCTTTTCTCACCTGTGTCCTGGCCGTG
CTGGGCGAGGGAGTCCCAGCCCCCCGCCACAAGCTGCAGCCCCCCGCCCAGCGGCTGGCC
CTGTTCCTAGTAGGCTCGGCCTTGGGCTCGAGCTGTGGGTGCCCCATCAACCCCGCCGAG
GACCTGGGCCCGCGGGCGTTCGCGGCAGTGGCCGGCTGGGGGCTGGAAGTCTTCAGGGTT
GGAAACCACTGGTGGTGGATCCCCGTCCTGGGGCCCCTGGTGGGCGCCCTGCTCGGGGCC
TCCGTGTACCACCATGCCCTGGCCCTACCTGTTCTGCTCTCGGGGAGAAAGAAAACTGCG
GCC---

>Echidna_AQP13
ATGGCCGGACTTAGC---------------------CCGGTCTGGGGCAAACAAAGTGTC

CACCTGCGCTGTGGCCTGGCTGAGTTCTGGGGACTTTTGTGCTGATTCTGCTCGGCTGT
GGCTGGGTGGCCCAGGCTGAGCTGCGAGGCCTGGCCCACATGCGACTTCTCGGCATGGAC
AAGCCCCTCCGGTTCGTGGTCTCCGCAGGAGCCTACATAGGCTCCGGAGCCTCAGGTACT
CACCTGAACCCCGCGGTGTCCCTGGCCATGTGCTTCCTGCGGCGACTGGACTGGAACCTC
CTGCCCACATTGTGCCTAGCCCAACTGGCCGGGGCCTTCTGCGGCGCGGCCACCGTCTTC
GCCTGGCACTATGACGCTCTCCAAGCCTTCAGCGCT---GGTGCCTGGGCAGTGGCCGGA
CCCAAT---GCCACAGCTGGGATCTTTGCTTCCTACCCCTCCGGGCAGCAGAGTTCACTC
AGCAGCTTTGCTGACCAGGTGATGGCGTCGGCAGCGTTCCTCACCTGCGTCCTGGCCGTG
CTGGGAGAGGGAGCCCCAGCCCCCCGCCACAAGCTGCAGCCCCCCGCCCGGCGGCTGGCC
CTGTTCCTCATTGGCTCTGCCTTGGGCTCGAGCTGTGGGTGCCCCATCAACCCCGCCGAG
GACCTGGGCCCAAGGGTGTTCGCGGCAGTGGCTGGCTGGGGGCTGGAAGTCTTCAGGGTT
GGAAACCACTGGTGGTGGATCCCAGTCCTGGGGCCCCTGGTGGGTGCCCTGCTCGGGGCC
TCCGTGTACCACCATGCCCTGGCCCTGCCTGCTCTGCTCTCGGGAAGAAAGAAAGCTGCG
GCC---

>Inshore_hagfish_Aqp13
ATGGAGTCATGCAGCAGAGATGCCGCAAAGATGCTCAACAATCTGAGGACCAGCAATGAG
TCCATACGTTTGTTTATGGCGGAGCTGCTTGCGACAGCAATGTTGGTGCTCATTGGTTGT
GCAGCTGTTGCTCAGGGGGTGTTGTCAGGTGGTGGTGTTGCTGCCACTCTGAGCATTAAT
TTGGCCTTTGGATTGGCTGTGATGATGTCTGCGCACATCGCAATGGGAGTTTCAGGTGCT
CACATGAATCCTGCGGTTACCCTGGCGATGTGCATCTTGGACCGATTTCCATGGAAACGG
CTTCCTCTCTATTGGATCTCACAGTTCTTGGGTGCATTCATTGGTGCTCTAGGGGTCTAT
GCCCTGTATCATGAGGGGATCCAAGCATACGAAGGT---GGGGAGCTGACTGTTGTCGGG
CCTCAT---GCCACGGCCGGAATATTTGCCACCTACCCCAACTCTCACCTCTCCACTGGA
GGAGGAGTTATTGATCAGGTGATTGGAACCGCTATTTTGCTTTGCCTCATTCTTGCACTT
ACGGACGAATCGAACTGTGCAATGCCACGCTATCTGCAACCCTTGGGTTTTGGTTTAGTG
GTGGTTGCTATCGGGCAAGCTACGAACTTCAACTGTGGTTATGCCATTAATCCAGCCCGG
GATTTGGGGCCCAGGATTTTCAGCGCGCTGGTTGGATATGGATCTGAGGTGTTTTGGGCG
AAGGACTGTTGGTGGTGGGTCCCTGTGCTCGCTCCTCTCATTGGATCTGTTCTCGGATCT
TTGTGTTACCAACTCCTGATCGCACTCCATTACCCGAGGAGCACTGACAGGGAAAATGAG
AAACCACCTTCGCTTCTGCAAAATGACCATGATAACCTC---------------------
------------------GATGGTTCACAGGTG---GACAAGATGTCTGAAGTTCACGAG
ATTTCTGAGGAATTG---

>Japanese_treefrog_AQP9
------------ATGAGGGAAAGAAGAAGCTGTTTAGAAAAACTTGCTCTGAGGAACAGC
CTGGCAAGGGAAACCTTGTCTGAGTTTTTGGGACATGCCTGTTGGTAACCTTCACATGT
TGTAGTATCGCTACAGCAGTTCTCAACTATGGATCTTCTGGAGGAACCTTGGGTGCTACT
GTTGGCTGTTCATTGGCAGTCACTATGGCAATCTACGCAAGTGGAGGAGTGTCAGGAGGC
CACGTCAATCCAGCAGTTTCGTTTGCGATGAGTATGACTGGAAAGCTGCCATGGGTCAAA
CTTCCTTTCTACATAACAGCACAGTTCCTTGGAGCTATCACCGGATCTGCCGCTGTTTTT
GGTGTTTATTATGATGCATTAATGGCATACTCAGGA---GGAGTGTTTAGAGTAACTGGG
CCAAAT---GCTACAGCTCAAATTTTTGCAACATATCCATCTCCGTATCTGTCAACATTG
AATGGACTTGTTGATCAAATGATGTCTACAGCTCTGTTGCTCATGCTGATCTTTGCCATA
TTTGACAAAAAGAACATGCCAGCACCAAAGGGACTAGAGCCAGTTGCTGTTGGGCTCCTC
ATTCTAACATTAGCCCTGTCTCTAGGATCCAACTGTGGAGCCGCTATGAACCCAGCCAGA
GACTTAGGCCCAAGAATCTTTACAGCTCTGGCTGGCTGGGGCCCTGAAGTTTTCACTGCT
GGTGGTAGCTTTTGGTGGATCCCTGTGGTCGGACCAATGCTTGGAGCTGTAATCGGGTCC
TACATATACATTCTTTGCATTGACATTCATCACAAGAAAGAGCCAGAT---------CAT
---GAA----------------ATGGATCCTGATCACTTT---------------------
------------------GAAAAACATGAACTT---GCCAACATGACTGAAAAGCCTAAA
ACTCGTTGC---

>Southern_gray_treefrog_AQP9
AGCACTTTAGACATGAGGGAAAGAAGAAGCTGTTTAGAAAAACTTGTTCTGAGGAACAGC
CTGGCAAGGGAAACCTTGTCTGAGTTTTTCGGGACATGGCTGTTGGTAACCTTCACATGT
TGTAGTATCGCTACAGCTGTTCTCAACTATGGATCTTCTGGAGGAACCTTGGGTGCTGCT
GTTGGCTGTTCATTGGCAGTCACTATGGCAATCTACGCAAGTGGAGGAGTTTCAGGAGGC
CATGTCAATCCAGCAGTTTCTTTTGCGATGAGTATGACTGGAAAGCTGCCATGGGTTAAA

CTTCCTTTCTACATAACGGCACAGTTCCTGGGAGCTATCACTGGATCTGCCTTTGTTTTT
GGTGTCTATTACGATGCATTAATGGCATACTCAGGA---GGAGTGCTTAGAGTAACTGGG
CCAAAT---GCTACAGCTCAAATTTTTGCAACATATCCATCTCCGTATCTGTCAACATTG
AATGGACTTGTTGATCAAATGATGTCTACAGCTCTGCTGCTCATGCTGATCTTTGCCATA
TTTGACAAAAAGAACATGCCAGCACCAAAGGGACTAGAGCCAATTGCTGTTGGGCTCCTC
ATTCTAACATTAGCGGTGTCTCTAGGATCTAACTGTGGAGCCGCTATGAACCCAGCCAGA
GACTTAGGCCCAAGAATATTTACAGCTCTGGCTGGCTGGGGCCCTGAAGTTTTTACTGCT
GGTGGTAGCTTTTGGTGGATCCCTGTGGTTGGACCAATGCTTGGAGCCGTAATCGGGTCC
TACATATACATTCTGTGCATTGACATTCATCACAAGAAAGAACCAGAT---------CAT
---GAC---------------ATGGATCCTGATCACTTT---------------------
------------------GAAAAACATGAACTT---GCCAACATGACTGAAAAGCCTAAA
AATCGTTGC---

>Hourglass_treefrog_AQP9
------------ATGAAGGAAAGAATGAGCTGTTTAGAAAGAATTGCTCTGAAGAACAGT
CTGGCAAAGGAAACCCTCTCAGAATTTTTCGGGACATGTCTGTTAGTAATCTTCACATGT
TGTAGTATTGCCACAGCTGTTCTCAACCATGCATCATCTGGCGGAGTCCTGGGTGCCACT
GTTGGCTGTTCACTGGCAGTGACCATGGCCGTCTACGCGGCTGGAGGAGTGTCAGGAGGC
CATGTCAATCCAGCCGTTTCTTTTGCCATGTGTATGACTGGAAAACTGCCATGGGTCAAG
CTTCCTTTCTATGTGACTGCTCAGTTCTTGGGAGCCTTCACTGGATCTGCAGCAACTTTT
GGTGTATATTATGATGCATTAATGAAATACTCAGGA---GGAGTGCTTACAGTCACTGGA
CCAAAT---GCTACAGCTCAAATTTTTGCAACGTATCCATCTCCATATCTTTCACAATTC
AATGGACTTATAGATCAAATGTTGTCTACAGCTCTGCTGCTTACTATGATCTTTGCCATT
TTTGACAAAAAGAACATGCCAGCTCCAAAGGGACTAGAGCCAGTTGCTATCGGGCTCCTG
ATTCTAACATTAGCGCTATCTCTAGGATCCAACTCTGGAGCTGCTATGAACCCAGCTAGA
GACCTAGGTCCAAGAATCTTTACAGCTCTGGCCGGCTGGGGACTTGAAGTTTTTACGGCT
GGCAATAGCTTTTGGTGGATCCCAGTGGTTGGACCAATGTTTGGAGCTGTAATTGGGGCC
TACATATACATACTATGCATTGAACTTCATCACAAAAAGGAACCAGACACCAAGAATCAC
---AAC---------------CTTGATATTGATCACTGT---------------------
------------------GAAAAACATGAACTT---GCCAACATGGTT------------

>Argentine_toad_AQP9
------------ATGAGGGAAAAAAGGAGCTGTTTGGAACGAATTGCTCTGAGGAATACA
CTGGCAAAGGAAACCCTTGCTGAATTTTTCGGGACATTTCTGCTGGTAACCTTCACATGC
TGTAGTATTGCCACATCTGTTCTCAACTATGGAACATCTGCAGGAACTCTGGGCGCTGTT
GTTGGCTGTTCATTGGGAGTCACAATGGCCATCTATGCAAGTGGAGGAGTGTCAGGTGGC
CACGTCAATCCAGCAGTGTCTTTTGCCATGTGTATAACTGGGAAACTACCATGGGTTAAA
CTTCCTTTCTACGTGAGTGCACAGTTCCTGGGAGCCATCGCTGGATCTGCAGTTGTTTTT
GGTGTTTATTATGATGCATTAAAAACATACTCGGGA---GGAGTGCTTACAGTAACTGGC
CCGAAT---GCGACAGCACACATTTTTGCAACATATCCATCTCCATATCTTACAACACTA
AATGGACTTGTAGATCAAATCATGTCCACTGCTCTGCTGCTCATTATGATTTTTGCCATG
TTTGACAAAAAGAACATGCCAGCTCCAAGTGGACTAGAGCCAATTGCTGTTGGGCTCCTC
ATTCTAGCATTGGTGCTAGCACTAGGAACTAACTGTGGAGCTGCTATGAATCCAGCTAGA
GATTTAGGTCCAAGAATCTTTACCGCTGTGGCTGGCTGGGGCTTTGAAGTTTTCACTGCT
GGCAATAGCTTTTGGTGGATCCCTGTGATTGGCCCAATGATTGGCGCTGTAATTGGGTCC
TACATATACATTCTATTCATTGAAGCTCATCACAAAAAGGAACCATTCGCCTTGGATCAA
---ACC---------------CTTGATACTGATCACTTT---------------------
------------------GAAAAACATGAACTT---GCCAACATGGCG------------

>Himalaya_frog_AQP9
AGCACAATAGCCATGCAAAAGGACAGAAGCTGTTTAGGAAAACTGAAACTAAGGAACAGC
CTGGCGAAGGAAACCCTGTCTGAGTTTTTCGGCACATGTTTGTTGATTACTTTAGGCTGT
TCGTGTGTAGCATCTTCTGTTCTCAGTGGGGGAAAGGCTGGAGGTCACTTAACTAATAAT
CTTGGCTTTGCAATGGCAGTCGCTATGGCAGTCTACGCTACTGGAGGAGTGTCTGGGGGC
CACATTAATCCTGCAGTTTCATTTGCCATGTGTTTAACTGGAAGATTAGAATGGGTCAAG
CTCCCCTTCTATGTGAGTGCACAGTTTGTGGGTGCCATTACTGGGTCTGCTTTAGTTTTT
GGTGTTTATTATGAAGCAATAATCAAATATTCTGGT---GGAGTTCTTACAGTAGATGGT
CCAAAT---GCCACAGCTCATATTTTTGCAACCTATCCTGCTCCATATCTGTCAACAATA
AATGGACTTGGAGATCAAATACTGTCTACTGCACTTCTGCTCATTTTGGTATTTGCCATA

TTTGACAAGAAAAACATTGCAGCCCCTAAGGGATTGGAGCCGATTGCAATTGGCCTTCTG
ATTATGCTTCTAGGATTAGCTCTGGGAATGAATTGTGGCGGTGCTATGAACCCAGCTAGA
GACTTAGGTCCACGAATCTTCACAGCAGTGGCTGGATGGGGTTATGATGTTTTCACAGCT
GGCAATAACTTTTGGTGGATTCCTGTAGTTGGCCCAATGGCTGGTGGAGCACTTGGAGCT
TACATATATATTCTCTGCATAGAAGCTCATCATCACAATGGACAAGATAGCATGCATCCT
----------------------GTTGATACTGATCAGTAT---------------------
-------------------GAAAAGCATGAACTT---ACCAATATGGCA------------
----

>African_bullfrog_AQP9
---ATGGTAGCCAAACAGGAGGAAAGAAGCTGTTTAAACAGACTGAAACTAAGGAATAGC
CTGGCAAAGGAAACTCTGTCTGAGTTTTTCGGTACATGTTTGTTGATTACCTTAGGCTGC
TCGTGTGTAGCATCTTCAGTTCTCAGTGATGGAAAGGCTGGAGGATTCTTGACTAATAAT
CTTGGCTTTGCAATGGCAGTGACTATGGCAATCTATGCTACTGGAGGAGTATCTGGGGGC
CACATTAATCCTGCAGTTTCATTTGCCATGTGTATAACTGGAAGATTAGAATGGGTCAAG
CTTCCCTTCTATGTCAGTGCACAGTTTTTGGGTGCCATCACTGGTTCTGCTTTAGTTTTT
GGTGTCTATTATGATTCATTAATGAAGTACACTGGT---GGCGTTTTTACAGTGGATGGT
CCAAAT---GCCACAGCTCATATCTTTGCAACCTATCCTGCTCCATATTTATCAACAATA
AATGGACTTGCAGACCAAATACTGTCTACTGCACTTTTGCTCATTATGGTATTTGCCATA
TTTGATAAAAAGAACATTGCAGCCCCTAAGGGACTGGAGCCAATTGCAGTCGGCCTTCTC
ATTATGCTTCTTGGACTCTCTCTTGGATCTAATTGTGGAGGTGCTATGAACCCAGCTAGA
GACTTAGGACCACGCATCTTCACAGCAGCAGCTGGATGGGGTTATGAGGTTTTCACAACT
GGCAATAACTTCTGGTGGATTCCTGTGGTTGGACCTCTGGTTGGTGGAGCACTTGGGGCA
TACGTATACATCCTCTGCATTGAAGCCCATCATCACAATGGACAAGAGAGCACAAATCAG
---GAT----------------GTTGATACTGAACAATTT---------------------
-------------------GAAAAGCATGAACTC---GCCAATATGCCA------------
----

>American_bullfrog_AQP9
AGTACGATAGCCATGCAGGAAGACAGAAGCTGTTTGGGCAAGCTGAAACTAAGGAACAGC
CTGGCGAAGGAAACTCTGTCTGAGTTTTTTGGCACATGTTTGTTGATTACTTTAGGCTGC
TCCTGTGTAGCATCTTCTGTTCTCAGTAATGGAAAGGCTGGAGGATTTTTGACTAATAAT
CTTGGCTTTGCAATGGCAGTCACAATGGCAATCTACGCTACTGGAGGAGTGTCTGGTGGG
CACATTAATCCTGCAGTTTCATTTGCCATGTGTTTAACTGGAAGACTAGATTGGGTTAAG
CTCCCTTTTTATGTGACTGCACAGTTTTTGGGTGCCATCACTGGATCTGCTTTAGTTTTT
GGCGTTTATTATGATGCATTAATCAAGTACACTGGT---GGAGTTTTTACAGTAGATGGT
CCAAAT---GCCACAGCTCATATTTTTGCAACCTATCCCGCTCCATATCTATCAACAATA
AATGGACTTGCAGATCAAATACTGTCTACTGCACTTCTGCTCATAATGGTATTTGCCATA
TTTGACAAGAAAAATCTTGCAGCCCCCAAGGGCCTGGAGCCAGTAGCAGTCGGCCTTCTC
ATTCTGCTTCTAGGGCTATGTCTGGGAATGAATTGTGGCGGTGCTATGAACCCGGCTAGA
GACTTAGGTCCACGCGTTTTTACAGCAGCGGCTGGCTGGGGTTATGAAGTTTTCACAGCT
GGAAATAACTTTTGGTGGATTCCTGTGATTGGACCAATGGCTGGTGGAACACTTGGAGCT
TACATATACATCCTCTGCATTGAAGCTCATCACCACAACGGACAAGATAGCATAAATAAC
---GAT----------------GTTAACACCTATCACTGT---------------------
-------------------GAAAAGCATGAACTT---GCCAATATGGCA------------
----

>Eastern_banjo_frog_AQP9
AGCACCTTAGACCTGACAGAAAAAAGGAGCTGTTTAGAGAAATTAGCTCTGAGGAACAGC
CTGGCAAAAGAAACTCTGTCTGAATTTTTCGGCACATGTTTATTGATAACTATGGGGTGT
GGCTGTGTTGCCTCATCTATTCTCAGCAGTGGAGCTGCTGGAGGACACCTTACTAATAAT
CTTGGCTTTGCGATGGCAGTCACAATGGCGATATATGCAACTGGAGGAGTGTCAGGTGGC
CATATCAATCCTGCAGTTTCATTGGCCATGTGTGCAACTGGACGGCTACCTTGGGTTAAA
CTTCCTTTCTATATTGCTGCCCAGATGTTGGGAGCTATTACAGGATCTGCAGCTGTTTTT
GGGGTCTACTATGACGCATTGCTCATGCATACAGGT---GGAGTTTTTACAGTGTCTGGT
CCAAAT---TCAACAGCACAAATTTTTGCAACGTATCCATCGCCATATCTTTCAACAATG
AATGGACTTGTAGATCAAATTATGTCTACTGCTCTGTTGCTTATTTTGATCTTTGCCATA
TTTGACAAAAAGAACATAGCAGTTCCAAAGGGACTAGAGCCTATTGCAATTGGTCTCCTC
ATTCTGGTGCTAGGACTATCATTTGGATTTAATTGTGGAGGAGCTATGAACCCAGCTAGA
GACTTAGGGCCACGGATTTTCACAGCATTGGCTGGATGGGGTCTTGAAGTTTTCAAGGCT
GGAAATAGCTTTTGGTGGATTCCTGTTGTTGGACCAATGATTGGCGCTGTACTTGGATCC

TACATATACATTCTGTGTATTGAGGTTCATCACAATAAGGAGCCTGATAGTATTAAACAT
---AAT----------------CTTGATACTGACCACTTT---------------------
------------------GAAAAACATGAACTG---GCTAACATGGCA------------

>cetFrog_AQP9
AGTACGATAGCCATGCAGGAAGACAGAAGCTGTTTGGGCAAGCTGAAACTAAGGAACAGC
CTGGCGAAGGAAACCCTGTCTGAGTTTTTTGGCACATGTTTGTTGATTACTTTAGGCTGT
TCCTGTGTAGCATCTTCTGTTCTCAGTGAAGGAAAGGCTGGAGGACTTTTGACTAATAAT
CTTGGCTTTGCAATGGCAGTCGCTATGGCAATCTATGCTTCTGGAGGAGTGTCTGGTGGG
CACATTAATCCTGCAGTTTCATTTGCCATGTGTTTAACTGGAAGACTAGATTGGGTCAAG
CTCCCTTTTTATGTGAGTGCACAGATTTTGGGTGCCATCACTGGATCTGCTTTAGTTTTT
GGTGTTTATCATGATGCATTAATCAAGTACACTGGT---GGAGTTTTTACAGTAGATGGT
CCAAAT---GCCACAGCTCATATTTTTGCAACCTATCCTGCTCCATATCTGTCAGCAATA
AATGGACTTGCAGATCAAATACTGTCTACTGCACTTCTGCTCATACTGGTGTTTGCCATA
TTTGACAAGAAAAATCTTGCAGCCCCTAAGGGACTGGAGCCGATTGCAGTTGGCCTTCTC
ATTCTGCTTCTAGGACTATCTCTGGGAATGAATTGTGGAGGTGCTATGAACCCGGCTAGA
GACTTAGGTCCACGCATCTTCACAGCGGTGGCTGGCTGGGGTTATGAAGTTTTCACAGCT
GGAAATAACTTTTGGTGGATTCCTGTGGTTGGACCAATGGCTGGTGGAGCACTTGGAGCT
TACATATACATCCTCTGCATTGAAGCTCATCACCACAATGGACAAGATATAATGAATAAC
---GAT----------------GCTAACACTGATCACTAT---------------------
------------------GAAAAGCATGAACTT---GCCAATATGGCA------------

>Common_frog_AQP9
AGTGTGATAGCCATGCAGGAAGACAGAAGCTGTTTGGGCAAGCTGAAATTAAGGAACAGC
CTGGCGAAGGAAACTCTGTCTGAGTTTTTTGGCACATGTTTGTTGATTACTTTAGGCTGC
TCCTGTGTAGCATCTTCTGTTCTCAGTGAAGGAAAGGCTGGAGGATTTTTGACTAATAAT
CTTGGCTTTGCAATGGCAGTCACGATGGCAATCTATGCTACTGGAGGAGTGTCTGGTGGG
CACATTAATCCTGCAGTTTCATTAGCCATGTCTTTAACTGGAAGATTAGATTGGGTCAAG
CTCCCTTTTTATGTGACTGCACAGTTTTTGGGCGCCATCACTGGGTCTGCTTTAGTTTTT
GGTGTTTATCATGATGCATTAATCAAGTACACTGGT---GGAGTATTTACAATAGATGGT
CCAAAT---GCCACAGCTCATATTTTTGCAACCTATCCTGCTCCATATCTGTCAACAATA
AATGGACTTGCAGATCAAATACTGTCTACTGCACTTCTGCTGATAATGGTATTTGCCATA
TTTGACAAGAAAAATCTTGCAGCCCCCAAGGGCCTGGAGCCAGTTGCAGTTGGCCTTCTC
ATTCTGCTTCTAGGGCTATGTCTCGGAATGAATTGTGGAGGTGCTATGAACCCGGCTAGA
GACTTAGGGCCACGCATCTTTACAGCGGTGGCTGGCTGGGGTTATGAAGTTTTCACAGCT
GGAAATAACTTTTGGTGGATTCCTGTGGTTGGACCAATGGCTGGAGGAGCACTTGGAGCT
TACATATACATCCTCTGCATTGAAGCTCATCACCACAATGGACAAGATAACATAAATAGC
---GAT----------------GTTAACACTGATCACTAT---------------------
------------------GAAAAGCATGAACTT---GCCAATATGGCA------------

>Ornate_chorus_frog_AQP9
------------ATGCAGAAGAACAGAGGCTTCCTTGAGAGAATCAGGATCCGAAATGAC
CTGGCAAAAGAAACCTTGGCCGAGTTCTTCGGGACGTGTGTGCTGATAACTCTTGGCTGC
TCATCCGTAGCGTCTGCGGTACTCAGTGAAGGGAAAGTCGGATCTCATCTCACTGTCATT
CTTGGCTTCTCCATGGCAGTCACTATGGGCGTCTATGCAGCTGGAGGAGTATCTGGTGGC
CACATCAATCCTGCAGTTTCATTTGCTATGTGTGTAACAGGAAGATTGCCCTGGATCAAG
CTCCCTTTTTATGTGGGTGCACAGCTTTTGGGTGCCATGTTTGGCTCTGTTTTAGTATAT
GGCGTCTATTATGAGGCGCTGATTAATTACACCGGC---GGAGTGCTGACAGTACATGGG
CCAAAC---GCCACCGCACACATCTTCGCTACCTACCCATCTTCATACCTGTCTGCATGG
AATGGGCTTGGAGATCAGATATTGGCTACAGCACTGCTGCTTATCATGATATTTGCCATA
TTTGACAAAAAGAATATCGGAGCTCCTAAGGGTCTGGAGCCAGTTGTAATCGGTCTTCTT
ATTATGCTCCTGGCACTGGCATTGGGAATGAATTGTGGGGGTGCAATGAACCCAGCCAGA
GACCTTGGACCGCGAGTCTTCACCGCAATGGCTGGATGGGGCTATGATGTCTTCACAGCT
GGCAATAACTTTTGGTGGATCCCTGTGATTGGCCCAATGATCGGGGGAGCCCTTGGAGGC
TACCTGTACATCCTGTGCATTGAAGCTCACCATACCCCAGATGAGGAACCCATTAGTAAA
CCGGGT----------------ATTGACCTTGATGACTTTGAGGTGAAT------------
------------------GAAAAACACGAACTC---GCCAGCAAGGCA------------

```
---------------
>WC_frog_AQP9
AACAGTCTGACTATAAGAGTTTACAGAAAGTTTAAGCATAAAATAGCTCTGAAGAACAGC
CTGGCAAAAGAGACACTCTCTGAGTTTTTCGCCACATGTTTACTGATAATTCTTGGCTGT
GGTTGTGTTGCAACCTCTGTCCTAAGC---AATACTTCAGATGCCTACCTCACAAATAAC
CTGGGTTTTGCAATGGCAGTGACTATAGCAGTTTATGTCTCTGGAGGGGTCTCAGGTGGC
CATATAAACCCAGCTGTCTCCTTTGCCATGTGTTTAACAGGAAGACTAAAGTGGGCTAAG
TTTCCTTTCTATGTAAGTGCACAGTTTTTGGGTGCAATTGCTGGATCTGCAGCAGTTTTT
GGCATCTATTATGATGCACTTTATAATTACACAGGA---GGGATCCTTACAGTTGATGGT
CCAAAT---GCTACAGCATACATTTTTGCAACATATCCCAAGCCTTATCTGTCTATTATG
GGTGGATTTGTAGATCAGGTAATGTCTACTGCTCTCCTACTCATTGGAGTGCTTGCAATA
TTTGACAACAAGAATTTGGGAACTCCCAAGGGGCTAGAACCCATTGCGGTTGGTCTACTC
ATTCTGCTTTTAGGCTTGTCACTTGGAATGAATTCCGGATGTGCAATGAATCCTGCAAGA
GATCTGGGACCAAGGATCTTTACAGCTATGGCTGGATGGGCATGGAAGTATTCACATCT
GGAAATAGCTGGTGGTGGGTTCCTGTCGTGGGGCCTATGCTTGGAGCAGCTATTGGTGCC
TTCATTTATGTTCTCTGCATAGAAGCTCATTTCAGTGATGACATGGAAGATGTACATAAT
---AGC---------------CTGGAAGCAGATCATTAT---------------------
------------------GAGAAACAAGAACTT---ACCAGCATGGCG------------
------------------------------------------------------------
---------------
>AC_frog_AQP9
AACAGTCTGACTATAAGAGTTTACAGAAAGTTTAAGCATAAAATAGCTCTGAAGAACAGC
CTGGCAAAAGAGACACTCTCTGAGTTTTTCGCCACATGTTTACTGATAATTCTTGGCTGT
GGTTGTGTTGCAACCTCTGTCCTAAGC---AATACTTCAGATGCCTACCTCACAAATAAC
CTGGGTTTTGCAATGGCAGTGACTATAGCAGTTTATGTCTCTGGAGGGGTCTCAGGTGGC
CATATAAACCCAGCTGTCTCCTTTGCCATGTGTTTAACAGGAAGACTAAAGTGGGCTAAG
TTTCCTTTCTATGTAAGTGCACAGTTTTTGGGTGCAATTGCTGGATCTGCAGCAGTTTTT
GGCATCTATTATGATGCACTTTATAATTACACAGGA---GGGATCCTTACAGTTGATGGT
CCAAAT---GCTACAGCATACATTTTTGCAACATATCCCAAGCCTTATCTGTCTATTATG
GGTGGATTTGTAGATCAGGTAATGTCTACTGCTCTCCTACTCATTGGAGTGCTTGCAATA
TTTGACAACAAGAATTTGGGAACTCCCAAGGGGCTAGAACCCATTGCGGTTGGTCTACTC
ATTCTGCTTTTAGGCTTGTCACTTGGAATGAATTCCGGATGTGCAATGAATCCTGCAAGA
GATCTGGGACCAAGGATCTTTACAGCTATGGCTGGATGGGCATGGAAGTATTCACATCT
GGAAATAGCTGGTGGTGGGTTCCTGTCGTGGGGCCTATGCTTGGAGCAGCTATTGGTGCC
TTCATTTATGTTCTCTGCATAGAAGCTCATTTCAGTGATGACATGGAAGATGTACATAAT
---AGC---------------CTGGAAGCAGATCATTAT---------------------
------------------GAGAAACAAGAACTT---ACCAGCATGGCG------------
------------------------------------------------------------
---------------
>Fire_salamander_AQP9
------ATGGGCAGCAAAGCTAAAAAGAGCTTGAAAAACAGACTGGCTCTGAAGAGCAGC
CTGGCTAAAGAGACACTTGCAGAGTTCGTGGCCACGTTTGTGTTGATTGTACTCGGATGT
GGATGTGTGGCCCAAGCTGTCCTTAGCAGAGGAGTTGCTGGTAATATTGTAACCATAAAT
ATTGGTTTTGCGCTAGCAGTGACAATGGCAGTATATGTTGCAGGTGGTGTTTCTGGTGGA
CATGTAAACCCTGCCATTTCCTTGGCTATGTGTGCAACTGGGAGGATGGCCTGGTGTAAA
TTCCCTCTCTATGTATGTGCACAGATGCTGGGTGCTACGGCAGGAGCAGCAGCGATTTTT
GGAATTTATTATGATGCATTCATGTCCTTTGCTGAT---GGAACGCTTGCAGTCTCAGGA
CCAAAC---GCAACTGCACATATTTTTGCCACATATCCCGCACCGTTTCTTTCCATCACA
AACGGATTTATAGAGCAAGTGGCATCAACAGCTCTCCTCGTTGTTTCAGTGTTTGCTATT
TTCGACCCCAAAAATAAAGGAGCACCTAACGGATTGGAGCCGGTGGCAGTCGGTCTGCTC
ATTTTCCTTTTAGGCTGTTCCTTGGGGATGAACTCTGGCTGTGCCATGAACCCAGCAAGG
GACCTTGGTCCAAGAATGTTCACTGCAGTGGCAGGATGGGGCCTTGAAGTTTTCACAGCC
GGTAACAACTGGTGGTGGATTCCCGTAGTGGCCCCTATGGTTGGAGGAATTATTGGCGCG
TACATCTATATTTTGTCTATCGAAATGCATCATGAAAAACAGCCAGAAGAAACAGAG---
---------------------CAAGATGGCGAGACGTAT---------------------
------------------GGAAAATATGAACTT---ACAAACACCGCAGAGATTCATCTA
GGTGCTTCCGAGACCTCATATGCA------------------------------------
---------------
>Wenxian_knobby_newt_AQP9
------ATGGGCCAAGAAGCCAAAAAGAGCTTGAAAACCAGACTAGCTCTGAAGAGCAGC
CTGGCTAAAGAGACGCTTGCCGAGTTCGTGGCCACATTTGTATTGATTGTACTCGGATGT
```

GGATGTGTTGCCCAAGCTGTCCTTAGCAGAGGGGTTGCTGGCAATGTTGTAACCATAAAT
ATTGGTTTTGCGCTGGCAGTGACAATGGCAGTATATGTTGCAGGTGGTGTTTCTGGTGGA
CATGTAAACCCTGCCGTTTCCTTAGCGATGTGTGCAACTGGGAGGATGGCCTGGTGTAAA
TTCCCCTTGTATGTATGTGCGCAGATGCTGGGTGCCATGGCTGGAGCAGCAGCAATTTTT
GGAATTTATTATGATGCATTCATGTCTTTTGCTGAT---GGAAACCTTGCAGTATCCGGA
CCAAAC---GCAACGGCACATATTTTTGCCACATATCCTGCACCGTTTCTTTCCATCACA
AATGGATTTGCAGAGCAGGTGGCATCAACAGCTCTTCTCGTTATTTCTGTGTTTGCTATT
TTCGACCCCAAGAATAAAGGGGCACCTCAGGGATTGGAGCCAGTGGTAGTCGGTTTGCTC
ATTTTTCTTCTAGGCTGTTCCCTAGGGATGAACTCTGGCTGTGCCATGAACCCAGCCAGG
GACCTTGGCCCAAGAATGTTCACTGCAGTGGCAGGATGGGGACTTGAAGTTTTCAGTGCT
GGTAACAACTGGTGGTGGGTTCCCGTAGTGGCCCCTATGGTTGGAGGAATTATTGGCGCG
TACATCTATGTGTTGTCTATCGAAATCCATCATCAAAATCAGATAGAAGATACAGAA---
---------------------CAAGATGGCGATACATAC---------------------
------------------GGAAAATATGAACTT---ACGAACAGCGCAGATATTCACCTC
GGTTCTTCCGAGACTTCATGTGCA------------------------------------

>ymSalamander_AQP9
------ATGGCTAACGCAAATAAAAAGACCTTGAAACAAAAGCTGTCTCTGAAGAGCACA
GTGGCCAGAGAGACGATGGCGGAATTTGCAGCAACGTTTGTGCTAATAGTCCTTGGGTGC
GGCTGTGTTGCCGAAGCTGTCCTCAGCAGAGGTGGCGCTGGAAATATTGTTACAGTATAC
ATCGGTTTTTCACTAGCAGTGACTATGGCAGTGTATCTTGCAGGTGGTATTTCCGGTGGG
CATGTAAACCCGGCTATTTCATTGGCCATGTGTGTAACTGGAAGGATGACCTGGGATAAA
TTTCCTTTTTATGTTTGTGCTCAGATGCTGGGTGCCTTTCTAGGATCAGCAGCAACGTTT
GGTATTTATCATGATGCATTCATGGCCTTTGCTGAT---GGAACACTTGCAGTTACAGGC
CCAAAT---GCAACTGCGCACATATTTGCCACATATCCTGCACCACATCTTTCCATCACA
AATGGAGTTGGAGAGCAAGTGGCAGCTACAGCTCTGCTGGTGTTTTTATTGTTTGCAGTT
TTTGACAAAAAGAATAAAGGAGCCCCAGAAGGGTTAGAGCCTATAGCGCTCGGGCTGCTC
ATTTTCCTTATATCCTGTTCCTTGGGAATGAACTCCGGCTGTGCCATGAATCCGGCCAGG
GACCTTGGGCCACGAATATTCACCGCAATGGCAGGATGGGGTATTGAGGTCTTCACGGCT
GGTCATCATTGGTGGTGGGTTCCTGTTGTGGCTCCGATGATTGGAGGGGTTATTGGTGCA
TATTTGTATGTCCTGCTTATCGAAATTCATGAGCAGAAACCAACAGAAGACAAAAAACCC
---AAG----------------AAAGATGATGAAATATGT---------------------
------------------GAAAAAGTTGAACTT---ATGAACACTTCTGAGTACTCACGT
GCA---

>Hokkaido_salamander_AQP9
------ATGAGTAACAAAAATAAGAGAAGCTTGAGGGAGCGACTAGCGCTGAAGAGCAGC
CTGGCCAAGTCAACGCTCGCAGAGTTTGTGGCTACATTTGTATTGATTGTACTTGGATGT
TGCAGTGTTGCCCAAGCTGTCCTAAGCAGAGGGCTTGCTGGTAATTTTATAACCGTAAAT
ATTGGCTTTGCGATGGCAGTGGCGATGGCAGTATATGTTGCAGGTGGTGTTTCCGGTGGT
CACGTAAACCCTGCCATTACATTGGCCATGTGTGCAACTGGAAGATTGGACTGGTGTAAG
TTTCCTTTTTATGTATGTGCACAGATGTTGGGTGCCATGGCTGGAGCAGCAGCAGTTTTT
GGAATCTATTATGATGCATTCATGGCCTTTGCTGAT---GGGATGCTTGAAGTCTCTGGT
CCAAAT---GCAACTGCACATATATTTGCCACGTATCCCTCACCATTTCTTTCAATCACA
AATGGATTTGCAGAGCAGGTGACATCAACTGCTCTGCTAGTTTTGGCTGTATTTGCAATT
CTTGACTCCAAAAATATAGGCGTACCGAAAGGATTGGAACCTGTGGCCATTGGTCTACTT
ATTTTTCTGCTATGTTGTTCCTTGGGCCTGAACTCTGGTTGCGCCATGAACCCAGCCAGG
GATCTTGGTCCACGAATGTTCACCGCAGTGGCCGGATGGGGTCTTGAGGTATTCACAGCT
GGGAATAATTGGTGGTGGATCCCTGTAGTTGCACCTATGGTTGGAGCAGTTATTGGCGCC
TTTATATACATCGTGACTATTGAAATGCATCATGAAAGGGAGCCAAAAGGCACAAAGTCA
---GAA----------------GAAGATGACGAAATGCTT---------------------
------------------GAGAAATATGAACTT---ACCAACACTGCA------------

>Axolotl_AQP9
------ATGACCCAAACAACCCATAAGAGCTTGCGAGACAGGCTGGCACTGAAGAACAGC
CTGTCAAAGGAGATCGTCGCTGAGTTCCTGGCCACATTTGTGTTAATTGTACTTGGATGT
GGAAGTGTTGCCCAAGCTGTCCTGAGCAGAGGAGTTGCTGGCAATGCTGTAACCATAAAT
GCTGGGTTTGCGATGGCAGTCACTATGGCCGTCTACGTCGCAGGTGGGGTTTCTGGGGGC
CATGTAAATCCTGCCATTTCTTTGGCCATGTGTGCAACTGGAAAGATGAGGTGGTGTAAA
TTCCCTTTTTATGTCTGTGCACAGATGCTGGGAGCCATGGCTGGGGCAGCAGCCATTTTT

GGAATATATTATGATGCATTCATGTCCTTTGCTGAG---GGGACGCTTGCAGTCTCAGGT
CCAAAT---GCAACAGCACATATATTTGCCACATACCCAGCATCGTTTCTTTCCACCACA
AATGGATTTGCAGAGCAAGTGGCATCGACTGCTCTCTTGGTTATTTCTGTATTTGCCATT
TTCGACAACAAAAACATAGGAGTGCCTGCAGGGCTGGAACCGGTGGTAGTTGGCCTGCTC
ATTTTTCTTTTAAGTTGTTCTTTGGGGATGAACTCTGGCTGCGCTATGAATCCAGCAAGG
GACCTAGGTCCAAGGATGTTCACCGCTGTGGCAGGATGGGGTCTTGAAGTCTTCACTGCT
GGTAATAATTGGTGGTGGGTTCCCGTAGTGGCCCCGATGATTGGAGGACTTATTGGTGCT
TATGTCTACATCCTGGCCATCGAATTGCATCATCAGAAACCGCTAGAAGACACAAAGTCA
---GAACAA---------GAGGAAGATGGATCACTATAT---------------------
------------------GGGAAATATGAACTT---ACGAATAGTGTA------------

>2Lcaecilian_AQP9
------ATGAATGCAAAGAGCAGAAGAAGCTTGAAAGATAAGATTTCACTTAAGAACAGC
CTGGTAAAAGAAACCCTGTCCGAGTTCTTTGCAACTTCTTTACTGATTGTACTTGGGTGC
GGCTGTATTGCTCAGTCTGTCCTGAGTCAAGGGGCTGCTGGGAATATTATCACGATAAGT
GCTGGGTTTGCAATGGCAGTGACTATGGCAGTGTATGTAGCAGGAGGTGTATCTGGTGGT
CACATAAACCCAGCGGTTTCATTTGCTATGTGCTTAACTGGAAAGTTAAAATGGAGAAAA
TTTCCTTTTTATGTATTGGCGCAAATGTTGGGTGCCATGGCTGGAGCAGCAGCAGTTTTC
GGGGTCTATTATGATGCATTGATGCATTACACAGGT---GGAAATCTTACCGTCAGTGGT
CCAAAT---GCAACAGCCCAGATTTTTGCAACATACCCTGCTCCGTTCCTGTCCACCATG
AATGGATTTGTAGACCAAGTGATGGCCACGGCTTTGTTAGTAATTGCTATTTTTGCTATC
TTTGATAGCAAAAATATAGGTGTACCCAAGGGGTTGGAACCAGTTGCAGTGGGTTTACTC
ATTATGCTTCTATGTTTCTCCCTGGGGCTTAACTCAGGCTGTGCCATAAACCCAGCTAGA
GATCTAGGGCCAAGGATCTTCACCGCAGCAGCAGGATGGGGACTTGATGTATTCAGGGCT
GGAAATAGTTGGTGGTGGATTCCTGTTGTTGGACCTATGATTGGAGCTGCTATTGGAGCA
TTTGCCTATATGATGTGCATTGAAATTCACCATAGTAAACAATCTGAAGATGTAAAGTCA
---GAA---------------CAAAATGATGGAATGTAT---------------------
------------------GAAAAATCTGAACTG---ACCTCTATG---------------

>tcCaecilian_AQP9
------ATGAATACAAAAGGCAGACGAAGCTGGAGAGATAGAATTGCGCTAAAGAGCAGC
CTTGCAAAAGAAGCCCTTTCTGAGTTCTTTGCAATGTCTTTACTGATTGCTTTTGGATGT
GGTTGTGTCGCTCAGACTGTTCTGAGTCGAGGACAAGCTGGGAATGTTGTAACAGTCAGT
GCTGGGTTTGCAATGGCAGTAACTATGGCAGTGTATGTAGCAGGAGGAGTATCTGGTGGT
CATGTAAACCCAGCTGTTTCATTTGCTATGTGCTTAATGGGAAAGCTTGAATGGACTAAA
TTTCCTTTCTATGTGCTGGCACAGCTTTTGGGTGCTATCGCTGGATCAGCAGTGGTCTTT
GGTGTCTATTATGATGCACTGATGGCATACACAGGT---GGAAATTTTATGACCACTGGT
CCGAAT---GCAACAGCTCAAATCTTCGCCACATACCCTTCTGAATACCTCTCCATCATG
AATGGATTTGCAGACCAAGTAATGGGAACGGCTCTGCTAGTGATCGTTATTTTTGCTATT
TTTGATAACAAAAATATAGGCGCACCCAAAGGGTTGGAACCAATTGCAGTTGGTCTCCTC
ATTATGGTTCTAAGCTTTTCTATGGGGCTAAACGCAAGCTGTGCCATAAACCCAGCCAGG
GATCTAGGGCCAAGACTCTTCTCAGCAGCAGCAGGATGGGGACTTGATGTATTCACAGCT
GGAAATAGCTGGTGGTGGGTTCCTGTTGTGGCACCTATGGTTGGAGCAGCCATTGGAACT
TTCGCCTATGTGCTATGTATTGAAGTTCATCACCCTAAACAACCAGAAAGTAAAAAGTCA
---GAG---------------CATAATTCTGACATGTAT---------------------
------------------GAAAAATCTGAACTG---ACCAATATG---------------

>Gaboon_caecilian_AQP9
------ATGACCACAGCAAGCAGACGAAGCTTGAGAGACAGAATTGCGCTGAAGAACAGC
CTTACAAAAGAAGTCTTGGCTGAGTTCTTTGCGACGTCGTTACTGATTGTTTTTGGATGT
GGTTGCGTTGCTCAGGCTGTTCTGAGTCGAGGAGCTGCTGGGAATATTGTATCAATCAGT
GCTGGGTTTGCAATGGCAGTAACTATGGCAGTGTATGTAGCAGGCGGCGTATCTGGTGGT
CACGTAAACCCAGCTGTATCATTTGCTATGTGCTTAATGGGAAAGCTTCCATGGAGAAAA
CTTCCTTTCTACATACTGGCACAGTTGCTGGGTGCCATAGCTGGATCAGCAGTGGTCTTT
GGTGTCTATCATGATGCACTGATGGAATACACGGGT---GGAGAACTTACTACCAGCGGT
CCGAAT---GCAACAGCTCATATCTTTGCCACATATCCATCTCAATTCCTCTCTACTATG
AATGGATTTGCAGACCAAGTAATAGGAACGGCTCTGTTGGTGATTGCTATTTTTGCTATT
TTTGATAGCAAAAATATAGGCGCACCCCGAGGGTTGGAACCAATTGCAGTCGGTCTGCTC

GTTATGCTTCTAAGCTTCTCGATGGGGCTAAACTCAGGCTGTGCCGTAAATCCAGCCAGA
GATCTAGGGCCAAGGCTCTTCACAGCAGCAGCAGGATGGGGACTTGATGTATTCAGAGCT
GGAAATAGCTGGTGGTGGATTCCTGTTGTGGCACCTATGGTTGGAGGAGCCGTTGGCGCT
TTCGTCTACATGATATGCATCGAAGTCCACCACAGTAAACAATCAGACAATAAGAAGTCA
---GAA---------------CACAATTCTGACATGTAT---------------------
------------------GAAAAATGTGAACTG---ACCAATATG---------------

>bCaecilian_AQP9
------ATGAATATAAAAAGCAAACGAAGCTTAAAAGACAGAATTGCACTGAAAAACACC
CTGGCAAAAGAAGCCCTGTCTGAGTTCTTTGCAACGTGTCTACTGATTGTTTTTGGATGT
GGGTGTGTCGCTCAGAGTGTTCTGAGTCGAGATGCTGCTGGAAATGTTGTGACAGTCAGT
GTTGGGTTTGCAATGGCCGTGGCTATGGGAGTCTATGTAGCAGGTGGCGTATCCGGTGGC
CACATAAACCCAGCTGTTTCATTTGCTATGAGCTTAACAGGAAGGCTTCCATGGAGTAAA
TTTCCTGTCTATGTACTGGCGCAGATATTGGGTGCCATAGTTGGATCAGCAGTGGTCTTT
GGTGTCTATCATGATGCACTGATGGAGTACACAGGT---GGAGTACTTACAACCACTGGT
CCAAAT---GCAACAGCCCATATATTTGCCACATATCCTGCTCCATTCCTCTCCACCGTA
AATGGATTTGCAGACCAAGTACTAGGAACGGCTCTGTTAGTGATTGTTGTTTTTGCTATG
TTTGATAGCAAAAATTTAGGCGTACCCAGAGGGTTGGAACCAATTGCAGTTGGTCTCCTC
ATTATGCTCCTGTGTTTCTCCATGGGACTAAATGCGGGCTGTGCAATAAACCCAGCCAGG
GATCTAGGGCCAAGGCTCTTCACAGCAGCAGCAGGATGGGGACTGGATGTATTCACAGCT
GGAAATAACTGGTGGTGGGTTCCTGTTGTGGCACCTATGATTGGAGGAGCCATTGGAGCT
TTCACCTATATACTATGTATTGAAATCCACCACAGTAAACCATCAGATGATATGAAG---
---GAG---------------CAAAATTCTGAAATGTAT---------------------
------------------GAAAAATCTGAACTG---ACCAATATG---------------

>B_bambooshark_Aqp9
ATGGAGACACAG---AGCCAGAAGCACAGCCTGATAGACAAATGCAAACTAAAGAATAGA
TGGATTAAAGAAGGGCTCGCCGAATTTTTGGGAACATTTATACTGATTCTGTTTGGTTGT
GGTTCACTTGCCCAATCAATACTGAGCAGAGGAGTATATGGCAACATGCTGACAGTTGCC
ATTGGCTTTCCATTTGGAATCACCATAGCTGTTTATGCTACCATAGGAGTATCAGGAGCA
CATCTGAATCCTGCTATTTCCCTTTCTATGTGTGTTCTTGGACGGCTTCAGTGGTTGAAA
CTGCCGGTCTACTGTTTTTCTCAGCTTGTCGGGGCCTTTGTTGGTTCAGCAGCTGTTTTT
GGACTTTACTATGATGCGTTCATGGCTTTTGATGAT---GGGAACTTCGCAATAACTGGT
CAAAAT---GCAACAGCACAAATCTTCTCCTCTTATCCAGCCTCACATTTGTCGTTTGCA
AATGGCTTCGCAGACCAGGTAGTGGGTACTGCTGCCCTTCTCTTCTCCATTCTGGCCATT
TTAGACTCTAAGAATAATTGCGTCCCCAAAGGTTTGGAGCCAGTGGTGATTGGCCTCATC
ATCATGGTCATTGGTCTTTCGATGGGGTACAACTGTGGTGGTCCCATCAACCCAGCTCGT
GATCTTGGACCACGACTCTTCACAGCCGTGGCTGGATGGGGACTGGAGGTTTTCACGGCT
GGTAATGGATGGTGGTGGGTTCCTGTCATCGCACCACTGATTGGAGGAGTTCTCGGGACT
GCCATTTATGTACTGATAATTGAGCTGCACCACATGGACACAACCCTGAAGGAGAACCAC
TGTACCTCA---------GAACAACAGGCGGAAGGGAAG---------------------
------------------GCAAAATATGAAATG---ATTACAATACAGTGCAACAAA---

>W_bambooshark_Aqp9
ATGGAGACACAG---AGCCAGAAGCACAGCCTGATAGACAAATGCAAACTAAAGAATAGA
TGGATTAAAGAAGGGCTCGCCGAATTTTTGGGAACATTTATACTGATTCTGTTTGGTTGT
GGTTCACTTGCCCAATCAATACTGAGCAGAGGAGTATATGGCAACATGCTGACAGTTGCC
ATTGGCTTTCCATTTGGAATCACCATAGCTGTTTATGCTACCATAGGAGTATCAGGAGCA
CATCTGAATCCTGCTATTTCCCTTTCTATGTGTGTTCTTGGACGGCTTCAGTGGTTGAAA
CTGCCGGTCTACTGTTTTTCTCAGCTTGTCGGGGCCTTTGTTGGTTCAGCAGCTGTGTTT
GGACTTTACTATGATGCGTTCATGGCTTTTGATGAT---GGGAACTTCGCAATAACTGGT
CAAAAT---GCAACAGCACAAATCTTCTCCTCTTATCCAGCCTCACATTTGTCGTTTGCA
AATGGCTTCGCAGACCAGATGATGGGTACTGCTGCCCTTCTCTTCTCCATTCTGGCCATT
TTAGACTCTAAGAATAATTGCGTCCCCAAAGGTTTGGAGCCAGTGGTGATTGGCCTCATC
ATCATGGTCATTGGTCTTTCGATGGGGTACAACTGTGGTGGTCCCATCAACCCAGCTCGT
GATCTTGGACCACGACTCTTCACAGCCGTGGCTGGATGGGGACTGGAGGTTTTTACGGCT
GGTAATGGATGGTGGTGGGTTCCTGTCATCGCACCACTGATTGGAGGAGTTCTCGGGACT
GCCATTTATGTTCTGATAATTGAGCTGCACCACATGGACACAACCCTGAAGGAGAATCAC

TGTACCTCA---------GAACAACAGGCGGAAGGGAAG--------------------
------------------GCAAAATATGAAATG---ATTACAATACAGTGCAACAAA---

>Whale_shark_Aqp9
ATGGAGACACAG---GCTCAGAAGCACAGCCTGATAGACAAATGCAAATTAAAGAATAGA
TGGATCAAAGAAGGGCTCGCCGAATTTCTGGGAACATTTATACTGATTCTATTTGGTTGT
GGTTCACTTGCCCAATCAATACTGAGCAGAGGAGTAAATGGCAACATGCTGACAGTTGCC
ATTGGCTTTCCATTTGGAATCACCATAGCTGTTTATGCTACCATAGGAGTATCAGGAGCA
CATCTGAATCCTGCTATTTCCCTTTCTATGTGTGTTCTTGGACGGCTTCAGTGGTTGAAA
CTGCCAGTCTACTGCTTTTCTCAGCTTGTCGGGGCTTTTATTGGCTCAGCAGCCGTTTTT
GGACTTTACTATGATGCGTTCATGGCTTTTGATGGT---GGGAACTTGGCAATAACTGGT
CAAAAT---GCAACAGCACAAATTTTCTCCTCTTATCCAGCTCCGCATTTGTCTTTTGCA
AATGGCTTTGTAGACCAGGTAGTGGGTACTGCTGCTCTACTCTTCTCCATTCTGGCTATT
TTGGACTCTAAGAATGATTGCGTCCCCAAAGGTTTGGAGCCAGTGGTGATTGGCCTCATC
ATCATGGTCATTGGTCTTTCGATGGGGTTCAACTGTGGTGGCCCCATCAATCCAGCGCGT
GATCTTGGACCACGACTCTTCACAGCTGTGGCTGGATGGGGATTTGAGGTTTTCACGGCT
GGTGATGGATGGTGGTGGGTCCCTATCATCGCACCACTGATTGGAGGAGTTCTCGGGACT
GCCATTTATGTTCTGATAATTGAGCTTCACCACAAGGAGACAGCCCCAAAGGAGAACCAC
TGTACCTCA---------GAACAACAGATGGAGGGGAAG--------------------
------------------GCAAAATATGAAATG---ACTTCAATACAGTGCAACAAA---

>Zebra_bullhead_shark_Aqp9
ATGGAAACACAG---AAGCAGAAACAGAGTCTGGTCGACAAATGCAAATTAAAGAATAGC
TGGATTAAAGAAGGACTCGCCGAATTTCTGGGAACATTTATACTGATTCTGTTTGGTTGT
GGTTCACTTGCCCAATCAATACTGAGTAGAGGAGTAAGTGGCAACATGCTGACTGTATCC
ATTGGCTTTTCATTTGGAATCACCATAGCTGTTTATGCTACCATAGGAGTATCAGGGGCA
CATCTGAATCCTGCTATTTCCCTCTCTATGTGTGTGCTGGGTCGGCTTCAATGGTTGAAA
CTTCCCATCTACTGTGTTGCTCAGTTTGTCGGGGCCTTTGTTGGTTCAGCAGCAGTTTTT
GGGCTTTATTATGATGCATTCATGGCTTTTGATGGT---GGGAACTTGGCAATAACTGGT
CAAAAT---GCAACCGCGCAGATCTTCTCCTCTTATCCAGCTCCACATTTGTCTTTTGCG
AATGGCTTTGCAGACCAGATTGTGGGTGCTGCTGCACTGCTCTTCTCTATCCTGGCCATT
CTGGACTCGAAGAATGATTGTGTACCCAAAGGTTTGGAGCCAGTGGTGATTGGCCTGATC
CTCATGGTTATCAGTCTTTCAATGGGCTTCAACTGCGGTGGTCCTATAAACCCAGCTCGT
GATCTCGGACCCAGGCTCTTTACAGCTGTGGCTGGATGGGGATTTGAGGTTTTCAGGGCT
GGTGATGGATGGTGGTGGGTTCCTGTCATCGCACCAATGATTGGCGGAGTTCTCGGGACG
GCCATTTACGTTCTGATTATTGAGCTGCACCATAAAGACATAACGCCAAAGGAGATCCGC
TGTACTTCA---------GAACCGCAAAGTGAAGGGAAG--------------------
------------------GCAAAATATGAAATG---ATTTCAGTACAATGTAACAAA---

>Cloudy_catshark_Aqp9
ATGGAAACAGAA---CCCCAGAAACACAGCCTGACAGACAAATACAAGTTAAAGAACAGC
TGGATAAAACAAGGGCTCGCCGAGTTCGTGGGGACCTTTATGTTGATTCTCTTTGGTTGC
GGTTCACTTGCCCAATCAGTACTGAGCAGAGGCGTTAGTGGCAACATGCTGACTGGTTCC
ATTGGCTTCTCATTTGGACTCACCATAGCTGTTTATGCTACCATAGGAGTGTCAGGGGCA
CATCTGAATCCTGCTATTTCCCTTTCCATGTGTGTGTTGGGAAGGCTCCAATGGTTGAAA
CTGCCTGTCTACTGCATTGCTCAGTTTATTGGAGCCTTTGTCGGTTCAGCGACAGTCTTT
GGGCTTTATTACGATGCATTCATGGCTTTTGATGGC---GGAAACTTGGCAATAACGGGC
CAAAAT---GCAACAGCGCAGATTTTCTCCTCTTATCCAGCTCCACATTTATCCTTTGCG
AATGGCTTTGCAGACCAGATCATAGGTACGGCAGCTCTGCTCTTCTCCATTCTGGCCATC
TTGGACTCGAAGAATGACGGTGTACCCAAGGGTTTGGAGCCGGTGGTCATTGGCCTGATC
ATCATGGTCATCGGTTTTTCAATGAGCTTCAACTGTGGTGGTCCTATCAACCCAGCTCGT
GACCTTGCGCCGCGGCTCTTCACAGCCATGGCGGGGTGGGATTTGGGGTATTCAGGGCC
GGCGACGGGTGGTGGTGGGTTCCTGTCATCGCACCAATGATCGGCGGAGTTGTTGGGACT
CTTGTTTATGTCCTGATCATCGAGCTGCACCACAACGAGACAGTCCCAAAGGAGAGGTCC
TGTACCTCA---------GAACAGCAACTGGAGGCGAAG--------------------
------------------GCAAAATACGAAATG---ATTTCTGTGCAGTGTAACAAA---

>Great_white_shark_Aqp9
ATGGAAACGCAG---ATTCAGAAGCACAGCCTGATAGACAAGTGCAAATTAAAGAACAGC
TGGATAAAACAAGGGCTCGCCGAATTTCTGGGGACATTTGCATTGATTCTGTTTGGTTGT
GGTTCAGTTGCCCAAGCAGTATTGAGCAGAGGAGTAAGTGGCAACCTGCTGACTGTTTCC
ATTGGCTTTCCATTTGGAATCACCATAGCTGCTTATGCTACCATAGGAGTATCAGGGGCA
CATCTGAATCCTGCTATTTCCTTTTCTATGTGTGTGCTGGGACGGCTTCAATGGTTGAAA
CTGCCTGTCTACTGCCTTGCTCAGCTTGTAGGAGCCTTTGCTGGTTCAGCAGCAGTTTTT
GGGCTTTATTATGATGCATTCATGGCTTTTGATGGT---GGGAACTTGGCAATAACTGGT
GAAAAT---GCAACAGCGCAGATTTTCTCCTCTTATCCAGCTCCACATTTGTCTCTTGCA
AATGGCTTTGCAGACCAGATCGTGGGCACTGCTGCTCTGCTCTTCGCTATCCTTGCCATT
TTGGACTCCAAGAATAATGCAGTGCCCAAAGGTTTGGAGCCAGTGGTGATTGGCCTGATC
ATCATGGTTATCAGTCTTTCAATGGGCTTCAACTGTGGCTGTCCTATCAACCCAGCTCGT
GACCTTGGGCCGCGGCTCTTCACAGCTGTGGCTGGATGGGGATTTGAGGTTTTCAGGGTC
AGCGATGGGTGGTGGTGGGTTCCTGTCATCGCACCAATGATCGGTGGAGTCCTTGGGACC
GCCATTTATGTTCTGACAATCGAGCTGCACCATGAAGAGACAACTCCAAAGGAGAGCCAC
ACTACCTCA---------GGACAGCAAGTGGAAGGGAAG---------------------
------------------GCAAAATATGAAATG---ATTTCAGTGCAGAGTAACAAA---

>Spiny_dogfish_Aqp9
ATGGAGCCGCAG---ATCCAGAAGAAGAGTCTGATAGACAGATGCAAATTAAAGAACCGC
TTCCTAAAAGAAGGGCTGGCCGAATTTTTGGGAACATTCATATTGATTCTGTTTGGTTGT
GGTTCACTCGCCCAATCAATACTGAGCAGAGGAGTAAGTGGTAATATACTGACCTCTTCC
ATTGGCTTTCCATTTGGAATTACCATAGCTGTTTATGCTACAATGGGAGTTTCAGGGGGA
CACCTGAATCCTGCGATTTCCCTTTCTATGTGTGTGCTGGGACGGCTTCAATGGTTGAAA
CTTCCTGTCTACTGTGTTGCTCAGCTTTTCGGAGCCTTTATCGGTTCAGCGGCAGTTTTT
GGGCTTTATTACGATGCCTTCATGGCTTTCGATGAC---GGGAACTTGACAATAACCGGT
CAAAAT---GCAACAGCGCAGATTTTCTCCTCTTATCCAAGTCCACATTTATCATTTGCG
AATGGCTTTGCAGATCAGATTGTGGGCGCTGCTGCACTGCTCTTCTCTATTCTGGCCATT
TTGGACTCTAAGAATGACGGTGTACCCAAAGGTTTGGAGCCAGTGGTGATTGGCCTGATC
GTCATGGTTATCGGTGTTTCAATGGGCTACAACTGTGGTTGTCCCATAAACCCAGCTCGT
GACCTTGGACCCCGGCTCTTTACAGCTGTGGCCGGATGGGGATTTGAGGTTTTCAGTGCG
GGCGATGGCTGGTGGTGGGTTCCTGTCATCGCACCAATGATTGGAGGAATGCTCGGGACT
TCCATTTATGTTCTGATAATTGAGCTGCACCACAAAGAGTCGATCCCAGAGGAGAGCCGC
TGTATTCCA---------GAACCGCAAATCGAAGGGAAG---------------------
------------------GCAAAATATGAAATG---ATTTCAATACAGTGTAACAAA---

>Little_skate_Aqp9
ATGGAAGCTCTG---GTCCAGAAGAAGTCTTTGGTGGATAAATGCAAGCTGAAGAACAGC
TGGATCAAAGAAGGGCTCGCCGAATTTCTCGGGACATTTATTCTAATTTTGTTTGGTTGC
GGTTCTATAGCTCAGTCAGTGCTGAGCAGAGGAGCGAGTGGCAATATGCTGACCTCTTCT
ATTGGTTTTCCATTTGGTGTTACGATCGGTGTTTATGCAACAATAGGGGTTTCAGGGGCG
CACCTGAATCCTGCAATTTCGCTGTCAATGTGTGTACTGGGCCGTCTTCAGTGGTTGAAA
CTTCCCATCTACTGCACTGCTCAGCTCATAGGAGCTTTTATTGGTGCCGCAGCAGTATTT
GGTCTTTATTATGATGCCTTCATGAGTTATGATGGC---GGAAACTTGACAATCACCGGT
CAAAAT---GCAACTGCACACATCTTCTCCTCTTATCCTGCTCCACATCTATCCTTTGCA
AATGGATTT------CAGATTGTGGGCACGGCCGCGCTACTTTTCTCCATTTTGGCCATT
TTGGACTCCAAGAACGATCGTGTGCCCAAGGGTCTGGAGCCGGTGGTGATCGGTCTGATC
CTCATGGTGATCGGACTATCGATGGGATACAACTGCGGTGGTCCCATCAACCCAGCGCGT
GACCTGGGTCCTCGGCTCTTCACTGCTGTGGCCGGATGGGGAATTGAGGTTTTCAGGGCT
GGTAATGGCTGGTGGTGGGTCCCTGTCATTGCGCCTTTCATCGGGGGAGTCCTGGGCACT
GCCATTTACGTTCTGTTCATTGAACTACATCACAAAGAGACGGTCTTGGAGGAGGCCCGG
TGCGTTAAA---------GAACAGCAGGCGGAGGGGAAA---------------------
------------------ACAAAATACGAAATG---ATTGATATACAGATAAACAAA---

>Winter_skate_Aqp9
ATGGAAGCTCTG---GTCCAGAAGAAGTCTTTGGTGGATAAATGCAAACTGAAGAACAGC
TGGATCAAAGAAGGGCTCGCCGAATTTCTCGGGACATTTATTCTAATTTTGTTTGGTTGC
GGTTCTATAGCTCAGTCAGTGCTGAGCAGAGGAGCGAGTGGCAATATGCTGACCTCTTCT

ATTGGTTTTCCATTTGGTGTTACCATCGGTGTTTATGCAACAATAGGGGTGTCAGGGGCG
CACCTGAATCCTGCAATTTCGCTGTCAATGTGTGTACTGGGCCGGCTTCAGTGGTTGAAA
CTTCCCATCTACTGCACTGCTCAGCTCATAGGAGCTTTTATTGGTTCTGCAGCAGTATTT
GGCCTTTATTATGATGCCTTCATGAGTTATGATGGC---GGAAACTTGACAATCACCGGC
CAAAAT---GCAACTGCACACATCTTCTCCTCTTATCCTGCTCCACATCTATCCTTTGCA
AATGGATTTGCCGACCAGATTGTGGGCACGGCCGCGCTACTTTTCTCCATTTTGGCCATT
TTGGACTCCAAGAACGATCGTGTGCCCAAGGGTCTGGAGCCGGTGGTGATCGGTCTGATC
CTCATGGTGATCGGACTATCGATGGGCTACAACTGCGGTGGTCCCATCAACCCAGCGCGT
GACCTGGGTCCTCGGCTCTTCACTGCTGTGGCCGGATGGGGAATTGAGGTTTTCAGAGCT
GGTAATGGCTGGTGGTGGGTTCCTGTCATTGCGCCTTTCATCGGGGGAGTCCTGGGCACT
GCCATTTACGTTCTGTTCATTGAACTACATCACAAAGAGACGGTCTTGGAGGAGGCCCGG
TGCGTTAAA---------GAACAGCAGGTGGAGGGGAAA---------------------
------------------ACAAAATATGAAATG---ATTGATATACAGATAAACAAA---

>Thorny_skate_Aqp9
ATGGAAGCTCAG---ATCCAGAGGAAGACTTTGGTGGATAAATGCAAACTGAAGAACAGC
TGGATCAAAGAAGGGCTCGCCGAATTTCTGGGGACATTTATTCTAATCTTGTTTGGTTGC
GGATCTATAGCTCAGTCAGTGCTGAGCAGAGGAACGAGTGGCAATATGTTGACCTCTTCT
ATTGGTTTTCCATTTGGAGTTACCATCGGTGCTTATGCGACAATAGGGGTGTCAGGGGCG
CACCTGAATCCTGCAATTTCGCTGTCAATGTGTGTACTGGGCCGGCTTCAGTGGTTGAAA
CTTCCCATCTACTGCACTGCTCAGCTCATAGGAGCTTTTATTGGTTCCGCAGCAGTATTT
GGCCTTTATTATGATGCCTTCATGAGTTATGATGGC---GGAAACTTGACAATCACCGGT
CAAAAT---GCAACTGCACACATCTTCTCCTCTTATCCTGCTCCACATCTATCCTTTGCA
AATGGATTTGCGGACCAGATTGTGGGCACGGCCGCTCTACTTTTCTCCATTTTGGCCATT
TTGGACTCCAAGAACGATCGTGTGCCCAAGGGTCTGGAGCCGGTGGTGATCGGTCTGATC
CTCATGGTGATCGGACTATCGATGGGCTACAACTGCGGTGGTCCCATCAACCCAGCACGT
GACCTGGGTCCTCGGCTCTTCACTGCTGTGGCCGGATGGGGATTTGAGGTTTTCAGGGCT
GGTAATGGCTGGTGGTGGGTCCCTGTCATTGCGCCTTTCATCGGGGGAGTCCTGGGCACT
GCCATTTACGTTCTGTTCATTGAACTACATCACAAAGAGACGGTCTTGGAGGAGGCCCGG
TGCGTTAAA---------GAACAGCAGGCGGAGGGGAAA---------------------
------------------ACAAAATATGAAATG---ATTGATATACAGTGTAACAAA---

>Smalltooth_sawfish_Aqp9
ATGCAATCACAG---ATCCAGAAGAAGAGTTTGGTAGATACATGCAAACTCAAGAACAGC
TGGATAAAAGAAGGGCTCGCCGAATTTTTGGGAACATTTATTCTGATTCTGTTTGGTTGT
GGGTCTATCGCCCAGTCAGTGCTGAGCAGAGGAGTAAGTGGCAACATGTTGACTTCTTCT
ATTGGCTTTCCATTTGGCGTTACCATTGCTGTTTATGCTACAATAGGAGTGTCAGGGGGA
CACCTGAACCCTGCTATTTCACTGTCAATGTGTGTGCTGGGGCGGCTTCAGTGGCTGAAA
CTTCCTGTCTACTGCTTCGCTCAGTTCGTCGGAGCTTTTATTGGTTCAGCGGCAGTTTTT
GGTCTTTATTATGATGCCTTCATGACTTTTGATGGT---GGGAACTTGACAATAACTGGT
CAAAAT---GCAACAGCGCAGATTTTCTCCTCTTATCCAGCTCCACATCTGTCCTTTGTA
AATGGATTTGCAGACCAGATTGTGGGCACAGCCGCACTGCTTTTCTCCATTTTGGCCATC
TTGGACTCCAAGAACAATCGTGTTCCCAAAGGTCTCGAGCCAGTAGTGATTGGTCTGATC
ATTATGGTTATCAGTCTTTCAATGGGCTTCAACTGCGGTGGTCCCATAAATCCAGCTCGT
GACCTGGGACCACGTCTCTTCACTGCTGTAGCCGGATGGGGAATTGAGGTTTTCAGGGCT
GGTGACGGCTGGTGGTGGGTTCCCGTCATTGCACCCTTGATCGGGGGCATCCTGGGCGCT
TCCATATATATTCTGTTCATTGAACTTCACCACAAAGAAAAGGTCCTAGAGGAGGTCCGC
TGTACTAAA---------GAGCAGCAAGCCGAGGGAAAG---------------------
------------------TCAAAATATGAAATG---ATTTCTGTACACTGTAACAAA---

>Ghost_shark_Aqp9
ATGGAG---------AAACAAAAGACAAGTTGGAAAGAGAAATGTAAATTAAAGAACGGT
TTGTTAAAAGAAGGGCTTGCAGAGTGCTTCGGGACATTCATACTAATTCTCTTTGGTTGT
AGCTCAAATGCCCAAACAGTGCTGAGCAGAGGAGTGAATGGTAACATAATTACCTCTTCC
GTTGGCTTTTCATTTGGAATAACCATTGCTGCTTATGCAACAATAGGAGTATCAGGGGCA
CACCTGAATCCTGCTATTTCTCTTTCTATGTGTGTGCTGGGTCAGCTTCAATGGTTGAAG
TTTCCAATTTACTGCATTGCTCAGTTTGTCGGAGCCTTTGTGGGTTCAGCAGCTGTCTTT
GGGCTCTATTATGACGCTTTCATGGCATTCGATGGT---GGAAACCTGACAATTACCGGT

GAAAAT---GCGACAGCACAAATCTTCTCTTCTTACCCGTCTCCGCATTTATCCTTCGTG
AATGGCTTTGCAGACCAGATAGTTGGCACAGCTGCACTGCTCTTCTCCGTGCTAGCCATT
CTGGACTCCAAAAACGACAGGGTGCCGAAAGGCTTGGAGCCGGTGGTGATTGGAATCATC
ATTTTGGTCCTCGGCTGTTCAATGAGCTTCAACTGCGGTGGCTCCATCAATCCAGCTCGC
GACCTTGGGCCACGCCTCTTCACGGCAGTGGCAGGATGGGGGCTGGAGGTGTTCAGG---
---GATGGCTGGTGGTGGGTTCCTGTCTTGGCACCGATGATTGGTGGAGTTGTCGGCACT
TCATTTTACCTTCTCATCATTGAACTCCACCACAAGGAATCAGTCACTGAGCAGTGTGCT
ATAGAA---------------CCACAACAATCCCCGAAG---------------------
------------------AAAAACTACGAACTG---GTTGCCATAAAGGGCAACATT---

>Smalleyed_rabbitfish_Aqp9
ATGGAA---------AAACAAATCACAAGTTGGAAAGAGAGATGCAAATTAAGGAACAGT
TTGGTAAAAGAAGGGCTTGCAGAGTTCATAGGGACATTTATACTAATTCTCTTTGGTTGC
AGTTCAAATGCCCAATCAATCCTGAGCCGAGGAGTGAACGGCAACATAATTACCTCTTCC
ATTGGCTTTTCATTTGGAATTATGATTGCTGCTTACGCAACGATAGGCGTATCAGGGGCA
CACCTGAATCCTGCTATTTCTCTTTCCATGTGTGCGCTGGGTCAGCTTCAATGGTTGAAA
TTTCCGATCTACTGCGTGGCTCAGATTGTCGGAGCCTTTGCAGGTTCAGCCGCAGTCTTT
GGGCTCTATTATGATGCTTTCATGGCATTCGATGGT---GGAAACCTGGCAATTACCGGC
GAGAAT---GCGACGGCACAGATTTTCTCTTCTTACCCGGCTCCGCATTTGTCCTTCGTC
AATGGCTTTGCAGACCAGATAGTTGGCACAGCTGCTCTGCTCTTCTCTGTGCTGGCCATT
CTGGACTCCAAAAATGACAGGGTGCCGAAAGGCTTGGAGCCGGTGGTGATTGGAATCGTC
ATTTTGGTCATCGGCTCTTCAATGAGCTACAACTGCGGTGGCTCCATCAATCCAGCTCGC
GACCTTGGGCCACGCCTCTTCACAGCAGTGGCAGGATGGGGGCTGGGGGTATTCAGGTTT
AGAGATGGCTGGTGGTGGGTTCCTGTAGTTGCACCGATGATTGGAGGATTTCTCGGCACT
TCAATTTACCTTCTCGTCATTGAACTCCACCACAAAGAAATAGCCGTTGAGAAGTCGGAG
TTTGCTATA---------GATCCACAATCATCCGCAAAG---------------------
------------------AAAAACTATGAGCTC---GTTGCCATAAAGGGCAACATT---

>Arctic_lamprey_Aqp9_13L1
ATGTGCTCCCCCCGGCACGCTCCGACGCGCCTGGCGGCCCGGATCAAGGCATCGAACCCG
ATCGTGAGGGAGGCTCTGGCTGAGTTCCTCGGCACGTTCGTGCTCATCGTGTTCGGATGT
GGTTCCGTGGCTCAGGTAGAACTGAGCCACCACTCCGCCGGAGAGACTCTCACCATCAAC
CTCGCCTTCGCATTTGGGGTGGTCATGGGCGCTTACCTGTCCTGGGGCATCTCAGGTGCA
CACCTGAACCCCGCCGTCTCGTTCTCCATGTCGCTGATCGGACGCTTCCACTGGTGGAAG
CTGCCCATTTTCTGCCTCGCGCAGTTCCTGGGCGCGTTCACAGCGGCCGCCACCGTCTAC
GGCCTCTACCACGAGGCCCTCATGGCGTTCAACGGT---GGGAACCTGACTGTGACTGGC
CCCGGT---GCCACGGCCGCGATATTCGCCACCTATCCCTCGGAACACCTGTCCATCGCC
GGTGGCTTCCTTGACCAGGTGCTGGGCACGGCGCTCCTCCTGCTGTGCGTGATGGCGCTG
CTGGACCCCAAGAACAACGCGGTGCCGCGCGGCCTCGAGCCACTGCTCGTGGGGCTCGTG
GTGCTCGTCATCGGCCTCTCCATGGGCTTCAACGCCGGCTATGCCATCAACCCCGCGCGC
GACCTCGGCCCCCGCGTCTTCACCGCCATGGTCGGATACGGCAGCGAAGTGTTCACGACG
GGCCCCCACTGGTGGTGGGTTCCCGTGGTGGCCCCCCTCGTGGGGGGCCCGCTGGGCACA
ATGTGCTACATGTTCTTCGTGGACCTGCACCACCCCGCCCCGCCGGAGCTGTCCACCGGC
AAAGCCAAGCCGCTGGTGGACAGCGACAGCGACCACCCCAAG------------------
------------------GCCAACTACGCCGTGGCGGTGAAGATGGAGCAGCAGGCA---

>Sea_lamprey_Aqp9_13L1
ATGTGCTCCCCCCGGCACGCTCCGCCGCGCCTGGCGGCCCGGATCAAGGCGTCAAACCCG
ATCGTGAGGGAGGCTCTGGCTGAGTTCCTCGGCACGTTCGTGCTCATCGTGTTCGGATGT
GGTTCCGTGGCTCAGGTGGAGCTGAGCCACCACTCCGCCGGAGAGACTCTCACCATCAAC
CTCGCCTTCGCATTTGGGGTGGTCATGGGCGCTTACCTATCCTGGGGCATCTCAGGTGCA
CACCTGAACCCCGCCGTCTCCTTCTCCATGTCGCTCATCGGACGCTTTCACTGGTGGAAG
CTGCCCATTTTCTGCCTCGCTCAGTTCCTGGGCGCGTTCACAGCAGCCGCCACCGTCTAC
GGCCTCTACCACGAGGCCCTCATGGCGTTCAACGGT---GGGAACCTGACGGTGACCGGC
CCCGGC---GCCACGGCCGCGATCTTCGCCACCTATCCCTCGGAACACCTGTCCATCGCC
GGTGGCTTCCTTGACCAGGTGCTGGGTACGGCGCTCCTCCTGCTGTGCGTGATGGCGCTG
CTGGACCCCAAGAACAACGCGGTGCCGCGCGGCCTCGAGCCACTGCTCGTGGGGCTCGTG
GTGCTCGTCATCGGCCTCTCCATGGGCTTCAACGCCGGCTATGCCATCAACCCCGCGCGC

GACCTCGGCCCCCGCGTCTTCACCGCCATGGTCGGATACGGCAGCGAAGTGTTCACGATG
GGGCCCCACTGGTGGTGGGTCCCCGTAGTGGCCCCCCTCGTGGGGGGCCCGCTGGGCACA
ATGTGCTACATATTCTTCGTGGACCTGCACCACCCCGCCCCGCCGGAGCTGTCCACCGGC
AAAGCCAAGTCGCTGGTGACCAGCGACAGCGAGCACGCCAAG------------------
------------------GCCAACTATGCCGTGGTGGTGAAGATGGAGCAGCAGGCA---

>Arctic_lamprey_Aqp9_13L2
ATGGGCTCCCCAGGGCCGCTCTACTCGCGCCTG---TCGCGGGTCAAAGTGTCCAATCCC
GTGCTGCGAGAAGCCCTCGCGGAGTTCATCGGCGTCTTTATTCTCATCCTGTTCGGCTGC
GGCTCAGTGGCCCAGGTGCATCTGAGCCGCCACTCCGCCGGGCAGACTCTCGGCATCAAC
TTCGCCTTCGCCTTCGGGGTGGTCATGGGGGCCCACCTGGCCTGGGGAGTCTCGGGTGCG
CACCTGAACCCCGCCGTCTCGCTCTCCATGTCGCTGCTCGGCCGCTTACCCTGGAGGAAG
CTCCCCGTGTTCTGCCTCGCGCAGTTCCTGGGCTCGTTCGTGGCGGCTGCCACCGTCTAC
GGCCTCTACTACGATGCCCTCATGGCGTTCAACGGC---GGGAACCTGACGGTGACCGGC
CCCGGT---GCCACGGCCGCGATCTTCGCCACTTATCCCTCGGAACACCTGTCCATCGCC
GGCGGCTTCTTTGACCAGGTGCTGGGCACGGCGGCGCTGCTGCTGTGCATCATGGCACTC
CTCGATTCCAAGAACAACGCCGTGCCGCGCGGCCTGGAGCCGCTGCTCATCGGCCTGGCG
GTGTTCGGCATCAGCCTCTCCATGGCCTTCAACTCGGGGTGCGCCATCAACCCCGCGCGA
GACTTCGGCCCCCGCCTCTTCACCGCCATGGCGGGATACGGCAGCGAGGTGTTCACGGTG
GGCCCCCATTGGTGGTGGGTCCCCATCATCGCCCCCCTCGTGGGGGGCCCGCTGGGTGCA
CTGTGCTACATTTTGTTCGTGGACCTGCATCACATCGCCCCGCCGGAGCTGCCCCCAATG
GCGCTGGAGCCGCTGGTGTCCGACGAGAACCACCACAGCAAG------------------
------------------GGCAGCTACGTCGCC---GTGAAGACGGGGCAGCAGCCA---

>Sea_lamprey_Aqp9_13L2
ATGGGCTACCCAGGGCCGCTCTACTCCCGCCTG---TCGCGGGTCAAAGTGTCCAATCCC
GTGTTGCGAGAAGCTCTCGCGGAGTTCATCGGCGTCTTTATTCTAATCCTGTTCGGCTGC
GGTTCAGTGGCCCAGGTGCATCTGAGCCGCCACTCCGCCGGGCAGACTCTTGGCATCAAC
TTCGCCTTCGCCTTCGGGGTGGTCATGGGGGCATACCTCGCCTGGGGGATCTCGGGTGCG
CACCTGAACCCCGCCGTTTCGCTCTCCATGTCGCTGCTCGGCCGCTTACCCTGGAGGAAG
CTCCCCGCGTTCTGCCTCGCTCAGTTCCTCGGCTCGTTTTTGGCAGCTGCCACCGTCTAC
GGCCTCTACTACGAGGCCCTCATGGCGTTCAACGGT---GGGAACCTGACGGTGACCGGC
CCCGGC---GCCACGGCCGCAATCTTCGCCACCTATCCCTCTGAACACCTGTCCATCGCC
GGTGGCTTCTTTGACCAGGTGCTGGGCACGGCGGCGCTGCTGCTGTGCATCATGGCGCTC
CTCGACTCCAAGAACAACGCCGTGCCGCGCGGCCTGGAGCCGCTACTCATCGGCCTGGCG
GTGTTCGGCATCAGCCTCTCCATGGCCTTCAACTCCGGGTGTGCCATCAACCCCGCGCGA
GACTTCGGCCCCCGCCTCTTCACTGCCATGGCGGGATACGGCAGAGAAGTGTTCACGGTG
GGCCCCCACTGGTGGTGGGTCCCCATCATTGCCCCCCTCATGGGGGGCCCGCTGGGTGCC
CTGTGCTACATATTATTCGTGGACCTGCATCACCTCGCCCCGCCGGAGCTGCCCGCAATA
GTGCTGGAGCCGCTGGTGTCCGACGAGAACAACCACAGCAAG------------------
------------------GGCAGCTACGTCACA---GTGAAGACGGAG------------

>Hourglass_treefrog_AQP10
ATGGCCCCCTGG---GACCTCCGAGAGCGAACACAGGAACTGCTCCGCATCAGGAACCCC
CTTATCCGCGAATGTCTGGCCGAATTTCTGGGGGTCTTTGTACTGCTTTTCATCACCACC
GCGGCTGTGGCCCAAGGCGTCACCAGCTCTGAGACCAAAGGCAACTTCTTCTGCATGTAC
CTGGCAGGGGCCATCGCTGTGGTCATCGCTATATATGTGTCCGGAGGAGTGTCAGGTGGA
CACCTCAACCCGGCCTACTCACTCAGCTTGTGCGTGCTCGGACGCTTCCCTTGGCGGAAA
TTACCCCTTTATGTTTTGATTCAGCTGCTGGGGTCATTTATTGGGGCAGCAGCAACCTTC
GCTCTGTACTATGACGCCATTCAGCATTACACCAGG---GGTAACCTAACCGTGTATGGA
CCCAGA---GAGACGGCTTCCATATTCTCCACCTACCCGGCCCCATATCTGAGTATAATG
AACGGCTTTCTGGATCAGGTGATGGGAACGGCCATGCTAATGACGGGGATCCTGGCCATC
TGTGACTCCAAAAACAAACCAGTCCCTAAAGGGCTAGAACCGGTGGTGGTTGGGATGTTG
GTGTTTTCCATTGGTTTGTCTATGGGAGCCAATTGTGGATACCCCATCAACCCTAGCAGA
GACCTGGGACCACGGCTCTTCACCTACGTGGCTGGCTGGGGGGTGGATGTGTTTAGGGCC
GGTAACAACTGGTGGTGGGTCCCCGTGGTGGCGCCATGTGTTGGAGGAGTTCTCGGCTCT
ATGATCTACCAGGTTTTAGTGGAATTGCACCATCCTGTAGAAGACGTCAAGAACGAGAAG
GAAGCG---------------AAGGATGTG------------------------------

------------------AAGGACGTCTCACCA---GAAAAGTGGAAAGAGATTCCAATA
TTCACTATCAATATG------------------------------------AACAATTCC
CTGTCCCACCGCTTA
>Himalaya_frog_AQP10
ATGGCACGCTGG---CCTGCCCGGGAGAGGTTGCAGAACAGGCTGAGACTCAGGAACACT
CTAGCCCGGGAATGCCTCGCCGAATTTCTTGGAGTTTTCGTCCTGCTTTTGATCACGGTT
GCGGCCACTGCGCAGGGGGTCACAAGCCAAAACACCAAGGGCAACTTCTTTTGCATGTAT
CTGGGCGGAGCCATTGGTGTCACTATGGCGATTTATGTGTCAGGGGGTGTGTCAGGTGGG
CATCTAAATCCAGCATACTCCCTCAGCCTGTGTGTCCTGGGCCGATTCCCATGGTGGAAA
TTGCCCATCTATGTCATCATCCAAATGGTGGGGTCTTTTGCTGGAGCAGCAGCTGTGTTC
GCGCTGTACTACGATGCCATCCAGAATTACACCGGC---GGCAACCTGACTGTGCATGGA
CCACGA---GAGACGGCCTCCATATTTTCTTCGTACCCCGCCCCCTACCTGAGCATTGCA
AACGGATTTCTGGATCAGGTGATGGGCACAGCAATGTTGATGATTGGCATCTTGGCCATT
ATTGATTCCAGCAACAAGCCAGTCCCAAAGGGTCTTGAGCCAATCGCGGTGGGGATGCTG
GTCTTCTCCATCGGCCTTTCCATGGGGGCAAACTGCGGCTATCCGATCAACCCAAGTCGA
GACCTGGGGCCACGGCTCTTCACCTATGTGGCGGGATGGGGCTCAGACGTCTTCAGGGCC
GGGAACAACTGGTGGTGGGTCCCCATCGTAGCACCATGCTTTGGGGGCGTTTTTGGCACG
CTGCTGTACCAGATCTTGGTGGCGATCCACCACCCAGCACAGGAAACCAGAGCCGAGGAG
GCC------------------ACCAATGTC------------------------------
---------------------GAGACCCCAGCG---GAGAAAATGAAGGAGATTCCGGTC
TTCACAATACATTTG------------------------------------GACAACTCT
TTGTCTCATCGGCTT
>African_bullfrog_AQP10
ATGGCGTGCTGG---CATGCACGGGAGAAGCTGCACAACTGGCTGAGGCTCCGGAACACC
CTGGCCCGGGAATCCCTCGCCGAATTCCTGGGGGTTTTCGTTCTGCTTCTGATCACCGTT
GCAGCCACGGCGCAGGGAGTCACCAGCCAAAGCACAAAGGGGAACTTCTTTTGCATGTAC
CTGGCTGGAGCCATTGCGGTCACCATAGCGATTTATGTGTCAGGGGGCGTTTCGGGTGCA
CATCTGAATCCGGCATACTCTCTCAGCCTGTGTGTCCTGGGCCGCTTCCCTTGGTGGAAA
CTTCCCATCTACGTCATCATCCAGATGGTGGCGTCGTTTGCTGGAGCGGCAGCTGCGTTC
GCTCTGTACTACGATGCCATCCAAAATTACACTGGT---GGCAACCTGACCGTGTATGGA
CCGCGA---GAGACGGCCTCCATTTTTTGCTCGTACCCCGCCCCTTACCTGAGCATCAGG
AATGGTTTTCTGGATCAGGTGATGGGGACGGCAATGTTGATGGTTGGCATCTTGGCTATT
CTTGATTCCAGGAACAAGCCAGTCCCAAGGGGTCTGGAGCCAATCGTGGTGGGGGCGCTG
GTCTTCTCCATTGGCCTATCCATGGGGGCAAACTGTGGCTATCCGATTAACCCCAGTCGG
GACCTTGGACCGCGGCTCTTCACCTACGTGGCGGGATGGGGTTCGGAAGTCTTCAGAGCC
GGAAACAACTGGTGGTGGGTGCCCGTGGTGGCGCCATGTGTGGGTGGAGTTTTTGGCTCG
CTGCTCTACCAGATCTTGGTGGAGATCCACCACCCGCCACTGGAGACCAAAATCGAGGAG
GCT------------------CCCAATGTG------------------------------
---------------------GAGACCACGGCG---GAAAAAAGGAAAGAGATTCCGATC
TTCACAATAAATTTG------------------------------------GACAACGCT
CTGTCCCATCGCCTT
>Argentine_toad_AQP10

------------------------AGCACCGAAACCAAAGGCAACTTCTTCTGTATGTAC
CTGGCTGGAGCCATCGCGGTCGTCATAGCCATATACGTGTCCGGAGGGGTGTCAGGTGGA
CACCTGAACCCGGCCTACTCCCTCAGCCTGTGCATGCTCGGCCGCTTCCCTTGGTGGAAA
CTGCCCCTTTATTGTCTAATTCAAATGGTGGCGTCATTTATTGGGGCAGCGGCAACATAC
GCTCTGTACTATGACGCCATTCAACATTACACTAAC---GGTAACCTGACCGTTTATGGA
CCCAGA---GAGACGGCTTCCATATTTTCTACATATCCAGCCCCGTATCTAAGCATTAGG
AATGGTTTTTTGGATCAGGTGATGGGAACGGCCATGTTGATGATTGGTATCCTGGCCATT
TGTGATTCTAAAAACAAACCC---------------------------------------

>LaPaz_rubber_frog_AQP10

ATGAAGTCTTGG---CATCTCCGGGAATGGGCGCAGAAACGCCTAAGACTCAGGAACACC
GTCCTGCGGGAATGTCTGGCTGAATTTCTGGGGGTCTTTGTGCTGCTTTTCATCACCACC
GCCGCTGTGGCCCAAGGTGTCACCAGCTTCGAGACCAAAGGGAACTTCTTCTGTATGTAC
CTGGCTGGGGCCATCGCCGTCGTGATCGCCATATACGTGTCTGGAGGAGTGTCAGGTGGG
CACCTGAACCCGGCGTATTCCCTCAGCCTGTGCGTGCTGGGCCGCTTCCCCTGGTGGAAA
TTACCCCTTTATTTCTAATTCAGATGCTGGCGTCATTTACCGGGGCAGCAGCAACGTTT
GCGCTATATTACGATGCCATTCAATATTATACCAAC---GGTAACCTGACCGTGTATGGA
CCCAGA---GAGACGGCGTCCATATTTTCCACGTACCCAGCCCCATACCTGACGATAAGG
AACGGCTTTCTGGATCAGGTGATGGGGACGGCCATGTTAATGATTGGGATCTTGGCCATC
TGTGACTCTAAAAACAAACCTGTCCCTAAAGGGCTCGAACCGATTGTCGTTGGCATGTTG
GTCTTTTCCATTGGTCTGTCAATGGGCTCTAATTGTGGATACCCGATCAACCCAAGCCGA
GACCTGGGCCCTCGTATCTTCACCTATGTGGCTGGCTGGGGGGCCGATGTGTTCAGAGCT
GGTAACAACTGGTGGTGGGTCCCTGTGGTGGCGCCGTGTGTTGGGGCAGTCCTCGGATCT
TTGATCTATGAGGTTTTAGTGGAGATTCATCATCCCGTGGAAAGCATGAAGAACGAGGAG
CTT------------------------GTG------------------------------
------------------AAGGACCTTCCACCA---GAGAAGTGGAAAGAAATTCCAATT
TTCACCATCAATATG------------------------------------GACAGTTCT
CTATCTCACCGTTTA
>Eastern_banjo_frog_AQP10
ATGGCACAATGG---AGCGTTCTACAGAGGGTACAGGGTGGTCTACGGCTGAAGAGTCCC
ATCCTACGGGAATGTCTGGCAGAATTTCTTGGGGTATTTGTGCTGCTTCTGATCACCGTT
GGAGCCACCGCTCAGGGAGTCACCAGCTCCAACACCAAAGGAAACTTCTTCTGTATGTAC
CTGGGTGGAGCCATTGCCGTCACCATAGCCATCTACGTGTCAGGAGGCATCTCCGGAGGG
CACCTGAATCCGGCATACTCCCTCAGCCTGTGCGTCCTGGGCCGGTTCCCCTGGAGGAAG
CTTCCTCTCTACTTCCTCATCCAGTTGGTCGGCTCATTCGCCGGCTCCGCCGCCGTGTTT
GCTTTATATTATGACGCCATCCAAAGTTACACCAAC---GGCAATCTGACTGTTTTTGGA
CCAAGA---GAGACGGCTTCCATATTCACCTCGTACCCCGCCCCCTATCTGAGCATCTGG
AATGGGTTCCTGGATCAGGTCATGGGGACGGCCATGTTGATGATCGGGATTTTGGCCATC
TGTGATTCCAGAAATAAACCTGTCCCCAAGGGTCTGGAACCGGTCGTAGTGGGCATGCTG
GTGTTCTCGATCGGCCTATCCATGGGAGCCAACTGTGGGTACCCCATCAACCCAACCCGA
GACCTGGGGCCCAGGCTGTTTACCTATGTGGCTGGATGGGGAGCCGATGTGTTCCGGGCT
GGTAACAGCTGGTGGTGGGTCCCGGTGGTGGCCCCCTGTGTTGGGGCAGTTCTTGGCTCT
CTGCTCTACCAGGTATTTGTGGAATTCCACCACCCACCACTGGAGGATGACATCGGAGAG
AAG---
---------------------AAATTCCCTGCG---GAGGACTGGAAAGAAATTCCTATC
TTTACAGTAAACTTG------------------------------------GACAATTCC
ATTTCCCACCGGCTG
>WC_frog_AQP10
ATGGCATGGTGG---CGCTCTCGGCGGGACCTACGGAGCTGTCTGAGGCTCAGGAACCCC
CTGGCACGGGAATGTCTCGCTGAGTTCCTCGGGGTGTTTGTGCTGCTTTTAATCACAGTC
GCAGCCACTGCTCAAGGGGTCACCAGCAATGAGACCAGGGGCAACTTCTTCTGTATGTAC
CTGGCAGGGGCAATTGCTGTCGTTCTGGCCATATACATATCTGGGGGAGTGTCAGGAGGA
CACCTGAACCCCGCCTACTCCCTCAGCATGTGTATCCTGGGGCGCTTCCCGTGGTGGAAA
CTTCCTCTCTACGCTCTCATCCAGCTGGTGGGCTCATTCGCTGGGGCGGCAGCTGCCTTT
GCTTTGTATTATGATGCCATCCGAGACTACACTAAA---GGAAACCTGACCGTCTTTGGA
CCTAGA---GAGACTGCGTCAATTTTTAGCTCTTACCCGGCCCCATACCTGAGCATTGGC
AACGGCTTCCTAGACCAGGTGATGGGAACGGCCATGCTAATGGTTGGCATCTTGGCTATC
GTGGACTCCAAGAACAAACCGGTTCCACGGGGCCTGGAACCTATCGTGGTGGGAATGCTT
GTCTTCTGCATTGGGCTGTCGATGGGCGCAAACTGCGGATACCCCATCAATCCTACACGG
GACTTGGGGCCCCGACTTTTCACTGCTGTGGCAGGTTGGGGGTTAGATGTCTTCAGAGCT
GGTAACAACTGGTGGTGGGTTCCGGTGGTGGCACCCTGTGTTGGCGCTGTTCTGGGATCG
ATTTTGTACCAGATTCTTGTGGAAATACATCATCCATTGGCTGAGAGCGAAGAGGAGCCC
CCC-------------------AAGGAGAAG-----------------------------
---------------------GAAGCCCTACAG---GAAGACACAAAGGAGGTGCAAGTG
TTCTCCATCAACCTG------------------------------------GATCATTCA
CTCTCCCACCGCCTG
>AC_frog_AQP10

--TTAATCACTGTG
GCAGCAACTGCTCAAGGGGTCACCAGCAATGAGACTAAGGGCAACTTCTTCTGCATGTAC
CTGGCAGGGGCTATTGCGGTGGTTCTGGCCATACACGTATCTGGGGGAGTGTCAGGAGGT

CACCTGAACCCTGCCTACTCCCTCAGTATGTGCCTCCTGGGGCGCTTCCCATGGTGGAAA
CTTCCTCTCTACACCCTCATCCAGCTGGTGGGCTCATTCGCTGGGGCGGCAGCTGCCTTT
GCCTTGTATTATGATGCCATCCAAGACTACACTAAA---GGAAACCTGACCATCTTTGGA
CCTAGA---GAGACAGCGTCCATCTTTTGCTCTTACCCAGCCCCATACCTGAGCATTGCC
AACGGCTTCCTTGACCAGGTGATGGGAACGGCAATGCTGTTAGTCGGTATCTTGGCTATT
GTGGACTCCAAGAATAAACCGGTTCCTCAAGGCCTGGAACCTATTGTAGTAGGAATGCTT
GTCTTCTCTATTGGGCTGTCAATGGGAGCAAACTGTAGTTACCCCATAAATCCTACGCGG
GACCTAGGGCCGAGGCTCTTCTCTGCTGTGGCAGGTTGGGGTCTAGATGTCTTCAGAGCT
GGTAACAATTGGTGGTGGGTTCCTGTTGTGGCACCATGTGTTGGTGCTGTTTTGGGTGCA
ATTCTCTACCAAATCTTTGTGGAAATACATCACCCACTGGCTAAAGATGAAGAGGAGTCC
CCC-------------------AAAGAGAAA------------------------------
---------------------GAAGCCCAGCGG---GGAGACATGAAGGAGGCACAAGTG
TTTTCCATCAGCCTG------------------------------------GATAATCCC
CTCTCCCACCGTCTG
>Fire_salamander_AQP10
ATGGCC---------CGCGCCTTGAACCGAATCAGGGCCTTGCTGCGCATAGAGCACCCT
CTCGCCCGGGAGTGCATGGCAGAGTTCCTGGGGGTCTTCGTCCTGATTCTGATGACCAAT
GGCGCAACGGCGCAGGCTGTGACCAGCTTCCACACAAAAGGTGGCTATTTCACCATGTAC
TTGGGCGGTGCGCTTGCTGTCACCGTGGCAATCTACGTCTCTGGCGGTGTCTCAGGGGGA
CACCTGAATCCAGCCTTCTCGCTCAGCATGTGCCTTCTAGGACGTTTCCAGTGGAGGAAA
CTACCGCTCTTCGTCATCATACAGATTGTTGCATCCTTTACAGCTGCCGGAACTGTCTAT
GCTCTTTATTATGACGCCATCCAACACTACTGCAGA---GGAAACCTGACTGTCACTGGC
CCCACG---GAGACCGCCTCCATCTTCGCTACGTACCCTGCCGGTTACCTCACTGTCTGG
AATGGCTTCCTGGACCAGGTTATTGGGACAGCCATGCTTCTTGTTGGCATCTTGGCAATT
GTGGACTCCAAGAACAAACCAGTGCCAAAAGGCCTGGAACCACTGGTAGTGGGATTACTA
GTTCTTTCCATTGGCCTCTCTATGGGTTCCAACTGCGGCTATCCCATCAACCCTGCCCGG
GACCTTGGCCCACGACTCTTCACTTTTGTGGCAGGTTGGGGCCCAGAAGTGTTTAGTGCC
GGGAACAACTATTGGTGGATACCAATTGCGGCACCATTAGTTGGGGCAGTCATTGGGACC
ATTGTGTATGAGGTCTTTGTGGAGTTCCATCACCTTGATAACCCCACTGAACAGCTGGCA
TGCTCG---------------GTCAATAAGGAC---------ATGGAGAAGGGTCTG---
------------------CCAGAGTCCAAGAAC---ATGAAACTAGAAGTGACCCCGGTC
TTCACCATTAACACTTACATGGAGGGATGTACGGAGCGTTCCGGAGACACAGAAGGCTAC
TTAAGCCACAGGTTA
>ymSalamander_AQP10

---------------GCAATACATAATTATTGCAAC---GGAAATCTGACTGTCACCGGC
CCCAGG---GAGACTGCATCCATCTTTGCCACTTATCCTGCTGGTTACCTCACCGTCTGG
AATGGCTTTCTGGACCAGGTCATCGGGACAGCCATGCTCCTCGTTGGCATCTTGTCAATT
GTAGATGCGAAGAATAAACCGGTACCCAAGGGCCTGGAGCCTGTGGTGGTGGGCTTACTA
GTCCTGTCCATAGGTCTCTCCATGGGCTCAAACTGTGGCTACCCCATCAACCCAGCTCGG
GACCTCGGCCCTCGACTCTTCACCTATGCAGCAGGCTGGGGTCCGGAAGTTTTTAGTGCC
GGGAACAGCTGGTGGTGGATCCCGATCGTAGCACCAATTGTTGGAGCGGTTGTTGGGTCA
TTTATATACATGATCTGCGTGGAGCTGCACCATCCCGATAAATCTTCAGAACTGCTGGAC
ATTTCT---------------GTTCACAAGAACCTTTCTTACACGGACAAA---------
---------------------AGGGCAAAGGAT---AATCAGCTGGAGGCAGTCCAGGTG
TTTAGCATTAACAACTACATG---------------------------------------

>Wenxian_knobby_newt_AQP10

---ATGACCAAT
GGCTCAACGGCGCAGGCTGTAACAAGCTTCCATACAAAAGGAGGCTATTTCACCATGTAC
TTGGGCGGTGCCCTTGCTGTTACCGTGGCAATCTACGTCTCAGGTGGCGTCTCAGGAGGG
CACCTAAATCCTGCCTTCTCGCTCAGCATGTGCCTTCTAGGACGCTTCCAGTGGAAGAAA
CTACCGCTGTTCATCGTCATACAGATTGTTGCATCATTTGCAGCTGCTGGAACAGTCTAT
GCTCTTTATTATGACGCCATCCAACACTACTGCAAC---GGAAACCTGACCGTCACTGGC
CCCACG---GAGACTGCCTCCATCTTCGCCACATACCCTGCTGGATACCTCACCGTCTGG

AATGGCTTCCTGGACCAGGTGATTGGGACAGCTATGCTTCTGGTTGGCATCTTGGCAATT
GTGGACTCCAAGAACAAACCAGTGCCGAAGGGCCTCGAACCACTGGTAGTGGGGTTACTG
GTTCTCTCCATTGGCCTCTCAATGGGTTCCAACTGCGGCTATCCCATAAACCCTGCCCGG
GACCTTGGCCCACGACTCTTCACGTTTCTGGCAGGCTGGGGTCCAGAAGTCTTCAGTGCC
GGGAACCACTATTGGTGGATACCAGTCGTCGCTCCATTATTTGGGGCAGTCATTGGGTCT
GTTGTGTATGAGGTCTTTGTGGAGTTCCACCACCTTGATAACCTCAATGAACAGCTGGCA
GGCTCC----------------GACAACAAGGAC---------ATGGAGAAGGGGATG---
-------------------TCAGAGCCCAAGATC---AAGAAACTGGAAGTGACACCAATT
TTCACAATTGACACCTACATGGAGGGATGTACAGAGTGTCCCAGAGACACAGAAGGCTAC
TTAAGCCACAGGTTA
>Axolotl_AQP10
ATGGCC---------CGCGCCATCCACCTTGCCAGGGCCCTGCTGCGCATAGAGCACCCC
CTGGCCCGCGAGTGCATGGCAGAGTTCCTGGGTGTCTTTGTGCTGATTCTGATAACCAAT
GGCGCGACGGCGCAGGCTGTGACCAGCTTACAAACAAAGGGCGGCTATTTCACTATGTAC
CTGGGAGGTGCCCTGGCGGTTACCGTAGCAATCTACGTCTCGGGTGGCGTCTCAGGAGGG
CACCTGAACCCGGCCTACACGCTCAGCATGTGCCTTCTTGGC---TTCCAGTGGAGGAAG
ATGCCGCTATTCGTCTTCATCCAGATCCTCGCCTCCTTTGCAGCTGCCGGAACTGCATAC
GCTCTTTATTACGATGCAATCCAGCAGTATTGCAAT---GGAAGCTTGACTGTAACCGGC
CCTAGG---GAGACTGCATCAATCTTCGCCACATACCCTGCCGACTACCTCACCTTATGG
AACGGCTTCCTGGACCAGGTGATTGGGACGGCCATGCTGCTTGTTGGCATCTTGGCAATC
GTGGACTCTAAGAACAAACCAGTGCCAAAAGGCCTGGAGCCGCTCCTGGTGGGCTTACTC
GTTCTCTCCATCGGCCTTTCTATGGGCTCCAACTGTGGCTATCCCATCAACCCTGCTCGG
GATCTTGGCCCGCGTCTATTTACGTTTGTGGCA------------------TTCAGTGCA
GGGAACAATTGGTGGTGGATACCGATCGTAGCACCGCTACTTGGCGCAGTCATCGGATCA
GTCGTATATGAGGTCTTTGTAGAGTTCCATCATCCTGATAACCCCTCTGAACCTTTGGCT
TCGTCA----------------ACCAGCAAAGACCACTTGTACGTGGAGAAGTGGACA---
-------------------CCGGAAACTAAGAAG---AAGAAACTAGAGATGGAGCCGGTC
TTTACTGTGGACACATACATGGAGGGATGCACAGAGAGATCCAGGGAGACAGAGGGCTAC
TTGAGCCACAGGTTG
>2Lcaecilian_Aqp10_2
ATGGCCAAGGTT---CCCACACTAGACCAAGCCAGGCCCCTGCTGCGAATCGAAAATGCC
ACTGTTCGGCAGTGCCTGGCTGAGTTCCTGGGGGTGTTTCTGCTGATCCTAATCACCATT
GGTGCAACGGCTCAAAGCGTGACCAGCTTTGACAGGAAGGGTGGCTACTTCCCCATGTGC
CTGGCAGGAGCCCTTGCTGTTACCATGGCAATTTATGTCTCAGGAGGAGTCTCAGGGGGC
CACCTGAACCCTGCCTACTCTCTGAGCCTGTGCCTGCTGGGGCGCTTCCAGTGGACAAAG
CTACCCTTGTTCTTTCTTGTCCAGACTTCGGCAGCCTTTCTCGCTGCAGCGGGGGCCTAC
GCCCTGTATTACGATGCGATCCACAACTATTGCAGT---GGGAATCTGACTGTCACTGGT
CCCCGG---GAAACTGCCTCCATTTTTGCCACCTACCCTGCCAGTTACCTCTCAGCTTGG
AATGGATTCCTGGATCAGGTGATTGGAACCGCCACCCTCCTGCTCAGCATCCTGAGTTTG
GTGGACTCCAAGAACAAGCCTGTCCCAAAAGGCCTGGAGCCCGTGGTTGTGGGCATGGTG
GTTCTCTCCATTGGCCTTTCGATGGGCTCCAACTGTGGTTACCCCATCAACCCAGCCCGG
GACCTGGGGCCCAGACTTTTCACGTGGCTGGCTGGCTGGGGCCCGGAGGTTTTCAGGGCT
GGCAATAACTGGTGGTGGATACCTATAGTGGCACCGTTAGTAGGCGCTGTTATTGGGTCA
ACTTTGTATGAGCTGATGATCGAGTTCCACCATCCGGAAACCCAGAGTGAGCTGACGGAC
GCCGGC----------------AAGGAGAGCCACTGCTTGGAGGCAGAGAAGGGACGG---
-------------------CCTCCTGTGGAGGCC---ATAAAAGCTGGAGAAGTGCCAGTT
TTCACCATTGACACATACATGGAGGGCTTCATGCAAAGGGAGGGACACAGGAAGAAAGTT
ATAAGTCACAGGCTG
>B_bambooshark_Aqp10C1
ATGGGTAGAGCAGCCACAATCCTGGTCAAAGTCCACGATGCGTTTCGATTGAAGAACAAA
CTCTTCAGAGAGTGTCTGGCAGAGTTCCTGGGGGTCTGCATGTTGATTCTGTTTGGCTGT
GGAGCTGTAGCGCAGATGGTAGTCAGTAACACGACACGTGGTGAATTCCTGTCGGTCAAT
CTCGGCTTTGGACTCGGGGCAACGTTTGGAATCTACATCTCCGGAGGGATCTCAGGT---

---------------CAGTTGATTGGCACCGGCACCCTCCTCCTGTGCATCTTCGCGGTG
GTGGACTCCCAGAACTACGGCGCTCCCAAGATCCTTCAGCCCATCTTCATCGGCCTTTCG
GTGGTGGGCATCGGGATGTCCATGGGCTCCAATTCTGGTTACGCCATCAATCCCGCCCGG
GACTTCGGGCCACGTCTGCTCACCCTGGCCGCGGGATGGGGCACCGAGGTCTTCACGGCC

GGGAACGGTTGGTGGTGGATCCCCATTGTGGCGCCCATGGTGGGCGCGGTGCTGGGGGCC
CTGGTCTACGAGTTGCTGGTGGAGCTGCACCATCTGGAGGCCAAGTCAGGTCCCACCAAC
TAC------------------GCCGATGAGGAGACGCTGAAGGGGACGGATGCCAGG---
------GGCAAGGACGGCCCGGCCATACGGAGG---GACAAGGCCGGAGCAGATGACCAG
TTCGTCATGGCGATG---

>W_bambooshark_Aqp10C1
ATGGGGAGAGCAGCCACAATCCTGGTCAAAGTCCACGATGCATTTCGATTGAAGAACAAA
CTCTTCAGAGAGTGTCTGGCAGAGTTCCTGGGGGTCTGCATGTTGATTCTGTTTGGATGT
GGAGCTGTCGCGCAGATGGTCGTCAGTAACACGACACGTGGTGAATTCCTGTCGGTCAAT
CTCGGCTTCGGACTCGGGGCAACGTTCGGAATTTACATCTCCGGAGGGATCTCAGGGGGC
CATCTGAATCCGGCCGTGTCCTTCAGTCTGTGCCTGCTTGGCCGGTTCCAATGGAAGAAG
TTGCCCTTTTACATGTTCTTCCAGACCCTGGGGGGGTTTGTCGGAGCGGCTGTAGTCTAT
GGGGTGCATCACGATGGGATCCATGCTGTGAACAAC---GGGACTTTGTCTGTCACCGGG
CCACGT---GCCACCGCCTTCATTTTTGGCACCTACCCTGCACCGTTCCTCACCCTTCCC
AACGGCTTTATAGACCAGTTGATTGGCACCGGCACCCTCCTCCTGTGCATCTTCGCGGTG
GTGGACTCCCAGAACTACGGCGCTCCCAAGATCCTTCAGCCCATCTTCATCGGCCTGTCG
GTGGTGGGCATCGGGATGTCCATGGGCTCCAATTCCGGTTACGCCATCAATCCCGCCCGG
GACTTCGGGCCGCGTCTGCTCACCCTGGCCGCGGGATGGGGCACCGAGGTCTTCACGGCC
GGGAACGGTTGGTGGTGGATCCCCATTGTGGCGCCCATGGTGGGCGCGGTGCTGGGGGCC
CTGGTCTACGAGTTGCTGGTGGAGCTGCACCACCTGGAGGCCAAGTCGGGTGCCGCCAAC
TAC------------------GCCGACGAGGAGACGCTGAAGGGGACGGGTGCCGGG---
------GGCAAGGACGGCCCGGCCGTGCGGAGG---GACAAGGCCGGAGCAGATGACCAG
TTCGTCATGGCGATG---

>Whale_shark_Aqp10C1
ATGGGCCAAGCAGCCACAATCCAAGAGAAGGTCCTTGATGCGTTTCGACTGAAGAATCAA
CTGTTCAGAGAATGTTTGGCTGAGTTTCTGGGAGTCTGCATGTTGATCCTGTTTGGGTGT
GGGGCAGTGGCACAGATGGTGGTCAGTAACACAACACGTGGTGAATTTCTGTCGGTCAAT
CTCGGCTTCGGGCTTGGTGCCACGTTTGGGATCTACATCTCCGGCGGGATCTCAGGGGGC
CATCTGAACCCGGCTGTGTCCTTCAGTCTGTGTTTACTCGGGAGGTTCCAGTGGAAGAAA
TTGCCCTTTTACATGTTCTTCCAGACCCTGGGAGGGTTTGTCGGAGCTGCTGTAGTCTAT
GGGGTACATCAC--

---GCC
GGGAACAGTTGGTGGTGGGTCCCCATCGTGGCTCCCATGGTGGGCGCGGTGCTGGGGGCA
TTGGTGTACGAGCTCCTGGTGGAACTACACCATCTGCAGGCCAAGGCAGTGCCCACAGAC
GACGAC---------------ATGATGGAGCAGAAGCTGAAGGAGGTCGGGGGCCGGGGC
ACAGCGGGCAAGGATGGCCCGTCGGTGCGGAAC---GACAAGGCCAGAGCGGACGACGAA
TTTGTCATGGCAATG---

>Great_white_shark_Aqp10C1
ATGGGGACAACCGAGATGGTGTTACAAAAGGTGCATGGTGCACTGCGATTGAAGAATCAC
CTCGTGAGGGAGTGCTTCGCCGAGTTTCTGGGAGTCTGCATGCTTATCCTCTTCGGATGT
GGTGCCGTGGCACAAATGGTGGTGAGTAAGACAACCCGCGGTGAATTCCTGTCCGTCAAC
CTGGGCTTCGGACTCGGAGCCACGTTTGGCATCTACATTTCGGGCGGCATCTCTGGTGGC
CATCTGAACCCAGCTGTATCCTTCAGCTTGTGTTTACTCGGACGGTTCCCATGGAAGAAG
CTCCCATTTTACATGTTCTTCCAAACTCTGGGAGGATTTGTTGGTGCTGCCATCGTCTAC
GGAGTTCATCACGATGGGATACACTCTGTTGACAAT---GGGACCTTATCTGTCACCGGA
CCACGA---GCCACTGCATTTATCTTTGGCACCTACCCCGCACCGTTCCTCAGCCTTTCG
AACGGCTTCATTGACCAGGTAATTGGCACTGGCACCTTGCTCCTGTGCATCTTCGCCGTG
GTGGACACCCAGAACTATGGGGCCCCCAAGATCTTGCAGCCCATCTTCATCGGCCTGTCG
ATCGTGGGCATCGGGATGTCGATGGGATCCAACTCCGGCTATGCCATCAACCCCGCCCGG
GATTTCGGGCCCCGCCTGTTGACCCTCGCTGCCGGCTGGGGGACCGAGGTTTTCACG---


```
------------------------------------------------------------
---------------
>Zebra_bullhead_shark_Aqp10C1
ATGGGGAGAGTAGAGACGTTTTTCAGAGCACTACATGATGGGTTGCAGTTGAAGAATCGC
CTCTTCAGGGAATGCTTAGCGGAGTTTCTAGGAGTTTGCATGCTGATTTTGTTTGGCTGT
GGTGCCGTGGCACAGATGGTGACGAGCCACACAACCCGCGGAGAATTCCTGTCTGTCAAC
CTAGGATTCGGCCTTGGCGCAACCTTTGGCATCTACATCTCAGGTGGCATCTCGGGTGGC
CATCTGAACCCGGCCGTGTCCTTCAGTTTGTGTTTGCTGGGAAGGTTCCAGTGGAAGAAG
CTGCCATTCTACATGTTCTTCCAGACACTTGGGGGTTTTGTTGGTGCTGCCATAGTCTTT
GGGGTCCACCATGATGGTATCTACGCTGTTGATAAC---GGGACCTTGTCCGTCACCGGA
CCGCGG---GCCACTGCATTTATTTTTGGCACGTATCCCGCAGCATACCTCACCCTTCCG
AATGGCTTCATTGACCAGCTGATTGGCACCGGCACTCTGCTCCTCTGTATTTTTGCCGTG
GTGGATGCCCGGAACTACGGAGCCCCGAAGATCTTACAGCCCATCTTCATCGGTCTGTCG
GTGGTTGGCATCGGGATGTCCATGGGCTCCAACTCTGGCTATGCCATCAACCCCGCCCGT
GATTTTGGGCCCCGCCTGTTAACCCTGGCCGCCGGCTGGGGCACCGAAGTCTTTACGGCT
GGAGGCGGGTGGTGGTGGATCCCCATCGTGGCGCCGATGGTTGGCGCGGTCCTTGGCGCC
CTGGCCTACGAGCTGCTGATCGAGCTTCACCACCTGCAGGCCAGGGACGGGCCCAGCGAG
AGGGAC---------------GTGACGGAGCAGAAGCTGAAGGAGATGGGCAATCCGGCC
GCCGGCTGCAAGGAGGGCCCGGCCTGGCGGAAC---GAGGCGGCGGGCGCGGGTGACCAG
TTCGCCGTGGCGATG---------------------------------------------
---------------
>Cloudy_catshark_Aqp10C1
ATGGGGAGAGCTGCAGCAATAGCACAGACGGTGCACAATGCTCTGCAGCTGAGGAACCGT
CTCTTTCGCGAATGCTTGGCTGAGTTTCTGGGGGTGTGCATGCTGATCCTGTTCGGGTGC
GGGGCCGTGGCACAGATGGTGGTCAGCAACACTACCCGTGGGGAATTCCTCTCCGTCAAC
CTGGGCTTTGGACTGGGCGCCACGTTCGGCATCTACATCGCTGGTGGGATCTCAGGT---
------------------------------------------------------------
------------------------------------------------------------
------------GATGGGATTCACGCTGTCAATAAC---GGGACCTTATCTGTCACTGGG
CCACGA---GCAACTGCCTTCATCTTTGGCACCTATCCAGCACCATTCCTCACCCTTTCG
AATGGCTTCATTGACCAGCTAATCGGCACCGCCACTCTACTGCTTTGCATCTTCGCTGTC
CTGGACGGCAAGAACAACGGCGCCCCCAAAGTCCTGCAGCCTATCTTCATTGGCCTGTCG
GTTGTCGGCATCGGAATGTCCATGGGATCCAACTCCGGCTACGCCATCAACCCCGCCCGA
GATTTCGGACCCCGCCTGCTGACCCTCGCCGCGGGCTGGGGGACTGAGGTCTTCACGGCT
GGCAACTCCTGGTGGTGGATACCCATCGTGGCGCCCATGGTGGGCGCCGTCCTGGGCGCC
CTGGCGTACGAGCTGCTGGTTGAACTGCACCACCTGCAGGCCCAGGACGAGTGCGAGGGG
AGGGAG---------------CAGACGGACCAGAAGCTGAAGGAGGTGGGC---------
---GGCTGCAAGGAGGGCCCCGCCTGCCGGAGA---GACCCGGACAGCTCGGGGGACCAG
TTCGTCATGGCGATG---------------------------------------------
---------------
>Little_skate_Aqp10C1
ATGGAGCAGGTCCGGGCCATCTGCAGCTCGGTTCGCGAATCACTAAAGCTGAAGAACCGC
CTGTTCCGTGAGTGTTTGGCTGAATTTCTGGGAGTTTGCGTTTTGATT------------
------------------------------------------------------------
------------------------------------------------------GGTGCA
CATCTGAATCCAGCCGTGTCCTTCAGTCTGTGTCTGCTGGGAAGATTCCAATGGAAGAAA
CTACCGTTTTACATGTTCTTCCAGACACTCGGAGGGTTTGTTGGAGCTGCCATAGTCTAC
GGAGTCCACCAC------------------------------------------------
------------------------------------------------------------
---------------CAGCTGATCGGCACGGGAACGCTGCTGCTCTGTATCCTGGCTGTG
GTGGACAGCAAGAACAATGGGGCTCCGAAGGCCCTTCAGCCCATCTTCATAGGACTGTCC
GTGCTGGCCATCGGGATGTCCATGGGATCCAACTCGGGATACGCCATCAATCCGGCCCGA
GACTTCGGACCACGGCTGCTCACACTCGCTGCTGGGTGGGGAACTGAGGTCTTCACG---
------------------------------------------------------------
------------------------------------------------------------
------------------------------------------------------------
------------------------------------------------------------
------------------------------------------------------------
---------------
>Thorny_skate_Aqp10C1
ATGGAGCGGGTCCGGGCCATCTTCAGCCCGGTTCGCGAATCACTAAAGCTGAAGAACCGC
```

CTGTTCCGTGAGTGTTTGGCTGAATTTCTGGGAGTTTGCATTTTGATTTTATTTGGCTGT
GGGGCAGTGGCCCAGATGGTCACCAGCCACACCACCCGCGGGGAATTCCTCTCCGTCAAC
CTCGGCTTCGGGATCGGAGCCACATTCGGAGTTTGTATCGCGGGAGAAATCTCCGGAGCG
CATCTGAACCCAGCCGTGTCCTTCAGCCTGTGTCTGCTCGGCCGGTTCCAATGGAAGAAA
CTACCTTTCTACATGTTCTTCCAGACGCTCGGAGGGTTTGTTGGAGCTGCCATAGTCTAC
GGAGTCCACCACGATGGGATACATGCCGTCAACAAC---GGAACTCTCTCTGTCACTGGC
CCCAAT---GCCACTGCT--

>Smalltooth_sawfish_Aqp10C1
ATGGAGAGGGTCGGGGCACTGGGGAGAAGTTTGCACCGAGCTCTGCAGCTCAAGAGTCGC
CTCTTCAGGGAATGTTTGGCCGAGTTTCTTGGAGTCTGCATTCTGGTTCTGTTCGGCTGC
GGGGCCGTCGCGCAGATGGTCACCAGTCACACCAACGCCGGGAAGTTCCTGTCCGTCAAC
CTGGGATTCGGGCTGGGAGCCACCTTCGGGGTCTACATCGCCGGGGGAATCTCCGGAGCC
CATCTGAATCCAGCCGTGTCCTTCAGCATGTGTTTGCTGGGAAGGTTCCAGTGGAGGAAG
CTGCCGTTTTATATGTTCTTCCAGACCCTCGGGGGATTCGTTGGCGCTGCGGTCGTGTAC
GGTGTGCATCACGATGGCATCTACGCGGTGAACAAT---GGCACCCTCTCTGTCACCGGC
CCAAGA---GCCACCGCCTTCATTTTCGGCACCTACCCTGCGCCCTTCCTGACCCTCCTG
AATGGCTTCATTGACCAGCTGATCGGGACGGGGACGCTTCTGCTCTGTATCCTGGCCGTG
GGGGACACCCAGAACAACGGAGCTCCCAAGGTGCTTCAGCCCATCTTCATCGGCCTATCC
ATCGTGGCCATCGGAATGTCCATGGGCTCCAACTCAGGATACGCCATCAACCCCGCCCGA
GACCTTGGACCTCGCCTCCTGACCCTGGTGGCGGGTTGGGGGACAGAAGTCTTCACGGCC
GGGGGAGGCTGGTGGTGGGTGCCGATCGTGGCGCCGATGGTGGGGGCCATCCTGGGCTCC
GCGGTCTACGAGCTGCTGATCGAGTTCCACCACTTGGAGGACCAGAGGAGGGCCGAGGAG
CTGGGT---------------CTGAAGGAGGCACGGAGGCAGAGGTCGGAGGGCGAGGAG
GCGAGGCAGCCGCAGACGGTCGTCTACAAGAAG---GAGGAGGAGGAGGGGGGGCACTAC
ATG---

>B_bambooshark_Aqp10C2
---------------------ATGAGGAGCCTCCGTCAGAAGCTGCAGATCAGGAACAGG
CTGGCCCGGGAATGCCTGGCCGAATTCTTCGGGGAATACATGCTCATTCTCATGGGCACA
GCGGCAGTGGCTCAAGTGGTGACAAACTTCGATCAGAAAGGGACCTATTTATCGATTAAC
ATTGGCTATGCTGCTGGAGTTCTGTTTGGGATCTACGCCTCAGTTGGAGTCTCAGGGGCT
CACCTGAACCCGGCGGTGACCTTCAGCCTGTGCGTCCTGGGACGGTTTCCCTGGAAAAAG
CTTCCGTTCTACACCATCTCCGAGTGCCTGGGGTCATTCGTCGCCTCGGCAACCACGTTC
ACCCTCTACTACGACGCCATCCAAGAGTTCTCTGGT---GGCAATCTGACCGTGCGTGGG
CCGAGG---GGAACAGCTGGCATCTTTGCGACGTATCCCGTGGAGTATCTGTCTGTCCGC
AACGGCTTCATAACTGAGGTAATTGGCACCGCTGTTTTGTTGATTTGCGTCTTGAGTGTT
GGCGACGCCAAAAATGCTGGTGCCCCGCCTTTCATCCAGCCGTTGCTGATCTCGGTCTCG
GTACTCGTCATCGGTGCTGCTATGGGTGCCAACACCGGCTACGCCATCAACCCAGCGAGA
GACCTCGGACCCAGACTCTTCACCTTCGTGGCTGGCTGGGGGACTGAAGTTTTCAAGGCC
GGGAATGGCTGGTGGTGGATCCCTATCGTCGCACCCCTGATTGGCGGTGTACTAGGCAGC
CTGGCCTACACGCTCCTCATCGACCTGCACCACGCGGAGCCCGTCTCGGCCAAAGAGGAG
GTGAAGGAC------------GTCAAGGCTGAA---------------------------
------------------CCTCAAGCTGAGTCA---GAGGAGGCCGCGGAGGAGCCTGTT

>W_bambooshark_Aqp10C2
---------------------ATGAGGAGTCTCCGTCAGAAGCTGCAGATCAGGAACAGG
CTGGCCCGGGAATGCCTGGCCGAATTCCTCGGGGAATACATGCTCATTCTCATGGGCACA
GCGGCAGTGGCTCAGGTGGTGACAAACTTCGATCAGAAAGGGACCTATTTATCGATTAAC
ATTGGCTATGCTGCTGGAGTTCTGTTTGGGATCTACGCCTCAGTTGGAGTCTCAGGGGCT
CACCTGAACCCGGCGGTGACCTTCAGCCTGTGCGTCCTGGGCCGGTTTCCCTGGAGAAAG

```
CTTCCGTTCTACACCATCTCCGAGTGCCTGGGGTCATTCGTCGCCTCGGCAACCACGTTC
ACCCTCTACTACGACGCCATCCACGAGTTCTCTGGT---GGCAATCTGACCGTGCGTGGG
CCGAGG---GGAACAGCTGGCATCTTTGCGACGTATCCCGTCGAGTACCTGTCTGTCCGC
AACGGCTTCATAACTGAGGTAATTGGCACCGCTGTTTTGTTGATTTGCGTCTTGAGTGTC
GGCGACGCCAAAAATGCTGGTGCCCCGGCTTTCCTCCAGCCGTTGCTGATCTCGGTCTCG
GTACTCGTCATCGGTGCTGCTATGGGTGCCAACACCGGCTATGCCATCAACCCAGCGAGA
GACCTTGGACCCAGACTCTTCACATTCGTGGCTGGCTGGGGACTGAAGTTTTCAAGGCC
GGGAACGGCTGGTGGTGGATCCCTATCGTCGCGCCCCTGATTGGCGGCGTAATAGGCAGC
CTGGCCTACACGCTCCTCATCGAACTGCACCACGCGGAACCCGTGTCGGCCAAAGAGGAG
GTGAAGGAC------------GTCAAGGCCGAA---------------------------
------------------CCTCAAGCTGAAACA---GAGGAGGCCGGGGAGGAGCCTGTT
------------------------------------------------------------
---------------
>Whale_shark_Aqp10C2
---------------------ATGAAGTCCCTCCGTCGGAAGCTGCAGATCCGGAACCAG
CTCGCCCGGGAATGCCTCGCCGAATTCCTCGGGGAATACATGCTGATT------------
------------------------------------------------------------
------------------------------------------------------------
------------------------------------------------------------
------------------------------------------------------------
------------GATGCCATCCAGGAATTTTCCGGG---GGCAATCTGACCGTGCGTGGA
CCAAGG---GGAACGGCCGGAATCTTCGCAACGTATCCTGTTGAGTACCTGTCCATCCGG
AATGGGTTCCTG---CAGTTGATTGCCACCGCTGTTTTGTTGATCTGCATCATGTGTGTC
GGCGACGCCAAAAACGCCGGTGCTCCGGCTTTCCTGCACCCCCTGCTGGTCTCGGTCGCC
GTGCTCGTCATCGGTGCCACCATGGGTGCTAACACCGGCTATGCCATCAACCCCGCACGG
GACCTTGGACCCAGGCTCTTCACCTTCATAGCTGGATGGGGCACTGAGGTGTTCAGG---
------------------------------------------------------------
------------------------------------------------------------
------------------------------------------------------------
------------------------------------------------------------
------------------------------------------------------------
---------------
>Zebra_bullhead_shark_Aqp10C2
------------------------------------------------------------
------------------------------------------------------------
------------------------------------------------------------
---------------------------------------------------TCAGGTGCT
CACTTGAACCCTGCCGTCAGTCTATGCCTCTGCGTGCTCGGCCGCTTCCCGTGGAAGAAG
CTGCCGTTTTACACCTTGGCCGAGTGTCTGGGGTCCTTCACCGCCGCGGCCACCACCTTC
TGCCTCTATTACGATGCAATACAGGAATTTTCTCAA---GGGAATTTAACTGTTCGTGGC
CCAAGA---GGCACAGCAGGTTTATTCGCCACCTACCCTGTTGAAAACCTCAGCGTTCGT
AATGGATTCATCACGGAGGTGATAGGCACCGCTGTTTTGCTAATCTGTATTCTGGCTATC
GGTGATGCCAAAAACGCGGGCGCTCCCGCTTTCCTGCAGCCCCCCCTGGTCGCCATTTCG
GTTTTCGCCATTGGCGTTGGGTTGGGTGCCAACACTGGCTATGCCATCAACCCAGCCAGA
GACATAGGA---------------------------------------------------
------------------------------------------------------------
------------------------------------------------------------
------------------------------------------------------------
------------------------------------------------------------
------------------------------------------------------------
---------------
>Cloudy_catshark_Aqp10C2
---------------------ATGAACAGTTTGAAGAAAAAACTCCAGATTAAGAACCTG
CTCGTACGACAGTGTCTGGCTGAGTTTCTGGGTGTTTACTTGCTTATGTTGATGGGGACA
GCATCTGTGGCTCAGGTTGTAACAAACTTTGACCAGAAGGGAACATTCTTATCTATTAAC
TTTGGCTATGCTGCCGGAGTCCTGTTTGGCGTCTACGCATCAGTCGGTGTCTCAGGTGCC
CATTTGAACCCCGCCGTCTCGTTGAGCCTGTGCATAATTGGCCGGTTCCCGTGGAAGAAG
CTGCCTTTCTACATCATCGCCGAGTGCCTGGGGGCCTTTATTGCCGCGGCTACCACCTTC
TGCCTTTACTACGATGCGATACACGAATTCTCTCGA---GGGAATTTCACGGTCCGAGGT
CCGCGG---GGCACAGCCGGGATCTTCGCCACCTACCCCGTTGAATACCTCTCGACCAGC
AATGGCTTCATCACCGAGGTGATTGCCACCGCCGTTTTGCTAATCTGCATCCTGTGCGTT
```

```
TTGGACAGCAAAAACGCGGCTGTGCCGCCATTTTTACAGCCGCCCATGATCGCGGTGTCA
GTGCTGGTCATTGGACTTTCCATGGGCGCCAACACTGGCTACGCCATCAATCCAGCCAGA
GATTTCGGCCCGAGGATGTTCACCTTTGTGGCTGGGTGGGCTCCGAGGTTTTCACGGCT
GGTCACGGATGGTGGTGGGTTCCGATCGTCGCCCCCATGATCGGAGGTGTCGCCGGTACC
CTAGTCTACATGCTCCTCATCGATCTGCACCACGAGGATACCAAGGAGCTTGAATCAGAA
GCCAAATTG------------ATCAAGCAGGAC---------------------------
------------------ACGGAAGATGAGCCC---GAACCGATCAGCGAGGGGCCTGTG
------------------------------------------------------------
---------------
>Great_white_shark_Aqp10C2
---------------------ATGAAGAGACTTCGGAAGAAACTCCGGATTAAGAACAAG
CTCATTCGGGAATGCCTGGCAGAATTCTTCGGCGTTTACCTGCTGATTTTGATGGGCTCG
GCGTCAGTGGCACAGGTTGTATTGTTTTTCGACCGGAAGGGCGAGTACCTGTCAATTGCC
TTTGGATACGCTTGCGGGGTCCTGTTCGGCATCTATGCATCAAGAGGAATCTCAGGTGCT
CATTTGAACCCCGCCGTCACCTTCAGTCTGTGCCTGCTGGGCCGGTGCCCATGGAAGAAG
CTGCCTTTTTACACCATCGCTGAGTGCCTGGGCTCCTTCACTGCCGCAGCGACCACCTTC
TGCCTTTATTAC------------------------------------------------
------------------------------------------------------------
---------------CAGATCATTGGTACCGCTGTTTTGTTAATCTGCATCCTGTGCGTT
GGGGATGCCAAAAATGCTGGGGCTCCGGCATTCTTACAACCCCCTCTTGTCGCCACCTCA
GTGTTTATCATTGGCATTTGCATGGGTGCCAACACTGGTTACGCCATCAACCCAGCAAGG
GACTTCGGACCTCGCTTGTTTACCTATGTAGCTGGGTGGGCACTGAGGTGTTCACGGCC
GGCAACAATTGGTGGTGGATCCCGATCGTCGCCCCGGTCCTGGGGGGAGCGCTGGGCAGC
CTGGCCTACGTTCTCCTCATCGAGATGCACCACGAGGACCCCAAGCCACTCAAA---GAT
GTCAAGGAC---------------------------------------------------
------------------GAGGCAGTCGATGAC---------------------------
GCG---------------------------------------------------------
---------------
>Little_skate_Aqp10C2
ATGAACCCGCTGAGCGGGGGATGGGTCGCGGTGAGGAGGAAACTACGCGTGAAGAACAGG
CTCCTGCGCGAATGTATGGCCGAGTTCATCGGAGAATACATGCTGATCTTGTTCGGGTCA
GCGGCTGTTGCCCAGGTGGTCACCAACTACGACAGGAAGGGGATGTATCTCTCCATCAAC
CTGGGATACGCCATTGGCGTGCTGTTTGGCATTTACGTCTCAGCCGGTGTGTCAGGGGGT
CATTTGAACCCAGCCGTGACGTTCTGCTTGTGTGTTCTGGGCCGCTGCCCGTGGTATAAG
CTGCCCCTCTACACGTTGTCCGAGTGCCTGGGGTCTTTCATGGCAGCGGCTACCACCTTC
GCCGTGTACTAC------------------------------------------------
------------------------------------------------------------
------------------------------------------------------------
------------------------------------------------------------
------------------------------------------------------------
---------------------------------------------------------GCT
GGTAATGGCTGGTGGTGGGTCCCTGTCATTGCGCCTTTCATCGGGGGAGTCCTGGGCACT
GCCATTTACGTTCTGTTCATTGAACTACATCACAAAGAGACG---GTCTTG---------
------------------------------------------------------------
------------------------------------------------------------
------------------------------------------------------------
---------------
>Smalltooth_sawfish_Aqp10C2
CTAGACAAGGCGGCGACGGGGGTGAAGGCGTTCCGGAGGCACCTACGGGTGAAGAACGAG
CTACTGCGACAGTGCCTGGCCGAATTCTTGGGGGAGTACATACTGATCTTAATGGGATCG
GCGACTGTGGCACAGGTCATCACCAACTATGACCGGAAAGGGACCTACCTGTCGATCAAC
ATGGGCTATGCCATCGGAGTCCTGTTCGGAATCTACATGTCAAGTGGAGTCTCAGGTGCA
CACCTGAATCCGGCGGTCACCTTGAGCCTGTGCGCCCTGGGTCGATTCCCCTGGCAGAAG
ATGCCCTTCTACACGCTGGCGGAATGCCTGGGCTCCTTCGTGGCAGCGGCCACCACCTAC
GCTCTTTACTACGATTCTATCCACGAGTTTTCCAAC---GGGAGTCTGACTGTGCTGGGA
CCCAGG---GGGACGGCCGGAATCTTCGCCACCTACCCCGCGGAACACATCACCACCCGC
AACGGCTTCATCACCGAGGTGATAGCCACCGGGGTGTTGTTGATCTGCATCCTGGCGATC
GGCGATTCCCGGAACGCCATGTTGCCCGACTTCCTCAGGCCGCTGGTGACCTCCATCTGC
GTGCTCACCATCGGGATGGGCATGGGAGCCAACACTGGCTACGCCATTAACCCCGCGAGG
GACATCGGGCCACGCATGTTCACCTTCGTCGCTGGCTGGGGCTCCGAAGTATTCACGGCT
GGGAACAGCTGGTGGTGGATCCCACTGGTCGCCCCGATGTTGGGGGGTCTCCTGGGGACG
```

CTGATCTACGAGCTGCTCATTGAGTTCCACCACGAGGACGCCAGGGCGGCCGAGGAGGAG
GCGCGGCTG------------GCACAGGAAGAT---------------------------
------------------CCC---GTG---------------------------------

>Ghost_shark_Aqp10C2b
CTGAGGAGCCTCCAGGCTTTGCCTGTCCAGATGCAGAGGAAACTCCACCTGAAGAACAAG
CTCCTGCGGGAATGTTTGGCCGAATTTCTGGGTGTCTTCCTGTTGATTTTTATCGGAGGT
GCGGCTGTAGCTCAGGTCCAGACCACA------GGAAAAGGTTCGTATCTGTCCATCAAC
ATCGGTTACGGTATCGGTGTTATGTTCGCCATTTACGCAGCCGGAGGCGTCTCAGGAGCT
CACCTGAACCCGGCCGTTTCCATCTCTTTCTGTGTTTTGGGGAAACTGATTTGGTGGAAA
GTCCCATTTTATATCTTCTCCCAAACGTTCGGAGCCTTCACCGCGGCGGCCGTCATCTTC
ACCATGTACTACGATTCCATCATGCACTTCACGGGG---GGGCAGCTGATCGCCGATGGG
GGAAACCTGGCAACGGGGGGAATATTCGCCACATATCCGGCCTCGTTCCTCACCACACGG
AACGGCTTCATTGACCAGATCGTGGCGACGGGGATCTTGCTCCTGGTTATCCTGAGCCTG
AACGACTCGCGGAACAATGAGCCCCCGGACTTCCTGAAGCCCCTCCTGGTGGGGGCTCTG
GTGCTGGTGATCGGAGTGGCCATGGGGTCCAATTGCGGTTACCCCATCAACCCGGCCCGC
GACATCGGCCCCCGACTCTTCTCCTACCTGGCGGGGTACGGACCCCAAGTCTTCACGGCT
GGTGATCACTGGTGGTGGGTCCCCATTGTAGCCCCAGTTATTGGGGGACTCGTTGGTTGT
TTCTTCTACAAGATCCTGATCGAGATCCACCACGACGACTTCGAGGTGGAGGAGATAGAA
CTAGAG---------------AAAGACCCCGAGAGA------------------------
------------------CCCCGAGCCGACAAT---TCATCTGAAGAGAAA---------

>Ghost_shark_Aqp10C2a
ATGGCCAGTGTGTCTGCCATCCTGAAACAAGTGAAGAGGAGGCTCCATTTGAAGAACTGT
CTCGTGCGGGAATGCTTGGCGGAGTTCCTGGGAACTTTCCTGGTTATTTTTATAGGAGGA
GCAGCGGTTGCTCAGGTACAGACCACG------AGCAAAGGTGCATACCTGTCCATTAAC
ATTGGCTATGGTGTGGGCGTCATGTTTGGCATTTATGCAGCTGGAGGCGTCTCAGGTGCT
CACTTGAACCCAGCCATCTCCATCTGCTTCTGTGTGTTGGGGAAGCTGCCGTGGTGGAAG
CTGCCATTTTACATCCTGTCTCAGACCTTTGGAGCTTTCGTAGGTGCAGCTGTGATCTTT
ACCATGTATTACGATTCCATCATGCACTTCACTGGG---GGCGAGCTGATAGCTGACGGT
GCAACGCTGGCTACTGGGGGGATATTTGCCACCTACCCTGCCGTGTACCTCACCACACGT
AATGGCTTCATTGACCAGGTTGTGGCCACTGCAGTTCTGCTGCTTGTCATCCTGAGTTTA
AACGACTCCCGGAATAATGAGCCCCCGGCGTTCCTCAAGCCCATCCTGATTGGGACGCTG
GTACTTGTGATAGGTGTGGCCATGGGCTCCAACTGTGGCTATGCCATCAATCCCGCCCGA
GACTTCGGCCCACGCCTCTTCTCCTATCTTGTTGGTTATGGCAGTCAGGTATTCACGGCT
GGGGACAGCTGGTGGTGGGTACCTATCGTGGCCCCGGTGTTGGGAGGGCTGCTTGGCTGT
TTCTTGTACAAGATCCTAATCGAGATCCACCACGATGACCCTGAGGAGCATAGCCAGCAG
CCAGCC---------------GCAGACCTCGAACTG------------------------
------------------CCAACCATAGACAGT---TCATCCAGAGAGCAG---------

>Smalleyed_rabbitfish_Aqp10C2
ATGACGAGTTTG---ACCTTCCTGAAACAAATCCGGAGGAAACTCCACGTGAAGAACAGA
CTCTTGCGGGAATGCTTGGCAGAGTTTCTGGGTGTTTTCCTGCTTGTTATTATAGGAGGA
GCAACAGTAGCCCAGGTACAGACGACA------GGAAAAGGCTCGTACCTGTCCATCAAC
ATCGGTTACGGATTGGGAGTGATGTTCGGAATTTATGCATCTGGAGGCATCTCAGGTGCT
CACCTCAACCCAGCGGTCTCAATCTCCTTCTGTGTGTTAGGAAAGCTATCTTGGTGGAAG
GTTCCATTCTACATTCTCTCCCAGATCCTCGGAGCATTTGTAGCGGCTGCCGTCATCTTC
GCCATTTATTACGATTCCATCATGCACTTTACTGGC---GGGGAGCTGATTGCTGACGGT
GGACCCCTCGCAACCGGTGGGATATTTTCCACCTATCCTGCCAAGTATCTCACCACACGC
AATGGTTTCATTGACCAGGTGGTGGCTACTGGAATTCTGCTGCTCGTCATCCTGAGTTTA
AACGACTCCTGGAATAATGAAGCCCCAGAATTTCTGAAGCCCATCCTGATTGGAGCACTG
GTGCTGGTGATAGGGGTTGCAATGGGCTCCAACTGTGGCTACCCCATCAACCCAGCACGA
GACTTCGGACCGCGCCTCTTCTCCTATTTCGCTGGCTACGGCAGTCAGGTCTTCACGGCA
GGTGACAATTGGTGGTGGGTGCCTCTTGTGGCCCCAGTTCTTGGAGGACTTATTGGCTGT
TTCTTCTACAAGATCCTAATCGAGATGCACCATGATGAGACTGAGGAAGAGGGGCAGCAA
GCA------------------GCTGACCTAAAGCAG------------------------
------------------CCAAAAGCAGATGTT---TCATCCAAAGAGAACCAGCTCCAT
GCC---

```
---------------
>Himalaya_frog_AQP7
ATGAAGATGGGGACATCCTTCATCCAATCGATCCACGCCGCCATCTCCATCCGCAACCAA
TGGGCTCGGGAGGCCATGGCCGAGATGCTGTCCACCTTCATTATGATGCTGTTTGGTTTG
GGCTCAGTGGCGCAGGTGGTTCTGGGTAAGAAGCACTATGGACAATACTTCAGCATTAAC
CTTTCCTTTGGAATCGGTGTCACGATGGGGATCCATGTGGCCGGGGGGGTATCCGGAGCT
CACATGAACTGGGCCGTGTCCTTAACGAATTGCGTCTTGGGGAATCTTCCATGGAAGAAA
CTTCCCCTTTATATGGTATCACAGGTCGCCGGATCCTTCCTGGCCGCTGCGGTGGTGTTC
TTCGTCTACTATGAGGCCCTGGACAACTACTGCGGG---GGGAATTTCACTGTAACTGGA
GATTTG---GAAACCGCCAGTATATTTGCCACCTACCCACAGCCGTACCTGTCTATAGGG
GGCGGATTCCTGGACCAGGTGGTGGGAACGGCGGCACTTATGTTATGTATTTTGGCCATC
GGTGATAAGAAGAACAGCCCAGCCCTGGATGGCATGCAGGCCGTGGTAGTCGGGCTCTTG
GTAACCGTCATTGGAATGTCTATGGGCATGAATTCCGGATACGCCATCAACCCTGCCAGG
GACCTGGGACCCCGAATCTTCACTGCCGCTGCAGGGTGGGGGCTGGAAGTGTTCAGGGCT
GGGAACTACTGGTTCTGGATACCTATTGTGGCCCCTCTAGTGGGCGCCCTGACCGGGGCT
TTCCTGTACAAGCTGCTGGTGGGACTTCACCATCAGAAAGACCAGGGGGAGAAAAAGTTA
CAGGAG---------------GTGGAGAAG------------------------------
------------------ACGAACACAGAGATG---GTGAACTGTGAACACATG------
------------------------------------------------------------
---------------
>African_bullfrog_AQP7
ATGAAGTCGGGGACGTCCATTATCAAAAGGATCCGCGCCGCCATCTCTATCCGCAACAGA
TGGGCCCGGGAGGGCATGGCCGAACTGCTGTCCACGTTTATCATGATGCTGTTTGGTTTG
GGATCCGTGGCACAAGTGGTTCTGGGTAAGAAGGAATATGGACAATACCTTAGCATTAAC
CTGTCCTTCGGAATTGGTGTCACCATGGGTATCCACGTGGCTGGAGGGGTTTCCGGAGCT
CACATGAACTGCGCCGTGTCACTGACAAACTGCATCTTAGGGAAGCTTCTGTGGAAGAAA
CTTCCCCTGTACATGTTGTGCCAAATCGCCGGAGCCTTCCTGGCAGCTGTGGTGGTGTTC
TTCCTATACTATGAGGCCCTGAACAACTACTGCGGG---GGAAATTTTACCGTAACTGGG
GACCTG---GAAACAGCCAGTATATTTGCCACCTACCCCCAGCCTTACCTGTCCATAGGC
GGAGGGTTCCTGGACCAGGTGGTGGGAACAGCTGCTCTCATGCTATGTATTTTGGCCATT
AATGATAAGAAGAACAGCCCGGCCATGAATGGCACTCAGGCCGTGGTAGTCGGACTCCTG
GTCACTGTCATTGGAATGTCTATGGGAATGAATTCTGGATATGCCATCAACCCTGCCAGG
GACCTTGGCCCCAGACTTTTCACTGCCGCTGCAGGATGGGGGCTGGAAGTGTTCAGGGCA
GGGAACTACTGGTGCTGGATACCTATTGTGGCCCCTCTAGTGGGCGCCGTAACTGGAGCC
TTCCTGTACAAACTACTAGTGGGACTTCACCATCATACAGACCAGGAGGAGGAAAAATTA
CAGGAC---------------ATGGAGAAA------------------------------
------------------GGGAACACAGAGATG---GTGAACTCTGAATACATG------
------------------------------------------------------------
---------------
>Hourglass_treefrog_AQP7
ATGAAAACTGGAAGCTTCATAGCCAGCAAGATCCGCTCCGCTATATCTATCCGCAACCAG
TGGGTACGAGAGGCCATGGCAGAACTGCTCTCCACATTTGTCATGATGATGTTCGGTTTG
GGAGCGGTGGCGCAAGTGGTTCTGGGTAAGAACGCGTTTGGAGAATACCTGAGCATCAAT
TTATCTTTCGGAATTGGAGTCACAATGGGAATGCACGTGGCTGGGGGAGTGTCTGGAGCC
CACATGAACTGTGCCGTGTCACTGACTAACTGCGTCCTGGGAAAACTTTCTTGGAAAAAA
CTCCCAATTTACATGATTTCTCAAATCGCTGGAGCGTTCCTGGCGGCTGTGATTATATAC
TTTCTATACTATGAGGCTTTGCATACTTACTGCAAA---GGAAACTTGACTGTGACGGGT
CCTTTG---GAAACAGCCAGTATATTTGCCACCTACCCGCAACCATATCTATCTATAGGT
GGGGGCTTTCTAGATCAGGTGATTGGCACGGGAGCCCTGTTGTTATGTATCCTAGCTATA
CATGATAAGAAGAACAATAGCGCTCTGAATGGGACACAAGCTGTGGTTGTCGGTCTCCTG
GTTACAGTTATCGGGATGTCTATGGGGATGAATTCTGGATATGCCATCAACCCTGCCAGG
GATTTGGGGCCACGTCTCTTCACTGCAGCGGCTGGATGGGGAATGGAAGTATTCAGGGCC
GGGAATTACTGGTGCTGGGTGCCGATTGTGGCCCCTATGGTGGGCGGCCTGACCGGGGCC
TTCCTGTACAAGCTGTTAGTGGGGCTACATCACAAGCCA---AACCCGGAGAAGGTTATA
TGTGAG---------------ATGGAGAAG------------------------------
------------------GGGAATATAGAGATG---GTGAATGCGGAATATATG------
------------------------------------------------------------
---------------
>Eastern_banjo_frog_AQP7
ATGAAGACTGGCAGCTCCATAGCCAACAGGATCCGCAGGGCCATTTCCATACATAACATA
TGGGTGAGAGAGGCCATGGCTGAGCTGTTGTCCACGTTCGTCATGATGACGTTCGGCTTG
```

GGCTCTGTCGCGCAGGTGGTTCTGGGGAAGAAAGAGTATGGACAGTACCTG---------
---AGCGGAGCC
CACATGAACTGCGCTGTGTCTCTAACAAACTGCATCCTGGGGAAGCTGCCGTGGAGAAAA
CTGCCGGTCTACATGTTATCTCAGATCGTGGGCGCGTTCTTGGCAGCCATGGTGGTGTAT
TTTCTATACTACGAGGCCTTTATCACCTTCAGCGGA---GGAGAATATATTGTGACTGGG
CCCAAT---GAAACTGCCAGTATATTTGCCACGTACCCACAGCCGTACCTGTCCATAGGG
GGCGGGTTCCTGGACCAGGTGGTCGGTACAGCCGCCCTGTTGCTCTGCATCCTGGCTATC
AATGACAAGAACAATAACGCGGCTCTGGATGGGACACAGGCCGTGGTGGTTGGGCTCCTG
GTGACGGTTATCGGAATGTCCATGGGAATGAACTCCGGCTACGCCATCAATCCAGCCAGA
GACCTGGGGCCGCGTCTCTTTACCGCGGTCGCCGGATGGGGAGTGGAAGTCTTCAGGGCT
GGGAATTACTGGTGTTGGGTGCCGATCGTGGCCCCTATGGTGGGCAGTGTGACCGGCGCC
TTCCTTTACCAGCTTCTGGTGGGACTTCACAGCCAGCCG---GTGGAAGAGAAGAAGATG
GATGAG---------------ATGGAGAAA------------------------------
-------------------GGGAACGCAGACATG---GAGACCTCCGAATACATG------
----

>Argentine_toad_AQP7
ATGAAGACTGGGCGCTTCATCGCCAACAAGATCCGCTCAGCTATATCTATCCGCAACCAA
TGGGTGCGAGAGGCCATGGCCGAGCTGCTGTCCACCTTTGTGATGATGATGTTCGGGCTG
GGAGCTGTAGCTCAGGTGGTTTTGGGTAAGAACGCATTCGGACAATACCTGAGCATTAAC
TTATCTTTCGGAATCGGAGTGACAATGGGAATCCATGTGGCCGGAGGAGTGTCTGGCGCT
CACATGAACTGTGCTGTGTCCCTAGTCAACTGCGTTCTGGGGAAACTTCCATGGAGAAAA
CTACCAATTTACATGATTTCTCAAATTGCTGGAGCATTCCTGGCTGCTGTCATTATATAC
TTTCTATACTACGAGGCTCTGTTTAATTACTGCAAA---GGAAACTTCACTGTGACAGGT
CCTTTG---GAAACAGCCAGCATATTTGCCACCTACCCGCAGCCTTACCTGTCTATAGGT
GGTGGCTTTCTAGATCAGGTGATTGGCACAGGAGCGCTGCTGCTCTGTATCCTGGCTATA
AATGATAAGAAAAACTTTGGCGCTCTGGATGGGACACAATCTGTGGTGGTCGGCCTCCTG
GTTACAGTGGTTGGGATGTCTATGGGTATGAATTCTGGATATGCTATCAACCCCGCCAGG
GATTTGGGACCACGTCTCTTCACTGCAGCTGCCGGATGGGGAGTGGAAGTATTCAGGGCT
GGGAACTATTGGTGCTGGGTGCCAATTGTAGCCCCCATGGTGGGTGGCCTGACCGGAGCC
TTCCTATACAAGCTGCTGGTGGGACTACATCTCCAGCACAACGAGGAAGAGAAGAAGCTA
TGTGAG---------------ATGGAGAAG------------------------------
-------------------GGAAATCCAGAGATG---GTGGCCACTGAATATATG------
----

>Strawberry_poison_frog_AQP7

---ACGTTCGGTTTG
GGAGCGGTGGCGCAGGTGGTTCTCGGTAAGAACGCATTCGGACAATACCTGAGCATTAAT
TTATCTTTCGGAATTGGAGTCACAATGGGAATTCACGTGGCCGGGGGAGTATCTGGAGCC
CATATGAACTGCGCCGTCTCACTAACTAACTGCATCCTGGGAAAACTTTCCTGGAAAAAA
CTCCCAGTTTACATGATTTCTCAAATTGCTGGAGCATTCCTGGCTGCTGTGATTGTATAC
TTCTTATACTATGAGGCTCTGTACAACTATTGCAAA---GGAAACTTTACTGTGACAGGT
CCTCTG---GAAACAGCCAGTATATTTGCCACCTACCCACAGCCGTACCTTTCCATAGGG
GGTGGCTTTTTGGATCAGGTGATTGGCACTGGAGCCTTGCTGCTATGTATCCTGGCTATA
AATGATAAGAAAAACAATAGCGCTCTGGACGGGACACAAGCTGTGGTTGTCGGCCTCCTG
GTTACAGTTATTGGGATGTCTATGGGCATGAATTCAGGATATGCTATCAACCCTGCCAGG
GATCTGGGGCCACGTCTCTTCACTGCGGCTGCTGGATGGGGACTGGAAGTTTTCAGGGCT
GGGAATTACTGGTGCTGGGTGCCAATTGTAGCCCCTATGGTGGGTGGCCTGACCGGGGCC
TTCCTGTACAAGCTGCTAGTGGGGCTCCATCACCAGCCGAAAGTGGAGGAGAAGAAGCTA
TGTGAG---------------ATGGAGAAG------------------------------
-------------------GGGAATGCAGAGATG---GAGACCTGTGACTATATG------
----

>LaPaz_rubber_frog_AQP7
ATGAAAACGGGGAGAGTCATCGCCAACAAGATCCGCTCAGCTATATCTATCCGCAACGCC
TGGGTACGAGAGGCCATGGCGGAGCTGCTGTCCACCTTCGTGATGATGATGTTCGGTTTG
GGAGCCGTGGCGCAGGTGGTTCTGGGTAAGCACGCATACGGACAATACCTGAGCATTAAT
TTATCCTTCGGAATTGGAGTCACAATGGGAATCCACGTGGCCGGAGGAGTGTCTGGAGCC
CACATGAACTGTGCCGTATCACTCACCAACTGTGTTCTGGGGAAGCTTTCCTGGAAAAAA
CTTCCAGTTTACATGATTTCTCAGATCACTGGATCTTTCCTGGCTGCGATGATTGTATAC

TTTCTATATTATGAGGCTCTGTATAATTACTGCAAG---GGAAACTTCACGGTGACGGGT
CCTTTG---GAAACAGCCAGTATATTTGCCACCTACCCACAGCCGTACCTGTCCATTGGG
GGTGGCTTTCTAGATCAGGTGATTGGTACGGAGCTCTGCTTCTCTGCATCCTGGCTATA
AATGATAAGAAAAACAGTGGCGCCCTGGATGGGACACAAGCTGTGGTCGTCGGCCTCCTG
GTTACAGTTATTGGCATGTCTATGGGAATGAATTCGGGATATGCTATCAACCCCGCCAGG
GATTTGGGGCCACGTCTCTTCACCGCAGCTGCCGGATGGGGAGTGGAAGTCTTCAGGGCT
GGGAATTACTGGTGCTGGGTGCCGATTGTAGCCCCTATGGTGGGTGCGCTGACCGGTGCC
TTCCTCTACAAGCTGTTAGTGGGACTTCATCACCAGCCCAAAGTGGAAGAGAAGAAGACG
AGCGAG----------------ATGGAGAAG-------------------------------
-------------------GGGAATTCAGAAATG---GGGATGTATGAATATATG------
----

>Ornate_chorus_frog_AQP7
ATGAAGACCAGCCGATGCCTCCTGCGCAGGATCCGCGCCGCCGTCTCCATCCGCAACCTG
TGGGCGCGCCAAGCCATGGCCGAGCTGCTGGCCACCTTCATCATGATGCTCTTCGGCTTG
GCGTCGGTGGCACAGGTGGTTCTGGGCAGGAAGCAGTATGGTCAATACCTTAGCATTAAC
CTGTCCTTCGGATTTGGCGTCACGATGGGCATCCATGTGGCCGGCGGCGTCTCTGGCGCT
CACATGAATTGCTCCATCTCCATCACCGAGTGTGTCCTGGGGAGACTCCCGTGGAAGAAG
CTTCCGGTCTATATGGTGTCGCAGATCGCTGGAGCTTTCCTGGCTGCTGCTGTGGTCTAC
TTCCTCTACTACGATGCTCTGCACCAGTACAGCGGG---GGGCAACTCCTCGTGACCGGA
CCCAAT---GAGACCGCCAGTATATTCGCCACCTACCCTCAACCCTACCTGACTCTGGGC
GGGGGCATCTTAGATCAGGTGGTCGGCACGGCGGCTCTCATGCTTTGTATCCTGGCCATC
AATGATCAGAAGAATAACCCCGCGCTGCGCGGCACACAGGCCGTGGTCATAGGGCTACTG
GTGACCCTCATTGGAATGTCCATGGGGATGAATTCCGGATACGCCATCAACCCGGCCAGA
GACCTGGGGCCCCGACTCTTCACCGCAGCCGCTGGATGGGGACTGGAGGTGTTCAGGGCT
GGAAACTACTGGTGCTGGGTTCCCATCGTCGCCCCCATCATCGGCAGCCTGACGGGCGCC
TTCCTCTACAAGCTGCTGGTCGGCCTCCACCATCAGAGCGAAGAGGAGGAGAAGCAGATG
GATGAG----------------CTGGAGAAG-------------------------------
-------------------GGGAATATGGAGATG---CCGCCATACCCCCCTGCACCC---
----

>AC_frog_AQP7
ATGAAGACCTACGGCAAAATCTTCTGCAAAATCCGCAGAGTCATTTCCATCCGCAACAGG
TGGGCACGGGAAGCCCTGGCAGAGCTCCTATCCACCTTCATCATGATGCTCTTCGGACTG
GGATCTGTGGCTCAAGTGGTTTTAGGAAGACATCAGTTTGGCCAATATCTCAGCATTAAT
CTCTCGTTCGGGTTTGGAGTCACAATGGGAATCCATGTGGCCGGAGGAATTTCTGGTGCT
CATATGAACTCAGCTGTGTCACTAACAAATGCCGTTCTGGGAAATCTGCCTTGGAAAAAA
CTGCCAGTTTATGTACTGGCCCAAATGCTCGGCTCCTTCCTGGCAGCTGTAGTGGTGTAT
TGTCTCTATTCTGAGGCCATGTACAACTACTGCGGC---GGCAACTTCACAGTGACAGGT
CCAAAT---GAGACAGCCAGTATTTTTGCCACATACCCACAGCCCTACCTCTCCATTGGA
GGAGGGTTCTTGGACCAGGTCATTGGCACAGGAGCCTTGTTACTTTGCCTCCTTGCAATT
GGTGACATTAGGAATAGCCCGGCCCTGAGGGGAACTGAAGCTCTTATAGTAGGACTGTTG
GTCACTGTTATTGGGATGTCCATGGGAATGAATTCCGGATATGCCATCAACCCAGCAAGG
GATCTTGGGCCCCGAGTCTTTACTGCCATTGCTGGATGGGGAATAGAAGTATTCCGAGCC
GGACATTACTGGAGCTGGGTGCCTATTGTGGCTCCCTGTGTTGGTGGCCTGACTGGTGCC
TTTCTGTACAAGCTGCTAGTTGGGCTACACCATCAGCCAGAAGAAGAAGAGACCAAAGTA
GAGGAG----------------ATGGAGAAA-------------------------------
-------------------GGAAACCAAGAGACT------TTCAGCGAGTTCATG------
----

>Fire_salamander_AQP7
ATGACGGTGGCAGGCACAATGCGTGCAAAACTTAGGAAAAGTTTAAGAATCAAGAACCAG
ACGGTCAGGGAAGCAATGGCCGAGATGCTGTCCACCTTCGTCATGATGAGTTTTGGCTTG
GGCTCGGTTGCCCAGGTTGTTCTTGGAAAAAAGGAATTTGGGCAGTATTTAAGTATTAAT
TTGTCCTTCGGATTTGGTGTGACGATGGGGATACATGTGGCTGGTGGAGTTTCAGGAGCG
CACATGAATACGTCTGTCTCCCTAACAAACTGCATCCTGGGGAACCTTCCCTGGAGGAAG
CTGCCAGTCTACGCCCTCGCCCAGTGTGTGGGGTCCTTCCTTGCTGCCGCCGTGGTCTAC
TGCCTCTATTATGAGGCACTGATTAACTACTGTGGA---GGAAACCTTACAGTGACCGGG
CCTTGT---GCTACTGCAGGCATCTTTTCTACCTACCCTGAACCGTACTTGTCTGTGGGA
GGCGGGTTTGTAGACCAGGTGATTGGCACAGCAGCGCTCTTGCTCTGTATCCTGGCCATC
AACGACCGCAGGAACAGCCCGGCTTTGATTGGGACTCACGCTCTTCTTATTGGGATCCTA

GTGGTATTAATTGGCATGGCCATGGGGATGAATTCAGGATACGCTATCAACCCAGCCAGA
GATCTTCCTCCGAGGATTTTCACTGCCATAGCTGGCTGGGGCCTGGAAGTCTTTTGGGCT
GGGGATTACTGGTGGTGGGTCCCAGTGGTGGCCCCAATGGTGGGAAGTGTCACGGGTGCA
TTAATCTACACACTTCTCATAGAATTACATCACCCACCTGAAGCATCCGAAAAACCGGTA
TGTCAA---------------GAGACCAAG------------------------------
------------------GACGTTCACGACACTGATATTATGTGTCAACATATG------

>Axolotl_AQP7
ATGACGACAGCCGGCAAGATGCGCGCCAAAATCAGGACTCGCCTGAGGATCGGCAACCAG
GCCGCGCGGGAAGCCGCCGCCGAGCTGCTCTCCACCTTCGTCATGATGACTTTTGGATTA
GGTTCGGTTGCTCAAGCGGTTCTGGGCAGAAAAGAATATGGGCAGAATTTAAGCATCAAT
CTGTCTTTCGGATTTGGCGTGACAATGGGGATCCATGTAGCTGGAGGGGTTTCAGGAGCG
CATATGAATACTTCTGTATCCTTAACAAACTGCATCCTGGGAAACCTTCCATGGAGAAAG
CTGCCATTGTATGCACTGGCACAGTGCCTGGGTTCGTTCCTCGCAGCTGTTGTGGTATAC
TGTCTCTATCATGAAGCATTGTTGGACTACTGTGGG---GGGAACCTCACAGTAACAGGC
CCTTGC---GCTACAGCCGGCATATTTGCAACATACCCAGCACCGTATTTGTCTATCGGA
GGCGGGTTTGTAGACCAGGTGATCGGCACAGCAGCACTCTTGCTCTGCATTCTTGCGATC
AATGACAAGAGGAACAGCCCGGCCTTGAACGGGACTCAGGGCCTTCTGGTTGGGATCCTA
GTGGTGCTAATTGGTATGTCTATGGGTATGAATTCTGGATATGCCATTAACCCAGCCAGA
GATCTACCTCCAAGGATATTCACCGCCATGGCAGGCTGGGGACTGGAAGTATTTTGGGCT
GGTAATAACTGGTGGTGGGTTCCAGTGGTTGCTCCTTTTGTTGGGAGTGTCATAGGAGCG
TTCATCTACAAACTCCTCATCGAATTACACCATCCACCCGAGCCAGAAGAAACAAAG---
TGTCAA---------------CTATCCAAG------------------------------
------------------GACATTCAAGACAATGACGTTATGTGTCAACATATG------

>2Lcaecilian_Aqp7
ATGGCAAAAAAG---ATACGGCTCATGAAAATAAAACAAAAATGCATAATAAGAAACGGA
ACTGTCAGGGAGACGCTGGCTGAGGCTCTGGCGACCTTTGTCATGATGAGTTTTGGTTTA
GGTTCTGTTGCCCAGGTCGTCCTAGGAAAAAAGGAATACGGGGAGTATTTGAGCATCAAT
CTCGCTTTTGGATTTGGTGTTACCATGGGCATTCACGTGGCTGGAAACATTTCCGGAGCC
CACATGAATACTTCTGTTTCGCTTACAAACTGCATCTTGGGTTACCTGCCTTGGAGAAAA
CTGCCATGTTACGCCTTGGGACAGTTCATTGGCTCCTTTCTCGCAGCTGCGTTGGTATTC
TGTTTATATTATGAGGCGTTGTACGACTATTGTGGC---GGGAACCTGACTGTCACAGGG
CCTTAT---GCTACAGCAGGGATATTTTCCACCTATCCTGCTCCTCATATGTCTGCAGGA
GGAGGATTTCTAGCTGAGGTTGTTGCCACGGGAATGCTTCTGCTGTGCATCCTTGCCATT
ACCGACAAGAAGAACAACGCAGCCTTGGACGGGACGCAGGCTCTTCTGGTTGGAATCCTT
GTTATTGTGATTGGTTTGGCGATGGGAATGAACACCGGCTATGCCATAAACCCAGCCAGG
GACCTTCCTCCCAGGATCTTCACTGCAATCGCAGGATGGGGCCTGGAGGTCTTCAGGGCT
GGGAATTACTGGTGGTGGATCCCAGTAGTAGCGCCATTCATAGGAAGTATCTGCGGTGCC
TTAATCTATAAACTTTTAATAGCCTTGCACAATAAGATCGAGCCAGAAGATGTGATC---
GGCGAA---------------GACGTGAAA------------------------------
------------------GGAAATCAAGAAAAA---GATCAAGACCAATATATG------

>tcCaecilian_AQP7
ATGAAGACAGAAAAGAAACAGCTCATGAAAATGAAGAAGAAATGTATCATCAAGAACGGA
ACGGTCAGGGAGATGCTGGCCGAGGCCCTGGGGACCTTTGTAATGATGAGTTTTGGTTTA
GGTTCTGTTGCACAGGTTGTCCTTGGAAAAAAGGAATATGGACAGTATTTGAGCATTAAT
CTCTCGTTTGGATTTGGTGTCACCATGGGGATTCATGTAGCTGGAGGAATTTCCGGTGCC
CATATGAATACTTCTGTATCACTAACAAACTGCATTTTGGGACACCTGCCCTGGAGAAAA
TTACCGTTTTATGCCTTAGGACAATTCATTGGCTCCTTCCTCGCAGCCGCGTTGGTGTAT
TGTGTATATTATGACGCGTTGTATGATTACTGTGGC---GGTAACCTGACTGTAACAGGA
CCTTAT---GCAACAGCAGGGATATTTTCCACCTATCCTGCAGCCTACATGACTCCAGGA
GGAGGATTTCTAACTGAGTTTGTTGCCACTGGAATGCTTTTGCTCTGCATCCTTGCCATC
AACGACAAGAAAAACTTTGCCACCCTGGATGGAACTCAAGCTCTTCTGGTTGGAATCCTT
GTTATAGTAATTGGTATGGCAATGGGAATGAATACTGGATATGCCATAAATCCAGCCAGG
GATCTTCCTCCCAGAATCTTTACCGCAATAGCAGGTTGGGGAGTGGAGGTCTTCAGGGCT
GGAAATTATTGGTGCTGGATCCCAGTAGTAGCTCCCTTGGCAGGAAGTATTACTGGCGCC
TTTATCTATAAACTTGTAATAGCTTTGCACAATCAGACTGTGTCAGACGACGCGGAG---

TGTGAA----------------GACACAAAA------------------------------
-------------------GAAAATTCAGATATTAACGTGTCAAGTCAGTATATG------
----

>Gaboon_caecilian_AQP7
ATGAAGACAGAAAGGAAGCAGTTTATGAAAATCAAACAAAAATGCATCCTCAAAAATGGA
ACTGCCAGGGAGATGCTGGCGGAGGCTCTGGGGACCTTTGTAATGATGAGTTTTGGTTTA
GGTTCTGTTGCACAGGTTGTCCTAGGAAAAAAGGAGTACGGACAGTATTTGAGTATTAAT
CTCTCATTTGGATTTGGTGTCACCATGGGAATTCATGTAGCCGGAGGAATCTCTGGAGCC
CACATGAACACTTCTGTATCACTGACAAACTGCATTTTGGGACACCTGCCCTGGAGAAAA
TTACCATTTTACGCCTTAGGACAATTCACTGGCTCCTTCCTCGCAGCCGCGTTGGTTTAC
TGTGTGTATTATGATGCATTGTATGATTACTGTGGC---GGTAACCTGACTGTAACAGGG
CCTTAT---GCAACAGCGGGGATATTTTCCACCTATCCTGCGGCCTATATGACTCCGGGA
GGAGGATTTCTAACTCAGTTTGTTGCCACTGGAATGCTTTTGCTTTGTATCCTTGCCATC
AACGACAAGAAGAACTTTGCCGCCCTGGATGGAACTCAAGCTCTTCTGGTTGGAATCCTT
GTTATAGTAATTGGTATGGCGATGGGAATGAATACCGGATATGCCATAAATCCAGCCAGG
GATCTTCCTCCCAGAATCTTTACTGCAATAGCAGGTTGGGGAGTTGAGGTCTTCAGGGCT
GGAAATTATTGGTGCTGGATTCCAGTAGTGGCTCCATTGGCAGGAAGTATTACTGGTGCC
TTTATCTATAAACTCCTAATAGCACTGCACAATCAGGCCGTGCCAGATGATGTGAAA---
TGTGAA----------------GATACGAAA------------------------------
-------------------GAAAATCCAGATATTGTCATGACTGGTCAGTATATG------
----

>bCaecilian_AQP7
ATGAAGACAGAA---AAACTGCTCATGAAAATCAAACAAAAGTTCAGCATAAAAAAATAGA
ACTGTCCAAGAAACACTGGCGGAGGCCCTGGGGACCTTTGTAATGATGTGTTTTGGTTTA
GGTGGTGTTGCACAGGTCGTCCTAGGAAAAAACGAATTTGGACAATATTTGAGCATAAAT
CTCGCTTTTGGTTTTGGTGTCACCATGGGGATTCATGTAGCTGGAGGAATCTCTGGAGCC
CACATGAATACTGCTGTATCGCTAACAAACTGCGTTTTGGGATACCTGCCCTGGAGAAAA
TTACCATTTTATGCCTTAGGACAATTCATTGGCTCCTTCCTTGCAGCTGCGTTGGTATAC
TGCATATATAATGAGGCGTTGCATAATTACTGTGGC---GGGAACCTGACTGTAACAGGG
CCTTGT---GCAACAGCAGGAATATTTGCTACCTACCCTGCTGCCTACATGTCTTCAGGA
GGAGGATTTCTATGTGAGTTTGTTGCCACTGGAATACTTTTGCTTTGTATCCTTGCCATC
AACGACAAGAAGAATTGTGCCGCCCTGGATGGTACTCAAGCTATTGTGGTTGGAATCCTT
GTTCTAGTAATTGGTATAACAATGGGAATGAATACCGGATATGCCATAAATCCAGCCAGG
GATCTTCCTCCCAGAATCTTTACTGCAATAGCTGGTTGGGGAATAGAGGTCTTCAGAGCT
GGAAATTATTGGTGCTGGATCCCAATAGTAGCTCCGATGACAGGAGGTATTATTGGTGCT
TTTATCTATAAACTTGTAATTGCATTGCACCATCAGACTGTGGAAGAAGATGTGAAT---
TGTGAA----------------GACACAAAA------------------------------
-------------------GAAAATCTAGATGTTGGCGTGACTATG---------------
----

File S4: Alignment for Fig. 2, Fig. S3
>nhsSquirt_Glp
GCGTCACTACTTCGCATTGAAAACAATCTGGTGCGTGAAATGCTGGCGGAATTCCTAGGC
ACATTTATTCTCATGGTTTTTGGCAATGGTTCCGTTGCACAAAGCGTGTTAAGTCGGGAA
GCT---AAAGGC---ACCTTCATTTCAATCAACTGGGCATTCGGCCTCGGCGTTACCATG
GCGATCTACGTCACTGGAAGTGTTTCTGGAGCTCATATCAACCCAGCAGTGTCGACGGCA
TTATGTTTGTTCGGAAAGTTACCTCTGTACAAGTTGCCATGTTATATTCTATCTCAAGTA
TTCGGCGCATTTGTGTCTGGGGCAGCGGTTTACTCGATTTACTACGACGCATTGAATGCT
TTTGATGGTGGAGTAAGGCAAGTGTTGGGACCA---AACGGGACCGGTGGGATTTTCTGT
ACT-------------TACCCAGCTGATTATCTCGCGATTACTGGCGGTTTATGGGATCAG
GTTTTTGCAACCGCTATGTTGGTTGGAATTGTGTTTGCTGTAACGGATGAGCGGAACAAC
GACGTGGTTGGTGGATTGCAACCAATCATCATCGGCTTCGTGGTTTTCGTTCTAGGTCTG
TCATACGGAGTGAACTGTGGGTACGCTATTAACCCCGCCCGAGACTTTGGACCCCGGTTA
TTCACCTACTTTGCCGGATGGGGCAACGCAGTGTTT---ACGGAGCCGGGTGGCATGCAT
TGGTGGTGGGTCCCAATTGTGGGTCCTCTCATAGGAGGGTCCCTTGGAGCTGTGATATAC
AAACTTGTCGTAGGAGTTCACTTACCCCGAAAAACCCGTCAT
>BsSquirt_Glp
GCTTCGCTACTTCGCATCGAAAACAAGCTTGTGTGCGAAATGTTGGCGGAGTTCCTTGGT

ACTTTTATTTTGATGGTATTCGGAAACGGTTCGGTCGCACAAAGTGTTTTAAGTCGAGAA
GCC---AAGGGC---ACCTTCATTTCAATCAATTGGGCATTCGGTCTCGGCGTTACCATG
GCTATTTACGTCACTGGAAGTGTCTCAGGAGCTCATATTAATCCAGCAGTGTCAACAGCT
ATGTGTTTGTTTGGGAAGCTTCCTCTGTACAAACTACCCTGCTACATCATGGCGCAGGTG
TTTGGTGCATTTGTGTCGGGCGCAGCTGTCTACTCCATTTACTACGACGCATTGAATGCC
TTTGATGGTGGTGTAAGGCAAGTCCTTGGAGCA---AATGGAACTGGTGGAATTTTCTGC
ACT------------TATCCATCTGAATATCTGACAATAACTGGCGGATTGTGGGACCAG
GTTTTTGCCACTGCGTTGCTTGTTGGTATCGTGTTTTCAGTGACGGATGAGCGGAACAAC
GATATCGTCGGCGGACTGCAGCCAATTATTATCGGATTCGTCGTTTTTGTGCTCGGTCTG
TCTTACGGCGTCAACTGTGGATACGCCATCAACCCTGCCCGGGACTTCGGCCCCCGGTTG
TTCACCTATTTTGCTGGATGGGGTAACGCTGTGTTC---ACGGAACCTGGTGGCATGCAT
TGGTGGTGGGTGCCTATCGTGGGCCCGCTTATTGGAGGGTCTCTTGGTGCAGTCATATAT
AAACTTACGGTTGGAATTCATTTGCCTCGGAAAACCCGACGA
>vTunicate_Glp
TCATCTTTAGTTCGAATTGAAAACGTCCTTTTGAGGGAAATGTTGGCGGAGTTTCTGGGG
ACGTTCATACTGCTTGTTTTTGGCAATGGAGCGGTCGCACAAAAAGTATTAAGCAGAGAC
ACG---TTGGGC---ACCACACTTTCAATAAACTGGGCGTATGGTTTTGGTGTCACTATG
GCAGTATATGTTACTGGAAAAGTTTCAGGTGCACATATAAACCCAGCTGTATCGGTAGCA
CAATGTGCATTTGGGAACTTACCATTATACAAGCTGCCTTGTTACATTTTCTCACAAGTC
TTTGGTGGTTTTGTTTCCGGGGCGGCTGTTTATTCAATCTATTATGAGGCACTTAACGCT
TTTGACGGTGGACAACGTTCTGTTTTGGGTCCA---AATGGAACCGGGGGTATCTTCGCA
ACA------------TACCCGCAAGATTACCTTTCCATTAACAACGGTCTTTGGGACCAG
GTCTTCGGCACTGCACTACTAGTTGGTATTATATTTGCTGTTACTGACAACAAAAACAAT
ACAATTGCTGATGGTCTTACTCCTATAATAATCGGCCTGCTTGTCTTTATACTAGGTACA
TCGTTCGGACTCAACTGTGGCTATGCTATCAACCCGGCTCGCGATTTTGGACCGCGCTTA
TTTACTTTTGCCGCTGGCTGGGGCCCGGGAGTCTTT---ACAGAGCCAAATGGCATGTCG
TGGTGGTGGGTGCCTATCGTGGGCCCTATTATAGGCGGGTTGACTGGAGCAATATTGTAT
AAGTTAATGGTTGGAACTCATTTACCATCGGGAAGTCAAAGT
>Csavigni_Glp
TCATCAGCTATACGGATCAAAAACGTCTTGATTAGAGAAATGTTGGCGGAATTTTTCGGC
ACATTTATACTACTGGTTTTCGGAAATGGGGCTGTTGCCCAAAAAGTGCTAAGCAGAGAA
ACT---TTAGGC---ACTACACTGTCGATAAACTGGGCATACGGTTTGGGCGTTACAATG
GCAATTTATGTAACTGGCAAAGTATCAGGAGCGCACATTAATCCTGCTGTCTCCGTCGCT
TTATGTGCATTTGGAAAACTTCCTCTGTACAAACTTCCTTGCTATGTAATTTCCCAGGTC
TTTGGTGGTTTCGTATCGGGTGCCGCTGTTTATTCGGTTTATTACGATGCAATAAACGAA
TTCGACGGTGGTAAACGTTATGTTCTGGGACCA---AACGGCACTGGCGGGATATTTGCT
ACA------------TACCCACAAGATTACCTTTCACTTGGAAGCGGTTTGTGGGATCAG
GTGTTCGGAACAGCTCTACTTGTTGGAATTATCTTCGCTGTAACGGATGACAAAAATAAT
ACAATTGCTGATGGTTTAACTCCCATTATTATAGGACTACTGGTATTTATTTTAGGTACG
TCATTTGGACTGAACTGCGGATATGCTATCAATCCTGCGCGAGATTTAGGGCCGAGGTTG
TTTACTTTTGCCGCTGGGTGGGGCACAGAAGTTTTT---ACGGAGCCAAATGGAATGTCA
TGGTGGTGGGTTCCAATTGTTGGACCTATTCTCGGGGGGCTAACAGGCGCAGTCATATAC
AAGTTATTTGTGGGCACTCATTTTCCACCAGATGATGATGAC
>SalThomson_Glp
CTGGATCGACTGAAAGTAGAAAATTCCCTAATCCGAGAAATGTTTGCCGAATTTCTCGGT
GTCCTCATCTTAGTGACGTTTGGAAATGGAGCAGTTGCACAAAGTGTGTTGAGTAAGAAG
TCG---CAAGGT---GAAACTATTTCCATCAATTGGGCTTACGGAATGGGAGTTACAATG
GCAATCTATGTGGCAGGAAACGTATCAGGTGCACACTTGAATCCTGCCATTTCAATCCTC
TTCTCAGTCATGGGTCGTCTCTCGTGGCTAAAACTCCCTTTTTATATTTTGGCCCAGATG
TTGGGCGGATTTGTCTCCGGTGCGGTTGTGTACTCCACTTACTACGATGCATTGAACAGT
TTTGACGGAGGGGTACGGAGTGTATACGGACATGTAAACGCCACAGCTGGAATATTCGCC
ACA------------TATCCACAAGATTATCTCTCCATAACAAACGGATTGTGGGATCAG
GTGGTCGGTACAGCTTTACTGGCCAGCATGCTCCTAGCCATCACGTCGGAT------AAC
AAGATAGTCCATGGATTTGTGCCCTTATTAGTTGGACTGTTGGTGTTTTCGATTGGCTTG
TCATATGGGTTCAATTGTGGATATGCGATTAATCCCGCACGTGACATGGGTCCTCGACTC
TTCACGTTTGCTGTTGGATACGGAAGTGGTGTTTTC---ACGGAGCCGAACGGTTTGTGC
TGGTGCTGGGTGCCTGTCGTCGGGCCAATAGTGGGCGCTCTTGTGGGTGGACTCATCTAC
AAGATGTTGGTTGGTCATCATCTTCCCGAAGAGGAGGAA---
>gsTunicate_Glp

---------------GTTTTTGGGAATGGATCCGTCGCACAAACTGTATTGAGCAATGGA

GCT---GATGGA---AATTTTTTGACGATATATTGGGCATATGGTATTGGAGTAACAATG
GGAGTGTACATTGCCGGTGGAGTATCAGGCGCTCACTTGAATCCAGCGGTCAGCGTTTCG
ATGAGTGCGTTGGGTAAATTGCCGTGGAAAAAGTTACCGGTGTTTGTGACTGCGCAACTG
CTTGGTGCATTTTTATCGGACCTGTTGTATACGGAATTTATTACGATGCCCTTAACGAA
TACGACGGCGGAATAAGGTACGTGAGTGGAGTA---AATCAAACTGCATCAATTTTCGCC
ACA------------TATCCAAAAGACTACCTTAGCATTTCCAATGGATTTTTCGACCAG
GTCTTTGCGACAGCAGTTTTATTGGGAGGAATCCTCGCTATTACAGATGAGAAAAACAAT
AAGCCACCCGCCGGGATGGAACCGCTGTTGGTCGGTCTGCTGGTATTTGCTATAGGCAAC
TCCTACGGAACAAACTGCGGTTACGCCATCAATCCTGCAAGGGATCTTGGACCTCGCATA
TTCACAGCTATCGCTGGATGGGGAGGGGAAGTGTTC---ACG---CCAGACGGAGCTTAT
TGGTTCTGGGTACCGATCGTGGCACCGATACTCGGTGGCCTCATCGGTAGTTTCGTATAT
GAAATGATGATCGGACTGCACTTGCCATCACGTGACACAACG
>Colonial_sea_squirt_Glp
TCAGCAAAATTGCGCACAGAAAATGTTCGAATACGGGAATTTTTGGCGGAATGTATGGGA
ATATTTATGTTGATGGTGCTAGGCAATGGATCCGTGGCACAGACGGTATTGAGCAGAGGA
GTA---GACGGC---AATTTTCTTACGATATATTGGGCGTATGGCATGGGAGTTACGATG
GGTGTATACATCGCAGGAGGCGTTTCAGGCGCACATTTGAATCCAGCAGTAAGCGTTTCC
ATGTGCGTGCTTGGAAAATTACCTTGGAAAAAGTTACCCGTATACGNAACCGCTCAGCTC
CTTGGCGCTTTTCTATCCGGGCCAGTCGTTTACGGAATCTACTACGATGCATTGAACGAA
TTCGATGGAGGAGTTCGAGAGGTTTCCGGCGAT---AACGCTACGGCTTCAATATTTGCG
ACA------------TACCCGAAGGACTACTTGTCCATTTTGAACGGATTTTTTGATCAG
GTTTTTGCAACTGCTCTGTTGCTGGCTGGCATTCTAGCTATCAACGACGACAGAAACAAC
AAACCACCGTCTGGATTGGAACCGCTGTTAGTGGGTTTGTTGGTGTTTGCTTTGGGTGAC
GCGTACGGCACTAACTGCGGCTATGCCATCAATCCTGCTAGAGATCTTGGACCTCGTATA
TTCACTAGCATCGCTGGTTGGGGAACAGAAGTTTTT---ACTGCGCCTGATGGTAAGTGT
TGGTTCTGGGTTCCGATTGTAGCACCAATCGTCGGCGGTATACTCGGCAGCCTTGCATAC
GTCGCTGTGATCGAGCTGCACTGGCCCCAACGTGATACCAGG
>Sea_peach_Glp
AGTGATAAAGTTGCTAAGAAGAGAGCTTTGCTTCACGAGTGTTTAGCAGAATGCCTTGGA
ACATTCATACTCATGGTATTCGGCAATGGTTCAGTAGCGCAGTCAACACTGAGCAGAGGG
GCA---AGTGGA---AATTTTCTCACTATTTACTGGTCTTACGGGTTGGGTGTCACTATG
GCTGTTTACGTTGCTGGTGGAGTATCAGGGGGACACATCAATCCTGCAGTAAGCTTTGCG
CAGACTGTGATTGGACGACTACCTTTGATAAAACTTCCGTTTTATGTCCTGTCACAAACT
CTTGGTGCTTTTCTTTCTGGGCCTGTTGTGTATGGGATTTACTACGATGCTTTAAACTCA
TTCGACGGCGGTGTGCGGCAGGTTTTGGGTGAA---AACGGAACTGCTGGGATATTCGCT
ACT------------TACCCTCAACCGCATCTGTCAATTTTAAACGGATTTTTTGATCAG
GTATTTGCAACATCACTACTTCTTATCGGAATTCTTGCGATAAATGATGATAAAAACATG
AAGCCACCGAAAGGATTGGAACCATTGATGGTTGGATTTTTAGTATTCATAATAGGTGGG
GCATACGGTTACAACTGCGGATACCCTATAAACCCGGCTCGAGATTTTGGGCCGAGATTA
TTCACTTTTTTCGCCGGTTGGGGAACTGAAGTGTTT---ACG------GCAGGAAATAAC
TGGTGGTGGGTACCGATCGTCGCACCGTTGGTCGGTGGCGTTATAGGCGCGGTCGTCTAC
TTGTTAGTAATCGAATTCCCGCGAGTTAAGACAACGAAGAGT
>S_pineapple_Glp
AATAATAAAGTTGCTAAGAAGAGAGCTTTGTTTCACGAGTGTTTAGCAGAATGCCTTGGA
ACGTTCATACTCATGGTATTCGGCAATGGTTCAGTGGCGCAGTCAACACTAAGCAGAGGG
GAA---AGTGGA---AATTTTCTCACTATTTACTGGTCTTACGGTTTGGGTGTAACTATG
GCTGTTTACGTTGCTGGGGGAGTATCAGGGGGACACATCAATCCTGCAGTAAGCCTTGCG
CAGACTGTAATTGGGCGACTTCCTTTGATAAAACTGCCGTTTTATGTCCTCTCACAAACT
CTTGGTGCTTTCCTTTCTGGGCCTGTTGTATATGGGATTTACTACGATGCTTTAAACTCA
TTTGACGGCGGCGTGAGGCAGGTTTTAGGTGAA---AACGGAACTGCGGGGATATTCGCT
ACT------------TATCCACAACCGCACCTATCAATTCTAAACGGATTTTTTGATCAG
GTATTTGCAACATCACTTCTTCTTATTGGAATTCTTGCGATAAACGATGATAAAAACATG
AAGCCACCGAAAGGATTGGAACCGCTGATGGTTGGATTTTTAGTATTCATAATAGGTGGG
GCATACGGATACAACTGCGGATACGCCATAAACCCAGCTCGAGATTTCGGGCCGAGGTTA
TTCACTTTTTTCGCAGGCTGGGGAACTGAAGTATTC---ACG------GCAGGAAATTAC
TGGTGGTGGGTACCGATCGTCGCACCGTTAGTCGGAGGCGTCACAGGTGCGGTCGTCTAC
TTGTTAGTAATCGAATTTCCTCGAGATAAGGAACCAACGAGC
>Bfloridae_Glp1a
GTAGGCGCTCTTCGGGTGAGGAGGTTCGTATTCCGGGCGGCGCTGGCTGAACTACTCGGC
ACATTTCTGCTGGTGACGATGGGAAACGGATCCGTGGCGCAGGTCGTGCTGAGTCGGGAG
GAC---AAAGGA---ACGTTTTTATCCATTAACTGGGGGTACGGCATCGGGGTAGTCATC

GGAGTCTACGCCTCCTGGGGTGTCTCAGGAGCCCATCTAAACCCTGCGGTATCCTTAACC
ATGGCAGTCCTGGGGAAGCTGCGCTGGGTCTATCTGCCTTGTTACGTCTTGGCGCAGATG
TTAGGGGCCTTCTTGTCAGCAGTTTGTGTGTACATAGTGTACTATGATGCATTAGCAAAC
TTTGACGGCGGGACCCGGGCAGTTTTAGGTGTG---AACGGGACCGGAGGAATCTTCAGT
ACG------------TACCCGCAAGACTACCTGTCTATTGGCAGTGGAGTCCTGGACCAG
GTGGTGGGCACTGGCCTGTTACTCTGCGGTGTTCTGGCCCTGACTGACTCCAGGAACAAC
AAGGTAACGGCGGGGATGGAGCCGCTGCTTGTCGGCCTGCTGGTGCTCGCTATCGGCACG
TCCTTCGGGTTTAACTGCGGCTACGCCATCAACCCAGCCCGAGACCTGGGCCCGAGAATC
TTCACGGCAATGGCAGGTTGGGGCATAGAAGTCTTC---AGG------GCGGGAAACCAT
TGGTGGTGGGTGCCGATAGTCGGACCGTTGATCGGCGGGCTGGTTGGAGGTCTGGTCTAC
ACGCTTATGGTGGCGCTGCATCATCCGGAGGAAGAGGATGAC
>Bfloridae_Glp1b
GTGGGCACTCTTCGGGTGAAGAGGTTCATAGTCCGGGCAGCTATGGCAGAAATGCTCGGC
ACATTCCTCCTGATGACGATAGGAAACGGTTCCGTGGCACAGGTCGTGCTGAGTCGGGAG
GAC---AAGGGA---ACGTTCTTTACAATCAACTGGGGGTACGGTATCGGGGTGGTCATC
GGAGCATACGCCTCCTGGGGTGTCTCAGGAGCCCATCTAAACCCTGCCGTATCCCTGACC
ATGGCAGTCCTGGGGAAGCTACGCTGGGTCTACCTGCCGTGTTACGTCATGGCACAGATG
ATAGGGGCCTTCCTGTCAGCAGCCTGTGTGTATGCTGTGTACTATGATGCGTTAGCAAAC
TTTGACGGTGGGACCCGGGCAGTTTTAGGTGTT---AACGGGACCGGAGGCATCTTCAGT
ACG------------TACCCCCAAGACTACCTGTCTATTGGCAGTGGAGTGCTGGACCAG
GTGGTGGGCACTGGCCTGTTACTCTGCGGTGTTCTGGCCCTGACTGACTCAAGGAACAAC
AAGGTAACAGCAGGGATGGAGCCGCTGCTTGTCGGCCTACTGGTGTTCGCTATTGGCACG
TCCTTCGGGCTCAACTGTGGGTACCCTATCAACCCAGCCCGGGACCTGGGGCCGAGAATA
TTCACGGCAATGGCAGGCTGGGGCCTGGATGTCTTC---AGG------GCTGGAAACCAT
TGGTGGTGGGTCCCTATAGTCGGACCATTGATTGGTGGGCCGGTCGGAGGTTTGGTATAC
ACCCTGATGGTGGGGCTGCACCATCCAGAGGAAGATGATGAC
>Bbelcheri_Glp
GTGAGCGCTCTTCGGGTGAGGAGGTTCTTGGTTCGGGCGGCATTGGCTGAACTTCTCGGC
ACATTTCTCCTGGTGACGATCGGAAACGGCTCCGTGGCGCAGGTCGTGTTGAGTCGGGAG
GCT---AAGGGG---ACGTTCTTGTCCATCAACTGGGCGTACGGCATCGGGGTAGTCATC
GGCGTGTACGCCTCCTGGGGGGTCTCAGGAGCCCATCTGAACCCTGCGGTCTCCCTGACC
ATGGCAGTCCTGGGTAAACTACGCTGGGTGTACCTGCCGTGTTACGTCATGGCTCAGATG
ATAGGGGCCTTCTTGTCAGCGGTTTGTGTGTACATTGTGTACTATGATGCATTGGGTAAC
TTTGACGGTGGTACCCGAGCAGTTGTAGGTGTG---AACGGGACGGGAGGCATCTTCAGT
ACC------------TACCCGCAAGACTATCTGTCTATCGGCAGTGGAGTGCTGGACCAG
GTGGTGGGCACTGCCCTGTTGCTCTGCGGTGTTCTGGCTCTGACGGACTCCAAGAACAAC
AAGGTGGCGGCCGGGATGGAGCCTCTCCTGGTCGGCCTGCTGGTGTTCGCCATCGGCACG
TCCTTCGGGTTCAACTGCGGGTACGCCATCAACCCTGCCCGAGACCTGGGCCCGAGACTC
TTCACGGCGATGGCAGGCTGGGGCCTGGAAGTCTTC---AGG------GCGGGAAACCAC
TGGTGGTGGGTGCCGATAGTCGGCCCGTTAATCGGCGGGCTGGTCGGAGGGTTGGTCTAC
ACGCTGATGGTGGCGCTGCATCACCCGGAGGATGAAGACGAC
>Amphioxus_Glp
GTGAGCGCTCTTCGGGTGAGGAGATTTGTAATCCGGGCGACGTTGGCAGAGCTACTCGGC
ACATTTCTCCTGGTGACGTTTGGAAACGGATCGGTGGCGCAGGTGGTGCTGAGTCGTGAG
GAC---AAGGGA---ACGTTCTTATCCATCAACTGGGCGTACGGCATCGGGGTGGTCATC
GGCGTGTACGCCTCCTGGGGTGTCTCAGGAGCCCATCTGAACCCTGCGGTATCCCTGACC
ATGGCAGTCCTGGGGAAGTTACGCTGGGTGTACCTGCCGTGTTACGTCATGGCTCAGATG
ATAGGCGCCTTCTTGTCAGCAGTTTGTGTGTACATTGTGTACTATGATGCATTGGTGAAC
TTTGACGGCGGGACCCGGGCAGTGTTGGGTGTG---AACGGGACCGGAGGAATCTTCTGT
ACG------------TACCCGCAAGACTACCTGTCTATCGGCAGTGGAGTCCTTGACCAG
GTAGTGGGCACCGCTCTGTTACTCTGCGGTGTCCTGGCTCTGACTGACGCTAAGAACAAC
AAGGTAACGGCGGGGATGGAGCCGCTGCTGGTCGGCCTGCTGGTGTTCGCCATCGGGACG
TCCTTCGGGTTCAACTGCGGATACGCCATCAACCCTGCCCGAGACCTGGGCCCGAGGATC
TTCACGGCGATGGCAGGCTGGGGCCTGGAGGTCTTC---AGG------GCGGGAAACCAT
TGGTGGTGGGTGCCGATAGTCGGACCGTTGATCGGCGGGCTGGTCGGGGGTTTGGTCTAC
ACGCTGATGGTGGCGCTGCATCATCCAGAGGAAGAAGATAAC
>psUrchin_Glp1a1
CTAAATTCGTTAACAATTCGGAATGAGCTTGGACGATATCTCATCTGCGAGTTCTACTCG
ACGTTCATTGTATCGGCCTTCGTACATGCGGCTGTCGCTCAGGATGTGGTATCTAGG---
------ACAGGA---TCGGGATTCAACGTCGCCTTGGCCGCCGGTATGGGGGTCATGTTC
GGCATCTACTCCGGGTTCGGTGTCTCAGGGGGCCACGTCAACCTGGCTCTATCGATAGGA

ATAGCTGCTATAGGAATATTCCCTTGGAGAAGGATTCCTCTCTATTTCTTAGCCGAACTA
GCGGGCGGGTTCGCCGGAGCCTGGGTCGTCTATCGAGTTTACGAAGACGCCTTTGATCAT
TTTGACGGTGGAATGCGTCAAGCGTTTGGCGAG---AACGGCACGGCGGGGATCTTTGCC
ACG------------TTCCCACAACCTTACCTGTCTGTTACGACCGGGTTCCTCGAACAG
GTATTGAACACGGCGCTATTATTAGCCGTTATAGGTGCGATGATGGACCGACGAAACAAC
CCTCCTCCTCTCAGCTTCGCACCTTTCTTCTTTGGTTTGATTGTATTCACCATCCTCATG
AGCTATGGATATAATGCAGGAGCACCCCTGAATCCTTCGATAGACCTATCAGGACGTCTT
CTTCTGGCTGCAATGGGCTACGGCAAGGAGGTCTGG---ACG---CCCGGTGGTGTTCAT
TGGTGGTGGATACCCATCTTAGGACCCGCCATCGGGAGTGCTCTCGGATCCTGGGCCTAC
TACCTCGCTATCTCCATACACCATCCTCCTCTTGATGTGAAA
>gsUrchin_Glp1a1
CTGAATTCGATAACAATTCGGAGTGAGCTTGGACGATATCTCATCTGCGAGTTCTACTCG
ACGTTCATTCTATCGGCCTTCGTACACGCGGCTGTCGCTCAGGATGTCGTATCTAGG---
------ACAGGA---TCGGGATTCAACATCGCCTTGGCCGCCGGTATGGGGGTCATGTTC
GCCGTCTACTCCGGCTTCGGTGTCTCAGGAGGCCACGTCAACCTTGCTCTATCGATAGGA
ATAGCTGTTATAGGAATATTCCCTTGGAAAAGGATTCCTCTCTATTTCTTAGCCGAACTA
GCGGGCGGTTTCGCCGGATCCTGGGTCGTCTATCGAGTTTACGAAGACGCCTTTGATCAT
TTTGATGGTGGAACGCGTCAAGCGTTTGGCGAG---AACGGGACGGCAGGGATCTTTGCT
ACA------------TTCCCACAACCGTACCTGTCTGTTACGACCGGGTTTCTC---CAG
GTATTGAACACAGCGCTATTATTAGCCGTTATAGGTGCGGTAATGGACCGACGCAACAAC
CCTCCCCCTCTCAGCTTCGCGCCTTTCTTCTTTGGTTTGATTGTGTTCACCGTCCTCATG
AGCTATGGATATAATGCAGGAGCACCCTTGAACCCTTCAGTAGACCTGTCAGGACGCCTT
CTTCTGGCTGCTATGGGCTACGGCAAGGAGGTCTGG---ACG---CCCGGTGGTGTTCAT
TGGTGGTGGATACCCATCTTAGGACCCGCCCTCGGGAGCGCTCTCGGATCCTGGGCCTAC
TACCTCGCTATCACCCTCCACCATCCTCCTCTCGATGGGAAA
>Kina_Glp1a1
TTGAACATGATAACAGTTCGAAATGAGCTTGCACGACACCTCATCTGCGAATTCTATGCG
ACTTGCGTTTTCGCGCTATTCGTACGTGGAGCCATCGCTCAGGATGTCACATCGGGG---
------ACGGGA---TCAACGTTCAACATCGCTATCGCCGTCGGTATGGCGGCCTGTTTT
GGCATCTATCAAGGCTTTGGGGTCTCAGGTGGTCACGTCAACCTGTCTATTACGATAGGC
CTAGCTGCAATAGGGTTATTTCCCTGGAAAAGAGTACCTATCTATTTCGTGATTCAAGTA
GCTGCTAGCTTCGTCGCCACTTTGTTCGTCTATCTCGTTTACGAAGACGCTTTCGATTAT
ATTGATGGTGGAACACGAGTGGCGTTTGGTGAG---AACGGAACGGCGGGAATCTTCGCT
TCC------------TTTCCGCAACCATACTTGTCTGTTACGACCGGCTTTTTCGAGCAG
GTATTGAACACTGGTCTGCTCTTAGCATGTGTAGGGGCGATGTTGGATAGTCGTAACAAC
CCACCCCCACTCAGCCTAGCACCTTTCTTCTTCGGTTTGATTATCTTCACAATCATACTG
ACCTTTGGTCATAACGCTGGAGCACCTCTGAATCCTTCTCTAGACCTAGCTGGTCGTCTT
CTGATGTCTGCAGTCGGGTATGACAAGGAGGTCTGG---GTA---CCCGAAGGTATTCAT
TGGTGGTGGATACCTATCGTAGGACCTTCTGTTGGGAGTGCTCTAGGATCGTGGGCCTAT
TACCTGTCAGTCACCTTACACCATCCTCCTCTAGAAGGCAAG
>ChilUrchin_Glp1a1
TTGAATTTGATAACTGTTCGAAATGAGCTCGGGCGATTCCTCATCTGCGAGTTCTACTCG
ACGTTCATTTTATCGGTGTTTGTGCATGCAGCCGTCGCTCAAGATGTCATATCACGT---
------ACGGGA---TCAGGCTTCAACATCGCTATCTCCGCGGGTATGGGAGTCAGTCTG
GCTGTCTACTCCGGCTTTGGTGTCTCAGGGGGTCATATCAACCTGGCACTGTCGATAGGA
ATAGCTGTTATAGGAATATTCCCTTGGAAGAGGATACCCTTCTACTTCTTGGCCCAAGTA
GCCGGCGGGTTCGTCGGGGCTTTAGTCGTCTATCTAGTTTACGAAGATGCTTTTGATGAG
TTTGATGGCGGAACGCGGCAGGTATTTGGTGAG---AACGGGACAGCAGGAATCTTCGCG
ACG------------TTCCCGCAACCATACTTGTCTATTACCACTGGATTCTTCGAGCAG
GTGTTGAACACAGCGCTGTTATTAGCAGCTATTGGGGCGGTGATGGACCGACGTAACAAC
CCTCCCCCGCTCAGTCTAGCCCCCTTCTTCTTTGGTTTGATCGTATTCACCATCCTAATG
AGTTATGGCTACAATGCTGGAGCACCCCTCAATCCTTCTGTTGACTTGTCTGGACGTCTT
CTTCTGACAGCAATGGGGTACGGCAAGGAGGTCTGG---ACA---CCGGGAGGCGTTCAT
TGGTGGTGGATACCAATCGTCGGACCAGCCATAGGAAGCGCTCTGGGATCGTGGGCTTAC
TACCTTGCTATCACCATACACCATCCTCCTCTCGAAGGCGAA
>psUrchin_Glp1a2
CTGAGTGTGATAACAATTCGGAGTGACCTCGGACGATGTCTCATGGCCGAGTTCTACTCG
ACGTTCATGCTATCGGTCTTCTTGCGTGCGGCCATCGCTCAGGATCTCACTTCAGGA---
------ACGGGA---TCAGTATTCAACATCGCTTTCACCGCCGGTATGGGGGTCGCTTTC
GCCATCTACTCCAGTTTCGGCGTCTCAGGGGGTCACATCAACCTCGCCGTATCGATAGGA
GTAGCTGTTTTAGGAAAATTACCATGGAAAAGGGTTCCTGTCTATTACTTAGCCCAACTA

GCGGGCGGGTTTGTCGGAGCTGTGCTTGTCTATGCTGTTTACCAAGATGCCTTCGATGAT
TTCGACGGTGGAGAGCGTCAGGCGTTTGGCCCG---AATGGGACGGCAGCGATATTTGCT
ACA------------TTCCCCCAACTGTATCTGTCTGTGACGACCGGGTTTTTCGAACAG
GTATTGAGCACAGCGCTATTTTTAGCCATCATAGGCGCAGTGTTGGACCATCGCAACGTC
CCGCCCCCTCTCAACTGGGCCCCTTTCTTCTTTGGTTTGATTATTTTAGCCCTCGTCATG
ACCTTTGGTCATAATGCAGGGGCACCCCTGAATCCTTCGATAGACCTTTCGGGACGTCTT
TGTCTGGGTGTCATGGGGTACGGCGCGGAGGTCTGG---GTGCTGCACGATGGTGTTCAT
TGGTGGTTGATACCCATCTTTGGACCCGCCATTGGGGGCGCTGTGGGATCCTGGGCCTAC
TACCTCGCAATCGAACTCCACCATCCTCCTGTTGATGAGAAA
>gsUrchin_Glp1a2
CTGAGTGTGATAACGATTCGGAGTGACCTCGGACGTTGTCTCATGGCCGAGTTCTATTCG
ACGTTCCTTCTATCGGTCTTCTTACGTGCGGCCATAGCTCAGGATCTCACTTCAGGA---
------ACGGGA---TCAGTATTCAACGTCGCTTTCACCGCCGGTATGGGGGTCGCTTTC
GCCATCTACTCCAGCTTCGGTGTCTCAGGGGGTCACATCAACCTCGCTGTATCAATAGGA
GTAGCTGTTTTAGGAAAATTGTCATGGAAAAGGGTTCCTCTCTATTACATAGCCCAACTA
GCGGGTGGGTTCATTGGAGCTGTGCTCGTCTATCTCGTTTACCAAGACGCCTTCGATGAT
TTCGACGGTGGAACGCGTGAAGCTTTTGGCCCG---AATGGGACGGCGGCGATCTTTGCT
ACA------------TTCCCGCAACTGTATCTGTCTTTGACGACCGGGTTCTTCGAACAG
GTATTGAGCACAGCGCTATTTTTAGCCATTATAGGTGCGGTGTTGGACCAACGCAACGTC
CCGCCCCCTCTCAACTTCGCCCCTTTCTTCTTTGGTTTGATCATATTTGCCCTCGTCATG
ACCTTTGGTCATAATGCAGGAGCACCCCTGAATCCTTCGATAGACCTTTCGGGACGTCTT
TGTCTGGCTGCAATGGGGTACGGCGCGGAGGTCTGG---GTACTGCACGATGGTGTTCAT
TGGTGGTTGATACCCATCTTTGGACCCGCCATTGGGGGCGCTCTGGGATCCTGGGTCTAC
TACCTCGCTATCACCCTCCACCATCCTCCTCCTCCTTATGTG
>rsUrchin_Glp1a2

---GTGTCAAGA---
------ACAGGA---TCAGTATTCAACATCGCTTTCACCGCCGGTTTGGGGGTCGTTTTC
GCCATCTACTCCGGCTTCGGGGTCTCAGGGGGTCACATCAACCTTGCCGTATCAATAGGA
GTAGCTGTTTTAGGAAAAGTATCATGGAAAAGGGTTCCTCTCTACTACTTAGCCCAACTA
GCGGGTGGGTTCATCGGAGCTTGGCTGGTCTATCAAGTTTACCAAGACGCCTTTGATCAT
TTTGACGGTGGATCGCGTCAAGCGTTTGGCGAG---AATGGGACGGCAGGAATCTTTGCC
ACA------------TTCCCGCAACCTTACCTGTCTCTTAAGACCGGATTCTTTGAATCG
GTTTTGTGCACAGCGCTATTTTTAGCCGCTATAGGTGCGGTGTTGGACCATCGCAATACC
CCTCCCCCTCTCAGCTTCGCACCTTTCTTCTTTGGTTTGATAATATTGGCCCTCATCATG
AGCCTTGGTCATAATGCGGGAGGAAACCTGAATCCTTCGATAGACCTATCGGGACGTCTT
CTTCTGGCTGCAGTGGGGTACGGCAAGGAGGTCTGG---GTA------------------

>GVSurchin_Glp1a2
CTTAATTCGATACGAATTCGTAGCGAGCTTGGACGACATGTCATCTGCGAGTTCTATGCG
ACTTTCATTCTATCAGTCATCGTCCATGCTGCCGTGGCTCAGGATGTGGTATCGAGG---
------ACAGGG---TCTGCATTCAACGTCGCTTTGACGGTCGGGATGGGGGTCGTTCTC
GCAGTCTACGCTGGATTCGGTGTAACAGGGGCTCACATCAACCTGGCTGTATCAATAGGA
ATAGCTGTTATAGGACAATTCCCATGGCGGAGGATACCTGTATATTTCCTAGCCCAGCTT
GTCGGTGGGTTCGCCGGGGCATGGGTAGTATATCGAGTTTATGAAGATGGTTTTGATCAT
TTTGATGGTGGAACTCGTCAGGTTTTGGGTGAG---AATGGTACAGCTGGAGTCTTTGCT
ACA------------TTCCCGCAGCCGTACTTGTCAATGACGACTGGGTTTTTTGAGCAG
GTATTGAATACAGCATTGTTATTAGCCTCTATAGGAGCTGTGATGGATCAGCGAAACAAC
GCCGCCCCTACTCGCGTAGCCCCGTTTTTCTTTGGGTTGATTGTGTTTACTATTCTAATG
AGCTACGGATATAACGCTGGCGCCCCTCTCAATCCTTCAATCGATCTATCCGGACGTCTT
TTTCTCACCGCTGCTGGGTACGGAAGAGAGGTCTGG---ACG---CCCGATGGCGTTCAC
TGGTGGTGGATACCAATTGTAGGACCAATTGTTGGGGCTTCTCTCGGGTCCTGGGCCTAC
TTCCTCGCTATCATCATACACCATCCACCAGAGGAGATC---
>Sg_psUrchin_Glp1a2
CTAAATTCGATACGAATTCGTAGCGAGCTTGGACGACATGTCATCTGCGAGTTCTATGCG
ACTTTCGTTTTATCGGTCATCGTCCATGCAGCTGTGGCCCAGGATATCGTATCACGG---
------ACAGGG---TCCCCATTCAACGTCGCTTTGACCGTCGGTATGGGAGTCGTTCTC
GCAGTCTACGCCGGCTTCGGTGTAACAGGTGCTCACATCAACCTGGCTGTATCAGTAGGA
GTAGCTGTTATAGGAGGATTCCCATGGAAAAGGATTCCCGCGTTTTTCTTAGCTCAGCTT
ACCGGAGGATTCGCCGGGGCTTGGGTTGTCTATAGAGTTTACGAAGACGGTTTTGATCAT

TTTGATGGTGGGACTCGTCAGGCGTTTGGTGAG---AATGGGACTGCTGGAATATTTGCT
ACA------------TTTCCGCAACCATACCTGTCGATGACGACTGGCTTTTTTGAACAG
GTATTAAACACAGCATTACTGTTAGCCACTATAGGCGCTGTGATGGATGATCGCAACAAC
GCTCCCCCTGTCCGCGTGGTGCCGTTCTTCTTTGGGTTGACCGTGTTTACTATTCTAATG
AGCTACGGATATAATGCTGGGGCACCCCTCAATCCTTCCATAGATCTATCGGGACGTCTG
TTTCTGACTGCCGTGGGGTACGGCAGAGAGGTCTGG---ACT---CCTGATGGTGTTCAC
TGGTGGTGGATACCAATCATAGGACCAATTTGTGGTGCTGCCCTCGGATCATGGGCCTAC
TTTCTCGCTATCATCATACACCATCCCCCAGAGGAT------
>cUrchin_Glp1a2b
TTGGACTCCATAACGATAAGAAATGACCTCTTCAAGTGTTTCATTGCCGAATGCTATGGA
ACATTTCTTATGGCGGCATTGACGCGCGCAGCACTTGCACAGGATACGGTTTCACGA---
------ACAGGT---TCAACTTTTAACATCTCAATAACAATTGGTTTGGGTTTGATGTTT
TCAATCTACTCAGCATTCGGAGTATCAGGCGGCCATATAAACCTGTCTATTTCAATCGGC
TTGGCTGCAGGCGGATCGTTTCCATGGAAACGAATACCAGTCTATTTCGTTGCCCAACTT
ATTGGAGGATTTTTAGGAGCTGCTGTGGTATATTGCATTTATAAAGAAACTTTCGATGAA
TTTGATGGAGGTACAAGACAGGCTTTCGGTGAG---AATGGAACGGCGGGAATCTTTGCT
ACT------------TTTCCGCAACCTTATCTCTCATTCACTACTGGTTTCTTAGAACAG
GTTTTCAACACGGGTCTCATGCTTGCCCTCACCCTGGCTGTAACCGACAAGATGAACAGC
GCCCCACCTTCTTACGTTCAACCAGTCCTATTTGGGCTAACTGTTTTTGTAATCTTGATG
AGTTATTCATTCAATGCTGGATCTCCTCTTAACCCATCCCTTGATCTGTCGGGAAGAACC
CTCTTAGCTGCTGCTGGATATGGAAAGGAGGTTTGG---AAT---CCTGGTGGTCGTCAC
TGGTGGTGGATACCTATTCTAGGACCAGCCACTGGGAGCGCTCTGGGATCCTGGTCTTAC
ATACTCGCTATAAGACTACATCATCATCAACCTTCGAAAGAA
>cUrchin_Glp1a2a
GTGGACAAAATGAGAATAAGAAATGATCTGGTCAGATGTTGTGTTGCTGAATGTTATGGA
ACATTTCTCATGGCGTCTTTCACACGTGGAGCTCTCGCCCAGGATACCGTATCTCGA---
------ACAGGA---TCAACGTTTAACATCTCAATCACAATTGGTTTAGGATTAATGTTT
TCAATCTATTCAGCATTCGGAGTATCGGGGGGTCATGTAAACCCGTCTATCTCAATCGGC
TTAGCAGCGGGAGGATTGTTTCCATGGAAACGAGTACCAATATACATCATCTCTCAAGTC
ACTGGAGGATTTTTAGGAGCTGCCTGTGTGTATGGAATTTATCAAGAAACTTTTGATGAG
TTTGATGGAGGTACGAGACAGGCGTTCGGTGAG---AATGGAACGGCGGGAATTTTTGCT
ACA------------TTCCCTCAACCATATATCTCATTCACTACAGGCTTCCTTGAACAG
ATTTTCAACACAGGTCTCATGCTCGCCCTCACCCTAGCTGTATCAGATCAGAGGAACAGT
GCCCCACCTCCATACGTTCAACCAGTCCTATTTGGTATCAGTGTTTTCATCATCTTAATG
AGTTATTCATTCAATGCCGGATCTCCTCTTAACCCATCTCTTGATCTTTCTGGAAGAACA
CTCTTAGCTATTGCTGGATATGGAAAAGAGGCTTGG---AAA---CCTGGTGATCGTCAC
TGGTGGTGGATACCTCACTTAGGACCAGCCATAGGGAGCGCACTAGGGTCTTGGTCTTAC
GTACTCGCTATAAGACTACATCATGTTCCCGATCATCATCAT
>ChilUrchin_Glp1a2
TTGGACACGATAACAATACAAAATGAACTGATCAGATGTTGTGTTGCCGAATGTTATGGA
ACATTTCTCTTTGCGGCATTTACACGCGCAGCGATCGCTCAGGATACTGTGTCTCGC---
------ACAGGT---TCACATTTCAACGTCGCCATCACAGTCAGTTTAGGTATAATGTTG
TCAATCTACTCAACATTCGGAGTATCAGGTGGTCATCTAAACCTCTCTCTCTCGGTTGGC
GTGGCAGCGGCAGGATTGTTTCCATGGAAACGGATACCAATATATTTCATTTCCCAACTT
ATTGGAGGATTTGCAGGAGCTGCTGTGGTATATGGCATTTACCAAGAGGCTTTCGATGAA
TTTGACGGAGGTACGAGAGAGCCTTTTGGTGAA---AATGGAACCGCCGGAATCTTTGCT
ACT------------TTCCCGCAACCATATCTCTCCTTCACTACTGGTTTCATTGAACAG
GTTTTCAACACGGGTCTTCTGCTCGCCCTGATCCATACTCTACTTGACAAGAAGAACACT
GCCCCACCTTCATACCTTCAACCAGCCTTCTTTGGGCTTAGTGTATTCACCATCTTGATG
AGTTATGCGTACAATTCCGGGTCTCCTCTTAATCCATCCCTTGATCTCTCTGGAAGAACC
TTCTTAGCTGTCGCTGGATACGGAAAGGATGTTTGG---ATA---CCTGGTGGTATGCAG
TGGTGGTGGGTACCTGTTCTAGGACCAGCCATTGGTAGCGCGCTGGGGACCTGGGCCTAC
ATACTCGCCATACAATTACACCATCCTTCAAAAGAGAACCCA
>spUrchin_Glp1
AAGAATACGTTTCGAATACGGAATGAATTGGCGCGGTTTCTGCTGTGTGAATTTTACGCT
ACATTTCTTCTGTCGTTGTTGTGCCATGCCGCTGTGGCCCAAGAGTTCGTATCAAGA---
------TCATAT---TCGATATTCAATATAGCGATTGCCGTCGGAATGTCAGTATGCTTC
GCAATCTACACCGGATTTGGTGTATCGGGTGGCCACATCAACCCAGCCATCTCCGTGGGG
ATAGCAGCGGCCGGTCATTTCCCATGGAGGAGGGTCCCGCTATACAGCCTGGCACAAACT
GCTGGAGCTTTTGTCGGGGCTGCCTTGGTGTACGCTATTTACAAAGAGGCATTTGATGTG
TTTGACGGTGGGACCCGTCAGGCGTTCGGCGAG---AACGGAACGGCTTACATATTTACG

ACG------------TTTCCACAACCGTATCTCTCTGTTACAACCGGCTTCTTGGAACAG
GTTTTAAACACAGCCATCCTCCTAGCCATCATTGGACCCATCATGGATACGAGAAACAGC
GCAGCGCCACTCAACGTGGCACCCATTTCTTCGGGCTCATAGTCTTCACCATCCTGCTA
ACGTACAATTACAACGCCTCATCACCGCTGAACCCAGCATTAGACTTGTCCGGTCGAATG
CTGCTATCTGCTGCTGGATACGGCAGAGAGGTGTGG---ATG---CCTAGAGGCATCCAT
TGGTGGTGGATACCTATCGTAGGACCACTGATTGGAGGTGCTGTAGGATCCTGGGTTTAC
ATCGCATTTGTAGAATTTCACCATCCTCCACTGGATGAAAGT
>jsCucumber_Glp1a1
AAGTGTAAGCTCCGAGTGAAGAGCGATTTAATACGATGTCTCATGGCCGAATTCATCGGG
ACTTTTGTGTTGGTTGCTCTTGTAGATGGCGGCGGGGCAGGGGTGATAACTAGTAGATTA
AGTGGTTTGGAG---AACTACCTTTGGTTGTCTTTCGGATCTGGATTTGGCGTTGGCTTT
GGCATCTGGACTGCGTATGGTATTTCAGGTGGTCACGTGAATCCAGCGGTAACTCTTGGT
TTGACAATGACAGGAAAGTTTCCATGGCGACGAGTACCATTTTACTTCCTGGCTCAACTA
TGTGGCGCTTTCTGCGCATGTGTAGTTTGTTTCCTTGTCTACTTTGATGGTATTAACAAC
ATTGACGGAGGTACCAGGATAGCGGTCGACCATCCCAACGCGACTTGTGGTATCTTTACC
ACG------------TTCCCTGCTGCATACGTATCTCTCAGTACTGGATTCTTTGAACAG
ATCGTGAATACTGGTATTATGCTCGGTCTTATCCACGTGATCGGTGACATGCGCAATGCA
GGCCCTCCATTCAACCTTGCCCCTCTCTTCTACGGGCTGATTGTCTTCTCCGTTATCCTG
TCATATGGTACGAATGCGGGCGCTCCTCTAAACCCCGCCCGCGACTTCGCCGGTAGACTG
ATGTGCGCTATCGCTGGATATGGACCAAAAGTTTGG---GCA---CCGTACGGTGTCCAG
TGGTGGTGGATACCAACCTTTGGTCCCCTGGTAGGGGCAGCTGTCGGTTCATTGGTCTAT
CTGTTCCTCATTGAAATTCACCATCCACCGGAAGAGAAATGC
>gosCucumber_Glp1a1
AAGTGTAAGCTTCGCGTGAAGAGCGATTTAATACGATGTCTCATGGCCGAATTCATCGGG
ACTTTTGTGTTGGTTGCTCTTGTAGATGGCGGCGGGGCAGGGGTGATAACTAGTAGATTA
AGTGGTTTGGAG---AACTACCTCTGGTTGTCTTTCGGATCTGCATTTGGCGTTGGCTTT
GGCATCTGGACTGCGTATGGTATTTCAGGTGGTCACGTGAATCCAGCGGTAACTCTTGGT
TTGACAATGACAGGAAAGTTTCCATGGCGACGAGTACCATTTTACTTCCTGGCTCAGCTA
TGTGGCGCTTTCTGCGCATGTGTAGTTTGTTTCCTTGTCTACTTTGATGGTATTAACAAC
ATTGACGGAGGTACCAGGATAGCGGTCGACCACCCTAATGCGACTTGTGGTATCTTTACC
ACT------------TTCCCTGCTGCATACGTATCTCTCACTACTGGATTCTTTGAACAG
ATCGTTAATACTGGTATTATGCTTGGTCTTATCCACGTGATCGGTGACATGCGCAATGCA
GGCCCTCCATTCAACCTTGCCCCACTCTTTTACGGGCTGATTGTCTTCTCCGTTATCCTG
TCATATGGTACGAATGCGGGCGCTCCTCTTAACCCCGCCCGCGACTTCGCCGGTAGACTG
ATGTGCGCTATCGCTGGTTATGGACCAAAAGTTTGG---GCG---CCGTACGGTGTCCAG
TGGTGGTGGATACCAACCTTTGGTCCTCTGGTAGGGGCAGCTGTCGGTTCATTGGTCTAT
CTGTTCCTCATTGAAATTCACCATCCACCGGAAGAGAAATGC
>wsCucumber_Glp1a1
AAGTGTAAGCTCCGCGTGAAGAGCGATTTAATACGATGTCTCATGGCCGAATTCATTGGG
ACGTTTGTGTTGGTT---

------------------------TCAGGTGGTCACGTGAATCCAGCGGTAACTCTTGGT
TTGACAATGACAGGAAAGTTTCCATGGCGACGAGTACCATTTTACTTCCTGGCTCAGCTA
TGTGGCGCTTTCTGCGCATGTGTAGTTTGTTTCCTTGTCTACTTTGATGGTATTAACAAC
ATTGACGGAGGTACCAGGATAGCAGTCGACCACCCCAATGCGACTTGTGGTATCTTTACC
ACA------------TTTCCTGCTGCATACGTATCTCTCACTACTGGATTCTTTGAACAG
ATCGTTAATACTGGTATTATGCTTGGTCTTATCCACGTGATCGGTGACATGCGCAATGCA
GGCCCTCCATTCAACCTTGCCCCTCTCTTCTACGGGCTGATTGTCTTCTCCGTTATCCTG
TCATATGGTACGAATGCGGGCGCTCCTCTAAACCCCGCCCGCGACTTCGCCGGTAGACTG
ATGTGCGCTATCGCTGGTTATGGACCAAAAGTTTGG---GCG---CCGTACGGTGTCCAG
TGGTGGTGGATACCAACCTTTGGTCCTCTGGTAGGGGCAGCTGTCGGTTCATTGGTCTAT
CTGTTCCTCATTGAAATTCACCATCCACCGGAAGAGAAATGC
>shWCucumber_Glp1a1
AAATGTAAACTTGGAGTTGAGAACGAGCTGATACGGTGTCTTTTGGCAGAATTCATCGGG
ACTTTTATACTCGTGGCTCTCGTCGATGGTGGCGCAGGAGGAGTTATAACAAGCCGATTA
AGTGGCTTAGAG---AACTACCTATGGCTGAGCTTTGGATCTGGCTTCGGTGTTGGCTTT
GGGATCTGGACCGCTTATGGTGTATCAGGAGGCCATGTTAATCCATCAGTAACTATTGGT
TTAACAATGACGGGAAAGTTCCCTTGGCGACGTGTACCGTTTTATTTCTTGGCCCAGTTT
TGTGGAGCTTTCTGCGCAGGGGCCGTATGTTTTTGGTCTATTATGATGGTATCAACGAA
ATAGACGGAGGCGTGAGGATAGCGGTCGATCACCCAAATGCCACTTGTGGTATCTTCACA
ACA------------TTCCCTGCTGAGTACGTCACAGTGACCACAGGGTTCTGTGAACAG

ATCGTCAACACTGGTATTATGCTATTTCTCATTCACGTGATCGGTGACATGCGTAATGCG
GGACCTCCATTCAACCTAGCTCCCCTCTTTTACGGCCTGATTGTCTTCTCTGTGATCCTG
TCGTACGGTACCAACGCCGGGGCTCCTCTTAACCCCGCCCGGGACTTCTCCGGCAGACTG
ATGTGCGCAATCGCTGGTTATGGATCGAAAGTTTGG---GCG---CCGTACGGTGTCCAG
TGGTGGTGGATACCAACCTTTGGTCCCCTGGTGGGGGCAGCCGTCGGGTCACTGGTGTAC
CTGTTGACCATAGAAATTCACCATCCTCCGGAAGAGAGATGT
>fCucumber_Glp1a1

----------------------------GGTGGTCACGTGAATCCAGCTATATCTATCGGT
CTGGCGATGACAGGGAAGTTTCCATGGAGGAGATTCCCCCTCTACGTCATGGCGCAAGTA
TCGGGGGCGTTTGCAGCCTGCGTTGTGTGTTTCCTGGTTTACTACGATGGAATCAACCAT
CTCGACGGTGGTGACCGACAAGTGTTTGACCACGAGAACGCTACTTGTATCATCTTTACT
ACC------------TTCCCTGCTGAATACGTCACTCTCACCACAGGATTCTTTGAACAG
GTCGTCAACACTGGTCTTATGCTTGGACTGGTTCACGCCATCGGGGACCAACGTAACGCC
GGCCCTGTCCCCAGTCTGGCCCCGTTCTTCTATGGGTTAATAGTGTTCTCTGTCATATTA
TCGTATGGTGTCAACGCGGGTGCTCCACTCAACCCATCCCGGGACTTTGCCGGTAGACTC
ATGTGTGCCATCGCTGGATATGGGTCCGAGGTTTGG---GCC---CCATACGGGGTCCAA
TGGTGGTGGATCCCCACCTTTGGTCCACTGGTCGGTGGTGCCGTGGGGTCGTTGATCTAT
CTGCTGTTTATTGAGATCCATCACCCCGTTGCCAAC------
>hsCucumber_Glp1a1

----------------------------GGGGGTCACGTGAACCCAGCCATATCGATTGGT
TTGGCGGTCACAGGGAAATTTCCCTGGAGAAGGTTTCCTTTTTACGTTCTGGCTCAGCTA
GCAGGGGCGTTCACCGCGTGTGTCGTCTGCTTCATGATTTACTTTGATGGAATCAACAAT
CTTGACGGTGGTGATCGAGCTGTGTTTGATCATGAAAATGCTACTTGTGTGATTTTCACG
ACA------------TTTCCTGCTGAATACGTAACGCTCACAACAGGATTCTTCGAGCAG
ATCGTCAACACTGGTCTGATGCTGGCACTGGTCCACACCATCGGGGACCAGTGTAACGCC
GGTCCTGTTCCCAATCTAGCCCCCCTATTCTATGGTCTGATAGTATTCTCTGTTATACTG
TCTTATGGCATAAACACCGGGGCACCGCTCAAC---------------------------

>jsCucumber_Glp1a2
AAGGGCAAGTTGGCGATACAAAATGACCTATTCAGATGTCTATTGTCAGAATTTCTGGGG
ACATTTATCTTAGTGGTACTTGCAGATGGCGCTCTTGCAAGTCTTGCCATTAGTGGA---
------TCCGAT---AATCATCTGTGGGCGGCTTTTGGAGTTGGGTTTGCGGTGGCGTTT
GGTATTTGGACTGCTTTAGGAGTGTCAGGTGGACACGTAAACCCAGCCGTAAGTCTAGGC
CTGGCTGTTACTGGTAAATTCCCCATGAAGCGATTTTTACCTTATTGTTTAGCCCAATGC
TCAGGTGCGTTCGTAGCCAGCGTCGTGTGTTACTCTGTATACTATGATGGAGTAAAAAAC
GCTGTACCATTAGAC------GATGTTAACCAT---ACAGCAGCATGTGGCATCTTCACC
ACA------------TTCCCTGGAGAGAGTGTAGGGTATACAACTGCCTTTTTTGATCAG
ATAATTACCACTGGGCTGTTGATTGGACTTCTTCTCGCATTGTTAGACAAACGGAATATG
GCACCTGACCTCACCCTCGTACCCTTGTCTGCTGGCATCCTTGTTTCTAGTATCATTCTG
TCCTATGGTATGAACACTAGTGCACCTCTTAATCCAGCACGTGATTTCGCAGGACGAATC
ATGTGCACACTGGCTAACTACGGACCGGACGTATGG---CTG---GATGACGGAAAACAT
TGGTGGTTTATTCCTACATTTGGTCCATTGGTTGGTTCTGTAGTCGGTGCCTGGACATAT
ATGCTCTGTATTGAGATCCACCATCCTAACGAAGAAACAGAG
>wsCucumber_Glp1a2
AAGGGCAAGTTGGCGATACAAAATGACCTATGCAGATGTCTATTGTCAGAATTTCTGGGG
ACATTTATCTTAGTGGTTCTTGCAGATGGCGCTCTTGCAAGTCTTGCCATTAGTGGA---
------TCAGAT---AATTTTCTGTGGGCGGCTTTTGGAGTTGGGTTTGCGGTGGCCTTT
GGTATTTGGACTGCTTTAGGAGTGTCAGGTGGACACGTAAACCCAGCCGTAAGTCTAGGC
CTGGCTGTTACTGGTAAATTCCCCATGAAGCGATTTTTACCTTATTGTTTAGCCCAATGC
TCAGGTGCGTTCGTAGCCAGCGTCGTGTGTTACTCTGTATACTATGATGGAGTTAAGAAC
GCGGTACCATTAGAC------GAGGTTAACCAT---ACAGCAGCGTGTGGCATCTTCACC
ACA------------TTCCCTGCAGAGAGTGTAGGGTATACAACTGCCTTTTTTGATCAG
ATAATCACCACTGGGCTGTTGATTGGACTTCTTCTCGTATTGTTAGACAAACGGAACATG

GCACCTGACCTAACCCTCGTACCCTTGTCTGCTGGCGTCCTTGTTTCTAGTATCATTCTG
TCCTACGGTATGAACACTAGTGCACCTCTTAATCCAGCACGTGATTTCGCAGGACGAATC
ATGTGCACACTGGCTAACTACGGACCGGAAGTGTGG---CTG---GATGACGGTAAACAT
TGGTGGTTGATTCCTACATTTGGTCCATTGGTTGGTTCTGTAGTCGGTGCCTGGACATAT
ATGCTCTGTATTGAGATCCACCATCCTAACGAAGAAACAGAG
>gosCucumber_Glp1a2
AAGGGCAAGTTGGCGATACAAAATGACCTATGCAGATGTCTATTGTCAGAATTTCTGGGG
ACATTTGTCTTAGTGGTGCTTGCAGATGGCGCTCTTGCAAGTCTTGCCATTAGTGGA---
------TCCGAT---AATTTTCTGTGGGCGGCTTTTGGAGTTGGGTTTGCAGTAGCCTTT
GGAATTTGGACTGCTATAGGAGTGTCAGGTGGACACGTAAACCCAGCCGTAAGTCTAGGC
CTGGCTGTTACTGGTAAATTCCCCATGAAGCGATTTTTACCTTACTGTTTAGCCCAATGC
TCAGGTGCGTTCGTAGCCAGCGTCGTGTGTTACTCTGTCTACTATGATGGAGTAAAGAAC
GCTGTACCATTAGAC------GTTGTTAACCAT---ACAGCAGCATGTGGCATCTTCACC
ACA------------TTCCCTGCAGAGAGTGTAGGATATACAACTGCCTTTTTTGATCAG
ATAATCACCACTGGGATATTGATTGGACTTCTTCTCGCATTGTTAGACAAACGGAATATG
GCACCTGACCTAACCCTCGTACCCTTGGCTGCTGGCGTCGTTGTTTCTAGTATCATTCTG
TCCTACGGTATGAACACTAGTGCACCTCTTAATCCAGCACGTGATTTCGCAGGACGAATC
ATGTGCACACTGGCTAACTACGGACCGGAAGTGTGG---CTG---GATGACGGTAAACAT
TGGTGGTTGATTCCTACATTTGGTCCATTGGTTGGTTCTGTGGTCGGTGCCTGGACATAT
ATGCTCTGTATTGAGATCCACCATCCTAACGAAGAAACAGAG
>shWCucumber_Glp1a2
AAGTCCAAATTGGTCATCAGGAATGAATTGATAAGATGTATGCTGTCTGAATTTCTGGGA
ACATTTCTCTTAGCGCTACTAGCAGACGGTGCTATCGCTGGGAATTTAATCTCAGAA---
------TCCAAC---AATCATGTGTGGGTAGCTACAGGCGTCGGGTTAGCTGTTGCCTTC
GGGGTGTGGGTTGGATTCGGGGTCTCAGGTGGTCACGTCAATCCAGCCGTAACAATCGGT
TTGGCTGCATCTGGTAAGTTCCCCTTTAGGAGGCTTCCGTGGTACTGTCTGGCACAGTTC
GCTGGTGGGTTTTTCGGTGGCCTGGTGTGCTTTCTTACGTATTTCGAGGGAATCGTGACT
CTTCAA---------------GAGACT---------AACTCAACTTGCAGTATCTTTACA
ACA------------TTCCCTGCAGAATACACTGGATTTGTGACTGGCTTCGTCGATCAG
ATAGTCACCGCGGGGTTGTTGATTGGACTAATTCTCATGTTGGTAGATAAACGCAATTCA
GCTCCGGACGCGAGTCTTGCACCGTTCTTCGTTGGCATCTTGGTGACTAGTATTATCCTT
TCCTACGGTGTCAGCACTGGGGCTCCACTTAACCCAGCTCGTGACTTTACCGGCAGAATC
TTGTGCAGCATTACCGAAAGCCAACCCAACGCATGG---ATT---GTGGATGGTGGCCAC
TGGTGGTTCGTTCCGACTGTCGGCCCGTTGATAGGAGGTATCGTTGGTGCGTGGATATAC
CTGCTGTTCATTGAAATTCACCATCCTAAAGGAGAGGCGGAC
>Batstar_Glp1a2
CGCAGCTGCATCACGGTGAAGCATGAGATAGCCAGGGGAATGCTCGCCGAAATGGCTGGA
ATATTTGTCCTCGTACTCTTCGTGGATGGCGGTCTTGCCGGGGGTATCGTCAGCGGATGG
ATC---TCGGGC---ACTCCAGTCTCGACCGCCTTCTCCTCCGGAATGGGGGTGGCATTC
GGCATATACACCGGTCTTGCAGTGTCGGGTGGTCACGTGAACCCGGCGATTACCGTGGGC
TTAGCGTCCGTTGGTAAGTTTCCATGGCGACGGGTGCCACTCTGGATCCTGGCTCAACTT
ATCGGAGCCTTCTGCGGCGCTTCGGTCGTCTTCGGTATTTACTACGAGAGCATCGATGCG
CTCGATGGAGGTAACCGAACGGTCTTTGGACCC---ACAGGGACCGGCGGGATCTTCTGT
ACC------------TACCCACAGGAGTATTTCGGCATGGGTCCTGGATTTGTGGAACAG
ATCATCAACACAGGACTGCTCTTGCACTGCACCCTGATGTTTTTCGACGAGAGAAACGGC
AAACCGACCAAAGGGATGGAGCCGTTCTTTGTGGGTCTGTCTGTGGCCGTGATCATCCTG
GCCTGGGGACACAACGCCGGGGCACCCATGAACCCAGCCAGGGACTTGGCTGGTCGGCTA
TTGACATGGCTGGCTGGCTACGGAGCTGAAGTCTGG---GTG---CCTAGGGGCGTCCAC
TGGTGGTGGATTCCCACGCTGGTTCCGCCCCTCGGAGGGATCGTCGGAGCCAATTTCTAC
TACTTGTTTGTGGAGCTGCACCACCCTGCTGAGGACGGGGAC
>Blue_batstar_Glp1a2
CGCAGCTGCATTACGGTGAAGCATGAGATAGCCAGGGGAATGCTCGCCGAAATGGCTGGA
ATATTTGTCCTCGTACTCTTTGTGGATGGCGGTCTTGCCGGGGGTATCGTCAGCGGATGG
ATC---TCGGGG---ACTCCAGTCTCGACCGCCTTCTCCTCCGGAATGGGGGTGGCATTC
GGCATATACACCGGTCTTGCAGTGTCAGGTGGTCACGTGAACCCGGCGATTACCGTGGGC
TTAGCGTCCGTTGGTAAGTTTCCATGGCGACGGGTGCCACTCTGGATCCTGGCTCAACTT
ATCGGAGCCTTCTGCGGCGCTTCGGTCGTCTTCGGTATTTACTACCAGAGCATCGATGCG
TTCGATGGAGGTAACCGAACGGTCTTTGGACCC---ACAGGGACCGGCGGGATCTTCTGT
ACC------------TACCCGCAGGAGTATTTCGGCATGGGTCCTGGATTTGTGGAACAG
ATCATCAACACAGGTCTACTCTTGCACTGCACCCTGATGTTTTTCGACGAGAGAAACGGC
AAACCGACCAAAGGGATGGAGCCGTTCTTTGTGGGTCTGTCTGTGGCCGTGATCATCCTG

GCCTGGGGACACAACGCCGGGGCGCCCATGAACCCAGCCAGGGACTTGGCTGGTCGGCTG
TTGACATGGCTGGTTGGCTACGGAGCTGAAGTCTGG---GTA---CCTAGGGGCGTCCAC
TGGTGGTGGATTCCCACGCTGGTTCCGCCCCTTGGAGGGATCGTCGGGGCCAATTTCTAC
TACTTGTTTGTGGAGCTGCACCACCCTGCTGAGGACGGGGAC
>cotStarfish_Glp1a2
CGGAACTGCATCACGGTTAAGAATGAGATCGTACGGGGAATGCTTGCAGAAATGGCTGGG
ATATTCGTACTCGTACTCTTTGTTGACGGGGGTCTCGCCGCGGGTATCGTTAGTGGATGG
ATT---TCAGGG---ACACCGGTGTCTACTGCCGTCACGTCCGGAATGGGCGTTGCATTC
GGGATATACACCGGCGTGGCTGTATCAGGTGGTCACGTGAATCCCTCGATTACAGTCGGC
TTAGCGTCCGTCGGCAAGTTTCCATGGCGACGGGTACCACTCTGGATCCTTGCTCAACTC
GTTGGCGCCTTGTGCGGTGCTTCGGTTGTCTTTGCAATATACTATGAGGGCATCAATGCG
CTGGATGGGGGTAACAGGACTGTCTTCGGTGAC---ACGGGCACCGGTGGAATCTTCTGT
ACT------------TACCCGCAGGAGTACTTCGGGATGGGTCCTGGATTTGTGGAGCAG
ATCATTAACACGGGCCTCCTCCTGCACTGCACCTTAATGTTTTTCGACGAGAAGAACGGC
AAACCGACCAAAGGGATGGAGCCGTTCTTCGTGGGTCTGTCAGTGGCTATGATTATCCTA
GCGTGGGGCCACAACGCAGGCTCACCCATGAACCCAGCCAGGGACTTCGCTGGCAGGTTT
CTTACATGGATAGCTGGCTACGGATCTGAAGTCTGG---GTG---CCTCGGGGAGTCCAC
TGGTGGTGGATTCCCACGTTCGTCCCGCCCCTTGGGGGAATCGTCGGGGCCAATTTCTAC
TACCTGTTCGTCGAGCTGCACCACCCGAGCGATAGCGAGGTG
>Purple_sea_star_Glp1a2
CGTCGCCGACTCACAGCAAAGAATGAAATCATCCGTGGAGTTCTGGCAGAAATGGCTGGA
ATATTCGTTCTGGTGCTTTTTGTTGACGGTGGCCTGGCCGGGGGTATAGTAAGCGGCTGG
GTG---TCGGGT---ACACCCGTATCTACGGCCTTCACCTCTGGTATGGGAGTCGCATTT
GGTATCTACACAGGCATAGCGGTGTCAGGTGGTCATGTCAACCCAGCGATAACAATTGGT
CTAGCTTCAGTCGGCAAGTTTCCATGGCGACGCGTTCCTTTGTGGATTATGGCTCAATTA
GTGGGAGCCTTTTGCGGTGCCTCTGTTGTCTTCGGGTTATATTACGCGGCCATTCACAAT
TTCGACGGGGGAAACAGGACCGTGTTTGGGCCT---ACCGGTACCGGTGGCATCTTCTGT
ACC------------TACCCACAGCCATACTTTGGTGTAGGTGTCGGTACCGTGGAACAG
ATTGTCAACACAGGGTTGCTGCTTTACTGCACTCTCGTCTTCTTCGATGAGAAAAACGGC
AAGCCGACCAAAGGAATGGAGCCTTTTTCGTGGGTCTGGCCGTAGCAATGATTATCCTG
GCCTGGGGGCACAACGCCGGCTCGCCCATGAATCCCGCTCGAGACTTCGCCGGTAGGCTC
TTGACGTGGATTGTCGGGTATGGAAATCAAGTATGG---ATG---CCCAATGGAATTCAC
TGGTGGTGGATACCTTTGTTTGTTCCACCCATTGGGGGCATCGTTGGTGCCAACTTCTAC
TACCTCATGGTTGAGATCCACCACCCTACAGGGGACCAGGAA
>BroodStar_Glp1a2

---TGG
GTG---TCGGGT---ACACCCGTATCTACGGCCTTCACCTCTGGTATGGGAGTTGCATTT
GGTATCTACACAGGCATAGCTGTGTCAGGTGGTCACGTCAACCCAGCGATAACTATTGGT
CTAGCTTCAGTCGGCAAGTTTCCATGGCGACGCGTTCCTTTGTGGATTATGGCTCAGTTG
GTCGGTGCCTTCTGCGCTGCTTCTGTCGTCTTCGGGATATATTACCCGGCCATTCACAAT
TTTGACGGGGGCAATAGGACGGTGTTTGGGCCT---ACCGGTACCGGTGGAATATTCTGT
ACC------------TACCCGCAGCCATACTTTGGTGTAGGTGTCGGTACCGTGGAACAG
ATTGTCAACACAGGGTTGCTGCTTTACTGCACTCTCGTCTTCTTCGATGATAAAAACGGC
AAACCGACCAAGGGAATGGAGCCCCTTTTCGTGGGTCTGGCCGTGGCAGTGATTATCCTA
GCTTGGGGTCACAATGCCGGCTCTCCCATGAATCCTGCTCGAGACTTCGCCGGTAGGCTC
TTGACGTGGATTGTCGGTTATGGAAATGAAGTATGG---ATG---CCCAATGGAGTTCAC
TGGTGGTGGATCCCTCTGTTTATT------------------------------------

>eStarfish_Glp1a2
CGTCGCCGAATCACAACAAAGAATGAAATCCTTCGTGGAATTTTGGCTGAAATGGCGGGA
ATATTCGTTCTGGTGCTTTTTGTTGACGGCGGCCTAGCCGGTGGTATAGTGAGCGGCTGG
GTG---TCAGGT---ACACCCGTCTCTACGGCCTTCACCTCCGGTATGGGAGTCGCATTT
GGTATCTACACAGGAGTAGCTGTGTCAGGTGGTCATGTCAACCCAGCGATAACTATTGGT
CTAGCTTCGGTCGGCAAGTTTCCATGGCGACGTGTTCCTTTGTGGATTATGGCTCAGTTA
GTAGGTGCTTTCTGCGGTGCCTCCGTCGTCTTCGGGCTATATTACGCGGCCATTGAACAT
TTTGACGGGGGAAATAGGACGGTATTTGGGCCT---ACTGGTACCGGTGGAATCTTCTGT
ACT------------TACCCACAGCCGTACTTTGGTGTAGGGGTCGGTACCGTGGAGCAG
ATTGTCAACACTGGGTTGCTGCTTTATTGCACCCTCGTCTTCTTCGATGAGAAAAACGGC
AAACCGACGAAAGGAATGGAGCCTTTTTCGTGGGTCTGGCCGTGGCAATGATCATCCTG
GCTTGGGGTCATAATGCCGGCTCGCCCATGAACCCAGCTCGGGACTTTGCCGGTAGGCTC

```
TTGACGTGGATTGCTGGTTATGGGAATGAAGTGTGG---ATG---CCAAGAGGAGTTCAC
TGGTGGTGGATACCTCTGTTTATTCCACCCATTGGAGGCATCGTTGGTGCCAACTTCTAC
TGCCTCATGGTTGAGATGCATCACCCTACAGGCGACGAAGAC
>sasStarfish_Glp1a2
CTAAAATGTCTCACGACGAGGAATGAAATAGTGCGCTGCATTCTGGCAGAAATGACGGGA
ACTTTTGTGCTTGTGTTGTTTGTTGACGGCGGCCTGGCTGGGGGCATTATTAGCGGCTGG
GTG---TCCGGC---ACGCCAGTGTCTACAGCTTTCTCATCTGGTATGGGCGTGGCCTTT
GGGATATACACGGGGCTCGCTGTCTCAGGTGGTCATGTTAATCCCGCCATTTCGATTGGT
CTAGCGTCCGTTGGCAAGTTTCCATGGAAACGAGTGCCTGTGTGGATTATAGCGCAGCTT
ATTGGCGCCTTTTGTGGTGCCTCTGTCGTCTTCGGCATATATTACCAGGGTATAACCGGG
TATGACGGTGGAACCAGAGCAGTGTTTGGCCCG---AACGGCACGGGTGGAATCTTCTGT
ACG------------TACCCCCAACCTTACTTCGGGTTAGCGGTTGGCTTTATTGAACAG
ATAATTAACACCGGCCTGCTGCTGCACTGCACACTGATGTTTTTCGACGACAAGAACGCC
AAACCGGCCAAGGGACTGGAACCGTTCTTCGTGGGACTGTCCGTAGCTATCATCATCCTG
GCATGGGGACATAACGCCGGAGCTCCTATGAACCCTGCTCGGGACCTAGCCGGTAGATTC
CTAACGTGGCTTGCTGGTTATGGCTCCGAAGTCTGG---GCA---CCTGGTGGTGTCCAG
TGGTGGTGGATCCCAACCCTAGTCCCACCAATCGGGGGGATCGTCGGTGCAAACTTCTAC
TACCTCTTCGTAGAGATGCATCATCCTGGTAATCAGGAAGAT
>sssStarfish_Glp1a2
------------------------------------------------------------
------GTGCTTGTGTTGTTTGTAGACGGTGGGCTGGCTGGGGGCATTATCAGCGGCTGG
GTG---TCCGGC---ACGCCAGTGTCTACAGCCTTTTCATCTGGTATGGGCGTCGCTTTT
GGGATATACACGGGGCTTGCCGTGTCAGGTGGTCATGTAAATCCCGCTATTTCGATTGGT
CTGGCGTCCGTTGGCAAGTTTCCATGGAAACGAGTGCCTTTGTGGATTATAGCGCAGCTT
ATTGGTGCCTTCTGTGGGGCGTCTGTCGTCTTCGGTATATATTACCAGGGTATAACTAAG
TACGACGGTGGGACGAGAGCAGTGTTTGGCCCA---AACGGCACGGGTGGAATCTTCTGT
ACG------------TACCCGCAACCGTACTTTGGGTTAGTGACTGGCTTCATTGAACAG
ATAATCAACACCGGCCTCCTGCTGCACTGCACACTGATGTTTTTTGACGAAAAGAACGCC
AAACCGGCCAAAGGTTTGGAACCGTTCTTTGTGGGGCTGTCTGTAGCCGTTATCATCCTT
TCATGGGGACATAACGCCGGAGCACCCATGAATCCTGCTCGAGATTTAGCCGGTCGATTC
CTGACGTGGCTTGCTGGTTATGGCTCCCAAGTCTGG---GCA---CCCGGTGGGGTCCAT
TGGTGGTGGATTCCAACCCTAGTCCCACCG------------------------------
------------------------------------------
>Batstar_Glp1a1p
CACTGCGATATCGTCGTAAGAAACGGCTTGGCTCGAGGCATGTTGGCCGAGATGGCGGGA
ACTTTCATCTTAGTCCTATTCGTGGATGGTGGGCTAGCTCAAGGCGTGGTCAGCGGCTGG
ACA---TCAGGA---ACACCCGTCTCGACTTCGCTTACCACAGGGATGGGCGTGGCTTTT
GGAATCTACACTAGTATCGGGATATCAGGAGGTCACGTGAATCCATCTATTACCATTGGC
CTGGCGTCAGTGGGGACGTTTGCCTGGCGAAGGGTTCCTCTGTGGATTACAGCCCAACTG
ATAGGCGCACTGGCCGCGGCAGCAGTGGTGTTCGGAATATATTAC---------------
------------------------------------------------------------
------------------------------------------------------------
------------------------------------------------------------
------------------------------------------------------------
------------------------------------------------------------
------------------------------------------------------------
------------------------------------------------------------
------------------------------------------
>Blue_batstar_Glp1a1
---------------------------TTTTGTATAGTTATTTTGGCCGAGATGGCGGGA
ACTTTCATCTTAGTCTTATTCGTGGATGGCGGGCTAGCTCAAGGCGTGGTCAGCGGCTGG
ACA---TCAGGA---ACACCCGTCTCGACTTCGCTTACCACAGGGATGGGCGTGGCTTTT
GGAATCTACACTAGTATCGGGATATCAGGAGGTCACGTGAATCCATCTATTACCATTGGC
CTGGCGTCAGTGGGGAAGTTTGCCTGGCGAAGGGTTCCTCTGTGGATTACAGCCCAACTG
ATAGGCGCACTGGCCGCGGCAGCAGTGGTGTTCGGAATATATTACGCTGCTATTGAAAAC
CTGGACGGTGGAAACCGGACAGTATTCGGTCCG---ACCGGAACAGGGGTCATTTTCTGC
ACG------------TACCCACAACCTTACCTTGGTGTCTGGGTGGGGCTGGTAGAACAG
ATACTCAACACGGGTCTCCTGTTGCACTGCACGATGATGTTTTTCGACGAGAGAAACGGC
AAACCGACCAAAGGGATGGAGCCGTTCTTTGTGGGTCTGTCTGTGGCCGTGATCATCCTG
GCCTGGGGACACAACGCCGGGGCGCCCATGAACCCAGCCAGGGACTTGGCTGGTAGGTTC
CTGTCATGGCTGGTCGGCTACGGAAGCGAAGTATGG---ATG---CCTTACAAAGCGCAC
```

TGGTGGTTGGTTTCTACCTTCGTGCCGCTAGCCGGCGGAATCTGCGGTGCCTACTTTTAC
GTTTTGTTCGTTAAGAGGCATCACCCGGGCAATCTCGCCCAG
>cotStarfish_Glp1a1
TGCAACCGGCTCGTAGTAAGAAACGACCTGATTCGCAGCATGCTTGCTGAGACAGCGGGA
ACATTCACACTAGTCATATTTGTGGATGGTGGATTAGCCCAGGGCGTTGTGAGCGGCTGG
ACA---TCCGGG---ACACCTGTCTCGACTGCGTTTACCACAGGACTGGGTGTTTCGTTC
GGGATCTTTACCGGCCTCGGAGTGTCAGGAGGTCACGTGAATCCATCCGTCACCATTGGA
TTAGCGTCAGTGGGGAAGTTTGACTGGCGAAGGGTGCCGCTGTGGATTACCGCACAGCTA
ATTGGAGCCCTCGCCGCAGCAGTTGTAGTATTTGGAATATATTACGCTGGCATCAACAAC
TTGGATGGTGGGAATCGGACAGTATTCGGCCCT---ACGGGTACAGGAGTAATTTTTTGC
TCA------------TATCCACAACCGTACTTCGGAATCTGGTTGGACTGGCAGAACAGG
ATAATCAGCGCGGGACTTCTGTTGCACTGCACATTGGTATTTTTCGACGAGAAAAACAGC
AAGCCGACCCAAGGTTTGGAAGCGTGCTTCTGTGGACTGGCTGTGACCCTCATTATCCTT
GCGTGGGGCCACGACGCTGGCGCGCCAATGAACCCGGCCAGGGACTTGACTGGAAGGCTC
CTGTCATGGATGGCCGGGTATGGAAACGAAGTATGG---ATG---CCTCGCGAAGCGCCT
TGGTGGTTGGTGGCTACTATCGTGCCGCTGGTAGGAGGAATATGCGGCGCAAACTTCTAC
GTCTTCTTTGTTGAAATGCACCATCCAGAAACTATGGCCGAG
>Batstar_Glp1b
CGCCGGCATCTGGCCGTCAGGAGCGACATAGCACGAGGCATGCTGGCTGAAATGGCAGGA
ACCTTCATACTGGTTTTATTCGTGGATGGCGGACTAGCCCAAGGTATCGTCAGTGGCTGG
ACA---TCCGGG---ACACCCGTTTCCACCGCATTGACCTCAGGCATGGGCGTGGCCTTC
GGGATCTACACAGGTTTAGGGGTGTCAGGAGGGCACGTGAATCCATCCATTACTCTGGGC
TTGGCTTCTGTCGGCAAGTTTGCCTGGCGAAGGGCTCCGCTGTGGATACTCGCCCAGTTT
CTAGGCGCATTCGCTGCAGCGTCTGTTGTATTTGGAATATACTACGCTGGGTTGGATAAT
TTTGACGGAGGCAACCGGACGGTTTTCGGGCCG---ACCGGGACGGGAGGGGTTTTTTGC
ACT------------TATCCGCAGCCGTACTTTGGGCTCTGGGCCGGCCTGGCTGAACAG
ATACTCAACACGGGTCTCCTGTTGCACTGCACGATGATGTTTTTCGACGAGAGAAACGGC
AAACCGACCAAAGGGATGGAGCCGTTCTTTGTGGGTCTGTCTGTGGCCGTGATCATCCTG
GCCTGGGGACACAACGCCGGGGCGCCCATGAACCCAGCCAGGGACTTGGCTGGTAGGTTC
CTGTCATGGCTGGTTGGCTACGGAAGCGAAGTGTGG---ATG---CCTCGCGGAGTTCAC
TGGTGGTGGGTGCCAACCTTTGTACCGCTGTTAGGGGGAGTATGCGGGGCCAACTTTTAC
TTTCTGCTCGTGGAAATTCATCACCCGAAGGAGATGGGCGAT
>Blue_batstar_Glp1b
CGCCGGCATCTGGCCGTCAGGAGTGACATAGCACGAGGCATGCTGGCTGAAATGGCAGGA
ACCTTCATACTGGTTTTATTCGTGGATGGCGGACTAGCCCAAGGTATCGTCAGTGGCTGG
ACA---TCCGGG---ACACCCGTTTCCACCGCACTGACCTCAGGCATGGGCGTGGCCTTC
GGTATCTACACAGGTTTAGGGGTATCAGGAGGGCACGTGAATCCATCCATTACTCTGGGC
TTGGCTTCTGTCGGCAAGTTTGCCTGGCGAAGGGCTCCGCTGTGGATACTCGCCCAATTT
CTGGGCGCATTCGCTGCAGCATCTGTTGTATTTGGAATATACAACCCTGGGTTGGATAAT
TTTGACGGAGGCAACCGGACGGTTTTCGGGCCG---ACCGGGACGGGAGGGATTTTTTGC
ACT------------TATCCGCAGCCGTACTTTGGGCTCTGGGCCGGCCTGGCTGAACAG
ATACTCAACACGGGTCTCCTGTTGCACTGCACGATGATGTTTTTCGACGAGAAAAACGGC
AAACCGACCAAAGGGATGGAGCCGTTCTTTGTGGGTCTGTCTGTGGCCGTGATCATCCTG
GCCTGGGGACACAACGCCGGGGCGCCCATGAACCCAGCCAGGGACTTGGCTGGTCGGTTC
CTGTCATGGCTGGTCGGCTACGGAAGCGAAGTGTGG---ATA---CCTCGCGGAGTGCAC
TGGTGGTGGGTGCCAACCTTTGTACCGCTGTTAGGGGGAGTATGCGGGGCCAACTTTTAC
TTTCTGCTCGTGGAAATTCATCACCCGAAGGAGATGGACGAT
>cotStarfish_Glp1b
CGTAGGCATTTAGCCATTAAAAGTAACTTGGCACGTGGAATTCTCGCTGAGATGGCCGGA
ACCTTCATACTTGTTTTATTCGTGGATGGCGGATTAGCCCAAGCCGCAGTGAGCGGCTAC
ACA---TCCGGC---ACACGTGTCTCCACCGCGCTGACCTCAGGCATGGGCGTGGCATTT
GGGATCTACACTGGTGTAGGGGTGTCAGGAGGCCACGTGAATCCATCAATTACTGTGGGC
CTGGCATCGGTTGGCAAGTTTGCGTGGCGACGGGCCCCACTCTGGATAGCAGCTCAATTC
ATCGGCGCATTCGTCGCAGCATATGTGGTGTATGGGATATACTACGCTGGGCTGGATCAT
TTTGACAACGGGAACCGGACAGTTTTTGGACCT---ACTGGGACAGGAGGGATCTTCTGC
ACC------------TATCCGCAGCCATACTTTGGGCTCTGGGTAGGTCTCGCTGAACAG
ATTATTAACACCGGTCTCTTGCTGCACTGCACCCTGATGTTTTTCGACGAGCGAAATGGT
AAACCGACTAAAGGGATGGAGCCGTTCTTTGTGGGTCTGTCCGTGGCAGTTATCATTCTG
GCATGGGGACACAACGCTGGAGCGCCCATGAATCCAGCCAGGGACTTGGCCGGTCGGTTG
CTGTCCTGGCAGGTCGGATACGGAGCTGATGTGTGG---CAG---CCTCATGGAATGCAC
TGGTGGTGGGTGCCAACCTTTGTACCGCTACTAGGGGGGCTGTGCGGGGCCAACTTTTAC

TTCTTATTCGTGGAAATGCACCACCCAAAGAATGTCGGCGAA
>Purple_sea_star_Glp1b
AAACGGTATCTCACCATACAGAGCGACCTGGTGCGTGGCATGCTGGCCGAGTTGGCGGGA
ACATTTATATTAGTTTTATTTGTAGATGGCGGATTGGCCCAAGCCTTTGTGAGTGGTTGG
ACA---TCCGGG---ACACCTGTATCAACCGCCCTGTCATCGGGGATGGGTGTGGCTTTT
GGAATTTACACGGGAGTTGGAATCTCAGGTGGTCACGTCAACCCGTCTATAACCATCGGC
TTGGCTTCAGTTGGAAAATTTCCTTGGAAGAGGGCGCCCTTGTGGATTGCTGCTCAACTT
GTTGGCGCATTCAGTGCGGCGTTTGTTGTTTATGGAATATACTCTGCTGGTATCGCTAAT
CTGGACGGAGGGAATCGGACGGTGTTTGGCTCT---ACTGGAACGGCGGGTATCTTTTCT
ACG------------TACCCACAGCCATATTTTGGTCTCTGGGTCGGATTAGCTGAGCAA
ATAATAAATACTGGTCTCTTACTGCACTGTACATTGGTCTTTTTTGATGAGAAAAACGGC
AAACCACCCAAAGGAATGGAACCCTTTTTCGTGGGTCTATCGGTAGCCATGATTTTACTG
ACATGGGGTCACAACGCGGGAGCTCCTATGAATCCGGCCCGGGACTTCACCGGTAGACTC
CTCACATGGTTGGTCGGTTATGGAGATCAAGTTTGG---ATG---CCACGTGGAATCCAC
TGGTGGTGGATACCGACCTTTGTACCGCTTCTGGGAGGCATCTGTGGGGCTCACTTTTAC
GTTCTTTTTGTCGAGAGGCATCACCCAAAAGACACCCGGGAT
>BroodStar_Glp1b
AAACGGTATCTCACAATACAGAGTGACCTGGTGCGCGGCATGCTGGCAGAGTTGGCGGGA
ACATTTATACTAGTATTATTTGTAGATGGCGGATTGGCCCAAGCTTTTGTGAGTGGTTGG
ACA---TCCGGG---ACACCTGTATCGACCGCCCTGTCATCAGGGATGGGTGTGGCTTTT
GGAATTTACACGGGAGTTGGAGTCTCAGGTGGTCACGTCAACCCGTCTATAACCATCGGC
TTGGCTTCAGTTGGAAAATTTCCTTGGAAGAGGGCGCCCTTGTGGATTGCTGCTCAACTT
GTTGGTGCATTCAGTGCCGCGTTTGTTGTTTATGGAATATACTATCCTGGTATCGATAAT
CTGGACGGAGGGAACCGGACGGTATTTGGCTCT---ACAGGAACGGCGGGTATCTTTTCC
ACG------------TACCCACAGCCATATTTTGGTCTCTGGGTCGGACTAGCTGAAGAA
ATAATAAATACCGCCCTCTTACTGCACTGTACATTGGTCTTTTTTGATGAGAAAAACGGC
AAACCACCTAAAGGAATGGAACCCTTTTTCGTGGGTCTATCGGTAGCCATGATTTTACTG
ACATGGGGTCACAACGCGGGCGCTCCCATGAATCCTGCCCGGGACTTCGCAGGTAGACTT
CTCACCTGGTTGGTCGGTTATGGAAATCAAGTTTGG---ATG---CCACGTGGACTCCAT
TGGTGGTGGATACCGACCTTTGTACCGCTGCTGGGAGGCATATGTGGGGCTCACTTCTAC
GTTCTTTTTGTGGAGAGGCACCACCCAACGGACACACAGGAT
>eStarfish_Glp1b
AAACGGTGTCTCACCATACAGAGCGACCTGGTGCGTGGAATGCTGGCAGAGTTGGCTGGA
ACATTTATATTAGTGTTATTTGTTGACGGCGGATTGGCCCAAGCCTTTGTGAGTGGTTGG
ACA---TCCGGG---ACACCTGTATCGACAGCCCTGACATCGGGGATGGGCGTGGCTTTT
GGAATATATACGGGAGTGGGCGTCTCAGGTGGCCACGTCAACCCGTCCATAACCATTGGC
TTGGCTTCAGTTGGGAAGTTTCCCTGGAAGAGGGCGCCCTTGTGGATCGCTGCTCAGCTC
GTTGGTGCATTCAGTGCGGCGTTTGTTGTTTACGGAATATACTATGCTGGAATCGACAAT
CTGGACGGAGGGAACCGGACGATGTTTGGTTCT---ACAGGAACGGCGGGTATCTTTTCC
ACA------------TACCCTCAGCAGTACTTTGGTCTTTGGGTCGGACTAGCTGAACAA
ATAATAAATACAGGTCTCTTACTGCATTGTACATTGGTCTTTTTTGATGAGAAAAACGGC
AAACCACCTAAAGGAATGGAACCCTTTTTCGTGGGTCTATCCGTATCCATGATCTTACTG
ACGTGGGGTCACAACGCTGGTGCCCCTATGAATCCGGCCCGTGACTTCGCTGGTAGACTT
CTCACCTGGGTGGTCGGTTATGGAAATCAAGTTTGG---ACG---CCACGAGGACTTCAC
TGGTGGTGGATACCAACCTTTGTACCGCTACTCGGAGGCATATGTGGGGCTCACTTTTAC
GTCCTTTTCGTCGAGAGGCACCATCCGAAAGACACACACGGC
>shWCucumber_Glp3
ATTGACAAGTTTAGGATCAGAAATCACCTGGCAAGAGAAATTTTGGCAGAGTTCATCGGA
ACATGGATTCTTTTGGTGTTTGGAGATGGAGTTGTCGCTCAAACAGTACTGAGCAGGGGC
GCC---AATGGC---AGCTCACTTTCAATTAACTGGGCCTGGGCCATGGGAGTGGTGATG
GGTATCTACTTCGCTGGTGGTATATCAGGAGCACATATTAATCCCGCCGTGTCGATCACT
ATGGCAACCATCGGGCGATTTCCGTGGAAGAAAGTCCCTTTCTATATTATAGCACAGTTT
TTAGGTGCATTCATGGCGGCCGCCTGTGTATTTGGAGTTTATCATGATGCAATCGACAAT
TTTGACGGTGGTCAACGTCAAGTGTACGGACCG---AACGCGACTGCGGGCATATTTGCA
ACC------------TACCCTCAGGAATTTCTCTCTGTCTCGAGTGGTTTCGCTGATCAG
GTGTTCGGTACCGCTCTTCTCCTGGGGTGCATTCTGGCGATCTCCGATCAGAAAAACGGC
CCCCCTCCAAAGGGCCTTGCTGCCTTGATGGTCGGTCTCGTCGTCTTCGTCATCGGTATG
ACGTTCGGCTTCAACTGTGGCTATGCAATCAACCCCGCCAGGGATTTCGGTCCCCGGGTT
TTCACCGCAATGGCTGGCTACGGCTATGAAGTTTGGTTGACG---CCCCATGGACAGCAC
TGGTGGTGGGTGCCAATTTTAGGACCAATCATCGGGGGCATCTGCGGTGCTTTCATGTAC
ATCGTCTTCGTTGAGATGCACCACGAACCGGAAAACAAA---

>jsCucumber_Glp3
ATAGACAAGTTCAGAATCAGGAATCATCTCGCTAGGGAAATCTTGGCAGAATTTATTGGA
ACATGGATTCTTCTGGTATTTGGAGACGGTGTAGTCGCTCAAACAGTACTCAGTCGTGGG
GCT---AATGGG---TCTGCTCTCTCCATTAATTGGGCGTGGGGCATGGCAGTGGTGATG
GGTATCTATTTCGCTGGTGGTATATCAGGAGCACATATAAATCCCGCAGTGACGGTAACC
ATGGCAACGATTGGACGGTTTCCCTGGAAGAAAGTTCCTTTCTATATTATAGCACAATTT
CTAGGCGCATTCATGGCAGCCGCGTGTGTATTTGGAGTTTATCATGATGCAATCCAGAAC
TATGACGGAGGCGAAAGACAAGTATATGGACCC---AACGCTACTGCTGGAATATTTGCC
ACT------------TATCCACAAGACTTTCTTTCAGTTGGAAGTGGCTTTGCTGATCAG
GTATTTGGTACTGCTCTTCTTCTGGCGTGTATTCTAGCCATCTCCGATTCGCGGAATGGT
CCTCCACCACCAGGCATGGGTGCGCTGATGGTCGGTCTCGTGGTATTCGTCATTGGAATG
ACATTTGGCTTCAACTGCGGCTACGCTATCAATCCTGCCAGGGACTTTGGTCCTCGAGTC
TTTACAGCTATGGCTGGATATGGGCAGGAAGTTTGGTTCACG---CGCGATGGCAAGCAT
TGGTGGTGGGTGCCTATATTAGGCCCCATCGTAGGGGGTATCTGTGGTGCTCTCATGTAC
ATTGTCTTCGTGGAAATGCACCATGAACCAGAGAAG------
>wsCucumber_Glp3
ATAGACAAGTTCAGAATCAGGAATCATCTCGCTAGGGAAATCTTGGCAGAATTTATTGGA
ACATGGATTCTTCTGGTATTTGGAGACGGTGTAGTCGCTCAAACAGTACTCAGTCGTGGG
GCT---AATGGC---TCTGCGCTTTCCATTAATTGGGCATGGGGCATGGCAGTGGTGATG
GGTATCTATTTCGCTGGTGGTATATCAGGAGCACATATAAATCCCGCAGTGACGGTAACC
ATGGCAACAATTGGACGGTTTCCCTGGAAGAAAGTTCCTTTCTATATTATAGCACAATTT
CTAGGTGCATTCATGGCAGCCGCGTGTGTATTTGGAGTTTATCATGATGCAATCCAGAAC
TATGACGGAGGCGAAAGACAAGTATATGGACCC---AACGCTACTGCTGGAATATTTGCC
ACT------------TATCCACAAGACTTTCTTTCAGTTGGAAGTGGCTTTGCTGATCAG
GTATTTGGTACCGCTCTTCTTCTGGCGTGTATTCTAGCCATCTCCGATTCGAAGAATGGT
CCTCCGCCACCAGGCATGGGTGCGCTGATGGTCGGCCTCGTGGTATTCGTCATTGGAATG
ACATTTGGCTTCAACTGCGGCTACGCTATCAATCCTGCCAGGGACTTCGGTCCTCGAGTA
TTTACAGCTATGGCTGGATATGGGCAGGAAGTTTGGTTTACG---CGCGATGGCAAGCAT
TGGTGGTGGGTGCCTATATTAGGCCCCATCGTAGGGGGTATCTGTGGTGCTCTCATGTAC
ATTGTCTTCGTGGAAATGCACCATGAACCAGAGAAG------
>gosCucumber_Glp3
ATAGACAAGTTCAGAATCAGGAATCATCTCGCTAGGGAAATCTTGGCAGAATTTATTGGA
ACATGGATTCTTCTGGTATTTGGAGACGGTGTAGTCGCTCAAACAGTACTCAGTCGTGGG
GCT---AATGGC---TCTGCGCTTTCCATTAATTGGGCATGGGGCATGGCAGTGGTGATG
GGTATCTATTTCGCTGGTGGTATATCAGGAGCACATATAAATCCCGCAGTGACGGTAACC
ATGGCAACGATTGGACGGTTTCCCTGGAAGAAAGTTCCTTTCTATATTATAGCACAATTT
CTAGGTGCATTTATGGCAGCCGCGTGTGTATTTGGAGTTTATCATGATGCAATCCAGAAC
TATGACGGAGGCGAAAGACAAGTATATGGACCC---AACGCTACTGCTGGAATATTTGCC
ACT------------TATCCACAAGACTTTCTTTCAGTTGGAAGTGGCTTTGCTGATCAG
GTATTTGGTACCGCTCTTCTTCTGGCGTGTATTCTAGCCATCTCCGATTCGCGGAATGGT
CCTCCGCCGCCAGGCATGGGTGCGCTGATGGTCGGCCTCGTGGTATTCGTCATTGGAATG
ACATTTGGCTTCAACTGCGGCTACGCTATCAACCCTGCCAGGGATTTTGGTCCTCGAGTC
TTTACAGCTATGGCTGGATATGGGCAGGAAGTTTGGTTCACG---CGCGATGGCAAGCAT
TGGTGGTGGGTGCCTATATTAGGCCCCATCGTAGGGGGTATCTGTGGTGCTCTCATGTAC
ATTGTCTTCGTGGAAATGCACCATGAACCAGAGAAG------
>gcsCucumber_Glp3
ATAGACAAGTTCAGAATCAGGAATCATCTCGCTAGGGAAATCTTGGCAGAATTTATTGGA
ACATGGATTCTTCTGGTATTTGGAGACGGTGTAGTCGCTCAAACAGTACTCAGTCGTGGG
GCA---AATGGC---TCTGCGCTTTCCATTAATTGGGCATGGGGCATGGCAGTGGTGATG
GGTATCTATTTCGCTGGTGGTATATCAGGGGCACATATTAATCCCGCAGTGACGGTAACC
ATGGCAACGATTGGACGGTTTCCCTGGAAGAAAGTTCCTTTCTATATTATAGCACAATTT
CTAGGTGCATTCATGGCAGCCGCGTGTGTATTTGGAGTTTATCATGATGCAATCCAGAAC
TATGACGGAGGCGAAAGACAAGTATATGGACCC---AACGCTACTGCTGGAATATTTGCC
ACT------------TATCCACAAGACTTTCTTTCAGTTGGAAGTGGCTTTGCTGATCAG
GTATTTGGTACCGCTCTTCTTCTGGCGTGTATTCTAGCCATCTCCGATTCGAAGAAT---
CCTCCCCCACCAGGCATGGGTGCGCTGATGGTCGGCCTCGTGGTATTCGTCATTGGAATG
ACATTTGGCTTCAACTGCGGCTACGCTATCAATCCTGCCAGGGACTTCGGTCCTCGAGTA
TTTACAGCTATGGCTGGATATGGGCAGGAAGTTTGGTTCACG---CGCGATGGCAAGCAT
TGGTGGTGGGTGCCTATATTAGGCCCCATCGTAGGGGGTATCTGTGGTGCTCTCATGTAC
ATTGTCTTCGTGGAAATGCACCATGAACCAGAGAAG------
>prsCucumber_Glp3m

ATTGACACTTTCAGGATACGGAATCATTTGGCGAAAGAAATCCTCGCGGAATTCATCGGG
ACCTGGATTTTATTGGTTTTTGGCGATGGCGTAGTTGCTCAGACGGTGCTGAGTCGGGGC
GCA---AATGGA---TCCTCCCTCTCTATCAACTGGGCGTGGGGTATGGGCGTCACCATG
GGTATATTCTTCGCTGGCGGTATTTCAGGTGCTCACATAAATCCAGCTGTCTCGATCGCC
TTGGCAACCATCGGGAGATTCCCCTGGAAGAAAGTACCATTTTACATCATCGCCCAGTTT
TTAGGGGCTTTCATGGCAGCAGCTTGTGTATTCGGTGTTTATCATGACGCTATAGATAAT
TTCGATGGTGGCGTTCGTCAGGTGTTTGGGGAG---AATGGCACTGCCGGCATTTTCGCC
ACC------------TACCCCCAGGGTTTCCTTTCAATTGGCAGTGGCTTTGCTGATCAG
GTTTTCGGTACCACGCTGCTCTTGGGCTGTATCATGGCTATCAATGACCAGCGTAATGGC
CCCCCTACCAAGGGGATGGCCGCTCTCATGGTGGGGCTGGTGGTGTTTGTGATTGGTATG
ACCTTCGGCTTCAACTGCGGCTACGCCATCAATCCTGCCAGGGACTTCGGGCCCAGGGTC
TTCACTGCTATGGCTGGATATGGACCCGATGTATGGCTCACG---CCTGATGGAGCTCAT
TGGTGGTGGGTCCCTATTCTAGGGCCTATCATTGGCGGGATCTGCGGGTGCCTCCTCTAC
CTCGTATTCATTGAGATGCACCACGACCCGGAGGAAGAGAAA
>CssCucumber_Glp3
ATGGAAACATGCAGGGTACGAAATCAACTGGCGAAAGAAATACTAGCTGAATTCATCGGA
ACTTGGATTTTAATTATTTTTGGTGATGGTGTAGTAGCACAGACCGTCCTGAGCAGAGGC
GCC---AATGGT---TCGTCGCTCTCGATTAACTGGGCTTGGGGTATGGGAGTGACAATG
GGTATATTCTTCGCTGGCGGTATATCAGGGGCCCATATAAACCCCGCTGTTACAGTCGCC
ATGGCAACCATTGGAAGGTTCCCTTGGAAAAAAGTAGGATTCTACATCCTTGCGCAGTTC
TTGGGTGCTTTCATGGCCGCAGCCTGTGTATTTGGAGTTTATCGTGATGCTTTAGATAAT
TTCGATGGCGGCGTCCGTCAAGTTTTTGGAGTA---AATGGAACTGGTGGTATATTTGCA
ACT------------TATCCACAGGAATATCTTTCTATAAGTAGTGGCCTGGCGGATCAG
ATCTTTGGTACTGCTATCCTGTTGGGCTGTATTTTGGCCATTAATGACCAACGTAATGGA
CCCCCTACTAAAGGCATGGCGGCCCTTATGGTCGGCCTGGTCGTCTTCGCGATCGGGATG
ACATATGGCCTTAACTGCGGCTACGCCATCAACCCGGCCAGGGACTTTGGGCCAAGAGTA
TTTACAGCGATGGCCGGATATGGTTCTGATGTCTGGAAAGGG---CCAAATGGAGAGCAG
TGGTGGTGGGTTCCTATCATAGGGCCGCTGATTGGTGCTATATGTGGGGCCCTCATGTAC
ATCGTATTCATCGAGATGCACCATGAACCAGAACGTGAACCA
>hsCucumber_Glp3
TTCGATAGCTTCCGGATACGGAATCACCTGGCGAAAGAAATCCTTGCAGAATTCATCGGA
ACATGGATTTTACTGATTTTCGGTGACGGCGTGGTTGCTCAGACAGTGCTTAGCCGGGGG
GCA---AATGGA---TCCTCCCTCTCTATCAACTGGGCTTGGGGCATGGCGGTCACCATG
GGTATATTCTTCGCTGGCGGTATCTCAGGTGCACACATCAATCCAGCCGTCACGGTCGCC
CTGGCAACCATCGGCAGATTTCCTTGGAAGAAAGTTCCGTTTTATGTCGTCGCCCAGTTC
TTGGGAGCTTTCATGGCCGCTGCGTGTGTGTTCGGTGTTTATCATGATGCTATAGACAAT
TTCGACGGTGGCGTTCGTCAGGTTTTCGGCGAG---AATGCCACTGCTGGGATATTCGCC
ACG------------TACCCCCAGCCCTTCCTTTCCGTCAGAAGTGGCTTCGCAGACCAG
ATATTCGGCACCCTGCTCCTACTGGGTTGCATCATGGCCATCAATGACCAACGAAACGGA
CCCCCTACCAAAGGAATGGCATCCCTCATGGTTGGGTTGGTGGTCTTTGTGATCGGGATG
ACGTTCGGCTTCAACTGTGGCTACGCCATCAATCCCGCCAGGGACTTCGGGCCCAGGGTG
TTCACCGCTATGGCTGGATATGGATCAGATGTATGGCTCAGC---CCGAACGGCCCCCAC
TGGTGGTGGGTACCTATTCTAGGGCCGCTAATTGGCGGCATCTGCGGCTGCTTCCTGTAC
ATCGTATTTGTTGAGATGCACCATGACCCGGAAGATGAGAAG
>PtyFlava_Glpc
CGAGACCGATTTCAGATCAACAATATGCTGATACGAGAAACTTTGGCCGAGTTTATAGGG
ACCTTTATTTTTGTTTCGTTTGGTTGCGCCTCGATCGCTCAGGTTGTCTTGAGTCGAGGG
GAA---TTCGGG---ACGACTCTCAACATAAACTGGGGTTGGGGTTTCGGCATCGCATTA
GGAGTGCAGTGGGCATTCAACATCTCAGGGGCACATTTAAACCCAGCCATATCACTGATC
ATGGGAATACTTAGGGTCGTCAATTGGAAGAGGGTTTTGCTATTCACTTGTGCCCAGATT
CTAGGCGCTTTCGTAGCGGCGGGCTGCATCTATGGAATATACTTAGAGTCGATCAACAAC
CTAGATGGCGGGAAACGTAGTGTACTCGGTGTC---AATGCAACGGCGAATATCTTTGCG
ACA------------TATCCCCAAGAACATGTAACCCTAGGCGGTGGTTTGGCTGACCAG
ATATTTGGTACTATGATGCTCGCCAGTTGTATATTAGCCATAATCGACCCTGATAATTAC
AGACCGACAAAAGGGCTGGAACCGTTACTTATCGGGATAGTTGTGTTCACCCTGGGAAAC
ACCTTTTCATTCAACTGTGGCGGCGCTCTCAATCCAGCGAGAGACTTAGGGCCCAGACTT
TTCACATATATGGATGGATATGGACGAGAAGTATGG---ACA---CCCCGGGGGATACAC
TGGTGGTGGGTTCCTATCGTTGGACCATACCTTGGCAGCATTCTCGGCGGTGTACTTTAT
GCGACGTTTGTGGGATACCACATCAAGAGGGCGCCAAACCAT
>Sk_Acorn_worm_Glpc
GGACCACGACTGAGAATTCACAGCCCATTGGCAAGACAAGCTGTTGCCGAATTTCTTGGG

ACGTTCGTCTTGATATCATTTGGTTGCAGTGGAACAGCTCAGTATATCCTCAGTGACGGG
GAG---AATGGG---AATGTGCTCACTGCTAACTGGGGTTGGGGAATTGGTTTGTGTATG
GGCATGTACGTTGCATTTGGACCGTCGGGTGCACATTTCAACCCCGCTGTTTCTGTGGCA
TTTGCGATAGTTGGCAGATTTCCATGGCGTTCTGTTCCAGTCTATATCCTGGTTCAATTC
CTAGCGTCCTTTGTAGCATCTATAGTAGTCTATGGGGTGTATTACGATGCACTGAATGAT
TTTGATGGGGGAGTTCGAGTTGTAATTGGTCCG---AATGCCACAGCTAGTATTTGGGCT
ACA------------TATCCACAAGAGTATCTGTCGGTGGGAAATGCTATATGGGACCAG
CTGTTTGCAACTTATATTGTTATATTGTGTGTGACTGCGGTTATTGACGAGAGAAATATT
CGCCCACCGCCTGGAATGGAACCATTTATTGTCGGGCTCCTTATATTCACTCTGGGCAAT
TGTTTGTGTTACAACGCTGGTGGCGCTATTAATCCAACGAGGGATTTCGCACCAAGGCTG
TTTACTTATATGGTGGGGTACGGTAACGAGGTGTGG---ACG---CCATATGGTTGGCAC
TTTTGGTGGATTCCTATTGTAATGCCATTGATAGGAGCAACACTGGGTGCATTAACGTAT
ATATTTTTGGTAGAAATTCATCATCCACCGCTTGGGACAGCT
>Sk_Acorn_worm_Glpa
ATTAAAGCTATTGGTATTCGGAATCAGGTTGTCCGTGAATCGTTGGCTGAGTTCATTGGC
ACGTTTATTCTTATTACATTCGGAGATGGTTCGGTGGCACAGTCGGTTCTCAGTCGAGGT
GAA---AAAGGC---GAATATCTTTCCATCAACTGGGGATGGGGTATAGCTGTTGTTATG
GGAGTACATTTCGCCTCTGGCGTATCAGGTGCTCATCTAAATCCTGCCGTCACCTTGGCC
AAAGCCAGCATTGGAAGGTTTCCATGGGTGAAGTTACCGCTGTATTGGGCGATGCAGTTT
ATTGGAGCCTTCGCTGCAGCTGCATGTTTATATGGGATATATTATGATGCAATAGAGGAT
TTTGATGGCGGCGAACGACAAGTATATGGTCCT---AACGCCACTGCCGCTATATGGGCA
ACG------------TATCCACAAGATTATGTTACCATAGAAACAGGTTTAGGCGATCAG
ATTTTTGGAACCATGTTGCTGCTGGGTTGTATAATGGCAATAGTGGACACGAGAAACAAC
AAGCCGCCACATGGTATGGAGCCTCTGCTGATTGGGTTGGCAGTCTTTGTTATAGGATTG
GCTTTTGGTCATAACTGTGACTATGCTATCAACCCAGCAAGGGATTTGGCACCACGTACT
TTCTCATACTGGGTCGGATATGGGGCAGAGGTTTGG---ACG---CCTAATGGTATAAAC
TGGTGGTGGGTGCCAGTTGTAGGTCCACTAATAGGAGGTGTATGTGGGGCGCTTATTTAT
ATTGTATTCGTAGAAGCCCATCATCCTGATGACAAGGATTCG
>Sk_Acorn_worm_Glpb
ATTAAAGCTATTGGTATTCGGAATCAGGTTGTCCGTGAATCGTTGGCTGAGTTCATTGGC
ACGTTTATTCTTATTACATTCGGAGATGGTTCGGTGGCACAGTCGGTTCTCAGTCGAGGT
GAA---AAAGGC---GAATATCTTTCCATCAACTGGGGATGGGGTATAGCTGTTGTTATG
GGAGTACATTTCGCCTCTGGCGTATCAGGAGCTCACATCAATCCAGCTGTGACTTTAGCT
ATGGCAACTATCGGAAGATTTGATTGGGTAAAAGTTCCCCTTTATTGGTTGATGCAATTT
CTCGGTTCATTTGCAGCGTGTGGATGTTTATACGGAGTATATTATGATGCAATAGAGGAT
TTTGATGGCGGCGAACGACAAGTATATGGTCCT---AACGCCACTGCCGCTATATGGGCA
ACG------------TATCCACAAGATTATGTTACCATAGAAACAGGTTTAGGCGATCAG
ATTTTTGGAACCATGTTGCTGCTGGGTTGTATAATGGCAATAGTGGACACGAGAAACAAC
AAGCCGCCACATGGTATGGAGCCTCTGCTGATTGGGTTGGCAGTCTTTGTTATAGGATTG
GCTTTTGGTCATAACTGTGACTATGCTATCAACCCAGCAAGGGATTTGGCACCACGTACT
TTCTCATACTGGGTCGGATATGGGGCAGAGGTTTGG---ACG---CCTAATGGTATAAAC
TGGTGGTGGGTGCCAGTTGTAGGTCCACTAATAGGAGGTGTATGTGGGGCGCTTATTTAT
ATTGTATTCGTAGAAGCCCATCATCCTGATGACAAGGATTCG
>Sk_Acorn_worm_Glpd

GTGTTTGGAACAATGCTGTTAGTGGCATGTATAATGGCTATTACCGATGAGCGAAATAAC
AAACCACCATCCGGCATGGAGCCTTTTCTCATTGGCTTAGTGGTATTTGTAATTGGGTTA
TCATTTGGTCTGAACTGTGGATATGCAATCAATCCAGCAAGGGATCTGTCCCCGCGTTTT
TTCTCATATTGCGTTGGTTATGGGGCAGAGGTGTGG---ACG---CCTTACGGTATTAAT
TGGTGGTGGGTGCCAATAGTCGGGCCGCTAATTGGCGGTGTCTGTGGAGCACTCGTCTAC
ATTACCATGATTGAAGCACACCATGTTAACGAAGATGATGTA
>PtyFlava_Glpa
ACCAATGCTATCGCGATCAAGAATCGTCTGGTTCGGGAGTCCTTAGCGGAATTCATCGGA
ACTTTCATACTTATGATTTTTGGAAATGGATCTGAAGCCCAAAGCATTCTTAGCCGTGGA

GCA---TACGGT---GAATATCTTTCCGTCAACTGGGCCTGGGGTATCGCCGTTACCATG
GGCATTCACTTTGCGTCGAGTGTCTCAGGTGCTCACATTAACCCGGCGGTGACGTTAGCT
AAAGCAGTGGTTGGTAGATTTCCATGGTACAAAGTTATCCCCTACTGGATTATGCAGTGC
TTGGGGGCGTTCGCTGCCTCGGCATGTGTTTACGGAGTCTATTATGACGCCATCAATGCC
TTCGACGACGGTATCAGACAAGTCACCGGTCCA---AACGCAACGGCCGCTATCTTTGCG
ACA------------TATCCGGGCCAGTATCTGAGTATCGCGAGCGGACTTGGAGATCAG
ATTGTGGGCACGATGCTATTGCTGTCTTGCATATTTGCTATCATCGATGAAAGAAACGCC
AAGCCACCGACTGGAATGGAACCATTCCTGATTGGCTTAGCGGTGTTCGTGATCGGTTTG
TCGTTCGGCGCAAACTGTGGCTACCCGTTGAATCCGGCTCGTGATTTCCCGCCGAGACTG
TTCTCGTACATGGTCGGCTACCCTGACGAAGTCTGG---ACG---CCCAATGGTGTACAC
TGGTGGTGGGTTCCCATAGTTGGACCTTTCATCGGTGGGATTTGTGGAGCTGTAGTTTAC
ATCATTCTCATTGAAGCTCATCATCCCAAAGTTAATGAACTT
>PtyFlava_Glpb
ATCAAAGCGATCAGAATCCCTAATCAGCTGGTCCGGGAGTCGTTGGCAGAGTTCATCGGG
ACATTCATACTCATCGTTTTGGGGATGGTTCTGTGGCTCAAAGTGTCCTCAGCGACAAA
GCA---TATGGT---GAATTTTTGTCAATCAACTGGGCCTGGGGTGTTGGCGTCACCATG
GCCGTTTACTTTGCAAACGGGGTCTCAGGTGCACATATAAATCCAGCAGTGACCTTGGCC
ATGGCGATAGTCGGACGATTTCCCTGGTACAAAGTAATCTTTTACTGGTTGATGCAGTTT
CTTGGCGCCTTTGCTGCTGCCGCCTGTGTCTTTGGAGTTTATTACGATGCTATCAATGAC
TTCGATGGAGGTCAAAGACAGGTCACGGGACCG---AATGCGACGGCGGGCATTTTTGCG
ACA------------TACCCGGCTGATTTTTTGAGAATTGAAAGTGGACTCGGAGATCAG
ATATTCGCCACCATGTTATTGGTGGCTTGCATCATGGCAATCACCGATAAAAGGAACACC
AAACCACCGGCGGGAATGGAGCCTTTCCTCATAGGTTTGGTGGTGTTCGTCATCGGCTTG
GCCTTCGGTTTCAACTGCGGCTACGCCATCAACCCGGCCCGAGACTTCTCACCAAGGCTC
TTCACGGCCATTGCAGGCTGGGGCTCTGAAGTCTGG---ACG---CCGAATGGTATGAGC
TGGTGGTGGGTTCCTATAGTGGGACCTTTCATCGGGGGAATATGTGGAGCGTTTGTCTAC
ATTGTGTTCATCGAGGCTCACCACCCACCAGCACAGGAATAT
>Batstar_Glp3
ACGCAGCATCTGAGGATCAAGAACCAGTGGGTCAAGGAATTCTTGGCGGAGTTTTTAGGA
ACTTTTGTTTTGATGTTATTTGGAGATGGCAACGTAGCACAAAGCGTCCTGAGCAGAGAG
GCA---TACGGA---GACTTTCTCTCTATCAACCTGGGTTGGTGGGTGGCTGTCACCATG
GGAATCTACGCGTCGGCTGGGATATCTGGGGGTCACATCAACCCAGCCGTCTCCCTGGCC
CTGGCCGTGGTGGGCAAGTTTAGCTGGAAGAAACTGCCCGTGTATGTGCTGGCCCAGTTC
ATTGGCGCGTTTGCTGCCTCGGCCTGTCTTTACGGAGTCTACCTTGATGCACTGAACGCT
TTTGACGGTGGTAATCGGATGGTGTTAGGCGTT---AATGCCTCCGCTATGATCTGGGCA
ACA------------TATCCCCAGGAATATCTCAGCCTACAGGGCGGCTTGGGAGACCAG
ATTCTTGGTACGGCGCTACTAATGCTTAGCGTGTTGGCCATCACCGATAAGCGGAACAAT
GGTGCACCAGCAGGGATGGTGGCTATCATGGTGGGGTTGAGTGTGCTTGGTATCGGCTTG
AGCTTCGGGTCCAATTGCGGCTACGCTATCAATCCGGCACGTGACTTCCCTCCCAGGCTC
TTTACCTACTGCGCCGGTTGGGGTACTGAAGTTTGG---ACG---CCCCGCGGTATGCAC
TGGTGGTTTGTGCCGATCGTTGGGCCGTGCATCGGAGCGATAGGCGGCGCCCTGTTGTAC
ATCCTATGCGTGGAGGCCCATCACACACCGGAACATGAAATG
>Blue_batstar_Glp3
ACGCGGCATCTGAGGATCAAGAACCAGTGGGTCAAGGAATTCTTGGCGGAGTTTCTGGGG
ACTTTTGTTTTGATGTTATTTGGAGATGGCAACGTAGCACAAAGCGTCCTGAGCAGAGAA
GCA---TATGGG---GACTTTCTCTCTATCAACCTGGGATGGTGGGTGGCTGTCACCATG
GGAGTCTACGCGTCGGCTGGGATATCTGGGGGTCACATCAATCCAGCCGTCTCTCTGGCC
CTGGCCGTGGTGGGCAAGTTTAGCTGGAAGAAACTGCCCGTGTATGTGCTGGCCCAGTTC
ATTGGCGCGTTTGCTGCCTCAGCCTGTCTTTACGGAGTCTACCTTGATGCACTGAACGCT
TTTGACGGTGGTAATCGGATGGTGTTAGGCGTT---AATGCCTCCGCTATGATCTGGGCT
ACA------------TACCCCCAGGAATATCTCACACTACAGGGCGGCTTGGGAGACCAG
ATTCTTGGTACGGCGCTACTAATGCTCAGCGTGTTGGCCATCACCGATAAGCGGAACAAT
GGGGCACCAGCAGGGATGGCGGCTATCATGGTGGGGTTGAGCGTGCTTGGTATCGGCTTG
AGCTTCGGGTCCAATTGCGGCTACGCTATCAATCCGGCACGGGACTTCCCTCCCAGGCTT
TTTACCTACTGCGCCGGTTGGGGTACTGAAGTTTGG---ACC---CCCCGCGGTATGCAC
TGGTGGTTTGTGCCGATCGTTGGGCCGTGCATCGGAGCGATAGGTGGCGCACTGTTGTAC
ATCTTGTGCGTGGAGGCCCATCACACACCGGAACATGAAATG
>NZccStar_Glp3
ACACAGCATCTGAGGATCAAGAACCAATGGGTCAAGGAATTCCTGGCGGAGTTTTTGGGG
ACTTTTGTTTTGATGATATTTGGAGATGGCAACGTGGCGCAGAGCGTCCTCAGCAAAGAA
GCA---TACGGC---GACTTCCTCTCTATCAACCTGGGTTGGTGGGTGGCTGTCACCATG

GGAGTCTATGCCTCGGCTGGGATATCTGGTGGTCACATCAACCCAGCCGTCTCCCTGGCC
CTGGCTGTGGTGGGGAAGTTCAGCTGGAAGAAACTGCCCATGTACGTGCTGGCCCAGTTC
ATCGGTGCGTTTGCTGCTTCGGCCTGCCTTTACGGAGTCTACCTCGATGCACTGAACGCT
TTCGACGGGGGCAATCGGATTGTTTTGGGCGAA---AATGCCACCGCGATGATCTGGGCC
ACA------------TACCCCAAGGAATTCCTCAGCTTACAGAGTGGATTGGGAGACCAG
GTACTCGGG------CTACTGATGCTCTGCGTGTTGGCCATCACCGATAAGCAGAACAAC
GGGGCACCGCCTGGAATGGTGGCTGTTATGGTGGGGCTGAGCGTGCTTGGTATCGGGTTG
AGCTTCGGCTATAACTGCGGCTACGCCATCAATCCGGCGCGGGATTTTCCTCCTAGGCTC
TTTACTTACTGCGCCGGTTGGGGCACTGAAGTGTGG---ATG------------ATGCAT
TGGTGGTTTGTGCCGATTGTTGGCCCGTGCATCGGAGCGATAGGTGGCGCCCTGCTGTAC
ATCCTGTGCGTGGAAGCCCATCACATACCAGAACATGAAAAG
>cotStarfish_Glp3
ACGCGGCATCTCAGGATAAGGAACCAGTGGGTGCGGGAATTCTTGGCAGAATTTCTGGGG
ACGTTTATTTTGATGGTTTTTGGAGATGGCAACGTAGCGCAGAGCGTCCTCAGCAGCAGT
GCT---GCCGGT---GAATTCCTCTCCATCAACGTTGGCTGGTGGGTTGCAGTTACCATG
GGCGTGTATGCGTCGGCCGGAGTCTCTGGTGGCCACATCAATCCGGCCGTGTCTTTAGCC
CTGGCTGTGGTGGGCAAGTTCAGTTGGATCAAGCTTCCCATGTACGTGTTGGCCCAGTTC
CTCGGCTCTTTTGCCGCATCAGCTTGTCTCTACGGAGTCTACGCAGACGCACTGAATGCT
TATGATGGAGGCAATCGCACGGTAATAGGTCCT---AAAGGCACTGCCGGCATCTGGGCC
ACT------------TATCCCCAAGATTTCCTTTCACTGCAGGGCGGCCTAGGAGACCAG
ATTTTTGCCACGGGGCTGCTTCTGGTCTGTGTGCTGGCCATCACGGACAAGAGGAACGAC
GGGGCACCCAGCGGTATGGCAGCCATCATGGTTGGTCTGAGCGTGCTTGGAATCGGTGTG
AGCTTCGGTTTCAACTGCGGCTACGCCATCAATCCAGCGCGAGACTTCCCGCCGAGACTC
TTCACCTACTGCGCTGGTTGGGGCGTTGATGTTTGG---GTG---CCCAACGGAATGCAT
TGGTGGTGGGTGCCGATCGTTGGGCCCTGCATTGGAGCGGTACTGGGCGCCCTGTTGTAC
ATTCTCTGTGTGGAGGCCCATCATACCCCGGAAGACGAATTG
>BroodStar_Glp3
TCAAGGCATCTACGAATAAGACAGCAGTGGATCAAGGAGTTTCTAGCAGAGTTCCTGGGG
ACTTTTATTTTAATGATATTTGGAGATGGCAACGTAGCCCAGAGCGTATTAAGTAGGAAA
GAG---TACGGA---GAGTTTCTCTCTATCAACCTAGGATGGTTTGTTGCAGTCACAATG
GGTGTATATGCATCCGCGGGAGTTTCAGGAGGACACATCAACCCTGCTGTCTCGTTGGCC
CTCGCTGTAGTTGGTAAATTCAGCTGGAAGAAACTTCCACTCTATATCGTAGCACAGATG
TTGGGTTCGTTTGCAGCTTCGGCTGCTCTGTATGGCGTTTATCTGGACGCATTGAACAAT
TTTGATGGCGGTGTCAGGCAGGTACTTGGTGTG---AACGGTACAGCTGGTATATGGGCG
ACC------------TACCCAAAGCCTTTCCTCAGTATAGAGTCGGGTCTTGGCGACCAG
ATTCTCGGCACTGGTCTGCTCATGTGCTGCGTCATGGCCGTCACGGATAAACGCAACGAC
GGAGCACCAAAGGGTCTAACGGCCATGATGGTCGGACTGTCGGTGCTGGGCATCGGCTTG
GCATTCGGGTACAACTGCGGCTATGCCATCAACCCAGCCCGTGACCTGGCTCCGAGGCTG
TTCACGTACTGTGCTGGATGGGGAGTGGATGTTTGG---GTA---CCCGATGGTATGCAT
TGGTGGTGGGTGCCTATAGTTGGCCCTTGCCTAGGGGCGATAGCGGGCGCTATGACGTAC
ATGCTTTGCGTGGAACTCCATCACACACCTGGGGGTGAAATG
>Purple_sea_star_Glp3
TCAAGGCACCTACGAATAAGACACCAGTGGATCAAGGAGTTTCTAGCAGAGTTCCTGGGG
ACGTTTCTTTTAATG---

--TTAGACGCATTGAACAAT
TTTGATGGCGGTGTCAGACAGGTACTTGGTGAA---AACGGTTCAGCTGGTATATGGGCG
ACC------------TACCCAAAGCCTTACCTTAGCCTACAGACGGGTCTTGGCGACCAG
ATTCTGGGCACAGGTCTGCTCATGTGCTGCGTCATGGCCATCACGGATAAACGCAACGAC
GGAGCACCAAAGGGTATGACGGCAATGATGGTCGGACTGTCAGTGCTGGGTATCGGCTTG
TCATTCGGGTACAACTGCGGCTACGCCATCAACCCAGCCCGGGACCTGGCTCCAAGGCTG
TTCACGTACTGTGCTGGATGGGGAGTGGAGGTTTGG---GTG---CCCAATGGAATGCAT
TGGTGGTGGGTACCTATAGTTGGCCCCTGCCTTGGGGCGATAGCGGGCGCTATGACGTAC
ATGCTTTGCGTGGAACTCCATCACACACCGGAGGCTGAAATG
>eStarfish_Glp3
TCACGGTATCTACGAATAAAACAACAGTGGATTAAAGAGTTTCTCGCCGAGTTTCTGGGA
ACATTTCTGCTAATGTTGTTTGGAGATGGCAACGTAGCCCAGAGCGTGCTAAGCGGGAAA
GAG---TACGGG---GAGTTTCTCTCTATCAACCTAGGATGGTTTGTTGCCGTCACAATG
GGTGTATATGCATCGGCGGGAGTTTCAGGAGGACACATCAACCCTGCTGTCTCGTTGGCC

CTGGCTGTGGTCGGTAAATTCAGCTGGAAGAAAGTTCCACACTACATTGTGGCACAGATG
CTTGGGTCATTCGCAGCTTCAGCTGCTCTCTATGGCGTGTATCTGGACGCATTGAATGAT
TTCGATGGTGGTGTCAGACAGGTACTGGGCGAG---AACGGTACAGCCGGTATATGGGCG
ACC------------TACCCAAAGCCTTTCCTCAGTTTGCAGACGGGTCTTGGTGATCAG
ATTCTCGGCACCGGTTTGCTTGTGTGCTGCGTCTTGGCCGTCACTGATAAACGCAACGAC
GGAGCACCGAAGGGTATGACGGCCTTGATGGTGGGGTTATCGGTGCTAGGCATCGGCTTG
GCATTCGGGTACAACTGCGGCTACGCCATCAACCCGGCCCGTGACTTGGCACCGAGGCTG
TTCACGTACATCGCTGGATGGGGGGTGGAGGTTTGG---GTG---CCCAATGGAATACAT
TGGTGGTGGGTTCCTATAGTTGGGCCCTGCCTCGGGGCAATAGCGGGCGCTCTGACCTAC
ATGCTCTGTGTGGAACTCCATCATACACCGGAGGATGAAAAA
>ChilUrchin_Glp3
TTGAACCCGTTGCAAATTCGCAACAAGCTTGCAAAGGAGATTATAGCAGAAGCTATCGGG
ACCTACATTTTGATCGTGTTTGGCGATGCCTCGGTAGCACAATCAGTGCTTAGCAAGGGA
GAA---AATGGC---GGTTTTCTCTCCATCAACTGGGGCTGGGCTGTCGGTGTTATGCTT
GGTGTTTACTGGGCATCTGGTGTATCAGGAGCTCATATCAACCCCGCTGTGACCCTAGCC
TCGGCCTGCCTAGGAAGGTTTCCGTGGAAGAAGGTCCCATTTTATATGTTGGCCCAGATG
GTGGGGGCCTTCGTAGCTGCGGCCTGTGTATATGGAGTGTATTCAGACGCCATCAATGAC
TTTGACGGTGGAACTCGTGCAGTACTCGGGGAG---AATGGTACAGCTGGTATATTTGCG
ACC------------TATCCGAAGGATTTTCTCTCTATCTGGAGTGGACTTGGTGATCAG
GTTTTAGGTACTGCTTTATTGATGTCTTGCATCCTGGCCATCACAGACAAGAGAAACAAC
TCGCCCCCTAACGGTATGGAACCCCTTCTAATCGGCCTCATCGTCTTCAACATCGGTATC
TGCTTCGGCTACAATTGCGGCTATGCAATCAACCCGGCGAGAGATCTGGGGCCGAGGTTA
TTCACCGCTTGCGCAGGTTACGGCGCGGATGTGTGG---ACT---CCCAATGGTATGCAT
TGGTGGTGGGTGCCAATAATTGGACCGTTTGTGGGCGCCATCTTGGGAGGTTATCTCTAC
GTGTTCGCCATTGAACTCCATCACGAGACCGACACGGCTTCG
>cUrchin_Glp3

---------------------------------------AATCCCGCAGTGACCCTCGCT
TCGGCCTGTCTTGGAAGGTTTCCCTGGAAGAAAGTCCCATTTTACATGTTGGCTCAAATG
GTGGGGGCGTTTGTTGCAGCAGCATGTGTCTATGGAGTGTATATAGATGCCATCAATGAC
TTTGACGGTGGAACTCGTGCAGTACTTGGGGAG---ACTGGTACTGCTGGAATCTTTTCC
ACC------------TATCCGAAGGACTTCCTCTCTATCTGGAGTGGACTTGGGGATCAG
ATATTAGGTACTGCATTATTGATGGGCTGCATCCTAGCTATCACAGACAAGCGAAACAAC
TCGCCCCCTAACGGTATGGAACCTCTTCTAATCGGCCTTATTGTATTCAATATTGGTATC
TGCTTTGGCTACAATTGCGGCTATGCCATCAATCCGGCGAGAGATCTGGGGCCGAGGTTA
TTCACTGCTTGTGCAGGTTACGGCAAAGATGTGTGG---ACT---CCCAATGGTATGCAT
TGGTGGTGGGTACCAATAGTTGGACCGTGTTTGGGCGCCATCTTGGGAGGTTATCTCTAT
GTATTCACCATTGAACTTCATCACGAGACTGAGACGGCATCA
>Kina_Glp3
TTGAGCCCGTTGCAGATTCGCAACAAGCTAGCGAAGGAGATCTTAGCAGAAGCAATCGGG
ACCTACATTCTGATCGTGTTTGGCGATGCCTCGGTAGCACAGTCGGTGCTGAGTAAAGGA
GAG---AATGGC---GGGTTTCTCTCTATCAACTGGGGCTGGGGTGTCGGTGTTACACTT
GCTGTTTACTTCGCATCTGGTGTATCAGGAGCTCACATTAACCCTGCAGTAACACTAGCC
TTCGCATGCCTTGGACGGTTTCCATGGAAGAAGGTCCCCTTTTACATGTTGGCCCAGATG
GTAGGAGCTTTCGTTGCGGCAGCCTGTGTCTTTGGGGTGTATTCAGATGCCATCAATGAC
TTTGACGGTGGAACTCGAGCCGTCCTTGGGGAG---AATGGGACGGCTGGCATTTTTGCG
ACC------------TATCCGAAGGACTTCCTATCAATTTGGAGTGGATTTGGTGATCAG
ATATTAGGTACTGCTCTTTTGATGTCCTGTATATTGGCTATCACCGACAAGAGAAACAAC
TCGCCCCCGAATGGTATGGAACCCCTTCTAATCGGCTTCATCGTATTCAACATCGGTATT
TGCTTCGGCTACAATTGCGGCTACGCTATCAACCCTGCCAGAGATCTGGGGCCGAGGTTA
TTCACCGCATGTGCGGGTTACGGCCAAGATGTGTGG---ACT---CCAAATGGTATGCAT
TGGTGGTGGGTGCCCATAGTAGGACCTTGTCTTGGCGCCATCTTGGGAGGCTATCTCTAC
GTGCTCGCCATCGAACTCCACCACGACACCGAAACGGCATCG
>rsUrchin_Glp3
CTGAGCCCGCTGCAAATTCGCAACAAGCTTGCGAAGGAGATCTTAGCCGAGGCAATCGGG
ACCTTCATTCTCATTGTGTTTGGCGATGCCTCGGTAGCACAGTCGGTGCTAAGCAATGGA
GCT---AATGGC---GGTTTTCTCTCCATCAACTGGGGATGGGGTGTCGGTGTAATACTT
GCTGTCTACTTCGCTGCAGGTGTATCAGGAGCCCACATCAACCCTGCGGTGACCTTAGCC
TTCGCTTGCTTAGGACGGTTCCCATGGAAGAAGGTCCCGTTTTACATCTTAGCCCAGATG

GTTGGTGCCTTCGTGGCAGCAGCTTGTGTATATGGGGTGTATTCAGATGCTATCAATGAT
TTTGACGGTGGAATTCGAGCTGTACTTGGGGAG---AATGGGACAGCCGGCATCTTTGCA
ACC------------TATCCGAAGGACTTCCTTTCAATCTGGAGTGGACTCGGAGATCAG
ATATTAGGCACTGCTCTATTGATGTCCTGTATATTGGCCATCAGTGACAAAAGAAACAAC
TCGCCTCCTAACGGTATGGAGCCCCTTCTCATCGGTCTCGTTGTATTTAACATCGGTATT
TGCTTCGGATACAACTGCGGCTATGCCATCAACCCTGCCAGAGATCTGGGGCCGAGGTTG
TTCACCGCCTGTGCGGGTTACGGTCAAGATGTCTGG---ACT---CCCAATGGTATGCAT
TGGTGGTGGGTGCCTATAGTAGGACCGATTGTGGGCGCCATC------------------

>psUrchin_Glp3
CTGAGCCCGCTGCAAATTCGCAACAAGCTTGCGAAGGAGATCTTAGCCGAGGCAATTGCG
ACCTTCATTCTCATTGTGTTTGGCGATGCCTCGGTAGCACAGTCGGTGCTAAGCAATGGA
GCT---AATGGT---GGTTTTCTCTCCATCAACTGGGGATGGGGTGTCGGTGTTATACTT
GGTGTTTACTTCGCAGCAGGTGTATCAGGAGCCCACCTCAACCCTGCGGTGACCTTGGCC
TTCGCTTGCTTAGGACGGTTCCCGTGGAAGAAGGTCCCCTTCTACATCTTAGCCCAGATG
GTCGGTGCCTTCATAGCTGCAGCCTGTGTATTTGGGGTGTATTCGGATGCTATTAATGAT
TTTGACGGTGGAGTTCGAGCTGTACTCGGGGAG---AATGGGACAGCCGGCATCTTTGCT
ACC------------TATCCGAAGGACTTTCTCTCAATCTGGAGTGGACTTGGAGATCAG
ATATTAGGCACTGCTCTGTTGATGTCCTGTATATTGGCCATCACTGACAAGAGAAACAAC
TCTCCTCCTAACGGCATGGAACCCCTTCTTATCGGTCTCGTTGTATTCAACATCGGTATT
TGCTTCGGATACAACTGCGGCTATGCCATCAACCCTGCCAGGGATCTGGGGCCGAGGTTG
TTCACCGCCTGCGCGGGTTACGGCCAAGATGTTTGG---ACG---CCCAATGGTATGCAT
TGGTGGTGGGTGCCTATAGTGGGACCGATATTGGGCGCCATCTTGGGCGGATATCTTTAC
TTGTTCGCTATCGAACTCCACCATGACACGGACACGGCATCG
>gsUrchin_Glp3
CTCAGCCCGCTGCAAATTCGCAACAAGCTTGCGAAGGAGATCTTAGCCGAGGCAATCGGG
ACATTCATTCTCATTGTTTTTGGCGATGCCTCGGTAGCACAGTCGGTGCTAAGCAATGGA
GCT---AATGGC---GGCTTTCTCTCCATCAACTGGGGGTGGGGTGTCGGTGTTATGCTT
GGTGTTTACTTCGCTGCAGGTGTATCAGGAGCCCACCTCAACCCTGCGGTGACCTTGGCC
TTCGCTTGCCTAGGACGGTTTCCGTGGAAGAAGGTTCCGTTTTACATCTTAGCCCAAATG
GTTGGTTCCTTCATAGCTGCAGCTTGTGTATTTGGGGTATATTCG---------------

ATATTAGGTACTGCTTTGTTAATGGCCTGTATATTGGCCATCAGTGACAAGAGAAACAAC
TCGCCTCCTAACGGCATGGAACCCTTTTTTATCGGTCTCGTTGTATTTAACATCGGTATT
TGCTTCGGATACAACTGCGGCTATGCCATCAACCCTGCCAGGGATCTGGGGCCGAGGTTG
TTCACCGCCTGCGCGGGTTACGGTCAAGATGTTTGG---ACG---CCCAATGGTATGCAT
TGGTGGTGGGTGCCTATAGTGGGACCGATTGTGGGCGCCATCTTGGGCGGATACCTTTAC
GTGTTTGCCATCGAACTCCATCACGACACGGACACGGCTTCG
>GVSurchin_Glp3
TTGAGCCCGCTGCAAATTCGCAACAAGCTTGCGAAGGAGATCCTAGCCGAGGCAATCGGT
ACCTTCATTTTAATTGTATTTGGTGACGCATCGGTAGCACAGTCTGTTCTGAGTAGCGGG
GCT---AATGGC---GGCTTTCTCTCAATCAATTGGGGATGGGGAATCGCCGTCACACTC
GCCGTTTATTTCGCATCTGGTGTGTCAGGTGCCCACATAAACCCAGCAGTGACCTTGGCC
TTTGCCTGCTTGGGACGGTTTCCTTGGAAGAAGGTCCCGTTTTACATTTTAGCTCAGATG
GTCGGTGCCTTTGTCGCAGCTGCCTGTGTGTTCGGAGTGTATTCAGATGCTATCGATGCC
TTTGACGGAGGAACTCGAGCAGTACTCGGCGAA---AACGGGACTGCTGGTATTTTTGCA
ACC------------TATCCGAAGGACTTCCTTTCTATCTGGAGTGGACTCGGCGATCAG
ATACTAGGCACTGCTCTTTTGATGTCCTGCATATTGGCTATCACGGACAAGAGAAACAAC
TCGCCTCCTAACGGGATGGAACCCCTTCTCATTGGTCTCGTTGTGTTCAATATTGGTATT
TGCTTCGGGTTCAACTGCGGCTACGCCATTAACCCAGCCAGGGACCTGGGCCCTAGGTTA
TTCACCGCGTGCGCAGGTTATGGTCAAGATGTATGG---ACG---CCCAATGGTATGCAT
TGGTGGTGGGTGCCCATAGTAGGACCCTGTGTGGGCGCCATCTTGGGTGGATATCTCTAC
GTGTTCACTGTCGAGCTTCATCACGACACAGAAACAGCATCG
>Sg_psUrchin_Glp3
TTGAGCCCGCTGCAAATTCGCAACAAGTTTGCGAAGGAAATCCTCGCCGAGGCAATCGGG
ACCTTCATTCTAATCGTATTTGGCGACGCCTCTGTAGCACAATCTGTTCTGAGTGGCGAG
GCT---AATGGC---GGCTTTCTCTCAATAAACTGGGGATGGGGTATCGCCGTGACACTT
GCCGTTTATTTCGCATCTGGCGTATCAGGTGCCCACATCAACCCAGCAGTGACCTTGGCT
TTTGCCTGCTTGGGACGGTTTCCATGGAAGAAGGTCCCATTTTACGTCTTAGCCCAGATG
GTTGGTGCCTTTGTCGCAGCTGCCTGTGTCTTCGGAGTGTATGCAGATGCTATCAATGCC

TTTGACGGAGGAACTCGTGCAGTACTCGGTGAA---AATGGGACAGCTGGTATTTTTGCA
ACC------------TATCCGAAGGACTTCCTTTCAATCTGGAGTGGACTTGGCGATCAG
ATATTAGGCACAGCTCTTTTGATGTCCTGTATATTGGCCATCACAGACAAGAGAAACAAC
TCACCTCCTAATGGAATGGAACCCCTTCTCATTGGTCTCGTTGTATTCAATATTGGTATT
TGCTTCGGTTTTAACTGCGGCTACGCCATCAACCCAGCCAGGGATCTAGGGCCCAGGTTA
TTCACCGCCTGTGCAGGTTACGGCCAAGATGTATGG---ACT---CCCAATGGTATGCAT
TGGTGGTGGGTGCCCATAGTAGGACCTTGTGTGGGTGCCATCTTGGGCGGATACCTCTAC
GTGTTCACTGTCGAACTTCATCATGACACAGAAACAGCATCG
>PctUrchin_Glp3

------------------------------AAAGTGCCCTGCTATATCCTGGCCCAGATG
GTGGGGGCGTTTATCGCTGCAGCCTGCGTGTATGGAGTCTACTCAGACGCCATCAACGCC
TTCGACGGTGGTGTTCGGACGGTGCTCGGAGAG---AATGGCACAGCCGGTATCTTCGCT
ACC------------TACCCACAGGACTTTCTCTCAGTGTGGAGTGGCCTCGGTGATCAG
ATCTTCGGCACGGCGCTCTTGATGTCCTGTATCTTGGCCATCACCGACAAGAAGAACAAC
TCACCTCCGAATGGCATGGAGCCCCTTCTCATCGGCCTCGTCGTCTTCAACATCGGCATC
TGCTTCGGCTTCAACTGCGGCTACGCCATCAACCCCGCCCGCGACTTAGGGCCCCGCCTC
CTGACGGCCTGCGCTGGATACGGGCAGGAGGTCTGG---ACG---CCCAACGGCATGCAT
TGGTGGTGGGTGCCCATTGTGGGCCCATGTCTCGGGGCCATCATGGGGGCCTTCATGTAC
GTCTTCGCCATCGAGCTCCACCACGACCCCTCCCCAACCTCC
>csDollar_Glp3
TTCGAGGCTCTACGAATCCGCAATCCGCTCGCCCGGGAGATTCTCGCTGAATTCATCGGC
ACATTCATCCTAATCGCCTTCGGGGATGCGTCGGTAGCACAGTCAGTCCTGAGCAATGGA
ACG---AAAGGC---GGTTTCCTCTCCATCAACTGGGGCTGGGGCGTGGGCGTCATTTTG
GGGGCGTACTTTGCATCTGGAGTCTCAGGTGCTCACATCAACCCGGCAGTGACATTAGCC
TTCGCATCCTTGGGGCGGTTCCCCTGGAAGAAGGTTCCATTCTACATATTCGCTCAGATG
ATCGGCGCCTTCATAGCGGCTGCATGTGTATACGGTGTCTACGCAGATGCGATCAACGCG
TTTGACGGGGGAACGAGGCAGGTTCTAGGTGAG---AACGGTACGGCGGGAATCTTCGCT
ACC------------TATCCGCAGGACTTCCTGTCGATCTGGAGTGGACTCGGGGACCAG
ATTTTCGGTACGGCCCTGCTGATGGGATGCATTCTCGCCATCACGGACAAGAAGAACAAT
GCGCCCCCTAGTGGCATGGAACCACTGCTGATCGGCCTCGTCGTCTTCAATATCGGGATC
TGCTTCGGGTTCAACTGCGGCTACGCCATCAACCCCGCTCGGGATCTGGGACCAAGGCTA
CTCACCGCGGCAGCTGGTTACGGGCATGAGGTCTGG---ACT---CCTAATGGCATGCAT
TGGTGGTGGGTGCCCATCGTTGGCCCGTGCATCGGTGCAGTACTGGGTGGCTACCTCTAC
GTGCTGGTGATCGAGCTCCATCACGATCGCGAGCCATCGGAA
>spUrchin_Glp3
TTCGATACGCTTCGAATTCGAAATCATTTGGCGAGGGAAATCCTGGCTGAATTCATCGGT
ACATTTATCCTACTAGTATTCGGTGATGGGTCAGTGGCGCAATCTGTTCTTAGCAGTGGA
GTG---AAGGGA---GAATTCCTGTCAATCAACTGGGGGTGGGGTGTTGGTGTCACATTA
GCTGTTTATTTTGCATCAGGAATCTCAGGAGCCCATATTAACCCTGCAGTGACGTTGGCC
TTTGCGTGTATCGGTCGTTTTCCATGGAGGAAGGTCCCTCTCTACATCTTGGCTCAGATG
GTCGGAGCCTTCGTGGCGGCTGGTTGCGTCTTTGGAGTATATATAGATGCGATCAACGCC
TTTGATGGAGGCACACGAGCCGTCCTTGGGCCA---ACTGGAACAGCGGGTATCTTTGCT
ACT------------TATCCCCAAGATTTCTTGTCTATGTGGAGTGGCTTTGGCGATCAG
ATCTTTGCAACCGCTCTGCTGATGTCTTGCATCCTGGCGATCACGGATAAGAAGAACAAC
GCGCCCCCAAGCGGCATGGAACCACTACTGATTGGGCTCATAGTCTTCAATATCGGTATC
TGTTTTGGATTCAACTGCGGCTATGCGATCAACCCAGCTCGTGACTTGGGACCCAGATTA
TTCACAGCTTGCGCTGGTTACGGACAAGACGTCTGG---ACG------------------

>jCrinoid_Glp3
ATGTCAGTATTAAGAATTCGAAACCAGATAGTTCGCGAATTCATGGCTGAATTTATTGGG
ACATTTATTTTGATCGCTTTTGGTGACGGTTCAGTTGCGCAGTCGGTTTTAAGCAATTCG
GAA---AAAGGA---GGATTCTTGTCAATCAACTGGGCATGGGGTGTCGGCGTTACCATA
GGTGTATATTTTGCATCCGGGATATCAGGTGGGCATCTTAACCCAGCAGTTACGCTAGCG
TTCGCTAGCATCGGCCGTCTGTCTTGGAAGAAAGTACCCTTCTACATGCTTGCACAATTG
CTTGGCGCTTTTACAGCAGCAGCGTGTGTATTTGGAGTCTATTCAGAGGCGATTGATGCA
TTTGATGGCGGTAACCGATCCGTGATAGGACCC---AAAGCTACAGCAGGAATATTTGCC

ACT------------TACCCGGCTGAATATCTGTCGGTGTGGGGCGGGTTTGGAGACCAG
GTGTTTGCAACAGCACTCTTGATGGCTTGTATTCTTGCCATCACAGACAAGGATAACAAT
AAACCTCCAAATGGTATGGAGCCATTTATCGTAGGTCTAGTCGTCTTCGCCATCGGGCTT
GCCTATGGGCTTAACTGTGGCTACGCAATTAACCCCGCTAGGGACTTTGGCCCCAGACTG
TTAACAGCCTGTGCGGGATATGGCAAGGAAATTTGG---ACG---CCAAGGGGCATACAT
TGGTGGTGGGTTCCGATAGTCGGGCCGTTCGTTGGCGCAATCGTCGGGGCAATTACCTAC
CTTATCGTCATCGAAATACACAACACTGAAGATAACGAGGAC
>sBrittleStar_Glp3
GCAGATAAATTCAGAGTAAGGAACCAGTATCTGCGAGAATTCCTGGCTGAATTGTTGGGC
ACGTTTGTTTTGCTCGCGTTTGGTAATGGAACAGTTGCACAAGTGGTGTTAAGTGGAGAA
GTG---AAAGGA---GGGTTCATAACCATTAACTGGTGTTGGGCTCTTGCTGTTGCACTT
GCAGTTTACAGTTGTGGCGGAGTCTCAGGTGCGCACATCAATCCAGCAGTGACGGTAGCA
TTTGCTAGTATTGGGCGTTTTCCATGGGTCAAAGTGCCTTTATTTGTTGGAGCTCAACTG
TTGGGTGCTTTCCTTGGTGCAGCATGTGTATATGGTGTCTATATTGATGCAATCAACGCA
TTCGATGGTGGTAATCGGCAGGTAATTGGTCCA---AATGCAACTGCAGGGATCTTTGCC
ACA------------TATCCCCAGGATTTCTTGTCAATATGGAGTGGATTAGGGGACCAG
ATATTTGCAACAGCTTTATTAATGCTTCTTATTATGGCTATCGTCGACAAACGTAACAGT
AGTCCGCCAAAAGGAATGGAGCCTTTCATGATAGGACTCGTGGTCGGAGCCATAGGAATG
AGTTTTGGCTTCAATTGCGGTTATGCTATCAATCCTGCAAGAGATTTGGGACCAAGGTTA
TTCACAGCTTTAGCTGGTTACGGCGAAGAAATTTGG---GTG---TATCAAGGAATGCAT
TGGTGGTGGGTGCCAATCGTTGGCCCAACAATTGGAGCAATTGCAGGTGCCGTTATGTAT
ATCACATTTATTGAGTTACATCATTCACCTGAGGAGCCTCCG
>smBrittleStar_Glp3
----------------------------TACGTCAGGGAATTTATGGCTGAATTTCTCGGA
ACTTTTGTCTTGTGTGTTTTGGGCGATGGTTCTATTGCCCAGGTGGTCCTCAGCAGAGGA
GCA---AAAGGA---GGGTTTCTCACGATAAATTTCTGCTGGGGCTTGGCTGTGGCACTT
GGGGCGTATTGCTGTGCAGGAGTTTCAGGTGCCCATATAAATCCAGCGGTAACTGTTGCC
TTCGCAACGATTGGTCGTTTTCCTTGGCTTAAAGTTCCGCTATTCGTTGGTGCTCAAATG
CTTGGTGCTTTTCTTGCTGCTGCCTGTGTCTTTGGTGTATATCATGACTCAATCAACGCA
TTTGACGGTGGTGTCCGGCAGGTCGTAGGGGAA---AATGCAACAGCAGGTATCTTTGCA
ACA------------TATCCACAAAACTATTTATCGATTTGGAGTGGACTTGGTGATCAG
TTTTTTGGCACACTTCTACTCATGCTTTGCATCATGGCCATCATCGATAAGCGTAATAGC
AGCCCTCCAGATGGTATGGAACCGTTCATCATTGGTCTCGTAGTAGCAGCAATTGGTATG
AGTTTTGGCTATAACTGCGGATATGCCATTAATCCAGCAAGAGATTTTGGGCCAAGATTA
TTCACGGCTATGGCTGGATACGGTCGTGAGATTTGG---GTA---TACAATGACATGCAT
TGGTGGATAGCCCCAATTGTAGGGCCGACCTTAGGCGCCATAGTTGGCGGTATCATCTAC
GTCTCTCTCATCGAATTACATCATTCACCCGAAGAACCACCT
>pUrchin_Glp2
AAAGAAAGGCTTCACATCGGTAACTGGTGGATGAGGGTATTTCTATCCGAACTTTTTGGA
ACTTTCATGTTGGTGTTTATTACAGATGGCGCTGTAGCAAGAACCGTTCTGAGCCGTGGA
GCA---GCGGGT---GGTGCTCTGTCACTTAACATTGGTGCCTCTCTTGCTGTCACCGTG
TGTATTTACATGACCGGGGGAGTGTCAGGTGGTCACATAAATCCGGCCGTAACTCTCTCG
ATGTGTAGCCTTGGCCGTCTGAGATGGTTAGCTCTGCCTGTCTACTGGTTTGCTCAGTTT
ATCGGTGCTTTCCTTGGTGCTGCTGTAGTCTATGGTATCTACCTAGATGGTATCAACAGT
TTTGAAGGAGGGCCTTCTAATCGATCT---------CTAGCCACCGCAGTAATCTTTGCC
ACATACCCCAATGGATACCCCGAATCTTTCCTAAGTGTACCAGGAGGAGTCATGGACCAG
CTTGTGGGTTCGGCCCTTCTCGTTGGCGGTATTTTCGCCATCTTTGATAAACACAACATC
AAACCACCTGCGGGGCTTGAACCAATAGCAGTAGGTCTACTTCTTCTCGTCGTTAATATA
GCATACGGCTACAACGCGGGTGCAGCGGTCAACCCTGCAAGGGATTTCAGCCCGAGACTG
TTCACCGCTTGTGCTGGATACGGAAAGGACATCTGGGTGACG---CCATCTGGTGACCAT
TTTTGGTGGATACCGCTGTTTGTACCCTTGGTCGGGGGCCCGATAGGAGGATGGGTCTAC
TATCTCACTATAGAAGTCCATCATCCACATAAAATTAGTAGC
>gsUrchin_Glp2
AAGGAAAAGCTTCACATCGATAGCTGGTGGATGAGGGTATTTCTATCCGAACTTTTTGGA
ACTTTTATGTTGGTGTTTATTACAGATGGCGCTGTAGCGAGAACCGTTCTGAGCCGTGGA
GCA---GGGGGC---GGTGCTCTATCACTTAACATTGGTGCCTCTCTTGCTGTCACCGTG
TGTATTTACATGACCGGGGGAGTGTCAGGT------------------------------

--TTAGATGGCATCAACACT
TTCGAAGGAGGGCCTTCTAATCGATCT---------TTAGCCACCGCCGTAATCTTTGCC
ACATACCCCAATGGGTACCCCGAATCTTTCCTCAGTGTACCGGGAGGAGTCATGGACCAG

CTTGTGGGTTCGGCCCTTCTCGTTGGCGGTATTTTCGCCATCTTTGATAAGTACAACATC
AAACCGCCGGCGGGTCTTGAACCAATAGCAGTAGGATTACTTCTTCTCGTTGTTAATATA
GCATACGGCTACAACGCGGGTGCAGCGGTCAACCCTGCAAGGGATTTCAGCCCGAGACTG
TTTACCGCTTGTGCTGGATACGGACAGGACATCTGGGTGACG---CCATCTGGGGACCAT
TTCTGGTGGATACCGCTGTTTGTACCCTTGATCGGGGGCCCGATTGGAGGGTGGCTCTAC
TATCTCACTATAGAAGTCCATCATCCACATAAAATCAGCAGA
>rsUrchin_Glp2
AAGGGAAAGCTTCAAATCAATAACTGGTGGATCAGAGTATTTCTATCGGAACTTATTGGA
ACTTTTGCTTTGGTGTTTATTACAGATGGCGCTATAGCAAGAACCGTACTGAGCCATGGA
GCA---GCAGGC---GGTGCTCTATCAGCTAACATTGGTGCAGCTCTTGCTGTCACCGTG
TGTATTTACATTACCGGGGGAGTTTCAGGCGGTCATATAAATCCAGCCGTAACTCTCTCG
ATGTGTACCCTTGGCCGTCTGCGATGGTTAGCTCTACCTGTCTACTGGATTGCTCAGTTT
ATCGGTGCTTTCCTTGGCGCTGCTCTAGTTTATGGGATCTACCTGGATGGTATAAACAGT
GTCGAAGGAGGGCCTTCTAATCGATCT---------CTAGCCACCGCAGGAATCTTTGCT
ACA------------TACCCCGAATCTTTCCTAACTATACCAGGAAACGTCATGGACCAG
CTTGTGGGTACGGCCCTTCTCGTCGGCGGTATTTTCGCCATCTTTGATAAGCACAACATC
AAACCACCGGGGGGTCTTGAACCGATAGCAGTAGGTTTACTTCTTCTCGTTGTTAATATA
TCATACGGCTACAATGCGGGTGCTGCAGTGAACCCTGCAAGGGATTTCAGCCCGAGACTC
TTCACCGCTTGCGTTGGCTACGGAGAGGACGTCTGGTTGACA---CCATCTGGTGACCAT
TTTTGGTGGATACCGCTGTTTGTACCCTTCATCGGGGGCCCGATTGGAGGATGGCTGTAC
TATCTCACTATAGAAGTTCATCATGCCCATACAAGCAGCGCA
>GVSurchin_Glp2
AAAGAACGACTTCATATCAATAATTGGTGGATCAAAGTGTTTCTATCAGAACTTGTTGGA
ACGTTTACATTGGTGTTCATTACAGATGGCGCTATAGCAAGGACCGTCCTGAGCGGTGGA
AAA---GCGGGT---GGTGCTCTGTCAGCTAACATTGGTTCTGCTCTTGCTGTAACTGTG
TCCATTTATATTACCGGAGGAGTTTCAGGTGGTCACATTAATCCTGCGGTAACCCTATCG
ATGTGTAGCCTTGGTCGGCTAAGATGGCTAGCTCTACCGGTCTACTGGATAGCTCAGTTT
ATAGGTGCTTTCCTTGGGGCTGCCCTAGTTTATGGGATCTACATCGATGGTATAAACCAT
ATTGAGGGCGGACAGGCTAATCGTTCT---------CTTGCTACCGCGGGAATCTTTGCA
ACA------------TACCCCGAACCTTTTTAACAATACAAGCAGGAGTTGCAGATCAG
CTTGTGGGTACAGCTCTTCTGGTCGGTGGAATATTTGCAATCTTTGATAAACAAAACATC
AAGCCAGCCGTTGGTCTTGAACCGATAGCGGTTGGTTTGCTTCTTCTTGCTGTCAATATA
TCATACGGCTACAATGCAGGTGCAGCAGTCAACCCTGCAAGAGATTTTAGCCCGAGACTC
TTCACCGCTGTTGTCGGCTACGGAGACGACATTTGGTTGACG---GCATCTGGCGACGAA
TTTTGGTGGATACCGCTATTTATTCCATTCATCGGTGGCCCTATTGGAGCATGGTTGTAT
TATATAACAATAGAAGTTCACCACAGACCAGCAGAAAAAGCA
>ChilUrchin_Glp2
CATGAAAAACTTCAAATCAATAATTGGTGGATCAAAGTATTTATATCGGAGCTTATCGGA
ACTTTCTTTTTGGTGTTTATGACAGATGGCGCTATAGCAAGAACGGTTCTGAGCGATGGA
GAA---GCGGGT---GGGGCGCTATCCACCAACATTGGAACCGCCTTTGCCGTCACTATT
TCTATCTATATCAGCGGGGGCGTTTCAGGTGGTCATATAAATCCCGCCGTGTCTTTATCG
ATGTGTACCCTTGGTCGACTGCGGTGGCTAGCTCTACCAGTCTACTGGGTGGCTCAGTTT
ATTGGTGCTTTCCTCGGGGCAGCTCTGGTTTATGGTATCTACTTAGATGGTATAAACAGC
GTTGAAGGTGGGCCTGCTAATCGAACC---------CTAGCCACGGCAGGAATCTTTGCT
ACA------------TACCCCCAACCATTTGTGACGATACCGGGTTTAGTCATTGACCAG
CTTGTAGGTACAACCCTCCTCGTTTCGGGCATTTTCGCTATATTTGATAAGCATAATATC
AAACCACCGACGGGTCTTGAACCGATAGCAGTAGGTCTACTCCTTCTCGTGGTTAATATA
GCATACGGCTATAATGCGGGAGCAGCAGTTAATCCGGCCAGAGACTTCAGCCCAAGACTT
TTCACCGCTCTCGTAGGCTACGGCGAGGACATCTGGTTTACA---CCATCTGGTGAGCAC
TTTTGGTGGATACCACTATTTATTCCCCTTATTGGGGGCCCCGTTGGAGGCTGGCTCTAC
TACCTCACTATAGAAGTGCATCATGCGTATAAAAGCAGAGCA
>Kina_Glp2
---------CTTCAAATCAACAATTGGTGGGTCAAAGTGTTTCTAGCTGAGCTCATTGGA
ACTTTCACTTTAGTGTTTGTAACAGATGGCGCTATAGCAAGAACGGTTCTTAGCGATGGG
GCA---GCGGGG---GACGCTCTATCCGCCAACATTGGTGCCGCCTTTGCTGTCACTGTT
GGAATCTACATAAGCGGGGGAGTTTCAGGTGGTCATATAAATCCGGCCGTGACTCTATCG
ATGTGTATTATGGGTCGGCTGAGATGGCTAGCTCTACCCGTCTATTGGGTTGCTCAGTTT
ATTGGTGCTTTCCTTGGGGCTACTGTAGTGTATGGTATTTACCTGGATGGTATAAACAGC
GTTGAAGGCGGGCCTTCTAACCGAACA---------TTAGCCACAGCAGGAATTTTCGCA
ACA------------TACCCCGCACCTTTTGTAACTATACCGGGAGAAGTCATGGACCAG
CTTGTAGGTACAGCGCTCCTGGTTGCCGGTATTTTCGCCATCTTTGATAAGCACAACTTC

AAACCACCAACGGGTCTAGAACCAATATCAGTTGGTCTACTTCTTCTTGTCGTCAATATA
TCATATGGATACAATGCTGGCGCAGCAGTCAACCCTGCTAGAGATTTAGTCCTAGATTA
TTCACCGCTCTGGTAGGCTACGGGGAGGACATTTGGATCACC---CCATCTGGTGACCAT
TTCTGGTGGATACCACTGCTTGTCCCTTTTATCGGTGGCCCTATAGGTGCCTGGCTATAC
TACCTTACTATAGAAGTTCACCATGGTTATACTGGCAGAGCA
>spUrchin_Glp2

---------------TTCATAGGTGATGGCGCCATTGCACAAGTCAAACTCAGCAAGTCG
GAG---AATGGA---ACCTTCCTGTCAATCAACTTTGGTTACGCCTTAGCGCTCATGATT
GCCATATACGTCTCCGGTGGAGTATCAGGTGGTCACGTAAATCCCGCTATTTCCCTCGCC
ATGGCAACCCTGGGACGTCTGCGGTTAATCGCCCTGCCTGTCTACTGGGCTGCTCAGTTC
TTAGGGGCGTTTGTTGGAGCCGTCTGTGTCTATGGAATATATCTTGATGGCATAAATCAT
TTGGAGGGAGGGATTCACAATCGAACC---------AATTTGACAGCTGGAATATTTGCA
ACC------------TATCCTGCGCCATTTTTGACGCTAGCCGAAGGATTCTTTGATCAG
GTTGCCGGGACAGCTCTCTTAGCGGGTGGAATCATGGCTGCTTTCGATAAGGATAACATC
AAACCTCCAAAAGGTCTGGAGCCCATGCTTGTCGGTTTCACCCTCCTCGCTATCGGCCTG
TCTTACGGGCTCAATTTCGGATACGCCCTCAACCCTGCCCGAGACTTCAGTCCCCGAGTC
TTTACTGCGATGGCCGGTTATGGCAACGAAGTATGG---ATTCTGCCAAACGGTGATCAC
TTCTGGTGGATTCCGATTGTCGGGCCTTTAGTCGGTGCAACGTTGGGGGCGTGGATCTAC
TACCTGGCCATTGAAGCTCACCACAAAAAAGACCAGGACGGA
>Batstar_Glp2
CTGGACAACCTCCGTCTCAGGAACGAGCTTATCCGTCAATTTTGCGCCGAGCTCCTGGGG
ACGTTCATGCTAGTGTTGATTGGTGATGGAGCCATAGCGCAGATGAAGTTCTTCGGTGGG
ACAGGCACTGGGGCAGGTTTTCTGAACGTCAACATCGGTTATGCCTTCGGCTTGATGCTG
GGGGTGTATTTCACAGCATCAGTGTCAGGTGGTCATTTGAATCCGGCGGTGTCACTTGCC
TTCTGTACCTTGGGCAAGCTGAAGTGGCTTGCCTTCCCGGTCTACATGTTGGCTCAGTTC
ATCGGTGCCTTCCTGGCCGCGGCCATGGTCTTCGCCGTCTATTACGATTCCATTGACAAA
GTAGAC---CACAATAGGACGGTGGTGTTACCG---AACAGTACGGCGGGCATTTTTGCT
ACT------------TACCCCACACCGGGGCTGTCCTGGGGTGTCGGATTCGTCGACCAG
CTCTTGGGCACAGCCCTCCTGATGGGTGGCATCATGGCCGTGACAGACAGCAAGAACTCC
AAGCCACCCAGCGGTCTGGAACCCATCTTCGTTGCCTTGACTTTCTTCGCTGTGGGCATC
AGCTTCGGCTACAACTTTGGCTATGGCATCAACCCTGCCAGGGACTTCGGACCGCGTGTC
TTCACCGCCCTTGCCGGCTATGGGTCCGATGTCTGG---ACG---CAAGCTGGGGTTCAC
TGGTGGATCATCCCGACCTTTGTGCCCTTCCTCGGGGCACCGATAGGAGCCTGGGTGTAC
TACCTCGGTATCGGCATGCACTGCGAGTCGGCCTACAAACGA
>Blue_batstar_Glp2
CTGGACAACCTCCGTCTCAGGAACGAGCTTATCCGTCAATTTTGCGCCGAGCTCCTGGGG
ACGTTCATGCTAGTGTTGATTGGTGATGGAGCCATTGCGCAGATGAAGTTCTTCGGTGGG
ACGGGCACTGGGGCAGGTTTTCTGAACGTCAACATCGGCTATGCCTTCGGCTTGATGCTG
GGGGTGTATTTCACAGCATCAGTGTCAGGTGGTCATTTGAATCCGGCGGTGTCACTTGCC
TTCTGTACCTTGGGCAAGCTGAAGTGGCTTGCCTTCCCGGTCTACATGTTGGCTCAGTTC
ATCGGTGCCTTCCTGGCCGCTGCCATGGTCTTCGCCGTCTATTACGATTCCATTGACAAA
GTAGAC---CACAATAGGACAGTGGTATTACCG---ACCAGTACGGCGGGCATTTTTGCT
ACT------------TACCCCGCACCGGGGCTGTCCTGGGGTGTCGGATTCGTCGACCAG
CTCTTGGGCACGGCCCTCCTGATGGGTGGCATCATGGCCGTGACAGACAGCAAGAACTCC
AAGCCACCCAGCGGTCTGGAACCCATCTTCGTTGCCTTGACTTTCTTCGCTGTAGGCATC
AGCTTCGGCTACAACTTTGGCTACGGCATCAACCCTGCCAGGGACTTCGGACCGCGTGTC
TTCACCGCCCTTGCAGGCTATGGATCCGATGTCTGG---ACA---CAAGCTGGGGTTCAC
TGGTGGATCATCCCGACCTTTGTGCCGTTCCTCGGGGCACCGATAGGAGCCTGGGTGTAC
TACCTTGCTATCGGCATGCACTGCGAGTCGGCCTACAAACGA
>NZccStar_Glp2
CTGGACAACCTCCGTCTCAGGAATGAGCTTCTCCGCCAATTCTCTGCCGAGCTCCTTGGG
ACGTTCATGCTAGTGTTGATAGGTGATGGTGCCATAGCACAGATGAAGTTCTTCGGTGGG
ACGGGGACCGGGGCAGGCTTCCTGAACGTCAACATCGGCTATTCCTTCGGCCTAATGTTG
GGGGTGTATTTCACAGCATCAGTCTCAGGTGGTCATTTGAATCCGGCGGTGTCACTTGCC
TTCTGCACCTTGGGCAAGCTGAAGTGGATTGCCTTCCCGGTTTACATGCTGGCCCAGTTC
ATCGGCGCCTTCTTGGCTGCAGCCATGGTGTTTGCCGTATATTACGATGCTATTGACAAA
GTAGAC---CACAATAGGACAGTGGTAGGACCG---ACTAGTACGGCGGGTATTTTTGCT
ACC------------TACCCCACACCCGAGCTGTCATGGGGTGTCGGATTTATCGACCAG
CTCTTGGGCACAGCTCTCTTGATGGGTGGCATCATGGCCGTTACAGACAGCAAAAACTCC
AAGCCACCTAGCGGTCTGGAACCCATCTTCGTTGCCTTGACTTTCTTCTCTGTCGGCATC

AGCTTCGGCTACAACTTTGGCTACGGCATCAACCCTGCGAGGGACTTCGGACCGCGTGTC
TTCACCGCCCTGGCCGGCTATGGAGCCGATGTCTGG---ACG---CAAGGTGGCATTCAG
TGGTGGATCATACCGACCTTTGTGCCGTTCCTTGGGGCGCCGATAGGAGCATGGGTCTAC
TATCTCGCCATTGGCATGCACTGCGAGTCAGCTTACAAAAGG
>cotStarfish_Glp2
CTGGACAACCTCCGTCTCAGAAATGAACTCGTGCGCCAGTTCTTTGCCGAGCTCCTGGGA
ACTTTTATGCTGGTGTTGATCGGTGATGGAGCTATTGCGCAGATGCAGTTCTTTGGCGGA
ACAGCATTAGGGGCTGGCTTTCTGAATGTGAACATCGGGTATGCCTTCGGGTTGATGCTT
GGGGTATATTTTACAGCTTCAGTATCAGGTGGTCACTTGAATCCGGCCGTTTCACTGGCC
TTCTGTACCTTGGGGAAGTTGAGATGGATCGCCTTCCCTGTCTACATGCTTGCCCAGTTT
ATCGGAGCCTTCCTGGCGGCTGTCATGGTGTTTGCTGTCTATTATGATGCCATCGACAAG
CATGAC---CACAACAGGACGGTGGTAGGA---------GGTACGGCGGGCATATTCGCC
ACC------------TACCCCAAAGCAGAGCTGTCCTGGGGAGTTGGATTCGTGGACCAG
CTCTTGGGCACGGCCCTCTTGATGGCGGGCATCATGGCGGTGACGGACAACAAGAACTCC
AAGCCCCCCAACGGTCTGGAGCCCATCTTCGTGGCCCTGACGTTCTTCGCCGTGGGCATC
AGCTTCGGCTACAACTTCGGATACGGCATCAACCCAGCGAGAGACTTCGGCCCTCGGGTC
TTTACTGCTATAGCTGGCTACGGATCTGACGTTTGG---ACG---CTGAACGGGATTCAG
TGGTGGATCATCCCCACATTCGTGCCTTTCCTGGGGGCACCGGTTGGCGCCTGGGTCTAC
TACCTGGCGATCGGCATGCACTGCGAGTCGCCCCACCGAGGT
>sasStarfish_Glp2
CTTGACTACATTCGACTAAAGCACGAGCTTCCACGGCAGTTTTGCGCCGAGCTAATTGGG
ACGTTCATGCTAGTGCTAATCGGCGATGGTGCTATTGCTCAGTGGCAATTCTTTGGTGCC
AAC---AATGGAGCTGGCTTTCTTAACGTCAACATCGGATATGCATTTGGTCTCATGCTT
GGAATATTTTTCACGGCTTCCGTGTCTGGAGGTCATTTAAACCCAGCCGTTTCCTTGGCC
TTTTGCTCTTTGGGCAAGATGAGACTGATAGCCTTCCCAGTCTACCTGCTAGCTCAATTC
ATTGGAGCGTTTATCGCCGCTGCTATGGTGTATGGTGTATATTATGACACCATTGACGAG
CTTGAT---CCAAACCGCACAGCG---------------AGGACATCGGGAATCTTTGCA
ACT------------TATCCCAATCCCCAATTGGGAGCAGGAGCTGCGTTTGTTGATCAG
TTGCTTGGCACAGCTATCCTGATGGCCGGCATTCTAGCCATAACCGACAAGAAGAATTCC
AAGCCACCGAGCGGTCTGGAACCCATCTTCATTGCACTGACCTTCTTTGCTGTAGGGATC
AGCTTTGGCTATAACTACGGCTACGGCATCAATCCGGCCAGGGATTTTGGCCCCAGACTC
TTCACAGCTATGGCTGGCTATGGGAAGGAGGTTTGG---GTG---ATCAATGGCGGCCAC
TGGTGGCTGATTCCTACATTGGTTCCATTTATTGGTGCACCAATCGGTGGCTGGATCTAT
TACTTACTGGTCGGTCTCCACTGCGAATCACCGTACGGCCAT
>sssStarfish_Glp2
CTTGACTACATCCGATTAAAGCACGAGCTCCCACGCCAGTTTTGCGCCGAGCTAATCGGA
ACGTTCATGCTGGTGCTAATCGGTGATGGCGCTATTGCTCAATGGCAGTTCTTTGGCGCC
AAC---AACGGTGCCGGTTTTCTAAACGTCAACATTGGATATGCCTTCGGCCTCATGCTG
GGAATATATTTCACGGCTTCGGTGTCGGGTGGTCATTTAAACCCCGCCGTTTCCTTGGCC
TTCTGCTCCTTGGGCAAGATGAGACTGATAGCCTTGCCAATCTACATAATAGCTCAGTTT
CTGGGAGCTTTTATTGCCGCTGCTATGGTGTATGCTGTATATTATGATGCCATTGACTTG
CGTGAT---CCAAACCGCACAGTGACCGAGCCT---TACAGTACGGCTGGAATCTTCGCC
ACT------------TATCCCAAACCAGAACTGGGAGCAGGAGTTGGTTTTGTTGATCAG
TTGCTTGGTACAGCTCTCTTGATGGCCGGCATTCTAGCCATGACCGACAAGAAGAACAGC
AAGCCACCTAGCGGTCTGGAACCCATCTTCATTGCATTGACCTTCTTTGCTATAGGAATC
AGCTTTGGCTATAACTACGGCTACGGCATCAATCCTGCTAGGGATTTTGGCCCCAGACTC
TTCACAGCGATGGCTGGCTATGGGAAGGAGGTTTGG---GTG---ATCAATGGCATCCAG
TGGTGGTTTATTCCAACATTGGTTCCATTTATCGGCGCACCCATTGGCGGATGGATTTAT
TACCTACTAGTCGGACTCCACTGTGAATCACCGTACGGCAGA
>eStarfish_Glp2
TTGGATCATGTCCGACTTAGAAGTCTTCTCGGCCGGCAATTTTTGGCCGAACTTATAGGG
ACGTTCATGCTGGTGATGATCGGTGATGGTGCCATAGCACAACGACAGTTCTTTGGTAAC
GAT---ACGGGGGCGGGCTTCCTGAACGTTAACGTCGGCTATGCCTTCGCCCTCATGCTC
GGTGTTTACTTCACTGCCTCGGTCTCAGGAGGTCATTTAAACCCAGCCGTATCTCTAGCC
CTCTGTACCCTTGGTCAGATGAAATGGATTGCACTGCCTGTCTACGCGTTGGCCCAGCTC
ATTGGGGCCTTCTTGGGTGCCGCCACGGTGTTTGCTGTATACTACGATACCATCAACAAG
ATCGATGGAGGTAACCGAACCGCCTTTGGACCT---ACCGGAACGGCCGGTATATTTGCC
ACC------------TATCCGGCCGAAGGAGTGACGATGGGCGCAGGATTTCTAGACCAG
GTGGTAGGCACCGCCATCTTAGTGGCAGGCATCATGGCCGTTACAGACAAGAAGAACTCT
AAGCCACCGAGTGGTTTGGAGCCTCTGTTCGTGGGCTTGACATTCTTTGCCGTCGGCGTC
AGTTTCGGCTATAACTACGGCTATGGTATCAACCCGGCCCGGGACTTCGGTCCCCGTCTG

TTTACCGCCATCGCTGGGTACGGACCGGACATCTGG---GCG---CCCGGTGGTATGCAG
TGGTGGTGGATACCCATCGTGGGTCCATTAGTGGGTGGACCATTAGGTGGTTGGATCTAC
TATCTGATGGTCAGTATGCACATCGATACATCCTACAATAAG
>fStarfish_Glp2
TTGGATCATGTCCGACTTAGAAGTCTTCTCGGCCGGCAATTTTTGGCCGAACTGATAGGG
ACGTTCATGCTGGTGATGATCGGTGATGGTGCCATAGCACAACGACAGTTCTTTGGTAAC
GAT---ACGGGGGCGGGCTTCCTGAACGTTAACATCGGCTATGCCTTCGCCCTCATGCTC
GGTGTTTACTTCACTGCCTCGGTCTCAGGAGGTCATTTAAACCCAGCCGTATCTCTAGCC
CTCTGTACCCTTGGTCAGATGAAATGGATTGCACTGCCTGTCTACGCGTTGGCCCAGCTC
ACTGGGGCCTTCTTGGGTGCCGCCACGGTGTTTGCTGTATACTACGACACCATCAACAAG
ATCGATGGAGGTAACCGAACCGCCTTTGGACCT---ACCGGAACGGCCGGTATATTTGCC
ACC------------TATCCGGCCGAAGGAGTGACGATGGGCGCAGGATTTCTAGACCAG
GTGGTAGGCACCGCCATCTTAGTGGCAGGCATCATGGCCGTCACAGACAAGAGGAACTCT
AAGCCACCGAGTGGTTTGGAGCCTCTGTTCGTGGGCTTGACATTCTTTGCCGTCGGCGTC
AGTTTCGGCTATAACTACGGCTATGGTATCAACCCGGCCCGGGACTTCGGTCCCCGTCTG
TTTACCGCCATCGCTGGGTACGGACCGGACATCTGG---GCA---CCCGGTGGTATGCAG
TGGTGGTGGATACCCATCGTGGGTCCATTAGTGGGTGGACCATTAGGTGGTTGGATTTAC
TATCTGATGGTCAGTATGCACATCGATACATCCTACAATAAG
>npsStarfish_Glp2

---------------ATGATCGGTGATGGCGCCATAGCACAACGACAGTTCTTTGGTAAC
GAT---ACGGGGGCGGGCTTCCTGAACGTTAACATCGGCTATGCCTTCGCCCTCATGCTC
GGTGTTTACTTCACTGCCTCGGTCTCAGGT------------------------------

---TTCGGTCCCCGTCTG
TTTACCGCCATCGCTGGGTACGGACCGGACATCTGG---GCA---CCCGGTGGTATGCAG
TGGTGGTGGATACCCATCGTGGGTCCATTAGTGGGTGGGCCATTAGGTGGTTGGATCTAC
TATCTGATGGTCAGTATGCACATCGATACATCCTACAATAAG
>psStarfish_Glp2
TTGGATCATGTCCGAATTAGGAGTCTTCTTGGCCGGCAATTCTGCGCCGAAATCTTAGGG
ACGTTCATGCTGGTGATGATCGGTGATGGCGCTATAGCCCAGCGGCAATTCTTTGGTAAT
GAT---ACGGGAGCGGGCTTCCTGAACGTCAATATCGGCTACGCCTTCGCCCTTATGCTC
GGTGTGTTCTTCACTGCCTCGGTATCAGGAGGTCATTTGAACCCAGCCGTATCTCTGGCC
TTCTGTACCCTTGGTCAGATGAAATGGGTTGCACTGCCTGTCTACATGTTGGCCCAGCTC
ATTGGGGCCTTCTTGGGTGCCGCTACGGTGTTTGCTGTGTACTATGACACTATCAACAAG
ATCGATGGAGGTAACAGAACTGCACTGGGGCCT---ACCGGAACGGCGGGGATATTTGCC
ACC------------TATCCGGCCGAAGGAGTGACGATGGGTGAAGGATTTCTGGACCAG
GTGGTGGGCACTGCCATCTTGGTGGCAGGCATCATGGCCGTGACAGACAAGAAGAATTCT
AAGCCACCGAGTGGCTTGGAGCCTCTGTTCGTGGGCTTGACTTTCTTTGCTGTTGGCATC
AGTTTCGGTTATAACTACGGTTACGGTATCAACCCAGCTCGGGATTTCGGACCCCGCCTA
TTCACTGCCTTCGCTGGTTACGGAGCGGACATCTGG---GCG---CCCGGTGGTCTCCAG
TGGTGGTGGATACCTATAGTGGGTCCCTTCATAGGTGGACCGCTGGGAGGTTGGATCTAC
TATCTGTTGATCAGCATGCATATCGACACATCCTACACAAGA
>BroodStar_Glp2
TTGGATCATGTTCGTATCAGAAGTCTTCTTGGCCGGCAATTCTGCGCCGAAATCCTAGGG
ACGTTCATGCTGGTGATGATCGGTGATGGCGCCATAGCTCAGCGGCAATTCTTCGGTAAT
GAT---ACGGGGGCGGGCTTCCTGAACGTCAACATCGGCTACGCCTTCGCACTCATGCTT
GGTGTGTTCTTCACAGCCTCGGTGTCAGGAGGTCATTTGAACCCAGCCGTATCTTTGGCC
TTCTGTACCCTTGGGCAGATGAAATGGGTTGCACTGCCAGTCTACATGTTGGCCCAGCTC
ATCGGGGCCTTCTTGGGTGCCGCCACGGTGTTTGCTGTGTACTAT---------------

GTGGTGGGCACCGCCATTCTAGTGGCAGGCATCATGGCCGTGACAGACAAGAAGAATTCT
AAGCCACCGAGTGGATTGGAGCCTCTGTTCGTGGGTTTGACGTTCTTTGCTGTCGGCATC
AGTTTCGGCTATAACTACGGTTACGGTATCAACCCCGCCAGGGATTTCGGACCCCGCCTG
TTCACTGCCGTCGCTGGGTACGGACCGGAAGTCTGG---GCA---CCCGGTGGTCTCCAG

TGGTGGTGGATACCCATCGTGGGTCCCTTCATAGGTGGACCGTTGGGAGGATGGATCTAC
TACCTGTTGATCAGCATGCATATTGATACATCTTACAAAGAG
>sBrittleStar_Glp2a
CTACAACGGATTAAGATCAGAAGTCTTCTTGGACGCCAGTTTCTTGCTGAGTTATTGGGA
ACATTTATCCTGGTGATTTTTGGCGACGGAGCTGTGGCACAATCCGTGCTAAGCAGTTCT
AAC---AATGGA---ACATTCCTCTCCATCAACTGGGGCTTCATGGTTGGACTTATGCTT
GGTGTCTATTTTTCAGCTGGAATATCAGGTGGTCATCTGAATCCAGCTGTCTCTCTAGCA
ATGGCGTCTCTGGGTAAAATGCCATGGATTGCTCTACCAGTTTATGCATTGGCTCAGTTT
CTTGGTGCATTTTTGGCATCTGTTATGATATATGCTGTCTATATTGATGCAATAAATGAC
TTTGACGGGGGCACCAGAACAGTAACAGGAACC---AATGCCACTGCCGGAATATGGGCT
TCC------------TATCCAGCCCCATTTGTTACACTTCAAGAAGGATTTCTAGATCAG
GTTTAGGGACAGCTCTTCTTGTTGCATGTATTTGTGCAATCACTGACAAACGTAACTCT
AAGCCAGTCAATGGAATGGAACCACTGATCGTGGGCTTCACTCTCTTCGGAATCGGTGTA
GCATATGGGATGAACTGTGGATTTCCTGTCAACCCGGCAAGGGATTTGGCTCCGCGTCTA
TTTACTTATATTGCACAATATGGGTCTGATGTATGG---TGG---TCATATGACAAGCAA
TGGTGGTGGGTTCCAGTTTTGGGCCCTTTCTTGGGCGCTTTAATCGGAGGCTGGACATAC
TTTCTCATGATTGAGTTACATCATCCACGTGATACTAATTAC
>smBrittleStar_Glp2a
---------------------AGTCTACTTGGTAGACAGTTTCTTGCCGAGATGTTGGGA
ACTTTTATCTTAGTAATTTTTGGTGATGGTGCTGTCGCACAGTTTATAGTGAGTGGTTCG
CAT---CCGGCG---GCATTTTTGTCCGTCAACTGGGGTTTTGCAGCTGGTCTTTTACTT
GGAGTATACTTTTCTGCAGGAGTATCAGGTGGACATCTAAATCCCGCAGTGTCTTTGGCC
ATGGCTTCATTAGGTAAAATGCCATGGATTGCTCTCCCCGTGTATTGTCTTGCCCAGTTT
CTTGGTGCTTTCTTTGCATCGGTTATGATATATGCCGTATACATTGATGCAATAAACCAT
CTTGACGGAGGCAAGAGACAAGTAACAGGGGAA---TTTGCAACTGCTGGTATATGGGCC
TCA------------TATCCACCGGATTACGTCACAATTCAGGAAGGCTTTCTTGATCAA
GTACTTGGCACAGCTCTCCTAGTTGCTTGCATATGTGCAATTACGGATAAAAGAAACTCC
AAACCAGTGAACGGAATGGAACCATTAATCGTTGGCTTGGCTCTACTTGCTATTGGAGTG
GCATATGGATTCAACTGTGGTTTCCCAGTCAATCCAGCAAGGGATTTGGCACCGCGGCTT
TTCACCTACATCGCACAATATGGCTCCGAAGTTTGG---GCA---CCAAACGGCAGACAG
TGGTGGTGGGCTCCGATATTCGGACCTTTACTTGGCGGTTTAATAGGAGGCTGGACATAC
TATCTGATGGTTGAGTTACATCATCCGCGTGAATTAACCTAC
>sOphiocoma_Glp2a
CTGCAACGCCTCCGGATACGGAGTCTACTTGGAAGGCAATTTCTTGCCGAGATGTTGGGA

------------------GAACCTTTGATAGTTGGTTTTGCTCTACTCGCCATCGGCGTA
GCATTCGGGTTTAACTGTGGTTTCCCTGTAAATCCAGCGAGGGATTTAGCACCGCGTCTT
TTCACATACATTGCGCAATATGGCTCTGATGTCTGG---GTA---CCCGGCGGCAAGCAA
TGGTGGTGGGTACCAATCCTTGGTCCCTTTTTAGGAGCTTTAATAGGAGGTTGGTGTTAC
TATCTAATGATAGAGCTGCATCATCCAAGAGATTCAAGTTAC
>sBrittleStar_Glp2b
TTACAGTCGATTCGGATCCGTAGTCTGCTGGGTAGACAGTTTCTCGCAGAATTCTTCGGT
ACATTTATACTAGTGATATTTGGTAATGGATCTGTCGCTCAAACGGTGCTAAGTAAGTCG
GCA---AATGGC---ACGTTCCTGTCTATCGATTGGGGATTCGGAATTGGATTACTTCTG
GGGGTGTATTTCTCAGCTGGCGTATCAGGTGGTCATTTGAATCCAGCCGTTTCATTGGCC
TTTGCAACGCTTGGTAAAATGCCATGGATTGCCTTACCCGTGTACGCCGCTGCACAATTC
CTTGGTGCCTTTTTTGCTTCTGTTATAATCTATGCTGTTTATATTGATGCTATCAATGCA
TACGATGGTGGTATACGAGCAGTGACTGGACCA---AATGGTACGGCTGGAATCTGGGGT
ACA------------TACCCGCAAGAGTTTCTCAGCTTGCAGGAAGGCTTCTTTGACCAG
CTTGTTGGGGCTGCTCTACTCATGGCCTGTATATTGGCTGTTACGGACAAGAAAAATTCG
AAGCCAGTAGGGGGCATGGAACCATTGATCGTGGGTCTTATCCTGTTTGGAATTGGCTTA
GCATATGGGTTCAACTGTGGGTTTGCAGTGAACCCTGCCAGAGACTTTGCACCACGTTTG
TTTACTTACATTGCACAGTATGGATCAGAAGTTTGG---ACG---CCTTACGGAAGGCAT
TGGTGGTGGGTACCAATCCTAGGACCATTCGTTGGGGCTGTAATGGGAGGATGGTTCTAT

TACCTGTTGATTGAATTGCATCATCCAGATGAAGAGGGGGAC
>smBrittleStar_Glp2b
CTTCAACGTCTTAGAATTCGAAGCCTTCTTGGAAGACAGTTCTTAGCAGAGTTCTTTGGT
ACTTTTATACTTGTCATTTTCGGAAATGGCTCAGTGGCTCAATCAGTGCTCAGTAACGGC
AAC---AGAGGT---GAATTTTTGTCTATCGATTGGGGATTTGGAGTAGGATTACTTCTT
GGGGTCTACTTCTCGGCTGGAATTTCAGGTGGCCATCTTAATCCGGCAGTATCATTGGCA
ATGGCGACATTGGGTAAAATGCCATGGAGGGCGTTGCCTGTTTATGCTGCTGCCCAGTTT
TGTGGCGCTTTCTTTGCTTCAGTAATTATATACGGAGTTTATGCTGATGCTATCAACGCT
TTTGATGGAGGCGAGAGAATGGTATCGGGACCA---ACTGGTACTGCTGGAATCTGGGGC
ACA------------TACCCACAGGAGTTCCTCACACTTCAAGAAGGTTTCCTCGATCAG
ATCGTTGGTACCGCACTTCTGGTAGCATGTATTCTGGCCATTACCGACAAGAAGAACTGT
AAGCCAGTGGGAGGCATGGAACCATTTATTGTTGGTCTGGCCTTGTTTGGAATCGGTTTG
GCTTATGGATTCAACTGTGGTTTTGCTGTCAATCCTGCAAGAGATTTCGCACCACGTCTG
TTTACCGCCATCGCACAGTATGGCTCAGACGTCTGG---ACA---CCCAACGGAAAACAC
TGGTGGTGGGTTCCGATATTGGGCCCATTTATTGGGGGTCTAATTGGAGGATGGTTCTAC
TACTTATTGATTGAATTACATCATCCACCAGAAGACGAGGAT
>sOphiocoma_Glp2b

-------------------------------------TTGAATCCGGCTGTGTCTCTAGCT
ATGGCTACCCTGGGTAAAATGCCTTGGATAGCACTACCAGTATACGCTCTTGCACAATTT
GCGGGTGCCTTCTTCGCTTCTATTATAATATATGGGGTATATATTGAGGCCCTTAACAAA
TTTGACGGCGGGGTGAGGCAAGTAACCGGACCA---AATGGTACCGCTGGAATCTGGGGC
ACC------------ATTCCACAGGAGTACCTAAGTCTCCAAGAAGGATTCTTTGACCAG
ATCGTAGGCACCGCACTTTTGGTGGCATGTATATTGGCCATTACGGACAAGAAGAACTCG
AAACCCGTGGGTGCAATGGAACCTTTCATAGTCGGACTAGTACTGTTCGGAATCGGTTTG
GCCTATGGATATAACTGTGGATTTGCCGTCAATCCTGCTAGGGATTTAGCTCCACGTCTT
TTCACATATATTGCACAATACGGCTCG---GTTTGG---ACG---CCTTACGGAAAGCAC
TGGTGGTGGGTTCCAGTGCTCGGTCCATTCATTGGTGCTTTGATTGGAGGATGGTTCTAC
TACCTCTTAATTGAACTACACCATCCTGATGAGGAGGCGGAA
>sBrittleStar_Glp2c
GTTCAGCCTTTGAGAGTCAAGAGTAAGTTAGGGAGGAGTTGTTTAGCAGAATTCATTGGC
ATGTTTGTCTTTGTGATGTTTCATAATGGCTCCATTGCTCAATCCATATTAAGTGGTAGT
---------------TCATTTCTCTCTATTAATTGGGGTGGTGGAGTTGGCCTTTTCCTC
GGAGTCTACTTTTCTGCAGGAATATCAGGTGGTCATCTTAATCCAGCCGTGTCATTAGGA
TTTACTCTACTGGGAAAACTCAAACTATCCTGGCTTTTGTTTTACACAATTTTTCAACTC
CTTGGCGCCTTTTGTGCCTCGGTATTTATCTACGGTGTTTATGTCGAAGCCATCAACAAT
TTCGACCAGGGTAATAGAACAGTGCTTCCTCGTGGCAATGCCACGGCAGGAATATTTGCA
ACA------------TACCCACAAGAGTATCTGTCTTTGCAGGAAGGGTTATTTGATCAA
ATAATAGGTACAGCCTTGTTGATGGGTTGCATTGCAGCTGTTACCGATGAGCGCAATTCT
AAGCCAGTCAAAGGCATGGAACCTTACATCATTGGACTCATCCTATTTGGTGTTGGTTTA
AGCTATGGTTACAACTGTGGCTACTCATTGAATCCAGCCAAAGATCTGGGGCCACGACTC
TTTTCTTACTTCATA---TATGGACCCGAGGTTTGG---ACG---CCATATGGGATCCAG
TGGTGGTGGGTTCCAATCGTTGGGCCTCTAATTGGTGGTTCGATTGGTTCTACGATATAT
TTCTTCTTTATACAATTACATCATCCAAAAGAGAGTTACACA
>smBrittleStar_Glp2c
TTGCAACCCCTCCGAATTAAGAGTAAATTAGGCAGAGCATGTCTGGCAGAGTTCATCGGT
ATCTTTATCCTTGTGATCTTCCATCATGGAGCAATTGCGCAAGTGATCTTAAGTAGGGGA
GGC---AGTGGC---ACATTTCTCTCCATCAACTGGGGCGGTGGAGTTGGTCTCTTTCTC
GGCGTTTATTACTCGGCTGGAATATCAGGTGGACACCTCAATCCTGCCGTATCATTGGGT
TTTACTCTTCTGGGCAAACTCAGATGGGCATGGTTACCATGTTACATGATGGCCCAGTTT
TTTGGTGCTTTCTGTGCTTCCATCGTTTTATATGGCGTATATATAGATGGCATTAACAAT
ATTGATGGTGGTACGAGACAAGTAACTGGTCCA---AATGCAACAGCCGGGATATTTGGA
ACC------------TATCCACAAGAATTCTTATCTCTTGGGGAAGGTGTATTTGACCAA
ATCGTAGGCACAGCGTTACTAATGGGCACTATTGTAGCAGTCACAGATGAACGTAATCAC
AAACCAGTAAAAGGGATGGAACCATATATTATTGGTATGATATTGTTTGGAGTTGGTTTA
GCCTTTGGATTTAATTGTGGTTATTCATTGAATCCAGCTAAAGATCTTGGACCACGCCTG
TTTGCTTACATCGCACAATATGGGCCAAAAGTATGG---GCA---CCCTATGGTAAGCAG
TGGTGGTGGGTGCCGATCGTGGGGCCACTTATCGGTGGTCCAGTTGGAGCCTGGACATAC
TACTTAATTATAGGACTACATCATCCACCTGTGTATAAAGCG

>sOphiocoma_Glp2c
TTGCAACCTCTCCGAATCCGGAGTAAATTGGGCAGAAGCTTTCTAGCGGAATTCATTGGT
ACTTTCATTTTTGTGATGTTTCATCACGGATCTATTGCTCAATCTATTCTAAGTAGATCA
GGC---AACGGT---ACTTTCTTGTCCATCAACTGGGGCGGCGGAGTAGGACTTTTTCTT
GGTGTCTACTACTCAGCGGGAATATCTGGTGGCCATCTAAATCCTGCCGTATCATTGGGC
TTTACTCTTCTTGGCAAACTGCGATGGGCGTGGTTACCAATTTACATTTGTGCACAGTTT
CTAGGTGCATTTTGTGCCTCTATTATACTATACGGTGTTTACATTGATGGTATCAATCAC
TTTGACGGCGGTAACAGACAGGTTATTGGCGAA---AATGCAACAGCTGGAATATTTGGA
ACC------------TATCCACAGGAATGGTTGACACTTCAAGAAGGGCTTTTTGATCAA
ATTGTAGGTACGGCTTTGCTAATGGGGACGATTGCAGCTGTGACGGATGAACGCAATTTG
AAGCCAGTGAAAGGAATGGAACCGTACATCATTGGTCTGATTCTATTTGGAGTTGGTTTA
AGTTTTGGCTTTAACTGCGGCTATTCCTTGAATCCTGCTAAGGATCTAGCACCACGCCTG
TTTTCTTACATTGCACAATATGGTCCAAAGGTTTGG---GCG---CCTTATGGTAAACAA
TGGTGGTGGGTGCCAGTAGTAGGTCCACTTATCGGCGGTTCAATTGGAGCATGGTTGTAT
TTCCTGATAATACAGTTACATCATCCGACCCAATCCGAAGAA

File S5: Alignment for Fig. S4
>Human_Aqp7p1
ATGGTTCAAGCATCTGGGCACAGG---CGGTCCACCCGTGGCTCCAAAATGGTCTCCTGG
TCCGTGATAGCAAAGATCCAGGAAATATGGTGCGAGGAAGATGAGAGGAAGATGGCGCGA
GAGTTCCTGGCCGAGTTCATGAGCACATATGTCATGATGGTATTCGGCCTTGGTTCCGTG
GCCCATATGCTTCTAAAT---AAAACATTTGGGAGCTACCTTGGTGTCAACTTGGGTTTT
GGCTTCGGAGTCACCATGGGAGTGCACATGGCAGGCCGCACCTCTGGAGCCCACATGAAC
GCAGCTGTGAGCTTCACTAACTGTGCACTGGGCCGTGTGCCCTGGAGGAAGTTTCCAGTC
TATGTGCTGGGGCAGTTCCTGGGCTCCTTCCTGGCGGCTGCCACCATCTAC---AGTCTC
TTCTACACGGCCATTCTCCACTTTTCGGGTGGAGAGCTGATGGTGACCGGTCCCGTTGCT
ACAGCTGGCATTTTTGCCACCTACCTTCCTGATCACATGACATTGTGGCGGGGCTTCCTG
AATGAGGAGTGGCTGACCGGGATGCTCCAGCTGTGTCTCTTCGCCATCGTGGACCAGGAG
AACAACCCAGCACTGCCAGGAACACACGCACTGGTGATAGGCATCCTCGTGGTCATCATC
AGGGTGTACCATGGCATGAACACAGGATATGCCATCAATCCGTCCCGGGACCTGCCCCCC
---CCCATCTTCACCTTCATTGCTGGTTGGGGCAAACTGGTCTTCAGCGATGGGGAGAAC
TTGTGGTGGGTGCCAGTGGTGGCACCACTTCTGGGTGCCTCTCTAGGTGGCATCATCTAC
CTGGTCTTCATTGGCTCCACCATCCCACGGGAGCCCCTGAAATTGGAGGACTCTGTGGCA
TATGAAGACCACGGGATAACCGTATTGCCCAAGATGGGATCTCATGAACCCATGATCTCT
CCCCTTACCCTCATCTCCGTGAGCCCTGCCAACAGATCTTCAGTCCACCCTGCCCCACCC
TTACATGAATCCATGGCCCTAGAGCACTTC
>Human_AQP7p2
ATGGTTCAAGCATCTGGGCACAGG---CGGTCCACCCGTGGCTCCAAAATGGTCTCCTGG
TCCGTGATAGCAAAGATCCAGGAAATA---TGCGAGGAAGATGAGAGGAAGATGGCGCGA
GAGTTCCTGGCCGAGTTCATGAGCACATATGTCATGATGGTATTCGGCCTTGGTTCCGTG
GCCCATATGCTTCTAAAT---AAAACATTTGGGAGCTACCTTGGTGTCAACTTGGGTTTT
GGCTTCGGAGTCACCATGGGAGTGCACATGGCAGGCCGCACCTCTGGAGCCCACATGAAC
GCAGCTGTGAGCCTCACTAACTGTGCACTGGGCCGTGTGCCCTGGAGGAAGTTTCCAGTC
TATGTGCTGGGGCAGTTCCTGGGCTCCTTCCTGGCAGCTGCCACCATCTAC---AGTCTC
TTCTACACGGCCATTCTCCACTTTTCGGGTGGAGAGCTGATGGTGACCGGTCCCGTTGCT
ACAGCTGGCATTTTTGCCAACTACCTTCCTGATCACATGACATTGTGGCGGGGCTTCCTG
AATGAGGAGTGGCTGACCGGGATGCTCCAGCTGTGTCTCTTCGCCATCGTGGACCAGGAG
AACAACCCAGCACTGCCAGGAACACACGCACTGGTGATAGGCATCCTCGTGGTCATCATC
AGGGTGTACCATGGCATGAACACAGGATATGCCATCAATCCGTCCCGGGACCTGCCCCCC
---CCG--

>Human_Aqp7p3
ATGGTTCAAGCATCTGGGCACAGG---CGGTCCACCCGTGGCTCCAAAATGGTCTCCTGG
TCCGTGATAGCAAAGATCCAGGAAATA---TGTGAG---------AGGAAGATGGCGCGA
GAGTTCCTGGCCGAGTTCATGAGCACATATGTCATGATGGTATTCGGCCTTGGTTCCGTG
GCCCATATGCTTCTAAAT---AAAACATTTGGGAGCTACCTTGGTGTCAACTTGGGTTTT
GGCTTCGGAGTCACCATGGGAGTGCACATGGCAGGCCGCACCTCTGGAGCCCACATGAAC

GCAGCTGTGAGCCTCACTAACTGTGCACTGGGCCGTGTGCCCTGGAGGAAGTTTCCAGTC
TATGTGCTGGGGCAGTTCCTGGGCTCCTTCCTGGCAGCTGCCACCATCTAC---AGTCTC
TTCTACACGGCCATTCTCCACTTTTCGGGTGGAGAGCTGATGGTGACCGGTCCCGTTGCT
ACAGTTGGCATTTTTGCCACCTACCTTCCTGATCACATGACATTGTGGCGGGGCTTCCTG
AATGAGGAGTGGCTGACCGGGATGCTCCAGCTGTGTCTCTTCGCCATCGTGGACCAGGAG
AACAACCCAGCACTGCCAGGAACACACGCACTGGTGATAGGCATCCTCGTGGTCATCATC
AGGGTGTACCATGGCATGAACACAGGATATGCCATCAATCCGTCCCGGGACCTGCCCCCC
---CGCATCTTCACCTTCATTGCTGGTTGGGGCAAACTGGTCTTCAGCGATGGGGAGAAC
TTGTGGTGGGTGCCAGTGGTGGCACCACTTCTGGGTGCCTCTCTAGGTGGCATCATCTAC
CTGGTCTTCATTGGCTCCACCATCCCACGGGAGCCCCTGAAATTGGAGGACTCTGTGGCA
TATGAAGACCACGGGATAACCGTATTGCCCAAGATGGGATCTCATGAACCCATGATCTCT
CCCCTTACCCTCATCTCCGTGAGCCCTGCCAACAGATCTTCAGTCCACCCTGCCCCACCC
TTACATGAATCCATGGCCCTAGAGCACTTC
>Human_Aqp7p4

------------------------------------ATGGTATTCGGCCTTGGTTCCGTG
GCCCATATGCTTCTAAAT---AAAACATTTGGGAGCTACCTTGGTGTCAACTTGGGTTTT
GGCTTCGGAGTCACCATGGGAGTGCACATGGCAGGCCGCACCTCTGGAGCCCACATGAAC
GCAGCTGTGAGCCTCACTAACTGTGCACTGGGCCGTGTGCCCTGGAGGAAGTTTCCAGTA
TATGTGCTGGGGCAGTTCCTGGGCTCCTTCCTGGCAGCTGCCACCATCTAC---AGTCTC
TTCTACACGGCCATTCTCCACTTTTCGGGTGGAGAGCTGATGGTGACCGGTCCCGTTGCT
ACAGCTGGCATTTTTGCCACCTACCTTCCTGATCACATGACATTGTGGCGGGGCTTCCTG
AATGAGGAGTGGCTGACCGGGATGCTCCAGCTGTGTCTCTTCGCCATCGTGGACCAGGAG
AACAACCCAGCACTGCCAGGAACACACGCACTGGTGATAGGCATCCTCGTGGTCATCATC
AGGGTGTACCATGGCATGAACACAGGATATGCCATCAATCCGTCCCGGGACCTGCCCCCC
---CGCATCTTCACCTTCATTGCTGGTTGGGGCAAACTGGTCTTCAGCGATGGGGAGAAC
TTGTGGTGGGTGCCAGTGGTGGCACCACTTCTGGGTGCCTCTCTAGGTGGCATCATCTAC
CTGGTCTTCATTGGCTCCACCATCCCACGGGAGCCCCTGAAATTGGAGGACTCTGTGGCA
TATGAAGACCACGGGATAACCGTATTGCCCAAGATGGGATCTCATGAACCCATGATCTCT
CCCCTTACCCTCATCTCCGTGAGCCCTGCCAACAGATCTTCAGTCCACCCTGCCCCACCC
TTACATGAATCCATGGCCCTAGAGCACTTC
>Human_AQP7p5
ATGGTTCAAGCATCTGGGCACAGG------TCCACCCGTGGCTCCAAAATGGTCTCCTGG
TCCGTGATAGCAAAGATCCAGGAAATA---TGCGAGGAAGATGAGAGGAAGATGGCGCGA
GAGTTCGTGGCCAAGTTCATGAGCACATATGTCATGATGGTATTCGGCCTTGGTTCCGTG
GCCCATATGCTTCTAAAT---AAAACATTTGGGAGCTACCTTGGTGTCAACTTGGGTTTT
GGCTTCGGAGTCACCATGGGAGTGCACATGGCAGGCCGCACCTCTGGAGCCCACATGAAC
GCAGCTGTGAGCCTCACTAACTGTGCACTGGGCCGTGTGCCCTGGAGGAAGTTTCCAGTA
TATGTGCTGGGGCAGTTCCTGGGCTCCTTCCTGGCAGCTGCCACCATCTAC---AGTCTC
TTCTACACGGCCATTCTCCACTTTTCGGGTGGAGAGCTGATGGTGACCGGTCCCGTTGCT
ACAGCTGGCATTTTTGCCACCTACCTTCCTGATCACATGACATTGTGGCGGGGCTTCCTG
AATGAGGAGTGGCTGACCGGGATGCTCCAGCTGTGTCTCTTCGCCATCACGGACCAGGAG
AACAACCCAGCACTGCCAGGAACACACGCACTGGTGATAGGCATCCTCGTGGTCATCATC
AGGGTGTACCATGGCATGAACACAGGATATGCCATCAATCCGTCCCGGGACCTGCCCCCC
---CGCATCTTCACCTTCATTGCTGGTTGGGGCAAACTGGTCTTCAGCGATGGGGAGAAC
TTGTGGTGGGTGCCAGTGGTGGCACCACTTCTGGGTGCCTCTCTAGGTGGCATCATCTAC
CTGGTCTTCATTGGCTCCACCATCCCACGGGAGCCCCTGAAATTGGAGGACTCTGTGGCA
TATGAAGACCACGGGATAACCGTATTGCCCAAGATGGGATCTCATGAACCCATGATCTCT
CCCCTTACCCTCATCTCCGTGAGCCCTGCCAACAGATCTTCAGTCCACCCTGCCCCACCC
TTACATGAATCCATGGCCCTAGAGCACTTC
>Chimpanzee_AQP7L2
------ATGGGCTCGGGCCACTGTATCAGGTCCACCCGTGGCTCCAAAATGGTCTCCTGG
TCCATGATAGCAAAGATCCAGGAAATATGGTGCGAGGAAGATGAGAGGAAGATAGCGCGA
GAGTTCCTGGCCGAGTTCATGAGCACATATGTCATGATGGTATTCGGCCTTGGTTCCGTG
GCCCATATGCTTCTAAAT---AAAACATTTGGGAGCTACCTTGGTGTCAACTTGGGTTTC
GGCTTCGGAGTCACCATGGGAGTGCACGTGGCAGGCTGCATCTCTGGAGCCCACATGAAC
GCAGCTGTGAGCTTCACTAACTGTGCACTGGGCCGTGTGCCCTGGAGGAAGTTTCCAGTC
TATGTGCTGGGGCAGTTCCTGGGCTCCTTCCTGGCGGCTGCCACCATCTAC---AGTCTC
TTCTACAGTGCCATTCTCCACTTTTCGGGTGGAGAGCTGATGGTGACCGGTCCCATTGCT
ACAGCTGGCATTTTTGCCACCTACCTTCCTGATCACATGACATTGTGGCGGGGCTTCCTG

AATGAGGAGTGGCTGACCGGGATGCTCCAGCTGTGTCTCTTTGCCATCACGGACCAGGAG
AACAACCCAGCACTGCCAGGAACACACACGCTGGTGATAGGCATCCTCGTGGTCATTATC
AGGGTGTCCCATGGCATGAACACAGGATATGCCATCAATCCGTCCTGGGACCTGCCCCCC
CCCCGCATCTTCACCTTCATTGCTGGTTGGGGCAAACAGGTCTTCAGCGATGGGGAGAAC
TTGTGGTGGGTGCCAGTGGTGGCACCACTTCTGGGTGCCTCTCTAGGTGGCATCATCTAC
CTGGTCTTCATTGGCTCCACCATCCCACGGGAGCCCCTGAAATTGGAGGACTCTGTGGCA
TATGAAGACCACGGGATAACCGTATTGCCCAAGATGGGATCTCATGAACCCATGATCTCT
CCCCTTACCCTCATCTCCGTGAGCCCTGCCAACGGATCTTCAGTCCACCCTGCCCCACCC
TTACATGAATCCATGGCCCTAGAGCACTTC
>Bonoobo_AQP7L2

--GTATTCGGCCTTGGTTCCGTG
GCCCATATGCTTCTAAAT---AAAACATTTGGGAGCTACCTTGGTGTCAACTTGGGTTTC
GGCTTCGGAGTCACCATGGGAGTGCACGTGGCAGGTTGCATCTCTGGAGCCCACATGAAT
GCAGCTGTGAGCTTCACTAACTGTGCACTGGGCCGTGTGCCCTGGAGGAAGTTTCCAGTC
TATGTGCTGGGGCAGTTCCTGGGCTCCTTCCTGGCGGCTGCCACCATCTAC---AGTCTC
TTCTACAGTGCCATTCTCCACTTTTCGGGTGGAGAGCTGATGGTGACCGGTCCCATTGCT
ACAGCTGGCATTTTTGCCACCTACCTTCCTGATCACATGACATTGTGGCAGGGCTTCCTG
AATGAGGAGTGGCTGACCGGGATGCTCCAGCTGTGTCTCTTTGCCATCACGGACCAGGAG
AACAACCCAGCACTGCCAGGAACACACACGCTGGTGATAGGCATCCTCGTGGTCATCATC
AGGGTGTCCCATGGCATGAACACAGGATATGCCATCAATCCGTCCTGGGACCTGCCCCCC
CCCCGCATCTTCACCTTCATTGCTGGTTGGGGCAAACAGGTCTTCAGCGATGGGGAGAAC
TTGTGGTGGGTGCCAGTGGTGGCACCACTTCTGGGTGCCTCTCTAGGTGGCATCATCTAC
CTGGTCTTCATTGGCTCCACCATCCCACGGGAGCCCCTGAAATTGGAGGACTCTGTGGCA
TATGAAGACCACGGGATAACCGTATTGCCCAAGATGGGATCTCATGAACCCATGATCTCT
CCCCTTACCCTCATCTCCGTGAGCCCTGCCAACAGATCTTCAGTCCACCCCGCCCCACCC
TTACATGAATCCATGGCCCTAGAGCACTTC
>Human_AQP7L1
ATGGTTCAAGCATCTGGGCACAGG---CGGTCCACCCGTGGCTCCAAAATGGTCTCCTGG
TCCGTGATAGCAAAGATCCAGGAAATATGGTGCGAGGAAGATGAGAGGAAGATGGTGCGA
GAGTTCTTGGCCGAGTTCATGAGCACATATGTCATGATGGTATTCGGCCTTGGTTCTGTG
GCCCATATGGTTCTAAAT---AAAACATATGGGAGCTACCTTGGTGTCAACTTGGGTTTT
GGCTTCGGGGTCACCATGGGAGTCCACGTGGCAGGCCGCATCTCTGGAGCCCACATGAAT
GCAGCTGTGACCTTCACTAACTGTGCGCTGGGCCGCGTGCCCTGGAGGAAGTTTCCAGTC
CATGTGCTGGGGCAGTTCCTGGGCTCCTTCCTGGCAGCTGCCACCATCTACATCTGTGTC
TCCGCAGCGGCCATTCTCCACTTTTCGGGTGGAGAGCTGATGGTGACCGGTCCCTTTGCT
ACAGCTGGCATTTTTGCCACCTACCTTCCTGATCACATGACATTGTGGCGGGGCTTCCTG
AATGAGGAGTGGCTGACCAGGATGCTCCAGCTGTGTCTCTTCACCATCACGGACCAGGAG
AACAACCCAGCACTGCCAGGAACACACGCGCTGGTGATAAGCATCCTCGTGGTCATCATC
AGGGTGTCCCATGGCATAAACACAGGATATGCCATCAATCCATCCCGGGACCCGCCCCCC
---AGCATCTTCACCTTCATTGCTGGCTGGGGCAAACAGGTCTTCAGCGATGGGGAGAAC
TGGTGGTGGGTGCCAGTGGTGGCACCACTTCTGGGTGCCTCTCTAGGTGGCATCATCTAC
CTGGTCTTCATTGGCTCCACCATCCCACGGGAGCCCCTGAAATTGGAGGACTCTGTGGCG
TATGAAGACCACGGGATAACCGTATTGCCCAAGATGGGATCTCATGAACCCATGATCTCT
CCCCTCACCCTCATCTCCGTGAGCCTTGCCAACAGATCTTCAGTCCACTCTGCCCCACCC
TTACATGAATCCATGGCCCTAGAGCACTTC
>Chimpanzee_AQP7L1
---------------------------AGGTCCACCCGTGGCTCCAAAATGGTCTCCTCA
TCCGTGATAGCAAAGATCCAGGAAATATGGTGCGAGGAAGATGAGAGGAAGATGGTGCGA
GAGTTCCTGGCCGAGTTCATGAGCACATATGTCATGATGATATTCGGCCTTGGTTCTGTG
GCCCATATGGTTCTAAAT---AAAACATATGGGAGCTACCTTGGTGTCAACTTGGGTTTT
GGCTTCGGAGTCACCATGGGAGTCCACGTGGCAGGCCGCATCTCTGGAGCCCACATGAAT
GCAGCTGTGACCTTCACTAACTGTGCACTGGGCCGCGTGCCC---AGGAAGTTTCCAGTC
TATGTGCTGGGGCAGTTCCTGGGCTCCTTCCTGGTGGCTGCCACCATCTACGTCTGTGTC
TCCGCAGCGGCCATTCTCCACTTTTTGGGTGGAGAGCTGATGGTGACCGGTCCCTTTGCT
ACAGCTGGCATTTTTGCCACCTACCTTCCTGATCACATGACATTGTGGCGGGGCTTCCTG
AATGAGGAGTGGCTGACCAGGGTGCTCCAGCTGTGTCTCTTCACCATCACGGACCAGGAG
AACAACCCAGCACTGCCAGGAACACACGCGCTGGTGATAAGCATCCTCGTGGTCATCATC
GGGGTGTCCCATGGCATAAACACAGGATATGCCATCAATCCATCCCGGGACCCACCCCCC
---AGCATCTTCACCTTCATTGCTGGTTGGGGCAAACAGGTCTTCAGCGATGGGGAGAAC

TGGTGGTGGGTGCCAGTGGTGGCACCACTTCTGGGTGCCTCTCTAGGTGGCATCATCTAC
CTGGTCTTCATTGGCTCCACCATCCCACGGGAGCCCCTGAAATTGGAGGACTCTGTGGTG
TATGAAGACCAGGGGTTAACCGTATTGCCCAAGATGGGATCTCATGAACCCATGATCTCT
CCCCTCACCCTCATCTCCGTGAGCCTTGCCAACAGATCTTCAGTCCACTCTGCCCCACCC
TTACATGAATCCATGGCCCTAGAGCACTTC
>Bonoobo_AQP7L1

---GTCTGTGTC
TCCGCAGCGGCCATTCTCCACTTTTCGGGTGGAGAGCTGATGGTGACCGGTCCCTTTGCT
ACAGCTGGCATTTTTGCCACCTACCTTCCTGATCACATGACATTGTGGCGGGGCTTCCTG
AATGAGGAGTGGCTGACCAGGATGCTCCAGCTGTGTCTCTTCACCATCACGGACCAGGAG
AACAACCCAGCACTGCCAGGAACACACGCGCTGGTGATAAGCATCCTCGTGGTCATCATC
GGGGTGTCCCATGGCATAAACACAGGATATGCCATCAATCCATCCCGGGACCCACCCCCC
---AGCATCTTCACCTTCATTGCTGGTTGGGGCAAACAGGTCTTC---------------

>Gorilla_AQP7L1
------ATGGGCTCGGGCCACTGTATCTGGTCCACCCGTGGCTCCAAAATGGTCTCCTGG
TCCGTGATAGCAAAGATCCAGGAAATATGGTGCGAGGAAGATGAGAGGAAGATGGTGCGA
GAGTTCCTGGCTGAGTTCATGAGCACATATGTCATGATGGTATTCGGCCTTGGTTCCGTG
GCCCATATGGTTCTAAAT---AAAAAATATGGGAGCTACCTTGGTGTCAACTTGGGTTTT
GGCTTTGGAGTCACCATGGGATTGCACATGGCAGGCCGCATCTCTGGAGCCCACATGAAC
ACAGCTGTGAGCTTCGCTAACTGTGCACTGGGCCACGTGCCCTGGAGGAAGTTTCCAGTC
TATGTGCTGGGGCAGTTCCTGGGCTCCTTCCTGGCGGCTGCCACC------ATCTGTGTC
TCCACAGAGGCCATTCTCCACTTTTCGGGTGGAGAGCTGATGGTGACCGGTCCCGTTGCT
ACAGCTGGCATTTTCGCCACCTACCTTCCTGATCACATGACATTGTGGCGGGGCTTCCTG
AATGAGGAGTGGCTGACCGGGATGCTCCAGCTGTGTCTCTTCGCCATCACGGACCAGGAG
AACAATGCAGCACTGCCAGGAACACAAGCACTGGTGATAGGCATCCTCGTGGTCATCATC
GGGGTGTCCCATGGCATAAACACAGGATATGCCATCAATCCGTCCTGGGACCTGCCCCCC
---AGCACCTTCACCTTCATTGCTGGTTGGGGCAAACAGGTCTTCAGCGATGCGGAGAAC
TGGTGGTGGGTGCCAGTGGTGGCACCACTTCTGGGTGCCTCTCTAGGTGGCATCATCTAC
CTGGTCTTCATTGGCTCCACCATCCCACGGGAGCCCCTGAAATTGGAGGACTCTGTGGTG
---GAAGACCACGGGATAACCGTATTGCCCAAGATGGGATCTCATGAACCCATGATCTCT
CCCCTCACCCTCATCTCCGTGAGCCTTGCCAACAGATCTTCAGTCCACTCTGCCCCACCC
TTACATGAATCCATGGCCCTAGAGCACTTC
>Human_AQP7
------ATGGGCTCGGGCCACTGTCTTAGGTCCACCCGTGGCTCCAAAATGGTCTCCTGG
TCCGTGATAGCAAAGATCCAGGAAATACTG------------CAGAGGAAGATGGTGCGA
GAGTTCCTGGCCGAGTTCATGAGCACATATGTCATGATGGTATTCGGCCTTGGTTCCGTG
GCCCATATGGTTCTAAAT---AAAAAATATGGGAGCTACCTTGGTGTCAACTTGGGTTTT
GGCTTCGGAGTCACCATGGGAGTGCACGTGGCAGGCCGCATCTCTGGAGCCCACATGAAC
GCAGCTGTGACCTTTGCTAACTGTGCGCTGGGCCGCGTGCCCTGGAGGAAGTTTCCGGTC
TATGTGCTGGGGCAGTTCCTGGGCTCCTTCCTGGCGGCTGCCACCATCTAC---AGTCTC
TTCTACACGGCCATTCTCCACTTTTCGGGTGGACAGCTGATGGTGACCGGTCCCGTCGCT
ACAGCTGGCATTTTTGCCACCTACCTTCCTGATCACATGACATTGTGGCGGGGCTTCCTG
AATGAGGCGTGGCTGACCGGGATGCTCCAGCTGTGTCTCTTCGCCATCACGGACCAGGAG
AACAACCCAGCACTGCCAGGAACAGAGGCGCTGGTGATAGGCATCCTCGTGGTCATCATC
GGGGTGTCCCTTGGCATGAACACAGGATATGCCATCAACCCGTCCCGGGACCTGCCCCCC
---CGCATCTTCACCTTCATTGCTGGTTGGGGCAAACAGGTCTTCAGCAATGGGGAGAAC
TGGTGGTGGGTGCCAGTGGTGGCACCACTTCTGGGTGCCTATCTAGGTGGCATCATCTAC
CTGGTCTTCATTGGCTCCACCATCCCACGGGAGCCCCTGAAATTGGAGGATTCTGTGGCG
TATGAAGACCACGGGATAACCGTATTGCCCAAGATGGGATCTCATGAACCCACGATCTCT
CCCCTCACCCCCGTCTCTGTGAGCCCTGCCAACAGATCTTCAGTCCACCCTGCCCCACCC

TTACATGAATCCATGGCCCTAGAGCACTTC
>Gorilla_AQP7
------------------CACTGTCTCAGGTCCACCCGTGGCTCCAAAATGGTCTCCTGG
TCCGTGATAGCAAAGATCCAGGAAATACTG------------CAGAGGAAGATGGTGCGA
GAGTTCCTGGCTGAGTTCATGAGCACATATGTCATGATGGTATTCGGCCTTGGTTCCGTG
GCCCATATGGTTCTAAAT---AAAAAATATGGGAGCTACCTTGGTGTCAACTTGGGTTTT
GGCTTCGGAGTCACCATGGGAGTGCACGTGGCAGGCCGCATCTCTGGAGCCCACATGAAT
GCAGCTGTGACCTTTGCTAACTGTGCGCTGGGCCGCGTGCCCTGGAGGAAGTTTCCGGTC
TATGTGCTGGGGCAGTTCCTGGGCTCCTTCCTGGCGGCTGCCACCATCTAC---AGTCTC
TTCTACAGTGCCATTCTCCACTTTTCGGGTGGACAGCTGACGGTGACCGGTCCCGTCGCT
ACAGCTGGCATTTTTGCCACCTACCTTCCTGATCACATGACATTGTGGCGGGGCTTCCTG
AATGAGGTCTGGCTGACCGGGATGCTCCAGCTGTGTCTCTTCGCCATCACGGACCAGGAG
AACAACCCAGCACTGCCAGGAACAGAGGCGCTGGTGATAGGCATCCTCGTGGTCATCATC
GGGGTGTCCCTTGGCATGAACACAGGATATGCCATCAACCCGTCCCGGGACCTGCCCCCT
---CGCATCTTCACCTTCATTGCTGGTTGGGGCAAACAGGTCTTCAGCAATGGGGAGAAC
TGGTGGTGGGTGCCAGTGGTGGCACCACTTCTGGGTGCCTATCTAGGTGGCATCATCTAC
CTGGTCTTCATTGGCTCCACCATCCCACGGGAGCCCCTGAAATTGGAGGACTCTGTGGCG
TATGAAGACCACGGGATAACCGTATTGCCCAAGATGGGATCTCATGAACCCACGATCTCT
CCCCTCACCCCCGTCTCTGTGAGCCCTGCCAACAGATCTTCAGTCCACCCTGCCCCACCC
TTACATGAATCCATGGCCCTAGAGCACTTC
>Chimpanzee_AQP7
------ATGGGCTCGGGCCACTGTCTCAGGTCCACCCGTGGCTCCAAAATGGTCTCCTGG
TCCGTGATAGCAAAGATCCAGGAAATACTG------------CAGAGGAAGATGGTGCGA
GAGTTCCTGGCCGAGTTCATGAGCACATATGTCATGATGGTATTCGGCCTTGGTTCCGTG
GCCCATATGGTTCTAAAT---AAAACATATGGGAGCTACCTTGGTGTCAACTTGGGTTTT
GGCTTCGGAGTCACCATGGGAGTGCACGTGGCAGGCCGCATCTCTGGAGCCCACATGAAT
GCAGCTGTGACCTTTGCTAACTGTGCGCTGGGCCGCGTGCCCTGGAGGAAGTTTCCGGTC
TATGTGCTGGGGCAGTTCCTGGGCTCCTTCCTGGCGGCTGCCACCATCTAC---AGTCTC
TTCTACAGTGCCATTCTCCACTTTTCGGGTGGAGAGCTGATGGTGACCGGTCCCGTCGCT
ACAGCTGGCATTTTTGCCACCTACCTTCCTGATCACATGACATTGTGGCGGGGCTTCCTG
AATGAGGTGTGGCTGACCGGGATGCTCCAGCTGTGTCTCTTCGCCATCACGGACCAGGAG
AACAACCCAGCACTGCCAGGAACAGAGGCGCTGGTGATAGGCATCCTCGTGGTCATCATC
GGGGTGTCCCTTGGCATGAACACAGGATATGCCATCAACCCATCCCGGGACCTGCCCCCC
---CGCATCTTCACCTTCGTTGCTGGTTGGGGCAAACAGGTCTTCAGCAATGGGGAGAAC
TGGTGGTGGGTGCCAGTGGTGGCACCACTTCTGGGTGCCTATCTAGGTGGCATCATCTAC
CTGGTCTTCATTGGCTCCACCATCCCACGGGAGCCCCTGAAATTGGAGGACTCTGTGGCA
TATGAAGACCACGGGATAACCGTATTGCCCAAGATGGGATCTCATGAACCCACGATCTCT
CCCCTCACCCCCGTCTCCGTGAGCCCTGCCAACAGATCTTCAGTCCACCCTGCCCCACCC
TTACATGAATCCATGGCCCTAGAGCACTTC
>Bonobo_AQP7
------ATGGGCTCGGGCCACTGTCTCAGGTCCACCCGTGGCTCCAAAATGGTCTCCTGG
TCCGTGATAGCAAAGATCCAGGAAATACTG------------CAGAGGAAGATGGTGCGA
GAGTTCCTGGCCGAGTTCATGAGCACATATGTCATGATGATATTCGGCCTTGGTTCTGTG
GCCCATATGGTTCTAAAT---AAAACATATGGGAGCTACCTTGGTGTCAACTTGGGTTTT
GGCTTTGGAGTCACCATGGGAGTCCACGTGGCAGGCCGCATCTCTGGAGCCCACATGAAT
GCAGCTGTGACCTTCACTAACTGTGCACTGGGCCGCGTGCCC---AGGAAGTTTCCAGTC
TATGTGCTGGGGCAGTTCCTGGGCTCCTTCCTGGTGGCTGCCACCATCTAC---AGTCTC
TTCTACAGCGCCATTCTCCACTTTTCGGGTGGAGAGCTGATGGTGACCGGTCCCGTCGCT
ACAGCTGGCATTTTTGCCACCTACCTTCCTGATCACATGACATTGTGGCGGGGCTTCCTG
AATGAGGCGTGGCTGACCGGGATGCTCCAGCTGTGTCTCTTCGCCATCACGGACCAGGAG
AACAACCCAGCACTGCCAGGAACAGAGGCGCTGGTGATAGGCATCCTCGTGGTCATCATC
GGGGTGTCCCTTGGCATGAACACAGGATATGCCATCAACCCATCCCGGGACCCTCCCCCC
---CGCATCTTCACCTTCGTTGCTGGTTGGGGCAAACAGGTCTTC------GGGGAGAAC
TGGTGGTGGGTGCCAGTGGTGGCACCA---------------CTAGGTGGCATCATCTAC
CTGGTCTTCATTGGCTCCACCATCCCACGGGAGCCCCTGAAATTGGAGGACTCTGTGGCA
TATGAAGACCACGGGATAACCGTATTGCCCAAGATGGGATCTCATGAACCCACGATCTCT
CCCCTCACCCCCGTCTCCGTGAGCCCTGCCAACAGATCTTCAGTCCACCCTGCCCCACCC
TTACATGAATCCATGGCCCTAGAGCACTTC
>Sumatran_orangutan_AQP7
------ATGGGCTCGGGCCACTGTCTCAGGTCCACCCGTGGCTCCAAAATGGTCTCCTGG
CCCGTGATAGCAAAGATCCAGCATATACTG------------CAGAGGAAGATGGTGCGA

GAGTTCCTGGCCGAGTTCATGAGCACATATGTCATGATGGTATTCGGCCTTGGTTCCGTG
GCCCATATGATTCTAAAT---AAAAACTATGGGAGCTACCTTGCTGTCAACTTGGGTTTT
GGCTTCGGAGTCACCATGGGAGTGCACGTGGCAGGCCGCATCTCTGGAGCCCACATGAAC
GCAGCTGTGACCTTCGCTAACTGTGCGCTGGGCCGCGTGCCCTGGAGGAAGTTTCCAGTC
TATGTGCTGGGGCAGTTCCTGGGCTCCTTCCTGGCGGCTGCCACCATCTAC---AGTCTC
TTCTACAGTGCCATTCTCCGCTTTTCGGGTGGACAGCTGATGGTGACCGGTCCCGTCGCT
ACAGCTGGCATTTTTGCCACCTACCTTCCTGATCACATGACATTGTGGCGGGGCTTCCTG
AATGAGGCGTGGCTGACCGGGATGCTCCAGCTGTGTCTCTTCGCCATCACGGACCAGGAG
AACAACCCAGCACTGCCAGGAACACAGGCGCTGGTGATAGGCATCCTCGTGGTCATCATC
GGGGTGTCCCTCGGCATGAACACAGGATATGCCATCAACCCGTCCCGGGACCTGCCCCCC
---CGCATCTTCACCTTCATTGCTGGTTGGGGCAAACAGGTCTTCAGCGATGGGGAGAAC
TGGTGGTGGGTGCCAGTGGTGGCACCACTTCTGGGTGCCTATCTAGGTGGCATCATCTAC
CTGGTCTTCATTGGCTCCACCATCCCACGGGAGCCCCTGAAATTGGAGAACTCTGTGGCG
TATGAAGACCACGGGATAACCGTATTGCCTAAGATGGGATCTCATGAACCCACAATCTCT
CCCCTCACCCCCGTCTCCGTGAGCCCTGCCAACAGATCTTCAGTCCGCCCTGCCCCACCC
TTACATGACTCCATGGCCCTAGAGCACTTC
>Northern_white_cheeked_gibbon_AQP7
------ATGGGCTCGGGCCACTGTCTCAGGTCCACCCGTGGCTCCAAAATGGTCTCCTGG
TCCGTGATAGCAAAGATCCAGGAAATACTG------------CAGAGGAAGATGGTGCGA
GAGTTCCTGGCCGAGTTCATGAGCACATATGTCATGATGGTATTCGGCCTTGGTTCCGTG
GCCCATATGGTTCTAAAT---AAAAAATACGGGAGCTACCTTGGTGTCAACTTGGGTTTT
GGCTTCGGAGTCACCATGGGAGTGCACGTGGCAGGCCGCATCTCTGGAGCCCACATGAAC
GCAGCTGTGACCTTCGCTAACTGTGCACTGGGCCGCGTGCCCTGGAGGAAGTTTCCGGTC
TATGTGCTGGGGCAGTTCCTGGGCTCCTTCCTGGCAGCTGCCACCATTTAC---AGTCTC
TTCTACAGTGCCATTCTCCACTTTTCGGGTGGACAGCTGATGGTGACCGGTCCCGTCGCT
ACAGCTGGCATTTTTGCCACCTACCTTCCTGATCACATGACATTGTGGCGGGGCTTCCTG
AATGAGGCGTGGCTGACCGGGATGCTCCAGCTGTGTCTCTTCGCCATCACGGACCAGGAG
AACAACCCAGCACTGCCAGGAACACAGGCGCTGGTGATAGGCATCCTCGTGGTCATCATC
GGGGTGTCCCTCGGCATGAACACAGGATATGCCATCAACCCGTCCCGGGACCTGCCCCCC
---CGCATCTTTACCTTCATTGCTGGTTGGGGCAAACAGGTCTTCAGCGATGGGGAGAAC
TGGTGGTGGGTGCCAGTGGTGGCACCACTTCTGGGTGCCTATCTAGGTGGCATCATCTAC
CTGGTCTTCATTGGCTCCACCATCCCACGGGAGCCCCTGAAACTGGAGGACTCTGTGGCG
TATGAAGACCACGGGATAACCGTATTGCGCAAGACGGGATCTCATGAACCCACGATCTCT
CCCCTCACCCTTGTCTCCGTGAGCCCTGCCAACAGATCTTCAGTCCACCCTGCCCCACCC
TTACATGAATCCATGGCCCTAGAGCACCCC
>Silvery_gibbon_AQP7
------ATGGGCTCGGGCCACTGTCTCAGGTCCACCCGTGGCTCCAAAATGGTCTCCTGG
TCCGTGATAGCAAAGATCCAGGAAATACTG------------CAGAGGAAGATGGTGCGA
GAGTTCCTGGCCGAGTTCATGAGCACATATGTCATGATGGTATTCGGCCTTGGTTCCGTG
GCCCATATGGTTCTAAAT---AAAAAATACGGGAGCTACCTTGGTGTCAACTTGGGTTTT
GGCTTCGGAGTCACCATGGGAGTGCACGTGGCAGGCCGCATCTCTGGAGCCCACATGAAC
GCAGCTGTGACCTTCGCTAACTGTGCACTGGGCCGCGTGCCCTGGAGGAAGTTTCCAGTC
TATGTGCTGGGGCAGTTCCTGGGCTCCTTCCTGGCAGCTGCCACCATCTAC---AGTCTC
TTCTACAGTGCCATTCTCCACTTTTCGGGTGGACAGCTGATGGTGACCGGTCCCGTCGCT
ACAGCTGGCATTTTTGCCACCTACCTTCCTGATCACATGACATTGTGGCGGGGCTTCCTG
AATGAGGCGTGGCTGACTGGGATGCTCCAGCTGTGTCTCTTCGCCATCACGGACCAGGAG
AACAACCCAGCACTGCCAGGAACACAGGCACTGGTGATAGGCATCCTCGTGGTCATCATC
GGGGTGTCCCTCGGCATGAACACAGGATATGCCATCAACCCATCCCGGGACCTGCCCCCC
---CGCATCTTTACCTTCATTGCTGGTTGGGGCAAACAGGTCTTCAGCGATGGGGAGAAC
TGGTGGTGGGTGCCAGTGGTGGCACCACTTCTGGGTGCCTATCTAGGTGGCATCATCTAC
CTGGTCTTCATTGGCTCCACCATCCCACGGGAGCCCCTGAAACTGGAGGACTCTGTGGCG
TATGAAGACCACGGGATAACCGTATTGCACAAGATGGGATCTCATGAACCCACGATCTCT
CCCCTCACCCTTGTCTCCGTGAGCCCTGCCAACAGATCTTCAGTCCACCCTGCCCCACCC
TTACATGAATCCATGGCCCTAGAGCACCCC
>Olive_baboon_AQP7
ATGGTTCAAACATCCAGGCACAGA---CGGTCCACCCGTGGCTCCAAAATGGTCTCCTGG
TCCGTGATAGCAAAGATCCAGGAAATACTG------------CAGAAGAAGATGGTGCGA
GAGTTCCTGGCCGAGTTCATGAGCACGTATGTCATGATGGTGTTCGGCCTTGGTTCCGTG
GCCCATATGGTTCTAAAT---AAAAAATATGGGAGCTACCTTGGTGTCAACTTGGGTTTT
GGCTTCGGAGTCACCATGGGAGTGCACGTGGCAGGCCACATCTCTGGGGCCCACATGAAC
GCAGCTGTGACCTTCGCTAACTGTGCACTGGGCCGTGTGCCCTGGAGGAAGTTTCCGGTC

TATGTGCTGGGGCAGTTCCTGGGCTCCTTCCTGGCAGCTGCCACCATCTAC---ACCCTC
TTCTACACGGCCATTCTCCACTTTTCGGGTGGACAGCTGATGGTGACCGGTCCCGTCGCT
ACAGCTGGCATTTTTGCCACCTACCTTCCTGATCACATGACACTGTGGCGGGGCTTCCTG
AATGAGGCGTGGCTGACCGGGATGCTCCAGCTGTGTCTCTTCGCCATCACGGACCAGGAG
AACAACGCAGCACTGCCAGGAACACAGGCGCTGGTGATAGGCATCCTCGTGGTCATCATT
GGGGTGTCCCTCGGTATGAACACAGGATATGCCATCAACCCGTCCCGGGACCTGCCCCCT
---CGCGTCTTCACCTTCATTGCTGGCTGGGGCAAAGAGGTGTTCAGTGAAGGGGAGAAC
TGGTGGTGGGTGCCAGTGGTGGCACCACTTCTGGGTGCCTGTCTAGGTGGCATCATCTAC
CTGGTCTTCATTGGCTCCACCATCCCACGGGAACCCCTGAAATTGGAGGACTCTGTGGCG
TACGAAGACCACGGGATAACCGTATTGCCCAAGATGGGATCTCACGAACCCACGATCTCT
CCCCTCACCCCCGTCTCCGTGAGCCCTGCCAACAGATCTTCAGTCCACCCTGCCCCGCCC
TTACATGAATCCATGGCCCTAGAGCACTTC
>Sooty_mangabey_AQP7
ATGGTTCAAACATCCAGGCACAGG---CGGTCCACCCGTGGCTCCAAAATGGTCTCCTGG
TCCGTGATAGCAAAGATCCAGGAAATACTG------------CAGAAGAAGATGGTTCGA
GAGTTCCTGGCCGAGTTCATGAGCACGTATGTCATGATGGTGTTCGGCCTTGGTTCCGTG
GCCCATATGGTTCTAAAT---AAAAAATATGGGAGCTACCTTGGTGTCAACTTGGGTTTT
GGCTTCGGAGTCACCATGGGAGTGCACGTGGCAGGCCACATCTCTGGGGCCCACATGAAC
GCCGCTGTGACCTTCGCTAACTGTGCACTGGGCCGTGTGCCCTGGAGGAAGTTTCCGGTC
TATGTGCTGGGGCAGTTCCTGGGCTCCTTCCTGGCAGCTGCCACCATCTAC---ACCCTC
TTCTACACGGCCATTCTCCACTTTTCGGGTGGACAGCTGATGGTGACCGGTCCCGTCGCT
ACAGCTGGCATTTTTGCCACCTACCTTCCTGATCACATGACACTGTGGCGGGGCTTCCTG
AATGAGGCGTGGCTGACCGGGATGCTCCAGCTGTGTCTCTTCGCCATCACGGACCAGGAG
AACAACGCAGCACTGCCAGGAACACAGGCGCTGGTGATAGGCATCCTCGTGGTCATCATT
GGGGTGTCCCTCGGTATGAACACAGGATATGCCATCAACCCGTCCCGGGACCTGCCCCCT
---CGCGTCTTCACCTTCATTGCTGGCTGGGGCAAAGAGGTCTTCAGTGAAGGGGAGAAC
TGGTGGTGGGTGCCAGTGGTGGCACCACTTCTGGGTGCCTGTCTAGGTGGCATCATCTAC
CTGGTCTTCATTGGCTCCACCATCCCACGGGAACCCCTGAAATTGGAGGACTCTGTGGCG
TACGAAGACCACGGGATAACCGTATTGCCCAAGATGGGATCTCACGAACCCACGATCTCT
CCCCTCACCCCCGTCTCCGTGAGCCCTGCCAACAGATCTTCAGTCCACCCTGCCCCGCCC
TTACATGAATCCATGGCCCTAGAGCACTTC
>Green_monkey_AQP7
ATGGTTCAAACATCCAGGCACAGG---CGGTCCACCCGTGGCTCCAAAATGGTCTCCTGG
TCCGTGACAGCAAAGATCCAGGAAATACTG------------CAGAAGAAGATGGTGCGA
GAGTTCCTGGCCGAGTTCATGAGCACGTATGTCATGATGGTGTTCGGCCTTGGTTCCGTG
GCCCATATGGTTCTAAAT---AAAAAATACGGGAGCTACCTTGGTGTCAACTTGGGTTTT
GGCTTCGGAGTCACCATGGGAGTGCACGTGGCAGGCCACATCTCTGGGGCCCACATGAAC
GCAGCTGTGACCTTCGCTAACTGTGCACTGGGCCGTGTGCCCTGGAGGAAGTTTCCGGTC
TATGTGCTGGGGCAGTTCCTGGGCTCCTTCCTGGCAGCTGCCACCATCTAC---ACCCTC
TTCTACACGGCCATTCTCCACTTTTCGGGTGGACAGCTGATGGTGACCGGTCCCGTTGCT
ACAGCTGGCATTTTTGCCACCTACCTTCCTGATCACATGACACTGTGGCGGGGCTTCCTG
AATGAGGCGTGGCTGACCGGGATGCTCCAGCTGTGTCTCTTCGCCATCACGGACCAGGAG
AACAACCCAGCACTGCCAGGAACACAGGCGCTGGTGATAGGCATCCTCGTGGTCATCATT
GGGGTGTCCCTCGGTATGAACACAGGATATGCCATCAACCCGTCCCGGGACCTGCCCCCT
---CGCGTCTTCACCTTCATTGCTGGCTGGGGCAAACAGGTCTTCAGTGAAGGGGAGAAC
TGGTGGTGGGTGCCAGTGGTGGCACCACTTCTGGGTGCCTGTCTAGGTGGCATCATCTAC
CTGGTCTTCATTGGCTCCACCATCCCACGGGAACCCCTGAAATTGGAGGACTCTGTGGCG
TACGAAGACTACGGGATAACCGTATTGCCCAAGATGGGATCTCACGAACCCACGATCTCT
CCCCTCACCCCTGTCTCCGTGAGCCCTGCCAACAGATCTTCAGTCCACCCTGCCCCACCC
TTACATGAATCCATGGCCCTAGAGCACTTC
>Gelada_AQP7
ATGGTTCAAACATCCAGGCACAGG---CGGTCCACCCGTGGCTCCAAAATGGTCTCCTGG
TCCGTGATAGCAAAGATCCAGGAAATACTG------------CAGAAGAAGATGGTGCGA
GAGTTCCTGGCCGAGTTCATGAGCACGTATGTCATGATGGTGTTCGGCCTTGGTTCCGTG
GCCCATATGGTTCTAAAT---AAAAAATATGGGAGCTACCTTGGTGTCAACTTGGGTTTT
GGCTTCGGAGTCACCATGGGAGTGCACGTGGCAGGCCACATCTCTGGGGCCCACATGAAC
GCAGCTGTGACCTTCGCTAACTGTGCACTGGGCCGTGTGCCCTGGAGGAAGTTTCCGGTC
TATGTGCTGGGGCAGTTCCTGGGCTCCTTCCTGGCAGCTGCCACCATCTAC---ACCCTC
TTCTACACGGCCATTCTCCACTTTTCGGGTGGACAGCTGATGGTGACCGGTCCCGTCGCT
ACAGCTGGCATTTTTGCCACCTACCTTCCTGATCACATGACACTGTGGCGGGGCTTCCTG
AATGAGGCGTGGCTGACCGGGATGCTCCAGCTGTGTCTCTTCGCCATCACGGACCAGGAG

AACAACGCAGCACTGCCAGGAACACAGGCGCTGGTGATAGGCATCCTCGTGGTCATTATT
GGGGTGTCCCTCGGTATGAACACAGGATATGCCATCAACCCGTCCCGGGACCTGCCCCCT
---CGCGTCTTCACCTTCATTGCTGGCTGGGGCAAAGAGGTGTTCAGTGAAGGGGAGAAC
TGGTGGTGGGTGCCAGTGGTGGCACCACTTCTGGGTGCCTGTCTAGGTGGCATCATCTAC
CTGGTCTTCATTGGCTCCACCATCCCACGGGAACCCCTGAAATTGGAGGACTCTGTGGCG
TACGAAGACCACGGGATAACCGTATTGCCCAAGATGGGATCTCACGAACCCGCGATCTCT
CCCCTCACCCCCGTCTCCGTGAGCCCTGCCAACAGATCTTCAGTCCACCCTGCCCCGCCC
TTACATGAATCCATGGCCCTAGAGCACTTC
>Drill_AQP7
ATGGTTCAAACATCCAGGCACAGG---CGGTCCACCCGTGGCTCCAAAATGGTCTCCTGG
TCCGTGATAGCAAAGATCCAGGAAATACTG------------CAGAAGAAGATGGTGCGA
GAGTTCCTGGCCGAGTTCATGAGCACGTATGTCATGATGGTGTTCGGCCTTGGTTCCGTG
GCCCATATGGTTCTAAAT---AAAAAATATGGGAGCTACCTTGGTGTCAACTTGGGTTTT
GGCTTCGGAGTCACCATGGGAGTGCACGTGGCAGGCCACATCTCTGGGGCCCACATGAAC
GCAGCTGTGACCTTCGCTAACTGTGCACTGGGCCGTGTGCCCTGGAGGAAGTTTCCGGTC
TATGTGCTGGGGCAGTTCCTGGGCTCCTTCCTGGCAGCTGCCACCATCTAC---ACCCTC
TTCTACACGGCCATTCTCCACTTTTCGGGTGGACAGCTGATGGTGACCGGTCCCGTCGCT
ACAGCTGGCATTTTTGCCACCTACCTTCCTGATCACATGACACTGTGGCGGGGCTTCCTG
AACGAGGCGTGGCTGACCGGGATGCTCCAACTGTGTCTCTTCGCCATCACGGACCAGGAG
AACAACGCAGCACTGCCAGGAACACAGGCGCTGGTGATAGGCATCCTCGTGGTCATCATT
GGGGTGTCCCTCGGTATGAACACAGGATATGCCATCAACCCGTCCCGGGACCTGCCCCCT
---CGCGTCTTCACCTTCATTGCTGGCTGGGGCAAAGAGGTCTTCAGTGAAGGGGAGAAC
TGGTGGTGGGTGCCAGTGGTGGCACCACTTCTGGGTGCCTGTCTAGGTGGCATCATCTAC
CTGGTCTTCATTGGCTCTACCATCCCACGGGAACCCCTGAAATTGGAGGACTCTGTGGCG
TACGAAGACCACGGGATAACCGTATTGCCCAAGATGGGATCTCACGAACCCACGATCTCT
CCCCTCACCCCCGTCTCCGTGAGCCCTGCCAACAGATCTTCAGTCCACCCTGCCCCGCCC
TTATATGAATCCATGGCCCTAGAACACTTC
>Rhesus_monkey_AQP7
ATGGTTCAAACATCCAGGCACAGG---CGGTCCACCCGTGGCTCCAAAATGGTCTCCTGG
TCCGTGATGGCAAAGATCCAGGAAATACTG------------CAGAAGAAGATGGTGCGA
GAGTTCCTGGCCGAGTTCATGAGCACGTATGTCATGATGGTGTTCGGCCTTGGTTCCGTG
GCCCATATGGTTCTAAAT---AAAAAATATGGGAGCTACCTTGGTGTCAACTTGGGTTTT
GGCTTCGGAGTCACCATGGGAGTGCACGTGGCAGGCCACATCTCTGGGGCCCACATGAAC
GCAGCTGTGACCTTCGCTAACTGTGCACTGGGCCGTGTGCCCTGGAGGAAGTTTCCGGTC
TATGTGCTGGGGCAGTTCCTGGGCTCCTTCCTGGCAGCTGCCACCATCTAC---ACCCTC
TTCTACACGGCCATTCTCCACTTTTCGGGTGGACAGCTGATGGTGACCGGTCCCGTCGCT
ACAGCTGGCATTTTTGCCACCTACCTTCCTGATCACATGACACTGTGGCGGGGCTTCCTG
AATGAGGCGTGGCTGACCGGGATGCTCCAGCTGTGTCTCTTCGCCATCACGGACCAGGAG
AACAACGCAGCACTGCCAGGAACACAGGCGCTGGTGATAGGCATCCTCGTGGTCATCATT
GGGGTGTCCCTCGGTATGAACACAGGATATGCCATCAACCCGTCCCGGGACCTGCCCCCT
---CGCGTCTTCACCTTCATTGCTGGCTGGGGCAAAGAGGTCTTCAGTGAAGGGGAGAAC
TGGTGGTGGGTGCCAGTGGTGGCACCACTTCTGGGTGCCTGTCTAGGTGGCATCATCTAC
CTGGTCTTCATTGGCTCCACCACCCCACGGGAACCCCTGAAATTGGAGGACTCTGTGGCG
TACGAAGACCACGGGATAACCGTATTGCCCAAGATGGGATCTCACGAACCCACGATCTCT
CCCCTCACCCCCGTCTCCGTGAGCCCTGCCAACAGATCTTCAGTCCACCCTGCCCCGCCC
TTACATGAATCCATGGCCCTAGAGCACTTC
>Crab_eating_macaque_AQP7
ATGGTTCAAACATCCAGGCACAGG---CGGTCCACCCGTGGCTCCAAAATGGTCTCCTGG
TCCGTGATGGCAAAGATCCAGGAAATACTG------------CAGAAGAAGATGGTGCGA
GAGTTCCTGGCCGAGTTCATGAGCACGTATGTCATGATGGTGTTCGGCCTTGGTTCCGTG
GCCCATATGGTTCTAAAT---AAAAAATATGGGAGCTACCTTGGTGTCAACTTGGGTTTT
GGCTTCGGAGTCACCATGGGAGTGCACGTGGCAGGCCACATCTCTGGGGCCCACATGAAT
GCAGCTGTGACCTTCGCTAACTGTGCACTGGGCCGTGTGCCCTGGAGGAAGTTTCCGGTC
TATGTGCTGGGGCAGTTCCTGGGCTCCTTCCTGGCAGCTGCCACCATCTAC---ACCCTC
TTCTACACGGCCATTCTCCACTTTTCGGGTGGACAGCTGATGGTGACCGGTCCCGTCGCT
ACAGCTGGCATTTTTGCCACCTACCTTCCTGATCACATGACACTGTGGCGGGGCTTCCTG
AATGAGGCGTGGCTGACCGGGATGCTCCAGCTGTGTCTCTTCGCCATCACGGACCAGGAG
AACAACGCAGCACTGCCAGGAACACAGGCGCTGGTGATAGGCATCCTCGTGGTCATCATT
GGGGTGTCCCTCGGTATGAACACAGGATATGCCATCAACCCGTCCCGGGACCTGCCCCCT
---CGCGTCTTCACCTTCATTGCTGGCTGGGGCAAAGAGGTCTTCAGTGAAGGGGAGAAC
TGGTGGTGGGTGCCAGTGGTGGCACCACTTCTGGGTGCCTGTCTAGGTGGCATCATCTAC

CTGGTCTTCATTGGCTCCACCACCCCACGGGAACCCCTGAAATTGGAGGACTCTGTGGCG
TACGAAGACCACGGGATAACCGTATTGCCCAAGATGGGATCTCACGAACCCACGATCTCT
CCCCTCACCCCCGTCTCCGTGAGCCCTGCCAACAGATCTTCAGTCCGCCCTGCCCCGCCC
TTACATGAATCCATGGCCCTAGGGCACTTC
>Pig_tailed_macaque_AQP7
ATGGTTCAAACATCCAGGCACAGG---CGGTCCACCCGTGGCTCCAAAATGGTCTCCTGG
TCCGTGATGGCAAAGATCCAGGAAATACTG------------CATAAGAAGATGGTGCGA
GAGTTCCTGGCCGAGTTCATGAGCACGTATGTCATGATGGTGTTCGGCCTTGGTTCCGTG
GCCCATATGGTTCTAAAT---AAAAAATATGGGAGCTACCTTGGTGTCAACTTGGGTTTT
GGCTTCGGAGTCACCATGGGAGTGCACGTGGCAGGCCACATCTCTGGGGCCCACATGAAC
GCAGCTGTGACCTTCGCTAACTGTGCACTGGGCCGTGTGCCCTGGAGGAAGTTTCCGGTC
TATGTGCTGGGGCAGTTCCTGGGCTCCTTCCTGGCAGCTGCCACCATCTAC---ACCCTC
TTCTACACGGCCATTCTCCACTTTTCGGGTGGACAGCTGATGGTGACCGGTCCCGTCGCT
ACAGCTGGCATTTTTGCCACCTACCTTCCTGATCACATGACACTGTGGCGGGGCTTCCTG
AATGAGGCGTGGCTGACCGGGATGCTCCAGCTGTGTCTCTTCGCCATCACGGACCAGGAG
AACAACGCAGCACTGCCAGGAACACAGGCGCTGGTGATAGGCATCCTCGTGGTCATCATT
GGGGTGTCCCTCGGTATGAACACAGGATATGCCATCAACCCGTCCCGGGACCTGCCCCCT
---CGCGTCTTCACCTTCATTGCTGGCTGGGGCAAAGAGGTCTTCAGTGAAGGGGAGAAC
TGGTGGTGGGTGCCAGTGGTGGCACCACTTCTGGGTGCCTGTCTAGGTGGCATCATCTAC
CTGGTCTTCATTGGCTCCACCACCCCACGGGAACCCCTGAAATTGGAGGACTCTGTGGCG
TACGAAGACCACGGGATAACCGTATTGCCCAAGATGGGATCTCATGAACCCACGATCTCT
CCCCTCACCCCCGTCTCCGTGAGCCCTGCCAACAGATCTTCAGTCCACCCTGCCCCGCCC
TTACATGAATCCATGGCCCTAGAGCACTTC
>Francoiss_langur_AQP7
ATGGTTCAAACATCCAGGCACAGG---CGGTCCACCCGTGGCTCCAAAATGGTCTCGTGG
TCCGTGATAGCAAAGATCCAGAACACACTG------------CAGAAGAAGATGGTGCGA
GAGTTCCTGGCTGAGTTCATGAGCACGTATGTCATGATGGTGTTCGGCCTTGGTTCCGTG
GCCCATATGGTTCTAAAT---AAAAAATATGGGAGCTACCTTGGTGTCAACTTGGGTTTT
GGCTTCGGAGTCACCATGGGAGTGCACGTGGCAGGCCACATCTCTGGGGCCCACATGAAC
GCAGCTGTGACCTTCGCTAACTGTGCACTGGGCCGCGTGCCCTGGAGGAAGTTTCCGGTC
TATGTGCTGGGGCAGTTCCTGGGCTCCTTCCTGGCAGCTGCCACCATCTAC---ACCCTC
TTCTACACGGCCATTCTCCACTTTTCGGGTGGACAGCTGATGGTGACCGGTCCCGTCGCT
ACAGCTGGCATTTTTGCCACCTACCTTCCTGATCACATGACATTGTGGCGGGGCTTCCTG
AACGAGGCGTGGCTGACCGGGATGCTCCAGCTGTGTCTCTTCGCCATCACGGACCAGGAG
AACAACCCAGCACTGCCAGGAACACAGGCGCTGGTGATAGGCATCCTCGTGGTTATCATT
GGGGTGTCCCTCGGTATGAACACAGGATATGCCATCAACCCGTCCCGGGACCTGCCCCCT
---CGCGTCTTCACCTTCATTGCTGGCTGGGGCAAACAGGTCTTCAGCGAAGGGGAGAAC
TGGTGGTGGGTGCCAGTGGTGGCACCACTTCTGGGTGCCTGTCTAGGTGGCATCATCTAC
CTGGTCTTCATTGGCTCCACCATCCCACGGGAACCCCTGAAATTGGAGGACTCTGTGGCG
TACGAAGACCACGGGATAACCGTATTGCCCAAGATGGGATCTCATGAACCCACGATCTCT
CCCCTCACCCCCGTCTCCGTGAGCCCTGCCATCAGATCTTCAGTCCACCCTGCCCCGCCC
TTACATGAATCCATGGCCCTAGAGCACTTC
>Golden_snub_nosed_monkey_AQP7
ATGGTTCAAACATCCAGGCACAGG---CGGTCCACCCGTGGCTCCAAAATGGTCTCGTGG
TCCGTGATAGCAAAGATCCAGAACACACTG------------CAGAAGAAGATGGTGCGA
GAGTTCCTGGCTGAGTTCATGAGCACGTATGTCATGATGGTGTTCGGCCTTGGTTCCGTG
GCCCATATGGTTCTAAAT---AAAAAATATGGGAGCTACCTTGGTGTCAACTTGGGTTTT
GGCTTCGGAGTCACCATGGGAGTGCACGTGGCAGGCCACATCTCTGGGGCCCACATGAAC
GCAGCTGTGACCTTCGCTAACTGTGCACTGGGCCGCGTGCCCTGGAGGAAGTTTCCGGTC
TATGTGCTGGGGCAGTTCCTGGGCTCCTTCCTGGCAGCTGCCACCATCTAC---ACCCTC
TTCTACACGGCCATTCTTCACTTTTCGGGTGGACAGCTGATGGTGACCGGTCCTGTCGCT
ACAGCTGGCATTTTTGCCACCTACCTTCCTGATCACATGACATTGTGGCGGGGCTTCCTG
AATGAGGCGTGGCTGACCGGGATGCTCCAGCTGTGTCTCTTCGCCATCACGGACCAGGAG
AACAACCCAGCACTGCCAGGAACACAGGCGCTGGTGATAGGCATCCTCGTGGTCATCATT
GGGGTGTCCCTCGGTATGAACACAGGATATGCCATCAACCCGTCCCGGGACCTGCCCCCT
---CGTGTCTTCACCTTCATTGCTGGCTGGGGCAAACAGGTCTTCAGCGAAGGGGAGAAC
TGGTGGTGGGTGCCAGTGGTGGCACCACTTCTGGGTGCCTGTCTAGGTGGCATCATCTAC
CTGGTCTTCATTGGCTCCACCATCCCACGGGAACCCCTGAAATTGGAGGACTCTGTGGCG
TACGAAGACCACGGGATAACCGTATTGCCCAAGATGGGATCTCATGAACCCACGATCTCT
CCCCTCACCCCCGTCTCCGTGAGCCCTGCCATCAGATCTTCAGTCCACCCTGCCCCGCCC
TTACATGAATCCATGGCCCTAGAGCACTTC

>Black_snub_nosed_monkey_AQP7
ATGGTTCAAACATCCAGGCACAGG---CGGTCCACCCGTGGCTCCAAAATGGTCTCGTGG
TCCGTGATAGCAAAGATCCAGAACACACTG------------CAGAAGAAGATGGTGCGA
GAGTTCCTGGCTGAGTTCATGAGCACGTATGTCATGATGGTGTTCGGCCTTGGTTCCGTG
GCCCATATGGTTCTAAAT---AAAAAATATGGGAGCTACCTTGGTGTCAACTTGGGTTTT
GGCTTCGGAGTCACCATGGGAGTGCACGTGGCAGGCCACATCTCTGGGGCCCACATGAAC
GCAGCTGTGACCTTCGCTAACTGTGCACTGGGCCGCGTGCCCTGGAGGAAGTTTCCGGTC
TATGTGCTGGGGCAGTTCCTGGGCTCCTTCCTGGCAGCTGCCACCATCTAC---ACCCTC
TTCTACACGGCCATTCTTCACTTTTCGGGTGGACAGCTGATGGTGACCGGTCCTGTCGCT
ACAGCTGGCATTTTTGCCACCTACCTTCCTGATCACATGACATTGTGGCGGGGCTTCCTG
AATGAGGCGTGGCTGACCGGGATGCTCCAGCTGTGTCTCTTCGCCATCACGGACCAGGAG
AACAACCCAGCACTGCCAGGAACACAGGCGCTGGTGATAGGCATCCTCGTGGTCATCATT
GGGGTGTCCCTCGGTATGAACACAGGATATGCCATCAACCCGTCCCGGGACCTGCCCCCT
---CGCGTCTTCACCTTCATTGCTGGCTGGGGCAAACAGGTCTTCAGCGAAGGGGAGAAC
TGGTGGTGGGTGCCAGTGGTGGCACCACTTCTGGGTGCCTGTCTAGGTGGCATCATCTAC
CTGGTCTTCATTGGCTCCACCATCCCACGGGAACCCCTGAAATTGGAGGACTCTGTGGCG
TACGAAGACCACGGGATAACCGTATTGCCCAAGATGGGATCTCATGAATCCACGATCTCT
CCCCTCACCCCCGTCTCCGTGAGCCCTGCCATCAGATCTTCAGTCCACCCTGCCCCGCCC
TTACATGAATCCATGGCCCTAGAGCACTTC
>Ugandan_red_colobus_AQP7
ATGGTTCAAACATCCAGGCACAGG---CGGTCCACCCGTGGCTCCAAAATGGTCTCGTGG
TCCGTGATAGCAAAGATCCAGAACACACTG------------CAGAAGAAGATGGTGCGA
GAGTTCCTGGCTGAGTTCATGAGCACGTATGTCATGATGGTGTTCGGCCTTGGTTCCGTG
GCCCATATGGTTCTAAGT---AAAAAATATGGGAGCTACCTTGGTGTCAACTTGGGTTTT
GGCTTCGGAGTCACCATGGGAGTGCACGTGGCAGGCCACATTTCTGGGGCCCACATGAAC
GCAGCTGTGACCTTCGCTAACTGTGCACTGGGCCGCGTGCCCTGGAGGAAGTTTCCGGTC
TATGTGCTGGGGCAGTTCCTGGGCTCCTTCCTGGCAGCTGCCACCATCTAC---ACCCTC
TTCTACACGGCCATTCTCCACTTTTCGGGTGGACAGCTGATGGTGACCGGTCCCGTCGCT
ACAGCTGGCATTTTTGCCACCTACCTTCCTGATCACATGACATTGTGGCGGGGCTTCCTG
AATGAGGCGTGGCTGACCGGGATGCTCCAGCTGTGTCTCTTCGCCATCACGGACCAGGAG
AACAACCCAGCACTGCCAGGAACACAGGCGCTGGTGATAGGCATCCTCGTGGTCATCATT
GGGGTGTCCCTCGGTATGAACACAGGATATGCCATCAACCCGTCCCGGGACCTGCCCCCT
---CGCGTCTTCACCTTCATTGCTGGCTGGGGCAAACAGGTCTTCAGCGAAGGGGAGAAC
TGGTGGTGGGTGCCAGTGGTGGCACCACTTCTGGGTGCCTGTCTAGGTGGCATCATCTAC
CTGGTCTTCATTGGCTCCACCATCCCACGGGAACCCCTGAAATTGGAGGACTCTGTGGCA
CGCGAAGACCACGGGATAACCGTATTGCCCAAGATGGGATCTCATGAACCCACGATCTCT
CCCCTCACCCCCGTCTCCGTGAGCCCTGCCATCAGATCTTCACTCCACCCTGCCCCGCCC
TTACATGAATCCATGGCCCTGGAGCACTTC
>Angolan_colobus_AQP7
ATGGTTCAAACATCCAGGCACAGG---CGGTCCACCCGTGGCTCCAAAATGGTCTTGTGG
TCCGTGATAGCAAAGATCCAGAACACACTG------------CAGAAGAAGATGGTGCAA
GAGTTCCTGGCTGAGTTCATGAGCACGTATGTCATGATGGTGTTCGGCCTTGGTTCCGTG
GCCCATATGGTTCTAAAT---CAAAAATATGGGAGCTACCTTGGTGTCAACTTGGGTTTT
GGCTTCGGAGTCACCATGGGAGTGCACGTGGCAGGCCACATCTCTGGGGCCCACATGAAC
GCAGCTGTGACCTTCGCTAACTGTGCACTGGGCCGCGTGCCCTGGAGGAAGTTTCCGGTC
TATGTGCTGGGGCAGTTCCTGGGCTCCTTCCTGGCAGCTGCCACCATCTAC---ACCCTC
TTCTACACGGCCATTCTCCACTTTTCGGGTGGACAGCTGATGGTGACCGGTCCCGTCGCT
ACAGCTGGCATTTTTGCCACCTACCTTCCTGATCACATGACATTGTGGCGGGGCTTCCTG
AATGAGGCGTGGCTGACCGGGATGCTCCAGCTGTGTCTCTTCGCCATCACGGACCAGGAG
AACAACCCAGCACTGCCAGGAACACAGGCGCTGGTGATAGGCATCCTCGTGGTCATCATT
GGGGTGTCCCTCGGTATGAACACAGGATATGCCATCAACCCGTCCCGGGACCTGCCCCCT
---CGCGTCTTCACCTTCATTGCTGGCTGGGGCAAACAGGTCTTCAGCGAAGGGGAGAAC
TGGTGGTGGGTGCCAGTGGTGGCACCACTTCTGGGTGCCTGTCTAGGTGGCATCATCTAC
CTGGTCTTCATTGGCTCCACCATCCCACGGGAACCCCTGAAATTGGAGGACTCTGTGGCA
TACGAAGACCACGGGATAACCGTATTGCCCAAGATGGGATCTCATGAACCCACGATCTCT
CCCCTCACCCCCGTCTCCGTGAGCCCTGCCATCAGATCTTCAGTCCACCCTGCCCCGCCC
TTACATGAATCCATGGCCCTAGAGCACTTC
>Mas_night_monkey_AQP7
ATGATTCACACATCCAAGCACAGG---CGGTCCACCCGCAGCTCCAAAATGGTCTCCTGG
TCCGGGATAACAAGGATACAGCAAATACTG------------CAGAAGAAGTTGGTGCGA
GAGTTCCTGGCCGAGTTCATGAGCACGTATGTCATGATGGTGTTTGGCCTTGGTTCCGTG

GCCCATATGGTTCTAAGT---AAAAAACATGGGAGCTACCTTGCTGTCAACTTGGCTTTT
GGCTTCGGTGTCACCATGGGAGTGCATGTGGCAGGCAACATCTCTGGGGCCCACATGAAT
GCAGCTGTGACCTTCGCTAACTGCGCGCTGGGCCGCCTGCCCTGGAGGAAGTTTCCGGTC
TACGTGCTGGGTCAGTGCCTCGGCTCCTTCCTGGCGGCTGCCACCATCTAC---ACCCTC
TTCTACACGGCTATTCTCCACTTTTCGGGTGGACAGCTGATGGTGACCGGTCCTGTAGCG
ACGGCTGGCATTTTTGCCACCTACCTTCCTGAATACATGACATTGTGGCGGGGCTTCCTG
AATGAGATGTGGCTGACTGGGATGCTCCAGTTGTGTCTCTTCGCCATCACGGACCAGGAA
AACAACCCAGCACTGCCGGGAACACAGGCGCTGGTGATAGGCATACTCGTGGTCACCATC
GGGATGTCCCTTGGCATGAACACAGGATATGCCATCAACCCATCCCGGGACCTGCCCCCA
---CGTATCTTCACCTTTATTGCTGGCTGGGGCAAACAGGTCTTCAGTGATGGGGAGAAC
TGGTGGTGGGTGCCAGTGGTGGCACCACCTCTGGGTGCCTATCTAGGTGGCATCATCTAC
GTGGTCTTCATTGGCTCCACCATCCCACGGGAGCCCCCGAAATTGGAGGACTCTGTGGTG
TATGAAGATCACAGGATAACCGTATCGTCCAAGATGGAACCTCATGCATCCGTGGCCTCT
CCCCTCACCCCCGTCTCCGTGAGCCCTGACAACAGACCTTCAGTCCACCCGGCCCCCACC
TTACAGGAATCCGTGGCCCAAGAACACTTC
>White_tufted_ear_marmoset_AQP7
ATGATTCACGCATCCAAGCACAGG---CGGTCCACCCGCAGCTCCAAAATGGTTTCCTGG
TCCGGGATAACAAGGATACAGCAAATACTG------------CAGAAGAAGTTGGTGCGA
GAGTTCCTGGCCGAGTTCATGAGCACGTATGTCATGATGGTGTTTGGCCTTGGTTCCGTG
GCCCATATGGTTCTAAGT---AAAAAACATGGGAGCTACCTTGCTGTCAACTTGGCTTTT
GGCTTCGGTGTCACCATGGGAGTGCATATGGCAGGCAACATCTCTGGGGCCCACATGAAT
GCAGCTGTGACCTTTGCTAACTGCGCGCTGGGCCGCCTGCCCTGGAGGAAGTTTCCGGTC
TATGTGCTGGGTCAGTGCCTCGGCTCCTTTCTGGCGGCTGCCACCATCCAC---ACCCTC
TTCTACACGGCCATTCTCCACTTTTCGGGTGGACAGCTGATGGTGACCGGTCCTGTAGCG
ACAGCTGGCATTTTTGCCACCTACCTTCCTGATTACATGACATTGTGGCGGGGCTTCCTG
AATGAGGTGTGGCTGACCGGGATGCTCCAGCTGTGTCTCTTTGCCATCACGGACCAGGAG
AACAACCCAGCACTGCCGGGAACACAGGCGCTGGTGATAGGCATACTCGTGGTCACCATT
GGGATGTCCCTTGGCATGAACACAGGATATGCCATCAACCCATCCCGGGACCTGCCCCCC
---CGTATCTTCACCTTCATTGCTGGCTGGGGCAAACAGGTCTTCAGCGATGGGGAGAAC
TGGTGGTGGGTGCCAGTGGTGGCACCACCTCTGGGTGCCTATCTAGGTGGCATCATCTAT
GTGGTCTTCATTGGCTCCACCATCCCACGGGAACCCCCAAAATTGGAGGACCCTGTGACG
TATGAAGATCACAGGATAACCGTATCGACCAAGATGGAACCTCATGCATCTGTGGCCTCT
CCCCTCACCCCTGTCTCCGTGAGCCCTGACAACAGACCTTCAGTCCGCCTGGCCCCCACC
TTACAGGAATCCATGGCCCAAGAACACTTC
>Bolivian_squirrel_monkey_AQP7v1
ATGATTCACGCATCCAAGCACAGG---CGGTCCACCCGCAGCTCCAAAATGGTCTCCTGG
TCTGGGATAACAAGGACACAGCAAATACTG------------CAGAAGAAGTTGGTGCGA
GAGTTCCTGGCCGAGTTCATGAGCACATATGTCATGATGGTGTTTGGCCTTGGTTCCGTG
GCCCATATGGTTCTAAGT---AAAACACATGGGAGCTACCTTGCTGTCAACTTGGCTTTT
GGCTTCGGTGTCACCATGGGAGTGCATGTGGCAGGCAACATCTCTGGGGCCCACATGAAT
GCAGCTGTGACCTTCGCTAACTGCGCACTGGGCCGCCTGCCCTGGAGGAAGTTTCCGGTC
TACGTGCTGGGTCAGTGCCTCGGCTCCTTCCTGGCGGCTGCCACCATCTAC---GCCCTC
TTCTACACGGCCATTCTACACTTTTCGGGTGGACAGCTGATGGTGACCGGACCCGTAGCG
ACGGCTGGCATTTTTGCCACCTACCTTCCTGATTACATGACATTGTGGTGGGGCTTCCTG
AATGAGGTGTGGCTGACTGGGATGCTCCAGCTGTGTCTCTTCGCCATCACGGACCAGGAG
AACAACCCAGCACTGCCGGGCACACAGGCGCTGGTGATAGGCATACTTGTGGTCACCATC
GGGATGTCCCTTGGCATGAACACAGGATATGCCATCAACCCATCCCGGGACCTGCCCCCC
---CGTATCTTCACCTTCATTGCCGGCTGGGGCAAACAGGTCTTCAGCGATGGGGAGAAC
TGGTGGTGGGTGCCAGTGGTGGCGCCACCTCTGGGTGCCTATCTAGGTGGCATCATCTAT
GTGGTCTTCATTGGCTCCACCATCCCACGGGAGCCCCCAAAAGTGGAGGACTCTGTGGCG
TATGAAGATCACAGGATAACCGTATTATCCAAGATGGAACCTCATGCATCCATGGCCTCT
CCCCTCACCCCTGTCTCCGTGAGCCCTGACAACAGACCTGCAGTCCGCCTGGCCCCCACC
TTACAGGAATCCATGGCCCAAGAACACTTC
>Panamanian_white_faced_capuchin_AQP7
ATGATTCAGGCATCCAAGCACAGG---CGGTCCACCCGCAGCTCCAAAATGGTCTCCTGG
TCCTGGAAAACAAGGACACAGCAAATACTG------------CAGAAGAAGTTGGTGCGA
GAGTTCCTGGCCGAGTTCATGAGCACGTATGTCATGATGGTGTTTGGCCTTGGTTCCGTG
GCCCATATGGTTCTAAGT---AAAAAACATGGGAGCTACCTTGCTGTCAACTTGGCTTTT
GGCTTCGGTGTCGCCATGGGAGTGCATGTGGCAGGCAACATCTCTGGGGCCCACATGAAT
GCAGCTGTGACCTTCGCTAACTGCGCGCTGGGCCGCCTGCCCTGGAGGAAGTTTCCGGTC
TACGTGCTGGGTCAGTTCCTCGGCTCCTTCCTGGCGGCTGCCACCATCTAC---ACCCTC

TTCTACACGGCCATTCTCCACTTTTCGGGTGGACAGCTGATGGTGACCGGTCCCGTAGCG
ACGGCTGGCATTTTTGCCACCTACCTTCCTGATTACATGACATTGTGGCGGGGCTTCCTG
AATGAGGTGTGGCTGACAGGGATGCTCCAGCTGTGTCTCTTCGCCATCACGGACAAGGAG
AACAACCCAGCACTGCCGGGCACACAGGCGCTGGTGATAGGCATACTCGTGGTCACCATC
GGGATGTCCCTTGGCATGAACACAGGATATGCTATCAACCCATCCCGGGACCTGCCCCCC
---CGTATCTTCACCTTCATTGCTGGCTGGGGCAAACAGGTCTTCAGCGATGGGGAGAAC
TGGTGGTGGGTGCCAGTGGTGGCGCCACTTCTGGGTGCCTATCTAGGTGGCATCATCTAT
GTGGTCTTCATTGGCTCCACCATCCCACGGGAGCCCCCAAAATTGGAAGACTCTGTGGCA
TATGAAGATAACAGGATAACCGTATCGTCCAAGATGGAACCTCACACATCCATGGCCTCT
CGCCTCACCCCCGTCTCCATGAGCCCTGACAACAGACCTTCAGTCCGCCTGGCCCCCACC
TTACAGGAATCCATGGCCCAAGAACAGTTC
>Tufted_capuchin_AQP7
ATGATTCAGGCATCCAAGCACAGG---CGGTCCACCCGCAGCTCCAAAATGGTCTCCTGG
TCCTGGAAAACAAGGACACAGCAAATACTG------------CAGAAGAAGTTGGTGCGA
GAGTTCCTGGCCGAGTTCATGAGCACGTATGTCATGATGGTGTTTGGCCTTGGTTCCGTG
GCCCATATGGTTCTAAGT---AAAAAACATGGGAGCTACCTTGCTGTCAACTTGGCTTTT
GGCTTCGGTGTCGCCATGGGAGTGCATGTGGCAGGCAACATCTCTGGGGCCCACATGAAT
GCAGCTGTGACCTTCGCTAACTGCGCGCTGGGCCGCCTGCCCTGGAGGAAGTTTCCGGTC
TACGTGCTGGGTCAGTTCCTCGGCTCCTTCCTGGCGGCTGCCACCATCTAC---ACCCTC
TTCTACACGGCCATTCTCCACTTTTCGGGTGGACAGCTGATGGTGACCGGTCCCGTAGCG
ACGGCTGGCATTTTTGCCACCTACCTTCCTGATTACATGACATTGTGGCGGGGCTTCCTG
AATGAGGTGTGGCTGACAGGGATGCTCCAGCTGTGTCTCTTCGCCATCACGGACAAGGAG
AACAACCCAGCACTGCCGGGCACACAGGCGCTGGTGATAGGCATACTCGTGGTCACCATC
GGGATGTCCCTTGGCATGAACACAGGATATGCTATCAACCCATCCCGGGACCTGCCCCCC
---CGTATCTTCACCTTCATTGCTGGCTGGGGCAAACAGGTCTTCAGCGATGGGGAGAAC
TGGTGGTGGGTGCCAGTGGTGGCGCCACTTCTGGGTGCCTATCTAGGTGGCATCATCTAT
GTGGTCTTCATTGGCTCCACCATCCCACGGGAGCCCCCGAAATTGGAGGACTCTGTGGCA
TATGAAGATAACAGGATAACCGTATCGTCCAAGATGGAACCTCACACATCCATGGCCTCT
CGCCTCACCCCCGTCTCCATGAACCCTGACAACAGACCTTCAGTCCGCCTGGCCCCCACC
TTACAGGAATCCATGGCGCAAGAACAGTTC
>Bolivian_squirrel_monkey_AQP7v2
------ATGGGCTCGGGCTGCTGTCTCAGGTCCACCCGCAGCTCCAAAATGGTCTCCTGG
TCTGGGATAACAAGGACACAGCAAATACTG------------CAGAAGAAGTTGGTGCGA
GAGTTCCTGGCCGAGTTCATGAGCACATATGTCATGATGGTGTTTGGCCTTGGTTCCGTG
GCCCATATGGTTCTAAGT---AAAACACATGGGAGCTACCTTGCTGTCAACTTGGCTTTT
GGCTTCGGTGTCACCATGGGAGTGCATGTGGCAGGCAACATCTCTGGGGCCCACATGAAT
GCAGCTGTGACCTTCGCTAACTGCGCACTGGGCCGCCTGCCCTGGAGGAAGTTTCCGGTC
TACGTGCTGGGTCAGTGCCTCGGCTCCTTCCTGGCGGCTGCCACCATCTAC---GCCCTC
TTCTACACGGCCATTCTACACTTTTCGGGTGGACAGCTGATGGTGACCGGACCCGTAGCG
ACGGCTGGCATTTTTGCCACCTACCTTCCTGATTACATGACATTGTGGTGGGGCTTCCTG
AATGAGGTGTGGCTGACTGGGATGCTCCAGCTGTGTCTCTTCGCCATCACGGACCAGGAG
AACAACCCAGCACTGCCGGGCACACAGGCGCTGGTGATAGGCATACTTGTGGTCACCATC
GGGATGTCCCTTGGCATGAACACAGGATATGCCATCAACCCATCCCGGGACCTGCCCCCC
---CGTATCTTCACCTTCATTGCCGGCTGGGGCAAACAGGTCTTCAGCGATGGGGAGAAC
TGGTGGTGGGTGCCAGTGGTGGCGCCACCTCTGGGTGCCTATCTAGGTGGCATCATCTAT
GTGGTCTTCATTGGCTCCACCATCCCACGGGAGCCCCCAAAAGTGGAGGACTCTGTGGCG
TATGAAGATCACAGGATAACCGTATTATCCAAGATGGAACCTCATGCATCCATGGCCTCT
CCCCTCACCCCTGTCTCCGTGAGCCCTGACAACAGACCTGCAGTCCGCCTGGCCCCCACC
TTACAGGAATCCATGGCCCAAGAACACTTC
>Philippine_tarsier_AQP7
------ATGGGCTCGGGCTGCTGCTTCAGGCCCACCTGCAGCTCCAAGATGGTCTCCTGG
TCTGTGATAACGAGAACACAAGCAATACTG------------CAGAGGAAGATGGTGCGA
GAATTTGTGGCTGAGTTCATGAGCACATATGTCATGATGGTGTTTGGCCTTGGTTCTGTG
GCCCACATGGTTCTAGGAGATAAAAAATATGGGAGCTTCCTCGGTGTCAACTTGGGTTTT
GGCTTCGGAGTCACCATGGGCGTGCACGTGGCAGGCAACATCTCTGGGGCCCACATGAAC
GCGGCTGTGACCTTCACCAACTGTGCACTAGGCCGCATGTCCTGGAAGAAGTTTCCAGTA
TATGTGCTGGGTCAGTTCCTGGGCTCCTTCACAGCTGCTGCCACCATCTAT---GGCCTC
TTCTACCCGGCCATTCTCCACTTCTCGGGTGGACAGTTGATGGTGACCGGTCCCACAGCC
ACCGCTGGCATTTTTGCCACCTACCTTCCTGATCACATGACACTGTGGTGGGGCTTCCTG
AATGAGGTGTTACTGACAGGGATGCTGCAGCTATGTCTCTTCGCCATCACGGACAAGGAG
AACAACCCAGCGCTGCAAGGGACACAGGCGCTGGTGATCGGCATCCTCGTTGTCAGCATG

GGGGCGTCCCTAGGCATGAACACAGGCTATGCCATCAACCCATCCCGGGACCTGCCCCCT
---CGCTTCTTCACCTTCATTGCTGGCTGGGGCAAACCGGTCTTCAGGGCTGGGGAGAAC
TGGTGGTGGGTGCCAGTGGTGGCACCAATTCTGGGTGCCTACATAGGTGGCATCATCTAC
CTGGTCTTCATCGGCTCCAGCACCCCACAGGAGCCCCAGAGACTGGAGGACCCGGAGGCA
TATGAAGACCCCAAGACGACTGTGTCACCCAAGCCCGAATCTCACCTGTCCATGACCTCT
CCTCTCCCCCTTGTCTCTGTGACCCCTACTAACAGATCTTCCACCCAGCCGGTCCCACTC
TTACATGACCCCATGCCCCTAGAGCACTTC
>Gray_mouse_lemur_AQP7
ATGTCTCAAGTAATCGGGCAGAGG---CGGTCCACCCGCAGCACCAGGATGAACTCCAGT
TCGGTGATTACGTCGGTGCATACAACACTG------------AGGAGGCAGATGGTACAG
GAGTTCCTGGCCGAGTGCCTGAGCACATATGTCATGATGGTGTTTGGCCTGGGTTCCGTG
GCCCATATGGTTCTAGGAAATAAAACATTTGGAGCTACCTCGGTGTCAACTTGGGTTTT
GGCTTCGGAGTCACCATGGGAGTGTACATAGCAGGCAACATCTCTGGGGCCCACATGAAT
TCGGCAGTGACCTTCACTAGTTGTGCACTAGGTCGCATGCCCTGGAAGAAGTTTCCTGTG
TACGTGCTGGGTCAGTTCCTGGGCTCCTTCATGGCTGCTGCCACCATCTAC---GGCCTC
TTCTACCCAGCCATTATCCACTTCTCGAGTGGAGAGCTGACGGTGACTGGTCCCACAGCC
ACTGGTGGCATTTTTGCCACCTACCTTCCAGATCACATGACACTGTGGCGGGGCTTCGTG
GATGAGGTGATAGTGACGGGGATGCTCCAGCTGTGTCTCTGCGCCATCACGGACAAGGAT
AAC---CCAGAACATCAAGGGACACAGCCCCTGGTGATTGGCTTCCTTATTGTCATCATT
GGGGTGGGCCTAGGCATGAACTCAGGATATGCTATCAATCCCTCCCGGGACCTGCCCCCC
---CGCTTCTTCACCTACCTTGCCGGCTGGGGCACGCAAGTCTTCAGGGCTGGGGATAAC
TGGTGGTGGGTGCCAGTGGTGGCGCCACCTCTGGGTGCTTACCTAGGTGGCATAGTCTAC
CTGGTCTTCATTGGCTCCAGCACCTCACGAAAGCCCAAGAAATTGGAGGACCCTGCGGCA
TATGAAGACCACAGGATATCTTCATTGCCCAAGACCAGCCATGATCCATCCATGATGTCT
TCCCTCACCCCTGCCTCTGTGGCCCCTACCACCAGATCTTCATTCCAGCCTGCCCCACCT
GTACGTGACTCTGTGAATGAAGAGCACTTC
>Rabbit_AQP7
------ATGGGCTCGGGCTGCTGCTTCAGGTCCCCCTGCAGCTCCCAGATGGTCTCCTGG
CCTGTGCTAGTGCGGATCCACGCAGTCCTG------------CAGAAGGAGACGGTGCGA
GAGTTCCTGGCAGAGTTCCTGAGCACGTACGTCATGATGGTGTTTGGTCTTGGTTCTGTG
GCCCATATGGTTCTAGGAGGCCAAAAATTCGGGAGCTTCCTCGCTGTCAACTTGGGTTTT
GGCTTTGGAGTCACCATGGGCGTGCACGTGGCAGGCAACATCTCTGGGGCCCACATGAAC
GCTGCCGTGAGCTTCACCGCCTGCGCGCTCGGCCGCATGTCCTGGAAGAAGTTTCCCGTG
TACGTGTTGGGTCAGTTCCTGGGCTCCTTCACCGCGGCTGCCACCATCTAC---GGCCTC
TTCTACACGGCCATTCTGCACTTCTCCGGCGGACATCTGGCAGTGACCGGGCCCACAGCC
ACTGCGAACATTTTTGCCACCTATCTTCCTGACCACATGACGCTGTGGTGGGGCTTCCTC
AATGAGGTGGTACTGACGGGGATGCTCCAGCTGTGTCTCTTAGCCATCACCGATAAGGAG
AATAACCCAGCGCTGCCGGGGACACAGGCCCTGGTCACCGGCATCCTTGTTGTCATCATT
GGGGCATCCCTGGGCATGAACACAGGATATGCTATCAACCCATCTCGGGACCTGCCTCCC
---CGCTTCTTCACCTTCATTGCCGGCTGGGGCAAACAGGTGTTCAGGGCTGGGGAGAAC
TGGTGGTGGGTGCCAGTGGTGGCACCAATTGTAGGTGCCTACCTGGGAGGAATCGTCTAT
CTGGTCTTCATTGGCTCCGGCATCCCACGGGAGCCCCAGGAATCAGAGGACTCGGCCATG
AATGAAGAACACAGGATAACCACATTGTCCAAGACAAGTGCTCACCTATCCGTGACCTCT
CACCTCGACCCTGTCTCCAGAGTCCCTGCCAACACATCTTCAGTCCAGCCTGTCCCACCT
GTGAATGGCTCTATACTCTTAGAGAGCTTC

File S6: Alignment for Fig. 3A, Fig. S5
>Tiger_rockfish_Aqp3b
ATGGGAAAACAGAGAGACGTTTTGGAGAAACTGGCAGGGACCTTTCAGATCCGACATGTG
CTCCTCCGCCAGGCCCTGGCTGAGTGCCTGGGAACCTTTATTCTGGTGATGTTTGGTTGT
GGCGCAGTAGCACAGCTGGTGCTAAGCGGAGGCTCCCATGGCACGTTCCTCAGCGTCAAC
TTTGCTTTTGGTTTTGCTGTAACGTTGGGCATCCTTGTGAGCGGACAGATCTCAGGTGGT
CATCTGAACCCAGCGGTGACCTTCTCGCTGTGTTTACTCGGGAGAGAGCCCTGGAGGAAG
TTCCCGCTGTTCTTTTTCTTCCAGACCCTCGGAGCCTTTCTGGGTGCAGCGATTGTATTT
GGCATGTATTTT--

----------------------CAGGTTATTGGAACGGCTGCGTTGATAATGTGCATCCTG
GCCATAGTTGATCCTCACAACAATCCCGTCCCGAGTGGTCTAGCACCCTTCAYCGTGGGC
TTTGTGGTGTTGGTCATCGGCCTGTCAATGGGCTTCAACGCTGGCTATGCCGTCAACCCA
GCCAGGGACCTGGGGCCGCGCATCTTCACGGCTATGGCAGGCTGGGGTACAGAGGTTTTC
ACGGCGAACACCTACTGGTTCTTTGTGCCCATCTGTGCACCCTTCTTGGGTGCAGTGGTG

GGTGTGCTGATATACCAGCTAATGATTGGATATCATTTAGAAGGAGAAGTGCAAGAGAAG
CAGAAGAAGGAG---------GAGGAGGAGGAAAGGTTCAAACTTTCCAATATGACAACC
---AACGAAGATGCA---
>Rougheye_rockfish_Aqp3b
ATGGGAAAACAGAGAGACGTTTTGGAGAAACTGGCAGGGACCTTTCAGATCCGACATGTG
CTCCTCCGCCAGGCCCTGGCTGAGTGCCTGGGAACCTTTATTCTGGTGATGTTTGGTTGT
GGCGCAGTAGCACAGCTGGTGCTAAGCGGAGGCTCCCATGGCACGTTCCTCAGCGTCAAC
TTTGCTTTTGGTTTTGCTGTAACGTTGGGCATCCTTGTGAGTGGACAGATCTCAGGTGGT
CATCTGAACCCAGCGGTGACCTTCTCGCTGTGTTTACTCGGGAGAGAGCCCTGGAGGAAG
TTCCCGCTGTTCTTTTTCTTCCAGACCCTCGGAGCCTTTCTGGGTGCAGCGATTGTATTC
GGCATGTATTTTGATGCATTATGGGACTTTAGCCCCGGG------------GAGCTGATC
GTGGTGGGRGAGAATGCCACAGCTGGGATTTTTGCCACATATCCATCCAAACATCTCACC
CTGGTTAATGGGTTCTTTGATCAGGTTATTGGAACGGCTGCGTTGATAATGTGCATCCTG
GCCATAGTTGATCCTCACAACAATCCCGTCCCGAGTGGTCTAGCACCCTTCACCGTGGGC
TTTGTGGTGTTGGTCATCGGCCTGTCAATGGGCTTCAACGCTGGCTATGCCGTCAACCCA
GCCAGGGACCTGGGGCCGCGCATCTTCACGGCTATGGCAGGCTGGGGTACAGAGGTTTTC
ACGGCGAACACCTACTGGTTCTTTGTGCCCATCTGTGCACCCTTCTTGGGTGCAGTGGTG
GGTGTGCTGATATACCAGCTAATGATTGGATATCATTTAGAAGGAGAAGTGCAAGAGAAG
CAGAAGAAGGAGGAG------GAGGAGGAGGAAAGGTTCAAACTTTCCAATATGACAACC
---AACGAAGATGCA---
>Flag_rockfish_Aqp3b
ATGGGAAAACAGAGAGACGTTTTGGAGAAACTGGCAGGGACCTTTCAGATCCGACATGTG
CTCCTCCGCCAGGCCCTGGCTGAGTGCCTGGGAACCTTTATTCTGGTGATGTTTGGTTGT
GGCGCAGTAGCACAGCTGGTGCTAAGCGGAGGCTCCCATAGCACGTTCCTCAGCGTCAAC
TTTGCTTTTGGTTTTGCTGTAACGTTGGGCATCCTTGTGAGTGGACAGATCTCAGGTGGT
CATCTGAACCCAGCGGTGACCTTCTCGCTGTGTTTACTCGGGAGAGAGCCCTGGAGGAAG
TTCCCGTTGTTCTTTCTCTTCCAGACCCTCGGAGCCTTTCTGGGTGCAGCGATTGTATTT
GGCATGTATTTTGATGCATTATGGGACTTTAGCCCCGGG------------GAGCTGATC
GTGGTGGGAGAGAATGCCACAGCTGGGATTTTTGCCACATATCCATCCAAACATCTCACC
CTGGTTAATGGGTTCTTTGATCAGGTTATTGGAACGGCTGCGTTGATAATGTGCATCCTG
GCCATAGTTGATCCTCACAACAATCCTGTCCCGAGTGGTCTAGCACCCTTCACCGTGGGC
TTTGTGGTGTTGGTCATCGGCCTGTCAATGGGCTTCAACGCTGGCTATGCCGTCAACCCA
GCCAGGGACCTGGGGCCGCGCATCTTCACGGCTATGGCAGGCTGGGGTACAGAGGTTTTC
ACGGCGAACACCTACTGGTTCTTTGTGCCCATCTGTGCACCCTTCTTGGGTGCAGTGGTG
GGTGTGCTGATATACCAGCTAATGATTGGATATCATTTAGAAGGAGAAGTGCAAGAGAAG
CAGAAGAAGGAGGAG------GAGGAGGAGGAAAGGTTCAAACTTTCCAATATGACAACC
---AACAAAGATGCA---
>Minor_flag_rockfish_Aqp3b

--ATGTTTGGTTGT
GGCGCAGTAGCACAGCTGGTGCTAAGCGGAGGCTCCCATGGCACGTTCCTCAGCGTCAAC
TTTGCTTTTGGTTTTGCTGTAACGTTGGGCATCCTTGTGAGTGGACAGATCTCAGGT---

------------GATGCATTATGGGACTTTAGCCCCGGG------------GAGCTGATC
GTGGTGGGGGAGAATGCCACAGCTGGGATTTTTGCCACATATCCATCCAAACATCTCACC
CTGGTTAATGGGTTCTTTGATCAGGTTATTGGAACGGCTGCGTTGATCATGTGCATCCTT
GCCATAGTTGATCCACACAACAATCCTGTCCCGAGTGGTCTAGCACCCTTCACTGTGGGC
TTTGTGGTGTTGATCATCGGCCTGTCAATGGGCTTCAACGCTGGCTATGCCGTCAACCCA
GCCAGGGACCTGGGGCCGCGCATCTTCACGGCCATGGCAGGCTGGGGTACAGAGGTTTTC
ACGGCGAACACCTACTGGTTCTTTGTGCCCATCTGTGCACCCTTCTTGGGTGCAGTGGTG
GGTGTGCTGGTATACCAGCTAATGATTGGATATCATTTAGAAGGAGAAGTGCAAGAGAAG
CAGAAGAAGGAAGAG------GAGGAGGAGGAAAGGTTCAAACTTTCCAATATGAAAACC
---AACGAAGATGCA---
>Korean_rockfish_Aqp3b
ATGGAAATACAGAGAGACGTTTTGGAGAAACTGGCAGGGACCTTTCAGATCCGACATGTG
CTCCTCCGCCAGGCCCTGGCTGAGTGCCTGGGAACCTTTATTCTGGTG------------

------------GCGGTGACCTTCTCTCTGTGTTTACTCGGGAGAGAGCCCTGGAGGAAG
TTCCCGCTGTTCTTTTTCTTCCAGACCCTCGGAGCCTTTCTGGGTGCAGCGATTGTATTT

GGCATGTATTTTGATGCATTATGGGACTTTAGCCCCGGG------------GAGCTGATC
GTGGTGGGAGAGAATGCCACAGCTGGGATTTTTGCCACATATCCATCCAAACATCTCACC
CTGGTTAATGGGTTCTTTGATCAGGTTATTGGAACGGCTGCGTTGATCATGTGCATCCTT
GCCATAGTTGATCCACACAACAATCCCGTCCCGAGTGGTCTAGCACCCTTCACTGTGGGC
TTTGTGGTGTTGATCATCGGCCTGTCAATGGGCTTCAACGCTGGCTATGCCGTCAACCCA
GCCAGGGACCTGGGGCCGCGCATCTTCACGGCCATGGCAGGCTGGGGTACAGAGGTTTTC
ACGGCGAACACCTACTGGTTCTTTGTGCCCATCTGTGCACCCTTCTTGGGTGCAGTGGTG
GGTGTGCTGGTATACCAGCTAATGATTGGATATCATTTAGAAGGAGAAGTGCAAGAGAAG
CAGAAGAAGGAAGAG------GAGGAGGAGGAAAGGTTCAAACTTTCCAATATGACAGCC
---AACGAAGATGCA---
>Blackfin_icefish_Aqp3b
ATGGGAAAACAAAGAGACATTTTGAAGAAACTTGCAGGGACCTTTCAGATCCGACATGTG
CTCCTCCGCCAGGCCATGGCAGAGTGCCTGGGGACCTTCATTCTGGTGATGTTTGGGTGT
GGGTCAGTAGCCCAGGCGGTGCTGAGCGGAGGCTCCCACGGCTCCTTCCTCACGGTCAAC
TTTGCTTTTGGCTTTGCTGCGACTTTGGGCATCCTAGTGAGTGGACAGATCTCAGGTGGT
CATCTGAACCCAGCTGTGACCTTCTCACTGTGTTTACTTGGGAGAGAGCCCTGGAGGAAG
TTCCCGCTGTTCTTTTTCTTCCAGACTCTCGGTGCCTTTCTGGGGGCAGCAGTTGTATTT
GGCATGTATTTTGATGCATTATGGGACATTAGCCAGGGA------------GAGCTGATC
GTAGTGGGGAATAATGCCACAGCTGGGATTTTTGCCACATATCCATCCAAACATCTCTCG
TTGGTGAATGGATTCTTTGATCAGATTATTGGAACAGCTGCATTGATCGTGTGTATCCTG
GCCATAGTGGACCCTCGAAACAACCCCGTCCCTCCGGGTCTGGAGCCCCTCACAGTCGGC
CTCGTGGTGCTGGTCATCGGCCTGTCGATGGGCTTCAACTCTGGCTATGCTGTGAACCCA
GCCCGGGACCTGGGGCCACGCATCTTCACCGCTCTGGCAGGGTGGGGAAGAGAGGTTTTC
ACGGCAAACACCTACTGGTTCTTTGTGCCCCTCTGTGCCCCCTTCGTGGGGGCAGCCGTG
GGGGTGCTGATCTACCAGCTCCTGATCGGATACCATTTGGAAGGAGAAGTGCAAAGAAAG
CAGAAG---------------GAGGAGGAGGAAAGGTCCAAACGATCCAATATGACAACC
---AACGAAGAAGCA---
>Black_rockcod_Aqp3b
ATGGGAAAACAAAGAGACATTTTGAAGAAACTGGCAGGGACCTTTCAGATCCGACATGTG
CTCCTCCGGCAGGCCATGGCAGAGTGCCTGGGAACCTTCATTCTGGTGATGTTTGGGTGT
GGGTCAGTCGCCCAGGTGGTGCTGAGCGGAGGCTCCCACGGCTCCTTCCTCACGGTCAAC
TTTGCTTTTGGTTTTGCTGCGACTTTGGGCATCCTAGTGAGTGGACAGATCTCAGGTGGT
CATCTGAACCCAGCTGTGACCTTCTCACTGTGTTTACTTGGGAGAGAGCCCTGGAGGAAG
TTCCCGCTGTTCTTTTTCTTTCAGACTCTCGGTGCCTTTCTGGGGGCAGCAGTTGTATTT
TGCATGTATTTTGATGCATTATGGGACATTAGCCAGGGA------------GAGCTGATC
GTAGTGGGGAATAATGCCACGGCTGGGATTTTTGCCACATATCCATCCAAACATCTCTCC
TTGGTTAATGGATTCTTTGATCAGATTATTGGAACAGCTGCATTGATCGTCTGTATCCTG
GCCATAGTGGACCCTCGAAACAACCCCGTCCCTCGGGGTCTGGAGCCCCTCACAGTGGGC
CTTGTGGTGCTGGTCATCGGCCTGTCGATGGGCTTCAACTCCGGCTATGCGGTGAACCCA
GCCCGGGACCTCGGGCCACGCATCTTCACCGCTCTGGCAGGGTGGGGAAGAGAGGTTTTC
ACGGCAAACACCTACTGGTTCTTTGTGCCCCTCTGTGCCCCCTTCCTGGGGGCAGCCGTG
GGGGTGCTGATCTACCAGCTCCTGATCGGATACCATTTAGAAGGAGAAATGCAAAAAAAA
CAGGAGGAGGAGGAGGAGGAGGAGGAGGAGGAGGAAAGGTCCAAACGATCCAACATGACAACC
---AACGAAGAAGCA---
>Sablefish_Aqp3b

--ATGTTTGGTTGT
GGTGCAGTAGCACAGCTGGTGCTGAGCGGAGGCTCCCATGGCACATTCCTCACCGTCAAC
CTTGCTTTTGGTTTTGCTGCAACATTAGGCATCCTTGTGAGCGGACAGATCTCAGGTGGT
CATCTGAATCCAGCCGTGACCTTCTCACTGTGTTTACTCAGGAGAGAGCCTTGGAGGAAG
TTCCCGCTCTTCTTTTTCTTCCAGACTCTCGGAGCCTTTCTGGGTGCAGCGGTTGTATTC
GGCATGTATATCGATGCATTATGGGACTTTGGCCAGGGG------------GAGCTGATT
GTGGTGGGGACAAATGCCACAGCTGGGATTTTTGCCACATATCCATCCAAACATCTCACG
CTGGTTAATGGATTCTTTGATCAGATTATTGGAACAGCTGCATTGATTGTGTGCATCCTG
GCCATAGTCGATCCAAACAACAACCCCGTCCCTCGAGGTCTGGAGCCCTTCACTGTTGGC
TTTGTGGTGTTGGTCATCGGCCTGTCGATGGGTTTCAACTGTGGCTATGCCGTCAATCCA
GCCAGGGACCTTGGACCACGCATCTTCACGGCTCTTGCAGGCTGGGGTGGAGAGGTTTTC
ACGGCAAACACCTACTGGTTCTTTGTGCCCATCTGTGCACCCTTTTTGGGTGCAGTGGTG
GGTGTGCTGATTTACCAGCTGATGATTGGATACCATTTAGAAAGAGAAGTGCAGGAGAGG
CAAAAG---------------GAGGAGGAGGAAAGGTTCAAACTTTCCAGTATGACAAAC
---AACGAAAATGCA---

>Rhine_sculpin_Aqp3b
ATGGGAAAACGGGGAGACATGTTGGAGAAACTGGCGAAGACCTTTCAGATCCGACACGCT
CTCCTCCGGCAGGGCCTGGCCGAGTGCCTGGGCACCTTCGTTCTGGTGATGTTTGGTTGT
GGCGCAGTAGCACAGCTGGTGCTGAGCAGAGGCTCCCACGGCACGTTCCTCACCGTCAAC
CTGGCTTTTGGATTTGCTGCAACGTTGGGCATCCTCGTGAGCGGGCAGATCTCAGGCGGT
CATCTGAACCCAGTCGTGGCCTTCTCGCTGTGCCTT------------------------

------------GATGCATTATGGGACTTTAGCCACAGG------------GAGCTGATT
GTGGTGGGGACGAATGCCACGGCCGGGATTTTCGCCACGTATCCATCCAAACATCTCACC
CTGGTTAACGGATTCTTTGATCAGATCATTGGAACAGCTGCATTGATCGTGTGCATCCTG
GCCATAGTGGATCCCAACAACAACCCGGTCCCTGGAGGGCTGGAGCCCTTCACCGTGGGC
TTTGTGGTGCTGGTCATCGGCCTGTCGATGGGCTTCAACTCCGGCTACGGCGTCAACCCG
GCCCGGGACCTGGGGCCGCGCATCTTCACGGCTGTGGCGGGCTGGGGCGGAGAGGTCTTC
ACGGCAAACACCTACTGGTTCTTTGTGCCCATCTGTGCAACCTTCTTGGGTGCAGTGGTG
GGTGTGCTGATTTACCAGCTGATGATTGGATACCACTTAGAAGGAGAAGTCAAAGAGAAG
CAGAAG---------------GAGGAGGAGGAAAGGTTAAAACCGTCCAGTATGACAACC
---AACGAAGAAGAC---
>Channel_bull_blenny_Aqp3b
ATGGGAAAACAAAGAGACACTTTAAAGAAACTGGCAAGGACCTTTCAGATCCGACATGTG
CTTCTCTGCCAGGCCCTGGCAGAGTGCCTGGGAACCCTAATTCTGGTGATGTTTGGTTGC
GGTGCAGTAGCACAGATGGTGCTAAGCGGAGGCTCCCATGGCACGTTCCTCACAGTCAAC
TTTGCTTTTGGTTTTGCTGCAACATTGGGAATCCTTGTGAGCGGACAGATCTCAGGTGGT
CATCTGAACCCAGCGGTGACCTTCTCCCTGTGTTTACTTGGGAGGGAGCCTTGGAGGAAG
TTCCCGCTGTTCTTTTTCTATCAGACTGTCGGGGCCTTTTTGGGTGCAGCGGTCGTATTC
GGCATGTATTTTGATGCATTATGGGACTTTAGCCAGGGG------------GAGCTGATC
GTGGTGGGGAAGAACGCCACAGCTGGGATTTTTGCCACATATCCATCCAAACATCTCACC
CTGGTTAATGGATTCTTTGATCAGATTATTGGAACAGCCGCGCTGATCGTGTGTATCCTG
GCCATAGTTGATCCACACAACCGTCCAGTGGCGAGAGGTCTGGAGCCCTTCACCGTAGGC
TGTGTGGTGTTGGTCATCGGCCTGTCAATGGGATTCAACTCTGGCTATGCTGTCAACCCA
GCCAGGGACCTGGGGCCGCGCGTCTTCACGGCTCTCGCAGGCTGGGGCAGAGAGGTCTTC
ACGGCAAACACCTACTGGTTCGTTGTGCCCATCTGTGCGCCCTTCTTGGGTGCAGTGGTG
GGTGTGCTGATTTACCAGCTCATGATTGGATACCATTTAGAAGGAGAAGTGCAAGAAAAG
CAGAGGGAGGAGGAGGAGGACGAGGAGGAGGAAAGGTTCAAACGTTCCAATATGACAACC
---AACGAAGATGCA---
>White_bass_Aqp3b
ATGGGAAGACAAAACAACATTTTGGAAAAACTGGCTGAGACCTTTCAGATTCGTCATGTG
CTCCTCCGCCAGGCCCTGGCAGAGTGCCTGGGAACCCTTATTCTGGTGATGTTTGGTTGT
GGTGCAGTAGCACAGATGGTCCTAAGTGGAGGCTCCCATGGCACGTTCCTCACCGTCAAC
TTTGCTTTTGGTTTTGCCACAACTTTGGGCATCCTTTTGAGTGGGCAGATCTCAGGTGGT
CATCTGAACCCAGCGGTGACATTCACACTGTGTTTACTTGGAAGAGAGCCTTGGAGGAAG
TTCCCGTTGTTCTTTTTGTTCCAGACTCTTGGAGCCTTTCTGGGTGCAGCAATTGTATTT
GCCATGTATTTTGATGCATTATCGGACTTCAGCCAGGGG------------GAGCTG---
GTAGTGGGGAAGAATGCCACAGCTGGGATTTTTGCCACATATCCTTCCAAACATCTCACC
CTGATTAATGGATTCTTTGATCAGATAATTGGAACAGCAGCACTGATCGTGTGCATCCTG
GCGATAGTGGATCCACACAACAATCCAATCCCTAGAGGTCTGGAGCCCTTCACTGTTGGT
TTTGTGGTGCTGGTCATCGGGCTGTCGATGGGCTTCAACTCTGGCTATGCTGTCAACCCA
GCCAGGGACCTGGGACCACGCATCTTCACGGCTCTTGCTGGTTGGGGTGGAGAGGTTTTC
ACGGCACACACCTACTGGTTCTTTGTGCCCATCTGCGCCCCCTTCCTGGGAGCAGTGGTG
GGTGTGCTGATGTATCAGCTAATGATCGGATATCATTTGGAAAGAGAAGTGCAAGAGAAG
CAGAAGAAG------------GAGGAGGAGGACAGGTTTACACTTTCCAATATTACAACC
---AATGAAGATGCA---
>Striped_seabass_Aqp3b
ATGGGAAGACAAAACAACATTTTGGAAAAACTGGCTGAGACCTTTCAGATCCGTCATGTG
CTCCTCCGCCAGGCCCTGGCAGAGTGCCTGGGAACCCTTATTCTGGTGATGTTTGGTTGT
GGTGCAGTAGCACAGATGGTCCTAAGCGGAGGCTCCCATGGCACGTTCCTCACCGTCAAC
TTTGCTTTTGGTTTTGCCGCAACTTTGGGCATCCTTGTTAGTGGGCAGATCTCAGGT---
TATCTGAACCCAGCGGTGACATTCACACTGTGTTTTCTTGGAAGAGAGCCTTGGAGGAAG
TTCCCGTTGTTCTTTTTGTTCCAGACTCTTGGAGCCTTTCTGGGTGCAGCAATTGTATTT
GCCAGGTATTTTGATGCATTATCGGACTTCAGCCAGGGG------------GAGCTG---
GTAGTGGGGAAGAATGCCACAGCTGGGATTTTTGCCACATATCCTTCCAAACATCTCACC
CTGATTAATGGATTCTTTGATCAGATAATTGGAACAGCAGCACTGATTGTGTGCATCCTG

GCGATAGTGGATCCATACAATAATCCAATCCCTAGAGGTCTGGAGCCCTTCACTGTTGGT
TTTGTGGTGCTGGTCATCGGGCTGTCGATGGGCTTCAACTCTGGCTATGCTGTCAACCCA
GCCAGGGACCTGGGACCACGCATCTTCACGGCTCTTGCTGGTTGGGGTGGAGAGGTTTTC
ACGGCACACACCTACTGGTTCTTTGTGCCCATCTGCGCCCCCTTCCTGGGAGCAGTGGTA
GGTGTGCTGATGTACCAGCTAATAATCGGATATCATTTGGAAAGAGAAGTGCAAGAGAAG
CAGAAGAAG------------GAGGAGGAGGACAGGTTTAAACTTTCCAATATTACAACC
---AATGAAGATGCA---
>European_seabass_Aqp3b
ATGGGAAGACAAAACAACATTTTGGAGAAACTGGCAGAGACCTTTCAGATCCGTCATGTG
CTCCTCCGCCAGGCCCTGGCAGAGTGCCTGGGAACCCTGATTCTGGTGATGTTTGGTTGT
GGTGCAGTAGCACAGATGGTCCTAAGCGGGGGCACCCATGGTACGTTCCTCACCGTCAAC
TTTGCTTTTGGTTTTGCCGCAACTTTGGGAATCCTTGTGAGTGGGCAGATCTCAGGTGGT
CATCTGAACCCAGCGGTGACATTCACACTGTGTTTACTTGGAAGAGAGCCTTGGAGGAAG
TTCCCGTTGTTCTTTTTGTTCCAGACTCTTGGAGCCTTTCTGGGTGCAGCGATTGTATTT
GCCATGTATTTTGATGCATTATCGGACTTCAGCCAGGGG------------GAGCTGATG
GTGGTGGGGAAGAATGCCACAGCTGGGATTTTTGCCACATATCCTTCTAAACATCTCACA
ATGATTAATGGATTCTTTGATCAGATAATTGGAACAGCAGCACTGATAGTGTGCATCCTG
GCCATAGTGGATCCACACAACAATCCAATCCCTAGAGGTCTGGAGCCCTTCACTGTTGGT
TTTGTGGTGCTGGTCATCGGGCTGTCGATGGGCTTCAATTCTGGCTATGCTGTCAACCCA
GCCAGGGACCTGGGACCGCGCATCTTCACGGCTCTTGCTGGTTGGGGTGGAGAGGTTTTC
ACGGCACACACCTACTGGTTCTTTGTGCCCATCTGCGCCCCCTTCCTGGGAGCAGTGGTT
GGTGTGCTGATGTACCAGCTAATGATCGGATATCATTTGGAAAGAGAAGTGCAAGAGAAG
CAGAAGAAG------------GAGGAGGAGGACAGATTTGAACTTTCCAATATTACAACC
---AATGAAGATGCA---
>Gilthead_seabream_Aqp3b
ATGGGAAAACAAAAAGAAGTCTTGGAGAAACTGGCAGAGGACTTCCAGATCCGACATGTG
CTGCTCCGCCAGGCCCTGGCAGAATGCCTGGGAACCCTTATTCTGGTGATGTTTGGTTGC
GGTGCTGTCGCTCAGCTGGTGCTGAGCCGAGGCTCTCATGGCACGTTCCTTTCTGTCAAC
TTGGCTTTTGGTTTCGCTGCAACCTTGGGCGTCCTCGTGAGTGGACAGGTCTCAGGTGGC
CATCTGAACCCAGCGGTGACCTTCGCGGTGTGTTTACTGGGGAGAGAGCCTTGGAGGAAG
TTCCCGCTGTTCTTTTTCTTCCAGACTCTCGGAGCCTTTCTGGGTGCAGCGGTCGTGTTC
GGCTTGTATTTTGATGCATTGCAGGACTTCAGCCAAGGG------------GAGCTGGTC
GTGGTGGGAAAGAACGCCACAGCCGGGATTTTCGCCACGTATCCATCCAAACATCTCAGC
CCGGTTAACGGATTCTTTGATCAGGTAGTTGGGACGGCGGCGTTGATCGTGTGCGTCCTG
GCCATAATCGATCCACGCAACAGTCCGGTCCCTCCAGGCCTGGAGCCCTTCACTGTAGGC
CTCGTGGTGTTGGTCATCGGCCTGTCGATGGGCTTCAACTCTGGCTACGCCGTCAACCCA
GCCAGGGACCTCGGGCCGCGCGTCTTCACGGCTCTTGCAGGCTGGGGCGGGGAGGTTTTC
ACGGCAAACACCTACTGGTTCTTTGTGCCCATCTGTGCCCCGTTCTTGGGTGCAGTGGTG
GGTGTGCTGATGTACCAGCTGATGATTGGATATCATTTAGAAAGAGACGCCCAGGAGAAG
CAGAAGAGGAAGAAGGAGGAGGAGGAGGAGGAGAGGCTCAAACTCTCCGATATTACAACC
---AACGAGGATGCA---
>Red_seabream_Aqp3b
ATGGGAAAACAGAAAGAAGTCTTGGGGAAACTGGCAGAGGCCTTCCAGATCCGACATGTG
CTCCTCCGCCAGGCCCTGGCAGAATGCCTGGGAACCCTTATTCTGGTGATGTTTGGTTGC
GGTGCAGTCGCGCAGCTGGTGCTGAGCAGAGGCTCCCATGGCACGTTCCTCACTGTCAAC
TTGGCTTTTGGTTTTGCTGCAACCTTGGGCATCCTTGTGAGTGGACAGGTCTCAGGTGGT
CATGTGAACCCAGCGGTGACCTTCGCGCTGTGTTTACTTGGTAGAGAGCCTTGGAGGAAG
TTCCCGTTGTTCTTTTTCTTCCAGACTCTCGGAGCCTTTCTGGGTGCAGCGATTGTATTT
GGCTTGTATTTTGATGCATTGCAGGACTTCAGCCAGGGG------------GAGCTGGTC
GTGGTGGGAGAGAATGCCACAGCCGGGATTTTCGCCACGTATCCATCCAAACATCTCAGC
CCGCTTAATGGATTCTTTGATCAGGTTATTGGGACAGCGGCATTGATCATGTGCATCCTG
GCCATAATTGATCCAAAGAACAATCCAGTCCTTCAGGGCCTGGAGCCCTTCACTGTTGGC
TTCGTGGTGTTGGTCATCGGCCTGTCGATGGGCTTCAACTCCGGCTACGCTGTCAACCCA
GCCAGGGACCTCGGACCGCGC---------------------------------------
---GCAAACACCTACTGGTTCTTTGTGCCCATCTGTGCCCCCTTCTTGGGTGCAGTGGTG
GGTGTGCTGATGTATCAGCTGATGATTGGATATCATTTAGGAAGAGACGTCCAGGAGAAG
CATAAGAAGAAGAAGAAGGAGGAGGAGGAGGAAAGATTCAAACTCTCCAATATTACAACC
---AACGAGGATGCA---
>Large_yellow_croaker_Aqp3b
ATGGGAAAACAAAAAGACATTTCGGAGAAACTGGCAGAGACCTTCCAGATCCGACATGTG
CTCCTCCGCCAGGCCCTGGCAGAGTGCCTGGGAACCCTGATTCTGGTGATGTTTGGTTGT

GGTGCGGTGGCACAGTTCGTGCTGAGCAGAGGATCTCACGGCACGTTCCTCACCGTCAAC
TTTGCTTTTGGTTTCGCCGCAACTTTGGGCATCGTCGTGAGTGGACAGATCTCAGGTGGT
CATCTGAACCCGGCGGTGACCTTCACGCTGTGTTTACTTGGAAGAGAGCCTTGGAGGAAG
TTCCCGTTGTTCTTTTTCTTCCAGACTCTCGGAGCCTTCGTGGGTGCAGCGATTATATTC
GCCTTGTATTTTGATGCATTATGGGACTTCAACCAGGGG------------GAGCTGATC
GTGGTGGGGTCCAACGCCACGGCGGGGATTTTTGCCACGTATCCGTCCAAACACCTCACC
ATGGTTAATGGATTCTTTGACCAGATTATTGGAACGGCTGCATTGATCGTGTGTATCCTG
GCCATAGTTGATCCGCACAACAATGCAGTCCCCAGAGGTCTGGAGCCCTTCGCTGTAGGC
TTTGTGGTCTTAGCCATCGGCCTGTCGATGGGCTTCAACTCTGGCTATGCTGTCAACCCG
GCGAGGGACCTGGGACCACGTATCTTCACCGCTCTTGCAGGCTGGGGTGGAGAGGTTTTC
ACAGCAAACACCTACTGGTTTTTTGTGCCCATCTGTGCCCCCTTCTTGGGCGCATTGGTG
GGTGTGCTGATGTACCAGCTAATGATTGGATATCATCTAGAAAGAGAAGTGCAAGAAAAG
CTGAAGAAGGAGGAG------GAGGAGGAGGAGAGGCTCAAACTTTCCAGTATTCCAACC
---AATGAAGATGCA---
>Miiuy_croaker_Aqp3b
ATGGGAAAACAAAAAGACATTTTGGAGAAACTGGCAGAGACCTTCCAGATCCGACATGTG
CTCCTCCGCCAGGCCCTGGCAGAATGCCTGGGATCCCTGATTCTGGTGATGTTTGGTTGC
GGTGCAGTGGCACAGTTCGTGCTAAGCAGAGGATCTCATGGCACGTTCCTCACCGTCAAC
TTTGCTTTTGGTTTCGCCGCAACTTTGGGCATCCTCGTGAGTGGACAGATCTCAGGTGGT
CATCTGAACCCGGCGGTGACCTTCACGCTGTGTTTACTTGGAAGAGAGCCTTGGAGGAAG
TTCCCGTTGTTCTTTTTCTTCCAGACTCTTGGAGCCTTCGTGGGTGCAGCGATTATATTT
GCCTTGTATTTTGATGCATTACGGGACTTCAACCAGGGG------------GAGCTGATC
GTGGTGGGGACCAACGCCACGGCAGGAATTTTTGCCACATATCCGTCCAAACACCTCACC
GCGGTTAATGGATTCTTTGACCAGATTATTGGAACGGCTGCATTGATCGTGTGTATCCTG
GCCATAGTTGATCCGCACAACAATGCAGTCCCCAGAGGTCTGGAGCCCTTCGCTGTAGGC
TTTGTGGTGTTAGTCATCGGCCTGTCGATGGGCTTCAACTCTGGCTATGCCGTCAACCCG
GCGAGGGACCTGGGGCCACGTATCTTCACCGCTCTTGCAGGCTGGGGTGGGGAGGTTTTC
ACGGCAAACACCTACTGGTTTTTTGTGCCCATCTGTGCCCCCTTCTTGGGTGCATTGGTG
GGTGTGCTGATGTACCAGCTAATGATTGGATATCATCTAGAAAGAGAAGTGCAAGAAAAG
CTGAAGAAGGAGGATGAGGAGGAAGAGGAGGAAAGGTTCAAACTTTCCAGTATTCCAACC
---AATGAAGATGCA---
>Barred_knifejaw_Aqp3b
ATGGGAAAACAAAAAGACATTTTGGAGAAACTGGCAGGGACCTTCCAGATCCGACATGTG
CTCCTCCGCCAGGCCCTGGCAGAATGCCTGGGAACCCCTTATTCTGGTGATGTTTGGTTGT
GGTGCAGTAGCACAGCTGGTGCTAAGCGGAGGCTCCCATGGCACGTTCCTCACCGTCAAC
TTTGCTTTTGGTTTCGCTGCCACTTTGGGCATCCTTGTGAGTGGACAGATCTCAGGTGGC
CATTTGAACCCAGCCGTGACCTTCACACTGTGTTTACTTGGGAGAGAGCCCTGGAGGAAG
TTCCCATTGTTCTTTTTCTTCCAGACTCTAGGAGCCTTTCTGGGTGCAGCGATTGTATTT
GGCATGTATTTTGATGCATTATGGGACTTCAGCCAGGGG------------GAGCTGATC
GTGGTGGGGAAGAATGCCACAGCTGGGATTTTTGCCACATATCCATCCAAACATCTCACC
CTGGTTAATGGATTCTTTGATCAGATTATTGGAACAGCTGCATTGATCGTGTGCATCCTG
GCCATAGTTGATCCACACAACAATCCAATCCCTAGAGGTCTGGAGCCCTTCACTGTAGGC
TTTGTGGTGTTGGTCATCGGCCTGTCAATGGGCTTCAACTCTGGCTATGCGGTCAACCCA
GCCAGGGACCTAGGACCACGCATCTTCACGGCTCTTGCAGGCTGGGGTAGAGAGGTTTTC
ACGGCAAACACCTACTGGTTCTTTGTGCCCATCTGTGCCCCCTTCTTGGGTGCATTGGTG
GGTGTGCTGATGTACCAGCTAATGATTGGATACCATTTAGAAGGAGAAGTGCAAGAGAAG
---AAGAAG---------------GAGGAGGAAAGGCTCAAACTTTCCAACATTACAACC
---AACGAAAATGCA---
>Murray_cod_Aqp3b
ATGGGAAAACAAAAAGACATTGTGGAGAATCTGGCAGGGACCTTTCAGATCCGACATGTG
CTCCTCCGCCAGGCCCTGGCAGAATGCCTGGGAACCCCTTATTCTGGTGATGTTTGGTTGT
GGTGCAGTAGCT---------CTAAGCGGAGGCTCCCATGGCACGTTCCTCACTGTCAAC
TTTGCTTTTGGTTTCGCCGCAACATTGGGAATCCTTGTGATTGGACAGGCCTCAGGTGGC
CATCTGAACCCAGCAGTGACCTTCACACTGTGTTTACTTGGGAGAGAGCCTTGGAGGAAG
TTCCCGTTGTTCTTTTTCTTCCAGACTCTTGGAGCCTTTCTGGGTGCAGCGATTGTATTT
GGCATGTATTTT---ACATTATGGGACTTCAGCCCGGGG------------GAGCTGATG
GTGGTGGGGAAGAATGCCACAGCTGGGATTTTTGCCACATATCCATCCAAACATCTCACC
CTGGTTAATGGATTCTTTGATCAGATTATTGGAACAGCTGCATTGATCGTGTGCATCCTG
GCCATAGTTGATCTACACAACAATCCAATCCCCAGGGGTCTGGAGCCCTTCACTGTAGGC
TTTGTGGTGTTGGTCATCGGCCTGTCAATGGGCTTCAACTCTGGCTATGCTGTCAACCCA
GCCAGGGACCTGGGACCACGCATCTTCACGGCTCTTGCTGGCTGGGGTAGAGAGGTTTTC

ATGGCAAACACCTACTGGTTCTTTGTGCCTATCTGTGTCCCCTTCTTGGGTGCACTGGTG
GGTGCGCTGATGTACCAGCTAATGATTGGA---CATTTAGAAGGAGAAGTGCAAGAGAAG
CAGAAGAAG------------GAAGAGGATGAAAGGTTCAAACTTTCCAATATTACAACC
---AACGAAGATGCA---
>Ballan_wrasse_Aqp3b
ATGGGAGAACAGAAGCACATCGTGGCCAAACTGGCGGCTGCCTTTCAGGTCCGACATGTC
CTCCTGCGTCAGGCGCTGGCTGAATGTCTCGGGACTCTGATTCTGGTGATGTTTGGTTGT
GCTTCCGTTGCGCAGCTGGTGCTAAGCCGAGGGTCCCACAGCACGTTCCTCAGCGTCAAC
TTGGCCTTTGGTTTCGCTGCGACTTTGGGGATTCTTGTGAGCGGTCAGATCTCAGGCGGT
CATCTGAACCCGGCGGTGACCTTCGCTCTGTGTTTACTCAGGAGGGAGCCCTGGAGGAAC
TTCCCCTTGTACTTTTTCTTTCAGACCCTCGGAGCGTTTCTGGGTGCAGCGGTCGTGTTC
GGCCTTTATTCAGATGCGTTGCTGGACTTCAGCCAGGGC------------GAGCTGATC
GTGGTGGGGGAAAACGCTACAGCCGGAATTTTTGCCACGTATCCATCACAACATCTCAGC
GTGGTTAACGGAGTCCTCGATCAGGTTATTGGAACAGCTGCATTGATCGTCTGCATCCTG
GCCATAGTTGATCCACACAACAATCCAACTCCCAAAGGTCTGGAGGCGTTCACTGTAGGC
TTTGTTGTGCTGGTCATCGGCCTGTCAATGGGCTTCAACTCTGGCTATGCTGTGAATCCA
GCCAGAGACCTTGGACCGCGCATCTTCACAGCTATGGCAGGCTGGGGTGGAGAGGTTTTT
ACGGCGAACTCCTACTGGTTCTTTGTGCCCGCCTGTGCCCCCTTCTTGGGTGCAGCGGTG
GGTGTGCTGATATATCAGCTAATGATTGGACACCACTTAGAAGGAGAAGTGCAAGAGGAG
CAGAAGAAGGAGGAA------GCGGAGGAGGAAAGGTTGAAACTTTCAAGTACTGCAACT
---GACCAAGATGTG---
>Corkwing_Aqp3b
ATGGGAGAACAGAAACGCATGGCGGCCAAACTGGCGGCGGCCTTTCAGGTCCGACACGTC
CTTGTGCGTCAGGCGCTGGCTGAATGTCTCGGGACTCTGATTCTGGTGATGTTTGGCTGT
GCTTCCGTAGCACAGCTGGTGCTAAGCCGAGGGTCCCACGGCACGTTCCTCAGCGTCAAC
CTGGCTTTTGGTTTCGCCGCAACTTTGGGGATTCTCGTGAGCGGTCAGATCTCAGGCGGT
CATCTGAACCCAGCGGTGACCTTCGCTCTGTGTTTGCTCAGGAGGGAGCCCTGGAGGAAC
TTCCCCTTGTACTTCTTCTTCCAGACTCTCGGAGCGTTTCTGGGTGCAGCGACTGTATTC
GGCCTTTACTCAGATGCGTTGCTGGACTTCAGCCAGGGG------------GAGCTGATC
GTGGTGGGGAAAAACGCGACAGCTGGCATTTTTGCCACTTATCCATCGCAACATCTCAGC
CTGGTGAACGGAGTCTTTGATCAGGTTATTGGAACAGCGTCATTGATCGTGTGCATCCTG
GCCATCGTTGATCCACGCAACAACCCGACTCCCAAAGGTCTGGAGGCGTTCACCGTCGGC
TTCGTTGTGTTGGTCATCGGCCTGTCGATGGGCTTCAACTCTGGCTATGCTGTGAATCCA
GCCAGAGACCTGGGACCACGAATCTTCACAGCTGTGGCAGGCTGGGGTGGAGAGGTTTTT
ACGGCGAACACCTACTGGTTCTTTGTGCCCGCCTGTGCCCCCTTCTTGGGTGCAGCGGTG
GGTGTGCTGATATATCAGCTGATGATTGGATACCATTTAGAAGGAGAAGTGCAAGAGGAG
CAGAAGGAGGAGGAA------GCGGAGGAGGAGAGGTTGAAACTTTCAAATGCTGCAAAC
---AACCAAGATGTA---
>Pacific_bluefin_tuna_Aqp3b
ATGGGAAAACAAAAAGACATTTTGAAGAAACTGGCAGGGACCTTTCAGATCCGACATGTG
CTCCTCCGCCAGGCCCTGGCAGAATGCCTGGGAACCCTTATTCTGGTGATGTTTGGTTGT
GGTGCTGTGGCAAAGTTGGTGCTCAGTGGAGGCTCCCATGGCACCTTCCTCACTGTTAAC
TTGGCTTTTGGATTTGCTGCAACATTGGGCGTCCTTGTGAGTGGACAGGTCTCAGGTGGC
CATCTGAACCCAGCGGTGACCTTCGCATTATGTTTACTTGGAAGAGAGCCTTGGAGGAAG
TTCCCATTGTTCTTTCTCTTCCAGACTCTTGGAGCCTTCTTGGGTGCAGTGATTATATTT
GGCATGTATTTTGATGCCTTATGGGATTTTGGCCAGGGG------------GAGCTGGTC
GTGGTGGGGAAGAATGCCACAGCTGGGATTTTTGCCACATATCCGTCCAAACATCTCAGC
TTGGTTAACGGATTCTTTGATCAGATTATTGGAACAGCTGCATTGATCGTGTGCATCCTG
GCCATCGCTGATCCACATAAC---CCTATCCCTCGAGGTCTTGAGGCCTTCACAGTAGGT
TTTGTGGTGTTGGTCATCGGCCTTTCAATGGGCTTCAACTCTGGCTATGCTGTCAACCCA
GCCAGGGACCTGGGACCACGCATATTCACGGCTCTTGCAGGCTGGGGTGGCGAGGTTTTC
ACGGCAAAGACCTACTGGTTCTTTGTGCCTATCTGTGCCCCGTTCTTGGGTGCAGTGGTG
GGTTTGATGGTGTACCAGTTCATGGTTGGATACCATTTAGAGGGAGAAGTACAAGTGAAG
CAGAAGAAG------------GGGGAGGACGAAAGGTTCAACCTATCTAATATTACAACC
---AACGAAGAGGCA---
>Atlantic_bluefin_tuna_Aqp3b
ATGGGAAAACAAAAAGACATTTTGAAGAAACTGGCAGGGACCTTTCAGATCCGACATGTG
CTCCTCCGCCAGGCCCTGGCAGAATGCCTGGGAACCCTTATTCTGGTGATGTTTGGTTGT
GGTGCTGTGGCACAGTTGGTGCTCAGTGGAGGCTCCCATGGCACCTTCCTCACTGTTAAC
TTGGCTTTTGGATTTGCTGCAACATTGGGCGTCCTTGTGAGTGGACAGGTCTCAGGT---
CATCTGAACCCAGCGGTGACCTTCGCATTATGTTTACTTGGAAGAGAGCCTTGGAGGAAG

TTCCCATTGTTCTTTCTCTTCCAGACTCTTGGAGCCTTCTTGGGTGCAGTGATTATATTT
GGCATGTATTTTGATGCCTTATGGGATTTTGGCCAGGGG------------GAGCTGGTC
GTGTTGGGGAAGAATGCCACAGCTGGGATTTTTGCCACATATCCGTCCAAACATCTCAGC
TTGGTTAACGGATTCTTTGATCAGATTATTGGAACAGCTGCATTGATCGTGTGCATCCTG
GCCATCGCTGATCCACATAAC---CCTATCCCTCGAGGTCTTGAGGCCTTCACAGTAGGT
TTTGTGGTGTTGGTCATCGGCCTTTCAATGGGCTTCAACTCTGGCTATGCTGTCAACCCA
GCCAGGGACCTGGGACCACGCATATTCACGGCTCTTGCAGGCTGGGGTGGTGAGGTTTTC
ACGGCAAAGACCTACTGGTTCTTTGTGCCTATCTGTGCCCCATTCTTGGGTGCAGTGGTG
GGTTTGATGGTGTACCAGTTCATGGTTGGATACCATTTAGAGGGAGAAGTACAAGTGAAG
CAGAAGAAG------------GGGGAGGACGAAAGGTTCAACCTATCTAATGTTACAACC
---AACGAAGAGGCA---
>Yellowfin_tuna_Aqp3b
ATGGGAAAACAAAAAGACATTTTGAAGAAACTGGCAGGGACCTTTCAGATCCGACATGTG
CTCCTCCGCCAGGCCCTGGCAGAATGCCTGGGAACCCTTATTCTGGTGATGTTTGGTTGT
GGTGCTGTGGCACAGTTGGTGCTCAGTGGAGGCTCCCATGGCACCTTCCTCACTGTTAAC
TTGGCTTTTGGATTTGCTGCAACATTGGGCGTCCTTGTGAGTGGACAGGTCTCAGGTGGC
CATCTGAACCCAGCGGTGACCTTCGCATTATGTTTACTTGGAAGAGAGCCTTGGAGGAAG
TTCCCATTGTTCTTTCTCTTCCAGACTCTTGGAGCCTTCTTGGGTGCAGTGATTATATTT
GGCATGTATTTTGATGCCTTATTGGATTTTGGCCATGGG------------GAACTGGTC
GTGTTGGGGAAGAATGCCACAGCTGGGATTTTTGCCACATATCCGTCCAAACATCTCAGC
TTGGTTAACGGATTCTTTGATCAGATTATTGGAACAGCTTCATTGATCGTGTGCATCCTG
GCCATCGCTGATCCACATAACAACCCAATCCCTCGAGGTCTTGAGGCCTTCACAGTAGGT
TTTGTGGTGTTGGTCATCGGCCTTTCAATGGGCTTCAACTCTGGCTATGCTATCAACCCA
GCCAGGGACCTGGGACCACGCATCTTCACGGCTCTTGCAGGCTGGGGTGGCGAGGTTTTC
ACGGCAAAGACCTACTGGTTCTTTGTGCCTATCTGTGCCCCATTCTTGGGTGCAGTGGTG
GGTTTGATGGTGTACCAGTTCATGGTTGGATACCATTTAGAGGGAGAAGTACAAGTGAAG
CAGAAGAAG------------GGGGAGGACGAAAGGTTCAACCTTTCTAATATTACAACC
---AACGAAGAGGCA---
>Silver_pomfret_Aqp3b

--ATGTTTGGTTGT
GGTGCTGTGGCACAGTTGGTGCTCAGCAGAGGCTCCCACGGCAGCTTCCTCACCGTCAAC
TTGGCTTTTGGATTTGCTGCAACATTGGGCATCCTTGTAAGTGGACAGATCTCA------
---GAGCCTTGGAAGAAG
TTCCCCTTCTTCTTTTTCTTCCAGACTCTTGGAGCCTTCTTGGGTGCAGCGATCGTATTC
GGTTTGTATTTTGATGCCTTATGGGACTTTGGCCAAGGG------------GAGCTGGTC
GTGGTGGGGAGGAATGCCACAGCTGGGATTTTTGCCACATATCCATCCGAACATCTCACG
TTGGTTAACGGATTCTTTGATCAGATTATTGGAACAGCTGCATTGATTATGTGCATCCTG
GCCATCATTGATCCACACAACAATCCAATCCCTCGAGGTCTGGAAGCCTTCACAGTAGGT
TTTGTGGTGCTGGTCATTGGCCTGTCAATGGGCTTCAACTGTGGCTATGCCGTCAACCCA
GCCAGGGACCTGGGACCCCGCGTCTTCACGGCTCTTGCAGGCTGG---------------

>Clown_anemonefish_Aqp3b
ATGCGAGGACAAAAAGAGATTTTAGAGCAACTGGCAGGGACCTTCCACATCCAGCATGTG
CTGCTCCGCCAGGCCCTGGCAGAATGCCTGGGAACGCTTATTCTGGTGATGTTTGGTTGT
GGCTCGGTGGCACAGATGCTGCTAAGTGAAGGATCTCATGGCACATTCCTCTCTGTTAAC
CTGGCTTTTGGTTTTGCTGTAACACTGGCCATCCTTGTGAGTGGACAGATCTCAGGTGGT
CATCTGAACCCAGCGGTGACCTTCACACTGTGTTTACTTGGGAGAGAGCCTTGGAGGAAG
TTCCTGCTGTTCTGTTTCTTCCAGACGCTTGGAGCCTTTCTGGGTGCGGCCGTTGTATTT
GGAATGTATTATGATGCACTGTGGGACTTTGGCCAGGGG------------CAGCTGATT
GTGGTGGGGAAGAATGCCACAGCCGGGATTTTTGCCACATACCCAGCCAAGCATCTCACC
CTGGTTAATGGATTCTTTGATCAGATTATTGGAACAGCTGCACTGATCGTGTGTATCCTG
GCCATAGTTGATCCACACAACAACCCAGTCCCTAAAGGTCTGGAGGCCTTCACAGTGGGC
TTTGTTGTGTTGGTCATCGGCCTGTCAATGGGCTTCAACTCTGGCTATGCTGTCAATCCA
GCTAGGGATTTAGGACCACGCATCTTTACAGCTGTTGCCGGCTGGGGTGGAGAGGTTTTC
ACGGCGAACACCTACTGGTTCTTTGTGCCCATCTGTGCTCCATTCTTTGGCGCAGTGGTG
GGTGTGCTGATGTACCAGCTGATGATTGGATACCATCTAGAAGGAGAAGAGAAACAGGAG
CAGAGG---------------GAGGAGGAGGAAAGGTTCAAACTTTCCAATATCACAACC

---AACAATGATGCA---
>Orange_clownish_Aqp3b
ATGCGAGGACAAAAAGAGATTTTAGAGCAACTGGCAGGGACCTTCCACATCCAGCATGTG
CTGCTCCGCCAGGCCCTGGCAGAATGCCTGGGAACGCTTATTCTGGTGATGTTTGGTTGT
GGCTCGGTGGCACAGATGCTGCTAAGTGAAGGATCTCATGGCACATTCCTCTCTGTTAAC
CTGGCTTTTGGTTTTGCTGTAACACTGGCCATCCTTGTGAGTGGACAGATCTCAGGTGGT
CATCTGAACCCAGCGGTGACCTTCACACTGTGTTTACTTGGGAGAGAGCCTTGGAGGAAG
TTCCTGCTGTTCTGTTTCTTCCAGACGCTTGGAGCCTTTCTGGGTGCGGCCGTTGTATTT
GGAATGTATTATGATGCACTGTGGGACTTTGGCCAGGGG------------CAGCTGATT
GTGGTGGGAAAGAATGCCACAGCCGGGATTTTTGCCACATACCCAGCCAAGCATCTCACC
CTGGTTAATGGATTCTTTGATCAGATTATTGGAACAGCTGCACTGATCGTGTGTATCCTG
GCCATAGCTGATCCACACAACAACCCAGTCCCTAAAGGTCTGGAGGCCTTCACAGTGGGC
TTTGTTGTGTTGGTCATCGGCCTGTCAATGGGCTTCAACTCTGGCTATGCTGTCAATCCA
GCTAGGGATTTAGGACCACGCATCTTTACAGCTGTTGCCGGCTGGGGTGGAGAGGTTTTC
ACGGCGAACACCTACTGGTTCTTTGTGCCCATCTGTGCTCCATTCTTTGGCGCAGTGGTG
GGTGTGCTGATGTACCAGCTGATGATTGGATACCATCTAGAAGGAGAAGAGAAACAGGAG
CAGAGG---------------GAGGAGGAGGAAAGGTTCAAACTTTCCAATATCACAACC
---AACAATGATGCA---
>Twoband_anemonefish_Aqp3b
ATGCGAGGACAAAAAGAGCTTTTAGAGCAACTGGCAGGAACCTTCCACATCCAGCATGTG
CTGCTCCGCCAGGCCCTGGCAGAATGCCTGGGAACGCTTATTCTGGTGATGTTTGGTTGT
GGCTCGGCGGCGCAGATGCTGCTAAGTGAAGGATCTCATGGCACATTCCTCTCTGTTAAC
CTGGCTTTTGGTTTTGCTGTAACCCTGGCCATCCTTGTGAGTGGACAGATCTCAGGTGGT
CATCTGAACCCAGCGGTGACCTTCACACTGTGTTTACTTGGGAGAGAGCCTTGGAGGAAG
TTCCTGCTGTTCTATTTCTTCCAGACGCTTGGAGCCTTTCTGGGTGCGGCCGTTGTATTT
GGAATGTATTATGATGCATTGTGGGACTTTGGCCAGGGG------------CAGCTGACT
GTGGTGGGGAAGAATGCCACAGCCGGGATTTTTGCCACATACCCAGCCAAGCATCTCACC
CTGGTTAATGGATTCTTTGATCAGATTATTGGAACAGCTGCACTGATCGTGTGTATCCTG
GCCATAGTTGATCCACACAACAACCCAGTCCCTAAAGGTCTGGAGGCCTTCACAGTGGGC
TTTGTTGTGTTGGTCATTGGCCTGTCAATGGGCTTCAACTCTGGCTATGCTGTCAATCCA
GCTAGGGATTTAGGACCACGCATCTTTACAGCTGTTGCCGGCTGGGGTGGAGAGGTTTTC
ACGGCGAACACCTACTGGTTCTTTGTGCCCATCTGTGCTCCATTCTTTGGCGCAGTGGTG
GGTGTGCTGATGTACCAGCTGATGATTGGATACCATCTC---------------------

>Spiny_chromis_Aqp3b
ATGCGAGGACAAAAAGAGATTTTAGAGCAACTGGTAGGGACCTTCCACATCCAGCATGTG
CTGCTCCGCCAGGCCCTGGCAGAATGCCTGGGAACGCTTATTTTGGTGATGTTTGGTTGT
TGCTCGGTGGTGCAGATGGTGCTAAGTGAAGGATCCCATGGCACGTTCCTCTCTGTTAAC
CTGGCTTTTGGTTTTGCTGTAACACTGGCCATCCTTGTGAGTGGACAGATCTCAGGTGGT
CATCTGAAGCCACCGGTGACCTTCACACTGTGTTTACTTGGGAGAGAGCCTTGGAGGAAG
TTCCCGCTGTTCTATTTCTTCCAGACGCTTGAAGCCTTTCTGGGTGCGGCCGTTGTATTT
GGAATGAATTATGATGCATTGTGGGACTTTGGCCAGGGG------------CAGCTG---
---GTTGGGAAGAATGCCACAGCTGGGATTTTTGCCACATACCCAGCCAAGCAGCTCACC
CTGGTTAATGGATTCTTTGATCAGATTATTGGAACAGCTGCACTGATCGTGTGCATCCTG
GTCATAGCTGATCCACACAACAACCCAGTCCCTAAAGGCCTGGAGTCCTTCACCGTGGGC
TTTGTTGTGTTGGTCATCGGCCTGTCAATGGGCTTCAACTCTGGCTATGCTGTCAATCCA
GCCAGGGATTTAGGATCACGCATCTTTACAGCTATTGCTGGCTGGGGTGGAGAGGTTTTC
ATGGCGAACACCTACTGGTTCTTTGTGCCCATCTGTGCTCCATTCTTTGGTGCAGTGGTG
GGTGTGCTGATGTACCAGCTGATGATTGGATACCATCTACAAGGAGAAGTGAAAGAGGAG
CAGAGG---------------GAGGAGGAGGAAAGGTTCAAACTTTCCAATATCACAAAC
---AACAATGATGCA---
>Bicolor_damselfish_Aqp3b
ATGAGAGGACAAAAAGAGATTTTAGAGAAACTGGCAGGGACCTTCCACATCCAGCATGTG
CTGCTCCGCCAGGCCCTGGCAGAATGCCTGGGAACGCTTATTTTAGTGATGTTTGGTTGT
GGTGCGGTGGCGCAGATGGTGCTGAGTGAAGGATCTCATGGCACATTTCTCTCTGTTAAC
CTCGCTTTTGGTTTCGCTGCAACTCTAGGCATCCTTGTGAGTGGAAAGGTCTCAGGTGGT
CATCTGAACCCAGCATTGACATTCACGCTGTGTTTACTTGGGAGAGAGCCTTGGAGGAAG
TTCCCGCTGTTCTTTTTCTTCCAGACGCTTGGAGCCTTCCTGGGTGCGGCCGTCGTATTT
GGAATGTATTTCGAAGCATTGTGGGACTTCGGCCAGGGA------------CAGCTGATT
GTGGTGGGGAAGAATGCCACAGCTGGGATTTTTGCAACTTACCCAGCCAAACATCTCACC

CTGGTTAATGGATTCTTTGATCAGATTATTGGAACAGCTGCTCTGATCGTGTGCATCCTG
GCCATAGCTGATCCATGCAACAGTCCAGTCCCTAGAGGTCTGGAGGCCTTCGCAGTGGGC
TTTGTTGTGTTGGTCATCGGCCTGTCAATGGGCTTCAACTCTGGCTATGCCGTCAACCCA
GCCAGGGATTTAGGACCACGCATCTTTACGGCGGTTGCAGGCTGGGGTGGAGAGGTTTTC
ACAGCGAACACCTACTGGTTCTTTGTGCCCATCTGTGCTCCATTCTTGGGCGCAGTGGTG
GGTGTGTTGATGTACCAGCTGATGATTGGATACCATCTACAAGGAGTAGTGCAAGAGGAG
CAGAGG---------------GAGGAGGAGGAAAGGTTCAAACTCTCCAATATCACAACC
---AACGAAGATGCA---
>Rockpool_blenny_Aqp3bp
ATGAGAACACAAAAACAAATTTTACAGAAACTGGCAGCGACATTTCGCATCCGGTGGGTG
CTCCTCCAGCAGGCCCTGGCAGAATGCCTGGGAACCCTCATTTTGCTGATGTTTGGCTGC
AGTCCGGTGGCACAAACGATGTTGGCTTCAGGATCCCACGGCACATTTCTCACCGTGAAC
TTTGCGTTTGGTTTGGCTGTGATGCTGGCTGTTATTGCAAGTGGACAGGTCTCAGGTGAC
TATTTGAACCCTGCAGTGACCTTCACGCTGTGTCTGCTCGGGAGGGAGCCTTGGAAGAAG
TTT------TTCTTTGCCTTCCAGTCGCTTAGAGCTTTCCTGTGTGCAGCCATCGTGTTC
TGTGTGTATTTTGATGCATTACGGGACTTCGGCCAGGGG------------CAGCTCATT
GTGGTGGGAAAGAATGCCACAGCTGGGATATTTGCCACATACCCAGCCAAACATCTTGCC
CTGATTAATGGATTCTTTGATCAG------------------------------------
---------GACCCG------------GTTCCTCCGGATCTGCAGGCCGTCGCTGTCGGC
TCGGTTGTGTTAGTAGTCGGCCTGTCAATGGGCTACAACACTGGCTACACCATCAACCCG
GCCAGACATTTAGGATCGCGCCTCTTCACGGCCTTTGTGGGCTGGGGTTTGGAGGTTTTT
ACGGCAAACGCCTACTGGTCCTTCGTGCCCATCTGTGCCCCATTCCTGGGTGCAGTGGTG
GGTGTGCTGATATACCAGCTAATGACTGGATGCCATCGAGAAGGAGACGCGAGAGAGATG
TCAAAG---------------GAAGAGGAGGAAAAGGTAAAGTTT---------------

>Derbio_Aqp3b
ATGGCGAAACAGAAAGACATTTTGGAGAAACTGGCAGGGACCTTTCAGATCCGACATGTG
CTCCTCCGCCAGGCCCTGGCTGAATGCCTGGGAACCCTCATTCTGGTGATGTTTGGTTGT
GGTGCAGTAGCACAGCTGGTGCTAAGCCGAGGTTCCCATGGCACATTCCTCACTGTTAAT
CTTGCTTTTGGATTTGCCGCAACGTTGGGCGTCCTTGTGAGTGGACAGGTCTCAGGTGGT
CATCTGAACCCAGCGGTGACCTTCACACTGTGTTTACTTGGGAGAGAGCCCTGGAAGAAG
CTCCCATTGTTCTTTTTCTTCCAGACTCTTGGGGCCTTCCTGGGTGCAGCGATCATATTT
GCCATGTATTCCGATGCATTATGGGACTTTGGTCATGGG------------GAGCTGGTT
GTGGTGGGGAAGAATGCCACAGCTGGAATTTTTGCTACATATCCATCCAGACATCTCACC
CTGCTAAATGGATTCTTTGATCAGATTATTGGAACAGCTGCATTGATCGTGTGCATCCTG
GCCATAGTTGATCCACACAACAATCCCATCCCTCCAGGTCTGGAGGCCTTCACTGTAGGC
TTTGTGGTGCTGGTCATCGGCTTGTCAATGGGCTACAACTCTGGGTATGCTGTCAACCCA
GCGAGGGACCTGGGACCACGCGTCTTCACAGCTCTTGCAGGCTGGGGTGGAGAGGTTTTC
ACGGCGTACACCTACTGGTTCTTCGTGCCCATCTGTGCCCCCTTCTTGGGTGCAGTGGTG
GGTGTGCTGACGTACCAGCTAATGATTGGACACCATTTAGAAGGAGAAGTGCAGGAGAAG
CAGAAGGAG------------GAGGAGGAGGAAAGGGTCAAACTTTCCAGTGTTCCAACC
---AATGAAGATGCA---
>Japanese_amberjack_Aqp3b
ATGGGGAAACAAAAAGACATTTTGGAGAAGCTGGCAGGGATCTTTCAGATCCGACATGTG
CTCCTCCGCCAGGCCCTGGCTGAATGCCTGGGAACCCTCATTCTGGTGATGTTTGGTTGT
GGTGCAGTAGCACAGCTGGTGCTAAGCCGAGGATCCCATGGCACATTCCTCTCTGTTAAC
TTTGCTTTTGGATTTGCTGCAACATTGGGCATCCTTGTGAGTGGACAGGTCTCAGGTGGT
CATCTGAACCCGGCAGTCACCTTCACACTGTGCTTACTGGGGAGAGAGCCCTGGAGGAAG
TTCCCGTTGTTCTTCCTCTTCCAGACGCTCGGGGCCTTCCTGGGTGCAGCGATCGTATTT
GGCATGTATTCCGATGCATTATGGGACTTTGGCAAGGGG------------GAGTTGATT
GTGGTGGGGAAGAATGCCACAGCTGGAATTTTTGCCACATATCCTTCCAAACACCTCACC
CTGCTTAATGGAGTCTTTGATCAGATTATTGGAACAGCTGCATTGATTGTGTGCATCCTG
GCCATAGTTGATCCACACAACAACCCAGTCCCTCGAGGTCTGGAGGCCTTCACTGTCGGC
TTTGTGGTGTTGGTCATCGGCCTGTCAATGGGCTTCAACTCCGGGTATGCCGTCAACCCA
GCCAGGGACCTGGGACCGCGCATCTTCACGGCTCTTGCAGGCTGGGGTGGAGAGGTTTTC
ACGGCAAACACCTACTGGTTCTTCGTGCCCATCTGTGCCCCCTTCTTGGGCGCAGTGGTG
GGTGTGCTGATGTACCAGCTAATGATTGGACACCATTTAGAAGGAGAAGTGCAAGAGAAG
CAGAAGGAAGAGGAG------GAGGAGGAGGAAAGGTTCAAACTTTCCAACATGACAACC
---AATGAAGATGCA---
>Greater_amberjack_Aqp3b
ATGGGGAAACAAAAAGACATTTTGGAGAAGCTGGCAGGGATCTTTCAGATCCGACATGTG

CTCCTCCGCCAGGCCCTGGCTGAATGCCTGGGAACCCTCATTCTGGTGATGTTTGGTTGT
GGTGCAGTAGCACAGCTGGTGCTAAGCCGAGGATCCCATGGCACATTCCTCTCTGTTAAC
TTTGCTTTTGGATTTGCTGCAACACTGGGCATCCTTGTGAGTGGACAGGTCTCAGGTGGT
CATCTGAACCCGGCGGTCACTTTCACACTGTGCTTACTGGGGAGAGAGCCTTGGAGGAAG
TTCCCGTTGTTCTTCTTCTTCCAGACGCTCGGGGCCTTCCTGGGTGCAGCGATCGTATTT
GGCATGTATTCCGATGCATTATGGGACTTTGGCAAGGGG------------GAGTTGATT
GTGGTGGGGAAGAATGCCACAGCTGGAATTTTTGCCACATATCCATCCAAACACCTCACC
CTGCTTAATGGAGTCTTTGATCAGATTATTGGAACAGCTGCATTGATCGTGTGCATCCTG
GCCATAGTTGATCCACACAACAACCCAGTCCCTCGAGGTCTGGAGGCCTTCACTGTCGGC
TTTGTGGTGTTGGTCATCGGCCTGTCAATGGGCTTCAACTCCGGGTATGCTGTCAACCCA
GCCAGGGACCTGGGCCCGCGCATCTTCACGGCTCTTGCAGGCTGGGGTGGAGAGGTTTTC
ACGGCAAACACCTACTGGTTCTTCGTGCCCATCTGTGCCCCCTTCTTGGGCGCAGTGGTG
GGTGTGCTGATGTACCAGCTAATGATTGGACGCCATTTAGAAGGAGAAGTGCAAGAGAAG
CAGAAGGAAGAGGAG------GAGGAGGAGGAAAGGTTCAAACTTTCCAACATGACAACC
---AATGAAGATGCA---
>Barramundi_Aqp3b
ATGGGAAAACAAAAAGATATTTTGGAGAAACTGGCAGGGATCTTTCAGATTCGACATGTG
CTCCTCCGCCAGGCCCTGGCAGAATGCCTGGGAACCCTTATTCTGGTGATGTTTGGTTGT
GGTGCAGTTGCACAGCTGGTGCTAAGCAGAGGATCCCATGGCACATTCCTCAGTGTTAAC
TTTGCTTTTGGATTAGCTGCAACTCTGGGCATCCTTGTGAGTGGACAGGTCTCAGGTGGT
CATCTGAACCCAGCGGTGACCTTCACACTTTGTTTACTTGGGAGAGAGCCTTGGAGGAAG
TTCCCATTGTTCTTTTTCTTCCAGACTCTCGGGGCCTTCCTGGGTGCATCGATCGTATTT
GGCATGTATTCTGATGCATTATGGGACTTTGGCCATGGG------------AAGCTGATT
GTAATAGGGAAGAATGCCACAGCGGGAATTTTTGCCACATATCCATCAAAACACCTCACC
CTGGTTAATGGATTCTTTGATCAGATCACTGGAACAGCTGCACTGATCGTGTGCATCCTG
GCCATAGTTGATCCACACAACAATTCAGTCCCTCGAGGCCTAGAGGCCTTCACTGTAGGC
TTTGTGGTGTTGGTCATCGGCCTGTCAATGGGATTCAACTCTGGGTATGCTGTCAACCCA
GCCAGGGACCTGGGACCACGTATCTTCACTGCTCTTGCAGGCTGGGGTGCTGAGGTTTTC
ATGGCAAACACCTACTGGTTCTTTGTGCCCATCTGTGCCCCCTTCTTGGGTGCAGTGGTG
GGAGTGCTGATGTACCAGCTAATGATTGGGCACCATTTAGAAGGAGACGTGCAAGAGAAG
CAGAAGGAG------------GAGGCGGTGGAAAGACTCAGACTTTCCAATATTACAACC
---AATGAAGATGAA---
>Bearded_brotula_Aqp3b
ATGGGGAAACAAAAAGACATCCTGGAGAAACTGGCAACAACATTCCAGATCCGGCACGTT
CTCCTTCGCCAGGGCCTGGCAGAATGCCTGGGAACGCTTGTTCTGGTGATGTTTGGCTGT
GGCTCGGTGGCACAGCTGGTCCTCAGCCATGGCTCTCACGCCGTGTTCCTCACTGTTAAT
TTTGCTTTTGGGTTCGCTGTAACACTGGGGATACTCATCAGTGGACAAATCTCAGGTGGT
CATCTGAACCCAGCAGTAACTTTCACGCTGTGTTTACTTGGGAGAGAACCTTGGAAGAAA
TTCCCTGTGTTTTTCTTCTTCCAAACAGTGGGTGCTTTCCTGGGTGCAGCGATCATTTTT
GGCATGTACTATGATGCATTATGGGACTACGGCCAGGGA------------GAGCTCATT
GTAGTGGGGAAGAATGCCACAGCGGGAATTTTTGCCACATATCCATCCAAACACCTGGGC
CTCATTAATGGAGTCTTTGACCAGATCATTGGGACGGCTGCATTGATCGTGTGTATCCTG
GCTATAGTTGATCCACATAACAATTCCGTTCCCAGAGGTCTGGAGGCTTTCACTGTGGGC
TTTGTGGTGTTGGTCATCGGTCTGTCAATGGGCTTCAACTCTGGCTATGCTGTAAATCCA
GCCAGGGACCTGGGACCTCGAATTTTTACAGCTCTTGCAGGTTGGGGTGGTGAGGTTTTC
ACGGCAAACACCTACTGGTTCTTTGTGCCAACCTGTGCCCCTTTCGTGGGTGCAGTGATG
GGTGTGATGGTCTACCAGCTGATGATTGGATATCATTCAGAAGGAGAAGCTAAAGAGAAG
CAAAAGAAA---------------GAAGAGGAAATATTCAAACTTTCCAATAGTACAGCC
---AGTGAAGATGTT---
>Legless_cuskeel_Aqp3b
ATGGGAAAACAAAGAGACATTTTGGAGAAACTTGCATGGACCTTTCAGATCCAGCACACG
CTGCTGCGCCAGGTCCTGGCAGAATGCCTGGGAACTCTTATTCTGGTGGTGTTTGGCTGT
GGCGCTGTGGCACAGCTGGTCCTGAGTCGAGGTTCTCATGGCACGTTCCTCACTGTTAAT
TTTGCGTTTGGCTTTGCCGTAACGCTGGGGATTCTCGTGAGTGGACAGATCTCAGGTGGT
CATCTGAACCCAGCCGTAACGTTCACGCTGTGTTTACTTGGTAGAGAACCGTGGAAGAAG
TTCCCTGCGTTTTTCTTTTTCCAAACAGTGGGTGCCTTCCTGGGTTCAGCGATCGTATTT
GGCATGTATTTTGATGCATTATGGGACTATGGCCAGGGA------------CAACTCATT
GTTGTGGGGGAAAATGCCACAGCTGGAATTTTTGCTACGTATCCATCCAAGCACCTGAGC
CTGGCGAATGGAGTCTTTGATCAGATTATTGGAACAGCTGCATTGATCGTATGTATCCTG
GCTATAGTTGATCCACACAACAATCCAGTCCCCAGAGGTCTGGAAGCCTTCACTGTGGGC
TTTGTGGTGCTGGTCATTGGCCTGTCAATGGGCTTCAACTCTGGCTATGCTGTGAACCCA

GCCAGGGACCTGGGACCCCGCATCTTTACTGCTCTTGCAGGTTGGGGTGGTGAGGTATTC
ACGGCAAATGCCTACTGGTTCTTTGTGCCCCTCTGCGCCCCCTTTGTGGGTGCCATAGTG
GGTGTGATGATATACCAACTAATGATCGGATATCATTTAGAAGGAGAAGCAAGAGAGAAG
CAAAAGAAT------------GAGGAAGAAGAAAGATTCAAACTTTCCAGTGTTACAGCT
---AATGAGGATGCA---
>Sammara_squirrelfish_Aqp3b
ATGGGAAAACAAAAGGACATTTTGGAGAAACTGGCGCGAACCTTTAAGATCCGACACAAG
CTCCTGCGCCAGGCCCTGGCAGAGTGTCTGGGAACCCTTATCCTGGTGATGTTTGGCTGT
GGTGCAGTGGCACAGTTGGTGCTGAGCGGGGGCTCCCATGGCATATTCCTGACCGTTAAT
TTTGCCTTTGGATTTGCTGCCACACTGGGCATCCTTGTGAGTGGACAGGTCTCAGGTGGC
CATCTGAACCCAGTGGTGACCTTCGCCCTTTGCTTACTTGGGAGAGAGCCTTGGAGGAAA
TTCCCTGTGTTTTTCTTCTTTCAGACACTGGGTGCCTTCCTGGGGTCAGCAATCATATTT
GGCATGTATTTTGATGCATTGTGGGACTACGGTCAGGGG------------GAACTCACT
GTGGTGGGGAAGAATTCTACAGCTGGGATTTTTGCCACATATCCATCCAAACATCTCACC
CTGCTTAATGGATTCTTTGATCAGATTATTGGAACAGCTGCTTTGATTGTGTGCATCCTG
GCTATAGTTGATCCGCACAACAATCCAGTCCCCAGAGGTCTGGAGGCCTTCACTGTTGGC
TTTGTGGTGCTGGTCATCGGCCTTTCCATGGGCTTCAACTCAGGCTATGCTGTCAACCCG
GCCCGGGACCTGGGACCACGCATCTTTACCGCTGTTGCAGGCTGGGGTGCTGACGTTTTC
ATGGCAAGAGACTACTGGTTCCTCGTACCCATCTTTGCCCCCTTCTTGGGTGCAGTAGTG
GGTGTGATTTTGTACCAGCTGATGGTGGGATACCATGTGGAAGGGGAAGCACGAGAGGAG
GAGGAG---------------GAAGAAGAGGAAAGGTTCAAACTTTCCAATATTACAACC
---AAAGGAGATGCA---
>Longspine_squirrelfish_Aqp3b
ATGGCAAGACAAAAGGACATTTTGGAGAAACTGGCACAAACCTTTCAGATCCGACACAAG
CTCCTGCGCCAGGCCCTGGCAGAATGTCTGGGAACCCTCATACTGGTGATGTTTGGATGT
GGTGCAGTGGCACAGTTGGTGCTAAGTGGGGGCTCCCATGGCATATTCCTGACTGTTAAT
TTTGCCTTTGGATTTGCTGCAACACTGGGCATCCTTGTGAGCGGACAGGTCTCAGGTGGC
CATCTGAACCCAGTAGTGACCTTCGCCCTTTGTTTACTTGGGCGAGAGCCTTGGAGGAAG
TTCCCTGTGTTTTTCTTCTTTCAGACACTGGGCGCCTTCCTGGGGTCAGCAATCATATTT
GGCATGTATTTTGATGCATTATGGGACTATGGTCAGGGG------------GAACTCACT
GTGGTGGGGAAGAATTCTACGGCTGGGATTTTTGCCACATATCCATCCAAACATCTCACC
CTGCTTAATGGATTCTTTGATCAGATTATTGGAACAGCTGCTTTGATCGTGTGCATCCTG
GCTATAGTTGATCCATATAACAATCCAGTCCCTCGAGGTCTGGAGGCCTTCACTGTGGGT
TTTGTGGTGCTGGTCATCGGCCTTTCCATGGGCTTCAACTCAGGCTATGCTGTCAACCCG
GCCCGGGACCTGGGACCACGCATCTTTACCGCTGTGGCAGGCTGGGGTGCTGAGGTTTTC
ATGGCAAGAGACTACTGGTTCCTCGTGCCCGTCTTTGCTCCCTTCTTGGGTGCAGTAGTG
GGTGTGATTTTGTACCAGCTGATGGTGGGATGCCATGTCGAAGGGGAAGCACGAGAGAAG
GAGGAG---------------GAAGAAGAGGAAAGGCTCAAACTTTCCAATATTACAACC
---ACAGGAGATGCA---
>Blackbar_soldierfish_Aqp3b
ATGGGAAAACAGAAGGTCATTTTGGAGAAACTGGCACAAACCTTTCAGATCCGTCACGTG
CTCCTCCGCCAGGCTCTGGCAGAATGTCTGGGAACCCTCATACTGGTGATGTTTGGCTGT
GGTGCCGTGGCACAGCTGGTACTGAGCGGCGGCTCCCACGGCACATTCCTCACCGTTAAT
TTTGCCTTTGGATTTGCTGCAACACTGGGCATCCTCGTGAGCGGACAGGTCTCAGGTGGC
CATCTGAATCCAGCAGTGACCTTTGCCCTGTGCTTGCTTGGGCGAGAGCCTTGGACCAAG
TTCCCCGTGTTTTTCTTCTCTCAGACTTTAGGAGCCTTCCTGGGTTCAGCACTTATATTT
GGCATGTATTTTGATGCATTGTGGGACTACGGACAGGGG------------GAACTCATT
GTGGTGGGGAGCAATTCCACGGCCGGGATTTTTGCCACATACCCATCCAAACATCTGACC
CTGGTTAATGGATTCTTTGATCAGATTATCGGGACAGCTGCACTGATCGTGTGCATCCTG
GCTATAGTCGATCCATACAACAATCCGGTCCCCAGAGGTCTGGAGGCCTTCACCGTCGGC
TTGGTGGTGCTGGTCATCGGACTCTCAATGGGCTTCAACGCAGGCTATGCCATCAACCCA
GCCCGGGATCTGGGACCACGCATCTTTACAGCTGTGGCCGGCTGGGGTGCTGAGGTTTTC
ATGGCAAGAGAATACTGGTTCTTCGTGCCCGTCTGCGCCCCCTTCTTGGGTGCAGTGGTG
GGGGTGGTTTTGTACCAGCTAATGGTGGGATACCACGTAGAGGGGGAGGCACGAGAGAAG
---------------------CACGAAGAGGAAAAGCTTGGAGGTGCCAGTGTTACAACC
---AAAGTAGATGCA---
>Splendid_alfonsino_Aqp3b
ATGGGGAAACAACAGGACATGCTGGAGAAGCTGGCGCGGACCTTTCGGATCCGACACACG
CTCCTCCGCCAGGCCCTGGCAGAATGTCTGGGAACCCTTATTTTGGTGATGTTTGGCTGC
GGTGCGGTGGCACAGCTGGTACTGAGTGGGGGCTCCCACGGCATGTTCCTCACCGTTAAC
TTTGCTTTTGGATTTGCTGCAACACTGGGCATCCTTGTGAGCGGACAGGTCTCAGGTGGT

CATCTGAACCCCGCAGTGACCTTCGCTCTGTGTGTTCTTGGGAGAGAGCCTTGGAGGAAG
TTCCCTGTGTTCTTCCTCTTCCAGACTCTGGGTGCCTTCCTGGGTTCAGGGATCGTTTTT
GGCCTGTATTTTGATGCATTATGGGACTATGGCCAGGGG------------GAACTCATC
GTGGTGGGGAAGAACTCCACAGCTGGGATTTTTGCCACATATCCATCCAAACATCTCACC
CTGGTTAATGGATTCTTTGATCAGATGATCGGAACAGCTGCGTTGATCGTGTGTATCCTG
GCTATAGTGGATCCATACAACAATCCAATCCCTCGAGGTCTGGAGGCTTTCACTGTAGGC
TTCGTGGTATTGGTCATCGGCCTTTCAATGGGCTTCAACTCAGGTTATGCTGTCAACCCA
GCCAGGGACCTGGGACCGCGCCTCTTTACGGCTCTTGCAGGCTGGGGTGGTGAGGTTTTC
ACGGCAAAAGCCTACTGGTCCTTCGTGCCCATCTGTGCCCCCTTCCTGGGTGCAGTAGTG
GGTGTGATGGTGTACCAGCTCATGGTGGGATACCATGTGGAAGGGGAAGCGCGAGAGAAG
GAGGAGGAGGAGGAGAAG---GAAGAAGAGGAACGACTCAAACTGTCCAATATTACAACC
---AAAGAAGATGCC---
>Redmouth_whalefish_Aqp3b

--ATGTTTGGCTGT
GGTGCGGTGGCACAGCTGGTACTGAGTGGGGGCTCCCATGGCATG------ACCGTTAAT
TTTGCCTTTGGATTTGCTGCAACACTGGGCATCCTTGTGAGTGGACAGGTCTCAGGTGGT
CATCTGAACCCAACAGTGACCTTTGCACTGTGTTTACCTGGGAGAGAGCCTTGGAGGAAG
TTCCCTGTGTTTTTCTTCTTTCAGAATCTGGGTGCCTTCCTGGGTTCAGGGATCATATTT
GGCCTGTATTTT--

>Pricklfish_Aqp3bp
GTGGGAAAACAAAAGGACATTCTGGAGAAACTGGTATGGACCTTT---ATCCGACACGTG
CTCCTTCGC---

--GGTGGT
CATCTGAACCCAACAGCGACCTTCGCACTGTGTTTACTTGGGAGAGAGCCTTGGAGGAAG
TTCCCT------------------TCCCTGGGTGCCTACCTGGGTTCAGTGATCATATTT
GGCCTGTATTTT--

>Pinconefish_Aqp3b
--ATTCAGATCCCTCACAAG
CTCCTTCGCCAGGCCCTGGCAGAATGCCTGGGAACCCTTATTCTGGTGATGTTTGGCTGT
GGTGCGGTGGCACAGCTGGTGCTGAGTCGGGGCTCCCATGGCATGTTCCTCACCGTTAAT
TTTGCCTTTGGATTTGCTGCAACACTGGGCATCCTTGTGAGTGGACAGGTCTCAGGT---
---------------------------CTGTGTTTACTTGGGAGAGAGCCTTGGAGGAAG
TTCCCTGTGTTTTTCTTCTTTCAGACTCTGGGTGCCTTTCTTGGTTCAGGGATCATATTT
GGCATGTACTTTGATGCATTATGGGACTATGGCCAGGTG------------TCACTCATC
GTAGTGGGGGAGAATGCCACTGCTGGGATTTTTGCCACATATCCATCCAAACATCTCACG
CTGGTTAATGGAGTCTTTGATCAGATTATTGGCACAGCCGCATTGATTGTGTGCATCCTG
GCTATAGTTGATCCATACAACAATCCAATCCCTAGAGGTCTGGAGGCCTTCACTGTAGGC
TTTGTGGTATTGGTCATCGGCCTGTCAATGGGCTTCAACTCAGGCTATGCTGTCAACCCA
GCCAGGGACCTGGGACCTCGCATCTTTACAGCTCTCGCAGGCTGGGGTAGTGAGGTTTTC
ACG---

>Highfin_tadpole_fish_Aqp3b

---ATGTTTGGCTGC
GGTGCCCTGGCACAGCTGGTGCTGAGCGGAGGGTCCCATGGCACATTCCTCACTGTCAAC
CTAGCCTTCGGTTCTGCGGCGACACTGGGCGTTCTCGTGAGCGGACAGGTCTCAGGTGTG
CGTCTGACCCCGGCCGTGACCTTTGCACTGTGGTTACTTGGGAGCGTGCCGTGGAGGAAG
TTGCCTGTGTTTTTCCTTTCCCAAACTTTGGGGGCCTTCCTGGGTTCAGGGATCATATTT
GCCATGTATTTCGATGCATTATGGGACTACGGCCAGGGT------------GCCCTCACT
GTGGTGGGGAAGAATGCCACAGCAGGGATTTTTGCAACTTATCCATCCAAACACCTCACC
CCGGTGAACGGATTCTTTGATCAGGTGATTGGAACAGCAGCACTGATTGTATGTATCCTG
GCTATCGTTGACCCATATAACAACCCCGTCTCCAGCGGTCTGGAGGCCTTCACCGTGGGC
TTTGTGGTTCTGGTCATTGGCTTATCAATGGGCTTCAACTCAGGCTACGCTGTCAATCCA
GCCAGGGACCTAGGACCCCGCATCTTCACGGCTCTGGCAGGCTGGGGCCCCGAGGTCTTT
ACGGCAAAAACCTGCTGGTCCTTCGTGCCCATCTGTGCCCCCTTCCTGGGAGCTATTGTG
GGGGTGATATTGTACCAGCTGATGGTGGGCTACCACCTGGAAGGCACCCCACGAGAGGAC
AAGGAGAAAGAG---------GAGGAAGAGAGAAGGGTGAAGCTTCCCAACATCACAACC
TCT---
>Atlantic cod_Aqp3b2
ATGGATAAACTGAAGGCAGTTCTTGAGAAGCTGGCACAGATCTTCCACATCAGGCACCGG
ATTCTCCAGCAGGGCCTGGCAGAGTGCCTGGGGACCCTGGTTCTTGTGACGTTTGGCTGT
GGCTCGGTGGCCCAGATGCTTCTCAGCAGAGGTTCCCACGGCAGGTTCCTCAGCGTCAAC
CTGGCCTTTGGAGTCGCCACCACGCTCGGGATCCTCATCAGTGGACAGGTGTCTGGTGGT
CACCTGAACCCGACCGTGACCTTTGCCCTCTGCCTCCTTGGGAGAGTGCCATGGAGGAAG
TTCCCGGTTTACTTCCTCTTCCAGACATTAGGGGCCTTCCTGGGGGCCGGGATCATATTT
GGCATGTATTCTGATGCACTGTGGGCTTACGGCCAGGGG------------ACGCTGTCT
GTGGAGGGGACCAACGCCACGGCGGGGATCTTCGCTACGTATCCCTCCCCACATCTCAGT
CTACTCAATGGATTCTTCGACCAGGTAATCGGAACAGCCGCATTAATTGTGTGTATCCTG
GCCATCGTGGACCCCCACAACACCTCGGTCCCGGCGGGTCTGGAGGCCTTCACGGTCGGC
CTGGTGGTGCTGGTGATCGGCCTCTCCATGGGCTTCAACTCGGGCTACGCCGTGAACCCC
GCCAGGGACCTGGGGCCGCGCCTCTTCACCGCGCTGGCGGGCTGGGGCAGCGGGGTGTTC
ACGGTGAAGTGCTACTGGTTCTTTGTGCCCCTGTTCGCCCCGTTCCTAGGGGCAGTGGTG
GGGGTGGTGATTTACCAGCTGATGGTGGGCTATAACGTGGAAGGAGAGGCACGCATCCAG
GGAAGACGA------------GAGGAAGAGGGAAGCCTC---CTGAGTACA---ACCACC
ATCAATGAAGAGGCA---
>Walleye_pollock_Aqp3b2
ATGGATAAACAGAAGGCAGTTCTTGAGAAGCTGGCACAGATCTTCCACATCAGGCACCGG
ATTCTTCAGCAGGGCCTGGCAGAGTGCCTGGGGACCCTGGTTCTTGTGACGTTTGGCTGT
GGCTCGGTGGCCCAGATGCTTCTCAGCAGAGGTTCCCACGGCAGGTTCCTCAGCGTCAAC
CTGGCCTTTGGAGTCGCCACCACGCTCGGGATCCTCATCAGTCGACAGGTGTCTGGTGGT
CACCTGAACCCGACCGTGACCTTTGCTCTCTGCCTCCTTGGGAGAGTGCCATGGAGGAAG
TTCCCGGTTTACTTCCTCTTCCAGACATTAGGGGCCTTCCTGGGGGCCGGGATCATATTT
GGCATGTATTCTGATGCACTGTGGGCTTACGGCCAGGGG------------ACGCTGTCT
GTGGAGGGGACCAACGCCACGGCGGGGATCTTCGCTACGTATCCCTCCCCACATCTCAGT
CTACTCAATGGATTCTTCGACCAGGTGATCGGAACGGCCACATTAATCGTGTGTATCCTG
GCCATCGTGGACCCCCACAACACCCCGGTCCCGGCGGGTCTGGAGGCCTTCGCGTTGGGG
CTGGTGGTGCTGGTGATCGGCCTCTCCATGGGCTTCAACTCGGGCTACGCCGTGAACCCC
GCCCGGGACCTTGGGCCGCGCCTCTTCACCGCGCTGGCGGGCTGGGGCAGCGGGGTGTTC
ACGGTGAAGGGCTACTGGTTCTTGGTGCCCCTGTTCGCCCCGTTCCTAGGGGCAGTGGTG
GGGGTGGTGATTTACCAGCTGATGGTGGGCTATAACGTGGAAGGAGAGGCACGCATCCAG
GGAAGACGA------------GAGGAAGAGGAAAGCCTC---CTAAGTACA---ACCACC
ATCAATGAAGAGGCA---
>Arctic_cod_Aqp3b2
AAGGATAAACAGAAGGCAGTTCTTGAGAAGCTGGCACAGATCTTCCACATCAGGCACCGG
ATTCTTCAGCAGGGCCTGGCAGAGTGCCTGGGGACCCTGGTTCTTGTGACGTTTGGCTGT
GGCTCGGTGGCCCAGATGCTTCTCAGCAGAGGTTCCCACGGCAGGTTCCTCAGCGTCAAC
CTGGCCTTTGGAGTCGCCACCACGCTCGGGATCCTCATCAGTGGACATGTGTCTGGTGGT
CACCTGAACCCGACCGTGACCTTTGCCCTCTGCCTCCTTGGGAGAGTGCCATGGAGGAAG
TTCCCGGTTTACTTCCTCTTCCAGACATTAGGGGCCTTCCTGGGGGCCGGGATCATATTT
GGCATGTATTCTGATGCGCTGTGGGACTACGGCCAGGGG------------ACGCTGTCT

GTGGAGGGGACCAACGCCACGGCGGAGATCTTCGCTACGTATCCCTCCCCACATCTCAGT
CTACTCAATGGATTCTTCGACCAGGTGATCGGAACGGCCGCATTAATCATGTGTATCCTG
GCCATCGTGGACCCCCACAACACCCCGGTCCCGGCGAGTCTGGAGGCCTTCACGGTGGGG
CTGGTGGTGCTGGTGATCGGCCTCTCCATGGGCTTCAACTCGGGCTACGCCGTGAACCCC
GCCCGGGACCTGGGGCCGCGCCTCTTCACCGCGCTGGCGGGCTGGGGCAGCGGGGTGTTC
ACGGTGAAGGGCTACTGGTTCTTGGTGCCCCTGTTCGCCCCGTTCCTAGGGGCAGTGGTG
GGGGTGGTGATTTACCAGCTGATGGTGGGCTATAACGTGGAAGGAGAGGCAAGCATCCAG
GGAAGACGA------------GAGGAAGAGGAAAGCCTA---CTAACTACA---ACCACC
ATCAATGAAGAGGCA---
>Whiting_Aqp3b2
ATGGATAAACAGAAGGCAGTTCTTGAGAAGCTGGCACAGATCTTCCACATCAGGCACCTG
CTTCTCCAGCAGGGCCTGGCAGAGTGCCTGGGGACCCTGGTTCTGGTGATGTTTGGCTGC
GGCTCGGTGGCCCAGATGCTTCTCAGCAGAGGTTCCCACGGCGTGTTCCTCAGCGTCAAC
CTGGCCTTTGGGATCGCCACCACGCTCGGGATCCTCATCAGTGGACAGGTGTCAGGTGGT
CACCTGAATCCGACCGTGACTTTTGCCCTCTGCCTCCTTGGGAGAGTGCCATGGAGGAAG
TTCCCGGTTTACTTCCTCTTCCAGACATTAGGGGCCTTCCTGGGGGCCGGGATCATATTT
GGCATGTATTCTGATGCGCTGTGGGCGTATGGCCAGGGG------------ACGCTGTCT
GTGGACGGGACCAATGCCACGGCGGGGATCTTCGCTACGTATCCCTCCCCACATCTCAGT
CTACTCAACGGATTCTTCGACCAGGTAATCGGAACAGCCGCATTAATCGCGTGTATCCTG
GCCATCGTGGACCCCCACAACACCCCGGTCCCGGCGGGTCTAGAGGCCTTCACGGTGGGG
CTGGTGGTGCTGGTGATCGGCCTCTCCATGGGCTTCAACTCGGGCTACGCCGTGAACCCT
GCCCGGGACCTGGGGCCACGCCTCTTCACCGCGCTGGCGGGCTGGGGCAGCGGGGTGTTC
ACGGTGAATGGGTACTGGTTCTTGGTGCCCCTGTTCGCCCCGTTCCTAGGGGCAGTGGTG
GGGATGGTGATTTACCAGCTGATGGTGGGCTACAACATGGAAGGAGAGGCACGCATCCAG
GGAAGACGA------------------GAGGAAAGCCTC---CTAACTACA---ACCAGC
ATCTATGCAGAGGCA---
>Haddock_Aqp3b2
ATGGCTAAACAGAAGGCAGTTCTTGAGAAGCTGGCACAGATCTTCCACATCAGGCACCTG
CTTCTTCAGCAGGGCCTGGCAGAGTGCCTGGGGACCCTGGTTCTGGTGATGTTTGGCTGC
GGCTCGGTGGCCCAGATGCTTCTCAGCAGAGGTTCCCACGGCGCGTTCCTCAGCGTCAAC
CTGGCCTTTGGTGTCTCCACCACGCTTGGGATCCTCATCAGTGGACATGTGTCAGGTGGT
CATCTGAACCCGACCGTGACTTTTGCCCTCTGCCTCCTTGGGAGAGTGCCATGGAGGAAG
TTCCCGGTCTACTTCCTCTTCCAGACGTTGGGGGCCTTCCTGGGGGCCGGGATCATATTT
GGCATGTATTCTGATGCGCTGTGGGAATACGGCCAGGGG------------ACGCTGTCT
GTGGATGGGACCAACGCCACAGCGGGGATCTTCGCTACGTATCCCTCCCCACATCTCAGT
CTACTCAACGGATTCTTCGACCAGGTGATCGGAACGGCCGCATTAATCGTGTGTTTCCTG
GCCATCGTGGACCCCCACAACACCCCAGTCCCGACTGGTCTGGAGGCCTTCACGGTGGGG
CTGGTGGTGCTGGTGATCGGCCTCTCCATGGGCTTCAACTCGGGCTACGCCGTGAACCCC
GCCCGGGACCTGGGGCCTCGCCTCTTCACCGCGCTGGCGGGCTGGGGCAGCGCGGTGTTC
ACGGTGAAGGGCTACTGGTTCTTGGTACCCCTGTTCGCCCCGTTCCTAGGGGTGGTGATG
GGGGTGGTGATTTACCAGCTGATGGTGGGCTACAACGTGGAAGGAGAGGCACACATCCAG
GGAAGA---------------------GAGGAAAGCCTC---CTAGCTACA---ACCACC
ATCTATGAAGAGGCA---
>Atlantic_cod_Aqp3b1
ATGGAAAAACAGAAGGCAGTCCTTGAGATGCTGGCACAGATCTTCCACATCAGGCACCTG
CTTCTCCAGCAGGGCCTGGCAGAGTGCCTGGGGACCCTGGTTCTGGTGACGTTTGGCTGC
GGCTCGGTGGCCCAGATGCTTCTCAGCGGAGGTTCCCACGGCGCGTTCCTCACCGTCAAC
CTGGCCTTTGGGTTCGCCGCCACGCTGGGGATCCTCATCAGTGGACAGGTGTCAGGTGGT
CACCTGAACCCGGCCGTGACCTTTGCCCTCTGTCTCCTGGGGAGGGAGCCATGGAGGAAG
CTCCCGGTCTACTTCCTCTTCCAGACGTTGGGGGCCTTCCTGGGCGCCGGAATCATATTT
GGCATGTATTCTGATGCACTGTGGGACTACGGCCAGGGG------------ACGCTGTCC
GTGGACGGGACCAACGCCACTGCGGGGATCTTCGCTACGTATCCCTCCCCACATCTCAGC
CTGCTCAACGGATTCTTCGACCAGGTAATCGGAACGGCCGCATTAATTGTGTGTATCCTG
GCCATCGTGGACCCCCACAACACCCCGGTCCCAGCGGGTCTGGAAGCCTTCACGGTGGGG
CTGGTGGTGCTGGTGATCGGCCTCTCCATGGGCTTCAACTCGGGCTACGCCGTGAACCCC
GCCCGGGACCTGGGGCCGCGCCTCTTCACCGCGCTGGCGGGCTGGGGCAGCGGGGTGTTC
ACGGTGAAGGGCTACTGGTTCTTGGTGCCCCTGTTCGCCCCGTTCCTAGGGGCGGTGGTG
GGGGTGGTGATTTACCAGCTGATGGTGGGCTACAACGTGGAAGGAGAGGCACGCATCCAG
AGAAGACGA------------GAGGAAGAGGAAAGCCTAAAATATGCAACTATAACCACC
ATCAATGAAGAGGCA---
>Walleye_pollock_Aqp3b1

ATGCAAAAACAGAAGGCAGTTCTTGAGAAGGCGGCACAGATCTTCCACATCAGGCACCTG
CTTTTCCAGCAGGGCCTGGCAGAGTGCCTGGGGACCCTGGTTCTGGTGACGTTTGGCTGT
GGCTCGGTGGCCCAGATGCTTCTCAGCGGAGGTTCCCACGGCGCGTTCCTCACCGTCAAC
CTGGCCTTTGGGTTCGCCGCCACGCTGGGGATCCTCATCAGTGGACAGGTGTCAGGTGGT
CACCTGAACCCGGCCGTGACCTTTGCCCTCTGTCTCCTGGGGAGGGAGCCATGGAGGAAG
TTCCCGGTCTACTTCCTCTTCCAGACGTTGGGGGCCTTCCTGGGCGCCGGAATCATATTT
GGCATGTATTCTGATGCGCTGTGGGACTACGGCCAGGGG------------ACGCTGTCT
GTGGACGGGACCAACGCCACGGCGGGGATCTTCGCTACGTATCCCTCCCCACATCTCAGC
CTGCTCAACGGATTCTTCGACCAGCTGATCGGAACGGCCGCATTAATTGTGTGTATCCTG
GCCATCGTGGACCCCCACAACACCCCGGTCCCGGCGGGTCTGGAAGCCTTCACGGTCGGC
CTGGTGGTGCTGGTGATCGGCCTCTCCATGGGCTTCAACTCGGGCTACGCCGTGAACCCC
GCCCGGGACCTGGGGCCGCGCCTCTTCACCGCGCTGGCGGGCTGGGGCAGCGGGGTGTTC
ACGGTGAAGGGCTACTGGTTCTTGGTGCCCTTGTTCGCCCCGTTCCTAGGGGCGGTGGTG
GGGGTGGTGATTTACCAGCTGATGGTGGGCTACAACGTGGAAGGAGAGGCACGCATCCAG
AGAAGACGA------------GAGGAAGAGGAAAGCCTCAAATATGCAACTATAACCACC
ATCAATGAAGAGGCA---
>Arctic_cod_Aqp3b1
ATGGAAAAACAGAAGGCAGTTCTTGAGAAGGCGGCACAGATCTTCCACATCAGGCACCTG
CTTCTCCAGCAGGGCCTGGCAGAGTGCCTGGGGACCCTGGTTCTGGTGACGTTTGGCTGC
GGCTCGGTGGCCCAGATGCTTCTCAGCGGAGGTTCCCACGGCGCGTTCCTCACCGTCAAC
CTGGCCTTTGGCTTCGCCGCCACGCTGGGGATCCTCATCAGTGGACAGGTGTCAGGTGGT
CACCTGAACCCGGCCGTGACCTTTGCCCTCTGTCTCCTGGGGAGGGAGCCATGGAGGAAG
TTCCCGGTCTACTTCCTCTTCCAGACGTTGGGGGCCTTCCTGGGCGCCGGAATCATATTT
GGCATGTATTCTGATGCGCTGTGGGACTACGGCCAGGGG------------ACGCTGTCT
GTGGACGGGACCAACGCCACGGCGGGGATCTTCGCCACGTATCCCTCCCCACATCTCAGC
CTGCTCAACGGATTCTTCGACCAGCTGATCGGAACGGCCGCATTAATTGTGTGTATCCTG
GCCATCGTGGACCCCCACAACACCCCGGTCCCGGCGGGTCTGGAAGCCTTCACGGTCGGC
CTGGTGGTGCTGGTGATCGGCCTCTCCATGGGCTTCAACTCGGGCTACGCCGTGAACCCC
GCCCGGGACCTGGGGCCGCGCCTCTTCACCGCGCTGGCGGGCTGGGGCAGCGGGGTGTTC
ACGGTGAAGGGCTACTGGTTCTTGGTGCCCCTGTTCGCCCCGTTCCTAGGGGCGGTGGTG
GGGGTGGTGGTGTACCAGCTGATGGTGGGCTACAACGTGGAAGGAGAGGCACGCATCCAG
AGAAGACGA------------GAGGAAGAGGAAAGCCTCAAATATGCAACTATAACCACC
ATCAATGAAGAGGCA---
>Whiting_Aqp3b1
ATGGAAAAACAGAAGGCAGTTCTTGAGAAGCTGGCACAGATCTTCCACATCAGGCACCTG
CTTCTCCAGCAGGGCCTAGCAGAGTGCCTGGGGACCCTGGTTCTGGTGACGTTTGGCTGC
GGCTCGGTGGCCCAGATGCTTCTCAGCGGAGGTTCCCACGGCGCGTTCCTCACCGTCAAC
CTGGCCTTTGGGTTCGCCGCCACGCTGGGGATCCTCATCAGTGGACAGGTGTCAGGTGGT
CACCTGAACCCGGCCGTGACCTTTGCCCTCTGCCTCCTGGGGAGAGTGCCATGGAGGAAG
TTCCCGGTCTACTTCCTCTTCCAGACGTTGGGGGCCTTCCTGGGTGCCGGAATCATATTT
GGCATGTATTCTGATGCGCTGTGGGACTACGGCCGGGGG------------ACGCTGTCT
GTGGACGGGACCAACGCCACGGCGGGGATCTTCGCTACGTATCCCTCCCCACATCTCAGC
CTGCTCAACGGATTCTTCGACCAGGTAATCGGAACGGCCGCATTAATCGTGTGTATCCTG
GCCATCGTGGACCCCCACAACACCCCGGTCCCGGCGGGTCTGGAAGCCTTCACAGTGGGG
CTGGTGGTGCTGGTGATCGGCCTCTCCATGGGCTTCAACTCGGGCTACGCCGTTAACCCC
GCCCGGGACCTGGGGCCGCGCCTCTTCACCGCACTGGCGGGCTGGGGCAGCGGGGTGTTC
ACGGTGAAGGGCTACTGGTTCTTGGTGCCCCTGTTCGCCCCGTTCCTAGGGGCGGTGGTG
GGGGCGATGGTGTACCAGCTGATGGTGGGCTACAACGTGGAAGGAGAGGCACGCATCCAG
AGAAGACGA------------GAGGAAGAGGAAAGCCTCAAATATACAGCTATAACAACC
ATCAATCAAGAGGCA---
>Haddock_Aqp3b1
ATGGAAAAACAGAAGGCAATTCTTGAGAAGCTGGCACAGATCTTCCACATCAGGCACCTG
CTTCTCCAGCAGGGCCTGGCAGAGTGCCTGGGGACCCTGGTTCTGGTGACGTTTGGCTGC
GGCTCGGTCGCCCAGATGCTTCTCAGCGGAGGTTCCCACGGCGCGTTCCTCACCGTCAAC
CTGGCCTTTGGGTTCGCCGCCACGCTGGGGATCCTCATCAGTGGACAGGTGTCAGGGGGT
CACCTGAACCCGGCCGTGACCTTTGCCCTCTGCCTCCTGGGGAGGGAGCCATGGAGGAAG
TTCCCGGTCTACTTCCTCTTCCAGACGTTGGGGGCCTTCCTGGGGGCCGGTATCATATTC
GGCATGTATTCTGATGCGCTGTGGGACTACGGCCAGGGG------------ATGCTGTCT
GTGGACGGGACCAATGCCACGGCGGGGATCTTCGCTACGTATCCCTCCCCACATCTCAGC
CTGCTCAACGGATTCTTCGACCAGTTGATCGGAACGGCCGCGTTAATTGTGTGTATCCTG
GCCATCGTGGACCCCCACAACACACCGGTTCCGGCGGGTCTGGAGGCCTTCACGGTGGGG

CTGGTGGTGCTGGTGATCGGCCTCTCCATGGGCTTCAACTCGGGCTACGCCGTGAACCCC
GCCCGGGACCTGGGGCCTCGCCTCTTCACCGCGCTGGCGGGCTGGGGCAGCGGGGTGTTC
ACGGTGAAGGGCTACTGGTTCTTAGTGCCCCTGTTCGCCCCGTTCCTAGGGGCGGTGGTG
GGGGTGGTGGTGTACCAGCTGATGGTGGGCTACAACGTGGAAGGAGAGGCACACGTCCAG
AAAAGACGA------------GAGGAAGAGGAAAGCCTCAACTATACAGCTATAACC---
ATCAATCAAGAGGCA---
>Poor_cod_Aqp3b1

------------GATGCGCTGTGGGCCTACGGCCAGGGG------------ACGCTGTCT
GTGGACGGGACCAACGCCACGGCGGGGATCTTCGCTACGTATCCCTCCCCACATCTCAGC
CTGCTCAACGGATTCTTCGACCAGGTGATTGGAACGGCCGCACTAATGGTGTGTATCCTG
GCCATCGTGGACCCCCACAACAGCGCGGTCCCGGCGGGCCTGGAGGCCTTCACGGTGGGC
CTGGTGGTGCTGGTGGTCGGCCTGTCCATGGGCTTCAACTCGGGCTACGCCGTGAACCCC
GCCCGGGACCTGGGGCCGCGCCTCTTCACGGCACTGGCGGGCTGGGGCGGCGGCGTGTTC
ACGGTGAAGGGCTACTGGTTCTTGGTGCCCTTGTTGGCCCCGTTCCTGGGGGCCGTGGTG
GGGGTGGGGGTGTACCAGCTGATGGTGGGCTACAACGTGGAAGGAGAGGCACGCATCCAG
AGGAGACGA------------GAGGAAGAGGAAAGCCTCAAATGTACGACTGTAACCACC
ATCAATTTAGAGGCA---
>Greater_forkbeard_Aqp3b
ATGGAAAGACAGAAGGTAATTCTTGAGAAGCTGGCACAGATCTTCCACATCAAAAACCTG
CTTGTTCAACAGGGCCTCGCAGAATGCCTGGGAACCCTTGTTCTGGTGATGTTTGGCTGC
GGGGCGGTGGCCCAGATGTTGCTGAGCGGGGGGTCCCACGGCACGTTCCTGACGGTCAAC
CTGGCCTTTGGGTTCGCCACCACTCTGGGGATCCTCGTCAGTGGACAGGTGTCAGGGGGT
CACCTGAACCCGGCCGTGACCTTCGCCCTCTGCTCCCTGGGGAGGGAGCCGTGGAAGAAG
TTCCCGGTTTACCTGCTCTTCCAGACGTTGGGAGCGTTCCTGGGCGCCGGGATCATCTTC
GGACTCTACTCGGATGCATTGTGGGAGTACGGCCAGGGG------------ACGCTGTCC
GTGGAGGGGCCCAACGCCACCGCGGGGATCTTCGCCACGTACCCCTCCAAACACCTCTCT
CTGCTCAATGGGTTCTTTGACCAGGTGATCGGAACGGCCGCCCTCCTCGTGTGCGTGCTG
GCCATCGCCGACCCCCACAACAACCCGGTCCCCGCCGGCCTGGAGGCCTTCACGGTGGGC
CTGGCCGTGCTGGCGGTGGGCCTCTCCATGGGCTTCAACGCGGGCTACGCCGTGAACCCG
GCCCGGGACCTGGGGCCCCGGATGCTCACGGCGCTGGCCGGCTGGGGCAGCGGCGTCTTC
ACGGTGCAAGACCACTGGTTCCTCGTGCCTTTGTTCGCCCCGTTCCTGGGGGCGGCGGTG
GGGGTGGCGGTGTACCAGCTGGCGGTGGGCCTCAGCGTGGAAGGAGAGGCCCGCAATAAG
AAAGGACCA------------CAGGGGGAGGAAGGC---CAC------------------

>Forkbeard_Aqp3b
ATGGAAAGACAGAAGGTAATTCTTGAGAAGCTGGCACAGATCTTCCACATCAAAAACCTG
CTTGTTCAACAGGGCCTCGCAGAATGCCTGGGAACCCTTGTTCTGGTGATGTTTGGCTGC
GGGGCGGTGGCCCAGATGTTGCTGAGCGGGGGGTCCCACGGCACGTTCCTCACGGTCAAC
CTGGCCTTTGGGTTCGCCGCCACTCTGGGGATCCTCGTCAGTGGACAGGTGTCAGGTGGT
CACCTGAACCCGGCCGTGACCTTCGCCCTCTGCTCCCTGGGGAGGGAGCCGTGGAGGAAG
TTCCCGGTCTTCCTTCTGTTCCAGACGTTGGGGGCGTTCCTGGGCGCCGGGATCATCTTC
GGAATGTACTCGGATGCATTGTGGGAGTACGGCCAGGGG------------ACGCTGTCT
GTGGAGGGGCCCAACGCCACCGCGGGGATCTTCGCCACGTACCCCTCCAGACACCTCTCT
CTGCTCAATGGATTCTTTGACCAG------------------------------------

------------CACTGGTTCGTCGTGCCTTTGTTGGCGCCCTTCCTGGGGGCCGCGGTG
GGGGTCGCGGTGTACCAGCTGGCGGTGGGCTTCAGCGCAGAAGGAGAGGCGCGAAACAAG
AAGCGACCA------------CAGGAA---------------------------------

>Tusk_Aqp3b
ATGGAAAAACAGAAGGTAATTATTGAGAAGCTGGCACAGACCTTCCACATCAGACACCTG
CTTCTTCAACAGGGCCTGGCAGAATGCCTCGGGACCCTGGTTCTGGTGACGTTTGGCTGT
GGGGCGGTGGCCCAGATGGTTCTCAGCGGAGGTTCCCATGGCATGTTCCTCACCGTCAAC

CTGGCCTTTGGGTTCGCCGCAACGCTGGGAATCCTCGTCAGTGGACAGGTGTCAGGTGGT
CATCTGAACCCGGCCGTGACCTTTGCCCTCTGCCTCCTGAGGAGGGAGCCTTGGAAGAAG
TTCCCCGTCTTCTTTATCTTCCAAACGTTTGGCGCGTTCCTGGGGGCCGGGATCATATTC
GGCATGTATTCTGATGCGCTGAGGGATTATGGCCAGGGG------------ACGCTGTTT
GTGGACGGGACCAACGCCACAGCAGGGATTTTCGCTACATATCCCTCCAAACATCTCACT
CTGCTCAACGGATTCTTTGACCAGGTGATTGGAACAGCCGCATTAATCGTGTGCATCCTT
GCTATCGTGGACCCCCACAACAACCCCGTCCCCGCGGGCCTGGAGGCCTTCACGGTGGGC
CTCGTGGTGCTGGTGATCGGCCTCTCCATGGGCTTCAACTCGGGCTACGCCGTGAACCCG
GCCAGGGACCTGGGGCCCCGGCTCTTCACCGCGCTGGCGGGCTGGGGCAGCGAGGTGTTC
ACGGTGAAAGGCTACTGGTTCCTGGTGCCCTTGTTTGCCCCGTTCCTGGGGGCAGTGGTG
GGGGTGGTGGTGTACCAGCTGATGGTGGGCTACAACGTGGAAGGAGAGGCACGCATCAAG
AAAAGACGA------------GAGGAAGAGGAAAGCCTCAAATGTACCACTATAACCACC
ATCAATGAAGAGGCA---
>Ling_Aqp3b
ATGGAAAAACAGAAGGTAATTATTGAGAAGCTGGCACAGATCTTCCACATCAAGCAGCTG
CTTCTTCAACAGGGCCTGGCAGAGTGCCTTGGGACCCTGGTTCTGGTGACGTTTGGCTGC
GGCTCGGTGGCCCAGATGGTGCTCAGCAGAGGATCCCATGGCATGTTCCTCACGGTCAAC
CTGGCCTTTGGATTCGCCGCAACGCTGGGAGTCCTCGTCAGTGGACAGGTGTCAGGTGGT
CATCTGAACCCGGCCATGACCTTTGCCCTCTGCCTCCTGGGGAGGGAGCCTTGGAAGAAG
TTCCCCGTCTTCTTTCTCTTCCAAACGTTGGGCGCGTTCCTGGGCGCCGGGATCATATTT
GGCATGTATTCTGATGCACTGTGGAATTATGGCCAGGGG------------ACGCTGTTT
GTGGATGGGACGAACGCCACAGCAGGGATTTTCGCTACGTATCCCTCCAAACATCTCACT
CTGCTCAACGGATTCTTTGACCAGGTGATCGGAACAGCCGCATTAATCGTGTGTATCCTG
GCTATCGTGGACCCCCACAACAACCCCATCCCTGCGGGCCTGGAGGCCTTCACGGTGGGC
CTGGTGGTGCTGGTGATTGGCCTCTCCATGGGCTTCAACTCGGGCTACGCGGTGAACCCG
GCCCGGGACCTGGGGCCTCGGCTCTTCACCGCGCTGGCGGGATGGGGCAGCGAGGTGTTC
ACGGTGAAAGGCTACTGGTTCTTGGTGCCCTTGTTTGCCCCGTTCCTGGGGGCGGTGTTG
GGGGTGGGGGTGTACCAGCTGATGGTGGGCTACAACGTGGAAGGAGAGGCCCGCATCAAG
AAAAGACGA------------GAGGAAGAGGAAAGCCTCAAATGTACAACTATAACCACC
ATCAATGAAGAGGCA---
>Vaillants_grenadier_Aqp3b
ATGGAAAGACAGAAGGAGGTTCTAGAGAAGCTGGCGCAGATCTTCCACGTCAGGCCGCAG
CTCCTTCAGCAGGGCCTCGCAGAATGCCTGGGAACCCTGGTTCTGGTGATGTTTGGCTGC
GGAGCAGTGGCCCAGGTGGTGTTGAGCGGAGGTTCCCATGGCATGTTCTTCACGGTAAAC
CTGGCGTTTGGATTCGCCGCAACGCTCGGTATCCTCGTCAGTGGCCAGGTGTCAGGTGGT
CATCTGAACCCAGTGGTGACCTTTGCACTCTGCTTCCTGGGGAGGGAGCCTTGGAAGAAG
TTCCCTGTGTTCTTTCTCTTCCAAACGTTGGGCGCTTTCCTGGGCGCTGGGATCATATTT
GGCATGTATTTTGATGCACTGTGGGATTATGGCCAAGGG------------TCTCTGTTT
GTGGATGGGAGGAACGCCACAGCAGGGATCTTCGCTACATATCCATCCAAACATCTCACT
CTGCTCAACGGATTCTTCGACCAGATGATCGGAACAGCAGCGTTAATCGTGTGTATCCTA
GCTATCGTCGATCCTCACAACAACCCCATCCCCAGGGGTCTGGAGGCCTTCACCGTAGGC
TTTGTGGTGTTAGTGATTGGCCTTTCAATGGGCTTCAACTCGGGCTACGCTGTGAACCCG
GCCAGGGACCTGGGGCCGCGGATCTTCACCGCTCTGGCCGGCTGGGGCAGCGAGGTCTTC
ATGGTGAAGGGCTACTGGTTCCTTGTGCCCCTGTTTGCCCCGTTCTTGGGCGCGTTGGTT
GGGGTGGTGGTGTACCAGCTCATGGTGGGTTACAACGTGGAAGGAGAGGCCCGCAACAAG
AAAAGACGA------------GAGGAAGAGGAAAGCCTCAAATGTACAGACACAGCCACC
ACCAAAGAAGAGCCG---
>Roughsnout_grenadier_Aqp3b
ATGGAAAAACAGAAGGAACTCCTCCAGCGGCTGGGGCAGACCTTCCACACCAAGCACCTG
CTTCTTCAGCAGGGCCTCGCAGAATGCCTGGGAACCCTTGTTCTGGTGATGTTTGGCTGC
GGTGCCGTGGCCCAGGTGGTCCTAAGCGGGGGCTCCCATGGCATGTTCCTCACGGTAAAC
CTGGCGTTCGGATTCGCCGCAACGCTCGGCATCCTCGTCAGCGGCCAGGTGTCCGGTGGT
CACCTGAACCCGGCCGTGACCTTTGCCCTCTGCTTCCTGGGGAGGGAGCCCTGGAAGAAA
TTCCCCGTGTTCTTTCTCTCCCAAACGTTGGGCGCTTTCCTGGGCGCCGGGATCATATTT
GGCATGTATTTCGATGCATTGTGGGATTACGGCCAGGGG------------TCCCTGTTT
GTGGACGGGCAGAACGCCACAGCGGGCATCTTCGCGACGTATCCGTCCAAACATCTCACT
CTGCTCAACGGATTCTTCGACCAGGTGATCGGAACAGCCGCATTAATCGTGTGTATCCTG
GCTATCGTGGACCCTCACAACAACCCCATCCCCAGGGGTCTGGAGGCCTTCACCGTGGGC
TTTGTAGTGCTAGTGATCGGCCTTTCAATGGGATTCAACTCAGGCTACGCCGTGAACCCA
GCCAGGGACCTGGGGCCACGGCTCTTCAGCGCTCTGGCGGGCTGGGGCACCGAGGTCTTC
ACGGTGAAGGGCTACTGGTTCCTAGTGCCCTTGTTTGCCCCATTCGTGGGTGCAGTGTTG

GGCGTGGTGGTCTACCAGCTCATGGTGGGCTACAACCTGGAAGGAGAGGCTCGCAACAGG
AGAAGACGA------------GAGGAAGAGGAAAGCCTGAGATGTACAGAGATAACCACC
GCCAAAGAGGAGGTTCAT--
>Roughnose_grenadier_Aqp3b
ATGGAAAAACAGAAGGAACTTCTCGAGCGGCTGGGGCAGACCTTCCATACCAAGCACCTG
CTTCTTCAACAGGGCCTCGCAGAATGCCTGGGAACCCTTGTTCTGGTGATGTTTGGCTGC
GGTGCGGTGGCCCAGGTGGTGTTAAGCGGAGGCTCCCATGGCATGTTCCTCACGGTAAAC
CTGGCCTTCGGATTCGCCGCAACGCTCGGCATCCTGGTCAGCGGCCAGGTGTCCGGTGGT
CATCTGAACCCGGCTGTGACCTTTGCCCTCTGCTTCCTGGGGAGGGAGCCTTGGAAGAAG
TTCCCCGTGTTCTTTCTCTCCCAAACGTTGGGCGCTTTCCTGGGCGCCGGGATCATATTT
GGCATGTATTTTGATGCATTGTGGGATTACGGCCAGGGG------------TCGCTGTTT
GTGGATGGGCAGAACGCCACAGCGGGCATCTTCGCTACGTATCCGTCCAAACATCTCACT
CTGCTCAACGGATTCTTCGACCAGGTGATCGGAACAGCCGCATTAATCGTGTGTATCCTG
GCTATCGTGGACCCTCACAACAACCCCATCCCCGGGGTCTGGAGGCCTTCACGGTGGGC
TTTGTAGTGCTAGTGATCGGCCTTTCAATGGGATTCAACTCGGGCTACGCTGTGAACCCA
GCCAGGGACCTGGGGCCGCGGCTCTTCAGCGCTCTGGCAGGCTGGGGCACCGAGGTCTTC
ACGGTGAAGGGCTACTGGTTCCTAGTGCCCTTGTTTGCCCCATTCTTGGGGTCAGTGGTG
GGCGTGGTGGTCTACCAGCTCATGGTGGGCTACAACCTGGAAGGAGAGGCTCGCAACAGG
AGAAGACGA------------GAGGACGAGGAAAGCCTCAAATGTACAGAGATAACC---
---AAAGAGGAGGTTCAT--
>Cape_hake_Aqp3b
ATGGAAAAACAGAAGGAAGTTCTTGAGAAGCTGGTGCAGATCTTCCACATCAAGCACCTG
CTTCTTCAACAGGGCCTCGCTGAATGCCTGGGAACCCTTATTCTGGTGATGTTTGGCTGC
GGCGCGGTGGCCCAGGTGGTGTTAAGCGGAGGTTCCCATGGCCTGTTCCTCACGGTAAAC
CTGGCGTTTGGATTTGCCGCAACGCTCGGCATCCTCGTCAGTGGCCAGGTGTCGGGTGGT
CATCTGAACCCAGCGGTGACCTTTGCACTCTGCTTCCTGGGAAGGGAGCCTTGGAAGAAG
TTCGCCACGTACTTTATCTTCCAAACTCTGGGTGCTTTCCTGGGCGCCGGGATCATATTT
GGCATGTATTTTGATGCACTGCAGGATTATGGGCAGGGG------------GCGCTGTTT
GTGGATGGGAACAACGCCACAGCAGGGATCTTCGCTACGTATCCCTCTAAACATCTCACT
CTGCTCAACGGATTCTTCGACCAGGTGATCGGAACAGCTGCATTAATCGTGTGTATCCTG
GCTATCGTCGACCCTCACAACAACCCCATCCCCCGGGGTCTGGAGGCCTTCACGGTGGGC
TTTGTGGTGCTAGTGATTGGCCTTTCGATGGGCTTCAACTCGGGCTACGCCGTGAACCCT
GCCAGGGACCTGGGGCCGCGGCTCTTCACCGCTATGGCCGGCTGGGGCAGCGAGGTCTTC
ACGGTGAACGGCTACTGGTTCTTGGTGCCCTTGTTTGCCCCGTTCCCGGGGGCCGTGGTG
GGGGCGGCGGTGTACCAGCTCATGGTGGGCTACAACGTGGAAGTGGAGGCTCGCAACAAG
AAAAGACGA------------GAGGAAGAGGAAAGCCTTAAATGTACAGATATAACCACC
ACCGAAGAAGAAGTTCAC--
>Benguela_hake_Aqp3b
ATGGAAAAACAGAAGGAAGTTCTTGAGAAGCTGGCGCAGATCTTCCACATCAAGCACCTG
CTTCTTCAACAGGGCCTCGCTGAATGCCTGGGAACCCTTATTCTGGTGATGTTTGGCTGC
GGCGCGGTGGCCCAGGTGGTGTTAAGCGGAGGTTCCCATGGCCTGTTCCTCACGGTAAAC
CTGGCGTTTGGATTCGCCGCAACGCTCGGCATCCTCGTCAGTGGCCAGGTGTCGGGTGGT
CATCTGAACCCAGCGGTGACCTTTGCACTCTGCTTCCTGGGAAGGGAGCCTTGGAAGAAG
TTCGCCACGTACTTTATCTTCCAAACTCTGGGTGCTTTCCTGGGCGCCGGGATCATATTT
GGCATGTATTTTGATGCACTGCAGGATTATGGGCAGGGG------------GCGCTGTTT
GTGGATGGGAACAACGCCACAGCAGGGATCTTCGCTACGTATCCCTCTAAACATCTCACT
CTGCTCAACGGATTCTTCGACCAGGTGATCGGAACCGCTGCATTGATCGTGTGTATCCTG
GCTATCGTCGACCCTCACAACAACCCCATCCCCCGGGGTCTGGAGGCCTTCACGGTGGGC
TTTGTGGTGCTAGTGATCGGCCTTTCGATGGGCTTCAACTCGGGCTACGCCGTGAACCCT
GCCAGGGACCTGGGGCCGCGGCTCTTCACCGCTATGGCCGGCTGGGGCGGCGAGGTCTTC
ACGGTGAAAGGCTACTGGTTCTTGGTGCCCTTGTTTGCCCCGTTCCTGGGGGCCGTGGTG
GGGGCGGCGGTGTACCAGCTCATGGTGGGCTACAACGTGGAAGGGGAGGCTCGCAACAAG
AAAAGACGA------------GAGGAAGAGGAAAGCCTCAAATGTACAGATATAACCACC
ACCCAAGAAGAAGTTCAC--
>European_hake_Aqp3b
ATGGAAAAACAGAAGGAAGTTCTTGAGAAGCTGGTGCAGATCTTCCACATCAAGCACCTG
CTTCTTCAACAGGGCCTCGCTGAATGCCTGGGAACCCTTATTCTGGTGATGTTTGGCTGC
GGCGCCGTGGCCCAGGTGGTGTTAAGCGGAGGTTCCCATGGCCTGTTCCTCACGGTAAAC
CTGGCGTTTGGATTCGCCGCAACGCTCGGCATCCTCGTCAGTGGCCAGGTGTCGGGTGGT
CATCTGAACCCAGCGGTGACCTTTGCACTCTGCTTCCTGGGAAGGGAGCCTTGGAAGAAG
TTCGCCACGTACTTTATCTTCCAAACTCTGGGTGCTTTCCTGGGCGCCGGGATCATATTT

```
GGCATGTATTTTGATGCACTGCAGGATTATTGGCAGGGG------------GCGCTGTTT
GTGGATGGGAACAACGCCACAGCAGGGATCTTCGCTACGTATCCCTCTAAACATCTCACT
CTGCTCAACGGATTCTTCGACCAGGTGATCGGAACAGCTGCATTAATCGTGTGTATCCTG
GCTATCGTCGACCCTCACAACAACCCCATCCCCCGGGGTCTGGAGGCCTTCACGGTGGGC
TTTGTGGTGCTAGTGATCGGCCTTTCGATGGGCTTCAACTCGGGCTACGCCGTGAACCCT
GCCAGGGACCTGGGGCCGCGGCTCTTCACCGCTATGGCCGGCTGGGGCAGCGAGGTCTTC
ACGGTGAACGGCTACTGGTTCTTGGTGCCCTTGTTTGCCCCGTTCCCGGGGGCCGTGGTG
GGGGCGGCGGTGTACCAGCTCATGGTGGGCTACAACGTGGAAGGGGAGGCTCGCAACAAG
AAAAGACGA------------GAGGAAGAGGAAAGCCTCAAATGTACAGATATAACCACC
ACCGAAGAAGAAGTTCAC------------------------------------------
>Common_mora_Aqp3b
ATGGAAAAACAGAAGGAAGTTCTTGAAAAGCTGGCGCAGATCTTCCACATCAAGCACCTG
CTCCTTCAACAGGGCCTCGCAGAATGCCTGGGAACCCTTGTTCTGGTG------------
------------------------------------------------------------
------------------------------------------------------GGTGGT
CACCTGAACCCAGCGGTGACCTTTGCACTCTGCCTCCTGGGGCGGGAGCCTTGGAGGAAG
TTCCCCGTGTTCTTCCTCTTCCAGACGGTGGGCGCTTTCCTCGGCGCCGGGATCATATTC
GGCATGTATTTTGACGCCCTGTGGGATTACGGCCAGGGG------------TGGCTTCTT
GTGGATGGGAAGAACGCCACGGCGGGGATCTTCGCTACGTATCCGTCCAAACATCTCACT
CTGCTCAACGGCTTCTTCGACCAGGTGATCGGAACAGCCGCATTAATAGTGTGCATCCTG
GCTATCGTGGACCCTCACAACAACCCCATCCCCAGGGGTCTGGAGGCCTTCACGGTGGGC
TTTGTGGTTCTGGTGATTGGCCTTTCAATGGGCTTCAACTCGGGCTACGCTGTGAACCCG
GCCAGGGACCTGGGGCCACGGCTCTTCACCGCTCTGGCGGGCTGGGGCAGCGAGGTCTTC
ACG---------------------------------------------------------
------------------------------------------------------------
------------------------------------------------------------
------------------------------------------------------------
>Guinean_codling_Aqp3b
ATGGAAAAACAGAAGGAACTTCTTGAGAAGCTGGCACAGATCTTCCACATCAAGCACCTG
CTTCTTCAACAGGGCCTCGCAGAATGCCTGGGAACCCTTGTTCTGGTGATGTTTGGCTGC
GGTGCGGTGGCCCAGGTGGTGTTAAGCGGAGGTTCCCACGGCACGTTCTTCACGGTGAAC
CTGGCGTTTGGATTCGCCGCAACGCTCGGCGTCCTCGTCAGTGGCCAGGTGTCAGGTGGT
CATCTGAACCCGGCGGTGACCTTTGCCCTCTGCTTCCTGGGGAGGGAGCCATGGAAGAAG
TTCCCCGTGTTCTTTGTCTTCCAAACGGTGGGCGCCTTCCTGGGCGCCGGGATCATATTC
GGCCTGTATTTTGATGCACTGTGGGATTATGGCCAGGGG------------GGGCTTGTT
GTGGATGGGAAGAACGCCACAGCGGGGATCTTCGCCACGTATCCATCCAAACATCTCACT
CTGCTCAACGGATTCTTCGACCAGGTGATCGGAACAGCCGCATTAATCGTGTGCATCCTG
GCTATCGCCGACCCTCACAACAACCCCATCCCCAGGGGTCTGGAGGCCTTCACGGTGGGC
TTAGTGGTGCTAGTGATTGGCCTTTCAATGGGCTTCAACTCGGGCTACGCTGTGAACCCA
GCCAGGGACCTGGGGCCGCGGCTCTTCACCGCTCTGGCGGGCTGGGGCAGCAAGGTCTTC
ACGGTGAACAGCTACTGGTTCTTTGTGCCCTTGTTTGCCCCATTCTTGGGGGCAGTGGTG
GGGGTGATGGTGTACCAGCTCTTGGTGGGCTACAACGTGGAAGGAGAGGCTCGCAACAAG
AAAAGACGA------------GAGGAAGAGGAAAGCCTCAAATGTACAGATATAACCACC
ACCAAAGAAGAGGCA---------------------------------------------
>Arrowtail_Aqp3b
ATGGAAAAACAGAAGGAAGTTCTTGAGAGGCTGGCGCAGATCTTCCACATCAAGGCGCTG
CTTCTTCAACAGGGCCTTGCAGAATGCCTGGGAACCCTTGTTCTGGTG------------
------------------------------------------------------------
------------------------------------------------------GGTGGT
CACCTGAACCCGGCGGTGACCTTTGCACTCTGCGTCCTGGGGAGGGAGCCTTGGAAGAAG
TTCCCCGTGTTCTTTCTTTTCCAAACGTTGGGCGCTTTCCTGGGCGCTGGGATCATATTT
GGCATGTATTTCGATGCACTGTGGGATTACGGCCAGGGG------------TCGCTGTTT
GTGGACGGGAAGAACGCCACGGCGGGGATCTTCGCCACGTATCCGTCCAAACATCTCACT
CTGCTCAACGGATTCTTCGACCAGGTGATCGGAACCGCCGCGTTAATCGTGTGTATCCTG
GCTATCGTCGACCCTCACAACAACCCCGTCCCCAGGGGTCTGGAGGCCTTCACCGTGGGC
TTCGTGGTGCTGGTGATCGGCCTTTCAATGGGCTTTAACTCGGGCTACGCCGTGAACCCC
GCCAGGGACCTGGGGCCGCGGCTCTTCACCGCTGTGGCGGGCTGGGGCCTCGAGGTCTTC
ACG---AAAGGCTGCTGGTCCCTGGTGCCCTTGTTGGCCCCGTTCGTGGGGGCGGTGCTG
GGGGTGGGGGTGTACCAGCTCACCGTGGGCTACAGCGTGGAAGGAGAGGCTCGCAACAAG
AGAAGACGA------------GAGGAAGAGGAAAGCCTCAGATGTGCGAATATAACCGCC
ACCGAAGAAGAGGCA---------------------------------------------
```

>Glacier_lantern_fish_Aqp3b
ATGGGAAGACAACAGGACATGTTAGACCGGCTGGCACAGAGCTTCCAGACACAACACCTT
CTGCTGCGCCAGGCCCTGGCGGAATGTCTGGGCACTCTCATCCTGGTGATGTTTGGATGT
GGTGCAGTGGCACAGATGGTGTTAAGTGGCGGCTCTCATGGCATGTTCCTGAGTGTCAAT
CTGGCCTTTGGCTTTGCTGCAACGCTGGGCGTCCTGGTGAGCGGACAGGTGTCAGGAGGA
CATTTGAACCCGGCAGTGACATTTGCAGTGTGTTTACTGGGCAGAGAGCCTTGGAGGAAG
TTCCCTGTGTTTTTCTGCTCCCAGACGCTGGGTGCTTTCCTGGCTTCTGCCATCATATTC
TGCATGTATTTTGATGCATTATGTGACTTTGGCCAGGGT------------GTATTCATC
ATAGAGGGGGTGAATGCTACTGCTGGTATATTTGCCACCTACCCATCCAGTCACGTCAGC
TTGCTCAACGGAGTCTTTGATCAGGTGATTGGAACGGCATCGCTGGTGGTGTGCATCCTG
GCTATAATGGACCCTCATAACAACCCCGTTCCCAGAGGACTGGAGGCCTTTAGTGTGGGC
CTGGTGGTGCTGGTGCTTGGTCTGTCCATGGGCTTCAATGCAGGTTATGCATTCAACCCA
GCCAGGGACCTAGGACCACGCCTCTTCACGGCGCTGGCGGGCTGGGGTGACCAGGTCTTC
ACGACCAGATCCTACTGGTCTCTGGTCCCCCTGTTTGTCCCATTCCTGGGTGCAGCAGTC
GGGGTGATGGTGTACCAGCTGATGGTTGGATGTCAGGTGGAGGTGGAGTTGAAGCCAGAG
GAGGGAAGA------------GTGGGGGTTCAGACTGTAAAACTTTCCAATATCACCACA

>Atlantic_greeneye_Aqp3b
ATGGGAAAACAAAAGGTACTTCTGGACAAGATGGCAAGGGCTTTTCAGATCCAACACATG
CTCCTCCGCCAGGCCCTGGCAGAATGCCTAGGAACCCTGATTCTTGTGATGTTTGGCTGT
GGAGCAGTGGCACAGTTGGTGCTAAGTGGAGGCTCCCATGGCATGTTCCTCACAGTTAAC
TTTGCCTTTGGCTTCGCTGCGACGCTGGGCATCCTCGTGAGTGGGCAGGTCTCAGGTGGT
CACTTGAACCCAGCGGTGACCTTTGCACTCTGCTTACTTGGGAGAGAACCTTGGAGGAAG
TTCCCTGTGTTTTTCTTCTTCCAGACACTTGGTGCCTTCCTGGGCTCAGGGATTATATTT
GGCATGTATTTTGATGCATTATGGGACTATGGCCAAGGC------------ACGCTCATC
GTGGTGGGGAAGAATGCCACAGCTGGGATTTTTGCTACCTACCCATCAAAACATCTCACA
CTGGTTAATGGATTCTTTGATCAGATGATTGGAACTGCTGCACTGATTGTGTGTATCCTG
GCTATAGTGGACCCTTATAACAACCCAATCCCCAGGGGCCTGGAGGCCTTCACGGTGGGC
TTCGTGGTCTTGGTCATCGGTCTGTCTATGGGCTTCAACTCAGGCTATGCCGTGAATCCA
GCCAGGGACCTGGGACCACGCATCTTCACAGCTCTGGCTGGCTGGGGCAGTGAGGTCTTC
ACGGCCAAAGCCTACTGGTTCTTTGTACCCATCTTTGCTCCCTTCCTGGGTGCAGTAGTG
GGAGTGATGGTGTACCAGCTGATGGTGGGATACCATGTAGAGGGGGACGCCCGAGAGAAA
GAGGTGAAAGAGGAA------GATGAGAAGGAAAGGCTCAAACTTTCAAGTATCACAACC
---AAAGAAGATGCA---
>Ayu_Aqp3b
ATGGGAAGACAGAAGGTTCTTTTGGACAAGGTGGCGCGGAGCTTCCAGATTCGTCATATG
CTCCTTCGACAGGCCCTGGCTGAATGTCTGGGAACCCTGATCCTGGTGATGTTTGGCTGC
GGAGCTGTTGCACAGCTGGTGTTGAGCGGGGGCTCACATGGCATGTTCCTCACTGTCAAC
TTTGCCTTCGGATTCGCCGCCACTTTAGGCATCCTCGTGAGTGGACAGGTGTCAGGTGGT
CATCTAAACCCAACAGTCACCTTTGCTCTGTGTTTGCTGGGGAGAGAGCCTTGGAGGAAG
TTTCCTGTGTTTTTCTTCTTCCAGACGCTCGGTGCCTTTCTAGGGTCTGGCATCATATTT
GGCATGTATTTTGATGCATTGTGGGATTACGGCCAAGGC------------ACACTTATC
GTGGTGGGGAATAACGCCACAGCTGGCATTTTTGCCACCTACCCCTCAAAGCATCTCACC
CTTCTTAACGGATTCTTTGATCAGATGATTGGCACAGCAGCCCTTATTGTGTGCATTCTG
GCCATTGTGGATCCCTATAACAACCCTATTCCCAGAGGACTTGAGGCCTTCACTGTGGGT
TTTGTGGTGCTGGTCATTGGCCTATCCATGGGCTTCAACTCGGGCTACGCTGTAAACCCG
GCCAGGGATTTTGGCCCGCGCCTCTTCACCGCTTTGGCAGGGTGGGGGGGTGAGGTTTTC
ACAGCCAGCGGCTATTGGTTCTTCGTGCCTATCTTTGCCCCTTTCCTGGGTGCCGTGGTG
GGGGTGATGGTGTACCAGCTGATGGTGGGATACCACGTGGAAGGAGAAGCGCGGGAGAGG
AAGGAGGAGGAG---AAGGGGGAGAGAGAGGAGAGGCTTAAACTATCCAGCCTCACCACC
---AAGGACTGTGCA---
>European_smelt_Aqp3b
ATGGGAAGACAGAAGGCTATTTTGGACAAGCTGGCGCGGAGCTTCCAGATTCGTCACATG
CTTCTCCGACAGGCCTTGGCTGAATGCTTGGGAACCCTGATCCTGGTGATGTTTGGCTGT
GGAGCCGTTGCACAGCTGGTGCTGAGCGGAGGCTCTCATGGCATGTTCCTCACTGTCAAC
TTTGCGTTCGGATTCGCCGCCACTTTAGGCATCCTCGTGAGTGGCCAGGTTTCAGGCGGG
CACCTAAACCCAACAGTGACCTTTGCCCTGTGTTTGCTGGGGAGAGAGCCTTGGAGGAAG
TTCCCAGTGTTCTTCTTCTTCCAGACCCTTGGTGCCTTTCTAGGATCTGGCATCATATTT
GGCCTGTATTTTGATGCATTGTGGGACTACGGCCAAGGC------------ACACTTATC
GTGGTGGGGAATAACGCGACAGCCGGCATTTTTGCCACCTACCCCTCAAAACATCTCACC
CTTCTTAACGGATTCTTTGATCAGATGATTGGCACCGCAGCCCTGATTGTGTGCATCCTG

GCCATCGTGGATCCCTACAACAACCCTATCCCCAGAGGACTTGAGGCCTTCACTGTAGGC
TTTGTGGTGCTGGTCATCGGCCTATCGATGGGCTTCAACTCGGGCTATGCTGTGAACCCG
GCCAGGGACTTGGGCCCGCGCCTCTTCACCGCGTTGGCAGGGTGGGGGGTGAGGTATTC
ACGGCCTGTAGCTATTGGTTCTTCGTGCCGATCTTCGCCCCCTTCCTGGGTGCCGTGGTG
GGGGTGATGGTGTACCAGCTGATGGTGGGCTACCACGTGGAAGGAGAAGCGCGGGAGAGG
ATGGAGGAGGAGGAGAAGGGTGAGAGAGAGGAGAGGCTCAAACTATCCAGCCTCACCGCC
---AAGGACTGTGCG---
>Large_eye_snaggletooth_Aqp3b
ATGGGAAGACAGAAGGCACTCATGGACAAGCTGGCACGGACCTTCCAGATCCGTCACACA
CTCCTCCGCCAGGCCCTGGCAGAGTGTTTGGGGACCCTGATCCTGGTGATGTTTGGCTGC
GGCGCGGTGGCACAGCTGGTGCTTAGCGGCGGCTCCCACGGCATGTTCCTCACGGTCAAC
TTTGCGTTCGGCTTCGCCGCCACCCTGGGCATCCTCGTGAGCGGACAGGTCTCAGGTGGC
CACCTGAACCCAACTGTGACCTTTGCACTGTGTTTACTGGGGAGAGAGCCTTGGAGGAAG
TTCCCAGTATTCTTTTTCTTCCAGACTCTGGGTGCCTTTCTAGGATCTGGGATCATATTT
GGAATGTATTTTGATGCATTGTGGGATTACGGCCAGGGC------------ACGCTCATT
GTGGAGGGGAAGAATGCGACGGCTGGGATTTTTGCCACTTACCCCTCCAAACATCTCACC
CTGATTAACGGATTCTTTGATCAGATGATCGGCACAGCAGCGCTGATCGTGTGTATACTG
GCCATCGTGGACCCCTACAACAACCCCATCCCCAGGGGCCTGGAGGCCTTCACGGTGGGC
TTTGTGGTGCTGGTCATTGGCCTGTCTATGGGCTTCAACTCGGGCTACGCCGTCAACCCC

---GCCAAATCCTATTGGTTCTTTGTGCCCATCTTTGCCCCCTTCTTGGGTGCGATGGTG
GGGGTGATGGTGTACCAACTGATGGTGGGATACCATGTAGAGGGAGAGGCTCGAGAGAAG
AAGGAGGAAGAGGAGAAGAAGGAGGAAGAGGAGAGGCTCAAGCTATCCAGCATCACCACC
---AAGGAGAGCGCA---
>Coho_salmon_Aqp3b1
ATGGGAAAACAGAAGGCGCTTATGGACAAACTCGCAAGGACCTTTCAGGTCCGTCACATG
CTGCTACGCCAGGCTCTGGCAGAGTGCCTGGGAACCCTGATACTGGTGATGTTTGGCTGT
GGTGCGGTGGCCCAGCTGGTGCTAAGTGGTGGCTCCCATGGAATGTTCCTCACCGTCAAC
TTTGCCTTTGGCTTCGCTGCTACACTGGGCATCCTGGTCAGCGGACAGATCTCAGGAGGC
CACCTGAACCCAACGGTGACCTTTGCCCTCTGTCTATTGGGGAGAGAACCTTGGAGGAAA
TTCCCTGTGTTTTTCTTGTTCCAGACTATAGGGGCCTTCCTGGGAGCTGGGATCATATTT
GGCATGTATTTTGATGCGTTGTGGGACTACGGCCAAGGA------------ACACTGATC
GTTGTGGGGGAGAATGCCACTGCTGGGATCTTTGCCACATATCCCTCCAAACATCTCACT
CTGGTTAATGGATTCTTTGACCAGATTATAGGCACAGCAGCTCTGATCGTGTGTATCCTG
GCCATTGTGGACCCCTACAACAACCCCATCCCCCGTGGCCTGGAGGCCTTCACGGTGGGG
TTTGTGGTGCTGGTCATTGGCCTGTCAATGGGCTTCAACTCAGGCTACGCTGTCAACCCT
GCCAGGGACCTGGGCCCACGCATCTTTACTGCCCTGGCTGGCTGGGGTGGTGAGGTCTTC
ACGGCTAGTGCCTACTGGTTCTTTGTGCCAATCTTTGCCCCCTTCATTGGTGCAGTGTTG
GGGGTGATGGTGTACCAGCTGATGGTAGGGTACCATGTGGAGGGAGAAGCCCGAGAGAAG
AAGGAAGAGGAGGAGAAGGAGGAGCAAGATGAAAGGCTCAAATTGTCCAGCCTAACTACC
---AAGGACAGTGCA---
>Chinook_salmon_Aqp3b1
ATGGGAAAACAGAAGGCGCTTATGGACAAGCTCGCAAGGACCTTTCAGGTCCGTCACATG
CTGCTACGCCAGGCTCTGGCAGAGTGCCTGGGAACCCTGATACTGGTGATGTTTGGCTGT
GGTGCGGTGGCCCAGCTGGTGCTAAGTGGTGGCTCCCATGGAATGTTCCTCACCGTCAAC
TTTGCCTTTGGCTTCGCTGCTACACTGGGCATCCTGGTCAGCGGACAGATCTCAGGAGGC
CACCTGAACCCAACGGTGACCTTCGCCCTCTGTCTATTGGGGAGAGAACCTTGGAGGAAA
TTCCCTGTGTTTTTCTTGTTCCAGACTATAGGGGCCTTCCTGGGAGCTGGGATCATATTT
GGCATGTATTTTGATGCGTTGTGGGACTACGGCCAAGGA------------ACACTGATC
GTTGTGGGGGAGAATGCCACTGCTGGGATCTTTGCCACATATCCCTCCAAACATCTCACT
CTGGTTAATGGATTCTTTGACCAGATTATAGGCACAGCAGCTCTGATCGTGTGTATCCTG
GCCATCGTGGACCCCTACAACAACCCCATCCCCCGGGGCCTGGAGGCTTTCACGGTGGGG
TTTGTGGTGCTGGTCATCGGCCTGTCAATGGGCTTCAACTCGGGCTACGCTGTCAACCCT
GCCAGGGACCTGGGCCCACGCATCTTTACTGCCCTGGCTGGCTGGGGTGGTGAGGTCTTC
ACGGCTAGTGCCTACTGGTTCTTTGTGCCAATCTTTGCCCCCTTCATTGGTGCAGTGTTG
GGGGTGATGGTGTACCAGCTGATGGTAGGGTACCATGTGGAGGGAGAAGCACGAGAGAAG
AAGGAAGAGGAGGAGAAGGAGGAGCAAGATGAAAGGCTCAAATTGTCCAGCCTAACTACC
---AAGGACAGTGCA---
>Rainbow_trout_Aqp3b1
ATGGGAAAACAGAAGGCGCTTATGGACAAGCTCGCAAGGACCTTTCAGGTCCGTCACATG
CTGCTACGCCAGGCTCTGGCAGAGTGCCTAGGAACCCTGATACTGGTGATGTTTGGCTGT

GGTGCGGTGGCCCAGCTGGTGCTAAGTGGTGGCTCCCATGGAATGTTCCTTACCGTCAAC
TTTGCCTTTGGCTTCGCAGCTACACTGGGCATCCTGGTCAGCGGACAGATCTCAGGAGGC
CACCTGAACCCAACGGTGACCTTCGCCCTCTGTCTATTGGGGAGAGAACCTTGGAGGAAA
TTCCCTGTGTTTTTCTTGTTCCAGACTATAGGGGCTTTCCTGGGAGCTGGGATCATATTT
GGCATGTATTTTGATGCGTTGTGGGACTACGGCCAAGGA------------ACACTGATC
GTTGTGGGGGAGAATTCCACTGCTGGGATCTTTGCCACATATCCCTCCAAACATCTCACT
CTGGTTAATGGATTCTTTGACCAGATTATAGGCACAGCAGCTCTGATCGTGTGTATCCTG
GCCATCGTGGACCCCTACAACAACCCCATCCCCCGGGGCCTGGAGGCCTTCACGGTGGGG
TTTGTGGTGCTGGTCATCGGCCTGTCAATGGGCTTCAACTCGGGCTACGCTGTCAACCCT
GCCAGGGACCTGGGCCCACGCATCTTTACTGCCCTGGCTGGCTGGGGTGGTGAGGTCTTC
ACGGCTAATGCCTACTGGTTCTTTGTGCCAATCTTTGCCCCCTTCATTGGTGCAGTGTTG
GGGGTGATGGTGTACCAGCTGATGGTGGGGTACCATGTGGAGGGAGAAGCACGAGAGAAG
AAGGAAGAGGAGGAGAAGGAGGAGCAAGATGAAAGGCTCAAATTGTCCAGCCTAACTACC
---AAGGACAGTGCA---
>Brown_trout_Aqp3b1
ATGGGAAAACAGAAGGCGCTTATGGACAAGCTCGCAAGGACCTTTCAGATCCGTCACATG
CTGCTACGCCAGGCTCTGGCAGAGTGCCTGGGAACCCTGATACTGGTGATGTTTGGCTGT
GGTGCGGTGGCCCAGCTGGTGCTAAGTGGTGGCTCCCATGGGATGTTCCTCACCGTCAAC
TTTGCCTTTGGCTTCGCTGCTACACTGGGCATCCTGGTCAGCGGACAGATCTCAGGAGGC
CACCTGAACCCAACGGTGACCTTCGCCCTCTGTCTATTGGGCAGAGAACCTTGGAGGAAA
TTCCCTGTGTTTTTCTTGTTCCAGACTATAGGGGCCTTCCTGGGAGCTGGGATCATATTT
GGCATGTATTTTGATGCGTTGTGGGACTACGGCCAAGGA------------ACACTGATC
GTTGTGGGGGAGAATGCCACTGCTGGGATCTTTGCCACATATCCCTCCAAACATCTCACT
CTGGTTAATGGATTCTTTGACCAGATTATAGGCACAGCAGCTCTGATCGTGTGTATCCTG
GCCATC--

>Atlantic_salmon_Aqp3b1
ATGGGAAAACAGAAGGCGCTTATGGACAAGCTCGCAAGGAACTTTCAGATCCGTCACATG
CTGCTACGCCAGGCTCTGGCAGAGTGCCTGGGAACCCTGATACTGGTGATGTTTGGCTGT
GGTGCGGTGGCCCAGTTGGTGCTAAGTGGTGGCTCCCATGGAATGTTCCTCACCGTCAAC
TTTGCCTTTGGCTTCGCTGCTACACTGGGCATCCTGGTCAGCGGACAGATCTCAGGAGGC
CACCTGAACCCAACGGTGACCTTCGCCCTCTGTCTATTGGGGAGAGAACCTTGGAGGAAA
TTCCCTGTGTTTTTCTTGTTCCAGACTATAGGGGCCTTCCTGGGAGCTGGGATCATATTT
GGCATGTATTTTGATGCGTTGTGGGACTACGGCCAAGGA------------ACACTGATC
GTTGTGGGGGAGAATGCCACTGCTGGGATCTTTGCCACATATCCCTCCAAACATCTCACT
CTGGTTAATGGATTCTTTGACCAGATTATAGGCACAGCAGCTCTGATCGTGTGTATCCTG
GCCATCGTGGACCCCTACAACAACCCCATCCCCCGGGGCCTGGAGGCCTTCACGGTGGGG
TTCGTGGTGCTGGTCATCGGCCTGTCAATGGGCTTCAACTCGGGCTATGCTGTCAACCCT
GCCAGGGACCTGGGCCCACGCATCTTTACTGCCCTGGCTGGCTGGGGTGGTGAGGTCTTC
ACGGTTAATGCCTACTGGTTCTTTGTGCCAATCTTTGCCCCCTTCATTGGTGCGGTGTTG
GGGGTGATGGTGTACCAGCTGATGGTTGGGTACCATGTGGAGGGAGAAGCACGGGAGAAG
AAGGAAGAGGAGGAGAAGGAGGAGCAAGATGAAAGGCTCAAACTGTCCAGCCTAACTACC
---AAGGACAGTGCA---
>Brook_trout_Aqp3b1
ATGGGAAAACAGAAGGCGCTTATGGACAAGCTCGCAAGGACCTTTCAGATCCGTCACATG
CTGCTACGCCAGGCTCTGGCAGAGTGCCTGGGAACCCTGATACTGGTGATGTTTGGCTGT
GGTGCAGTGGCCCAGCTGGTGCTAAGTGGTGGCTCCCATGGAATGTTCCTCACCGTCAAC
TTTGCCTTTGGCTTCGCAGCTACACTGGGCATCCTGGTCAGCGGACAGATCTCAGGAGGC
CACCTGAACCCAACGGTGACCTTCGCCCTCTGTCTATTGGGGAGAGAACCTTGGAGGAAA
TTCCCTGTGTTTTTCTTGTTCCAGACTATAGGGGCCTTCCTGGGAGCTGGGATCATATTT
GGCATGTATTTTGATGCGTTATGGGACTACGGCCAAGGA------------ACACTGATC
GTTGTGGGGGAGAATGCCACTGCTGGGATCTTTGCCACATATCCCTCCAAACATCTCACT
CTGGTTAATGGATTCTTTGACCAGATTATAGGCACAGCAGCTCTGATCGTGTGTATCCTG
GCCATCGTGGACCCCTACAACAACCCCATCCCCCGGGGCCTGGAGGCCTTCACAGTGGGG
TTCGTGGTGCTGGTCATCGGCCTGTCAATGGGCTTCAACTCAGGCTACGCTGTCAACCCT
GCCAGGGACCTGGGCCCACGCATCTTTACTGCCCTGGCTGGCTGGGGTGGTGAGGTCTTC

ACGGCTAATGCCTACTGGTTCTTTGTGCCAATCTTTGCCCCCTTCATTGGTGCAGTGTTG
GGGGTGATGGTGTACCAGCTGATGGTGGGGTACCATGTGGAGGGAGAAGCACGGGAGAAG
AAGGAAGAGGAGGAGAAGGAGGAGCAAGATGAAAGGCTCAAACTGTCCAGCCTAACTATC
---AAGGACAGTGCA---
>Arctic_charr_Aqp3b1
ATGGGAAAACAGAAGGCGCTTATGGACAAGCTCGCAAGGACCTTTCAGATCCGTCACATG
CTGCTACGCCAGGCTCTGGCAGAGTGCCTGGGAACCCTGATACTGGTGATGTTTGGCTGT
GGTGCAGTGGCCCAGCTGGTGCTAAGTGGTGGCTCCCATGGAATGTTCCTCACCGTCAAC
TTTGCCTTTGGCTTCGCAGCTACACTGGGCATCCTGGTCAGCGGACAGATCTCAGGAGGC
CACCTGAACCCAACGGTGACCTTCGCCCTCTGTCTATTGGGGAGAGAACCTTGGAGGAAA
TTCCCTGTGTTTTTCTTGTTCCAGACTATAGGGGCCTTCCTGGGAGCTGGGATCATATTT
GGCATGTATTTTGATGCGTTGTGGGACTACGGCCAAGGA------------ACACTGATC
GTTGTGGGGGAGAATGCCACTGCTGGGATCTTTGCCACATATCCCTCCAAACATCTCACT
CTGGTTAATGGATTCTTTGACCAGATTATAGGCACAGCAGCTCTGATCGTGTGTATCCTG
GCCATTGTGGACCCCTACAACAACCCCATCCCCCGGGGCCTGGAGGCCTTCACAGTGGGG
TTCGTGGTGCTGGTCATCGGCCTGTCAATGGGCTTCAACTCAGGCTACGCTGTCAACCCT
GCCAGGGACCTGGGCCCACGCATCTTTACTGCCCTGGCTGGCTGGGGTGGTGAGGTCTTC
ACGGCTAATGCCTACTGGTTCTTTGTGCCAATCTTTGCCCCCTTCATTGGTGCAGTGTTG
GGGGTGATGGTGTACCAGCTGATGGTGGGGTACCATGTGGAGGGAGAAGCACGGGAGAAG
AAGGAAGAGGAGGAGAAGGAGGAGCAAGATGAAAGGCTCAAACTGTGCAGCCTAACTATC
---AAGGACAGTGCA---
>Huchen_Aqp3b1
ATGGGAAAACAGAAGGCACTTATGGACAAGCTCGCAAGGACCTTTCAGATCCGTCACATG
CTGCTACGCCAGGCTCTGGCAGAGTGCCTGGGAACCCTGATACTGGTGATGTTTGGCTGT
GGTGCAGTGGCCCAGCTGGTGCTAAGTGGTGGCTCCCATGGAATGTTCCTCACTGTCAAC
TTTGCCTTTGGTTTCGCTGCTACACTGGGCATCCTGGTCAGTGGACAGATCTCAGGAGGC
CACCTTAACCCAACGGTGACCTTCGCCCTCTGTCTATTGGGGAGAGAACCTTGGAGGAAA
TTCCCTGTGTTTTTCTTGTTCCAGACTATAGGGGCCTTCCTGGGAGCTGGGATCATATTT
GGCATGTATTTTGATGCGTTGTGGGACTACGGCCAAGGA------------ACACTGATC
GTTGTGGGGGAGAATGCCACTGCTGGGATCTTTGCCACGTATCCCTCCAAACATCTCACT
CTGGTTAATGGATTCTTTGACCAGATTATAGGCACAGCCGCTCTGATCGTGTGTATCCTG
GCCATCGTGGACCCCTACAACAACCCCATCCCCCGGGGCCTGGAGGCCTTCACGGTGGGG
TTCGTGGTGCTGGTCATCGGCCTGTCAATGGGCTTCAACTCGGGGTACGCTGTCAACCCT
GCCAGGGACCTGGGCCCACGCATCTTTACTGCCCTGGCTGGCTGGGGTGGTGAGGTCTTC
ACGGCTAATGCCTACTGGTTCTTTGTGCCCATCTTTGCCCCCTTCATTGGTGCGGTGTTG
GGGGTGATGGTGTACCAGCTGATGGTGGGGTACCATGTGGAGGGAGAAGCAAGGGAGAAG
AAGGAAGAGGAGGAGAAGGAGGAGCAAGATGAAAGGCTCAAACTGTCCAGCCTAACTACC
---AAGGACAGTGCA---
>Taimen_Aqp3b1

---CTACGCCAGGCTCTGGCAGAGTGCCTGGGAACCCTGATACTGGTGATGTTTGGCTGT
GGTGCAGTGGCCCAGCTGGTGCTAAGTGGTGGCTCCCATGGAATGTTCCTCACTGTCAAC
TTTGCCTTTGGTTTCGCTGCTACACTGGGCATCCTGGTCAGTGGACAGATCTCAGGAGGC
CACCTTAACCCAACGGTGACCTTCGCCCTCTGTCTATTGGGGAGAGAACCTTGGAGGAAA
TTCCCTGTGTTTTTCTTGTTCCAGACTATAGGGGCCTTCCTGGGAGCTGGGATCATATTT
GGCATGTATTTTGATGCGTTGTGGGACTACGGCCAAGGA------------ACACTGATC
GTTGTGGGGGAGAATGCCACTGCTGGGACATTTGCCACGTATCCCTCCAAACATCTCACT
CTGGTTAATGGATTCTTTGACCAGATTATAGGCACAGCCGCTCTAATCGTGTGTATCCTG
GCCATCGTGGACCCCTACAACAACCCCATCCCCCGGGGCCTGGAGGCCTTCACGGTGGGG
TTCGTGGTGCTGGTCATCGGCCTGTCAATGGGCTTCAACTCGGGGTACGCTGTCAACCCT
GCCAGGGACCTGGGCCCACGCATCTTTACTGCCCTGGCTGGCTGGGGTGGTGAGGTCTTC
ACGGCTAATGCCTACTGGTTCTTTGTGCCCATCTTTGCCCCCTTCATTGGTGCGGTGTTG
GGGGTGATGGTGTACCAGCTGATGGTGGGGTACCATGTGGAGGGAGAAGCAAGGGAGAAG
AAGGAAGAGGAGGAGAAGGAGGAGCAAGATGAAAGGCTCAAACTGTCCAGCCTAACTACC
---AAGGACAGTGCA---
>Grayling_Aqp3b1
ATGGGAAAACAGAAGGCACTTATGGACAAGCTCGCGAGGACCTTTCAGATCCGTCACATG
CTGCTACGCCAGGCTCTGGCAGAGTGCCTGGGAACCCTGATACTGGTGATGTTTGGCTGT
GGTGCAGTGGCCCAGCTGGTGCTGAGTGGTGGCTCCCATGGAATGTTCCTCACCGTCAAC
TTTTCCTTTGGCTTCGCTGCCACACTGGGCATCCTGGTCAGCGGACAGATCTCAGGAGGC
CACCTTAACCCAACGGTGACCTTCGCCCTCTGTCTATTGGGGAGAGAACCTTGGAGGAAA

TTCCCTGTGTTTTTCTTGTTCCAGACTATAGGGGCCTTCCTGGGAGCTGGGATCATATTT
GGCATGTATTTGATGCATTGTGGGACTACGGCCAAGGA------------ACACTGATT
GTTGTGGGGGAGAATTCCACTGCTGGAATCTTTGCCACATATCCCTCCAAACATCTCACT
CTGGTTAATGGATTCTTTGACCAGATGATAGGCACAGCAGCTCTGATCGTGTGTATCCTG
GCCATCGTGGACCCCTACAACAACCCCATCCCCCGGGGCCTGGAGGCCTTCACGGTGGGG
TTCGTGGTGCTGGTCATCGGCCTGTCAATGGGCTTCAACTCGGGCTACGCTGTCAACCCT
GCCAGGGACCTGGGCCCACGCGTCTTTACTGCTCTAGCTGGCTGGGGTAGTGAGGTCTTC
ACGGCTAATGCCTACTGGTTCTTCGTGCCCATCGTTGCCCCCTTCATTGGTGCGGGGGTG
GGGGTGATGGTGTACCAGCTGATGGTGGGGTACCATGTGGAGGGAGAAGCACGGGAGAAG
AAGGAAGAGGAGGAGAAGGAGGAGCAAGAGGAAAGGATCAAACTGTCCAGCCTAACTACA
---AAGGACAGTGCA---
>Coho_salmon_Aqp3b2p
ATGGGAAAACAGAAGGCACTTATGGACAAGCTAGTGAGGACCTTTCAGATCCGCCACATG
TTGCTACGCCAGGCTCTGGCAGAGTGCTTGGGAACCCTGATTCTCATGATGTTTGGCTGT
GGTACGGTGGCCCAGCTGGTGCTGAGTGGTGGCTCCCATGGAGTGTTCCTCACCGTCAAC
TTTGCCTTTGGCTTCGCTGCCACATTGGGGATAATGGTCAGCGGACAGGTCTCAGGT---
------ACCCCAACTGTAACCTTCGCTCTCTGTCTATTGGGGAGAGAACCTTGGAGGAAG
TTCCATGTGTTTTTCTTATTCCAGACTCTGGGGGCCTTCCTGGGATCTGGGATCATATTT
GGCATGTATTTGATGCATTGTGGGACTACGGCCAAGGA------------ACACTGATC
GTT---GGGGAGAATGCCACTGCTGGGATCTTTGCTACCTATCCCTCCAAACATCTCACT
CTGGTTAATGAATTCTTTGACCAGATGATAGGCATGGCTGCCCTGATCATGTGTATCCTG
GCCATTGTGGACCCCTACAACAACCCTATTCACCGGGGTTTGGAGACCTTCACGGTGGGC
TTTGTGGTGCTGGTCATCGGTCTGTCCATGGGCTTC------------------AACCCT
GCCAGAGAC------CCA--
------------TACTGGTTCTTTGTGCCCATCTTTGCCCCCTTCATTGGTGTGGTGGTG
GGGATGACGGTGTACCAGCTGATGGTGGGT------------------------------
---TTTACAACC
---AAGGAGAGCGCC---
>Chinook_salmon_Aqp3b2p
ATGGGAAAACAGAAGGCACTTATGGACAAGCTAGTGAGGACCTTTCAGATCCGCCACATG
TTGCTACGCCAGGCTCTGGCAGAGTGCTTGGGAACCCTGATTCTCGTGATGTTTGGCTGT
GGTGCAGTGGCCCAGCTGGTGCTGAGTGGTGGCTCCCATGGAGTGCTCCTCACCGTCAAC
TTTGCCTTTGGCTTCGCTGCCACATTGGGCATACTGGTCAGCGGACAGGTCTCAGGT---
------ACCCCAACTGTAACCTTCGCTCTCTGTCTATTGGGGAGAGAACCTTGGAGGAAG
TTCCCTGTGTTTTTCTTATTCCAGACTCTGGGGGCCTTCCTGGGATCTGGGATCATATTT
GGCATGTATTTGATGCATTGTGGGACTACGGCCAAGGA------------ACACTGATC
GTT---GGGGAGAATGCCACTGCTGGGATCTTTGCTACCTATCCCTCCAAACATCTCACT
CTGGTTAATGGGTTCTTTGACCAGATGATAGGCATGGCTGCCCTGATCATGTGTATCCTG
GCCATTGTGGACCCCTACAACAACCCTATTCACCGGGGATTGGAGGCCTTCACGGTGGGC
TATGTGGTGCTGGTCATCGGTCTGTCCATGGGCTTC------------------AACCCT
GCCAGAGACCTGGGCCCACGCATATTTACTGCCCTGGCTGGCTGGGGTAGCGAGGTCTTC
ACGGCTTATTCCTACTGGTTCTTTGTGCCCATCTTTGCCCCCTTCATTGGTGCGGTGGTG
GGGATGACGGTGTACCAGCTGATGGTGGGGTCCCACGTGGAGGGAGAGGCACGGGAGAAG
AGGGAAGAGGAAGAAAAGGAGGAGCAAGAGGAGATGCTTAAACTGTCCAGCCTTTACAAC
---CAA--
>Rainbow_trout_Aqp3b2p
ATGGGAAAACAGAAGGCACTTATGGACAAGCTAGTGAGGACCTTTCAGATCCGCCACATG
TTGCTACGCCAGGCTCTGGCAGAGTGCTTGGGAACCCTGATTCTCGTGATGTTTAGCTGT
GGTGAGGTGGCCCAGCTGGTGCTGAGTGGTGGCTCCCATGGAATGTTCCTCACCATCAAC
TTTGCCTTTGGTTTCGCTGCCACATTGGGTATACTGGTCAGCGGACAGGTCTCAGGAGGC
CACCTGAACCCAACTGTAACCTTCCCTCTCTGTATATTGGGGAGAGAACCTTGGAGGAAG
TTCCCTGTGTTTTTCTTATTCCAGACTCTGGGGGCCTTCCTGGGATCTGGTATCATATTT
GGCATGTATTCTGATGCATTGTGGGACTATGGCCAAGGA------------ACACTGATC
GTT---GGGGAGAATGCCACTGCTGGGATCTTTGCTACCTATCCCTCCAAACATCTCACT
CTGGTTAATGGATTCTTTGACCAGATGATAGGCACGGCTGCCCTGATCATGTGTATCCTG
GCCATCGTGGACCCCTACAACAACCCTATTCACCGGGGATTGGAGGCCTTCACGGTGGGC
TTTGTGGTGCTGGTCATCGGTCTGTCCATGGGCTTC------------------AACCCT
GCCAGAGACCTGGGCCCACGCATATTTACTGCCCTGGCTGGCTGGGGTAGCGAGGTCTTC
ACGGCTTATTCCTACTGGTTCTTTGTGCCCATCTTTGCCTCCTTCATTGGTGTGGTGGTG
GGCATGACGGTGTACCAGCTGATGGTGGGGTACCACGTGGAGGGAGAGGCACAGGAGAAT
AGGGAAGAGGAAGAAAAGGAGGAGCAAGAGGAGATGCTTAAAATGTCCAGCCTCTATGTA

---AAACCCTCTATG---
>Arctic_charr_Aqp3b2
ATGGGAAAACAGAAGGCACTTATGGACAAGCTAGTGAGGACCTTTCAGATCCGCCACATG
TTGCTACGCCAGGCTCTTGCAGAGTGCTTGGGAACCCTGATACTCGTAATGTTTGGCTGT
GGTGCAGTGGCCCAGCTGGTGCTGAGTGGTGGCTCCCATGGAATGTTCCTCACCGTCAAC
TTTGCCTTTGGCTTCGCTGCCACATTGGGCATACTGGTCACCGGACAGGTCTCAGGAGGC
CACCTGAACCCAGCTGTAACCTTCGCTCTCTGTCTATTGGGGAGAGAACCTTGGAGGAAG
TTCCCTGTGTTTTTCTTATTCCAGACTCTGGGGGCCTTCCTGGGATCTGGGATCATATTT
GGCATGTATTTTGATGCATTGTGGGACTACGGCCAAGGA------------ACACTGATC
GTTGTGGGGGAGAATGCCACTGCTGGGATCTTTGCTACCTACCCCTCCAAACATCTCACT
CTGGTTAATGGATTCTTTGACCAGATGATAGGCACGGCTGCCCTGATCATGTGTATCCTG
GCCATCGTGGACCCCTACAACAACCCTATTCCCCGGGGTTTGGAGGTTTTCACGGTGGGC
TTTGTGGTGCTGGTCATCGGTCTGTCCATGGGCTTCAACTCGGGCTATGCTGTCAACCCT
GCCAGAGACCTGGGCCCACGCATATTTACTGCCCTGGCTGGCTGGGGTAGCGAGGTCTTC
ACGGCTTATTCCTACTGGTTCTTTGTGCCTATCTTTACCCCCTTCATTGGTGCGGTGGTG
GGGATGATGGTGTACCAGCTGATGGTGGGGTACCACGTGGAGGGAGAGGCACGGGAGAAG
AGGGAAGAGGAAGAAAAGGAGGAGCAAGAGGAGATGCTTAAACTGTCCAGCCTTACAACC
---AAGGAGAGCGCC---
>Atlantic_salmon_Aqp3bS2p
ATGGGAAGACAGAAGGCACTTATGGACAAGCTAGTGAGGACCTTTCAGATCCGCCACATA
TTGCTACGCCAGGCTCTGGCAGAGTGCTTGGGAACCCTGATACTC---ATGTTTGGCTGT
GGTGCAGTAGCCCAGCTGGTGCTGAGTGGTGGCTCCCATGGAATGGTCCTCATCGTCAAC
TTTGCCTTTGGCTTCGCTGCCACATTGGGCATAGGTGAGGGGGGCACATTATCAGGAGGC
CACCTGAACCCAGCTGTAACCTTCACCCTCTGTCTATTGGGGAGAGAACCTTCGAGGAAG
TTCCCTGTGCTTTTCTTATTCCAGACTCTGGGGGCCTTCCTGGGATCT---ATCATATTT
GGCATGTATTTTGATGCATTGTGGGACTACAGCAAAGGA------------ACACTGATC
GTTGTGGGGGAGAATGGCACTACTGGGATCTTTGCTACCTATCCCTCCAAACATCTCACT
CTGGTTAATGGATTCTTTGACCAGGTAGAAGGCACGGCTACCCTGATCATGTGTATCCTG
GCCATCGTGGACCCCTACAACAACCCTATTCCCCGGGGTTTGGAGGCG---CAG---GGT
------------------------AGCCGTGCCTTCAACTCGGGCTATGCTGTCAACCCT
GCCAGAGACCTGGGCCCATGCATATTTACTGCCCTGGCTGGCTGGGGTAGCGAGGTCTTC
ACGGCTTATTCCTACTGGTTCTTTGTGCCCATCTTTGCCCCCTTCATTGGTGCGGTGGTG
GGGATGATGGTGTACCAGCTGATGGTGGGGTACCACGTGGAGGGAAAGGCACGGGAGAAG
AGGGAAGAGGAAGAGAAGGAGGAGCAAGAGGAGATGCTTAAACTGTCCAGCCTTACAACC
---AAGGAGAGCGCC---
>Huchen_Aqp3b2p
---------CAGAAGGCACTTATGGACAAGCTAGTGAGGACCTTTCAGATCCGCCACATG
TTGCTACGCCAGGCTCTGGAAGAGTGCTTGGGAACCCTGATACTCGTGATGTTTGGCTGT
GGTGCAGTAGCCCAGCTGGTGCTGAGTGGTGGCTCCCATGGAATGTTCCTCACCGTCAAC
TTTGCCTTTGGCTTCGCTGCCACATTGGGCATACTGGTCAGTGGACAGATCTCAGGAGGC
CACCTGAACCCAACTGTAACCTTCGCCCTCTGTCTATTGGGGAGAGAAACTTGGAGGAAG
TTTACTATGTTTTTCTTATTCCAGACTCTGGGGGCCTTCCTGGGATCTGGGATCATATTT
GGCATGTATTTTGATGCATTGTGGGACTACGGCCAAGGA------------ACACTGATC
GTTGTGGGAGAGAATGCCACTGCTGGGATTTTTGCTACCTATCCCTCCAAACATCTCACT
CTGGTTAATGGAATCTTTGATCAGATGATAGGCACAGCTGCCCTGATCATGTGTATCCTG
GCCATCGTGGACCCCTACAAAAACCCTATTCCCCGGGGCTTGGAGGCCTTCACGGTGGGC
TTTGTGGTGCTGGTCATTGGTCTGTCCATGGGCTTCAACTCGGGCTATGCTGTCAACCCT
GCCAGAGACCTGGGCCCACGCATATTTACTGCCCTGGCTGGCTGGGGTAGCGAGGTCTTC
ACGGCTAATTCCTACTGGTTCTTTGTGCCCATCTTTGCCCCCTTCATTGGTGCGGTGGTG
GGGATGATGGTGTACCAGCTGATGGTGGGGTACCACGTGGAGGGAGAGGCACGGGAGAAG
AGGGAAGAGGAAGAGGAGGAGGAGCAAGAGGAGATGCTTAAA---ACTGTCCAGCCAAGG
---CCTGAGTGCATG---
>Anshui_blind_cavefish_Aqp3b1
ATGGGAAGACAGAAGGTGATTCTGGAAAAGATGGCTCGGATCTTTCATGTTCGGAACGTG
CTAATACGACAGGCACTGGCAGAATGCCTGGGCACTCTCATTCTAGTGATGTTTGGATGT
GGTGCCGTCGCCCAGTTGATTCTAAGTGGAGGCTCTCATGGAATGTTTCTGACGGTGAAT
TTCGCTTTTGGTTTCGCTGCTACATTGGGAATCCTAGTTTGTGGGCAAGTCTCAGGAGGT
CACATAAACCCTACTGTAACCTTTTCCCTTTGCTTGTTGGGGAGAGAGCCCTGGAGGAAA
TTTCCCGTGTACTTCCTGGCCCAGACTCTAGGGGCTTTTCTTGGCTCAGGAATAATATTT
GGCATGTATATTGATGCAATTTGGGACTATGGACAAGGT------------TCTCTAGTC
GTTGTAGGGGACAATGCAACGGCTGGAATCTTTGCTACATACCCTTCTAAACACCTTACT

TTGCTCAATGGCTTTTTTGATCAGATGATTGGCACGGCAGCTCTCATAGTTTGTATCCTA
GCCATTGTCGACCCTTACAATAATCCCATCCCACAAGGACTGGAGGCCTTCACTGTGGGC
TTTGTAGTTCTAGTGATTGGTCAGTCCATGGGCTTTAACTCAGGTTATGCTGTAAACCCA
GCCAGAGACTTGGGACCTCGGATCTTCACAGCACTTGCTGGATGGGGCTCAGAGGTGTTC
TCGGCAAACTCTTACTGGTCCTTTGTGCCCATCTTTGCCCCGTTCATTGGTGCCATGTTG
GGTGTGATGGTGTATCAGCTGATGGTGGGATACCATGTGGAAGGAGAAGCAAGAGATAAA
AGGGAAGCAGAGGAAAGA---GAAGAGAAGGAAAGACTCAAACTCTCTGCTGTTTCTAAG
---AAGGAGACAGCG---
>Horned_golden_line_barbel_Aqp3b1
ATGGGAAGACAGAAGGTGATTCTGGAAAAGATGGCTCGGATCTTTTATGTTCGGAACGTG
CTGATACGACAGGCACTGGCAGAATGCCTGGGCACTCTCATTCTAGTGATGTTTGGATGT
GGTGCCGTCGCCCAGTTGATTCTAAGTGGAGGCTCTCATGGAATGTTTCTGACGGTGAAT
TTCGCTTTTGGTTTCGCTGCTACATTGGGAATCCTAGTTTGTGGGCAAGTCTCAGGAGGT
CACATAAACCCTACTGTAACCTTTTCCCTTTGCTTGTTGGGGAGAGAGCCCTGGAGGAAA
TTTCCCGTGTACTTCCTGGCCCAGACTCTGGGGGCTTTTCTTGGCTCAGGAATAATATTT
GGCATGTATATTGATGCAATTTGGGACTATGGACAAGGT------------TCTCTAGTC
GTTGTAGGGGACAATGCAACGGCTGGAATCTTTGCTACATACCCTTCTAAACACCTTACT
TTGCTCAATGGCTTCTTTGATCAGATGATTGGCACGGCAGCTCTCATAGTTTGTATCCTC
GCCATTGTCGACCCTTACAATAATCCCATCCCACAAGGACTGGAGGCCTTCACTGTGGGC
TTTGTAGTTCTAGTGATTGGTCAGTCCATGGGCTTTAACTCAGGTTATGCTGTAAACCCA
GCCAGAGACTTGGGACCTCGGATCTTCACCGCACTTGCTGGATGGGGCTCAGAGGTGTTC
TCAGCAAACTCTTACTGGTCCTTTGTGCCCATCTTTGCCCCATTCATTGGTGCCATGTTG
GGTGTGATGGTGTATCAGCTGATGGTGGGATACCATGTGGAAGGAGAAGCAAGAGATAAA
AGGGAAGCAGAGGAAAGA---GAAGAGAAGGAAAGACTCAAACTCTCTGCTGTTTCTAAG
---AAGGAGACAGCG---
>Golden_line_fish_Aqp3b1
ATGGGAAGACAGAAGGTGATTCTGGAAAAGATGGCTCGGATCTTTCATGTTCGGAACATG
CTGATACGACAGGCACTGGCAGAATGCCTGGGCACTCTCATTCTAGTGATGTTTGGATGT
GGTGCTGTCGCCCAGTTGATTCTAAGTGGAGGCTCTCATGGAATGTTTCTGACGGTGAAT
TTCGCTTTTGGTTTCGCTGCTACATTGGGAATCCTAGTTTGTGGGCAAGTCTCAGGAGGC
CACATAAACCCTACTGTAACCTTTTCCCTTTGCTTGTTGGGGAGAGAGCCCTGGAGGAAA
TTTCCCGTGTACTTCCTTGCCCAGACTCTAGGGGCTTTTCTTGGCTCAGGAATAATATTT
GGCATGTATATTGATGCAATTTGGGACTATGGACAAGGT------------TCTCTAGTC
GTTGTAGGGGACAATGCAACGGCTGGAATCTTTGCTACATACCCTTCTAAACACCTTACT
TTGCTCAATGGCTTCTTTGATCAGATGATTGGCACGGCAGCTCTCATAGTTTGTATCCTT
GCCATTGTTGACCCTTACAATAATCCCATCCCACAAGGACTGGAGGCCTTCACTGTGGGC
TTTGTAGTTCTAGTGATTGGTCAGTCCATGGGTTTTAACTCAGGTTATGCTGTAAACCCA
GCCAGAGACTTGGGACCTCGGATCTTCACCGCACTTGCTGGATGGGGCTCAGAGGTGTTC
TCGGCAAACTCTTACTGGTCCTTTGTGCCCATCTTTGCTCCATTCATTGGTGCCATGTTG
GGTGTGATGGTGTATCAGCTGATGGTGGGATACCATGTGGAAGGAGAAGCAAGAGATAAA
AGGGAAGCAGAGGAAAGA---GAAGAGAAGGAAAGACTCAAACTCTCTGCTGTTTCTAAG
---AAGGAGACAGCG---
>Common_carp_Aqp3b1
ATGGGAAGACAGAAGGTGATTCTGGAAAAGATGGCTCGGATCTTTCATGTTCGGAATGTG
CTGATACGACAGGCACTGGCAGAATGCCTGGGCACTCTCATTCTAGTGATGTTTGGATGT
GGTGCTGTCGCCCAGTTGATTCTAAGTGGAGGCTCTCATGGAATGTTTCTGACGGTGAAT
TTCGCTTTTGGGTTCGCTGCTACATTGGGAATCCTAGTTTGTGGGCAAGTTTCAGGAGGT
CACATAAACCCTACTGTAACATTTTCCCTTTGCTTGTTGGGGAGAGAGCCCTGGAGGAAA
TTTCCCGTGTTCTTCCTGGCCCAGACTCTAGGGGCTTTTCTTGGCTCAGGAATAATATTT
GGCATGTATATTGATGCAATTTGGGACTATGGACAAGGT------------TCTCTAGTC
GTTGTAGGGGACAATGCAACGGCGGGAATCTTTGCTACATACCCTTCTAAACACCTTACT
TTGCTCAATGGCTTCTTTGATCAGATGATTGGCACGGCAGCTCTCATAGTTTGTATCCTC
GCCATTGTCGACCCTTACAATAACCCCATCCCACAAGGACTGGAGGCCTTCACTGTGGGC
TTTGTAGTTCTGGTGATTGGTCAGTCCATGGGTTTTAACTCAGGTTATGCTGTAAACCCA
GCCAGAGACTTGGGACCTCGGCTCTTCACCGCACTTGCTGGATGGGGCTCAGAGGTGTTC
TCGGCAAACTCTTACTGGTCCTTTGTGCCCATCTTTGCCCCATTCATTGGTGCCATGTTG
GGTGTGATGGTGTATCAGCTGATGGTGGGATACCATGTGGAAGGAGAAGCAAGGGATAAA
AGGGAAGCAGAGGAAAGA---GAAGAGAAGGAAAGACTCAAACTCTCTTCTGTTTCTGAG
---AAGGAGACAGCG---
>Goldfish_Aqp3b1
ATGGGAAGACAGAAGGTGATCCTGGAAAAGATGGCTCGGATCTTTCAGGTCCGGAACGTC

CTGATACGACAGGCACTGGCAGAATGCCTGGGCACTCTCATTCTAGTGATGTTTGGATGT
GGTGCCGTCGCCCAGCTGATACTAAGTGGAGGCTCTCATGGAATGTTTCTGACGGTGAAT
TTTGCTTTTGGGTTCGCTGCAACATTGGGAATCCTAGTTTGTGGGCAAATTTCAGGAGGT
CACATAAACCCTACTGTGACCTTTTCCCTTTGCTTGTTGGGAAGAGAGCCCTGGAGGAAA
TTTCCCGTGTACTTCCTGGCCCAGACTCTAGGGGCTTTTCTTGGCTCAGGAATAATATTT
GGCATGTATATTGATGCAATTTGGGACTATGGACAAGGT------------TCTCTAGTC
GTTGTAGGGGACAATGCAACAGCTGGAATCTTTGCTACATACCCTTCCAAACACCTTACT
TTGCTCAATGGCTTCTTTGATCAGATGATTGGCACGGCAGCTCTCATAGTTTGTATCCTC
GCCATTGTCGACCCTTACAATAATCCCATCCCACAAGGACTGGAGGCCTTCACTGTGGGC
TTTGTAGTTTTGGTGATTGGTCAGTCCATGGGTTTTAACTCCGGTTATGCTGTAAACCCA
GCCAGAGACTTGGGACCTCGGATCTTCACCGCACTTGCTGGATGGGGCTCAGAGGTGTTC
TCAGCAAACTCTTACTGGTCCTTTGTGCCCATCTTTGCCCCATTCATTGGTGCCATGTTG
GGTGTGATGGTGTATCAGCTGATGGTGGGATACCATGTGGAAGGAGAAGCAAGAGATAAA
------GCAGAGGAAAGA---GAAGAGAAGGAAAGACTCAAACTCTCTGCTGTTTCTGAG
---AAGGAGACAGCA---
>Anshui_blind_cavefish_Aqp3b2
ATGGGAAGACAGAAGGTGATCCTGGAAAAGATGGCTCGGATCTTTCAGGTTCGGAATGTG
CTGATACGACAGGCACTGGCAGAATGCCTGGGCACTCTCATTCTAGTGATGTTTGGATGT
GGTGCTGTCGCCCAGTTGATTCTAAGCGGAGGCTCTCATGGAATGTTTCTGATGGTGAAT
TTCGCTTTTGGGTTCGCTGCTACATTGGGAATCCTAGTTTGTGGGCAAGTCTCAGGAGGT
CACATAAACCCTACTGTGACCTTTTCCCTTTGTTTGTTGGGGAGAGAGCCCTGGAGGAAA
TTTCCCGTGTACTTCCTGGCCCAGACTCTGGGGGCTTTTCTTGGCTCAGGAATAATATTT
GGCATGTATATTGATGCAATTTGGGACTATGGACAAGGT------------TCTCTAGTT
GTTGTAGGGGACAATGCAACGGCTGGAATCTTTGCTACATACCCTTCTAAACACCTTACT
TTGCTCAATGGCTTCTTTGATCAGATGATTGGCACGGCAGCTCTCATAGTTTGTATCCTC
GCCATTGTCGACCCTTACAATAATCCCATTCCACAAGGACTGGAGGCCTTTACTGTGGGC
TTTGTAGTTCTAGTGATTGGTCAGTCCATGGGTTTTAACTCCGGTTATGCTGTAAACCCA
GCCAGAGACTTGGGACCTCGGATCTTCACCGCAATTGCTGGATGGGGCTCAGAGGTGTTC
TCGGCAAACTCTTACTGGTCCTTTGTACCCATCTTTGCCCCATTCATTGGTGCCATGTTC
GGTGTGATGGTGTATCAGCTGATGGTGGGATGCCATGTGGAAGGAGAAGCAAGAGATAAA
AGTGAAACAGACGAAAAA---GAAGAGAAGGAAAGACTAAAACTCTCTGCTGTTTCTGAG
---AAGGAGACAGTA---
>Horned_golden_line_barbel_Aqp3b2
ATGGGAAGACAGAAGGTGATCCTGGAAAAGATGGCTCGGATCTTTCAGGTTCGGAATGTG
CTGATACGACAGGCACTGGCAGAATGCCTGGGCACTCTCATTCTAGTGATGTTTGGATGT
GGTGCTGTCGCCCAGTTGATTCTAAGCGGAGGCTCTCATGGAATGTTTCTGACGGTGAAT
TTCGCTTTTGGGTTCGCTGCTACATTGGGAATCCTAGTTTGTGGGCAAGTCTCAGGAGGT
CACATAAACCCTACTGTGACCTTTTCCCTTTGTTTGTTGGGGAGAGAACCCTGGAGGAAA
TTTCCCGTGTACTTCCTGGCCCAGACTCTGGGGGCTTTTCTTGGCTCAGGAATAATATTT
GGCATGTATATTGATGCAATTTGGGACTATGGACAAGGT------------TCTCTAGTT
GTTGTAGGGGACAATGCAACGGCTGGAATCTTTGCTACATACCCTTCTAAACACCTTACT
TTGCTCAATGGCTTCTTTGATCAGATGACTGGCACGGCAGCTCTCATAGTTTGTATCCTC
GCCATTGTCGACCCTTACAATAACCCCATTCCACAAGGACTGGAGGCCTTTACTGTGGGC
TTTGTAGTTCTAGTGATTGGTCAGTCCATGGGTTTTAACTCAGGTTATGCTGTAAATCCA
GCCAGAGACTTGAGACCTCGGATCTTCACCGCAATTGCTGGATGGGGCTCAGAGGTGTTC
TCAGCAAACTCTTACTGGTCCTTTGTACCCATCTTTGCCCCATTCATTGGTGCCATGTTC
GGTGTGATGGTGTATCAGCTGATGGTGGGATGCCATGTGGAAGGAGAAGCAAGAGATAAA
AGTAAAACAGAGGAAAAA---GAAGAGAAGGAAAGATTCAAACTCTCTGCTGTTTCTGAG
---AAGGAGACAGCA---
>Golden_line_fish_Aqp3b2
ATGGGAAAACAGAAGGTGATCCTGGAAAAGATGGCTCGGATCTTTCGGGTTCGGAATGTG
CTGATACGACAGGCACTGGCAGAATGCCTGGGCACTCTCATTCTAGTGATGTTTGGATGT
GGTGCTGTCGCCCAGTTGATTCTAAGCGGAGGCTCTCATGGAATGTTTCTGACGGTGAAT
TTCGCTTTTGGGTTCGCTGCTACATTGGGAATCCTAGTTTGTGGGCAAGTCTCAGGAGGT
CACATAAACCCTACTGTGACCTTTTCCCTTTGTTTGTTGGGGAGAGAGCCCTGGAGGAAA
TTTCCCGTGTACTTCCTGGCCCAGACTCTGGGGGCTTTTCTTGGCTCAGGAATAATATTT
GGCATGTATATTGATGCAATTTGGGACTATGGACAAGGT------------TCTCTAGTT
GTTGTAGGGGACAATGCAACGGCTGGAATCTTTGCTACATACCCTTCTAAACACCTTACT
TTGCTCAATGGCTTCTTTGATCAGATGATTGGCACGGCAGCTCTCATAGTTTGTATCCTC
GCCATTGTCGACCCTTACAATAACCCCATTCCACAAGGACTGGAGGCCTTTACTGTGGGC
TTTGTAGTTCTAGTGATTGGTCAGTCCATGGGTTTTAACTCAGGTTATGCTGTAAACCCA

GCCAGAGACTTGGGACCTCGGATCTTCACCGCAATTGCTGGATGGGGCTCAGAGGTGTTC
TCGGCAAACTCTTACTGGTCCTTTGTACCCATCTTTGCCCCATTCATTGGTGCCTTGTTC
GGTGTGATGGTGTATCAGCTGATGGTGGGATGCCATGTGGAAGGAGAAGCAAGAGATAAA
AGGGAAACAGAGGAAAAA---GAAGAGAAGGAAAGACTCAAACTCTCTGCTGTTTCTGAG
---AAGGAGACAGCA---
>Common_carp_Aqp3b2
ATGGGAAGACAGAAGGTGATCCTGGAAAAGATGGCTCGGATCTTTCAGGTTCGGAATGTG
CTGATACGACAGGCACTGGCAGAATGCCTGGGCACTCTTATTCTAGTGATGTTTGGATGT
GGTTCTGTCGCCCAGTTGATTCTAAGCAGAGGCTCTCATGGAATGTTTCTGACTGTGAAT
TTTGCTTTTGGGTTTGCTGCTACATTGGGAATCCTTGTTTGTGGGCAAGTCTCAGGAGGT
CACATAAACCCTACTGTGACCTTTGCCCTTTGTTTGTTGGGGAGAGAGCCCTGGAGGAAA
TTTCCTGTGTACTTCCTGGCCCAGACTCTGGGGGCTTTTCTTGGCTCAGGAATAATATTT
GGAATGTATTTTGATGCAATTTGGGACTATGGACAAGGT------------TCTTTAGTT
GTTGTAGGGGACAATGCAACGGCTGGAATCTTTGCTACATACCCTTCTAAACACCTTACT
TTGCTCAATGGCTTCTTTGATCAGATGATTGGCACGGCAGCTCTCATAGTTTGTATCCTC
GCCATTGTCGACCCTTACAATAACCCCATTCCACAAGGACTGGAGGCCTTTACTGTGGGC
TTTGTAGTTCTAGTGATTGGTCAGTCCATGGGTTTAACTCAGGTTATGCTGTAAACCCA
GCCAGAGACTTGGGACCTCGGATCTTCACCGCAATTGCTGGATGGGGATCAGAGGTGTTC
TCGGCAAACTCTTATTGGTCCTTTGTACCCATCTTTGCCCCATTCATTGGTGCCTTGCTC
GGTGTGATGGTGTATCAGCTGATGGTGGGATACCATGTGGAAGGAGAAGCAAGAGATAAA
AGGGAAACAGAGGAAAAA---GAAAAGAAGGAAAGATTCAGTCTCTCTGCTGTTTCTGAG
---AAGGAAACACCG---
>Goldfish_Aqp3b2
ATGGGAAGACAGAAGGTGATCCTGGAAAAGATGGCTCGGATGTTTCATGTTCGGAATGAG
CTGATACGACAGGCACTGGCCGAATGCCTGGGCACTCTCATTCTAGTGATGTTTGGATGT
GGTGCGGTCGCCCAGTTGATTCTTAGCAGAGGCTCTCATGGAATGTTTCTGACGGTGAAT
TTTGCTTTTGGGTTCGCTGCTACACTGGGAATCCTAGTTTGTGGGCAAGTTTCAGGAGGT
CATATAAACCCTACTGTGACCTTTTCCCTTTGTTTGTTGGGGAGAGAACCCTGGAGGAAA
TTTCCCGTGTACTTCCTGGCCCAGACTCTGGGGGCTTTTCTTGGCTCAGGAATAATATTT
GGCATGTATATTGATGCAATTTGGGACTATGGACAAGGT------------TCTCTAGTT
GTTGTAGGGGACAATGCAACGGCTGGAATCTTTGCTACATACCCTTCTAAACACCTTACT
TTGCTCAATGGCTTCTTTGATCAGGTGATTGGCACAGCAGCTCTCATAGTTTGTATCCTC
GCCATTGTCGACCCTTACAATAACCCAATTCCACAAGGACTGGAGGCCTTCACTGTGGGC
TTTGTAGTTCTAGTGATTGGTCAGTCTATGGGTTTAACTCGGGTTATGCTGTAAACCCA
GCCAGAGACTTGGGGCCTCGGATCTTCACCGCAATTGCTGGATGGGGCTCAGAAGTGTTC
TCAGCAAACTCTTACTGGTCTTTTGTACCCATCTTTGCCCCATTCATTGGTGCCATGTTG
GGTGTGATGGTGTATCAGCTGATGGTGGGATACCATGTGGAAGGAGAAGCAAGAGATAAA
AGGGAAACAGAGGAAAAA---GAAGAGAAGGAAAGACTCAAACTCTCTCCTATTTCTGAG
---AAGGAGACAGCG---
>Iberian_chubb_Aqp3b

--ATGTTTGGCTGC
GGTGCTGTCGCCCAGTTGGTTTTAAGCGGAGGCTCTCATGGAATGTTTCTGACGGTGAAT
TTTGCATTTGGGTTTGCCGCTATGTTGGGAATCCTTGTTTGTGGGCAAGTCTCAGGAGGT
CACATAAACCCTACTGTGACCTTTGCCCTCTGTTTGTTGGGGAGAGAGCCCTGGAGGAAA
TTTCCCGTGTACTTCCTGGCCCAGACTCTGGGGGCTTTTCTTGGCTCAGGAATAATATTT
GGCATGTATATTGATGCAATTTGGGACTTTGGACATGGT------------TCTCTGATC
GTTGTAGGTGACAATGCAACAGCTGGAATCTTTGCTACATACCCTTCTAAACACCTTACT
TTGCTCAACGGCTTCTTCGATCAGATAATTGGCACGGCAGCTCTGATTGTGTGTATCCTT
GCCATTGTTGACCCTTATAATAACCCCATCCCACGAGGACTGGAGGCCTTTACTGTGGGC
TTTGTGGTTCTGGTGATTGGTCAGTCCATGGGTTTAACTCAGGTTATGCTGTAAACCCA
GCAAGAGACTTGGGACCTCGGATCTTCACTGCAATTGCTGGATGGGGCTCAGAGGTGTTC
TCTGCAAACTCTTACTGGTCCTTTGTGCCAATCTTTGCCCCATTCATTGGTGCCGTGTTC
GGCGTGATGGTGTATCAGCTGATGGTGGGATGCCATGTGGAAGGAGAAGAAAGAGACAAA
AGGGAAGCAGTGGAAAGG---GAACAGAAGGACAGACTCAAACTCACTGCTGTTTCTGAG
---AAGGAGACAGCA---
>Amur_ide_Aqp3b
ATGGGAAGACAGAAGGTGCTTCTGGAAAAGATGTCTCGGACCTTGCAGGTGCGGAACATG
CTGATGCGACAGGCACTGGCAGAATGTCTTGGCACCCTCATTCTAGTGATGTTTGGCTGC
GGTGCTGTCGCCCAGTTGGTTTTAAGCGGAGGCTCTCATGGAATGTTTCTGACGGTGAAT
TTTGCATTTGGGTTTGCCGCTATGTTGGGAATCCTAGTTTGTGGGCAAGTCTCAGGAGGT

CACATAAACCCCACTGTGACCTTTGCCCTCTGTTTGTTGGGGAGAGAGCCCTGGAGGAAA
TTTCCCGTTTACTTCCTGGCCCAGACTCTGGGGGCTTTTCTTGGCTCAGGAATAATATTT
GGCATGTATATTGATGCAATTTGGGACTTTGGACAAGGT------------TCTCTGATC
GTTGTAGGGGACAATGCAACAGCTGGAATCTTTGCTACATACCCTTCAAAACACCTTACT
TTGCTCAACGGCTTCTTCGATCAGATAATTGGCACGGCAGCTCTGATTGTGTGTATCCTT
GCCATTGTTGACCCTTACAATAACCCCATCCCACGAGGACTGGAGGCCTTTACTGTGGGC
TTTGTGGTTCTGGTGATTGGTCAGTCCATGGGTTTTAACTCGGGTTATGCTGTAAACCCA
GCAAGAGACTTGGGACCTCGGATCTTCACTGCAATTGCTGGATGGGGCTCAGAGGTGTTC
TCGGCAAACTCTTACTGGTCCTTTGTGCCAATCTTTGCCCCATTCATTGGTGCCGTGTTC
GGTGTGATGGTGTATCAGCTGATGGTGGGATGCCATGTGGAAGGAGAAGAAAGAGACAAA
AGGGAAGCAGTGGAAAGA---GAACAGACGGACAGACTCAAACTCACTGCTGTTTCTGAG
---AAGGAGACA--
>Fathead_minnow_Aqp3b
ATGGGAAGACAGAAGGTGCTTCTGGAAAAGATGTCTCGGACCTTGCAGGTGCGGAACCTG
CTGATGCGACAGGCACTGGCAGAATGTCTAGGCACCCTCATTCTAGTGATGTTTGGCTGT
GGTGCTGTCGCTCAGTTGGTTTTAAGCGGAGGATCTCATGGAATGTTTCTGACGGTGAAT
TTTGCATTTGGGTTTGCCGCTACGTTGGGAATCCTTGTTTGTGGGCAAGTCTCAGGAGGC
CACATAAACCCCACTGTGACCTTTGCCCTCTGTGTGTTGGGGAGAGAGCCCTGGAGAAAA
TTTCCCGTGTACTTCCTGGCCCAGACTCTGGGGTCTTTTCTTGGCTCAGGAATAATATTT
GGCTTGTATATAGATGCAATTTGGGACTTTGGACATGGT------------TCTCTAATC
GTTGTAGGGGACAATGCAACAGCCGGAATCTTTGCTACATACCCTTCTAAACACCTTACT
TTGCTCAACGGCTTCTTCGATCAGATGATTGGCACGGCAGCTCTGATTGTGTGTATCCTT
GCCATTGTTGACCCTTACAATAACCCCATCCCTCGCGGACTGGAGGCCTTTACTGTGGGC
TTTGTGGTTCTGGTGATTGGTCAGTCCATGGGTTTTAACTCGGGTTATGCTGTAAACCCA
GCAAGAGACTTGGGACCTCGGATCTTCACCGCAATTGCTGGATGGGGCTCAGAGGTGTTC
TCGGCAAACTCTTACTGGTCCTTTGTGCCAATCTTTGCCCCATTCATTGGTGCTGTTTTC
GGTGTGATGGTGTATCAGCTGATGGTGGGATGCCATGTGGAAGGAGAAGAAAGAGACAAA
AGGGAAGCATTGGAAAAA---GAACAGAAGGACAGATTCAAACTCACTGCTGTTTCTGAG
---AAGGACACACCA---
>Grass_carp_Aqp3b

--ATGTTTGGCTGT
GGTGCTGTCGCCCAGTTGGTTCTAAGCGGAGGCTCTCATGGAATGTTTCTGACAGTGAAT
TTTGCGTTTGGGTTTGCTGCTACATTGGGAATCCTAGTTTGTGGGCAAGTCTCAGGAGGT
CACATAAACCCTGCTGTGACCTTTTCCCTCTGTTTGTTGGGGAGAGAGCCCTGGAGGAAA
TTTCCCGTGTACTTCCTGTCCCAGACTCTGGGGGCTTTTCTTGGCTCAGGAATAATATTT
GGCATGTATATCGATGCAATTTGGGACTTTGGACAAGGT------------TCTCTAGTC
GTTATAGGGGACAATGCAACAGCTGGAATCTTTGCTACATACCCTTCTAAACACCTTACT
TTGCTCAACGGCTTCTTTGATCAGATAATTGGCACGGCAGCTCTGATCGTGTGTATCCTT
GCCATTGTTGACCCTTACAATAACCCCATCCCACGAGGTCTGGAGGCCTTTACTGTGGGC
TTTGTGGTTCTGGTGATTGGTCAGTCCATGGGTTTTAACTCAGGTTATGCTGTAAATCCA
GCGAGAGACTTGGGACCTCGGATCTTCACCGCAATTGCTGGATGGGGCTCAGAGGTGTTC
TCAGCAAACTCTTACTGGTCCTTTGTGCCCATCTTTGCCCCATTCATTGGTGCCGTGTTC
GGTGTAATGGTGTATCAGCTGATGGTGGGATACCATGTGGAAGGAGAAGAAAGAGATAAA
AGGGAAGCAGTGGAAAGA---GAACAGAAGGAGAGACTCAAACTCTCTGCTGTTTCTGAG
---AAGGAGACAGCA---
>Zebrafish_Aqp3b
ATGGGAAGACAGAAGGTAATCCTGGAAAAGATGGCTCGGATCTTTCAGATTCGGAACATG
CTGATGAGACAAGCACTGGCAGAATGCCTGGGCACCCTCATTCTAGTGATGTTTGGTTGT
GGTGCTCTTGCCCAGCATATTTTAAGCGGAGGCTCTCATGGAATGTTTCTGACAGTGAAT
TTTGCATTTGGATTCGCTGCTACATTGGGAATCCTGGTTTGTGGGCAAGTCTCAGGAGGT
CACATAAACCCTACTGTGACCTTTTCTCTCTGTTTGTTGGGGAGGGAGCCCTGGAGGAAA
TTTCCCGTTTACTTTCTGGCCCAGACTGTGGGGGCTTTTCTTGGAGCTGGAATAATATTT
GGCATGTATTTTGATGCAATTTGGAAATTTGGACAAGGT------------TCTCTTGAT
GTTGATGGGGTAAATGCAACTGCTGGAATCTTTGCTACGTACCCTTCTAAACACCTTACT
TTGCTAAATGGATTCTTTGATCAGATGATCGGCACGGCAGCTCTGATCGTGTGTATCCTT
GCCATTGTTGACCCTTACAATAACCCCATCCCGCAAGGACTGGAGGCCTTCACTGTGGGC
TTTGTGGTTCTGGTGATTGGTCTGTCTATGGGATTTAACTCAGGCTATGCTGTAAACCCA
GCCAGAGACTTGGGACCACGGATCTTCACTGCAATTGCTGGATGGGGCTCAAAAGTGTTC
TCAGCGGAGTCTTACTGGTCCTTTGTGCCAGTCTTTGCCCCATTCATTGGTGCTGTGTTC
GGTGTGATGGTGTATCAGTTGATGGTGGGATGCCATGTGAAAGGAGAAGAAAGAGATAAA

AGAGAAGCGGTGGAAAGA---GAAGAGAAGGAAAGACTCAAACTATCTGCCGTTTCTGAT
---AAAGATGCAGCA---
>White_sucker_Aqp3b
ATGGGAAGACAGAAAGTGCTCCTGGAAAAGATAGCTCGGATCTTTCAGGTTCGGAATATG
CTGATGCGACAGGCACTGGCAGAATGCCTGGGCACCCTCGTTCTAGTGATGTTTGGTTGT
GGTGCTGTTGCCCAGTTGATTCTTAGTGGAGGCTCTCATGGAATGTTCCTGACAGTGAAT
TTTGCTTTTGGATTTGCTGCTACTTTGGGGATCCTAGTTTGTGGCCAAATTTCAGGAGGT
CATATAAATCCTACTGTGACCTTTTCCCTGTGTTTGTTGGGGAGAGAGCCCTGGAGGAAG
TTTCCTGTCTACTTCCTGTCCCAGACTCTGGGGGCCTTTCTTGGCTCAGGAATAATATTT
GGCATGTATTTTGATGCACTGTGGGACTATGGACAAGGC------------TCTTTCGTC
GTTGTAGGGGACAATGCAACAGCTGGAATTTTTGCGACATACCCCTCTAAGCACCTTAAT
TTGCTCAATGGCTTTTTTGATCAGATGATTGGCACTGCAGCTTTGATCGTATGTATCCTA
GCCATTGTCGACCCCTACAATAACCCAATCCCACGAGGACTGGAGGCTTTCACTGTGGGG
TTTGTGGTTCTGGTGATTGGTCTGGCCATGGGTTTAACTCAGGTTATGCTGTAAACCCA
GCCAGAGACCTGGGACCTCGGATCTTTACGGCACTTGCTGGATGGGGCTCAGAGGTGTTC
TCAGCAAACTCTTACTGGTCCTTTGTACCCATCTTTGCCCCATTCATTGGTGCCATGATT
GGTGTGATGGTGTATCAGCTGATGGTGGGATATCATGTGGAAGGAGAAGCGAGAGATAAA
AGGGAAGCTGTTGAAAGA---GAAGACAAGGAAAGACTCAAACTCTCTGCTATTTCTGAG
---AAGGAGACAGCA---
>Oriental_weatherfish_Aqp3b
ATGGGAATACAGAAGGTGTTGCTGGAAAAAATAGCTCGGAACTTACAGATCCGCGACAAG
CTTCTGCGACAGGCGCTGGCAGAATGTCTGGGCACCCTCATTTTAGTGATGTTTGGTTGT
GGCGCTGTTGCCCAGTTGGTTCTTAGCGGAGGTTCCCATGGAATGTTTCTGACGGTAAAT
TTCGCTTTCGGGTTCGCTGCAACTCTGGGTATCCTAGTTTGTGGGCAGGTTTCAGGTGGT
CACATAAACCCCACTGTGACTTTTTCTCTATGCCTGTTGGGGAGGGAACCATGGAAGAAG
TTCCCCGTTTATTTCCTAGCCCAGACTATCGGTGGTTTTCTTGGCTCAGGGATAATATTT
GGCATGTATTTTGATGCACTGTGGGAATACGGGGAAGGG------------TCTCTAATC
GTTGTCGGGGACAATGCAACAGCTGGGATTTTTGCTACGTACCCATCAAAACACCTTACT
GTAGTCAATGGCTGTTTTGATCAGATGATTGGCACTGCCGCTCTGATCGTGTGTATTCTA
GCAATTGTCGACCCTTACAATAACCCTATCCCACAAGGACTGGAGGCATTCACTGTGGGC
TTTGTAGTACTGGTGATTGGTCAGTCAATGGGTTTCAATTCAGGTTATGCTGTAAACCCA
GCCAGAGACTTGGGACCTCGGCTCTTTACTGCAATTGCTGGATGGGGCACAGAAGTCTTC
ACAGCAAACTCTTTCTGGTCTCTTGTACCCGTCTTTGTCCCATTCATTGGTGCTGTGATT
GGTGTTATTGTGTATCAGCTGATGGTGGGATTCCATGTGGAAGGAGAAGAAATGGATAAA
AGAGAAGCTGTGGAAAGA---GAAGAAAGAGAAAGAATCAAACTCTCTGCTCTTACTGAG
---AAAGAGTTTGCA---
>Channel_catfish_Aqp3b
ATGGGAAGGCAGAAGGTTTTCATGGACAAAATGATGCAGATGTTCCACATACGAAACCTC
TTGATGCGTCAGGCTCTTGCAGAATGCCTGGGCACCCTCATCCTGGTGATGTTTGGCTGT
GGTGCTGTTGCTCAGTTGGTCCTGAGTGAAGGATCGCATGGAATGTTCCTAACAGTGAAT
TTCGCATTTGGTTTCGCTGCCACACTTGGGATTTTGGTGTGTGGCCAGGTTTCAGGTGGC
CACCTGAACCCAGCTGTGACCTTTGCCCTTTGCATCCTGGGTAGGGAACCATGGAGGAAG
TTCCCTGTTTACTTCATATTTCAGACTGTGGGTGCCTTCCTTGGATCTGGAATAATATTT
GGCATGTATTTTGATGCACTCTGGGAGTATCGGAAAGGC------------AGTCTGATT
GTGTTGGGGGAAAATGCGACAGCTGGAATATTCGCCACGTATCCGTCCAAACATCTCAGC
CTGTTTAACGGCTTCTTTGACCAAGTGATTGGCACAGCAGCGCTGATCGTGTGTATACTC
GCCATTGTGGACCCGTATAACAACCCAATCCCACGTGGGCTAGAAGCCTTCACTGTAGGC
TTCGTGGTTTTGGTTATTGGCCTTTCTATGGGATTTAACTCAGGCTACGCCGTGAACCCC
GCCAGAGACCTCGGACCCCGAATCTTTACCTCACTTGCAGGCTGGGGCGGTGAAGTCTTC
ACGGCAAATGATTACTGGTTCTTTGTTCCGATCTTTGCACCATTTATCGGTGCCATGGTG
GGCGTTTTGGTGTACCAGCTGATGGTGGGATACCATTTGGAGGGCGAAGCCCAAGACAGG
GAAGAAGCAGTGACCCGC---GAAGAGAAAGAAAGACTGAAGTCATGCAAAGTGTCTGGA

>Striped_catfish_Aqp3b
ATGGGAAGGCAGAAGGTTTTCATGGACAAAATGATGCAAATGTTCCACATCCGAAACCTG
CTAATACGTCAGGCTCTTGCGGAATGCCTGGGCACGCTCATCCTGGTGATGTTTGGCTGT
GGTGCTGTTGCTCAGTTAGTCCTGAGCGAAGGATCGCATGGAATGTTCCTGACGGTGAAC
TTCGCATTTGGTTTCGCTGCCACACTTGGGATTTTGGTGTGTGGCCAGATTTCAGGTGGC
CACCTGAACCCAGCTGTGACCTTTGCCCTGTGCATCCTGGGAAGGGAACCATGGAGGAAG
TTCCCTGTGTATTTCATATTTCAGACTGTGGGTGCATTCCTTGGCTCTGGGATAATATTT
GGCATGTATTTTGATGCACTTTGGGAGTATCGGAAGGGC------------AGTCTGATT

GTGTTGGGGGAAAATGCTACAGCTGGAATATTTGCCACGTATCCATCCAGACATCTCAGC
CTGGTCAACGGCTTCTTTGACCAAGTGATTGGCACAGCAGCTCTGATCGTGTGTATACTC
GCCATCGTGGACCCGTATAACAACCCAATCCCACGTGGCCTAGAAGCCTTCACTGTAGGC
TTTGTGGTTTTGGTTATTGGTCTTTCTATGGGATTTAACTCAGGCTATGCCGTGAACCCT
GCCAGAGACCTCGGACCCCGAATCTTTACCTCACTTGCAGGCTGGGGCAGTGAAGTCTTC
ACGGCAAATGACTACTGGTTCTTTGTTCCAATCTTTGCACCATTTATTGGTGCCATGGTG
GGCGTTTTGGTGTACCAACTGATGGTGGGATACCATTTGGAGGGAGAAGCCCAAGACAGG
GCAGAAGCGGAAGCCCGA---GAAGAGAAAGAAAGGCTGAAGTCATGCAAAGTGTCTGGG

>Yellow_catfish_Aqp3b
ATGGGATGGCAAAAGGTTTTCACGGACAAAATGATGCAAACGTTCCACATCCGAAACCTA
CTGATACGCCAGGCTCTTGCGGAATGCCTGGGCACCCTTATCCTGGTGATGTTTGGCTGT
GGTGCTGTTGCTCAGCTAGTCCTGAGTGAAGGATCGCATGGAATGTTCCTGACAGTGAAT
TTCGCATTTGGTTTCGCTGCAACGCTTGGGATTTTGGTGTGTGGCCAGGTTTCAGGTGGA
CACCTGAACCCAGCTGTGACCTTTGCCCTTTGCATCCTTGGTAGGGAACCATGGAGGAAG
TTCCCTGTATTCTTCATATTTCAGACTATAGGTGCCTTTCTTGGATCTGCAATCATATTT
GGCATGTATTTTGATGCACTTTGGGAGTATCGGAAAGGC------------AGTCTGATT
GTGTTGGGTGAAAATGCTACAGCTGGAATATTTGCCACGTATCCATCCAATCATCTTAGC
CTGGTCAACGGCTTCTTTGATCAAGTGATTGGCACAGCAGCACTGATCGTGTGTATACTT
GCCATCGTGGACCCGTATAACAACCCAATCCCACGTGGGCTAGAAGCCTTCACTGTAGGC
TTCGTGGTTTTGGTTATTGGCCTTTCTATGGGATTTAACTCTGGTTATGCCGTAAACCCA
GCCAGAGACCTCGGACCTCGAATCTTTACATCGATTGCAGGCTGGGGCAGTGAAGTCTTC
ACAGTGAATGATTACTGGTTCTTTGTTCCAATCTTCGCACCATTTATCGGTGCCCTAGTG
GGCGTTTTGGTGTATCAGCTGATGGTGGGATACCATTTGGAGGGAGAAGCCCAAGAAAGG
GAAGAAGCAGAAGACCCC---AAAGAGAAAGTAAGGCTGAAGTCATGCAAAGTGTCTGGG

>Walking_catfish_Aqp3b
ATGGGAAGGCAAAAGATTTTCTTGGACAAAATTATGCACATTTTCCGAATCCGAAACCTC
CTAGCACGCCAAGCTCTGGCGGAGTGCCTGGGTACCCTCATCCTGGTGATGTTTGGCTGT
GGTGCTGTAGCTCAGTTAGTTCTGAGTGAAGGATCGCATGGAATGTTCCTGACAGTGAAT
TTCGCATTTGGTTTTGCAGCTACACTTGGGATATTGGTGTGTGGCCAGGTTTCAGGTGGT
CACTTGAACCCAACTGTGACCTTTGCCCTTTGCATTCTGGGTAGAGAACCATGGAAGAAG
TTCCCTGTGTACTTTCTGTTTCAGACTGTTGGGGCATTCCTTGGCTCTGGAATAATATTT
GGCATGTATTATGACGCACTTTGGGAGTATAAGAAAGGC------------AGTCTGATT
GTGATGGGAGAAAATGGTACAGCTGGAATATTTGCCACGTATCCATCCGAACATCTTAGT
CTTGTCAACGGCTTCTTTGACCAAGTGATTGGCACAGCAGCTTTGATCGTGTGTATCCTT
GCCATCGTGGACCCCTATAACAACCCCATCCCACGTGGGCTAGAAGCCTTTACTGTTGGC
TTCGTGGTATTGGTTATTGGCCTTTCAATGGGATTTAACTCAGGCTATGCCGTGAACCCC
GCCAGAGACCTCGGACCCCGAATCTTTACTGCAATTGCAGGCTGGGGCGCTGAAGTCTTC
ACGGTGAACAATTACTGGTTCTTTGTCCCGATCTTTGCACCGTTTATCGGTGCCATGGTG
GGTGTTTTGGTGTACCAGCTGATGGTTGGATATCATCTGGAAGGAGAAGCCCAAGACAGG
GGACAAGCTGAGGCCCAG---GAAGAGAAAGTAAGACTGAAGGCATATAAAGTGTCTGGG

>Bottlenose_catfish_Aqp3b
ATGGGAAGGCAGAAGGTTTTCATGGAAAAAATTATGCAAATGTTCCACGTCCGAAACCTT
CTGATGCGTCAGGCTCTCGCAGAATGCCTGGGCACACTCATCCTGGTGATGTTTGGCTGT
GGTGCTGTGGCACAGTTAGTACTGAGTGAAGGATCGCATGGAATGTTCCTGACAGTGAAT
TTTGCATTTGGTTTTGCTGCAACACTTGGGATTTTGGTGTGTGGCCAGGTTTCAGGTGGC
CACCTGAACCCAGCTGTGACTTTTGCCCTTTGCATCCTGGGTAGGGAACCATGGAAGAAG
TTCCCTGTGTACTTCTTATTTCAGACTGTTGGTGCTTTTTTTGGATCTGGAATAATATTT
GGCATGTATTTTGATGCATTTTGGGAGTTTCGGAAAGGC------------AGTCTGATT
GTGGTGGGGGAAAATGCAACAGCTGGAATATTTGCCACATATCCATCCACCCATCTCAGC
CTGGTCAACGGCTTCTTTGACCAAGTGATTGGAACAGCAGCTCTTATCGTGTGTATACTT
GCCATCGTGGACCCGTATAACAACCCAATCCCACGCGGACTAGAAGCCTTCACTGTAGGC
TTTGTGGTTTTGGTTATTGGKCTTTCTATGGGATTTAACTCAGGCTATGCTGTGAACCCA
GCCAGAGATCTCGGACCTCGAATCTTTACCTCACTTGCAGGCTGGGGCAGTGAAGTTTTC
ACGGCAAATGATTACTGGTTCTTTGTTCCAATCTTTGCACCATTTATTGGTGCAATGGTG
GGCGTTATGGTTTACCAGCTGATGGTGGGATACCATTTGGAGGGAGAAGCCCACAATAGG
GAGGAAGCACTGGCCCGT---GAAGAGAAACAAAGGCTGAAATCATGCAAAGTGTCTGGA

>Pencil_catfish_Aqp3b

ATGGGATGGCAGAAGGCTTTTTGGACAAAATGATGCAAAATCTTCGGATCCGAAACCTT
TTGATGCGTCAGGCTCTGGCCGAATGCCTGGGTACCCTCATTCTGGTGATGTTTGGCTGT
GGTGCAGTTGCTCAGTTGGTTCTGAGTGAAGGATCTCATGGAATGTTCCTGACAGTGAAT
TTCGCATTTGGTTTTGCTGCCACGCTTGGGATATTGGTGTGTGGTCAAGTTTCAGGTGGC
CACCTGAACCCGACCGTTACTTTCGCACTTTGCATTTTGGGTAGAGAACCATGGAGGAAG
TTTCCGGTGTATTTTCTGTTTCAGACTCTGGGCGCTTTCCTTGGATCAGGGATCATATTC
GGCATGTATTTTGATGCCCTGTGGGAGTACCGTAAGGGC------------AGCCTGATT
GTGGTGGGGGAAAATTCTACAGCTGGCATATTTGCCACGTATCCGTCCAGCCATCTCAGC
GTGGTCAACGGCTTCTTCGACCAAGTGATTGGCACAGCGGCTTTAATCCTGTGTATTCTG
GCCATCGTGGACCCATTTAACAACCCAATCCCACGCGGGCTGGAGGCTTTCACTGTGGGC
TTGGTGGTTTTGGTGATCGGCCTTTCCATGGGCTTCAACTCGGGCTACGCTGTCAATCCC
GCCAGAGACCTCGGACCTCGGATCTTCACATCAATCGCAGGCTGGGGCACCGAAGTCTTC
ACAGTGAACGAATACTGGTTCTTCATTCCAATTTTTGCCCCATTTATCGGTGCCGTGTTT
GGCGCTTTGGTGTACCAGCTGATGGTGGGTTGCCACACTGAAGGAGAAGCTCACGACAAA
AAAAGAAGC---

>Glass_knifefish_Aqp3b
ATGGGAAAGCAGAAGATGCTCCTGGACAAGCTGGCGCAGATGTTCCAGATCCGGAACCTT
CTAGTCCGCCAGGCCCTGGCCGAGTGTCTGGGGACCCTCATACTGGTGATGTTTGGTTGT
GGGGCAGTGGCTCAGCTGGTCCTCAGCGAGGGATCCCATGGGATGTTCCTCACCGTGAAC
TTTGCCTTTGGATTCGCCGCCATGCTGGGGATTCTAGTGTGTGGCCAAGTCTCAGGTGGG
CACCTTAATCCAACAGTGACCTTTGCCCTCTGCATGCTGGGTCGAGAACCATGGAGGAAG
TTTCCAGTGTACTTCTTGTTTCAGACGATAGGGTCCTTCTTCGGGGCAGCGATCATATTT
GGCATGTATTACGATGCTCTCTGGGTGTATCAGAAGAAC------AGTGACACTCTCCTC
GTGGTGGGCGAGAACGCCACAGCTGGGATCTTCGCCACGTACCCCTCCAAACACCTCAGT
CTTGTGAACGGCTTCTTTGACCAGATGATCGGCACCGCGGCCTTGATTGTGTGCGTCCTC
GCAATCGTGGACCCCTACAACAATCCCATCCCCCGCGGCCTGGAGGCCTTCACCGTGGGG
TTCGTGGTGCTGGTCATCGGCCTATCCATGGGCTTCAACTCAGGCTACGCCGTGAACCCT
GCCCGCGACCTCGGCCCCCGCATCTTCACCTCCATCGCTGGCTGGGGCAGTGAAGTCTTC
ACGGTTAACGAGTACTGGTTCTTTGTGCCAATCTTCGCTCCGTTCTTTGGCACGCTGGTG
GGGGTGCTGGTGTACCAGCTGATGGTGGGTTACCACGTGGAGGGTGAGAACCGCGCCCGG
GAGATGGCGCGGGCGCAA---GGAGAGAAGGAGAGAATGAAGCTC---------ACGGAG
AAGAGTGATTTGGCA---
>Electric_eel_Aqp3b
ATGGGTAGGCAGAAGATGCTTCTGGACAAAATGTCCCAGACGTTCCAGATCCAGAACCTT
CTGATGAGGCAGGCGCTGGCCGAGTGCCTCGGGACCCTCATACTGGTGATGTTTGGCTGT
GGGGCAGTGGCCCAACTGGTCCTCAGCGAGGGATCTCATGGAATGTTCCTCACCGTGAAC
TTTGCCTTTGGTTTTGCTGCCACGCTGGGGATCCTGGTGTGTGGTCAAGTCTCAGGTGGG
CACCTGAACCCGACAGTGACCTTTGCCCTTTGCCTGCTGGGTCGAGAGCCATGGAGGAAG
TTTCCTATGTATTTCCTGTTTCAGACAATAGGAGCCTTCTTTGGAGCGGCGACCATATTT
GGCATGTACTATGATGCTCTCTGGGCATATCAGAAGAAC------------GGTCTGCTC
GTGGTGGGCGAGAACGCCACAGCAGGAATG------------------------------

---GTTAACGAGTGCTGGTTCTTCGTGCCAATCTTTGCTCCATTTTTCGGCACGCTGGTG
GGCGTGCTGGTATACCAGCTGATGGTGGGCTACCACGTGGAGGGCGAGAGCCGTGCCCGG
GAGATGGCACAGGCTCAG---GGGGAGAAGGAGAGACTGAAGCTT---------GCGGAG
AAGAGCGACTTGGCA---
>Mexican_tetra_Aqp3b
ATGGGCAAGCAGAAGGAGCTCCTGGACAAACTGGCACGCATGTTCCAGATCAAGAACCTG
CTGGTGCGCCAGGCGCTGGCCGAGTGCCTGGGAACCCTCATACTGGTGATGTTTGGTTGT
GGAGCGGTTGCTCAGTTGGTTCTCAGCGAGGGTTCTCATGGAATGTTCCTGACAGTGAAC
TTCGCTTTCGGTTTTGCTGCCATGCTGGGAATTCTAGTGTGTGGTCAAGTCTCAGGTGGC
CACCTGAACCCGGCCGTCACATTTTCCCTGTGCATTCTGGGCCGAGAGCCATGGAAGAAG
TTCCCTGTGTACTTTCTGTTCCAGACATTAGGAGCCTTCCTTGGATCAGGGATCATATTT
GGCATGTATTTTGATGCTCTGTGGGAGTTTCGTAAGGGA------------AGTCTGATT
GTGGTGGGAGAGAACGCTACCGCTGGAATCTTTGCTACCTACCCCTCCACTCATCTTAGC
TTGATCAACGGCTTCTTTGACCAGATGATTGGCACAGCAGCTCTGATCGTGTGTATCCTG
GCCATCGTTGACCCCTATAACAACCCCATCCCGCGGGGGCTGGAGGCTTTTACTGTGGGT

TTTGTGGTGCTGGTGATCGGCCTGTCGATGGGCTTCAACTCTGGGTATGCTGTTAATCCT
GCCAGAGATCTGGGACCCCGCATCTTCACTGCACTCGCAGGCTGGGGCAGTGAGGTCTTC
ACGGCTGGAGATTACTGGTTCTTAGTGCCAATCTTCGCCCCTTTCTTTGGCTCACTGGTG
GGAGTACTGGTGTACCAGCTGATGGTGGGCTTCCATGTGGAGGGAGAAGCCCGAGACAAA
GCGGCGGCAGCTTCACGG---GAAGAGAAAGAGAGACTAAAGCTCTCCAGCATGACAGAG
AAGAACGAGATGGCA---
>Redbellied_piranha_Aqp3b
ATGGGCAGGCAGAAGGCGCTCCTGGACAAGCTGGCGCAGACCTTCCAGATCCGGAGCCTG
CTGGTGCGCCAGGCGCTGGCTGAATGTCTTGGCACCCTCATACTGGTGATGTTTGGCTGT
GGTGCAGTTGCCCAGTTGGTCCTCAGTGAGGGATCTCATGGAATATTCCTGACGGTGAAC
TTCGCCTTCGGTTTTGCCGCCATGCTGGGAATCCTGGTCTGTGGGCAAGTCTCAGGTGGC
CACCTGAACCCAACTGTGACATTTGCCCTTTGCATGCTGGGTCGAGAGCCATGGAAGAAG
TTTCCTGTGTACTTTCTGTTCCAGACACTAGGAGCCTTCCTCGGATCAGGGATCATATTT
GGCATGTATTTTGATGCTCTGTGGGAGTTTCGTAAGGGC------------AGTCTGATT
GTGGTGGGGGAGAACGCCACAGCTGGAATCTTTGCCACGTACCCCTCCAACCATCTCAGC
CTGGTCAACGGCTTCTTCGACCAAATGATTGGCACAGCAGCTCTGATTGTGTGCATCCTG
GCCATCATAGACCCCTACAACAACCCCATCCCTCGGGGGCTGGAGGCCTTCACTGTGGGT
TTTGTAGTGTTGGTGATCGGCTTGTCAATGGGCTTCAACTCTGGGTATGCCGTCAATCCT
GCCAGGGACCTGGGACCTCGCATCTTTACTGCAATCGCAGGCTGGGGGAGTGAAGTTTTC
ACGGCTAACGATTATTGGTTCTTTGTACCAATCTTCGCCCCATTCTTTGGTACGGTGGTG
GGCGTGCTGGTGTACCAGCTGATGGTGGGCTTCCATGTGGAAGGAGAGGCCCGAGACAGA
GGGGAAGCAGTGGCTCGG---GAGGAGAAAGACAGGCTAAAGCTCTCCAATGTCACAGAG
AAGAATGAAATGGCA---
>Denticle_herring_Aqp3b
ATGGGCAGACAGAAGGTCATCCTGGACAAGATTGGCCGCTCCTTACGAATCCGCAACCTG
CTGGTTCGCCAGGCTCTGGCGGAATGCTTGGGCACCCTCGTCCTGGTGATGTTCGGTTGC
GGCGCAGTCGCACAGTTGGTGTTGAGCGGAGGCACCCATGGAATGTTCCTCACCGTCAAT
TTTGCGTTTGGCTTAGCAGCCACGCTGGGGATCCTGGTGTGTGGACAAATCTCAGGAGGC
CATCTGAACCCAAGTGTCACTTTGGCCCTTTGCATTCTGGGCAGGGAGCCCTGGAAGAAA
TTCCCAGTTTATTTCTTGTTTCAGACACTAGGTGCATTTCTTGGGTCAGGGGTCATATTT
GGAATGTACTTTGATGCCTTACAGGACTATGGGAATGGC------------TTATTTCTG
GTTGAAGGGCAAAATGCCACTGCTGACATTTTTGCTACATACCCCTCCCAACATCTTTCT
CTTGTTAATGGGTTCTTTGACCAAGTAATAGGCACGGCTGCCCTGATTGTGTGCATCCTG
GCCATCGTGGACCCTCACAACAACTCAGTCCCTAAAGGCCTGGAGGCTTTCACTGTAGGC
TTTGTGGTGCTGGTCATTGGACTCTCTATGGGCTTCAACTCAGGCTACGCAGTTAACCCA
GCCAGAGACCTGGGACCACGTATTTTTACTGCAATTGCAGGATGGGGCAGTGCAGTTTTC
ACGGCAAAGGACTACTGGTTCTTTGTGCCAATCTTTGCTCCTTTCATTGGGACTCTTGTT
GGTGTGCTTGTGTACCAGCTTATGGTAGGTTATCATGTAGAGGGGGAAGCAAGGGAGAAC
ACATCAGTTAAAGATGAA---GAGGACAAGGAGCGCCTCAAATTGTCTAACATCAGCACC
---AGAGATGCTGTG---
>Torafugu_Aqp3a
ATGGGCAGACAGAAGGTGTACTTAGAGAAACTGTCTCACTTCTTCCAGATTCGTAACCTG
CTGATCCGTCAGGGCCTGGCAGAGTGTCTCGGCACCCTCATCCTGGTGATGTTTGGCTGT
GGTGCTGTGGCCCAGCTGGTGTTGAGCAAGGGTACACACGGCATGTTCCTGACCGTCAAC
TTTGCTTTTGGCTTTGCTGCCACCTTAGGCATTCTGGTGTGTGGCCAGATATCAGGCGGC
CACCTGAACCCCGCGGTCACCTTCGCCCTGTGTTTGCTGGGACGAGAGCGCTGGAGGAAG
TTTCCTGTGTACTTCTTCTTTCAGACCATCGGTGCCTTTTTTGGTGCTGCCATTATATTT
GGCATGTATTACGATGCCCTGTTCGACTTCCCCGGAGCT---------------TTCAAT
ATGTCTGGAAAAAATTTCACAGGGGGAATCTTTGCTACGTACCCCGGAAAACACCTCACC
CTCGTCAACGGCTTCTTTGACCAGATCATTGGTACGGCAGCACTCATAGTTTGTATTCTG
GCTATCGTGGATCCCTACAACAACCCAATCCCCCAGGGTCTGGAGGCCTTCACGGTTGGG
TTTGTGGTTCTGGTCATTGGATTGTCCATGGGCTTCAACTCTGGCTATGCTGTTAACCCT
GCCAGAGACCTCGGGCCGCGTATTTTCACAGCAATGGCGGGATGGGGCGGTGAAGTTTTC
ACCGCTAGAAACGGGTGGTTCCTTGTGCCCACCTTTGCACCGTTCCTGGGCACCATCATT
GGCGTGGTCATCTACCAGCTGATGGTGGGCTTCCACGTGGAGGGAGAGGCGCGCGACAAA
AAGAGCCGA---------------GAGGAGGAGAACCTCTACCTCACCAACATCTCTGCC
---AACGAAAACGCC------AAACAC---ACCAAAGACATCCTG---------------
>Sansaifugu_Aqp3a
ATGGGCAGACAGAAGGTGTACTTAGAGAAACTGTCTCACTTCTTCCAGATTCGTAACCTG
CTGATCCGTCAGGGCCTGGCAGAGTGTCTCGGCACCCTCATCCTGGTGATGTTTGGCTGT
GGTGCTGTGGCCCAGCTGGTGTTGAGCAAGGGTACACACGGCATGTTCCTGACCGTCAAC

TTTGCTTTTGGCTTTGCTGCCACCTTAGGCATTCTGGTGTGTGGCCAGATATCAGGCGGC
CACCTGAACCCCGCGGTCACCTTCGCCCTGTGTTTGCTGGGACGAGAGCGCTGGAGGAAG
TTTCCTGTGTACTTCTTCTTTCAGACCATCGGTGCCTTTTTTGGTGCTGCCATTATATTT
GGCATGTACTACGATGCCCTGTTCGACTTCCCCGGAGCT---------------TTCAAC
ATGTCTGGAAAAAATTTCACAGGGGGAATCTTTGCTACGTACCCCGGAAAACACCTCACC
CTCGTCAACGGCTTCTTTGACCAGATCATTGGTACGGCAGCACTCATAGTTTGTATTCTG
GCTATCGTGGATCCCTACAACAACCCAATCCCCCAGGGTCTGGAGGCCTTCACGGTTGGG
TTTGTGGTTCTGGTCATTGGATTGTCCATGGGCTTCAACTCTGGCTATGCTGTTAACCCT
GCCAGAGACCTCGGGCCGCGTATTTTCACAGCAATGGCGGGATGGGGCGGTGAAGTTTTC
ACGGCTAGAAACGGGTGGTTCCTTGTGCCCACCTTTGCACCGTTCCTGGGCACCATCATT
GGCGTGGTCATCTACCAGCTGATGGTGGGCTTCCACGTGGAGGGAGAGGCGCGCGACAAA
AAGAGCCGA---------------GAGGAGGAGAACCTCTACCTCACCAACGTCTCTGCC
---AACGAAAACGCC------AAACAC---ACCAAAGACATCCTG---------------
>Mefugu_Aqp3a
ATGGGCAGACATAAGGTGTACTTAGAGAAACTGTCTCACTTCTTCCAGATTCGTAACCTG
CTATTCCGTCAGGGCCTGGCAGAGTGTCTCGGCACCCTAATCCTGGTGATGTTTGGCTGT
GGTGCTGTGGCCCAGCTGGTGTTGAGCAAGGGTACACACGGCATGTTCCTGACGGTCAAC
TTTGCTTTTGGCTTTGCTGCCACCTTAGGCATTCTGGTGTGTGGCCAGATATCAGGCGGC
CACCTGAACCCCGCGGTCACCTTCGCCCTGTGTTTGCTGGGACGAGAGCCCTGGAGGAAG
TTTCCTGTGTACTTCTTGTTTCAGACCATTGGTGCCTTTTTTGGTGCTGCCATTATATTT
GGCATGTACTACGATGCACTGTTCGACTTCCCCGGAGCT---------------TTCAAT
ATGACTGGACGAAATTTCACAGGGGGAATCTTTGCTACGTACCCCGGGAAACACCTCACC
CTCGTCAACGGCTTCTTTGACCAGATCATTGGTACGGCAGCACTCATAGTTTGTATTCTG
GCTATCGTGGATCCCTACAACAACCCAATCCCCCAGGGTCTGGAGGCCTTCACGGTTGGG
TTTGTGGTTCTGGTCATTGGATTGTCCATGGGCTTCAACTCTGGCTATGCTGTTAACCCT
GCCAGAGACCTCGGGCCGCGTATTTTCACAGCAATGGCGGGATGGGGCGGTCAAGTTTTC
ACCGCTAGAAACGGGTGGTTCCTCGTGCCCACCTTTGCACCGTTCCTGGGCACCATCATT
GGCGTGGTCATCTACCAGCTGATGGTGGGCTTCCACGTGGAGGGAGAGGCGCGCGACAAA
AAGAGCCGA---------------GAGGAGGAGAACCTCCACCTCACCAACGTCTCTGCC
---AACGAAAACGCC------AAACAC---ACCCCAGACGTCCTG---------------
>Green_spotted_pufferfish_Aqp3a
ATGGGCAGACAGAAGGTGTATTTGGAGAAACTCTCTCACTTCTTCCAGATTCGTAACCTG
CTGATCCGTCAGGGCCTGGCAGAGTGTCTTGGCACCCTCGTCCTGGTGATGTTTGGCTGT
GGTGCTGTGGCTCAGCTGGTGTTGAGCAAGGGGACACATGGAATGTTCCTGACTGTCAAC
TTTGCTTTTGGCTTTGCCGCCATGTTAGGCATTCTGGTGTGCGGCCAGATATCAGGGGGC
CACCTGAATCCCGCAGTCACCTTTTCTCTGTGTTTGCTGGGGAGAGAGCCCTGGAGGAAG
TTTCCCATGTATTTTCTGTTTCAGACAATCGGTTCCTTTTTTGGTGCTGCAATCATATTC
GGCATGTACTATGATGCCTTGTTCGATTTTCCTGGAGCT---------------TTCGAC
ATGTCTGGAGAAAATTCCACAGCTGGAATCTTTGCCACATACCCTGGAAAACATCTCACC
ATTTTAAATGGCTTCTTTGACCAGATCATTGGCACAGCAGCGCTCATCGTTTGCATTTTG
GCTATCGTGGATCCCTACAACAACCCCATCCCCCAAGGACTGGAGGCCTTCACTGTGGGC
TTTGTGGTTCTGGTCATTGGACTGTCCATGGGCTTCAACTCGGGCTACGCTGTTAACCCC
GCCAGGGACCTCGGGCCACGTCTTTTTACAGCAATGGCTGGGTGGGGCTCCGAAGTTTTC
ACAACTAGAAACGGCTGGTTCCTGGTGCCCACTTTCGCACCATTCCTGGGCTCCATCATT
GGCGTGATCATCTACCAGCTGATGGTGGGCTTCCACGTGGAGGGAGAGGTGCGCGACAGG
AACATGAGG---------------GAGGCAGAGAACATCGGCCTCACCGGCGTTCCTGCG
------GAAAACTCC------AAAAAC---ACCAAACAAATCCTC---------------
>Ocean_sunfish_Aqp3a
ATGAGCAGACAGAAGTTTTACTTGGAGAAGCTGTCCTGCTTCTTCCAGATCCGCAACCTG
CTGCTTCGTCAGGCCCTGGCAGAATGTTTGGGCACCCTCATCCTTGTGATGTTTGGCTGT
GGTGCTGTGGCCCAGCTGGTGTTGAGTGGTGGTAGCCATGGCATGTTCCTCACCGTCAAC
TTCGCCTTTGGTTTTGCTGCCACCTTGGGCATCCTGGTGTGTGGCCAGGTATCAGGTGGC
CATCTGAACCCCGCAGTGACCTTTGCCCTGTGCTTGCTTGGAAGAGAGCGCTGGAAAAAG
TTTCCCGTTTACTTCCTCTTTCAGACAATCGGTGCTTTTTTTGGTGCTGCGATAATTTTT
GGAATGTACTATGATGCCCTGTGGGACTTTCCTAGAGCT---------------TTCAAT
ATAACTGGCAAAGATTCCACAGCTGGAATCTTTGCTACCTACCCCGGCAAACATCTCACC
ATCGTCAATGGCTTCTTTGATCAGATAATTGGCACAGCTGCACTCATAGTTTGCATTCTG
GCTATTGTGGATCCATACAACAACCCCATCCCCCAAGGACTGGAGGCTTTCACTGTGGGA
TTTGTGGTTCTGGTCATTGGATTGTCCATGGGCTTTAACTCCGGTTATGCTGTTAACCCT
GCCAGAGACCTTGGGCCACGTATTTTTACTGCTATAGCTGGGTGGGGCTCAGATGTTTTC
ACGGCTCGGCAAGGCTGGTTCCTGGTGCCCATTTTTGCCCCGTTCCTCGGCACCATTATT

GGCGTGATGATCTACCAGCTGATGGTTGGCTTCCATGTGGAGGGAGAGGTGCGTGACAAG
GAAACTGCG---------------GCGCAGGAGAATGTGCGCCTCACCAACATCAGCAGC
---AGCGACAACTTA------AAAAAT---GCCAAAAAA---------------------
>Striated_frogfish_Aqp3a
ATGGGCAGACAGAAGATCTACTTGGAGAAACTCTCTCGGTTCTTCCAGATCCGCAACCTG
CTGCTTCGTCAGGCCATGGCTGAATGCCTTGGGACCCTCATCCTGGTGATGTTTGGTTGC
GGTGCTGTGGCCCAGCAGGTTTTGAGCAGCGGTTCCCATGGCATGTTCCTCACTGTCAAC
TTTGCTTTCGGATTCGCCGCCACTTTAGGCATCCTGGTGTGTGGCCAGATATCAGGTGGC
CATCTGAACCCTGCAGTGACCTTTGCCCTGTGTCTGCTTGGAAGAGAGCGCTGGAGGAAG
TTCCCCATGTTCTTCCTCTTTCAGACAATCGGCGCCTTTTTCGGTGCTGCTATCATTTTT
GGCATGTACTACGATGCTCTGTGGGACCATCCCGGAAAC---------------TTCCAT
GTAAGCGGACCAAATGCCACGGCCGGCATCTTCGCTACCTACCCTGGGAAACATCTCACC
ATCGTCAACGGCTTCTTTGATCAGATAATTGGCACAGCGGCGCTTATTGTCTGTATTCTG
GCCATTGTGGATCCGTACAACAACCCCATCCCACAAGGCCTGGAAGCCTTCACTGTGGGC
TTTGTGGTTCTGGTCATCGGCTTGTCTATGGGCTTCAACTCCGGTTATGCTGTCAACCCT
GCCAGAGACCTCGGACCACGTATTTTTACCGCTATGGCTGGGTGGGGCACGGATGTTTTC
ACGGCTAGGAGAGGTTGGTTCCTGGTGCCCATTTTTGCACCGTTTCTGGGCACCATCATC
GGCGTGATGATCTACCAGTTCATGGTTGGCTTCCACGTGGAAGGAGAAGTACGTGATCAG
AAGGAGGCA---------------GAGGAGGAGAACGTCCGACTCACTAACATCAGCACC
---AAAGATAACCCC------AAAAAC---TCCAAAGAAGCATTC---------------
>Gilthead_seabream_Aqp3a
ATGGGCAGACAGAAGGTGTATTTGGACAAACTGGCCCGGTCCTTCCAGGTCCGGAACCTG
CTGCTCCGTCAGGCCCTGGCAGAGTGTCTCGGGACCCTCATCCTTGTGATGTTTGGCTGC
GGTGCTGTGGCCCAGCTGGTTTTGAGCGGAGGTTCCCATGGCATGTTCCTCACTGTCAAC
TTTGCCTTCGGCTTCGCTGCCACCTTAGGCATCCTAGTCTGTGGCCAGGTCTCAGGTGGC
CATCTGAACCCCGCAGTGACCTTTTCCCTGTGTCTGCTCGGAAGAGAGCGCTGGAGAAAG
TTCCCCATGTACTTCCTCTTTCAGACAATCGGAGCCTTTTTTGGTGCTGCAGTCATTTTT
GGCATGTACTACGATGCCCTGTGGGACATTCCTGGATGT---------------TTCAAT
GTGACTGGGCCTAAGGCCACAGCTGGTATCTTTGCTACCTACCCCGGAAAACATCTCAGC
ATTGTCAATGGCTTCTTTGACCAGATTATTGGCACAGCAGCACTCATTGTTTGTATTCTG
GCTATTGTGGATCCATACAACAACCCCATCCCCCAAGGTCTGGAGGCCTTCACTGTGGGA
TTTGTGGTTCTAGTCATTGGACTGTCTATGGGCTTCAACTCTGGTTATGCTGTCAACCCT
GCCAGAGACCTCGGGCCACGTACTTTCACCTCTATGGCTGGCTGGGGCACCGAAGTTTTC
ACGATTAGAAATGGCTGGTTCCTGGTGCCCGTTTTTGCCCCGTTCCTTGGTGCCATCATC
GGTACGATGATCTACCAGGTCATGGTCGGCTTCCACGTGGAGGGAGAAATACGTGACCAG
AAGAGCGCA---------------GCGGAGGAGAACGTCCGACTCACCAATGTCGCCAGC
---AACGACAACTCC------CTAAAGGCCACCAAGGAAATGCAA---------------
>Red_seabream_Aqp3a
ATGGGCAGACAGAAGGTGTATTTGGACAAACTGGCCCGGTTCTTCCAGATCCGGAACCTG
CTGCTTCGTCAGGCCCTGGCAGAGTGTCTCGGGACCCTCATCCTTGTGATGTTTGGCTGT
GGTGCTGTGGCCCAGCTGGTTTTGAGCGGAGGTTCCCATGGCATGTTTCTAACTGTCAAC
TTCGCCTTCGGCTTCGCTGCCACCTTAGGCATCCTAGTCTGTGGCCAGGTATCAGGTGGG
CATCTGAACCCCGCAGTGACGTTTTCCCTGTGTCTGCTTGGAAGAGAGCGCTGGAGAAAG
TTCCCCATGTACTTCCTCTTTCAGACAATCGGTGCTTTTTTCGGTGCTGCAGTCATTTTT
GGCATGTACTACGATGCCCTGTGGGACATTCCTGGATGT---------------TTCAAT
GTGACTGGGCCTAAGGCCACAGCTGGTATCTTTGCTACCTACCCTGGAAAACATCTCAGC
ATTGTCAATGGCTTCTTTGACCAGATTATTGGCACAGCAGCGCTCATTGTTTGTATTCTG
GCTATTGTGGATCCATACAACAACCCAATCCCCCAAGGTCTGGAGGCCTTCACTGTGGGA
TTTGTGGTTCTGGTTATTGGACTGTCTATGGGTTTCAACTCTGGCTATGCTGTCAACCCT
GCCAGAGACCTCGGGCCACGTCTTTTCACCGCTATGGCTGGCTGGGGCACCGAAGTTTTC
ACGATTAGAAACGGCTGGTTCCTGGTGCCCGTTTTTGCTCCGTTCCTCGGCGCCATCATT
GGTACGATGATCTACCAGATCATGGTCGGCTTCCATGTGGAGGGAGAAATACGTGACCAG
AAGAGCGCA---------------GCGGAGGAGAATGTCCGACTCACCAATGTCACCAGC
---AACGACAACTCC------AAAGACGGCACCAAAGAAATGCAC---------------
>Crimson_snapper_Aqp3a
ATGGGCAGACAGAAGGTGTACTTGGACAAACTGGCCCGGTTCTTCCAGATCCGTAACCTG
CTGCTCCGTCAGGCCCTGGCAGAGTGTCTTGGCACCCTCATCCTTGTGATGTTTGGCTGC
GGTGCTGTGGCACAGCTAGTATTGAGCGGCGGTTCCCATGGCATGTTCCTCACTGTCAAC
TTTGCCTTCGGCTTTGCTGCAACCCTAGGCATCCTGGTTTGTGGCCAGGTATCAGGTGGC
CATCTGAACCCTGCAGTGACCTTTGCCCTGTGCCTGCTTGGAAGAGAGCGCTGGAGAAAG
TTCCCCATGTACTTCCTTTTTCAGACAATTGGTGCCTTTTTCGGTGCTGCAGTCATTTTT

GGCATGTACTACGATGCCCTGTGGGACCATCCTGGATGT---------------TTCAAT
GTGACTGTAGAGAGGGCCACAGCAGGCATCTTTGCTACCTACCCTGGAAAACATCTCACC
ATCGTCAATGGCTTCTTTGATCAGATCATTGGCACAGCAGCGCTGATAGTTTGCATTCTG
GCTATTGTGGATCCATACAACAACCCCATCCCCCAGGGACTGGAGGCTTTCACTGTGGGA
TTTGTGGTTTTGGTCATTGGACTGTCTATGGGCTTCAACTCCGGCTATGCTGTCAACCCT
GCCAGAGACCTTGGCCACGTCTTTTCACTGCTATGGCTGGCTGGGGAGTTGAAGTTTTC
ACGGCTAGAAGAGGCTGGTTCCTGGTGCCCATTTTCGCCCCATTCCTCGGCACCATCATC
GGTGTGATGATCTACCAGTTAATGGTCGGCTTCCATGTGGAGGGTGAAATACGCGACCAG
AAGAGCGCA---------------GAGGAGGAGAATGTCCGACTCACTAACGTCACCTCC
---AACGAAAACTCC------AAAAACGCTAACAAAGAAATGCAC---------------
>Large_yellow_croaker_Aqp3a
ATGGGCAGGCAGAAGTTGTATTTGGAGAAACTGTCCCGCTTCTTCCAGATCCGTAACCTG
CTTCTTCGTCAGGCCCTGGCAGAGTGTCTTGGCACCCTCATCCTTGTGATGTTTGGCTGC
GGTGCTGTGGCCCAGCTGAAGTTGAGCGGTGGTAGCCATGGCATGTTCCTGACTGTCAAC
TTTGCCTTCGGCTTCGCTGCCACATTAGGCATCCTGGTCTGTGGTCAGGTATCAGGTGGA
CATCTGAACCCTGCAGTGACCTTTGCCCTGTGTCTGCTTGGAAGAGAGCGCTGGAGAAAG
TTCCCCATGTACTTCCTCTTTCAGACAATCGGTGCCTTTTTCGGTGCTGCGATCATTTTC
GGCATGTACTACGATGCCTTGTGGGACTTACCTGGAAGT---------------TACAAT
ATGACTGGGCCAAATGCCACCGCTGGTATCTTTGCTACCTACCCTAGAGAACATCTCACC
CTTGTGAATGGCTTCTTTGATCAGATCATTGGCACAGCAGCACTGATAGTTTGTATTCTG
GCTATTTTGGATCCATACAACAATCCCATCCCCCAAGGACTGGAGGCCTTCACTGTGGGA
TTTGTGGTTTTGGTCATTGGATTGTCTATGGGCTTTAACTCTGGTTATGCTGTCAACCCT
GCTAGAGACCTTGGACCACGTCTTTTCACTGCTATGGCTGGGTGGGGCAGTGAAGTTTTC
ACGGCTAGAAATGGCTGGTTCCTGATTCCAGTTTTTGCCCCATTCCTTGGCACCATCATT
GGTGTGATGATCTACCAGCTGATGGTTGGTTTCCATGTGGAGGGAGAAGTACGCGATAGA
AAGAGCACA---------------GAGGAGGAGAATGTCGGACTCACCAATGTCACCAGC
---AATGACAACTCC------AAAGACAAAAACAAAGAAATGCAC---------------
>White_flower_croaker_Aqp3a
ATGGGCAGGCAGAAGCTGTATTTGGACAAACTGTCCCACTTCTTCCAGATCCGTAACCTG
CTTCTTCGTCAGGCCCTGGCAGAGTGTCTTGGCACCCTCATCCTCGTGATGTTTGGCTGT
GGTGCTGTGGCCCAGCTGGTGTTGAGCGGTGGTAGTCATGGCATGTTCCTCACTGTCAAC
TTTGCCTTTGGCTTCGCTGCCACATTAGGCATCCTGGTCTGCGGTCAGGTGTCAGGTGGA
CATCTGAACCCTGCAGTGACCTTTGCCCTGTGTCTGCTTGGAAGAGAGCGCTGGAGAAAG
TTCCCCATGTACTTCCTCTTTCAGACAATCGGTGCCTTTTTCGGTGCTGCGATCATTTTC
GGCATGTACTACGATGCCCTGTGGGACTTTCCTGGAAGC---------------TTCAAT
ATGACTGGGCCAAACTTCACCGCTAACATCTTTGCTACCTACCCTAGAAAAGATCTCACC
ATTGTGAATGGCTTCTTTGATCAGATCATTGGCACAGCAGCACTGATAGTTTGTATTCTC
GCTATTGTGGATCCGTACAACAACCCCATCCCCCAAGGACTGGAGGCCTTCACTGTGGGA
TTTGTGGTTTTGGTCATTGGATTGTCTATGGGCTTTAACTCTGGTTATGCTGTCAACCCT
GCTAGAGACCTTGGACCACGTCTTTTCACTGCCATGGCTGGGTGGGGCAGTGACGTTTTC
ACGCTTAGAAATGGCTGGTTCCTGATTCCACTTCTTGCCCCATTCATTGGCACCGTCATC
GGTGTGATAATCTACCAGCTGATGGTTGGTTTCCACGTGGAGGGAGAAATACGTGACCAG
AAGAGCACA---------------GAGCAGGAGAACGTCCGACTCACCAATGTCACCAGC
---AACGACAACTCC------AAAGACAAAAACAAAGAAATGCAC---------------
>Miiuy_croaker_Aqp3a
ATGGGCAGGCAGAAGGTGTATTTGGAGAAACTGTCCCGCTTCTTCCAGATCCGTAACCTG
CTTCTTCGTCAGGCCCTGGCAGAGTGTCTTGGCACCCTCATCCTTGTGATGTTTGGCTGC
GGTGCTGTGGCCCAGCTGGTGTTGAGCGGTGGTACCCATGGCATGTTCCTCACTGTCAAC
TTTGCCTTCGGCTTCGCTGCCACATTAGGCATCCTGGTCTGTGGTCAGGTATCAGGTGGA
CATCTGAACCCTGCAGTGACCTTTGCCCTGTGTCTGCTTGGAAGAGAGCGCTGGAGAAAG
TTCCCCATGTACTTCCTCTTTCAGACAATCGGTGCCTTTTTCGGTTCTGCAATCATTTTC
GGCATGTACTACGATGCCATGTGGGACTTTCCTGGAAGT---------------TTCAAT
GTGACTGGGCCAAAAGCCACCGCTGGCATCTTTGCTACCTACCCTGGAAAACATCTCACC
ATTGTGAATGGCTTCTTTGATCAGATCATTGGCACAGCAGCACTGATAGTTTGTATTCTG
GCTATTGTGGATCCATACAACAACCCCATCCCCCAAGGACTGGAGGCCTTCACTGTGGGA
TTTGTGGTTTTGGTCATTGGATTGTCTATGGGCTTTAACTCTGGTTATGCTGTCAACCCT
GCTAGAGACCTTGGACCACGTCTTTTCACTGCTTTGGCTGGGTGGGGCAGTGAAGTTTTC
ACGGTTAGAAATGGCTGGTTCCTGGTTCCAGTTTTTGCCCCATTCCTTGGCACCATCATT
GGTGTGATGATCTACCAGCTGATGGTTGGTTTCCATGTGGAGGGAGAAATACGTGATCAG
AAGAGCACA---------------GAGGAGGAGAATGTCCGACTCACCAATGTCACCAGC
---AATGACAACTCC------AAAGACAAAAACAAAGAAATGCAC---------------

>Meagre_Aqp3a
ATGGGCAGGCAGAAGGTGTATTTGGAGAAACTGTCCCGCTTCTTCCAGATCCGTAACCTG
CTTCTTCGTCAGGCCCTGGCAGAGTGTCTTGGCACCCTCATCCTTGTGATGTTTGGCTGC
GGTGCCGTAGCCCAGCTGGTGTTGAGCGGTGGTACCCATGGCATGTTCCTCACTGTCAAC
TTTGCCTTCGGCTTCGCTGCCACATTAGGCATCCTGGTCTGTGGTCAGGTATCAGGTGGA
CATCTGAACCCTGCAGTGACCTTTGCCCTGTGTCTGCTTGGAAGAGAGCGCTGGAGAAAG
TTCCCCATGTACTTCCTCTTTCAGACAATCGGTGCCTTTTTCGGTGCTGCAATCATTTTC
GGCATGTACTATGATGCCATGTGGGACTTTCCTGGAAGT---------------TTCAAT
GTGACTGGGCCAAAAGCCACCGCTGGCATCTTTGCTACCTACCCTGGAAAACATCTCACC
ATTGTGAATGGCTTCTTTGATCAGATCATTGGCACAGCAGCACTGATAGTTTGTATTCTG
GCTATTGTGGATCCATACAACAACCCCATCCCCCAAGGACTGGAGGCCTTCACTGTGGGA
TTTGTGGTTTTGGTCATTGGATTGTCTATGGGCTTTAACTCTGGTTATGCTGTCAACCCT
GCTAGAGACCTTGGACCACGTCTTTTCACTGCTATGGCTGGGTGGGGCACTGAAGTTTTC
ACGATTAGAAATGGCTGGTTCCTGGTTCCAGTTTTTGCCCCATTCCTTGGCACCATCATT
GGTGTGATGATCTACCAGCTGATGGTTGGTTTCCATGTGGAGGGAGAAATACGTGATCAG
AAGAGCATA---------------GAGGAGGAGAATGTCCGACTCACCAATGTCACCAGC
---AATGACAACTCC------AAAGACAAAAACAAAGAAATGCAC---------------
>White_bass_Aqp3a
ATGGGCAGACATAAGGTGTATCTGGACAAACTGTCCCGGTTCTTCCAGATTCGTCACCTG
CTGCTTCGTCAGGCCCTGGCAGAGTGTCTTGGCACCCTCGTCCTTGTGATGTTTGGCTGC
GGTGCTGTGGCCCAGCTAGTGTTGAGCGGTGGTTCCCATGGCATGTTCCTAACTGTCAAC
TTTGCCTTTGGCTTCGCTGCCACCTTAGGCATCCTGGTCTGTGGCCAAGTATCAGGTGGC
CATCTGAACCCTGCAGTGACCTTTGCCCTGTGCCTGCTTGGAAGAGAGCGCTGGAGAAAG
TTCCCCACGTACTTCCTCTTTCAGACAATCGGTGGTTTTTTTGGTGCTGCAATCATTTTT
GGCATGTACTATGATGCTCTGTGGGACCACCCTGGATGT---------------TTCAAT
GTGACTGGACCTGATGCCACAGCTGGCATCTTTGCTACCTACCCTGGAAAACATCTCACC
ATTCTCAATGGCTTCTTTGATCAGATAATTGGCACAGCAGCACTGATAGTTTGTATTCTG
GCTATTGTGGATCCATACAACAACCCCATCCCCCAAGGGCTGGAGGCCTTTACTGTGGGA
TTTGTGGTTCTGGCCATTGGATTGTCTATGGGCTTTAACTCTGGTTATGCTGTGAATCCT
GCCAGAGACCTTGGACCACGTATTTTCACAGCTATGGCTGGGTGGGGTAGTGAGGTTTTC
ACGGTCAGAAATGGCTGGTTCCTGGTGCCAGTTTTAGCCCCATTCCTTGGCACCATCGTC
GGTGTGATGATCTACCAGTTAATGGTTGGCTTCCATGTGGAGGGAGAAGTACGCGACCGT
AAGAGCACA---------------GAGGAGGAGAATGTCCAACTCAGCAATGTCACCAGC
---AAAGACAACTCC------AAAGAAGCTACCAAAGAAATGTAC---------------
>Striped_seabass_Aqp3a
ATGGGCAGACATAAGGTGTATCTGGACAAACTGTCCCGGTTCTTCCAGATCCGTCACCTG
CTGCTTCGTCAGGCCCTGGCAGAGTGTCTTGGCACCCTCATCCTTGTGATGTTTGGCTGT
GGTGCTGTGGCCCAGCTAGTGTTGAGCGGTGGTTCCCATGGCATGTTCCTAACTGTCAAC
TTTGCCTTCGGCTTCGCTGCCACCTTAGGCATCCTGGTCTGTGGCCAAGTATCAGGTGGC
CATCTGAACCCTGCAGTGACCTTTGCCCTGTGCCTGCTTGGAAGAGAGCGCTGGAGAAAG
TTCCCCACGTACTTCCTCTTTCAGACAATCGGTGCTTTTTTTGGTGCTGCAATCATTTTT
GGCATGTACTATGATGCCCTGTGGGACTTCCCTGGAAGT---------------TTCAAT
GTGACTGGACCTGGTGCCACAGCTGGCATCTTTGCTACCTACCCTGGAAACCATCTCACC
ATTGTCAATGGCTTCTTTGATCAGATAATTGGCACAGCAGCACTGATAGTTTGTATTCTG
GCTATTGTGGATCCATACAACAACCCCATCCCCCAAGGGCTGGAGGCCTTTACTGTGGGA
TTTGTGGTTCTGGTCATTGGATTGTCTATGGGCTTTAACTCTGGTTATGCTGTGAATCCT
GCCAGAGACCTTGGACCACGTATTTTCACAGCTATGGCTGGGTGGGGTAGTGAGGTTTTC
ACGGTCAGAAATGGCTGGTTCCTGGTGCCAGTTTTAGCCCCATTCCTTGGCACCATCATC
GGTGTGATGATCTACCAGTTAATGGTTGGCTTCCATGTGGAGGGAGAAGTACGCGACCGT
AAGAGCACA---------------GAGGAGGAGAATGTCCGACTCAGAAATGTCACCAGC
---AACGACAACTCC------AAAGAACTTACCAAAGAAATGCAC---------------
>European_seabass_Aqp3a
ATGGGCAGACATAAGGTGTATCTGGACAAACTGTCCCGGTTCTTCCAGATCCGTCACCTG
CTGCTTCGTCAGGCCCTGGCAGAGTGTCTTGGCACCCTCATCCTTGTGATGTTTGGCTGC
GGTGCTGTGGCCCAGCTAGTGTTGAGCGGTGGTTCCCATGGCATGTTCCTAACGGTCAAC
TTTGCCTTCGGCTTCGCTGCCACCTTAGGCATCCTGGTCTGTGGCCAGGTATCAGGTGGC
CATCTGAACCCTGCAGTGACCTTTGCACTGTGTCTGCTTGGAAGAGAGCGCTGGAGAAAG
TTCCCCATGTACTTCCTCTTTCAGACAATCGGTGCTTTTTTTGGTGCTGCAATCATTTTT
GGCATGTACTACGATGCCCTGTGGGACCACCCTGGATGT---------------TTCAAT
GTGACTGGACCTAATGCCACAGCTGGCATCTTTGCTACCTACCCTGGAAAACATCTCACC
CTTGTCAATGGCTTCTTTGATCAGATAATTGGCACAGCAGCACTGATAGTTTGTATTCTG

GCTATTGTGGATCCATACAACAACCCCATCCCCCAAGGGCTGGAGGCCTTCACTGTGGGC
TTTGTGGTTCTGGTGATTGGATTGTCTATGGGCTTTAACTCTGGCTATGCTGTGAATCCT
GCCAGAGACCTTGGACCACGTATTTTCACAGCTTTGGCTGGGTGGGGCAGCGATGTTTTC
ACGGTTAGAAATGGCTGGTTCCTGGTGCCAGTTTGTGCCCCATTCCTTGGCACCATCATC
GGTGTGATGATCTACCAGTTAATGGTTGGTTTCCATGTGGAGGGAGAAGTACGTGACCAA
AAGAGCAGA---------------GACCAGGAGAATGTCCGACTCACTAATGTCTCCAGC
---AACGACAACTCC------AAAGAAGATACCAAAGAAATGCAC---------------
>3_spine_stickleback_Aqp3a
ATGGGACGACACAAGTTCTATTTGGACAAACTGTCCCGGTTCTTCCAGATCCGCAACCTG
CTCCTTCGCCAAGCCCTGGCAGAGTGTCTTGGCACTCTCATCCTTGTGATGTTTGGTTGC
GGTTCTGTGGCCCAGCTGGTGTTGAGCGGCGGTTCCCATGGCATGTTCATCACCGTCAAC
TTTGCCTTTGGCTTTGCCGCCACATTAGGCATCCTGGTCTGTGGCCAAGTGTCAGGCGGC
CATCTGAACCCTGCAGTGACCTTTGCTCTTTGCCTGCTTGGAAGAGAGCCCTGGAGAAAG
TTCCCCATGTACTTCCTCTTTCAGACAATTGGTGGTTTTTTCGGAGCTGCTATCATTTTC
GGCATGTACTACGATGCCCTGTGGGACCATCCGGGATCT---------------TACGAA
GTATCCGGGCCCAATGCCACAGCTGGCATTTTTGCTACCTATCCCGGAAACCATCTCACT
ATTGTGAATGGATTCTTTGATCAGATAATTGGCACAGCTGCGCTGATTGTGTGTGTCCTG
GCTATTGTGGACCCGTACAACAACCCCATCCCCCAGGGACTGGAGGCCTTCACCGTGGGA
TTCGTGGTTCTGGTCATTGGATTGTCCATGGGCTTTAATTCTGGTTACGCTGTCAATCCC
GCCAGAGACCTCGGACCGCGTCTTTTCACCGCCATAGCTGGGTGGGGCGTTGAAGTTTTT
ACAGTTAGAAATGGGTGGTTCCTGGTTCCTGTCTGTGCCCCATTCCTTGGAACCATCGTG
GGTGTGGTGATCTACCAGCTGATGGTTGGCTTCCACGTGGAAGGAGAAGCGCGTGACTTT
AAAAGCAAG---------------CTGGAGGAGAGTGTCGCACTCACTGATGTCCCCAAA
---AATGAGAAAACC------AATGAAAAAAACAAAAACATGCAC---------------
>9_spine_stickleback_Aqp3a
ATGGGCAGACAGAAGCTCTATTTGGACAAACTGGCCCGGTTCTTCCAGATCCGTAACCTG
CTCCTCCGCCAGGCCCTGGCAGAGTGTCTTGGAACTCTCATCCTTGTGATGTTTGGTTGC
GGTGCGGTGGCCCAGCTGGTGTTGAGCGGTGGTTCCCATGGCATGTTCCTCACTGTCAAC
TTTGCCTTTGGCTTTGCGGCCACATTAGGCATCCTGGTCTGTGGCCAAGTGTCAGGTGGA
CATCTGAACCCTGCAGTGACCTTTGCTCTGTGCCTGCTCGGAAGAGAGCGCTGGAGAAAG
TTCCCCATGTTCTTCCTCTTTCAGACAATTGGTAGTTTTTTCGGAGCTGCTATCATTTTC
GCCATGTACTACGATGCCCTGTGGGACCATCCAGGATCT---------------TTCGAA
GTGACTGGGCCTAATGCCACAGCTGGCATTTTTGCCACCTACCCCGGAAAACATCTCACT
CTTCTCAATGGATTCTTTGACCAGATCATTGGCACAGCGGCGCTGATTGTGTGTATCCTG
GCTATTGTTGATCCGTACAACAACCCCATCCCCAAAGGACTGGAGGCCTTCACTGTGGGG
TTTGTGGTTCTGGTCATTGGATTGTCCATGGGCTTTAACTCTGGTTATGCTGTCAATCCC
GCCAGAGACCTCGGACCACGTCTTTTCACCGCTATAGCTGGGTGGGGCGGTGAAGTTTTT
ACGGCTGGAAATGGGTGGTTCATGGTGCCTGTCTTTGTCCCATTCCTTGGAACCATCGTG
GGTGTGATAATCTACCAGCTGATGGTTGGCTTCCATGTGGAAGGAGAAGCGCGTGACTTT
AACAGCAAG---------------CTGGAGGAGAGTGTCGCACTCGCTGATGTCCCCAAC
---AATGACAAAACC------AATGACAAAAACAAATCCATGTAC---------------
>Sablefish_Aqp3a1
ATGGGCAGACACAAGGTGTATTTGGAAAAACTGTCCCGGTTCTTCCAGGTCCGTAACCTG
CTGCTTCGCCAGGCCCTGGCAGAGTGTCTTGGCACTCTCATCCTTGTGATGTTTGGTTGT
GGTGCTGTGGCCCAGCTGGTGTTGAGTGGTGGTTCCCATGGCATGTTCCTCACTGTCAAC
TTTGCCTTCGGCTTCGCTGCCATGTTAGGCATCCTGGTCTGTGGCCAGGTATCAGGTGGC
CATCTGAACCCTGCAGTGACCTTTGCCCTGTGCCTGCTCGGAAGAGAGCGCTGGAGAAAG
TTCCCCATGTACTTCCTCTTTCAGACAATCGGCGGGTTCTTTGGTGCTGCTATCATTTTC
GGCATGTACTACGATGCCCTGTGGGACCGCCCTGGAGCC---------------TTCAAT
GTGACTGGGCCTAATGCCACAGCTGGCATTTTTGCTACCTACCCTGGAAAACATCTCACT
ATCGTGAATGGATTCTTTGACCAGATCATCGGCACAGCAGCGCTGATTGTGTGTATTCTG
GCTATTGTTGATCCATACAACAACCCCATCCCCCAAGGACTGGAGGCCTTCACTGTGGGA
TTTGTGGTTCTGGTCATTGGATTGTCTATGGGCTTTAACTCTGGCTATGCTGTCAATCCT
GCCAGAGACCTCGGACCACGTCTTTTCACCGCTATTGCTGGGTGGGGCAGCGAGGTTTTC
ACGGTTAGAAACGGCTGGTTCCTGGTGCCTGTTTTTGCCCCATTCCTTGGCACCATTATC
GGTGTGATAATCTACCAGCTGATGGTTGGCTTCCATGTGGAGGGAGAAGCGCGTGACCAG
AAAAGCCAGGCG------------GAGGAGGAGAATGTCCAACTCACCAATGTCTCCGCC
---AATGACAAAACC------AACGACAAAACCAAAGACATACAC---------------
>Sablefish_Aqp3a2
ATGGGAAAACAAAGAGACATTTTGGACAAACTGGCAGGGACCTTTCAGATCCGACATGCG
CTCCTCCGACAGGCCCTGGCAGAGTGCCTGGGAACCTTTATTCTGGTGATGTTTGGTTGT

GGTGCTGTGGCCCAGCTGGTGTTGAGTGGTGGTTCCCATGGCATGTTCCTCACTGTCAAC
TTTGCCTTCGGCTTCGCTGCCATGTTAGGCATCCTGGTCTGTGGCCAGGTATCAGGTGGC
CATCTGAACCCTGCAGTGACCTTTGCCCTGTGCCTGCTCGGAAGAGAGCGCTGGAGAAAG
TTCCCCATGTACTTCCTCTTTCAGACAATCGGCGGGTTCTTTGGTGCTGCTATCATTTTC
GGCATGTACTACGATGCCCTGTGGGACCGCCCTGGAGCC---------------TTCAAT
GTGACTGGGCCTAATGCCACAGCTGGCATTTTTGCTACCTACCCTGGAAAACATCTCACT
ATCGTGAATGGATTCTTTGACCAGATCATCGGCACAGCAGCGCTGATTGTGTGTATTCTG
GCTATTGTTGATCCATACAACAACCCCATCCCCCAAGGACTGGAGGCCTTCACTGTGGGA
TTTGTGGTTCTGGTCATTGGATTGTCTATGGGCTTTAACTCTGGCTATGCTGTCAATCCT
GCCAGAGACCTCGGACCACGTCTTTTCACCGCTATTGCTGGGTGGGGCAGCGAGGTTTTC
ACGGTTAGAAACGGCTGGTTCCTGGTGCCTGTTTTTGCCCCATTCCTTGGCACCATTATC
GGTGTGATAATCTACCAGCTGATGGTTGGCTTCCATGTGGAGGGAGAAGCGCGTGACCAG
AAAAGCCAGGCG------------GAGGAGGAGAATGTCCAACTCACCAATGTCTCCGCC
---AATGACAAAACC------AACGACAAAACCAAAGACATACAC---------------
>Rhine_sculpin_Aqp3a
ATGGGCAGACATAAAGTGTATTTGGACAAACTTTCCCGGTTCTTCCAGATCCGTCACCTG
CTGCTTCGCCAGGCCATGGCAGAGTGTCTTGGCACCCTCATCCTTGTGATGTTTGGTTGT
GGTGCTGTGGCTCAGCTGGTGCTGAGTGGTGGTTCCCATGGCATGTTCCTCACAGTCAAC
TTTGCCTTCGGCTTCGCTGCCATGTTAGGCATCCTGGTCTGTGGCCAGGTGTCAGGTGGC
CATCTGAACCCTGCAGTGACCTTTGCCCTGTGCCTGCTCGGAAGAGAGCGCTGGAGAAAG
TTCCCCATGTACTTCCTCTTTCAGACAATCGGTGGTTTTTTTGGTGCTGCTATCATTTTT
GGCATGTACTACGATGCCCTGTGGGACCATCCTGGGAGT---------------TTCAAT
GTGACTGGGCCTGATGCCACAGCTGGCATTTTTGCTACCTACCCTGGAAACCATCTCACC
ATTCTCAATGGATTCTTTGATCAGATCATCGGCACAGCAGCGCTGATTGTGTGTATTCTG
GCTATTGTGGATCCACACAACAACCCCATCCCCCAAGGACTGGAGGCCTTCACTGTGGGA
TTTGTGGTTCTGGTCATTGGACTGTCTATGGGCTTTAACTCTGGCTATGCTGTCAATCCT
GCCCGAGACCTCGGACCACGTCTTTTCACCGCAGTAGCTGGGTGGGGCAGCGAAGTTTTC
ACGGTTAGAAATGGCTGGTTCCTGGTGCCTGTTTTTGCCCCATTCATTGGCACCATCATC
GGTGTGATAATCTACCAGCTGATGGTCGGCTTCCATGTGGAGGGAGAAGTGCGTGACCAG
AAAAGTCGA---------------GAGGAGGAGAATGTCCAACTCACCAATGTCCACACC
---AATGAGAAAGCC------AATGACAAAACC------ATGCAC---------------
>Channel_bull_blenny_Aqp3a
ATGGGTAGACAAAAGGTGTATTTGGACAAACTGGCCCGGTTCTTCCAGATCCGTAACCTG
CTGCTTCGCCAGGCCCTGGCAGAGTGTCTTGGCACCCTCATCCTTGTGATGTTTGGCTGT
GGTGCTGTGGCGCAGCTGGTGTTGAGCGGTGGTTCCCATGGCATGTTCTTAACAGTCAAC
TTTGCCTTCGGCTTCGCTGCAATGTTAGGCATCCTGGTCTGCGGCCAGGTATCAGGTGGA
CATCTGAACCCGGCAGTGACCTTTGCCCTGTGCCTGCTCGGAAGAGAGCGCTGGAGAAAG
TTCCCCATGTACTTCCTCTTTCAGACAATCGGTGCCTTTTTTGGCGCTGCTATCATTTTC
GCCATGTACTACGATGCCATGTGGGACCATCCTGGATGT---------------TTCAAT
GTGACTGGGCCTAATGCCACAGCTGGCATTTTTGCTACCTACCCTGGAAAACATCTCACC
CTCGTCAATGGCTTCTTTGATCAGATAATCGGAACAGCAGCGCTGATTGTTTGTATTCTG
GCTATTGTGGATCCATACAACAACCCCATCCCTAAAGGGCTGGAGGCCTTCACTGTGGGG
TTTGTGGTTCTGGTCATTGGATTGTCTATGGGCTTTAACTCCGGCTATGCTGTGAATCCT
GCCAGAGATCTCGGACCACGTATTTTCACCGCTATGGCTGGGTGGGGCAGCGAAGTCTTC
ACGGCTAGAAACGGCTGGTTCCTTGTGCCCGTCTTTGCCCCTTTCCTTGGCACCATCATC
GGTGTGATAATCTACCAGCTGATGGTTGGCTTTCATATGGAGGGAGAAGAACGTGACGTA
AGAGATGCA---------------GAGGAGGAGAACGTCCGACTCACCAATATCAACACC
---AATGACAAACCC------AAAGACACTAGCAAAGAAGTTATC---------------
>Blackfin_iceish_Aqp3a

---ATGTTCGGCTGT
GGTGCCGTGGCCCAGCAGGTGTTGAGCAGTGGTTCCCATGGCATGTTCCTCACAGTCAAC
TTTGCCTTCGGTTTTGCTGCCATGCTAGGCATTCTGGTCTGTGGCCAGATATCAGGGGGG
CATTTAAATCCAGCAGTGACCTTTGCCATGTGCCTGCTCGGAAGAGAGCCCTGGAGAAAG
TTCCCCATGTACTTCCTCTTTCAGACAATCGGTGCTTACTTCGGCGCAGCTATCATATTC
GGCATGTACTACGATGCCCTGTGGGACATTCCTGGATGT---------------TTTAAT
GTGACTGGGCCTAGCGCCACCGCTAGCATTTTTGCTACATACCCTGGAAAACATCTCACC
CTTGTCAATGGCTTCTTTGATCAGATAATCGGCACAGCAGCACTAATTGTTTGCATTCTG
GCAATTGTGGATCCACACAACAACCCCATCCCCCAGGGGCTGGAGGCCTTCACTGTGGGA
TTTGTGGTTCTGGTCATTGGATTGTCCATGGGCTTTAACTCTGGCTATGCTGTAAATCCT
GCCAGAGATCTTGGACCACGTATTTTCACCGCTATGGCTGGGTGGGGCAGTGAGGTTTTC

ACGGTTAGAAATGGCTGGTTCCTTGTACCTGTTTTCGCCCCTTTCATTGGCACCATTGTT
GGTGTGATAATCTACCAGTTGATGGTCGGCTTTCATACGGAGGGAGAATATCGTGACCAG
AAAAGGTCA---------------ACGGAGGAGAATGTCCGACTCACCAATGTCAACTCC
---AATGACAAAACA------AATAACACTAGCAAAGAACATCTG---------------
>Black_rockcod_Aqp3a
ATGGGCAGACAAAAGATGTATTTGGACAAACTATCCCGGTTCTTTCAGATCCGAAACCTG
CTGCTGCGTCAGGCCCTGGCCGAATGTCTTGGAACCCTCATCCTTGTGATGTTCGGCTGT
GGTGCCGTGGCCCAGCTGGTGTTGAGCGGTGGTTCCCATGGCATGTTCCTCACAGTCAAC
TTTGCCTTCGGCTTTGCTGCCATGCTAGGCATTCTGGTCTCTGGCCAGATATCAGGGGGC
CATTTAAATCCAGCAGTGACCTTTGCCATGTGCCTGCTCGGAAGAGAGCCCTGGAGAAAG
TTCCCCATGTACTTCCTCTTTCAGACAATCGGTGCTTACTTCGGCGCAGCTATCATATTC
GGCATGTACTACGATGCCCTGTGGGACATTCCTGGAAGT---------------TTTAAT
GTGACTGGGCCTAGAGCCACCGCTGGCATTTTTGCTACATACCCTGGAAAACATCTCACC
CTTGTCAATGGCTTCTTTGATCAGATAATCGGCACAGCAGCACTGATTGTTTGCATTCTG
GCAATTGTGGATCCACACAACAACCCCATCCCCCAGGGGCTGGAGGCCTTCACTGTGGGA
TTTGTGGTTCTGGTCATTGGATTGTCCATGGGCTTTAACTCTGGCTATGCTGTAAATCCT
GCCAGAGATCTTGGACCACGTCTTTTCACCGCTATGGCTGGGTGGGGCAGTGAGGTTTTC
ACGGTTAGAAAAGGCTGGTTCCTTGTACCTGTTTTCGCCCCTTTCATTGGCACCATTGTT
GGTGTGATAATCTACCAGGTGATGGTCGGCTTTCATACGGAGGGAGAATATCGTGACCAG
AAAAGGTCA---------------GAGGAGGAG---GTCTCACTCACCAATGTCAACTCC
---AATGAAAAAACA------AATAACACTAGCAAAGAACATCTG---------------
>Emerald_rockcod_Aqp3a
ATGGGCAGACAGAAGGTGTATTTGGAAAAAATATCCCAGTTCTTTCAGATCCGAAACCTG
CTGCTGCGCCAGGCCCTGGCCGAATGTCTTGGAACCCTCATCCTTGTGATGTTCGGCTGT
GGTGCTGTGGCCCAGCTGGTGTTGAGCGGTGGTTCCCATGGCATGTTCCTCACAGTCAAC
TTTGCCTTCGGCTTTGCTGCCATGCTAGGCATTCTGGTCTGTGGCCAGATATCAGGGGGC
CATTTAAATCCAGCAGTGACCTTTGCCATGTGCCTGCTCGGAAGAGAGCCCTGGAGAAAG
TTCCCCATGTACTTCCTCTTTCAGACAATCGGTGCTTACTTCGGCGCAGCTATCATATTC
GGCATGTACTACGATGCCCTGTGGGACATTCCTGGAAGT---------------TTTAAT
GTGACTGGGCCTAGAGCCACCGCTGGCATTTTTGCTACATACCCTGGAAAACATCTCACC
CTTGTCAATGGCTTCTTTGATCAGATAATCGGCACAGCAGCACTGATTGTTTGCATTCTG
GCAATTGTGGATCCACACAACAACCCCATCCCCCAGGGGCTGGAGGCCTTCACTGTGGGA
TTTGTGGTTCTGGTCATTGGGTTGTCCATGGGCTTTAACTCTGGCTATGCTGTAAATCCT
GCCAGAGATCTTGGACCACGTATTTTCACCGCTATGGCTGGGTGGGGCAGTGAGGTTTTC
ACGGTTAGAAACGGCTGGTTCCTTGTACCTGTTTTCGCCCCTTTCATTGGCACCATTGTT
GGTGTGATAATCTACCAGGTGATGGTCGGCTTTCATACGGAGGGAGAATATCGTGACCAG
AAAAGGTCA---------------AAGGAGGAGAATGTCCGACTCACCAATGTCAACTCC
---AATGACAAAACA------AATAACACTAGCAAAGAACATCTC---------------
>European_perch_Aqp3a
ATGGGCAGACATAAATTGTTTTTGGCCAAACTGGCCCGGTCCTGCCAGATCCGAAACCTG
CTGCTTCGCCAGGCCATGGCAGAGTGTCTGGGCACCCTCATCCTTGTGATGTTTGGCTGT
GGTGCTGTGGCCCAGCTGGTTTTGAGCGATGGTACCCATGGCATGTTCCTCACAGTCAAC
TTTGCATTCGGCTTCGCTGCCATATTAGGCATCCTGGTCTGTGGCCAGGTATCAGGTGGC
CATCTGAACCCTGCGGTGACCTTTGCCCTGTGCCTGCTTGGAAGAGAGCGCTGGAGAAAG
TTCCCCATTTTCTTCCTCTTTCAGACGATCGGTGCTTTCTATGGTGCTGCCATCATTTTC
AGCATGTATTTTGACGCCCTGATAAAACATCCTGGATCT---------------TTCAAT
GTCTCTGGTCTTAATGCGACAGCTGGGATCTTTGCTACCTACCCTGGAGAACATCTCACC
CTTCTCATGGGCTTCTTTGATCAGATGATCGGCACAGCAGCGCTCATTGTTTGTATCCTG
GCTATTGTGGATCCATACAACACCCCCATCCCCCAAGGGCTAGAGGCCTTCACTGTGGGA
TTTGTAGTTCTGTTCATAGGATTGTCTATGGGCTTTAACTCTGGCTATGCTGTCAATCCT
GCTCGAGACTTCGGACCACGTCTTTTCACCGCTATGGCTGGGTGGGGCGGCGAAGTTTTC
ACGGCTGGAAACGGCTGGGTCCTGGTACCCATTTTTGCCCCATTCTTTGGCAGCTTCATC
GGTGCGTTTATCTACCAGCTGATGGTTGGCTTCCATACGGAGGGAGAAGCACGTGACCAG
AAAATCGCA---------------ATAGAAGAGAGTGTCCGACTTACCAATGTCACCACC
---AATGATAAAACC------AAAGACAATGCCAAAGAAATACAC---------------
>Flag_rockfish_Aqp3a
ATGGGCAGACAGAAGGTGTATTTGAACAAACTGGCCCGGTTCTTCCAGATCCGTAATTTG
CTGCTTCGCCAGGCCCTGGCGGAGTGTCTCGGCACCCTCATCCTTGTGATGTTTGGCTGT
GGTGCGGTGGCCCAGCTGGTGTTGAGCAAAGGCTCCCATGGCATGTTCCTCACTGTCAAC
TTTGCCTTCGGCTTCGCTGCCATGTTAGGCATCCTGGTCTGTGGCCAGGTATCAGGTGGC
CATTTGAACCCTGCGGTGACCTTTGCTATGTGCCTGCTCGGAAGAGAGCCCTGGAAAAAG

TTCCCCATGTACTTCCTCTTTCAGACAATCGGTGGTTTTTTCGGTGCTGCCATCATTTTC
GGCATGTACTACGATGCCCTGTGGGACCTCCCTGGATGT---------------TTCAAT
GTGACTGGGGTTAATAATACAGCTAAGATCTTTGCTACCTACCCTGGACCACATCTCACC
ATTGTCAACGGCTTCTTTGATCAGATAATCGGCACAGCAGCGCTGATTGTTTGCATTCTG
GCTATTGTGGATCCATACAACAACCCCATCCCCCAGGGGCTGGAGGCCTTCACTGTGGGA
TTTGTGGTTCTGGTTATTGGATTGTCTATGGGCTTTAACTCTGGCTACGCTGTCAATCCT
GCCAGAGACCTTGGACCACGTCTTTTCACCGCTATGGCTGGATGGGGCAGCGACGTTTTC
ACGGTTAGAAACGGCTGGTTCCTGGTGCCCCTTTTCGCCCCGTTCCTTGGCACCATCATC
GGCGTGCTAATTTACCAGCTGATGGTCGGCTTCCATGTGGAGGGAGAAGTACGTGACCAG
AAGAGCGCA---------------GAGGAGGAGAATGTCCGACTCACCAACGTCACCAAC
---AATGACAGACCC------AAAGACGCTACCAAAGAAATGCAC---------------
>Tiger_rockfish_Aqp3a
ATGGGCAGACAGAAGGTGTATTTGAACAAACTGGCCCGGTTCTTCCAGATCCGTAATTTG
CTGCTTCGCCAGGCCCTGGCGGAGTGTCTCGGCACCCTCATCCTTGTGATGTTTGGCTGT
GGTGCGGTGGCCCAGCTGGTGTTGAGCAAAGGCTCCCATGGCATGTTCCTCACTGTCAAC
TTTGCCTTCGGCTTCGCTGCCATGTTAGGCATCCTGGTCTGTGGCCAGGTATCAGGTGGC
CATCTGAACCCTGCGGTGACCTTTGCTATGTGCCTGCTCGGAAGAGAGCCCTGGAAAAAG
TTCCCCATGTACTTCCTCTTTCAGACAATYGGTGGTTTTTTCGGTGCTGCCATCATTTTC
GGCATGTACTACGATGCCCTGTGGGACCTCCCTGGATGT---------------TTCAAT
GTGACTGGGGTTAATAATACAGCTAAGATCTTTGCTACCTACCCTGGACCACATCTCACC
ATTGTCAACGGCTTCTTTGATCAGATAATCGGCACAGCAGCGCTGATTGTTTGCATTCTG
GCTATTGTGGATCCATACAACAACCCCATCCCCCAGGGGCTGGAGGCCTTCACTGTGGGA
TTTGTGGTTCTGGTTATTGGATTGTCTATGGGCTTTAACTCTGGCTACGCTGTCAATCCT
GCCAGAGACCTTGGACCACGTCTTTTCACCGCTATGGCTGGATGGGGCAGCGACGTTTTC
ACGGTTAGAAACGGCTGGTTCCTGGTGCCCCTTTTCGCCCCGTTCCTTGGCACCATCATC
GGCGTRGTAATTTACCAGCTGATGGTCGGCTTCCATGTGGAGGGAGAAGTACGTGACCAG
AAGAGCGCA---------------GAGGAGGAGAATGTCCGACTCACYAACGTCACCAAC
---AATGACAGACCC------AAAGACGCTACCAAAGAAATGCAC---------------
>Copper_rockfish_Aqp3a
ATGGGCAGACAGAAGGTGTATTTGAACAAACTGGCCCGGTTCTTCCAGATCCGTAATTTG
CTGCTTCGCCAGGCCCTGGCAGAGTGTCTCGGCACCCTCATCCTTGTGATGTTTGGCTGT
GGTGCGGTGGCCCAGCTGGTGTTGAGCAAAGGCTCCCATGGCATGTTCCTCACTGTCAAC
TTTGCCTTCGGCTTCGCTGCCATGTTAGGCATCCTGGTCTGTGGCCAGGTATCAGGTGGC
CATCTGAACCCTGCGGTGACCTTTGCTATGTGCCTGCTCGGAAGAGAGCCCTGGAAAAAG
TTCCCCATGTACTTCCTCTTTCAGACAATTGGTGGTTTTTTCGGTGCTGCCATCATTTTC
GGCATGTACTACGATGCCCTGTGGGACCTCCCTGGATGT---------------TTCAAT
GTGACTGGGGTTAATAATACAGCTAAGATCTTTGCTACCTACCCTGGACCACATCTCACC
ATTGTCAACGGCTTCTTTGATCAGATAATCGGCACAGCAGCGCTGATTGTTTGCATTCTG
GCTATTGTGGATCCATACAACAACCCCATCCCCCAGGGGCTGGAGGCCTTCACTGTGGGA
TTTGTGGTTCTGGTTATTGGATTGTCTATGGGCTTTAACTCTGGCTACGCTGTCAATCCT
GCCAGAGACCTTGGACCACGTCTTTTCACCGCTATGGCTGGATGGGGCAGCGACGTTTTC
ACGGTTAGAAATGGCTGGTTCCTGGTGCCCCTTTTCGCCCCGTTCCTTGGCACCATCATC
GGCGTGGTAATTTACCAGCTGATGGTCGGCTTCCATGTGGAGGGAGAAGTACGTGACCAG
AAGAGCGCA---------------GAGGAGGAGAATGTCCGACTCACCAACGTCACCAAC
---AATGACAGACCC------AAAGATGCTACCAAAGAAATGCAC---------------
>Korean_rockfish_Aqp3a
ATGGGCAGACAGAAGGTGTATTTGAACAAACTGGCCCGGTTCTTCCAGATCCGTAATTTG
CTGCTTCGCCAGGCCCTGGCAGAGTGTCTCGGCACCCTCATCCTTGTGATGTTTGGCTGT
GGTGCGGTGGCCCAGCTGGTGTTGAGCAAAGGCTCCCATGGCATGTTCCTCACTGTCAAC
TTTGCCTTCGGCTTCGCTGCCATGTTAGGCATCCTGGTCTGTGGCCAGGTATCAGGTGGC
CATCTGAACCCTGCGGTGACCTTTGCCATGTGCCTGCTCGGAAGAGAGCCCTGGAAAAAG
TTCCCCATGTACTTCCTCTTTCAGACAATCGGTGGTTTTTTCGGTGCTGCCATCATTTTC
GGCATGTACTACGATGCCCTGTGGGACCTCCCTGGATGT---------------TTCAAT
GTGACTGGGGTTAATAATACAGCTAAGATCTTTGCTACCTACCCTGGACCACATCTCACC
ATTGTCAACGGCTTCTTTGATCAGATAATCGGCACAGCAGCGCTGATTGTTTGCATTCTG
GCTATTGTGGATCCATACAACAACCCCATCCCCCAGGGGCTGGAGGCCTTCACTGTGGGA
TTTGTGGTTCTGGTTATTGGATTGTCTATGGGCTTTAACTCTGGCTACGCTGTCAATCCT
GCCAGAGACCTTGGACCACGTCTTTTCACCGCTATGGCTGGATGGGGCAGCGACGTTTTC
ACGGTTAGAAACGGCTGGTTCCTGGTGCCCCTTTTCGCCCCGTTCCTTGGCACCATCATC
GGCGTGGTAATTTACCAGCTGATGGTCGGCTTCCATGTGGAGGGAGAAGTACGTGACCAG
AAGAGCGCA---------------GAGGAGGAGAATGTCCGACTCACCAACGTCACCAAC

---AATGACAGACCC------AAAGATGCTACCAAAGAAATGCAC---------------
>Minor_flag_rockfish_Aqp3a
ATGGGCAGACAGAAGGTGTATTTGAACAAACTGGCCCAGTTCTTCCAGATCCGTAATTTG
CTGCTTCGC---------------------------------------ATGTTTGGCTGT
GGTGCGGTGGCCCAGCTGGTGTTGAGCAAAGGCTCCCATGGCATGTTCCTCACTGTCAAC
TTTGCCTTCGGCTTCGCTGCCATGTTAGGCATCCTGGTCTGTGGCCAGGTATCAGGTGGC
CATCTGAACCCTGCGGTGACCTTTGCCATGTGCCTGCTCGGAAGAGAGCCCTGGAAAAAG
TTCCCCATGTACTTCCTCTTTCAGACAATCGGTAGTTTTTTCGGTGCTGCCATCATTTTC
GGCATGTACTACGATGCCCTGTGGGACCTCCCTGGATGT---------------TTCAAT
GTGACTGGGGTTAATAATACAGCTAAGATCTTTGCTACCTACCCTGGACCACATCTCACC
ATTGTCAACGGCTTCTTTGATCAGATAATCGGCACAGCAGCGCTGATTGTTTGCATTCTG
GCTATTGTGGATCCATACAACAACCCCATCCCCCAGGGGCTGGAGGCCTTCACTGTGGGA
TTTGTGGTTCTGGTTATTGGATTGTCTATGGGCTTTAACTCTGGCTACGCTGTCAATCCT
GCCAGAGACCTTGGACCACGTCTTTTCACCGCTATAGCTGGATGGGGCAGCGACGTTTTC
ACGGTTAGAAACGGCTGGTTCCTGGTGCCCCTTTTCGCCCCGTTCCTTGGCACCATCATC
GGCGTGGTGATTTACCAGCTGATGGTCGGCTTCCATGTGGAGGGAGAAGTACGTGACCAG
AAGAGCGCA---------------GAGGAGGAGAATGTCCGACTCACCAACGTCACCAAC
---AATGACAGACCC------AAAGATGCTACCAAAGAAATGCAC---------------
>Rougheye_rockfish_Aqp3a
ATGGGCAGACAGAAGGTGTATTTGAACAAACTGGCCCGGTTCTTCCAGATCCGTAATTTG
CTGCTTCGCCAGGCCCTGGCAGAGTGTCTCGGCACCCTCATCCTTGTGATGTTTGGCTGT
GGTGCGGTGGCCCAGCTGGTGTTGAGCAAAGGCTCCCATGGCATGTTCCTCACTGTCAAC
TTTGCCTTCGGCTTCGCTGCCATGTTAGGCATCCTGGTCTGTGGCCAGGTATCAGGTGGC
CATCTGAACCCTGCGGTGACCTTTGCCATGTGCCTGCTCGGAAGAGAGCCCTGGAGAAAG
TTCCCCATGTACTTCCTCTTTCAGACAATCGGTGGTTTTTTCGGTGCTGCCATCATTTTC
GGCATGTACTACGATGCCCTGTGGGACCTCCCTGGATGT---------------TTCAAT
GTGACTGGGGTTAATAATACAGCTAAGATCTTTGCTACCTACCCTGGACCACATCTCACC
ATTGTCAACGGCTTCTTTGATCAGATAATCGGCACAGCAGCGCTGATTGTTTGCATTCTG
GCTATTGTGGATCCATACAACAACCCCATCCCCCAGGGGCTGGAGGCCTTCACTGTGGGA
TTTGTGGTTCTGGTTATTGGATTGTCTATGGGCTTTAACTCTGGCTACGCTGTCAATCCT
GCCAGAGACCTTGGACCACGTCTTTTCACCGCTATGGCTGGGTGGGGCAGCGAGGTTTTC
ACGGTTAGAAACGGCTGGTTCCTGGTGCCCCTTTTCGCCCCGTTCCTTGGCACCATCATC
GGCGTGGTGATTTACCAGCTGATGGTCGGCTTCCATGTGGAGGGAGAAGTACGTGACCAG
AAGAGCGCA---------------GAGGAGGAGAATGTCCGACTCACCAACGTCACCAAC
---AATGACAGACCC------AAAGACGCTACCAAAGAAATGCAC---------------
>Amazon_molly_Aqp3a
ATGGGTCGACAGAAGTTTTATTTGGACAAACTGGCCCGCTTCTTTCAGATCCGTAACCTG
CTGCTCCGCCAGGCCCTGGCAGAGTGCCTTGGCACTCTCATCCTTGTGATGTTTGGCTGC
GGCGCCGTGGCCCAAAGCGTGCTGAGCTACGGTTCCCACGGCAGGTTCCTCACTGTCAAC
TTTGCCTTTGGCTTTGCTGCCACATTAGGCATTCTGGTCTGCGGCCAAATATCAGGTGGA
CATTTGAATCCTGCAGTGACCTTTTCACTTTGCCTGCTTGGAAGAGAGCGCTGGAGAAAG
TTCCCCATGTACTTCCTCTTTCAGACAATCGGAGCTTTTTTTGGTGCTTCCATTATCTTT
GGCATGTACTACGATGCCTTGTGGGACTTTCCAGGATGC---------------TTCAAT
CTCACTAGCGAATATAACACTGCTGGCATTTTTGCCACCTATCCCGCAAAACATCTCACA
ATTGTGAATGGATTCTTTGATCAGATTATTGGCACGGCAGCCCTCATCGTTTGCATTCTG
GCGATTGTGGACCCATACAACAACCCGATCCCACAGGGTTTGGAGGCCTTCACTGTGGGA
TTTGTGGTTCTGGTCATTGGACTGTCAATGGGGTTCAATTCTGGTTACGCTGTCAATCCT
GCCAGAGATTTCGGACCACGTCTTTTCACTGCTATCGCTGGCTGGGGAACTGGAGTTTTC
ACGGCTAGAGACGGCTGGTTTCTGGTGCCGATTTTCGCCCCATTCCTTGGTGCCATTATT
GGCACGATGGTTTACCAGCTAATGGTCGGCTTCCATGTGGAGGGAGAAGTGCGTGAAAGG
GAAGAGAGAAAG---------AACGAAGAGGAAAATCTACAACTCACC---GTCAACTCC
---AACCCCAAATCT------AATTCCAACGGCAAAGAGGCTAACTGC------------
>Sailfin_molly_Aqp3a
ATGGGTCGACAGAAGTTTTATTTGGACAAACTGGCCCGCTTCTTTCAGATCCGTAACCTG
CTGCTCCGCCAGGCCCTGGCAGAGTGCCTTGGCACTCTCATCCTTGTGATGTTTGGCTGC
GGCGCCGTGGCCCAAAGCGTGCTGAGCTACGGTTCCCACGGCAGGTTCCTCACTGTCAAC
TTTGCCTTTGGCTTTGCTGCCACATTAGGCATTCTGGTCTGCGGCCAAATATCAGGTGGA
CATTTGAATCCTGCAGTGACCTTTTCACTTTGCCTGCTTGGAAGAGAGCGCTGGAGAAAG
TTCCCCATGTACTTCCTCTTTCAGACAATCGGAGCTTTTTTTGGTGCTTCCATTATCTTT
GGCATGTACTACGATGCCTTGTGGGACTTTCCAGGATGC---------------TTCAAT
CTCACTAGCGAATATAACACTGCTGGCATTTTTGCCACCTATCCCGCAAAACATCTCACA

ATTGTGAATGGATTCTTTGATCAGATTATTGGCACGGCAGCCCTCATCGTTTGCATTCTG
GCGATTGTGGACCCATACAACAACCCGATCCCACAGGGTTTGGAGGCCTTCACTGTGGGA
TTTGTGGTTCTGGTCATTGGACTGTCAATGGGGTTCAATTCTGGTTACGCTGTCAATCCT
GCCAGAGATTTCGGACCACGTCTTTTCACTGCTATCGCTGGCTGGGGAACTGGAGTTTTC
ACGGCTAGAGACGGCTGGTTTCTGGTGCCGATTTTCGCCCCATTCCTTGGTGCCATTATT
GGCACGATGGTTTACCAGCTAATGGTCGGCTTCCATGTGGAGGGAGAAGTGCGTGAAAGG
GAAGAGAGAAAG---------AACGAAGAGGAAAATCTACAACTCACC---GTCAACTCC
---AACCCCAAATCT------AATTCCAACGGCAAAGAGGCTAACTGC------------
>Shortfin_molly_Aqp3a
ATGGGTCGACAGAAGTTTTATTTGGACAAACTGGCCCGCTTCTTTCAGATCCGTAACCTG
CTGCTCCGCCAGGCCCTGGCAGAGTGCCTTGGCACTCTCATCCTTGTGATGTTTGGCTGC
GGCGCCGTGGCCCAAAGCGTGCTGAGCTACGGTTCCCACGGCAGGTTCCTCACTGTCAAC
TTTGCCTTTGGCTTTGCTGCCACATTAGGCATTCTGGTCTGCGGCCAAATATCAGGTGGA
CATTTGAATCCTGCAGTGACCTTTTCACTTTGCCTGCTTGGAAGAGAGCGCTGGAGAAAG
TTCCCCATGTACTTCCTCTTTCAGACAATCGGAGCTTTTTTTGGTGCTTCCATTATCTTT
GGCATGTACTACGATGCCTTGTGGGACTTTCCAGGATGC---------------TTCAAT
CTCACTAGCGAATATAACACTGCTGGCATTTTTGCCACCTATCCCGCAAAACATCTCACA
ATTGTGAATGGATTCTTTGATCAGATTATTGGCACGGCAGCCCTCATCGTTTGCATTCTG
GCGATTGTGGACCCATACAACAACCCGATCCCACAGGGTTTGGAGGCCTTCACTGTGGGA
TTTGTGGTTCTGGTCATTGGACTGTCAATGGGGTTCAATTCTGGTTACGCTGTCAATCCT
GCCAGAGATTTCGGACCACGTCTTTTCACTGCTATCGCTGGCTGGGGAACTGGAGTTTTC
ACGTATAGAGACGGCTGGTTTCTGGTGCCGATTTTCGCCCCATTCCTTGGTGCCATTATT
GGCACGATGGTTTACCAGCTAATGGTCGGCTTCCATGTGGAGGGAGAAGTGCGTGAAAAG
GAAGAGAGAAAG---------AACGAAGAGGAAAATCTACAACTCACC---GTCAACTCC
---AACCCCAAATCT------AATTCCAACGGCAAAGAGGCTAACTGC------------
>Guppy_Aqp3a
ATGGGTCGACAGAAGTTTTATTTGGACAAACTGGCCCGCTTCTTTCAGATCCGTAACCTG
CTGCTCCGCCAGGCCCTGGCAGAGTGCCTTGGCACTCTCATCCTTGTGATGTTTGGCTGC
GGCGCCGTGGCCCAAAGTGTGCTCAGCTACGGTACCCACGGCAGGTTCCTCACTGTCAAC
TTTGCCTTTGGCTTTGCTGCCACATTAGGCATTCTGGTCTGCGGCCAAGTATCAGGTGGA
CATTTGAATCCTGCAGTGACCTTTTCACTTTGCCTGCTTGGAAGAGACCGCTGGAGAAAG
TTCCCCATGTACTTCCTCTTTCAGACAATCGGAGCCTTTTTTGGTGCTTCCGTTATCTTT
GCCATGTACTACGATGCCTTGTGGGACTTTCCAGGATGT---------------TTCAAT
CTCACCAGCGATTCTTCCACTGCTGGCATTTTTGCCACCTATCCTGCAAAACATCTCACT
ATTGTGAATGGATTCTTTGATCAGATTATTGGCACGGCAGCCCTCATCGTTTGCGTTCTG
GCGATTGTGGACCCATACAACAACCCGATCCCGCAGGGCTTGGAGGCCTTCACTGTGGGA
TTTGTGGTTCTGGTCATTGGACTGTCAATGGGCTTCAATTCTGGTTACGCTGTCAATCCT
GCCAGAGATCTCGGACCACGTCTTTTCACTGCTATCGCTGGCTGGGGATCTGAAGTTTTC
ACGTATGGAAATGGCTGGTTTCTGGTGCCGATTTTCGCCCCATTCCTTGGTGCCATTATT
GGCACGATGATTTACCAGCTAATGGTCGGCTTCCATGTGGAGGGCGAAGTGCGTGACAAG
AAAGAGAACAAG---------AACGAAGAGGAAAATCTACAACTCACC---GTCAACTCC
---AACCCCAAATCT------AAATCCAACGGCAAAGAGGCTAACTGC------------
>Green_swordtail_Aqp3a
ATGGGTCTACAGAAGTTTATTTTGGACAAACTGGCCCGCTTCTTTCAGATCCGTAACCTG
CTGCTGCGCCAGGCCCTGGCAGAGTGCCTTGGCACTCTCATCCTTGTGATGTTTGGCTGC
GGCGCTGTGGCCCAAAGTGTGCTGAGCGGTGGCTCCCACGGCAGGTTCCTCTCTGTCAAC
TTTGCCTTTGGCTTTGCTGCCATGTTAGGCATTCTGATCTGCGGCCAAGTATCAGGTGGA
CATTTGAATCCTGCAGTGACATTTTCACTTTGCCTGCTTGGAAGAGAGCGCTGGAGAAAG
TTCCCCATGTATTTCCTCTTTCAGACAATCGGAGCTTTTTTTGGTGCTTCCATTATCTTT
GCCATGTACTACGATGCCCTGTGGGACTTTCCAGGATGT---------------TTCAAT
CTCACTGACGATTCTTCCACTGCTGGCATTTTTGCCACCTATCCCGCAAATCACCTCTCA
ATTGTGAATGGATTCTTTGATCAGATTATTGGCACGGCAGCCCTCATTGTTTGCATTCTG
GCGATTGTGGACCCGTACAACAACCCAATCCCCCAGGGCTTGGAGGCCTTCACTGTGGGA
TTTGTGGTTCTTGTCATTGGACAGTCAATGGGCTTCAATTCTGGTTACGCTGTCAATCCT
GCCAGAGATCTCGGACCACGTCTTTTCTCTGCTATCGCTGGCTGGGGAACTGCAGTTTTC
ACGTTTAAAGATGGCTGGTTTCTGGTGCCGACTTTAGCCCCATTCATTGGTGCCATTATT
GGCACGATGATTTATCAGCTAATGGTTGGCTTCCATGTGGAGGGAGAAGTGCGTGACCGG
AAAGAGAACAAA---------AACAAAGAGGAAAATCTGCAACTCAAC---ATCAACTCC
---AACCCCAAATCT------AATTATAACGACAAAGATGCTAATTGC------------
>Monterrey_platyfish_Aqp3a
ATGGGTCTACAGAAGTTTATTTTGGACAAACTGGCCCGCTTCTTTCAGATCCGTAACCTG

CTGCTGCGCCAGGCCCTGGCAGAGTGCCTTGGCACTCTCATCCTTGTGATGTTTGGCTGC
GGCGCTGTGGCCCAAAGTGTGCTGAGCGGTGGCACCCACGGCAGGTTCCTCTCTGTCAAC
TTTGCCTTTGGCTTTGCTGCCATGTTAGGCATTCTGATCTGCGGCCAAGTATCAGGTGGA
CATTTGAATCCTGCAGTGACATTTTCACTTTGCCTGCTTGGAAGAGAGCGCTGGAGAAAG
TTCCCCATGTATTTCCTCTTTCAGACAATCGGAGCTTTTTTTGGTGCTTCCATTATCTTT
GCCATGTACTACGATGCCCTGTGGGACTTTCCAGGATGT---------------TTCAAT
CTCACTGACGATTCTTCCACTGCTGGCATTTTTGCCACCTATCCCGGAAATCACCTCTCA
ATTGTGAATGGATTCTTTGATCAGATTATTGGCACGGCAGCCCTCATTGTTTGCATTCTG
GCGATTGTGGACCCGTACAACAACCCAATCCCCCAGGGCTTGGAGGCCTTCACTGTGGGA
TTTGTGGTTCTTGTCATTGGACAGTCAATGGGCTTCAATTCTGGTTACGCTGTCAATCCT
GCCAGAGATCTCGGACCACGTCTTTTCTCTGCTATCGCTGGCTGGGGAACTGCAGTTTTC
ACGTTTAAAGATGGCTGGTTCCTGGTGCCGATTTTCGCCCCATTCATTGGTGCCATTATT
GGCACGATGATTTATCAGCTAATGGTTGGCTTCCATGTGGAGGGAGAAGTGCGTGACCGG
GAAGAGAACAAA---------AACAAAGAGGAAAATCTACAACTCAAC---ATCAACTCC
---AACCCCAAATCT------AATTATAACGACAAAGAGGCTAATTGC------------
>Southern_platyfish_Aqp3a
ATGGGTCTACAGAAGTTTTATTTGGACAAACTGGCCCGCTTCTTTCAGATCCGTAACCTG
CTGCTGCGCCAGGCCCTGGCAGAGTGCCTTGGCACTCTCATCCTTGTGATGTTTGGCTGC
GGCGCTGTGGCCCAAAGTGTGCTGAGCGGTGGTACCCACGGCAGGTTCCTCTCTGTCAAC
TTTGCCTTTGGCTTTGCTGCCATGTTAGGCATTCTGATCTGCGGCCAAGTATCAGGTGGA
CATTTGAATCCTGCAGTGACATTTTCACTTTGCCTGCTTGGAAGAGAGCGCTGGAGAAAG
TTCCCCATGTATTTCCTCTTTCAGACAATCGGAGCTTTTTTTGGTGCTTCCATTATCTTT
GCCATGTACTACGATGCCCTGTGGGACTTTCCAGGATGT---------------TTCAAT
CTCACTGACGATTCTTCCACTGCTGGCATTTTTGCCACCTATCCCGCAAATCACCTCTCA
ATTGTGAATGGATTCTTTGATCAGATTATTGGCACGGCAGCCCTCATTGTTTGCATTCTG
GCGATTGTGGACCCGTACAACAACCCAATCCCCCAGGGCTTGGAGGCCTTCACTGTGGGA
TTTGTGGTTCTTGTCATTGGACAGTCAATGGGCTTCAATTCTGGTTACGCTGTCAATCCT
GCCAGAGATCTCGGACCACGTCTTTTCTCTGCTATCGCTGGCTGGGGAACTGCAGTTTTC
ACGTTTAAAAACGGCTGGTTTCTGGTGCCGACTTTCTCCCCATTCATTGGTGCCATTATT
GGCACGATGATTTATCAGCTAATGGTTGGCTTCCATGTGGAGGGAGAAGTGCGTGACCGG
AAAGAGAACAAA---------AACAAAGAGGAAAATCTGCAACTCAAC---ATCAACTCC
---AACCCCAAATCT------AATTATAACGACAAAGAGGCTAATTGC------------
>Blackstripe_livebearer_Aqp3a
ATGGGTCGACAGAAGGTTTATTTGGACAAACTGGCCCGCTTCTTTCAGATCCGTAACCTG
CTGCTCCGCCAGGCCCTGGCAGAGTGCCTAGGCACTCTCATCCTTGTGATGTTTGGCTGC
GGCTCTGTAGCCCAAACTGTGCTGAGCGGTGGCACCCACGGCATGTTCCTCACCATCAAC
TTTGCCTTTGGCTTTGCTGCCACATTAGGTATTCTGGTCTGCGGCCAAGTATCAGGTGGA
CATTTGAATCCTGCAGTGACCTTTTCGCTTTGTCTGCTTGGAAGAGATCGCTGGAGAAAG
TTCCCCATGTACTTCCTCTTTCAGACAATCGGAGCTTTTTTTGGTGCTTCCATTATCTTT
GGCATGTATTACGATGCCTTGTGGGACTTTCCAGGATGT---------------TTCAAT
CTCACTGACGATGCTAACACTGCTGGCATTTTTGCCACGTATCCTGCAAAACATCTCACT
CTTGTGAATGGATTCTTTGATCAGGTTATTGGCACGACAGCCCTCATCGTTTGCATTCTG
GCGATTGTGGACCCGTACAACAACCCAATCCCCCAGGGCTTGGAGGCCTTCACTGTGGGA
TTTGTGGTTCTGGTCATTGGACAGTCAATGGGCTTCAATTCTGGTTACGCTGTCAACCCT
GCCAGAGATTTCGGACCACGTCTTTTCTCTGCTATCGCTGGCTGGGGATCTGACGTTTTC
ACGTACAGAGATGGCTGGTTTCTGGTGCCGATTTTCGCCCCATTCATTGGTGCCATTATT
GGCACGATGATTTATCAGCTAATGGTCGGCTTCCATGTGGAAGGAGAAGCACGTGAGCTG
GAAGAGAAAAAG---------AACAAAGAGGAAAATCTACAACTCAAC---GTCCACTCC
---AACCCCAAATCT------AATTCCAACGGCAAAGAGGCTAATTGC------------
>Western_mosquitofish_Aqp3a
ATGGGTCGACAGAAGTTTTATTTGGACAAACTGGCCCGCTTCTTTCAGATCCGTAACCTG
CTGCTCCGCCAGGCCCTGGCAGAGTGTCTAGGCACTCTCATCCTTGTGATGTTTGGCTGC
GGCGCCGTGGCCCAAAGTGTGCTGAGCGGCGGCACCCATGGCAGGTTCCTCACTATCAAC
TTTGCCTTTGGCTTTGCTGCCATGTTAGGCATTCTGATCTGCGGCCAAATATCAGGTGGA
CATTTGAATCCTGCAGTGACCTTTGCGCTTTGCCTGCTTGGAAGAGAGCGCTGGAGAAAG
TTCCCCATGTACTTCCTCTTTCAGACAATCGGAGGTTTTTTCGGCGCTGCCGTTATCTTT
GGCATGTACTACGATGCCTTGTGGGATTTCCCAGGATGT---------------TTTAAT
CTCACTGACGATTCTTCCACTGCTGGCATTTTTGCCACCTATCCCGCAAAACACCTCACA
CTTGTGAATGGATTCTTTGACCAGATTATTGGCACGGCAGCCCTCATTGTTTGCATTCTG
GCGATTGTGGACCCGTTCAACAACCCAATCCCCCAGGGCTTGGAGGCCTTCACTGTGGGA
TTTGTGGTTCTGGTCATTGGACAGTCGATGGGCTTCAATTCTGGTTACGCTGTCAATCCT

GCCAGAGATCTCGGACCACGTCTTTTCTCTGCCATCGCTGGTTGGGGAACTGCAGTTTTC
ACGTTTAGAGATGGCTGGTTTCTGGTGCCGACTTTCGCTCCATTCCTTGGTGCCATTACT
GGCACGATGATTTATCAGCTAATGGTTGGCTTCCACTTGGAGGGAGAAGTGCGTGACCGG
GAAGAGAACAAG---------AACAAAGAGGAAAATCTACAACTCACC---GTCAGCTCC
---AACCCCAAATCT------AATTCCAATGGCAAAGAGGCTAACTGC------------
>Amargosa_pupfish_Aqp3a
ATGAGTCGACAGAAGATTTATTTGGACAAATTGGCCCGCTTCTTTCAGATCCGAAACAAG
CTGCTCCGCCAGGCCCTGGCAGAGTGCCTTGGCACTCTCATCCTTGTGATGTTTGGCTGT
GGTTCTGTGGCCCAATTTTCACTCAGCAAAGGTACCCATGGACAGTTCCTTACTGTCAAC
TTTGCCTTTGGCTTTGCTGCCACTCTAGGGATCCTTGTCTGTGGCCAAGTATCAGGTGGC
CATTTGAACCCTGCAGTGACTTTTTCACTTTGCTTGCTTGGAAGAGATAAGTGGAGAAAG
TTCCCCATGTTCTTCCTCTTTCAGACAATTGGAGCCTTTTTTGCTGCCGCTGTGATTTTT
GGCATGTATTATGATGCCTTGTGGGAC---ACCGGATGT---------------TTCACT
GTTGATGGGGAAAATTCCACCGCTGGAATCTTTGCTACTTACCCAGGGAAACATCTCACT
------------------------ATTATTGGCACAACAGCCCTCATAGTCTGCATTCTG
GCAATTGTGGACCCATACAACAACCCAATCCCACAAGGTTTGGAGGCCTTCACAGTGGGA
TTTGTGGTTCTGGTCATTGGATTGGCCATGGGCTTTAACTCTGGTTACGCTGTCAATCCT
GCAAGAGACTTTGGACCACGTCTTTTTACTTCCATGGCTGGCTGGGGAAGTGATGTTTTC
ACGTTTAGAAATGGATGGTTTCTGGTGCCGCTTTGTGCACCATTCCTTGGAGCCATTATT
GGCACCATGATTTACCAGGTAATGGTTGGTTTCCACACCGAGGGAGAATTTCGGGATAGG
CAGGAGGCGGAG---------CAACAGGAGGAGAATCTACAACTCACC---GTCACAACC
---AACTCCAAATCT------AATTCCAACGGCAAAGAGGCTAACTGC------------
>Sheepshead_minnow_Aqp3a
ATGAGTCGACAGAAGATTTATTTGGACAAATTGGCCCGCTTCTTTCAGATCCGAAACAAG
CTGCTCCGCCAGGCCCTGGCAGAGTGCCTTGGCACTCTCATCCTTGTGATGTTTGGCTGT
GGTTCTGTGGCCCAATTTTCACTCAGCAAAGGTACCCATGGACAGTTCCTTACTGTCAAC
TTTGCCTTTGGCTTTGCTGCCACTCTAGGGATCCTTGTCTGTGGCCAAGTATCAGGTGGC
CATTTGAACCCTGCAGTGACTTTTTCACTTTGCTTGCTTGGAAGAGATAAGTGGAGAAAG
TTCCCCATGTTCTTCCTCTTTCAGACAATTGGAGCCTTTTTTGCTGCCGCTGTGATTTTT
GGCATGTATTATGATGCCTTGTGGGAC---ACCGGATGT---------------TTCACT
GTTGATGGGGAAAATGCCACCGCTGGAATCTTTGCTACTTACCCAGGGAAACATCTCACT
CTTGTGAATGGTTTCTTTGATCAGATTATTGGCACAGCAGCCCTCATAGTCTGCATTCTG
GCAATTGTGGACCCATACAACAACCCAATCCCACAAGGTTTGGAGGCCTTCACAGTGGGA
TTTGTGGTTCTGGTCATTGGATTGGCCATGGGCTTTAACTCTGGTTACGCTGTCAATCCT
GCAAGAGACTTTGGACCACGTCTTTTTACTGCCATGGCTGGCTGGGGAAGTGATGTTTTC
ACGTTTAGAAATGGATGGTTTCTGGTGCCGCTTTGTGCGCCATTCCTTGGAGCCATTATT
GGCACCATGATTTACCAGGTAATGGTTGGTTTCCACATCGAGGGAGAATTTCGGGATAGG
CAGGAGGCGAAA---------GAACAGGAGGAGAATCTACAACTCACC---GTCACAACC
---AACTCCAAATCT------AATTCCAACGGCAAAGAGGCTAACTGC------------
>Common_mummichog_Aqp3a
ATGGGTCGACAGAAGATTTATTTGGACAAACTGGCCCGCTTCTTTCAGATCCGTCACCTG
TTGCTCCGCCAGGCCCTGGCAGAGTGCCTTGGCACTCTCATCCTTGTGATGTTTGGCTGT
GGCGCTGTGGCCCAAAGCGTGCTGAGTCGTGGTTCCCATGGCAGGTTCCTCACTATCAAC
TTTGCCTTTGGCTTTGGCGCCACCTTAGGGATCCTGGTCTGTGGCCAAGTATCAGGTGGC
CATCTGAACCCTGCAGTGACCTTTTCACTTTGCCTGCTTGGAAGAGAGCGCTGGAGAAAG
TTCCCCATGTACTTCCTCTTTCAGACAATCGGAGCATTTTTCGGCGCTGCTATTATCTTT
GGCATGTATTATGATGCCTTTTGGGACTTTCCCGGGTGC---------------TTTAAT
GTTACTGGGGATTCTTCCACTGCTGGCGTCTTTGCCACCTACCCCGGAAAGCATCTTACT
CTTGTGAATGGATTCTTTGATCAGATTATTGGCACGGCAGCCCTCATAGTGTGCATTCTG
GCAATTGTGGACCCATACAACAACCCCATTCCCCAAGGGTTGGAGGCCTTCACTGTGGGA
TTTGTGGTTTTGGTCATTGGATTGTCCATGGGCTTCAATTCCGGTTATGCTGTTAACCCT
GCCAGAGACATCGGACCACGCCTTTTCACTGCTATAGCTGGCTGGGGAACTGATGTTTTT
ACGGCTAGAAATGGCTGGTTCTTGGTGCCACTCTGCGTCCCATTCCTTGGTGCCCTTATT
GGCACGATAATTTATCAGCTGATGGTTGGTTTCCATGTGGAAGGAGAAGCACGTGATCAG
AAAAAGAACACG---------------GAAGAGAGTCTACAACTTACC---ATCAACCCC
---GACTCAAAATCT------AACGCCAACGGCAAAGAGGCTAACTGC------------
>Mangrove_rivulus_Aqp3a
ATGGGTCGACAGAAGATATACCTGGACAAACTGGCCCGGTTCTTTCAGATCCGTAACATG
CTGCTTCGTCAGGCGCTGGCAGAGTGCCTCGGCACCCTCATCCTTGTGATGTTCGGCTGC
GGCGCCGTGGCACAACTGGTGCTGAGCGGGGGTTCGCATGGCATGTTCCTCACTGTCAAC
TTCGCCTTCGGCTTCGCTGCCACCTTGGGCATCCTGGTCTGTGGCCAAGTATCAGGTGGC

CATCTGAACCCCGCAGTGACCTTTTCACTTTGTCTGCTTGGAAGAGAGCGCTGGAGAAAG
TTCCCCATGTACTTCCTCTTTCAGACAATTGGTGGCTTTTTCGGTGCCGCTGTGATTTTT
GGCATGTACTACGATGCCTTGTGGGACCATCCCGGAAGT---------------TTCAAT
GTGACTGGGCCGTCTGCCACAGCTGGCATCTTTGCCACATATCCTGGAAACCACCTCACC
ATTGTCAATGGCTTCTTTGATCAGATTATTGGCACAGCAGCCCTGATAGTTTGCATTCTG
GCTATCGTGGATCCATACAACAACCCCATCCCCCAAGGGCTGGAGGCGTTCACTGTGGGC
TTTGTGGTTCTGGTCATTGGACTGTCCATGGGCTTTAATTCCGGTTATGCTGTCAATCCT
GCCAGAGACCTTGGACCACGTCTTTTCACCGCTTTAGCCGGCTGGGGAAGTGAAGTTTTC
ACGGTTAGAAACGGCTGGTTCCTGGTGCCAGTTTTGGCCCCTTTCCTCGGCGCCATCATC
GGAACAATGATCTACCAGCTAATGGTTGGTTTTCACGTGGAGGGAGAAGTACGTGACAGG
GCCGAACAAACA---------------GAGGAGAACGTCCAACTAACCAACACCAACTCC
---AATGCCAACTCA------AAAGCCCACGCCAAAGAAATGGCC---------------
>Rio_pearlfish_Aqp3a
ATGGGTCAACAGAAGATATATCTGGACAAGCTGGCCCGGTTCTTTCAGATCCGTAACCTG
CTGCTTCGTCAGGCCCTTGCAGAGTGCCTTGGCACTCTTATCCTCGTGATGTTTGGCTGT
GGCGCTCTGGCCCAACATGTGCTGAGTGGTGGATCACATGGAATGTTCCTCACTGTCAAC
TTTGCCTTCGGCTTTGCTGCCACCTTAGGCATCTTGGTCTGTGGCCAGGTATCAGGTGGC
CATCTGAACCCTGCAGTGACATTTTCACTTTGTCTGCTTGGAAGAGAGCGCTGGAGAAAG
TTCCCCATGTACTTCCTCTTTCAGACAATTGGTGCATTTTTCGGCGCTGCTGTGATTTTT
GGCATGTACTACGATGCCTTGTGGGACCATCCTGGAAGT---------------TTCAAC
GTGACTGGGCCTGATGCCACAGCTGGCATCTTTGCCACATATCCTGGAAAACACCTCACC
ATTGTCAATGGCTTCTTTGATCAGATTATTGGCACGACAGCGCTGATAGTTTGCATTCTG
GCAATCGTGGATCCATACAACAACCCAATCCCGAAAGGGCTGGAAGCGTTCACTGTGGGA
TTTGTGGTTCTGGTCATTGGACTGTCCATGGGCTTTAACTCTGGTTATGCTGTCAATCCT
GCCAGAGACCTTGGACCACGTCTTTTCACCGCTATAGCCGGCTGGGGAAGTGAGGTTTTC
ACGGTCAACAATGGCTGGTTCTTGGTGCCCGTTTTTGCCCCTTTCCTTGGTTCCATTATC
GGCACCATAATCTACCAGCTGATGGTTGGTTTCCATGTGGAGGGAGAAGTACGCGATAAG
GCTGAAAAAACA------------GAGGAGGAGAATGTCCGACTAACTAATGTCAACTCC
---AATGCCAACTCA------AAG---------AAATGTCCCGAG---------------
>Annual_killifish_Aqp3a
ATGGGTCGACAGAAGATCTATCTGGAGAAATTGTCCCAGTTCTTTCAGCTCCGTAACCTG
CTGCTCCGCCAGGCCCTGGCAGAATGCCTTGGCACTCTCATCCTTGTGATGTTTGGCTGT
GGCGCTGTGGCCCAGCAGGTGCTGAGTGGCGGTTCACATGGCATGTTCCTCACCGTTAAC
TTTGCCTTCGGCTTCGCTGCCACCTTAGGCATTCTGGTCTGTGGCCAGGTATCAGGTGGT
CATCTGAACCCTGCGGTGACATTTTCACTTTGTCTGCTTGGAAGAGAACGCTGGAGAAAG
TTCCCCATGTACTTCCTCTTTCAGACAATCGGTGCATTTTTTGGCGCTGCTGTAATTTTT
GGCATGTACTACGATGCCTTATGGGACTTCCCCGGAAGT---------------TTTAAT
GTGACAGGACCTGATGCAACAGCTGGCATTTTTGCCACATATCCTGCAAGTCATCTGACC
ATTGTCAATGGCTTTTTTGATCAGCTTATTGGCACAGCAGCATTGATAGTTTGCGTTCTG
GCCATCGTGGATCCATACAACAACCCCATCCCACAAGGGCTGGAGGCGTTCACTGTGGGT
TTTGTGGTTCTGGTCATTGGACTGTCCATGGGCTTTAATTCTGGTTATGCTGTCAATCCT
GCCAGAGACTTGGGACCACGTCTTTTCACCGCTTTAGCTGGCTGGGGAAGTGAGGTTTTC
ACGGTTGGAAATGGCTGGTTCCTGGTGCCCATTTTTGCCCCTTTCCTTGGTGCCATCATC
GGCACAATGATCTACCAGATGATGGTTGGTTTTCACGTGGAGGGAGAAGTACGTGATAGA
ACCGCGAATCGC------------GAGAGGGAGAATGTCCCACTTACCGATACCAACTCC
---AATGCC------------AAG------TCTAAAGAAATGCCT---------------
>Beira_killifish_Aqp3a
ATGGGTCAACAGAAGGCATTCCTAGACAATCTGTCCCGGTTCTTTCAGATCCGTAATAAG
TTGCTTCGCCAGTCATTGGCAGAGTGTCTCGGCACCCTCGTCCTTGTGATGTTTGGCTGT
GGTGCTCTGGCACAACATGTGCTGAGCTCTGGCTCGCATGCTATATTCCTCACCGTCAAC
TTTGCTTTCGGCTTCGGTGCCACCTTAGGCATCCTGGTCTGTGGCCAGGTTTCAGGTGGT
CATCTGAACCCTGCTGTGACCTTTTCGCTGTGCCTGCTTGGAAGAGAACGCTGGAGAAAG
TTCCCCATGTACTTCCTGTTTCAGACAATTGGTGCATTTTTTGGTGCTGCTGTGATTTTT
GGCATGTACTATGATGCCTTGTGGGATTTTGAGGGAGCG---------------TTCAAT
GTTACTGGCTCTGGTGTCACCGCTGGCATCTTTGCTACATACCCTGGAACCCACCTCACT
ACTCTCAATGGCTTCTTTGACCAGCTTATTGGCACAGCAGCACTGATCGTTTGCATTCTG
GCTATTGTGGATCCATACAACAATCCCATCCCCCAAGGGCTGGAGGCCTTCACTGTGGGG
TTTGTGGTTCTGGTCATTGGACTTTCCATGGGCTTTAATTCTGGCTATGCTGTTAACCCT
GCTAGAGATTTCGGTCCACGTCTTTTCACCGCCATAGCTGGCTGGGGAGGCGAAGTGTTC
ACGGTTGGAAACGGCTGGTTCCTGGTGCCTATCATTGCCCCGTTCATTGGCGCCATCATT
GGCACAGTCATTTATCAGTTGATGGTTGGTATCCACGTGGAGGGAGAAGCACGCGAAAAG

AAGGAGAGAGCG------------GAGAGGGAGAACCTTCGACTGGCA------------
---AATGCCAAC---------AAG---------AAAGAAGTGCAC---------------
>Turquoise_killifish_Aqp3a
ATGGGTCGACAGAAGGCATTCCTAGACAAACTGTCCCGGTTCTTTCAGATCCGTAATAAG
TTGCTTCGCCAGTCATTGGCAGAGTGTCTCGGCACCCTCGTCCTTGTGATGTTTGGCTGT
GGTGCTCTGGCACAACATGTGCTGAGCTCTGGCTCACATGCTATATTCCTCACTGTCAAC
TTCGCTTTTGGCTTCGGTGCCACCTTAGGCATCCTGGTCTGTGGCCAGGTTTCAGGTGGT
CATCTGAACCCTGCTGTGACCTTTTCGCTGTGCCTGCTTGGAAGAGAACGCTGGAGAAAG
TTCCCCATGTACTTCCTGTTTCAGACAATTGGTGCATTTTTTGGTGCTGCTGTGATTTTT
GGCATGTACTATGATGCCTTGTGGGATTTTGAGGGAGCG---------------TTCAAT
GTTACTGGCTCTGGTGTCACCGCTGGCATCTTTGCTACTTACCCTGGAACCCACCTCACT
ACTCTCAATGGCTTCTTTGACCAGCTTATTGGCACAGCAGCACTGATCGTTTGCATTCTG
GCTATTGTGGATCCATACAACAATCCCATCCCCCAAGGGCTGGAGGCCTTCACTGTGGGG
TTTGTGGTTCTGGTCATTGGACTTTCCATGGGCTTTAATTCTGGCTATGCTGTTAACCCT
GCCAGAGATTTCGGTCCACGTCTTTTCACCGCCATAGCTGGCTGGGGAGGCGAAGTGTTC
ACGGTTGGAAACGGCTGGTTCCTGGTGCCTATCATTGCCCCGTTCATTGGCGCCATCATT
GGCACAGTCATTTATCAGTTGATGGTTGGTATCCACGTGGAGGGAGAAGCACGCGAAAAG
AAGGAGAGAGCG------------GAGGGGGAGAACGTTCGACTGGCA------------
---AATGCCAAC---------AAG---------AAAGACGTGCCC---------------
>Indian_ricefish_Aqp3a
ATGAGTCGTCAAAAAATAATGTTGGACAAACTAGCGCGGACCTTTCAGATCCGCAACAAG
CTGCTTCGACAGTCTCTTGCAGAATGCCTCGGGACTCTCATCCTCGTGATGTTTGGCTGT
GGTGCTGTGGCACAGCTGGTGCTAAGTGGGGGCACCCACGCCCGTTTCATGACGGTTAAC
TTCGCGTTCGGCTTTGGGGCCACCTTAGGCATCCTGGTGTGTGGCCAGGTATCAGGTGGC
CATCTGAATCCCGCAGTGACCTTTGCCCTCTGCTTGCTTGGAAGAGAGCGCTGGAGAAAG
TTCCCCATGTATTTCCTCTTTCAAACAATCGGAGCTTTCTTTGGGGCTGCCATCATTTTC
GGCATGTATTACGATGCATTGTGGGACTTTCCTGAGGCT---------------TTTAAC
ATGACTGATAGTAATCCCACGGCAGGCATCTTTGCCACTTACCCAGCAAAACATCTAACC
CTCCTCAATGGTTTCTTTGATCAGTTCATTGGCACAGCAGCATTGATAGTTTGCATTCTG
GCTATTGTGGATCCCTACAACAATCCCATCCCTCAAGGTCTTGAGGCCTTCACTGTGGGG
TTTGTGGTTTTGGTGATTGGGTTATCAATGGGCTTCAATTCCGGCTATGCAGTCAATCCT
GCCAGAGACCTTGGTCCTCGTATTTTCACCGCTATGGCCGGCTGGGGGATGGAAGTTTTC
ACAATGAGACAGGGCTGGTTCCTGGTCCCCATCTTTGCTCCTTTCCTTGGCACCATTGTC
GGCACGATGATCTACCAGTTAATGGTTGGTTTCCACGTGGAGGGAGAGGTTCGGGACAGG
AAAGAAGAA---------------GAGGAAAGAAATGTCAGGCTAACAAACGTTTCATCA
---------------------AAAGAGGAAAACAAAGATACCTAC---------------
>Inidan_medaka_Aqp3a
ATGAGTCGTCAAAAAATAATGTTGGACAAACTAGCGCGGACCTTTCAGATCCGCAACAAG
CTGCTTCGACAGTCTCTTGCAGAATGCCTCGGGACTCTCATCCTCGTGATGTTTGGCTGT
GGTGCTGTGGCACAGCTGGTGCTAAGTGGGGGCACCCACGCCCGTTTCATGACGGTTAAC
TTCGCGTTCGGCTTTGGGGCCACCTTAGGCATCCTGGTGTGTGGCCAGGTGTCAGGTGGC
CATCTGAATCCCGCAGTGACCTTTGCCCTCTGCTTGCTTGGAAGAGAGCGCTGGAGAAAG
TTCCCCATGTATTTCCTCTTTCAAACAATCGGAGCTTTCTTTGGGGCTGCCATCATTTTC
GGCATGTATTACGATGCATTGTGGGACTTTCCTGAGGCT---------------TTTAAC
ATGACTGATAGTAATCCCACGGCAGGCATCTTTGCCACTTACCCAGCAAAACATCTAACC
CTCCTCAATGGTTTCTTTGATCAGTTCATTGGCACAGCAGCATTGATAGTTTGCATTCTG
GCTATTGTGGATCCCTACAACAATCCCATCCCTCAAGGTCTTGAGGCCTTCACTGTGGGG
TTTGTGGTTTTGGTGATTGGGTTATCTATGGGCTTCAATTCCGGCTATGCAGTCAATCCT
GCCAGAGACCTTGGTCCTCGTATTTTCACCGCTATGGCCGGCTGGGGGGTGGAAGTTTTC
ACAATGAGACAGGGCTGGTTCCTGGTCCCCATCTTTGCTCCTTTCCTTGGCACCATTGTC
GGCACGATGATCTACCAGTTAATGGTTGGTTTCCACGTGGAGGGAGAGGTTCGGGACAGG
AAAGAAGAA---------------GAGGAAAGAAATGTCAGGCTAACAAACGTTTCATCA
---------------------AAAGAGGAAAACAAAGATACCTAC---------------
>Japanese_medaka_Aqp3a
ATGAGTCGTCAAAAGATCATATTGGACAAACTAGCGCGGACCTTTCAGATCCGCAACAAG
TTGCTTCGTCAGTCTCTTGCAGAATGCCTCGGGACCCTCATCCTTGTGATGTTTGGCTGC
GGTGCCTGTGCACAGCATGTGCTAAGTGAAGGCAGCCATGGCCGTTTCATGACGGTTAAC
TTTGCGTTCGGCTTTGGGGCCACCTTAGGCATCCTGGTGTGTGGCCAGGTGTCAGGCGGC
CATCTGAATCCTGCAGTGACCTTTGCCCTCTGCTTGCTTGGGAGAGAGCGCTGGAGAAAG
TTCCCCATGTATTTCCTCTTTCAAACAATTGGAGCTTTCTTTGGGGCTGCCATCATTTTT
GGCATGTATTATGATGCATTGTGGGACTTTCCTGAGAGT---------------TTTAAC

ATGAATGTGACCAGTCCCACTGCGGGCATCTTTTCCACTTACCCAGCAAAACATCTGACC
CTGCTTAATGGCTTCTTTGATCAGTTTATTGGCACATCAGCACTGATTGTTTGCATTCTG
GCTATTGTGGATCCCTACAATAATCCCATCCCTCAAGGGCTTGAGGCCTTCACTGTGGGA
TTTGTGGTTTTGGTGATTGGGTTGTCTATGGGCTTTAACTCTGGCTACGCAGTCAATCCT
GCCAGAGACCTCGGACCTCGTATTTTCACCGCCATGGCAGGCTGGGGGATGGAAGTTTTC
ACAGCCAGAGATGGGTGGTTCCTGATACCGATCTTTGCTCCTTTCCTTGGCGCCATTTTT
GGTACGATGATCTACCAGTTAATGGTTGGTTTCCATGTGGAGGGAGAAGTTCGTGACAAA
AGAGAAAAAATG------------GAGGAACAAAATGTCAAGCTAACAAATGTTACATCA
---------------------AAGGAGGACAACAAAGAACTCTAC---------------
>Javanese_ricefish_Aqp3a
ATGAGTCGTCAAAAGATGATATTGGACAAACTAGCGCGGACCTTTCAGATTCGCAACAAG
CTGCTTCGTCAGTCTCTTGCAGAATGCCTCGGGACCCTCATCCTTGTGATGTTTGGTTGT
GGTGCCGTGGCACAGCTGGTGCTAAGTGGCGGCACCCATGGCCGTTTCATGACGGTTAAC
TTTGCCTTCGGCTTTGGGGCCACCTTAGGCATCCTGGTGTGTGGCCAGGTGTCAGGTGGC
CATCTGAATCCCGCAGTGACCTTTGCCCTCTGCTTGCTTGGGAGAGAGCGCTGGAGAAAG
TTCCCCATGTATTTCCTCTTTCAAACAATCGGAGCTTTCTTTGGGGCTGCCATCATTTTC
GGCATGTATTACGATGCTTTGTGGGACTTTCCTGAGGCT---------------TTTAAC
ATGACTGTGAATAATCCCACGGCGGGCATCTTTGCCACTTACCCAGGAAAACATCTAACC
CTCCTCAATGGTTTCTTTGATCAGTTCATTGGCACAGCAGCACTGATAGTTTGCATTCTG
GCAATTGTGGATCCCTACAACAATCCCATCCCTCAAGGGCTTGAGGCCTTCACTGTGGGG
TTTGTGGTTTTGGTGATTGGGTTATCTATGGGCTTCAACTCCGGCTATGCAGTCAATCCT
GCCAGAGACCTCGGTCCTCGTCTTTTCACCTCTATAGCTGGCTGGGGGTCGGACGTTTTC
ACGATGAGACAGGGGTGGTTCCTGGTTCCCATCTTTGCTCCTTTCCTTGGCACCATTGTC
GGCGCGATGATCTACCAGTTAATGGTTGGTTTCCACGTGGAGGGAGAGGTTCGGGACAAG
CAAGAAGAA---------------GAGGATAAAAAAGTCAGACTAACGAATGTTACATCA
---------------------AAAGAGGAAAACAAAGAAACCTAC---------------
>Atlantic_silverside_Aqp3a
ATGGGTCGACAGAAGATATATCTGGACAAACTGGCCCGCACTTTCCAGATCCGCAACCTG
CTACTTCGCCAGGCCCTGGCAGAATGTCTCGGCACTCTCATCCTCGTGATGTTCGGCTGT
GGTGCTGTGGCCCAACTAGTCCTGAGTAAAGGTTCTCACGGCATGTTCCTGACTGTCAAT
TTTGCCTTTGGCTTTGCTGCCACTCTGGGCATTCTGGTCTGTGGCCAGGTATCAGGTGGA
CATCTGAACCCTGCAGTGACCTTTGCACTGTGCCTGCTTGGAAGAGAGCGCTGGAGAAAG
TTCCCCATGTTCTTCCTCTTTCAGACAATCGGTGCTTTTTTGGGCGCTGCTATCATTTTT
GGCATGTATTACGATGCCCTATGGGACTTTCCTGGGGCT---------------TTCAAT
GTGACTGGACCCAACGCCACAGCTGGCATCTTTGCCACCTATCCTGGAAAACATCTGACT
CTTGTCAATGGCTTCTTTGATCAGATTATTGGCACAGCAGCTCTGATCGTCTGCATTCTT
GCTATTGTGGATCCGTACAACAATCCCATTCCCCAAGGGTTGGAGGCCTTCACTGTGGGA
TTTGTGGTTTTGGTCATTGGACTGTCCATGGGCTTTAATTCTGGCTATGCCGTAAACCCA
GCTAGAGACCTTGGCCCACGTCTTTTCACTGCTATGGCTGGATGGGGGATTGACGTCTTC
---ATA---AATGGATGGTTCCTTGTACCGATTTTGGCCCCATTCCTGGGTACGATCGTT
GGCGTGATTATCTACCAGATGATGGTTGGCTTCCATGTGGAGGGAGAAGTACGCGATAGA
ACACAGAGA---------------GAAAAGGAGAATGTCCCACTAACGAATGTCAACTCT
---------------------AAAGAGGCCAGCAAAGAAATTATC---------------
>Clown_anemonefish_Aqp3a
ATGGGTAGACAGAAGGTGTATTTGGAAAAACTTGCCCGGTTCTTCCAGATCCGAAACCTG
CTGCTTCGTCAGGCCCTGGCAGAGTGTCTTGGTACTCTCATCCTTGTGATGTTTGGCTGT
GGCGCTGTGGCCCAGCTGGTGCTGAGTGGCGGTTCCCATGGCATGTTCCTCACTGTCAAC
TTTGCCTTCGGCTTTGCAGCCACTTTAGGGATCCTGGTGTGTGGCCAGGTATCAGGTGGC
CATCTGAACCCTGCAGTGACCTTTGCCCTTTGCCTGCTCGGAAGAGAGCGCTGGAGGAAG
TTCCCCATGTACTTCCTCTTTCAGACCATTGGTGCTTTTTTCGGTGCTGCCGTCATTTTT
GGCATGTACTACGATGCCCTGTGGGACCATCCTGGATGT---------------TTCAAT
GTGACTGGTCCTAAGGCAACAGCTGGCATCTTTGCTACCTACCCTGGAAGGCATCTCACT
CTTGTCAATGGCTTCTTTGATCAGATTATTGGCACAGCAGCCCTGATAGTTTGTATTCTG
GCTATTGTGGATCCATACAACAACCCCATCCCCCAGGGACTCGAGGCCTTCACTGTGGGG
TTTGTGGTTCTGGTCATTGGACTGTCTATGGGATTTAATTCTGGCTATGCTGTCAATCCT
GCCAGAGACCTCGGACCACGTCTTTTCACTGCCATGGCTGGATGGGGTGTTGAAGTTTTT
ACGGTTAGAAATGGCTGGTTCCTCGTGCCTGTTTTTGCCCCATTCCTCGGCACCATCATT
GGCGTGATGGTCTACCAATTAATGGTTGGTTTCCATGTGGAGGGAGAAGTACGGGACCAG
AAAGAGGGCACA------------GAGCAGGAGAACGTCCGACTGACCAATGTCCACTCC
---AACAACGACTCG------AAAGAGGCCAACAAAGAAATGCAC---------------
>Orange_clownish_Aqp3a

ATGGGTAGACAGAAGGTGTATTTGGAAAAACTTGCCCGGTTCTTCCAGATCCGAAACCTG
CTGCTTCGTCAGGCCCTGGCAGAGTGTCTTGGTACTCTCATCCTTGTGATGTTTGGCTGT
GGCGCTGTGGCCCAGCTGGTGCTGAGTGGCGGTTCCCATGGCATGTTCCTCACTGTCAAC
TTTGCCTTCGGCTTTGCAGCCACTTTAGGGATCCTGGTGTGTGGCCAGGTATCAGGTGGC
CATCTGAACCCTGCAGTGACCTTTGCTCTTTGCCTGCTCGGAAGAGAGCGCTGGAGGAAG
TTCCCCATGTACTTCCTCTTTCAGACCATTGGTGCTTTTTTTGGTGCTGCCGTCATTTTT
GGCATGTACTACGATGCCCTGTGGGACCATCCTGGATGT---------------TTCAAT
GTGACTGGTCCTAAGGCAACAGCTGGCATCTTTGCTACCTACCCTGGAAGGCATCTCACT
CTTGTCAATGGCTTCTTTGATCAGATTATTGGCACAGCAGCCCTGATAGTTTGTATTCTG
GCTATTGTGGATCCATACAACAACCCCATCCCCCAGGGACTCGAGGCCTTCACTGTGGGG
TTTGTGGTTCTGGTCATTGGACTGTCTATGGGATTTAATTCTGGCTATGCTGTCAATCCT
GCCAGAGACCTCGGACCACGTCTTTTCACTGCCATGGCTGGATGGGGTGTTGAAGTTTTT
ACGGTTAGAAATGGCTGGTTCCTCGTGCCTGTTTTTGCCCCATTCCTCGGCACCATCATT
GGCGTGATGGTCTACCAATTAATGGTTGGTTTCCATGTGGAGGGAGAAGTACGGGACCAG
AAAGAGGGCACA------------GAGCAGGAGAACGTCCGACTGACCAATGTCCACTCC
---AACAACGACTCA------AAAGAGGCCAACAAAGAAATGCAC---------------
>Twoband_anemonefish_Aqp3a
ATGGGTAGACACAAGGTGTATTTGGAAAAACTGGCCCGGTTCTTCCAGATCCGAAACCTG
CTGCTTCGTCAGGCCCTGGCAGAGTGTCTTGGTACTCTCATCCTTGTGATGTTTGGCTGT
GGTGCTGTGGCCCAGCTGGTACTGAGTGGCGGTTCTCATGGCATGTTCCTCACTGTCAAC
TTTGCCTTCGGCTTTGCAGCCACTTTAGGGATCCTGGTGTGTGGCCAGGTGTCAGGTGGC
CATCTGAACCCTGCAGTGACCTTTGCCCTTTGCCTACTCGGGAGAGAGCGCTGGAGGAAG
TTCCCCATGTACTTCCTCTTTCAGACCATTGGTGCTTTTTTCGGTGCTGCCGTCATTTTT
GGCATGTACTACGATGCCCTGTGGGACCATCCTGGATGT---------------TTCAAT
GTAACTGGTCCTAAGGCAACAGCTGGCATCTTTGCTACCTACCCTGGAAGGCATCTCACT
CTTGTCAATGGCTTCTTTGATCAGATTATTGGCACAGCAGCCCTGATAGTTTGTATTCTG
GCTATTGTGGATCCATACAACAACCCCATCCCCCAGGGACTGGAGGCCTTCACTGTGGGG
TTTGTGGTTCTGGTCATTGGACTGTCTATGGGATTTAATTCTGGCTATGCTGTCAATCCT
GCCAGAGACCTCGGACCACGTCTTTTCACTGCCATGGCTGGATGGGGTGTTGAAGTTTTT
ACGGTTAGAAATGGCTGGTTCCTCGTGCCTGTTTTTGCCCCATTCCTCGGCACCATCATT
GGCGTGATGGTCTACCAATTGATGGTTGGTTTCCATGTGGAGGGAGAAGTACGGGACCAG
AAAGAGGGCACA------------GAGCAGGAGAACGTCCGACTGACCAATGTCCACTCC
---AACAACGACTCG------AAAGAGGCCAACAAAGAAATGCAC---------------
>Spiny_chromis_Aqp3a
ATGGGTAGACAGAAGGTGTATTTGGACAAACTGGCCCGGTTCTTCCAGATCCGTAACCTG
CTGCTTCGTCAGGCCCTGGCAGAGTGTCTTGGTACTCTCATCCTTGTGATGTTTGGCTGT
GGTGCTGTGGCCCAGCTGGTACTGAGCGGCGGTTCCCATGGCATGTTCCTCACTGTCAAC
TTTGCCTTTGGCTTTGCAGCCACCTTAGGGATCCTGGTGTGTGGCCAGGTATCAGGTGGC
CATCTGAACCCTGCAGTGACCTTTGCCCTTTGTCTGCTTGGAAGAGAGCGCTGGAGGAAG
TTCCCCATGTACTTCCTCTTTCAGACCATTGGCGCTTTTTTCGGTGCTGCCATCATTTTT
GGCATGTACTACGATGCCCTGTGGGACCATCCTGGGAAT---------------TTCAAC
GTGACTGGTCCTAAGGCAACAGCTGGCATCTTTGCTACCTACCCTGGAAAGCATCTCACT
ATTGTCAATGGCTTCTTTGATCAGATTATTGGCACAGCAGCCCTGATAGTTTGTATTCTG
GCTATTGTGGATCCATACAACAACCCCATCCCCCAGGGACTGGAGGCTTTCACTGTGGGG
TTTGTGGTTCTGGTCATTGGGCTGTCTATGGGATTTAATTCTGGCTATGCTGTCAATCCT
GCCAGAGACCTCGGACCACGTATTTTCACTGCCATGGCTGGATGGGGTGTTGACGTTTTT
ACGGTTAGAAATGGCTGGTTCCTCGTGCCTATTTTTGCCCCGTTCCTCGGTACCATCATT
GGCGTGATGGTCTACCAATTGATGGTTGGTTTCCATGTGGAGGGAGAAGTACGCGACCAG
AAAGAGGGCACA------------GAGCAGGAGAACGTCCGACTGACCAATGTCCACTCC
---AACAATGACTCA------AAAGAGGCCAGCAAAGAAATGCAC---------------
>Bicolor_damselfish_Aqp3a
ATGGGTAGACAGAAGGTGTATTTGGACAAACTGGCCCGGTTCTTCCAGATCCGAAACCTG
CTGCTCCGTCAGGCCCTTGCAGAGTGTCTTGGTACTTTCATCCTTGTGATGTTTGGCTGT
GGTGCTGTGGCTCAGCTGGTATTGAGTTCAGGTTCCCATGGCATGTTCCTCACTGTCAAC
TTTGCCTTTGGCTTTGCAGCCACCCTAGGCATCCTGGTCTGTGGCCAGGTGTCAGGTGGC
CATTTGAACCCCGCGGTGACCTTTGCCCTGTGCCTGCTTGGAAGAGAGCGCTGGAGGAAG
TTCCCCATGTACTTCCTCTTCCAGACCATTGGTGCTTTTTTCGGTGCTGCTGTGATTTTC
GGCATGTACTACGATGCCTTGTGGGACCATCCTGGATGT---------------TTCAAT
GTGACTGGACCTACTGCCACAGCTGGCATCTTTGCTACCTACCCCGGAAAGCATCTCACG
CTTGTCAACGGCTTCTTTGATCAGATTATTGGCACAGCAGCCCTGATTGTTTGTATTCTG
GCTATTGTGGACCCATACAACAACCCCATCCCTCAAGGCCTGGAGGCCTTCACTGTGGGA

TTTGTGGTTCTGGTCATTGGATTGTCAATGGGCTTTAACTCTGGCTATGCTGTCAATCCT
GCCAGAGACCTCGGACCACGTCTTTTCACCGCCATGGCCGGGTGGGGTGTTGAAGTTTTC
ACGGTTAGAAATGGATGGTTCCTCGTGCCTATTTTTGCCCCCTTCCTTGGCACCATCATC
GGCGTGATGATCTACCAGTTGATGGTTGGCTTCCACGTGGAGGGAGAAGTACGAGACCAG
AAAGAGGGCACA------------GAGCAGGAGAATGTCCGACTGACCAACGTCCACTCC
---AACGACAACTCA------AAAGAGGCCACCAAAGAAATGCAC---------------
>Zebra_mbuna_Aqp3a2
ATGGGCAGACAAAAGGAGTATCTGGGTAAACTGTCCCGCTTCTTCCAGATCCGCAACCTG
CTGCTTCGTCAAGCCTTAGCAGAGTGTCTTGGCACGCTCATCCTTGTAATGTTTGGCTGT
GGTGCTGTGGCCCAGCGGGTGTTGAGCGGTGGTTCCCATGGCTTGTTCCTTACTGTCAAC
TTTGCCTTTGGCTTTGCTGCCATGTTAGGCATCTTGGTCTGTGGCCAAGTGTCAGGCGGC
CATCTCAACCCAGCAGTAACTTTTGCCTTGTGTCTGCTTGGAAGAGAGCGCTGGAGAAAG
TTTCCCATGTACTTCCTCTTTCAGACAATTGGTGCTTTTTTTGGTTCCGCTATTATTTTT
GGCATGTACTATGATGCTCTGTTGCTCCGTCCTGGAAGT---------------TTTAAT
TTTACTAGCATTAACAACACGGCTGGCATATTTTCAACTTACCCTGCGAGGCATCTCACA
CTTGTCAATGGTTTCTTTGATCAGATTATTGGCACAACAGCGCTCATAGTTTGTGTTCTG
GCCATTGTGGATCCTTACAACAACCCTATCCCCCAAGGGCTGGAAGCCTTCACTGTGGGA
TTTGTGGTTCTTGTCATTGGATTGTCCATGGGCTTTAATTCTGGCTATGCAGTCAATCCT
GCCAGAGACTTTGGACCACGTCTTTTCACCTCTATGTCTGGGTGGGGGGGTGCTGTTTTC
ACGGCTAGAGACTGCTGGTTCCTGGTCCCTATTTTTGCCCCATTCCTTGGTTCCATTCTT
GGCGTAGTGATTTACCAGTTGATGGTTGGTTTCCACACAGAGGGAGAAGCACGTGACAAG
AAACAGGGGACA------------GTCCAGCAGAACCTCCAACTTGCCAATGTTGCTTCC
---TCAAACAACTCT------AAAGAGGCTACCAAAGAAATTTAC---------------
>Eastern_happy_Aqp3a2
ATGGGCAGACAAAAGGAGTATCTGGGTAAACTGTCCCGCTTCTTCCAGATCCGCAACCTG
CTGCTTCGTCAAGCCTTAGCAGAGTGTCTTGGCACGCTCATCCTTGTAATGTTTGGCTGT
GGTGCTGTGGCCCAGCGGGTGTTGAGCGGTGGTTCCCATGGCTTGTTCCTTACTGTCAAC
TTTGCCTTTGGCTTTGCTGCCATGTTAGGCATCTTGGTCTGTGGCCAAGTGTCAGGCGGC
CATCTCAACCCAGCAGTAACTTTTGCCTTGTGTCTGCTTGGAAGAGAGCGCTGGAGAAAG
TTTCCCATGTACTTCCTCTTTCAGACAATTGGTGCTTTTTTTGGTGCCGCTATTATTTTT
GGCATGTACTATGATGCTCTGTTGCTCCGTCCTGGAAGT---------------TTTAAT
TTTACTAGCATTAACAACACGGCTGGCATATTTTCAACTTACCCTGCGAGGCATCTCACA
CTTGTCAATGGTTTCTTTGATCAGATTATTGGCACAACAGCGCTCATAGTTTGTGTTCTG
GCCATTGTGGATCCTTACAACAACCCTATCCCCCAAGGGCTGGAAGCCTTCACTGTGGGA
TTTGTGGTTCTTGTCATTGGATTGTCCATGGGCTTTAATTCTGGCTATGCAGTCAATCCT
GCCAGAGACTTTGGACCACGTCTTTTCACCTCTATGTCTGGGTGGGGGGGTGCTGTTTTC
ACGGCTAGAGACTGCTGGTTCCTGGTCCCTATTTTTGCCCCATTCCTTGGTTCCATTCTT
GGCGTAGTGATTTACCAGTTGATGGTTGGTTTCCACACAGAGGGAGAAGCACGTGACAAG
AAACAGGGGACA------------GTCCAGGAGAACCTTCAACTTGCCAATGTTGCTTCC
---TCAAACAACTCT------AAAGAGGCTACCAAAGAAATTTAC---------------
>Red_mwanza_Aqp3a2
ATGGGCAGACAAAAGGAGTATCTGAGTAAACTGTCCCGCTTCTTCCAGATCCGCAACCTG
CTGCTTCGTCAAGCCTTAGCAGAGTGTCTTGGCACGCTCATCCTTGTAATGTTTGGCTGT
GGTGCTGTGGCCCAGCGGGTGTTGAGCGGTGGTTCCCATGGCTTGTTCCTTACTGTCAAC
TTTGCCTTTGGCTTTGCTGCCATGTTAGGCATCTTGGTCTGTGGCCAAGTGTCAGGCGGC
CATCTCAACCCAGCAGTAACTTTTGCCTTGTGTCTGCTTGGAAGAGAGCGCTGGAGAAAG
TTTCCCATGTACTTCCTCTTTCAGACAATTGGTGCTTTTTTTGGTGCCGCTATTATTTTT
GGCATGTACTATGATGCTCTGTTGCTCCGTCCTGGAAGT---------------TTTAAT
TTTACTAGCATTAACAACACGGCTGGCATATTTTCAACTTACCCTGCGAGGCATCTCACA
CTTGTCAATGGTTTCTTTGATCAGATTATTGGCACAACAGCGCTCATAGTTTGTGTTCTG
GCCATTGTGGATCCTTACAACAACCCTATCCCCCAAGGGCTGGAAGCCTTCACTGTGGGA
TTTGTGGTTCTTGTCATTGGATTGTCCATGGGCTTTAATTCTGGCTATGCAGTCAATCCT
GCCAGAGACTTTGGACCACGTCTTTTCACCTCTATGTCTGGGTGGGGGGGTGCTGTTTTC
ACGGCTAGAGACTGCTGGTTCCTGGTCCCTATTTTTGCCCCATTCCTTGGTTCCATTATG
GGCGTAGTGATTTACCAGTTGATGGTTGGTTTCCACACAGAGGGAGAAGCACGTGACAAG
AAACAGGGGACA------------GTCCAGGAGAACCTCCAACTTGCCAATGTTGCTTCC
---TCAAACAACTCT------AAAGAGGCTACCAAAGAAATTTAC---------------
>Burtons_mouthbrooder_Aqp3a2
ATGGGCAGACAAAAGGAGTATCTGAGTAAACTGTCCCGCTTCTTCCAGATCCGCAACCTG
CTGCTTCGTCAAGCCTTAGCAGAGTGTCTTGGCACGCTCATCCTTGTAATGTTTGGCTGT
GGCGCTGTGGCCCAGCGGGTGTTGAGCGGTGGTTCCCATGGCTTGTTCCTTACTGTCAAC

TTTGCCTTTGGCTTTGCTGCCATGTTAGGCATCTTGGTCTGTGGCCAAGTGTCAGGCGGC
CATCTCAACCCAGCAGTAACTTTTGCCTTGTGTCTGCTTGGAAGAGAGCGCTGGAGAAAG
TTTCCCATGTACTTCCTCTTTCAGACAATTGGTGCTTTTTTTGGTGCCGCTATTATTTTT
GGCATGTACTATGATGCTCTGTTGCTCCGTCCTGGAAGT---------------TTTAAT
TTTACTAGCATTAACAACACGGCTGGCATATTTTCAACTTACCCTGCGAGGCATCTCACA
CTTGTCAATGGTTTCTTTGATCAGATTATTGGCACAACAGCGCTCATAGTTTGTGTTCTG
GCCATTGTGGATCCTTACAACAACCCTATCCCCCAAGGGCTGGAAGCCTTCACTGTGGGA
TTTGTGGTTCTTGTCATTGGATTGTCCATGGGCTTTAATTCTGGCTATGCAGTCAATCCT
GCCAGAGACTTTGGACCACGTCTTTTCACCTCTATGTCTGGGTGGGGGGGTGCTGTTTTC
ACGGCTAGAGACTGCTGGTTCCTGGTCCCTATTTTTGCCCCATTCCTTGGTTCCATTATT
GGCGTAGTGATTTACCAGTTGATGGTTGGTTTCCACACAGAGGGAGAAGCACGTGACAAG
AAACAGGGGACA------------GTCCAGGAGAACCTCCAACTTGCCAATGTTGCATCC
---TCAAACAACTCT------AAAGAGGCTACCAAAGAAATTTAC---------------
>Lyretail_cichlid_Aqp3a2
ATGGGCAGACAAAAGGAGTATCTGGATAAACTGTCCCGCTTCTTCCAGATCCGCAACCTG
CTGCTTCGTCAAGCCTTAGCAGAGTGTCTTGGCACGCTCATCCTTGTGATGTTTGGCTGT
GGCGCTGTGGCCCAGCGGGTGTTGAGCGGTGGTTCCCATGGCTTGTTCCTTACTGTCAAC
TTTGCCTTTGGCTTTGCTGCCATGTTAGGCATCTTGGTCTGTGGCCAAGTGTCAGGAGGC
CATCTCAACCCAGCAGTAACTTTTGCCTTGTGTCTGCTTGGAAGAGAGCGCTGGAGAAAG
TTTCCCATGTACTTCCTCTTTCAGACAATTGGTGCTTTTTTTGGTGCTGCTATTATTTAT
GGCATGTACTATGATGCTCTGTTGCTCCGTCCTGGAAGT---------------TTTAAT
TTTACTAGCATTAACAACACGGCTGGCATATTTTCAACTTACCCTGCGAGGCATCTCACA
CTTGTCAATGGTTTCTTTGATCAGATTATTGGCACAACAGCGCTCATAGTTTGTGTTCTG
GCCATTGTGGATCCTCACAACAACCCCATCCCCCAAGGGCTGGAAGCCTTCACTGTGGGA
TTTGTGGTTCTTGTCATTGGATTGTCCATGGGCTTTAATTCTGGCTATGCAGTCAATCCT
GCCAGAGACTTTGGACCACGTCTTTTCACCTCTATGTCTGGGTGGGGGGGTGCTGTTTTC
ACGGCTAGAGACTGCTGGTTCCTGGTCCCTATTTTTGTCCCATTCCTTGGCTCCATTCTT
GGCGTAGTGATTTACCAGTTGATGGTTGGTTTCCACACAGAGGGAGAAGCACGTGACAAG
AAACAGGGGACA------------TTCCAGGAGAACCTCCAACTCGCCAATGTTGCTTCC
---TCAAACAACTCT------AAAGAGGCTACCAAAGAACTTTAC---------------
>Nile_tilapia_Aqp3a2
ATGGGCAGACAAAAGGAGTATCTGGATAAACTGTCCCGCTTCTTCCAGATCCGCAACCTG
CTGCTTCGTCAAGCCTTAGCAGAGTGTCTTGGCACGCTCATCCTTGTAATGTTTGGCTGT
GGCGCTGTGGCCCAGCGGGTGTTGAGCGGTGGTTCCCATGGCTTGTTCCTTACTGTCAAC
TTTGCCTTTGGCTTTGCTGCCATGTTAGGCATCTTGGTCTGTGGCCAAGTGTCAGGCGGC
CATCTCAACCCAGCAGTAACTTTTGCCTTGTGTCTGCTTGGAAGAGAGCGCTGGAGAAAG
TTTCCCATGTACTTCCTCTTTCAGACAATTGGTGCTTTTTTTGGTTCTGCTATCATTTTT
GGCATGTACTATGATGCTTTGTTGCTCCGTCCTGGAAGT---------------TTTAAT
TTGACTAGCACTAACAACACGGCTGGCATATTTGCAACTTACCCTGCGAGGCATCTCACA
CTTGTCAATGGTTTCTTTGATCAGATTATTGGCACAACAGCACTCATAGTTTGTGTTCTG
GCCATTGTGGATCCTTACAACAACCCCATCCCCCAAGGGCTGGAGGCCTTCACTGTGGGA
TTTGTGGTTCTGGTCATTGGATTGTCTATGGGTTTTAATTCTGGCTATGCAGTCAATCCT
GCCAGAGACTTTGGACCACGTCTTTTCACCTCTATGTCTGGGTGGGGGGGTGCTGTTTTC
ACGGCTAGAGACTGCTGGTTCCTGGTCCCTATTTTTGCCCCATTCCTTGGCGCCATTCTT
GGTGTGCTGATTTACCAGTTGATGGTTGGTTTCCACACAGAGGGAGAAGCACGTGACAAG
AAGCAGGGGACA------------GTCCAGGAGAACCTCCAACTCGCCAATGTTGCTTCC
---TCAAACAACTCT------AAAGAGGCTACCAAAGAAATTTAC---------------
>Mozambique_tilapia_Aqp3a2
ATGGGCAGACAAAAGGAGTATCTGGATAAACTGTCCCGCTTCTTCCAGATCCGCAACCTG
CTGCTTCGTCAAGCCTTAGCAGAGTGTCTTGGCACGCTCATCCTTGTAATGTTTGGCTGT
GGCGCTGTGGCCCAGCGGGTGTTGAGCGGTGGTTCCCATGGCTTGTTCCTTACTGTCAAC
TTTGCCTTTGGCTTTGCTGCCATGTTAGGCATCTTGGTCTGTGGCCAAGTGTCAGGCGGC
CATCTCAACCCAGCAGTAACTTTTGCCTTGTGTCTGCTTGGAAGAGAGCGCTGGAGAAAG
TTTCCCATGTACTTCCTCTTTCAGACAATTGGTGCTTTTTTTGGTTCTGCTATCATTTTT
GGCATGTACTATGATGCTTTGTTGCTCCGTCCTGGAAGT---------------TTTAAT
TTGACTAGCACTAACAACACGGCTGGCATATTTGCAACTTACCCTGCGAGGCATCTCACA
CTTGTCAATGGTTTCTTTGATCAGATTATTGGCACAACAGCACTCATAGTTTGTGTTCTG
GCCATTGTGGATCCTTTCAACAACCCCATCCCCCAAGGGCTGGAGGCCTTCACTGTGGGA
TTTGTGGTTCTGGTCATTGGATTGTCTATGGGCTTTAACTCCGGCTACGCAGTCAATCCT
GCCAGAGACTTTGGACCACGTCTTTTCACCTCTATGTCTGGGTGGGGGGGTGCTGTTTTC
ACGGCTAGAGATTGCTGGTTCCTGGTCCCTATTTTTGCCCCATTCCTTGGCTCCATTCTT

GGTGTGGTGATTTACCAGTTGATGGTTGGTTTCCACACGGAGGGAGAAGCACGTGACAAG
AAGCAGGGGACA------------GTCCAGGAGAACCTCCAACTCACCAATGTTGCTTCC
---TCAAACAACTCT------AAAGAGGCTACCAAAGAAATTTAC---------------
>Blackchin_tilapia_Aqp3a2
ATGGGCAGACAAAAGGACTATCTGGAAAAACTGTCCTGCTTCTTCCAGATCCGCAACCTG
CTGCTTCGTCAAGCCCTGGCAGAGTGCCTTGGCACTCTCATCCTTGTGATGTTTGGCTGT
GGTGCTGTGGCCCAGCGGGTGTTGAGCGGTGGTTCCCATGGCTTGTTCCTTACTGGCAAC
TTTGCCTTCGGCTTTGCTGCCATATTAGGCATCTTGGTCTGTGGCCAAATATCAGGTGGC
CACCTCAACCCAGCAGTGACGTTTTCCTTGTGTCTGCTTGGAAGAGAGCGCTGGAGAAAG
TTTCCCATGTACTTCCTCTTTCAGACAATTGGTGCTTTTTTTGGTTCTGCAATTATTTAT
GGCATGTACTATGATGCTCTTTTGCTCCGTCCTGGAAGT---------------TTTAAT
GTGACTGGACCTAATAACACAGCTGGCATCTTTGCTACTTACCCTGCGAGGCATCTCACT
CTTGTCAACGGTTTCTTTGACCAGATTATTGGTACAACAGCACTGATAGTTTGTATTCTG
GCCATTGTGGATCCTTTCAACAATCCCATCCCTCAAGGGCTGGAGGCCTTCACTGTGGGA
TTTGTGGTTTTGGTCATTGGATTGTCTATGGGCTTTAATTCTGGCTATGCAGTGAATCCT
GCCAGAGACTTTGGACCACGTCTTTTCACTGCTATGTCTGGGTGGGGGGTTGCTGTTTTC
ACGGCTAGAGATTGCTGGTTCCTGGTCCCTATTTTTGCCCCATTCCTTGGCTCCATTCTT
GGTGTAATGATCTACCAGTTGATGGTTGGTTTCCACACGGAGGGAGAGGCGCGTGACAAG
AAACAGGGCACA------------GTCCAGGAGAATGTCCAACTTGCCAATGTTACATCC
---ACAAACAACTCT------AAAGAGGCTACCAAAGAAATTTAC---------------
>Midas_cichlid_Aqp3a2
ATGGGGAGGCAAAAGGAGTGTCTGGAAAAACTTTCCCGCTTCTTCCAGATCCGTAACCTG
CTGCTTCGTCAAGCCCTGGCAGAGTGTCTCGGCACTCTCATCCTTGTGATGTTTGGCTGT
GGTGCTGTGGCCCAGAGGGTGTTGAGTGGTGGTTCTCACGGCTTGTTCCTTACTGTCAAC
TTTGCCTTCGGCTTTGCTGCCATGTTAGGCATCTTGGTCTGTGGCCAAATATCAGGTGGT
CATCTCAACCCAGCAGTGACTTTTGCTTTGTGTCTGCTTGGAAGAGAGCGCTGGAGAAAG
TTCCCCATGTACTTCCTCTTTCAGACAATTGGYGCTTTTTTTGGYGCTGCTATTATTTTT
GGCATGTACTATGATGCCCTGTGGGACCATCCTGGAAGT---------------TTTAAT
GTGACTGGACCTAGAGCCACAGCTGGCATCTTTGCTACCTACCCTGCAAAGCATCTCACC
CTTGTCAACGGTTTCTTTGATCAGATTATCGGCACAGCAGCACTGATAGTTTGCATTCTA
GCTATTGTGGATCCTTATAACAACCCCATCCCCCAAGGGCTGGAGGCCTTCACTGTGGGA
TTTGTGGTTCTGGTCATTGGATTGTCTATGGGCTTTAATTCTGGCTATGCAGTAAATCCT
GCCAGRGACCTTGGACCGCGTCTTTTCACTGCCATGGCTGGGTGGGGGAGTGCCGTCTTC
ACGGCTAGAGACTGCTGGTTCTTGATCCCCATTTTTGTCCCATTCCTTGGCGCCACCCTT
GGTGTAATAATCTACCAGTTGATGGTTGGTTTCCACACGGAGGGAGAGGCCCGTGACAAG
AAAAAGCAGTCA------------GGCCAGGAGAATGTCCAACTCACCAATGTTGCTTCT
---AAAGACAACTCT------AAAGAGGCGTCCAAAGAAATATAC---------------
>Midas_cichlid_Aqp3a1
ATGGGGAGACAAAAGGAGTATCTGGATAAACTATCCCGCTTTTTCCAGATCCGTAACCTC
CTGCTTCGACAAGCCCTGGCAGAGTGCCTCGGCACTCTCATCCTTGTGATGTTTGGCTGT
GGTGCTGTGGCCCAGAGGGTGTTGAGTGGTGGTTCTCATGGCTTGTTCCTTACTGTCAAC
TTTGCCTTCGGCTTTGCTGCCATGTTAGGCATCTTGGTCTGTGGCCAAATATCAGGTGGC
CATCTCAACCCAGCAGTGACTTTTGCTTTGTGTCTGCTTGGAAGAGAGCGCTGGAGAAAG
TTCCCCATGTACTTCCTCTTTCAGACAATTGGCGCTTTTTTTGGCGCTGCTATTATTTTT
GGCATGTACTATGATGCCCTGTGGGACCATCCTGGAAGT---------------TTCAAT
GTGACTGGACCTAGAGCCACAGCTGGCATCTTTGCTACCTACCCTGGAAAGCATCTCAAC
CTTGTCAACGGTTTCTTTGATCAGATAATCGGCACAGCAGCACTGATAGTTTGCATTCTG
GCTATTGTGGATCCTTACAACAACCCCATCCCCCAAGGGCTGGAGGCCTTCACTGTGGGA
TTTGTGGTTCTGGTCATTGGATTGTCTATGGGCTTTAATTCTGGCTATGCAGTAAATCCT
GCCAGAGACCTTGGACCGCGTCTTTTCACTGCCATGGCTGGGTGGGGGAGTGCCGTTTTC
ACGGCTAGAGACTGCTGGTTCCTGGTCCCTATTTTTGCCCCATTCCTTGGCGCCCTCATT
GGTGTAATAATCTACCAATTAATGGTTGGTTTCCACACGGAGGGAGAGGCCCGTGACAAG
ACAGACGGCACA------------GCCCAGGAGAATGTCCGACTCACCAATGTTGCTTCT
---AAAGACAACTCT------AAAGAGGCGTCCAAAGAAATGCATGCTAGAAATGAGTGT
>Zebra_mbuna_Aqp3a1
ATGGGCTGGCAAAAGCATTATCTGGATAAACTGTCCCGCTTCTTCCAGATCCGCAACCTG
CTGCTTCGTCAAGCCCTGGCAGAGTGTCTTGGCACTCTCATCCTTGTGATGTTTGGCTGC
GGTTCTGTGGCCCAGTTGGTGTTGAGCGGTGGTTCCCATGGTATGTTCCTTACTGTCAAC
TTTGCCTTCGGCTTTGCTGCTACGTTAGGCATCTTGGTCTGTGGCCAAATATCAGGCGGC
CATCTCAACCCAGCAGTAACTTTTGCCTTGTGTCTGCTTGGAAGAGAGCGCTGGAGAAAG
TTTCCCATGTACTTCCTCTTTCAGACAATTGGTGCTTTCTTTGGTGCTGCTATTATTTTT

```
GGCATGTACTATGATGCCCTGTGGGACCATCCTGCAAGT---------------TTTAAT
GTGACTGGACCTGACGCCACAGCTGGCATCTTTGCTACTTATCCTGGAAACCATCTCACC
CTTGTCAACGGTTTCTTTGATCAGATTATTGGCACAGCAGCACTGATAGTTTGCATTCTG
GCCATTGTGGATCCTCACAACAACCCTATCCCCCAAGGGCTGGAGGCCTTCACTGTGGGA
TTTGTGGTTCTGGTCATTGGATTGTCTATGGGCTTTAATTCTGGCTATGCAGTGAATCCT
GCCAGAGACCTCGGACCACGTCTTTTCACCGCTATAGCTGGTTGGGGGAGTGAGGTTTTC
ACGGTTAGACAAGGCTGGTTCCTGGTCCCTGTTTTTGCTCCATTCCTCGGTACCTTCATT
GGTGTAATGATATACCAGCTGATGGTTGGTTTCCACATGGAAGGGGAAGTACGTGACAGG
AAAGAGAGCACA------------GAGCAGGAGACTGTCCGACTCACCAATGTGACTTCC
---AAAGACAACTCC------AGAGAGGCTGTGAAAGAA---------AGAAATGAGTGT
>Eastern_happy_Aqp3a1
ATGGGCTGGCAAAAGCATTATCTGGATAAACTGTCCCGCTTCTTCCAGATCCGCAACCTG
CTGCTTCGTCAAGCCCTGGCAGAGTGTCTTGGCACTCTCATCCTTGTGATGTTTGGCTGC
GGTTCTGTGGCCCAGTTGGTGTTGAGCGGTGGTTCCCATGGTATGTTCCTTACTGTCAAC
TTTGCCTTCGGCTTTGCTGCTACGTTAGGCATCTTGGTCTGTGGCCAAATATCAGGCGGC
CATCTCAACCCAGCAGTAACTTTTGCCTTGTGTCTGCTTGGAAGAGAGCGCTGGAGAAAG
TTTCCCATGTACTTCCTCTTTCAGACAATTGGTGCTTTCTTTGGTGCTGCTATTATTTTT
GGCATGTACTATGATGCCCTGTGGGACCATCCTGCAAGT---------------TTTAAT
GTGACTGGACCTGACGCCACAGCTGGCATCTTTGCTACTTATCCTGGAAACCATCTCACC
CTTGTCAACGGTTTCTTTGATCAGATTATTGGCACAGCAGCACTGATAGTTTGCATTCTG
GCCATTGTGGATCCTCACAACAACCCTATCCCCCAAGGGCTGGAGGCCTTCACTGTGGGA
TTTGTGGTTCTGGTCATTGGATTGTCTATGGGCTTTAATTCTGGCTATGCAGTGAATCCT
GCCAGAGACCTCGGACCACGTCTTTTCACCGCTATAGCTGGTTGGGGGAGTGAGGTTTTC
ACGGTTAGACAAGGCTGGTTCCTGGTCCCTGTTTTTGCTCCATTCCTCGGTACCTTCATT
GGTGTAATGATATACCAGCTGATGGTTGGTTTCCACATGGAAGGGGAAGTACGTGACAGG
AAAGAGAGCACA------------GAGCAGGAGACTGTCCGACTCACCAATGTGACTTCC
---AAAGACAACTCC------AGAGAGGCTGTGAAAGAA---------AGAAATGAGTGT
>Red_mwanza_Aqp3a1
ATGGGCTGGCAAAAGCATTATCAGGATAAACTGTCCCGCTTCTTCCAGATCCGCAACCTG
CTGCTTCGTCAAGCCCTGGCAGAGTGTCTTGGCACTCTCATCCTTGTGATGTTTGGCTGC
GGTTCTGTGGCCCAGTTGGTGTTGAGCGGTGGTTCCCATGGTATGTTCCTTACTGTCAAC
TTTGCCTTCGGCTTTGCTGCTACGTTAGGCATCTTGGTCTGTGGCCAAATATCAGGCGGC
CATCTCAACCCAGCAGTAACTTTTGCCTTGTGTCTGCTTGGAAGAGAGCGCTGGAGAAAG
TTTCCCATGTACTTCCTCTTTCAGACAATTGGTGCATTTAAAAAAAAACATCTTATTATT
TGGATCCCATTAGATGCCCTGTGGGACCATCCTGCAAGT---------------TTTAAT
GTGACTGGACCTGACGCCACAGCTGGCATCTTTGCTACTTATCCTGGAAACCATCTCACC
CTTGTCAACGGTTTCTTTGATCAGATTATTGGCACAGCAGCACTGATAGTTTGCATTCTG
GCCATTGTGGATCCTCACAACAACCCTATCCCCCAAGGGCTGGAGGCCTTCACTGTGGGA
TTTGTGGTTCTGGTCATTGGATTGTCTATGGGCTTTAATTCTGGCTATGCAGTGAATCCT
GCCAGAGACCTCGGACCACGTCTTTTCACCGCTATAGCTGGTTGGGGGAGTGAGGTTTTC
ACGGTTAGACAAGGCTGGTTCCTGGTCCCTGTTTTTGCTCCATTCCTCGGTACCTTCATT
GGTGTAATGATATACCAGCTGATGGTTGGTTTCCACATGGAAGGGGAAGTACGTGACAGG
AAAGAGAGCACA------------GAGCAGGAGACTGTCCGACTCACCAATGTGACTTCC
---AAAGACAACTCC------AGAGAGGCTGTGAAAGAA---------AGAAATGAGTGT
>Burtons_mouthbrooder_Aqp3a1
ATGGGCTGGCAAAAGCATTATCTGGATAAACTGTCCCGCTTCTTCCAGATCCGCAACCTG
CTGCTTCGTCAAGCCCTGGCAGAGTGTCTTGGCACTCTCATCCTTGTGATGTTTGGCTGC
GGTTCTGTGGCCCAGTTGGTGTTGAGCGGTGGTTCCCATGGTATGTTCCTTACTGTCAAC
TTTGCCTTCGGCTTTGCTGCTACGTTAGGCATCTTGGTCTGTGGCCAAATATCAGGCGGC
CATCTCAACCCAGCAGTAACTTTTGCCTTGTGTCTGCTTGGAAGAGAGTGCTGGAGAAAG
TTTCCCGTGTTCTTCCTCTTTCAGACAATTGGTGCTTTCTTTGGTGCTGCTATTATTTTT
GGCATGTACTATGATGCCCTGTGGGACCATCCTGCAAGT---------------TTTAAT
GTGACTGGACCTGATGCCACAGCTGGCATCTTTGCTACTTATCCTGGAAACCATCTCACC
CTTGTCAACGGTTTCTTTGATCAGATTATTGGCACAGCAGCACTGATAGTTTGCATTCTG
GCCATTGTGGATCCTCACAACAACCCCATTCCCCAAGGGCTGGAGGCCTTCACTGTGGGA
TTTGTGGTTCTGGTCATTGGATTGTCTATGGGCTTTAATTCTGGCTATGCAGTGAATCCT
GCCAGAGACCTCGGACCACGTCTTTTCACCGCTATAGCTGGTTGGGGGAGTGAGGTTTTC
ACGGTTAGACAAGGCTGGTTCCTGGTCCCTGTTTTTGCTCCATTCCTTGGTACCTTCATT
GGTGTAATGATATACCAGCTGATGGTTGGTTTCCACATGGAAGGGGAAGTACGTGACAGG
AAAGAGAGCACA------------GAGCAGGAGACTGTCCGACTCACCAATGTGACTTCC
---AAAGACAACTCC------AGAGAGGCTGTGAAAGAA---------AGAAACGAGTGT
```

>Lyretail_cichlid_Aqp3a1
ATGGGCTGGCAAAAGCATTATCTGGATAAACTGTCCCGCTTCTTCCAGATCCGCAACCTG
CTGCTTCGTCAAGCCCTGGCAGAGTGTCTTGGCACGCTCATCCTTGTGATGTTTGGCTGC
GGTTCTGTCGCCCAGTTGGTGTTGAGCGGTGGTTCCCATGGTATGTTCCTTACTGTCAAC
TTTGCCTTCGGCTTTGCTGCTACGTTAGGCATCTTGGTCTGTGGCCAAATATCAGGCGGC
CATCTCAACCCAGCAGTAACTTTTGCCTTGTGTCTGCTTGGAAGAGAGCGCTGGAGAAAG
TTTCCCATGTACTTCCTCTTTCAGACAATTGGTGCTTTTTTTGGTGCTGCTATTATTTTT
GGCATGTACTATGATGCCCTGTGGGACCATCCTGCAAGT---------------TTTAAT
GTGACTGGACCTGACGCCACAGCCGGCATCTTTGCTACTTATCCTGGAAACCATCTCACC
CTTGTCAACGGTTTCTTTGATCAGATTATTGGCACAGCAGCACTGATAGTTTGCATTCTG
GCCATTGTGGATCCTCACAACAACCCCATCCCCCAAGGGCTGGAGGCCTTCACTGTGGGA
TTTGTGGTTCTGGTCATTGGATTGTCTATGGGCTTTAATTCTGGCTATGCAGTGAATCCT
GCCAGAGACCTTGGACCACGTCTTTTCACCGCTATAGCTGGTTGGGGGAGTGAGGTTTTC
ACGGTTAGACAAGGCTGGTTCCTGGTCCCTGTTTTTGCTCCATTCCTTGGTACCTTCATT
GGTGTAATGATATACCAGCTGATGGTTGGTTTCCACATGGAAGGGGAAGTACGTGACAGG
AAAGAGAGCACA------------GAGCAGGAGACTGTCCGACTCACCAATGTGACTTCC
---AAAGACAACTCC------AGAGAGGCTGTGAAAGAA---------AGAAATGAGTGT
>Nile_tilapia_Aqp3a1
ATGGGCTGGCAAAAGCATTATCTGGATAAACTGTCCCGCTTCTTCCAGATCCGCAACCTG
CTGCTTCGTCAAGCCCTGGCAGAGTGTCTTGGCACTCTCATCCTTGTGATGTTTGGCTGT
GGTTCTGTGGCCCAGTTGGTGTTGAGCGGTGGTTCTCATGGTATGTTCCTTACTGTCAAC
TTTGCCTTCGGCTTTGCTGCTACGTTAGGCATCTTGGTCTGTGGCCAAATATCAGGCGGC
CATCTCAACCCAGCAGTAACTTTTGCCTTGTGTCTGCTTGGAAGAGAGCGCTGGAGAAAG
TTTCCCATGTACTTCCTCTTTCAGACAATTGGTGCTTTTTTTGGTGCTGCTATTATTTTT
GGCATGTACTATGATGCCCTGTGGGACCATCCTGGAAGT---------------TTTAAT
GTGACTGGACCTGACGCCACAGCTGGCATCTTTGCTACTTATCCTGGAACCCATCTCACC
CTTGTCAATGGTTTCTTTGATCAGATTATTGGCACAGCAGCACTGATAGTTTGCATTCTG
GCCATTGTGGATCCTTACAACAACCCCATCCCTCAAGGGCTGGAGGCCTTCACTGTGGGA
TTTGTGGTTCTGGTCATTGGATTGTCTATGGGCTTTAATTCTGGCTATGCAGTCAATCCT
GCCAGAGACCTCGGACCACGTCTTTTCACCGCTATAGCTGGTTGGGGGAGTGAGGTTTTC
ACGGCTAGCCCAGGCTGGTTCCTGGTCCCTGTTTTTGCTCCATTCCTTGGTACCCTCATT
GGTGTAATGATCTACCAGCTGATGGTTGGTTTCCACATGGAAGGGGAAGTACGTGACAGA
AAAGAGAGCACA------------GAGCAGGAGACTGTCCGACTCACCAATGTGACTTCC
---AAAGACAACTCC------AGAGAGGCTGTGAAAGAA---------AGAAATGAGTGT
>Indian_glassy_fish_Aqp3a
ATGGGTAGGCAGAAGGTGTATTTGGACAAACTGGCTCGGCTCTTCCAGATCCGCAACCTG
TTGCTCCGTCAGGCCCTTGCAGAGTGTCTTGGCACTCTCATCCTTGTGATGTTTGGCACT
GGTTCTGTGGCACAGCTGGTATTGAGCGGTGGTAGTCATGGCATGTTCCTCACTGTCAAT
TTTGCCTTTGGCTTCGCCGCAACTTTGGGCATCCTGGTCTGTGGCCAAGTATCAGGTGGT
CATCTGAACCCTGCAGTCACTTTCGCCCTGTGTCTTCTTGGAAGGGAGCGCTGGAGAAAA
TTCCCTATGTACTTCCTCTTTCAGACTATTGGTGCTTTCTTTGGTGCCGCCATCATTTTT
GCCATGTACTACGATGCCTTATGGGACCTTCCTTCAAGT---------------TTCAAC
ATGACTGATCCTACATTAACAGCAGGTATCTTCGCTACCTACCCTGCAAAGCATCTCACC
CTTCTCAATGGCATCTTTGACCAGATCATTGGCACAGCATCGCTGATTGTCTGTATTTTG
GCTATTGTGGATCCATACAACAACCCCATCCCCCAGGGCCTGGAGGCCTTCACTGTAGGG
TTTGTGGTTCTGGTCATTGGATTGGCTATGGGCTTTAATTCTGGCTATGCTGTCAATCCT
GCCAGAGACCTCGGACCACGTGTTTTCACTGCTATGGCTGGATGGGGGGGTGAAGTCTTC
ACGTCGGGGAATGGCTGGTTCCTGGTGCCAGTATTTGCCCCATTCTTCGGCACCCTGATT
GGTGTGATTATTTATCAGCTGATGGTGGGTTTTCATGTGGAGGGAGAAGTGCGCGACAGG
AAAGAGAGGACA------------GAGCACGAGAACGTCCGACTCACCAACGTCACATCC
---AATGACAACGCC------AAAGAGGTCGGCAAAGAAATGCAC---------------
>Peacock_blenny_Aqp3a
ATGGGCCGCCAAAAGACGTACTTGGATAAGCTCTCCCGGTTCTTCCAGATCCGAAACCTG
CTGCTTCGCCAGGCCCTGGCAGAGTGTCTGGGCACTCTCATCCTTGTGATGTTTGGCTGC
GGTGCAGTGGCACAGCTGGTGTTGAGCGGCGGCTCCCATGGCATGTTCCTCACTGTCAAC
TTTGCTTTTGGCTTCGCTGCTACCTTAGGAATTCTGGTCTGCGGTCAGGTATCAGGTGGA
CATTTGAACCCTGCCGTGACCTTTGCCCTGTGTCTTCTTGGGCGAGAGCGTTGGAGGAAG
TTCCCCACCTACTTTCTCTTTCAGACGATTGGAGCCTTTTTCGGTGCTGCTATCATTTTT
GGCATGTACTACGACGCCCTGTGGGACCACGAGGGTGCT---------------TTTAAC
GTGACCGGACCCAAAGCAACAGCTGGCATCTTTGCTACATACCCTGGAAAACATCTCACT
CTCGTCAATGCCTTCTTTGATCAGATCATTGGCACAGCAGCACTGATCGTGTGTATCCTG

GCTATTGTGGATCCATACAACAACCCCGTCCCACAAGGGTTAGAGGCCTTCACCGTGGGA
TTTGTGGTTCTGGTGATTGGACTGTCGATGGGCTTCAATTCTGGATACGCAGTGAATCCC
GCCAGGGACCTCGGGCCGCGTATTTTTACCGCTTTAGCCGGGTGGGGGACTGACGTTTTC
ACGGCGAGAAACGGCTGGTTCCTGGTGCCCATTTTTGCCCCGTTCCTCGGCACCATCATC
GGCGTGATGATCTACCAGCTGATGGTCGGTTTCCACGTGGAGGGAGAAGTACGCGACCGC
AAAGAAGGCACA------------GCGCAGGAGAACGTTCGACTCACCAACGTCACCTCA
---AAGGACAACCCC------AAAGAGACCAACAAAGAAATGCAC---------------
>Rockpool_blenny_Aqp3a
ATGGGCCGACACAAGACGTACTTGGATAAGCTGTCCCGGTTCTTCCAGATCCGAAACCTG
CTGCTTCGCCAGGCCCTGGCAGAGTGTCTCGGCACTCTCATCCTCGTGATGTTTGGCTGC
GGTGCAGTGGCACAGCTGGTGTTGAGCGGCGGCTCCCATGGCATGTTCCTCACTGTCAAC
TTTGCTTTTGGCTTCGCTGCCACCTTAGGAATTCTGGTCTGCGGTCAGGTATCAGGTGGA
CATTTGAACCCTGCCGTGACCTTTGCCCTTTGCCTTCTTGGAAGAGAGCGCTGGAGGAAG
TTCCCCACCTACTTTCTCTTTCAGACAGTTGGTGCCTTTTTTGGTGCTGCTGTCATTTTT
GGCATGTACTACGACGCTCTGTGGGACCACGAGGGTGCT---------------TTTAAT
GTGACCGGACCCAAAGCAACAGCTGGCATCTTTGCTACATACCCTGGAAAACATCTCACT
CTTGTCAACGCCTTCTTTGATCAGATCATTGGCACAGCAGCACTAATTGTGTGTATCCTG
GCTATTGTGGATCCATACAACAACCCCATCCCACAAGGGTTAGAGGCCTTCACCGTGGGA
TTTGTGGTTCTGGTGATCGGACTGTCGATGGGTTTCAATTCTGGATATGCAGTGAATCCC
GCCAGGGACCTCGGGCCGCGTCTTTTTACCGCTATGGCCGGGTGGGGGACTGAAGTTTTC
ACGGCGAGAAATGGTTGGTTCCTGGTGCCCATTTTTGCCCCATTCCTCGGGACCATCATT
GGCGTGATGATCTATCAGCTGATGGTCGGTTTCCACGTGGAGGGAGAAGTACGCGACCGC
AAAGAAGGCACA------------GAGCAGGAGAACGTTCGACTCACCAACGTCACCTCA
---AACGACAACCCC------AAAGAGACCAACAAAGAAATGCAC---------------
>Blunt_snouted_clingfish_Aqp3a
ATGGGTTTACATAAGACATACTTGGACAAACTGTCTCGTTTCTTCCAGATCCGTAACCTG
CTTCTCCGCCAGGCCTTGGCAGAGTGTCTAGGGACTCTCATCCTTGTGATGTTTGGTTGT
GGTGCGGTGGCCCAGTTAGTATTGAGCGGCGGTTCCCACGGCATGTTCCTCACTGTAAAT
TTCGCCTTCGGCTTTGCTGCAACCTTGGGCATTCTAGTATGCGGTCAGGTATCAGGAGGT
CATCTGAACCCTGCAGTCACCTTTGCCCTTTGCCTGCTGGGAAGAGAGCGCTGGAGGAAG
TTCCCCACCTATTTTCTTTTTCAGACGATTGGTGGCTTTTTTGGTGCTGCCATAATCTTT
GGCATGTATTATGATGCCTTGTGGGATCATCCTGGAGCT---------------TTTGAT
GTGACTGGGCCAAACGCGACGGCTGGTATCTTTGCCACCTACCCTGGAAAACATCTCACT
CTCGTCAACGCCTTTTTTGACCAGATCATTGGCACTGCAGCACTCATAGTTTGCATTCTT
GCCATTGTGGATCCATACAACAACCCCATTCCTCAGGGGCTGGAAGCCTTCACTGTTGGA
TTTGTAGTTCTGGTCATTGGATTGTCCATGGGCTTCAACTCTGGCTATGCAGTAAACCCT
GCTAGAGATCTTGGACCACGTCTGTTCACCGCCATAGCTGGGTGGGGCACTGATGTTTTC
ACGGCGAGGAAATGCTGGTTCCTGGTGCCTATTTTTGCTCCATTCCTTGGCACCATCATT
GGTGTGATGATTTACCAGGTGATGGTTGGCTTTCATGTGGAGGGAGAGGTACGCGATCGC
AAAGAGGGAATAACG---------GAGCACGAGAACGTCCGACTTACCAACGTCACCTCT
---AACGACAACCCC------AAAGAAGAAAACAAGGAGAAGAGC---------------
>Tigertail_seahorse_Aqp3a
ATGGGCAGACAAAAGATCTACTTGGAGAAACTGTCCACGACCTTCCAGATCCGCAACCTG
CTGCTTCGCCAGGCCTTGGCAGAATGTCTGGGCACCCTCATCCTCGTGATGTTTGGCTGC
GGTGCCGTGGCCCAATTGGTGCTGAGTAGTGGTTCTCATGGTTTGTTTCTGACTGTCAAC
TTTGCCTTTGGCTTTGCGGCCACCTTGGGGATTCTGGTCTGTGGCCAAATATCGGGAGGA
CATCTGAACCCCGCAGTGACGTTTGCCCTATGCCTGCTCGGGAGAGAACGCTGGAGAAAG
TTCCCCATGTACTTCCTCTTTCAAACCATCGGCGGTTTCTTGGGTGCAGCCATCATTTTT
GGCATGTACTATGATGCCCTGTGGGACCGTCCCGGTGCG---------------TTCAAT
GTCACTGGCCCGAATGCCACAGCTGGAATATTTGCCACCTATCCTGGAAAACATCTCACT
CTTGTCAACGGCTTCTTTGACCAGATTATTGGCACGGCGGCACTGATCGTTTGTATCCTG
GCCATCGTTGATCCCTACAATAATCCCATCCCACAAGGATTGGAGGCCTTCACTGTGGGC
TTTGTGGTTCTGGTCATCGGCCTGTCGATGGGCTTCAACTCGGGCTACGCAGTCAATCCC
GCCAGAGACCTCGGACCTCGTCTTTTTACCGCAATGGCCGGCTGGGGAGTAGAAGTCTTC
ACCGTCAGAAACGGTTGGTTCCTGGTGCCGGTTTTCGCCCCCTTCCTCGGCACCTTCATC
GGCGTGGTCATCTACCAAATGATGGTCGGCTTCCACGAGGAGGGAGAAGTACGTGACCGC
AGGAACGCCGAG------------GCGGAGGAGAACTTGCGGCTCACCAGCGTCGCTGCT
---AACGACAAGTCG------AAAGAGGCCACCAAACAAGTGCAC---------------
>Gulf_pipefish_Aqp3a
ATGGGTAGACAAAAGATTTATTTGGAAAAATTGTCCAAGACCTTCCAGATCCGCAACCTA
CTGCTTCGCCAGGCTTTGGCAGAATGTCTGGGCACCCTCATCCTTGTGATGTTCGGCTGC

GGTGCCGTGGCCCAGTTGGTGTTGAGTAGTGGTTCTCATGGTCTGTTCTTGACGGTGAAC
TTTGCCTTCGGCTTTGCGGCCACCTTAGGAATCCTGGTCTGCGGCCAAATATCAGGAGGA
CATCTGAACCCCGCAGTGACCTTTGCCCTCTGCTTGCTTGGGAGAGAACGCTGGAGAAAG
TTTCCAATGTACTTCCTTTTTCAGACAATCGGCGGTTTCTTTGGTGCGGCCATCATTTTT
GGAATGTATTATGATGCCCTGTGGGACCATCCTGGTGCG---------------TTCAAT
GTCACTGGCCCAAATGCCACAGCTGGAATCTTTGCCACCTATCCCGGAAAACATCTCACC
CTTGTCAACGGCTTCTTTGATCAGATTATTGGCACGGCAGCACTGATTGTTTGCATCCTG
GCCATTGTGGATCCCTACAATAATCCCATCCCACAAGGACTGGAGGCCTTCACTGTGGGC
TTTGTGGTTCTGGTCATTGGCCTGTCTATGGGATTCAACTCTGGCTACGCCGTCAATCCC
GCCAGAGACCTCGGACCTCGTCTTTTTACCGCAATGGCCGGATGGGGTGTTGAAGTCTTC
ACTGTCCGAAACGGTTGGTTCCTGGTGCCCGTTTTTGCCCCCTTCCTGGGCACCATCATC
GGCGTGATCATCTACCAAATGATGGTCGGCTTCCACGAGGAGGGCGAAGTACGCGACAAG
AGGAACGCAGAC------------GCCGAGGAGAACCTGCGACTCACCAACGTCGCCGCC
---AACGACAAATCG------ACAGAGGCCACCAAACAAGTGCAT---------------
>Ballan_wrasse_Aqp3a
ATGGGCAGACAGAAGGTGTATTTGGACAAACTTTCCCGGTTCTTCCAGATCCGTAACTTG
CTGCTTCGCCAGGCCCTGGCAGAGTGTCTTGGCACCCTCGTCCTTGTGATGTTTGGCTGT
GGTGCTGTGGCCCAGTTGGTGTTGAGTGGTGGTTCTCATGGCATGTTCTTTACAGTCAAC
TTTGCCTTTGGCTTTGCTGCCACTTTAGGTATCCTGGTCTGTGGCCAAGTATCAGGTGGC
CATCTGAACCCTGCAGTGACCTTTGCCCTGTGTCTGCTCGGAAGAGAGCGCTGGAGAAAG
TTCCCCATGTTCTTCCTCTTTCAGACAATCGGTGCTTTCTTTGGTGCTGCTGTCATTTTT
GGCATGTACTATGATGCACTGTGGGATCATCCTGGAAGC---------------TTCAAT
GTGTATGGACCTAATGCCACAGCGGGAATTTTTGCTACCTACCCTGGAAAACATCTAACA
CTTGTTAATGGATTCTTTGATCAGATAATTGGCACAGCAGCACTCATAGTTTGCATTCTG
GCCATCGTGGATCCATACAACAACTCCATTCCCCAGGGGCTGGAGGCCTTCACTGTGGGA
TTTGTGGTTCTGGTTATTGGACTGTCGATGGGCTTTAACTCTGGCTATGCTGTCAATCCT
GCCAGAGACCTTGGACCACGTATTTTCACCGCAATTGCTGGCTGGGGCAGTGAAGTTTTC
ACGGTTAGAAATGGCTGGTTCCTGGTGCCTTTGTGTGCCCCGTTCATCGGCACCATCATC
GGTGTGATGATCTATCAGCTGATGGTCGGCTTCCATCAGGAGGGAGAAGCTCGGGACACA
AAGTGCGCA---------------GAGCAGGAGAGCGTACGACTCACCAATGTCAACGCC
---AAGGGCAACTCC------AACGACGCAACCAAAGAAGTGTAC---------------
>Corkwing_Aqp3a
ATGGGCAGACATAAGATGTATTTGGACAAACTGTCCCGGTTCTTCCAGATCCGTAACCTG
CTGCTTCGCCAAGCCCTGGCAGAGTGTCTTGGAACCCTCATCCTTGTGATGTTTGGCTGT
GGTGCTGTGGCCCAGCTGGTGTTGAGTGGTGGTTCTCATGGCATGTTCCTTACAGTCAAC
TTTGCCTTCGGCTTTGCTGCCACCTTAGGTATCCTGGTCTGTGGCCAAGTATCAGGTGGC
CATCTGAACCCTGCAGTGACCTTTGCCCTGTGTTTACTCGGAAGGGAGCGCTGGAGAAAG
TTCCCCATGTTCTTCCTCTTTCAGACAATCGGTGCTTTCTTTGGAGCTGCTATCATTTTT
GGCATGTACTACGATGCATTGTGGGATCATCCTGGATGC---------------TTCAAT
GTTACCGGACCTAAGGCCACAGCGGGAATTTTTGCTACCTACCCTGGAAAACATCTAACA
CTTGTTAATGGATTTTTTGATCAGATAATTGGCACAGCAGCACTAATAGTTTGCATTCTG
GCCATCGTGGATCCATACAACAACTCCATTCCCCAGGGACTGGAGGCCTTCACTGTGGGA
TTTGTGGTTCTGGTTATTGGACTGTCGATGGGCTTTAACTCTGGTTATGCTGTCAATCCT
GCCAGAGACCTCGGACCACGTATTTTCACCGCAATAGCTGGCTGGGGCAGTGAAGTTTTC
ACGGTTAGAAAAGGCTGGTTCCTGGTGCCTGTGTTCGCCCCGTTCCTCGGCACCATCATC
GGCGTGATGATTTACCAGCTGATGGTCGGCTTCCATCAGGAGGGAGAATATCGGGACAGT
AAGTGCGCA---------------GAGGAGGAGAACGTGCGCCTTACCAACGTCAATGCC
---AACACCAACCCC------AAAGAACCAACCAAAGAAATGTAC---------------
>Marbled_flounder_Aqp3a
ATGGGCAGACAGAAGGTGTATCTGGACAAACTGTCCCGCTTCTTCCAGATCCGCAACCTG
TTGCTTCGGCAGGCGCTAGCAGAGTGTCTTGGCACCCTCATCCTAGTGATGTTTGGCTGT
GGTGCTGTGGCCCAGCTGGTGTTGAGCGGCGGTTCCCATGGCATGTTCCTCACCGTCAAC
TTTGCCTTTGGCTTTGCTGCCACCTTAGGCATCCTGGTCTGTGGACAAGTATCAGGAGGC
CATCTGAACCCTGCAGTGACTTTTGCCCTGTGTCTACTTGGAAGAGAACCCTGGAAGAAA
TTCCCCATGTATTTCCTCTTTCAGACAATCGGCGCTTTCTTTGGTTCTGCCATCATTTTC
GCCATGTACTACGATGCTCTGTGGGACCATCCCGGAAAT---------------TTCCAT
GTGAGTGGTCCTAATGCCACAGCTGGCATCTTTGCCACGTACCCTGGAAAACATCTCACC
ATCGTCAATGGCTTCTTTGATCAGATAATTGGCACGTCAGCGCTAATCGTTTGTATTCTG
GCTATCGTGGACCCAAACAACAACTCCATCCCCCAGGGGCTGGAGGCCTTCACTGTGGGA
TTTGTGGTCCTGGTTATTGGACTGTCAATGGGCTTTAACTCTGGATATGCTGTCAATCCT
GCCAGAGACCTCGGACCACGTATTTTCACTGCGATGGCTGGGTGGGGCATTGAGGTCTTC

ACGGCGAGAAACGGCTGGTTCCTTGTGCCCATATTCGCCCCGTTCCTCGGCACCATCATC
GGCGTGATGATCTACCAGCTGATGGTTGGCTTCCACGTGGAAGGAGAAGCACGTGACAGA
AAAAGCGCAGAG------------CAGTCAGAAAACGTCCGACTCACCAACGTCGACTCC
---AAC---CATGCC------AAGGAAGTG------GAC---TGT---------------
>Japanese_flounder_Aqp3a
ATGGGCAGACAGAAGGTGTATTTGGACAAACTGTCCCGCTTCTTCCAGATCCGCAACCTG
TTGCTTCGGCAGGCGCTGGCAGAGTGTCTTGGCACCCTCATCCTTGTGATGTTTGGCTGC
GGTGCTGTGGCCCAGCTGGTGTTGAGCAGCGGTTCCCATGGCATGTTCCTCACTGTCAAC
TTTGCCTTCGGCTTTGCTGCCACCTTAGGCATCCTGGTCTGTGGTCAAGTATCAGGTGGC
CATCTGAACCCCGCAGTGACTTTTGCCCTGTGTCTACTTGGAAGAGAGCCCTGGAAGAAA
TTCCCAATGTACTTCCTCTTTCAGACAATCGGTGCTTTCCTTGGTTCTGCCATCATTTTT
GGCATGTACTACGACGCTCTGTGGGACCATCCTGGAAAT---------------TTCAAT
GTGACTGGTCCTAGAGCCACAGCTGGCATCTTTGCCACCTACCCTGGAAAGCATCTCACT
ATTGTCAATGGCTTCTTTGATCAGATAATTGGCACAGCAGCCCTAATCGTTTGTATTCTG
GCTATCGTGGACCCATACAACAACCCCATCCCCCAGGGGCTGGAGGCCTTCACTGTGGGA
TTTGTGGTTCTGGTTATTGGACTGTCTATGGGCTTTAACTCTGGATATGCTGTCAATCCT
GCCAGAGACCTTGGACCACGTATTTTCACTGCAATGGCTGGGTGGGGCACTGAGGTCTTC
ACGACGAGAAACGGCTGGTTCCTTGTGCCCATTTTTGCCCCGTTCCTTGGCACCATCATC
GGTGTGATGATCTACCAGCTGATGGTTGGCTACCACATGGAGGGAGAAGCACGTGACAGA
AAGAGCCAG---------------GAGCAGGAAAACGTCGGACTCACCAACGTCAACACC
---AAC---AACGCC------AAAGACGGG------------CAC---------------
>Turbot_Aqp3a
ATGGGCAGACAGAAGGTGTATTTGGACAAATTGTCCCGGTTCTTCCAGATCCGTAACCTG
TTGCTTCGGCAGGCTCTGGCAGAGTGTCTCGGCACCCTCATCCTTGTGATGTTTGGCTGC
GGTGCTGTGGCCCAGCTGGTTTTGAGCGGTGGTTCTCATGGCATGTTCCTCACTGTCAAC
TTTGCATTCGGCTTTGCTGCCACCTTAGGCATCCTGGTCTGTGGCCAGGTATCAGGTGGC
CATCTGAACCCCGCCGTGACCTTTGCCCTCTGTCTTCTCGGAAGAGAGCCCTGGAAAAAA
TTCCCCATGTACTTCCTCTTTCAGACAATCGGTGCTTTCTTTGGATCGGCCATCATTTTT
GGCATGTACTACGATGCCCTTTGGGATCATCCTGGGAAT---------------TTCAAT
GTGACTGGGCCCAGGTCCACAGCCGGCATCTTCGCCACCTACCCTGGAAAACATCTCACC
ATTGTCAACGGCTTCTTTGATCAGATAATTGGCACAGCAGCTCTTATAGTCTGTATCCTG
GCTATTGTGGACCCGTACAACAACCCCATACCGCAAGGGCTGGAGGCCTTCACTGTGGGA
TTTGTGGTTCTGGTCATTGGCCTGTCTATGGGCTTCAACTCAGGTTATGCTGTCAATCCG
GCCAGAGACCTCGGACCACGTATTTTCACTGCCATGGCCGGGTGGGGCGTTGAAGTTTTC
ACG---AGAAATGGATGGTTCCTGGTGCCCATTTTTGCCCCATTCCTTGGCACCATTATC
GGTGTGGTAATCTATCAGATGATGGTTGGCTACCACCAGGAGGGAGAAGTGCGTGACCAG
AGCGAGAGGGAT------------GAGCAGGAAAATGTCCGACTCACAAATGTCAACACC
---AAC---AACTCC------AAAGACGGG------------CAC---------------
>Barramundi_Aqp3a
ATGGGCAGACACAAGATGTATTTGGACAAACTGTCCCGGTTCTTCCAGATCCGTAACCTG
TTGCTTCGGCAGGCTTTGGCAGAGTGTCTTGGCACCCTCATCCTTGTGATGTTTGGCTGT
GGTGCCGTGGCCCAGCAGGTGTTGAGCGATGGTTCCCATGGCATGTTCCTCACCGTCAAC
TTTGCCTTCGGCTTCGCTGCCACTTTAGGCATCCTGGTCTGTGGCCAGGTATCAGGTGGC
CATCTGAACCCTGCAGTGACCTTTGCCCTGTGTCTACTTGGAAGAGAGCGCTGGAGAAAA
TTCCCCATGTACTTCCTCTTTCAGACAATCGGTGCTTTTTTTGGTGCTGCCATCATTTTT
GGCATGTACTACGATGCTCTGTGGGACTTCCCGGGAGCT---------------TTCAAT
GTGACTGGGCCTGGAGCTACAGCCGGCATCTTTGCCACCTATCCTGGAAAACACCTTACC
CTTGTCAATGGCTTCTTTGATCAGATAATTGGCACATCAGCATTGATAGTTTGCATTCTG
GCTATTGTGGATCCATACAACAACCCAGTCCCCCAAGGGCTGGAGGCTTTCACAGTGGGA
TTTGTGGTTCTGGTTATTGGATTGTCTATGGGCTTTAACTCAGGCTATGCTGTCAATCCT
GCCAGAGACCTCGGACCACGTATTTTCACAGCTATGGCTGGGTGGGGCTCTGAGGTTTTC
ACGACTAGAAATGGCTGGTTCCTGGTGCCCATATTTGCCCCATTCCTTGGCACCATCATT
GGTGTGATGATCTACCAGCTGATGGTTGGCTTCCACGTGGAGGGAGAAATACGTGACAAA
AAGAGTTCA---------------GAGGAGGAAAATGTCCGACTCACCAATGTCAACACC
---AAC---AACTCC------AAAGGTGCTAATAAAGAGGTGCAC---------------
>Derbio_Aqp3a
ATGGGCAGACAGAAGGTGTATTTGGACAAACTGTCCCGGTTCTTCCAGATCCGAAACCTC
CTGCTTCGACAGGCGCTGGCAGAGTGTCTTGGCACCCTCATCCTTGTGATGTTCGGCTGC
GGTGCTGTGGCCCAGCTGGTGTTGAGCGGTGGTTCCCATGGCATGTTCCTCACTGTCAAC
TTTGCCTTCGGCTTTGCTGCCATGTTAGGCATCCTGGTCTGTGGCCAGGTATCAGGTGGC
CATCTGAACCCTGCTGTGACCTTTGCACTGTGTCTACTCGGAAGAGAGCGCTGGAGAAAA

TTCCCCATGTACTTCCTCTTTCAGACAATTGGTGCATTTTTTGGTGCTGCCGTCATTTTC
GGCATGTACTACGATGCCCTGTGGGACCATCCTGGATGT---------------TTCAAT
GTGACTGGGCCTAAAGCCACAGCTGGCATCTTTGCCACCTACCCTGGAAAACATCTCACC
ATTGTCAATGGCTTCTTTGATCAGATCATTGGCACAGCAGCCCTGATAGTTTGTATTCTG
GCTATTGTGGATCCATACAACAACCCCATTCCCCAAGGGCTGGAGGCCTTCACTGTGGGA
TTTGTGGTTCTGGTTATTGGGTTGTCTATGGGCTTTAACTCAGGCTATGCTGTCAATCCT
GCCAGAGATCTCGGACCACGTCTTTTCACTGCTATGGCTGGGTGGGGCAGCGAAGTTTTC
ACGGCCAGAAATGGCTGGTTCCTGGTGCCCATTTTTGCCCCATTCCTTGGGACCATCATT
GGTGTGATAATCTACCAGCTGATGGTTGGCTTCCATGTGGAGGGAGAAGTACGTGACAGA
AAGAACTCA---------------GAGGAAGAAAATGTCAGACTCACCAATGTCAACAGC
---AAC---AATACC------AAAGAGGGTACCAAAGAAGTGCAC---------------
>Japanese_amberjack_Aqp3a
ATGGGCAGACAGAAGTTGTATTTGGACAAACTGTCCCGGTTCTTCCAGATCCGTAATATG
CTGCTTCGGCAAGCGTTGGCAGAGTGTCTTGGCACCCTCATCCTTGTGATGTTTGGCTGC
GGTGCTGTGGCCCAGCTGGTGTTGAGTGGCGGTTCACATGGCATGTTCCTCACCGTCAAC
TTTGCCTTCGGCTTCGCTGCAACCCTAGGCATCCTGGTCTGTGGCCAGGTATCAGGTGGC
CATCTGAACCCCGCAGTGACCTTTGCCCTGTGTCTACTTGGAAGAGAGCGCTGGAGAAAA
TTCCCCATGTACTTCCTCTTTCAGACAATCGGTGCATTTTTTGGTGCCGCCATCATTTTT
GGCATGTACTACGATGCCCTGTGGGACCATCCTGGATGT---------------TTCAAT
GTGACTGGGCCTAATGCCACAGCTGGCATCTTTGCCACCTACCCTGGAAAACATCTCACC
ATTGTCAATGGCTTCTTTGATCAGATAATTGGCACAGCAGCGCTAATAGTTTGTATTCTG
GCTATTGTGGATCCGTACAACAACCCCATCCCCCAAGGGCTGGAGGCTTTCACTGTGGGA
TTTGTGGTTCTGGTTATTGGATTGTCTATGGGCTTTAACTCAGGCTACGCTGTCAATCCT
GCCAGAGATCTCGGACCACGTATTTTCACCTCTATGGCTGGGTGGGGCAGTGAAGTTTTC
ACGGTTAGAAAGGGCTGGTTCCTGGTGCCCACTTTTGCCCCATTCCTTGGCACCTTCATT
GGCGTGATCATCTACCAGTTGATGGTTGGCTTCCATGTGGAGGGAGAAGTACGTGACAAA
AAGACCTTA---------------GAGGAGGAAAATGTCCGACTCACCAATGTCAACGCC
---AAC---AACACC------AAG---GCTACCAAAGAAATGCAC---------------
>Greater_amberjack_Aqp3a
ATGGGCAGACAGAAGGTGTATTTGGACAAACTGTCCCGGTTCTTCCAGATCCGTAATGTG
CTCCTTCGGCAAGCGTTGGCAGAGTGTCTTGGCACCCTCATCCTTGTGATGTTTGGCTGC
GGTGCTGTGGCCCAGCTGGTGTTGAGCGGCGGTTCACATGGCATGTTCCTCACCGTCAAC
TTTGCCTTCGGCTTTGCTGCCACCTTAGGCATCCTGGTCTGTGGCCAGGTATCAGGTGGC
CATCTGAACCCCGCAGTGACCTTTGCCCTGTGTCTACTTGGAAGAGAGCGCTGGAGAAAA
TTCCCCATGTACTTCCTCTTTCAGACAATCGGTGCATTTTTTGGAGCCGCCATCATTTTT
GGCATGTACTACGATGCCCTGTGGGACCATCCTGGATGT---------------TTCAAT
GTGACTGGGCCTAAAGCCACAGCTGGCATCTTTGCCACCTACCCTGGAAAACATCTCACC
ATTGTCAATGGCTTCTTTGATCAGATAATTGGCACAGCAGCGCTAATAGTTTGTATTCTG
GCTATTGTGGATCCATACAACAACCCCATCCCCCAAGGGCTGGAGGCTTTCACTGTGGGA
TTTGTGGTTCTGGTTATTGGATTGTCTATGGGCTTTAACTCAGGCTACGCTGTCAATCCT
GCCAGAGATCTCGGACCACGTATTTTCACCTCTATGGCTGGGTGGGGCAGTGAAGTTTTC
ACGGTTAGAAATGGCTGGTTCCTGGTGCCCATTTTTGCCCCATTCCTTGGCACCTTCATT
GGTGTGATCATCTACCAGTTGATGGTTGGCTTCCATGTGGAGGGAGAAATACGTGACAAA
AAGACCTTA---------------GAGGAGGAAAATGTCCGACTCACCAATGTCAACGCC
---AAC---AACGCC------AAG---GCTACCAAAGAAATGCAC---------------
>Spotted_snakehead_Aqp3a
ATGGGGAGACAGAAGATGTACCTGGAGAAAATGGCCCAGGTCTTGCACCTCCGTAACAAG
CTGCTTCGGCAGTCCCTGGCCGAGTGTCTGGGCACCCTCATCCTTGTGATGTTTGGTTGT
GGATCTGTGGCCCAACACGTACTGAGTGAAGGTTCCCACGCCATTTTTCTCACTGTCAAC
TTTGCCTTCGGCTTCGGTGCCACATTAGGGATCTTGGTGTGTGGACAGGTGTCAGGTGGT
CATCTGAATCCTGCCGTGACCTTTGCCCTGTGTCTGCTTGGACGAGAACGATGGAGGAAA
TTTCCTATGTACTTTCTCTTTCAGACAATCGGTGCTTTTTTTCGGGGCTAGCATAATTTTT
GCCATGTACTATGATGCTATATCAACCTACCCTATGGGT---------------TTCAAC
ATGACTGCGACCACCAACTTAGCAGGCATCTTTGCCACCTACCCGGGAAACCATCTCACT
CTTTTCAATGGCTTCTTTGATCAGCTAATTGGCACGGCAGCCCTGATAGTTTGCATTCTG
GCTATTGTGGATCCATACAACAACCCCATTCCTCAAGGCCTGGAGGCCTTCACTGTGGGG
TTTGTGGTTCTGGTCATTGGATTATCTATGGGCTTTAACTCAGGCTACGCAGTCAATCCT
GCCAGAGACTTTGGACCACGTCTTTTCACAGCTATGGCCGGGTGGGGTGGTGAAGTTTTC
ACGACAAGCAATGGCTGGTTCCTGGTGCCCGTTTTAGTCCCTTTCCTCGGCACCATCATT
GGTGTGATAGTCTACCAGTTCATGGTCGGCTTCCATGTGGAGGTAGAATACCGTTTGAAA
AAAAGCAAC---------------GAGCAGGAGAGCGCTCGACTCTCAAACATCAACACC

---AAC---AACTCC------AAAGAGGCCAGCAAAGAAATGCTC---------------
>Northern_snakehead_Aqp3a
ATGGGGAGACAGAAGATGTACTTGGAGAAAATGGCCCAGGCCTTGCACCTCCGTAACAAG
CTGCTTCGGCAGTCCCTGGCAGAGTGTCTAGGCACCCCTCATCCTTGTGATGTTTGGTTGT
GGAGCTGTGGCCCAACATGTTTTGAGTGGTGGTTCCCATGCTATTTTCCTCACTGTCAAC
TTTGCCTTTGGCTTTGGTGCCACCTTAGGCATCCTGGTGTGTGGCCAGGTATCAGGTGGT
CATCTGAACCCTGCCGTGACCTTTGCACTGTGTCTGCTTGGAAGGGAGCGCTGGAGAAAA
TTCCCTATGTACTTTCTCTTTCAGACAATCGGTGCTTTTTTCGGCGCTAGCATAATTTAT
GCCATGTACTATGATGCCCTGTCAGACAAACCTGATAGT---------------TTCAAC
ATGAGTTCATCTGCTAACACAGCTGGCATCTTTGCTACCTACCCAGCAGATCATCTCACC
CTTTTCAATGGCTTCTTTGATCAGCTAATCGGCACAGCAGCACTGATAGTTTGTATTCTG
GCTATTGTGGATCCATACAACAACCCCATTCCCCAAGGGCTGGAGGCCTTCACTGTGGGA
TTTGTGGTTCTGGTCATTGGATTATCTATGGGCTTTAACTCAGGCTATGCTGTCAATCCT
GCCAGAGACTTTGGACCACGTCTTTTCACATCTATTGCTGGGTGGGGTGGTGAAGTTTTT
ACGACTCGAAGTGGCTGGTTCCTGGTGCCTATTTGTGTCCCATTCCTTGGGACCATCGTC
GGTGTGATAGTCTACCAGTTCATGGTTGGATTCCACGTGGAGGTAGAAGCAAAAATGAGG
AAGAACAGT---------------GAGCAGGAGAATGTCCGACTCACTAATGTCAACACC
---AAC---AACTCC------AAAGAGGGTACCAAAGAAGTGCAC---------------
>Kissing_gourami_Aqp3a
ATGGGCAGACAGAAAATGTGTTTGGACAAAATTGCCAAGTTCTTTCGCATTCGAAACCTG
CTGCTTCGGCAGTCCCTAGCAGAGTGTCTTGGCACCCCTCATCCTCGTGATGTTTGGCTGT
GGTGCTGTGGCACAGCTGGTGTTGAGCAATGGATCCCATGGCCTTTTCCTTACTGTCAAT
CTTGCCTTTGGCTTTGCTGCCACACTGGGTATCCTGGTCTGCGGCCAGGTGTCAGGTGGT
CATCTGAACCCCGCAGTGACCTTTGCCCTGTGTTTACTTGGAAGAGAACCCTGGAAAAAA
TTCCCCATGTACTTCCTCTTTCAGACAATTGGTTCTTTTTTTGGTGCTGCCATAATTTTT
GGCATGTACTTTGATGCCCTGTCGGAGTATCCTGGAGGT---------------TTTGAT
ATGTCTGGACCTAATTCCACAGCTGGCATCTTTGCTACCTTCCCTGGAGCCCATCTCTCC
ATTATCAATGGCGTCTTTGATCAGTTAATTGGCACAGCAGCACTGATAGTTTGTATTCTA
GCTATTGTGGATCCATACAACAACCCCATCCCCCAAGGGCTGGAGGCCTTCACTGTGGGA
TTTGTGGTTCTGGCCATTGGATTATCTATGGGCTTTAACTCAGGCTATGCTGTCAATCCT
GCCAGAGACTTCGGGCCACGTCTTTTCACTGCTTTGGCCGGGTGGGGCGGTGAAGTTTTC
ACG------AACGGCTGGTTCCTGGTGCCAGTTCTTGTCCCATTTATAGGCACCGCTGTT
GGTGTGTTCATCTACCAGTTGATGGTTGGCTTCCATGTGGAGGGAGAAGCACTTGACAGG
AGGAGCTCA---------------GGGCAGGAGAATGTCCGACTCACCAATGTCAGCAGC
---AAC---AACTCC------AAAGAGGCTACCAAAGAAGTGCAT---------------
>Climbing_perch_Aqp3a
ATGGGCAGACAGAAAGTGCTGTTGGACAAAATGGCAAGGTTCTTTCGCATCCGAAACCTG
CTGCTTCGGCAGTCCCTGGCAGAGTGTCTAGGCACCCCTCATCCTTGTGATGTTCGGCTGT
GGCGCTGTGGCCCAGCTGGTGTTGAGCAATGGTTCTCACGGCCTTTTCCTCACTGTCAAT
CTTGCCTTTGGCTTTGCTGCCACCTTGGGCATCCTGGTCTGTGGCCAGGTATCAGGTGGC
CATCTGAACCCTGCAGTAACCTTCGCCCTGTGTGTACTGGGAAGAGAGCCTTGGAAAAAA
TTCCCAATGTACTTCCTCTTTCAGACAATTGGTTCTTTTTTTGGTGCTGCCGTAATTTAT
GCCATGTACTATGATGCTCTGTGCGAATACACCGGAGGT---------------TTTGAT
ATTAATGGGCCCAACTCCACAGCTGGCATCTTTGCTACTTATCCTGGAACCCATCTCTCC
CTTATCAATGGCTTCTTTGACCAGCTAATTGGCACAGCAGCACTGATAGTTTGTATTCTG
GCTATTGTGGATCCATACAACAACCCCATCCCCCAAGGGCTGGAGGCCTTCACAGTGGGA
TTTGTGGTTCTGGCCATTGGATTATCTATGGGCTTTAACTCAGGGTATGCTGTCAATCCT
GCCAGAGACATCGGCCCACGTCTTTTCACTGCCATAGCTGGGTGGGGCGGCGGCGTTTTC
ACGATTGGAAGTGGCTGGTTCCTGGTGCCCATTTTTGTCCCATTTATTGGCACCATGCTT
GGTGTGCTAATCTACCAGTTCATGGTTGGGTTCCACGTGGAGGGAGAAGCACGGGACAAG
AGAGGCTCA---------------GGGCCGGAGAATGTCCGACTCACCAACGTCAACAGC
---AAC---AACTCC------AAAGAGGCTACCAAAGAAGTGAAC---------------
>Siamese_fighting_fish_Aqp3a
ATGGGCAGACAGAAAGTGTTTTTGGACAAAATGGTCAGCACCTTCCGCATCCGGAACCTG
CTGCTGCGCCAGTCCCTGGCCGAGTGCCTGGGCACCCCTCATCCTGGTGATGTTTGGCTGC
GGAGCTCTGGCCCAGCTGGAGCTGAGCAAAGGGTCTCACGGCATTTTCCTCACCGTCAAT
CTGGCCTTTGGCTTCGCTGCCACCTTGGGCATCCTGGTGTGTGGCCAAGTATCAGGGGGG
CATCTGAACCCTGCCGTGACCTTTGCCCTGTGTCTGCTTGGAAGGGAGCGCTGGAGAAAG
TTCCCCATGTTCTTCCTCTTTCAGATAATTGGGGCCTTCTTTGGTGCCGCTGTCATTTAT
GCGATGTACTATGACGCCCTGTGTGAGGTCCATGGAGGT---------------TTTGAT
ACGACTGGGCCTAATTCTACTGCTGGCATCTTTGCTACCTACCCCGGAATCCACCTCTCT

TTTATGAATGGCGTGTTTGATCAGCTGATCGGCACAGCAGCACTGATCGTCTGTATTCTG
GCTATTGTGGATCCATACAACAACCCCATCCCCCAGGGCCTGGAGGCCTTCACTGTGGGC
TTTGTGGTTCTGGCCATTGGGCTATCGATGGGCTTTAACTCAGGCTACGCTGTCAACCCT
GCCAGAGACCTTGGGCCACGTCTCTTCACTTCTATAGCCGGGTGGGGTTACGAAGTTTTC
ACGATTGGCAGCGGCTGGTTCCTGATCCCCGTCTTCGTCCCGTTTTTCGGCACCATCATC
GGCGTGGTCCTCTACCAGATGATGGTGGGACTACACGTGGAGGCGGAGGTACGCGAGAGG
AGGAGCTCC---------------GAGCAGGAGAACGTCCGGCTCACCAACGTCAACAGC
---AAC---AACTCC------AAGGGGGGAACCAAAGAAGTGAAC---------------
>Swamp_eel_Aqp3a
ATGGGCAAGCAGAAGATGATATTGGACAAATTAGCCAGGACCGTCCAAGTCCGCAACCTG
CTGCTACGGCAGGCCCTGGCTGAGTGTCTTGGCACCCTCATCCTTGTGATGTTTGGCTGT
GGTGCTGTGGCCCAAGTGGTGTTGAGTGGTGGTACCCACGGCATGTTCCTCACTGTCAAT
CTTGCCTTCGGCTTTGCTGCCACATTAGGAATCCTAGTCTGTGGCCAGGTATCAGGTGGC
CATCTGAACCCCGCAGTGACCTTTGCTGCGTGTCTGCTTGGAAGAGAGCGCTGGAGAAAA
TTCCCCATGTACTTCCTCTTTCAGACAATTGGTGCTTTTTTTGGAGCTTCCATAATTTAT
GCCATGTACTATGATGCCCTGGTGAGCTATCCTGGATCT---------------TTCAAT
ATGACTGGGCCCAATCACACAGCTGGCATCTTTGCTACCTATCCTGCAGAGCACCTCTCC
ATTTTCAATGGCTTCTTTGATCAGTTAATTGGAACAGCAGCACTTATAGTTTGTATTCTG
GCTATTGTGGATCCCTACAACAACCCCATACCCCAAGGGCTGGAGGCCTTCACTGTGGGA
TTTGTGGTTCTCGTCATTGGATTAGCAATGGGCTTTAACTCAGGCTATGCTGTCAATCCT
GCCAGAGATTTCGGACCACGTCTTTTCACTGCTATGGCTGGGTGGGGCGGCGGAGTTTTC
ACGGTGAGCAGTGGCTGGTTCCTGGTGCCCATTTTTGTCCCATTTATTGGCACCATCATC
GGGGTGTTGGTCTACCAGTTGATGGTTGGCTTCCATGTGGAGGGAGAAGTACGTGACAGG
AAGAGCTCA---------------GAGCAGGAAAATGTCCGACTCACCAATGTCAACACC
---AAC---AACTCC------AAAGAGGGTACCAAAGAAGAGCAC---------------
>Zigzag_eel_Aqp3a
ATGGGCAGACAGAAGATGTTTTTGGAAAAAATGTCCAAGTCCCTCCAAATCCGTAGCCTG
CTGCTTCGGCAGGCCCTGGCAGAGTGTCTTGGAACCCTCATCCTTGTGATGTTTGGCTGT
GGTGCTGTGGCCCAACTGGTGTTAAGCGATGGTTCCCATGGCATGTTCCTCACTGTCAAC
CTTGCCTTTGGCTTCTCTGCCACCTTAGGCATCCTGGTCTGTGGCCAAGTATCAGGCGGA
CATCTGAATCCTGCGGTGACCCTTGCCCTGTGTCTGCTTGGAAGAGAACGCTGGAGAAAA
TTTCCCATGTACTTCCTCTTTCAGACAATTGGTGCTTTTTTTGGCTCGGCAATCATTTTT
GGCATGTACTATGATGCCCTGTGGGACCGTCCTGGATGT---------------TTCAAT
GTTACTGGGCCAGGTGCCACAGCTGGCATCTTTGCTACCTACCCTGCCTCCCATCTCACT
CTTCTCAATGGCTTCTTTGATCAGTTAATTGGCACAGCAGCACTTGTAGTTTGCATTCTG
GCTATTGTGGATCCATACAACAACCCCATCCCCCAAGGACTGGAGGCCTTCACTGTGGGA
TTTGTGGTTCTGGTCATTGGATTGTCTATGGGCTTTAACTCAGGCTATGCTGTCAATCCT
GCCAGAGATTTTGGACCACGTCTTTTCACTGCTATGGCTGGGTGGGGCAGGGATGTGTTC
ATATATGGCGACGGCTGGTTCCTGGTGCCTATTTTTGCTCCATTTCTTGGCAGCTTTATT
GGTGTGGTGATCTACCAGTTGATGGTTGGCTTCCATGTGGAGGGTGACGCACGTGACAAA
AAGAGCTCA---------------GAGCAGGAAAATGTCCGGCTCACCAATGTCAACGCC
---AAT---AACTCC------AAAGAGGCTATGAAAGACGCAGCC---------------
>Florida_bass_Aqp3a
ATGGGCAGACAGAAGGTGTGTTTGGAAAAACTGTGCCGGTTCTTCCACATCCGGAACCAG
CTGCTTCGTCAGGCCCTTGCAGAGTGTCTTGGCACCCTCATTCTTGTGATGTTTGGCTGT
GGTGCTGTGGCCCAGCTGGTGTTGAGTGGAGGTTCCCACGGCTTGTTCATCACTGTCAAC
TTTGCCTTCGGCTTTGGTGCCACCTTAGGCATCCTGGTCTGTGGCCAGGTATCAGGTGGC
CATCTGAACCCTGCAGTGACATTTGCCCTCTGTTTGCTTGGAAGAGAGCGCTGGAGAAAG
TTCCCTATGTACTTCCTTTTTCAGACAATCGGCGCTTTTTTTGGTGCTGCCATAATTTTT
GGCATGTATTACGATGCCCTGTTTGACTTTCCTGAATCT---------------TTCAAT
ATTACTGGGCCT------ACTGCTGGCATCTTTGCTACCTACCCTGCAACCCATCTCACA
ATTGTCAACGGCTTCTTTGATCAGTTAATTGGCACAGCAGCGCTGATAGTTTGTATTCTG
GCTATTGTGGATCCATACAACAACCCCATCCCCCAAGGACTGGAGGCCTTCACTGTGGGA
TTTGTGGTTCTGGTCATTGGATTGTCTATGGGATTTAACTCTGGTTATGCTGTCAATCCT
GCCCGAGACTTCGGACCACGTCTTTTCACCTCTATGGCAGGGTGGGGCAGTGATGTTTTC
ACGGCTAGAGACGGCTGGTTCCTGGTGCCCATTACTTCCCCATTCATTGGCTCCTTCATC
GGTGTGATAGTCTATCAATTGATGGTTGGCTTCCATGTGGAGGCAGAAGCAAAGTACAAG
ATGAGTAGA---------------GAGCAGGAGAATGTCCGACTCACCAATGTTGCCGCC
---AACGACAACTCC------AAAGAGGATACAAAAGAAATGCAC---------------
>Northern_largemouth_bass_Aqp3a
ATGGGCAGACAGAAGGTGTGTTTGGAAAAACTGTGCCGGTTCTTCCACATCCGGAACCAG

CTGCTTCGTCAGGCCCTTGCAGAGTGTCTTGGCACCCTCATTCTTGTGATGTTTGGCTGT
GGTGCTGTGGCCCAGCTGGTGTTGAGTGGAGGTTCCCACGGCTTGTTCATCACTGTCAAC
TTTGCCTTCGGCTTCGGTGCCACCTTAGGCATCCTGGTCTGTGGCCAGGTATCAGGTGGC
CATCTGAACCCTGCAGTGACATTTGCCCTCTGTTTGCTTGGAAGAGAGCGCTGGAGAAAG
TTCCCTATGTACTTCCTTTTTCAGACAATCGGCGCTTTTTTTGGTGCTGCCATAATTTTT
GGCATGTACTACGATGCCCTGTTTGACTTTCCTGAATCT---------------TTCAAT
ATTACTGGGCCT------ACTGCTAGCATCTTTGCTACCTACCCTGCAACCCATCTCACA
ATTGTCAACGGCTTCTTTGATCAGTTAATTGGCACAGCAGCGCTGATAGTTTGTATTCTG
GCTATTGTGGATCCATACAACAACCCCATCCCCCAAGGACTGGAGGCCTTCACTGTGGGA
TTTGTGGTTCTGGTCATTGGATTGTCTATGGGATTTAACTCTGGTTATGCTGTCAATCCT
GCCCGAGACTTCGGACCACGTCTTTTCACCTCTATGGCAGGGTGGGGCAGTGATGTTTTC
ACGGCTAGAGACGGCTGGTTCCTGGTGCCCATTACTTCCCCATTCATTGGCTCCTTCATC
GGTGTGATAGTCTATCAATTGATGGTTGGCTTCCATGTGGAGGCAGAAGCAAAGTACAAG
ATGAGTAGA---------------GAGCAGGAGAATGTCCGACTCACCAATGTTGCCGCC
---AACGACAACTCC------AAAGAGGATACAAAAGAAATGCAC---------------
>Bluegill_Aqp3a
ATGGGTAGACAGAAGGCGTGTTTGGAAAGACTGTGCCGTGTCTTCCACATCCGGAACCTG
CTGCTTCGTCAGGCCCTTGCAGAGTGTCTTGGCACCCTCGTTCTTGTGATGTTTGGCTGT
GGTGCTCTGGCCCAGCATGTGTTGAGTGGAGGTTCCCACGGCAGGTTCATCACTGTTAAC
TTTGCCTTTGGCTTCGGTGCCACC------------------------------------

------------GATGCCCTGTTTGACTATCCTGAATCT---------------TTCAAT
ATTACTGGGCCT------ACTGCTGGCATCTTTGCTACCTACCCTGGACCCCACCTCACA
ACTGTCAATGGCTTCTTTGATCAGTTAATCGGCACAGCGGCGCTGATAGTTTGTATTCTG
GCTATTGTGGATCCGTACAACAACCCAATCCCCCAAGGGCTGGAGGCCTTCACTGTGGGA
TTTGTGATTCTGGTCATTGGATTGTCTATGGGATTTAATTCTGGTTATGCTGTCAATCCT
GCCCGAGACTTC---CCACGTCTTTTCACCTCTATGGCAGGGTGGGGGAGTGATGTTTTC
ACGGTTAGAGACGGCTGGTTCCTGGTGCCCATCATTTCCCCATTCATTGGCTCCTTCATC
GGTGTGATAGTGTATCAGGTGATGGTTGGCTTCCATGTGGAGGCAGAAGCAAAGGCCATA
AAGAGTAGA---------------GAGCAGGAGAATGTCCGACTTACCAATGTCGCCGCC
---AATGACAACTCC------AAAGAGGATACAAAAGAAATGCAC---------------
>Murray_cod_Aqp3a
ATGGGCAGACAGAAGGTGTATTTGGACAAACTGTCCCGGTTCTTCCATCTCCGGAACCTG
CTGCTTCGTCAGGCCCTGGCAGAGTGTCTTGGCACCCTCATCCTTGTGATGTTTGGCTGT
GGTTCTGTGGCGCAGCATGTTTTGAGCGATGGTTCCCATGGTGTGTTCCTCACTGTCAAC
TTTGCTTTCGGCTTCGCTGCCACTTTAGGCATCCTGGTCTGTGGCCAGGTATCAGGTGGC
CATCTGAACCCTGCAGTGACCTTTGCCCTGTGCATGCTTGGAAGAGAGCGCTGGAGAAAG
TTCCCCATGTACTTCCTCTTTCAGACAATCGGTGCCTTTTTTGGTGCCGCCATCATTTAT
GCCATGTACTACGATGCCCTGTGCGATCGTCCTGGGAGT---------------TTCAAT
ATGACCGGGCCTAATACCACAGCTGGCATCTTTGCTACCTACCCTGGAAATCATCTTACC
ATTGTCAATGGCTTCTTTGATCAGATAATTGGCACAGCAGCGCTGATAGTTTGTATTCTG
GCTATTGTGGATCCATACAACAACCCCATCCCCCAAGGGCTGGAGGCCTTCACTGTGGGA
TTTGTGGTCCTGGTCATTGGATTGTCTATGGGATTTAACTCTGGCTATGCTGTCAACCCT
GCCAGAGACATCGGACCACGTCTTTTCACTGCTATGGCAGGGTGGGGCGGCGAAGTTTTC
ACGGCTAACAACGGCTGGTTCCTGGTGCCTTTTTTTGCTCCATTCATTGGCACCATCATT
GGTGTGATAGTCTACCAGTTGATGGTTGGCTTCCATGTGGAGGGAGAAGTACGTGACAAG
AAGAGCAGA---------------GAG---GAGAATGTCCGACTCACCAATGTCGCCGCC
---AGCGACAACTCC------AAAGAGGCTACCAAAGAAATGCAT---------------
>Barred_knifejaw_Aqp3a
ATGGGCAGACAGAAGGTGTATTTGGACAAACTGTCCCGGTTCTTCCAGATCCGTAACCTG
CTGCTTCGTCAGGCCCTGGCAGAATGTCTTGGCACCCTCATCCTTGTGATGTTTGGCTGT
GGTGCTGTGGCCCAGCTGGTGTTGAGCGGTGGTTCCCATGGCATGTTCCTCACTGTCAAC
TTCGCCTTCGGCTTCGCTGCCACCTTAGGCATCCTGGTCTGTGGCCAAGTATCAGGTGGC
CATCTGAACCCTGCAGTGACCTTTGCCCTGTGTCTGCTTGGAAGAGAGCGCTGGAGAAAG
TTCCCCATGTACTTCCTCTTTCAGACAATCGGTGCTTTTTTTGGTGCTGCGGTCATTTTT
GGCATGTACTACGATGCCCTGTGGGACCACCCTGGATGC---------------TTCAAT
GTGACTGGGCCTCGTGCCACTGCCGGCATCTTTGCTACCTACCCCGGAAAACATCTCACC
ATTGTCAATGGCTTCTTTGATCAGATAATTGGCACAGCAGCGCTGATAGTTTGTATTCTG
GCTATTGTGGATCCATACAACAACCCCATCCCCCAAGGACTGGAGGCCTTCACTGTGGGA
TTTGTGGTTCTGGTCATTGGATTGTCTATGGGCTTTAACTCTGGCTATGCCGTCAATCCT

GCCAGAGACCTCGGACCACGTCTTTTCACCGCTATGGCTGGGTGGGGCAGCGAAGTTTTC
ACGGCTAGAAAAGGCTGGTTCCTGGTACCCGTTTTTGCCCCATTCCTTGGCACCATCATC
GGTGTGATAATCTACCAGCTGATGGTTGGCTTCCATGTGGAGGGAGAAGTACGTGACCAG
AAGAGCACA---------------GAGCAGGAGAATGTCCGACTCACCAACGTCACCGCC
---AACGACAACTCC------AAAGAGGCTACCAAAGAAATGCAC---------------
>Korean_spotted_seabass_Aqp3a
ATGGGCAGACAGAAGGTGTATCTGGACAAATTGGCTCGGTTCTTTCAGATCCGTCACCTG
CTGCTTCGCCAGGCCCTGGCAGAGTGTCTTGGCACCCTCATCCTTGTGATGTTTGGCTGT
GGTGCTGTGGCCCAGCTGGTGTTGACG---GGTTCCCATGGCATGTTTCTCACTGTCAAC
TTTGCCTTCGGCTTTGCTGCTACCTTAGGCATCCTGGTCTGTGGCCAGGTATCAGGTGGC
CATCTGAACCCTGCAGTGACATTTGCCCTGTGCCTGCTTGGAAGAGAGCGCTGGAGGAAG
TTCCCCATGTACTTCCTCTTTCAGACAATCGGTGCTTTTTTTGGTGCTGCCATAATTTTT
GGGATGTACTACGATGCCCTGTGGGACCGTCCTGGATGT---------------TTCAGT
GTGAGTGGGGCTAATTCCACAACTGGCATCTTTGCTACCTACCCTGGAAAGCATCTCACC
ATTGTCAACGGCTTCTTTGATCAGATAATTGGCACAGCGGCGCTGATAGTTTGTATCCTG
GCTATTGTGGATCCCTACAACAACCCCATCCCCCAAGGACTGGAGGCCTTTACTGTGGGA
TTTGTGGTTCTGGTCATTGGATTGTCTATGGGCTTTAACTCTGGCTATGCTGTCAATCCT
GCCAGAGACCTCGGACCACGGCTTTTCACCGCTATGGCTGGGTGGGGCGTTGAAGTTTTC
ACGGCTAGAGGCGGCTGGTTCCTGGTGCCCATTTGTGCCCCATTCCTTGGCGCCATCATC
GGTGTGATGATCTACCAGTTGATGGTTGGCTTCCACGTGGAGGGAGAAGTACGCGACCAG
AAGAGCACA---------------GAGCAGGAGAATGTCCGACTCACCAATGTCAGCGCC
---AACGACAATCCC------AAAGGGGCTACCAAAGAAATGCAC---------------
>Japanese_seabass_Aqp3a
ATGGGCAGACAGAAGGTGTATCTGGACAAATTGGCTCGGTTCTTTCAGATCCGTCACCTG
CTGCTTCGTCAGGCCCTGGCAGAGTGTCTTGGCACCCTCATCCTTGTGATGTTTGGCTGT
GGTGCTGTGGCCCAGCTGGTGTTGAGCGATGGTTCCCATGGCATGTTTCTCACTGTCAAC
TTTGCCTTCGGCTTTGCTGCTACCTTAGGCATCCTGGTCTGTGGCCAGGTATCAGGTGGC
CATCTGAACCCTGCAGTGACATTTGCCCTGTGCCTGCTTGGAAGAGAGCGCTGGAGGAAG
TTCCCCATGTACTTCCTCTTTCAGACAATCGGTGCTTTTTTTGGTGCTGCCATAATTTTT
GGGATGTACTACGATGCCCTGTGGGACCGTCCTGGATGT---------------TTCAGT
GTGAGTGGGGCTAATTCCACAGCTGGCATCTTTGCTACCTACCCTGGAAAGCATCTCACC
ATTGTCAACGGCTTCTTTGATCAGATAATTGGCACAGCGGCGCTGATAGTTTGTATCCTG
GCTATTGTGGATCCCTACAACAACCCCATCCCCCAAGGACTGGAGGCCTTTACTGTGGGA
TTTGTGGTTCTGGTCATTGGATTGTCTATGGGCTTTAACTCTGGCTATGCTGTCAATCCT
GCCAGAGACCTCGGACCACGGCTTTTCACCGCTATGGCTGGGTGGGGCGTTGAAGTTTTC
ACGGCTAGAGGCGGCTGGTTCCTGGTGCCCATTTGTGCCCCATTCCTTGGCGCCATCATC
GGTGTGATGATCTACCAGTTGATGGTTGGCTTCCACGTGGAGGGAGAAGTACGCGACCAG
AAGAGCACA---------------GAGCAGGAGAATGTCCGCCTCACCAATGTCAGCGCC
---AACGACAATCCC------AAAGGGGCTACCAAAGAAATGCAC---------------
>Pacific_bluefin_tuna_Aqp3a
ATGAGCAGACAGAAGGTGTATTTGGAAAAACTGGCCCGGTTCTTCCAGATCCGTAACCTC
CTGCTTCGTCAGGCCCTGGCAGAGTGTCTTGGCACCCTCATCCTTGTGATGTTTGGCTGT
GGTGCTGTGGCGCAGCTGGTGTTGAGCGGTGGTTCCCATGGTATGTTTCTTACTGTCAAC
TTCGCCTTCGGCTTCGCTGCCACCTTGGGCATCCTGGTCTGTGGCCAGGTATCAGGTGGC
CATCTGAACCCTGCAGTGACCTTTGCCCTGTGCCTGCTTGGAAGAGAGCGCTGGAGAAAG
TTCCCCATGTACTTTCTATTTCAGACAATTGGTGCTTTCTTTGGGGCTGCCATCATTTTT
GGCATGTATTACGATGCCCTGTGGGACCATCCTGGGAGT---------------TTCAAT
GTGACTGGGCCACATGCCACAGCTGGCATCTTTGCTACCTACCCTGGAAAACATCTCACC
CTTGTCAATGCCTTCTTTGATCAGATTATTGGCACATCAGCGCTGATCGTTTGTATCCTG
GCTATTGTCGATCCATACAACAACCCCATCCCCCCAGGGCTGGAAGCCTTCACTGTGGGA
TTTGTGGTTCTGGTCATTGGATTGTCTATGGGTTTTAACTCTGGCTATGCTGTCAATCCT
GCCAGAGACCTCGGACCACGTCTTTTCACCGCTATAGCTGGATGGGGCAGCGGGGTTTTC
ACGGTCAGAAACGGCTGGTTCTTGGTGCCCATTTTTGCCCCATTCCTTGGCACCATCATT
GGTGTGATGATCTACCAGGTGATGGTTGGCTTCCACGTAGAGGGAGAAGTACGTGACCGG
AACAGCAAG---------------GAGCAGGAGAATGTCCGGCTCACCAATGTCACTGCC
---AATGACAACTCT------AAAGAGGCTACCAAAGAAATGTAC---------------
>Atlantic_bluefin_tuna_Aqp3a
ATGAGCAGACAGAAGGTGTATTTGGAAAAACTGGCCCGGTTCTTCCAGATCCGTAACCTC
CTGCTTCGTCAGGCCCTGGCAGAGTGTCTTGGCACCCTCATCCTTGTGATGTTTGGCTGT
GGTGCTGTGGCGCAGCTGGTGTTGAGCGGTGGTTCCCATGGTATGTTTCTTACTGTCAAC
TTCGCCTTCGGCTTCGCTGCCACCTTGGGCATCCTGGTCTGTGGCCAGGTATCAGGTGGC

```
CATCTGAACCCTGCAGTGACCTTTGCCCTGTGCCTGCTTGGAAGAGAGCGCTGGAGAAAG
TTCCCCATGTACTTTCTATTTCAGACAATTGGTGCTTTCTTTGGGGCTGCCATCATTTTT
GGCATGTATTACGATGCCCTGTGGGACCATCCTGGGAGT---------------TTCAAT
GTGACTGGGCCACATGCCACAGCTGGCATCTTTGCTACCTACCCTGGAAAACATCTCACC
CTTGTCAATGCCTTCTTTGATCAGATTATTGGCACATCAGCGCTGATCGTTTGTATCCTG
GCTATTGTCGATCCATACAACAACCCCATCCCCCCAGGGCTGGAAGCCTTCACTGTGGGA
TTTGTGGTTCTGGTCATTGGATTGTCTATGGGTTTAACTCTGGCTATGCTGTCAATCCT
GCCAGAGACCTCGGACCACGTCTTTTCACCGCTATAGCTGGATGGGGCAGCGGGGTTTTC
ACGGTCAGAAACGGCTGGTTCTTGGTGCCCATTTTTGCCCCATTCCTTGGCACCATCATT
GGTGTGATGATCTACCAGGTGATGGTTGGCTTCCACGTAGAGGGAGAAGTACGTGACCGG
AACAGCAAG---------------GAGCAGGAGAATGTCCGGCTCACCAATGTCACTGCC
---AATGACAACTCT------AAAGAGGCTACCAAAGAAATGTAC---------------
>Yellowfin_tuna_Aqp3a
ATGAGCAGACAGAAGGTGTATTTGGAAAAACTGGCCCGGTTCTTCCAGATCCGTAACCTC
CTGCTTCGTCAGGCCCTGGCAGAGTGTCTTGGCACCCTCATCCTTGTGATGTTTGGCTGT
GGTGCTGTGGCGCAGCTGGTGTTGAGCGGTGGTTCCCATGGTATGTTTCTTACTGTCAAC
TTCGCCTTCGGCTTCGCTGCCACCTTGGGCATCCTGGTCTGTGGCCAGGTATCAGGTGGC
CATCTGAACCCTGCAGTGACCTTTGCCCTGTGCCTGCTTGGAAGAGAGCGCTGGAGAAAG
TTCCCCATGTACTTTCTATTTCAGACAATTGGTGCTTTCTTTGGGGCTGCCATCATTTTT
GGCATGTATTACGATGCCCTGTGGGACCATCCTGGGAGT---------------TTCAAT
GTGACTGGGCCACATGCCACAGCTGGCATCTTTGCTACCTACCCTGGAAAACATCTCACC
CTTGTCAATGCCTTCTTTGATCAGATTATTGGCACATCAGCGCTGATCGTTTGTATCCTG
GCTATTGTCGATCCATACAACAACCCCATCCCCCCAGGGCTGGAAGCCTTCACTGTGGGA
TTTGTGGTTCTGGTCATTGGATTGTCTATGGGTTTAACTCTGGCTATGCTGTCAATCCT
GCCAGAGACCTCGGACCACGTCTTTTCACCGCTATAGCTGGATGGGGCAGCGGGGTTTTC
ACGGTCAGAAACGGCTGGTTCTTGGTGCCCATTTTTGCCCCATTCCTTGGCACCATCATT
GGTGTGATGATCTACCAGGTGATGGTTGGCTTCCACGTAGAGGGAGAAGTACGTGACCGG
AACAGCAAG---------------GAGCAGGAGAATGTCCGGCTCACCAGTGTCACTGCC
---AATGACAACTCT------AAAGAGGCTACCAAAGAAATGTAC---------------
>Silver_pomfret_Aqp3a
ATGGGCAGACAAAAGGTGTATTTGGAAAAACTCTCTCGGTTCTTCCAGATCCGTAATCTG
CTGCTTCGTCAGGCCCTGGCAGAGTGTCTTGGCACCCTC---------------------
------------------------------------------------------------
------------------------------------------------------GGTGGC
CATCTTAACCCTGCAGTGACCTTTGCCCTGTGCCTGCTTGGGAGAGAGCGTTGGAGAAAG
TTCCCCATGTACTTTCTATTTCAGACAATTGGTGCTTTCTTTGGTGCTGCCATTATTTTT
GGCATGTACTACGATGCCCTGTGGGACTTTCCCGGGAGT---------------TTCAAT
ATGACTGGACCAGGTAACACTGCTGGTATATTTGCTACCTACCCTGGAAAACATCTTACC
ATTGTCAATGGCTTCTTTGATCAGATTATTGGTACAGCAGCACTCATCGTTTGTATCCTG
GCTATTGTCGATCCATACAACAACCCTATCCCACAAGGGCTTGAGGCCTTTACTGTGGGA
TTTGTGGTTCTGGTCATTGGACTGTCTATGGGTTTCAACTCTGGCTATGCTGTCAATCCC
GCCAGAGACCTCGGACCACGTCTTTTCAGTGCTATGGCTGGATGGGGTTCCGGGGTTTTC
ACGATTAGAAAGGGCTGGTTCCTGGTGCCCATTTTTGCTCCATTCCTTGGTACCATCATT
GGTGTGATGATCTACCAGTTGATGGTCGGCTTCCACGTAGAGGGAGAAGTGCGTGACAAG
TTGAGCATG---------------GAGGAGGAGAATGTCCGACTCGCCAATGTCAACACC
---AAAGACAACACT------AAAGAGGCTACCAAAGAAATATAC---------------
>Walking_goby_Aqp3a
ATGGGTGCGCAAAAGGTGGCCTTGGAGAAACTATCATACTTCTTCCAGATCCGTAACCTG
CTGCTTCGACAGGCTCTAGCGGAGTGTCTGGGCACCCTCATCCTTGTGATGTTTGGTACG
GGTGCGGTGGCTCAGTATGTGTTGAGCGGCGGAACTCATGGAATGTTTATTACAGTCAAC
TTTGCCTTTGGTTTTGCTGCTACCTTAGGCATCCTCGTCTGCGGTCAAATATCAGGAGGA
CACCTTAATCCCGCCGTGACCTTTGCCCTCTGCCTCCTGGGACGAGAGCGCTGGAGAAAG
TTGCCCATGTTTTTCCTCTTCCAGACGATCGGCGCATTCTTCGGCGCTGCCATTATCTTC
GGCATGTACTATGATGCCCTGTGGGACTACCCTGGATGT---------------TTCAAC
ATGACCGGCAACTCAAGTACAGCTGGTATCTTTGCCACTTACCCCGGGAAGCACTTGACT
ATTGTGAATGGCTTTTTTGACCAGGTCATTGGCACTGCTGCCCTTATAGTGTGTATCTTG
GCAATTGTGGATCCATACAACAACCCCATCCCACAGGGTCTGGAGGCCTTCACTGTCGGC
TTTGTGGTTCTGGTCATCGGACTCTCCATGGGCTTTAACTCCGGTTACGCCGTCAACCCG
GCCAGAGACCTCGGACCTCGTGTTTTCTCTGCCATGGCCGGTTGGGGCCTTGAGGTTTTT
AGGTTTAGAAACGGGTGGTTCTTGGTGCCCACGTTTGCCCCGTTCATCGGCACTTTCATC
GGGGTGGTGGTGTATCAGATAATGGTGGGCTTCCACGTGGAGGGTGAAGTACGCGATCGC
```

AAAGCTGCACAG---------CATGAGAACGAGAGAGTCAGGCTAACAAATGTAACTACC
AACAACGACCACAGC------AAAGAGAGCAGCAAAGAACTGCAC---------------
>gbsMudskipper_Aqp3a
ATGGGCGCGCAAAAGGTGGCATTGGAAAAACTATCACATTTCTTCCAGCTCCGTAACCTG
TTGCTTCGACAGGCTCTGGCAGAGTGTCTGGGCACCCTCATCCTTGTGATGTTTGGCACG
GGTGCAGTGGCTCAGTATGTGTTGAGTGGCGGTACTCATGGAATGTTTATTACAGTCAAC
TTTGCCTTTGGGTTTGCTGCTACCTTAGGCATTCTGGTCTGTGGCCAGATATCAGGAGGA
CATCTTAATCCTGCTGTGACCTTTGCCCTGTGCCTCCTTGGACGAGAGCGCTGGAGAAAG
TTGCCCATGTTTTTCTCTTCCAGACGATTGGTGCATTCTTCGGCGCTGCCATTATCTTT
GGCATGTACTATGATGCCCTGTGGGACTACCCTGGATGT---------------TTCAAC
ATGACCGGCAACTCAAGTACAGCTGGTATCTTTGCCACTTACCCCGGGAAGCACCTGACA
ATTGTGAATGGCTTTTTTGACCAGGTCATTGGGACTGCTGCCCTTATAGTGTGTATCTTG
GCTATTGTGGATCCATACAACAACCCCATCCCACAGGGGCTGGAGGCCTTCACTGTGGGT
TTTGTGGTTCTGGTCATTGGACTCTCCATGGGCTTTAACTCTGGTTATGCTGTCAACCCA
GCCAGAGACCTTGGACCTCGTGTTTTCTCTGCCATGGCTGGTTGGGGTGTTGATGTTTTT
ACGTTTAGAAATGGGTGGTTCCTGGTGCCCACATTTGCCCCATTCATTGGCACTTTCATC
GGTGTAATGGTGTATCAGCTGATGGTAGGCTTCCATGTGGAGGGTGAAGTACGTGATCGC
AAAGCTGCACAG---------CAAGAGAATGAGAAAGTCAGGTTAACAAATGTAACTACC
AACAATGACCACAGC------------------AAAGAACTGCAC---------------
>Atlantic_mudskipper_Aqp3a
ATGGGTGTGCAAAAGGTGTACTTGGAGAAACTAGCATGCTTCTTCCAGATCCGTCACCTG
TTGCTTCGACAGGCTTTGGCAGAGTGTCTGGGCACCCTCATCCTTGTGATGTTTGGCACG
GGTGCAGTGGCCCAGCTGGTGTTGAGTGGCGGGACTCACGGAATGTTTCTCACTGTTAAC
TTCGCCTTCGGGTTCGCTGCTACCTTGGGCATCCTGGTCTGCGGCCAAATATCAGGAGGA
CATCTTAATCCTGCCGTGACCTTTGCCCTCTGCCTCCTTGGACGAGAGCGCTGGAGGAAG
TTCCCCATGTTCTTTCTCTTCCAGACGATCGGCGCATTCTTTGGTGCTGCCATTATCTTC
GGCATGTACTATGATGCCCTGTGGGACTTCCCTGGATGT---------------TTCAAC
ATGACCGGCAACTCACCTACAGCTGGTATCTTCGCCACGTACCCCGGGAAGCATCTGACT
ATCGTGAATGGCTTTTTTGACCAGGTCATCGGGACAGCTGCTCTTATAGTCTGCATCTTG
GCTATTGTGGACCCATACAACAACCCCATCCCACAGGGTCTGGAGGCCTTCACCGTGGGC
TTTGTGGTTCTGGCCATTGGACTGTCCATGGGTTTTAACTCTGGTTACGCCGTCAACCCC
GCCAGAGACCTCGGACCTCGTGTTTTCTCTGCGATGGCTGGTTGGGGCGTTGACGTTTTT
ACGTTCAGAAATGGGTGGTTCCTGGTGCCCACGTTTGCCCCGTTCATTGGCACTCTCATC
GGGGTAATGGTGTATCAGGTGATGGTGGGTTTCCATGTGGAGGGCGAAGTGCGTGATCGC
AAAGCTGCACAG---------CAAGAGAACGAGAGAGTCAGGCTAACAAACGTAACCACC
AACAACGACCACAGC------AAAGAGAGCAACAAAGAACTGCAT---------------
>Giant_udskipper_Aqp3a
ATGGGTGTGCAAAAGGTTTACTTGGAGAAACTAGCATGCTTCTTCCAGATCCGTCACCTG
TTGCTTCGACAGGCTTTGGCAGAGTGTCTGGGCACCCTCATCCTCGTGATGTTTGGCACG
GGTGCAGTGGCCCAGCTGGTGTTGAGTGGCGGTACTCACGGAATGTTTCTGACAGTTAAC
TTTGCCTTTGGGTTTGCTGCTACCTTAGGCATCCTGGTCTGTGGCCAGATATCAGGAGGA
CATCTTAATCCTGCCGTGACCTTTGCCCTCTGCCTCCTTGGACGAGAGCGCTGGAGAAAG
TTCCCCATGTTCTTTCTCTTCCAGACGATTGGTGCATTCTTTGGCGCTGCCATTATCTTT
GGCATGTACTATGATGCCCTGTGGGACTTCCCTGGATGT---------------TTCAAC
ATGACCGGCAACGCAGCTACAGCTGGTATCTTTGCCACTTACCCCGGGAAGCACCTGACT
ATCGTGAATGGCTTTTTTGACCAGGTCATTGGGACAGCTGCCCTTATAGTCTGTATCTTG
GCTATTGTGGATCCATACAACAACCCCATCCCACAGGGGCTGGAGGCCTTCACCGTGGGC
TTTGTGGTTCTGGTCATTGGACTGTCCATGGGCTTTAACTCTGGTTATGCTGTCAACCCC
GCCAGAGACCTTGGACCTCGTGTCTTCTCTGCGATGGCTGGTTGGGGAGTTGACGTTTTT
ACGTTTAGAAATGGGTGGTTCCTGGTGCCCACATTTGCCCCGTTCATTGGCACTATCATT
GGTGTAATGGTGTATCAGCTGATGGTGGGCTTCCATGTGGAGGGCGAAGTACGTGATCGC
AAAGCTGCAGAG---------CAAGAGAATGAGAGAGTCAGGCTAACAAATGTAACTACC
AACAACGACCACAGC------AAAGAGAGCAACAAAGAACTGCAC---------------
>Pearlfish_Aqp3a
ATGGGCAGACACAAGGAGTATCTCTACAAGCTGTCGCGCTTCTTCCAGATCCGCAACCTG
CTGCTGCGCCAGGCCATGGGCGAGTGTCTGGGCACCCTCATCCTCGTGATGTTTGGCTGC
GGCGCGGTGGCGCAGCTGGTGTTGAGCGGCGGCTCCCATGGAATGTTCCTCACCGTCAAC
TTCGCTTTTGGCTTTGCGGCCACCTTGGGCATCCTGGTGTGCGGCCAAGTGTCAGGAGGA
CATCTGAACCCTGCCGTGACCTTTGCCCTCTGCCTGCTTGGAAGGGAGCGCTGGAGGAAA
TTCCCCACCTACTTTCTCTTCCAGACCATTGGAGGCTTCCTCGGTGCCGCCGTGATCTTT
GGGATGTACTATGACGCCCTGTGGGACCATCCTGGGTGT---------------TTCAAT

GTTACCGGACCAAAAGCCACAGCAGGGATCTTTGCGACCTATCCCGGCAAACACCTCACC
CTCGTCAACGGCTTCTTCGATCAGATCATCGGCACAGCGGCGCTTATCGTCTGCATCCTT
GCGATCGTGGACCCGTACAACAACCCCATCCCTCAGGGCCTGGAGGCTTTCACGGTGGGA
TTCGTGGTTCTGGTCATCGGACTGTCGATGGGTTTCAACTCCGGCTATGCCGTCAATCCT
GCCAGGGACTTGGGACCTCGGATTTTCACCGCCGTGGCTGGCTGGGGTGTGGATGTTTTC
ACGGTTCGCAACGGCTGGTTCCTGGTCCCTGTCTTCGCGCCGTTCCTCGGCAGCTTCATC
GGAGTGGTCATCTACCAGCTGATGGTTGGCTTCCACGTGGAGGGTGAAGTGCGAGACCGC
AAGAACGCCGAG---------CTGGAGAACGAGAGGGTCCGACTGACCAATGTCACCACC
---AACGACAACCCC------AAGGAGGTCACCAAGGATATGTGC---------------
>Legless_cuskeel_Aqp3a
ATGGGCAGGCTTAAGGAGTATTTGGACAAACTGCCACACTTCTTCCAGATCCGCAACCTG
CTGCTTCGTCAGGCCATGGGAGAGTGTCTCGGCACCCTCATCCTTGTGATGTTTGGCTGC
GGTGCTGTGGCCCAGCTGGTGTTGAGCGGTGGCTCCCATGGCATGTTCCTCACCGTCAAC
TTCGCTTTCGGCTTCGCTGCCACCTTAGGCATCCTGGTCTGCGGCCAGGTGTCAGGGGGG
CATCTGAACCCTGCAGTGACTTTTGCCCTGTGCCTCCTTGGAAGAGAGCGCTGGAGAAAA
TTCCCCATGTACTTTGCCTTCCAGACCTTGGGTGGCTTCCTGGGTGCTGCCGTCATTTTT
GGCATGTACTACGATGCACTGTGGGACCATCCTGGGTGT---------------TTCAAT
GTGACTGGGCCAAATGCCACAGCAGGGATCTTTGCTACCTACCCTGGGAAACATCTCACC
ATTGTCAATGGTTTCTTTGATCAGATCATTGGCACAGCAGCACTAATCGTGTGTATCTTG
GCAATTGTGGATCCGCATAACAACCCCATACCCCAGGGCCTAGAGGCCTTCACAGTGGGA
TTTGTGGTTCTGGTCATTGGACTGTCAATGGGTTTTAACTCTGGTTACGCTGTCAACCCT
GCCAGAGACTTGGGACCACGGCTTTTCACTGCTATGGCTGGCTGGGGCATGGAAGTTTTC
ACGGCTCGTAATGGCTGGTTCCTGGTGCCCGTCTTTGCTCCGTTCCTTGGCACCATTATC
GGTGTGGTGATCTATCAGCTGATGGTCGGCTTCCATGTGGAGGGTGAGGTACGTGACCGG
AAGATTGCAGAG---------CAGGAGAGCGAGAGAGTCAGACTCACCAATGTCACTACC
---AACGACAACCCC------AAAGAGGCCACCAAAGAGATGTTG---------------
>Bearded_brotula_Aqp3a
ATGGGCAAACAGAAGGAGTACTTGGACAAACTGGCCCACTTCTTCCAGATCCGCAACCTG
CTGCTTCGCCAGGCCTTGGGAGAGTGTCTGGGAACCCTCATCCTTGTGATGTTCGGCTGC
GGTGCCGTGGCCCAGCTGGTTTTGAGCGACGGCTCCCACGGGATGTTCCTCACTGTCAAC
TTTGCTTTTGGCTTCGCTGCCACCTTAGGCATTCTGGTGTGTGGCCAAGTGTCAGGAGGC
CATCTGAACCCCGCGGTGACCTTTGCTCTGTGCCTGCTCGGGCGGGAACGCTGGAGAAAA
TTCCCAGTTTACTTCGCCTTCCAGACATTGGGCGCTTTCCTGGGCGCTGCCATCATTTTT
GGCATGTACTATGACGCCCTGTGGGATTTTCCTGGATGT---------------TTCAAT
ATGACGGGACCAACCAACACAGCCGGGATCTTTGCCACCTACCCTGGAAAACATCTCACC
ATTGTCAATGGTTTCTTCGATCAGATCATTGGCACCGCTGCACTGATCGTGTGTATCCTG
GCGATCGTGGATCCGTACAATAATCCCATTCCCAAGGGTCTGGAGGCCTTCACTGTGGGA
TTTGTGGTTCTGGTCATTGGACTGTCCATGGGTTTTAACTCTGGTTATGCTGTCAACCCT
GCCAGGGACCTGGGTCCGCGAATTTTCACTGCTATGGCTGGCTGGGGCACTGAGGTTTTC
ACGGTTCGTAGAGGCTGGTTCCTGGTGCCCGTCTTTGCACCCTTCATCGGCACCTTCATC
GGCGTGGTGATCTACCAGACGATGGTCGGCTTCCATATACAGGGTGAGGTACGCGACCAA
AAGATGAGAGAG---------GAGGAGAACGAGCGAGTCAGACTCACCAATGTGACCACC
---AACGACAACCCC------AAAAGTGCCCCTAAGGAGGTCATC---------------
>Gulf_toadfish_Aqp3a
---------------GTATATTTGGAGAAACTGGCCCGGTTCTTCCAGATCCGCAACCTG
CTGCTTCGCCAGGCACTGGCAGAATGTCTCGGCACCCTCATCCTAGTGATGTTTGGCTGT
GGCGCACTAGCACAGCTAGTGTTGAGCAGCGGCAGCCACGGCATGTTTCTCACTGTCAAC
TTTGCTTTTGGATTCGCAGCCACCTTAGGCATCCTGGTGTGTGGCCAAATATCAGGTGGA
CATCTAAATCCTGCAGTGACTTTTGCCCTGTGCCTGCTTGGCAGAGAGCGTTGGAGAAAG
TTCCCCATGTATTTTGCCTTCCAGACACTGGGTGCTTATCTGGGCGCTGCCGTCATTTTT
GGCATGTACTATGATGCCTTGTGGGACCTTCCCGGGAGT---------------TTCGAC
ATAACAGGGCCAAACGCCACAGCTGGCATCTTTGCTACCTACCCTGGAAAACATCTGACA
ATTGTCAATGGCTTCTTTGATCAGATCATTGGCACAGCAGCGCTGATCGTGTGCATTCTG
GCTATTGTGGATCCGTACAACAACCCCATTCCCCAGGGCCTGGAGGCCTTCACTGTGGGA
TTTGTGGTTTTGGTAATTGGACTGTCTATGGGGTTCAACTCTGGCTATGCTGTCAATCCT
GCCAGGGACTTGGGCCCACGTCTTTTCACTGCTATGGCTGGCTGGGGCAGCAAGGTTTTC
ACTACTAGGCGCTGCTGGTTCCTGGTGCCCATTTTTGCCCCGTTCCTCGGAAGCTTCATC
GGTGTGATGATCTACCAAGTGATGGTTGGCTGGCACATGGAGGGAGAGGTACGTGACCGT
CAGAGGCTGCAG---------CTGGAGAAGGAGAGAATCGGACTCACAGATGTGACCACC
---AACGACAACTCC------AAAGAGGCCAGCAAAGACAATCAC---------------
>Plainfin_midshipman_Aqp3a

ATGGGCAGACAGAAGGTGATTTTAGAGAAACTGGCACGGTTCTTCCAGATCCGCAACTTG
CTGCTTCGCCAGGCGCTGGCAGAATGTCTTGGCACCCTCATCTTGGTGATGTTTGGCTGC
GGCGCACTCGCACAGCTCGTATTGAGCAGCGGCAGCCACGGCATGTTTCTCACTGTCAAC
TTTGCATTTGGATTCGCAGCCACCTTAGGCATCCTGGTGTGTGGCCAAGTATCAGGTGGA
CATCTAAATCCTGCAGTAACGTTTGCCCTGTGCGTGCTTGGGAGAGAGCGCTGGAGAAAG
TTCCCCATGTATTTTGCCTTTCAG------------------------GCCATCATTTTT
GGCATGTACTATGATGCCTTGTGGGACCTTCCTGGGAGT---------------TTCGAC
CTAACAGGACCAAACGCCACAGCTGGCATCTTTGCTACCTACCCAGGAAAGCATCTGACA
ATTGTCAATGGCTTCTTTGACCAGATCATTGGCACAGCAGCACTGATCGTATGCATTCTG
GCTATTGTGGATCCCTACAACAACCCCATCCCCCCAGGCCTTGAGGCCTTCACTGTGGGA
TTTGTGGTGTTGGTCATTGGACTGTCTATGGGGTTCAACTCTGGCTATGCTGTCAATCCT
GCCAGGGATCTGGGCCCACGTCTTTTCACTGCTATGGCTGGTTGGGGCAGCAAGGTTTTC
ACTGTTAGGCGCGGCTGGTTCCTGGTGCCCATTTTTGCCCCGTTCATCGGAAGCCTCATC
GGTGTGATGATCTACCAAGTAATGGTTGGCTGGCACGTGGAAGGAGAGGTACGTGACCGT
GAGAGGGAGCAG---------CTGGAGAAGGAGAGAATGCGACTCACAGACGTGACCACC
---AACGACAACTCC------AAAATGGGCAGCAAAGACCATCAC---------------
>Pony_toadfish_Aqp3a
ATGGGCAGACAGAAGGTGTATTTGGACAAGCTGGCACGGTTCTTCCAGATCCGCCACCTG
CTGCTTCGCCAGGCGCTGGCAGAATGTCTCGGCACCCTCATTCTAGTGATGTTCGGCTGT
GGTGCTTTAGCCCAGCACGTGTTAAGCGGCGGCACCCATGGCGTGTTTCTCACTGTCAAC
TTTGCCTTTGGATTCGGTGCCACCTTAGGCATCCTGGTGTGTGGCCAAATATCAGGTGGA
CATCTAAACCCTGCGGTGACCTTTTCCCTGTGCTTGCTCGGGAGAGAGCGCTGGAGAAAG
TTCCCTATGTACTTTGCCTTCCAGACACTGGGTGCTTTTCTGGGCGCTGCCGTCATTTTT
GGCATGTACTATGATGCCTTGTGGGACCTTCCTGGGAGT---------------TTCAAC
ATGACAGGGCCAAATGCCACAGCTGGCATCTTTGCAACTTACCCTGGAAAACATCTGACA
ATCGTCAATGGCTTCTTTGATCAGATCATTGGCACAGCAGCGCTGATTGTGTGCATTCTG
GCTATTGTGGATCCGTACAACAACCCCATCCCCCAGGGCCTGGAAGCCTTCACTGTGGGA
TTCGTGGTTTTGGTCATTGGACTGTCTATGGGGTTTAACTCCGGCTATGCTGTCAATCCT
GCCAGGGATCTGGGCCCACGTCTCTTCACCGCTATGGCTGGCTGGGGCAGCAAGGTTTTC
ACGACTAGGAACTGCTGGTTCCTGGTGCCCATTGTTGCCCCATTCTTGGGAAGCATCATC
GGCGTGATGATCTACCAGGTGATGGTTGGCTGGCATGTGGAGGGAGAGGTACGTGACCGT
CAGAGGCCGCAG---------CCAGAGAACGAGAGAGTCCGACTCACAGACGTAACCGCC
---AATGACAACCCC------AAAGAGGTCAAGAAAGACATGCAC---------------
>Longspine_squirrelfish_Aqp3a
ATGGGCAGACAGAAGGTGTACCTGGAAAAGCTCTCACAGTTCTTCCAGATCCGCAACCTG
TTGCTTCGCCAGGCCCTGGCAGAGTGTCTTGGTACCCTCATCCTGGTGATGTTTGGCTGT
GGTGCGGTAGCTCAGATGAAGCTGAGCAGCGGTTCCCATGGCATGTTCCTCACTGTCAAC
TTTGCCTTTGGCTTCGCTGCCATGTTAGGCATCCTGGTCTGTGGCCAAGTGTCAGGAGGC
CATCTGAACCCTGCTGTGACCTTTGCCCTGTGCCTGCTTGGGAGAGAGCGCTGGAGAAAG
TTCCCCATGTACTTTGCCTTCCAGACACTCGGTGCTTTCCTGGGCGCAGCCGTCATCTTT
GGCATGTACTATGATGCCCTGTGGGATCATCCTGGGAGT---------------TTCGAT
ATGACTGGGCCAAACAACACAGCTGGCATCTTCGCTACGTACCCTGGAAAACATCTCACC
CTTGTCAATGGCTTCTTTGATCAGATCATTGGCACAGCTGCGCTGATCGTGTGTATCCTG
GCTATTGTGGATCCATACAACAACCCCATTCCTCAGGGACTGGAGGCCTTCACTGTGGGC
TTCGTCGTCCTGGTCATTGGACTGTCTATGGGTTTCAACTCTGGCTATGCTGTCAACCCT
GCCAGGGACCTCGGACCACGTCTTTTCACCGCTATGGCTGGCTGGGGCAGTTCAGTTTTT
ACGGCAAGGAACTGCTGGTTCCTGGTGCCCATCTTTGCCCCATTCCTCGGCACCATCATT
GGAGTGATGGTCTACCAGCTGATGGTTGGTTTCCATGTGGAGGGAGAAGCACGAGACAGT
GAGAATGCAATG---------CAGGAGAAGGAGAGAGTCCGACTCACCAATGTCACCACC
---AATGACATCTCC------AAAGAAGGCACCAAGGAGATGTCA---------------
>Sammara_squirrelfish_Aqp3a
ATGGGCAGACAGAAGGTGTACCTAGAAAAGCTCTCACAGTTCTTCCAGATCCGCAACCTG
TTGCTTCGCCAGGCCCTGGCAGAGTGTCTTGGTACCCTCATCCTGGTGATGTTTGGCTGT
GGCGCAGTAGCTCAAATGAAGCTGAGCAGTGGTTCCCATGGCATGTTCCTCACTGTCAAC
TTTGCCTTTGGCTTCGCTGCCATGTTAGGCATCCTGGTCTGTGGCCAAGTATCAGGAGGC
CATCTGAATCCTGCTGTGACTTTTGCCCAATGCCTGCTTGGGAGAGAGCGCTGGAGAAAG
TTCCCCATGTACTTTGCCTTCCAGACACTCGGTGCTTTCCTAGGCGCAGCCGTCATCTTT
GGCATGTACTATGATGCCCTGTGGGACCATCCTGGGAGT---------------TTCGAT
ATGACTGGGCCAAACAATACAGCTGGCATCTTCGCTACGTACCCTGGAAAACATCTCACC
CTTGTCAACGGCTTCTTTGATCAGATCATTGGCACAGCAGCGCTGATCGTGTGTATCCTG
GCTATTGTGGATCCACACAACAACCCCATTCCTCAGGGACTGGAGGCCTTCACTGTGGGC

TTCGTCGTCCTGGTCATTGGCCTGTCTATGGGCTTCAACTCTGGCTATGCTGTCAACCCT
GCCAGGGATCTCGGACCACGTCTTTTCACCGCTATGGCTGGCTGGGGCAGTTCAGTTTTT
ACGGCTAGGAACTGCTGGTTCCTGGTGCCCATCTTTGCCCCATTCCTTGGCACCATCATT
GGTGTGATGGTCTACCAGCTGATGGTTGGTTTCCATGTGGAGGGAGAAGCACGCGACCGT
GAGAATGCAAAC---------CAGGAGGCTGAGAGAGTCCGACTCACCAAGGTCACCACC
---AACGACATCTCC------AAAGAGTGCACCAAGGAGATGTCA---------------
>Blackbar_soldierfish_Aqp3a
ATGGGCAGACAGAAGATGTACTTAGAGAAGCTCTCACATTTCTTCCAGATCCGCAACCTG
TTGCTTCGCCAAGCCCTGGCAGAATGTCTCGGCACCCTCATCCTGGTGATGTTTGGCTGT
GGCGCGGTGGCTCAGATGAAGTTGAGCAGTGGCTCCCATGGCATGTTCCTCACTGTCAAC
TTTGCTTTTGGCTTCGCTGCCACCTTAGGCATCCTGGTCTGTGGCCAAGTATCAGGAGGC
CATCTGAACCCTGCTGTGACCTTTGCTCAGTGCCTGCTGGGCAGAGAGCGCTGGAGAAAG
TTCCCCATGTACTTTGCCTTCCAGACACTGGGTGCTTTCCTGGGCTCAGCCATCATCTTT
GGCATGTACTATGATGCCCTGTGGGACCATCCTGGGAGT---------------TTCGAT
ATGACTGGGCCAAACAACACAGCTGGTATCTTCGCTACATACCCTGGAAAGCATCTCACC
ATTGTCAACGGCTTCTTTGATCAGATCATTGGCACAACGGCGCTGATCGTGTGTATCCTG
GCTATTGTGGACCCATACAACAACCCTATTCCTCCAGGGTTGGAGGCCTTCACCGTGGGC
TTCGTGGTCCTGGTCATTGGACTGTCTATGGGCTTCAACTCTGGCTATGCCGTCAACCCT
GCCAGGGACCTGGGACCTCGCATCTTCACCGCTATGGCTGGCTGGGGCACAGACGTTTTT
ACGGCGAGGAAATGTTGGTTCCTGGTGCCCGTCTTTGCCCCATTCATTGGCACCATCATT
GGTGTGATGATTTACCAGCTGATGGTTGGTTTCCATGTGGAAGGAGAAGCACGCGACCGG
GAGAACGCAAAG---------CAGGAGAATGAGAGAGTCCGGCTCACCAATGTTACCACC
---AACGACATCTCT------AAAGAGGGCACCAAGGAGATGTCA---------------
>Pineconefish_Aqp3a
ATGGGCATACATAAAATATACTTGGAAAAGCTGGCACAGTTCTTCCAGATCCGCCACTTG
CTGCTTCGCCAGGCCCTGGCAGAGTGTCTTGGCACCCTCATCCTGGTGATGTTTGGCTGT
GGTGCTGTAGCCCAGGTGGTGCTGAGCAGTGGCTCCCATGGCGTGTTCCTCACTGTCAAC
TTTGCTTTTGGATTTGCTGCTACATTAGGCATCCTAGTGTGTGGCCAAGTGTCAGGAGGC
CATCTGAACCCTGCTGTGACCTTTGCCCTGTGCCTGCTTGGCCGAGAGCGCTGGAGAAAG
TTCCCCACGTACTTTGCCTTCCAGACACTGGGTTCTTTCCTGGGCGCTGCCATCATCTAT
GGCATGTACTATGATGCCCTGTGTGACCATCCTGGCAAT---------------TTTGCT
GTGAGTGGGGTCAATTCCACAGCTAAAATCTTTGCCACCTACCCTGGAAAACATCTCACC
ATTGTCAACGGCTTCTTTGATCAGATCATTGGCACAGCAGCGCTGATCGTGTGTATCCTG
GCTATTGTGGATCCATACAACAATCCCATCCCCCAGGGACTGGAGGCCTTCACTGTGGGC
TTTGTGGTTCTGGTCATTGGCCTGTCTATGGGCTTCAACTCTGGCTATGCTGTCAACCCT
GCCAGGGACCTGGGACCACGTCTTTTCACTGCTATTGCTGGCTGGGGCAGTGAAGTTTTC
ACGACAAGGAACTGCTGGTTCCTGGTGCCCACGTTAGCCCCATTTCTTGGCACCATCATC
GGTGTGATGGTCTACCAGCTGTTTGTTGGCTTCCATGTGGAGGGAGAGGCACGCGACCGG
AAGAACAGAGAG---------CAGGAAAATGAGACGGTGCGACTCACCAACGTCACCACC
---AATGACATATCC------AAAGAGTCCACCAAGGAGATGTCA---------------
>Redmouth_whalefish_Aqp3a
ATGGGCAGACAGAAGGTGTACTTGGACAAGCTGGCACACTTCTTCCAGATCCGCAACCTG
CTGCTTCGCCAGGCCCTGGCAGAGTGTCTCGGCACCCTCATCCTGGTGATGTTTGGCTGT
GGTGCTGTAGCCCAGCTGGTGCTGAGCGGTGGCTCCCATGGCATGTTCCTCACTGTCAAC
TTTGCTTTCGGCTTCGCTGCCACCTTAGGCATCCTGGTCTGTGGCCAGGTGTCAGGAGGC
CATCTGAACCCTGCTGTGACCTTTGCCCTGTGCCTGCTTGGGAGAGAGCGCTGGAGAAAG
TTCCCCATGTACTTTGCCTTCCAGACACTCGGTTCATTCCTGGGCGCCGCCATAATCTTT
GGCATGTACTATGATGCCCTGTGGGACCGTCCTGGGAGT---------------TTCAAT
GTGACTGGGCCAAATGCCACAGCTGGCATCTTTGCTACCTACCCTGGAAAACATCTCGCC
ATTGTCAACGGCTTCTTTGATCAGATCATTGGCACAGCAGCGCTGATCGTGTGTATCCTG
GCTATTGTGGATCCATACAACAACCCCATCCCCCAGGGACTGGAGGCCTTCACTGTGGGC
TTTGTGGTTCTGGTCATTGGACTGTCTATGGGCTTCAACTCTGGCTATGCTGTCAACCCT
GCCAGGGACCTGGGACCACGTCTTTTCACCGCTATGGCCGGCTGGGGCAGGGAAGTTTTC
ACGGCTAGGAACTGTTGGTTCCTGGTGCCCATCTTTGCCCCATTCCTTGGCACCATCATT
GGTGTGATTGTCTACCAGCTGTTGGTTGGCTTCCATGTGGAGGGAGAGGCACGCGACCGG
GAGAGCACAGAG---------CAGGAGAACGAGAGAGTCCGACTCACCAACGTCACCACC
---AACGACATCCCC------AAAGAGGGCACCAAGGAGATGTCA---------------
>Pricklefish_Aqp3a
ATGGGCATACAGAAGGTGTACTTGGACAAGCTGGCACGGTTCTTCCAGATCCGCAACCTG
CTGCTTCGCCAGGCCCTGGCAGAGTGTCTCGGCACCCTCATCCTGGTGATGTTTGGCTGT
GGTGCTGTGGCCCAGCTGGTGCTGAGCGGCGGCTCCCATGGCATGTTCCTCACTGTCAAC

TTTGCTTTCGGCTTCGCCGCCACCTTAGGCATCCTGGTCTGTGGCCAAGTGTCAGGAGGC
CATCTGAACCCTGCTGTGACCTTTGCCCTGTGCCTGCTTGGGAGAGAGCGCTGGAGAAAG
TTCCCCATGTACTTTGCCTTCCAGACACTCGGTTCATTCCTGGGCGCCGCCATAATCTTT
GGCATGTACTATGATGCCCTGTGGGACCATCCTGGGAAT---------------TTCAAT
GTGACTGGGCCAAATGCCACAGCTGGCATCTTCGCTACCTACCCAGGAAAACATCTCACC
ATTGTCAACGGCTTCTTTGATCAGATCATTGGCACAGCAGCGCTGATCGTGTGTATCCTG
GCTATTGTGGATCCATACAACAACCCCATCCCCCAGGGACTGGAGGCCTTCACTGTGGGC
TTTGTGGTTCTGGTCATTGGACTGGCTATGGGCTTCAACTCTGGCTATGCTGTCAACCCT
GCCAGGGACCTGGGACCACGTATTTTCACCGCTATGGCTGGCTGGGGCATGGAAGTTTTC
ACGGTTAGGAACTGTTGGTTCCTGGTGCCCATCTTCGCCCCATTCCTTGGCACCATCATC
GGCGTGGTTGTCTACCAGCTGTTGGTTGGCTTCCACGTAGAGGGAGAGGCACGCGACCGG
GAGAGCGCACAG---------CAGGAGGACGAGAGAGTCCGACTCACCAACGTCACCACC
---AACGACATCCCC------AAAGAGGGCACCAAGGAGATGTCA---------------
>Splendid_alfonsino_Aqp3a

---ATGTTTGGCTGT
GGTGCTGTAGCCCAGCTGGTGCTGAGCAGTGGCTCCCATGGCATGTTCCTCACTGTCAAC
TTTGCTTTCGGCTTCGCTGCCACCTTAGGCATCCTGGTCTGTGGCCAAGTGTCAGGTGGC
CATCTGAACCCTGCTGTGACCTTTGCCCTGTGCCTGCTTGGGAGAGAGCGCTGGAGAAAG
TTCCCCATGTACTTTGCCTTCCAGACACTCGGTGCTTTCCTGGGCGCTGCCATAATCTTC
AGCATGTACTATGATGCCCTGTGGGATCATCCTGGGAAT---------------TTCAAT
GTGACTGGGCCAAATGCCACAGCTGGCATCTTTGCTACTTATCCTGGAAAACATCTCACC
CTTGTCAACGGCTTCTTTGATCAGATCATTGGCACAGCAGCGCTGATCGTGTGTATCCTG
GCTATTGTGGATCCACACAACAACCCCATCCCCCAGGGACTGGAGGCCTTCACTGTGGGC
TTTGTGGTTCTGGTCATTGGACTGGCTATGGGCTTCAACTCTGGCTATGCTGTCAACCCT
GCCAGGGACCTGGGACCACGTCTTTTCACCGCTATGGCTGGCTGGGGCATGGATGTTTTC
ACGGCTAGGAACTGCTGGTTCCTGGTGCCCATCTTCGCCCCATTCCTCGGCACCATCATT
GGTGTGATGGTCTACCAGCTCATGGTTGGCTTTCACGTTGAGGGAGAGGTGCGCGACCGG
GAGGAGAAAGCG---------CAGGAGAGCGAGAGAGTCCGACTCACCAACATCACCACC
---AACGACAACCCC------AAAGAAGGCACCAAGGAA---------------------
>Opah_Aqp3a
ATGGGCATACAAAAGGTGTACCTGGAAAAGATGGCAGGGTTCTTCCAGATCCGCAACCTT
CTGCTCCGCCAGGCCTTGGCAGAGTGTCTTGGCACCCTCATCCTAGTGATGTTTGGCTGT
GGTGCTGTAGCCCAGCTGGTTCTGAGTGGAGGATCCCATGGCATGTTTCTCACTGTTAAC
TTCGCTTTTGGCTTCGCTGCCACCCTTGGCATCCTGGTCTGTGGCCAAGTGTCTGGAGGC
CATCTGAACCCTGCTGTGACCTTTGCCCTGTGCCTGCTTGGGAGAGAGCGCTGGAGAAAG
TTCCCTGTGTATTTTGCCTTCCAGACGCTGGGGGCTTTTCTTGGCGCCGCAATAATCTTC
GGCATGTACCATGATGCCTTGTGGGACTTTCCTGGAAGT---------------TTCAAT
ATTACCGGGCCAAAAGCCACAGCTGGCATCTTCGCTACCTACCCTGGAAAACATCTCACC
ATTCTCAATGGCTTCTTCGATCAGATCATTGGCACGGCAGCCCTGATTGTGTGTATCCTG
GCCATTGTGGACCCGTACAACAACCCCATCCCACAGGGTCTGGAGGCCTTCACTGTTGGC
TTCGTTGTTCTGGTCATTGGACTTTCTATGGGCTTCAACTCTGGCTATGCTGTAAACCCC
GCCAGGGACCTGGGACCACGCCTTTTCACTGCCATAGCTGGCTGGGGAAAAGATGTTTTT
ACGGTGAGGCAATGCTGGTTCCTGGTGCCCATCTTTGCCCCATTCCTTGGAACCATCATT
GGAGTGATGGTCTACCAGCTGATGGTTGGCTTCCATGTGGAAGGAGAGGCGCGGGACAAA
GAGAACCAACAG---------CAGGAAAACGAGAGAGTTCGGCTCACCAACATCACCACA
---AACGACAACCTC------AAAGAGACCACCAAGGAAATGCCG---------------
>King_of_herrings_Aqp3a
ATGGGCAGACAAAAGCTGTACCTGGAAAAGCTGTCGCAGCTGTTCCAGATCCGGAACCTT
CTCCTTCGACAGGCCTTGGCTGAATGTCTTGGCACCCTCATCTTAGTGATGTTTGGCTGT
GGTGCTGTAGCCCAGCTGGTTCTGAGTGGAGGATCCCATGGCATGTTTCTCACTGTAAAC
TTTGCATTCGGCTTTGCTGCCACCCTTGGCATCCTGGTCTGTGGCCAAGTGTCAGGAGGC
CACCTGAACCCAGCTGTGACCTTTGCCCTGTGTCTGCTCGGGAGAGAGCGCTGGAGAAAG
TTCCCCGTGTATTTTGCCTTTCAGACGCTGGGGGCTTTCCTTGGAGCCGCAATCATCTTC
GCCATGTACTATGATGCCTTGTGGGACTTTCCTGGAAGT---------------TTCAAT
ATTACCGGGGCAAAAAACACAGCTGGCATCTTCGCTACCTACCCAGGAAAACATCTCACT
ATTCTCAACGGCTTCTTCGATCAGATCATTGGCACAGCAGCCCTGATCGTTTGCATCCTG
GCCATTGTGGACCCGTACAACAACCCCATCCCACAGGGTCTGGAGGCCTTCACTGTTGGC
TTTGTTGTTCTGGTCATCGGACTCTCTATGGGCTTCAACTCCGGCTATGCGGTAAACCCC
GCCAGGGACCTGGGCCCACGCCTTTTCACTGCCATGGCTGGCTGGGGAGCAGATGTTTTT
ACGGTGAGGCAATGCTGGTTCCTGGTGCCCATCTTCGCCCCGTTCCTTGGGACCATCATT

GGAGTTATGGTCTACCAGGTGATGGTTGGCTTCCATGTGGAAGGAGAAGCCCGGGACAAA
GCGAGGCAAGAG---------GAAGAAAACGAGAGGGTCCAACTCACCAACATCACCACA
---AACGACAACCTG------AAAGAGGCCCACAAGGAGATGTCA---------------
>Atlantic_cod_Aqp3a
ATGGGAAGACAGAAGGTATATTTGGACAAGCTGTCCAGAACCTGCCAGATCCGCAACCTG
TTGCTTCGCCAGGCCCTGGCAGAATGTCTTGGCACACTCATCCTTGTGATGTTTGGCTGC
GGCGCTGTAGCTCAACTAGTGCTCAGCGGAGGATCCCATGGCATGTTTCTAACTGTCAAC
TTTTCTTTCGGCTTTGCGGCCACCCTGGGCATTCTAGTCTGTGGCCAAGTGTCGGGTGGC
CATCTGAATCCGGCTGTGACCTTTGCCCTTTGCCTACTCGGAAGAGAACCGTGGAGAAAG
TTCCCAGTATACTTTGCCTTTCAGACACTGGGAGCATTCCTGGGTTCTGCAATAATCTTT
GGCATGTACTATGATGCCCTGTGGGACCATCCTGACAAC---------------TTTAAT
GTGACGGGAAATCGGTCTACTGCCGGCATCTTCGCTACCTATCCTGGAAAACATCTCACG
ATTCTCAATGGCTTCTTTGATCAGCTCATTGGAACGGCAGCCCTGATCGTATGCATCCTG
GCCATTGTGGATCCATACAACAACCCCATACCCCAGGGGCTTGAGGCCTTCACTGTGGGC
TTTGTGGTTCTGGTCATTGGACTGGCGATGGGCTTTAACTCTGGCTACGCCGTCAACCCA
GCCAGGGATCTGGGGCCACGTATCTTCACTTCGATGGCTGGCTGGGGCATGGAGGTCTTC
ACGGCTCGAAAATGCTGGTTCCTGGTGCCCATCTTTGCCCCGTTTCTTGGGGCTATCATC
GGGGTGGTGGTCTACCAGATGATGGTGGGTTACCATTTTGAGGGCGAAGTACGCGACCGG
CGTGAGGCGCAG---------CAACAGAACGAAAGAGAGCGACTCAACAGCATCAGCACT
---AACGACCACAAC------AAAGAG------AAGGAGGTGTCA---------------
>Walleye_pollock_Aqp3a
ATGGGAAGACAGAAGGTGTATTTGGACAAGCTGTCCAGAACCTGCCAGGTCCGCAACCTG
TTGCTTCGCCAGGCCCTGGCAGAATGTCTTGGCACACTCATCCTTGTGATGTTTGGCTGC
GGCGCTGTAGCTCAACTAGTGCTCAGCGGAGGATCCCATGGCATGTTTCTAACTGTCAAC
TTTTCCTTCGGCTTTGCGGCCACCCTGGGCATTCTAGTCTGTGGCCAAGTGTCGGGAGGC
CATCTGAATCCGGCTGTGACCTTTGCCCTTTGCCTACTCGGAAGAGAACCGTGGAGAAAG
TTCCCAGTATACTTTGCCTTTCAGACACTGGGAGCATTCCTGGGTTCTGCAATAATCTTT
GGCATGTACTATGATGCCCTGTGGGACCATCCTGACAAC---------------TTTAAC
GTGACGGGAAATCGGTCTACTGCCGGCATCTTCGCTACCTATCCTGGAAAACATCTCACG
ATTCTCAATGGCTTCTTTGATCAGCTCATTGGAACGGCAGCCCTGATCGTATGCATCCTG
GCCATTGTGGATCCATACAACAACCCCATACCCCAGGGGCTCGAGGCCTTCACTGTGGGC
TTTGTGGTTCTGGTCATTGGACTGGCGATGGGCTTTAACTCTGGCTATGCCGTCAACCCT
GCCAGGGATCTGGGGCCACGTATCTTCACCTCGATGGCTGGCTGGGGCATGGAGGTCTTC
ACGGCTCGAAACTGCTGGTTCCTGGTGCCCATCTTTGCCCCGTTTCTTGGGGCTATCATC
GGGGTGGTGGTCTACCAGATGATGGTGGGTTACCATATGGAGGGCGAAGTACGCGACCGG
CGTGAGGCGCAG---------CAACAGAACGAAAGAGAGCGACTCAACAGCATCAGCACT
---AACGACCACAAC------AAAGAG------AAGGAGGTGTCA---------------
>Polar_cod_Aqp3a
ATGGGAAGACAGAAGGTATTTTTGGACAAGCTGTCCAGAACCTGCCAGATCCGCAACCTG
TTGCTTCGCCAGGCCCTGGCAGAATGTCTTGGCACACTCATCCTTGTGATGTTTGGCTGC
GGTGCTGTAGCTCAACTAGTGCTCAGCGGAGGATCCCATGGCATGTTTCTAACTGTCAAC
TTTTCCTTCGGCTTTGCGGCCACCCTGGGCATTCTAGTCTGTGGCCAAGTGTCGGGAGGC
CATCTGAATCCG---AAG
TTCCCAGTATACTTTGCCTTTCAGACACTGGGGGCATTCCTGGGTTCTGCAATAATCTTT
GGCATGTACTATGATGCCCTGTGGGACCATCCTGACAAC---------------TTTAAT
GTGATGGGAAATCGGTCTACTGCCGGCATCTTCGCTACCTATCCTGGAAAACATCTCACA
ATTCTCAATGGCTTCTTTGATCAGCTCATTGGAACGGCAGCCCTGATTGTATGCATCCTG
GCCATTGTGGATCCATACAACAACCCCATACCCCAGGGGCTTGAGGCCTTCACTGTGGGC
TTTGTGGTTCTGGTCATTGGACTGGCGATGGGCTTTAACTCTGGCTATGCCGTCAACCCA
GCCAGGGATCTGGGGCCACGTATCTTCACTTCGATGGCTGGCTGGGGCATGGAGGTCTTC
ACGGCTCGAAACTGCTGGTTCCTGGTGCCCATCTTTGCCCCGTTTCTTGGGTCTATCATC
GGGGTGGTGGTCTACCAGATGATGGTGGGTTACCATATGGAGGGCGAAGTACGCGACCGG
CGTGAGGCGCAA---------CAACAGAACGAAAGAGAGCGACTCAACAGCATCAGCACC
---AACGACCACAAC------AAAGAG------AAGGAGGTGTCA---------------
>Arctic_cod_Aqp3a
ATGGGAAGACAGAAGGTATATTTGGACAAGCTGTCCAGAACCTGCCAGATCCGCAACCTG
TTGCTTCGCCAGGCCCTGGCAGAATGTCTTGGCACACTCATCCTTGTGATGTTTGGCTGC
GGTGCTGTAGCTCAACTAGTGCTCAGCGGAGGATCCCATGGCATGTTTCTAACTGTCAAC
TTTTCCTTCGGCTTTGCGGCCACCCTGGGCATTCTAGTCTGTGGCCAAGTGTCGGGAGGC
CATCTTAATCCGGCTGTGACCTTTGCCCTTTGCCTACTCGGAAGAGAACCGTGGAGAAAG
TTCCCAGTATACTTTGTCTTTCAGACACTGGGGGCATTCCTGGGTTCTGCAATAATCTTT

GGCATGTACTATGATGCCCTGTGGGACCATCCTGACAAC---------------TTTAAT
GTGACGGGAAATCGGTCTACTGCCGGCATCTTCGCTACCTATCCTGGAAATCATCTCACA
ATTCTCAATGGCTTCTTTGATCAGCTCATTGGAACGGCAGCCCTGATTGTATGCATCCTG
GCCATTGTGGATCCATACAACAACCCCATACCCCAGGGGCTTGAGGCCTTCACTGTGGGC
TTTGTGGTTCTGGTCATTGGACTGGCGATGGGCTTTAACTCTGGCTATGCCGTCAACCCA
GCCAGGGATCTGGGGCCACGTATCTTCACTTCGATGGCTGGCTGGGGCATGGAGGTCTTC
ACGGCTCGAAACTGCTGGTTCCTGGTGCCCATCTTTGCCCCGTTTCTTGGGTCTATCATC
GGGGTGGTGGTCTACCAGATGATGGTGGGTTACCATGTGGAGGGCGAAGTACGCGACCGG
CGTGAGGCGCAA---------CAACAGAACGAAAGAGAGCGACTCAACAGCATCAGCACT
---AACGACCACAAC------AAAGAG------AAGGAGGTGTCA---------------
>Whiting_Aqp3a

---ATGTTTGGCTGC
GGCGCTGTAGCTCAACTAGTGCTCAGTGGAGGATCCCATGGCATGTTTCTAACTGTCAAC
TTTTCCTTCGGCTTTGCGGCCACCCTAGGCATTCTAGTTTGTGGCCAAGTGTCGGGAGGC
CATCTGAATCCGGCTGTGACCTTTGCCCTTTGCCTACTCGGAAGAGAACCCTGGAGAAAG
TTCCCTGTATACTTTGCCTTTCAGACACTGGGGGCATTCCTGGGTTCTGCAATAATCTTC
GGCATGTACTATGATGCCCTGTGGGACCATCCTGACAAC---------------TTTAAT
GTGATGGGAAATCGGTCTACTGCCGGCATCTTCGCTACCTATCCTGGAAAACATCTCACA
ATTCTCAATGGCTTCTTTGATCAGCTCATTGGAACGGCAGCCCTGATCGTATGCATCCTG
GCCATTGTGGATCCATACAACAACCCCATACCCCAGGGGCTGGAGGCCTTCACTGTGGGC
TTTGTGGTTCTGGTCATTGGACTGGCAATGGGCTTTAACTCTGGCTATGCCGTCAACCCA
GCCAGGGATCTGGGACCACGTATTTTCACTTCGATGGCTGGCTGGGGCATGGGGGTCTTC
ACGGCTCGAAACTGCTGGTTCCTGGTTCCCATCTTTGCCCCGTTTCTTGGGGCTATCATC
GGGGTGTTGGTCTACCAGATGATGGTGGGTTACCATATGGAGGGCGAAGTACGCGATCGG
CGTGAGGCGCAA---------CAACAGAATGAAAGAGAGCGACTCAACAGCATCAGCACT
---AACGACCACAAC------AAAGAG------AAGGAGGTGTCA---------------
>Haddock_Aqp3a
ATGGGAAGACAGAAGGTATATTTGGACAAGCTGTCCAGAACCTGCCAGATCCGCAACCTG
TTGCTTCGCCAGGCCCTGGCAGAATGTCTTGGCACACTCATCCTTGTGATGTTTGGCTGC
GGCGCTGTAGCTCAACTAGTGCTCAGCGGAGGATCCCATGGCATGTTTCTAACTGTCAAC
TTTTCCTTCGGCTTTGCGGCCACTCTGGGCATTCTAGTCTGTGGCCAAGTGTCGGGAGGC
CATCTGAATCCGGCTGTGACCTTTGCCCTTTGCCTACTCGGAAGAGAACCCTGGAAAAAG
TTCCCAGTATACTTTGCGTTTCAGACACTGGGGGCATTCCTGGGTTCTGCAATAATCTTC
GGCATGTACTATGATGCCCTGTGGGACCATCCAGACAAC---------------TTTAAT
GTGATGGGAAATCGGTCTACTGCCGGCATCTTCGCTACCTATCCTGGAAAACATCTCACA
ATTCTCAATGGCTTCTTTGATCAGCTCATTGGAACGGCAGCCCTGATCGTATGCATCCTG
GCCATTGTGGATCCATACAACAACCCCATACCCCAGGGCCTGGAGGCCTTCACTGTGGGC
TTTGTGGTTCTGGTCATTGGACTGGCAATGGGCTTTAACTCTGGCTATGCCGTCAACCCA
GCCAGGGATCTGGGGCCACGTATTTTCACTTCGATGGCTGGCTGGGGCATGGGGGTCTTC
TCGGCTCGAAACTGCTGGTTCCTGGTGCCCATCTTTGCCCCGTTTCTCGGGGCTATCATC
GGGGTGGTGGTCTACCAGATGATGGTGGGTTACCATATGGAGGGCGAAGTACGCGACCGG
CGTGAGTTGCAA---------CAACAGAACGAAAGAGAGCGACTTAACAGCATCAGCACT
---AACGACCACAAC------AAAGAG------AAGGAGGTGTCA---------------
>Saithe_Aqp3a
ATGGGAAGACAGAAGGTATATTTGGACAAGTTGTCCAGAAGCTGCCAGATCCGCAACCTG
TTGCTTCGCCAGGCCCTGGCAGAATGTCTTGGCACACTCATTCTTGTGATGTTTGGCTGC
GGCGCTGTAGCTCAACTAGTGCTCAGCGGAGGATCCCATGGCATGTTTCTAACTGTCAAC
TTTTCCTTCGGCTTTGCGGCCACCTTGGGCATTCTAGTCTGTGGCCAAGTGTCGGGAGGC
CATCTGAATCCAGCTGTGACCTTTGCCCTTTGCCTACTCGGAAGAGAACCCTGGAGAAAG
TTCCCAGTATACTTTGCCTTTCAGACACTGGGGGCATTCCTGGGTTCTGCAATAATCTTT
GGCATGTACTATGATGCCCTGTGGGACAATCCTGACAAC---------------TTTAAT
GTGATGGGAAATCGGTCTACTGCCGGCATCTTCGCTACCTATCCTGGAAAACATCTCACA
ATTCTCAATGGCTTCTTTGATCAGCTCATTGGAACGGCAGCCCTGATCGTATGCATCCTG
GCTATTGTGGATCCATACAACAACCCCATACCCCAGGGGCTTGAGGCCTTCACCGTGGGC
TTTGTGGTTCTGGTCATTGGACTGGCGATGGGCTTTAACTCTGGCTACGCCGTCAACCCA
GCCAGGGATCTGGGGCCGCGCATTTTCACTTCGATGGCTGGCTGGGGCATGGGGGTCTTC
ACGGCTCGAAACTGCTGGTTCCTGGTGCCCATCTTTGCCCCGTTTCTTGGGTCTATCATC
GGGGTGGTGGTCTACCAGACGATGGTGGGTTACCATATGGAGGGCGAAGTACGCGACCGG
CGTGAGGCACAG---------CAACAGAACGAAACAGAGCGACTCAACAGCATCAGCACT
---AACGACCACAAC------AAAGAG------AAGGAGGTGTCA---------------

```
>Silvery_cod_Aqp3a
ATGGGAAGACAGAAGGTATATTTGGACAAGCTGTCCAGAACCTTCCAGATCCGCAACTTG
TTGCTTCGCCAGGCCCTGGCAGAATGTCTGGGCACACTCATCCTTGTGATGTTCGGCTGT
GGAGCTGTAGCTCAACTAGTTCTCAGCGGCGGATCTCATGGCATGTTTCTAACGGTCAAC
TTTTCCTTCGGCTTTGCGGCCACCCTGGGCATTTTAGTCTGTGGCCAAGTGTCGGGAGGC
CATCTGAATCCGGCTGTGACCTTTGCCCTGTGCCTACTCGGAAGAGAACCATGGAGAAAG
TTCCCAGTATACTTTGCCTTTCAGACACTGGGTGCATTCCTGGGTTCTGCAATCATCTTC
GGCATGTACTATGATGCCCTGTGGGACGAGCCTGAATTC---------------TTTAAC
GTGACGGGAGCTCGGTCTACTGCTGGCATCTTCGCGACCTATCCTGGAAAACATCTCACA
ATCCTCAATGGCTTCTTTGATCAGCTCATTGGAACGGCCGCCCTGATTGTGTGCATCCTG
GCCATCGTGGATCCATACAACAACCCCATACCCCAGGGGCTGGAGGCCTTCACTGTGGGC
TTTGTGGTTCTGGTCATTGGACTGGCCATGGGCTTTAACTCTGGCTACGCCGTCAACCCA
GCCAGAGATCTGGGGCCACGTATTTTCACTTCGATGGCTGGCTGGGGCTCGGAGGTCTTC
ACGGCGCGAAGGTGCTGGTTCCTGGTGCCCATCTTCGCCCCGTTTCTCGGGGCTATCATC
GGGGTGATGATCTACCAGACGATGGTGGGTTACCATGTGGAGGGCGAAGTCCGTGACCGG
AGCGAAGCGCAA---------CTGCAGAACGAAAGCGAGAGACTCAACAGCATCAGCACT
---AACGACCACAAC------AAAGAG------AAGGAGGTGTCA---------------
>Poor_cod_Aqp3a
ATGGGAAGACAGAAGGTCTATTTGGACAAGCTGTCCAGAAGCTTCCAGATCCGTAACCTG
CTGCTCCGCCAGGCGCTGGCAGAATGTCTGGGGACGCTCATCCTCGTGATGTTTGGCTGT
GGCGCTGTAGCTCAGCTAGTTCTCAGCGGAGGGTCCCATGGCATGTTTCTAACGGTCAAC
TTTTCCTTCGGCTTTGCGGCCACCCTGGGCATTCTAGTCTGTGGCCAAGTGTCTGGAGGC
CATCTGAATCCGGCTGTGACCTTTGCCCTGTGCCTACTTGGAAGAGAACCCTGGAGAAAG
TTCCCTGTATACTTTGCCTTTCAGACACTGGGGGCATTCCTGGGTTCAGCAATAATCTTC
GGCATGTACTACGACGCCCTGTGGGACCATCCTGACAAC---------------TTTAAT
GTGACGGGAATCCGGTCGACTGCCGGCATCTTCGCAACGTATCCTGGAAAACATCTCACA
ATTCTCAATGGCTTCTTTGATCAGCTCATTGGAACGGCCGCCCTGATCGTGTGCATCTTG
GCCATCGTGGATCCATACAACAACCCCATCCCCCAGGGGCTGGAGGCCTTCACCGTGGGC
TTCGTGGTGCTGGTCATCGGACTGGCCATGGGCTTTAACTCTGGCTATGCCGTCAACCCA
GCCAGGGACCTGGGGCCACGTATCTTCACCTCCATGGCTGGCTGGGGTGCGGAGGTCTTC
ACGGCTCGAAGGTGCTGGTTCCTGGTGCCCATCTTTGCCCCGTTTCTTGGGGCTATCATC
GGG------------------------GGATACCACGTGGAGGGAGAAGTCCGCGACCGG
CAAGAGGCACAG---------CTGCAGAACGAAAGAGAGCGCCTCAACAGCATCAGCACC
---AACGACCACAAC------AAAGAG------AAGGAGGTGTCA---------------
>Tusk_Aqp3a
ATGGGAAGACAGAAGGTATATTTGGACAAGCTGTCCAGAACATTCCAGATCCGTAACCAG
TTGCTTCGCCAGGCCCTGGCAGAATGTCTGGGCACACTCATCCTTGTGATGTTTGGCTGT
GGGGCTGTAGCTCAACTAGTGCTCAGCGGTGGATCCCATGGCATGTTTCTAACTGTCAAC
TTTTCCTTTGGTTTTGCCGCCACCCTGGGCATTCTAGTCTGTGGCCAAGTGTCTGGTGGC
CATCTGAATCCGGCTGTGACCTTTGCCCTGTGCCTACTCGGAAGAGAACACTGGAGAAAG
TTCCCAGTATACTTTTTCTTTCAGACACTGGGTGCATTCCTGGGTTCTGCAATAATCTTT
GGCATGTACTATGATGCCCTGTGGGACCATCCTGACAAC---------------TTTAAT
GTGACTGGAGTACGGTCTACAGCTGGCATCTTCGCTACCTATCCTGGAAAACATCTCACA
ATTCTCAATGGCTTCTTTGATCAGCTCATTGGGACGGCAGCCCTGATCGTGTGCATCCTG
GCCATTGTGGATCCATACAACAACCCCATACCCCAGGGGCTCGAAGCGTTCACCGTGGGC
TTCGTGGTTCTGGTCATTGGACTGGCTATGGGCTTTAACTCTGGCTATGCAGTCAACCCA
GCCAGGGATCTGGGCCCACGCATTTTCACTTCGATGGCTGGCTGGGGCGTAGAGGTCTTC
ACGGTTCGAAACGGCTGGTTCCTGGTGCCCATCTTTGCCCCGTTTCTTGGGGCTGTCATC
GGGGTGGTTGTCTACCAGATGATGGTGGGTTTCCATGTGGAGGGCGAAGTCCGCGACAGA
CGGGAATTGGAG---------CAGCAGAACGAAAGAGTGCGTCTCAACAGCATCAGCACT
---AACGACCACAAC------AAAGAG------AAGGAGGTGTCA---------------
>Ling_Aqp3a
ATGGGAAGACAGAAGGTATATTTGGACAAGCTGTCCAAAACCTTCACGATCCGCAACCAG
TTGCTTCGCCAGGCCCTGGCAGAATGTCTGGGCACACTCATCCTTGTGATGTTTGGCTGT
GGGGCTGTAGCTCAACTAGTGCTCAGCGGTGGATCCCATGGCATGTTTCTAACTGTCAAC
TTTTCCTTTGGCTTTGCCGCTACCCTGGGCATTCTAGTCTGTGGCCAAGTGTCGGGAGGC
CATCTGAATCCGGCTGTGACCTTTGCCCTGTGCCTACTCGGAAGAGAACGCTGGAGAAAG
TTCCCAGTATACTTTGCCTTTCAGACACTGGGTGCATTCCTGGGTTCTGCAATAATCTTT
GGCATGTACTATGATGCCCTGTGGGACCAACCTGACTTC---------------TTTAAT
GTGACGGGAGTACGGTCTACAGCTGGCATCTTCGCTACCTATCCTGGAAAACATCTCACA
ATTCTCAATGGCTTCTTTGATCAGCTCATTGGAACGGCAGCCCTGATCGTGTGCATCCTG
```

GCCATTGTGGATCCATACAACAACCCCATACCCGAGGGGCTTGAAGCATTCACCGTGGGC
TTTGTGGTTCTGGTCATTGGACTGGCTATGGGCTTTAACTCTGGCTATGCAGTCAACCCA
GCCAGGGATCTGGGTCCACGTATTTTCACTTCGATGGCTGGCTGGGGCATGGAGGTCTTC
ACGGCTCGAAAATGCTGGTTCCTGGTGCCCATCTTTGCCCCGTTTCTTGGGGCTGTCATC
GGGGTGATTGTCTACCAGTTCATGGTGGGTTACCATACGGAGGGCGAATTCCGCGACAGG
CAGGAATTGGAG---------CAGCAGAACGAAAGAGTGCGTCTCAACAGCATCAGCACT
---AACGACCACAAC------AAAGAG------AAGGAGGTGTCA---------------
>Burbot_Aqp3a
ATGGGAAGACATAAGATATATTTGGACAAGCTGTCCAGAACCTTCCAGGTCCGCAACCAG
TTGCTGCGCCAGGGCCTGGCAGAATGTCTGGGCACACTCATCCTTGTGATGTTTGGCTGT
GGGGCTGTAGCTCAACTAGTGCTCAGTGGTGGATCTCATGGCATGTTTCTAACTGTCAAC
TTTTCCTTTGGCTTTGCCGCCACCCTGGGTATTCTAGTCTGTGGCCAAGTGTCGGGAGGC
CATCTGAATCCAGCTGTGACCTTTGCCCTGTGCCTACTCGGAAGAGAACGCTGGAGAAAG
TTCCCAATATACTTTGCCTTTCAGACACTGGGTGCATTCCTGGGTTCTGCAATAATCTTT
GGCATGTACTATGATGCCCTGTGGGACCATCCTGAGAAC---------------TTCAAT
ATGTCAGATACACAGTCTACAGCTGGCATCTTCGCTACCTATCCTGGACCTCATCTCACA
ATTCTCAATGGCTTCTTTGATCAGCTCATTGGAACGGCAGCCCTGATCGTGTGCATCCTG
GCCATCGTGGATCCGTACAACAACCCCATACCCCAGGGGCTCGAAGCGTTCACCGTGGGC
TTTGTGGTTCTGGTCATTGGACTGGCTATGGGCTTTAACTCTGGCTATGCAGTCAACCCA
GCCAGGGATCTGGGGCCACGTATTTTCACTGCGATGGCTGGCTGGGGCATGGAGGTCTTC
ACGACTCGAAACTGCTGGTTCCTGGTGCCCATCACTGCCCCGTTTCTTGGGACTATCATC
GGGGTGATTGTCTACCAGATGATGGTCGGCTTCCATGTGGAGGGTGACGTCCGCGACAAG
CGGGAAGCGGCG---------CAGCAGAACGAAAGAGTGCGTCTCAACAGCATCAGCACT
---AACGACCACAAC------AAAGAG------AAGGAGGTGTCA---------------
>Greater_forkbeard_Aqp3a
ATGGGGAAACAAAAGATGTATTTGGACAAGCTGTCCAAAATCTTCCAGATCCGCAATCTT
TTGCTTCGCCAGGCCCTGGCAGAATGTCTGGGCACGCTCATCCTTGTTATGTTCGGCTGT
GGGGCTGTAGCTCAACTCGTGCTCAGCGGTGGATCCCACGGCATGTTTCTAACTGTCAAC
TTTTCCTTCGGCTTCGCTGCCACCCTGGGCATTCTAGTCTGTGGCCAAGTGTCAGGAGGC
CATCTGAATCCCGCCGTGACCTTTGCCCTGTGCCTACTCGGGAGAGAGCGCTGGAGAAAG
TTCCCAGTATACTTTGCCTTTCAGACACTGGGCGCATTCCTTGGTTCTGCAATAATCTTC
GGCATGTACTATGATGCGCTGTGGGACCACCCTGACAAC---------------TTCAAC
GTGACGGGAGTGCGGTCTACAGCTGGCATCTTCGCTACCTATCCTGGAAAACATCTCACA
ATTGTCAATGGCTTCTTTGATCAGCTCATCGGAACGGCAGCCCTGATCGTGTGCATCCTG
GCCATCGTGGATCCATACAACAACCCCGTGCCCCAGGGGCTGGAGGCGTTCACCGTGGGC
TTTGTGGTTCTGGTCATCGGACTGGCTATGGGCTTTAACTCTGGCTACGCGGTCAACCCG
GCCAGGGATCTGGGGCCCCGCATCTTCACCGCGATGGCTGGCTGGGGCGTGGAGGTCTTC
ACGGCGCGGAACTGCTGGTTCCTGGTGCCCATCTTCGCCCCGTTCCTGGGGACCGTCATC
GGGGTGGTCGTGTACCAGATGATGGTGGGCTTCCACGTGGAGGGCGATGTCAGGGACAAG
CTGCAAGCGGAG---------CAGCTGAACGAAAGGGTGCGGCTCACCGGCATCACCACC
---AATGACCACAAC------AAAGAG------AAGGAGCAGTCA---------------
>Forkbeard_Aqp3a
ATGGGGAAACAAAAGATGTATTTGGACAAGCTGTCCAAAATCTTCCAGATCCGCAATCTT
TTGCTTCGCCAGGCCCTGGCAGAATGTCTGGGCACGCTCATCCTTGTTATGTTTGGCTGT
GGGGCCGTAGCTCAACTTGTTCTCAGCGGTGGATCCCACGGCATGTTTCTAACTGTCAAC
TTTTCCTTCGGCTTCGCTGCCACCCTGGGCATTCTAGTCTGTGGCCAAGTGTCAGGAGGC
CATCTGAATCCTGCCGTGACCTTTGCCCTGTGCCTACTCGGGAGAGAGCGCTGGAGAAAG
TTCCCAGTATACTTTGCCTTTCAGACACTGGGCGCATTCCTTGGTTCTGCAATAATCTTT
GGCATGTACTATGATGCGCTGTGGGACCACCCTGACAAC---------------TTCAAC
GTGACGGGAGTGCGGTCTACAGCTGGCATCTTCGCTACCTATCCTGGAAAACATCTCACA
ATTGTCAATGGCTTCTTTGATCAGCTCATCGGAACGGCCGCCCTGATTGTGTGCATCCTG
GCCATCGTGGATCCATACAACAACCCCATACCCCAGGGGCTGGAGGCGTTCACCGTGGGC
TTTGTGGTTCTGGTCATTGGACTGGCCATGGGCTTCAACTCTGGCTACGCGGTCAACCCG
GCCAGGGATCTGGGGCCCCGCATCTTCACCGCGATGGCTGGCTGGGGCGTAGAGGTCTTC
ACGGCTCGGAACTGCTGGTTCCTGGTGCCCATCTTTGCCCCGTTCCTGGGGACCGTCATC
GGGGTGGTCGTGTACCAGATGATGGTGGGCTTCCACGTGGAGGGCGAGGTCAGGGACAAG
CAGCAAGCGGAG---------CAGCTGAATGAAAGAGTGCGTCTCACCGGCATCACCACC
---AACGACCACAAC------AAAGAG------AAGGAGCAGTCA---------------
>Common_mora_Aqp3a
ATGGGAAGACAGAAGCTGTATTTGGACAAGCTGGCCAGAATCTTCCAGATTCGCAACCTG
TTGCTTCGCCAGGCACTGGCAGAATGTCTGGGCACCCTCATCCTTGTGATGTTTGGCTGT

GGGGCCGTAGCTCAACTGGTGCTCAGCGGTGGATCCCATGGCATGTTTCTAACCGTCAAC
TTTTCCTTCGGCTTCGCTGCCACCCTGGGTATTCTAGTCTGTGGCCAAGTGTCGGGAGGC
CATCTGAATCCTGCTGTGACCTTTGCCCTGTGCCTACTCGGGAGAGATCGCTGGAGAAAG
TTCCCAGTATACTTTGCCTTTCAGACACTGGGTGCATTCCTGGGTTCTGCAATAATCTTT
GGCATGTACTATGATGCCCTGTGGGATCATCCTGATAAC---------------TTCAAT
GTGACGGGAGTACGGTCTACAGCTGGCATCTTCGCTACCTACCCTGGAAAACATCTCACA
ATTGTCAATGGCTTCTTTGATCAGCTCATTGGAACGGCAGCCCTGATCGTGTGCATCCTG
GCCATTGTGGATCCATACAACAACCCCATCCCCCAGGGACTCGAGGCGTTCACCGTGGGC
TTTGTGGTTCTGGTCATTGGACTGGCTATGGGCTTCAACTCTGGCTATGCAGTCAACCCG
GCCAGGGATCTGGGGCCACGTATTTTCACTGCGATGGCTGGCTGGGGCATGGAGGTCTTC
ACGGTTCGAAACTGCTGGTTCCTGGTGCCCATCTTTGCCCCGTTTCTCGGGACTATCATC
GGGGTGGTTGTCTACCAGATGATGGTGGGCTTCCATATGGAGGGCGAGGTCCGCGACAAG
CGGGAGGCGGAG---------CAGCAGAACGAAAGAGTGCACCTCACCAACGTCACCACC
---AACGACCACAAC------AAAGAG------AAGGAGGCGTCA---------------
>Marbled_moray_cod_Aqp3a
ATGGGAAGACATAAGGTATATTTGGACAAGCTGTCCAGAACCTTCCAGGTCCGCAACCTG
TTGCTTCGCCAGGGCCTGGCAGAATGTCTAGGCACCCTCATCCTTGTCATGTTCGGCTGT
GGGGCCGTAGCTCAACTGGTCCTCAGCGGTGGATCCCATGGCATGTTCTTAACCGTCAAC
TTTTCCTTTGGCTTTGCCGCCACCCTGGGCATTCTAGTCTGTGGCCAAGTGTCGGGGGGC
CACCTGAATCCTGCTGTGACCTTTGCCCTGTGCCTGCTCGGGAGAGACCGCTGGAGAAAG
TTCCCAGTATACTTTGCCTTTCAGACACTGGGTGCATTCCTGGGTTCTGCAATAATCTTT
GGCATGTACTATGATGCCCTTTGGGACCATCCCGATAAC---------------TTCAAT
GTGACGGGAGTGCGGTCTACAGCTGGCATCTTCGCTACCTACCCCGGAAAACATCTCACA
ATTGTCAATGGCTTCTTTGATCAGCTCATTGGAACGGCAGCCCTGATCGTGTGCATCCTG
GCCATTGTGGATCCGCACAACAACGCCATCCCCCAGGGACTCGAGGCGTTCACCGTGGGC
TTTGTGGTTCTGGTCATCGGACTGGCCATGGGCTTTAACTCTGGCTACGCAGTAAACCCA
GCCAGGGATCTGGGGCCGCGAATTTTCACTGCAATGGCCGGCTGGGGCATGGAGGTCTTC
ACGACTCGAAACTGCTGGTTCCTGGTGCCCATCTTTGCCCCGTTTCTCGGGACCATCATC
GGGGTGATCGTCTACCAGATGATGGTGGGCTTCCATATCGAGGGAAAGGTCCGTGACCAG
CAGGAGCTGGCC---------GAGCGTAACGAGAGAGTGCACCTCACCAACGTCACAACT
---AACGACCACAAT------AAAGAG------AAGGAGTCATCA---------------
>Guinean_codling_Aqp3a
ATGGGAAGACAGAAGATATATTTGGACAAGCTGTCCAGAATCTTCCAGATTCGCAACCTG
TTGCTTCGCCAGGCACTGGCAGAATGTCTAGGCACCCTCATCCTTGTGATGTTTGGCTGC
GGGGCCGTAGCTCAGCTGGTGCTCAGCGGCGGATCCCATGGCATGTTTCTGACCGTCAAC
TTTTCCTTCGGCTTTGCAGCCACCCTGGGCATCCTAGTCTGTGGCCAAGTGTCGGGAGGC
CATCTGAATCCTGCCGTGACCTTTGCCCTGTGCCTACTCGGGAGAGATCGCTGGAGAAAG
TTCCCAGTATACTTTGCCTTTCAGACGCTGGGTGCATTCCTGGGTTCTGCAATAATCTTT
GGCATGTACTATGATGCCCTGTGGGACCATCCTGACAAC---------------TTCAAT
GTGACGGGAGTACGGTCTACAGCTGGCATCTTCGCTACCTACCCTGGAAAGCATCTCACA
ATTGTCAATGGCTTCTTTGATCAGCTCATTGGAACGGCAGCCCTGATCGTGTGCATCCTG
GCCATTGTGGATCCATACAACAACCCCATCCCCCAGGGACTGGAGGCGTTCACCGTGGGC
TTTGTGGTTCTGGTCATTGGACTGGCTATGGGCTTTAACTCTGGCTATGCAGTCAACCCG
GCCAGGGATCTGGGGCCACGGATTTTCACTGCGATGGCTGGCTGGGGAATGGAGGTCTTC
ACGGCTCGAAACTGCTGGTTCCTGGTGCCCATCTTCGCCCCGTTTCTTGGGACTATCATC
GGGGTGGTTGTCTACCAGATGATGGTGGGCTTCCATATGGAGGGCGAAGCCCGTGACAAG
CAGGAAGTGGAG---------CAGCAGAACGAAAGAATGCGCCTCACCAACGTCACCACT
---AACGACCACAAC------AAAGAG------AAGGAGGTGTCA---------------
>European_hake_Aqp3a
ATGGGAAGACAGAAGTTATATTTGGACAAGCTGTCCAGAATCTTCCAGATCCGCAACCTG
TTGCTTCGCCAGGCACTGGCAGAATGTCTGGGCACCCTCATCCTTGTGATGTTTGGCTGT
GGGGCCGTAGCTCAACTAGTGCTCAGCAAAGGATCCCATGGCATGTTTCTAACCGTTAAC
TTTTCCTTCGGCTTTGCTGCCACTCTGGGCATTCTCGTTTGTGGCCAAGTGTCGGGAGGC
CATCTGAATCCTGCTGTGACTTTTGCCCTGTGCCTACTCGGGAGAGATCGCTGGAGAAAG
TTCCCAGTATACTTTGCCTTTCAGACACTGGGCGCATTCCTGGGTTCTGCAATAATCTTT
GGCATGTACTATGATGCCCTGTGGGACACACCTGACAGC---------------TTCAAT
GTGACGGGAGAACATTCTACAGCTGGCATCTTCGCTACCTACCCTGGAAAACATCTCACG
ACTGTCAATGGCTTCTTTGATCAGCTTATTGGAACTGCAGCCCTGATCGTGTGCATCCTG
GCCATTGTGGATCCATACAACAACCCCATCCCCCAGGGACTCGAGGCATTCACCGTGGGC
TTTGTGGTTCTGGTCATTGGACTGGCTATGGGCTTTAACTCTGGCTATGCAGTCAACCCA
GCCAGAGATCTGGGGCCACGTATTTTCACTGCGATGGCTGGCTGGGGCATGGAGGTCTTC

ACGGTTCGAAACTGCTGGTTCCTGGTGCCCATCTTTGCCCCGTTTCTTGGAACCGTCATT
GGGGTGGTTGTCTACCAGATGATGGTGGGCTTCCATGTGGAGGGCGAGGTCCGCGACAAG
CTGGAAGCGGAG---------GCGCAGAACGAAAGAGTGCGTCTCACCAACGTCACCACC
---AACGACCACAAC------AAAGAG------AAGGAGGTGTCA---------------
>Benguela_hake_Aqp3a
ATGGGAAGACAGAAGGTATATTTGGACAAGCTGTCCAGAATCTTCCAGATCCGCAACCTG
TTGCTTCGTCAGGCACTGGCAGAATGTCTGGGCACCCTCATCCTTGTGATGTTTGGCTGT
GGGGCCGTAGCTCAACTAGTGCTCAGCAAAGGATCCCATGGCATGTTTCTAACCGTTAAC
TTTTCCTTCGGCTTTGCTGCCACTCTGGGCATTCTCGTCTGTGGCCAAGTGTCGGGAGGC
CATCTGAATCCTGCTGTGACTTTTGCCCTGTGCCTACTCGGGAGAGATCGCTGGAGAAAG
TTCCCAGTATACTTTGCCTTTCAGACACTGGGCGCATTCCTGGGTTCTGCAATAATCTTT
GGCATGTACTATGATGCCCTGTGGGACACACCTGACAGC---------------TTCAAT
GTGACGGGAGAACATTCTACAGCTGGCATCTTCGCTACCTACCCTGGAAAACATCTCACG
ACTGTCAATGGCTTCTTTGATCAGCTTATTGGAACTGCAGCCCTGATCGTGTGCATCCTG
GCCATTGTGGATCCGTACAACAACCCCATCCCCCAGGGACTCGAGGCGTTCACCGTGGGC
TTTGTGGTTCTGGTCATTGGACTGGCTATGGGCTTTAACTCTGGCTATGCAGTCAACCCA
GCCAGAGATCTGGGGCCACGTATTTTCACTGCGATGGCTGGCTGGGGCATGGAGGTCTTC
ACGGTTCGAAACTGCTGGTTCCTGGTGCCCATCTTCGCCCCGTTTCTTGGAACCGTCATT
GGGGTGGTTGTCTACCAGATGATGGTGGGCTTCCATGTGGAGGGCGAGGTCCGCGACAAG
CTGGAAGCGGAG---------GCGCAGAACGAAAGAGTGCGTCTCACCAACGTCACCACC
---AACGACCACAAC------AAAGAG------AAGGAGGTGTCA---------------
>Cape_hake_Aqp3a
ATGGGAAGACAGAAGATATATTTGGACAAGCTGTCCAGAATCTTCCAGATCCGCAACCTG
TTGCTTCGCCAGGCACTGGCAGAATGTCTGGGCACCCTCATCCTTGTGATGTTTGGCTGT
GGGGCCGTAGCTCAACTAGTGCTCAGCAAAGGATCCCATGGCATGTTTCTAACCGTTAAC
TTTTCCTTCGGCTTTGCTGCCACTCTGGGCATTCTCGTTTGTGGCCAAGTGTCGGGAGGC
CATCTGAATCCTGCTGTGACTTTTGCCCTGTGCCTACTCGGGAGAGATCGCTGGAGAAAG
TTCCCAGTATACTTTGCCTTTCAGACACTGGGCGCATTCCTGGGTTCTGCAATAATCTTT
GGCATGTACTATGATGCCCTGTGGGACACACCTGACAGC---------------TTCAAT
GTGACGGGAGAACATTCTACAGCTGGCATCTTCGCTACCTACCCTGGAAAACATCTCACG
ACTGTCAATGGCTTCTTTGATCAGCTTATTGGAACTGCAGCCCTGATCGTGTGTATCCTG
GCCATTGTGGATCCATACAACAACCCCATCCCCCAGGGACTCGAGGCGTTCACCGTGGGC
TTTGTGGTTCTGGTCATTGGACTGGCTATGGGCTTTAACTCTGGCTATGCAGTCAACCCA
GCCAGAGATCTGGGGCCACGTATTTTCACTGCGATGGCTGGCTGGGGCATGGAGGTCTTC
ACGGTTCGAAACTGCTGGTTCCTGGTGCCCATCTTCGCCCCGTTTCTTGGAACCATCATT
GGAGTGGTTGTCTACCAGATGATGGTGGGCTTCCATGTAGAGGGCGAGGTCCGCGACAAG
CTGGAAGCGGAG---------GCGCAGAACGAAAGAGTGCGTCTCACCAACATCACCACC
---AACGACCACAAC------AAAGAG------AAGGAGGTGTCA---------------
>Arrowtail_Aqp3a
ATGGGAAGACAGAAGGTATATTTGGACAAGCTGGCCAAAAGCTTCCAGGTCCGTAACCTG
TTGCTTCGCCAGGCGCTGGCAGAATGCCTGGGCACCCTCATCCTTGTGATGTTTGGCTGT
GGGGCTGTAGCGCAACTAGTGCTCAGCGGTGGATCCCATGGCATGTTTCTAACTGTCAAC
TTTTCCTTCGGCTTCGCCGCCACCCTGGGCATTCTCGTCTGTGGCCAAGTGTCGGGAGGC
CATCTGAATCCTGCTGTGACCTTTGCCCTGTGCCTACTCGGGAGAGAACGCTGGAGAAAG
TTCCCTGTATACTTTGCCTTTCAGACACTGGGTGCATTCCTGGGTTCTGCAATAATCTTT
GGCATGTATTATGATGCCCTGTGGGACAATCCTGAGAAC---------------TTCAAC
GTGACGGGAATACGGTCTACAGCTGGCGTCTTTGCTACCTACCCTGGAAAACATCTCTCA
ATTGTCAATGGCTTCTTTGATCAGCTCATCGGAACAGCAGCCCTGATCGTGTGCATCCTG
GCCATTGTGGATCCATACAACAACCCCATCCCGCAGGGACTCGAGGCGTTCACCGTGGGC
TTTGTGGTTCTGGTCATTGGACTGGCTATGGGCTTCAACTCCGGCTATGCGGTCAACCCG
GCCAGGGATCTGGGGCCGCGTATTTTCACGGCGATGGCTGGCTGGGGCATGGAGGTGTTC
ACGGCTCGAAACTGTTGGTTCCTGGTGCCCATCTTTGCCCCATTTCTTGGGACCATCATC
GGAGTGATTGTCTACCAGATGATGGTGGGCTGGCATATGGAGGGCGAGGTCCGCGACAGG
CTGGAAGAGGAG---------CAGCAGAACGAGAGAGTGCGCCTCACCAACATCACCACT
---AACGACCACAAC------AAAGAG------AAGGAGGTGTCA---------------
>Roughsnout_grenadier_Aqp3a
ATGGGAAGACAGAAGGTATATTTGGACAAGCTGTCCAGAATCTTCCAGGTCCGCAACCTG
TTGCTTCGCCAGGCACTGGCAGAATGTCTGGGCACCCTCATCCTTGTGATGTTTGGCTGT
GGGGCTGTAGCTCAACTAGTGCTCAGCGGTGGAACCCATGGCATGTTTCTAACTGTCAAC
TTTTCCTTCGGCTTTGGTGCCACCCTGGGCATTCTAGTCTGTGGCCAAGTGTCAGGAGGC
CATCTGAATCCTGCTGTGACCTTTGCCCTGTGCCTACTCGGGAGAGATCGCTGGAGAAAG

TTCCCAGTATTCTTTGCCTTTCAAACACTGGGTGCATTCCTGGGTTCTGCAATAATCTTT
GGCATGTACTATGATGCCCTGTGGGATAATCCTGAGAAC---------------TTCAAT
GTGACCGGACTAAAGTCCACAGCTGGCATCTTCGCTACCTACCCTGGAAAACATCTCACA
ATTGTCAACGGCTTCTTTGATCAGCTCATCGGAACGGCAGCCCTGATCGTGTGCATCCTG
GCCATTGTGGATCCGCACAACAACGCCATCCCCCAGGGCCTGGAGGCGTTCACCGTGGGA
TTTGTGGTTCTGGTCATTGGACTGGCTATGGGCTTTAACTCTGGCTATGCAGTCAACCCG
GCCAGGGATCTGGGGCCACGTATTTTCACTGCGATGGCTGGCTGGGGCACAGAGGTCTTC
ACGGTTCGAAACTGCTGGTTCCTGGTGCCCGTCTTTGCCCCGTTTCTTGGGACTATCATC
GGGGTGTTTGTCTACCAGATGATGGTAGGCTTCCACACGGAGGGAGAGGTCCGCGACAAG
CAGGAAGCAGAG---------CAGCAGAGCGAAAGAGTGCGTCTCACCAGCATCACCACT
---AACGACCACAAC------AAAGAG------AAGGAGGTGTCA---------------
>Roughnose_grenadier_Aqp3a
ATGGGAAGACATAAGGAATATTTGGACAAGCTGTCCAGAATATTCCAGGTCCGCAACCTG
TTGCTTCGCCAGGCACTGGCAGAATGTCTGGGCACCCTCATCCTTGTGATGTTTGGCTGT
GGGGCTGTAGCTCAACTAGTGCTCAGCGGTGGAACCCATGGCATGTTTCTAACTGTCAAC
TTCTCCTTCGGCTTTGGTGCCACCCTGGGCATTCTAGTCTGTGGCCAAGTGTCAGGAGGC
CATCTGAATCCTGCTGTGACCTTTGCCCTGTGCCTACTCGGGAGAGATCGCTGGAGAAAG
TTCCCAGTATACTTTGCCTTTCAAACACTGGGTGCATTCCTGGGTTCTGCAATAATCTTT
GGCATGTACTATGATGCCCTGTGGGATAATCCTGAGAAC---------------TTCAAT
GTGACCGGACTAAAGTCCACAGCTGGCATCTTCGCTACCTACCCTGGAAAACATCTCACA
ATTGTCAACGGCTTCTTTGATCAGCTCATCGGAACGGCAGCCCTGATCGTGTGCATCCTG
GCCATTGTGGATCCGCACAACAACGCCATCCCCCAGGGACTGGAGGCGTTCACCGTGGGA
TTTGTGGTTCTGGTCATTGGACTGGCTATGGGCTTTAACTCTGGCTATGCAGTCAACCCG
GCCAGGGATCTGGGGCCACGTATTTTCACTGCGATGGCTGGCTGGGGCTCAGAGGTCTTC
ACGGCTCGAAACTGCTGGTTCCTGGTGCCCATCTTTGCCCCGTTTCTTGGGACTATCATC
GGGGTGTTTGTCTACCAGATGATGGTAGGCTTCCACACGGAGGGCGAGGTCCGCGACAAG
CGGGAAGCGGAG---------CAGCAGAGCGAAAGAGTGCGTCTCACCAGCATCACCACT
---AACGACCACAAC------AAAGAG------AAGGAGGTGTCA---------------
>Roundnose_grenadier_Aqp3a
ATGGGAAGACAGAAGTTCTATTTGGACAAGCTCTCCAAAACCTTCCAGATCCGCAACCTG
CTGCTTCGCCAGGCACTGGCCGAATGTCTGGGCACCCTCATCCTGGTGATGTTTGGCTGT
GGCGCCGTAGCGCAACATGTGCTGAGCGGCGGATCCCATGGCTTGTTTATCACCGTCAAC
TTTGCATTCGGGTTCGCCGCCACCCTGGGCATTCTCGTCTGTGGCCAAGTGTCGGGTGGC
CATCTGAATCCTGCTGTGACCTTTGCCCTCTGCCTCCTCGGGAGAGATCGCTGGAGAAAG
TTCCCAGTATTCTTTGCCTTTCAGACACTGGGCGCATTCCTGGGTTCTGCAATAATCTTT
GGCATGTACTATGATGCCCTGTGGGACAGTCCGGAGAAC---------------TTCAAC
GTGATGGGAAACCGGTCCACAGCTGGCATCTTCGCGACCTACCCTGGAAAGCATCTCACA
ATCGTCAATGGCTTCTTTGATCAGCTCATCGGAACGGCGGCCCTGATCGTATGCATCCTA
GCCATTGTGGATCCGTACAACAACCCCATCCCCCAGGGACTCGAGGCCTTCACCGTGGGC
TTCGTGGTCCTGGTCATCGGCCTGTCGATGGGCTTCAACTCCGGCTACGCCGTCAACCCG
GCCAGGGACCTGGGCCCACGTATTTTCTCCGCCATGGCGGGCTGGGGCATGCAGGTCTTC
ACGGTTCGAAACTGCTGGTTCCTGGTGCCCATCTTCGCCCCGTTCCTGGGGACGATCATC
GGCGTGGTGGTCTACCAGATGATGGTGGGCTTCCACATGGAGGGCGAGTTCCGCGACAAG
CGGGAGGCGGAG---------GAGCAGAACGAGAGAGTGCGTCTCACCAGCATCACCGCC
---AACGACCACAAC------AAAGAG------AAGGAGGTGTCA---------------
>Onion_eye_grenadier_Aqp3a
ATGGGAAGACATAAGTTCTATTTGGACAAGCTGTCCAAAACCTTCCAGGTCCGCAACCTG
TTGCTTCGCCAGGCACTGGCCGAATGTCTGGGCACCCTCATCCTGGTGATGTTTGGCTGT
GGCGCCGTGGCACAGTTTAGTCTGAGCAGTGGAACCCATGGCTTGTTTATCACCGTCAAC
TTTGCCTTCGGCTTCGCCGCCACCCTGGGCATTCTCGTCTGTGGCCAAGTGTCAGGAGGC
CATCTGAATCCCGCGGTGACCTTTGCCCTGTGTCTCCTCGGGAGAGATCGCTGGAGAAAG
TTCCCAGTATTCTTCGCCTTTCAGACACTGGGCGCATTCCTGGGTTCTGCAATAATCTTT
GGCATGTACTATGATGCCCTGTGGGACAGTCCAGAGAAC---------------TTCAAC
GTGTTGGGAAACCGGTCCACAGCTGGCATCTTCGCGACCTACCCTGGAAAACATCTCACA
ATCGTCAATGGCTTCTTTGATCAGCTCATCGGAACGGCGGCCCTGATCGTGTGCATCCTG
GCCATTGTGGATCCCTACAACAACCCCATCCCCCAGGGACTCGAGGCCTTCACCGTGGGC
TTCGTGGTGCTGGTCATCGGCCTGTCCATGGGCTTCAACTCTGGCTATGCGGTCAACCCG
GCCAGGGACCTGGGCCCTCGTATTTTCACCGCCATGGCAGGCTGGGGCGTAGATGTCTTC
ACGGTTAGAAAGGGCTGGTTCCTGGTGCCCATCTTCGCCCCGTTCCTGGGGGCCATCATC
GGCACTGTGATCTACCAGATGATGGTGGGCTTCCACGTGGAGGGCGAGGCCCGGGACAAG
AAGGAGTTGGAG---------CAGCAGAACGAGAGAGTGCGTCTCACCAGCATCACCACC

---AACGACCACAAC------AAAGAG------AAGGAGGTGTCA---------------
>Western_softhead_grenadier_Aqp3a

--ATGTTTGGTTGT
GGTGCTGTAGCGCAACATGTACTGAGCGGCGGATCCCATGGCTTGTTTATCACCGTCAAC
TTTGCCTTCGGCTTCGCCGCCACCCTGGGCATTCTCGTGTGCGGCCAAGTGTCGGGAGGC
CACCTGAATCCCGCTGTGACCTTCGCCCTGTGCCTCCTCGGGAGGGATCGCTGGAGAAAG
TTCCCAGTATTCTTTGCCTTTCAGACGCTGGGTGCATTCCTGGGATCTGCAATCATCTTT
GGCATGTACTATGATGCCCTGTGGGACAGTCCTGAGAAC---------------TTCAAT
ATGACGGGAACCCGTTCTACAGCTGGCATTTTTGCGACCTACCCTGGAAAACATCTCACG
ATCGTCAATGGCTTCTTTGATCAGCTCATCGGAACGGCGGCCCTGATCGTGTGCATTCTG
GCCATTGTGGATCCGTACAACAACCCCATCCCCCAGGGCCTCGAGGCCTTCACCGTGGGC
TTTGTGGTCCTGGTCATCGGCCTGTCGATGGGCTTCAACTCTGGCTATGCTGTCAACCCG
GCCAGGGATCTGGGCCCGCGTATTTTCTCGGCGATGGCGGGCTGGGGCATGGAGGTCTTC
ACGATTCGAAAGGGCTGGTTCCTGGTGCCCATCTTTGCCCCTTTCCTCGGGACGGTCATC
GGCGTGGTGGTCTACCAGATGATGGTGGGCTTCCACGTGGAGGGCGAGGCCAGAGACAAG
CGAGAGGAGGAG---------CAGCACAACGAGAGAGTGCGTCTCACCAGCATCACCACT
---AACGACCACAAC------AAAGAG------AAGGAGGTGTCA---------------
>Vaillants_grenadier_Aqp3a
ATGGGAAGACAGAAGTTATATTTGGACAAGCTGTCCAGAATCTTCCAGGTCCGAAACCTG
TTGCTTCGCCAGGGACTGGCAGAATGTCTGGGCACCCTCATCCTTGTGATGTTTGGCTGT
GGCGCTGTAGCTCAGAAAGTGCTCAGCGGGGGATCCCATGGCTTGTTTATTACTGTCAAC
TTTGCCTTCGGCTTTGCTGCCACCCTGGGCATTCTAGTCTGTGGCCAAGTGTCGGGAGGC
CATCTGAATCCTGCTGTGACCTTTGCCCTGTGCCTACTCGGGAGGGATCGCTGGAGAAAG
TTCCCTGTATACTTTGCCTTTCAGACACTGGGTGCATTCCTGGGTTCTGCAATAATCTTC
GGCATGTACTATGACGCCCTGTGGGACAGTCCTGAGAAC---------------TTCAAT
GTGACGGGACCACGGTCTACAGCTGGCATCTTCGCTACCTACCCTGGAAAACATCTCACA
ATTGTCAATGGCTTCTTTGATCAGCTCATTGGAACGGCAGCCCTGATCGTGTGCATCCTG
GCCATTGTGGATCCATACAACAACCCCATCCCCCAGGGGCTTGAGGCGTTCACCGTGGGC
TTTGTAGTTCTGGTCATTGGACTGTCTATGGGCTTTAACTCTGGCTATGCAGTCAACCCG
GCCAGGGATCTGGGGCCACGTATTTTCACTGCAATGGCTGGCTGGGGCATGGATGTCTTC
ACGGTTCGAAACTGCTGGTTCCTGGTGCCCATCTTTGCCCCATTTCTTGGGACTGTCATC
GGCGTGGTTGTCTACCAGATGATGGTGGGCTTCCATATGGAGGGCGATGTCCGCGACAAG
CGGGAAGCGGAG---------CAGCAGAAGGAAAGAGTGCGTCTCACCAACATCACCACT
---AACGACCACAAC------AAAGAG------AAAGAGGTGTCA---------------
>Tubeeye_Aqp3a

--ATGTTTGGCTGT
GGTGCTGTAGCTCAACTAGTGCTGAGCAAAGGAACCCATGGCATGTTCCTAACTGTTAAC
TTTGCCTTCGGCTTCGCCGCAACCCTTGGCATTCTGGTCTGTGGCCAAGTATCAGGAGGC
CATCTGAACCCCGCTGTGACCTTTGCCCTGTGCCTACTCGGGAGAGAGCGCTGGAGAAAG
TTCCCAGTATACTTTGCCTTTCAGACGCTGGGTGCATTCCTGGGCTCTGCTATAATCTTT
GCCATGTACTATGATGCCTTGTGGGACGATCCTACCCGC---------------TTCAAT
GTGACAGGCCCAAATAATACTGCTATTATCTTCGCTACCTACCCTGGAAAACATCTCACA
CTTGTGAACGGCTTCTTTGATCAGATCATTGGAACTACAGCCTTGATTGTGTGTATCCTG
GCTATTGTGGATCCGCACAACAACCCCATCCCCCCGGGACTTGAGGCGTTCACTGTTGGC
TTTGTGGTCCTGGTCATTGGACTGTCCATGGGCTTTAACTCTGGCTACGCCGTCAACCCT
GCCAGGGATCTGGGACCACGAATCTTCACCGCAATGGCTGGCTGGGGAAGTCAGGTTTTC
ACGGCTGGTAATTGCTGGTTCCTGGTGCCCATCTTTGCCCCGTTTATTGGTACCATCATT
GGCGTGCTTGTCTACCAACTTATGGTGGGCTTTCACGTGGAAGGAGAGGCCCGCGACAAG
AAGGATGCAGAG---------CAGCACAACGAAAGAGTCCACCTTACCGCCGTCAACACT
---AACGACGGCCTC------AAAGAGAACACAAAGGAGATGTCA---------------
>John_dory_Aqp3a
ATGGGGCGACAGAAGATTTATCTGGAGAAGCTGGCAAAGTTCTTCCAGATCCGCAACCTG
CTGCTTCGCCAGGCCCTGGCCGAGTGCCTGGGCACCCTCATCCTGGTGATGTTTGGCTGC
GGAGCCGTAGCACAGCTTGTGCTGAGCAGCGGATCCCACGGCTTGTTCCTCACTGTCAAC
TTTGCCTTCGGCTTTGCTGCAACTCTCGGCATTCTGGTCTGCGGCCAGGTGTCAGGAGGC
CATCTCAATCCCGCTGTGACCTTTGCTCTATGCCTGCTCGGGAGGGAGCGCTGGAGAAAA
TTCCCAGTGTACTTTGCCTTCCAGACACTGGGTGCATTCCTGGGCTCCGCAATCATCTTT
GGCATGTACTACGATGCCCTGTGGGACCGCCCTGGGGAT---------------TTCAAT
GTGACTGGACCAAACAGCACAGCCGGCATCTTTGCTACGTACCCTGGAAAACATCTCACG

ATCCTCAACGGCTTCTTCGATCAGATCATTGGAACAACAGCCCTGATTGTCTGCATCCTG
GCCATTGTGGATCCATACAACAACCCCATTCCTCCGGGGCTGGAGGCGTTCACTGTGGGC
TTTGTGGTTCTGGTCATCGGACTTTCCATGGGCTTCAACTCCGGCTATGCCGTCAACCCT
GCCAGGGATCTGGGACCACGTATCTTCACTGCAATGGCTGGCTGGGGCATTGAAGTTTTC
ACGGTCAGAAATGGCTGGTTCCTGGTGCCCGTCATTGCCCCCTTCATTGGGACCGTCATT
GGGGTAATTATCTATCAGCTGATGGTCGGCTTCCACGTGGAGGGCGAGGCTCACGACAAG
AAGCTGAGAGAC---------CAGCAGAACGAGAGAGTCCACCTCACCAACGTCACCAGC
---AACAACGCCCCC------AAAGAGGACAACAAGGAGATGTCATCA------------
>Rosy_dory_Aqp3a
ATGGGCCGACAGAAGGTTTATCTGGACAAGCTGACGAAGTTCTTCCAGATCCGCAACATG
CTGCTTCGCCAGGCCCTGGCCGAGTGTCTGGGCACCCTCATCCTGGTGATGTTTGGCTGT
GGAGCCGTAGCGCAGCTTGTGCTGAGCAGCGGATCCCATGGCATGTTCCTAACCGTCAAC
TTTGCCTTTGGCTTTAGCGCTACCCTCGGCATTCTGGTCTGCGGCCAGGTATCAGGAGGC
CATCTCAACCCCGCGGTGACCTTTGCCTTATGCCTGCTCGGGAGGGAGCGCTGGAGAAAA
TTCCCAGTGTACTTTGCCTTCCAGACATTGGGCGCATTCCTGGGCTCCGCAATCATCTTT
GGCATGTACTATGATGCCCTGTGGGACCACCCTGGGAAT---------------TTCAAT
GTGTCTGGACCAAACAGCACAGCTGGCATCTTTGCTACCTACCCTGGAAAACATCTCACT
ATCGTCAACGGCTTCTTCGATCAGATCATTGGAACAACAGCCTTGATTGTCTGCATCCTG
GCCATTGTGGACCCGTACAACAACCCCATTCCTCAGGGACTGGAGGCGTTCACTGTGGGC
TTTGTGGTTCTGGTCATCGGACTTTCCATGGGCTTCAACTCCGGCTATGCCGTCAACCCT
GCCAGGGATCTGGGACCGCGTATCTTCACTGCAATGGCTGGCTGGGGCACTGAAGTTTTC
ACGGTCAGAAAGTGCTGGTTCCTGGTGCCCATCGTTGCCCCCTTCATTGGGACCATCATT
GGCGTGATTGTCTATCAGATGATGGTCGGCTTCCACGTGGAGGGCGAGGCCCACGACAGG
AAGCTGAAAGAA---------CAGCAGAACGAGAGAATCCACCTCACCAACGTCACCAGC
---AACGACGCCTCC------AAAGAG---AACAAGGAGATGTCCTCA------------
>Sand_roller_Aqp3a
ATGGGCAGACAAAAGATATATCTGGAGAAACTGGCTGCAACCTTCCGGATCCACAACCTA
CTGATGCGACAGGCACTGGCTGAGTGTCTGGGCACCCTTATTTTGGTGATGTTTGGTTGT
GGTGCTATTGCCCAGAACGTGCTGAGTGGTGGAAGTCATGGTTTATTCCTCGCTGTAAAC
TTTGCTTTTGGCTTCGCCGCCACCCTGGGCATTCTTGTCTGTGGCCAAGTGTCAGGAGGT
CATCTAAACCCTGCCGTGACCTTTGCCCTATGTCTACTTGGGAGGGAGCGCTGGAGAAAG
TTTCCTATATACTTTTTGTTTCAGACACTGGGGGCTTTCCTAGGCTCTGGGATTGTCTTT
GCCATGTACTACGATGCACACTGGGACCATCCAGGTAGC---------------TATAAT
GTGACTGGAGAACACGCCACAGCTGGAATCTTTGCTACTTATCCTGGACCTCATCTCACC
CTTCTTAATGGTTTATTTGATCAGATCATTGGCACAGCTGCTCTGATTGTGTGCATCCTG
GCTATTGTGGACCCATTCAACAACCCCATCCCTCAAGGGCTGGAGGCCTTCACTGTGGGC
TTTGTGGTTTTGGTCATTGGACTGTCAATGGGCTTTAACTCTGGCTATGCTGTCAACCCG
GCCAGGGACTTGGGACCACGTATTTTCTCTGCTATGGCTGGCTGGGGCAGTGAAGTTTTC
ACG---

>Southern_cavefish_Aqp3a
ATGGGAAGACAAAAGATATATCTGGAGAAAATTGCTGCAACCGCCCGGATCCGCAACCTA
CTGCTTCGACAGGGACTAGCTGAGTGTCTGGGCACCCTTATTTTGGTGATGTTTGGTTGT
GGTGCTATAGCCCAGCATGTCCTAAGCAGTGGAAGTCATGGTTTATTCCTTGCTGTAAAC
TTTGCTTTTGGCTTTGCCGCCACCCTTGGCATTCTGGTCTGCGGCCAAGTGTCAGGTGGT
CATCTAAACCCTGCTGTGACCTTTGCACTGTGTCTACTCGGGAGGGAGCGCTGGAGAAAG
CTCCCTGTATACTTTTTCTTCCAGACACTGGGCGCTTTCTTGGGCTCTGGGATTGTCTTC
GTTATGTACTATGATGCACACTGGGACCATCCTGGGAGC---------------TTTAAT
GTGACTGGAGATGTGAACACAGCTGGCATTTTTGCTACTTATCCTGGACCTCACCTCACC
CTTATCAATGGATTATTTGACCAGATCATTGGCACAGCTGCTCTGATTGTGTGCATCCTG
GCTATTTTGGACCCATTCAACAATCCCATCCCTAAAGGCCTTGAGGCCTTCACTGTGGGC
TTTGTGGTTCTGGTCATTGGCCTGTCGATGGGCTTCAACTCTGGCTATGCTGTCAACCCT
GCCAGGGACTTGGGCCCACGTATTTTCACCTCTATAGCTGGCTGGGGCAGTGAAGTATTT
ACGGCTGGCCACTGCTGGTTCCTCATACCCATCTTCGCCCCATTCATTGGCAGCTTTATT
GGTTCGGTCATCTACCAGCTGATGGTTGGCTTTCATGTGGAGGGAGAGGCCCGTGACAGG
GAGAGAAGAACCCCA------CAGGGGACAGAGGGTTTCCCACTTGCCAAGGTCACCTCC
---AATGAT------------AAAGGA------AAAGACATGTCA---------------
>Glacier_lantern_fish_Aqp3a
ATGGGTAGACAGAAGGTGTACCTGGACAAACTGGCACAGACCTTCAGGCTGCGCAACCTA

CTGCTTCGTCAGGCCCTGGCTGAATGTCTCGGAACCCTCATCCTGGTTATGTTTGGTTGT
GGTGCCGTGGCCCAGCTGGTGCTGAGCAAGGGAACCCACGGCATGTTCCTCACTGTCAAC
TTTGCCTTCGGCTTCGCCGCCACCTTGGGCATCCTGGTCTGCGGCCAAGTTTCAGGAGGC
CATCTGAACCCTGCAGTGACCTTTGCCCTGTGTCTGCTTGGGAGGGAGCGGTGGAGGAAG
TTCCCAGTATACTTTGCGTTTCAGACGCTGGGTGCTTTCTTGGGTTCTGCCATCATCTTC
GCCATGTACTATGATGCACTGTGGGACCTCCCTGGAAGT---------------TTCAGT
CTGACTGGGCCCAACGCCACAGCTGGCATCTTTGCCACCTACCCTGGAAAGCATCTCACC
CTCGTCAACGGCTTCTTTGATCAGATCATCGGGACAACGGCTCTCATAGTCTGTATCCTG
GCGATCGTGGACCCGCACAACAACCCCATTCCTCAGGGCCTGGAGGCCTTCACAGTGGGT
TTCGTGGTTCTGGTCATTGGACTGTCCATGGGCTTCAACTCCGGTTACGCCGTCAACCCT
GCCAGAGACCTGGGACCACGTATTTTCACTGCCATGGCTGGCTGGGGCACCGGAGTCTTC
ACG---GGGAACTGCTGGTTCCTGGTGCCAATCTTCGCTCCGTTCCTGGGCACCATCCTC
GGCGTGATCATCTACCAGCTGATGGTCGGCTTCCACGTGGAGGGAGAGTTCCGTGACCGG
CAGGAGAAGGAG---------CAGGATACCGAGAGAGTCCGGCTCACCAACGTCACGACC
---AACGACAACTCT------AAAGACGTGACCAAGGAGATGGCG---------------
>Atlantic_greeneye_Aqp3a
ATGGGCAGACAGAAGGTGTACCTGGACAAGCTGTCACATACCTTCCAGATCCGCAACCTG
CTACTTCGCCAGGCCCTGGGAGAATGTCTTGGCACCCTCATCCTGGTGATGTTTGGTTGT
GGTGCAGTGGCCCAGCTGGTGCTGAGTTCAGGGTCCCATGGCATGTTCCTCACTGTCAAC
TTTGCTTTTGGCTTCGCTGCCACCTTGGGCATCTTGGTCTGCGGCCAAGTATCAGGAGGC
CATCTGAACCCTGCTGTGACCTTTGCCCTCTGCCTGCTTGGGAGAGAACGCTGGAGAAAG
TTCCCAGTGTACTTTGCCTTCCAGACACTCGGCGCTTTCCTGGGCTCTGCCATCATCTTC
GGCATGTACTATGATGCTCTGTGGGATCATCCTGGAGCC---------------TTCACT
GTTCTGGGGAAAAACTCTACAGCTGGAATCTTTGCTACATACCCTGGAGAACACCTCACC
CTTGTCAACGGTTTCTTTGATCAGATCATCGGTACAGCTGCTCTGATCGTGTGTATCCTG
GCCATCGTGGACCCATACAACAACCCTATCCCCCAGGGACTGGAGGCCTTCACTGTGGGC
TTTGTGGTTCTGGTCATTGGACTTTCCATGGGCTTCAACTCTGGCTACGCTGTCAACCCT
GCCAGGGACCTGGGACCACGTATTTTCACCGCTATGGCTGGCTGGGGCACAGAGGTCTTC
ACGGCTAGAAGGTGCTGGTTCCTGGTTCCCATCTTTGCCCCGTTCCTGGGCACCATCATT
GGCGTGATCGTCTACCAGATGATGGTTGGTTTCCATGTGGAGGGAGAGGTCCGCGACAAG
CAGAGCCTTGAG---------CAGGAGAACGAGAGGGTCAAACTCACCAACGTCACCACC
---AACGACCTCCCC------AAGGAGCCTGCCAAGGAGTCTTTT---------------
>Highfin_tadpole_fish_Aqp3a
ATGGGCAGACAGAAGGTGTACCTGGACAAACTGGCACGGTCCTTCCAGATTCGCAACCTG
CTCCTTCGCCAGGCCCTGGCTGAATGTCTCGGCACCCTCGTTCTGGTGATGTTCGGCTGT
GGTGGGGTGGCCCAGGTGGTACTGAGTGGTGGTACCCATGGCTTGTTCCTCACGGTCAAC
TTTGCTTTTGGCTTTGCTGCCACCCTGGGTATCCTCGTCTGCGGTCAAGTATCAGGAGGC
CATCTGAACCCCGCTGTGACCTTTGCCCTTTGCCTGCTTGGGAGAGAGCGCTGGAGAAAG
TTCCCAGTGTACTTTCTCTTTCAGACGCTGGGTGCTTTCTTGGGGTCAGCAATCATTTAC
GGGATGTACTACGACGCTATTTGGAGCTATGCTGGTCAG---------------TTGTAC
GTTACTGGGCCAAATTCTACAGCTAAGATCTTTGCTACCTACCCTGGGGAACATCTTACC
ATCATCAACGGTTTCTTTGATCAGATCATTGGCACAGCAGCTCTGATCGTGTGCATCCTG
GCCATCGTGGACCCGTACAACAACCCTATTCCTCAGGGGCTGGAGGCTTTCACAGTGGGC
TTTGTGGTTCTGGTCATCGGATTGTCTATGGGCTTCAACTCTGGCTACGCTGTCAACCCT
GCCAGAGATCTGGGGCCACGTCTCTTCACGGCTCTGGCTGGATGGGGCTCTGAAGTATTC
ACGGCTCGAAGCTGCTGGTTCCTGGTGCCCATCTTTGCTCCATTCTTGGGCACCATCATC
GGTGTCCTTGTCTACCAGCTGATGGTTGGTTTCCACATGGAGGGAGATTTCCGCGATAAG
AGGGCCAAGGAGCAG------GAGGAGAATGAGATCGTCAGGCTGACAGAGGTCACGTCC
---AATGACCACCCC------------------AAAGAGATGTCA---------------
>European_smelt_Aqp3a
ATGGGAAGACAGAAGCTTTACCTGGAGAAGTTGTCTCAGTCCTTCCAGATCCGCAACCTG
CTCCTTCGCCAGGCCCTGGCCGAGTGTCTGGGCACGCTCATCCTGGTGATGTTTGGTTGT
GGTGCCGTGGCCCAGCTGGTGCTTAGCGGGGGATCTCACGGGTTGTTCCTCACCGTCAAC
TTTGCGTTCGGCTTCGCTGCCACCCTGGGGATCCTTGTCTGTGGGCAGGTATCAGGAGGC
CATCTGAACCCGGCGGTGACCTTTGCCCTCTGCCTGCTGGGGAGAGAGCGCTGGATCAAG
TTCCCAGTGTACTTCGCCTTCCAAACGCTCGGCGCTTTCCTAGGCTCAGGGGTTATCTTC
GGCCTGTACTACGATGCTCTGTGGGACTTCAAAGGAGTT---------------CTTGCC
GTAACTGGAGATAACGCTACGGCTGGTATCTTCGCTACGTACCCTGGCAAACACCTGACT
CTCGTCAATGGCTTCTTTGACCAGATGATTGGCACCGCGGCGCTGATCGTGTGTATCCTG
GCCATCGTGGACCCCTACAACAACCCCATCCCCACGGGTCTGGAAGCTTTCACTGTGGGC
TTTGTGGTGCTGGTCATCGGCCTGTCCATGGGCCTCAACTCGGGCTACGCCGTCAACCCC

GCCAGGGACCTGGGCCCCCGCCTCTTCACCGCCATAGCAGGCTGGGGCTCGGAGGTCTTC
AGTGCTCATGACTACTGGTTCCTGGTCCCGATCTTCGCTCCGTTCCTCGGTACCATCTTT
GGAGTGATGGTCTACCAGTTTATGGTTGGATTTCATCAGGAAGGTGAGGTCCGCGACAAG
---GCCAGGATG---------TCCGAC---GAAAGGGTGAAACTGACCAACGTCTCCTCC
AGCAACGACCAGTCG------AAAGGTTCTTCCAAGGAAATGGAA---------------
>Ayu_Aqp3a
ATGGGAAGGCAGAAGTATTACCTGGAGAAGATGTCTCAAACCTTCCAGATCCGCAACTTG
CTGCTACGTCAGGCCCTGGCGGAGTGTCTAGGAACCCTCATCCTGGTGATGTTTGGCTGT
GGTGCTGTTGCCCAGCTGGTGCTCAGCCAGGGATCTCATGGCTTGTTCCTCACCGTCAAC
TTTGCATTTGGCTTTGCTGCCACCCTGGGGATCCTTGTCTGTGGCCAGGTATCAGGTGGC
CATCTGAACCCGGCGGTGACCTTTGCCCTCTGCTTGCTGGGGAGAGAGCGCTGGATAAAG
TTCCCAGTGTACTTCGCCTTCCAAACGCTTGGGGCTTTCCTAGGCTCAGGGATTATCTTC
GGCCTGTACTACGATGCTCTGTGGGACTTTAAAGGAGTT---------------CTCGCA
GTAACTGGAGAAAATGCTACTGCTGGTATCTTTGCCACGTACCCTGGAAAACACCTGTCT
CTCATCAATGGCTTCTTTGACCAGATGATCGGCACGGCGGCGCTTATCGTGTGCATCCTG
GCTATCGTGGACCCCTACAACAACCCTATCCCCCAGGGCCTGGAGGCTTTCACTGTGGGG
TTTGTGGTGCTGGTCATTGGCCTGTCCATGGGCCTCAACTCTGGCTACGCCGTCAACCCC
GCCAGGGACTTGGGCCCTCGCCTCTTCACTTCCATCGCAGGCTGGGGGTCTGAAGTGTTC
AGTGCCTATGATTACTGGTTCCTGGTCCCGATCTTCGCCCCGTTCCTAGGCACCATCTTT
GGAGTGCTTGTCTACCAGTTCATGGTGGGATATCACCAGGAAGGGGAGTTTCGTGATAAG
---GCCAGGCAG---------GCCGAA---GAAAGGGTCAAACTGACCAACATCTCCTCC
GGCAATGATCAGTCG------AAAGGATGTTCCAAGGAAATGGAA---------------
>Large_eye_snaggletooth_Aqp3a
ATGGGACGGCAGAAAATTTACCTGAGCAAGCTGGCACAGATGTTCCAGATCCGCAACATT
CTGCTTCGCCAGGCCCTGGCAGAGTGTCTGGGCACCCTCATCCTGGTGATGTTTGGTTGT
GGTGCTGTGGCCCAGCTGGTGCTGAGTGGAGGATCTCATGGAATGTTCCTCACCGTCAAC
TTTGCCTTCGGCTTCGCTGCCACCCTCGGGATCCTGGTCTGTGGCCAGGTGTCAGGGGGC
CATCTCAACCCTGCAGTGACCTTTGCCCTCTGCCTGCTGGGTAGAGAGCGCTGGAGGAAG
TTCCCAATGTTCTTTTTCTTCCAAACGCTGGGCGCTTTCCTCGGCTCTGGGATCATCTTT
GGTCTCTACTATGACGCTCTGTGGGACTATGCAGGAAAG---------------CTCATT
GTGACTGGGCCAAACGCCACGGCTGGGATCTTCGCCACCTACCCCGGGAAACACCTGACC
CTCGTCAACGGCCTTTTTGACCAGATCATCGGCACGGCTGCGCTGATAGTGTGCATCCTG
GCCATCGTGGACCCCTACAACAACCCCATCCCCCAGGGCCTAGAGGCCTTCACAGTGGGC
TTTGTGGTGCTGGTCATCGGCCTGTCTATGGGCTTCAACTCAGGCTACGCTGTCAACCCC
GCCAGGGACCTGGGACCCCGCCTCTTCACCTCTCTGGCAGGCTGGGGCACTGAGGTTTTC
ACGGCGAGGAACTGCTGGTTCCTGGTGCCAATCTTCGCCCCGTTCCTGGGCACCATCATT
GGGGTTGTGGTCTACCAGCTGATGGTGGGCTTCCATGTGGAGGGAGAGGCTCGCGACAGG
GAGGCCAACCAGCATCAG---GAGGACCAGGTCAAACTTAAACTTATCAATGTCACCTCC
AACAATGACAACCCC------AAGGAGCCCACCAAAGAAATGGAA---------------
>Coho_salmon_Aqp3a1a
ATGGGAAAACAGAAGATATTCATGGACAAGCTGGCACAGACTTTCCAGATCCGTAACTTG
CTGCTGCGCCAGGCCATGGCAGAGTGTCTGGGTACCCTCATCCTGGTGATGTTTGGTTGT
GGTGCAGTGGCTCAACTGGTGCTTAGTGGAGGATCTCATGGCATGTTCCTAACTGTCAAC
TTTGCATTCGGCTTCGCTGCCACCCTAGGGATCCTGGTCTGTGGCCAGGTATCAGGAGGC
CATCTCAATCCGGCAGTAACCTTTGCCCTCTGCCTGCTTGGGAGAGATCGCTGGAGAAAG
TTTCCAGTCTACTTTGTCTTCCAGACACTGGGAGCTTTCCTGGGCTCCGGGATCATCTTT
GGTCTGTACTATGACGCTCTGTTGAACTATGCTGGAGAA---------------CTCATT
GTTACCGGGCCCAACGCCACCGCTGGCATCTTCGCCACCTACCCTGGCAAACATCTCACA
TTGGTCAACGGCTTCTTCGACCAGACGATTGGCACTGCAGCATTGATCGTCTGTATCCTG
GCCATCGTGGACCCCCATAACAATCCCATTCCCCAGGGCCTGGAGGCCTTCACGGTGGGC
TTTGTGGTGCTGGTCATCGGCCTGTCCATGGGCTTCAACTCTGGCTACGCTGTCAACCCT
GCCAGGGACCTGGGGCCACGCCTCTTTACCGCTCTGGCAGGCTGGGGCACCGAGGTCTTC
ACGGCCAACAAGTGCTGGTTCCTGGTGCCCATCTTCGCCCCATTCCTGGGCGCCATCATT
GGTGTGGTGGTCTATCAGCTGATGGTTGGCTTTCATCAGGAGGGTGAGGTTCGTGACAGG
AAGAGC---------------CAGGAT---GAGAGAGTCAAACTGACCAACATCAACTCA
---AAGGACGCCCTA------AAAGAA---------GAGATGTTA---------------
>Chinook_salmon_Aqp3a1a
ATGGGAAAACAGAAGATATTCATGGACAAGCTGGCACAGACTTTCCAGATCCGTAACTTG
CTGCTGCGCCAGGCCATGGCAGAGTGTCTGGGTACCCTCATCCTGGTGATGTTTGGTTGT
GGTGCAGTGGCTCAACTGGTGCTTAGTGGAGGATCTCATGGCATGTTCCTAACTGTCAAC
TTTGCTTTCGGCTTCGCTGCCACCCTAGGGATCCTGGTCTGTGGCCAGGTATCAGGAGGC

CATCTCAATCCGGCAGTAACCTTTGCCCTCTGCCTGCTTGGGAGAGATCGCTGGAGAAAG
TTTCCAGTCTACTTCGTCTTCCAGACACTGGGAGCTTTCCTGGGCTCCGGGATCATCTTT
GGTCTGTACTATGACGCTCTATTGAACTATGCTGGAGAA---------------CTCATT
GTTACCGGGCCCAACGCCACCGCTGGCATCTTCGCCACCTACCCTGGCAAACATCTCACA
TTGGTCAATGGCTTCTTCGACCAGACGATTGGCACTGCAGCATTGATCGTCTGTATCCTG
GCCATCGTGGACCCCCATAACAATCCCATTCCCCAGGGCCTGGAGGCCTTCACGGTGGGC
TTTGTGGTGCTGGTCATCGGCCTGTCCATGGGCTTCAACTCTGGCTACGCTGTCAACCCT
GCCAGGGACCTGGGGCCACGCCTCTTTACCGCTCTGGCAGGCTGGGGCACCGAGGTCTTC
ACGGCCAACAAGTGCTGGTTCCTGGTGCCCATCTTCGCCCCATTCCTGGGCGCCATCATT
GGTGTGGTGGTCTATCAGCTGATGGTTGGCTTTCATCAGGAGGGAGAGGTTCGTGACAGG
ATGAGC----------------CAGGAT---GAGAGAGTCAAACCGACCAACATCAACTCA
---AAGGACGCCCTA------AAGGAA---------GAGATGTTA---------------
>Rainbow_trout_Aqp3a1a
ATGGGAAAACAGAAGATATTCATGGACAAGCTGGCACATACTTTCCAGATCCGTAACTTG
CTGCTGCGCCAGGCCATGGCAGAGTGTCTGGGTACCCTCATCCTGGTGATGTTTGGTTGT
GGTGCAGTGGCTCAACTGGTGCTTAGTGGAGGATCTCATGGCATGTTCCTAACTGTCAAC
TTTGCTTTCGGCTTCGCTGCCACCCTAGGGATCCTGGTCTGTGGCCAGGTATCAGGTGGC
CATCTCAATCCGGCAGTAACCTTTGCCCTCTGCCTGCTTGGGAGAGATCGCTGGAGAAAG
TTTCCAGTCTACTTCGTCTTCCAGACACTGGGAGCTTTCCTGGGCTCCGGGATCATCTTT
GGTCTGTACTATGACGCTCTGTTGAACTATGCTGGAGAA---------------CTCATT
GTTACCGGGCCCAACGCCACCGCTGGCATCTTCGCCACCTACCCTGGCAAACATCTCACA
TTGGTCAACGGCTTCTTCGACCAGACGATTGGCACTGCAGCATTGATCGTCTGTATCCTG
GCCATCGTGGACCCCCATAACAATCCCATTCCCCAGGGCCTGGAGGCCTTCACGGTGGGC
TTTGTGGTGCTGGTCATCGGCCTGTCCATGGGCTTCAACTCTGGCTACGCTGTCAACCCT
GCCAGGGACCTGGGGCCACGCCTCTTTACCGCTCTGGCAGGCTGGGGCACCGAGGTCTTC
ACGGCCAACAAGTGCTGGTTCCTGGTGCCCATCTTCGCCCCATTCCTGGGCGCCATCATT
GGTGTGGTGGTCTATCAGCTGATGGTTGGCTTTCATCAGGAGGGAGAGGTTCGTGACAGG
AAGAGC----------------CAGGAT---GAGAGAGTTAAACTGACCAACATCAACTCA
---AAGGACGTCCTA------AAGGAA---------GAGATGTTA---------------
>Arctic_charr_Aqp3a1a
ATGGGAAAACAGAAGGTATTCATGGACAAGCTGGCACAGACTTTCCAGATCCGTAACTTG
CTGCTGCGCCAAGCCATGGCAGAGTGTCTGGGTACCCTCATCCTGGTGATGTTTGGTTGT
GGTGCAGTGGCTCAGCTGGTGCTTAGTGGAGGATCTCATGGCATGTTCCTAACTGTCAAC
TTTGCTTTCGGCTTCGCTGCCACCCTAGGGATCCTGGTCTGTGGCCAGGTATCAGGTGGC
CATCTCAATCCAGCAGTAACCTTTGCCCTCTGCCTGCTTGGGAGAGATCGCTGGAGAAAG
TTCCCAGTCTACTTCTTCTTCCAGACACTGGGAGCTTTCCTGGGCTCCGGGATCATCTTT
GGTCTGTACTATGACGCTCTGTTGAACTATGCTGGAGAA---------------CTCATT
GTTACCGGACCCAACGCCACCGCTGGCATCTTCGCCACCTACCCTGGCAAACATCTCACA
TTGGTCAACGGCTTCTTCGACCAGACGATTGGCACTGCAGCATTGATCGTGTGTATCCTG
GCCATCGTGGACCCCCATAACAATCCCATTCCCCAGGGCCTGGAGGCCTTCACGGTGGGC
TTCGTGGTGCTGGTCATCGGCCTGTCCATGGGCTTCAACTCTGGCTACGCTGTCAACCCT
GCCAGGGACCTGGGGCCACGCCTCTTTACTGCTCTGGCAGGCTGGGGCACCGAGGTCTTC
ACGGCCAACAAGTGCTGGTTCCTGGTGCCCATCTTCGCCCCATTCCTGGGCGCCATCATT
GGTGTGGTGGTCTATCAGCTGATGGTTGGCTTTCATCAGGAGGGAGAGGTTCGTGACCGG
AAGAGC----------------CAGGAG---GAGAGAGTCAAACTGACCAACATTAACCCA
---AAGGATGTTCGA------AAGGAA---------GAGATGTTA---------------
>Brook_trout_Aqp3a1a
ATGGGAAAACAGAAGATATGGTTGGACAAGCTGGCACAGAACTTCCAGATCCGTAACCTG
CTGCTGCGCCAGGCTCTGGCAGAGTGTCTGGGTACCCTCATCCTGGTGATGTTTGGTTGT
GGTGCGGTGGCTCAGCTGGTACTTAGTGGAGGATCTCATGGCATGTTCCTAACTGTCAAC
TTTGCTTTCGGCTTTGCTGCCACCCTAGGGATCCTGGTCTGTGGCCAAGTATCAGGAGGC
CATCTTAACCCGGCGGTGACCTTCGCCCTCTGCCTGCTTGGGAGAGAACGCTGGAGAAAG
TTCCCAGTGTTCTTTGCCTTCCAGACACTGGGCGCTTTCCTGGGCTCCGGGATCATCTTT
GGTCTGTACTATGACGCTCTGTGGGGCTTTGCTGGAAAG---------------CTCATT
GTTACCGGGCCCAACGCCACCGCTGGCATCTTCGCCACCTACCCTGGCAAACATCTCACA
TTGGTCAACGGCTTCTTCGACCAGACGATTGGCACTGCCGCATTGATCGTGTGTATCCTG
GCCATCGTGGACCCCCATAACAATCCCATTCCCCAGGGCCTGGAGGCCTTCACGGTGGGC
TTTGTGGTGCTGGTCATCGGCCTGTCCATGGGCTTCAACTCTGGCTACGCTGTCAACCCT
GCCAGGGACCTGGGGCCACGCCTCTTTACCGCTCTGGCAGGCTGGGGCACCGAGGTCTTC
ACTGCCAACAAGTGCTGGTTCCTGGTGCCCATCTTCGCCCCATTCCTGGGCGCCATCATT
GGTGTGGTGGTCTATCAGCTGATGGTTGGCTTTCATCAGGAGGGAGAGGTTCGTGACCGG

AAGAGC----------------CAGGAG---GAGAGAGTCAAACTGACCAACATTAACCCA
---AAGGATGCTCGA------AAGGAA---------GAGATGTTA---------------
>Brown_trout_Aqp3a1a
ATGGGAAAACAGAAGATATTCATGGACAAGCTGGCACAGACTTTCCAGATCCGTAACTTG
CTGCTGCGCCAGGCCATGGCAGAGTGTCTGGGTACCCTCATCCTGGTGATGTTTGGTTGT
GGTGCGGTGGCTCAGCTGGTGCTTAGTGGAGGATCTCATGGCATGTTCCTAACCGTCAAC
TTTGCTTTCGGCTTCGCTGCCACCCTAGGGATCCTGGTCTGTGGCCAGGTATCAGGAGGA
CATCTCAATCCGGCAGTGACCTTTGCCCTCTGCCTGCTTGGGAGAGAACGCTGGAGAAAG
TTCCCAGTCTACTTCGTCTTCCAGACACTGGGAGCTTTCCTGGGCTCCGGGATCATCTTT
GGTCTGTACTATGACGCTCTGTTGAACTATGCTGGAGAA----------------CTCATT
GTTACCGGGCCCAACGCCACCGCTGGCATCTTCGCCACCTACCCTGGCAAACATCTCACA
TTGGTCAACGGCTTCTTCGACCAGACGATTGGCACTGCAGCATTGATCGTGTGTATCCTG
GCCATCGTGGACCCCCATAACAATCCCATTCCCCAGGGCCTGGAGGCCTTCACGGTGGGC
TTTGTGGTGCTGGTCATCGGCCTGTCCATGGGCTTCAACTCTGGCTACGCTGTCAACCCT
GCCAGGGACCTGGGGCCACGCCTCTTTACCTCTCTGGCAGGCTGGGGTACCGAGGTCTTC
ACTGCCAATAAGTGCTGGTTCCTGGTGCCCATCTTCGCCCCATTCCTGGGAGCCATCATT
GGTGTGGTGGTCTATCAGCTGATGGTTGGCTTTCATCAGGAGGGAGAGGTTCGTGACAGG
AAGAGC----------------CAGGAG---GAGAGAGTCAAACTGGCCAACATGAACCCA
---AAGGATGCCCTA------AAGGAA---------GAGATGTTA---------------
>Atlantic_salmon_Aqp3a1a
ATGGGAAAACAGAAGATATTCATGGACAAGCTGGCACAGACTTTCCAGATCCGTAACTTG
CTGCTGCGCCAGGCCATGGCAGAGTGTCTGGGTACCCTCATCCTGGTGATGTTTGGTTGT
GGTGCAGTGGCTCAGCTGGTGCTTAGTGGAGGATCTCATGGCATGTTCCTAACCGTCAAC
TTTGCTTTCGGCTTCGCTGCCACCCTAGGGATCCTGGTCTGTGGCCAGGTATCAGGAGGC
CATCTCAATCCGGCAGTGACCTTTGCCCTCTGCCTGCTTGGGAGAGATCGCTGGAGAAAG
TTCCCAGTCTACTTCGTCTTCCAGACACTGGGAGCTTTCCTGGGCTCCGGGATCATCTTT
GGTCTGTACTATGACGCTCTGTTGAACTATGCTGGAGAA----------------CTCATT
GTTACCGGGCCCAACGCCACCGCTGGCATCTTCGCCACCTACCCTGGCAAACATCTCACA
TTGGTCAACGGCTTCTTCGACCAGACGATTGGCACTGCAGCATTGATCGTGTGTATCCTG
GCCATCGTGGACCCCCATAACAATCCCATTCCCCAGGGCCTGGAGGCCTTCACGGTGGGC
TTCGTGGTGCTGGTCATCGGCCTGTCCATGGGCTTCAACTCTGGCTACGCTGTCAACCCT
GCCAGGGACCTGGGGCCACGCCTCTTTACCTCTCTGGCAGGCTGGGGTACCGAGGTCTTC
ACTGCCAATAAGTGCTGGTCCCTGGTGCCCATCTTCGCCCCATTCCTGGGAGCCATCATT
GGTGTGGTGGTCTATCAGCTGATGGTTGGCTTTCATCAGGAGGGAGAGGTTCGTGACAGG
AAGAGC----------------CAGGAG---GAGAGAGTCAAACTGGCCAACATGAACCCA
---AAAGATGCCCTA------AAGGAA---------GAGATGTTA---------------
>Huchen_Aqp3a1a
ATGGGAAAACAGAAGATATTCATGGACAAGCTGGCACAGACTTTCCAGATCCGTAACTTG
TTGCTGCGCCAGGCCCTGGCAGAGTGTCTGGGTACCCTCATCCTGGTGATGTTTGGTTGT
GGTGCAGTGGCTCAGCTGGTGCTTAGTGGAGGATCTCATGGCATGTTCCTAACCGTCAAC
TTTGCATTCGGCTTCGCTGCCACCCTAGGGATCCTGGTCTGTGGCCAGATATCAGGAGGC
CATCTCAATCCGGCAGTGACCTTTGCCCTCTGCCTTCTTGGGAGAGATCACTGGAGAAAG
TTCCCAGTCTACTTTGTCTTCCAGACACTGGGAGCATTCCTGGGCTCCGGGATCATCTTT
GGTCTGTACTATGACGCTCTGTTGAACTTTGCTGGAGAA----------------CTCATT
GTTACCGGGCCCAACGCCACCGCTAGCATCTTCGCCACCTACCCTGGCATACATCTCACA
TTGGTCAACGGCTTCTTTGACCAGACGATTGGCACTGCAGCATTGATCGTGTGTATCCTG
GCCATCGTGGACCCCCATAACAATCCCATTCCCCAGGGCCTGGAGGCCTTCACGGTGGGC
TTCGTGGTGCTGGTCATCGGCCTTTCCATGGGCTTCAACTCTGGCTACGCTGTCAACCCT
GCCAGGGACCTGGGGCCACGCCTCTTTACCTCTCTGGCAGGCTGGGGCACGGAGGTCTTC
ACGGCCAATGAGTACTGGTTCCTGGTGCCCATCTTCGCCCCGTTCCTGGGGGCCATCATT
GGTGTGGTGGTCTATCAGCTGATGGTTGGCTTTCATCAGGAGGGAGAGGTTCGCGACAGG
AAGAGC----------------CAGAAA---GAGAGAGTCAAACTGACCAACATCAACCCA
---AAGGATGCCCTA------AAGGAA---------GATATGATA---------------
>Common_whitefish_Aqp3a1a
ATGGGAAAACAGAAGATATTAATGGACAAGCTGTCACAGACCTTCCAGATCCGTAACTTG
CTGCTGCGCCAGGCCCTGGCAGAGTGTCTGGGTACCCTCATTCTGGTGATGTTTGGTTGC
GGTGCGGTGGCTCAGCTGGTGCTTAGTGGAGGATCTCATGGCATGTTCCTAACCGTCAAC
TTTGCTTTTGGCTTCGCTGCCACCCTAGGGATCCTGGTCTGTGGCCAGGTATCAGGAGGC
CATCTCAATCCGGCGGTGACCTTTGCCCTCTGCCTGCTTGGGAGAGAACGATGGAGAAAG
TTCCCTGTCTACTTTTTCTTCCAGACACTGGGAGCTTTCCTGGGCTCCGGGATCATCTTT
GGTCTGTACTATGACGCTCTGTTGGACTATGCTGGAGAA----------------CTCATT

GTTACCGGGCCCAACGCCACCGCTGGCATCTTCGCCACCTACCCTAGCAAACATCTCACA
TTGGTCAACGGCTTCTTCGACCAGACGATTGGCACTGCAGCATTGATCGTGTGTATCCTG
GCCATCGTGGACCCCCATAACAACCCCATCCCCCAGGGCCTGGAGGCCTTCACGGTGGGC
TTTGTGGTGCTGGTCATCGGCCTGTCCATGGGCTTCAACTCTGGCTACGCTGTCAACCCT
GCCAGGGACCTGGGGCCACGCATCTTTACCGCTCTGGCAGGCTGGGGCACTGAGGTCTTC
ACTGCCAATGAGTGCTGGTTCCTGGTGCCAATCTTCACGCCGTTCCTGGGCGCCATCATT
GGTGTGGTGGTCTATCAGCTGATGGTTGGCTTCCATCAGGAGGGAGAGGTTCGCGACAGG
AAGAGT---------------CGGGAG---GAGAGAGTCAAACTGACCAACATCAACCCA
---AAGGACGCCCTA------AAGGAA---------GAGATGGTA---------------
>Lake_whitefish_Aqp3a1a
ATGGGAAAACAGAAGATATTAATGGACAAGCTGTCACAGACCTTCCAGATCCGTAACTTG
CTGCTGCGCCAGGCCCTGGCAGAGTGTCTGGGTACCCTCATTCTGGTGATGTTTGGTTGC
GGTGCAGTGGCTCAGCTGGTGCTTAGTGGAGGATCTCATGGCATGTTCCTAACCGTCAAC
TTTGCTTTTGGCTTCGCTGCCACCCTAGGGATCCTGGTCTGTGGCCAGGTATCAGGAGGC
CATCTCAATCCGGCGGTGACCTTTGCCCTCTGCCTGCTTGGGAGAGATCGATGGAGAAAG
TTCCCAGTCTACTTTTTCTTCCAGACACTGGGAGCTTTCCTGGGCTCCGGGATCATCTTT
GGTCTGTACTATGACGCTCTGTTGGACTATGCTGGAGAA---------------CTCATT
GTTACCGGGCCCAACGCCACCGCTGGCATCTTCGCCACCTACCCTAGCAAACATCTCACA
TTGGTCAACGGCTTCTTCGACCAGACGATTGGCACTGCAGCATTGATCGTGTGTATCCTG
GCCATCGTGGACCCCCATAACAACCCCATCCCCCAGGGCCTGGAGGCCTTCACGGTGGGC
TTTGTGGTGCTGGTCATCGGCCTGTCCATGGGCTTCAACTCTGGCTACGCTGTCAACCCT
GCCAGGGACCTGGGGCCACGCATCTTTACCGCTCTGGCAGGCTGGGGCACCGAGGTCTTC
ACTGCCAATGAGTGCTGGTTCCTGGTGCCAATCTTCACGCCGTTCCTGGGCGCCATCATT
GGTGTGGTGGTCTATCAGCTGATGGTTGGCTTCCATCAGGAGGGAGAGGTTCGCGACAGG
AAGAGT---------------CGGGAG---GAGAGAGTCAAACTGACCAACATCAACCCA
---AAGGACGCCCTA------AAGGAA---------GAGATGGTA---------------
>Grayling_Aqp3a1a
ATGGGAAAACAGAAGATATTCATGGACAAGCTGGCACGGACCTTCCAGATCCGTAATGTG
CTGCTGCGCCAAGCCCTGGCAGAGTGTCTGGGTACCCTCATCCTGGTGATGTTTGGTTGC
GGTGCAGTGGCTCAGCTGGTGCTTAGTGGAGGATCTCATGGCATGTTCCTCACCGTCAAC
TTTGCATTTGGCTTCGCTGCCACCCTAGGGATCCTGGTCTGTGGCCAGGTATCAGGAGGC
CATCTCAATCCGGCTGTGACCTTTGCCCTCTGCCTGCTTGGGAGAGAACGCTGGAGAAAG
TTCCCAGTCTACTTTTTCTCCCAGACTATGGGCGCTTTCCTGGGCGCCGGGATCATCTTT
GGTCTGTACTATGATGCTCTGTGGGACTTTGCTGGAAAG---------------CTCGTT
GTTATCGGGCCCAACTCCACCGCTGACATCTTCGCCACCTACCCTGGCAACCATCTCACA
CTGGTCAACGGCTTCTTTGACCAGATGATTGGCACTGCATCATTGATCGTGTGTGTCCTG
GCCATCGTGGACCCCCATAACAACCCCATCCCCCAGGGCCTGGAGGCCTTCACGGTGGGC
TTCGTGGTGCTGGTTATCGGCCTGTCCATGGGCTTCAACTCTGGCTACGCCGTCAACCCT
GCCAGGGACCTTGGGCCACGCCTTTTCACCGCTGTGGCAGGCTGGGGCATGGAGGTCTTT
ACTGCCAAAGATTGCTGGTTCCTGGTGCCCATCTTTGCCCCGTTCCTGGGCGCCATCATT
GGTGTGATGGTCTATCAGCTGATGGTTGGTTTCCATCAGGAGGGAGAGGTTCGCGACAGG
AAGAACCAG------------GTGGAGGAGGAGAGAGTCAAACTGACCAATTCCAACCCA
---AAGGACACCCTA------AAGGAA---------GAGATGGTA---------------
>Coho_salmon_Aqp3a2a
ATGGGAAAACAGAAGATATGGTTGGACAAGCTGGCACGGAACTTCCAGATCCGTAACCTG
CTGCTGCGCCAGGCTCTGGCAGAGTGTCTGGGTACCCTCATCCTGGTGATGTTTGGTTGT
GGTGCGGTGGCTCAGCTGGTGCTTAGTGGAGGATCTCATGGCATGTTCCTCACCGTGAAC
TTTGCTTTCGGCTTCGCTGCCACCCTAGGGATCCTGGTCTGTGGCCAAGTATCAGGAGGC
CATCTAAACCCGGCGGTGACCTTTGCCCTCTGCCTGCTTGGGAGAGAACGCTGGAGAAAG
TTCCCAATGTACTTTGCCTTCCAGACACTGGGCGCTTTCCTGGGCTCCGGGATCATCTTT
GGTCTGTACTTTGACGCTCTGTGGGGCTTTGCTGGAAAG---------------CTCATC
GTTACCGGGCCCAACGCCACCGCTGGCATCTTCGCCACCTACCCTGGCGAACATCTCAAC
CTGCTCAACGGCTTCTTTGACCAGGTGATTGGCACGGCAGCGTTGGTCGTGTGTATCCTG
GCCATCGTAGACCCCTACAACAACCCCATCCCCCAGGGCCTGGAGGCCTTCACGGTGGGC
TTCGTGGTGCTGGTCATTGGCCTGTCCATGGGCTTCAACTCCGGCTACGCCGTCAATCCT
GCCAGGGACCTGGGGCCTCGTCTCTTCACCTCCCTGGCAGGCTGGGGCCCCGAGGTCTTC
ACGGCCGGTAAGTGCTGGTTCCTGGTGCCCATCTTTGCCCCGTTTATAGGCTCCACCTTC
GGGGTGATAGTCTATCAGCTGATGGTGGGCTACCATCAAGAGGGAGAGGCTCGCGACAAG
AAGAGGAGGGAGGATGAGGAGAAAGAGGAAGCCAGAGTTAGACTCACTAATATCACCAAA
---AAGGATGCCCTA------AAGGAA---------GAGATGGTA---------------
>Chinook_salmon_Aqp3a2a

ATGGGAAAACAGAAGATATGGTTGGACAAGCTGGCACGGAACTTCCAGATCCGTAACCTG
CTGCTGCGCCAGGCTCTGGCAGAGTGTCTGGGTACCCTCATCCTGGTGATGTTTGGTTGT
GGTGCGGTGGCTCAGCTGGTGCTTAGTGGAGGATCTCATGGCATGTTCCTCACCGTGAAC
TTTGCTTTCGGCTTCGCTGCCACCCTAGGGATCCTGGTCTGTGGCCAAGTATCAGGAGGC
CATCTAAACCCGGCGGTGACCTTTGCCCTCTGCCTGCTTGGGAGAGAACGCTGGAGAAAG
TTCCCAATGTACTTTGCCTTCCAGACACTGGGCGCTTTCCTGGGCTCCGGGATCATCTTT
GGTCTGTACTTTGACGCTCTGTGGGGCTTTGCTGGAAAG---------------CTCATC
GTTACCGGGCCCAACGCCACCGCTGGCATCTTCGCCACCTACCCTGGCGAACATCTCAAC
CTGCTCAATGGCTTCTTTGACCAGGTGATTGGCACGGCAGCGTTGGTCGTGTGTATCCTG
GCCATCGTAGACCCCTACAACAACCCCATCCCCCAGGGCCTGGAGGCCTTCACGGTGGGC
TTTGTGGTGCTGGTCATTGGCCTGTCCATGGGCTTCAACTCCGGCTACGCCGTCAATCCT
GCCAGGGACCTGGGGCCTCGTCTCTTCACCTCCCTGGCAGGCTGGGGCACCGAGGTCTTC
ACGGCCGGTAAGTGCTGGTTCCTGGTGCCCATCTTTGCCCCGTTTATAGGCTCCACCTTC
GGGGTGATAGTCTATCAGCTGATGGTGGGCTACCATCAAGAGGGAGAGGCTCGTGACAAG
AAGAGGAGGGAGGATGAGGAGAAAGAGGAAGCCAGAGTTAGACTCACTAATATCACCAAA
---AAGGATGTCCTA------AAGGAA---------GAGATGGTA---------------
>Pink_salmon_Aqp3a2a

--TGTGGCCAAGTATCAGGAGGC
CATCTAAACCCGGCGGTGACCTTTGCCCTCTGCCTGCTTGGGAGAGAACGCTGGAGAAAG
TTCCCAATGTACTTTGCCTTCCAGACACTGGGCGCTTTCCTGGGCTCCGGGATCATCTTT
GGTCTGTACTTTGACGCTCTGTGGGGCTTTGCTGGAAAG---------------CTCATC
GTTACCGGGCCCAACGCCATCGCTGGCATCTTCGCCACCTTCCCTGGC------------

---------------------AACCCCATCCCCCAGGGCCTGGAGGCCTTCACGGTGGGC
TTCGTGGTGCTGGTCATTGGCCTGTCCATGGGCTTCAACTCCGGCTACGCCGTCAATCCT
GCCAGGGACCTGGGGCCTCGTCTCTTCACCTCCCTGGCAGGCTGGGGCACCGAGGTCTTC
ACTGCCGGTAAGTGCTGGTTCCTGGTGCCCATCTTTGCCCCGTTCATAGGCTCCACCTTC
GGGGTGATAGTCTATCAACTGATGGTGGGCTACCATCAAGAGGGAGAGGCTCGCGACAAG
AAGAGGAGGGAGGATGAGGAGAAAGAGGAAGTCAGAGTCAGACTCACTAATATCACC---

>Rainbow_trout_Aqp3a2a
ATGGGAAAACAGAAGATATGGTTGGACAAGCTGGCACGGAACTTCCAGATCCGTAACCTG
CTGCTGCGCCAGGCTCTGGCAGAGTGTCTGGGTACCCTCATCCTGGTGATGTTTGGTTGT
GGTGCGGTGGCTCAGCTGGTGCTTAGTGGAGGATCTCATGGCATGTTCCTCACCGTGAAC
TTTGCTTTCGGCTTCGCTGCCACCCTAGGGATCCTGGTCTGTGGCCAAGTATCAGGAGGC
CATCTAAACCCGGCGGTGACCTTCGCCCTCTGCCTGCTTGGGAGAGAACGCTGGAGAAAG
TTCCCAATGTACTTTGCCTTCCAGACACTGGGCGCTTTCCTGGGCTCCGGGATCATCTTT
GGCCTGTACTTTGACGCTCTGTGGGGCTTTGCTGGAAAG---------------CTCATC
GTTACCGGGCCCAACGCCACTGCTGGCATCTTCGCCACCTACCCTGGCGAACATCTCAAC
CTGCTCAATGGCTTCTTTGACCAGGTGATTGGCACGGCGGCGTTGGTCGTGTGTATCCTG
GCTATCGTAGACCCCTACAACAACCCCATCCCCCAGGGCCTGGAGGCCTTCACGGTGGGC
TTCGTGGTGCTGGTCATTGGCCTGTCCATGGGCTTCAACTCCGGCTACGCCGTCAATCCT
GCCAGGGACCTGGGGCCTCGTCTCTTCACCTCCCTGGCAGGCTGGGGCACCGAGGTCTTC
ACGGCCGGTAAGTGCTGGTTCCTGGTGCCCATCTTTGCCCCGTTTATAGGCTCCACCTTT
GGGGTGATAGTCTATCAGCTGATGGTGGGCTACCATCAAGAGGGAGAGGCTCGCGACAAG
AAGAGGAGGGAGGATGAGGAGAAAGAGGAAGTCAGAGTCAGACTCACTAATATCACCAAA
---AAGGATGCCCTA------AAGGAA---------GAGATGGTA---------------
>Brook_trout_Aqp3a2a
ATGGGAAAACAGAAGGTATTCATGGACAAGCTGGCACAGACTTTCCAGATCCGTAACTTA
CTGCTGCGCCAGGCCATGGCAGAGTGTCTGGGTACCCTCATCCTGGTGATGTTTGGTTGT
GGTGCGGTGGCTCAGCTGGTACTTAGTGGAGGATCTCATGGCATGTTCCTAACTGTCAAC
TTTGCTTTCGGCTTTGCTGCCACCCTAGGGATCCTGGTCTGTGGCCAAGTATCAGGAGGC
CATCTCAATCCAGCAGTAACCTTTGCCCTCTGCCTGCTTGGGAGAGATCGCTGGAGAAAG
TTCCCAGTCTACTTCTTCTTCCAGACACTGGGAGCTTTCCTGGGCTCCGGGATCATCTTT
GGTCTGTACTATGACGCTCTGTGGGGCTTTGCTGGAAAG---------------CTCATT
GTTACCGGGCCCAACGCCACCGCTGGCATCTTCGCCACCTACCCTGGCGAACATCTCAAC
CTGCTCAATGGCTTCTTTGACCAGGTGATTGGCACGGCAGCGTTGGTTGTGTGTATCCTG
GCAATCGTAGACCCCTACAACAACCCCATCCCCCAGGGCCTGGAGGCCTTCACGGTGGGC

TTCGTGGTGCTGGTCATTGGCCTGTCCATGGGCTTCAACTCCGGCTACGCCGTCAATCCT
GCCAGGGACCTGGGGCCTCGTCTCTTCACCTCCCTGGCAGGCTGGGGCACCGAGGTCTTC
ACTGCCGGTAAGTGCTGGTTCCTGGTGCCCATCTTTGCCCCGTTTATAGGCTCCACCTTC
GGGGTGATAGTCTACCAGCTGATGGTGGGCTACCATCAAGAGGGAGAGGCTCGCGACAAG
AAGAGGAGGGAGGACGAGGAGAAAGAGGAAGTCAGAGTCAGACTCACTAATATCACCAAA
---AAGGATGCCCTA------AAGGAA---------GAGATGGTA---------------
>Arctic_charr_Aqp3a2a
ATGGGAAAACAGAAGATATGGTTGGACAAGCTGGCACGGAACTTCCAGATCCGTAACCTG
CTGCTGCGCCAGGCTCTGGCAGAGTGTCTGGGTACCCTCATCCTGGTGATGTTTGGTTGT
GGTGCGGTGGCTCAGCTGGTGCTTAGTGGAGGATCACATGGCATGTTCCTCACCGTGAAC
TTTGCTTTCGGCTTCGCTGCCACCCTAGGGATCCTGGTCTGTGGCCAAGTATCAGGAGGC
CATCTAAACCCRGCGGTGACCTTCGCTCTCTGCCTGCTTGGGAGAGAACGCTGGAGAAAG
TTCCCAGTGTTCTTTGCCTTCCAGACACTGGGCGCTTTCCTGGGCTCCGGGATCATCTTT
GGTCTGTACTTTGACGCTCTGTGGGGCTTTGCTGGAAAG---------------CTCATT
GTTACCGGGCCCAACGCCACCGCTGGCATCTTCGCCACCTACCCTGGGGAACATCTCAAC
CTGCTCAATGGCTTCTTTGACCAGGTGATTGGCACGGCAGCGTTGGTCGTGTGTATCCTG
GCAATCGTAGACCCCTACAACAACCCCATCCCCCAGGGCCTGGAGGCCTTCACGGTGGGC
TTTGTGGTGCTGGTCATTGGCCTGTCCATGGGCTTCAACTCCGGCTACGCCGTCAATCCT
GCCAGGGACCTGGGGCCTCGTCTCTTCACCTCCCTGGCAGGCTGGGGCACCGAGGTCTTC
ACGGCCGGTAAGTGCTGGTTCCTGGTGCCCATCTTTGCCCCGTTTATAGGCTCCACCTTC
GGGGTGATAGTCTACCAGCTGATGGTGGGCTACCATCAAGAGGGAGAGGCTCGCGACAAG
AAGAGGAGGGAGGACGAGGAGAAAGAGGAAGTCAGAGTCAGACTCACTAATATCACCAAA
---AAGGATGCCCTA------AAGGAA---------GAGATGGTA---------------
>Brown_trout_Aqp3a2a
ATGGGAAAACAGAAGATATTCATGGACAAGCTGGCACAGACTTTCCAGATCCGTAACTTG
CTGCTGCGCCAGGCCATGGCAGAGTGTCTGGGTACCCTCATCCTGGTGATGTTTGGTTGT
GGTGCGGTGGCTCAGCTGGTGCTTAGTGGAGGATCTCATGGCATGTTCCTCACCGTGAAC
TTTGCTTTTGGCTTCGCTGCCACCCTAGGGATCCTGGTCTGTGGCCAGGTATCAGGAGGC
CATCTAAACCCGGCGGTGACCTTCGCCCTCTGCCTGCTTGGGAGAGAACGCTGGAGAAAG
TTCCCAGTGTACTTTGCCTTCCAGACACTGGGCGCTTTCCTGGGCTCTGGGATCATCTTT
GGTCTGTACTTTGACGCTCTGTGGGGCTATGCTGGAAAG---------------CTCATT
GTTACCGGGCCCAACGCCACCGCTGGCATCTTCGCCACCTACCCTGGAGAACATCTCAAC
CTGCTCAATGGCTTCTTTGACCAGGTGATTGGCACGGCAGCGTTGGTCGTGTGTATCCTG
GCCATCGTGGACCCCTATAACAACCCCATCCCCCAGGGCCTGGAGGCCTTCACGGTGGGC
TTCGTGGTGCTGGTCATTGGCCTGTCCATGGGCTTCAACTCTGGCTACGCCGTCAATCCT
GCCAGGGACCTGGGGCCTCGTCTCTTCACCTCCCTGGCAGGCTGGGGCACCGAGGTCTTC
ACTGCCGGTAAGTGCTGGTTCCTGGTGCCCATCTTTGCCCCGTTTATAGGCTCCACCTTC
GGGGTGATAGTCTACCAGCTGATGGTGGGCTACCATCAAGAGGGAGAGGCTCGGGACAGG
AAGAGGAGGGAGGAAGAGGAGAAAGAGGAAGTCAGAGTTAGACTCACTAATATCACCAAA
---AAGGATGCCCTA------AAGGAA---------GAGATGGTA---------------
>Atlantic_salmon_Aqp3a2a
ATGGGAAAACAGAAGATATGGTTGGACAAGCTGGCACGGAACTTCCAGATCCGTAACCTG
CTGCTGCGCCAGGCTCTGGCAGAGTGTCTGGGTACCCTCATCCTGGTGATGTTTGGTTGT
GGTGCGGTGGCTCAGCTGGTGCTTAGTGGAGGATCTCATGGCATGTTCCTCACCGTGAAC
TTTGCTTTTGGCTTCGCTGCCACCCTAGGGATCCTGGTCTGTGGCCAGGTATCAGGAGGC
CATCTAAACCCGGCGGTGACCTTCGCCCTCTGCCTGCTTGGGAGAGAACGCTGGAGAAAG
TTCCCAGTGTACTTTGCCTTCCAGACACTGGGCGCTTTCCTGGGCTCTGGGATCATCTTT
GGTCTGTACTTTGACGCTCTGTGGGGCTATGCTGGACAG---------------CTCATT
GTTACCGGGCCCAACGCCACTGCTGGCATCTTCGCCACCTACCCTGGAGAACATCTCAAC
CTGCTCAATGGCTTCTTTGACCAGGTGATTGGCACGGCAGCGTTGGTCGTGTGTATCCTG
GCCATCGTGGACCCCTATAACAACCCCATCCCCCAGGGCCTGGAGGCCTTCACGGTGGGC
TTCGTGGTGCTGGTCATTGGCCTGTCCATGGGCTTCAACTCCGGCTACGCCGTCAATCCT
GCCAGGGACCTGGGGCCTCGTCTCTTTACCTCCCTGGCAGGCTGGGGCACCGAGGTCTTC
ACTGCCGGTAAGTGCTGGTTCCTGGTGCCCATCTTTGCCCCGTTTATAGGCTCCACCTTC
GGGGTGATAGTCTACCAGCTGATGGTGGGCTACCATCAAGAGGGAGAGGCTCGCGACAAG
AAGAGGAGGGAGGAAGAGGAGAAAGAGGAAGTCAGAGTCAGACTCACTAATATCACCAAA
---AAGGATGCCCTA------AAGGAA---------GAGATGGTA---------------
>Huchen_Aqp3a2a
ATGGGAAAACAGAAGATATGGTTGGACAAGCTGGCACGGAACTTCCAGATCCGTAACCTG
CTGCTGCGCCAGGCTCTGGCAGAGTGTCTGGGTACCCTCATCCTGGTGATGTTTGGTTGT
GGTGCGGTGGCTCAGCTGGTGCTTAGTGGAGGATCTCATGGCATGTTCCTCACCGTGAAC

TTTGCTTTCGGCTTCGCGGCCACCCTAGGGATCCTGGTCTGTGGCCAGGTATCAGGAGGA
CATCTAAACCCAGCGGTGACCTTCGCCCTCTGCCTGCTTGGGAGAGAACGCTGGAGAAAG
TTCCCAGTGTACTTTGCCTTCCAGACACTAGGCGCTTTCCTGGGCTCCGGGATCATCTTT
GGTCTGTACTTTGACGCTCTGTGGGGCTTTGCTGGAGAG---------------CTCATT
GTTACCGGGCCCAACGCCACCGCTGGCATCTTCGCCACCTACCCTGGCGAACATCTCAAC
CTGCTCAATGGCTTCTTTGACCAGGTGATTGGCACGGCAGCGTTGGTCGTGTGTATCCTG
GCCATCGTGGACCCCTACAACAACCCCATCCCCCAGGGCCTAGAGGCCTTCACGGTGGGC
TTCGTGGTGCTGGTCATTGGCCTGTCCATGGGCTTCAACTCCGGCTACGCAGTCAATCCT
GCCAGGGACCTGGGGCCTCGTCTCTTCACCTCCCTGGCAGGCTGGGGCTCCGAGGTCTTC
ACGGCCGGTAAGTGCTGGTTCCTGGTGCCCATCTTTGCCCCGTTTATAGGCTCCACCTTC
GGGGTGATAGTCTACCAGCTGATGGTGGGCTACCATCAAGAGGGAGAGGCTCGCGACAAG
AAGAAGAGGGAGGAAGAGGAGAAAGAGGAGGTCAGAGTCAGACTCACTAATATCACCAAA
---AAGGACCCCCTA------AAGGAA---------GAGATGGTA---------------
>Taimen_Aqp3a2a
ATGGGAAAACAGAAGATATGGTTGGACAAGCTGGCACGGAACTTCCAGATCCGTAACCTG
CTGCTGCGCCAGGCTCTGGCAGAGTGTCTGGGTACCCTCATCCTGGTGATGTTTGGTTGT
GGTGCGGTGGCTCAGCTGGTGCTTAGTGGAGGATCTCATGGCATGTTCCTCACCGTGAAC
TTTGCTTTCGGCTTCGCGGCCACCCTAGGGATCCTGGTCTGTGGCCAGGTATCAGGAGGC
CATCTAAACCCAGCGGTGACCTTCGCCCTCTGCCTGCTTGGGAGAGAACGCTGGAGAAAG
TTCCCAGTGTACTTTGCCTTCCAGACACTAGGCGCTTTCCTGGGCTCCGGGATCATCTTT
GGTCTGTACTTTGACGCTCTGTGGGGCTTTGCTGGAGAG---------------CTCATT
GTTACCGGGCCCAACGCCACCGCTGGCATCTTCGCCACCTACCCTGGCGAACATCTCAAC
CTGCTCAATGGCTTCTTTGACCAGGTGATTGGCACGGCAGCGTTGGTCGTGTGTATCCTG
GCCATCGTGGACCCCTACAACAACCCCATCCCCCAGGGCCTAGAGGCCTTCACGGTGGGC
TTCGTGGTGCTGGTCATTGGCCTGTCCATGGGCTTCAACTCCGGCTACGCAGTCAATCCT
GCCAGGGACCTGGGGCCTCGTCTCTTCACCTCCCTGGCAGGCTGGGGCTCCGAGGTCTTC
ACTGCCGGTAAGTGCTGGTTCCTGGTGCCCATCTTTGCCCCGTTTATAGGCTCCACCTTC
GGGGTGATAGTCTACCAGCTGATGGTGGGCTACCATCAAGAGGGAGAGGCTCGCGACAAG
AAGAAGAGGGAGGAAGAGGAGAAAGAGGAGGTCAGAGTCAGACTCACTAATATCACCAAA
---AAGGACCCCCTA------AAGGAA---------GAGATGGTA---------------
>Common_whitefish_Aqp3a2a
ATGGGAAAACAGAAGACATGGTTGAAAAAGCTGGCACAGAACTTCCAGATCCGTAACCTG
CTGCTGCGCCAGGCTCTGGCAGAGTGTCTGGGTACCCTCATCCTGGTGATGTTTGGTTGT
GGTGCGGTGGCTCAGCTGGTGCTTAGTGGAGGATCTCATGGAATGTTCCTCACCGTCAAC
TTTGCTTTTGGCTTCGCTGCCACCCTAGGGATCCTGGTCTGTGGCCAGGTATCAGGAGGC
CATCTCAACCCGGCAGTGACCTACGCCCTCTGCCTGCTTGGGAGAGAACGCTGGAGAAAG
TTCCCAGTGTACTTTGCCTTCCAGACACTGGGCGCTTTCCTGGGCTCCGGGGTCATCTTT
GGTCTGTACTATGATGCTCTGTGGGGCTTTGCTGGAAAG---------------CTCATT
GTTACTGGGCCCAACGCCACCGCTGGCATCTTCGCCACCTACCCTGGCGGTCATCTCACC
CTAATCAACGGCTTCTTCGACCAGATGATTGGCACGGCAGCGTTGGTCGTGTGTGTCCTG
GCCATTGTGGACCCCTACAACAACCCCATCCCCCAGGGTCTGGAGGCCTTCACAGTGGGC
TTTGTGGTGCTGGTCATTGGCCTGTCCATGGGCTTCAACTCCGGCTATGCCGTCAACCCT
GCCAGGGACCTGGGGCCCCGTCTCTTCACTTCCCTGGCAGGCTGGGGCTCCGAGGTCTTC
ACTGCCGGTCAGTGCTGGTTCCTGGTGCCCATCTTTGCCCCGTTTCTAGGTGCCACCTTC
GGGACGATAGTCTACCAGCTGATGGTGGGCTTCCATCAAGAGGGAGAGGCTCACGACAGG
AAGAAGAGGGAGCAGGAGGAGAAAGAGGAGGACAGAGTCAGACTCACTAATGTCACCACA
---AAGGACGCCCTA------AAGGAT---------GAGATGGTA---------------
>Lake_whitefish_Aqp3a2a
ATGGGAAAACAGAAGACATGGTTGAAAAAGCTGGCACAGAACTTCCAGATCCGTAACCTG
CTGCTGCGCCAGGCTCTGGCAGAGTGTCTGGGTACCCTCATCCTGGTGATGTTTGGTTGT
GGTGCGGTGGCTCAGCTGGTGCTTAGTGGAGGATCTCATGGAATGTTCCTCACCGTCAAC
TTTGCTTTTGGCTTCGCTGCCACCCTAGGGATCCTGGTCTGTGGCCAGGTATCAGGAGGC
CATCTCAACCCGGCAGTGACCTACGCCCTCTGCCTGCTTGGGAGAGAACGCTGGAGAAAG
TTCCCAGTGTACTTTGCCTTCCAGACACTGGGCGCTTTCCTGGGCTCCGGGGTCATCTTT
GGTCTGTACTATGATGCTCTGTGGGGCTTTGCTGGAAAG---------------CTCATT
GTTACTGGGCCCAACGGCACCGCTGGCATCTTCGCCACCTACCCTGGCGATCATCTCACC
CTAATCAACGGCTTCTTCGACCAGATGATTGGCACGGCAGCGTTGGTCGTGTGTGTCCTG
GCCATTGTGGACCCCTACAACAACCCCATCCCCCAGGGCCTGGAGGCCTTCACAGTGGGC
TTCGTGGTGCTGGTCATTGGCCTGTCCATGGGCTTCAACTCCGGCTACGCCGTCAACCCT
GCCAGGGACCTGGGGCCCCGTCTCTTCACTTCCCTGGCAGGCTGGGGCTCCGAGGTCTTC
ACTGCCGGTCAGTGCTGGTTCCTGGTGCCCATCTTTGCCCCGTTTCTAGGTGCCACCTTC

GGGACGATAGTCTACCAGCTGATGGTGGGCTTCCATCAAGAGGGAGAGGCTTACGACAGG
AAGAAGAGGGAGCAGGAGGAGAAAGAGGAGGACAGAGTCAGACTCACTAATGTCACCACA
---AAGGACGCCCTA------AAGGAT---------GAGATGGTA---------------
>Grayling_Aqp3a2a
ATGGGACATCAGAAGATATGGTTGGACAAGCTGGCACGGAACTTCCAGATCCGTAACCTG
CTGCTGCGCCAGGCTCTGGCAGAGTGTCTGGGTACCCTCATCCTGGTGATGTTTGGTTGT
GGTGCGGTGGCTCAGCTGGTGCTTAGTGAAGGATCTCATGGCATGTTCCTCACCGTCAAC
TTTGCTTTCGGCTTCGCTGCCACCCTAGGGATCCTGGTCTGTGGCCAGGTATCAGGAGGC
CATCTCAACCCGGCGGTGACCTTTGCCCTCTGCCTGCTTGGGAGAGAACGCTGGAGAAAG
TTCCCAATGTTCTTTGCCTTCCAGACACTGGGCGCTTTCCTGGGGTCTGGGGTCATCTTT
GGTCTGTACTATGACGCTCTGTGGGCCTTTGCTGGAAAG---------------CTCATT
GTTACCGGGCCCAACTCCACCGCTGGCATCTTCGCCACCTATCCTGCCGGTCATCTCACC
ATAGTCAATGGCTTCTTCGACCAGATGATTGGCACGACAGCGTTGATCGTGTGTATCCTG
GCCATCGTGGACCCCTACAACAACCCCATCCCTCAGGGCCTGGAGGCCTTCACGGTGGGC
TTTGTGGTGCTGGTCATTGGTCTGTCCATGGGCTTAAACTCTGGCTACGCCGTCAACCCT
GCAAGGGACCTAGGGCCCCGTCTCTTCACCTCCCTGGCAGGCTGGGGCTCCGAGGTCTTC
ACTGCTGGTCAGTGCTGGTTCCTGGTGCCCATCATTGCCCCGTTTATAGGTGCCACCATC
GGGGTGATAATCTACCAGCTGATGGTGGGCTTCCATCAAGAGGGAGAGGCTCGTGACAAG
AAGAGGAAGGCGCAGGAGGAGAAAGAGGAGGACAGAGTCAGACTCACTAATGTCACCTCA
---AAGGATGCCCTA------AAGGAA---------GAGATGGTA---------------
>Northern_pike_Aqp3a1a
ATGGGAAACAGAAGATATGGATAGACAAGCTGATGCGGACCTTCCAGATCCGTAACCTG
CTGTTGCGTCAGGCTTTGGCTGAATGCCTGGGCACCCTCATCCTGGTGATGTTCGGTTGT
GGCGCGTTGGCTCAACTGGTGCTCAGCAGAGGGTCCCATGGCCGCTTTCTCACCGTCAAC
TTTGCTTTCGGTTTCGCTGCTACCCTGGGCATCCTGGTTTGTGGCCAGATATCAGGAGGC
CATCTTAACCCTGCAGTGACCTTTGCCCTCTGCCTACTTGGAAGAGAACGCTGGAGAAAG
TTTCCGGTGTACTTTGCGGCCCAGACATTGGGCGCTTTCCTGGGCTCTGCGGTCATCTTC
GGCCTGTACTATGACGCCCTGTGGGACCATGCCGGGAAG---------------CTCCTG
GTTGAGGGTGAAAACGCCACCGCCGGCATCTTTGCCACCTACCCCGGCAGTCATCTCACG
CTGGTCAACGGCTTCTTCGATCAGTTGATTGGCACCGCAGCGTTGATTGTGTGCATCCTG
GCCATTGTGGACCCCTACAACAACCCCATCCCTCAGGGTCTGGAGGCTTTCACGGTGGGC
TTCGTGGTGCTGGTCATCGGCCTGTCCATGGGCTTCAACTCCGGCTACGCCGTCAATCCT
GCCAGGGACTTTGGGCCCCGTCTCTTCACAGCCATTGCGGGCTGGGGCGGGGAAGTGTTC
ACGGCTCGTCAGGGCTGGTTCCTCATACCCATCTTTGCCCCGTTCCTGGGTTCGTCCATT
GGTGTGATGGTCTACCAGCTGATGGTAGGCTACCATCAGGAGGGCGAGGTTCGTGACAGG
AAAAGCAAAGAGTTGGAGGAGAGGGATGAGGGCAGAGTCAAACTCACCAATGTCACTACA
---AAGGACCCCTTA------AAGGAA---------GACATGGTA---------------
>Northern_pike_Aqp3a1b
ATGGGGAAACAGAAT------ATAGACAAGCTGACGCGGACCTTCCAGATCCGTAACCTG
CTGTTGCGTCAGGCTTTGGCTGAATGCCTGGGCACCCTCATCCTAGTGATGTTCGGTTGT
GGCGCGGTGGCTCAACTGGTGCTCAGCAGAGGGTCCCATGGCAGCTTTCTCTCCGTCAAC
TTTGCTTTTGGTTTAGCTGCTACCCTGGGCATCCTGGTTTGTGGCCAGGTATCAGGAGGC
CATCTTAACCCTGCAGTGACCTTTGCCCTCTGCTTACTTGGAAGAGAACGCTGGAGAAAG
TTTCTGGTGTACTTTGCGGCCCAGACAGTGGGCGCTTTCCTGGGCTCCGCGATCATCTTC
GGCCTGTACTATGGTGCCCTATGGAACCATGCCGGGGAG---------------CTCCTG
GTTGAGGGTGAAAACGCCACGGCCGGCATCTTTGCTACCTACCCTGGCAGTCATCTCACG
CTGGTCAACGGCTTCTTCGATCAGATGATTGGCACCACAGCGTTGATCGGGTGTATCTTG
GCCATTGTGGACCCCTACAACAACCCCATCCCTCAGGGTCTGGAGGCTTTCACGGTGGGC
TTCGTTGTGCTGGTCATCGGCCAGTCTATGGGCTTCAATTCTGGCTACGCCGTCAATCCT
GCCAGGGACTTGGGGCCCCGTCTCTTCACAGCCATTGCGGGCTGGGGCGGGGAAGTATTC
ACGACTCGTCAGGGCTGGTTCCTCATACCCATCTTTGCCCCGTTCCTGGGTTCGTCCATT
GGTGTGATGGTCTACCAGCTGATGGTAGGCTACCATCAGGAGGGCGAGGTTCGTGACAGG
AAAAACAAAGAGTTGGAGGAGAGGGAC---GGCAGAGTCAAACTCACCAATGTCACTCCA
---AAGGACCCCTTA------AAGGAA---------GACATGGTA---------------
>Eastern_mudminnow_Aqp3a
ATGGGGAAACAAAAGATATTTATAGACAAACTGTCGAGGTCCTTTCAGTGCCGTAACCTG
CTGCTTCGTCAGGCCCTGGCTGAATGTCTGGGTACTCTGATCCTTGTGATGTTTGGTTGT
GGTGCACTGGCTCAGCTGGTGCTTAGCAGAGGGTCCCATGGGCGCTTTCTCACCGTCAAC
TTTGCTTTCGGTTTTGCTGCTACCCTGGGACTCCTGGTCTGTGGCCAGGTATCAGGAGGA
CATATTAACCCTGCAGTGACTTTTGCCCTGTGCCTGTTGGGGAGAGAACCTTGGATAAAG
TTCCCTGTGTACTTTGCAGCACAGACAATTGGTGCTTTCCTTGGCTCTGGGGTAATCTTT

GGCCTGTACTATGATGCTCTGTGGAGCCATGGTGGGTCG---------------CTCATG
GTTGAAGGTGAAAATGCCACTGCAGGCATATTTGCCACCTACCCTGGTAGTCATCTTACA
TTGGTCAACGGCTTCTTCGATCAGTTGATCGGCACGACGGCACTAATCGTATGTGTCCTC
GCCATTGTGGACCCTTTCAACAACCCTATCCCTCAAGGCCTGGAGGCTTTCACGGTGGGC
TTTGTGGTGCTGGTTATCGGCTTGTCCATGGGCTTCAACTCTGGTTATGCCGTCAACCCT
GCCAGGGACTTAGGGCCCCGTCTCTTCACAGCCATTGCAGGCTGGGGCGGGGAAGTCTTC
ACTTCCCGGAATTGCTGGTTCCTGATACCAATCTTCGCCCCGTTCCTGGGTTCCTTTATC
GGGGTCTTGATCTACCAGTTCATGGTTGGCATCCATCAGGAAGGAGAGGTTCGTGACAAG
AAGATCAGAGAGTTAGAGGAGAGGGATGAGGGCAGGGTCAAACTCACCAACGTCACACCC
---AAGGACCCCTTA------AAGGAA---------GACATGGTA---------------
>Anshui_blind_cavefish_Aqp3a2
ATGGGTTGGCAGAAGGTCATGCTGGAAAAGCTTGCACACACTTTCCAGATTCGCAACAAG
CTACTGCGCCAGGGACTGGCTGAATGCTTGGGAACTCTCATCCTGGTGATGTTTGGCTGT
GGTGCAGTGGCCCAAATGGTACTAAGTGGAGAAAGTCATGGTCGCTTTCTCTCTGTAAAC
CTTGCTTTTGGTTTTGCTGCTACCCTTGGAATCTTGGTTTGTGGCCAGGTGTCAGGCGGA
CATTTAAATCCAGCAGTTACATTTGCTCTCTGCCTCTTGGGAAGAGAAAAATGGAGAAAG
TTCCCTGTGTACTTCATGTTCCAAACAATTGGAGCCTTCTTTGGTGCCGCGATCATCTTT
GCTGAATACCATGATGCAATGTATGATTATGCTGGAGAA------AAAAACGAGTTGCTC
GTAACTGGGGAAAATGCCACTGCTGGCATTTTTGCTACTTACCCAAACCCACATCTCACC
ATCTTGAATGGCTTTTTTGACCAGGTGATAGGCACAGCATCTCTGATTGTCTGCATCCTG
GCCATTGTGGACCCCTACAACAACCCAATCCCCCAAGGTCTTGAAGCCTTCACAGTGGGA
TTCAGTGTCCTTATCATTGGACTCTCCATGGGCTTTAATTCTGGTTATGCAGTCAACCCA
GCTAGGGATTTCGGGCCTCGTCTTTTCACTGCCATGGCTGGTTGGGGTAGTGAAGTCTTC
ACGACCAGGGAGTGTTGGTTTTTGGTGCCATTCTTCGCCCCCTTCCTGGGATCCATTATT
GGTGCGATTGTGTACCAGCTGATGGTGGGGTGGCATGTGGAGGGAGAAGTACGGGACAAT
AAGAATAAAGCT---------AGGGAAGAG---TCTTTGAAACTTAATGACATCACCAGC
---AAGGAG---
>Horned_golden_line_barbel_Aqp3a2
ATGGGTTGGCAGAAGGCCATGCTGGAAAAGCTTGCACATACTTTCCAGATTCGCAACAAG
CTACTGCGCCAGGGACTGGCTGAATGCTTGGGAACTCTCATCCTGGTGATGTTTGGCTGT
GGTGCGGTGGCCCAAATGGTACTAAGTGGAGAAAGTCATGGTCGCTTTCTCTCTGTAAAC
CTTGCTTTTGGTTTTGCTGCTACCCTTGGAATCTTGGTTTGTGGCCAGGTGTCAGGCGGA
CATTTAAATCCAGCAGTTACATTTGCTCTCTGCCTCTTGGGAAGAGAAAAATGGAGAAAG
TTCCCTGTGTACTTCATGTTCCAAACAATTGGAGCATTCTTTGGTGCCGCAATCATCTTT
GCTGAATACCATGATGCAATGTATGATTATGCTGGAGAA------AAAAACGAGTTGCTC
GTAACTGGGGAAAATGCCACTGCTGGCATTTTTGCTACTTACCCAAACCCACATCTCACC
ATCTTGAATGGCTTTTTTGACCAGGTGATAGGCACAGCATCTCTGATTGTCTGCATCCTG
GCCATTGTGGACCCCTACAACAACACAGTCCCCCAAGGTCTTGAAGCCTTCACGGTGGGA
TTCAGTGTCCTTATCATTGGACTCTCCATGGGCTTCAATTCTGGTTATGCAGTCAACCCA
GCTAGGGATTTCGGGCCTCGTCTTTTCACTGCCATGGCTGGTTGGGGTAGTGAAGTCTTC
ACTACCAGGGAGTGTTGGTTTTTGGTGCCATTCTTCGCCCCCTTCCTGGGATCCATTATT
GGTGCGATTGTGTACCAGCTGATGGTGGGGTGGCATGTGGAGGGAGAAGTACGGGACAAT
AAGAATAAAGCT---------AGGGAAGAG---TCTTTGAAACTTAATGACATTACCAGC
---AAGGAG---
>Golden_line_fish_Aqp3a2
ATGGGTTGGCAGAAGGCCATGCTCGAAAAGCTTGCACACACTTTCCAGATCCGCAACAAG
CTACTGCGCCAGGGACTGGCTGAATGCTTGGGAACTCTCATCCTGGTGATGTTTGGCTGT
GGTGCGGTGGCCCAAATGATACTAAGTGGAGAAAGTCATGGTCGCTTTCTCTCTGTAAAC
CTTGCTTTTGGTTTTGCTGCTACCCTTGGAATCTTGGTTTGTGGCCAGGTGTCAGGCGGA
CATTTAAATCCAGCAGTTACATTTGCTCTTTGCCTCTTGGGAAGAGAAAAATGGAGAAAG
TTCCCTGTGTACTTCATGTTCCAAACAATTGGAGCCTTCTTTGGTGCCGCAATCATCTTT
GCTGAATACCATGATGCAATGTATGATTATGCTGGAGAA------AAAAACGAGTTGCTC
GTAACTGGGGAAAATGCCACTGCTGGCATTTTTGCTACTTACCCAAGCCCACATCTCACC
ATCTTGAATGGCTTTTTTGACCAGGTGATAGGCACAGCATCTCTGATTGTCTGCATCCTG
GCCATTGTGGACCCCTACAACAACCCAATCCCCCAAGGTCTTGAAGCCTTCACGGTGGGA
TTCAGTATCCTTATCATTGGACTCTCCATGGGCTTCAATTCTGGTTATGCAGTCAACCCA
GCTAGGGATTTTGGGCCTCGTCTTTTCACTGCCATGGCTGGTTGGGGTAGTGAAGTCTTC
ACGACCAGAGGGTGTTGGTTTTTGGTGCCATTCTTCGCCCCCTTCCTGGGATCCTTTATT
GGTGCGATTGTGTACCAGCTGATGGTGGGGTGGCATGTGGAGGGAGAAGTACGGGACAAT
AAGAATAAAGCT---------AGGGAAGAG---TCTTTGAAACTTAATGACATCACCAGC
---AAGGAG---

>Common_carp_Aqp3a2
ATGGGTTGGCAGAAGGACATGCTGGAAAAGCTTGCACACACTTTCCAGATCCGCAACAAG
CTACTGCGCCAGGGACTGGCTGAATGCTTGGGAACTCTCATCCTTGTGATGTTTGGCTGT
GGTGCGGTGGCTCAGATGGTACTAAGTAGAGAAACTCACGGTCGCTTTCTCTCTGTAAAC
CTTGCTTTTGGTTTTGCTGCTACCCTTGGAATCTTGGTTTGTGGCCAGGTGTCAGGTGGA
CATTTAAATCCAGCAGTTACATTTGCTCTCTGTCTCTTGGGAAGAGAAAAATGGAGAAAG
TTCCCTGTGTACTTTATGTTCCAAACACTTGGAGCCTTCTTGGGTGCCGCGATCATCTTT
GCTGAATACCATGATGCAATGTATGATTATTCTGGAGAA------AACAATGTGTTGCTC
GTAACTGGGGAAAATGCCACTGCTGGTATTTTTGCTACTTACCCAAACCCACATCTCACC
ATCTTGAATGGCTTTTTTGACCAGGTGATAGGCACAACATCTCTAATTGTCTGCATCCTG
GCCATTGTGGACCCCTACAACAACCCAATCCCCCAAGGTCTTGAAGCCTTCACGGTGGGA
TTCAGTGTCCTTATCATTGGACTCTCCATGGGCTTCAATTCTGGTTATGCAGTCAACCCA
GCTAGGGATTTCGGGCCTCGTCTTTTCACTGCCATGGCTGGTTGGGGTGGTGAAGTCTTC
ACGACCAGGGAGTGTTGGTTTTTGGTGCCATTCTTCGCCCCCTTCCTGGGATCCATTATT
GGTGTGATTGTGTACCAGCTGATGGTGGGGTGGCATGTGGAGGGAGAAGTAAAGAACAAT
AAGAACAAAGCT---------AGGGAAGAG---ACTTTGAAACTTAATGACATCACCAGC
---AAGGAG---
>Goldfish_Aqp3a2
ATGGGTTGGCAGAAGTCCATGCTGGAAAAGCTTGCACACACTTTCCAGATCCGCAACAAG
CTACTGCGCCAGGGACTGGCTGAATGCTTGGGGACTCTCATCCTGGTGATGTTTGGCTGT
GGTGCAGTGGCCCAAATGGTACTAAGTAGAGAAAGTCATGGTCGCTTTCTCTCTGTAAAC
CTTGCTTTTGGTTTCGCTGCTACCCTTGGAATCTTGGTTTGTGGCCAGGTGTCAGGCGGA
CATTTAAATCCAGCAGTTACATTTGCTCTCTGCCTCTTGGGAAGAGAAAAATGGAGAAAG
TTCCCTGTTTACTTTTTCTTTCAAACAATTGGAGCTTTCTTTGGTGCCGCGATCATCTTT
GCTGAATACCATGATGCAATGTATGATTATGCTGGAGAA------GATAATTTATTGCTC
GTAACTGGGGAAAATGCCACTGCTGGCATTTTTGCTACTTATCCAAGCCCACATCTCACC
ATCTTGAATGGCTTTTTTGACCAGGTGATAGGCACAACATCTCTGATTGTCTGCATCCTG
GCCATTGTAGACCCCTACAACAACCCAATCCCCCAAGGTCTTGAAGCCTTCACGGTGGGA
TTCAGTGTCCTTATCATTGGACTCTCCATGGGCTTCAATTCTGGTTATGCAGTCAACCCA
GCTAGGGATTTCGGGCCTCGTCTTTTCACTGCCATGGCTGGTTGGGGTGGTGAAGTCTTC
ACTACCAGGGATTGTTGGTTTTTGGTGCCATTCTTCGCCCCCTTCCTGGGATCCATTATT
GGTGTGATTGTGTACCAGCTGATGGTGGGGTGGCATGTGGAGGGAGAAGTAAAGGACAAA
AAGAATAAACCT---------GGGGAAGAG---TCTATGAAACTTAATGACATCACCAGC
---AAGGAGTGCCCTGCG--
>Anshui_blind_cavefish_Aqp3a1
ATGGGTTGGCAGAAGGCCATGCTGGATAAGCTTGCACACACTTTCCAGATCCGCAACAAG
CTGCTGCGCCAGGGACTGGCTGAATGCTTGGGAACTCTCTTGCTAGTGATGTTTGGCTGT
GGTGCGGTGGCCCAAATGGTACTAAGTGGAGAAAGCCATGGTCGCTTTCTCTCTGTAAAC
CTTGCTTTTGGGTTTGCTGCTACCCTTGGAATCTTGGTTTGTGGCCAGGTGTCAGGCGGA
CATTTAAATCCAGCAGTTACATTTGCTCTCTGCCTCTTGGGAAGAGAAAAATGGAGAAAG
TTCCCTGTGTACTTTCTGTTCCAAACAATTGGAGCCTTCTTGGGTGCCGCAATTATCTTT
GCTGAATACCATGATGCAATGTATGATTATGCTGGAGAA------AAAAATGAGTTGCTT
GTAATTGGGGATAAAGCCACTGCTGGTATTTTTGCTACGTACCCAAACCCTCATCTCACC
ATCTTGAATGGATTTTTTGACCAGGTGATAGGCACAGCATCTCTGATTGTCTGCATCCTG
GCCATTGTGGACCCCTACAACAACCCAATCCCTCAAGGTCTTGAGGCCTTCACGGTGGGA
TTCAGCGTCCTTATCATTGGACTCTCCATGGGCTTCAATTCTGGTTATGCAGTCAACCCA
GCTAGGGATTTCGGGCCTCGTCTTTTCACTGCCATGGCTGGTTGGGGTGGTGAAGTCTTC
ACGACCAGACAGTGTTGGTTTTTGGTGCCCATCTTTGCCCCCTTCCTGGGATCCATTATT
GGTGTGCTTGTGTACCAGCTGATGGTGGGGTGGCATGTGGAGGGAGAAGTACGGGACAAT
AAGAATAAAGCT---------AAGGAAGAG---TCTTTGAAACTTAATGATGTCACCAGC
---AAGGAT---
>Horned_golden_line_barbel_Aqp3a1
ATGGGTTGGCAGAAGGTCATGCTGGATAAGCTTGCACACACTTTCCAGATCCGCAACAAG
CTGCTGCGCCAGGGACTGGCTGAATGCTTGGGAACTCTCTTGCTAGTGATGTTTGGCTGT
GGTGCGGTGGCCCAAATGGTACTAAGTGGAGAAAGCCATGGTCGCTTTCTCTCTGTAAAC
CTTGCTTTTGGGTTTGCTGCTACCCTTGGAATCTTGGTTTGTGGCCAGGTGTCAGGCGGA
CATTTAAATCCAGCAGTTACATTTGCTCTCTGCCTCTTGGGAAGAGAAAAATGGAGAAAG
TTCCCTGTGTACTTTCTGTTCCAAACAATTGGAGCCTTCTTCGGTGCCGCAATTATCTTT
GCGGAATACCATGATGCAATGTATGATTATGCTGGAGAA------AAAAATGAGTTGCTT
GTAATTGGGGATAAAGCCACTGCTGGTATTTTTGCTACGTACCCAAACCCTCATCTCACC
ATCTTGAATGGATTTTTTGACCAGGTGATAGGCACAGCATCTCTGATTGTCTGCATCCTG

GCCATTGTGGACCCCTACAACAACCCAATCCCTCAAGGTCTTGAGGCCTTCACGGTGGGA
TTCAGCGTCCTTATCATTGGACTCTCCATGGGCTTCAATTCTGGTTATGCAGTCAACCCA
GCTAGGGATTTCGGGCCTCGTCTTTTCACTGCCATGGCTGGTTGGGGTGGTGAAGTCTTC
ACTACCAGACAGTGTTGGTTTTTGGTGCCCATCTTTGCCCCCTTCCTGGGATCCATTATT
GGTGTGCTTGTGTACCAGCTGATGGTGGGGTGGCATGTGGAGGGAGAAGTACGGGACAAT
AAGAATAAAGCT---------AAGGAAGAG---TCTTTGAAACTTAATGACGTCACCAGC
---AAGGAT---
>Golden_line_fish_Aqp3a1
ATGGGTTGGCAGAAGGCCATGCTGGATAAGCTTGCACACACTTTCCAGATCCGCAACAAG
CTGCTGCGCCAGGGACTGGCTGAATGCTTGGGAACTCTCTTGCTAGTGATGTTTGGCTGT
GGTGCGGTGGCCCAAATGGTACTAAGTGGAGAAAGCCACGGTCGCTTTCTCTCTGTAAAC
CTTGCTTTTGGGTTTGCTGCTACCCTTGGAATCTTGGTTTGTGGCCAGGTGTCAGGCGGA
CATTTAAATCCAGCAGTTACATTTGCTCTCTGCCTCTTGGGAAGAGAAAAATGGAGAAAG
TTCCCTGTGTACTTTCTGTTCCAAACAATTGGAGCCTTCTTGGGTGCCGCAATTATCTTT
GCTGAATACCATGATGCAATGTATGATTATGCTGGAGAA------AAAAATGAGTTGCTT
GTAACTGGGGAGAAAGCCACTGCTGGTATTTTTGCTACGTACCCAAACCCTCATCTCACC
ATCTTGAATGGATTTTTTGACCAGGTGATAGGCACAGCATCTCTGATTGTCTGCATCCTG
GCCATTGTGGACCCCTACAACAACCCAATCCCTCAAGGTCTTGAGGCCTTCACGGTGGGA
TTCAGCGTCCTTATCATTGGACTCTCCATGGGCTTCAATTCTGGTTATGCAGTCAACCCA
GCTAGGGATTTCGGGCCTCGTCTTTTCACTGCCATGGCTGGTTGGGGTGGTGAAGTCTTC
ACGACCAGACAGTGTTGGTTTTTGGTGCCCATCTTTGCCCCCTTCCTGGGATCCATTATT
GGTGTGCTTGTGTACCAGCTGATGGTGGGGTGGCATGTGGAGGGAGAAGTACGGGACAAT
AAGAATAAAGCT---------AAGGAAGAG---TCTATGAAACTTAATGACGTCACCAGC
---AAGGAT---
>Gara_mabienska_Aqp3a1
ATGGGTTGGCAGAAGGCCATGCTGGATAAGCTTGCACACACTTTCCAGATCCGCAACAAG
CTGCTGCGCCAGGGACTGGCTGAATGCTTGGGAACTCTCTTGCTAGTGATGTTTGGCTGT
GGTGCGGTGGCCCAAATGGTACTAAGTGGTGAAAGCCATGGTCGCTTTCTCTCTGTAAAC
CTTGCTTTTGGGTTTGCTGCTACCCTTGGAATCTTGGTTTGTGGCCAGGTGTCAGGCGGA
CATTTAAATCCAGCAGTTACATTTGCTCTCTGCCTTTTGGGAAGAGAAAAATGGAGAAAG
TTCCCTGTGTACTTTCTGTTCCAAACAATTGGAGCCTTCTTGGGTGCCGCAATTATCTTT
GCTGAATACCATGATGCAATGTATGATTATGCTGGAGAA------AAAAATGAGTTGCTT
GTAACTGGGGAGAAAGCCACTGCTGGTATTTTTGCTACGTACCCAAACCCTCATCTCACC
ATCTTGAATGGATTTTTTGACCAGGTGATAGGCACAGCATCTCTGATTGTCTGCATCCTG
GCCATTGTGGACCCCTACAACAACCCAATCCCTCAAGGTCTTGAGGCCTTCACGGTGGGA
TTCAGCGTCCTTATCATTGGACTCTCCATGGGCTTCAATTCTGGTTATGCAGTCAACCCA
GCTAGGGATTTCGGGCCTCGTCTTTTCACTGCCATGGCTGGTTGGGGTGGTGAAGTCTTC
ACTACCAGACAGTGTTGGTTTTTGGTGCCCATCTTTGCCCCCTTCCTGGGATCCATTATT
GGTGCGCTTGTGTACCAGCTGATGGTGGGGTGGCATGTGGAAGGAGAAGTACGGGACAAT
AAGAATAAAGCT---------AAGGAAGAG---TCTATGAAACTTAATGACGTCACCAGC
---AAGGAT---
>Common_carp_Aqp3a1
ATGGGTTGGCAGAAGGCCATGCTGGATAAGCTTGCACACACTTTCCAGATCCGCAACAAG
CTGCTGCGCCAGGGACTGGCTGAATGCTTGGGAACTCTCATCCTGGTGATGTTTGGCTGT
GGTGCGGTGGCCCAAATGGTACTAAGTGAAAAAAGTCATGGTCGCTTTCTCTCTGTAAAC
CTTGCTTTTGGGTTTGCTGCTACCCTTGGAATCTTGGTTTGTGGCCAGGTGTCAGGTGGA
CATTTAAATCCAGCAGTTACATTTGCTCTCTGCCTCTTGGGAAGAGAAAAATGGAGAAAG
TTCCCTGTGTACTTTCTGTTCCAAACAATTGGAGCCTTCTTGGGTGCCGCAATTATCTTT
GCTGAATACCATGATGCAATGTATGAATATGCTGGAGAA------AAAAATGAGTTGCTT
GTAACTGGGGATAAAGCCACTGCTGGTATTTTTGCTACGTACCCAGGCCCACATCTCACC
ATCTTGAATGGATTTTTT---CAGGTGATAGGCACAGCAGCTCTGATTGTCTGCATCCTA
GCCATTGTGGACCCCTACAACAACCCAATCCCTCAAGGTCTTGAGGCCTTCACGGTGGGA
TTCAGCGTCCTTATCATTGGACTCTCCATGGGCTTCAATTCTGGTTATGCAATCAACCCA
GCTAGGGATTTCGGGCCTCGTCTTTTCACTTCCATGGCTGGTTGGGGTGGTGAAGTCTTC
ACGAACAGACAGTGTTGGTTTTTGGTGCCCATGTTTGCCCCCTTCCTGGGATCCATTATT
GGTGTGCTTGTGTACCAGCTGATGGTGGGGTGGCATGTGGAGGGAGAAGTACGGGACAAT
AAGAATAAAGCT---------AAGGAAGAG---TCTTTGAAACTTAATGACGTCACCAGC
---AAGGAT---
>Goldfish_Aqp3a1
ATGGGTTGGCAGAAGGCCACGCTGGATAAGCTTGCACACACTTTCCAGATCCGCAACAAG
CTGCTGCGCCAGGGACTGGCTGAATGTTTGGGAACTCTCATCCTGGTGATGTTCGGCTGT

GGTGCGGTTGCCCAAATGGTACTTAGTGAAAAAAGCCATGGCCGTTTTCTCTCTGTAAAC
CTTGCTTTTGGGTTTGCTGCTACCCTTGGAATCTTGGTTTGTGGCCAGGTGTCAGGTGGA
CATTTAAATCCAGCAGTTACATTTGCTCTCTGCCTCTTGGGAAGGGAAAAATGGAGAAAG
TTCCCTGTGTACTTTCTGTTCCAAACAATTGGAGCCTTCTTGGGTGCCGCAATTATCTTT
GCTGAATACCATGATGCAATGTATGATTATGCTGGAGAA------AAAAATGAGTTGCTT
GTAACTGGGGACAAAGCCACTGCTGGTATTTTTGCTACATACCCAAGCCCACATCTCACC
ATCTTGAATGGATTTTTTGACCAGGTGATCGGCACAGCAGCTCTGATTGTCTGCATCCTG
GCCATTGTGGACCCCTACAACAACCCAATCCCTCAAGGTCTTGAGGCCTTCACAGTGGGA
TTCAGCGTCCTGATCATTGGACTCTCCATGGGCTTCAATTCTGGTTATGCCATCAACCCA
GCTAGGGATTTCGGGCCTCGTCTTTTCACTGCCATGGCTGGTTGGGGTGGTGAAGTCTTC
ACTAACAGACAGTGCTGGTTTTTGGTGCCCTTTTTTGCCCCCTTCCTTGGATCCATTATT
GGTGTGATTGTGTACCAGTTGATGGTCGGGTGGCATGTGGAGGGAGAAGTACGAGACAAT
AAGAATAAAGCC---------AAGGAAGAG---TCTTTGAAACTTAACGACGTCAGCAGC
---AAGGAA---
>Iberian_chubb_Aqp3a
ATGGGTTGGCAGAAGGTCATGCTGGATAAGCTTGCACACACTTTCCATATCCGCAACAAG
CTGCTGCGCCAGGGATTGGCTGAGTGCTTGGGAACTCTCATCCTGGTGATGTTTGGCTGT
GGTGCAGTGGCTCAAGTGGTACTAAGTGAAGGCTCTCACGGACGCTTTCTCACTATAAAC
CTTGCTTTTGCGTTTGCCGCTACCCTTGGAATCTTGGTTTGCGGCCAGGTGTCAGGCGGA
CATTTAAATCCAGCAGTTACATTTGCTCTCTGCCTCTTGGGACGTGAAAAATGGAGAAAG
TTCCCTGTATACTTTCTGTTTCAAACAATTGGATCCTTCTTGGGTGCCGCAATTATCTTT
GCTGAATACCATGATGCAATGTATGAGTATGCTGGAGAA------TCAAATCTGTTGCTT
GTAAATGGGCCAAAAGCCACTGCTGGGATTTTTGCTACATACCCCAACCCTCATCTCACC
ATCTTAAATGGATTCTTTGACCAGGTGATAGGCACTGCATCTCTTATTGTCTGCATCCTG
GCCATTGTGGACCCCTACAACAACCCGATCCCCCAAGGTCTTGAGGCCTTCACGGTGGGG
TTTAGCATTCTTGTCATTGGACTCTCCATGGGCTTCAATTCTGGTTATGCAGTTAACCCA
GCTAGGGATTTCGGGCCTCGTCTTTTCACCGCCATGGCTGGTTGGGGTGGTGAAGTCTTT
ACTACCAGACAGTGTTGGTTTTTGGTGCCCATCTTCGCCCCCTTCCTGGGATCCATTATT
GGTGTGATTGTGTACCAGCTAATGGTGGGGTGGCACGTGGAGGGAGAGGCACGGGAGAAG
AATAATAAAGCT---------AGGGAAGAG---TCTTTGAAACTTAATGACGTCACCAGC
---AAGGAA---
>Roach_Aqp3a
ATGGGTTGGCAGAAGGTCATGCTGGATAAGCTTGCACACACTTTCCATATCCGCAACAAG
CTGCTGCGCCAGGGATTGGCTGAGTGCTTGGGAACTCTCATCCTGGTGATGTTTGGCTGT
GGTGCAGTGGCTCAAGTGGTACTAAGTGAAGGATCTCACGGACGCTTTCTCACTATAAAC
CTTGCTTTTGGGTTTTCCGCTACCCTTGGAATCTTGGTTTGCGGCCAGGTGTCAGGCGGA
CATTTAAATCCAGCAGTTACATTTGCTCTCTGCCTCTTGGGACGTGAAAAATGGAGAAAG
TTCCCTGTGTACTTTCTGTTTCAAACAATTGGATCCTTCTTGGGTGCCGCAATTATCTTT
GCTGAATACCATGATGCAATGTATGAGTATGCTGGAGAA------TCAAATCTGTTGCTT
GTAACTGGGCCAAAAGCCACTGCTGGGATTTTTGCTACATACCCCAACCCTCATCTCACC
ATCTTAAATGGATTCTTTGACCAGGTGATAGGCACTGCATCTCTTATTGTCTGCATCCTG
GCCATTGTGGACCCCTACAACAACCCGATCCCCCAAGGTCTTGAGGCCTTCACGGTGGGG
TTCAGCATTCTTGTCATTGGACTCTCCATGGGCTTCAATTCTGGTTATGCAGTTAACCCA
GCTAGGGATTTCGGGCCTCGTCTTTTCACCGCCATTGCTGGTTGGGGTGGTGAAGTCTTT
ACTACCAGAGAGTGTTGGTTTTTGGTGCCCATCTTCGCCCCCTTCCTGGGATCCATTATT
GGTGTGATTGTGTACCAGCTAATGGTGGGGTGGCACGTGGAGGGAGAGGCACGGGACAAG
AATAATAAAGCT---------AGGGATGAG---TCTTTGAAACTTAATGACGTCACCAGC
---AAGGAC---
>Amur_ide_Aqp3a
ATGGGTTGGCAGAAGGCCATGCTGGATAAGCTTGCACATACTTTCCATATCCGCAACAAG
CTGCTGCGCCAGGGATTGGCTGAGTGCTTGGGAACTCTCATCCTGGTGATGTTTGGCTGT
GGTGCAGTGGCTCAAGTGATACTAAGTGAAGGATCTCACGGACGCTTTCTCACTATAAAC
CTTGCTTTTGGGTTTTCCGCTACCCTTGGAATCTTGGTTTGCGGCCAGGTGTCAGGCGGA
CATTTAAATCCAGCCGTTACATTTGCTCTCTGCCTGTTGGGACGTGAAAAATGGAGAAAG
TTCCCTGTGTACTTTCTGTTTCAAACAATTGGATCCTTCTTGGGTGCCGCAATTATCTTT
GCTGAATACCATGATGCAATGTATGAGTTTGCTGGAGAA------TCAAATCTGTTGCTT
GTAACTGGGCCAAAAGCCACTGCTGGGATTTTTGCTACATACCCCAACCCTCATCTCACC
ATCTTAAATGGATTCTTTGACCAGGTGATAGGCACTGCATCTCTTATTGTCTGCTTCCTG
GCCATTGTGGACCCCTACAACAACCCGATCCCCCAAGGTCTTGAGGCCTTCACGGTGGGG
TTCAGCATTCTGGTCATTGGACTCTCCATGGGCTTCAATTCTGGTTATGCAGTTAACCCA
GCTAGGGATTTCGGGCCTCGTCTTTTCACCGCCATGGCTGGTTGGGGTGGTGAAGTCTTT

ACGACCAGACAGTGTTGGTTTTTGGTGCCCATCTTCGCCCCCTTCCTGGGATCCATTATT
GGTGTGTTTGTGTACCAGCTAATGGTGGGGTGGCACGTGGAGGGAGAGGCACGGGACAAG
AAGGATAAAGCT---------AGGGAAGAG---TCTTTGAAACTTAATGACGTCACCAGC
---AAGGAT---
>Big_scaled_redfin_Aqp3a
ATGGGTTGGCAGAAGGCCATGCTGGATAAGCTTGCACAAACTTTCCGTATCCGCAACAAG
CTGCTGCGCCAGGGATTGGCTGAATGCTTGGGAACTCTCATCCTGGTGATGTTTGGCTGT
GGTTCAGTGGCTCAAATGGTACTAAGTGGTGGTTCTCACGGACGCTTTCTCACTATAAAC
CTTGCTTTTGGGTTTGCCGCTACCCTTGGAATCTTGGTTTGCGGCCAGGTGTCAGGCGGA
CATTTAAATCCAGCAGTTACATTTGCTCTCTGCCTCTTGGGACGTGCAAAATGGAGAAAG
TTCCCTGTGTACTTTCTGTTTCAAACAATTGGATCCTTCTTGGGTGCCGCAATTATCTTT
GCTGAATACCATGATGCAATGTATGATTTTGCTGGAGAA------ACAAATCAGTTGCTT
GTATCTGGGCCAAAAGCCACTGCTGGGATTTTTGCTACCTACCCCAACCCTCATCTCACC
ATCTTAAATGGATTCTTTGACCAGGTGATAGGCACTGCGTCTCTGATTGTCTGCGTCCTG
GCCATTGTGGACCCCTACAACAACCCGGTCCCCCCAGGTCTTGAGGCCTTCACGGTGGGG
TTCAGCATTCTTCTCATTGGACTCTCCATGGGCTTCAATTCTGGTTATGCAGTTAACCCA
GCTAGGGATTTCGGGCCTCGTCTTTTCACCGCCATTGCTGGTTGGGGTAGTGAAGTCTTT
ACTACCAGACAGTGTTGGTTTTTGGTGCCCTTCTTCGCCCCCTTCCTGGGATCCATTATT
GGCGTGATTGTGTACCAGCTAATGGTGGGGTGGTACTTGGAGGCAGAGGCACGGGACAAG
ACGAATAAAGAT---------ATGGAAGAG---TCTTTGAAACTTAATGACGTCACCGGC
---AAGAAT---
>Fathead_minnow_Aqp3a
ATGGGTTGGCAGAAGGCCATGCTGGATAAGCTTTCACACACTTTCCATATCCGCAACAAG
CTGCTGCGCCAGGGATTGGCTGAATGCTTGGGAACTCTCATCCTGGTGATGTTTGGCTGT
GGTGCAGTGGCTCAAGTGATACTAAGTGAAGGATCTCACGGACGCTTTCTCACTATAAAC
CTTGCTTTCGCGTTTGCCGCTACTCTTGGAATCTTGGTTTGCGGCCAGGTGTCAGGCGGA
CATTTAAATCCAGCAGTTACATTTGCTCTCTGCCTCCTGGGACGTGAAAAAATGGAGAAAG
TTCCCAGTGTACTTTCTGTTCCAAACAATTGGATCCTTCTTGGGTGCTGCAATTATCTTT
GCTGAATACCATGATGCAATGTATGATTATGCTGGAGAA------TCAAATGAGTTGCTT
GTTGTTGGGGAAAAAGCCACTGCTGGGATTTTCGCCACCTACCCCAGCCCCCATCTCACC
ATCTTAAATGGATTCTTTGACCAGGTGATAGGCACTGCGTCCCTGATTGTCTGCATCCTG
GCCATCGTGGACCCCTACAACAACCCGATCCCACAAGGTCTTGAGGCCTTCACGGTGGGG
TTCAGCATTCTTGTCATTGGACTTTCCATGGGCTTCAATTCTGGTTATGCAGTTAACCCA
GCTAGGGATTTCGGGCCTCGTCTTTTCACCGCCATGGCTGGTTGGGGTAGTGAAGTCTTT
ACTACCAGACAGTGTTGGTTTTTGGTGCCCATCTTCGCCCCCTTCCTGGGATCCGTTATT
GGTGCGATTGTGTACCAGCTAATGGTGGGGTGGCACGTGGAGGGAGAGGCACGGGACAAG
ATGAATAAAGCT---------AGAGAAGAG---TCTTTGAAACTTAATGACGTCTCCAGC
---AAGGAG---
>Grass_carp_Aqp3a
ATGGGTTGGCAGAAGGCTATGCTGGATAAGCTTGCACACACTTTCCAGATCCGCAACAAG
CTGCTGCGCCAGGGATTGGCTGAATGCTTGGGAACTCTCATCCTGGTGATGTTTGGCTGT
GGTGCAGTGGCCCAAGTGGTACTAAGTGAAGGATCTCACGGACTCTTTCTCACTGTAAAC
CTTGCTTTTGGGTTTGCCGCTACCCTTGGAATCTTGGTTTGCGGCCAGGTGTCAGGCGGA
CATTTAAATCCAGCAGTTACATTTGCTCTTTGCCTCTTGGGACGAGAACAGTGGAGAAAG
TTTCCTGTGTACTTTCTGTTCCAAACACTTGGATCCTTCTTGGGTGCCGCAATTATCTTT
GCTGAATACCATGATGCAATGTATGATTATGCTGGAGAA------TCAAATGAGTTGATT
GTAATTGGGCCAAAAGCCACTGCTGGGATTTTTGCTACTTACCCAAGCCCGCATCTCACC
ATCTTAAATGGATTTTTTGACCAGGTGATAGGCACATCGTCTCTGATTGTCTGCATCCTG
GCCATTGTGGACCCCTACAACAACCCGATTCCCCAAGGTCTTGAGGCCTTCACGGTGGGA
TTTAGCGTTCTTGTCATTGGCCTCTCCATGGGCTTCAATTCTGGTTATGCAGTTAACCCA
GCTAGAGATTTCGGGCCTCGTCTTTTCACCGCCATTGCTGGATGGGGTGGTGAAGTCTTC
ACTGCCAGGGATTGTTGGTTTTTGGTGCCCATCTTCGCCCCCTTCATCGGATCCGTTATT
GGTGTGCTTGTGTACCAGCTGATGGTGGGGTGGCATGTGGAGGGAGAGGCACGGGACAAG
AAGAATAAATCT---------AGGGAAGAA---TCTTTGAAACTTAATGATGTCACCAGC
---AAGGAG---
>Kanglang_fish_Aqp3a
ATGGGTTGGCAGAAGGCTATGCTGGATAAGCTTGCACACACTTTCCAGATCCGCAACAAG
CTGCTGCGCCAGGGATTGGCTGAATGCTTGGGAACTCTCATCCTGGTGATGTTTGGCTGT
GGTGCAGTGGCCCAAGTGGTACTAAGTGAAGGATCTCACGGACTCTTTCTCACTGTAAAC
CTTGCTTTTGGGTTTGCAGCTACCCTTGGAATCTTGGTTTGCGGCCAGGTGTCAGGCGGA
CATTTAAATCCAGCAGTTACATTTGCTCTTTGCCTCTTGGGACGAGAAAAATGGAGAAAG

TTTCCTGTGTACTTTCTGTTCCAAACACTTGGATCCTTCTTGGGTGCCGCAATTATCTTT
GCTGAATACCATGATGCAATGTATGATTATGCTGGAGAA------TCAAATGAGTTGCTT
GTAGTTGGGCCAAAAGCCACTGCTGGGATTTTTGCTACTTACCCCAGCGCGCATCTCACC
ATCTTAAATGGATTTTTTGACCAGGTGATAGGCACATCATCTCTGATTGTCTGCATCCTG
GCCATTGTGGACCCCTACAACAACCCGATTCCCCAAGGTCTTGAGGCCTTCACGGTGGGA
TTCAGCGTTCTTGTCATTGGCCTCTCCATGGGCTTCAATTCTGGTTATGCAGTTAACCCA
GCTAGAGATTTCGGGCCTCGTCTTTTCACCGCCATTGCTGGATGGGGTGGTGAAGTCTTC
ACTGCCAGGGAATGTTGGTTTTTGGTTCCCATCTTCGCTCCCTTCATCGGATCCGTTATT
GGTGTGCTTGTGTACCAGCTGATGGTGGGGTGGCATGTGGAGGGAGAGGCACGGGACAAG
AAGAATAAAGCT---------ACGGAAGAG---TCTTTGAAACTTAATGACATCGCCAGC
---AAGGAT---
>Zebrafish_Aqp3a
ATGGGTTGGCAGAAAAGCGTTCTGGATAAGCTTGCGCAGACTTTCCAAATCCGCAACAAG
TTACTGCGCCAGGGATTGGCTGAATGCTTAGGAACTCTCATCCTGGTGATGTTTGGCTGT
GGTTCATTGGCCCAGTTGAAACTAAGCGAAGGTTCTCATGGTCTCTTTCTCACTGCAAAC
CTTGCTTTTGGGTTTGGTGCTACTCTTGGAATCTTGGTTTGCGGCCAGGTGTCAGGCGGA
CATTTAAATCCTGCTGTTACATTTGCTCTCTGCCTCTTGGGAAGAGAAAAATGGAGAAAG
TTTCCTGTGTACTTTCTGTTCCAAACACTCGGATCCTTCTTGGGTGCTGCTATTATCTTT
GCCGAATACCATGACGCAATTTATGATTATGCTGGAGAA------TCAAATGAGTTGCTT
GTACTGGGTGAAAAAGAAACAGCTGGGATTTTTGCTACATACCCAAGCAAATATCTCACC
CCCCTAAATGGATTTTTTGACCAGGTGATAGGCACAGCATCCCTGATTGTGTGCATCCTG
GCCATTGTGGACCCCTACAACAACCCGATCCCTCAAGGTCTTGAGGCCTTCACAGTGGGA
TTCAGCGTCCTTATCATTGGTCTCTCCATGGGCTTCAATTCTGGCTATGCAGTAAACCCA
GCTCGAGATTTTGGACCTCGTCTTTTTACTGCCATGGCTGGTTGGGGTAGTGAAGTCTTC
ACAGCCAGGGATTATTGGTTTTTGGTGCCCATCTTTGCTCCGTTCATTGGAGCCGTTATT
GGTGTGATTGTGTACCAGCTGATGGTGGGATGGCATGTGGAAGGAGAGGCACGAGATAAG
AAAGCTAAAGCT---------AGAGAGGAG---GTGATGAACCTCAATGACGTCGCCAGC
---AAGGAA---
>Oriental_weatherfish_Aqp3a
ATGGGAACGCAGAAGGCCATGCTGGAAAAGATTATGCAGACTATGCATGTTCGCAACAAG
CTACTGCGCCAGGGATTGGCTGAATGTTTGGGAACTCTAATCTTAGTGATGTTTGGTTGT
GGTGCCGTGGCCCAAGTCACACTGAGTGAAGGATCCCACGGTCTCTTTCTGACTGTAAAC
CTGGCTTTTGGGTTTGCCGCCACCCTTGGAATTTTGGTCTGTGGCCAAGTGTCAGGGGGC
CATTTAAATCCAGCTGTTACATTTGCTCTCTGCCTCTTGGGAAGAGAAAAATGGAGAAAG
TTCCCTGTTTACTTCTTCTTCCAAACATTAGGATCATTCTTGGCTGCTGCAATTATTTTT
GCTGAGTACCATGATGCAATGTATGACTATGCTGGTGAA------TCAAATGAATTGGTT
GTAATTGGAAATCATTCCACTGCTGGGATTTTTGCTACGTACCCCAGCAAGCATCTTACC
CTCCTAAATGGATTTTTTGACCAGGTTATAGGCACATCGTCTCTCATCGTCTGCATCCTA
GCCATTGTTGATCCGTACAACAACCCAATCCCTCAAGGTCTCGAGGCATTCACTGTTGGA
TTTAGCGTCCTTGTTATTGGCCTCTCCATGGGATTCAATTCCGGTTATGCTGTGAATCCA
GCCAGAGATTTTGGACCCCGCCTTTTCACCTCCATGGCCGGCTGGGGCCTCGAAGTCTTT
ACTGCCAGGGATTGTTGGTTTTTGGTGCCCATCTTTGCTCCCTTCCTGGGATCCATCATT
GGCGTGATTGTGTACCAGTTGATGGTGGGGTGGCACGCGGAGGCAGAAGCGTGGATCAAG
AAAAATAAATCT---------ATGGAGGAG---AATGTGAAGCTTAATGATGTCACCAGT
---AAGGATGGATCT------AGCCTTCTC------------------------------
>Chinese_weather_loach_Aqp3a
ATGGGAACGCAGAAGGCCATGCTGGAAAAGATTATGCAGACTATGCATGTTCGCAACAAG
CTACTGCGCCAGGGATTGGCTGAATGTTTGGGAACTCTAATCTTAGTGATGTTTGGTTGT
GGTGCCGTGGCCCAAGTCACACTGAGTGAAGGATCCCACGGTCTCTTTCTGACTGTAAAC
CTGGCTTTTGGGTTTGCCGCCACCCTTGGAATTTTGGTCTGTGGCCAAGTGTCAGGGGGC
CATTTAAATCCAGCTGTTACATTTGCTCTCTGCCTCTTGGGAAGAGAAAAATGGAGAAAG
TTCCCTGTTTACTTCTTCTTCCAAACATTAGGATCATTCTTGGCTGCTGCAATCATTTTC
GCTGAGTACCATGATGCAATGTATGACTATGCTGGTGAA------TCAAATGAATTGGTT
GTAATTGGAAATCATTCCACTGCTGGGATTTTTGCTACGTACCCCAGCAAGCATCTTACC
CTCCTAAATGGATTTTTTGACCAGGTTATAGGCACATCGTCTCTCATCGTCTGCATCCTA
GCCATTGTTGATCCGTACAACAACCCAATCCCTCAAGGTCTCGAGGCCTTCACCGTTGGA
TTTAGCGTCCTTGTCATTGGCCTCTCCATGGGATTCAATTCCGGTTATGCTGTGAATCCA
GCCAGAGATTTTGGACCCCGCCTTTTCACCTCCATGGCCGGCTGGGGCCTCGAAGTCTTT
ACTGCCAGGGATTGTTGGTTTTTGGTGCCCATCTTTGCTCCCTTCCTGGGATCCATCATT
GGCGTGATTGTGTACCAGTTGATGGTGGGGTGGCACGCGGAGGCAGAAGCGTGGATCAAG
AAAAATAAATCT---------ATGGAGGAG---AATGTGAAGCTTAATGATGTCACCAGT

---AAGGATGGATCT------AGCCTTCTC------------------------------
>Spined_loach_Aqp3a
ATGGGAAGACAGAAGGCCATGCTGGAAAAGATTATGCAGACTTTGCGGATCCGCAACAAG
CTACTGCGCCAGGGATTGGCTGAATGTTTGGGAACTCTAATCTTAGTGATGTTTGGTTGT
GGTGCTGTGGCCCAAGTTGTACTAAGTGAAGGATCTCATGGTCTCTTTCTGACTGTAAAC
CTGGCTTTTGGGTTTGCCGCCACCCTTGGAATTTTGGTCTGTGGCCAAGTGTCAGGGGGG
CATTTAAATCCAGCTGTTACATTTGCTCTCTGCCTCTTGGGAAGAGAAAAATGGAGAAAG
TTCCCTGTTTACTTCTTCTTCCAAACATTTGGATCATTCTTGGGTGCTGCAATTATCTTT
GCTGAGTACCATGATGCAATGTATGACTATGCTGGTGAA------ACAAATGAATTGGTT
GTAATTGGAAATAAATCCACTGCTGAGATTTTTGCTACATACCCCAGCAAGCATCTTACC
CTCCTAAATGGATTTTTTGACCAGGTTATAGGCACAGCGGCTCTCATTGTCTGCATCCTA
GCCATTGTTGATCCGTACAACAACCCAATCCCTCAAGGTCTCGAGGCCTTCACTGTTGGA
TTTAGTGTCCTTGTTATTGGCCTCTCCATGGGTTTCAATTCCGGTTATGCAGTCAACCCA
GCCAGAGATTTTGGACCCCGCCTTTTCACCTCCATAGCTGGCTGGGGTCCGGAAGTCTTC
ACTGCCAGGGATTGTTGGTTTTTGGTGCCCATCTTTGCTCCCTTCCTGGGATCCATCATT
GGCGTGATTGTGTACCAGATGATGGTGGGGTGGCACGCAGAGGCAGAAGCGTGGGACAAG
AAGAATAAAGCT---------ATGGAGGAG---AATATGAGGCTTAATGATGTGACCAGT
---AAGGATGGA---------TCTGGG---------------------------------
>White_sucker_Aqp3a

------CGGCAGGGATTGGCTGAATGCTTGGGAACTCTCATTCTGGTGATGTTTGGCTGT
GGTGCAGTGGCCCAAGTTGTACTAAGTCAAGGATCTCATGGTCTCTTTCTCACCGTAAAC
CTTGCTTTTGGGTTTGCGGCTACCCTTGGAATCTTGGTTTGTGGCCAGGTGTCAGGAGGC
CATTTAAATCCTGCAGTTACATTTGCCCTCTGCCTCTTGGGAAGAGAAAAATGGAGAAAG
TTCCCTGTGTACTTTTTCTTCCAGACACTTGGATCCTTCTTGGGTGCAGCAATTATCTTT
GCTGAATACCATGATGCAATGTATGACTTTGCTGGAGAC------ACAAACGAGTTGCTT
ATAGAAGGAGATAAAGCCACTGCTGGGATTTTTGCTACATACCCCAGCAAGCATCTCACT
ATCTTAAATGGATTTTTTGACCAGGTGATAGGCACAGCGTCTTTGATTGTCTGCATCCTG
GCCATTGTGGACCCATATAACAATCCAATACCCCAAGGTCTTGAGGCCTTCACTGTGGGG
TTCAGCGTGTTGGTCATTGGCCTCTCCATGGGTTTCAATTCTGGTTATGCAGTCAACCCA
GCAAGAGATTTTGGACCTCGCCTCTTCACCGCCATAGCAGGCTGGGGTGGGGAAGTCTTT
ACTGCCAGGGAATGTTGGTTTTTGGTGCCCATCTTTGCCCCCTTCCTGGGATCCATAATT
GGTGTGATTGTGTACCAGCTAATGGTAGGTTGGCATGCGGAGGGAGATGCACGTGACAAG
AGGAACAAAGCT---------ACGGAAGAG---TCTATCAAGCTCAATGACGTTACCAGC
---AAGGATGGA---------TCTGCG---------------------------------
>Channel_catfish_Aqp3a
ATGGGTAGGCAAAAGATTCTGCTGGATAGGATTGCTCGCACCTTCCAAATTCGTAATAAG
CTACTGCGCCAGGCTTTGGCTGAATGCTTGGGCACACTAATCCTGGTAATGTTTGGCTGT
GGTTCAGTGGCTCAGTTAATGCTCAGTTCAGGAACTCATGGCATCTTCCTCGCTGTAAAC
TTAGCCTTTGGCTTTGCAGCCACACTTGGAATCTTGGTGTGCGGCCAAGTCTCAGGAGGT
CATCTGAACCCAGCTGTAACTTTCGCCCTCTGCCTTCTTGGAAGGGAGAAATGGAGAAAG
TTTCCAGTGTACTTTCTCTTCCAGACACTTGGTGGATTTTTTGGGGCTGGTATCATCTTT
GGCATGTATTATGATGCTATCCAGACATACCATAAAAGA------GAAAATGAACTA---
------------------CCAATGGGAATCTTCGCCACTTACCCCAATGAGCACGTTAGC
ACTGCCAATGGCTTTTTTGATCAGGTGATAGGCACAGCTGCACTGATTGTGTGTGTCCTG
GCGATTGTAGACCCTTTCAACAACCCCATCCCACAAGGACTTGAGGCCTTCACTGTGGGT
TTCACTGTACTAGTCATTGGGTTGTCTATGGGCTTCAATTGTGGCTATGCTGTAAATCCA
GCCAGAGACCTGGGACCTCGTCTTTTCACTGCCATTGCAGGCTGGGGCAGCATAGTCTTC
ACTCATAAGAACTACTGGTTCCTGGTGCCCATCTTTGCCCCATTCCTGGGTACCATCATC
GGAGTGATTTTGTACCAATTGATGGTGGGCTGGCATACAGAAGGGGATACACGGGACAAG
CAA---AAGGCA---------GCAGAAGAATCGAATGTCAAGCTGAATGATATG------
------GAT---TCT------TCAGTTCCC------------------------------
>Bullhead_Aqp3a
ATGGGTAGGCAAAAAATTCTGCTGGATAAGATTGCTCGCACTTTACAAATTCGTAATAAG
CTACTGCGCCAGGCTTTGGCTGAATGCTTGGGCACACTAATCCTGGTGATGTTTGGCTGT
GGTTCAGTGGCTCAGTTAATGCTCAGTTCAGGAACTCATGGCATCTTCCTCGCTGTAAAC
CTAGCCTTCGGCTTTGCAGCCACACTTGGAATCTTGGTGTGTGGCCAAGTCTCAGGAGGT
CATCTGAACCCAGCTGTAACTTTTGCCCTCTGCCTTCTCGGAAGAGAGAAATGGAGAAAG
TTTCCGGTGTACTTTCTCTTCCAGACAATTGGTGGATTTTTTGGGGCTGGTATCATCTTT
GGCATGTATTATGATGCTATCCAGACATATCATACAAAA------GAAAAAGAACTA---
------------------CCAATGGGAATCTTTGCCACTTACCCCAATGAGCACGTTAGC

ACCGCCAATGGCTTTTTTGATCAGGTGATAGGCACAGCTGCACTGATTGTGTGCGTCCTG
GCAATTGTAGACCCTTTCAACAACCCCATCCCACCAGGACTTGAGGCATTCACTGTGGGA
TTCACTGTACTAGTCATTGGGTTGTCTATGGGCTTCAATTGTGGCTATGCTGTAAATCCA
GCCAGAGACCTGGGACCTCGTCTTTTCACTGCCATTGCAGGCTGGGGCAGCATAGTCTTC
ACTCATAAGAACTACTGGTTCCTAGTGCCCATCTTTGCCCCATTCCTGGGTACCATCATT
GGAGTGATTTTGTACCAACTGATGGTGGGCTGGCATACAGAAGGGGATGCACGGGACAAG
CAA---AAGACA---------GCAGAAGAATCGAATGTCAAGCTGAATGATATG------
------GAT---TCT------TCAGTTCCC------------------------------
>Striped_catfish_Aqp3a
ATGGGCAGGCAAAAGATAATCCTGGATAAGATAGCTCGCACCTTGCAAATTCGTAATAAG
CTAGTGCGTCAGGGACTGGCTGAATGCTTGGGCACACTAATCCTAGTGATGTTTGGCTGC
GGTGCAGTGGCTCAGTTAGTGCTCAGTTCAGGATCTCACGGCATCTTCCTTGCTGTAAAC
TTAGCCTTTGGCTTTGCAGCTACACTGGGGATCTTGGTGTGTGGCCAAGTTTCAGGAGGT
CATCTGAACCCAGCTGTAACCTTTGCCCTCTGCCTTCTTGGAAGGGCAAAATGGAGAAAA
TTTCTGGTGTACTTTCTCTTCCAGACACTTGGTGCCTTTTTTGGAGCTGGTATCATCTTT
GGCATGTATTATGATGCTATCCAGACATACTATAAAAAA------GAAAACCAACTA---
------------------CCACTTGGAATTTTTGCCACCTACCCCAACCTGCACCTTAGT
ACCGCCAATGGCTTTTTTGATCAGGTGATAGGCACAGCTGCACTGATTGTGTGCATCCTG
GCCATTGTAGACCCCTTCAACAACCCCATCCCAGCAGGACTTGAGGCCTTCACTGTGGGA
TTCACTGTACTTGTCATTGGGTTATCTATGGGCTTCAACTGTGGCTATGCCGTAAACCCA
GCCAGGGACCTGGGGCCTCGTCTTTTCACCGCCATTGCAGGCTGGGGTGGTCAAGTCTTC
ACTCATGGGAATTACTGGTTCCTGGTGCCCATCTTTGCCCCATTCCTGGGTACCATCATT
GGGGTGATTTTGTACCAACTGTTGGTGGGCTGGCATACAGAAGGGGAGGCACGGGACAAG
CAA---AAGGCA---------ACAGAAGAGCAGAATGTCAAGCTGAATGATATT------
------GAT---TCT------TCAGCTCCC------------------------------
>Yellow_catfish_Aqp3a
ATGGGCAGGCAAAAGATTTTGCTGGATAAGATAGCTCATGCCTTACAAATCCGTAATAAG
TTATTGCGCCAGGGACTGGCTGAACTATTGGGTACCCTAATACTTGTGATGTTTGGCTCT
GGTGCAGTGGCTCAATTAATCCTCAGTGAAGGATCTCACGGCATCTTCCTCGCTGTAAAC
TTGGCCTTTGGCTTTGCAGCCACACTTGGAATCTTGGTGTGTGGTCAAGTCTCAGGAGGA
CATCTGAACCCAGCTGTAACTTTTGCCCTGTGTCTACTTGGAAGGGACAAATGGAGAAAA
TTTCTGGTGTACTTTTTGTGCCAGACAATTGGTTCCTTTTTTGGGGCTGGTATTATCTTT
GGCATGTATTATGATGCTATCCAGGTATACCATAAAAAA------GAAGGCAAACTA---
------------------CCATATGGAATTTTTGCCACCTACCCCGGCGAACACCTTAGT
ACCGCAAATGGGTTTTTTGATCAGGTGATCGGCACAGCTGCACTGATCGTGTGCATCCTG
GCAATTGTAGACCCATACAACAACCCTGTCCCACAAGGACTCGAGGCCTTCACTGTTGGA
TTCACTGTACTTGTCATTGGTTTATCTATGGGCTTTAATTCTGGCTATGCTGTAAACCCA
GCCAGAGATCTGGGACCACGTCTTTTTACCTCCATTTCAGGCTGGGGTTCCCAAGTTTTC
ACTATGGGGAACTACTGGTTCCTGGTGCCTATCTTTGCCCCATTCCTGGGTACAATCATT
GGGGTGATTTTGTATCAACTGCTGGTGGGCTGGCACACAGAAGGGGAGGCACGAGAGAAG
CAAAATAAA------------ACAGAAGAGCTGAATGTCAAGCTGAATGATATT------
------AAT---TCT------TCAGCTCCA------------------------------
>Bottlenose_catfish_Aqp3a
ATGGGCAGGCAAAAGATKATTCTGGATAAGATAGCTCGCACCTTACAAATTCGTAATAAG
TTAGTGCGCCAGGGACTGGCTGAATGCTTGGGCACACTAATCCTGGTGATGTTTGGCTGT
GGTTCAGTGGCTCAGTTAGTGCTCAGTTCAGGATCTCATGGCATCTTCCTCGCTGTAAAC
TTAGCCTTTGGTTTTGCAGCCACACTTGGAATCTTGGTGTGTGGCCAAGTCTCAGGCGGT
CATCTGAACCCAGCTGTAACCTTTGCCCTCTGCATTTTGGGAAGGGAAAAAATGGAGAAAA
TTTCTGGTGTACTTTCTCTTCCAGACCCTTGGTGCATTTTTTGGGGCTGGTATCATCTTT
GGCATGTATTATGATGCTATCCAGGCATACTATAAAAAA------GAAAATGAGCTA---
------------------CCAGTTGGAATTTTTGCCACCTACCCTGGCCAACATCTGAGT
ACSGCCAATGGCTTTTTTGATCAGGTTATTGGCACAGCTGCACTGATTGTATGCGTCCTG
GCCATTGTAGACCCTTACAACAATCCCATCCCACAAGGACTTGAGGCCTTCACTGTAGGA
TTCACTGTACTCGTCATTGGGTTGTCAATGGGCTTCAATTCTGGCTATGCTGTAAATCCA
GCCAGGGACCTGGGACCTCGTCTTTTTACTTCCATTGCAGGCTGGGGTAGCTTTGTTTTC
ACGCATGGGAACTATTGGTTCCTGGTGCCCATCTTTGCCCCGTTCCTGGGTACCATCTTG
GGTGTGATCATTTATCAACTGATGGTGGGATGGCATACAGAAGGGGAAGCACGAGACMAG
CAA---AAGGCA---------ACAGAAGGGCTGAATGTCAAGCTGAATGATGTT------
------GAT---TCC------TCGGTTCCC------------------------------
>Walking_catfish_Aqp3a
ATGGGCAGGCAAAAGGCTTTCCTGGATAAGATCGCTAGCACCTTACAAATTCGTAATAAG

CTAGTGCGCCAGGGACTGGCTGAATGCTTAGGGACACTAATCCTGGTGATGTTTGGCTGT
GGTGCAGTAGCTCAGAATACACTCAGTGACGGAGCTTACGGCAGGTTCCTCAGCGTGAAC
TTAGCCTTTGGATTTGCAGCCACACTTGGAATCTTGGTGTGTGGTCAGGTCTCAGGAGGT
CATCTAAACCCAGCTGTAACTTTTGCCTTATGCCTTCTCGGGAGATCAAAATGGAGAAAA
TTTCCAGTGTACTTTCTTAGCCAGACAATTGGTGCCTTTTTTGGGGCTGGTATAATCTTT
GGCATGTATTATGATGCAATCCAGACATTCCAGAAAAAA------TCTAACGACCTT---
------------------CCCCTTGGAATTTTTGCCACCTATCCTAACGAACACCTTACT
ACCGCAAATGGCTTTTTTGATCAGTTTATAGGAACAGCTGCACTGATTGTGTGCGTCCTG
GCGATTGTAGACCCCTACAACAACCCCATCCCACAAGGACTCGAGGCCTTCACTGTGGGA
TTCACTATACTTGTCATTGGGTTATCTATGGGCTTCAATTCTGGCTATGCTGTAAACCCA
GCCAGGGACCTGGGGCCTCGTCTTTTCACTTCAATGGCAGGCTGGGGCAGCAAGGTCTTC
ACTCATAAAAACTATTGGTTCCTGGTACCTATCTTTGCCCCATTCCTGGGTGCCATCGTT
GGAGTGATTGTGTACCAACTGATGGTGGGCTGGCATACACAAGGGGAGGCACAGGAAAGG
AAA---AAGGAA---------ACAGAGGGGATGAATGTCAAGTTGAATGATATT------
------GAT---TCT------TCAGTTCCC------------------------------
>Stinging_catfish_Aqp3a
ATGGGCAAGCAACAGGTTCTCCTGGATAAGATAGCTCAAACCTTGCATATTCGTAATAAG
CTAGTACGCCAGGGACTGGCTGAATGCTTGGGCACACTAATCCTGGTGATGTTTGGCTGC
GGTGCAGTGGCTCAGGTAACGCTCAGTGAGGGATCACATGGCATGTTCCTGACTGTAAAC
TTAGCCTTTGGCTTTGCAGCCACACTTGGAGTCTTGGTGTGTGGCCAGGTGTCAGGAGGT
CATCTGAACCCAGCTGTAACTTTTGCCCTCTGCATTCTCGGAAGGTCCAAATGGAGAAAA
TTTCCTGTGTACTTTCTTAGCCAGACAATCGGTGCCTTTTTTGGAGCTGGTATAATTTTT
GGCATGTATTATGATGCTATCCAGACCTACCAACAAAAA------TCAGGTAAACTA---
------------------CCCTACGGAATTTTTGCCACCTACCCTAATGATCACCTTACT
ACCGCTAATGGCTTTTTTGATCAGTTTATAGGAACAGCTGCACTGATCGTGTGCATCCTG
GCCATTGTGGACCCCTACAACAACCCCATCCCACAAGGACTTGAGGCCTTCACTGTGGGG
TTCACTATCCTTGTCATTGGGCTTTCTATGGGCTTTAATTCTGGCTATGCTGTGAACCCA
GCCAGGGATCTGGGGCCTCGTATTTTCACTTCAATGGCAGGCTGGGGCAGCAACGTCTTC
ACTCACAAAAACTATTGGTTCCTGGTGCCTATCTTTGCCCCCTTCCTGGGTAGCATCATT
GGAGTTATTATGTACCAACTGATGGTAGGCTGGCATACGGAAGTGGAGGCAAAGAGCAAG
AAA---ATGGAA---------GCAGAAGGGATGAATGTCAAGTTAAATGATATT------
------GAT---TCT------TCAGTTCCT------------------------------
>Pencil_catfish_Aqp3a
ATGGGCATGCAAAAGGTTTTCCTGGAAAAAGTATCTCAAGCTTTACAGATTCGTAACCAA
CTGCTGCGACAAGGACTGGCAGAATGCCTGGGCACACTTATACTGGTGATGTTTGGCTGT
GGTGCGGTGGCTCAACTAAAGCTCAGTGATGGAACTCATGGAATCTTTCTTGCTGTGAAC
TTAGCCTTTGGTTTTGCAGCGACACTTGGAATTTTGGTGTGCGGCCAAGTCTCAGGAGGC
CATCTAAATCCAGCTGTAACTTTTGCTGTCTGCCTTCTTGGAAGAGAAAAAATGGAGAAAA
TTTCCAGTGTACTTCTTCTTCCAGACTTTGGGTGCCTTTTTGGGGTCTGCTATCATCTAC
GGCATGTACCATGATGCCTTCATGGTGCAACATAACAAG------AGCCAGCAGCTT---
------------------CCAATGGGAATCTTTGCTACTTATCCCAACGAGCACCTCAGT
CTTGCCAACGGCTTTTTTGATCAGGTGATTGGCACTGCTGCACTAATTGTTTGCATCCTA
GCCATTGTGGACCCCTACAACAACCCTATCCCACAAGGACTGGAGGCTTTCACTGTGGGA
TTCACTGTACTGGTCATTGGGTTATCCATGGGCTTCAACTCTGGCTACGCCGTAAACCCT
GCCAGAGACTTCGGACCTCGTCTTTTCACTGCCATTGCAGGCTGGGGTTCACAAGTTTTC
ACGCTTGGACACTACTGGTTTTTCGTGCCCATCTTCGCTCCATTTATTGGTAGCGTCATT
GGAGTGCTTGTGTACCAGCTAATGGTGGGTATCCACGTAGAAGGAGAAGCACGGGACAAA
AAG---AAAGCA---------ACTGAGGAC---ACTGTTAAACTTAACGATATGAAT---
------GAT---------------GCT---------------------------------
>Brown_ghost_knifefish_Aqp3a
ATGGGCAGGCAGAAGATCTTCCTGGATAAAGTATCACGCACCCTGCACATTCAGAACAAG
CTTCTGCGACAGGGGTTGGCTGAGTGCCTGGGCACTTTCATCCTGGTGATGTTTGGTTGC
GGCTCTGTGGCTCAGCTGGTGCTGAGTGAAGGTAGTCACGGCATCTTCCTTGCTGTAAAT
TTAGCCTTCGGGTTTGCAGCCACACTTGGAATCCTGGTTTGTGGCCAGGTGTCAGGAGGC
CATCTAAACCCAGCTGTAACCTTTGCCCTTTGTCTGCTTGGAAGGGAGAAATGGAGAAAA
TTCCCTGTATACTTTTTCTTCCAGACACTTGGTGCCTTTTTGGGTGCTGGTGTTATCTTT
GGCATGTACTATGATGCCATGCATATGCGAGCTGTAAGG------GATAATAAGCTC---
------------------CCACTGGGAATTTTCGCTACCTATCCCAGTGAGCACCTTAGC
ATTGTGAACGGCTTTTTTGATCAGCTGATAGGCACCGCTGCACTGATCATTTGTGTCCTG
GCCATTGTGGACCCATACAACAACCCAATCCCACAAGGACTTGAGGCTTTCACAGTGGGA
TTCACAGTGCTAGTCATTGGTTTATCCATGGGCTTCAACTCTGGCTACGCTGTAAACCCA

GCCAGGGACTTTGGACCTCGATTATTCACTGCCATTGCTGGCTGGGGCACCCAAGTCTTC
ACTTATGGGAACTGCTGGTTCCTGGTGCCCATCTTTTCCCCATTCTTGGGGGCCACCATC
GGGACTCTTGTGTACCAGCTGATGGTGGGCTGGCATGTTGAAGGCGAGGCGCGAGACAAA
AAG---AGGGCG---------GCGGAGGAC---AATGTCAAACTCAATGATATTAAT---
------GATGGCTTG------TCAGCTCCC------------------------------
>Black_ghost_Aqp3a
ATGGGCAGGCAGAAGATCTTCCTGGATAAAGTATCACGCACCCTGCACATTCAGAACAAG
CTTCTGCGACAGGGGTTGGCTGAGTGCCTGGGCACTTTCATCCTGGTGATGTTTGGTTGT
GGCTCTGTGGCTCAGCTGGTGCTGAGTGAAGGTAGTCACAGCATCTTCCTTGCTGTAAAT
TTAGCCTTTGGGTTTGCAGCCACACTTGGAATCCTGGTTTGTGGCCAGGTGTCAGGAGGC
CATCTGAACCCAGCTGTAACCTTTTCCCTTTGTCTGCTTGGAAGGGAGAAATGGAGAAAA
TTCCCTGTATACTTTTTCTTCCAGACCCTTGGTGCCTTTTTGGGTGCTGGTGTTATCTTT
GGCATGTACTATGATGCCATGCATATACGAGCTGCAAGG------GATAATAAGCTC---
------------------CCAATGGGAATTTTCGCTACCTATCCCAATGAGCACCTTAGC
ATTGTGAACGGCTTTTTTGATCAGCTGATAGGCACCGCTGCACTGATCATTTGTGTCCTG
GCCATTGTGGACCCATACAACAACCCAGTCCCACAAGGACTTGAGGCTTTCACAGTGGGA
TTCACAGTGCTAGTCATTGGTTTATCCATGGGCTTCAACTCTGGCTACGCTGTAAACCCA
GCCAGGGACTTTGGACCTCGATTATTCACTGCCATTGCTGGCTGGGGCTCCAAAGTCTTC
ACTTTCGGGAACTGCTGGTTCCTGGTGCCCATCTTTTCCCCATTCTTGGGCGCCACCATC
GGGACACTTGTGTACCAGCTGATGGTGGGCTGGCATGTTGAAGGGGAGGCGCGAGAGAAA
AAG---AGGGCG---------GCGGAGGAC---AACGTCAAACTCAATGATATTAAT---
------GATGGCTTG------TCGGCTCCC------------------------------
>Glass_knifefish_Aqp3a
ATGGGCAAGCAGAAGATCTTCCTGGATAAAGTTTCGCGCGCCCTGCAAATTCAAAACCTG
CTGCTGCGTCAGGGCCTGGCTGAATGTCTGGGCACTCTCATCCTGGTGATGTTTGGCTGT
GGTTCAGTGGCTCAGCTGGTGCTCAGTGATGGTAGCCACGGCATCTTCCTTGCTGTAAAT
CTAGCTTTCGGGTTTGCAGCCACACTTGGAATCCTGGTTTGCGGCCAGGTCTCAGGTGGC
CATCTGAACCCTGCCGTGACCTTTTCCCTTTGTCTGCTTGGAAGGGAAAAAATGGAGAAAA
TTCCCTGTATACTTTTTCTTTCAGACACTTGGTGCCTTTTTGGGTGCCGGTATCATCTTT
GGCATGTACTATGATGCCATGCAGAAACGTTTTTCAAAG------GACAACCATCTT---
------------------CCGACTGGAATTTTCGCTACCTATCCCAGTGAACACCTTAGT
ATTGTGAATGGCTTTTTTGATCAGGTGATAGGTACTGCTGCGTTGATTATTTGTATTCTG
GCCATTGTGGACCCATACAACAACCCAATCCCACAAGGACTTGAGGCTTTCACTGTGGGA
TTCACAGTGCTCGTAATTGGCCTGTCTATGGGCTTCAACTCTGGCTATGCTGTAAACCCA
GCCAGGGATTTTGGACCTCGTCTTTTCACTTCCATTGCTGGCTGGGGTTCTGAGGTTTTC
ACTAGAGGGAACTGCTGGTTCCTGGTTCCCATCTTTGCCCCATTCCTGGGTTCCCTGATT
GGGACGCTTGTGTACCAGCTGATGGTGGGCTGGCACGTGGAAGGGGAGGCGCGGGACAAA
AGA---AAGGTG---------ACTGAGGAC---AATGTCAAACTCAATGATATTAAC---
------GATGGCTCT------TCAGTTCCC------------------------------
>Electric_eel_Aqp3a
ATGGGCAAGCAGAAGATCTTCCTGGATAAAATATCACGTGCCCTGCACATTCAGAACAAG
CTGCTACGACAGGGGCTGGCTGAATGCTTGGGCACCCTTATCCTGGTGATGTTTGGCTGT
GGCTCTGTGGCTCAGCTGGTGCTCAGTGAAGGTAGTCATGGCATCTTCCTTGCTGTAAAT
TTAGCCTTTGGGTTCGCAGTCACACTTGGAATCCTGGTTTGCGGCCAAGTCTCAGGAGGT
CATCTGAATCCAGCTGTAACTTTTGCCCTGTGTCTCCTTGGAAGGGAAAAAATGGAGAAAA
TTCCCTGTATACTTCTTCTTCCAGACACTTGGTGCCTTTTTGGGTGCAGGCATCATCTTT
GGCATGTATTATGATGCCATGCATATACGTGCTGACAGG------GACAAGAAGCTT---
------------------CCAATCGGAATTTTTGCTACCTACCCCAGTGAGCACCTTAGT
ATTGTGAACGGATTTTTTGATCAGCTAATAGGCACTGCTGCACTGATTGTTTGTATCCTG
GCTATTGTGGACCCATACAACAACCCAGTTCCACAAGGACTGGAGGCCTTCACTGTGGGA
TTCACAGTGCTTGTCATTGGCTTATCCATGGGCTTCAACTCTGGCTATGCTGTAAACCCA
GCCCGGGACTTTGGTCCTCGTCTTTTCACTGCCATTGCTGGCTGGGGCTCCCAAGTGTTC
ACCAATGGGAACTGCTGGTTCCTGGTGCCCATCTTCGCCCCATTGCTGGGTGCCGTGGTT
GGGACGCTTGTGTACCAGCTGATGGTGGGCTGGCATGTGGAAGGGGAGGCACGGGACAAA
AAG---AGGGCA---------ACAGAGGAC---AGTGTCAAACTCAATGATATTAAC---
------GATGGCTCT------ACAGTTCCC------------------------------
>Redbellied_piranha_Aqp3a
ATGGGCAAGCAGAAGATTTTCCTGGATAAGCTGTCCCGTAGCTTGCAGATTCGCAACAAG
CTGCTGCGTCAAGGACTTGCAGAATGCTTGGGCACCCTCATCCTGGTGATGTTTGGCTGT
GGTTCGGTGGCTCAGCTAAAGCTTAGCGACGAAAAGCACGGCATCTTCCTAGGTGTGAAC
TTAGCCTTCGGATTCGCGGCCACTCTCGGAATCCTGGTGTGTGGACAGGTCTCAGGAGGC

CATCTAAACCCAGCTGTCACCTTTGCCCTCTGCCTCCTTGGGAGGGAAAAATGGAGGAAG
TTCCCTGTGTACTTTCTCTTCCAGACCCTTGGTGCCTTTTTGGGTGCTGGTATCATCTTT
GGCATGTACCATGATGCTCTGGTGACATATGAACACACA------CATAAAGAATTA---
-------------------CCTGTTGGAATTTTCGCTACCTACCCCGGCGAGCATCTGAGT
ATTGTCAATGGATTTTTTGATCAGCTGATCGGCACAGCTGCACTGATAGTTTGTGTCCTG
GCCATCGTGGACCCCTTCAACAACCCCATCCCACAAGGACTTGAGGCTTTCACTGTGGGG
TTCACTGTACTTGTCATTGGTCTATCCATGGGCTTCAACTCTGGTTATGCTGTAAACCCA
GCCAGAGACCTGGGACCTCGGCTTTTTACTGCCATTGCAGGCTGGGGCTATGAAGTCTTT
GTTAAAGGGAACTGCTGGTTCCTGGTGCCCATCTTTGCCCCGTTCCTGGGTACCATCGTC
GGAGTCATTGTGTACCAGCTGATGGTGGGCTGGCATGTGGAGGGCGAGGTGCGAGACAAA
AAG---AAGACA---------ACCGAGGAC---AATGTGAAACTGAATGATGTGAAT---
------GACATC---------ACGGCTCCC-------------------------------
>Tambaq_Aqp3a

---------------------TTTGCCCTCTGCCTCCTCGGGAGGGAAAAATGGAGGAAG
TTCCCTGTGTACTTTCTCTTCCAGACCCTTGGTGCCTTTTTGGGTGCTGGTATCATCTTT
GGCATGTACCATGATGCTCTGGTGAAATATGAACAGGTT------CATAATAAATTA---
-------------------CCTGTTGGAATTTTCGCTACCTACCCCGGCGAGCACCTGAGT
ATCGTCAATGGATTTTTTGATCAGCTGATCGGCACAGCTGCACTGATCGTTTGTGTTCTG
GCCATTGTGGACCCCTTCAACAACCCCATCCCACAAGGACTTGAGGCTTTCACTGTGGGG
TTCACTGTACTTGTCATTGGTCTATCCATGGGCTTCAACTCTGGTTATGCTGTAAACCCA
GCCAGAGACCTGGGACCTCGCCTTTTCACTGCCATTGCAGGCTGGGGCCACGAAGTCTTT
GTTAAAGGGAACTGCTGGTTCCTGGTGCCCATCTTTGCCCCGTTCCTGGGTACCATCGTT
GGAGTCATTGTGTACCAGCTGATGGTGGGCTGGCATGTGGAGGGCGAGGTGCGGGACAAA
AAG---AAGGCA---------ACCGAGGAC---AATGTGAAACTGAATGATATGAAT---
------GATGTC---------TCGGCTCCC-------------------------------
>Mexican_tetra_Aqp3a
ATGGGCAAGCAGAAGATTTTCTTGGACAAGTTGTCCCAGTCGTTGCAGATTCGCAACCTG
CTGGTGCGGCAAGGACTTGCTGAGTGCTTGGGCACCCTCATCCTGGTGATGTTTGGCTGT
GGTTCAGTGGCCCAGTTCACGCTCAGCGGTGGAACTCACGGCATCTTCCTCGCTGTGAAC
TTAGCGTTCGGCTTTGCGGCCACCCTCGGAATCCTCGTGTGCGGCCAGGTCTCAGGCGGT
CATCTAAACCCAGCGGTTACCTTTGCTCTCTGCCTCCTGGGAAGGGAAAAATGGAGGAAA
TTCCCAGTGTACTTCCTCTTCCAGACACTTGGTGCCTTTTTGGGTGCTGGGGTCATCTTT
GGCTTGTACTTCGATGCTCTGCAGAGAAAAGTAGCAGAAACACCGAATAATGATTTA---
-------------------CCAATTGGGATCTTTGCTACCTACCCTGGCGAACACCTGAGT
ACTGTCAATGGATTTTTTGACCAGGTTATTGGAACAGCTGCCCTGATCGTTTGTATCCTG
GCCATCGTGGACCCATTCAACAACCCAATCCCACAAGGACTTGAGGCGTTTACCGTGGGA
TTCACTGTTCTTGTCATTGGTTTATCCATGGGCTTCAACTCTGGCTACGCTGTCAACCCA
GCCAGGGACCTGGGACCTCGGCTCTTCACTTCCATCGCAGGCTGGGGCTCCAGAGTCTTC
ACGAATGGCAATTACTGGTTCCTGGTGCCCATCTTTGCCCCATTCCTGGGGGCCATTGTT
GGGGTGATCGTGTACCAGCTGATGGTGGGCTGGCATGTGGAAGGTGAGGTACGAGACAAA
AAG---AGGGCA---------ACAGAGGAC---AATGTCAAACTGAATGACATGAAT---
------GAA---------------GCT---------------------------------
>Hilsa_shad_Aqp3a
ATGGGCAGACAGCAGTACTACCTGGATAAGCTGTCCCGCACCTTCCAGATCCGCAACAAG
CTCCTTCGGCAGGCCATGGCTGAATGTCTTGGTACCCTCATTCTGGTGATGTTTGGCTGC
GGTGCTGTGGCACAGCTGACTCTGAGCAAGGGCACACATGGCATGTTCTTCACCGTTAAC
TTCGCCTTTGGCTTTGCTGCCATGCTGGGAATCTTGGTGTCTGGCCAGGTGTCAGGTGGG
CATCTCAACCCTGCGGTGACGTTCGCCCTGTGCCTCCTGGGCCGAGAGAAATGGAGGAAG
TTCCCGGTCTACTTCCTAGCACAGACCATTGGTTCCTTTTTAGGCGCTGCTATTATTTTT
GGTCTCTATTATGATGCCATGTGGGACTTTGCTGGACTTTTGCCA---GATGAGCTATTG
GTTGTTGGTGAAAAGGCCACAGCTGGAATATTTGCTACATACCCTGCCAATCATCTCACT
ATTGTTAATGGTTTCTTTGACCAGACCATTGGCACTGCAGCTCTGATAGTGTGTATCCTG
GCTATTGTGGACCCCTACAACAACCCCATCCCCCAGGGCTTGGAGGCCTTCACTGTGGGC
TTTGCTGTTCTGGTGATCGGCCTCTCCATGGGTTTCAACTCCGGCTACGCTGTCAACCCA
GCGCGTGACTTGGGACCGCGCCTCTTCACTGCCATTGCCGGCTGGGGCTCAGAGGTGTTC
ACGGCCGGTAATTGTTGGTTCCTAGTGCCAATCTTTGCTCCTTTCCTTGGCACCATTGTT
GGGGTGATAGTCTACCAGTTGATGGTGGGCTTCCATGTCGAGGGGGAGGTGCGTGACAGG

AGG---ACTTCG---------ATCGAGGAG---GCAGTCAAGCTGAACGATGTCACCACA
TCAAAGGAGGGCAAAGACGGCACAGTTGCA------------------------------
>Allis_shad_Aqp3a
ATGGGAAGACAACAGTACTACCTGGATAAGCTCTCCCGCACCTTCCAGATCCGCAACAAG
CTCTTGCGTCAGGCCCTGGCCGAATGTCTCGGGACCCTGATTCTCGTGATGTTTGGCTGC
GGTTCAGTGGCACAACTGACGCTGAGCAAGGGCACACATGGCATGTTCTTCACCGTTAAC
TTCGCCTTTGGCTTCGCCGCCATGCTGGGAATCTTGGTGTCTGGCCAGGTGTCAGGCGGG
CACCTCAACCCTGCTGTGACATTCGCTCTTTGCCTCCTGGGCCGAGAGAAATGGAGGAAG
TTTCCCATCTACTTCCTGGCCCAGACCATTGGTTCCTTCTTGGGTGCCGCTGTAATCTTT
GGTCTCTATTATGATGCCATTTGGGACTTTGCTGGAAAGATGCCA---GATGAGCTGTTC
GTTGAGGGTGAGAAGGCCACTGCTGGGATATTTGCTACATACCCGGGCCCGCACCTTGAC
ATTGTTAATGGTTTCTTTGATCAGACCATTGGCACTGCGGCTCTGATCGTGTGCATTCTA
GCCATCGTGGATCCCTACAACAACCCCGTCCCTCCAGGCCTGGAGGCCTTCACCGTGGGC
TTCGCCGTCCTGGTCATCGGCCTCTCCATGGGCTTCAACTCCGGCTACGCCGTCAACCCG
GCACGTGACCTGGGACCGCGCCTCTTCACCGCCATTGCTGGCTGGGGCACGGAGGTGTTC
ACGGTAGGGAAGTGTTGGTTCCTGGTGCCAATCTTTGCCCCTTTCCTTGGCTCCATCGTT
GGGGTCATGGTCTACCAGCTGATGGTGGGCTTCCATGAGGAAGGGAAGGTGCGCGACAAC
AAA---GCTGCT---------ATCGAGGAG---ACGGTCAAACTGAACGACATCACCACA
GAAAAGGACGGGAAAGACGGCACTGTCGCA------------------------------
>Alewife_Aqp3a
ATGGGAAGACAACAGTACTACCTGGATAAGCTCTCCCGCACCTTCCAGATCCGCAACAAG
CTCTTGCGTCAGGCCCTGGCCGAATGTCTCGGGACCCTGATTCTCGTGATGTTTGGCTGC
GGTTCAGTGGCACAACTGACGCTGAGCAAGGGCACACATGGCATGTTCTTCACCGTTAAC
TTCGCCTTTGGCTTCGCCGCCATGCTGGGAATCTTGGTGTCTGGCCAGGTGTCAGGCGGG
CACCTCAACCCTGCTGTGACATTCGCTCTTTGCCTCCTGGGCCGAGAGAAATGGAGGAAG
TTTCCCGTCTACTTCCTGGCCCAGACCATTGGTTCCTTCTTGGGTGCCGCTATAATCTTT
GGTCTCTATTATGATGCCATTTGGGACTTTGCTGGAAAGATGCCA---GATGAGCTGTTC
GTTGTGGGTGAGACGGCCACGGCTGGGATATTTGCTACATACCCGGGCCCGCACCTTGAC
------------------GATCAGACCATTGGCACTGCGGCTCTGATCGTGTGCATTCTG
GCC---

------------------------TTCACCGCCATTGCTGGCTGGGGCACGGAGGTGTTC
ACGGTAGGGAAGTGTTGGTTCCTGGTGCCTATCTTTGCCCCTTTCCTTGGCTCCATCGTT
GGGGTCATGGTCTACCAGCTGATGGTGGGCTTCCATGAGGAAGGGGAGGTGCGCGACAAG
AGA---GCTGCT---------ATCGAGGAG---ACGGTCAAACTGAACGACATCACCACA
GAAAAGGACGGGAAAGACGGCACCGTCGCA------------------------------
>Japanese_grenadier_anchovy_Aqp3a

--CCA---GATGAGCTGCTG
GTTGAGGGGGAAAAGGCCACAGCTGGAATATTTGCTACGTATCCTGGCAAGCACCTTACC
ATTGTTAATGGTTTCTTTGACCAGACCATTGGCACCGCGTCTCTAATCGTCTGCATCTTG
GCCATCGTGGACCCCCATAACAACCCCATCCCCCAGGGCCTGGAGGCCTTCACCGTGGGC
TTCGCCGTCCTGGTCATCGGCCTGTCCATGGGCTTCAACTCCGGCTACGCCGTCAACCCG
GCCCGTGACCTGGGGCCGCGCCTCTTCACCGCCATAGCAGGCTGGGGCCCGGAGGTGTTT
ACGGTGGGGCAGTGTTGGTTCCTGGTGCCCATCTTTGCCCCCTTCCTGGGCACCATCGTT
GGGGTGATGGTGTACCAGCTGATGGTGGGCTTCCACGTGGAGGGGGAGGCGCGCGACAGG
GCG---GCCGCC---------ATCGAGGAGGAGTCGGTCAAACTGAACGACATCACCACC
---AAGGATGACAAAAATGGCACAAATGCA------------------------------
>Konoshiro_gizzard_shad_Aqp3a
ATGGGCAGACATCAGTACTACCTGGATAAGCTGTCCCGCACCTTCCAGATCCGCAACAAG
CTCCTTCGGCAGGCCCTGGCTGAATGTCTTGGTACCCTCATTCTGGTGATGTTTGGCTGC
GGTGCAGTGGCTCAGCTGACTCTGAGCAAGGGCACACACGGCATGTTCTTCACTGTTAAC
TTCGCCTTTGGATTCGCTGCCATGCTGGGAATCTTGGTGTCTGGCCAGGTGTCAGGTGGG
CATCTCAACCCTGCGGTGACATTCGCTCTGTGCCTCCTGGGTCGAGAGAAATGGAGGAAG
TTCCCCGTCTACTTCCTAGCGCAGACCATTGGTTCCTTTTTAGGTGCCGCTATCATTTTC
GGTCTCTATTATGATGCTATTTGGGACTATGCTGGAAAGATGCCA---GATGAGCTGATT

GTTATAGGTGAAAAGGCCACGGCTGGAATATTTGCTACATACCCTGCCAAACATCTCACC
ATTGTTAATGGTTTCTTTGACCAGACCATTGGCACTGCAGCTCTGATAGTGTGTGTCCTG
GCTATTGTGGACCCCTACAACAACCCCATCCCCCCGGGCTTGGAGGCCTTCACCGTGGGC
TTTGCTGTCTTGGTGATCGGCCTCTCCATGGGTTTCAACTCCGGCTACGCCGTCAACCCA
GCGCGTGACCTGGGACCACGCCTCTTCACTGCCATTGCCGGCTGGGGCCCGGAGGTGTTC
ACAGCCATGAATTGTTGGTTCCTAGTGCCAATCTTTGCTCCATTCCTTGGCACCATTGTT
GGGGTGATGGTCTACCAGCTGATGGTGGGCTTCCATGTCGAGGGGGAGGCGCGTGACAAG
AGG---GCT---

>Sardine_Aqp3a
ATGGGAAGACATCAGTACTACCTGGATAAGCTGTCCCGCACCTTCCAGATCCGCAACAAG
CTCATGCGTCAGGCCCTGGCTGAGTGTCTCGGGACCCTGATTCTTGTGATGTTTGGCTGC
GGTTCCGTGGCACAGCTGACGCTGAGCAAGGGCACACATGGCATGTTCTTCACCGTTAAC
TTCGCCTTTGGCTTCGCCGCCATGCTGGGAATCTTGGTGTCTGGCCAGGTGTCAGGCGGC
CACCTCAACCCCGCTGTGACATTCGCCCTTTGCCTCCTGGGCCGAGAGAGATGGAGGAAG
TTCCCCGTCTACTTCCTGGCCCAGACCATCGGTTCCTTCTTGGGCGCCGCTATAATCTTT
GGTCTCTACTATGATGCCATTTGGGACTATGCTGGAAAGATGCCA---GATGAACTGTTG
GTTGTGGGTGAGAAGGCCACTGCTGGGATATTTGCTACATACCCGGCCAAGCACCTTACC
GTCGTTAATGGTTTCTTTGATCAGGTGATTGGCACTGCGGCTCTGATCGTGTGCATTCTG
GCCATCGTGGACCCCCACAACAACGCCGTCCCCCCCGGCCTGGAAGCCTTCACCGTGGGC
TTCGCCGTCCTGTCCATCGGCCTCTCCATGGGCTTCAACTCCGGCTACGCCGTCAACCCG
GCGCGTGACCTGGGGCCGCGCCTCTTCACCGCCATCGCCGGCTGGGGCCCGGAGGTGTTC
ACGGCGAGGAACTGTTGGTTCCTGGTGCCAATCTTTGCCCCTTTCCTCGGCTCCATCGTT
GGCGTCATGGTCTACCAGCTGATGGTGGGTTTCCATCAGGAGGGCCAGTCGCGCGACGAG
AGA---GCTACT---------ATCGAGGAG---ACGGTCAAACTGAACGACGTCAGCACC
GAAAAGGACGGCAAAGACGGCACCGTGGCA------------------------------
>Atlantic_herring_Aqp3a
ATGGGCAGGCATCAGTACTACCTGGAAAAGCTGTCTCGCACCTTCCAAATCCGCAACAAA
CTTCTGCGCCAGGCCCTGGCTGAATGTCTCGGCACCCTCATTCTGGTGATGTTTGGCTGC
GGCGCCGTGGCACAGCTGGTACTTAGCGGTGGCACACACGGCATGTTCCTCACCGTCAAC
TTCGCCTTTGGCTTCGCCGCCATGCTAGGAATATTGGTGTGTGGCCAGGTATCAGGTGGG
CATCTCAACCCAGCTGTGACATTTGCTCTTTGCCTCCTGGGTCGAGAGCAATGGAGGAAG
TTTCCTGTCTACTTCCTCTTCCAGACCATTGGTGCCTTCCTGGGTGCCGCTATAATCTTT
GGTCTCTACTTTGATGCCCTGTGGGACTATGCTGGAAAGGTGCCA---GATGAGCTGCTT
GTTGTGGGTGACAAAGCCACGGCTGGAATATTTGCTACATACCCTGGCAAACACCTTACC
ATTGTAAATGGTTTCTTTGACCAGGTCATTGGCACAGCAGCTCTGATTGTATGCATCCTG
GCCATCGTGGACCCTCACAATAACCCCATCCCCCAGGGCCTGGAGGCCTTCACCGTGGGC
TTCGTCGTCCTGGTCATCGGCCTCTCCATGGGTTTTAACTCCGGCTATGCCGTCAACCCG
GCACGTGACCTGGGACCGCGCCTCTTCACTGCCATAGCCGGCTGGGGCCCGGAGGTGTTC
ACGGCCGGACGATGTTGGTTCCTGGTGCCGGTATTTGCCCCTTTCCTTGGCACCATCGTT
GGGGTGATGGTGTACCAGCTGATGGTGGGCTTCCATATGGAAGGGGAGGCGCGTGATAAA
GCGAATGATAAT---------ATCGAGGAG---TCAGTGAAACTGAACGATGTCACCACC
---AAGGACAGCAAAGACGGCACACACGCA------------------------------
>Denticle_herring_Aqp3a
ATGAGCAGGCAGAAGGCTTTCCTCAATAAGGTAGCACATTCCATCCACATTAAGAACAAG
TTGCTCCGCCAGGGTCTAGCTGAATGTCTGGGCACGCTTATTTTGGTGATGTTTGGCTGT
GGCTCTGTGGCCCAGGTGATACTAAGTGAAGGCTCCCATGGCAGGTTTCTCACAGTCAAC
CTGGCCTTTGGTTTTGCTGCCACTCTTGGAATCTTAGTGTGTGGTCAAATATCAGGTGGG
CATCTCAACCCTGCTGTGACATTTGCTCTCTGTCTTCTGGGAAGAGACAAATGGAGAAAA
TTCCCAGTGTATTTCCTCTTCCAAACTCTGGGTGCCTTCTTAGGGGCTGGTATCATCTAT
GGCCTGTACTTTGATGCTTTGTGGAACTTTGCGGGAATT---------GATGGACTGGTT
GTTGATGGGAACAAATCCACTGCAGGAATTTTTGCTACATACCCTGCAGCACATCTTTCC
CTTGTCAATGGCTTCTTCGACCAGCTTATTGGAACAGCGGCTCTGGTTGTTTGTGTCCTT
GCCATTGTGGACCCATATAACAACCCCATCCCTCAAGGACTGGAAGCATTCACCGTTGGC
TTTGTTGTTTTGGTGGTTGGTCTGTCTATGGGTTTGAATTCAGGCTATGCTGTTAACCCC
GCCCGTGACTTTGGACCACGCCTGTTTACAGCTATTGCCGGTTGGGGCGGTGAAGTCTTC
ACGAACAGAAATGGTTGGTTCTTGGTGCCCATCTTTGCTCCCTTCCTGGGCTCCTTTGTT
GGTGCGTTGGTTTACCAGCTGATGGTCGGTTTCCATACAGAGGCTGAAGCACAAACCGCC
AAA---AGGAAC---------ACTGAAGAG---AATGTTAAGCTAAAGGATGTCCGTAGT
---GCTGATGGCAAAGAT--
>Asian_arowana_Aqp3b2

ATGGGAAGGCAGAAGTACTACCTGGACAAGCTGTCACGCTCGTTCCACATCCGCAACCTA
TTGGTCCGCCAAGCCCTGGCTGAGTGCTTGGGAACTCTCATTTTAGTGATGTTTGGCTGT
GGATCGCTGGCCCAAGTGGTACTCAGCGGTGGATCTCATGGTTTGTTCATCACTGTCAAC
TTTGCTTTTGGGTTTGGTGCAACGCTGGGGATCCTGGTGTGCGGACAAGTGTCAGGAGGC
CATCTGAACCCTGCTGTGACCTTGGCCCTGTGCATACTGGGAAGAGAGAAGTGGAGGAAG
TTTCCAGTGTATTTCGCCTTTCAAACTCTGGGAGCCTTCCTGGGTGCTGGAATCATTTTT
GGCCTCTACTTCGATGCCATCTGGGACTTT---GGCAAA---------GGGAACCTTATT
GTTATGGGGGAAAATTCCACGGCAGGAATCTTTGCTACATACCCTTCACCTCACCTCACA
CCCATCAATGGATTTTTTGACCAGATGATTGGTACGGCTGCTCTCATCGTTTGCATCTTG
GCCATCGTGGACCCCTACAACAACCCCATCCCCCAAGGACTGGAAGCATTCACAGTTGGC
TTTGTGGTGCTGGTCATTGGCCTTTCAATGGGCTTCAACTCGGGATACGCTGTTAACCCT
GCCAGAGACCTGGGCCCTCGTCTTTTCACTGCTATTGCAGGCTATGGTGGTGAAGTCTTC
TCGGCTGGTTCCTACTGGTTCTTCGTGCCAATTTTCGGTCCTTTCATCGGAGCATTTTTT
GGGGTAGTGGTATACCAGCTCATGGTGGGATTCCATGTTGAGCAAGAGAGCCGGCAGAAG
CAACAG----------------GCAGAAGAAGAGCGCCTTAAACTGTCCAATGTTGCATCT
---AAC---ACT--
>Silver_arowana_Aqp3b2
ATGGGAAGGCAGAAGTACTACCTGGAGAAACTGTCACGCGCGGTGCACATCCGCAACCTG
CTGGTGCGCCAGGCGCTGGCTGAGTGCTTGGGAACTCTCATCTTAGTGATGTTCGGCTGC
GGATCGGTGGCCCAGCTGACGCTCAGCTTTGGATCCCACGGCTTGTTCCTCACCGTCAAC
TTTGCTTTCGGTTTCGCCGCAACATTGGGGATCCTGGTGTGTGGCCAAGTGTCAGGAGGC
CATCTGAACCCGGCTGTGACCTTTGCCGTCTGCCTACTGGGAAGAGAGAAGTGGAGGAAG
TTTCCCGTGTATTTTGCCTTCCAAACCCTGGGAGCCTTCCTGGGTGCCGCAGTAATTTTC
GCCCTGTACTTTGATGCCATATGGGACTTT---GGCAAA---------GGGAACCTTTAT
GTTCAGGGGGAAAATGCCACAGCAGGAATCTTCGCCACGTACCCGTCAATCCACCTCACG
CCCATCAATGGATTTTTTGACCAGATGATCGGCACGGCTGCTCTCATCGTTTGCATCTTG
GCTATCGTGGACCCCTACAACAACCCCACCCCGCAAGGACTGGAGGCTTTCACGGTCGGC
TTCGTCGTGCTCGTCATTGGCCTTTCGATGGGCTTCAACTCGGGATACGCCGTCAACCCA
GCCAGGGACCTGGGCCCTCGTCTCTTCACCGCCATTGCGGGCTACGGCGGCGAAGTCTTC
AGTGCTGGTTCCTACTGGTTCTTCGTGCCCATCTTTGCCCCTTTCTTCGGAGCAATTCTC
GGGACGATCGTGTACCAGCTCATGGTGGGATGCCATACTGAACAAGAGGCCCGGCAGAAG
AAGGAG----------------GTACAGGAAGAGCAGCTTAAACTGTCCAATGTTGCATCT
---AAC---GCT--
>Pirarucu_Aqp3b2
ATGGGAAAGCAGAAGTACTACCTGGAGAAATTGTCACAGTCGTTCCACATCCGCAACCTG
TTGATCCGACAGGCTCTGGCAGAATGCCTGGGAACCCTCATTTTAGTGATGTTTGGCTGT
GGTTCAGTGGCCCAGCTGGTGCTCAGTGGAGGGTCTCACGGCTTGTTCCTCACTGTTAAT
TTTGCTTTTGGCTTTGGTGCCACCTTGGGGATCCTGGTCTGTGGTCAGGTGTCAGGAGGC
CATCTGAACCCTGCTGTGACCTTTGCTTTGTGCTTACTGGGAAGAGAGAAGTGGAGAAAG
TTTCCAGTGTATTTTGCCTTTCAGACTCTGGGCTCCTTCCTGGGTGCCGCAATCATCTTT
GGCCTGTACTATGATGCCATCTGGGATTTT---GGCAAA---------GGATCCTTCTAT
GTTGAAGGGCAAAATTCCACTGCTGGGATCTTTGCCACTTACCCTTCAGCCCACCTCACG
TCCATCAATGGTTTCTTTGACCAGATGATCGGCACAGCAGCCCTCATTGTCTGCATCTTG
GCTATCGTGGACCCTTATAACAACCCCATCCCCCAAGGACTGGAAGCTTTCACAGTTGGC
TTTGTAGTGCTGGTCATTGGACTCTCAATGGGCTTCAATTCTGGATACGCTGTTAACCCT
GCCAGAGACCTGGGCCCTCGTCTCTTCACTGCTATTGCAGGCTACGGTGGCCAAGTCTTC
TCGGTTGGCTCCTACTGGTTCTTCGTCCCAATTTTTGCTCCATTCCTGGGGGCGTTCCTT
GGAGTAGTGATATACCAGCTCATGGTGGGCTTCCACACCGAGCAAGAAGCCCGTCAGAAG
AAGGGG-------------------GGAGAGGAAAACATTAAATTGTCTAATGTTGCATCT
---AAT---GCT--
>Elephantnose_fish_Aqp3b2
ATGGGAAGGCAGAAGTTCCTTCTGGACAAACTGTCACAGTCCTTCTTCATCCGCAACCTG
CTGATACGGCAGGCTCTAGCTGAGTGCCTGGGGACCCTTATCCTGGTGATGTTTGGCTGC
GGAGCAGTAGCCCAAGTGGTGCTGAGCAATGGATCCCACGGCATGTTTCTAACTGTCAAT
TTTGCCTTTGGCTTTGCTGCCACCTTGGGAATCCTGGTTTGTGGTCAGGTATCAGGAGGT
CACCTGAACCCCGCAGTCACCTTTAGTTTATGTCTACTTGGGAGGGAAAAAATGGAGGAAG
TTTCCAGTATATTTTGCCTTTCAAACTCTTGGTGCCTTCTTAGGAGCTGGCATCATTTTT
GGCCTCTACTTTGATGCCATTTGGGATTTG---AACAAA---------GCGGAACTCCTT
GTCACGGGAGTAAATTCCACAGCTGGAATCTTTGCCACATACCCCTCAAACCACCTTACA
ATCGTTAATGGGCTGTTTGACCAGCTGATTGGCACAGCTGCCCTGGTTGTGTGTGTGCTG
GCTATTGTGGACCCATATAATAACCCCATCCCTCGAGGCCTGGAGGCTTTCACAGTGGGC

TTTGTAGTTCTGGTCATCGGCCTTTCCATGGGCTTCAACTCTGGATATGCTGTCAACCCC
GCCAGAGATCTTGGCCCTCGTATCTTCACATCGATCGCAGGCTGGGGAACTGAAGTCTAC
TCGGCTGGCAGATACTGGTTCCTCGTCCCAGTTTTTGTTCCCTTCCTGGGAGCATTTTTT
GGGACTGTGGTATACCAGTTCATGGTGGGATTCCATGTGGAGCAGGAGTCGAAG---CGA
AGGGAG------------------GAGGATGAGAACCTCAAACTGACCAACATGGCCTCC
---AAT---GCC--
>Mormyrid_electric_fish_Aqp3b2
ATGGGAAGGCAGAAGTTCCTTCTGGACAAAATATCACGGTCCTTCTTCATCCGCAACCTG
CTGTTACGGCAGGGTCTTGCTGAGTGCCTGGGGACCCTTATCCTGGTGATGTTTGGCTGC
GGAGCAGTAGCCCAGGTGGTGCTGAGCGGTGGATCCCACGGCATGTTTCTAACTGTCAAT
TTTGCCTTTGGCTTTGCTGCCACCTTGGGAATCCTGGTTTGTGGGCAGGTATCAGGAGGT
CACCTGAACCCCGCAGTCACCTTTAGTTTATGTCTACTTGGGAGGGAAAAATGGAGGAAG
TTTCCAGTATATTTTGCCTTTCAAACTCTTGGTGCCTTCTTAGGAGCTGGCATAATTTTT
GGCCTCTACTTTGATGCCATTTGGAATTTG---AACAAA---------ACAGAACTCCGT
GTCGTGGGAGAAAATGCCACTGCTGGGATCTTCGCCACATACCCCTCAAATCACCTTACA
ATCGTCAATGGGTTGTTTGACCAGCTGATTGGCACAGCTGCCCTGGTTGTGTGTGTGCTG
GCTATTGTGGACCCATATAACAACCCCATCCCTCGAGGACTGGAGGCTTTCACAGTGGGC
TTCGTGGTGCTGGTCATTGGCCTTTCCATGGGCTTCAACTCTGGATATGCTGTAAACCCT
GCCAGAGATCTTGGCCCTCGTATCTTCACTTCAATCGCAGGCTGGGGAACTGAAGTCTAC
TCGGCTGGCAGATACTGGTTCCTTGTCCCAGTTTTTGTTCCCTTCCTGGGAGCATTTTTT
GGGACTGTGGTATACCAGTTCATGGTGGGATTCCATGTGGAGCAGGAATCGAAG---CGG
AAGGAG------------------GAGGATGAGAATCTCAAACTGACCAACATGGCCTCC
---AAT---GCC--
>Freshwater_butteflyfish_Aqp3b2
ATGGGAAAACAAAAGGCTTTCCTGGACAAGATTTCCTTCTTATTCCACATTCGCAACCTG
CTGATTCGTCAGGCTTTAGCAGAATGCCTTGGAACTCTTATTCTGGTGATGTTTGGTTGC
GGTGCTGTGGCCCAGATGAAGCTCAGTGACAGGAATTTTGGCAGATTCCTCTCTGTCAAT
TTTGCTTTTGGCTTTGGAGCCACCTTGGGGATCTTGGTTTGTGGACAAGTCTCAGGAGGC
CACCTTAACCCTGCTGTCACCTTTAGTCTCTGCTTACTTGGACGAGAGAAATGGAGGAAG
TTCCCCGTGTACTTTGCTGCCCAAATTCTTGGTGCATTCATGGGTTCAGGGATCATCTTT
GGACTCTACTTTGATGCTCTATTTGCGTTT---GGCAAT---------GGCTCCCTTTCA
GTCTTGGGGGAAAACGGTACTGCGGGGATTTTTGCTACTTACCCCTCACCTCATCTCTCC
TTAGTCAATGGTTTCTTTGACCAGCTAATTGGAACAGCTGCCCTCATTGTTTGCATCCTG
GCTATTGTTGATCCCTACAACAATCCTATCCCCCGGGGGCTGGAGGCCTTCACGGTTGGG
TTTGTAGTGCTTGTCATTGGTCTCTCCATGGGCTTCAACTCTGGATATGCTGTGAATCCT
GCCAGGGACTTTGGCCCTCGCCTCTTCACTTCCATTGCCGGCTGGGGTAGTGAAGTTTTT
ACAGCCCGAGACTACTGGTTCTTCATTCCTATCTTTGCCCCCTTTTTGGGGGCTTTCTTT
GGTGCTGTGGTGTACCAATTCATGGTAGGATTTCACGTTGAACAAGAGGCTCGTCAGAAG
AGGCCA---------------CAGGAAGACGAGAACTTAAAACTGACCAACGTCTCATCT
---AAT---GCA--
>American_eel_Aqp3b2
ATGGGGAAGCAGAAGTTCTACCTGGACAAACTGGCCCGGACGTTCCAGATCCGCAACCGG
CTGCTTCGGCAAGGCCTGGCCGAGTGCCTGGGGACCCTCATCCTGGTGATGTTCGGCTGT
GGCGCGGTGGCCCAAGTGGTGCTGAGCGCTGGCTCTCATGGCATGTTCCTCACGGTGAAC
TTCGCCTTCGGCTTCGCCGCCACTCTGGGTATCCTGGTCTGCGGACAGGTTTCAGGTGGC
CATCTGAACCCCACTGTGACCTTCGCCCAGTGCCTACTCGGGAGAGAACCATGGATAAAG
TTCCCAGTGTACTTCCTGTTTCAAACCCTGGGGGCCTTCTTGGGTTCTGGAGTCATTTTC
GGCTTGTACTACGATGCAATGTGGGACTTC---GGCAAA---------AACGATCTCATT
GTTGTTGGGGAAAAAGCCACAGCCGGGATCTTTGCCACCTACCCTTCAAATCATCTGACC
CTCCTCAATGGTTTCTTTGACCAGCTGATTGGCACGGCGGCACTGATCGTGTGCATCCTG
GCGATCGTGGACCCTTACAACAACCCCATCCCCCGGGGCCTGGAGGCCTTCACCGTGGGC
TTTGTGGTGCTGGTCATCGGCCTGTCCATGGGCTTCAACTCTGGATACGCCGTCAACCCG
GCCAGGGACTTCGGACCCCGTCTCTTCACCGCTCTGGCAGGCTGGGGCAGGGAGGTGTTC
ACGGCCAATTCATACTGGTTCTTCGTGCCCATTTTTGCCCCCTTCCTGGGCACAGTGGTA
GGGGTGCTGGTGTACCAGCTGATGGTGGGTTTCCACGTGGAGGGAGAGGCGCGGGACAAG
ATGGAGGCG------------ACAGAAGACGAGCGTATCAAGCTGTCGAACGTCGCGACC
---AAAGATGCGGCG---
>European_eel_Aqp3b2
ATGGGGAAGCAGAAGTTCTACCTGGACAAACTGGCCCGGACGTTCCAGATCCGCAACCGG
CTGCTTCGGCAAGGCCTGGCCGAGTGCCTGGGGACCCTCATCCTGGTGATGTTCGGCTGT
GGCGCGGTGGCCCAGGTGGTGCTGAGCGCTGGCTCTCATGGCATGTTCCTCACGGTGAAC

TTCGCCTTCGGCTTCGGCGCCACTCTGGGTATCCTGGTCTGCGGACAGGTTTCAGGTGGC
CATCTGAACCCCACTGTGACCTTCGCCCAGTGCCTACTCGGGAGAGAACCATGGATAAAG
TTCCCAGTGTACTTCCTGTTTCAAACCCTGGGGGCCTTCTTGGGTTCTGGAGTCATTTTC
GGCTTGTACTACGATGCAATGTGGGACTTC---GGCAAA---------AACGATCTCATT
GTTGTTGGGGAAAAAGCCACAGCCGGGATCTTTGCCACCTACCCTTCAAATCATCTGACC
CTCCTCAATGGTTTCTTTGACCAGCTGATTGGCACGGCGGCACTGATCGTGTGCATCCTG
GCGATCGTGGACCCTTACAACAACCCCATCCCCCGGGGCCTGGAGGCCTTCACCGTGGGC
TTTGTGGTGCTGGTCATCGGCTTGTCCATGGGCTTCAACTCTGGATACGCCGTCAACCCG
GCCAGGGACTTCGGACCCCGTCTCTTCACCGCTCTGGCAGGCTGGGGCAGGGAGGTGTTC
ACGGCCCATTCATACTGGTTCTTCGTGCCCATTTTTGCCCCCTTCCTGGGCACAGTGGTA
GGGGTGCTGGTGTACCAGCTGACGGTGGGTTTCCACGTGGAGGGAGAGGCGCGGGACAAG
ATGGAGGCG------------ACAGAAGACGAGCGTATCAAGCTGTCGAACGTCGCGACC
---AAAGATGCGGCG---
>Japanese_eel_Aqp3b2
ATGGGGAAGCAGAAGTTCTACCTGGACAAAGTGGCCCGGATGTTTCAGATCCGCAACCGG
CTGCTTCGGCAAGGCCTGGCCGAGTGCCTGGGGACCCTCATCCTGGTGATGTTCGGCTGT
GGCGCGGTGGCCCAGGTGGTGCTGAGCGCTGGCTCTCACGGCATGTTCCTCACGGTGAAC
TTCGCCTTCGGCTTCGCCGCCACTCTGGGTATCCTGGTCTCCGGACAGGTTTCAGGTGGC
CATCTGAACCCCACTGTGACCTTCGCCCAGTGCCTACTCGGGAGAGAACCATGGATAAAG
TTCCCAGTGTACTTCCTGTTTCAAACCCTGGGGGCCTTCTTGGGTTCTGGAGTCATTTTC
GGCTTGTACTACGATGCATTGTGGAATTTC---GGCAAA---------AACGATCTCATT
GTTGTTGGGGTAAACGCCACAGCCGGGATCTTTGCCACCTACCCTTCAGATCATCTGACC
ATCGTCAATGGTTTCTTTGACCAGCTGATTGGCACGGCGGCACTGATCGTGTGCATCCTG
GCGATCGTGGACCCTTACAACAACCCCATTCCCCGGGGCCTGGAGGCCTTCACCGTGGGC
TTTGTGGTGCTGGTCATCGGCCTGTCCATGGGCTTCAACTCTGGATACGCCGTCAACCCA
GCCAGGGACTTCGGACCCCGTCTCTTCACCGCTCTGGCAGGCTGGGGCAGGGAGGTGTTC
ACGGCCAAATCATACTGGTTCTTCGTGCCCATTTTTGCCCCCTTCCTGGGCACAGTGGTA
GGGGTGCTGGTGTACCAGCTGATGGTGGGTTTCCACGTGGAGGGAGAGGCGCGGGACAAG
ATGGATGCG------------ACAGAAGACGAGCGTATCAAGCTGTCGAACGTCGGGACC
---AAAGATGCGGCG---
>Asian_arowana_Aqp3b1
ATGGGGAAACAGAAGGTTTACCTGGAGAAGCTGGCACGCGTTGTGCAGGTCCGTAACTTG
CTCATGCGACAGGCTCTGGCTGAATGCCTGGGGACCCTAATCTTGGTGATGTTTGGCTGC
GGATCTGTGGCCCAGCTGGTGCTCAGTGGCGGCTCCCACGGAATGTTCCTTACCGTCAAC
TTCGCTTTCGGCTTTGCTGCCATGCTTGGGATCCTCGTTTGTGGACAGATTTCAGGAGGC
CATCTCAACCCTACTGTGACCCTTGCCTTGTGCATACTTGGGAGAGAACCGTGGAGGAAG
TTCCCAATATTTTTTCTGTCTCAAACACTGGGTGGCTTTCTGGGCTCTGGAATCATTTTT
GGAATGTACTATGATGCTTTGACAGACTAT---AGCCAA---------GGAGTGTTTAAG
GTTTCTGGAGAAAATGCCACAGCATCAATCTTCGCCACATACCCCTCCAAGCATCTGACT
CTTGTCAATGGCTTTTTTGATCAGATGATAGGAACTGCTGCACTCATCATCTGCATCCTC
GCCATAGTGGATCCCTACAACAATCCCATCCCAAGAGGCCTTGAGGCCTTCACGGTAGGC
TTTGTTGTGCTAGTCATCGGCTTGTCCATGGGCTTCAATTCTGGGTATGCTGTCAACCCT
GCCAGAGACCTGGGTCCTCGTCTCTTCACTGCAATAGCCGGCTGGGGAACTGAAGTCTTC
ACGTCCGACTCTTACTGGTTTTTCGTGCCCGTTTTTGCCCCCTTCCTGGGAACAGTTGTT
GGCGTGCTGGTGTATCAGCTCATGGTGGGCTTCCATGTGGAGGGGGAGGCCCGGGAGAAA
GCAGAGCAA------------GCGGCAGAA---AGCGTTCATCTGTCCAACATGACTTCC
---AAGGATACAGCC---
>Silver_arowana_Aqp3b1
ATGGGAAAGCAGAAGGTCTACCTGGAGAAGCTGGCTCGCATTGTGCAGGTCCGCAACTTG
CTCATGCGGCAGGCTTTGGCTGAATGCCTGGGAACCCTAATCCTTGTGATGTTTGGCTGC
GGGTCTCTGGCCCAGCTGGTGCTCAGTGGCGGCTCCCATGGAATGTTCCTCACCGTCAAC
TTCGCTTTCGGCTTTGCTGCCATGCTTGGGATCCTAGTGTGTGGACAGGTTTCAGGAGGC
CATCTCAACCCTGCTGTGACCTTTGCCTTGTGCATACTTGGGAGAGAACCTTGGAGGAAG
TTCCCAGTATTTTTCCTGTCTCAAACATTGGGTGGCTTTCTGGGTGCTGCGATCATTTTT
GGAATGTACTATGATGCTTTGAGTGACTAT---AGCCAA---------GGAGTGTTTAGG
GTTACTGGAGAAAATGCCACAGCACCAATCTTCGCCACATACCCCTCCAACCATCTGACT
CTTGCCAATGGCTTTTTTGATCAGATGATAGGAACTGCTGCACTCATCACCTGCATCCTC
GCCATAGTGGATCCCTACAACAATCCGATCCCAAGGGGCCTTGAGGCCTTCACGGTCGGC
TTTGTTGTGCTGGTCATCGGCTTGTCCATGGGCTTCAACTCTGGATATGCTGTCAACCCT
GCCAGAGACCTGGGTCCTCGTCTCTTCACTGCAATAGCCGGCTGGGGCACTGAAGTCTTC
ACGTCTTACTCTTACTGGTTTTTCGTGCCCATTTTTGCCCCCTTCTTGGGGACAGTTGTT

GGCGTGCTGGTGTATCAGTTCATGGTGGGCTTCCATGTGGAAGGGGAGGCCCGGGACAAA
GCAGAGAGA------------GCAGCAGAA---AGTGTTCATCTGTCCAACATGACTTCC
---AAGAACACAGCC---
>Pirarucu_Aqp3b1
ATGGGCAAACAGAAGATCTACCTGGAGAAGCTAGCCCGCATCGTGCAAGTCCGCCACTTG
CTCATGCGACAGGCTCTGGCTGAATGTCTGGGAACCCTAATCCTGGTGATGTTTGGCTGC
GGATCTGTAGCCCAGCTGGTGCTCAGCGGCGGCTCCCACGGATTGTTCCTCACTGTTAAC
TTCGCTTTCGGCTTCGCCGCCATGCTCGGGATCCTGGTGTGCGGACAGGTTTCAGGTGGT
CATCTCAACCCTACTGTGACCCTCGCCTTATGCATACTTGGTCGCGAACCTTGGAGGAAG
TTTCCAGTCTTTTTCTTATCTCAGACTCTGGGTGCCTTCCTGGGCTCAGGAATCGTTTTT
GGAATGTACTACGATGCTTTGAGAGAATAT---AGTCAA---------GGAGTTTTTAGG
GTTTCTGGAGAAAACGCCACAGCGTCAATCTTCGCCACTTACCCCTCCAAACATCTCAGC
ATGGTCAATGGCTTTTTTGATCAGATGATAGGAACTGCTGCTCTCATCGTCTGCATCCTC
GCCATAGTGGATCCTTACAACAATCCGATCCCAAGAGGCCTAGAGGCCTTTACAGTGGGT
TTTGCTGTGCTAGTCATCGGGTTGTCCATGGGTTTCAACTCTGGGTATGCGGTGAACCCC
GCCAGAGACCTGGGTCCTCGCCTCTTCACTGCAATATCTGGCTGGGGCACTGAAGTCTTC
ACGTCTGACTCTTACTGGTTTCTTATACCCATTTTCGCCCCCTTCCTGGGGACAATTGTT
GGCGTGCTGGTGTATCAGCTCATGGTGGGCTTCCACGTGGAGGGGGAAGCCCGCAGAAAA
GCAGAGCAG------------GCGGCAGAA---AGTGTTAATCTGTCCAACATCACCTCC
---AAGGACGCAGCC---
>Elephantnose_fish_Aqp3b1
ATGGGAAAACAGAAGGTCTTCCTGGACAAGCTTTTACACACCTGCCACATCCGCAACTTG
CTGATACGTCAGGCTCTGGCTGAATGTCTGGGAACACTGATCCTGGTGATGTTTGGTTGT
GGTTCTGTGGCCCAGCTGGTCCTCAGCAATGGATCCCATGGGCTGTTCCTTACCGTCAAC
TTCGCCTTCGGCTTTGCTGCTACTCTTGGGATCTTGGTATGTGGACAGGTTTCAGGAGGA
CATTTAAACCCTGCTGTGACATTTGCCTTATGCATACTTGGGAGAGAACCTTGGAGGAAG
TTTCCAATATATTTCTTATTTCAGACTATAGGTGCCTTCCTGGGTTCAGGAATAGTTTAT
GGAATGTACCATGATGCATTGTTGGAATTT---AATCCA---------GGTGCTCTCAGG
GTTGTAGGAGACAATGCCACCGCAGGAATCTTTGCTACATACCCCTCCACACACCTGACA
ATGATCAACGGCTTCTTTGACCAGATGATTGGAACTGCCGCTCTCATTGTGTGTATTCTG
GCCATCGTGGATCCATACAACAACCCTATTCCTCATGGGCTAGAAGCCTTCACAGTGGGC
TTTGTAGTGCTTGTCATTGGCTTGTCCATGGGCTTCAACTCTGGGTATGCCGTCAATCCT
GCCAGAGACCTGGGTCCTCGTCTCTTCACTGCAATGGCTGGCTGGGGCGCAGAGGTCTTT
ACGGCTCAGTCTTACTGGTTCTTTGTACCAATATTTGCACCTTTCCTGGGGACAGTATTT
GGCGTTCTGGTTTATCAGTTCATGGTGGGCTTCCATGTAGAGGTAGAGGCCCGAGAGAAG
GCACAGAAC------------GAAGCAGAG---AGTATGAACCTGTCCAACATAAACTCC
---AAGGAAATGGCG---
>Mormyrid_electric_fish_Aqp3b1
ATGGGAAAACAGAAGGTCTTCCTGGACAAGCTTTTACACACCTGCCACATCCGCAACTTG
CTGATACGTCAGGCTCTGGCTGAATGTCTGGGAACACTGATCCTGGTGATGTTTGGTTGT
GGCTCAGTGGCCCAGCTGGTGCTCAGCAATGGATCCCATGGGCTGTTCCTTACCGTCAAC
TTCGCCTTCGGCTTTGCTGCTACTCTTGGGATCTTGGTATGTGGACAGGTTTCAGGAGGA
CATTTAAACCCTGCTGTGACATTTGCCTTATGCATACTTGGGAGAGAACCTTGGAGGAAG
TTTCCAATATATTTCTTATTTCAGACTTTAGGTGCCTTCCTGGGTTCAGGAATAGTTTAT
GGAATGTACCATGATGCATTGTGGGAATTT---AACCCA---------GGTGCTCTCAGG
GTTTTAGGAGACAATGCCACCGCAGGAATCTTTGCTACATACCCCTCCACACACCTGACG
ATGATCAACGGCTTCTTTGACCAGATGATTGGAACTGCTGCCCTCATTGTGTGTATTCTG
GCCATCGTGGATCCATACAACAACCCTATTCCTCATGGGCTAGAAGCCTTCACAGTGGGC
TTTGTAGTGCTTGTCATTGGCTTGTCCATGGGCTTCAACTCTGGGTATGCCGTCAATCCT
GCCAGAGACCTAGGTCCTCGTCTCTTCACTGCAATGGCTGGCTGGGGCGCAGAGGTCTTT
ACGGCTCAGTCTTACTGGTTCCTTGTACCAATATTTGCACCTTTCCTGGGGACAGTATTT
GGCGTTCTGGTTTATCAGTTCATGGTGGGCTTCCATGTAGAGGTAGAGGCCCGAGAGAAG
GCACAGAAC------------GAAGCAGAG---CGTATGAACCTGACCAACATAAACTCC
---AAGGAAATGGCG---
>Freshwater_butterflyfish_Aqp3b1
ATGGGGAAGCAGAAGATGTATTTGGACAAGCTGTCCCGCATGCTGCAAGTGCGCAACCTG
CTCTTACGACAGGCTCTCGCAGAGTGTCTGGGCACAATGATCCTGGTGATGTTTGGCTGT
GGATCTGTGGCCCAACTGGTCCTCAGTGGTGGAAGTCATGGCATGTTCCTCACTGTCAAC
TTTGCTTTCGGCTTTGCCGCCACCCTGGGCATCCTAGTGTGTGGACAGGTTTCCGGCGGC
CATCTCAATCCTGCTGTGACCCTGGCTGTTTGTCTACTCGGGAGAGAACCTTGGAAAAAG
TTCCCTGTCTACTTTCTTGCTCAGACCGTGGGTGCGTTCTTGGCCTCGGCAATCATTTTT

GGAATGTATTATGATGCACTGATGCAATAT---GGAAAG---------GGCTCTCTAACT
ATT---GGAGAAAATGGCACTGCAGGGATCTTTGCCACCTTCCCTTCCAGCCACCTGTCG
TCACTCAATGGATTCTTTGACCAGATGATTGGCACTGCTGCTCTGATAGTCTGCATTCTT
GCCATTGTGGATCCCTACAACAACCCCATCCCGAGTCACCTAGAGGCCTTCACCGTTGGC
TTTGTTGTGTTGGTCATTGGCCTTTCCATGGGCTTCAATTCTGGCTATGCAGTCAACCCT
GCACGAGACCTGGGACCTCGTATCTTCACCGCCATAGCCGGCTGGGGCGGTCAGGTCTTC
ACGACTAACTCCTACTGGTTCCTCATTCCTATTTTTGCTCCTTTCCTGGGCTCTCTGATC
GGAGTGGTGTTGTACCAGCTGATGGTTGGCTTTCACGTAGAAGGAGATGCTCGGGACAAG
AAGGGCCAG------------GAAGCAGAG---AGTGTCAATCTCTCCAACATCACCTCC
---AAGGACGCAGCC---
>American_eel_Aqp3b1
ATGGGAAAGCAGAAGATCATCCTGGACAAGTTGGCACGGATCTTCTATATCCGTAATTTG
CTTCTGCGCCAGGCTCTAGCTGAGTGTCTGGGGACCCTCATCTTGGTGATGTTTGGCTGT
GGTGCAGTGGCCCAGCTGGTACTCAGCGGTGGCTCCCATGGGATGTTCCTTACTGTGAAC
TTTGCTTTTGGTTTTGCGGCCACCTTGGGCATCCTGGTATGTGGACAGGTTTCAGGAGGT
CACCTCAACCCTGCTGTGACCTTTGCCCTGTGTATTCTTGGGAGAGATCCTTGGAGGAAG
TTTCCGGTCTTTTTCTTCTTTCAGACTCTGGGTGCCTTCCTGGGCTCAGGAATAATCTTT
GGCATGTACTTTGATGCACTGTGGGACTTT---GGACAA---------GGGAAGCTGATT
GTGGTGGGACAGAATGCCACAGCCGGGATCTTTGCCACATACCCCTCCAAGCATCTCACT
CTGGTGAATGGCTTCTTCGATCAGATGATTGGTACTGCTGCCCTCATTGTGTGCATATTG
GCCATTGTGGATCCATACAATAACCCTATACCTCGGGGCCTGGAGGCCTTTACTGTGGGA
TTTGTGGTGCTGGTCATTGGCCTCTCAATGGGATTCAACTCTGGATATGCAGTCAATCCT
GCGAGAGACCTAGGGCCTCGCCTGTTCACTGCTCTTGCTGGATGGGGCACTGAAGTCTTC
ACGGCCAAATCCTGCTGGTTCTATGTGCCCGTTATTGCTCCATTGCTGGGGGCGTTTATC
GGTGTGTTGGTGTATCAGCTGATGGTGGGCTACCATGTAGAGGGAGAGGTGCGGGACAAG
GCAGAGCTG------------GAGGAGGAG---AGTGTCAAGCTGTCCGATGTGAGCACC
---AAGGAAGCTGAT---
>European_eel_Aqp3b1
ATGGGAAAGCAGAAGATCATCCTGGACAAGTTGGCACGGATCTTCTATATCCGTAATTTG
CTTCTGCGCCAGGCTCTAGCTGAGTGTCTGGGGACCCTCATCTTGGTGATGTTTGGCTGT
GGTGCAGTGGCCCAGCTGGTACTCAGCGGTGGCTCCCATGGGATGTTCCTTACTGTGAAC
TTTGCTTTTGGTTTTGCGGCCACCTTGGGCATCCTGGTATGTGGACAGGTTTCAGGAGGT
CACCTCAACCCTGCTGTGACCTTTGCCCTGTGTATTCTTGGGAGAGATCCTTGGAGGAAG
TTTCCGGTCTTTTTCTTCTTTCAGACTCTGGGTGCCTTCCTCGGCTCAGGAATAATCTTT
GGCATGTACTTTGATGCACTGTGGGACTTT---GGACAA---------GGGAAGCTGATT
GTGGTGGGACAGAATGCCACAGCCGGGATCTTTGCCACATACCCCTCCAAGCATCTCACT
CTGGTGAATGGCTTCTTCGATCAGATGATTGGTACTGCTGCCCTCATTGTGTGCATATTG
GCCATTGTGGATCCATACAATAACCCTATACCTCGGGGCCTGGAGGCCTTTACTGTGGGA
TTTGTGGTGCTGGTCATTGGCCTCTCAATGGGATTCAACTCTGGATATGCAGTCAATCCT
GCGAGAGACCTAGGGCCTCGCCTGTTCACTGCTCTTGCTGGATGGGGCACTGAAGTCTTC
ACGGCCAAATCCTACTGGTTCTATGTGCCCGTTATTGCTCCATTGCTGGGGGCGTTTATC
GGTGTGTTGGTGTATCAGCTGATGGTGGGCTACCATGTAGAGGGAGAGGTGCGGGACAAG
GCAGAGCTG------------GAGGAGGAG---AGTGTCAAGCTGTCCGATGTGAGCACC
---AAGGAAGCTGAT---
>Japanese_eel_Aqp3b1
ATGGGAAAGCAGAAGATCATCCTGGACAAGTTGGCACGGATCTTCTATATCCGTAATTTG
CTTCTGCGCCAGGCTCTAGCAGAGTGTCTGGGGACCCTCATCTTGGTGATGTTTGGCTGT
GGTGCAGTGGCCCAGCTGGTACTCAGCGGTGGCTCCCATGGGATGTTCCTTACTGTGAAC
TTTGCTTTTGGTTTTGCGGCCACCTTGGGCATCCTGGTATGTGGACAGGTTTCAGGAGGT
CACCTCAACCCTGCTGTGACCTTTGCCCTGTGTATTCTTGGGAGAGATCCTTGGAGGAAG
TTTCCGGTCTTTTTCTTCTTTCAGACTCTGGGTGCCTTTCTGGGCTCAGGAATAATCTTT
GGCATGTACTTTGATGCACTGTGGGACTTT---GGACAA---------GGGAAGCTGATT
GTGGTGGGACAGAATGCCACAGCCGGGATCTTTGCCACATACCCCTCCAAGCATCTCACT
CTGGTGAATGGCTTCTTCGATCAGATGATTGGTACTGCTGCCCTCATTGTGTGCATATTG
GCCATTGTGGATCCATACAATAACCCTATACCTCGGGGCCTGGAGGCCTTTACTGTGGGA
TTTGTGGTGCTGGTCATTGGCCTTTCAATGGGATTCAACTCTGGATATGCAGTCAATCCT
GCGAGGGACCTAGGGCCTCGCCTGTTCACTGCTCTTGCTGGATGGGGCTCTGAAGTCTTC
ACGGCCAAATCCTACTGGTTCTATGTGCCCGTTATTGCTCCATTGCTGGGGGCGTTTATC
GGTGTGTTGGTGTATCAGCTGATGGTGGGCTACCATGTAGAGGGAGAGGTGCGGGACAAG
GCAGAGCTG------------GAGGAGGAG---AGTGTCAAGCTGTCCGATGTGAGCACC
---AAGGAAGCTGAT---

```
>Bowfin_Aqp3
ATGGGCAGACAGAAGTTTTACCTGGACAAGCTGGCTCGCACCTTCCAGGTCAGGAACATG
CTCATACGCCAGTTTCTGGCTGAGTGTCTGGGGACCCTCATTCTTGTGATGTTCGGCTGT
GGTGGCGTTGCCCAGGTGATCCTCAGTGGTGGCACACACGGCAGGTTCTTGACTGTCAAC
TTTGCTTTCGGCTTTGGAGCCACCCTAGGCATCCTCATCTGCGGACAAGTTTCAGGAGGG
CACCTGAACCCCGCTGTGACCTTTGCCCAGTGCCTGCTAGGGAGAGACCCCTGGAAGAAG
TTCCCAGTTTACTTTGCAGCTCAGACCCTGGGTTCCTTTCTGGGCGCTGGTATCATCTTT
GGCCTGTACTTTGATGCCATTTGGGGCTTT---GGCCAA---------GGACAGCTGTTT
GTGGTTGGCGAAAACGCCACTGCAGGCATTTTTGCCACCTACCCATCTGACCATCTGACC
CTCCTCAATGGCTTGTTTGACCAGCTGATCGGTACTGCCGCCCTGGTGGTATGTATCCTG
GCTATCGTTGATCCGTACAACAACCCCATCCCACGTGGGTTGGAGGCCTTCACAGTGGGC
TTCGTGGTGCTGGTCATTGGCCTGTCCATGGGCTTCAACTCTGGCTACGCTGTCAACCCT
GCCAGGGACCTGGGGCCCCGTCTCTTCACTGCCATAGCTGGCTGGGGTGGTGAGGTGTTT
ACTGCCAAAAACTACTGGTTCTTTGTGCCAATCTTCGCCCCCTTCCTGGGTTCTTTTGTG
GGCGTATTGGTATACCAGCTAATGGTGGGATACCATGTGGAGGGGGAGGCCCGGGAGAAG
GCAAAGCAG------------GATGGAGAGGATAGGATCAAACTGTCCAACACTAAC---
---AAGGAGCTCCTG---------------------------------------------
>Gar_Aqp3
ATGGGCAGGCAGAAGTACTACATTGACAAGCTGGCGCGCGCCTTCCAGATCAGGAACCTG
CTGCTGCGCCAGGCCCTGGCAGAGTGCCTGGGGACCCTCATTCTCGTGATGTTCGGCTGT
GGTGCAGTGGCTCAGCTGGTGCTTAGCGGTGGCTCTCATGGTATGTTCATGACCGTGAAC
TTTGCTTTCGGCTTTGCGGCTACCCTAGGAATCCTGATCTGCGGACAGGTGTCGGGAGGA
CATCTTAACCCGGCGGTGACTTTTGCCCTGTGCCTGCTTGGGAGGGATCCCTGGAGGAAA
CTCCCTGCTTACTTTCTGGCCCAGACACTGGGAGCCTTCCTGGGCTCTGGGATCATCTTC
GGCCTGTACTTTGATGCCCTGTGGGATTTC---GCTGGT---------GGGGATCTGCTG
GTGACCGGAACAAATGCGACTGCTGGCATCTTCGCCACCTACCCCTCCAACCACCTCACC
CTGATCAACGGCTTATTTGACCAGCTGATTGGTACTGCCGCCCTGGTGGTGTGTATCCTG
GCTATCGTGGACCCCTACAACAACCCCATCCCACGCGGGCTGGAAGCCTTCACGGTGGGC
TTCGTGGTGCTGGTTATCGGCCTGTCCATGGGCTTCAACTCCGGCTATGCAGTCAACCCT
GCCAGAGACCTCGGCCCGCGGCTCTTCACCGCCATAGCTGGCTGGGGCAGCGAGGTCTTC
ACGGCTAATGACTACTGGTTCTTTGTGCCCATCTTCGCCCCCTTCATTGGCTCGTTTGTG
GGTGTGGTGGTGTACCAGCTGATGGTTGGCTACCACGTGGAGGGAGAGGTTCGGGACAGA
GAGGAGAGA------------GGCGGGGAGGAGGGAGCCAAGCTGTCCAACTTCAACCCC
---AAGGAGACGGCG---------------------------------------------
>Sterlet_Aqp3
ATGGGCAGACAGAAGCAGGCTCTGACCAGGATCTCTGAGCTGTTTTATATCCAGAACTTG
CTGCTGAGGCAAGCTCTGGCAGAATGTCTGGGGACCCTCGTTCTAGTGATGTTCGGCTGT
GGCTCTGTCGCTCAGCTAGTTCTCAGTGGTGGATCTCATGGACAGTTCCTGACTGTGAAC
TTCGCTTTCGGCTTTGCTGCGACCCTGGGTGTCCTGGTGTCTGGGCAGGTGTCAGGTGGC
CATCTGAACCCGGCTCTGACCTTTGCCCTCTGTCTGCTGGGGCGGGAGCCGTGGCGCAAG
ATGCCGGTGTACTTCTTGGCGCAGACGGTGGGTGCCTTCCTGGGGGCTGGGGTCATCTTT
GGAATGTACTTCGATGCCTTGTGGTTCTTT---GGGAAT---------GACAATCTGTTA
GTGTCA------AACGGTACCGCCGGGATCTTTGCTACCTATCCATCTCAGCACTTGACC
CTGCTCAATGGCTTCTTTGACCAGATGATTGGTACTGCTGCCCTGATCGTGTGCCTCCTG
GCCATCGTGGACCCCTACAACAACCCCATCCCCAGGGGTCTGGAGGCCTTTACTGTGGGC
TTTGTCGTCTTGGTCATCGGTCTGTCCATGGGCTTCAACTCTGGCTACGCCGTCAACCCT
GCCAGAGACTTTGGTCCTCGCCTCTTCACCGCCATAGCCGGCTGGGGCACAGAGGTCTTC
ACGACTGGACCTCACTGGTGGTTGGTGCCCATCTTTGCCCCCTTCCTGGGTGCCGTCATC
GGGGTTCTGGTGTACCAGTTCATGGTGGGCTTCCACACTGAGGGCGAGGCCAAAGACCGC
GCAAGGGAG------------GACAATGAGGAGAACGTCAAGCTGTCCAACATTCACTCC
---AAACAGACAGCT---------------------------------------------
>Common_sturgeon_Aqp3
ATGGGCAGACAGAAGCAGGCTCTGACCAGGATCTCTGAGCTGTTTTATATCCAGAACTTG
CTGCTGAGGCAAGCTCTGGCAGAATGTCTGGGGACCCTCGTTCTAGTGATGTTCGGCTGT
GGCTCTGTCGCTCAGCTAGTTCTCAGTGGTGGATCTCATGGACAGTTCCTGACTGTGAAC
TTCGCTTTCGGCTTTGCTGCGACCCTGGGTGTCCTGGTGTCTGGGCAGGTGTCAGGTGGC
CATCTGAACCCGGCTCTGACCTTTGCCCTCTGTCTGCTGGGGCGGGAGCCGTGGCGCAAG
ATGCCGGTGTACTTCTTGGCGCAGACGGTGGGTGCCTTCCTGGGGGCTGGGGTCATCTTT
GGAATGTACTTCGATGCCTTGTGGTTCTTT---GGGAAT---------GACAATCTGTTA
GTGTCA------AACGGTACCGCCGGGATCTTTGCTACCTATCCATCTCAGCACTTGACC
CTGCTCAATGGCTTCTTTGACCAGATGATTGGTACTGCTGCCCTGATCGTGTGCCTCCTG
```

GCCATCGTGGACCCCTACAACAACCCCATCCCCAGGGGTCTGGAGGCCTTTACTGTGGGC
TTTGTCGTCTTGGTCATCGGTCTGTCCATGGGCTTCAACTCTGGCTACGCCGTCAACCCT
GCCAGAGACTTTGGTCCTCGCCTCTTCACCGCCATAGCCGGCTGGGGCACAGAGGTCTTC
ACCACTGGATCTCACTGGTGGTGGGTGCCCATCTTTGCCCCCTTCCTGGGTGCCTTCATC
GGGGTTCTGGTGTACCAGTTCATGGTGGGCTTCCACACGGAGGGTGAGGCCAAAGACAAA
GCAAGGGAG------------GACAATGAGGAGAACGTCAAGCTGTCCAACATCCACTCT
---AAACAGATCGCT---
>Atlantic_sturgeon_Aqp3
ATGGGCAGACAGAAGCAGGCTCTGACCAGGATCTCTGAGATGTTTTACATCCAGAACTTG
CTGATTAGGCAAGCTCTGGCAGAATGTCTGGGGACCCTCGTTCTAGTGATGTTTGGCTGT
GGCTCTGTCGCTCAGCTAGTTCTCAGTGGTGGATCTCATGGACAGTTCCTGACTGTGAAC
TTCGCTTTCGGCTTTGCTGCGACCCTGGGTGTCCTGGTGTCTGGGCAGGTGTCAGGTGGC
CATCTGAACCCGGCTCTGACCTTTGCCCTCTGTCTGCTGGGGCGGGAGCGGTGGCTCAAG
CTGCCGGTGTACTTCTTGGCGCAGACGGTGGGTGCCTTCCTGGGGGCTGGGGTCATCTTC
GGAATGTACTTCGATGCCTTGTGGATGTTT---GCGAAT---------AACGATCTGTTA
GTGACAGGAAAAAACAGTACCGCTGGGATCTTCGCTACCTATCCATCTCAGCACTTGACC
CTGCTCAATGGCTTCTTTGACCAGATGATTGGTACTGCTGCCCTGATCGTGTGCCTCCTG
GCCATCGTGGACCCCTTCAACAACCCCATCCCCAGGGGTCTGGAGGCCTTTACTGTGGGC
TTCGCCGTCTTGGTCATCGGTCTGTCCATGGGCTTCAACTCTGGCTATGCCGTCAACCCT
GCCAGAGACTTTGGTCCTCGCCTCTTCACCGCCTTAGCCGGCTGGGGCACAGAGGTCTTC
ACCACTGGATCTCACTGGTGGTTGGTGCCCATCTTTGCCCCCTTCCTGGGTGCCTTCATC
GGGGTTCTGGTGTACCAGTTCATGGTGGGCTTCCACACGGAGGGCGAGGCCAAAGACAGC
GCAAGGGAG------------GACAATGAGGAGAACGTCAAGCTGTCCAACATCCACTCT
---AAACAGATCGCT---
>Chinese_sturgeon_Aqp3
ATGGGCAGACAGAAGCAGGCTCTGACCAGGATCTCTGAGCTGTTTTATATCCAGAACTTG
ATGCTGAGGCAAGCTCTGGCAGAATGTCTGGGGACCCTCGTTCTAGTGATGTTCGGCTGT
GGCTCTGTCGCTCAGCTAGTTCTCAGTGGTGGATCTCATGGACAGTTCCTGACTGTGAAC
TTCGCTTTCGGCTTTGCTGCGACCCTGGGTGTCCTGGTGTCTGGGCAGGTGTCAGGTGGC
CATCTGAACCCGGCTCTGACCTTTGCCCTCTGTCTGCTGGGGCGGGAGCCGTGGCTCAAG
ATGCCGGTGTACTTCTTGGCGCAGACGGTGGGTGCCTTCCTGGGGGCTGGGGTCATCTTC
GGAATGTACTTCGATGCCTTGTGGATGTTT---GGGAAT---------AACGATCTGTTA
GTGTCAGGAGCAAACAGCACCGCCGGGATCTTCGCTACCTATCCATCTCAGCACTTGACT
CTGCTCAATGGCTTCTTTGACCAGATGATTGGTACTGCTGCCCTGATCGTGTGCCTCCTG
GCCATCGTGGACCCCTACAACAACCCCATCCCCAGGGGTCTGGAGGCCTTTACTGTGGGC
TTCGTCGTCTTGGTCATCGGTCTGTCCATGGGCTTCAACTCTGGCTACGCCGTCAACCCT
GCCAGAGACTTTGGTCCTCGCCTCTTCACCGCCATAGCCGGCTGGGGCACAGAGGTCTTC
ACCACTGGATCTCACTGGTGGTTGGTGCCCATCTTTGCCCCCTTCCTGGGTGCCTTCGTC
GGGGTTCTGGTGTACCAGTTCATGGTGGGCTTCCACACGGAGGGCGAGGCCAAAGACAAA
GCAAGGGAG------------GACAATGAGGAGAACGTCAAGCTGTCCAACATCCACTCT
---AAACAGATCGCT---
>Russian_sturgeon_Aqp3
ATGGGCAGACAGAAGCAGGCTCTGGCCAGGATCTCTGAGCTGTTTTATATCCAGAACTTG
CTGCTGAGGCAGGCTCTGGCAGAATGTCTGGGGACCCTCGTTCTAGTGATGTTCGGCTGT
GGCTCTGTCGCTCAGCTAGTTCTCAGTGGTGGATCTCATGGACAGTTCCTGACTGTGAAC
TTCGCTTTCGGCTTTGCTGCGACCCTGGGTGTCCTGGTGTCTGGGCAGGTGTCAGGTGGC
CATCTGAACCCGGCTCTGACCTTTGCCCTCTGTCTGCTGGGGCGGGAGCCGTGGCGCAAG
ATGCCGGTGTACTTCTTGGCGCAGACGGTGGGTGCCTTCCTGGGGGCTGGGGTCATCTTT
GGAATGTACTTCGATGCCTTGTGGTTCTTT---GGGAAT---------GACAATCTGTTA
GTGTCA------AACGGTACCGCCGGGATCTTTGCTACCTATCCATCTCAGCACTTGACC
CTGCTCAATGGCTTCTTTGACCAGATGATTGGTACTGCTGCCCTGATCGTGTGCCTCCTG
GCCATCGTGGACCCCTACAACAACCCCATCCCCAGGGGTCTGGAGGCCTTTACTGTGGGC
TTTGTCGTCTTGGTCATCGGTCTGTCCATGGGCTTCAACTCTGGCTACGCCGTCAACCCT
GCCAGAGACTTTGGTCCTCGCCTCTTCACCGCCATAGCCGGCTGGGGCACAGAGGTCTTC
ACCACTGGACCTCACTGGTGGTTGGTGCCCATCTTTGCCCCCTTCCTGGGTGCCTTCATC
GGGGTTCTGGTGTACCAGTTCATGGTGGGCTTCCACACTGAGGGCGAGGCCAAAGACCGC
GCAAGGGAG------------GACAATGAGGAGAACGTCAAGCTGTCCAACATTCACTCC
---AAACAGACAGCT---
>Reedfish_Aqp3
ATGGAAAGACAAAAGCGTTTCCTGGCCTGGATGGGACAGATGTTCTACATTAAGAATAAA
CTTTTAAGGCAATCACTAGCTGAGTGTTTGGGCACCCTAATCTTGGTGATGTTTGGCTGT

GGCTCTGTGGCTCAGCTGGTCCTTAGTGGTGGCACCCATGGCCAATTTCTTACAGTCAAC
TTTGCATTTGGTTTTGCAGTCACACTCGGCATACTTATTTCTGGACAGGTGTCAGGTGGA
CATCTCAACCCTGCTGTGACCTTTTCTCTTTGCCTTCTGGGACGTGAGCCTTGGATCAAA
TTCCCAGTGTACTTTCTGGCTCAGACCCTAGGATCCTTCCTTGGGGCTGGAATAATCTTT
GGACTGTACTTTGATGCCCTTTGGGCCTAT---GGAGGT---------AATCATCTCCTT
GTGACTGGGTCAAACGCCACTGCAGGAATCTTTGCTACCTTCCCCTCAGAGCATCTTACT
CTTGTAAATGGTTTTTTTGACCAGGTAATTGGCACTGCTGCTCTCATTGTGTGTATCTTG
GCAATTGTTGACCCATACAACAACCCCATTCCACAAGGCCTGGAGGCCTTCACTGTGGGT
TTTGTAGTTCTTGTTATTGGACTTTCAATGGGCTTCAACTCTGGATATGCAGTTAACCCA
GCCAGGGACTTTGGGCCAAGACTGTTCACTTCAATTGCAGGCTGGGGTTCCGAGGTCTTC
ACGTCTGGAAATAATTGGTGGTGGGTACCAATTCTTGCCCCCTTCTTCGGAGCCTTCGCT
GGAGTAATTGTGTATCAGTTCATGGTTGGCTTTCATGTGGAAGGGATGTCAAAGCAAAAG
AAA-------------------CAAAGTGAAGAAAACATCAAATTGTCCAATACAAGCTCT
---AAAGATATGGCA---

File S7: Alignment for Fig. 3B, Fig. S6
>Torafugu_Aqp7
ATGAAGGAAATGGCAGAATCTATGGAACTGGGCGTC---TCTCAGCAGAGGGGAGCCACT
GGAAGCAGACCCAGAGCTTGGCTC---CAGAATGAGTGTGTGCGCGTGGGACTCGCTGAA
ACCCTCTGCACATATATCATGATGGCATTTGGGCTGGGGTCTGTGGCACAGGTAGTGACG
GGGCAGGGGGCGTACGGCCAATACGTGAGCATCAACCTGGGCTTTGGTCTGGGCGTTGCT
ATGGGGATTCATGTTGGAGGCAAAGTCTCAGGGGCTCACATGAACGCAGCAGTGACACTC
ACCATGTGCACATTTGGCCGCCTTGCGTGGAAGATGTTGCCCCTTTATGTTTTTGCACAG
TTTTTGGGCTCTTTCCTCGCCGCGGGGACGATCTATGCTGTCTACTATGAAGCTATTTAT
GATTACTGTGGAGGGAACATGACCGTGACGGGCGTGAAGGCCACAGCTGGTATTTTTGCC
ACCTATCCTGCGCCATACCTCTCCTTGCTGGGGGGATTTATTGACCAGGTGTTTGGGACG
GCCATGCTTCTGCTCTGCATCATGGCTCTGTCCGACCAGAAGAACAAACCGGCCCCAGCG
GGCAGCGAACCCGTTGCTGTGGGTCTCCTGGTGCTGCTCATTGGGATTTCTCTGGGCAGC
AACAGCGGCTACGCCATCAACCCCACCAGGGATATCGCACCGAGGATCTTCACCGCCATT
GCTGGCTGGGGGGTTGACGTTTTCAGGTCTGGAAATGGGTGGTGGTGGGTGCCTATAACT
GCTCCCTTCATCGGAGGATTATTGGGTGCAGGGCTTTACAAGATCATGGTGGAACTGCAC
CACCCAGGAACCTGCACGAGGAGAGAAGGGCCG---GTG---------GAGGAGGAGAGC
GCCCCTCTCGGGAACCAAGAAAACACC---AGCAATAATGTT------TGTGTG------

>Sansaifugu_Aqp7
ATGAAGGAAAAGGCAGAATCTATGGAACTGGGCGTC---TCTCAGCAGAGGGGAGCCACT
GGAAGCAGACCCAGAGCTTGGCTC---CAGAATGAGTGTGTGCGCGTGGGACTCGCTGAA
ACCCTCTGCACATATATCATGATGGCATTTGGGCTGGGGTCTGTGGCACAGGTAGTGACG
GGGCAGGGGGCGTACGGCCAATACGTGAGCATCAACCTGGGCTTTGGTCTGGGCGTTGCT
ATGGGGATTCATGTTGGAGGCAAAGTCTCAGGGGCTCACATGAACGCAGCAGTGACACTC
ACCATGTGCACATTTGGCCGCCTTGCGTGGAAGATGTTGCCCCTTTATGTTTTTGCACAG
TTTTTGGGCTCTTTCCTCGCCGCGGGGACGATCTATGCTGTCTACTATGAAGCTATTTAT
GATTACTGTGGAGGGAACATGACCGTGACGGGCGTGAAGGCCACAGCTGGTATTTTTGCC
ACCTATCCTGCGCCATACCTCTCCTTGCTGGGGGGATTTATTGACCAGGTGTTTGGGACG
GCCATGCTTCTGCTCTGCATCATGGCTCTGTCCGACCAGAAGAACAAACCGGCCCCAGCG
GGCAGCGAACCCGTTGCTGTGGGTCTCCTGGTGCTGCTCATTGGGATTTCTCTGGGCAGC
AACAGCGGCTACGCCATCAACCCCACCAGGGATATCGCACCGAGGATCTTCACCGCCATT
GCTGGCTGGGGGGTTGACGTTTTCAGGTCTGGAAATGGGTGGTGGTGGGTGCCTATAACT
GCTCCCTTCGTCGGAGGATTATTGGGTGCAGGGCTTTACAAGATCATGGTGGAACTGCAC
CACCCAGGAACCTGCACGAGGAGAGAAGGGCCG---GTG---------GAGGAGGAGAGC
GCCCCTCTCGGGAACCAAGAAAACACC---AGCAATAATGTT------TGTGTG------

>Mefugu_Aqp7
ATGAAGGAAATGGCAGAATCTATGGAACTGGGCGTC---TCTCAGCAGAGGGGAGCCACT
GGAAGCAGACCCAGAGCTTGGCTC---CAGAATGAGTGTGTGCGCGTGGGACTCGCTGAA
ACCCTCTGCACGTATATCATGATGGCATTTGGGCTGGGGTCTGTGGCACAGGTAGTGACG
GGGCAGGGGGCGTACGGCCAATACGTGAGCATCAACCTGGGCTTTGGTCTGGGCGTTGCT
ATGGGGATTCATGTTGGAGGCAAAGTCTCAGGGGCTCACATGAACGCAGCAGTGACACTC
ACCATGTGCGCATTTGGCCGCCTTGCGTGGAAGATGTTGCCCCTTTATGTTTTTGCACAG
TTTTTGGGCTCTTTCCTCGCCGCGGGGACGATCTATGCTGTCTACTATGAAGCTATATAT
GATTACTGTGGAGGGAACATGACCGTGACGGGCGTGAAGGCCACAGCTGGTATTTTTGCC

ACCTATCCTGCGCCATACCTCTCCTTGCTGGGGGGATTTATTGACCAGGTGTTTGGGACG
GCCATGCTTCTGCTCTGCATCATGGCTCTGTCCGACCAGAAGAACAAACCGGCCCCAGCG
GGCAGCGAACCCATTGCTGTGGGTCTCCTGGTGCTGCTCATTGGGATTTCTCTGGGCAGC
AACAGCGGCTACGCCATCAACCCCACCAGGGATATCGCACCGAGGATCTTCACCGCCATT
GCTGGCTGGGGGGTTGACGTTTTCAGGTCTGGAAATGGGTGGTGGTGGGTGCCTATAACT
GCTCCCTTCATCGGAGGATTATTGGGTGCAGGGCTTTACAAGATCATGGTGGAACTGCCC
CACCCAGGAACCTCCACGAGGAGAGAAGGGCCG---GTG---------GAGGAGGAGAGC
GCCCCTCTCGGGAACCAAGAAAACACC---AGCAATAATGTT------TGTGTG------

>GSPufferfish_Aqp7
ATGAAGGACACGGCAGAATCTGTGGAACTGGGGGTC---TCTCAGCACAGGGGGGCCGCG
GGAGGCAGGACCAGATCTTGGCTC---CAGAGTGAGGTCGTGCGCGTTGGACTGGCTGAA
ACCCTCTGCACGTACATCATGATGGCATTTGGGCTGGGCTCCGTGGCGCAGGTAGTGACG
GGGCAGGGGGCTTTCGGCCAGTATGTGAGCATCAACCTGGGCTTTGGCCTGGGCGTTGCC
ATGGGGATTCACGTTGGAGGCCAAGTCTCAGGGGCCCACATGAACGCAGCAGTGTCGCTC
ACCATGTGCGTGTTCGGCCGCCTTGCGTGGAGGATGCTGCCCCTTTATGTTTTGGCACAG
TTTTTGGGCTCATTTCTCGCTGCGGCGACGATCTATGCCGTCTACTACGAGGCTATTTAC
GATTATTGCGGAGGGAACATGACAGTGACGGGCGTGAAGGCCACAGCTGGAATTTTCGCC
ACCTACCCCGCGCCGTACCTGTCCTTGCTCGGTGGATTTATTGACCAGGTTTTTGGGACC
GCCATGCTCCTGCTCTGCATCATGGCTCTGTCCGACCAGAAGAACAAACCGGCCCCGGCA
GGCAGCGAACCCATCACTGTGGGTCTCCTGGTCCTGCTTATCGGGATTTCTCTGGGCAGC
AATAGCGGCTACGCCATCAACCCCACCAGAGACATTGCGCCGAGGATCTTCACCGCCATT
GCTGGCTGGGGGGTGGATGTATTTAGGTCCGGAAATGGGTGGTGGTGGGTGCCCATAACC
GCCCCCTTCATCGGAGGATTGTTGGGCGCAGGAATTTACAGGATCATGGTGGAAATGCAC
CATCCAGTTACCTCCGAGAAGGAGGAAGGGCTG---GCG---------AAGGAGGAGAGC
ATCCCTCTTGGAAAACAAGAGAACGCC---GGTGCTAATGTT------TGCGTC------

>Ocean_sunfish_Aqp7
---------ATGGTGGAGGCGGTAGAACTCGGGTTG---CCTCGGCAGAGAGGAGCTACT
GGAACTCGGCTCAAAGCTTGGCTC---AGGAATGAATATGTCCGTGTGGGACTTGCCGAA
ACTCTTTGCACATATGTCATGATGGTGTTTGGCCTGGGTTCCGTGGCCCAGGTCGTGACA
GGAAAGGGTGCGTTTGGACACTACATCAGCATCAACCTGGGTTTTGGACTGGGTGTTGCA
ATGGGGTGTCACGTTGGCGGGAAAGTTTCAGGGGCTCACATGAATGCAGCGGTGTCGTTC
ACCATGTGCGCTTTTGGCCGCCTTGCCTGGAAGTTTTTGCCTCTGTATGTTTGCGCACAG
TTTTTGGGTTCATTCCTTGCAGCTGGGACAGTTTATGCTGTCTATTACGAAGCCATATAT
GACTACTGTGGAGGCAACCTGACGGTGACCGGTGTGAGAGCCACAGCTGGCATCTTTGCC
ACCTATCCTGCACCATACCTCTCCTTGATGTCTGGATTCGCTGATCAGGTGTTTGGTACA
GCCATGCTGCTGCTCTGCCTATGGGCCCTGTTCGACCAGAGGAACAAACCGGCTCTTGAG
GGCAGCGAGCCTGTCGCGGTGGGTCTCCTGGTGCTGCTCATTGGGATGTCTCTGGGCAGC
AACAGTGGCTATGCCATCAACCCCACCAGAGACTTGGGACCCAGGATCTTCACTGCCATA
GCAGGCTGGGGGACTGACGTGTTCAGGTCTGGAAATGGGTGGTGGTGGGTGCCTGTAATC
GCCCCCCTCGTTGGAGGAGTATTGGGTGCGGGGATCTACACGGCCATGGTGGAACTGCAC
CACCCTCCGAGCTCTAAACAGAGCTGTGAGGGG---GTG---------GAGGAGGAGTCT
GCCCCTCTAGGAAAACAAGAGAACATC---TGTGCTGATGTA------TGTGTG------

>Striated_frogfish_Aqp7
ATGAAAGATCTAGTGGAGTCGGTGGAACTGGGGGCC---TCACAGCAAAAAAGAGTGAAC
ATGACCAGACCCAGAGTTTGGATA---AAGAACGAATTTGTTCGTGTGGGACTTGCTGAA
ATTCTTTGCACGTATGTCATGATGGTGTTCGGCCTGGGTTCTGTGGCCCAGGTAGTCACT
GGACAGGGAGCATTCGGCCACTACATTAGCATTAACCTGGGTTTTGGATTAGGTGTTGCT
ATGGGGGTTCACGTTGGAGGGAAAGTCTCAGGGGCTCATATGAATGCAGCAGTGTCTGTG
TCTATGTGCGCGTTCGGCCGCCTTGCATGGAAGATGTTGCCCCTGTATGTTTTTGCGCAG
TTTTTGGGGTCATTGCTGGCAGCTGGGACAATTTATGCTGTGTATTATGATGCCATATTC
AACTATTGTGGAGGAAACTTTACTGTAACTGGTGCAAGAGCCACAGCTGGCATTTTTGCC
ACCTACCCAGCACCATACCTTTCCGTGCTGTCTGGATTTGTTGACCAGGTCTTTGGCACA
GCTATGTTGCTGTTGTGCCTGATGGCCTTGACAGATCAGAAGAACAAACCGCCGCCGGTG
GGTGGTGAGCCAATAGCTGTGGGTCTTCTGGTGCTGCTTATTGGCATTTCTCTGGGCAGC
AACAGCGGCTATGCCATCAACCCCACCAGGGACATTGCACCCAGGATCTTCACTGCCATT
GCAGGCTGGGGAACTGAGGTGTTCAGGGCTGGAAACGGATGGTGGTGGGTGCCTCTAGTT
GCACCCACCATAGGAGGAGTATTGGGTGGAGGGCTGTACAAAGTCTTGGTGGAAATGCAC
CACCCTCCCCTCTCGAAACAGGGT----------------------------GAGGAGGTC

ACCCCTCTGGGAAAACAGGAAAACATC---TGTGATAATGTT------TGTGTA------

>Florida_bass_Aqp7
ATGAAGGACTTGGTGGAGTCAGTAGAACTCGGGGTC---TCTCAGCGGAAAGGAGTTATC
ATAACTAAACCCAAAGTTTGGCTA---AAGAATGAATATGTTCGTGTGGGATTTGCTGAA
TCCCTTTGCACATATGTCATGATGGTGTTTGGCCTGGGGTCTGTGGCCCAGGTAGTGACA
GGACAGGGAGCATTCGGAGAGTATCTCAGCATCAACCTGGGTTTTGGACTCAGTGTTGCT
ATGGGGGTTCATGTTGGAGGGAAGGTCTCAGGGGCTCATATGAATGCAGCAGTGACACTC
ACGATGTGCGCATTTGGCCGCCTTGCGTGGAAGATGCTGCCTCTGTATGTTTTTGCACAG
CTATTGGGGTCATTTCTGGCAGCGGGGACAATCTATGCTGTCTATTATGAAGCCATATAT
GACTTTTGTGGAGGAAACCTGACTGTAACCGGTGTAAAGGCCACTGCTGGTATCTTTGCC
ACCTATCCTGCACCGTACCTCTCCTTGCTGGCTGGATTCATTGACCAGGTGATCGGCACA
GCTATGCTGCTGCTGTGCCTGATGGCTCTGTCCGACCAGAAGAACAAACCGGCCGCAGCG
GGCAGCGAGCCTGTCGCAGTGGGTCTCCTGGTGCTGCTCATTGGCATTTCTCTAGGCAGC
AACAGCGGCTATGCTATCAACCCCACCAGAGACATCGCACCCAGGGTCTTCACTGCCATA
GCAGGCTGGGGGGTTGATGTGTTCAGGTCTGGAAATGGGTGGTGGTGGGTGCCCCTAGTT
GCCCCTCCCATTGGAGGACTATTGGGTGCGGGTCTCTACAAAGCCTTGGTGGAAATGCAC
CACTCTCTCTTCTCTGAACAGGGAGAGGAGTTG---GTG---------GAGGAGACGACT
ACCCCTCTGGGGAAACAGGACAACATC---TGTGCTAATGTA------TGTGTC------

>Northern_largemouth_bass_Aqp7
ATGAAGGACTTGGTGGAGTCAGTAGAACTCGGGGTC---TCTCAGCGGAAAGGAGTTATC
ATAACTAAACCCAAAGTTTGGCTA---AAGAATGAATATGTTCGTGTGGGATTTGCTGAA
TCCCTTTGCACATATGTCATGATGGTGTTTGGCCTGGGGTCTGTGGCCCAGGTAGTGACA
GGACAGGGAGCATTCGGAGAGTATCTCAGCATCAACCTGGGTTTTGGACTCAGTGTTGCT
ATGGGGGTTCATGTTGGAGGGAAGGTCTCAGGGGCTCATATGAATGCAGCAGTGACACTC
ACGATGTGCGCGTTTGGCCGCCTTGCGTGGAAGATGCTGCCTCTGTATGTTTTTGCACAG
CTATTGGGGTCATTTCTGGCAGCGGGGACAATCTATGCTGTCTATTATGAAGCCATATAT
GACTTTTGTGGAGGAAACCTGACTGTAACCGGTGTAAAGGCCACTGCTGGTATCTTTGCC
ACCTATCCTGCACCGTACCTCTCCTTGCTGGCTGGATTCATTGACCAGGTGATCGGCACA
GCTATGCTGCTGCTGTGCCTGATGGCTCTGTCCGACCAGAAGAACAAACCGGCCGCAGCG
GGCAGCGAGCCTGTCGCAGTGGGTCTCCTGGTGCTGCTCATTGGCATTTCTCTAGGCAGC
AACAGCGGCTATGCTATCAACCCCACCAGAGACATCGCACCCAGGGTCTTCACTGCCATA
GCAGGCTGGGGGGTTGATGTGTTCAGGTCTGGAAATGGGTGGTGGTGGGTGCCCCTAGTT
GCCCCTCCCATTGGAGGACTATTGGGTGCGGGTCTCTACAAAGCCTTGGTGGAAATGCAC
CACTCTCTCTTCTCTGAACAGGGAGAGGAGTTG---GTG---------GAGGAGACGACT
ACCCCTCTGGGGAAACAGGACAACATC---TGTGCTAATGTA------TGTGTC------

>Smallmouth_bass_Aqp7

---------CATGTTGGAGGGAAGGTCTCAGGGGCTCATATGAATGCAGCAGTGACACTC
ACGATGTGCGCGTTTGGCCGCCTTGCGTGGAAGATGCTGCCTCTATATGTTTTTGCACAG
CTATTGGGGTCATTTCTGGCAGCGGGGACAATCTATGCTGTCTATTATGAAGCCATACAT
GACTTTTGTGGAGGAAACCTGACTGTAACCGGTGTAAAGGCCACTGCTGGTATCTTTGCC
ACCTATCCTGCACCGTACCTCTCCTTGCTGGCTGGATTCATTGACCAGGTGATCGGCACA
GCTATGCTGCTGCTGTGCCTGATGGCTCTGTCCGACCAGAAGAACAAACCGGCCGCAGCG
GGCAGCGAGCCTGTCGCAGTGGGTCTCCTGGTGCTGCTCATTGGCATTTCTCTAGGCAGC
AACAGCGGCTATGCCATCAACCCCACCAGAGACATCGCACCCAGGGTCTTCACTGCCATA
GCAGGCTGGGGGGTTGATGTGTTCAGGTCTGGAAATGGGTGGTGGTGGGTGCCCCTAGTT
GCCCCTCCCATTGGAGGACTATTGGGTGCGGGTCTCTACAAAGCCTTGGTGGAAATGCAC
CACTCTCCCTTCTCTGAACAGGGAGAGGAGTTG---GTG------------GAGGAGACT
ACCCCTCTGGGGAAACAGGACAACATC---TGTGCTAATGTA------TGTGTC------

>Bluegill_Aqp7
ATGAAGGACTTGGTGGAGTCAGTAGAACTCGGGGTC---TCTCAGCGGAAAGGAGTTATC
GTAACTAGACCCAAAGTTTGGCTA---AAGAATGAATATGTTCGTGTGGGATTTGCTGAA
TCCCTTTGCACATATGTCATGATGGTGTTTGGCCTGGGGTCTGTGGCCCAGGTAGTGACA
GGACAGGGAGCATTTGGAGAGTACCTCAGCATCAACCTGGGTTTTGGACTCAGTGTTGCT

ATGGGGGTTCATGTTGGAGGGAAGGTCTCAGGGGCTCATATGAATGCAGCAGTGACACTC
ACGATGTGTGCGTTTGGCCGCCTTGCGTGGAAGATGCTGCCTCTATATGTTTTTGCGCAG
CTATTGGGGTCATTTCTGGCAGCGGGAACAATCTATGCGGTCTATTATGAAGCCATATAT
GACTTTTGTGGAGGAAACCTGACTGTAACCGGTGTGAAGGCCACAGCTGGTATCTTTGCC
ACCTATCCTGCACCGTACCTCTCCTTGCTGGCTGGATTCATTGACCAGGTGATCGGCACA
GCTATGCTGCTGCTGTGCCTGATGGCTCTGTCCGACCAGAAGAACAAACCGGCTGCAGCG
GGCAGTGAGCCTGTTACAGTTGGTCTCCTGGTGCTGCTCATTGGCCTTTCTCTAGGCAGC
AACAGCGGCTATGCTATCAACCCCACCAGAGACATTGCACCCAGGGTTTTCACTGCCATA
GCAGGCTGGGGGGCTGATGTGTTCAGGTCTGGAAATGGGTGGTGGTGGGTGCCCGTAGTT
GCCCCTCCCATTGGAGGACTATTGGGTGCGGGTCTCTACAAGGCCTTGGTGGAAATGCAC
CACTCTCCCTTCTCTGAACAGGGAGAGGAGTTG---GTG---------GAGGAGGGGACT
ACCCCTCTGGGGAAACAGAACAACATC---TGTGCTAATGTA------TGTGTC------

>Murray_cod_Aqp7
ATGAAGGACTTAGTGGAGTCAGTAGAACTCGGGGTC---TGTCAACATAAAGGAGTTAAC
ATAACTCGACCCAAAGTTTGGCTA---AAGAATGAACTTGTTCGTGTGGGACTTGCTGAA
TTCCTCTGCACGTATGTCATGATGGTGTTTGGCCTGGGGTCTGTGGCCCAGGTAGTGACA
GGAGAGGGGGCGTTCGGACAGTACCTCAGCATCAACCTGGGTTTTGGACTGGGTGTTGCT
ATGGGGGTTCACGTCGGAGGGAACGTCTCAGGGGCTCATATGAATGCAGCAGTGTCACTC
ACGATGTGCACGTTTGGCCGCCTTGCGTGGAAGATGCTGCCTCTGTATGTTTTTGCACAG
CTGTTGGGGTCATTTCTGGCAGCAGGGACAATTTATGGTGTCTATTATGAAGCCATATAT
GACTATTGTGGAGGAAACCTGACTGTAACTGGTGTAAAGGCCACAGCTGGCATCTTTGCC
ACCTATCCTGCACCGTACCTCTCCATGCTGGCTGGATTCATTGACCAGGTGTTCGGCACG
GCCATGCTGCTGCTGTGCCTGATGGCTCTGTCCGACCAGAAGAACAAGCCGGCCGCGGCG
GGCAGCGAGCCTGTCGCAGTGGGTCTCCTGGTGCTGCTCATCGGCATTTCTCTGGGCAGT
AACAGCGGCTATGCTATCAACCCCACCAGAGACATTGCACCCAGGGTCTTCACCGCCATA
GCAGGCTGGGGGGCTGACGTGTTCAGGTCTGGAAATGGCTGGTGGTGGGTGCCTCTAGTT
GCCCCCCCCATTGGAGGAGTATTGGGTGCGGGGCTCTACAAGGCCTTGGTGGAAATGCAC
CACCCTCCCCTCTCTGAACAGGGTGAGGGGCTG---GTG---------GAGGAAGAGACT
GCCCCCCTGGGGAAACAGGACAACATC---TGTGCTAATGTC------------------

>Barred_knifejaw_Aqp7
ATGAAGGACTTGGTGGAGTCAGTGGAACTGGGGGTC---TCTCAGAGGAAAGGAGTTAAC
GTGACTCGACCCAAAGTTTGGCTA---AAGAATGAACTTGTTCGTGTGGGACTTGCTGAA
TCCCTTTGCACATATGTTATGATGGTGTTTGGCCTGGGGTCTGTGGCCCAGGTAGTGACA
GGACAGGGAGCGTTCGGACAGTACCTCAGCATCAACCTGGGCTTTGGACTGGGTGTTGCT
ATGGGGGTTCATGTTGGAGGGAAGGTCTCAGGGGCTCATATGAATGCAGCAGTGTCACTC
ACAATGTGTGCGTTTGGCCGCCTTGCGTGGAAGATGCTGCCTCTGTATGTTTTTGCGCAG
CTATTGGGGTCATTTCTGGCAGCAGGGACAATTTATGCCGTCTATTATGAAGCCATATAT
GACTATTGTGGAGGAAACCTGACTGTAACTGGTGTAAAGGCCACAGCTGGTATCTTTGCC
ACCTATCCTGCACCGTACCTCTCCTTGCTGGCTGGATTCATTGACCAGGTGTTCGGGACA
GCTATGCTGCTGCTGTGCCTGATGGCTCTGTCCGACCAGAAGAACAAACCGGCCGCAGCG
GGCAGCGAGCCTGTCGCAGTGGGTCTCCTGGTGCTGCTCATTGGCATTTCTCTGGGCAGC
AACAGCGGCTATGCCATCAACCCCACCAGAGACATTGCACCCAGGGTCTTCACTGCCATA
GCAGGCTGGGGGGCTGACGTGTTCAGGTCTGGAAATGGATGGTGGTGGGTGCCTCTAGTT
GCCCCTCCCATTGGAGGAGTATTGGGTGCAGGGCTCTACAAGGCCATGGTGGAAATGCAC
CACCCTCCCCTCTCTGAATGGGGTGAAGGGCTG---GTG---------GAGGAGGAGACT
GCCCCTCTGGGGAAACAGGACAACATC---TGTGCTAATGTA------TGTGTC------

>Korean_spotted_seabass_Aqp7
ATGAAGGACTTGGCTCAGTCAGTAGAACTTGGGGTC---TCTCAGCGGAAAGGAGTTAAT
GCAACTCGACCCAAAGTTTGGCTA---AAGAATGAACTTGTTCGCGTGGGACTTGCTGAA
TCCCTTTGCACTTATGTCATGATGGTGTTTGGCCTGGGGTCTGTGGCCCAGGTAGTGACA
GGACAGGGAGCGTTCGGACAGTACATCAGCATCAACCTGGGTTTTGGACTGGGTGTTACT
ATGGGGGTTCATGTTGGAGGGAAGGTCTCAGGGGCTCATATGAATGCAGCAGTGTCGCTC
ACAATGTGCGCGTTTGGCCGCCTTGCGTGGAAGATGCTGCCTCTGTATGTTTTTGCACAG
CTATTGGGGTCATTTCTGGCAGCAGGGACAATTTATGCCGTCTATTATGAAGCCATATAT
GACTATTGTGGAGGAAACCTGACTGTGACTGGTGTAAAGGCCACAGCTGGTATCTTTGCC
ACCTATCCTGCACCGTACCTCTCTTTGCTGGCTGGATTCATTGACCAGGTGTTTGGCACA
GCAATGCTGCTGCTGTGCCTGATGGCTCTGTCCGACCAGAGG---AAACCGGCCCCGGCG
GGCAGCGAGCCTGTCGCAGTGGGTCTCCTGGTGCTGCTCATTGGCATTTCTCTGGGCAGC

AACAGCGGCTATGCTATCAACCCCACCAGAGACATCGCACCCAGGGTCTTCACCGCCATA
GCAGGCTGGGGGGCGGACGTGTTCAGGTCTGGAAATGGGTGGTGGTGGGTGCCTCTAGTT
GCCCCCCCTATTGGAGGAGTATTGGGTGGGGGGCTCTACAAGGCCTTGGTGGAAATGCAC
CACCCTTCCCTCTCTGAACAGGATGACGGGCTG---ATG---------GAGGAGGAGGCC
GCCCCTCTTGGGAAACAGGAAAACATC---TGTGCCAATTTA------TGTGTC------

>Japanese_seabass_Aqp7

-------------------------GTGTTTGGCCTGGGGTCTGTGGCCCAGGTAGTGACA
GGACAGGGAGCGTTCGGACAGTACATCAGCATCAACCTGGGTTTTGGACTGGGTGTTACT
ATGGGGGTTCATGTTGGAGGGAAGGTCTCAGGGGCTCATATGAATGCAGCAGTGTCGCTC
ACAATGTGCGCGTTTGGCCGCCTTGCGTGGAAGATGCTGCCTCTGTATGTTTTTGCACAG
CTATTGGGGTCATTTCTGGCAGCAGGGACAATTTATGCCGTCTATTATGAAGCCATATAT
GACTATTGTGGAGGAAACCTGACTGTGACTGGTGTAAAGGCCACAGCTGGTATCTTTGCC
ACCTATCCTGCACCGTACCTCTCCTTGCTGGCTGGATTCATTGACCAGGTGTTTGGCACA
GCAATGCTGCTGCTGTGCCTGATGGCTCTGTCCGACCAGAGGAACAAACCGGCCCCGGCG
GGCAGCGAGCCTGTCGCAGTGGGTCTCCTGGTGCTGCTCATTGGCATTTCTCTGGGCAGC
AACAGCGGCTATGCTATCAACCCCACCAGAGACATCGCACCCAGGGTCTTCACCGCCATA
GCAGGCTGGGGGGCGGACGTGTTCAGGTCTGGAAATGGGTGGTGGTGGGTGCCTCTAGTT
GCCCCCCCTATTGGAGGAGTATTGGGTGGGGGGCTCTACAAGGCCTTGGTGGAAATGCAC
CACCCTTCCCTCTCTGAACAGGATGACGGGCTG---ATG---------GAGGAGGAGGCC
GCCCCTCTTGGGAAACAGGAAAACATC---TGTGCCAATTTA------TGTGTC------

>Ballan_wrasse_Aqp7
ATGAAGGACTTGGTGCAGTCAGTAGAACTTGGGGTC---TCTCAGCGGAAAGGAGTTAAA
GTAAGTCGACCCAAAGCTTGGCTC---CAGAATGAAGTGGTTCGTGTGGGACTTGCTGAA
TCCCTTTGCACTTACGTCATGATGGTTTTTGGCCTGGGGTCTGTGGCCCAGGTAGTGACA
GGACAGGGAGCGTTTGGACAGTACATCAGCATCAACCTGGGGTTTGGACTGGGTGTTGCT
ATGGGGGTTCATGTTGGAGGGAAGGTCTCAGGGGCTCATATGAATGCAGCAGTGTCCATC
ACAATGTGCGCGTTTGGTCGCCTTGCATGGAAGATGCTGCCTCTGTATGTTTTTGCACAG
CTATTGGGTTCATTTTTGGCTGCAGGGACAATTTATGGTGTCTATTATGAAGCTATTTTT
GACTACTGTGGAGGGAACCTGACTGTGACTGGTGCCAGGGCCACAGCCGGTATTTTCGCC
ACCTATCCTGCACCGTACCTCTCCTTGTTGGCTGGATTTATTGACCAGGTATTCGGCACA
GCTATACTGCTGCTGTGTCTGATGGCGCTGGGCGACCAGAAGAACAAACCAGCTGCAGCA
GGAAGCGAGCCTGTCGCAGTGGGCCTCCTAGTGCTGCTTATTGGCATTTCTCTGGGCAGC
AATAGCGGATATGCTATCAACCCCACCAGAGACATCGCACCCAGGGTCTTCACCGCCATA
GCAGGCTGGGGGGCAGATGTGTTCAGGTCTGGTAATGGTTGGTGGTGGGTGCCTCTTGTT
GCACCCCCAATTGGAGGAATATTGGGTGCAGGGCTCTACAAGGCGTTTGTGGAGATGCTC
CACCCTCCCCTTTCTGAACAGAGTGAGGGGCTG---TTG---------GAGGAGGAGACT
GTTGCTCTGGAGAAACGGGAAAACAAC---TGTGCAAATGTA------TGTGTC------

>Corkwing_Aqp7
ATGAAAGACTTAGTGCAGTCAGTAGAAGTTGGGGTC---TCTCAGCGGAAAGGAGTTAAA
TTAAGTCGACCCAAAGTTTGGCTT---CAGAATGAAGTGGTTCGTGTGGGACTTGCTGAA
TCCCTTTGCACTTACGTCATGATGGTTTTTGGCTTGGGCTCTGTGGCCCAGGTAGTGACA
GGACAGGGAGCGTTTGGACAGTACATCAGCATCAACCTGGGGTTTGGACTGGGTGTTGCT
ATGGGGGTTCATGTTGGAGGGAAGGTCTCAGGGGCTCATATGAACGCAGCAGTGTCCATC
ACAATGTGCGCATTTGGTCGTCTTGCGTGGAAGATGCTGCCTCTGTATGTTTTTGCACAA
CTATTGGGTTCATTTCTGGCTGCAGGGACAATTTATGCTGTTTATTATGAAGCTATATTT
GACTACTGTGGAGGGAACTTGACTGTGACTGGTGCCAGGTCCACAGCTGGTATCTTTGCC
ACCTATCCTGCACCGTACCTCTCCTTGATGGCTGGATTTATTGATCAGGTGTTTGGCACA
GCTATGCTGCTGCTGTGTCTGATGGCGCTGAGCGACCAGAAGAACAAACCAGCCGCAGCA
GGAAACGAGCCTGTCGCAGTGGGCCTCCTGGTGCTGCTTATTGGCATTTCTCTGGGCAGC
AACAGCGGCTATGCTATCAACCCCACCAGAGACATCGCACCCAGGGTCTTCACCGCCATA
GCAGGCTGGGGGGCCGATGTGTTCAGGTCTGGTAATGGTTGGTGGTGGGTGCCTCTTGTT
GCACCCCCAATTGGAGGAATATTGGGTGCAGGGCTCTACAAGGCCTTGGTGGAGATGCAC
CACCCTCCCGTTTCTGAGCAGAGTGAGGGACTG---TTG---------GAAGAGGAGACG
ATTGCTCTGGAGAAACAGGAAAACAAC---TGTGCAAATATA------TGCGTC------

>Large_yellow_croaker_Aqp7

ATGAAGGACTTGGTGCAGTCAGTAGAACTAGGGGTC---TCTCAACGGAAAGGCGTTAAT
GTAACTCGACCCAGAGTTTGGCTA---AAGAATGAATTTGTTCGTGTGGGACTTGCTGAA
TCCCTTTGCACGTATGTCATGATGGCGTTGGGCCTGGGGTCTGTGGCCCAGGTAGTGACA
GGACAGGGCGCATTTGGACAATACCTCAGCATCAACCTGGGTTTTGGACTGGGTGTTGCT
ATGGGGGTTCATGTTGGAGGAAAGGTCTCAGGGGCTCATATGAATGCAGCAGTGTCACTT
ACAATGTGCGTGTTTGGCCGCCTTGCATGGAAGATGCTGCCTTTGTATGTTTTCGCGCAG
CTATTGGGCTCATTTCTGGCAGCAGGGACAATTTATGCTGTCTATAATGAAGCCATATAT
GACTATTGTGGAGGAAACCTGACTGTAACTGGTGTAAAGGCCACAGCTGGTATCTTTGCC
ACCTATCCAGCACCGTACCTCTCCTTGTTTGCTGGATTCATTGACCAGGTGTTTGGCACA
GCTATGCTGCTGCTGTGCCTGATGGCTCTGTCCGACCAGAAGAACAAACCGGCCGCAGCG
GGTAGTGAGCCTGTCGTAGTGGGTCTCCTGGTGCTCCTCATTGGCATTTCCCTGGGCAGC
AACAGCGGCTATGCCATCAACCCCACCAGAGACATTGGACCCAGGGTGTTCACTGCCATA
GCAGGCTGGGGGACTGATGTGTTCAGGGCTGGAAATGGGTGGTGGTGGGTGCCTCTAGTT
GCCCCTTGCATTGGAGGAGTATTGGGTGCAGGGATCTACAAGGCCTTGGTGGAAATGCAC
CACCCTCCCCTCTCTGAACAGGGTGAGAGGCTG---GTGGAGGAGTTGGCGGAAGAGACT
TCCCCTCTGGGAAAACAAGGAAACTGT---GAAACTAATGTA------TGTGTC------

>Yellow_croaker_Aqp7
ATGAAGGACTTGGTGCAGTCAGTAGAACTAGGGGTC---TCTCAACGGAAAGGCGTTAAT
GTAACTCGACCCAGAGTTTGGCTA---AAGAATGAATTTGTTCGCGTGGGACTTGCTGAA
TCCCTT--

--CAGGTGTTTGGCACA
GCTATGCTGCTGTTGTGCCTGATGGCTCTGTCCGACCAGAAGAACAAACCGGCCGCAGCG
GGTAGCGAGCCTGTCGTAGTGGGTCTCCTGGTGCTCCTCATTGGCATTTCCCTGGGCAGC
AACAGCGGCTATGCCATCAACCCCACCAGAGACATTGGACCCAGGGTGTTCACTGCCATA
GCAGGCTGGGGGGCTGATGTGTTCAGGGCTGGAAATGGGTGGTGGTGGGTGCCTCTAGTT
GCCCCTTGCATTGGAGGAGTATTGGGTGCAGGGATCTACAAGGCCTTGGTGGAAATGCAC
CACCCTCCCCTCTCTGAACAGGGTGAGAAATTG---GTGGAGGAGTTGGAGGAAGAGACT
TTCCCTCTGGTAAAACAAGGAAACTGT---GAAACTAATGTA------TGTGTC------

>Miiuy_croaker_Aqp7
ATGAAGGACTTGGTGCAGTCAGTAGAACTAGGGGTC---TCTCAACGGAAAGGCGTTAAT
GTAACTCGACCCAGAGTTTGGCTA---AAGAATGAATTTGTTCGTGTGGCACTTGCTGAA
TCCCTTTGCACGTATGTCATGATGGCGTTGGGCCTGGGGTCTGTGGCCCAGGTAGTGACA
GGACAGGGTGCATTTGGACAATACCTCAGCATCAACCTGGGTTTTGGACTGGGTGTTGCT
ATGGGGGTTCATGTTGGAGGAAAGGTCTCAGGGGCTCACATGAATGCAGCAGTGTCACTC
ACAATGTGCGTGTTTGGCCGCCTTGCATGGAAGATGCTGCCTTTGTATGTTTTTGCGCAG
CTATTGGGGTCATTTCTGGCAGCAGGGACAATTTTTGCTGTCTATTATGAAGCCATATAT
GACTATTGTGGAGGAAACCTGACTGTAACTGGTGTAAAGGCCACAGCTGGTATCTTTGCC
ACCTATCCAGCACCGTACCTCTCCTTGTTTGCTGGATTCATTGACCAGGTGTTTGGCACA
GCTATGCTGCTGCTGTGCCTGATGGCTCTGTCCGACCAGAAGAACAAACCGGCCGCAGCG
GGTAGCGAGCCTGTCGTAGTGGGTCTCCTGGTGCTCCTCATTGGCATTTCCCTGGGCAGC
AACAGTGGCTATGCCATCAACCCCACCAGAGACATTGGACCCAGGGTGTTCACTGCCATA
GCAGGCTGGGGGGCTGATGTGTTCAGGGCTGGAAATGGGTGGTGGTGGGTGCCTCTAGTT
GCCCCCTGCATTGGAGGAGTATTGGGTGCAGGCATCTACAAGGCCTTGGTGGAAATGCAC
CACCCTCCCCTCTCTGAACAGGGTGAGAGGCTG---GTGGAGGAGTTGGCAGAAGAGACT
TCCCCTCTGGGAAAACAAGAAAAC------TGTGCTAATGTA------TGTGTC------

>White_flower_croaker_Aqp7
ATGAAGGACTTGGTGCAGTCAGTAGAACTAGGGGTC---TCTCAACGGAAAGGCGTTAAT
GTAACTCGACCCAGAGTTTGGCTC---AAGAATGAATTTGTTCGTGTGGGACTTGCTGAA
TCCCTCTGCACGTATGTCATGATGGCTTTAGGACTGGGGTCTGTGGCCCAGGTAGTGACA
GGACAGGGAGCATTTGGACAATACCTCAGCATCAACCTGGGTTTTGGACTGGGTGTTGCT
ATGGGAGTTCATGTTGGAGGAAAGGTCTCAGGGGCTCATATGAATGCAGCAGTGTCACTC
ACAATGTGTGTGTTTGGCCGCCTTGCATGGAAGATGCTGCCTCTGTATGTTTTTGCACAG
CTGTTGGGGTCATTTCTGGCAGCAGGGTCAATTTATGCTGTCTATTATGAAGCCATACAT

GACTACTGTGGAGGAAACCTGACTGTAACTGGTGTAAAGGCCACAGCTGGTATCTTTGCC
ACCTATCCAGCACCGTACCTCTCCTTGTTTGGTGGATTCATTGACCAGGTGTTTGGCACA
GCTATGCTGCTGCTGTGCCTGATGGCTCTGTCCGACCAGAAGAACAAACCGGCCGCAGCG
GGTAGCGAGCCTGTCGTAGTGGGTCTTCTGGTGCTCCTCATTGGCATTTCCCTGGGCAGC
AACAGCGGCTATGCCATCAACCCCACCAGAGACATTGGACCCAGGGTGTTCACTGCCATA
GCAGGCTGGGGAACTGATGTGTTTAGGGCTGGAAATGGGTGGTGGTGGGTGCCTCTAGTT
GCCCCCTGCATTGGAGGAGTATTGGGTGCAGGGATCTACAAGGCCTTGGTAGAAATGCAC
CACCCTCCCCTCTCTGAACAGGGTGAGAGGCTG---GTGGAGGAGTTGGCGGAAGAGACT
TCCCCTCTGGGAAAACAAGAAAAC------TGTGCTAACGTA------TGTGTC------

>Meagre_Aqp7
ATGAAGGACTTGGTGCAGTCAGTAGAACTAGGGGTC---TCTCAACGGAAAGGCGTTAAT
GTAACCCGACCCAGAGTTTGGCTA---AAGAATGAATTTGTTCGTGTGGGACTTGCTGAA
TCCCTTTGCACGTATGTCATGATGGCGTTTGGCCTGGGGTCTGTGGCACAGGTAGTAACA
GGACAGGGCGCGTTTGGACAATACCTCAGCATCAACCTGGGTTTTGGACTGGGTGTTGCT
ATGGGGGTTCATGTTGGAGGAAAGGTCTCAGGGGCTCATATGAACGCAGCAGTGTCACTC
ACAATGTGTGTGTTTGGCCGCCTTGCATGGAAGATGCTGCCTTTGTATGTTTTTGCGCAG
CTATTGGGGTCATTTCTTGCAGCAGGGACAATTTATGCTGTCTATTATGAAGCCATATAT
GACTATTGTGGAGGAAACCTGACTGTAACTGGTGTAAAGGCCACAGCTGGTATCTTTGCC
ACCTATCCAGCACCGTACCTCTCCTTGTTGGCTGGATTCATTGACCAGGTGTTTGGCACA
GCTATGCTGCTGCTGTGCCTGATGGCTCTGTCCGACCAGAAGAACAAACCGGCCGCAGCG
GGTAGCGAGCCTGTCGTAGTGGGTCTCCTGGTCCTCCTCATTGGCATTTCCCTGGGCAGC
AACAGCGGCTATGCCATCAACCCCTCCAGAGACATTGGACCCAGGGTGTTCACTGCCATA
GCAGGCTGGGGGGCTGATGTGTTCAGGGCTGGAAATGGGTGGTGGTGGGTGCCTCTAGTT
GCCCCCTGCATTGGAGGAGTATTGGGTGCAGGGATCTACAAGGCCTTGGTGGAAATGCAC
CACCCTCCCCTCTCTGAACAGGGTGAGAGGCTG---GTGGAGGAGTTGGCGGAAGAGACT
TCCCCTCTGGGAAAACAAGAAAAC------TGTGCTAATGTA------TGTGTC------

>Gilthead_seabream_Aqp7
ATGAAGGACTTGGTGCAGTCAATAGAACTAGGGGTT---TCTCACCAGCGGGGA---GGA
GTAACTCGACCCAGAGCTTGGCTA---CAGAATGAAGTAGTTCGTGTGGGACTCGCTGAA
CTCCTTTGCACATATGTCATGATGGTGTTTGGCCTAGGATCCGTGGCCCAGGTAGTGACA
GGACAGGGAGCGTTTGGAGAGTACATCAGCATCAACCTGGGTTTTGGACTGGGTGTTGCC
TTGGGGGTCCATGTCGGAGGGAAGGTCTCAGGGGCTCACATGAATGCGGCTGTGTCATTC
ACAATGTGCGCCTACGGCCGCCTTGCGTGGAAGATGCTGCCTCTGTATATTTTTGCGCAG
CTATTGGGGTCATTTCTGGCAGCAGGGACGATTTATGCTGTATATTACGAAGCCATATAT
GATTACTGTGGAGGAAACCTGACGGTTACTGGTGTGAAGGCCACAGCGGGTATCTTTGCC
ACATACCCTGCATCGTACCTCTCCTTGCTGGCTGGATTCATTGACCAGGTGTTTGGCACA
GCCATGCTGCTGCTGTGCCTCACTGCTCTGTCCGACCAGAGGAACAAACCTGCGGCAGCG
GGCAGCGAGCCCGTCGCAGTGGGTCTCCTGGTGCTGCTCATTGGCATTTCCCTGGGCAGC
AACAGCGGCTATGCCATCAACCCCACCAGAGACATCGCACCCAGGGTGTTCACTGCCATA
GCAGGCTGGGGCGCTGATGTGTTCAGGGCTGGAAATGGGTGGTGGTGGGTGCCTCTAGTT
GCCCCCCCAATCGGTGGAGTATTGGGTGCTGGGATCTATAAGGTGTTTGTGGAAATGCAC
CACCGTGCCTTCTCTGAGCATGACGAGGCGCTG---GTG---------GAGGAGGAGACT
GCCCCCCTGGGGAAGAAAGAAAACATC---TGTGCAAACGAA------TGTGTC------

>Red_seabream_Aqp7
ATGAAGGACTTGGTGCAGTCAATAGAACTAGGAGTT---TCTCACCAGCAGAAA---GGA
GTAACTCGACCCAAAGTGTGGCTA---CAGAATGAAGTAGTTCGCGTGGGACTCGCTGAA
ATCCTTTGCACATATGTCATGATGGTGTTTGGCCTGGGATCCGTGGCCCAGGTCGTGACA
GGACGGGGAGCGTTTGGAGAATACATCAGCATCAACCTGGGTTTTGGACTGGGTGTTGCT
ATGGGGGTCCACATTGGAGGGAAGGTCTCAGGGGCTCATATGAATGCAGCCGTGTCACTC
ACAATGTGCGCCTATGGCCGCCTTGCGTGGAAGATGCTGCCTCTGTATGTTTTCGCGCAG
CTATTGGGGTCATTTCTGGCAGCAGGGACAATTTATGCTATCTATTACGAGGCCATATAT
GACTACTGTGGAGGAAACCTGACTGTAACTGGTGTTAAGGCCACAGCTGGTATCTTTGCC
ACATATCCTGCACCGTACCTCTCTTTGTTGGCTGGATTCATTGACCAGGTGTTTGGCACA
GCCATGCTGCTGATGTGCCTCATGGCTCTGTCTGACCAGAGGAACAAACCGGCTGCAGCG
GGCAGCGAGCCCATCGCAGTGGGTCTCCTGGTGCTGCTCATTGGTATTTCCCTGGGCAGC
AACAGCGGCTACGCCATCAACCCCACCAGAGACATTGCACCCAGGGTGTTCACTGCCATA
GCAGGCTGGGGGACCGACGTGTTCAGGGCTGGAAATGGGTGGTGGTGGGTGCCTCTAGTT
GCCCCCCCAATCGGAGGAGTATTGGGTGCAGGGATCTATAAGGTCTTTGTGGAAATGCAC

CACCTTCCCTTCTCTGAGCAGGGCGAGGTGCTG---GTG---------GAGGAGGAGACT
GCCCCTCTGGCGAAAAAAGAAAACATC---TGTGCAAACGAA------TGTGTC------

>White_bass_Aqp7
ATGAAGGACTTGGTGCAGTCAGTAGAACTAGGGGTC---TCTCAGCAGAGAGGAGTTAAC
GTAACTCGACCAAAAGTTTGGCTA---AAGAATGAAGTGGTTCGTGTGGGACTTGCTGAA
TCCCTTTGCACATATGTCATGATGGTGTTTGGCCTGGGGTCTGTGGCCCAGGTAGTGACA
GGACAGGGAGCGTTTGGAGAGTACCTCAGCATCAACCTGGGTTTTGGACTGGGTGTTGCA
ATGGGGGTTCATGTTGGAGGGAAGGTCTCAGGGGCTCATATGAATGCGGCAGTGTCGCTC
ACAATGTGCGCGTTTGGTCGCCTTGCATGGAAGATGCTGCCTGTGTATGTTTTTGCACAG
CTATTGGGGTCATTTCTGGCAGCGGGGACAATTTATGCTGTCTATTACGAAGCCATTTAT
GACTATTGTGGAGGAAACCTGACTGTGACTGGTGTGAAGGCCACAGCTGGTATCTTTGCC
ACCTATCCTGCACCGTACCTCTCCTTGCTGGCTGGATTCATTGACCAGGTGTTTGGCACA
GCTATGCTGCTGCTGTGCCTGATGGCTCTGTCCGACCAGAAGAACAAACCGGCCGCGGCG
GGCAGTGAGCCTGTTGCAGTGGGTCTCCTAGTGCTGCTCATTGGCATTTCCCTGGGCAGC
AACAGTGGCTATGCCATCAACCCCACCAGAGACATCGCACCCAGGATCTTCACTGCAATA
GCAGGCTGGGGGTCCGATGTGTTCAGGGCTGGAAATGGGTGGTGGTGGGTGCCTCTGGCT
GCACCGCCCATTGGTGGAGTATTGGGTGCGGGGCTCTACAAGGCCTTGGTGGAAATGCAC
CACCCACACCTCTCTGAACAGGGTGAGGGGCTG---CTG---------AAGGAGGAGACT
GCCCCTTTGAGGAAACAAGAAAACATC---TGTGCTAATGTA------TGTGTT------

>Striped_seabass_Aqp7
ATGAAGGACTTGGTGCAGTCAGTAGAACTAGGGGTC---TCTCAGCGGAAAGGAGTTAAC
GTAACTCGACCAAAAGTTTGGCTA---AAGAATGAAGTGATTCGTGTGGGACTTGCTGAA
TCCCTTTGCACATATGTCATGATGGTGTTTGGCCTGGGGTCTGTGGCCCAGGTAGTGACA
GGACAGGGAGCGTTCGGAGAGTACCTCAGCATCAACCTGGGTTTTGGACTGGGTGTTGCA
ATGGGGGTTCATGTTGGAGGGAAGGTCTCAGGGGCTCATATGAATGCGGCAGTGTCGCTC
ACAATGTGCGCGTTTGGTCGCCTTGCATGGAAGATGCTGCCTGTGTATGTTTTTGCACAG
CTATTGGGGTCATTTCTGGCAGCGGGGACAATTTATGCTGTCTATTACGAAGCCATTTAT
GACTATTGTGGAGGAAACCTGACTGTGACTGGTGTAAAGGCCACAGCTGGTATCTTTGCC
ACCTATCCTGCACCGTACCTCTCCTTGCTGGCTGGATTCATTGACCAGGTGTTTGGCACA
GCTATGCTGCTGCTGTGCCTGATGGCTCTGTCCGACCAGAAGAACAAACCGGCCGCAGCG
GGCAGTGAGCCTGTTGCAGTGGGTCTCCTAGTGCTGCTCATTGGCATTTCCCTGGGCAGC
AACAGTGGCTATGCCATCAACCCCACCAGAGACATCGCACCTAGGATCTTCACTGCTATA
GCAGGCTGGGGGTCCGATGTGTTCAGGGTTGGAAATGGGTGGTGGTGGGTGCCTCTGGCT
GCACCTCCCATTGGTGGAGTATTGGGTGCGGGGCTCTACAAGGCCTTGGTGGAAATGCAC
CACCCACCCCTCTCTGAACAGGGTGAGGGGCTG---CTG---------AAGGAGGAGACT
GCCCCTTTGAGGAAACAAGAAAACATC---TGTGCTAATGTA------TGTGTT------

>European_seabass_Aqp7
ATGAAGGACTTAATGCAGTCAGTAGAACTAGGAGTC---TCTCAGCGGAAAGGAGTTAAC
GTAACTGGACCAAAAGTTTGGCTA---AAGAATGAAGTGGTTCGTGTGGGACTTGCTGAA
TCCCTTTGCACATATGTCATGATGGTGTTTGGCCTGGGGTCTGTGGCCCAGGTAGTGACA
GGACAGGGAGCGTTCGGAGAGTACCTCAGCATCAACCTGGGTTTTGGACTGGGTGTTGCA
ATGGGGGTTCATGTTGGAGGGAAGGTCTCAGGGGCTCATATGAATGCGGCAGTGTCTCTC
ACAATGTGCACATTTGGCCGCCTTGCATGGAAGATGCTGCCTGTGTATGTTTTTGCACAG
CTATTGGGGTCATTTCTGGCAGCGGGGACAATTTATGCTGTCTATTATGAAGCCATTTAT
GACTATTGTGGAGGAAACCTGACTGTGACTGGTGTAAAGGCCACAGCTGGTATCTTTGCC
ACCTATCCTGCACCGTACCTCTCCTTGCTGGCTGGATTCATTGACCAGGTGTTTGGCACA
GCTATGCTGCTGCTGTGCCTGATGGCTCTGTCCGACCAGAAGAACAAACCGGCCGCAGCA
GGCAGTGAGCCTGTTGCAGTGGGTCTCCTAGTGCTACTCATCGGCATTTCCCTAGGCAGC
AACAGTGGCTATGCCATCAACCCCACCAGAGACATCGCACCGAGGGTCTTCACTGCCATA
GCAGGCTGGGGGTCCGATGTGTTCAGGGCTGGAAATGGGTGGTGGTGGGTGCCTCTGGCT
GCACCTCCCATTGGTGGAGTATTGGGTGCGGGGCTCTACAAGGCCTTGGTGGAAATGCAC
CACCCACCCCTCTCTGAACAGGGTGAGGGGCTG---CTC---------AAGGAGGAGACT
GCCCCTTTGAGGAAACAAGAAAACATC---TGTGCTAATGTA------TGTGTT------

>Flag_rockfish_Aqp7
ATGAAGGACTTTGTGCAGTCAGTAGAACTGGGGGTC---TCTCAGCGGAAACGACTCAAC
GTAACTCGACCCAGAGTTTGGCTG---AGGAATGAATTTGTTCGTGTGGGACTTGCTGAA
TCTCTTTGCACATATGTCATGATGGTGTTTGGCCTGGGGTCTGTGGCCCAGGTGGTGACG

GGACAGGGAGAGTTCGGACAGTACATCAGCATCAACCTGGGTTTTGGACTGGGTGTTGCT
ATGGGCGTTCATGTCGGAGGGAAGGTCTCAGGGGCTCACATGAATGCAGCAGTGTCATTC
ACAATGTGCACGTTTGGCCGCCTTGGGTGGAAGATGCTGCCTCTGTATATTTTTGCACAA
CTATTGGGGTCATTTCTGGCGGCGGGGACAATCTATGCTGTCTATTATGACGCCATACAT
GACTATTGTGGAGGAAACCTGACTGTAATAGGAGTGAAGGCCACAGCAGGTATCTTTGCC
ACCTATCCTGCACCGTACCTCTCCTTGCTGGCTGGATTCATTGATCAGGTGTTTGGCACA
GCTATGCTGCTGCTTTGCTTGATGGCTCTGTCCGACCAGAAGAACAAGCCGGCCGCAGCG
GGCAGTGAGCCCGTCGCAGTGGGTCTCCTGGTGCTGCTCATTGGCATTTCTCTGGGCAGC
AACAGCGGCTATGCTATCAACCCCACCAGAGACATCGCACCCAGGGTCTTCACTGCCATA
GCAGGCTGGGGGCCCGACGTATTCAGGGCTGGAAATGGCTGGTGGTGGGTGCCTCTAGTA
GCCCCCCCTATTGGAGGAGTATTGGGTGCGGGGCTCTACAGAGTCTTGGTGGAAATGCAC
CACCCTCCCGTCTACGAACAGGGTGAGGGGTCA---GTGGAGAAGTTTGAGGAGGAGACC
GCCCCACTGGAAAAACAGGAAAACATC---TGTGCTAATGCG------TATGTA------

>Tiger_rockfish_Aqp7

------------------------GTGTTTGGCCTGGGGTCTGTGGCCCAGGTGGTGACG
GGACAGGGAGAGTTCGGACAGTACATCAGCATCAACCTGGGTTTTGGACTGGGTGTTGCT
ATGGGCGTTCATGTTGGAGGGAAGGTCTCAGGGGCTCACATGAATGCAGCAGTGTCATTC
ACAATGTGCACGTTTGGCCGCCTTGGGTGGAAGATGCTGCCTCTGTATATTTTTGCACAA
CTATTGGGGTCATTTCTGGCGGCGGGGACAATCTATGCTGTCTATTATGACGCCATACAT
GACTATTGTGGAGGAAACCTGACTGTAATAGGAGTGAAGGCCACAGCAGGTATCTTTGCC
ACGTATCCTGCACCGTACCTCTCCTTGCTGGCTGGATTCATTGATCAGGTGTTTGGCACA
GCTATGCTGCTGCTTTGCTTGATGGCTCTGTCCGACCAGAAGAACAAGCCGGCCGCAGCG
GGCAGTGAGCCCGTCGCAGTGGGTCTCCTGGTGCTGCTCATTGGCATTTCTCTGGGCAGC
AACAGCGGCTATGCTATCAACCCCACCAGAGACATCGCACCCAGGGTCTTCACTGCCATA
GCAGGCTGGGGGCCCGACGTATTCAGGGCTGGAAATGGCTGGTGGTGGGTGCCTCTAGTT
GCCCCCCCTATTGGAGGAGTATTGGGTGCGGGGCTCTACAGAGTCTTGGTGGAAATGCAC
CACCCTCCCGTCTACGAACAGGGTGAGGGGTCA---GTGGAGAAGTTTGAGGAGGAGACC
GCCCCACTGGAAAAACAGGAAAACATC---TGTGCTAATGCG------TATGTA------

>Copper_rockfish_Aqp7
ATGAAGGACTTTGTGCAGTCAGTAGAACTGGGGGTC---TCTCAGCGGAAAAGACTCAAC
GTAACTCGACCCAGAGTTTGGCTG---AGGAATGAATTTGTTCGTGTGGGACTTGCTGAA
TCTCTTTGCACATATGTCATGATGGTGTTTGGCCTGGGGTCTGTGGCCCAGGTGGTGACG
GGACAGGGAGAGTTCGGACAGTACATCAGCATCAACCTGGGTTTTGGACTGGGTGTTGCT
ATGGGCGTTCATGTCGGAGGGAAGGTCTCAGGTGCTCACATGAATGCAGCAGTGTCATTC
ACAATGTGCACGTTTGGCCGCCTTGGGTGGAAGATGCTGCCTCTGTATATTTTTGCACAA
CTATTGGGGTCATTTCTGGCAGCGGGGACAATCTATGCTGTCTATTATGACGCCATACAT
GACTATTGTGGAGGAAACCTGACTGTAATAGGAGTGAAGGCCACAGCAGGTATCTTTGCC
ACCTATCCTGCACCGTACCTCTCCTTGCTGGCTGGATTCATTGATCAGGTGTTTGGCACA
GCTATGCTGCTGCTTTGCTTGATGGCTCTGTCCGACCAGAAGAACAAGCCGGCCGCAGCG
GGCAGTGAGCCCGTCGCAGTGGGTCTCCTGGTGCTGCTCATTGGCATTTCTCTGGGCAGC
AACAGCGGCTATGCTATCAACCCCACCAGAGACATCGCACCCAGGGTCTTCACTGCCATA
GCAGGCTGGGGGCCCGACGTATTCAGGGCTGGAAATGGCTGGTGGTGGGTGCCTCTAGTT
GCCCCCCCTATTGGAGGAGTATTGGGTGCGGGGCTCTACAGAGTCTTGGTGGAAATGCAC
CACCCTCCCGTCTACGAACAGGGTGAGGGGTCA---GTGGAGAAGTTTGAGGAGGAGACC
GCCCCACTGGAAAAACAGGAAAACATC---TGTGCTAATGCG------TATGTA------

>mfRockfish_Aqp7
ATGAAGGACTTTGTGCAGTCAGTAGAACTGGGGGTC---TCTCAGCGGAAAAGACTCAAC
GTAACTCGACCCAGAGTTTGGCTG---AGGAATGAATTTGTTCGTGTGGGACTTGCTGAA
TCTCTTTGCACATATGTCATGATGGTGTTTGGCCTGGGGTCTGTGGCCCAGGTGGTGACG
GGACAGGGAGAGTTCGGACAGTACATCAGCATCAACCTGGGTTTTGGACTGGGTGTTGCT
ATGGGCGTTCATGTCGGAGGGAAGGTCTCAGGGGCTCACATGAATGCAGCAGTGTCATTC
ACAATGTGCACGTTTGGCCGCCTTGGGTGGAAGATGCTGCCTCTGTATATTTTTGCACAA
CTATTGGGGTCATTTCTGGCGGCGGGGACAATCTATGCTGTATATTATGATGCCATACAT
GACTATTGTGGAGGAAACCTGACTGTAATAGGAGTGAAGGCCACAGCAGGTATCTTTGCC
ACCTATCCTGCACCGTACCTCTCCTTGCTGGCTGGATTCATTGATCAGGTGTTTGGCACA
GCTATGCTGCTGCTTTGCTTGATGGCTCTGTCCGACCAGAAGAACAACCCGGCCGCAGCG

GGCAGTGAGCCCGTCGCAGTGGGTCTCCTGGTGCTGCTCATTGGCATTTCTCTGGGAAGC
AACAGCGGCTATGCTATCAACCCCACCAGAGACATCGCACCCAGGGTCTTCACTGCCATA
GCAGGCTGGGGGCCCGACGTATTCAGGGCTGGAAATGGCTGGTGGTGGGTGCCTCTAGTT
GCCCCCCCTATTGGAGGAGTATTGGGTGCGGGGCTCTACAGAGTCTTGGTGGAAATGCAC
CACCCTCCTGTCTACGAACAGGGTGAGGGGTCA---GTGGAGAAGTTTGAGGAGGAGACC
GCCCCACTGGAAAAACAGGAAAACATC---TGTGCTAATGCG------TATGTA------

>Korean_rockfish_Aqp7
ATGAAGGACTTTGTGCAGTCAGTAGAACTGGGGGTC---TCTCAGCGGAAAAGACTCAAC
GTAACTCGACCCAGAGTTTGGCTG---AGGAATGAATTTGTTCGTGTGGGACTTGCTGAA
TCTCTTTGCACATATGTCATGATGGTGTTTGGCCTGGGGTCTGTGGCCCAGGTGGTGACG
GGACAGGGAGAGTTCGGACAGTACATCAGCATCAACCTGGGTTTTGGACTGGGTGTTGCT
ATGGGCGTTCATGTCGGAGGGAAGGTCTCAGGGGCTCACATGAATGCAGCAGTGTCATTC
ACAATGTGCACGTTTGGCCGCCTTGGGTGGAAGATGCTGCCTCTGTATATTTTTGCACAA
CTATTGGGGTCATTTCTGGCGGCGGGGACAATCTATGCTGTCTATTATGATGCCATACAT
GACTATTGTGGAGGAAACCTGACTGTAATAGGAGTGAAGGCCACAGCAGGTATCTTTGCC
ACCTATCCTGCACCGTACCTCTCCTTGCTGGCTGGATTCATTGATCAGGTGTTTGGCACA
GCTATGCTGCTGCTTTGCTTGATGGCTCTGTCCGACCAGAAGAACAAGCCGGCCGCAGCG
GGCAGTGAGCCCGTCGCAGTGGGTCTCCTGGTGCTGCTCATTGGCATTTCTCTGGGCAGC
AACAGCGGCTACGCTATCAACCCCACCAGAGACATCGCACCCAGGGTCTTCACTGCCATA
GCAGGCTGGGGGCCCGACGTGTTCAGGGCTGGAAATGGCTGGTGGTGGGTGCCTCTAGTT
GCTCCCCCTATTGGAGGAGTATTGGGTGCGGGGCTCTACAGAGTCTTGGTGGAAATGCAC
CACCCTCCCGTCTACGAACAGGGTGAGGGGTCA---GTGGAGAAGTTTGAGGAGGAGACC
GCCCCACTGGAAAAACAGGAAAACATC---TGTGCTAATGCG------TATGTA------

>Rougheye_rockfish_Aqp7
ATGAAGGACTTTGTGCAGTCAGTAGAACTGGGGGTC---TCTCAGCGGAAAAGACTCAAC
GTAACTCGACCCAGAGTTTGGCTG---AGGAATGAATTTGTTCGTGTGGGACTTGCTGAA
TCTCTTTGCACATATGTCATGATGGTGTTTGGCCTGGGGTCTGTGGCCCAGGTGGTGACG
GGACAGGGAGAATTCGGACAGTACATCAGCATCAACCTTGGTTTTGGACTGGGTGTTGCT
ATGGGCGTTCATGTTGGAGGGAAGGTCTCAGGGGCTCACATGAATGCAGCAGTGTCATTC
ACAATGTGCACGTTTGGCCGCCTTGGGTGGAAGATGCTGCCTCTGTATATTTTTGCCCAA
CTATTGGGGTCATTTCTGGCGGCGGGGACAATCTATGCTGTATATTATGATGCCATACAT
GACTATTGTGGAGGAAACCTGACTGTAATAGGAGTGAAGGCCACAGCAGGTATCTTTGCC
ACSTATCCTGCACCGTACCTCTCCTTGCTGGCTGGATTCATTGATCAGGTGTTTGGCACA
GCTATGCTGCTGCTTTGCTTGATGGCTCTGTCCGACCAGAAGAACAAGCCGGCCGCGGCG
GGCAGCGAGCCCATCGCAGTGGGTCTCCTGGTGCTGCTCATTGGCATTTCTCTGGGCAGC
AACAGCGGCTACGCTATCAACCCCACCAGAGACATCGCACCCAGGGTCTTCACTGCCATA
GCAGGCTGGGGGCCCGACGTGTTCAGGGCTGGAAATGGCTGGTGGTGGGTGCCTCTAGTT
GCCCCCCCTATTGGAGGAGTATTGGGTGCGGGGCTCTACAGAGTCTTGGTGGAAATGCAC
CACCCTCCCGTCTACGAACAGGGTGAGGGGTCA---GTGGAGAAGTTTGAGGAGGAGACC
GCCCCACTGGAAAAACAGGAAAACATC---TGTGCTAATGCG------TATGTA------

>3_spine_stickleback_Aqp7
ATGAAGGATTTGGTGCAGTCAGTCGAACTAGGGGTC---TCTCAACGAAAAAGAGTGAGT
GGG------------------CTA---AAGAATGAACTTGTTCGAGTGGGACTGGCTGAA
TCCCTTTGCACATATGTCATGATGGTGTTCGGCCTGGGGTCCGTGGCCCAGGTAGTGACA
GGACAGGGAGAGTTTGGCCAGTACATCAGCATCAATCTGGGTTTTGGACTGGGTGTTGCT
ATGGGGGTTCATGTTGGAGGGAAGGTCTCAGGGGCTCACATGAATGCGGCAGTGTCATTC
ACCATGTGCACGTTTGGCCGCCTTGGGTGGAAGATGCTGCCTCTGTATGTTTTTGCACAA
CTATTTGGGTCATTCCTGGCCGCAGCGACAATCTATGGCGTCTACTATGAAGCCATACAC
GACTATTGTGGAGGTAACCTGACTGTAACAGGACCAAAGGCCACAGCAGGTATCTTTGCC
ACTTATCCTGCACCGTACCTCTCCTTGCATGCCGGATTCATTGATCAGGTGATCGGCACA
GCCATGCTGCTGCTTTGCTTGATGGCTTTGTCAGATCAGAAGAACAAACCGGCGGCAGCG
GGAAGCGAGCCCGTCGCAGTGGGCCTCCTGGTGCTGCTCATTGGCATTTCTCTGGGCAGC
AACAGTGGCTACGCCATCAACCCGACCAGAGACATCGCACCCAGGGTCTTCACTGCCATA
GCGGGCTGGGGGGGTGATGTGTTCAGGTCTGGAAATGGGTGGTGGTGGGTGCCTCTAGTT
GCCCCCCCCCATTGGAGGAATATTGGGTGCAGGGCTCTACAAGGTGTTGGTGGAAATGCAC
CATCAACCT------GAACAGAAAGAGCGGCTG---GTGGAGGAGTTC---GAGGAGAGA
TTAGCGCTGGAG--

>9_spine_stickleback_Aqp7
ATGAAGGACTTGGTGCAGTCGGTTGAACTAGGGGTC---TCTCAGCGAAAAAGAGCGCGT
GGA------CCTAGAGTTTGGCTA---AAGAATGAACTTGTTCGGGTGGGACTGGCTGAA
TCCCTTTGCACATATGTCATGATGGTGTTCGGCCTGGGGTCCGTGGCCCAGGTAGTGACA
GGACAGGGAGAGTTCGGACAGTACATCAGCATCAATCTGGGTTTTGCACTGGGTGTTGCC
ATGGGGGTTCATGTCGGAGGGAAGGTGTCAGGGGCTCACATGAATGCCGCGGTGTCATTC
ACCATGTGCACGTTTGGCCGCCTTGGGTGGAAGATGCTGCCTCTGTATGTTTTTGCACAA
CTACTTGGGTCATTCCTGGCCGCAGTGACCATCTATGGCGTTTACTATGAAGCCATACAC
GACTATTGTGGAGGTAACCTGACTGTGACAGGATCAAAGGCCACAGCAGGTATCTTTGCC
ACTTATCCTGCACCGTACCTCTCCTTGCATGCTGGATTCCTTGATCAGGTGATCGGCACA
GCCATGCTGCTGCTTTGCCTGATGGCTTTGTCGGATCAGAAGAACAAACCGGCGGCGGCG
GGAAGCGAGCCTGTCGCAGTGGGTCTCCTGGTGCTGCTCATTGGCATTTCCCTGGGCAGC
AACAGTGGCTACGCCATCAACCCAACCAGAGACATCGCACCCAGGGTCTTCACCGCCATA
GCGGGCTGGGGGGGTGATGTGTTCAGGTCTGGAAATGGGTGGTGGTGGGTGCCTCTGGTT
GCCCCCCCCATTGGAGGAATACTGGGTGCAGGGCTCTACAAGGCGTTGGTGGAAATGCAC
CATCCACCTCTCCTTGAACAGAAAGAGCAGCTG---GTGGAGGAGTTT---GAGGAGAGT
TTGGCGCTGGAGAAACAAGAAAACATC---GGTGCCAATGTG------GCCGTC------

>Rhine_sculpin_Aqp7
ATGAAAGACTTGGTGCAGTCAGTCGAACTCGGGGTC---TCTCGGCGGAAAAGAGTCAAT
GGAGCTGGACCCAGAGTTTGGATC---AAGAATGAAGTTGTTCGAGTGGGACTCGCTGAA
TCCCTCAGCACATACGTCATGATGGTGTTTGGCTTGGGGTCGGTGGCCCAGGTAGTGACG
GGACAGGGAGCGTTCGGACAGTACCTCAGCATCAATGTGGGGTTTGGACTGGGCGTCGCT
ATGGGGGTTCACGTTGGAGGGAAGGTCTCAGGGGCTCACATGAATGCAGCCGTGTCATTC
ACCATGTGCACGTTTGGCCGCCTCGGCTGGAAGATGCTGCCTCTCTATGTTTTTGCGCAA
CTATTGGGGTCATTCCTGGCTGCAGGAACAATCTATGGTGTCTATTATGAAGCCATACAT
CACTATTGTGGAGGAAACCTGACTGTGACCGGAGCAAAGGCCACAGCAGGTATCTTTGCC
ACCTATCCTGCACCGTACCTCTCCTTGCTGGCTGGATTCATTGATCAGGTA---------

---CTGGGCAGC
AACAGTGGCTATGCCATCAACCCCACCAGAGACATCGCACCCAGGGTCTTCACTGCCATA
GCAGGCTGGGGGGGTGACGTGTTCAGG---------------------------------
------------GGAGGAGTATTGGGTGCAGGGCTCTACAGGGTGTTGGTAGAAATGCAC
CACCCCCCCCTCTCTGAACAAGACGAGCGGCTG---GTGGAGAAGTTT---GAGGAGAGT
GTCCCTCTGGAGAAACAAGAAAACATC---TGCGCTAATGTG------TGTGTC------

>Sablefish_Aqp7
ATGAAGGACATGGGGCAGTCAGTGGAACTAGGGGTC---TCTCAGCGGAAAAGAGTCAAT
GTAACTCGCCCCAAAGTTTGGCTA---AAGAATAAAATTGTTCGAGTGGGACTTGCTGAA
TCGCTCTGCACATACGTCATGATGGTGTTTGGCCTGGGGTCGGTGGCACAGGTAGTGACG
GGACAGGGAGCGTTCGGACAGTACATCAGCATCAATCTGGGTTTTGGACTGGGTGTTGCT
ATGGGGGTTCATGTTGGAGGGAAGGTCTCAGGGGCTCACATGAATGCAGCGGTGTCATTC
ACCATGTGCACGTTTGGCCGTCTTGGCTGGAAGATGCTGCCGCTTTATGTTTTTGCGCAA
CTACTGGGGTCATTCCTGGCCGCAGGGACAATCTATGCTGTCTATTATGATGCCATATAT
GACTATTGTGGAGGAAACCTCACAGTGACAGGAGTTAGGGCCACAGCAGGTATCTTTGCC
ACTTACCCTGCTCCATACCTCTCCTTGCTGGCTGGATTCATTGATCAGGTGTTCGGCACA
GCCATGCTTCTGCTTTGCTTGATGGCTTTGTCGGATCAGAGGAACAAACCGGCGCCGGCG
GGCAGCGAGCCTTTCGCAGTGGGTCTCCTGGTGGTGCTAATTGGCATTTCTCTGGGCAGC
AACAGCGGCTATGCCATCAACCCCACCAGAGACATCGCACCCAGGGTCTTCACTGCCATA
GCGGGCTGGGGGGTTGATGTGTTCAGGGCTGGAAATGGGTGGTGGTGGGTGCCTCTAGTT
GCCCCCCCCATTGGAGGAGTATTGGGTGCAGGACTCTACAGGGCATTGGTGGAAATGCAC
CATCCCCCTCTCTCTGAAAAGGATAAAGGGCTG---GTGGAGGAGTTT---GAAGAGAGT
GTCCCTCTGGAGAAACTAGAGAAAATC---TGTGCTACTGTC------------------

>Emerald_rockcod_Aqp7
ATGAAGGATTTGGAGCAGGCATTAGAACTGGGGGTC---TCTCAGCAGAAAAGATTCAAC
GCAACTCAACCCAAAGTTTGGCTA---AGGAATAAATGTGTTCGTGTTGGACTTGCTGAA
ACCCTCTGCACATATGTCATGATGGTGTTTGGTCTGGGGTCTGTGGCCCAGGTAGTGACG
GGGCAGGGAGCGTTCGGAGAGTACCTCAGCATCAACCTGGGCTTTGGACTGGGTGTTGCT
ATGGGGGTTCATGTTGGAGGGAAAGTCTCAGGGGCTCACATGAATGCAGCAGTGTCATTC
ACAATGTGTATGTTCGGCCGGCTCGGATGGAAGATGCTGCCTCTGTATATTTTTGCACAA

CTATTGGGGTCATTTCTGGCAGCAGGGACCATATATGGCGTCTATTACGATGCCATACAT
GAATATTGTGGAGGAAACCTGACTGTAACCGGGGAACTGGCCACAGCTGGTATCTTTGCC
ACCTACCCCGCACCATACCTCTCCTTGATGGGGGGATTCATTGATCAGGTGTTGGGCACA
GCCTTGCTTCTGCTTTGCTTGATGGCGTTGTCCGACCAGAGAAACAAACCGGCAGCAGTG
GGCACGGAGCCCATCGCAGTGGGTCTGCTGGTGCTGCTCATCGGCATCTCTATGGGCAGA
AACAGCGGATATGCTATCAACCCCACCAGAGACATCGCCCCCAGGCTCTTCACTGCCGTA
GCAGGCTGGGGGATGGACGTGTTCAGGGCTGGAAATGGTTGGTGGTGGGTGCCACTGGTT
GGGCCCCCGATTGGAGGAGTACTGGGGGCAGGACTGTACAAGGTGTTGGTGGAAATGCAC
CACCCCCACGTCTCTGAGCCGGTTGATGGGCTA---ATGGAGGAGTTTGATGAGGAGGCT
ACCCCCCTGAAGAAACAGAAGATAATC---TGTGCCAATGTA------TGTGTC------

>Yellow_notie_Aqp7
ATGAAGGATTTGGAGCAGGCATTAGAACTGGGGGTC---TCTCAGCAGAAAAGATTCAAC
GCAACTCAACCCAAAGTTTGGCTA---AGGAATGAATGTGTTCGTGTTGGACTTGCTGAA
ACCCTCTGCACATATGTCATGATGGTGTTTGGTCTGGGGTCTGTGGCCCAGGTAGTGACG
GGGCAGGGAGCGTTCGGAGAGTACCTCAGCATCAACCTGGGCTTTGGACTGGGTGTTGCT
ATGGGGGTTCATGTTGGAGGGAAAGTCTCAGGGGCTCACATGAATGGAGCAGTGTCATTC
ACAATGTGTATGTTCGGCCGGCTCGGATGGAAGATGCTGCCTCTGTATATTTTTGCACAA
CTATTGGGGTCATTTCTGGCAGCAGGGACCATATATGGCGTCTATTACGATGCCATACAT
GAATATTGTGGAGGAAACCTGACTGTAACCGGGGAACTGGCCACAGCTGGTATCTTTGCC
ACCTACCCCGCACCATACCTCTCCTTGATGGGGGGATTCATTGATCAGGTGTTGGGCACA
GCCTTGCTTCTGCTTTGCTCGATGGCGTTGTCCGACCAGAGGAACAAACCGGCAGCAGCG
AGCACGGAGCCCATCGCAGTGGGTCTGCTGGTGTTGCTCATCGGCATCTCTATGGGAAGA
AACAGCGGATATGCTATCAACCCCACCAGAGACATCGCCCCCAGGCTCTTCACTGCCGTA
GCAGGCTGGGGGATGGACGTGTTCAGGGCTGGAAATGGTTGGTGGTGGGTGCCACTGGTT
GGGCCCCCGATTGGAGGAGTACTGGGGGCAGGACTGTACAAGGTGTTGGTGGAAATGCAC
CACCCCCACGTCTCTGAGCCGGTTGATGGGCTG---ATGGAGGAGTTTGATGAGGAGGCT
GCCCCCGTGAAGAAACAGAAGATAATC---TGTGCCAATGTA------TGTGTC------

>Black_rockcod_Aqp7
ATGAAGGATTTGGAGCAGGCATTAGAACTGGGGGTC---TCTCAGCAGAAAAGATTCAAC
GCAACTCAACCCAAAGTTTGGCTA---AGGAATGAATGTGTTCGTGTTGGACTTGCTGAA
ACCCTCTGCACATATGTCATGATGGTGTTTGGTCTGGGGTCTGTGGCCCAGGTAGTGACG
GGGCAGGGAGCGTTCGGAGAGTACCTCAGTATCAACCTGGGCTTTGGACTGGGTGTTGCT
ATGGGGGTTCATGTTGGAGGGAAAGTCTCAGGGGCTCACATGAATGCAGCAGTGTCATTC
ACAATGTGTATGTTCGGCCGGCTCGGATGGAAGATGCTGCCTCTGTATATTTTTGCACAA
CTATTGGGGTCATTTCTGGCAGCAGGGACCATATATGGTGTATATTATGATGCCATACAT
GAATATTGTGGAGGAAACCTGACTGTAACCGGGGAACTGGCCACAGCTGGTATCTTTGCC
ACCTACCCCGCACCATACCTCTCCTTGATGGGGGGATTCATTGATCAGGTGTTGGGCACA
GCCTTGCTTCTGCTTTGCTTGATGGCGTTGTCCGACCAGAGGAACAAACCGGCAGCAGCG
GGCACGGAGCCCATCGCAGTTGGTCTGCTGGTGCTGCTCATCGGCATCTCTATGGGAAGA
AACAGCGGATATGCTATCAACCCCACCAGAGACATCGCCCCCAGGCTCTTCACTGCCGTA
GCAGGCTGGGGGATGGACGTGTTCAGGGCTGGAAATGGTTGGTGGTGGGTGCCACTGGTT
GCGCCCCCGATTGGAGGAGTACTAGGTGCAGGACTGTACAAGGTGTTGGTGGAAATGCAC
CACCCCCACGTCTCTGAGCCGGTTGATGGGCTG---ATGGAGGAGTTTGATGAGGAGGCT
GCCCCCCTGAAGAAACAGAAGATAATC---TGTGCCAATGTA------TGTGTC------

>Antarctic_icefish_Aqp7
ATGAAGGATTTGGAGCAGGCATTAGAACTCGGGGAC---TCTCAGCAGAAAAGATTCAAT
GCAACTCAACCCAAAGTTTGGCTA---AGGAATGAATGTGTTCGTGTTGGACTTGCTGAA
ACCCTATGCACATATGTCATGATGGTGTTTGGTCTGGGGTCTGTGGCCCAGGTAGTGACG
GGGCACGGAGCGTTTGGAGAGTACCTCAGCATCAACCTGGGCTTTGGACTGGGTGTTGCT
ATGGGGGTTCATGTTGGAGGGAAAGTCTCAGGGGCTCACATGAATGCAGCAGTGTCATTC
ACAATGTGTATGTTCGGCCGGCTCGGATGGAAGATGCTGCCTCTGTATATTTGTGCACAA
CTATTGGGGTCATTTCTGGCAGCAGGGACCATATATGGCGTCTATTACGATGCCATACAT
GAATATTGTGGAGGAAACCTGACTGTAACCGGGGAACTGGCCACAGCCGGTATCTTTGCC
ACTTACCCCGCACCATACCTCTCCTTGATGGGGGGATTCATTGATCAGGTGTTGGGCACA
GCCTTGCTTCTGCTTTGCTTGATGGCGTTGTCCGACCAGAGGAACAAACCGGCAGCAGCG
GGCACGGAGCCCATCGCAGTTGGTCTGCTGGTGCTGCTCATCGGCATCTCTATCGGAAGA
AACAGCGGATATGCTATCAACCCCACCAGAGACATCGCACCCAGGCTCTTCACTGCCGTA
GCAGGCTGGGGGATGGACGTGTTCAGGGCTGGAAATGGTTGGTGGTGGGTGCCACTGGTT

GCGCCCCCGATTGGAGGAGTACTGGGTGCAGGACTGTACAAGGTGTTGGTGGAAATGCAG
CACCCCCACGTCTCTGAGCCGGGTGATGGGCTG---ATGGAGGAGTTTGATGAGGAGGCT
GCCTCCCTGAAGAAACAGAAGATAATC---TGTGGCAATGTA------TGTGTC------

>Blackfin_icefish_Aqp7
ATGAAGGATTTGGAGCAGGCATTAGAACTGGGAGAC---TCTCAGCAGAAAAGATTCAAT
GCAACTCAACCCAAAGTTTGGCTA---AGGAATGAATGTGTTCGTGTTGGACTTGCTGAA
ACCCTATGCACATATGTCATGATGGTGTTTGGTCTGGGGTCTGTGGCCCAGGTAGTGACG
GGGCAGGGCGCGTTTGGAGAGTACCTCAGCATCAACCTGGGCTTTGGACTGGGTGTTGCT
ATGGGGGTTCATGTTGGAGGGAAAGTCTCAGGGGCTCACATGAATGCAGCAGTGTCATTC
ACAATGTGTATGTTCGGCCGGCTCGGATGGAAGATGCTGCCTCTGTATATTTGTGCACAA
CTATTGGGGTCATTTCTGGCAGCAGGGACCATATATGGCGTCTATTACGATGCCATACAT
GAATATTGTGGAGGAAACCTGACTGTAACCGGGGAACTGGCCACAGCCGGTATCTTTGCC
ACTTACCCCGCACCATACCTCTCCTTGATGGGGGGATTCATTGATCAGGTGTTGGGCACA
GCCTTGCTTCTGCTTTGCTTGATGGCGTTGTCCGACCAGAGGAACAAACCGGCAGCAGCG
GGCACGGAGCCCATCGCAGTTGGTCTGCTGGTGCTGCTCATCGGCATCTCTATCGGAAGA
AACAGCGGATATGCTATCAACCCCACCAGAGACATCGCACCCAGGCTCTTCACTGCCGTA
GCAGGCTGGGGGATGGACGTGTTCAGGGCTGGAAATGGTTGGTGGTGGGTGCCACTGGTT
GCGCCCCCGATTGGAGGAGTACTGGGTGCAGGACTGTACAAGGTGTTGGTGGAAATGCAG
CACCTCCACGTCTCTGAGCCGGGTGATGGGCTG---ATAGAGGAGTTTGATGAGGAGGCT
GCCTCCCTGAAGAAACAGAAGATAATC---TGTGCCAATGTA------TGTGTC------

>Channel_bull_blenny_Aqp7
ATGAAGGACGTGGGGCAGTTAGTAGAACTAGGGGGC---TCTCAGAGGAAAAAA------
------CGACCCAAAGTTTGGTTG---AGGAATGAATCTGTTCGTGTGGGACTTGCTGAA
ACCCTTTGCACATATGTCATGATGGTGTTTGGCCTGGGGTCTGTAGCCCAGGTAGTGACA
GGGCAGGGAGAGTTCGGACAGTACCTCAGCATCAACCTGGGCTTTGGACTGGGTGTTGCT
ATGGGGGTTCATGTTGGAGGGAAGGTCTCAGGGGCTCATATGAATGCAGCAGTGTCATTC
ACAATGTGCACGTTTGGACGCCTGGGGTGGAAGATGCTGCCTCTATATATTTTTGCGCAA
CTATTGGGGTCATTTCTGGCAGCAGGGACAATTTATGCTGTCTATTATGAAGCCATATAT
GACTATTGTGGAGGAAACCTGACTGTAACAGGGGCAACGGCCACAGCTGGTATCTTTGCC
ACCTATCCTGCACCGTACCTCTCCTTGATGGCTGGATTCATTGATCAGGTATTTGGCACA
GCCATGCTGCTGCTGTGCTTGATGGCTCTGTCCGACCAGAAGAACAAACCGGCGGCAGCG
GGCAGTGAGCCTGTCGCAGTGGGTCTCCTGGTGCTGCTCATTGGCATTTCTCTTGGCAGC
AACAGTGGCTATGCTATCAACCCCACTAGAGACATTGCACCCAGGATCTTCACTGCCATA
GCAGGCTGGGGGACTGACGTGTTCAGGGCTGGAAATGGGTGGTGGTGGGTGCCACTAGTT
GCGCCCTTGATTGGAGGAGTATTGGGTGCAGGGCTCTACAGGGCCTTGGTGGAGATGCAC
CACCCTCACCTCTCCGAACAGGGTGAGGGGCTG---ATGGGGGAGTTGGATGAGGAGACT
GCATCTATGGAGAAACAG---AACATC---TGTGGTCGTGTA------TGTGTC------

>European_perch_Aqp7
ATGAAGGACTTGGTGCAGTCAGTAGAACTAGGGGTC---TCTCAGCGAAAAAGACTCAAC
GTATCTCAACCCAAAGTTTGGCTA---AAGAATGAACTTGTTCGTGTGGGACTTGCTGAA
TCCCTTTGCACATATGTCATGATGGTGCTTGGCCTAGGGTCTGTGGCCCAGGTAGTGACA
GGACAGGGAGTGTTCGGCGAGTACCTCAGCATCAACCTGGGTTTTGGCCTGGGTGTTGCT
ATGGGGGTTCATGTTGGAGGGAAGGTCTCAGGGGCTCACATGAATGCAGCAGTGTCATTC
ACAATGTGCACGTTTGGCCGCCTGGGGTGGAAGATGCTGCCTCTCTATGTTTTTGCGCAG
CTATTGGGGTCATTTCTGGCGGCAGGGACAATTTATGTTGTCTATTATGAAGCCATATAT
GACTATTGTGGAGGAAACCTGACTGTAACAGGTGTAAAGGCCACAGCTGGTATTTTTGCC
ACCTATCCTGCGCCGTACCTCTCCTTGCTGGCTGGATTCATTGATCAGGTGTTTGGCACA
GCAATGCTGCTGCTGTGCTTGATGGCTCTGTCTGACCAGAAGAACAAACCGGCTGCAGTG
GGCAGCGAGCCTGTCGCAGTGGGTCTCCTGGTGCTGCTCATTGGCATTTCTCTGGGAAGC
AACAGCGGCTATGCTATCAACCCCACCAGAGACATCGCACCCAGAGTCTTTACTGCCATA
GCAGGCTGGGGTGCTGACGTGTTCAGGTCTGGAAATGGGTGGTGGTGGGTGCCTCTAGTT
GCCCCCCCCATTGGAGGAGTATTGGGTGCGGGGCTGTACAGGGTCTTGGTGGAAATGCAC
CACCCTCCCCTCTCTGAACAGGATGGGGGGCTG---GTG---------GAGGAGGAGACT
GCCTCTCTGGAGAAACAGGGAAACATC---TGTGCTAATATA------TGTGTC------

>BmGrouper_Aqp7
ATGAAGGACTTGGTGCAGTCGGTAGAACTAGGGGTC---TCTCAGCGGAAAAGAGTCAAC
GTAACACAACCCAGAGTTTGGCTG---AAGAATGAAATTGTTCGGGTGGGACTCGCTGAA

TGCCTTTGCACATATGTCATGATGGCGTTTGGCCTGGGGTCGGTGGCTCAGGTTGTGACA
GGTCAAGGAGCGTTTGGACAATACCTCAGCATCAACTTGGGTTTTGGACTGGGTGTTGCC
ATGGGGGTCCATGTTGGAGGGAAGGTCTCAGGGGCTCATATGAATGCAGCAGTTTCATTC
ACAATGTGCACATTTGGCCGTCTTGGGTGGAAGATGCTGCCTCTGTATGTTTTTGCACAG
TTTTTGGGGTCGTTTCTGGCTGCAGCAACAATTTACGCTGTCTATTACGAAGCCATATTT
GACTATTGTGGAGGAAACCTGACTGTAACAGGAGTTAAAGCCACAGCTGGTATTTTTGCC
ACCTATCCTGCACCGTACCTCTCCTTGATGGCGGGATTCGTTGATCAGGTGTTTGGCACA
GCTATGCTGCTGCTGTGCTTGATGGCTCTGTCCGACCAGAAGAACAAACCAGCCGCGGCG
GGAAGCGAGCCTGTCGTAGTGGGTCTCCTGGTGCTGCTCATTGGCATTTCTCTGGGCAGC
AACAGCGGCTATGCTATCAACCCCACCAGAGACATCGGACCCAGGATCTTCACTGCTATA
GCAGGCTGGGGGGCTGATGTGTTCAGGGCTGGAAACGGGTGGTGGTGGGTGCCTATAGTT
GCCCCCCTTATTGGAGGAGTACTGGGTGCAGGGGTCTACAAGGCCTTGGTGGAATTGCAC
CACCCTCCCCTCTCTGAGCAAGGTGAGCGGGTG---GTGAAGGACTTTGAAGAGGAGACT
ACCCCTCTGGAGAAACAGGAAAACATC---TTTGCTAATGAA------TGTGTC------

>Marbled_flounder_Aqp7
ATGAAGGACTTG------TCAGTAGAGGTCGGGGTC---TCTCAGCGGAAAGGAGATAAA
CTTACTCGACCCAAAGTTTGGCTA---AAGAACCAACTTGTTCGAGTGGGACTTGCTGAA
TGTCTTTGCACGTATGTCATGATGGCGTTCGGCCTGGGGTCTGTGGCCCAGGTAGTGACA
GGACAGGGGGCGTTCGGAGAGTACCTCAGCATCAACCTGGGTTTTGCACTGGGTGTCACT
ATGGGGGCGCATGTCGGAGGGAAAGTCTCAGGGGCTCATATGAACGCTGCGGTGTCGATC
ACAATGTGCACGTATGGTCGCCTTGCATGGAGGATGTTGCCTGTGTATGTTTTTGCCCAG
CTGTTGGGATCATTTCTGGCAGCAGGGACAATTTATGCCGTCTATTATGATGCTATATTT
GAGTATAGTGGAGGAAACCTGACAGTAACTGGTGAAAGGGCCACTGCTGGTATCTTTGCC
ACCTATCCAGCACCATACCTCTCTGTGATGTCTGGATTCATTGACCAGGTGTTTGGCACG
GCCATGCTGCTGCTGTGCCTGATGGCTCTGTCCGACCAGAGGAACAAGCCGGCCCCAGCA
GGGGGCGAGCCTGCCGCGGTGGGACTCCTGGTGCTGCTCATCGGCATGTCTCTGGGCAGC
AACAGCGGCTACGCCATCAACCCCAACAGAGACATCGCACCCAGGGTCTTCAGTGCCATA
GCAGGCTGGGGAACCGATGTGTTCAGGGCTGGAAATGGGTGGTGGTGGGTGCCGCTAGTT
GCTCCCCCCATTGGCGGGGTGCTGGGTGCAGGGATCTACAAGGCTTTGGTGGAATTGCAC
CACCCACACGTGGATGAACTGGGTGAGGGGTTG---CTGGAGGAGTTAAAGGAGGACTCT
GTT------------CATAAATTCAAA---ATTGAAAATGCA------GTTGTG------

>Starry_flounder_Aqp7
ATGAAGGACTTG------TCAGGAGAAGTCGGGGTC---TCTCAGCGGAAAGGAGATCAA
CTCACTCGACCCAAAGTTTGGCTA---AAGAACAAACTTGTTCGAGTGGGACTTGCTGAA
TGTCTTTGCACGTATGTCATGATGGCGTTCGGCCTGGGGTCTGTGGCCCAGGTAGTGACA
GGACAGGGAGCGTTCGGAGAGTACCTCAGCATCAACCTGGGTTTTGCACTGGGTGTCACT
ATGGGGGCGCATGTCGGAGGGAAAGTCTCAGGGGCTCACATGAACGCTGCGGTGTCGATC
ACAATGTGCACGTATGGTCGCCTTGCATGGAGGATGTTGCCTGTGTATGTTTTTGCACAG
CTGCTGGGATCATTTCTGGCAGCAGTGACAATTTATGGCGTCTATTATGATGCTATATTT
GAGTATTGTGGAGGAAACCTGACAGTGACTGGTGAAAGGGCCACCGCTGGGATCTTTGCC
ACCTATCCAGCACCATACCTCTCTGTGATGTCTGGATTCATGGACCAGGTGTTTGGCACG
GCCATGCTGCTGCTGTGCCTGATGGCTCTGTCCGACCAGAGGAACAAGCCGGCCCCAGCA
GGTGGCGAGCCGGCCGCGGTGGGACTCCTGGTGCTGCTCATCGGCATTTCTCTGGGCAGC
AACAGCGGCTACGCCATCAACCCCAACAGAGACATCGCACCCAGGGTCTTCAGTGCCATA
GCAGGCTGGGGAACTGATGTGTTCAGGGCTGGAAATGGGTGGTGGTGGGTGCCGCTAGTC
GCTCCCCCCATTGGAGGGGTGCTGGGTGCAGGGATCTACAAGGCTTTGGTGGAATTGCAC
CACCCACACGTGGAGGAACTGGGTGAGGGGTTG---CTGGAGGAGTCAAAGGAGGACTCT
GTT------------CATAAATTCAAA---ATTGAAAATGCA------ATTGTG------

>Atlantic_halibut_Aqp7
ATGAAGAACTTG------TCAGTAGAAGTCGGGGTC---TCTCAGCGGAAAGGAGATAAA
GTAACTCGACCCAAAGTC---------------AAATTTGTTCGAGTGGGACTTGCTGAA
TGTCTTTGCACGTATGTCATGATGGCGTTCGGCCTGGGGTCTGTGGCCCAGGTAGTGACA
GGACAGGGAGCGTTCGGAGAGTACCTCAGCATCAACCTGGGTTTTGCACTGGGTGTCACT
ATGGGGGCGCATGTCGGAGGGAAAGTCTCAGGGGCACATATGAACGCTGCGGTGTCGATC
ACAATGTGCACGTATGGTCGCCTTGCATGGAGGATGTTGCCTGTGTATGTTTTTGCACAG
CTGTTGGGATCATTTCTGGCAGCAGGGACAATTTATGCTGTCTATTATGATGCTATACTT
GAGTATTCTGGAGGAAACCTGACAGTGACTGGGGAAAGGGCCACCGCTGGTATCTTTGCC
ACATATCCAGCACCATACCTCTCTGTGATGTCTGGATTCATTGACCAGGTGTTTGGCACA

GCCATGCTGCTGCTGTGCCTGATGGCTCTGTCCGACCACAGGAACAAGCCGGCCCCAGCA
GGTGGCGAGCCAGCCGCGGTGGGACTCCTGGTGCTGCTCATCGGCATTTCTTTGGGCAGC
AACAGCGGCTACGCCATCAACCCCAACAGAGACATCGCACCCAGGGTCTTCAGTGCCATA
GCAGGCTGGGGAACTGATGTGTTCAGGGCTGGAAATGGGTGGTGGGTGCCGCTAGTT
GCTCCCCCCATTGGAGGGGTGCTGGGTGCAGGGATCTACAAGGCCTTGGTGGAATTGCAC
CACCCACACGTGGATGAACTGGGTGAGGGGTTG---CTGGAGGAGTTAAAG---GACACT
GTT------------CATAAAAACAAT---AGTGAAAACGCA------ATTGTG------

>Japanese_flounder_Aqp7
ATGAAGGACTTG------TCAGTAGAAGTCGGGGTC---TCTCAGCGGAAAGGAGCTAAA
GTAACTCGACCCAGAGTTTGGCTG---AAGAACAACCTTGTTCGAGTTGGACTTGCTGAA
TGTCTTTGCACGTTTGTCATGATGGCGTTTGGCCTGGGGTCTGTGGCCCAGGTAGTGACA
GGACAGGGAGCGTTCGGACAGTACCTCAGCATCAACCTGGGTTTTGCACTTGGTGTCACT
ATGGGGGCACATGTTGGCGGTAAAGTCTCAGGGGCACATATGAACGCTGCGGTATCGATC
ACAATGTGCACGTATGGTCGCCTTGCATGGAAGATGTTGCCTGTGTATGTCTTTGCGCAG
CTGTTGGGATCATTTCTGGCAGCAGGGACAATTTATGCTGTCTATTATGATGCTATCCAT
GAGTACTGTGGAGGAAACCTGACAGTAACTGGTGACAGGGCCACTGCTGGTATCTTTGCC
ACATATCCAGCACCATACCTCTCTGTGATGTCTGGATTCATTGACCAGGTGTTTGGCACA
GCCATGCTGCTGCTGTGCCTGATGGCTCTGTCCGACCAGAGGAACAAGCCGGCCGCAGCA
GGCAGCGAACCTGCCGTGGTGGGTCTCCTGGTGCTGCTCATCGGCATCTCTCTGGGCAGC
AACAGCGGCTACGCCATCAACCCCAACAGAGATATCGCACCCAGGGTCTTCACTGCCATA
GCAGGCTGGGGAACTGATGTGTTCAGGGCTGGAAATGGATGGTGGTGGGTGCCCCTAGTT
GCTCCCCCCATTGGAGGTGTGCTGGGTGCAGGGATCTACAGGACCTTGGTGGAATTACAC
CACCCACACGAGGCTGAACAGGGTGAGGGGTTG---CTGGAGGAGTTGAATGAGGACACT
GTT------------CATAAAAACAAT---TGTGAAAATGCA------ATTGTG------

>Turbot_Aqp7
ATGAAGGACTTGGTGAATTCAATAGAACTGGGGGTC---TCTCAGAGGAAAGGGGAGAGA
GTCACTCGACCCAAGGTTTGGCTA---AAGAACGAATTTGTTCGAGTGGGACTTGCTGAA
TATCTTTGCACATATGTCATGATGGTGTTTGGCCTGGGCTCTGTGGCCCAAGTAGTGACA
GGACAGGGAGAATTCGGACAGTACATCAGCATCAACCTGGGCTTCGCGCTGGGTGTCACC
ATGGGGGTGCACGTTGGGGGGAAAGTCTCAGGGGCGCACATGAATGCCGCGGTATCAATC
ACAATGTGCACATTTGGTCGCCTCGCGTGGAAGATGTTGCCTGTGTACATTTTCGCGCAG
CTGTTGGGATCGTTTCTGGCGGCGGGGACAATTTATGCCGTCTACTACGAGGCCATACAT
GATTATTGTGGCGGAAACCTGACTGTGACTGGTGACAGAGCCACTGCTGGTATCTTCGCC
ACCTATCCGGCACCATACCTCTCCTTGCCAGCTGGATTCTTCGACCAGGTGTTTGGCACA
GCCATGCTGCTGATGTGCCTGATGGCTCTGTCCGACCAGAAGAACAAGCCGGCCGCAGCA
GGCAGCGAGCCTGTCGCCGTGGGTCTCCTGGTGCTGCTCATTGGAATTTCTCTGGGCAGC
AACAGCGGCTACGCTATCAACCCCAACAGAGACATTGCACCCAGGGTCTTCACTGCCATA
GCAGGCTGGGGAGCTGATGTGTTCAGGTCTGGAAATGGTTGGTGGTGGGTGCCTCTAGTT
GCTCCGCCCATTGGAGGCATATTGGGTGCGGGGATGTACAGGGCTTTGGTGGAATTGCAC
CACCCACCTGTCTATGAACAGGGTGAGGGGTTG---TTGGAGGAGTTGAAGGAG---TTG
AAGGAGGAGACGGATCATAAAAACAAC---TGTGAGAATGCA------TGTGTG------

>Dover_sole_Aqp7
ATGAAGGAGTTG---------GGGGAAATGGCGGTC---TCCCTGAGCAGAGGAGACAAA
GTAACACGACCCAAAGTTTGGCTT---AAGAATGAATTTGTTCGAGTGGGACTCGCTGAA
TTCCTTTGCACATATGTCATGATGACGTTTGGCCTGGGGTCTGTAGCCCAGGTAGTGACG
GGACAGGGAGCATTTGGACAGTACCTGAGCATCAACATGGGTTTTGCACTGGGTGTCACT
ATGGGGGTGCACGTTGGAGGGAAGGTCTCAGGGGCACACATGAACGCTGCAGTGACAATC
ACGATGTGCACGTTTGGTCGCCTGGCCTGGAGGATGTTGCCTGTG---TTGTTTGCACAG
TTGTTGGGATCTTTTCTGGCAGCAGGGACTATTTATGCGGTCTACTATGAGGCCATACAT
GATTATTGTGGAGGAAACCTGACTGTAGTTGGTGATAGGGCCACTGCTGGTATCTTTGCC
ACCTATCCTGCACCATACCTCTCGCTGCAGGCTGGATTCATTGACCAGGTGTTTGGTACA
GGTATGCTGCTGCTGTGCCTCATGGCTCTGTCTGACCAGAAGAACAAACCAGCCGCGGCA
GGCAGCGAACCCGTCGCTGTCGGCCTCCTTGTGCTGCTCATTGGCATTTCTCTGGGCAGC
AATAGCGGCTACGCCATTAACCCCAACAGAGACATTGTACCGAGGGTCCTCACCGCCATA
GCAGGCTGGGGAGCTGATGTGTTCAGGGCTGGACATGGTTGGTGGTGGGTTCCTCTGGTT
GCTCCCCCCATTGGAGGTGTACTGGGTGCAGGGCTCTACAAGGCTTTAGTGGAATTGCAT
CACCCATTTCTCTCTGAACAGGGTGAGGGTTTA---CTGGATGAGTCAAAAGAGGACATT
GTCCCCCTAGAAAATCATAAAAACAAC---TGTGCAGATGCA------TGTGTG------

>Tongue_sole_Aqp7
ATGAAGGACTTG---------GCAGAAGCGGGGGTC---TCTCTGCGCAAAGGGGGCAAA
GTTACTCGCCCCAAAGTTTGGATA---AAGAGTGAGCTTGTTCGTGTGGGACTTGCTGAA
TGCCTTTGCACATATGTCATGATGGCGTTTGGCCTGGGGTCTGTAGCCCAGGTGGTGACA
GGGCAGGGAGCCTTTGGACAGTACCTCAGCATCAACCTGGGTTTTGCACTGGGAGTTGCT
ATGGGCATCCATGTTGGAGGAAACGTTTCAGGGGCACACATGAACGCTGCAGTATCATTC
ACCATGTGCACTTTTGGTCGCCTTGCATGGAAGATGTTACCTGTATATTTATTTGCACAG
CTTGTGGGTTCCTTCGTGGCAGCTGGGACCATTTATGCTGTTTACTACGAAGCCATACAT
GATTACTGTGGAGGAAACCTGACAGTAACTGGCGACAGAGCCACTGCTGGTATCTTTGCC
ACCTATCCTGCACCATATCTGTCCCTAATGGCTGGATTCATTGACCAGGTTTTTGGCACT
GCCATGCTGCTGTTGGGCCTGATGGCTCTGTCAGACCAGAGGAACAAACCGGCCGCTGCA
GGCAGTGAGCCTGCTGCTGTGGGACTCCTGGTGCTACTTATTGGTATTTCCTTGGGCAGC
AACAGCGGCTATGCAATAAACCCCAACAGAGACATTGGACCGAGGGTCTTTACTGCAATA
GCAGGCTGGGGAGCTGATGTGTTTAGGGCTGGACGTGGTTGGTGGTGGGTACCCCTGATT
GCTCCCCCTATTGGAGGAGTATTGGGTGCAGGGCTCTACAAGGCCTTAGTGGAATTGCAC
CATCCACCACTGCCTCCTCTTGGTGAGGGCTTA---TTT---------GAGGAGGACGTT
GTCCAAGAGGAAAATCCTAAAAACAAC---TTTGCAGACGCG------TGTGTG------

>Barramundi_Aqp7
ATGAAGGACTTGGTACAGTCTGTAGAACTGGGGGTC---ACCCAGCGGAAAGGAGTTAAA
GTAACTCGACCCAAAGTCTGGATA---AAGAATGAACTTGTTCGTGTGGGACTCGCTGAA
TGCCTTTGCACATATGTCATGATGGCGTTTGGCCTGGGGTCTGTGGCCCAGGTAGTGACA
GGACAGGGGGCATTTGGACAGTACCTCAGCATCAACCTGGGTTTTGGACTGGGTGTTGCT
ATGGGGGTGCATGTTGGAGGGAAGGTCTCAGGGGCTCACATGAATGCTGCAGTGTCATTC
ACAATGTGCACGTTTGGTCGCCTTGCGTGGAAGATGTTGCCTCTGTATGTTTTTGCGCAG
CTGTTGGGATCCTTTCTGGCAGCTGGGACAATTTATGCTGTCTATTACGAAGCCATACAT
GCTTACTGTGGGGGAAACCTGACTGTAACTGGGATAAAGGCCACAGCTGGTATCTTTGCC
ACTTATCCGGCACCATACCTCTCCTTGTTGGCTGGATTCATTGACCAGGTGTTTGGCACG
GCTATGCTGCTGCTGTGCCTGATGGCTCTGTCCGACCAGAGGAACAAACCGGCCGCAGCA
GGCAGTGAGCCTGTCGCTGTGGGTCTCCTGGTGCTGCTCATTGGCATCTCCCTAGGCAGC
AACAGCGGCTACGCTATCAATCCCACCAGAGACATCGCACCCAGGGTCTTCACCGCCATA
GCAGGCTGGGGAGCTGATGTGTTCAGGGCTGGAAATGGGTGGTGGTGGGTGCCTCTGGTT
GCTCCTCCCATTGGAGGTGTACTGGGTGCAGGGATTTACAAGGCCTTGGTGGAACTGCAC
CACCCACGCCTCTCTGAACAGGGTGGGGGGCTG---GTGGAGAAGCTGGAGGAGGAGAAT
GCTCCTCTCGAGAAACAGAAAAACAGC---TGTGAAAACGTA------TGTGTG------

>Greater_amberjack_Aqp7
ATGAAGGACTTGGTGCAGTCAGTAGAACTAGGAGTC---TCTCAGCGGAAAGGCATTAAA
GCAACTCGACCCAAAGTTTGGCTA---AAAAATGAACTTGTTCGTGTTGGACTTGCTGAA
TCCCTTTGCACATATGTCATGATGGTGTTTGGCCTGGGGTCTGTGGCCCAGGTAGTGACA
GGACAGGGAGCATTCGGACAGTACCTCAGCATAAACCTGGGTTTTGGACTGGGTGTCGCT
ATGGGGATACATGTTGGAGGGAAAGTTTCAGGGGCTCATATGAATGCTGCAGTGTCATTC
ACAATGTGCACATTTGGTCGCCTTACGTGGAGGATGTTGCCTGTGTATGTTTTTGCACAG
CTATTGGGATCATTTCTGGCAGCAGGGACAGTTTATGCTGTCTATTATGAGGCCATACAT
GATTATTGTGGAGGAAACCTGACTGTAACTGGTGTAAAGGCCACAGCTGGTATTTTTGCC
ACCTATCCTGCACCGTACCTCTCCTTGCTGGCTGGATTCATCGATCAGGTGTTTGGCACA
GCTATGCTGCTGCTGTGCCTGATGGCTCTGTCCGACCAGAAGAACAAACCGGCTGCAGCA
GGCAGCGAGCCTGTAGCGGTGGGTCTCCTGGTTCTGCTCATTGGCATTTCTCTGGGTAGC
AACAGCGGCTACGCTATCAACCCCACGAGAGACATCGCACCCAGGGTCTTCACTGCTGTA
GCAGGCTGGGGAGCTGATGTGTTCAGGGCTGGAAATGGGTGGTGGTGGGTGCCTCTCGTT
GCTCCCCCCATTGGAGGTGTATTGGGTGCGGGAGTCTACAAGGCCCTGGTGGAATTGCAC
CACCCACCCCTCTCTGAACAGGATGGGGGGTTG---GTGGAGGAGTTGGAGGAGGAGACT
GCTCCTCTGGAGAAACCGAAAAACATC---TGTGCAAATGTA------TGTGTG------

>Japanese_amberjack_Aqp7
ATGAAGGACTTGGTGCAGTCAGTAGAACTAGGAGTC---TCTCAGCGGAAAGGCATTAAA
GCAACTCGACCCAAAGTTTGGCTA---AAAAATGAACTTGTTCGTGTTGGACTTGCTGAA
TCCCTTTGCACATATGTCATGATGGTGTTTGGCCTGGGGTCTGTGGCCCAGGTAGTGACA
GGGCAGGGAGCATTCGGACAGTACCTCAGCATAAACCTGGGTTTTGGACTGGGTGTCGCT
ATGGGGATACATGTTGGAGGGAAAGTCTCAGGGGCTCATATGAATGCTGCAGTGTCATTC

ACAATGTGCACATTTGGTCGCCTTACGTGGAGGATGTTGCCTGTGTATGTTTTTGCGCAG
CTATTGGGATCATTTCTGGCAGCAGGGACAGTTTATGCTGTCTATTATGAGGCCATACAT
GATTATTGTGGAGGAAACCTGACTGTAACTGGTGTAAAGGCCACAGCTGGTATTTTTGCC
ACCTATCCTGCACCGTACCTCTCCTTGCTGGCTGGATTCATCGACCAGGTGTTTGGCACA
GCTATGCTGCTGCTGTGCCTGATGGCTCTGTCCGACCAGAAGAACAAACCGGCCGCAGCA
GGCAGCGAGCCTGTAGCGGTGGGTCTCCTGGTTCTGCTCATTGGCATTTCTCTGGGCAGC
AACAGCGGCTATGCTATCAACCCCACCAGAGACATCGCACCCAGGGTCTTCACTGCCATA
GCAGGCTGGGGAGCTGATGTGTTCAGGTCTGGAAATGGGTGGTGGTGGGTGCCTCTAGTT
GCTCCCCCCATTGGAGGTGTATTGGGTGCGGGAGTCTACAAGGCCCTGGTGGAAATGCAC
CACCCACCCCTCTCTGAACAGGGTGGGGGGTTG---GTGGAGGAGTTGGAGGAGGAGACT
GCTCCTCTGGAGAAACCGAAAAACATC---TGTGCAAATGTA------TGTGTG------

>Derbio_Aqp7
ATGAAGGACTTGGTGCCATCAGTAGAACGAGGAGTC---TCTCAGCGGAAAGGAGTTAAA
GTAACTCAACCCAAAGTTTGGCTA---AAAAATGAACTTGTTCGTGTTGGACTTGCTGAA
TCCCTTTGCACATATGTCATGATGGTGTTTGGCCTGGGCTCTGTGGCCCAGGTAGTGACA
GGACAGGGAGCATTTGGGCAGTACCTCAGCATCAACCTGGGTTTTGGACTGGGTGTCGCT
ATGGGGGTACACGTTGGAGGGAAAGTCTCAGGGGCACACATGAATGCTGCAGTGTCATTC
ACAATGTGCACGTTTGGTCGCCTTGCGTGGAGGATGTTGCCTGTGTATATTTTTGCACAG
CTATTGGGATCATTTCTGGCAGCAGGGACAGTTTATGCTGTCTATTATGAAGCCATTCAT
GATTATTGTGGAGGAAACCTGACTGTTACTGGTGTAAAGGCCACAGCTGGTATTTTTGCC
ACCTATCCCGCACCGTACCTCTCCTTGCTGGCTGGATTCATCGACCAGGTGTTTGGCACA
GCTATGCTGCTGCTGTGCCTGATGGCCCTGTCTGACCAGAAGAACAAACCGGCTGCAGCA
GGCAGCGAGCCTGTTGCGGTGGGTCTCCTGGTGCTGCTCATTGGCATTTCTCTGGGCAGC
AACAGCGGCTATGCTATCAACCCCACCAGAGACATTGCACCCAGGGTCTTCACTGCCATA
GCAGGGTGGGGAGCAGATGTGTTCAGGTCTGGAAATGGGTGGTGGTGGGTGCCTCTAGTT
GCTCCCCCCATTGGAGGTGTATTGGGAGCAGGGGTGTACAAGGCCTTGGTGGAATTGCAT
CACCCACCCCTCTCTGAACAGGGTGACGGGTTG---GTGGAGGAGTTGGAGGAGGAGACT
GCTCCTCTGGAGAAACAGAAAAACATC---TGTGCAAATGTA------TGTGTG------

>Snubnose_pompano_Aqp7
ATGAAGGACTTGGTGCCATCAGTAGAACGAGGAGTC---TCTCAGCGGAAAGGAGTTAAA
GTAACTCAACCCAAAGTTTGGCTA---AAAAATGAACTTGTTCGTGTTGGACTTGCTGAA
TCCCTTTGCACATATGTCATGATGGTGTTTGGCCTGGGCTCTGTGGCCCAGGTAGTGACA
GGACAGGGAGCATTTGGGCAGTACCTCAGCATCAACCTGGGTTTTGGACTGGGTGTCGCT
ATGGGGGTACACGTTGGAGGGAAAGTCTCAGGGGCACACATGAATGCTGCAGTGTCATTC
ACAATGTGCACGTTTGGTCGCCTTGCGTGGAGGATGTTGCCTGTGTATATTTTTGCACAG
CTATTGGGATCATTTCTGGCAGCAGGGACAGTTTATGCTGTCTATTATGAAGCCATTCAT
GATTATTGTGGAGGAAACCTGACTGTTACTGGTGTAAAGGCCACAGCTGGTATTTTTGCC
ACCTATCCCGCACCGTACCTCTCCTTGCTGGCTGGATTCATCGACCAGGTGTTTGGCACA
GCTATGCTGCTGCTGTGCCTGATGGCCCTGTCTGACCAGAAGAACAAACCGGCTGCAGCA
GGCAGCGAGCCTGTTGCGGTGGGTCTCCTGGTGCTGCTCATTGGCATTTCTCTGGGCAGC
AACAGCGGCTATGCTATCAACCCCACCAGAGACATTGCACCCAGGGTCTTCACTGCCATA
GCAGGGTGGGGAGCAGATGTGTTCAGGTCTGGAAATGGGTGGTGGTGGGTGCCTCTAGTT
GCTCCCCCCATTGGAGGTGTATTGGGAGCAGGGGTGTACAAGGCCTTGGTGGAATTGCAT
CACCCACCCCTCTCTGAACAGGGTGACGGGTTG---GTGGAGGAGTTGGAGGAGGAGACT
GCTCCTCTGGAGAAACAGAAAAACATC---TGTGCAAATGTA------TGTGTG------

>Climbing_perch_Aqp7
ATGAGGGACTATGTGCAGTCTGTGGAACTTGGGGTC---TCTCAGCAAAAAGGAGTCAAC
GTAACTCAACCCAAAGTTTGGCTA---AAGAATGAATTTGTGCGTGTCGGACTTGCTGAA
TCCCTTTGCACATATGTCATGATGGTGTTTGGCTTGGGGTCCGTGGCCCAGGTAGTGACA
GGTGAGGGAGCATTTGGACAGTACCTCAGCATAAATCTGGGTTTTGCGCTGGGTGTTGCT
ATGGGGGTGCATGTCGGAGGGAAAGTCTCAGGGGCTCACATGAATGCAGCTGTGTCGTTC
ACAATGTGTACATACGGTCGCCTTGCATGGAAGATGTTGCCCGTTTATGTTTTTGCGCAG
CTATTGGGATCATTTTTGGCAGCAGGGACGATTTATGCCGTCTACTATGAAGCCATCCAT
GATTACTGTGGCGGAAACCTGACTGTGACTGGTGTTAAGGCCACAGCCGGTATCTTTGCC
ACATATCCTGCACCATACCTCTCTTTGTTGGCTGGGTTCTTTGACCAGGTATGTGGCACT
GCAATGCTTCTGCTGTGTCTGATGGCTCTGTCCGACCAAAGGAACAAACCGGCTGCAGCT
GGCAGCGAGCCTCTTGCAGTGGGTCTCCTGGTGCTGCTAATTGGCATTTCTCTTGGTAGC
AACAGTGGCTATGCTATCAACCCCACCAGAGACATCGCACCCAGGGTCTTCACTGCCATA

GCAGGCTGGGGAGCTGATGTGTTCAGGTCTGGAAATGGGTGGTGGTGGGTGCCTCTAGTT
GCTCCCCCGATTGGAGGAGTGTTAGGTGCTGGGATATACAAGGTCTTTGTGGAAATGCAC
CATCAACCTGTCTCTGGACAGGGTGGACAACTG---GTGGAGGAGTTACAAGAGGAGAAT
ACGCCTCTGGAGAAACAAAAATACATC---TCATCTGGA---------------------

>Siamese_fightingfish_Aqp7
ATGAAGGACTTG---------GCAGAACTTGGAGCG---TCTCAGCAGAAAGGAGTTAAA
GTAGCGCGGCCCAAAGTTTGGCTG---AAGAGTGAAGCGGTTCGCGTGGGACTTGCTGAA
TCCCTCTGCACATATGTCATGATGGTGTTTGGTCTGGGGTCCGTGGCGCAGGTAGAGACA
GGACAAGGGGCCTTCGGAGAGTACCTCAGCATAAACCTGGGCTTTGGCCTGGGCGTCGCT
ATGGGGGTGCACGTCGGGGGGAAAGTCTCAGGGGCTCACATGAACGCAGCCGTGTCCCTC
GCCATGTGCACATACGGGCGTCTGGCGTGGAGGATGCTGCCTGTCTACGTCTGTGCCCAG
CTGATGGGATCCTTTCTGGCGGCAGGGACAATTTATGCCGTCTATTACGAAGCCATCCAT
GATTACTGTGGAGGAAACCTGACGGTGAGCGGGGAAAAGGCCACCGCCGGCATCTTTGCC
ACCTACCCTGCACCGTATCTCTCTCTGCTGGCTGGATTCTTTGACCAGGTGGTGGGCACT
GCAATGCTTCTGCTGTGCCTGATGGCTCTGTCCGACCAGAGGAACAAACCGGCCGCCGTG
GGCAGCGAGCCGGTTGCAGTAGGTCTGCTGGTGCTGCTCATTGGCATTTCTCTGGGAAGC
AACAGTGGCTACGCTATCAACCCCACCAGAGACATCGCACCCAGGGTCTTCACTGCCGTA
GCTGGCTGGGGGGCGGACGTGTTCAGGTTTGGAAACGGGTGGTGGTGGGTGCCTCTCGTT
GCGCCCCCCATTGGAGGGGTGTTAGGCGCTGGGCTATACAAGGCCTTTGTGGAAATGCAC
CATCAACCCATCTCCGGACAGCTTGAGAGG------------GGGTTACAAGAGGAGAGC
GCCCCTCTGGAGAAACAACAGCACACC---TCTGTGAATGTA------TGTGTG------

>Kissing_gourami_Aqp7

---------------------CTA---AAGAATAAAGTTGTTCGTGTGGGACTTGCTGAA
ACTCTTTGCACATATGTCATGATGGTTTTTGGCCTGGGGTCTGTGGCCCAGGTAGTGACA
GGACAGGGAGCATTTGGACAGTACCTCAGCATAAACCTGGGTTTTGCACTGGGTGTTGCT
ATGGGGGTGCACGTCGGAGGTAAAGTCTCAGGGGCTCACATGAACGCAGCCGTGTCATTC
ACAATGTGCACATTTGGTCGTCTTGCATGGAAGATGCTGCCTGTGTATGTTTTTGCGCAG
CTAATGGGATCATTTCTGGCTGCAGGGACTATTTATACTGTCTATTACGAAGCCATCTAT
GATTACTGTGGAGGAAACCTGACTGTGACTGGTGTAAAGGCCACAGCTGGTATCTTTGCC
ACTTATCCTGCACCGTACCTCTCCTTGTTGGCTGGATTTGTTGACCAGGTGATTGGCACT
GCAATGCTCCTGCTGTGCTTGATGGCTCTTTCTGACCAGAGGAACAAACCGGCTGCAGAG
GGCAGCGAGCCTCTTGCAGTGGGTCTCCTGGTGCTGCTCATCGGCATTTCTTTGGGCAGC
AACAGCGGCTATGCTATCAACCCCACCAGAGACATCGCACCCAGGGTCTTCACTGCCATA
GCCGGCTGGGGACCTGATGTGTTCAGGTCTGGAAATGGGTGGTGGTGGGTGCCTGTAGTT
GCTCCCCCGATTGGAGGAGTGCTGGGAGCTGGACTATACAAGGCCTTTGTAGAAATGCAC
CACCAACCTGTTTCTGGACAAGGTGGAAAGCAG---GTGGAGGAATTACAAGAGGAGAGT
ACCCCTCTGGAGAAACAAAAAGCCTTC---TCTGCAAATGTA------TGTGTG------

>Northern_snakehead_Aqp7
ATGAAGGACATTGTGCAGTCTGTAGAACTTGGGGTC---TGTCAGCAAAAACAAGTGAAC
GTACCTCGACACAAAGTTTGGCTC---AAGAATGAACCCCTTCGTGTGGGACTTGCTGAA
ACCCTTTGCACATATGTCATGATGGTATTTGGTCTGGGATCTGTGGCCCAGGTAGTGACA
GGACAGGGAGCGTTTGGACAGTACCTCAGCATAAACCTGGGTTTTGCGCTGGGTGTTGCT
ATGGGGGTACATGTTGGAGGGAAAGTCTCAGGGGCTCACATGAATGCAGCAGTGTCATTT
ACAATGTGCACATTTGGTCGCCTTGCATGGAACATGTTCCCTGTGTATGTTTTTGCACAA
CTATTGGGATCGTTTCTGGCAGCAGGGACAATTTTTGCCGTCTATTATGAAGCCATCAAT
GATTATTGTGGAGGAAACCTAACTGTGACTGGTGTAAAGGCCACAGCTGGTATCTTTGCC
ACATATCCTGCACCATATCTCTCCTTGTTTGCTGGATTTATTGACCAGGTGTTTGGCACT
GCGATGCTTTTGCTGTGCCTTATGGCTCTATCTGACCAGAAGAACAAACCGGCTGCACCA
GGTAGTGAGCCTCTTGCAGTGGGTCTCTTGGTACTGCTTATTGGCATTTCTCTGGGCAGC
AACAGTGGCTATGCCATCAATCCCACCAGAGACATTGCACCCAGGGTCTTCACTGCCATA
GCAGGATGGGGAGCTGATGTATTCAGGTCTGGAAATGGGTGGTGGTGGGTTCCTCTGGTT
GCCCCCCCCATTGGAGGAGTGTTGGGTGCTGGGCTGTATAAGGTCTTTGTGGAAATGCAT
CACCAACCCCTGCCTGGGGAGGGTGGTGGGCTG---GCAGTGGAGTTGGAAGAGGACCGT
GCCCCTCTGGAGAAACAGAACAACAGC---TATGCAAATGTA------TGTGTG------

>Swamp_eel_Aqp7
ATG---GACTCTGTGCAGTCAGTGGAACTTGAGGTC---CCTCAGAAGAAAGGAGTTAAT

ATAACTAAACCCAGAGTTTGGCTA---AAGAACGAAGTTGTTCGTGTGGGACTTGCTGAA
TCTCTTTGCACATATGTCATGATGGTGTTTGGCCTGGGGTCTGTGGCCCAGGTAGTGACA
GGACAGGGAGCGTTTGGACAGTACCTCAGTATCAACCTGGGTTTTGGACTGGGTGTTGCT
ATGGGGGTACATGTTGGAGGGACAGTCTCAGGGGCTCACATGAATGCAGCAGTGTCATTC
ACAATGTGCACATTCGGCCGCCTTGCCTGGAAGATGTTGCCTGTGTATGTTTGTTCACAG
CTGTTGGGATCATTTTTGGCAGCAGGGACAATTTATGGAGTCTATTATGAAGCCATACAT
GATTATTGTGGAGGAAACCTGACAGTAACTGGTGTAAAGGCTACAGCCGGTATCTTTGCC
ACCTACCCTGCACCATACCTCTCCTTATTGGCTGGATTCATTGATCAGGTGATTGGTACT
GCGATGCTTCTGCTGTGCCTAATGGCTCTGTCTGACCAGAAGAACAAACCGGCTGCAATG
GGCAGCGAGCCCATCGCTGTGGGTCTCTTGGTGCTGCTTATTGGCATTTCTCTGGGGAGC
AACAGCAGCTATGCCATCAACCCCACCAGAGATATCGCACCCAGGGTCTTCACTGCCATC
GCAGGCTGGGGAGCTGATGTGTTCAGGTCTGGAAATGGTTGGTGGTGGGTGCCACTAGTT
GCTCCCCCCATTGGAGGAGTACTGGGTGGAGGGCTATATAAGGCCTTAGTGGAAATGCAC
CACCAAACCCTGCCTGAACAGGGTCAGGGCAAG---AACGAGAAATTAGAAGATGAGAGT
ATCCCTCTGGAGAAACTGAAAAACATC---TGTGCAGATGTA------TGTGTG------

>Zig_zag_eel_Aqp7
ATGAAGGACTTTGTGGAGTCAGTAGAACTTGGAGTG---TCTCAACGGGAAGGAGTTAAC
ATAACTCAGCCTAAAGTTTGGATA---AAGAATGAACTTGTTCGTGTGGGACTTGCGGAA
TCCCTTTGCACATATGTCATGATGGTTTTGGCCTAGGATCTGTAGCTCAGGTAGTGACA
GGAGAGGGAGCGTTCGGACAGTATCTCAGCATCAACTTGGGTTTTGCACTGGGTGTTGCT
ATGGGGGTACACATTGGAGGGAAAATATCAGGAGCTCACATGAATGCAGCAGTGTCATTC
ACAATGTGCACATTTGGTCGTCTTGCGTGGAAGATGTTCCCTGTGTATGTTTTCTCTCAG
TTACTGGGATCATTTCTGGCAGCAGGGACAATTTATGCCGTCTATTATGAAGCTATACAT
GATTATTGTGGAGGAAACCTGACTGTAACTGGTGTTAAAGCGACAGCTGGTATCTTTGCC
ACCTACCCTGCACCATACCTCTCCTTGTTGGGTGGATTCATTGACCAGGTGTTTGGCACA
GCAATGCTCCTGCTGTGCCTGATGGCTCTGTCTGACCAGAAGAACAAACCGGCCGCAGTG
GGCGGCGAGCCTGTTGCAGTGGGTCTCCTGGTGCTGCTCATCGGCATTTCTCTGGGCAGT
AACAGCGGCTATGCCATCAACCCTACCAGAGATATTGCACCTAGAGTCTTCACTGCCATT
GCAGGCTGGGGAGCTGATGTGTTCAGGTCTGGAAATGGTTGGTGGTGGGTGCCTCTAGTT
GCTCCCCCCATTGGGGGAGTATTGGGTGCGGGGCTTTACAAGGCCTTGGTGGAAATGCAC
CACCAACCCCTCTCTGAACAGGGTCGGGGGGTG---ATGCAGGAATCGGAAGAGGAAAGT
ACCCCTTTGGAAAAACAGAAGACCATC---TGTGCAGATGTA------TGTGTG------

>Amazon_molly_Aqp7
---------ATGGGGCAGTCAGTGGAACTGGAGGTC---TCCATGCGGGGCAAACTGGAA
GTAGGGCGACCCAGGATTTGGCTG------AATGAACTCTCACGAGTGGGACTTGCTGAG
TTTCTTTCAACGTATGTCATGATGACATTAGGTCTGGGTTCGGTGGCCCAGGTTGTGACT
GGTAAAGGAGGATTTGGGACCTTCTTCAGCATCAACATTGGCTTCGGCCTGGCTGTTGCA
ATGGGGGTTCATATTGGAGGAACTGTCTCCGGGGCTCATATGAATGGCGCAGTCTCCTTT
GCATTGTGTGTTTATGGCCAACTACCATGGAAGAGGCTCCCCTTGTACATTTTTGCTCAG
CTGTTTGGATCGTTTCTTGCAGCAGCAACAGTTTATGGTGTTTACTATGACGCCATTTCT
ACTTATGCTGGTGGAAACCTGAGTGTTACTGGAGAAAGAGCCACGGCTGGGATATTTGCC
ACTTATCCAGCACCATACCTCTCTGTGTTAGGTGGCTTTGTTGACCAGGTGTTTGGCACG
GCCATACTGATGCTGTGTCTGACAGCTCTAGGTGATGAAAAGAACAAGCCAGCGGCCAAA
GGGGCTGAATCTATCTTTGTGGGTCTTCTGGTGGTCCTCATTGGCATTTCTTTGGGCAGC
AACAGTGGTTATGCCATCAACCCCACCAGAGACTTTGGACCCAGGCTCTTCACTGCAATA
GCAGGCTGGGGTTTGGATGTATTCAGGGCTGGAAATGGCTGGTTCTGGGTCCCTATAGTG
GCTACTCCCATAGGTGCAGTGCTGGGAGCAGGGCTTTACAAGGTATGTATACAAATGCAC
CACCCAGCCGTCTCTGAACAGTACCAGGAGAAG---GATCCC------AGTGAGGAA---

>Sailfin_molly_Aqp7
---------ATGGGGCAGTCAGTGGAACTGGAGGTC---TCCATGCGGGGCAAACTGGAA
GTAGGGCGACCCAGGATTTGGCTG------AATGAACTCTCACGAGTGGGACTTGCTGAG
TTTCTTTCAACGTATGTCATGATGACATTAGGTCTGGGTTCGGTGGCCCAGGTTGTGACT
GGTAAAGGAGGATTCGGAACCTTCTTCAGCATCAACATTGGCTTCGGCCTGGCTGTTGCA
ATGGGGGTTCATATTGGAGGAACTGTCTCTGGGGCTCATATGAATGCCGCAGTCTCCTTT
GCATTGTGTGTTTATGGCCAACTACCATGGAAGAGGCTCCCCTTGTACATTTTTGCTCAG
CTGTTTGGATCGTTTCTTGCAGCAGCAACAGTTTATGGTGTTTACTATGACGCCATTTCT
ACTTATGCTGGTGGAAACCTGAGTGTTACTGGAGAAAGAGCCACGGCTGGGATATTTGCC

ACTTATCCAGCACCATACCTCTCTGTGTTAGGTGGCTTTGTTGACCAGGTGTTTGGCACG
GCCATACTGATGCTGTGTCTGACAGCTCTAGGTGATGAAAAGAACAAGCCAGCGGCCAAG
GGGGCTGAATCTATCTTTGTGGGTCTTCTGGTGGTCCTCATTGGCATTTCTTTGGGCAGC
AACAGTGGTTATGCCATCAACCCCACCAGAGACTTTGGACCCAGGCTCTTCACTGCAATA
GCAGGCTGGGGTTTGGATGTATTCAGGGCTGGAAATGGCTGGTTCTGGGTCCCTATAGTG
GCTACTCCCATAGGTGCAGTGCTGGGAGCAGGGCTTTACAAGGTATGTATACAAATGCAC
CACCCAGCCGTCTCTGAACAGTACCAGGAGAAG---GATCCC------AGTGAGGAA---

>Shortfin_molly_Aqp7
---------ATGGGGCAGTCAGTGGAACTGGAGGTC---TCCATGCGGGGCAAACTGGAA
GTAGGGCGACCCAGGATTTGGCTG------AATGAACTCTCACGAGTGGGACTTGCTGAG
TTTCTTGCAACGTATGTCATGATGACATTAGGTCTGGGTTCGGTGGCCCAGGTTGTGACT
GGTAAAGGAGGATTTGGACCTTCTTCAGCATCAACATTGGCTTCGGCCTGGCTGTTGCA
ATGGGGGTTCATATTGGAGGAACTGTCTCCGGGGCTCATATGAATGGCGCAGTCTCCTTT
GCATTGTGTGTTTATGGCCAACTACCATGGAAGAGGCTCCCCTTGTACATTTTTGCTCAG
CTGTTTGGATCGTTTCTTGCAGCAGCAACAGTTTATGGTGTTTACTATGACGCCATTTCT
ACTTATTCTGGTGGAAACCTGAGTGTTACTGGAGAAAGAGCCACGGCTGGGATATTTGCC
ACTTATCCAGCACCATACCTCTCTGTGTTAGGTGGCTTTGTTGACCAGGTGTTTGGCACG
GCCATACTGATGCTGTGTCTGACGGCTCTAGGTGACGAAAAGAACAAGCCAGCGGCCAAA
GGGGCTGAATCTATCTTTGTGGGTCTTCTGGTGGTCCTCATTGGCATTTCTTTGGGCAGC
AACAGTGGTTATGCCATCAACCCCACCAGAGACTTTGGACCCAGGCTCTTCACTGCAATA
GCAGGCTGGGGTTTGGATGTATTCAGGGCTGGAAATAGCTGGTTCTGGGTCCCTATAGTG
GCTACTCCCATAGGTGCAGTGCTGGGAGCAGGGCTTTACAGGGTATGTATACAAATGCAC
CACCCAGCCGTCTCTGAACAGTACCAGGAGAAG---GATCCC------AGTGAGGAA---

>Guppy_Aqp7
---------ATGGGGCAGTCAGTGGAACTGGAGGTC---TCCATGCGGGGCAAACTGGAA
GTAGGGCGACCCAGGATTTGGCTG------AATGAACTCTCACGAGTGGGACTTGCTGAG
TTTCTTTCAACGTATGTCATGATGACATTAGGTCTGGGTTCGGTGGCCCAGGTTGTGACT
GGTAAAGGAGTATTCGGAACGTTCTTCAGCATCAACATTGGCTTTGGCCTGGCTGTTGCA
ATGGGGGCTCATATTGGAGGAACTGTCTCCGGGGCTCATATGAATGCCGCAGTCTCCTTC
GCATTGTGTGTTTATGGCCAACTACCATGGAAGAGGCTCCCCTTGTACATTTTTGCTCAG
CTGTTTGGGTCGTTTCTTGGAGCAGCAACAGTTTATGGTGTTTACTATGATGCTATTTCT
ACTTATTCTGGTGGAAACCTGAGTGTTACTGGAGAAAGAGCCACGGCTGGGATCTTTGCC
ACTTATCCAGCACCATACCTCTCTGTGTTAGGTGGCTTTGTTGACCAGGTGTTTGGCACG
GCCATACTGATGCTGTGTCTGACGGCTCTAGGTGACGAAAAGAACAAACCAGCTGCCAAA
GGGGCTGAATCTATCTTTGTGGGTCTCCTGGTGGTCCTCATTGGCATTTCTTTGGGCAGC
AACAGTGGTTATGCCATCAACCCCACCAGAGACTTTGGACCCAGGCTCTTCACTGCAATC
GCAGGCTGGGGTTTGGATGTATTCAGGGCTGGAAATTTCTGGTTCTGGATCCCTATAGTG
GCTACTCCCATAGGTGCAGTGCTTGGTGCAGGGCTTTACAAGGTATGTATACAAATGCAC
CACCCAGCCGTCTCTGAACAGTACCAGAAGAAG---GATTCC------AGTGAGGAA---

>Blackstripe_livebearer_Aqp7
---------ATGGGGCGGTCAGTGGAACTGGAGGTC---GCCATGCAGGGCAAAATGGAA
ATAGGGCGACCGAAGACTTGGCTG------AATGAATTCTCACGAGTGGGAGTTGCTGAA
TTCCTTTCAACATATATCATGATGACATTAGGTCTGGGTTCGGTGGCCCAGGTTGTGACT
GGCAAAGGAGGATTTGGAACGTTCTTCAGCATCAACGTTGGCTTTGGCCTGGCTGTTGCA
ATGGGGGTTCATATTGGAGGAACTGTCTCCGGGGCTCATATGAATGCTGCAGTCTCCTTC
GCATTGTGTGTTTTCGGCCAGCTGCCATGGAAGAGGCTCCCCTTGTACGTTTTTGCTCAG
CTAATTGGGTCGTTTCTTGCTGCAGCAACAGTTTATGGTATTTACTATGATGCCATTTCT
TCTTATTCTGGTGGAAACCTGACTGTTACTGGAGAAAGAGCCACGGCTGGGATCTTTGCC
ACTTATCCAGCACCATACCTCTCTGTGTTTGGTGGCTTTGTTGACCAGGTGTTTGGCACG
GCCATACTGCTGCTGTGTCTGATGGCTCTATCTGACGAAAAGAACAAACCAGCTGCCAAA
GGGGCTGAATCTATCTTTGTGGGTCTCCTGGTGACCCTCATTGGCATTTCTTTGGGCAGC
AACAGTGGTTATGCCATCAACCCCACCAGAGACTTTGGACCTAGGCTCTTCACTGCAATG
GCAGGCTGGGGTTTTGAGGTATTCAGGGCTGGAAAGAGCTGGTTCTGGGTCCCTATACTG
GCTACTCCCCTAGGTGCAGTGCTGGGAGCAGGGGTTTACAAGGTATGTGTACAAATGCAC
CACCCACCCGTCTCTGAAGAGTACCTGGCGAAG---GATCCC------ATTGAGGAA---

```
------------------------------------------------------------
------------------
>Southern_platyfish_Aqp7
---------ATGGGGCCGTCAGTGGAACTGGAGGTC---TCCATGCAGGGCAAACTGGAA
GTAAGGCGACCCCAGATCCATCTG------AATGAACTCTCACGAGTGGCACTTGCTGAA
TTCCTTTCAACATATGTCATGATGACATTAGGTCTAGGTTCGGTGGCCCAGGTTGTGACT
GGTGAAGGAGGAAAAGGAACGTTCTTCAGCATCAACATTGGCTTTGGCCTGGCTGTTGCA
ATGGGGGTTCATATTGGAGGAACTGTCTCCGGGGCTCATATGAATGCCGCAGTCTCCTTC
GCATTGTGTGTTTTTGGCCAACTACCATGGAAGAGGCTCCCCTTGTACATTTGTGCTCAG
CTATTTGCGTCGTTTCTTGCAGCAGCAACAGTTTATGGTGTTTACTATGATGCCATTTAT
ACTTTTTCTGGTGGAAACCTGACTGTTACTGGAGAAAAAGCCACGGCTGGGATCTTTGCC
ACTTATCCAGCACCATACCTCTCTGTGTTCGGTGGCTTTGTTGACCAGGTGTTTGGCACG
GCCATACTGCTGATGTGTCTGATGGCTCTATCTGACGAAAAGAACAAACCAGCTGCCAAA
GGGACTGAATCTATGTTTGTGGGTGTCCTGGTGGTCCTCATTGGCATTTCTTTGGGCAGC
AACAGTGGTTATGCTATCAACCCCACCAGAGACTTGGGACCTAGGCTCTTCACTGCAGTT
GCAGGCTGGGGTTTGGATGTATTCAGGGCTGGAAAATTCTGGTTCTGGGTCCCTATAGTG
GCCACTCCCATAGGTGCAGTGCTGGGAGGAGGGCTTTACAAGGTATGTGTACAAATGCAC
CACCCAGCCCCCTCTGAACAGTACCAGGAGAAG---GATAAC------AGTGAGGAATCT
CTCCCGCCAAAGAAGAAGCGAAACATC---TGTTCCACG---------------------
------------------
>Monterrey_platyfish_Aqp7
---------ATGGGGCCGTCAGTGGAACTGGAGGTC---TCCATGCAGGGCAAACTGGAA
GTAAGGCGACCCGAGATCCGTCTG------AATGAACTCTCACGAGTGGCACTTGCTGAA
TTCCTTTCAACATATGTCATGATGACATTAGGTCTGGGTTCGGTGGCCCAGGTTGTGACT
GGTGAAGGAGGAAAAGGAACGTTCTTCAGCATCAACATTGGCTTTGGCCTGGCTGTTGCA
ATGGGGGTTCATATTGGAGGAACTGTCTCCGGGGCTCATATGAATGCCGCAGTCTCCTTC
GCATTGTGTGTTTTTGGCCAACTACCATGGAAGAGGCTCCCCTTGTACATTTTTGCTCAG
CTATTTGCGTCGTTTCTTGCAGCAGCAACAGTTTATGGTGTTTACTATGATGCCATTTAT
ACTTTTTCTGGTGGAAACCTGACTGTTACTGGAGAAAAAGCCACGGCTGGGATCTTTGCC
ACTTATCCAGCACCATACCTCTCTGTGTTCGGTGGCTTTGTTGACCAGGTGTTTGGCACG
GCCATACTGCTGATGTGTCTGATGGCTCTATCTGACGAAAAGAACAAACCAGCTGCCAAA
GGGACTGAATCTATGTTTGTGGGTGTCCTGGTGGTCCTCATTGGCATTTCTTTGGGCAGC
AACAGTGGTTATGCTATCAACCCCACCAGAGACTTGGGACCTAGGCTCTTCACTGCAGTT
GCAGGCTGGGGTTTGGATGTATTCAGGGCTGGAAAATTCTGGTTCTGGGTCCCTATAGTG
GCCACTCCCATAGGTGCAGTGCTGGGAGGAGGGCTTTACAAGGTATGTGTACAAATGCAC
CACCCAGCCCCCTCTGAACAGTACCAGGAGAAG---GATAAC------AGTGAGGAATCT
CTCCCGCCAAAGAAGAAGCGAAACATC---TGTTCCACG---------------------
------------------
>Green_swordtail_Aqp7
---------ATGGGGCCGTCAGTGGAACTGGAGGTC---TCCATGCAGGGCAAACTGGAA
GTAAGGCGACCCGAGATCCGTCTG------AATGAACTCTCACGAGTGGCACTTGCTGAA
TTCCTTTCAACATATGTCATGATGACATTAGGTCTGGGTTCGGTGGCCCAGGTTGTGACT
GGTGAAGGAGGAAAAGGAACGTTCTTCAGCATCAACATTGGCTTCGGCCTGGCTGTTGCA
ATGGGGGTTCATATTGGAGGAACTGTCTCCGGGGCTCATATGAATGCCGCAGTCTCCTTC
GCATTGTGTGTTTTTGGCCAACTACCATGGAAGAGGCTCCCCTTGTACATTTTTGCTCAG
CTACTTGCGTCGTTTCTTGCAGCAGCAACAGTTTATGGTGTTTACTATGATGCCATTTAT
ACTTTTTCTGGTGGAAACCTGACTGTTACTGGAGAAAAAGCCACGGCTGGGATCTTTGCC
ACTTATCCAGCACCATACCTCTCTGTGTTCGGTGGCTTTATTGACCAGGTGTTTGGCACG
GCCATACTGCTGCTGTGTCTGATGGCTCTATCTGACGAAAAGAACAAACCAGCTGCCAAA
GGGACTGAATCTATGTTTGTGGGTGTCCTAGTGGTCCTCATTGGCATTTCTTTGGGCAGC
AACAGTGGTTATGCTATCAACCCCACCAGAGACTTGGGACCTAGGCTCTTCACTGCAGTT
GCAGGCTGGGGTTTGGATGTATTCAGGGCTGGAAAATTCTGGTTCTGGGTCCCTATAGTG
GCCACTCCCATAGGTGCAGTGCTGGGAGGAGGGCTTTACAAGGTATGTGTACAAATGCAC
CACCCAGCTCCCTCTGAACAGTACCAGGAGAAG---GATAAC------AGTGAGGAATCT
CTCCCGCCAAAGAAGAAGCGAAACATC---TGTTCCACG---------------------
------------------
>Western_mosquitofish_Aqp7
---------ATGGGGCCGTCAGTGGAAGTGGAGGTC---TCCATACAGGGCAAAGTGGAA
GTAAGGCGACCCAAGATTCTTCTG------AATGAACTCTCACGAGTGGGACTTGCTGAA
TTCCTTGCAACGTATATCATGATGACATTAGGTCTGGGTTCGGTGGCCCAGGTTGTGACT
GGTGACGGAGTATTTGGAACGTTCTTCAGCATCAACATTGGCTTTGGCCTGGCTGTTGCA
```

ATGGGGGTTCATATTGGAGGAACTGTCTCCGGGGCTCATATGAATGCCGCCGTCTCCTTC
GCATTGTGTGTTTTTGGCCAACTACCATGGAAGAGACTCCCCTTGTACATTTTTGCTCAG
CTATTTGGGTCGTTTCTTGCAGCAGCAACAGTTTTTGCTGTTTACTATGACGCCATTTCT
GATTATTCTGGTGGAAACCTGACTGTTACTGGAGAAAAAGGCACGGCTGGGATCTTTGCC
ACTTATCCAGCACCATACCTCTCTGTGTTCGGTGGCTTTTTTGACCAGGTGTTTGGCACG
GCCATACTGCTGCTGTGTCTGATGGCTCTATCTGACGAAAAGAACAAACCAGCTGCGAAA
GGGACTGAATCTATCTTTGTGGGTCTCCTAGTGGTCCTCATTGGTATTTCTTTGGGCAGC
AACAGTGGTTATGCTATCAATCCCACCAGAGACTTTGGACCTAGGCTCTTCACTGCAATG
GCAGGCTGGGGTTTGGATGTATTCAGGGCCGGAAATTACTGGTTCTGGGTCCCTATAGTG
GCCACTCCCATAGGTGCAGTGCTGGGAGCAGGGCTTTACAAGATATGTGTGCAAATGCAC
CACCCAGACCCCTCTGAACAGTACCGGGAGAAG---GGTCCC------AGTGAGGAA---

>Sheapshead_minnow_Aqp7
ATGAAGGACTTGATGCAATCAGTAGAATTAGGGGTA---TCCCAGCGGAGTCAAGTGGAA
ATAAAGCGACCCAAAGTTTGGCTG---AGGAGTGAACTGTCACGAGTGGGACTTGCTGAA
TTTCTTTCAACATATGTCATGATGGCATTAGGTCTGGGTTCTGTGGCCCAGGTTGTGACC
GGTCAAGGAGTATTTGGACAGTACCTCAGCATCAACCTCGGCTTTGGACTAGCTGTTGCA
ATGGGGGTTCATGTTGGAGGAAATGTGTCTGGCGCTCATATGAATGGAGCAGTCTCCCTC
ACAATGTGTGTCTTTGGCCGCCTGCAATGGAAGAGGCTGCCCTTGTATATTTTTGTACAG
CTTTTGGGGTCCTTCCTCGGTGCAGCAACAATTTACGCTATTTACTACGAGGCCATTTAT
GACTATTGTGGTGGAAACCTGACTGTTACGGGTGAAAGGGCCACAGCTGGGATCTTTGCC
ACCTACCCTGCACCATACCTCTCCTTGATTGGTGGCTTCTTTGACCAGGTGTTTGGCACA
GCCATGCTGCTGCTGTGCCTGATGGCTCTATCCGACCAAAAGAACAAACCAGCTGCTAGA
GGGGCTGAACCCATCGCTGTGGGCCTCCTTGTTGTTCTTATTGGAATTTCTCTGGGCAGC
AACAGTGGTTATGCTATCAACCCAACAAGAGACATTGCACCAAGGATCTTCACTGCAATT
GCAGGCTGGGGATGGGATGTGTTCAGGTCTGGAAATGGGTGGTGGTGGGTCCCTCTAGTG
GCTACTCCCATTGGAGGAGTGCTGGGAGCTGGGCTGTATAAGGTTTTTGTAGAAATGCAC
CACCCACCGTCCTCTGGACAGGAGATGGAGCCT---GAC---------GAAGAAGAGTCT
TTCCCGCTGAAGAATAAAAGAAACATC---TGTTCTGATGTA------TGTGTGAAACCA
GAAACAAATGGAAACAAC
>Amargosa_pupfish_Aqp7
ATGAAGGACTTGATGCAATCAGTAGAATTAGGGGTA---TCCCAGCGGAGCCAAGTGGAA
ATAAAGCGACCCAAAGTTTGGCTG---AGGAGTGAACTGTCACGAGTGGGACTTGCTGAA
TTTCTTTCAACATATGTCATGATGGCATTAGGTCTGGGTTCTGTGGCCCAGGTTGTGACC
GGTCAAGGAGTATTCGGACAGTACCTCAGCATCAACCTCGGCTTTGGACTAGCTGTTGCA
ATGGGGGTTCATGTTGGAGGAAATGTGTCTGGCGCTCATATGAATGGAGCAGTCTCCCTC
ACAATGTGTGTCTTTGGCCGCCTGCAATGGAAGAGGCTGCCCTTGTATATTTTTGTACAG
CTTTTGGGGTCCTTCCTCGGTGCAGCAACAATTTACGCTATTTACTACGAGGCCATTTAT
GACTATTGTGGTGGAAACCTGACTGTTACGGGTGAAAGGGCCACAGCTGGGATCTTTGCC
ACCTACCCTGCACCATACCTCTCCTTGATTGGTGGCTTCTTTGACCAGGTGTTTGGCACA
GCCATGCTGCTGCTGTGCCTGATGGCTCTATCCGACCAAAAGAACAAACCAGCTGCTAGA
GGGGCTGAACCCATCGCTGTGGGTCTCCTTGTTGTTCTTATTGGAATTTCTCTGGGCAGC
AACAGTGGTTATGCTATCAACCCAACAAGAGACATTGCACCAAGGATCTTCACTGCAATT
GCAGGCTGG---
------------------GTGCTGGGAGCTGGGCTGTATAAGGTTTTTGTAGAAATGCAC
CACCCACCGTCCTCTGGACAGGAGATGGAGCCT---GAT---------GAAGAAGAGTCT
TTCCCGCTGAAGAATAAAAGAAACATC---TGT---------------------------

>Common_mummichog_Aqp7
ATGAAGGACTTTGTAGAGTCAGTGGAACTGGGGGTC---TCCCAGCGGAGCGCAGTGGAG
CTAAAACGACCCAAAGTTTGGCTG---AGGAGTGAACTCTCACGAGTGGGACTCGCTGAA
TTCCTTTCAACATATGTGATGATGGCATTAGGTCTTGGTTCTGTGGCCCAGGTCGTGACC
GGTCAAGGAGTATTCGGACAGTACCTTAGCATCAACCTTGGCTTTGGGCTGGCTGTCGCA
ATGGGGGTTCATGTTGGAGGAAATGTCTCTGGAGCTCATATGAACGGAGCAGTCTCCTTC
ACTATGTGTGTTTTTGGCCGCCTACAGTGGAAGAGGCTGCCCTTGTATATTTTCGTACAG
CTTTGCGGTTCGTTTCTTGCAGCAGCAACAATTTATGCTATTTACTACGAGGCCATTTAT
GACTATTGTGGGGGAAACCTGACTGTAAGTGGTGAAAGGGCCACAGCTGGGATCTTTGCC
ACATATCCGGCACCATACCTGTCCTTGATTGGTGGCTTTGTTGACCAGGTGTTTGGCACA
GCCATGCTGCTGCTGTGCCTGATGGCTCTGTCTGACCAAAAGAACAAACCAGCAGCCAGG
GGGGCTGAACCCATTGCTGTGGGTCTCCTGGTGGTTCTCATTGGCCTTTCTTTGGGCAGC

AACAGTGGTTATGCTATCAACCCCACCCGAGACATCGCACCAAGGGTCTTCACTGCTATT
GCAGGCTGGGGATGGGAAGTGTTCAGGTCTGGACATGGGTGGTGGTGGGTCCCTCTAGTA
GCTACTCCCATTGGAGGAGTGCTGGGAGCAGGGTTGTACAAGGCATTTGTAGAAATGCAC
CACCCCCTCTTCGCTGGACGGGATAGGGAGCCC---AGT---------GAGGAGGAGTCT
CTCCCGCTGAAGAAAGAGCGAAACATC---TGCTCTGATGTA------TGTGTGAAACCT
GACACAAATGGAAACAAC
>Mangrove_rivulus_Aqp7
ATGAAG------------------GAACACGGGGCC---TTTCAGCAGAGGAGGGTGGAT
GTAACCCGACTCAGAGCTTGGCTG---AAGAGTGAAGCTTCACGTGTTGGACTTGCTGAA
TTCCTCTGCACGTATGTGATGATGGCACTGGGGCTGGGCTGCGTGGCCCAGGTGGTGACC
GGACAGGGGGCCTTCGGGCACTTCCTCAGCATCAACATCGGCTTCGGCCTGGCTGTTGCT
ATGGGGGTTCATGTGGGAGGAAACGTCTCAGGAGCTCATATGAACGGAGCAGTGTCTTTC
ACAATGTGTGTTTTTGGCCGCCTCGCGTGGAAGAGGCTACCCCTGTACATTTCTGCGCAG
CTGCTCGCGTCCTTTCTCGCAGCAGTCACAATTTATGCAGTCTATTATGAGGCCATTTAT
GATTACTGTGGAGGAAACCTGACTGTAACTGGGCCAAACGCCACAGCTGGCATCTTTGCC
ACCTATCCTGCCCCATACCTCTCCCTGATGGGTGGATTTGTTGACCAGGTGTTTGGCACG
GCCATGCTGCTTCTGTGCCTGATGGCTCTGTCCGACCAGAGGAATAAACCAGCTGCCAGG
GGCGGGGAGCCTCTCACAGTGGGCCTGGTGGTGGTTCTCATCGGCCTATCTCTGGGCAGC
AACAGCGGCTACGCCATCAACCCCACCAGAGACATCGCACCCAGGGTCTTCACGGCCATA
GCAGGCTGGGGAGTGGACGTGTTCAGGTCTGGAAACGGGTGGTGGTGGGTACCTCTGGTG
GCGCCCCCCATCGGAGGAGTGCTGGGAGCAGGGGTGTACGAGGCGTTTGTGGAGCTGCAC
CACCCACTCCGCTCCGGGCTG---------------------------GAGGAGGAATCG
ATCCCTCTGAAGAAAGAGGAAAACAAC---TGTGCCGATGTC------TGTGCGAAACCT
CAAACAAACACAACTGTA
>Annual_killifish_Aqp7
ATGAAG------------------GAACAGGGGGTC---TCTCAGCGGAAGACAGTGGAC
GTAACCCGACCCAAAGTTTGGCTG---AGGAATGAACTTTTACGTGTCGGACTTGCTGAA
TTCCTATGCACATATGTGATGATGGCTCTGGGGATGGGCTGTGTGGCGCAGGTGGTGACT
GGACAGGGGGAATTTGGAAACTTCCTCAGCATCAACATAGGTTTCGGGCTGGCTGTTGCT
ATGGGGGTTCATGTTGGAGGAAATGTCTCAGGAGCTCATATGAACGGAGCTGTGTCGTTT
ACAATGTGTGTGTTTGGACGCTTAGCGTGGAAGAGGCTGCCTCTGTATATTCTCGCCCAG
CTGATCGCATCATTTCTTGCAGCAGCAACAATCTATGGAGTCTATTACGAGGCCATATCT
GACTACAGTGGAGGAAACCTGACTGTAACTGGGCCAAAGGCCACAGCTGGCATCTTTGCC
ACCTATCCTGCACCATACCTCTCCTTGATGGGTGGATTTATTGACCAGGTCTTTGGCACA
GCAATGCTGCTTCTCTGCCTGATGGCTCTGTCCGACCAGAGGAACAAACCAGCTGCTGGG
GGCGGGGAACCTCTTGTGGTGGGGCTGGTGGTGGTCCTCATTGGCATGTCTCTGGGCAGC
AACAGCGGCTACGCCATCAACCCCACCCGAGACATTGCTCCCAGGGTCTTCACAGCCATC
GCAGGCTGGGGAGTGGATGTGTTCAGGTCTGGAAATGGGTGGTGGTGGGTACCCCTTGTG
GCTCCGCCCATCGGAGGCGTGCTGGGAGCCGGGGTCTACAAGGCATTTGTGGAGCTGCAC
CACCGGCCCCTGTCCGGGCAG---------------------------GGGGAGGAATCT
ATCCCCCTGAAGAAAGAAGAAAACAAC---TGTGCTGATGTA------TGTGCAAAACCT
CAAACAAATACAAACATA
>Turquoise_killifish_Aqp7
ATGAAGGACTTGGTGCGATCAGTAGAGCAGGGGGTG---TCTCATCGGAGAAGAGTGGAT
ATAACCCGACCCAAAGTTTGGCTG---AAGAATGAGCTTTCACGCGTGGGACTGGCTGAA
TTCCTCTGCACATATGTGATGATGGTTCTGGGTCTGGGTTGTGTGGCCCAGGTCGTGACC
GGACAGGGAGCTTTTGGACACTACATCAGCATCAACATTGGTTTTGGGCTGGCTGTTGCT
ATGGGGGTTCATATTGGAGGAAATGTCTCAGGGGCTCACATGAACGGAGCGGTGTCTTTC
ACAATGTGTGTTTTTGGCCGCCTCGCGTGGAAGAGGCTACCCCTGTATATATTTGCACAG
CTATTTGGGTCATTTCTAGGTGCAGTAACAGTATATGCAGTTTACTATGAGGCCGTTAAT
GATTATTGTGGAGGAAACCTGACTGTAACTGGTGCGAAGGCCACAGCTGGGATCTTTGCC
ACATATCCTGCACCATACTTATCTCTGATGGGTGGATTTGTTGACCAGGTGATTGGAACG
GCAATGCTGCTTCTCTGCCTGATGGCGCTGTCCGACCAGAAGAACAAACCAGCTGTTGGG
AGCAGCGAACCTCTGACAGTGGGTCTTGTGGTGGTACTCATCGGCATGTCGCTGGGCAGC
AACAGTGGCTACGCCATCAATCCCACCAGGGACATCGCACCCAGGGTCTTCACAGCCATA
GCAGGCTGGGGACTTGATGTGTTCAGGTCTGGAAATGGTTGGTGGTGGGTACCTTTAGTG
GCTCCCCCCATTGGAGGCGTGCTGGGAGCTGGGCTATACAAGGCGTTTGTAGAACTACAC
CACCCACCCATCTCCGGACAG---------------------------CAGGAGAACTCT
ATCCCTTTGAAGAAAGGAGAAAACATC---TGCACTAATGTA------------------

>Red_stirped_killifish_Aqp7

ATGAAGGACTTGGTGCGGTCAGTAGAGCTGGGGTTG---TCTCAGCGGAGAAGAGTGGAT
GTAACCCGACCCAAAGTTTGGCTG---AAGAATGAACTTTCACGCGTGGGACTCGCTGAA
TTCCTTTGCACGTATGTGATGATGGTTCTGGGTCTGGGTTGTGTGGCCCAGGTGGTGACC
GGACAGGGAGCTTTTGGACAGTACATCAGCATTAACATTGGTTTTGGGCTGGCTGTTGCT
ATGGGGGTTCATGTTGGAGGAAACGTCTCAGGGGCTCACATGAACGGAGCAGTGTCTTTC
ACAATGTGTGTTTTTGGCCGCCTCGTGTGGAAGAGGCTACCCCTGTATATATTTGCACAG
CTGTGTGGGTCATTTCTAGGTGCAGTAACAGTATATGCAGTTTACTACGAGGCCATTAAT
GATTATTCTGGAGGAAACCTGACAGTGACTGGTGCGAAGGCCACAGCTGGCATCTTTGCC
ACCTATCCTGCACCATACCTATCTCTGATGGGTGGATTTGTTGACCAGGTGTGTGGGACG
GCGATGCTGCTTCTCTGCCTGATGGCTCTGTCCGACCGGAAGAACAAACCAGCTGTTGGG
AGCAGCGAGCCTCTTACAGTGGGTCTGGTGGTGGTGCTCATTGGCATGTCCCTGGGCAGC
AACAGCGGCTACGCCATCAACCCCACCAGAGACATCGCACCCAGGGTCTTCACAGCCATA
GCAGGCTGGGGATTAGACGTGTTTAGGTCTGGAAATGGTTGGTGGTGGGTACCTTTAGTG
GCTACCCCCATTGGAGGAGTGCTGGGAGGTGGGCTATACAAGGCGTTTGTAGAACTACAC
CACCCACCCACCCCCGGTCAG---------------------------GACGAAGACTCT
ATCCCTTTGAAGAAAGGAGAAAACATC---TGTGCTAATGTA------TGTGTGAAACCT
GAACCAAAGGCAAACATA
>Japanese_medaka_Aqp7
ATGAAGGACTTGGTGCCCTCTGCCAAGATTGGTGTC---TCTGAGCTGAAAGGACAGGTT
GGGACCCAACCAAGAGTCTGGATC---AAGAATGAAGCAGCCCGAGTGGGACTTGCTGAA
ACCCTCAGCACCTATGTCATGATGGCATTTGGTCTGGGTTCGGTGGCTCAGGTAGTGACG
GGACAGGGAGCTTTCGGACAGTACATCAGCATCAACATTGCATTTGGGCTTGCCGTTGCT
ATGGGTACTTATGTTGGAGGAAAGGTCTCAGGAGCTCACATGAATGGAGCTGTGTCATTT
ACAATGTGTGTATTTGGGCGTCTGACATGGAAAATGCTGCCGCTGTATATTTGTGCTCAG
TTACTCGGCTCTTTCCTTGCATCCGGAACAATATATGCGATCTACTACGAGGCCATTCAT
AACTATTGTGGAGGAAATCTGACCGTTACTGGTCCAAAGCCCACAGCTGGAATCTTTGCC
ACTTATCCTGCACCATATCTGTCACTGGTGGCTGGATTTGTTGACCAGGTGTTTGGAACA
GCGATGCTGTTGCTGTGCGTGATGGCTCTGTCTGACCAGAAGAACAAACCCCCTCCTGCT
GGCTCTGAGCCTGTCATCGTGGGTCTCCTGGTGATGCTTATCGGCATGTCTCTGGGCAGC
AATAGCGGCTATGCCATCAACCCCACCAGAGACATTGCACCCAGGTTCTTCACTGCCATA
GCAGGCTGGGGGTTTGAAGTGTTCAGGGCTGGAAACTGCTGGTGGTGGGTGCCTCTAGTT
GCTCCCCTCATCGGAGGCGTCCTGGGAGCAGGGGTGTACAAGGCCTTTGTAGAACTGCAC
CACCCACACCCCTCTGAACAGAACAGGGTGCTA---GGG---------GAAGATGTGCAC
ATTCCTTTGGTGAAAAGAGTG---------------------------------------

>javanese_Ricefish_Aqp7
ATGAAGGACTTGGTGCCATCTGCAAAGACTGAGGTC---TCTGAGCGGAAAGGACAAGAT
GGGACCCAACCAAGAGTCTGGATC---AAGAACGAAGCAGCCCGAGTGGGACTTGCTGAA
ACCCTCAGCACCTATGTCATGATGGCATTTGGTCTGGGCTCTGTGGCTCAGGTGGTGACG
GGACAGGGTGCATTCGGACAGTACATCAGCATCAACATTGCTTTTGGGCTTGCCGTTGCT
ATGGGTACACATGTTGGAGGAAAGGTCTCAGGTGCTCACATGAATGGAGCAGTGTCGTTT
ACAATGTGCGTGTTTGGGCGATTGACATGGAAAATGCTGCCGCTGTACATTTGTGGTCAG
TTACTCGGCTCCTTGCTTGCATCAGGAACAATATATGCGATCTACTACGAGGCTATTCAT
AACTACTGTGGAGGAAATCTTACTGTTACTGGTCCAAAGGCCACAGCTGGGATCTTTGCC
ACCTATCCTGCACCATACCTGTCACTGGTGGCCGGATTTGCTGACCAGGTGTTTGGAACA
GCGATGCTGCTGCTGTGCGTGATGGCTCTGTCTGACCAGAAGAACAAACCACCTCCTGCT
GGCTGCGAGCCCGTCATTGTGGGTCTGCTGGTGACGCTCATCGGCATGTCTCTGGGCAGC
AACAGTGGCTATGCCATTAACCCCACCAGAGACATTGCACCCAGGGTCTTCACTGCCATA
GCAGGCTGGGGGTCTGAAGTGTTCAGGGCTGGAAACTGCTGGTGGTGGGTGCCTCTCGTT
GCTCCGTCCATCGGAGGTGTCCTGGGAGCAGGGGTGTATAAGGCCTTTGTAGAACTGCAC
CACCCACACCCCTCTGGACAGAACAGGGTGCAA---GTA---------GAAGATGAGGTC
ATTCCTTTGGTGAAAAGAGTG---------------------------------------

>Indian_Medaka_Aqp7
ATGAAGGACTTGGTGACATCTGCTAAGACTGAGGTC---TCTGAGCAGAAAGGACAGGAT
GGGACCCAACGAAGAGTCTGGATC---AAGAGTGAAGCAGCTCGAGTGGGACTTGCTGAA
ACCCTCAGCACCTATGTCATGATGGCATTTGGCCTGGGCTCGGTGGCTCAGGTGGTGACG
GGACAGGGCGCATTCGGACAGTACATCAGCATCAACATTGCTTTTGGGCTTGCCGTTGCT
ATGGGTACTTATGTTGGAGGGAAGGTCTCAGGAGCTCATATGAATGGAGCGGTGTCGTTT
ACGATGTGTGTGTTTGGGCGCCTGACGTGGAAAATGCTGCCGCTGTACATTTGTGCTCAG
TTACTCGGCTCCTTCCTGGCATCAGGAACAATATATGCAATCTACTACGAGGCTATTCAT

AACTACTGTGGAGGAAATCTGACTGTTACTGGTCCAAAGGCCACAGCTGGGATCTTTGCC
ACCTATCCTGCACCATACCTGTCACTGGTGGCCGGATTTGTTGACCAGGTGTTTGGAACA
GCGATGCTGCTGCTGTGCGTGATGGCTCTGTCGGACCAGAAGAACAAACCACCTCCTGCT
GGCTGTGAGCCCGTCATTGTGGGTCTGCTGGTGATGCTCATCGGCATGTCTCTGGGCAGC
AACAGTGGTTATGCCATTAACCCCACCAGAGACATTGCACCCAGGGTCTTCACTGCCATA
GCAGGCTGGGGGTCTGAAGTGTTCAGGGCTGGACACTGCTGGTGGTGGGTGCCTCTCGTT
GCTCCGTCCGTCGGAGGTGTCCTGGGAGCAGGGGTGTACAAGGCCTTTGTTGAACTGCAC
CACCCACACCCCTCTGAACAGAACAGGGTGCAA---GTG---------GAAGATGAGGTC
ATTCCTTTGGTGAAAAGAGTG---------------------------------------

>Freshwater_silverside_Aqp7
ATGAAGGATATTGTGCAGTCAGTTGAACTAGGTGTC---TCTAAGCGGAAAGGAGCTCAT
GTGTCCCGACCCAAAGTTTGGCTA---AGGAATGAATTTGTTCGTGTGGGACTTGCTGAA
GCCCTTTGCACATATGTCATGATGGCGTTTGGCCTCGGGTCTGTGGCCCAGGTAGTGACC
GGGCAGGGAGAGTTCGGACAGTACATCAGCATCAACCTTGGTTTTGGGCTGGGTGTCGCC
ATGGGCGTGCACGTTGGAGGAAAGGTCTCAGGGGCTCATATGAATGCAGCAGTGTCATTC
GCAATGTGTGTCTTTGGCCGCCTAGCATGGAAGATGCTACCCTTATATGTTTTTGCACAA
CTATTAGGGACGTTTCTCGCATCAGGA---------------------------------

---ATGCTGCTGCTGTGCCTAATGGCAGTATCCGACCAGAGGAACCAACCGGCTGCTGCG
GGCAGTGAGCCTGTCATAGTGGGCCTTGTGGTGCTTCTCATTGGCATTTCTCTGGGCAGC
AACAGCGGCTATGCCATTAACCCCACCAGAGATTTTGGAGCCAGGGTCTTCACTGCCATA
GCAGGCTGGGGAGCTGATGTGTTCAGGGCTGGAAATGGATGGTGGTGGGTACCTGCAGTT
GCTCCCCTGATTGGAGGAGTGCTGGGAGCAGGGGTATACAAGGCCTTTGTAGAACTGCAC
CACCCACCTCTCACTGGACAGGGTGGGAGGCCA---GTG---------GAGGAGGAGTCT
GTCTCTCTGGAGAAAGAGCAAAACATC---TGTTCTAATGAA------TGTGTG------

>Atlantic_silverside_Aqp7

GTGTCCCGACCCAAAATTTGGCTG---AGGAATGAATTTCTTCGTGTGGGACTTGCTGAA
TCCCTCTGCACATATGTCATGATGGCCTTTGGCCTTGGGTCTGTGGCTCAGGTCGTGACC
GGACAGGGAGTGTTCGGACAGTACATAAGCATCAACCTTGGTTTTGGGCTAGGTGTTGCC
ATGGGGGTTCATGTTGGAGGAAAGGTCTCAGGAGCTCATATGAACGCAGCAGTGTCATTC
ACAATGTGTGTCTTTGGCCAGCTAGCATGGAAGATGCTACCCTTATATGTTTGTGCACAG
CTGTTGGGAACCTTTCTTGCATCGGGGACAGTTTATGCTGTTTACTATGAAGCCATTCAT
CATTACAGTGGAGGAAACCTGACTGTAACCGGGGAGAGGGCCACAGCTGGCATCTTTGCT
ACCTACCCTGCACCCTACCTCTCCTTGACAGGTGGATTTCTTGACCAGGTGTTTGGCACA
GCAATGCTGCTGCTGTGTCTGATGGCGCTGTCGGACCAGAGGAACACTCCGCCTGCCGCG
GGCACTCAGCCCCTCATAGTGGGCTTCTTGGTGATGCTCATTGGTATTTCCTTCGGCAGT
AACAGTGGCTATGCCATTAACCCCACCAGAGATATTGGAGCAAGGCTCTTCACTGCCATA
GCAGGCTGGGGAACTGATGTGTTCAGGGCTGGAAATGGGTGGTGGTGGGTACCTATAGTT
GCGCCCCTGATCGGAGGAGTGCTGGGAGGAGGGGTGTACAAGGCTCTGGTGGAACTGCTC
CACCCACCTGTATCTGCACAGGGTGAGGGGCCG---CTG---------GAAGAGGAGTTT
GTCCTTCAGGAGAAAGAGCAAAGCATC---TGTTCAAACAAA------------------

>Desert_rainbowfish_Aqp7
ATGAAGGACTTTGTACAGACAGTAGAGCTAGGGGTC---TCTCGGCGGAAAGGAATTGAT
GTTACCCGACCCAAAGTTTGGCTG---AGGAATGAATTTGTTCGTGTGGGACTTGCTGAA
TTCCTTTCCACATACGTCATGATGGCCTTTGGCCTCGGGTCTGTGGCCCAGGTGGTGACT
GGAAAAGGAGAGTTTGGTCATTACATAAGCATCAACGTTGGTTTTGCGCTTGGTGTTGCT
ATGGGGGTACATGTCGGTGGAAAAGTCTCAGGGGCTCATATGAATGGAGCAGTGTCTTTC
ACAATGTGTGTGTTTGGTCGCCTAGCATGGAAGATACTTCCAGTATATATTTTTGCACAG
CTGTTAGGCACATTTCTTGCATCAGGGACCATTTATGCAATCTACTATGAAGCAATTCAC
GATTACTGTGGGGGAAACCTGACTGTTACTGGAGCAAAGGCCACAGCTGGCATTTTTGCC
ACCTATCCTGCACCCTACCTCTCCTTGATGGCTGGATTTGCTGATCAGGTGTTTGGCACA
GCGATGCTGCTGCTGTGCCTGATGGCTCTTTTCGACCAGAGAAACCAACCAGCTGCTGCG
GGCAGTGAGCCTATCACAGTGGGTCTCGTGGTGCTACTCATTGGCATTTCTCTGGGTAGC
AACAGCGGCTGTGCCATCAACCCCACTAGAGACATTGGAGCCAGGGTCTTCACTGCTATT
GCAGGCTGGGGAGTCGATGTGTTCAGGGCTGGAAATGGGTGGTGGTGGGTACCTGTTGTT
GCTCCCACTATTGGAGGAGTGCTGGGAGCAGGAGTATACAAAGCCCTTGTAGAACTGCAC

CACCCACCCCTTTCTGGTCAGCGTAAGGAGCAA---GTG---------GAGGAAGAGTCT
TTCCCTCTGGAGAAAGAACAAACCATC---TGTTCTAATGAA------TGTGTA------

>Peacock_blenny_Aqp7
ATGAAGGACCTGGCACAGTCAGTAGAACTCGGGGTC---TCTCAGCGGAGAGCAGCGAGT
GTAACTCGACCCAGGGTTTGGCTA---AAAAATGAATTTGTTCGTGTGGGACTCTCTGAA
ACCCTTTCCACTTTTGTCATGATGGCGTTTGGCCTCGGCTCTGTGGCCCAGGTAGTGATT
GGACAGGGTGCATTCGGAGAGTACCTCAGCATTAACATTGGCTTTGCACTGGGTGTCGCT
ATGGGAGTTCATGTTGGAGGAAACGTCTCAGGGGCTCATATGAACGCGGCAGTGTCGATT
GCAATGTGTGTGTTCGGCCGACTGGCTTGGAGGATGTTACCCCTGTACATATTTTCACAG
TTCATAGGGTCATTTCTAGCAGCAGGAACCATCTATGGTGTCTACTATGAGGCTATTCAT
AACTACTGCGGAGGAAATCTGACTGTTACTGGAGTGAAGGCCACAGCTGGTATTTTTGCC
ACCTATCCTGCACCGTACCTCTCCTTGATGGGTGGATTTGTGGATCAGGTGTTTGGCACG
GCAATGCTGCTGCTGTGCCTGATGGCTCTATCGGACCAGAGGAACAAACCGCCTGCTCCG
GGCAGCGAGCCAGCCTTGGTGGGTCTGCTGGTGCTGCTCATTGGCATTTCTCTGGGCAGC
AATAGCGGCTATGCCATCAACCCCACTCGAGACATTGCACCCAGGGTCTTCACTGCCGTG
GCAGGCTGGGGAGTGGACGTATTCCGGGCTGGAAATGGGTGGTGGTGGGTGCCTCTAGTT
GCTCCATGTATTGGAGGAATCCTGGGAGCAGGGGTGTACAAGGTCCTGGTGGAACTGCAC
CATCCACCAGTGTCTGAGGAGGAGCGAGCGCTG---GCA---------GGGGAGGAGTCT
GTGCCTCTTGAGAAAGTGCAAAACGTA---TGCGGG------------------------

>Rockpool_blenny_Aqp7
ATGAAGGACCTGGCACAGTCAGTAGAACTCGGGGTC---TCTCAGCGGAGAGCAGCGAGT
GTAACGCGACCCAGGGTTTGGCTA---AAAAATGAATTTGTTCGTGTGGGACTTGCTGAA
ACCCTTTGCACTTTTGTCATGATGGCGTTTGGCCTCGGCTCTGTGGCCCAGGTAGTGATT
GGACAGGGTGCATTCGGAGAGTACCTCAGCATTAACATTGGCTTTGCACTGGGTGTCGCT
ATGGGAGTTCATGTTGGAGGAAACGTCTCAGGGGCTCATATGAACGCCGCAGTGTCAATC
GCAATGTGTGTGTTTGGTCGACTGGCTTGGAAGATGTTACCCCTGTACATAGTGTCACAG
TTCATAGGGTCATTTCTAGCAGCAGGAACCATCTACGGTGTCTACTATGAGGCTATTCAT
AACTACTGTGGAGGAAACCTGACTGTTACTGGAGTGAAGGCCACAGCTGGTATCTTTGCC
ACCTATCCTGCACCGTACCTCTCTTTGATGGGTGGATTTGTGGATCAGGTGTTTGGCACG
GCAATGCTGCTGCTGTGCCTGATGGCTCTATCGGACCAGAGGAACAAACCGCCTGCTCCA
GGCAGCGAGCCAGCCTTGGTGGGTCTGCTGGTGCTGCTCATTGGCATTTCTCTGGGCAGC
AATAGCGGCTATGCCATCAACCCCACTCGAGACATCGCACCCAGGGTCTTCACTGCCATA
GCAGGCTGGGGAGTCGATGTATTCCGGGCTGGAAATGGGTGGTGGTGGGTGCCTCTAGTT
GCTCCATCTATTGGAGGAATCCTGGGAGCAGGGGTGTACAAGGTCCTGGTGGAACTGCAT
CATACACCAGTGTCTGAGGAGGAGCGGGCGGTG---GCA---------GGGGAGGAGTCT
GTGCCTCTAGAGAAAGTGCAAAACGTA---TGCGTG------------------------

>Bluntsnouted_clingfish_Aqp7
ATGAAGGACTCGATGAAACCAGTACAACAGGGGGTT---TCAGAGCGGAGGGGAGTCCAT
GTGACTCGAACCAAAAATTGGCTG---AAGAATCAACTTGTTCGAGTTGGACTGGCAGAA
ACTCTTTGTACTTATGTCATGATGGCATTTGGTCTTGGCTCTGTAGCCCAAGTAGTGACT
GGACAGGGTGTGTTTGGGCAGTACCTCAGCATCAACATTGGTTTTGCACTTGGCGTTGCT
ATGGGAGTTCATGTTGGAGGAAATGTGTCAGGGGCCCACATGAATGGAGCAGTTTCTGTA
GCTATGTGTGTGTTTGGCCGCCTTGCCTGGAGGATGCTGCCCTTGTACATCTTTGCACAG
TTCATAGGGTCATTTCTTGCAGCTGGCACAATTTATGGCATCTATTACGAGGCTATTCAT
GACTACTGTGGGGGAAACCTGACTGTAAGTGGTGCAAGAGCCACAGCTGGCATCTTTGCC
ACGTATCCTGCACCGTACCTTTCCTTGATGGCTGGATTTGCCGATCAGGTGTTTGGCACC
GCAATGCTGCTGCTCTGTCTGATGGCTCTTTCCGATCAGAGGAACAAACCAGCCCCCGTG
GGCAATGAGCCAGTCTTAGTGGGTCTCCTGGTTTTACTCATTGGCATCTCTTTTGGAAGT
AATAGTGGCTATGCCATCAATCCCACTAGAGACATTGGACCCAGAGTGTTTACTGCTGTA
GCAGGCTGGGGTCTGGATGTGTTCAGGGCTGGAAACGGATGGTGGTGGGTACCACTAATC
GCTCCGACCCTAGGAGGAGTTCTAGGAGCTGGAGTGTACAAATTCTTTGTGGAGATGCAC
CACCCATCTGACAATGAACAAGGCAAGAGTCTG---GTG---------GGGGAGGAGTCT
GTACCACTGGAGAAAAAGCAAAATGTA---------------------TGCGTT------

>Clown_anemonefish_Aqp7
ATGAAGAACTTGATGCAGTCAGTAGAAGTGGGGGTC---TCTCAGCGGAGGGGAGTTAAT
GTAACTCGACCCAAAGTTTGGCTA---AAGAATGAACTTGTTCGTGTGGGACTTGCTGAA
ACTCTTTGCACATATGTCATGATGGTATTTGGTCTGGGGTCTGTGGCTCAGGTCGTGACT

GGACAGGGAGCGTTCGGACAGTACTTCAGCATCAATGTGGGTTTTGGGCTCGGTGTTGCT
ATGGGTGTTCACGTTGGAGGGAAGGTCTCAGGGGCTCATATGAATGGAGCAGTGTCATTC
ACAATGTGTGTGTTTGGCCGCCTTGCATGGAAAATGTTGCCCCTGTACATTTTTGCACAA
TTGTTGGGGTCATTTCTGGCAGCAGGGACAATTTATGGGATCTACTATGAGGCCATTCAT
GACTATTGTGGAGGAAACCTGACTGTAACTGGTGCAAAGGCCACAGCTGGTATCTTTGCC
ACCTATCCTGCACCATACCTCTCCTTGATGGCTGGATTTGTTGACCAGGTGTTTGGCACA
GCGATGCTCCTGCTGTGCCTCATGGCTCTATCTGACCAGAAGAACAAACCGGCTGCAGCG
GGTGGCGAGCCTGCTGCGGTTGGTCTCCTGGTGGTGCTCATTGGCATTTCTCTGGGCAGC
AACAGCGGCTACGCTATCAACCCCACCAGAGACATTGCACCCAGAATCTTTACTGCCATA
GCAGGCTGGGGAACTGATGTGTTCAGGGCTGGAAATGGGTGGTGGTGGGTGCCTCTGGTT
GCTACCCCCGTTGGAGGAGTACTGGGAGCAGGAATATACAAAGTGTTCGTGGAACTGCAC
CACCCACCCCACTCTGAACAGGCTGAGGGGTCA---GCA------------GAGGAGTCT
ATCCCTCTGGAGAAAGACCAAAACATC---TGTGCTAATGTA------TGTGTA------

>Orange_clownfish_Aqp7
ATGAAGAACTTGATGCAGTCAGTAGAAGTGGGGGTC---TCTCAGCGGAGGGGAGTTAAT
GTAACTCGACCCAAAGTTTGGCTA---AAGAATGAACTTGTTCGTGTGGGACTTGCTGAA
ACTCTTTGCACGTATGTCATGATGGTATTTGGTCTGGGGTCTGTGGCTCAGGTCGTGACT
GGACAGGGAGCGTTCGGACAGTACTTCAGCATCAATGTGGGTTTTGGGCTCGGTGTTGCT
ATGGGTGTTCATGTTGGAGGGAAGGTCTCAGGGGCTCATATGAATGGAGCAGTGTCATTC
ACAATGTGTGTGTTTGGCCGCCTTGCATGGAAAATGTTGCCCCTGTACATTTTTGCACAA
TTGTTGGGGTCATTTCTGGCAGCAGGGACAATTTATGGGATCTACTATGAGGCCATTCAT
GACTATTGTGGAGGAAACCTGACTGTAACTGGTGCAAAGGCCACAGCTGGTATCTTTGCC
ACCTATCCTGCACCATACCTCTCCTTGATGGCTGGATTTGTTGACCAGGTGTTTGGCACA
GCGATGCTCCTGCTGTGCCTCATGGCTCTATCTGACCAGAAGAACAAACCGGCTGCAGCG
GGTGGCGAGCCTGCTGCGGTCGGTCTCCTGGTGGTGCTCATTGGCATTTCTCTGGGCAGC
AACAGCGGCTACGCTATCAACCCCACCAGAGACATTGCACCCAGAATCTTTACTGCCATA
GCAGGCTGGGGAACTGATGTGTTCAGGGCTGGAAATGGGTGGTGGTGGGTGCCTCTGGTT
GCTACCCCCGTTGGAGGAGTACTGGGAGCAGGAATATACAAAGTGTTCGTGGAACTGCAC
CACCCACCCCACTCTGAACAGGCTGAGGGGTCA---GCA------------GAGGAGTCT
ATCCCTCTGGAGAAAGACCAAAACATC---TGTGCTAATGTA------TGTGTA------

>Twoband_anemonefish_Aqp7
ATGAAGGACTTGGTGCAGTCAGTAGAACTCGGGGTC---TCTCAGCGGAGGGGAGTTAAT
GTAACTCGACCCAAAGTTTGGCTA---AAGAATGAACTTGTTCGTGTGGGACTTGCTGAA
ACTCTTTGCACGTATGTCATGATGGTATTTGGTCTGGGGTCTGTGGCTCAGGTTGTGACC
GGACAGGGAGCGTTCGGACAGTACTTCAGCATCAATGTGGGTTTTGGGCTCGGTGTTGCT
ATGGGTGTTCACGTTGGAGGGAAGGTCTCAGGGGCTCATATGAATGGAGCAGTGTCATTC
ACGATGTGTGTGTTTGGCCGCCTTGCATGGAAAATGTTGCCCCTGTACATTTTTGCACAA
TTACTGGGGTCATTTCTGGCAGCAGGGACAATTTATGGGATCTACTATGAGGCCATTCAT
GACTATTGTGGAGGAAACCTGACTGTAACTGGTGCAAAGGCCACAGCTGGTATCTTTGCC
ACCTATCCTGCACCATACCTCTCCTTGATGGCTGGATTTGTTGACCAGGTGTTTGGCACA
GCGATGCTCCTGCTGTGCCTCATGGCTCTATCTGACCAGAAGAACAAACCGGCTGCAGCG
GGTGGCGAGCCTGCTGCGGTCGGTCTCCTGGTGGTGCTCATTGGCATTTCTCTGGGCAGC
AACAGCGGCTACGCTATCAACCCCACCAGAGACATTGCACCCAGAATCTTTACTGCAATA
GCAGGCTGGGGGACTGATGTGTTCAGGGCTGGAAATGGGTGGTGGTGGGTGCCTCTGGTT
GCTACCCCCGTTGGAGGAGTACTGGGAGCAGGAATATACAAAGTGTTCGTGGAACTGCAC
CACCCACCCCACTCTGAACAGGCTGAGGGGTCA---GCA------------GAGGAGTCT
ATCCCTCTGGAGAAAGACCAAAACATC---TGTGCTAATGTA------TGTGTA------

>Spiny_chromis_Aqp7
ATGAAGGACTTGGCGCAGTCAGTAGAACTGGGGGTC---TCTCAGCGGAGGGGAGTTTAT
GTAACTCGACCCAAAGTTTGGGTA---AAGAATGAACTTGTTCGTGTGGGACTTGCTGAA
ACTCTTTGCACATATGTCATGATGGTATTTGGTCTGGGGTCTGTGGCTCAGGTAGTGACC
GGACAGGGAGCGTTCGGACAGTACTTCAGCATCAATGTGGGCTTTGGACTCGGTGTTGCT
ATGGGTTTTCATGTTGGAGGGAAGGTCTCAGGAGCTCATATGAATGGAGCAGTGTCATTC
ACAATGTGTGTGTTTGGCCGCCTTGCATGGAAAATGTTGCCCCTGTACATTTTTGCACAA
CTATTGGGGTCATTTCTGGCAGCAGGAACAATTTATGGGATCTACTATGAGGCCATTCAT
GATTACTGTGACGGAAACCTTACTGTAACTGGTGCAAAGGCCACAGCTGGTATCTTTGCC
ACCTATCCTGCACCATACCTCTCCTTGATGGCTGGATTTGCTGACCAGGTGTTTGGCACA
GCGATGCTGCTGCTGTGCCTCATGGCTCTATCTGACCAGAAGAACAAACCGGCCGCAGCG

GGCAGTGAGCCTGCTGCAGTCGGTCTCCTGGTGGTGCTCATTGGCATTTCTCTGGGCAGC
AACAGTGGCTACGCTATCAACCCCACCAGAGACATTGCACCCAGGATCTTTACTGCCATA
GCAGGCTGGGGAACTGATGTGTTCAGGGCTGGAAATGGGTGGTGGTGGGTGCCTCTGGTT
GCTACCCCCATTGGAGGAGTACTGGGAGCAGGAATATACAAAGTCTTCGTGGAACTGCAC
CACCCACCCCACTCTGAACAGGCTGAGGGGTCA---GCA------------GAGGAGTCT
ATCCCTCTGGAGAAAAACCAAAACATC---AGTGTTAATGTA------TGTGTA------

>Bicolor_damselfish_Aqp7
ATGAAGGACTTGGTGCAGTCAGTAGAACTGGGGGTC---TCTCAGCGGAGGGGAGCAAAT
GTAACTCGACCCAAAGTTTGGCTA---AAGAATGAACTTGTTCGTGTGGGACTTGCTGAA
TCTCTTTGCACGTATGTCATGATGGCGTTTGGTCTGGGGTCTGTGGCCCAGGTAGTGACC
GGACAGGGAGCGTTTGGACAGTACTTAAGCATCAACGTGGGTTTTGGACTCGGTGTTGCT
ATGGGTGTTCATGTTGGAGGGAAGGTCTCAGGGGCTCATATGAATGGAGCAGTGTCATTT
ACAATGTGTGTGTTTGGCCGCCTTTCATGGAAAATGTTACCCTTGTACATTTTTGCACAA
CTATTGGGGTCATTTCTGGCAGCAGGGACGATTTATGGCATCTACTATGAGGCCATTCAT
GACTATTGTGGAGGAAACCTGACTGTAACTGGTGCGAGGGCCACAGCTGGTATCTTTGCC
ACCTATCCTGCACCATACCTCTCCTTGATGGCTGGATTTTTTGACCAGGTGTTTGGCACA
GCGATGCTGCTGCTGGGCCTCATGGCTCTATCTGACCAGAAGAACAAACCAGCTGCAGCG
GGCAGTGAGCCTGCTGCGGTGGGTCTCCTGGTGATACTCATTGGCATTTCTCTGGGCAGC
AACAGCGGCTATGCTATCAACCCTACCAGAGACATTGCACCCAGGGTCTTTACTGCCATA
GCAGGCTGGGGAACTGATGTGTTCAGGGCTGGAAATGGGTGGTGGTGGGTGCCTCTGGTT
GCTACCCCCATTGGAGGAGTACTGGGAGCAGGGATATACAAAGTCTTCGTGGAACTGCAC
CACCCACCCCTCTCTGAACAAGATGGGGGGTCA---GCA------------GAGGAGTCC
ATCCCTCTGGAGAAAGACCAAAACATC---TGTGCTAATGTA------TGTGTG------

>Zebra_mbuna_Aqp7
ATGAAGGACTTGGTGCAGTCAGTAAAACCCAGGGTG---TTTCAGCAGAGGGGAGGTAAA
GTAACTCGATCCAAAGTTTGGCTA---AAGAATGAACTCATTCGTGTGGGACTTGCTGAA
TCACTTAGCACATATGTCATGATGTCATTGGGCTTGGGGTCTGTGGCCCAGGTAGTGACT
GGTCAGGGAGCTTTTGGACAGTACCTCAGCATCAACCTGGGTTTTGGACTGGCTGTTGCC
ATGGGGTCTCATGTTGGAGGGAAGATCTCGGGGGCTCATATGAATGGCGCTGTATCATTC
ACAATGTGTGTGTTTCGCCGCCTCCCGTGGAAGATGCTACCTCTTTATATTTCGGCACAG
CTATTGGGGTCATTTCTTGCAGCAGGGACAATTTATGCTGTCTACTATGAAGCCATTCAT
GACTACTGTAGAGGAAACCTGACTGTGACTGGTGAGAAGGCCACTGCTGGTATCTTTGCC
ACCTATCCTGCTCCATACCTCTCTCTGATAGCTGGATTTTTTGACCAGGTATTTGGCACA
GCGATGCTACTACTGTGCCTTATGGCTCTATCCGACCAGAAGAACAAACCAGCGCCAGCA
GGAAGTGAGCCTGCATTTGCGGGTTTCCTGGTGCTTCTCATTGGCATTTCTTTGGGTAGC
AATAGTGGCTATGCCATCAACCCCACCAGAGACATCGCACCCAGGGTTTTCACTGCCATG
GCAGGCTGGGGATCTGATGTGTTCAGGGCTGGAAACGGATGGTGGTGGGTGCCTCTAGTT
GCCCCCCCTATTGGAGGAGTCCTTGGAGCAGGGCTATACAAGGCTGTTGTGGAACTGCAT
CACCCACACCTCTCTAAAGCGGGTGGAGAGATG---GTT------------GAAGAAGCT
GTCCCTCTGGATAAAGAGATAAATACC---ATTGAAAATATG------TGTGTG------

>Eastern_happy_Aqp7
ATGAAGGACTTGGTGCAGTCAGTAAAACCCAGGGTG---TTTCAGCAGAGGGGAGGTAAA
GTAACTCGATCCAAAGTTTGGCTA---AAGAATGAACTCATTCGTGTGGGACTTGCTGAA
TCACTTAGCACATATGTCATGATGTCATTGGGCTTGGGGTCTGTGGCCCAGGTAGTGACT
GGTCAGGGAGCTTTTGGACAGTACCTCAGCATCAACCTGGGTTTTGGACTGGCTGTTGCC
ATGGGGTCTCATGTTGGAGGGAAGATCTCGGGGGCTCATATGAATGGCGCTGTATCATTC
ACAATGTGTGTGTTTCGCCGCCTCCCGTGGAAGATGCTACCTCTTTATATTTCGGCACAG
CTATTGGGGTCATTTCTTGCAGCAGGGACAATTTATGCTGTCTACTATGAAGCCATTCAT
GACTACTGTAGAGGAAACCTGACTGTGACTGGTGAGAAGGCCACTGCTGGTATCTTTGCC
ACCTATCCTGCTCCATACCTCTCTCTGATAGCTGGATTTTTTGACCAGGTATTTGGCACA
GCGATGCTACTACTGTGCCTTATGGCTCTATCCGACCAGAAGAACAAACCAGCGCCAGCA
GGAAGTGAGCCTGCATTTGCGGGTTTCCTGGTGCTTCTCATTGGCATTTCTTTGGGTAGC
AATAGTGGCTATGCCATCAACCCCACCAGAGACATCGCACCCAGGGTTTTCACTGCCATG
GCAGGCTGGGGATCTGATGTGTTCAGGGCTGGAAACGGATGGTGGTGGGTGCCTCTAGTT
GCCCCCCCTATTGGAGGAGTCCTTGGAGCAGGGCTATACAAGGCTGTTGTGGAACTGCAT
CACCCACACCTCTCTAAAGCGGGTGGAGAGATG---GTT------------GAAGAAGCT
GTCCCTCTGGATAAAGAGATAAATACC---ATTGAAAATATG------TGTGTG------

>Red_mwanza_Aqp7
ATGAAGGACTTGGTGCAGTCAGTAAAACCCAGGGTG---TTTCAGCAGAGGGGAGGTAAA
GTAACTCGATCCAAAGTTTGGCTA---AAGAATGAACTCATTCGTGTGGGACTTGCTGAA
TCACTTAGCACATATGTCATGATGTCATTGGGCTTGGGGTCTGTGGCCCAGGTAGTGACT
GGTCAGGGAGCTTTTGGACAGTACCTCAGCATCAACCTGGGTTTTGGACTGGCTGTTGCC
ATGGGGTCTCATGTTGGAGGGAAGATCTCAGGGGCTCATATGAATGGCGCTGTATCATTC
ACAATGTGTGTGTTTCGCCGCCTCCCGTGGAAGATGCTACCTCTTTATATTTCGGCACAG
CTATTGGGGTCATTTCTTGCAGCAGGGACAATTTATGCTGTCTACTATGAAGCCATTTAT
GACTACTGTAGAGGAAACCTGACTGTGACTGGTGAGAAGGCCACTGCTGGTATCTTTGCC
ACCTATCCTGCTCCATACCTCTCTCTGATAGCTGGATTTTTTGACCAGGTATTTGGCACA
GCGATGCTACTACTGTGCCTTATGGCTCTATCCGACCAGAAGAACAAACCAGCGCCAGCA
GGAAGTGAGCCTGCATTTGCGGGTTTCCTGGTGCTTCTCATTGGCATTTCTTTGGGTAGC
AACAGTGGCTATGCCATCAACCCCACCAGAGACATCGCACCCAGGGTTTTCACTGCAATG
GCAGGCTGGGGATCTGATGTGTTCAGGGCTGGAAACGGATGGTGGTGGGTGCCTCTAGTT
GCCCCCCCTATTGGAGGAGTCCTTGGAGCAGGGCTATACAAGGCTGTTGTGGAACTGCAT
CACCCACACCTCTCTGAAGCGGGTGGAGAGATG---GTT------------GAAGAAGCT
GTCCCTCTGGATAAAGAGATAAATACC---ATTGAAAATATG------TGTGTG------

>Burtons_mouthbrooder_Aqp7
ATGAAGGACTTGGTGCAGTCAGTAAAACCCAGGGTG---TTTCAGCAGAAGGGAGGTAAA
GTAACTCGATCCAAAGTTTGGCTA---AAGAATGAACTCATTCGTGTGGGACTTGCTGAA
TCACTTAGCACATATGTCATGATGTCATTGGGCTTGGGGTCTGTGGCCCAGGTAGTGACT
GGTCAGGGAGCTTTTGGACAGTACCTCAGCATCAACCTGGGTTTTGGACTGGCTGTTGCC
ATGGGGTCTCATGTTGGAGGGAAGATCTCGGGGGCTCATATGAATGGAGCTGTATCATTC
ACAATGTGTGTGTTTCGCCGCCTCCCGTGGAAGATGTTACCTCTTTATATTTCGGCACAG
CTATTGGGGTCATTTCTTGCAGCAGGGACAATTTATGCTGTCTACTATGAAGCCATTTAT
GACTACTGTAGAGGAAACCTGACTGTGACTGGTGAGAAGGCCACTGCTGGTATCTTTGCC
ACCTATCCTGCTCCATACCTCTCTCTGATAGCTGGATTTTTTGACCAGGTATTTGGCACA
GCGATGCTACTACTGTGCCTTATGGCTCTATCCGACCAGAAGAACAAACCAGCGCCAGCA
GGAAGTGAGCCTGCATTTGCGGGTTTCCTGGTGCTTCTCATTGGCATTTCTTTGGGTAGC
AACAGTGGCTATGCCATCAACCCCACCAGAGACATCGCACCCAGGGTTTTCACTGCCATG
GCAGGCTGGGGATCTGATGTGTTCAGGGCTGGAAACGGATGGTGGTGGGTGCCTCTAGTT
GCCCCCCCTATTGGAGGAGTCCTTGGAGCAGGGCTATACAAGGCTGTTGTGGAACTGCAT
CACCCACACCTCTCTGAAGCGGGTGGAGAGATG---GTT------------GAAGAAGCT
GTCCCTCTGGATAAAGAGATAAATACC---ATTGAAAATATG------TGTGTG------

>Lyretail_cichlid_Aqp7
ATGAAGGACTTGGCGCAGTCAGTAAAACCCGGGGTG---TTTCAGCAGAGGGGAGGTAAA
GTAACTCGATCCAAAGTTTGGCTA---AAGAATGAACTCATTCGTGTGGGACTTGCTGAA
TCACTTAGCACATATGTCATGATGTCATTGGGTTTGGGGTCTGTGGCCCAGGTAGTGACT
GGTCAGGGAGCTTTTGGACAGTACCTCAGCATCAACCTGGGTTTTGGACTGGCTGTTGCC
ATGGGGTCTCATGTTGGAGGGAAGATCTCGGGGGCTCATATGAATGGAGCTGTATCATTC
ACAATGTGTGTGTTTCGCCGCCTCCCGTGGAAGATGCTACCTCTTTATATTTCGGCACAG
CTATTGGGATCATTTCTTGCAGCAGGGACAATTTATGCTGTCTACTATGAAGCCATTCAT
GACTACTGTAGAGGAAACCTGACTGTGACTGGTGAGAAGGCCACTGCTGGTATCTTTGCC
ACCTATCCTGCTCCATACCTCTCTCTGATAGCTGGATTTTTTGACCAGGTATTTGGCACA
GCGATGCTACTACTGTGCCTTATGGCTCTATCCGACCAGAAGAACAAACCAGCGCCAGCA
GGAAGTGAGCCTGCATTTGCGGGTTTCCTGGTGCTTCTCATTGGCATTTCTTTGGGTAGC
AACAGTGGCTATGCCATCAACCCCACCAGAGACATCGCACCCAGGGTTTTCACTGCCATG
GCAGGCTGGGGATCTGATGTGTTCAGGGCTGGAAATGGATGGTGGTGGGTGCCTCTAGTT
GCCCCCCCTATTGGAGGAGTCCTTGGAGCAGGGCTATACAAGGCTGTTGTGGAACTGCAT
CACCCACACCTCTCTGAAGCGGGTGGAGAGATG---GTT------------GAAGAAGCT
GTCCCTCTGGATAAAGAGATAAATACC---ATTGAAAATATG------TGTGTG------

>Nile_tilapia_Aqp7
ATGAAGGACTTGGCGCAGTCAGTAGAAGTTGGGGTG---TTTCAGCAGAGGGGAGGTAAA
GTAACTCGACCAAAAGTTTGGCTA---AAGAATGAACTCATTCGTGTGGGACTTGCTGAA
TCACTTAGCACATATGTCATGATGTCATTGGGCTTGGGGTCTGTGGCCCAGGTAGTGACC
GGTCAGGGAGCTTTTGGACAGTACCTCAGCATCAACCTGGGTTTTGGACTGGCTGTTGCC
ATGGGGTCTCATGTTGGAGGGAAGATCTCGGGGGCTCATATGAACGGAGCCGTATCATTC
ACAATGTGTGTGTTTCGCCGCCTCCCGTGGAAGATGCTACCTCTTTATATTTCGGCACAG

CTATTGGGGTCATTTCTGGCAGCAGGGACAATTTATGCTGTCTACTATGAAGCCATTCAT
GACTACTGTGGAGGGAACCTGACTGTGACTGGTGAGAAGGCCACAGCTGGTATCTTTGCC
ACCTATCCTGCTCCATACCTCTCTCTGATAGCTGGATTTTTGACCAGGTATTTGGCACA
GCGATGCTACTACTGTGCCTTATGGCTCTATCCGACCAGAAGAACAAACCAGCGCCAGCA
GGAAGTGAGCCTGCATTTGTGGGTTTCCTGGTGCTTCTCATTGGCATTTCTTTGGGTAGC
AACAGTGGCTATGCCATCAACCCCACCAGAGACATCGCACCCAGGGTTTTCACTGCCATG
GCAGGCTGGGGAACTGATGTGTTCAGGGTTGGAAATGGATGGTGGTGGGTGCCTCTAGTT
GCAACCCCTATTGGAGGAGTCCTTGGAGCAGGGCTATACAAGGCTGTTGTGGAACTGCAA
CACCCACACCTCTCTGAAGCAGGTGGAGAGATG---GTT------------GAAGAAGCT
GTCCCTCTGGATAAAGAGATAAATACC---AGTGAAAATGTG------TGTGTG------

>Midas_cichlid_Aqp7
ATGAAGGACTTGGTGCAGCCAGTAGAAGCAGGGGTC---TCTCAGCAGAGGGGAGGTAAT
CTAACTCGACCTAAAGTTTGGCTA---AAGAATAACCTCATTCGTGTGGGACTTGCTGAA
TGTCTTAGCACATATGTCATGATGTCATTTGGCCTGGGGTCTGTGGCCCAGGTAGTGACT
GGTCAGGGAGTTTTTGGACACTACCTCAGCATCAACCTGGGTTTTGGACTGGCTGTTGCC
ATGGGGGTTTACGTTGGAGGGAAGGTCTCAGGGGCTCACATGAATGGAGCAGTATCATTC
ACGATGTGTGTGTTTCATCGCCTTAAGTGGAAGATGCTACCTCTGTATCTTCTGGCACAG
CTATTGGGGTCGTTTCTGGCAGCAGGGACAATTTATGCTGTCTACTATGAAGCCATTCAT
GACTACTGTGGAGGAAACCTGACTGTGACTGGTGAGAAGGCCACAGCTGGTATCTTTGCC
ACCTATCCTGCACCATACCTCTCCTTGATGGCTGGATTTTTGACCAGGTATTTGGCACA
GGGATGCTGCTACTGTGCCTCATGGCTCTATCCGACCAGAAGAACAAACCGATCCCAGCA
GAAAGCGAGCCTGCAGCAGTAGGTCTCCTGGTGCTGCTCATTGGCATTTCTCTGGGCAGC
AACAGTGGGTATGCCATCAACCCCACCAGAGACATTGCACCCAGGGTCTTCACTGCCATG
GCAGGCTGGGGAACTGATGTGTTCAGGGCTGGAAATGGGTGGTGGTGGGTGCCTCTAGTT
GCTCCCCCCGTTGGAGGAGTACTTGGAGCGGGGCTATACAAGGGTTTGTGGAACTGCAT
CACTCACACCTCTCCGAACAGGATGGAGCAATG---GTG------------GAAGAGGCT
GTCCCTCTTGATAAAGAGACAAACACC---TGTGCAAATGTA------TGCGTG------

>Indian_glassy_fish_Aqp7
ATGAAGGACTTGGTGCAGTCTGTAGAGTTAGGGGTC---TCACAGCGGAGGGGGGGTGCT
GCAACACGACCCAAAGTTTGGCTA---AAAAATGAACTTGTTCGTGTTGGACTTGCTGAG
TTCCTTTGCACGTATGTCATGATGGCGTTTGGCCTGGGGTCTGTGGCCCAGGTAGTGACA
GGACAGGGAGCATTCGGACAGTACCTTAGCATCAACCTGGGGTTTGGGCTGGGTGTTGCT
ATGGGGGTTCATGTTGGAGGAAAGGTTTCAGGGGCTCATATGAATGGAGCAGTGTCATTC
ACAATGTGTGTGTTTGGCCGCCTAGCATGGAAGATGTTGCCTTTGTATCTTTCTGCACAG
CTTTCTGGCTCATTTTTGGCAGCAGGCACAATCTATGCTGTCTACTATGAGGCCATTAAT
GACTACTGTGGAGGAAACCTGACAGTGACTGGAGCGAAGGCCACAGCTGGTATCTTTGCC
ACTTACCCTGCACCATACCTCTCCGTGATGGCTGGATTTATTGACCAGGTGTTTGGCACA
GCCATGCTGCTGCTGTGCCTGATGGCTCTGTCTGATCAGAGGAACAAACCAGCAGTTGCT
GGCAGTGAGCCTGCTGCAGTGGGTTTTCTGGTGCTACTCATTGGTATTTCTCTGGGCAGC
AACAGTGGCTATGCTATCAACCCCACCAGAGACATTGCACCCAGGGTCTTTACTGCTATA
GCAGGCTGGGGAGCTGATGTGTTCAGGTCTGGCAATGGATGGTGGTGGGTGCCTCTAGTT
GCTCCCCCAATTGGAGGAGTATTAGGGGCAGGGGTGTACAAGGCCTTTGTGGAACTGCAC
CACCCACTGATCTCTGAACAGTGTGGGGGGCTG---ATGGAG---------GAGGAGTCT
GCCCCTCTGGAGAAAGAGCAAAACATC---TGTGCCAATGTA------TGTGTG------

>Pacific_bluefin_tuna_Aqp7
ATGAAGGACCTGGTGCAGTCAGTAGATGTGGGGGTC---TCTCAGTGGAAAGGAGTTCAC
GTAACTCGATCCAAAGTTTGGATA---AAGAATGAACTTGTTCGTGTTGGACTTGCTGAA
TTTCTTTGCACATATGTCATGATGGTGTTTGGTCTGGGGTCTTCAGCCCAGGTACTAACA
GGACAGGGAGCGTATGGACAGTACCTCAGCATCAACCTGGGTTTTGGACTGGGTGTCGCT
ATGGGGGTTCATGTTGGAGGGAAGGTGTCAGGAGCTCATATGAATGCAGCAGTGTCATTC
ACAATGTGTGTATTTGGCCGCCTTGCGTGGAAGATGCTGCCCCTGTATGTTTTTGCACAG
CTATTTGGGTCCTATCTGGCAGCAGGGACAATTTATGGTGTCTACTACGAAGCCATATAT
GACTATTGTGGAGGAAATCTCACTGTAAATGGTGTACGGGCCACAGCTGGTATCTTTGCC
ACCTATCCAGCACCATATCTCTCCTTGCTGGGTGGATTCATGGACCAGGTGTTTGGCACA
GCTATGCTGCTGCTGTGTCTGATGGCTCTGTCCGACCAGAAGAATAAACCGGCCGCAGCA
GGCAGCGAGCCTGTCGCAACGGGTCTCCTGGTGCTGCTCATTGGCATTTCTCTGGGCAGC
AACAGCGGCTACGCTATCAACCCAACCAGAGACATCGCACCTAGAGTCTTCACTGCCATT
GCAGGCTGGGGGGCTGACGTGTTCAGGTCTGGAAATGGGTGGTGGTGGGTGCCTCTAGTT

GCCCCTCCTATTGGAGGTGTACTGGGTGCTGGGCTGTACAAGGCCTTGGTGGAACTGCAC
CACCCTCCCCTCCCTGAACAGGTTGGGGGACTG---ATGGAGGAGTTGAAAGAGGAGACC
ATCCCTCTGGAAAAACAGAAAAACATC---TGTGCTGATGTG------TGTGTG------

>Atlantic_bluefin_tuna_Aqp7
ATGAAGGACCTGGTGCAGTCAGTAGATGTGGGGGTC---TCTCAGTGGAAAGGAGTTCAC
GTAACTCGATCCAAAGTTTGGATA---AAGAATGAACTTGTTCGTGTTGGACTTGCTGAA
TTTCTTTGCACATATGTCATGATGGTGTTTGGTCTGGGGTCTTCAGCCCAGGTACTAACA
GGACAGGGAGCGTATGGACAGTACCTCAGCATCAACCTGGGTTTTGGACTGGGTGTCGCT
ATGGGGGTTCATGTTGGAGGGAAGGTGTCAGGAGCTCATATGAATGCAGCAGTGTCATTC
ACAATGTGTGTATTTGGCCGCCTTGCGTGGAAGATGCTGCCCCTGTATGTTTTTGCACAG
CTATTTGGGTCCTATCTGGCAGCAGGGACAATTTATGGTGTCTACTACGAAGCCATATAT
GACTATTGTGGAGGAAATCTCACTGTAAATGGTGTACGGGCCACAGCTGGTATCTTTGCC
ACCTATCCAGCACCATATCTCTCCTTGCTGGGTGGATTCATGGACCAGGTGTTTGGCACA
GCTATGCTGCTGCTGTGTCTGATGGCTCTGTCCGACCAGAAGAATAAACCGGCCGCAGCA
GGCAGCGAGCCTGTCGCAACGGGTCTCCTGGTGCTGCTCATTGGCATTTCTCTGGGCAGC
AACAGCGGCTACGCTATCAACCCAACCAGAGACATCGCACCTAGAGTCTTCACTGCCATT
GCAGGCTGGGGGGCTGACGTGTTCAGGTCTGGAAATGGGTGGTGGTGGGTGCCTCTAGTT
GCCCCTCCTATTGGAGGTGTACTGGGTGCTGGGCTGTACAAGGCCTTGGTGGAACTGCAC
CACCCTCCCCTCCCTGAACAGGTTGGGGGACTG---ATGGAGGAGTTGAAAGAGGAGACC
ATCCCTCTGGAAAAACAGAAAAACATC---TGTGCTGATGTG------TGTGTG------

>Yellowfin_tuna_Aqp7
ATGAAGGACCTGGTGCAGTCAGTAGATGTGGGGGTC---TCTCAGTGGAAAGGAGTTCAC
GTAACTCGATCCAAAGTTTGGATA---AAGAATGAACTTGTTCGTGTTGGACTTGCTGAA
TTTCTTTGCACATATGTCATGATGGTGTTTGGTCTGGGGTCTTCAGCCCAGGTACTAACA
GGACAGGGAGCGTATGGACAGTACCTCAGCATCAACCTGGGTTTTGGACTGGGTGTCGCT
ATGGGGGTTCATGTTGGAGGGAAGGTCTCAGGAGCTCATATGAATGCAGCAGTGTCATTC
ACAATGTGTGTATTTGGCCGCCTTGCGTGGAAGATGCTGCCCCTGTATGTTTTTGCACAG
CTATTTGGGTCCTATCTGGCAGCAGGGACAATTTATGGTGTCTACTACGAAGCCATATAT
GACTATTGTGGAGGAAATCTCACTGTAAATGGTGTACGGGCCACAGCTGGTATCTTTGCC
ACCTATCCAGCACCATATCTCTCCTTGCTGGGTGGATTCATGGACCAGGTGTTTGGCACA
GCTATGCTGCTGCTGTGTCTGATGGCTCTGTCCGACCAGAAGAATAAACCGGCCGCAGCA
GGCAGCGAGCCTGTCGCAACGGGTCTCCTAGTGCTGCTCATTGGCATTTCTCTGGGCAGC
AACAGCGGCTACGCTATCAACCCAACCAGAGACATCGCACCTAGAGTCTTCACTGCCATT
GCAGGCTGGGGGGCTGACGTGTTCAGGTCTGGAAATGGGTGGTGGTGGGTGCCTCTAGTT
GCCCCTCCTATTGGAGGTGTACTGGGTGCTGGGCTGTACAAGGCCTTGGTGGAACTGCAC
CACCCTCCCCTCCCTGAACAGGTTGGGGGACTG---ATGGAGGAGTTGAAAGAGGAGACC
ATCCCTCTGGAAAAACAGAAAAACATC---TGTGCTGATGTG------TGTGTG------

>Atlantic_chubb_mackerel_Aqp7
ATGAAGGACTTGGTGCAGTCCGTAGACATGGGGGTC---TCTCAGTGGAAAGGAGTCAAT
GTAACTCGATCCAAAGGCTGGCTA---AAGAATGAATTTGTTCGGGTGGGACTTGCTGAA
CTTCTTTGCACATATATCATGATGGTGTTTGGGCTGGGGTCTGTAGCCCAGGTAGTGACA
GGGCAGGGAGCGTTTGGAGAGTACCTCAGCATCAACCTGGGCTTTGGACTGGGTGTTGCT
TTGGGAGTTCATGTTGGAGGGAAGGTCTCAGGAGCTCATATGAACGGAGCAGTGTCATTC
ACAATGTGCGCATTTGGCCGCCTAGCATGGAAAATGCTGCCCCTCTATGTTTTTGCACAG
CTTTTGGGGTCCTTTCTGGCAGCGGGGACAATTTATGGTGTATACTACGATGCCATATAT
GACTACTGTGGAGGAAATCTGACTGTAAATGGTGTACGGGCCACAGCTGGTATCTTTGCC
ACCTATCCAGCACCATATCTCTCCTTGCTGGGTGGATTCATTGACCAGGTGTTTGGCACA
GCTATGCTGCTGCTGTGTCTGATTGCACTGTCCGACCAGAGGAACAAACCGGCCGCAGCA
GGCAGTGAGCCTGTCACAGTGGGTCTCCTGGTGCTGCTCATTGGCATTTCTCTAGGCAGC
AACAGTGGCTACGCTATCAACCCGACCAGAGACATCGCACCCAGAGTCTTCACTGCCATC
GCAGGCTGGGGGCCTGATGTGTTCAGGGCTGGCAATGGGTGGTGGTGGGTGCCTCTAGTT
GCCCCCCCTATTGGAGGTGTACTGGGTGCAGGGTTATACAAAGCCTTAGTGGAACTGCAC
CACCCTCTCCTCCCTGAACAGGATGAAGGGGAG---ATGGAGGAGCTGAAAGAGGAGACT
GTCCCTCTGGAAAAACAGAAAAACATC---TGTGCTGATGTG------TGTGTA------

>Silver_pomfret_Aqp7
ATGAAGGACTTGGTGCAGTCAGTAGATCTGGGGGTG---TCTCGGCCAAAAGGAGTTAAC
GTAACTCGATCTAAAGTTTGGATA---AAGAATGAACTTGTTCGTGTGGGACTCGCTGAA

```
TCAATTTGCACATATGTCATGATGGTGTTCGGCCTGGGGTCTGTGGCCCAGGTAGTGACA
GGACAGGGAGCGTTTGGACAGTACCTCAGCATCAACCTGGGTTTTGGACTGGGTGTTGCT
ATGGGGGCTCATGTTGGAGGGAAAGTCTCAGGAGCTCATATGAACGCGGCAGTATCATTC
ACAATGTGTGCATTTGGCCGCCTTGCGTGGAAGATGCTGCCCCTGTACGTTTTTGCGCAG
CTGTTGGGATCCTTTCTGGCAGCGGGGACAATTTATGGTGTCTACTACGAAGCCATAAAT
GACTATTGTGGAGGAAATCTGACTGTAACTGGTGTTCGGGCCACAGCTGGTATCTTTGCC
ACATATCCGGCACCATATCTCTCCTTGCTGGCTGGATTCATTGACCAGGTGTTTGGCACA
GCTATGCTGCTGCTGTGTCTGATGGCTCTGTCCGACCAGAAGAACAAACCGGCCGCAGCG
GGCAGCGAGCCCTTCGCAGTTGGTCTCCTGGTGCTGCTGATTGGCATTTCTTTGGGAAGC
AATAGCGGCTACGCTATCAACCCAACCAGAGACTTTGCACCCAGAGTCTTCACCGCCATT
GCAGGCTGGGGGGTTGACGTGTTCAGGTCTGGAAATGGGTGGTGGTGGGTGCCTATAGTT
GCCCCCCCTATTGGAGGTGTACTGGGTGCAGGGCTATACAAGGTCTTGGTGGAACTGCAC
CACCCTGTCGTTCCTGAACAGGTTGGGAGGCTG---ACGCATGAATTGGAAGAAGAGACT
GCCCCTCTGGAAAAACATAAAAACATC---TGTGCTGATGTG------TGTGTG------
------------------
>Tigertail_seahorse_Aqp7
ATGAAGGACTTTGCACAGTCAGCAGATGGAGGGGTC---TCTCTGGGAAAAGCGTCTCTT
GTAACCCGA---TCTTTTCGATTA---AGGAATGAAATTGTTCGCGTGGGACTCGCCGAA
ACTCTTTGCACATACGTGATGATGGTATTTGGCCTCGGCTCTGTGGCGCAGGTCGTGACT
GGACAAGGAGCATTTGGACATTACATAAGCATCAATCTGGGCTTTGGGCTGGGTGTCGCG
CTCGGGTGTCATGTCGGAGGCAAAGTTTCAGGCGCTCACATGAACGCGGCGGTGTCCTTC
GCCATGTGCGTGTATGGCCGCCTCGCGTGGAAGATGCTCCCGCTCTACTTTTTGTCACAG
CTGCTGGGGTCCTTTTTAGCCGCGGGGTCGGTTTACGCTGTGTATTATGAGGCAATACAT
GACTATTGTGGAGGGAATCTGACTGCCACCGGTGCCAAGGCTACCGCTGCTATCTTTGCC
ACCTATCCGGCCCCGTATCTCTCTTTGTTTGGCGGATTCATTGACCAAGTATTTGGTACA
GCGATGCTTCTGCTGTGCCTGATGGCTTTGTCCGACCAGAAAAACAAACCGGCTGAAGCA
GGCAGCGAATCAACTCTCGTGGGTCTCCTGGTGCTCTTAATCGGCATTTCTCTGGGCAGC
AACAGCGGTTACGCAATCAACCCCACCAGAGACCTCGGACCGAGGCTCTTCACAGCCGTT
GCCGGCTGGGGGCTTGACGTGTTCAGGGCTGGAAACGGTTGGTGGTGGGTGCCCGTTATT
GCCCCGATGATCGGAGGCGTGCTGGGCGCGGGGCTCTACAAGCTCTTGGTGGAACTTCAC
CATCCTCCACGCTCGGAAAATTGTGCTGGACCC---ATTAAAACCTTA---GAGGAGACG
GCA---------------CAAAACACC---GACACTGAATTG------CGTGTG------
------------------
>Gulf_pipefish_Aqp7
ATGAAGGACTTTGCAAAGTCAGCAGATCAAGGAATC---CCTCTGCGAAAAGCACCTCTT
GTAACCCGA---GCTTTTCGATTA---AGGAATGAAATTGTTCGCGTGGGACTTGCTGAA
ACTCTTTGCACTTATGTAATGATGGTGTTTGGCCTTGGTTCTGTGGCGCAGGTCACGACC
GGAAAAGGAGAATTTGGACATTACATAAGCATCAATCTGGGCTTTGGGCTGGGTGTTGCA
ATTGGGAGTCATATAGGAGGGACAGTTTCAGGCGCTCATATGAACGCGGCGGTGTCCTTC
GCCATGTGTGTCTATGGCCGCCTGCTGTGGAGGATGTTCCCCCTCTATATTTTCTCACAG
ATTCTGGGGTCCTTTTTAGCCGCAGGGTCGGTTTATGCTGTGTATTACGAGGCAATACAC
GACTATTGTGGAGGGAATCTGACTGTAACTGGCGTCAAGGCCACTGCTAGTATCTTTGCC
ACCTATCCTGCCCATTACCTCTCCTTGTTGGGAGGATTCATTGACCAGGTATTTGGGACA
GCTTTGCTACTGCTGTGCCTGTTGGCATTGTCCGACCAGAAAAACAAACCAGCTGCAGCA
GGCACCGAGCCAACCGTCGTGGGTCTCTTGGTGCTATTAATCGGCATTTCTATGGGCAGT
AACAGTGGCTACGCGATCAATCCCACCAGAGACATCGGGCCGAGGATCTTCACGGCCATT
GCGGGTTGGGGGGCTGACGTGTTCAGGGCCGGAAATGGTTGGTGGTGGGTGCCCATTGTC
GCCCCGATGATTGGAGGCGTATTGGGTGCGGGAGTCTACAAGGTGTTGGTTGAACTTCAT
CATCCTCCACGCTCAGAAAATTGCACTGGACCG---ATAGAAAGAGTAGAGAAGGAGACT
GCATCACTGGAAAAA---CAAAACGCT---GACACTGATTTT------TGCGTG------
------------------
>Giant_mudskipper_Aqp7
---------ATGGTTCAGTCCATGGAGCTTGGGTCT---TCGGTCCAGAGGAAG------
ACCCTAAAGACGAGAGTCCGCATC---CAGAACCAGATACTGAGACTTGGACTCGCCGAG
ACCCTCTGCACCTACGTCATGATGGTTTTCGGGCTGTGCTCCGTGGCCCAGGTGGTCACA
GGTAAAGGAGAGTTCGGACACTACATCAGTATAAACCTGAGCTTTGGTCTCGCTGTCGCT
CTGGGAGTGCACGTGGGGGGCGGAGTCTCAGGAGCCCACATGAATGCGGCGGTCTCCTTC
TCTTCCTGCGTCTTTGGGACTCTCCGGTGGAGGCTCCTTCCACTTTACATCACAGCGCAG
TTCATTGGCTCGTTCCTCGCCGCAGCAACTGTTTACGCCGTTTACTACGAGGCCATCCAC
AGTTACTGCGGGGGGAACCTGACGGTGTCGGGCCCTGGGGCCACGGCCGGGATCTTCTCC
ACATATCCAGCTCCTTATCTGTCCCTGAGCGGAGGCTTCATCGACCAGGTATTCGGCACG
```

GCCATGCTCCTCCTTTGCCTGTCCGCTCTCTCTGACCGCAGGAACCAGCCGTGTCCTGCA
GGGGGCGAGCCTGTGGCCGTGGGGCTCCTGGTGCTGCTCATCGGGATGTCAGTGGGGAGC
AACAGTGGCTATGCCATCAACCCCACAAGAGACATCGGGCCCCGAGTGTTCACTGCCATC
GCAGGCTGGGGCCCCGAGGTCTTCAGGGCTGGCAACTGGTGGTGGTGGGTGCCATTGGTC
GCGCCCTGTCTTGGGGCGGTTGTGGGGACAGGACTCTACAAACTCCTGGTTCAGCTGCAC
CACCCCCTGCTCAGTGCGCCCTCTGCTGGAGAG---TCGGAGGAACCGCACAGGGAGACA
GAGCTACTGGAGAAACACAAC---------------------------TGTGTAGTC---

>Atlantic_mudskipper_Aqp7
---------ATGGTTCAGTCCATGGAGCTCGGGTCG---TCGGACCAGAGGAAG------
ACCCTAAAGACCAGAGTCCGCATC---CAGAACCAGATACTGAGACTTGGACTCGCCGAG
ACCCTCTCGACCTACGTCATGATGGTTTTCGGGCTGTGCTCCGTGGCCCAGGTGGTCACA
GGTAAAGGAGAGTTCGGACAATACATCAGTATAAACCTGAGCTTTGGCCTCGCCGTTGCT
CTGGGAGTGCACGTCGGTGGTGGAGTCTCAGGAGCCCACATGAACGCGGCGGTCTCCTTT
TCTTCTTGCGTCTTTGGGACTCTCCGGTGGAGGCTCCTTCCACTTTACGTCACAGCGCAG
TTCATTGGCTCGTTCCTCGCTGCAGCAACTGTCTACGCTGTTTACTACGAGTCCATCCAC
AGCTACTGCGGGGGGAACCTGACAGTGTCGGGCCCAGTGGCCACGGCCGGGATCTTCTCT
ACATATCCAGCCCCTTATCTGTCCCTGAGCGGAGGCTTTATTGACCAGGTATTCGGCACG
GCCATGCTCCTCCTCTGCCTGTCAGCTCTCTCTGACCGCAGGAACCAGCCGTGTCCTGCA
GGGGGCGAGCCTGTGGCTGTGGGGCTCCTAGTGCTGCTCATTGGGATGTCCGTGGGGAGC
AACAGCGGCTATGCCATCAACCCCACAAGAGACATCGGGCCCCGTGTGTTCACTGCCATC
GCAGGCTGGGGCCCCGAGGTCTTCAGGGCTGGTAACTGGTGGTGGTGGGTGCCATTGGTC
GCGCCCTGTGTTGGGGCAGTTGTGGGGACAGGACTCTACAAACTCCTGGTTCAGCTGCAC
CACCCCCTGCTCAGTGCGCCCTCTGCTGGAGAA---TCGGAGGAACCACACAGAGAGACA
GAACCACTGGAGAAACACAGTAAC------------------------TGTGTAGTA---

>gbsMudskipper_Aqp7
---------ATGGTTCAGTCGTTGGAGCTCGGGTCC---TCGGTCCAGAGGAAA------
ACCCTCAAGTCCAGAATCCAGATC---CAGAACCAGGTCCTGAGACTCGGACTCGCCGAG
ACACTCTGCACCTACGTCATGATGGTGTTCGGGCTGTGCTCCGTGGCTCAGGTGGTCACA
GGTAAAGGAGAGTTCGGTCACTACATCAGTATAAACCTGAGCTTCGGTCTCGCCGTCGCC
ATGGGCGTCCACGTGGGGGGCGGAGTCTCAGGCGCTCACATGAACGCCGCCGTCTCTTTC
TCTTCCTGCGTCTTCGGGACCCTGAGGTGGAGGCTCCTCCCACTTTACATCACGGCTCAG
TTCATTGGCTCCTTCCTCGCCGCGGCAACCGTCTACGCCGTGTACTACGAGGCCATCCAG
AGTTACTGCGGGGGGAACCTGACGGTCTCGGGCCCCGGGGCCACGGCCGGAATCTTCGCC
ACGTATCCGGCCCCGTACCTGTCCCTGAGCGGAGGCTTCAGCGACCAGGTGTTCGGGACG
GCCATGCTGCTGCTCTGTCTGTCGGCTCTGAGCGACCGCAGGAACCAGCCGTGTCCAGCA
GGGGGCGAGCCGGTGGCCGTGGGGCTCCTGGTGCTGCTCATCGGGATGTCGGCCGGGAGC
AACAGCGGCTACGCCATCAACCCCACCAGGGACATCGGGCCCCGCGTCTTCACCGCCGTC
GCCGGATGGGGCCCCGACGTCTTCAGG---------------------------------

>Walking_goby_Aqp7
---------ATGGTTCAGTCCGTGGAGCTGGGGTCC---TCGGTCCAGAGGAAG------
ACCCTGAAGACCAGAGTCCAGATC---CAGAACCAGATCCTGAGAATCGGACTCGCGGAG
ACTCTCTGCACCTACGTCATGATGGTGTTCGGTCTCTGCTCCGTGGCTCAGGTGGTCACA
GGTAAAGGAGCGTTCGGACATTACATCAGTATAAACCTGAGCTTCGGCCTCGCCGTGGCC
ATGGGCGTGCACGTGGGGGGTGGAGTCTCAGGCGCACACATGAATGCCGCCGTCTCTTTC
TCTTCCTGCGTCTTCGGGACCCTCAGGTGGAGGCTCCTCCCACTTTACGTCACGGCTCAG
TTCATTGGCTCATTCCTCGCCGCGGCAACCGTCTACACCGTTTACTACGAGGCCATCCAC
AGTCACTGCGGGGGGAACCTGACGGTCTGGGGCCCCGGGGCCACGGCCGGAATCTTCTCC
ACTTACCCAGCGCCATATCTGTCCCTGAGCGGAGGCTTCATCGACCAGGTGTTCGGCACG
GCCATGCTGCTCTTGTGCCTGTCGGCTCTGTCTGACCGCAGGAACCAGCCGTGTCCAGCA
GGGGGCGAGCCGGTGGCCGTGGGGCTCCTGGTTCTGCTCATCGGGATGTCGGCAGGAAGT
AACAGCGGCTACGCCATAAACCCCACCAGAGACATCGGGCCCCGAGTGTTCACCGCCATC
GCCGGGTGGGGCCCCGACGTCTTCAGG---------------------------------

>Javaline_goby_Aqp7
----------------------------ATGGGATCT---TCTGTCCAGAGGAAA------
ACACCCAGAACCAGAGTCTGGATC---CGGAACCAGAACCTGAGAGTGGCCCTCGCAGAG
ACTCTGTGCACCTACGTCATGATGGTGTTCGGCCTGGCCTCAGTGGCTCAGGTGGTCACA
GGTGAAGGACACTTCGGACACTACTTCAGTATAAACCTGAGCTTTGGCCTCGCTGTGACT
CTGGGGATTCATGTGGGAGGCGGAGTCTCAGGAGCGCACATGAACGCCGCCGTGTCGTTC
TCGTCGTGTGTCTTCGGGACGCTCCGGTGGAAACTACTTCCTGTTTACGTCGCAGCGCAA
TTCATCGGATCATTCCTCGCCGCTGGAACTGTCTACGGCGTTTACTACGAGGCCATAAAC
AGTTACTGCGGGGGGAACCTTACGGTGACTGGACCCAGAGCCACAGCCGGAATCTTCTCC
ACGTTTCCAGCTCCATATCTGTCTCTGACCGGGGGCTTCATTGACCAGGTGTTCGGTACG
GCCATGCTGCTGCTCGGGCTGGCGGCGCTCTCGGACCGCAGGAACCAGCCGTGTCCAGCA
GGAGGCGAGCCGCTGGCCGTGGGGCTCCTGGTGCTGCTCATCGGAATGTCTGCGGGGGCC
AACAGCGGCTACGCCATCAACCCCACCAGGGACATTGGGCCCCGAGTGTTCACCGCCGTG
GCAGGCTGGGGCCCCGACGTCTTCAGAGCTGGCGGCGGCTGGTGGTGGGTTCCGTTGGTG
GCGCCGTGTGTCGGGGCGGTGTTGGGGACAGGACTCTACAAAGTCCTGGTTCAAATGCAC
CACCCGTCACTCAGTGCGCCCCCTGCTGGACAC---TCTGAGGAC---GTGAGGGGGAAC
GAGTTGCTGGACAAACAC------------------------------TGTGTGGTG---

>Pony_toadfish_Aqp7
ATGAAGGACGTGGTGCAGTCG---------AGGGTC---CCTCAACAAAAAGGA------
GTGACCCGATGCAGAGTTTGGATG---AAGAATGATTTTGTTCGTGTGGGAGTTGCTGAA
TTGCTTTGCACTTATGTCATGATGGTCTTTGGCCTGGGGTCTGTGGCCCAGGTCGTGCTC
GGACAAGGTGCATTTGGAGAGTACATCAGCATCAACCTGGGTTTCGGTCTGGCCGTTGCT
ATGGGTATGCACGTCGGAGGGAAGGTCTCAGGTGCTCATATGAATGGAGCGGTGTCGTTC
ACAATGTGTGTGTTTGGCCGGCTTAGGTGGAAGATGTTGCCTCTGTACATTTTTTCACAG
CTATTGGGATCTTTCCTGGGAGCCATCACCATCTACGGTGTCTATTACGAAGCCATATAT
GATTATTCTGGAGGGAACCTGACTGTAACTGGTTTAAAGGCCACAGCTGGAATCTTTGCC
ACGTATCCTGCTCCGTAC---------------------------CAGGTGTGTGGCACA
GCCATGTTGCTGCTGAGCCTGATGGCTCTGGCCGACCAGAAGAACAAACCGGTGGCGGAA
GGCATTGAGCCTGTTGTTGTTGGTCTCCTGGTGACGCTCATTGGCATTTCTCTGGGGAGC
AACTGTGGCTATACCATCAACCCCACCAGGGATATTGCACCCAGGGTTTTTACCGCCATA
GCAGGCTGGGGGACAGACGTGTTCAGGGCTGGAAATGGTTGGTGGTGGGTGCCACTAGTT
GCTCCTCTCATTGGAGGTGTGACAGGTGCAGGGTTGTACAGGATGTTGGTGGAACTGCAC
CACCCACCCCTCCCTCGTGAGGACGGGGGGCTG---GGGAATGAACTACAGGGGGAGGGA
AGCCCTCTGGAGAAACACAAAAACAAC---TGCGCTGACATG------ATTGTG------

>Plainfin_midshipman_Aqp7
ATGAAGGACGCAGTGCAGTCG---------AGGGGG---ACTCAACAGAAACGT------
GGTGCCAGATGCAGATTTTTGATA---AAGAATCATCTTCTTCGTGTGGGATTGGCTGAA
TTTCTCAGCACATATGTCATGATGGTCTTTGGCCTGGGGTGCGTGGCCCAGGTCATAATT
GGACAGGGTGCATTTGGAGAGTGGATCAGCATAAACCTGGGTTTTGGTCTGGCTGTTGCT
ATGGGTATGCACGTCGGAGGGAAGGTCTCAGGCGCTCATATGAATGGAGCGGTGTCATTC
ACAATGTGTGTGTTTGGCCAGCTTCCATGGAAGATGCTGCCTCTGTACATTTTTGCACAG
CTATTGGGATCTTTCCTGGGAGCCTTCACAATCTATGGTGTCTATTACGAAGCCATACAT
GATTATTCTGGAGGAAATCTGACTGTAACTGGTCTAAAAGCCACAGCTGGGATCTTTGCC
ACATATCCTTCACCTTACCTCTCCATACAGGGTGGATTCATTGACCAGGTGGTTGGTACA
GCCATGTTGATGCTGAGCTTGATGGCTCTGTGCGATCAAAAGAACAAACCGGTGGCGGAA
GGTACTGAGCCTGTTGTTGTTGGTCTCCTGATAACGCTCATTGGCATTTCTTTGGGTATC
AACTGCGGCTATACCATCAACCCCACCAGAGATATTGCACCAAGGGTTTTTACTGCCATA
GCAGGCTGGGGGACAGAAGTGTTCAGGGCTGGAAATGGTTGGTGGTGGGTGCCACTAGTT
GCTCCTCTCATTGGAGGTGTGACAGGTGCAGGTTTGTACAGGATGTTGGTGGAACTGCAC
CACCCAGAGCCATCTCATAAGGACATCCTG------GGGGATGAGCTACAGCTGGAGAAA
AGCCTTGTAGACAAACCAACAAATATG---TCCGCTGACATG------ATTGTG------

>Pearlfish_Aqp7

ATGCCAAGGTCCAAACGCCGGCTG---GAGAGTGAAGTCCTCCGTGTGGGGCTTGCTGAG
ATCCTCTGCACCTACGTCATGATGGTGTTCGGGCTGGGATCTGTGGCCCAGGTGGTCACA
GGCCAAGGAGCCTTTGGACAGTACATCAGCATCAATCTTGGCTTTGGTCTGGGGGTCGCC
TTTGGGATCCATGTTGGTGGGAAGGTCTCGGGAGCTCACATGAATGCAGCAGTGTCCTTC

ACTATGTGTGTGTTTGGTCGCTTGGCCTGGAAGATGCTGCCTGTGTATGTTGCTGCGCAG
TTTCTTGGCTCTTTTCTGGCAGCTGCCACAATTTACGGTGTCTACTATGATGCCATCCAT
GATTACTGTGGCGGAAATCTGTCCGTGACTGGTCTGAAGGCGACAGCCGGGATCTTTGCC
ACCTATCCTGCTCCGTACCTCTCCACTCTGGCTGGCGTCATTGACCAGGTGTTTGGCACA
GCTATGCTGCTCTTGTGCCTGATGGCTCTGTCAGACCAGAGGAACAAACCTGCGATGGCC
AATGGGGAGCCAGTGGCTGTGGGTCTCCTGGTGCTGCTCATCGGCATCTCTCTAGGCAGC
AACAGCGGCTATGCTATCAACCCCACCAGAGACATTGGACCCAGAGTCTTCACTGCCATA
GCGGGCTGGGGGACAGGCGTTTTCAGGGCTGGAAACGGCTGGTGGTGGGTGCCCTTAGTT
GCTCCCCTTATCGGTGGCGTGGTAGGTGCAGCCGCGTACAAGATCTTAGTGGAGCTGCAC
CACCCGTCACCTCCAGCACAGGGCGGGGCAGTC---AGGGCAGAG---CCTGAGGAGTCT
TCCCCGCTTGAAAAACTCGATCAGAGC---TCCGCCAACGCG------TGTGTG------

>Bearded_brotula_Aqp7

ATGCCCAGATTCAGACGATGCCTG---AAGAGTGAACTTTTCCGGGTGGGACTTGCTGAA
ACCCTCTGCACGTATGTCATGATGGTGTTTGGTCTGGGATCCGTGGCCCAGGTGGTGACA
GGGCAGGGATCTTTCGGACAGTACCTCAGCATCAACCTGGGTTTTGGCCTGGGCGTCATG
ATGGGGATTCATGTCGGAGGGAAGGCCTCAGGGGCTCACATGAATGCAGCAGTGTCCTTC
ACTATGTGCGTGTTTCGTCGCCTTTCGTGGAAGATGCTGCCTCTGTACGTTTTCGCCCAG
TTTCTCGGGTCGTTTCTGGCAGCGGCCACGATACACGCTGTCTATTACGATGCCATCTAC
ACTTACTGTGGAGGAAATCTGACTGTAACTGGATTGAAGGCTACAGCTGGGATCTTTGCC
ACCTATCCTGCACCGTACCTCTCAATACTCTCTGGATTCATTGACCAGGTGTTTGGCACA
GCCATGCTGCTGTTGGGCCTGATGGCTCTGTCCGACCAGAGGAACAAACCGGCCGTTGCC
GGCACGGAGCCTGTGGCCGTGGGTCTGCTGGTCATACTCATTGGCATTTCTCTGGGCAGC
AACAGCGGCTACGCCATCAACCCCACCAGAGACATCGGACCCAGGACCTTCACGGCCATC
GCCGGCTGGGGGGCTGATGTCTTCAGGGCCGGAAATGGCTGGTGGTGGGTACCCATAATT
GCCCCCCTCCTTGGAGGTGTGATCGGTGCAGGGTTGTACCAGATCATGGTGGGACTGCAC
CACCCTTCACCCTCCTCCGGGGGTCATCAA------------------GGGGACGAGTCT
GTCCCTCTGGAGAAAGTCGAAAGGAAC---GCTGCTAATGAG------TGTGTG------

>Legless_cuskeel_Aqp7

ATGCCAAGATCCAAGCGCTGGCTA---AAGAATGAGCTTCTGCGCGTGGGATTTGCCGAG
ACGCTCTGCACGTACGTCATGATGGTGTTTGGTCTGGGATCTGTGGCCCAGGTCGTGACA
GGACAGGGAGCGTTCGGGCAGTACCTCAGCATCAACCTGGGTTTTGGTCTGGGCGTGGCT
ATGGGGGTTCACGTCGGAGGGAGCGTCTCAGGAGCTCACATGAACGCAGCAGTGTCCTTC
ACAATGTGTGTGTTTGGCCGCCTGGCGTGGAAGATGTTCCCCCTCTATGTTCTCGCACAG
CTTCTCGGGTCGTTTCTGGCAGCGAGCACCATTTATGCCGTCTACTATGATGCCATTAAT
GTTTATTCTGGAGGAAATCTGACTGTGACTGGTTTGAAGGCGACAGCCGGGATCTTTGCC
ACCTATCCTGCACCGTACCTCTCAATACTGGCCGGGTTCATTGACCAGGTGTTCGGCACA
GCCATGCTGCTGTTGTGCCTGATGGCTCTGTCTGACCAGAGGAACAAACCGGCGGCGGCA
GGCAGCGAGCCCGTGTCGGTGGGTCTCCTGGTGCTGCTCATTGGCATTTCTCTGGGCAGC
AACAGCGGCTACGCCATCAACCCCACCCGAGACATCGCGCCCAGGGTCTTCACCGCCATA
GCTGGCTGGGGAGCCGACGTCTTCAGGGCTGGAAACGGGTGGTGGTGGGTGCCCATAGTT
GCCCCCCTCATCGGAGGCGTGGTGGGTGCAGGGATGTACAAGATCTTGGTGGAGCTGCAC
CACCCTACCACCCCCGATCAGGGCGGGGCACCG---AGGGAGGGGCCAGAGGAGGAGCGT
GCCCCCCTGGAGAAACAGGAGAGGAAC---TGTGCCAACGCG------TGTGTG------

>Longspine_squirrelfish_Aqp7
ATGAAAGACTTGGTGCAGTCACTAGAACTGGGGGGC---CCTCAGCAGAAAGGGGGCTGT
AAAACTCGATCCAGACATTGGCTG---AGGAATGAGTTCTTCCGAGTGGGACTTGCTGAA
ACCCTCTGCACGTATGTCATGATGGTGTTTGGCCTGGGTTCTGTGGCCCAGGTAGTGACA
GGACAGGGCGCGTTTGGACAGTACCTCAGCATCAACCTGGGTTTTGGTCTGGGTGTCGCT
ATGGGGGTGCACGTTGGAGGGAAAGTCTCAGGGGCTCATATGAATGCAGCAGTGTCATTC
ACAATGTGTGTGTTTGGCCGACTCACGTGGAAGATGCTACCTCTCTATGTTTTCACTCAG
CTGTTGGGGTCATTCTTGGCCGCGGGGACAATTTACGTGGTGTACTATGATGCTATATAT
ACCTACTGTGGCGGGAATCTGACTGTGACTGGTGCAAAAGCTACTGCTGGGATCTTCGCC
ACCTACCCTGCACCGTACCTCTCCATACAGGCTGGATTCGTTGACCAGGTGTTTGGCACC
GCCATGCTGCTGCTGAGCCTGATGGCTCTGTCCGACCAGAGGAACAAACCGGCAGCTGCG
GGCAGCGAGCCTCTGGCTGTGGGTCTCCTGGTTCTACTCATTGGCATCTCTCTGGGAAGC
AACAGCGGCTATGCTATCAACCCCACCAGAGACCTGGCACCCAGGGTCTTCACTGCCATA

GCAGGCTGGGGGACTGATGTGTTCAGGGCTGGGAATGGGTGGTGGTGGGTGCCTCTACTT
GCCCCCCTTGTAGGTGGTGTACTGGGTGCCGGTGTGTACAAGGGCTTTGTGGAGATGCAC
CACCCTTCTGCCTCTGATCAGGGGGAGAAGCTG---CCAGATGATCAGGAGGAGGGGACG
GCCCCTCTGGAGAAACAGAAACACGCC---TCCGCTGACGTG------TGTGTG------

>Sammara_squirrelfish_Aqp7
ATGAAAGACTTGGTGCAGTCGTTAGAACTGGGGAGC---CCTCAGCAGAAAGGGGGCTGT
AAAACTCGATCCAGACATTGGCTG---AGGAATGAGTTTTTCCGAGTGGGACTTGCTGAA
ACCCTCTGCACATATGTCATGATGGTGTTTGGCCTGGGTTCTGTGGCCCAGGTAGTGACA
GGACAGGGGGCGTTTGGACAATACCTCAGCATCAACCTGGGTTTTGGTCTGGGTGTCGCT
ATGGGGGTGCACGTTGGAGGGAAAGTCTCAGGGGCTCATATGAACGCAGCAGTGTCATTC
ACAATGTGTGTATTTGGCCGACTTGCGTGGAAGATGCTACCTCTCTATGTTTTTACGCAG
CTGTTGGGGTCATTCTTGGCAGCAGGGACAATTTATGTGGTCTACTATGATGCTATATAT
ACCTACTGTGGCGGGAATCTGACTGTGACTGGTGCAAGAGCGACTGCTGGGATCTTTGCC
ACCTACCCTGCACCATACCTCTCCATACAGGCTGGATTCGTTGACCAGGTGTTTGGCACA
GCCATGCTGCTGCTGAGCCTGATGGCTCTGTCCGACCAGAGGAACAAACCAGCAGCTGCG
GGCAGCGAGCCTCTGGCCGTAGGTCTCCTGGTTTTACTCATTGGCATCTCTCTGGGAAGC
AACAGCGGCTATGCTATCAACCCCACCAGAGACCTGGCACCCAGGGCCTTCACTGCCATA
GCAGGCTGGGGGACTGACGTGTTCAGGGCTGGGAATGGGTGGTGGTGGGTGCCTCTACTT
GCCCCCCTTGTAGGTGGTGTACTGGGTGCAGGTGTGTACAAGGGCTTTGTGGAGATGCAC
CACCCTTCTGCCTCTGATCAGGGGGAAAAGCTG---CCAGAGGAGCCGGAGGACGAGACT
GCCCCTCTGGAGAAACAGAAACGCGCC---TCTGCTGACATG------TGTGTG------

>Blackbar_soldierfish_Aqp7
ATGAAAGACTTAGGGCAGTCATTAGAACTGAGGGGC---CCTCAGCAGAAAGGGGGCTGC
AAAACTCGACCCAGACTTTGGCTA---CAGAATGAACTTTTACGAGTTGGACTTGCTGAA
ACCCTGTGCACATATGTCATGATGGTGTTTGGCCTGGGTTCTGTGGCCCAGGTAGTGACA
GGACAGGGGGCGTTTGGAGAGTACCTCAGCATCAACCTGGGTTTTGGTTTGGGTGTCGCT
ATGGGGGTGCACGTTGGAGGCAAAGTCTCAGGGGCTCATATGAATGCAGCAGTGTCATTC
ACAATGTGTGTGTTTGGCCGACTTACATGGAAGATGCTACCTCTCTACGTTTTTACCCAG
CTGCTGGGGTCATTTATGGCAGCAGGGACAATTTACGTGGTCTACTATGATGCAATATAT
ACTTACTGTGGTGGGAACCTGACTGTGACTGGTGATAGGGCCACAGCTGGGATCTTTGCC
ACCTATCCTGCACCATACATCTCCATACAGGCTGGATTCATTGACCAGGTGTTTGGCACA
GCCATGCTGCTGCTGAGCCTGATGGCTCTGTCTGACCAGAGGAACAAGCCAGCTGTGGCG
GGCAGCGAGCCTCTGGCGGTGGGTCTTCTGGTCCTTCTCGTTGGCATCTCTCTGGGCAGC
AACAGCGGCTATGCTATCAACCCCACCAGAGACCTGGCACCCAGGGTCTTCACTGCCATA
GCAGGCTGGGGCTCTGATGTTTTCAGGGCTGGGAACGGGTGGTGGTGGGTGCCTCTACTC
GCCCCCCTTGTAGGGGGTGTACTGGGTGCAGGCGTGTACAAGGCCTTTGTCGAAATGCAC
CATCCTTCCCCTTCTGATCAGGAAGAAAAGCTG---GCACAGGAGCCAGAAGAGGAGACT
GCCCCTCTGGAGAAACAGAGCCGTATC---TGTGCTGATGTG------TGTGTG------

>Redmouth_whalefish_Aqp7
ATGAAAGAGTTGGTGCATTCACTAGAACTGGGGGGC---CCTCAGCAGAAAGGGGGCTGC
AAAACTCGATCCAGACTTTGGCTA---AAGAATGAGCTTTTTCGGGTAGGATTTGCTGAA
ACCCTCTGCACATATGTCATGATGGTGTTTGGCCTGGGTTCTGTGGCCCAGGTAGTGACA
GGACAGGGAGCGTTTGGACAATACCTCAGCATCAACCTGGGTTTTTGTCTGGGTGTTGCT
ATGGGGATGCACGTTGGAGGGAAGGTCTCAGGGGCTCATATGAACGCAGCAGTATCATTC
ACAATGTGTGTGTTTGGTCGTCTTGCGTGGAAGATGCTGCCTCTCTATGTTTTCACCCAG
CTGTTGGGGTCATTTCTGGCAGCGGGGACAATTTATATGATCTACTATGATGCCATATAT
GACTACAGCGGAGGGAATCTGACTGTGACTGGTTCGAGGGCCACAGCTGGGATCTTTGCC
ACCTACCCTGCACCGTACCTCTCCTTACAGGCTGGATTCATTGACCAGGTGTTTGGCACA
GCCATGCTACTGCTGTGCATAATGGCTCTGTCCGACCAGAGGAACAAGCCGGCCGCACCG
GGCAGCGAGCCTGTGGCAGTGGGTCTCCTGGTGCTGCTCATTGGTATCTCGCTGGGCAGC
AACAGCGGCTATGCCATCAACCCCACCAGAGACCTGGCACCCAGGGTCTTCACTGCCATA
GCAGGCTGGGGGACTGACGTGTTCAGGGCTGGGAATGGGTGGTGGTGGGTGCCTGTAGTT
GCCCCCCTTGTAGGTGGTCTACTGGGTGCAGGGCTGTACAAGGCCTTGGTGGAAATGCAC
CACCCTTCTCCCTCTGATCAGGGGGGAGAGCTG---ACAGAAGAGGCA---GAGGAGACT
GCCCCTCTGGAGAAACAGAAATGCATC---TGTGCTGACGTG------TCTGTG------

>Pricklefish_Aqp7
ATGAAAGAGCTGGTGCAGTCAGTAGAACTGGGGGGC---CCTCAGCAGAAAGGGGGCTGC

AAAACCCGATGCAAACTTTGGCTA---AAGAATGAGCTTCTTCGGGTGGGATTTGCTGAA
ACCCTCTGCACATATGTCATGATGGTGTTTGGCCTGGGTTCTGTGGCCCAGGTAGTGACA
GGACAGGGAGCATTTGGACAATACCTCAGCATCAACCTGGGTTTTTGTCTGGGTGTGGCT
ATGGGGATGCACGTTGGAGGGAAGGTCTCAGGGGCTCATATGAACGCAGCAGTGTCATTC
ACGATGTGTGTGTTTGGTCGTCTTGCGTGGAAGATGCTGCCTCTCTATGTTTTCACCCAG
CTGTTGGGGTCATTTCTGGCAGCGGGGACAATTTTTACGATCTACTATGATGCTATATAT
GACTACAGCGGAGGGAATCTCACTGTGACTGGTTCGAGGGCCACAGCTGGGATCTTTGCC
ACCTACCCTGCACCGTACCTCTCCTTACAGGCTGGAGTCATTGACCAGGTGTTTGGTACA
GCCATGCTACTGCTGTGCCTGATGGCTCTGTCCGACCAGAGGAACAAGCCGGCGGCGGCG
GGCAGCGAGCCTGTGGCAGTGGGTCTCCTGGTGCTGCTCATCGGTATCTCTCTGGGCAGC
AACAGCGGCTATGCCATCAACCCCACCAGAGACCTGGCACCCAGAATCTTCACTGCCATA
GCAGGCTGGGGGACTGACGTGTTCAGGGCTGGGAATGGGTGGTGGTGGGTGCCTGTAGTT
GCCCCCCTTGTAGGTGGTGTACTGGGTGCAGGGCTGTACAGGGCCTTGGTGGAACTGCAC
CACCCTTCCCCCTCTGATCAGGGGGGAGAGCTG---GTGGAAGAAGCAAAGGAGGAGACT
GCCCTTCTGGAGAAACAGAAATGCATC---TGTGCTGATGTG------ACTGTG------

>Splendid_alfonsino_Aqp7
ATGAAAGACTTGGTGCAGTCAGTGGAACTGGGGGCC---CCTCAGCAGAAAGGGGGCTGC
AAATCTCGATCCAAACTCCGGCTA---AAGAATGAACTTCTCCGTGTGGGACTTTCTGAA
ACCCTCTGCACATATGTCATGATGGTGTTTGGCCTGGGTTCTGTGGCCCAGGTAGTGACA
GGACAGGGGGCGTTTGGACAATACCTCAGCATCAACCTGGGTTTTTGTCTGGGTGTCGCT
ATGGGGGTGCACGTTGGAGGCAAGGTCTCAGGGGCCCACATGAACGCAGCAGTGTCATTC
ACAATGTGCGTGTTTGGTCGCCTTGCGTGGAAGATGCTGCCCGTCTATGTTTTCACCCAG
CTGTTGGGGTCATTTCTGGCAGCGGGGACAATTTATGTGATCTACTATGATGCCATATAT
GACTACAGTGGAGGGAATCTGACTGTGACTGGTGCGAGGGCCACAGCTGGGATCTTTGCC
ACCTACCCTGCACCATACCTCTCCTTGCAGGCTGGATTCATTGACCAGGTGTTTGGCACA
GCCATGCTGCTGCTGTGCCTGATGGCTCTGTCCGACCAGAGGAACAAGCCGGCGGCGGCG
GGCAGCGAGCCTGTGGCAGTGGGCCTCCTGGTGCTGCTCATTGGCATCTCTCTGGGCAGC
AACAGCGGCTATGCCATCAACCCCACCAGAGACCTGGGACCAAGGGTCTTCACTGCCATA
GCAGGCTGGGGGACTGACGTGTTCAGGGCTGGGAGAGGGTGGTGGTGGGTGCCTGTGGTT
GCCCCCCTTGTAGGAGGGGTACTGGGTGCGGGGCTGTACAAGGTCATGGTGGAACTGCAC
CACCCTTCAACCTCTGATCAGGGGGGAGAGCTG---GCGGAAGAGCCAGAGGAGGAGGCC
GCCCCTCTGGAGAAAAAGAAAAGCATC---TGTGCTGATGTG------TGCGTG------

>Pinecone_fish _Aqp7
ATGAAAGACTTGGTGCAGTCAGTAGAACTGGGGGGT---CCTCAGCAGAAAGGGGGCTGC
AAAACTCGATCCAAACTTTGCCTA---AAGAATGAGCTTTTACGTGTGGGACTTGCTGAA
ACCCTCTGCACATATGTCATGATGGTGTTTGGTCTGGGTTCTGTGGCCCAGGTAGTGACA
GGACAGGGAGCATTTGGACAATACCTCAGTATCAACCTGGGTTTTGGTCTGGGAGTTGCT
ATGGGAGTGCACATTGGAGGGAATGTCTCAGGGGCTCATATGAACGCAGCTGTGTCATTC
ACAATGTCTGTGTTTGGCCGTGTTGCGTGGAAGATGCTGCCTGTCTACGTTTTCGCCCAG
TTATTGGGATCATTTCTGGCAACAGGGACAATTTATGCTGTCTATTATGATGCCATTAAT
GACTACTGTGGGGGGAATTTGACTGTAACTGGTCCAAAAGCCACAGCTGGGATCTTTGCC
ACCTACCCTGCGCCATATCTTTCCTTACAGACTGGATTCATTGACCAGGTGCTTGGCACA
GCCATGCTACTGCTGTGCCTGATGGCGCTGTCTGACCAGAGGAACAAGCCGGCTGCAGAA
GGGAGCGAGCCTGTGGCAGTGGGTCTCCTGGTGCTCCTCATTGGCATCTCTCTGGGCAGC
AACAGCGGCTATGCCATCAACCCCACCAGAGACCTGGCACCCAGGGTCTTCACTGCCTTA
GCAGGCTGGGGAGCTGACGTGTTCAGGGCTGGGAATGGGTGGTGGTGGGTGCCAGTAGTT
GCCCCCCTTGTAGGTGGTGTAGTGGGTGCAGGGCTTTACAAGGTCTTTGTGGAACTGCAT
CACCCTTCCCCTTGTAATCTGAGAGGAGAGCTG---GCAGAAGAACCA---GAGGAGACT
GCCCCTCTGGAGAAAAGGAAAAGCATC---TTAGCTGATGTG------TGTGTA------

>Opah_Aqp7
ATGAAAGACACAGTGCAGTCGGTAGATTTAGGGGGT---GCTTGCTTGAAAGGGGGCAAC
AAATTGCCATCGAAGCTTTGGCTT---AAGAATGAGCTTTTCCGTGTGGGACTTGCTGAG
ACCCTCTGCACATATGTCATGATGGTGTTTGGCCTGGGCTCTGTGGCCCAGGTTGTGACA
GGACGAGGCGTTTTTGGACAATACCTCAGCATTAACCTGGGTTTTGGCCTTGGTGTCACC
ATGGGAGTGCACATTGGCGGAAAGGTCTCAGGGGCTCATATGAATGGAGCAGTGTCATTC
ACAATGTGTGTGTTTGGCCGCCTTGCATGGAGGATGCTGCCTCTCTATGTTTGTACACAG
CTGCTTGGCTCATTTCTTGCAGCCGGGACAATTTATGCTGTTTACTATGATGCCATATAT
GTCTACAGCGGTGGGAATTTAACAGTGACTGGCCCAAACGCCACAGCCGGAATCTTTGCC

ACTTACCCTGCACAGTACCTGTCCTTACATGCTGGATTCTTTGACCAGGTATTTGGCACA
GCCATGCTGCTGCTTTGTCTGATGGCTCTGTCTGACCAGAGGAACAAGCCAGCCGCAGCA
GGGAGTGAGCCAGTTGCAGTGGGCCTCCTTGTGTTGCTCATTGGCATTTCTTTGGGCAGC
AACAGTGGATACGCTATCAATCCAACTAGAGACCTAGCACCCAGGGTCTTCACTGCCATA
GCAGGCTGGGGGATTGATGTGTTCAGGGCTGGAAAAGGGTGGTGGTGGGTGCCTGTAGTT
GCCCCTCTTGTAGGAGGTCTATTGGGTGCAGGGGTCTACAAAGCCTTTGTGGAGCTCCAC
CACCCTTCAATTTGTGACCAAAACAAGAAGCAA---CCTGGAGTGCCA---GAAGAGACT
GCCCACCTGGAAAAACAAGATGGCATT---TTTGCTGACGAG------TGTGTG------

>King_of_herrings_Aqp7
ATGAAAGACACAGGCCCGTCCGTAGATATAGGGGGT---GCTACTTGGAAAAGGGGAAAC
GTATTGCCAGTAAAGCTTTGGCTT---AAGGACGAGCTCTTCCGTGTGGGACTGGCTGAG
ACTCTATGCACATATGTCATGATGGTGTTTGGCCTGGGCTCCGTGGCCCAGGTTGTGACG
GGAAAAGGGGCTTTTGGACAATACCTCAGCATCAACCTGGGCTTTGGCCTCGGTGTCACC
ATGGGAGTGCACATCGGTGGAACGGTCTCAGGTGCTCATATGAACGGAGCGGTGTCATTC
ACAATGTGTGCGTTTGGCCGCCTTGCGTGGAGGATGCTGCCTCTTTATATTTGCACACAG
CTGCTTGGCTCATTTCTTGCAGCCGGGACAATTTATTGTGTTTACTATGATGCCATATAC
GTCTACAGTGGTGGGAATTTAACAGTGACCGGCCCAAAAGCCACAGCTGGAATCTTTGCC
ACATACCCTGCACCGTACCTGTCCTTACATGCTGGATTCTTTGACCAGGTACTTGGCACA
GCCATGCTGCTGCTTTGTTTGATGGCTCTGTCTGACCAGAGGAACAAGCCAGCGGTAGCG
GGGACCGAGCCGGTCACAGTGGGTCTCCTTGTGTTGCTCATTGGCATTTCTTTGGGCAGC
AACAGCGGATACGCCATCAACCCGACTAGAGACCTAGCACCCAGGGTCTTCACTGCCATA
GCAGGCTGGGGGATTGATGTGTTCAGGGCGGGAAATGGGTGGTGGTGGGTGCCTTTAGTT
GCTCCTTTTATAGGAGGTCTATTGGGTGCAGGCCTCTACAAGGCCTTCGTGGAGTACCAC
CACCCTTCCATATGTGACCAAGACAAGAAGCTG---CCTGGAGTGCCAGAAGATGAGATC
ATCCACCTGGAAAAACAAGATGGCATT---TCTGCTGACGAG------TGTGTG------

>Atlantic_cod_Aqp7
ATGAAGGATTCAGTAGAATCAGTAGATCCGAGAGAG---GACCAGCAGGAAATGGGTCAC
AGATCTGGACCTATGGCTTGTCTG---CGCAGCAGGTTCATTCGGGTTGGACTGGCGGAG
GCCCTCTGCACCTATGTTATGATGGTGTTTGGCCTGGGCTCAGTGGCACAGGTGGTCACG
GGCCATGGAGCATTCGGACAATACCTCAGCATCAACATTGGCTTTGGCCTTGGAGTGACC
ATGGGAGTGCATGTTGGAGGTTCAGTCTCAGGGGCTCATATGAATGCAGCAGTCTCATTG
GCCATGTGTATGTTTGGCCGCCTTGCGTGGAAGATGCTCCCTGTCTACGTTTTTGCCCAA
TTTCTCGGGTCGTTTCTGGCTGCGGCGACAATCTATACCGTGTTCAATGATGCCATCTTT
GACTTTGGGGATGGGAACCTGACCACGACGGGCGCTAAAGCCACCGCTGGGATCTTCGCC
ACCTACCCTGCGCCCTACCTCTCTCTGCAATCTGGATTCATCGATCAGGTTTTGGGAACG
GCCATGCTCCTGCTGTGCCTGACGGCACTCTCTGACCAGAGGAACAAGCCGGCAGCGAGG
GGCAGCGAGAGCCTGATGGTGGGCCTCCTGGTGATGCTGATTGGCGTCTCCCTGGGCAGC
AACAGCGGCTACCCCATCAACCCCACCAGGGACCTGGCACCCAGACTCTTCACTGCTGTA
GCCGGCTGGGGCAACGAGGTCTTCAGGGCCGGAAACGGGTGGTGGTGGGTGCCTGTGGTA
GCGCCCTTGTTAGGAGGAGTGATGGGTGCAGGACTGTACAAGGCTCTCGTAGAGCTTCAT
CACCCGGCCATTGTGTGTGGCCAGAACAGCCAT---GGAGAG------CAGGAGGAGATG
GATCCTCTGGAGAAACAA------AGC---CACGCTGATGTG------TGTGTG------

>Walleye_pollock_Aqp7
ATGAAGGATTCAGTAGAATCAGTAGATCCGAGAGAG---GACCAGCAGGAAAGGGGTCAC
AGATCTGGACCTATGGCTTGTCTG---CGCAGCAGGTTCATTCGGGTTGGACTGGCGGAG
GCCCTCTGCACCTATGTTATGATGGTGTTTGGCCTGGGCTCAGTGGCACAGGTGGTCACG
GGCCATGGAGCATTCGGACAATACCTCAGCATCAACATTGGCTTTGGCCTTGGAGTGACC
ATGGGAGTGCATGTTGGAGGTTCAGTCTCAGGGGCTCATATGAATGCAGCAGTCTCATTG
GCCATGTGTATGTTTGGCCGCCTTGCGTGGAATATGCTCCCTGTCTATGTTTTTGCCCAA
TTTCTCGGGTCGTTTCTGGCTGCGGCGACAATCTATACCGTGTTCAATGATGCCATCTTT
GACTTTGGGGATGGGAACCTGACCACGACGGGCGCTAAAGCCACCGCTGGGATCTTCGCC
ACCTACCCTGCGCCCTACCTCTCTCTGCAATCTGGATTCATCGATCAGGTTTTGGGAACG
GCCATGCTCCTGCTGTGCCTGACGGCACTCTCTGACCAGAGGAACAAGCCGGCAGCGAGG
GGCAGCGAGAGCCTGATGGTGGGCCTCCTGGTGATGCTGATTGGCGTCTCCCTGGGCAGC
AACAGCGGCTACCCCATCAACCCCACCAGGGACCTGGCACCCAGACTCTTCACTGCTGTA
GCCGGCTGGGGCAACGAGGTCTTCAGGGCCGGAAACGGGTGGTGGTGGGTGCCTGTGGTG
GCGCCCTTGTTAGGAGGAGTGATGGGTGCAGGACTGTACAAGGCTCTCGTAGAGCTTCAT
CACCCGGCCATTGTGTGTAGCCAGAACAGCCAT---GGAGAG------CAGGAGGAGATG

GATCCTCTGGAGAAACAA------AGC---CACGCTGATGTG------TGTGTG------

>Arctic_cod_Aqp7
ATGAAGGATTCAGTA---------GAACCGAGAGAG---GATCAGCAGGAAAGGGGTCAC
AGATCTGGACCTATGGCTTGTCTG---CGCAGCAGGTTCATTCGGGTTGGACTGGCGGAG
GCCCTCTGCACCTATGTTATGATGGTGTTTGGCCTGGGCTCAGTGGCACAGGTGGTCACG
GGCCATGGAGCATTCGGACAATACCTCAGCATCAACATTGGCTTTGGCCTTGGAGTGACC
ATGGGAGTGCATGTTGGAGGTTCAGTCTCAGGGGCTCATATGAATGCAGCAGTCTCATTG
GCCATGTGTATGTTTGGCCGCCTTGCGTGGAAGATGCTCCCTGTCTATGTTTTCGCCCAA
TTTCTCGGGTCGTTTCTGGCTGCGGCGACAATCTATACCGTGTTCAATGATGCCATCTAT
GACTTTGGGGGTGGGAACCTGACCACGACGGGCGCTAAAGCCACCGCTGGGATCTTCGCC
ACCTACCCTGCGCCCTACCTCTCTCTGCAATCTGGATTCATCGATCAGGTTTTGGGAACG
GCCATGCTCCTGCTGTGCCTGACGGCACTCTCTGACCAGAGAAACAAGCCGGCAGCAAGG
GGCAGCGAGAGCCTGATGGTGGGCCTCCTGGTGATGCTGATTGGCGTCTCCCTGGGCAGC
AACAGCGGCTACCCCATCAACCCCACCAGGGACCTGGCACCGAGACTCTTCACTGCTGTA
GCCGGCTGGGGCAACGAGGTCTTCAGGGCCGGAAACGGGTGGTGGTGGGTGCCTGTGGTG
GCGCCCTTGTTAGGAGGAGTGATGGGTGCAGGACTGTACAAGGCTTTCGTAGAGCTTCAT
CACCCGGCCATTGTGTGTGGCCAGAACGGCCAT---GGAGAG------CAGGAGGAGATG
GATCTTCTGGAGAAACAA------AGC---CACGCTGATGTG------TGTGTG------

>Whiting_Aqp7
ATGAAGGATTCAGTAGAATCAGTAGAACCGAGAGAG---GATCAGCAGGAAAGGGGTCAC
AGATCTGGATCTATGGCTTGTGTG---CGCAGCAGGTCCATTCGGGTTGGACTGGCGGAG
GCCCTCTGCACCTATGTTATGATGGTGTTTGGCCTGGGCTCAGTGGCACAGGTGGTCACG
GGCCATGGAGCATTCGGACATTACCTCAGCATCAACATTGGCTTTGGCCTTGGAGTGACC
ATGGGAGTGCATGTTGGAGGTTCAGTCTCAGGGGCTCATATGAATGCAGCAGTCTCATTG
GCCATGTGTATGTTTGGCCGCCTTGCGTGGAAGATGCTCCCTGTCTATGTTTTCGCCCAA
TTTCTCGGGTCGTTTCTGGCTGCGGCAACAATCTATACCGTGTTCAATGATGCCATCTAT
GACTTTGGGGGTGGGAACCTGACCACGACGGGCGCTAAAGCCACCGCTGGGATCTTCGCC
ACCTACCCTGCGCCCTACCTCTCTCTGCAATCTGGATTCATCGATCAGGTTTTGGGAACG
GCCATGCTCCTGCTGTGCCTGACGGCACTCTCTGACCAGAGGAACAAGCCGGCAGCGAGG
GGCAGCGAGAGCGTGATGGTGGGCCTCCTGGTGATGCTAATTGGCGTCTCCCTGGGCAGC
AACAGCGGCTACCCCATCAACCCCACCAGGGACCTGGCACCCAGACTCTTTACTGCTGTA
GCCGGCTGGGGCAACGAGGTCTTCAGGGCCGGAAATGGGTGGTGGTGGGTGCCTGTGGTG
GCGCCATTGTTAGGAGGTGTGATGGGTGCAGGACTGTACAAGGCTCTCGTAGAGCTTCAT
CACCCGGCCATTGTGTGTGATCAGAACGGCCAT---GGAGAA------CAGGAGGAGATG
GATCCTCTGGAGAAACAA------AGC---CACGCTGATGTG------TGTGTG------

>Saithe_Aqp7
ATGAAGGATTCAGTAGAATCAGTAGAACCAAGAGAG---GATCAGCAGGAAAGGGGTCAC
AGATCTGGACCTATGGCTTGTCTG---CGCCGCAGGTTCATTCGGGTTGGACTGGCGGAG
GCCCTCTGCACCTATGTTATGATGGTGTTTGGCCTGGGCTCAGTGGCACAGGTGGTCACG
GGCCATGGAGCATTCGGACAATACCTCAGCATCAACATTGGCTTTGGCCTTGGAGTGACC
ATGGGAGTGCATGTTGGAGGTTCAGTCTCAGGGGCTCATATGAATGCAGCAGTCTCGTTG
GCCATGTGTGTGTTTGGCCGCCTTGCGTGGAAGATGCTGCCTGTCTATGTTTTTGCCCAA
TTTCTCGGGTCGTTTCTGGCTGCGGTGACCATCTATACCGTGTTCAATGATGCCATCTAT
GACTTTGGGGGTGGGAACCTGACCACGACGGGCGCTAAAGCCACCGCTGGGATCTTCGCC
ACCTACCCTGCGCCCTACCTCTCTCTGCAATCTGGATTCATTGATCAGGTTTTGGGAACG
GCCATGCTCCTGCTGTGCCTGACGGCACTCTCTGACCAGAGGAACAAGCCGGCAGCGAGG
GGCAGTGAGAGCGTGATGGTGGGCTTCCTGGTGATGCTGATTGGCGTCTCCCTGGGCAGC
AACAGCGGCTACCCCATCAACCCCACCAGGGACCTGGCACCCAGACTCTTCACTGCTGTA
GCCGGCTGGGGCCACGAGGTCTTCAGGGCCGGAAATGGGTGGTGGTGGGTGCCTGTGGTG
GCGCCCTTGTTAGGAGGTGTGATGGGTGCAGGACTGTACAAGGCTCTCATAGAGCTTCAT
CACCCAGCCATTGTGTGTGATCAGAACGGCCAT---GGAGAG------CAG---GAGATG
GATCCCCAGGAGAAACAA------AGC---CACGCTGATGTG------TGTGTG------

>Haddock_Aqp7
ATGAAGGATTCAGTAGAATCAGTAGAACCAAGAAAG---GATCAGCAGGAAAGGGTTCAC
AGATCTGGAACTATGGCTTGTCTG---AACAGCAGGTTCATTCGGGTTGGACTGGCGGAG
GCCCTCTGCACCTATGTTATGATGGTGTTTGGCCTGGGCTCAGTGGCACAGGTGGTCACG
GGCCATGGAGCATTCGGACATTATCTCAGCATCAACATTGGCTTTGGCCTTGGAGTGACC

ATGGGAGTGCATGTTGGAGGTTCAGTCTCAGGGGCTCATATGAATGCAGCAGTCTCATTG
GCCATGTGTATGTTTGGCCGCCTTGCGTGGAAGATGCTCCCTGTCTATGTTTTCGCCCAA
TTTCTCGGGTCGTTTCTGGCTGCGGCGACAATCTATACCGTGTTCAATGATGCCATCTAT
GACTTTGGGGGTGGGAACCTGACCACGACGGGTGCTAAAGCCACCGCTGGGATCTTCGCC
ACCTACCCTGCACCCTACCTCTCTCTGCAATCTGGATTCATCGATCAGGTTTTGGGAACG
GCCATGCTCCTGCTGTGCCTGACGGCACTCTCTGACCAGAGGAACAAGCCGGCAGCGAGG
GGCAGCGAGAGCGTGATGGTGGGCCTCCTGGTGATGCTGATTGGCGTCTCCCTGGGCAGC
AACAGCGGCTACCCCATCAACCCCACCAGGGACCTGGCACCCAGACTCTTCACCGCTGTA
GCCGGCTGGGGCAACGAGGTCTTCAGGGCTGGAAACGGGTGGTGGTGGGTGCCTGTTGTG
GCGCCCTTGTTGGGAGGTGTGATGGGTGCAGGACTGTACAAGGCTCTCATAGAGCTTCAT
CACCCGGCCATTGTGTGTGATCAGAACGGCCAT---GGAGAG------CAGGAGGAGATG
GATCCTCTGGAGAAACAA------AGC---CACGCTGATGTG------TGTGTG------

>Silvery_cod_Aqp7
ATGAAGGATTCAGTGGAATTAGTAGAACCGAGAGAG---GATCATCAGGAAAGGGGTCAC
AGATCTGGACCTATGGCTTGTCTG---CGCAGCAGGTTTATTCGGGTGGGACTGGCGGAG
GCCCTTTGCACCTATGTTATGATGGTGTTTGGTCTGGGCTCAGTAGCGCAGGTGGTCACG
GGCCACGGAGCATTCGGACAATACCTCAGCATCAACATTGGCTTTAGCCTGGGAGTGACC
ATGGGAGTGCATGTTGGAGGCTCAGTCTCAGGGGCTCATATGAACGCAGCAGTCTCGTTG
GCCATGTGTATGTTTGGCCGCCTTGCGTGGAAGATGCTCCCTGTCTATGTTTTCGCCCAA
CTACTCGGGTCGTTTCTGGCTGCGGCGACAATCTATACCGTGTTCAATGATGCCATCTAT
GACTTCGGGGGTGGGAACCTGACCGTGACGGGGGCTAAAGCCACCGCTGGGATCTTTGCC
ACCTACCCTGCGCCCTACCTCTCTCTGCACTCTGGCTTCATCGACCAGGTCTTCGGCACG
GCCATGCTCCTGCTGTGTCTGACGGCACTCTCTGACCAGAGGAACAAGCCGGCAGCGAGG
GGCAGCGAGAGTGTGATGGTGGGCCTCCTGGTGCTGCTGATCGGCGTTTCCCTGGGCAGC
AACAGCGGCTACGCCATCAACCCCACCAGGGACCTGGCACCCAGATTCTTCACTGCTGTA
GCCGGCTGGGGCAGTGAGGTCTTCAGGGCTGGAAACGGGTGGTGGTGGGTGCCTGTGGTG
GCTCCCTTGTTAGGAGGTGTGATGGGTGCAGGACTGTACAAGGCTCTCATAGAGCTTCAT
CACCCGGCCATCGTGTGTGATCAGAACGGCCAT---CAAGAG------CAG---GAGATG
GATCCTCTGGAGAAACAC------AGC---CACACTGATGCG------TGTGTG------

>Poor_cod_Aqp7
ATGAAGGATTCAGTTGAATCAGTAGAACCGAGAGCG---GATCAACAGGAAAGGGGTCAC
AGATCTGGACCCATGACTTGTCTG---CGCAGCAGGTTTATTCGGGTGGGACTGGCGGAG
GCCCTCTGCACCTATATTATGATGGTGTTTGGCCTGGGCTCAGTGGCACAGGTGGTAACG
GGCCACGGAGCATTCGGACAATACCTCAGCATCAACATCGGCTTTGGCCTGGGAGTGACC
ATGGGAGTGCATGTTGGAGGCACAGTCTCAGGGGCTCATATGAACGCAGCAGTCTCGTTG
GCCATGTGTACGTTTGGACGCCTTGCGTGGAAGATGCTCCCTGTCTATGTTTTCGCCCAA
CTACTCGGGTCGTTCCTGGCTGCCGCGACAATCTATACTGTGTTCAATGATGCCATCTAT
GACTTCGGGGGTGGGAACCTGACCGTGACGGGGGCTAAAGCCACCGCTGGGATCTTCGCC
ACCTACCCTGCGCCCTACCTCTCTCTGCACGCTGGATTCATCGACCAGGTGTTCGGCACG
GCCATGCTCCTGCTGTGCCTGATGGCGCTCTCTGACCAGAGGAATAAGCCGGCGGCGAGG
GGCAGCGAGAGCGTGATGGTGGGCCTCCTGGTGCTACTGATCGGCATTTCCCTGGGCAGC
AACAGCGGCTACGCCATCAACCCCACCAGGGACCTGGCACCCAGACTCTTCACAGCTGTA
GCCGGCTGGGGCAGCGAAGTCTTCAGGGCCGGAAACGGGTGGTGGTGGGTGCCTGTGGTG
GCACCCTTGTTAGGAGGGGTGATGGGTGCAGGACTGTACAAGGTTCTCATAGAGCTTCAT
CACCCAGCCATTGTGTGTGATCAGAATGGCCAT---CAAGAA------CAGGAAGAGATG
GATCCTCTGGAGAAACAC------AAC---CATGCTGATGTG------TGTGTG------

>Burbot_Aqp7
ATGAAGGATTCAGTGGAATCAGTAGAACCGAGAGAG---GATCAGCAGAAAAGGGGTCAC
AGATCTCGACCTATGGCTTGTCTG---CGCAACAGGTTTATTCGGGTGGGACTGGCGGAG
GCCCTCTGCACCTATGTTATGATGGTGTTTGGCCTGGGCTCAGTGGCGCAGGTGGTAACG
GGCCATGGAGAATTCGGACAGTACCTCAGCATCAACATTGGCTTTGGCCTGGGAGTGACC
ATGGGAGTGCATGTCGGAGGCACAGTCTCAGGGGCTCATATGAACGCGGCAGTCTCGTTG
GCCATGTGTATGTTTGGCCGCCTTGCGTGGAAGATGCTCCCCGTCTATGTTTTCGCCCAA
CTACTCGGGTCGTTTCTGGCTGCGGCAACAATCTATACCGTGTTCAATGATGCCATCTAT
GACTACGGAGGCGGGAACCTGACCGTGACGGGTGCTAAAGCCACCGCTGGGATCTTCGCC
ACCTACCCTGCGCCCTACCTCTCTTTGCATTCTGGATTCATCGACCAGGTGTTCGGCACG
GCCATGCTCCTGCTGTGCCTGATGGCGCTCTCTGACCAGAGAAACAAGCCGGCGGCGAAG
GGCAGCGAGAGCGTGATGGTGGGCCTCCTGGTGCTACTGATCGGCATTTCCCTGGGGAGC

AACAGCGGCTACGCCATTAACCCCACCAGGGACCTGGCACCCAGACTCTTCACTGCTGTA
GCCGGCTGGGGCAGCGAGGTCTTCAGGGCTGGAAACGGGTGGTGGTGGGTGCCTGTAATG
GCCCCCTTGTTAGGAGGTGTGATGGGTGCAGGACTGTACAAGGCTCTTGTAGAGCTTCAT
CACCCGGCCTTTGAGTGTGATCAGAATGGCCAT---CAAGAG------CAG---GAGATG
GATCCTCTGGAGAAACAC------AGC---CACGCTGATGTG------TGTGTG------

>Ling_Aqp7
ATGAAGGATTCAGTGGAATCAGTAGAACCGAGAGAG---GATCAGCAGAAAAGGGGTCAC
AGATCTCAACCTATGGCTTGTCTG---CGCAATAGGTTTATTCGGGTGGGACTGGCGGAG
GCCCTCTGCACCTATGTTATGATGGTGTTTGGCCTGGGCTCAGTGGCGCAGGTGGTAACG
GGCCATGGAGCATTCGGACAGTACCTCAGCATCAACATTGGCTTTGGCCTGGGAGTTACC
ATGGGAGTGCATGTTGGAGGCACAGCCTCAGGGGCTCATATGAACGCAGCAGTCTCGTTG
GCCATGTGTATGTTTGGCCGCCTTGCGTGGAAGATGCTCCCCGTCTATGTTTTCGCCCAA
CTACTCGGGTCGTTTCTGGCTGCGGCGACAATCTATACCGTGTTCAATGATGCCATCTAT
GACTACGGGGGTGGGAACCTGACCGTGACAGGTGCTAAAGCCACCGCTGGGATCTTCGCC
ACCTACCCTGCGCCCTACCTCTCTCTGCATTCTGGATTCATCGACCAGGTGTTTGGCACG
GCCATGCTCCTGCTGTGCCTGATGGCGCTCTCTGACCAGAGGAACAAGCCAGCGGCGAGG
GGCAGCGAGAGCGTGATGGTGGGCCTCCTGGTTCTGCTGATCGGCGTTTCCCTGGGGAGC
AACAGCGGCTACGCCATAAACCCCACCAGGGACCTGGCACCCAGACTCTTCACTGCTGTA
GCCGGCTGGGGCAGCGAGGTCTTCAGGGCCGGAAACGGGTGGTGGTGGGTGCCTGTGGTG
GCCCCCTTGTTAGGAGGTGTGATGGGTGCAGGACTGTACAAGGCTCTCGTAGAGCTTCAT
CACCCGGCCATCGTGTGTGATCAGAACGGCAAT---CAAGAA------GAGGAGGAGATG
AATCCTCTGGAGAAACAT------AGC---CACGCTGATGTG------TGTGTG------

>Tusk_Aqp7
ATGAAGGATTCAGTGGAATCAGTACAACCAGGAGAG---GGTCAGCAGAAAAGGGGTCAC
AGATCTCGACCTATGGCTTGTCTG---CACAACAGGTTTATTCGGGTGGGACTGGCGGAG
GCCCTCTGCACCTATGTTATGATGGTGTTTGGCCTGGGCTCAGTGGCGCAGGTGGTAACG
GGCCATGGAGCATTCGGACAGTACCTCAGCATCAACATTGGCTTTGGCCTGGGAGTTACC
ATGGGAGTGCATGTTGGAGGCACAGTCTCAGGGGCTCATATGAACGCAGCAGTCTCTTTG
GCCATGTGTGTGTTTGGCCGCCTTGCGTGGAAGATGCTCCCGGTCTATGTTTTCGCCCAA
CTACTCGGTTCGTTTCTGGCTGCGGCGACAATCTATACCGTGTTCAATGAGGCCATCTAT
GACTACGGGGGTGGGAACCTGACCGTTACGGGTGCTAACGCCACCGCTGGGATCTTCGCC
ACCTACCCTGCTCCCTACCTCTCTCTGCATTCTGGATTCATCGACCAGGTGTTCGGCACG
GCCATGCTCCTGCTGTGCCTGATGGCGCTCTCTGACCAGAGGAACAAGCCAGCGGCGAGG
GGCGGCGAGAGCGTGATGGTGGGCCTCCTGGTGCTGCTGATCGGCGTTTCCCTGGGGAGC
AATAGCGGCTACGCCATCAACCCCACCAGGGACCTTGCACCCAGACTCTTCACTGCTGTA
GCCGGCTGGGGCAGCGAGGTCTTCAGGGCCGGAAACGGGTGGTGGTGGGTGCCTGTGGTG
GCCCCCTTGTTAGGAGGTGTGATGGGTGCAGGACTGTACAAGGCTCTGGTAGAGCTTCAT
CACCCGGCCATCGTGTGTGATGAGAACGGCCAT---CAAGGG------CAGGAGGAGATG
GAGTCTCTGGAGATACAC------AGC---CACACTGATGCG------TGTGTG------

>Greater_forkbeard_Aqp7
ATGAAGGATTCAGTGGAATCAGTAGAACGGAGAGAA---GATCAGCAGAAAAGGGGTCCC
AGATGTCGACCTGTGGCTGGTCTA---CACAACAGGTTTATTCGGGTGGGACTGGCGGAG
GCTCTCTGCACCTATGTTATGATGGTGTTTGGCCTGGGCTCGGTGGCCCAGGTGGTCACG
GGCCGCGGAGCATTCGGACAGTACCTCAGCATCAACATTGGCTTTGGCCTGGGAGTCGCC
ATGGGAGTGCACGTCGGAGGCTCAGTCTCAGGGGCTCACATGAACGCCGCGGTCTCGTTG
GCCATGTGCGTGTTCGGCCGCCTGGCGTGGAAGATGCTCCCCGTGTATGTTCTCGCACAA
CTACTCGGGTCGTTTCTGGCCGCGGCGACCATCTACGCCGTGTACAACGATGCCATCTAT
GACTACGGGGGCGGGAACTTGACCGTGACGGGAGCTAAAGCCACCGCTGGGATCTTCGCC
ACCTACCCTGCGCCCTACCTCTCCCTGCATGCTGGCTTCATCGACCAGGTGTTCGGCACG
GCCGTGCTCCTGCTGTGCCTGACGGCGCTCTCCGACCAGAGGAACAAGCCGGCGGCGAGG
GGCTGCGAGAGCATCACGGTGGGCCTCCTGGTGCTGCTGATCGGCATTTCCCTGGGGAGC
AACAGCGGTTACGCCATCAACCCCACCAGGGACCTGGCACCCAGACTCTTCACTGCGGTA
GCCGGCTGGGGCGGCGAGGTGTTCAGGGCCGGAAACGGGTGGTGGTGGGTGCCTGTGACC
GCTCCCTTGTTAGGTGGTGTGATGGGTGCAGGACTGTACAAGATGCTTGTAGAGCTTCAT
CACCCGGCCCTCGTGTGTGAGCAGAAGGGCCTT---CAAGAG---------CAGGAAATG
GCCCCTCTGGAGAAACAC------AGC---TATGCTGACGTG------------------

>Forkbeard_Aqp7

ATGAAGGATTCAGTGGAATCAGTGGAAAGGAGAGAA---GATCAGCAGAAAAGGGGTCCC
AGATCCCGACCTTTGGCTTGTCTG---CACAACAGGTTTATCCGGGTGGGACTGGCGGAG
GCTCTCTGCACCTATGTTATGATGGTGTTTGGCCTGGGCTCGGTGGCCCAGGTGGTCACG
GGTCGCGGAGCGTTCGGACAGTACCTCAGCATCAACATTGGCTTTGGCCTGGGAGTCGCC
ATGGGAGTGCACGTTGGAGGCTCAGTCTCAGGGGCTCACATGAACGCCGCGGTCTCGTTG
GCCATGTGTGTGTTTGGCCGCCTGGCGTGGAAGATGCTCCCCGTGTATGTTCTCGCACAA
CTGCTCGGGTCGTTTCTGGCCGCGGCGACAATCTACGCCGTGTACAACGATGCCATCTAT
GACTACGCGGGTGGGAACTTGACCGTGACGGGCGCCAAAGCCACCGCGGGGATCTTCGCC
ACCTACCCTGCGCCCTACCTCTCCCTGCATTCTGGATTCATTGACCAGGTGTTCGGCACG
GCCATGCTCCTGCTGTGCCTGACGGCGCTCTCCGACCAGAGGAACAAGCCGGCGGCGAGG
GGCTGCGAGAGCATCATAGTGGGCCTCCTGGTGCTGCTGATCGGCATTTCCCTGGGGAGC
AACAGCGGCTACGCCATCAACCCCACCAGGGACCTGGCACCCAGACTCTTCACTGCAGTG
GCCGGCTGGGGCGGCGAGGTGTTCAGGGCCGGAAACGGGTGGTGGTGGGTGCCCGTGACG
GCCCCCTTGTTAGGTGCTGTGATGGGTGCAGGACTGTACAAGGTGCTCGTAGAGCTTCAT
CACCCGGCCCTGGTGTGTGAGCAGAAGGGCCAT---CAAGAG---------CAGGAAATG
GCCCCTCTGGAGAAACAC------AGC---TATGCTGACGTG------------------

>Cape_hake_Aqp7
ATGAAGGATTCAGTGGATTCAGTAGAACGGAGAAAA---GATCAGCGGGAGCGGGGTCAG
AGATCTCGACCTATGGCTTGCCTG---CAGAACAGGCTTATTCGGGTGGGATTGGCGGAG
GCACTCTGCACCTATGTCATGATGGTATTTGGCCTGGGCGCCGTGGCGCAGGTGGTAACG
GGACGCGGGGCGTTCGGACAGTACCTCAGCATCAACGTGGGCTTTGGCCTGGGAGTGACC
ATGGGAGTGCATGTTGGAGGCACAGTCTCAGGGGCTCATATGAACGCGGCCGTCTCGTTC
GCCATGTGTGTGTTTGGTCGCCTCGCTTGGAAGATGCTCCCCATCTACGTTCTCGCGCAA
CTCCTCGGCTCATTCATGGCCGCAGTGACTATCTACACCGTGTACAATGATGCCATATAT
GACTATGGTGGAGGGAACCTGACCGTGACGGGTGCTAAGGCCACCGCTGGGATCTTCGCC
ACCTACCCTGCACCCTACCTCTCGGTGCACGGCGGGTTCATCGACCAGGTGTTTGGCACG
GCCATTCTCCTGCTGTGTCTGATGGCGTTTGCTGACCAGAGGAACAGGCCGGCGGCGAGG
GGCAGCGAGGGCATCATGGCGGGCCTCCTGGTGCTGCTGATCGGCATTTCCCTGGGGAGC
AACAGCGGCTACCCCATCAACCCCACCAGAGACCTGGCCCCCAGAGTCTTCACCGCTGTG
GCGGGCTGGGGGAGCGAGGTGTTCAGGGCGGGAAACACGTGGTGGTGGGTGCCGGTGGTG
GCCCCCCTGCTGGGCGGCGTGATGGGCGCAGGACTGTACAACGCCTTCGTCGAGCTTTTT
CACCCGGCCCTGGTGTGCGACCAGAGCGGCCCT---CAAGAA------GTGCAGGAGCTG
GAGCCTCTGGAGGTCAGC------TCT---TTGGCCACG---------------------

>European_hake_Aqp7

------------------------------GGGGCTCATATGAACGCGGCCGTCTCGTTC
GCCATGTGTGTGTTTGGTCGCCTCGCTTGGAAGATGCTCCCCATCTACGTTCTCGCTCAA
CTCCTCGGCTCATTCATGGCCGCAGTGACTATCTACACCGTGTACAATGATGCCATATAT
GACTATGGTGGAGGGAACCTGACCGTGACGGGTGCTAAGGCCACCGCTGGGATCTTCGCC
ACCTACCCTGCACCCTACCTCTCGGTGCACGGCGGGTTCATCGACCAGGTGTTTGGCACG
GCCATTCTCCTGCTGTGTCTGATGGCGTTTGCTGACCAGAGGAACAGGCCGGCGGCGAGG
GGCAGCGAGGGCGTCATGGCGGGCCTTCTGGTGCTGCTGATCGGCATTTCCCTGGGGAGC
AACAGCGGCTACCCCATCAACCCCACCAGAGACCTGGCCCCCAGAGTCTTCACCGCTGTG
GCGGGCTGGGGGAGCGAGGTGTTCAGGGCGGGAAACACGTGGTGGTGGGTGCCAGTGGTG
GCCCCCCTGCTGGGCGGCGTGATGGGCGCAGGACTGTACAACGCCTTCGTCGAGCTTTTT
CACCCGGCCCTGGTGTGCGACCAGAGTGGCCCT---CAAGAA------GTGCAGGAGCTG
GAGCCTCTGGAGGTCAGC------TCG---TTGGCCACG---------------------

>Benguela_hake_Aqp7
ATGAAGGATTCAGTGGAGTCAGTAGAACGGAGAGAA---GATCAGCGGGAGCGGGGTCAG
AGATCTCGACCTATGGCTTGTCTG---CAGAACAGGCTTATTCGGGTGGGATTGGCGGAG
GCGCTCTGCACCTATGTCATGATGGTATTTGGCCTGGGCGCCGTGGCGCAGGTGGTAACG
GGACGCGGGGCGTTCGGACAGTACCTCAGCATCAACGTGGGCTTTGGCCTGGGAGTGACC
ATGGGAGTGCATGTTGGAGGCACAGTCTCAGGGGCTCATATGAACGCGGCCGTCTCGTTC
GCCATGTGTGTGTTTGGCCGCCTCGCTTGGAAGATGCTCCCCATCTACGTTCTCGCTCAA
CTCCTCGGCTCATTCATGGCCGCAGTGACCATCTACACCGTGTACAATGATGCCATATAT

GACTACGGTGGAGGGAACCTGACCGTGACGGGTGCTAAGGCCACTGCTGGGATCTTCGCC
ACCTACCCTGCACCCTACCTCTCGGTGCACGGCGGGTTCATCGACCAGGTGTTTGGCACG
GCCATTCTCCTGCTGTGTCTGATGGCGTTTGCTGACCAGAGGAACAGGCCGGCGGCAAGG
GGCAGCGAGGGCGTCATGGCGGGCCTCCTGGTGCTGCTGATCGGCATTTCCCTGGGGAGC
AACAGCGGCTACCCCATCAACCCCACCAGAGACCTGGCCCCCAGAGTCTTCACCGCTGTG
GCGGGCTGGGGGAGCGAGGTGTTCAGGGCGGAAACACGTGGTGGTGGGTGCCGGTGGTG
GCCCCCCTGCTGGGCGGCGTGATGGGCGCAGGACTGTACAAGGCCTTCGTCGAGCTTTTT
CACCCAGCCCTGGTGTGCGACCAGAACGGCCCT---CAAGAA------GTGCAGGAGCTG
GAGCCCCTGGAGGTCAGC------TCG---TTGGCCACG---------------------

>Common_mora_Aqp7
ATGAAGGATTCAGTGGAATCG---GAACAGAGAGAG---GATCAGCAGAAAAGGACTCAC
AGATGTCGACCTGTGGCTTGTCTG---TACAAAAGGTTTATTCGCGTGGGACTGGCGGAG
GCTCTCTGCACCTATGTCATGATGGTGTTTGGCCTGGGTGCGGTGGCACAGGTTGTAACG
GGACATGGAGCGTTCGGACAGTACCTCAGCATCAACTTGGGCTTTGGCCTGGGAGTTACC
ATGGGAGTGCATGTTGGAGGCGCGGTTTCAGGGGCTCATATGAATGCTGCAGTCTCGTTT
GCCATGTGTGTGTTTGGCCGCCTTGCTTGGAAGATGCTCCCTGTCTATGTTCTCGCCCAA
CTCCTCGGGTCGTTTCTGGCCGCGGTGACCATCTACACCGTGTTCAATGACGCCATATAC
GACTACGGGGGTGGGAACCTGACCGTGACGGGTGCTAAAGCCACCGCGGGGATCTTCGCC
ACCTACCCTGCACCCTACCTGTCCCTGTATTCTGGATTCATCGACCAGGTGTTTGGCACG
GCCATGCTCCTGCTGTGCCTGACGGCGCTCTCTGACCAGAGGAACAAACCGGCCGCGAGG
GGCAGTGAGGGCGTCATGGCGGGCCTACTGGTGCTGCTGATCGGCGTTTCCCTGGGGAGC
AACAGTGGCTACCCCATCAACCCCACCAGAGACCTGGCCCCCAGACTCTTCACTGCTGTA
GCAGGCTGGGGCTGCGAGGTGTTCAGGGCTGGAAACGGGTGGTGGTGGGTGCCCGTGGTG
GCGCCCTTGTTAGGAGGTGTGATGGGTGTGGGGCTGTACAAGGCCTTCGTACAGCTTCAT
CACCCGGCCCTGGTGTGTGATCAGAATGGCCAT------GAA------GAGCAGGAGATG
GATCCTCTGGAGAAACACGAGACCAGC---TACGTTGACGTG------------------

>Guinean_codling_Aqp7
ATGAAGGATTCAGTGGAGTCA---GAAAGGAGAGAA---GATCAACAGAAAAGGGCTCAC
AGATCTCGACCCGTGGCTTGTCTG---CATAACAGGTTTATTCGTGTGGGATTGGCGGAA
GCTCTCTGCACCTATGTGATGATGGTGTTTGGCCTGGGAGCAGTGGCCCAGGTTGTAACT
GGACATGGAGCATTCGGACAGTACCTCAGCATCAACCTGGGCTTTGGCCTGGGAGTTACC
ATGGGAGTGCATGTTGGAGGCACAGTCTCAGGGGCTCATATGAACGCGGCAGTCTCGTTG
GCCATGTGTGTGTTTGGCCACCTTGCCTGGAAGATGCTCCCCGTCTATGTTCTCGCCCAA
TTCCTCGGGTCGTTTCTTGCCGCGGTGACCATCTACACGGTGTACAACGACGCCATATAT
GACTACGGGGGTGGGAACCTGACCGTGACGGGTGCTAAAGCCACCGCAGGGATCTTCGCC
ACCTATCCCGCACCCTACCTGTCCCTACATTCAGGGTTCATCGACCAGGTGTTTGGCACG
GCCATGCTCCTGCTGTGCCTGACAGCGCTCTCTGACCAGAGGAACAAACCGGCGGCGAGG
GGCAGCGAGGGCGTCATGGCCGGCCTCCTGGTGCTGCTGGTCGGCGTGTCCCTAGGGAGC
AACAGCGGCTACCCCATCAACCCCACCAGGGACCTGGCGCCCAGACTCTTCACTGCTGTA
GCGGGCTGGGGGTGCGAGGTGTTCAGGGCTGGAAACGGGTGGTGGTGGGTGCCGGTGGTG
GCTCCGTTGATAGGAGGTGTGATGGGTGCGGGGCTGTACAAGGCCTTCATACAGCTTCAT
CACCCGGCCCTGGTGTGTGATCAGAAAGTCCAT------GAA------GAGCAGGAGATG
GATCCTCTGGAGAAACACACGACCAGC---TGCGCTGACGTG------------------

>Roughsnout_grenadier_Aqp7
---GAGGATTCAGTGGAGACAGTAGGACCGAGAGGA---GATCAGCGGAACAGGGCTCCC
AGGTCTCCTCCTATGGCCTGTCTG---CACAACAGGTTTATTCGGGTGGGCTTGGCGGAG
GGTCTCTGCACCTATGTCATGATGGTGTTTGGCCTGGGCTCGGTGGCGCAGGTGGTAACG
GGACACGGAGCATTCGGTCAGTACCTCAGCATCAACTTGGGCTTCGGCGTGGGAGTCACC
ATGGGCGTGCACGTCGGAGGCACGGTCTCAGGGGCTCACATGAACGCGGCGGTCTCGCTG
GCCTTGTGTGTGTTCGGCCGCCTCGCTTGGAGGATGCTCCCCGTCTACGTTCTCGCCCAG
CTACTGGGGTCGTTTCTGGCCGCGGTGACGGTCTTTACCGTGTACAGCGATGCCATATAT
GACTACGGGGGTGGGAATCTGACCGTGACGGGCGCCAAAGCCACCGCTGGGATCTTCGCC
ACCTACCCTGCACCCTACCTCTCCCTGCAATCTGGATTCATCGACCAGGTGTTCGGCACG
GCCATGCTCCTGCTCTGCTTGACGGCGCTCTCTGACCAGAGGAACAAACCGGCGGTGAGG
GGCGGTGAGGGCGTCATGGCGGGTCTCCTGGTGCTGCTGATCGGCGTTTCCCTGGGGAGC
AACAGTGGCTACCCCATCAACCCCACCAGAGACCTGGCGCCAAGACTCTTCACTGCTGTA
GCGGGCTGGGGGAGTGAGGTGTTCAGGGCTGGCAACGGGTGGTGGTGGGTGCCCGTGGTG
GCCCCTTTGTTAGGAGGTGTTACGGGCGCAGGACTGTACAAGTTCTTCGTCGAGCTTCAT

CATCCGGACCTCATGTGTGATCAGAACGGCCGT---CAAGAG------GAGGAGGAGATG
AATCCTCTGGACAAACACAAAACCAGC---CAGGCTGACGTG------CGTGACGTA---

>Roughnose_grenadier_Aqp7
---GAGGATTCAGTGGAGTCAGTAGGACCGAGAGGA---GATCAGCAGAACAGGGCTCCC
AGGTCTCCTCCTATGGCCTGTCTG---CACAACAGGTTTATTCGGGTGGGCTTGGCGGAG
GGTCTCTGCACCTATGTCATGATGGTGTTTGGCCTGGGCTCGGTGGCGCAGGTGGTAACG
GGACACGGAGCATTCGGTCAGTACCTCAGCATCAACTTGGGCTTCGGCGTGGGAGTCACC
ATGGGCGTGCACGTCGGAGGCACGGTCTCAGGGGCTCACATGAACGCGGCGGTCTCGCTG
GCCTTGTGTGTGTTTGGCCGCCTCGCTTGGAGGATGCTCCCCGTCTACGTTTTCGCCCAG
CTACTGGGGTCGTTTCTGGCCGCGGTGACGGTCTTTACCGTGTACAGCGATGCCATATAT
GACTACGGGGGTGGGAATCTGACCGTGACGGGCGCCAAAGCCACCGCTGGGATCTTCGCC
ACCTACCCTGCACCCTACCTCTCCCTGCAATCTGGATTCATCGACCAGGTGTTCGGCACG
GCCATGCTCCTGCTCTGCTTGACGGCGCTCTCTGACCAGAGGAACAAACCGGCGGTGAGG
GGCGGTGAGGGCGTCATGGCGGGTCTCCTGGTGCTGCTGATCGGCGTTTCCCTGGGGAGC
AACAGTGGCTACCCCATCAACCCCACCAGAGACCTGGCGCCAAGACTCTTCACTGCTGTA
GCGGGCTGGGGGAGCGAGGTGTTCAGGGCTGGAAACGGGTGGTGGTGGGTGCCCGTGATG
GCCCCTTTGTTAGGAGGTGTTACGGGTGCAGGACTGTACAAGGTCTTCGTAGAGCTTCAT
CATCCGGACCTCGTGTGTGATCAGAACGGCCGT---CAAGAG------GAGGAGGAGATG
AATCCTCTGGACAAACACAAGACCAGC---CAGGCTGAAGTG------TGTGACGTG---

>Onion_eye_grenadier_Aqp7

----------------------------TTTGGCCTGGGCTCCGTGGCGCAGGTGGTGACG
GGACACGGAGCCTTCGGGCAGTATATCAGCATCAACCTAGGCTTTGGCCTGGGCGTTACC
ATGGGAGTGCATATTGGAGGCACAGTCTCAGGGGCTCATATGAACGCAGCGGTCTCGTTG
GCCTTGTGTGTGTTTGGCCGCCTTGATTGGAAGATGCTCCCCGTCTATGTTGTCGCCCAG
CTACTGGGGTCGTTTCTGGCTGCGGCGACAATCTACACCGTGTACAACGATGCCATATAT
GAATACTGTGATGGGAACCTGACCGTGACGGGTGCTAAGGCCACCGCTGGGATCTTCGCC
ACCTACCCTGCGCCCTATCTCACCCTGCACTCTGGGTTCATCGACCAGGTTTTCGGCACG
GCCATGCTGCTGCTGTGCCTGACGGCGCTCTCGGACCAGAGGAACAAAGCGGCAGCACGG
GGCTGCGAGGGTGTCATGGCGGGCCTGCTGGTGCTGCTGATCGGCGTTTCCCTGGGGAGC
AACAGCGGCTACGCCATCAACCCCACCAGAGACCTGGCGCCCAGACTTTTCACTGCCGTA
GCGGGCTGGGGGGGCGAGGTGTTCAGGGCAGGGAACGGGTGGTGGTGGGTGCCCGTGGTG
GCCCCCCTGGTAGGCGGTGTGCTGGGTGCCGGACTTTACAAGGCCTTGGTAGAGCTTCAC
CACCCGGCCCTCGCTTGTGATCAGAATGACCAT---CGAGAG------CAGGAGGAAATG
GCTCCTCTGGAGAAACACAAGGCCAGC---TACGATGATGTG------TGCGTG------

>Roundnose_grenadier_Aqp7
ATGAAGGATTCAGTGGAGTCAATAGAACTGGGAGAA---GATGGGAAGAAGAGGCCTCGC
ACGTCTCGACCAATGGCGTGCCTG---CGCAACAAGTTTATTCGGGTGGGATTGGCCGAG
ACTCTCTGCACCTACGTCATGATGGTATTTGGCCTGGGCTCGGTGGCGCAGGTGGTGACG
GGACATGGGGCGTTCGGACAGTACTTCAGCATCAACCTGGGCTTTGGCCTGGGAGTTACC
ATGGGAGTGCATGTTGGAGGCACAGTCTCAGGGGCTCATATGAACGCAGCGGTCTCGTTG
GCCTTGTGTGTGTTTGGCCGCCTTGATTGGAAGATGCTCCCCGTCTATGTGGTCGCCCAA
CTGCTGGGGTCGTTTCTGGCCGCGGCGACAATCTTCACTGTGTACAACGATGCCATATTT
GACTACTGTGGCGGGAACCTGACCGTGACGGGTGCTAAGGCCACCGCTGGGATCTTCGCC
ACCTACCCTGCCCCCTACCTCTCGCTGCACTCTGGATTCATCGACCAGGTGTTCGGCACG
GCCATGCTGCTGCTGTGCCTGACGGCGCTCTCGGACCAGAGGAACAAAGCGGCGCCGAGG
GGCTGCGAGGGCGTCATGGCGGGCCTGCTGGTGCTCCTGATCGGCGTTTCCCTGGGGAGC
AACAGCGGCTACGCCATCAACCCCACCAGAGACTTGGCACCCAGATTCTTCACTGCCATA
GCGGGCTGGGGGAGCGAGGTGTTCAGGGCAGGGAACGGGTGGTGGTGGGTGCCCCTGGTG
GCCCCCTTGGTAGGCGGTGTGCTGGGTGTCGGACTGTATAAGGCCTTGGTAGAGCTTCAC
CACCCGGCCCTCGCGTGTGATCAGAACGGCCAT---CGAGAG------CAGGAGGAAATG
GTTCCTCTGGAGAAACACCAGCTACAA---------------------------------

>Western_softhead_grenadier_Aqp7
ATGAAGGATTCAGTGGAATCAGTAGAACGAGGAGAA---CATGAGAAGAAGAGGACTCAC
ACTTCGCGACTAATGGCCTGCCTG---CGCAACAGGTTTATTCGGGTGGGCTTGGCCGAG
ACTCTCAGCACCTATGTCATGATGGTATTTGGCCTGGGCTCGGTAGCGCAGGTAGTGACC

GGACATGGAGCGTTCGGACAGTACCTCAGCATCAACCTGGGCTTTGGCCTGGGAGTTACC
ATGGGAGTGCATGTTGGAGGCACAGTCTCAGGGGCTCATATGAACGCGGCGGTGTCGTTG
GCCTTGTGTGTGTTTGGCCAACTTGATTGGAAGATGCTCCCCATCTATGTTGTCGCCCAA
TTACTGGGCTCGTTTCTGGCCGCGGCGACAATCTATACCGTGTACAATGATGCCATATAT
GACTACTGTGGTGGGAACCTGACCGTGACTGGTGCTCAAGCCACGGCCGGGATCTTCGCC
ACCTACCCTGCGCCCTACCTGTCCCTGCACTCTGGGTTCATGGACCAGGTGTTCGGCACG
GCCATGCTGCTGCTGTGCCTGACGGCGCTCTCGGACCAGAGGAACAAACCGGCGGCGAGG
GGCTGCGAGGGCGTCATGGCGGGCCTGCTGGTGCTGCTGATCGGCGTTTCCCTGGGGAGC
AACAGCGGCTACCCCATCAACCCCACCAGAGACCTGGCGCCCCGACTCTTCACTGCCATC
GCGGGCTGGGGGAGCGAGGTGTTCAGGGCAGGAAACGGGTGGTGGTGGGTCCCCGTGGTG
GCCCCCTTGGTAGGCGGTGTGCTGGGTGCTGGACTGTACAAGACCCTGGTAGAGCTTCAT
CACCCGGCCCTCGCGTGCCATCAGAATGACCAT---CGAGAG------CTGGAGGAAATG
GCTTCTCTGGAGAAACACAACACAAGC---TATGTGGACGTG------TGTGTG------

>Vaillants_grenadier_Aqp7
ATGAAGTATCCCGTGGAATTAGAAGAACGGGGAGAA---GATGAGCAGAAGAGGAGTCAC
ACACCTCGACCAATAGCGTGTCTG---CACAACAGCTTCATTCGGGTGGGATTGGCCGAG
ACTCTCTGCACCTATGTAATGATGGTGTTTGGCCTGGGATCCGTGGCACAGGTGGTGACG
GGACACGGAGCATTCGGACAGTACGTCAGCATCAACGTGGGCTTTGGCCTGGGAGTTACC
ATGGGGGTGCACGTTGGAGGCACAGTCTCAGGGGCTCATATGAACGCAGCAGTCTCGTTG
GCCTTGTGTGTGTTCGGCCGCCTTGCTTGGAAGATGCTCCCCGTCTATGTTCTCGCACAA
CTACTTGGGTCATTTCTGGCCGCGGCGACAATCTATACCGTGTACAATGATGCCATATAT
GACTACGGGGGAGGGAACCTGACTGTGACGGGTGCTAAAGCCACTGCTGGGATCTTCGCC
ACCTACCCTGCACCCTACCTCTCCCTGCATTCTGGATTCATCGACCAGGTGTTTGGCACG
GCCATGCTTCTGCTGTGCCTGACAGCGCTCTCTGACCAGAGGAACAAGCCAGCGGTGAGG
GGTGGCGAGGGCGTCATGGCGGGCCTCCTGGTGCTGCTGATCGGCATTTCCCTGGGGAGC
AACTGTGGCTACCCCATCAACCCCACCCGAGACCTGGCACCCAGACTCTTCACTGCTGTA
GCAGGCTGGGGGAGCGAGGTGTTCAGGGCTGGAAACGGGTGGTGGTGGGTGCCCGTGGTG
GCCCCCTTGTTTGGCGGTGTGTTGGGTGCCGGACTGTACAAGGCCTTCGTGGAGCTTCAT
CACCCGGCCCTCGTGTGTGATCAGAGTGACCAT---CGAGAG------CAGGAGGAGATG
---GCTCTGGAGAAGCACAAGACCAGC---TACACTGACCTG------TGTGTG------

>Marbled_moray_cod_Aqp7

------------------------GTGTTCGGCCTGGGCTCGGTGGCCCAGGTGGTGACG
GGTGGCGGAGCGTTCGGTCAGTACCTCAGCATCAACCTGGGCTTCGGCCTTGGAGTCACC
ATGGGGGTGCATGTTGGAGGCGCCATCTCAGGGGCTCACATGAACGCAGCGGTGTCCCTG
GCCATGTGCGTGTTCGGCCGCCTGGCGTGGAAGATGTTCCCCGTCTACCTTCTGGCCCAG
CTGCTCGGCTCCTTTCTGGCCGCCGCCACCGTCTACGCCGTGTACAACGACGCCATATAC
GACTACAGCGGTGGGAACCTGACGGTGACGGGCGCTAGGGCCACAGCTGGGATCTTCGCC
ACCTACCCGGCGCCCTACCTCTCCCTGCATTCTGGATTCATCGACCAGGTGCTGGGCACG
GCCATGCTCCTGTTGTGCCTGACCGCGCTCTCCGACCAGAGGAACACGCCCGCGGTGAGG
GGCGGCGAGGGCCTGATGGCGGGCCTCCTGGTGCTGCTCATCGGCGTCTCCCTGGGGAGC
AACTGCGGCTACCCCATCAACCCCACCAGGGACCTGGGGCCCCGGGTCTTCACCGCCGTG
GCGGGCTGGGGGACCGAGGTGTTCAGGGCGGGCAGCGGGTGGTGGTGGGTCCCCATAGTG
GCGCCGTCCCTGGGAGGTGTGATGGGAGCCGGACTGTACAAGGCCCTGGTGGAGCTCCAT
CACCCCGAACTCGAGACCCATCAGAGCGACCGT---CGAGAG------CAGGACGAGATG
GATTCTCTGGAGAAGCACAAGGCCAGC---TACACAGACACG------TGTGTG------

>Arrowtail_Aqp7
ATGAAGGATTCAGTA---------GAACGGAGAGAA---GATCAGCAGGAAAGGGCTCAC
AGATCTCGACCTATGGCTTGCCTG---CGCAACAGGTTTATTCGGGTGGGATTGGCGGAG
GCTCTCTGCACCTATGTTATGATGGTATTTGGCCTTGGCGCGGTGGCGCAGGTGGTGACG
GGACGCGGAGCGTTTGGACAGTACCTCAGCATCAACTTGGGCTTTGGCCTGGGAGTCACC
ATGGGAGTGCATGTTGGAGGGGCGGTGTCAGGGGCTCACATGAACGCGGCAGTCTCGTTG
GCCATGTGTGTGTTTGGCCGTCTTGCGTGGAAGATGCTCCCCATCTATGTTCTCGCCCAA
CTGCTCGGGTCGTTTCTGGCTGCGGCAACAGTCTATATTGTGTACAATGATGCCATATAT
GAGTACGGGGGTGGCAACCTGACCGTGACGGGTGCTAAAGCCACCGCTGGCATCTTCGCC
ACCTACCCAGCACCCTACCTCTCCCTGCATTCTGGATTCATCGACCAGGTGTTCGGCACG
GCCATGCTCCTGCTGTGCCTGACCGCGCTCTCTGACCAGAGGAACAAACCGGCGGCGAGG

GGCGCAGAGGGCGTCATGGCAGGTCTCCTGGTGCTGCTGATTGGCATCTCCCTGGGGAGC
AACAGCGGCTACCCCATCAACCCCACCAGAGACCTGGCACCCAGACTCTTCACCGCTGTA
GCGGGCTGGGGGAGCGAGGTGTTCAGGGCCGGTAATGGGTGGTGGTGGGTGCCCGTGGTG
GCCCCCCTGCTAGGCGGTGTGATGGGTGCAGGACTGTACAAGGCCTTCATAGAGCTCCAT
CACCCGGCCCTCGTGTGTGAGCAGAACGGCCAC---CAGGAG------CAGGAGGAGATG
CATCCTCTGGAGAAACACAAGACCAGC---TATGCCGATGTG------TGTGTG------

>Tube_eye_Aqp7

------------------------GTCTTTGGCCTGGGCTCTGTGGCACAGGTGGTTACA
GGACGAGGGGCGTTTGGTCACTACCTCAGCATCAACGTAGGGTTTGGCCTGGGCGTTACC
ATGGGAGTGCACATAGGAGGGAACGTCTCAGGTGCTCATATGAATGCAGCCGTGTCATTG
GCCATGTGTGTATATGGCCGCCTTGCTTGGAAGATGTTGCCCGTCTATGTTTTCGCCCAA
CTTCTTGGGTCATTTCTCGCCTCAGCGACAATTTATACCGTTTACAATGATGCCATTTTT
CACTACGGTGACGGGAACTTGACTGTGACTGGTGAAAAAGCCACGGCGGGGATCTTCGCG
ACCTACCCTGCGCCATACCTCTCTGTGCATTCTGGATTCATTGACCAGGTCTTTGGAACA
GCCATACTTCTGCTATGCCTGATGGCTTTGTCTGACCAGAGGAACAAGCCAGCGGTAAGG
AGTGGCGAGGCCATCATGGTGGGTTCCCTGGTGCTGCTGATCGGCATCTCCCTGGGCAGC
AATAGTGGCTATGCCATCAATCCCACTAGAGACCTGGCTCCCAGACTCTTCACTGCCCTA
GCTGGCTGGGGCCTCGATGTGTTCAGGGCTGGAAACGGGTGGTGGTGGGTGCCTGTAGTG
GCCCCCTTGTTAGGTGGTGTCATAGGTGCAGGGCTTTACAGGGCGGTCGCGGAGCTCCAC
CACCCTTCCCTTTGTGACAAAAGCAGCGGA------CAA---------GAGGAAGAGACA
GGTCCTCTGGAGACGCGGAAAACCAGC---TATGCTGATGTG------TGCGTT------

>Rosy_dory_Aqp7
ATGACGGATTCCGTGGGATCAGAGGACCTCAAAAGA---CCTCACCAGAAAAGAGGCGGC
AAAGCCCCACGTACGATTTGGCTA---CAGAATATGTCTATTCGAGTGGGACTCGCCGAG
ACTCTCTGCACATATGTCATGATGGTGTTTGGCCTGGGCTCTGTGGCACAGGTCGTAACT
GGACATGGAGCATTTGGACAATACCTTAGCATCAATTTAGGATTTGGCCTTGGCGTTGCT
ATGGGAGTGCATGTTGGGGGACAGGTCTCAGGTGCTCACATGAATGCAGCAGTCTCTTTG
GCCATGTGTGTGTTTGGTCACCTTAGGTGGAAGATGCTCCCTCTCTATGTTTTCTCCCAG
TTGCTTGGGTCATTTTTGGCAGCAGGGACAGTTTATACAATTTACTATGATGCCATTTAT
GAGTACAGTGGTGGGAATTTTACTGTGACCGGTACCAAAGCCACAGCTGGGATCTTTGCC
ACCTACCCGGCATCATACCTTTCGGTGCATGCCGGATTCATTGACCAGGTGTTTGGCACA
GCCATGCTTCTGCTTTGCCTGATGGCTCTGTCTGACCAGAGGAACAAGCCGGCTGTGAAG
GGCAGCGAGCCAGTGATGGTCGGCCTCCTGGTGCTGCTCATCGGCATCTCACTGGGCAGC
AACAGTGGCTACGCCATCAACCCCACCAGAGACCTGGCTCCGAGAGTCTTTACTGCCATA
GCAGGCTGGGGGACTGATGTGTTCAGGGCTGGAAAAGGGTGGTGGTGGGTGCCCATCGTG
GCTCCGTCACTAGGAGGTGTAATGGGTGCAGGGCTATACAAGGCCTTTGTTGAGCTTCAT
CACCCTCCTCTTTGTGACCAAAGGGAG---------------------GAAGCGGAGATC
AAGCCGCTGGAGATACAGAAGAGCAGC---TGTGCTGATGTT------TGTGTG------

>John_dory_Aqp7

------------------------GTGTTTGGCCTGGGCTCTGTGGCGCAGGTCGTAACT
GGACACGGCGCATTTGGACAATACCTTAGCATCAACTTGGGATTTGGCCTCGGCGTTGCC
ATGGGAGTGCATATTGGGGGGAAGGTCTCAGGTGCTCACATGAATGCAGCAGTGTCTTTG
GCCATGTGTGTGTTCGGTCGTCTCAAGTGGAAGATGCTCCCTCTCTATGTTTTCTCCCAG
TTTCTTGGCTCATTTTTGGCAGCAGGGACAATTTATACAATTTACTTTGATGCCATTCAT
GAGTACAGTGGTGGGACTTTAACTGTGACCGGTACCAAAGCCACAGCCGGGATCTTTGCC
ACCTACCCGGCATCGTACCTTTCGGTGCATGCCGGGTTCATTGACCAGGTTTTTGGCACA
GCCATGCTTCTGCTTTGCCTGATGGCTCTGTCCGACCAGAGGAACAAGCCGGCCGCGGAG
GGCAGCGAGCCAGTCATGGTCGGGCTCCTGGTGCTGCTTGTCGGCATCTCACTGGGCAGC
AACAGTGGCTACGCCATCAACCCCACCAGAGACCTGGCTCCTCGAATCTTTACTGCCATA
GCTGGCTGGGGCACGGACGTATTCAGG------------TGGTGGTGGGTGCCCATCGTG
GCTCCGTCACTAGGAGGTGTCATGGGCGCAGGGCTGTACAAGGCCTTGGTTGAGCTTCAT
CACCCTCCTCTGTGTGACCAGGTGGGGGAC-------------------ACGGAGGAGACC
CACCAGCTGGAGATCCACAAGAGCAGC---CGTGCGGATGCT------TGTGTG------

>Sand_roller_Aqp7
ATGAAAGACACTGTAAGATCTGTAGAAGTCTTGGGC---CCTAACCACAAAAGAAGCTGG
AAAATAAAATTCAAGCTTTGGCTA---AAGAATAAACTTTTTCGGGTGGGGCTAGCTGAG
ACACTCTGCACGTATGTAATGATGGTGTTTGGTCTAGGCTCGGTGGCCCAGGTAGTAACA
GGACAGGGAGCGTTTGGGCAATACATCAGCATTAACTTAGGTTTTGGCCTCGGAGTTGCT
ATGGGAGTGCACGTTGGAGGGAAGGTCTCAGGGGCTCACATGAACGCAGCGGTGTCGTTT
ACAATGTGTGTGTTCGGCTGTCTCGCGTGGAGGAAGCTGCCCCTCTACGTTTTCTGCCAG
CTTCTGGGTTCA--
------------GGGAATTTGACGGTGACTGGTGCACAAGCCACAGCTGGGATTTTTGCC
ACCTATCCAGCACCGTACCTCTCGTTACATGCTGGATTCTTCGACCAGGTGTTTGGCACA
GCCATGCTACTGCTGTGCCTGAGGGCTTTGTCTGACCAGAGGAACAAGCCGGCTGTGGCG
GGCAGCGAGCCGGTGGCGGTGGGCCTCTTGGTGGCACTCATTGGAATCTCCCTGGGGAGC
AACAGTGGCTACGCCATCAATCTCACCAGAGACCTGGCACCCAGGGTCTTCACTGCTATA
GCGGGCTGGGGGGCCGATGTGTTCAGGGCCGCCAATGGGTGGTGGTGGGTGCCTGTAGTT
GCCCCTCTTCTAGGAGGTGTAATGGGTGCAGGGCTCTACAAGGCCTGTGTGGAGCTGCAC
CACCCCACAGAGGAGCCA------------------------------GGGGAGGAGACT
AGCCCTCTGGAGAAGCACAAAGACACC---TGTACTGACTGT------------------

>Southern_cavefish_Aqp7
ATG------------AGATCTGTAGAAGTCATAGGC---CCTAACTACAAAAGAAGCTGG
ACAATTAAATGCAAGCTTTGGCAA---AATAATACACTTTTTCGGGTGGGGCTTGCTGAG
ACACTCTGCACATATGTAATGATGGTGTTTGGTCTAGGCTCCGTGGCCCAAGTAGTAACC
GGACAGGGCGTGTTTGGACAATACATCAGCATTAACTTAGGTTTTGGCCTCGGAGTTGCC
ATGGGAGCGCACGTTGGTGGAAAGGTCTCAGGT---------------------------

>Atlantic_greeneye_Aqp7
ATGAAGGACTCAGTGTCGTCGATGGAACTTGGGATC---CCTCAAGAGAAAGGAGGGAAC
AGAACCCAACTCAGGCTTTGGTTA---AAGAACGAGATTGTTCGAGTAGGACTTGCTGAG
ACCCTCTGCACCTATGTCATGATGGTGTTCGGCCTGGGTTCTGTAGCCCAGGTGGTGACA
GGACAAGGAGCATTTGGAGAGTACATCAGTATCAACCTGGGCTTTGGCCTTGGTGTTGCC
ATGGGAGTGCACATCGGAGGGAAGGTGTCAGGGGCCCATATGAATGCAGCGGTGTCGTTC
ACGATGTGTGTGTTTGGCCGTCTTACTTGGAAGATGCTTCCCCTCTATGTTTTCACCCAG
TCGCTTGGGTCATTTCTGGCAGCGGGGACAATTTACACTGTTTACTATGATGCCATACAT
CACTACAGTGGAGGGAATCTGACTGTGGTTGGTTCCAAAGCCACGGCAGGTATCTTTGCC
ACCTACCCTGCCCCGTACCTTTCCCTGCATGCGGGATTCATCGACCAGGTGTTTGGCACA
GCCATTCTTCTGCTGTGCCTTATGGCTCTGTCAGACCAGAGGAACCAGCCTGCTGCGGTG
GGCAGTGAGCCTGTGGCGGTGGGACTCCTGGTGCTTCTGATTGGCATCTCTCTGGGCAGC
AACTGCGGCTATGCCATCAACCCCACCAGAGACCTGGCGCCCAGGGTCTTCAGTGCCATG
GCAGGCTGGGGCACCAACGTGTTCAGGGCTGGGAATGGGTGGTGGTGGGTGCCTGTAGTT
GCCCCCTTAGTGGGAGGAGTAATAGGAGCAGCCGTCTACAAGGCCTTTGTGGAAATGCAC
CACCCGAACCTTGGTGACCAGAGGGGGGACCTA---GAGGGAGAG---ACAGAGGAGACT
GCTCCACTGGAGAAAAAGAAGAGCATC---TGTGCTGATGGG------TGTGTG------

>Highfin_tadpole_fish_Aqp7

---CAGGTGTTTGGCACT
GCCATGCTACTCCTCGGCCTGATGGCCCTGGGAGATCAGAGGAACCAGCCAGCGCCAGCG
GGCACGGAGCCCGTGGCAGTGGGCTTGCTGGTACTCCTGATTGGCATCTCTCTGGGCAGC
AACAGTGGCTACGCCATCAACCCCAGCAGAGACCTTGGACCCAGGGTATTCACCGCCATC
GCCGGCTGGGGGATGGAGGTGTTCAGGGCCGGGAACTATTGGTGGTGGATACCGGTCGTA
GCCCCCCTGTTGGGCAGTGTAATGGGTGCGGCGGTCTACAAGGGTTTTGTGGAACTGCAT
CACCCTACCCTTGGTGACCAGCAGGAGATGCTG---AAGGGAGAG---CCGGAGGAGACA
GTAGCTCTGGAGAAGCAGCAGATCGTC---TGTGCTGATGTG------CGCGTG------

>European_smelt_Aqp7
ATGAAAGACTTGCAGAGGGCACCAGAAGACGAAGGA---TCTGAAAAGCATGGGAGTCAC
AGAGCACCATGCAGGTTATGGGTG---AATAATATATTGGTTCGAGTGGGACTTGCAGAG
ACTCTCAGCACTTATGTAATGATGGTATTTGGCTTGGGCTCTGTGGCGCAGGTAGTGACA
GGACAAGGAGCGTTCGGGGAATACCTCAGTATCAACTTGGGGTTTGGCCTGGGTGTGGCT
ATGGGAGTGCACATTGGCGGTAGGATCTCTGGGGCCCATATGAATGCAGCTGTATCATTT
ACCATGTGTATATTTGGCCGACTCAGTTGGAAGATGCTGCCCCTGTACATCCTCACTCAG
TTCATTGGTTCATTCTTAGCAGCTGGAACTGTTTATACTCTTTACTATGATGCCATTTAT
CACTATTGTGGAGGGAATTTTACTGTCACTGGACCCAAAGCCACAGCTGGGATCTTTTCC
ACCTACCCAGCACATTATCTCTCATTACACACTGGATTCCTTGACCAAGTGTTGGGCACT
GCCATGTTGTTGCTGTGTCTGATGGCTCTATCAGACCAGAAGAACCAGCCGGCTTCTTCT
GGTGGAGAACCTATAGCAGTGGGGCTACTGGTGCTCCTTGTAGGGATCTCCCTGGGCAGT
AATAGTGGCTATGCCATCAACCCCACTAGGGACCTCGGCCCAGAATCTTCACTGCGATT
GCAGGTTGGGGGCAAGAAGTTTTTAGGGCGGGTAAGGGTTGGTGGTGGGTACCTGTTGTA
GCCCCACTAGTGGGTGGAGTAATAGGTGCGGCCATCTACAAAGTGTTTGTGGAACTGCAT
CACCCTCCCAGCCATAACCAGAGGGAGCTAGTG---GAA---------CAGGAAGAGGCT
GACCCTCTG---AAGCAGAAGAGCATT---TGCACCGAAGTG------TGTGTG------

>Ayu_Aqp7
ATGAAAGACCTGCACAGGGCACCAGAAGAGGAAAAT---ACAGAGAGGCATGCGAGTCAC
GGATCTCCATGCAGGTTCTGGGTG---AATAATGTATTTGTTCGTGTGGGACTTGCAGAG
ACGCTCAGCACTTATGTCATGATGGTATTTGGCTTGGGCTCTGTGGCGCAGGTAGTGACA
GGACAAGGAGCGTTCGGGGAATACCTCAGTATCAACTTGGGGTTTGGCCTTGGCGTGGCT
ATGGGAGTACACATCGGCAGGAAAATCTCTGGGGCCCATATGAATGCAGCTGTGTCATTT
ACCATGTGTGTATTTGGTCGACTCAGTTGGAAGATGCTGCCCCTGTACATCCTCACTCAG
TTAATTGGTTCATTCTTGGCAGCTGGAACTATTTATACTCTGTACTATGATGCCATTTAT
TACTATTGTGAAGGGAATTTTACTGTCACTGGCCCCAAAGCCACAGCTGGGATCTTTTCC
ACCTACCCAGCACATTATCTGTCATTACACGCTGGATTCCTTGACCAAGTGTTGGGCACT
GCCATGCTGTTGCTGTGTCTGATGGCTCTATCGGACCAGAGGAACCAGCCAGCTTCTTCC
GGTGGAGAACCTATAGCAGTGGGGTTACTGGTGCTCCTTGTAGGGATTTCCCTAGGCAGT
AACAGTGGCTATGCCATCAACCCCACCAGGGACCTCGGCCCAGAATCTTCACTGCGATT
GCAGGGTGGGGGCCAGAAGTGTTCAGGGCAGGTAAGGGTTGGTGGTGGGTACCTGTTGTA
GCCCCGCTAGTGGGTGGAGTACTAGGTGCAGCCATCTACAAAGGGTTCGTGGAACTATAT
CATCCTTCCAGCCATAACCAGAGAGAGCAAGTG---GAA---------CAGGAAGAGGTT
TCTCATCTG---AAGCAGAGGAGCATT---TGCACTGAAGTG------TGTGTG------

>Sockeye_salmon_Aqp7_1
ATG------------CATGCGATGGAGCAGGGGAGC---GTTCAAGAGCATGGAAGAAGT
AGAAAACTCTCCAAGATCTGGGTGATA---AATGAACATGTTCGAGTGGGATTCGCAGAA
ACCCTCAGCACATATGTCATGATGGTCTTTGGCTTGGGCTCTGTGGCACAGGTCGTGACT
GGACGGGGTGCGTTCGGAGAGTACCTTAGTGTCAACCTGGGCTTCGGCCTGGGCGTGGCT
ATGGGAGTGCATATTGGCGGGAGGGTCTCAGGGGCCCATATGAACGCAGCAGTATCATTC
TCCATGTGTCTCTTTGGTCGTTTGGGTTGGAGGATGCTTCCCGTCTATATCCTCAGTCAG
CTGATAGGCTCCTTCTTGGCTGCTGGGACCATATACTCCCTTTACTATGACGCCATATAC
CACTACTGTGGAGGGAATCTGACTGTGTCTGGCCCTAAGGCTACCGCTGGGATCTTTGCT
ACCTACCCAGCACCCTACCTCTCGCTTCATGCTGGATTCTTAGACCAGGTGATCGGCACG
GCCATGTTGTTGCTGTGCCTGATGGCCCTGTCTGACCAAAGGAACCAGCCTGCTCCATCG
GGGGGTGAGCCCATCGCTGTGGGCCTGTTGGTACTGCTCATAGGAGTCTCTCTGGGGAGC
AACAGTGGATACGCTATCAACCCTAGCCGCGACCTGGGACCTCGAATCTTCACCGCTATA
GCAGGCTGGGGGCCAGAGGTGTTCCGGGCAGGTAACGGCTGGTGGTGGGTACCTGTACTT

GCCCCCATGGTGGGAGGAGTCACAGGGGCTTCCATCTACAAGGTCTTTGTGGAATTGCAC
CACCCCTCCCCCTGTGAACACAGGAGGGAGATA---GAGGACGAGGCAGAGGAGGAGGCT
GCGCCTCTGGATATGCAGAAAAACCTC---AGCCCAGAGGTG------TGTGTG------

>Coho_salmon_Aqp7_1
ATG------------CATGCGATGGAGCAGGGGAGC---GTTCAAGAGCATGGAAGAAGT
AGAAAACCCTCCAAGATCTGGGTGATA---AATGAACGTGTTCGGGTGGGACTCGCAGAA
GCCCTCAGCACCTATGTCATGATGGTGTTTGGCTTGGGCTCTGTGGCACAGGTCGTGACT
GGACGGGGTGCGTTCGGAGAGTACCTTAGTGTCAACCTGGGCTTCGGCCTGGGCGTGGCT
ATGGGAGTGCATATTGGCGGGAGGGTCTCAGGGGCCCATATGAACGCAGCTGTATCATTC
TCCATGTGTCTCTTTGGTCGTTTGGGTTGGAGGATGCTTCCCGTCTATATCCTCTGTCAG
CTGATAGGCTCCTTCTTGGCTGCTGGGACCATATACTCCCTTTACTATGACGCCATATAC
CACTACTGTGGAGGGAATCTGACTGTGTCTGGCCCTAAGGCTACAGCTGGGATCTTTGCT
ACCTACCCAGCACCCTACCTCTCGCTTCATGCTGGATTCTTAGACCAGGTGATCGGCACG
GCCATGTTGTTGCTGTGCCTGATGGCCCTGTCTGACCAAAGGAACCAGCCTGCTCCATCG
GGGGGTGAGCCCATCGCTGTGGGCCTGTTGGTACTGCTCATAGGAGTCTCTCTGGGGAGC
AACAGTGGATACGCTATCAACCCTAGCCGCGACCTGGGACCTCGAATCTTCACCGCTATA
GCAGGCTGGGGGCCAGAGGTGTTCCGGGCAGGTAACGGCTGGTGGTGGGTACCTGTACTT
GCCCCCATGGTGGGAGGAGTCACAGGGGCTTCCATCTACAAGGTCTTTGTGGAATTGCAC
CACCCTTCCCCCTGTGAACACAGGAGGGAGATA---GAGGACGAGGCAGAGGAGGAGGCT
GCGCCTCTGGATATGCAGAAAAACCTC---AGCCCAGAGGTG------TGTGTG------

>Chinook_salmon_Aqp7_1
ATG------------CATGTGATGGAGCAGGGGAGT---GTTCAAGAGCATGGAAGAAGT
AGAAAACTCTCCAAGATCTGGGTGATA---AATGAACGTGTTCGGGTGGGACTCGCAGAA
GCCCTCAGCACCTATGTCATGATGGTGTTTGGCTTGGGCTCTGTGGCACAGGTCGTGACT
GGACGGGGTGCGTTCGGAGAGTACCTTAGTGTCAACCTGGGCTTCGGCCTGGGCGTGGCT
ATGGGAGTGCATATTGGCGGGAGGGTCTCAGGGGCCCATATGAACGCAGCAGTATCATTC
TCCATGTGTCTCTTTGGTCGTTTGGGTTGGAGGATGCTTCCCGTCTATATCCTCTGTCAG
CTGATAGGCTCCTTCTTGGCTGCTGGGACCATATACTCCCTTTACTATGACGCCATATAC
CACTACTGTGGAGGGAATCTGACTGTGTCTGGCCCTAAGGCTACAGCTGGGATCTTTGCT
ACCTACCCAGCACCCTACCTCTCGCTTCATGCTGGATTCTTAGACCAGGTGATCGGCACG
GCCATGTTGTTGCTGTGCCTGATGGCCCTGTCTGACCAAAGAAACCAGCCTGCTCCATCG
GGGGGTGAGCCCATCACTGTGGGCCTGTTGGTACTGCTCATAGGAGTCTCTCTGGGGAGC
AACAGTGGATACGCTATCAACCCTAGCCGCGACCTGGGACCTCGAATCTTCACCGCTATA
GCAGGCTGGGGGCCAGAGGTGTTCCGGGCAGGTAACGGCTGGTGGTGGGTACCTGTACTT
GCCCCCATGGTGGGAGGAGTCACAGGGGCTTCCATCTACAAGGTCTTTGTGGAATTGCAC
CACCCCTCCCCCTGTGAACACAGGAGGGAGATA---GAGGATGAG------------GCT
GCGCCTCTGGATATGCAGAAAAACCTC---AGCCCAGAGGTG------TGTGTG------

>Pink_salmon_Aqp7_1
ATG------------CATGCGATGGAGCAGGGGAGC---GTTCAAGAGCATGGAAGAAGT
AGAAAACTCTCCAAGATTTGGGTGATA---AATGAACGCGTTCGAGTGGGACTCGCAGAA
ACCCTCAGCACCTATGTCATGATGGTGTTTGGCTTGGGCTCTGTGGCACAGGTCGTGACT
GGACGGGGTGCGTTCGGAGAGTACCTTAGTGTCAACCTGGGCTTCGGCCTGGGCGTGGCT
ATGGGAGTGCATATTGGCGGGAGGGTCTCAGGGGCCCATATGAACGCAGCAGTATCATTC
TCCATGTGTCTCTTTGGTCGTTTGGGTTGGAGGATGCTTCCCGTCTATATCCTCAGTCAG
CTGATAGGCTCCTTCTTGGCTGCTGGGACCATATACTCCCTTTACTATGACGCCATATAC
CACTACTGTGGAGGGAATCTGACTGTGTCTGGCCCTAAGGCTACCGCTGGGATCTTTGCT
ACCTACCCAGCACCCTACCTCTCGCTTCATGCTGGATTCTTAGACCAGGTGATCGGCACG
GCCATGTTGTTGCTGTGCCTGATGGCCCTGTCTGATCAAAGGAACCAGCCTGCTCCATCG
GGGGGTGAGCCCATCGCTGTGGGCCTGTTGGTACTGCTCATAGGAGTCTCTCTGGGGAGC
AACAGTGGATACGCTATCAACCCTAGCCGTGACCTGGGACCTCGAATCTTCACCGCTATA
GCAGGCTGGGGGCCAGAGGTGTTCCGGGCAGGTAACGGCTGGTGGTGGGTACCTGTACTT
GCCCCCATGGTGGGAGGAGTCACAGGGGCTTCCATCTACAAGGTCTTCGTGGAATTGCAC
CACCCCTCCCCCTGTGAACACAGGAGGGAGATA---GAGGACGAGGCAGAGGAGGAGGCT
GCGCCTCTGGATATGCAGAAA---------------AAGGTG------TGTGTG------

>Rainbow_trout_Aqp7_1
ATG------------CATGCGATGGAGCAGGGGAGC---GTTCAAGAGCATGGAAGAAGT
AGAAAACTCTCCAAGATCTGGGTGATA---AATGAACGTGTTCGAGTGGGACTCGCAGAA

TCCCTCAGCACCTATGTCATGATGGTGTTTGGCTTGGGCTCTGTGGCACAGGTCGTGACT
GGACGGGGTGCGTTCGGAGAGTACCTTAGTGTCAACCTGGGCTTCGGCCTGGGCGTGGCT
ATGGGAGTGCATATTGGCGGGAGGGTCTCAGGGGCCCATATGAATGCAGCAGTATCATTC
TCCATGTGTCTCTTTGGTCGTTTGGGTTGGAGGATGCTTCCCGTCTATATCCTCTGTCAG
CTGATAGGCTCCTTCTTGGCTGCTGGGACCATATACTCCCTTTACTATGACGCCATATAC
CACTACTGTGGAGGGAATCTGACTGTGTCTGGCCCTAAGGCTACAGCTGGGATCTTTGCT
ACCTACCCAGCACCCTACCTCTCGCTTCCTGCTGGATTCTTAGACCAGGTGATCGGCACG
GCCATGTTGTTGCTGTGCCTGATGGCCCTGTCTGACCAAAGGAACCAGCCTGCTCCATCG
GGGAGTGAGCCCATCGCTGTGGGCCTGTTGGTACTGCTCATAGGAGTCTCTCTGGGGAGC
AACAGTGGATACGCTATCAACCCTAGCCGCGACCTGGGACCACGAATCTTCACCGCTATA
GCAGGCTGGGGGCCAGAGGTGTTCCGGGCAGGTAACGGCTGGTGGTGGGTACCTGTACTT
GCCCCCATGGTGGGAGGAGTCACAGGGGCTTCCATCTACAAGGTCTTTGTGGAATTGCAC
CACCCCTCCCCCTGTGAACACAGGAGGGAGATA---GAGGACGAGGCAGAGGAGGAGGCT
GCGCCTCTGGATATGCAGAAAAACCTC---AGCCCAGAGGTG------TGTGTG------

>Arctic_charr_Aqp7_1
ATG------------CATGCGATGGAGCAGGAGAGC---GTTCAAGAGCATGGAAGAAGT
AGAAAACTGTCCAAGATCTGGGTGATA---AATGAACATGTTCGAGTGGGACTCGCAGAA
ACCCTCAGCACCTATGTCATGATGGTGTTTGGCTTGGGCTCTGTGGCACAGGTCGTGACT
GGACGGGGTGTATTCGGAGAGTACCTTAGTGTCAACCTGGGTTTCGGCCTGGGCGTGGCT
ATGGGAGTGCATATTGGCGGGAGGGTCTCAGGGGCCCATATGAACGCAGCAGTATCATTC
TCCATGTGTCTTTTTGGTCGTTTGGGTTGGAGGATGCTTCCAGTCTATATCCTCAGTCAG
CTGATAGGCTCCTTCTTGGCTGCTGGGACCATATATTCCCTTTACTATGATGCCATATAC
CACTACTGTGGAGGGAATCTGACTGTGTCTGGCCCTAAGGCTACAGCTGGGATCTTTGCT
ACCTACCCAGCAACCTACCTCTCACTTCATGCTGGATTCTTAGACCAGGTGATCGGCACG
GCCATGTTGTTGCTGTGCCTCATGGCTCTGTCTGACCAAAGGAACCAGCCTGCTCCATCG
GGGGGTGAGCCCATCGCTGTGGGCCTGTTGGTACTGCTCATAGGAGTCTCTCTGGGGAGC
AACAGCGGATACGCAATCAACCCTAGCCGTGACCTGGGACCTCGAATCTTCACCGCTATA
GCAGGCTGGGGGCCAGAGGTGTTCCGGGCAGGTAACGGCTGGTGGTGGGTACCTGTACTT
GCCCCCATGGTGGGAGGAGTCACAGGGGCTTCCATCTACAAGGTCTTTGTGGAACTGCAC
CACCCCTCCCCCTGTGAACACAGGAGGGAAATA---GAGGACGAGGCAGAGGAGGAAGCT
GCCCCTCTGGATATGCAGAAAACCCTC---AGCCCAGAGGTG------TGTGTG------

>Brook_trout_Aqp7_1
ATG------------CATGCGATGGAGCAGGGGAGC---GTTCAAGAGCATGGAAGAAGT
AGAAAACTGTCCAAGATCTGGGTGATA---AATGAACATGTTCGAGTGGGACTCGCAGAA
ACCCTCAGCACCTATGTCATGATGGTGTTTGGCTTGGGCTCTGTGGCACAGGTCGTGACT
GGACGGGGTGTATTCGGAGAGTACCTTAGTGTCAACCTGGGCTTCGGCCTGGGCGTGGCT
ATGGGAGTGCATATTGGCGGGAGGGGCTCCGGGGCCCATATGAACGCAGCAGTATCATTC
TCCATGTGTCTCTTTGGTCGTTTGGGTTGGAGGATGCTTCCAGTCTATATCCTCAGTCAG
CTGATAGGCTCCTTCTTGGCTGCTGGGACCATATATTCCCTTTACTATGATGCCATATAC
CACTACTGTGGAGGGAATCTGACTGTGTCTGGCCCTAAGGCTACAGCTGGGATCTTTGCT
ACCTACCCAGCAACCTACCTCTCACTTCATGCTGGATTCTTAGACCAGGTGATCGGCACG
GCCATGTTGTTGCTGTGCCTGATGGCTCTGTCTGACCAAAGGAACCAGCCTGCTCCATCG
GGGGGTGAGCCCATCGCTGTGGGCCTGTTGGTACTGCTCATAGGAGTCTCTCTGGGGAGC
AACAGCGGATACGCAATCAACCCTAGCCGTGACCTGGGACCTCGAATCTTCACCGCTATA
GCAGGCTGGGGGCCAGAGGTGTTCCGGGCAGGTAACGGCTGGTGGTGGGTACCTGTACTT
GCCCCCATGGTGGGAGGAGTCACAGGGGCTTCCATCTACAAGGTCTTTGTGGAACTGCAC
CACCCCTCCCCCTGTGAACACAGGAGGGAAATA---GAGGACGAGGCAGAGGAGGAAGCT
GCCCCTCTGGATATGCAGAAATACCTC---AGCCCAGAGGTG------TGTGTG------

>Brown_trout_Aqp7_1
ATG------------CATGCGATGGAGCAGGGGAGC---GTTCAAGAACATGGAAGAAGT
AGAAAACTGTCCAAGATCCGGGTGATA---AATGAACATGTTCGAGTGGGACTCGCAGAA
ACCCTCAGCACCTATGTCATGATGGTGTTTGGCTTGGGCTCTGTGGCACAGGTCGTGACT
GGACGGGGTGCGTTCGGAGAGTACCTTAGTGTCAACCTGGGCTTCGGCCTGGGTGTGGCT
ATGGGGGTGCATATTGGCGGGAGGGTCTCAGGGGCCCATATGAACGCAGCAGTATCATTC
TCCATGTGTCTCTTTGGTCGTTTGGGTTGGAGGATGCTTCCCGTCTATATCCTCAGTCAG
CTGATAGGCTCCTTCTTGGCTGCTGGGACCATATATTCCCTTTACTATGATGCCATATAC
CACTACTGTGGAGGGAATCTGACTGTGTCTGGCCCTAAGGCTACAGCTGGGATCTTTGCT
ACCTACCCAGCAACCTACCTCTCACTTCATGCTGGATTCTTAGACCAGGTGATCGGCACG

```
GCCATGTTGTTGCTGTGCCTGATGGCCCTGTCTGACCAAAGGAACCAGCCTGCTCCATCG
GGGGGTGAGCCCATCGCTGTGGGCCTGTTGGTACTGCTCATAGGAGTCTCTCTGGGGAGC
AACAGCGGATACGCTATCAACCCTAGCCGTGACCTGGGACCTCGAATCTTCACCGCTATA
GCAGGCTGGGGGCCAGAGGTGTTCCGGGCAGGTAACGGCTGGTGGTGGGTACCTGTACTT
GCCCCCATGGTGGGAGGTGTCACAGGGGCCTCCATCTACAAGGTGTTTGTGGAACTGCAC
CACCCCTCCCCCTGTGAACACAGGAGGGAGATA---GAGGACGAGGCA---GAGGAGGCT
GCACCTCTGGATATGCAGAAAAACCTC---AGCCCAGAGGTG------TGTGTG------
------------------
>Atlantic_salmon_Aqp7_1
ATG------------CATGCGATGGAGCAGGGGAGC---GTTCAAGAACATGGAAGAAGT
AGAAAACTGTCCAAGATCTGGGTGATA---AATGAACATGTTCGAGTGGGACTCGCAGAA
ACCCTCAGCACCTATGTCATGATGGTGTTTGGCTTGGGCTCTGTGGCACAGGTCGTGACT
GGACGGGGTGCGTTCGGAGAGTACCTTAGTGTCAACCTGGGCTTCGGCCTGGGTGTGGCT
ATGGGGGTGCATATTGGCGGGAGGGTCTCAGGGGCCCATATGAACGCAGCAGTATCATTC
TCCATGTGTCTCTTTGGTCGTTTGGGTTGGAGGATGCTTCCCGTCTACATCCTCAGTCAG
CTGATAGGCTCCTTCTTGGCTGCTGGGACCATATATTCCCTTTACTATGACGCCATATAC
CACTACTGTGGAGGGAATCTGACTGTGTCTGGCCCTAAGGCTACAGCTGGGATCTTTGCT
ACCTACCCAGCAACCTACCTCTCACTTCATGCTGGATTCTTAGACCAGGTGATCGGCACG
GCCATGTTGTTGCTGTGCCTGATGGCCCTGTCTGACCAAAGGAACCAGCCTGCTCCATCG
GGGGGTGAGCCCATCGCTGTGGGCCTGTTGGTACTGCTCATAGGAGTCTCTCTGGGGAGC
AACAGCGGATACGCTATCAATCCTAGCCGTGACCTGGGACCTCGAATCTTCACCGCTATA
GCAGGCTGGGGGCCAGAGGTGTTCCGGGCAGGTAACGGCTGGTGGTGGGTACCTGTACTT
GCCCCCATGGTAGGAGGAGTCACAGGGGCTTCCATCTACAAGGTGTTTGTGGAACTGCAC
CACCCCTCCCCCTGTGAACACAGGAGGGAGATA---GTGGACGAGGCA---GAGGAGGCT
TCACCTCTGGATATGCAGAAAAACCTC---AGCCCAGAGGTG------TGTGTG------
------------------
>Taimen_Aqp7_1
ATG------------CATGCGATGGAGCAGGGGAGT---GTTCAAGAGCATGGAAGAAGT
AGAAAACTGTCCAAGATCTGGGTGATA---AATGAACATGTTCGAGTGGGACTCGCAGAA
ACCCTCAGCACCTATGTCATGATGGTGTTTGGCTTGGGCTCTGTGGCACAGGTCGTGACT
GGACGGGGTGCGTTCGGAGAGTACCTTAGTGTCAACGTGGGCTTCGGCCTGGGCGTGGCT
ATGGGAGTGCATATTGGC------------------------------------------TTC
TCCATGTGTCTCTTTGGTCGTTTGGGTTGGAGGATGCTTCCCGTCTATATCCTCAGTCAG
CTGATAGGCTCCTTCTTGGCTGCTGGGACCATATACTCCCTTTACTATGACGCCATATAC
CACTACTGTGGAGGGAATCTGACTGTGTCTGGCCCTAAGGCTACAGCTGGGATCTTTGCT
ACCTACCCAGCACCCTACCTCTCACTTCATGCTGGATTCTTAGACCAGGTGATCGGC---
------------------------------------------------------------
------------------------------------------------------------
------------------------------------------------------------
------------------------------------------------------------
---------GTGGGAGGAGTCACAGGGGCTTCCATCTACAAGGTCTTTGTGGAACTGCAC
CACCCCTCCCCCTGTGAACACAGGAGGGAGCTA---GAGGACAAGGCAGAGGAGGAGGCT
GCCCCTCTGGATATGCAGAAAAACCTC---AGCCCAGAGGTG------TGTGTG------
------------------
>Huchen_Aqp7_1
ATG------------CATGCGATGGAGCAGGGGAGT---GTTCAAGAGCATGGAAGAAGT
AGAAAACTGTCCAAGATCTGGGTGATA---AATGAACATGTTCGGGTGGGACTCGCAGAA
ACCCTCAGCACCTATGTCATGATGGTGTTTGGCTTGGGCTCTGTGGCACAGGTCGTGACT
GGACGGGGTGCGTTCGGAGAGTACCTTAGTGTCAACGTGGGCTTCGGCCTGGGCGTGGCT
ATGGGAGTGCATATTGGCGGGAGGGTCTCAGGGGCCCATATGAACACAGCAGTATCATTC
TCCATGTGTCTCTTTGGTCGTTTGGGTTGGAGGATGCTTCCCGTCTATATCCTCAGTCAG
CTGATAGGCTCCTTCTTGGCTGCTGGGACCATATACTCCCTTTACTATGACGCCATATAC
CACTACTGTGGAGGGAATCTGACTGTGTCTGGCCCTAAGGCTACAGCTGGGATCTTTGCT
ACCTACCCAGCACCCTACCTCTCACTTCATGCTGGATTCTTAGACCAGGTGATCGGCACG
GCCATGTTGTTGTTGTGCCTTATGGCCCTGTCTGACCAAAGGAACCAGCCTGCTCCATCA
GGGGGTGAGCCCATCGCTGTGGGCCTGTTGGTACTACTCATAGGAGTCTCTCTGGGGAGC
AACAGCGGATACGCTATCAACCCTAGCCGCGACTTGGGACCTCGAATCTTTACCGCTATA
GCAGGCTGGGGGCCAGAGGTGTTCCGGGCAGGTAACGGCTGGTGGTGGGTACCTGTACTT
GCCCCCATGGTGGGAGGAGTCACAGGGGCTTCCATCTACAAGGTCTTTGTGGAACTGCAC
CACCCCTCCCCCTGTGAACACAGGAGGGAGCTA---GAGGACAAGGCAGAGGAGGAGGCT
GCCCCTCTGGATATGCAGAAAAACCTC---AGCCCAGAGGTG------TGTGTG------
```

>Tsinling_lenok_trout_Aqp7_1
ATG------------CATGCGATGGAGCAGGGGAGT---GTTCAAGAGCATGGAAGAAGT
AGAAAACTGTCCAAGATCTGGGTGATA---AATGAACATGTTCGAGTGGGACTCGCAGAA
ACCCTCAGCACCTATGTCATGATGGTGTTTGGCTTGGGCTCTGTGGCACAGGTCGTGACT
GGACGAGGTGCGTTCGGAGAGTACCTTAGTGTCAACGTGGGCTTCGGCCTGGGCGTGGCT
ATGGGAGTGCATATTGGCGGGAGGGTCTCAGGGGCCCATATGAACACAGCAGTATCATTC
TCCATGTGTCTCTTTGGTCGTTTGGGTTGGAGGATGCTTCCCGTCTATATCCTCAGTCAG
CTGATAGGCTCCTTCTTGGCTGCTGGGACCATATACTCCCTTTACTATGACGCCATATAC
CACTACTGTGGAGGGAATCTGACTGTGTCTGGCCCTAAGGCGACAGCTGCAATCTTTGCC
ACCTACCCAGCACCCTACCTCTCACTTCACGCTGGATTCCTAGACCAGGTGATCGGCACG
GCCATGTTGTTGTTGTGCCTTATGGCCCTGTCTGACCAAAGGAACCAGCCTGCTCCATCG
GGGGGTGAGCCCATCGCTGTGGGCCTGTTGGTACTACTCATAGGAGTCTCTCTGGGGAGC
AACAGCGGATACGCTATCAACCCTAGCCGCGACCTGGGACCTCGAATCTTTACCGCTATA
GCAGGCTGGGGGCCAGAGGTGTTCCGGGCAGGTAACGGCTGGTGGTGGGTACCTGTACTT
GCCCCCATGGTGGGAGGAGTCACAGGGGCTTCCATCTACAAGGTCTTTGTGGAACTGCAC
CACCCCTCCCCCTGTGAACACAGGAGGGAGCTA---GAGGACAAGGCAGAGGAGGAGGCT
GCCCCTCTGGATATGCAGAAAAACCTC---AGCCCAGAGGTG------TGTGTG------

>Lake_whitefish_Aqp7_1
ATG------------CATGCGATGGAGCAGGGGAGC---GTTCAAGAGCATGGAAGAAGT
AGAAAACTGTCCAAGATCTGGGTGATA---AATGAATATGTTCGAGTGGGACTCGCAGAA
ACCCTCAGCACCTATGTCATGATGGTGTTTGGCTTGGGCTCTGTGGCGCAGGTCGTGACT
GGACTGGGTGCGTTCGGAGAATACCTCAGTGTCAACCTGGGCTTCGGCCTGGGTGTGACT
ATGGGAGTGCATATTGGCGGGAGGGTCTCAGGGGCCCATATGAACGCAGCAGTATCATTT
TCCATGTGTCTCTTTGGTCGTTTGGGTTGGAGGATGCTGCCCGTCTATATCCTCAGTCAG
CTGATAGGCTCCTTCTTGGCGGCTGGGACCATATACTCCCTTTACTATGATGCCATTTAC
CACTACTGTGGAGGGAATCTGACTGTGTCTGGCCCTAAGGCTACAGCTGGGATCTTTGCT
ACCTACCCAGCACCATACCTCTCACTTCATGCTGGATTCCTAGACCAGGTGATCGGCACG
GCCATGTTGTTGCTGTGCCTGATGGCCCTGTCTGACCAAAGGAACCAGCCTGCTCCATCG
GGGGGTGAGCCCATCGCTGTGGGCCTGTTGGTACTGCTCATAGGAGTCTCTCTGGGGAGC
AACAGCGGATACGCTATCAACCCTAGCCGCGACCTGGGACCTCGAATCTTCACCGCTATA
GCAGGCTGGGGGCCAGAGGTGTTCCGGGCAGGTAACGGCTGGTGGTGGGTACCTGTACTT
GCCCCCCTGGTGGGAGGAGTCACAGGGGCTTCCGTCTACAAGGTCTTTGTGGAACTGCAC
CACCCCTCCCCCTGTGAACACAGGAGGGAGCTA---GAGGACGAGGCAGAGGAGGCGGCT
GTCCCTCTGGATATGCAGAAAAACCTC---GGCCCAGAGGTG------TGTGTG------

>Common_whitefish_Aqp7_1
ATG------------CATGCGATGGAGCAGGGGAGC---GTTCAAGAGCATGGAAGAAGT
AGAAAACTGTCCAAGATCTGGGTGATA---AATGAATATGTTCGAGTGGGACTCGCAGAA
ACCCTCAGCACCTATGTCATGATGGTGTTTGGCTTGGGCTCTGTGGCGCAGGTCGTGACT
GGACTGGGTGCGTTCGGAGAATACCTCAGTGTCAACCTGGGCTTCGGCCTGGGCGTGACT
ATGGGAGTGCATATTGGCGGGAGGGTCTCAGGGGCCCATATGAACGCAGCAGTATCATTT
TCCATGTGTCTCTTTGGTCGTTTGGGTTGGAGGATGCTGCCTGTCTATATCCTCAGTCAG
CTGATAGGCTCCTTCTTGGCGGCTGGGACCATATACTCCCTTTACTATGATGCCATTTAC
CACTACTGTGGAGGGAATCTGACTGTGTCTGGCCCTAAGGCTACAGCTGGGATCTTTGCT
ACCTACCCAGCACCCTACCTCTCACTTCATGCTGGATTCCTAGACCAGGTGATCGGCACG
GCCATGTTGTTGCTGTGCCTGATGGCCCTGTCTGACCAAAGGAACCAGCCTGCTCCATCG
GGGGGTGAGCCCATCGTTGTGGGCCTGTTGGTCCTGCTCATAGGAGTCTCTCTGGGGAGC
AACAGCGGATACGCTATCAACCCTAGCCGCGACCTGGGACCTCGAATCTTCACCGCTATA
GCAGGCTGGGGGCCAGAGGTGTTCCGGGCAGGTAACGGCTGGTGGTGGGTACCTGTACTT
GCCCCCCTGGTGGGAGGAGTCACAGGGGCTTCCGTCTACAAGGTCTTTGTGGAACTGCAC
CACCCCTCCCCCTGTGAACACAGAAGGGAGCTA---GAGGACGAGGCAGAGGAGGCGGCT
GTCCCTCTGGATATGCAGAAAAACCTC---GGCCCAGAGGTG------TGTGTG------

>Balchen_Aqp7_1b
ATG------------CATGCGATGGAGCAGGGGAGC---GTTCAAGAGCATGGAAGAAGT
AGAAAACTGTCCAAGATCTGGGTGATA---AATGAATATGTTCGAGTGGGACTCGCAGAA
ACCCTCAGCACCTATGTCATGATGGTGTTTGGCTTGGGCTCTGTGGCGCAGGTCGTGACT
GGACTGGGTGCGTTCGGAGAATACCTCAGTGTCAACCTGGGCTTCGGCCTGGGCGTGACT
ATGGGAGTGCATATTGGCGGGAGGGTCTCAGGGGCCCATATGAACGCAGCAGTATCATTT

TCCATGTGTCTCTTTGGTCGTTTGGGTTGGAGGATGCTGCCTGTCTATATCCTCAGTCAG
CTGATAGGCTCCTTCTTGGCGGCTGGGACCATATACTCCCTTTACTATGATGCCATTTAC
CACTACTGTGGAGGGAATCTGACTGTGTCTGGCCCTAAGGCTACAGCTGGGATCTTTGCT
ACCTACCCAGCACCCTACCTCTCACTTCATGCTGGATTCCTAGACCAGGTGATCGGCACG
GCCATGTTGTTGCTGTGCCTGATGGCCCTGTCTGACCAAAGGAACCAGCCTGCTCCATCG
GGGGGTGAGCCCATCGTTGTGGGCCTGTTGGTCCTGCTCATAGGAGTCTCTCTGGGGAGC
AACAGCGGATACGCTATCAACCCTAGCCGCGACCTGGGACCTCGAATCTTCACCGCTATA
GCAGGCTGGGGGCCAGAGGTGTTCCGGGCAGGTAACGGCTGGTGGTGGGTACCTGTACTT
GCCCCCCTGGTGGGAGGAGTCACAGGGGCTTCCGTCTACAAGGTCTTTGTGGAACTGCAC
CACCCCTCCCCCTGTGAACACAGAAGGGAGCTA---GAGGACGAGGCAGAGGAGGCGGCT
GTCCCTCTGGATATGCAGAAAAACCTC---GGCCCAGAGGTG------TGTGTG------

>Balchen_Aqp7_1ap

---GTGATCGGCACG
GCCATGTTGTTGCTGTGCCTGATGGCCCTGTCTGACCAAAGGAACCAGCCTGCTCCATCG
GGGGGTGAGCCCATCGCTGTGGGCCTGTTGGTACTGCTCATAGGAGTCTCTCTGGGGAGC
AACAGCGGATACGCTATCAACCCTAGCCGCGACCTGGGACCTCGAATCTTCACCGCTATA
GCATGCTGGGGGCCAGAGGTGTTCCGGGCAGGTAACGGCTGGTGGTGGGTACCTGTACTT
GCCCCCCTGGTGGGAGGAGTCACAGGGGCTTCCGTCTACAAGGTCTTTGTGGAACTGCAC
CACCCCTCCCCCTGTGAACACAGGAGGGAGCTA---GAGGACGAGGCAGAGGAG---GCT
GTCCCTCTGGATATGCAGAAAAACCTC---GGCCCAGGGGTG------TGTGTG------

>Grayling_Aqp7_1
ATG------------CATGCGATGGAGCAGGGGAGC---GTTCAAGAGCATGGAAAAACT
AGAAAACTGTCCAAGATCTGGGTGATA---AATGAATATGTTCGAGTGGGACTCGCAGAA
ACCCTCAGCACCTATGTCATGATGGTGTTTGGCTTGGGCTCTGTGGCACAGGTCGTGACT
GGACGGGGTGCGTTCGGAGACTACCTCAGTGTCAACCTGGGCTTCGGCCTGGGCGTGGCT
ATGGGAGTGCATATTGGCGGGAGGGTCTCAGGGGCCCATATGAATGCAGCAGTATCCTTC
TCCATGTGTCTCTTTGGTCGTTTGGGTTGGAGGAAGCTGCCCGTCTATATCCTCAGTCAG
CTGATAGGCTCATTCTTGGGAGCTGGGACCATATACTCCCTTTACTATGACGCCATATAC
CACTACTGTGGAGGGAATCTGACTGTGTCTGGCCCTAAGGCTACAGCTGGGATCTTTGCC
ACATACCCAGCACCCTACCTAACACTTCATGCTGGATTCCTAGACCAGGTGATCGGCACG
GCCATGTTGTTGCTGTGCCTTATGGCCCTGTCTGACCAAAGGAACCAGCCTGCTCCATCA
GGGGGTGAGCCCATCGCTGTGGGCCTGTTGGTACTGCTCATAGGAGTCTCTCTGGGGAGC
AACAGCGGATACGCTATCAACCCTAGCCGTGACCTAGGACCTCGAATCTTCACCGCTATA
GCAGGCTGGGGGCCTGAGGTGTTCCGGGCAGGTAACGGCTGGTGGTGGGTACCTGTACTT
GCCCCCCTGGTGGGAGGAGTCACAGGGGCTTCCGTCTACAAGGTCTTTGTGGAACTGCAC
CACCCCGCCCCCTGTGAACACAGGAGGGCGCTA---GAGGACGAGGCAGAGGAGGAGGAT
GCCCTTCTGGAAATGCAGAAAACCCTC---AGCCCAGAGGTG------TGTGTG------

>Atlantic_salmon_Aqp7_2p
ATG------------CTTGAGATGAAGCAGGGGAGT---GTACAAGCGCATGGAAGAAGT
AGAAAGCTGTCAATGATCTGGGTGATAAAGAATGAGTATGTTCGAATGGTACTTGCAGAA
ACCCTCAGCACCTACCTCATGATGGTA---------------------------------


```
------------------------------------------------------------
------------------------------------------------------------
------------------------------------------------------------
------------------------------------------------------------
------------------
>Huchen_Aqp7_2
ATG------------CTTGAGATGGAGCAGGGGAGT---GTACAAGCGCATGGAAGAAAT
AGAAAGCTGTCCAGGATTTGGGTGATAAAGAATGAGTGTGTTCGAATGGTACTCGCAGAA
ACCCTCAGCACCTACGTCATGATGGTATTTGGCTTGGGCTCTGTGGCGCAAGTCGTGACT
GGACGGGGTGCGTTCGGAGAATACCTCAGTATCAACCTGGGTTTCGGCCTGGGTGTGGCT
ATGGGAGTACATATTGGCGGGAGAGTCTCGGGGGCCCATATGAATTCCGCAGTATCATTC
ACCATGTGTCTGTTCGGCCGTCTGTGTTGGAGGGTGCTGCCCCTGTATATCCTCTCTCAA
CTTATAGGCTCCTTCTTGGCGGCTGGGACCATCTACTCACTTTACTACGATGCCATATGC
CACTACTGTGGAGGGAATCTGACTGTGTCTGGCCCTAAGGCGACAGCTGCAATCTTTGCC
ACCTACCCAGCACCCTACCTCTCACTTCACGCTGGGTTCCTAGACCAGTTGTTCGGCACG
GCCATGTTGTTGCTGTGCCTGATGGCCCTGTCTGACCAGAGGAACCAGCCTACTCCATCG
GGGGGTGAGCCCATCGCTGTGGGTCTACTTGTACTACTCATAGGAGTCTCTCTGGGGGGC
AACAGCGGCTACGCTATCAACCCTAGCCGCGACCTGGGACCTCGAGTCTTCACCGCTATA
GCAGGCTGGGGGCCAGAGGTGTTCAGGGCTGGTAACGGGTGGTGGTGGGTACCTGTTCTA
GCCCCCCTGGTGGGAGGAGTCACAGGGGCAGCCGTCTACAAGGTCTTTGTGGAGTTGCAC
CACACCTCCCCCCGTGACCAGAGGATGGGGCTA---GAGGACGAGACTGAGAAAGAGGCT
GCCCCTCTGGAGAAGCAGAAGAGCCTC---AGGCCCGAGGTG------TGTGTG------
------------------
>Tsinling_lenok_trout_Aqp7_2
------------------------------------------------------------
------------------------------------------------------------
------------------------------------------------------------
------------------------------------------------------------
------------------------------------------------------------
------------------------------------------------------------
------------------------------------------------------------
------------------------------------------------------------
------------------------------------------------------------
------------------------------------------------------------
------------------------------------------------------------
------------------------------------------------------------
GCAGGCTGGGGGCCAGAGGTGTTCAGGGCTGGTAACGGGTGGTGGTGGGTACCTGTTCTA
GCCCCCCTGGTGGGAGGAGTCACAGGGGCAGCCGTCTACAAGGTCTTTGTGGAGCTGCAC
CACACCTCCCCCCGTGACCAGAGGATGGGGCTA---GAGGACGAGACTGAGAAGGAGGCT
GCTGGAGAAGCAGAAGAGCCTCAGGCC---CGA---GGTGTG------TGTGTG------
------------------
>Balchen_Aqp7_2p
------------------------------------------------------------
------------------------------------------------------------
------------------------------------------------------------
------------------------------------------------------------
------------------------------------------------------------
------------------------------------------------------------
------------------------------------------------------------
------------------------------------------------------------
------------------------------------------------------------
------------------------------------------------------------
------------------------------------------------------------
------------------------------------GGCGCACGAGTCTTCACTGCTATA
GCAGGCTGGGGGGCAGAGGTGTTCAGGGCAGGTAACGGGTGGTGGTGGGTAACTGTTCTA
GCCCCCCTGGTGGGAGGAGTCACAGGGGCAGCC---------------GTAGAACTG---
------------------------------------------------------------
------------------------------------------------------------
------------------
>Grayling_Aqp7_2p
ATG------------CTTGAGATGGAGCAGGGGAGC---GTTCAAGCGCATGGAAGAAGT
```

AGAAAGCTATCCAAGATCTGGGTGATAAAGAATGATTATGTTCGAATGGGACTCTCAGAA
ACCCTCAGCAACTATGTCATGATGGTA---------------------------------

--GATGCCATATGC
CACTACTGTGGAGGGAATCTGACTGTGTCTGGCCCTAAGGAGACAGCTGCAATCTTTGCC
ACCTACCCAGCACCCTAT--

>Northern_pike_Aqp7p
---------------CATGCGATGGAGCTGGGGAGC---ATTCAAGAGCAGAGAAAAACA
AGAAGGCTGTCAAACATC---GTAATAAAGAAT---TATTTTCAAGTGGGACTTGCCGAA
ACCCTCAGCTCCTAC---
------------------GAATCCTTCAGTATCAACCTTGGCTTTGGCCTGGGCGTGGCC
ATGGGAGTGCAAATTGGCAGAAAGGTATCAGGGGCTCACATGAATGCGGCTGTATCATTC
ACCATGTGCATATCTGGTAATCTG------------------------GTAATCTGCCAG
CTGATAGGCTCCTTCCTGGCGGCTGGGACCATCTACTCCCTTTATTAC------------

>Eastern_mudminnow_Aqp7
ATG------------CCCGCAATTGAGCAGGGGAAT---ATTAATCAGCAAGGAGAAACG
AGAAGGCTGTCCAAGATCTGGGTGATAAAGAATGAATACGTTCGAGTCGGACTCGCTGAA
TCCCTCAGCACCTATGTCATGATGGTGTTTGGCTTGGGCTCGGTAGCGCAGGTCGTGACT
GGACGGGGTGCGTTCGGCGAATACCTCAGTATCAACCTGGGTTTTGGCCTGGGCGTGGCT
ATGGGAGTGCACATTGGTGGGAGGGTCTCAGGGGCTCATATGAACACAGCCGTGTCCTTC
ACCATGTGTCTGGTTGGTGGGCTGGACTGGAGGAAGCTGCCTCTCTATGTCCTCTCTCAG
CTGATTGGCTCGTTCCTGGCAGCTGGGACCATCTACTCCCTTTACTATGATGCCATATAC
CACTACTGTGAAGGGAATCTGACTGTATCCGGCCCAAAGGCCACGGCTGGCATCTTTGCC
ACCTACCCAGCACCCTATCTCTCACTTCATTCTGGATTCCTGGACCAGGTGTTTGGCACG
GCCATTTTGATGCTGTGCCTGATGGCCCTGTCGGACCAGAAGAACCAGCCAGCGCAATCC
GGGGGCCAGGCCATCGCTGTGGGTCTGCTGGTACTGCTCATAGGAGTGTCTCTGGGGAGC
AACAGTGGCTACGCTATTAACCCCAGCCGTGACCTGGGACCTCGAGTCTTCACCGCTTTC
GCAGGCTGGGGGCCAGAGGTGTTCAGGGCAGGTAACAACTGGTGGTGGGTGCCTGTACTG
GCACCCCTGGTGGGAGGAGTCACTGGGGCAGCCATCTACAAGGTGTTTGTGGAGCTGCAC
CACACCTCACCCATGGACCACAGCAGGGAGCCA---------------GAGGCCCAGGCA
ATCCCTCTGGAAAAACAGAAGAGCCTG---GGGCCTGAAGTA------TGCGTT------

>Anshui_blind_cavefish_Aqp7
---------------------ATGGAGGATGGTAGTAGGATACAAGGCCATATGGCACCT
ACTGTAGTCTCCAGGTTGAAG---ATAAAAAATGAATGCATTCGAGTGGTCCTGGCAGAG
ACCCTCTGCACGTTCATCATGATGGTGTTTGGCCTTGGCACTGTTGCACAAGTGGTCACA
GGAAATGGTGTTTTTGGAGGGTATTTCAGCATAAATGTAGGCTTTGGGCTGAGTGTGGCT
ATGGGTGTGCATGTTGGTGGAAAAGTTTCAGGAGCTCATATGAATGCAGCTGTTTCATTC
ACAATGTGCGTGTTTGGCCGTTTGCGCTGGAAGATGTTCCCACTGTATGTTTTTGCCCAG
TTTCTGGGTTCCTTCCTTGCTGCTGGGACCATTTTTTTACTTTATTATGATGCTATACAT
CATTACTGTGGGGGCAATTTCACTGTGTCGGGCCCCAAAGCAACAGCTGGGATCTTCGCT

ACATATCCAGCACCTTACATCTCAATCTACACTGGATTCTTTGATCAGGTTCTGGGCACT
GCCATGCTGTTGCTGTGTCTGATGGCTCTGGCAGACCAGAGAAACCAGCCGGTCCTGCCT
GGAGGTGAGCCTGTGGGTGTGGGGCTCCTGGTGCTCCTCATTGGTGTCTCTCTGGGGAGC
AACAGCGGCTATGCCATCAATCCAACACGAGATCTAGGGCCGAGAGTCTTCACACTGATG
GCAGGTTGGGGCATGGAGGTTTTCAGGGCCGGCAATGGCTGGTGGTGGGTACCTTTGGTG
GCCCCCTTTATTGGTGGAGTGACTGGGGCTTTAATCTACGAAGCATTTGTAGAGCTACAC
CACCCTGATCTTAAGAGCACTAAGACACAGTCT---GCAGAA------GATCCTGAATGT
GTTCCTCTGGAAAAGTGCAAGAATAGC---AGTACAGAGATG------TCTGTC------

>Horned_goldenline_barbel_Aqp7
---------------------ATGGAGGATGGTAGTAGGATACAAGGCCATATGGCACCT
ACTGTAGTTTCCAGGTTGAAG---ATAAAAAATGAATGCATTCGAGTGGTCCTGGCAGAG
ACCCTCTGCACGTTCATCATGATGGTGTTTGGCCTTGGCACTGTTGCACAAGTGGTCACA
GGAAATGGTGTTTTTGGAGAGTATTTCAGCATAAATGTAGGCTTTGGGCTGAGTGTGGCT
ATGGGTGTGCACGTTGGTGGAAAAGTGTCAGGAGCTCATATGAATGCAGCTGTTTCATTC
ACAATGTGCGTGTTTGGCCGTTTGCGTTGGAAGATGTTCCCACTGTACGTTTTTGCCCAG
TTTCTGGGTTCCTTCCTTGCTGCTGGGACCATTTTTTTACTTTATTATGATGCTATACAT
CATTACTGTGGGGGCAATTTCACTGTGTCGGGCCCCAAAGCAACAGCTGGGATCTTCGCT
ACATATCCAGCACCTTACATCTCAATCTACACTGGATTCTTCGATCAGGTTCTGGGCACT
GGCATGCTGTTGCTGTGTCTGATGGCTCTGGCTGACCAGAGAAACCAGCCGGTCGTGCCT
GGAGTTGAGCCTGTGGGTGTGGGGCTCCTGGTGCTCCTCATTGGTGTCTCTCTGGGGAGC
AACAGCGGCTATGCCATCAATCCAACACGAGATCTGGGGCCGAGAGTCTTCACACTGATG
GCAGGTTGGGGCATGGAGGTTTTCAGG---------------------------------

>Golden_line_fish_Aqp7
---------------------ATGGAGGATGGTAGTAGGATACAAGGCCATATGGCACCT
ACTGTAGCTTCCAGGTTGAAG---ATCAAAAATGAATGCATTCGAGTGGTCCTGGCAGAG
ACCCTCTGCACGTTCATCATGATGGTGTTTGGGCTTGGCACTGTTGCACAAGTGGTCACA
GGAAATGGTGTTTTTGGAGAGTATTTCAGCATAAATGTAGGCTTTGGGCTGAGTGTGGCT
ATGGGTGTGCACGTTGGTGGAAAAGTGTCAGGAGCTCATATGAATGCAGCTGTTTCATTC
ACAATGTGCGTGTTTGGCCGTTTGCGCTGGAAGATGTTCCCACTGTATGTTTTTGCCCAG
TTTCTGGGTTCCTTCCTTGCTGCTGGGACCATTTTTTTACTTTATTATGATGCTATACAT
CATTACTGTGGGGGCAATTTCACTGTGTCGGGCCCCAAAGCAACAGCTGGGATCTTCGCT
ACATATCCAGCACCTTTCATCTCAATCTACACTGGATTCTTTGATCAGGTTCTGGGCACT
GCCATGCTGTTGCTGTGTCTGATGGCTCTGGCAGACCAGAGAAACCAGCCGGTCGTGCCT
AGAGGTGAGCCTGTGGGTGTGGGGCTCCTGGTGCTCCTCATTGGTGTCTCTCTGGGAAGC
AACAGCGGCTATGCCATCAATCCAACACGAGATCTGGGGCCGAGAGTCTTCACACTGATG
GCAGGTTGGGGCATGGATGTTTTCAGGGCCGGCAATGGCTGGTGGTGGGTACCTTTGGTG
GCCCCCTTTATTGGTGGAGTGACTGGGGCTTTAATCTACGAAGCATTTGTAGAGCTACAC
CACCCTGATCTTAAGAGCACTAAGACACAGTCT---GCAGAA------GATCCTGAATGT
GTTCCTCTGGAAAAGTGCAAGAATAGC---AGTACAGAGATA------TCTGTC------

>Common_carp_Aqp7_1
---ATGGCACCT
ACTGAAGTTTTCAGGCTGAAG---ATCAAAAACGAATGCATTCGAGTGGCCCTGGCAGAG
ACCCTCTGCACGTTCATCATGATGGTGTTTGGCCTTGGCACTGTTGCACAAGTGGTCACA
GGAAATGGTGTTTTTGGAGAGTATTTCAGCATAAATGTAGGTTTTGGGCTGAGTGTGGCT
ATGGGTGTGCATGTTGGTGGAAAAGTGTCAGGAGCTCATATGAATGCAGCTGTTTCATTC
ACAATGTGCGTGTTTGGCCGTTTGCGCTGGAAGATGTTCCCACTGTATGTTTTTGCCCAG
TTTCTGGGTTCCTTCCTTGCTGCTGGGACCATTTTTTTACTTTATTATGATGCTATACAT
CATTACTGTGGGGGCAATTTCACCGTGTCGGGCCCCAAAGCAACAGCTGGGATCTTCGCT
ACATATCCAGCACCTTACATCTCAATCTACACTGGATTCTTTGATCAGGTTCTGGGCACT
GCCATGCTGTTGCTGTGTCTGATGGCTCTGGCAGACCAGAGAAACCAGCCGGTCATGTCT
GGAGGTGAGCCTGTGGGTGTGGGGCTCCTGGTGCTCCTCATTGGTGTCTCTCTGGGGAGC
AACAGCGGCTATGCCATCAATCCAACACGAGATCTGGGGCCGAGAGTTTTCACACTGATG
GCAGGTTGGGGCATGGAGGTTTTCAGGGCCGGCAATGGCTGGTGGTGGGTACCTTTGGTG
GCCCCCTTTATTGGTGGAGTGACTGGGGCTTTAATCTACGAAGCATTTGTAGAGCTACAC
CACCCTGATCTTAAGAGGACTAAGACACAGTCT---GCAGAA------GATCCTGAATGT

GTTCCTCTGGAAAAGTGCAAGAATGGC---AGTACAGAGATG------TCTGTC------

>Goldfish_Aqp7
ATGAAACAGGAAGTTAGCATAATGGAGGATGGTAGTAGGAGAGAAGACCATATGGCACCT
AATGTAGTTTCCAGGTTGAAG---ATCAAAAATGAATGCATTCGAGTGGTCCTGGCAGAG
ACGCTCTGCACGTTCATTATGATGGTGTTTGGCCTTGGCACTGTTGCACAAGTGGTCACA
GGAAATGGTGCTTTTGGAGAGTATTTCAGCATAAATGTAGGCTTTGGGCTGAGTGTGGCT
ATGGGTGTGCACGTTGGTGGAAAAGTGTCAGGAGCTCATATGAATGCAGCTGTTTCATTC
ACAATGTGTGTGTTTGGTCGTTTGCGCTGGAAGATGTTCCCACTGTATGTTTTTGCCCAG
TTTCTGGGTTCCTTCCTTGCTGCTGGGACCATTTTCTTACTTTATTATGATGCTATACAT
CATTACTGTGGGGGCAATTTCACTGTGTCGGGCCCCAAAGCAACAGCTGGGATCTTCGCT
ACATATCCAGCACCTTACATCTCAATCTACACTGGATTCTTTGATCAGGTTCTGGGCACT
GCCATGCTGTTGCTGTGTCTGATGGCTCTGGCAGACCGGAGAAACCAGCCGGTCGTGTCT
GGAGGTGAGCCTGTGGGTGTGGGGCTCCTGGTGCTCCTCATTGGTATCTCTATGGGGAGC
AACAGCGGCTATGCCATCAATCCAACACGAGATCTGGGGCCGAGAGTCTTCACACTGATG
GCAGGTTGGGGCATGGAGGTTTTCAGGGCAGGCAATGGCTGGTGGTGGGTACCTTTGGTG
GCCCCCTTTATTGGTGGAGTGACTGGGGCTTTAATCTACGAAGCATTTGTAGAGCTACAC
CACCCTGATCTTAAGAGCACTAAGACACAGTCT---GCAGAA------GACCCTGAATGT
GTTCCTCTGGAAAAGTGCAAGAATGGC---AGTACAGAGATG------TCTCTC------

>Ya_fish_Aqp7
ATGAAACAGGAAGTTAGCACAATGGAGGATGGTAGT---ATTCAAGGCCATATGGCACCT
ACTGTAGTTTCCAAGTTGAAG---ATCAAAAATAAATGCATTCGAGTGGTCCTGGCAGAG
ACCCTCTGCACATTCATCATGATGGTGTTTGGCCTTGGCACCGTTGCACAAGTGGTCACT
GGAAATGGTGTTTTTGGAGAGTATTTCAGCATAAATGTAGGCTTTGGGCTGGGTGTGGCT
ATGGGTGTGCACATTGGTGGAAAAGTGTCAGGAGCTCATATGAATGCAGCTGTTTCGTTC
ACAATGTGCGTGTTTGGCCGTTTGCGCTGGAAGATGTTCCCACTGTATGTTTTTGCCCAG
TTTCTGGGTTCCTTCCTTGCTGCTGGGACCATTTATTTACTTTATTATGATGCTATACAT
CATTACTGTGGGGGCAATTTCACTGTGTCGGGCCCCAAAGCAACAGCTGGGATCTTTGCT
ACATATCCAGCACCTTACATCTCAATTTACACTGGATTCTTTGATCAGGTTCTGGGCACT
GCCATGCTGTTGCTGTGTCTGATGGCTCTGGCAGACCAGAGGAACCAGCCGGTCGTGCCT
GGAGGTGAGCCTGTGGGTGTGGGGCTCCTGGTGCTTCTCATTGGTGTCTCTTTGGGGAGC
AACAGCGGCTATGCCATTAATCCTACACGAGATCTGGGGCCGAGAGTCTTCACACTGATG
GCAGGTTGGGGCATGGAGGTTTTCAGGGCCGGCAATGGCTGGTGGTGGGTACCTTTGGTG
GCCCCCTTTATTGGTGGAGTGACTGGGGCTTTAATCTACAAAGCATTTGTAGAGCTACAC
CACCCTGATCTTAAGAGCATGAAGACACAGTCT---GCAGAA------GATCCTGAATGG
GTTCCTCTGGAAAAGTGCAAGAATGGC---AGTACAGAGATG------TCTGTA------

>Catla_Aqp7

---GGTGTGCACATTGGTGGAAAAGTGTCAGGAGCTCATATGAATGCTGCTGTTTCATTC
ACAATGTGTGTGTTTGGACATCTGCGCTGGAAGATGCTCCCACTATATGTTTTCGCACAG
TTTCTGGGTTCCTTCCTTGCTGCTGGGACCATTTTTTTAATTTATCATGATGCTATATAT
CATTATTGTGGGGGCAATTTCACTGTGTCGGGCCCCAAAGCAACAGCTGGGATCTTCGCT
ACATATCCAGCACCTTACATCTCAATCTACACTGGATTCTTCGACCAGGTTCTGGGCACT
GCCATGCTGTTGCTGTGTCTGATGGCTCTGGCAGACCAGAGAAACCAGCCGGTTGTGCCT
GGAGGTGAGCCTGTGGGTGTGGGACTTGTAGTGCTGCTTATTGGTCTCTCTCTGGGGAGC
AACAGCGGTTATCCCATCAATCCTACACGAGATCTGGCGCCAAGAGTCTTCACACTGATG
GCAGGTTGGGGCATGGAAGTTTTCAGGGCAGGCAATGGCTGGTGGTGGGTACCTGTGGTG
GCCCCCTTTATTGGTGGAGTAACTGGGGCTGTAATCTACAAAGCATTTGTAGAACTGCAC
CACCCTGATCTTAAGAGCACCAAGACATCATCT---GCAGAA------GATCCTGAATGT
GTTCCCCTGGAAAAGTGCAAGAATGGC---AGTACAGAGATG------GTTGTG------

>Amur_ide_Aqp7
ATGAAACAGGAAGTCTACATAATGGAGGACGGT------TTTCAAGGCCATATGGCACCT
ACTGTGGTATCCATGTTGAAG---ATCAAGAATGAATGCATTCGAGTGGTCCTGGCAGAG
ACCCTCTGCACATTTGTCATGATGGTGTTCGGCCTTGGCTCTGTTGCACAAGTGGTCACA
GGAAATGGTTTCTTCGGAGATTACCTCAGCATGAATATAGGCTTTGGACTGAGTGTGGCT

```
ATGGGTGTGCACATTGGTGGAAAAGTGTCAGGAGCTCATATGAATGCAGCTGTTTCCTTC
ACAATGTGTGTGTTCGGCCGTTTGCGCTGGAAGATGCTGCCACTGTATGTTTTCGCCCAG
TTTCTGGGTTCCTTCCTCGCTGCCGGGACCATTTTTTCACTTTATTACGATGCTATACAT
CATTACTGTGGGGGCAATTTCACTGTGTCTGGTCCCAAAGCAACAGCTGGGATCTTTGCC
ACATATCCAGCACCTTACATCTCAATCTACACTGGATTCTTCGACCAGGTTGTGGGCACC
GCCATGCTGTTGCTGTGTCTGATGGCTCTGGCAGACCAGAGAAACCAGCCGGTTGTGGCT
GGAGGTGAGCCTGTTGGTGTGGGGCTCGTGGTGCTGCTCATTGGTGTCTCTATGGGGAGC
AACAGCGGCTATGCCATCAATCCCACACGAGATCTGGGGCCAAGAGTCTTCACACTGATG
GCAGGTTGGGGCATGGAGGTTTTCAGGGCAGGCAATGGATGGTGGTGGGTACCTTTGGTG
GCCCCCTTTATTGGTGGCGTGACTGGGGCTTCAATCTACAAAGCATTTGTAGAGCTCCAC
CACCCTGATCTTAAAAGCACTCAGACACAGTCT---ACAGGA------GATCCTGAATGT
GTTCCTCTGGAAAAGTGCAAGAATGGAGTATGTGAGACCATC------TCAAGT------
------------------
>Roach_Aqp7
------------------------------------------------------------
------------------AAG---ATCAAGAATGAATGCATTCGAGTGGTCCTGGCAGAG
ACCCTCTGCACATTTATCATGATGGTGTTCGGCCTTGGCTGTGTTGCACAAGTGGTCACA
GGAAATGGTTTCTTCGGAGATTACCTCAGCAGTAATATAGGCTTTGGACTGAGTGTGGCT
ATGGGTGTGCACATTGGTGGAAAAGTGTCAGGAGCTCATATGAATGCAGCTGTTTCGTTC
ACAATGTGTGTGTTCGGTCGTTTGCGCTGGAAGATGCTGCCACTGTATGTTTTCGCCCAG
TTTCTGGGCTCCTTCCTCGCTGCCGGGACCATTTTTTCACTTTATTACGATGCTATACAT
CATTACTGTGGGGGCAATTTCACTGTGTCTGGTCCCAAAGCAACAGCTGGGATCTTTGCC
ACATATCCAGCACCTTACATCTCAATCTACACTGGATTCTTCGACCAGGTTGTGGGCACC
GCCATGCTGTTGCTGTGTCTGATGGCTCTGGCAGACCAGAGAAACCAGCCGGCTGTGGCT
GGAGGTGAGCCTGTTGGTGTGGGGCTCGTGGTGCTGCTCATTGGTGTCTCTATGGGGAGC
AACAGCGGCTATGCCATCAATCCCACACGAGATCTAGGGCCAAGAGTCTTCACACTGATG
GCAGGTTGGGGCATGGAGGTTTTCAGGGCAGGCAATGGATGGTGGTGGGTACCTTTGGTG
GCCCCCTTTATTGGTGGAGTGACTGGGGCTTCAATCTACAAAGCATTTGTAGAGCTACAC
CACCCTGATCTTAAAAGCACTCAGACACAGTCT---ACAGGA------GATCCTGAATGT
GTTCCTTTGGAAAAGTGCAGGAATGGC---AGTACAGAGATA------TCTGTG------
------------------
>Fathead_minnow_Aqp7
ATGAAACAGGAAGTCAACATAATGGAGGACGGT------TTTCAAGGCCATAAGCCACCT
ACTTTCGTATGCATGTTAAAG---ATCAAGAATGAATGCGTTCGAGTGGTCCTGGCAGAG
ATCCTCTGCACATTTATCATGATGGTGTTCGGCCTTGGTTGTGTTGCACAAGTGGTCACA
GGAAATGGTTTTTTTGGAGATTACCTCAGCATAAATATAGGTTTTGGACTGAGTGTGGCT
ATGGGTGTGCACATTGGTGGAAAAGTGTCAGGAGCTCATATGAATGCAGCTGTTTCGTTC
ACAATGTGCGTGTTCGGCCGTTTGCGCTGGAAGATGCTGCCACTGTATGTTTTCGCCCAG
TTTCTGGGTTCATTCCTCGCTGCCGGGACCATTTTTTCACTTTATTACGATGCTATACAT
CATTATTGTGGGGGCAATTTCACTGTGTCTGGTCCCAAAGCAACAGCTGGGATCTTTGCC
ACATATCCAGCACCTTACATCTCAATCTACACTGGATTCCTCGACCAGGTTGTGGGCACC
GCCATGCTGTTGCTGTGTCTGATGGCTCTGGCGGACCAGAGAAACCAGCCGGTTGCGTCC
GGAGGTGAGCCTGTGGGTGTGGGGCTCGTGGTGCTGCTCATTGGTGTCTCTCTGGGGAGC
AACAGCGGCTATGCCATCAATCCCACACGAGATTTGGGCCAAGAGTCTTCACACTGATG
GCAGGTTGGGGCATGGAAGTTTTCAGGGCAGGAAATGGATGGTGGTGGGTACCTTTGGTG
GCCCCCTTTATTGGTGGAGTGATGGGGGCTTTAATCTACAAAGCATTTGTAGAGCTACAC
CACCCTGATCTTAAAAGCACTAAGACACAGTCT---CCTGAA------GATCCTGAATGT
GTTCCTCTGGAAAAGTGCAAGAATGGC---AGTGCAGAGATA------TCTGTG------
------------------
>Rare_gudgeon_Aqp7
ATGCAT------------------------------------------------------
------------------------------------------CGAGTGGTCCTGGCAGAG
ACCCTCTGCACATTCATCATGATGGTGTTCGGCCTTGGCTGTGTTGCACAAGTGGTCACA
GGAAATGGCTTTTTCGGAGAGTACCTCAGTATAAATATAGGCTTTGGACTGAGTGTGGCT
ATGGGTGTGCACATTGGTGGAAAAGTGTCAGGAGCTCATATGAATGCAGCGGTTACATTC
ACAATGTGCTTGTTGGGCCGTTTGCGCTGGAAGATGCTGCCTCTGTATGTTTTCGCCCAG
TTCCTGGGTTCCTTCCTAGCTGCTGGAACTGTTTTTTCACTTTATTATGATGCTATATAT
CATTACTGTGGGGGCAATTTCACCGTGTCTGGCCCCAAAGCAACAGCTGGGATCTTTGCC
ACATATCCGGCACCTTACATCTCAATCTACACTGGATTCTTCGACCAGGTTCTGGGCACC
GCCATGCTGTTGCTGTGTCTAATGGCTCTGGCAGACCAGAGAAACCAGCCGGTTGTGGCT
GGAGGTGAGCCTGTGGGTGTGGGGCTGGTGGTGCTGCTTATTGGTGTCTCTCTGGGGAGC
```

AACAGCGGCTATGCCATC--

>Grass_carp_Aqp7
ATGAAACAGGAAGTCACCATAATGGAGGACGGT------ATTCAAGGCCATATGGCACCT
ACTGTGGTCTCCATGTTGAAG---ATCAAGAATGAAAGCATTCGAGTGGTCCTGGCAGAG
ACCCTCTGCACATTCATCATGATGGTTTTCGGCCTTGGCTGTGTTGCACAAGTGGTCACA
GGAAATGGATTTTTTGGAGAGTACCTCAGCATAAATGTAGGCTTTGGGCTGAGTGTGGCT
ATGGGTGTGCACATTGGTGGAAAAGTGTCAGGAGCTCATATGAATGCAGCTGTTTCATTC
ACAATGTGCGTGTTTGGCCGTTTACGCTGGAAGATGCTGCCATTATATGTCTTAGCCCAG
TTTCTGGGTTCCTTCCTTGCTGCTGGGACCATTTTTTCACTTTATTACGATGCTATACAT
CATTACTGTGGGGGCAATTTCACTGTGTCTGGCCCCAAAGCAACAGCTGGGATCTTTGCC
ACATATCCAGCACCTTACATCTCAATCTACACTGGATTCTTCGACCAGGTTCTGGGCACC
GCCATGCTGTTGCTGTGTCTGATGGCTCTGTCAGACCAGAGAAACCAGCCGGTTGTGGCA
GGAGGTGAGCCTGTGGGAGTGGGGCTCGTGGTGCTGCTCATTGGTGTCTCTCTGGGGAGC
AACAGCGGCTATGCTATCAATCCCACCCGAGACCTGGGGCCAAGAGTCTTCACACTGATG
GCAGGATGGGGCATGGAGGTTTTCAGGGCAGGCAATGGATGGTGGTGGGTACCCTTGGTA
GCCCCCTTTATTGGTGGAGTGACTGGGGCTTTAATCTACAAAGCATTTGTAGAGCTACAC
CACCCTGATCTTAAGAGCACTAAGACACAGTCT---ACAGAA------GATCCTGAATGT
GTTCCTCTGGAAAAGTACAAGAATAGC---ACTACAGAGATA------TCTGTG------

>Zebrafish_Aqp7
---------------------ATGGAAGATGGCAGC---ATTCAAGGCCGCATGGCTCCA
AATGTTGGATCCATGTTGAAG---ATCAAGAATGAATACATTCGAGTGGCTTTGGCAGAA
AGCCTCTGCACATTCATCATGATGGTGTTTGGCCTTGGCACTGTTGCACAAGTGGTTACA
GGAGAAGGTTATTTTGGTGAATATCTCAGCATTAATATAGGCTTTGGGCTGGCAGTGGCT
ATGGGTGTGCATGTTGGTGGAAAAGTGTCAGGAGCTCATATGAACGCAGCTGTTTCATTC
ACAATGTGCGTGTTTGGCCGATTGCGCTGGAAGATGCTGCCGCTGTATGCTTTCGCTCAG
TTTCTGGGTTCATTCCTTGCCGCCGGGACCATTTTTTCACTTTATTATGATGCCATAAAT
CATTTCTGCGGGGGTAATTTGACTGTGTCCGGACCCAAAGCAACAGCTGGGATCTTCGCC
ACATATCCAGCACCCTATATCTCAGTCTACACTGGATTCTTTGATCAGGTTGCTGGCACG
GGCCTGCTGTTGTTGTGTCTGATGGCTCTGTCAGACCAAAGGAACCAGCCGCTGGTGTCT
GGAGGTGAAGCCGTCGGTGTGGGGCTTCTAGTGATGCTCATCGGCATCTCTATGGGGAGC
AACAGCGGTTACGCCATCAATCCCACACGGGACCTGGGGCCACGGCTCTTCACACTCATA
GCAGGATGGGGCACAGAGGTTTTTAGGGCAGGCAATTGCTGGTGGTGGGTACCCTTGGTG
GCTCCTTTTATTGGAGGAGTTTTAGGGGCTTTAATCTACAAAGCACTTGTAGAACTACAC
CACCCTGATCTTAAAAACACTACAACACGGCCA---GCAGTA------GATCCTGAATGC
ATTCCTCTGGACAAGTGCAAGAACGGC---AGAATAGAGATA------CCTGTG------

>White_sucker_Aqp7
---------------------ATGGGTGAAGGAAGT---ATTCAGGGCAATATGGCAGCT
ACAGTGGGATCCATTTTGAGG---ATTAAAAGTGAATGTATCCGAGTGATTCTGGCAGAG
ACCCTCTGTACGTTTATCATGATGATATTTGGTCTCGGCACTGTCGCCCAGGTAGTCACA
GGAAGTGGGGTATTTGGAGAGTACCTCAGCATAAATGTGGGGTTTGGGCTGGCCGTAGCT
ATGGGAGTGCACATTGGTGGAAAAGTGTCAGGAGCTCATATGAATGCTGCCGTTTCATTC
ACAATGTGTGTGTTTGGCCGTTTGCACTGGAAAATGCTCCCATTATATGTCCTCGCCCAG
TTTATGGGTTCCTTCCTTGCCGCTGGGACTATCTTTTCACTTTACTATGATGCTATATAT
CATTACTGTGGGGGCAATTTTACTGTGTCTGGCCCCAAAGCAACAGCTGGGATTTTTGCA
ACATATCCAGCCCCTTACATCTCAATCTACACTGGATTCTTTGATCAGGTTCTGGGCACT
GCCATGCTGCTGCTGTGTCTGATGGCCTTTGCAGATCAGAGAAATCAGCCAGTTGTTCGT
GGGGGTGAACCTGTGGGTGTCGGGCTTCTGGTGCTTCTGATCGGAATATCTCTGGGGAGC
AACAGTGGCTATGCCATCAATCCCACACGAGACCTGGGACCAAGAGTCTTTACACTGTTG
GCAGGTTGGGGCCCTGAGGTGTTTAGGGCAGGCAATGGCTGGTGGTGGGTACCGCTGGTG
GCACCCTTATTTGGAGGAGTGATAGGGGCTTTAATCTACAAAGCTTTTGTAGAGCTACAT
CATCCTGCTCGCAGTGACATAAAGAAACAATCC---ATAAGG------GATCCTGAATGT
GTTCCTCTGGATAAGCGCAAGCATATC---AGTACAGAGATA------TGCATG------

>Blue_catfish_Aqp7

---AAAGAGCATATAAGCATGCTGGAGGAGGGGAAG---GTCGAGGAGCTGATTAAGGCC
AGGAGAAGAACCACATGCTGG---ATTAGGAAGAAACATGTTCGAGTGGGACTAGCAGAA
ACTCTCAGCACGTTTGTCATGATGGTGTTTGGTTTAGGTTCTGTTGCTCAAGTGGTCACA
GGAGGTGGGGTTTTCGGAGATTACCTGAGCATTAATCTGGGTTTTGGTCTTGGTGTAGCA
ATGGGGGTGCATGCTGGTGGGAAAGTGTCAGGTGCCCACATGAACGCAGCAGTGACGTTT
ACCATGTGCGTAGTCGGCCGTCTGAGCTGGAAGATGCTTCCACTCTATGTAGTTTCACAA
CTTCTGGGCTCCTTTATCGCTGCGGGAACAGTTTTCACCCTGTACTACGATGCCATATAC
CACTTCAGTGAAGGAAACTTAACAGTGTTTGGCCCCAAAGCAACAGCGGGGATCTTTGCC
ACTTATCCAGCTCCTTATCTCTCAATACAAGCTGGATTTTTGGACCAGGTGCTTGGTACT
GCCATGCTGTTGCTATGTCTGATGGCTCTGGCAGATAAAAGGAACCAGCCTGCCCCCACT
GGTGGAGAGCCTGTGGCTGTTGGGGCTCTTGTATTGCTGATTGGAGTTTCCATGGGCAGC
AACAGTGGCTATGCCATCAATCCCACCCGAGACCTGGGGCCAAGAGTCTTTACAGCAATT
GCAGGCTGGGGATTAGAGGTATTCAGGGCAGGCAGATGTTGGTGGTGGGTGCCCTTGGTG
GCACCATTAGTCGGTGGAGTGACTGGAGCTTTGATATATAAAGCATGTGTGGAGTTGTTC
CATCCCAAACATAAGGATAGAAAACCTGAGCAC---AATGGA------CTATCAGAGAGC
ATAGCGCTGGATCAATGCAAGAATGAC---AACACAGATGAG------TGTGTGAACTGT

>Channel_catfish_Aqp7
---AAAGAGCATATAAGCATGCTGGAGGAGGGGAAG---GTCGAGGAGCTGATTAAGGCC
AGGAGAAGAACCACATGCTGG---ATTAGGAAGAAACATGTTCGAGTGGGGCTAGCAGAA
ACTCTCAGCACGTTTGTCATGATGGTGTTTGGTCTAGGTTCTGTTGCTCAAGTGGTCACA
GGAGGGGGTGTTTTTGGAGATTACCTGAGCATTAATCTGGGTTTTGGTCTTGGTGTAGCA
ATGGGGGTGCATGCTGGTGGGAAAGTGTCAGGTGCTCACATGAACGCAGCAGCGACGTTT
ACCATGTGCGTAGTCGGCCGTCTGAGCTGGAAGATGCTTCCACTCTATGTAGTTTCACAA
CTTCTGGGCTCCTTTATCGCTGCGGGAACAGTTTTCACCCTGTACTACGATGCCATATAC
CACTTCAGTGAAGGAAACTTAACAGTGTTTGGCCCCAAAGCAACAGCGGGGATCTTTGCC
ACTTATCCAGCTCCTTATCTCTCAATACAAGCTGGATTTTTGGACCAGGTGCTTGGTACT
GCCATGCTGTTGCTATGTCTGATGGCACTGGCAGATAAAAGGAACCAGCCTGCCCCCACT
GGTGGAGAGCCTGTGGCTGTTGGGGCTCTTGTATTGCTGATTGGAGTTTCCATGGGCAGC
AACAGTGGCTATGCCATCAATCCCACCCGAGACCTGGGGCCAAGAGTCTTTACAGCAATT
GCAGGCTGGGGATTAGAGGTATTCAGGGCAGGCAGATGTTGGTGGTGGGTGCCCTTGGTG
GCGCCATTAGTCGGTGGAGTGACTGGAGCTTTGATATATAAAGCATGTGTGGAGTTGTTC
CATCCCAAACATAAGGATAGAAAACTTGAGCAC---AATGGA------CTATCAGAGAGC
ATAGCGCTGGATCAGTGCAAGAATGAC---AACACAGATGAG------TGTGTGAACTGT

>Striped_catfish_Aqp7
------------------ATGCTGGAGGAGGGGAAG---GTTGAGGAGCCGAATAAGGCC
AGAAGAAGAACCACATGCTGG---ATTAGGAAGAAGCATGTTCGTGTAGGCCTAGCAGAA
ACTCTCAGCACATTTGTTATGATGGTGTTTGGTCTAGGTTCTGTGGCTCAGGTGGTCACA
GGAGGGGGGGTTTTTGGAGATTACCTTAGCATTAATCTGGGTTTTGGTCTTGGTGTAGCG
ATGGGGGTGCATGCTGGTGGGAAAGTGTCAGGTGCTCACATGAACGCAGCAGTGACGTTT
ACTATGTGCGTACTTGGCCGTCTGAGCTGGAAGATGCTTCCACTCTATGTAGTTGCACAA
CTTCTGGGCTCCTTTATTGCTGCAGGAACAGTTTTCACCCTGTACTACGATGCTATATAC
CACTACTGCAAAGGAAACTTCACGGTGTTTGGCCCCAAAGCAACAGCGGGGATCTTTGCC
ACATATCCGGCTCCTTATCTCTCAGTACAAGCTGGATTTTTGGACCAGGTGCTTGGCACT
GCCATGCTGTTACTATGTCTGATGGCTCTGGCAGATAAGAGGAACCAGCCTGCCCCATCT
GGTGGAGAGCCTGTGGCTGTTGGGGCTCTTGTATTGCTGATTGGAGTTTCCATGGGCAGC
AACAGTGGCTATGCCATCAATCCCACCCGAGACCTGGGGCCTAGAGTCTTTACAGCGATT
GCAGGCTGGGGATTAGATGTATTCAGGGCAGGCAGATGTTGGTGGTGGGTGCCCTTGGTA
GCGCCATTAGTGGGTGGAGTGACTGGAGCTTTGATATATAAAGCTTTCGTGGAACTGCTC
CATCCCAAGCATAAGGATAGAAAACCTGAGCAC---AGTGGA------TCATCAGAGTGC
ATACCGCTGGATCAGTGTAAGAAAGAC---AACGCAGATGAG------TGTGTGAACTGT

>Yellow_catfish_Aqp7
ATGAAGGAGCACCCAAGCATGCTGGAGGAGGGGAAG---TTTGAGCAGAGGAATAAGACT
AGGAGAAGAACCACATGCTGG---ATTAGAAAGAAGCATGTTCGAGTGGGCTTAGCAGAA
ACTCTCAGCACTTTTGTCATGATGGTGTTTGGTCTAGGTTCTGTGGCTCAGGTGGTCACA
GGAGGGGGGGTTTTTGGAGATTTCCTGAGCATTAATCTGGGTTTCGGTCTTGGTGTAGCG
ATGGGGGTACATGCTGGTGGGAAAGTGTCAGGTGCTCACATGAATGCAGCGGTGACGTTT
ACTATGTGCGTATTGGGCCGCCTTAGCTGGAAGATGCTCCCACTCTACGTAGTTGCACAA
CTGCTGGGCTCCTTTATTGCTGCGGGAACAATTTTCACCTTGTACTATGATTCCATAAAT

CATTATTGTGGAGGAAACCTCACAGTGTTTGGCCCCAAAGCAACAGCAGGGATCTTTGCC
ACTTATCCAGCTCCTTATCTCTCAATACAAACTGGATTCTTGGACCAGGTACTTGGCACT
GCCATGCTTTTGCTATGTCTGATGTCTCTGGCAGATAAGAGGAACCAGCCTGCCCCAACT
GGTGGAGAGCCTGTGGCTACTGGAGCTCTTGTATTGCTGATTGGAGTTTCCATGGGCAGC
AACAGTGGCTATGCAATCAATCCCACCCGAGACCTTGGGCCAAGGATTTTTACAGCAATT
GCAGGCTGGGGAATAGATGTTTTCAGGGCAGGTAACAATTGGTGGTGGGTGCCCTTGGTG
GCCCCATTAGTGGGTGGAGTGACTGGAGCTTTGATATATAAAGCATTTGTGGAGATGTTC
CATCCTAAG------GATAAAAAACCAAAGCCC---AATGGA------CCATCAGAGCAC
ATACCGCTGGATGAGTGCAAGAAAGAC---AACACTGAGGAG------TGTGTGAACTGT

>Walking_catfish_Aqp7
------------------ATGCTGGAGGAGGGGAAG---GTCGAGCAGCCGAATAAGGCC
GGGAGAAGGACCATGTGCTGG---ATTAAGAAGAAGCATGTCCGAGTTGGCCTAGCTGAA
ACACTCAGCACATTTGTTATGATGGTGTTTGGCCTAGGTTCTGTTGCTCAGGTGGTCACA
GGAGGGGGCGTTTTTGGAGATTACCTGAGCATTAATATGGGTTTTGGTCTTGGTGTAGCA
ATGGGAGTGCATGCTGGAGGGAGAGTGTCAGGGGCTCACATGAACGCAGCAGTGACGCTT
ACTATGTGTGTTCTTGGCCGTCTGAGCTGGAAAATGCTTCCTCTCTATGTAGCTGCACAA
TTTCTGGGCTCGTTTATTGCTGCGGGAACAGTTTTCACTCTGTACTATGATGCCATATAC
AACTATTGCGAAGGAAACTTCACAGTGATTGGCCCCAAAGCAACGGCAGGAATCTTTGCC
ACTTATCCCGCTCCTTATCTCTCAATACAAGCTGGATTTTTGGACCAGGTGCTTGGAACT
GCCATGCTGTTGCTATGCTTGATGGCGCTGGCAGATAAAAGGAACCAGCCTGCCCCAACT
GGTGGGGAACCTGTGGCTATTGGAGCTCTTGTATTGCTGATCGGAGTTTCCATGGGTAGC
AACAGTGGTTACGCCATCAATCCCACCCGAGACCTGGGGGCAAGAGTCTTTACAGCTATT
GCAGGCTGGGGATTAGATGTTTTCAGGGCAGGTAACAGCTGGTGGTGGGTACCCTTGGTG
GCACCATTTGTTGGCGGAGTAACTGGAGCTTTAATATATAAAGTATTTGTTGAACTCTTT
CATCCCAAGGATACGGATAGAAAATCTGAGCAC---AGTGGC------TCATCAGAGTGC
ATTCAACTAGCTGACTGCAAGAAAGAC------AGCGAA---------------------

>Bottlenose_catfish_Aqp7
GTGGAGGACACA---AGCATAACGGAGGAGGTGCAG---GTTAAGGAGCGGTATAAGGGC
AGGAAAAGATTCATATACTGG---ATTAAGAAGGAGAATGTTCGAGTGGGCCTCGCAGAA
GCTCTCTGCACATTTGTCATGATGGTGTTTGGTCTTGGTTCGGTTGCACAGGTGGTCACA
GGAGGAGGACTTTTTGGAGACTTCCTTAGCATTAATCTGGGTTTTGGTCTTGGTGTAGCA
ATGGGGGTGCATGCTGGTGGGAAAGTGTCAGGTGCTCACATGAACGCAGCAGTGACGTTT
ACTATGTGCGTAATTGGCCGCCTGAGCTGGAAGATGCTTCCACTCTATGTATTTGCACAA
CTTCTGGGCTCCTTTACTGCTGCGGGAACAATTTTCGCCCTGTACTATGATGCCATACAG
CATTTCAGTGAAGGAAACTTGACGGTGTTGGGCTCCAGAGCGACAGCTGGGATTTTTGCC
ACTTATCCAGCTCCTTATCTCTCAATACAGGCTGGATTTTTTGACCAGGTGCTTGGTACT
GCCATGCTGTTGCTATGTCTGATGGCTCTGGCGGATAAAAAGAACCAGCCTGCCCCAATT
GGTGGAGAGCCTTTGGCTGTTGGAGCTCTTATAGTGTTGATTGGCCTTTCCATGGGCAGC
AACAGTGGCTATGCCATCAATCCAACCCGAGACCTGGGACCAAGAATCTTTACAGCAATT
GCAGGCTGGGGATTAGATGTATTCAGGGCAGGCAGCAGTTGGTGGTGGGTGCCCTTGGTG
GCACCATTGGTGGGTGGAGTGACTGGAGCAATGATATATAAGGCATTTGTGGAGCTGCTC
CATCCCAAGCAGGAGATAAAGAATCCTGARCAA---AGTGGT------GAAACAGAGGTC
ATAGCGCTGGATCATTGCAAGAAAGAC---AACTCGAACGTA------------------

>Pencil_catfish_Aqp7
------------------ATGCCAGAGGAAGGCAAA------GCCAGCAAGGATAATACA
ATGGTTAGCTCCTCATGCTGT---TTTAGAAAGGAGTACGTCCGGGTCGGCCTGGCAGAA
ACTCTCAGCACGTTTGTAATGATGGTTTTTGGACTAGGTTCTGTTGCCCAGGTGGTCACA
GGAGGGGGGGTTTTTGGAGATTACTTGAGCATTAATCTTGGATTTGGTCTTGCTGTGGTG
ATGGGGGTGCATACTGGTGGAAAGGTATCAGGTGCTCACATGAATGCAGCGGTGTCCTTC
ACCATGTGTGTGTTTGGCCGCCTGAGCTGGAAAATGCTTCCACTTTACATAATTGCACAG
CTTCTAGGATCCTTCCTGGCTGCTGGAACAATTTTTACTCTCTATTATGATGCCATCTTC
AGTTACTGTGATGGTAACTTGACTGTGTCTGGAGCGAAAGCTACAGCAGGGATCTTTGCA
ACCTATCCAGCACCTTACCTCTCAATACAGTTTGGATTTTTAGACCAGGTGCTCGGTACG
GCCATGCTGCTGTTGTGTCTAATGGCTCTGGCAGATCAGAAAAAACCAGCCAGCAACACCC
GGTGGACAGCCCCTCGCCATAGGCGCTCTTGTACTTCTAATTGGAGTGTCCATGGGGAGC
AACAGTGGCTATGCCATCAATCCAACAAGAGACTTTGCACCGAGACTCTTCACAGCCATG
GCAGGCTGGGGATCAGAGGTATTCAGGGCGGCAACAGCTGGTGGTGGGTGCCTGTGGTG
GCACCGTTTGTAGGTGGAGTGACTGGCGCTTTAATGTATGAAACATTTGTGGAGCTGCTC

CATCCCACACATACTGACAAAGATCTTAAAGAG---AAAGAA------GCATCCGAGTCG
ACTCCACTGGATATGTGCAGTAAATGC---AATGCAGAGGTG------TCCGTG------

>Snow_pleco_Aqp7
------------------ATGACAGAGGAAGGAAAG---GCTGAGCAGACTGGG------
---GGAAGATCCATGTGCAGG---ATTAGGAAGGAGCATGTTCGGGTCGCTCTGGCAGAA
ACCCTCTGCACGTTTGTCATGATGGTGTTTGGACTAGGCTCTGTTGCCCAGGTGGTCACA
GGAGGGGGACTTTTTGGCGATTATTTTAGCATTAATGTCGGTTTCGGTCTTGGTGTTGCA
ATGGGGGTCCACACTGGAGGAAAGGTATCAGGAGCTCACATGAATGCAGCAGTGTCGTTC
ACCATGTGCGTTTTCCGCCGCCTGAGCTGGAAGCTACTTCCACTATACGTAATTGCGCAA
TTTTTGGGCTCCTTCCTTGCTACAGGAATAGTTTTTGTCCTTTACTATGATGCTATACAA
ACCTATAGTGGAGGTAACTTAACCGTGACTGGGCCGAAAGCAACAGCGGGGATCTTTGCT
ACTTATCCAGCACCTTACCTCTCCCTTCAGGCTGGATTCTTGGACCAGGTTCTTGGCACT
GCCATGCTGTTGCTGTGCCTGATGGCTTTCGCGGATCAGAGGAACCAGCCGGCTCCATCA
GGTGGCGAGCCCCTGGCCGTCGGGGCTCTTGTAATTCTAATCGGAATTTCCATGGGGAGC
AACAGCGGTTATGCGATCAACCCCACACGAGATCTAGGGGCCAGGGTCTTTACAGCCATC
GCAGGCTGGGGGACAGAGGTATTCAGGGCGGGCAATGGTTGGTGGTGGGTTCCCTTGGTA
GCACCAACGGTGGGTGGAGTAACTGGTGCGTTAATCTATAAGACTCTTGTGGAGCTGCTT
CATCCTCTTAAGAAA---AGAGAAATAACACAC---AAAGGA------CCAAGCGAGTGT
GTTCCCCTGGATCTGTGCGAGAACAAA------GCAGAATTG------TGTGTT------

>Redbellied_piranha_Aqp7
------------------ATGCTGGAGGAAGGCAAA---GCAGAAGAGCTGAGTAGGGCA
CAGGGACGCTTCATGTGCTTT---GTGAGGAAGAAGCATGCTAGAGTTGGACTAGCAGAA
ACTCTCAGCACGTTCGTCATGATGGTGTTTGGACTAGGCTCTGTTGCTCAAGTAGTCACA
GGAGGGGGGCTATTTGGAGATTATTTAAGCATTAATCTGGGTTTTGGTCTGGGGGTTGCC
ATGGGGGTGCACATTGGTGGGAAAGTATCAGGTGCTCATATGAACGCGGCTGTGTCGTTC
ACCATGTGCGTGCTTGGGCGGCTGGGCTGGAAGATGCTGCCACTCTATGTAGCTGCTCAG
CTTCTGGGCTCTTTTTTTGCTGCTGGAACAGTTTTTATTCTTTACTATGATGCTATATAC
CACTACTGTGAGGGTAACTTCACAGTGTCTGGCCCCAAAGCGACAGCTGGGATTTTTGCC
ACTTATCCAGCACCATATATACCAACACATGCTGGATTTCTGGACCAGGTGCTTGGCACT
GCAATGCTGTTGCTTTGTCTAACGGCTCTGGCAGACCAGAGGAACCAGCCTGCCCCATCT
GGTGGAGAGCCTGTGGCTGTGGGGGCTCTTGTACTTCTGATTGGAGTTTCCATGGGGAGC
AACAGCGGCTATGCCATCAACCCCACACGAGACCTGGGGCCAAGAATCTTTACAGCATTT
GCAGGCTGGGGATCGGAGGTATTCAGGGCAGGAAATGCTTGGTGGTGGGTGCCTGTGGTA
GCTCCATTAGTGGGTGGAGTGACTGGAGCTTTGATCTATAAGGCATTAGTGGAGCTGCTC
CATCCTGTTCATACCGACAAAGAACTAGAGCACAGAGAGGGG------TCATCAGAGTGT
GCTCCACTGGATCAGTGCAAGAAAGGC---ACAGCAGAAGTG------TGTGTG------

>Tambaq_Aqp7
ATGAAAGAACAGTTGAGCATGCTGGAGGAAGGCAAA---GCTGAAGAGCTGAGTAGGGCC
AGGGGACGATCCATGTGCTGT---GTGAGGAAGAAGCATGTTCGAGTTGGACTAGCAGAA
ACTCTCAGCACGTTCGTCATGATGGTGTTTGGACTAGGCTCTGTCGCTCAGGTGGTCACG
GGAGGGGGACTATTTGGAGATTATTTAAGTATTAATCTGGGTTTTGGTCTGGGGGTTGCC
ATGGGGGTGCACATTGGTGGGAAAGTATCAGGTGCTCATATGAACGCGGCCGTGTCGTTC
ACCATGTGCGTGCTTGGCCGCCTGGGCTGGAAGATGCTGCCACTCTATGTAGCTGCTCAG
CTTCTGGGCTCTTTTTTTGCTGCTGGAACAGTTTTTATTCTTTACTATGATGCCATATAC
CACTACTGTGAGGGTAACTTCACAGTATCAGGCCCCAAAGCAACAGCTGGGATATTTGCC
ACTTATCCAGCACCTTACATCCCAATACATGCTGGATTTCTGGACCAGGTGCTTGGCACT
GCCATGCTGTTGCTTTGTCTAACGGCTCTGGCAGACCAGAGGAACCAGCCCGCCCCATCT
GGTGGAGAGCCTGTGGCTGTGGGGGCTCTTGTACTGCTGATTGGAGTTTCCATGGGGAGC
AACAGTGGCTATGCCATCAACCCCACACGAGACTTGGGGCCAAGAGTCTTTACAGCACTT
GCAGGCTGGGGATCGGAGGTATTCAGGGCAGGTAATGCTTGGTGGTGGGTGCCTGTGGTA
GCTCCATTAGTGGGTGGAGTGACTGGAGCTTTGATCTATAAGGCATTAGTGGAGCTGCTC
CATCCTGTTTATACCAACAAAGAACTAGAGCATGGAGAGGGG------TCATCAGAGTGT
GCTCCACTGGATCAGTGCAAGAAAGGC---ACAGCAGAAGTG------TGCGTG------

>Mexican_tetra_Aqp7
ATGAAAGAGCAGAAGAGCATGCTGGGAGAGGGCAGA---GCTGGAGAAGTGTCAGGGGCC
AGAGGACGCTCTGTGTGCTGG---GGAGGGAGGGAGCATGTTCGAGTCGGCCTGGCAGAA
GCTCTCAGCACATTTGTCATGATGGTGTTTGGACTAGGCTCTGTAGCTCAGGTGGTCACA

GGAGGGGGACATTTTGGAGATTATTTAAGCATAAATCTAGGTTTTGGTCTGGCGGTGGCA
ATGGGGGTCCATGTTGGAGGGAAGGTATCAGGTGCTCACATGAACGCAGCAGTGTCCTTC
ACCATGTGTGTGCTCGGCCGGCTGAGCTGGAAGATGCTGCCTCTATATGTAGTTGCTCAG
CTTCTGGGCTCTTTTCTTGCAGCTGGAACAGTTTTTACACTTTACTATGATGCCATTCAC
CACTACTGTGATGGTAATTTCACAGTGTCTGGTCCCAAAGCAACAGCTGGCATCTTTGCC
ACTTATCCAGCACCATACCTCTCAATACCAGCTGGATTTCTGGACCAGGTGCTTGGCACT
GCCATGCTGTTGCTGTGTGTAACAGCTCTGGCAGACCAGAAGAACCAGCCTGCCCCATCT
GGTGGAGAGCCCGTGGCTGTGGGGGCTCTTGTACTGCTGATTGGGATTTCCATGGGGAGC
AACAGCGGCTATGCCATCAACCCCACACGAGACCTGGGGCCAAGAATCTTTACAGCCCTG
GCAGGCTGGGGATCAGAGGTATTCAGGGCAGGTAACGGCTGGTGGTGGGTTCCAGTGCTG
GCTCCGTTAGTTGGTGGAGTGATTGGGGCTTTGATCTACAAGGCTTTAGTGGAGCTGTTC
CATCCTGTTCATAACAACAAAGAACCAGAGCATGGAGAGCGA------TCATCAGAGCTC
GCTCCACTGGATCAGTGCAAGAAAGGA---AGTGCAGATGTC------TGCGTG------

>Alewife_Aqp7
--GAACAGAGGGAGAGC
AAAGGGGCCACCATGGCGTGG---GTCAGGAACGAGGTCGTCCGCGTGGGGCTAGCCGAG
ACCCTCGGCACATTCGTTATGATGGTGTTCGGGCTGGGCTCAGTGGCTCAGGTGGTCACT
GGGACAGGAGCATTTGGGGAGTTCCTGAGCATCAACCTAGGTTTTGGGCTAGGTGTGGCT
ATGGGTGTGCATGTCGCTGGCAAAGTTTCAGGTGCTCACATGAATGCAGCGGTCTCCTTT
ACTACGTGTGTCTTTGGACGTCTGAAGTGGACGATGCTGCCCTTATATATCGCATCCCAG
TTCCTGGGATCTTTTCTAGCGGCTGGAACAGTCTTTTCCCTCTACTATGATGCCATTTAT
CACTACAGTGGGGGGAACTTCACGGTGACTGGCCCTAAAGCAACAGCTGGGATTTTTGCC
ACCTACCCGGCCCCATACCTCTCTCTCCATGCTGGCTTCCTAGACCAGGTGCTGGGCACT
GCCATGCTGCTGCTTTGCCTGATGGCCCTGTCAGATCAGCGAAACCAGCCTGCACCGATC
GGTAGTGAACCTCTGGCTGCCGGCCTCCTGGTCATCCTGATTGGTATCTCTTTGGGCAGT
AACAGCGGCTATGCCATCAATCCCACGCGAGACTTGGCACCCCGCGTCTTCAGTGCCCTC
GCTGGATGGGGACCTGATGTGTTCAAGGCGGGCGGTGGA---------------------

>Allis_shad_Aqp7
ATGAGGGACTCTCAGACCATGCTGGAAGAGGGGCGA---GTGGGGGAACAGAGGGAGAGC
AAAGGGGCCACCATGGCGTGG---GTCAGGAACGAGGTCGTCCGCGTGGGGCTAGCCGAG
ACCCTCGGCACATTCGTTATGATGGTGTTCGGGCTGGGCTCAGTGGCTCAGGTGGTCACT
GGGACAGGAGCATTTGGGGAGTTCCTGAGCATCAACCTAGGTTTTGGGCTAGGTGTGGCT
ATGGGTGTGCATGTCGCTGGCAAAGTTTCAGGTGCTCACATGAATGCAGCGGTCTCCTTT
ACTATGTGTATCTTTGGACGTCTGAAGTGGACGATGCTGCCCTTGTATATCGCATCCCAG
TTCCTGGGATCTTTTCTAGCGGCTGGAACAGTCTTTTCCCTCTACTATGATGCCATTTAT
CACTACAGTGGGGGAAACTTCACGGTGACTGGCCCTAAAGCAACAGCTGGGATTTTTGCC
ACCTACCCGGCCCCATACCTCTCTCTCCATGCTGGCTTCCTAGACCAGGTGCTGGGCACT
GCCATGCTGCTGCTTTGCCTGATGGCCCTGTCAGATCAGCGAAACCAGCCTGCACCGATC
GGTAGTGAACCTCTGGCTGCCGGCCTCCTGGTCATCCTGATTGGTATCTCTTTGGGCAGT
AACAGCGGCTATGCCATCAATCCCACGCGAGACTTGGCACCCCGCGTCTTCAGTGCCCTC
GCTGGATGGGGACCTGATGTGTTCAAGGCAGGCGGTGGATGGTGGTGGGTCCCTGTCATG
GCCCCTTTGGTTGGTGGGGTCATTGGTGCCCTGATCTACAAAGTATTTGTGGAGATGCAT
CACCCCTCCGTGGACAAGCGTGGACTTGGGTCT---AATAAC------AAGCCTGAGAGT
GAGCCGCTGGACCAGCTGAAAAAAGCAGCTGCAGCTGAAATG------TGTGTG------

>Sardine_Aqp7
ATGAGGGACTCTCAGACCATGCTGGAAGAGGGGCGC---GCTGGGGAGCAGACGGAAAGC
AAAGGGGCCACCATGGCGTGG---GTCAGGAACGAGGTCGTCCGCGTGGGGCTGGCCGAG
ACCCTCAGCACGTTCGTTATGATGGTGTTCGGGCTGGGCTCTGTGGCTCAGGTGGTCACT
GGGACAGGAGCATTTGGGGAGTTCCTGAGCATCAACCTCGGTTTTGGGCTAGGTGTGGCT
ATGGGGGTGCATGTTGCTGGCAAAGTTTCAGGTGCCCATATGAATGCAGCGGTCTCCTTT
ACTATGTGTGTCTTTGGACGTCTGAAGTGGACAATGCTGCCCTTGTACATTGTGGCCCAG
TTCCTGGGATCTTTTCTAGCGGCTGGAACAGTCTTTACCCTCTACTATGATGCCATTCAT
CACTACAGTGGGGGAAACTTCACGGTGACTGGCCCTAAAGCGACAGCTGGGATTTTTGCC
ACCTACCCAGCCCCATACCTCTCTCTCCACGCTGGCTTCCTTGACCAGGTGGTTGGCACT
GCCATGCTGCTGCTTTGCCTGATGGCCTTGTCAGATCAGCGAAACCAGCCCGCGCCGATC

GGTAGTGAACCTCTGGCGGTCGGCCTCCTGGTCATCCTGATTGGTATCTCTCTGGGCAGT
AACAGCGGCTACGCCATCAATCCCACGCGAGACTTGGCGCCCCGCGTCTTCAGTGCCCTC
GCCGGATGGGGACCTGAGGTGTTCAGGGCTGGCGGTGGATGGTGGTGGGTTCCTGTCATG
GCCCCGTTGGTTGGTGGGGTCATTGGTGCCCTGATCTACAAAGTGTTTGTGGAGATGCAT
CACCCCTCCGTGGGCAAGCGTGGGCTTGGGTCT---AACAAC------CAGCCCGAGAGC
GAGCCGCTGGACCAGCTGAAAAAAGCAGCTGCTGCCGAAATG------TGTGTG------

>Hilsa_shad_Aqp7
ATGAGGGACTCCCAGACCATGCTGGAAGAGGGGTGC---ACAGGGGATCAGAGGCAGAGC
AAAGGGGCCATCATGGCGTGG---GTCAAGAATGAGGTCGTCCGCGTGGGGCTAGCCGAG
ACACTCAGCACGTTTGTTATGATGGTGTTTGGCCTGGGTTCGGTGGCTCAGGTGGTCACA
GGGACAGGAGCGTTTGGGGAGTTCCTCAGCATCAACCTGGGTTTTGGCCTAGGTGTGGCT
ATGGGGGTGCATGTGGCAGGCAAAGTTTCAGGTGCCCATATGAATGCAGCGGTCTCCTTT
ACGATGTGCGTCTTTGGTCGTCTGAAGTGGACAATGCTTCCCTTGTACATCACAGCCCAG
TTTCTAGGATCTTTTCTAGCAGCTGGGACAGTCTTTTCCCTCTACTATGATGCCATCTAC
CACTACAGTGAGGGAAACTTCACAGTGACTGGCCCAAAAGCAACAGCTGGAATTTTTGCT
ACCTACCCAGCCCCATATCTCTCTCTCCATGCTGGATTCCTGGACCAGGTGCTGGGCACT
GCCATGCTTCTGCTTTGCCTGATGGCCCTATCAGATCAACGAAACCAGCCTGCTCCAGTC
GGCAGTGAGCCTCTGACTGTCGGCCTCTTGGTCATACTGATTGGTATCTCTCTGGGCAGC
AACAGTGGCTACGCCATCAATCCCACTCGAGACTTGGCACCCCGAGTCTTCAGTGCCCTC
GCTGGATGGGGACCTGAGGTGTTCAAGGCAGGCAATGGGTGGTGGTGGGTCCCTGTCACA
GCCCCTTTGGTTGGTGGGGTTGTGGGTGCCCTGATCTACAAGTTGTTTGTGGAGATACAT
CACCCCTCTGTCAACAAAGGGGGCCGGGGATCC---CATGAG------GAGCCAGAGAGC
GAACCGCTGGACCAGTTGAAAAAGGCATCTGCTGCAGAAATG------TGTGTG------

>Konoshiro_gizzard_shad_Aqp7
ATGAGGGACTTGCAGACCATGCTGGAAGAGGGGCGC---ACAGGAAATCAGAGGGAGAGC
AAAGGGGCCACCATGGCGTGG---GTCAAGAATGAGGTCGTCCGCGTGGGGCTAGCCGAG
ACACTTGGCACGTTCGTTATGATGGTGTTTGGCCTGGGTTCGGTGGCTCAGGTGGTCACA
GGGACAGGAGCGTTTGGGGAG---------------------------------------

------------------------AAGTGGACAATGCTTCCCTTGTACATCACATCTCAG
TTCCTGGGGTCTTTTCTAGCAGCTGGGACAGTCTTTTCCCTCTACTATGATGCCATCTAT
CACTACAGTGAGGGAAACTTCACAGTGACCGGCCCAAAAGCAACAGCTGGAATTTTTGCC
ACCTACCCAGCCCCATACCTCTCTCTCCACGCCGGCTTCCTGGACCAGGTGCTGGGCACT
GCCATGCTTCTGCTTTGCCTGATGGCCCTGTCAGATCAACGAAACCAGCCTGCTCCGGCC
GGCAGTGAACCTCTGACTGTTGGCCTCTTGGTCATACTGATTGGTATCTCTCTGGGCAGC
AACAGTGGCTACGCCATCAATCCCACTCGAGACTTGGCACCCCGAGTCTTCAGCGCCCTA
GCTGGATGGGGACCTGAGGTGTTCAAGGCAGGCAATGGGTGGTGGTGGGTCCCTGTCGCG
GCCCCTTTGGTTGGTGGGGTTGTGGGTGCTCTGATCTACAAGGTGTTTGTGGAGATGCAT
CACCCCTCCGTCAACAGACGGGGCCAGGCATCC---CATGAG------GAGCCAGAGAGT
GAACCGCTGGAACAGCTGAAAAAGGCAGCTGCTGCAGAAATG------TGTGTG------

>Japanese_grenadier_anchovy_Aqp7
ATGAAGGACTCCCAGGGCACTGTGGAACAGGCACTC---GCTAGGAAAGCA---CAGCAG
AGGGGAGAC---ATGGCCTGG---GTCAGGAATGAGGTCGTCCGTGTGGGGTTGGCCGAG
ACTCTCAGCACATCGGTTATGATGGTGTTTGGCTTGGGTTCGGTGGCTCAGGTTGTCACA
GGGACTGGAGCATTTGGGGAGTACCTGAGCATCAACCTGGGCTTTGGCATTGGCGTGGCC
ATGGGGGTCCACGTAGCTGGCAAGGTTTCAGGAGCCCATATGAATGCAGCTGTCTCCTTT
ACGATGTGTGTCTTTGGTCGTCTGAAGTGGAAAATGCTTCCTTTGTATGTCACGGCTCAG
TTTCTGGGATCGTTTCTAGCAGCTGGAACGGTCTATTCTCTCTACTATGATGCCATTCAT
CACTACAGCGGAGGAAATCTCACAGTGTCTGGCCCCAAAGCGACAGCTGGAATTTTTGCC
ACATACCCTGCGCCATACCTTTCTGTCTATGCCGGCTTCCTGGATCAAGTGGTCGGTACT
TCTGTGCTCCTTCTCTGCTTGATGGCCTTGTCAGACCGGCGAAACCAGCCTGCACCAATT
GGCAGTGAACCCCTGGCAGCTGGACTCCTAGTACTGCTGATTGGCATCTCTCTGGGCAGT
AACAGTGGCTACGCCATCAATCCCACCCGAGACTTGGCACCCCGAGTGTTCAGTGCCCTT
GCTGGATGGGGACCTGAAGTGTTCAAGGCAGGCCACGCATGGTGGTGGGTGCCCGTCGTT
GCCCCTCTGGTTGGTGGTGTTGTCGGTGCCCTGATCTACAGGGTGTTTGTAGAGATGCAT
CACCCCTCTGTCCACAAGCGTGCCCGTGCTTAC---GAAAGC------GAGCCGGAGGGA
GAGCCTCTGGACAAGCTTGAAAAGCCAGGCCCCCCGGAAGTG------TGTGTG------

```
>Atlantic_herring_Aqp7
ATGAAGGATTCCCAGAGCATGCTGGAAGAGGGACGC---GCAGGGGAACAAAAGGGAAGC
AGAGAGGCCCCCATGGCCTGG---TTCAGGAATGAGGTTGTCCGGGTGGGGCTGGCCGAG
ACACTCGGCACATTCGTTATGATGGTTTTCGGGCTCGGTTCAGTGGCTCAGGTGGTCACA
GGGAAGGGAGCGTTTGGGGAGTACCTGAGCATCAACCTGGGTTTCGGGATAGGTGTGGCG
ATGGGGGTACACGTAGCTGGCAGCGTTTCTGGTGCCCATATGAATGCAGCGGTCTCCTTT
ACGATGTGTGTCTTTGGCCGTCTGAAGTGGGCGATGCTTCCTTTGTACATCACAGCACAG
TTTCTGGGATCTTTTCTAGCAGCTGGAACAGTCTTTGTCCTCTACTATGATGCCATTCAT
CATTACAGTGAGGGAAACTTCACAGTATCTGGCCCAAAAGCAACAGCTGGGATTTTTGCC
ACCTATCCAGCCCCATACCTCTCTCTCCACGCTGGCTTCCTAGACCAGGTGCTGGGCACT
GCCATGTTGCTTCTCTGCCTGATGGCCCTGTCAGATCAGCGAAACCAGCCGGCACCCAAG
GGCACCGAACCTCTGGCTTCCGGCCTCCTGGTACTGCTGATTGGCATCTCTCTGGGTAGC
AACAGTGGCTACGCCATCAATCCCACTCGAGACTTGGCACCCCGAGTCTTCAGTGCCCTC
GCTGGATGGGGACCTGAGGTGTTCAAGGCAGGCTCTGGATGGTGGTGGGTTCCTGTGGTG
GCCCCTTTGGTTGGGGGGGTCCTGGGCGCCCTGATCTACAGGGTGTTTGTGGAGATGCAT
CACCCCCGCATACGGGCCCAGGGGCCGGAAAAC---------------GAGCCAGAACGC
GAACCGCTGTACCAGCTGAAAAAAGCCGCTCCCGCTGAAATG------TGTGTG------
------------------
>Denticle_herring_Aqp7
ATGAAAGACTCAATGAGCAGGATAAAAAATGGC------ATTGAGGAGGGTGTGGAAGAC
AAG---GCACCACCTTTTGGG---GTGAGGAGCGAGGTGGTCCGTGTGGCACTGGCAGAG
ACACTCAGCACATTTGTCATGATGGTGTTTGGTCTAGGCAGCGTGGCCCAGGTAGTCACA
GGGCAGAGCATGTTTGGAGATTTCCTTAGCATCAACCTGGGATTTGGTCTCGGCGTTGCC
ATGGGAGTCCATGTTGCAGGAAAAGTGTCAGGTGCTCATATGAACGCGGCTGTATCGTTC
ACCATGTGTATTTTTGGCCGTCTGGACTGGTGGCTGCTGCCCCTGTATGTTGTGTGCCAG
TTCTTTGGATCTTTCCTGGCTGCTGGGACCATCTTCTCTTTGTACTATGACGCCATTCTG
CACTACAGTGGAGGTAATTTTACTGTGGCAGGCCCCAGGGGAACTGCTGGGATTTTTGCA
ACTTACCCCGCCCCCTACATCTCACTACACGTTGGCTTCTATGACCAGGTGCTGGGCACT
GCCATGCTGTTACTTTGCCTGATGGCACTCTCAGACCAACAGAACCAACCCGCGCCTGCT
GGAAGTGAGCCTGTGGCTGTCGGGCTCCTGGTCTTGCTGATTGGCATTTCTCTGGGCAGC
AACAGTGGCTACGCCATCAACCCCACTCGAGACCTGGCACCCAGAATCTTTTCCGCCTTG
ACAGGTTGGGGAATGGAAGTTTTCAGGGCTGGGAATGGATGGTGGTGGGTGCCAGTGGTT
GCCCCTCTGCTAGGTGGGGTGGTGGGAGCTCTGATTTACAAGGTCTTAGTGGAGCTCCAT
CACCCAACCCCTTCAAACAAGAAAACAGAGAAG---AAGGAG------AATATTGAGCAT
GTCCCAATGGAACAGCTGAAAAAAGAT---------GAGATG------TGTGTA------
------------------
>Mormyrid_electric_fish_Aqp7
ATGAAGTGCACTTTAAACTCAGTAGAAGAAAGTGAC---GGAGTTCAGCCTGCAGGGATT
CGGATCCTCCGCACATTTCAG---ATGAAGAATAAATTGGTCCGAGTCGGTTTGGCAGAA
ACCCTTAGTACTTATGTCATGATGGTCTTTGGCTTGGGCTCTGTGGCCCAGGTGGTGACT
GGTGGTGGGTTGTTTGGGGATTACCTGAGCATCAACCTCGGTTTTGGCTTAGGTGTCACC
ATGGGAGTGCACGTTGCCGGCCAGGTATCAGGTGCCCACATGAACACTGCCGTCTCCTTC
ACCATGTGTACATTTGGCCGTCTTGGCTGGAAGATGCTTCCTCTCTACATAACAGCACAA
ATGCTGGGCTCTTTCCTGGCAGCTGGA---------------------GATGCAATGTTT
AAGTTCTGCAGCGGTAATTTCACTGTGTCTGGGCCAAAAGCCACAGCTGGAATCTTTGCG
ACTTATCCTGCGCCATACATCTCCATCCATGTGGGCTTCTTAGACCAGGTGCTGGGCACA
GCCATGCTGCTGCTGTGCCTCATGGCCCTATCGGACCAGCAGAACTCCGCCCCTGCTCCC
GGCACTGAGCCCCTGCTTGTCGGGCTTTTAGTGCTACTCATTGGTATTTCCATGGGTAGC
AACAGTGGCTATGCTATTAACCCTTCGCGGGACCTGGCACCCCGTGTTTTTACGGCCATG
ACTGCCTGGGGACAGCAAGTGTTCAGGGCTGGAAACGGCTGGTGGTGGGTGCCAGTGGCA
GCCCCTCTGGTGGGAGGGCTTTTGGGAGGCGTGGTCTACAAGCTTTTTGTGGAGCTGCAT
CACCCACGGGAGGCACCTAGATGTACAGAAAGTGGTGTAGAG------GATAACAGGGGT
CACCCAGGGGAGGCTATACATATGGACAATCTGGGGCAAGTG------TGCCTA------
------------------
>Freshwater_butterflyfish_Aqp7
ATGAGTCCACAACCG------------------------TGCGAACGAATGAGGAGCAAC
---ATGCTC---GGTTTTCAG---ATCAGAAATCAGTATGTGCGTGTATGGCTGGCAGAG
GTGCTCTGTACCTACGTTATGATGGTGTTTGGCCTGGGCTCCGTGGCCCAGGTGGTGACG
GGCGGAGGAGTGTTTGGCGAGTTCCTCAGCATCAACCTGGCTTTCGGGCTAGGTGTGGCC
ATGGGGGTGCATGTCAGTGGACAGGTCTCAGGTGCACACATGAACGCGGCTGTGACGTTC
ACCATGTGTGTCATCGGACGCCTACCTTGGAAGATGTTTTTGGTTTACGTGAGTGCACAA
```

CTGCTGGGCTCCTTCCTTGCTGCTGGAAGCATCTTCTGTTTGTACTATGACGCGATGCTC
CATTACTGCGGGGGGAAATTCACTGTTACAGGCCCCAATGCCACAGCGGGTATATTTGCC
ACCTACCCTGCCCCTTACCTGTCCATACATGTGGGCTTCCTGGACCAGGTGATGGCAACG
GCTCTGCTGTTGTTGTGCCTCTTGGCGCTGTCAGACAAGAGGAACCAGGCTGCCCCGCCT
GGGTTCGAGCCGATAGCTGTGGGATTGTTGGTGGTGTTGATAGGCCTCTCGTTGGGAAGC
AACAGTGGATACGCCATCAACCCGACCCGGGACCTGGCGCCTCGCATCTTCACCGCTCTG
GCTGGGTGGGGCACAGATGTTTTCAGAGCGGGGAACAACTGGTGGTGGGTCCCAATTGTG
GCACCGCTGCTAGGTGGCGTTATTGGTGCGCTGGTCTATCAGGCATTTGTGGGACTCCAT
CACCCACCACCTCTACTCCCAGAGACACACCAG---------------GAA---GAGACC
TCAGACCTGGAGCTGCTCAAGACGAGTGAATCAATAAATGCG------------------

>European_eel_Aqp7
ATGAAGGACACG---------CTGAACTCATGGAAG---GACGGTCAGGCTGGGGGAGGA
AGAATGCCTTTCGCTTTTCGG---ATTAGAAACGAATGCTGCCGCTTGGCGCTGGCAGAA
ACGCTCAGCACCTATGTGATGATGGTATTCGGCCTGGGCACGGTGGCACAGGTGGTGACG
GGGGACGGGCTGTTTGGACGTTACCTGAGCATCAACCTGGGCTTCGGGCTGGGAGTGGCC
ATGGGAGTGCACATCGCCGGAAAGGTCTCAGGTGCCCACATGAACGCCGCCGTCTCCTTC
ACCATGTGCCTGTTCGGCCGGCTCGCCTGGAGGATGTTCCCCGTCTACGTCGCCGCGCAG
ATGCTGGGCTCCTTCATGGCGGCCGGCACTGTCTTCTCGCTG------GACGCCATGCTG
AATTACTGCGGGGGCAACTTCACCGTCGCTGGTCCCCGGGCCACCGCTGGGATCTTCGCC
ACCTACCCCGCCCCCTACCTGTCCTTACCTGGGGGCTTCCTGGACCAGGTGCTGGGGACG
GCCATGCTGCTCCTGTGCCTCATGGCGCTGTCGGACCAGCGGAACCAGCCGGCGCAGGCG
GGGTCCGAGCCCCTGGCGGTGGGCCTGCTGGTTCTGCTCCTCGGCGTCTCCATGGGGAGC
AACAGCGGCTACGCCATCAACCCGTCCCGCGACTTGCCCCCCCGAATATTCACCGCCCTG
GCCGGCTGGGGCCCGGAGGTCTTCAGGGCAGGGCAGAACTGGTGGTGGGTGCCCCTGCTG
GCACCCCTGATTGGCGGTGTACTGGGGGCCGGGATCTACAAGCTGTTCGTGGAGCTGCAT
CACCCCGCCCCTCCCCGGATCTACGGGGCCCAG---CCCAGG------------------
---GAGGCGGAGCTTGAGAAGAGCGCC---ACGTCAGAGCTG------TGCGTG------

>American_eel_Aqp7_2
ATGAAGGACACG---------CTGAACTCAGGGAAG---GACGGTCAGGCTGGGGGAGGA
AGAATGCCTTTCGCTTTTCGG---ATTAGAAACGAATGCTTCCGCTCGGCGCTGGCAGAA
ACGCTCAGCACCTATGTGATGATGGTATTCGGCCTGGGCACGGTGGCGCAGGTGGTGACG
GGGGACGGGCTGTTTGGACGTTACCTGAGCATCAACCTGGGCTTCGGGCTGGGAGTGGCC
ATGGGAGTGCACATCGCCGGAAAGGTCTCAGGTGCCCACATGAACGCCGCCGTCTCCTTC
ACCATGTGCCTGTTCGGCCGGCTCGCCTGGAGGATGTTCCCCGTCTACGTCGCCGCGCAG
ATGCTGGGCTCCTTCATGGCGGCCGGCACCGTCTTCTCGCTGTACTACGACGCCATGCTG
AATTACTGCGGGGGCAACTTCACCGTCGCTGGCCCCCGGGCCACCGCTGGGATCTTCGCC
ACCTACCCCGCCCCCTACCTGTCCTTACCTGGGGGCTTCCTGGACCAGGTGCTGGGGACG
GCCATGCTGCTCCTGTGCCTCATGGCGCTGTCGGACCAGCGGAACCAGCCGGCGCAGGCG
GGGTCCGAGCCCCTGGCGGTGGGCCTGCTGGTTCTGCTCCTCGGCGTCTCCATGGGGAGC
AACAGCGGCTACGCCATCAACCCGTCCCGCGACCTGCCCCCCCGAATATTCACCGCCCTG
GCCGGCTGGGGCCCGGAGGTCTTCAGGGCAGGGCAGAACTGGTGGTGGGTGCCCCTGCTG
GCACCCCTGATTGGCGGTGTAATGGGGGCCGGGATCTACAAGCTGTTCGTGGAGCTGCAT
CACCCCACCCCTCCCCGGATCTATGGGGCCCAG---CCTAGG------------------
---GAGGCGGAGCTTGAGAAGGGCGCC---ACGTCAGAGCTG------TGCGTG------

>American_eel_Aqp7_1
ATGAAGGACACG---------CTGAACTCATGGAAG---GACGGTCAGGCTGGGGGAGGA
AGAATGCCTTTCGCTTTTCGG---ATTAGAAACGAGTGCTTCCGCTTGGCGCTGGCAGAA
ACGCTCAGCACCTATGTGATGATGGTATTCGGCCTGGGCACGGTGGCGCAGGTGGTGACG
GGGGACGGGCTGTTCGGACGTTACCTGAGCATCAACCTGGGCTTCGGGCTGGGAGTGGCC
ATGGGAGTGCACATCGCCGGAAAGGTCTCAGGGGCCCACATGAACGCCGCCGTCTCCTTC
ACCATGTGCCTGTTCGGCCGGCTCGCCTGGAGGATGTTCCCCGTCTACGTCGCCGCGCAG
ATGCTGGGCTCCTTCATGGCGGCCGGCACCGTCTTCTCGCTGTACTACGACGCCATGCTG
AATTACTGCGGGGGCAACTTCACCGTCGCTGGCCCCCGGGCCACCGCTGGGATCTTCGCC
ACCTACCCCGCCCCCTACCTGTCCATACCTGGGGGCTTCCTGGACCAGGTGCTGGGGACG
GCCATGCTGCTCCTGTGCCTCATGGCGCTGTCGGACCAGCGGAACCAGCCGGCGCGGGCG
GGGTCCGAGCCCCTGGCGGTGGGCCTGCTGGTTCTGCTCCTCGGCGTCTCCATGGGGAGC
AACAGCGGCTACGCCATCAACCCGTCCCGCGACCTGCCCCCCCGAATATTCACCGCCCTG
GCCGGCTGGGGCCCAGAGGTCTTCAGGGCAGGGCAGAACTGGTGGTGGGTGCCCCTGCTG

GCGCCCCTGATTGGCGGTGTAATAGGGGCCGGGATCTACAAGCTGTTCGTGGAGCTGCAT
CACCCCGCCCCTCCCCGGATCTACGGGGCCCAG---CCAAGG------------------
---GAGGCGGAGCTTGAGAAGAGCGCC---ACGTCAGAGCTG------TGCGTG------

>Japanese_eel_Aqp7
ATGAAGGACACG---------TTGAACTCAGGGAAG---GACGGTCAGGCTGGGGGAGGA
AGAATGCCTTTCGCTTTTCGG---ATTAGAAATGAGTGCTTCCGCTTGGCGCTGGCAGAA
ACGCTGAGCACCTATGTGATGATGGTATTCGGCCTGGGCACGGTGGCGCAGGTGGTGACG
GGGGACGGGCTGTTTGGGCGTTACCTGAGCATCAACCTGGGCTTCGGCCTGGGAGTGGCC
ATGGGGGTGCACATCGCCGGAAAGGTCTCAGGT---------------GCCGTCTCCTTC
ACCATGTGCCTGTTCGGCCGGCTCGCCTGGAGGATGTTCCCCGTCTACGTCGCCGCGCAG
ATGCTCGGCTCCTTCATGGCGGCCGGCACCGTCTTCTCGCTGTACTACGACGCCATTCTT
AATTACTGCGGGGGCAACTTCACCGTCACTGGCCCCCGGGCCACCGCTGGGATCTTCGCC
ACCTACCCCGCCCCCTACCTGTCCATACCTGGGGGCTTCCTGGACCAGGTGCTGGGGACG
GCCATGCTGCTCCTGTGCCTCATGGCTCTGTCGGACCAGCGGAACCAGCCGGCGCAGGCG
GGGTCCGAGCCCCTGGCGGTGGGCCTGCTGGTTCTGCTCCTCGGCGTCTCCATGGGGAGC
AACAGCGGCTACGCCATCAACCCGTCCCGCGACCTGCCCCCCCGAATATTCACCGCCCTC
GCCGGCTGGGGCCCGGAGGTCTTCAGGGCAGGGCAGAGCTGGTGGTGGGTGCCCCTGCTG
GCACCCCTGATTGGCGGTGTAGTGGGAGCCGGGATCTACAAGCTGTTCGTGGAGCTGCAT
CACCCCGCCCCGCCCCGGATCTACGGGGCCCAG---CCCAGA------------------
---GAGGCGGAGCTTGAGAAGAGCGCC---ACATCAGAGCTG------TGCGTG------

>Daggertooth_pike_conger_Aqp7
ATGAAATGTGCG---------TTGAATTCAGGG------GAAGCTGGTCATACTGACAGA
AGAATGCCATTCACTTTTCGG---ATTAAAAACGAATGCTTCCGCATGGCGTTGGCAGAA
ATTCTTAGCACCTATGTCATGATGACATTCGGCCTCGGCACCGTGGCGCAGGTGGTCACG
GGGGAGGGGCTGTTTGGGGGTTACCTGAGCATCAACCTGGGCTTTGGGCTCGCAGTGGCC
ATGGGAGTGCACATTGCCGGCAAGGTGTCAGGTGCCCACATGAACGCGGCGGTCTCCTTC
ACCATGTGCCTGTTCGGCCGGCTTGCCTGGAAGATGCTCCCCGTCTACATTGCTTCTCAG
ATGCTGGGATCATTTCTGGCGGCCGGGACTGTTTTCTCCGTTTACTATGACGCCATGGTT
CATTACTGCGGGGGCAATTTCACCGTCTCTGGGCCCCGGGCCACGGCTGGGATCTTCGCC
ACCTACCCCGCCCCCTACCTCTCCATACCTGCAGGGTTCCTAGACCAGGTGCTGGGTACC
GCCATGCTGCTCCTGTGCATCATGGCGCTGTCGGACCAGAGGAACCTGCCCGCCCGACCG
GGAGCCGAGCCCATCGCGGTGGGCCTGCTGGTTCTCCTCATCGGGGTCTCCATGGGGAGC
AACAGCGGATATGCCATCAACCCGTCCCGCGACCTACCGCCCCGAATCTTCACCGCCCTG
GCAGGCTGGGGCCCGGAGGTCTTCAGGGCGGGGCACAGCTGGTGGTGGGTGCCCCTGCTG
GCGCCCCTCATCGGTGGGGTGATGGGGGCGGCCGTCTACAAGCTGTTCGTGGAGCTGCAT
CACCCCGCCCCGCCCCGCATCTACATGGCCCCT---CCCATG------------------
---GAGGCGGAGCTTGAGAAATGTGGC---ACCACAGAGGTC------TGTGTC------

>Siberian_sturgeon_Aqp7
ATGAAG---------------CTG---------------GACAAGCAGTTTGTATCCAAT
---GTAAAGAAAGCCATTCGA---ATCCGAAACGAATACCTGCGAGAGGCTCTGGCTGAG
ATACTCAGCACCTTTGTCATGATGGTTTTCGGTCTGGGCTCGGTAGCCCAGGTGGTCATG
GGAGGTGGTTCCTATGGTGACTACTTCAGTATAAATTTGGGCTTCGGCCTTGGGGTCACC
ATGGGTATTCACATCGCTGGGGGCGTCTCAGGAGCTCATATGAACACTGCAGTCACCTTC
TCCATGTGTGTGCTGGGGAGCCTGAGCTGGAGGAAGCTGCCGGTCTACGCTGCTGCCCAG
TTCCTCGGCTCCTTTATGGCTGCGGTCACCGTCTTCTGGGTCTACTATGATGCTCTGTTT
GAGTTCTGCGGAGGGAACTTCACAGTGACGGGCCCGAGAGCAACTGCTGGGATCTTCGCT
ACCTACCCGGCACCATACCTGTCAGTGGGAGGAGGCTTTCTGGACCAGGTGCTGGGCACT
GCCATGCTGCTGCTGTGTATCCTGGCGCTGAATGACCACAGGAACAGCCCGGCGCTCAGC
GGCACGCAGCCTCTGCTCATCGGCCTCCTGGTGGTGGTGATTGGCATCTCTCTGGGCAGC
AACAGCGGGTACGCCATCAACCCTGCCCGTGACCTGCCCCCGCGCTTCTTCACCTCCATG
GCAGGCTGGGGTCCTGACGTCTTCAGTGCTGGTAATGGCTGGTGGTGGATCCCTGTGGTG
GCGCCCATGGTTGGCAGTGTGACTGGCTCTCTGCTCTACAAACTCTTCATCGAGTATCAC
CATCCGTCTGAGGAGCATCTAGAGGAAGGGCTG---GGGGGA------------------
------------------------GTC---TGCTCTGATCAAAAACACTGCTTCCGCTTT

>Atlantic_sturgeon_Aqp7
ATGAAG---------------CTG---------------GACAAGCAGTTTGTATCCAAT
---GTAAAGAAATCCATTCGA---ATCCGAAACGAATACCTGCGAGAGGCTCTGGCTGAG

ATACTCAGCACCTTTGTCATGATGGTTTTCGGTCTGGGCTCGGTAGCCCAGGTGGTCATG
GGAGGTGGTTCCTATGGTGACTACTTCAGTATAAATTTGGGCTTCGGCCTTGGGGTCACC
ATGGGCATTCACATCGCTGGGGGCGTCTCAGGAGCTCATATGAACACTGCAGTCACCTTC
TCCATGTGTGTGCTGGGGAGCCTGAGCTGGAGGAAGCTGCCGGTCTACGCTGCTGCCCAG
TTCCTCGGCTCCTTTATGGCTGCGGTCACCGTCTTCTGGGTCTACTATGATGCTCTGTTT
GAGTTCTGCAGAGGGAACTTCACAGTGACGGGCCCAAGAGCAACTGCTGGGATCTTCGCT
ACCTACCCGGCGCCATACCTGTCAGTGGGAGGAGGCTTTCTGGACCAGGTGCTGGGCACT
GCCATGCTGCTGCTGTGTATCCTGGCGCTGAATGACCACAGGAACAGCCCGGCGCTCAGA
GGCACGCAGCCTCTGCTCATCGGCCTCCTGGTGGTGGTGATTGGCATCTCTCTGGGCAGC
AACAGCGGGTACGCCATCAACCCCGCCCGCGACCTGCCCCCACGCTTCTTCACCTCCATG
GCAGGCTGGGGTCCTGACGTCTTCAGTGCTGGTAATGGCTGGTGGTGGATCCCTGTGGTG
GCGCCCATGGTTGGCAGTGTGACTGGCTCTCTGCTCTACAAACTCTTCATCGAGTATCAC
CATCTGGCTGAGGAGCATCTAGAGGAGGGGCTG---GGGGGA------------------
------------------------GTC---TGCTCTGATCAAAAACACTGCTTCCGCTTT

>Common_sturgeon_Aqp7

------TGTGTGCTGGGGAGCCTGAGCTGGAGGAAGCTGCCGGTCTACGCTGCTGCCCAG
TTCCTCGGCTCCTTTATGGCTGCGGTCACTGTCTTCTGGGTCTACTATGATGCTCTGTTT
GAGTTCTGCGGAGGGAACTTCACAGTGACGGGCCCGAGAGCAACTGCTGGGATCTTCGCT
ACCTACCCGGCACCATACCTGTCAGTGGGAGGAGGCTTTCTGGACCAGGTGCTGGGTACT
GCCATGCTGCTGCTGTGTATCCTGGCGCTGAATGACCACAGGAACAGCCCGGCGCTCAGC
GGCACGCAGCCTCTGCTCATCGGCCTCCTGGTGGTGGTGATTGGCATCTCTCTGGGCAGC
AACAGCGGGTACGCCATCAACCCCGCCCGCGACCTGCCCCCGCGCTTCTTCACCTCCATG
GCAGGCTGGGGTCCTGGCGTCTTCAGTGCTGGTAATGGCTGGTGGTGGATCCCTGTGGTG
GCGCCCATGGTTGGCAGTGTGACTGGCTCTCTGCTCTACAAACTCTTCATCGAGTATCAC
CATCCGGCTGAGGAGCATCTAGAGGAGGGGCTG---GGGGGA------------------
------------------------GTC---TGCTCTGATCAAAAACACTGCTTCCGCTTT

>Chinese_sturgeon_Aqp7
ATGAAG---------------CTG---------------GACAAGCAGTTTGTATCCAAC
---GTAAAGAAAGCCATTCGA---ATCCGAAACGAATACCTGCGAGAGGCTCTGGCTGAG
ATACTCAGCACCTTTGTCATGATGGTTTTCGGTCTGGGCTCGGTAGCCCAGGTGGTCATG
GGAGGTGGTTCCTATGGTGACTACTTCAGTATAAATTTGGGCTTCGGCCTTGGGGTCACC
ATGGGTATTCACATCGCTGGGGGCGTCTCAGGAGCTCATATGAACACTGCAGTCACCTTC
TCCATGTGTGTGCTGGGGATCCTGAGCTGGAGGAAGCTGCCGGTCTACGCTGCTGCCCAG
TTCCTCGGCTCCTTTATGGCTGCGGTCACCGTCTTCTGGGTCTACTATGATGCTCTGTTT
GAGTTCTGCGGAGGGAACTTCACAGTGACGGGCCCGAGAGCAACTGCTGGGATCTTCGCT
ACCTACCCGGCACCATACCTGTCAGTGGGAGGAGGCTTTCTGGACCAGGTGCTGGGCACT
GCCATGCTGCTGCTGTGTATCCTGGCGCTGAATGACCACAGGAACAGCCCGGCGCTCAGC
GGCACGCAGCCTCTGCTCATCGGCCTCCTGGTGGTGGTGATTGGCATCTCTCTGGGCAGC
AACAGCGGGTACGCCATCAACCCCGCCCGCGACCTGCCCCCGCGCTTCTTCACCTCCGTG
GCAGGCTGGGGTCCTGACGTCTTCAGTGCTGGTAATGGCTGGTGGTGGATCCCTGTGGTG
GCGCCCATGGTTGGCAGTGTGACTGGCTCTCTGCTCTACAAACTCTTCATTGAGTATCAC
CATCCGGCTGAGGAGCATCTAGAGGAGGGGCTG---GGGGGA------------------
------------------------GTC---TGCTCTGATCAAAAACACTGCTTCCGCTTT

>Russian_sturgeon_Aqp7

------------GGGAACTTCACAGTGACGGGCCCGAGAGCAACTGCTGGGATCTTCGCT
ACCTACCCGGCACCATACCTGTCAGTGGGAGGAGGCTTTCTGGACCAGGTGCTGGGCACT

GCCATGCTGCTGCTGTGTATCCTGGCGCTGAATGACCACAGGAACAGCCCGGCGCTCAGC
GGCACGCAGCCTGTGCTCATCGGCCTCCTGGTGGTGGTGATTGGCATCTCTCTGGGCAGC
AACAGCGGGTACGCCATCAACCCCGCCCGC------------------------TCCATC
GCAGGCTGGGGTCCTGACGTCTTCAGTGCTGGTAATGGCTGGTGGTGGATCCCTGTGGTG
GCACCCATGGTTGGCAGTGTGACTGGCTCTCTGCTCTACAAACTCTTCATCGAGTATCAC
CATCCATCTGAGGAGCATCTAGAGGAGGGGCTT---GGGGGA------------------
------------------------GTC---TGCTCTTATCAAAAACACTGCTTCCGCTTT

>Sterlet_Aqp7
ATGAAG---------------CTG---------------GACAAGCAGTTTGTATCCAAT
---GTAAAGAAAGCCATTCGA---ATCCGA------------------------------
------------------------GTTTTCGGTCTGGGCTCGGTAGCCCAGGTGGTCATG
GGAGGTGGTTCCTATGGTGACTACTTCAGTATAAATTTGGGCTTCGGCCTTGGGGTCACC
ATGGGTATTCACATCGCTGGGGGCGTCTCAGGAGCTCATATGAACACTGCAGTCACCTTC
TCCATGTGTGTTCTGGGGAGCCTGAGCTGGAGGAAGCTGCCGGTCTACGCTGCTGCCCAG
TTCCTCGGCTCCTTTATGGCTGCGGTCACCGTCTTCTGGGTCTACTATGATGCTCTGTTT
GAGTTCTGCGGAGGGAACTTCACAGTGACTGGCCCGAGAGCAACTGCTGGGATCTTCGCT
ACCTACCCGGCACCATACCTGTCAGTGGGAGGAGGCTTTCTGGACCAGGTGCTGGGCACT
GCCATGCTGCTGCTGTGTATCCTGGCGCTGAATGACCACAGGAACAGCCCGGCGCTCAGC
GGCACGCAGCCTCTGCTCATCGGCCTCCTGGTGGTGGTGATTGGCATCTCTCTGGGCAGC
AACAGCGGGTACGCCATCAACCCCGCCCGTGACCTGCCCCCGCGCTTCTTCACCTCCATG
GCAGGCTGGGGTCCTGACGTCTTCAGGGCTGGTAATGGCTGGTGGTGGATCCCTGTGGTG
GCACCCATGGTTGGCAGTGTGACTGGCTCTCTGCTCTACAAACTCTTCATCGAGTATCAC
CATCCATCTGAGGAGCATCTAGAGGAGGGGCTG---GGGGGA------------------
------------------------GTC---TGCTCTGATCAAAAACACTGCTTCCGCTTT

>Reedfish_Aqp7

---------------ATGAAG---ATCACAAATCAATATATCCGAGAGTTTCTTTCAGAA
TTCTTAAGCACTTATATCATGATGCTCTTTGGCTTAGGCTCAGTTGCTCAGGTGGTCATG
GGAGGTGATTCCTATGGTAACTACTTAAGTATAAACCTGGGCTTTGGTCTTGGAGTCACC
ATGGGCATTCACATTGGTGGTGGGGTCTCAGGAGCTCACATGAACACTTCAGTGACATTT
GCAATGTGCCTAATAGGAAATCTGTTTTGGAAGAAGCTGCCAGTATATGCACTGGGACAA
TTACTGGGCTCCTTTTTGGCTGCAGTCACCATCTTCTGGCTTTATTATGATGCCTTACAA
GACTACTGTGGTGGAAACTTCACGGTGACTGGACCCAAAGCCACAGCGGGGATCTTTGCT
ACATATCCAGCTCCTTACTTGTCTGTAGCAGGAGGTTTTATAGACCAGGTGGTAGGAACA
GCGGTACTGCTGCTATGTATTCAAGCTATCAATGATCAGAAGAACTGCTCTGCTCTCAGT
GGGACATCTCCATTGGTGACTGGCCTGTTGGTGGCACTTATTGGTATCTCCTTGGGTAGT
AACAGTGGCTATCCTATCAATCCAGCCAGAGATCTTCCTCCCAGGATCTTCACTGCTATG
GCAGGTTGGGGGACTACTGTTTTCAGTGCAGGAAATAACTGGTGGTGGATCCCTGTTGTT
GCACCAATGTTTGGCAGTGTGACTGGAGTCCTAATCTACAAAGTGTTCATTGAAATGCAT
CATCCTTCAGTTGAGCAGCAGAAGAAACAACTT---CAAGAA------------------
------------------AAAAGCCCA---AGTACAGATCTTTCTCTTTGCTTT------

File S8: Alignment for Fig. 3C, Fig. S7
>Torafugu_Aqp9a
--ATGAGGAAACGCTGTGCCATC
AAACACGGAATTTTTAAGGAATTCCTGGCAGAATTTCTGGGGACGTTCGTCCTGGTCCTG
TTCGGCTGCGGCTCCGTGGCCCAGACCGTCCTCAGCCGGAACACCCTGGGGGAGCCGCTC
ACCGTGCACATCGGCTTCTCCGTGGGGCTGATGATGGCCGCGTATGTGGCCGGAGGGGTG
TCAGGGGGCCACGTGAACCCCGCGGTGTCTTTGGCGATGGTGATTCTGGGTAAGCTGAAG
ATCTGGAAGTTCCCCTTCTACGTCATCGCCCAGTTTCTCGGCGCCTTCGCCGGAGCCGCC
GCCGTGTTTGGGCTGTATTACGACGCCTTCATGGACTTCACCAGCGGGATCCTGTCGGTC
ACAGGCATCAATGCCACGGGTCACATCTTCGCCTCCTACCCTGCGCGGCACCTGTCCATC
CTCGGCGGCTTCATCGATCAAGTGGTGGGGACGGGCATGCTGGTCCTCTGCATCCTCGCC
ATCATCGACGGCGGAAACATCGGCGCTCCCAAAGGCGTGGAGCCGCTGGCCATCGGCCTG
ATCATCATGGCCATCGGCGTGTCCATGGGGCTGAACTGCGGCTACCCCCTGAACCCCGCC
AGAGACCTGGGCCCCCGCCTGTTCACCGCCGTGGCGGGGTGGGGCATGGAGGTCTTCAGC
ACTGCCGACTACTGGTGGTGGATCCCGGTGGCGGGCCCCATGGTGGGGGGCGTGGTCGCC
GCCGTGCTCTACTACCTGCTCATCGAGCTGCACCACCCCCGA------------------

---GACGAGCCCGAGAAGCCCCGCGAG---GAGGAGGAG------------------GAC
GAGGAGGACGAGGACGAGGACAGCAGCCTGAAGGACAAATACGAGATGATCACCATGAGC
>Sansaifugu_Aqp9a
--ATGAGGAAACGCTGTGCCATC
AAACACGGAATTTTTAAGGAATTCCTGGCAGAATTCCTGGGGACGTTCGTCCTGGTCCTG
TTCGGCTGCGGCTCCGTGGCCCAGACCGTCCTCAGCCGGAACACCCTGGGGGAGCCGCTC
ACCGTGCACATCGGCTTCTCCGTGGGGCTGATGATGGCCGCGTATGTGGCCGGAGGG---
---GGGGGCCACGTGAACCCCGCGGTGTCTTTGGCCATGGTGATTCTGGGTAAGCTGAAG
ATCTGGAAGTTCCCCTTCTACGTCATCGCCCAGTTTCTCGGCGCCTTCGCCGGAGCCGCC
GCCGTGTTTGGGCTGTATTACGACGCCTTCATGGACTTCACCAGCGGGATCCTGTCGGTC
ACAGGCATCAATGCCACGGGTCACATCTTCGCCTCCTACCCTGCGCGGCACCTGTCCGTC
CTCGGCGGCTTCATCGATCAAGTGGTGGGGACGGGCATGCTGGTCCTCTGCATCCTCGCC
ATCGTCGACGGCGGAAACATCGGCGCTCCCAAAGGCGTGGAGCCGCTGGCCATCGGCCTG
ATCATCATGGCCATCGGCGTGTCCATGGGGCTGAACTGCGGCTACCCCCTGAACCCCGCC
AGAGACCTGGGCCCCCGCCTGTTCACCGCCGTGGCGGGGTGGGCATGGAGGTCTTCAGG
ACTGCCGACTACTGGTGGTGGATCCCGGTGGCGGGCCCCATGGTGGGGGGCGTGGTCGGC
GCCGTGCTCTACTACCTGCTCATCGAGCTGCACCACCCCCGA------------------
---GACGAGCCCGAGAAGCCCCGCGAG---GAGGAGGAG------------------GAC
GAGGAGGACGAGGACGAGGACAGCAGCCTGAAGGACAAATACGAGATGATCACCATGAGC
>Green_spotted_pufferfish_Aqp9a
---------------ATGGAGCTGCAACACAGGACAAAGATGAGGAAACACTGCGCCATC
AAACATGGAATTTTAAAGGAATTCCTGGCAGAATTCCTGGGAACGTTTGTATTGGTCCTG
TTCGGCTGTGGCGCCGTCGCTCAGACCGTCCTCAGCCGGAACATGCTGGGGGAACCTCTC
ACCGTGCACATCGGCTTCTCCGTGGGGCTGATGATGGCGGCCTACGTGGCTGGAGGAGTG
TCAGGGGGCCACGTGAACCCTGCTGTGTCCTTGGCGATGGTGATTCTGGGGAAACTGAAG
ATCTGGAAGTTCCCTTTCTACGTCCTGGCTCAGTTTCTGGGCGCTTTCGCCGGCGCCGCT
GCCGTGTTCGGCCTTTATTACGACGCCTTCATGGACTTCACCAGCGGGATCCTGTCGGTC
ACAGGGATCAACGCCACCGGGCACATTTTCGCCTCCTACCCCGCGAGACACCTGTCGGTC
CTCGGCGGCTTCGTGGATCAAGTGGTGGGGACGGGCATGCTGGTCCTGTGCATCCTGGCC
ATCATCGACGGCGGGAACATCGGGGCTCCCAGGGGCGTGGAGCCGCTGGCCATCGGCCTG
ATCATCATGGCCATCGGCGTGTCCATGGGGCTGAACTGCGGCTATCCTCTGAACCCCGCC
AGGGACCTGGGCCCCCGGCTGTTCACCGCCCTGGCAGGGTGGGCATGGAGGTCTTCAGG
ACGGCTGGCTACTGGTGGTGGATCCCCGTGGCGGGGCCCATGGTGGGGGGCGTGGTGGCC
GCCGTGCTCTACTACCTGCTCATCCAACTGCACCACAGCCAC------------------
---GACGAGCCCGAGAAGCCCCACGAG---CAGGAGGAG------------------GAG
GAGGAGGAAGAGGACGAGGACAGCAGCCTGAAGGATAAATACGAGATGATCACCATGAGC
>Ocean_sunfish_Aqp9a
--ATGAGGCAACACTGTGCTCTC
AAACATGGAATAGTCAAGGAATTCCTGGCCGAATTCCTGGGGACTTTTGTCTTGGTTTTG
TTTGGCTGCGGCTCAGTCGCTCAGACCGTCCTCAGCCGGAACACCCTTGGAGAGCCTCTC
ACCGTGCACATCGGCTTCTCTGTGGGACTGATGATGGCAGCGTATGTGGCCGGCGGAGTG
TCGGGGGGCCACGTGAACCCTGCCGTGTCCCTGGCCATGGTGATTCTGGGTAAACTCAAG
ATCTGGAAGTTTCCCTTCTATGTCATCGCCCAGTTTCTTGGTGCTTTTGCTGGCGCTGCT
GCCGTCTTTGGGTTATACTATGATGCCTTCATGGACTTCACCAGTGGGATTCTGTCAGTG
ACGGGGATCAATGCAACAGGTCACATTTTTGCTTCCTACCCTGCGAGACACCTGTCGATC
CTCGGCGGCTTCATCGATCAAGTGGTGGGAACCGGCATGCTCGTCTTGTGCATCCTGGCC
ATCATCGATGGCGGAAACATCGGAGCTCCCAAAGGCGTGGAGCCGCTGGCCATCGGTCTG
ATCATCATGGCCATCGGTGTGTCTATGGGACTAAACTGCGGCTACCCCCTGAACCCCGCC
AGAGACCTGGGACCCCGGCTGTTCACCGCTGTAGCAGGATGGGGGATGGAGGTGTTCAGG
ACCGCAGGCTACTGGTGGTGGATCCCCGTGGCCGGGCCGATGGTGGGGGGCGTCATCGCA
GCCGTCATATACTACCTGCTCATTGAGCTGCACCACCACCGC------------------
---GACGAGCCCGACAAGCCCCACGAGGAGGAGGAGGAGTTGGATGAGGAG------GAG
GAGGACGAAGAAGATGATGACAGCAGTCTGAAGGACAAATATGAGATGATCACCATGAGC
>Striated_frogfish_Aqp9a
--ATGAGGAAACATTGTGCACTC
AAACATGGAATACTCAAGGAATTCCTGGCAGAATTCCTGGGGACATTTGTTTTGGTTTTG
TTTGGCTGTGGTTCCGTCGCTCAGACCGTCCTCAGTCGAAACACGCTGGGAGAGCCTCTG
ACCGTCCACATCGGCTTCTCTGTCGGACTGATGATGGCCTCGTACGTCGCTGGTGGAGTG
TCAGGGGGCCATGTGAACCCTGCTGTATCTCTGGCCATGGTGATTCTGGGCAAACTGAAG
ATCTGGAAGTTTCCCTTCTATGTCATCGCTCAGTTTCTTGGTGCTTTTGCTGGAGCTGCT
GCAGTCTTTGGGTTATACTATGATGCTTTCATGGACTTCACCAGTGGGATCCTGTCGGTG

ACGGGAATCAATGCAACAGGTCACATTTTGCATCCTACCCCGCAAGACACTTGTCAGTC
CTTGGCGGCTTCATTGATCAAGTGGTGGGGACAGGGATGCTGGTCCTGTGCATACTGGCT
ATCATTGATGGTGGAAACATCGGAGCTCCCAAAGGCGTGGAGCCGCTGGCGATAGGCCTG
ATCATCATGGCCATCGGTGTGTCCATGGGACTGAACTGTGGCTACCCGCTGAACCCCGCC
AGAGATCTGGGGCCTCGGCTGTTCACAGCTGTAGCAGGATGGGGCATGGAGGTCTTCAGG
ACCGCAGACTACTGGTGGTGGATCCCCGTGGCGGGGCCCATGGTGGGCGGCGTGGTCGCA
GCTGTCCTCTACTACATGCTCATCGAGCTGCATCACCGCCGC------------------
---GATGAGCCGGAGAAGCCCCACGAG---GAGGAAGAGGAAGATGAG---------GAT
GAAGATGATGAAGATGAGGACAGCAGTCTGAAGGACAAATATGAGATGATCACCATGAGC
>Crimson_snapper_Aqp9a
---------------------------------------ATGAGGCAACACTGTGCTCTC
AAACATGGAATATTCAAGGAATTCCTGGCAGAATTCCTGGGGACGTTTGTCTTGATTCTG
TTTGGCTGCGGCTCGGTCGCTCAGACCGTCCTCAGTCGAAACACCCTCGGCGAGCCTCTC
ACCGTCCACATCGGCTTCTCTGTAGGACTGACCATGGCGGCGTATGTGGCCGGTGGAGTG
TCAGGGGGCCATGTGAACCCTGCTGTGTCTCTGGCCATGGTGATTCTGGGCAAACTGAAG
ATCTGGAAGTTTCCCTTCTACGTCATCGCTCAGTTTCTTGGCGCCTTCGCCGGAGCCGCC
GCAGTCTTTGGGCTGTACTACGATGCTTTCATGGACTTCACCAGTGGGATTCTTTCCGTG
ACGGGAATCAACGCAACAGGTCACATTTTGCTTCCTACCCTGCGAGACACCTGTCGATC
CTCGGAGGCTTCATTGATCAGGTGGTGGGGACGGGCATGCTGGTCTTGTGCATCCTGGCG
ATCATTGATGGCGGAAACATCGGAGCTCCTAAAGGCGTGGAGCCGCTGGCCATCGGTCTG
ATCATCATGGCCATCGGCGTGTCCATGGGACTGAACTGTGGATACCCCCTGAACCCCGCC
AGAGACCTGGGACCCCGACTGTTCACGGCCGTAGCAGGATGGGGGATGGAGGTGTTCAGC
ACTGCAGACTACTGGTGGTGGATCCCCGTGGCGGGGCCCATGGTGGGCGGCGTGGTCGCA
GCCGTCATCTACTACCTGCTCGTTGAGATGCACCACCACCGC------------------
---GATGAGCCCGAGAAGCCCCACGAG---GAGGAGGAAGAGGAGGAG------------

>Gilthead_seabream_Aqp9a
---------------------------------------ATGAGGCAACACTGTGCACTC
AAACATGGAATATTCAAGGAATTCCTGGCAGAATTCCTGGGGACGTTTGTCTTGGTTCTG
TTTGGCTGTGGCTCAGTCGCTCAGACCGTCCTCAGTCGAAACACCCTGGGGGAGCCTCTC
ACCGTCCACATCGGCTTCTCCGTAGGACTGATGATGGCAGCGTATGTGGCCGGTGGAGTG
TCGGGTGGCCACGTGAACCCTGCTGTGTCTCTGGCTATGGTGATTCTGGGCAAACTGAAG
ATCTGGAAGTTTCCCTTCTATGTCATCGCTCAGTTTCTTGGTGCTTTTGCTGGAGCCGCT
GCAGTCTTCGGGCTGTACTACGATGCTTTCATGGACTTCACCAGTGGGATTCTGTCGGTG
ACGGGAATCAATGCAACAGGTCACATTTTGCTTCCTACCCTGCGAGACACCTGTCAATC
CTCGGTGGCTTCATTGACCAGGTGGTGGGGACAGGTATGCTGGTCCTGTGTATCCTGGCT
ATCATTGACGGTGGCAACATCGGAGCTCCCAAAGGCGTGGAGCCGCTGGCTATCGGCCTG
ATCATCATGGCCATTGGTGTGTCTATGGGACTGAATTGTGGCTACCCTCTGAACCCTGCC
AGAGACCTGGGACCCCGGCTGTTCACAGCTGTAGCAGGATGGGGGATGGAGGTCTTCAGG
ACTGCAGACTACTGGTGGTGGATCCCTGTGGCGGGGCCCATGGTGGGCGGAGTGGTGGCA
GCCGTCATCTACTACCTGCTGATCGAGCTGCACCATCACCGC------------------
---GATGAGCCTGAGAAGCCCCATGAG---GAGGAGGAGGAGGAG------------GAG
GAAGACGAAGAGGACGACGACAGCAGTCTGAAGGACAAATATGAGATGATCACCATGAGC
>Red_seabream_Aqp9a
---------------------------------------ATGAGGCAACACTGTGCACTC
AAACATGGAATATTCAAGGAATTCCTGGCAGAATTCCTGGGGACGTTTGTCTTGGTCCTG
TTTGGCTGTGGCTCAGTCGCTCAGACCGTTCTCAGTCGAAACACCCTGGGGGAGCCTCTC
ACCGTCCACATCGGCTTCTCCGTAGGACTGATGATGGCAGCGTATGTGGCCGGTGGAGTG
TCGGGGGGCCATGTGAACCCTGCTGTGTCTCTGGCCATGGTGATTCTGGGCAAACTGAAG
ATCTGGAAGTTTCCCTTCTATGTCATTGCTCAGTTTCTTGGTGCTTTTGCTGGAGCCGCT
GCAGTCTTTGGGCTGTACTACGATGCTTTCATGGACTTCACCAGTGGGATTCTGTCGGTG
ACGGGAATCAATGCAACAGGTCACATTTTGCTTCCTACCCTGCGAGACACCTGTCAATC
CTCGGTGGCTTCATCGATCAGGTGGTGGGGACAGGTATGCTGGTCCTGTGTATCCTGGCT
ATCATTGACGGTGGGAACATCGGCGCTCCCAAAGGTGTGGAGCCGCTGGCTATCGGTCTG
ATCATCATGGCCATCGGTGTGTCTATGGGACTAAACTGTGGCTACCCTCTGAACCCTGCC
AGAGACCTGGGACCCCGACTGTTCACAGCTGTAGCAGGATGGGGGATGGAGGTCTTCAGG
ACTGCAGACTACTGGTGGTGGATCCCCGTGGCGGGGCCCATGGTGGGCGGAGTGGTCGCA
GCCGTCATCTACTACCTGCTGATCGAGCTGCACCACCACCGT------------------
---GATGAGCCTGAGAAGCCCCATGAG---GAGGAGGAGGAGGAG------------GAG
GAAGACGAAGAGGACGACGACAGCAGTCTGAAGGACAAATATGAGATGATCACCATGAGC
>Minor_flag_rockfish_Aqp9a

---------------ATGGAAACACAACAAAAGAGAAGCATGAGGCAACACTGTGCTCTC
AAACATGGAATATTCAAGGAATTCCTGGCAGAATTCCTGGGAACGTTTGTCTTGGTTCTG
TTTGGCTGTGGCTCCGTCGCTCAGAGCGTCCTCAGTCGAAACACACTGGGTGAGCCTTTC
ACCGTCCACATCGGCTTCTCCGTGGGACTGATGATGGCGGCGTACGTGGCCGGTGGAGTG
TCAGGGGGCCACGTGAACCCTGCTGTGTCCCTGGCCATGGTGATCCTGGGCAAACTGAAG
ATCTGGAAGTTTCCCTTCTACGTCATCGCTCAGTTTCTTGGTGCTTTTGCTGGAGCTGCT
GCAGTCTTTGGATTATACCACGATGCTTTCATGGACTTCACCAGTGGGATTCTGTCCGTG
ACGGGAATCAATGCAACAGGTCACATTTTTGCATCCTACCCTGCGAGACACCTGTCAGTC
CTCGGCGGCTTCGTTGACCAGGTGGTGGGGACGGGTATGCTGGTCCTGTGTATCCTGGCT
ATTATTGATGGTGAAAACACCGGCACTCCCAAAGGCGTGCAGCCGCTGGCTATTGGTCTG
ATCATCATGGCCATCGGCGTCTCCATGGGACTGAACTGTGGCTACCCGCTGAACCCGGCC
AGAGACCTGGGACCCCGACTGTTCACAGCTGTGGCGGGCTGGGGGATGGAGGTCTTCAGG
ACTGCAAACTACTGGTGGTGGATCCCCGTGGCGGGGCCCATGGTGGGGGGCGTGGTCGCC
GCCGTCATCTACTATCTGTTCATCGAGCTGCACCACCACCGC------------------
---GAGGAGCCCGAGAAGCCCCACGAG---GAGGAGGAAGAGGAG------------GAG
GAAGACGAGGATGACGAGGACAGCAGCCTGAAGGACAAATATGAGATGATCACCATGAGC
>Flag_rockfish_Aqp9a
---------------ATGGAAACACAACAAAAGAGAAGCATGAGGCAACACTGTGCTCTC
AAACATGGAATACTCAAGGAATTCCTGGCAGAATTCCTGGGAACGTTTGTCTTGGTTCTG
TTTGGCTGTGGCTCCGTCGCTCAGAGCGTCCTCAGTCGAAACACACTGGGTGAGCCTCTC
ACCGTCCACATCGGCTTCTCCGTGGGACTGATGATGGCGGCGTACGTGGCCGGTGGAGTG
TCAGGGGGCCACGTGAACCCTGCTGTGTCCCTGGCCATGGTGATCCTGGGCAAACTGAAG
ATCTGGAAGTTTCCCTTCTACGTCATCGCTCAGTTTCTTGGTGCTTTTGCTGGAGCTGCT
GCAGTCTTTGGATTATACCACGATGCTTTCATGGACTTCACCAGTGGGATTCTGTCCGTG
ACGGGAATCAATGCAACAGGTCACATTTTTGCTTCCTACCCTGCGAGACACCTGTCAGTC
CTCGGCGGCTTCGTTGACCAGGTGGTGGGGACGGGTATGCTGGTCCTGTGTATCCTGGCT
ATTATTGATGGTGAAAACACCGGCACTCCCAAAGGCGTGCAGCCGCTGGCTATTGGTCTG
ATCATCATGGCCATCGGCGTCTCCATGGGACTGAACTGTGGCTACCCGCTGAACCCGGCC
AGAGACCTGGGACCCCGACTGTTCACAGCTGTGGCGGGCTGGGGGATGGAGGTCTTCAGG
ACTGCAAACTACTGGTGGTGGATCCCCGTGGCGGGGCCCATGGTGGGGGGCGTGGTCGCC
GCCGTCATCTACTATCTGTTCATCGAGCTGCACCACCACCGC------------------
---GAGGAGCCCGAGAAGCCCCACGAG---GAGGAGGAAGAGGAG------------GAG
GAAGACGAGGATGACGAGGACAGCAGCCTGAAGGACAAATATGAGATGATCACCATGAGC
>Tiger_rockfish_Aqp9a
---------------ATGGAAACACAACAAAAGAGAAGCATGAGGCAACACTGTGCTCTC
AAACATGGAATACTCAAGGAATTCCTGGCAGAATTCCTGGGAACGTTTGTCTTGGTTCTG
TTTGGCTGTGGCTCCGTCGCTCAGAGCGTCCTCAGTCGAAACACACTGGGTGAGCCTCTC
ACCGTCCACATCGGCTTCTCCGTGGGACTGATGATGGCGGCGTACGTGGCCGGTGGAGTG
TCAGGGGGCCACGTGAACCCTGCTGTGTCCCTGGCCATGGTGATCCTGGGCAAACTGAAG
ATCTGGAAGTTTCCCTTCTACGTCATCGCTCAGTTTCTTGGTGCTTTTGCTGGAGCTGCT
GCAGTSTTTGGATTATACCACGATGCTTTCATGGACTTCACCAGTGGGATTCTGTCCGTG
ACGGGAATCAATGCAACAGGTCACATTTTTGCTTCCTACCCTGCGAGACACCTGTCAGTC
CTCGGCGGCTTYGTTGACCAGGTGGTGGGGACGGGTATGCTGGTCCTGTGTATCCTGGCT
ATTATTGATGGTGAAAACACCGGCACTCCCAAAGGCGTGCAGCCGCTGGCTATTGGTCTG
ATCATCATGGCCATCGGCGTCTCCATGGGACTGAACTGTGGCTACCCGCTGAACCCGGCC
AGAGACCTGGGACCCCGACTGTTCACAGCTGTGGCGGGCTGGGGGATGGAGGTCTTCAGG
ACTGCAAACTACTGGTGGTGGATCCCCGTGGCGGGGCCCATGGTGGGGGGCGTGGTCGCC
GCCGTCATCTACTATCTGTTCATCGAGCTGCACCACCACCGC------------------
---GAGGAGCCCGAGAAGCCCCACGAG---GAGGAGGAAGAGGAG------------GAG
GAAGACGAGGATGACGAGGACAGCAGCCTGAAGGACAAATATGAGATGATCACCATGAGC
>3_spine_stickleback_Aqp9a
---------------ATGGAAATACAACACAAGAGAAGCATGAGGCAACACTGTGCGATC
AAACATGGAATACTCAAGGAATTCCTGGCAGAATTCCTGGGGACGTTTGTCTTGGTTTTG
TTTGGCTGTGGCTCAGTCGCTCAGACCGTCCTCAGCCGAAACAGCCTGGGCGAACCTCTC
ACCGTCCACATCGGCTTCTCCGTGGGACTGATGATGGCGGCGTACGTGGCCGGCGGAGTG
TCGGGGGGGCACGTGAACCCCGCTGTGTCCCTGGCCATGGTGGTTCTGGGCAAACTGAAG
ATTTGGAAGTTTCCCTTCTACGTCATCGCTCAGTTTCTTGGTGCTTTTGCGGGAGCGGCT
GCAGTCTTTGGATTGTACTACGATGCTTTCATGGACTTCACCAGTGGGATTCTGTCAGTG
ACGGGAATCAATGCAACGGGTCACATTTTTGCTTCCTACCCTGCGAGACACCTGTCAGTC
CTCGGCGGCTTCATAGATCAGGTGGTGGGCACGGGGATGCTGGTCCTGTGTATCTTGGCC
ATCATCGACGGGGAGAACATCGGCGCGCCCAAAGGCGTGCAGCCGCTGGCTATTGGTCTG

ATCATCATGGCCATTGGTGTTTCTATGGGGCTGAACTGCGGTTACCCCCTGAACCCTGCC
AGAGACCTGGGACCACGACTGTTCACCGCTGCAGCAGGATGGGGGATGGAGGTCTTCAGG
ACTGCAAACAACTGGTGGTGGATCCCTGTGGCGGGGCCCATGGTGGGGGGGGTGCTGGCG
GCCGCCATCTACTACCTGCTTATCGAGGTGCACCACCACCGC------------------
---GAAGCGCCCGAGAAGCCCCGCGAG---GAAGAGGAGGAG----------------GAG
GAAGAGGAGGAGGACGAGGACAGCAGTCTGAAGGACAAATACGAGATGATCACGATGAGC
>9_spine_stickleback_Aqp9a
---------------ATGGAAGTACAACACAAGAGAAGCATGAGGCGACACTGTGCGATC
AAGCATGGAATACTCAAGGAGTTCCTGGCAGAATTCCTGGGGACATTTGTCTTGGTTTTG
TTCGGCTGCGGCTCAGTCGCTCAGACCGTCCTCAGCCGAAACAGCCTGGGCGAACCCCTC
ACCGTCCACATCGGCTTCTCCGTGGGGCTGATGATGGCGGCGTACGTGGCCGGCGGAGTG
TCCGGGGGCCACGTGAACCCTGCCGTGTCCCTGGCCATGGTGGTTCTGGGCAAACTGAAG
ATTTGGAAGTTTCCCTTCTACGTCATGGCTCAGTTTCTCGGTGCTTTTGCGGGAGCCGCT
GCAGTCTTTGGGTTATACTACGATGCTTTCATGGACTTCACCAGTGGGATTCTGTCAGTG
ACGGGAATCAATGCAACGGGTCACATTTTTGCTTCCTACCCTGCGAGACACCTGTCAGTC
CTCGGCGGCTTAATTGATCAGGTGGTGGGCACCGGGATGCTGGTCCTGTGCATCTTGGCC
ATCATCGATGGGGAGAACATCGGCGCTCCCAAAGGCGTGCAGCCGCTGGCCATCGGTCTG
ATCATCATGGCCATTGGTGTGTCCATGGGGCTGAACTGCGGTTACCCCCTGAACCCCGCC
AGAGACCTGGGACCACGGCTGTTCACCGCTGCAGCCGGGTGGGGGATGGAGGTCTTCAGG
ACTGCAAACAACTGGTGGTGGATCCCTGTGGCGGGGCCCATGGTGGGGGGGGTGCTGGCG
GCCGTCGTCTACTACCTGCTCATCGAGGTGCACCACCACCGC------------------
---GAAGCGCCCGAGAAGCCCCGCGAG---GAAGAGGAGGAGGAG-------------GAA
GAGGAGGACGAGGACGAGGACAGCAGCCTGAAGGACAAATATGAGATGATCACGATGAGC
>Sablefish_Aqp9a
---------------ATGGAAGTACAACACAAGAGAAGCATGAGGCAACACTGTGCTCTC
AAACATGGAATACTCAAGGAATTCCTGGCAGAATTCCTGGGGACATTTGTCTTGGTTTTG
TTTGGCTGTGGCTCAGTCGCTCAGACCGTCCTCAGTCGAAACAGCCTGGGTGAGCCTCTC
ACCGTCCACATCGGCTTCTCCGTGGGACTGATGATGGCGGCGTACGTGGCCGGTGGAGTG
TCAGGGGGCCATGTGAACCCTGCCGTGTCTCTGGCCATGGTGATTCTGGGTAAACTGAAG
ATCTGGAAGTTTCCCTTCTATGTCATTGCTCAGTTTCTTGGTGCTTTTGCCGGAGCTGCT
GCGGTCTTTGGATTATACTATGATGCTTTCATGGACTTCACCAGTGGGATTCTGTCTGTG
ACGGGAATCAATGCAACAGGTCACATTTTTGCTTCCTACCCTGCGAGGCACCTGTCAATC
CTTGGCGGCTTCATTGATCAGGTGGTGGGTACAGGTATGCTGGTCCTGTGTATCTTGGCT
ATTATTGATGGTGAAAACATCGGAGCTCCAAAAGGCGTGCAGCCGCTGGCTATTGGTCTG
ATCATCATGGCCATCGGTGTGTCCATGGGGCTGAACTGCGGCTACCCGCTGAACCCCGCC
AGAGACCTGGGACCCCGACTGTTCACAGCTGCAGCAGGATGGGGGATGGAGGTCTTCAGG
ACTGCAGACAACTGGTGGTGGATCCCTGTGGCGGGGCCCATGGTGGGGGGCGTGGTGGCC
GCAGTCATCTACTACCTGCTCATCGAGGTGCACCACCACCGC------------------
---GACGCGCCCGAGAAGCCCAATGAG---GAAGAGGAGGAGGAG-------------GAG
GAAGATGAGGAGGACGAGGACAGCAGTCTGAAGGACAAATATGAGATGATCACGATGAGC
>Rhine_sculpin_Aqp9a
---------------ATGGAAACACAACACAAGAGAAGCATGAGGCAACACTGTGCTCTC
AAACATGGAATACTCAAGGAATTCCTGGCAGAATTCCTGGGGACATTTGTCTTGGTTTTG
TTCGGCTGTGGCTCCGTCGCTCAGACCGTC------------------------------

---GGGGGCCATGTGAACCCTGCTGTGTCTCTGGCCATGGTGATTCTGGGCAAGCTGAAG
ATTTGGAAGTTTCCCTTCTACGTCATTGCTCAGTTTCTTGGTGCTTTTGCGGGAGCTGCT
GCAGTCTTTGGATTATACTATGATGCTTTCATGGACTTCACCAGTGGGATTCTGTCAGTG
ACGGGAATCAATGCAACAGGTCACATTTTCGCTTCCTACCCTGCGAGACACCTGTCCATC
CTCGGCGGCTTCATCGATCAGGTGGTGGGGACAGGTATGCTGGTCCTGTGCATCTTGGCT
ATTATTGATGGTGAAAACATCGGAGCTCCCAAAGGCGTGCAGCCGCTGGCCATCGGCCTG
ATCATCATGGCCATCGGCGTGTCCATGGGTCTGAACTGTGGCTACCCCCTGAACCCCGCC
AGAGACCTGGGACCCCGACTGTTCACAGCTGCAGCCGGATGGGGGATGGAGGTCTTCAGG
ACTGCAGACAACTGGTGGTGGATCCCCGTGGCGGGGCCCATGGTGGGAGGCGTGGTGGCC
GCCGTCATCTACTACCTGTTCATCGAGGTGCACCACCACCGC------------------
---GATGCGCCAGAGAAACCCCTT---GTGGAGGAGGAGGAGGAG-------------GAG
GAAGACGAGGACGATGAGGACAGCAGTCTGAAGGACAAATATGAGATGATCACGATGAGC
>European_perch_Aqp9a
---ATGAGGCAACACTGTGCTATC
AAACATGGGATACTCAAGGAATTCCTGGCAGAATTCCTGGGGACATTTGTCTTGGTCTTG
TTTGGATGTGGCTCAGTCGCTCAGACCGTCCTTAGTCGAAACACCCTGGGTGAGCCTCTC

ACCGTCCACATCGGCTTCTCTGTGGGACTGATGATGGCAGCGTACGTGGCCGGTGGAGTG
TCAGGGGGGCATGTGAACCCTGCTGTGTCTCTGGCCATGGTGGTTCTGGGCAAACTGAAG
ATCTGGAAGTTTCCCTTCTATGTCATTGCTCAGTTTCTTGGTGCTTTTGTTGGGGCTGCT
GCAGTTTTTGGATTATACCATGATGCTTTCATGGACTTCACCAGTGGGATTTTGTCAGTG
ACGGGAATCAACGCAACAGGTCACATTTTTGCTTCCTACCCTTCGAGACACCTGTCAGTC
CTCGGAGGCTTTATCGATCAGGTGATGGGGACAGGTATGCTGGTCCTGTGTATCCTGGCT
ATTATTGATGGCGGAAACATTGGAGCTCCCAAAGGTGTGGAGCCGCTGGCTATTGGTCTG
GTCGTTATGGCCATCGGTGTGTCCATGGGACTGAACTGTGGCTACCCCCTGAACCCTGCC
AGAGACCTGGGACCCCGACTGTTCACAGCTGTAGCCGGATGGGGATGGAGGTCTTCAGC
ACTGCAGACAACTGGTGGTGGATCCCTGTGGCGGGGCCCATGGTGGGGGGGCTGGTTGCA
GCCCTCATCTACTATCTGCTCATTGAGCTGCACCACCAGCGC------------------
---GATGAGCCCGAAAAGCCCCAGGAG---GAGGAGTTGGAGGAA------------GAA
GAAGACGAGGATGAAGAGGACATCAGTCTGAAGGACAAATATGAGATGATCACCATGAGC
>Blackfin_icefish_Aqp9a
---------------ATGGAGAAGAAACAGAAGAAAAGCATGAGGCAACGCTGTGCTCTC
AAACATGGAATATTCAAGGAATTCCTGGCAGAATTCCTGGGGACATTTGTCTTGGTTTTG
TTCGGCTGTGGCTCAGTCGCTCAGACCGTCCTCAGTCGAAACACCCTGGGCGAGCCTCTC
ACCGTCCACATCGGCTTCTCCGTGGGCCTGATGATGGCGTCGTACGTGGCCGGTGGAGTG
TCAGGGGGCCATGTGAACCCTGCTGTTTCCCTGGCCATGGTGATTCTGGGCAAACTGAAG
ATCTGGAAGTTCCCCTTCTACGTCCTTGCTCAGTTTCTTGGTGCTTTTTCTGGAGCTGCT
GCAGTCTTTGGGTTATACCATGATTCTTTCATGGACTTCACCAGTGGGATTCTGTCAGTC
ACAGGAATCAATGCAACAGGCCACATTTTTGCTTCCTACCCTGCGAGACACCTGACGGTC
CTCGGCGGCTTCATCGATCAGGTGGTGGGGACGGGCATGCTGGTGCTGTGCATTCTGGCT
ATCATTGACGACGGGAACATCGGAGCTCCTAAAGGCGTGCAGCCGCTGGCCATCGGTCTG
ATCATCATGGCCATCGGTGTGTCCATGGATCTGAACTGTGGGTACCCCCTGAACCCCGCC
AGAGACCTGGGCCCCCGGCTCTTCACAGCCGTAGCAGGATGGGGATGGAGGTCTTCAGC
ACCGGAAACCACTGGTGGTGGATCCCTGTGGTGGGGCCCATGGTGGGGGGGGTGGTCGCC
GCCGTCGTCTACTACGCGTTCATCGAGCTGCACCACCCCCGC------------------
---GCCGAGCCCGAGCCGACCCACGAGGTGGAGGAGGAAGAGGAGGAA---------GAG
GAGGAAGACGATGATGAGGGGAACAGCATCAAGGACAAATATGAGATGATCACCATGAGC
>Black_rockcod_Aqp9a
---------------ATGGAGAAAAAACAGAAGAAAAGCATGAGGCAACGCTGTGCTCTC
AAACATGGAATATTCAAGGAATTCCTGGCAGAATTCCTGGGGACATTTGTCTTGGTTTTG
TTCGGCTGTGGCTCAGTCGCTCAGACCGTCCTCAGTCGAAACACCTTGGGCGAGCCTCTC
ACCGTCCACATCGGCTTCTCCGTGGGCCTGATGATGGCGGCGTACGTGGCCGGTGGAGTG
TCAGGGGGCCATGTGAACCCTGCTGTTTCCCTGGCCATGGTGATTCTGGGCAAACTGAAG
ATCTGGAAGTTCCCCTTCTACGTCCTTGCTCAGTTTCTTGGTGCTTTTGCTGGAGCTGCT
GCAGTCTTTGGATTATACTATGATTCTTTCATGGACTTCACCAGTGGGATTCTGTCAGTC
ACAGGAATCAATGCAACAGGCCACATTTTTGCTTCCTACCCTGCGAGACACCTGACGGTC
CTCGGCGGCTTCATCGATCAGGTGGTGGGGACGGGCATGCTGGTGCTGTGTATTCTGGCT
ATCATTGACGACGGGAACATCGGAGCTCCTAAAGGCGTGCAGCCGCTGGCCATCGGTATG
ATCATCATGGCCATCGGTGTGTCCATGGGGCTGAACTGTGGGTACCCCCTGAACCCCGCC
AGAGACCTGGGCCCCCGGCTCTTCACAGCCGTAGCAGGATGGGGATGGAGGTCTTCAGC
ACCGGAAACCACTGGTGGTGGATCCCTGTAGTGGGGCCCATGGTGGGGGGGGTGGTCGCC
GCCGTCGTCTACTACGCGTTCATCGAGCTGCACCACCCCCGC------------------
---ACCGAGCCCGAGCCGACCCACGAGGTGGAGGAGGAAGAGGAGGAA---------GAG
GAGGAAGAGGATGATGAGGGGAACAGCATCAAGGACAAATATGAGATGATCACCATGAGC
>Channel_bull_blenny_Aqp9a
---------------ATGGAAATAAAAGAGAAGAGAAGCATGAGGCAACACTGTGCTCTC
AAACATGGAATACTCAAGGAATTCCTGGCAGAATTCCTGGGGACATTTGTCTTGGTTTTG
TTTGGCTGTGGCTCAGTCGCTCAGACTGTCCTCAGTCGGAACACCCTGGGTGAGCCTCTC
ACCGTCCACATCGGCTTCTCTGTAGGACTGATGATGGCAGCGTACGTGGCCGGTGGAGTG
TCAGGGGGCCATGTGAACCCTGCTGTTTCTCTGGCCATGGTGATTCTGGGCAAACTGAAG
ATCTGGAAGTTTCCCTTCTATGTCATTGCTCAGTTTCTTGGTGCTTTTACTGGAGCTGCT
GCAGTATTTGCATTATACTATGATGCCTTCATGGACTTCACCAGTGGGATTCTGTCAGTG
ACGGGCATCAATGCAACAGGTCACATTTTTGCTTCCTACCCTGCGAGACACCTGTCAGTC
CTCGGCGGCTTCATCGATCAGGTGATGGGGACAGGTATGCTGGTGCTGTGTATTCTGGCA
ATTATTGATGGTGAAAACATCGGCGCTCCCAAAGGCGTGGAGCCGCTGGCTATCGGTCTG
ATCGTCATGGCCATCGGTGTGTCCATGGGGCTGAACTGTGGCTACCCCCTGAACCCCGCC
AGAGACCTGGGACCCCGACTGTTCACAGCTGTAGCAGGATGGGGATGGAGGTCTTCAGC
ACTGGAGACTACTGGTGGTGGATTCCTGTGGCGGGGCCCATGGTGGGGGGCGTGGTCGCA

GCCGTCATCTACTACCTGCTCATTGAGCTGCACCACCCCCGC------------------
---GAGGAGCCCGAGCAGCCCCATGAG---GAGGAGGAAGAAGAG------------GAG
GAAGAGGAGGACGAGGAGGAC---AGCATGAAGGACAAATATGAGATGATCACCATGAGC
>Ballan_wrasse_Aqp9a
---------------ATGGAAAAAAATCACAAGAGAAGCATGAGGCAACACTGTGCTCTC
AAACATGGAATACTTAAGGAATTCCTGGCAGAATTCCTGGGGACGTTTGTTTTGGTTCTG
TTCGGCTGTGGCTCCGTCGCTCAGACCGTCCTCAGTCGAAACACTCTGGGTGAGCCTCTC
ACCATCCACATCGGCTTCTCTGTGGGACTGATGATGGCGGCCTACGTGGCCGGTGGAGTG
TCAGGGGGCCATGTTAACCCTGCTGTGTCTCTGGCCATGGTGATTCTTGGAAAACTGAAG
ATCTGGAAGTTTCCCTTCTACGTCATAGCTCAATTTCTTGGCGCTTTCGCTGGAGCTGCT
GCAGTCTTTGGAATATACTATGATGCTTTCATGGACTTCACCAGTGGGATTCTGTCAGTG
ACAGGTATCAACGCAACAGGTCACATTTTTGCTTCTTACCCAGCGAGGCACCTGTCAGTC
CTAGGCGGCTTCATTGATCAGGTGGTGGGGACGGGCATGCTGGTCCTGTGTATCCTGGCT
ATCATCGACGGGGGAAACATTGGAGCTCCTAAAGGTGTGGAGCCGCTGGCCATCGGTCTG
ATCATCATGGCCATCGGTGTGTCCATGGGGCTGAACTGTGGGTACCCCCTGAACCCGGCC
AGAGACCTGGGGCCCCGACTGTTCACGGCGCTGGCAGGATGGGGGATGGAGGTGTTCAGG
ACCGGAGAGTACTGGTGGTGGATCCCCGTGGCCGGGCCCATGGTGGGCGGCGTGCTGGCA
GCCATCATCTACTACCTGCTCATCGAGCTGCACCACCAGCGC------------------
CATGACGAGCCTGAGAAGCCCCAT---GTGGAGGAGGAGGAAGAGGAG---------GAG
GAAGAGGAAGACGATGATGACAGCAGTCTTAAGGATAAATACGAGATGATCACCATGAGT
>Corkwing_Aqp9a
---------------ATGGAAAAACCCCACAAGAGAAGCATGAGGCAACATTGTGCTCTC
AAACATGGAATACTTAAGGAATTTCTGGCAGAATTCCTGGGGACGTTTGTTTTGGTTCTG
TTTGGCTGTGGCTCCGTCGCTCAGACCGTCCTCAGTCGAAACACTCTGGGTGAGCCTCTC
ACGATCCACATCGGCTTCTCTGTGGGACTGATGATGGCGGCTTACGTGGCCGGTGGAGTA
TCAGGGGGCCATGTAAACCCTGCTGTGTCTCTGGCCATGGTGATTCTTGGAAAGCTGAAG
ATCTGGAAGTTTCCCTTCTACGTCATCGCTCAGTTTCTTGGTGCTTTCGCTGGAGCCGCC
GCAGTCTTTGGAATATACTATGATGCTTTCATGGACTTCACCAGTGGGATTCTGTCAGTG
ACAGGAATCAATGCAACAGGTCACATTTTTGCTTCTTACCCAGCGAGGCACCTGTCAGTC
TTGGGCGGCTTCATTGATCAGGTGGTGGGGACGGGCATGCTGGTCCTGTGTATCCTGGCT
ATCATCGATGGGGGAAACATTGGAGCTCCTAAAGGTGTGGAGCCGCTGGCCATCGGTCTG
ATCATCATGGCCATCGGTGTGTCCATGGGGCTGAACTGTGGGTACCCCCTGAACCCAGCC
AGAGACCTGGGGCCCCGACTGTTTACAGCACTAGCAGGATGGGGGATGGAGGTGTTCAGG
ACGGGAGATAACTGGTGGTGGATCCCCGTGGCGGGGCCGATGGTGGGGGGCGTGCTGGCA
GCCGTGATCTACTACCTGCTCATCGAGCTGCACCACCATCAC------------------
CCCGACGAGCCTGAGAAGCCCCAT---GTTGAAGAAGAGGAAGAGGAG---------GAG
GAAGAGGAAGACGATGATGACAGCAGTCTTAAGGATAAATATGAGATGATCACCATGAGT
>White_bass_Aqp9a
--ATGAGGCAACACTGTGCTCTC
AAACATGGAATACTTAAGGAATTCCTGGCAGAATTCCTGGGAACATTTGTCTTGGTTCTG
TTTGGCTGTGGCTCAGTCGCTCAGACCGTCCTCAGTCGAAACAGCCTGGGTGAACCTCTC
ACCATCCACATCGGCTTCTCTGTGGGACTAATGATGGCAGCGTATGTGGCTGGTGGAGTG
TCAGGGGGCCATGTGAACCCTGCTGTGTCTCTGGCCATGGTGATTCTGGGAAAACTGAAG
ATCTGGAAGTTTCCCTTCTATGTCATCGCTCAGTTTCTTGGTGCTTTTGTTGGCGCTGCT
GCGGTCTTTGGGTTGTACTACGATGCTTTCATGGACTTCACTAGTGGGATTCTGTCAGTG
ACGGGAATCAATGCAACAGGTCACATTTTTGCTTCCTACCCTGCGAGACACCTGTCAGTC
CTCGGCGGCTTCATTGATCAGGTGATGGGGACGGGCATGCTGGTCTTGTGTATCCTGGCT
ATCATTGATGGTGGAAACATTGGAGCTCCCAAAGGCGTGGAACCGCTGGCCATTGGTCTG
ATCATTATGGCCATCGGTGTGTCCATGGGACTGAACTGTGGCTACCCCCTGAACCCTGCC
AGAGACCTGGGACCCCGACTGTTCACAGCTTTAGCAGGATGGGGGATGGAGGTCTTCAGC
ACTGCAGACTACTGGTGGTGGATCCCCGTGGCGGGGCCCATGGTGGGGGGCGTGGTCGCA
GCCATCATCTACTACCTGTTCATTGAGCTGCACCACCACCAT------------------
---GATGAGCCTGAGAAGCCC---------------------------------------

>Striped_seabass_Aqp9a
--ATGAGGCAACACTGTGCTCTC
AAACATGGAATACTTAAGGAATTCCTGGCAGAATTCCTGGGAACATTTGTCTTGGTTCTG
TTTGGCTGTGGCTCAGTCGCTCAGACCGTCCTCAGTCGAAACAGCCTTGGTGAACCTCTC
ACCATCCACATCGGCTTCTCTGTGGGACTAATGATGGCAGCGTATGTGGCTGGTGGAGTG
TCAGGGGGCCATGTGAACCCTGCTGTGTCTCTGGCCATGGTGATTCTGGGAAAACTGAAG
ATCTGGAAGTTTCCCTTCTATGTCATCGCTCAGTTTCTTGGTGCTTTTGTTGGCGCTGCT

GCGGTCTTTGGGTTGTACTATGATGCTTTCATGGACTTCACCAGTGGGATTCTGTCAGTG
ACGGGAATCAATGCAACAGGTCACATTTTTGCTTCCTACCCTGCGAGACACCTGTCAGTC
CTCGGTGGCTTCATCGATCAGGTGATGGGGACGGGTATGCTGGTCTTGTGTATCCTGGCT
ATCATTGATGGTGGAAACATTGGAGCTCCCAAAGGCGTGGAACCGCTGGCCATTGGTCTG
ATCATCATGGCCATCGGTGTGTCCATGGGACTGAACTGTGGCTACCCCCTGAACCCTGCC
AGAGACCTGGGACCCCGACTGTTCACAGCTTTAGCAGGATGGGGATGGAGGTCTTCAGG
ACTGCAGACTACTGGTGGTGGATCCCCGTGGCGGGGCCTATGGTGGGGGGCGTGGTCGCA
GCCATCATCTACTACCTGTTCATTGAGCTGCACCACCACCGT------------------
---GATGAGCCTGAGAAGCCCCATGAG---GAGGAGGAAGAAGAG------------GAG
GAAGATGAAGAGGACGAGGACAGCAGTCTGAAGGACAAATATGAGATGATCACCATGAGC
>European_seabass_Aqp9a
--ATGAGGCAACACTGTGCTCTC
AAACATGGAATATTTAAGGAATTCCTGGCAGAATTCCTAGGAACATTTGTCTTGGTTCTG
TTTGGCTGCGGCTCAGTCGCTCAGACCGTCCTCAGTCGAAACAGCCTGGGTGAACCTCTC
ACCGTCCACATCGGCTTCTCTGTGGGACTGATGATGGCAGCGTATGTGGCTGGTGGAGTG
TCAGGGGGCCATGTGAACCCTGCTGTGTCTCTGGCCATGGTGATTCTGGGAAAACTGAAG
ATCTGGAAGTTTCCCTTCTATGTCATCGCTCAGTTTCTTGGTGCTTTTGTTGGCGCTGCT
GCAGTCTTTGGGTTGTACTATGATGCTTTCATGGACTTCACCAGTGGGATTCTGTCAGTG
ACGGGAATCAATGCAACAGGTCACATTTTTGCTTCCTACCCTGCGAGACACCTGTCAGTC
ATCGGCGGCTTCATCGATCAGGTGATAGGGACGGGTATGCTGGTCTTGTGTATACTGGCT
ATCATTGATGGTGGAAATATCGGAGCTCCCAAAGGCGTGGAACCGCTTGCCATTGGTCTG
ATCATCATGGCCATCGGTGTGTCCATGGGGCTGAACTGTGGCTACCCCCTGAACCCTGCC
AGAGACCTGGGACCCCGACTGTTCACAGCTTTAGCAGGATGGGGAATGGAGGTCTTCAGG
ACTGCAGACTACTGGTGGTGGATCCCTGTGGCGGGGCCCATGGTGGGGGGCGTGGTCGCA
GCCATCATCTACTACCTGTTTATTGAGCTGCACCACCACCGT------------------
---GATGAGCCCGAGAACCCCCACGAG---GAGGAGGAAGAAGAG------------GAG
GAAGATGAAGAGGACGAGGACAGCAGTCTGAAGGACAAATATGAGATGATCACCATGAGC
>Large_yellow_croaker_Aqp9a
--ATGAGGCAACACTGTGCTCTC
AAACATGGAATACTCAAGGAATTCTTGGCAGAATTCCTGGGGACGTTTGTCTTGGTTTTG
TTTGGCTGTGGCTCGGTCGCTCAGACCGTCCTCAGTCGAAACACCCTCGGGGAGCCTCTC
ACCGTCCACATTGGCTTCTCTGTTGGACTGATGATGGCGGCCTATGTGGCTGGTGGAGTG
TCAGGGGGCCACGTGAACCCTGCTGTGTCCCTGGCTATGGTGATTCTGGGCAAACTGAAG
ATCTGGAAGTTTCCCATCTATGTCATCGCTCAGTTTCTTGGTGCTTTTGTTGGAGCTGCT
GCAGTCTTTGGGTTATACTATGATGCTTTCATGGACTTCACCAGCGGGATTCTGTCAGTG
ACAGGAATCAATGCAACAGGTCATATTTTTGCTTCCTACCCTGCGAGACACCTGTCAGTC
CTCGGCGGCTTCGCCGATCAGGTGATAGGGACAGGTATGCTGGTGTTGTGTATCTTGGCT
ATCATTGATGGTGGAAACATCGGAGCTCCCAAAGGTGTTGAACCGCTGGCCATTGGTCTG
ATCATCATGGCCATTGGTGTGTCGATGGGACTGAACTGTGGCTACCCACTGAACCCTGCC
AGAGACCTGGGACCCCGTGTGTTCACAGCTATAGCAGGATGGGGGTTTGAGGTCTTCAGC
CCCGCCGGCCACTGGTGGTGGATACCTGTGGCAGGGCCGATGGTGGGCGGCGTGGTCGCA
GCCGTCATTTACTATCTGTTCATTGAGCTGCACCACCACCGT------------------
---GATGAGCCTGAGAAGCCCCACGAG---GAAGAGGAAGAGGAG------------GAG
GAAGACGAAGAGGACGATGACAGCAGTCTGAAGGACAAATATGAGATGATCACCATGAGC
>Miiuy_croaker_Aqp9a
--ATGAGGCAGCACTGTGCTCTC
AAACATGGAATACTCAAGGAATTCTTGGCAGAATTCCTGGGGACGTTTGTCTTGGTTTTG
TTTGGCTGCGGCTCGGTCGCTCAGACCGTCCTCAGTCGAAACACCCTCGGTGAGCCTCTC
ACCGTCCACATTGGCTTCTCTGTTGGACTGATGATGGCAGCCTATGTGGCTGGCGGAGTG
TCAGGGGGCCATGTGAACCCTGCTGTGTCCCTGGCCATGGTGATTCTGGGCAAACTGAAG
ATCTGGAAGTTTCCCATCTATGTCATCGCTCAGTTTCTTGGTGCTTTTGCTGGAGCTGCT
GCAGTCTTTGGGTTATACTATGATGCTTTCATGGACTTCACCAGTGGGATTCTGTCAGTG
ACGGGAATCAATGCAACAGGTCATATTTTTGCTTCCTACCCTGCGAGACACCTGTCAGTC
CTCGGCGGCTTCGTCGATCAGGTGATAGGGACAGGTATGCTGGTGTTGTGTATCTTGGCT
ATCATTGATGGTGGAAACATCGGAGCTCCCAAAGGTGTTGAACCGCTGGCCATTGGTCTG
ATCATCATGGCCATTGGTGTGTCGATGGGACTGAACTGTGGCTACCCACTGAACCCTGCC
AGAGACCTGGGACCCCGTGTGTTCACAGCTATAGCAGGATGGGGGTTGGAGGTCTTCAGG
ACCGCAAACCACTGGTGGTGGATCCCTGTGGCAGGGCCGATGGTGGGCGGCGTGGTCGCA
GCCGTCATTTACTACCTGCTCATTGAGCTGCACCACCACCGT------------------
---GATGAGCCTGAGAAGCCCAACGGG---GAAGAGGAAGAGGAG------------GAT
GAAGACGAAGAGGACGATGACAGCAGTCTGAAAGACAAATATGAGATGATCACCATGAGC

>Florida_bass_Aqp9a
---------------ATGGAAATACAACAAAAGCCAAGCATGAGGCAACACTGTGCTCTC
AAACATGGAATACTCAAGGAATTCCTGGCAGAATTCCTGGGGACATTTGTCTTGGTTTTG
TTTGGCTGTGGCTCAGTCGCTCAGACTACCCTCAGTCGAAACACCCTGGGTGAGCCTCTC
ACCGTCCACATTGGCTTCTCTGTAGGGCTGATGATGGCAGCGTATGTTGCTGGTGGAGTG
TCAGGGGGCCATGTGAACCCTGCAGTGTCTCTGGCCATGGTGATTCTGGGCAAACTGAAG
ATCTGGAAGTTTCCCTTCTATGTCATCGCTCAGTTTCTTGGAGCTTTTGCTGGAGCTGCT
GCAGTCTTCGGGTTATACTATGATGCTTTCATGGACTTCACCAGTGGGGTTCTGTCAGTG
ACAGGAATCAATGCAACAGGTCACATCTTTGCTTCCTACCCTGCGAGACACCTGTCGGTC
CTCGGCGGCTTCATCGATCAGGTGGTGGGGACAGGTATGCTGGTCTTGTGCATCCTGGCT
ATCATTGATGATGAAAACACTGGAGCTCCCAAAGGTGTGCAGCCACTGGCTATTGGTCTG
ATCATCATGGCCATTGGTGTGTCCATGGGACTGAACTGTGGCTACCCCCTCAACCCTGCC
AGAGACCTGGGACCCCGACTGTTCACAGCTGTAGCAGGATGGGGCATGGAGGTCTTCAGG
ACTGCAGACAACTGGTGGTGGATCCCTGTGGCAGGGCCCATGGTGGGGGGCGTGGTCGCA
GCCGTCATCTACTACCTGCTCATTGAGCTGCACCACCACCGT------------------
---GACGAGCCTGAGAAGCCCCACGAG---GAGGAAGACGAG---------------GAG
GAAGACGAGGAGGACGAGGATATCAGTCTGAAGGACAAATATGAGATGATCACCATGAGC
>Murray_cod_Aqp9a
---------------ATGGAAATACAACACAAGAGAAGCATGAGACAACACTGTGCTCTC
AAACATGGAATACTCAAGGAATTCCTGGCAGAATTCCTGGGGACATTTGTCCTGGTTTTG
TTTGGCTGTGGCTCAGTCGCTCAGACCGTCCTCAGTCGAAACACCCTGGGTGAGCCTCTC
ACCGTCCACATCGGCTTCTCTGTAGGACTGATGATGGCAGCGTATGTGGCCGGTGGAGTG
TCAGGGGGCCATGTGAACCCTGCTGTGTCTCTGGCCATGGTGATTCTGGGCAAACTGAAG
ATCTGGAAGTTTCCCTTCTATGTCATCGCTCAGTTTCTTGGTGCTTTTGTTGGAGCGGCT
GCAGTCTTTGGGTTATACTACGATGCTTTCATGGACTTTACCAGTGGGATTCTGTCAGTG
ACGGGAATAAATGCAACAGGTCACATTTTTGCTTCCTACCCTGCGAGACACCTGTCTATC
CTCGGCGGCTTCATCGATCAGGTGGTGGGGACAGGTATGCTGGTCTTGTGTATCCTGGCT
ATCATTGATGGTGGAAACATTGGAGCTCCCAAAGGTGTGGAGCCGCTGGCTATTGGTTTG
ATCATTATGGCCATTGGCGTGTCCATGGGACTGAACTGTGGCTACCCCCTGAACCCTGCC
AGAGACCTGGGACCCCGACTGTTCACAGCTGTAGCAGGATGGGGGCTGGAGGTCTTCAGG
ACTGCAGACTACTGGTGGTGGATCCCTGTGGCGGGACCCATGGTGGGGGGCGTGGTCGCG
GCCGTCATCTACTACCTGCTCATTGAGCTGCACCACCACCGT------------------
---GATGAGCCTGAGAAGCCCCACGAG---GAGGAGGACGAGGAG------------GAG
GAAGAGGAGGAGGACGAGGACATCAGTCTGAAGGACAAATATGAGATGATCACCATGAGC
>Barred_knifejaw_Aqp9a
---------------ATGGAAATACAACACAAGAGAAGCATGAGGCAACACTGTGCTCTC
AAACATGGGATACTCAAGGAATTCCTGGCAGAATTCCTGGGGACATTTGTCTTGGTTTTG
TTTGGCTGTGGCTCAGTCGCTCAGACCGTCCTCAGTCGAAACACCCTGGGCGAGCCTCTC
ACCGTCCACATTGGCTTCTCTGTAGGACTGATGATGGCGGCGTATGTGGCTGGTGGAGTG
TCAGGGGGCCACGTGAACCCTGCTGTGTCTCTGGCCATGGTGATTCTGGGCAAGCTGAAG
ATCTGGAAGTTTCCCTTCTATGTCATCGCTCAGTTTCTTGGTGCTTTCGCTGGAGCTGCT
GCAGTCTTTGGGTTATACTACGATGCTTTCATGGACTTCACCAGTGGGATTCTGTCTGTG
ACGGGAATCAATGCAACAGGTCACATTTTTGCGTCCTACCCTGCGAGACACCTGTCAGTC
CTCGGCGGCTTCATCGATCAGGTGGTGGGGACGGGTATGCTGGTCTTGTGTATCCTGGCT
ATCATTGATGGCGGAAACATCGGAGCTCCCAAAGGTGTGGAGCCGCTGGCTATTGGTCTG
ATCATCATGGCCATTGGTGTGTCCATGGGACTGAACTGTGGCTACCCCCTGAACCCTGCC
AGGGACCTGGGACCCCGACTCTTCACAGCTGTAGCAGGATGGGGAATGGAGGTCTTCAGG
ACCGCAGACTACTGGTGGTGGATCCCTGTGGCGGGGCCCATGGTGGGTGGCGTGGTCGCA
GCCGTCATCTACTACCTGCTCATTGAGCTGCACCACCACCGC------------------
---GATGAGCCCGAGAAGCCCCATGAG---GAGGAGGAAGAG---------------GAG
GAAGACGAAGAAGACGACGACAGCAGTCTCAAGGACAAATACGAGATGATCACCATGAGC
>Korean_spotted_seabass_Aqp9a

-------------------------GGAATGATGATGGCAGCGTACGTGGCTGGTGGAGTT
TCAGGGGGCCATGTGAACCCTGCTGTGTCTCTGGCCATGGTGATTCTGGGAAAACTGAAG
ATCTGGAAGTTTCCCTTCTATGTCATCGCTCAGTTTCTTGGTGCTTTTGCTGGAGCTGCT
GCAGTTTTTGGATTATACTATGATGCTTTCATGGACTTCACTAGTGGGATTCTGTCAGTG
ACGGGAATCAATGCAACAGGTCACATTTTTGCTTCATACCCCGCGAGACACCTGTCAGTC
CTCGGCGGCTTCATTGATCAGGTGGTGGGCACAGGTATGTTGGTCTTGTGTATCCTGGCT

ATCATTGACGGTGGAAACATTGGAGCTCCCAAAGGTGTGGAGCCGCTGGCTATTGGTCTG
ATCATCATGGCAATTGGTGTGTCCATGGGACTGAACTGTGGCTACCCTCTGAACCCTGCA
AGAGACCTGGGACCCCGACTGTTCACAGCCGTAGCAGGATGGGGATGGAGGTCTTCAGC
ACTGCAGACTACTGGTGGTGGATCCCTGTGGCGGGGCCCATGGTGGGGGGCGTGGTCGCC
GCCGTCATCTACTACCTGCTCATCGAGCTGCACCACCAGCGC------------------
---GATGAGCCTGAGAAGCCCCACGAG---GAGGAGGAGGAGGAG---------------

>Amazon_molly_Aqp9a
---------------------------------------ATGAGGCAACACTGCGCGCTC
AAACACGGAATATTCAAGGAGTTCCTGGCTGAATTCCTGGGAACGTTCGTCTTGGTACTG
TTCGGCTGCGGCTCAGTTGCTCAGACGGTCCTGAGTCGAAACACGCTGGGCGAGCCGCTC
ACCGTCCACATCGGCTTCTCCGTGGGCCTCATGATGGCCGTGTACGTGGCTGGAGGGGTG
TCAGGAGGCCATGTGAACCCCGCCGTGTCTCTGGCCATGGTGATTCTGGGGAAACTAAAG
ATCTGGAAGTTTCCCTTCTACGTCATCGCTCAGTTTCTTGGTGCTTTTGCAGGAGCTGCA
GCTGTCTTCGGATTATATTATGACGCTTTCATGGACTTCACCAGCGGTATTCTGTCGGTG
ACAGGAATCAATGCGACAGGTCACATTTTTGCCTCCTACCCTGCCAGACACCTGTCAGTC
CTCGGCGGCTTCATCGATCAGGTGGTGGGGACAGGTATGCTGGTTCTCTGTATTCTGGCG
ATCATTGACGGTGGGAACATCGGCGCTCCTAAAGGCGTGGAGCCGCTGGCCATCGGTCTG
ATCATCATGGCCATCGGCGTCTCCATGGGACTCAACTGTGGCTACCCTCTGAACCCGGCC
AGAGACCTGGGGCCCCGGCTGTTCACCGCGGTGGCCGGGTGGGGATGGAGGTCTTCAGC
ACTGGAGGCTACTGGTGGTGGATCCCAGTGGCGGGGCCCATGGTGGGAGGCGTGGTGGCG
GCCGTCCTCTACTTCCTGCTCATCGAGCTGCACCACCCCCAC------------------
---GACGAGGACGAGAAGGCCCACGAGGTGGAGGAAGAAGAGGAG------------GAA
GAGGAGGAGGACGACGATGACAGCAGTCTTAAGGACAAATACGAGATGATCGCCATGAGC
>Sailfin_molly_Aqp9a
---------------------------------------ATGAGGCAACACTGCGCGCTC
AAACACGGAATATTCAAGGAGTTCCTGGCTGAATTCCTGGGAACGTTCGTCTTGGTACTG
TTCGGCTGCGGCTCAGTTGCTCAGACGGTCCTGAGTCGAAACACGCTGGGCGAGCCGCTC
ACCGTCCACATCGGCTTCTCCGTGGGCCTCATGATGGCCGTGTACGTGGCTGGAGGGGTG
TCAGGAGGCCATGTGAACCCCGCCGTGTCTCTGGCCATGGTGATTCTGGGGAAACTAAAG
ATCTGGAAGTTTCCCTTCTACGTCATCGCTCAGTTTCTTGGTGCTTTTGCAGGAGCTGCA
GCTGTCTTCGGATTATATTATGACGCTTTCATGGACTTCACCAGCGGTATTCTGTCGGTG
ACAGGAATCAATGCGACAGGTCACATTTTTGCCTCCTACCCTGCCAGACACCTGTCAGTC
CTCGGCGGCTTCATCGATCAGGTGGTGGGGACAGGTATGCTGGTTCTCTGTATTCTGGCG
ATCATTGACGGTGGGAACATCGGCGCTCCTAAAGGCGTGGAGCCGCTGGCCATCGGTCTG
ATCATCATGGCCATCGGCGTCTCCATGGGACTCAACTGTGGCTACCCTCTGAACCCGGCC
AGAGACCTGGGGCCCCGGCTGTTCACCGCGGTGGCCGGGTGGGGATGGAGGTCTTCAGC
ACTGGAGGCTACTGGTGGTGGATCCCAGTGGCGGGGCCCATGGTGGGAGGCGTGGTGGCG
GCCGTCCTCTACTTCCTGCTCATCGAGCTGCACCACCCCCAC------------------
---GACGAGGACGAGAAGGCCCACGAGGTGGAGGAAGAAGAGGAG------------GAA
GAGGAGGAGGACGACGATGACAGCAGTCTTAAGGACAAATACGAGATGATCGCCATGAGC
>Shortfin_molly_Aqp9a
---------------------------------------ATGAGGCAACACTGCGCGCTC
AAACACGGAATATTCAAGGAGTTCCTGGCTGAATTCCTGGGAACGTTCGTCTTGGTACTG
TTCGGCTGCGGCTCAGTTGCTCAGACGGTCCTGAGTCGAAACACGCTGGGCGAGCCGCTC
ACCGTCCACATCGGCTTCTCCGTGGGCCTCATGATGGCCGTGTACGTGGCTGGAGGGGTG
TCAGGAGGCCATGTGAACCCCGCCGTGTCTCTGGCCATGGTGATTCTGGGGAAACTAAAG
ATCTGGAAGTTTCCCTTCTACGTCATCGCTCAGTTTCTTGGTGCTTTTGCAGGAGCTGCA
GCCGTCTTCGGATTATATTATGACGCTTTCATGGACTTCACCAGCGGTATTCTGTCGGTG
ACAGGAATCAATGCGACAGGTCACATTTTTGCCTCCTACCCTGCCAGACACCTGTCAGTC
CTCGGCGGCTTCATCGATCAGGTGGTGGGGACAGGTATGCTGGTTCTCTGTATTCTGGCG
ATCATTGACGGTGGGAACATCGGCGCTCCCAAAGGCGTGGAGCCGCTGGCCATCGGTCTG
ATCATCATGGCCATCGGCGTCTCCATGGGACTCAACTGTGGCTACCCTCTGAACCCGGCC
AGAGACCTGGGGCCCCGGCTGTTCACCGCGGTGGCCGGGTGGGGATGGAGGTCTTCAGC
ACTGGAGGCTACTGGTGGTGGATCCCAGTGGCGGGGCCCATGGTGGGAGGCGTGGTGGCG
GCCGTCCTCTACTTCCTGCTCATCGAGCTGCACCACCCCCAC------------------
---GACGAGGACGAGAAGGCCCACGAGGTGGAGGAAGAAGAGGAG------------GAA
GAGGAGGAGGACGACGATGACAGCAGTCTTAAGGACAAATACGAGATGATCGCCATGAGC
>Guppy_Aqp9a
---------------------------------------ATGAGGCAACACTGCGCGCTC
AAACACGGAATATTCAAGGAGTTCCTGGCTGAATTCCTGGGAACGTTCGTCTTGGTACTG

TTCGGCTGCGGCTCAGTTGCTCAGACGGTGCTGAGTCGAAACACGCTGGGCGAGCCGCTC
ACCGTCCACATCGGCTTCTCCGTGGGCCTCATGATGGCCGTGTACGTGGCTGGAGGGGTG
TCAGGAGGCCATGTGAACCCCGCCGTGTCTCTGGCCATGGTGATTCTGGGGAAACTAAAG
ATCTGGAAGTTTCCCTTCTACGTCATCGCTCAGTTTCTTGGTGCTTTTGCAGGAGCTGCA
GCCGTCTTTGGATTATATTATGATGCTTTCATGGACTTCACCAGCGGTATTCTGTCGGTG
ACAGGAATCAATGCGACAGGTCACATTTTTGCCTCCTACCCTGCCAGACACCTGTCAGTC
CTGGGCGGCTTCATCGATCAGGTGGTGGGGACAGGTATGCTGGTTCTCTGCATCCTGGCG
ATCATTGACGGTGGGAACATCGGCGCTCCTAAAGGAGTGGAGCCGCTGGCCATCGGTCTG
ATCATCATGGCCATCGGCGTCTCCATGGGACTCAACTGTGGCTACCCTCTGAACCCGGCC
AGAGACCTGGGACCCCGGCTGTTCACCGCGGTGGCCGGGTGGGGATGGAGGTCTTCAGC
ACTGGAGGCTACTGGTGGTGGATCCCAGTGGCGGGGCCCATGGTGGGAGGCGTGGTGGCG
GCCGTCCTCTACTTCCTGCTCATCGAGCTGCACCACCCCCAC------------------
---GACGAGGACGAGAAGGCCCACGAGGTGGAGGAAGAGGAGGAG------------GAA
GAGGAGGAGGACGACGATGACAGCAGTCTTAAGGATAAATACGAGATGATCGCCATGAGC
>Monterrey_platyfish_Aqp9a
--ATGAGGCAACACTGCGCGCTC
AAACACGGAATATTCAAGGAGTTCCTGGCTGAATTCCTGGGAACGTTCGTCTTGGTACTG
TTTGGCTGCGGCTCAGTTGCTCAGACGGTCCTGAGTCGAAACACGCTGGGCGAGCCGCTC
ACCGTCCACATCGGCTTCTCCGTGGGCCTCATGATGGCCGTGTACGTGGCTGGAGGGGTG
TCAGGAGGCCATGTGAACCCCGCCGTGTCTCTGGCCATGGTGATTCTGGGGAAACTAAAG
ATCTGGAAGTTTCCCTTCTACGTCATCGCTCAGTTTCTTGGTGCTTTTGCAGGAGCTGCA
GCCATCTTTGGATTATATTATGACGCTTTCATGGACTTCACCAGCGGTATTCTGTCCGTG
ACAGGAATCAATGCGACAGGTCACATTTTTGCCTCCTACCCTGCCAGACACCTGTCAGTC
CTCGGCGGCTTCATCGATCAGGTGGTGGGGACAGGTATGCTGGTTCTCTGTATTTTGGCG
ATCATTGACGGCGGGAACATTGGCGCCCCTAAAGGCGTGGAGCCGCTGGCCATCGGTCTG
ATCATCATGGCCATCGGTGTCTCCATGGGGCTCAACTGTGGCTACCCTCTGAACCCCGCC
AGAGACCTGGGACCCCGGCTGTTCACGGCGGTGGCCGGGTGGGGATGGAGGTCTTCAGC
ACTGGAGGCTACTGGTGGTGGATCCCAGTGGCGGGGCCCATGGTGGGAGGCGTGGTGGCG
GCCGTCCTCTACTTCCTGCTCATCGAGCTGCACCACCCCCAT------------------
---GACGAGGACGAGAAGGCCCACGAGGTGGAGGAAGAGGAGGAG------------GAA
GAAGAGGAGGACGACGATGACAGCAGTCTTAAGGACAAATACGAGATGATCGCCATGAGC
>Southern_platyfish_Aqp9a
--ATGAGGCAACACTGCGCGCTC
AAACACGGAATATTCAAGGAGTTCCTGGCTGAATTCCTGGGAACGTTCGTCTTGGTACTG
TTTGGCTGCGGCTCAGTTGCTCAGACGGTCCTGAGTCGAAACACGCTGGGCGAGCCGCTC
ACCGTCCACATCGGCTTCTCCGTGGGCCTCATGATGGCCGTGTACGTGGCTGGAGGGGTG
TCAGGAGGCCATGTGAACCCCGCCGTGTCTCTGGCCATGGTGATTCTGGGGAAACTAAAG
ATCTGGAAGTTTCCCTTCTACGTCATCGCTCAGTTTCTTGGTGCTTTTGCAGGAGCTGCA
GCCATCTTTGGATTATATTATGACGCTTTCATGGACTTCACCAGCGGTATTCTGTCGGTG
ACAGGAATCAATGCGACAGGTCACATTTTTGCCTCCTACCCTGCCAGACACCTGTCAGTC
CTTGGCGGCTTCATCGATCAGGTGGTGGGGACAGGTATGCTGGTTCTCTGTATTTTGGCG
ATCATTGACGGTGGGAACATTGGCGCCCCTAAAGGCGTGGAGCCGCTGGCCATCGGTCTG
ATCATCATGGCCATCGGCGTCTCCATGGGGCTCAACTGTGGCTACCCTCTGAACCCCGCC
AGAGACCTGGGACCCCGGCTGTTCACGGCGGTGGCCGGGTGGGGATGGAGGTCTTCAGC
ACTGGAGGCTACTGGTGGTGGATCCCAGTGGCGGGGCCCATGGTGGGAGGCGTGGTGGCG
GCCGTCCTCTACTTCCTGCTCATCGAGCTCCACCACCCCCAT------------------
---GACAAGGATGAGAAGGCCCACGAGGTGGAGGAAGAGGAGGAG------------GAA
GAAGAGGAGGACGACGATGACAGCAGTCTTAAGGACAAATACGAGATGATCGCCATGAGC
>Green_swordtail_Aqp9a
--ATGAGGCAACACTGCGCGCTC
AAACACGGAATATTCAAGGAGTTCCTGGCTGAATTCCTGGGAACGTTCGTCTTGGTCCTG
TTTGGCTGCGGCTCAGTTGCTCAGACGGTCCTGAGTCGAAACACGCTGGGCGAGCCGCTC
ACCGTCCACATCGGCTTCTCCGTGGGCCTCATGATGGCCGTGTACGTGGCTGGAGGGGTG
TCAGGAGGCCATGTGAACCCCGCCGTGTCTCTGGCCATGGTGATTCTGGGGAAACTAAAG
ATCTGGAAGTTTCCCTTCTACGTCATCGCTCAGTTTCTTGGTGCTTTTGCAGGAGCTGCA
GCCGTCTTTGGATTATATTATGACGCTTTCATGGACTTCACCAGCGGTATTCTGTCGGTG
ACAGGAATCAATGCGACAGGTCACATTTTTGCCTCCTACCCTGCCAGACACCTGTCAGTC
CTCGGCGGCTTCATCGATCAGGTGGTGGGGACAGGTATGCTGGTTCTCTGTATTTTGGCG
ATCATTGACGGTGGGAACATTGGCGCCCCTAAAGGCGTGGAGCCCCTGGCCATCGGTCTG
ATCATCATGGCCATCGGCGTCTCCATGGGGCTCAACTGTGGCTACCCTCTGAACCCCGCC
AGAGACCTGGGACCCCGGCTGTTCACGGCGGTGGCCGGGTGGGGATGGAGGTCTTCAGG

ACTGGAGGCTACTGGTGGTGGATCCCAGTGGCGGGGCCCATGGTGGGAGGCGTGGTGGCG
GCCGTCCTCTACTTCCTGCTCATCGAGCTGCACCACCCCCAT------------------
---GACGAGGACGAGAAGGCCCACGAGGTGGAGGAAGAGGAGGAG------------GAA
GAAGAGGAGGACGACGATGACAGCAGTCTTAAGGACAAATACGAGATGATCGCCATGAGC
>Western_mosquitofish_Aqp9a
---------------------------------------ATGAGGCAACACTGCGCGCTC
AAACACGGAATATTCAAGGAGTTCCTGGCTGAATTCCTGGGAACGTTTGTCTTGGTGCTG
TTTGGCTGCGGCTCAGTTGCTCAGACGGTCCTGAGTCGAAACACGCTGGGCGAGCCGCTC
ACCGTCCACATCGGCTTCTCCGTGGGCCTCATGATGGCCGTGTACGTGGCTGGAGGGGTG
TCAGGAGGCCATGTGAACCCCGCCGTGTCTCTGGCCATGGTGATTCTGGGGAAACTAAAG
ATCTGGAAGTTTCCCTTTTACGTCATCGCTCAGTTTCTTGGTGCTTTTGCAGGAGCTGCA
GCCGTCTTTGGATTATATTATGACGCTTTCATGGACTTCACCAGCGGTATTCTGTCGGTG
ACAGGAATCAATGCGACCGGTCACATTTTTGCCTCCTACCCTGCCAGACACCTGTCAGTC
CTCGGCGGCTTCATCGATCAGGTGGTGGGGACAGGTATGCTGGTTCTCTGTATTCTGGCG
ATCATTGACGGTGGGAACATTGGCGCTCCTAAAGGCGTGGAGCCGCTGGCCATCGGTCTG
ATCATCATGGCCATCGGCGTCTCCATGGGACTGAACTGTGGCTACCCTCTGAACCCCGCC
AGAGACCTGGGACCCCGGCTGTTCACGGCGGTGGCCGGGTGGGGCATGGAGGTCTTCAGC
ACTGGAGGCTACTGGTGGTGGATCCCAGTGGCGGGGCCCATGGTGGGAGGCGTGGTGGCG
GCCGTCCTCTACTTCCTGCTCATCGAGCTGCACCACCCCCAT------------------
---GACGAGGATGAGAAGGCCCACGAGGTGGAGGAAGAGGAGGAG------------GAA
GAGGAGGAGGACGACGATGACAGCAGTCTTAAGGACAAATACGAGATGATCGCCATGAGC
>Blackstripe_livebearer_Aqp9a

---GTC
TCAGGAGGCCATGTGAACCCCGCCGTATCTCTGGCCATGGTGATTCTGGGGAAACTAAAG
ATCTGGAAGTTTCCCTTCTACGTCATCGCTCAGTTTCTTGGTGCTTTTGCAGGAGCTGCA
GCCGTCTTTGGATTATATTATGACGCTTTCATGGACTTCACCAGCGGTATTCTGTCAGTG
ACAGGAATCAATGCGACAGGTCACATTTTTGCCTCCTACCCTGCCAGACACCTGTCAGTC
CTCGGCGGCTTCATCGATCAGGTGGTGGGGACAGGTATGCTGGTTCTCTGTATTCTGGCG
ATCATCGATGGTGGGAACATTGGCGCTCCTAAAGGCGTGGAGCCGCTGGCCATCGGTCTG
ATCATCATGGCCATCGGCGTCTCCATGGGACTCAACTGCGGCTACCCTCTGAACCCGGCC
AGAGACCTGGGACCCCGGCTGTTCACGGCGGTGGCCGGCTGGGGGATGGAGGTCTTCAGC
ACTGGAGGCTACTGGTGGTGGATCCCAGTGGCGGGGCCCATGGTGGGAGGCGTGGTGGCG
GCGGTCCTCTACTTCCTGCTCATCGAGCTGCACCATCCCCAC------------------
---GACGAAGACGAGAAGGCCCACGAGGTGGAGGAAGAGGAGGAGGAA------------

>Amargosa_pupfish_Aqp9a
---------------------------------------ATGAGGCAGCGCTGCGCGCTC
AAACATGGAATATTCAAGGAATTCCTGGCAGAATTCCTGGGGACTTTTGTCTTGGTA---

------------GGCTTCTCTGTGGGCCTCATGATGGCAGTGTACGTGGCTGGTGGGGTT
TCAGGAGGCCATGTGAACCCTGCTGTGTCTCTGGCTATGGTGATTCTGGGCAAACTGAAG
ATCTGGAAGTTTCCCTTCTATGTCATGGCTCAGTTCCTTGGTGCTTTTGCAGGAGCTGCA
GCAGTCTTTGGATTGTATTATGATGCTTTCATGGACTTCACAAGTGGTATTCTGTCAGTG
ACAGGAATCAATGCCACAGGCCACATTTTCGCCTCCTACCCAGCCAGACACCTGTCGGTC
CTCGGAGGCTTCATTGATCAGGTGGTTGGGACAGGCATGCTGGTTCTCTGTATTCTGGCA
ATCATTGATGGCGGAAACATCGGAGCTCCCAAAGGCGTGGAGCCGCTCGCCATCGGCCTG
ATCATCATGGCCATCGGTGTGTCCATGGGGCTCAACTGTGGCTACCCGCTGAACCCTGCC
AGAGACCTGGGACCACGTCTGTTCACGGCGGCAGCAGGATGGGGGATGGAGGTCTTCAGG
ACTGGAGGCTACTGGTGGTGGATTCCAGTGGCAGGGCCCATGGTGGGAGGAGTGGTGGCC
GCCTTCCTCTACTTCCTGCTCATCGAGCTTCACCACCCCCAT------------------
------GAAGGGGAGGGGGCTCTCGAGGTGGAGGAAGAGGAGGAG------------GAG
GACGACGAAGAGGACGAGGACAGCAGTCTAAAGGACAAATATGAGATGATCACCATGAGT
>Sheepshead_minnow_Aqp9a
---------------------------------------ATGAGGCAGCGCTGCGCGCTC
AAACATGGAATATTGAAGGAATTCCTGGCAGAATTCCTGGGGACTTTTGTCTTGGTATTG
TTTGGCTGTGGCTCAGTTGCTCAGACTGTCCTGAGTCGAAACACGCTGGGGGAGCCTCTT
ACCGTCCATATCGGCTTCTCTGTGGGCCTCATGATGGCAGTGTACGTGGCTGGTGGGGTT
TCAGGAGGCCATGTGAACCCCGCCGTGTCTCTGGCTATGGTGATTCTGGGCAAACTGAAG

ATCTGGAAGTTTCCCTTCTATGTCATTGCTCAGTTCCTTGGTGCTTTTGCAGGAGCTGCA
GCAGTCTTTGGATTGTATTATGATGCTTTCATGGACTTCACAAGTGGTATTCTGTCAGTG
ACAGGAATCAATGCCACAGGCCACATTTTCGCCTCCTACCCAGCCAGACACCTGTCGGTC
CTCGGAGGCTTCATTGATCAGGTGGTTGGGACAGGCATGCTGGTTCTCTGTATTCTGGCC
ATCATTGATGGCGGAAACATCGGAGCTCCTAAAGGCGTGGAGCCGCTCGCCATCGGCCTG
ATCATCATGGCCATCGGTGTGTCCATGGGGCTCAACTGTGGCTACCCGCTGAACCCTGCC
AGAGACCTGGGACCACGTCTGTTCACGGCGGCAGCAGGATGGGGATGGAGGTCTTCAGG

>Common_mummichog_Aqp9a
--ATGAGGCAGCACTGCGCGCTC
AAACATGGAATATTCAAGGAATTCCTGGCTGAGTTTCTCGGGACTTTTGTCTTGGTATTG
TTTGGCTGTGGCTCAGTTGCTCAGACCGTCCTGAGTCGAAACACGCTGGGTGAGCCGCTC
ACCATCCACATCGGCTTCTCTGTGGGCCTCATGATGGCAGTGTACGTGTCTGGTGGGGTG
TCAGGAGGCCATGTGAACCCCGCTGTGTCTCTGGCCATGGTGATTCTGGGCAAACTGAAA
ATCTGGAAGTTTCCCTTCTACGTCATCGCTCAGTTTCTTGGTGCTTTTGCAGGAGCTGCG
GCGGTCTTCGGATTATATTACGATGCTTTCATGGACTTCACAAGTGGTATTTTGTCAGTG
ACAGGAATCAATGCGACAGGTCACATTTTTGCCTCTTACCCCGCCAGACACCTGTCAGTC
CTCGGAGGCTTCATTGATCAGGTGGTGGGGACGGGCATGCTGGTTCTCTGTATCCTGGCC
ATCATAGACGGCGGCAACATCGGCGCTCCTAAAGGCGTGGAGCCGCTGGCCATCGGCCTG
ATCATCATGGCCATCGGCGTGTCCATGGGACTCAACTGCGGCTACCCCCTGAACCCCGCC
AGAGACTTCGGACCCCGACTGTTCACCGCAGTGGCAGGATGGGGATGGAGGTCTTCAGG
ACTGGAGGCTACTGGTGGTGGATCCCGGTGGCGGGGCCCATGGTGGGAGGCGTGGTGGCC
GCCGTCCTCTACTTCCTGCTCATCGAGCTTCACCACCCCCAC------------------
---GACGAGGATGAGAAGGCCCACGAGGTGGAGGAAGAGGAA---------------GAA
GAGGAGGAAGACGACGAGGACAGCAGTCTTAAGGACAAATACGAGATGATCGCCATGAGT
>Mangrove_rivulusivulus_Aqp9a
--ATGAGGCAACACTGTGCTCTC
AAACATGGAATATTCAAGGAATTCCTGGCAGAATTCCTCGGGACATTTGTCTTGGTTCTG
TTCGGCTGCGGCTCAGTCGCTCAGACCGTCCTGAGTCGAAACACGCTGGGCGAGCCCATC
ACCGTCCACATCGGCTTCTCCGTGGGCCTCATGATGGCCGTGTACATCGCCGGCGGGGTG
TCAGGGGGCCACGTGAACCCTGCTGTCTCTCTGGCCATGGTGGTTTTGGGCAAACTGAAG
ATCTGGAAGTTTCCTTTTTATGTCATCGCTCAGTTTCTCGGAGCTTTTGCAGGAGCTGCT
GCAGTCTTTGGACTATACTACGATGCTTTCATGGAGTTCACGAGCGGGATTCTGTCGGTG
ACAGGAATCAACGCAACGGCTCACATTTTCGCCTCGTACCCTGCGCGGCACCTGTCGGTC
CTTGGCGGCTTCGTTGATCAGGTTGTGGGGACGGGCATGCTGGTTCTGTGCATTCTTGCC
ATCATCGACGGCGGAAACATTGGCGCTCCTAAAGGCGTGGAGCCGCTGGCCATCGGTCTG
ATCATCATGGCCATCAGCGTGTCCATGGGGCTTAACTGCGGGTACCCCGTGAACCCGGCT
CGAGACCTGGGACCCCGACTTTTCACAGCAGTGGCGGGGTGGGGGATGGAGGTCTTCAGC
ACCGCAGACAACTGGTGGTGGATCCCAGTGGCAGGGCCCATGGTGGGAGGCGTGGTCGCG
GCCGTCATCTACTACCTGCTCATTGAGCTGCACCACCCCCGC------------------
---GACGAGACCGAGAAGGCCCACGAGGAAGAAGAGGAGGAGGAG------------GAC
GACGAGGAAGAGGATGAGGACAGCAGCCTTAAGGACAAATACGAGATGATCGCCATGAGT
>Annual_killifish_Aqp9a
--ATGAGGCAACACTGTGCTCTG
AAACATGGAATATTCAAGGAGTTCCTGGCAGAATTTCTTGGGACTTTTGTTCTGGTTCTG
TTCGGCTGCGGCTCAGTGGCTCAGAACGTCCTGAGTCGAAACACTCTGGGCGAGCCGATC
ACCGTCCACATCGGCTTCTCCGTGGGCCTCATGATGGCCGTGTACGTGTCTGGGGGGATA
TCAGGGGGCCATGTGAACCCCGCTGTCTCTCTGGCCATGGTGGTTCTGGGCAAACTGAAG
ATCTGGAAGTTTCCCTTTTATGTCATCGCTCAGTTTCTGGGTGCTTTTGCAGGAGCTGCT
GCAGTCTTTGGACTTTACTACGATGCCTTCATGGAGTTCACGAGCGGGATTCTGTCGGTG
ACGGGGATCAACGCAACAGCTCATATTTTTGCCTCCTACCCTGCGAGACACCTGTCAGTT
CTTGGCGGCTTCATTGATCAGGTGGTGGGTACAGGCATGCTGGTTCTGTGTATTCTCGCC
ATCATCGATGGCGGGAATATCGGCGCTCCTAAAGGCGTGGAGCCGCTGGCCATCGGCCTG
ATCATCACGGCCATCAGCGTGTCCATGGGTCTAAACTGCGGTTACCCCGTGAACCCCGCT
CGAGACCTGGGACCCCGACTGTTCACGGCGGTGGCAGGGTGGGGGATGGAGGTCTTCAGC
ACGGGGGACAACTGGTGGTGGATCCCAGTGGCAGGGCCCATGGTGGGAGGCGTGGTCGCC
GCCATCATCTACTTCCTGCTGATCGAGCTGCACCATCCCCAC------------------
---GACGAGGCCGAGAAGGCCCACGAGGAGGAAGAGGAGGAG---------------GAG

GAGGAGGAAGAGGACGAGGACAGCAGCCTTAAAGACAAATATGAGATGATCGCCATGACT
>Turquoise_killifish_Aqp9a
--ATGAGGCAACACTGTGCCCTC
AAACATGGAATATTCAAAGAATTCCTGGCAGAATTCCTTGGGACTTTTGTCTTGGTTTTG
TTTGGCTGTGGCTCGGTAGCTCAGACCGTGCTAAGTCGAAACACTCTTGGAGAGCCGCTC
ACCATCCACATCGGCTTCTCCGTGGGCCTCATTATGGCCGTGTATGTGGCTGGTGGAGTC
TCAGGGGGCCACGTGAACCCTGCTGTGTCTCTGGCCATGGTGGTTTTGGGCAAGCTGAAG
ATCTGGAAGTTCCCCTTCTACGTGATGGCCCAGTTCCTCGGTGCTTTTGCGGGAGCCGCT
GCGGTCTTTGGATTATACTATGATTCATTTATGGACTTCACAAGCGGGATTCTGTCAGTG
ACGGGAATCAATGCAACTGCTCACATCTTTGCCTCATACCCTGCTAGGCACCTGTCAGTT
CTTGGAGGCTTCATCGATCAGGTTGTGGGCACAGGTATGCTCGTTCTCTGTATTCTTGCA
ATCATCGATGGTGGAAACATTGGAGCTCCGAAAGGCGTGGAGCCGCTGGCCATTGGCCTG
ATCATCATGGCCATTGGTGTGTCCATGGGACTGAACTGTGGCTACCCCCTTAACCCAGCC
CGAGACCTAGGACCCCGACTGTTCACGGCGGTGGCCGGATGGGGGATGGAGGTCTTCAGC
ACTGCAGACAACTGGTGGTGGATCCCAGTAGCAGGACCCATGGTGGGCGGCGTGGTCGCA
GCCGTCATCTACTTCCTGCTCATTGAGCTGCACCACGTCCAC------------------
---AATGAGCCCGAGAAAGCTCATGAGGAGGACGAAGAGGAG---------------GAG
GAGGAGGAAGAGGACGAGGACATCAGTCTGAAGGACAAATATGAGATGATCACCATGAGT
>Atlantic_silverside_Aqp9a
--ATGAGGCAACACTGTGCTATC
AAACATGGAATATTCAAGGAATTCCTGGCAGAATTCCTCGGGACTTTTGTCTTGGTTTTG
TTTGGGTGTGGCTCAGTGGCACAGACTGTCCTGAGTCGAAACACGCTGGGGGAGCCTCTG
ACCGTCCACATCGGCTTCTCCGTGGGCCTCATGATGGCTGCGTATGTGGCCGGCGGAGTC
TCAGGGGGCCACGTGAACCCTGCTGTGTCTCTGGCCATGGTGGTTCTGGGTAAACTGAAG
ATCTGGAAGTTTCCCTTCTACGTCATCGCCCAGTTTCTTGGTGCTTTTGCTGGAGCTGCT
GCAGTGTTCGGGTTGTATTACGATGCTTTCATGGACTTCACCAGCGGGATTCTGTCTGTG
ACTGGAATCAATGCAACAGCTCACATTTTTGCCTCATACCCTGCCAGACACCTGTCAGTC
CTCGGTGGTTTCATTGATCAGGTGGTGGGGACGGGCATGCTAGTTCTGTGCATTCTTGCT
ATCATTGATGGTGGAAACATCGGAGCTCCTAAAGGTGTGGAGCCGCTGGCTATTGGCCTG
ATCATTATGGCCATTGGTGTGTCCATGGGACTCAACTGTGGTTACCCCCTGAACCCGGCC
AGGGACCTGGGACCCCGGCTGTTCACGGCCGTGGCAGGATGGGGGATGGACGTCTTCAGC
ACGGCGGACTACTGGTGGTGGATCCCCGTGGCGGGGCCCATGGTCGGGGGAGTGGTCGCG
GCCGTCATCTACCTCCTCTTCATCGAGCTGCACCACCCCCGC------------------
---GACGAGTCCGAGAAA---CACCAAGAGGAGGAGGAGGAGGAG------------GAG
GAAGAGGAGGATGACGAGGACAGCAGTCTGAAGGACAAATACGAGATGATAGCCATGAGT
>Desert_rainbow_fish_Aqp9a
----------------ATGGAAGTACAAAACAAGAGAAGCATGAGGCAACACTGTGCTCTG
AAACATGGAATATTCAAGGAATTCCTGGCAGAATTCCTTGGGACTTTTGTCTTGGTTTTA
TTTGGATGTGGCTCAGTTGCGCAGACCGTCCTGAGTCGAAACACTTTGGGGGAACCGCTC
ACCGTCCACATTGGCTTCTCTGTGGGTCTCATGATGGCAGTGTATGTGGCTGGTGGAGTG
TCAGGGGGCCATGTGAACCCTGCTGTATCTCTGGCCATGGTGATTCTGGGCAAACTGAAG
ATCTGGAAGTTTCCCTTCTACGTCATCGCTCAGTTTCTTGGTGCTTTTGCAGGAGCTGCT
GCAGTGTTCGGGCTATACTACGATGCCTTCATGGATTTCACCAGTGGGATTCTGTCTGTG
ACGGGAATCAATGCAACAGGTCACATTTTTGCCTCATACCCTGCAAGACACCTGTCAGTC
CTCGGCGGCTTCATTGATCAGGTGGTGGGGACAGGCATGCTGGTTCTTTGTATTCTTGCT
ATCGTTGATGGTGGAAACATTGGAGCTCCTAAAGGCATGGAGCCACTGGCCATCGGCCTG
ATCATCATGGCCATCGGAGTGTCCATGGGGCTCAACTGTGGCTACCCTCTGAACCCTGCC
AGAGATCTGGGACCACGACTGTTCACAGCAGTGGCAGGATGGGGGATAGAAGTCTTCAGC
ACTGCAGACAACTGGTGGTGGATCCCAGTGGCAGGGCCCATGGTGGGGGGCGTGGTTGCT
GCCGTCATTTACTTCCTGCTCATTGAGCTGCACCACCCCCAC------------------
---GATGAGGCTGAGAAGCCC---ACTGAGGAGGAGGAAGAGGAC------------GAG
GAAGAAGAAGATGATGAGGACAGCAGTCTGAAGGATAAATATGAGATGATTGCCATGAGT
>Japanese_medaka_Aqp9a
--ATGAGGCAACACTGTGCTATC
AAACATGGAATATTCAAGGAAATCTTGGCAGAATTCCTTGGGACATTTGTCTTGGTTTTG
TTTGGCTGTGGCTCAGTTGCTCAGACCATCCTCAGTCGAAACACCCTGGGTGAGCCTTTG
ACCATCCACATTGGCTTCTCTGTAGGCCTCGCAATGGCAGTATATGTGGCTGGAGGAGTG
TCAGGAGGCCACGTCAACCCTGCTGTGTCTCTGGCTATGGTGATTCTGGGCAAACTGAAG
ATCTGGAAGTTTCCTTTCTACGTCATTGCTCAGTTTCTTGGTGCATTTGCAGGAGCTGCT
GCTGTCTTTGGGTTATACTATGATGCTTTTATGGACTTTACCAATGGGATTCTGTCAGTG
ACTGGAATCAATGCAACAGCTCACATTTTTGCCTCATACCCTGCAAGACACCTGTCAGTC

CTAGGCGGCTTCATCGATCAGGTGGTGGGGACTGGTATGTTAGTTCTGTGTATTCTTGCG
ATTATCGACGGTGGAAACATTGGTGCTCCTAAAGGTGTGGAACCTCTAGCAATCGGCTTG
ATCCTCATGGCCATCAGTGTTTCCATGGGACTCAACTGCGGCTACCCTCTGAACCCAGCC
AGAGACCTGGGACCCCGACTGTTCACGGCAGTGGCAGGTTGGGGATGGAGGTGTTCAGC
ACTGCAGATTACTGGTGGTGGATTCCAGTGGCAGGGCCCATGGTGGGCGGGGTGGTCGGT
GCTGTTCTCTATTTCCTGCTCGTCGAGATGCACCATGCCCAC------------------
---AATGAGACCGAGAAGCCGCACGAGGAGGAGGAGGAAGAGGAG------------GAA
GAGGAGGAGGATGAGGACGACAACAGCCTGAAGGACAAATATGAGATGATCACCATGGGT
>Javanese_ricefish_Aqp9a
---------------------------------------ATGAGGCAACACTGCGCCATC
AAACATGGAATATTCAAGGAAATCTTGGCAGAATTCCTTGGGACGTTTGTCTTGGTTTTG
TTTGGCTGTGGCTCAGTTGCTCAGACCATCCTCAGTCGAAACACCCTGGGCGAGCCTCTG
ACTATCCACATTGGCTTCTCTGTAGGCCTCACAATGGCCGTATATGTGGCTGGTGGAGTG
TCAGGAGGCCACGTCAACCCCGCTGTGTCTCTGGCTATGGTGATCCTGGGCAAACTGAAG
ATCTGGAAGTTTCCTTTCTACGTCATTGCTCAGTTTCTTGGTGCATTTGCAGGAGCTGCT
GCTGTCTTTGGATTATACTATGACGCTTTCATGGACTTCACCAACGGGATTCTGTCAGTG
ACTGGAATCAATGCAACAGCTCACATTTTTGCCTCGTACCCTGCAAGACACCTGTCAGTC
CTGGGCGGCTTCATCGATCAGGTGGTGGGGACCGGTATGTTAGTTCTGTGTATTCTTGCG
ATCATCGACGGTGGGAACATTGGCGCTCCCAAAGGTGTGGAACCTCTCGCAATCGGGTTA
ATCATCATGGCCATCAGTGTTTCCATGGGGCTCAACTGCGGCTACCCTCTGAACCCAGCC
AGAGACCTTGGCCCCCGACTGTTCACGGCAGTGGCCGGATGGGGATGGAGGTGTTCAGC
ACTGGGGATTACTGGTGGTGGATTCCAGTGGCGGGCCCCATGGTGGGCGGGGTGGTCGCC
GGCGTGCTCTATTTCCTGCTCATCGAGATGCACCATGCCCAC------------------
---GACGAGACCGAGAAGCCGCACGAGGAGGAGGAGGAAGAGGAG------------GAA
GAGGAGGAGGATGAGGACGACAACAGCCTGAAGGACAAATATGAGATGATCACCATGGGT
>Indian_medaka_Aqp9a
---------------------------------------ATGAGGCAACACTGCGCCATC
AAACATGGAATATTCAAGGAAATCTTGGCTGAATTCCTTGGGACGTTCGTCTTGGTTTTG
TTTGGCTGTGGCTCAGTTGCTCAGACCATCCTCAGTCGAAACACCCTGGGTGAGCCTCTG
ACTATCCACATTGGATTCTCTGTAGGCCTCATGATGGCAGTCTATGTGGCTGGTGGAGTG
TCAGGAGGCCACGTCAACCCCGCTGTGTCTCTGGCTATGGTGATCCTGGGCAAACTGAAG
ATCTGGAAGTTTCCTTTCTACGTCATTGCTCAGTTTCTTGGTGCATTTGCAGGAGCTGCT
GCTGTCTTTGGATTATACTATGACGCTTTCATGGACTTCACCAACGGGATTCTGTCAGTG
ACTGGAATCAATGCAACAGCTCACATTTTTGCCTCGTACCCTGCAAGACACCTGTCAGTC
CTGGGCGGCTTCATCGATCAGGTGGTGGGGACTGGTATGTTAGTTCTGTGTATTCTTGCA
ATCATCGACGGCGGGAACATTGGCGCCCCTAAAGGTGTGGAACCTCTCGCAATCGGCTTG
ATCATCATGGCCATCAGTGTTTCCATGGGGCTCAACTGCGGCTACCCTCTGAACCCAGCC
AGAGACCTGGGGCCTCGACTGTTCACGGCGGTGGCTGGATGGGGATGGAGGTGTTCAGC
ACTGGGGATTACTGGTGGTGGATTCCAGTGGCGGGACCCATGGTGGGCGGGGTGGTCGCC
GCCGTGCTCTATTTCCTGCTCATCGAGATGCACCATGCCCAC------------------
---GACGAGACCGAGAAGCCGCACGAGGAGGAGGAGGAAGAGGAG------------GAA
GAGGAGGAGGATGAGGACGACAACAGCCTGAAGGACAAATATGAG---------------
>Zebra_mbuna_Aqp9a
---------------ATGGAAGTACAAAACAAGAGAAGCATGAGGCAACACTGTGCTCTA
AAACATGGAATATTTAAGGAATTTCTGGCAGAATTCCTGGGAACGTTTGTCTTGGTTCTG
TTTGGCTGCGGCTCCGTCGCTCAGACCGTCCTCAGTCGAAACACCCTGGGCGAACCCCTC
ACCGTCCACATCGGCTTCTCTGTAGGCCTGATGATGGCAGCGTATGTGGCCGGTGGAGTT
TCAGGGGGCCATGTGAACCCCGCTGTGTCTTTGGCCATGGTAATTTTGGGCAAACTGAAG
ATCTGGAAGTTTCCTTTCTACGTCATCGCTCAGTTTCTTGGTGCTTTTGCAGGAGCTGCT
GCAGTCTTTGGATTATACTATGATGCCTTCATGGACTTCACCAGTGGGATTCTATCAGTG
ACGGGTATCAATGCAACGGGCCACATTTTTGCCTCATACCCCGGCAGACACCTGTCAGTC
CTCGGCGGCTTCATTGATCAGGTGGTGGGAACAGGTATGCTGGTTTTGTGCATTCTTGCT
ATCATCGACGGCGGAAACATCGGAGCTCCTAAAGGCGTCGAGCCGCTGGCCATCGGCCTG
ATCATCATGGCCATCGGCGTGTCCATGGGGCTGAACTGTGGCTACCCCCTGAACCCAGCC
AGAGACCTGGGACCCAGACTGTTCACAGCTGTGGCAGGATGGGGATGGAGGTCTTCAGC
ACCGCAGACAACTGGTGGTGGATCCCAGTGGCGGGACCGATGGTGGGGGGAGTGGTCGCG
GCCGTCATATACTACCTGTTCATTGAGCTGCACCACCCCCAC------------------
---GTCGAGCACGAGAAGCCC---CAGGAGGAAGAAGAGGAGGAC------------GAG
GAAGACGAAGAGGATGATGACAGCAGTCTGAAGGACAAATATGAGATGATCACGATGAGT
>Eastern_happy_Aqp9a
---------------ATGGAAGTACAAAACAAGAGAAGCATGAGGCAACACTGTGCTCTA

AAACATGGAATATTTAAGGAATTTCTGGCAGAATTCCTGGGAACGTTTGTCTTGGTTCTG
TTTGGCTGCGGCTCCGTCGCTCAGACCGTCCTCAGTCGAAACACCCTGGGCGAACCCCTC
ACCGTCCACATCGGCTTCTCTGTAGGCCTGATGATGGCAGCGTATGTGGCCGGTGGAGTT
TCAGGGGGCCATGTGAACCCCGCTGTGTCTTTGGCCATGGTAATTTTGGGCAAACTGAAG
ATCTGGAAGTTTCCTTTCTACGTCATCGCTCAGTTTCTTGGTGCTTTTGCAGGAGCTGCT
GCAGTCTTTGGATTATACTATGATGCCTTCATGGACTTCACCAGTGGGATTCTGTCAGTG
ACGGGTATCAATGCAACGGGCCACATTTTTGCCTCATACCCCGGCAGACACCTGTCAGTC
CTCGGCGGCTTCATTGATCAGGTGGTGGGAACAGGTATGCTGGTTTTGTGCATTCTTGCT
ATCATCGACGGCGGAAACATCGGAGCTCCTAAAGGCGTCGAGCCGCTGGCCATCGGCCTG
ATCATCATGGCCATCGGCGTGTCCATGGGGCTGAACTGTGGCTACCCCCTGAACCCAGCC
AGAGACCTGGGACCCAGACTGTTCACAGCTGTGGCAGGATGGGGGATGGAGGTCTTCAGC
ACCGCAGACAACTGGTGGTGGATCCCAGTGGCGGGACCGATGGTGGGGGGAGTGGTCGCG
GCCGTCATATACTACCTGTTCATTGAGCTGCACCACCCCCAC------------------
---GTCGAGCACGAGAAGCCC---CAGGAGGAAGAGGAGGAGGAC------------GAG
GAAGACGAAGAGGATGATGACAGCAGTCTGAAGGACAAATATGAGATGATCACGATGAGT
>Red_mwanza_Aqp9a
---------------ATGGAAGTACAAAACAAGAGAAGCATGAGGCAACACTGTGCTCTA
AAACATGGAATATTTAAGGAATTTCTGGCAGAATTCCTGGGAACGTTTGTCTTGGTTCTG
TTTGGCTGCGGCTCCGTCGCTCAGACCGTCCTCAGTCGAAACACCCTGGGCGAACCCCTC
ACCGTCCACATCGGCTTCTCTGTAGGCCTGATGATGGCAGCGTATGTGGCCGGTGGAGTT
TCAGGGGGCCATGTGAACCCCGCCGTGTCTTTGGCCATGGTAATTTTGGGCAAACTGAAG
ATCTGGAAGTTTCCTTTCTACGTCATCGCTCAGTTTCTTGGTGCTTTTGCAGGAGCTGCT
GCAGTCTTTGGATTATACTATGATGCCTTCATGGACTTCACCAGTGGGATTCTGTCAGTG
ACGGGTATCAATGCAACGGGCCACATTTTTGCCTCATACCCCGGCAGACACCTGTCAGTC
CTCGGCGGCTTCATTGATCAGGTGGTGGGAACAGGTATGCTGGTTTTGTGCATTCTTGCT
ATCATCGACGGCGGAAACATCGGAGCTCCTAAAGGCATCGAGCCGCTGGCCATCGGCCTG
ATCATTATGGCCATCGGCGTGTCCATGGGGCTGAACTGTGGCTACCCCCTGAACCCAGCC
AGAGATCTGGGACCCAGACTGTTCACAGCTGTGGCAGGATGGGGGATGGAGGTCTTCAGC
ACCGCAGACAACTGGTGGTGGATCCCAGTGGCGGGACCGATGGTGGGGGGAGTGGTCGCG
GCCGTCATATACTACCTGTTCATTGAGCTGCACCACCCCCAC------------------
---GTCGAGCACGAGAAGCCC---CAGGAGGAAGAAGAGGAGGAC------------GAG
GAAGACGAAGAGGATGATGACAGCAGTCTGAAGGACAAATATGAGATGATCACGATGAGT
>Burtons_mouthbrooder_Aqp9a
---------------ATGGAAGTACAAAACAAGAGAAGCATGAGGCAACACTGTGCTCTA
AAACATGGAATATTTAAGGAATTTCTGGCAGAATTCCTGGGAACGTTTGTCTTGGTTCTG
TTTGGCTGCGGCTCCGTCGCTCAGACCGTCCTCAGTCGAAACACCCTGGGCGAACCCCTC
ACCGTCCACATCGGCTTCTCTGTAGGCCTGATGATGGCAGCATATGTGGCCGGTGGAGTT
TCAGGGGGCCATGTGAACCCCGCTGTGTCTTTGGCCATGGTAATTTTGGGCAAACTGAAG
ATCTGGAAGTTTCCTTTCTACGTCATCGCTCAGTTTCTTGGTGCTTTTGCAGGAGCTGCT
GCAGTCTTTGGATTATACTATGATGCCTTCATGGACTTCACCAGTGGGATTCTGTCAGTG
ACGGGTATCAATGCAACGGGCCACATTTTTGCCTCATACCCCGGCAGACACCTGTCAGTC
CTCGGCGGCTTCATTGATCAGGTGGTGGGAACAGGTATGCTGGTTCTGTGCATTCTTGCT
ATCATCGACGGCGGAAACATCGGAGCTCCTAAAGGCGTCGAGCCGCTGGCCATCGGCCTG
ATCATCATGGCCATCGGCGTGTCCATGGGGCTGAACTGTGGCTACCCCCTGAACCCAGCC
AGAGACCTGGGACCCAGACTGTTCACAGCTGTGGCAGGATGGGGGATGGAGGTCTTCAGC
ACCGCAGACAACTGGTGGTGGATCCCAGTGGCGGGACCGATGGTGGGGGGAGTGGTCGCG
GCCGTCATATACTACCTGTTCATTGAGCTGCACCACCCCCAC------------------
---GTCGAGCACGAGAAGCCC---CAGGAGGAAGAGGAGGAGGAC------------GAG
GAAGACGAAGAGGATGATGACAGCAGTCTGAAGGACAAATATGAGATGATCACGATGAGT
>Lyretail_cichlid_Aqp9a
---------------ATGGAAGTACAAAACAAGAGAAGCATGAGGCAACACTGTGCTCTA
AAACATGGAATATTTAAGGAATTTCTGGCAGAATTCCTGGGAACGTTTGTCTTGGTTCTG
TTTGGCTGCGGCTCCGTCGCTCAGACCGTCCTCAGTCGAAACACCCTGGGCGAACCCCTC
ACTGTCCACATCGGCTTCTCTGTAGGCCTGATGATGGCAGCGTATGTGGCCGGTGGAGTT
TCAGGGGGCCATGTGAACCCCGCTGTGTCTTTGGCCATGGTAATTTTGGGAAAACTGAAG
ATCTGGAAGTTTCCTTTCTACGTCATCGCTCAGTTTCTTGGTGCTTTTGCAGGAGCTGCT
GCAGTCTTTGGATTATACTATGATGCCTTCATGGACTTCACGAGTGGGATTCTGTCAGTG
ACGGGTATCAATGCAACGGGCCACATTTTTGCCTCATACCCCGCCAGACACCTGTCAGTC
CTCGGCGGCTTCATTGATCAGGTGGTGGGAACAGGTATGCTGGTTTTGTGCATTCTTGCT
ATCATCGACGGCGGAAACATCGGAGCTCCTAAAGGCGTCGAGCCGCTGGCCATCGGCCTG
ATCATCATGGCCATCGGCGTGTCCATGGGACTGAACTGTGGCTACCCCCTGAACCCAGCC

AGAGACCTGGGACCCAGACTGTTCACAGCTGTGGCAGGATGGGGGATGGAGGTCTTCAGC
ACCGCAGACAACTGGTGGTGGATCCCAGTGGCGGGACCGATGGTGGGGGGAGTGGTCGCG
GCCGTCATCTACTACCTGTTCGTCGAGCTGCACCACCCCCAC------------------
---GCCGAGCACGAGAAGCCC---CAGGAGGAAGAGGAGGAGGAC------------GAG
GAAGACGAAGAGGATGATGACAGCAGTCTGAAGGACAAATATGAGATGATCACGATGAGT
>Nile_tilapia_Aqp9a
---------------ATGGAAGTACAAAACAAGAGAAGCATGAGGCAACACTGTGCTCTA
AAACATGGAATATTCAAGGAATTCCTGGCAGAATTCCTGGGAACCTTTGTCTTGGTTCTG
TTTGGCTGTGGCTCCGTTGCTCAGACCGTCCTCAGTCGAAACACCCTGGGCGAACCCCTC
ACCGTCCACATCGGCTTCTCTGTAGGCCTGATGATGGCAGCGTATGTGGCCGGTGGAGTT
TCAGGGGGCCATGTGAACCCCGCTGTGTCTTTGGCCATGGTAATTTTGGGCAAACTGAAG
ATCTGGAAGTTTCCGTTCTACATCATCGCTCAGTTTCTTGGTGCTTTTGCAGGAGCTGCT
GCAGTCTTTGGATTATACTATGATGCCTTCATGGACTTCACCAGTGGGATTCTGTCAGTG
ACGGGAATCAATGCAACGGGCCACATTTTTGCCTCATACCCCGCCAGACACCTGTCAGTC
CTCGGCGGCTTCATTGATCAGGTGGTGGGAACAGGTATGCTGGTTTTGTGCATTCTTGCT
ATCACTGACAGCGGAAACATCGGAGCTCCTAAAGGCATCGAGCCGCTGGCCATCGGCCTG
ATCATCATGGCCATCGGCGTGTCCATGGGGCTGAACTGTGGCTACCCCCTAAACCCAGCC
AGAGACCTGGGACCCAGACTGTTCACAGCTGTGGCAGGATGGGGGATGGAGGTCTTTAGG
ACCGCAGACAACTGGTGGTGGATCCCAGTGGCGGGACCGATGGTGGGGGGAGTGGTCGCG
GCCGTCATCTACTACCTGTTCATCGAGCTGCACCACCCCCAC------------------
---GCCGAGCACGAGAAGCCC---CAGGAGGAAGAGGAGGAGGAC------------GAG
GAAGACGAAGAGGATGATGACAGCAGTCTGAAGGACAAATATGAGATGATCACGATGAGT
>Midas_cichlid_Aqp9a
---------------ATGGAAGTACAAAACAAGAGAAGCATGAGGCAACACTGTGCTCTG
AAACATGGAATATTCAAGGAATTCCTTGCAGAATTTCTGGGAACATTTGTCTTGGTCCTG
TTTGGCTGCGGCTCCGTCGCTCAGACTGTCCTCAGTCGAAGCACCCTGGGCGAGCCCCTC
ACCGTCCACATCGGTTTCTCTGTAGGCCTGATGATGGCAGTGTATGTGGCCGGTGGAGTG
TCAGGGGGCCATGTGAACCCTGCTGTGTCCCTGGCCATGGTAATTCTGGGCAAACTGAAG
ATCTGGAAGTTTCCCTTCTATGTCATCGCACAGTTTCTTGGTGCTTTTGCAGGAGCTGCT
GCAGTCTTTGGATTATACTATGACGCCTTCATGGACTTCACCAGTGGGATTCTGTCAGTG
ACAGGAATTAATGCAACGGGCCACATTTTTGCCTCATACCCCGCGAGACACCTGTCAGTC
CTTGGCGGCTTCATTGATCAGGTGGTGGGAACAGGTATGCTGGTTTTGTGTATTCTCGCC
ATCATTGATGGTGGAAACATTGGAGCTCCTAAAGGTGTCGAGCCACTGGCCATTGGCCTG
ATCATCATGGCCATTGGTGTGTCCATGGGACTGAACTGTGGCTATCCCCTGAACCCTGCC
AGAGACCTGGGACCCAGATTGTTCACAGCTGTGGCAGGATGGGGGATGGAGGTCTTCAGG
ACTGCAGACAACTGGTGGTGGATCCCAGTGGCGGGACCGATGGTGGGGGGAGTGATAGCA
GCCGTCATCTACTACCTGTTCATCGAGCTGCACCACCCCCAT------------------
---GTCGAGCATGAGAAGCCC---CAGGAGGAAGATGACGAGGAC------------GAG
GACGACGAAGAGGACGAGGACAGCAGTCTGAAGGACAAATATGAGATGATCACAATGAGT
>Indian_glassy_fish_Aqp9a
---------------ATGGAAGTACAACACAAGAGAAGCATGAGGCAACACTGCGCTCTC
AAACATGGAATATTCAAGGAATTCCTGGCAGAATTCCTGGGCACTTTTGTCCTGGTGTTG
TTCGGCTGTGGTTCAGTTGCTCAGACTGTGCTGAGTCGAAATACTCTCGGTGAACCTCTG
ACCGTGCACATCGGCTTCTCTGTAGGCCTGACAATGGCAGCGTATGTGGCTGGTGGAGTG
TCAGGAGGCCATGTGAATCCTGCTGTGTCTCTGGCCATGGTGATCCTGGGCAAACTGAAG
ATCTGGAAGTTTCCCTTCTACGTCATTGCTCAGTTTCTTGGTGCTTTTGCAGGAGCTGCT
GCAGTCTTTGGATTGTACTATGATGCTTTCATGGACTTCACCAACGGGATTCTGTCAGTG
ACCGGAATCAATGCAACAGGTCACATTTTTGCCTCATACCCCGCCAGACACCTGTCAGTC
CTCGGTGGCTTCATCGATCAGGTGGTGGGCACAGGTATGCTGGTGTTGTGTATTCTTGCG
ATCATTGATGGCGGGAACATTGGAGCTCCTAAAGGCGTAGAGCCACTGGCCATCGGCCTG
ATCATTATGGCTATCGGCGTGTCCATGGGACTGAACTGTGGCTACCCACTCAACCCCGCC
AGAGACCTGGGACCCCGACTGTTCACTGCTGTGGCAGGGTGGGGGATGGAGGTCTTCAGC
ACTGCAGACTACTGGTGGTGGATCCCAGTGGCCGGGCCCATGGTGGGGGGTGTGGTGGCA
GCCGTCATTTACTACCTGCTCATCGAGCTGCACCACCCCCGC------------------
---GAGGAGCACGAGAAACCC---CAGGAGGAGGAAGAGGAGGAA---------------
GAAGAGGAAGACGACGAGGACAGCAGTCTGAAGGACAAATATGAGATGATCACCATGAGC
>Rockpool_blenny_Aqp9a
---------------ATGGAAGTACACCACAAGAGAAGCATGAGGCAACACTGTGCTCTG
AAACATGGAATATTCAAGGAATTCTTGGCAGAATTCCTGGGAACGTTTGTCTTGGTGCTG
TTTGGCTGTGGCTCCGTCGCTCAGACCGTCCTCAGTCGAAACACCCTGGGCGAGCCACTC
ACCGTCCACATCGGCTTCTCTGTGGGCCTGATGATGGCAGCGTATGTGGCTGGTGGTGTT

TCAGGGGGCCATGTGAACCCTGCTGTGTCTCTGGCCATGGTGATTCTGGGCAAACTGAAG
ATCTGGAAGTTTCCCTTCTATGTCATCGCTCAGTTTCTTGGAGCTTTTGCAGGGGCTGCT
GCTGTCTTTGGATTATACTATGATGCTTTCATGGACTTCACCAGTGGGATTCTGTCAGTG
ACGGGAATCAATGCAACAGGTCATATTTTTGCTTCGTATCCTGCGAGACACCTGTCGGTG
CTCGGCGGCTTCGTTGATCAGGTGGTGGGGACTGGTATGCTGGTGCTGTGTATTCTTGCC
ATCATCGATGGCGGGAACATCGGTGCTCCTAAAGGCGTGGAGCCGCTGGCCATCGGCCTG
ATCATTATGGCCATCGGCGTGTCTATGGGACTGAACTGTGGCTACCCTCTAAACCCTGCC
AGGGACCTGGGACCCCGACTCTTCACCGCCGTGGCAGGGTGGGGAATGGAGGTCTTCAGC
ACCGGGGACTACTGGTGGTGGATCCCGGTGGCAGGGCCCATGGTGGGCGGCGTGATTGCG
GCCGTCATCTACTACCTGCTCATCGAGCTCCACCATCCCCGC------------------
---GACGAGCAGGAGAAGCCCCATCAGGAGGAGGGGGAGGAGGAC------------GAA
GATGACGAGGACGATGATGACAGCAGTCTGAAGGACAAATATGAGATGATTACCATGAGT
>Clown_anemonefish_Aqp9a
---------------ATGGAAGTACAACACAAGAGAAGCATGAGGCAACACTGTGCTCTC
AAACATGGAATATTCAAGGAATTCCTGGCTGAATTCCTGGGAACATTTGTCTTGGTTTTG
TTTGGCTGTGGCTCAGTCGCTCAGACCGTCCTCAGTAGAAACACTCTGGGTGAACCTCTC
ACCGTCCACATTGGTTTCTCTGTAGGCCTGATGATGGCAGCGTATGTGGCCGGTGGAGTG
TCAGGGGGCCATGTGAACCCTGCTGTGTCTCTGGCCATGGTGATTCTGGGCAAACTGAAG
ATCTGGAAGTTTCCCTTTTATGTCATCGCTCAGTTTCTCGGTGCTTTTGCAGGAGCTGCT
GCAGTCTTTGGATTATACTACGATGCTTTTATGGACTTCACCAGTGGAATTCTGTCGGTG
ACGGGAATCAATGCAACAGGTCACATTTTTGCGTCGTACCCTGCAAGACACCTGTCAGTC
CTCGGCGGTTTCATTGATCAGGTGGTGGGAACAGGGATGCTGGTTCTGTGCATCCTCGCG
ATCATTGATGGTGGAAACATCGGCGCTCCTAAAGGTGTTGAGCCGCTGGCCATCGGTCTG
ATCATCATGGCCATCGGTGTGTCGATGGGTCTGAACTGTGGTTACCCCCTGAACCCTGCC
AGAGACCTGGGACCCCGACTGTTCACAGCCGTGGCAGGATGGGGGATGGAGGTGTTCAGG
ACCGCCGACTACTGGTGGTGGATCCCGGTGGCGGGGCCCATGGTGGGAGGTGTGGTCGCA
GCCGTCATCTACTACCTGCTGATCGAGCTGCACCACCCCTCAC------------------
---GATGAGCAGGAGAAGCCCCATGAGGAGGAAGAAGAG------------------GAG
GAAGATGAAGAGGACGACGACAGCAGTCTGAAGGACAAATATGAGATGATCACCATGAGT
>Orange_clownfish_Aqp9a
---------------ATGGAAGTACAACACAAGAGAAGCATGAGGCAACACTGTGCTCTC
AAACATGGAATATTCAAGGAATTCCTGGCTGAATTCCTGGGAACATTTGTCTTGGTTTTG
TTTGGCTGTGGCTCAGTCGCTCAGACCGTCCTCAGTAGAAACACTCTGGGTGAACCTCTC
ACCGTCCACATTGGTTTCTCTGTAGGCCTGATGATGGCAGCGTATGTGGCCGGTGGAGTG
TCAGGGGGCCATGTGAACCCTGCTGTGTCTCTGGCCATGGTGATTCTGGGCAAACTGAAG
ATCTGGAAGTTTCCCTTTTATGTCATCGCTCAGTTTCTCGGTGCTTTTGCAGGAGCTGCT
GCAGTCTTTGGATTATACTACGATGCTTTTATGGACTTCACCAGTGGAATTCTGTCGGTG
ACGGGAATCAATGCAACAGGTCACATTTTTGCGTCGTACCCTGCAAGACACCTGTCAGTC
CTCGGCGGTTTCATTGATCAGGTGGTGGGAACAGGGATGCTGGTTCTATGCATCCTCGCG
ATCATCGATGGTGGAAACATCGGCGCTCCTAAAGGTGTTGAGCCGCTGGCCATCGGTCTG
ATCGTCATGGCCATCGGTGTGTCGATGGGTCTGAACTGTGGTTACCCCCTGAACCCTGCC
AGAGACCTGGGACCCCGACTGTTCACAGCCGTGGCAGGATGGGGGATGGAGGTGTTCAGG
ACCGCCGACTACTGGTGGTGGATCCCGGTGGCGGGGCCCATGGTGGGAGGTGTGGTCGCA
GCCGTCCTCTACTACCTGCTGATCGAGCTGCACCACCCCTCAC------------------
---GATGAGCAGGAGAAGCCCCATGAGGAGGAAGAAGAG------------------GAG
GAAGATGAAGAGGACGACGACAGCAGTCTGAAGGACAAATATGAGATGATCACCATGAGT
>Twoband_anemonefish_Aqp9a

--GGCAAACTGAAG
ATCTGGAAGTTTCCCTTTTATGTCATCGCTCAGTTTCTCGGTGCTTTTGCAGGAGCTGCT
GCAGTCTTTGGATTATACTACGATGCTTTTATGGACTTCACCAGTGGAATTCTGTCGGTG
ACGGGAATCAATGCAACAGGTCACATTTTTGCGTCGTACCCTGCAAGACACTTGTCAGTC
CTCGGCGGTTTCATTGATCAGGTGGTAGGAACAGGGATGCTGGTTCTGTGCATCCTCGCG
ATCATCGATGGTGGAAACATCGGCGCTCCTAAAGGTGTTGAGCCGCTGGCCATAGGTCTG
ATCATCATGGCCATCGGTGTGTCGATGGGTCTGAACTGTGGTTACCCCCTGAACCCTGCC
AGAGACCTGGGACCCCGACTGTTCACAGCCGTGGCAGGATGGGGGATGGAGGTGTTCAGC
ACCGCCGACTACTGGTGGTGGATCCCGGTGGCGGGGCCCATGGTGGGAGGTGTGGTCGCA
GCCGTCATCTACTACCTGCTGATCGAGCTGCACCACCCCTCAC------------------

---GATGAGCAGGAGAAGCCCCATGAGGAGGAAGAAGAG------------------GAG
GAA---
>Spiny_chromis_Aqp9a
---------------ATGGAAGTACAACACAAGAGAAGCATGAGGCAACACTGTGCTCTC
AAACATGGAATATTCAAGGAATTCCTGGCAGAATTCCTGGGAACATTTGTCTTGGTGTTG
TTTGGCTGTGGCTCAGTCGCTCAGACCGTCCTCAGTAGAAACACTCTGGGTGAACCTCTC
ACCGTCCACATTGGTTTCTCTGTAGGCCTGATGATGGCAGCGTATGTGGCCGGTGGAGTG
TCAGGGGGCCATGTGAACCCTGCTGTGTCTCTGGCCATGGTGATTCTGGGCAAACTGAAG
ATCTGGAAGTTTCCCTTTTATGTCATCGCTCAGTTTCTCGGTGCTTTTGCAGGAGCTGCT
GCAGTCTTTGGATTATACTACGATGCTTTTATGGACTTCACCAATGGAATTCTGTCGGTG
ACGGGAATCAATGCAACAGGTCACATTTTTGCGTCGTACCCTGCAAGACACCTGTCAGTC
CTCGGCGGTTTCATTGATCAGGTTGTGGGGACAGGGATGCTGGTTCTGTGCATCCTCGCG
ATCATCGACGGTGGAAACATCGGCGCTCCTAAAGGTGTTGAGCCGCTGGCCATCGGTCTG
ATCATCATGGCCATCGGTGTGTCGATGGGCCTGAACTGTGGTTACCCCCTGAACCCTGCC
AGAGACCTGGGACCCCGACTGTTCACCGCCGTGGCGGGATGGGGATGGAGGTGTTCAGG
ACTGCGGACTACTGGTGGTGGATCCCGGTGGCGGGGCCCATGGTGGGAGGTGTGGTCGCG
GCCGTCATCTACTACCTGCTGATCGAGCTGCACCACCCTCAC------------------
---GATGAGCAGGAGAAGCCCCATGAGGAGGAGGAAGAG------------------GAG
GAAGATGAAGACGATGATGATAGCAGTCTGAAGGACAAATATGAGATGATCACCATGAGT
>Bicolor_damselfish_Aqp9a
---------------ATGGAAGTACAACACAAGAGAAGCATGAGGCAACACTGTGCTCTC
AAACATGGAATATTCAAGGAATTCCTGGCAGAATTCCTGGGAACGTTTGTCTTGGTTTTG
TTTGGCTGCGGCTCAGTCGCTCAGACCGTCCTCAGTCGAAACACCCTGGGCGAACCTCTC
ACCGTCCACATCGGCTTCTCTGTAGGCCTGATGATGGCAGCGTATGTGGCTGGTGGAGTG
TCAGGGGGCCATGTGAACCCTGCTGTGTCTCTGGCCATGGTGATTCTGGGCAAACTGAAG
ATCTGGAAGTTTCCCTTTTATGTCATCGCTCAGTTTCTTGGTGCTTTTGCAGGAGCTGCT
GCAGTCTTTGGATTATACTACGATGCTTTTATGGACTTCACCAGCGGGATTCTGTCAGTG
ACAGGAATCAATGCAACAGGTCACATTTTTGCTTCGTACCCTGCGAGACACCTGTCAGTC
CTCGGCGGCTTCATCGATCAGGTGGTGGGGACAGGAATGCTGGTTCTGTGTATTCTCGCC
ATCATCGACGGTGGAAACATCGGAGCTCCTAAAGGCGTTGAGCCGCTGGCCATCGGTCTG
ATCATCATGGCTATCGGCGTGTCCATGGGCCTGAACTGCGGCTATCCCCTGAACCCCGCC
AGAGACCTGGGACCCCGACTGTTCACAGCTGTGGCAGGATGGGGATGGAGGTGTTCAGC
ACCGCTGACTACTGGTGGTGGATCCCGGTGGCGGGGCCCATGGTGGGAGGTGTGGTCGCG
GCCGTCATCTACTACCTGCTCATCGAGCTGCACCACCCCCCAC------------------
---GAGGAGCAGGAGAAGCCCCACGAGGAGGAAGAGGAG------------------GAG
GAAGACGAAGAGGACGAGGACAGCAGTCTGAAGGACAAATACGAGATGATCACCATGAGT
>Tigertail_seahorse_Aqp9a
--ATGAGGAAACATTGTGCGATC
AAACATGGAATACTGAAGGAATTCCTGGCAGAATTCCTCGGGACCTTTGTTCTGGTTTTG
TTTGGTTGTGGCTCGGTGGCCCAGACGGTCCTCAGTCGCAACTCTTTGGGTGAGCCACTC
ACCGTGCACATCGGTTTCTCTGTAGGACTGATGATGGCGGCTTACGTAGCTGGTGGAGTA
TCAGGGGGGCACGTGAACCCTGCTGTGTCTCTGGCCATGGTGATCCTTGGCAAACTGAAG
ATCTGGAAGTTTCCTTTCTACGTCATCGCTCAGTTTCTTGGCGCGTTTGCTGGCGCTGCT
GCAGTATTTGGATTATACTATGATGCTTTCATGGACTTCACCAGCGGGATATTGTCGGTG
ACGGGAATAAATGCAACCGGACACATTTTCGCATCCTACCCGGCGAGACACCTGTCGATT
CTCGGCGGATTCATTGATCAGGTGGTGGGCACCGGCATGCTTGTTCTGTGCATTTTGGCC
ATCATCGACGGAGGAAACATCGGCGCTCCGAAAGGTGTGGAGCCACTGGCCATCGGCTTG
ATCATCATGGCCATCGGGGTGTCGATGGGATTGAACTGTGGCTACCCCTTGAATCCTGCC
AGGGATCTCGGGCCGAGACTGTTCACTGCTGTGGCTGGATGGGGAATGGACGTCTTCAGC
ACGGCAGGATACTGGTGGTGGATCCCAGTGGCGGGGCCCATGGTTGGGGCTGTGGTCGCG
GCCGTCCTGTACTACCTGCTCATCGAGCTTCACCATCCCCAC------------------
---GCCGAAGCCGAGAAGCCTCACGAGGAGGAGGGCGACGAAGAGGAG---------GAG
GAAGATGAGGAGGACGAAGACTGCAGTCTGAAGGACAAATATGAGATGATCACCATGAGT
>Gulf_pipefish_Aqp9a
--ATGAGGAAACACTGTGCGATC
AAACATGGAATACTTAAGGAATTCCTGGCAGAATTCCTTGGGACCTTTGTTTTGGTTTTG
TTTGGTTGCGGCTCAGTTGCCCAGACAGTCCTCAGTCGAAACTCTTTGGGTGAGCCGCTC
ACCGTACACATCGGCTTCTCTGTAGGATTGATGATGGCCGTTTACGTGGCCGGTGGAGTA
TCAGGGGGCCACGTAAACCCTGCCGTTTCTCTGGCCATGGTGATCCTCGGCAAACTCAAG
ATCTGGAAGTTTCCTTTCTATGTCATTGCTCAGTTTCTTGGTGCATTTGCTGGAGCTGCT
GCTGTATTTGGATTATACTACGATGCGTTCATGGACTTCACCAGCGGGATTTTGTCGGTG

ACAGGAATCAATGCCACCGGTCACATTTTCGCATCTTACCCAGCGAGACACCTCTCGATT
CTTGGCGGATTCATTGATCAGGTGGTGGGAACCGGCATGCTGGTTCTGTGCATTCTGGCC
ATCATTGATGGAGGAAACATTGGTGCTCCTAAAGGTGTGGAGCCACTGGCCATCGGGCTG
ATCATCATGGCCATTGGGGTATCCATGGGACTGAACTGTGGTTACCCCTTGAATCCTGCC
AGGGACCTTGGGCCCAGACTGTTCACAGCTGTGGCCGGATGGGGCATGGAGGTCTTCAGC
ACGGCAGGCTACTGGTGGTGGATCCCAGTAGCGGGGCCTATGGTGGGAGCTGTGGTCGCA
GCCGTCCTGTACTACCTGCTCGTCGAGCTTCACCACCCCCGT------------------
---GTGGAAGCCGAAAAGCCTCATGAGGAGGAGGGTGAGGAGGACGAG---------GAG
GAGGAAGAGGATGAAGAGGAC---------------------------------------
>Atlantic_halibut_Aqp9a
---------------ATGGCAATACCACTCAAGAGAATCATGAGGCAACGCTGTGCTCTC
AAACACGGAATACTCAAGGAATTCCTGGCAGAATTCCTGGGGACGTTTGTCTTGGTCTTG
TTCGGCTGTGGCTCAGTCGCTCAGACCGTCCTCAGTCGGAACACCCTGGGCGAACCTCTC
ACCGTCCACATCGGCTTCTCTGTGGGACTGATGATGGCAGCGTATGTGTCCGGGGGAGTG
TCAGGGGGCCACGTGAACCCCGCCGTCTCTCTGGCCATGGTGATTCTGGGCAAACTGAAG
ATTTGGAAATTTCCCTTCTACGTCATCGCTCAGTTTCTCGGTGCCTTTGCCGGAGCTGCT
GCAGTCTTTGGATTATACTATGATGCTTTCATGGACTTCACCAGCGGGATTCTGTCAGTG
ACTGGAATCAATGCAACAGGCCACATCTTTGCTTCCTACCCAGCGAGACACCTGTCAGTC
CTCGGGGGCTTCATCGACCAGGTGGTGGGGACAGGGATGCTGGTTTTGTGTATCCTCGCC
ATCATCGATGGTGGGAACATCGGAGCTCCTAAAGGCGTGGAGCCGCTGGCCATCGGACTC
GTCATCATGGCCATCGGGGTGTCCATGGGACTGAACTGCGGATACCCCCTGAACCCCGCC
AGAGACCTGGGACCCCGGCTGTTCACGGCCATGGCTGGATGGGGGATGGAGGTTTTTAGC
ACTGCAGACTACTGGTGGTGGATCCCTGTGGCGGGGCCCATGGTCGGGGGAGTGGTCGCG
GCCATCATCTACTACCTGCTCATCGAGCTGCACCACCCCCGC------------------
---GAAGAGCCCGAGAAGCCCCACGAGGAGGAGGAGGAGGAG---------------GAG
GAAGACGAGGAGGACGACGACAGCAGTCTGAAGGACAAATATGAGATGATCACCATGAGC
>Marbled_flounder_Aqp9a
---------------ATGGCAATACCACTCAAGGGAATCATGAGGCAACACTGTGCTCTC
AAACATGGAATACTCAAGGAGTTCCTGGCAGAATTCCTGGGGACGTTTGTCTTGGTCTTG
TTCGGCTGTGGGTCAGTCGCTCAGACCGTCCTCAGTCGAAACACCCTGGGTGAACCTCTG
ACCGTCCACATCGGCTTCTCTGTGGGACTGATGATGGCAGCATATGTGTCCGGTGGAGTT
TCAGGGGGCCACGTCAACCCCGCCGTCTCTCTGGCCATGGTGATTCTGGGCAAACTGAAG
ATTTGGAAATTTCCCTTCTACGTCATCGCTCAGTTTCTCGGTGCCTTTGCCGGAGCTGCT
GCAGTCTTTGGATTATACTATGATGCTTTCATGGACTTCACCAGTGGGATTCTGTCAGTG
ACTGGAATCAATGCAACAGGCCACATCTTCGCTTCCTACCCAGCGAGACACCTGTCAGTC
CTCGGTGGCTTCATCGACCAGGTG------------------------------------

--CCCCTGAACCCCGCC
AGAGACCTGGGACCCCGGCTGTTCACGGCCGTTGCTGGATGGGGGATGGAGGTTTTTAGG
ACTGCAAACTACTGGTGGTGGATCCCTGTGGCGGGGCCCATGGTCGGGGGAGTGGTCGCC
GCCATCATCTACTACCTGCTCATCGAGCTGCACCACCCCCGC------------------
---GAGGAGCCCGAGAAGCCCCACGAGGAGGAGGAGGAGGAG---------------GAG
GAAGACGAGGACGACGACGACCACAGTCTGAAGGACAAATATGAGATGATCACCATGAGC
>Japanese_flounder_Aqp9a
---------------ATGGCAATACAACACAAGAGAAGCATGAGGCAACACTGTGCAATC
AAACATGGAATACTCAAGGAATTCCTGGCAGAATTCCTGGGGACGTTTGTCTTGGTCTTG
TTCGGCTGTGGCTCAGTCGCTCAAACCGTTCTCAGTCGAAACACCCTGGGCGAACCTCTC
ACCGTCCACATCGGCTTCTCTGTGGGACTGATGATGGCAGCGTATGTGGCCGGTGGAGTG
TCAGGGGGCCACGTGAACCCCGCTGTCTCTCTGGCCATGGTGATTCTGGGCAAACTGAAG
ATTTGGAAATTTCCCTTCTACGTCATCGCTCAGTTTCTTGGTGCCTTTGCAGGAGCTGCT
GCAGTCTTTGGATTATACTATGATGCTTTCATGGACTTCACCAGTGGAATTCTGTCAGTG
ACTGGAATCAATGCAACAGGCCACATTTTTGCCTCCTACCCAGCGAGGCACCTGTCAGTC
CTTGGCGGCTTCATCGACCAGGTGGTGGGGACAGGGATGCTGGTTTTGTGTATCCTCGCC
ATCATCGATGGTGGGAACATTGGAGCTCCTAAAGGCGTGGAGCCGCTGGCCATCGGACTC
ATCGTCATGGCCATCGGGGTGTCCATGGGACTGAACTGCGGATACCCCCTGAACCCTGCC
AGAGACCTGGGACCCCGACTGTTCACGGCAGTGGCTGGATGGGGGATGGAGGTTTTTAGG
ACCGCAGACTACTGGTGGTGGATCCCTGTGGCGGGGCCCATGGTCGGGGGAGTGGTCGCG
GCCGTCATCTACTACCTGCTCATCGAGCTGCACCACCACCGC------------------
---GATGAGCCCGAGAAGCCCCACGAGGAGGAGGAGGACGAA---------------GAC
GAAGACGAGGACGAGGACGACAGCAGTCTGAAGGACAAATATGAGATGATCACCATGAGC
>Turbot_Aqp9a

---------------ATGGAGATAAAACACAAGAGAAGCATGAGGCAACACTGTGCTCTC
AAACATGGAATACTCAAGGAATTCCTGGCAGAATTCCTGGGGACGTTTGTCTTGGTCTTG
TTCGGCTGCGGCTCAGTCGCTCAGACCGTCCTCAGTCGAAACACCCTGGGCGAGCCCCTC
ACCGTGCACATCGGCTTCTCTGTGGGACTGATGATGGCAGCGTACGTGGCTGGTGGAGTG
TCGGGGGGCCACGTGAACCCTGCTGTGTCTCTGGCCATGGTGATTCTGGGCAAACTAAAG
ATTTGGAAGTTTCCCTTCTACGTCATCGCCCAATTTCTCGGTGCCTTTGTTGGAGCCGCT
GCAGTCTTTGGATTGTACTACGATGCGTTCATGGACTTCACCAGCGGGATCCTGTCAGTG
ACAGGAATCAATGCGACGGGTCACATCTTTGCGTCCTACCCTGCGAGACACCTGTCAATC
CTCGGCGGCTTCATCGATCAGGTGGTGGGGACAGGTATGCTGGTTTTGTGTATCCTCGCC
ATCATCGACGGCGGAAACATCGGAGCTCCCAAAGGCGTGGAGCCGCTGGCCATCGGGCTG
ATCATCATGGCCATCGGAGTGTCCATGGGACTGAACTGTGGCTACCCGCTGAACCCTGCC
AGGGACCTGGGACCCCGGCTGTTCACGGCCGTGGCGGGATGGGGATGGAGGTTTTTAGG
ACTGCAGATAACTGGTGGTGGATCCCTGTGGCGGGCCCCATGGTCGGGGGAGTCGTCGCC
GCCGTCATCTACTACCTGTTCATTGAGCTGCACCACCACCAC------------------
---GATGAGCCCGAGAAGCCCCACGAGGAGGAGGAGGAGGAGGAGGAG---------GAG
GAAGACGAGGAGGACGAGGACTGCAGTCTGAAGGACAAATACGAGATGATCACCATGAGC
>Greater_amberjack_Aqp9a
---------------ATGGAAATACAACACAAGAGAAGCATGAGGCAACACTGTGCTCTC
AAACATGGAATACTCAAGGAATTCCTGGCAGAGTTCCTGGGGACGTTCGTCTTGGTTTTG
TTCGGCTGTGGCTCAGTCGCTCAGACCGTCCTCAGTCGAAACACCCTTGGTGAGCCTCTC
ACCGTCCACATCGGCTTCTCTGTGGGACTGATGATGGCAGCGTACGTGGCTGGTGGAGTC
TCAGGGGGCCATGTGAACCCTGCTGTGTCTCTGGCCATGGTGATTCTAGGCAAACTGAAG
ATTTGGAAGTTTCCCTTCTATGTCATCGCCCAGTTTCTTGGTGCTTTCGTTGGAGCTGCT
GCAGTTTTTGGATTATACTATGATGCTTTCATGGACTTCACCAGCGGGATTCTGTCAGTC
ACAGGAATCAATGCAACAGGTCACATTTTTGCTTCCTACCCTGCGAGACACCTGTCAGTC
CTCGGCGGCTTCATCGATCAGGTGGTGGGCACGGGTATGCTGGTGTTGTGTATCCTTGCT
ATCATCGATGGCGGAAACATTGGAGCTCCTAAAGGCGTGGAGCCGCTGGCCATCGGGCTG
ATCATCATGGCCATCGGCGTGTCCATGGGACTGAACTGTGGCTACCCCCTGAACCCCGCC
AGAGACCTGGGACCCCGACTGTTCACGGCTGTAGCAGGATGGGGGATGGAGGTTTTTAGC
ACTGCAGACTACTGGTGGTGGATCCCCGTGGCCGGGCCCATGGTGGGGGGAGTTGTTGCG
GCCGTCATCTACTACCTGCTGATCGAGCTGCACCACCACCAC------------------
---GACGAGCCCGAGAAGCCCCAAGAGGAGGAGGAGGAGGAG---------------GAA
GAAGACGAGGATGACGAGGACAGCAGTCTGAAGGACAAATATGAGATGATCACCATGAGC
>Japanese_amberjack_Aqp9a
---------------ATGGAAATACAACACAAGAGAAGCATGAGGCAACACTGTGCTCTC
AAACATGGAATACTCAAGGAATTCCTGGCAGAGTTCCTGGGGACGTTCGTCTTGGTTTTG
TTCGGCTGTGGCTCAGTCGCTCAGACCGTCCTCAGTCGAAACACCCTTGGTGAGCCGCTC
ACCGTCCACATCGGCTTCTCTGTGGGACTGATGATGGCAGCGTACGTGGCTGGTGGAGTC
TCAGGGGGCCATGTGAACCCTGCTGTGTCTCTGGCCATGGTGCTTCTAGGCAAACTGAAG
ATTTGGAAGTTTCCCTTCTATGTCATTGCTCAGTTTCTTGGTGCTTTCGTTGGAGCTGCT
GCAGTTTTTGGATTATACTACGATGCTTTCATGGACTTCACCAGCGGGATTCTGTCGGTG
ACAGGAATCAATGCAACAGGTCACATTTTTGCTTCCTACCCTGCGAGACACCTGTCAGTC
CTCGGCGGCTTCATCGATCAGGTGGTGGGCACCGGTATGCTGGTGTTGTGTATCCTTGCT
ATCATCGATGGCGGAAACATTGGAGCTCCTAAAGGCGTGGAGCCGCTGGCCATTGGGCTG
ATCATCATGGCCATCGGCGTGTCCATGGGACTGAACTGTGGCTACCCCCTGAACCCCGCC
AGAGACCTGGGACCCCGACTGTTCACGGCTGTAGCAGGATGGGGGATGGAGGTTTTTAGC
ACTGCAGACTACTGGTGGTGGATCCCTGTGGCCGGGCCCATGGTGGGGGGAGTTGTTGCG
GCCGTCATCTACTACCTGCTGATCGAGCTGCACCACCACCAC------------------
---GACGAGCCCGAGAAGCCCCAAGAGGAGGAGGAGGAGGAG---------------GAA
GAAGACGAGGACGACGAGGACAGCAGTCTGAAGGACAAATATGAGATGATTACCATGAGC
>Barramundi_Aqp9a
---------------ATGGAAATACAACACAAGAGAAGCATGAGGCAACACTGTGCTCTC
AAACATGGAATACTCAAGGAATTCCTGGCAGAATTCCTGGGGACGTTTGTGTTGGTTTTG
TTCGGCTGTGGCTCAGTCGCTCAGACCGTCCTCAGTCGAAACACTCTGGGTGAGCCTCTC
ACCGTCCACATCGGCTTCTCTGTGGGACTCATGATGGCAGCATACGTGGCTGGTGGAGTG
TCAGGGGGCCATGTGAACCCTGCTGTGTCCCTGGCCATGGTGATTCTGGGAAAACTGAAG
ATTTGGAAGTTCCCCTTTTACGTCATCGCTCAGTTTCTTGGTGCTTTTGCCGGAGCTGCC
GCAGTCTTTGGATTATACTATGATGCCTTCATGGACTTCACCAGCGGGATTCTGTCGGTG
ACAGGAATCAATGCAACAGGTCACATTTTTGCTTCCTACCCTGGCAGACACCTGTCAGTA
CTCGGCGGCTTCATCGATCAGGTGGTGGGGACAGGCATGCTGGTTTTGTGTATCCTTGCC
ATCATTGATGGCGGAAACATTGGAGCTCCTAAAGGTGTGGAGCCACTGGCTATCGGCCTG

ATCATCATGGCCATCGGTGTGTCCATGGGCCTGAACTGTGGCTACCCCCTGAACCCCGCC
CGAGACCTTGGACCCCGACTGTTCACGGCTGTAGCAGGATGGGGATGGAGGTTTTAGC
ACTGCAGACTACTGGTGGTGGATCCCTGTGGCAGGGCCCATGGTGGGGGGAGTCGTAGCG
GCTGTCATCTACTACCTGCTCATCGAGCTGCACCACCACCGC------------------
---GACGAGCCGGAGAAGCCCCACGGGGGGGAGGGGGAGGAGGATGAG---------GAG
GAAGACGAGGAGGATGATGACAGCAGTCTGAAGGACAAATATGAGATGATCACCATGAGC
>Derbio_Aqp9a
---------------ATGGAAATACAACAGAAGAGAAGCATGAGGCAACACTGTGCTCTC
AAACATGGAATACTCAAGGAATTCCTGGCAGAGTTCCTGGGGACATTCGTCTTGGTTTTG
TTCGGCTGTGGCTCAGTCGCTCAGACCGTCCTCAGTAGGAACACCCTGGGTGAGCCTCTC
ACCGTCCACATCGGCTTCTCTGTAGGACTGATGATGGCAGCTTATGTGGCTGGTGGAGTC
TCAGGGGGCCATGTGAATCCTGCTGTGTCTCTGGCCATGGTGATTCTGGGCAAACTGAAG
ATTTGGAAGTTTCCCTTCTATGTCATCGCTCAGTTTCTTGGTGCTTTTGCTGGAGCTGCT
GCAGTCTTTGGATTGTACTATGATGCTTTCATGGACTTCACCAGCGGGATTCTGTCAGTG
ACAGGGATCAATGCAACAGGACACATTTTTGCTTCCTATCCTGCGAGACACCTGTCAGTC
CTCGGCGGCTTCATCGATCAGGTGGTGGGGACGGGTATGCTGGTGTTGTGTATCCTTGCT
ATCATCGATGGTGGAAACATTGGCGCTCCTAAAGGCGTGGAGCCGCTGGCCATCGGGCTG
ATCATCATGGCCATCGGTGTGTCCATGGGGCTGAACTGTGGTTACCCCCTGAACCCCGCC
AGAGACCTGGGACCCCGACTGTTCACGGCTGTAGCAGGATGGGGATGGAGGTTTTAGC
ACTGCAGACTACTGGTGGTGGATCCCTGTGGCGGGGCCCATGGTGGGGGGAGTTGTCGCT
GCTGTCATCTACTACCTGCTCATTGAGCTGCACCACCACCAC------------------
---GACGAGCCCGAGAAGTCCCATGAGGAGGAGGAGGAG------------------GAG
GAAGACGACGAGGACGAGGACAGCAGTCTGAAGGACAAATATGAGATGATCACCATGAGC
>Northern_snakehead_Aqp9a
---------------ATGGAACTGCAAGACAACAGAAACATGAGGCAACACTGTGCTCTC
AAACATGGGATCCTCAAGGAATTCCTCGCTGAA---------------------------

---CTGTCAGTG
ACAGGAATCAATGCAACAGGTCACATTTTCGCTTCCTACCCTGCAAGACACCTGTCAGTC
CTCGGTGGCTTCATCGATCAGGTGGTGGGGACAGGTATGCTGGTTTTGTGTATCCTTGCT
ATCATCGATGGTGGAAACATCGGAGCTCCTAAAGGCATGGAGCCGCTGGCCATCGGTCTG
ATCCTCATGGCCATCGGTGTGTCCATGGGACTGAACTGTGGGTACCCCCTGAACCCAGCC
AGAGACCTGGGACCCCGGCTGTTCACGGCGGTAGCAGGATGGGGACGGAGGTTTTCAGC
ACTGCAGACTACTGGTGGTGGATCCCTGTGGCGGGGCCCATGGTGGGGGGAGTCGTAGCG
GCCGTCCTCTACTACCTGCTTATCGAGCTGCACCACCGCCGC------------------
---GAGCAGCCTGAGAGGCCCCGTGAGGAGGAGGAAGAGGAAGACGAT---------GAG
GAGGATGAGGAGGATGAGGACAGCAGCCTGAAGGACAAGTACGAGATGATCACCATGAGC
>Swamp_eel_Aqp9a
---------------TTGGAATTACAACACAGAAGAAGCATGAGGCAGCGTTGTGTTCTT
AAACATGGAATACTCAAGGAATTCCTGGCAGAGTTCCTGGGAACATTTGTCTTGGTTTTA
TTCGGTTGTGGTTCAGTTGCTCAGACCGCCCTCAGTCGAAACACCCTCGGTGACCCGCTC
ACCATCCACATTGGCTTCTCTGTAGGACTGATGATGGCAGTGTATGTGGCTGGAGGAGTA
TCAGGGGGCCACGTGAACCCTGCTGTTTCTCTGGCCATGGTGATTCTGGGCAAACTAAAG
ATCTGGAAGTTTCCCTTCTATGTCATTGCTCAGTTTCTTGGTGCTTTTGCTGGAGCTGCT
GCAGTCTTTGGGTTATATTATGACGCCTTCATGGATTTCACAAGTGGGAGTCTGTCAGTG
ACAGGGATCAACGCAACAGGTCACATTTTTGCTTCCTACCCCGCGAGACACCTGTCAGTC
CTTGGCGGCTTCATCGATCAGGTGGTGGGGACAGGTATGCTGGTTTTGTGTATCCTTGCT
ATCATCGATGATGGAAACATTGGAGCTCCCAGAGGCATGGAGCCGCTGGCCATCGGCCTG
ATCTTCATGGCCATTGGTGTGTCCATGGGACTGAACTGTGGCTACCCCCTGAACCCTGCC
AGAGACCTGGGACCCCGACTGTTCACGGCTATAGCAGGATGGGGATGGAGGTCTTTAGC
ACTGCAGACTACTGGTGGTGGATCCCTGTGGCGGGACCCATGGTGGGGGGAGTTGCTGCA
GCCGTCATCTACTACCTGCTCATTGAGCAGCATCACCATCAC------------------
---GATGAGCCCCAAAAGCCCCAGGAGGATGAGGAGGAG------------------GAA
GACGAGGAGGAGGATGAGGACAGCAGTCTGAGGGACAAATACGAGATGATCTCCATGAAC
>Kissing_gourami_Aqp9a
---------------ATGGAAATACAGCACAAGAGAAGTATGAGGCAACACTGTGCTCTC
AAACATGGAATATTCAAGGAATTCCTGGCTGAATTCCTCGGGACATTTGTCTTGGTTTTA
TTTGGTTGTGGATCAGTTGCTCAGACTGTCCTCAGTCGAAACACCCTGGGTGAGCCCCTC

ACTGTCCACATCGGCTTCTCCGTGGGACTGATGATGGCGGCTTATGTGGCTGGAGGAGTA
TCAGGGGGCCATGTGAACCCTGCTGTGTCTCTGGCCATGGTGATTCTGGGCAAACTCAAG
ATTTGGAAATTTCCTTTCTATGTCATCGCTCAGTTTCTTGGTGCTTTCGCTGGAGCTGCC
GCAGTCTTTGGATTATATTATGATGCCTTCATGGACTTCACCAGTGGGATTCTGTCAGTG
ACAGGAATCAATGCAACAGGTCACATTTTTGCGTCCTACCCTGCGAGACACCTGTCAATC
CTCGGTGGCTTCATCGATCAGGTGGTGGGGACGGGGATGCTGGTTTTGTGTATCCTCGCT
ATCATTGATGGTGGAAACATCGGAGCTCCCAAAGGTGTGGAGCCACTGGCCATTGGCCTG
ATCATCATGGCCATCGGTGTGTCAATGGGTCTTAACTGTGGCTACCCCCTGAACCCTGCC
AGAGACCTGGGGCCCCGACTGTTCACAGCAGTAGCAGGATGGGGGATGGAGGTCTTCAGC
ACTGCAGACTACTGGTGGTGGATCCCTGTGGCCGGGCCCATGGTGGGGGGAGTTGTTGCA
GCCGTCATCTACTACCTGCTCATTGAGCTGCACCACCATCAT------------------
---GATGAGCTCGAGAAGCCCCATGAGGAGGAGGAGGAG------------------GAG
GAAGATGAGGAGGACGAGGACAGCAGCCTGAAGGACAAATACGAGATGATCACCATGAGC
>Climbing_perch_Aqp9a
---------------ATGGAAATACAACCCAAGAGAAGCATGAGGCAACACTGTGCTCTC
AAACATGGAATATTCAAGGAATTCCTGGCTGAATTCCTGGGAACATTTGTCTTGGTTTTG
TTTGGCTGTGGATCAGTTGCTCAGACCGTCCTCAGTCGAAACACCCTGGGTGAGCCTCTC
ACTGTCCACATCGGCTTCTCTGTAGGACTGATGATGGCGGTATACGTGGCTGGTGGAGTA
TCAGGGGGCCATGTGAACCCTGCTGTGTCTCTGGCCATGGTGATTCTGGGCAAACTGAAG
ATTTGGAAGTTTCCCTTTTACGTCATCGCTCAGTTTCTTGGTGCTTTTGCTGGAGCTGCT
GCAGTCTTTGGATTGTATTATGATGCTTTCATGGACTTCACCAGTGGGATTCTGTCGGTG
ACGGGAATCAATGCAACAGGTCACATTTTTGCTTCCTACCCTGCGAGACACCTGTCAATC
CTCGGTGGCTTCATCGATCAGGTGGTGGGGACAGGGATGCTGGTTTTGTGTATCCTCGCT
ATCATTGATGGTGGCAACATTGGAGCTCCAAAAGGCGTGGAGCCTCTGGCCATCGGCCTG
ATCATCATGGCCATCGGCGTGTCCATGGGACTTAACTGTGGGTACCCGCTGAACCCTGCT
AGAGACCTGGGACCCCGACTGTTCACAGCTGTGGCAGGATGGGGGATGGAGGTCTTCAGC
ACCGCAGACTACTGGTGGTGGATCCCTGTGGCGGGGCCCATGGTGGGGGGAGTTATTGCC
GCCGTCATCTACTACCTGCTTATTGAACTGCACCACCGCCGC------------------
---GATGAGCCCGAGAAGCCCCATGAGGAGGAGGAGGAG------------------GAC
GAAGATGAAGAGGATGAAGACAGCAGCCTGAAGGACAAATACGAGATGATCACCATGAGC
>Siamese_fighting_fish_Aqp9a
---------------ATGGAAAGACCACACAAGAGAAGCATGAGGCAACACTGTGCTCTC
AAACATGGAATATTCAAGGAATTCCTGGCAGAGTTCCTCGGGACATTTGTCTTAGTTTTA
TTCGGCTGCGGCTCAGTGGCTCAGACTGTGCTCAGTCGAAACACCCTGGGTGAGCCTCTC
ACCGTGCACATCGGCTTCTCCGTGGGTCTGATGATGGCGGCGTACGTGGCTGGTGGAGTG
TCAGGGGGCCATGTCAACCCGGCTGTGTCTCTGGCCATGGTGATTCTGGGCAAACTGAAG
ATCTGGAAGTTCCCCTTCTACGTCATCGCTCAGTTTCTGGGCGCCTTCGCCGGAGCCGCC
GCGGTGTTCGGCCTGTACTATGATGCCTTCATGGACTTCACCAGCGGGATTCTGTCCGTG
ACGGGAATCAACGCCACGGGTCACATCTTCGCTTCCTACCCAGCCAGGCACCTGTCCATC
CTGGGGGGCTTCATCGATCAGGTGGTGGGGACCGGCATGCTGGTCATGTGCATCCTCGCT
ATCATCGACGGTGGGAACATCGGCGCTCCTAAAGGCGTGGAGCCGCTGGCCATCGGCCTG
GTCGTCATGGCCATCGGCGTGTCCATGGGACTGAACTGTGGCTACCCCCTGAACCCGGCC
AGGGACCTGGGGCCTCGGCTCTTCACGGCTGTGGCAGGATGGGGGATGGAGGTGTTCAGC
ACGGGCGACTACTGGTGGTGGATCCCCGTGGCCGGGCCCATGGTGGGGGGGGTCGTCGCC
GCCATCATCTACTACCTGTTCATCGAGCTGCACCACCGCCGC------------------
---GACGAACCCGAGAAGCCCCGCAAGGAGGAGGAGGAGGAGGAGGAAGAT------GAG
GAAGATGAGGAGGACGAGGACAGCAGCCTGAAGGACAAATACGAGATGATCACCATGAGC
>Zigzag_eel_Aqp9a
---------------ATGGAAATACAACACACGAGAAGCATGAGGCAACACTGTGCTCTG
AAACACGGAATACTCAAGGAATTCCTGGCAGAATTCCTGGGCACATTTGTCTTGGTTTTG
TTTGGCTGTGGGTCAGTGGCTCAAACCGTCCTCAGTCGAAACACCCTGGGCGAGCCTCTC
ACTGTCCACATCGGCTTCTCCATAGGACTGATGATGGCTGTGTATGTGTCTGGTGGAGTG
TCAGGGGGCCACGTGAACCCTGCTGTGTCTCTGGCCATGGTGATTCTGGGCAAACTGAAG
ATCTGGAAGTTTCCCTTATATGTCATCGCTCAGTTTCTTGGTGCCTTTGCTGGAGCGGCT
GCAGTCTTTGGATTATATTATGACGCTTTCATGGATTTCACCAGTGGGGTCCTAACAGTG
ACGGGAATCAATGCAACAGGTCACATTTTTGCCTCCTATCCTGCGAGACACCTGTCAATC
CTTGGCGGCTTCATTGATCAGGTGGTGGGGACGGGTATGCTGGTTTTGTGCATACTTGCT
ATCATTGATGGTGGAAACATCGGAGCTCCTAAAGGCATGGAGCCCCTGGCCATTGGCCTG
ATCATCATGGCCATCGGTGTCTCCATGGGACTGAACTGTGGCTACCCCCTAAACCCTGCC
AGAGACCTGGGACCTCGACTGTTCACCGCTGTAGCAGGATGGGGGATGGAGGTCTTCAGC
ACCGCAGACTACTGGTGGTGGATCCCCGTGGCAGGGCCCATGGTGGGGGGAGTTGTCGCG

GCCATCATCTACTACATGCTCATCGAGCTCCATCACCACCAT------------------
---GATGAGACTGAGAAGCCCCACGACGAGGACGAGGAG------------------GAG
GAAGACGAGGAGGACGAGGACAGCAGTCTGAAAGACAAATACGAGATGATCGCCATGAGC
>Pacific_bluefin_tuna_Aqp9a
---------------ATGGAAATAAAACAGAAGAGAAGCATGAGGCAACACTGTGCTCTT
AAACATGGAATACTTAAGGAATTCCTGGCAGAATTCCTTGGGACCTTCGTCTTGGTTTTG
TTCGGCTGTGGCTCGGTCGCTCAAACTGTCCTCAGTCGAAACTCTCTGGGTGAGCCGCTC
ACCATCCACATCGGCTTCTCTGTAGGACTGATGATGGCTGCATATGTGGCTGGTGGAGTG
TCAGGGGGCCATGTGAACCCTGCTGTGTCTCTGGCCATGGTGATTCTGGGCAAACTGAAG
ATTTGGAAGTTTCCTTTCTATGTGATCGCTCAGTTTCTTGGTGCTTTTGCTGGAGCTGCT
GCAGTCTTTGGATTATATTATGATGCTTTCATGGACTTCACCAGTGGGATTCTGTCGGTG
ACGGGAATCAATGCAACAGGTCACATTTTTGCTTCCTACCCTGCGAGACACCTGTCAATT
ATCGGTGGCTTCGTCGATCAGGTGGTGGGGACAGGTATGCTGGTCCTGTGTATCCTGGCT
ATCATCGATGGTGGAAACATTGGCGCTCCTAAAGGCGTGGAGCCGCTGGCTATTGGCCTG
ATCATCATGGCCATCGGTGTGTCCATGGGTCTGAACTGTGGCTATCCCCTGAACCCTGCC
AGAGACCTGGGACCCCGACTGTTCACAGCTGTGGCAGGATGGGGGATGGAGGTCTTCAGT
ACTGCAGAATACTGGTGGTGGATCCCTGTGGCGGGTCCCATGGTGGGGGGCATGATCGCG
GCCGTCATCTACTACCTGCTCATCGAGCTGCACCACCACCGC------------------
---GATGAGCCCGAGAAGCCCCCTGAGGAGGAGGAGGAGGAG---------------GAG
GAAGACGAGGAGGATGAGGACAGCAGTCTGAAGGACAAATATGAGATGATCACCATGAGC
>Atlantic_bluefin_tuna_Aqp9a
---------------ATGGAAATAAAACAGAAGAGAAGCATGAGGCAACACTGTGCTCTT
AAACATGGAATACTTAAGGAATTCCTGGCAGAATTCCTTGGGACCTTCGTCTTGGTTTTG
TTCGGCTGTGGCTCGGTCGCTCAAACTGTCCTCAGTCGAAACTCTCTGGGTGAGCCGCTC
ACCATCCACATCGGCTTCTCTGTAGGACTGATGATGGCTGCGTATGTGGCTGGTGGAGTG
TCAGGGGGCCATGTGAACCCTGCTGTGTCTCTGGCCATGGTGATTCTGGGCAAACTGAAG
ATTTGGAAGTTTCCTTTCTATGTGATCGCTCAGTTTCTTGGTGCTTTTGCTGGAGCTGCT
GCAGTCTTTGGATTATATTATGATGCTTTCATGGACTTCACCAGTGGGATTCTGTCGGTG
ACGGGAATCAATGCAACAGGTCACATTTTTGCTTCCTACCCTGCGAGACACCTGTCAATT
ATCGGTGGCTTCGTCGATCAGGTGGTGGGGACAGGTATGCTGGTCCTGTGTATCCTGGCT
ATCATCGATGGTGGAAACATTGGCGCTCCTAAAGGCGTGGAGCCGCTGGCCATTGGCCTG
ATCATCATGGCCATCGGTGTGTCCATGGGTCTGAACTGTGGCTATCCCCTGAACCCTGCC
AGAGACCTGGGACCCCGACTGTTCACAGCTGTGGCAGGATGGGGGATGGAGGTCTTCAGG
ACTGCAGAATACTGGTGGTGGATCCCTGTGGCGGGTCCCATGGTGGGGGGCATGATCGCG
GCCGTCATCTACTACCTGCTCATCGAGCTGCACCACCACCGC------------------
---GATGAGCCCGAGAAGCCCCCTGAGGAGGAGGAGGAGGAG---------------GAG
GAAGACGAGGAGGATGAGGACAGCAGTCTGAAGGACAAATATGAGATGATCACCATGAGC
>Yellowfin_tuna_Aqp9a
---------------ATGGAAATAAAACAGAAGAGAAGCATGAGGCAACACTGTGCTCTT
AAACATGGAATACTTAAGGAATTCCTGGCAGAATTCCTTGGGACCTTCGTCTTGGTTTTG
TTCGGCTGTGGCTCGGTCGCTCAAACTGTCCTCAGTCGAAACTCTCTGGGTGAGCCGCTC
ACCATCCACATCGGCTTCTCTGTAGGACTGATGATGGCTGCGTATGTGGCCGGTGGAGTG
TCAGGGGGCCATGTGAACCCTGCTGTGTCTCTGGCCATGGTGATTCTGGGCAAACTGAAG
ATTTGGAAGTTTCCTTTCTATGTGATCGCTCAGTTTCTTGGTGCTTTTGCCGGAGCTGCT
GCAGTCTTTGGATTATATTATGATGCTTTCATGGACTTCACCAGTGGGATTCTGTCGGTG
ACGGGAATCAATGCAACAGGTCACATTTTTGCTTCCTACCCTGCGAGACACCTGTCAATT
ATCGGTGGCTTCGTCGATCAGGTGGTGGGGACAGGTATGCTGGTCCTGTGTATCCTGGCT
ATCATCGATGGTGGAAACATTGGCGCTCCTAAAGGCGTGGAGCCGCTGGCCATTGGCCTG
ATCATCATGGCCATCGGTGTGTCCATGGGTCTGAACTGTGGCTATCCCCTGAACCCTGCC
AGAGACCTGGGACCCCGACTGTTCACAGCTGTGGCAGGATGGGGGATGGAGGTCTTCAGG
ACTGCAGAATACTGGTGGTGGATCCCTGTGGCGGGTCCCATGGTGGGGGGCATGATCGCG
GCCGTCATCTACTACCTGCTCATCGAGCTGCACCACCACCGC------------------
---GATGAGCCCGAGAAGCCCCCTGAGGAGGAGGAGGAG------------------GAG
GAAGACGAGGAGGATGAGGACAGCAGTCTGAAGGACAAATATGAGATGATCACCATGAGC
>Silver_pomfret_Aqp9a

--CCCTTG
ACTATCCACATTGGTTTCACTCTGGGAGTCATGATGGCCGTCTATATGGCAGGAGGAGTG
TCAGGGGGCCATGTCAACCCTGCTGTGTCTCTGGCCATGGTGATTCTGGGCAAACTGAAG
ATTTGGAAGTTTCCTTTCTATGTCATCGCTCAGTTTCTTGGTGCTTTTGTTGGAGCTGCT

GCAGTCTTTGGATTATACTATGATGCTTTCATGGAGTTCACCAGTGGGATTCTGTCAGTG
ACGGGAATTAATGCGACAGGTCACATTTTTGCTTCTTACCCTGCGAGACACCTGTCAGTT
CTTGGCGGTTTCATCGATCAGGTCGTGGGGACAGGTATGCTGGTTTTGTGTATCCTGGCT
ATCATTGATGGAGGAAACATTGGAGCTCCTAAAGGCGTGGAGCCACTGGCCATTGGCCTG
ATCATCATGGCCATCGGTGTGTCGATGGGACTGAACTGTGGCTACCCACTGAATCCTGCC
AGAGACCTGGGACCCCGACTGTTCACGGCTGTGGCAGGATGGGGATGGAGGTCTTCAGG
ACTGCAGACTACTGGTGGTGGATCCCTGTGGCGGGGCCCATGGTGGGGGGCGTGGTCGCG
GCTGTAATTTACTACTTGCTCATCGAGCTGCACCACCACCGT------------------
---GATGAGCCCGAGAAGCCCCCTGAGGAGGAGGAAGAGGAG---------------GAG
GAAGATGAGGAGGATGAGGACAGCAGTCTGAAGGACAAATATGAGATGATCACCATGAGC
>gbsMudskipper_Aqp9a
---------------------------------------ATGAGGCGACACTGTGGGCTC
AAATATGGAATATGCAAGGAATTTCTTGCAGAATTCCTAGGGACATTTGTTTTGGTGTTG
TTTGGTTGTGGTTCTGTGGCCCAAACCGTTTTGAGTCGGAACCAACTGGGTGAACCCCTC
ACAGTCCATATTGGTTTCTCTGTTGGTCTCATGATGGGAGTCTATGTGGCAGGAGGAGTG
TCAGGGGGGCACGTGAACCCTGCTGTGTCCCTGGCCATGGTCATTCTGGGCAAACTGAAG
ATCTGGAAGTTCCCCATTTATGTCATTGCTCAGTTTCTTGGAGCCTTCGCCGGAGCAGCT
GCTGTCTTTGGGTTATATTACGACGCCTTCATGGACTTCACCAGTGGGATTCTGTCTGTA
ACCGGGATCAATGCCACAGGTCATATATTCGCTTCATATCCGGCCAGACACCTGTCCGTT
CTGGGGGGCTTCATTGACCAGGTGGTGGGGACCGGGATGTTGGTCTTGTGTATACTGGCT
ATCATTGATGGGGGGAACATAGGAGCACCAAAAGGAATGGAGCCACTAGCAATCGGACTT
ATTATCATGGCTATCGGAGTGTCTATGGGCCTGAACTGTGGCTACCCTCTGAACCCTGCC
CGCGACCTGGGACCTCGACTGTTCACCGCGGTGGCCGGGTGGGGCTTTGAGGTCTTCAGG
ACGGCAGGATACTGGTGGTGGATTCCTGTGGCGGGTCCAATGGTCGGGGGAGTAGTGGCT
GCTGTCCTGTACTACCTACTGATTGAAATGCACCACAAACGG------------------
---GACACCCCAGAGAAA---ACAGATGAAGAGGAGGAAGAG---------------GAG
GAGGACGACGAGGATGAAGACAGCAGCCTGAAGGACAAGTATGAGATGATCCTGATGAAC
>Walking_goby_Aqp9a
---------------------------------------ATGAGGCGACACTGTGGGCTC
AAATATGGAATATGCAAGGAGTTTCTTGCAGAATTCCTGGGGACATTTGTTTTGGTGTTG
TTTGGTTGTGGTTCTGTGGCCCAAACCGTCTTGAGTCGGAACCAACTGGGTGAACCCCTC
ACGGTCCATATTGGTTTCTCTGTTGGTCTCATGATGGGAGTCTATGTGGCTGGAGGAGTG
TCAGGGGGGCACGTGAACCCTGCTGTGTCCCTGGCTATGGTCATTCTGGGCAAACTGAAG
ATCTGGAAGTTCCCCATCTATGTCATTGCTCAGTTTCTTGGAGCCTTCGCCGGAGCAGCT
GCTGTCTTTGGGTTATATTACGATGCCTTCATGGACTTCACCAGTGGGATTCTGTCTGTA
ACCGGGATCAATGCCACAGGTCATATATTTGCTTCGTATCCGGCCAGACACCTGTCCATT
CTGGGGGGCTTCATCGATCAGGTGGTGGGGACCGGGATGCTGGTCTTGTGTATACTGGCC
ATCATCGACGGGGGGAACATAGGAGCACCAAAAGGAATGGAGCCACTGGCCATTGGACTT
ATTATCATGGCCATCGGGGTGTCCATGGGCCTAAACTGTGGCTACCCCCTGAACCCTGCC
CGTGACCTGGGACCTCGAGTGTTCACCGCTGTGGCCGGGTGGGGCTTTGAGGTCTTCAGG
ACGGCAGGATACTGGTGGTGGATTCCTGTGGCGGGTCCCATGGTCGGGGGCGTAGTGGCT
GCCGTCCTGTACTACCTCCTGATTGAAATGCACCACAAACGG------------------
---GACGCCCCAGAGAAA---ACAGACGAAGAAGAGGAGGAG---------------GAC
GACGAGGAGGAGGATGAAGACAGCAGCCTGAAGGACAAGTATGAGATGATCCTGATAAAC
>Atlantic_mudskipper_Aqp9a
---------------------------------------ATGAGGCGACACTGTGGGCTC
AAATATGGAATATGCAAGGAATTTCTTGCTGAATTCCTGGGAACATTTGTTTTGGTGTTG
TTTGGTTGTGGTTCGGTGGCCCAAACCGTCTTGAGTCGGAACCAGCTGGGAGAACCCCTC
ACGGTCCATATTGGTTTCTCTGTTGGACTCATGATGGGCGTGTATGTGGCTGGAGGAGTA
TCTGGGGGGCACGTGAACCCTGCTGTGTCCCTGGCTATGGTCATTCTGGGCAAACTGAAA
ATTTGGAAGTTCCCCATTTATGTGATTGCGCAGTTTCTTGGAGCATTTGCCGGAGCAGCT
GCCGTCTTTGGGTTATATTACGATGCCTTCATGGATTTCACCAGTGGGATTCTATCTGTA
ACTGGGATCAATGCCACAGGCCATATATTCGCTTCGTATCCAGCGCGACACCTGTCCATT
CTGGGAGGCTTCATTGACCAAGTGGTGGGGACCGGGATGCTGGTCTTGTGTATACTGGCT
ATCATTGACGGAGGAAACATAGGAGCACCAAAAGGAATGGAGCCGCTGGCGATTGGACTT
ATTATCATGGCTATCGGGGTGTCTATGGGCCTGAACTGTGGCTACCCCCTGAACCCTGCT
CGTGACCTGGGACCTCGAATGTTCACTGCTGTGGCTGGCTGGGGCTTTGAGGTCTTCAGG
ACGGCAGGATACTGGTGGTGGATTCCTGTGGCTGGTCCCATGGTCGGAGGCGTAGTGGCC
GCTGTCCTGTACTACCTGCTGATCGAAATGCACCACAAACGA------------------
---GATGCACCAGAGAAG---TCAGATGACGAGGAGGAAGAG---------------GAG
GATGAGGATGAAGATGAAGACAGCAGCCTGAAGGACAAGTATGAAATGATCCTGATGAAC

```
>Giant_mudskipper_Aqp9a
---------------------------------------ATGAGACGACACTGTGGGCTC
AAATATGGAATATGCAAGGAATTTCTTGCAGAATTCCTGGGAACATTTGTTTTGGTGTTG
TTTGGTTGTGGTTCGGTGGCCCAAACCGTGTTAAGTCGGAACCAGCTGGGAGAACCCCTC
ACGGTCCATATTGGTTTCTCAGTTGGACTCATGATGGGAGTGTATGTGGCTGGAGGAGTG
TCTGGGGGGCACGTGAACCCTGCTGTGTCCCTGGCCATGGTCATTCTGGGCAAACTGAAA
ATTTGGAAGTTCCCCATTTATGTGATTGCGCAGTTTCTTGGAGCATTTGCCGGAGCAGCT
GCCGTCTTTGGGTTATATTACGATGCCTTCATGGACTTCACCAGTGGGATTCTGTCTGTA
ACTGGAATCAATGCCACAGGCCATATATTCGCTTCGTATCCAGCCCGACACCTGTCCATT
CTGGGAGGCTTCATTGACCAAGTGGTGGGGACTGGGATGCTGGTCTTGTGTATACTGGCT
ATCATTGACGGTGGAAACATAGGAGCACCAAAAGGAATGGAGCCACTGGCAATTGGACTT
ATTATCATGGCTATTGGGGTGTCTATGGGCCTGAACTGTGGCTACCCCCTGAACCCTGCC
CGCGACCTGGGACCTCGAGTGTTCACTGCTGTGGCCGGGTGGGGCTTTGAGGTCTTCAGG
ACGGCAGGATACTGGTGGTGGATTCCTGTGGCTGGTCCCATGGTCGGAGGCGTAGTGGCC
GCTGTCCTGTACTACCTGCTGATCGAAATGCACCACAAACAA------------------
---GATGCACCAGAGAAG---ACAGATGAGGAGGAGGAAGAG---------------GAG
GATGAGGATGAAGATGAAGATAGCAGCCTGAAGGACAAGTATGAAATGATCCTGATGAAC
>Pearlfish_Aqp9a
---------------------------------------ATGAGGCGACGCTGTGCTCTT
AAACATGGAATACTCAAGGAATTTCTGGCGGAGTTCCTGGGAACGTTTGTTTTGGTTCTG
TTTGGCTGTGGGTCTGTTGCCCAGACAGTACTGAGTCGGAACGCACTGGGCGAACCTCTC
ACCGTCCACATCGGCTTCTCTGTGGGATTGACGATGGCGGTTTATGTGGCTGGCGGTGTG
TCAGGGGGCCACGTGAACCCTGCTGTGTCTCTTGCCATGGTGCTTCTGGGCAAATTAAAG
GTCTGGAAGTTTCCGTTCTACATCATGGCTCAGTTTCTTGGTGCCTTTGCCGGGGCCGCT
GCCGTCTTTGGATTGTACTACGATGCTTTCATGGATTTCACCAGTGGGATTCTATCTGTG
ATAGGAATCAACGCCACCGGCCACATCTTTGCTTCCTACCCAGCCAGACACTTGTCAGTC
CTTGGCGGCTTCATTGATCAGGTGGTTGGGACAGGGATGCTGGTGTTGTGTATCCTGGCG
ATCATGGATGGCGGAAACATGGGCGCTCCTCGGGGCGTGGAGCCGCTGGCCATCGGCCTG
ATCTTCATGGCCATTGGCGTGTCCATGGGTCTCAACTGTGGCTATCCACTGAACCCTGCC
CGGGACCTGGGGCCCCGCCTCTTCACCGCAGTGGCGGGATGGGGGATGGACGTGTTCAGG
ACCGCAGACTACTGGTGGTGGATCCCCGTGGCAGGGCCCATGGTCGGGGGTGTAGTTGCC
GCCGTCCTCTACTACCTGCTCATCGAGATGCACCATCGCCGC------------------
---GAAAAGCCTGACAATCACCAAGAGGAGATGGAGGAGCTGTGTGAGGAAGAGGAGGAG
GTGGAAGAAGAGGAAGATGACAGTAGCTTGAAGGACAAATACGAGATGATCAACATGAGC
>Legless_cuskeel_Aqp9a
---------------------------------------ATGAGGCGACGCTGTGCTCTC
AAACATGGAATACTCAAGGAATTTCTGGCAGAATTCCTGGGGACCTTTGTTTTGGTTTTG
TTTGGCTGTGGGTCCGTTGCTCAGACTGTCCTCAGTCGAAACGCTCTGGGTGAACCTCTC
ACCGTCCACATCGGCTTCTCTATAGGCCTGATGATGGCAGCTTACGTGGCCGGCGGCGTG
TCAGGGGGCCACGTGAACCCTGCTGTGTCTCTGGCCATGGTTATTCTGGGCAAACTGAAG
GTCTGGAAGTTTCCCTTCTACGTCATTGCTCAGTTTCTTGGTGCTTTTGTTGGAGCTGCT
GCAGTCTTCGGATTGTACTACGATGCTTTCATGGATTTCACAAGTGGGATTCTGTCCGTG
ACGGGAATCAATGCAACAGGCCACATCTTTGCTTCCTACCCTGCCAGACACCTGTCAGTC
CTTGGCGGCTTCATTGATCAGGTGGTTGGGACAGGTATGCTGGTCCTGTGTATCCTGGCG
ATCATCGATGGTGGGAACATTGGAGCTCCCAGAGGTGTGGAGCCGCTTGCCATCGGCCTC
ATCATCATGGCCATCGGTGTGTCCATGGGACTGAACTGTGGCTACCCACTGAACCCTGCC
AGGGACCTGGGGCCTCGGCTGTTCACTGCTGTGGCAGGATGGGGGATGGAGGTGTTCAGG
ACTGCAGACAACTGGTGGTGGATCCCCGTGGCGGGGCCCATGGTCGGGGGCGTGGTCGCG
GCCGTCATCTACTACCTGTTCATTGAATTGCACCACCACCGC------------------
---GACCAGCCCGAGAATCCCCAAGAGGAGGCAGAAGAGCTGGAAGAGGAGGAG---GAA
GAG---------------------------------------------------------
>Bearded_brotula_Aqp9a
---------------------------------------ATGAGGCGACGCTGTGCTCTC
AAACATGGAATACTCAAGGAGTTTCTGGCTGAATTCCTGGGCACCTTTGTTTTGGTTTTG
TTCGGCTGTGGGTCCGTGGCTCAGACGGTCCTCAGTCGAAACGCCCTGGGTGAACCTCTC
ACCGTCCACATCGGCTTCTCTGTGGGTCTGTCGATGGCGGTCTACGTGTCCGGTGGGGTG
TCAGGGGGCCACGTCAACCCTGCTGTGTCCCTGGCCATGGTTCTTCTGGGCAAACTGAAA
GTCTGGAAGTTTCCCTTCTACGTCATCGCTCAGTTCCTCGGCGCTTTTGTCGGCGCCGCT
GCCGTCTTTGGGCTGTACTACGACGCCTTCATGGATTTCACCAGTGGGATTCTGTCGGTG
ACAGGAATCAACGCCACAGGCCACATCTTTGCCTCCTACCCTGCCAGACACCTGTCAGTC
CTGGGAGGCTTCATCGATCAGGTGGTGGGTACAGGTATGTTGGTCTTGTGCATCCTGGCC
```

ATCATCGATGGGGGAAACATCGGAGCTCCCAAAGGCGTGGAGCCGCTGGCCATCGGCCTG
ATCATCATGGCCATCGGCGTGTCCATGGGCCTGAACTGTGGCTACCCTCTGAACCCTGCC
AGGGATCTGGGGCCCCGGGTGTTCACTGCTGTGGCAGGATGGGGATGGAGGTCTTCAGG
ACGGCGGGCTACTGGTGGTGGATCCCCGTGGCGGGGCCCATGGTAGGGGGCGTGGTCGCC
GCCGTCATCTACTACCTGTTCGTCGAACTCCACCACCGCCGG------------------
---GATGAGGCAGAGAAGCCCCAAGAGGAGGCGGAGGAGCTGGAGGAGGAGGAG---GAC
GAAGAAGAGGAGGACGATGACAGCAGTCTGAAGGACAAGTATGAGATGATCACCATGAGC
>Pony_toadfish_Aqp9a
---------------------------------------ATGAGGAAACGCTGTGCTCTC
AAACATGGAATATTCAAAGAATTCCTGGCAGAATTCCTCGGGACATTTGTCTATGTTCTC
TTTGGCTGTGGGTCAGTCGCCCAGACTGTCCTCAGCAGAAATGCTCTGGGTGAACCTCTC
ACCGTCCACATCGGCTTCTCTGTGGGACTGATGATGGCAACATACGTGGCTGGTGGTGTG
TCAGGGGGCCATGTGAACCCTGCTGTGTCCCTGGCCATGGTTATTCTGGGCAAACTGAAG
ATCTGGAAGTTTCCCTTCTATGTCATTGCTCAGTTTCTTGGAGCTTTTGCTGGAGCTGCT
GCAGTCTTTGGATTATACTACGATGCGTTTATGGACTTCACCAGTGGGATTCTGTCAGTG
ACAGGCATCAGTGCCACAGGGCACATCTTTGCTTCGTACCCTGCGAGACATCTGTCAGTC
CTTGGCGGTTTTATCGATCAGGTAGTAGGAACCGGTATGCTGGTGCTGTGCATCCTGGCT
ATCAATGATGGTGGAAACATTGGAGCTCCTAAAGGTATGGAGCCGCTGGGCGTCGGCCTG
ATCATCATGGCCATTGGTGTATCCATGGGACTGAACTGTGGATATCCACTGAATCCTGCC
CGGGACCTGGGACCCCGCCTGTTCACTGCTGTGGCAGGATGGGGATGGAAGTCTTCAGG
ACTGCAGACTACTGGTGGTGGATCCCTGTGGCGGGTCCCATGGTCGGTGGCGTGGTTGCC
GCCGTCATCTACTTCCTCTTTATCGAGCTGCACCATCACCAC------------------
CTCATTGAGTCAGAGAAGCCACGTGAGGAAGAGGAGGAGGAGGAG------------GAA
GAAGATGAGGAGGAAGATGACAGCAGCCTGAAGGACAAATATGAGATGATCACCATGAAC
>Plainfin_midshipman_Aqp9a

---GGGGGCCATGTGAACCCTGCTGTGTCCCTGGCCATGGTTATTCTGGGCAAACTGAAG
ATTTGGAAGTTTCCCTTCTATGTCATCGCTCAGTTTCTCGGAGGTTTTGCTGGAGCTGCT
GCAGTCTTTGGATTATACTACGATGCCTTCATGGACTTTACCAGTGGGATCCTGTCAGTG
ACGGGCATCAATGCCACGGGTCACATTTTTGCTTCCTACCCTGCGAGACATCTGTCAGTC
CTGGGCGGCTTTATCGATCAGGTG------------------------TGCATCCTGGCT
ATCAATGATGGTGGAAATATTGGAGCTCCTAAAGGTATGGAGCCGCTGGCCATTGGCCTG
ATCATCATGGCCACTGGTGTGTCCTTGGGAATGAACTGTGGATACCCACTAAATCCTGCA
CGGGACCTTGGACCCCGTCTTTTCACCGCTGTGGCAGGATGGGGATGGAAGTCTTCAGC
ACTGCAGACTACTGGTGGTGGATCCCTGTGGCAGGTCCCATGGTCGGTGGTGTGGTCGCC
GCTGTCATCTACTTCCTTTTCATTGAGTTGCACCATCATCAC------------------
CACAATGAGTCAGAGAAGCCCCATGAGGAAGAGGAGGAGGAGGAA---------------

>Longspine_squirrelfish_Aqp9a
---------------ATGGAAATAAAAAACAAGAGAAGTATGAGGCAACACTGTGCTCTG
AAACATGGAATACTCAAGGAATTCCTGGCTGAATTCCTGGGAACATTTGTCTTGGTTTTG
TTTGGCTGTGGGTCGGTCGCCCAGACTGTCCTCAGTCGCAACACCCTGGGGGAGCCTCTC
ACCGTCCACATCGGCTTCTCTGTAGGACTGACGATGGCAGCGTATGTGGCTGGTGGAGTG
TCGGGGGGCCATGTGAACCCTGCTGTGTCTCTGGCCATGGTTATTCTGGGCAAACTGAAG
ATCTGGAAGTTTCCCTTCTATGTCATTGCTCAATTTCTTGGCGCTTTTGCTGGAGCCGCT
GCCGTCTTTGGATTATACTATGATGCTTTCATGGACTTCACCAGTGGGATTCTGTCAGTG
ACAGGCATCAACGCAACAGGTCACATCTTTGCGTCCTACCCTGGGCGACACCTGTCAGTC
CTCGGCGGTTTCATAGATCAGGTTGTAGGGACGGGCATGCTGGTCTTGTGTATCCTGGCT
ATCATAGATGGAGGAAATATTGGAGCTCCCAAAGGGGTGGAGCCACTGGCCATTGGTCTG
ATCATCATGGCCATCGGTGTGTCCATGGGTCTAAACTGTGGGTACCCACTGAACCCGGCC
CGGGACCTGGGGCCACGACTGTTCACCGCTGTGGCCGGATGGGGCATGGAGGTCTTCAGG
ACCGCGGACTACTGGTGGTGGATCCCCGTGGCCGGGCCTATGGTGGGGGGCGTGGTCGCG
GCCGTGGTCTACTTCCTCCTCATCGAGCTGCACCACCACCGC------------------
---GAGGAGCCGGAGAAGCCCAGCGGGGAGGAGGAGGAGGAA---------------GAG
GAGGAGGAGGAGGACGAGGACAGCAGTCTGAAGGACAAATACGAGATGATTACCATGAGC
>Sammara_squirrelfish_Aqp9a
---------------ATGGAAATAAAAAACAAGAGAAGTATGAGGCAACACTGTGCTCTG
AAACATGGAATACTCAAGGAATTCCTGGCTGAATTCCTGGGAACATTTGTCTTGGTTTTG

TTTGGCTGTGGGTCGGTCGCCCAGACTGTCCTCAGTCGCAACACCCTGGGTGAGCCTCTC
ACCGTCCACATCGGCTTCTCTGTAGGACTGACGATGGCATCGTATGTGGCTGGTGGAGTG
TCGGGGGGCCATGTGAACCCTGCTGTGTCTCTGGCCATGGTTATTCTGGGTAAACTGAAG
ATCTGGAAGTTTCCCTTCTATGTCATTGCTCAATTTCTTGGTGCTTTTGCTGGAGCTGCC
GCCGTCTTTGGATTATACTATGATGCTTTCATGGACTTCACCAGTGGGATTCTGTCAGTG
ACAGGCATCAATGCAACAGGTCACATCTTTGCCTCCTACCCTGGCGACACCTGTCAGTC
CTTGGCGGTTTCATAGATCAGGTTGTGGGGACGGGTATGCTGGTCTTGTGTATCCTGGCT
ATCATAGATGGAGGAAATATTGGAGCTCCCAAAGGGGTGGAGCCACTGGCCATCGGTCTG
ATAATCATGGCCATCGGTGTGTCCATGGGACTGAACTGTGGCTACCCACTGAACCCAGCC
CGGGACCTGGGGCCCCGACTGTTCACTGCTGTGGCCGGATGGGGCATGGAGGTCTTCAGG
ACCGCAGACTACTGGTGGTGGATCCCCGTGGCAGGGCCTATGGTGGGGGGCGTGGTCGCG
GCCGTGGTCTACTACCTCCTCATCGAGCTGCACCACCACCGC------------------
---GAGGAGCCAGAGAAGCCCCATGGGGAGGAAGAGGAGGAAGAG------------GAA
GAGGACGAGGAGGACGAGGACAGCAGTCTGAAGGACAAATATGAGATGATTACCATGAGC
>Blackbar_soldierfish_Aqp9a
---------------ATGGAAATAAAACACAAGAGAAGCATGAGGCAACACTGTGCTCTC
AAATATGGAATACTCAAGGAATTCCTGGCTGAATTCCTGGGAACTTTTGTCTTGGTTTTG
TTTGGCTGCGGCTCAGTCGCCCAGACTGTCCTCAGTCGCAACACCCTGGGTGAGCCTCTC
ACCGTCCACATCGGCTTCTCTGTGGGACTGACGATGGCAGCGTATGTGGCTGGTGGAGTG
TCGGGGGGCCATGTGAACCCTGCTGTGTCTCTGGCCATGGTTATTCTGGGCAAACTGAAG
ATCTGGAAGTTTCCCTTCTATGTCATCGCTCAGTTTCTTGGTGCTTTTGCCGGAGCTGCT
GCAGTCTTTGGATTATACTATGATGCTTTCATGGACTTCACCAGCGGGATTCTGTCGGTG
ACAGGCATCAACGCAACAGGTCACATCTTTGCGTCCTATCCTGCGAGGCACCTGTCAGTC
CTTGGCGGCTTCATAGATCAGGTTGTGGGGTCTGGGATGCTGGTCTTGTGTATCCTGGCC
ATCATTGACGGGGGAAACATTGGCGCTCCCAAAGGGGTGGAGCCTCTGGCCATTGGCCTG
ATCATCATGGCCATCGGTGTGTCCATGGGACTGAACTGTGGCTACCCGCTGAACCCTGCC
AGGGACCTGGGGCCCCGGCTGTTCACCGCTGTGGCCGGATGGGGCATGGAGGTCTTCAGG
ACCGCGGGCTACTGGTGGTGGATCCCCGTGGCAGGGCCCATGGTGGGGGGTGTGGTTGCA
GCCGTCGTCTACTACCTCCTCATCGAGCTGCACCACCACCAT------------------
---GATGAGCCAGAGAAGCCCCAGGGGGAAGAAGAGGAG------------------GAG
GAGGACGAGGAGGATGAGGACAGCAGTCTGAAGGACAAATATGAGATGATCACCATGAGC
>Pricklefish_Aqp9a
--ATGAGGCAACACTGCGCCCTG
AAACATGGAATACTCAAGGAATTCCTGGCAGAATTCCTGGGAACCTTTGTCTTGGTTTTG
TTTGGCTGTGGGTCAGTCGCTCAGACTGTCCTCAGTCGCAACACCCTGGGTGAGCCTCTC
ACCGTCCACATCGGCTTCTCTGTCGGACTGACGATGGCTTCGTATGTGGCTGGCGGAGTG
TCAGGT--

---------------------GATGCTTTCATGGACTTCACCAGTGGGATTCTGTCAGTG
ACAGGCATCAATGCAACAGGTCACATCTTTGCTTCCTACCCTGCCAGACACCTGTCAGTC
CTGGGCGGCTTCATTGACCAGGTTGTGGGGACAGGCATGCTGGTCTTGTCTATCCTGGCT
ATCCTTGATGGGGGAAACATCGGAGCTCCCAAAGGTGTGGAGCCGCTGGCCATTGGCCTG
ATCATCATGGCCATCGGTGTGTCTATGGGACTGAACTGTGGCTACCCGTTGAACCCGGCC
AGGGACCTGGGACCCCGGCTGTTCACGGCCGTGGCAGGATGGGGCATGGAGGTCTTCAGG
ACTGCAGACTACTGGTGGTGGATCCCTGTGGCAGGGCCCATGGTGGGGGGCGTGGTCGCC
GCCGTCGTCTACTACCTCCTCATCGAGGTGCACCACCGCCGC------------------
CACGAAGAGCCAGAGAAGCCCTGTGAGGAGGAAGAGGAC------------------GAG
GAAGAGGACGAGGACGAGGACAGCAGTCTGAAGGACAAATATGAGATGATCACCATGAGC
>Splendid_alfonsino_Aqp9a
--ATGAGGCAACGCTGTGCTCTG
AAACATGGAATACTCAAGGAATTCCTGGCAGAATTCCTGGGAACTTTTGTCTTGGTTTTG
TTTGGCTGTGGGTCCGTCGCTCAGACCGTCCTCAGCCGCAACACCCTGGGCGAGCCGCTC
ACCGTCCACATCGGCTTCTCTGTAGGACTGATGATGGCATCGTATGTGGCTGGTGGAGTG
TCAGGAGGCCATGTGAACCCTGCTGTGTCTCTGGCCATGGTTATCCTGGGCAAACTGAAG
ATCTGGAAGTTTCCCCTCTATGTCATTGCTCAGTTTCTCGGTGCTTTTGCTGGAGCTGCT
GCAGTCTTTGGATTATACTACGATGCTTTCATGGACTTCACCAGCGGGATTCTGTCGGTG
ACGGGCATCAATGCAACAGGTCACATCTTTGCTTCCTACCCTGCCAGACACCTGTCGGTC
CTGGGCGGCTTCATTGATCAGGTTGTGGGAACAGGCATGCTGGTCATGTGCATCCTGGCA
ATCATTGATGGGGGAAACATTGGAGCTCCCAAAGGTGTGGAGCCGCTAGCCATCGGCCTG
ATCTTCATGGCCATCGGCGTGTCTATGGGACTGAACTGCGGCTACCCGCTGAACCCGGCC
AGAGACCTGGGACCCCGGGTGTTCACTGCCGTGGCAGGATGGGGCATGGAGGTGTTCAGG

ACTGCAGGCTACTGGTGGTGGATCCCGGTGGCGGGGCCCATGGTGGGGGGCGTGGTCGCC
GCCGTCGTCTACTACCTCCTCATCGAGATGCACCACCGTCGC------------------
CACGACGAGCCAGAGAAGCCC---GACGAGGAGGAA------------------------

>Redmouth_whalefish_Aqp9a
--ATGAGGCAACACTGTGCTCTG
AAACATGGAATACTCAAGGAATTCCTGGCAGAATTCCTGGGAACATTTGTCTTGGTTTTG
TTTGGCTGTGGGTCAGTCGCTCAGACTGTCCTCAGTCGCAACACCCTGGGTGAGCCTCTC
ACCGTCCACATTGGCTTCTCTGTAGGACTGATGATGGCTTCTTATGTGGCTGGTGGAGTG
TCAGGGGGCCACGTGAACCCTGCTGTGTCTCTGGCCATGGTTATCCTGGGCAAACTGAAG
ATCTGGAAGTTTCCCTTCTATGTCATTGCTCAGTTTCTTGGTGCTTTTGCTGGAGCTGCT
GCAGTCTTTGGATTATACTATGATGCTTTCATGGACTTCACCAGTGGGATTCTGTCAGTG
ACAGGCATCAATGCAACAGGTCACATCTTTGCTTCCTACCCTGCCAGACACCTGTCAGTC
CTGGGCGGCTTCATTGATCAGGTTGTGGGGACAGGCATGCTGGTCTTGTGTATCCTGGCT
ATCATTGATGGGGGAAACATTGGAGCTCCCAAAGGTGTGGAGCCGCTGGCCATTGGCCTG
ATCATCATGGCCATCGGTGTGTCTATGGGACTGAACTGTGGCTACCCGCTGAACCCGGCC
AGGGACCTGGGACCCCGGCTGTTCACTGCCATGGCAGGATGGGGCATGGAGGTCTTCAGG
ACTGCAGACTACTGGTGGTGGATCCCTGTGGCAGGGCCCATGGTGGGGGGCGTGGTCGCC
GCCATCATCTACTACCTCCTCATCGAGCTGCACCACCACCGC------------------
CACGAAGAGCCAGAGAAGCCCCATGAGGAGGAAGAGGAGGAG----------------GAG
GAAGAGGAGGAGGACGAGGACAGCAGTCTGAAGGACAAATATGAGATGATCACCATGAGC
>Pinecone_fish_Aqp9a
--ATGAGGCAACACTGTGCTCTC
AAACATGGAATACTCAAGGAATTCCTGGCAGAATTCCTGGGAACTTTTGTCTTGGTTTTA
TTTGGCTGTGGGTCAGTCGCCCAGACTGTCCTCAGTCGCAACACCTTGGGTGAGCCTCTC
ACCGTCCACATTGGATTCTCTGTAGGACTGATGATGGCAGCATATGTGGCTGGTGGAGTG
TCAGGGGGCCATGTGAACCCTGCTGTTTCTCTGGCCATGGTTATCCTGGGCAAACTGAAG
ATCTGGAAGTTTCCCTTCTACGTCATTGCTCAATTTCTTGGTGCTTTTGCTGGAGCTGCT
GCAGTCTTTGGGTTATACTATGATGCTTTCATGGACTTCACCAGTGGGATTCTGTCAGTC
ACAGGCATCAATGCAACAGGTCACATCTTCGCTTCCTACCCTGCCAGACACCTGTCAGTC
CTGGGCGGCTTCATCGATCAGGTTGTGGGGACTGGCATGTTGGTCTTGTGTATCCTGGCT
ATCATTGACGGTGGAAACATTGGGGCTCCCAGAGGGGTGGAGCCACTGGCCATTGGCCTG
ATCTTCATGGCCATCGGGGTGTCTATGGGACTGAACTGTGGTTACCCACTGAACCCTGCC
AGGGACCTGGGACCCAGGCTGTTCACTGCTGTGGCAGGATGGGGCATGGAGGTCTTCAGG
ACTGCAAACTACTGGTGGTGGATCCCTGTGGCAGGGCCCATGGTGGGGGGCGTGGTTGGG
GCTGTCATCTACTACCTCCTCATCGAGCTACACCACCACCGC------------------
TGTGATGAGCCAGAGAAGCCCCACGAGGAAGAAGAGGAG------------------GAG
GAAGATGAGGAGGACGAGGACAGCAGCCTCAAGGACAAATATGAGATGATCACCATGAGC
>Opah_Aqp9a
--ATGAGGCAACACTGTGCTCTC
AAACATGGAATACTCAAGGAATTCCTGGCAGAATTCCTCGGGACTTTTGTTTTGGTTTTG
TTCGGCTGTGGGTCCGTGGCCCAGACCGTCCTCAGTCGCAACACCCTGGGTGAGCCCCTC
ACCATCCACATCGGCTTCTCGGTAGGACTGATGATGGCAGCGTACATTGCCGGCGGAGTG
TCAGGGGGCCACGTAAACCCTGCTGTGTCTCTGGCCATGGTCGTCCTGGGCAAACTGAAG
ATATGGAAGTTTCCCTTCTACGTCATTGCCCAGTTCCTTGGGGCTTTTGCTGGAGCGGCT
GCGGTGTTTGGTCTCTACTACGATGCTTTCATGGACTTCACCAGCGGGAATCTGTCTGTA
ACAGGTATCAATGCAACCGCTCACATCTTTGCGTCCTACCCAGCGAGACACCTGTCGGTC
CTCGGTGGCTTCGCTGACCAGGTTGTAGGGACGGGAATGTTGGTCTTGTGTATCCTGGCC
ATCATCGATGGGGGGAACATCGGAGCTCCTAAAGGCCTGGAGCCGCTGGCCATCGGTCTG
ATCATCATGGCCATTGGAGTTTCCATGGGGCTGAACTGCGGCTACCCACTCAACCCTGCC
AGGGACCTGGGACCCAGGCTGTTCACCGCTTTGGCGGGATGGGGCATGGAGGTCTTCAGG
ACTGCAGACTACTGGTGGTGGATCCCCGTGGCAGGTCCAATGGTGGGGGGTGTGGTTGGG
GCCATCATCTACTACCTCCTCATCGAGCTGCACCACCACCAT------------------
CACGATGAGCCAGAGAAGCCACACGAGGAGGAAGAGGAC------------------CAG
GAAGATGAGGAGGACGAGGACTGCAGTCTTAAGGACAAATATGAGATGATCACCATGAGC
>King_of_herrings_Aqp9a
--ATGAGGCAACACTGTGTTCTC
AAACATGGAATACTCAAGGAATTCCTGGCAGAATTCCTCGGCACTTTTGTTTTGGTTTTG
TTTGGCTGTGGGTCTGTGGCTCAGACCGTCCTTAGTCGCAACACCCTGGGTGAGCCTCTC
ACCATCCACATAGGCTTCTCAGTAGGACTGATGATGGCAGTGTATATTGCTGGTGGAGTG
TCAGGGGGCCACGTAAACCCTGCTGTGTCTCTGGCCATGGTTGTTCTGGGCAAGCTGAAG

ATATGGAAGTTTCCCTTCTATGTAATTGCTCAGTTCCTTGGTGCTTTTGCTGGAGCTGCT
GCAGTGTTTGGTCTATACTACGATGCTTTCATGGACTTCACCAGCGGAAATCTGTCTGTA
ACGGGTATCAATGCAACCGGTCACATCTTTGCTTCCTACCCAGCAAGACACCTCTCAGTC
ATCGGAGGCTTCATTGACCAGGTTGTAGGGACAGGAATGTTGGTCTTGTGTATCCTGGCC
ATCATCGATGGGGGAAACATTGGAGCTCCTAGAGGGCTTGAGCCTCTGGCCATCGGCCTG
ATCATCATGGCCATTGGAGTATCCATGGGGCTGAACTGTGGCTACCCACTCAACCCTGCC
AGGGACCTGGGACCCAGGCTGTTCACTGCTTTGGCGGGATGGGGAATGGAGGTCTTTAGG
ACTGCAGACTACTGGTGGTGGATCCCCGTGGCAGGGCCAATGGCGGGGGGTTTGGTCGGG
GCTGTCATCTATTACCTGCTCATCCAGCTGCACCACCACCACCAC---------------
CATGATGAGCCAGAGCAGTCACAGGAGGAGGATGATGAA------------------GAG
GAAGATGAAGAGGATGAGGACTGCAGTCTTAAGGACAAATACGAGATGATCACCATGAGC
>Atlantic_greeneye_Aqp9a
---------------ATGGAAATAAATCACAGGAGAAGTATGAGGCAACGCTGCGCTCTT
AAACATGGAATACTCAAGGAATTCCTGGCAGAATTCCTGGGGACTTTTGTCTTAGTGTTG
TTTGGCTGTGGGTCGGTCGCTCAGACTGTCCTAAGTCGCAACACCCTGGGTGAGCCCCTC
ACTATCCACATCGGCTTCTCCGTAGGGCTGATGATGGCGGCATATGTGGCCGGTGGAGTA
TCAGGGGGGCATGTGAACCCTGCTGTATCTCTGGCTATGGTTATTCTGGGCAAACTGAAG
GTCTGGAAATTTCCCTTCTATGTCATTGCTCAGTTCCTCGGTGCTTTTGTTGGAGCGGCT
GCCGTCTTCGGGCTATACTATGATGCTTTCATGGACTTCACCAGTGGCATTCTGTCAGTG
ACAGGCATCAATGCCACAGGTCACATCTTTGCCTCCTATCCAGGGAGACACCTGTCCGTC
CTCGGTGGCTTCATCGACCAGGTTATGGGAACCGGCATGTTGGTCTTGTGCATCCTGGCG
ATCATCGATGGAGGTAACATCGGAGCCCCCAAAGGCGTGGAGCCCCTCGCCATCGGCCTG
ATCATCATGGCCATCGCGGTGTCAATGGGACTGAACTGTGGCTACCCGCTCAACCCGGCC
AGGGACCTGGGTCCTAGGCTCTTCACTGCCGTGGCAGGGTGGGGCATGGAGGTCTTCAGG
-------------------------------GCGGGGCCCATGGTAGGGGGCGTGGTCGGG
GCCATCCTCTACTTCCTGCTCATTGAGCTGCACCACCACCAC------------------
CCCGCCGAGCCGGAGAAGCCCCACGAGGAGGAAGACGAGGAG---------------GAG
GAAGACGAGGAGGATGAGGACAGCAGCCTGAAGGACAAATACGAAATGATCACCATGAGC
>Atlantic_cod_Aqp9a
---------------------------------------ATGAGGAGACACTGTGTTCTC
AAACATGGGATACTTAAGGAATTCCTGGCGGAGTTCCTGGGGACCTTCGTCTTGGTGCTG
TTTGGCTGCGGCGCCGCGGCCCAGACGGCCCTCAGCAGGAACACCCTGGGCGAGCCGCTC
ACCGTGCACATCGGCTTCTCCCTGGGCCTCACCATGGCCTGCTACGTGGCGGGAGGAGTC
TCAGGTGGTCATGTGAACCCGGCCGTCTCGCTGGCCATGGTGGTCCTTGGCAAACTCAAG
ATCTGGAAGTTCCCCATTTACGTCCTCGCACAATTCCTGGGTGCCTTTGCCGGGGCGGCA
GCCGTGTTCGGCTTATACTACGACGCCTTCATGGACTTCACCAGTGGGATCCTGTCAGTG
ACCGGCATCAACGCCACAGGTCACATCTTTGCCTCCTACCCTGGACGACACCTGTCAATC
CTGGGCGGCTTCCTCGATCAGGTCATCGGGACCGGGATGCTCGTGGTGTGCATCCTGGCC
ATCGTCGACGGGGGGAACATCGGAGCCCCTAAGGGCGTGGAGCCGCTGGCCATAGGCCTC
ATCGTCATGGCCATCTCCGTGTCCATGGGGCTCAACTGTGGCTACCCTGTGAACCCGGCC
CGCGACCTGGGCCCGCGCCTCTTCACAGCCGTGGCCGGCTGGGGCATGGAGGTCTTCAGG
ACCGGCGACTACTGGTGGTGGATCCCAGTGGCCGGGCCCCTGGTGGGGGGCCTGGTTGGT
GCGGCCCTCTACTACCTGCTCATCGAGCTCCACCACCCCCAGCAGCCCCCCCCCTCGAGC
CCCGAGGAGCCGGAGAAGACCCCGGAAGAGGAGGACGAG------------------GAG
GAGGAAGATGAGGAAGACGACAGT---CTCAAGGACAAATATGAGATGATCGCCATGAGC
>Walleye_pollock_Aqp9a
---------------------------------------ATGAGGAGACACTGTGTTCTC
AAACATGGGATACTTAAGGAATTCCTGGCGGAGTTCCTGGGGACCTTCGTCTTGGTG---

------------------CAGGTCATCGGGACCGGGATGCTCGTGGTGTGCATCCTGGCC
ATCGTCGACGGGGGGAACATCGGAGCCCCTAAGGGCGTGGAGCCGCTGGCCATAGGCCTC
ATCGTCATGGCCATCTCCGTGTCCATGGGGCTCAACTGTGGCTACCCCGTGAACCCGGCC
CGCGACCTGGGCCCGCGCCTCTTCACAGCCGTGGCCGGCTGGGGCATGGAGGTCTTCAGG
ACCGGCGACTACTGGTGGTGGATCCCAGTGGCCGGGCCCCTGGTGGGGGGCCTGGTTGGN
------------TACCTGCTCATCGAGCTCCACCACCCCCAGCAGCCCCCCCCCTCGAGC
CCCGAGGAGCCGGAGAAGACCCCGGAAGAGGAGGACGAG------------------GAG

GAGGAAGATGAGGAAGACGACAGT---CTCAAGGACAAATATGAGATGATCGCCATGAGC
>Arctic_cod_Aqp9a
--ATGAGGAGACACTGTGTTCTC
AAACATGGGATACTTAAGGAATTCCTGGCGGAGTTCCTGGGGACCTTCGTCTTGGTGCTG
TTTGGCTGTGGCGCCGCGGCCCAGACGGCCCTCAGCAGGAACACCCTGGGCGAGCCGCTC
ACCGTGCACATCGGCTTCTCCCTGGGCCTCACCATGGCCTGCTACGTGGCGGGGGGAGTC
TCAGGGGGTCATGTGAACCCGGCCGTCTCGCTGGCCATGGTGGTCCTTGGCAAACTCAAG
ATCTGGAAGTTCCCCATTTACGTCCTCGCACAATTCCTGGGTGCCTTTGCCGGGGCGGCA
GCCGTGTTCGGCTTATACTACGACGCCTTCATGGACTTCACCAGTGGGATCCTGTCAGTG
ACAGGCATCAACGCCACAGGTCACATCTTCGCCTCCTACCCTGGACGACACCTGTCAATC
CTGGGTGGCTTCCTCGATCAGGTCATCGGGACCGGGATACTCGTGGTGTGCATCCTGGCC
ATCGTCGACGGGGGGAACATCGGAGCCCCTAAGGGCGTGGAGCCGCTGGCCATAGGCCTC
ATCGTCATGGCCATCTCCGTGTCCATGGGGCTCAACTGTGGCTACCCCGTGAACCCGGCC
CGCGACCTGGGCCCGCGCCTCTTCACAGCCGTGGCCGGATGGGGCATGGAGGTCTTCAGG
ACCGGCGACTACTGGTGGTGGATCCCAGTGGCCGGGCCCCTGGTGGGGGGCCTGGTTGGT
GCGGCCCTCTACTACCTGCTCATCGAGCTCCACCACCCCCAGCAGCCCCCCGCCACGAGC
CCCGAGGAGCCGGAGAAGACCCCGGAAGAGGAGGAGGAG------------------GAG
GAGGAAGATGAGGAAGACGACAGT---CTCAAGGACAAATATGAGATGATCGCCATGAGC
>Saithe_Aqp9a
--ATGAGGAGACACTGTGTTCTC
AAACATGGGATACTTAAGGAATTCCTGGCGGAGTTCCTTGGGACCTTCGTCTTGGTGCTG
TTTGGCTGTGGCGCCGCGGCCCAGACGGCCCTCAGCAGGAACACACTGGGCGAGCCGCTC
ACCGTGCACATCGGCTTCTCCCTGGGCCTCACCATGGCCTGCTACGTGGCGGGGGGAGTC
TCAGGGGGTCATGTGAACCCGGCCGTCTCGCTGGCCATGGTGGTCCTCGGCAAACTCAAG
ATCTGGAAGTTCCCCATTTACGTCTTTGCACAATTCCTGGGTGCCTTTGCCGGGGCAGCA
GCCGTGTTCGGCTTATACTACGACGCCTTCATGGACTTCACCAGTGGGATCCTGTCAGTG
ACGGGCATCAACGCCACAGGTCACATCTTCGCCTCCTACCCTGGACGACACCTGTCAATC
CTGGGCGGCTTCCTCGATCAGGTCATCGGGACTGGGATGCTCGTGGTGTGCATCCTGGCC
ATCGTCGACGGGGGGAACATCGGAGCCCCCAAGGGCGTGGAGCCGCTGGCCATAGGCCTC
ATCGTCATGGCCATCTCCGTGTCCATGGGACTCAACTGTGGCTACCCCGTGAACCCGGCC
CGCGACCTGGGCCCGCGCCTCTTCACCGCCGTGGCCGGCTGGGGCATGGAGGTCTTCAGG
ACCGGCGACTACTGGTGGTGGATCCCAGTGGCCGGACCCCTGGTGGGGGGCCTGGTTGGT
GCGGCCCTCTACTACCTACTCATC------------------------------------
---------CCGGAGAAGACCGCGGAAGAGGAGGAGGAG------------------GAG
GAGGAAGATGAGGAAGACGACAGT---CTCAAGGACAAATATGAGATGATCGCCATGAGC
>Whiting_Aqp9a
--ATGAGGAGACACTGTGTTCTC
AAACATGGGATACTTAAGGAATTCCTGGCGGAGTTCCTGGGGACCTTCGTCTTGGTGCTG
TTTGGCTGCGGCGCCGCGGCCCAGACGGCCCTCAGCAGGAACACCCTGGGCGAACCGCTC
ACCGTGCACATCGGCTTCTCCCTGGGCCTCACCATGGCCTGCTACGTGGCGGGCGGAGTC
TCAGGGGGTCACGTGAACCCGGCCGTCTCGCTGGCCATGGTGGTCCTTGGCAAACTCAAG
ATCTGGAAGTTCCCCGTTTACGTCTTGGCACAACTCCTGGGTGCCTTTGCCGGGGCTGCA
GCCGTGTTCGGCTTATACTACGACGCCTTCATGGACTTCACCAGTGGGATCCTGTCAGTG
ACGGGCATCAACGCCACAGGTCACATCTTCGCCTCCTACCCTGGACGACACCTGTCCATC
CTGGGCGGCTTCCTTGATCAGGTCATCGGGACCGGGATGCTAGTGGTGTGCATCCTGGCC
ATCGTCGACGGGGGGAACATCGGGGCCCCTAAGGGCGTGGAGCCGCTGGCGATAGGCCTC
ATCGTCATGGCCATCTCCGTGTCCATGGGGCTCAACTGTGGCTACCCTGTGAACCCGGCC
CGCGACCTGGGCCCGCGCCTCTTCACAGCCGTGGCCGGCTGGGGCATGGAGGTCTTCAGG
ACCGGCGACTACTGGTGGTGGATCCCCGTGGCCGGGCCCCTGGTGGGGGGCCTGGTCGGT
GCGGCCCTCTACTACCTGCTCATCGAGCTCCACCACCCCCAGCAGCCCCCCACCTCGAGC
CCCGAGGAGCCGGAGAAGACCCCGGAAGAGGAGGAG---------------------GAG
GAGGAAGATGAGGAAGACGACAGT---CTCAAGGACAAATATGAGATGATCGCCATGAGC
>Haddock_Aqp9a
--ATGAGGAGACACTGTGTTCTC
AAACATGGGATACTTAAGGAATTCCTGGCGGAGTTCCTAGGGACCTTCGTCTTGGTGCTG
TTTGGCTGCGGCGCCGCGGCCCAGACGGCCCTCAGCAGGAACACCCTGGGCGAACCACTC
ACCGTGCACATCGGCTTCTCCCTGGGCCTCACCATGGCCTGCTACGTGGCGGGGGGAGTC
TCAGGGGGTCATGTGAACCCGGCCGTCTCGCTGGCCATGGTGGTCCTCGGCAAACTCAAG
ATCTGGAAGTTCCCCATTTACGTCTTCGCACAATTCCTGGGTGCCTTTGCCGGGGCCGCA
GCCGTGTTCGGCTTATACTACGACGCCTTCATGGACTTCACCAGTGGGATCCTGTCAGTG
ACGGGCATCAACGCCACAGGTCACATCTTCGCCTCCTACCCTGGACGACACCTGTCAATC

CTGGGCGGCTTCCTCGATCAGGTCATCGGGACCGGGATGCTTGTGGTGTGCATCCTGGCC
ATCGTCGACGGGGAGAACATCGGAGCCCCTAAGGGCGTGGAGCCGCTGGCCATAGGCCTA
ATCGTCATGGCCATCTCCGTGTCCATGGGGCTCAACTGTGGCTACCCTGTGAACCCGGCC
CGTGACCTGGGCCCGCGCCTCTTCACAGCCGTGGCCGGCTGGGGCATGGAGGTCTTCAGG
ACCGGCGACTACTGGTGGTGGATCCCAGTGGCCGGGCCCCTGGTAGGG------------

---------------ATGACCCCGGAGGAGGAGGAG---------------------GAG
GAGGAAGATGAGGAAAACGACAGT---CTCAAGGACAAATATGAGATGATCGCTATGAGC
>Silvery_cod_Aqp9a
---ATGAGGCGACACTGTGTTCTC
AAACATGGGATACTCAAGGAATTCCTGGCGGAGTTCCTGGGGACCTTCGTCCTGGTGCTG
CTGGGCTGCGGAGCGGCGGCGCAGACGGCCCTCAGCAGGAACACCCTGGGAGAGCCGCTC
ACCGTGCACATCGGCTTCTCCCTGGGCCTCACCATGGCCTGCTACGTGGCAGGGGGAGTC
TCAGGGGGTCATGTGAACCCGGCGGTCTCGCTGGCCATGGTGGTCCTTGGCAAACTCAAG
ATCTGGAAGTTCCCCGTTTACGTCCTCGCACAGTTCCTGGGCGCCTTTGCCGGGGCGGCG
GCCGTGTTCGGCTTATACTATGATGCCTTCATGGACTTCACCAGTGGGATTCTGTCAGTG
ACGGGCATTAACGCCACAGGTCACATCTTCGCCTCCTACCCTGGACGACACCTGTCAATC
CTAGGCGGCTTATTTGATCAGGTCATCGGGACCGGGATGCTGGTGGTGTGCATCCTGGCC
ATCGTTGATGGGGGGAACATTGGAGCCCCCAAGGGCATGGAGCCGCTGGCCATCGGCCTC
ATCCTCATGGCCATCTCGGTGTCCATGGGGCTGAACTGTGGCTACCCCGTGAACCCGGCC
CGCGACCTGGGCCCGCGCCTCTTCACTGCTGTGGCTGGCTGGGGCATGGAGGTCTTCAGG
ACCGGCGGCTACTGGTGGTGGATCCCCGTGGCGGGGCCCATGGTGGGGGGCCTGGTGGGA
GCGGTCCTGTACTACTTGCTCATCGAGCTTCACCACCCCCAGCAGCCCCTCCATCCCAGC
CCCCAGGAGCCGGAGAAGACCCCGGAGGAGGAGGAGGAGGAGGAGGAGGAGGAG---GAG
GAAGAAGAGGAGGAAGACGACAGT---CTCAAGGACAAATATGAGATGATCGCCATGAGC
>Tusk_Aqp9a
---ATGAGGCGACACTGTGTTCTC
AAACATGGAATACTCAAGGAGTTCCTGGCGGAGTTCCTGGGGACCTTCGTGCTGGTGCTG
TTTGGCTGCGGGTCGCTGGCCCAGACGGTGCTCAGCAGGAACACCCTGGGGGAACCGCTC
ACGGTGCACATCGGGTTCTCCGTGGGCCTCGCCATGGCCAGCTACGTGGCGGGGGGAGTC
TCAGGGGGCCATGTCAACCCCGCCGTGTCGCTGGCCATGGTGGTCCTGGGCAAACTGAAG
ATCTGGAAGTTCCCTGTTTACGTCCTCGCACAGTTCCTGGGTGCCTTCGCAGGGGCGGCC
GCGGTGTTTGGTTTATACTACGATGCCTTCATGGACTTCACCAGTGGGATTCTGTCAGTG
ACAGGCATCAACGCGACAGGTCACATCTTTGCTTCCTACCCTGGAAGACACCTGTCAATT
CTAGGCGGCTTCCTCGATCAGGTCATAGGGACGGGGATGCTGGTGGTCTGCATCCTGGCC
ATCCTGGACGGGGGGAACATCGGCGCCCCCAAGGGGGTGGAGCCGCTGGCCATCGGCCTG
ATCGTCATGGCGATCGCGGTGTCCATGGGGCTCAACTGTGGCTACCCTCTGAACCCGGCG
CGGGACCTGGGGCCCCGGCTGTTCACCGCGGTGGCCGGCTGGGGCATGGAGGTCTTCAGG
ACCGCAGACTACTGGTGGTGGATCCCAGTGGCCGGGCCCATGGTGGGGGGACTGGTGGGC
GCGGGCCTCTACTACCTGCTCATCGAGCTGCACCACCCCCAACAGCCC---CACCCGAGC
CACGACGGGCCGGAGAAGCCCCAGGACGAGGAAGAGGAGGAG---------------GAG
GAAGATGAGGAAGACGACGACAGCAGTCTCAAGGACAAATATGAGATGATCGCCATGAGC
>Ling_Aqp9a
---ATGAGGCGACACTGTATTCTC
AAACATGGAATACTCAAGGAGTTCTTGGCAGAGTTCCTGGGGACCTTTGTCTTGGTGTTG
TTTGGCTGCGGGTCTGTGGCCCAGACGGTCCTCAGCAGGAACACGCTGGGGGAACCTCTC
ACCGTGCACATCGGCTTCTCTGTGGGCCTCACCATGGCCAGCTACGTGGCGGGGGGAGTC
TCAGGGGGACATGTCAACCCTGCCGTGTCGCTGGCCATGGTGGTCCTGGGCAAACTGAAG
ATCTGGAAGTTCCCCATTTACGTCCTCGCACAGTTTCTGGGTGCCTTTGCAGGGGCGGCC
GCGGTGTTTGGTTTATACTATGATGCCTTCATGGACTTCACCAGTGGGATTCTGTCAGTG
ACAGGCATCAATGCGACAGGTCACATCTTTGCTTCCTACCCTGGAAGACACCTGTCAATC
CTAGGCGGCTTCCTCGATCAGGTTATAGGGACTGGAATGCTGGTGGTGTGCATCCTGGCC
ATCACTGACGGGGGCAACATCGGGGCCCCCAAGGGAGTGGAGCCGCTGGCCATCGGCCTG
ATCGTCATGGCCCTCGCGGTGTCCATGGGTCTCAACTGTGGCTACCCTCTGAACCCGGCC
CGGGACCTGGGGCCGCGGCTCTTCACCGCCGTGGCCGGGTGGGGCATGGAGGTCTTCAGG
ACCGCAGACTACTGGTGGTGGATCCCAGTGGCCGGGCCCATGGTCGGGGGACTGGTGGGC
GCGGCCCTCTACTACCTCCTCATCGAGCTCCACCACCCCCAACCGCCC---CAGCTGGGC
CACGAGGAGACGGAGAAGCCCCAAGACGAGGAGGAGGAGGAG---------------GAG
GAAGATGAGGAAGACGACGACAGCAGTCTCAAGGACAAATATGAGATGATCGCCATGAGC
>Burbot_Aqp9a
---ATGAGGAGACACTGTGTTCTC

AAACATGGAATACTCAAGGAATTCCTGGCAGAGTTCCTGGGGACCTTCGTCTTGGTGTTG
TTTGGCTGTGGGTCCGTGGCCCAGACGGTCCTCAGCAGGAACACTTTGGGGGAGCCCCTC
ACTGTGCACATTGGCTTCTCCGTGGGCCTCACCATGGCCAGCTACGTGGCGGGGGGAGTC
TCAGGT------------------------------ATGGTGGTCCTGGGCAAACTGAAG
ATCTGGAAGTTCCCCGTTTACGTCCTCGCACAGTTTCTCGGTGCCTTTGCAGGGGCGGCC
GCAGTGTTTGGTTTATACTATGACGCCTTCATGGACTTCACCAGTGGGATTCTGTCAGTG
ACAGGCATCAACGCGACGGGTCACATCTTTGCTTCCTACCCTGGAAGACACCTGTCAATC
CTAGGCGGCTTCATCGATCAGGTCATAGGGACTGGAATGCTGGTCGTGTGCATCTTGGCC
ATCGTTGACGGGGGAAACATCGGAGCGCCAAAGGGGGTCGAGCCACTGGCCATCGGCCTG
GTCGTCATGGCCCTCGCGGTATCCATGGGTCTCAACTGTGGCTACCCTCTGAACCCGGCC
CGGGACCTGGGGCCTCGGCTGTTCACCGCTGTCGCCGGGTGGGGCATGGAGGTCTTCAGG
ACCGCAGACTACTGGTGGTGGATCCCAGTGGCTGGGCCCATGGTCGGGGGATTGGTGGGC
GCGGCCCTCTACTACCTCCTCATCGAGCTCCACCACCCCCAACAGCCC---CACCCGAGC
CATGACGAGCCGGAGAAGCACCTAGACGAGGAAGAGGAG------------------GAG
GAAGATGAGGAAGACGACGACGGCAGTCTCAAGGACAAATATGAGATGATCGCCATGAGC
>Greater_forkbeard_Aqp9a
--ATGAGGCGGCACTGTGTTCTC
AAACATGGAATACTGAAGGAGTTCCTGGCCGAGCTGCTGGGGACCTTCGTCTTGGTGCTG
TTTGGCTGTGGCTCCGTGGCCCAGACGGTCCTCAGCAGGAACACTTTGGGGGAGCCGCTC
ACCGTGCACATCGGCTTCTCCGTGGGCCTGGCCATGGCCAGCTACGTGGCCGGGGGGATC
TCAGGGGGACATGTGAACCCAGCCGTGTCGCTGGCCATGGTGGTCCTGGGCAAACTGAAG
ATCTGGAAGTTCCCCATATACGTCCTGGCACAGTTTCTGGGTGCCTTTGCCGGGGCGGCC
GCAGTGTTTGGTTTATATTATGATGCCTTCATGGACTTCACCAGTGGGATTCTGTCAGTG
ACAGGCATCAACGCGACAGGTCACATCTTTGCTTCCTACCCTGGAAGACACCTGTCAATC
CTTGGCGGCTTCATCGATCAGGTTGTAGGGACCGGCATGCTGGTCTTGTGCATCTTGGCC
ATCGTCGACGGGGGGAACATCGGAGCACCCAAGGGCGTCGAGCCCCTGGCCATTGGCCTG
ATCGTCATGGCCATCGCAGTCTCCATGGGGCTCAACTGTGGCTACCCTGTGAACCCGGCC
CGGGACCTGGGGCCCCGGCTGTTCACCGCCGTGGCCGGCTGGGGGATGGAGGTCTTCAGG
ACCGCAGACTACTGGTGGTGGATCCCCGTAGCGGGGCCCATGGTGGGGGGCTTGGTGGGG
GCTGTCGTCTACTACCTCTTCATCGAGCTCCACCACCCCCAACAGCCC---CACCCGAGC
CCGGACGAGCCGGAGAAGCCCCAGGACGAGGAAGAGGAG------------------GAG
GAAGATGAGGAAGATGACGACAGCAGTCTCAAGGACAAATATGAGATGATCGCCATGAGC
>Forkbeard_Aqp9a
--ATGAGGCGGCACTGCGTTCTC
AAACATGGAATACTGAAGGAGTTCCTGGCCGAGCTGCTGGGGACCTTCGTCTTGGTGCTG
TTTGGCTGCGGCTCTGCAGCCCAGACGGTCCTCAGCAGGAACACCCTGGGGGAGCCCCTC
ACCGTGCACCTCGGCTTCTCCGTGGGGCTCGCCATGGCCAGCTACGTGGCCGGGGGGATC
TCAGGGGGACACGTGAACCCGGCCGTGTCGCTGGCCATGGTGATCCTGGGCAAACTGAAG
ATCTGGAAGTTCCCCATATACGTCCTGGCACAGTTTCTGGGTGCTTTCGCTGGGGCGGCC
GCAGTGTTTGGTTTATATTATGATGCCTTCATGGACTTCACCAGTGGGATTCTGTCGGTG
ACAGGCATCAACGCCACAGGTCACATCTTCGCTTCCTACCCTGGAAGACACCTGTCAACC
CTTGGCGGCTTCATCGATCAGGTTGTAGGGACCGGCATGCTGGTGTTGTGCATCTTGGCC
ATCGTCGACGGGGGAAACATCGGAGCGCCCAAGGGAGTCGAGCCTCTGGCCATTGGCCTG
ATCGTCATGGCCATCGCGGTCTCCATGGGGCTCAACTGTGGCTACCCTGTGAACCCGGCC
CGGGACCTGGGGCCTCGGCTGTTCACCGCCGTGGCCGGCTGGGGGATGGAGGTCTTCAGG
ACCGCAGACTACTGGTGGTGGATCCCCGTAGCCGGGCCCATGGTGGGGGGCTTGGTGGGG
GCGGTCCTCTACTACCTCTTCATCGAGCTCCACCACCCCCAGCAGCCC---CACCCGAGC
CCGGACGAGCCGGAGAAGCCCCAGGATGAGGAAGAGGAG------------------GAG
GAAGATGAGGAAGATGACGACAGCAGTCTCAAGGACAAATATGAGATGATCGCCATGAGC
>Marbled_moray_cod_Aqp9a
--ATGAGGCGACACTGTGTTCTC
AAACATGGAATACTCAAGGAGTTCCTGGCCGAGTTCCTGGGGACCTTCGTCTTGGTGTTG
TTTGGCTGCGGGTCCGTGGCCCAGACGGTCCTCAGCAGGGACACCCTGGGGGAGCCCCTC
ACTATGCACATCGGCTTCTCGGTGGGCCTCACCATGGCTACCTACGTGGCCGGGGGAGTC
TCAGGGGGACATGTGAACCCGGCCGTATCGCTGGCCATGGTGATCCTCGGCAAACTGAAG
ATCTGGAAGTTCCCCGTATACGTCCTTGCACAAATGCTCGGCGCTTTCACTGGCGCCGCC
GCAGTGTTTGGTTTATACTACGATGCCTTCATGAGCTACACCAGTGGGACTCTGTCTGTG
ACGGGCATCAACGCCACCGGTCACATCTTTGCTTCCTTCCCTGCGAACCACCTGTCAATC
CTAGGCGGCTTCGTCGATCAGGTGGTAGGGACTGGCATGCTGGTGCTGTGCATCTTGGCC
ATTCTGGACGAAGGAAACATGGGAGCGCCCAAGGGAGTCCAGCCTCTGGCCATCGGCCTG
ATCGTCATGGCCATTGGGGTGTCCATGGGCCTCAACTGCGGCTACCCGCTGAACCCGGCC

CGGGACCTGGGGCCTCGGCTGTTCACCGCCGTGGCCGGATGGGGCATGGAAGTCTTCAGG
ATCGCAAACTACTGGTGGTGGATCCCCGTGGCGGGTCCCATGGTAGGGGGCGTGGTGGGG
GCTGTCCTCTACTACCTCCTGGTCGAGCTCCACCACCCCCAGCAGCTC---CAC------
---GACGAGCTGGAGAAGCCCTGCGGGGACGAGGAAGAGGAGGAGGAGGAGGAGGAAGAG
GAGGATGAGGTGGAGGACGACAGCAGTCTAAAGGACAAATATGAGATGATCGCCATGAGC
>Arrowtail_Aqp9a
---------------------------------------ATGAGGCGACACTGTGTTCTC
AAACATGGAATACTCAAGGAATTCCTGGCAGAGTTCCTGGGGACCTTCGTCTTGGTGTTG
TTCGGCTGTGGGTCCGTGGCCCAGACAGTCCTCAGCAGAAACACCCTGGGGGAGCCGCTC
ACCGTGCACATCGGCTTCTCTGTGGGCCTCGCCATGGCTGTCTACGTGGCCGGGGGAGTC
TCCGGGGGACATGTGAACCCGGCCGTATCGCTGGCCATGGTGATCCTGGGCAAACTGAAG
ATCTGGAAGTTCCCCGTATATGTCCTTGCGCAATTTCTCGGTGGCTTCGCAGGGGCCGCT
GCAGTGTTTGGTTTATACTATGATGCCTTCATGGACTTCACCAGTGGGATTCTGTCAGTG
ACAGGCATCAACGCGACAGGTCACATCTTCGCTTCCTACCCTGCAAGACACCTGTCAATC
CTAGGCGGCTTCATCGATCAGGTTGTAGGGACCGGCATGCTGGTCTTGTGCATCTTGGCC
ATCGTTGACGGGGGAAACATCGGAGCACCCAAGGGAGTCGAGCCACTGGCCATCGGTCTG
ATCATGATGGCCATCGGGGTGTCCATGGGTCTCAACTGTGGCTACCCTCTGAACCCGGCC
CGGGACCTGGGGCCTCGGCTGTTCACCGCCGTGACCGGGTGGGGCATGGAGGTCTTCAGG
ACCGCAGACTACTGGTGGTGGATCCCTGTGGCGGGGCCCATGGTCGGGGGCGTGGTGGGG
GCG---CCC---CACCCAAGC
CCCGACGAGCCGGAGAAGCCCCAAGACGAGGAAGAGGAGGACGAG------------GAG
GAGGAGGAGGAGGACGATGACAGCAGTCTCAAGGACAAATATGAGATGATCGCCATGAGC
>European_hake_Aqp9a

---GGGGGACATGTGAACCCTGCCGTGTCGCTGGCCATGGTGATCCTGGGCAAACTGAAG
ATCTGGAAGTTCCCCATTTACGTGCTGGCGCAGTTTCTCGGTGCCTTCGCAGGGGCCGCC
GCAGTGTTCGGTTTATACTATGATGCCTTCATGGACTTCACCAGCGGGATTCTGTCGGTG
ACCGGCATCAACGCCACAGGTCACATCTTCGCTTCCTACCCTGCGAGACACCTGTCAGTC
CTAGGCGGCTTCATCGATCAGGTCGTGGGCACCGGCATGCTGGTCTTGTGCATATTGGCC
ATCGTGGACGGGGGAAACATCGGAGCGCCCAAGGGAGTCGAGCCGCTGGCCATCGGTCTG
ATCTTCATGGCCCTCGGCGTGTCCATGGGTCTCAACTGCGGCTACCCCCTGAACCCGGCC
CGGGACCTGGGGCCCCGGCTGTTCACCGCCCTGGCCGGGTGGGGCATGGAGGTCTTCAGG
ATCGCAGACTGCTGGTGGTGGATCCCGGTGGCCGGGCCCATGGTGGGCGGCCTGGTGGGG
GCGGTCCTCTACTACTTCCTCATCGAGCTGCACCACGCCCCGCTGCCC---CGCCCGAGC
CTCGACGCCACGGAGAAGCCCCTCGACCCGGAGGAGGAGGAGGAGGAG---------GAG
GAAGAGGAGGAGGACGACGACAGCAGCCTCAAGGACAAATACGAGATGATCGCCATGAGC
>Cape_hake_Aqp9a
---------------------------------------ATGAGGCGACACTGTGTGCTC
AAACATGGAATACTCAAGGAGTTCCTGGCTGAGTTCCTGGGGACCTTCGTCTTGGTGCTG
TTTGGCTGCGGGTCGGTGGCCCAGACGGTCCTCAGCAGGAACACTCTGGGAGAGCCGCTC
ACCGTCCACATCGGCTTCTCCGTGGGCCTCACCATGGCTTCCTTCGTGGCCGGGGGAGTC
TCCGGGGGACATGTGAACCCTGCCGTGTCGCTGGCCATGGTGATCCTGGGCAAACTGAAG
ATCTGGAAGTTCCCCATTTACGTGCTGGCGCAGTTTCTCGGTGCCTTCACAGGGGCCGCC
GCAGTGTTCGGTTTATACTATGATGCCTTCATGGACTTCACCAGCGGGATTCTGTCGGTG
ACCGGCATCAACGCCACAGGTCACATCTTCGCTTCCTACCCTGCGAGACACCTGTCAGTC
CTAGGCGGCTTCATCGATCAGGTCGTGGGCACCGGCATGCTGGTCTTGTGCATATTGGCC
ATCGTGGACGGGGGAAACATCGGAGCGCCCAAGGGAGTGGAGCCGCTGGCCATCGGCCTG
ATCTTCATGGCCCTCGGCGTGTCCATGGGCCTCAACTGCGGCTACCCCCTGAACCCGGCC
CGGGACCTGGGGCCCCGGCTGTTCACCGCCCTGGCCGGGTGGGGCATGGAGGTCTTCAGG
ATTGCAGACTGCTGGTGGTGGATCCCGGTGGCCGGGCCCATGGTGGGCGGCCTGGTGGGG
GCGGTCCTCTACTACTTGCTCATCGAGCTGCACCACGCCCCGCTGCCC---CGCCCGAGC
CTCGACGCCACGGAGAAGCCCCTCGACCCGGAGGAGGAGGAGGAG------------GAG
GAAGAGGAGGAGGACGACGACAGCAGCCTCAAGGACAAATACGAGATGATCGCCATGAGC
>Benguela_hake_Aqp9a
---------------------------------------ATGAGGCGACACTGTGTGCTC
AAACATGGAATACTCAAGGAGTTCCTGGCTGAGTTCCTGGGGACCTTCGTCTTGGTGCTG
TTTGGCTGCGGGTCGGTGGCCCAGACGGTCCTCAGCAGGAACACTCTGGGAGAGCCGCTC
ACCGTCCACATCGGCTTCTCCGTGGGCCTCACCATGGCTTCCTTCGTGGCCGGGGGAGTC

TCC---

----------------------GATGCCTTCATGGACTTCACCAGCGGGATTCTGTCGGTG
ACCGGCATCAACGCCACAGGTCACATCTTCGCTTCCTACCCTGCGAGACACCTGTCAGTC
CTAGGCGGCTTCATCGATCAGGTCGTGGGGACCGGCATGCTGGTCTTGTGCATATTGGCC
ATCGTGGACGGGGGAAACATCGGAGCGCCCAAGGGAGTCGAGCCGCTGGCCATCGGCCTG
ATCTTCATGGCCCTCGGCGTGTCCATGGGTCTCAACTGCGGCTACCCCCTGAACCCGGCC
CGGGACCTGGGGCCCCGGCTGTTCACCGCCCTGGCCGGGTGGGGCATGGAGGTCTTCAGG
ATCGCAGACTGCTGGTGGTGGATCCCGGTGGCCGGGCCCATGGTGGGCGGCCTGGTGGGG
GCGGTCCTCTACTACTTCCTCATCGAGCTGCACCACGCCCCGCTGCCC---CGCCCGAGC
CTCGACGCCACGGAGAAGCCCCTCGACCCGGAGGAGGAGGAGGAG------------GAG
GAAGAGGAGGAGGACGACGACAGCAGCCTCAAGGACAAATACGAGATGATCGCCATGAGC
>Roundnose_grenadier_Aqp9a
---------------------------------------ATGAGGCGACACTGTGTTCTC
AAACATGGAATACTCAAAGAGTTCCTGGCCGAGTTCCTGGGCACCTTCGTTCTGGTGCTG
TTTGGCTGCGGCTCGGTGGCCCAGACGGTGCTCAGCAGGAACACGCTGGGGGAGCCCCTC
ACTGTGCACATCGGCTTCTCCGTGGGCCTCGCTATGGCCTCCTACGTGGCCGGGGGCATC
TCAGGTGGCCATGTGAACCCTGCCGTGTCCCTGGCCATGGTGATCCTGGGCAAGCTGAAG
ATCTGGAAGTTCCCCGTGTACGTCCTGGCGCAATTTCTGGGCGCTTTTGCAGGGGCCGCC
GCGCTGTTTGGTTTATATTACGATGCCTTCATGGACTTCACCAGTGGGATTCTGTCAGTG
ACAGGCATCAATGCCACAGGTCACATCTTCGCTTCCTACCCTGCGAGACACCTGTCAGTC
CTAGGAGGCTTCATCGATCAGGTTGTGGGGACCGGCATGCTGGTCTTGTGCTTCTTAGCC
ATCGTGGACGGCGGGAACATCGGCGCTCCCAAGGGAGTGGAGCCGCTGGCCATCGGCCTG
ATCGTCATGGCCCTCGGCGTCTCCATGGGCCTCAACTGTGGCTACCCTCTGAACCCGGCC
CGGGACCTGGGGCCTCGGCTGTTCACCGCCGTGGCCGGCTGGGGCATGGAGGTCTTCAGG
ACCGCAAACTACTGGTGGTGGATCCCGGTGGCGGGGCCCATGGTGGGGGGCGTGGTGGGG
GCGGTCCTGTACTACCTCCTCATCGAGCTGCACCACCCGCAGCAGCCC---CGCCCAGCA
GGCGAGGAGCCGGAGAAGCCCTGCGACGAGGGGGAGGAGGAGGTGGAGGAGGAAGAGGAG
GAGGACGAGGAGGACGACGACAGCAGTCTCAAGGACAAATATGAGATGATCGCCATGAGC
>Onion_eye_grenadier_Aqp9a
---------------------------------------ATGAGGCGACACTGTGTTCTC
AAACATGGAATACTCAAGGAGTTCCTGGCAGAGTTCCTGGGCACCTTCGTCCTGGTGCTG
TTTGGCTGTGGCTCGGTGGCCCAGACGGTGCTCAGCAGGAACACTCTGGGGGAGCCTCTC
ACTGTGCACATCGGCTTCTCGGTGGGCCTCGCCATGGCCTCCTACGTGGCCGGGGGCATC
TCAGGGGGCCACGTGAACCCTGCCGTGTCCCTGGCCATGGTGATCCTGGGCAAGCTGAAG
ATCTGGAAGTTCCCTGTGTACGTGCTGGCGCAATTTTTGGGCGCCTTCGCAGGGGCCGCT
GCCCTGTTTGGTTTATACTACGATGCCTTCATGGACTTCACCAGTGGGATTCTGTCAGTG
ACAGGCATTAATGCAACAGGTCACATCTTTGCTTCCTACCCTGCCAGACACCTGTCCGTC
CTAGGAGGCTTCATCGATCAGGTTGTGGGGACTGGCATGCTGGTCTTGTGCTTCTTGGCC
ATCGTGGACGGCGGGAACATCGGTGCTCCCAATGGGGTGGAGCCGCTGGCCATCGGCCTG
ATCTTCATGGCCCTCGGTGTCTCCATGGGTCTCAACTGTGGCTACCCTCTGAACCCGGCC
CGGGACCTGGGGCCTCGACTCTTCACCGCCGTGGCCGGCTGGGGCATGGAGGTCTTCAGG

---------CCGGAGAAGCCCTGCAACGAGGGGGAGGAGGAGGAAGAGGAGGGGGAGGAG
GAGGACGACGACGACGACGACAGCAGTCTCAAGGACAAATACGAGATGATCGCCATGAGC
>Western_softhead_grenadier_Aqp9a
---------------------------------------ATGAGACGACACTGTGTTCTC
AAACATGGAATACTCAAGGAATTCCTGGCCGAGTTCCTGGGCACCTTCGTCCTGGTGCTG
TTTGGCTGCGGGTCGGTGGCCCAGACGGTGCTCAGCAGGAACACTTTGGGGGAGCCCCTG
ACCGTGCACATCGGCTTCTCCGTGGGCCTCGCCATGGCCTCCTATGTGGCCGGGGGCATC
TCAGGGGGCCACGTGAACCCTGCTGTGTCACTGGCCATGGTGATCCTGGGCAAGCTGAAG
ATCTGGAAGTTCCCCGTGTACGTCCTGGCGCAGTTCCTCGGAGCCTTTGCGGGGGCCGCC
GCCCTGTTTGGTTTATACTATGATGCCTTCATGGACTTCACCAGTGGCATTCTGTCAGTG
ACAGGCATCAATGCAACCGGGCACATCTTCGCTTCCTACCCTGCCAGACACCTGTCCATC
CTAGGAGGCTTCATCGATCAGGTTGTGGGGACTGGTATGCTGGTCTTGTGCTTCTTGGCC
ATCATCGATGGCGGGAACCTCGGAGCTCCCAAGGGAGTGGAGCCTCTGGCCATCGGCCTG
ATCGTCATGGCCCTCGGCGTGTCCATGGGTCTCAACTGTGGCTACCCTCTGAACCCGGCC
CGGGACCTGGGGCCTCGGCTGTTCACCGCCGTGGCCGGCTGGGGCATGGAGGTCTTCAGG
ACCGCAAACTACTGGTGGTGGATCCCGGTGGCGGGGCCCATGGTCGGGGGAGTGGTGGGG
GCCGTCCTTTACTACCTCCTCATCGAGCTCCACCACCCGCAGCAGCTC---CGCCCGGGC

AGCGAGGAGGCGGAGAAGCCCTGCGATGAGGGAGAGGAGGACGAGGAGGAAGAG---GAG
GACGAGGAAGAGGACGACGACAGCAGCCTCAAGGACAAATACGACATGATCGCCATGAGC
>Vaillants_grenadier_Aqp9a
--ATGAGGCGACACTGTGTTCTC
AAACATGGAATACTCAAGGAATTCCTGGCAGAGTTCCTGGGGACCTTCGTCCTGGTGCTG
TTTGGCTGCGGGTCCGTGGCCCAGACGGTCCTCAGCAGGAACACCCTGGGAGAGCCCCTC
ACCGTGCACATCGGCTTCTCTGTGGGCCTTGCCATGGCTTCCTACGTGGCCGGAGGCATC
TCAGGGGGACATGTGAATCCGGCCGTGTCGCTGGCCATGGTGATCCTGGGCAAACTGAAG
ATCTGGAAGTTCCCCGTGTACGTCCTGGCGCAATTCCTCGGCGCCTTCGCAGGGGCCGCT
GGAGTGTTTGGGTTGTACTATGATGCCTTCATGGACTTCACCAGTGGGATTCTGTCAGTG
ACAGGCATCAACGCGACAGGTCACATCTTCGCTTCCTACCCTGCAAGACACCTGTCAATC
CTAGGTGGCTTCATCGATCAGGTTGTAGGGACCGGCATGCTGGTCTTGTGCTTTTTGGCC
ATCGTTGACGGGGGAAACATCGGAGCACCCAAAGGAGTCGAGCCGCTGGCCATCGGCCTG
ATCATCATGGCCATCGGGGTGTCCATGGGTCTGAACTGTGGCTACCCTCTAAATCCGGCC
CGGGATCTGGGGCCTCGGCTGTTCACTGCCGTGGCCGGGTGGGGCATGGAGGTCTTCAGG
ACCGCAGACTACTGGTGGTGGATCCCTGTGGCGGGGCCCATGGTCGGGGGCGTGGTGGGG
GCGGTCCTCTACTACCTCCTCATCGAGCTCCATCACCCCCAACAGCCC---CTCCCGGGT
CACGACGAACCGGAGAAGCCCCACGACGAGGAAGAGGAGGAGGAGGAG---------GAA
GAAGAGGAGGAGGATGAAGACAGCAGTCTGAAGGACAAATATGAGATGATCGCCATGAGC
>Roughnose_grenadier_Aqp9a
--ATGAGGCGCCGCTGTGTTCTG
AAACATGGAATACTCAAGGAGTTCCTGGCAGAGTTCCTGGGGACCTTCGTCTTGGTGCTG
TTTGGCTGTGGGTCCGTGGCCCAGACGGTCCTCAGCAGGAACGCCCTGGGAGAGCCCCTC
ACCGTGCACATCGGCTTCTCTGTGGGCCTCGCCATGGCTTCCTACGTGGCCGGGGGAGTC
TCAGGGGGACATGTGAACCCTGCCGTGTCGCTGGCCATGGTGATCCTCGGCAAGCTGAAG
ATCTGGAAGTTCCCCATTTATGTCCTGGCACAATTTCTCGGTGCCTTTGTGGGGGCCGCC
GCGGTGTTTGGTTTATACTATGATGCCTTCATGGACTTCACCAGTGGGATTCTGTCAGTG
ACAGGCATCAACGCCACAGGTCACATCTTTGCTTCCTACCCTGCAAGACACCTGTCAGTC
CTAGGCGGCTTCATCGATCAGGTTGTAGGGACCGGCATGCTGGTCTTGTGCTTCTTGGCC
ATCGTTGACGGGGGAAACATCGGAGCACCCAAGGGAGTCGAGCCACTGGCCATCGGCCTG
ATCATCATGGCCATCGGGGTATCCATGGGTCTCAACTGTGGCTACCCTCTGAACCCGGCC
CGGGACCTGGGGCCTCGGCTGTTCACCGCCGTGGCCGGCTGGGGCATGGAGGTCTTCAGG
ACCGCAGACTACTGGTGGTGGATCCCCGTGGCGGGGCCCATGGTCGGGGGCGTGGTGGGG
GCGGTCCTCTACTACCTCCTCATCGAGCTCCACCACCCCCGACAGCCC---CAACCGAGC
CACGACGAGCCGGAGGATCCGCACGGCGAGGAACAGGAGGAG---------------GAA
GAGGAGGAGGAGGAGGACGACAGCAGTCTCAAGGACAAATATGATATGATCGCCATGAGC
>Roughsnout_grenadier_Aqp9a

---GGGGGACATGTGAACCCTGCCGTGTCGCTGGCCATGGTGATCCTCGGCAAGCTGAAG
ATCTGGAAGTTCCCCATTTATGTCCTGGCACAATTTCTCGGTGCCTTTGTGGGGGCCGCC
GCGGTGTTTGGTTTATACTATGATGCCTTCATGGACTTCACCAGTGGGATTCTGTCAGTG
ACAGGCATCAACGCCACAGGTCACATCTTTGCTTCCTACCCTGCAAGACACCTGTCAGTC
CTAGGCGGCTTCATCGATCAGGTTGTAGGGACCGGCGTGCTGGTCTTGTGCTTCTTGGCC
ATCGTTGACGGGGGAAACATCGGAGCACCCAAGGGAGTCGAGCCGCTGGCCATCGGCCTG
ATCATCATGGCCATCGGGGTATCCATGGGTCTCAACTGTGGCTACCCTCTGAACCCGGCC
CGGGACCTGGGGCCTCGGCTGTTCACCGCCGTGGCCGGCTGGGGCATGGAGGTCTTCAGG

------------TACCTCCTCATCGAGCTCCACCACCCCCGACAGCCC---CAACCGAGC
CGCGACGAGCCGGATGACACGCACGACGAGGAAGAGGAGGAG---------------GAA
GAGGAGGAGGAGGAGGACGACAGCAGTCTCAAGGACAAATATGATATGATCGCCATGAGC
>Common_mora_Aqp9a
--ATGAGGCGACACTGTGTTCTC
AAACATGGAATACTCAAGGAATTCCTGGCAGAGTTCCTGGGGACCTTCGTCTTGGTGTTG
TTTGGCTGTGGGTCTGTGGCCCAGACGGTCCTCAGCAGGAACACCCTGGGGGAGCCCCTC
ACCGTGCACATCGGCTTCTCTGTGGGCCTCGCCATGGCTTCCTACGTGGCTGGGGGAGTC
TCAGGAGGACATGTGAACCCCGCCGTGTCGCTGGCCATGGTGATCCTGGGCAAGCTGAAG
ATCTGGAAGTTCCCCTTGTACGTCCTCGCGCAGTTTCTGGGGGCCTTCGCGGGGGCCGCC
GCGGTGTTCGGTCTATACTATGATGCCTTCATGGACTTCACCAGTGGGATTCTGTCAGTG

ACAGGCATCAACGCGACAGGTCACATCTTTGCCTCTTACCCTGCGAGACACCTGTCAGTC
TTAGGCGGCTTCATCGATCAGGTTATAGGGACCGGCATGCTGGTCTTGTGCATCTTGGCC
ATCGTAGACGGGGGAAACATTGGAGCACCCAAGGGAGTCGAGCCGCTGGCCATCGGCGTG
ATCATGATGGCCATCGGGGTCTCCATGGGTCTCAATTGTGGATACCCTCTGAACCCGGCC
CGGGACCTGGGGCCTCGGCTGTTCACCGCCGTGGCCGGGTGGGGCGTAGAGGTCTTCAGG
ACCGCAGACTACTGGTGGTGGATCCCCGTGGCGGGGCCCATGGTCGGGGGCGTGGTGGGG
GCGGTCCTCTACTACCTCCTCATCGAGCTCCACCACCCCCAACAGCCC---CACCTGAGC
CAAGAAGAGCTGGAGAAGCCCCACGACGAGGAAGAGGAAGAG---------------GAG
GAAGAGGAGGAGGAGGACGACAGCAGTCTCAAGGACAAATATGAGATGATCGCCATGAGC
>Guinean_codling_Aqp9a

---------CACGTGAACCCTGCCGTGTCGCTGGCCATGGTGATCCTGGGCAAACTGAAG
ATCTGGAAGTTCCCCTTGTACGTCCTCGCGCAATTCCTCGGGGCGTTCGCCGGAGCCGCC
GCCGTGTTCGGGTTGTACTACGATGCCTTCATGGACTTCACCAGTGGGATTCTGTCGGTG
ACAGGCATCAACGCCACAGGTCACATCTTCGCTTCCTACCCTGCGAGACACCTGTCAGTC
TTAGGTGGCTTCATCGACCAGGTTGTAGGGGCCGGCATTCTGGTCTTGTGCTTCTTGGCC
ATCGTTGACGGGGGGAACATCGGAGCACCCAAGGGAGTCGAGCCGCTGGCCATCGGCCTG
ATCATCATGGCCATCGGGGTCTCCATGGGTCTCAATTGTGGTTACCCTCTGAACCCGGCC
CGGGACCTGGGGCCCCGGCTGTTCACCGCCGTGGCCGGGTGGGGCTTAGAGGTCTTCAGG
ACAGGGAACTACTGGTGGTGGATCCCCGTGGCAGGGCCCATGGTCGGGGGCGTGGTGGGG
GCGGTCCTCTACTACCTCCTCATCGAGCTCCACCACCCGCAACAGCCC---CACCTGAGC
CACGACGAGCTGGAGAAGCCCCACGACGAGGAAGAGGAGGAGGAGGAGGAG------GAA
GAGGAGGAGGAGGAGGACAACAGCAGTCTCAAGGACAAGTATGAGATGATCGCCATGAGC
>Tube_eye_Aqp9a
--ATGAGGCAACACTGTGTTCTC
AAACACGGAATACTCAAGGAATTCCTGGCAGAGTTCCTTGGGACGTTCGTCTTGGTTTTA
TTTGGCTGTGGGTCGGTGGCACAGACTGTACTCAGTCGTAATACTCTGGGTGAACCCCTG
ACCATCCACATTGGCTTCTCTGTGGGACTGATGATGGCCTCCTACGTTGCAGGTGGAGTG
TCAGGGGGCCATGTGAACCCTGCTGTGTCGCTGGCCATGGTCATTCTGGGAAAGCTGAAG
ATCTGGAAGTTTCCATTCTACGTCATCGCTCAGTTCCTCGGAGCCTTCGCAGGGGCTGCA
GCCGTTTTCGGGCTGTACTAC---------------------------------------

---------------------GTTGTGGGGACCGGTATGTTGGTCTTGTGCATCTTGGCC
ATCATTGACGGGGGTAACATTGGGGCTCCCAAAGGAGTCGAGCCGCTTGCCATCGGCCTG
ATTATTATGGCCATCGGGGTTTCCATGGGACTCAACTGTGGCTACCCGCTCAACCCAGCC
CGGGACCTGGGGCCTCGGCTGTTCACTGCTGTTGCCGGATGGGGCATGGAGGTCTTCAGG
ACCGCAGACTACTGGTGGTGGATCCCTGTGGCAGGGCCCATGGTCGGGGGCGTGGTGGGG
GCTGTCCTCTACTATCTCCTCGTCGAGCTCCACCACCATCGC---------CACGGCGCC
CACGACGAGGCCGAGAAGCCC---GACGAGGAGGACGAGGAAGAGGAGGAC------GAA
GACGACGACGACGACGACAACGACAGTCTCAAGGACAAATATGAGATGATCGCCGTGAGC
>Sand_roller_Aqp9a
--ATGAGGCGGCACTGTGTTCTC
AAGCATGGAATACTCAAGGAATTCCTGGCAGAATTCCTGGGCACCTTTGTGTTAGTCTTA
TTTGGCTGTGGGTCAGTGGCTCAGAATGTCCTGAGCCGAAACACTCTGGGGGAGCCTCTG
ACTGTCCACATTGGGTTCTCTATTGGGCTGATGATGGCAGCGTACATTGCTGGTGGGGTT
TCAGGGGGCCACGTGAACCCTGCCGTGTCGCTCGCCATGGTCATCCTGGGGAAGCTGAAG
CTCTGGAAGTTTCCTTTCTATGTCATCGCTCAGTTTCTGGGTGCTTTCGCTGGAGCGGCT
GCAGTGTTTGGGCTGTACTACGATTCGTTCATGGATTTTACCAGTGGGACTCTGTCAGTG
ACCGGCATCAGTGCAACAGGTCACATCTTTGCTTCCTACCCTGGCAGACACCTGTCAGTG
CTGGGCGGCTTTATTGATCAGGTTGTGGGGACAGGTATGTTGGTTTTGTGCATCCTTGCC
ATCATTGATGGCGGGAACATTGGTGCTCCCAAGGGGATGGAGCCACTGGCTATTGGCTTG
ATCATCATGGCCATTGGGGTTTCTATGGGACTGAACTGTGGCTACCCGCTCAACCCTGCC
AGGGATCTGGGACCCAGGCTGTTCACTGCCGTGGCAGGATGGGGCATGGAGGTCTTCAGG
ATTGCAGACCACTGGTGGTGGATTCCCGTGGCAGGACCCATGGTGGGGGGCGTGGTCGGG
GCGGGCGTCTACTACCTCTTCATCGAACTGCATCATCACCACCAC------------AAC
GATGACCCTGCACAGAAGCCCCAGGAGGGGGACGAGGAGGAG---------------GAA
GAGGAGGATGAGGATGAGGACAGCAGTCTGAAGGACAAATACGAGATGATCGCTATGAGC
>European_smelt_Aqp9a

---------------ATGGAGATAAAACACAAGAGAACGATGAGGCAGCATTGTGCTCTG
AAGCATGGGATACTCAAGGAGTTCTTGGCTGAATTTCTTGGGACTTTCGTCTTGGTTCTG
TTCGGCTGTGGCTCTGTGGCTCAGTCTGTCCTGAGTCGCAATGCCCTGGGGGAGCCGCTT
ACCATCCACATCGGCTTCTCTGTTGGACTGATGATGGCAGTATATGTGGCAGGTGGAGTG
TCAGGGGCCCACGTGAACCCTGCCGTGTCCCTCGCCATGGTCATCCTGGGAAAGCTCAAG
TTCTGGAAGTTTCCCCTCTATGTGATCGCTCAGTTGTTGGGGGCCTTCACTGGGGCTGCA
GCAGTCTTCGGACTGTACTATGATGCCTTTATGGAGTACACTAACGGGATCCTGTCAGTG
ACGGGGATCAACGCTACGGCTCACATCTTTGCCTCCTACCCTTCCAGGCACCTGTCCGTC
CTCGGGGGATTCATAGACCAGGTGGTGGGGACAGGGATGCTGGTGATGTGCATCCTGGCC
ATCATCGATGGCAAGAACATCGGAGCCCCCAGAGGAACGGAGCCACTGGCCATCGGCCTG
ATCATCATGGCCATTGGGGTGTCCATGGGCCTGAACTGTGGCTACCCCTTGAACCCGGCC
CGGGACCTCGGGCCCAGACTCTTCACTGCGGTGGCCGGATGGGGCATGGACGTCTTCAGG
------------TGGTGGTGGATTCCCGTGGCAGGGCCCATGGCCGGGGGTGTGGCAGGG
GCTCTTGTCTACTTCCTGTTCATCGAGCTGCACCACACACAG------------------
---------CCCGAGAAGCCCCACGAGGAGGAAGAGGAGGAG----------------GAC
GACGAGGAAGAGGACGAAGACAGCAGTCTCAAGGACAAATATGAGATGATCACCATGAGC
>Ayu_Aqp9a
---------------ATGGAGATTAAACACAAGAGAACGATGAGGCAACATTGTGCTCTG
AAACATGGCATACTCAAGGAATTCCTGGCGGAGTTTCTTGGGACTTTTGTCCTGGTTCTG
TTCGGCTGTGGCTCCGTGGCTCAGACGGTCCTAAGTCGCAACGCCCTGGGAGAGCCGCTC
ACCATCCACATAGGTTTCTCTGTTGGACTGATGATGGCTGTATATGTGGCTGGTGGAGTG
TCAGGGGCGCACGTGAACCCTGCCGTGTCTCTCGCCATGGTCATTCTGGGCAAACTGAAG
TTCTGGAAGTTTCCCATTTACGTGATCGCTCAGTTCTTGGGTGCGTTCGCCGGAGCTGCT
GCAGTCTTCGGACTGTACTATGATGCTTTTATGGAATACACTAACGGCATCCTGTCCGTT
ACCGGGATCAATGCCACGGCTCACATCTTCGCCTCCTACCCCGCCCGGCACCTGTCAGTA
CTTGGTGGCTTCATAGACCAGGTGGTGGGGACAGGGATGCTGGTCATGTGCATCCTAGCT
ATCATCGATGGGAAGAACATCGGGGCCCCCAGAGGAATGGAGCCGCTGGCTGTTGGTCTG
ATCATCATGGCCATCGGGGTGTCCATGGGTCTGAACTGTGGCTACCCTCTCAACCCAGCC
CGTGATCTTGGCCCCAGACTCTTTACTGCGGTGGCTGGCTGGGGCATGGATGTCTTCAGG
ACTGCAGACAACTGGTGGTGGATTCCTGTGGCAGGGCCCATGGCAGGGGGCGTGGCCGGG
GCTCTCGTTTACTTCCTGTTCATTGAGCTACATCACACGCAG------------------
---------CCAGAGAAACCCCACGAGGAGGAAGAGGAGGAG----------------GAT
GACGAGGAAGAGGAGGAAGACAGCAGC---------------------------------
>Large_eye_snaggletooth_Aqp9a
--ATGAGGCGCCGTTTCTCTCTG
AAGCATGGCATACTCAGGGAATTCCTGGCTGAATTTCTGGGGACTTTTGTCTTGGTGTTA
TTTGCCATTGGGTCTGTGGCTCAGACTGTCCTGAGTCGCAACGCCATGGGCGAGACCCTT
ACCATCCACATCGGGTTCTCTGTGGGACTGATGATGGCGATGTACGTGGCCGGTGGAGTC
TCAGGGGGCCATGTGAACCCTGCTGTGTCGTTGGCCATGGTGATCCTGGGCAAGCTGAAG
TTCTGGAAGTTCCCCATCTACGTCATGGCTCAGTTCCTCGGTGGCTTCGCTGGGGCCGCT
GCAGTCTTCGGGATGTACTACGATGCCTTCATGGAATACAGCAGTGGCATACTGTCGGTG
ACAGGGATCAATGCCACCGGCCACATCTTCGCCTCCTACCCCGCCAGGCACCTGTCGGTC
CTGGGTGGCTTCCTAGACCAGGTGGTTGGAACGGGAATGCTAGTGATGTGTACCCTGGCC
ATCATTGATGGAAAAAACATTGGAGCTCCCAGAGGGATGGAACCACTGTCCGTAGGGCTG
ATCATCATGGCCATAGGGGTGTCTATGGGTCTGAACTGTGGGTACCCCCTCAACCCTGCA
CGCGACCTCGGGCCCAGGCTCTTCTCTGCAGTGGCTGGGTGGGGCATGGAGGTCTTCAGG
ACTGGAGACTACTGGTGGTGGATCCCTGTGGCAGGGCCCATGGTCGGTGGTGTGGTCGGG
GCCGTGGTCTACTTCCTGTTCATCGAGCTGCACCACGCTCAG------------------
---------CCTGAGAAGCCCTGTGACGAGGAAGAGGAGCAGGAG-------------GAG
GAGGAGGAAGAGGATGAGGACAGCAGCCTCAAGGACAAATACGAGATGATCACCATGAGC
>Sockeye_salmon_Aqp9a1
---------------ATGGAGATGAAACACAGGAGAACTATGAGGCAGCGCTATTCTCTG
AAACATGGGATACTGAAGGAGTTCCTGGCTGAGTTCCTGGGGACTTTTATCTTGGTTTTG
TTTGGCGTTGGGTCTGTTGCTCAGACGGTCCTGAGTCGTAATGCCATGGGGGAGACTCTC
ACCATCCATATAGGCTTCTCTGTAGGACTGATGATGGCAGTGTACGTGGCCGGAGGAGTG
TCGGGAGCCCATGTGAACCCTGCTGTCTCCTTGGCCATGGTGATCCTGGGAAAACTCAAG
TTCTATAAATTCCCTGTCTATGTCATCGCTCAGTTCCTTGGTGCTTTCTCTGGAGCTGCT
GCAGTGTTTGGGCTGTATTATGATGCGTTCATGGACTTCACCAATGGGATTTTATCAGTG
ACTGGTATTAACGCCACCGGTCACATCTTTGCCTCTTACCCTGCCAGGTCCCTAACCATC
CTCGGAGGCATCATAGACCAGGCAGCAGGAACAGGGATGCTGGTGTTGTGTATCCTGGCC
ATCAGCGATGGGAAGAACATCGGCGCCCCTAGAGGCATGGAGCCACTGTGCATCGGCCTG

ATCATCATGGCTATCGGGGTGTCCATGGGGCTGAACTGTGGGTACCCCCTCAACCCTGCC
AGGGACCTGGGGCCACGACTCTTTACTGCTGTAGCAGGCTGGGGTTGGGAGGTGTTCAGG
ACTTCAGACTACTGGTGGTGGGTCCCGGTGGCGGGGCCGATGATAGGGGGCGTGGTCGGG
GCTCTGGTCTACTTCCTGTTCATCGAGATGCATCACAAACAG------------------
---------CCTGAGAAACCCCACGAGCAGGAAGAGGAGGAGGAGGACGAAGAG---GAG
GAAGAAGATCTGGAGGAGGACAGCAGTCTTAAGGACAAATATGAGATGATTACCATGAGT
>Coho_salmon_Aqp9a1
---------------ATGGAGATGAAACACAGGAGAACTATGAGGCAACGCTATGCTCTA
AAACATGGGATACTGAAGGAGTTCCTGGCGGAGTTCCTGGGGACTTTTATCTTGGTTTTG
TTTGGCGTTGGGTCTGTTGCTCAGACGGTCCTGAGTCGTAATGCCATGGGGGAGACTCTC
ACCATCCATATAGGCTTCTCTGTAGGACTGATGATGGCAGTGTACGTGGCCGGAGGAGTG
TCAGGAGCCCATGTGAACCCTGCTGTCTCCTTGGCCATGGTGATCCTGGGAAAACTCAAG
TTCTATAAATTCCCTGTCTATGTCATCGCTCAGTTCCTTGGTGCTTTCTCTGGAGCTGCT
GCAGTGTTTGGGCTGTATTATGATGCGTTCATGGACTTCACCAATGGGATTTTATCAGTG
ACTGGTATTAATGCCACCGGTCACATCTTTGCCTCTTACCCTGCCAGGTCCCTAACCATC
CTCGGAGGCATCATAGACCAGGCAGCGGGAACAGGCATGCTGGTGTTGTGTATCCTGGCC
ATCAGCGATGGGAAGAACATCGGCGCCCCTAGAGGCATGGAGCCACTGTGCATCGGCCTG
ATCATCATGGCTATCGGAGTGTCCATGGGGCTGAACTGTGGGTACCCCCTCAACCCTGCC
AGGGACCTGGGGCCACGACTCTTTACTGCTGTAGCAGGCTGGGGTTGGGAGGTGTTCAGG
ACTTCAGACTACTGGTGGTGGGTCCCGGTGGCGGGGCCGATGATAGGGGGCGTGGTCGGG
GCTCTGGTCTACTTCCTGTTCATCGAGATGCATCACAAACAG------------------
---------CCTGAGAAACCCCACGAGGAGGAAGAGGAAGAGGACGAAGAG------GAG
GAAGAAGATCTGGAGGAGGACAGCAGTCTTAAGGACAAATATGAGATGATTACCATGAGT
>Chinook_salmon_Aqp9a1
---------------ATGGAGATGAAACACAGGAGAACTATGAGGCAACGCTATGCTCTA
AAACATGGGATACTGAAGGAGTTCCTGGCGGAGTTCCTGGGGACTTTTATCTTGGTT---

---GGAGCCCATGTGAACCCTGCTGTCTCCTTGGCCATGGTGATCCTGGGAAAACTCAAG
TTCTATAAATTCCCTGTCTATGTCATCGCTCAGTTCCTTGGTGCTTTCTCTGGAGCTGCT
GCAGTGTTTGGGCTGTATTAT---------------------------------------

------------------CAGGCAGCGGGAACAGGCATGCTGGTGTTGTGTATCCTGGCC
ATCAGCGATGGGAAGAACATCGGCGCCCCTAGAGGCATGGAGCCACTGTGCATCGGCCTG
ATCATCATGGCTATCGGAGTGTCCATGGGGCTGAACTGTGGGTACCCCCTCAACCCTGCC
AGGGACCTGGGGCCACGACTCTTTACTGCTGTAGCAGGCTGGGGTTGGGAGGTGTTCAGG
ACTTCAGACTACTGGTGGTGGGTCCCGGTGGCGGGGCCGATGATAGGGGGCGTGGTCGGG
GCTCTGGTCTACTTCCTGTTCATCGAGATGCATCACAAACAG------------------
---------CCTGAGAAACCCCACGAGGAGGAAGAGGAGGAGGACGAAGAG------GAG
GAAGAAGATCTGGAGGAGGACAGCAGTCTTAAGGACAAATATGAGATGATTACCATGAGT
>Rainbow_trout_Aqp9a1
---------------ATGGAGATGAAACACAGGAGAACTATGAGGCAACGCTATGCTCTG
AAACATGGGATACTGAAGGAGTTCCTGGCTGAGTTCCTGGGGACTTTTATCTTGGTTTTG
TTTGGCGTTGGGTCTGTTGCTCAGACGGTCCTGAGTCGTAATGCCATGGGGGAGACTCTC
ACCATCCATATAGGCTTCTCTGTAGGACTGATGATGGCAGTGTACGTGGCCGGAGGAGTG
TCAGGAGCCCATGTGAACCCTGCTGTCTCCTTGGCCATGGTGATCCTGGGAAAACTCAAG
TTCTATAAATTCCCTGTCTATGTCATCGCTCAGTTCCTTGGTGCTTTCTCTGGAGCTGCT
GCAGTGTTTGGGCTGTATTACGATGCGTTCATGGACTTCACCAATGGGATTTTATCAGTG
ACTGGTATTAATGCCACCGGCCACATCTTTGCCTCTTACCCTGCCAGGTCCCTAACCATC
CTCGGAGGCATCATAGACCAGGCAGCAGGAACAGGCATGCTGGTGTTGTGTATCCTGGCC
ATCAGCGATGGGAAGAACATCGGCGCCCCTAGAGGCATGGAGCCACTGTGCATCGGCCTG
ATCATCATGGCTATCGGAGTGTCCATGGGGCTGAACTGTGGGTACCCCCTCAACCCTGCC
AGGGACCTGGGGCCACGACTCTTTACTGCTGTAGCAGGCTGGGGTTGGGAGGTGTTCAGG
ACTTCAGACTACTGGTGGTGGGTCCCGGTGGCGGGGCCGATGATAGGAGGCGTGGTCGGG
GCTCTGGTCTACTTCCTGTTCATCGAGATGCATCACAAACAG------------------
---------CCTGAGAAACCCCACGAGGAGGAAGAGGAGGAGGACGAAGAG------GAG
GAAGAAGATCTGGAGGAGGACAGCAGTCTTAAGGACAAATATGAGATGATTACCATGAGT
>Arctic_charr_Aqp9a1
---------------ATGGAGATGAAACACAGGAGAACTATGAGGCAACGCTATGCTCTA
AAACATGGGATACTCAAGGAGTTCCTGGCTGAGTTTCTGGGAACTTTTATCTTGGTTTTG
TTTGGCGTTGGGTCTGTTGCTCAGACGGTCCTGAGTCGTAATGCCATGGGGGAGACTCTC

ACCATCCATATAGGCTTCTCTGTAGGACTGATGATGGCAGTGTACGTGGCCGGAGGAGTG
TCAGGAGCCCATGTGAACCCTGCTGTCTCCTTGGCCATGGTGATCCTGGGAAAACTCAAG
TTCTATAAGTTTCCCGTCTATGTCATCGCTCAGTTCCTTGGTGCTTTCGCTGGAGCTGCT
GCAGTCTTTGGGCTGTATTATGATGCGTTCATGGACTTCACCAATGGGATTTTATCAGTG
ACTGGTATTAACGCCACCGGTCACATCTTTGCCTCTTACCCTGCCAGGTCCCTAACCATC
CTCGGAGGCCTCATAGACCAGGCAGCGGGAACAGGCATGCTGGTGTTGTGTATCCTGGCC
ATCAGCGATGGGAAGAACATCGGCGCCCCTAGAGGCATGGAGCCACTGTGCATCGGCCTG
ATCATCATGGCTATCGGGGTGTCCATGGGGCTGAACTGTGGGTACCCCCTCAACCCTGCC
AGGGACCTGGGGCCACGGCTCTTTACTGCTGTAGCAGGCTGGGGCTGGGAGGTGTTCAGG
ACGTCAGACTACTGGTGGTGGGTCCCGGTGGCGGGGCCGATGATAGGGGGCGTGGTCGGG
GCCCTGGTCTACTTCCTGTTCATCGAGATGCATCACAAACAG------------------
---------CCTGAGAAACCCCACGAGGAGGAAGAGGAGGAGGACGAAGAG------GAG
GAAGAGGATCTGGAGGAGGACAGCAGTCTTAAGGACAAATATGAGATGATTACCATGAGT
>Brown_trout_Aqp9a1
---------------ATGGAGATGAAACACAAGAGAACTATGAGGCAACGCTATGCTCTG
AAACATGGGATACTCAAGGAGTTCCTGGCTGAGTTCCTGGGCACTTTTATCTTGGTTTTG
TTTGGCGTTGGGTCTGTTGCTCAGACGGTCCTGAGTCGTAATGCCATGGGGGAGACTCTC
ACCATCCATATAGGCTTCTCTGTAGGACTGATGATGGCAGTGTACGTGGCCGGAGGAGTG
TCAGGAGCCCACGTGAACCCTGCTGTCTCCTTGGCCATGGTGATCCTGGGAAAACTCAAG
TTCTATAAATTCCCTGTCTATGTCAGCGCTCAGTTCCTTGGTGCTTTCGCTGGAGCTGCT
GCAGTGTTTGGCCTGTATTATGATGCGTTCATGGACTTCACCAATGGGATTTTATCAGTG
ACTGGCATTAACGCCACCGGTCACATCTTTGCCTCTTACCCTGCCAGGTCCCTAACCATC
CTCGGAGGCCTCATAGACCAGGCAGCGGGAACAGGCATGCTGGTGTTGTGTATCCTGGCC
ATCAACGATGGGAAGAACATCGGTGCCCCTAGGGGCATGGAGCCGCTGTGCATCGGCCTG
ATCATCATGGCTATCGGGGTGTCCATGGGGCTGAACTGTGGGTACCCCCTCAACCCTGCC
AGGGACCTGGGGCCACGGCTCTTTACTGCTGTAGCAGGCTGGGGCTGGGAGGTGTTCAGC
ACGTCAGACTACTGGTGGTGGGTCCCGGTGGCGGGGCCGATGATAGGGGGTGTGGTCGGG
GCCCTGGTCTACTTCCTGTTCATCGAGATGCATCACAAACAG------------------
---------CCTGAGAAACCCCACGAGGAGGAAGAGGAGGAGGACGAAGAG------GAG
GAAGAAGATCTGGAGGAGGACAGCAGTCTTAAGGACAAATATGAGATGATTACCATGAGT
>Atlantic_salmon_Aqp9a1_v1
---------------ATGGAGATGAAACACAAGAGAACTATGAGACAACGCTATGCTCTG
AAACATGGGATACTCAAGGAGTTCCTGGCTGAGTTCCTGGGGACTTTTATCTTGGTTTTG
TTTGGCGTTGGGTCTGTTGCTCAGACGGTCCTGAGTCGTAATGCCATGGGGGAGACTCTC
ACCATCCATATAGGCTTCTCTGTAGGACTGATGATGGCAGTGTACGTGGCCGGAGGAGTG
TCAGGAGCCCATGTGAACCCTGCTGTCTCCTTGGCCATGGTGATCCTGGGAAAACTCAAG
TTCTATAAATTCCCTGTCTATGTCAGCGCTCAGTTCCTTGGTGCTTTCGCTGGAGCTGCG
GCAGTGTTTGGCCTGTATTATGATGCGTTCATGGACTTCACCAATGGGATTTTATCAGTG
ACTGGTATTAACGCCACCGGTCACATCTTTGCCTCTTACCCTGCCAGGTCCCTAACCATC
CTCGGAGGCCTCATAGACCAGGCAGCGGGAACAGGCATGCTGGTGTTGTGTATCCTGGCC
ATCAACGATGGGAAGAACATCGGCGCCCCTAGGGGCATGGAGCCGCTGTGCATCGGCCTG
ATCATCATGGCTATCGGGGTGTCCATGGGGCTGAACTGTGGGTACCCCCTCAACCCTGCC
AGGGACCTGGGGCCACGGCTCTTTACTGCTGTAGCAGGCTGGGGCTGGGAGGTGTTCAGC
ACGTCAGACTACTGGTGGTGGGTCCCGGTGGCGGGGCCGATGATAGGGGGTGTGGTCGGG
GCCCTGGTCTACTTCCTGTTCATCGAGATGCATCACAAACAG------------------
---------CCTGAGAAACCCCACGAGGAGGAAGAGGAGGAGGACGAAGAG------GAG
GAAGAAGATCTGGAGGAGGACAGCAGTCTTAAGGACAAATATGAGATGATTACCATGAGT
>Atlantic_salmon_Aqp9a1_v2
---------------ATGGAGATGAAACACAAGAGAACTATGAGACAACGCTATGCTCTG
AAACATGGGATACTCAAGGAGTTCCTGGCTGAGTTCCTGGGGACTTTTATCTTGGTTTTG
TTTGGCGTTGGGTCTGTTGCTCAGACGGTCCTGAGTCGTAATGCCATGGGGGAGACTCTC
ACCATCCATATAGGCTTCTCTGTAGGACTGATGATGGCAGTGTACGTGGCCGGAGGAGTG
TCA---

---------------------GATGCGTTCATGGACTTCACCAATGGGATTTTATCAGTG
ACTGGTATTAACGCCACCGGTCACATCTTTGCCTCTTACCCTGCCAGGTCCCTAACCATC
CTCGGAGGCCTCATAGACCAGGCAGCGGGAACAGGCATGCTGGTGTTGTGTATCCTGGCC
ATCAACGATGGGAAGAACATCGGCGCCCCTAGGGGCATGGAGCCGCTGTGCATCGGCCTG
ATCATCATGGCTATCGGGGTGTCCATGGGGCTGAACTGTGGGTACCCCCTCAACCCTGCC
AGGGACCTGGGGCCACGGCTCTTTACTGCTGTAGCAGGCTGGGGCTGGGAGGTGTTCAGC
ACGTCAGACTACTGGTGGTGGGTCCCGGTGGCGGGGCCGATGATAGGGGGTGTGGTCGGG

GCCCTGGTCTACTTCCTGTTCATCGAGATGCATCACAAACAG------------------
---------CCTGAGAAACCCCACGAGGAGGAAGAGGAGGAGGACGAAGAG------GAG
GAAGAAGATCTGGAGGAGGACAGCAGTCTTAAGGACAAATATGAGATGATTACCATGAGT
>Taimen_Aqp9a1
---------------ATGGAGATGAAACACAGGAGAACTATGAGGCAACGCTATGCTCTG
AAACATGGGATACTCAAGGAGTTCCTGGCTGAGTTCCTGGGGACTTTTATCTTGGTTTTG
TTTGGCGTTGGGTCTGTTGCTCAGACGGTCCTGAGTCGTAATGCCATG------------

------------------------GTCTCCTTGGCCATGGTGATCCTGGGAAAACTCAAG
TTCTATAAGTTCCCTGTCTATGTCATCGCTCAGTTCCTTGGTGCTTTCGCTGGAGCTGCT
GCAGTCTTTGGGCTGTATTATGATGCGTTCATGGACTTCACCAGTGGGATTTTATCAGTG
ACTGGTATTAACGCCACCGGTCACATCTTTGCCTCTTACCCTGCCAGGTCCCTAACCATC
CTCGGAGGCCTCATAGACCAGGCAGTGGGAACAGGCATGCTGGTGTTGTGTATCCTGGCC
ATCAACGATGGGAAG---

---GGG
GCCCTGGTCTACTTCCTGTTCATCGAGATGCATCACAAACAG------------------
---------CCTGAGAAACCCCACGAGGAGGAAGAGGAGGACGACGAAGAG------GAG
GAAGAAGATCTGGAGGAGGACAGCAGTCTTAAGGACAAATACGAGATGATTACCATGAGT
>Huchen_Aqp9a1
---------------ATGGAGATGAAACACAGGAGAACTATGAGGCAACGCTATGCTCTG
AAACATGGGATACTCAAGGAGTTCCTGGCTGAGTTCCTGGGGACTTTTATCTTGGTTTTG
TTTGGCGTTGGGTCTGTTGCTCAGACGGTCCTGAGTCGTAATGCCATGGGGGAGACTCTC
ACCATCCACATAGGCTTCTCTGTAGGACTGATGATGGCAGTGTACGTGGCTGGAGGAGTG
TCAGGAGCCCATGTGAACCCTGCTGTCTCCTTGGCCATGGTGATCCTGGGAAAACTCAAG
TTCTATAAGTTCCCTGTCTATGTCATCGCTCAGTTCCTTGGTGCTTTCGCTGGAGCTGCT
GCAGTCTTTGGGCTGTATTATGATGCGTTCATGGACTTCACCAGTGGGATTTTATCAGTG
ACTGGTATTAACGCCACCGGTCACATCTTTGCCTCTTACCCTGCCAGGTCCCTAACCATC
CTCGGAGGCCTCATAGACCAGGCAGTGGGAACAGGCATGCTGGTGTTGTGTATCCTGGCC
ATCAACGATGGGAAGAACATCGGCGCCCCTAGTGGCATGGAGCCACTGTGCATCGGCCTG
GTTATCATGGCTATCGGGGTGTCCATGGGGCTGAACTGTGGGTACCCCCTCAACCCTGCC
AGGGACCTGGGGCCACGGCTCTTTACTGCTGTAGCAGGCTGGGGCTGGGAGGTGTTCAGC
ACGTCCGACTACTGGTGGTGGGTCCCGGTGGCGGGGCCGATGATAGGGGGCGTGGTCGGG
GCCCTGGTCTACTTCCTGTTCATCGAGATGCATCACAAACAG------------------
---------CCTGAGAAACCCCACGAGGAGGAAGAGGAGGACGACGAAGAG------GAG
GAAGAAGATCTGGAGGAGGACAGCAGTCTTAAGGACAAATACGAGATGATTACCATGAGT
>Tsinling_lenok_trout_Aqp9a1

------------------CAGGCAGTGGGAACAGGCATGCTGGTGTTGTGTATCCTGGCC
ATCAACGATGGGAAGAACATCGGCGCCCCTAGTGGCATGGAGCCACTGTGCATCGGCCTG
GTTATCATGGCTATCGGGGTGTCCATGGGGCTGAACTGTGGGTACCCCCTCAACCCTGCC
AGGGACCTGGGGCCACGGCTCTTTACTGCTGTAGCAGGCTGGGGCTGGGAGGTGTTCAGG

>Common_whitefish_Aqp9a1
---------------ATGGAGATGAAACACAGGAGAACTATGAGGCAACGCTATGCTCTG
AAACATGGGATACTCAAGGAGTTCCTGGCTGAGTTCCTGGGGACTTTTATCTTGGTTTTG
TTTGGCGTCGGGTCTGTTGCTCAGACGGTCCTGAGTCGTAATACCATGGGTGAGACGCTC
ACCATCCACATAGGCTTCTCTGTAGGACTGATGATGGCAGTGTACGTGGCCGGAGGAGTG
TCAGGTGCCCATGTGAACCCTGCTGTCTCCCTGGCCATGGTGATCCTGGGAAAACTCAAG
TTCTATAAGTTCCCCGTCTATGTCGTCGCTCAGTTCCTCGGTGCTTTCTCTGGAGCTGCT

GCAGTGTTTGGGCTTTATTACGATGCTTTTATGGACTTCACCAATGGAATATTATCAGTG
ACTGGTATTAACGCCACCGGTCACATCTTTGCCTCTTACCCTGCCAGGTCCCTGACCATC
CTCGGAGGCCTCATAGACCAGGCAGTGGGAACAGGCATGCTGGTGCTGTGTATCCTGGCC
ATCATCGATGGGAAGAACATCGGCGCCCCTAGGGGCATGGAGCCACTGTGCATCGGCCTG
ATCATCATGGCTATCGGGGTGTCCATGGGGCTGAACTGTGGGTATCCCCTCAACCCTGCC
AGGGACCTGGGGCCACGGGTCTTCACTGCTGTAGCAGGCTGGGGCTGGGAGGTGTTCAGC
ACATCAGACTACTGGTGGTGGGTCCCCGTGGCTGGGCCGATGGTAGGGGGCGTAGTTGGG
GCCCTGGTCTACTTCCTGTTCATCGAGATGCATCACAAACAG------------------
---------CCGGAGAAACCCCACGAGGTGGAGGAGGAGGATGAAGAGGAG------GAG
GAGGAAGATCTGGAGGAGGACAGCAGTCTTAAGGACAAATACGAGATGATACCCATGAGT
>Balchen_Aqp9a2
---------------ATGGAGATGAAACGCATGAGAACTATGAGGCAACGCTATGCTCTG
AAACATGGGATACTCAAGGAGTTCCTGGCTGAGTTCTTGGGGACATTTATCTTGGTTTTG
TTTGGCGTCGGGTCTGTTGCTCAGACGGTCCTGAGTCGTAATGCGATGGGGGAGACGCTC
ACCATCCACATAGGCTTCTCTGTAGGACTGATGATGGCAGTGTACGTTGCCGGAGGAGTG
TCAGGTGCCCATGTGAACCCTGCTGTCTCCCTGGCCATGGTGATCCTGGGAAAACTCAAG
TTCTATAAGTTCCCCGTCTATGTCGTGGCCCAGTTCCTCGGTGCTTTCTCTGGAGCTGCT
GCAGTGTTTGGGCTGTATTACGATGTGTTCATGGACTTCACCAATGGGATTTTATCAGTG
ACTGGTATTAACGCCACCGGTCACATCTTTGCCTCCTACCCTGTCAGGTGCCTGACCATC
CTCGGAGGCCTCATAGACCAGGTAGTGGGAACCGGCATGCTGGTGCTGTGTATCCTGGCC
ATCATCGATGGGAAGAACATCGGCGCCCCTAGGGGCATGGAGCCACTGTGCATCGGCCTG
GTCATCATGGCTATCGGGGTGTCCATGGGGCTGAACTGTGGGTATCCCCTCAACCCTGCC
AGGGACCTGGGGCCACGGGTCTTCACTGCTGTAGCAGGCTGGGGCTGGGAGGTGTTCAGG
ACGGCAGACTACTGGTGGTGGGTCCCCGTGGCGGGGCCGATGCTAGGGGGCGTTGTCGGG
GCCCTGGTCTACTTCCTGTTTATCGAGATGCATCACAAACAG------------------
---------CCGGAGAAACCCCACAAGGAGGAAGAGGAGGATGAGGAAGAG------GAG
GATGAAGACCTGGAGGAGGACAGCAGTCTCAAAGACAAATACGAGATGATTACCATGAGT
>Grayling_Aqp9a1b
---------------ATGGAGATGAAACACAGGAGAACTATGAGGCAACGCTATGCTCTG
AAACATGGGATACTCAAGGAGTTCCTGGCTGAGTTCCTGGGGACTTTTATCTTGGTTTTG
TTTGGGGTTGGGTCTGTTGCTCAGACGGTCCTGAGTCGTAATGCCATGGGGGAGACTCTC
ACCATCCACATAGGCTTCTCTGTAGGACTGATGATGGCAGTGTACGTGGCTGGAGGAGTG
TCAGGAGCCCATGTGAACCCTGCTGTCTCCTTGGCCATGGTGATCCTGGGAAAACTCAAG
TTCTATAAGTTCCCTGTGTATGTCATCGCTCAGTTCCTTGGTGCTTTCGCTGGAGCTGCT
GCAGTCTTTGGGCTGTATTATGATGCTTTTATGGACTTCACCAATGGAATTTTATCAGTG
ACTGGTATTAACGCCACCGGTCACATCTTTGCCTCTTACCCTACAAGGTCCCTGACCATA
CTCGGAGGCCTCATAGACCAGGCAGTGGGAACAGGCATGCTGGTGTTGTGTATACTTGCC
ATCATTGATGGGAAGAACATCGGCGCCCCTAGGGGCATGGAGCCACTGTGCATCGGCCTG
ATCATCATGGCTATCGGGGTGTCCATGGGCCTGAACTGTGGGTACCCCCTCAACCCTGCC
AGGGACCTGGGGCCACGGGTCTTTACTGCTGTAGCAGGCTGGGGCTGGGAGGTGTTCAGG
ACATCAGACTACTGGTGGTGGGTCCCGGTGGCGGGGCCGATGGTAGGGGGCGTGGTCGGG
GCCCTGGTCTACTTCCTGTTCATCGAGATGCATCACAAACAG------------------
---------ACGGAG---------GATGAAGAGGAGGATGAAGAGGAGGAGGAAGAGGAG
GAGGAAGATCTGGAGGAGGACAGTAGTCTTAAGGACAAATATGAGATGATTACCATGAGT
>Brown_trout_Aqp9a2p
---------------ATGGAGATGAAACACAGGAGAACTATGAGGCATCGCTATGCTCTG
AAACATGGGATACTCAAGGAGTTCCTGGCTGAGTTCCTGGGGACTTTTATCTTGGTTTTG
TTTGGCGTTGGGTCTGTTGCTCAGACAGTCCTGAGTCGTAATGCCATCGGGGAGACTCTC
ACCATCCACATAGGCTTCTCTGTAGGACTGATGATGGCAGTGTACGTGGCCGGAGGAGTG
TCAGGTGCCCACGTGAACCCTGCTGTCTCCCTGGCCATGGTGATCGTGGGAAAACTCCAA
TTCTATAAGTTCCCCGTTTATGTCAGCGCTCAGTTCCTTGGTGCTTTCACTGGATCTGCT
GCTGTGTTTGGGCTGTATTATGACGTGTTGATGGACTTCACCAGTGGGATTTTATCAGTG
ACTGGTATTAACGCCACCGGTCACATCTTTGCCTCTTACCCTGTCAGGTGCCTGACCATC
CTCGGAGGCCTCGTAGACCAGGTAGTGGGAACAGGTATGCTGGTGCTGTGCATTCTGGCC
ATCATCGATGGGAAGAACATCGGCGCCCCTAGAGGCATGGAGCCTCTCTGCATCGGCCTG
GTCATCTTGGCTATCGGGGTGTCCATGGGACTGAACTGTGGGTACCCCCTCAACCCTGCC
AGGGACCTGGGGCCACGGATGTTCACTGCTGTAGCAGGCTGGGGCTGGGAGGTGTTCAGG
ACGGCAGACTACTGGTGGTGGGTCCCGGTGGCGGGGCCGATGCTAGGGGGCGTGGTCGGG
GCCCTGGTCTACTTCCTGTTTATCGAGATGCATCACAAAGAG------------------
---------CAAGAGAAACCCCACGAGGAGGAGGAAGAAGAGGAGGAGGAAGAAGAGGAG
GATGAAGGCCTGGAGGAGGACAGCAGTCTCAAAGACAAATCCCAGATGATTACCATGAGT

>Atlantic_salmon_Aqp9a2p
---------------ATGGAGATAAAACACAGGAGAACTATGAGGCAACGCTATGCTCTG
AAACATGGGATACTCAAGGAGTTCCTGGCTGAGTTCCTGGGGACTTTTATCTTGGTTTTG
TTTGGCGTTGGGTCCGTTGCTCAGACGGTCCTGAGTCGTAATGCCATGGGGGAGACTCTC
ACCATCCACATAGGCTTCTCTGTAGGACTGATGATGGCAGTGTACGTGGCCGGAGGAGTG
TCAGGTGCCCATGTGAACCCTGCTGTCTCCCTGGCCATGGTGATCCTGGGAAAACTCAAG
TTCTATAAGTTCCCCGTTTATGTCAGCGCTCAGTTCCTTGGTGCTTTCACTGGATCTGCT
GCAGTGTTTGGGCTGTATTATGACGTGTTGATGGGCTTCACCAGTGGGATTTTATCAGTG
ACTGGTATTAACGCCACCGGTCACATCTTTGCCTCTTACCCTGTCAGGTGCCTGACCATC
CTCGGAGGCCTCGGAGACCAGGTAGTGGGAACAGGTATGCTGGTGCTGTGTATCCTGGCC
ATCATCGATGGGAAGAACATCGGCGCCCCTAGAGGCATGGAGCCTCTGTGCATCGGCCTG
GTCATCTTGGCTATCGGGGTGTCCATGGGGCTGAACTGTGGGTACCCGCTCAACCCTGCC
AGGGACCTGGGGCCACGGATCTTCACTGCTGTAGCAGGCTGGGGCTGGGAGGTGTTCAGG
ACGGCAGACTACTGGTGGTGGGTCCCGGTGGCGGGGCCGATGCTAGGGGGCGTGGTCGGG
GCCCTGGTCTACTTCCTGTTTATCGAGATGCATCACAAAGAG------------------
---------CAAGAGAAACCCCATGAGGAGGAAGAAGAAGAG---------------GAG
GATGAAGGCCTGGAGGAGGACAGCAGTCTCAAAGACAAATCCCAGATGATTACCATGAGT
>Huchen_Aqp9a2p
---------------ATGGAGATGAAACACAGGAGAACTATGAGGCAACGCTATGCTCTG
AAACATGGGATACTCAAGGAGTTCCTGGCTGAGTTCCTGGGTACTTTTCTCTTGGTTTTG
TTTGGCGTTGGGTCTGTTGCTCAGACGGTCCTGAGTCGTAATGCCATGGGGGAGACTCTC
ACCATCCACATAGGCTTCTCTGTAGGACTGATGATGGCAGTGTACGTGGCCGGAGGAGTG
TCAGGTGCCCATGTGAACCCTGCTGTCTCCCTGGCCATGGTGATCCTGGGAAAACTACAG
TTCTATAAGTTTCCCGTTTATGTCATCGCCCAGTTCCTTGGTGCTTTCGCTGGAGCTGCT
GCAGTGTTTGGGCTGTATTAT---------------------------------------

---------------------GTAGTAGGAACAGGCATGCTGGTGCTGTGTATCCTGGCC
ATCATCGATGGGAAGAACATTGGCGCCCCGAGGGGCATGGAGCCTCTGTGCATCGGCCTG
GTAATCATGGCTATCGGGGTGTCCATGGGGCTGAACTGTGGGTACCCCCTCAACCCTGCC
AGGGACCTGGGGCCACGGCTCTTCACCGCTGTAGCAGGCTGGGGCTGGGAGGTGTTCAGG
ACGGCAGACTACTGGTGGTGGGTCCCGGTGGTGGGGCCGATGCTAGGGGGCGTGGTCGGG
GCCCTGGTCTACTTCCTGTTTATCGAGATGCATCACAAAGAG------------------
---------CTGGAGAAACCCCAAGAGGAGGAAGAGGAGGAGGAGGAGGAGGAGGAAGAA
GATGAAGGCCTGGAAGAGGACAGCAGTCTCAAAGACAAATACGAGATGATTACCATGAGT
>Northern_pike_Aqp9a
---------------ATGGTAACTGAATCCAGTATAACCATGAGCCAACACTTTGCTCTG
AAACATGGGATATTCAAGGAGTTCCTGGCAGAATTTCTGGGGACTTTTGTCTTGGTTCTG
TTTGGCGTCGGGTCCGTTGCTCAGACAGTCCTGAGTCGAAACGCCATGGGGGAGACTTTG
ACCATCCACATAGGTTTCTCCGTGGGACTGATGATGGCTGTGTTCGTGGCCGGAGGAGTG
TCAGGTGCCCATGTCAATCCTGCTGTCTCCTTGGCAATGACGGTCCTGGGAAAACTCAAG
TTCTATAAGTTCCCTATCTATGTCATCGCTCAGTTCCTTGGGGCCTTCGCTGGAGCCGCT
GCAGTCTTTGGCTTGTACTATGACGCTTTCATGGACTTCACCAATGGGATTCTATCGGTG
ACTGGAATCAACGCCACTGGACACATCTTCGCCTCTTATCCTGTCAGGTCCTTGACCCTC
CTAGGAGGCTTTGTTGACCAGGTGGTGGGTACTGGAATGTTAGTGCTGTGTATCCTGGCC
ATCATCGACGGGGAGAACATCGGTGCCCCAAGAGGCATGGAGCCCCTGTGCATCGGCCTG
ATCGTCATGGCGATCGGAGTGTCCATGGGGCTGAACTCCGGGTACCCTCTCAACCCGGCC
AGGGACCTGGGGCCTCGGCTCTTCACCGCTGTGGCCGGCTGGGGCTGGGAAGTGTTCAGG
ACATCAGACTACTGGTGGTGGATCCCAGTGGCAGGGCCCATGGTAGGAGGGCTGGTCGGC
GCCCTGGTCTACTTCCTTTTTATTGAAATGCATCACAAACAG------------------
---------CCCGAGAAACCATGCGAAGAAGAGGAGGAAGAG---------------GAT
GATGAAGATTTGGAGGAAGACAGCAGTCTCAAAGACAAATATGAGATGATCACCATGAGC
>Anshui_blind_cavefish_Aqp9a1
--ATGAAGCAGCACTGCGTCATC
AAACACAGCGTCCTCAAAGAGTTCCTGGCCGAGTTCCTCGGGACCTTTGTGCTGGTGCTG
TTCGGCTGCGGCTCGGTGGCTCAGACGGTGTTGAGCAGAAACACTCTCGGGGAACCGCTG
ACCGTCCACATCGGCTTCAGCACCGGACTCATGATGGGCATCTATGTGTCCGGCGACGTG
TCGGGCGGTCACCTGAACCCCGCCGTGTCTCTGGCCATGGTCATCCTGGGGAAGCTGAAG
ATCTGGAAGTTTCCCATCTACGTGACGGCGCAGATGTTGGGCGCGTTTGTGGGTGCGGCT
GCAGTCTTTGGATTGTATTACGATGCCTTCATGGACTTCACCAGCGGGATTCTGTCAGTC
ACAGGAATTAACGCCACAGGACACATCTTTGCCTCATACCCAGGCAGACACCTGACAGTG
CTGGGTGGTTTCGTGGATCAGGTGGTGGGG------ATG---------TGTATTCTAGCG

GTTGTGGACGGCAGGAACATCGGTCCTCCTCGAGGCGTGGAGCCACTGGCTGTGTGTCTG
ATCCTGCTGGGCATCAGCGTCTCTATGGGACTGAACTGCGGTTACCCTCTGAACCCCGCC
AGAGACCTGGGCCCTCGCCTCTTCACCGCCGCCGCCGGATGGGGCATGGAGGTGTTCAGC
ACTGCAGATTACTGGTGGTGGATTCCTGTCGCCGGGCCGTTGGTAGGGGGCATCGCCGGT
GCTGTGATCTACTTCCTGTTGATCGAGCTTCATCACACCAAC------------------
------CACAACGACAGACCTCACGAAGAACCCGAGGAAGAG---------------GAG
GATGAGGACGAAGACGAGGACAGCAGCCTGAAGGACAAATACGAGATGATCAACATGAGC
>Horned_golden_line_barbel_Aqp9a1
--ATGAAGCAGCACTGCGCCATC
AAACACAGCGTCCTCAAAGAGTTCCTGGCCGAGTTCCTCGGGACCTTGGTGCTGGTGCTG
TTCGGCTGCGGCTCGGTGGCTCAGACGGTGTTGAGCAGAAACACTCTCGGGGAACCGCTG
ACCGTCCACATCGGCTTCAGCACCGGACTCATGATGGGCGTCTATGTGTCCGGCGGCGTG
TCGGGTGGTCACCTGAACCCCGCCGTGTCTCTGGCCATGGTCATCCTGGGGAAGCTGAAG
ATCTGGAAGTTTCCCATCTACGTGACGGCGCAGATGTTGGGCGCGTTTGTGGGAGCGGCT
GCAGTCTTTGGATTGTATTACGATGCCTTCATGGACTTCACCAGCGGTATTCTGTCAGTC
ACAGGAATTAACGCCACAGGACACATCTTCGCCTCATACCCAGGCAGACACCTGACAGTG
CTGGGCGGTTTCGTGGATCAGGTGGTGGGGACGGGAATGCTGGTCCTCTGTATTCTAGCG
GTTGTGGATGGCAGGAACATCGGTGCTCCACGAGGCGTGGAGCCGCTGGCTGTGGGTCTG
ATCCTGCTGGGCATCAGCGTCTCAATGGGACTGAACTGCGGTTACCCTCTGAACCCCGCC
AGAGACCTGGGCCCTCGCCTCTTCACCGCCGCCGCCGGATGGGGCATGGAGGTGTTCAGC
ACTGCAGATTACTGGTGGTGGATTCCTGTCGCCGGGCCGTTGGTAGGGGGCATCGCCGGT
GCTGTGATCTACTGCCTGTTGATCGAGCTTCATCACACCAAC------------------
------CACAACGACAGACCTCACGAAGAACCCGAGGAAGAG---------------GAG
GATGAGGACGAAGACGAGGACAGCAGCCTGAAGGACAAATACGAGATGATCAACATGAGC
>Golden_line_fish_Aqp9a1
--ATGAAGCAGCACTGCGCCATC
AAACACAGCGTCCTCAAAGAGTTCCTGGCCGAGTTCCTCGGGACCTTTGTGCTGGTGCTG
TTCGGCTGCGGCTCGGTGGCTCAGACGGTGTTGAGCAGAAACACTCTCGGGGAACCGCTG
ACTGTCCACATCGGCTTCAGCACCGGACTCATGATGGGCGTCTATGTGTCCGGCGGCGTG
TCGGGCGGTCACCTGAACCCCGCCGTGTCTCTGGCCATGGTCATCCTGGGGAAGCTGAAG
ATCTGGAAGTTTCCCATCTACGTGATGGCGCAGATGTTGGGCGCGTTTGTGGGAGCGGCT
GCAGTCTTTGGATTGTATTACGATGCCTTCATGGACTTCACCAGCGGGATTCTGTCAGTC
ACAGGAATTAACGCTACAGGACACATCTTCGCCTCATACCCAGGCAGACACCTGACAGTG
CTGGGCGGTTTCGTGGATCAGGTGGTGGGGACGGGAATGCTGGTCCTCTGTATTCTAGCG
GTTGTGGACGGCAGGAACATCGGTGCTCCTCGAGGCGTGGAGCCGCTGGCTGTGGGTCTG
ATCCTGCTGGGCATCAGTGTCTCTATGGGACTGAACTGCGGTTACCCTCTGAACCCCGCC
AGAGACTTGGGCCCTCGCCTCTTCACCGCCGCTGCCGGATGGGGCATGGAGGTGTTCAGC
ACTGCAGATTACTGGTGGTGGATTCCTGTCGCCGGGCCGTTGGTAGGGGGCATCGCCGGT
GCTGTGATCTACTTCCTGTTGATCGAGCTTCATCACACCAAC------------------
------CACAACGACAGACCTCACGAAGAACCCGAGGAAGAGGAGGAT---------GAG
GACGAGGACGAAGACGAGGACAGCAGCCTGAAGGACAAATACGAGATGATCAACATGAGC
>Common_carp_Aqp9a1
--ATGAAGCAGCACTGTGCCATC
AAAAACAGTGTCTTCAAAGAGTTCCTGGCCGAGTTCCTTGGGACCTTTGTGCTGGTGCTG
TTCGGCTGCGGCTCAGTGGCTCAGACGGTGTTGAGCAGAAACACTCTCGGGGAACCGCTG
ACCGTTCACATTGGCTTCAGCACCGGACTCATGATGGGCGTCTATGTGGCCGGCGGCGTG
TCGGGTGGTCACCTGAACCCCGCCGTGTCTCTGGCCATGGTCATCCTGGGGAAGCTGAAG
ATCTGGAAGTTTCCCATCTATGTGACAGCACAGATGTTGGGCGCATTTGTGGGAGCAGCT
GCAGTCTTTGGATTGTATTACGATGCCTTCATGGACTTCACCAGCGGGATTCTGTCAGTC
ACAGGAATTAACGCCACTGGACACATCTTTGCCTCATACCCTGGCAGACACCTGACAGTC
CTGGGCGGTTTCGTGGATCAGGTGGTGGGGACAGGAATGCTGGTCCTGTGTATTCTAGCG
GTTGTGGACGGCAGGAACATCGGTGCTCCTCGAGGCGTGGAGCCGCTGGCTGTGGGTCTG
ATCCTGCTGGGCATCAGCGTCTCTATGGGACTGAACTGCGGTTACCCTCTGAACCCCGCC
AGAGACCTAGGCCCTCGCCTCTTCACTGCCGCTGCTGGATGGGGCATGGAGGTGTTCAGG
ACTGCAGATTACTGGTGGTGGATCCCTGTTGCCGGGCCATTGGTAGGGGGCGTCGCCGGT
GCTGTGATCTACTTCCTGTTGATCGAGCTTCATCACACCAAC------------------
------CACAGCGACAAACCTCACGAAGAACCCGAGGAGGAGGAGGAG---------GAG
GATGAAGACGAAGATGAGGACAGCAGCCTGAAGGACAAATACGAAATGATCAACATGAGC
>Goldfish_Aqp9a1
--ATGAAGCAGCACTGCGTCATC
AAACACAGCGTCTTCAAAGAGTTCCTGGCCGAGTTCCTCGGGACCTTTGTGCTGGTGCTG

TTCGGCTGTGGCTCGGTGGCTCAGACGGTGTTGAGCAGAAACACTCTCGGGGAACCACTG
ACCGTCCACATTGGCTTCAGCACTGGACTCATGATGGGTGTCTATGTGTCCGGCGGTGTC
TCTGGTGGTCACCTGAACCCTGCCGTATCTCTGGCCATGGTCATCCTGGGGAAGCTGAAG
ATCTGGAAGTTTCCCATCTACGTGATGGCGCAGATGTTGGGTGCATTTGTGGGAGCGGCT
GCAGTCTTTGGATTGTATTACGATGCCTTCATGGACTTCACCAATGGGATCCTGTCAGTC
ACAGGAATTAACGCCACAGGACACATCTTTGCCTCATACCCAGGCAGACACCTGACAGTC
CTGGGCGGTTTCGTGGATCAGGTGGTGGGGACAGGAATGCTGGTCTTGTGTATTCTAGCG
GTTGTGGACGGCAGGAACATCGGTGCTCCTCGAGGCGTGGAGCCACTGGCTGTGGGTCTA
ATCCTGCTGGGCATCAGTGTTTCTATGGGGCTGAACTGTGGTTACCCTCTGAACCCCGCC
AGAGACCTGGGCCCTCGCCTCTTTACTGCTGCTGCCGGATGGGGAATGGAGGTGTTCAGC
ACTGCAGATTACTGGTGGTGGATACCTGTCGCTGGGCCGTTGGTAGGGGGCATCGCTGGT
GCTGTGATCTACTTCCTGTTAATCGAGCTTCATCACACCAAC------------------
------CACAATGACAAACCTCACGAAGAACCTGAGGAAGAA----------------GAG
GATGAGGAGGAAGACGAGGACAGCAGCCTGAAAGACAAATACGAGATGATCAACATGAGC
>Anshui_blind_cavefish_Aqp9a2
--ATGAAGCATCACTGCGCCATC
AAACACAGCGTCTTCAAAGAGTTCCTGGCCGAGTTCCTCGGGACTTTGTGCTGGTGCTG
TTCGGCTGCGGCTCGGTGGCTCAGACGGTGTTGAGCAGAAACACTCTCGGTGAACCGCTG
ACCGTCCACATCGGCTTCAGCACCGGACTCATGATGGGTGTCTATGTGTCCGGCGGCGTG
TCGGGCGGTCACCTGAACCCCGCCGTGTCTCTGGCCATGGTCATCCTGGGGAAGCTGAAG
ATCTGGAAGTTTCCCGTCTACGTGACGGCGCAGATGTTGGGCGCATTTGTCGGAGCGGCT
GCAGTCTTTGGATTGTATTACGATGCCTTCATGGACTTCACCAGCGGGATCCTGTCGGTC
ACAGGAATTAACGTCACAGGACACATCTTCGCCTCGTACCCAGGCAGACACCTGACGGTC
CTGGGCGGATTCGTGGATCAGGTGGTGGGGACGGGAATGCTGGTCCTCTGTATTCTGGCG
GTTGTGGACGGCAGGAACATGGGTGCTCCTCGAGGCGTGGAGCCGCTGGCTGTGGGTCTG
ATCCTGCTGGGCATCAGCGTCTCCATGGGGCTGAACTGCGGTTACCCTCTGAACCCCGCC
AGAGACCTGGGCCCGCGCCTCTTCACCGCCGCTGCCGGGTGGGGCATGGAGGTGTTCAGC
ACTGCAGATTACTGGTGGTGGATCCCTGTTGCCGGGCCGTTGGTAGGGGGCGTCGCCGGT
GCTGTGATCTACTTTCTGTTGATTGAGCTTCATCACTCCAAC------------------
------CACAACGACAAATCTCACAAAGAACCTGAGGAAGAG----------------GAG
GATGAGGAGGAAGATGAGGACAGCAGCCTAAAGGACAAATACGAGATGATCAACATGAGC
>Horned_golden_line_barbel_Aqp9a2

------------------------------------ATGGTCATCCTGGGGAAGCTGAAG
ATCTGGAAGTTCCCCATCTACGTGACGGCGCAGATGTTGGGCGCATTTGTCGGAGCGGCT
GCAGTCTTTGGATTGTATTACGATGCCTTCATGGACTTCACCAGCGGGATCCTGTCGGTC
ACAGGAATTAACGCCACAGGACACATCTTCGCCTCGTACCCAGGCAGACACCTGACGGTC
CTGGGCGGATTCGTGGATCAGGTGGTGGGGACGGGAATGCTGGTCCTCTGTATTCTGGCG
GTTGTGGACGGCAGGAACATGGGTGCTCCTCGAGGCGTGGAGCCGCTGGCTGTGGGTCTG
ATCCTGCTGGGCATCAGCGTCTCCATGGGGCTGAACTGCGGTTACCCTCTGAACCCCGCC
AGAGACCTGGGCCCGCGCCTCTTCACCGCCGCCGCTGGGTGGGGCATGGAGGTGTTCAGC
ACTGCAGATTACTGGTGGTGGATCCCTGTTGCCGGGCCGTTGGTAGGGGGCGTCGCCGGT
GCTGTGATCTACTTCCTGTTGATTGAGCTTCATCACTCCAAC------------------
------CACAACGACAAATCTCACAAAGAACCTGAGGAAGAG----------------GAG
GATGAGGAGGAAGATGAGGACAGCAGCCTGAAGGACAAATACGAGATGATCAACATGAGC
>Golden_line_fish_Aqp9a2
--ATGAAGCATCACTGCGCCATC
AAACACAACATCTTCAAAGAGTTCCTGGCCGAGTTCCTCGGGACCTTTGTGCTGGTGCTG
TTCGGCTGCGGCTCGGTGGCTCAGACGGTGTTGAGCAGAAACACTCTCGGTGAACCGCTG
ACCGTCCACATCGGCTTCAGCACTGGACTCATGATGGGTGTCTATGTGGCCGGTGGCGTG
TCGGGCGGTCACCTGAACCCCGCTGTGTCTCTGGCCATGGTCATCCTGGGGAAGCTGAAG
ATCTGGAAGTTTCCCGTCTACGTGATGGCGCAGATGTTGGGCGCATTTGTCGGAGCGGCT
GCAGTCTTTGGATTGTATTACGATGCCTTCATGGACTTCACCAGCGGGATCCTGTCGGTC
ACAGGAATTAACGCCACAGGACACATCTTCGCCTCGTACCCAGGCAGACACCTGACGGTC
CTGGGCGGATTCGTGGATCAGGTGGTGGGGACGGGAATGCTGGTCCTCTGTATTCTGGCG
GTTGTGGACGGCAGGAACATGGGTGCTCCTCGAGGCGTGGAGCCGCTGGCTGTGGGTCTG
ATCCTGCTGGGCATCAGCGTCTCCATGGGGCTGAACTGCGGTTACCCTCTGAACCCCGCC
AGAGACCTGGGTCCGCGCCTCTTCACCGCCGCCGCTGGGTGGGGCATGGAGGTGTTCAGC

ACTGCAGATTACTGGTGGTGGATCCCTGTTGCCGGGCCGTTGGTAGGGGGCGTCGCCGGT
GCTGTGATCTACTTCCTGTTGATTGAGCTTCATCACTCCAAC------------------
------CACAACGACAAATCTCACAAAGAACCTGAGGAAGAG---------------GAG
GATGAGGAGGAAGATGAGGACAGCAGCCTAAAGGACAAATACGAGATGATCAACATGAGC
>Common_carp_Aqp9a2
---------------------------------------ATGAAGCATCACTGCGCCATC
AAACACGGCATCTTCAAAGAGTTCCTGGCCGAGTTCCTCGGGACCTTTGTGCTGGTGTTG
TTCGGCTGCGGGTCGGTGGCTCAGACGGTGTTGAGCAGAAACACTCTTGGTGAACCGCTG
ACTGTCCACATCGGCTTCAGCACCGGACTCATGATGGGTGTCTATGTGGCCGGCGGCGTG
TCGGGCGGTCATCTGAACCCCGCCGTGTCTCTGGCCATGGTCATCCTGGGGAAGCTGAAG
ATCTGGAAGTTTCCCGTCTACGTGACGGCGCAGATGTTGGGTGCGTTTGTCGGAGCGGCT
GCAGTCTTTGGATTGTATTATGATGCCTTCATGGACTTCACCAGCGGGATCCTGTCGGTC
ACAGGAATTAATGCCACAGGACACATCTTCGCCTCATACCCAGGCAGACACCTGACGGTC
CTGGGCGGTTTCGTGGATCAGGTGGTGGGGACGGGGATGCTGGTCCTCTGTATTCTGGCG
GTTGTGGACGGCAGGAACATGGGTGCTCCTCGAGGCATGGAGCCACTGGCG---GGG---
---------GGN------------ATGGGGTTGAACTGCGGTTACCCTCTGAACCCCGCC
AGAGACTTGGGCCCGCGCCTCTTCACCGCTGCCGCCGGGTGGGGCATGGAGGTGTTCAGG
ACTGCAGATTACTGGTGGTGGATCCCTGTTGCCGGGCCGTTGGTAGGGGGTGTCGCCGGT
GCTGTGATCTACTTCCTGTTGATTGAGCTTCATCACTCCAAC------------------
------CACAATGACAAATCTCACAAAGAACCTGAGGAAGAG------------------
GTTGAGGAGGAAGACGAGGACAGCAGCCTGAAGGACAAATACGAGATGATCAACATGAGC
>Goldfish_Aqp9a2
---------------------------------------ATGAAGCATCACTGCGCCATC
AAACACGGCATCTTCAAAGAGTTCCTGGCCGAGTTCCTCGGGACCTTTGTGCTGGTGTTG
TTTGGCTGTGGGTCGGTGGCTCAGACGGTGTTGAGCAGAAACACTCTTGGTGAACCGCTG
ACCGTCCACATCGGCTTCAGCACCGGACTCATGATGGGAGTCTATGTGGCTGGCGGTGTA
TCGGGCGGTCACCTGAACCCCGCCGTGTCTCTGGCCATGGTCATCCTGGGGAAGCTGAAG
ATCTGGAAGTTTCCCATCTACGTGACGGCACAGATGTTGGGCGCGTTTGTCGGAGCGGCT
GCAGTCTTTGGATTGTATTACGATGCGTTCATGGACTTCACCAGTGGGATCCTGTCGGTC
ACGGGGATTAACGCCACAGGACACATCTTTGCGTCATACCCAGGCAGACACCTGACGGTC
CTGGGTGGGTTTGTGGATCAGGTGGTGGGGACAGGAATGCTGGTCCTCTGTATTCTGGCG
GTGGTGGACGGCAGGAACATGGGTGCTCCTCGAGGCGTGGAGCCGCTGGCCGTGGGTCTG
ATCCTGCTGGGCATCAGTGTCTCCATGGGGCTGAACTGTGGCTATCCTCTAAACCCGGCC
AGAGACCTGGGCCCGCGCCTCTTCACCGCCGTCGCTGGATGGGGCATGGAGGTGTTCAGC
ACTGCAGATTACTGGTGGTGGATCCCTGTTGCCGGGCCGTTGGTAGGGGGCGTTGCCGGT
GCTGTGATCTACTTCCTGTTGATTGAGCTTCATCACTCCAAC------------------
------CACAATGACAAATCTCACAAAGAACCTGAGGAAGAG---------------GAG
GACGAGGAGGAAGATGAGGACAGCAGCCTGAAGGACAAATATGAGATGATCAACATGAGC
>Amur_ide_Aqp9am
---------------------------------------ATGAAGCATCACTGCGCCATC
AAACACAGCATCTTCAAAGAGTTCCTGGCCGAGTTCCTCGGGACCTTCGTGCTGGTGTTG
TTCGGCTGCGGGTCGGTGGCCCAGACGGTGTTGAGCAGAAACACTCTCGGCGAGCCGCTG
ACGGTCCACATCGGCTTCAGCACTGGACTCATGATGGGTGTCTACGTGTCCGGCGGCGTG
TCGGGCGGTCACCTCAACCCGGCCGTGTCCCTGGCAATGGTGATTCTGGGGAAGCTGAAG
ATCTGGAAGTTTCCCATCTATGTGATGGCGCAGATGCTCGGGGCGTTCGTCGGGGCCGCC
GCAGTGTTCGGACTGTATTACGATGCCTTCATGGAGTTCACCAGCGGGATCCTCTCGGTG
---GGGATCAACGCCACAGGACACATCTTCGCCTCGTATCCGGGGAGACACCTGACCGTC
CTGGGTGGATTCGTGGATCAGGTGGTGGGAACGGGAATGCTGGTTCTCTGTATTCTGGCC
ATCGTGGACGGGAGGAACATCGGCGCCCCTAGAGGCGTGGAGCCGCTGGCCGTGGGCCTG
ATCCTGCTGGGCATCAGTGTGTCGATGGGCCTGAACTGCGGATACCCCCTGAACCCCGCG
CGAGACCTCGGCCCGAGACTCTTCACTGCTGTGGCCGGGTGGGGGATGGAGGTGTTC---

>Fathead_minnow_Aqp9a
---------------------------------------ATGAAGCATCACTGCGCCATC
AAACACAGCGTCTTCAAAGAGTTCCTGGCCGAGTTCCTCGGGACCTTCGTGCTGGTGTTG
TTCGGCTGCGGGTCGGTGGCCCAGACGGTGTTGAGCAGAAACACTCTCGGCGAGCCACTG
ACGGTCCACATCGGCTTCAGCACTGGACTCATGATGGGCGTCTACGTGTCCGGCGGCGTG
TCGGGTGGTCACCTCAACCCGGCCGTGTCTCTGGCGATGGTGATCCTGGGGAAGCTGAAG

ATCTGGAAGTTTCCCATCTACGTGACGGCGCAGATGCTCGGGGCGTTCGTCGGGGCCGCC
GCAGTGTTCGGGCTGTATTACGATGCCTTCATGGAGTTCACCAGCGGGATCCTCTCGGTG
ACGGGGATCAACGCCACAGGACACATCTTCGCATCATATCCGGGGAGACACCTGACCGTC
CTGGGCGGATTCGTGGATCAGGTGGTGGGAACGGGAATGCTGGTTCTCTGTATTCTGGCC
ATCGTGGACGGGAGGAACATCGGCGCCCCTAGAGGCGTGGAGCCGCTGGCCGTGGGCCTG
ATCCTGCTGGGCATCAGTGTGTCGATGGGCCTGAACTGCGGATACCCCATGAACCCGGCG
CGAGACCTCGGCCCGAGACTCTTCACTGCAGCGGCCGGCTGGGGATGGAGGTGTTCAGT
ACTGCAGATTACTGGTGGTGGATCCCTGTTGCCGGGCCGATGGTGGGCGGGGTCGTTGGC
GCTGTGATCTACTTCCTGCTGATCGAGCTTCATCACCCCAAA------------------
------CACACTGACAAACCTCTGGAGGAACCCGAGGAAGAG----------------GAG
GATGAGGAGGAGGACGAGGACAGCAGCCTGAAGGACAAATACGAGATGATCAACATGAGC
>Grass_carp_Aqp9a

----------------------CAGACGGTGTTGAGCAGAAACACTCTGGGAGAACCGCTG
ACCGTCCACATCGGCTTCAGCACCGGACTCATGATGGGCGTCTACGTGTCCGGCGGCGTG
TCGGGCGGTCACCTCAATCCGGCCGTGTCTCTGGCAATGGTGATCCTGGGGAAGCTGAAG
ATCTGGAAGTTTCCCATCTATGTGACGGCGCAGATGCTCGGGGCGTTCGTTGGCGCGGCC
GCGGTGTTCGGACTGTATTACGATGCCTTCATGGATTTCACCAGCGGGATCCTTTCCGTG
ACGGGAATCAACGCCACAGGACACATCTTTGCGTCGTATCCGGGGAGACACCTGACCGTC
CTGGGCGGATTCGTGGATCAGGTGGTGGGAACGGGAATGCTGGTGCTCTGTATCCTGGCC
ATCGTGGACGGGAGGAACATCGGCGCTCCGAAAGGCGTGGAGCCGCTGGCCGTGGGTCTG
ATCCTCCTGGGCATCAGTGTGTCGATGGGGCTGAACTGCGGTTACCCTCTGAACCCGGCG
CGAGACCTCGGCCCGCGCCTCTTCACCGCCGTTGCTGGATGGGGGATGGAGGTGTTCAGC
ACTGCTGATTACTGGTGGTGGATCCCCGTCGCCGGGCCGCTGGTGGGGGGCGTCGCCGGC
GCTGTAATCTACTTCCTGTTGATCGAGCTGCATCACTCCAAC------------------
------CACAACGACAAACCTCACGAAGAGCCCGAGGAAGAG----------------GAG
GACGAGGAGGAAGAGGAGGACAGCAGCCTGAAGGACAAATACGAGATGATCAACATGAGC
>Zebrafish_Aqp9a
--ATGAAGCAGCACTGCGCGCTC
AAACAGCGGCTCTTCAAGGAGTTTCTGGCCGAGTTTTTGGGGACCTTCGTGCTGGTGCTG
TTCGGCTGTGGGTCGGTGGCTCAGACAGTGTTGAGCAGAAACACACTCGGTGAACCGCTG
ACCATCCACATCGGCTTCAGCACCGGCCTCATGATGGGCGTCTATGTGTCCGGCGGCGTC
TCAGGCGGGCACCTGAACCCGGCCGTGTCTCTGGCGATGGTGATTCTGGGGAAGCTGAAG
ATCTGGAAGTTCCCGGTGTACGTGATCGCGCAGATGCTCGGAGCGTTTGCAGGAGCGGCC
GCTGTGTTCGGCCTGTACTACGACGCCTTCATGGAGTTCACCAGCGGGATCCTGTCAGTG
ACGGGCATTAACGCCACAGGACACATCTTCTCCTCGTACCCGGCAGACACCTGACGGTC
CTGGGCGGGTTCGTGGATCAGGTGGTGGGCACAGGGATGCTGGTGCTCTGTATTCTCGCT
ATAGTGGACGGCAGGAACATCGGCGCCCCCAGAGGTGTGGAGCCGCTGGCTGTGGGTGTG
GTGCTGCTGGGCATCAGCGTCTCCATGGGCCTGAACTGCGGATACCCCCTGAACCCGGCC
CGAGACCTGGGGCCCAGACTCTTCACCGCACTCGCCGGATGGGGGATGGAGGTGTTCAGC
ACTGCAGATTACTGGTGGTGGATCCCAGTTGCCGGGCCGCTGGTGGGGGGTGTCGTCGGA
GCGGTGATCTACTTCCTGTTAATCGAGCTTCATCACTCCAAT------------------
------CACAACGACACACCACAGGAGGAGCCTGAGGAGGAG----------------GAG
GATGAGGATGAAGAAGAGGACAGCAGCCTGAAGGACAAATACGAGATGATCAACATGAGC
>Mexican_tetra_Aqp9a
--ATGAAGCAGCACTGTGCTCTG
AAACATGGCATTTTTAAAGAGTTCTTGGCGGAATTTCTTGGAACGTTCGTGTTGGTGTTA
TTTGGCTGTGGATCGGTTGCTCAGACAGTCCTGAGCAGGAATACACTGGGGGAGCCTCTC
ACTATTCACCTTGGTTTCACTACCGGCCTCATGATGGGTGTCTACGTGGCTGGTGGAGTT
TCAGGAGGTCATTTGAACCCAGCCGTGTCACTGGCCATGGTCATACTGGGAAAACTGAAG
ATCTGGAAGTTCCCTATTTATGTTATCGCTCAGTTCCTGGGTGCCTTTGCTGGAGCTGCT
GGAGTTTTTGGGCTGTATTATGATGCCTTCATGGATTTTACCAGTGGGATACTGTCAGTG
ACTGGCATCAATGCTACAGGTCACATTTTTGCCTCCTATCCTGGAAGACATCTTACTGTA
CTTGGAGGATTCATTGACCAGGTGTTTGGAACAGGAATGCTGGTGCTGTGCATCCTGGCC
ATAACCGATGGCAAGAATATAGGAGCCCCTAAAGGTGTGGAGCCTCTAGCCATTGGTCTC
ATCATCCTGGGCATTAGCGTCTCAATGGGGATGAACTGTGGATACCCACTGAACCCTGCC
AGGGATTTGGGGCCTAGACTCTTTACCGCTGTGGCTGGCTGGGGCATGGAGGTGTTCAGC
ACTGCAGATTACTGGTGGTGGATCCCAGTGGCCGGACCATTGATAGGGGGAATCACAGGA
GCTGTTGTCTACTTTCTCCTGATAGAGCTCCATCACTCTGAC------------------
------CACTCAGAGAAAGCCCACGAGGAGCCTGAGGAAGAGGAG-------------GAA

GACGATGAAGAGGAAGAGGACAGCAGCCTGAAGGACAAATATGAAATGATCACCATGAGC
>Redbellied_piranha_Aqp9a
--ATGAAGCAACACTGCGCTCTT
AAACATGGCATTTTGAAAGAGTTCTTGGCGGAGTTCCTTGGAACGTTTGTGTTAGTGTTG
TTTGGCTGTGGATCTGTGGCTCAGACAGTCTTGAGCAGGAATACGCTGGGCGAATCTCTC
ACTATTCACATTGGCTTCACTACCGGACTAATGATGGGTGTCTATGTAGCTGGTGGAGTT
TCAGGAGGCCATTTGAATCCAGCTGTTTCTTTGGCCATGGTCGTACTGGGTAAACTGAAG
ATCTGGAAGTTTCCCATTTATGTCATAGCACAGTTTTTGGGTGCCTTTGCTGGAGCAGCT
GGGGTTTTTGGACTATATTATGATGCCTTTATGGATTTTACTAGTGGAATTCTGTCAGTG
ACTGGCATCAATGCTACTGGACACATTTTTGCTTCTTATCCTGGACGACATCTCACAGTA
CTTGGAGGATTCATTGATCAGGTGATTGGAACAGGTATGCTGGTGCTCTGTATTCTGGCC
ATAACTGACAGCAAGAATATAGGAGCACCTAAAGGAATGGAGCCTCTGGCCATTGGCCTG
GTCATCCTGGGCATTAGCGTCTCTATGGGGATGAACTGTGGATACCCACTGAACCCTGCC
AGGGACCTGGGGCCCAGACTCTTCACTGCTGTGGCTGGTTGGGGCATGGAGGTGTTCAGC
ACTGCTGAATACTGGTGGTGGATCCCGGTGGCTGGTCCATTGATAGGGGGAATCACAGGA
GCTGTGGTGTACTTCCTTCTGATCGAGCTCCATCACTCCAAC------------------
------CACACAGAGAAAACCAACCAGGAGCCAGAAGAAGAA----------------GAG
GAGGATGAAGAGGAAGAGGACAGCAGCCTGAAGGACAAATACGAAATGATCACCATGAGC
>Brown_ghost_knifefish_Aqp9a

---TTG
TTTGGATGTGGGTCTGTAGCTCAGACTGTGCTGAGCAGGAATACGCTGGGGGAGCCTCTC
ACTATCCACCTTGGCTTTACCATTGGACTCATGATGGGTGTTTACATCTCTGGTGGGGTC
TCAGGGGGTCATTTGAACCCAGCAGTGTCCTTGGCCATGGTCATACTGGGTAAACTGAAG
ATATGGAAGTTTCCCGTTTATGTTCTCGCCCAGCTCCTGGGAGGATTTGTTGGGGCTGCT
GGAGTTTTTGGACTGTATTATGACGCCTTCATGGATTTTACCAGTGGAATACTGTCAGTA
ACAGGCATTAATGCGACTGGACACATTTTTGCTTCCTATCCTGGACGACACCTCACAGTG
CTCGGAGGGTTCATGGATCAGGTGATTGGAACAGGCTCTCTGGTGTTGTGCATCTTAGCA
ATAACTGATGGCAAGAATGTAGGAGCTCCTAAAGGAGTGGAGCCCCTGGCCATTGGTCTG
ATCATCCTGGGCATCAGTGTCTCTATGGGGATGAACTGTGGCTACCCACTGAACCCTGCC
AGGGACCTGGGGCCCAGACTCTTCACTGCCATGGCTGGCTGGGGCATGGAGGTGTTCAGC
ACTGCGGAGTACTGGTGGTGGATCCCAGTTGCTGGGCCTTTGATAGGGGGTGTTACAGGA
GCTGTAGTGTACTTCCTCCTGATCGAGCTCCACCATCCCAAC------------------
------CACTCCAAGAAAGCAAGTGAGGAGGTTGAGGAAGAG----------------GAG
GATGAAGATGAGGAG---
>Black_ghost_Aqp9a

---TTG
TTTGGATGTGGGTCTGTAGCTCAGACTGTGCTGAGCAGGAATACGCTGGGGGAGCCTCTC
ACTATCCACCTTGGCTTTACCATTGGACTCATGATGGGTGTTTACATCTCTGGTGGGGTC
TCAGGGGGTCATTTGAACCCAGCAGTGTCCTTGGCCATGGTCATACTGGGTAAACTGAAG
ATATGGAAGTTTCCCGTTTATGTTCTCGCCCAGCTCCTGGGAGGATTTGTTGGGGCTGCT
GGAGTTTTTGGACTGTATTATGACGCCTTCATGGATTTTACCAGTGGAATACTGTCAGTA
ACAGGCATTAATGCGACTGGACACATTTTTGCTTCCTATCCTGGACGACACCTCACAGTG
CTCGGAGGGTTCATGGATCAGGTGATTGGAACAGGCTCTCTGGTGTTGTGCATCTTAGCA
ATAACTGATGGCAAGAATGTAGGAGCTCCTAAAGGAGTGGAGCCCCTGGCCATTGGTCTG
ATCATCCTGGGCATCAGTGTCTCTATGGGGATGAACTGTGGCTACCCACTGAACCCTGCC
AGGGACCTGGGGCCCAGACTCTTCACTGCCATGGCTGGCTGGGGCATGGAGGTGTTCAGC
ACTGCGGAGTACTGGTGGTGGATCCCAGTTGCTGGGCCTTTGATAGGGGGTGTTACAGGA
GCTGTAGTGTACTTCCTCCTGATCGAGCTCCACCATCCCAAC------------------
------CACTCCAAGAAAGCAAGTGAGGAGGTTGAGGAAGAG----------------GAG
GATGAAGATGAGGAG---
>Glass_knifefish_Aqp9a
--ATGAAGCAGTGCTGCACTCTT
CAACATGGCATTTTTAAAGAGTTCTTGGCGGAGTTCCTCGGGACGTTTGTGTTGGTGCTG
TTTGGCTGTGGGTCGGTAGCCCAGACCGTCCTGAGCAGAAACACACTGGGTGAGCCTCTC
ACTATCCACCTGGGCTTCACCACCGGACTCATGATGGGCGTCTACATCTCTGGTGGGGTC
TCAGGAGGTCATTTGAACCCAGCAGTGTCCCTGGCCATGGTCATACTGGGAAAACTGAAG
ATATGGAAATTTCCCTTTTATGTCCTGGCGCAGCTACTGGGAGGATTCGTGGGAGCTGCT
GGAGTTTTTGGACTGTATTATGACGCCTTCATGGATTTTACCAGTGGAATACTGTCAGTA
ACAGGCATCAACGCCACTGGACACATTTTTGCCTCCTACCCTGGACGACACCTCACGGTT

CTCGGAGGGTTCGTGGACCAGGTGATTGGAACAGGATCGCTGGTGCTGTGCATCCTAGCC
ATAACCGATGAGAAGAATATCGGCGCTCCTAAAGGAGTGGAGCCCCTGGCTGTTGGCCTG
GTCATCCTGGGCATCAGTGTCTCCATGGGAATGAACTGTGGCTACCCACTGAACCCTGCC
CGAGACCTGGGGCCCAGGCTGTTCACCGCTGTGGCTGGCTGGGGAATGGAGGTGTTCAGC
ACTGCGGACTACTGGTGGTGGATTCCTGTGGCTGGGCCTTTGATAGGGGGAGTCACAGGA
GCTGTTGTGTACTTCCTGCTGATCGAGCTCCATCGCTCCAAC------------------
------CATACCGAGAAAACAAGTGAATCA---GAGGAAGAG---------------GAG
GATGAAGATGAGGAAGAAGACAGCAGTCTGAAGGACAAATATGAAATGATCGCTATGAGC
>Electric_eel_Aqp9a
--ATGATGCAGCACTATGCTCTT
AAACATGGAATTTTTAAAGAGTTCTTGGCGGAGTTTCTTGGGACCTTTGTGTTGGTGTTG
TTTGGCTGTGGGTCTGTAGCTCAGACCGTGCTGAGCAGGAATATGCTGAGTGAGTCTCTT
ACTATCCACCTTGGCTTCACCATTGGACTCATGATGGGCATTTACGTCTCTGGTGGGGTC
TCAGGAGGTCATTTGAACCCAGCAGTGTCCCTGGCCATGGTTATACTGGGTAGACTGAAG
ATCTGGAAGTTTCCAATTTATGTCCTCGCACAGCTCCTGGGAGGGTTTGTCGGGGCTGCT
GGAGTGTTTGGACTGTATTATGATGCCTTCATGGATTTTACAGGTGGAATACTGTCAGTA
ACAGGCATCAGTGCCACTGGACACATTTTTCCTCCTACCCTGGACGACATCTGACAGTG
CTTGGAGGGTTCGTGGACCAGGTGATTGGAACAGGCTCGCTGGTGTTGTGCATCCTAGCC
ATAACTGATGGCAAAAATATAGGAGCTCCAAAAGGAGTGGAGCCCCTGGCTATTGGCCTG
ATCATCCTGGGCATCAGTGTCTCCATGGGGATGAACTGTGGCTACCCAGTGAACCCTGCC
AGGGACTTGGGGCCCAGGCTCTTCACGGCTGTGGCTGGCTGGGGCATGGAGGTGTTCAGC
ACTGCGGATTACTGGTGGTGGATCCCAGTGGCCGGACCTTTGACAGGGGGCATTACAGGA
GCTGTAGTATACTTCCTCCTGATCGAGCTTCATCATTCCAAC------------------
------CACTCCGAGAAAGCAAGTGAGGAGTCTGAGAAAGAT---------------GAG
GATGAAGATGAGGAAGAAGACAGCAGCCTGAAGGACAAATATGAAATGATCACTATGAGT
>Channel_catfish_Aqp9a
--ATGAAGCATCACTGCACTCTT
AAACATGACATTTTTAAAGAGTTCTTGGCTGAGTTTCTTGGGACATTTGTGCTGGTGTTG
TTTGGCTGTGGCTCTGTAGCTCAGACTGTCCTAAGCAGGAATACACTGGGTGAACCACTC
ACTATCCACCTCGGCTTCACCACTGGACTCGTGATGGGTGTCTATATCGCTGGTGGGGTT
TCAGGGGGTCATTTGAACCCAGCAGTATCTCTGGCCATGGTCATACTGGGTAAACTGAAG
ATCTGGAAATTTCCAGTTTACGTCTTTGCACAGCTCCTGGGAGCCTTTACTGGAGCTGCT
GGAGTGTTTGGACTATATTATGATGCGTTTATGGAGTTTACCAGTGGACTTCTGTCAGTA
ACAGGCATTAATGCCACCGGACATATTTTTGCCTCTTACCCTGGACGACATCTCACAATA
CTTGGAGGGTTTGTGGACCAGGTGATTGGAACAGGCATGCTGGTGTTGTGTATCCTGGCC
ATAACCGATAACAGGAATATTGGAGCTCCTAAAGGAGTGGAGCCTCTGGCCATTGGCTTG
ATCATCCTGGGCATCAGCGTGTCCATGGGCATGAACTGTGGATACCCACTGAACCCTGCC
AGAGACCTTGGTCCCCGACTCTTCACTGCTATGGCTGGTTGGGGCATGGAGGTGTTCAGC
ACTGCAGATCACTGGTGGTGGATCCCTGTGTTCGGGCCTTTGGTAGGAGGCATCACCGGA
GCTGTAGTGTACTTCCTCCTGATCGAGCTGCACCACTCGGAC------------------
------CCCACAGAGAAATCCCAGCAAAAACCTGAGGAAGAG---------------GAG
GATGAAGATGAGGATGAAGACAGCAGCCTGAGGGACAAATATGAAATGATAACGATGGGC
>Striped_catfish_Aqp9a
---------------ATGGAATCGGAGCACAAAAGAACCATGAAGCATCACTGCACTCTT
AAACATGACATTTTTAAAGAGTTCTTGGCGGAGTTTCTTGGGACATTTGTGCTGGTGGTG
TTTGGCTGTGGCTCTGTAGCTCAGACAGTCCTAAGCAGGAATACTCTGGGTGAGCCACTC
ACTATCCACCTCGGCTTCACCACCGGACTCATGATGGGTGTCTATATCGCTGGTGGGGTT
TCAGGGGGTCATTTGAACCCAGCTGTGTCTCTGGCCATGGTCATACTGGGTAAACTGAAG
ATCTGGAAATTTCCAATTTACGTCATTGCACAGCTCCTGGGAGCCTTTGCTGGAGCTGCT
GGAGTGTTTGGACTGTATTATGATGCGTTTATGGAGTTTACTAGTGGAATTCTGTCAGTA
ACAGGCATTAATGCCACTGGACACATTTTTGCCTCTTACCCTGGACGACATCTCACAATA
CTTGGAGGGTTTGTGGATCAGGTGATTGGAACAGGCATGTTGGTGCTGTGCATCCTGGCC
ATAACCGATAGCAGGAATATTGGAGCTCCTAAAGGAGTGGAGCCTCTGGCCATTGGCTTG
ATCATCTTGGGCATCAGCGTGTCCATGGGCATGAACTGTGGATACCCACTGAACCCTGCC
AGAGACCTTGGTCCCCGACTCTTCACTGCTATGGCTGGTTGGGGCGTGGAGGTGTTCAGC
ACTGCAGATCACTGGTGGTGGATCCCGGTGTTCGGGCCTTTGATAGGAGGCATCACCGGA
GCTGTAGTGTACTTCCTCTTGATCGAGCTGCACCACTCCGAC------------------
------CCATCAGAGAAATCCCAGCAAAAAACTGAGGAAGAG---------------GAG
GATGAAGAAGAGGATGAGGACAGCAGCCTGAGGGACAAATATGAAATGATAACTATGGGC
>Yellow_catfish_Aqp9a
--ATGAAGCATCACTGCACTCTT

AAACATGACATTTTTAAAGAGTTCCTGGCGGAGTTTCTCGGGACATTTGTGCTGGTGTTG
TTCGGCTGTGGCTCCGTAGCTCAGACGGTCCTTAGCAGGAACACACTGGGAGAACCTCTC
ACCATCCACCTCGGCTTCACCACCGGACTCGTGATGGGAGTCTATATCGCTGGTGGGGTT
TCAGGAGGTCATTTAAACCCGGCAGTGTCACTGGCTATGGTCATACTGGGTAAACTGAAG
ATCTGGAAATTTCCAGTTTACGTGATTGCACAACTACTGGGAGCGTTTGCTGGAGCCGCT
GGAGTGTTTGGACTGTATTATGATGCGTTTATGGAGTTCACCAGTGGAATTCTGTCAGTA
ACTGGGATTAACGCCACTGGACACATTTTTGCCTCTTACCCTGGAAGACATCTTACAATT
CTTGGAGGGTTTGTGGACCAGGTGATTGGAACAGGCATGCTGGTGCTGTGTATCCTGGCT
ATAACCGATAGCAGGAATATTGGTGCTCCTAAAGGAGTGGAGCCTCTGGCCATTGGTCTG
ATCATCCTGGGCATCAGTGTGTCCATGGGCATGAACTCTGGATACCCTCTGAACCCTGCA
AGAGACCTCGGACCACGGCTCTTCACTGCTATGGCCGGTTGGGGCATGGAGGTGTTCAGC
ACTGCAGATCACTGGTGGTGGATCCCCGTGTTCGGGCCTTTGATAGGAGGCATCACCGGC
GCTGTAGTGTACTTCTTGTTGATTGAGCTGCACCACACTGAC------------------
------CACTCTGAGAAATCCCAGCAAAAACCTGAGGAAGAG---------------GAG
GATGAAGAAGACGACGAGGACAGCAGCCTGAGGGACAAATATGAGATGATAACTATGGGC
>Walking_catfish_Aqp9a
--ATGAAGCATCACTGCACTCTT
AAACATGACATCTTTAAAGAGTTCTTGGCAGAGTTTCTTGGGACATTTGTGCTTGTGTTG
TTTGGCTGCAGCTCTGTAGCTCAGACAGTCCTCAGCAGGAATACACTGGGTGAGCCACTC
ACTATTCACCTTGGCTTCACCACCGGACTCATTATGGGTGTCTATATCGCTGGTGGTGTT
TCAGGGGGTCATTTGAACCCAGCAGTGTCTCTGGCCATGGTCATGCTGGGTAAGCTGAAG
ATTTGGAAATTTCCAATTTACGTCATTGCACAGTTCCTGGGAGCCTTCGCTGGAGCTGCT
GGAGTGTTTGGATTGTATTATGATGCGTTCATGGACTTTACCAGTGGAATTCTGTCTGTA
ACGGGCATTAATGCTACTGCACACATTTTTGCCTCTTACCCTGGACGTCATCTCACAATA
CTTGGAGGGTTTGTGGACCAGGTGATTGGAACAGGCATGCTGGTGCTGTGTATCCTGGCC
ATAACCGATACCAGGAATATTGGAGCTCCTAAAGGAGTGGAGCCTGTGGCCATTGGCCTA
ATTATCCTGGGCATCAGCGTATCCATGGGTATGAACTGTGGATACCCTCTGAACCCTGCA
AGAGACCTTGGTCCCAGACTCTTCACTGCTATAGCTGGTTGGGGCATGGAGGTCTTCAGT
ACTGCAGGTCACTGGTGGTGGATCCCAGTATTTGGCCCTTTGATAGGAGGCATCACCGGC
GCTGTAGTGTACTTCATCCTGATCGAGCTGCACCATGCCGAC------------------
------CCCTCTGAGAAATCCCAGGAAAAACCTGAGGAAGAG---------------GAG
GATGAAGAAGAGGATGAGGAAAGCAGCCTGAGGGACAAATATGAAATGATAGCTATGGGC
>Bottlenose_catfish_Aqp9a
--ATGAAGCATCACTGCACTCTT
AAACATGACATTTTTAAAGAGTTCTTGGCAGAATTTCTTGGGACGTTCGTGCTTGTGTTG
TTTGGCTGTGGCTCTGTAGCTCAGACAGTCCTAAGCAGGAATACACTGGGTGAGCCACTC
ACTATCAACCTCGGCTTCACCACCGGACTCATGATGGGTGTCTATATCGCCGGTGGGGTT
TCAGGGGGTCATTTGAACCCAGCAGTATCTCTGGCCATGGTCATACTGGGTAAATTGAAG
ATCTGGAAATTTCCAATTTACATCATTGCACAGCTTGTGGGAGCCTTTGCTGGAGCTGCT
GGAGTGTTTGGACTATATTATGATGCGTTTATGGAGTTTACCAGTGGAATTCTGTCAGTA
ACAGGCATTAATGCAACTGGACACATTTTTGCCTCTTACCCTGGACGACATCTCACAATA
CTTGGAGGGTTTGTGGATCAGGTGATTGGAACAGGCATGCTGGTGCTGTGCATCCTGGCC
ATAACTGATAGCAGGAATATTGGAGCTCCTAAAGGAGTGGAGCCTCTGGCCATTGGCTTA
ATCATCCTGGGCATCAGTGTGTCTATGGGTATGAACTGTGGATATCCTCTGAACCCTGCC
AGAGACCTCGGTCCCCGACTCTTCACTGCTGTGGCTGGTTGGGGCATGGAGGTGTTCAGC
ACTGCAGATCACTGGTGGTGGATCCCAGTGTTCGGGCCTTTGATAGGAGGCATCACTGGA
GCTGTAGTGTACTTCCTCCTGATCGAGCTGCACCACTCTGAA------------------
------CCCTCAGAGAAATCCCAGCAAMAGCCAGATGAAGAC---------------GAG
GATGAAGAAGAGGATGAGGACAGCAGCCTGAGGGACAAATAYGATATGATAACTATGGGC
>Pencil_catfish_Aqp9a

------------------------TTGGCAGAATTTCTCGGGACATTTGTGCTAGTGCTG
TTTGGCTGTGGCTCTGTAGCTCAGACTGTTCTGAGCAGAAATACCCTAGGTGAGCCTCTC
ACCATTCACCTTGGCTTTACCACTGGACTTATGATGGGTGTATATGTAGCTGGAGGGGTT
TCAGGAGGTCATCTAAACCCAGCAGTTTCTCTGGCCATGGTCATACTGGGTAAATTAAAG
ATTTGGAAATTTCCTATTTATGTCCTTGCGCAGTTCCTTGGAGCATTTGCTGGAGCTGCA
GGAGTCTTCGGGCTGTATTATGATGCATTTATGGAGTTTACCAGTGGAATACTGTCAGTG
ACGGGCATTAATGCCACTGCACATATTTTTGCCTCTTACCCTGGAAGGCATCTTTCAATA
CTTGGAGGATTTATGGACCAGGTTATTGGAACTGCTACGCTTGTGCTGTGCATCCTGGCC
ATAACTGATGACAAAAATATAGGAGCTCCCAAAGGAATGGAGCCCCTGGCTGTAGGATTG
ATAATTCTGGGCATCAGTGTGTCCATGGGCATGAACTGTGGGTACCCTCTAAACCCTGCC

```
AGGGATCTGGGGCCCAGACTCTTTACTGCAATCTCTGGTTGGGGGATGGAGGTGTTCAGC
ACTGCAGATTATTGGTGGTGGATCCCAGTGGCTGCACCTTTGATAGGGGGTATCACAGGT
GCTGTAGTGTACTTCCTCCTAATTGAGCTGCACCACTCTGAC------------------
------CCA---GATAAAGCCCAGCAGAACCCGGAGAAAGAC----------------GAA
GATGATGACGACGATGAAGACAGCAGCCTAAAAGACAAATACGAAATGATCCATATGGGA
>Sardine_Aqp9a
----------------------------------------ATGAGCCGCAAGTGTGCCCTC
AAGCACGGCATCTTCAAGGAGTTCCTGGCCGAGCTGCTGGGGACGTTCGTCCTGGTGTTG
TTTGGCTGTGGTTCCGTGGCCCAGACGGTTCTAAGCAGGAACACGCTGGGCGAGAGCCTC
ACCATCCACATCGGCTTCACCACGGGCCTCATCATGGGGGTGTACGTGTCTGGAGGAGTA
TCAGGAGGCCATTTAAATCCAGCGGTTTCCTTGGCGATGGTTCTGCTGGGTAAGCTGAAG
TTCTATAAGTTCCCTGTCTACGTTCTGGCCCAGTTCATCGGGGCCTTCGCTGGTGCTGCC
GCAGTCTTCGGCCTCTACTACGATGCCTTCATGGACTTCACGAGTGGCATCCTGTCGGTG
ACGGGCATCAACGCCACGGCGCACATCTTCGCCTCGTACCCAGGACGACACCTGTCCATC
CTCAACGGCATCATCGATCAGGTGGTGGGGACGGGCATGCTGGTGTTGTGCATCTTGGCG
ATCACGGACGGGAAGAACATCGGGGCTCCGCGGGGGGTGGAGCCTCTGGCAGTGGGCCTG
GCGCTGCTGGCCATCAGCGTGTCCATGGGCCTCAACTGCGGCTACCCCATCAACCCCGCC
CGCGACCTGGGCCCACGCCTCTTCACCTTCCTCACCGGCTGGGGCTGGGAGGTGTTCAGG
ACTGCAAATTACTGGTGGTGGATCCCGGTGGCAGGGCCAATGGGAGGGGGTGTGGTCGGC
GCCATCATCTACTTCCTGTTTATCAAGATGCACCACTCC---------------------
------CACACTGAGAAACCTCACGAGGAGCAGGAGGAA------------------GAG
GAGGAGGAGGAAGAGGATGATAACAGCCTGAAGGACAAGTATGATATGATCACTGTGAGC
>Alewife_Aqp9a
------------------------------------------------------------
---------------------------------------------------------TTG
TTTGGCTGTGGTTCCGTGGCCCAGACGGTTCTAAGCAGGAACACGCTAGGGGAGAGCCTC
ACCATCCACATCGGCTTCACCACTGGCCTCATCATGGGGGTGTACGTGTCTGGAGGAGTA
TCAGGAGGCCATTTAAACCCAGCGGTGTCCTTGGCGATGGTTCTACTGGGGAAGCTGAAG
TTCTATAAGTTCCCCATCTACGTTCTGGCCCAGTTCATCGGAGCCTTCGCTGGCGCCGCC
GCTGTCTTTGGTCTCTACTACGACGCCTTCATGGACTTCACGAGTGGGATCCTGTCGGTG
ACGGGCATCAACGCCACGGCTCACATCTTCGCCTCCTATCCAGGAAGACACCTGTCCATC
CTCAACGGTATCATCGATCAGGTG------ACAGGA------------------------
------------------------------------------------------------
------------------------------------------------------------
------------------------------------------------------------
------------------------------------------------------------
------------------------------------------------------------
------------------------------------------------------------
------------------------------------------------------------
>Allis_shad_Aqp9a
----------------------------------------ATGACCCGCAAGTGTGCCCTC
AAGCATGGCATCTTCAAGGAGTTCCTGGCCGAGCTGCTGGGCACGTTCGTGCTGGTGTTG
TTTGGCTGTGGTTCCGTGGCCCAGACGGTTCTAAGCAGGAACACGCTAGGGGAGAGCCTC
ACCATCCACATCGGCTTCACCACTGGCCTCATCATGGGGGTGTACGTGTCTGGAGGAGTA
TCAGGAGGCCATTTAAACCCAGCGGTGTCCTTGGCGATGGTTCTACTGGGGAAGCTGAAG
TTCTATAAGTTCCCCATCTACGTTCTGGCCCAGTTCATCGGAGCCTTCGCTGGCGCCGCC
GCTGTCTTTGGTCTCTACTACGACGCCTTCATGGACTTCACGAGTGGGATCCTGTCGGTG
ACGGGCATCAACGCCACGGCTCACATCTTCGCCTCCTATCCAGGAAGACACCTGTCCATC
CTCAACGGCATCATCGATCAGGTGGTGGGGACGGGTATGCTGGTGTTGTGCATCTTGGCG
ATCACAGATGGGAAGAACATCGGGGCACCCCATGGTGTGGAGCCGCTGGCCGTGGGCTTG
GCCCTGCTGGCCATCAGTGTGTCCATGGGCATGAACTGTGGCTACCCCATCAACCCCGCC
CGCGACCTGGGACCACGCCTCTTCACCTTTGTGGCCGGATGGGGCTTGGAGGTGTTCAGC
ACTGCAGATTACTGGTGGTGGATCCCTGTGGCGGGGCCAATGGGAGGGGGTGTGGTTGGT
GCCATCATCTACTTCCTGTTTATCAAGATGCACCACTCC---------------------
------CATACCGAGAAACCTCATGAGGAGCCGGAGGAA------------------GAG
GAAGAGGAGGAAGAGGATGATAACAGCCTGAAGGACAAG---------------------
>Hilsa_shad_Aqp9a
----------------------------------------ATGACCCGTCAGTGCTGCCTC
AAGCATCGCATCTTCAAAGAGTTCCTGGCCGAGCTGCTGGGCACCTTTGTGTTGGTGTTG
TTTGGCTGTGGTTCTGTGGCTCAGACGGTTCTAAGCAGGAACACGCTAGGAGAGAACCTC
ACCATCCACATCGGTTTCACCACCGGGCTCATTATGGGGGTGTATGTGGCTGGGGGAGTC
```

```
TCAGGTGGGCACCTAAACCCAGCCGTGTCGCTGGCGATGGTTCTGCTGGGGAAGCTCAAG
TTCTACAAGTTCCCAATCTATGTTCTGGCCCAGTTCATCGGAGCCTTTGCTGGGGCCGCA
GCTGTCTTCGGCCTCTACTATGATGCCTTCATGGACTTCACCAGTGGCATTCTGTCGGTT
ACGGGGATTAATGCAACAGCTCACATCTTTGCCTCCTATCCAGGAAGACACTTGTCCATC
CTGAACGGCATCATTGATCAGGTGGTGGGTGCGGGCATGTTGGTACTGTGTATCCTGGCA
ATCACGGATGGAAAGAACATCGGGGCGCCTCATGGTGTGGAGCCCCTGGCTGTGGGCTTG
GCTCTGCTGGCCATCAGCGTGTCCATGGGCCTCAACTGCGGCTATCCCATAAACCCCGCC
CGGGACCTCGGCCCGCGGCTCTTCACCTTTGTGGCTGGCTGGGGCATTGAGGTGTTCAGG
ACTGCGGATAACTGGTGGTGGATTCCGGTGGCAGGGCCAATGGGAGGGGGCGTGGCTGGG
GCCCTCATCTACTTCCTGTTTGTAAAGATGCACCACGCC---------------------
------CACAATGAGAAACCCCATGAGGAGCCTGAGGAA------------------GAG
GAGGAGGAGGAAGAGGATGACAACAGCCTGAAGGACAAGTATGAAATGATTACTGTCAGC
>Atlantic_herring_Aqp9a
---------------------------------------ATGACCCGCCAGTGC------
AAGCATGGCATCTTCAAGGAGTTCCTGGCCGAGTTCCTGGGCACGTTTGTGCTCGTGCTG
TTTGGCTGTGGAGCGGTGGCTCAGACGGTTCTAAGCAGGAACACACTGGGAGAGAACCTC
ACCATCCACATCGGCTTTGCCACTGGACTCACGATGGGCGTGTACGTGTCTGGAGGAGTA
TCAGGTGGGCACTTAAACCCAGCGGTGTCCCTGGCGATGGTTCTCCTGGGGAAGCTGAAG
TTCTATAAGTTCCCTGTGTATGTTCTGGCCCAGTTCCTGGGAGCCTTTGCTGGAGCTGCA
GCCGTCTTCGGCCTCTACTACGATGCCTTCATGGATTTCACCAGTGGGATCCTGTCAGTG
ACAGGAATCAACGCCACTGCACACATCTTCGCCTCCTACCCAGGAAGACACCTTTCCATC
ATCAACGGAATCATCGATCAGGTGGTTGGGACAGGCATGCTGGTGTTGTGTATCCTGGCG
ATCACGGACGGGAAGAACATTGGTGCGCCTCATGGGGTGGAGCCTCTGTCGGTGGGCTTG
GCTCTGCTGGCTATCAGCGTGTCCATGGGGCTGAACTGCGGCTACCCCATCAACCCTGCC
CGAGACCTGGGCCCGCGTGTCTTCACCTTCGTCGCCGGCTGGGGCATGGAGGTGTTCAGG
ACTGCAAATTACTGGTGGTGGATCCCTGTGGCAGGGCCAATGGGTGGGGGCGTGGCTGGG
GCCCTCATCTACTTCCTGTTTGTCAAAATGCACCACTCC---------------------
------CATGCCGAGAAGCCACACGAGGAGCTTGAGGAAGAGGAGGAG---------GAG
GAGGATGAGGAAGAGGATGACAACAGCCTGAAGGACAAGTATGACATGATCACCGTGAGC
>Denticle_herring_Aqp9a
---------------------------------------ATGACGCACCGGTGTGCCATC
AAACACAGCATCTTTAAGGAGTTCCTGGCTGAATTCCTTGGTACATTTGTCTTAGTGCTG
TTCGGCTGTGGAGCTGTGGCACAGACCGTCCTGAGCCGCAACACCCTGGGCGAGAGTTTG
ACCATACACATCGGCTTCACCATTGGACTCACCATGGGGGTCTATGTCTCTGGAGGAGTT
TCAGGGGGTCATTTGAACCCAGCTGTTTCACTGGCCATGGTCATTCTGGGGAAATTGAAG
ATCTATAAATTTCCCATCTATGTTATCGCCCAGTTCATGGGGGCTTTTGCGGGAGCTGCT
GCCGTCTTCGGGCTTTATTATGATGCCTTCATGGATTTTACCAGTGGAATACTGTCAGTG
ACAGGAATTAATGCCACTGGACACATTTTTGCCTCCTACCCCGGAAGGCATCTGTCCGTC
CTTAATGGCTTCATAGACCAGGTGATTGGGACAGGGATGCTGGTTCTGTGCATCCTGGCC
ATCACGGATGGGCACAACATTGGCGCACCAAAGGGTGTGGAGCCACTGGCTATTGGTCTT
GTCCTCATGGCCATCAGTGTTTCCATGGGTCTGAACTGCGGCTACCCTCTGAACCCTGCG
CGAGACCTGGGGCCCAGGCTCTTTACCGCTGTGGCTGGCTGGGGCATGGAAGTGTTCAGG
ACTGCAAACAACTGGTGGTGGATCCCAGTGGCCGGACCCCTGGTGGGGGGTGTAGCTGGA
GCAGTGATTTACTTCTTATTTGTTGAGCTCCACCACGCT---------------------
------GAGCCAGAGAAGCCCCAGGAGGAGCCAGAGGAGGAGGAA------------GAG
GAGGAGGAGGAGGATGAAGACAGCAGCCTGAAGGACAAGTACGAAATGATCACTATGAGC
>Asian_arowana_Aqp9a
---------------ATGGACGTGGAGCGCAAGAGGAACCTGCGGCAGCGCTGCGCTCTG
CGCCAGCCCATCCTCAGGGAGTTCCTGGCCGAGTTCCTGGGCACCTTCGTCTTGGTTCTG
TTTGGGTGTGGCTCCGTAGCCCAGACGGTGTTGAGTCGTGGGGACCTGGGTGAGAATCTC
ACCATACACATTGGATTCACACTGGGAGTGATGATGGGTGTCTATGTGGCGGGTGGAGTG
TCAGGGGCCCACATCAATCCGGCTGTGTCACTTGCTATGGTCATTTTGGGTAAGCTCAAG
GTGGCCAAGTTCCTGGTGTATGTCATAGCACAGTTCCTGGGTGCCTTCGCTGGGGCTGCT
GCTGTCTTTGGGCTCTATTACGATGCCTTCATGGATTTCACAAGTGGAATACTGTCAGTG
ACTGGCATAAATGCCACAGCGCACATCTTCGCATCCTATCCCGCTAGGCACCTCTCGGTC
CTCAACGGATTCATAGACCAGGTAATAGGGACTGGTGCCCTTGTCCTGTGCATCCTGGCC
ATCATTGATGGGAAGAACATTGGAGCACCCAGAGGAATGGAGCCGCTGGCTATTGGCTTG
ATCGTCATGGCCATTGGGGTTTCCATGGGGCTGAACTGCGGCTACCCCGTGAACCCTGCC
CGTGACCTTGGCCCCAGGCTCTTCACCGCTGTGGCTGGATGGGGCATGGAGGTGTTCAGG
ACAGCGGATTACTGGTGGTGGATCCCAGTGGCGGGACCTCTCGTCGGAGGGGTGGTGGGG
GCAGTCATCTACTTCCTCTTCGTTGAATTGCACCACACA---------------------
```

------GAGCCCGAGAAGCAGCAG------------------------------------
---------GAGGACGAGGACAGCAGCGTCAAGGACAAGTACGAGATGATCACCATGAGC
>Silver_arawana_Aqp9a
---------------ATGGACGTGGAGCCCAAGAGGAACCTGCGGCAGCGCTGCGCCCTG
CGGCAGCCCATCCTCAGAGAGTTCCTGGCCGAGTTCCTGGGCACCTTCGTCTTGGTTCTG
TTTGGGTGCGGTTCCGTAGCCCAGACGGTGTTGAGTCGTGGGGACCTGGGTGAGAATCTC
ACCATACACATCGGATTCACGCTGGGAGTCATGATGGCTGTCTATGTGGCGGGCGGAGTG
TCAGGAGCCCACATCAATCCGGCCGTGTCACTTGCCATGGTCATTTTGGGCAAGCTCAAG
GTGGCCAAGTTCCCCGTGTATGTCATAGCGCAGTTCCTGGGTGCCTTTGCCGGGGCAGCC
GCTGTCTTTGGGCTCTATTACGATGCCTTCATGGACTTCACGAGTGGAATACTGTCAGTG
ACTGGCATAAATGCCACAGCGCACATCTTCGCGTCCTATCCGGCTCGGCACCTCTCAGTC
CTCAACGGATTCATAGACCAGGTCATAGGGACCGGAGCCCTTGTCCTGTGCATCCTGGCC
ATCGTTGATGGGAAGAACATTGGAGCACCCAGAGGAATGGAGCCACTGGCTATTGGCTTG
ATCGTCATGGCCATTGGGGTTTCCATGGGGCTGAACTGCGGCTACCCCGTGAACCCTGCC
CGTGACCTTGGCCCCAGGCTCTTCACCGCTGTGGCTGGCTGGGGCATGGAGGTGTTCAGC
ACGGCGGATTACTGGTGGTGGATCCCAGTGGCGGGACCTCTCGTTGGAGGGGTGGTGGGG
GCAGTTATCTACTTCCTCTTCGTCGAATTGCACCACGCG---------------------
------GAGCCCGAGAAGCAGCAG------------------------------------
---------GAGGACGAGGACAGCAGCGTCAAGGACAAGTACGAGATGATCCCCATGGGC
>Pirarucu_Aqp9a
---------------ATGGACCTAGAGCCCAAGAGGAGCCTGCGGCAGCGGTGCGCCCTG
CGCCACCCGCTCCTCAGGGAGTTCCTGGCCGAGTTCTTGGGCACCTTCGTCCTGGTGTTG
TTTGGGTGCGGTTCTGTAGCCCAGATGGTGTTGAGTCGTGGGGCCCAGGGTGAAACTCTC
ACCGTACATGTCGGATTCACGCTGGGAGTGATGATGGCCGTTTACGTGGCAGGCGGAGTG
TCAGGGGCCCACATCAATCCCGCTGTGTCACTTGCTATGGTCATTTTGGGCAGGCTCAAG
GTGGTCAAGTTCCCCGTATATGTCATAGCACAGTTCCTGGGTGCCTTTGCTGGGGCTGCC
GCTGTCTTCGGGCTCTATTACGATGCCTTCATGGACTTCACAAGTGGAATACTGTCAGTG
ACCGGCATTAACGCCACAGCACACATCTTTGCCTCCTATCCGACTCGGCACCTCTCAGTC
CTCAATGGATTCATAGACCAGGTGATAGGGACTGGTGCCCTAGTCCTCTGCATCCTGGCC
ATCATTGATGGGAAGAACATTGGAGCACCCAGAGGAATGGAGCCGCTGGCTATTGGCTTG
ATCATCATGGCCATTGGTGTGTCCATGGGTCTGAACTGTGGCTACCCTCTGAACCCTGCT
CGTGACCTGGGCCCCAGGCTCTTCACCGCTGTGGCTGGATGGGGCTTCGAGGTGTTCAGG
ACTGCGGGATACTGGTGGTGGATCCCAGTGGCAGGACCCCTGGTCGGAGGGGTGGTGGGG
GCAGTGATCTACTTCCTCTTCATTGAGCTGCACCACCCT---------------------
------GAGCCCGAGAACCAGCAGCAG---------------------------------
------GAGGAGGATGAGGACAGCAGCATCAAGGACAAGTACGAGATGATCACCATGAGC
>Elephantnose_fish_Aqp9a
---------------ATGGAGACTGAGCGCAAAAGGACAATGAAAGAGTGCTGCGTGGTG
CGTAACCGCATCTTCAGGGAGTTTCTGGCCGAATTTCTCGGAACATTTATATTAATTCTG
TTCGGCTGCGGCTCCGTGGCACAGACGGTGTTGAGTCGCGGTGCCCTGGGAGAGCCGCTC
ACCATCCACATCGGCTTCACTCTGGGAGTGATGATGGCGGTCTACGTGGCTGGAGGGGTG
TCAGGTGCCCACATTAACCCTGCCGTGTCCCTGGCTATGGTCGTCCTGGGTCGGCTGAAA
CTGGTGAAGTTCCCCGTCTATGTCATCGCGCAGTTCCTGGGAGCCTTCGTCGGAGCGGCT
GCTGTCTTCGGCCTGTACTACGATGCCTTTATGGACTTCACGAGTGGAATACTGTCAGTG
ACTGGCATCAACGCCACAGCACACATCTTTGCCTCCTACCCTTCTAGGCACCTCTCAATA
ATCAACGGATTCATTGACCAGGTGTTTGGGACGGGCATCTTGGTCTTGTGCATCCTGGCC
ATCATCGACGGCAAGAACATTGGTGCACCCAGAGGAATGGAGCCCTTGGTCATCGGCTTG
ATCATCCTGGGGATTGGCGTGTCCATGGGCTTGAACTGTGGTTATCCTCTGAACCCTGCC
CGCGACCTGGGCCCCAGGGTCTTCACGGCAGTGGCTGGGTGGGGCATGGAGGTGTTCAGC
ACGGCAGATTACTGGTGGTGGATCCCAGTGGCGGGGCCACTCGTCGGGGGTGTGGTGGGC
GGGGCGATCTACTTCCTTTTCATCGAGCTGCACCATGAAAGT------------------
------GAGCCCGTGAAGGAACAG------------------------------------
------GAGGAAGAGGAAAGCAGCAGCGTCCAGGACAAGTATGAAATGATCGCCGTGAGC
>Mormyrid_electric_fish_Aqp9a
---------------ATGGAGACTGAGCGCAAAAGGACAATGAAAGAGTGCTGCGTGGTG
CGTAACCGCATCTTCAGGGAGTTTCTGGCCGAATTTCTCGGAACATTTATATTAATTCTG
TTCGGCTGCGGCTCCGTGGCACAGACAGTGTTGAGTCGCGGTGCCCTGGGAGAGCCGCTC
ACCATCCACATCGGCTTCACTCTGGGAGTGATGATGGCGGTCTACGTGGCAGGAGGGGTG
TCAGGTGCCCACATTAACCCTGCCGTGTCCCTGGCTATGGTCGTCCTGGGTCGGCTGAAA
CTGGTGAAGTTCCCCGTCTATGTCATGGCGCAGTTCCTGGGAGCCTTCGTTGGCGCGGCT
GCTGTCTTCTGCCTGTACTACGATGCCTTTATGGACTTCACGAGTGGAATACTGTCAGTG

ACTGGCATCAACGCCACAGCACACATCTTTGCCTCCTACCCTTCTAGGCACCTCTCAATA
ATCAACGGATTCATTGACCAGGTGTTTGGGACGGGCATCCTGGTCTTGTGCATCCTGGCC
ATTATCGACGGGAAGAACATTGGTGCACCCAGAGGAATGGAGCCCTTGGTCATCGGCTTG
ATCATCCTGGGGATTGGCGTGTCCATGGGCTTGAACTGTGGTTATCCTCTGAACCCCGCC
CGCGACCTGGGCCCCAGGGTCTTCACGGCAGTGGCTGGGTGGGGCATGGAGGTGTTCAGG
ACAGCAGATTACTGGTGGTGGATCCCAGTGGCGGGGCCCCTCGTCGGGGGTGTGGTGGGC
GGGGCGATCTACTTCCTCTTCATCGAGCTGCACCATGAGAGT------------------
------GAGCCCGTGACGGAACAG------------------------------------
------GAGGAAGAGGAAAGCAGCAGCATCCAGGACAAGTATGAGATGATCGCCGTGAGC
>Aba_Aqp9a
---------------ATGGAGACCGAGCGCAAGAAAAGCCTGAAAGCCCGCTGTGCCTTG
AGGCGCGGCATCCTGAGGGAATTTCTGGCCGAATTCCTCGGGACGTTTATATTAATACTG
TTTGGGTGTGGCTCCATGGCACAGATGGTGCTCAGCCGCGAAGCGCTGGGAGAGCCCCTC
ACCGTCCACATTGGTTTCACTTTGGGAGTGGTGATGGCCATTTACGTGGCAGGAGGAGTG
TCAGGAGCCCACATCAACCCTGCTGTGTCCCTGGCTATGGTCATCCTGGGTAAGCTGAAG
CTGGCCAAGTTGCCCGTGTATGTCCTGGCACAGTTCCTGGGTGCCTTCACTGGTGCTGCC
GCGGTCTTCTGCTTGTACTATGATGCCTTCATGGACTTCACGAACGGAATACTGTCAGTG
ACCGGCATCAACGCCACAGCTCACATCTTTGCCTCCTATCCTTCTAGACACCTCTCAGCA
CTCAATGGATTCATAGACCAGGTGATTGGGACTGGTACCCTGGTCCTGTGCATCCTGGCT
ATCATTGATGGGAAGAACATTGGTGCACCCAGAGGAATGGAGCCCTTGGCCATTGGGTTG
ATTATCATGGGGATCAGCGTGTCCATGGGTTTGAACTGTGGCTGTCCACTCAACCCTGCC
CGGGACCTGGGCCCCAGGCTCTTCATGGCAGTGGCAGGATGGGGCATGGAGGTGTTCAGG
ACAGCGCATTACTGGTGGTGGATCCCAGTCGTGGGGCCCCTTGTTGGGGGTGTGGTGGGG
GCGGTGATCTACTTCCTCTTTATTAAGCTGCACCACCAAACG------------------
------GAGCTCACGAAGGAAGAA------------------------------------
------GAGGATGAGGAAGACAGCAGTGTCAAGGACAAATATGATATGATCACCATGAGC
>American_eel_Aqp9a
---------------ATGGAGCTGGAACACAAGAAACCCAGAAAATCCCGCTGTGGGTTC
AGACACAGCATTATCAAAGAATTCTTGGCAGAATTTCTCGGGACATTCGTATTAGTTCTA
TTCGGGTGTGGGTCAGCTGCCCAGACGGTGCTGAGCCGGGGGGCTTTGGGGGAGACCTTC
ACCATTCACGTCGGCTTCACCTTGGGGTTGATGATGGCGGTCTACATAGCCGGGGGAGTC
TCAGGCGGCCACGTCAACCCTGCTGTGTCCCTGGCGATGGTTATCCTGGGCAAGCTGAAG
GTGGTAAAGTTTCCAGTCTACGTGGTCGCACAGTTCCTTGGCGCGTTTGCGGGCGCCGCT
GCCGTCTTTGGCCTTTATTACGATGCCTTCATGGACTATACCAGTGGAATACTGTCAGTG
ACAGGCATTAATGCCACAGCTCACATCTTTGCCTCCTATCCTGCCAGACACCTCTCGGTA
CTCAATGGATTCATAGACCAGGTGATCGGGACGGGCATTTTGGTCCTTTGCATTCTGGCG
ATCATTGATGGGAGGAACATCGGAGCGCCCAGGGGGATGGAGCCTTTGGCCATAGGTCTT
ACCGTCATGGCGATTGGGGTGTCCATGGGCTTGAACTGTGGCTATCCCATCAACCCCGCC
CGGGACCTGGGCCCTCGACTGTTCACCGCAGTGGCAGGCTGGGGCATGGACGTGTTCAGG
ACAGCAGACTACTGGTGGTGGATCCCTGTGGCTGGGCCCATGGTGGGGGGCATTGTGGGC
GCAGTGGTGTACTTCCTCTTCATTGAGCTGCACCATGCCAGA------------------
---------CCTGAGGAGCAACAACAGGAGGAGGAGGAGGAG---------------GAG
GAAGAGGAGGAAGAGGATGAC------ATCAAGGACAAGTATGAGATGATCACCATGAGC
>European_eel_Aqp9a
---------------ATGGAGTTGGAACACAAGAAACCCAGAAAAGCCCGCTGTGGGTTC
AGACACAGCATTATCAAAGAATTCCTGGCAGAATTTCTCGGGACATTCGTATTAGTTCTA
TTCGGGTGTGGGTCAGCTGCCCAGACGGTGCTGAGCCGGGGGGCTTTGGGGGAGACCTTC
ACCATTCACATCGGCTTCACCATGGGGTTGATGATGGCGGTCTACATAGCCGGGGGAGTC
TCAGGCGGCCACGTCAACCCTGCTGTGTCCCTGGCGATGGTTATCCTGGGCAAGCTGAAG
GTGGTAAAGTTTCCCGTCTACGTGGTCGCACAGTTCCTTGGCGCGTTTGCGGGCGCCGCT
GCCGTCTTTGGCCTTTATTACGATGCCTTTATGGACTATACCAGTGGAATACTGTCAGTG
ACAGGCATTAATGCCACAGCTCACATCTTTGCCTCCTATCCTGCCAGACACCTCTCGGTA
CTCAATGGATTCATAGACCAGGTGATCGGGACGGGCATTTTGGTCCTTTGCATTCTGGCG
ATCATTGATGGGAGGAACATCGGAGCGCCCAGGGGGATGGAGCCTTTGGCCATAGGTCTT
ACCGTCATGGCGATTGGGGTGTCCATGGGCTTGAACTGTGGCTATCCCATCAACCCCGCC
CGGGACCTGGGCCCTCGCCTGTTCACCGCAGTGGCAGGCTGGGGCATGGACGTGTTCAGG
ACAGCAGACTACTGGTGGTGGATCCCTGTGGCTGGGCCCATGGTGGGGGGCATTGTGGGC
GCAGTGGTGTACTTCCTCTTCATTGAGCTGCACCATGCCAGA------------------
---------CCTGAGGAGCAACAACAGGAGGAGGAGGAGGAG------------------
GAAGAGGAGGAAGAGGATGAC------ATCAAGGACAAGTATGAGATGATCACCATGAGC
>Japanese_eel_Aqp9a

---------------ATGGAGCTGGAACACAAGAAACCCAGAAAATCCCGCTGTGGGTTC
AGACACAGCATTATCAAAGAATTCTTGGCAGAATTTCTCGGGACATTCGTATTAGTTCTA
TTCGGGTGTGGGTCAGCAGCCCAGACGGTGCTGAGCCGGGGGCTTTGGGGGAGACCTTC
ACCATTCACGTCGGCTTCACCATGGGGTTGATGATGGCGGTCTACATAGCCGGGGGAGTC
TCAGGCGGCCACGTGAACCCTGCTGTGTCCCTGGCGATGGTTATCCTGGGCAAGCTGAAG
GTGGTAAAGTTTCCCGTCTACGTGGTCGCGCAGTTCCTTGGTGCGTTTGCGGGCGCCGCT
GCCGTCTTTGGCCTTTATTACGATGCCTTCATGGACTATACCAGTGGAATACTGTCAGTG
ACAGGCATTAATGCCACAGCTCACATCTTTGCCTCCTATCCTGCCAGACACCTCTCGGTA
CTCAATGGATTCATAGACCAGGTGATCGGGACGGGCATTTTGGTCCTTTGCATTCTGGCG
ATCATTGATGGGAGGAACATCGGAGCGCCCAGGGGGATGGAGCCTTTGGCCATAGGTCTT
ACCGTCATGGCGATTGGGGTGTCCATGGGCTTGAACTGTGGCTATCCCATCAACCCCGCC
CGGGACCTGGGCCCTCGCCTGTTCACTGCAGTGGCAGGCTGGGGCATGGACGTGTTCAGG
ACAGCAGACTACTGGTGGTGGATCCCTGTGGCTGGGCCCATGGTGGGGGGCATTGTGGGC
GCAGTGGTGTACTTCCTCTTCATTGAGCTGCACCACGCCAGA------------------
---------CCTGAGGAGCAACAACAGGAGGAGGAG------------------------
GAAGAGGAGGAAGAGGATGAC------ATCAAGGACAAGTATGAGATGATCACCATGAGC
>Torafugu_Aqp9b
---------------ATGGAAAGCGAGAGCAAGAGGAAGATGAAGGAGAGATTTGGCCTG
AGGCGGAACATCTTTAAGGAGTTCCTGGCGGAATTTCTGGGGATATTTGTCCTGATACTC
TTCGGATGTGGTTCAGTAGCCCAGACGGTCCTGAGCAGAGGGGCTCTCGGGGAACCCCTG
ACCATCCACGTCGGTTTCACCCTGGGAGTCATGATGGCTGTTTACATGGCAGGAGGAGTG
TCAGGAGCCCACGTCAACCCGGCGGTTTCTCTGGCCATGGTCATCTTGGGGAAGCTGCCG
CTGAAGAAGTTCCCCGTGTACGTGCTGGCGCAGTTCCTGGGGGCCTTCGCCGGTTCCTGT
GCTGTCTACGGGCTGTATTATGATGCGTTGATGGAATACACGAAGGGCGAATTCGTCGTC
ACCGGCGAGAACGCCACAGCCAACATATTTGCGTCTTACCCCGCGAAACACCTCTCGCTC
CTCAACGGGTTTGTTGACCAGGTAATTGGAACCGGGGCGCTCGTCCTGTGCATCCTGGCC
ATCACTGACAGGAAGAACATCGGCGCCCCAAAAGGTATGGAGCCTCTGTGCATCGGCCTG
GTCATCATGGCCATCGCCGTGTCCATGGGGCTGAACTGCGGCTACCCCATCAACCCGGCA
CGAGACCTCGGGCCGCGGTTCTTCACAGCCGTGGCCGGGTGGGGCATGGACGTGTTCAGA
GCCGGGGGGTGCTGGTGGTGGATCCCTGTGGCCGGGCCCATGGTGGGCGGAGCGGTGGGG
GCCGGACTTTACTTCCTGTTCATTGATTTGCACCAGCCGGAG------------------
---------CCGGAAAAACAG---------------------------------------
---------GAGGAGAACAAAAGTTCCGTCCAAGACAAATATGAAATCATGACCATGACC
>Sansaifugu_Aqp9b
---------------ATGGAAAGCGACAGCAAGAGGAAGATGAAGGAGAGATTTGGCCTG
AGGCGGAACATCTTTAAGGAGTTCCTGGCGGAATTTCTGGGGATATTTGTCCTGATACTC
TTCGGATGTGGTTCAGTNGCCCAGACGGTCCTGAGCAGAGGGGCTCTCGGGGAACCCCTG
ACCATCCACGTCGGTTTCACCCTGGGAGTCATGATGGCTGTTTACATGGCAGGAGGAGTG
TCAGGAGCCCACGTCAACCCGGCGGTTTCTCTGGCCATGGTCATCTTGGGGAAGCTGCCG
CTGAAGAAGTTCCCCGTGTACGTGCTGGCGCAGTTCCTGGGGGCCTTCGCCGGTTCCTGT
GCTGTCTACGGGCTGTATTATGATGCCTTGATGGAATACACGAAGGGCGAATTCGTCGTC
ACCGGCGAGAACGCCACAGCCAACATATTTGCGTCTTACCCCGCGAAACACCTCTCGCTC
CTCAACGGGTTTGTTGACCAGGTAATTGGAACCGGGGCGCTCGTCCTGTGCATCCTGGCC
ATCACTGACAGGAAGAACATCGGCGCCCCAAAAGGTATGGAGCCTCTGTGCATCGGCCTG
GTCATCATGGCCATCGCCGTGTCCATGGGGCTGAACTGCGGCTACCCCATCAACCCGGCA
CGAGACCTCGGG---CGGTTCTTCACAGCCGTGGCCGGGTGGGGCATGGACGTGTTCAGG
GCCGGGGGGTGCTGGTGGTGGATCCCTGTGGCCGGGCCCATGGTGGGCGGAGCGGTGGGG
GCCGGACTTTACTTCCTGTTCATTGATTTGCACCAGCCGGAG------------------
---------CCGGAAAAACAG---------------------------------------
---------GAGGAGAACAAAAGTTCCGTCCAAGACAAATATGAAATCATGACCATGACC
>Green_spotted_pufferfish_Aqp9b
---------------ATGGAAGATGAGAGCAAGAGGAAGATGAAAGAGAAATTTGGCCTG
AGGCGGGACATCTTTAAGGAGTTCCTGGCGGAATTTCTGGGGATATTTGTCCTGATACTC
TTCGGATGCGGTTCAGTGGCCCAGACGGTGCTGAGTAAAGGGGCTCTGGGGGAGCCTCTG
ACCATCCACGTTGGTTTCACCCTGGGAGTCATGATGGCTGTTTACATGGCAGGAGGAGTG
TCAGGAGCCCACGTCAACCCCGCCGTTTCTCTGGCCATGCTCATCCTGGGGAAGCTGCCT
CTCAAGAAGTTCCCCGTCTACGTGCTGGCACAGTTCCTGGGGGCCTTCGCCGGCTCCTGT
GCCGTCTACGGGCTCTACTATGACGCTTTGATGGAATACACGAACGGAGAATTCATCGTC
ACCGGTCAAAACGCCACCGCTGGCATATTTGCGTCCTACCCTGCAAAACACCTCTCGGTC
CTCAACGGCTTTGTTGATCAGGTCATTGGGACTGGGGCACTCATCCTGTGCATCCTGGCC
ATCACTGACAAGAGGAACATGGGGGCCCCCAAAGGGATGGAGCCTCTGTGCATCGGCCTG

GCCATCATGGCCATCGCCGTGTCCATGGGCCTGAACTGCGGCTACCCCATCAACCCGGCC
AGAGACCTCGGGCCGCGGTTCTTCACGGCTGTGGCTGGCTGGGGCATGGACGTGTTCAGG
GCTGGCGGTTGTTGGTGGTGGATCCCCGTGGCCGGGCCCATGGTGGGTGGAGCGTTTGGA
GCCGGCGTTTACTTCCTGTTCATAGAGTTGCACCAGCCAGAG------------------
---------CCGGAAAAGCAG---------------------------------------
------------GAGAACAAATGCTCCGTCCAAGACAAATATGAAATCATGACCATGACC
>Ocean_sunfish_Aqp9b
---------------ATGGAAAATGAGAGCAAGAGGAAGATGAAGGAGAGATTTGGCCTG
AGGCGGGACATCTTGAAGGAGTTCCTGGCGGAATTTCTGGGGATATTTGTCCTGATACTC
TTCGGATGTGGTTCGGTGGCCCAGACGGTGCTGAGTAAGGGGGCTCTCGGCGAGCCCCTG
ACCATCCACATTGGCTTTACTCTGGGAGTCATGATGGCTGTTTACGTGGCAGGGGGGGTG
TCAGGTGCGCACGTCAACCCCGCAGTTTCTCTGGCCATGGTGATCCTGGGGAAGCTGCCT
GTGAGGAAGTTCCCTGTGTACGTGCTGGCACAGTTCCTGGGGGCTTTTGCTGGATCCTGT
GCTGTCTATGGGCTGTATTTTGATGCTTTGATGGAATACACAAATGGAGAACTTGTTGTT
ACCGGGGCAAATGCCACAGCCAACATATTTGCTTCTTACCCTGCCAAACATCTCTCAGTC
CTAAATGGGTTTGTTGACCAGGTGATTGGAACTGGTGCACTCATCCTGTGTATCCTGGCC
ATCACAGACAGGAGGAATATTGGTGCTCCGAAAGGCATGGAGCCTCTGTGCATCGGCCTG
ACCATCATGGCCATCGCCGTGTCCATGGGTCTGAACTGCGGATATCCCATCAACCCGGCA
CGAGACCTTGGCCCACGCCTCTTCACGGCTGTGGCTGGGTGGGGCATGGACGTGTTCAGG
GCTGGAGATTGCTGGTGGTGGATCCCTGTGGCGGGGCCCATGGTGGGTGGAGCAGTTGGA
GCTGGCATTTACTTCCTCTTCATCGAGCTGCATCACCCTGAG------------------
---------CCAGAAAGGAAG---------------------------------------
---------GAGGAGAACAAC------GTCCAGGACAAATATGAAATCGTAACTATGACT
>Striated_frogfish_Aqp9b
---------------ATGGAAAATGAAAGCAAGAGGAAGATGAAGGAGAGATTTGGCCTG
AGGCGGGACATCTTTAAGGAGTTCCTGGCGGAATTTCTGGGCATATTCGTGCTGATACTC
TTCGGATGTGGATCAGTGGCCCAGACGGTGCTGAGCAAAGGGACTCTGGGCGAGCCCCTG
ACCATCCATGTGGGCTTCACTCTGGGTGTGATGATGGCTGTTTACATGGCAGGGGGAGTC
TCAGGAGCCCACGTGAACCCTGCAGTCTCTCTGGCCATGGTGATCCTGGGGAAGCTACCA
CTGAAGAAGTTCCCCGTGTATGTTGTGGCCCAGTTCCTGGGGGCTTTTGCTGGCTCCTGT
GCTGTCTATGGGTTGTACTATGATGCTTTGATGGAATATACTAATGGAGACTTTGCTGTT
ACTGGTGTGAATGCCACAGCCAACATATTTGCATCTTACCCTGCAAAACATCTGTCAGTC
CTAAATGGGTTTGTTGATCAGGTCATTGGCACTGGCGTGTTGATCCTGTGCATCCTGGCC
ATCACTGACCGGAGGAACATCGGTGCTCCTAAAGGCATGGAGCCTCTGTGCATCGGTCTG
ACTATCATGGCCATCGGAGTGGCCATCGGTCTGAACTGTGGTTATCCAATCAACCCAGCG
AGAGACCTCGGCCCCCGGTTATTCACAGCTGTGGCCGGGTGGGGCATGGATGTATTCAGG
GCTGGAGGGTGCTGGTGGTGGATCCCTGTGGCCGGTCCGATGGTGGGTGGAGCAGTCGGA
GCAGCTGTTTATTTCTTTTTCATAGAGCTGCACCATCCAGAC------------------
---------AATGAGAAACAG---------------------------------------
---------GAGGACAACGAC------GTCCAGGACAAATATGAAATGGTAACAGTGAGT
>Gilthead_seabream_Aqp9b
---------------ATGGAAAATGAGAGCAAGAGAAAGATGAAGGAGAGATTCGGCCTG
AGGCGGGACATCTTTAAGGAGTTCCTGGCGGAATTTCTGGGGATATTTGTCCTGATCCTC
TTTGGATGTGGTTCAGTGGCCCAGACGGTGCTGAGTAAAGGGGCTCTCGGGGAGCCCCTG
ACCATCCACGTTGGCTTCACTCTAGGAGTCATGATGGCTGTTTACATGGCGGGGGGAGTG
TCAGGAGCTCACGTGAACCCTGCCGTCTCTCTGGCCATGGTGATCCTCGGGAAGCTCCCT
CTGAAGAAGTTCCCTGTGTACGTGCTGGCACAGTTCCTGGGGGCTTTTGCAGGATCCTGC
GCCGTCTATGGGTTGTATTATGATGCTCTGATGGAATATACCAATGGAGAATTTGTTGTC
ACTGGTGTAAATGCCACCGCCAACATTTTTGCATCTTACCCTGCAAAACATCTCTCAGTC
CTGAACGGGTCTGTCGATCAGGTAATCGCCACTGGAGCTCTGATCCTGTGCATCCTGGCC
ATCACTGACAGGAGGAATATCGGCGCTCCAAAGGGCATGGAGCCTCTGTGCATCGGCCTG
ATCATCATGGCCATCGGAGTGTCCATGGGTCTGAACTGTGGCTATCCGATCAACCCAGCG
CGAGATCTCGGCCCGCGCTTCTTCACCGCTGTGGCCGGCTGGGGCATGGATGTATTCAGA
GCCGGAGGTTGCTGGTGGTGGATTCCCGTGGCGGGGCCGATGGTCGGCGGAGCAGTCGGA
GCGGGCGTTTACTTTCTCTTCATTGAGCTGCACCACGCCGAG------------------
---------CCTGAAAAGCAG---------------------------------------
---------GAGGAGAAAAAC------GTCCAAGACAAATATGAGATAATAACCATGAGT
>Red_seabream_Aqp9b
---------------ATGGAAAATGAGAGCAAGAGGAAGATGAAGGAGAGATTTGGCCTG
AGGCGGGACATCTTTAAGGAGTTCCTGGCGGAATTTCTGGGGATATTTGTCCTGATCCTC
TTTGGATGTGGTTCAGTGGCCCAGACGGTGCTGAGTAAAGGGGCTCTCGGGGAGCCCCTG

ACCATCCACGTTGGTTTCACTCTGGGAGTCATGATGGCTGTTTACATGGCGGGGGGAGTG
TCAGGAGCTCACGTGAACCCTGCGGTCTCTCTGGCCATGGTGATCCTTGGGAAGCTACCT
CTGAAGAAGTTCCCTGTGTACGTGCTGGCACAGTTCCTGGGGGCTTTTGCTGGATCCTGT
GCTGTCTATGGGTTGTATTATGATGCTTTGATGGAATATACCGATGGAGAATTTGTTGTT
ACTGGTGTGAATGCCACTGCCAACATATTCGCGTCTTATCCTGCAAAACATCTCTCAGTC
CTGAATGGGTTTGTTGATCAGGTAATTGCCACTGGAGCTCTGATCCTGTGCATCCTGGCC
ATCACCGACAGGAAGAATATTGGCGCTCCAAAAGGCATGGAGCCTCTGTGCATCGGCCTG
ATCATCATGGCCATCGGAGTGTCCATGGGTCTGAACTGTGGCTATCCAATCAACCCGGCA
CGAGACCTCGGCCCGCGCTTCTTCACGGCTGTGGCCGGGTGGGGCATGGATGTATTCAGG
GCTGGAGGTTGCTGGTGGTGGATCCCTGTGGCAGGGCCCATGGTCGGCGGAGCAGTTGGA
GCTGGCATTTACTTTCTCTTCATTGAGCTGCACCACGCCGAG------------------
---------CCTGAAAAACAG---------------------------------------
---------GAGGAGAAAAAC------GTCCAGGACAAATACGAGATAATAACTATGAGT
>White_bass_Aqp9b

---------ATTTTTAAGGAGTTCCTGGCGGAATTTCTGGGGATATTTGTCCTGATACTC
TTTGGATGTGGTTCAGTGGCCCAGACGGTGCTGAGTAAAGGGGCTCTAGGGGAGCCCTTG
ACCATCCACGTTGGTTTTACTCTGGGAGTCATGATGGCTGTCTACATGGCAGGGGGAGTG
TCAGGAGCCCACGTTAACCCTGCAGTCTCTCTGGCCATGGTGATCCTGGGGAAGCTTCCT
CTGAAGAAGTTCCCTGTGTATGTGGTGGCACAGTTCCTGGGGGCTTTTGCTGGATCCTGT
GCTGTCTATGGGTTGTATTACGATGCTTTGATGGAATACACCAATGGAGAATTTGTTGTT
ACTGGTGCAAATGCTACAGCCAACATATTTGCATCTTATCCAGCAAAACAT---------

--GGCTATCCAATCAACCCAGCA
CGAGACCTCGGCCCACGGTTCTTCACCGCTGTGGCTGGGTGGGGCATGGACGTATTCAGA
GCTGGAGGTTGCTGGTGGTGGATCCCTGTGGCAGGACCCATGGTGGGCGGAGCAGTTGGA
GCAGGCGTTTACTTCCTCTTCATTGAGTTGCACCACCCTGAG------------------
---------CCTGAAAAACAG---------------------------------------
---------GAGGAGAACAAT------GTTCAGGACAAATATGAAATGATAACTATGAGT
>Striped_seabass_Aqp9b
---------------ATGGAAAATGAGAACAAGAAGAAGATGAAGGAGAGATTTGGCCTG
AGGCGGGACATTTTTAAGGAGTTCCTGGCGGAATTTCTGGGGATATTTGTCCTGATACTC
TTTGGATGTGGTTCAGTGGCCCAGACGGTGCTGAGTAAAGGGGCTCTAGGGGAGCCCTTG
ACCATCCACATTGGTTTTACTCTGGGAGTCATGATGGCTGTCTACATGGCAGGGGGAGTG
TCAGGAGCCCACGTTAACCCTGCAGTCTCTCTGGCCATGGTGATTCTGGGGAAGCTTCCT
CTGAAGAAGTTCCCTGTGTATGTGGTGGCACAGTTCCTGGGGGCTTTTGCTGGATCCTGT
GCTGTCTATGGGTTGTATTACGATGCTTTGATGGAATACACCAATGGAGAATTTGTTGTT
ACTGGTGCAAATGCTACAGCCAACATATTTGCATCTTATCCTGCAAAACATCTCTCAGTC
CTTAATGGGTTTGTTGATCAGGTAATTGCAACTGGTGCGCTGATCCTGTGCATCCTGGCC
ATCACTGACAGGAAGAATATCGGTGCTCCAAAAGGTATGGAGCCTCTGTGCATCGGCCTG
ATTATTATGGCCATTGGAGTGTCCATGGGTCTGAACTGTGGCTATCCAATCAACCCAGCA
CGAGACCTCGGCCCACGGTTCTTCACCGCTGTGGCTGGGTGGGGCATGGACGTATTCAGG
GCTGGAGGTTGCTGGTGGTGGATCCCTGTGGCAGGACCCATGGTGGGCGGAGCAGTTGGA
GCAGGCGTTTACTTCCTCTTCATAGAGTTGCACCACCCTGAG------------------
---------CCTGAAAAACAG---------------------------------------
---------GAGGAGAACAAT------GTTCAGGACAAATATGAAATGATAACTATGAGT
>European_seabass_Aqp9b
---------------ATGGAAAATGAGAGCAAGAGGAAGATGAAGGAGAGATTTGGCCTG
AGGCGGGACATTTTTAAGGAGTTCCTGGCGGAATTTCTGGGGATATTTGTCCTGATACTC
TTTGGATGTGGTTCAGTGGCCCAGACAGTGCTGAGTAAAGGGGCTCTAGGGGAGCCCTTG
ACCATCCACGTTGGTTTCACTCTGGGAGTCATGATGGCTGTCTACATGGCAGGGGGAGTG
TCAGGAGCCCACGTTAACCCTGCAGTCTCTCTGGCCATGGTGATCCTGGGGAAGCTTCCT
CTGAAGAAGTTCCCTGTGTATGTGATGGCACAGTTCCTGGGGGCTTTTGCTGGATCCTGT
GCTGTCTATGGGTTGTATTACGATGCTTTGATGGAATACACTGATGGAGAATTTGTTGTT
ACTGGTGCAAATGCTACAGCCAACATATTTGCATCTTATCCTGCAAAACATCTCTCAGTC
CTTAATGGGTTTGTTGATCAGGTAATTGCAACTGGTGCGCTGATCCTGTGCATCCTGGCC
ATCACTGACAGGAAGAATATCGGTGCTCCAAAAGGTATGGAGCCTCTGTGCATCGGCCTG
ATCATTATGGCCATTGGAGTGTCCATGGGTCTTAACTGCGGCTATCCAATCAACCCAGCG
CGAGACCTCGGCCCACGGTTCTTCACGGCTGTGGCTGGGTGGGGCATGGACGTATTCAGG
GCTGGAGGTTGCTGGTGGTGGATCCCTGTGGCAGGACCCATGGTGGGCGGAGCAGTTGGA

GCAGGCGTTTACTTCCTCTTCATTGAGTTGCACCACCCTGAG------------------
---------CCTGAAAAACAG---------------------------------------
---------GAGGAGAACAAT------GTTCAGGACAAATATGAAATGATAACTATGAGT
>Ballan_wrasse_Aqp9b
---------------ATGGAAAATGAGAAGAAGAGGAAGCTGAAGGAGAGATTTGGCCTG
AGGCGGGACATCTTAAAGGAGTTCCTGGCGGAATTTCTGGGAATATTCGTCCTGATACTC
TTTGGATGTGGTTCAGTGGCCCAGACGGTGCTGAGTAAAGGGGGTCTTGGGGAACCTTTG
ACTATCCACATAGGTTTCACTCTGGGAGTCATGATGGCCGTCTACATGGCAGGGGGAGTG
TCAGGTGCTCACGTGAACCCTGCCGTCTCTCTGGCCATGGTGATCCTGGGGAAGTTGCCT
CTGAAGAAGTTCCCTGTGTACGTGGTGGCGCAGTTCCTGGGGGCTTTTGCTGGATCCTGT
GCCGTCTATGGGTTGTATTATGATGCTTTGATGGAATTTACCAATGGACAATTCCTTGTT
ACTGGTGCAAATGCTACAGCCAACATATTTGCATCCTATCCTGCAAAACACCTCTCAGTC
CTAAATGGGTTTGTTGATCAGGTAATTGCAACAGGCGCGTTGATCCTGTGCATCCTGGCC
ATCACTGACAGGAGGAACATCGGGGCTCCAAAAGGTGTAGAGCCGCTTTGCATTGGCCTG
ATCATCATGGCCATCGGAGTGTCCATGGGTCTGAACTGCGGTTATCCCATCAACCCAGCA
CGGGACCTCGGCCCACGCTTCTTCACCGCTGTGGCGGGATGGGGCATGGAAGTTTTCAGG
GCTGGAGGTTGCTGGTGGTGGATCCCTGTGGCAGGACCAATGGTGGGCGGAGCAGTCGGA
GCAGGAATCTACTTTCTCTTCATAGAGTTGCACCATGCTGAG------------------
---------CCTGAGAAACAA---------------------------------------
---------GAGGAAAACAAT------GTCCAGGACAAGTATGAAATGATAACTGTAAGT
>Corkwing_Aqp9b
---------------ATGGAAAATGAA---AAG---AAGCTGAAGGAGAGATTTGGCCTG
AGGCGGGACATCTTAAAGGAGTTCCTGGCGGAATTTCTGGGAATATTCGTTCTGATACTC
TTTGGATGTGGTTCAGTGGCCCAGACGGTGCTGAGTAAAGGGGGTCTTGGGGAACCTTTG
ACTATCCACATAGGTTTCACTCTGGGAGTCATGATGGCCGTCTACATGGCAGGGGGAGTG
TCAGGAGCTCATGTGAACCCTGCAGTCTCTCTGGCTATGGTGATCCTGGGGAAGTTGCCT
CTGAAGAAGTTCCCTGTGTACGTGGTGGCACAGTTCCTGGGGGCTTTTGCTGGATCTTGT
GCCGTCTACGGGTTGTATTATGATGCTTTGATGGATTTTACCAATGGACAATTCCTCGTT
ACTGGTGCAAATGCTACAGCCAACATATTTGCATCTTATCCTGCAAAACACCTCTCAGTC
CTAAACGGGTTTGTTGATCAGGTAATTGCAACTGGCGCTTTGATCCTGTGCATCCTGGCA
ATCACTGACAAGAAGAACATTGGGGCTCCAAAAGGTGTGGAGCCACTTTGCATTGGCCTG
ATCATCATGGCCATCGGAGTGTCCATGGGTCTGAACTGCGGGTATCCCATCAACCCAGCA
CGGGACCTCGGCCCAAGGTTCTTCACCGCAGTAGCTGGGTGGGGCATGGAAGTTTTCAGG
GCTGGAGGTTGCTGGTGGTGGATCCCTGTGGCAGGACCAATGGTGGGCGGAGCAGTCGGA
GCAGGGATCTACTTTCTCTTCATAGAGTTACACCACACTGAG------------------
---------CCTGACAAACAA---------------------------------------
---------GAGGAAAACAAT------GTCCAGGACAAGTATGAAATGATAACTGTGAGT
>Crimson_snapper_Aqp9b
---------------ATGGAAAATGTGAGCAAGAGGAAGATGAAGGAGAGATTTGGCCTG
AGGCGGGACATCTTTAAGGAGTTCCTGGCGGAATTTCTTGGGATATTTGTCCTGATACTC
TTTGGATGTGGTTCAGTGGCCCAGACGGTGCTGAGTAAAGGGGCTCTCGGGGAGCCCTTG
ACCATCCACATTGGTTTCACCCTGGGAGTCATGATGGCTGTTTACATGGCAGGAGGAGTG
TCAGGAGCCCACGTG---

------------------------------AAAGGCATGGAGCCTCTGTGCATCGGCCTG
ATCATCTTGGCCATCGGCGTGTCCATGGGTCTGAACTGTGGCTACCCAATCAACCCAGCA
CGAGACCTCGGCCCGCGCTTCTTCACGGCTGTGGCTGGGTGGGGCATGGACGTATTCAGA
GCTGGAGGTTGCTGGTGGTGGATCCCTGTGGCAGGACCCATGGTGGGTGGAGCTGTTGGA
GCGGGCGTTTACTTTCTCTTCGTTGAGTTACACCACCCTGAG------------------

>Florida_bass_Aqp9b
---------------ATGGAA---------------AAGATGAAGGAGAGATTTGCCCTG
AGGCGGGACATTTTTAAGGAGTTCCTGGCGGAATTTCTGGGGATATTTGTCCTGATACTC
TTTGGATGTGGTTCAGTGGCCCAGACAGTGCTGAGTAAAGGGGCTCTCGGGGAGCCCTTG
ACCATCCACATTGGTTTTACTCTGGGAGTCATGATGGCCGTCTACATGGCAGGGGGAGTG
TCAGGAGCCCACGTGAACCCTGCAGTCTCTCTGGCCATGGTGATCCTGAAGAAACTACCT
CTCAAGAAGTTCCCTGTGTATGTGGTGGCACAATTCCTGGGGGCTTTTGCTGGATCCTGT

GCTGTTTACGGGTTTTATTATGATGCTTTGATGGAATATACCAATGGAGAATTTGTTGTT
ACTGGTGCAAATGCCACTGCCAATATATTTGCATCTTATCCCGCAAAACATCTCTCAGTC
CTAAATGGGTTTGTTGATCAGGTAATTGCAACTGCCGCACTGATCCTGTGCATCCTGGCT
ATCACTGACAGGAAGAATATTGGCGCTCCAAAAGGCATGGAGCCTCTGTGCATCGGCCTG
ATCATCATGGCCATCGGGGTGTCCATGGGTCTGAACTGTGGCTATCCAATCAACCCGGCA
CGAGACCTCAGCCCACGGTTCTTCACAGCTGTGGCGGGATGGGGCATGGAAGTATTCAGG
GCTGGAGGCTGCTGGTGGTGGATCCCTGTGGCAGGGCCTATGGTGGGTGGAGCAGTTGGA
GCGGGCGTTTACTTTCTCTTCATTGAGTTGCACCACGCTGAG------------------
---------CCTGAAAAACAG---------------------------------------
---------GAGGAGAACAAC------GTCCAGGACAAATATGAAGTCATAACTATGAGT
>Smallmouth_bass_Aqp9b

---CTC
TTTGGATGTGGTTCAGTGGCCCAGACAGTGCTGAGTAAAGGGGCTCTCGGGGAGCCCTTG
ACC--GTT
TCAGGAGCCCACGTGAACCCTGCAGTCTCTCTGGCCATGGTGATCTTGAAGAAACTACCT
CTCAAGAAGTTCCCTGTGTATGTGGTGGCACAATTCCTGGGGGCTTTTGCTGGATCCTGT
GCTGTTTATGGGTTTTATTAT---------------------------------------

---------------------GTAATTGCAACTGCCGCACTGATCCTGTGCATCCTGGCT
ATCACTGACAGGAAGAATATTGGCGCTCCAAAAGGCATGGAGCCTCTGTGCATCGGCCTG
ATCATCATGGCCATCGGGGTGTCCATGGGTCTGAACTGTGGCTATCCAATCAACCCGGCA
CGAGACCTCAGCCCACGGTTCTTCACAGCTGTGGCGGGATGGGGCATGGAAGTATTCAGG
---------------------------------GGGCCTATGGTGGGTGCAGCAGTTGGA
GCGGGCGTTTACTTTCTCTTCATTGAGTTGCACCACGCTGAG------------------
---------CCTGAAAAACAG---------------------------------------
---------GAGGAGAACAAC------GTCCAGGACAAATATGAAGTCATAACTATGAGT
>Barred_knifejaw_Aqp9b
---------------ATGGAAAATGAGAGCAAGAGGAAGATGAAGGAGAGATTTGGCCTG
AGGCGGGACATCTTTAAGGAGTTCCTGGCGGAATTTCTGGGGATATTTGTCCTGATACTC
TTTGGATGTGGTTCAGTGGCCCAGACGGTGCTGAGCAAAGGGGCTCTCGGGGAGCCCTTG
ACCATCCACGTTGGTTTCACTCTGGGAGTCATGATGGCCGTCTACATGGCCGGGGGAGTG
TCAGGAGCCCACGTGAACCCTGCAGTCTCTCTGGCCATGGTGATCCTGGGGAAGCTGCCT
CTGAAAAAGTTCCCTGTGTATGTGGTGGCACAATTCCTGGGGGCTTTTGCTGGATCCTGT
GCTGTCTATGGGTTGTATTATGATGCTTTGATGGAATATACCAATGGAGAGTTTGTTGTT
ACTGGTGCCAATGCCACAGCCAACATATTTGCATCTTATCCTGCAAAACATCTCTCAGTC
CTAAATGGGTTTGTTGATCAGGTCATTGCAACTGCCGCACTGATCCTGTGCATCCTGGCC
ATCACTGACAGGAAGAATATCGGCGCTCCAAAAGGCATGGAGCCTCTCTGCATCGGCCTG
ATCATCATGGCCATCGGAGTGTCCATGGGTCTGAACTGCGGCTACCCAATCAACCCAGCA
CGAGACCTCGGCCCACGGTTCTTCACGGCTGTGGCCGGGTGGGGCATGGACGTATTCAGG
GCTGGAGGTTGCTGGTGGTGGATCCCTGTGGCAGGGCCCATGGTGGGCAGTGCAGTTGGA
GCGGGCGTTTACTTTCTCTTCATTGAGTTGCACCACCCTGAG------------------
---------CCTGAGAAACAG---------------------------------------
------------GAGAACAAC------GTCCAGGACAAATATGAAATGATAACCATGAGT
>Murray_cod_Aqp9b
---------------ATGGAAAATATGAGCAAGAGGAAGATGAAGGAGAGATTTAGCCTG
AGGCGGGACATCTTTAAGGAGTTCCTGGCGGAATTTCTGGGGATATTTGTGCTGATACTC
TTTGGATGTGGTTCAGTGGCCCAGATGGTGCTGAGCAAAGGGGTTCTCGGGGAGCCCTTA
ACTGTCCACATTGGTTTCACTCTGGGTGTCATGATGGCTGTCTACATGGCAGGGGGAGTG
TCAGGAGCCCACGTGAACCCTGCAGTCTCTCTGGCCATGGTGATCCTGGGGAAGCTACCT
CTGAAGAAGTTCCCAGTGTATGTGGTGGCACAATTCCTGGGGGCTTTTGCAGGATCCTGT
GCTGTCTATGGGTTTTATTATGATGCTTTGATGGAATATACCAATGGAGAATTTGTTGTT
ACTGGTGCAAATGCCACAGCCAACATATTTGCATCTTATCCTGCAAAACATCTCTCAGTC
CTAAATGGGTTTGTTGATCAGGTAATTGGAACTGGCGCGCTGGTCCTGTGCATCCTGGCC
ATCACTGACAGGAAGAATATCGGTGCTCCAAAAGGCATGGAGCCTCTGTGCATCGGCCTG
ATCATCATGGCCATCGGAGTGTCCATGGGTCTGAACTGCGGCTATCCAATCAACCCGGCA
CGAGACCTCGGCCCACGGTTCTTCACGGCCGTGGCTGGGTGGGGCATGGAAGTATTCAGG
GCTGGAGGCTGCTGGTGGTGGATCCCTGTGGCAGGGCCCATGGTGGGCGGAGCAGTTGGA
GCGGGCGTTTACTTCCTCTTAATTGAGTTGCACCACCCTGAG------------------
---------CCTGAAAAACAG---------------------------------------
------------GAGAACAAC------GTCCAGGACAAATATGAAATGGTAACTATGAGT

```
>Flag_rockfish_Aqp9b
---------------ATGGAGAATGAGAGCAAGAGGAAGATGAAGGAGAGATTTGGCCTG
AGGCGGGACATCTTTAAGGAGTTCCTGGCTGAGTTTCTGGGGATATTTGTCCTGATACTC
TTTGGATGTGGTTCAGTGGCCCAGACGGTGCTGAGTAGAGGGACTCTCGGGGAGCCCTTG
ACCATCCACATTGGTTTCACTCTGGGAGTCATGATGGCTGTCTACCTGGCAGGGGGAGTG
TCAGGAGCCCACTTGAACCCTGCAGTCTCTCTGGCCATGGTGATCCTGGGGAAGCTTCCT
CTGAAGAAGTTCCCTGTGTATGTGGTGGCACAATTCCTGGGGGCTTTTGCTGGATCCTGT
GCTGTCTATGGGTTGTATTATGATGCTTTGATGGACTATACCGATGGAGAATTTGCTGTT
ATTGGTGTAAATGCCACGGCCAACATATTTGCCTCGTATCCTGCAAAACATCTCTCAGTC
GTAAATGGCCTTGTTGATCAGGTGATTGCAACTGCTGCGCTGATCCTGTGCATCCTGGCC
ATCACTGACAGGAAGAATATCGGTGCTCCAAAAGGCATGGAGCCTCTGTGCATCGGCCTG
ATCATCATGGCCATCGGAGTGTCCATGGGTCTGAACTGCGGCTATCCAATCAACCCGGCA
CGAGACCTTGGCCCACGGTTGTTCACGGCTGTGGCCGGGTGGGGATGGACGTATTCAGG
GCCGGAGGTTGCTGGTGGTGGATCCCTGTGGCAGGACCCATGGTGGGCGGAGCAGTAGGA
GCGGGCATTTACTTCCTCTTCATTGAGTTGCACCATCCTGAG------------------
---------CCTGAAAAACAG---------------------------------------
---------GAGGAGAACAAC------GTCCAGGACAAATATGAAATGATAACTATGAGT
>Rougheye_rockfish_Aqp9b
---------------ATGGAGAATGAGAGCAAGAGGAAGATGAAGGAGAGATTTGGCCTG
AGGCGGGACATCTTTAAGGAGTTCCTGGCTGAGTTTCTGGGGATATTTGTCCTGATACTC
TTTGGATGTGGTTCAGTGGCCCAGACGGTGCTGAGTAGAGGGACTCTCGGGGAGCCCTTG
ACCATCCACATTGGTTTCACTCTGGGAGTCATGATGGCTGTCTACCTGGCAGGGGGAGTG
TCAGGAGCCCACTTGAACCCTGCAGTCTCTCTGGCCATGGTGATCCTGGGGAAGCTTCCT
CTGAAGAAGTTCCCTGTGTATGTGGTGGCACAATTCCTGGGGGCTTTTGCTGGATCCTGT
GCTGTCTATGGGTTGTATTATGATGCTTTGATGGACTATACCGATGGAGAATTTGCTGTT
ATTGGTGTAAATGCCACGGCCAACATATTTGCCTCGTATCCTGCAAAACATCTCTCAGTC
GTAAATGGCCTTGTTGATCAGGTGATTGCAACTGCCGCGCTGATCCTGTGCATCCTGGCC
ATCACTGACAGGAAGAATATCGGTGCTCCAAAAGGCATGGAGCCTCTGTGCATCGGCCTG
ATCATCATGGCCATCGGAGTGTCCATGGGTCTGAACTGCGGCTATCCAATCAACCCGGCA
CGAGACCTTGGCCCACGGTTCTTCACGGCTGTGGCCGGGTGGGGATGGACGTATTCAGG
GCCGGAGGTTGCTGGTGGTGGATCCCTGTGGCAGGACCCATGGTGGGCGGAGCAGTAGGA
GCGGGCATTTACTTCCTCTTCATTGAGTTGCACCATCCTGAG------------------
---------CCTGAAAAACAG---------------------------------------
---------GAGGAGAACAAC------GTCCAGGACAAATATGAAATGATAACTATGAGT
>Korean_rockfish_Aqp9b
---------------ATGGAGAATGAGAGCAAGAGGAAGATGAAGGAGAGATTTGGCCTG
AGGCGGGACATCTTTAAGGAGTTCCTGGCTGAGTTTCTGGGGATATTTGTCCTGATACTC
TTTGGATGTGGTTCAGTGGCCCAGACGGTGCTGAGTAGAGGGACTCTCGGGGAGCCCTTG
ACCATCCACATTGGTTTCACTCTGGGAGTCATGATGGCTGTCTACCTGGCAGGGGGAGTG
TCAGGT------------------------------------------------------
------------------------------------------------------------
---------------------GATGCTTTGATGGACTATACCGATGGAGAATTTGCTGTT
ATTGGTGTAAATGCCACAGCCAACATATTTGCCTCGTATCCTGCAAAACATCTCTCAGTC
GTAAATGGCCTTGTTGATCAGGTGATTGCAACTGCTGCGCTGATCCTGTGCATCCTGGCC
ATCACTGACAGGAAGAATATCGGTGCTCCAAAAGGCATGGAGCCTCTGTGCATCGGCCTG
ATCATCATGGCCATCGGAGTGTCCATGGGTCTGAACTGCGGCTATCCAATCAACCCGGCA
CGAGACCTTGGCCCACGGTTGTTCACGGCTGTGGCCGGGTGGGGATGGACGTATTCAGG
GCCGGAGGTTGCTGGTGGTGGATCCCTGTGGCAGGACCCATGGTGGGCGGAGCAGTAGGA
GCGGGCATTTACTTCCTCTTCATTGAGTTGCACCATCCTGAG------------------
---------CCTGAAAAACAG---------------------------------------
---------GAGGAGAACAAC------GTCCAGGACAAATATGAAATGATAACTATGAGT
>Minor_flag_rockfish_Aqp9b
---------------ATGGAGAATGAGAGCAAGAGGAAGATGAAGGAGAGATTTGGCCTG
AGGCGGGACATCTTTAAGGAGTTCCTGGCTGAGTTTCTGGGGATATTTGTCCTGATACTC
TTTGGATGTGGTTCAGTGGCCCAGACGGTGCTGAGTAGAGGGACTCTCGGGGAGCCCTTG
ACCATCCACATTGGTTTCACTCTGGGAGTCATGATGGCTGTCTACCTGGCAGGGGGAGTG
TCAGGAGCCCATTTGAACCCTGCAGTCTCTCTGGCCATGGTGATCCTGGGGAAGCTTCCT
CTGAAGAAGTTCCCTGTGTATGTGGCGGCACAATTCCTGGGGGCTTTTGCTGGATCCTGT
GCTGTCTATGGGTTGTATTATGATGCTTTGATGGACTATACCGATGGAGAATTTGCTGTT
ATTGGTGCAAATGCCACAGCCAACATATTTGCCTCGTATCCTGCAAAACATCTCTCAGTC
GTAAATGGCCTTGTTGATCAGGTGATTGCAACTGCTGCGCTGATCCTGTGCATCCTGGCC
```

ATCACTGACAGGAAGAATATCGGTGCTCCAAAAGGCATGGAGCCTCTGTGCATCGGCCTG
ATCATCATGGCCATCGGAGTGTCCATGGGTCTGAACTGCGGCTATCCAATCAACCCGGCA
CGAGACCTTGGCCCACGGTTGTTCACGGCTGTGGCCGGGTGGGGATGGACGTATTCAGG
GCCGGAGGTTGCTGGTGGTGGATCCCTGTGGCAGGACCCATGGTGGGCGGAGCAGTAGGA
GCGGGCATTTACTTCCTCTTCATTGAGTTGCACCATCCTGAG------------------
---------CCTGAAAAACAG---------------------------------------
---------GAGGAGAACAAC------GTCCAGGACAAATATGAAATGATAACTATGAGT
>Tiger_rockfish_Aqp9b
---------------ATGGAGAATGAGAGCAAGAGGAAGATGAAGGAGAGATTTGGCCTG
AGGCGGGACATCTTTAAGGAGTTCCTGGCTGAGTTTCTGGGGATATTTGTCCTGATACTC
TTTGGATGTGGTTCAGTGGCCCAGACGGTGCTGAGTAGAGGGACTCTCGGGGAGCCCTTG
ACCATCCACATTGGTTTCACTCTGGGAGTCATGATGGCTGTCTACCTGGCAGGGGGAGTG
TCAGGAGCACACTTGAACCCTGCAGTCTCTCTGGCCATGGTGATCCTGGGGAAGCTTCCT
CTGAAGAAGTTCCCTGTGTATGTGGTGGCACAATTCCTGGGGGCTTTTGCTGGATCCTGT
GCTGTCTATGGGTTGTATTATGATGCTTTGATGGACTATACCGATGGAGAATTTGCTGTT
ATTGGTGCAAATGCCACAGCCAACATATTTGCCTCGTATCCTGCAAAACATCTCTCAGTC
GTAAATGGCCTTGTTGATCAGGTG------------------------------------

---AGA
GCCGGAGGTTGCTGGTGGTGGATCCCTGTGGCAGGACCCATGGTGGGCGGAGCAGTAGGA
GCGGGCATTTACTTCCTCTTCATTGAGTTGCACCATCCTGAG------------------
---------CCTGAAAAACAG---------------------------------------
---------GAGGAGAACAAC------GTCCAGGACAAATATGAAATGATAACTATGAGT
>Yellow_notie_Aqp9b

------------CCTGTGTATGTGCTGGCACAATTCCTGGGGGCTTTTGCTGGATCCTGT
GCTGTCTATGGGTTGTACTATGATGCTTTGATGGAATATACCGATGGAGAATTTGCTGTT
ACTGGTGCAAATGCCACAGCCAACATATTTGCATCGTATCCTGCAAAACATCTCTCTGTC
CTAAATGGGTTTGTTGATCAGGTGATTGCCACAGCCGCTCTGATCCTGTGCATCCTGGCC
ATCACCGACAAGAGGAACATCGGTGCTCCTAAAGGCATGGAGCCTCTGTGCATCGGCCTC
ATCATCATGGCCATCGGGGTGTCCATGGGTCTGAACTGCGGATATCCCATCAACCCGGCA
CGGGACCTCGGACCCCGGTTCTTCACAGCTGTGGCCGGGTGGGGATGGACGTATTCAGA
GCCGGAGGGTGCTGGTGGTGGATCCCCGTGGCAGGACCCATGGTGGGCGGAGCCGTCGGA
GCAGGTGTTTACTTCCTGTTCATTGAGTTGCACCACCCCCAG------------------
---------CCTGAAAAACTG---------------------------------------
---------GACGAGATAAAT------GACCAGGACAAATATGAAATAATAACTATGAGT
>Blackfin_icefish_Aqp9b
---------------ATGGAAGATGAGAGCAAGATGAAGATGAAGGAGAGATTTGGCCTG
AGGCGGGACATCTTTAAGGAGTTCCTGGCGGAATTTCTGGGGACATTTGTCCTGATACTC
TTTGGATGTGGTTCAGTGGCCCAGACGGTGCTGAGTAGAGGGGCTCTCGGGGAGCCTTTG
ACCATCCACATTGGTTTCACTCTGGGAGTCATGATGGCCGTCTACATGGCAGGGGGGGTG
TCAGGAGCCCACCTGAACCCTGCAGTCTCTCTGGCCATGGTGATCCTGGGGAAGCTTCAT
ATTAAGAAGTTCCCTGTGTATGTGCTGGCACAATTCCTGGGGGCTTTTGCTGGATCCTGT
GCTGTCTATGGGTTGTACTATGATGCTTTGATGGAATATACCGATGGAGAATTTGCTGTT
ACTGGTGTAAATGCCACAGCCAACATATTTGCATCGTATCCTGCAAAACATCTCTCTGTC
CTAAATGGGTTTGTTGATCAGGTGATTGCCACAGCCGCTCTGATCCTGTGCATCCTGGCC
ATCACCGACAAGAGGAACATTGGTGCTCCTAAAGGCATGGAGCCTCTGTGCATCGGCCTC
ATCATCATGGCCATCGGGGTGTCCATGGGTCTGAACTGCGGATATCCCATCAACCCGGCA
CGGGACCTCGGACCCCGGTTCTTCACAGCTGTGGCCGGGTGGGGATGGACGTATTCAGG
GCCGGAGGGTGCTGGTGGTGGATCCCAGTGGCAGGACCCATGGTGGGCGGAGCCGTCGGA
GCAGGTGTTTATTTCCTGTTCATTGAGCTGCACCACCCCCAG------------------
---------CCTGAAAAACTG---------------------------------------
---------GACGAGAAAAAT------GCCCAGGACCAATATGAAATAATAACTATGAGT
>Channel_bull_blenny_Aqp9b
---------------ATGGAAAATGATAGCAAGAGGAAGATGAAGGAGAGATTTGGCCTG
AGGCAGGACATCTTTAAGGAATTCCTGGCGGAATTTCTGGGAATATTTGTCCTGATACTC

TTTGGATGTGGTTCAGTGGCCCAGACGGTGCTGAGTAAAGGGGCTCTCGGGGAGCCCTTA
ACCATCCACATCGGTTTCACTCTGGGCGTCATGATGGCCGTCTACATGGCAGGGGGAGTG
TCAGGAGCCCACGTGAACCCTGCAGTCTCTCTGGCCATGGTGATCCTGGGGAAGCTTCCT
CTGAAGAAGTTCCCTGTGTATGTGTTGGCACAATTCCTGGGGGCTTTTGCTGGATCCTGT
GCTGTCTATGGGTTGTATTATGATGCTTTGATGGAATATACCAATGGAGAATTTGCTGTT
ACCGGTGCAAATGCCACGGCCAACATATTTGCATCTTATCCTGCAAAACATCTCTCAGTC
CTAAATGGGTTTGTTGATCAGGTAATTGCAACTGCTGCGCTGATCCTATGCATCCTGGCC
ATCACTGACAGGAAGAATATGGGTGCTCCAAAAGGCATGGAGCCTCTGTGCATCGGCCTG
ATCATCATGGCCATCGGGGTGTCCATGGGTCTGAACTGCGGCTATCCAATCAACCCGGCA
CGAGACCTCGGCCCGCGGTTCTTCACGGCTGTGGCCGGGTGGGGATGGATGTATTCAGG
GCTGGAGGTTGCTGGTGGTGGATCCCAGTGGCAGGGCCCATGGTGGGCGGAGCAGTAGGA
GCGGGTGTTTACTTCCTGTTTATTGAGTTGCACCACCCCGAG------------------
---------CCTGAAAAACAG---------------------------------------
---------GATGACAACAAC------GTCCAGGACAAATATGAAATTATAACTATGAGT
>Black_rockcod_Aqp9b
---------------ATGGAAGATGAGAGCAAGATGAAGATGAAGGAGAGATTTGGCCTG
AGGCGGGACATCTTTAAGGAGTTCCTGGCGGAATTTCTGGGGACATTTGTCCTGATACTC
TTTGGATGTGGTTCAGTGGCCCAGACGGTGCTGAGTAGAGGGGCTCTCGGGGAGCCTTTG
ACCATCCACATTGGTTTCACTCTGGGAGTCATGATGGCCGTCTACATGGCAGGGGGGGTG
TCAGGAGCCCACCTGAACCCTGCAGTCTCTCTGGCCATGGTGATCGTGGGGAAGCTTCAT
ATTAAGAAGTTCCCTGTGTATGTGCTGGCACAATTCCTGGGGGCTTTTGCTGGATCCTGT
GCTGTGTATGGATTGTACTATGATGCTTTGATGGAATATACCGATGGAGAATTTGCTGTT
ACTGGTGCAAATGCCACAGCCAACATATTCGCATCGTATCCTGCAAAACATCTCTCCGTC
CTAAATGGGTTTGTTGATCAGGTGATTGCCACAGCCGCTCTGATCCTGTGCATCCTGGCC
ATCACCGACAAGAGGAACATCGGTGCTCCTAAAGGCATGGAGCCTCTCTGCATCGGGCTC
ATCATCATGGCCATCGGGGTGTCCATGGGTCTGAACTGCGGATATCCCATCAACCCGGCA
CGGGACCTCGGACCACGGTTCTTCACAGCTGTGGCCGGGTGGGGATGGACGTATTCAGA
GCCGGAGGGTGCTGGTGGTGGATCCCCGTGGCAGGACCCATGGTGGGCGGAGCCGTCGGA
GCAGGTGTTTACTTCCTGTTCATTGAGTTGCACCACACCCAG------------------
---------CCTGAAAAACTG---------------------------------------
---------GATGAGAAAAAT------GCCCAGGACAAATATGAAATAATAACTATGAGT
>European_perch_Aqp9b
---------------ATGGAAAATGAGAGCAAGAGGAAGATGAAGGAGAGATTTGGCCTG
AGGCGGGACATCTTTAAGGAGTTCCTGGCGGAATTTCTAGGGATATTTGTCCTGATACTC
TTTGGATGTGGTTCAGTGGCCCAGACGGTGCTGAGTAAAGGGGCTCTCGGGGAGCCCTTA
ACAATCCATGTTGGTTTCACTCTGGGAGTCATGATGGCTGTCTACATTGCAGGGGGAGTG
TCAGGAGCCCACGTCAACCCTGCAGTCTCTCTGGCCATGGTAATCCTGGGGAAGCTTCCT
CTGAAGAAGTTTCCTGTGTATGTGGTGGCACAATTCATGGGGGCTTTTGCTGGATCCTGT
GCTGTCTATGGGTTTTATTATGATGCTTTGATGGAATATACCAATGGAGAATTTGCTGTT
ACTGGTGCAAATGCTACAGCCAACATATTTGCATCCTATCCTGCAAAACATCTCTCAGTC
CTAAATGGGTTTGTTGATCAGGTAATTGCAACTGCTGCGCTGATCCTGTGCATCCTGGCC
ATCACCGACAGGAAGAATATTGGTGCTCCAAAAGGCATGGAGCCTCTGTGCATCGGCCTG
ATCATCATGGCCATCGGAGTGTCTATGGGTCTGAACTGCGGCTATCCAATCAACCCGGCA
CGAGACCTTGGCCCACGGTTCTTCACGGCTGTGGCCGGGTGGGGATGGACGTATTCAGA
GCTGGAGGTTGCTGGTGGTGGATCCCTGTGGCAGGGCCCATGGTGGGCGGAGCAGTAGGA
GCGGGCATTTACTTCCTGTTCATTGAGTTGCACCACCACGAG------------------
---------CCTGAAAAACAG---------------------------------------
---------GAGGAGAACAAC------GTCCAGGACAAGTATGAAATGATAACTATGAGT
>3_spine_stickleback_Aqp9b
---------------ATGGAAGATGAGAGCAAGAGGAAGATGAAGGAGAGATTTGGCCTG
AGGCAGGACATCTTTAAGGAGTTCCTGGCAGAATTTCTGGGGATATTTGTCCTGATACTC
TTTGGATGTGGTTCAGTGGCCCAGACGGTGCTGAGTAAAGGGGCTCTCGGGGAGCCCTTG
ACCATCCACATTGGTTTTACTCTGGGAGTCATGATGGCCGTCTACGTGGCAGGGGGAGTG
TCAGGAGCCCATGTGAACCCTGCAGTCTCTCTGGCCATGGTGATCCTGGGGAAGCTTCCT
CTAAAGAAGTTCCCTGTCTATGTGGTGGCACAATTCCTCGGGGCTTTTGCTGGATCCTGT
GCTGTCTATGGGTTGTATTATGATGCTTTGATGGAATACACCAATGGAGAATTCACTGTT
ACCGGTGCAAATGCCACGGCTAACATATTTGCATCTTATCCATCAAAACACCTCTCAATC
CTTAATGGGTTTGTTGACCAGGTAATTGCGACCGCAGCACTGATCCTGTGCATCCTGGCC
ATTACTGACAAGAGGAATATCGGTGCTCCAAAAGGGATGGAGCCTCTGTGCATTGGCCTG
ATCATCATGGCCATCGGTGTGTCCATGGGTCTGAACTGCGGCTATCCAATAAACCCGGCG
CGAGACCTCGGCCCGCGGTTCTTCACGGCCGTGGCTGGCTGGGGGGTGGACGTATTCAGA

```
GCTGGGGGTTGCTGGTGGTGGATCCCTGTGGCGGGGCCTATGGTAGGCGGCGCTGTGGGG
GCGGGCATATACTTCCTCTTTATTGAGTTGCACCACACCGAG------------------
---------CCTGAAAAACAG---------------------------------------
---------GAGGAGAAAAAC------ATCCAGGACAAATATGAAATGATAACTCTGAGT
>9_spine_stickleback_Aqp9b
---------------ATGGAAAATGAGAGCAAGAGGAAGATGAAGGAGAGATTTGGCCTG
AGGCAGGACATCTTTAAGGAGTTCCTGGCGGAATTTCTGGGGATATTTGTCCTGATACTC
TTTGGATGTGGTTCAGTGGCCCAGACAGTGCTGAGCAAAGGGGCTCTCGGGGAGCCTTTG
ACCATCCACATTGGTTTTACTCTGGGAGTCATGATGGCCGTCTACATGGCGGGGGGAGTG
TCAGGAGCCCATGTAAACCCTGCAGTCTCTCTGGCCATGGTGATCCTGGGGAAGCTTCCT
CTAAAGAAGTTCCCTGTCTATGTGGTGGCACAATTCCTGGGGGCTTTTGCTGGATCCTGT
GCTGTCTATGGGTTGTATTATGATGCTTTGATGGAATACACCAATGGGGAATTCACTGTT
ACTGGTGCAAATTCCACGGCCAACATATTTGCATCATATCCAGCAAAACACCTCTCGATC
CTTAACGGGTTTGTTGACCAGGTAATTGCGACCGCTGCACTGATCCTGTGCATCTTGGCC
ATCACTGACAAGAAGAATATCGGTGCTCCAAAAGGCATGGAGCCTCTGTGCATTGGCCTG
ATCATCATGGCCATCGGAGTGTCCATGGGTCTCAACTGCGGCTATCCGATAAACCCAGCA
CGAGACCTCGGCCCGCGGTTCTTCACGGCTGTGGCTGGGTGGGGGGTGGACGTATTCAGG
GCTGGAGGTTGCTGGTGGTGGATCCCTGTAGCAGGGCCTATGGTAGGCGGAGCTGTGGGA
GCGGGCATATACTTCCTCTTTATTGAGTTGCACCACACTGAG------------------
---------CCTGAAAAACAG---------------------------------------
---------GAGGAGAACAAC------GTCCAGGACAAATATGAAATGATAACTCTGAGT
>Sablefish_Aqp9b
---------------ATGGAAAGTGAAAGCAAGAGGAAGATGAAGGAGAGATTTGCCCTG
AGGCGGGACATCTTTAAGGAATTCCTGGCAGAATTTCTGGGGATATTTGTCCTGATACTC
TTTGGATGTGGTTCAGTGGCCCAGACGGTGCTGAGTAAAGGGGCTCTCGGGGAGCCCTTG
ACTATCCACATTGGTTTCACTCTGGGAGTCATGATGGCCGTCTTCATGGCAGGGGGAGTC
TCAGGAGCCCATGTGAACCCTGCAGTCTCTCTGGCTATGGTGATCCTGGGGAAGCTTCCT
CTGAAGAAGTTCCCTGTGTATGTGGTGGCACAATTCCTGGGGGCTTTTGCTGGATCCTGT
GCTGTCTACGTGTTGTATTATGATGCTTTGATGGAATATACCAATGGAGAATTTGCTGTT
ACTGGTGCAAATGCCACGGCCAACATATTTGCATCTTATCCAGCAAAACATCTCTCAGTT
CTTAATGGGTTTGTTGATCAGGTAATTGCAACTGCTGCACTGATCCTGTGCATCCTGGCC
ATCACTGATAGGAGGAATATTGGTGCTCCAAAGGGCATGGAGCCTTTGTGCATCGGCCTG
ATCATCATGGCCATCGGAGTGTCCATGGGTCTGAACTGCGGCTATCCAATCAACCCGGCA
CGAGACCTTGGCCCACGTTTCTTCACGGCTGTAGCTGGGTGGGGGATGGACGTATTCAGG
GCTGGAGGTTGCTGGTGGTGGATCCCTGTGGCAGGGCCAATGGTAGGCGGAGCTGTAGGA
GCAGGTGTTTACTTCCTCTGCATTGAGTTGCACCATCCCGAG------------------
---------CCTGAAAAACAG---------------------------------------
------------GAGAACAAC------ATCCAGGACAAATATGAAATGATAACTATGAGT
>Rhine_sculpin_Aqp9b
---------------ATGGAAAATGAGAGCAAGAGGAAGATGAAGGAGAGATTCAGCCTG
AGGCGGGACATCTTTAAGGAGTTCCTGGCGGAATTTCTGGGGATATTTGTCCTGATACTC
TTTGGATGTGGTTCAGTGGCCCAGACGGTGCTGAGTAAAGGGGCTCTCGGGGAGCCCTTG
ACCATCCACATTGGTTTCACTCTGGGAGTCATGATGGCTGTCTACATGGCAGGGGGAGTG
TCAGGAGCCCACGTGAACCCTGCAGTCTCTCTGGCCATGGTGATCCTGGGGAAGCTTCCT
CTGAAGAAGTTCCCTGTGTATGTGTTGGCACAATTCCTGGGGGCTTTTGCTGGGTCCTGT
GTTGTCTATGGATTGTACTATGATGCTTTGATGGAATATACCAATGGAGAATTTGCTGTT
ACTGGTGCAAATGCCACTGCCAACATATTTGCATCTTACCCAGCAAAACATCTCTCAGTC
CTGAATGGGTTTGTTGATCAGGTAATTGCAACTGCTGCTCTGATCCTGTGCATCCTGGCC
ATCACTGACAAGAAGAACATCGGTGCTCCAAAAGGCATGGAGCCTCTTTGCATTGGCCTG
ATCATCATGGCCATCGGAGTGTCCATGGGTCTGAATTGCGGCTATCCAATCAACCCGGCA
CGAGACCTCGGCCCACGGCTCTTCACGTCTGTAGCTGGGTGGGGAATGGACGTATTCAGG
GCTGGAGGTTGCTGGTGGTGGATCCCTGTGGCAGGGCCTATGGTAGGCGGAGCAGTAGGA
GCATGCCTTTACTTTCTCTTCATTGAGTTGCACCATCCGGAG------------------
---------CCTGAAAAACAA---------------------------------------
---------GAGGAGAACAAC------GTCCAGGACAAATATGAAATGATAACTATGAGT
>Korean_spotted_seabass_Aqp9b
---------------ATGGAAAATGATAGCAAGAGGAAGATGAAGGAGAGATTTGGCCTG
AGGCGGGACATCTTTAAGGAGTTCCTGGCGGAATTTCTGGGGATATTTGTCCTGATACTC
TTTGGATGT---TCAGTGGCCCAGACGGTGCTGAGTAAAGGGGCTCTCGGGGAGCCCTTG
ACCATCCACATTGGTTTCACTCTGGGAGTCATGATGGCCGTCTACATG------------
-------------------------------------------ATCCTAGGGAAACTACCG
```

TTGAAGAAGTTCCCTGTGTATGTTGTGGCACAATTCCTGGGGGCTTTTGTTGGATCCTGT
GCTGTCTATGGGTTCTATTATGATGCTTTGATGGAGTATACCAATGGAGAATTTTCTGTC
ACTGGTGCAAATGCCACAGCCAACATATTTGCTTCTTATCCTGCAAAACATCTCTCAGTC
CTAAATGGGTTTGTTGATCAGGTAATTGCAACTGGCGCACTGATCCTGTGCATCCTGGCA
ATCACTGACAAGAGGAATATCGGTGCTCCAAAAGGCATGGAGCCCCTGTGCATCGGCCTG
ATCATCATGGCCATTGGAGTGTCCATGGGCCTGAACTGCGGCTATCCAATTAACCCGGCA
CGGGACCTCGGTCCGCGGTTCTTCACGGCTGTGGCCGGGTGGGGCATGGACGTATTCAGA
GCTGGAGGTTGCTGGTGGTGGATCCCTGTGGCAGGGCCGATGGTGGGCGGAGCAGTCGGA
GCGGGCATTTACTTCCTCTTCATTGAGTTGCACCACCCTGAG------------------
---------CCTGAAAAACAG---------------------------------------
---------GAGGAGAACAAT------GTC------------------------------
>Atlantic_halibut_Aqp9b
---------------ATGGATAGCGAGAACAAGAGGAGGCTGAGGGAGAGGTTTGGCCTG
AGGAAGGACATCTTCAAGGAGTTCCTGGCGGAGTTTCTGGGGATATTTGTCCTGATACTC
TTTGGATGTGGTTCAGTGGCCCAGGCGGTGCTCAGTAAAGGGGCTCTCGGGGAGCCCTTG
ACCATCCACATAGGCTTCACTCTGGGAGTCATGATGGCCGTCTTCATGGCAGGAGGAGTG
TCAGGAGGCCACGTGAACCCTGCAGTCTCTCTGGCCATGGTGATCCTGGGGAAGCTGCCA
CTGAAGAAGTTCCCTGTGTACCTGGTGGCTCAGTTCCTGGGTGCTTTCGCTGGCTCCTGT
GCCGTCTATGGGTTGTACTATGATGCTTTGATGGACTTTAGCGACGGAGAATTACTTGTT
ACTGGTGCCAATGCCACAGCCAACATCTTTGCATCTTATCCTGCCAAACATCTCTCCATC
CTAAATGGGTTTGTGGATCAGGTCATCGCAACTGCTGCTCTGGTCCTGTGCATCCTGGCC
ATCACTGACAAGAAGAACATTGGTGCCCCAAAAGGCATGGAGCCTCTGTGCATCGGCTTG
GCCATCCTGGGCATCGGAGTGGCCATGGGACTGAACTCCGGCTATCCCATCAACCCAGCC
CGAGACCTCGGCCCACGGTTCTTCACGGCTGTGGCCGGCTGGGGCATGGATGTGTTCAGG
GCTGGAGGCTGCTGGTGGTGGATCCCTGTGGTTGGGCCCATGGTTGGCGGAGCAGTCGGA
GCCGGCATTTACTTTCTCTTCATTGAGTTGCACCACCAAGAG------------------
---------CCGGAAAAACTG---------------------------------------
---------GAGGAGACCAAC------ATGCAGGACAAATATGAAATGATAACCATGAGT
>Japanese_flounder_Aqp9b
---------------ATGGAGAGCGAGAACAAGAGGAAGATGAGGGAGAGGTTTGGCCTG
AGGAGGGACATCTTCAAGGAGTTCCTGGCGGAGTTTCTGGGGACATTTGTCCTGATACTC
TTTGGATGTGGTTCAGTGGCCCAGACGGTGCTCAGTAAAGGGGCTCTCGGGGAACCCTTG
ACCATCCACGTAGGCTTCACTCTGGGAGTCATGATGGCCGTCTACATGGCAGGAGGAGTG
TCAGGAGCCCACGTGAACCCTGCAGTCTCTCTGGCCATGGTGATCCTGGGGAAGCTGCCA
CTGAAGAAGTTTCCTGTGTACGTGGTGGCTCAGTTCCTGGGTGCTTTCGCTGGATCCTGT
GCCGTTTATGGGCTGTACTATGATGCTTTGATGGACTATACCAACGGAGAATTTGTTGTT
TCTGGTGCCAATGCCACAGCCAACATCTTTGCATCTTATCCAGCCAAACATCTCTCCATC
CTAAATGGGTTTGTTGATCAGGTCATCGCAACTGGCGCTCTGATCCTTTGCATCCTGGCC
ATCACTGACAAGAAGAACATTGGTGCTCCAAAAGGCATGGAGCCTCTGTGCATCGGCTTG
ATCATCCTGGCCATCGGAGTGTCCATGGGTCTGAACTGCGGCTATCCCATCAACCCAGCT
CGAGACCTCGGCCCACGGTTCTTCACGGCTGTGGCTGGCTGGGGCATGGATGTGTTCAGA
GCTGGAGGCTGCTGGTGGTGGATCCCTGTGGCAGGGCCCATGGTTGGTGGAGCAGTTGGA
GCGGGCATTTACTTTCTCTTCATTGAGTTGCACCACCCAGAA------------------
---------CCGGAAAAACAG---------------------------------------
---------GAGGAGAACAAC------CTCCAGGACAAATATGAAATGATAACCATGAGT
>Turbot_Aqp9b
---------------ATGGAAAACGACAATACGAGGAAGATGAAGGAGAGATTTGGCCTG
AGGCGGGACATCTTCAAGGAGTTCCTGGCGGAATTTCTGGGGATATTTGTCCTGATACTC
TTTGGATGTGGTTCAGTGGCCCAGACGGTGCTCAGCAAAGGGGCTCTCGGGGAGCCCTTG
ACCATCCATGTGGGTTTCACTCTGGGAGTCATGATGGCCGTCTTCATTGCAGGGGGAGTG
TCAGGAGCCCACGTGAACCCTGCAGTCTCTCTGGCCATGGTGATCCTGGGGAAGCTACCG
CTGAAGAAGTTTCCTGTGTATGTGGTTGCTCAATTCCTGGGGGCTTTTGCTGGATCCTGC
GCCGTCTATGGGTTGTATTATGATGCTTTGATGGAATACACCAATGGAGAATTTTCTGTT
ACTGGTGCCAATGCCACAGCCAACATATTTGCATCTTATCCTGCCAAACATCTCTCAGTC
CTCAATGGGTTCGTTGATCAGGTATGTGCCACTGCTGCTCTCATCCTTTGCATCCTGGCC
ATCACTGACAGGAAGAATATCGGTGCTCCGAAGGGCATGGAACCTCTGTGCATCGGCCTG
ATCATCATGGCCATCGGAGTGTCCATGGGTCTGAACTGCGGCTATCCAATCAACCCAGCC
CGTGACCTTGGGCCTCGGTTCTTCACGGCTGTGGCCGGCTGGGGCATGGATGTATTCAGG
GCTGGAGGTTGCTGGTGGTGGATCCCTGTGGCAGGACCCATGGTGGGGGGAGCAGTTGGA
GCAGGGATTTACTTTCTCTTCATTGAGTTGCACCAGCCCGAG------------------
---------CCTGAAAAACAG---------------------------------------

---------GAGGAGAACAAC------ATCCAGGACAAATATGAAATGATTACTATGAGT
>Tongue_sole_Aqp9b
---------------ATGGAAGACGAGGGGACGAGGAAGATGAAGGAGAAATTTGGCCTG
AGGCGGGACATCTTGAAGGAGTTCCTGGCGGAATTTCTGGGGATATTTGTCCTGATCCTT
TTTGGATGCGGTTCAGTGGCCCAGACGGTGCTCAGTAAAGGGGCTCTCGGCGAGCCTTTG
ACCATCCATCTGGGTTTCACCCTGGGAGTCATGATGGCCGTGTACATGGCAGGGGGTGTG
TCAGGAGCCCACGTCAACCCTGCAGTCTCTCTGGCCATGGTGATCCTGAAGAAGCTGCCA
CTGAAGAAGTTTCCTGTGTATGTGGCAGCTCAATTCCTCGGAGCCTTTGCTGGATCCTGT
GTAGTTTATGGATTGTATTATGATGCTCTAATGGCTTTCACCAATGGAGAGTTCATTGTC
ACTGGTGCCAATGCCACAGCCAACATATTTGCGTCCTATCCTGCCAAACATCTCTCTGTG
CTAAATGGATTTGTTGATCAGGTTATTGGAACTGCTGCTCTGATCCTGTGCATCCTGGCC
ATCACTGATAGGAAGAACATTGGAGCTCCCAAAGGCATGGAGCCTCTATGCATTGGTCTC
ATCATCATGGCCATTGGAGTGTCCATGGGTCTGAACTGCGGTTATCCAGTCAACCCGGCT
CGAGACCTTGGCCCTCGCTTCTTCACAGCTGTGGCAGGCTGGGGTGTGGATGTCTTCAGG
GCTGGTGGCTGCTGGTGGTGGATCCCAGTTGCCGGTCCAATGGTGGGTGGAGCAGTCGGA
GCTGGGGTTTACTTCCTCTTTATCGAGCTGCACCACCCTGAA------------------
---------CCTGAGAAACAA---------------------------------------
---------GAGGAGAAGAAC------ATTCAGGACAAATATGAAATGGTAACAATGAGT
>Barramundi_Aqp9b
---------------ATGGAAAACGAGAATAAGAGGAAGATGAAGGAGAGATTTGGCCTG
AGGCGAGACATCTTTAAAGAGTTCCTGGCGGAATTTCTGGGGATCTTTGTCCTGATACTC
TTTGGATGTGGTTCAGTGGCCCAGACAGTGCTGAGTAAAGGGGCTCTTGGGGAGCCTCTC
ACCATCCATGTTGGTTTCACTCTGGGAGTCATGATGGCCGTCTACATGGCAGGAGGAGTG
TCAGGAGCTCATGTCAACCCTGCAGTCTCTCTGGCCATGCTGATCCTGGGGAAACTACCA
CTGAAGAAGTTTCCTGTGTATGTGGCGGCACAGTTCCTGGGTGCCTTTGCTGGATCCTGT
GCTGTCTATGGGCTGTACTACGATGCTTTGTTGGAATTTACTAAAGGAGAATTTGCTGTT
ACTGGAGCCAATGCCACAGCCAACATCTTTGCATCTTATCCTGCCAAACATCTCTCAGTC
CTGAATGGGTTTGTAGATCAGGTGATTGCGACCGGTGCTCTGATCCTGTGCATCCTAGCC
ATCACTGACAGGAAGAACATCGGAGCTCCTAAAGGCATGGAGCCTCTGTGCATCGGCCTT
ATCATTATGGCCATCGGAGTGTCCATGGGTCTGAACTGCGGCTATCCCATCAACCCGGCT
CGAGACCTGGGTCCGCGGTTCTTCACGGCTGTGGCCGGCTGGGGCATGGACGTGTTCAGA
GCTGGAGGTTGTTGGTGGTGGATTCCTGTGGCAGGGCCTATGGTTGGTGGAGCGGTCGGA
GCGGGTGTTTACTTCCTCTTCATTGAGCTGCATCACCCTGAG------------------
---------CCTGAGAAACAG---------------------------------------
------------GAGAACAAC------ATCCAAGACAAATATGAAATGATAACTATGAGT
>Derbio_Aqp9b
---------------ATGGAGAACCACAGCAAGAGGAAGATGAAGGAGAGATTTGGCCTG
AGGCGGGACATCTTTAAGGAGTTCCTGGCGGAATTTCTGGGGATATTTGTCCTGATACTC
TTTGGATGTGGTTCAGTGGCCCAGACGGTGCTGAGTAAAGGGGCTCTCGGTGAGCCCTTG
ACCATCCACGCTGGTTTCACTCTAGGAGTCATGATGGCGGTCTACATGGCAGGGGGAGTG
TCAGGCGCCCACGTGAACCCAGCAGTCTCTCTGGCCATGGTGATCCTGGGGAAGCTACCA
CTGAAGAAGTTTCCTGTGTATGTGGTGGCACAATTCCTGGGTGCTTTTGCTGGATCTTGT
GCTGTCTATGGATTGTATTACGATGCTTTGATGGAATATACCAATGGTGAATTTGCTGTT
ACTGGTGCTAATGCCACAGCCAACATATTTGCATCTTATCCTGCAAAACATCTCTCAGTC
CTAAATGGGTTTGTTGATCAGGTAATTGCAACTGGCGCTCTGATCCTGTGCATCCTGGCC
ATCACTGACAGGAAGAATATTGGTGCTCCAAAGGGCATGGAGCCTCTGTGCATTGGCCTG
ATCATCATGGCCATTGGAGTGTCCATGGGTCTGAACTGCGGCTATCCAATCAACCCAGCT
CGAGACCTTGGACCACGGTTCTTCACAGCTGTGGCTGGATGGGGCATGGACGTATTCAGG
GCTGGAGGTTGTTGGTGGTGGATCCCTGTAGCAGGGCCCATGGTGGGTGGAGCAGTTGGA
GCGGGCATTTACTTTCTCTTCATTGAGTTGCACCACCCTGAG------------------
---------CCTGAAAAACAG---------------------------------------
---------GAGGAGAACAAC------ATCCAGGACAAATATGAAATAGTAACTATGAGT
>Greater_amberjack_Aqp9b
---------------ATGGACAGCGACAGCAAGAGCAAGATGAAGGAGAGATTTGGCCTG
AGGCGGGACATCTTTAAGGAGTTCCTGGCGGAATTTCTGGGGATATTTGTCCTGATACTC
TTTGGATGTGGTTCAGTGGCCCAGACGGTGCTGAGTAAAGGGGCTCTCGGGGAACCCTTG
ACCATCCACGTTGGTTTCACTCTGGGAGTCATGATGGCCGTCTACATGGCAGGGGGAGTG
TCAGGAGCCCATGTGAACCCGGCGGTCTCTCTGGCCATGGTGATCCTGGGGAAGCTACCG
CTGAAGAAGTTTCCTGTGTATGTGGTGGCACAATTCCTGGGTGCTTTTGCTGGATCTTGT
GCTGTCTATGGGTTGTATTATGATGCTTTGATGGAATATACCAATGGAGAATTTGCTGTT
ACTGGTGCCAATGCCACAGCCAACATATTTGCATCTTATCCTGCACAACATCTCTCAGTC

ATAAATGGGTTTGTTGATCAGGTAATTGCAACTGGCGCTCTGATCCTGTGCATCCTGGCC
ATCACTGACAGGAAGAATATTGGTGCTCCAAAAGGCATGGAGCCTCTGTGCATCGGCCTG
ATCATCATGGCCATCGGAGTGTCCATGGGTCTGAACTGCGGCTATCCAATCAACCCGGCT
CGAGACCTCGGCCCACGGTTCTTCACGGCTGTGGCCGGGTGGGCATGGACGTATTCAGG
GCTGGAGGTTGCTGGTGGTGGATCCCTGTGGCAGGGCCGATGGTGGGCGGAGCAGTTGGA
GCGGGCATTTACTTTCTCTTCATTGAGTTGCACCACCCTGAG------------------
---------CCTGAAAAACAG---------------------------------------
---------GAGGAGAACAAC------ATCCAGGACAAATATGAAATAGTAACTATGAGT
>Japanese_amberjack_Aqp9b
---------------ATGGACAGCGACAGCAAGAGCAAGATGAAGGAGAGATTTGGCCTG
AGGCGGGACATCTTTAAGGAGTTCCTGGCGGAATTTCTGGGGATATTTGTCCTGATACTC
TTTGGATGTGGTTCAGTGGCCCAGACGGTGCTGAGTAAAGGGGCTCTCGGGGAACCCTTG
ACCATCCACGTTGGTTTCACTCTGGGAGTCATGATGGCCGTCTACATGGCAGGGGGAGTG
TCAGGAGCCCATGTGAACCCGGCGGTCTCTCTGGCCATGGTGATCCTGGGGAAGCTACCG
CTGAAGAAGTTTCCTGTGTATGTGGTGGCACAATTCCTGGGTGCTTTTGCTGGATCTTGT
GCTGTCTATGGGTTGTATTATGATGCTTTGATGGAATATACCAATGGAGAATTTGCTGTT
ACTGGTGCCAATGCCACAGCCAACATATTTGCATCTTATCCTGCACAACATCTCTCAGTC
ATAAATGGGTTTGTTGATCAGGTAATTGCAACTGGCGCTCTGATCCTGTGCATCCTGGCC
ATCACTGACAGGAAGAATATTGGTGCTCCAAAAGGCATGGAGCCTCTGTGCATCGGCCTG
ATCATCATGGCCATCGGAGTGTCCATGGGTCTGAACTGTGGCTATCCAATCAACCCGGCT
CGAGACCTCGGCCCGCGGTTCTTCACGGCTGTGGCCGGGTGGGCATGGACGTATTCAGG
GCTGGAGGTTGCTGGTGGTGGATCCCTGTGGCAGGGCCCATGGTGGGCGGAGCAGTTGGA
GCGGGCATTTACTTTCTCTTCATTGAGTTGCACCACCCTGAG------------------
---------CCTGAAAAACAG---------------------------------------
---------GAGGAGAACAAC------ATCCAGGACAAATATGAAATAGTAACTATGAGT
>Snubnose_pompano_Aqp9b
---------------ATGGAGAACCACAGCAAGAGGAAGATGAAGGAGAGATTTGGCCTG
AGGCGGGACATCTTTAAGGAGTTCCTGGCGGAATTTCTGGGGATATTTGTCCTGATACTC
TTTGGATGTGGTTCAGTGGCCCAGACGGTGCTGAGTAAAGGGGCTCTCGGTGAGCCCTTG
ACCATCCACGCTGGTTTCACTCTAGGAGTCATGATGGCGGTCTACATGGCAGGGGGAGTG
TCAGGCGCCCACGTGAACCCAGCAGTCTCTCTGGCCATGGTGATCCTGGGGAAGCTACCA
CTGAAGAAGTTTCCTGTGTATGTGGTGGCACAATTCCTGGGTGCTTTTGCTGGATCTTGT
GCTGTCTATGGATTGTATTACGATGCTTTGATGGAATATACCAATGGTGAATTTGCTGTT
ACTGGTGCTAATGCCACAGCCAACATATTTGCATCTTATCCTGCAAAACATCTCTCAGTC
CTAAATGGGTTTGTTGATCAGGTAATTGCAACTGGCGCTCTGATCCTGTGCATCCTGGCC
ATCACTGACAGGAAGAATATTGGTGCTCCAAAGGGCATGGAGCCTCTGTGCATTGGCCTG
ATCATCATGGCCATTGGAGTGTCCATGGGTCTGAACTGCGGCTATCCAATCAACCCAGCT
CGAGACCTTGGACCACGGTTCTTCACAGCTGTGGCTGGATGGGGCATGGACGTATTCAGA
GCTGGAGGTTGTTGGTGGTGGATCCCTGTAGCAGGGCCCATGGTGGGTGGAGCAGTTGGA
GCGGGCATTTACTTTCTCTTCATTGAGTTGCACCACCCTGAG------------------
---------CCTGAAAAACAG---------------------------------------
---------GAGGAGAACAAC------ATCCAGGACAAATATGAAATAGTAACTATGAGT
>Large_yellow_croaker_Aqp9b
---------------ATGGAAAATGAGAGCAAGAGGAAGATGAAGGAGAGATTTGGCCTG
AGGCGGGACATCTTTAAGGAGTTCCTGGCGGAATTTCTGGGGATATTTGTCCTGATACTC
TTTGGATGTGGTTCAGTGGCCCAGACGGTGCTGAGTAAAGGGGCTCTCGGGGAACCTTTG
ACCATCCATGTTGGTTTCACTCTCGGAGTCATGATGGCTGTTTACATGGCAGGGGGAGTG
TCAGGAGCCCATGTGAACCCTGCGGTCTCTCTGGCCATGGTGATCTTGGGGAAGCTTCCT
CTGAAGAAGTTTCCTGTGTATGTGGTGGCACAATTCCTGGGGGCCTTTGCTGGATCCTGT
GCGGTCTACGGGTTGTATTATGATGCTTTGATGGACTACACCAAAGGAGAACTTTCTGTT
ACTGGTGCAAATGCCACAGCCAACATATTTGCATCTTATCCTGCAAAACATCTCTCAGTC
CTCAATGGGTTTGTTGATCAGGTTATTGCAACTGGTGCTCTGATCCTGTGCATCCTGGCC
ATCACTGACAGAAAGAATATCGGTGCTCCCAAAGGCATGGAGCCTCTGTGCATCGGCCTG
ATCATCATGGCCATTGGAGTGTCCATGGGTCTCAACTGCGGCTATCCAATCAACCCGGCA
CGAGACCTTGGCCCACGGTTCTTCACAGCTGTGGCTGGATGGGGCATGGACGTATTCAGA
GCTGGAGGTTGCTGGTGGTGGATTCCTGTGGCAGGGCCCATGGTGGGTGGAGTGGTTGGA
GCTGGCGTTTACTTCCTCTTCATTGAGCTGCACCACCATGAG------------------
---------CCTGAAAAACAG---------------------------------------
------------GAGAACAAC------GTCCAGGACAAATATGAAATGGTAACTATGAGT
>Miiuy_croaker_Aqp9b
---------------ATGGAAAATGAGAGCAAGAGGAAGATGAAGGAGAGATTTGGCCTG

AGGCGGGACATCTTTAAGGAGTTCCTGGCGGAATTTCTGGGGATATTTGTCCTGATACTC
TTTGGATGTGGTTCAGTGGCCCAGACGGTGCTGAGTAAAGGGGCTCTCGGGGAGCCTTTG
ACCATCCATGTTGGTTTCACTCTCGGAGTCATGATGGCTGTTTACATGGCAGGGGGAGTG
TCAGGAGCCCACGTGAACCCTGCAGTCTCTCTGGCCATGGTGATCTTGGGGAAGCTTCCT
CTGAAGAAGTTTCCTGTGTATGTGGTGGCACAATTCCTGGGGGCTTTTGCTGGATCCTGT
GCTGTCTACGGGTTGTATTATGATGCTTTGATGGACTACACCAAAGGAGAACTTTCTGTT
ACTGGTGCAAATGCCACAGCCAACATATTTGCATCTTATCCTGCAAAACATCTCTCAGTC
CTAAATGGGTTTGTTGATCAGGTTATTGCAACTGGTGCTCTGATCCTGTGCATCCTGGCC
ATCACTGACAGGAAGAATATCGGTGCTCCCAAAGGCATGGAGCCTCTGTGCATCGGCCTG
ATCATCATGGCCATCGGAGTGTCCATGGGTCTCAACTGCGGCTATCCAATCAACCCGGCA
CGAGACCTTGGCCCACGGTTCTTCACGGCTGTGGCTGGATGGGGCATGGACGTATTCAGG
GCTGGAGGTTGCTGGTGGTGGATTCCTGTGGCAGGGCCCATGGTGGGTGGAGCGGTTGGA
GCTGGCGTTTACTTCCTCTTCATTGAGCTGCACCACCCTGAG------------------
---------CCTGAAAAACAG---------------------------------------
------------GAGAACAAT------GTCCAGGACAAATATGAAATGGTAACTATGAGT
>Meagre_Aqp9b
---------------ATGGAAAATGAGAGCAAGAGGAAGATGAAGGAGAGATTTGGCCTG
AGGCGGGACATCTTTAAGGAGTTCCTGGCGGAATTTCTGGGGATATTTGTCCTGATACTC
TTTGGATGTGGTTCAGTGGCCCAGACGGTGCTGAGTAAAGGGGCTCTCGGGGAGCCTTTG
ACCATCCATGTTGGTTTCACTCTCGGAGTTATGATGGCTGTTTACATGGCAGGGGGAGTG
TCAGGAGCCCACGTGAACCCTGCGGTCTCTCTGGCCATGGTGATCTTGGGGAAGCTTCCT
CTGAAGAAGTTTCCTGTGTATGTGGTGGCACAATTCCTGGGGGCTTTTGCTGGATCCTGT
GCTGTCTACGGGTTGTATTATGATGCTTTGATGGACTACACCAAAGGAGAACTTTCTGTT
ACTGGTGCAAATGCCACAGCCAACATATTTGCATCTTATCCTGCAAAACATCTCTCAGTC
CTAAATGGGTTTGTTGACCAGGTTATTGCAACTGGTGTTCTGATCCTGTGCATCCTGGCC
ATCACTGACAGGAAGAATATCGGTGCTCCCAAAGGCATGGAGCCTCTGTGCATCGGCCTG
ATCATCATGGCCATCGGAGTGTCCATGGGTCTCAACTGTGGCTATCCAATCAACCCGGCA
CGAGACCTCGGCCCACGGTTCTTCACGGCTGTGGCTGGATGGGGCATGGACGTATTCAGA
GCTGGAGGTTGCTGGTGGTGGATTCCTGTGGCAGGGCCCATGGTGGGTGGAGCGGTTGGA
GCTGGCGTTTACTTCCTCTTCATTGAGCTGCACCACCCTCAG------------------
---------CCTGAAAAACAG---------------------------------------
------------GAGAACAAC------GTCCAGGACAAATATGAAATGGTAACTATGAGT
>Amazon_molly_Aqp9b
---------------ATGGAAGAAGAGAGCAGGAGGAAGATGAAGGAGAAATTTGGCCTG
AGGCGGGACATCGTGAAGGAGTTCCTGGCGGAGTTTCTGGGGATCTTTGTCCTGATCCTC
TTTGGATGCGGTTCAGTGGCCCAGACGGTGCTGAGTAAAGGGGCTCTGGGAGAGCCGCTC
ACCATCCACGTCGGTTTCACCCTGGGAGTCATGATGGCCGTCTACATGGCGGGGGGAGTG
TCAGGAGGCCATGTGAACCCGGCCGTGTCTTTGGCCATGGTGATTCTGGGGAAGCTGCCT
CTGAAGAAGTTTCCCGTCTACGTGGCGGCACAGTTCCTCGGAGCATTCGCCGGTTCCTGT
GCCGTCTTTGGCTTGTACTACGATGCTTTGATGGACTACACCAGTGGAGATCTTGCAGTT
ACTGGTGAAAATGCAACGGCCAACATATTTGCATCCTATCCTGCAAAACATCTGTCGGTG
CTAAACGGGTTTGTTGACCAGGTTATTGCCACCGGAGCGCTGGTCCTGTGCATCCTGGCC
ATCACTGACAGGAAGAACATCGGCGCCCCAAAGGGCATGGAGCCGCTCTGCATCGGCCTG
ACCATCATGGCCATCGGCGTCTCCATGGGTCTGAACTGCGGCTATCCCATAAACCCGGCT
CGAGATCTGGGCCCCCGGCTCTTCACTGCTGTGGCCGGCTGGGGCATGGAGGTGTTCAGA
GCTGGAGGCTGCTGGTGGTGGATCCCTGTGGCCGGACCCATGGTGGGCGGAGCGGTCGGA
GCCGGGATTTACTTTCTCTTCATTGACTTGCACCAACCTGAG------------------
---------CCGCAGAAACAG---------------------------------------
------------GACAACAAC------GTTCAGGACAAATACGAAATCATTACCATGACT
>Sailfin_molly_Aqp9b
---------------ATGGAAGAAGAGAGCAGGAGGAAGATGAAGGAGAAATTTGGCCTG
AGGCGGGACATCGTGAAGGAGTTCCTGGCGGAGTTTCTGGGGATCTTTGTCCTGATCCTC
TTTGGATGCGGTTCAGTGGCCCAGACGGTGCTGAGTAAAGGGGCTCTGGGAGAGCCGCTC
ACCATCCACGTCGGTTTCACCCTGGGAGTCATGATGGCCGTCTACATGGCGGGGGGAGTG
TCAGGAGGCCATGTGAACCCGGCCGTGTCTTTGGCCATGGTGATTCTGGGGAAGCTGCCT
CTGAAGAAGTTTCCCGTCTACGTGGCGGCGCAGTTCCTCGGAGCATTCGCCGGTTCCTGT
GCCGTCTTTGGCTTGTACTACGATGCTTTGATGGACTACACCAGTGGAGATCTTGCAGTT
ACTGGTGAAAATGCAACGGCCAACATATTTGCATCCTATCCTGCAAAACATCTGTCGGTG
CTAAACGGGTTTGTTGACCAGGTTATTGCCACCGGAGCCCTGGTCCTGTGCATCCTGGCC
ATCACTGACAGGAAGAACATCGGCGCCCCAAAGGGCATGGAGCCGCTCTGCATCGGCCTG
ACCATCATGGCCATCGGCGTCTCCATGGGTCTGAACTGCGGCTATCCCATAAACCCGGCT

CGAGATCTGGGCCCCCGGCTCTTCACTGCTGTGGCCGGCTGGGGCATGGAGGTGTTCAGA
GCTGGAGGCTGCTGGTGGTGGATCCCTGTGGCCGGACCCATGGTGGGCGGAGCGGTCGGA
GCCGGGATTTACTTTCTCTTCATCGACTTGCACCAACCTGAG------------------
---------CCGCAGAAACAG---------------------------------------
------------GACAACAAC------GTTCAGGACAAATACGAAATCATTACCATGACT
>Shortfin_molly_Aqp9b
---------------ATGGAAGAAGAGAGCAGGAGGAAGATGAAGGAGAAATTTGGCCTG
AGGCGGGACATCGTGAAGGAGTTCCTGGCGGAGTTTCTGGGGATCTTTGTCCTGATCCTC
TTTGGATGCGGTTCAGTGGCCCAGACGGTGCTGAGTAAAGGGGCTCTGGGAGAGCCGCTC
ACCATCCACGTCGGTTTCACCCTGGGAGTCATGATGGCCGTCTACATGGCGGGGGGAGTG
TCAGGAGCCCATGTGAACCCGGCCGTGTCTTTGGCCATGGTGATTCTGGGGAAGCTGCCT
CTGAAGAAGTTTCCCGTCTACGTGGCGGCACAGTTCCTCGGAGCATTCGCCGGTTCCTGT
GCCGTCTTTGGCTTGTACTACGATGCTTTGATGGACTACACCAGTGGAGATCTTGCAGTT
ACTGGTGAAAATGCAACGGCCAACATATTTGCATCCTATCCTGCAAAACATCTGTCGGTG
CTAAACGGGTTTGTTGACCAGGTTATTGCCACCGGAGCCCTGGTCCTGTGCATCCTGGCC
ATCACTGACAGGAAGAACATCGGCGCCCCAAAGGGCATGGAGCCGCTCTGCATCGGCCTG
ACCATCATGGCCATCGGCGTCTCCATGGGTCTGAACTGCGGCTATCCCATAAACCCGGCT
CGAGATCTGGGCCCCCGGCTCTTCACTGCTGTGGCCGGCTGGGGCATGGAGGTGTTCAGG

>Guppy_Aqp9b
---------------ATGGAAGAAGAGAGCAGGAGGAAGATGAAGGAGAAATTTGGCCTG
AGGCGGGACATCTTGAAGGAGTTCCTGGCGGAGTTTCTGGGGATCTTCGTCCTGATCCTC
TTTGGATGCGGTTCAGTGGCCCAGACGGTGCTGAGTAAAGGGGCTCTGGGAGAGCCGCTC
ACCATCCACGTCGGTTTCACCCTGGGAGTCATGATGGCCGTCTACATGGCGGGGGGAGTG
TCAGGAGCCCATGTGAACCCGGCCGTGTCTTTGGCCATGGTGATTCTGGGGAAGCTGCCT
CTGAAGAAGTTCCCCGTCTACGTGGCGGCGCAGTTCCTCGGAGCATTCGCCGGTTCTTGT
GCTGTCTATGGCTTGTACTACGATGCATTGATGGACTACACCGGTGGAGATCTTGCAGTT
ACTGGTGAAAATGCAACGGCCAACATATTTGCATCTTATCCTGCAAAACATCTGTCGGTG
CTAAACGGGTTTGTTGACCAGGTTATTGCCACTGGAGCGCTGGTCCTGTGCATCCTGGCC
ATCACTGACAGGAAGAACATCGGCGCCCCAAAGGGCATGGAGCCGCTCTGCATCGGCCTG
ACCATCATGGCCATCGGCGTCTCCATGGGTCTGAACTGCGGCTATCCCATAAACCCGGCT
CGAGATCTGGGCCCCCGGCTCTTCACCGCGATGGCCGGCTGGGGCATGGAGGTGTTCAGA
GCTGGAGGCTGCTGGTGGTGGATCCCTGTGGCCGGACCCATGGTGGGCGGAGCGGTCGGA
GCCGGGATTTACTTTCTCTTCATCGACTTGCACCAACCTGAG------------------
---------CCGCAGAAACAG---------------------------------------
---------GACGACAACAAC------GTTCAGGACAAATACGAAATCATTACCATGACT
>Monterrey_platyfish_Aqp9b
---------------ATGGAAGAAGAGAGCAGGAGGAAGATGAAGGAGAAATTTGGCCTG
AGGCGGGACATGTCCAAGGAGTTCCTGGCGGAGTTTCTGGGGATTTTTGTCCTGATCCTC
TTTGGATGCGGTTCAGTGGCCCAGACGGTGCTGAGTAAAGGGGCTCTGGGAGAGCCGCTC
ACCATCCACGTCGGTTTCACCCTGGGAGTCATGATGGCCGTCTACATGGCGGGGGGAGTG
TCAGGAGCCCATGTGAACCCGGCTGTGTCTTTGGCCATGGTGATTCTGGGGAAGCTGCCT
CTGAAGAAGTTCCCCGTCTACGTGGTGGCGCAGTTCCTCGGAGCATTCGCCGGTTCCTGT
GCCGTCTTTGGGTTGTATTACGATGCTTTGATGGACTACACCGGTGGAGATCTTGCAGTT
ACTGGTGAAAATGCAACAGCCAACATATTTGCATCTTATCCTGCAAAACATCTGTCAGTG
CTAAACGGGTTTGTTGACCAGGTTATTGCCACTGGAGCGCTGGTCCTGTGCATCCTGGCC
ATCACTGACAGGAAGAACATCGGCGCCCCAAAAGGCATGGAGCCGCTCTGCATCGGCCTG
ACCATCATGGCCATCGGCGTCTCCATGGGTCTGAACTGCGGCTATCCCATAAACCCGGCT
CGAGATCTCGGCCCGAGGCTCTTCACCGCTGTGGCTGGCTGGGGGATGGAGGTGTTCAGG
GCTGGAGGCTGCTGGTGGTGGATCCCTGTGGCCGGGCCCATGGTGGGCGGAGCGGTCGGA
GCCGGGATTTACTTTCTCTTCATCGACTTGCACCAACCTGAG------------------
---------CCGCAGAAACAG---------------------------------------
---------GACGACAACAAT------GTTCAGGACAAATACGAAATCATTGCCATGACT
>Southern_platyfish_Aqp9b
---------------ATGGAAGAAGAGAGCAGGAGGAAGATGAAGGAGAAATTTGGCCTG
AGGCGGGACATCTCCAAGGAGTTCCTGGCGGAGTTTCTGGGGATTTTTGTCCTGATCCTC
TTTGGATGCGGTTCAGTGGCCCAGACGGTGCTGAGTAAAGGGGCTCTGGGAGAGCCGCTC
ACCATCCACGTCGGTTTCACCCTGGGAGTCATGATGGCCATCTACATGGCGGGGGGAGTG

TCAGGAGCCCATGTGAACCCGGCTGTGTCTTTGGCCATGGTGATTCTGGGGAAGCTGCCT
CTGAAGAAGTTCCCCATCTACGTGGTGGCGCAGTTCCTCGGAGCATTCGCCGGTTCCTGT
GCCGTCTTTGGGTTGTATTACGATGCTTTGATGGACTACACCGGTGGAGATCTTGCAGTT
ACTGGTGAAAATGCAACAGCCAACATATTTGCATCTTATCCTGCAAAACATCTGTCGGTG
CTAAACGGGTTTGTTGACCAGGTTATTGCCACTGGAGCGCTGGTCCTGTGCATCCTGGCC
ATCACTGACAGGAAGAACATCGGCGCCCCAAAAGGCATGGAGCCGCTCTGCATCGGCCTG
ACCATCATGGCCATCGGCGTCTCCATGGGTCTGAACTGCGGCTATCCCATAAACCCGGCT
CGAGATCTCGGCCCGAGGCTCTTCACCGCTGTGGCTGGCTGGGGCATGGAGGTGTTCAGG
GCTGGAGGCTGCTGGTGGTGGATCCCTGTGGCCGGGCCCATGGTGGGCGGAGCGGTCGGA
GCCGGGATTTACTTTCTCTTCATCGACTTGCACCAACCTGAG------------------
---------CCGCAGAAACAG---------------------------------------
---------GACGACAACAAT------GTTCAGGACAAATATGAAATCATTGCCATGACT
>Green_swordtail_Aqp9b
---------------ATGGAAGAAGAGAGCAGGAGGAAGATGAAGGAGAAATTTGGCCTG
AGGCGGGACATCTCCAAGGAGTTCCTGGCGGAGTTTCTGGGGATTTTTGTCCTGATC---

---GGAGCCCATGTGAACCCGGCTGTGTCTTTGGCCATGGTGATTCTGGGGAAGCTGCCT
CTGAAGAAGTTCCCCGTCTACGTGGTGGCGCAGTTCCTCGGAGCATTCGCCGGTTCCTGT
GTCGTCTTTGGGTTGTATTACGATGCTTTGATGGACTACACCGGTGGAGATCTTGCAGTT
ACTGGTGAAAATGCAACAGCCAACATATTTGCATCTTATCCTGCAAAACATCTGTCGGTG
CTAAACGGGTTTGTTGACCAGGTGATTGCCACTGGAGCGCTGGTCCTGTGCATCCTGGCC
ATCACTGACAGGAAGAACATCGGCGCCCCAAAAGGGATGGAGCCGCTCTGCATCGGCCTG
ACCATCATGGCCATCGGCGTCTCCATGGGTCTGAACTGCGGCTATCCCATAAACCCGGCT
CGAGATCTCGGCCCGAGGCTCTTCACCGCTGTGGCTGGCTGGGGCATGGAGGTGTTCAGG
GCTGGAGGCTGCTGGTGGTGGATCCCTGTGGCCGGGCCCATGGTGGGCGGAGCGGTCGGA
GCCGGGATTTACTTTCTCTTCATCGACTTGCACCAACCTGAG------------------
---------CCGCAGAAACAG---------------------------------------
---------GACGACAACAAT------GTTCAGGACAAATACGAAATCATTACCATGACT
>Western_mosquitofish_Aqp9b
---------------ATGGAAGAAGAGAGCAGGAGGAAGATGAAGGAGAAATTTGGCCTG
AGGCGGGACATCTTGAAGGAGTTCCTGGCGGAGTTTCTGGGGATTTTTGTCCTGATCCTC
TTTGGATGCGGTTCAGTGGCCCAGACGGTGCTGAGTAAAGGGGCTCTGGGAGAGCCGCTC
ACCATCCACGTCGGTTTCACCCTGGGAGTCATGATGGCCGTCTACATGGCGGGGGGAGTG
TCAGGAGCCCATGTGAACCCGGCTGTGTCTTTGGCCATGGTGATTCTGGGGAAGCTGCCT
CTGAAGAAGTTCCCCGTCTATGTGGCGGCGCAGTTCCTCGGAGCGTTCGCCGGTTCCTGT
GCCGTCTTTGGGTTGTATTACGATGCTTTGATGGACTACACCGGTGGAGATCTTGCAGTT
ACTGGTGAAAATGCAACAGCCAACATATTTGCATCTTATCCTGCAAAACATCTGTCGGTG
CTAAACGGGTTTGTTGACCAGGTTATTGCCACTGGAGCGCTGGTCCTGTGCATCCTGGCC
ATCACCGACAAGAAGAACATCGGCGCCCCAAAAGGCATGGAGCCGCTCTGCATCGGCCTG
ACCATCATGGCCATCGGCGTCTCCATGGGTCTGAACTGCGGCTATCCCATAAACCCGGCT
CGAGATCTCGGCCCGAGGCTCTTCACCGCTGTGGCTGGCTGGGGCAGGGAGGTGTTCAGG
GCTGGAGGCTGCTGGTGGTGGATCCCTGTGGCCGGGCCCATGGTGGGCGGAGCGGTCGGA
GCCGGGATTTACTTTCTCTTCATCGACTTGCACCAACCTGAG------------------
---------CCGCAGAAACAG---------------------------------------
---------GACGACAACAAT------GTACAGGACAAATACGAAATCATTACCATGACT
>Common_mummichog_Aqp9b
---------------ATGGAAGATGAGAGGAGGAGGAAGATGAAGGAGAAATTTGGCCTG
AGGCGGGACGTCCTGAAGGAGTTCCTGGCGGAGTTTCTGGGGATCTTTGTCCTGATACTC
TTTGGATGCGGTTCAGTGGCCCAGACGGTGCTGAGCAAAGGGGCTCTGGGAGAGCCGCTC
ACCATCCACATCGGTTTCACCCTGGGAGTCATGATGGCCGTCTACATGGCGGGGGGAGTG
TCAGGAGCCCATGTGAACCCGGCCGTCTCTTTGGCCATGGTGATTCTGGGGAAGCTACCT
GTGAAGAAGTTCCCGGTGTATGTGGTGGCACAGTTCCTCGGAGCATTTGCTGGCTCCTGT
GCTGTCTTTGGGTTGTATTACGATGCTTTGATGGAGTACACCGGTGGGGTGCTTGCAGTT
ACTGGTGAAAATGCAACGGCCAACATATTTGCCTCTTATCCTGCAAAACATCTCTCGGTG
CTAAATGGATTTGTTGACCAGGTCATTGCAACCGGCGCTCTGATTCTGTGCATCCTGGCC
ATCACTGACAGGAAGAACATCGGCGCCCCAAAAGGCATGGAGCCGCTGTGCATCGGCTTG
ATCATCATGGCCATCGGCGTCTCCATGGGTCTGAACTGCGGCTATCCCATCAACCCGGCG
CGAGACCTCGGCCCACGGTTCTTCACTGCCGTGGCCGGGTGGGGCATGGAGGTGTTCAGG
GCCGGAGACTGCTGGTGGTGGATCCCTGTGGCGGGACCGATGGTGGGTGGAGCGGTTGGA
GCTGGGATTTACTTTCTCTTCATTGACTTGCACCAACCTGAG------------------

---------CCTCAGAAACAC---------------------------------------
---------GAGGACAACAAC------GTTCAGGACAAATATGACATCATTACTATGACT
>Amargosa_pupfish_Aqp9b
---------------ATGGAGGGGGAAAGAGGAAGGAAGATGAAGGAGAAATTTGGCCTG
AGGCATGCCATCCTGAGGGAGTTCCTGGCAGAATTTCTGGGGATATTTGTCCTGATACTC
TTTGGATGCGGTTCAGTGGCCCAGACCGTGCTGAGTAAAGGGGCTCTGGGAGAGCCGCTA
ACCATCCACATTGGTTTTACCCTGGGAGTTATGATGGCGGTCTACATGGCTGGGGGAGTG
TCAGGAGCTCATGTGAACCCTGCTGTCTCAATAGCCATGGTGATTCTGGGGAAACTTCCT
TTGAAGAAATTCCCAGTGTATGTGGTGGCACAGTTCCTTGGAGCATTTGTTGGTTCCTGT
GCTGTCTTTGGTTTATATTATGATGCTTTGATGGACTACACTGGTGGAAAACTCGAAGTT
ACTGGTGAAAATGCAACAGCCAACATTTTTGCATCTTACCCTGCGAAACATCTCTCACTG
CTAAATGGGTTTGTAGACCAGGTG------------------------------------
---ACTGACAGGAAAAACATCGGCGCCCCAAAAGGCATGGAACCACTCTGCATTGGCTTG
ATCATAATGGCCATCGGAGTTTCTATGGGTCTGAACTGTGGCTATCCCATAAACCCTGCA
CGAGACCTTGGCCCACGATTCTTCACCGCTGTGGCTGGGTGGGGCACTGATGTGTTCAGG
GCCGGGGGCTGCTGGTGGTGGATCCCTTTAGTAGGACCCATG------------------

>Sheepshead_minnow_Aqp9b
---------------ATGGAGGGGGAAAGAGGAAGGAAGATGAAGGAGAAATTTGGCCTG
AGGCATGCCATCCTGAGGGAGTTCCTGGCAGAATTTCTGGGGATATTCGTCCTGATACTC
TTTGGATGCGGTTCAGTGGCCCAGACCGTGCTGAGTAAAGGGGCTCTGGGAGAGCCGCTA
ACCATCCACATTGGTTTTACCCTGGGAGTTATGATGGCGGTCTACATGGCTGGGGGAGTG
TCAGGAGCTCATGTGAACCCTGCTGTCTCAATAGCCATGGTGATTTTGGGGAAACTTCCT
TTGAAGAAATTCCCAGTGTATGTGGTGGCACAGTTCCTTGGAGCATTTGTTGGTTCCTGT
GCTGTCTTTGGTTTATATTATGATGCTTTGATGGACTACACTGGTGGAAAACTCGAAGTT
ACTGGTGAAAATGCAACAGCCAACATTTTTGCATCTTACCCTGCGAAACATCTCTCACTG
CTAAATGGGTTTGTAGACCAGGTCATAGCAACTGGTGCTCTGATTTTGTGCATCCTGGCC
ATCACTGACAGGAAAAACATCGGCGCCCCAAAAGGCATGGAACCACTCTGCATTGGCTTG
ATCATAATGGCCATCGGAGTTTCTATGGGTCTGAACTGCGGCTATCCCATAAACCCTGCA
CGAGACCTTGGCCCACGATTCTTCACCGCTGTGGCTGGGTGGGGCACTGATGTGTTCAGA
GCCGGGGGCTGCTGGTGGTGGATCCCTGTAGTAGGACCCATGGTTGGTGGAGCTGTTGGT
GCTGGGATTTACTTCCTCCTCATTGATTTGCACCAACCGGAG------------------
---------CCTCAGAAACAG---------------------------------------
------------GAAAACAAC------ATTCGGGACAAATATGAAAGCATTACTCTGACT
>Atlantic_silverside_Aqp9b
---------------ATGGAAAATGAAAGCCTGAGGAAGATGAAGGAGAAATTTGGCCTG
AGGCGGGACATCTTGAAGGAGTTCCTCGCTGAATTTCTGGGGATATTTGTCCTGATACTC
TTTGGATGCGGTTCAGTGGCCCAGACAGTGCTGAGTAAAGGGGCTCTCGGGGAGCCTCTG
ACCATCCACATTGGTTTCACTCTGGGAGTCATGATGGCTGTCTACATGGCGGGGGGAGTG
TCAGGAGCCCATGTGAACCCGGCAGTCTCTCTGGCCATGGTGATTCTGGGCAAGCTGCCT
CTGAAGAAGTTCCCCGTGTATGTGGTGGCACAGTTCCTGGGGGCTTTTGCTGGGTCCTGT
GCAGTCTATGTGTTGTACTATGATGCTTTGATGAACTACACCAAAGGAGAGCTGGCTGTT
ACTGGTGAAAATGCCACAGCCAACATATTTGCCTCTTATCCTGCAGAACACCTCTCAGTA
CTGAACGGGTTTGTTGATCAGGTAATTGCAACCGGGGCCCTGATTTTGTGCATCCTGGCC
ATCACTGACAGGAAGAATATCGGCGCCCCAAAAGGCATGGAGCCCCTGTGCATCGGCCTG
ATCATCATGGCCATCGGAGTGTCCATGGGCCTGAACTGTGGCTATCCCATAAACCCGGCG
CGAGACCTCGGACCCCGGATCTTCACCGCTGTGGCTGGGTGGGGCATGGAGGTGTTCAGG
GCTGGAGACTGCTGGTGGTGGATTCCTGTGGCAGGACCCATGGTGGGCGGAGCGGTTGGA
GCCGGAATTTACTTTCTCCTCATTGACTTGCACCAGCCTGAA------------------
---------CCTGACCGACGA---------------------------------------
---------GCACAGACGAAT------GTCCAGGACAAATACGAAATCATTACTATGAGT
>Mangrove_rivulus_Aqp9b
---------------ATGGAAGACGAGAGCAGGAGGAAGATGAAGGAGAAATTTGGCCTG
AGGCAGGACATCTTTAAGGAGTTCCTGGCTGAATTTCTGGGGATATTTGTCCTGATACTC
TTTGGATGCGGTTCAGTGGCCCAGACGGTGCTGAGTCGAGAAACTCTCGGAGAGCCGCTG
ACCATCCACATTGGTTTCACTCTGGGAGTTATGATGGCTGTCTACATGGCAGGAGGAGTC
TCAGGAGCCCATGTGAACCCTGCTGTCTCTCTGGCCATGGTGATTCTGGGGAAGCTACCT
CTGAAGAAGTTCCCCGTGTACGTGCTGGCGCAGTTCCTGGGAGCGTTTGCTGGATCCTGT
GCTGTCTTTGGGTTGTATTACGATGCTTTGATAGACTACACCAATGGAGAGTTCAGAGTT

ACTGGAGCAAATGCTACGGCTAATATATTCGCATCTTATCCTGCACAACATCTCTCAGTC
TTAAACGGGTTTGTTGATCAGGTTATTTCAACTGGTGTACTGATCCTGTGCATCCTGGCC
ATCACTGACAGGAAGAACATTGGCGCCCCGAAAGGCATGGAGCCCCTGTGTATCGGCCTG
ACCATCATGGCCATCGGGGTTTCCATGGGTCTGAACTGTGGCTACCCAATAAACCCGGCT
CGAGACCTCGGCCCGCGCCTTTTCACAGCTGTGGCCGGGTGGGGCATGGAGGTGTTCAGA
GCTGGAGGCTGCTGGTGGTGGATTCCTGTGGCAGGACCCATGGTGGGTGGAGCGGTCGGA
GCTGGGATTTACTTTGTCTTCATCGAGCTGCACCAACATGAA------------------
---------CCTGAGAGACAG---------------------------------------
---------GCGGACAACAAC------ATTCAGGACAAATATGAAGTTATTGCACTGAGT
>Rio_pearlfish_Aqp9b
---------------ATGGAAGATGAGAGCCGGAGGAAGATGAAGGCGAAGTTTGGCCTG
AGGCAGGACATCTTCAAGGAGTTCCTGGCAGAATTTCTGGGGATATTTGTCCTGATACTC
TTTGGATGTGGCTCAGTGGCCCAGACGGTGCTGAGTGGAGGGAGTCTCGGAGACTCGCTC
ACCATCCACATTGGTTTCACTCTGGGAGTCATGATGGCCGTCTACATGGCAGGGGGGGTC
TCAGGAGCCCATGTGAACCCTGCTGTCTCTCTGGCCATGGTGATTCTGGGGAAGCTCCCG
CTGAAGAAGTTCCCCGTGTACGTGCTGGCACAGTTCCTGGGAGCTTTTACTGGCTCCTGC
GCCGTCTTTGGGTTGTACTACGATGCTTTAATAGAATACACCAACGGAGAGTTTACAGTT
ACTGGAGCAAACGCCACTGCCAATATATTTGCATCTTATCCTGCGAATCACCTCTCAGTC
CTCAACGGGTTTGTTGATCAGGTAATTGCAACTGGTGCACTGATCCTGTGCATTCTGGCC
ATCACTGACAAGAAGAATATTGGCGCCCCGAAAGGCATGGAGCCCCTGTGCATCGGCCTG
ACCATCATGGCCATCGGGGTTTCCATGGGCCTGAACTGTGGCTATCCCATAAACCCGGCA
CGAGACCTCGGACCACGCCTTTTCACAGCTGTGGCTGGGTGGGGCATTGACGTTTTCAGT
GCTGGAGGCTGCTGGTGGTGGATTCCTGTCGCAGGACCCATGGTGGGGGGAGCCGTCGGA
GCCGGGGTCTACTTTCTCTTCATCGAGCTGCACCAAGCCGAC------------------
---------TCTGAGAAACAG---------------------------------------
---------GAGGACAACAAC------ATCCAGGACAAATATGAAGTTATTGCACTGAGT
>Annual_killi_Aqp9b
---------------ATGGAAGATGAGAGCAGGAGGAAGCTGAAGGAGAAATTTGGCCTG
AGGCAGGACATCTTTAAGGAGTTCCTGGCAGAATTTCTGGGGATATTTGTCCTGATACTC
TTTGGATGCGGTTCAGTGGCCCAGACCGTGCTGAGCAGAGGGGCTCTYGGAGAGCCGCTG
ACCGTCCACATTGGTTTCACCCTGGGAGTCATGATGGCCGTCTACATGGCAGGAGGAGTC
TCAGGAGCCCATGTGAACCCTGCTGTCTCTCTGGCCATGGTGATTCTGGGGAAGCTACCG
TTGAAAAAGTTTCCTGTGTACGTCTTGGCACAGTTCCTGGGAGCTTTTGCTGGATCCTGT
GCTGTCTTTGGATTATATTATGATGCTTTAATGGATTACACCAATGGAGAGTTTGCCGTA
ACTGGAGTAAATGCCACAGCCAATATATTTGCATCTTATCCTGCAAGTCATCTCTCAGTC
CTCAATGGGTTTGTTGATCAGGTAATTGCGACAGGTGCGCTGATCCTGTGCATCCTGGCC
ATCACGGACAGGAAGAATATTGGCGCCCCGAAAGGCATGGAGCCCCTGTGCATCGGGCTG
ATCATCATGGCCATCGGGGTTTCCATGGGTCTGAACTGTGGCTATCCCATAAACCCGGCC
CGGGACCTCGGCCCGCGCCTTTTCACAGCTGTGGCTGGGTGGGGCATGGAGGTGTTCAGA
GCCGGGGGCTGCTGGTGGTGGATCCCTGTGGCAGGACCCATGGTGGGTGGAGCGGTTGGA
GCAGTAGTTTACTTTGTCTTCATTGATCTGCACCAACATGAA------------------
---------CCTGAGAGACGG---------------------------------------
---------GCGGACAAT---------GTCCAGGACAAATATGAAGTTATTGCACTCAGT
>Turquoise_killifish_Aqp9b
---------------ATGGAAGAGGAGAGCAGGAGGAAGATGAAGGAGAAGTTCGGCTTG
AGGCAGAACATCTTTAAAGAGTTCCTGGCGGAATTTCTGGGGATCTTTGTCCTGATACTC
TTTGGTTGCGGTGCAGTGGCCCAGACTGTGCTGAGCAAAGGTGCTCTGGGAGAGCCACTC
ACCATCCACGTTGGTTTCACTCTGGGGGTCATGATGGCTGTCTACATGGCAGGGGGAGTT
TCAGGAGCTCATGTGAACCCTGCAGTCTCTCTGGCCATGGTGATTCTGGGGAAATTACCT
CTGAAGAAGTTTCCCGTGTACGTGGTGGCACAGTTCCTGGGTGCTTTTGCTGGGTCCTGT
GCTGTTTTTGTGTTGTATTATGATGCTCTAATGGACCACACCAACGGACAGTTTGAAGTT
ACTGGTGCAAACGCCACCGCTAACATCTTTGCATCTTATCCTGCTAAACACCTCTCTGTT
CTCAACGGGTTTGTTGATCAGGTAATCGCAACTGGTGCACTGATCCTGTGCATTCTGGCA
ATCACCGACAGGAAGAATATCGGCGCCCCTAAAGGAATGGAGCCTCTGTGTATTGGACTG
ATCATCATGGCCATTGGGGTTTCCATGGGTCTGAACTGTGGCTATCCCATAAACCCGGCA
CGGGACCTCGGGCCGCGTCTTTTCACAGCAGTGGCAGGGTGGGGCATGGAGGTGTTCAGT
GCTGGAGGCTGTTGGTGGTGGATCCCTGTTGCAGGACCCATGGTGGGCGGAGCCATTGGA
GCTGGGATTTACTTTGTCTTCATTGAGCTGCACCAACATGAA------------------
---------CCTGAGAGACAA---------------------------------------
---------GTAGACAACAAC------GTCCAGGACAAATATGAAGTTATTGCACTGAGT
>Japanese_medaka_Aqp9b

---------------ATGGGAGATGACAGCAGGAGGAAGATGAAGGAGAAATTTGGCCTG
AGACGGGACATCTTTAAAGAGTTCCTGGCGGAATTTCTTGGGATATTTGTCCTGATACTC
TTTGGATGCGGTTCAGTGGCTCAGGCGGTGCTGAGTAAAGGGGCTCTTGGGGAGCCGCTC
ACCATCCATGTCGGTTTTACCCTGGGAGTCATGATGGCCGTCTACATTGCAGGAGGAGTG
TCAGGAGGTCATGTGAACCCTGCTGTCTCCCTGGCCATGGTCATTTTAGGAAAGCTACCA
CTGAAGAAGTTTCCAGTGTATGTAGCAGCTCAGTTCTTGGGAGCTTTTGCTGGATCCTGT
GCAGTCTTTGGCTTGTATTATGATGCTTTGAGGGAATTCACCAATGGAGAGTTTGCAGTA
ACTGGTGCCAATGCAACAGCTGGTATATTTGCATCGTATCCTGCAAAACATCTTTCTGTC
ATTAATGGATTTGTTGATCAGGTTATTGCAACTGCTGCACTGGTCCTCTGCATCCTGGCC
ATCACTGACAGAAAGAATATCGGTGCCCCCAAAGGCATGGAGCCTTTGTGCATCGGTTTG
ATCATCTTGGCCATTGGGGTGTCCATGGGTCTGAACTGTGGGTATCCCATAAACCCAGCA
CGAGACCTTGGCCCGCGGCTCTTCACGGCTGTTGCTGGATGGGGCATGGATGTGTTCAGG
GCTGGAGGCTGCTGGTGGTGGATTCCTGTGGCAGGGCCCATGGTGGGTGGAGCAGTTGGA
GCTGGAATTTACTTTCTTATCATTGACCTGCACCAACCAGAG------------------
---------TCCGACAGACAG---------------------------------------
---------GTGGATAACAAC------GTCCAGGACAAATATGAAATTGTTACCATGAAT
>Javanese_ricefish_Aqp9b
---------------ATGGAAGATGAGAGCAGGAGGAAGATGAAGGAGAAATTTGGCCTG
AGACGGGACATCTTTAAAGAGTTCCTGGCGGAATTTCTTGGAATATTTGTCCTGATACTC
TTTGGATGCGGTTCAGTGGCTCAGGCAGTGCTGAGCAAAGGGGCTCTCGGGGAGCCACTC
ACCATCCATGTGGGTTTTACTCTGGGAGTCATGATGGCCGTCTACGTTGCAGGGGGAGTG
TCAGGTGGTCACGTAAACCCTGCTGTCTCCCTCGCCATGGTCATTTTAGGAAAGCTGCCA
CTGAAGAAGTTTCCAGTGTATGTTGCAGCTCAGTTCTTGGGGGCTTTTGCTGGATCCTGT
GCAGTGTTTGGATTGTATTATGATGCTTTGATGGAATTCACCAATGGAGAGTTTGCAGTA
ACTGGAGCCAATGCAACAGCTGGTATATTTGCATCCTATCCTGCAAAACATCTTTCTGTC
CTTAATGGATTTGTTGATCAGGTCATTGCAACGGCTGCACTGGTCCTGTGCATCCTGGCC
ATCACCGACAGAAAGAATATTGGTGCCCCCAAAGGCATGGAGCCTCTGTGCATCGGCTTG
ATCATTTTGGCCATTGGAGTGTCCATGGGTCTGAACTGTGGATATCCCATAAACCCAGCA
CGAGACCTCGGCCCGCGGCTCTTCACAGCGGTTGCTGGGTGGGGCATGGATGTGTTCAGG
GCTGGAGGCTGCTGGTGGTGGATTCCTGTGGCAGGACCCATGGTGGGTGGAGCGGTTGGA
GCTGGCATTTACTTTCTTATGATTGACCTGCACCAGCCGGAG------------------
---------TCTGAAAGACAG---------------------------------------
---------GTGGACAACAAC------GTCCAGGACAAATATGAAATTGTTACTATGAAC
>Indian_medaka_Aqp9b
---------------ATGGAAGATGAGAGCAGGAGGAAGATGAAGGAGAAATTTGGCCTG
AGACGGGACATCTTTAAAGAGTTCCTGGCGGAATTTCTTGGGATATTTATCCTGATACTC
TTTGGATGCGGTTCAGTGGCTCAGGCGGTGCTGAGCAAAGGGGCTCTCGGGGAGCCACTC
ACCATCCATGTGGGTTTTACTCTGGGAGTCATGATGGCCGTCTACGTTGCAGGGGGAGTG
TCAGGAGGTCACGTGAACCCTGCTGTCTCCCTGGCCATGGTCATTTTAGGAAAGCTGCCA
CTGAAGAAGTTTCCAGTGTACGTCGCAGCTCAGTTCTTGGGGGCTTTTGCTGGATCCTGT
GCAGTCTTTGGGTTGTATTATGATGCTTTGATGGAATTCACCAATGGAGAGTTTGCAGTG
ACTGGAGCCAATGCAACAGCTGGAATATTTGCATCATATCCTGCAAAACATCTTTCTGTC
CTTAATGGATTCGTTGATCAGGTCATTGCAACTGCTTCATTGGTCCTGTGCATCCTGGCC
ATCACCGACAGAAAGAATATTGGTGCCCCCAAAGGCATGGAACCTCTGTGCATCGGCTTG
ATCATCTTGGCCATTGGAGTGTCCATGGGTCTGAACTGTGGATATCCCATAAACCCAGCA
CGAGACCTCGGCCCGCGGCTCTTTACAGCGGTTGCTGGGTGGGGCATGGATGTGTTCAGG
GCTGGAGGCTGCTGGTGGTGGATTCCTGTGGCAGGACCCATGGTGGGTGGAGCGGTTGGA
GCTGGGATTTACTTTCTTATCATTGACCTGCACCAACCTGAG------------------
---------TCTGACAGACAG---------------------------------------
---------GTGGACAACAAC------GTTCAGGACAAATATGAAATTGTTACTATGAAC
>Desert_rainbowfish_Aqp9b
---------------ATGGAAAATGAGAGCAGAAGAAAAATGAAGGAGAAATTTGGCCTG
AGGCGGGACATCTTTAAGGAGTTCCTGGCGGAATTTCTGGGGATATTTGTCCTGATACTC
TTTGGATGCGGTTCAGTGGCCCAGACGGTGCTGAGTAAAGGGGCGCTCGGGGAGCCGCTG
ACCATCCATGTTGGTTTTACTCTGGGAGTCATGATGGCCGTCTTCATGGCAGGGGGAGTG
TCAGGAGGCCATGTAAACCCTGCTGTCTCTCTGGCGATGGTTATTCTGGGGAAGCTGCCT
CTCAAGAAGTTCCCCGTGTATGTGGCAGCACAGTTCCTTGGGGCTTTTGCTGGATCCTGC
GCTGTTTTTGGTTTGTATTACGATGCATTGATGGACTACACCAAAGGAGAATTTGCAGTT
ACTGGTCCAAATGCCACCGCCAACATATTCGCATCTTATCCTGCAGAGCATCTCTCAGTC
ATAAATGGGTTTGTTGATCAGGTAATTGCAACTGGAGCACTGATCCTGTGCATCTTGGCA
ATCACTGACAGGAAGAATATTGGTGCCCCGAAAGGCATGGAGCCTCTGTGCGTTGGCCTG

ATCATCATGGCTATCGGAGTGTCCATGGGCCTAAACTGTGGCTATCCAATAAATCCGGCA
CGAGACCTCGGCCCACGGTTCTTCACAGCTGTGGCTGGGTGGGGCATGGAAGTGTTCAGA
GCTGGGGGCTGTTGGTGGTGGATCCCTGTGGCAGGACCCATGGTGGGTGGAGCAGTTGGA
GCTGGAATTTACTTTCTCTTCATTGACTTACACCAACCTGAG------------------
---------CCTCAAAAACAG---------------------------------------
---------GAGGACAACAAT------GTCCAGGACAAATATGAAATAATTACTATGAGT
>Clown_anemonefish_Aqp9b
---------------ATGGAAAATGAGAGCAAGAGGAAGATGAAGGAGAGATTTGGCCTG
AGGCGGGACATCTTTAAGGAGTTCCTGGCGGAGTTTCTGGGGATATTTGTCCTGATACTC
TTTGGATGCGGTTCAGTGGCCCAGACGGTGCTGAGTAAAGGGGCTCTCGGGGAGCCGCTG
ACCATCCACGTTGGCTTCACTCTGGGTGTCATGATGGCCGTCTACATGGCCGGGGGAGTG
TCAGGAGCCCATGTGAACCCTGCAGTCTCTCTGGCCATGGTGATCCTGGGGAAGCTGCCT
CTGAAGAAGTTCCCTGTGTATGTGGTAGCACAGTTCCTGGGGGCTTTTGCTGGATCCTGT
GCTGTCTATGGGTTGTATTATGATGCTTTGATGGACTATACTAATGGAGAATTTGCAGTT
ACTGGTGCAAACGCCACAGCCAACATATTTGCATCTTATCCTGCAAAACATCTTTCAGTC
CTCAATGGGTTCGTGGATCAGGTAATTGCAACTGGCGCACTGATCCTGTGCATCCTGGCC
ATCACTGACAGGAAGAATATTGGTGCTCCCAAAGGGATGGAGCCTCTGTGCATCGGCCTC
ATTATCATGGCCATCGGAGTGTCCATGGGTCTGAACTGTGGTTACCCAATCAACCCGGCC
CGAGACCTCGGCCCGCGCTTCTTCACAGCCGTAGCTGGGTGGGGCATGGAAGTATTCAGG
GCCGGAGGCTGCTGGTGGTGGATTCCTGTGGCAGGTCCTATGGTGGGTGGAGCAGTCGGA
GCAGGCATTTACTTTCTCTTCATTGAGCTGCACCATCCAGAG------------------
---------CCTGAAAAACAG---------------------------------------
---------GAGGAGAACAAC------GTCCAGGACAAATATGAAATTATAACTATGAGC
>Orange_clownfish_Aqp9b
---------------ATGGAAAATGAGAGCAAGAGGAAGATGAAGGAGAGATTTGGCCTG
AGGCGGGACATCTTTAAGGAGTTCCTGGCGGAATTTCTGGGGATATTTGTCCTGATACTC
TTTGGATGCGGTTCAGTGGCCCAGACGGTGCTGAGTAAAGGGGCTCTCGGGGAGCCGCTG
ACCATCCACGTTGGCTTCACACTGGGTGTCATGATGGCCGTCTACATGGCCGGGGGAGTG
TCAGGAGCCCATGTGAACCCTGCAGTCTCTCTGGCCATGGTGATCCTGGGGAAGCTGCCT
CTGAAGAAGTTCCCTGTGTATGTGGTAGCACAATTCCTGGGGGCTTTTGCTGGATCCTGT
GCTGTCTATGGGTTGTATTATGATGCTTTGATGGACTATACTAATGGAGAATTTGCAGTT
ACTGGTGCAAACGCCACAGCCAACATATTTGCATCTTATCCTGCAAAACATCTTTCAGTC
CTCAATGGGTTCGTGGATCAGGTAATTGCAACTGGCGCACTGATCCTGTGCATCCTGGCC
ATCACTGACAGGAAGAATATTGGTGCTCCCAAAGGGATGGAGCCTCTGTGCATCGGCCTC
ATTATCATGGCCATCGGAGTGTCCATGGGTCTGAACTGTGGTTACCCAATCAACCCGGCC
CGAGACCTCGGCCCGCGCTTCTTCACAGCCGTAGCTGGGTGGGGCATGGAAGTATTCAGG
GCCGGAGGCTGCTGGTGGTGGATTCCTGTGGCAGGTCCTATGGTGGGTGGAGCAGTCGGA
GCAGGCATTTACTTTCTCTTCATTGAGCTGCACCATCCAGAG------------------
---------CCTGAAAAACAG---------------------------------------
---------GAGGAGAACAAC------GTCCAGGACAAATATGAAATTATAACTATGAGC
>Twoband_anemonefish_Aqp9b
---------------ATGGAAAATGAGAGCAAGAGGAAGATGAAGGAGAGATTTGGCCTG
AGGCGGGACATCTTTAAGGAGTTCCTGGCGGAGTTTCTGGGGATATTTGTCCTGATACTC
TTCGGATGTGGTTCAGTGGCCCAGACGGTGCTGAGTAAAGGGGCTCTCGGGGAGCCGCTG
ACCATCCACGTTGGCTTCACTCTGGGTGTCATGATGGCTGTCTACATGGCCGGGGGAGTG
TCAGGAGCCCATGTGAACCCTGCAGTCTCTCTGGCCATGGTGATCCTGGGGAAGCTGCCT
CTGAAGAAGTTCCCTGTGTATGTGGTAGCACAGTTCCTGGGGGCTTTTGCTGGATCCTGT
GCTGTCTATGGGTTGTATTATGATGCTTTGATGGACTACACTAACGGAGAATTTGCAGTT
ACTGGTGCAAACGCCACAGCCAACATATTTGCATCTTATCCTGCAAAACATCTGTCAGTC
CTCAATGGGTTCGTGGATCAGGTAATTGCAACTGGCGCACTGATCCTGTGCATCCTGGCC
ATCACTGACAGGAAGAATATCGGTGCTCCCAAAGGGATGGAGCCTCTGTGCATCGGCCTC
ATTATCATGGCCATCGGAGTGTCCATGGGTCTGAACTGTGGTTACCCAATCAACCCAGCC
CGAGACCTCGGCCCACGCTTCTTCACGGCCGTAGCTGGGTGGGGCATGGAAGTATTCAGA
GCCGGAGGCTGCTGGTGGTGGATTCCTGTGGCAGGGCCCATGGTGGGTGGAGCAGTCGGA
GCAGGCATTTACTTTCTCTTCATTGAGCTGCACCATCCAGAG------------------
---------CCTGAAAAACAG---------------------------------------
---------GAGGAGAACAAC------GTCCAGGACAAATATGAAATTATAACTATGAGC
>Spiny_chromis_Aqp9b
---------------ATGGAAAATGAGAGCAAGAGGAAGATGAAGGAGAGATTTGGCCTG
AGGCGAGACATCTTTAAGGAGTTCCTGGCGGAGTTTCTGGGGATATTTATCCTGATACTC
TTTGGATGTGGTTCAGTGGCCCAGACGGTGCTGAGTAAAGGGGCTCTGGGGGAGCCGCTG

ACCATCCATGTTGGCTTCACTCTGGGAGTCATGATGGCCGTCTACATGGCTGGGGGAGTG
TCAGGAGCCCATGTGAACCCTGCAGTCTCTCTGGCCATGGTGATCCTGGGGAAGCTGCCT
CTGAAGAAGTTCCCTGTGTATGTGGTAGCACAGTTCCTGGGGGCTTTTGCTGGATCCTGT
GCTGTCTATGGGTTGTATTATGATGCTTTGATGGACTACACTAACGGAGAATTTGCAGTT
ACTGGTGCAAACGCCACAGCCAACATATTTGCATCTTATCCTGCAAAACATCTCTCAGTC
CTAAATGGGTTCGTGGATCAGGTAATTGCAACTGGCGCACTGATCCTGTGCATCCTGGCC
ATCACTGACAGGAAGAATATTGGTGCTCCCAAAGGGATGGAGCCTCTGTGCATCGGCCTC
ATCATCATGGCCATCGGAGTGTCCATGGGTCTGAACTGTGGTTACCCGATCAACCCGGCC
CGAGACCTCGGCCCGCGCTTCTTCACGGCCGTAGCTGGGTGGGCATGGAAGTATTCAGG
GCTGGAGGCTGCTGGTGGTGGATTCCTGTGGCAGGGCCCATGGTGGGTGGAGCAGTCGGA
GCTGGCATTTACTTTCTCTTCATTGAGCTGCACCATCCAGAG------------------
---------CCTGAAAAACAG---------------------------------------
---------GAGGAGAACAAC------GTCCAGGACAAATATGAAATTATAACTATGAGC
>Bicolor_damselffish_Aqp9b
----------------ATGGAAAATGAGAGCAAGAGGAAGCTGAAGGAGAGATTTGGCCTG
GAGCGGGACATCATAAAGGAGTTCCTGGCGGAGTTTCTGGGGATATTCGTCCTGATACTC
TTCGGATGTGGTTCAGTGGCCCAGACGGTGCTGAGTAAAGGGGCTCTCGGGGAGCCGCTG
ACCATCCACGTCGGCTTCACTCTGGGAGTCATGATGGCCGTCTACATGGCCGGGGGAGTG
TCAGGAGCCCACGTGAACCCTGCGGTCTCTCTGGCCATGGTGATCCTGGGGAAGCTGCCG
CTGAAGAAGTTCCCCGTGTACGTGGCAGCGCAGTTCCTGGGGGCTTTTGTTGGATCCGGC
GTCGTCTATGGCTTGTACTATGATGCTCTGATGGACTACACTAACGGAGAATTTGCAGTG
ACTGGTGCAAATGCCACAGCCAACATATTTGCATCTTATCCTGCAAAACATCTCTCGGTC
CTCAATGGATTCATGGATCAGGTAATTGCAACCGGCGCACTGATCCTGTGCATCCTGGCC
ATCACCGACAGGAAGAACATCGGCGCTCCAAAAGGCATGGAGCCTCTGTGCATCGGCCTC
ATCATCATGGCCATCGGAGTGTCCATGGGTCTGAACTGCGGTTACCCAATCAACCCGGCC
CGAGACCTCGGCCCGCGCTTCTTCACCGCCGTGGCTGGGTGGGCATGGAGGTGTTCAGA
GCTGGAAGCTGCTGGTGGTGGATTCCCGTGGCGGGGCCCATGGTGGGTGGAGCGGTCGGA
GCCGCCGTTTACTTTCTCTTCATTGAGCTGCACCATCCCGAG------------------
---------CCTGAAAAACAG---------------------------------------
---------GAGGAGAACAAC------GTCCAGGACAAATACGAAATCATAACCATGAAC
>Zebra_mbuna_Aqp9b
----------------ATGGAAAACGAATACAGGAGGAAGATGAAGGAGAAACTTGGCCTG
AGGCGAGACATCTTTAAGGAGTTCCTGGCGGAGTTTCTGGGGATATTTTTCCTGATACTC
TTTGGATGTGGTTCGGTGGCCCAGATGGTCCTGAGCAGAGCAGGTCTCGGTGACATATTG
AGCGTCCACATTGGTTTCACTCTTGGAGTCATGATGGCCGTCTATATTGCAGGGGGAGTG
TCAGGAGCCCATGTGAACCCGGCAGTCTCTCTGGCCATGCTGATCCTGGGGAAGCTGCCT
CTAAAGAAGTTCCCCATATACGTGGCAGCACAATTCCTGGGGGCTTTTGTTGGATCATGT
GCCGTCTATGGATTGTATTATGATGCTTTGATGGACTTTACCAAAGGAGAATTTATTGTT
ACAGGTGAAAATGCCACAGCCACCATATTTGCAAGCTATCCTTCAAAACATCTGTCTGTC
CTAAACGGGCTTGGTGATCAGGTAATTGCAACCGCTGCACTGGTCATATGCATCCTGGCT
ATCACTGACAGGAAGAATATCGGCGCTCCAAAGGGCATGGAGCCTCTGAGTATTGGCCTG
ATCATCGCAGGCATTGGAGTGTCCATGAGTCTGAACTGTGGCTATCCAATCAACCCGGCT
CGAGACCTTGGCCCCCGTTTCTTCACAGCTTTGGCTGGATGGGGCATGGAAGTTTTCAGA
GCTGGAGGCTGCTGGTGGTGGATTCCTGTGGTAGGGCCCATGGTGGGTGGTGCAGTGGGA
GCAGGTGTTTACCTTATCTTCATTGAGCTGCACCACCATGAA------------------
---------CCTGAAAAACAA---------------------------------------
---------GAGGAGAACAAT------GTCCAGGACAAATATGAGATCGTAACTATGACT
>Eastern_happy_Aqp9b
----------------ATGGAAAACGAATACAGGAGGAAGATGAAGGAGAAACTTGGCCTG
AGGCGAGACATCTTTAAGGAGTTCCTGGCGGAGTTTCTGGGGATATTTTTCCTGATACTC
TTTGGATGTGGTTCGGTGGCCCAGATGGTCCTGAGCAGAGCAGGTCTCGGTGACATATTG
AGCGTCCACATTGGTTTCACTCTTGGAGTCATGATGGCCGTCTATATTGCAGGGGGAGTG
TCAGGAGCCCATGTGAACCCGGCAGTCTCTCTGGCCATGCTGATCCTGGGGAAGCTGCCT
CTAAAGAAGTTCCCCATATACGTGGCAGCACAATTCCTGGGGGCTTTTGTTGGATCATGT
GCCGTCTATGGATTGTATTATGATGCTTTGATGGACTTTACCAAAGGAGAATTTATTGTT
ACAGGTGAAAATGCCACAGCCACCATATTTGCAAGCTATCCTTCAAAACATCTGTCTGTC
CTAAACGGGCTTGGTGATCAGGTAATTGCAACCGCTGCACTGGTCATATGCATCCTGGCT
ATCACTGACAGGAAGAATATCGGCGCTCCAAAGGGCATGGAGCCTCTGAGTATTGGCCTG
ATCATCGCAGGCATTGGAGTGTCCATGAGTCTGAACTGTGGCTATCCAATCAACCCGGCT
CGAGACCTTGGCCCCCGTTTCTTCACAGCTTTGGCTGGATGGGGCATGGAAGTTTTCAGA
GCTGGAGGCTGCTGGTGGTGGATTCCTGTGGTAGGGCCCATGGTGGGTGGCGCAGTGGGA

GCAGGTGTTTACCTTATCTTCATTGAGCTGCACCACCCTGAA------------------
---------CCTGAAAAACAA--
---------GAGCAGAACAAT------GTCCAGGACAAATATGAGATCGTAACTATGACT
>Red_mwanza_Aqp9b
---------------ATGGAAAACGAATACAGGAGGAAGATGAAGGAGAAACTTGGCCTG
AGGCGAGACATCTTTAAGGAGTTCCTGGCGGAGTTTCTGGGGATATTTTTCCTGATACTC
TTTGGATGTGGTTCGGTGGCCCAGATGGTCCTGAGCAGAGCAGGTCTCGGTGACATATTG
AGCGTCCACATTGGTTTCACTCTTGGAGTCATGATGGCCGTCTATATTGCAGGGGGAGTG
TCAGGAGCCCATGTGAACCCGGCAGTCTCTCTGGCCATGCTGATCCTGGGGAAGCTGCCT
CTAAAGAAGTTCCCCATATACGTGGCAGCACAATTCCTGGGGGCTTTTGTTGGATCATGT
GCCGTCTATGGATTGTATTATGATGCTTTGATGGACTTTACCAAAGGAGAATTTATTGTT
ACAGGTGAAAATGCCACAGCCACCATATTTGCAAGCTATCCTTCAAAACATCTGTCTGTC
CTAAACGGGCTTGGTGATCAGGTAATTGCAACCGCTGCACTGGTCATATGCATCCTGGCT
ATCACTGACAGGAAGAATATCGGCGCTCCAAAGGGCATGGAGCCTCTGAGTATTGGCCTG
ATCATCGCAGGCATTGGAGTGTCCATGAGTCTGAACTGTGGCTATCCAATCAACCCGGCT
CGAGACCTTGGCCCCCGTTTCTTCACAGCTTTGGCTGGATGGGGCATGGAAGTTTTCAGA
GCTGGAGGCTGCTGGTGGTGGATTCCTGTGGTAGGGCCCATGGTGGGTGGCGCAGTGGGA
GCAGGTGTTTACCTTATCTTCATTGAGCTGCACCACCCTGAA------------------
---------CCTGAAAAACAA--
---------GAGGAGAACAAT------GTCCAGGACAAATATGAGATCGTAACTATGACT
>Burtons_mouthbrooder_Aqp9b
---------------ATGGAAAACGAATACAGGAGGAAGATGAAGGAGAAACTTGGCCTG
AGGCGAGACATCTTTAAGGAGTTCCTGGCGGAGTTTCTGGGGATATTTTTCCTGATACTC
TTTGGATGCGGTTCGGTGGCCCAGATGGTCCTGAGCAGAGCAGGTCTCGGTGACATATTG
AGCGTCCACATTGGTTTCACTCTTGGAGTCATGATGGCCGTCTATATTGCAGGGGGAGTG
TCAGGAGCCCATGTGAACCCAGCAGTCTCTCTGGCCATGCTGATCCTGGGGAAGCTGCCT
CTAAAGAAGTTCCCCATATACGTGGCAGCACAATTCCTGGGGGCTTTTGTTGGATCATGT
GCCGTCTATGGATTGTATTATGATGCTTTGATGGACTTTACCAAAGGAGAATTTATTGTT
ACAGGTGAAAATGCCACAGCCACCATATTTGCAAGCTATCCTTCAAAACATCTGTCTGTC
CTAAACGGGCTTGGTGATCAGGTAATTGCAACCGCTGCACTGGTCATATGCATCCTGGCT
ATCACTGACAGGAAGAATATTGGCGCTCCAAAGGGCATGGAGCCTCTGAGCATTGGCCTG
ATCATCGCAGGCATTGGAGTGTCCATGAGTCTGAACTGTGGCTATCCAATCAACCCGGCT
CGAGACCTTGGCCCCCGTTTCTTCACAGCTTTGGCTGGATGGGGCATGGAAGTTTTCAGA
GCTGGAGGCTGCTGGTGGTGGATTCCTGTGGTAGGGCCCATGGTGGGTGGCGCAGTGGGA
GCAGGTGTTTACCTCATCTTCATTGAGCTGCACCACCCTGAA------------------
---------CCTGAAAAACAA--
---------GAGGAGAACAAT------GTCCAGGACAAATATGAGATCGTAACTATGACT
>Lyretail_cichlid_Aqp9b
---------------ATGGAAAACGAATACAGGAGGAAGATGAAGGCGAAACTTGGCCTG
AGGCGAGACATCTTTAAGGAGTTCCTGGCGGAGTTTCTGGGGATATTTTTCCTGATACTC
TTTGGATGCGGTTCGGTGGCCCAGATGGTCCTGAGCAGAGCACGTCTCGGTGACATACTG
AGCGTCCACATTGGTTTCACTCTTGGAGTCATGATGGCCGTCTATATTGCAGGGGGAGTG
TCAGGAGCCCATGTGAACCCGGCAGTCTCTCTGGCCATGCTGATCCTGGGGAAGCTGCCT
CTAAAGAAGTTCCCCATATACGTGGCAGCACAATTCCTGGGGGCTTTTGTTGGATCATGT
GCCGTCTATGGATTGTATTACGATGCTTTGATGGACTTTACCAAAGGAGAATTTATTGTT
ACAGGTGAAAATGCCACAGCCACCATATTTGCAAGCTATCCTGCAAAACATCTGTCTGTC
CTAAACGGGCTTGGTGATCAGGTAATTGCAACCGCCACACTGGTCATATGCATCCTGGCT
ATCACTGATAGGAAGAATATTGGCGCTCCAAAGGGCATGGAGCCTCTGAGCATTGGCCTG
ATCATCGCAGGCATTGGAGTGTCCATGAGTCTGAACTGTGGCTATCCAATCAACCCGGCT
CGAGACCTTGGCCCCCGTTTCTTCACAGCTTTGGCTGGATGGGGCATGGAAGTTTTCAGA
GCTGGAGGCTGCTGGTGGTGGATTCCTGTGGTAGGGCCCATGGTGGGTGGCGCAGTTGGA
GCAGGTGTTTACCTTATCTTCATTGAGCTGCACCACCCTGAA------------------
---------CCTGAAAAACAA--
---------GAGGAGAACAAT------GTCCAGGACAAATATGAGATCGTAACTATGACT
>Nile_tilapia_Aqp9b
---------------ATGGAAAACGAATACAGGAGGAAGATGAAAGAGAAACTTGGCCTG
AGGCGAGACATCTTTAAGGAGTTCCTGGCGGAGTTTCTGGGGATATTTTTCCTGATACTC
TTTGGATGCGGTTCGGTGGCCCAGATGGTCCTGAGCAGAGCAGGTCCTGGTGACATACTG
AGCGTCCACATTGGTTTCACTCTTGGAGTCATGATGGCCGTCTATATTGCAGGGGGAGTG
TCAGGAGCCCACGTGAACCCGGCAGTCTCTCTGGCCATGCTGATCCTGGGGAAGCTGCCT
CTAAAGAAGTTCCCCATATACGTGGCAGCACAGTTCCTGGGGGCTTTTGCTGGATCATGT

GCCGTCTATGGATTGTATTATGATGCTTTGATGGACTTTACCAAAGGAGAATTTATTGTT
ACAGGTGAAAATGCCACAGCCACCATATTTGCAAGTTATCCTGCAAAACATCTGTCTGTC
CTAAACGGGCTTGGTGATCAGGTAATTGCAACCGCTGCACTGGTCATATGCATCCTGGCT
ATCACTGACAGGAAGAATATTGGCGCTCCAAAGGGCATGGAGCCTCTGTGCATTGGCCTG
ATCATCGCGGCCATTGGAGTGTCCATGAATCTGAACTGTGGCTATCCAATCAACCCGGCT
CGAGACCTCGGCCCCCGTTTCTTCACGGCTTTGGCCGGATGGGGCATGGAAGTTTTCAGG
GCTGGAGGCTGCTGGTGGTGGATTCCTGTGGTAGGACCCATGGTGGGTGGCGCAGTTGGA
GCAGGTGTTTACCTTATCTTCATTGAGCTGCACCACCCTGAA------------------
---------CCTGAAAAACAA---------------------------------------
---------GAGGAGAACAAT------GTCCAGGACAAATATGAGATTGTAACTATGACT
>Midas_cichlid_Aqp9b
---------------ATGGAAAACGAATACAGGAAGAAGATAAAAGAGAAACTTGGCCTG
AGGCGGGACATCTTTAAGGAGTTCTTGGCGGAGTTTCTGGGCATATTTTTCTTGATACTC
TTTGGGTGTGGCTCAGTGGCCCAGATGGTGCTGAGCAGAACACATCTCTGTGACATACTG
AGCGTCCACATTGGCTTCACTCTTGGAGTCATGATGGCCGTCTACATCGCAGGGGGAGTG
TCAGGAGGCCATGTGAACCCTGCGGTCTCTCTGGCCATGGTGATCCTGGGGAAGCTGCCT
CTGAAGAAATTCCCTGTGTATGTGGCGGCACAGTTCCTGGGGGCTTTTGCTGGATCTTGT
GCCGTCTATGGATTATATTATGATGCTTTGATGGACTTTACCAAAGGAGAATTTATTGTT
ACAGGTGAAAATGCCACAGCCACCATATTTGCAAGTTACCCCTTAAAACATCTCTCTGTC
CTAAACGGGCTTGTTGATCAGGTAATTGCAACCGCTGCGCTGGTCATATGCATCCTGGCT
ATCACTGACAGGAAGAATATTGGTGCTCCAAAAGGCATGGAGCCTCTGTGCATTGGTCTG
ATCATCATGGGCATCGGAGTGTCCATGAGCCTGAACTGTGGTTATCCAATCAACCCGGCA
CGAGACCTCAGCCCTCGTTTCTTCACCGCTTTGGCTGGATGGGGCATGGAAGTATTCAGG
GCTGAAGGCTGCTGGTGGTGGATTCCTGTGGTAGGGCCCATGGTGGGTGGCGCAGTTGGA
GCAGGCGTTTATGCTCTCTTCATTGAGCTGCACCACCCTGAG------------------
---------CCTGAAAAACAA---------------------------------------
---------GAGGAGAACAAT------GTCCAGGACAAATATGAGATCATAACTATGACT
>Indian_glassy_fish_Aqp9b
---------------ATGGAAAGTGAGAGCAGGAGGAAGATGAAGGAGAAATTTGGCCTG
AGGCGGGACATCTTTAAGGAGTTCCTGGCTGAATTTCTGGGGATATTCGTGCTGATACTC
TTTGGATGTGGTTCAGTGGCCCAGACGGTGCTGAGTAAAGGGGCTCTCGGGGAGCCGTTG
ACCATCCACATCGGTTTCACTCTGGGAGTCATGATGGCCGTCTACATGGCAGGGGGAGTG
TCAGGAGCCCATGTGAACCCTGCCGTCTCTCTGGCCATGGTGATCCTGGGGAAACTACCT
CTGAAGAAGTTCCCTGTGTATGTGGCAGCACAGTTCCTGGGGGCTTTTGCCGGTTCCTGT
GCTGTCTATGGGTTGTATTACGATGCTTTGATGGACTACACCAATGGAGAATTTGCAGTT
ACTGGTGCAAACGCCACAGCCAATATTTTTGCATCGTATCCTGCAAAACATCTGTCAGTG
CTCAATGGGTTTGTTGATCAGGTAATTGCAACCGGCGCACTGATCCTCTGCATCCTGGCC
ATCACTGACAGGAAGAACATTGGTGCACCAAAAGGCATGGAGCCTCTGTGCATCGGTCTG
ATCATCATGGCCATCGGTGTGTCCATGGGTCTGAACTGTGGCTATCCAATCAACCCGGCA
CGAGACCTCGGCCCACGGATCTTCACGGCTGCGGCTGGGTGGGGCATGGACGTATTCAGA
GCTGGAGGCTGCTGGTGGTGGGTTCCTGTGGCCGGGCCCATGGTGGGTGGAGCAGTTGGA
GCAGGTGTTTACTTTCTGTTTGTTGAGCTGCATCACACTGAG------------------
---------CCTGAAAAACAG---------------------------------------
---------GAGGACAACCAC------GTCCAGGACAAATACGAAATCGTAACTATGACT
>Rockpool_blenny_Aqp9b
---------------ATGGAAAGTGAGAAGAAGAGGAAGATGAAGGAGAAATTTGGCCTG
AGGCGGGACATCTTTAAGGAGTTCCTGGCGGAATCTCTGGGGATATTCGTCCTGATACTC
TTTGGATGTGGTTCAGTGGCCCAGACCGTGCTGAGTAAAGGGGCTGTCGGCGAGCCACTC
ACCATCCACATTGGTTTCACCCTGGGAGTCATGATGGCCGTTTACATGGCAGGTGGAGTG
TCAGGAGCTCATGTGAACCCTGCAGTCTCTCTGGCTATGGTGATCCTGGGGAAGCTCCCC
CTGAAGAAGTTTCCTGTGTATGTGTCGGCACAGTTCCTGGGTGCTTTTGTTGGATCCTGT
GCAGTCTACGGATTGTATTACGATGCTTTGATGGATTATACCAATGGTCAATTTGAAGTT
ACTGGAGCAAACGCTACAGCCAACATATTTGCATCCTACCCAGCAAAACATCTCTCAGTC
GTCAATGGGTTTGTTGACCAGGTAATTGCCACAGGTGCACTGATCCTGTGCATCCTGGCC
ATCACTGACAGGAAGAACATTGGCGCTCCAAAAGGTATGGAGCCTTTGTGCATTGGCCTG
GTTATCATGGCCATTGGCGTGTCCATGGGCCTAAACTGCGGATATCCGATCAACCCAGCA
CGAGACCTCGGCCCGCGGCTCTTCACAGCGGTGGCGGGGTGGGGCATGGAAGTATTCAGG
GCTGGAGGCTGCTGGTGGTGGATCCCTGTCGCAGGGCCCATGGTGGGTGGAGCGGTCGGA
GCAGGCATTTACTTTCTGTTTGTTGAGCTGCACCACCCGGAG------------------
---------CCTAAAGCACAG---------------------------------------
---------GAGGAGAACAAC------GTCCAGGACAAATATGAAATCATAACTATGAGT

>Peacock_blenny_Aqp9b
---------------ATGGAAAGTGAGAAGAAGAGGAAGATGAAGGAGAAATTTGGCCTG
AGGCGGGACATCTTTAAGGAGTTCCTGGCGGAATCTCTGGGGATATTCGTCCTGATACTC
TTTGGATGTGGTTCAGTGGCCCAGACCGTGCTGAGTAAAGGGGCTGTCGGCGAGCCGCTC
ACAATCCACATTGGTTTCACTCTGGGAGTCATGATGGCTGTTTACATGGCAGGGGGAGTG
TCAGGAGCTCATGTGAACCCTGCAGTCTCTCTGGCCATGGTGATCCTGGGTAAGCTCCCC
CTGAAGAAGTTTCCTGTGTATGTGGCGGCACAGTTCCTGGGTGCTTTTGTTGGATCCTGT
GCAGTCTACGGATTGTATTACGATGCTTTGATGGATTATACCAATGGTCAATTCGAAGTT
ACTGGAGCAAACGCTACAGCCAACATATTTGCATCCTACCCAGCAAAACATCTCTCGGTC
ATCAATGGGTTTGTTGATCAGGTAATTGCCACAGGTGCACTGATCCTGTGCATCCTGGCC
ATCACTGACAGGAAGAACATTGGCGCTCCAAAAGGCATGGAGCCTTTGTGCATTGGCCTG
GTCATCATGGCCATTGGCGTGTCCATGGGACTGAACTGCGGATATCCTATCAACCCAGCG
CGAGACCTCGGCCCGCGGCTCTTCACAGCGGTGGCGGGATGGGGCATGGAAGTATTCAGG
GCTGGAGGCTGCTGGTGGTGGATCCCTGTCGCGGGGCCCATGGTGGGTGGAGCGGTCGGA
GCAGGCATTTACTTTCTGTTCGTTGAGCTGCACCACCCGGAG------------------
---------CCTAAAGCACAG---------------------------------------
---------GAGGAGAACAAC------GTCCAGGACAAATATGAAATCATTACTATGAGT
>Black_faced_blenny_Aqp9b
---------------ATGGAAAATGAGAGAAGGAGGAAGATGAAGGAGAAATTTGGCCTG
AGGCGGGACATCTTTAAGGAGTTCCTGGCGGAATTTCTGGGAATATTTGTCCTGATACTC
TTTGGATGTGGTTCAGTGGCCCAGACGGTGCTGAGTAGAGGGGCTCTCGGGGAGCCGCTG
ACCATCCATATTGGTTTCACCCTGGGAGTGATGATGGCTGTCTACATGGCAGGGGGAGTG
TCAGGAGCTCATGTGAACCCTGCGGTCTCTCTGGCTATGGTGATCCTGGGGAAGTTGCCT
CTGAAGAAGTTTCCTGTGTACGTGGCGGCACAGTTCCTGGGGGCTTTTGCTGGATCTTGT
GCCGTCTACGGATTGTATTACGATGCGTTGATGGAATATACCAATGGAGAATTTGAAGTT
ACTGGAGTTAATGCCACAGCCAACATTTTTGCATCGTATCCTGCAAAGCATCTCTCAGTC
CTTAACGGGTTTGTTGATCAGGTAATTGCCACCGGTGCACTGATCCTGTGCATCCTGGCC
ATCACCGACAAGAAGAATATTGGTGCTCCAAAAGGCGTAGAGCCTCTGTGCATCGGCCTG
ATCATCCTGGCCATCGGCGTGTCCATGGGTCTGAACTGTGGATATCCCATCAACCCCGCA
CGAGACCTTGGCCCGCGCTTCTTCACGGCTGTGGCCGGGTGGGGCACGGAA---------

>Blunt_snouted_clingfish_Aqp9b
---------------ATGGAGAATGAGAGGATGAGGAAGATAAAGGAGAGATTTGGCCTG
AGGCGGGACATCTTGAAGGAGTTCCTGGCGGAGTTTGTGGGGATATTTGTGCTGATACTC
TTTGGATGTGGTTCAGTGGCTCAGACGGTGCTGAGCAGAGGGACGCTGGGGGAGCCACTG
ACCATTCACGTTGGTTTCACTCTGGGAGTCATGATGGCTGTCTACATGGCCGGGGGAGTG
TCAGGAGCTCATGTGAACCCTGCAGTTTCTTTGGCCATGGTGATGTTGGGGAAGCTCCCT
CTAAAGAAGTTTCCCATCTATGTGCTGGCACAGTTTCTGGGAGCTTTCGCTGGATCCTGT
GCTGTATTTGGACTGTATTATGATGCTTTGATGGATTTTACAAATGGAGAATTTGAAGTT
ACAGGTGCCAATGCCACAGCCAACATATTTGCCTCGTATCCTGCTAAACACCTGTCTGTC
CTAAATGGGTTTGTGGACCAGGTGATTGCCACTGGTGCACTGATTCTGTGCATCCTGGCC
ATCACTGACAGGAAGAATATTGGCGCTCCCAAAGGCATGGAACCTCTCTGCATAGGCCTG
ATCATCATGGCCATCGGTGTGTCCATGGGACTCAACTGTGGATATCCAATCAACCCGGCA
CGAGACCTCGGCCCCCGTTTATTTACAGCATTGGCTGGGTGGGGACTGGACGTGTTCAGG
GCTGGAGGCTGCTGGTGGTGGATTCCTGTGGCAGGACCCATGGTGGGCGGAGCGGTCGGA
GCAGGCATTTACTTCCTCTTCGTTGATTTGCACCAACAAGAA------------------
---------CCTGACAAGCAG---------------------------------------
---------GAGGAGAACAAC------GTGCAGGAAAAATATGAAATTGTAACCATGAGT
>gbsMudskipper_Aqp9b
---------------ATGGAAGACGAGAACAAGAAGAGGATGAAGGAGAAATTTGGACTG
AGGCGGGATATCTTTAAAGAGTTCCTGGCTGAGTTTCTGGGGATATTTGTCTTGATACTT
TTCGGATGTGGTTCAGTGGCCCAGACCGTGCTGAGTAAAGGGGCTCTGGGGGAGCCCTTG
ACCATCCACATTGGCTTCACTCTGGGAGTCATGATGGCTGTTTACATGGCCGGAGGAGTG
TCAGGAGGTCATGTAAATCCCGCTGTTTCTCTGGCCATGGTGATCCTGGGGAAACTTTCT
GTCAAGAAGTTCCCTATTTATGTGGCAGCCCAGTTCCTTGGAGCATTTGTCGGATCCTGT
GCTGTCTTTGGGCTCTACTATGATGCTCTGATGGAGTACACCAATGGAGAATTCATGGTT
ACTGGTCCAAATGCTACAGCCAACATATTTGCTTCATATCCAGCGAAACATCTGTCAGTC
CTTAATGGATTTATTGATCAGGTGATTGGAACTGCAGCACTGATCCTCTGTATCTTGGCC

ATCACTGACAAGAGGAATATTGGGGCTCCTAAAGGCATGGAGCCCCTGTGCATTGGCTTG
GCCATTACTGCCATTGCGGTGTCTATGGGTCTGAACTGTGGTTATCCCATCAACCCTGCG
CGAGACCTGGGCCCACGGTTCTTTACTGCTGTGGCCGGCTGGGGCATGGATGTGTTCAGG
GCTGGGGGCTGCTGGTGGTGGATTCCAGTGGCGGGGCCTATGGTGGGTGGAGCTGTGGGA
GCTGGAGTTTACTTCCTGTGTGTGGAGCTGCATCACCCTGAA------------------
---------CCTGAGAAACAG---------------------------------------
---------GCTGAGAACAAC------ATCCCTGACAAGTATGAGATGGTCACCATGAGT
>Atlantic_mudskipper_Aqp9b
---------------ATGGAGGACGATAACAAGAAGAGGATGAAGGAGAAATTTGGCCTG
AGGCGGGATATCTTTAAAGAGTTCTTGGCGGAGTTTCTGGGGATATTTGTCTTGATACTC
TTTGGATGTGGTTCAGTGGCCCAGACTGTGCTGAGTAAAGGGGCTCTGGGGGAGCCCTTG
ACCATCCACATTGGCTTCACTCTGGGAGTCATGATGGCCGTCTACATGGCAGGAGGAGTG
TCAGGAGGTCATGTAAACCCTGCTGTTTCCCTGGCCATGGTGATCCTGGGTAAACTTCCT
GTCAAGAAGTTCCCTATTTATGTAGCAGCCCAGTTCCTTGGAGCATTTGCAGGATCCTGT
GCTGTCTTTGGGCTCTACTATGATGCTCTGATGGAGTACACCACTGGAGAATTTGCAGTT
ACTGGTCCAAATGCAACAGCCAACATATTTGCTTCATATCCAGCGAAACATCTGTCAGTC
CTTAATGGATTTGTTGATCAGGTAATTGGAACTGCAGCACTGATCCTCTGTATCTTGGCC
ATCACTGACAAGAGGAATCTTGGGGCTCCTAAAGGCATGGAGCCGCTGTGCATTGGCTTG
GCCATTACTGCCATTGCAGTGTCTATGGGCCTGAACTGTGGTTATCCCATCAACCCGGCC
AGAGACCTGGGCCCGCGCTTCTTTACTGCTGTGGCCGGCTGGGGCATGGATGTGTTCAGG
GCTGGGGGCTGCTGGTGGTGGATTCCAGTGGCGGGGCCGATGGTAGGTGGAGCTGTGGGA
GCTGGAGTTTACTTCCTGTTTGTGGAGCTGCACCACCCTGAA------------------
---------CCCGAGAAACAG---------------------------------------
---------GCCGAGAACAAC------ATCTCTGACAAGTATGAGATGGTCACCATGAGT
>Walking_goby_Aqp9b
---------------ATGGAAGACGAGAACAAGAAGAGGATGAAGGAGAGATTTGGCCTG
AGGCGGGATATCTTTAAAGAGTTCCTGGCGGAGTTTCTCGGGATATTTGTCTTGATACTT
TTCGGATGTGGTTCAGTGGCCCAGACCGTGCTGAGTAAAGGGGCTCTGGGGGAGCCTTTG
ACCATCCACATCGGCTTCACTCTGGGAGTCATGATGGCTGTTTACATGGCAGGAGGAGTG
TCAGGAGGTCATGTCAATCCTGCTGTTTCTCTGGCCATGGTGATCCTGGGGAAACTTCCT
GTCAAGAAGTTCCCTATTTATGTGGCAGCCCAGTTCCTTGGAGCATTTGCTGGATCCTGT
GCTGTATTTGGGCTCTACTATGATGCTCTGATGGAGTACACGAATGGAGAATTCATGGTT
ATTGGTCCAAATGCTACAGCCAACATATTTGCTTCATATCCAGGGAAACATCTGTCAGTC
CTTAATGGATTTGTGGATCAGGTGATTGGAACTGCAGCACTGATCCTCTGTATCTTGGCC
ATCACCGACAAAAGGAATATTGGGGCTCCTAAAGGCATGGAGCCACTGTGTATCGGCTTG
GCCATTACTGCCATTGCAGTGTCTATGGGTCTGAACTGTGGTTATCCCATCAACCCTGCC
CGAGACCTCGGCCCACGGTTCTTTACTGCTGTTGCCGGCTGGGGCATGGATGTGTTCAGG
GTCGGCGGCTGCTGGTGGTGGATTCCAGTGGCGGGACCTATGGTAGGTGGAGCTGTGGGA
GCTGGAGTTTACTTCCTGTGTGTGGAGCTGCACCACCCTGAA------------------
---------CCTGAGAAACAG---------------------------------------
---------CCAGAGAACAAC------ATCCCTGACAAGTATGAGATGGTCACCATGAGT
>Giant_mudskipper_Aqp9b
---------------ATGGAGGACGATAACAAGAAGAGGATGAAGGAGAAATTTGGCCTG
AGGCGGGATATCTTTAAAGAGTTCTTGGCGGAGTTTCTGGGGATATTTGTCTTGATACTC
TTTGGATGTGGTTCAGTGGCCCAGACTGTGCTGAGTAAAGGGGCTCTGGGGGAGCCCTTG
ACCATCCATATTGGTTTCACTCTGGGAGTCATGATGGCCGTCTACATGGCAGGAGGAGTG
TCAGGAGGTCATGTAAACCCTGCTGTTTCTCTGGCCATGGTGATCCTGGGTAAACTTCCT
GTCAAGAAGTTCCCTATTTATGTAGCAGCCCAGTTCCTTGGAGCATTTGCCGGATCCTGT
GCTGTCTTTGGGCTCTACTATGATGCTCTGATGGAGTACACCACTGGAGAATTCACGGTT
ACTGGTCCAAATGCTACAGCCAACATATTTGCTTCATATCCAGCGAAACATCTGTCTGTC
CTTAATGGATTTGTTGATCAGGTAATTGGAACTGCAGCACTGATCCTCTGTATCTTGGCC
ATCACTGACAAGAGGAATATTGGTGCTCCTAAAGGCATGGAGCCGCTGTGCATTGGCTTG
GCCATTACTGCCATTGCAGTGTCTATGGGTCTGAACTGTGGTTATCCCATCAACCCGGCC
CGAGACCTGGGCCCGCGCTTCTTTACTGCTGTGGCCGGCTGGGGCATGGATGTGTTCAGG
GCTGGGGGTTGCTGGTGGTGGATTCCAGTGGCGGGGCCGATGGTGGGCGGAGCTGTGGGA
GCTGGAGTTTACTTCCTGTGTGTGGAGCTGCACCACCCTGAA------------------
---------CCCGAGAAACAG---------------------------------------
---------GCCGAAAACAAC------ATCTCTGACAAGTATGAGATGGTCACCATGAGT
>Spotted_snakehead_Aqp9b
---------------ATGGACCACGAGCTCAAGAAGAAGATGAAGGAGAAACTCGGCCTG
AGGCGAAACGTCTGGAAGGAGTTTCTGGCGGAGTTTCTGGGGACATTTGTCCTGATACTT

TTCGGATGCGGTTCAGTGGCCCAGGCGGTGCTCAGCAGAGGGTCACAGGGGGAGCCCCTG
ACCATCCACATCGGCTTCACCCTGGGAGTCACGATGGCCGTCTACACGGCAGGAGGAGTG
TCAGGAGCCCACGTGAACCCTGCCGTCTCTCTGGCCATGCTGCTCCTGGGGAAGCTTCCT
CTGAAGAAGTTTCCAGTCTACGTGTCGGCCCAGTTCCTGGGGGCGTTTGTTGGATCCTGC
GTCGTCTTCGGGTTTTATTACGATGCTTTGATGGATTTTTCCGAGGGGAAATTTGTTGTT
ACCGGTGAAAATGCCACAGCCCAAATATTTGCCTCATATCCTGCAAAACATCTGTCCCTC
CTGAATGGGTTTGTGGATCAGGTGACTGCCACCGCGGCTCTGATCCTCTGCGTCCTGGCC
GTGACGGACCGGAGGAACCTCGGCGCCCCGAGGGGCCTGGAGCCGCCGTGCGTCGGCCTC
GTCGTCATGGCCGTGGGCGTGTCCATGGGTCTGAACTGC---------------------
----------------------------------GCCGGCTGGGGCCTGGACGTGTTCAGA
GCTGGATGCTGCTGGTGGTGGATCCCTGTGGTCGGGCCCATGGTGGGCGGAGCCGTGGGA
GCGGCCGTTTACTTCTTTTCATCGAGCTTCATCACCCCGAA------------------
---------CCTGAAAAGAAG---------------------------------------
---------GAGGAGAACAGC------GTCCAGGACAAATACGAAATGATAAACGTGAGC
>Kissing_gourami_Aqp9b
---------------ATGGAAAACGAGTTCAAGAGGAAGATAAAAGAGAGATTTGGCCTG
AGGCGGGACATCTTTAAGGAGTTCCTGGCGGAATTTCTGGGGATTTTTGTGTTGATAGTT
TTTGGATGTGGTTCAGTGGCCCAGACGGTGCTCAGTAAAGGGGCACTGGGGGAGCCCCTG
ACGATCCACATTGGTTTCTCTTTGGGAGTCATGATGGCTGTCTACATGGCTGGAGGAGTG
TCAGGAGCCCATGTGAATCCTGCAGTCTCTCTGGCCATGGTGATCCTGGGGAAGCTTTCT
CTAAAGAAGTTTCCTGTATATGTGTTGGCACAGTTCCTGGGGGCCTTTGCTGGATCCTGT
GTTGTCTTTGGATTTTATTATGATGCTTTGATGGATTTTACCCAGGGAGAATTTGCGGTT
ACTGGTGCAAATGCTACAGCCAACATATTTGCATCATATCCTGCAAAACATCTCTCGGTC
CTGAACGGCTTTTTTGACCAGGTAATTGCAACTGCAGCACTGATCCTGTGCATCCTGGCC
ATCACAGACCGGAGGAATATCGGTGCTCCAAAAGGCATGGAGCCGCTGTGTATTGGCCTG
ATCATCATGGCCATCGGAGTGTCCATGGGTCTAAACTGTGGCTATCCAATCAACCCGGCA
CGAGACCTTGGACCACGATTGTTCACAGCTGTGGCCGGGTGGGGAATGGAAGTATTCAGG
GCTGGAGGTTGCTGGTGGTGGATCCCAGTGGCAGGGCCCATGGTGGGTGGAGTGGTCGGA
GCAGGCGTTTACTTTCTCTTCGTTGAGTTGCATCAACCGGAG------------------
---------CCTGAAAAGCAG---------------------------------------
------------GAGAACAAC------GTACCAGACAAATATGAAATGGTAACAATGAGT
>Climbing_perch_Aqp9b
---------------ATGGAAAACGAGTTTAAGAGGAAGATAAAGGAGAGATTTGGCCTG
AGGCGGGACATCTTTAAGGAGTTCCTAGCGGAATTTCTGGGGATATTTGTCCTGATACTT
TTTGGATGTGGTTCAGTGGCCCAGACGGTGCTCAGTAAAGGGGAACTAGGAGAGCCCCTG
ACGATCCACATTGGTTTCACTCTGGGAGTCATGATGGCCGTCTACTTGGCGGGGGGAGTG
TCAGGAGCCCACGTGAACCCTGCAGTCTCTCTGGCCATGGTGATCCTGGGCAAACTTCCT
CTAAAGAAGTTTCCTGTGTATGTGTTGGCACAGTTTCTGGGGGCTTTTGCTGGATCCTGT
GCTGTCTTTGGGTTTTATTATGATGCTTTGATGGAGTACACCAAGGGAGAATTTGCTGTG
ACTGGTGAAAATGCCACAGCCAACATATTTGCATCTTATCCTGCAAAACATCTCTCAGTC
CTGAATGGGTTTCTTGATCAGGTAATTGCGACGGCTGCTCTGATCCTGTGCATCTTGGCC
ATAACTGACAGGAGGAACATCGGGGCTCCAAAAGGCATGGAGCCTCTGTGTATTGGCCTG
ATCATCATGGCTATCGGAGTGTCCATGGGTCTAAACTGCGGCTATCCAATCAACCCGGCA
CGAGACCTCGGACCACGCTTGTTCACAGCTGTGGCCGGATGGGGCATGGAAGTGTTCAGA
GCTGGAGGTTGCTGGTGGTGGATCCCTGTGGCGGGGCCCATACTGGGCGGAGCAGTCGGA
GCAGGCGTTTACTTTGTCTTCATTGAGTTGCACCATCCTGAG------------------
---------CCTGAAAAGCAG---------------------------------------
---------GAGGAGAACAAC------ATCCCGGACAAATACGAAATGGTAACTATGAGT
>Siamese_fighting_fish_Aqp9b
---------------ATGGAAAACGAGTTCAGGAGGAAGATAAAGGAGAGATTTGGCCTG
AGGCGGGACATCTTCAAGGAGTTCCTGGCGGAATTTCTGGGAATCTTTGTGCTGATACTT
TTTGGATGTGGTTCAGTGGCCCAGACGGTGCTCAGTAGAGGGGCTCAGGGGGAGCCCCTC
ACCATCCACATCGGTTTCACCCTGGGAGTGATGATGGCCGTCTACATGGCGGGGGGAGTG
TCAGGAGCTCACGTCAACCCCGCCGTCTCTCTGGCCATGCTGATCCTGGGCAAGCTTCCG
CTCAAGAAGTTCCCCGTCTACGTGTTGGCGCAGTTCCTGGGGGCGTTCGCTGGCTCGTGC
GCCGTCTTCGGCTTTTATTACGATGCTTTCATGGATTACACTGAGGGACAATTTGCTGTG
ACTGGTGCAAATGCCACCGCCAAGATATTTGCATCGTATCCTGAAAAGCATCTCTCAGTC
CTGAACGGGGTCGTCGACCAGGTAATTGCCACCGCCGCTCTGGTCCTGTGCATCCTGGCC
ATCACCGACAGGAGGAACATCGGCGCCCCAAAGGGCATGGAGCCCTTGTGCATCGGCCTG
ATCATCATGGCCATCGGCGTGTCCATGGGGCTGAACTGCGGCTACCCCATCAACCCGGCG
CGGGACCTCGGGCCCCGTGTGTTCACGGCGGTGGCCGGCTGGGGCATGGAGGTTTTCAGG

GCCGGAGGCTGCTGGTGGTGGATCCCCGTGGTGGGGCCTATGGCGGGCGGGGCGGTCGGA
GCAGCCGTTTACTTCGTCTTCGTCGAGCTGCACCACCCCGAG------------------
---------CCGGAAAAGCAG---------------------------------------
---------GAGGAGAACAAC------ATCCCAGACAAATATGAAATGGTAACAATGAGT
>Swamp_eel_Aqp9b
---------------ATGAAGAACGAGACCAAAAGGAAAATGAAGGAGAGATTTGGCCTG
AGGCGGGACATCTTTAAGGAGTTCCTGGCGGAATTTCTGGGGATATTTGTCCTGATACTT
TTTGGATGTGGTTCAGTGGCCCAGGCAGTGCTGAGTAAAGGGTCGCTCGGGGAGCCCTTG
ACTATCCACATCGGTTTCACTCTAGGAGTCATGATGGCTGTTTACATGGCAGGCGGGGTG
TCAGGAGCGCACGTGAACCCTGCAGTTTCTCTGGCCATGGTGATCCTGGGGAAGCTCCCT
CTGAAGAAGTTTCCTGTGTATGTGGTGGCACAATTTCTGGGGGCCTTTACTGGATCCTGC
ATTGTCTTTGGGTTGTATTATGATGCTTTGATGGACTACACCTATGGAGAACTTGCTGTT
GCTGGTGAAAATGCAACAGCCAAAATATTTGCATCTTATCCTGCAAAACATCTGTCAGTC
CTGAATGGATTTGCTGATCAAGTAATTGCAACTGCCGCACTGATCCTGTGCATCCTGGCC
ATCACTGACAAGAAGAACATTGGTGCTCCTACAGGCATGGAGCCTCTGTGCATCGGCCTG
GTCATCATGGCCATTGGAGTGTCCATGGGTCTGAACTGTGGCTATCCAATCAACCCGGCA
CGAGACCTTGGCCCACGGTTCTTCACGGCTCTGGCCGGGTGGGGCATGGACGTATTCAGG
GCTGGAGGTTGCTGGTGGTGGATCCCTGTGACAGGGCCTATGGTGGGTGGGGCAGTTGGA
GCAGGTATTTACTTTCTCTTCATTGAGTTGCACCACCCTGAG------------------
---------CCTGAAAAACAG---------------------------------------
---------GAGGAAAACAGT------GTCCAGGACAAATATGAAATGGTAACCATGAGC
>Zigzag_eel_Aqp9b
---------------ATGGAAAGCGAGAGCAAAAGGAAGATAAAGGAGAAATTTGGCCTG
AAGCGAGACATCTTTAAGGAGTTCCTGGCGGAATTTCTGGGAATATTTGTCCTGATACTT
TTTGGATGTGGTTCAGTGGCCCAGACTGTGCTGAGTAAAGGGGTGCTCGGGGAGCCCTTG
ACCATTCATGTTGGGTTCACCCTCGGAGTCATGATGGCCGTTTACATGGCAGGGGGAGTG
TCAGGAGCCCATGTGAACCCTGCAGTCTCTTTGGCCATGGTGATCCTGGGAAAACTACCT
CTGAAGAAATTTCCTGTGTATGTGGTGGCACAGTTCCTGGGGGCCTTTGCTGGATCCTGC
TCTGTCTTTGGGTTGTATTATGATGCTTTGATGGAATATACCAATGGGGAATTTGCTGTT
ACTGGTCCAAATGCCACAGCCAACATATTTGCATCTTATCCTGCAAAACACCTCTCAGTC
CTAAATGGGTTTTTTGATCAGGTAATTGCAACTGGAGCACTGGTCCTGTGCATCCTGGCC
ATCACTGACAGGAAGAATATTGGTGCTCCCAAAGGCATGGAGCCCCTGTGCATCGGCCTG
ATCATCATGGCCATCGGGGTGTCGATGGGTCTGAACTGCGGTTATCCAATCAACCCGGCA
CGAGACCTGGGCCCACGCTTCTTCACGGCTGTTGCTGGGTGGGGCATGGAGGTATTCAGA
GCCGGAGGCTGCTGGTGGTGGATCCCTGTGGCAGGACCAATGCTGGGAGGAGCAGTTGGA
GCGGGAGTTTACTTTGTCTTCATTGAGCTGCACCATCCTGAA------------------
---------CCTGAAAAACAG---------------------------------------
------------GAGAACAAC------GTCCAGGACAAATATGAAATGGTAACTATCAGT
>Pacific_bluefin_tuna_Aqp9b
---------------ATGGAAAATGAGAGCAAGAGAAAAATGAAGGAGAAATTTGCCCTG
AGGCGGGACATCTTTAAGGAGTTCCTGGCGGAATTTCTGGGGATATTTGTCCTAATACTC
TTTGGATGTGGTTCAGTGGCCCAGACAGTGCTGAGTAAAGGGGCTCTCGGGGAGCCCTTG
ACTATCCACATTGGTTTCACTCTGGGAGTCATGATGGCCGTCTACATGGCAGGGGGAGTG
TCAGGAGCCCACGTCAACCCCGCTGTCTCGCTCGCCATGGTGATCCTGGGGAAGCTACCG
GTGAAGAAGTTCCCTGTGTATGTGGTGGCACAGTTCCTGGGAGCTTTTGCTGGATCTTGT
GCTGTTTATGGCCTTTATTATGATGCTTTGATGGACTACACCAATGGAGAATTTGCTGTT
ACTGGTGTGAATGCCACAGCCAACATATTTGCATCTTATCCTGCCAAACATCTCTCAGTC
TTAAATGGATTTGTTGATCAGGTGATTGCAACTGGTGCATTGATCCTGTGCATCCTGGCC
ATTACTGACAGGAAGAATATCGGTGCTCCAAAAGGCATGGAGCCTCTGTGCATCGGCCTG
ATCATCATGGCCATCGGAGTGTCAATGGGCCTGAACTGCGGCTATCCAATCAACCCGGCA
CGAGACCTCGGCCCACGGTTCTTCACTGCGGTGGCCGGGTGGGGCATGGATGTGTTCAGG
GCTGGGGGATGCTGGTGGTGGATCCCAGTGGCGGGACCCATGGTGGGCGGAGCAGTTGGA
GCTGGTGTTTACTTTCTCTTCATTGAGTTGCACCACCCCGAG------------------
---------CCTGAAAAACAA---------------------------------------
---------GAGGAAAACAAC------GTCCAGGACAAATATGAAATGATAACGATGAGT
>Atlantic_bluefin_tuna_Aqp9b
---------------ATGGAAAATGAGAGCAAGAGAAAAATGAAGGAGAAATTTGCCCTG
AGGCGGGACATCTTTAAGGAGTTCCTGGCGGAATTTCTGGGGATATTTGTCCTAATACTC
TTTGGATGTGGTTCAGTGGCCCAGACAGTGCTGAGTAAAGGGGCTCTCGGGGAGCCCTTG
ACTATCCACATTGGTTTCACTCTGGGAGTCATGATGGCCGTCTACATGGCAGGGGGAGTG
TCAGGAGCCCACGTCAACCCCGCTGTCTCGCTCGCCATGGTGATCCTGGGGAAGCTACCG

GTGAAGAAGTTCCCTGTGTATGTGGTGGCACAGTTCCTGGGAGCTTTTGCTGGATCTTGT
GCTGTTTATGGCCTTTATTATGATGCTTTGATGGACTACACCAATGGAGAATTTGCTGTT
ACTGGTGTGAATGCCACAGCCAACATATTTGCATCTTATCCTGCCAAACATCTCTCAGTC
TTAAATGGATTTGTTGATCAGGTGATTGCAACTGGTGCGTTGATCCTGTGCATCCTGGCC
ATTACTGACAGGAAGAATATCGGTGCTCCAAAAGGCATGGAGCCTCTGTGCATCGGCCTG
ATCATCATGGCCATCGGAGTGTCAATGGGCCTGAACTGCGGCTATCCAATCAACCCGGCA
CGAGACCTCGGCCCACGGTTCTTCACTGCGGTGGCCGGGTGGGGCATGGATGTGTTCAGG
GCTGGGGGATGCTGGTGGTGGATCCCAGTGGCGGGACCCATGGTGGGCGGAGCAGTTGGA
GCTGGTGTTTACTTTCTCTTCATTGAGTTGCACCACCCCGAG------------------
---------CCTGAAAAACAA---------------------------------------
---------GAGGAAAACAAC------GTCCAGGACAAATATGAAATGATAACGATGAGT
>Yellowfin_tuna_Aqp9b
---------------ATGGAAAATGAGAGCAAGAGAAAAATGAAGGAGAAATTTGCCCTG
AGGCGGGACATCTTTAAGGAGTTCCTGGCGGAATTTCTGGGGATATTTGTCCTAATACTC
TTTGGATGTGGTTCAGTGGCCCAGACAGTGCTGAGTAAAGGGGCTCTCGGGGAGCCCTTG
ACTATCCACATTGGTTTCACTCTGGGAGTCATGATGGCCGTCTACATGGCAGGGGGAGTG
TCAGGAGCCCACGTCAACCCCGCTGTCTCGCTCGCCATGGTGATCCTGGGGAAGCTACCG
GTGAAGAAGTTCCCTGTGTATGTGGTGGCACAGTTCCTGGGAGCTTTTGCTGGATCTTGT
GCTGTTTATGGCCTTTATTATGATGCTTTGATGGACTACACCAATGGAGAATTTGCTGTT
ACTGGTGTGAATGCCACAGCCAACATATTTGCATCTTATCCTGCCAAACATCTCTCAGTC
TTAAATGGATTTGTTGATCAGGTGATTGCAACTGGTGCGTTGATCCTGTGCATCCTGGCC
ATTACTGACAGGAAGAATATCGGTGCTCCAAAAGGCATGGAGCCTCTGTGCATCGGCCTG
ATCATCATGGCCATCGGAGTGTCAATGGGCCTGAACTGCGGCTATCCAATCAACCCGGCA
CGAGACCTCGGCCCACGGTTCTTCACTGCGGTGGCCGGGTGGGGCATGGATGTGTTCAGG
GCTGGGGGATGCTGGTGGTGGATCCCAGTGGCGGGACCCATGGTGGGCGGAGCAGTTGGA
GCTGGTGTTTACTTTCTCTTCATTGAGTTGCACCACCCCGAG------------------
---------CCTGAAAAACAA---------------------------------------
---------GAGGAAAACAAC------GTCCAGGACAAATATGAAATGATAACGATGAGT
>Atlantic_chubb_mackerel_Aqp9b
---------------ATGGAAAATGAGAGCAAGAGAAAAATGAAGGAGAAATTTGGCCTG
AGGCGGGACATCTTTAAGGAGTTCCTGGCGGAATTTATGGGGATATTTGTCCTAATACTC
TTTGGATGTGGTTCAGTGGCCCAAACGGTGCTGAGTAAAGGGGCTCTCGGGGAGCCCTTA
ACTATCCACATTGGTTTCACTCTGGGAGTCATGATGGCCGTCTACATGGCAGGGGGAGTG
TCAGGGGCTCACGTGAACCCTGCCGTCTCGCTTGCCATGGTGATACTTGGGAAACTACCA
GTGAAGAAGTTCCCTGTGTATGTGGTGGCACAGTTCCTGGGGGCTTTTGCTGGATCTTGT
GCTGTCTATGGGCTTTATTATGATGCTTTGATGGAATACACCAATGGAGAATTTGCCGTT
ACTGGTGTGAATGCCACAGCCAACATATTTGCATCTTATCCTGCTAAACACCTCTCAGTT
GTAAATGGATTTGTTGACCAGGTGATTGCAACCGCTGCACTGATCCTGTGCATCCTGGCC
ATTACTGACAAGAAGAATATTGGTGCTCCACAAGGCATGGAGCCTCTGTGCATCGGCCTG
ATCATCATGGCTATCGGAGTGTCAATGGGTCTGAACTGTGGCTATCCAATCAACCCAGCA
AGAGACCTCGGCCCACGGTTCTTCACTGCGGTGGCTGGATGGGGCATGGATGTGTTCAGA
GCTGGGGGATGCTGGTGGTGGATCCCTGTGGCGGGGCCCATGGTGGGTGGAGCAGTTGGA
GCTGGTATTTACTTTCTCTTCATTGAGCTGCACCATCCCGAG------------------
---------CCTGAAAAACAA---------------------------------------
---------GAGGAAAACAAC------GTCCAGGACAAATATGAAATGATAACGATGAGT
>Silver_promfret_Aqp9b
---------------ATGGAAAATGAAGGCAAGAGAAAAATGAAGGAGAGATTTGGCCTG
AGGCGGGACATCTTTAAGGAGTTCCTGGCGGAGTTTCTGGGGATATTTGTCCTAATACTC
TTTGGATGTGGTTCAGTGGCCCAGACGGTGCTGAGTAAAGGGGCTCTCGGGGAGCCCTTG
ACTATCCACATTGGTTTCACTCTGGGAGTCATGATGGCCGTCTATATGGCAGGGGGAGTG
TCAGGAGCCCACGTGAATCCCGCTGTCTCGCTCGCCATGGTGATCCTGGGGAAGCTACCT
GTGAAGAAGTTCCCTGTGTATGTGGCGGCACAGTTCCTGGGGGCTTTTGCTGGATCTTGT
GCTGTCTATGGGTTTTATTATGACGCTTTGATGGAATACACCAACGGAGAATTTGCTGTT
ACTGGTGCGAATGCCACAGCCAACATATTTGCATCTTATCCTGCTAAACATCTCTCAGTC
TTAAATGGATTTGTTGATCAGGTGATTGCAACTGGTGCACTGATCTTGTGCATCTTGGCC
ATTACTGACAGGAAGAATATCGGTGCGCCAAAAGGCATGGAGCCTCTGTGCATCGGCCTA
GTCATCATGGCTATCGGAGTGTCAATGGGCCTGAACTGTGGCTATCCAATCAACCCGGCA
CGAGACCTCGGCCCACGGTTCTTCACTGCAGTAGCCGGTTGGGGCATGGATGTGTTCAGA
GCTGGAGGATGCTGGTGGTGGATCCCAGTGGCGGGGCCCATGGTGGGCGGAGCAGTTGGA
GCTGGTGTTTACTTTCTCTTCATTGAGCTGCACCACCCTGAG------------------
---------CCTGAAAAACAA---------------------------------------

---------GAGGACAACAAC------GTTCAGGACAAATATGAAATGATAACGATGAGT
>Opah_Aqp9b

---CTC
TTTGGATGCGGCTCGGTGGCCCAGACCATTCTCAGTAAGGGGGCCCTCGGGGAGCCCCTG
ACCATCCACATCGGCTTCACCCTGGGGGTCATGATGGCCGTCTACATGGCGGGGGGGGTG
TCAGGAGCCCATGTGAACCCTGCCGTGTCTCTGGCCATGGTGCTCTTGGGCAAACTTCCC
TTGAAAAAGTTCCCCGTTTATGTGGCGGCTCAGTTTCTGGGGGCATTTGCTGGCTCGTGT
GCAGTTTATGGGTTGTATTATGATGCTTTGATGGAATATACCAGTGGAGAGTTTTCTGTT
ACCGGTGTGAACGCCACGGCCAACATATTTGCATCCTACCCTGCCAAACACCTCTCAGTC
CTCAACGGCTTCGTAGATCAGGTCATTGCGACTGGTGCTCTGATCCTATGCATCCTGGCC
ATCACAGATGGAAAGAACATCGGCGCTCCAAAGGGAATGGAGCCTCTGTGCATCGGCCTG
ATTATCATGGCCATTGGCGTGTCCATGGGACTGAACTGTGGCTATCCCATCAACCCTGCA
CGCGACCTGGGCCCGAGGTTCTTCACTGCTGTGGCCGGCTGGGGCATGGAAGTGTTCAGG
GCTGGAGGTTGCTGGTGGTGGATCCCGGTGGCGGGGCCCATGGTGGGGGGAGCGGTGGGG
GCCGCTATTTACTTCCTGTTCATCGAGCTGCACCACGTGGAA------------------
---------GCTGAAAAACAG---------------------------------------
---------AAGGAGAACAAC------GTCAAGGACAAATACGAAATGATAACGATGAGT
>King_of_herrings_Aqp9b

---CTT
TTCGGATGCGGCTCAGTGGCCCAGGCCGTCCTCAGTAAGGGGGCCCTCGGGGAGCCCCTG
ACCATCCATATCGGCTTCACCCTGGGGGTCATGATGGCCGTCTACATGGCCGGAGGGGTG
TCAGGT--

----------------------GATGCTTTGGTGGACTACAGCAGCGGGGACTTGTCGGTC
ACCGGCGTGAATGCCACAGCCAACATCTTTGCCTCCTACCCTGCCAAACATCTGTCAGTC
GTCAACGGGCTCGTGGATCAGGTCATTGCGACTGGTGCGCTGATCCTGTGCATCCTGGCC
ATCACAGATGGGAGGAACATCGGTGCTCCAAAGGGAATGGAGCCTCTGTGCATCGGGCTG
ATTATCATGGCCATCGGCGTGTCCATGGGACTGAACTGTGGCTATCCCATCAACCCTGCA
CGGGACCTGGGCCCCAGGGTCTTCACTGCTGTGGCTGGCTGGGGCACGGAGGTG------
GCTGGAGGTTGTTGGTGGTGGATCCCGGTAGCAGGGCCCATGGTCGGGGGAGCAGTGGGA
GCTGCCATTTACTTCCTCTTCATTGAACTGCACCACACAGAA------------------
---------GCTGAACAAGAT---------------------------------------
---------ACAGAGAACAAC------GTCAAGGACAAATATGAAATGGTGACGATGAGT
>Plainfin_midshipman_Aqp9b
----------------ATGGATCCTGACACCAGGAGGAAAATGAAGAATAAGTTTGGCCTG
AAGAAGGACATCTTCAGGGAGTTCCTGGCTGAATTCCTGGGCATCTTTATGCTGATACTC
TTTGGATGTGGTTCAGTGGCCCAGACGGTGCTGAGCAGAGGAACGATGGGAGAGCCCCTG
ACAATCCACATTGGGTTTACTCTTGGAGTCATGATCGCCGTTTACGTATCAGGAGGCGTG
TCAGGGGGCCATGTGAATCCTGCAGTTTCTCTCGCTATGGTGATTCTGGGGCGGCTACCT
GTGAAGAAGTTTCCTGTGTACATGGCAGCACAGTTTCTGGGGGCTTTTGCTGGATCCTGT
GCAGTCTACGGATTGTATTATGATGCCCTGATGGAGTTTACCAATGGAGAATTTGCTATT
ATGGGTGTGAATGCCACAGCCAACATTTTTGCATCCTACCCTGCCAAACATCTCTCAGTC
CTAAATGGATTTGGCGATCAGGTAATTGCAACTGGAGCATTGATCCTGTCCATTTTAGCC
ATCACTGACAGGAAGAACATAGGTGCTCCAAGAGGCATGGAGCCTCTGTGCATTGGCCTC
ATCATCTTGGCCATTGGAGTGTCTATGGGGTTCAATTGTGGCTATCCCATCAACCCAGCA
CGAGATCTCAGCCCACGGATCTTCACTGCAGTGGCTGGTTGGGGAATGGATGTCTTCAGA
GCTGGAGGATGCTGGTGGTGGATCCCAGTGGCAGGGCCCATGGTTGGTGGTGCACTTGGA
GCAGGGATTTACTTTCTCTTCACTGAATTGCACCAACCTGAG------------------
---------GCTGAGAAACAG---------------------------------------
---------GACGGGAACAAT------GTCCAGGACAAATATGAAATGGTAACTGAAAAT
>Pony_toadfish_Aqp9b

---CTC
TTTGGATGTGGTTCAGTGGCCCAGACGGTGCTGAGCAGAGGAATGATGGGAGAGCTCCTG
ACAATCCACATTGGTTTTACTCTTGGAGTCATGATCGGCGTCTACATATCAGGAGGCGTG
TCAGGAGGCCATGTGAACCCTGCAGTCTCTCTCGCTATGGTGATTCTGGGGCGGTTACCT
GTGAAGAAGTTCCCTGTGTACGTGGCAGCACAGTTTCTGGGGGCTTTTGTTGGATCCTGT
GCAGTCTATGGATTGTATTACGATGCCCTGATGGCATATACCAATGGAGAATTTGCTGTT
ACAGGTGTCAATGCCACGGCCAATATTTTTGCATCCTACCCTGCCAAACATCTCTCTGTC

CTAAATGGATTTGGTGATCAGGTAATTGCAACTGGAGCATTGATCCTGTCCATCTTAGCA
ATCACTGACAGGAAGAACATAGGTGCTCCGAGAGGCATGGAGCCTCTGTGCATTGGCCTC
ATCATCATGGCCATCGGCGTGTCCATGGGCCTGAACTGCGGATATCCAATCAACCCGGCA
CGAGATCTCAGCCCACGGATCTTCACTGCAGTGGCTGGTTGGGGAATGGATGTATTCAGG
GCAGGAGGTTGCTGGTGGTGGATCCCAGTGGCAGGGCCCATGGTCGGTGGCGCACTCGGA
GCAGGCGTTTACTTTCTCTTCACCGAGTTGCACCAACCTGAG------------------
---------GCTGAGAAACAG---------------------------------------
---------GAGGAGAACAAT------GTCCAGGACAAATATGAAATGGTAACTGAGAAT
>Pearlfish_Aqp9b
---------------ATGGAAAATGACCACATAAGGAACATGAAGGAACGTTTCGCCTTG
AGGCACGATATCTTCAAAGAATTCCTGGCGGAATTCCTCGGAATATTCGTCCTAATACTC
TTCGGATGTGGGTCGGTGGCCCAGGCAGTGCTAAGTAAAGGAGCTCTTGGAGAACCACTA
ACCATCCACATTGGTTTCACCCTGGGACTCATGATGGCCGTATACACGGCAGGCGGCGTG
TCAGGAGCCCATGTGAACCCAGCAGTCTCTCTAGCCATGGTGATCCTGGGAAACTCCCC
TTGAAAAAGTTCCCAATTTATGTAGCAGCACAATTCCTTGGAGCTTTTTGTGGATCTTGT
GCCGTCTTTGTATTATATTATGATGTTTTGATGGAACATACTAAAGGTGACTTTGCTGTC
ACTGGTGAGAATGCCACAGCCAACATATTCGCATCATATCCCGCACAACACCTCTCAGTA
CTTAATGGATTTCTTGATCAGGTAACTGGAACAGGTGCATTGATCTTGTGTATCCTTGCC
ATTACTGATAAGAAGAACATTGGTGCTCCAAAGGGCATGGAACCCTTCTGTATTGGGTTG
ATCATCATGGCCATTGGTGTGTCTATGGGCCTGAACTGTGGATACCCACTCAATCCTGCA
CGAGATCTTGCCCCCCGAGTATTCACAGCTGTGGCTGGGTGGGGTACGGATGTATTCAGG
GCTGGAGGTTGCTGGTGGTGGATTCCTGTAATGGGGCCAATGGTGGGCGGAGCGATTGGA
GCGGGCTTGTATTTTCTCTTCATTGAGCTGCATCATGCAATG------------------
---------CCTGAGAAATCA---------------------------------------
---------GAACGGAGCAAC------ACCCAGGACAAATATGAAATTGTCACCATGAGT
>Legless_cuskeel_Aqp9b
---------------ATGGAAAATGACACCAAGAGAAAAAATGAAGGAACGCTTCGCCCTG
AGGCAGGACGTCTTCAAAGAATTCCTAGCGGAATTACTGGGAGTATTCATCCTAATACTC
TTTGGATGTGGGTCGGTGGCCCAGACGGTGCTGAGTAAAGGGGCTCTTGGGGAGCCACTG
ACCATCCACATTGGTTTCACTTTGGGGCTCATGATGGCTGTCTACATGGCAGGCGGAGTA
TCAGGAGCCCACGTGAACCCTGCAGTTTCTCTTGCCATGGTGATCTCGGGGAAGCTCCCT
GTGAAAAAGTTCCCTGTTTATGTAGCAGCACAATTCCTGGGGGCTTTTGCAGGATCCTGT
GCAGTCTTTGGGTTGTATTATGATGCTTTGATGGAACATACTAAAGGTGAATTTGCTGTC
ACTGGTGTGAATGCCACAGCCAACATATTTGCATCCTATCCAGCCAAACACCTCTCAGTC
CTAAATGGATTCATTGATCAGGTCACTGGAACCGGTGCACTGATCTTGTGCATCCTTGCC
ATCACTGATAAGAAGAATATTGGTGCTCCAAAGGGCATGGAGCCTCTGTGCATCGGCCTG
ATCATCATGGCCATCGGCGTGTCCATGGGCCTGAACTGCGGCTACCCGCTCAACCCTGCG
CGAGACCTCGGCCCCCGGGTCTTCACTGCTGTGGCTGGGTGGGGCATGGATGTATTCAGG
GCTGGAGGTTGCTGGTGGTGGATTCCCGTGGCGGGGCCCATGGTGGGCGGAGCGGTTGGA
GCAGGTGTGTACTTTCTCTTCATTGAGCTGCACCATGCTGCA------------------
---------CCTGAGAAACGG---------------------------------------
---------GAAGGGAACAAC------GTCCAGGAAAAATATGAAATCATCACTATGAGT
>Bearded_brotula_Aqp9b
---------------ATGGAAAATGACAACAAGAGGAAAATGAAGGAACGATTCGCCCTC
AGGCAGGACATCTTCAAAGAATTCCTGGCGGAGTTCCTGGGAATATTCGTCCTAATACTC
TTTGGATGTGGGTCGGTGGCCCAGACGGTGCTGAGCAAGGGGGCTCTCGGGGAGCCACTG
ACCATCCACATTGGTTTCACTCTGGGGATCATGATGGCCGTCTACGTGGCAGGCGGCGTT
TCAGGAGCCCACGTGAATCCCGCTGTGTCTCTCGCCATGGTGATCTTGGGGAAGCTCCCT
TTGAAAAAGTTCCCTGTGTACGTAGTAGCACAGTTCCTGGGGGCTTTTGCTGGATCCTGT
GCAGTCTTTGTGTTGTACTATGATGCGCTGATGGAACACACTAAAGGAGAATTTGCTGTG
ACCGGTGTGAATGCCACGGCCAACATATTTGCATCGTATCCTGCCAAACACCTCTCAGTC
CTAAATGGATTCATGGATCAGGTTATTGGGACTGCTGCGCTGCTCCTGTGCATCCTGGCC
ATCACCGATAAGAAGAATATCGGCGCTCCGAAGGGCATGGAGCCTCTGTGCATCGGCCTG
ATCATCATGGCCATCGGCGTGTCCATGGGCCTGAACTGCGGCTACCCACTCAACCCCGCA
CGAGACCTTGGTCCCCGGGTCTTCACGGCTGTGGCTGGGTGGGGCATCGATGTATTCAGG
GCTGGAGGCTGCTGGTGGTGGATTCCCGTGGCGGGGCCCATGGTGGGCGGAGTCCTGGGA
GCAGGCGTGTACTTTCTCTTCATCGAGCTTCACCACGCTGCT------------------
---------CCTGAGAAACAG---------------------------------------
---------GAAGAGAACAAC------GTGCAGGACAAATACGAAATGATCACCATGAGC
>Sammara_squirrelfish_Aqp9b
---------------ATGGATCATGAGAGGAAGAGAAAAATGAAGGAAAGATTTGCCTTG

AGGCAAGACATCTTCAAAGAGTTCCTGGCTGAATTCCTGGGAATATTCGTCCTAATACTC
TTTGGATGTGGGTCGGTGGCCCAGACGGTGCTGAGTAAAGGGGCGCTGGGGGAGCCTTTG
ACTATCCACATTGGCTTCACTCTGGGAGTCATGATGGCTGTCTACATGGCAGGGGGAGTA
TCAGGAGCCCACGTGAACCCTGCAGTCTCTCTGGCTATGGTGATCTTAGGGAAGCTCCCT
CTGAAGAAGTTCCCTGTTTATGTGGTGGCACAATTCCTGGGTGCCTTCGCTGGATCTTGT
GCTGTTTATGGGCTGTATTATGATGCTTTGATGGAATATACCAATGGTGAATTTGTTGTT
ACTGGTGTGAATGCCACAGCCAACATATTTGCATCCTATCCTGCCAAACATCTCTCCGTT
GTAAATGGATTTGTAGATCAGGTAATTGCGACTGGTGCACTGATTCTGTGCATCCTAGCC
ATCACTGATGGGAAGAATATTGGCGCTCCAAAAGGCATGGAGCCTCTGTGCATCGGCCTG
ATCATCTTGGCTATCGGAGTATCTATGGGTCTGAACTGCGGCTATCCAATCAACCCTGCG
CGAGACCTCGGCCCGAGGTTCTTCACTGCTGTGGCCGGCTGGGGCATGGAAGTATTCAGG
GCTGGAGGTTGCTGGTGGTGGATCCCTGTGGCAGGGCCCATGGTGGGTGGAGTGGTCGGT
GCTGCCATATACTTTGTTTTCATCGACTTGCACCACTCTGAG------------------
---------CCTGAAATACAG---------------------------------------
---------GAGGAGAACAAC------GTCAAGGACAAATACGAGATGATCACTATGAGT
>Longspine_squirrelfish_Aqp9b
---------------ATGGACCATGAGAGGAAGAGAAAAATGAAGGAAAGATTTGCCTTG
AGGCATGATATCATCAAAGAGTTCCTGGCTGAATTCCTGGGAATATTCGTCCTCATACTC
TTTGGATGTGGGTCGGTGGCCCAGACGGTGCTGAGTAAAGGGGCGCTGGGGGAACCTTTG
ACTATCCACATTGGCTTCACTCTGGGAGTCATGATGGCTGTTTACATGGCAGGGGGAGTA
TCAGGAGCCCACGTGAACCCTGCAGTCTCTCTGGCTATGGTGATCTTAGGGAAGCTCCCT
CTGAAGAAGTTCCCTGTATATGTGGTGGCACAATTTCTTGGTGCCTTCGCTGGATCTTGT
GCTGTTTACGGGTTGTATTATGATGCTTTGATGGAATATACCGATGGAGAATTTGTTGTT
TCGGGTGTGAACGCCACAGCCAACATATTTGCATCCTATCCTGCCAAACATCTCTCAGTC
GTAAATGGATTTGTAGATCAGGTAATTGCAACCGGTGCACTGATTCTGTGCATCCTAGCC
ATCACTGATGGGAAGAATATTGGTGCTCCAAAAGGCATGGAGCCTCTGTGCATCGGCCTG
ATCATTTTGGCCATCGGAGTGTCTATGGGCCTGAACTGCGGCTATCCAATCAACCCTGCA
CGAGACCTCGGCCCAAGGTTCTTCACTGCTGTGGCTGGCTGGGGCATGGAAGTATTCAGG
GCTGGAGGCTGCTGGTGGTGGATCCCTGTGGCAGGG------------------------
---GCTATATACTTTGTTTTCATCGACTTGCACCACTCTGAG------------------
---------CCTGAGAAACAG---------------------------------------
---------GAGGAGAACAAC------GTCAAGGACAAATACGAGATGATCACTATGAGT
>Blackbar_soldierfish_Aqp9b
---------------ATGGATCATGAGAGGAAGAGAAAAATCAAGGAGAGATTTGCCCTG
AGGCATGACATCTTCAAAGAGTTCCTGGCTGAATTCCTGGGGATCTTCGTCCTAATACTC
TTCGGATGTGGGTCGGTGGCCCAGACGGTGCTGAGTAAAGGGGCTCTCGGGGAGCCCCTG
ACCATCCACATTGGCTTCACTCTGGGGGTCATGATGGCTGTCTACATGGCAGGGGGAGTA
TCAGGGGCCCATGTGAACCCTGCGGTTTCTCTGGCTATGATGATCTTGGGAAAGCTACCT
GTGAAGAAGTTCCCAGTGTATGTGGTGGCACAGTTCCTGGGTGCCTTTGCCGGGTCTTGT
GCTGTCTATGGGCTTTATTACGATGCTTTGATGGAGTATACCAATGGGGAATTCGCTGTT
ACTGGCGTGAATGCCACAGCCAACATATTTGCATCCTACCCTGCCAAACATCTGTCAGTT
ATAAATGGATTTGTTGATCAGGTTATTGCAACTGGTGCACTGATTCTGTGCATCCTAGCC
ATCACTGACGGGAAGAATATCGGCGCTCCAAAAGGCATGGAGCCTCTGTGCATCGGCCTG
ATCATCCTGGCCATCGGAGTGTCCATGGGCCTGAACTGCGGCTATCCAATCAACCCTGCA
CGGGACCTCGGCCCGAGGTTCTTCACTGCTGTGGCCGGCTGGGGCATGGAAGTGTTCAGG
GCTGGAGGCTGCTGGTGGTGGATCCCTGTGGCAGGGCCTATGGTGGGCGGAGCGGTCGGC
GCTGCCATATACTTTGTCTTCGTTGACTTGCACCACTCCGAG------------------
---------CCTGAAAAACAA---------------------------------------
---------GAGGAGAACAAC------GTCAAGGACAAATACGAGATGATCACTATGAGT
>Splendid_alfonsino_Aqp9b
---------------ATGGAACAGGAGAGCAAGAGGAACATAAAGGAGAGATTCGCCCTG
AGGCAGGACATCTTCAAAGAGTTCCTGGCTGAGTTCCTGGGGATATTCGTCCTAATACTC
TTTGGATGTGGGTCGGTGGCCCAGACGGTGTTGAGTAAAGGGGCTCTCGGGGAGCCGCTG
ACTATCCACATCGGCTTCACCCTGGGGGTCATGATGGCGGTCTACATGGCAGGAGGAGTG
TCAGGAGCCCATGTGAACCCGGCAGTCTCTCTGGCCATGGTGATCTTGGGCAAGCTGCCT
ATAAAGAAGTTCCCTGTGTACGTGGCTGCACAGTTTCTGGGTGCCTTTGCTGGATCCTGT
GCCGTCTATGGCTTGTATTATGATGCGCTGATGGAATATACCAATGGAGAACTCGCTGTC
ACCGGTGTGAATGCCACAGCCAACATATTTGCGTCCTATCCTGCCAAACATCTCTCGGTC
CTAAATGGATTCGTAGATCAGGTAATTGCAACTGGGGCACTGATCCTGTGCATCCTAGCC
ATCACTGACGGGAAGAACATCGGTGCCCCAAAGGGCATGGAGCCTCTGTGCATCGGGCTG
ATCATCATGGCCATCGGAGTGTCGATGGGCCTGAACTGCGGCTATCCAATCAACCCTGCC

AGAGACCTCGGCCCGAGGTTCTTCACTGCTGTGGCTGGGTGGGGCATGGACGTTTTCAGG
GCTGGAGGGTGCTGGTGGTGGATCCCTGTGGCGGGGCCCATGGTGGGCGGAGCGGTGGGC
GCCGCCGTGTACTTTGTCTTCATTGAGCTGCACCACCCCGAG------------------
---------CCTGAAAAACAG---------------------------------------
---------GAGGAGAACAAC------GTCAAGGACAAATACGAGATGATCACTATGAGC
>Redmouth_whalefish_Aqp9b
---------------ATGGAACATGAGCGCAAGAGAAACATAAAGGAAAGATTTGCCCTG
CGGCAGGACATCTTCAAAGAGTTCCTGGCAGAATTTCTGGGGATATTCGTCCTAATACTC
TTCGGGTGTGGGTCGGTGGCCCAGACGGTGCTGAGTAAAGGGGCTCTGGGCGAGCCCTTG
ACTATCCACATTGGCTTCACTCTGGGAGTCATGATGGCCGTCTACATGGCAGGAGGAGTG
TCAGGAGCCCACGTGAACCCTGCAGTCTCTCTGGCCATGGTGATCTTGGGCAAGCTACCT
GTAAAGAAGTTCCCTGTGTATGTGGTGGCACAATTCCTCGGTGCCTTTGTTGGATCTTGT
GCTGTCTATGGGTTGTATTATGATGCTTTGATGGAATATACCAATGGAGAACTCTCTGTT
ACCGGTGTGAATGCCACAGCCAACATATTTGCATCCTATCCTGCCAAACATCTCTCAGTC
CTAAACGGATTCGTGGATCAGGTAATTGCAACTGGTGCACTGATCCTGTGCATCCTAGCC
ATCACTGATGGGAAGAATATTGGTGCTCCAAAAGGCATGGAGCCTCTGTGCATCGGGCTG
ATCGTCATGGCCATTGGAGTGTCTATGGGCCTGAACTGCGGCTATCCAATCAACCCTGCA
CGAGACCTCGGCCCAAGGCTCTTCACTGCTGTGGCTGGGTGGGGCATGGACGTATTCAGG
GCTGGAGATTGCTGGTGGTGGATTCCTGTTGTAGGGCCCATGGTGGGTGGAGTGGTGGGC
GCTGCCATATACTTTGTCTTCATTGAGCTGCACCACCCTGAG------------------
---------CCTGAAAAGCAG---------------------------------------
---------GAGGAGAGCAAT------GTCAAGGACAAATATGAAATGATAACTATGAGT
>Pricklefish_Aqp9b
---------------ATGGAACACGAGGGACGGAGAAAGATGAAGGAGCGGTTCGCCCTG
CGGCAGGACATCTTCAAAGACTTCCTGGCCGAGTTCCTGGGGACGTTGGTCCTGATACTC
TTTGGATGTGGGTCGGTGGCCCAGGCGGTGCTGAGTAAAGGGGCTCTGGGCGAGCCCTTG
ACTATCCACATCGGCTTCACTCTGGGAGTCATGATGGCTGTCTACATAGCAGGAGGAGTG
TCAGGAGCCCACGTGAACCCTGCAGTCTCTCTTGCCATGGTGATGTTGGGCAAGCTACCT
GTGAAGAAGTTCCCTGTGTATGTGGTGGCACAATTCCTGGGTGCCTTTGCTGGATCTTGT
ACTGTCTATGGGTTGTATTATGATGCTTTGATGGAATATACCAATGGAGAACTCTCTGTT
ACCGGTGTGAATGCCACAGCCAACATATTTGCAACCTATCCTGCCAAACATCTCTCAGTC
CTAAATGGATTTGTAGATCAGGTAATTGCAACTAGTGCACTGATCCTGTGCATCCTAGCC
ATCACTGATGGGAAGAATATGGGTGCTCCTAAAGGCATGGAACCTCTGTGCATCGGGCTG
ATCATCATGGTCATTGGAGTGTCTATGGGCCTGAACTGCGGCTATCCAATCAATCCTGCA
CGAGACCTCGGCCCAAGGTTGTTCACTGCTGTGGCTGGGTGGGGTATGGACGTATTCAGG
GCTGGGGGCTGCTGGTGGTGGATTCCTGTGGTAGGGCCCATGGTGGGTGGAGTGCTGGGC
GCTGCCATATACTTTGTCTTCATTGAGTTGCACCACCCTGAG------------------
---------CCTGAAAAGCAG---------------------------------------
---------GAGGATAGCAAC------GTCAAGGACAATTATGAAATGATAACTATGAGT
>Pinecone_fish_Aqp9b
---------------ATGGAGCTTGAGAGCAAGAAAAAACTAAAGGAAAAATTTGGCCTG
AGGCAGGACTTCATCAAAGAGTTCTTGGCTGAATTTCTGGGGATATTCGTCCTAATACTC
TTTGGATGTGGGTCGGTGGCCCAGACGGTGCTGAGCAAGGGGGCTCTAGGGGAGCCCTTG
ACTATCCACATTGGCTTCACTCTGGGAGTCATGATGGCTGTTTATATGGCAGGGGGTATA
TCAGGGGCTCACGTGAACCCTGCAGTGTCTCTGGCTATGGTGATCTTGGGCAAGCTACCT
GTGAAGAAGTTCCCTGTGTATGTGGTGGCACAGTTCCTGGGGGCTTTTGCTGGATCTTGC
GCTGTCTATGGGTTGTATTATGATGCTTTGATGGACTATACCAATGGAGAACTCTCTGTT
ACGGGTGTGAATGCCACAGCCAACATATTTGCGTCCTATCCTGCCAAACATCTCTCAGTC
CTAAATGGGTTCATAGATCAGGTAATTGCAACTGGTGCGCTGATCCTGTGCATCCTAGCC
ATCACTGATGGGAAGAATATTGGTGCTCCGAAAGGCATGGAACCTCTATGTATCGGCCTG
ATCATCATGGCCATTGGAGTGTCCATGGGCCTGAACTGCGGCTATCCAATCAACCCTGCA
CGAGACCTAGGCCCAAGGTTCTTCACTGCTGTGGCTGGGTGGGGCATGGAAGTATTCAGG
GCTGGGGGTTGCTGGTGGTGGATTCCTGTGGCAGGGCCCATGGTGGGTGGAGTGCTGGGG
GCTGCCATATACTTTTTCTTCATTGAGTTGCACCACCCTGAG------------------
---------CCGGAAAAACAG---------------------------------------
---------GAGGAGAACAAC------GTCAAGGACAAATATGAAATGATAACTATGAGT
>Atlantic_greeneye_Aqp9b
---------------ATGGAAAATGAGAGGAAGAGAAAATTTAAGGAAAGATGTGCCCTG
AGGCATGACATCATCAAAGAGTTCCTGGCAGAATTTCTGGGGATATTCGTCTTAATACTC
TTTGGGTGTGGGTCGGTGGCCCAGACAGTGCTGAGTAAGGGGGCTCTTGGGGAGCCCTTG
ACTATCCATATAGGCTTTACTCTGGGAGTCATGATGGCCGTCTACATGGCAGGGGGAGTG

TCAGGAGCCCATGTGAACCCTGCAGTGTCTCTGGCTATGGTGGTCCTGGGCAAGCTACCT
CTGAAGAAGTTCCCTGTGTATGTGGTGGCACAGTTCCTGGGGGCCTTCGCTGGATCTTGT
GCTGTCTATGGGTTATATTAT---------------------------------------

---------------------GTAATTGCAACAGGTGCACTAATTCTGTGCATCCTAGCC
ATCACTGATGGGAAGAACATCGGTGCTCCAAAAGGTATGGAGCCTCTGTGCATCGGCCTC
ATCATCATGGCTATTGGAGTGTCTATGGGCCTGAACTGCGGCTATCCCATCAACCCTGCA
CGTGACCTGGGCCCAAGGTTCTTTACTGCTGTGGCTGGCTGGGGCATGGAAGTATTCAGG
GCTGGAGGGTGCTGGTGGTGGATACCAGTGGCAGGACCCATGGTGGGTGGAGTGGTTGGG
GCTGCCATATACTTCCTCTTTGTTGAGTTGCACCACCCTGAG------------------
---------CCGGAAAAACAG---------------------------------------
---------GAGGAAAATAAC------ATCAAGGACAAATATGAGATGGTTACAATGAGT
>Atlantic_cod_Aqp9b
---------------ATGGGACACGAAAGCATGACCAAAATCAAGGAGAAGTTCTCCCTG
AAGCACGCCATCATCAAGGAGTTCCTGGCCGAGTTTCTGGGGATCTTCGTCTTAATTCTG
TTTGGCTGCGGGGCGGTGGCCCAGGCTGTCCTCAGCAAGGGGGCCCTGGGGGGCCCCCTG
ACCATCCACATCGGCTTCACCCTGGGGGTCATGATGGCGGTGTTCGTGGCCGGGGGGGTG
TCTGGCGCCCACGTGAACCCGGCCGTCTCTCTGGCCATGGTCCTCCTGGGGAAGCTGCCG
CTCAGGAAGTTCCCGGTGTACGTCCTGGCCCAGTTCCTGGGGGCCTTCGCGGGCTCCTGC
GCGGTGTACGCCCTGTACTACGATGCCCTGATGGCGTTCACCAACGGAGACCTGATGGTT
ACCGGGGACAACGCCACCGCCAACATCTTTGCTTCGTACCCGGCGAAGCATCTCTCCACT
CTCAACGGCTTTGTGGATCAGGTGATCGCCACGGCCGCCCTGATCCTGTGCATCCTGGCG
ATCACCGACGCCAAGAACCTGGGCGCGCCGCGGGGGATGGAGCCGCTCTGCATCGGCCTG
GTCATCATGGCGATCGGCGTCTCCATGGGCTTCAACTGCGGCTACCCCATCAACCCCGCC
CGCGACCTGGGACCGCGCCTCTTCACCGCCGTGGCCGGCTGGGGGCTGGACGTGTTCAGG
GCTGGGGGGTGCTGGTGGTGGGTCCCGGTGGCGGGCCCCATGGTGGGGGGCGTGGTCGGG
GCCTCGCTCTACTTCCTGCTGGTGGAGCTGCACCACTCGGAG------------------
---------CCGGACGCCCCC---------------------------------------
---------GAGGAGACCGGC------ACCAAGGACAAGTACGAGATGATCACCGTGAGC
>Walleye_pollock_Aqp9b
---------------ATGGGACACGAAAGCATGACCAAAATCAAGGAGAAGTTCTCCCTG
AAGCACGCCATCATCAAGGAGTTCTTGGCCGAGTTTCTGGGGATCTTCGTCTTAATTCTG
TTTGGCTGCGGGGCGGTGGCCCAGGCTGTCCTCAGCAAGGGGGCCCTGGGGGGGCCCCTG
ACCATCCACATCGGCTTCACCCTGGGGGTCATGATGGCGGTGTTCGTGGCCGGGGGGGTG
TCTGGT--

------------------CAGGTGATCGCCACGGCCGCCCTGATCCTGTGCATCCTGGCA
ATCACCGACGCCAAGAACCTGGGCGCGCCGCGGGGGATGGAGCCGCTCTGCATCGGCCTG
GTCGTCATGGCGATCGCCGTCTCCATGGGCTTCAACTGCGGCTACCCCATCAACCCCGCC
CGCGACCTGGGACCGCGCCTCTTCACCGCCGTGGCCGGCTGGGGGCTGGACGTGTTCAGG

>Arctic_cod_Aqp9b
---------------ATGGGACACGAAAGCATGACCAAAATCAAGGAGAAGTTCTCCCTG
AAGCACGCCATCATCAAGGAGTTCCTGGCCGAGTTTCTGGGGATCTTCGTCTTAATTCTG
TTTGGCTGCGGGGCGGTGGCCCAGGCTGTCCTCAGCAAGGGGGCCCTGGGGGGGCCCCTG
ACCATCCACATCGGCTTCACCCTGGGGGTCATGATGGCGGTGTTCGTGGCCGGGGGGGTG
TCTGGCGCCCACGTGAACCCGGCCGTCTCTCTGGCCATGGTCCTCCTGGGGAAGCTGCCG
CTCAGGAAGTTCCCGGTGTACGTCCTGGCCCAGTTCCTGGGGGCCTTCGCGGGCTCCTGC
GCCGTGTACGCCCTGTACTACGACGCCCTGATGGCGTTCACCGACGGAGACCTGATGGTA
ACCGGGGACAACGCCACCGCCAACATCTTTGCTTCGTACCCGGCGAAGCATCTCTCCACT
CTCAACGGCTTCGTGGATCAGGTAATC---------------------------------
--GGCCTG
GTCGTCATGGCGATCGGCGTCTCCATGGGCTTCAACTGCGGCTACCCCATCAACCCCGCC
CGCGACCTGGGGCCCCGCCTCTTCACCGCCGTGGCCGGCTGGGGGCTGGACGTGTTCAGG

>Saithe_Aqp9b
---------------ATGGGACACGAAAGCATGACCAAATTCAAGGAGAAGTTCTCCCTG
AGGCACGCCATCATCAAGGAGTTCCTGGCCGAGTTTCTGGGGATCTTCGTCTTAATTCTG
TTTGGCTGCGGGGCGGTGGCCCAGGCTGTCCTCAGCAAAGGGGCCCTGGGGGGGCCCCTG
ACCATCCACATCGGCTTCACCCTGGGGGTCATGATGGCGGTGTTCATGGCGGGGGGGGTG
TCTGGAGCCCACGTGAACCCGGCCGTCTCTCTGGCCATGGTCCTCCTGGGGAAGCTGCCG
CTCAGGAAGTTCCCGGTGTACGTCCTGGCCCAGTTCCTGGGGGCCTTCGCGGGCTCCTGC
GCGGTGTACGCCCTGTACCACGATGCCCTGATGGCGTTCACCAACGGAGACCTGATGGTT
ACCGGGGACAACGCCACCGCCAACATCTTTGCTTCGTACCCAGCGAAGCATCTCTCCACT
CTCAACGGCTTCGTGGATCAGGTGATCGCCACGGCCGCCCTGATCCTGTGCATCCTGGCC
ATCACCGACGCTAAGAACCTGGGCGCGCCGCGGGGCATGGAGCCGCTCTGCATCGGCCTG
GTCGTCATGGCGATCGGCGTCTCCATGGGCTTCAACTGCGGCTACCCCATCAACCCCGCC
CGCGACCTGGGGCCGCGCTTCTTCACCGCCGTGGCCGGCTGGGGGCTGGACGTGTTCAGG

>Whiting_Aqp9b
---------------ATGGGACACGAAAGCATGACCAAAATCAAGGAGAAGTTCTCCCTG
AAGCACGCCATCATCAAGGAGTTCCTTGCCGAATTTCTGGGGATCTTCGTCTTAATTCTG
TTTGGCTGCGGGGCGGTGGCCCAGGCTATCCTCAGCAAGGGGGCCCTTGGGGGGCCCCTG
ACCATCCACATCGGCTTCACCCTGGGGGTCATGATGGCGGTGTTCATGGCGGGGGGGGTG
TCTGGCGCCCACGTGAACCCGGCCGTCTCTCTGGCCATGGTCCTTCTGGGGAAGCTGCCG
CTCAGGAAGTTCCCGGTGTACGTCTTGGCCCAGTTCCTGGGGGCCTTCGCGGGCTCCTGC
GCCGTGTACGCCCTGTACTACGATGCCCTGATGGCGTTCACCAACGGAGACCTGTTGGTT
ACCGGGGACAACGCCACCGCCAACATCTTTGCTTCATACCCAGCGAAGCATCTCTCCACT
CTCAACGGCTTCGTGGATCAGGTA------------------------------------

>Haddock_Aqp9b
---------------ATGGGACACGAAAGCATGACCAAAATCAAGGAGAAGTTCTCCCTG
AAGCACGCCATCATCAAGGAGTTCCTTGCCGAGTTTCTGGGGATCTTCGTCTTAATTCTG
TTTGGCTGCGGGGCGGTGGCCCAGGCTATCCTCAGCAAGGGGGCCCTTGGGGGGCCCCTG
ACCATCCACATCGGCTTCACCCTGGGGGTCATGATGGCGGTGTTCATGGCGGGGGGGGTG
TCTGGCGCCCACGTGAACCCGGCCGTCTCTCTGGCCATGGTCCTCCTGGGGAAGCTGCCC
CTCAGGAAGTTCCCGGTGTACGTCTTGGCCCAGTTCCTGGGGGCCTTCGCGGGCTCCTGC
GCCGTGTACGCTCTGTACTACGATGCCCTGATGGCGTTCACCAACGGAGACCTGATGGTT
ACCGGGGACAACGCCACCGCCAACATCTTTGCTTCGTACCCAGCGAAGCACCTCTCCACT
CTCAACGGCTTCGTGGATCAGGTGATCGCCACGGCCGCCCTGATCCTGTGCATCCTGGCG
ATCACCGACGCTAAGAACCTGGGCGCCCCGAGGGGCATGGAGCCGCTCTGCATCGGCCTG
GTCGTCATGGCGATCGGCGTCTCCATGGGCTTCAACTGCGGCTACCCCATCAACCCCGCC
CGCGACCTGGGGCCGCGCCTCTTCACCGCCGTGGCCGGCTGGGGGCTGGACGTGTTCAGG
GCTGGGGGGTGCTGGTGGTGGGTCCCGGTGGCGGGCCCCATGGTGGGGGGCGTGGTCGGG
GCCTCGCTCTACTTCCTGCTGGTGGAGCTGCACCACGCGGAG------------------
---------CCGGAGGGCCCC---------------------------------------
---------GAGGAGAACAGC------ACCAAGGACAAGTACGAGATGATCACCGTGAGC
>Poor_cod_Aqp9b
---------------ATGGGACACGAAAGCATGACCAAAATCAAGGAGAAGTTCGCTCTG
AAG--CTG
TTTGGCTGCGGGTCGGTGGCCCAGGCCGTCCTCAGCAAGGGGACCCAGGGGGGGCCCCTC
ACCATCCACATCGGCTTCACCCTGGGGGTCATGATGGCGGTGTTCATCGCGGCGGGGGTG
TCTGGCGCCCACGTGAACCCGGCCGTCTCTCTGGCCATGGTCCTGCTGGGGAAGATGCCC
CTCAAGAAGTTCCCCGTGTACGTCCTGGCCCAGTTCCTGGGGGCCTTCGCGGGCTCCGGC
GCGGTCTACGCTCTGTACTACGATGCACTGATGGCGTTCACCAACGGAGACCTGATGGTG

ACCGGGGACAACGCCACGGCCAACATCTTTGCCTCCTACCCAGCGACGCACCTCTCCACG
CTGAACGGCTTTGTGGATCAGGTGGTTGCCACGGCGGCGCTGATCCTGTGCATCCTGGCG
ATCACCGACACCCAGAACCGGGGCGCTCCGCGGGGCATGGAGCCGCTCTGCATCGGCCTG
GTCGTCATGGCGATCGGCGTCTCCATGGGCTTAAACTGCGGCTACCCCATCAACCCCGCC
CGCGACCTGGGGCCCCGCCTCTTCACCGCCGTGGCCGGCTGGGGCCTGGACGTGTTCAGG

>Silvery_cod_Aqp9b
---------------ATGGGACACGAAAACATGACCAAAATCAAGGAGAAGTTTGCCCTG
AAGCACGCCATCATCAAGGAGTTCCTGGCCGAGTTCCTGGGGATCTTCGTCTTAATTCTG
TTTGGCTGCGGCTCGGTGGCCCAGGCGGTCCTCAGCAAGGGGGCCCTGGGGGGCCCCCTC
ACCATCCACATCGGCTTCACCCTGGGGGTCATGATGGCCGTGTTCATGGCGGCGGGAGTA
TCCGGGGCCCACGTGAACCCGGCCGTCTCTCTGGCCATGGTCCTCCTGGGGAAGCTGCCC
ATCAAGAAGTTTCCGGTGTACGTCTTGGCCCAGTTCCTGGGGGCCTTCGCCGGCTCCTGC
GCGGTCTACGCCCTGTACTACGATGCTCTGATGGCGTTCACCAACGGAGATCTGATGGTG
ACCGGGGAGAACGCCACCGCCAGTATCTTTGCTTCCTACCCTGCGAAGCACCTCTCCACC
CTCAACGGCTTCGTGGATCAGGTGATTGCGACGGCTGCGCTGATCCTGTGCATCCTGGCG
ATCACCGACACCAAGAACCTGGGCGCACCGCGGGGCATGGAGCCGCTCTGCATCGGCTTG
GTCGTCATGGCGATCGGCGTGTCCATGGGCTTCAACTGTGGCTACCCCATCAACCCCGCC
CGCGACCTGGGGCCACGCCTCTTCACCGCCGTGGCCGGCTGGGGCCTGGACGTGTTCAGG
GCTGGCGGGTGCTGGTGGTGGATCCCGGTGGCAGGCCCCATGGTGGGGGGCGTGGTGGGG
GCCTCGCTGTACTTCCTGCTGGTGGAGCTCCACCACAAGGAG------------------
---------CCGGAGGTGGCG---------------------------------------
---------GAGGAGAGCAGC------ACCAAGGACAAGTACGAGATGATCACTGTGAGC
>Tusk_Aqp9b
---------------ATGGGACTCGATAGTATGACAAAAATAAAGGAGAAGTGTGCCCTT
AAGCACGACATCATCAAAGAGTTTTTGGCAGAATTTCTGGGGATATTCGTCTTAATTCTG
TTTGGCTGTGGGTCGGTGGCCCAGACCGTGCTCAGCAAGGGGGCCCTTGGGGGGCCCCTC
ACCATCCACATTGGCTTCACCCTGGGGGTCATGATGGCTGTGTTCATGGCCGGGGGGGTG
TCCGGAGCCCACGTGAACCCAGCGGTCTCTCTAGCCATGGTCCTCCTCGGAAAGCTGCCT
CTGAGGAAGTTCCCAGTGTATGTGCTGGCCCAGTTCCTCGGTGCCTTCGCCGGATCCTGC
GCTGTGTATGCCCTGTATTACGATGCTTTGATGGACTTCACAAACGGAGATCTGATGGTT
ACGGGTGGGAATGCTACAGCCAATATCTTTGCGTCCTACCCTGCGAAACACCTTTCAACT
CTAAACGGGTTTGTAGATCAGGTTATTGCAACTGCTGCCCTCGTCCTATGCATTCTGGCA
ATTACTGATGGGAAAAATATCGGGGCCCCGAAAGGAATGGAACCCCTTTGCATTGGTCTG
GTCATCATGGCCATCGGAGTGTCGATGGGCTTCAACTGTGGCTATCCAATCAACCCAGCA
CGGGACCTGGGCCCGAGGATCTTCACAGCTGTGGCTGGCTGGGGCATGGACGTGTTCAGG

>Ling_Aqp9b
---------------ATGGGACACGATAGTATGACAAAAATAAAGGAGAAGTGTGCCCTT
AGGCACGACATCATCAAAGAGTTTTTGGCTGAATTTCTGGGGACATTCGTCTTAATTCTG
TTTGGCTGTGGGGCGGTGGCCCAGACCCTGCTCAGCAAGGGGGCCTCTGGGGGGCCCCTC
ACCATCCACATTGGCTTCAGCCTGGGGGTCATGATGGCTGTGTTCATGGCCGGGGGGGTG
TCCGGAGCCCACGTGAACCCAGCGGTCTCTTTAGCCATGGTTCTCCTCGGGAGGCTGCCT
CTGAGGAAGTTCCCAGTGTATGTGCTGGCCCAGTTCCTCGGTGCCTTCGCCGGATCCTGC
GCTGTGTACGCCCTGTATTACGATGCTTTGATGAACTACACAAACGGAGATTTGATGGTT
ACGGGTCAGAATGCTACAGCCAATATCTTTGCGTCCTACCCTGCGAAACACCTTTCAACT
CTAAATGGGTTTGTAGATCAGGTTATTGCAACTGCTGCCCTCATCCTATGCATTCTGGCA
ATTACTGATGGGAAGAATATCGGGGCCCCGAAAGGAATGGAGCCCCTTTGCATTGGTCTG
GTCATCATGGCCATCGGAGTGTCAATGGACTTCAACTGTGGCTATCCCATCAACCCAGCG
CGGGACCTGGGCCCCAGGGTGTTCACAGCTGTGGCCGGCTGGGGCGTGGACGTGTTCAGG

------------------CTGGTTGAGCTGCACCACGTGGAG------------------
---------CCGGAGACGCTG---------------------------------------
---------GAGGTGAACAAC------GTGAACGACAAATATGAAATGATCACCGTGAGT
>Burbot_Aqp9b

```
---------------ATGGGACACGATAGTATGACAAAAATAAAGGAGAAGTGTGCCCTT
AGGCACAGCATCATCAAAGAGTTTTTGGCAGAATTTCTGGGGATATTCATCTTAATTCTG
TTTGGCTGTGGGTCGGTGGCCCAGACCGTACTCAGCAAGGGGGCCCTTGGGGGGCCCCTC
ACCATCCACATTGGCTTCACCCTGGGGGTCATGATGGCTGTGTTCATGGCCGGGGGAGTG
TCCGGAGCCCACGTGAACCCAGCGGTCTCTTTAGCCATGGTCCTCCTTGGAAGGCTGCCT
CTGAGGAAGTTCCCAGTGTATGTGCTGGCCCAGTTCCTCGGTGCCTTTGCCGGATCCTGT
GTTGTGTATGCCCTGTATTACGATGCTTTGATGGACTACACAAACGGAGATTTGATGGTT
ACGGGTGAGAATGCTACAGCCAATATCTTTGCGTCCTACCCTGCGAAACACCTTTCGACT
CTAAACGGGTTTGTAGATCAGGTTATTGCGACTGCTTCCCTCATCCTATGCATTCTGGCA
ATTACTGATGGGAAAAATATCGGGGCCCCGAAAGGAATGGAACCCCTTTGCATTGGTCTG
ATCATCATGGCCATCGGAGTGTCGATGGGCTTCAACTGTGGCTATCCAATTAACCCAGCA
CGGGACCTGGGCCCGAGGTTCTTCACTGCTGTCGCCGGCTGGGGCATGGACGTGTTCAGG
---GGG---TGCTGGTGGTGGATCCCCGTGGTGGGGCCCATGGTGGGGGCCGTGGTAGGG
GCCTCTGTGTACTTCCTGCTGGTTGAGCTGCACCACACGGAG------------------
---------CCGGAGACGCTG---------------------------------------
---------GAGGTGAACAAC------GTGAAGGACAAATATGAAATGATCACCGTGAGT
>Forkbeard_Aqp9b
---------------ATGGGACACGACAGCATGGCAAAAATAAAGGAAAAGTGTGCCCTG
AGGCATGACATCATCAAAGAGTTTTTGGCTGAATTTCTGGGGATATTCGTCTTAATTCTG
TTCGGCTGTGGGTCAGTGGCCCAGACCGTGCTCAGCAAAGGAGACCTGGGAGGCCCCCTC
ACCGTCCACATCGGCTTCACCCTGGGCGTCATGATGGCCGTGTACATGGCCGGGGGGGTG
TCCGGGGCTCACGTGAACCCGGCGGTGTCTTTAGCCATGGTCCTCCTCGGCAAGCTGCCC
CTGAGGAAGTTCCCGGTGTATGTGCTGGCGCAGTTCCTGGGTGCCTTCGTGGGATCCTGC
GCCGTGTACGGCCTGTATTACGATGGTCTGATGGCCTACACCAACGGAGACCTGGTGGTG
ACGGGCGAGAACGCCACAGCCAATATCTTTGCGTCGTACCCGGCGAAACACCTTTCCACG
CTGAACGGATTCGTAGATCAGGTTATTGCGACTGCTGCCCTCATCCTATGCATTCTGGCA
ATCACCGATCGCAAAAACATCGGGGCCCCTAAAGGCATGGAGCCCCTTTGCATTGGCCTG
ATCATCATGGCCATCGGCGTGTCCATGGGCTACAACTGTGGCTATCCCATCAACCCAGCG
CGGGACCTGGGCCCCAGGTTCTTCACCGCCGTCGCCGGCTGGGGAATGGAGGTGTTCAGG
------------------------------------------------------------
------------------------------------------------------------
------------------------------------------------------------
------------------------------------------------------------
>European_hake_Aqp9b
---------------ATGGGACACGATAATATGAACAAAATAAAGGAAAAGTGTGCCCTG
AGGCAGGGCATCATCAAAGAGTTTTTGGCTGAATTTCTGGGGATATTCGTCTTAATTCTA
TTCGGCTGTGGGTCGGTGGCCCAGACGGTGCTCAGCAAGGGAGCGCTGGGGGAACCCCTG
ACCATCCACATTGGCTTCAGCCTGGGGGTCATGATGGCTGTGTACATGGCCGGGGGGGTG
TCCGGAGCTCACGTGAACCCAGCAGTTTCTTTAGCCATGGTTCTCCTTGGCAAGCTGCCT
CTGAAGAAGTTCCCGGTGTATGTGCTGGCACAGTTCCTCGGGGCCTTCGCTGGATCCTGT
GCAGTGTATGGCCTGTATTACGATGCTTTGATGGACTATACAAGCGGAGATTTGATGGTC
ACGGGTGAAAATGCTACAGCCAATATATTTGCATCCTACCCTGCAAAACACCTTTCGACT
CTGAACGGATTTGTAGATCAGGTTATTGCAACTGCTGCACTCATCCTATGCATTCTGGCA
ATCACTGATGGGAAAAATATTGGCGCCCCCAAAGGAATGGAACCGCTTTGCATTGGTCTG
ATCATTATGGCCATCGGCGTGTCGATGGGCTTCAACTGTGGCTATCCAATCAATCCGGCG
CGGGACCTTGGCCCGAGGTTCTTCACCGCCGTAGCCGGCTGGGGTATGGATGTGTTCAGG
GCTGGAGGGTGCTGGTGGTGGATCCCTGTGGTGGGGCCCATGGTGGGGGGCACTGTGGGG
GCCGGTGTGTACTTCCTGCTGATCGAGCTGCATCACCCAGAG------------------
---------CCGGACCCGTTG---------------------------------------
---------GAGGAGAACATG------GTGAAGGACAAATATGAAATGATCACTGTGAGT
>Benguela_hake_Aqp9b
---------------ATGGGACACGATAGTATGAACAAAATAAAGGAAAAGTGTGCCCTG
AGGCAGGGCATCATCAAAGAGTTTTTGGCTGAATTTCTGGGGATATTCGTCTTAATTCTA
TTCGGCTGTGGGTCGGTGGCCCAGACGGTGCTCAGCAAGGGAGCGCTGGGGGAACCCCTG
ACCATCCACATTGGCTTCAGCCTGGGGGTCATGATGGCTGTGTACATGGCCGGGGGGGTG
TCCGGAGCTCACGTGAACCCAGCAGTTTCTTTAGCCATGGTTCTCCTTGGCAAGCTGCCT
CTGAAAAAGTTCCCAGTGTATGTGCTGGCACAGTTCCTCGGGGCCTTCGCTGGATCCTGT
GCAGTGTATGGCCTGTATTACGATGCTTTGATGGACTATACAAGCGGAGATTTGATGGTC
ACGGGTGAAAATGCTACAGCCAATATATTTGCATCCTACCCTGCAAAACACCTTTCGACT
CTGAACGGATTTGTAGATCAGGTTATTGCAACTGCTGCACTCATCCTATGCATTCTGGCA
ATCACTGATGGGAAAAATATTGGCGCCCCCAAAGGAATGGAACCGCTCTGCATTGGTCTG
```

ATCATTATGGCCATCGGCGTGTCGATGGGCTTCAACTGTGGCTATCCAATCAATCCGGCG
CGGGACCTGGGCCCGAGGTTCTTCACCGCCGTAGCCGGCTGGGGTATGGACGTGTTCAGG
GCTGGAGGGTGCTGGTGGTGGATCCCTGTGGTGGGGCCCATGGTGGGGGGCACTGTGGGG
GCCGGTGTGTACTTCCTGCTGATCGAGCTGCATCACCCAGAG------------------
---------CCGGACCCGGTG---------------------------------------
---------GAGGAGAACATT------GTGAAGGACAAATATGAAATGATCACTGTGAGT
>Cape_hake_Aqp9b
---------------ATGGGACACGATAATATGAACAAAATAAAGGAAAAGTGTGCCCTG
AGGCAGGGCATCATCAAAGAGTTTTTGGCTGAATTTCTGGGGATATTCGTCTTAATTCTA
TTCGGCTGTGGGTCGGTGGCCCAGACGGTGCTCAGCAAGGGAGCGCTGGGGGAACCCCTG
ACCATCCACATTGGCTTCAGCCTGGGGGTCATGATGGCTGTGTACATGGCCGGGGGGGTG
TCCGGAGCTCACGTGAACCCAGCAGTTTCTTTAGCCATGGTTCTCCTGGGCAAGCTGCCT
CTGAAGAAGTTCCCGGTGTATGTGCTGGCACAGTTCCTCGGGGCCTTCGCTGGATCCTGT
GCAGTGTATGGCCTGTATTACGATGCTTTGATGGACTATACAAGCGGAGATTTGATGGTC
ACGGGTGAAAATGCTACAGCCAATATATTTGCATCCTACCCTGCAAAACACCTTTCGACT
CTGAACGGATTTGTAGATCAGGTTATTGCAACTGCTGCACTCATCCTATGCATTCTGGCA
ATCACTGATGGGAAAAATATTGGCGCCCCCAAAGGAATGGAACCACTTTGCATTGGTCTG
ATCATTATGGCCATCGGCGTGTCGATGGGCTTCAACTGTGGCTATCCAATCAATCCGGCG
CGGGACCTGGGCCCGAGGTTCTTCACCGCCGTAGCCGGCTGGGGTATGGACGTGTTCAGG
GCTGGAGGGTGCTGGTGGTGGATCCCTGTGGTGGGGCCCATGGTGGGGGGCACTGTGGGG
GCCGGTGTGTACTTCCTGCTGATCGAGCTGCATCACCCAGAG------------------
---------CCAGACCCGTTG---------------------------------------
---------GAGGAGAACATG------GTGAAGGACAAATATGAAATGATCACTGTGAGT
>Common_mora_Aqp9b
---------------ATGGGACACGATAGTATGAGAAAAATGAAGGAAAAGTGTGCTCTG
AGGCACGACATCATCAAAGAGTTTTTGGCTGAATTCCTGGGGATATTCGTCTTAATTCTG
TTTGGCTGTGGGTCGGTGGCCCAGATGGTGCTCAGCAGGGGAGCTCTGGGGGAGCCCCTC
ACCATCCACATCGGCTTCACCCTGGGGGTCATGATGGCTGTGTACCTGGCCGGGGGGGTG
TCCGGCGCTCACGTGAACCCAGCGGTTTCTTTAGCCATGGTTATCCTTGGCAAGCTGCCT
CTGAAGAAGTTCCCAGTGTATGTGCTGGCACAGTTCCTCGGTGCCTTCGCCGGATCCTGC
GCAGTGTACGGCCTGTATTACGATGCTTTGATGGACTACACAAACGGAGATCTGATGGTC
ACGGGCGAGAATGCGACAGCCAATATATTTGCGACCTACCCTGCGAAACACCTTTCGACT
CTGAACGGATTGGTAGATCAGGTAATCGCAACTGCTGCCCTCGTCCTATGCATCCTGGCA
ATCACTGACGGGAAAAATATTGGCGCCCCCAAAGGAATGGAGCCCCTCTGTATCGGTCTG
ATCGTTATGGCCATCGGCGTGTCGATGGGCTTCAACTGTGGCTATCCCATCAACCCGGCG
CGGGACCTGGGCCCGAGGTTCTTCACGGCTGTAGCCGGCTGGGGCATGGACGTGTTCAGG
GCCGGAGGGTGCTGGTGGTGGATCCCTGTGGCGGGGCCCATGGTGGGGGGCGTGGTGGGG
GCCGGCGTGTACTTCCTGCTGATCGAGCTGCATCACGCGGAG------------------
---------CCAGAGACGGTG---------------------------------------
---------GAGGAGAACAAC------GTGAAGGACAAATACGAAATGATCACCGTGAGT
>Guinean_codling_Aqp9b
---------------ATGGGACACGATAGCATGAGAAAACTTAAGGAAAAGTGTGCCCTG
AGGCACGACATCATCAAAGAGTTTTTGGCTGAATTCCTGGGGATATTCGTCTTAATTCTG
TTTGGCTGTGGGTCGGTGGCCCAGACGGTACTCAGCAAGGGAGCCCAGGGGGAGCCCCTC
ACCGTCCACATCGGCTTCACCCTGGGGGTCATGATGGCTGTGTACATGGCCGGGGGGGTG
TCCGGCGCCCATGTGAACCCAGCGGTTTCCTTAGCCATGGTCATCCTTGGCAAGCTGCCT
CTGAAGAAGTTCCCAGTGTATGTGCTGGCCCAGTTCCTCGGTGCCTTCGCTGGATCCTGT
GCAGTGTATGGCCTGTATTACGATGCGTTGATGGACTACACAAACGGATATTTGGTGGTC
ACGGGTGAGAATGCTACAGCCAATATATTCGCGTCCTACCCTGCGAAACACCTTTCGACT
CTGAACGGATTCGTAGATCAGGTAATGGCAACCGCTGCGCTCATCCTATGCATTCTGGCA
ATCACCGACGGGAAAAACATCGGCGCCCCCAAGGGGATGGAACCCCTCTGCATCGGTCTG
ATCATCATGGCCATCGGCGTGTCGATGGGCTTCAACTGCGGCTACCCCATCAACCCGGCG
CGGGACCTGGGCCCGAGGTTCTTCACAGCCGTGGCCGGCTGGGGTATGGAGGTGTTCAGG

>Arrowtail_Aqp9b
---------------ATGGGACACGATAGTATGAAAAAAATAAAGGAAAAGTGTGCCCTG
AGGCAGAACGTCATCAAAGAGTTTTTGGCTGAATTTCTGGGGATATTCGTCTTAATTCTG
TTTGGCTGTGGGTCGGTGGCCCAG------------------------------------

---GGAGCTCACGTGAACCCAGCGGTGTCTCTAGCCATGGTGGTCCTGGGCAAGCTGCCC
CTGAACAAGTTCCCGGTGTACGTTCTGGCTCAGTTCCTCGGTGCCTTCGCCGGATCCTGT
GCCGTGTACGCCGTGTACTACGATGCTCTGATGGACTACGCAGACGGAGATTTGATGGTC
ACGGGTGAGAATGCTACAGCCAATATATTTGCGTCCTACCCTGCGCGACACCTCTCGGCT
CTGAACGGATTCGGAGATCAGGTTATTGCAACTGCTGCACTCATCCTATGCATTCTGGCA
ATTACTGATGGGAAAAATATTGGCGCCCCAAAAGGAATGGAGCCCCTTTGTATTGGTCTG
AGCCTCATGGCCATCGCCGTGTCCATGGGCTTCAACTGCGGGTATCCGGTCAACCCGGCG
CGGGACCTGGGCCCCAGGTTCTTCACAGCGGTGGCCGGCTGGGGCATGGACGTGTTCAGG

>Roundnose_grenadier_Aqp9b
---------------ATGGGACAGGACAGTATGAACAAAATAAAGCAGAAGTGTCGCCTG
AGACACGCCGTCACCAGAGAGTTCCTGGCTGAGTTTCTGGGGATATTTTCGGTCCTGCTG
TTTGGCTGTGGGTCGGTGGCCCAGACGGTTCTCAGCAAGGGAGCCCTGGGTGAGCCCCTC
ACCGTCCACATTGGCTTCACCCTGGGGGTGATGATGGCTGTCTACATGGCCGGGGGGGTG
TCTGGTGCTCACGTGAACCCAGCAGTTTCTCTAGCCATGGTGGTCCTGGGCAAGCTGCCT
CTGAAGAAGTTCCCGGTGTATGTGCTGGCCCAGTTCCTGGGGGCCTTTGCTGGATCTTGC
GCCGTGTACGGCCTCTACTACGATGCACTGATGGACTTTGCCAATGGAGAGCTGGTGGTG
ACGGGGCAGAATGCTACAGCTAACATATTTGCGTCCTACCCTGCGAAACACCTTTCCACT
CTGAACGGGTTTGTAGATCAGGTTATTGCCACTGCTGCGCTCATCCTGTGCATCCTGGCG
ATCACCGATGGGAAGAACATTGGCGCTCCCAGAGGCATGGAGCCCCTCTGCATCGGTCTG
GTCATCATGGCCATCGCCGTGTCCATGGGCTTCAACTGCGGCTACCCCATCAACCCGGCG
CGGGACCTGGGCCCTCGCTTGTTCACTGCGGTGGCCGGCTGGGGCATGGACGTGTTCAGG
GCCGGGGGCTGCTGGTGGTGGATCCCGTGGTGGGGCCCAGTGGTGGGGCCCATGGTGGGG
GCCGCTGTGTACTTCCTGTTGGTGGAGCTGCACCACGCCCAA------------------
---------CCAGAGACCACGGCGGAG---------------------------------
---------CCCGAGAGCAGC------GCCAAGGACAAGTATGAGATGACCACCGTGAGT
>Western_softhead_grenadier_Aqp9b

---CTG
TTTGGCTGTGGGTCGGTGGCCCAGACGGTGCTCAGCAAGGGAGCTCTGGGAGAGCCTCTC
ACCATCCACATTGGCTTCACCCTGGGGGTGATGATGGCTGTGTACATGGCCGGGGGGGTG
TCTGGAGCGCACGTGAACCCAGCAGTTTCTCTCGCCATGGTTGTCCTGGGCAAGCTGCCT
CTGAAGAAGTTCCCGGTGTACGTGCTGGCCCAGTTCCTGGGTGCCTTTGCTGGGTCCTGT
GCGGTGTACAGCCTCTACTATGATGCGTTGATGGACTACACCAGCGGAGAGCTGCTGGTG
ACGGGGCAGAATGCTACAGCCAACATATTTGCGTCCTACCCTGCGAAGCACCTTTCCACT
CTGAACGGGTTTCTAGATCAGGTTATTGCCACTGCTGCGCTCATCCTTTGCATTCTGGCG
ATCACCGATGGGAAGAACATTGGTGCGCCCAAAGGCATGGAGCCCCTCTGCATCGGTCTG
GTCATCATGGCCATCGGAGTGTCCATGGGCTTCAACTGCGGCTACCCCATCAACCCAGCG
CGGGACCTGGGCCCCCGCTTTTTCACAGCGGTAGCCGGCTGGGGCATGGATGTGTTCAGG

>Roughnose_grenadier_Aqp9b
---------------ATGGGACGCGATAGTATGAAAAAAATTAAGGAAAAGTGTGCCCTG
AAGCACGGCTTCATCAAAGAGTTTTTGGCAGAATTTCTGGGGATGTTCATCTTAATTCTG
TTTGGCTGTGGGTCGGTGGCCCAGACGGTGCTCAGCAAGGGAGCCCTGGGCGAGCCTCTC
ACCATCCACATCGGCTTCACCCTGGGGGTCATGATGGCTGTGTACATGGCCGGGGGGGTG
TCCGGAGCCCACGTGAACCCAGCTGTTTCTTTAGCCATGGTTATTCTTGGCAAGCTGCCT
CTGAAGAAGTTCCCGGTGTACGTGCTGGCACAGTTCCTGGGTGCCTTCGCTGGGTCCTGT
GCAGTGTATGGCCTGTACTACGATGCTTTGATGGACTACACAAGCGGAGATTTGATGGTC
ACGGGTGAGAACGCAACAGCCAATATATTTGCGTCCTACCCTGCGAAACACCTTTCGACT
CTAAACGGATTCGTAGATCAGGTTATTGCAACTGCTGCACTCATCCTATGCATTCTGGCA
ATTACCGATGGGAAAAATATTGGCGCCCCAAAAGGAATGGAGCCCCTTTGTATCGGTCTG
ATCATCATGGCCATCGGCGTGTCCATGGGCTTCAACTGTGGCTATCCAATCAACCCGGCC
CGGGACCTGGGCCCCAGGTTCTTCACGGCTGTAGCCGGCTGGGGTATGGACGTGTTCAGG
GCTGGAGGCTGCTGGTGGTGGGTCCCTGTGGTAGGGCCCATGCTGGGGGGCATGGTGGGG

GCCGGCGTGTACTTCCTGCTGATTGCGCTGCATCACGAGGAG------------------
---------CCAGAGACGGGG---------------------------------------
---------GAGGAGAACAAC------GTGAAGGACAAATATGAAATGATCACTTTGAGC
>Vaillants_grenadier_Aqp9b
---------------ATGGGACACGACAGTATGAAAAAAATAAAGGAAAAGTGTGCCCTG
AGGCGCGACATCATCAAAGAGTTTTTGGCTGAATTTCTGGGGATATTCGTCTTAATTCTG
TTTGGCTGTGGGTCGGTGGCCCAGACAGTGCTCAGCAAGGGAGCCCTGGGGGAGCCCCTC
ACCATCCACATTGGCTTCACCCTGGGGGTCATGATGGCTGTGTACATGGCCGGGGGGGTG
TCCGGAGCTCACGTGAACCCAGCAGTTTCTTTAGCCATGGTTATCCTTGGCAAGCTGCCT
CTGAAGAAGTTCCCAGTGTATGTGCTGGCACAGTTCCTGGGTGCCTTTGCTGGATCCTGT
GCAGTGTATGGCCTGTATTACGATGCTTTGATGGACTACACAAACGGAGATTTGATGGTG
ACGGGTGAGAATGCTACGGCCAACATATTTGCGTCCTACCCTGCGAAACACCTTTCGACT
CTGAACGGATTTGTAGATCAGGTTATTGCAACCGCTGCACTCATCCTATGCATTCTGGCA
ATTACTGATGGGAAAAATATTGGCGCCCCGAAAGGAATGGAACCCCTTTGTATCGGTCTG
ATCATCATGGCCATCGGAGTGTCAATGGGCTTCAACTGTGGCTATCCAATCAACCCAGCG
CGGGACCTGGGCCCGAGGTTCTTCACAGCTGTAGCCGGCTGGGGCATGGATGTGTTCAGG
GCCGGAGGGTGCTGGTGGTGGATCCCTGTGGCAGGGCCCATGGTGGGGGGCGTGGTGGGG
GCCGGCGTGTACTTCCTGCTGGTGGAGCTGCATCACGCAGAG------------------
---------CCGGAGACGCCAGCA------------------------------------
---------GAGGAGACCAAC------GAGAAGGACAAATATGAAATGATCACCGTGAGT
>Onion_eye_grenadier_Aqp9b
------------------------------ATGGACAAACTGAAGCTAAAGTGCGCCCTG
AGGCACGACATCATCAGGGAGTTTCTGGCGGAGTTCCTGGGGACATGCGTCTTGATTCTG
TTTGGCTGTGGGTCGGTGGCTCAGACAGTGCTCAGCAAAGGAGCCCTAGGAGAGCCCCTC
ACCATCAACCTTGGCTTCACCCTGGGGGTGACGATGGCTGTCTTCATGGCCGGGGGCGTG
TCTGGAGCTCACTTGAACCCAGCGGTTTCTCTAGCCATGGTGGTCCTGGGCAAGCTGCCT
CTGAAGAAGTTCCCGGTGTACGTGCTGGCCCAGTTCCTGGGGGCCTTCGCTGGATCCTGC
GTCGTGTACGTCCTCTACTACGATGCACTGATGGACTACACCAACGGAGAGCTGCTGGTG
ACGGGGGAGAATGCTACAGCCAACATATTTGCGTCCTACCCTGCAAAACACCTTTCCACT
CTGAATGGGTTTTTAGATCAGGTTATTGCCACTGCTGCTCTCATCCTGTGCATCCTGGCG
ATCACCGATGGGAAGAACCTTGGTGCTCCCAAAGGCATGGAGCCCCTCTGCATCGGCCTG
GTCATCATGGGCATCGGCGTGTCCATGGGCTTCAACTGCGGCTACCCCATCAACCCGGCC
CGGGACCTGGCCCCCCGCTTCTTCACTGCCGTGGCCGGCTGGGGCATGGACGTGTTCAGG
GCCGGGGGCTGCTGGTGGTGGATCCCCGTGGTGGGGCCCATGCTGGGGGGCGTGGTGGGG
GCCGCTGTGTACGTCCTGCTGGTGGAGCTGCACCACACCCAG------------------
---------CCAGAGAGCACGGAGGAG---------------------------------
---------CCAGAGACCAGT------GTGAAGGACAAGTATGAGATGATCACCGTCAGT
>Roughsnot_grenadier_Aqp9b
---------------ATGGGACGCGTTAGTATGAAACAAATTAAGGAAAAGTGTGCCCTG
AAGCACAACTTCATCAAAGAGTTTTTGGCAGAATTTCTGGGGATGTTCGTCTTAATTCTG
TTTGGCTGTGGGTCGGTGGCCCAGTCGGTGCTCAGCAAGGGAACCCAGGGCGAGCCTCTC
ACCGTCCACATCGGCTTCACCCTGGGGGTCATGATGGCTGTGTACATGGCCGGGGGGGTG
TCCGGAGCCCACGTGAACCCAGCTGTTTCTTTAGCCATGGTTATTCTTGGCAAGCTGCCC
CTGAAGAAGTTCCCGGTGTACGTGCTGGCACAGTTCCTGGGTGCCTTCGCTGGGTCCTGT
GCAGTGTATGGCCTGTACTACGATGCTTTGATGGACTACACAAGCGGAGATTTGATGGTC
ACGGGTGAGAACGCTACAGCCAATATATTTGCGTCCTACCCTGCGAAACACCTTTCGACT
CTAAACGGATTCGTAGATCAGGTTATTGCAACTGCTGCACTCATCCTATGCATTCTGGCA
ATTACCGATGGGAAAAATATTGGCGCCCCAAAAGGAATGGAGCCCCTTTGTATCGGTCTG
ATCATCATGGCCATCGGCGTGTCCATGGGCTTCAACTGTGGCTATCCAATCAACCCGGCC
CGGGACCTGGGCCCCAGGTTCTTCACGGCTATAGCCGGCTGGGGTATGGACGTGTTCAGG

>Marbled_moray_cod_Aqp9b
---------------ATGGGACACGCCAGTATGCACAAAATAAGGGAAAAGTGTGCCCTG
AGGCACGACATCATCAAGGAGTTTTTGGCAGAGTTTCTGGGGATATTCGTCTTAATTCTG
TTTGGCTGTGGGTCGGTGGCCCAGACGGTGCTCAGCAAGGGTGCCCTGGGGGAGCCCCTC
ACTATCCACATTGGCTTCAGCCTGGGGGTCATGATGGCCGTGTACCTGGCTGGGGGGGTG
TCCGGAGCTCACGTCAACCCGGCCGTTTCTTTAGCCATGGTCATCCTTGGCAAGCTGCCA
GTGAAGAAGTTCCCAGTGTACGTGCTGGCCCAGTTCCTCGGAGCCTTCGCCGGGTCGTGC

GCCGTGTACGGCCTGTACTACGATGCTTTGATGGATTACACCAACGGGGATCTGATGGTT
ACGGGGGAGAACGCCACAGCCAATATATTTGCGTCCTACCCTGCGAAACACCTGTCGACT
CTCAACGGATTTGTAGACCAGATTATTGCAACTGCTGCACTCATCGTATGCATTCTGGCA
ATTACTGATGGGAAAAATATCGGGGCCCCAAAAGGAATGGAACCCCTTTGTATCGGTCTG
GTCATTATGGCCATCGGCGTGTCGATGGGCTTCAACTGTGGCTACCCAATCAACCCGGCA
CGCGACCTGGGGCCCAGACTCTTCACCGCCGTAGCCGGCTGGGGCATGGATGTGTTCAGG
GCTGGAGGGTGCTGGTGGTGGATCCCTGTGGCAGGGCCCATGGTGGGGGGTGTGGTGGGG
GCCGGCGTGTACTTCCTGTTGATCGAGCTGCACCACGCGGAG------------------
---------CCGGAG---ACAGAG------------------------------------
---------GAGGAGAGCAAC------GTGAAGGACAAATATGAGATGATCACCGTGAGT
>Tube_eye_Aqp9b
---------------ATGGAACATGATAGTATGAAAAAAATCAAGGAAAAGTGTGCCCTG
AGGCATGCCATCATCAAAGAGTTCCTGGCAGAATTTCTGGGAATATTCGTCTTAATTCTC
TTTGGTTGTGGATCAGTGGCCCAGACAGTGCTAAGCAAGGGGGCCCTTGGGGAGCCCCTC
ACCATCCACATCGGCTTCACCCTGGGGGTCATGATGGCTGTGTACATGGCTGGGGGGGTG
TCAGGGGCCCACGTTAACCCAGCCGTCTCTTTAGCCATGGTGGTCCTTGGCAAGTTACCT
CTGAAGAAGTTCCCCGTCTATGTGCTGGCACAGTTTCTGGGGGCCTTTGCTGGTTCCTGT
GCTGTTTATGGCTTGTATTATGATGCTTTGATGGACTATACAAATGGAGATCTTATTGTT
ACGGGTGAGAATGCTACAGCCAATATATTTGCATCCTATCCTGCCAAACACCTTTCGACT
CTAAATGGATTTGTAGATCAGGTTATTGCTACTGGTGCACTAATCCTGTGCATTCTGGCA
ATTACTGATGGGAAAAATATTGGTGCACCGAAAGGAATGGAACCCCTTTGTATTGGCTTG
ATCATCATGGCCATTGGCGTTTCAATGGGCTTGAACTGCGGCTATCCGATCAACCCTGCT
CGTGACCTGGGTCCTAGGTTCTTCACTGCCGTCGCTGGCTGGGGAATGGATGTGTTCAGG

>John_dory_Aqp9b
---------------ATGGAGCACGACAGGATGAGGAGATTCAGGGAGCGCTGTGCCCTG
AGGCAGGGCATCATCAAAGAGTTCCTGGCGGAATTCCTGGGGATTTTCCTCCTAATTCTT
TTTGGATGCGGGTCCGTGGCCCAGGCTGTGCTCAGCAAGGGAGCCCTGGGGGAGCCGCTG
ACCATCCACATTGGCTTCACCCTGGGCGTCATGATGGCTGTGTACGTGGCGGGTGGGGTG
TCAGGAGGTCACGTGAATCCCGCGGTCTCGTTGGCCATGGTGATTTTGGGAAAGTTACCT
CTCAAGAAGTTACCCGTGTACGTGCTCGCTCAGTTCCTGGGAGCCTTCGCCGGATCCTGT
GTTGTTTATTGCTTGTATTATGATGCTTTGATGGACTACACCAATGGAGAACTGGCTGTC
ACCGGTGTGAACGCCACGGCCAATATATTTGCGTCCTATCCTTCCAAACATCTTTCACCT
CTAAATGGATTTCTAGACCAGGTGATTGCAACCGCCGCGCTCATCCTTTGTATCCTGGCC
ATCACCGACCGTAAAAACATCGGCGCGCCGAAGGGGATGGAGCCTCTCGCCGTGGGCCTG
ATCATCCTGGCCATCGGCGTGTCGATGGGCCTGAACTGCGGCTACCCCATCAACCCTGCA
CGAGACCTGGCGCCCAGGTTCTTCACCGCCGTCGCCGGCTGGGGCATGGACGTGTTCAGG
TCTGGAGGCTGCTGGTGGTGGATCCCGGTGGCAGGTCCCATGGTGGGTGGCGTGGTGGGC
GCCGCCATATACCTCTTCTTCATTGACTTGCACCACGCCGAG------------------
---------CCCGAAAAACAA---------------------------------------
---------GAGGAT------------GTTAAGGACAAATATGAAATTATCACCATGAGT
>Rosy_dory_Aqp9b
---------------ATGGAGCACGACAAGATGAGGAGGTTCCGGCAGCGCTGCGCCCTG
AGGCAGGGCATCATCAAAGAGTTCCTGGCGGAATTCCTGGGGATTTTCCTCCTAATTCTT
TTTGGATGTGGGTCCGTGGCCCAGGCGGTGCTCAGCAAGGGAGCTCTGGGGGAGCCGCTG
ACCATCCACGTTGGCTTCACCCTCGGTGTCATGATGGCTGTGTACGTGGCAGGGGGTGTG
TCAGGAGGCCATGTGAATCCCGCGGTCTCGTTGGCCATGGTGATTTTGGGAAAGTTACCT
CTCAAGAAGTTCCCTGTGTATGTGCTCGCTCAGTTCCTGGGGGCCTTCGCTGGATCCTGT
GTTGTTTATTGCTTGTATTATGATGCTCTGATGGACTACACCAATGGAGAACTTGCTGTC
ACCGGTGTGAACGCCACGGCCAATATATTTGCGTCCTATCCTTCCAAACATCTTTCAGCT
GTAAATGGATTTCTAGACCAGGTGATTGCAACTGCCGCGCTGATCCTTTGCATTCTGGCG
ATCACCGATCGTAAAAACATCGGCGCTCCGAAGGGAATGGAACCTCTCGGCGTGGGCCTG
ATCATCATGGCCATCGGCGTGTCGATGGGCCTGAACTGCGGCTACCCCATCAACCCCGCA
CGAGACCTGGGACCCAGGTTCTTCACCGCTGTCGCCGGCTGGGGCATGGATGTGTTCAGG
GCTGGAGGCTGCTGGTGGTGGATCCCCGTGGCAGGTCCCATGGTGGGTGGCGTGGTGGGC
GCTGCCATATACCTTTTCTTCATTGAGTTGCACCACGCCGAG------------------
---------CCCGAAAAACAAGAG------------------------------------
---------GAGGAC------------GTTCGTGACAAATATGAAATTATTGCTATGAGT

```
>Southern_cavefish_Aqp9b
---------------ATGGAACTTGAGAGCAAAAGAAAATTAAAGGAAAGATGTGCCTTG
AGGCAAGACATCATCAAAGAGTTCTTGGCTGAATTTCTAGGAATATTCATCCTAATACTC
TTTGGATGTGGCTCGGTGGCCCAGTCAGTGCTCAGTAAAGGGGCAATGGGGGAGCAACTG
ACTATCCATATTGGTTTCACTCTGGGAGTTATGATGGCAGTCTACTTGGCAGGGGGGGTG
TCAGGCGCCCACGTTAATCCGGCAGTCTCTCTGGCTATGGTGATCTTGGGAAAATTACCT
CTGAAGAAGTTTCCTGTTTACGTGCTAGCACAGTTCCTGGGTGCCTTTGTTGGATCTTGT
GTTGTTTATGGCTTGTATTATGATGCTTTGATGGAGTATACCAATGGAGAACTTTATGTA
ACCGGTGCCAATGCCACTGCCAATATTTTTGCATCTTATCCTGCCAAACACCTCTCAGTT
CTAAATGGCTTTGTTGATCAGGTAATTGGTACTGGTGTTCTGATCTTGTGCATCCTGGCC
ATAACTGATGGGAATAACATTGGAGCTCCAAAAGGAATGGAACCTCTGTGCATTGGTTTG
ATCATCATGGCCATCGGGGTGTCCATGGGTTTCAACTGTGGTTATCCCATCAACCCTGCA
CGTGACTTGGGTCCAAGGTTGTTCACTGCTGTTGCTGGCTGGGGCATGGATGTGTTCAGG
GCTGGAGGGTGCTGGTGGTGGATCCCTGTAGCAGGGCCAATGGTGGGTGGTGTAGTGGGG
GCAGCCATCTACTTCCTGTTCATTGAGTTGCACCACCCCGAG------------------
---------CCTGAGAAACAA---------------------------------------
---------GAGGAGACCAAC------CTCAACGACAAGTATGAAATGGTAACTATGAGT
>Sand_roller_Aqp9b
---------------ATGGAATTTGAGAGCAAGAGAAAATTAAAGGAAAGATGTGCCCTG
AGGCAAGACATCATCAAAGAGTTCTTGGCTGAATTTCTGGGAATATTCGTTTTAATACTC
TTTGGATGTGGCTCGGTGGCCCAGACCGTGCTCAGTAAAGGAGCTCTGGGGGAGCCACTG
ACCATCCACATTGGTTTCACTCTGGGAGTTATGATGGCGGTCTACATGGCAGGGGGAGTG
TCAGGCGCCCATGTGAATCCTGCAGTCTCTCTGGCAATGGTGATCCTGGGAAAATTACCT
CTGAAGAAGTTCCCTGTTTACGTGCTGGCTCAGTTCCTGGGTGCCTTTGCTGGATCTTGT
GTTGTTTATGGCTTGTACTATGATGCTTTGATGGAATATACCAATGGAGAACTTTTTGTT
ACCGGTGTAAATGCCACTGCCAATATATTTGCATCCTATCCTGCCAAACACCTGTCGGTT
CTAAATGGCTTTGTAGATCAGGTGATTGCGACTGGCGCTCTGATCTTGTGCATCCTGGCA
ATTACTGATGGGAAGAACATTGGAGCTCCAAAAGGAATGGAACCGCTCTGCATTGGCCTG
ACCATCATGGCCATCGGGGTGTCCATGGGTCTGAACTGTGGTTATCCCATCAACCCGGCA
CGTGACCTGGGTCCAAGGTTCTTCACTGCTGTTGCTGGCTGGGGCATGGATGTGTTCAGG
GCTGGAGGTTGCTGGTGGTGGATCCCTGTGGCAGGGCCCATGGTGGGTGGCGTAGTGGGG
GCAGCCATCTACTTTGTGTTCATTGAGCTGCACCACCCCGAG------------------
---------CCTGAGAAACAG---------------------------------------
---------GAGGAGACCAAC------ATGAAGGAGAAGTATGAAATGGTTACTATGAGT
>European_smelt_Aqp9b
---------------ATGGAAGAAGAAAGCAAGAGAAAATTAAAGGAGCGATGCGCCCTG
AGGCAGGATATCATCAAAGAGTTCTTGGCGGAGTTTTTGGGAATATTTGTATTAATACTC
TTCGGGTGTGGCTCAGTGGCCCAGACGGTGCTCAGCAAGGGGGCTCAGGGAGAGCCCCTC
ACCATCCACATCGGCTTCACTCTGGGAGTGATGATGGCTGTCTACGTGGCTGGGGGAGTG
TCAGGAGCCCATGTCAACCCTGCTGTCTCTCTGGCTATGGTGATCCTGGGTAAGCTGCCC
ATCAAGAAGTTCCCAGTCTATGTGGTGGCACAGTTTCTGGGAGCCTTCGCTGGATCCTGT
GCAGTCTATGGGCTGTACTATGATGCCCTCATGGTCTACACGGGTGGAGTTCTGTCAGTT
ACTGGGCCAAATGCTACAGCTAACATATTTGCATCCTACCCTGCGCCACACCTCTCAGTC
CTCAACGGATTCGTAGATCAGGTAATCGCAACAGGTGCCTTGATCCTGTGCATCCTAGCC
ATCACTGATGGGAAGAACATCGGTGCTCCCAAAGGGATGGAGCCCCTCTGCATCAGTCTG
ATCATCATGGCCATCGGAGTGTCCATGGGCCTGAACTGTGGCTACCCCATAAACCCTGCT
CGTGACCTGGCCCCGAGACTGTTTACTGCCGTGGCTGGCTGGGGCATGGAAGTGTTCAGG
GCTGGAGGATGCTGGTGGTGGATTCCAGTGGCTGGGCCCATGGTGGGAGGCCTGGTAGGA
GCTGCCATCTACTTCCTCTTCATAGAGCTGCACCACCCTGAG------------------
---------CCCGAAAAACAG---------------------------------------
---------GAGGAGAACAAC------GTCCAGGACAAATATGAGATGATCACCATGAGT
>Rainbow_smelt_Aqp9b
---------------ATGGAAGAAGAAAGCAAGAGAAAATTAAGGGAGCGATGCGCCCTG
AGGCAGGATATCATCAAAGAGTTCTTGGCGGAGTTTTTGGGAATATTTGTATTAATACTC
TTCGGGTGTGGCTCAGTGGCCCAGACGGTGCTCAGCAAGGGGGCTCAGGGAGAGCCCCTC
ACCATCCACATCGGCTTCACTCTGGGAGTGATGATGGCTGTCTACGTGGCTGGGGGAGTG
TCAGGAGCCCATGTCAACCCTGCTGTCTCTCTGGCTATGGTGATCCTGGGTAAGCTGCCC
ATCAAGAAGTTCCCAGTCTATGTGGTGGCACAGTTTCTGGGAGCCTTCGCTGGATCCTGT
GCAGTCTATGGGCTGTACTATGATGCTCTCATGGTCTACACGGGTGGAGTTCTGTCAGTT
ACTGGGCCAAATGCTACAGCTAACATATTTGCATCCTACCCTGCGCCACACCTCTCAGTC
CTCAACGGATTCGTAGATCAGGTAATCGCAACAGGTGCCTTGATCCTGTGCATCCTAGCC
```

ATCACTGATGGGAAGAACATCGGTGCTCCCAAAGGGATGGAGCCCCTCTGCATCAGTCTG
ATCATCATGGCCATCGGAGTGTCCATGGGCCTGAACTGTGGCTACCCCATAAACCCTGCT
CGTGACCTGGCCCCGAGACTGTTCACTGCCGTGGCAGGCTGGGCATGGAAGTGTTCAGT
GTTGGAGGTTGCTGGTGGTGGATTCCAGTGGCTGGGCCCATGGTGGGAGGCCTGGTAGGA
GCTGCCATCTACTTCCTCTTCATAGAGCTGCACCACCCTGAG------------------
---------CCCGAGAAACAG---------------------------------------
---------GAGGAGAACAAC------GTCCAGGACAAATATGAGATGATCACCATGAGT
>Ayu_Aqp9b
----------------ATGGAAGAAGAAAGCAAGAGAAAATTAAAGGAGCGATGCGCCCTG
AGGCAGGATATCATCAAAGAGTTCTTGGCGGAGTTTCTGGGAATATTTGTATTAATACTC
TTCGGGTGTGGATCAGTGGCCCAGACGGTGCTCAGTAAGGGAGCTCTGGGAGAGCCCCTC
ACCATCCACATCGGCTTCACTCTGGGAGTAATGATGGCCGTCTACATGGCTGGGGGAGTG
TCAGGAGCCCATGTCAACCCCGCTGTCTCTCTGGCTATGGTGATCCTGGGCAAGCTACCC
ATCAAGAAGTTCCCCTTCTATGTGGTGGCGCAGTTTCTGGGAGCCTTCGCTGGATCGTGC
GCAGTTTATGGGCTGTACTATGATGCTCTCATGGACTACACGGGTGGAGTTCTGTCAGTG
ACTGGGGCAAATGCCACAGCTAACATTTTTGCATCCTACCCTGCGCCACACCTCTCAGTC
CTCAATGGATTCGTCGATCAGGTCATTGCAACAGGTGCCTTGATCCTTTGCATCCTGGCC
ATCACTGATGGGAAGAACATCGGTGCCCCCAAAGGGATGGAGCCCCTCTGCATCGGTCTG
ATCATCATGGCTATCGGAGTGTCCATGGGCCTAAACTGTGGCTATCCCATAAACCCTGCT
CGTGACCTGGCCCCGAGACTGTTCACTGCTGTGGCCGGCTGGGGAATGGAAGTGTTCAGT
TCTGGAGGATGCTGGTGGTGGATTCCGGTGGCTGGGCCCATGGTGGGAGGCCTGGTAGGA
GCAGCCATCTACTTCCTCTTCATAGAGCTGCACCACCCTGAG------------------
---------CCTGAGAAACAG---------------------------------------
---------GAAGAGAACAAC------GTCAAGGACAAATATGAGATGATCACCATGAGT
>Large_eye_snaggletooth_Aqp9b
----------------ATGGGGGACGAAAGACAGAGAAAAATAAAGCAGCGCTGTGGCTTG
AGGCAGGATATCATCAAAGAGTTCTTGGCGGAATGTCTGGGGATATTCGTATTAATACTC
TTTGGATGTGGTTCAGTGGCCCAGACGGTGCTGAGTAAAGGGGCTCTGGGAGAGCCGCTC
ACCATACACATCGGCTTCACGCTGGGGGTGATGATGGCCGTCTACGTGGCTGGAGGAGTG
TCAGGAGCCCATGTGAACCCTGCCGTCTCTCTGGCGATGGTCATCCTGGGGAAGCTGCCT
GTGAAGAAGTTCCCGGTCTACGTGGTCGCTCAGTTCCTGGGCGCCTTCGCTGGATCCTGT
GCCGTCTACGGACTGTATTATGACGCTCTAATGGAATACACTGGTGGAGTTCTGTCGGTT
ACTGGGGCCAATGCCACTGCTAATATATTTGCATCCTATCCTGCCCCGCACCTCTCTGTC
CTCAACGGATTTGTAGATCAGGTGATTGCAACGGGTGCCTTGATCCTGTGCATCCTGGCC
ATCACCGATAGCAAGAACATCGGGGCACCTAAAGGCATGGAGCCGCTATGCATCGGTCTG
ATCATCATGGCCATCGGGGTGTCCATGGGGCTGAACTGTGGCTATCCCATAAATCCCGCG
CGTGACCTGGGCCCCAGGCTCTTCACCGCAGTGGCCGGCTGGGGCATGGAGGTGTTCAGG
GCTGGAGGTTGCTGGTGGTGGATCCCAGTGGCGGGGCCCATGGTGGGAGGACTGGTGGGG
GCCGCCATCTACTTCCTGTTCATCGAGCTGCACCACCCCGAG------------------
---------TCCGAGAAACAA---------------------------------------
---------GAGGAGAACAAC------GTCAAGGAGAAATACGAGATGATCCCCTTGAGC
>Sockeye_salmon_Aqp9b2
ATGTCCGCGGGAAATATGGACCATGAGAGGAAAAGAAAAATGAAGGAGCAATGTGCCCTG
AGGAAGGACATCATCAAAGAGTTCCTGGCAGAATTTCTGGGGATATTCGTATTAATACTA
TTTGGATGTGGCTCGGTGGCCCAGACGGTGCTGAGTAAGGGAGCTCTGGGAGAACCCCTG
ACCATCCACATCGGCTTCACTCTGGGGGTGATGATGGCCGTCTACGTGGCTGGGGGAGTT
TCAGGGGCCCATGTGAACCCAGCAGTCTCCCTGGCCATGGTGGTCCTGGGCAAGCTGCCA
ATAAAGAAGTTCCCTGTCTACGTGGCAGCACAGTTCCTGGGTGCCTTTGCTGGATCTTGT
GCTGTCTATGGGTTGTATTATGATGCCTTAATGGAACACACAGGAGGAGTTATGCAAGTT
ACTGGCATCAATGCCACAGCTAATATATGGGCATCCTACCCTGCCAAGCACATCTCAGTC
CTTGGAGGATTCGTAGACCAGGTAATAGGAACAGCTGCCTTGATCCTGTGCATTCTGGCC
ATTATTGATGGGAAGAACATTGGAGCTCCTAAAGGGATGGAGCCACTGTGCATTGGCCTG
ATCATCATGGCCATCGGCGTGTCCATGGGACTCAACTGTGGCTATCCCATCAACCCTGCT
AGAGACCTGGGCCCAAGGCTCTTCACTGCTGTGGCCGGATGGGGCATGGAGGTGTTCCGG
GCTGGAGGCTGCTGGTGGTGGATCCCAGTAGTGGGGCCCATGGTGGGAGGAGTGGTGGGA
GCCGCCGTTTACTTCCTGTTCATCGAGCTGCACCATGCCGAG------------------
---------CCTGAGAAACAG---------------------------------------
---------GGGGAGAACAAC------ATCAAGGACAAATACGAGATGGTCACCCTGAGT
>Coho_salmon_Aqp9b2
ATGTCCGCGGGAAATATGGACCATGAGAGGAAAAGAAAAATGAAGGAGCAATGTGCCCTG
AGGAAGGACATCATCAAAGAGTTCCTGGCAGAATTTCTGGGGATATTCGTATTAATACTC

TTTGGATGTGGCTCGGTGGCCCAGACGGTGCTGAGTAAGGGAGCTCTGGGAGAACCCCTG
ACCATCCACATCGGCTTCACTCTGGGGGTGATGATGGCCGTCTACGTAGCTGGGGGAGTT
TCAGGGGCCCATGTGAACCCAGCAGTCTCCCTGGCCATGGTGGTCCTGGGCAAGCTGCCA
ATAAAGAAGTTCCCTGTCTACGTGGCAGCACAGTTCCTGGGTGCCTTTGCTGGATCTTGT
GCTGTCTATGGGTTGTATTATGATGCCTTAATGGAACACACAGGAGGAGTTATGCAAGTT
ACTGGCATCAATGCCACAGCTAATATATGGGCATCCTACCCTGCCAAGCACATCTCAGTC
CTTGGAGGATTCGTAGACCAGGTAATAGGAACAGCTGCCTTGATCCTGTGCATTCTGGCC
ATTATTGATGGGAAGAACATTGGAGCTCCTAAAGGGATGGAGCCACTGTGCATTGGCCTG
ATCATCATGGCCATCGGAGTGTCCATGGGACTCAACTGTGGCTATCCCATCAACCCTGCT
AGAGACCTGGGCCCAAGGCTCTTCACTGCTGTGGCCGGTTGGGGCATGGAGGTGTTCCGG
GCTGGAGGCTGCTGGTGGTGGATCCCAGTAGTGGGCCCAATGGTGGGAGGAGTGGTGGGA
GCTGCCGTTTACTTCCTGTTCATCGAGCTGCACCATGCCGAG------------------
---------GCTGAGAAACAG---------------------------------------
---------GGGGAGAACAAC------ATCAAGGACAAATATGAGATGGTCACCCTGAGT
>Chinook_salmon_Aqp9b2
ATGTCCGCGGGAAATATGGACCATGAGAGGAAAAGAAAAATGAAGGAGCAATGTGCCCTG
AGGAAGGACATCATCAAAGAGTTCCTGGCAGAATTTCTGGGGATATTCGTATTAATACTA
TTTGGATGTGGCTCTGTGGCCCAGACGGTGCTGAGTAAGGGAGCTCTGGGAGAACCCCTG
ACCATCCATATCGGCTTCACTCTGGGGGTGATGATGGCCGTCTACGTAGCTGGGGGAGTT
TCAGGGGCCCATGTGAACCCAGCAGTCTCCCTGGCCATGGTGGTCCTGGGCAAGCTGCCA
ATAAAGAAGTTCCCTGTCTACGTGGCAGCACAGTTCCTGGGTGCCTTTGCTGGATCTTGT
GCTGTCTATGGGTTGTATTATGATGCCTTAATGGAACACACAGGAGGAGTTATGCAAGTT
ACTGGCATCAATGCCACAGCTAATATATGGGCATCCTACCCTGCCAAGCACATCTCAGTC
CTTGGAGGATTCGTAGACCAGGTAATAGGAACAGCTGCCTTGATCCTGTGCATTCTGGCC
ATTATTGATGGGAAGAACATTGGAGCTCCCAAAGGGATGGAGCCACTGTGCATTGGCCTG
ATCATCATGGCCATCGGAGTGTCCATGGGACTCAACTGTGGCTATCCCATCAACCCTGCT
AGAGACCTGGGCCCAAGGCTCTTCACTGCTGTGGCCGGTTGGGGCATGGAGGTGTTCCGG
GCTGGAGGCTGCTGGTGGTGGATCCCAGTAGTGGGGCCCATGGTGGGAGGAGTGGTGGGA
GCCGCCATTTACTTCCTGTTCATCGAGCTGCACCATGCCGAG------------------
---------CCTGAGAAACAG---------------------------------------
---------AGGGAGAACAAC------ATCAAGGACAAATACGAGATGGTCACCCTGAGT
>Rainbow_trout_Aqp9b2
ATGTCCGCGGGAAATATGGACCATGAGAGGAAAAGAAAAATGAAGGAGCAATGTGCCCTG
AGGAAGGACATCATCAAAGAGTTCCTGGCAGAATTTCTGGGGATATTCGTATTAATACTA
TTTGGATGTGGCTCGGTGGCCCAGACGGTGCTGAGTAAGGGAGCTCTGGGAGAACCCCTG
ACCATCCACATCGGCTTCACTCTGGGGGTGATGATGGCCGTCTACGTGGCTGGGGGAGTT
TCAGGGGCCCATGTGAACCCAGCAGTCTCCCTGGCCATGGTGGTCCTGGGCAAGCTGCCA
ATAAAGAAGTTCCCTGTCTACGTGGCAGCACAGTTCCTGGGTGCCTTTGCTGGATCTTGT
GCTGTCTATGGGTTGTATTATGATGCCTTAATGGAACACACAGGAGGAGTTATGCAAGTT
ACTGGCGTCAATGCCACAGCTAATATATGGGCATCCTACCCTGCCAAGCACATCTCAGTC
CTTGGAGGATTCGTAGACCAGGTAATAGGAACAGCTGCCTTGATCCTGTGCATTCTGGCC
ATTATTGATGGGAAGAACATTGGAGCTCCTAAAGGGATGGAGCCACTGTGCATTGGCCTG
ATCATCATGGCCATCGGAGTGTCCATGGGACTCAACTGTGGCTATCCCATCAACCCTGCT
AGAGACCTGGGCCCAAGGCTCTTCACTGCTGTGGCCGGTTGGGGCATGGAGGTGTTCCGG
GCTGGAGGCTGCTGGTGGTGGATCCCAGTAGTGGGGCCCATGGTGGGAGGAGTGGTGGGA
GCCGCCGTTTACTTCCTGTTCATCGAGCTGCACCATGCCGAG------------------
---------CCTGAGAAACAG---------------------------------------
---------GGGGAGAACAAC------ATCAAGGACAAATACGAGATGGTCACCCTGAGT
>Arctic_charr_Aqp9b2
---TCCGCGGGAAATATGGACCTTGAGAGGAAAAGAAAAATGAAGGAGCAATGTGCCCTG
AGGAAGGACATCATCAAAGAGTTCCTGGCAGAATTTCTGGGGATATTCGTATTAATACTC
TTTGGATGTGGCTCGGTGGCCCAGACGGTGCTGAGTAAGGGAGCTCTGGGAGAACCCCTG
ACCATCCACATCGGCTTCACTCTGGGGGTGATGATGGCCGTCTACGTGGCTGGGGGAGTT
TCAGGGGCTCATGTGAACCCAGCAGTCTCCCTGGCCATGGTGGTCCTGGGCAAGCTGCCT
ATAAAGAAGTTCCCTGTCTACGTGGCAGCACAGTTCCTGGGTGCCTTCGCTGGATCTTGT
GCTGTCTATGGGTTGTATTATGATGCCTTAATGGAACACACAGGAGGAGTTATGCAAGTT
ACTGGCGTCAATGCCACAGCTAATATATGGGCATCCTACCCTGCCAAGCACATCTCAGTC
CTTGGAGGATTCGTAGACCAGGTGATAGGAACAGCTGCCTTGATCCTGTGCATTCTGGCC
ATTATTGATGGGAAGAACATTGGAGCTCCTAAAGGGATGGAGCCACTGTGCATTGGCCTG
ATCATCATGGCCATCGGAGTRTCCATGGGACTCAACTGTGGCTATCCCATCAACCCTGCC
AGAGACCTGGGCCCAAGGCTCTTCACTGCTGTGGCCGGATGGGGCATGGAGGTGTTCCGG

GCTGGAGGCTGCTGGTGGTGGATCCCAGTAGCGGGGCCCATGGTGGGAGGAGTGGTGGGG
GCCGCCGTTTACTTCCTGTTCATCGAGCTGCACCATTCCGAG------------------
---------CCTGAGAAACAG---------------------------------------
---------GGGGAGAACAAC------GTCAAGGACAAATATGAGATGGTCACCCTGAGT
>Brown_trout_Aqp9b2
ATGTCCTCGGGAAATATGGACCTTGAGAGGAAAAGAAAAATGAAGGAGCAATGTGCCCTG
AGGAAGGACATCATCAAAGAGTTCCTGGCAGAATTTCTGGGGATATTCGTATTAATACTC
TTTGGATGTGGCTCGGTGGCCCAGACAGTGCTGAGTAAGGGAGCTCTGGGAGAACCCCTG
ACCATCCACATCGGCTTCACTCTGGGGGTGATGATGGCCGTCTACGTGGCTGGGGGAGTG
TCAGGGGCTCATGTGAACCCAGCAGTCTCCCTGGCCATGGTGGTCCTGGGCAAGCTGCCT
ATAAAGAAGTTCCCTGTCTACGTGGCAGCACAGTTCCTGGGTGCCTTTGCTGGATCTTGT
GCTGTCTATGGGTTGTATTATGATGCCTTAATGGAACACACAGGAGGAGTTATGCAAGTT
ACTGGTGTCAATGCCACAGCTAATATATGGGCATCCTACCCTGCCAAGCACATCTCAGTC
CTTGGAGGATTCGTAGACCAGGTAATAGGAACAGCTGCCTTGATCCTGTGCATTCTGGCC
ATTATTGATGGGAAGAACATTGGAGCTCCTAAAGGGATGGAGCCACTGTGCATTGGCCTG
ATCATCATGGCCATCGGAGTGTCCATGGGACTCAACTGTGGCTATCCCATCAACCCTGCC
AGAGACCTGGGCCCAAGGCTCTTCACTGCTGTGGCCGGATGGGGCATGGAGGTGTTCCGG
GCTGGAGGCTGCTGGTGGTGGATCCCAGTAGCGGGGCCCATGGTGGGAGGAGTGGTGGGG
GCCGGCGTTTACTTCCTGTTCATCGAGCTGCACCATGCCGAG------------------
---------CCTGAGAAACAG---------------------------------------
---------GGGGAGAACAAC------GTCAAGGACAAATATGAGATGGTCACCCTGAGT
>Atlantic_salmon_Aqp9b2
ATGTCCTCGGGAAATATGGACCTTGAGAGGAAAAGAAAAATGAAGGAGCAATGTGCCCTG
AGGAAGGACATCATCAAAGAGTTCCTCGCAGAATTTCTGGGGATATTCGTATTAATACTC
TTTGGATGTGGCTCGGTGGCCCAGACAGTGCTGAGTAAGGGAGCTCTGGGAGAACCCCTA
ACCATACACATCGGCTTCACTCTGGGGGTGATGATGGCCGTCTACGTGGCTGGGGGAGTG
TCAGGGGCTCATGTGAACCCAGCAGTCTCCCTGGCCATGGTGGTCCTGGGCAAGCTGCCT
ATAAAGAAGTTCCCTGTCTACGTGGCAGCACAGTTCCTGGGTGCCTTCGCTGGATCTTGT
GCTGTCTATGGGTTGTATTATGATGCCTTAATGGAACACACAGGAGGAGTTATGCAAGTT
ACTGGTGTCAATGCCACAGCTAATATATGGGCATCCTACCCTGCCAAGCACATCTCAGTC
CTTGGAGGATTCGTAGACCAGGTAATAGGAACAGCTGCCTTGATCTTGTGCATTCTGGCC
ATTATTGATGGGAAGAACATTGGAGCTCCTAAAGGGATGGAGCCACTGTGCATTGGCTTG
ATCATCATGGCCATCGGAGTGTCCATGGGACTCAACTGTGGCTATCCCATCAACCCTGCC
AGAGACCTGGGCCCAAGGCTCTTCACTGCTGTGGCCGGATGGGGCATGGAGGTGTTCCGT
GCTGGAGGCTGCTGGTGGTGGATCCCAGTAGCGGGGCCCATGGTGGGAGGAGTGGTGGGG
GCCGGCGTTTACTTCCTGTTCATCGAGCTGCACCATGCCGAG------------------
---------CCTGAGAAACAG---------------------------------------
---------GGGGAGAACAAC------GTCAAGGACAAATACGAGATGGTCACCCTGAGT
>Tsinling_lenok_trout_Aqp9b2
---------------ATGGAACTTGAGAGCAAAAGAAAAATGAAGGAGCAATGTGCCCTG
AGGAAGGACATCATCAAAGAGTTTCTGGCAGAATTTCTGGGGATATTCGTATTAATACTA
TTTGGATGTGGCTCGGTGGCCCAGACAGTGCTGAGTAAGGGAGCTCTGGGAGAACCCCTG
ACCATCCACATCGGCTTCACTCTGGGGGTGATGATGGCCGTCTACGTGGCTGGGGGAGTT
TCAGGGGCTCATGTGAACCCAGCAGTCTCTCTGGCCATGGTGGTCCTGGGCAAGCTGCCT
ATAAAGAAGTTCCCTGTCTACGTGGCAGCACAGTTCCTGGGTGCCTTCGCTGGATCTTGT
GCTGTCTATGGGTTGTATTATGATGCGTTAATGGAACACACAGGAGGAGTTATGCAAGTT
ACTGGCGTCAATGCCACAGCTAATATATGGGCATCCTACCCTGCCAAGCACATCTCAGTC
CTTGGAGGATTCGTAGACCAGGTGATAGGAACAGCTGCCTTGATCCTGTGCATTCTGGCC
ATTATTGATGGGAAGAACATTGGAGCTCCTAAAGGGATGGAGCCACTGTGCATTGGCCTG
ATCATCATGGCCATCGGAGTGTCCATGGGACTCAACTGTGGCTATCCCATCAACCCTGCC
AGAGACCTGGGTCCAAGGATCTTCACTGCTGTGGCCGGATGGGGCATGGAGGTGTTTCGT
GCTGGAGGCTGCTGGTGGTGGATCCCAGTAGCGGGGCCCATGGTGGGAGGAGTGGTGGGG
GCCGCCGTTTACTTCCTGTTCATCGAGCTACACCATGCAGAG------------------
---------CCTGAGAAACAG---------------------------------------
---------GGGGAGAACAAC------GTCAAGGACAAATACGAGATGGTCACCCTGAGT
>Taimen_Aqp9b2
ATGTCCACGGGAAATATGGAACTTGAGAGCAAAAGAAAAATGAAGGAGCAATGTGCCCTG
AGGAAGGACATCATCAAAGAGTTTCTGGCAGAATTTCTGGGGATATTCGTATTAATACTA
TTTGGATGTGGCTCGGTGGCCCAGACAGTGCTGAGTAAGGGAGCTCTGGGAGAA------
---GGGGGAGTT
TCAGGGGCTCATGTGAACCCAGCAGTCTCTCTGGCCATGGTGGTCCTGGGCAAGCTGCCT

ATAAAGAAGTTCCCTGTCTACGTGGCAGCACAGTTCCTGGGTGCCTTCGCTGGATCTTGT
GCTGTCTATGGGTTGTATTATGATGCGTTAATGGAACACACAGGAGGAGTTATGCAAGTT
ACTGGCGTCAATGCCACAGCTAATATATGGGCATCCTACCCTGCCAAGCACATCTCAGTC
CTTGGAGGATTCGTAGACCAGGTAATAGGAACAGCTGCCTTGATCCTGTGCATTCTGGCC
ATTATTGATGGGAAGAACATTGGAGCTCCTAAAGGGATGGAACCACTGTGCATTGGCCTG
ATCATCATGGCCATCGGAGTGTCCATGGGACTCAACTGTGGCTATCCCATCAACCCTGCC
AGAGACCTGGGTCCAAGGATCTTCACTGCTGTGGCCGGATGGGGCATGGAGGTGTTCCGG
----------------------------------GGGCCCATGGTGGGAGGAGTGGTGGGG
GCCGCCGTTTACTTCCTGTTCATCGAGCTGCACCATGCCGAG------------------
---------CCTGAGAAACAG---------------------------------------
---------GGGGAGAACAAC------GTCAAGGACAAATATGAGATGGTCACCCTGAGT
>Huchen_Aqp9b2
ATGTCCACGGGAAATATGGAACTTGAGAGCAAAAGAAAAATGAAGGAGCAATGTGCCCTG
AGGAAGGACATAATCAAAGAGTTTCTGGCAGAATTTCTGGGGATATTCGTATTAATACTA
TTTGGATGTGGCTCGGTGGCCCAGACAGTGCTGAGTAAGGGAGCTCTGGGAGAACCCCTG
ACCATCCACATCGGCTTCACTCTGGGGGTGATGATGGCCGTCTACGTGGCTGGGGGAGTT
TCAGGGGCTCATGTGAACCCAGCAGTCTCTCTGGCCATGGTGGTCCTGGGCAAGCTGCCT
ATAAAGAAGTTCCCTGTCTACGTGGCAGCACAGTTCCTGGGTGCCTTCGCTGGATCTTGT
GCTGTCTATGGGTTGTATTATGATGCGTTAATGGAACACACAGGAGGAGTTATGCAAGTT
ACTGGCGTCAATGCCACAGCTAATATATGGGCATCCTACCCTGCCAAGCACATCTCAGTC
CTTGGAGGATTCGTAGACCAGGTAATAGGAACAGCTGCCTTGATCCTGTGCATTCTGGCC
ATTATTGATGGGAAGAACATTGGAGCTCCTAAAGGGATGGAACCACTGTGCATTGGCCTG
ATCATCATGGCCATCGGAGTGTCCATGGGACTCAACTGTGGCTATCCCATCAACCCTGCC
AGAGACCTGGGTCCAAGGATCTTCACTGCTGTGGCCGGATGGGGCATGGAGGTGTTCCGG
GCTGGAGGCTGCTGGTGGTGGATCCCAGTAGCGGGGCCCATGGTGGGAGGAGTGGTGGGG
GCCGCCGTTTACTTCCTGTTCATCGAGCTGCACCATGCCGAG------------------
---------CCTGAGAAACAG---------------------------------------
---------GGGGAGAACAAC------GTCAAGGACAAATATGAGATGGTCACCCTGAGT
>Grayling_Aqp9b2
---------------ATGGAACTTGAGGGCAAAACAAAAATGAAGGAGCAATGTTCCCTG
AGGAAGGACATCATCAAAGAGTTTCTGGCAGAATTTCTGGGGATATTCGTATTAATACTC
TTTGGATGTGGCTCGGTGGCCCAGGCCGTGCTGAGTAAGGGGGCTCTGGGAGAACCCCTG
ACCATTCACATCGGCTTCACCCTGGGGGTGATGATGGCAGTCTACGTGGCTGGGGGAGTG
TCAGGGGCTCATGTGAACCCAGCAGTCTCTCTGGCCATGGTGGTCCTGGGCAAACTGCCT
ATAAAGAAGTTCCCTGTCTACGTGGCAGCACAGTTCCTGGGTGCCTTCGCTGGATCTTGT
GCTGTCTATGGGTTGTATTATGATGCCTTAATGGAACACACAGGAGGAGTTATGCAAGTT
ACTGGCGTCAATGCCACAGCTAATATATGGGCATCCTACCCTGCCAAGAACCTCTCAGTC
CTTGGAGGATTTGTAGACCAGGTGATAGGAACAGCTGCCTTGATCCTGTGCATTCTGGCC
ATTATTGATGGGAAGAACAATGGAGCTCCTAAAGGGGTGGAGCCACTGTGCATTGGCCTG
ATCATCATGGCCATCGGAGTGTCCATGGGACTCAACTGTGGCTATCCCATCAACCCTGCC
AGAGACCTGGCCCCAAGGCTCTTCACTGCTGTGGCCGGATGGGGCATGGAGGTGTTCAGG
GCTGGAGACTGCTGGTGGTGGATCCCAGTAGCGGGGCCCATGGTGGGAGGAGTGGTGGGG
GCTGCCGTTTACTTCCTGTTCATTGAGCTGCACCATGCCGAA------------------
---------CCTGAGAAACAG---------------------------------------
---------GGGGAGAACAAC------GTCAAGGACAAATACGAGATGGTCACCCTGAGT
>Sockeye_salmon_Aqp9b1
ATGTCCGCGGGAAATATGGAACTTGAGGGAAAAAGAAAAATGAAGGAGCAATGTGCCCTG
AGGAAGGACATCATCAAAGAGTTCCTGGCAGAATTTCTGGGGATATTCGTATTAATACTC
TTTGGATGTGGCTCGGTGGCCCAGACAGTGCTGAGTAAGGGAGCTCTGGGAGAACCCCTG
ACCATCCACATCGGCTTCACTCTGGGGGTGATGATGGCCGTCTACGTGGCTGGGGGAGTG
TCAGGGGCCCATGTGAACCCAGCAGTCTCCCTGGCCATGGTGGTCCTGGGCAAGCTGCCT
ATAAAGAAGTTCCCTGTCTACGTGGCAGCACAGTTCCTGGGTGCCTTCGCTGGATCTTGT
GCTGTCTATGGGTTGTATTACGATGCCTTAATGGAACACACAGGAGGAGTTATGCAAGTT
ACTGGCATCAATGCCACAGCTAATATATGGGCATCCTACCCTGCCAAGCACGTCTCAGTC
CTTGGAGGATTCGTAGACCAGGTGATAGGAACAGCTGCCTTGATCCTGTGCATTCTGGCC
ATTATTGATGGGAAGAACATTGGAGCTCCTAAAGGGATGGAGCCACTGTGCATTGGCCTG
ATCATCATGGCCATCGGCGTGTCCATGGGACTCAACTGTGGCTATCCCATCAACCCTGCT
AGAGACCTGGGCCCAAGGCTCTTCACTGCTGTGGCCGGATGGGGCATGGAGGTGTTCCGG
GCTGGAGGCTGCTGGTGGTGGATCCCAGTAGCGGGGCCCATGGTGGGAGGAGTGGTGGGA
GCCGCCATTTACTTCCTGTTCATCGAGCTGCACCATGCCGAG------------------
---------CCTGAGAAACAG---------------------------------------

```
---------GGGGAGAACAAC------GTCAAGGACAAATACGAGATGGTCACCCTGAGT
>Coho_salmon_Aqp9b1
ATGTCCGCGGGAAATATGGAACTTGAGGGAAAAAGAAAAATTAAGGAGCAATGTGCCCTG
AGGAAGGACATCATCAAAGAGTTCCTGGCAGAATTTCTGGGGATATTCGTATTAATACTC
TTTGGATGTGGCTCGGTGGCCCAGACAGTGCTGAGTAAGGGAGCTCTGGGAGAACCCTTG
ACCATCCACATCGGCTTCACTCTGGGTGTGATGATGGCCGTCTACGTGGCTGGGGGAGTG
TCAGGGGCCCATGTGAACCCAGCAGTCTCTCTGGCCATGGTGGTCCTGGGCAAACTGCCT
ATAAAGAAGTTCCCTGTCTACGTGGCAGCACAGTTCCTGGGTGCCTTCGCTGGATCTTGT
GCTGTCTATGGGTTGTATTACGATGCCTTAATGGAACACACAGGAGGAGTTATGCAAGTT
ACTGGCATCAATGCCACAGCTAATATATGGGCATCCTACCCTGCCAAGCACATCTCAGTC
CTTGGAGGATTCGTAGACCAGGTGATAGGAACAGCTGCCTTGATCCTGTGCATTCTGGCC
ATTATTGATGGGAAGAACATTGGCGCTCCTAAAGGGATGGAACCACTGTGCATTGGCCTG
ATCATAATGGCCATCGGAGTGTCCATGGGACTCAACTGTGGCTATCCCATCAACCCTGCT
AGAGACCTGGGCCCAAGGCTCTTCACTGCTGTGGCCGGATGGGGCATGGAGGTGTTCCGG
GCTGGAGGCTGCTGGTGGTGGATCCCAGTAGCGGGGCCCATGGTGGGAGGAGTGGTGGGA
GCCGCCATTTACTTCCTGTTCATCGAGCTGCACCATGCCGAG------------------
---------CCTGAGAAACAG---------------------------------------
---------GGGGAGAACAAC------GTCAAGGACAAATACGAGATGGTCACCCTGAGT
>Chinook_salmon_Aqp9b1
ATGTCCGCGGGAAATATGGAACTTGAGGGAAAAAGAAAAATGAAGGAGCAATGTGCCCTG
AGGAAGGACATCATCAAAGAGTTCCTGGCAGAATTTCTGGGGATATTCGTATTAATACTC
TTTGGATGTGGCTCGGTGGCCCAGACAGTGCTGAGTAAGGGAGCTCTGGGAGAACCCCTG
ACCATCCACATCGGCTTCACTCTGGGGGTGATGATGGCCGTCTACGTGGCTGGGGGAGTG
TCAGGGGCCCATGTGAACCCAGCAGTCTCTCTGGCCATGGTGGTCCTGGGCAAGCTGCCT
ATAAAGAAGTTCCCTGTCTACGTGGCAGCACAGTTCCTGGGTGCCTTCGCTGGATCTTGT
GCTGTCTATGGGTTGTATTACGATGCCTTAATGGAACACACAGGAGGAGTTATGCAAGTT
ACTGGCATCAATGCCACAGCTAATATATGGGCATCCTACCCTGCCAAGCACATCTCAGTC
CTTGGAGGATTCGTAGACCAGGTGATAGGAACAGCTGCCTTGATCCTGTGCATTCTGGCC
ATTATTGATGGGAAGAACATTGGCGCTCCTAAAGGGATGGAGCCACTGTGCATTGGCCTG
ATCATAATGGCCATCGGAGTGTCCATGGGACTCAACTGTGGCTATCCCATCAACCCTGCT
AGAGACCTGGGCCCAAGGCTCTTCACTGCTGTGGCCGGATGGGGCATGGAGGTGTTCCGG
GCTGGAGGCTGCTGGTGGTGGATCCCAGTAGCGGGGCCAATGGTGGGAGGAGTGGTGGGA
GCCGCCATTTACTTCCTGTTCATCGAGCTGCACCATGCCGAG------------------
---------CCTGAGAAACAG---------------------------------------
---------GGGGAGAACAAC------GTCAAGGACAAATACGAGATGGTCACCCTGAGT
>Rainbow_trout_Aqp9b1
ATGTCCGCAGGAAATATGGAACTTGAGGGAAAAATAAAAATGAAGGAGCAATGTGCCCTG
AGGAAGGACATCATCAAAGAGTTCCTGGCAGAATTTCTGGGGATATTCGTATTAATACTC
TTTGGATGTGGCTCGGTGGCCCAGACAGTGCTGAGTAAGGGAGCTCTGGGAGAACCCCTG
ACCATCCACATCGGCTTCACTCTGGGTGTGATGATGGCCGTCTACGTGGCTGGGGGAGTG
TCAGGGGCTCATGTGAACCCAGCAGTCTCTCTGGCCATGGTGGTCCTGGGCAAGCTGCCT
ATAAAGAAGTTCCCTGTCTACATGGCAGCACAGTTCCTGGGTGCCTTCGCTGGATCTTGT
GCTGTCTATGGGTTGTATTACGATGCCTTAATGGAACACACAGGAGGAGTTATGCAAGTT
ACTGGCATCAATGCCACAGCTAATATATGGGCATCCTACCCTGCCAAGCACATCTCAGTC
CTTGGAGGATTCGTAGACCAGGTGATAGGAACAGCTGCCTTGATCCTGTGCATTCTGGCC
ATTATTGATGGGAAGAACATTGGAGCTCCTAAAGGGATGGAGCCACTGTGCATTGGCCTG
ATCATAATGGCCATCGGAGTGTCCATGGGACTCAACTGTGGCTATCCCATCAACCCTGCT
AGAGACCTGGGCCCAAGGCTCTTCACTGCTGTGGCCGGATGGGGCATGGAGGTGTTCCGG
GCTGGAGGCTGCTGGTGGTGGATCCCAGTAGCGGGGCCCATGGTGGGAGGAGTGGTGGGA
GCCGCCATTTACTTCCTGTTCATCGAGCTGCACCATGCCAAG------------------
---------CCTGAGAAACAG---------------------------------------
---------GGGGAGAACAAC------GTCAAGGACAAATATGAGATGGTCACCCTGAGT
>Arctic_charr_Aqp9b1
ATGTCCGCGGGAAATATGGAACTCGAGGGCAAAAGAAAAATGAAGGAGCAATGTGCCCTA
AGGAAGGACATCATCAAAGAGTTCCTGGCAGAATTTCTGGGGATATTCGTATTAATACTC
TTTGGATGTGGCTCGGTGGCCCAGACAGTGCTGAGTAAGGGAGCTCTGGGAGAACCCCTG
ACCATCCACATCGGCTTCACTCTGGGGGTGATGATGGCCGTCTACGTGGCTGGGGGAGTT
TCAGGGGCTCATGTGAACCCAGCAGTCTCTCTGGCCATGGTGGTCCTGGGCAAGCTGCCT
ATAAAGAAGTTCCCTGTCTACGTGGCAGCACAGTTCCTGGGTGCCTTCGCTGGATCTTGT
GCTGTCTATGGGTTGTATTAT---------------------------------------
------------------------------------------------------------
```

---------------------GTGATAGGAACAGCTGCCTTGATCCTGTGCATTCTGGCC
ATTATTGATGGGAAGAACATTGGAGCTCCTAAAGGGATGGAGCCACTGTGCATTGGCCTG
ATCATCATGGCCATCGGAGTRTCCATGGGACTCAACTGTGGCTATCCCATCAACCCTGCC
AGAGACCTGGGCCCAAGGCTCTTCACTGCTGTGGCCGGATGGGGCATGGAGGTGTTCCGG
GCTGGAGGCTGCTGGTGGTGGATCCCAGTAGCGGGGCCCATGGTGGGAGGAGTGGTGGGG
GCCGCCGTTTACTTCCTGTTCATTGAGCTGCACCATGCCGAG------------------
---------CCTGAGAAACAG---------------------------------------
---------GGGGAGAACAAC------GTCAAGGACAAATATGAGATGGTCACCCTGAGT
>Brook_trout_Aqp9b1
ATGTCCGCGGGAAATATGGAACTCGAGGGCAAAAGAAAAATGAAGGAGCAATGTGCCCTG
AGGAAGGACATCATCAAAGAGTTCCTGGCAGAATTTCTGGGGATATTCGTATTAATACTC
TTTGGATGTGGCTCGGTGGCCCAGACGGTGCTGAGTAAGGGAGCTCTGGGAGAACCCCTG
ACCATCCACATCGGCTTCACTCTGGGGGTGATGATGGCCGTCTACGTGGCTGGGGGAGTT
TCAGGGGCTCATGTGAACCCAGCAGTCTCCCTGGCCATGGTGGTCCTGGGCAAGCTGCCT
ATAAAGAAGTTCCCTGTCTACGTGGCAGCACAGTTCCTGGGTGCCTTCGCTGGATCTTGT
GCTGTCTATGGGTTGTATTATGATGCCTTAATGGAACACACAGGAGGAGTTATGCAAGTT
ACTGGCATCAATGCCACAGCTAATATATGGGCATCCTACCCTGCCAAGCACATCTCAGTT
CTTGGAGGATTCGTAGACCAGGTAATAGGAACAGCTGCCTTGATCCTGTGCATTCTGGCC
ATTATTGATGGGAAGAACATTGGAGCTCCTAAAGGGATGGAGCCATTGTGCATTGGCCTG
ATCATCATGGCCATCGGAGTATCCATGGGACTCAACTGTGGCTATCCCATCAACCCTGCC
AGAGACCTGGGCCCAAGGCTCTTCACTGCTGTGGCCGGATGGGGCATGGAGGTGTTCCGT
GCTGGAGGCTGCTGGTGGTGGATCCCAGTAGCGGGGCCCATGGTGGGAGGAGTGGTGGGG
GCCGCCGTTTACTTCCTGTTCATCGAGCTGCACCATTCCGAG------------------
---------CCTGAGAAACAG---------------------------------------
---------GGGGAGAACAAC------GTCAAGGACAAATATGAGATGGTCACCCTGAGT
>Brown_trout_Aqp9b1
ATGTCCGCGGGAAATATGGAACTTGAGGGCAAAAGAAAAATGAAGGAGCAATGTGCCCTG
AGGAAGGACATCATCAAAGAGTTCCTGGCAGAATTTCTGGGGATATTCGTATTAATACTC
TTTGGATGTGGCTCGGTGGCCCAGACAGTGCTGAGTAAGGGAGCTCTGGGAGAACCCCTA
ACCATCCACATCGGCTTCACTCTGGGGGTGATGATGGCCGTCTACGTGGCTGGGGGAGTG
TCAGGGGCTCATGTGAACCCAGCAGTCTCTCTGGCCATGGTGGTCCTGGGCAAGCTGCCT
ATAAAGAAGTTCCCTGTCTACGTGGCAGCACAGTTCCTGGGTGCCTTCGCTGGATCTTGT
GCTGTCTATGGGTTGTATTATGATGCCTTAATGGAACACACAGGAGGAGTTATGCAAGTT
ACTGGCGTCAATGCCACAGCTAATATATGGGCATCCTACCCTGCCAAGCACATCTCAGTC
CTTGGAGGATTCGTAGACCAGGTGATAGGAACAGCTGCCTTGATCCTGTGCATTCTGGCC
ATTATTGATGGGAAGAACATTGGAGCTCCTAAAGGGATGGAGCCACTGTGCATTGGCCTG
ATCATTATGGCCATCGGAGTGTCCATGGGACTCAACTGTGGCTATCCCATCAACCCTGCC
AGAGATCTGGGCCCAAGGCTCTTCACTGCTGTGGCTGGATGGGGCATGGAGGTGTTCCGG
GCTGGAGGCTGCTGGTGGTGGATCCCAGTAGCGGGGCCCATGGTGGGAGGAGTGGTGGGG
GCTGCCGTTTACTTCCTGTTCATCGAGGTGCACCATGCCGAG------------------
---------CCTGAGGAACAG---------------------------------------
---------GGGGAGAACAAC------GTCAAGGACAAATACGAGATGGTCACCCTGAAT
>Atlantic_salmon_Aqp9b1
ATGTCTGCGGGAAATATGGAACTTGAGGGCAAAAGAAAAATGAAGGAGCAATGTGCCCTG
AGGAAGGACATCATCAAAGAGTTCCTGGCAGAATTTCTGGGGATATTCGTATTAATACTC
TTTGGATGTGGCTCGGTGGCCCAGACAGTGCTGAGTAAGGGAGCTCTGGGAGAACCCCTG
ACCATCCACATCGACTTCACTCTGGGGGTGATGATGGCCGTCTACGTGGCTGGGGGAGTG
TCAGGGGTCCATGTGAACCCAGCAGTCTCTCTGGCCATGGTGGTCCTGGGCAAGCTGCCT
ATAAAGAAGTTCCCTGTCTACGTGGCAGCACAGTTCCTGGGTGCCTTCGCTGGATCTTGT
GCTGTCTATGGGTTGTATTATGATGCCTTAATGGAACACACAGGAGGAGTTATGCAAGTT
ACTGGCGTCAATGCCACAGCTAATATATGGGCATCCTACCCTGCCAAGCACATCTCAGTC
CTTGGAGGATTCGTAGACCAGGTGATAGGAACAGCTGCCTTGATCCTGTGCATTCTGGCC
ATTATTGATGGGAAGAACATTGGAGCTCCTAAAGGGATGGAGCCACTGTGCATTGGCCTG
ATCATTATGGCCATCGGAGTGTCCATGGGACTCAACTGTGGCTATCCCATCAACCCTGCC
AGAGATCTGGGCCCAAGGCTCTTCACTGCTGTGGCTGGATGGGGCATGGAGGTGTTCCGT
GCTGGAGGCTGCTGGTGGTGGATCCCAGTAGCGGGGCCCATGGTGGGAGGAGTGGTGGGG
GCTGCCGTTTACTTCCTGTTCATCGAGCTGCACCATGCCGAG------------------
---------CCTGAGGAACAG---------------------------------------
---------GGCGAGAACAAC------GTCAAGGACAAATACGAGATGGTCACCCTGAAT
>Huchen_Aqp9b1
ATGTCCGCGGGAAATATGGAACTTGAGGGCAAAAGAAAAATGAAGGAGCAATGTGCCCTG

AGGAAGGACATCATCAAAGAGTTCCTGGCAGAATCTTTGGGGATATTCGTATTAATACTC
TTTGGATGTGGCTCGGTGGCCCAGACGGTGCTGAGTAAGGGGGCTCTGGGAGAACCCCTG
ACCATCCACATCGGTTTCACTCTGGGGGTGATGATGGCCGTCTACGTGGCTGGGGGAGTG
TCAGGGGCTCATGTGAACCCAGCAGTCTCTCTGGCCATGGTGGTCCTGGGCAAGCTGCCT
ATAAAGAAGTTCCCTGTCTACGTGGCAGCACAGTTCCTGGGTGCCTTTGCTGGATCTTGT
GCTGTCTATGGGCTGTATTATGATGCCTTAATGGAACACACAGGAGGAGTTATGCAAGTT
ACTGGCGTCAATGCCACAGCTAATATATGGGCATCCTACCCTGCCAAGCACATCTCAGTC
CTTGGAGGATTCGTAGACCAGGTGATAGGAACAGCTGCCTTGATCCTGTGCATTCTGGCC
ATTATTGATGGGAAGAACATTGGAGCTCCTAAAGGGATGGAGCCACTGTGCATTGGCCTG
ATCATCATGGCCATCGGGGTGTCCATGGGACTCAACTGTGGCTATCCCATCAACCCTGCC
AGAGACCTGGGCCCAAGGCTCTTCACTGCTGTGGCTGGATGGGGCATGGAGGTGTTCCGG
GCTGGAGGCTGCTGGTGGTGGATCCCAGTAGCGGGGCCCATGGTGGGAGGAGTGGTGGGG
GCCGCCGTTTACTTCCTGTTCATCGAGCTGCACCATGCCAAG------------------
---------CCTGAGAAACAG---------------------------------------
---------GGGGAGAACAAC------GTCAAGGACAAATACGAGATGGCCACCCTGAGT
>Lake_whitefish_Aqp9b1
---------------ATGGAATTTGAGGGCAAAAGAAAAATGAAGGAGCTATGTGCCCTG
AGGAAGGACATCATCAAAGAGTTCCTGGCAGAATTTCTGGGGATATTCGTATTAATACTC
TTTGGTTGTGGCTCGGTGGCCCAGACGGTGCTGAGTAAAGGGGCTCTGGGAGAACCCCTG
ACCATCCACATCGGCTTCACTCTGGGGGTGATGATGGCCGTCTACGTGGCTGGGGGAGTG
TCAGGGGCTCATGTGAACCCAGCAGTCTCTCTGGCCATGGTGGTCCTGGGCAAACTGCCT
ATAAAGAAGTTCCCTGTCTACGTGGCAGCACAGTTCCTCGGTGCCTTCGCTGGTTCTTGT
GCTGTCTATGGGTTGTATTATGATGCCTTAATGGAACACACAGGAGGAGTTATGCAAGTT
ACTGGCGTCAATGCCACAGCTAATATATGGGCATCCTACCCTGCCAAGCACATCTCAGTC
ATTGGAGGATTCGTAGACCAGGTTATAGGAACAGCTGCCTTGATCCTGTGCATTCTGGCC
ATTATTGATGGGAAGAACATTGGAGCTCCTAAAGGGATGGAGCCACTGTGCATTGGCCTG
ATCATCATGGCCATTGGAGTGTCCATGGGACTCAACTGTGGCTATCCCATCAACCCTGCC
AGAGACCTGGGCCCAAGGCTCTTCACTGCTGTGGCTGGATGGGGCATGGAGGTGTTCAGT
GCTGGAGGCTGCTGGTGGTGGATCCCAGTAGCAGGGCCCATGGTGGGAGGAGTGGTGGGG
GCCGCCGTTTACTTCCTGTTCATCGAGCTGCACCATGCCGAG------------------
---------CCTGAGAAACAG---------------------------------------
---------GGGGAGAACAAC------GTCAAGGACAAGTACGAGATGGTCACCCTGAGT
>Common_whitefish_Aqp9b1
---------------ATGGAATTTGAGGGCAAAAGAAAAATGAAGGAGCAATGTGCCCTG
AGGAAGGACATCATCAAAGAGTTCCTGGCAGAATTTCTGGGGATATTCGTATTAATACTC
TTTGGTTGTGGCTCGGTGGCCCAGACGGTGCTGAGTAAAGGGGCTCTGGGAGAACCCCTG
ACCATCCACATCGGCTTCACTCTGGGGGTGATGATGGCCGTCTACGTGGCTGGGGGAGTG
TCAGGGGCTCATGTGAACCCAGCAGTCTCTCTGGCCATGGTGGTCCTGGGCAAACTGCCT
ATAAAGAAGTTCCCTGTCTACGTGGCAGCACAGTTCCTGGGTGCCTTCGCTGGTTCTTGT
GCTGTCTATGGGTTGTATTATGATGCCTTAATGGAACACACAGGAGGAGTTATGCAAGTT
ACTGGCGTCAATGCCACAGCTAATATATGGGCATCCTACCCTGCCAAGCACATCTCAGTC
CTTGGAGGATTCGTAGACCAGGTTATAGGAACAGCTGCCTTGATCCTGTGCATTCTGGCC
ATTATTGATGGGAAGAACATTGGAGCTCCTAAAGGGATGGAGCCACTGTGCATTGGCCTG
ATCATCATGGCCATTGGAGTGTCCATGGGACTCAACTGTGGCTATCCCATCAACCCTGCC
AGAGACCTGGGCCCAAGGCTCTTCACTGCTGTGGCTGGATGGGGCATGGAGGTGTTCAGT
GCTGGAGGCTGCTGGTGGTGGATCCCAGTAGCAGGGCCCATGGTGGGAGGAGTGGTGGGG
GCCGCCGTTTACTTCCTGTTCATCGAGCTGCACCATGCCGAG------------------
---------CCTGAGAAACAG---------------------------------------
---------GGGGAGAACAAC------GTCAAGGACAAATACGAGATGGTCACCCTGAGT
>Balchen_Aqp9b1
---------------ATGGAATTTGAGGGCAAAAGAAAAATGAAGGAGCTATGTGCCCTG
AGGAAGGACATCATCAAAGAGTTCCTGGCAGAATTTCTGGGGATATTCGTATTAATACTC
TTTGGTTGTGGCTCGGTGGCCCAGACGGTGCTGAGTAAAGGGGCTCTGGGAGAACCCCTG
ACCATCCACATCGGCTTCACTCTGGGGGTGATGATGGCCGTCTACGTGGCTGGGGGAGTG
TCAGGGGCTCATGTGAACCCAGCAGTCTCTCTGGCCATGGTGGTCCTGGGCAAACTGCCT
ATAAAGAAGTTCCCTGTCTACGTGGCAGCACAGTTCCTGGGTGCCTTCGCTGGTTCTTGT
GCTGTCTATGGGTTGTATTATGATGCCTTAATGGAACACACAGGAGGAGTTATGCAAGTT
ACTGGCGTCAATGCCACAGCTAATATATGGGCATCCTACCCTGCCAAGCACATCTCAGTC
CTTGGAGGATTCGTAGACCAGGTTATAGGAACAGCTGCCTTGATCCTGTGCATTCTGGCC
ATTATTGATGGGAAGAACATTGGAGCTCCTAAAGGGATGGAGCCACTGTGCATTGGCCTG
ATCATCATGGCCATTGGAGTGTCCATGGGACTCAACTGTGGCTATCCCATCAACCCTGCC

AGAGACCTGGGCCCAAGGCTCTTCACTGCTGTGGCTGGATGGGGCATGGAGGTGTTCAGG
GCTGGAGGCTGCTGGTGGTGGATCCCAGTAGCGGGGCCCATGGTGGGAGGAGTGGTGGGG
GCCGCCATTTACTACCTGTTCATCGAGCTGCACCATGCCGAG------------------

>Grayling_Aqp9b1
---------------ATGGAACTTGAGGGCAAAAAGAAAATGAAGGAGCAATGTGCCCTG
AGGAAGGACATCATCAAAGAGTTCTTGGCAGAATTTCTAGGGATATTCGTATTAATACTC
TTTGGATGTGGCTCGGTGGCCCAGACAGTGCTGAGTAAAGGGGCTCTGGGAGAACCCCTG
ACCATTCACATCGGCTTCACCCTGGGGGTGATGATGGCTGTCTACGTGGCTGGGGGAGTG
TCAGGGGCTCATGTGAACCCAGCAGTCTCTCTGGCCATGGTGGTCCTGGGCAAACTGCCT
ATAAAGAAGTTCCCTGTCTACGTGGCAGCACAGTTCCTGGGTGCCTTCGCTGGATCTTGT
GCTGTCTATGGATTGTATTATGATGCCTTAATGGAACACACAGGAGGAGTTATGCAAGTT
ACTGGCGTCAATGCCACAGCTAATATATGGGCATCCTACCCTGCCAAGCACCTCTCAGTC
CTTGGAGGATTCGTAGACCAGGTGATAGGAACTGCTGCCTTGATCCTGTGCATTCTGGCC
ATTATTGATGGGAAGAACATTGGAGCTCCTAAAGGGGTGGAGCCACTGTGCATTGGCCTG
ATCATCATGGCCATCGGAGTCTCCATGGGCCTCAACTGTGGCTATCCCATCAACCCTGCC
AGAGACCTAGGCCCAAGGCTCTTCACTGCTGTGGCCGGATGGGGCATGGAGGTGTTCAGG
GCTGGAGGCTGCTGGTGGTGGATCCCAGTAGCGGGGCCCATGGTGGGAGGAGTGGTGGGG
GCCGCCGTTTACTTCCTGTTCATTGAGCTGCACCATGCCGAA------------------
---------CCTGAGAAACAG---------------------------------------
---------GGGGAGAACAAC------GTCAAGGACAAATACGAGATGGTCACCCTGAGT
>Northern_pike_Aqp9b
---------------ATGGATCTTGAGAGCAGAAGAAAGATTAAGAAAAGATGTGCGCTG
AAGAAGGACATAATAAAAGAGTTCCTTGCAGAATTTCTGGGGATATTTGTGTTAATACTC
TTTGGCTGTGGCTCGGTGGCTCAGACGGTGCTGAGTAAGGGGGCTCTGGGTGAACCCCTG
ACCATTCACATTGGCTTCACTCTGGGGGTGATGATGGCAGCATACGTTGCTGGGGGCGTG
TCAGGGGCTCACGTGAACCCAGCTGTTTCTCTGGCTATGGTGGTTCTGGGCAAACTGCCT
ATAAAGAAGTTCCCAGTCTACGTAGCTGCACAGTTCCTGGGTGCCTTTGTAGGGTCGTGT
GCTGTCTACGGCTTGTATTATGATGCCTTAATGGAACACACAGGAGGAGTTCTGCAAGTT
ACTGGCGTCAATGCTACAGCTAACATATGGGCATCCTATCCAGCCAAGCACCTATCAATC
CTTGGAGGATTTGTAGACCAGGTGATTGGAACAGCGGCCCTGATCCTCTGCATCCTAGCG
ATTATTGACGGGAAGAACATTGGCGCTCCAAAAGGAATGGAGCCTCTGTGCATTGGGCTG
ATCATCATGGCCATTGGAGTGTCCATGGGACACAACTGTGGCTATCCCATCAACCCTGCC
AGAGACCTGGGCCCCAGGCTCTTCACTGCTGTGGCTGGATGGGGTATGGAAGTGTTCAGG
GCTGGAGGATGCTGGTGGTGGGTCCCGGTGGCAGGGCCTATGGTGGGAGGAGTGCTGGGG
GCTGTCATGTACTTCCTGTTCATCGAGCTGCATCACGCTGAA------------------
---------CCTGAGAAACAG---------------------------------------
---------GGGGAAAACAAC------ATCAAGGACAAATACGAGATGGTCACCATGAGT
>Eastern_mudminnow_Aqp9b
---------------ATGGAACTTGAGGGCAGAAGAAAAATTCAAGAGAAATGTGCCCTG
AAGAAGGACATTATAAAAGAGTTTCTAGCTGAATTCTTGGGGATATTTGTATTAATACTC
TTTGGATGTGGCTCAGTCGCCCAGACGGTGCTAAGCAAGGGGGCTCTTGGTGAACCCCTG
ACCATCCACATCGGCTTCACACTGGGGGTGATGATGGCTGTTTACATCGCTGGGGGAGTG
TCAGGCGCTCACGTGAACCCTGCTGTCTCCCTGGCTATGGTGGTTCTGGGAAAACTGCCT
ATTAGAAAGTTCCCAGTCTACGTGGCGGCACAGTTCCTGGGTGCCTTTGCTGGATCGTGT
GCTGTCTATGGGTTGTATTACGATGCCCTAATGGAACACAGTGGAGGAGTTCTGCAGGTT
ACAGGGATCAATGCTACTGCTAATATATGGGCATCTTACCCAGCCAAACATATCTCAGTG
CTTGGTGGATTTGTAGATCAGGTGATTGGAACAGCATCCTTGATCCTGTGCATCCTAGCG
ATTATCGATGGGAAAAACATTGGTGCTCCTAAAGGGATGGAGCCGCTGTGCATTGGGCTG
ATCATCATGGCCATCGGAGTGTCCATGGGATTCAACTGTGGCTATCCCATCAACCCTGCT
AGAGACCTGGGCCCCAGGCTGTTCACTGCTGTGGCTGGATGGGGTATGGAAGTGTTCAGA
GCTGGGGGCTGCTGGTGGTGGATCCCAGTGATCGGACCCATGCTCGGAGGAGTGGTGGGG
GCTGCCATTTATTTCCTGTTCATCGAGCTGCACCATACCGAA------------------
---------CCTGAGAAACAA---------------------------------------
---------GGGGAGAACAAC------GTCAAAGATAAATACGAGATGGTCACCATGAGT
>Anshui_blind_cavefish_Aqp9b1
---------------ATGGAGCTCGAAAACTTCAGAAATTTGAGGGAGAGGTGCAGCGTG
AGGCGGGACATCATCAGAGAGTTTCTGGCTGAATTACTAGGGACATTCGTACTAATACTC
TTCGGTTGTGGTTCAGTGGCCCAGACTATTCTCAGCAGAGAAAAACATGGAGAAAATCTT
ACCATCCACTTTGGCTTTACTCTAGGGGTGATGCTGGCTGTGTACATGGCAGGAGGCGTG

TCAGGTGGACATGTGAACCCCGCTGTTTCTCTGGCTATGGTCGTTTTGGGGAAACTCCCA
TTAAAGAAATTTCCTGTCTATGTGGCAGCCCAATTTCTAGGTGCTTTTGCAGGGTCTTGT
GCTGTCTTCTGTCTGTACTATGGTGCCTTCGCAAATTTTGCTGATGGAAAACAAATTGTA
GTTGGTGAAAACGCCACAGCAGGTATCTTTGCCTCATATCCACGTGAAAATCTCTCATTG
TTAAATGGATTCATTGATCAGGTGATTGGCACAGGCGCCCTGGTCCTCTGTATTTTAGCC
ATTGTTGATAAGAAGAATATTGGGGCACCTAAAGGAATGGAGCCACTGGTTATTGGTCTG
AGCATCCTGGCTATCGGAGTGTCCATGGCTCTGAACTGTGGCTATCCCATCAACCCTGCC
AGAGACTTGGGACCTCGGCTCTTCACTGCTATGGCAGGATGGGGACTAGAGGTGTTCAGG
GCTGGAAATGGCTGGTGGTGGGTTCCAGTGGTCGGACCGATGGTGGGCGGTGTGGCCGGG
GCAGTGATCTACTTCCTGATGATTGAGCTGCATCACCCTGAG------------------
---------CTTGAGAAAAACCTTGAA-----------------------------------
---------GATGACAACAGC------ATCAAAGATAAATATGAATTGAACACCGTAAAC
>Horned_golden_line_barbel_Aqp9b1
---------------ATGGAGCTCGAAAACTTCAGAAATTTGAGGGAGAGGTGCAGCGTG
AGGCGGGACATCATCAGAGAGTTTCTGGCGGAATTACTAGGGACATTCGTACTAATACTC
TTCGGTTGTGGTTCAGTGGCCCAGACTATTCTCAGCAGAGAAAAACATGGAGAAAATCTT
ACCATCCACTTTGGCTTTACTCTAGGGGTGATGCTGGCTGTGTACATGGCAGGAGGCGTG
TCAGGT--

-------------------------GCCTTCGCAAATTTTGCTGATGGAAAACAAATTGTA
GTTGGTGAAAATGCCACAGCAGGTATCTTTGCCTCATATCCACGTGAAAATCTCTCATTG
TTAAATGGATTCATTGATCAGGTGATTGGCACAGGCGCCCTGGTCCTCTGTATTTTAGCC
ATTGTTGATAAGAAGAATATTGGGGCACCTAAAGGAATGGAGCCACTGGTTATTGGTCTG
AGCATCCTGGCTATCGGAGTGTCCATGGCTCTGAACTGTGGCTATCCCATCAACCCTGCC
AGAGACTTGGGACCTCGGCTCTTCACTGCTATGGCAGGATGGGGACTAGAGGTGTTCAGG
GCTGGAAATGGCTGGTGGTGGGTTCCAGTGGTCGGGCCGATGGTGGGCGGTGTGGCCGGG
GCAGTGATCTACTTCCTGATGATTGAGCTGCATCACCCTGAG------------------
---------CTTGAGAAAAACCTTGAA-----------------------------------
---------GATGACAACAGC------ATCAAAGATAAATATGAATTGAACACCGTGAAC
>Golden_line_fish_Aqp9b1
---------------ATGGAGCTCGAAAACTTCAGAAATTTGAGGGAGAGGTGCAGCGTG
AGGCGGGACATCATCAGAGAGTTTCTGGCGGAATTACTAGGGACATTCGTACTAATACTC
TTCGGTTGTGGTTCAGTGGCCCAGACTATTCTCAGCAGAGAAAAACATGGAGAAAATCTT
ACCATCCACTTTGGCTTTACTCTAGGGGTGATGCTGGCTGTGTACATGGCAGGAGGCGTG
TCAGGTGGACATGTGAACCCCGCTGTTTCTCTGGCTATGGTTGTCTTGGGGAAACTCCCA
TTAAAGAAATTTCCTGTCTATGTGGCAGCCCAATTTCTAGGTGCTTTTGCAGGGTCTTGT
GCTGTCTTCTGTCTGTACTATGGTGCCTTTGCAAATTTTGCTGATGGAAAACAAATTGTA
GTTGGTGAAAATGCCACAGCAGGTATCTTTGCCTCATACCCACGTGAAAATCTCTCATTG
TTAAATGGATTCATTGATCAGGTGATTGGCACAGGCGCCCTGGTCCTCTGTATTTTAGCC
ATTGTTGATAAGAAGAATATTGGGGCACCTAAAGGAATGGAGCCACTGGTTATTGGTCTG
AGCATCCTGGCTATCGGTGTGTCCATGGCTCTGAACTGTGGCTATCCCATCAACCCTGCC
AGAGACTTGGGACCTCGACTCTTCACTGCTATGGCAGGATGGGGACTAGAGGTGTTCAGG
GCTGGAAATGGCTGGTGGTGGGTTCCAGTGGTTGGGCCGATGGTGGGCGGTGTGGCCGGG
GCAGTGATCTACTTCCTGATGATTGAGCTGCATCACCCTGAG------------------
---------CTTGAGAAAAACCTTGAA-----------------------------------
---------GATGACAACAGC------ATCAAAGATAAATATGAATTGAACACCGTAAAC
>Common_carp_Aqp9b1
---------------ATGGAGCTCGAAAACTTTCGAAATTTGAGGGAGAGATGCACCTTG
AGGCGGGACATCATCAGAGAGTTTCTGGCGGAATTGCTAGGGACATTCGTACTAATACTC
TTCGGTTGTGGTTCAGTGGCCCAGACTATTCTTAGCAGAGAAAAACAAGGAGAAAATCTC
ACCATCCACTTTGGCTTTACTCTAGGGGTGATGCTAGCCGTGTACATGGCAGGAGGCGTG
TCAGGTGGACATGTGAACCCAGCTGTTTCTCTGGCTATGGTCGTCTTGGGGAAACTCCCA
TTAAAGAAATTTCCTGTCTATGTGGCAGCCCAATTTCTAGGTGCCTTTGCAGGGTCTTGT
GCTGTCTTCTGTCTGTACTATGGTGCATTCGCAAATTTTGCTGATGGAAAACAAATTGTA
GTTGGTGAAAATGCCACAGCGGGTATCTTTGCCTCATATCCACGTGAAGATCTCTCATTG
TTAAATGGATTCATTGATCAGGTGATTGGCACAGGTGCCCTGGTCCTCTGTATTTTAGCC
ATTGTTGATAAGAAGAATATTGGGGCACCTAAAGGAATGGAGCCACTGGTTATTGGTCTG
AGCATCCTGGCTATCGGTGTGTCCATGGCGCTGAACTGTGGCTATCCCATCAACCCTGCC
AGAGACTTGGGACCTCGGCTCTTCACTGCTATGGCAGGATGGGGACTAGAGGTGTTCAGG
GCTGGAAATGGCTGGTGGTGGGTTCCAGTGGTCGGGCCGATGGTGGGCGGTGTGGCCGGG
GCAGTGATCTACTTCCTGATGATTGAGCTGCATCACCCTGAG------------------

---------CTTGAGAAAAACCTTGAA---------------------------------
---------GATGACAACAGC------ATCAAAGATAAATATGAACTGAACACCGTGAAT
>Goldfish_Aqp9b1
---------------ATGGAGCTCGAAAACTTTAGAAATCTGAGGGAGAGGTGCACCTTG
AGGCAGGACATCATCAGAGAGTTTCTGGCAGAATTACTAGGGACATTCGTACTAATACTC
TTCGGTTGTGGTTCAGTGGCCCAGACTATTCTTAGCAGAGAAAAACAAGGAGAAAATCTT
ACCATCCACTTTGGCTTTACTCTAGGGGTGATGCTGGCTGTGTACATGGCAGGAGGCGTG
TCAGGTGGACATGTGAACCCCGCTGTTTCTCTGGCTATGGTTGTCTTGGGGAAACTCCAA
TTAAAGAAATTTCCTGTCTATGTGGCAGCCCAATTTCTAGGTGCCTTTGCAGGGTCTTGT
GCTGTCTATTGTCTGTACTATGATGCCTTTGCAAATTTTGCTGATGGAAAACTAATTGTA
GTTGGTGAAAACGCCACAGCAGGTGTCTTCGCCTCATATCCACGTGAAGATCTCTCATTG
TTAAATGGATTCATTGATCAGGTGATTGGCACAGGTGCCCTGGTCCTCTGTATTTTAGCC
ATTGTTGATAAGAAGAATATTGGGGCACCTAAAGGAATGGAGCCACTGGTTATTGGTCTG
AGCATCCTGGCTATCGGAGTGTCCATGGCGCTGAACTGTGGGTATCCCATCAACCCTGCC
AGAGACTTGGGACCTCGGCTCTTCACTGCTATGGCAGGATGGGGACTAGAGGTGTTCAGC
GCTGGAAATGGCTGGTGGTGGGTTCCAGTGGTCGGGCCAATGGTGGGCGGTGTGGCCGGG
GCAGTGATCTACTTCCTGATGATTGAGCTTCATCACCCTGAG------------------
---------CTTGAGAAAAACCCTGAA---------------------------------
---------GATGACAACAGC------ATCAAAGATAAATATGAACTGAACACCGTGAAC
>Anshui_blind_cavefish_Aqp9b2
---------------ATGGAGATCGAAAACATTAGAAATTTGAGGGAGAGATGCACCGTG
AGGCGGGACATCATCAGAGAGTTTCTGGCAGAATTATTGGGGACATTCATATTAATACTC
TTCGGCTGTGGTTCAGTGGCCCAGACCATTCTCAGCAGAGAAAAACAAGGAGAAAATCTC
ACCATCCATTTTGGCTTTACTTTAGGGGTAATGCTGGCTGTGTACATGGCAGGAGGTGTG
TCAGGCGGACATGTGAACCCAGCTGTTTCTCTGGCTATGGTTGTCTTGGGGAAACTCCCA
TTAAAGAAATTTCCTGTCTATGTGGTAGCCCAGTTTCTAGGTGCCTTTGCGGGGTCTTGT
GCTGTCTTCTGTCTGTACTATGGTGCCTTCGCAAATTTTGCTGGTGGAAAACGGATTGTA
ATTGGTGAAAATGCCACAGCAGGTATCTTTGCCTCATATCCACACGAAGATCTTTCATTG
TTAAATGGATTAATTGATCAGGTGATTGGCACAGGTGCCCTGGTCCTCTGTATTTTAGCC
ATCGTAGATAAGAAGAATATTGGGGCCCCTAAAGGAATGGAGCCACTGGTTATTGGTCTG
AGCATCCTGGCTATCGGAGTGTCCATGGCGCTGAACTGCGGCTATCCCATCAACCCTGCC
AGAGACTTGGGACCTCGGCTCTTCACTGCTATGGCAGGATGGGGACTAGATGTGTTCAGG
GCTGGAAATGGCTGGTGGTGGGTTCCAGTGGTCGGGCCGATGGTGGGTGCTGTGGCTGGG
GCAGTGATCTACTTCCTGATGATTGAGCTGCATCACCCCCAG------------------
---------CCTGAGAAGAACCTTGAA---------------------------------
---------GACGACAACAGC------ATCAAAGATAAATATGAATTGAACTCCGTAAAC
>Horned_golden_line_barbel_Aqp9b2
---------------ATGGAGCTCGAAAACAGTAGAAATTTGAGGGAGAGATGCACCGTG
AGGCAGGACATCATCAGAGAGTTTCTGGCAGAATTATTGGGGACATTCATATTAATACTC
TTCGGCTGTGGTTCAGTGGCCCAGACCATTCTCAGCAGAGAAAAACAAGGAGAAAACCTC
ACCATCCATTTTGGCTTTACTTTAGGGGTAATGCTGGCTGTGTACATGGCAGGAGGAGTG
TCAGGTGGACATGTGAACCCAGCAGTTTCTCTGGCTATGGTTGTCTTGGGGAAACTCCCA
TTAAAGAAATTTCCTTTCTATGTGGTAGCCCAGTTTCTAGGTGCCTTTGCGGGGTCTTGT
GCTGTCTTCTGTCTGTACTATGGTGCCTTCGCAAATTTTGCTGGTGGAAAACGGATTGTA
ATTGGTGAAAATGCCACAGCAGGTATCTTTGCCTCATATCCACACGAAGATCTTTCATTG
TTAAATGGATTAATTGATCAGGTGATTGGCACAGGTGCCCTGGTCCTCTGTATTTTAGCC
ATCGTAGATAAGAAGAATATTGGGGCCCCTAAAGGAATGGAGCCACTGGTTATTGGTCTG
AGCATCCTGGCTATCGGAGTGTCCATGGCGCTGAACTGCGGCTATCCCATCAACCCTGCC
AGAGACTTGGGACCTCGGCTCTTCACTGCTATGGCAGGATGGGGACTAGATGTGTTCAGG
GCTGGAAATGGCTGGTGGTGGGTTCCAGTGGTCGGGCCGATGGTGGGTGGTGTGGCTGGG
GCAGTGATCTACTTCCTGATGATTGAGCTGCATCACCCCGAG------------------
---------CCTGAGAAGAACCTTGAA---------------------------------
---------GACGACAACAGC------ATCAAAGATAAATATGAATTGAACACCGTAAAC
>Golden_line_fish_Aqp9b2
---------------ATGGAGCTCGAAAACATTAGAAATTTGAGGGAGAGATGCACCGTG
AGGCGGGACATCATCAGAGAGTTCCTGGCAGAATTATTGGGGACATTCATATTAATACTC
TTCGGCTGTGGTTCAGTGGCCCAGACCATTCTCAGCAGAGAAAAACAAGGAGAAAATCTC
ACCATTCATTTTGGCTTTACTTTAGGGGTAATGCTGGCTGTGTACATGGCAGGAGGCGTG
TCAGGTGGACATGTGAACCCAGCTGTTTCTCTGGCTATGGTTGTCTTGGGGAAACTCCCA
TTAAAGAAATTTCCTGTCTATGTGGTAGCCCAGTTTCTAGGTGCCTTTGCGGGGTCTTGT
GCTGTCTTCTGTCTGTACTATGGTGCCTTCGCAAATTTTGCTGGAGGAAAACGGATTGTA

ATTGGTGAAAATGCCACAGCAGGTATCTTTGCCTCATATCCACACGAAGATCTTTCATTG
TTAAATGGATTAATTGATCAGGTGATTGGCACAGGTGCCCTGGTCCTCTGTATTTTAGCC
ATCGTAGATAAGAAGAATATTGGGGCCCCTAAAGGAATGGAGCCACTGGTTATTGGTCTG
AGCATCCTGGCTATCGGAGTGTCCATGGCGCTGAACTGCGGCTATCCCATCAACCCTGCC
AGAGACTTGGGACCTCGGCTCTTCACTGCTATGGCAGGATGGGGACTAGATGTGTTCAGG
GCTGGAAATGGCTGGTGGTGGGTTCCAGTGGTCGGGCCGATGGTGGGCGGTGTGGCCGGG
GCAGTGATCTACTTCCTGATGATTGAGCTGCATCACCCTGAG------------------
---------CTTGAGAAAAACCTTGAA---------------------------------
---------GATGACAACAGC------ATCAAAGATAAATATGAATTGAACACCGTAAAC
>Common_carp_Aqp9b2
---------------ATGGAGCTCGAACACATTAGAAATTTGAGGGAGAGATGCACCGTG
AGGCGGGACATCATCAGAGAGTTTCTGGCGGAATTGCTGGGGACATTCGTATTGATACTA
TTTGGTTGTGGTTCAGTGGCCCAGACTATTCTCAGCAGAGAAAAACAAGGAGAAAATCTC
ACCATCCACTTTGGCTTTACTCTAGGGGTGATGCTGGCTGTGTACATGGCAGGAGGCGTG
TCAGGTGGACATGTAAACCCAGCTGTTTCTCTGGCTATGGTTGTCTTGGGGAAACTCCCA
TTAAAGAAATTTCCTGTCTATGTGGTAGCCCAATTTCTAGGTGCCTTTGTGGGGTCTTGT
GCTGTCTTCTGTCTGTACTATGGTGCCTTTGCAAATTTTGCTGATGGAAAACGGATTGTA
AATGGTGAAAATGCCACAGCAGGTATCTTTGCCTCATATCCACGCGAAGATCTATCATTG
TTAAATGGATTAATTGATCAGGTGATTGGCACAGGTGCCCTGGTCCTTTGTATTTTAGCC
ATTGTAGATAACAAGAATATTGGGGCCCCTAAAGGAATGGAGCCACTGGTTATTGGTCTT
AGCATCCTGGCTATTGGAGTGTCCATGGCGCTGAACTGCGGCTATCCCATCAACCCCGCC
AGAGACTTGGGACCTCGGCTCTTCACTGCTATGGCAGGATGGGGACTAGATGTGTTCAGG
GCTGGAAATGGTTGGTGGTGGGTTCCAGTGGTGGGGCCGATGGTGGGTGGTGTGCTTGGG
GCAGTGATCTACTTCCTGATGATTGAGCTGCACCACCCCGAG------------------
---------CCTGAGAAGAATCTTGAA---------------------------------
---------GACGACAACAGC------ATCAAAGATAAATATGAATTGAATACCGTAAAC
>Goldfish_Aqp9b2
---------------ATGGAGCTCGAAAACATTAGAAATTTGAGGGAGAGATGCAGCGTG
AGGCGGGACATCGTCAGAGAGTTTCTGGCGGAATTACTGGGGACATTCGTATTGATACTC
TTTGGTTGTGGTTCAGTGGCCCAGACTATTCTCAGCAGAGAAAAACAAGGAGAAAATCTC
ACCATCCACTTTGGCTTTACTCTAGGGGTGATGCTGGCCGTGTATATGGCGGGAGGAGTG
TCAGGTGGACATGTAAACCCAGCTGTTTCTCTGGCTATGGTTGTCTTGGGGAAACTCCCA
TTAAAGAAATTTCCTGTCTATGTCGTAGCCCAATTTCTAGGTGCCTTTCTGGGGTCTTGT
GCTGTCTTCTGTCTGTACTATGGTGCCTTCGCAAATTTTGCTGACGGAAAACGGATTGTA
ATTGGTGAAAATGCCACAGCAGGTATCTTTGCCTCATATCCACGTGAAGATCTTTCATTG
TTAAATGGATTGATTGATCAGGTGATTGGTACAGGTGCCCTGGTCCTTTGTATTTTAGCT
ATTGTAGATAACAAGAATATTGGGGCCCCTAAAGGAATGGAGCCACTGGTTATTGGTCTG
AGCATCCTGGCTATTGGAGTGTCCATGGCGCTGAACTGTGGCTATCCCATCAACCCTGCC
AGAGATTTGGGACCTCGTCTCTTCACTGCTATGGCAGGATGGGGACTAGATGTGTTCAGG
GCTGGAAATTATTGGTGGTGGGTTCCAGTGGTCGGGCCGATGATGGGTGGTGTGGTTGGG
GCAGTGATCTACTTCCTGATGATTGAGCTGCACCACCCCGAG------------------
---------CCTGAGAAGAACCTTGAA---------------------------------
---------GATGACAACAGC------ATCAAAGATAAT---GAATTGAACACCGTAAAC
>Roach_Aqp9b
---------------ATGGAGCTCGAAAACATGAGAAAACTGAGGGGCAAATGCATGCTG
AGGCGGGACATCATAAGAGAGTTTCTGGCAGAATTACTGGGGACATTCGTATTAATACTG
TTCGGTTGTGGTTCAGTGGCCCAAAAAGTCCTGAGCAGAGGCGCACTTGGAGAAGATCTG
ACCGTCCACTTTGGCTTTACTCTAGGGGTGATGCTGGCTGTGTACATGGCAGGAGGCGTT
TCAGGTGGACATGTGAACCCAGCTGTTTCTCTGGCTATGGTTGTCGTGGGGAAACTCCCA
ATAAAGAAATTTCCTGTCTATGTGGCAGCTCAATTTCTTGGTGCCTTTGCCGGGTCTTGT
GCTGTGTATGGTCTGTACTATGGTGCCTTTGCCAATTTCTCTGATGGACATCTGCGTGTT
ACTGGTCAAAATGCCACAGCAGGTATCTTTGCTTCATATCCACGTGAAGATCTCTCATTG
TTAAATGGATTTGTTGATCAGGTGATTGGCACAGCTGCCCTGGTCCTCTGTATTTTAGCC
ATAGTAGATAAGAAAAATATCGGAGCACCTAAAGGGATGGAGCCTCTGGTTATTGGTCTG
AGCATCCTGGCTATCGGAGTGTCCATGGCGCTGAACTGCGGCTATCCGATCAACCCTGCC
AGAGACTTGGGACCACGGCTTTTCACTGCTGTAGCAGGATGGGGCTTAGAAGTGTTCAGT
GCTGGAAATGGCTGGTGGTGGGTTCCAGTGGTCGGGCCGATGGTGGGAGGTGTGGTTGGG
GCAGGGATCTACTTCCTAATGATTGAGCTGCACCATCCTGAA------------------
---------CCTGAGAAGAACCTTGAA---------------------------------
---------GATGACAACAGC------GTCAAAGACAAATATGAATTAAACACCGTCAAC
>Amur_ide_Aqp9b

---------------ATGGAGCTCGAAAACATGAGAAAATTGAGGGGCAAATGCATGTTG
AGGCGGGACATCATAAGAGAGTTTCTGGCAGAATTACTGGGGACATTCGTATTAATACTG
TTCGGTTGTGGTTCAGTGGCCCAAAAAGTCCTGAGCAGAGGCGCGCTTGGAGAAGATCTC
ACCATCCACTTTGGCTTTACTCTAGGGGTGATGCTGGCTGTGTACATGGCAGGAGGGGTT
TCAGGTGGACATGTGAACCCAGCTGTTTCTCTGGCTATGGTTGTCGTGGGGAAACTCCCA
ATAAAGAAATTTCCTGTCTATGTGGCAGCTCAATTTCTTGGTGCCTTTGCCGGGTCTTGT
GCTGTGTATGGTCTGTACTATGGTGCCTTTGCCAATTTCTCTGATGGACATCTGAGTGTT
ACTGGTCACAATGCCACAGCTGGAATCTTTGCTTCATATCCACGTGAAGATCTCTCATTG
TTAAATGGATTTGTTGATCAGGTGATTGGCACAGCTGCCCTGGTCCTCTGTATTTTAGCC
ATAATAGATAAGAAGAATATCGGGGCACCTAAAGGGATGGAGCCTTTGGTTATTGGTCTG
AGCATCCTGGCTATCGGAGTGTCCATGGCGCTGAACTGCGGCTATCCGATCAACCCTGCC
AGGGACTTGGGACCACGGCTTTTCACTGCTATAGCAGGATGGGGCTTAGACGTGTTCAGG
GCTGGAAATGGCTGGTGGTGGGTTCCAGTGGCCGGGCCCATGGTGGGAGGTGTGGTTGGG
GCAGGGATCTACTTCCTAATGATTGAGCTGCACCATCCTGAA------------------
---------CCTGCGAAGAACCTTGAA----------------------------------
---------GATGACAACAGC------GTCAAAGACAAATATGAATTAAACACCGTCAAC
>Fathead_minnow_Aqp9b
---------------ATGGAGCTCGAAAAGATGAGAAAATTGAGGGGCAAATGCATGTTG
AGGCGGGACATCATAAGAGAGTTTCTGGCAGAATTACTGGGGACATTCGTATTAATACTG
TTCGGTTGTGGTTCAGTGGCCCAAAAGGTCCTGAGCAGAGGCACACAAGGAGAAGATCTC
ACCATCCACTTTGGCTTTACTCTAGGGGTGATGCTGGCTGTCTACATGGCAGGAGGCGTG
TCAGGTGGACATGTGAACCCAGCTGTTTCTCTGAGTATGGTTGTCTTGGGGAAACTCCCA
ATTAAGAAATTTCCTGTCTATGTGGCAGCTCAATTTCTTGGTGCCTTTGCTGGGTCTTGT
GCTGTCTATGCTCTGTACTATGGTGCCTTTGCCAATTTCTCTGAGGGACATCTGCATGTT
ACTGGTCAAAATGCCACAGCAGGCATCTTTGCTTCATATCCACGTGAAGGTCTCTCATTG
TTAAATGGATTTGTTGATCAGGTGATCGGCACAGGTGCCCTTGTCCTCTGTATTTTAGCC
ATAGTAGATAAGAAGAATATCGGGGCACCTAAAGGGATGGAGCCTCTGGTTGTTGGTCTA
AGTATCCTGGCTATCGGAGTGTCCATGGCGCTGAACTGCGGCTATCCGATCAACCCTGCC
AGGGACTTGGGACCACGGCTTTTCACCGCTATAGCAGGATGGGGCTTAGATGTGTTCAGT
GCTGGAAATGGCTGGTGGTGGGTTCCAGTGGTCGGGCCGATGGTGGGAGGTGTGGTTGGG
GCAGTGATCTACTTCTTCATGATTGAGCTGCACCATCCTGAA------------------
---------CCCGAGAAGTACCTTGAA----------------------------------
---------GATGACAACAGC------GTCAAAGACAAATATGAATTAAACACCGTCAAC
>Grass_carp_Aqp9b
---------------ATGGAGCTCGAAAACATCAGAAATTTGAGGGGGAAATGCATGGTG
AGGCGGGACATCATAAGAGAGTTTCTGGCGGAATTACTGGGGACATTCGTATTAATACTG
TTCGGTTGTGGTTCAGTGGCCCAAACTGTCCTCAGCAGAGGCACACATGGAGAACTTCTC
ACCATCCACTTTGGCTTTACTCTAGGGGTGATGCTGGCTGTGTACATGGCAGGAGGCGTG
TCAGGTGGACATGTGAACCCAGCTGTTTCTCTGGCCATGGTTGTTGTTGGGAAACTCCCA
ATAAAGAAATTTCCTGTCTATGTGGCAGCTCAATTTCTTGGTGCCTTTGCCGGGTCTTGT
GCTGTCTACTGTCTGTACTATGGTGCCTTTGCAAATTTTGCTAATGGACTTCTGCTTGTA
ACTGGTGTAAATGCCACAGCAAATATCTTTGCTTCATATCCACGTGAAGATCTCTCATTG
TTAAATGGATTTATTGATCAGGTGATTGGCACAGGTGCCCTGGTCCTCTGTATTTTAGCC
ATAGTAGATAAGAAGAATATCGGGGCACCTAAAGGCATGGAGCCTCTGGTTATTGGTCTG
AGCATCCTGGCTATCGGAGTGTCAATGGCGCTGAACTGCGGCTATCCGATCAACCCTGCC
AGAGACTTGGGACCGCGGCTCTTCACTGCTCTAGCAGGATGGGGATTAGATGTGTTCAGA
GCTGGAGGAGGCTGGTGTTGGGTTCCAGTGGTCGGACCAATGGTGGGAGGTGTGGTTGGG
GCAGTGATCTACTTCCTAATGATTGAGATGCACCATCCTGAG------------------
---------CCTGAGAAGAACCTTGAA----------------------------------
---------GACAACAACAGC------ATCAAAGATAAATATGAATTAAACACCGTCAAC
>Zebrafish_Aqp9b
---------------ATGGAGCTCGAGAACATCCGAAATCTGAGGGGGAGATGCGTCCTG
AGGCGCGACATCATCCGAGAGTTTCTGGCAGAATTACTCGGGACATTCGTGTTAATACTT
TTCGGTTGCGGTTCAGTGGCCCAGACTGTCCTCAGCAGAGAAGCAAAAGGACAGCTTCTC
ACCATCCATTTTGGCTTTACTCTAGGGGTGATGCTGGCCGTCTACATGGCAGGAGGCGTG
TCAGGAGGACATGTGAACCCTGCTGTTTCTTTGGCTATGGTTGTCCTGAGGAAACTCCCA
CTAAAGAAGTTCCCTGTGTATGTGTTGGCCCAATTTCTAGGTGCCTTTTTTGGGTCTTGT
GCCGTCTACTGTCTTTACTATGATGCCTTTACAGAATTTGCTAATGGAGAGCTAGCTGTA
ACTGGCCCAAATGTCACAGCAGGTATCTTTGCATCATATCCACGTGAAGGACTCTCATTG
TTAAATGGATTCATTGATCAGGTGATTGGTGCAGGTGCCCTGGTCCTCTGTATTTTAGCT
GTTGTAGATAAGAAGAACATTGGAGCACCTAAAGGAATGGAGCCTCTGCTTGTCGGTCTG

AGCATCCTGGCTATTGGAGTGTCAATGGCACTAAACTGTGGATATCCTATAAACCCTGCC
AGAGACTTGGGACCTCGGCTGTTCACTGCCATTGCAGGATGGGGATTAACGGTGTTCAGT
GCTGGCAATGGCTGGTGGTGGGTTCCAGTGGTGGGGCCAATGGTGGGCGGAGTGGTTGGT
GCTGCTATCTACTTCCTGATGATCGAGATGCATCACCCTGAG------------------
---------AACGACAAGAACCTGGAA---------------------------------
---------GACGACAACAGC------CTTAAAGACAAATATGAGCTGAACACCGTCAAC
>Channel_catfish_Aqp9b
---------------ATGGAGCTGGACAGCCTGAGGAGTCTGAGAGAGAAATTCACACTT
CGGCATGCCATCATCAGAGAGTTTCTAGCGGAACTCCTCGGGACATTTGTACTTATACTT
TTTGGCTGTGGCTCGGTGGCACAGGCTGTACTCAGCAGAGGAGCTCTGGGTGAACCGCTC
ACCATCCACATTGGCTTCACCACAGGAGTGATGCTGGCTGTCTATGTGTCAGGAGGCGTT
TCAGGAGGACATGTAAACCCAGCTGTATCTCTAGCCATGGCAGTCTTAGGGAAGTTTCCC
ATTAAAAAATTCCCTGTGTATGTGGTTGCCCAGTTCCTTGGTGCTTTTGCAGGCTCTTGT
GCTGTCTTTGGACTGTACTATGATGCCTTCATGAACTACTCCAATGGAGAGTTGCTGGTC
ACAGGTGAAAACTCCACTGCAAACATCTTTGCCACCTATCCAGCAAAGCATCTCTCAGCC
CTCAATGGGTTTTTTGATCAGGTGATTGCCACTGCAGCTTTGGTGCTATGTGTCCTGGCC
ATCGTGGACAAGAAAAACATAGGAGCACCTAAAGGAATGGAGCCACTGCTCATTGGTTTC
ACCATCCTGGCCATCTCAGTGGCCATGGGTTTCAACTGCGGCTATCCCATCAACCCTGCC
CGAGACTTGGGACCCCGGCTCTTTACTGCAGTGGCAGGCTGGGGGGTGGAGGTTTTCAGG
GCCGGGGGCTGCTGGTGGTGGATCCCAGTTGGTGGTCCGATGGTGGGAGGTGTGCTGGGA
GCAATGATCTACTACCTGCTGATTGAGCTACACCACATAGAG------------------
---------CCAGAGAAAAAGCATGAG---------------------------------
---------GAGAACAACACC------GTTAAGGACAAGTATGAAATAATCACCATGAGC
>Striped_catfish_Aqp9b
---------------ATGGAGCTGGACAGCCTGAGGAGTCTGAGAGAGAAAGTCACACTC
CGGCATGCCATCATCAGAGAGTTTCTAGCTGAATTCCTCGGGACATTTGTGCTTATACTT
TTTGGCTGTGGCTCGGTGGCACAGGCTGTGCTCAGCAGAGGAGCTCTGGGTGAACCGCTC
ACCATCCACATTGGCTTCACCACGGGAGTGATGCTGGCTGTCTATGTGTCAGGAGGTGTA
TCAGGAGGTCATGTAAACCCAGCTGTATCTCTAGCCATGGCAGTCTTAGGGAAGTTTCCC
ATTAAAAAATTCCCAGTGTACGTGGCTGCCCAGTTCATTGGTGCTTTTGCAGGTTCTTGT
ACTGTCTTTGGACTGTACTATGATGCCTTCATGGACTACTCCAATGGAGAGCTGCTGGTC
ACAGGTGAAAATGCCACCGCACACATCTTCGCCACCTATCCAGCAAAGCATCTCTCAGGC
CTCAATGGGTTTTTTGATCAGGTGATTGCCACTGCCACTTTGGTGCTGTGTGTCTTGGCC
ATCGTGGACAAAAAAAACATAGGAGCCCCTAAAGGAATGGAGCCACTGCTCATTGGTTTC
ACCATCCTGGCCATCGCTGTGTCCATGGGATTGAACTGCGGCTATCCCATCAACCCTGCC
CGAGACCTGGGGCCCCGGCTCTTTACCGCTGTGGCAGGCTGGGGGGTAGAGGTGTTCAGA
GCCGGGGGCTGCTGGTGGTGGATCCCAGTTGCTGGTCCGATGGTGGGGGGTGTATTGGGA
GCGATGATCTACTACCTGCTGATCGAGCTACACCACGTGGAG------------------
---------CCAGAGAAAAAGCATGAG---------------------------------
---------GAGAAGAACAAT------GTTAAGGACAAGTATGAAATGATCACCACGAGC
>Yellow_catfish_Aqp9b
---------------ATGGAGCTGGACAGCTTGAGGGGTCTGAGAGAGAAATTCACACTT
CGTCATGCTATCATCAGAGAGTTTCTAGCAGAATTCCTCGGGACATTTGTGCTTATACTT
TTTGGCTGTGGCTCAGTGGCACAGGCTGTGCTCAGCAGAGGAGCTGTGGGTGAACCGCTC
ACCATCCACATTGGCTTCACCACAGGATTGATACTAGCTGTCTATGTGTCAGGAGGTGTA
TCAGGAGGACATGTAAACCCAGCCGTATCTCTAGCCATGGCAGTCTTAGGGAAGTTTCCC
ATTAAGAAATTTCCAGTATATGTGGCTGCCCAGTTCATTGGTGGTTTTGCAGGCTCTTGT
ACTGTCTTTGGACTATACTACGATGCCCTCATGGACTACTCCAATGGAGAGCTGCTGGTC
ACAGGTGAAAATGCCACCGCGAACATCTTTGCCAGCTATCCAGCAAAGCATCTTTCAGTC
CTTAATGGGTTTTTTGATCAGGTCGTTGCCACTGCAGCTTTAGTGCTGTGCATCCTGGCC
ATTGTGGACAAGAAAAACATCGGAGCCCCTAAAGGAATGGAGCCACTTCTCATTGGTTTC
ACCATCCTGGCCATCGCAGTGGCCATGGGCCTAAACTGTGGCTATCCCATCAACCCTGCA
CGAGACTTGGGACCCCGTCTCTTTACTGCTGTGGCAGGCTGGGGGGTGGAGGTGTTCAGG
GCCGGTGGTGGCTGGTGGTGGATCCCAGTTGCTGGTCCAATGGTGGGCGGGGTGTTGGGA
GCGATGATCTACTACCTGCTGATTGAGCTGCACCACCTG---------------------
---------CCAGAGGAAAAGCATGAG---------------------------------
---------GAGAAGAGCAAT------GTTAAGGACAAGTATGAAATGATAACCATGAGC
>Walking_catfish_Aqp9b
---------------ATGGAGCTGGACAGCATGAGGAGCCTGACAGAGAAATTCACACTT
CGGCATGCCATCATCAGAGAGTTTCTAGCTGAATTCCTTGGGACATTTGTGCTTATACTT
TTCGGCTGTGGCTCGGTGGCGCAGGCTGTCCTCAGCAGAGGAGCTCTGGGGGAACCGCTC

ACCGTCCACATTGGCTTCACCACAGGAGTGATGCTGGCCGTCTACGTGTCAGGAGGTGTA
TCAGGAGGACATTTAAACCCGGCTGTATCTCTCGCCTTGGCAGTCTTAAGGAAGTTTCCC
ATTCAAAAATTCCCAGTGTACGTGGCTGCCCAGTTCATTGGTGCCTTCGTAGGCTCGTGT
GCAGTCTTCGGATTGTACTATGATGCCATTATGGACTACTCCAATGGAGAACTGCTGGTC
ACTGGTGAAAATGCTACCGCAAACATCTTCGCCACCTATCCAGCAAAGCATCTCTCAAGG
TTTAATGGGTTTTTGACCAGGTGATTGCCACTGCAGCTTTGGTGCTGGGTGTCCTGGCC
ATCCTGGATAAGAAGAACATAGGAGCCCCAAAAGGACTGGAGCCACTATTCATAGGTTTC
ACCATCCTGGCCATCGCAGTAGCCATGGGTTTGAACTGCGGCTGTCCCATCAACCCTGCC
CGAGACCTGGGACCCCGGCTCTTTACAGCTGTGGCAGGCTGGGGATGGAGGTGTTCAGG
GCCGGGGGCTGCTGGTGGTGGATCCCAGTTGCTGGTCCGATGGTGGGGGGGGTCCTGGGA
GCGATGATCTACTACCTGCTAATCGAGCTACACCATGTGCAG------------------
---------CCAGAGAAAACGCTTGAA---------------------------------
---------GAAAAGAACAAT------GTTAAGGACAAGTATGAAATGGTCACTATGAGC
>Bottlenose_catfish_Aqp9b
---------------ATGCAGGTGGACAGACTGAAGAGTGTAAGAGAGAAATTCACACTT
CAACATGCCATCATCAGAGAGTTTCTAGCGGAATTCCTTGGGACATTTGTGCTTATACTT
TTTGGCTGTGGCTCAGTGGCACAGGCTGTGCTCAGCAGAGGAGCTCTGGGTGAACCGCTC
ACCATCCACATTGGCTTCACCACAGGAGTGACGCTGGCTGTCTATGTGTCAGGAGGTGTT
TCAGGAGGACATGTAAACCCAGCTATATCTCTAGCCATGGCAGTCTTAGGGAAGTTTCCC
ATTAAAAAATTCCCAGTGTACGTGGCTGCTCAGTTCATTGGTGCCTTTGTAGGCTCTTGT
GCTGTCTTTGGACTGTACTATGATGCCTTCATGGACTACTCTAATGGAGAGTTGCTGGTC
ACAGGTAAAAATGCCACAGCAAACATTTTTGCCTCCTATCCAGCAAAGCATCTCTCAAGC
CTCAATGGGTTTTTGGATCAGGTGATTGCCACTGCAGCTTTGGTGCTGTGTGTCCTGGCC
ATTGTGGACAAGAAAAACATAGGAGCCCCTAAAGGAATGGAGCCACTGCTCATCGGTTTC
ACCGTGCTGGCCATCGCAGTGGCCATGGGTTTGAATTGCGGCTATCCCATAAACCCTGCC
CGAGACCTGGGACCCCGGCTCTTTACTGCACTAGCAGGCTGGGGATGGAGGTGTTCAGG
GCYGGGGGCTGCTGGTGGTGGATCCCAGTTGCTGGTCCAATGGTGGGGGGTGTCCTGGGA
GCSATGGTCTACTACATGCTGATTGAGCTACACCACATAGAG------------------
---------CCAGAGAAAAAGCATGAA---------------------------------
---------GAGAAGAACAAT------GTTAAGGACAAATATGAAATGATCACTATGAGC
>Snow_pleco_Aqp9b
---------------ATGGAA------ACCTGGTCCAGTCTGAGAGAAAAGTTCACCATC
AGACGTCTCATCATCAGAGAATTTCTAGCAGAGTTTCTTGGGACTTTTCTACTCATTCTC
TTTGGCTGCGGTTCGGTGGCTCAGACCGTACTCAGCAGAGGTGCTATGGGGGAGATGCTC
ACCATACACATTGGCTTCACCATGGGAGTGATGCTGGCCGTGTATGTAGCAGGAGGGGTG
TCAGGAGCACATTTGAACCCAGCTGTGTCGCTGGCCATGGTCATCCTAGGAAAGCTTCAC
ATTAAAAAATTCCCAGTGTATGTGGCTGCTCAGTTCATTGGGGCTTTTACAGGCTCTTGT
GCTGTCTATGGGCTGTACCATGATGCCTTTAAGGAGTACACCAAAGGAGTGCTGCTGGTT
ACAGGTGAAAATGCCACCGCAAACATCTTTGCATCCTATCCTGGGAGGCATCTGTCAGTC
CTCAATGGATTTGTTGATCAGGTCATTGGCACTGGAGCGTTGATCCTATGCATCCTGGCC
ATTGTGGACAAGAAAAACACTGGTGCTCCTAAAGGCATGGAGCCACTGCTCATCGGCCTC
ACCATAATGGCCATCGGAGTGTCCATGGGTCTGAACTGTGGTTATCCAATCAACCCAGCG
CGAGACCTGGGACCCCGGATCTTCACAGCCATGGCCGGCTGGGGAATGGAGGTGTTCAGA
GCTGGAGGTTGCTGGTGGTGGATCCCTGTTGCCGGGCCCATGGTAGGAGGGGTGGTGGGC
GCGGTGATCTACTTCCTCTTGATCGAGCTGCACCTTCCAGAA------------------
---------CCAGAGAAAGAGCATGAC---------------------------------
---------AAGGACAATAAC------ATCCGAGAAAAGTACGAGATGATTACCATG---
>Black_ghost_Aqp9b
---------------ATGGATCTTGAGAACGCGAGGAATTTAAAGTGGAAATGGGCTCTC
AGGCAGCACGTGATCAAAGAATTTCTTGCAGAATTGCTCGGAACGTTCGTGTTAATAGTG
TTTGGCTGTGGCTCGGTGGCTCAGTCTGTCCTGAGCAGAGGGACCTTAGGGGAGCCTCTC
ACCATCCACATCGGCTTCACCATGGGAGTGATGCTGGCCATCTATGTGGCAGGAGGCGTG
TCGGGTGGGCACGTGAACCCGGCCGTGTCGCTGGCTATGGTCGTCCTAGGGAAGCTTCCT
GTTAAAAAGTTCCCGGTGTACGTGGTTGCACAGTTCAGTGGCGCGTTCGCAGGATCCTGC
GCTGTCTTTGGCCTGTACTACGATGCCTTCATGGACTACAGCAATGGAGTGTTGATGGTG
ACAGGTGAAAATGCCACAGCAAACATCTTTGCCTCGTATCCAGCAAAGCACCTCTCGATA
CTCAATGGGTTTGTTGATCAGGTAATTGGCACAGGGGCACTGGTGATGTGTATTCTGGCC
ATAGTGGACAAGAAAAACATGGGTGCCCCTAAGGGGATGGAGCCGCTGGTCATCGGCCTG
ACCGTCCTGGCTGTTGGGGTGTCCATGGGGCTAAACTGTGGCTACCCCATCAACCCAGCC
CGAGACCTGGGACCCAGACTCTTCACCGCTGTGGCAGGCTGGGGGATGGATGTGTTCAGA
GCTGCAGGCTGCTGGTGGTGGATCCCAGTTGCCGGGCCAATGGTGGGGGGCGTGGTGGGT

GCTGTGATCTACTTCCTGATGATCGAGCTCCATCACCCGGAG------------------
---------CTGGAGAAACAACGAACA---------------------------------
---------GAGGAGAACATC------ATCAAGGACAAGTATGAGATGATCACCATGAGC
>Electric_eel_Aqp9b
---------------ATGGAGCTTGGGAACGCAAGGAATTTAAGGGGGAAATGTGCTCTC
AGACAGCACATCATCAGAGAGTTTCTGGCAGAATTGCTTGGAACATTCGTATTAATACTC
TTTGGCTGTGGCTCTGTCGCCCAGGCTGTCCTGAGCAGAGGAACCTTGGGGGAGCCATTC
ACCATCCACATTGGCTTCACCATGGGAGTGATGTTGGCCATTTACGTGGCCGGAGGGGTG
TCGGGCGGGCACGTGAACCCAGCTGTATCGCTGGCTATGGTCATCCTGGGGAAGCTTCCT
GTTAAAAAATTCCCGGTGTATGTGGCTGCACAGTTCAGTGGGGCTTTCGTGGGCTCCTGT
GCTGTCTTCGGCCTGTACTACGATGCCTTCATGGACTACAGCAATGGAGTGTTGATGGTA
ACAGGTGAAAATGCCACAGCAAACATTTTTGCTTCCTATCCAGCAAAGCACCTCTCGGTC
CTCAATGGATTTGCTGATCAGGTAATTGGCACAGGGGCACTGGTGATGTGTGTTCTGGCT
ATAATGGACAAGAAGAACATGGGTGCACCTAAGGGGATGGAGCCTCTGGTCATCAGCCTG
ACCATCCTGGCCATTGGCGTGTCCATGGGCTTGAACTGCGGCTATCCCATCAACCCAGCT
CGAGACCTGGGTCCCAGGCTCTTCACCGCTGTGGCAGGGTGGGGGGTGGAGGTGTTCAGA
GCTGGGGGCTACTGGTGGTGGATCCCGGTTGCCGGGCCAATGGTTGGGGGCGTGCTGGGT
GCTATGATCTATCTCCTGCTTATTGAGCTACACCACCCAGAG------------------
---------CTGGGGAAACGGGGTGCG---------------------------------
---------GAGGAGAACAAC------ATCAAGGACAAGTACGAGATGATCACCATGAGC
>Redbellied_piranha_Aqp9b
---------------ATGGAGCTGGAGAACAGGAGGAACCTGAGGGAGAAATGCGCCCTC
AAGCAGGACATCCTTAAAGAGTTCCTGGCTGAGATCCTCGGAACCTTCGTGCTGATACTC
TTTGGCTGTGGCTCTGTGGCCCAGACTGTCCTGAGCCGAGGAGCTCTGGGGGAACCACTT
ACCATCCACATCGGCTTTACCACCGGAGTGATGTTGGCCGTCTACATGGCCGGAGGCGTG
TCAGGAGGGCATGTTAACCCAGCTGTATCGCTGGCTATGGTGGTCCTGGGGAAGCTATCG
CTGAAGAAATTCCCAGTGTATGTGGCTGCACAGTTTATCGGGGCTTTTGCTGGATCCTGT
GCTGTCTTTGGGCTTTACTATGATGCCCTGATGGACTACACGAATGGAGCGCTGCTGGTA
ACAGGTGAAAACGCCACAGCAAGCATATTTGCCTCCTATCCAGCAAAACATCTCTCAGTA
CTAAATGGATTTATTGATCAGGTGATCGGTACAGGGGCGCTGGTGTTGTGCATTCTGGCC
ATAGTGGACAGGAAGAACATGGGCGCTCCTAAGGGGATGGAACCACTGATCATTGGTCTG
ACCATCCTGGCTATCGGAGTGTCCATGGGACTGAACTGCGGATATCCTATCAACCCTGCG
CGAGACCTGGGCCCCAGACTCTTCACAGCCGTGGCAGGCTGGGGGGTGGATGTTTTCAGG
GCGGGGGGCTGCTGGTGGTGGATCCCAGTCGCCGGGCCGATGGTTGGCGGAGTGGTGGGA
GCTGTGATCTACTTCTTACTGATCGAGTTACACCATCCAGAG------------------
---------CCCGAGAAACAGAACGAG---------------------------------
---------GAGGAGAACAAG------GTCAAGGACAAATACGAGATGATCACCATGAGC
>Tambaq_Aqp9b
---------------ATGGAGCTGGAGAGCAGGAGGAACCTGAGGGAGAAATGCGCCCTC
AAACAGGACATCCTCAAAGAGTTCCTGGCCGAGATCCTCGGAACCTTCGTGCTGATACTC
TTTGGCTGCGGCTCCGTGGCCCAGACTGTCCTGAGCCGAGGGACTCTGGGGGAACCACTT
ACCATCCACATCGGCTTCACCACCGGAGTGATGCTGGCCGTCTACATGGCCGGAGGCGTG
TCAGGAGGGCATGTTAACCCGGCTGTATCACTGGCTATGGTGGTCCTGGGGAAGCTATCG
CTGAAGAAATTCCCAGTGTATGTGGCTGCACAGTTTATCGGAGCTTTTGCTGGATCCTGT
GCTGTCTTTGGGCTTTACTATGATGCCTTGATGGACTACACGAATGGAGCGCTGCTGGTA
ACAGGTGAAAACGCCACAGCAAATATATTTGCCTCCTATCCAGGAAAACACCTCTCAGTA
CTAAATGGATTTATTGATCAGGTGATCGGCACAGGGGCGCTGGTGCTGTGCATTCTGGCC
ATAGTGGACAGGAAGAACATGGGTGCTCCTAAGGGGATGGAACCGCTGATCATTGGTCTG
ACCATCCTGGCCATCGGAGTGTCCATGGGGCTGAACTGCGGATATCCTATTAACCCTGCA
CGAGACCTGGGCCCCAGGCTCTTCACAGCCGTGGCGGGCTGGGGGATGGATGTGTTCAGG
GCGGGGGGCTGCTGGTGGTGGATCCCAGTGGCCGGGCCGATGGTTGGCGGAGTGGTGGGA
GCTGTGATTTACTTCTTACTGATCGAGTTGCACCATCCGGAG------------------
---------CCTGAGAAAGAAAACGAG---------------------------------
---------GAGGAGAACAAG------GTCAAGGACAAATACGAGATGATCACCATGAGC
>Mexican_tetra_Aqp9b
---------------ATGGAGCTGGACAGCAGGAGGAACCTGAGGGAGAGATGCGCCCTG
AAGCAGGACATCCTCAGAGAGTTCCTGGCGGAGATCCTGGGAACCTTCGTGCTGATACTC
TTCGGCTGTGGTTCCGTGGCCCAGACTGTGCTGAGCAAAGGGGCTCTGGGAGAACCACTC
ACCATCCATATTGGCTTCACCACTGGAGTGATGCTGGCCGTCTACATGGCAGGAGGAGTG
TCAGGAGGCCATGTTAACCCAGCTGTATCTTTGGCTATGGTGGTCCTCGGGAAGCTTCCA
CTGAAGAAATTCCCTGTGTACGTGGTTGCACAGTTCATTGGTGCGTTTGCAGGCTCCTGT

GCTGTGTTCGGGCTGTACTATGATGCCTTGATGGACCACACCAATGGTGTTCTGCTGGTC
ACGGGTGAAAATGCCACTGCAAACATATTTGCCTCCTATCCAGCAAAACATCTCTCAGTA
CTTAATGGATTCATTGATCAGGTGGTGGGCACAGGGGCCTTGGTGCTGTGTATTCTGGCG
ATAGTGGACAGGAAGAACATGGGAGCACCAAAAGGGATGGAGCCGCTGATCATCGGTCTG
ACCATCCTGGCCATTGGAGTTTCCATGGGGCTGAACTGTGGATATCCCATCAACCCCGCC
AGAGACCTGGGCCCCAGACTCTTCACCGCCATCGCAGGGTGGGGATGGACGTGTTTAGG
GCGGGGGGCTGCTGGTGGTGGATCCCGGTGGCCGGGCCAATGGTTGGCGGGGTGGTGGGA
GCGGTCATATACTTCCTGGTGATAGAGCTGCACCACCCAGAG------------------
---------CCCGAGAAACAGAGCGAA---------------------------------
---------GAGGAGAATAAC------GTTAAGGACAAATACGAGATGATCACCATGAGC
>Allis_shad_Aqp9b
---------------ATGGAG---GGAAACAAAAGAACTATGAGGGAGCGATGCACCCTG
AGGAAAGACATCATCAAAGAGTTTTTGGCGGAATTTCTTGGAACGTTCATCTTAATACTC
TTCGGCTGTGGTTCAGTGGCCCAGACGGTGTTGAGTCGGGGAGCACTGGGGGAGCCCCTC
ACCATCCACATCGGCTTCACACTGGGGGTCATGCTGGCCGTTTACGTGGCTGGGGGAGTG
TCAGGAGGCCATGTGAACCCTGCGGTGTCCTTGGCGATGGTGGTCCTGGGGAAGCTGCCT
ATTAGGAAGTTCCCCTTGTACGTAACGGCTCAGTTCCTGGGGGCCTTCGCTGGGGCCTGC
GCCGTCTTCGGCCTCTACTACGACGCCTTTATGGATTACACGGGCGGAGTGCTCATCGTC
ACCGGAGAAAACGCCACCGCCAACATCTTTGCCTCGTACCCGGCCAAGCACCTGTCCGTC
CTCAACGGCTTTGTGGACCAGGTGATCGGCGCTGCAGCTCTGATTCTGTGCATCTTGGCG
ATCACCGATAAGAAGAACACTGGTGCTCCTAAGGGCATGGAGCCGCTGGCCGTGGGCCTG
ACCATCCTGGCCATCGGTGTGTCCATGGGCCTCAACTGTGGCTATCCCATCAACCCTGCC
CGAGACCTGGCGCCCCGACTCTTCACTGCCATGGCCGGCTGGGGCTTCGAGGTGTTCAGT
GCTGGGAACTGCTGGTTCTGGATCCCAGTGGCAGGCCCTCTGGTGGGGGGTGTGGTCGGG
GCCACCATCTACTTCCTGCTCATCGAGCTGCACCACCCCGAA------------------
---------CCCCAGAGACACGCGCAA---------------------------------
---------GATGACAACAAC------GTCAAGGAGAAA---------------------
>Hilsa_shad_Aqp9b
---------------ATGGAG---GGTAACAAGCGGAGTTTGAGGGAGCGCTGTGCCGTG
AGAAAAGACATCATCAAAGAGTTTTTGGCAGAATTTCTGGGGACGTTCGTCTTAATACTC
TTTGGCTGTGGCTCTGTGGCCCAGACGGTTCTTAGTCGGGGGGCACTGGGGGAACCCCTC
ACCATCCACATCGGCTTCACTCTGGGCGTCATGCTTGCTGTCTACATGGCTGGAGGAGTG
TCAGGAGGCCATGTCAACCCTGCTGTGTCTCTGGCGATGGTGGTCTTGGGGAAGCTGCCC
ATACACAAGTTCCCCGTCTACGTGGCGGCTCAGTTCCTGGGGGCCTTCGCTGGATCCTGT
GCCGTCTTTGGACTCTACTATGATGCCTTCATGGAGTACACTGGTGGAGTGCTCACCATC
ACCGGAGAGAACGCCACCGCCAACATCTTCGCCTCGTACCCCGCCAAGCACCTGTCCGTC
CTCAACGGCTTTGTGGACCAGGTGATTGGTGCTGGGGCTCTGATCCTGTGCATCCTGGCC
ATCACGGACAAGAGGAACATCGGCGCTCCTAAAGGCATGGAGCCTCTGGCGGTGGGCCTC
ACCATCCTGGCCATCGGCGTCTCCATGGGGCTCAACTGCGGCTATCCCATCAACCCCGCA
CGCGACCTGGGCCCACGACTCTTCACCGCCGTCGCTGGCTGGGGCTTCGAGGTGTTCAGG
GCTGGGAACTGCTGGTTCTGGATCCCTGTGGCGGGCCCTCTGGTGGGGGGCGTTGTCGGG
GCAACCATCTACTTCCTGCTCATCGAGCTGCACCACCCTGAG------------------
---------CCTCAGAGACACACACAG---------------------------------
---------GACGAGAACAAC------GTCAAGGACAAATACGAGATTGTCACCATGAGC
>Sardine_Aqp9b
---------------ATGGAG---GGAAACAAAAGAACTATGAGGGAACGATGCGCCCTG
AGGAAAGACATCATCAAAGAGTTTTTGGCGGAATTTCTTGGAACGTTCGTCTTAATATTG
TTTGGCTGTGGCTCGGTGGCCCAGACGGTGTTGAGTCGGGGGGCGCTGGGGGAGCCCCTC
ACCATCCACATCGGCTTCACTCTCGGGGTCATGCTGGCCGTCTACATGGCCGGGGGAGTG
TCAGGGGGCCACGTGAACCCCGCGGTGTCGTTGGCCATGGTGGTGCTGGGGAAGTTGCCC
ATTAAGAAGTTCCCCGTCTACGTAGCAGCTCAGTTCCTGGGCGCCTTCGCCGGGTCCTGC
GCTGTCTTCGGCCTCTACTACGACGCCTTCATGGACTACACGGGCGGAGTGTTGACCGTC
ACTGGAGAGAACGCCACCGCCAACATCTTCGCCTCGTACCCCGCCAAGCACCTGTCCGTC
CTCAACGGCTTCGTGGACCAGGTGATTGGCGCAGCGGCTCTGATTCTGTGCATCCTGGCG
ATCACGGATAAGAGGAACATCGGCGCCCCTAAGGGCATGGAGCCGCTGGCCGTGGGGCTG
ACCATCCTGGCCATCGGCGTGTCCATGGGCCTCAACTGCGGCTATCCCATCAACCCCGCC
CGCGACCTGGCCCCCCGACTCTTCACCTTCGTGGCCGGCTGGGGCTTCGAGGTGTTCAGT
GCTGGGAACTGCTGGTTCTGGATCCCTGTGGCGGGCCCTCTGGTGGGGGGCGTGGTCGGC
GCCACCATCTACTTCCTGTTCATCGAGCTGCACCACCCCGAA------------------
---------CCCCAGAGACTAGCGCAG---------------------------------
---------GACGACAACAAC------GTCAGGGATAAATACGAGATCGTCACCATGAGC

>Japanese_grenadier_anchovy_Aqp9b
---------------ATGGAG---GGAAACAAGAGGACTCTGAGAGAGCGATGTGCTCTG
AGGAAAGACATCATCAAAGAGTTTCTGGCGGAATTTCTGGGCACGTTTGTCTTAATACTA
TTTGGCTGTGGTTCTGTGGCCCAGACGGTTCTGAGTCGGGGTGCGCTGGGGGAACCCCTC
ACCATCCACATCGGCTTCACACTCGGGGTCATGCTGGCTGTCTACGTCGCCGGAGGAGTG
TCAGGAGGCCATCTGAACCCTGCGGTGTCCTTGGCGATGGTGGTGCTGGGGAAGCTGCCT
GTGAGGAAGTTACCCGTCTACATAGCAGCCCAGCTCCTGGGGGCCTTCGCCGGGTCCTGC
GCTGTATTTGGCCTGTACTATGATGCCTTCATGGATTACACTAACGGCGTGTTCTCTGTC
ACGGGCGAGAACGCCACGGCCAACATCTTTGCCTCCTACCCCTCCAAGCACCTGTCCGTG
CTCAACGGCTTTGTGGACCAGGTGATGGGCACTGCTGCCCTGGTGCTCTGTATCCTGGCC
ATCACAGACGGGCGTAATCTGGGGGCCCCTAAGGGCCTGGAGCCGCTGGTGGTGGGGCTG
AGCATCATGGCCATCGGAGTCTCCATGGGGCTCAACTGCGGCTACCCCATCAACCCCGCA
CGAGACCTGGGACCCCGGCTCTTCACCGCACTCGCCGGGTGGGGCGTGGAAGTCTTCAGT
GCTGGGAACTGCTGGTGGTGGATTCCTGTGGCGGGTCCTCTGGTGGGGGGCGTGGTCGGC
GCCACCGTCTACTTCCTGCTCATCGAGCTCCATCACCCCGAA------------------
---------CCCCAGACACACGCCCAG---------------------------------
---------GACCACAACGAC------GTCAAAGACAAATACGAGATCGTTACTATGACC
>Atlantic_herring_Aqp9b
---------------ATGGAG---GGAAACAAGAGGACTATGAGGGAGCGCTGCGCCCTG
AAGCAAGACATCATCAAAGAGTTTTTGGCCGAATTTCTGGGGACGTTCGTCTTAATACTC
TTGGGCTGTGGGTCAGTGGCTCAGACGGTGTTGAGTCGGGGTGCGTTGGGAGAACCCCTC
ACCATCCACATCGGCTTCACACTGGGGGTCATGCTGGCCGTCTACATGGCTGGGGGAGTG
TCAGGAGGCCATGTAAACCCTGCCGTGTCTCTGGCCATGGTGGTCCTGGGGAAGCTGCCA
CTGAAGAAGTTTCCTGTCTATGTGGCGGCTCAGTTCCTGGGCGCCTTTGCTGGGTCGTGC
GCTGTCTACGGCCTCTACTATGACGCCTTAATCGATTACACCAACGGAGTGTTAACCGTC
ACGGGAGAGAATGCCACAGCCAACATCTTCGCTTCCTACCCGGCCAAGCACCTGTCTGTC
CTCAACGGCTTTGCGGACCAGGTGATTGGTGCTGCGACGCTGGTCCTGTGTATCCTGGCA
ATCACCGACAAGAAGAACATCGGAGCACCTAAAGGCATGGAGCCGCTGGTCATCAGCCTG
TCCATCCTCGCCATCGGCGTGTCCATGGGCCTCAACTGCGGCTATCCCATTAACCCTGCC
CGTGACCTGGGGCCACGGCTCTTCACTGCCGTGGCTGGGTGGGGCTTCGAGGTGTTCAGG
GCTGGGAACTGCTGGTGGTGGATCCCTGTGGCCGGTCCTCTGGTTGGGGGTGTGGTGGGA
GCCAGCATCTACTTCCTGCTCATCGAGCTGCACCATCCCGAA------------------
---------CCACAGAGGCACACAGAG---------------------------------
---------GACGAGAACAAC------GTCAAGGACAAATACGAGATCATCACCATGAGC
>Denticle_herring_Aqp9b
---------------ATGGAG---GACGGCAGGAAAAGAATGAAAGAGCTCTGCACCCTC
AGGAAAGACATCATCAAAGAGTTCCTGGCGGAATTTCTGGGCACCTTCGTGTTAATACTC
TTCGGCTGCGGGTCGGTGGCCCAGACGGTGCTGAGCCGGGGGGCTCTGGGTGAGCCACTT
ACGATCCACATCGGCTTCACTCTGGGGGTCATGCTGGCCGTTTACATGGCGGGGGGAGTG
TCAGGCGGCCATGTGAACCCGGCGGTGTCCCTGGCTATGGTGGTCCTGGGAAAGCTGCCG
TTGAAGAAGTTCCCGGTCTACGTGGCCGCACAGTTCCTGGGAGCTTTTGTGGGATCCTGC
GCTGTTTTCGGGTTGTACTATGATGCTTTCATGGACTTCAATAATGGCACCTTCGTGGTC
ACAGGGGAAAACGCCACTGCCGGGATCTTTGCTTCCTACCCAGCAAAGCACCTGTCAGTC
CTTAACGGCTTTATTGATCAGGTGATCGGGGCCGGCGCGCTGGTGCTGTGTATCCTGGCC
ATTATTGATAAAAAGAACATCGGAGCTCCAAAAGGCACGGAGCCATTGCTGATTGGCCTG
AGCATCTTGGCGATCGGAGTGTCCATGGGGTTCAATTGCGGATATCCCATCAACCCTGCT
CGGGACCTGGGGCCACGGGCATTCACTGCAGCGGCAGGGTGGGGCATGGAGGTGTTCAAG
GCGGGGGGCTGCTGGTGGTGGATCCCAGTGGCAGGTCCCATGGTTGGAGGTGTGGTGGGG
GCCAGCATATATTTCCTTCTGATCGAACTGCACCATCCTGAA------------------
---------CCTCAGAAACACCCAGAA---------------------------------
---------GAAGACAACAAC------GTCAAGGAAAAATATGAGATCATAACCATGAGC
>American_eel_Aqp9b
---------------ATGGAGATAGAAAACAAGAGGAAACTGAAAGAGCGTTGTGCCCTG
AGGCGAAACATCGCCAAAGAATTTTTAGCAGAATTTCTCGGGACATTCGTATTAATTCTG
TTTGGGTGTGGATCCGTTGCCCAGACGGTGCTGAGTCGTGGGACACAGGGGGAGCTCTTG
ACCATTCACATTGGCTTCACTCTGGGAGTGATGATGGCTGTCTACATTGCAGGGGGGGTG
TCAGGAGCCCATGTGAATCCCGCTGTGTCTCTAGCCATGGTTGTCCTGGGCAGGCTGAAG
GTGGCAAAATTCCCTGTGTATGTACTAGCACAGTTCCTTGGTGCCTTTGCTGGAGCCACA
GCAGTCTTTGGGATGTATTATGATGCCTTCATGGACTACTCCGGTGGAGTCCTGACCGTG
ACTGGTAGCAACGCCACAGCTAACATCTTTGCCTCTTATCCAGCCAAACACTTATCAATC
CTGAATGGATTCGTAGACCAGGTTATCGGAACAGGTTCTCTGGTCATGTGCATCCTGGCC

ATCCTTGATGGGAAGAACATTGGGGCGCCCAGGGGGATGGAACCCCTGGCCATCGGTCTG
ATCATCATGGTGATCGGGGTGTCCATGGGGCTCAACTGCGGATATCCCATCAACCCTGCC
AGGGACCTGGGCCCCAGGCTCTTCACTGCCGTAGCTGGGTGGGGCAGAGAAGTCTTCAGG
GCGGCGGGACACTGGTGGTGGATTCCCGTGGTGGGGCCCATGGTGGGGGGCGTGGTGGGA
GCGGTGGTGTACCTCCTCTTCATCGAGCTGCACCACACCGAG------------------
---------CCAGAGAAGCACCAGGAG---------------------------------
---------GAGGAGAACAAC------GTCAAGGACAAATACGAGATTATCACCATGAGC
>European_eel_Aqp9b
---------------ATGGAGATAGAAAACAAGAGGAAACTGAAAGAGCGTTGTGCCCTG
AGGCGAAACATCGCCAAAGAATTTTTAGCAGAATTTCTCGGGACATTCGTATTAATTCTG
TTTGGGTGTGGATCCGTTGCCCAGACGGTGCTGAGTCGTGGGACACAGGGGGAGCTCTTG
ACCATTCACATTGGCTTCACTCTGGGAGTGATGATGGCTGTCTACATTGCAGGGGGGGTG
TCAGGAGCCCATGTGAATCCCGCTGTGTCTCTAGCCATGGTTGTCCTGGGCAGGCTGAAG
GTGGCAAAATTCCCTGTGTATGTACTAGCACAGTTCCTTGGTGCCTTTGCTGGAGCCACA
GCAGTCTTTGGGATGTATTATGATGCCTTCATGGACTACTCCGGTGGAGTCCTGACCGTG
ACTGGTAGCAACGCCACAGCTAACATCTTTGCCTCTTATCCAGCCAAACACTTATCAATC
CTGAATGGATTCGTAGACCAGGTTATCGGAACAGGTTCTCTAGTCATGTGCATCCTGGCC
ATCCTTGATGGGAAGAACATTGGGGCGCCCAGGGGGATGGAACCCCTGGTCATCGGTCTG
ATCATCATGGCGATCGGGGTGTCCATGGGGCTCAACTGCGGATATCCCATCAACCCTGCC
AGGGACCTGGGCCCCAGGCTCTTCACTGCCGTAGCTGGGTGGGGCAGAGAAGTCTTCAGG
GCGGCGGGACACTGGTGGTGGATTCCCGTGGTGGGGCCCATGGTGGGGGGCGTGGTGGGA
GCGGTGGTGTACCTCCTCTTCATCGAGCTGCACCACACCGAG------------------
---------CCAGAGAAGCACCAGGAG---------------------------------
---------GAGGAGAACAAC------GTCAAGGACAAATACGAGATTATCACCATGAGC
>Japanese_eel_Aqp9b
---------------ATGGAGATAGAAAACAAGAGGAAACTGAAAGAGCGTTGTGCCCTG
AGGCGAAACATCGCCAAAGAATTTTTAGCAGAATTTCTCGGGACATTCGTATTAATTCTG
TTTGGGTGTGGATCCGTTGCCCAGACGGTGCTGAGTCGTGGGACACTGGGGGAGCCCTTG
ACCATTCACATCGGCTTCACTCTGGGAGTGATGATGGCTGTCTACATTGCAGGGGGGGTG
TCAGGAGCCCATGTGAATCCCGCTGTGTCTCTAGCCATGGTTGTCCTAGGCAGGCTGAAG
GTGGCAAAATTCCCTGTATATGTACTAGCACAGTTCCTTGGTGCCTTTGCTGGAGCCACA
GCAGTCTTTGGGATGTATTATGATGCCTTCATGGACTACTCCGGTGGAGTCCTGGCAGTG
ACTGGTAGCAACGCCACAGCTAACATCTTTGCCTCTTATCCAGCCAAACACTTATCAATC
CTGAATGGATTCGTAGACCAGGTTATCGCAACAGGTTGTCTGGTCATGTGCATCCTGGCC
ATCCTTGATGGGAAGAACATTGGGGCGCCCAGGGGGATGGAACCCCTGGTCATCGGTCTG
ATCATCATGGCGATTGGGGTGTCCATGGGGCTCAACTGCGGATATCCCATCAACCCTGCC
AGGGACCTGGGCCCCAGGCTCTTCACTGCCATGGCTGGGTGGGGCAGAGAAGTCTTCAGG
GCGGCGGGACACTGGTGGTGGATTCCCGCGGTGGGGCCCATGGTGGGGGGCGTGGTGGGA
GCGGTCGTGTACCTCCTCTTCATCGAGCTGCACCACACCGAG------------------
---------CCAGAGAAGCATCAGGAG---------------------------------
---------GAGGAGAACAAC------GTCAAGGACAAATACGAGATTATCACCATGAGC
>Bowfin_Aqp9
---------------ATGGAGAGGAAAAATAAGAGGAACCTGAAGGAGCGATTTGCCATG
AGAAACAGCATCGTCAAGGAGGCTTTGGCAGAGTTTCTGGGGACATTCGTGCTCATTCTC
TTTGGCTGCGGGTCGGTGGCCCAGACGGTTCTGAGCCGGGGTGCCGTGGGAGAGGTGCTG
ACGATCCACATTGGCTTCACTCTGGGAGTGACAATGGCGGTGTATGTGGCTGGTGGAGTG
TCAGGGGCTCATGTTAACCCAGCAGTCTCCCTGGCCATGCTGGTCCTGGGCAAGCTGAAG
CTGGTGAAGTTCCCAGTGTACGTAGTGGCCCAGTTTCTTGGGGCGTTTGCTGGAGCGGCC
GCCGTGTACGGGCTCTACTATGATGCCTTTATGGATTATACCAACGGCATTCTGACTGTT
ACTGGCCCCAATGCTACTGCACAGATTTTTGCTTCGTACCCAGGAAGACATCTGTCAATC
CTGAATGGCTTCATCGATCAGGTCATAGGTACTGGTGCCTTGGTTCTCTGTATTCTGGCC
ATCTTGGACGGAAAGAACATTGGGGCCCCCAAAGGCATGGAGCCCCTGGTGATTGGCCTG
ATCATCATGGCTATCGGCGTCTCCATGAACCTGAACTGCGGATACCCCATCAACCCCGCC
CGTGACCTGGGGCCGCGGCTTTTCACCGCAGTGGCAGGGTGGGGCACCGCTGTCTTCAGT
GCTGGCAATAACTGGTGGTGGATTCCCGTGGCCGGGCCCTTGGTGGGGGGGATCGTGGGA
GCTGTGCTGTACCTGCTCTTCATCGAGCTACACCACACTGAG------------------
---------CCGCAGAAACACTTGGAG---------------------------------
---------GAGGAGAACAAC------GTCAAGGACAAGTACGAAATGATCACCATGAGT
>Spotted_gar_Aqp9
---------------ATGGAGAGGAAAACTAGGAGGAACCTGCGGGAGCGCTTCGCCCTG
AAGAACAGCATTGTCAAGGAAGCCTTGGCAGAATTCCTCGGGACATTCGTACTCATCCTT

TTCGGCTGTGGGTCAGTTGCCCAGACCGTTTTGAGTCGCGGTGCCGTGGGAGAGATGCTG
ACCATCCACATCGGATTCACTCTGGGTGTGACCATGGCGGTGTACGTGGCCGGAGGAGTG
TCAGGGGCACACGTGAACCCCGCGGTGTCCCTCGCCATGCTGGTGCTGGGCAAGTTGAAG
CTGGTGAAGTTCCCGGTGTACGTGCTCGCACAGTTCCTCGGCGCCTTTGCCGGGGCTGCA
GCCGTGTACGGGCTGTATTACGATGCCTTTATGGATTACACCAATGGAATACTAATTGTT
ACTGGTCCCAACGCTACAGCACATATCTTTGCATCATATCCGGGAAGACACTTGTCAATC
CTAAATGGATTCATAGATCAGGTGATAGGGACGGGCGCCCTGGTGCTTTGTATTCTGGCC
ATTGTGGATGGGAAGAACAATGGGGCTCCCAAAGGAATGGAGCCTTTAGTGATTGGGCTG
ATCATCATGGCTATTGGGGTCTCCATGGGACTGAACTGTGGGTACCCTATAAATCCTGCC
CGGGACCTCGGCCCACGCCTGTTCACTGCAGTGGCAGGATGGGGAAGAGATGTCTTCAGG
GCTGGGAGGAACTGGTGGTGGATTCCAGTGTCTGGCCCGTTGGTGGGCGGGGTTGTAGGA
GCTGTGTTGTACCTGTTGTTCATTGAGCTGCACCACGCCGAG------------------
---------CCGCAGAAGCACCTGGAA---------------------------------
---------GAAGAGAACAGC------GTCAAGGAAAAGTATGAAATGATA---ATGAGC
>Atlantic_sturgeon_Aqp9
---------------ATGGAAAATGAAAACAAGAAGAGCATTAAAGAGAGGTTTGCTCTG
AGGAACAGCCTGGTGAAGGAAGCCTTAGCAGAATTCCTTGGGACATTTCTGCTCATTCTT
TTCGGCGTCGGGTCAGTTGCCCAGACTGTCCTGAGCAGAGGTTCCATGGGAGATCCACTC
ACCATACACATTGGCTTTACACTCGCAGTCACCATGGCTGTGTATGTGGCGGGAGGAGTT
TCAGGCGCTCACATAAACCCTGCTGTCTCTCTGGCGATGTGTGTGCTGGGGAGGCTGAGC
CTCTGCAAATTCCCAGTCTACGCCGTGTCTCAGTTCCTTGGTGCTTTTATTGGCGCCGCA
GCAGTCTATGGACTTTATTATGATGCCTTCCTGGATTTTACAGGTGGAGTGTTCACAGTT
ACTGGCCCCAATGCAACAGCACATATTTTTCATCCTACCCTGGTAAACATCTTTCAATC
TTAAATGGATTTATAGATCAGGTTATAGGAACTGGTGCATTGCTCCTCTGCATCCTTGCC
ATCTTGGACAACAAGAACAAGGGCACCCCGAAAGGAATGGAGCCCTTGATGATTGGTCTC
ATCATCATGGTTATCGGGGTCTCCATGGGCCTGAACTGCGGCTACCCAATCAACCCAGCG
CGTGATCTCGGCCCCAGGCTCTTCACTGCCATAGCAGGGTGGGGCACTGAAGTTTTCAGC
GCTGGAAGTCACTGGTGGTGGATTCCTGTGGCTGGGCCGCTGGTTGGAGGGATGGCCGGA
GCTGTGATCTATGTGCTCTTCATCGAGCTTCACCACGCGGAC------------------
---------CCTCAGAAGACTCCAGAA---------------------------------
---------GAAGAAAACAGC------ATGAAGGATAAATACGAGATGATTGCAATGAGC
>Chinese_sturgeon_Aqp9
---------------ATGGAAAATGAAAACAACAAGAGAATTAAAGAGAGGTTTGCTCTG
AGGAACAGCCTGGTGAAGGAAGCCTTAGCAGAATTCCTTGGGACATTTCTGCTCATTCTT
TTCGGCGTCGGGTCAGTTGCCCAGACTGTCCTGAGCAGAGGTTCCATGGGAGATCCACTC
ACCATACACATTGGCTTTACCCTCGCAGTCACCATGGCTGTGTATGTGGCGGGAGGAGTT
TCAGGCGCTCACATAAACCCTGCTGTCTCTCTGGCGATGTGTGTGCTGGGGAGGCTGAGC
CTCTGTAAATTCCCAGTCTACGCTGTGTCTCAGTTCCTTGGTGCTTTTATTGGCGCCGCA
GCAGTCTATGGACTTTATTATGATGCCTTCCTGGATTATACAGGTGGAGTGCTGACAGTT
ACTGGCCCCAATGCAACAGCACACATTTTTCATCCTACCCTGGTAAACATCTTTCAATC
TTAAATGGATTTATAGACCAGGTTATAGGAACTGGTGCATTGCTCCTCTGCATCCTTGCC
ATCTTGGACAACAAGAACAAGGGCGCCCCGAAAGGAATGGAGCCCTTGATGATTGGTCTC
ATCATCATGGTTATCGGGGTCTCCATGGGCCTGAACTGCGGCTACCCAATTAACCCAGCA
CGTGATCTCGGCCCCAGGCTCTTCACTGCCATAGCAGGGTGGGGCACTGAAGTTTTCAGC
GCTGGAAGCCACTGGTGGTGGATTCCTGTGGCTGGGCCGCTGGTTGGAGGGATGGTCGGA
GCTGTGATCTATGTGCTCTTCATCGAGCTTCACCACGCGGAC------------------
---------CCTCAGAAGACTCCAGAA---------------------------------
---------GAAGAAAACAGC------ATGAAGGATAAATATGAAATTATTGCAATGAGC
>Russian_sturgeon_Aqp9
---------------ATGGAAAATGAAAACAAGAAGAGCATTAAAGAGAGGTTTGCTCTG
AGGAACAGCCTGGTGAAGGAAGCATTAGCAGAATTCCTTGGGACATTTCTGCTCATTCTT
TTCGGCGTCGGGTCAGTTGCCCAAACTGTCCTGAGCAGAGGTTCCATGGGAGATCCACTC
ACCATACACATTGGCTTTACACTCGCAGTCACCATGGCTGTGTATGTGGCGGGAGGAGTT
TCAGGCGCTCACATAAACCCTGCTGTCTCTCTGGCGATGTGTGTGCTGGGGAGGCTGAGC
CTCCGCAAATTCCCAGTCTACGCCGTGTCTCAGTTCCTTGGTGCTTTTATTGGCGCCGCA
GCAGTCTATGGACTTTATTATGATGCCTTCCTGGATTATACAGGTGGAGTGCTGACAGTT
ACTGGCCCCAATGCAACAGCACACATTTTTCATCCTACCCTGGTAAACATCTTTCAATC
TTAAATGGATTTATAGATCAGGTTATAGGAACTGGTGCATTGCTCCTCTGCATCCTTGCC
ATCTTGGACAACAAGAACAAGGGCGCCCCGAAAGGAATGGAGCCCTTGATGATTGGTCTC
ATCATCATGGTTATCGGGGTCTCCATGGGCCTGAACTGCGGCTATCCAATCAACCCAGCG
CGTGACCTCGGCCCCAGGCTCTTCACTGCCATAGCAGGGTGGGGCACTGAAGTTTTCAGC

GCTGGAAGCCACTGGTGGTGGATTCCTGTGGCTGGGCCGCTGGTTGGAGGGATGGTCGGT
GCTGTGATCTATGTGCTCTTCATCGAGCTTCACCACGCGGAC------------------
---------CCTCAGAAGACTCCAGAA---------------------------------
---------GAAGAAAACAGC------ATGAAGGATAAATACGAAATGATTGCAATGAGC
>Siberian_sturgeon_Aqp9
---------------ATGGAAAATGAAAACAAGAAGAGCATTAAAGAGAGGTTTGCTCTG
AGGAACAGCCTGGTGAAGGAAGCATTAGCAGAATTCCTTGGAACATTTCTGCTCATTCTT
TTCGGCGTCGGGTCAGTTGCCCAAACTGTCCTGAGCAGAGGTTCCATGGGAGATCCACTC
ACTATACACATTGGCTTTACACTCGCAGTCACCATGGCTGTGTATGTGGCGGGAGGAGTT
TCAGGCGCTCACATAAACCCTGCTGTCTCTCTGGCGATGTGTGTGCTGGGGAGGCTGAGC
CTCTGTAAATTCCCAGTCTACGTCGTGTCTCAGTTCCTTGGTGCTTTTATTGGCGCCGCA
GCAGTCTATGGACTTTATTATGATGCCTTCCTGGATTATACAGGTGGAGTGCTGACAGTT
ACTGGCCCCAATGCAACAGCACACATTTTTTCATCCTACCCTGGTAAACATCTTTCAATC
TTAAATGGATTTATAGATCAGGTTATAGGAACTGGTGCATTGCTCCTCTGCATCCTTGCC
ATCTTGGACAACAAGAACAAGGGCGCCCCGAAAGGAATGGAGCCCTTGATGATTGGTCTC
ATCATCATGGTTATCGGGGTCTCCATGGGCCTGAACTGCGGCTATCCAATCAACCCAGCG
CGTGATCTCGGCCCCAGGCTCTTCACTGCCATAGCAGGGTGGGGCACTGAAGTTTTCAGC
GCTGGAAGCCACTGGTGGTGGATTCCTGTGGCTGGGCCGCTGGTTGGAGGGATGGTCGGA
GCTGTGATCTATGTGCTCTTCATCGAGCTTCACCACGCGGAC------------------
---------CCTCAGAAGACTCCAGAA---------------------------------
---------GCAGAAAACAGC------ATGAAGGATAAATACGAAATGATTGCAATGAGC
>Sterlet_Aqp9_1
---------------ATGGAAAATGAAAACAAGAAGAGCATTAAAGAGAGGGTTGCTCTG
AGGAACAGCCTGGTGAAGGAAGCCTTAGCAGAATTCCTTGGGACATTTCTGCTCATTCTT
TTCGGCGTCGGGTCAGTTGCCCAGACTGTCCTGAGCAGAGGTTCCATGGGAGATCCACTC
ACTATACACATTGGCTTTACACTCGCAGTCACCATGGCTGTGTATGTGGCGGGAGGAGTT
TCAGGCGCTCACATAAACCCTGCTGTCTCTCTGGCGATGTGTGTGCTGGGGAGGCTGAGC
CTCTGCAAATTCCCAGTCTACGCCGTGTGTCAGTTCCTTGGTGCTTTCATTGGTGCCGCA
GCAGTCTATGGACTTTATTATGATGCCTTCCTGGATTATACAGGTGGAGTGCTGACAGTT
ACTGGCCCCAATGCAACAGCACACATTTTTTCATCCTACCCTGGTAAACATCTTTCAATC
TTAAATGGATTTATAGATCAGGTTATAGGAACTGGTGCATTGCTCCTCTGCATCCTTGCC
ATCTTGGACAACAAGAACAAGGGCGCCCCGAAAGGAATAGAGCCCTTGATGATTGGTCTC
ATCATCATGGTTATCGGGGTCTCCATGGGCCTGAACTGCGGCTACCCAGTCAACCCAGCG
CGTGATCTCGGCCCCAGGCTCTTCACTGCCATAGCAGGGTGGGGCACTGAAGTTTTCAGG
GCTGGAAGCCACTGGTGGTGGATTCCTGTGGCTGGGCCGCTAGTTGGAGGGATGGTCGGA
GCTGTGATCTATGTGCTTTTCATCGAGCTTCACCACGTGGAC------------------
---------CCTCAGAAGACTCCAGAA---------------------------------
---------GAAGAAAACAGC------ATGAAGGATAAATACGAAATGATTGCAATGAGC
>Sterlet_Aqp9_2
---------------ATGGAAAATGAAAACAAGAAGAGCATTAAAGAGAGGTTTGCTCTG
AGGAACAGCCTGGTGAAGGAAGCATTAGCAGAATTCCTTGGAACATTTCTGCTCATTCTT
TTCGGCGTCGGGTCAGTTGCCCAAACTGTCCTGAGCAGAGGTTCCATGGGAGATCCACTC
ACTATACACATTGGCTTTACACTCGCAGTCACCATGGCTGTGTATGTGGCGGGAGGAGTT
TCAGGCGCTCACATAAACCCTGCTGTCTCTCTGGCGATGTGTGTGCTGGGGAGGCTGAGC
CTCTGTAAATTCCCAGTCTACGTCGTGTCTCAGTTCCTTGGTGCTTTTATTGGTGCCGCA
GCAGTCTATGGACTTTATTATGATGCCTTCCTGGATTATACAGGTGGAGTGCTGACAGTT
ACTGGCCCCAATGCAACAGCACACATTTTTTCATCCTACCCTGGTAAACATCTTTCAATC
TTAAATGGATTTATAGATCAGGTTATAGGAACTGGTGCATTGCTCCTCTGCATCCTTGCC
ATCTTGGACAACAAGAACAAGGGCGCCCCGAAAGGAATGGAGCCCTTGATGATTGGTCTC
ATCATCATGGTTATCGGGGTCTCCATGGGCCTGAACTGCGGCTACCCAGTCAACCCAGCG
CGTGATCTCGGCCCCAGGCTCTTCACTGCCATAGCAGGGTGGGGCACTGAAGTTTTCAGG
GCTGGAAGCCACTGGTGGTGGATTCCTGTGGCTGGGCCGCTGGTTGGAGGGATGGTCGGA
GCTGTGATCTATGTGCTCTTCATCGAGCTTCACCACGCGGAC------------------
---------CCTCAGAAGACTCCAGAA---------------------------------
---------GCAGAAAACAGC------ATGAAGGATAAATATGAAATGATTGCAATGAGC
>Reedfish_Aqp9
---------------ATGGAAGCGGAGAATAAA---AGTTTTAAGAAGAGAATTGCTGTG
AGAAGCAAAATCCTAAGGGAAGCGCTCGCAGAATTCCTTGGAACATTTATACTAATTATT
TTTGGCTGTGGCTCAGTCGCCCAGACCGTCCTCAGCAGCGGGGCTTTTGGTGAGATGCTG
ACTATTCACATTGGCTTCACTATTGGTGTCATAATGGCAGTATACGTGGCAGGGGGGATT
TCAGGTGCACATGTGAATCCTGCAGTTTCTCTTGCCATGTGTGTTACGGGCAGGTTGAGC

TTCTTGAAGTTCCCAGTCTATGTCCTGTCTCAGTTCCTTGGGGCTTTCACTGGTGCAGCA
GCAGTGTATGGACTCTACTATGACGCATTTCTGAACTACACAGGAGGCGTGCTCACTGTC
ACCGGCCCTAATGCAAGTGCACAGATATTTGCAACTTACCCTGCAACATATCTTTCAATA
TTTAATGGCTTCATGGATCAGGTGGTAAGTACCGGAGCTTTGGTGCTCTGCATCTTTGCC
ATCACTGACAAGAAGAACAACGGTGCCCCAAAAGGAATGGAGCCACTTCTCATTGGTTTA
ATTATCATGGTAATTGGGGTCTCTATGGGCCTGAACTGTGGCTACCCCATCAATCCAGCT
CGAGATCTTGGCCCTCGCCTTTTTACCTTCATTGCAGGGTGGGGTGTTGAAGTTTTCAGG
GCTGGGAATAACTGGTGGTGGGTCCCTGTGGCAGGCCCAATGGTTGGTGGATTGTTGGT
GCCTTGATCTACGTTGCATTTATTGAGCTGCACCATCACGAC------------------
---------CCACAGAAGCAATCTGAA---------------------------------
---------GAAGATCACAAT------GTCCAAGATAAATATGAGATGATTACAATGAAC

File S9: Alignment for Fig. 3D, Fig. S8
>Torafugu_Aqp10aa
---------------ATGAAGGCTTTGAAAACCAGGAACGCTCTGGTGAGAGAGTGCATG
GCTGAGCTCCTGGGAACCTTCGTTTTACTGCTGTTCGGATGTTCGGCCACAGCGCAGGTG
AAGACCAGCAGGGAGACGAAAGGCCAGTATCTGTCAGTCAACATGGCCTTTTCTGTGGGT
GTCATGTCTGCCATGTATCTCACCAAAGGCATCTCAGGTGCTCATCTCAACCCAGCGGTG
ACGCTGAGCTTCTGTGCACTGGGCCAAGTGTCCTGGGACAGACTGGTGCCCTACTGCCTC
TCCCAGTTGCTGGGGGCTTACATCGCATCAGGGCTTGTTTACCTGGTCTATTACGATGCT
ATAATGAACTTCAGCGGGGGGGTGCTGACTGTGTATGGTCCGAATGAAACAGCATCGATA
TTCGCCACCTATCCATCAGAGTTCCTGAGCTTGGGCAGCAGTTTCCTGGACCAGGTAGTT
GGCACCAGCATGCTGATGCTGTGCATTCTGGGCCTGGGTGAACAGAGGAACACTCCGGCT
CCCTCGGACCTGATCCCGGCGATAGTGGCGGTCATCGTCCTTGGGATCTCCATGTCCATG
TCCGGCAACTGCGGCGCTGCCATAAATCCCGCTCGAGATCTGGGGCCCCGTCTGTTCACG
CTGACCGCGGGCTGGGGCCCGGAGGTCTTCACGTGTTACAACTACTGGTTCTGGGTTCCT
CTGGTGGCCCCGTGTATCGGGGCCCTCATCGGCACTTTACTGTATCAGATCTTAATTTCT
TTGCACCTCCCAGATCCAGCGGAG---------CCTGAAAGGGTGTGT---TGTCATGTC
TCCACC------ATCACAATGGCAGTA------------------------AAACAGCCT
GAACCAGTG---TGGGACCATGACAAAGGACTGAAGCTGATCCGATCC------------

>Sansaifugu_Aqp10aa
ATGGCGACA------ATGAAGGCTTTGAAAACCAGGAACGCTCTGGTGAGAGAGTGCATG
GCTGAGCTCCTGGGAACCTTCGTTTTACTGCTGTTCGGATGTTCGGCCGCAGCGCAGGTG
AAGACCAGCAGGGAGACGAAAGGCCAGTATCTGTCAGTCAACATGGCCTTTTCTGTGGGC
GTCATGTCTGCCATGTACCTCACCAAAGGCATCTCAGGTGCTCATCTCAACCCAGCGGTG
ACGCTGAGCTTCTGTGCACTGGGCCAAGTGTCCTGGGACAGACTGGTGCCCTACTGCCTC
TCCCAGTTGCTGGGGGCTTACATCGCATCAGGGCTTGTTTACCTGGTCTATTACGATGCT
ATAATGAACTTCAGCGGGGGGGTGCTGACTGTGTATGGTCCGAATGAAACAGCATCGATA
TTCGCCACCTATCCATCAGAGTTCCTGAGCTTGGGCAGCAGTTTCCTGGACCAGGTAGTT
GGCACCAGCATGCTGATGCTGTGCATTCTGGGCCTGGGTGAACAGAGGAACACTCCGGCT
CCCTCGGACCTGATCCCGGCGATAGTGGCGGTCATCGTGCTTGGGATCTCCATGTCCATG
TCCGGCAACTGCGGCGCTGCCATAAATCCCGCTCGAGATCTGGGGCCCCGTCTGTTCACG
CTGACCGCGGGCTGGGGCCCGGAGGTCTTCACGTGTTACAACTACTGGTTCTGGGTTCCT
CTGGTGGCCCCGTGTATCGGGGCCCTCATCGGCACTTTACTGTATCAGATCTTAATTTCT
TTGCACCTCCCAGACCCAGCGGAG---------CCTGAAAGGGTGTGT---TGTCATGTC
TCCACC------ATCACAATGGCAGTA------------------------AAACAGCCT
GAACCAGTG---TGGGACCATGACAAAGGACTGAAGTTGGAACACGAA------------

>Green_spotted_pufferfish__Aqp10aa2
---------------ATGAGGGCTTTGAGGGTGAGGAACGCCCTGGTGAGAGAGTGCATG
GCTGAGTTCCTGGGAACCTTTGTTTTGTTGCTCTTCGGCTGTAAGGCCGCGGCGCAGGTG
AAGACCAGCAGGGAGTCGAAAGGCCAGTATCTGTCGGTGAACATGGCCTTCTCCGAGGGC
GTCATGTCTGCCATGCATCTCAGCAAAGGCATCTCAGGCGCTCACCTGAACCCAGCGGTG
ACTCTGAGCTTCTGTGTACTGGGCCACGTGTCCTGGGACAGACTGGTGCCCTACAGCCTC
TCCCAGTTGCTGGGGGCCTACATGGCGTCAGGGCTCGTTTACCTGGTGTATTACGATGCC
ATAATGAACTTCAGCGAGGGGGTGCTGACTGTGTACGGTCCAAATGAAACAGCTTCCATA
TTTGCCACCTATCCGTCAGAGTTCTTGAGCTTGGGCAGGAGTTTCCTGGACCAGGTGGTG
GGCACTGGCATGTTGATGCTGTGCATCCTGGGCCTGGACGAGCAGAGAAACACTCCGGCT

CCCACGCAGCTGATTCCGGCCATAGTGGCGGCCGTCGTGCTGGGGATCTCCATGTCCATG
TCCGGCAACTGCGGCGCTGCCATAAATCCGGCTCGAGATCTGGGGCCTCGTCTCTTCACG
CTGACAGCGGGGTGGGCACAGAGGTCTTCACGTGTTACAACTATTGGTTCTGGGTCCCT
CTGGTGGCCCCGCCAATCGGGGCCCTCGTGGGCACTTTTCTGTATCAGATTCTTATTTCT
TGGCACCTCCCAGACCCAAAACAT---------CCTGAAAGAGTC------TCTCACGTC
TCCACC------ATCACGACAGCAGTA-----------------------ACACAGCCT
GAACCAGCC---CAGGAAAAGGGATCCGAGTTAGGACACATCTGCTGT------------

>Green_spotted_pufferfish__Aqp10aa1
---------------ATGAGGGCTTTGAGGGTGAGGAACGCCCTGGTGAGAGAGTGCATG
GCTGAGTTCCTGGGAACCTTTGTTTTGTTGCTCTTCGGCTGTTCGGCCGCGGCGCAGGTG
AAGACCAGCAGGGAGTCGAAAGGCCAGTATCTGTCGGTGAACATGGCCTTCTCCGTGGGC
GTCATGTCTGCCATGTATCTCAGCAAAGGCATCTCAGGCGCTCACCTGAACCCAGCGGTG
ACTCTGAGCTTCTGTGTACTGGGCCACGTGTCCTGGGACAGACTGGTGCCCTACAGCCTC
TCCCAGTTGCTGGGGGCCTACATGGCGTCAGGGCTCGTTTACCTGGTGTATTACGATGCC
ATAATGAACTTCAGCGAGGGGGTGCTGACTGTGTACAGTCCAAATGAAACAGCTTCCATA
TTTGCCACCTATCCGTCAGAGTTCTTGAGCTTGGGCAGGAGTTTCCTGGACCAGGTGGTG
GGCACTGGCATGTTGATGCTGTGCATCCTGGGCCTGGACGAGCAGAGAAACACTCCGGCT
CCCACGCAGCTGATTCCGGCCATAGTGGCGGCCGTGGTGCTGGGGATCTCCATGTCCATG
TCCGGCAACTGCGGCGCTGCCATAAATCCGGCTCGAGATCTGGGGCCTCGTCTCTTCACG
CTGACAGCGGGGTGGGCACAGAGGTCTTCACGTGTTACAACTATTGGTTCTGGGTCCCT
CTGGTGGCCCCGCCAATCGGGGCCCTCGTGGGCACTTTTCTGTATCAGATTCTTATTTCT
TGGCACCTCCCAGACCCAAAACAT---------CCTGAAAGAGTC------TCTCACGTC
TCCACC------ATCACGACAGCAGTA-----------------------ACACAGCCT
GAACCAGCC---CAGGAAAAGGGATCCGAGTTAGGACACATCTGCTGT------------

>Ocean_sunfish_Aqp10aa
ATGTTGAAG------CTACGTGTCTTGACAGTGAGAAATGCACTGGTGCGAGAGTGCATG
GCTGAGCTCTTGGCAACTTTTGTTTTGCTGCTCTTCGGCTGCTCCGCTGCAGCGCAGGTG
AAGACGAGCCGAGAGACTAAAGGCCAGTACCTGTCGGTCAACATGGCCTTCTCTGTGGGC
GTGATGTCAGCCATGTATCTCTCCAAAGGCATATCAGGTGCCCATCTGAACCCAGCAGTG
ACTCTGAGTTTCTGTGTGTTGGGTCGGTCGCCCTGGGACAGGCTGGTACCCTTTTGCCTC
TCCCAGCTGCTGGGGGCTTACATGGCATCAGCGCTTGTCTACCTGGTCTACTATGATGCT
ATCATGGACTTCAGTGGAGGAGTGCTGACTGTTTATGGCCAAAATGAGACGGCATCTATA
TTTGCCACATATCCCTCAGAGTTCTTGACTTTAGGCAGAAGTTTCCTTGACCAGATAGTT
GGCACCGGCATGCTGATGTTGTGCATCCTGAGTTTAAATGAGAAAAGAAATACCCCCGCT
CCCACAGAGCTGATTCCTGCTATAGTGGGGGTGATCGTCCTGGGGATCTCTGTGTCAATG
ACTGGTAACTGTGGCGGTGCAATAAATCCTGCACGCGACCTGGGGCCTCGTCTCTTTATG
CTGACTGCAGGCTGGGGCACAGAGGTCTTCACGTGTTACAACTACTGGTTCTGGGTCCCT
TTAGTGGCCCCGCTTATAGGAGGGCTCATTGGTACTTTCCTGTATCTGATCTTTATCGAC
TGGCATCTGCCCGACCCGGACCAA---------CCTGAGAACCTC------TCCAATGCC
CACGAC------GTCAATGACAAAATA-----------------------AAA------
---GCAACA---TGGGACAGTGGAGCGGAATTGAGGGAGCATAATTTC------------

>Striated_frogfish_Aqp10aa
ATGTTGACA------CACCGTGCTCTGAGAGTGAGGAACGCTCTGGTGCGAGAGTGCATG
GCTGAGTGCTTAGGGACGTTCGTTTTGCTGGTCTTTGGCTGCTCTGCTGCAGCACAGGTG
AAGACAAGCAGAGAGACTAAAGGCCAGTTCCTGTCAGTCAACATGGCCTTCTCTGTGGGC
GTGATGTCAGCTATGTATCTCACCAAGGGCATTTCAGGGGCCCATCTGAACCCAGCCTTA
ACACTGAGTTTCTGTGTGTCGGGTCATGCATCCTGGGGAAGACTGGTACCCTACTGTCTC
TCGCAGTTGCTGGGGGCTTACCTGGCATCAGGACTGGTTTTCCTGGTCTATTATGATGCT
ATAATGGACTATAGTGGAGGAGTACTGACTGTATATGGCCCAAATGAGACAGCATCAATA
TTTGCCACGTATCCCTCAGAGTACCTGACTGTTGGCAGAAGTTTCCTGGACCAGGTTGTG
GGAACTGCCATGTTGTTGTTGTGCATCATGGGTTTGGATGAAAAGAGAAACACCCCCTGCT
CCCCAAAAGCTGATTCCTGCGATTGTAGCTGCAATTGTCCTGGGGATCTCTATGTCAATG
TCTGGTAATTGCGGTGCAGCAATAAATCCTGCTCGAGACCTTGGGCCACGCCTCTTTACG
TTGACTGCAGGCTGGGGCACGGAGGTCTTCACGTGTTACAACTACTGGTTCTGGGTACCC
TTGGTGGCCCCACCTATTGGAGGTGTCATAGGGACTGTTTTGTATTTGACTTTCATCGAC

TGGCAGCTTCCTGACCAGGAGCAA---------CCCGAGAGTCCG------TCCTCTGTC
TTCAAC------ATTTGTGAGAAAATC------------------------ATACAGCCC
AGTATCACG---TGGGACAATGGAGGAGAATTGAAGGCTGCACATTTC------------

>Gilthead_seabream_Aqp10aa
ATGAAGAAG------CTACGAGCTCTGAGAGTGAGGAACGCTCTGGTGCGCGAGTGCATG
GCTGAGTTTTTGGGAACTTTCATCTTGTTGCTCTTTGGCTGCTCCGCCGCGGCGCAGGTG
AAGACGAGCAGAGAGACAAAGGGCCAGTTCCTGTCAGTAAACATGGCCTTCTCCGTGGGC
GTGATGTCGGCCATGTATCTCACCAAGGGCATCACAGGTGCTCATCTGAACCCGGCAGTG
AGTCTGAGTTTCTGTGTGCTGGGACAGGTGCAATGGGGAAGGCTGGTGCCCTACTGCCTC
TCCCAGCTGCTGGGGGCATACGTGGCGTCAGCGCTCGTCTACCTGGTCTACTATGATGCT
ATAATGGACTTCAGTGGAGGAGATTTGACTGTATATGGCCCAAATGAGACAGCGTCTATA
TTTGCCACGTATCCCTCAGAGTACATGACTTTAGGCAGAAGTTTCCTTGACCAGGTCGTG
GGCACTGGCATGCTGATGTTGTGCATCCTGTGTTTGGGTGAAAAGAGGAATACCCCGGCT
CCCGCAGAGCTGATCCCTCCGATAGTGGCAGTGATTGTCCTGGGGATCTCCATGTCAATG
TCCGGTAACTGCGGCGCTGCAATCAATCCTGCGAGGGACCTGGGGCCGCGCCTCTTTACC
CTGACTGCCGGCTGGGGCACAGAGGTTTTCACGTGTTACGACTACTGGTTCTGGGTACCC
CTGGTGGCTCCACCTATAGGAGGTGTGATGGGCACTTTTATGTATCTGATTTTCATCGAA
TGGCACCTGCCTGACCCGGACCAA---------CCTGACAACGAC------TCCGCTCTC
TCC---------ATTAGCGAGACAATA------------------------AAGCAGCCC
GGCCCGACG---TGGGAGAAAGATGTGGAATTAAAGTCTTCACATTTC------------

>Red_seabream_Aqp10aa
ATGATGAAG------CTACGAGCTCTGAGAGTGAGGAACGCTCTGGTGCGAGAGTGCATG
GCTGAGTTTTTGGGAACTTTTGTCCTGCTGCTCTTTGGCTGCTCCGCTGCGGCGCAGGTG
AAGACGAGCAGAGAGACAAAGGGCCAGTTCCTGTCAGTAAACATGGCCTTCTCTGTGGGC
GTGATGTCAGCTATGTATCTCACCAAGGGCATCACAGGTGCTCATCTGAACCCGGCGGTG
AGTCTGAGTTTCTGTGTGCTGGGACAGGTGCGATGGGGAAGGCTGGTGCCCTACTGCCTC
TCCCAGCTGCTGGGGGCGTACGTGGCATCAGCGCTCGTCTACCTGGTCTACTATGATGCT
ATAATGGACTTCAGTGGAGGAGATTTGACTGTATATGGCCCAAATGAGACGGCGTCTATA
TTTGCCACGTATCCCTCACAGTACATGACTTTGGGCAGAAGTTTCCTTGACCAGGTCGTG
GGCACTGGCATGCTGATGTTGTGCATCCTGTGTTTGGGTGAAAAGAGGAATACCCCGGCT
CCCACAGAGCTGATCCCTGCGATAGTGGCAGTGATCGTCCTGGGGATCTCCATGTCAATG
TCTGGTAACTGCGGTGCCGCAATCAATCCTGCGAGGGACCTGGGGCCGCGCCTCTTTACC
CTGACTGCAGGCTGGGGCACAGAGGTCTTCACGTGTTACAACTACTGGTTCTGGGTACCC
CTGGTGGCTCCACCTATAGGAGCTGTAACAGGCACTTTCATGTATTTGATTTTCATCGAA
TGGCAGCTGCCTGACCCGGACCAA---------CCTGAAAACCTC------TCAACTCTC
TCCTCC------ATTAGTGAGACAATA------------------------AAGCAGCCC
AGCTCCACA---TGGGAGAAAGATGTGGAGTTAAAGTCTTCACATTTC------------

>Miiuy_croaker_Aqp10aa
ATGTTGAAG------CTACGTGCTGTGAGAGTGAAGAATGCTCTGGTGCGAGAGTGCATG
GCTGAGTTCTTGGGAACTTTTGTCTTACTGCTCTTTGGCTGCTCCGCTGCAGCACAGGTA
AAGACAAGCAGAGGAACTAAAGGCCAGTTCCTGTCAGTCAACATGGCTTTCTCTGTGGGT
GTGATGTCAGCTATGTATCTCACCAAGGGCATCACAGGTGCTCATCTGAACCCAGCAGTG
TCTTTGAGTTTTTGTGTGTTGGGCCAGGTACCCTGGGAAAGGCTGCTGCCCTACTGCCTC
TCCCAGCTGATAGGGGCTTATGTGGCATCAGGGCTTGTCTACCTGGTCTACTATGATGCT
ATAATGGACTTTAGTGGAGGAGTATTGACTGTATATGGCCCAAATGAGACAGCATCAATA
TTTGCCACATATCCCTCAGAGCACATGACTTTGGGCAGAAGTTTTCTTGATCAGGTAGTT
GGCACTGGCATGCTGATGTTGTGCATCCTGTGCTTGGGTGAAAAGAGGAATACACCGGCT
CCAACTGAGCTGATTCCTCCTATAGTGGCAGTGATTGTCCTCGGGATCTCGATGTCGATG
TCAGGTAACTGTGGTGCTGCAATAAATCCTGCACGGGACCTGGGGCCACGCCTCTTTACG
CTGATGGCAGGCTGGGGCACAGAGGTCTTCACGTGTTACAACTACTGGTTCTGGGTTCCC
ATCGTGGCCCCACCTATCGGAGGCGTTATAGGTTCTTTTATGTATTTGATCTTCATCGAA
TGGCACCTGCCTGACCCAGACCAA---------CTGGAGAACCCC------TCAACTTTC
CCCACC------ATCAGTGACAAAATC------------------------AAGCAGCCC
AGCAGT---------------GGAGTAGAGTTAAAGGTTGCACGTTTC------------

>Large_yellow_croaker_Aqp10aa
ATGTTGAAG------CTACGTGCTGTGAGGGTGAAGAATGCTCTGGTGCGAGAGTGCATG
GCTGAGTTCTTGGGGACTTTTGTTTTACTGCTCTTTGGCTGCTCTGCTGCAGCACAGGTA
AAGACAAGCAGAGAAACTAAAGGCCAGTTCCTGTCAGTCAACATGGCTTTCTCTGTGGGT
GTGATGTCAGCTATGTATCTCACCAAGGGCATCACAGGTGCTCATCTGAACCCAGCAGTG
TCTCTGAGTTTTTGTGTGTTGGGCCAGGTACCCTGGGATAGGCTGCTGCCCTACTGCCTC
TCCCAGCTGATAGGGGCTTATGTGGCATCAGGGCTTGTCTACCTGGTCTACTATGATGCT
ATAATGGACTTTAGTGGAGGAGTATTGACTGTATATGGCCCAAATGAGACAGCATCAATA
TTTGCCACATATCCCTCAGAGTACATGACTTTGGGCAGAAGTTTTCTTGATCAGGTAGTT
GGCACTGGGATGCTGATGTTGTGCATCCTGTGCTTGGGTGAAAAGAGGAATACACCGGCT
CCAACTGAGCTGATTCCTCCTATAGTGGCAGTGATTGTCCTCGGGATCTCGATGTCAATG
TCAGGTAACTGTGGTGCTGCAATAAATCCTGCACGGGACCTGGGGCCACGCCTCTTTACG
CTGACGGCAGGCTGGGGCACAGAGGTCTTTACGTGTTACAACTACTGGTTCTGGGTTCCC
CTCGTGGCCCCACCTATCGGAGGCGTTATAGGTTCTTTCATGTATGTGATCTTCATCGAA
TGGCACCTGCCTGACCCAGACCAA---------CTGGAGAACCCC------TCAGCTTTT
CCCACC------ATCAGTGACAAAATC------------------------AAGCAGCCC
AGCAGT---------------GGAGTAGAGTTAAAGGATGCACGTTTC------------

>Meagre_Aqp10aa
ATGTTGAAG------CTACGTGCTGTGAGAGTGAAGAACGCTCTGGTGCGAGAGTGCATG
GCTGAGTTCTTGGGAACTTTTGTCTTGCTGCTCTTTGGCTGCTCCGCTGCAGCGCAGGTA
AAGACAAGCAGAGAAACTAAAGGCCAGTTCCTGTCAGTCAACATGGCTTTCTCTGTGGGT
GTGATGTCAGCTATGTATCTCACCAAGGGCATTACAGGTGCTCATCTGAACCCAGCAGTG
TCTCTGAGTTTTTGTGTGTTGGGCCAGGTACCCTGGGGAAGGCTGCTGCCCTACTGCCTC
TCCCAGCTGACAGGGGCTTATGTGGCATCCGGGCTTGTCTACCTGGTCTACTATGATGCT
ATAATGGACTTTAGTGGAGGAGTATTGGCTGTATATGGCCCAAATGAGACAGCATCAATA
TTTGCCACATATCCTTCAGAGTACATGACTTTGGGCAGAAGTTTTCTTGATCAGGTAGTT
GGCACTGGCATGCTGATGTTGTGCATCCTGTGCTTGGGTGAAAAGAGGAATACACCGGCT
CCAACTGAGCTGATTCCTCCTATAGTGGCAGTGATTGTCCTCGGGATCTCGATGTCGATG
TCAGGTAACTGTGGTGCTGCAATAAATCCTGCACGGGACCTGGGGCCACGCCTCTTTACG
CTGACGGCAGGCTGGGGCACAGAGGTCTTCACGTGTTACAACTACTGGTTCTGGGTTCCC
CTCGTGGCCCCACCTATCGGCGGCGTTATAGGTTCTTTCATGTATTTGATCTTCATCGAA
TGGCACCTGTCTGACCCAGACGAA---------CTGGAGAACCCC------TCAACTTTC
CCCACC------ATCAGTGACAAAATC------------------------AAGCAGCCC
AGCAGT---------------GGAGTAGAGTTAAAGGTTGCACGTTTC------------

>White_bass_Aqp10aa
ATGTTAAAG------CTACGTGCTCTGAGAGTGAGAAATGCTCTGGTGCGAGAGTGCATG
GCTGAGTTCTTGGGAACATTTGTCTTGTTGCTCTTTGGCTGCTCTGCCGCAGCGCAGGTA
AAAACAAGCAGAGAGACTAAAGGCCAGTTCCTGTCGGTCAACATGGCCTTCTCTGTTGGC
GTGATGTCAGCTATGTACCTCACCAAGGGCATCACAGGTGCTCATCTAAACCCAGCAGTG
ACTCTGAGTTTCTGTGTGTTGGGCCAGGTGCCCTGGGGACGGCTGGTTCCCTATTGCCTC
TCCCAACTGCTGGGGGCTTACATGGCATCAGGGCTTGTCTACTTGGTCTACTATGATGCT
ATAATGGACTTTAGTGGAGGAGTATTGACTGTATATGGCCCAAATGAGACAGCATCTATA
TTTGCCACATATCCCTCAGAGTACATGACTTTGGGCAGAAGTTTTCTTGACCAGGTCGTG
GGCACTGGCATGCTGATGTTGTGCATCCTGTGTTTGGGGGAAAAGAGGAATACCCCAGCT
CCCTCAAAGCTGATTCCTGCTATAGTGGCAGTGATCGTCCTGGGGATCTCCATGTCAATG
TCAGGTAATTGTGGTGCTGCAATAAATCCTGCTCGGGACCTGGGGCCACGCCTCTTTACG
CTGACGGCAGGCTGGGGCACAGAGGTCTTCACGTGTTACAACTACTGGTTCTGGGTACCC
CTGGTAGCCCCACCTATCGGAGGTGTCATAGGTACTTTCATGTATTTGATCTTCATCGAG
TGGCAACTGCCTGACCCGGACCAG---------CCTGAAAGCCTC------TCCACTCTA
TCCACC------ATCAGTGACAAAATC------------------------CAGCAGCCC
AGCACAACG---TGGGACAATGGAACAGGATTAAAGACTGCACATTTC------------

>Striped_seabass_Aqp10aa
ATGTTAAAG------CTACGTGCTCTGAGAGTGAGAAATGCTCTGGTGCGAGAGTGCATG
GCTGAGTTCTTGGGAACATTTGTCTTGTTGCTCTTTGGCTGCTCTGCCGCAGCGCAGGTA

AAAACAAGCAGAGAGACTAAAGGCCAGTTCCTGTCGGTCAATATGGCCTTCTCTGTGGGC
GTGATGTCAGCTATGTACCTCACCAAGGGCATCACAGGTGCTCATCTAAACCCAGCAGTG
ACTCTGAGTTTCTGTGTGTTGGGCCAGGTGCCCTGGGGACGGCTGGTTCCCTACTGCCTC
TCCCAACTGCTGGGGGCTTACGTGGCATCAGGGCTTGTCTACTTGGTCTACTATGATGCT
ATAATGGACTTTAGTGGAGGAGTATTGACTGTATATGGCCCAAATGAGACAGCATCTATA
TTTGCCACATATCCCTCAGAGTACATGACTTTGGGCAGAAGTTTCCTTGACCAGGTCGTG
GGCACTGGCATGCTGATGTTGTGCATCCTGTGTTTGGGGGAAAAGAGGAATACCCCAGCT
CCCTCAAAGCTGATTCCTGCTATAGTGGCAGTTATCGTCCTGGGGATCTCCATGTCAATG
TCAGGTAATTGTGGTGCTGCAATAAATCCTGCTCGGGACCTGGGGCCACGCCTCTTTACG
CTGACGGCAGGCTGGGGCACAGAGGTCTTCACGTGTTACAACTACTGGTTTTGGGTACCC
CTGGTAGCCCCACCTATCGGAGGTGTCATAGGTACTTTCATGTATTTGATCTTCATCGAG
TGGCAACTGCCTGACCCGGACCAG---------CCTGAAAGCCTC------TCCACTCTA
TCCACC------ATCAGTGACAAAATC------------------------CAGCAGCCC
GGCACAACG---TGGGACAATGGAACAGGATTAAAGACTGCACATTTC------------

>European_seabass_Aqp10aa
ATGTTAAAG------CTACGTGCTCTGAGAGTGAGAAATGCTCTGGTGCGAGAGTGCATG
GCTGAGTTCTTGGGAACCTTTGTCTTGTTGCTCTTTGGCTGCTCTGCCGCAGCGCAGGTA
AAAACAAGCAGGGAGACTAAAGGCCAGTTCCTGTCGGTTAACATGGCCTTCTCTGTGGGC
GTGATGTCAGCTATGTACCTCACCAAGGGCATCACAGGTGCTCATCTGAACCCAGCGGTG
ACTCTGAGTTTCTGTGTGTTGGGCCAGGTGCCCTGGGGACGGCTGGTTCCCTACTGCCTC
TCCCAGCTGCTGGGGGCTTACATGGCATCAGCGCTTGTCTACCTGGTGTACTATGATGCT
ATAATGGACTTTAGTGGAGGAGTATTGACTGTATATGGCCCAAATGAGACAGCATCTATA
TTTGCCACATATCCCTCAGAGTACATAACTTTGGGCAGAAGTTTCCTCGACCAGGTCGTG
GGCACTGGCATGCTGATGTTGTGCATCCTGTGTTTGGGGGAAAAGAGGAATACCCCAGCT
CCCTCAGAGCTGATTCCTCCTATAGTGGCAGTGATCGTCCTGGGGATCTCCATGTCAATG
TCAGGTAACTGTGGTGCTGCAATAAATCCTGCTCGGGACCTGGGGCCACGCCTCTTTACG
CTGACGGCAGGCTGGGGCACAGAGGTCTTCACGTGTTACAACTACTGGTTCTGGGTACCC
CTGGTAGCCCCACCTATCGGAGGTGTCATAGGTACTTTCATGTATTTGATCTTCATCGAC
TGGCACCTGCCTGACCCGGACCAG---------CCTGAAAGCCTC------TCCATTCTA
TCCACC------ATCAGTGACAAAATC------------------------CAGCAGCCC
GGCACAACG---TGGGACAAAGGAACAGGGTTAAAGACTGCACATTTC------------

>Blackfin_icefish_Aqp10aa
ATGTTGAAC------CGCCGAGTTCTGAGAGTGAGGAATCCTCTGATACGAGAGTGCATG
GCTGAGTTCTTGGGAACATTTGTTTTGCTGCTCTTCGGCTGCTCTGCTGCGGCGCAGGTG
AAGACGAGCAGAGAAACTAAAGGTCAGTTCCTGTCAGTCAACATGGCCTTCTCTGTGGGC
GTGATGTCGGCTATGTACCTCACCAAGGGCATCACAGGAGCTCACCTGAACCCGGCAGTG
TCTCTGAGTTTCTGTGTGTTGGGCCAGGTGCCCTGGGGAAGGCTGGTGCCCTACTTCCTC
TCCCAGCTGTTGGGGGCTTATGTGGCATCAGGACTCGTCTACCTGGTCTACTATGATGCT
ATAATGGAGTTTAGTGGAGGAGTATTGACTGTCTATGGCCCAAATGAAACAGCATCTATA
TTTGCCACATACCCCTCAGAGTACATCTCTTTGAGCAGCAGTTTCCTTGACCAGGTAGTT
GGCACTGGCATGCTGATGTTGTGCATCCTGAGTTTGGGTGAGAAGAGAAACACCCCGGCC
CCCCCGGAGCTGATTCCTGCGATAGTGGCAGCGATCGTCCTGGGGATCTCCATGTCAATG
TCAGCCAACTGTGGTGGTGCAATAAATCCAGCTCGGGATCTGGGGCCACGCCTCTTTACA
CTGACTGCAGGCTGGGGCACTGAGGTCTTCACGTGTTACAACTACTGGTTCTGGGTGCCA
CTGGTGGCCCCACTTGTAGGAGGTGTTTAGGGACATTCATGTACTTGATCTTCATCCAC
TGGCACCTGCCTGACCCCGACCCT---------CCGGAGAGCCTC------ACCCCTCTC
TCTACC------ATCAGTGATAAAGTC------------------------CTGCAGCCC
AGCACAATG---TGGGACAACAGAGAGATGTTAAAGGCTGCACGTCTC------------

>Black_rockcod_Aqp10aa
ATGTTGAAC------CGCCGAGTTCTGAGAGTGAGGAATCCTCTGATACGAGAGTGCATG
GCTGAGTTCTTAGGAACATTTGTTTTGCTGCTCTTCGGCTGCTCTGCTGCGGCGCAGGTG
AAGACGAGCAGAGAAACTAAAGGCCAGTTCCTGTCAGTCAACATGGCCTTCTCTGTGGGC
GTGATGTCGGCTATGTACCTCACCAAGGGCATCACAGGAGCTCACCTGAACCCGGCAGTG
TCTCTGAGCTTCTGTGTGTTGGGCCAGGTGCCCTGGGGAAGGCTGGTGCCCTACTTCCTC
TCCCAGCTGTTGGGGGCTTATGTGGCATCAGGACTCGTCTACCTGGTCTACTATGATGCT

ATAATGGAGTTTAGTGGAGGAGTATTGACTGTCTATGGCCCAAATGAAACAGCATCAATA
TTTGCCACATACCCCTCAGAGTACATCTCTTTGGGCAGCAGTTTCCTTGACCAGGTAGTT
GGCACTGGCATGCTGATGTTGTGCATCCTGAGTTTGGGTGAGAAGAGAAACACCCCGGCC
CCCCCGGAACTGATTCCTGCGATAGTGGCAGCGATCGTCCTGGGGATCTCCATGTCAATG
TCAGCCAACTGTGGTGGTGCAATAAATCCAGCTCGGGATCTGGGGCCACGCCTCTTTACA
CTGACTGCAGGCTGGGGCACTGAGGTCTTCACGTGTTACAACTACTGGTTCTGGGTGCCC
CTGGTGGCCCCACTTATAGGAGGTGTTTTAGGGACATTCATGTACTTGATCTTCATCCAC
TGGCACCTGCCTGACCCCGACCCT---------CCGGAGAGCCTC------ACCCCTCTC
TCTACC------ATCAGTGATAAAGTC------------------------CTGCAGCCC
AGCACAATG---TGGAACAACGGAGAGGTGTTAAAGGCTGCACGTCTC------------

>Channel_bull_blenny_Aqp10aa
ATGTTAAAC------CGACGGGTTGTGAGAGTGAGGAATGTCCTGGTGCGAGAGTGCATG
GCTGAGTTCTTGGGAACTTTTATCTTGCTGCTCTTCGGCTGCTCTGCCGCAGCTCAGGTA
AAGACAAGCAGAGAAACTAAAGGTCAGTTCCTGTCAGTCAACATGGCCTTCTCTGTGGGC
GTGATGTCAGCAATGTACCTCACCAAGGGCATCACAGGTGCTCATCTGAACCCGGCGGTG
ACTCTGAGTTTCTGTGTGTTGGGTCAGGTGCCCTGGGGAAGGCTGGTGCCATACTGCCTC
TCCCAGCTGCTGGGGGCTTATCTGGCATCAGCGCTCGTCTATCTGGTCTACTATGACGCT
ATAATGGCGTTTAGTGGAGGAGTTTTGACTGTCTATGGCCCAAATGAGACAGCATCTATA
TTTGCCACATATCCCTCAGAGTACATGACTTTGGGCAGAAGTTTCCTTGACCAGGTGGTG
GGCACTGGCATGCTGATGTTGTGCATCCTGTGTTTGGGTGAAAAGAGAAACACTCCAGCT
CCCCCGGAACTGATTCCTGCTATAGTGGCAGCGATCGTCCTGGGGATCTCCATGTCAATG
TCAGCCAACTGTGGGGGTGCGATAAATCCTGCTCGGGATCTGGGGCCACGCCTCTTTACA
CTGACTGCAGGCTGGGGCACTGAAGTGTTCACGTGTTACAACTACTGGTTCTGGGTGCCC
CTGGTGGCCCCACTTATCGGAGGAGTTATAGGGACATTCACATATTTGATCTTCATCCAC
TGGCACCTGCCTGATCCAGACCCT---------CCTGAGAGCCTC------TCCTCTCTC
TCTACC------ATCAGTGACAAAATC------------------------AAGCAGCCC
AGCACAATG---TGGGACAATGAAGTAGAGCTAAAGGCTGCACGTCTC------------

>European_perch_Aqp10aa
ATGTTGAAC------CGACGTGTCGGGAGAGTGAGGAATGCCCTGGTGCGAGAGTGCATG
GCTGAGTTCTTGGGAACTTTTGTGTTGCTGCTCTTTGGCTGCTCTGCTGCAGCGCAAGTA
AAGACAAGCAGAGAGACTAAAGGCCAGTTCCTGTCAGTCAATTTGGCCTTCTCCGTGGGT
GTGATGTCAGCTATGTACCTCACCAAGAACATCTCAGGTGCTCATTTCAATCCAGCGGTG
ACTCTGAGTTTCTGTGTGTTGGGCCAGGTCCCCTGGGGAAAGCTGGTGCCCTACTGCCTC
TCCCAGGTGCTGGGGGCTTATCTGGCATCAGGGCTCGTCTACTTGGTCTACTATGATGCC
ATAATGGAGTTTAGTGGAGGAGTATTGACTGTCTATGGCCCAAATGAGACCGCGTCTATA
TTTGCCACATATCCCTCAGAGTACATGACTTTGGGTAGAAGTTTCCTTGACCAGGTAGTG
GGCACTGGCATGCTGATGTTGTGCATACTGTCTTTGGAAGAAAAGAGGAACACCCCGGCT
CCCTCAGAGCTGATTCCTGCTATAGTAGCAGTGATCGTCCTGGGGATCTCTATGTCAATG
TCAGCCAACTGTGGTGCTGCAATAAACCCTGCTCGGGACCTGGGGCCACGCCTCTTAACA
CTGACTGCAGGCTGGGGCACTGAGGTCTTTACGTGTTACAACTACTGGTTCTGGGTGCCA
CTGGTGGCCCCACTTATTGGAGGTGTTATAGGTACTTTCATATATTTGTTCTTCATCCAC
TGGCACCTGCCTGACCCAGACCCT---------CCTGAGAGACTC------TCCTCTCTC
TCCACT------ATCAGTGACAAAATC------------------------AAGCAGCCC
TGCACAATG---TGGAACAATGGAGTAGAGTTAAAGGCTGCA------------------

>3_spine_stickleback_Aqp10aa
---------------CACCGTGTTGTGAGGGTGAGGAATGCCCTGTTGAGGGAGTGCATG
GCTGAGATATTGGGAACTTTTGTCTTGCTGCTCTTTGGCTGCGCTGCTGGGGCTCAGGTG
AAGACGAGCAGGGAATCTAAAGGCCAGTTCCTGTCGGCCAACATGGCCTTCTCTGTGGGT
GTGATGTCAGCTACGTACCTCACCAAGGGCATCTCAGGTGCTCACCTGAACCCGGCGGTG
ACTCTGAGTTTCTGCGTGCTGGGCCAGGTGCCGTGGGGAAAGCTGGTGCCCTACTGCCTC
AGCCAGGTGCTGGGGGCCTATCTGGCATCAGCCCTCGTCTTCCTGGTCTACTATGATGCC
ATAATGGAGTTTAGTGGAGGAGTATTGACTGTTTATGGCGCAAACGAGACAGCGTCTATA
TTTGCCACGTATCCGTCAGAGTACCTGAGTTTAGGACGAAGTTTTCTGGACCAGGTAGTG
GGCACTGGCATGCTGATGCTGTGCATCCTGGGTTTGGAGGAAAAGAGGAACACCCCGGCT
CCCTCAAAGCTGATTGCTCCCATAGTGGCAGCGATCGTCTTGGGCATCTCCATCTCCATG

TCAGCCAACTGTGGTGCTGCAATAAATCCTGCTCGGGACCTGGGACCTCGCCTCTTCACA
CTGACAGCAGGCTGGGGCACTGAGGTATTCACGTGTTACAACTACTGGTTCTGGGTACCC
TTGGTGGCCCCACTTGTGGGAGGCGTAATGGGGACTTTCATGTATTTGGTGTTCATCCAC
TGGCATCTGCCTGATCCGGATCCC---------GCGGAGAGCTTC------TCCTCC---
------------AGCGGGGACGTGATC------------------------AAGCAGCCC
AGCATCCAG---CAGGAAAACGGAGCGGAGTTAAAGGCTACACATCTC------------

>9_spine_stickleback_Aqp10aa
ATGCTGAAT------AGACGCGTTGTGAGGGTGAGGAATGCCCTGGTGCGAGAGTGCATG
GCCGAGTTCTTGGGGACTTTTGTCTTGATGCTCTTTGGCTGCTCTGCTGCAGCTCAGGTG
AAGACGAGCAGGGAATCTAAAGGCCAGTTCCTGTCGGTCAACATGGCCTTCTCTGTGGGT
GTGATGTCAGCTATGTACCTCACCAAGGGCATCACAGGCGCTCACCTGAACCCGGCGGTG
ACTCTGAGTTTCTGCGTGCTGGGCCAGGTGCCGTGGGGGAGGCTGGTGCCCTACTGCCTC
AGCCAGGTGCTGGGGGCCTATCTGGCATCAGCCCTCGTCTTCCTGGTCTACTACGATGCT
ATAATGGAGTTTAGTGGAGGAGTATTGACTATTTATGGCCCAAACGAGACAGCGTCTATA
TTTGCCACGTATCCGTCAGAGTACATGACTTTAGGAAGAAGTTTCCTGGACCAGGTAGTG
GGCACTGGCATGCTGCTGTTGTGCATCCTGGGATTGGATGAAAAGAGGAACACCCCGGCT
CCTTCACAGCTGATTCCTCCCATAGTGGCAGTGATCGTCCTGGGGATCTCCATGTCAATG
TCAGCCAACTGTGGTGGTGCAATAAACCCTGCTCGGGACCTGGGACCTCGCCTCTTCTCT
CTGACTGCGGGCTGGGGCACTGAGGTGTTCACGTGTTACAACTACTGGTTCTGGGTACCC
TTGGTGGCCCCACTTGTGGGAGGTCTTATGGGGACTTTCATGTATTTGGTCTTCATCCAC
TGGCATCTGCCTGACCCAGATCCC---------TCAGAGAGCTTC------TCCTCC---
------------ATCGGGGACATCATC------------------------AAGCAGCCC
AGCATCCAG---CCGGACAACGGAGTGGAGTTAAAGGCTACTCACCTG------------

>Rhine_sculpin_Aqp10aa
ATGTTGAAC------AGACGTGTGGCGAGAGTCAGGAACGCCCTGGTGCGAGAGTGCCTG
GCTGAGCTCTTGGGAACGTTCGTCTTGCTGCTCTTTGGCTGCTCTGCTGCAGCGCAGGTG
AAGACAAGCAGAGAAACTAAAGGCCAGTTCCTGTCAGTCAACATGGCCTTTTCCGTGGGT
GTGATGTCAGCTATGTACCTCACCAAGGGCATCACAGGAGCTCATCTGAACCCGGCGGTG
ACGCTGAGTTTCTGTGTGTTGGGCCAGGTGCCCTGGGGGAGGCTGGTGCCCTACGCCCTC
AGCCAGGTGCTGGGGGCTTATCTGGCCTCGGGCCTCGTCTACCTGGTCTACTATGATGCT
ATAATGGAGTTTAGTGGAGGAGTATTGACTGTTTATGGCCCAAATGAAACAGCGTCTATA
TTTGCCACATATCCTTCAGAGTACATGAGCTTGGGCAGAAGTTTCCTTGACCAGGTGGTG
GGCACTGGCATGCTGATGTTGTGCATCCTGTGTTTGGATGAAAAGAGGAACACTCCGGCT
CCCTCAAAGCTGATTCCTCCCATCGTGGCAGTGATCGTCCTGGGGATCTCCATGTCAATG
TCAGCCAACTGTGGCGGTGCAATAAATCCTGCACGGGACCTGGGACCACGCCTCTTTACA
CTGACGGCTGGCTGGGGCACTGAGGTCTTCACGTGTTACAACTACTGGTTCTGGGTGCCC
TTGGTGGCCCCACTTATTGGAGGTGTTACAGGTTCTTTCATGTACCTGATCTTCATTCAC
TGGCACCTGCCTGACCCAGATCCC---------CCTGAGGGCCTC------TCCATTCTC
TCCACC------ATCAGTGACAAAATC------------------------AAGCAGCCC
AGCGTCACG---TGGGAAGACGGAGTGGAGTTAAAGGCTTCACATCTC------------

>Sablefish_Aqp10aa
ATGTTGAAC------CGACGTGTTGTGAGAGTGAGGAATGCCCTGGTGCGAGAGTGCCTG
GCTGAACTCTTGGGAACTTTCGTCTTGCTGCTCTTTGGCTGCTCTGCTGCAGCGCAGGTA
AAGACAAGCAGAGAGACCAAAGGTCAGTTCCTGTCAGTCAACATGGCCTTCTCTGTGGGT
GTCATGTCAGCTATGTACCTCACCAAGAACATCACAGGTGCTCATCTGAACCCAGCGGTG
ACTCTGAGTTTCTGTGTGTTGGGCCAGGTGCCCTGGGGAAGGCTGGTGCCCTACTCCCTC
TCCCAGCTGTTGGGGGCTTATCTAGCATCAGGCCTCGTCTACCTTATCTACTATGATGCT
ATAATGGAGTTTAGTGGAGGAGTATTGACTGTTTATGGTCCAAATGAGACAGCGTCTATA
TTCGCCACATATCCTTCAGAGTACATGACTTTGGGCAGAAGTTTCCTCGACCAGGTAGTG
GGCACTGGCATGCTGATGTTGTGCATCCTGGGTTTGAATGAAAAGAGGAACACCCCGGCT
CCCTCAAAGCTAATTCCTCCTATAGTGGCAGCCATCGTCCTGGGAATCTCGACGTCAATG
TCAGCCAACTGTGGTGGTGCAATAAATCCTGCTCGGGACTTGGGACCACGCCTCTTTACT
CTGACTGCAGGCTGGGGCACTGAGGTCTTTACGTGTTACAACTACTGGTTCTGGGTGCCC
TTGGTGGCCCCACTTATCGGCGGTGTTGTAGGTACTTTCATCTATCTGATCTTCATCCAC
TGGCACCTGCCTGACCCAGATCCC---------CCTGAGAGACTC------TCCACTGTC

```
CCCACT------ATCAGTGAAAAAATC------------------------CGGCAGCCC
AGCACCACG---TGGGACAACGGAGTGGAGTTAAAGTCTGCACATCTC------------
------------------------------------------------------------
------------------------------------------------
>Minor_flag_rockfish_Aqp10aa
ATGTTGAAC------CGACGTCTTGTGAGAGTGAGGAATGCCCTGGTGCGAGAGTGCATG
GCTGAGTTCTTGGGAACTTTCGTCTTGCTGCTCTTCGGCTGCTCTGCTGCAGCGCAGGTG
AAGACGAGCAGAGAGACTAAAGGCCAGTTCCTGTCGGTCAACATGGCCTTCTCTGTGGGC
GTCATGACGGCTATGTACCTCACCAAGGGCATCACAGGTGCTCATCTGAACCCGGCGGTG
ACTCTGAGTTTCTGTGTGCTGGGTCAGGTGCCCTGGGGAAGACTGGTGCCCTACTGCCTC
TCCCAGGTGCTGGGGGCTTATCTGGCATCAGGGCTCGTCTACCTGGTCTACTATGATGCT
ATAATGGAGTTTAGTGGAGGAGTCTTGACTGTATACGGCCCAAATGAGACAGCGTCTATA
TTTGCCACATATCCTTCAGAATACCTTACTTTG---------------------GTCGTG
GGCACTGGCATGCTGATGTTGTGCATCCTGTGTCTGGGTGAAAAGAGGAATACCCCGGCC
CCCTCTGAGCTGATTCCCCCTATAGTGGCAGTGATCGTCCTTGGGATCTCCATGTCAATG
TCAGCCAACTGTGGCGCTGCAATAAATCCTGCTCGGGACCTGGGGCCGCGACTCTTCACG
CTGACTGCAGGCTGGGGCACTGAAGTCTTCACGTGTTACAACTACTGGTTCTGGGTGCCC
CTGGTTGCCCCACTTATCGGGGGTGTTATAGGTTCTTTCATGTATTTGATCTTCATTCAC
TGGCACCTGCCTGACCCCGACCCC---------CCCGAGAGCCTC------TCCCCTCTC
TCCACC------AGCAGTGAAAAAATG------------------------AATGAGCCC
AGCACCACG---TGGGACAACGGAGTACAGTTAAAGTCTGCACATCTC------------
------------------------------------------------------------
------------------------------------------------
>Flag_rockfish_Aqp10aa
ATGTTGAAC------CGACGTCTTGTGAGAGTGAGGAATGCCCTGGTGCGAGAGTGCATG
GCTGAGTTCTTGGGAACTTTCATCTTGCTGCTCTTTGGCTGCTCTGCTGCAGCGCAGGTG
AAGACGAGCAGAGAGACTAAAGGCCAGTTCCTGTCGGTCAATATGGCCTTCTCTGTGGGC
GTCATGACGGCTATGTACCTCACCAAGGGCATCACAGGTGCTCATCTGAACCCGGCGGTG
ACTCTGAGTTTCTGTGTGCTGGGTCAGGTGCCCTGGGGAAGACTGGTGCCCTACTGCCTC
TCCCAGGTGCTGGGGGCTTATCTGGCATCAGGGCTCGTCTACCTGGTCTACTATGATGCT
ATAATGGAGTTTAGTGGAGGAGTCTTGACTGTATACGGCCCAAATGAGACAGCGTCTATA
TTTGCCACATATCCTTCAGAGTACCTTACTTTGGGCAGGAGTTTCCTTGACCAGGTCGTG
GGCACTGGCATGCTGATGTTGTGCATCCTGTGTCTGGGTGAAAAGAGGAATACCCCGGCT
CCCTCTGAGCTGATTCCCCCTATAGTGGCAGTGATCGTCCTTGGGATCTCCATGTCAATG
TCAGCCAACTGTGGCGCTGCAATAAATCCTGCTCGGGACCTGGGGCCACGACTCTTCACG
CTGACTGCAGGCTGGGGCACTGAAGTCTTCACGTGTTACAACTACTGGTTCTGGGTGCCC
GTGGTTGCCCCACTTATCGGGGGTGTTATAGGTTCTTTCATGTATTTGATCTTCATTCAC
TGGCACCTGCCTGACCCCGACCCC---------CCCGAGAGCCTC------TCCCCTCTC
TCCACC------AGCAATGAAAAAATG------------------------AATGAGCCC
AGCACCACG---TGGGACAACGGAGTAGAGTTTAAGTCTGCACATCTC------------
------------------------------------------------------------
------------------------------------------------
>Rougheye_rockfish_Aqp10aa
------------------------------------------------------------
-------------------------------CTCTTCGGCTGCTCTGCTGCAGCGCAGGTG
AAGACGAGCAGAGAGACTAAAGGCCAGTTCCTGTCGGTCAACATGGCCTTCTCTGTGGGC
GTCATGACGGCTATGTACCTCACCAAGGGCATCACAGGTGCTCATCTGAACCCGGCGGTG
ACTCTGAGTTTCTGTGTGCTGGGTCAGGTGCCCTGGGGAAGACTGGTGCCCTACTGCCTC
TCCCAGGTGCTGGGGGCTTATCTGGCATCAGGGCTCGTCTACCTGGTCTACTATGATGCT
ATAATGGAGTTTAGTGGAGGAGTCTTGACTGTATACGGCCCAAATGAGACAGCGTCTATA
TTTGCCACATATCCTTCAGAGTACCTTACTTTGGGCAGGAGTTTCCTTGACCAGGTCGTG
GGCACTGGCATGCTGATGTTGTGCATCCTGTGTCTGGGTGAAAAGAGGAATACCCCGGCT
CCCTCTGAGCTGATTCCCCCTATAGTGGCAGTGATCGTCCTTGGGATCTCAATGTCAATG
TCAGCCAACTGTGGCGCTGCAATAAATCCTGCTCGGGACCTGGGGCCGCGACTCTTCACG
CTGACTGCAGGCTGGGGCACTGAAGTCTTCACGTGTTACAACTACTGGTTCTGGGTGCCA
CTGGTTGCCCCACTTATCGGGGGTGTTATAGGTTCTTTCATGTATTTGATCTTCATTCAC
TGGCACCTGCCTGACCCCGACCCC---------CCCGAGAGCCTC------TCCCCTCTC
TCCACC------AGCAATGAAAAAATG------------------------AATGAGCCC
AGCACCACG---TGGGACAACGGAGTAGAGTTTAAGTCTGCACATCTC------------
------------------------------------------------------------
------------------------------------------------
```

>Tiger_rockfish_Aqp10aa
ATGTTGAAC------CGACGTCTTGTGAGAGTGAGGAATGCCCTGGTGCGAGAGTGCATG
GCTGAGTTCTTGGGAACTTTCGTCTTGCTGCTCTTTGGCTGCTCTGCTGCAGCGCAGGTG
AAGACGAGCAGAGAGACTAAAGGCCAGTTCCTGTCGGTCAATATGGCCTTCTCTGTGGGC
GTCATGACGGCTATGTACCTCACCAAGGGCATCACAGGTGCTCATCTGAACCCGGCGGTG
ACTCTGAGTTTCTGTGTGCTGGGTCAGGTGCCCTGGGAAGACTGGTGCCCTACTGCCTC
TCCCAGGTGCTGGGGGCTTATCTGGCATCAGGGCTCGTCTACCTGGTCTACTATGATGCT
ATAATGGAGTTTAGTGGAGGAGTCTTGACTGTATACGGCCCAAATGAGACAGCGTCTATA
TTTGCCACATATCCTTCAGAGTACCTTACTTTGGGCAGGAGTTTCCTTGACCAGGTCGTG
GGCACTGGCATGCTGATGTTGTGCATCCTGTGTCTGGGTGAAAAGAGGAATACCCCGGCT
CCCTCTGAGCTGATTCCCCCTATAGTGGCAGTGATCGTCCTTGGGATCTCCATGTCAATG
TCAGCCAACTGTGGCGCTGCAATAAATCCTGCTCGGGACCTGGGGCCACGACTCTTCACG
CTGACTGCAGGCTGGGGCACTGAAGTCTTCACG---------------------------

>Florida_bass_Aqp10aa
ATGTTGAAG------CAACGTGTTATGAAGGTGAGGAATGCCCTGGTGCGAGAGTGCTTG
GCTGAGTTCTTGGGAACATTTGTATTGCTGCTTTTTGGCTGCGCTGCTGCAGCCCAGGTG
AAGACAAGCAGAGAGACCAAAGGCCAGTTCCTGTCAGTCAACATGGCCTTCTCTGTGGGT
GTGATGTCAGCTATGTACCTCACCAAGGGTATCTCAGGTGCTCATCTGAACCCAGCGGTG
ACTCTGAGCTTCTGTGTGTTAGGCCAAGTGCCCTGGGGAAGTCTGTTGCCCTACTGCCTC
TCCCAGATGCTTGGGGCGTACATGGCGTCAGGGCTTGTCTACCTGGTCTACTATGATGCT
ATAATGGAGTTTAGTGGAGGACTACTGACTGTGTACGGCCCAAACGAGACAGCGTCTATT
TTTGCCACATACCCCTCAGAGTACATGACTTTGAGCAGAAGTTTCCTTGACCAAGTAGTG
GGCACCGGCATGCTGATGTTGTGCATCCTGTGTTTGGGTGAAAAGAGGAATACTCCGGCT
CCCTCTGATCTGCTTCCTCCTATAGTGGCAGTGATTGTCTTGGGGATTTCCATGTCAATG
TCAGCTAACTGTGGTGGTGCAATAAATCCTGCTCGAGACCTGGGGCCACGCCTCTTTACA
CTGACGGCAGGCTGGGGCACTGAGGTTTTCACGTGTTACAACTACTGGTTCTGGGTACCC
GTGGTGGCCCCACTTATTGGGGGTGTTATAGGTACTTTCATGTATTTGGTCTTCATCCAC
TGGCACCTGCCTGACCCAGACCTC---------CCTGAGAGCCTC------TCCACTCTC
TCCACC------ATCAGTGACAAAATA------------------------AAGCAGCCC
AGCACCACG---TGGGACAAGGGAGTAGAGTTAAAGGCTGCAAATTTT------------

>Northern_largemouth_bass_Aqp10aa
ATGTTGAAG------CAACGTGTTATGAAGGTGAGGAATGCCCTGGTGCGAGAGTGCTTG
GCTGAGTTCTTGGGAACATTTGTATTGCTGCTTTTTGGCTGCGCTGCTGCAGCCCAGGTG
AAGACAAGCAGAGAGACCAAAGGCCAGTTCCTGTCAGTCAACATGGCCTTCTCTGTGGGT
GTGATGTCAGCTATGTACCTCACCAAGGGTATCTCAGGTGCTCATCTGAACCCAGCGGTG
ACTCTGAGCTTCTGTGTGTTAGGCCAAGTGCCCTGGGGAAGTCTGTTGCCCTACTGCCTC
TCCCAGATGCTTGGGGCGTACATGGCGTCAGGGCTTGTCTACCTGGTCTACTATGATGCT
ATAATGGAGTTTAGTGGAGGACTACTGACTGTGTACGGCCCAAACGAGACAGCGTCTATT
TTTGCCACATACCCCTCAGAGTACATGACTTTGAGCAGAAGTTTCCTTGACCAAGTAGTG
GGCACCGGCATGCTGATGTTGTGCATCCTGTGTTTGGGTGAAAAGAGGAATACTCCGGCT
CCCTCTGATCTGCTTCCTCCTATAGTGGCAGTGATTGTCTTGGGGATTTCCATGTCAATG
TCAGCTAACTGTGGTGGTGCAATAAATCCTGCTCGGGACCTGGGGCCACGCCTCTTTACA
CTGACGGCAGGCTGGGGCACTGAGGTTTTCACGTGTTACAACTACTGGTTCTGGGTACCC
GTGGTGGCCCCACTTATTGGGGGTGTTATAGGTACTTTCATGTATTTGGTCTTCATCGAC
TGGCACCTGCCTGACCCAGACCTC---------CCTGAGAGCCTC------TCCACTCTC
TCCACC------ATCAGTGACAAAATA------------------------AAGCAGCCC
AGCACCACG---TGGGACAACGGAGTAGAGTTAAAGGCTGCAAATTTT------------

>Barred_knifejaw_Aqp10aa
ATGTTGAAG------CTACGTGTTCTGAGAGTGAGGAATGCTCTGGTGCGAGAGTGCATG
GCTGAGTTCTTGGGAACTTTTGTCTTGCTGCTCTTTGGCTGCTCTGCTGCAGCGCAGGTG
AAGACAAGCAGGGAGACAAAAGGCCAGTTCCTGTCAGTCAACATGGCTTTCTCTGTGGGC

GTGATGTCAGCTATGTACCTCACTAAGGGCATCACAGGTGCTCATCTGAACCCAGCGGTG
ACTCTGAGTTTCTGTGTGTTGGGCCAGGTGCCCTGGGGAAGGCTGGTGCCCTACTGCCTC
TCCCAGCTGCTGGGGGCTTATGTGGCGTCAGGGCTTGTCTACCTGGTCTACTATGATGCT
ATAATGGAGTTTAGCGGCGGAGTGTTGACTGTATATGGCCCAAATGAGACAGCGTCCATA
TTTGCCACATATCCCTCAGAGTACATGACTTTGGGCCGAAGTTTCCTTGACCAGGTGGTG
GGAACCGGGATGCTGATGTTGTGCATCCTATGTTTGGATGAGAAGAGGAATACCCCCGCT
CCCTCAGAGCTGATTCCTCCTATAGTGGCAGCAATCGTACTGGGGATCTCCATGTCAATG
TCCGCTAACTGTGGTGCTGCAATAAATCCTGCTCGGGACCTGGGGCCACGCCTCTTTACA
CTGACTGCAGGCTGGGGCATAGAGGTGTTCACGTGTTACAACTACTGGTTCTGGGTACCC
CTGGTGGCCCCACCTATTGGAGGTGTCTTAGGTACTTTCATGTATTTGGTCTTCATCCAC
TGGCACCTGCCTGACCCAGACCCC---------CCTGAGAGTCTC------TCCAGTCTC
TCCACC------ATCAGTGACAAAATC------------------------AAGCAGCCC
AGCACAAAG---TGGGACAATGGAGTCGAGTTAAAGGCTGCACATTTC------------

>Murray_cod_Aqp10aa
ATGTTGGGA------CGACGTGATCTGAGAGTGAGGAATGCCCTGGTGCGAGAGTGCATG
GCTGAGTTCTTGGGAACTTTCGTCTTGCTGCTCTTTGGCTGCTCTGCTGCAGCGCAGGTG
AAGACAAGCAGAGAGACTAAAGGCCAGTTCCTATCAGTCAACATGGCCTTCTCTGTGGGC
GTGATGTCAGCTATGTACCTCACCAAGGGCATCTCAGGTGCTCATCTGAACCCAGCAGTG
ACTCTGAGTTTGTGTGTGTTGGACCAGGTGCCCTGGGGAAGGCTGGTGCCCTACTGTCTC
TCCCAGCTGCTGGGGGCGTACATGGCATCAGGGCTTGTCTACCTGGTCTATTATGATGCT
ATAATGGAGTTTAGTGGAGGAGTATTGACTGTTTTTGGCCCAAATGAGACAGCATCTATA
TTTGCCACATATCCCTCAGAGTACGTGAGTTTGGGCAGAAACTTCCTTGACCAGGTAGTG
GGCACTGGCATGCTGATGTTGTGCATCCTGTGTTTGGATGAAAAGAGGAACACCCCCGCT
CCCTCTGAGCTGATGCCTGCTATAGTGGCAGTGATCGTCCTGGGGATCTCCATGTCAATG
TCAGCTAACTGTGGAGCTGCAATAAATCCTGCTCGGGACCTGGGGCCGCGCCTCTTTACA
CTGACTGCAGGCTGGGGCACGGAGGTCTTCACGTGTTACAACTACTTTTTCTGGGTACCA
GTGGTGGCCCCACTTATCGGAGGACTTATAGGTACTTTCATGTATTTGATCTTCATCCAT
TGGCACCTTCCTGACCCAGACCCC---------CTTGAGAGCCTC------TCCACTCTC
TCCACC------ATCAGTGACAAAATC------------------------AAGCAGCCC
AGCACCACA---------AACAAAGTAGAGTTAAAGGCAGTACATCTG------------

>Ballan_wrasse_Aqp10aa
---ATGGTGAGAGAGTGCATG
GCTGAGTTCTTGGGAACTTTTGTCTTACTGCTGTTTGGCTGTTCTGCTGCAGCTCAAGTG
AAGACAAGCAGAGACACTAAAGGTCAGTTCCTGTCAGTCAACATGGCCTTCTCTGTGGGT
GTGATGTCGGCTATGTACCTCACCAAGGGCATCTCAGGTGCTCATCTGAACCCAGCAGTG
ACTATGAGTTTCTGTGTGTTGGGCCGGGTGCCCTGGGGCCGGCTGGTGCCCTACAGCCTC
TCCCAGCTGCTGGGGGCTTATATGGCATCAGGCCTCGTCTACCTGGTGTACTATGATGCC
ATAATGGAGTTTAGTGGAGGAGTATTGACTGTTTATGGTCAAAATGAGACAGCTTCCATA
TTCGCCACATATCCATCTGAGTACATCACTTTGGGCAGAAGTTTCCTTGACCAGGTAGTG
GGTACAAGCATGCTCATGTTGTGCATCCTGGCTTTGGACGAAAAGAGGAATACACCTGCT
CCCTCAGAGCTGATTCCCCCAATAGTAGCTGTGATCGTCCTGGGGATCTCCATGTCAATG
TCTGCTAACTGTGGTGCTGCAATCAATCCAGCTCGAGACCTAGGGCCGCGACTCTTTACG
CTGACTGCAGGCTGGGGAACTGAGGTCTTCACGTGTTACAACTACTGGTTCTGGGTACCG
CTGGTGGCCCCACCTATTGGAGGTGTCTTAGGATCTTTGATGTATTTGGTCTTTATCGAC
TGGCACCTCCCTGAACCAGACCCT---------CCTGAGAGTCTC------TCCACTCTT
TCCACC------ATCAGTGATAAAATT------------------------CAGCAGCCA
AGAATCACA---TGGGACAATGGAGAAGAGTTAAAGGCTGCATATTTC------------

>Corkwing_Aqp10aa
ATGTTGAAG------AAAGGTGCCCTGAGATTGAGGAATGCCATGGTGAGAGAGTGCATG
GCGGAGTTCTTGGGAACTTTTGTCTTACTGCTGTTTGGCTGCTCTGCTGCAGCTCAAGTG
AAAACAAGCAGAGACACTAAAGGTCAGTTCCTGTCAGTCAACATGGCCTTCTCCGTGGGT
GTGATGTCAGCTATGTACCTCACCAAGGGCATCTCAGGTGCTCATCTAAACCCAGCAGTG
ACGATGAGTTTCTGTGTGTTGGGCCGGGTGCCCTGGGGACGGCTGGTGCCCTATAGCCTC
TCCCAGCTGCTTGGGGCTTATTTGGCATCAGGCCTCGTCTACCTTGTGTACTATGATGCC
ATAATGGAGTTTAGTGGAGGAGTTTTGACTGTCTATGGTCAAAATGAGACAGCTTCCATA

TTCGCCACATATCCCTCTGAGTACCTAACTTTGGGCAGAAGTTTTCTTGACCAGGTAGTG
GGAACAGGCATGCTCATGTTGTGCATCCTGGCTTTGGACGAAAAGAGGAATACCCCTGCC
CCCTCAGAGCTGATTCCCCCAATAGTAGCTGTGGTCGTCCTGGGGATTTCCATGTCAATG
TCAGCTAACTGTGGTGCTGCAATAAATCCAGCTCGAGACCTAGGGCCACGCCTCTTTACA
CTGACTGCAGGCTGGGGAACTGAGGTCTTCACGTGTTACAACTACTGGTTCTGGGTACCG
CTGGTTGCCCCACCTATTGGAGGAGTCTTAGGATCTTTGATGTATTTGGTCTTTATCGAT
TGGCACCTGCCTGATCCAGACCCT---------TGTGACAGTCTCCCC---CCCACTCTC
TCCACC------ATCAGTGATAAAATT-----------------------CAGCAGCCA
AGCACCACA---TGGAACAATGTAGCAGGGTTAAAGGCGGCATATTTA------------

>Amazon_molly_Aqp10aa1
ATGCTGAAT------CCACGTTTGTTGAGAGTGAGGAGCGCTCTGATCCGACAATGCATG
GCTGAGTTCTTGGGAACGTTCGTCTTAATTCTCTTTGGCTTATCAGCAGCAGCGCAGGTA
AAAACCAGCAGAGAGACTAAAGGCCAAACTCTGTCGATCCACCTGTCTTGGGCCGTGGGA
GTCATGTCAGCCACGTATCTCACCAAGGGGATCTCAGGTGCTCACCTGAACCCCGCTGTG
TCGCTGTGTTTCTGCATTTTGGGGAAGCTGCGGTGGGGACAGCTGTTGCCCTACTGTGTG
TCACAGATCTTTGGGGCTTATGTGGGAGCAGCGCTGGTCTACGCGCTCTACTACGATGCT
ATAATGGAGTTTAGTGGAGGCGTGTTGACCGTCTATGGCCCAAGCGAAACATCATCTATA
TTTGCCACGTATCCAACTGAGTACCTCCCGTTAGGAGGGGGTTTTCTGGACCAAGTAGTG
GGCACTGGAACGCTGCTGTTGTGCATCCTGAGTTTGACCGAAAAGAGGAACGCCCCCGCT
CCCCCTGATCTGATTGCTCCCATAGTTGCAGGCGTCGTGCTCGGCATCATCATGTCCATG
TCGGCTAACTGTGGCGCTGCTTTAAATCCCGCTCGAGACCTGGGGCCACGCCTCCTCACG
CTGACTGCAGGCTGGGGAACCGAAGTCTTTACGTCTTTCAACTACTGGTTCTGGGTACCA
ATTGTGGCTCCGATTATTGGGGCTCTTACAGGCACTTCTTTGTATTTGATCTTCATCGAA
TGGCAGCTGCCTGAT---GAACCA---------CAGGAGAGTCAC------CCAGAAATA
TTCTCC------ATCAGTGCGAGCATC-----------------------AAGCAGTCC
GTCGCTTTG---TGGGAAAAAGGAGAGGAACCAAAGTCGACACATTTT------------

>Sailfin_molly_Aqp10aa1
ATGCTGAAT------CCACGTTTGTTGAGAGTGAGGAGCGCTCTGATCCGACAATGCATG
GCTGAGTTCTTGGGAACGTTCGTCTTAATTCTCTTTGGCTTATCAGCAGCAGCGCAGGTA
AAAACCAGCAGAGAGACTAAAGGCCAAACTCTGTCGATCCACCTGTCTTGGGCCGTGGGA
GTCATGTCAGCCACGTATCTCACCAAGGGGATCTCAGGTGCTCACCTGAACCCCGCTGTG
TCGCTGTGTTTCTGCATTTTGGGGAAGCTGCGGTGGGGACAGCTGTTGCCCTACTGTGTG
TCACAGATCTTTGGGGCTTATGTGGGAGCAGCGCTGGTCTACGCGCTCTACTACGATGCT
ATAATGGAGTTTAGTGGAGGCGTGTTGACCGTCTATGGCCCAAGCGAAACAGCATCTATA
TTTGCCACGTATCCAACTGAGTACCTCCCGTTAGGAGGGGGTTTTCTGGACCAAGTAGTG
GGCACTGGAACGCTGCTGTTGTGCATCCTGAGTTTGACCGAAAAGAGGAACGCCCCCGCT
CCCCCTGATCTGATTGCTCCCATAGTTGCAGGCGTCGTGCTCGGCATCATCATGTCCATG
TCGGCTAACTGTGGCGCTGCTTTAAATCCCGCTCGAGACCTGGGGCCACGCCTCCTCACG
CTGACTGCAGGCTGGGGAACCGAAGTCTTTACGTCTTTCAACTACTGGTTCTGGGTACCA
ATTGTGGCTCCGATTATTGGGGCTCTTACAGGCACTTCTTTGTATTTGATCTTCATCGAA
TGGCAGCTGCCTGAT---GAACCA---------CAGGAGAGTCAC------CCAGAAATA
TTCTCC------ATCAGTCCGAGCATC-----------------------AAGCAGTCC
GTCGCTTTG---TGGGAAAAAGAAGAGGAACCAAAGTCGACATGTTTT------------

>Shortfin_molly_Aqp10aa1
ATGCTGAAT------CCACGTTTGTTGAGAGTGAGGAGCGCTCTGATCCGACAATGCATG
GCTGAGTTCTTGGGAACGTTCGTCTTAATTCTCTTTGGCTTATCAGCAGCAGCGCAGGTA
AAAACCAGCAGAGAGACTAAAGGCCAAACTCTGTCGATCCACCTGTCTTGGGCCGTGGGA
GTCATGTCAGCCACGTATCTCACCAAGGGGATCTCAGGTGCTCACCTGAACCCCGCTGTG
TCGCTGTGTTTCTGTATTTTGGGGAAGCTGAGGTGGGGACAGCTGTTGCCCTACTGTGTG
TCACAGATCTTTGGGGCTTATGTGGGAGCAGCGCTGGTCTACGCGCTCTACTACGATGCT
ATAATGGAGTTTAGTGGAGGCGTGTTGACCGTCTATGGCCCAAGCGAAACAGCATCTATA
TTTGCCACGTATCCAACTGAGTACCTCCCGTTAGGAGGGGGTTTTCTGGACCAAGTAGTG
GGCACTGGAACGCTGCTGTTGTGCATCCTGAGTTTGACCGAAAAGAGGAACGCCCCCGCT
CCCCCTGATCTGATTGCTCCCATAGTTGCAGGCGTCGTGCTCGGCATCATCATGTCCATG
TCGGCTAACTGTGGCGCTGCTTTAAATCCCGCTCGAGACCTGGGGCCACGCCTCCTCACG

CTGACTGCAGGCTGGGGAACCGAAGTCTTTACGTCTTTCAACTACTGGTTCTGGGTACCA
ATTGTGGCTCCGATTATTGGGGCTCTTACAGGCACTTCTTTGTATTTGATCTTCATCGAA
TGGCAGCTGCCTGAT---GAACCG---------CAGGAGAGTCAC------CCAGAAATA
TTCTCC------ATCAGTGCGAGCATC------------------------AAGCAGTCC
GTCGCTTTG---TGGGAAAAAGGAGAGGAACCAAAGTCGACACATTTT------------

>Guppy_Aqp10aa1
ATGCTGAAT------CCACGCTTGCTGAGAGTGAGGAGCGCTCTGACCCGACAATGCATG
GCTGAGTTCTTGGGAACATTCGTCTTGATTCTCTTTGGCTTATCAGCAGCAGCGCAGGTA
AAAACCAGCAGAGAGACTAAAGGCCAAACTCTGTCGATCCACCTGTCTTGGGCCGTGGGA
GTCATGTCAGCCACGTATCTCACCAAGGGGATCTCAGGTGCCCACCTGAACCCCGCTGTG
TCGCTGTGTTTCTGTATTTTGGGGAAGCTGCGGTGGGGACAGCTGTTGCCGTATTGTGTG
TCACAGCTCTTAGGGGCGTATGTGGGAGCAGCGATGGTCTACGTGCTCTACTACGATGCT
ATAATGGAGTTTAGTGGAGGCGTGTTGACCGTCTATGGCCCAAACGAAACAGCATCTATA
TTTGCCACGTATCCAACTGAGTACCTCCCATTAGGAGGGGGTTTTCTGGACCAAGTAGTG
GGCACCGGAACGCTGCTGTTGTGCATCCTGAGTTTGACCGAAAAGAGGAACACCCCCGCA
CCCCCTGATCTGATTGCTCCCATAGTTGCGGGCGTCGTGCTCGGCATCATCATGTCCATG
TCGGCTAACTGCGGCGCTGCTTTAAATCCCGCTCGAGACCTGGGGCCACGCCTCCTCACG
CTGACTGCGGGCTGGGGAACCGAAGTCTTTACGTGTTACAACTACTGGTTCTGGGTACCA
ATTGTGGCTCCGATTATTGGGGCTCTTACAGGCACTTCTTTGTATTTGGTCTTCATCGAA
TGGCAGCTGCCTGAT---GAACCG---------CAGGAGAGTCAC------CCAGAAATC
TACTCC------ATCAGTGGGAGCATC------------------------AAGCCGTCC
GTCGCTTTG---TTGGAAAAAGTAGAGGAACCAAAG------------------------

>Southern_platyfish_Aqp10aa1
ATGCTCAAT------TCACGTTCGCTGAGAGTGAGGAGCGCTCTGATCCGACAATGCATG
GCTGAGTTCTTGGGAACATTCGTCTTGCTTCTCTTTGGCTTATCAGCAGCAGCGCAGGTA
AAAACCAGCAGAGAGACTAAAGGCCAAACTCTGTCGATCCACCTGTCTTGGGCCATGGGA
GTTATGTCAGCCACGTATCTCACCAAGGGGATCTCAGGTGCCCACCTGAACCCCGCGGTG
TCTCTGAGTTTCTGTATTTTGGGGAAGCTGCGGTGGGGACAGCTGTTGCCCTATTGTGTG
TCACAGATCTTTGGGGCGTATGTGGGAGCAGCACTGGTCTATTTGCTCTACTACGATGCT
ATAATGGAGTTTAGTGGAGGCGTGTTGACCGTCTATGGGCCAAACGAAACAGCATCTATA
TTTGCCACTTATCCATCTGAGTACCTCCCATTAGGAAAGGGCTTTCTGGACCAAGTGGTG
GGCACTGGAACGCTGCTGCTGTGCCTCCTGAGTTTGACCGAAAAGAGGAACACCCCTGCT
CCTCCTGATCTGATTGCTCCCATAGTTGCAGGCATCGTGCTCGGCATCATCATGTCCATG
TCGGCTAACTGTGGCGCTGCTTTAAATCCCGCTCGAGACCTGGGGCCACGTCTCCTCACG
CTGACTGCAGGCTGGGGAACCGAAGTCTTTACGTGTTACAACTACTGGTTCTGGGTACCG
ATTGTGGCTCCGATTATTGGGGCTCTTACAGGCACTTCTTTGTATTTGATCTTCATCGAA
TGGCAGCTGCCTGAT---GAACCG---------CAGGAGAGTCAC------CCTAAAATC
TGTTCA------GTGGGAGCATCAAGC------------------------CGTCCG---

>Monterrey_platyfish_Aqp10aa1
ATGCTCAGT------TCACGTTCGCTGAGAGTGAGGAGCGCTCTGATCCGACAATGCATG
GCTGAGTTCTTGGGAACATTCGTCTTGCTTCTCTTTGGCTTATCAGCAGCAGCGCAGGTA
AAAACCAGCAGAGAGACTAAAGGCCAAACTCTGTCGATCCACCTGTCTTGGGCCATGGGA
GTTATGTCAGCCACGTATCTCACCAAGGGGATCTCAGGTGCCCACCTGAACCCCGCGGTG
TCTCTGAGTTTCTGTATTTTGGGAAAGCTGCGGTGGGGACAGCTGTTGCCCTATTGTGTG
TCACAGATCTTTGGGGCGTATGTGGGAGCAGCACTGGTCTATTTGCTCTACTACGATGCT
ATAATGGAGTTTAGTGGGGGCGTGTTGACCGTCTATGGGCCAAACGAAACAGCATCTATA
TTTGCCACTTATCCATCTGAGTACCTCCCATTAGGAAAGGGCTTTCTGGACCAAGTGGTG
GGCACCGGAATGCTGCTGCTGTGCCTCCTGAGTTTGACCGAAAAGAGGAACACCCCTGCT
CCTCCTGATCTGATTGCTCCCATAGTTGCAGGCATCGTGCTCGGCATCATCATGTCCATG
TCGGCTAACTGTGGCGCTGCTTTAAATCCCGCTCGAGACCTGGGGCCACGCCTCCTCACG
CTGACTGCAGGCTGGGGAACCGAAGTCTTTACGTGTTACAACTACTGGTTCTGGGTACCA
ATTGTGGCTCCGATTATTGGGGCTCTTACAGGCACTTCTTTGTATTTGATCTTCATCGAA
TGGCAGCTGCCTGAT---GAACCG---------CAGGAGAGTCAC------CCTAAAATC
TGTTCA------GTGGGAGCATCAAGC------------------------TGTCCGTCG

CTATGTGGG---AAAAAGGAGAGGAATCAAAGTAAACATATTTTTAATTAC---------

>Green_swordtail_Aqp10aa1
ATGCTCAAT------TCACGTTCGCTGAGAGTGAGGAGCGCTCTGATCCGACAATGCATG
GCTGAGTTCTTGGGAACATTCGTCTTGCTTCTCTTTGGCTTATCAGCAGCAGCGCAGGTA
AAAACCAGCAGAGAGACTAAAGGCCAAACTCTGTCGATCCACCTGTCTTGGGCCATGGGA
GTTATGTCAGCCACTTATCTCACCAAGGGGATCTCAGGTGCCCACCTGAACCCCGCGGTG
TCTCTGAGTTTCTGTATTTTGGGGAAGCTGCGGTGGGGACAGCTGTTGCCCTATTGTGTG
TCACAGATCTTTGGGGCGTATGTGGGAGCAGCACTGGTCTATTTGCTCTACTACGATGCT
ATAATGGAGTTTAGTGGAGGTGTGTTGACCGTCTATGGGCCAAATGAAACAGCATCTATA
TTTGCCACTTATCCATCTGAGTACCTCCCATTAGGAAAGGGCTTTCTGGACCAAGTGGTG
GGCACTGGAACGCTGCTGCTGTGCCTCCTGAGTTTGACCGAAAAGAGGAACACCCCTGCT
CCTCCTGATCTGATTGCTCCCATAGTTGCAGGCATCGTGCTCGGCATCATCATGTCCATG
TCGGCTAACTGTGGCGCTGCTTTAAATCCCGCTCGAGACCTGGGGCCACGCCTCCTCACG
CTGACTGCAGGCTGGGGAACCGAAGTCTTTACGTGTTACAACTACTGGTTCTGGGTACCA
ATTGTGGCTCCGATTATTGGGGCTCTTACAGGCACTTCTTTGTATTTGATCTTCATCGAA
TGGCAGCTGCCTGAT---GAACCG---------CAGGAGAGTCAC------CCTAAAATC
TATTCA------GTGGGAGCATCAAGC------------------------TGTCCGTCT
CTATGTGGG---AAAAAGGAGAGGAATCAAAGTAAACAAAGTTTTAATTAC---------

>Common_mummichog_Aqp10aa1
ATGCTGAAT------CCACGTTTGCTGAGAGTGAGAAGTGCCCTGATGCGACAATGCATG
GCTGAGTTCCTGGGAACGCTTGTCTTGATTCTCTTTGGCTTATCTGCAGCAGCACAGGTG
ACGACCAGCAGACAGACTAAAGGCCAAACTCTTTCGATCCACCTGTCATGGGCTGTGGGA
GTTATGTCGGCCACATTTCTCACCAAGGGGATCTCGGGTGCCCACCTGAACCCTGCTGTG
TCTCTGTGTTTCTGTATTTTGGGGAAGTTGCGGTGGGGGCAGCTGCTGCCCTACTGTCTC
TCCCAGCTCCTCGGGGCGTATGTGGCAGCAGCTCTGGTCTTTGCCCTCTACTATGATGCT
ATAATGGATTTAGTGGAGGCGTGTTGACCGTTTATGGCCCCAATGAAACAGCATCTATA
TTTGCTACGTATCCGTCTGAGTACCTCACGTTAGGAGGTAGTTTTCTTGACCAAGTAGTG
GGTACTGGAACGCTGTTGCTGTGCATCCTGAGCTTGACCGAAAAGAGGAACACCCCGGCT
CCTCCTGACCTGATTGCTCCCATAGTTGCAGGGATCGTGCTCGGCATTATCATGTCCATG
TCGGCTAACTGTGGTGCTGCTTTAAATCCTGCTCGCGACCTTGGGCCACGTCTCCTCATG
TTGACTGCAGGCTGGGGGACTGAAGTCTTTACGTGTTATAATTATTGGTTCTGGGTACCA
ATTGTGGCTCCGATTACCGGGGCTCTTGCAGGAACTTCCTTGTATTTGTTCTTCATCGAA
TGGCAGCTTCCTGAT---GAACCT---------CTGGAGAAGCTC------CCAACAATC
TCTACC------ATCAGTGACAGCATC------------------------AAGCAGTCC
TCCACCATG---TGGGAAAAGGGAGATGAACTAAAGGCTACACATTTA------------

>Amargosa_pupfish_Aqp10aa1p
ATGCTAAAT------CCAAGTTTGCTGAGAGTGAAGAATGCCCTGATTCGACAATGTATG
GCTGAGTTCTTAGGAACGTTTGTCTTGATTCTCTTTGGTTTAGCTGCAGCAGCACAGGTG
AAAACAAGCAGAGAGACCAAGGGCCAAACTTTGTCAATCCATTTGTCGTGGGCTGTAGGG
GTGATGTCTGCCACATTCCTTACAAAGGGGATTTCAGGTGCCCACCTAAACCCTGCTGTG
TCTCTGTGTTTCTGTATTTTGGGGAAGCTGCAATGGGGTTACCTGGTGCCCTACTTTCTC
TCCCAGGTTGTTGGGGCCTATGTGGGAGCGGCTCTGGTCTATGCACTATACTAT------

---------------------------------------GAAAAGAGGAACACCCCGGCT
CCCCCTGACCTT--

---------------------------------TGTTACAATTATTGGTTTTGGGTACCA
ATAGTAGCTCCAATAACTGGGGCTCTGGCAGGCACTTCCTTGTATTTGTTTTTCATCGAA
---CAGCTGCCTGAC---GAACCA---------ATGGAGAAGCTC------TCAACAATC
TCTACT------GTC---------ATC------------------------AAAAACTCT
TCTGCCATA---TTGGAGAAAGAAGTTGTCGTGAAGTCAACACACTTC------------

>Sheepshead_minnow_Aqp10aa1

ATGCTGAAT------CCACGTTTGCTGAGAGTGAAGAATGCCCTGATTCGACAATGTATG
GCTGAGTTCTTGGGAACGTTTGTCTTGATTCTCTTTGGTTTAGCTGCAGCAGCACAGGTG
AAAACAAGCAGAGAGACCAAGGGCCAAACTTTGTCAATCCATTTGTCGTGGGCTGTTGGG
GTGATGTCTGCCACATTCCTTACAAAGGGGATTTCAGGTGCCCACCTAAACCCTGCTGTG
TCTCTGTGTTTCTGTATATTGGGGAGGCTGCAATGGGGTTACCTGGTGCCCTACTTTCTC
TCCCAGGTTGTTGGGGCCTATGTGGGAGCGGCTCTGGTCTATGCACTATACTATGATGCT
ATAATGGAGTTTAGTGAAGGAGTTTTGACTGTTTATGGCCCTAATGAAACAGCGTCTATA
TTTGCTACATATCCATCTGAATACCTTTCATTAGCAGGGAGTTTTCTTGACCAGGTAGTG
GGCACTGGCACACTGTTATTGTGCATCCTGAGCCTGACCGAAAAGAGGAACACCCCGGCT
CCCCCTGACCTGATTGCACCCATAGTTGCAGGGATTGTGCTTGGCATTATAATGTCCATG
TCGGCCAACTGTGGTGCCGCCTTAAATCCTGCCCGCGACCTTGGACCACGCCTCCTCACA
CTGACTGCAGGCTGGGAACTGAAGTCTTCACGTGTTACAATTATTGGTTTTGGGTACCA
ATAGTAGCTCCGATAACTGGGGCTCTTGCAGGCACTTCCTTGTATTTGTTCTTCATCGAA
TGGCAACTGCCTGAC---GAACCA---------ATGGAGAAGCTC------CCAACAATC
TCTACC------GTC---------ATC------------------------AAAAACTCT
[truncated: 1,028,727 more chars]
